# The Eukaryotic Ancestor Had a Complex Ubiquitin Signaling System of Archaeal Origin

**DOI:** 10.1093/molbev/msu334

**Published:** 2014-12-17

**Authors:** Xavier Grau-Bové, Arnau Sebé-Pedrós, Iñaki Ruiz-Trillo

**Affiliations:** ^1^Institut de Biologia Evolutiva (CSIC-Universitat Pompeu Fabra), Barcelona, Spain; ^2^Departament de Genètica, Universitat de Barcelona, Barcelona, Spain; ^3^Institució Catalana de Recerca i Estudis Avançats (ICREA), Barcelona, Spain

**Keywords:** ubiquitin, SUMO, Ufm1, post-translational signaling, multicellularity, eukaryogenesis, LECA, FECA

## Abstract

The origin of the eukaryotic cell is one of the most important transitions in the history of life. However, the emergence and early evolution of eukaryotes remains poorly understood. Recent data have shown that the last eukaryotic common ancestor (LECA) was much more complex than previously thought. The LECA already had the genetic machinery encoding the endomembrane apparatus, spliceosome, nuclear pore, and myosin and kinesin cytoskeletal motors. It is unclear, however, when the functional regulation of these cellular components evolved. Here, we address this question by analyzing the origin and evolution of the ubiquitin (Ub) signaling system, one of the most important regulatory layers in eukaryotes. We delineated the evolution of the whole Ub, Small-Ub-related MOdifier (SUMO), and Ub-fold modifier 1 (Ufm1) signaling networks by analyzing representatives from all major eukaryotic, bacterial, and archaeal lineages. We found that the Ub toolkit had a pre-eukaryotic origin and is present in three extant archaeal groups. The pre-eukaryotic Ub toolkit greatly expanded during eukaryogenesis, through massive gene innovation and diversification of protein domain architectures. This resulted in a LECA with essentially all of the Ub-related genes, including the SUMO and Ufm1 Ub-like systems. Ub and SUMO signaling further expanded during eukaryotic evolution, especially labeling and delabeling enzymes responsible for substrate selection. Additionally, we analyzed protein domain architecture evolution and found that multicellular lineages have the most complex Ub systems in terms of domain architectures. Together, we demonstrate that the Ub system predates the origin of eukaryotes and that a burst of innovation during eukaryogenesis led to a LECA with complex posttranslational regulation.

## Introduction

Of the three domains of life, eukaryotes have the most complex forms of cell organization. Understanding the emergence and early evolution of the eukaryotic cell is a major challenge for evolutionary biology. Recent findings have profoundly changed our long-held view of a simple last eukaryotic common ancestor (LECA) (Cavalier-Smith 1987, 1991), pointing instead to an ancestor that was already equipped with the machinery required for many of the cellular processes occurring in extant eukaryotes. These include, for instance, the cell division machinery (Makarova et al. 2010), the endomembrane apparatus (Brighouse et al. 2010), the spliceosome (Collins and Penny 2005), nuclear pores (Mans et al. 2004), a wide repertoire of transcription factors (de Mendoza et al. 2013), the RNA interference machinery (Shabalina and Koonin 2008), and cytoskeletal motors (Wickstead and Gull 2011; Sebé-Pedrós et al. 2014). It is unclear, however, whether the LECA already used tightly regulated signaling pathways to control these cellular processes.

We know that signaling systems are crucial in complex cells, as they provide the basis for finely tuned regulation of processes such as transcription (Aravind et al. 2006; Turjanski et al. 2007; Whitmarsh 2007), the cell cycle (Harashima et al. 2013), interactions with the milieu (Seger and Krebs 1995; Deshmukh et al. 2010; Suga et al. 2012), and localization of components within the cell (Field and Dacks 2009; Brighouse et al. 2010). Many of these functions rely on kinase activity and posttranslational protein modification, two signaling strategies of prokaryotic origin that gained importance at the origin of eukaryotes (Aravind et al. 2006). In eukaryotes, posttranslational protein modification by ubiquitin (Ub) constitutes a major source of proteome regulation (Hochstrasser 2009). Thus, understanding the evolution of Ub signaling can provide clues not only into how the LECA regulated its cellular processes but also into the role of signaling systems during the origin and early evolution of eukaryotes. Despite some evolutionary studies devoted to specific gene families (Gagne et al. 2002; Marín 2009a, 2009b, 2010a, 2010b, 2010c, 2013; Eme et al. 2011; Grau-Bové et al. 2013), however, a global picture of the evolution of Ub posttranslational signaling in eukaryotes is still missing.

Ubiquitination consists of the posttranslational modification of proteins by the covalent attachment of Ub, a 76-residue peptide (Hochstrasser 2000). Ub can be linked to proteins in various ways: Monoubiquitination (tagging a single Lys residue of the substrate), multiubiquitination (tagging multiple Lys), and polyubiquitination (Ub chain linked by isopeptide bonds between specific Lys residues) (Hochstrasser 2009). The type of ubiquitination regulates the function of the substrate. For example, poly-ubiquitinated proteins are typically degraded at the 26S proteasomal complex, whereas mono/multiubiquitinated proteins are involved in endocytosis, membrane trafficking, regulation of kinase signaling, DNA repair, and chromatin regulation (Mukhopadhyay and Riezman 2007). Ubiquitination involves a sequential enzymatic cascade: An activating enzyme (E1) for the label, a conjugating enzyme (E2), and a ligating enzyme (E3) that covalently binds the label to the target protein. Moreover, there are specific peptidases (deubiquitinases [DUB]) that reverse the action of E3 ligases (Hochstrasser 2000).

Since the discovery of Ub, other posttranslational signaling pathways, collectively known as Ub-like systems, have been characterized. These systems use different labeling peptides, which often do not have significant sequence similarity with Ub but nonetheless have the same tertiary structure (a β-grasp fold [Hochstrasser 2000]). Ub-like systems share a common enzymatic cascade structure, although most of the specific proteins involved differ between systems (van der Veen and Ploegh 2012). Small-Ub-related MOdifier (SUMO) and Ub-fold modifier 1 (Ufm1) are two of the most relevant Ub-like systems. The SUMO peptide is 100 residues long and shares approximately 18% sequence identity with Ub (Bayer et al. 1998). SUMO acts on a wide range of proteins from various organisms and is involved in ribosomal biogenesis and nuclear functions such as transcription, chromosome organization, DNA repair, or nuclear transport (Johnson 2004; Kerscher et al. 2006; Gareau and Lima 2010). Ufm1 has no significant sequence identity with Ub (Komatsu et al. 2004). It has a narrower range of possible substrates (Hochstrasser 2009) and is involved in the regulation of the endoplasmic reticulum activity and membrane transport, as well as animal development (Komatsu et al. 2004; Tatsumi et al. 2011).

The three systems share the same E1 and E2 enzymes, both of which belong to ancient protein families present in Eukaryota, Bacteria, and Archaea. The prokaryotic E1s and E2s are involved in other signaling systems and were co-opted into new functions with the emergence of the early Ub system (Iyer et al. 2006; Burroughs et al. 2008, 2009; Michelle et al. 2009). Unlike E1s and E2s, there are numerous protein families acting as E3 ligases. A first division can be drawn between HECT and RING protein families, with different and independently evolved catalytic mechanisms (Deshaies and Joazeiro 2009; Rotin and Kumar 2009). RINGs can be further classified into two canonical protein families (C3H2C3, defined by the zf-RING_2 domain, and C3HC4 RINGs, represented by the zf-C3HC4, zf-C3HC4_2, and zf-C3HC4_3 domains) and many unconventional ones (U-box, zf-RING_LisH, RINGv, FANCL, IBR/RBR, and Sina). There are also multiprotein complexes with E3 activity, known as Cullin-RING ligases (CRLs). CRLs are composed of a specific RING type (zf-rbx1), a Cullin subunit (structural backbone of the complex), and different adaptor and target recognition subunits (Cardozo and Pagano 2004; Willems et al. 2004; Petroski and Deshaies 2005; Stone et al. 2005; Deshaies and Joazeiro 2009).

The ligase activity of E3s can be reversed by DUBs, isopeptidase enzymes that cleave Ub chains after the C-terminus of the peptide label (Amerik and Hochstrasser 2004). Some DUBs are specific to a particular kind of Ub linkage (usually Lys48 or Lys63) but most are unspecific and promiscuous (Komander et al. 2009). According to their catalytic mechanism, DUBs are divided into cysteine proteases (UCH, USP, OTU, and Josephin) and metalloproteases (JAB). Finally, the SUMO and Ufm1 systems employ specific E3 and peptidase protein families. There are two E3s (zf-MIZ, RINGs, and IR1-M) and three peptidases (ULP/SENP, WLM, and C97) in SUMO; and one E3 (DUF2042) and one peptidase (C78) in Ufm1.

In this work, we use comparative genomics to decipher the origin and evolution of three Ub-like systems: Ub itself, SUMO, and Ufm1. Our reconstruction shows that the ubiquitination toolkit of the LECA was as complex as that of most modern eukaryotes, in terms of diversity of gene families. Furthermore, various species of Archaea belonging to three different lineages (Euryarchaeota, Crenarchaeota, and Aigarchaeota) already had a minimal but complete ubiquitination toolkit. Thus, Ub signaling existed prior to the origin of eukaryotes and underwent a profound process of innovation during eukaryogenesis, resulting in a complex Ub system in the LECA. Analysis of the subsequent evolution of the Ub-like posttranslational systems in eukaryotes shows that E1 and E2 predate the LECA and underwent little innovation during early eukaryotic evolution, whereas most E3 families appeared concomitantly with eukaryotes and underwent multiple lineage-specific expansions and diversifications of protein domain architectures. We also describe two independent expansions of the Ub signaling system at the origins of multicellularity in animals and plants. Overall, we show that the complexity of the LECA involved the capacity to perform posttranslational regulation of different cell processes by Ub and Ub-like systems. This suggests that Ub signaling was key to the origin of eukaryotes and was later expanded in some specific, mostly multicellular, lineages.

## Results

### A Comparative Survey of the Ub System Reveals an Archaeal Origin and a Complex Toolkit in the LECA

**Fig. 1.**
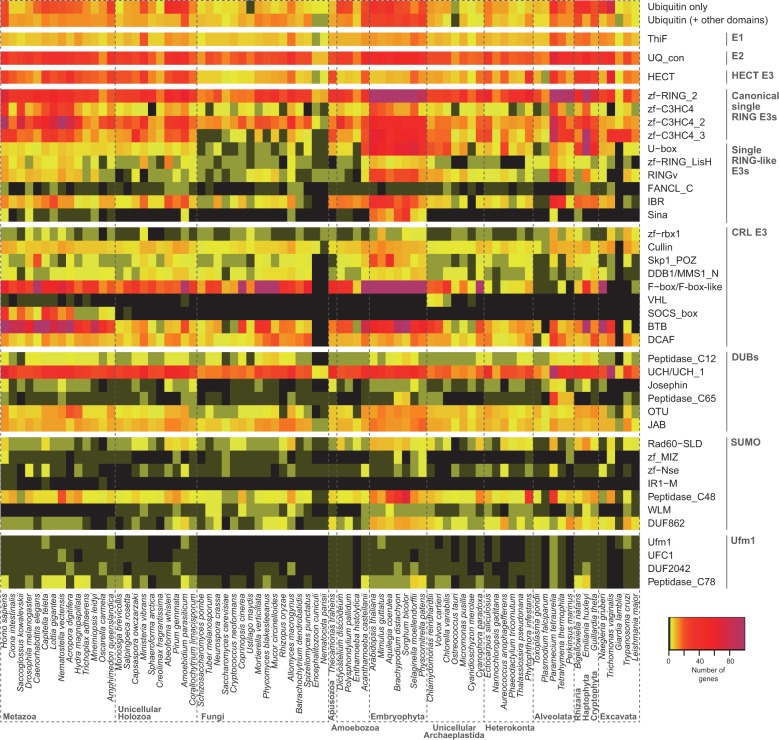
Presence and abundance of the different components of the Ub, SUMO, and Ufm1 systems in eukaryotes. The heat map depicts absolute protein counts in each of the sampled genomes, according to the color scale. The Ub domain is divided into Ub-only (which includes Ub labels and poly-Ub peptides) and Ub + other domains (which includes proteins which make use of Ub domains for functions other than protein labeling).

**Fig. 2.**
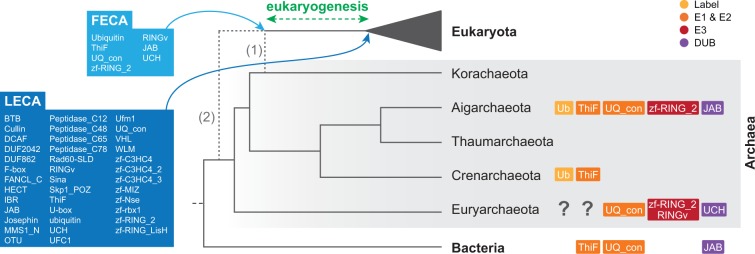
Pre-eukaryotic evolution of Ub and Ub-like systems. The dashed lines indicate two possible phylogenetic scenarios: the Eocyte hypothesis for the origin of eukaryotes within Archaea (1) (Williams et al. 2012) and the “three domains” hypothesis for the relationships among Eukaryota, Bacteria and Archaea (2) (Woese et al. 1990). The reconstruction is the same with both hypotheses. The Ub, SUMO, and Ufm1 toolkits before and after the eukaryogenesis process (i.e., the FECA and the LECA) are shown. Boxes to the right of the cladogram represent the components of the Ub toolkit found in each archaeal group.

To elucidate the origin and evolution of Ub-like systems, we first examined the presence and abundance of 40 protein families related to Ub, SUMO, and Ufm1 signaling in a broad range of eukaryotic genomes (see supplementary table S1, Supplementary Material online, and Materials and Methods). Specifically, we surveyed the generalist E1 and E2 enzyme protein families, 27 specific components of the Ub system (including the peptide label, E3s, and peptidases), 7 families related to SUMO, and 4 related to Ufm1 (see supplementary table S2, Supplementary Material online, and Materials and Methods). Our survey revealed that 38 of these 40 protein families are widespread among eukaryotic groups (fig. 1). We found that complete toolkits for Ub, SUMO, and Ufm1 systems exist in all the main groups of eukaryotes except for Fungi, in which Ufm1 is missing (see below). This phylogenetic distribution indicates that Ub, SUMO, and Ufm1 are ancient systems that were already present in the LECA (fig. 2).

To trace back the origin of the different signaling systems, we also examined a comprehensive database of prokaryotic genomes (see Materials and Methods). Although none of the analyzed bacterial genomes contained a complete Ub toolkit, many bacteria were found to possess signaling systems that employ JAB peptidases, and E1 and E2 enzymes akin to the ones acting in ubiquitination (Iyer et al. 2006; Hochstrasser 2009; Humbard et al. 2010). These bacterial homologs act in functional contexts unrelated to protein labeling, such as molybdopterin and thyamin biosynthesis (ThiF E1) and siderophora biosynthesis (JAB) (Iyer et al. 2006; Koonin 2006). We also found F-box, U-box, and DUB enzymes in a few genomes of obligate intracellular parasitic bacteria, such as *Agrobacterium tumefaciens*, *Legionella pneumophila*, *Candidatus* Amoebophilus asiaticus, or various Chlamydiae, probably as a result of independent horizontal gene transfer (HGT) events (Koonin et al. 2001; Spallek et al. 2009; Schmitz-Esser et al. 2010). Despite lacking Ub systems of their own, these pathogens exploit their hosts’ by mimicking various signaling effectors (Spallek et al. 2009). Overall, the Ub-specific components analyzed clearly evolved after the origin of bacteria.

Unlike in bacteria, Ub-specific protein families were observed in many Archaea. Previous work by Nunoura et al. (2011) identified a bona fide eukaryotic-like Ub peptide and an E3 ligase in the Archaea *Caldiarchaeum subterraneum.* In our survey, we found evidence of eukaryotic-like Ub toolkits in three independent Archaea lineages: Crenarchaeota (including eight environmental genomes from the YNPFFA candidate group with Ub labels), Euryarchaeota (one environmental genome with a UCH DUB, C3H2C3s and a RINGv E3: marine group ii euryarchaeote SCGC AB-629-J06), and Aigarchaeota (11 environmental genomes from the pSL4 candidate group, seven of them with complete ubiquitination toolkits, and *C. subterraneum,* also with a complete toolkit) (fig. 2). Interestingly, the number of Ub-related genes in some of these genomes was found to be quite high, including nine C3H2C3 RING (zf_RING_2 domain) E3s in an aigarchaeote and up to six C3H2C3 RING plus a RINGv in the euryarchaeote. In addition, C3H2C3 RING genes have also been detected in two unclassified archaea (fig. 2 and supplementary file S1, Supplementary Material online).

To determine whether HGT of eukaryotic sequences into prokaryotic genomes could have occurred, we conducted Basic Local Alignment Search Tool (BLAST) similarity searches for all the protein families present in Archaea and phylogenetic analyses of Ub, UQ_con, and UCH (see Materials and Methods for details). None of the prokaryotic genes were found to be unexpectedly similar to eukaryotic sequences according to these methods. Thus, under the current taxon sampling, we can rule out a HGT origin for the archaeal toolkit (supplementary figs. S6 and S7 and file S3, Supplementary Material online).

In contrast, both SUMO and Ufm1 were found to be absent from Archaea and Bacteria. Thus, extant archaeal genomes contain a complete Ub toolkit that includes Ub label, E1 ThiF enzyme, E2 UQ_con enzyme, two different E3 ligases (C3H2C3 RING and RINGv), and two different DUBs (JAB and USP) (fig. 2), whereas SUMO and Ufm1 are specific to eukaryotes.

### Evolution of Ub Signaling in Eukaryotes: Massive Secondary Losses, Few Gains, and Expansion of Gene Families

**Fig. 3.**
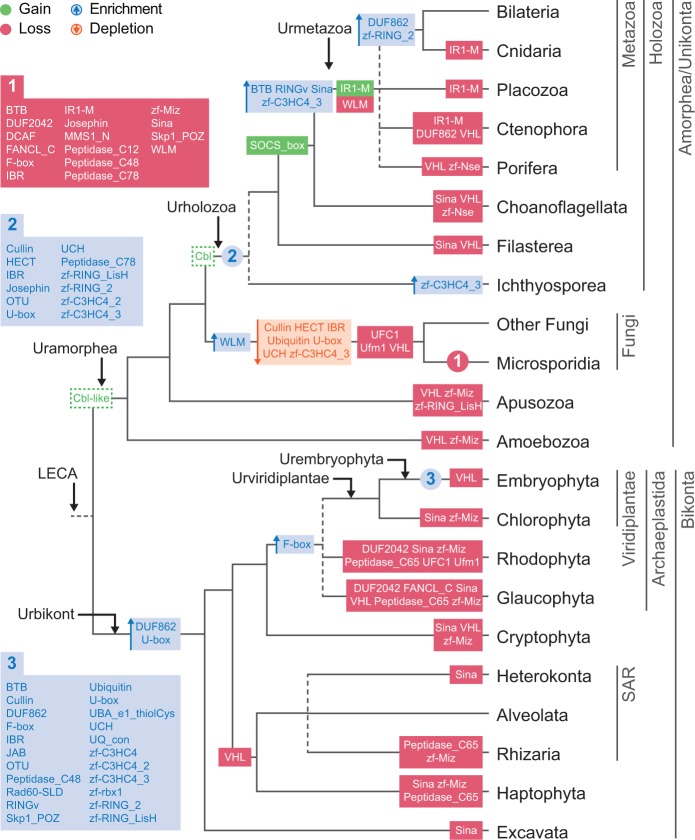
Pattern of gains/losses and enrichments/depletions of the components of the Ub, SUMO, and Ufm1 systems in eukaryote evolution. The modified “unikont-bikont” hypothesis for the root of eukaryotes is assumed (Derelle and Lang 2012). See supplementary figure S1*A*, Supplementary Material online, for an alternative reconstruction with Excavata as the earliest branching lineage (He et al. 2014) and supplementary figure S1*B* and *C*, Supplementary Material online, for gain/loss reconstructions based on likelihood methods. Solid green and red boxes indicate gains and losses of gene families, respectively. Shaded blue and orange boxes indicate significant quantitative enrichments and depletions in the number of genes, respectively. Eukaryotic ancestors reconstructed in figure 5 are indicated by black arrows at the tree's nodes.

To better understand the evolution of the Ub system in eukaryotes, we examined the counts of two generalist gene families (E1 and E2 enzymes) and 38 protein families that are specific to a particular Ub-like system (peptide labels, E3 ligases, and peptidases) (fig. 1). We then reconstructed the patterns of gains and losses of each Ub-like signaling toolkit across eukaryotes using information of the phylogenetic distribution of each protein family (fig. 3). Finally, we also checked for statistically significant gene enrichments and depletions between eukaryotic groups (fig. 3), that is, significant quantitative changes in the number of proteins of a particular family. In contrast, gains and losses are defined as zero-to-one or one-to-zero state changes.

Our analysis indicates that the LECA already had most of the surveyed gene families, independently of whether we root eukaryotes between unikonts/amorpheans and bikonts (Derelle and Lang 2012) or between excavates and the rest (He et al. 2014). In particular, under the modified “unikont-bikont” hypothesis for the root of eukaryotes (fig. 3), we identified only two gains: SOCS-box and IR1-M gene families (part of the Ub and SUMO E3 toolkits, respectively). Under the assumption of the “Excavata-first” hypothesis, the sole difference was the appearance of Sina E3s after the divergence of excavates (supplementary fig. S1*A*, Supplementary Material online). Finally, using likelihood-based gain/loss reconstruction (supplementary fig. S1*B* and *C*, Supplementary Material online), we obtained a similar result compared with the parsimony-based analysis (33 and 36 gene families in the LECA, respectively, under the “unikont-bikont” hypothesis for the root of eukaryotes). This shows that the recruitment of novel machinery in Ub-like systems is a relatively exceptional event during eukaryotic evolution, especially when compared with the frequent losses of individual system-specific gene families.

Among Ub-like signaling systems, we found that ubiquitination is the most gene-rich pathway in most of the examined eukaryotes, followed by SUMO and Ufm1 (supplementary fig. S2, Supplementary Material online). Indeed, the proportion of Ub-related genes can add up to approximately 5% in some plant genomes (Smalle and Vierstra 2004; Stone et al. 2005), making it one of the most expanded gene toolkits in several eukaryotes. In the supplementary information, Supplementary Material online, we describe our findings for specific components of the system.

### The Diversification of the Eukaryotic Ub System Is Driven by Architectural Rearrangements

To further analyze the diversification of Ub-like systems in eukaryotes, we used the array of domain architectures of each protein family as a proxy to assess the diversity and versatility of the Ub, SUMO, and Ufm1 toolkits. In particular, we compared the number of different protein domains that co-occur alongside the core protein domain of each protein family (see Materials and Methods). The most abundant families (e.g., canonical RINGs, F-box, BTB, DUBs, and deSUMOylases) are also the most diverse in terms of architectures (fig. 1 and supplementary fig. S4, Supplementary Material online), thereby implying a functionally diversifying gene expansion process.

**Fig. 4.**
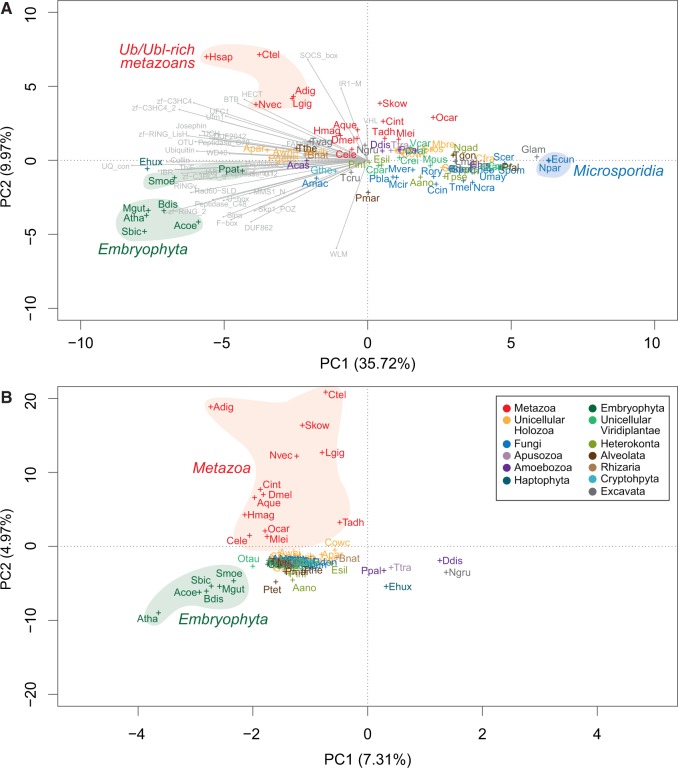
Phylogenetic patterns in the composition of the Ub-like systems. (*A*) Clustering of 78 eukaryotic genomes in a multidimensional space for the gene count of the Ub, SUMO, and Ufm1 systems, using a PCA. The two first principal components are displayed, accounting for 33.97% and 10.09% of the variation, respectively. (*B*) Clustering of 78 eukaryotic genomes in a multidimensional space for the counts of the number of each different domain architectures in the Ub, SUMO, and Ufm1 systems, using a PCA. The two first principal components are displayed, accounting for 7.31% and 4.97% of the variation, respectively. Organisms names are color coded according to taxonomic assignment (see label). Shades indicate particular groups of genomes referred to in the main text. See supplementary table S1, Supplementary Material online, for a list of organism acronyms and Materials and Methods for details on the PCA analysis.

To test whether there are phylogenetic patterns in the profiles of gene counts and architectural diversity of the Ub-like systems, we performed principal component analyses (PCA, see Materials and Methods for details) (fig. 4). The PCA based on gene counts revealed that embryophytes and metazoans have gene content profiles that differ from those of other eukaryotes (fig. 4*A*). In particular, we found that the principal component 1 identified a group of genomes rich in genes related to Ub-like signaling systems, including embryophytes, many animals (especially eumetazoans: *Homo sapiens, Capitella teleta,* or *Nematostella vectensis*) and ichthyosporeans (*Abeoforma whisleri*, *Pirum gemmata,* and *Amoebidium parasiticum*). Furthermore, PC2 differentiated most holozoans from embryophytes, which both clustered separately from the rest of the eukaryotes due to the loadings of many protein families that appeared or expanded in holozoans (e.g., HECT, BTB, SOCS-box, IR1-M, and C3HC4 RINGs) and plants (e.g., F-box, U-box, and C3H2C3 RINGs), respectively. The distinction between plants and holozoans (particularly animals) was also recovered by the PCA based on protein architectures (fig. 4*B*): Plants and animals, while sharing all the surveyed protein families, had specific sets of protein architectures that distinguished them from the rest of the eukaryotes.

### The Ancestral Ub Toolkit Revealed by Domain Networks

**Fig. 5.**
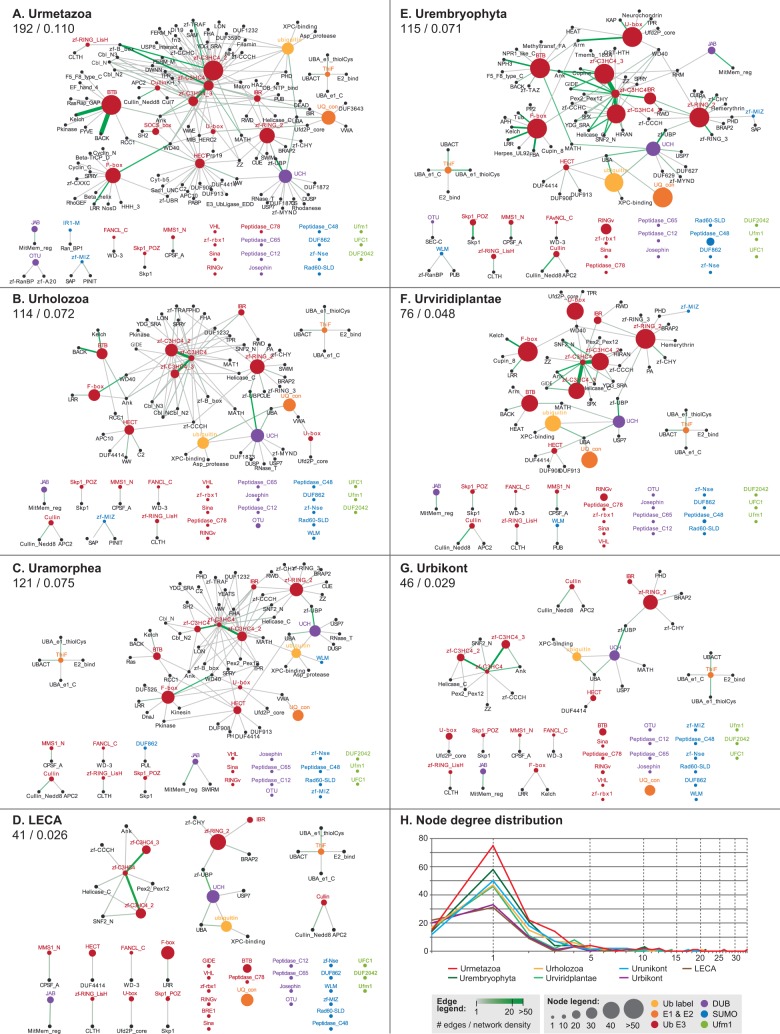
Reconstruction of the ancestral networks of accessory domains of Ub, SUMO, and Ufm1 systems. The systems are reconstructed at the last common ancestors of (*A*) Metazoa, (*B*) Holozoa, (*C*) Amorphea, (*D*) Eukaryota, (*E*) Embryophyta, (*F*) Viridiplantae, and (*G*) Bikonta. Colored nodes represent core protein family domains, and black nodes represent their inferred accessory domains. The size of colored nodes is an estimation of the gene content of each ancestor. Edges link core with accessory domains and core domains between them and are color- and width coded according to the inferred number of such concurrences in each ancestor. For each network, the network density index, the number of edges, and the node degree distribution (*H*) are shown (see Materials and Methods). See figure 3 for the phylogenetic position of the reconstructed nodes.

To gain insight into the complexity of Ub-like signaling during eukaryotic evolution, we used the protein domain architectures of extant species to reconstruct ancestral domain networks at various ancestral nodes of the eukaryotic tree (fig. 5) (see Materials and Methods). In particular, we inferred the network of accessory domains of genes related to Ub signaling in the urmetazoan, urholozoan, uramorphean, LECA, urembryophyte, urviridiplantae, and urbikont (fig. 5*A*–*G*, see fig. 3 for the phylogenetic positions of the reconstructed nodes).

We inferred that many Ub-related genes already employed multiple accessory protein domains (in black) in several Ub-related genes in the LECA (fig. 5*D*), although less than in most extant eukaryotes. For example, the LECA’s Ub toolkit used highly promiscuous domains such as Ankyrin repeats (linked to C3HC4 RINGs), UBA (Ub-associated domain, linked to USPs and Ub), and LRR (linked to F-box). Architectural diversification during eukaryogenesis also led to specific domain combinations in E1 and E2 protein families, which use exclusive sets of accessory domains (e.g., E1s have UBA_e1_thiolCys, UBACT, and UBA_e1_C domains) and have little interconnection with other nodes. These E1 and E2 types are conserved in all the other ancestral nodes and characterize the eukaryotic Ub network. Also, the usage of multidomain proteins in the early eukaryote appeared as an important difference compared with archaeal systems, in which all genes encode single-domain proteins.

Since the origin of eukaryotes, the connectivity and network density of Ub and SUMO toolkits independently increased in Amorphea and Bikonta, although to a lesser extent in Bikonta. This led to rich signaling systems in multicellular animals and plants (fig. 5*A*–*C* and *E*–*G*), confirmed by the PCA based on domain architectures (fig. 4*B*). Nevertheless, we found that the network structure of the deep ancestors influenced later ancestors and extant organisms. For example, the urembryophyte’s less extensively connected domain network could be traced back to the urbikont (fig. 5*E*–*G*). This phylogenetic inertia constrained the Ub and SUMO systems of plants, whose expansion was not accompanied by a significant increase of protein architectures. Conversely, the diversified toolkits of animals were recapitulated in the denser domain networks of the urmetazoan, the urholozoan, and the uramorphean (fig. 5*A*–*C*).

Despite these differences in network density, patterns common to all the ancestral networks emerged (fig. 5*A*–*C* and *E*–*G*). The most abundant catalytic machinery of Ub signaling employed a similar core of highly connected nodes in all the post-LECA ancestors. This included the C3HC4 variants (which shared most of their accessory domains and often co-occurred themselves), C3H2C3/zf-RING_2 (highly connected but not directly linked to other RINGs), IBR, or U-box. The CRL substrate recognition subunits BTB and F-box were both highly connected, particularly to protein-binding domains. In contrast, BTB and F-box shared few nodes, thus suggesting independent diversifications. For example, F-box often co-occurred with Kelch (in plants), LRR, and WD40, whereas BTB used Ankyrin, Kelch, BACK, and NPH3 (a signal-transducing motif that appears at the origin of plants).

## Discussion

### The Ancient Ub System and the Origin of Eukaryotes

Our data show that the core components of the eukaryote Ub system originated in Archaea and predate the process of eukaryogenesis that led to the LECA. In particular, the core Ub toolkit inferred from extant Archaea includes Ub, E1s, E2s, two different RING E3s, and two different DUBs (fig. 2). Interestingly, ubiquitination has been hypothesized to be a key mechanism for the symbiogenic origin of eukaryotes, during which it would be needed to act as a barrier against aberrant proteins resulting from the massive invasion of bacterial Group II introns into the host archaeal genome (Koonin 2006, 2011). Thus, our results are consistent with the presence of a complete Ub signaling toolkit in the theoretical proto-eukaryote, termed the first eukaryotic common ancestor (FECA) (Koonin 2011; Koumandou et al. 2013).

The initial toolkit was expanded during the stem phase of eukaryotic evolution with the addition of numerous new types of enzymes and an increase in the number of genes in some families (fig. 2). Similarly, the network of accessory domains of the LECA (fig. 5*D*) reveals that eukaryotic Ub-like systems switched to the use of multidomain protein families during their early evolution, whereas archaeal toolkits consist only of the catalytic protein domains. The presence of accessory domains within protein families reflect their ability to physically interact with other cellular components (Basu et al. 2008), which indicates that the rise of new protein families during eukaryogenesis was accompanied by an increasingly connected Ub domain architecture network. Interestingly, this increase in the LECA’s regulatory potential was concomitant with the appearance of eukaryote-specific cellular functions regulated by ubiquitination, such as endocytosis, vesicle trafficking, and histone modification, as well as nuclei-specific DNA repair machinery. Altogether, we find that Ub signaling expanded in multiple ways as the first complex eukaryotes evolved.

Overall, our analyses indicate that the LECA had a rich and complex repertoire of Ub signaling genes, generating an extensive ancestral core machinery shared by most of the extant eukaryotic lineages. Given that some gene families were also secondarily, and recurrently, lost during eukaryotic evolution (fig. 3), our results suggest that there were two phases in the evolution of Ub signaling: 1) an initial period of rapid innovation during eukaryogenesis, in which the minimal FECA toolkit was enriched with new gene families exclusive to eukaryotes and 2) a long process of toolkit contraction (loss of gene families) in various eukaryotic lineages. These findings fit the biphasic model of reductive genome evolution proposed by Wolf and Koonin (2013) and strengthen the idea of eukaryogenesis as a burst of innovation in the history of life.

### Diversification of Ub Signaling and the Origins of Multicellularity

Our data show that the core machineries of Ub, SUMO, and Ufm1 signaling were already present in the LECA (fig. 2). Subsequently, each eukaryotic group developed Ub-like systems. This dynamic evolutionary history was mainly driven by lineage-specific gene expansions, architectural diversification of protein domains, occasional recruitment of new machinery, and abundant gene losses.

Gene expansions mostly affected E3 ligases and peptidases of Ub and SUMO toolkits, that is the effector enzymes responsible for substrate selection. Also, we found that the most enriched E3 and peptidase families often made use of promiscuous protein-binding domains, namely RINGs (canonical, IBR and U-box) and CRLs’ substrate selector subunits (BTB and F-box), HECTs, and USPs. Likewise, HECTs are also rich in motifs that bind to lipids, complex sugars, and poly-A tails of RNA (Grau-Bové et al. 2013). The presence of such domains in the effector enzymes increases the substrate specificity and fine-tuned localization of Ub and SUMO (Tordai et al. 2005; Bhattacharyya et al. 2006; Di Roberto and Peisajovich 2013). Thus, the expansions of Ub and SUMO signaling brought an increased regulatory accuracy and functional diversification.

**Fig. 6.**
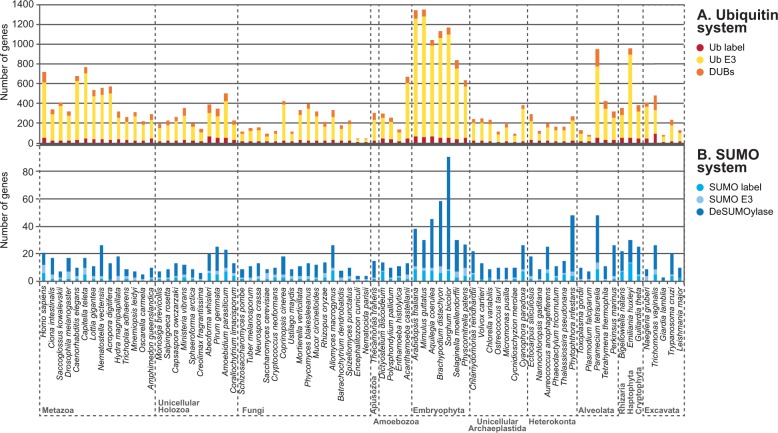
Composition of Ub and SUMO toolkits. Number of Ub- and SUMO-related proteins (upper and lower charts, respectively), including the label itself, E3s, and peptidases. Note that specific deSUMOylases are consistently more abundant than SUMO E3s in most eukaryotes, whereas the opposite is true for Ub-related enzymes.

Our analysis also reveals that deSUMOylases are more abundant and diverse than SUMO E3s in most eukaryotes. The opposite pattern is found in ubiquitination, where Ub E3s outnumber DUBs (fig. 6). We therefore propose that two different strategies underlie the specificity of SUMO and Ub labeling in eukaryotes: SUMO relies on postlabeling regulation mediated by peptidases, whereas Ub depends on directed E3 activity. Consistent with this hypothesis, the expansion of SUMO peptidases in *Arabidopsis thaliana* entailed sub- and neofunctionalization events, whereas its E3s are often redundant (Chosed et al. 2006; Colby et al. 2006). In addition, humans, yeast, and *Ar. thaliana* can tune SUMOylation using a substrate-specific SUMO paralogs and paralog-specific peptidases (Saitoh and Hinchey 2000; Mukhopadhyay and Dasso 2007; Hickey et al. 2012). We also know that SUMO E2s can directly affect signaling in a nonspecific manner, without using E3s (Reverter and Lima 2005). We see how, from an identical pathway in the early eukaryote, different modes of posttranslational signaling regulation evolved for SUMO and Ub.

Comparing the two structural types of Ub E3s, we see that RING families are more abundant and architecturally diverse than HECTs in all eukaryotes (fig. 1 and supplementary fig. S4, Supplementary Material online). This might be explained by the fact that HECTs’ tertiary structure is intrinsically constrained, as they require their catalytic site to be at the C-terminus to be active (Huang et al. 1999; Verdecia et al. 2003; Rotin and Kumar 2009). Consequently, they do not undergo C-terminal domain shuffling in any eukaryote (Grau-Bové et al. 2013). Also, the evolvability of RING-based catalysts was further increased by the emergence of CRLs, a combinatorial system of modular subunits with specific functions (e.g., interaction with E2s and substrates). Thus, historical and protein structural constraints explain the prevalence of RING-based catalysts in eukaryotes.

The greatest sophistication of Ub-like signaling systems is found in embryophytes and metazoans. These groups have the richest and most diverse Ub and SUMO systems among all eukaryotes (fig. 1). Moreover, the reconstruction of domain networks of ancestral Ub toolkits reveal that extensive innovation occurred at the origin of both animals and plants, probably through processes of domain shuffling that made use of already-in-place molecular machineries (fig. 5). Although most of the surveyed protein families existed prior to the origins of animals and plants, we find that ubiquitination diversified extensively in these multicellular contexts through new domain combinations and gene number expansions (fig. 1 and supplementary fig. S4, Supplementary Material online). This may be due to the complex multicellularity of plants and animals, which requires fine-tuned regulation of cellular functions. Indeed, parallel to this complexification of posttranslational regulation, animals and plants are known to have a rich transcriptional regulation machinery, probably related to their complex development (de Mendoza et al. 2013).

Despite their similarities, the expansions of Ub-like signaling in multicellular animals and plants were independent: Each lineage expanded different protein families (fig. 4*A*) and diversified its toolkit with different accessory domains (fig. 5). This lack of protein architecture conservation among eukaryotes is common in other multidomain protein families (Basu et al. 2008, 2009). The rise and diversification of multidomain protein families by shuffling is also recurrent in animal genomes (Tordai et al. 2005) and is regarded as a key genomic event to explain the origin of multicellularity (King et al. 2008). Shuffling of ubiquitous and promiscuous domains is a major source of evolvability in eukaryotic signaling networks (Basu et al. 2008), as exemplified by tyrosine kinases (Deshmukh et al. 2010; Suga et al. 2012), Notch (King et al. 2008; Gazave et al. 2009), or Hedgehog toolkits (Snell et al. 2006; Adamska et al. 2007). Here, we identify independent bursts of innovation by domain shuffling underlying the complex Ub and SUMO systems of both animals and plants.

## Conclusions

In summary, we found that Ub signaling predates the origin of eukaryotes, as core components of the pathway are present in three different archaeal groups: Aigarchaeota, Crenarchaeota, and Euryarchaeota. The Ub machinery of the earliest eukaryotes thus consisted of E1 and E2 enzymes (common to all three domains of life), two RING E3 types (canonical C3H2C3 and RINGv), and two peptidases (USP and JAB). This early Ub system underwent an important process of innovation during the eukaryogenic phase that led to the LECA.

We propose that three processes shaped Ub signaling during early eukaryotic evolution. First, almost all the Ub-related gene families seen in extant eukaryotes emerged at that time. This includes new catalytic mechanisms (e.g., HECTs and new peptidases) and, most importantly, two eukaryote-specific signaling systems (SUMO and Ufm1). Second, some gene families underwent massive expansions (e.g., RINGs and the highly versatile multisubunit CRLs). Finally, new and diverse protein domain architectures were acquired in both ancient and new enzyme families (e.g., E1s and CRLs’ substrate selectors BTB and F-box). Altogether, these events identify the stem phase of eukaryotic evolution as a period of rapid and intense innovation in posttranslational signaling.

After the initial eukaryotic radiation, the Ub and Ub-like systems further evolved by protein family expansion and domain architectural diversification, in a largely lineage-specific manner. There was, however, little protein family innovation, with only IR1-M (animal SUMO E3s) and SOCS-box selectors (holozoan CRLs) evolving later on. These diversification processes particularly affected E3s ligases (in the case of the Ub system) and delabeling peptidases (in the case of the SUMO system) probably because they are in charge of the target selection specificity. In this sense, the diversification of domain architectures in these families is related to the substrate specificity, with new accompanying domains allowing selective interaction with other proteins, complex sugars, lipids or nucleic acids. This process of architectural innovation was especially intense at the origin of animals and plants, coinciding with their need for a precise regulation of multicellularity-related protein products and processes. Thus, alongside the eukaryogenic phase of Ub expansion, the origins of multicellular animals and plants represent the main bursts of innovation in Ub systems in eukaryotes.

Overall, our investigation into the diversity of early eukaryotic Ub signaling clearly points to an important burst of evolutionary innovation at the origin of eukaryotes. This suggests that the LECA was much more complex than previously thought, not only in terms of cellular machineries but also in terms of elaborate regulation systems such as Ub signaling.

## Materials and Methods

We obtained all the proteins related to Ub, SUMO, and Ufm1 systems from a selection of 78 eukaryotic proteomes, the nonredundant Archaea and Bacteria protein database from National Center for Biotechnology Information (NCBI), and genomic data from the Microbial Dark Matter project (Rinke et al. 2013) (supplementary table S1, Supplementary Material online). The selection of eukaryotic taxa includes 14 animals, 10 unicellular holozoans, 16 fungi, 1 apusozoan, 4 amoebozoans, 7 embryophytes, 7 unicellular algae (chlorophytes, rhodophytes, and glaucophytes), 6 heterokonts/stramenopiles, 5 alveolates, 1 rhizarian, 1 haptophyte, 1 cryptophyte, and 5 excavates. We obtained the proteomes from publicly available databases, with the exception of *Oscarella carmela* and *Mnemiopsis leidyi*, kindly provided by Scott A. Nichols (University of Denver) and Andy Baxevanis (National Human Genome Research Institute), respectively. We also used RNA-Seq data generated in-house (*Ministeria vibrans*, *P. gemmata*, *Abeoforma whisleri*, *A. parasiticum,* and *Corallochytrium limacisporum*) (de Mendoza et al. 2013). We performed a Pfamscan on all eukaryotic proteomes and transcriptomes using Pfam A version 26 and selecting the gathering threshold as a conservative approach to minimize false positives (Punta et al. 2012). The identification of bacterial and archaeal sequences was done using HMMER (Eddy 1998), searching the hmm profiles of all the domains (supplementary table S2, Supplementary Material online) against the NCBI Bacteria and Archaea databases and the Microbial Dark Matter project database (Rinke et al. 2013).

We unambiguously assigned each protein of interest (including labeling peptides and E1, E2, E3, and delabeling enzymes) to a certain Pfam domain, referred to as the core defining domains of each protein family (see supplementary table S2, Supplementary Material online, for a complete list of protein families, associated Pfam domains, and examples of specific genes in model organisms). The ThiF, zf-MIZ, and DCAF protein families were identified, refining the domain search with specific amino acid motifs. Specifically, proteins with ThiF and Moez/MoeB catalytic motifs do not have E1 activity and were discarded (Burroughs et al. 2009); zf-MIZ were selected by picking those architectures involving this domain combined with PINIT and/or SAP motifs; and DCAFs were identified by selecting proteins composed of WD40 domains and then retaining those that had a DWD motif (He et al. 2006; Hua and Vierstra 2011) with the following logo: [D|E] XXXX [I|L|V] [W|Y] [D] [I|L|V|M] [R|K].

Using R (R Development Core Team 2008), we built heat maps based on 1) the number of proteins involving a given core domain in each genome and 2) the number of accessory domains (i.e., total number of different domains that appear with a particular core domain in the same predicted ORF). Additional heat maps of the domain architectures in which each core domain is involved were built (supplementary fig. S5, Supplementary Material online). Statistical analyses were performed using R to detect enrichments or depletions in gene content in different lineages, using the Wilcoxon rank sum tests with a significance threshold of *P* < 0.01.

We used the BLAST (Camacho et al. 2009) to look for a potential HGT origin for the archaeal Ub, UQ_con, zf-RING_2, RINGv, and UCH proteins (supplementary fig. S7 and table S3, Supplementary Material online). We searched all the archaeal sequences (identified by HMMER searches, see above) with a cut-off value of 10^−^^5^ and against a combined database including the full NCBI nonredundant protein database, the Microbial Dark Matter database, and the full genomes and transcriptomes included in this study. We took the top 50 hits and searched them back to the same combined database, with a cut-off value of 10^−^^10^. The network visualizations of this reciprocal BLAST analyses were generated using Cytoscape 3.1.1 (Smoot et al. 2011). We included the raw BLAST outputs in supplementary file S3, Supplementary Material online. Additionally, we performed phylogenetic analyses with UQ_con, UCH, and Ub families (zf-RING_2 and RINGv are not suitable for phylogenetic analysis because they are defined by short and poorly-conserved amino acid motifs). For these analyses, we used 1) all the Pfamscan-identified proteins from our selection of eukaryotes, 2) the identified archaeal sequences from NCBI and the Microbial Dark Matter databases, and 3) the top 100 hits from the BLAST searches in these databases. The alignments were performed using the Mafft L-INS-i algorithm, optimized for local sequence homology (Katoh and Standley 2013), and inspected and manually revised. We used the matched-pairs test of symmetry (Ababneh et al. 2006), implemented in Homo 1.2 for amino acids (http://www.csiro.au/Homo last accessed 1 October 2014), to determine whether the aligned sequences of amino acids are consistent with evolved under time-reversible conditions (assumed by most model-based phylogenetic programs). Based on the PP plots shown in supplementary figure S6*A*, Supplementary Material online, it was concluded that the data did not violate this assumption. The phylogenetic trees of UQ_con, UCH, and Ub were estimated using the Le and Gascuel (LG; 2008) evolutionary model with a discrete gamma (Γ) distribution of among-site variation rates (four categories), according to the respective analyses performed with ProtTest 3.4 (Darriba et al. 2011). The LG+Γ model with four categories was used in 1) maximum likelihood (ML) phylogenetic trees estimated with RaxML 7.2.8, using 100 bootstrap replicates as statistical support for the bipartitions (Stamatakis 2006) and 2) Bayesian inference trees calculated with PhyloBayes 3.3 (Lartillot et al. 2009), using two parallel runs for 500,000 generations and sampling every 100; and using Bayesian posterior probabilities as statistical support.

The reconstruction of ancestral states of each core element was inferred with Mesquite 2.75 using both a parsimony criterion and the AsymmMk likelihood model (http://mesquiteproject.org, last accessed 1 October 2014). We assumed two scenarios for the root of eukaryotes: 1) the modified “unikont-bikont” hypothesis (Derelle and Lang 2012) but renaming Unikonta as Amorphea (Adl et al. 2012) and 2) the “Discoba-first” hypothesis (He et al. 2014). For the relationships between Eukaryota, Bacteria, and Archaea, we contemplated both the “Eocyte” (eukaryotes root within Archaea) (Williams et al. 2012; Williams et al. 2013) and “three domains” hypotheses (Woese et al. 1990). The AsymmMk model was implemented with bias of 0.1 between gain and loss rates, with rates of change estimated by the model and taking into account branch lengths. To estimate the branch lengths, we built a multiprotein alignment with Hsp90, Hsp70, and actin homologs using Mafft L-INS-i (Katoh and Standley 2013), which was manually inspected. The matched-pairs test of symmetry performed using Homo showed that these sequences did not violate the time-reversibility assumption (supplementary fig. S1*D*, Supplementary Material online). In this case, ProtTest showed that the best evolutionary model for our data set was LG with a Γ distribution of four discrete categories and a proportion of invariable sites (LG+Γ+I). Using this model (PROTGMMAILG), we used RAxML with a fixed topology (consensus eukaryotic phylogeny, as in fig. 3 and supplementary fig. S1*A*, Supplementary Material online).

A PCA was performed using built-in R *prcomp* function, using scaling (so that all variables have unit variance before the analysis takes place) and a covariance matrix, and plotted using *bpca* R package. We used scaling because our data, although presenting the same units (counts of number of genes), show very different ranges of values (with some families having hundreds of genes and others just one or two). The PCA of the protein counts (fig. 4*A*) was based on the number of genes of each family in each species. In the PCA of protein domain architectures (fig. 4*B*), instead, the species were clustered based on the number of proteins with a particular domain architecture. To this end, we first created a list of all the existing protein domain architectures (for all protein families) and then counted how many proteins (with each particular architecture) each species has. These raw counts can be visualized in supplementary figure S5, Supplementary Material online.

Finally, we inferred the accessory protein domains of each protein family at ancestral nodes of the eukaryotic tree by comparing domain architectures (same raw data as for the PCA in fig. 4*B* and supplementary fig. S5, Supplementary Material online) within the corresponding clades. We represented these reconstructions as networks of co-occurring domains using Cytoscape 3.1.1 (Smoot et al. 2011). Our criterion linked core domains (central nodes, listed in supplementary table S2, Supplementary Material online) to accessory domains (other protein domains that co-occur with a core domain in the same protein) if such co-occurrence existed in at least the earliest-branching lineage of a clade and another internal taxon. We used a nested approach, first reconstructing the most external nodes and proceeding inward (e.g., first Bilateria, then Eumetazoa, followed by Metazoa, Holozoa, etc.). The abundance of each core domain (represented by the size of the node) at the reconstructed ancestors of particular clades was estimated with the median gene count of all the analyzed species in that clade (e.g., in the Urmetazoan in fig. 5*A*, the median of the counts of a particular core domain in all animals included in this study). The frequency of each domain co-occurrence (represented by the thickness of the edge between nodes) was estimated analogously. We calculated the network density index of each reconstructed ancestor using the Cytoscape NetworkAnalyzer module (Assenov et al. 2008).

## Supplementary Material

Supplementary figures S1–S7, files S1–S3, and tables S1 and S2 are available at *Molecular Biology and Evolution* online (http://www.mbe.oxfordjournals.org/).

Supplementary Data

## References

[msu334-B1] Ababneh F, Jermiin LS, Ma C, Robinson J (2006). Matched-pairs tests of homogeneity with applications to homologous nucleotide sequences. Bioinformatics.

[msu334-B2] Adamska M, Matus DQ, Adamski M, Green K, Rokhsar DS, Martindale MQ, Degnan BM (2007). The evolutionary origin of hedgehog proteins. Curr Biol..

[msu334-B3] Adl SM, Simpson AG, Lane CE, Lukeš J, Bass D, Bowser SS, Brown MW, Burki F, Dunthorn M, Hampl V (2012). The revised classification of eukaryotes. J Cell Biol..

[msu334-B4] Amerik AY, Hochstrasser M (2004). Mechanism and function of deubiquitinating enzymes. Biochim Biophys Acta..

[msu334-B5] Aravind L, Iyer LM, Koonin EV (2006). Comparative genomics and structural biology of the molecular innovations of eukaryotes. Curr Opin Struct Biol..

[msu334-B6] Assenov Y, Ramírez F, Schelhorn S-E, Lengauer T, Albrecht M (2008). Computing topological parameters of biological networks. Bioinformatics.

[msu334-B7] Basu MK, Carmel L, Rogozin IB, Koonin EV (2008). Evolution of protein domain promiscuity in eukaryotes. Genome Res..

[msu334-B8] Basu MK, Poliakov E, Rogozin IB (2009). Domain mobility in proteins: functional and evolutionary implications. Brief Bioinform..

[msu334-B9] Bayer P, Arndt A, Metzger S, Mahajan R, Melchior F, Jaenicke R, Becker J (1998). Structure determination of the small ubiquitin-related modifier SUMO-1. J Mol Biol..

[msu334-B10] Bhattacharyya RP, Reményi A, Yeh BJ, Lim WA (2006). Domains, motifs, and scaffolds: the role of modular interactions in the evolution and wiring of cell signaling circuits. Annu Rev Biochem..

[msu334-B11] Brighouse A, Dacks JB, Field MC (2010). Rab protein evolution and the history of the eukaryotic endomembrane system. Cell Mol Life Sci..

[msu334-B12] Burroughs AM, Iyer LM, Aravind L (2009). Natural history of the E1-like superfamily: implication for adenylation, sulfur transfer, and ubiquitin conjugation. Proteins.

[msu334-B13] Burroughs AM, Jaffee M, Iyer LM, Aravind L (2008). Anatomy of the E2 ligase fold: implications for enzymology and evolution of ubiquitin/Ub-like protein conjugation. J Struct Biol..

[msu334-B14] Camacho C, Coulouris G, Avagyan V, Ma N, Papadopoulos J, Bealer K, Madden TL (2009). BLAST+: architecture and applications. BMC Bioinformatics.

[msu334-B15] Cardozo T, Pagano M (2004). The SCF ubiquitin ligase: insights into a molecular machine. Nat Rev Mol Cell Biol..

[msu334-B16] Cavalier-Smith T (1987). Eukaryotes with no mitochondria. Nature.

[msu334-B17] Cavalier-Smith T (1991). Archamoebae: the ancestral eukaryotes?. Biosystems.

[msu334-B18] Chosed R, Mukherjee S, Lois LM, Orth K (2006). Evolution of a signalling system that incorporates both redundancy and diversity: *Arabidopsis* SUMOylation. Biochem J..

[msu334-B19] Colby T, Matthäi A, Boeckelmann A, Stuible H-P (2006). SUMO-conjugating and SUMO-deconjugating enzymes from *Arabidopsis*. Plant Physiol..

[msu334-B20] Collins L, Penny D (2005). Complex spliceosomal organization ancestral to extant eukaryotes. Mol Biol Evol..

[msu334-B21] Darriba D, Taboada GL, Doallo R, Posada D (2011). ProtTest 3: fast selection of best-fit models of protein evolution. Bioinformatics.

[msu334-B22] de Mendoza A, Sebé-Pedrós A, Sestak MS, Matejcic M, Torruella G, Domazet-Lošo T, Ruiz-Trillo I (2013). Transcription factor evolution in eukaryotes and the assembly of the regulatory toolkit in multicellular lineages. Proc Natl Acad Sci U S A..

[msu334-B23] Derelle R, Lang BF (2012). Rooting the eukaryotic tree with mitochondrial and bacterial proteins. Mol Biol Evol..

[msu334-B24] Deshaies RJ, Joazeiro CA (2009). RING domain E3 ubiquitin ligases. Annu Rev Biochem..

[msu334-B25] Deshmukh K, Anamika K, Srinivasan N (2010). Evolution of domain combinations in protein kinases and its implications for functional diversity. Prog Biophys Mol Biol..

[msu334-B26] Di Roberto RB, Peisajovich SG (2013). The role of domain shuffling in the evolution of signaling networks. J Exp Zool B Mol Dev Evol..

[msu334-B27] Eddy SR (1998). Profile hidden Markov models. Bioinformatics.

[msu334-B28] Eme L, Trilles A, Moreira D, Brochier-Armanet C (2011). The phylogenomic analysis of the anaphase promoting complex and its targets points to complex and modern-like control of the cell cycle in the last common ancestor of eukaryotes. BMC Evol Biol..

[msu334-B29] Field MC, Dacks JB (2009). First and last ancestors: reconstructing evolution of the endomembrane system with ESCRTs, vesicle coat proteins, and nuclear pore complexes. Curr Opin Cell Biol..

[msu334-B30] Gagne JM, Downes BP, Shiu S-H, Durski AM, Vierstra RD (2002). The F-box subunit of the SCF E3 complex is encoded by a diverse superfamily of genes in *Arabidopsis*. Proc Natl Acad Sci U S A..

[msu334-B31] Gareau JR, Lima CD (2010). The SUMO pathway: emerging mechanisms that shape specificity, conjugation and recognition. Nat Rev Mol Cell Biol..

[msu334-B32] Gazave E, Lapébie P, Richards GS, Brunet F, Ereskovsky A V, Degnan BM, Borchiellini C, Vervoort M, Renard E (2009). Origin and evolution of the Notch signalling pathway: an overview from eukaryotic genomes. BMC Evol Biol..

[msu334-B33] Grau-Bové X, Sebé-Pedrós A, Ruiz-Trillo I (2013). A genomic survey of HECT ubiquitin ligases in eukaryotes reveals independent expansions of the HECT system in several lineages. Genome Biol Evol..

[msu334-B34] Harashima H, Dissmeyer N, Schnittger A (2013). Cell cycle control across the eukaryotic kingdom. Trends Cell Biol..

[msu334-B35] He D, Fiz-Palacios O, Fu C-J, Fehling J, Tsai C-C, Baldauf SL (2014). An alternative root for the eukaryote tree of life. Curr Biol..

[msu334-B36] He YJ, McCall CM, Hu J, Zeng Y, Xiong Y (2006). DDB1 functions as a linker to recruit receptor WD40 proteins to CUL4-ROC1 ubiquitin ligases. Genes Dev..

[msu334-B37] Hickey CM, Wilson NR, Hochstrasser M (2012). Function and regulation of SUMO proteases. Nat Rev Mol Cell Biol..

[msu334-B38] Hochstrasser M (2000). Evolution and function of ubiquitin-like protein-conjugation systems. Nat Cell Biol..

[msu334-B39] Hochstrasser M (2009). Origin and function of ubiquitin-like proteins. Nature.

[msu334-B40] Hua Z, Vierstra RD (2011). The cullin-RING ubiquitin-protein ligases. Annu Rev Plant Biol..

[msu334-B41] Huang L, Kinnucan E, Wang G, Beaudenon S, Howley PM, Huibregtse JM, Pavletich NP (1999). Structure of an E6AP-UbcH7 complex: insights into ubiquitination by the E2-E3 enzyme cascade. Science.

[msu334-B42] Humbard MA, Miranda H V, Lim J-M, Krause DJ, Pritz JR, Zhou G, Chen S, Wells L, Maupin-Furlow JA (2010). Ubiquitin-like small archaeal modifier proteins (SAMPs) in *Haloferax volcanii*. Nature.

[msu334-B43] Iyer LM, Burroughs AM, Aravind L (2006). The prokaryotic antecedents of the ubiquitin-signaling system and the early evolution of ubiquitin-like beta-grasp domains. Genome Biol..

[msu334-B44] Johnson ES (2004). Protein modification by SUMO. Annu Rev Biochem..

[msu334-B45] Katoh K, Standley D (2013). MAFFT multiple sequence alignment software version 7: improvements in performance and usability. Mol Biol Evol..

[msu334-B46] Kerscher O, Felberbaum R, Hochstrasser M (2006). Modification of proteins by ubiquitin and ubiquitin-like proteins. Annu Rev Cell Dev Biol..

[msu334-B47] King N, Westbrook MJ, Young SL, Kuo A, Abedin M, Chapman J, Fairclough S, Hellsten U, Isogai Y, Letunic I (2008). The genome of the choanoflagellate *Monosiga brevicollis* and the origin of metazoans. Nature.

[msu334-B48] Komander D, Reyes-Turcu F, Licchesi JD, Odenwaelder P, Wilkinson KD, Barford D (2009). Molecular discrimination of structurally equivalent Lys 63-linked and linear polyubiquitin chains. EMBO Rep..

[msu334-B49] Komatsu M, Chiba T, Tatsumi K, Iemura S, Tanida I, Okazaki N, Ueno T, Kominami E, Natsume T, Tanaka (2004). A novel protein-conjugating system for Ufm1, a ubiquitin-fold modifier. EMBO J..

[msu334-B50] Koonin EV (2006). The origin of introns and their role in eukaryogenesis: a compromise solution to the introns-early versus introns-late debate?. Biol Direct..

[msu334-B51] Koonin EV (2011). The logic of chance: the nature and origin of biological evolution.

[msu334-B52] Koonin EV, Makarova KS, Aravind L (2001). Horizontal gene transfer in prokaryotes: quantification and classification. Annu Rev Microbiol..

[msu334-B53] Koumandou VL, Wickstead B, Ginger ML, van der Giezen M, Dacks JB, Field MC (2013). Molecular paleontology and complexity in the last eukaryotic common ancestor. Crit Rev Biochem Mol Biol..

[msu334-B54] Lartillot N, Lepage T, Blanquart S (2009). PhyloBayes 3: a Bayesian software package for phylogenetic reconstruction and molecular dating. Bioinformatics.

[msu334-B55] Le SQ, Gascuel O (2008). An improved general amino acid replacement matrix. Mol Biol Evol..

[msu334-B56] Makarova KS, Yutin N, Bell SD, Koonin E V (2010). Evolution of diverse cell division and vesicle formation systems in Archaea. Nat Rev Microbiol..

[msu334-B57] Mans BJ, Anantharaman V, Aravind L, Koonin EV (2004). Comparative genomics, evolution and origins of the nuclear envelope and nuclear pore complex. Cell Cycle.

[msu334-B58] Marín I (2009a). RBR ubiquitin ligases: diversification and streamlining in animal lineages. J Mol Evol..

[msu334-B59] Marín I (2009b). Diversification of the cullin family. BMC Evol Biol..

[msu334-B60] Marín I (2010a). Animal HECT ubiquitin ligases: evolution and functional implications. BMC Evol Biol..

[msu334-B61] Marín I (2010b). Diversification and specialization of plant RBR ubiquitin ligases. PLoS One.

[msu334-B62] Marín I (2010c). Ancient origin of animal U-box ubiquitin ligases. BMC Evol Biol..

[msu334-B63] Marín I (2013). Evolution of plant HECT ubiquitin ligases. PLoS One.

[msu334-B64] Michelle C, Vourc’h P, Mignon L, Andres CR (2009). What was the set of ubiquitin and ubiquitin-like conjugating enzymes in the eukaryote common ancestor?. J Mol Evol..

[msu334-B65] Mukhopadhyay D, Dasso M (2007). Modification in reverse: the SUMO proteases. Trends Biochem Sci..

[msu334-B66] Mukhopadhyay D, Riezman H (2007). Proteasome-independent functions of ubiquitin in endocytosis and signaling. Science.

[msu334-B67] Nunoura T, Takaki Y, Kakuta J, Nishi S, Sugahara J, Kazama H, Chee GJ, Hattori M, Kanai A, Atomi H (2011). Insights into the evolution of Archaea and eukaryotic protein modifier systems revealed by the genome of a novel archaeal group. Nucleic Acids Res..

[msu334-B68] Petroski MD, Deshaies RJ (2005). Function and regulation of cullin-RING ubiquitin ligases. Nat Rev Mol Cell Biol..

[msu334-B69] Punta M, Coggill PC, Eberhardt RY, Mistry J, Tate J, Boursnell C, Pang N, Forslund K, Ceric G, Clements J (2012). The Pfam protein families database. Nucleic Acids Res..

[msu334-B70] R Development Core Team (2008). http://www.R-project.org.

[msu334-B71] Reverter D, Lima CD (2005). Insights into E3 ligase activity revealed by a SUMO-RanGAP1-Ubc9-Nup358 complex. Nature.

[msu334-B72] Rinke C, Schwientek P, Sczyrba A, Ivanova NN, Anderson IJ, Cheng JF, Darling A, Malfatti S, Swan BK, Gies EA (2013). Insights into the phylogeny and coding potential of microbial dark matter. Nature.

[msu334-B73] Rotin D, Kumar S (2009). Physiological functions of the HECT family of ubiquitin ligases. Nat Rev Mol Cell Biol..

[msu334-B74] Saitoh H, Hinchey J (2000). Functional heterogeneity of small ubiquitin-related protein modifiers SUMO-1 versus SUMO-2/3. J Biol Chem..

[msu334-B75] Schmitz-Esser S, Tischler P, Arnold R, Montanaro J, Wagner M, Rattei T, Horn M (2010). The genome of the amoeba symbiont “*Candidatus* Amoebophilus asiaticus” reveals common mechanisms for host cell interaction among amoeba-associated bacteria. J Bacteriol..

[msu334-B76] Sebé-Pedrós A, Grau-Bové X, Richards TA, Ruiz-Trillo I (2014). Evolution and classification of myosins, a paneukaryotic whole genome approach. Genome Biol Evol..

[msu334-B77] Seger R, Krebs E (1995). The MAPK signaling cascade. FASEB J..

[msu334-B78] Shabalina SA, Koonin E V (2008). Origins and evolution of eukaryotic RNA interference. Trends Ecol Evol..

[msu334-B79] Smalle J, Vierstra RD (2004). The ubiquitin 26S proteasome proteolytic pathway. Annu Rev Plant Biol..

[msu334-B80] Smoot ME, Ono K, Ruscheinski J, Wang P-L, Ideker T (2011). Cytoscape 2.8: new features for data integration and network visualization. Bioinformatics.

[msu334-B81] Snell EA, Brooke NM, Taylor WR, Casane D, Philippe H, Holland PWH (2006). An unusual choanoflagellate protein released by Hedgehog autocatalytic processing. Proc R Soc B Biol Sci..

[msu334-B82] Spallek T, Robatzek S, Göhre V (2009). How microbes utilize host ubiquitination. Cell Microbiol..

[msu334-B83] Stamatakis A (2006). RAxML-VI-HPC: maximum likelihood-based phylogenetic analyses with thousands of taxa and mixed models. Bioinformatics.

[msu334-B84] Stone S, Hauksdóttir H, Troy A (2005). Functional analysis of the RING-type ubiquitin ligase family of *Arabidopsis*. Plant Physiol..

[msu334-B85] Suga H, Dacre M, de Mendoza A, Shalchian-Tabrizi K, Manning G, Ruiz-Trillo I (2012). Genomic survey of premetazoans shows deep conservation of cytoplasmic tyrosine kinases and multiple radiations of receptor tyrosine kinases. Sci Signal..

[msu334-B86] Tatsumi K, Yamamoto-Mukai H, Shimizu R, Waguri S, Sou YS, Sakamoto A, Taya C, Shitara H, Hara T, Chung CH (2011). The Ufm1-activating enzyme Uba5 is indispensable for erythroid differentiation in mice. Nat Commun..

[msu334-B87] Tordai H, Nagy A, Farkas K, Bányai L, Patthy L (2005). Modules, multidomain proteins and organismic complexity. FEBS J..

[msu334-B88] Turjanski AG, Vaqué JP, Gutkind JS (2007). MAP kinases and the control of nuclear events. Oncogene.

[msu334-B89] van der Veen AG, Ploegh HL (2012). Ubiquitin-like proteins. Annu Rev Biochem..

[msu334-B90] Verdecia MA, Joazeiro CA, Wells NJ, Ferrer J-L, Bowman ME, Hunter T, Noel JP (2003). Conformational flexibility underlies ubiquitin ligation mediated by the WWP1 HECT domain E3 ligase. Mol Cell..

[msu334-B91] Whitmarsh AJ (2007). Regulation of gene transcription by mitogen-activated protein kinase signaling pathways. Biochim Biophys Acta..

[msu334-B92] Wickstead B, Gull K (2011). The evolution of the cytoskeleton. J Cell Biol..

[msu334-B93] Willems AR, Schwab M, Tyers M (2004). A hitchhiker’s guide to the cullin ubiquitin ligases: SCF and its kin. Biochim Biophys Acta..

[msu334-B94] Williams TA, Foster PG, Cox CJ, Embley TM (2013). An archaeal origin of eukaryotes supports only two primary domains of life. Nature.

[msu334-B95] Williams TA, Foster PG, Nye TM, Cox CJ, Embley TM (2012). A congruent phylogenomic signal places eukaryotes within the Archaea. Proc R Soc B Biol Sci..

[msu334-B96] Woese CR, Kandler O, Wheelis ML (1990). Towards a natural system of organisms: proposal for the domains Archaea, Bacteria, and Eucarya. Proc Natl Acad Sci U S A..

[msu334-B97] Wolf YI, Koonin EV (2013). Genome reduction as the dominant mode of evolution. Bioessays.

