# Supplementary material for: The Eukaryotic Ancestor Had a Complex Ubiquitin Signaling System of Archaeal Origin
Source: Mol Biol Evol. 2014 Dec 17;32(3):726–39. doi: 10.1093/molbev/msu334 (PMC4327156; doi:10.1093/molbev/msu334)
Supplement: Supplementary Data [file supp_32_3_726__index.html]

The eukaryotic ancestor had a complex ubiquitin signalling system of archaeal origin — The Eukaryotic Ancestor Had a Complex Ubiquitin Signaling System of Archaeal Origin — The Eukaryotic Ancestor Had a Complex Ubiquitin Signaling System of Archaeal Origin — Supplementary Data 

# The Eukaryotic Ancestor Had a Complex Ubiquitin Signaling System of Archaeal Origin

## Supplementary Data

files

**Files in this Data Supplement:**

- Supplementary Data - pdf file
- Supplementary Data - pdf file
- Supplementary Data - pdf file
- Supplementary Data - pdf file
- Supplementary Data - xls file
