# Supplementary material for: The Eukaryotic Ancestor Had a Complex Ubiquitin Signaling System of Archaeal Origin
Source: Mol Biol Evol. 2014 Dec 17;32(3):726–39. doi: 10.1093/molbev/msu334 (PMC4327156; doi:10.1093/molbev/msu334)
Supplement: Supplementary Data [file supp_msu334_Supplementary1_Figure_Legends_and_Text.pdf]

Supplementary files

Table S1. Taxon sampling used in this study, including the taxonomic classification, the acronym used in the present study (in brackets) and the source of the proteome data.

Table S2. List of elements7. of the ubiquitin, SUMO and Ufm1 pathways sampled in our study, including their defining Pfam domain IDs. Examples of each element in the model systems *Homo sapiens, Saccharomyces cerevisiae* and *Arabidopsis thaliana* are also included.

Fig. S1. A. Pattern of gains/losses and quantitative enrichments/depletions for the components of the Ub, SUMO and Ufm1 systems in eukaryote evolution, inferred using parsimony. The "Excavata-first" hypothesis for the root of eukaryotes is assumed. **B.** Pattern of gains/losses inferred using AssymMk likelihood model, as implemented in Mesquite (see Materials and Methods). The "unikont-bikont" hypothesis for the root of eukaryotes is assumed. **C.** Pattern of gains/losses inferred using AssymMk likelihood model, as implemented in Mesquite (see Materials and Methods). The "Excavata-first" hypothesis for the root of eukaryotes is assumed. Solid green and red boxes indicate gains and losses of gene families, respectively. Shaded blue and orange boxes indicate significant enrichments and depletions, respectively.

D. Plot of the observed and expected P-values of the matched-pairs symmetry tests (P-P plot) performed on the protein alignment of Hsp70, Hsp90 and Actin. Expected values were obtained from the uniform distribution. The lowest observed P-values for each test was 0.001588116, with less than 1% of the 3003 tests being under a 0.01 threshold. This result implies that there is no evidence that any dataset evolved under non-homogeneous, non-stationary and non-time-reversible conditions (see Ababneh et al. 2006 for details).

Fig. S2. Number of proteins containing specific Ub-, SUMO-, or Ufm1-related domains, including the label itself, E3 ligases and de-labelling enzymes (left chart). Proportion (%) of each proteome represented by these proteins (right chart).

Fig. S3. Proportion (%) of each type of specific Ub E3s in each genome (left chart). Total number of Ub E3s in each genome (right chart).

Fig. S4. Protein domain architecture diversity of the different components of the Ub, SUMO and Ufm1 systems in eukaryotes. The heatmap represents the number of concurring domains that appear together with each of the core gene family domains (including themselves) in each of the sampled genomes, according to the colour scale.

Fig. S5. Architecture diversity of the different components of the Ub, SUMO and Ufm1 systems in eukaryotes. Each line represents a protein domain architecture; the heatmap is color-coded according to the number of occurrences of such protein type in a given genome.

A. Architecture diversity of ThiF-containing proteins in each of the sampled genomes. Most E1s are single-ThiF enzymes. We detect an eukaryotic-specific E1 type with a particular protein domain architecture consisting of ThiF (catalytic domain), UBA_e1_thiolCys (C-terminal part of the E1 active site), UBACT (conserved tandem domain) and/or UBA_e1_C (uncharacterized, C-terminal domain). Note a group of proteins in the ciliates *T. thermophila* and *P. tetraurelia* with both ThiF (and other E1-related domains) and UQ_con domains, thereby joining their E1 and E2 enzymes in a single gene. Yet, both organisms keep their canonical ThiF-only and UQ_con-only enzymes. Other ThiF prokaryotic families whose eukaryotic cognates are not related to ubiquitin-like signaling (i.e. the MoeZ/MoeB family) are excluded.

B. Architecture diversity of UQ_con-containing proteins in each of the sampled genomes. UQ_con identifies E2s from Ub and SUMO pathways. This domain is present in all the surveyed eukaryotes in constant numbers, mostly in form of single-domain enzymes. Alternative architectures are present but not abundant. Proteins with UQ_con and UBA (UBA_3 in fungi), such as the human UBE2K, represent the second most abundant architecture in most lineages. Note the E1-E2 fusion enzymes in the ciliates *T. thermophila* and *P. tetraurelia* (Additional file 5).

C. Architecture diversity of HECT-containing proteins in each of the sampled genomes. N-terminal domain rearrangements are common in many eukaryotic lineages. These often involve protein-binding motifs (WW, UBA, Ankyrin and RCC1 repeats, MIB_HERC2, SPRY, zinc fingers…), but also to other molecules such as lipids (C2), complex sugars (Laminin_G_3) and poly-A tails (PABP). The expansion of HECTs in holozoans (Fig. 3) is followed by an increase in architectural diversity in animals. Convergences in domain composition are observed between holozoans and heterokonts.

D. Architecture diversity of zf-RING_2-containing proteins (also canonical C3H2C3 or H2 RINGs) in each of the sampled genomes. C3H2C3 RINGs have an extremely plastic set of concurrent domains organized in a vast repertoire of architectures, variable among lineages. The most common architecture in eukaryotes is a single zf-RING_2 domain, but is also frequently combined with zf-CHY, BRAP2 and zf-UBP, CUE, or zf-RING_3 domains, especially in plants and holozoans. Animals have exclusive RING types such as TRIMs, a vertebrate family of E3s with architectures based on zf-RING_2, zf-B_box (involved in protein binding) and various other domains (Filamin, NHL, PHD fingers, PRY, SPRY…). Land plants, chlorophytes, rhodophytes and glaucophytes have a family of RINGs with cation-binding hemerythrin domains, involved in iron-responsive protein ubiquitination and degradation.

E. Architecture diversity of zf-C3HC4-containing proteins (subtype of the canonical C3HC4 RINGs) in each of the sampled genomes. This domain is commonly found in IBR E3s from many eukaryotes, especially land plants and ciliates (*P. tetraurelia* and *T. thermophila*). TNF-receptor associated factors (TRAFs) are a group of RING E3s closely related to TRIM proteins, which show a zf-C3HC4-zf-TRAF-MATH architecture exclusive to animals. Other TRIM-like E3s based on zf-

B_box architectures are present in many unikonts (animals, apusozoans and amoebozoans), the rhizarian *B. natans* and two excavates. Land plants have a group of exclusive architectures, such as histone-binding RINGs (also with YDG_SRA domains).

F. Architecture diversity of zf-C3HC4_2-containing proteins (subtype of the canonical C3HC4 RINGs) in each of the sampled genomes. The most common form is a single zf-C3HC4_2 domain. There is a wide set of paneukaryotically-distributed accompanying domains, e.g. zf-CCCH, Pex2_Pex12, IBR (see below), WD40, SPX, zf-TRAF and FHA. These domains are often present in architecture types present in both plants and fungi, such as SNF2s. Animals expanded some types also present in unicellular holozoans, such as TRIM- and TRAF-like E3s (Additional file 9), a histone-binding E3 (with YDG_SRA domains; also found in the chytrid fungi *Mortierella verticillata*) and a family that acts as constituent elements of the eukaryotic peroxisomes (with Pex2_Pex12 domains; also found in some plants and fungi). Similarly, land plants also have a group of exclusive architectures that are sometimes present in chlorophytes, such as histone-binding RINGs (also with YDG_SRA domains; Additional file 9). Fungi have a specific family with BRE1 domains, involved in histone H2B ubiquitination.

G. Architecture diversity of zf-C3HC4_3-containing proteins (subtype of the canonical C3HC4 RINGs) in each of the sampled genomes. A single zf-C3HC4_3 domain is the most common and abundant in most eukaryotes (although there are no alternative paneukaryotically-distributed architectures). Lineage-specific architecture diversifications are particularly common in animals and plants. There are various E3s involved in animal Notch signaling: a family of RINGs with Neuralized domains that regulate developmental processes, and a group of homologs to the human MIB (mind bomb) with ZZ zinc fingers flanked by MIB_HERC2 and Ankyrin domains. Animals have RINGs with FERM domains (involved in the degradation of myosin regulatory light chain). Plants have a set of architectures with Ankyrin repeats, Kinesin (microtubule-binding, probably related to cytoskeletal regulation) and Copine domains. Note also the Cbl-like architectures of holozoans and dictyostelid amoebozoans (Additional file 12).

H. Architecture diversity of Cbl_N-containing proteins (subtype of the canonical C3HC4 RINGs) in each of the sampled genomes. Canonical holozoan Cbls have a complete N-terminal RTK-binding region (Cbl_N, Cbl_N2, Cbl_N3) followed by a zf-C3C4_3 domain. This is likely the ancestral architecture. UBA domains were later acquired an are present at the C-terminus of choanoflagellate and animal Cbls. A Cbl-like homolog is present in dictyostelid amoebozoans.

I. Architecture diversity of U-box-containing proteins in each of the sampled genomes. It exists in three major forms: a single U-box domain, Ufd2P_core domain+U-box (core domain of the Ub elongation factor and takes part in multi-Ub chain elongation) and TPR repeats (protein binding) +U-box. This family is very diverse in terms of architectures, which often correlates with lineage-specific expansions – particularly in bikont lineages (Fig. 3). Land plants have expanded their U-box repertoire with specific architectures with Armadillo and KAP domains, WD40 repeats and

protein kinase domains. The holozoan U-box expansion entails comparatively less domain diversification than in plants (most holozoan U-box belong to the three major types). Animals have acquired various protein-binding domains (e.g., WD40 or WWE domains with SAM_1). Lineage-specific expansions are common in heterokonts, alveolates, rhizarians, haptophytes and cryptophytes.

J. Architecture diversity of zf-RING_LisH-containing proteins in each of the sampled genomes. The most common architecture includes a CTLH protein-binding motif, essential for the assembly of a multisubunit E3 complex TRIM-like proteins with zf-RING_LisH domains instead of canonical RINGs exist in many animals, the amoebozoan *Entamoeba histolytica* and the alveolates *P. tetraurelia* and *T. thermophila*. Since both canonical RINGs and zf-RING_LisH existed at the LECA, the recruitment of RING-like motifs, zf-B_box and coiled-coils to create TRIM-like architectures is likely a convergence.

K. Architecture diversity of RINGv-containing proteins in each of the sampled genomes. A single RINGv motif is the most abundant architecture in most eukaryotes. Many plants and green algae's RINGv are associated to domains of unknown function, and alveolates and heterokonts have a exclusive architecture with FHA (phosphopeptide-binding motif). The lycophyte *Selaginella moellendorffii, E. huxleyi* (haptophyte) and *G. theta* (cryptophyte) have specific architectures with canonical RINGs.

L. Architecture diversity of FANCL_C-containing proteins in each of the sampled genomes. Two main forms of FANCL exist: a single FANCL_C domain and FANCL_C+WD-3 (a domain involved in the FA complex assembly). These forms are not ubiquitious in eukaryotes, since FANCL E3s have been lost many times along eukaryotic evolution (e.g. glaucophytes and various Fungi such as microsporidians). FANCL with endonuclease GIY-YIG domains are found in some opisthokonts.

M. Architecture diversity of IBR-containing proteins in each of the sampled genomes. Canonical IBRs have one or two IBR domains flanked by a N-terminal canonical RING (either zf-RING_2, zf-C3HC4 or zf-C3HC4_2) and an ill-conserved C-terminal RING. Animal and embryophyte IBR families are enriched with ubiquitin binding (UIM, UBA, CUE), protein binding (RWD, Ankyrin…), and nucleic acid binding domains (DEAD, HA2, RRM). Other eukaryotes such as *Thecamonas trahens*, amoebozoans*, Naegleria gruberi* and *Paramecium tetraurelia* are also enriched with binding motifs. Animals, angiosperms and *P. tetraurelia* have a type of nucleic acid-binding IBRs (with Helicase_C domains) involved in RNA metabolism. We also find a family of angiosperm IBRs with RNA-dependent DNA polymerase domains (RVT_3), typical of retrotransposons.

N. Architecture diversity of Cullin-containing proteins in each of the sampled genomes. Cullins have a N-terminal Cullin domain, which interacts with different adaptor proteins, followed by a Cullin_Nedd8 domain (which its neddylation site). We detected two non-CRL proteins with Cullin domains, although both participate in ubiquitin ligation: APC2 (subunit of the Anaphase Promoting Complex, with APC2 and Cullin domains) and PARC (p53-associated Parkin-like cytoplasmic

protein, with a C-terminal IBR). PARC-like architectures are present in the sponge *Amphimedon queenslandica*, thus revealing a much older origin than previously thought.

O. Architecture diversity of F-box-containing proteins in each of the sampled genomes. A single F-box domain is the most common architecture in all species, followed by the combination of F-box with the protein-binding WD40 and LRR_6 domains. Plants display the largest number of lineage-specific F-box domain configurations; among them, the most abundant have FBD, FBA and Kelch protein-binding domains. We find that several plant F-box proteins are associated with transposons, a common feature for other ubiquitin system components. Metazoans also have some specific F-box domain architectures (including a combination with Hemerythrin domain, in the FBXL5 gene, involved in iron homeostasis), but always in low numbers, as it is also the case for amoebozoan- and *N. gruberi*-specific configurations.

P. Architecture diversity of SOCS_box-containing proteins in each of the sampled genomes. . It has several different architectures, including a combination with Y-phosphate interacting SH2 domain. Presumably, the ancestral form was a SOCS_box domain with N-terminal Ankyrin repeats, as this form is found in the choanoflagellate and sponges (*A. queenslandica* and *O.carmela*).

Q. Architecture diversity of BTB-containing proteins in each of the sampled genomes. BTB co-occurs with a huge variety of accessory domains, being one of the most architecturally diverse protein families algonside with F-box and canonical RINGs. Its most common forms are a single BTB domain and BTB with MATH or BACK domains. Metazoans show higher BTB diversification than plants, mostly because of lineage-specific diversifications, but also because of higher counts of paneukaryotic architectural types. In plants, BTB is often associated with NPH3 domain; NPH3 gene is involved in phototransduction. Surprisingly, three metazoans (the hemichordate *S. kowalevskii*, the annelid *C. teleta* and the mollusk *Crassostrea gigas* (data not shown)) have *bona fide* NPH3 genes, whose function remains a mystery. Finally, the excavate *N. gruberi*, the haptophyte *E. huxleyi* and the amoebozoan *Acanthamoeba castellanii* present lineage-specific expansions of BTB architectures.

R. Architecture diversity of UCH and UCH_1-containing proteins (also known as USPs) in each of the sampled genomes. USPs are diverse in terms of accompanying domains. The most abundant domain configurations are a single UCH domain or zf-UBP+UCH. Fungi have a specific group of USPs with including the RPT domain. Other architectures have a scattered taxonomic distribution, but often include domains such as zf-MYND, DUSP (USP-specific N-terminal region) or Ub binding domains like UIM and UBA. RNA-binding domains like Piwi, Tudor-knot o G-patch are also found in USPs. Transposon-associated motifs such as MULE (transposase) or DDE_3 (endonuclease) are also found.

S. Architecture diversity of Josephin-containing proteins in each of the sampled genomes. The most common architecture in most eukaryotes is a single Josephin domain. Two alternative

architectures exist, using UIM (Ub-binding motifs) and UBX (motif present in many regulators of the ubiquitin pathway).

T. Architecture diversity of OTU-containing proteins (including OTUs *sensu stricto* and A20-like enzymes) in each of the sampled genomes. The most common domain configuration involves a single OTU domain. A20-like OTUs typically have C-terminal zf-A20 domains (exclusive to bilaterians, cnidarians and poriferans) and N-terminal tandems of zf-RanBP (present in many animals, the apusozoan *T. trahens* and the excavate *L. major*). This includes the well-known Cezanne family. We also find OTUs with rve, an integrase domain typical of transposable elements.Other particular innovations include OTUs with nucleic acid-binding domains (SEC-C) in many bikonts, or lipid-binding OTUs in ichthyosporeans.

U. Architecture diversity of Peptidase_C65-containing proteins (Otubains) in each of the sampled genomes. The vast majority of them have a single catalytic domain.

V. Architecture diversity of JAB-containing proteins in each of the sampled genomes (also JAMMs). Eukaryotic JABs are divided in two main groups: peptidases with a single JAB domain, and a regulatory subunit of the 26S proteasome with MitMem_reg domains. We also find a group of JABs with SWIRM and/or Myb_DNA-binding domains, present in various animals (its human homolog is histone H2A-specific DUB), unicellular holozoans, fungi, amoebozoans and chlorophytes.

W. Architecture diversity of PIAS proteins in each of the sampled genomes. PIAS are defined by a zf-MIZ RING-like domain combined with a PINIT and/or a N-terminal SAP domain. These combinations are the most abundant architectures in most eukaryotes. Some plant PIAS have an exclusive PHD zinc finger for protein recognition instead of the PINIT motif.

X. Architecture diversity of zf-Nse-containing proteins in each of the sampled genomes. Most common forms of zf-Nse lack accessory domains.

Y. Architecture diversity of Peptidase_C48-containing proteins (ULPs) in each of the sampled genomes. The most common form of ULP consists of a single Peptidase_C48 domain. Besides that, there is a wide variety of ULPs with transposon-related motifs (e.g., PMD, MULE or rve) in several animals, land plants, the filasterean *C. owczarzaki* and the oomycete *P. infestans*.

Z. Architecture diversity of WLM-containing proteins in each of the sampled genomes. WLMs typically have a single domain, but forms with PUB domains (ATPase-binding) are common in plants, many unicellular bikonts (*V. cartieri*, *N. gaditana*, *G. theta…*) and some holozoans (*M. brevicollis* and *C. owczarzaki*).

AA. Architecture diversity of DUF862-containing proteins (also known as Peptidase_C97) in each of the sampled genomes. DUF862/Peptidase_C97 domains are often found with ubiquitin-associated motifs (PUL, PUB, UBA…). Fungi have Thioredoxin-containing peptidases with

disulphide oxidoreductase activity that regulate the arrangement of disulphide bonds in protein folding. Similar architectures are present in some alveolates and the haptophyte *E. huxleyi*.

AB. Architecture diversity of Peptidase_C78-containing proteins in each of the sampled genomes. Most eukaryotes have just a single-domain Peptidase_C78 enzyme. Some sparsely distributed eukaryotes have lineage-specific acquistions of various zinc finger motifs.

Fig. S6. P-P plots of the alignments of UCH, UQ_con and ubiquitin proteins (see Materials and Methods), with their corresponding phylogenetic trees using maximum likelihood (ML) and Bayesian inference (BI).

A. Plots of the observed and expected P-values of the matched-pairs symmetry tests (P-P plot) performed on ubiquitin, UCH and UQ_con protein alignments. Expected values were obtained from the uniform distribution. Lowest observed P-values for each test were 0.01327626 for ubiquitin (1669878 tests), 0.04912427 for UCH (733866 tests) and 0.04375998 for UQ_con (511566 tests), none of them being under a 0.01 threshold. This result implies that there is no evidence that any dataset evolved under non-homogeneous, non-stationary and non-time-reversible conditions (see Ababneh et al. 2006 for details).

B. Ubiquitin proteins ML analysis. **C.** Ubiquitin proteins BI analysis. **D.** UCH proteins ML analysis. **E.** UCH proteins BI analysis. **F.** UQ_con proteins ML analysis. **G.** UQ_con proteins BI analysis. The trees were rooted using the midpoint-rooted tree option. Bayesian Posterior Probabilities are indicated in the BI trees (C, E, G) and bootstrap supports are indicated in the ML trees (B, D, F). Archaeal sequences highlighted in red, bacterial sequences in yellow and metagenomic sequences in green. Note that none of the archaeal sequences cluster with support to any eukaryotic sequences.

Fig. S7. Reciprocal BLAST hit networks of the gene families found in Archaea (see Materials and Methods). **A.** Ubiquitin label (all nodes). **B.** UQ_con E2 (only first neighbors to archaeal and bacterial sequences). **C.** zf-RING_2 E3 (only first neighbors to archaeal sequences). **D.** RINGv E3 (all nodes). **E.** UCH DUB (only first neighbors to archaeal sequences). Red nodes indicate archaeal sequences, yellow nodes bacterial sequences, gray nodes eukaryotic sequences and green nodes metagenomic sequences. The intensity of the edge color is proportional to the significance (e-value). Note that none of the archaeal sequences appear strongly related to any eukaryotic sequences (see also supplementary File S3).

Supplementary File S1. Archaeal protein sequences identified in this study.

Supplementary File S2. Eukaryotic protein sequences identified in this study.

Supplementary File S3. List of blast hits of each archaeal sequence (included in supplementary File S1) against NCBI non-redundant and other databases (see Materials and Methods). For a summary of the complete reciprocal blast analyses, see supplementary fig. S7.

Supplementary text

The generalist toolkit: E1 and E2

E1 activating and E2 conjugating enzymes belong to two ancient and broad gene families present in Eukaryota, Bacteria and Archaea. We found the catalytic domains of E1 and E2 enzymes (ThiF and UQ_con, respectively) in all the surveyed eukaryotes (fig. 1), and E2 enzymes were significantly enriched in embryophytes (fig. 3). Although some bacteria (which lack ubiquitin signalling) have eukaryotic-like E2s, this is likely due to one or more horizontal gene transfer events (Arcas et al. 2013).

Ubiquitin system

A detailed look at the variety of ubiquitin E3 gene families revealed very different evolutionary histories. For instance, HECTs appeared at the origin of eukaryotes and are present in all the surveyed genomes (fig. 1). Our data confirm those of previous studies in which HECTs were found to be expanded in holozoans (animals and their closest unicellular relatives) and depleted in fungi (fig. 3) (Rotin and Kumar 2009; Grau-Bové et al. 2013). By contrast, RINGs (*really interesting new gene*), the other main group of E3s, appeared in Archaea before the divergence of eukaryotes. These genes are defined by the RING finger, a cysteine-rich region that coordinates zinc atoms and has catalytic ligase activity (Freemont et al. 1991; Borden and Freemont 1996; Lorick 1999). According to the spacing between metal ligands and the sequence of this cysteine-rich region, RINGs are divided into two canonical forms: C3H2C3 (also known as H2 and represented by the zf-RING_2 Pfam domain) and C3HC4 (also known as HC and represented by the zf-C3HC4, zf-C3HC4_2 and zf-C3HC4_3 domains). Also, there is a variety of non-canonical RINGs (U-box, zf-RING_LisH, RINGv, FANCL_C, IBR and Sina) (supplementary table S2) (Cardozo and Pagano 2004; Willems et al. 2004; Petroski and Deshaies 2005; Deshaies and Joazeiro 2009). We therefore analysed these multiple gene families separately.

Canonical C3H2C3 and C3HC4 RINGs were found in all eukaryotic lineages and represented the most abundant type of E3s in most eukaryotes (supplementary fig. S3). Our results also indicate that they underwent several linage-specific expansions, such as at the origin of embryophytes (involving zf-RING_2, zf-C3HC4, zf-C3HC4_2 and zf-C3HC4_3) and at the origins of holozoans (id. except zf-C3HC4), ichthyosporeans and animals (zf-C3HC4_3), and eumetazoans (zf-RING_2) (fig. 3). In contrast, zf-C3HC4_3 RINGs were depleted in fungi (fig. 3).

Our data show that most non-canonical RING families are also widespread in eukaryotes (fig. 1), and gene family expansions are found in holozoans and embryophytes (fig. 3). For example, the U-box, one of the most common modified RING domains (Aravind and Koonin 2000; Ohi et al. 2003), was present in all surveyed eukaryotes and expanded in Holozoa, consistent with the pre-

metazoan origin of most animal U-box families (Marín 2010). It is worth noting that RINGv (*RING variant*, also RING-CH) (Dodd et al. 2004), which was present in all sampled eukaryotes, was also found in an euryarchaeote Archaea (fig. 2), which shows that this modified RING family appeared early in the evolution of the ubiquitin system.

Both FANCL (subunit of the Fanconi Anemia complex) and Sina (named after the *Drosophila* protein *seven in absentia*) E3s were found to be less common than the RING-like families. Their phylogenetic distribution revealed multiple losses (fig. 1 and 3). For instance, we did not find FANCL homologs in most Fungi (including microsporidian parasites) nor in glaucophytes, whereas Sina was only found to be conserved in animals, embryophytes and some patchily distributed eukaryotes (e.g. two dictyostelids and the ciliate *Paramecium tetraurelia*). Notably, Sina was absent in excavates, which, under the "Excavata-first" hypothesis for the root of eukaryotes would imply that it appeared after the post-LECA early radiation (supplementary fig. S1).

Our analyses revealed that the gene families that constitute Cullin-RING ligase complexes (CRLs) are eukaryotic innovations (fig. 1 and 2). CRL complexes include a catalytic subunit that can be combined with adaptors and selectors within a structural backbone. Due to their combinatorial nature, CRLs have a huge evolvability potential (Sarikas et al. 2011) and are indeed the most abundant E3 types (fig. 1 and supplementary fig. S3). Cullin (backbone) and zf-rbx1 (RING ligase), the two core components of CRLs, were present in almost all eukaryotic species examined and showed little variation in terms of absolute number of genes (fig. 1). Similarly, Skp1 and DDB1 adaptors were also widespread and showed little variation. In sharp contrast, substrate selector subunits (such as F-box and BTB) were found to have a much more dynamic evolutionary history. For example, we identified massive lineage-specific gene enrichments in both F-box (in archaeplastids, i.e. plants and unicellular algae; and further enriched in plants) and BTB (in animals and plants) (fig. 3). Furthermore, we found that VHL and SOCS-box have restricted taxonomic distributions (fig. 1), and that the SOCS-box is specific to animals and *Salpingoeca rosetta* (a choanoflagellate, sister-group to animals). Finally, many parasites had a reduced CRL system, the most extreme cases being microsporidian fungi (only zf-rbx1 and Cullin), and the diplomonad *Giardia lamblia* (only zf-rbx1).

Interestingly, the gene counts for DUBs were consistently lower than those for E3 ligases (fig. 1), despite both enzyme types being ubiquitin-specific. The most abundant DUB gene families in all sampled eukaryotes were USPs (Ubiquitin-Specific cysteine Proteases, represented by Pfam domains UCH and UCH_1) and OTUs (including OTUs *sensu stricto* and A20-like enzymes (Nijman et al. 2005; Komander et al. 2009)). Both USP and OTU were expanded in plants and holozoans, and USP was depleted in fungi (fig. 3). In addition, UCH (ubiquitin C-terminal hydrolases, Peptidase_C12 domain) and Josephin families, which have similar catalytic domains (Amerik and Hochstrasser 2004; Komander et al. 2009), were found to be present in most examined genomes in low numbers. Both families have been lost numerous times in eukaryotic

evolution, e.g. in microsporidians and excavates (*G. lamblia* lacks both). Otubains (with Peptidase_C65 domains, similar to OTU) presented a similar scenario, in which they were lost in rhodophytes, glaucophytes, rhizarians and haptophytes (fig. 3). Finally, our data show that JAB metalloproteases (also known as JAMM) are an ancient gene family present in all examined eukaryotes, Archaea and Bacteria (fig. 1 and 2), where they are involved in ubiquitin-like systems (Iyer et al. 2006).

SUMO system

Our analyses show that the SUMO system is specific to eukaryotes (fig. 1 and 2). SUMO labels are defined by the Rad60-SLD domain, which we found in all surveyed eukaryotes, except *Oscarella carmela* (homoscleromorph sponge) and *O. tauri* (chlorophyte), which nonetheless have SUMO-specific gene families (fig. 1). The SUMO label was found to be expanded in embryophytes, vertebrates (with a conserved set of 3-4 paralogs (Hickey et al. 2012)), ichthyosporeans and the haptophyte *E. huxleyi* (fig. 3). Compared to ubiquitin, the number of SUMO peptides was lower in all the surveyed eukaryotes except *Ustilago maydis* (Fungi) and *Thalassiosira pseudonana* (heterokont) (fig. 1 and 6).

Although the SUMO system employs the same E1 and E2 domains as ubiquitin, it also has SUMO-specific enzymes, e.g. the E2 Ubc9, which directly conjugates SUMO moieties to its substrates without needing an E3 ligase (Reverter and Lima 2005). Consistent with this lack of requirement for E3, we could not detect SUMO-specific E3s in some eukaryotes, namely the coral *Acropora digitifera*, the diplomonad *G. lamblia* and the green algae *Chlorella variabilis* and *O. tauri*. The main SUMO E3 family, PIAS (with a RING-like zf-MIZ domain) (Duval et al. 2003), was present in holozoans (except filastereans), fungi (except microsporidians), embryophytes, *Perkinsus marinus* (alveolate), *Phytophthora infestans* (oomycete) and *Trichomonas vaginalis* (excavate) (fig. 1). The zf-Nse motif, a variant of zf-MIZ (Zhao and Blobel 2005), was present in all major eukaryotic lineages, although it has been lost many times as well, e.g. in choanoflagellates and sponges (fig. 1). We also identified the RanBP2/Nup358 E3 family, with IR1-M domains, in bilaterians and sponges. IR1-M, which does not resemble neither HECT nor RING ligases (Pichler et al. 2002; Pichler et al. 2004), is therefore an animal innovation.

Remarkably, and in contrast to ubiquitin, deSUMOylases were much more abundant than SUMO E3s in all examined genomes. ULPs were ubiquitous (known as SENPs in animals, with Peptidase_C48 domains) and represented the most common deSUMOylase family, particularly in plants (fig. 1 and 3). WLMs were present in all eukaryotic groups except animals, and were significantly enriched in fungi. Finally, the DUF862 domain (also C97 peptidases) was found to represent a paneukaryotic peptidase that was occasionally lost in some lineages (fig. 1 and 3).

Ufm1 system

The Ufm1 toolkit is also specific to eukaryotes (Burroughs et al. 2007) (fig. 1 and 2). Nearly all examined genomes had a single gene encoding the Ufm1 label, which was always present as a single-domain peptide of around 100 residues. According to our reconstruction, Ufm1 was secondarily lost in Fungi, as well as in other eukaryotes such as some ichthyosporeans and heterokonts (fig. 3). Contrary to previous hypotheses (Komatsu et al. 2004), we found that it is common in unicellular lineages. Ufm1 employs specific E2 enzymes with UFC1 domains (not UQ_con). Our data suggest that this gene family probably appeared during the early eukaryotic evolution as a paralog of a canonical E2 (Komatsu et al. 2004; Burroughs et al. 2008).

Proteins with DUF2042 and Peptidase_C78 domains have been identified as Ufm1-specific E3s and isopeptidases, respectively (Kang et al. 2007; Tatsumi et al. 2010). However, our survey shows that both domains are present in fungi that lack the Ufm1 peptide and its specific E2 (fig. 1). Thus, our data suggest that these enzymes may not be Ufm1-specific (either by co-option into new substrates in fungi or cryptic non-Ufm1 catalysis in other organisms).

Lineage-specific diversifications of the ubiquitin toolkit

To gain insight into the evolution of specific phylogenetic patterns, we undertook a detailed analysis of the diversity of ubiquitin, SUMO and Ufm1 toolkits across eukaryotes (supplementary fig. S5), especially in Holozoa (animals, choanoflagellates, filastereans and ichthyosporeans) and embryophytes. Analysis of the ubiquitin toolkit revealed multiple expansions within Holozoa, mostly involving E3s and DUBs: 12 at the origin of holozoans, four at the origin of metazoans and two at the origin of eumetazoans (bilaterians and cnidarians) (fig. 3). All holozoans have a core of prevailing protein families, with canonical RINGs (up to ~50%) and HECTs (up to ~10%) comprising the most extensive groups in most species (supplementary fig. S3). Within these gene families, the acquisition of new domain architectures appeared to be an important source of innovation (supplementary fig. S7-S11). A good example is that of Cbl, a family of C3HC4 RINGs present in holozoans and dictyostelid amoebozoans (supplementary fig. S5H). Cbl is a proto-oncogene that regulates signals from receptor tyrosine kinases (RTKs) (Thien and Langdon 2001; Schmidt and Dikic 2005; Lipkowitz and Weissman 2011). A canonical Cbl is composed of a multi-domain N-terminal RTK-binding region and a RING. In contrast, the proto-Cbl found in dictyostelids is involved in STAT signalling (Langenick et al. 2008) and only contains part of the RTK-binding region. Canonical Cbls with complete RTK-binding regions were found in ichthyosporeans and filastereans, which shows that these domain acquisitions were concomitant with the emergence of RTK signalling in unicellular holozoans (Manning et al. 2008; Suga et al. 2012). In animals, we also found Cbl paralogs with UBA domains (involved in oligomerization and ubiquitin binding (Schmidt and Dikic 2005)). Overall, our data suggest that the Cbl system of RTK-based signal transduction, a crucial innovation that controls cell proliferation in metazoans, was established in a step-wise manner long before the origin of animal multicellularity. In a similar vein, the HECT expansion in holozoans (fig. 3) was also followed by pervasive domain shuffling in the transition from unicellular

holozoans to animal, as previously observed (Grau-Bové et al. 2013). Most of these domains bind to proteins (WW, UBA, Ankyrin and RCC1 repeats, MIB_HERC2, SPRY, zinc fingers…) and other molecules such as lipids (C2), complex sugars (Laminin_G_3) and poly-A tails (PABP) (supplementary fig. S5C). These gene family expansions accompanied by acquisition of binding domains imply a functional diversification of the signalling system.

Besides domain innovation, we also detected shifts in the usage of particular E3 families between holozoan groups. For instance, BTB was the main CRL substrate recognition subunit in animals (~20-30% in most genomes) but not in unicellular holozoans (generally less than ~5%), which preferentially use F-box (~10-30%). Similarly, we only found SOCS-box genes in animals and the choanoflagellate *Salpingoeca rosetta*, which further differentiates multicellular from unicellular holozoans (fig. 3 and supplementary fig. S3).

We also detected expansions in embryophytes, which have the most abundant and diverse ubiquitin and SUMO systems among eukaryotes. Previous studies already reported a huge expansion of ubiquitin-related genes in *Arabidopsis thaliana*, which add up to ~5% of its genome (Lespinet et al. 2002). We found similar enrichments in the other examined angiosperms, and, to a lesser extent, the early-branching plants *Selaginella moellendorffii* (lycophyte) and *Physcomitrella patens* (bryophyte) (fig. 1 and supplementary fig. S2). In contrast, chlorophytes and rhodophytes, the unicellular relatives of plants, are generally poorer in genes related to ubiquitin-like signalling (~2% of the genome, with smaller genomes; supplementary fig. S2). Specifically, we detected 22 toolkit enrichments in embryophytes, mostly due to RING, RING-like and CRL gene families (fig. 3). These expansions often entail important diversifications of protein architectures, e.g. the multiple U-box enrichments in bikonts (fig. 3). Most eukaryotic U-box consist of either a single U-box domain, an U-box with elongation factors (involved in ubiquitin chain elongation (Tu et al. 2007)) or an U-box with TPR repeats (supplementary fig. S5I). Nevertheless, the U-box repertoire of embryophytes includes many other specific protein domain architectures, with WD40, Armadillo, KAP and protein kinase domains. Thus, again, the significant expansion of some gene families observed in embryophytes was mainly driven by the acquisition and shuffling of protein domains.

We also found that the expanded RING content of embryophytes' genomes (as well as holozoans') is particularly rich in architectures involving protein-binding domains (TPR repeats, Filamin, NHL, SPRY…) (supplementary fig. S5D-G). A similar pattern appears in the substrate selector subunits of the CRL machinery, mainly BTB (in animals) and F-box (in plants) (supplementary fig. S3 and S5). RING and CRL diversifications also occurred in other taxa, such as the cryptophyte *Guillardia theta* (C3H2C3 RINGs), the haptophyte *Emiliania huxleyi* (canonical RINGs, BTB), dictyostelids (C3H2C3, F-box), heterokonts (canonical RINGs) and excavates such as *Naegleria gruberi* (BTB and F-box). Altogether, this reinforces the view of canonical RINGs as plastic E3s with potential to take part in a huge variety of protein domain arrangements, which link them to very diverse functions.

In contrast to animals and plants, fungi were found to have a downsized ubiquitin system with multiple depletions, including HECT, U-box, a C3HC4 RING family and the ubiquitin domain itself (fig. 3). This trend was even clearer in the microsporidians, a group of obligate intracellular parasites that were found to have lost 18 toolkit families, including all the CRLs adaptor and recognition subunits (fig. 3). Also, the Ufm1 pathway was secondarily lost in fungi, where we could not identify any proteins containing signal peptides or Ufm1-specific E2. However, the whole SUMO toolkit was conserved in almost all examined fungi and its gene families were not depleted (fig. 1). Overall, our data show that fungi have a much simpler ubiquitin signalling network than other multicellular lineages, such as plants and animals.

Finally, we detected transposon-related domains in five gene families: IBR, F-box (ubiquitin E3s), USP, OTU (DUBs) and ULP (deSUMOylase). These motifs are often associated with lineage-specific diversifications of protein architectures in narrow phylogenetic contexts, such as plants (IBR, F-box and ULP), animals (OTU, USP and ULP), the filasterean *Capsaspora owczarzaki* or the oomycete *Phytophthora infestans* (both ULP) (supplementary fig. S5M, O, R, T and Y). These domains have transposon-related catalytic activities, such as transposase (MULE), integrase (rve), endonuclease (DDE) and DNA polymerase (RVT_3). This is a signal of transduplication, i.e. gene duplication resulting from the incorporation of gene fragments into DNA transposons. We propose that transduplication is as a minor but interesting source of diversification in these five gene families. Indeed, previous studies in ULPs show that, if the coding region remains functional, transduplication can be a source of protein diversification (van Leeuwen et al. 2007; Böhne et al. 2012), sometimes involving gene neo-functionalization by acquisition of new domains.

References

Amerik AY, Hochstrasser M. 2004. Mechanism and function of deubiquitinating enzymes. Biochim. Biophys. Acta 1695:189–207.

Aravind L, Koonin E V. 2000. The U box is a modified RING finger - a common domain in ubiquitination. Curr. Biol. 10:R132–4.

Arcas A, Cases I, Rojas AM. 2013. Serine/threonine kinases and E2-ubiquitin conjugating enzymes in Planctomycetes: unexpected findings. Antonie Van Leeuwenhoek 104:509–520.

Böhne A, Zhou Q, Darras A, Schmidt C, Schartl M, Galiana-Arnoux D, Volff J-N. 2012. Zisupton--a novel superfamily of DNA transposable elements recently active in fish. Mol. Biol. Evol. 29:631–645.

Borden KL, Freemont PS. 1996. The RING finger domain: a recent example of a sequence-structure family. Curr. Opin. Struct. Biol. 6:395–401.

Burroughs AM, Balaji S, Iyer LM, Aravind L. 2007. Small but versatile: the extraordinary functional and structural diversity of the beta-grasp fold. Biol. Direct 2:18.

Burroughs AM, Jaffee M, Iyer LM, Aravind L. 2008. Anatomy of the E2 ligase fold: implications for enzymology and evolution of ubiquitin/Ub-like protein conjugation. J. Struct. Biol. 162:205–218.

Cardozo T, Pagano M. 2004. The SCF ubiquitin ligase: insights into a molecular machine. Nat. Rev. Mol. Cell Biol. 5:739–751.

Deshaies RJ, Joazeiro C a P. 2009. RING domain E3 ubiquitin ligases. Annu. Rev. Biochem. 78:399–434.

Dodd RB, Allen MD, Brown SE, Sanderson CM, Duncan LM, Lehner PJ, Bycroft M, Read RJ. 2004. Solution structure of the Kaposi's sarcoma-associated herpesvirus K3 N-terminal domain reveals a Novel E2-binding C4HC3-type RING domain. J. Biol. Chem. 279:53840–53847.

Duval D, Duval G, Kedinger C, Poch O, Boeuf H. 2003. The "PINIT" motif, of a newly identified conserved domain of the PIAS protein family, is essential for nuclear retention of PIAS3L. FEBS Lett. 554:111–118.

Freemont PS, Hanson IM, Trowsdale J. 1991. A novel cysteine-rich sequence motif. Cell 64:483–484.

Grau-Bové X, Sebé-Pedrós A, Ruiz-Trillo I. 2013. A genomic survey of HECT ubiquitin ligases in eukaryotes reveals independent expansions of the HECT system in several lineages. Genome Biol. Evol. 5:833–847.

Hickey CM, Wilson NR, Hochstrasser M. 2012. Function and regulation of SUMO proteases. Nat. Rev. Mol. Cell Biol. 13:755–766.

Iyer LM, Burroughs AM, Aravind L. 2006. The prokaryotic antecedents of the ubiquitin-signaling system and the early evolution of ubiquitin-like beta-grasp domains. Genome Biol. 7:R60.

Kang SH, Kim GR, Seong M, et al. 2007. Two novel ubiquitin-fold modifier 1 (Ufm1)-specific proteases, UfSP1 and UfSP2. J. Biol. Chem. 282:5256–5262.

Komander D, Clague MJ, Urbé S. 2009. Breaking the chains: structure and function of the deubiquitinases. Nat. Rev. Mol. Cell Biol. 10:550–563.

Komatsu M, Chiba T, Tatsumi K, et al. 2004. A novel protein-conjugating system for Ufm1, a ubiquitin-fold modifier. EMBO J. 23:1977–1986.

Langenick J, Araki T, Yamada Y, Williams JG. 2008. A Dictyostelium homologue of the metazoan Cbl proteins regulates STAT signalling. J. Cell Sci. 121:3524–3530.

Van Leeuwen H, Monfort A, Puigdomenech P. 2007. Mutator-like elements identified in melon, Arabidopsis and rice contain ULP1 protease domains. Mol. Genet. Genomics 277:357–364.

Lespinet O, Wolf YI, Koonin E V, Aravind L. 2002. The role of lineage-specific gene family expansion in the evolution of eukaryotes. Genome Res. 12:1048–1059.

Lipkowitz S, Weissman AM. 2011. RINGs of good and evil: RING finger ubiquitin ligases at the crossroads of tumour suppression and oncogenesis. Nat. Rev. Cancer 11:629–643.

Lorick KL. 1999. RING fingers mediate ubiquitin-conjugating enzyme (E2)-dependent ubiquitination. Proc. Natl. Acad. Sci. U. S. A. 96:11364–11369.

Manning G, Young SL, Miller WT, Zhai Y. 2008. The protist, Monosiga brevicollis, has a tyrosine kinase signaling network more elaborate and diverse than found in any known metazoan. Proc. Natl. Acad. Sci. U. S. A. 105:9674–9679.

Marín I. 2010. Ancient origin of animal U-box ubiquitin ligases. BMC Evol. Biol. 10:331.

Nijman SMB, Luna-Vargas MP a, Velds A, Brummelkamp TR, Dirac AMG, Sixma TK, Bernards R. 2005. A genomic and functional inventory of deubiquitinating enzymes. Cell 123:773–786.

Ohi MD, Vander Kooi CW, Rosenberg J a, Chazin WJ, Gould KL. 2003. Structural insights into the U-box, a domain associated with multi-ubiquitination. Nat. Struct. Biol. 10:250–255.

Petroski MD, Deshaies RJ. 2005. Function and regulation of cullin-RING ubiquitin ligases. Nat. Rev. Mol. Cell Biol. 6:9–20.

Pichler A, Gast A, Seeler JS, Dejean A, Melchior F. 2002. The nucleoporin RanBP2 has SUMO1 E3 ligase activity. Cell 108:109–120.

Pichler A, Knipscheer P, Saitoh H, Sixma TK, Melchior F. 2004. The RanBP2 SUMO E3 ligase is neither HECT- nor RING-type. Nat. Struct. Mol. Biol. 11:984–991.

Reverter D, Lima CD. 2005. Insights into E3 ligase activity revealed by a SUMO-RanGAP1-Ubc9-Nup358 complex. Nature 435:687–692.

Rotin D, Kumar S. 2009. Physiological functions of the HECT family of ubiquitin ligases. Nat. Rev. Mol. Cell Biol. 10:398–409.

Sarikas A, Hartmann T, Pan Z-Q. 2011. The cullin protein family. Genome Biol. 12:220.

Schmidt MHH, Dikic I. 2005. The Cbl interactome and its functions. Nat. Rev. Mol. Cell Biol. 6:907–918.

Suga H, Dacre M, de Mendoza A, Shalchian-Tabrizi K, Manning G, Ruiz-Trillo I. 2012. Genomic Survey of Premetazoans Shows Deep Conservation of Cytoplasmic Tyrosine Kinases and Multiple Radiations of Receptor Tyrosine Kinases. Sci. Signal. 5:ra35–ra35.

Tatsumi K, Sou Y, Tada N, et al. 2010. A novel type of E3 ligase for the Ufm1 conjugation system. J. Biol. Chem. 285:5417–5427.

Thien CB, Langdon WY. 2001. Cbl: many adaptations to regulate protein tyrosine kinases. Nat. Rev. Mol. Cell Biol. 2:294–307.

Tu D, Li W, Ye Y, Brunger AT. 2007. Structure and function of the yeast U-box-containing ubiquitin ligase Ufd2p. Proc. Natl. Acad. Sci. U. S. A. 104:15599–15606.

Willems AR, Schwab M, Tyers M. 2004. A hitchhiker's guide to the cullin ubiquitin ligases: SCF and its kin. Biochim. Biophys. Acta 1695:133–170.

Zhao X, Blobel G. 2005. A SUMO ligase is part of a nuclear multiprotein complex that affects DNA repair and chromosomal organization. Proc. Natl. Acad. Sci. U. S. A. 102:4777–4782.
