# Supplementary material for: The Eukaryotic Ancestor Had a Complex Ubiquitin Signaling System of Archaeal Origin
Source: Mol Biol Evol. 2014 Dec 17;32(3):726–39. doi: 10.1093/molbev/msu334 (PMC4327156; doi:10.1093/molbev/msu334)
Supplement: Supplementary Data [file supp_msu334_Supplementary3_FileS1.pdf]

*******JAB***********
>315426919_Candidatus_Caldiarchaeum_subterraneum
MRVRIYPLALAKVVKHAASSLQREVAGLLVGKSAGKVLEIWDAVTGEQYGTPAYVQLDEMVMAKVAEELS
KSDKNLYIVGWYHSHPGLDVFLSPTDIDTQKRYQAMFSKAVALVVDPVDYAKTRRISSLKFKVFQISKEG
RVVSLPVSIGVHRAKLLESTFHALSTFDFMHILGESSGKTRDKPLSEEQESLLGKAKKLFGA

>315426996_Candidatus_Caldiarchaeum_subterraneum
MRVRIYPLALAKVVKHAASSLQREVAGLLVGKSAGKVLEIWDAVTGEQYGTPAYVQLDEMVMAKVAEELS
KSDKNLYIVGWYHSHPGLDVFLSPTDIDTQKRYQAMFSKAVALVVDPVDYAKTRRISSLKFKVFQISKEG
RVVSLPVSIGVHRAKLLESTFHALSTFDFMHILGESSGKTRDKPLSEEQESLLGKAKKLFGA

>315428083_Candidatus_Caldiarchaeum_subterraneum
MRVRIYPLALAKVVKHAASSLQREVAGLLVGKSAGKVLEIWDAVTGEQYGTPAYVQLDEMVMAKVAEELS
KSDKNLYIVGWYHSHPGLDVFLSPTDIDTQKRYQAMFSKAVALVVDPVDYAKTRRISSLKFKVFQISKEG
RVVSLPVSIGVHRAKLLESTFHALSTFDFMHILGESSGKTRDKPLSEEQESLLGKAKKLFGA

>343485670_Candidatus_Caldiarchaeum_subterraneum
MRVRIYPLALAKVVKHAASSLQREVAGLLVGKSAGKVLEIWDAVTGEQYGTPAYVQLDEMVMAKVAEELS
KSDKNLYIVGWYHSHPGLDVFLSPTDIDTQKRYQAMFSKAVALVVDPVDYAKTRRISSLKFKVFQISKEG
RVVSLPVSIGVHRAKLLESTFHALSTFDFMHILGESSGKTRDKPLSEEQESLLGKAKKLFGA

>526887488_Candidatus_Caldiarchaeum_subterraneum
MRVRIYPLALAKVVKHAASSLQREVAGLLVGKSAGKVLEIWDAVTGEQYGTPAYVQLDEMVMAKVAEELS
KSDKNLYIVGWYHSHPGLDVFLSPTDIDTQKRYQAMFSKAVALVVDPVDYAKTRRISSLKFKVFQISKEG
RVVSLPVSIGVHRAKLLESTFHALSTFDFMHILGESSGKTRDKPLSEEQESLLGKAKKLFGA

>557694900_Candidatus_Caldiarchaeum_subterraneum
MRVRIYPLALAKVVKHAASSLQREVAGLLVGKSAGKVLEIWDAVTGEQYGTPAYVQLDEMVMAKVAEELS
KSDKNLYIVGWYHSHPGLDVFLSPTDIDTQKRYQAMFSKAVALVVDPVDYAKTRRISSLKFKVFQISKEG
RVVSLPVSIGVHRAKLLESTFHALSTFDFMHILGESSGKTRDKPLSEEQESLLGKAKKLFGA

>2265088697_candidate_division_pSL4_archaeon_JGI_0000001-A7_GBS-A_001_112
MITQVSEALARFSRIGAMVSFLGAIHSLSLLLTWFSINYDNVGRSLISGY
TFSEPLIISLVAGGIAGVAGILASSLRQISKIRVVLPTLSFTSAGLALFS
PLYTYLVKLPSFQLSYTPEFGMFGALITGVAITGSAVLATVASIRTKSTV
YLGPPPPWATQELELARGPGEGGVEGQEVQVPEVWEETTRVETTQPMPSQ
PSSQAPTGGNCLICGDTIPSGDLATCKGCGATFHKDCMNVWIDLGNRCPS
CNAELTS

>2527530172_candidate_division_pSL4_archaeon_SCGC_AAA471-E14_Combined_Assembly_p
SL4_3__pSL4
MITQVSEALARFSRIGAMVSFLGAIHSLSLLLTWFSINYDNVGRSLISGY
TFSEPLIISLVAGGIAGVAGILASSLRQISKIRVVLPTLSFTSAGLALFS
PLYTYLVKLPSFQLSYTPEFGMFGALITGVAITGSAVLATVASIRTKSTV
YLGPPPPWATQELELARGPGEGGVEGQEVQVPEVWEETTRVETTQPMPSQ
PSSQAPTGGNCLICGDTIPSGDLATCKGCGATFHKDCMNVWIDLGNRCPS
CNAELTS

>2265091066_candidate_division_pSL4_archaeon_SCGC_AAA471-G05_GBS-N_001_14
MLVMGTDVRDALVRLIRIGALISVLGAIHVLTLMLDWASLLNDNVVVRGI
QGFVLNWSFTLSLLAGLLAGGAGVLASLSSNVRMLRFAVPSLAIAGSALA
ILSPLYVLLHHLPSLNIQFRPEVGAIAALFTGVAMAGGGFISAMIGFVVL
RPSSGPSAPPAIAYAPVQTPVIEAQQEIALGYEEPSLSHEEEVFEEILRQ
EEASIRAAAQPVPAEGTSPAPTTVPPQRPQTAAQECAICGEPIPSNSLRF
CNTCGAPMHRECVETWRDIGGKCPSCGTPLV

>2527526036_candidate_division_pSL4_archaeon_SCGC_AAA471-B22_Combined_Assembly_p
SL4_1__pSL4
MLVMGTDVRDALVRLIRIGALISVLGAIHVLTLMLDWASLLNDNVVVRGI
QGFVLNWSFTLSLLAGLLAGGAGVLASLSSNVRMLRFAVPSLAIAGSALA
ILSPLYVLLHHLPSLNIQFRPEVGAIAALFTGVAMAGGGFISAMIGFVVL

RPSSGPSAPPAIAYAPVQTPVIEAQQEIALGYEEPSLSHEEEVFEEILRQ
EEASIRAAAQPVPAEGTSPAPTTVPPQRPQTAAQECAICGEPIPSNSLRF
CNTCGAPMHRECVETWRDIGGKCPSCGTPLV

>2265089058_candidate_division_pSL4_archaeon_JGI_0000001-B8_GBS-A_001_113
VRRFRTPVVVEGRKAGEWFWGVICDSCLLEIDPSHTTTKCPHCGATFHSF
CYTAILSSKGRCPKCKMELA

>2527529566_candidate_division_pSL4_archaeon_SCGC_AAA471-D15_Combined_Assembly_pSL4_2__pSL4
VRRFRTPVVVEGRKAGEWFWGVICDSCLLEIDPSHTTTKCPHCGATFHSF
CYTAILSSKGRCPKCKMELA

>2265087851_candidate_division_pSL4_archaeon_SCGC_AAA471-E14_GBS-N_001_10
VLKVRSGRVLGRWEWGQPMCDACLLEIEEGVEPLKCENCGANFHPDCYTS
LKNTKAVCPKCKVTLE

>2265088700_candidate_division_pSL4_archaeon_JGI_0000001-A7_GBS-A_001_112
VLKVRSGRVLGRWEWGQPMCDACLLEIEEGVEPLKCENCGANFHPDCYTS
LKNTKAVCPKCKVTLE

>2527530169_candidate_division_pSL4_archaeon_SCGC_AAA471-E14_Combined_Assembly_pSL4_3__pSL4
VLKVRSGRVLGRWEWGQPMCDACLLEIEEGVEPLKCENCGANFHPDCYTS
LKNTKAVCPKCKVTLE

*******RINGv***********
>516675795_MULTISPECIES_hypothetical_protein_Archaea
MDNCCKYCLEDSKESEHPLIYPCQCADGVHPNCLAIWLLVRPDSNDRCRCEICHVNYIGVFIPPSTPPPP
PSPLLQPLSDEEEDDVVVAVPPPPPQPRLQQRNNNVDLDFICCQCQGLEAGSYILGTILGLSGSILTTQP
GYNHRSDIDIAFKVFIGLCISLYLMGLICTSRRCYKRCINGRRVYDNGGV

>516676027_hypothetical_protein_Marine_Group_II_euryarchaeote_SCGC_AB-629-J06
MENVKECRICMTEGSEESLIQPCRCNTAYVHESCLQKWRSENTDNEKYRKCEICQADYVILQDHPKETFK
IYAKKIKGCPLFCMYTIYLFIGSFATTLIDTFSNQQSLVILNGGNTNITLGIDIDDIDSLSWFIYYLSYT
SYIYGMIFFVFIFFGLISSVHRKQFYIKRTRCTFSAYFIMSWTYFYNFYIFYKTMHRLDLYMAASLASIP
INFFIMKRVAQIHDNIIRELNTNNVETIISVRYNPLIEITTIEDEAEN

*******ThiF***********
>315425344_Candidatus_Caldiarchaeum_subterraneum
MKVRALTTETVSLSFEEIKRYGRHLIIPEVGMAGQKKLKAAKVLVVGAGGLGSPISLYLAAAGVGKIGLV
DFDLVDESNLQRQVLYTTRDVKRPKLEVAKERLTALNPHIEVETYETRLTSENALDIIKDYDIVVDGTDN
FPTRYLVNDACVLLGKPNVYGSIFRFDGQVSVFDARRGPCYRCLYPEPPPPGLVPSCAEGGVLGVLPGVI
GALQAMETIKLIIGIGEPLVGRLLLFDGLHMSFTELKLRKDPNCVICGPNPRIRELIDYEAFCGVTPSAD
TSMHITPEELHEKLQKGEKVFLLDVREPVEYEICHLENALLIPLSKLPEHVNKLSLTDEIVAYCHTGVRS
SMAVKLLRDLGFRRVRNLAGGIDAWAERIDPSMPRY

>343485333_Candidatus_Caldiarchaeum_subterraneum
MKVRALTTETVSLSFEEIKRYGRHLIIPEVGMAGQKKLKAAKVLVVGAGGLGSPISLYLAAAGVGKIGLV
DFDLVDESNLQRQVLYTTRDVKRPKLEVAKERLTALNPHIEVETYETRLTSENALDIIKDYDIVVDGTDN
FPTRYLVNDACVLLGKPNVYGSIFRFDGQVSVFDARRGPCYRCLYPEPPPPGLVPSCAEGGVLGVLPGVI
GALQAMETIKLIIGIGEPLVGRLLLFDGLHMSFTELKLRKDPNCVICGPNPRIRELIDYEAFCGVTPSAD
TSMHITPEELHEKLQKGEKVFLLDVREPVEYEICHLENALLIPLSKLPEHVNKLSLTDEIVAYCHTGVRS
SMAVKLLRDLGFRRVRNLAGGIDAWAERIDPSMPRY

>526886305_Candidatus_Caldiarchaeum_subterraneum
MKVRALTTETVSLSFEEIKRYGRHLIIPEVGMAGQKKLKAAKVLVVGAGGLGSPISLYLAAAGVGKIGLV
DFDLVDESNLQRQVLYTTRDVKRPKLEVAKERLTALNPHIEVETYETRLTSENALDIIKDYDIVVDGTDN
FPTRYLVNDACVLLGKPNVYGSIFRFDGQVSVFDARRGPCYRCLYPEPPPPGLVPSCAEGGVLGVLPGVI

```
GALQAMETIKLIIGIGEPLVGRLLLFDGLHMSFTELKLRKDPNCVICGPNPRIRELIDYEAFCGVTPSAD
TSMHITPEELHEKLQKGEKVFLLDVREPVEYEICHLENALLIPLSKLPEHVNKLSLTDEIVAYCHTGVRS
SMAVKLLRDLGFRRVRNLAGGIDAWAERIDPSMPRY

>557694563_Candidatus_Caldiarchaeum_subterraneum
MKVRALTTETVSLSFEEIKRYGRHLIIPEVGMAGQKKLKAAKVLVVGAGGLGSPISLYLAAAGVGKIGLV
DFDLVDESNLQRQVLYTTRDVKRPKLEVAKERLTALNPHIEVETYETRLTSENALDIIKDYDIVVDGTDN
FPTRYLVNDACVLLGKPNVYGSIFRFDGQVSVFDARRGPCYRCLYPEPPPPGLVPSCAEGGVLGVLPGVI
GALQAMETIKLIIGIGEPLVGRLLLFDGLHMSFTELKLRKDPNCVICGPNPRIRELIDYEAFCGVTPSAD
TSMHITPEELHEKLQKGEKVFLLDVREPVEYEICHLENALLIPLSKLPEHVNKLSLTDEIVAYCHTGVRS
SMAVKLLRDLGFRRVRNLAGGIDAWAERIDPSMPRY

>519109680_Aigarchaeota_archaeon_SCGC_AAA471-G05
MGKALDEGLRERYDRQMRIEGWDQHLVSGSSVLIAGVGALGCEVAKNLALSGVGRLVLVDRDVVELSNLN
RQMLFDESDIGLRKAEVAAAKLRAMNPHVRVESYASDLREVPEEVFESVDVICSCLDSWGIRRWLNSVAV
LKRKPLVDGAIEGMVGNVQVVIPGRTACLECHGTTLIPQEERLAECTLRRRRPEELLEDLKAQGVEVSLE
EARTLFRYNLKTVFDLKYTPPESVPDQGVRELLNSLRERLKPKMPAVQSVSSVIAGIVSTEVLKLIHRGS
IGKPLRGLLVYDASNSRFTRVPLKRMEGCIVCGQVDVSPPEVVVGDDGTVYRLKELIAERFGIPDAEVIH
GTKVLGEDARLSSVLGPSGEGIVYVVTSRRYEPLPLVVRLSDFKDPSLSGKGDIPREATGSG

>516975882_candidate_division_YNPFFA
MFNADEYYSRLYVLKNIGKEGINILKRKTAAVVGLGGLGSVIAYLLTSNGIGKIKLIDQDIVELSNINRQ
FLYDINDLRYPKVEVAYKKLKDVNPEVKIEPIAENLNSDNAEEILGDVDIILDGLDNLKTRYIVNYIAVK
KKIPYVFGSVLEYTGNVSVFYPPETPCLNCIFEEINDSELPTCETVGIINTATSLVASIEVNEAIKLLIN
KSSNLLSKLLLIDLGNLIFDFIDIFKNENCKVCNNILSYKPKKIELIWQCGRDIVNINPRKKVNMNLNNI
KEKIEKYFKIILVSDFIIVFKYEDKEISIFKNGRMLIKNVKNEEEAKNVYEKIDKLINI

>2265088526_candidate_division_pSL4_archaeon_JGI_0000001A7_GBSA_001_112
VVGLTERQVLNPLDHVSLTMQEIRRYGRHLIMPEVGMTGQKRLKAARVLV
VGTGGLGAPVSLYLAAAGVGTIGLVDFDEVDETNLQRQVIFTTEDVGEKK
VEVAKRRLLSLNPYIKVETYGEAINSQNALEIIGEYDVVVDATDNFPTRY
LLNDACVFLKKPLVYGSIFRFDGQLSVFYAGHGPCYRCLYPEPPPPGLVP
SCAEGGVLGVLPGIVGSLQANEALKLILGVGQPLIGRLLLIDALSTGFRE
LHVQRDKNCPVCGENPTIRELIDYEQFCGVRITGEDPSSRATSIAPEQLK
EMLDRGEEIQLIDVREPVEWEICRLPGARLIPLGQLTSRLHEIDQTKKVI
VYCHSGQRSALAVKLLRDLGLTNTFNLAGGIDAYAERIDQSIPRY

>2265088061_candidate_division_pSL4_archaeon_SCGC_AAA471E14_GBSN_001_10
MKQLERYERQIRIDGWDQEKLFSATVFVAGVGALGCEVAKNLAMMGVGRL
MIADYDRVELSNLSRQLLFRDSDIGRGKAEAAAERLREINPHVEVEAFEG
DIRELGEETYRKADVLLSCLDNWASRRWYNSMAVYLGKPLVDGAMNGFYG
NVQIVLPGRTACLECQSSLLIPREERAAECTLRRRRPQDLVDELKQHGLS
ITLEEAENLFKLNIKTVYDLKYFRPEKSNQEISEETLRLIDGLRASLVPR
MPALQSVAATIAGIVSTHTLQILHGGSLGAVPTSLIVYDGLNSRLTRVKI
KRDPMCIVCGENEGEPITLRFDTSRTVYEFKEVIASKLGLPDPEILYISR
RLDDSQTLESAGVKNGDVVYVSTTRLYEPLAIKVVADEGDTASLG

>2265090260_candidate_division_pSL4_archaeon_SCGC_AAA471F17_GBSN_001_13
LGKALDEGLRERYDRQMRIEGWDQHLVSGSSVLIAGVGALGCEVAKNLAL
SGVGRLVLVDRDVVELSNLNRQMLFDESDIGLRKAEVAAAKLRAMNPHVR
VESYASDLREVPEEVFESVDVICSCLDSWGIRRWLNSVAVLKRKPLVDGA
IEGMVGNVQVVIPGRTACLECHGTTLIPQEERLAECTLRRRRPEELLEDL
RAQGVEVSLEEARTLFRYNLKTVFDLKYTPPESVPDQGVRELLNSLRERL
KPKMPAVQSVSSVIAGIVSTEVLKLIHRGSIGKPLRGLLVYDASNSRFTR
VPLKRMEGCIVCGQVDVSPPEVVVGDDGTVYRLKELIAERFGIPDAEVIH
GTKVLGEDVRLSSVLGPSGEGIVYVVTSRRYEPLPLVVRLSDFKDPSLSG
KGDIPREATGSG

>2265090995_candidate_division_pSL4_archaeon_SCGC_AAA471G05_GBSN_001_14
LGKALDEGLRERYDRQMRIEGWDQHLVSGSSVLIAGVGALGCEVAKNLAL
SGVGRLVLVDRDVVELSNLNRQMLFDESDIGLRKAEVAAAKLRAMNPHVR
VESYASDLREVPEEVFESVDVICSCLDSWGIRRWLNSVAVLKRKPLVDGA
```

```
IEGMVGNVQVVIPGRTACLECHGTTLIPQEERLAECTLRRRRPEELLEDL
KAQGVEVSLEEARTLFRYNLKTVFDLKYTPPESVPDQGVRELLNSLRERL
KPKMPAVQSVSSVIAGIVSTEVLKLIHRGSIGKPLRGLLVYDASNSRFTR
VPLKRMEGCIVCGQVDVSPPEVVVGDDGTVYRLKELIAERFGIPDAEVIH
GTKVLGEDARLSSVLGPSGEGIVYVVTSRRYEPLPLVVRLSDFKDPSLSG
KGDIPREATGSG

>2265092360_candidate_division_pSL4_archaeon_SCGC_AAA471J08_GBSN_001_19
LGKALDEGLRERYDRQMRIEGWDQHLVSGSSVLIAGVGALGCEVAKNLAL
SGVGRLVLVDRDVVELSNLNRQMLFDESDIGLRKAEVAAAKLRAMNPHVR
VESYASDLREVPEEVFESVDVICSCLDSWGIRRWLNSVAVLKRKPLVDGA
IEGMVGNVQVVIPGKTACLECHGTTLIPQEERLAECTLRRRRPEELLEDL
RAQGVEVSLEEARTLFRYNLKTVFDLKYTPPESVPDQGVRELLNSLRERL
KPKMPAVQSVSSVIAGIVSTEVLKLIHRGSIGKPLRGLLVYDASNSRFTR
VPLKRMEGCIVCGQVDVSPPEVVVGDDGTVYRLKELIAERFGIPDAEVIH
GTKVLGEDVRLSSVLGPSGEGIVYVVTSRRYEPLPLVVRLSDFKDPSLSG
KGDIPREATGSG

>2265094365_candidate_division_pSL4_archaeon_SCGC_AAA471D15_GBSN_001_9
LGGALDDVLRERYDRQIRIEGWDQQLVSGSSVLIAGVGALGCEVAKNLAL
SGVGRLVLVDRDVVELSNLNRQMLFDESDIGLRKAEVAAAKLRAMNPYIR
VESYASDLREVPEEVFESVDVICSCLDSWGIRRWLNSVAVLKRKPLVDGA
IEGMVGNVQVVIPGRTACLECHGTTLIPQEERLAECTLRRRRPEELMEDL
RAQGVEISLEEARALFRYNLKTVFDLKYTPPESIPDQGVRELLNSLRERL
KPKMPAVQSVSSVIAGIVSTEVLKLIHRGSIGKPLRGLLVYDASNSRFTR
VPLKRMEGCIVCGQVDVSPPEVVVGDDGTVYRLKELIAERFGIPDAEVIY
GTRVLGEDVQLSSVLGPSGEGVVYVVTSRRYEPLPLKVRLHSSTGSSLSG
KGDIPREGTGSG

>2527526327_candidate_division_pSL4_archaeon_SCGC_AAA471B22_Combined_Assembly_pS
L4_1__pSL4
LLKNASVMVGGAGALGNEIIKNLVMLGFGTIYVVDFDTVVRSNLNRCVLY
RTIDAELKTFKAEAIAKRAKEIDPYRYIEIIPIVEEIGPQGINYRNRLFT
EKNIXLIFGAFDNVASRIHMTTIAYYHGIPYVDGGMWGXIGNVFVMNPPX
TACYVCSLSEESWTEMLKRLQCSMKGTLESMEMPSLPTTSSIVAAIQVQE
ALKILFSKRNPGGSSLGVPSLGKMISFNLITNDWIVYEVPKRPDCPVCSN
VGRG

>2527529149_candidate_division_pSL4_archaeon_SCGC_AAA471D15_Combined_Assembly_pS
L4_2__pSL4
LGGALDDVLRERYDRQIRIEGWDQQLVSGSSVLIAGVGALGCEVAKNLAL
SGVGRLVLVDRDVVELSNLNRQMLFDESDIGLRKAEVAAAKLRAMNPYIR
VESYASDLREVPEEVFESVDVICSCLDSWGIRRWLNSVAVLKRKPLVDGA
IEGMVGNVQVVIPGRTACLECHGTTLIPQEERLAECTLRRRRPEELMEDL
RAQGVEISLEEARALFRYNLKTVFDLKYTPPESIPDQGVRELLNSLRERL
KPKMPAVQSVSSVIAGIVSTEVLKLIHRGSIGKPLRGLLVYDASNSRFTR
VPLKRMEGCIVCGQVDVSPPEVVVGDDGTVYRLKELIAERFGIPDAEVIY
GTRVLGEDVQLSSVLGPSGEGVVYVVTSRRYEPLPLKVRLHSSTGSSLSG
KGDIPREGTGSG

>2527530458_candidate_division_pSL4_archaeon_SCGC_AAA471E14_Combined_Assembly_pS
L4_3__pSL4
VVGLTERQVLNPLDHVSLTMQEIRRYGRHLIMPEVGMTGQKRLKAARVLV
VGTGGLGAPVSLYLAAAGVGTIGLVDFDEVDETNLQRQVIFTTEDVGEKK
VEVAKRRLLSLNPYIKVETYGEAINSQNALEIIGEYDVVVDATDNFPTRY
LLNDACVFLKKPLVYGSIFRFDGQLSVFYAGHGPCYRCLYPEPPPPGLVP
SCAEGGVLGVLPGIVGSLQANEALKLILGVGQPLIGRLLLIDALSTGFRE
LHVQRDKNCPVCGENPTIRELIDYEQFCGVRITGEDPSSRATSIAPEQLK
EMLDRGEEIQLIDVREPVEWEICRLPGARLIPLGQLTSRLHEIDQTKKVI
VYCHSGQRSALAVKLLRDLGLTNTFNLAGGIDAYAERIDQSIPRY

>2264867790_candidate_division_YNPFFA_archaeon_SCGC_AAA471O08_GBSN_001_29
```

MFNADEYYSRLYVLKNIGKEGINILKRKTAAVVGLGGLGSVIAYLLTSNG
IGKIKLIDQDIVELSNINRQFLYDINDLRYPKVEVAYKKLKDVNPEVKIE
PIAENLNSDNAEEILGDVDIILDGLDNLKTRYIVNYIAVKKKIPYVFGSV
LEYTGNVSVFYPPETPCLNCIFEEINDSELPTCETVGIINTATSLVASIE
VNEAIKLLINKSSNLLSKLLLIDLGNLIFDFIDIFKNENCKVCNNILSYK
PKKIELIWQCGRDIVNINPRKKVNMNLNNIKEKIEKYFKIILVSDFIIVF
KYEDKEISIFKNGRMLIKNVKNEEEAKNVYEKIDKLINI

>2264869056_candidate_division_YNPFFA_archaeon_SCGC_AAA471B05_GBSN_001_2
MFNADEYYSRLYVLKNIGKEGINILKRKTAAVVGLGGLGSVIAYLLTSNG
IGKIKLIDQDIVELSNINRQFLYDINDLRYPKVEVAYKKLKDVNPEVKIE
PIAENLNSDNAEEILGDVDIILDGLDNLKTRYIVNYIAVKKKIPYVFGSV
LEYTGNVSVFYPPETPCLNCIFEEINDSELPTCETVGIINTATSLVASIE
VNEAIKLLINKSSNLLSKLLLIDLGNLIFDFIDIFKNENCKVCNNILSYK
PKKIELIWQCGRDIVNINPRKKVNMNLNNIKEKIEKYFKIILVSDFIIVF
KYEDKEISIFKNGRMLIKNVKNEEEAKNVYEKIDKLINI

>2265120287_candidate_division_YNPFFA_archaeon_SCGC_AAA471L13_GBSN_001_22
MFNADEYYSRLYVLKNIGKEGINILKRKTAAVVGLGGLGSVIAYLLTSNG
IGKIKLIDQDIVELSNINRQFLYDINDLRYPKVEVAYKKLKDVNPEVKIE
PIAENLNSDNAEEILGDVDIILDGLDNLKTRYIVNYIAVKKKIPYVFGSV
LEYTGNVSVFYPPETPCLNCIFEEINDSELPTCETVGIINTATSLVASIE
VNEAIKLLINKSSNLLSKLLLIDLGNLIFDFIDIFKNENCKVCNNILSYK
PKKIELIWQCGRDIVNINPRKKVNMNLNNIKEKIEKYFKIILVSDFIIVF
KYEDKEISIFKNGRMLIKNVKNEEEAKNVYEKIDKLINI

>2265121764_candidate_division_YNPFFA_archaeon_SCGC_AAA471L14_GBSN_001_23
MFNADEYYSRLYVLKNIGKEGINILKRKTAAVVGLGGLGSVIAYLLTSNG
IGKIKLIDQDIVELSNINRQFLYDINDLRYPKVEVAYKKLKDVNPEVKIE
PIAENLNSDNAEEILGDVDIILDGLDNLKTRYIVNYIAVKKKIPYVFGSV
LEYTGNVSVFYPPETPCLNCIFEEINDSELPTCETVGIINTATSLVASIE
VNEAIKLLINKSSNLLSKLLLIDLGNLIFDFIDIFKNENCKVCNNILSYK
PKKIELIWQCGRDIVNINPRKKVNMNLNNIKEKIEKYFKIILVSDFIIVF
KYEDKEISIFKNGRMLIKNVKNEEEAKNVYEKIDKLINI

>2527532002_candidate_division_YNPFFA_archaeon_SCGC_AAA471B05_Combined_Assembly_
YNPFFA_1__YNPFFA
MFNADEYYSRLYVLKNIGKEGINILKRKTAAVVGLGGLGSVIAYLLTSNG
IGKIKLIDQDIVELSNINRQFLYDINDLRYPKVEVAYKKLKDVNPEVKIE
PIAENLNSDNAEEILGDVDIILDGLDNLKTRYIVNYIAVKKKIPYVFGSV
LEYTGNVSVFYPPETPCLNCIFEEINDSELPTCETVGIINTATSLVASIE
VNEAIKLLINKSSNLLSKLLLIDLGNLIFDFIDIFKNENCKVCNNILSYK
PKKIELIWQCGRDIVNINPRKKVNMNLNNIKEKIEKYFKIILVSDFIIVF
KYEDKEISIFKNGRMLIKNVKNEEEAKNVYEKIDKLINI

*******UCH***********
>516675686_Marine_Group_II_euryarchaeote_SCGC_AB-629-J06
MADKYKDKGLTGLANLGNTCFMNTTLQCLSHTYSFNDFLQHGTYKQKIKKKPESLVLMEWDKLRLMVWSE
NCIISPGGFLTSVQKVAKIKEKHLFTGFAQNDLPEFLTFIIDCFHTAIMREVNMKIKGNILTDKDKVAKK
CFDMMKNMYKKEYSEILDIFYGIHVSCVKGEDGDTLSCNPEPFLMLDLPVPDKRNVSLINCFDEYTKKEV
LDDDNQYINDEGKKVVAKQIEFWKFPDVLIVTLKRFTNSIRKNQCLVDFPFDNLDLSKYVVGYDPHSFNY
ELYGICNHSGGVMGGHYYAYVKNANNKWYDFNDARVQEIKEADLKTPYAYCFFYRKKK

*******UQ_con***********
>2265088662_candidate_division_pSL4_archaeon_JGI_0000001-A7_GBS-A_001_112
MHVPEAGYLPEPRWAKRLAQEYQLMKNYEPTFDAKDGDLTHYKGTIIGTG
IFEGGVFVVEILLPREFPFVPPQVVWHTRIWHPNFTDEVPARVCESIFNK
DWFPNMHVVSVIEALKNLLANPNPDDPLNVWAAYEMKYYPERFVARVREY

IRLYATPEKALSLL

>2265090259_candidate_division_pSL4_archaeon_SCGC_AAA471-F17_GBS-N_001_13
LQREVQEQIGFLPEQLWFKRLALEYQLIRNYEPTFDAVDGDLTHYKGVIV
GTGYYEGGFFLVEIFLDRTFPYTPPRVVWHTRIWHPNFTDESPARICESI
LTKDWEPNTNVIAVIEALKNLLSNPNPDDPLNAWAAYEMKNSFHTFLARV
RQYIELYASPEKVLRQG

>2265090996_candidate_division_pSL4_archaeon_SCGC_AAA471-G05_GBS-N_001_14
LQREVQEQIGFLPEQLWFKRLALEYQLIRNYEPTFDAVDGDLTHYKGVIV
GTGYYEGGFFLVEIFLDRTFPYTPPRVVWHTRIWHPNFTDESPARICESI
LTKDWEPNTNVIAVIEALKNLLSNPNPDDPLNAWAAYEMKNSFHTFLARV
RQYIELYASPEKVLRQG

>2265092361_candidate_division_pSL4_archaeon_SCGC_AAA471-J08_GBS-N_001_19
LQREVQEQIGFLPEQLWFKRLALEYQLIRNYEPTFDAVDGDLTHYKGVIV
GTGYYEGGFFLVEIFLDRTFPYTPPRVVWHTRIWHPNFTDESPARICESI
LTKDWEPNTNVIAVIEALKNLLSNPNPDDPLNAWAAYEMKNSFHTFLARV
RQYIELYASPEKVLRQG

>2265094366_candidate_division_pSL4_archaeon_SCGC_AAA471-D15_GBS-N_001_9
LQREVQEQIGFLPEQLWFKRLALEYQLIRNYEPTFDAVDGDLTHYKGVIV
GTGYYEGGFFLVEIFLDRTFPYTPPRVVWHTRIWHPNFTDESPARICESI
LTKDWEPNTNVIAVIEALKNLLSNPNPDDPLNAWAAYEMKNSFHTFLARV
RQYIELYASPEKVLRQG

>2527529148_candidate_division_pSL4_archaeon_SCGC_AAA471-D15_Combined_Assembly_p
SL4_2__pSL4
LQREVQEQIGFLPEQLWFKRLALEYQLIRNYEPTFDAVDGDLTHYKGVIV
GTGYYEGGFFLVEIFLDRTFPYTPPRVVWHTRIWHPNFTDESPARICESI
LTKDWEPNTNVIAVIEALKNLLSNPNPDDPLNAWAAYEMKNSFHTFLARV
RQYIELYASPEKVLRQG

>2527525289_candidate_division_pSL4_archaeon_SCGC_AAA471-B22_Combined_Assembly_p
SL4_1__pSL4
VDGDLTHYKGVIVGTGYYEGGFFXVEIFLDRTFPYTPPRVVWHTRIWHPN
FTDESPARICESILTKDWEPNTNVIAVIEALKNLLSNPNPDDPLNAWAAY
EMKNSFHTFLARVRQYIELYASPEKVLRQG

>2265088060_candidate_division_pSL4_archaeon_SCGC_AAA471-E14_GBS-N_001_10
MHVPEAGYLPEPRWAKRLAQEYQLMKNYEPTFDAKDGDLTHYKGTIIGTG
IXEGGVFVVEILLPREFPFVPPQVVWHTRIWHPNFTDEVPARVCESIFNK
DWFPNMHVVSVIEALKNLLANPNPDDPLNVWAAYEMKYYPERFVARVREY
IRLYATPEKALSLL

>2527530434_candidate_division_pSL4_archaeon_SCGC_AAA471-E14_Combined_Assembly_p
SL4_3__pSL4
MHVPEAGYLPEPRWAKRLAQEYQLMKNYEPTFDAKDGDLTHYKGTIIGTG
IXEGGVFVVEILLPREFPFVPPQVVWHTRIWHPNFTDEVPARVCESIFNK
DWFPNMHVVSVIEALKNLLANPNPDDPLNVWAAYEMKYYPERFVARVREY
IRLYATPEKALSLL

>516675903_Marine_Group_II_euryarchaeote_SCGC_AB-629-J06
MSTAQRIAKELENLTADPPANCSAGPLEDDIFHWQATLMGPQDSPYEGGIFIMNIKFPGDYPFKPPKVTF
ETKIFHPNINSSGGICLDILKEAWSPALTISKVLLSICSLLCDPNPDDPLVPDIARMYKHDRDKYNRTAQ
LWTIQFAVSE

>315426917_Candidatus_Caldiarchaeum_subterraneum
MESQYVELPENAWYRRLALEYALIQENEPTFTPVENDLTHYEGVIVGSGEYEGGFFRVEIIIPRSYPYFP
PDVIWHTRIWHPNFSDSVPARVCESIFKDHWSPSLRIVAVIESLRNLLTNPNPEDPLNPVAAFEYKNRPD
LFYSRVRQFVETYATPEQAFGKKRWKGL

>315426998_Candidatus_Caldiarchaeum_subterraneum
MESQYVELPENAWYRRLALEYALIQENEPTFTPVENDLTHYEGVIVGSGEYEGGFFRVEIIIPRSYPYFP
PDVIWHTRIWHPNFSDSVPARVCESIFKDHWSPSLRIVAVIESLRNLLTNPNPEDPLNPVAAFEYKNRPD
LFYSRVRQFVETYATPEQAFGKKRWKGL

>315428085_Candidatus_Caldiarchaeum_subterraneum
MESQYVELPENAWYRRLALEYALIQENEPTFTPVENDLTHYEGVIVGSGEYEGGFFRVEIIIPRSYPYFP
PDVIWHTRIWHPNFSDSVPARVCESIFKDHWSPSLRIVAVIESLRNLLTNPNPEDPLNPVAAFEYKNRPD
LFYSRVRQFVETYATPEQAFGKKRWKGL

>343485672_Candidatus_Caldiarchaeum_subterraneum
MESQYVELPENAWYRRLALEYALIQENEPTFTPVENDLTHYEGVIVGSGEYEGGFFRVEIIIPRSYPYFP
PDVIWHTRIWHPNFSDSVPARVCESIFKDHWSPSLRIVAVIESLRNLLTNPNPEDPLNPVAAFEYKNRPD
LFYSRVRQFVETYATPEQAFGKKRWKGL

>526887490_Candidatus_Caldiarchaeum_subterraneum
MESQYVELPENAWYRRLALEYALIQENEPTFTPVENDLTHYEGVIVGSGEYEGGFFRVEIIIPRSYPYFP
PDVIWHTRIWHPNFSDSVPARVCESIFKDHWSPSLRIVAVIESLRNLLTNPNPEDPLNPVAAFEYKNRPD
LFYSRVRQFVETYATPEQAFGKKRWKGL

>557694902_Candidatus_Caldiarchaeum_subterraneum
MESQYVELPENAWYRRLALEYALIQENEPTFTPVENDLTHYEGVIVGSGEYEGGFFRVEIIIPRSYPYFP
PDVIWHTRIWHPNFSDSVPARVCESIFKDHWSPSLRIVAVIESLRNLLTNPNPEDPLNPVAAFEYKNRPD
LFYSRVRQFVETYATPEQAFGKKRWKGL

>516675970_Marine_Group_II_euryarchaeote_SCGC_AB-629-J06
MSSSSKFMPPKNQKRLLRDVVRLIKSPLTSNGIYYSHDEDNMLKGYAVIFGPDDSLYRYGAYMFEFNYPT
EYPFVPPKLTYLTNDGKTRFNPNLYRNGKVCISLLNTWKGEQWTSCQTIESILLSLVALLHNEPLLNEPG
IKKTHRDFKSYNSIIQYKNYETAILGILTQKILPSTFSGFFPIIKKHFKEHEKFILAELAQLEQSKKNRQ
EFKTSLYNMNISANYTDLMEKMKIAFKEFLN

>519109681_Aigarchaeota_archaeon_SCGC_AAA471-G05
MQREVQEQIGFLPEQLWFKRLALEYQLIRNYEPTFDAVDGDLTHYKGVIVGTGYYEGGFFLVEIFLDRTF
PYTPPRVVWHTRIWHPNFTDESPARICESILTKDWEPNTNVIAVIEALKNLLSNPNPDDPLNAWAAYEMK
NSFHTFLARVRQYIELYASPEKVLRQG

>518007549_Methanomassiliicoccus_luminyensis
MPLPADILVIRLRNELSACRSYIRDMPDLSAPSRVRFPVEVEVELAKVPGPIIEEGKVGSTYLHRFSISI
GKNYPFEKPTVRWMSPIFHPNIMMPDDGGHVCTKLLEEWGFNSTLISFIKGVESLVMNPNPASPFGTDSC
TAAAEHFNCAEVKLPPMLKPPAPRVVRP

*******ubiquitin***********
>2265090997_candidate_division_pSL4_archaeon_SCGC_AAA471-G05_GBS-N_001_14
MKVRIVPAVGGGPPLEIDVPPSTTIGAIKLRVCAMKKLRPDDTRLTYKNR
ALADTETLESAGVSDGDKLVLVTRTVGG

>2265092362_candidate_division_pSL4_archaeon_SCGC_AAA471-J08_GBS-N_001_19
MKVRIVPAVGGGPPLEIDVPPSTTIGAIKLRVCAMKKLRPDDTRLTYKNR
ALADTETLESAGVSDGDKLVLVTRTVGG

>2265090258_candidate_division_pSL4_archaeon_SCGC_AAA471-F17_GBS-N_001_13
MKVRIVPAVGGGPPLEIDVPPSTTIGAIKLRVCAMKKLRPDDTRLTYKNR
ALADTETLESAGVSEGDKLVLVTRTVGG

>2265094367_candidate_division_pSL4_archaeon_SCGC_AAA471-D15_GBS-N_001_9
MKVRIVPAVGGGPPLEIDVPPSTTIGAIKLRVCAMKKLRPDDTRLTYKNR
ALADTETLESAGVSEGDKLVLVTRTVGG

>2527525290_candidate_division_pSL4_archaeon_SCGC_AAA471-B22_Combined_Assembly_p
SL4_1_pSL4

MKVRIVPAVGGGPPLEIDVPPSTTIGAIKLRVCAMKKLRPDDTRLTYKNR
ALADTETLESAGVSEGDKLVLVTRTVGG

>2527529147_candidate_division_pSL4_archaeon_SCGC_AAA471-D15_Combined_Assembly_p
SL4_2_pSL4
MKVRIVPAVGGGPPLEIDVPPSTTIGAIKLRVCAMKKLRPDDTRLTYKNR
ALADTETLESAGVSEGDKLVLVTRTVGG

>2265088059_candidate_division_pSL4_archaeon_SCGC_AAA471-E14_GBS-N_001_10
MKIRVVPAVGGGPPLELDVPPNASIGAVKMRVCTIKKLRPEDARLTYKGR
ALGDNETLASAGVVEGDKLVLITRTVGG

>2265088663_candidate_division_pSL4_archaeon_JGI_0000001-A7_GBS-A_001_112
MKIRVVPAVGGGPPLELDVPPNASIGAVKMRVCTIKKLRPEDARLTYKGR
ALGDNETLASAGVVEGDKLVLITRTVGG

>2527530435_candidate_division_pSL4_archaeon_SCGC_AAA471-E14_Combined_Assembly_p
SL4_3_pSL4
MKIRVVPAVGGGPPLELDVPPNASIGAVKMRVCTIKKLRPEDARLTYKGR
ALGDNETLASAGVVEGDKLVLITRTVGG

>2264867171_candidate_division_YNPFFA_archaeon_SCGC_AAA471-O08_GBS-N_001_29
MKVKINETFIFEEKPFFSLSKFWKKKIVTINIDNNSKIYEVKKAILKRYN
LNNSIDLFKIVYKNKELNDNLSISELGIKENETLMIFCKNKKELEDILRK
KNIL

>2264869028_candidate_division_YNPFFA_archaeon_SCGC_AAA471-B05_GBS-N_001_2
MKVKINETFIFEEKPFFSLSKFWKKKIVTINIDNNSKIYEVKKAILKRYN
LNNSIDLFKIVYKNKELNDNLSISELGIKENETLMIFCKNKKELEDILRK
KNIL

>2264869402_candidate_division_YNPFFA_archaeon_SCGC_AAA471-B23_GBS-N_001_5
MKVKINETFIFEEKPFFSLSKFWKKKIVTINIDNNSKIYEVKKAILKRYN
LNNSIDLFKIVYKNKELNDNLSISELGIKENETLMIFCKNKKELEDILRK
KNIL

>2264870547_candidate_division_YNPFFA_archaeon_SCGC_AAA471-C03_GBS-N_001_6
MKVKINETFIFEEKPFFSLSKFWKKKIVTINIDNNSKIYEVKKAILKRYN
LNNSIDLFKIVYKNKELNDNLSISELGIKENETLMIFCKNKKELEDILRK
KNIL

>2265120433_candidate_division_YNPFFA_archaeon_SCGC_AAA471-L13_GBS-N_001_22
MKVKINETFIFEEKPFFSLSKFWKKKIVTINIDNNSKIYEVKKAILKRYN
LNNSIDLFKIVYKNKELNDNLSISELGIKENETLMIFCKNKKELEDILRK
KNIL

>2265121746_candidate_division_YNPFFA_archaeon_SCGC_AAA471-L14_GBS-N_001_23
MKVKINETFIFEEKPFFSLSKFWKKKIVTINIDNNSKIYEVKKAILKRYN
LNNSIDLFKIVYKNKELNDNLSISELGIKENETLMIFCKNKKELEDILRK
KNIL

>2527531181_candidate_division_YNPFFA_archaeon_SCGC_AAA471-B05_Combined_Assembly
_YNPFFA_1_YNPFFA _
MKVKINETFIFEEKPFFSLSKFWKKKIVTINIDNNSKIYEVKKAILKRYN
LNNSIDLFKIVYKNKELNDNLSISELGIKENETLMIFCKNKKELEDILRK
KNIL

>315426918_Candidatus_Caldiarchaeum_subterraneum
MKIKIVPAVGGGSPLELEVAPNATVGAVRTKVCAMKKLPPDTTRLTYKGRALKDTETLESLGVADGDKFV
LITRTVGGCGEPIRRAA

>315426997_Candidatus_Caldiarchaeum_subterraneum

MKIKIVPAVGGGSPLELEVAPNATVGAVRTKVCAMKKLPPDTTRLTYKGRALKDTETLESLGVADGDKFV
LITRTVGGCGEPIRRAA

>315428084_Candidatus_Caldiarchaeum_subterraneum
MKIKIVPAVGGGSPLELEVAPNATVGAVRTKVCAMKKLPPDTTRLTYKGRALKDTETLESLGVADGDKFV
LITRTVGGCGEPIRRAA

>343485671_Candidatus_Caldiarchaeum_subterraneum
MKIKIVPAVGGGSPLELEVAPNATVGAVRTKVCAMKKLPPDTTRLTYKGRALKDTETLESLGVADGDKFV
LITRTVGGCGEPIRRAA

>526887489_Candidatus_Caldiarchaeum_subterraneum
MKIKIVPAVGGGSPLELEVAPNATVGAVRTKVCAMKKLPPDTTRLTYKGRALKDTETLESLGVADGDKFV
LITRTVGGCGEPIRRAA

>557694901_Candidatus_Caldiarchaeum_subterraneum
MKIKIVPAVGGGSPLELEVAPNATVGAVRTKVCAMKKLPPDTTRLTYKGRALKDTETLESLGVADGDKFV
LITRTVGGCGEPIRRAA

>519109682_Aigarchaeota_archaeon_SCGC_AAA471-G05
MKVRIVPAVGGGPPLEIDVPPSTTIGAIKLRVCAMKKLRPDDTRLTYKNRALADTETLESAGVSDGDKLV
LVTRTVGG

>516977482_candidate_division_YNPFFA
MKVKINETFIFEEKPFFSLSKFWKKKIVTINIDNNSKIYEVKKAILKRYNLNNSIDLFKIVYKNKELNDN
LSISELGIKENETLMIFCKNKKELEDILRKKNIL

*******zf-RING_2***********
>2265088697_candidate_division_pSL4_archaeon_JGI_0000001-A7_GBS-A_001_112
MITQVSEALARFSRIGAMVSFLGAIHSLSLLLTWFSINYDNVGRSLISGY
TFSEPLIISLVAGGIAGVAGILASSLRQISKIRVVLPTLSFTSAGLALFS
PLYTYLVKLPSFQLSYTPEFGMFGALITGVAITGSAVLATVASIRTKSTV
YLGPPPPWATQELELARGPGEGGVEGQEVQVPEVWEETTRVETTQPMPSQ
PSSQAPTGGNCLICGDTIPSGDLATCKGCGATFHKDCMNVWIDLGNRCPS
CNAELTS

>2527530172_candidate_division_pSL4_archaeon_SCGC_AAA471-E14_Combined_Assembly_p
SL4_3__pSL4
MITQVSEALARFSRIGAMVSFLGAIHSLSLLLTWFSINYDNVGRSLISGY
TFSEPLIISLVAGGIAGVAGILASSLRQISKIRVVLPTLSFTSAGLALFS
PLYTYLVKLPSFQLSYTPEFGMFGALITGVAITGSAVLATVASIRTKSTV
YLGPPPPWATQELELARGPGEGGVEGQEVQVPEVWEETTRVETTQPMPSQ
PSSQAPTGGNCLICGDTIPSGDLATCKGCGATFHKDCMNVWIDLGNRCPS
CNAELTS

>2265091066_candidate_division_pSL4_archaeon_SCGC_AAA471-G05_GBS-N_001_14
MLVMGTDVRDALVRLIRIGALISVLGAIHVLTLMLDWASLLNDNVVVRGI
QGFVLNWSFTLSLLAGLLAGGAGVLASLSSNVRMLRFAVPSLAIAGSALA
ILSPLYVLLHHLPSLNIQFRPEVGAIAALFTGVAMAGGGFISAMIGFVVL
RPSSGPSAPPAIAYAPVQTPVIEAQQEIALGYEEPSLSHEEEVFEEILRQ
EEASIRAAAQPVPAEGTSPAPTTVPPQRPQTAAQECAICGEPIPSNSLRF
CNTCGAPMHRECVETWRDIGGKCPSCGTPLV

>2527526036_candidate_division_pSL4_archaeon_SCGC_AAA471-B22_Combined_Assembly_p
SL4_1__pSL4
MLVMGTDVRDALVRLIRIGALISVLGAIHVLTLMLDWASLLNDNVVVRGI
QGFVLNWSFTLSLLAGLLAGGAGVLASLSSNVRMLRFAVPSLAIAGSALA
ILSPLYVLLHHLPSLNIQFRPEVGAIAALFTGVAMAGGGFISAMIGFVVL
RPSSGPSAPPAIAYAPVQTPVIEAQQEIALGYEEPSLSHEEEVFEEILRQ
EEASIRAAAQPVPAEGTSPAPTTVPPQRPQTAAQECAICGEPIPSNSLRF

CNTCGAPMHRECVETWRDIGGKCPSCGTPLV

>2265089058_candidate_division_pSL4_archaeon_JGI_0000001-B8_GBS-A_001_113
VRRFRTPVVVEGRKAGEWFWGVICDSCLLEIDPSHTTTKCPHCGATFHSF
CYTAILSSKGRCPKCKMELA

>2527529566_candidate_division_pSL4_archaeon_SCGC_AAA471-D15_Combined_Assembly_p
SL4_2__pSL4
VRRFRTPVVVEGRKAGEWFWGVICDSCLLEIDPSHTTTKCPHCGATFHSF
CYTAILSSKGRCPKCKMELA

>2265087851_candidate_division_pSL4_archaeon_SCGC_AAA471-E14_GBS-N_001_10
VLKVRSGRVLGRWEWGQPMCDACLLEIEEGVEPLKCENCGANFHPDCYTS
LKNTKAVCPKCKVTLE

>2265088700_candidate_division_pSL4_archaeon_JGI_0000001-A7_GBS-A_001_112
VLKVRSGRVLGRWEWGQPMCDACLLEIEEGVEPLKCENCGANFHPDCYTS
LKNTKAVCPKCKVTLE

>2527530169_candidate_division_pSL4_archaeon_SCGC_AAA471-E14_Combined_Assembly_p
SL4_3__pSL4
VLKVRSGRVLGRWEWGQPMCDACLLEIEEGVEPLKCENCGANFHPDCYTS
LKNTKAVCPKCKVTLE

>315427002_Candidatus_Caldiarchaeum_subterraneum
MFKGFRGACFYKVGHLGLIGMVVSERRLLVRFFQIGSVLALAGSIHVLTLLLPWYTVRADSVSTSVLSGY
LLPETLALSVAGGVLAGLSLLVTSFSQRPMAVRTVLVVLSLLGGVLAMVSPLYLGLVRVPALSVAGEPGI
GFFIALFSAIVILALGGVALMTRPRVVEIPYQGYGGVSGATVSSTQPMETTSFEVAGEVEEGVVCPICYT
SVEAENAVRCSSCGVVFHSGCLDAYVNINGTCPNCGRAVV

>315428087_Candidatus_Caldiarchaeum_subterraneum
MRLVIREVNAGFWNPFEEPKPRPKQENCVICGLEMGNEKTYSCPHCGAVGHMSCFDDWLVVKQTCPLCRR
PLVEM

>315428090_Candidatus_Caldiarchaeum_subterraneum
MVVSERRLLVRFFQIGSVLALAGSIHVLTLLLPWYTVRADSVSTSVLSGYLLPETLALSVAGGVLAGLSL
LVTSFSQRPMAVRTVLVVLSLLGGVLAMVSPLYLGLVRVPALSVAGEPGIGFFIALFSAIVILALGGVAL
MTRPRVVEIPYQGYGGVSGATVSSTQPMETTSFEVAGEVEEGVVCPICYTSVEAENAVRCSSCGVVFHSG
CLDAYVNINGTCPNCGRAVV

>343485674_Candidatus_Caldiarchaeum_subterraneum
MRLVIREVNAGFWNPFEEPKPRPKQENCVICGLEMGNEKTYSCPHCGAVGHMSCFDDWLVVKQTCPLCRR
PLVEM

>343485677_Candidatus_Caldiarchaeum_subterraneum
MFKGFRGACFYKVGHLGLIGMVVSERRLLVRFFQIGSVLALAGSIHVLTLLLPWYTVRADSVSTSVLSGY
LLPETLALSVAGGVLAGLSLLVTSFSQRPMAVRTVLVVLSLLGGVLAMVSPLYLGLVRVPALSVAGEPGI
GFFIALFSAIVILALGGVALMTRPRVVEIPYQGYGGVSGATVSSTQPMETTSFEVAGEVEEGVVCPICYT
SVEAENAVRCSSCGVVFHSGCLDAYVNINGTCPNCGRAVV

>516675792_Marine_Group_II_euryarchaeote_SCGC_AB-629-J06
MTCPICHNSITDINKVITECNHVFHFTCIYKNLKTNISTGEQCPLCRKSFNAPTTSFSLCPPGYQSTSSL
TNFGEQQMRIIQNLYRRPQIPRSQNTLIRMINERRRSRPVPTYTEPQRVRRREIKRQIAKLSFSHLKNKL
REKGVSSRGYLRDTLEKRLFDKMIAE

>516675835_Archaea
MSTSITKETQTSVEKQVKEKASEKAEEKAEEKAEEQTCGICYTDLNDKNTVITPCNHAYCTSCFFKWLGR
KETCALCRKVLLFDTIVEERLTDLQDVQAELMDNYRCLSVLKKNIKKKKCKKKNLTDDINSLISRQIRMR
CLLEQTRSACRETLAHSRALKQAIELQRESLDLMKNYRSEWEELYTPLPLPPAEEAEEEEEEINIVNMTV
ALDNMVRLESRRARTELRRLREAERAIAETIEVENSDDGDTASEADTVVVEDDMEEDDDEEGIEVDLTVF
GTGIPTFDFTPAARRTVTVPTARNHTQSPMFVFGTNPMPSAITSAFEIPTESQDPPVIEMTRDPVELTDS
IQESSFEWTPINLEMSDAMTRLVARPEMEPASTEEEITDE

>516675873_Marine_Group_II_euryarchaeote_SCGC_AB-629-J06
MSCLYNIFFKTNKIYAIDCEPYETEYSTNECAICLDSLNSQILTISCGHTYHSDCLLNWFEYNMTCPVCR
TKFIWGKRKKKKRRHRQRNSRLL

>516675915_Marine_Group_II_euryarchaeote_SCGC_AB-629-J06
MATQQQFITDLYRGNESDEGTIKYYKSRMVPSGTEFVYPVICIKRYNTFEWVRFNDLSAALWMRYRNNME
PEWISKSIFSGEKVKTDNKLVTSLLATLTIEQRKKWLTENSEYKDKICVECGAFEPEIKKCIHHDCSGMC
ATCFDIKNKPGFENCACCLKKQEMTCPICQEDFTTDKLVKSEQCSHHICWSCFGRSVKSSRPLSHCPLCR
GVFCEKLIDIEDYDLDDIPGLEDDDDMPPLIEIDDDDWSEQDERFAMARAQEGMDFEAIIAAIANNFVTV
RDRTVDGLEV

>516675918_Marine_Group_II_euryarchaeote_SCGC_AB-629-J06
MAANILEGPPLEEDVDKCIICLENLSAEPEYCLPECSHKFHQNCIMHWFRGGNCKCPLCNNLGINDKMDQ
VSGTSWGWWRGGKYRYKMIRQYSRKKEAPAKLKKEIAKLKKLEKKKSELSKSIKDFKSKTGVWKDLHKQW
GKLRRDRWRLDSNIRRRKMSIANFNIVPIIIAKKVNIP

>516676043_Archaea
MSTDLQLLRESIKTNISATTNVYDITGQWLPPGNYYFLINYTGLGCVKGELNCRGFKAEYTFASKDIIKM
MACAQARLARRANIRDDDMPTYNSPITSPTANYPPNLVSHRYGNVQPPHTSPTMRTHRRRLPTINTRPTC
TICLDVLDDNPKILSCQHKFHLACITRWYATRQTCPICRRRISIGLGTNRRHSRYVVVNRHVTTYSRQTR
ERTQNNRRPRPRAMPRRGYRNQQQSLPSLIS

>516676225_Marine_Group_II_euryarchaeote_SCGC_AB-629-J06
MDNSYNCVICLKDLDMNNGVLTPCNHRYHSECFFRWILKKRTCPLCRKELIAQPDIEERASLYELRRQID
WESTLYNTLRNNVDTLERHILTKKNELENLNLQVHLKQKQLVTIINRYKQFMRNSQRNQRRRKRGLLF

>516676312_Marine_Group_II_euryarchaeote_SCGC_AB-629-J06
MSIDPSNNNIQESLINNTPSMETLDFFQYVMNRTMDIEHDMPNRNFISSFLNHLDNSRNSTNMENIFSPP
PPPVQWTNNILSSFVSDTSSNSTDNDSSNNINYDNTNLYMRFIEDILQLPPLTSADNNNIRTLLRETLSE
DKNPIKYVLSADGEQKIETVEFDPEIYPDINCCPITIKEFKKGDMISKLPCNHLFNTDAILKWLKEEKAE
CPICRFKLESIEKKFEKRDSSYNTIQNGRVMSTRIIPPRIPGTNTRLPYGFGPPRMPRRRHNTHLRRLML
SRHEQEEHEELQAALLASLEEQYMPGHKTPKEDASDDASDIDSDTNDMETVD

>516676354_Marine_Group_II_euryarchaeote_SCGC_AB-629-J06
MSKRPADNNKSDEQVLKKTKMSLPKLKSKEEQEQQIQKKIENDRCAICLQPLNDGRPLKYCDSVSHIPTK
ENPRAHVHIFHKECINAWLRANNKDNNSCPECGSQILCLDPDKYIEEIVNAPDPLRYNQDPDNYNPDDIP
SDDEEMNTEPKDNEGVDIDVDIWNQEHPRGPWWRVIKCETPAQNCPYDENYPNHHGKNFQWFLSHEPEDV
ELTDAAQATGWESIDGYKICPVCANADIRDYCEQCGEGYFFPEEHLTHVDDELLCDECLEQTRSATRGES
KSRHGGRSRRKTPHKKNTQEKK

>526887492_Candidatus_Caldiarchaeum_subterraneum
MRLVIREVNAGFWNPFEEPKPRPKQENCVICGLEMGNEKTYSCPHCGAVGHMSCFDDWLVVKQTCPLCRR
PLVEM

>526887495_Candidatus_Caldiarchaeum_subterraneum
MFKGFRGACFYKVGHLGLIGMVVSERRLLVRFFQIGSVLALAGSIHVLTLLLPWYTVRADSVSTSVLSGY
LLPETLALSVAGGVLAGLSLLVTSFSQRPMAVRTVLVVLSLLGGVLAMVSPLYLGLVRVPALSVAGEPGI
GFFIALFSAIVILALGGVALMTRPRVVEIPYQGYGGVSGATVSSTQPMETTSFEVAGEVEEGVVCPICYT
SVEAENAVRCSSCGVVFHSGCLDAYVNINGTCPNCGRAVV

>557694904_Candidatus_Caldiarchaeum_subterraneum
MRLVIREVNAGFWNPFEEPKPRPKQENCVICGLEMGNEKTYSCPHCGAVGHMSCFDDWLVVKQTCPLCRR
PLVEM

>557694907_Candidatus_Caldiarchaeum_subterraneum
MFKGFRGACFYKVGHLGLIGMVVSERRLLVRFFQIGSVLALAGSIHVLTLLLPWYTVRADSVSTSVLSGY
LLPETLALSVAGGVLAGLSLLVTSFSQRPMAVRTVLVVLSLLGGVLAMVSPLYLGLVRVPALSVAGEPGI
GFFIALFSAIVILALGGVALMTRPRVVEIPYQGYGGVSGATVSSTQPMETTSFEVAGEVEEGVVCPICYT
SVEAENAVRCSSCGVVFHSGCLDAYVNINGTCPNCGRAVV
