# Supplementary material for: The Eukaryotic Ancestor Had a Complex Ubiquitin Signaling System of Archaeal Origin
Source: Mol Biol Evol. 2014 Dec 17;32(3):726–39. doi: 10.1093/molbev/msu334 (PMC4327156; doi:10.1093/molbev/msu334)
Supplement: Supplementary Data [file supp_msu334_Supplementary2_Fig_S1-7_TablesS1-S2.pdf]

Figure S1A

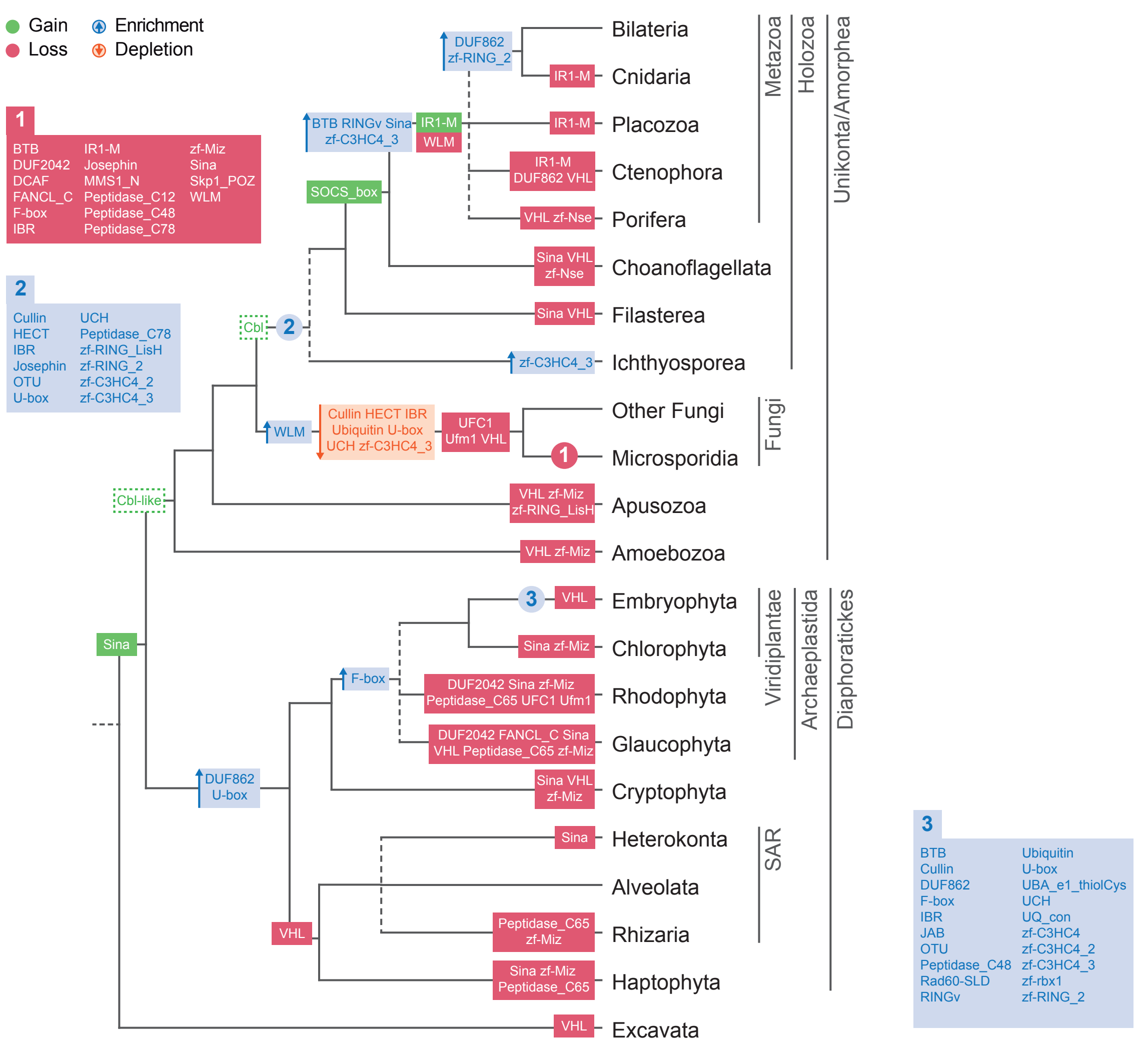

Figure S1B

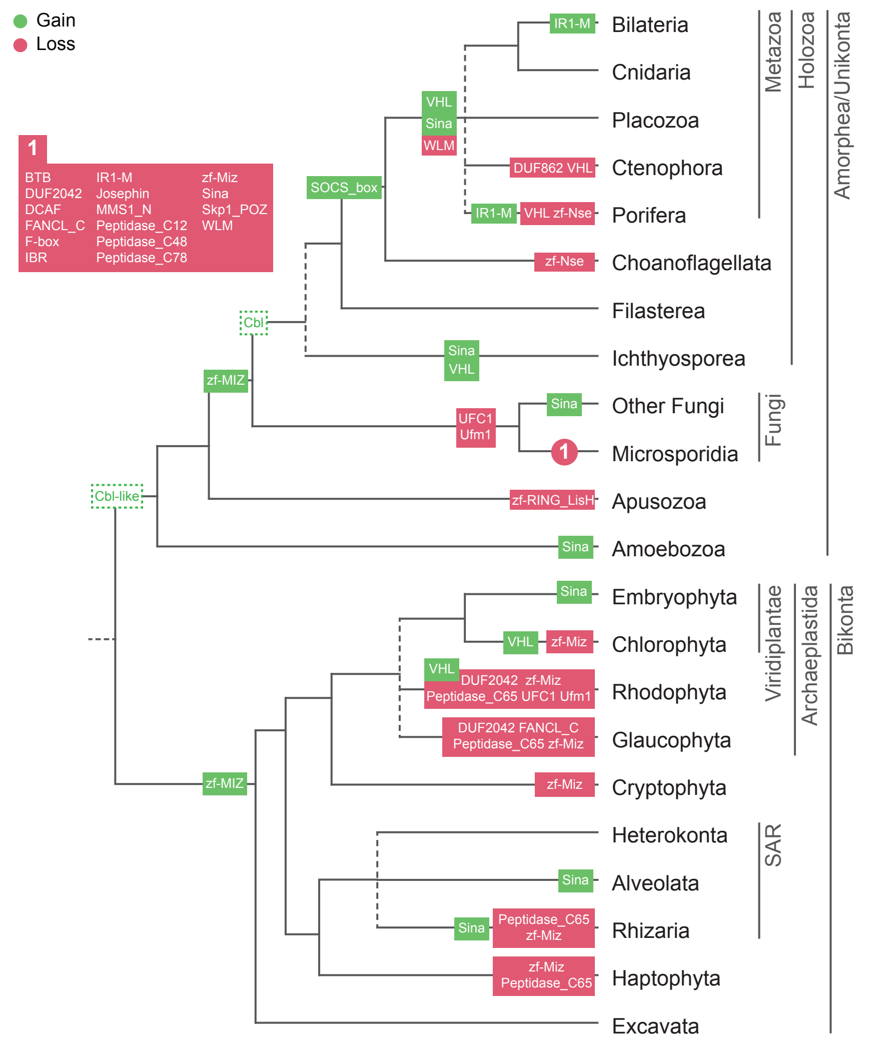

Figure S1C

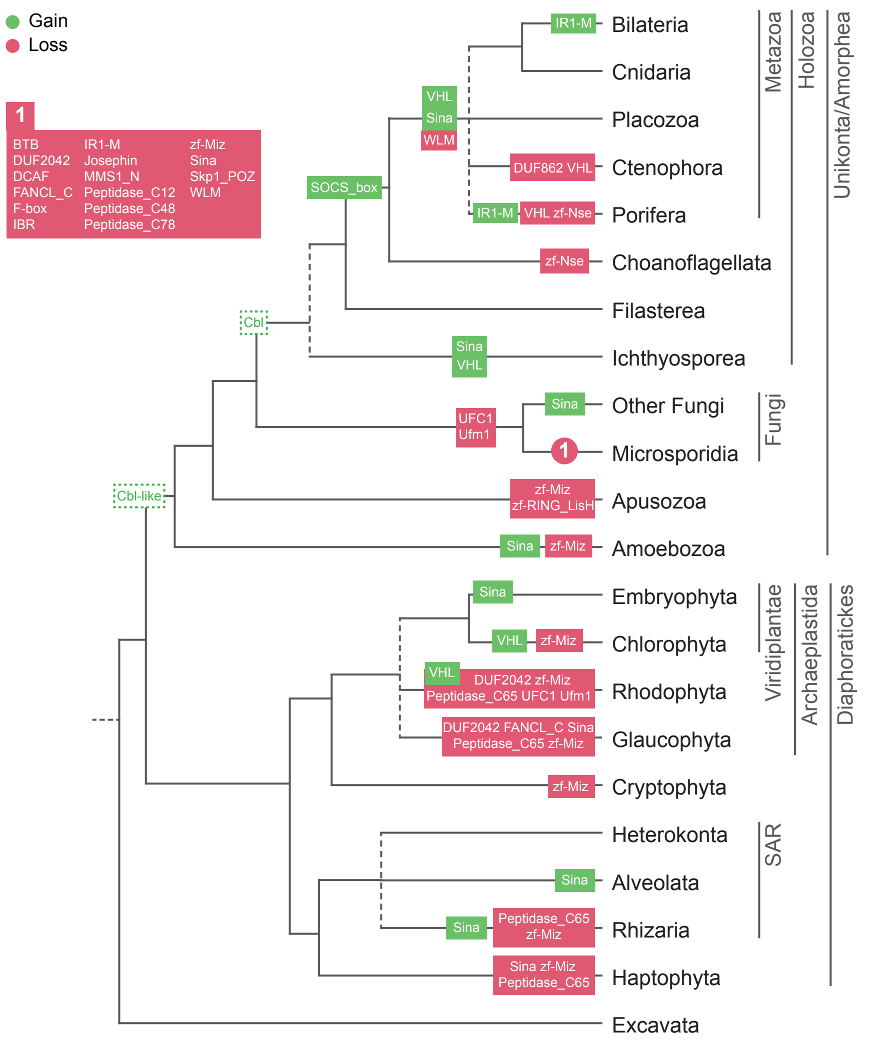

Figure S1D

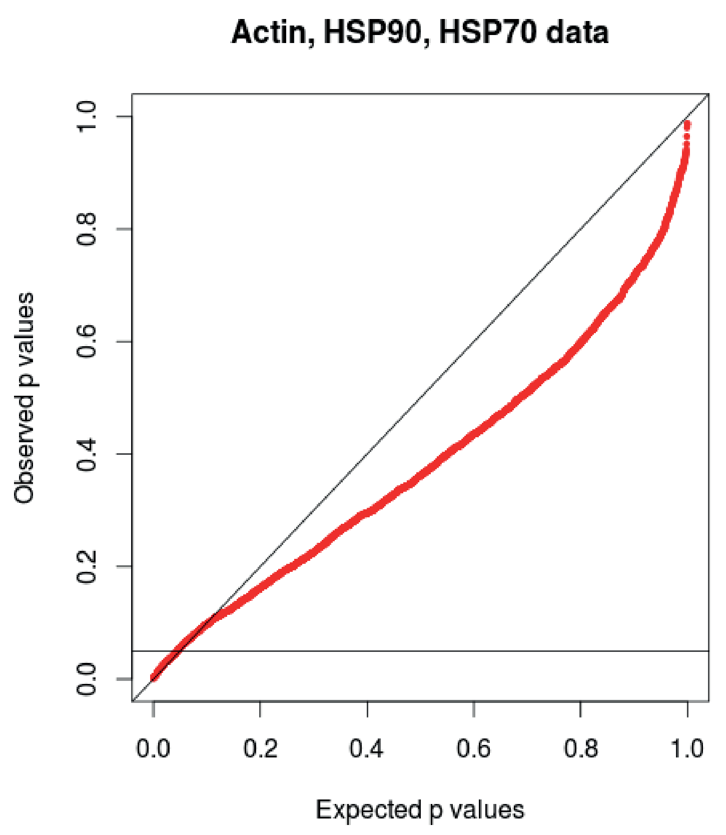

Figure S2

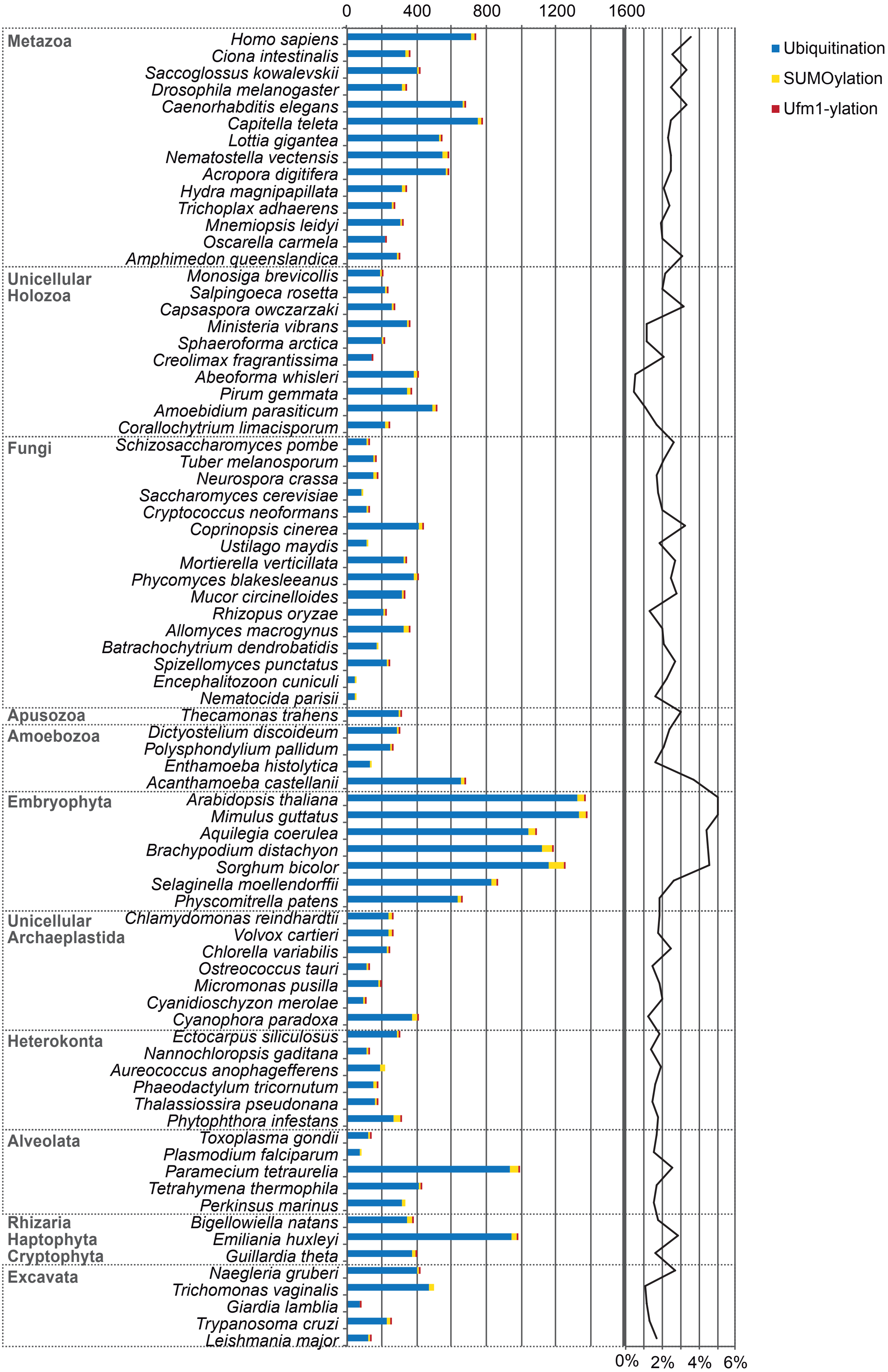
0
400
800
1200
1600
Ubiquitination
SUMOylation
Ufm1-ylation
Metazoa
Homo sapiens
Ciona intestinalis
Saccoglossus kowalevskii
Drosophila melanogaster
Caenorhabditis elegans
Capitella teleta
Lottia gigantea
Nematostella vectensis
Acropora digitifera
Hydra magnipapillata
Trichoplax adhaerens
Mnemiopsis leidyi
Oscarella carmela
Amphimedon queenslandica
Unicellular Holozoa
Monosiga brevicollis
Salpingoeca rosetta
Capsaspora owczarzaki
Ministeria vibrans
Sphaeroforma arctica
Creolimax fragrantissima
Abeoforma whisleri
Pirum gemmata
Amoebidium parasiticum
Corallochytrium limacisporum
Fungi
Schizosaccharomyces pombe
Tuber melanosporum
Neurospora crassa
Saccharomyces cerevisiae
Cryptococcus neoformans
Coprinopsis cinerea
Ustilago maydis
Mortierella verticillata
Phycomyces blakesleeanus
Mucor circinelloides
Rhizopus oryzae
Allomyces macrogynus
Batrachochytrium dendrobatidis
Spizellomyces punctatus
Encephalitozoon cuniculi
Nematocida parisii
Apusozoa
Thecamonas trahens
Amoebozoa
Dictyostelium discoideum
Polysphondylium pallidum
Enthamoeba histolytica
Acanthamoeba castellanii
Embryophyta
Arabidopsis thaliana
Mimulus guttatus
Aquilegia coerulea
Brachypodium distachyon
Sorghum bicolor
Selaginella moellendorffii
Physcomitrella patens
Unicellular Archaeplastida
Chlamydomonas reindhardtii
Volvox cartieri
Chlorella variabilis
Ostreococcus tauri
Micromonas pusilla
Cyanidioschyzon merolae
Cyanophora paradoxa
Heterokonta
Ectocarpus siliculosus
Nannochloropsis gaditana
Aureococcus anophagefferens
Phaeodactylum tricornutum
Thalassiossira pseudonana
Phytophthora infestans
Alveolata
Toxoplasma gondii
Plasmodium falciparum
Paramecium tetraurelia
Tetrahymena thermophila
Perkinsus marinus
Rhizaria
Haptophyta
Cryptophyta
Bigelowiella natans
Emiliania huxleyi
Guillardia theta
Excavata
Naegleria gruberi
Trichomonas vaginalis
Giardia lamblia
Trypanosoma cruzi
Leishmania major
0%
2%
4%
6%

Figure S3

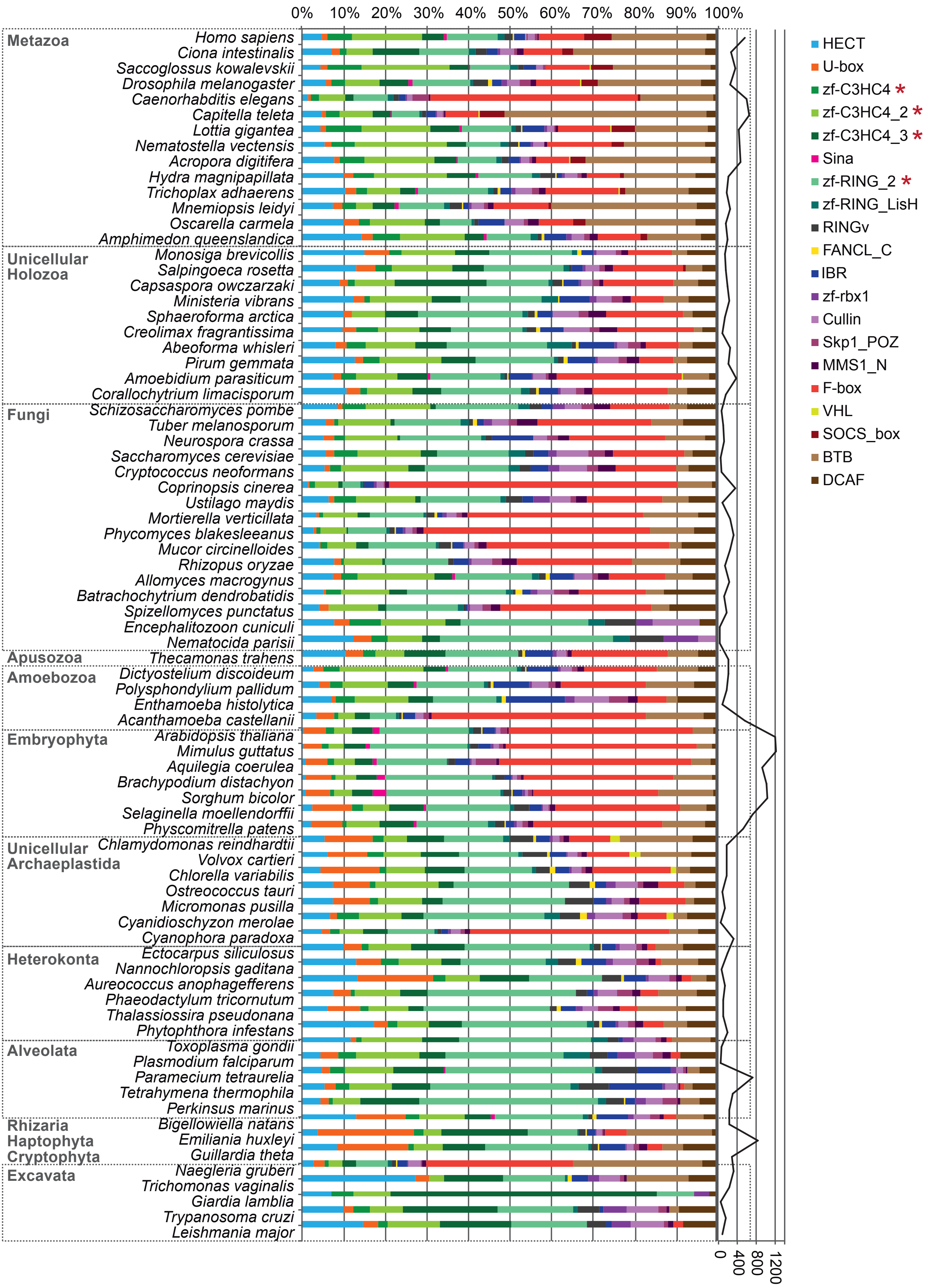

0%
10%
20%
30%
40%
50%
60%
70%
80%
90%
100%
Metazoa
Homo sapiens
Ciona intestinalis
Saccoglossus kowalevskii
Drosophila melanogaster
Caenorhabditis elegans
Capitella teleta
Lottia gigantea
Nematostella vectensis
Acropora digitifera
Hydra magnipapillata
Trichoplax adhaerens
Mnemiopsis leidyi
Oscarella carmela
Amphimedon queenslandica
Unicellular Holozoa
Monosiga brevicollis
Salpingoeca rosetta
Capsaspora owczarzaki
Ministeria vibrans
Sphaeroforma arctica
Creolimax fragrantissima
Abeoforma whisleri
Pirum gemmata
Amoebidium parasiticum
Corallochytrium limacisporum
Fungi
Schizosaccharomyces pombe
Tuber melanosporum
Neurospora crassa
Saccharomyces cerevisiae
Cryptococcus neoformans
Coprinopsis cinerea
Ustilago maydis
Mortierella verticillata
Phycomyces blakesleeanus
Mucor circinelloides
Rhizopus oryzae
Allomyces macrogynus
Batrachochytrium dendrobatidis
Spizellomyces punctatus
Encephalitozoon cuniculi
Nematocida parisii
Apusozoa
Thecamonas trahens
Amoebozoa
Dictyostelium discoideum
Polysphondylium pallidum
Enthamoeba histolytica
Acanthamoeba castellanii
Embryophyta
Arabidopsis thaliana
Mimulus guttatus
Aquilegia coerulea
Brachypodium distachyon
Sorghum bicolor
Selaginella moellendorffii
Physcomitrella patens
Unicellular Archaeplastida
Chlamydomonas reindhardtii
Volvox cartieri
Chlorella variabilis
Ostreococcus tauri
Micromonas pusilla
Cyanidioschyzon merolae
Cyanophora paradoxa
Heterokonta
Ectocarpus siliculosus
Nannochloropsis gaditana
Aureococcus anophagefferens
Phaeodactylum tricornutum
Thalassiossira pseudonana
Phytophthora infestans
Alveolata
Toxoplasma gondii
Plasmodium falciparum
Paramecium tetraurelia
Tetrahymena thermophila
Perkinsus marinus
Rhizaria
Haptophyta
Cryptophyta
Bigellowiella natans
Emiliania huxleyi
Guillardia theta
Excavata
Naegleria gruberi
Trichomonas vaginalis
Giardia lamblia
Trypanosoma cruzi
Leishmania major
0
400
800
1200
HECT
U-box
zf-C3HC4 *
zf-C3HC4_2 *
zf-C3HC4_3 *
Sina
zf-RING_2 *
zf-RING_LisH
RINGv
FANCL_C
IBR
zf-rbx1
Cullin
Skp1_POZ
MMS1_N
F-box
VHL
SOCS_box
BTB
DCAF

Figure S4

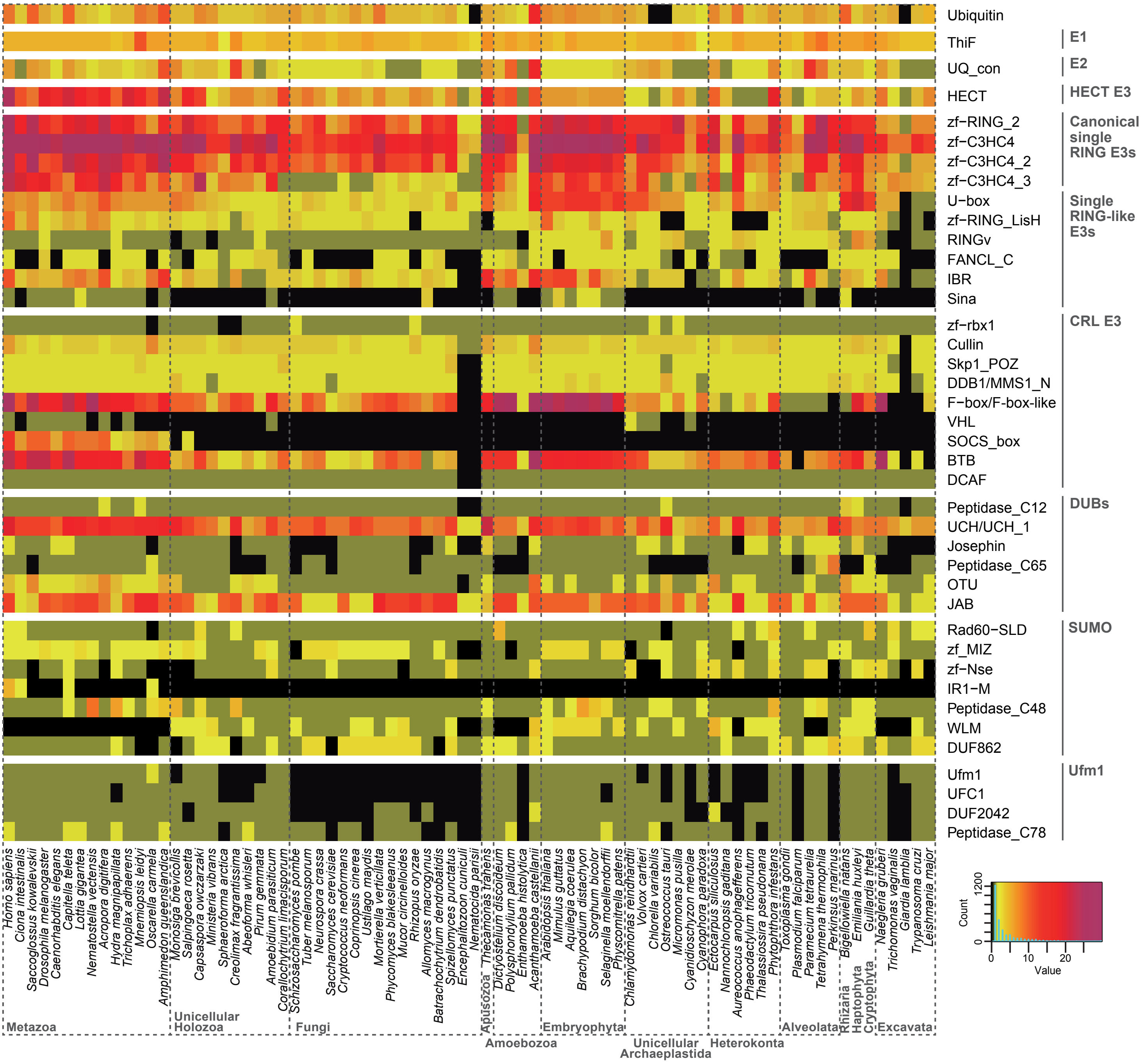
Ubiquitin
ThiF
E1
UQ_con
E2
HECT
HECT E3
zf−RING_2
zf−C3HC4
zf−C3HC4_2
zf−C3HC4_3
Canonical single RING E3s
U−box
zf−RING_LisH
RINGv
FANCL_C
IBR
Sina
Single RING-like E3s
zf−rbx1
Cullin
Skp1_POZ
DDB1/MMS1_N
F−box/F−box-like
VHL
SOCS_box
BTB
DCAF
CRL E3
Peptidase_C12
UCH/UCH_1
Josephin
Peptidase_C65
OTU
JAB
DUBs
Rad60−SLD
zf_MIZ
zf−Nse
IR1−M
Peptidase_C48
WLM
DUF862
SUMO
Ufm1
UFC1
DUF2042
Peptidase_C78
Ufm1
Homo sapiens
Ciona intestinalis
Saccoglossus kowalevskii
Drosophila melanogaster
Caenorhabditis elegans
Capitella teleta
Lottia gigantea
Nematostella vectensis
Acropora digitifera
Hydra magnipapillata
Trichoplax adhaerens
Mnemiopsis leidyi
Oscarella carmela
Amphimedon queenslandica
Monosiga brevicollis
Salpingoeca rosetta
Capsaspora owczarzaki
Ministeria vibrans
Sphaeroforma arctica
Creolimax fragrantissima
Abeoforma whisleri
Pirum gemmata
Amoebidium parasiticum
Corallochytrium limacisporum
Schizosaccharomyces pombe
Tuber melanosporum
Neurospora crassa
Saccharomyces cerevisiae
Cryptococcus neoformans
Coprinopsis cinerea
Ustilago maydis
Mortierella verticillata
Phycomyces blakesleeanus
Mucor circinelloides
Rhizopus oryzae
Allomyces macrogynus
Batrachochytrium dendrobatidis
Spizellomyces punctatus
Encephalitozoon cuniculi
Nematocida parisii
Thecamonas trahens
Dictyostelium discoideum
Polysphondylium pallidum
Enthamoeba histolytica
Acanthamoeba castellanii
Arabidopsis thaliana
Mimulus guttatus
Aquilegia coerulea
Brachypodium distachyon
Sorghum bicolor
Selaginella moellendorffii
Physcomitrella patens
Chlamydomonas reindhardtii
Volvox carteri
Chlorella variabilis
Ostreococcus tauri
Micromonas pusilla
Cyanidioschyzon merolae
Cyanophora paradoxa
Ectocarpus siliculosus
Nannochloropsis gaditana
Aureococcus anophagefferens
Phaeodactylum tricornutum
Thalassiosira pseudonana
Phytophthora infestans
Toxoplasma gondii
Plasmodium falciparum
Paramecium tetraurelia
Tetrahymena thermophila
Perkinsus marinus
Bigelowiella natans
Emiliania huxleyi
Guillardia theta
Naegleria gruberi
Trichomonas vaginalis
Giardia lamblia
Trypanosoma cruzi
Leishmania major
Metazoa
Unicellular Holozoa
Fungi
Apusozoa
Amoebozoa
Embryophyta
Unicellular Archaeplastida
Heterokonta
Alveolata
Rhizaria
Haptophyta
Cryptophyta
Excavata
Count
1200
0
0
10
20
Value

Figure S5A – ThiF

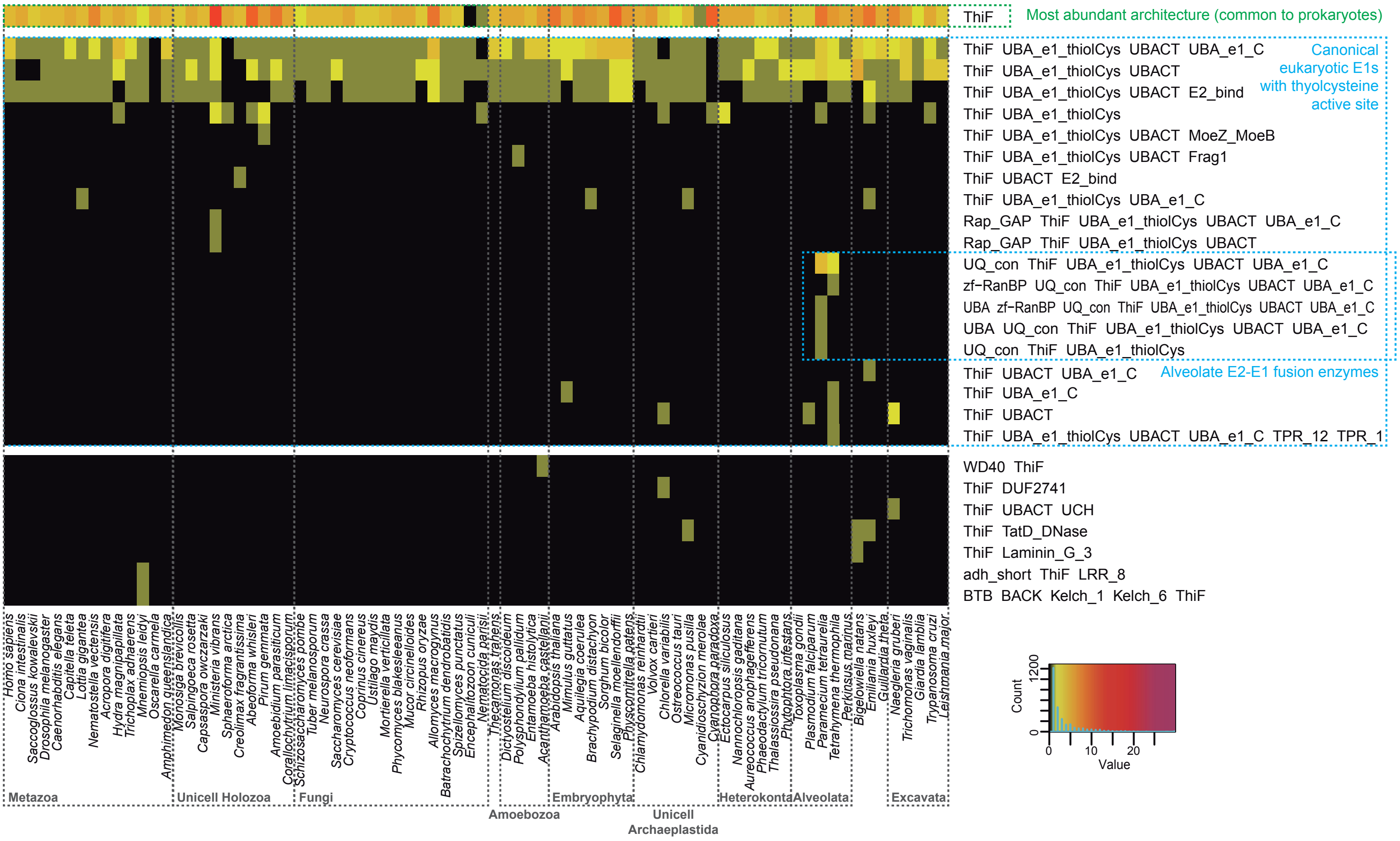

Figure S5B – UQ_con

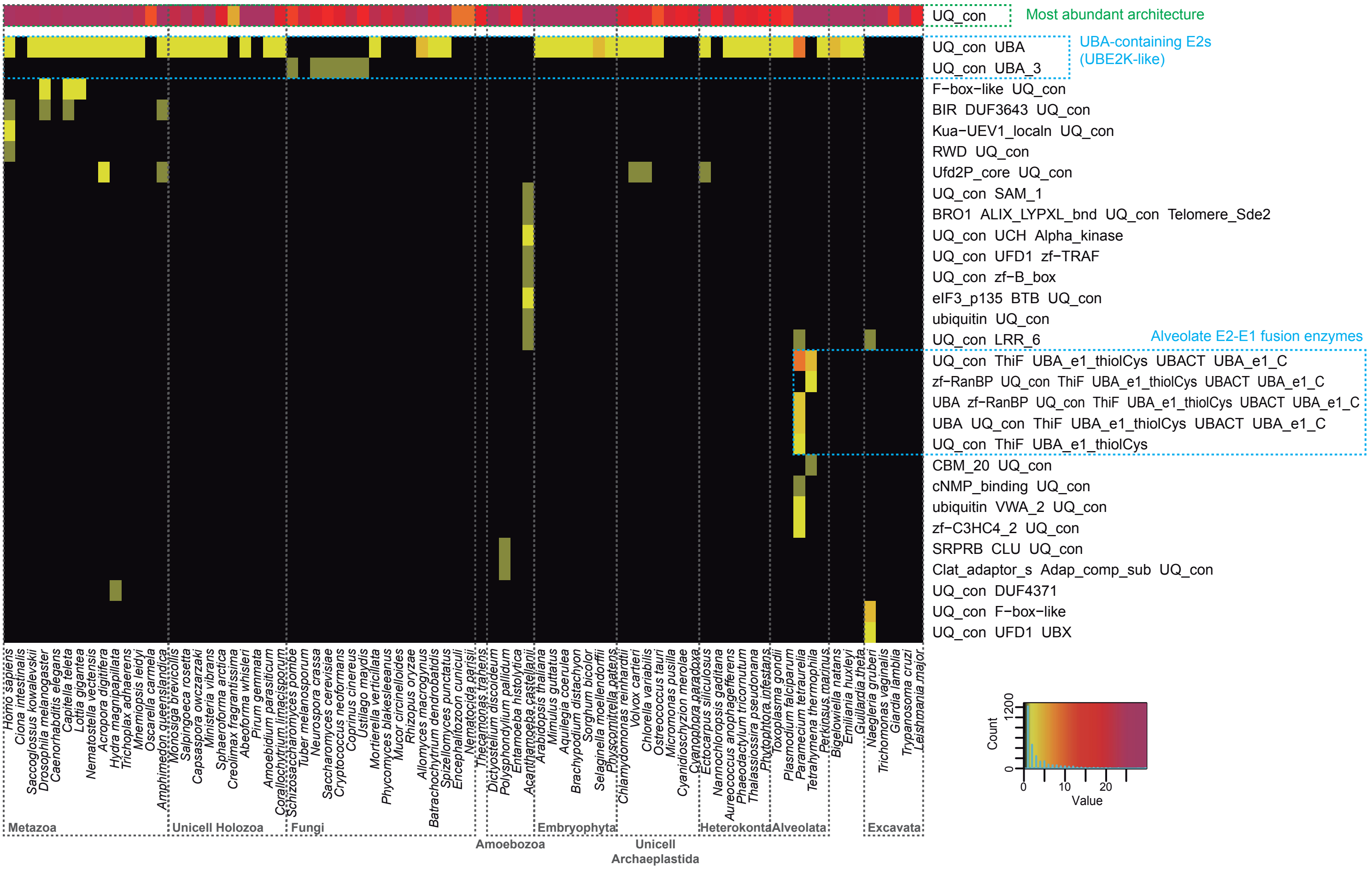

Figure S5C – HECT

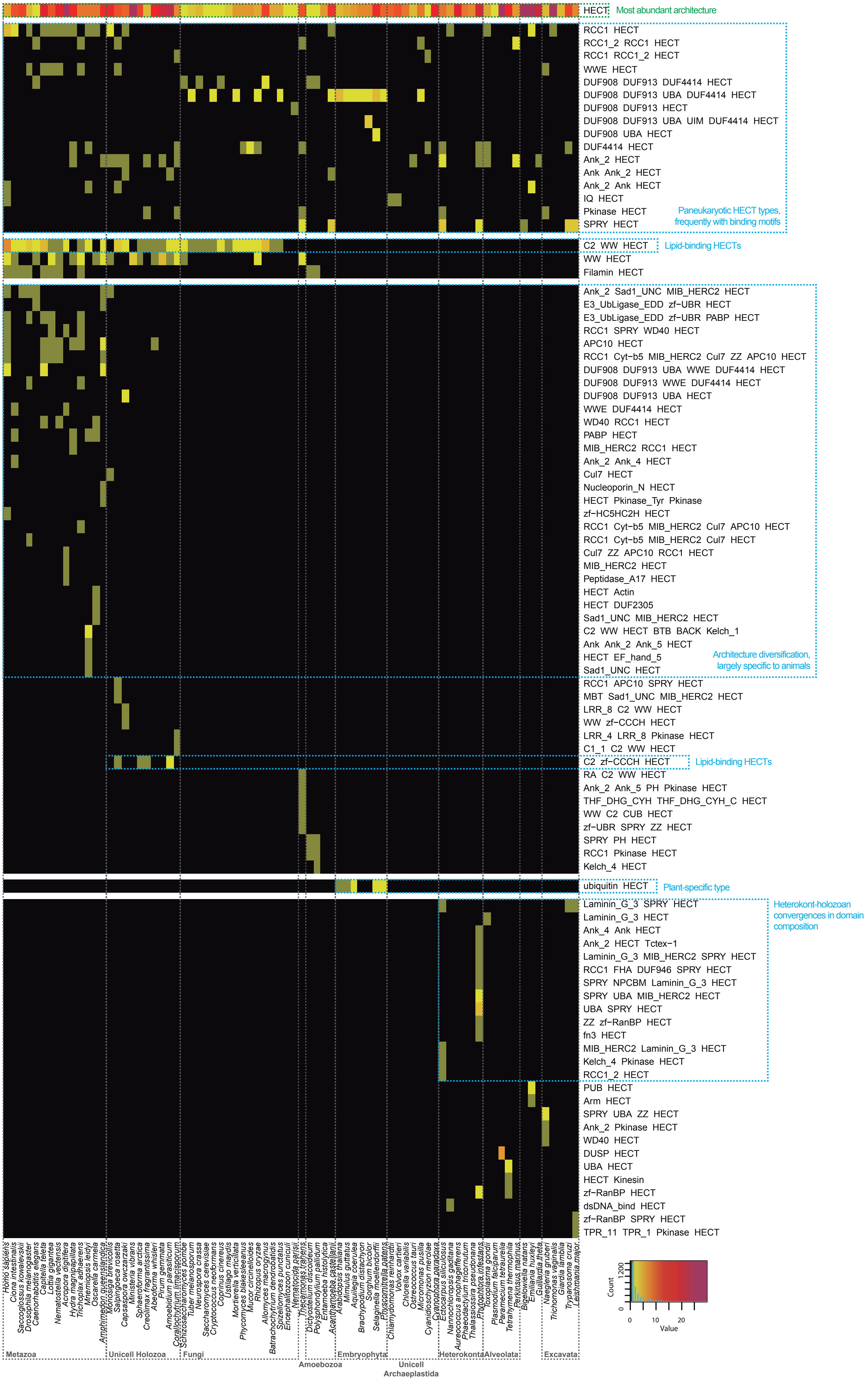

Figure S5D – zf-RING_2

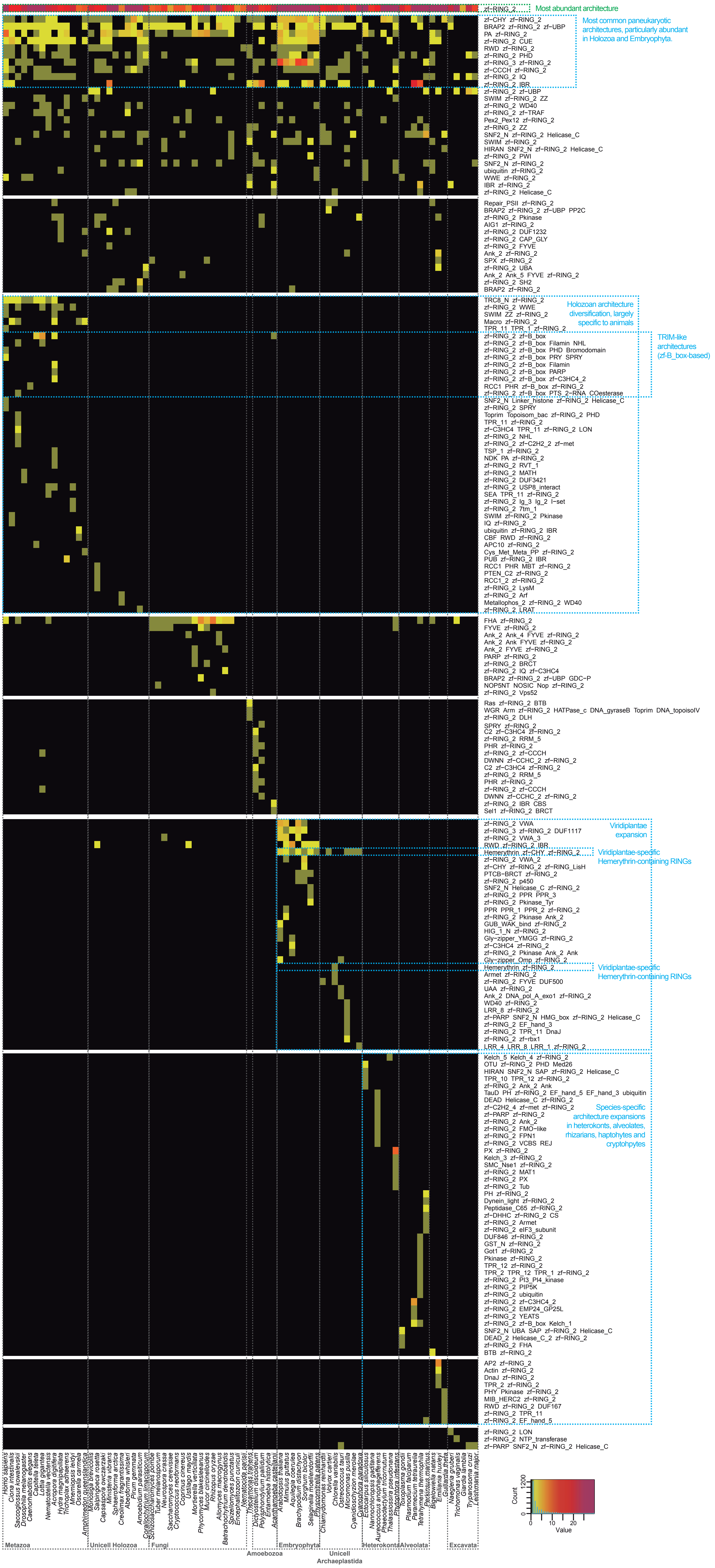

Figure S5E – zf-C3HC4

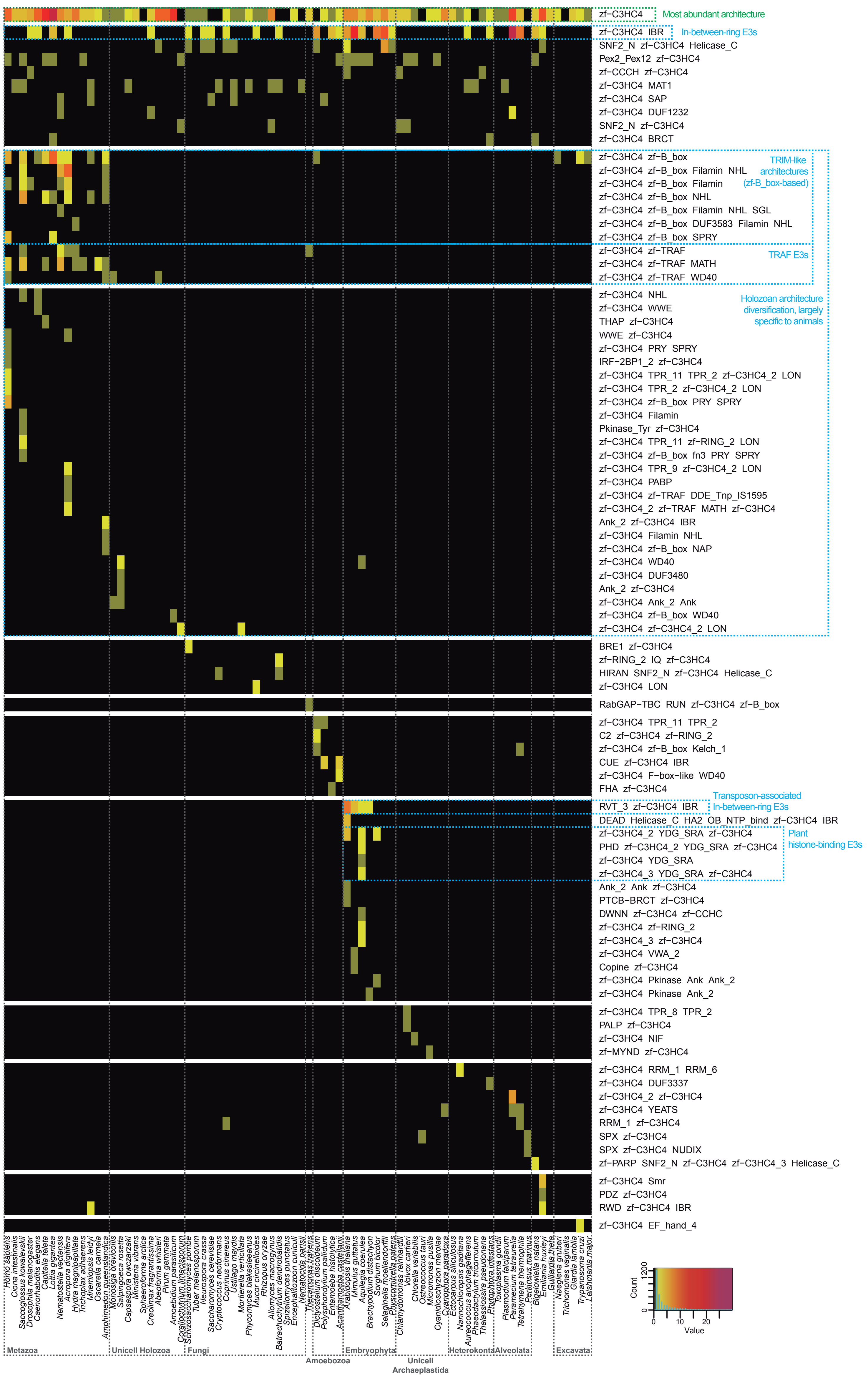

Figure S5F – zf-C3HC4_2

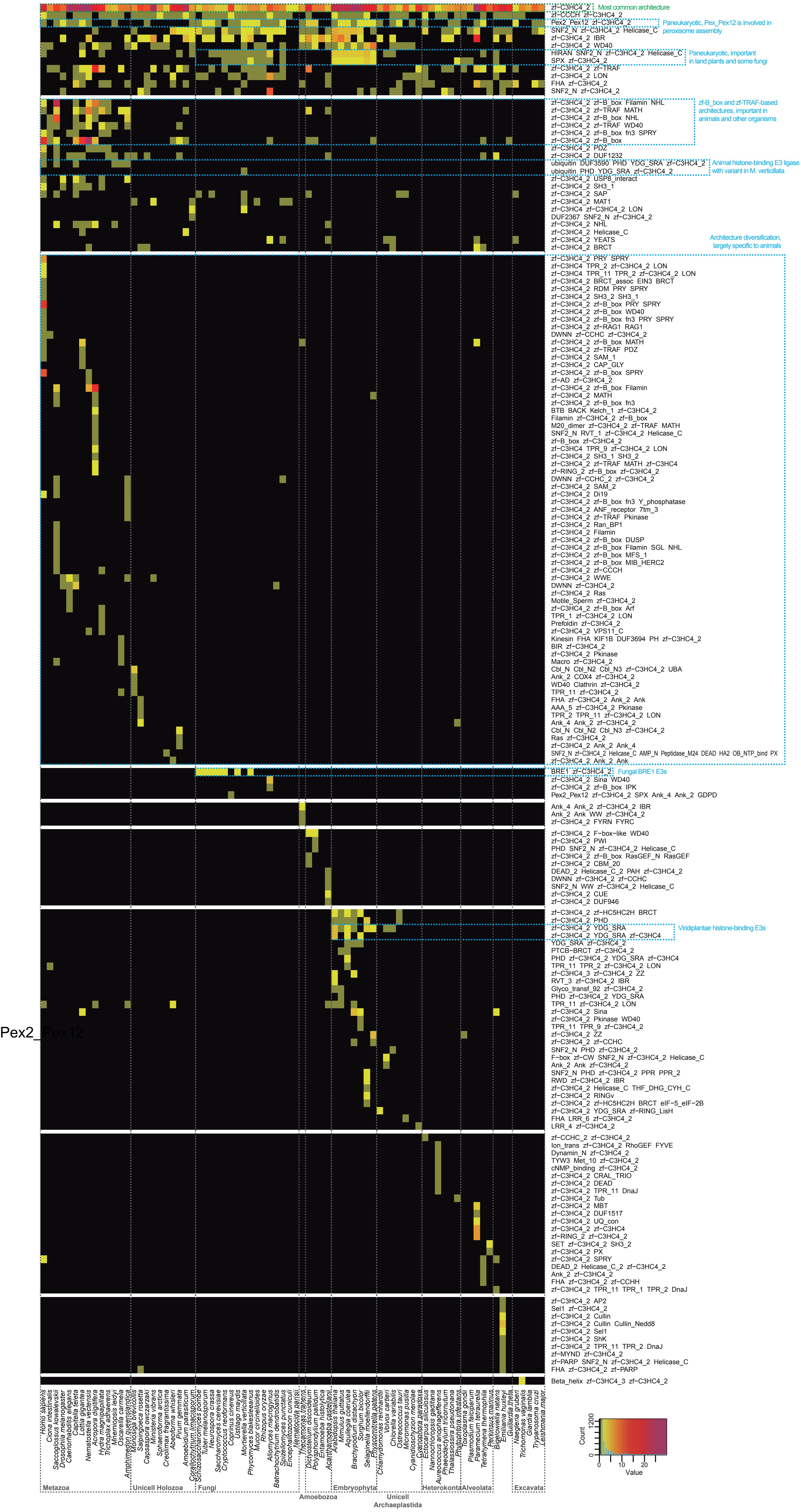

Figure S5G – zf-C3HC4_3

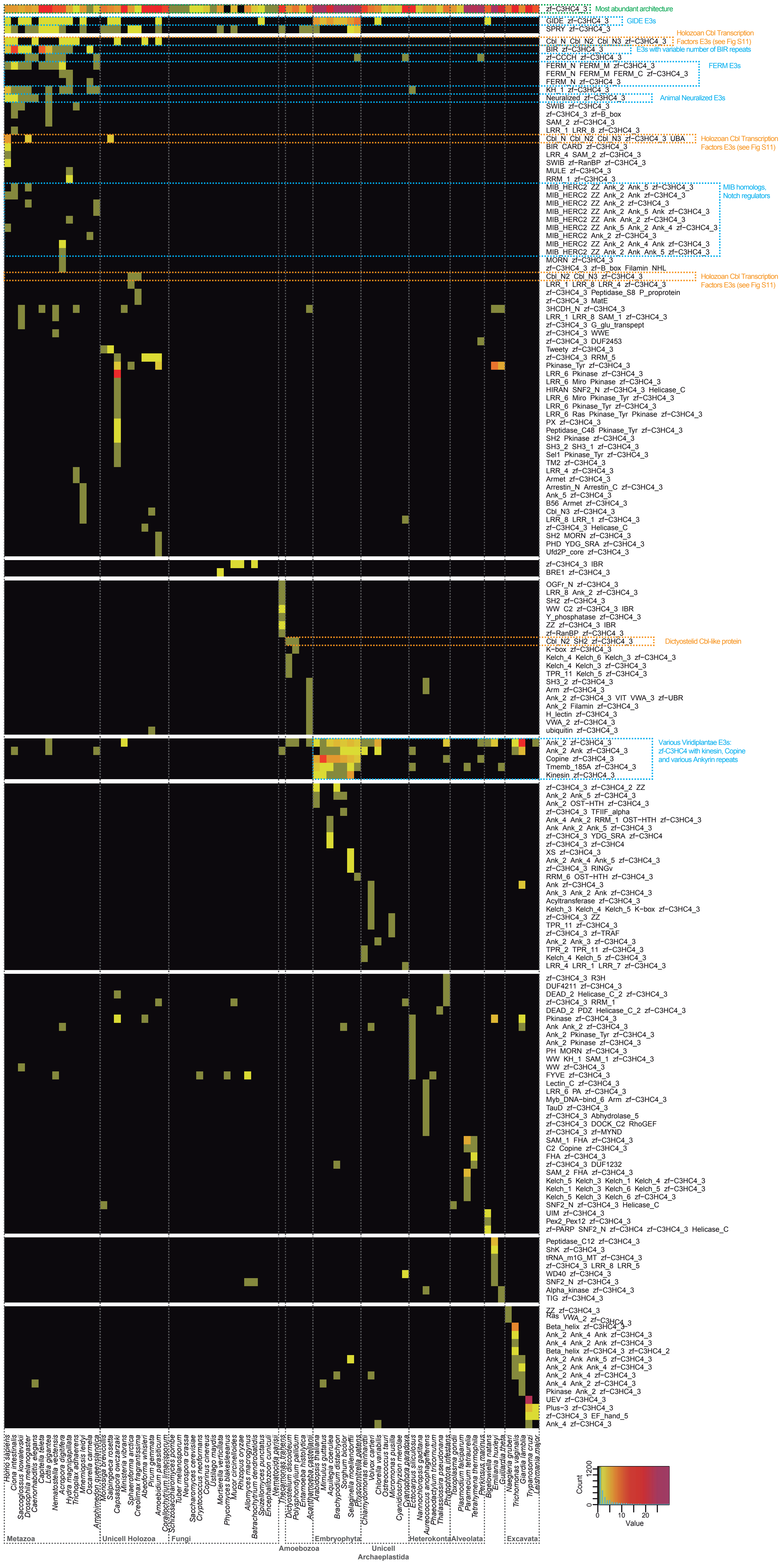

Figure S5H – Cbl_N

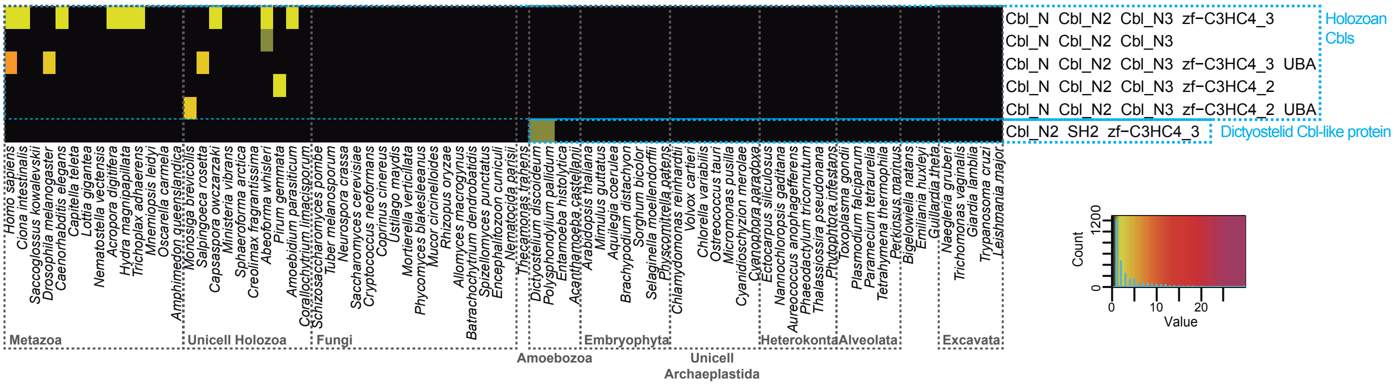

Figure S5I – U-box

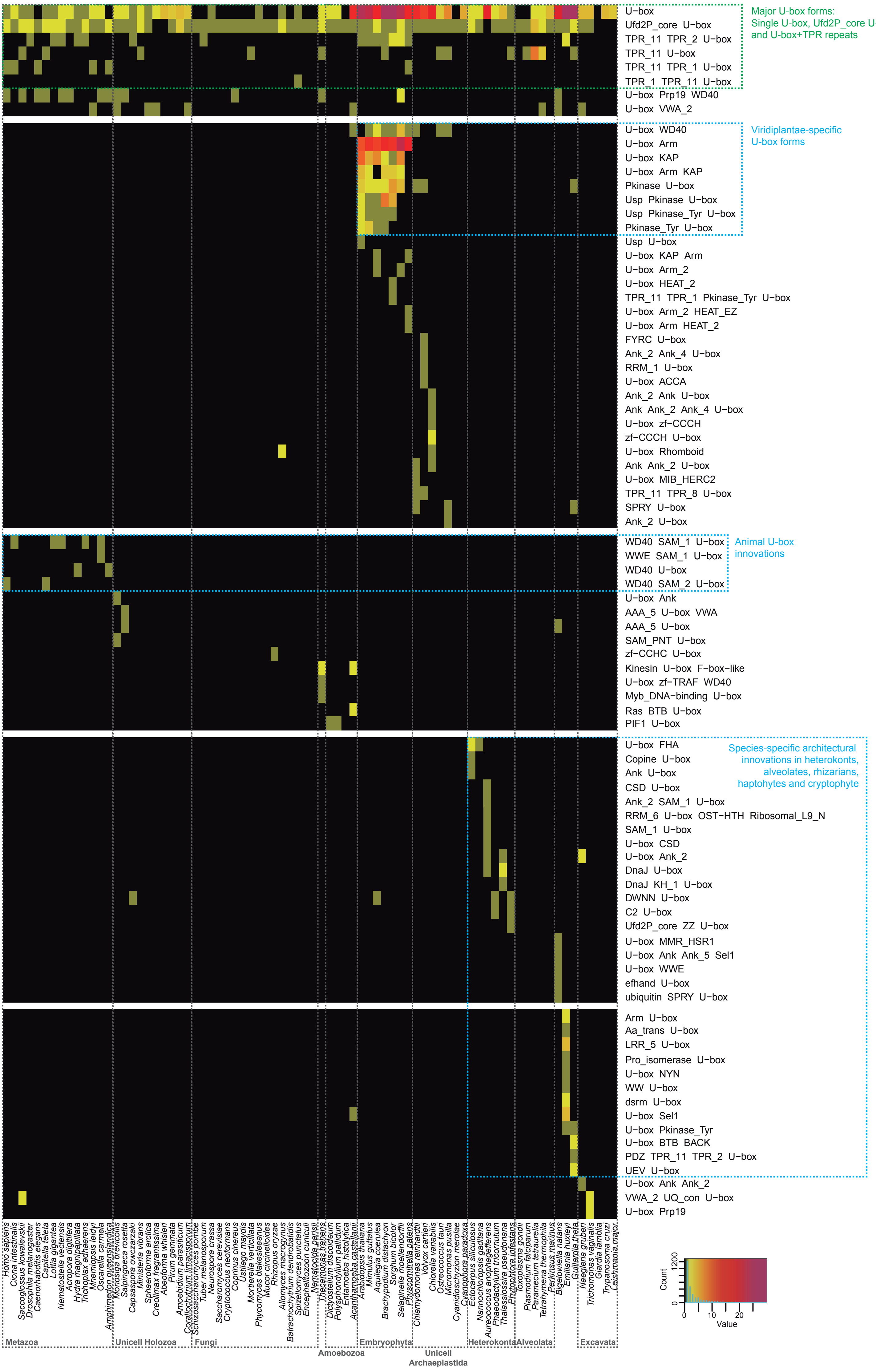

Figure S5J – zf-RING_LisH

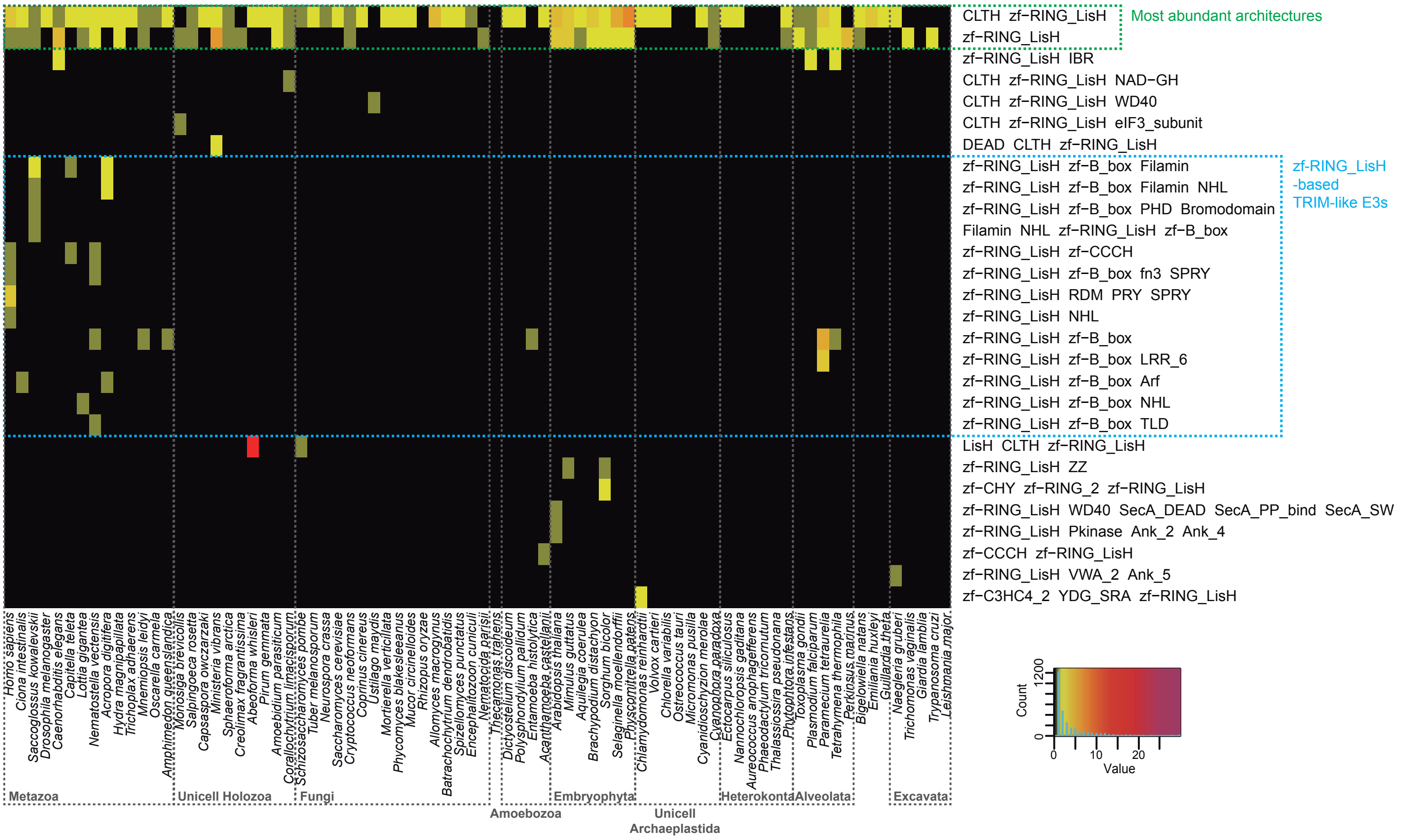

Figure S5K – RINGv

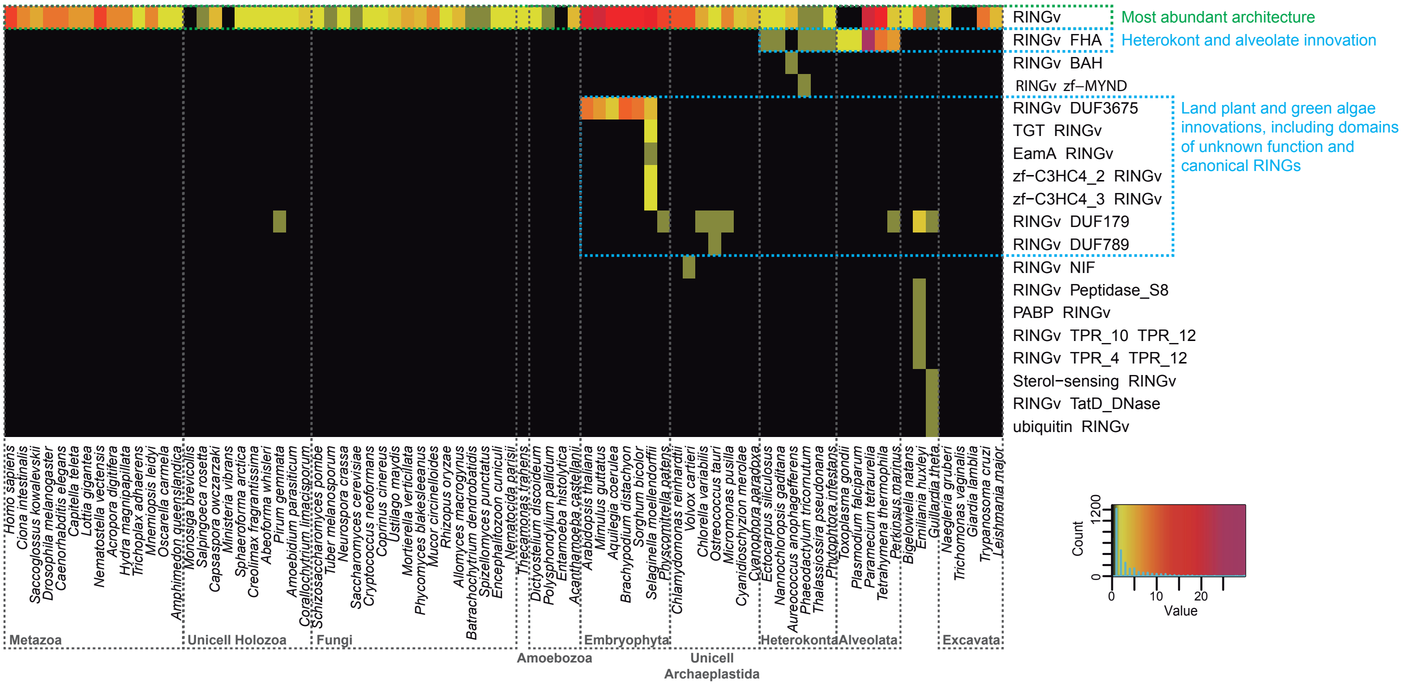

Figure S5L – FANCL_C

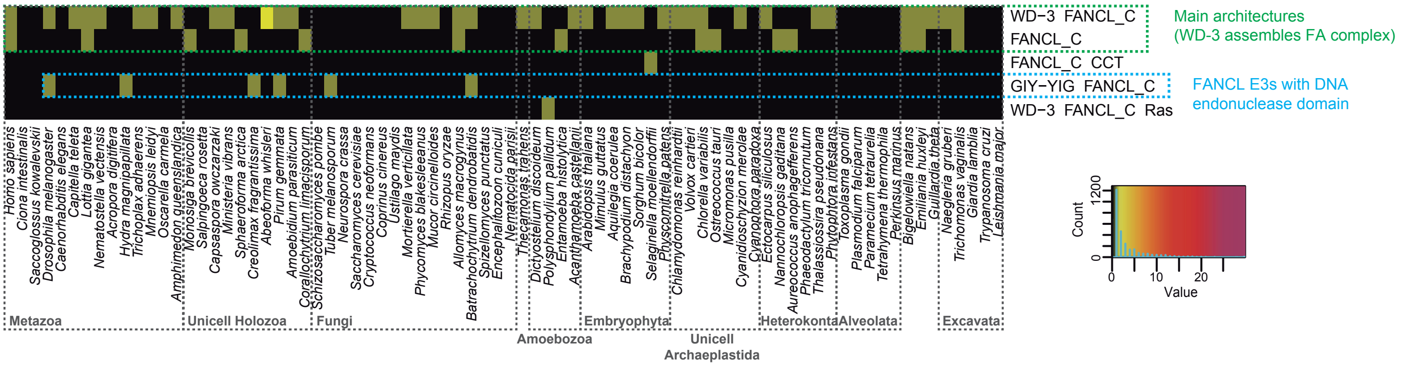

Figure S5M – IBR

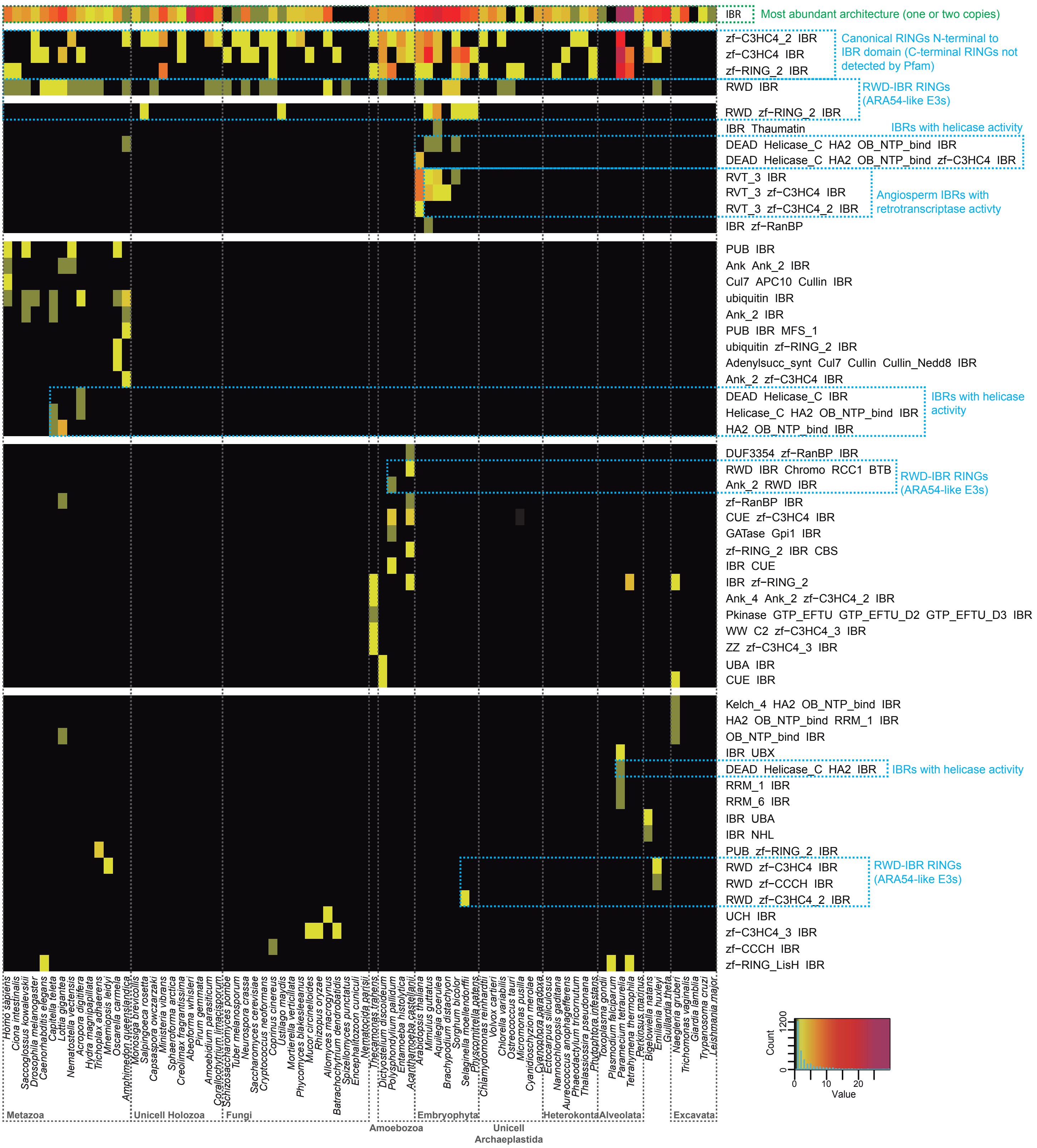

Figure S5N – Cullin

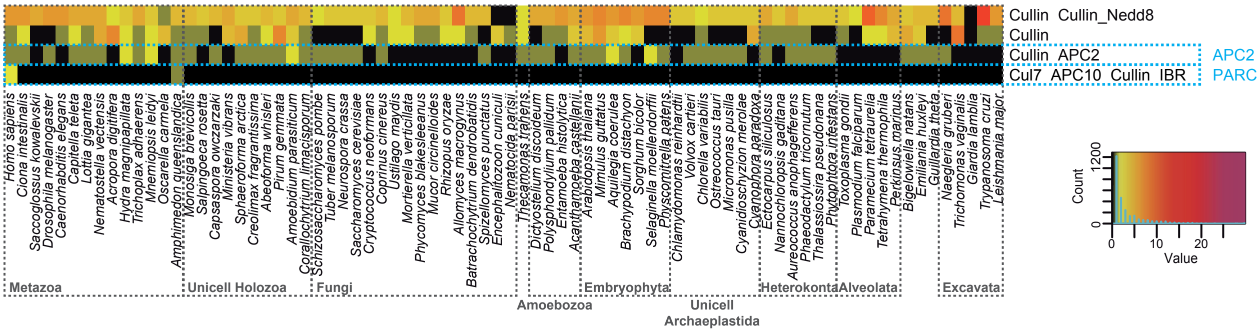

Figure S5O – F-box/F-box-like

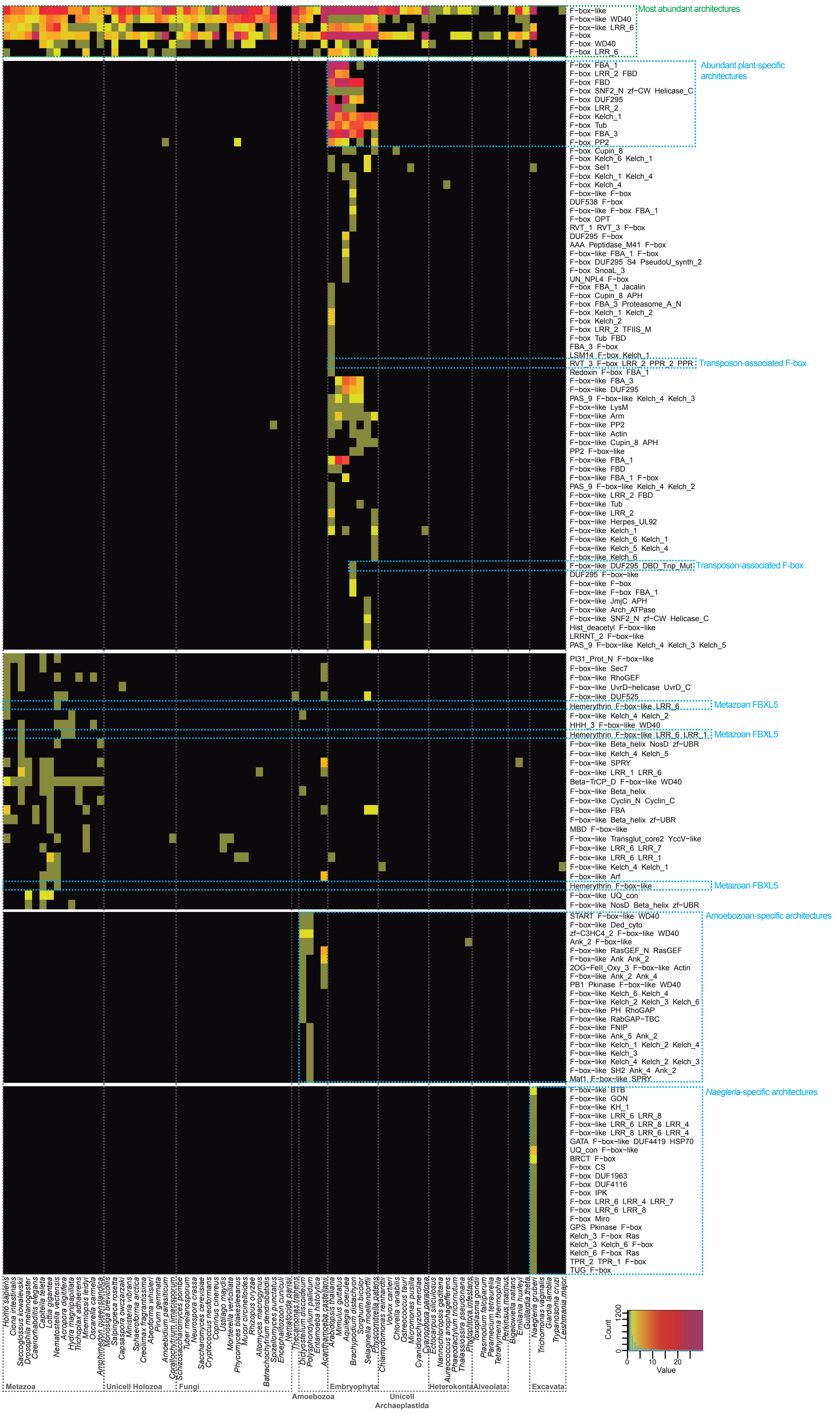

Figure S5P – SOCS_box

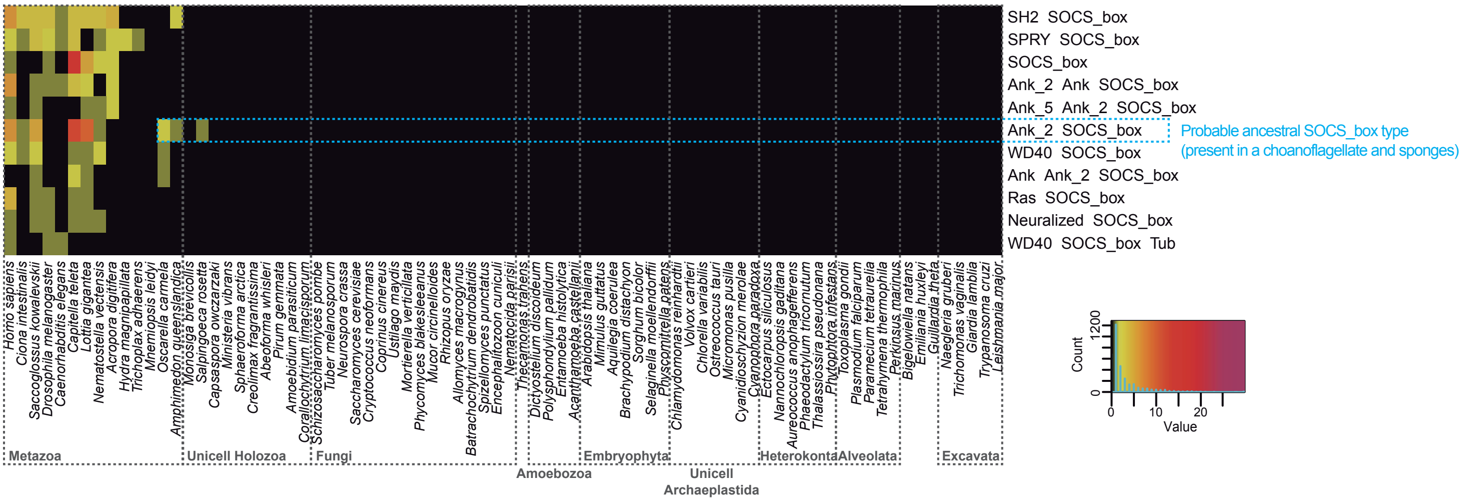

Figure S5Q – BTB

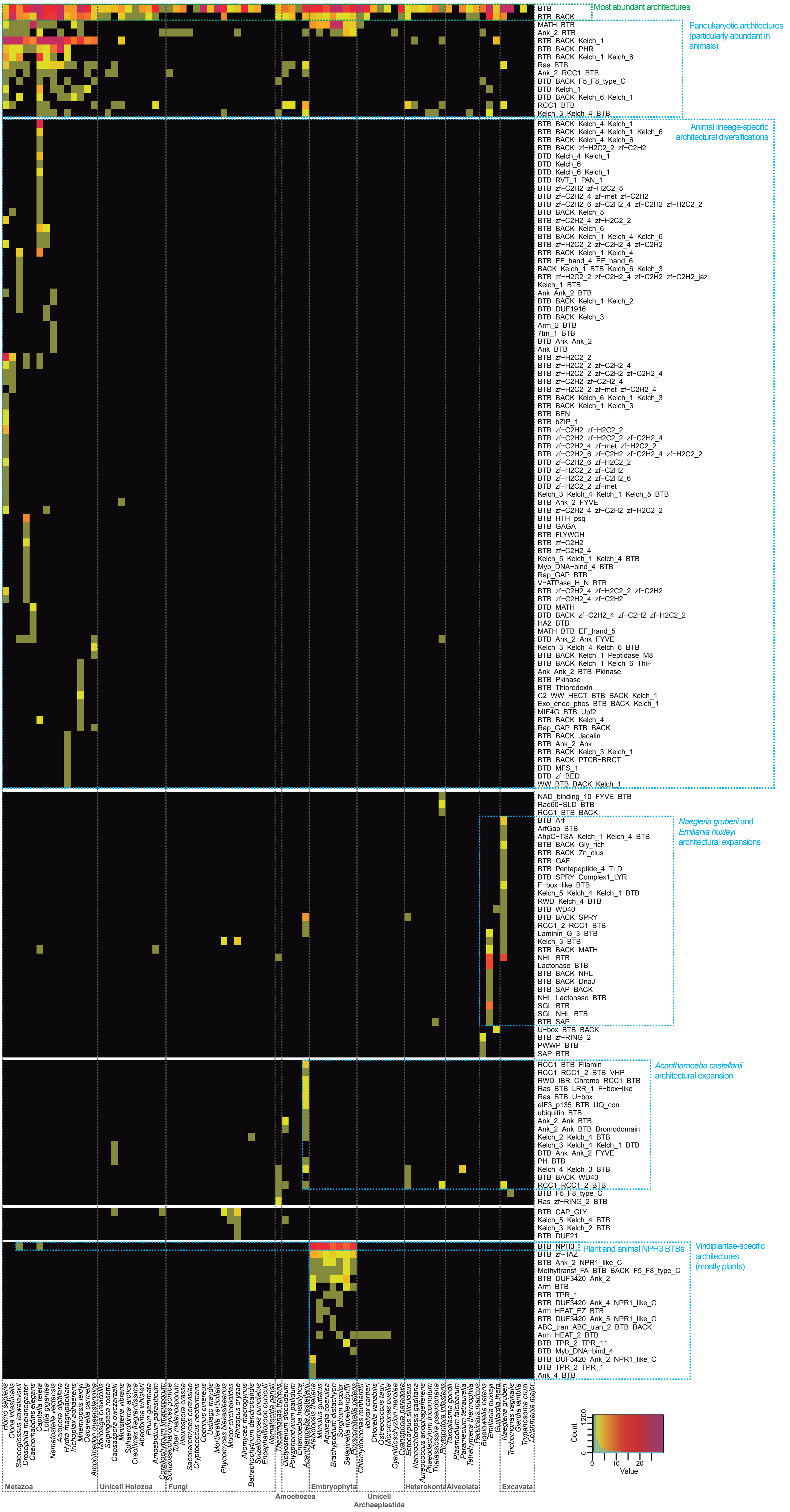

Figure S5R – UCH/UCH_1

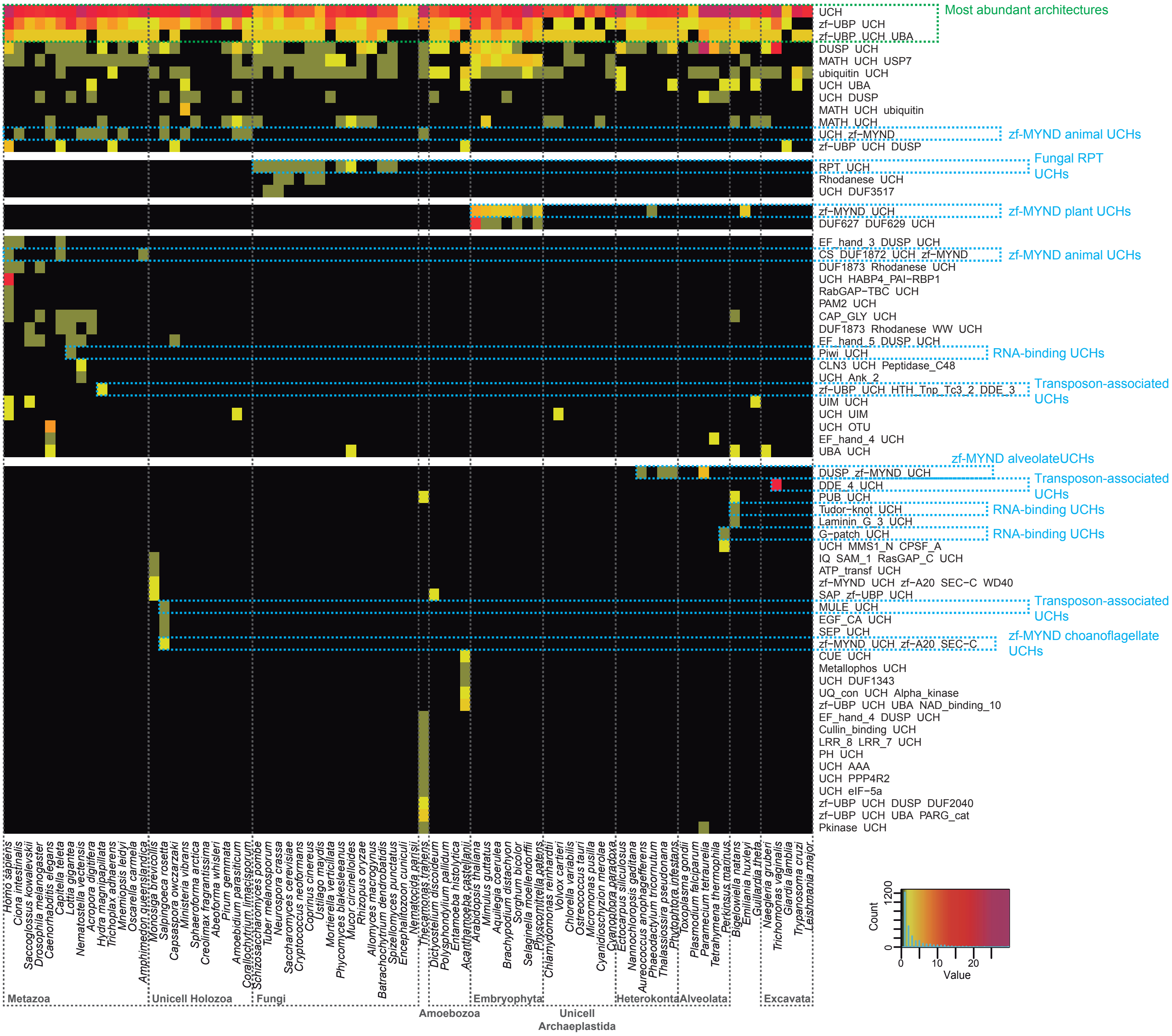

Figure S5S – Josephin

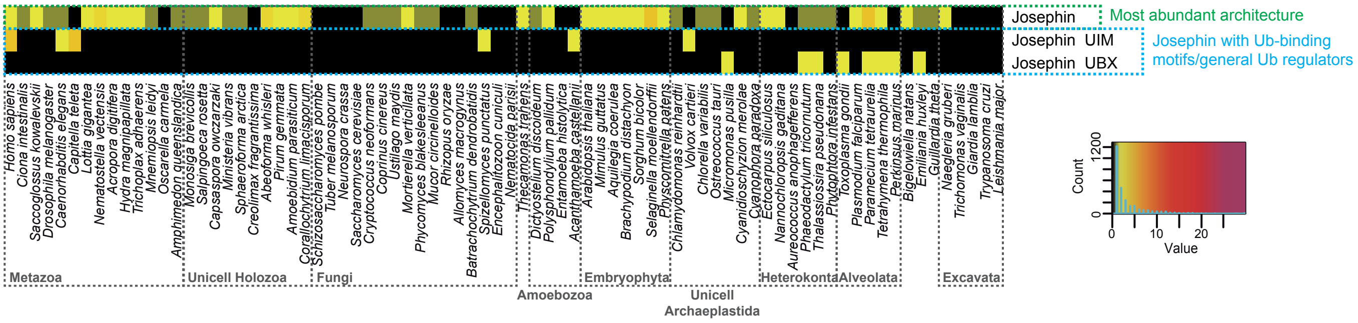

Figure S5T – OTU

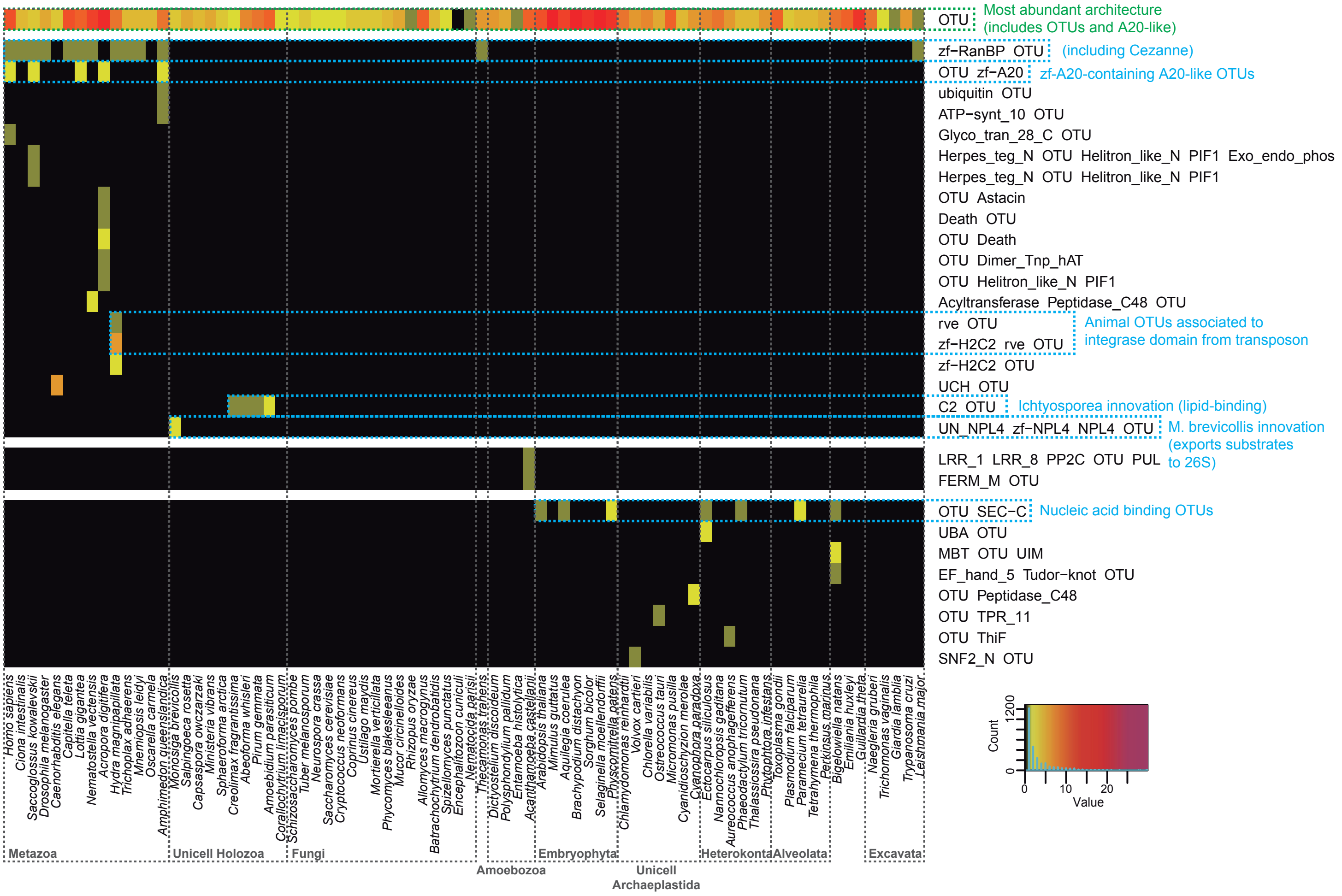

Figure S5U – Peptidase_C65

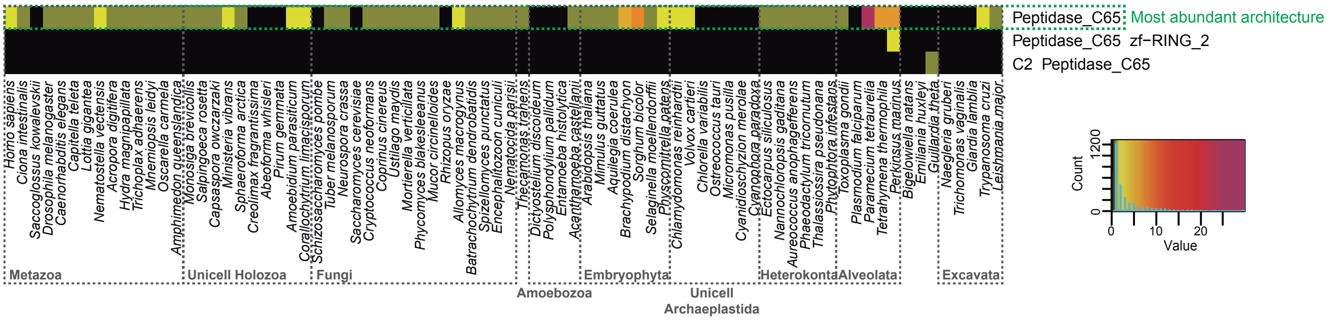

Figure S5V – JAB

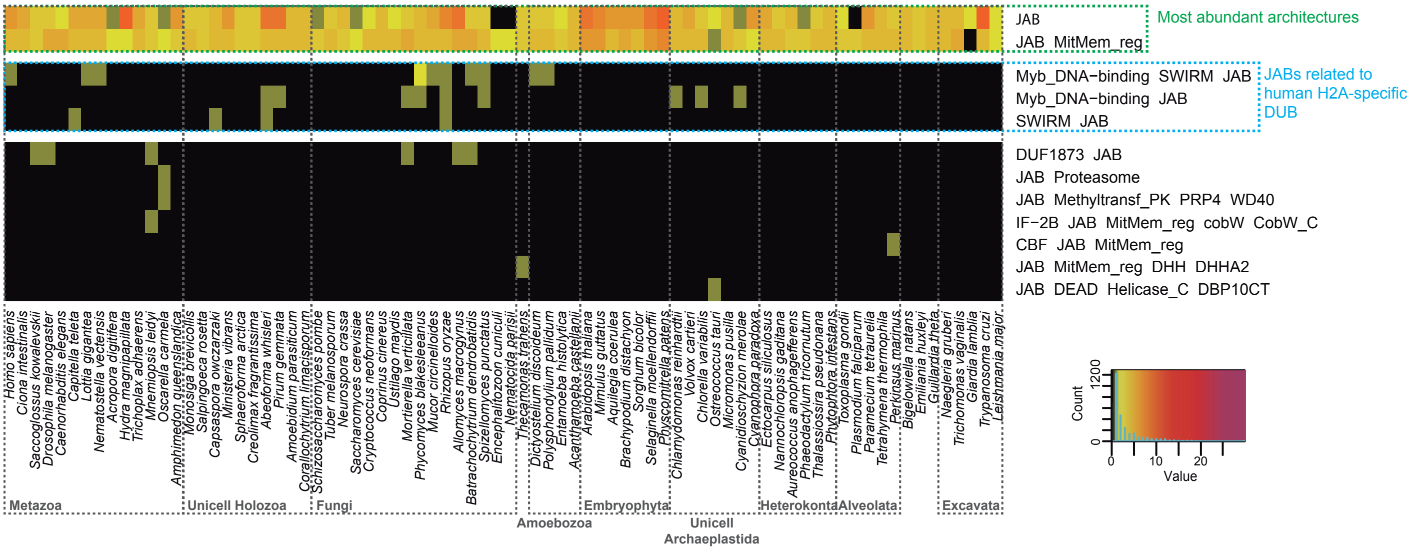

Figure S5W – zf-MIZ

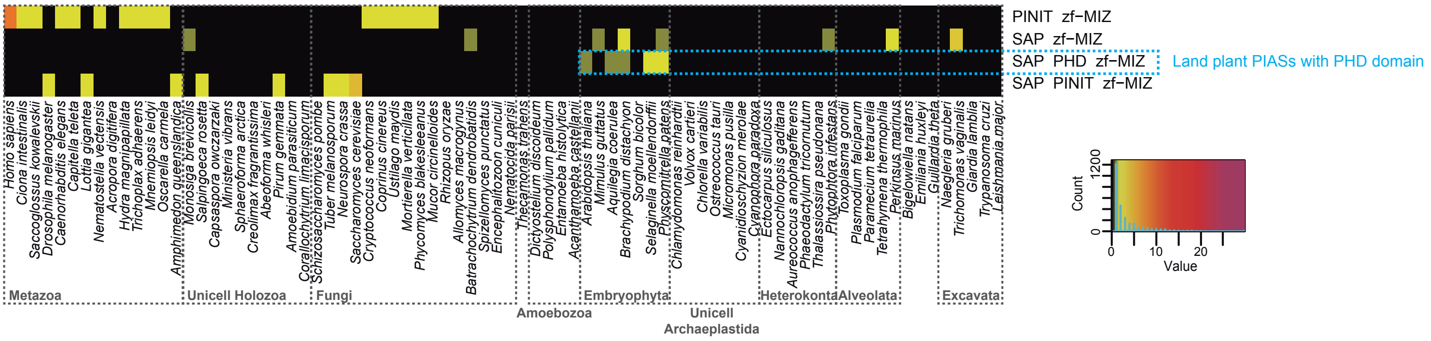

Figure S5X – zf-Nse

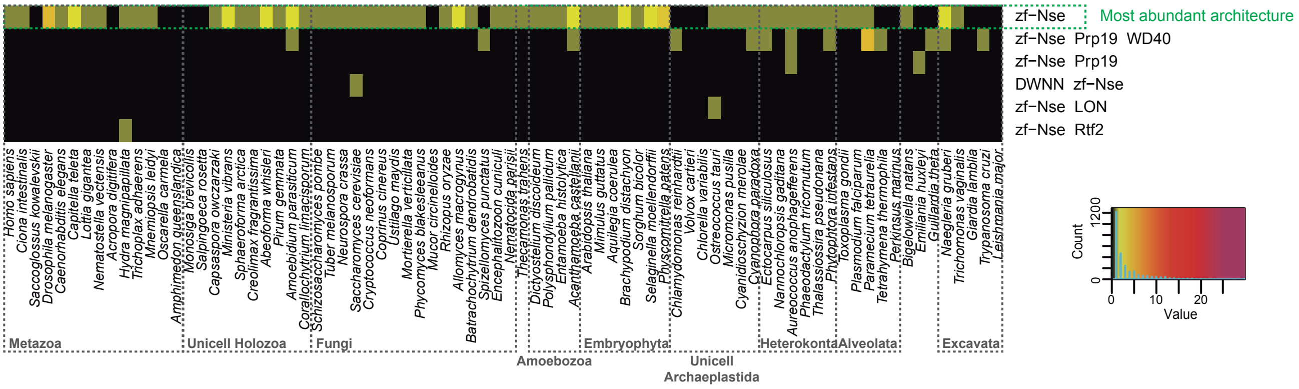

Figure S5Y – Peptidase_C48

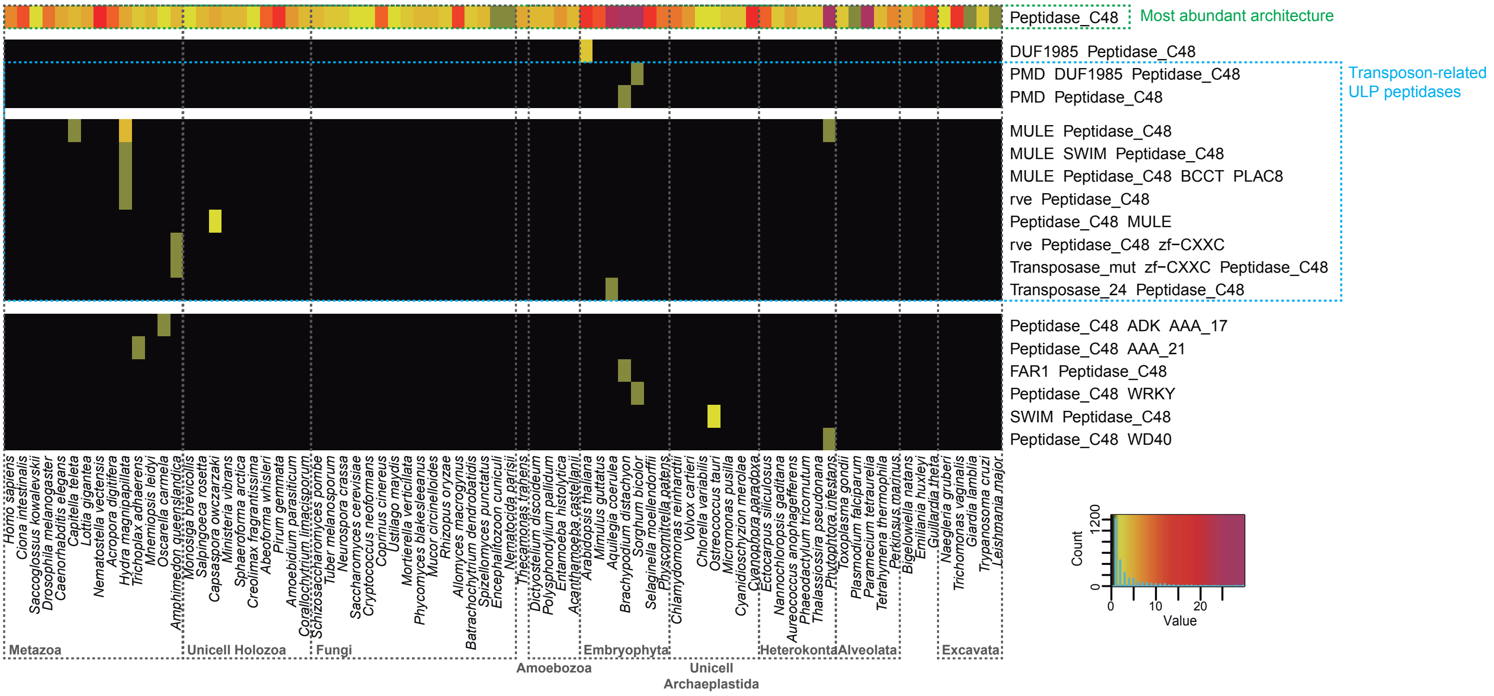

Figure S5Z – WLM

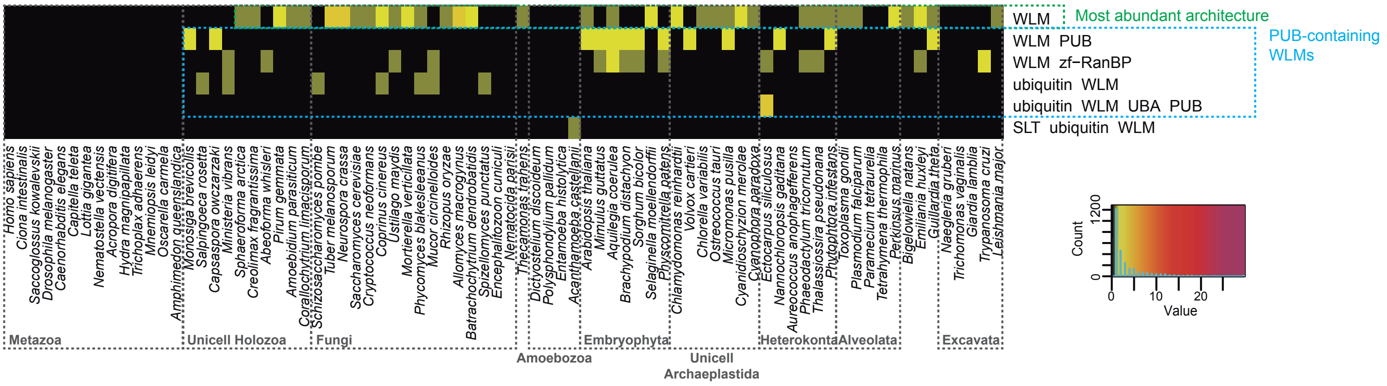

Figure S5AA – DUF862

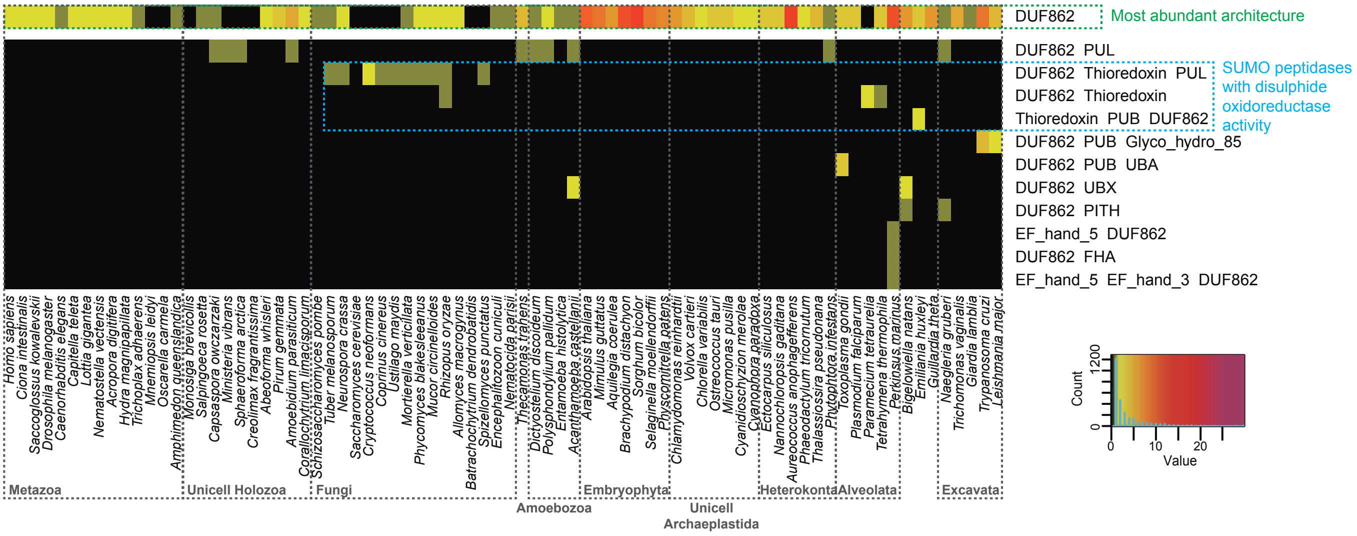

Figure S5AB – Peptidase_C78

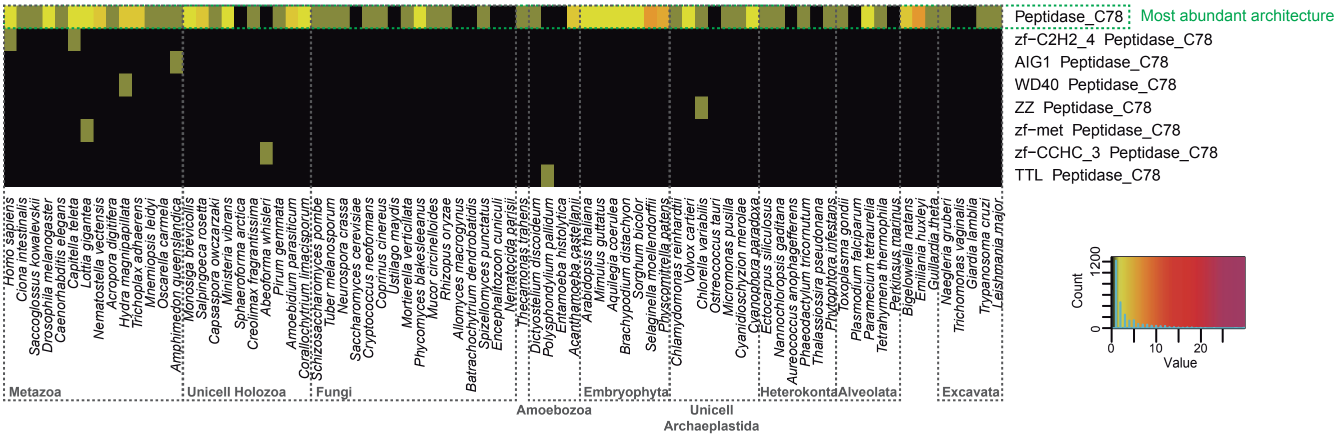

Fig. S6A
P-P plots of
matched-pairs symmetry tests

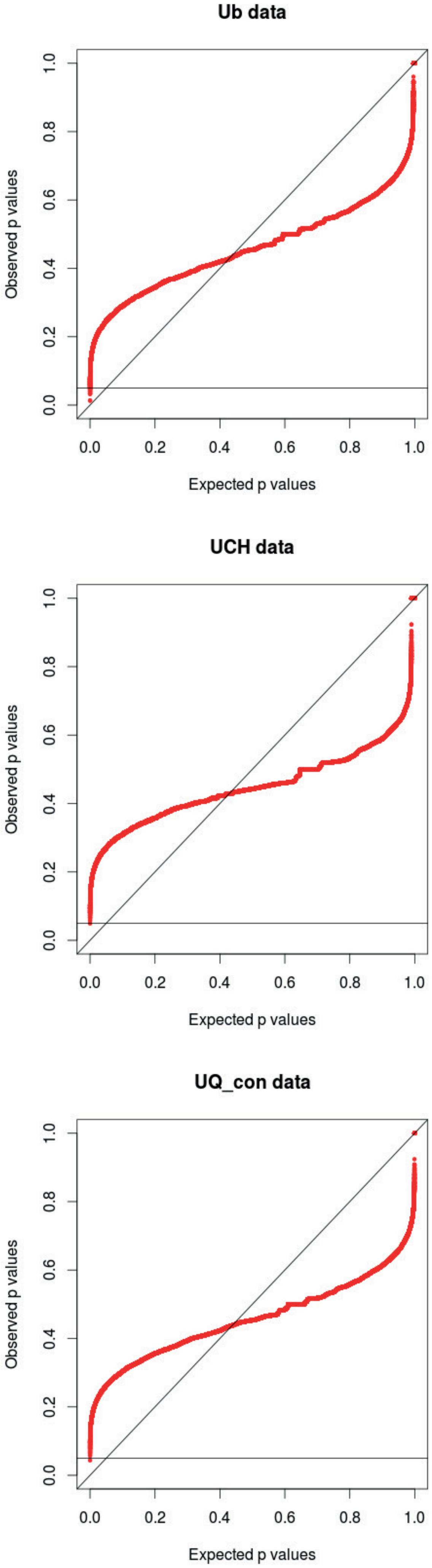

Fig. S6B Ubiquitin ML

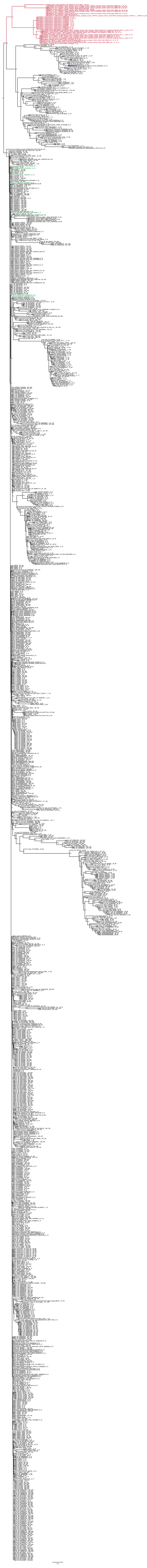

Fig. S6C Ubiquitin PB

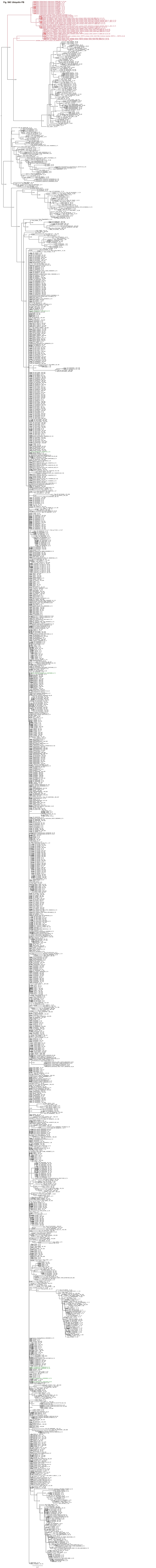

Fig. S6D UCH ML

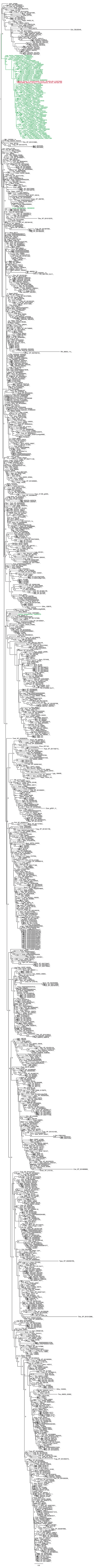

Fig. S6E UCH PB

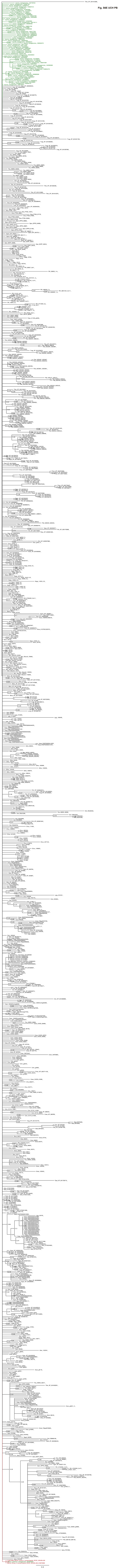

Fig. S6F UQ_con ML

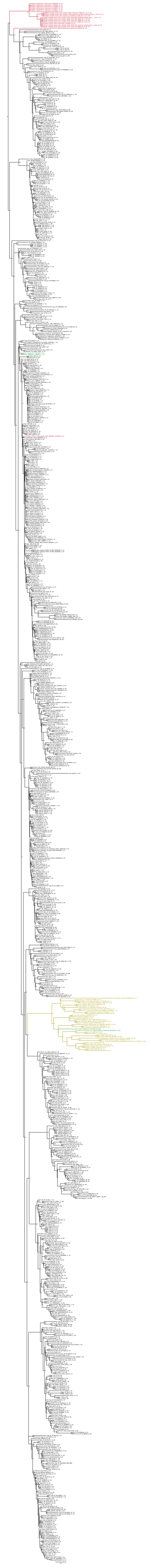

Fig. S6G UQ_con PB

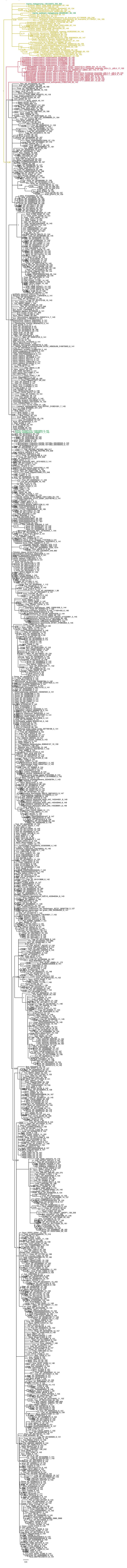

Figure S7

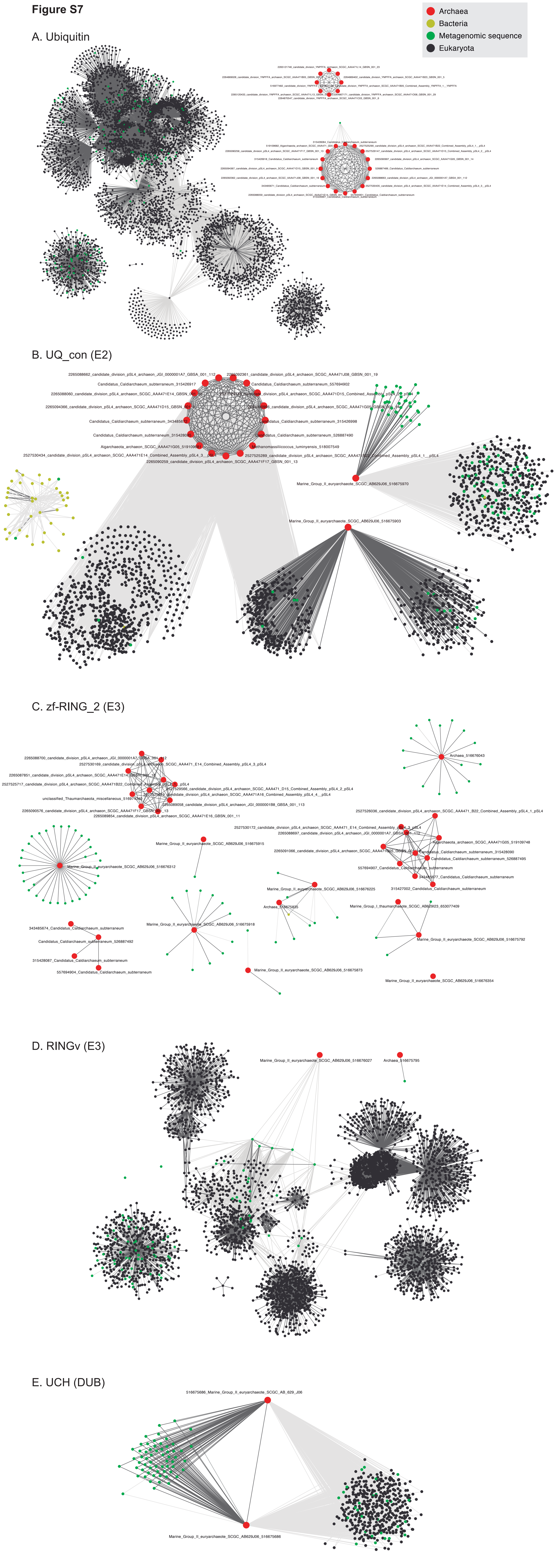

Archaea
Bacteria
Metagenomic sequence
Eukaryota
A. Ubiquitin
B. UQ_con (E2)
Marine_Group_II_euryarchaeote_SCGC_AB629J06_516675970
Marine_Group_II_euryarchaeote_SCGC_AB629J06_516675903
C. zf-RING_2 (E3)
Archaea_516676043
Marine_Group_II_euryarchaeote_SCGC_AB629J06_516676312
Marine_Group_II_euryarchaeote_SCGC_AB629J06_516675915
Marine_Group_II_euryarchaeote_SCGC_AB629J06_516675918
Marine_Group_II_euryarchaeote_SCGC_AB629J06_516675873
Marine_Group_II_euryarchaeote_SCGC_AB629J06_516675792
Marine_Group_II_euryarchaeote_SCGC_AB629J06_516676354
D. RINGv (E3)
Marine_Group_II_euryarchaeote_SCGC_AB629J06_516676027
Archaea_516675795
E. UCH (DUB)
516675686_Marine_Group_II_euryarchaeote_SCGC_AB_629_J06
Marine_Group_II_euryarchaeote_SCGC_AB629J06_516675686

Table S1. Taxon sampling used in this study, including the taxonomic classification, the acronym used in the present study (between brackets) and the source of the proteome data.

Taxonomic classification		Species	Source
**METAZOA**		*Homo sapiens (Hsap)*	Ensembl
		Ciona intestinalis (Cint)	Ensembl
		Saccoglossus kowalevskii (Skow)	NCBI
		Drosophila melanogaster (Dmel)	Flybase *http://flybase.org/*
		Caenorhabditis elegans (Cele)	Ensembl
		Lottia gigantea (Lgig)	JGI *http://genome.jgi.doe.gov/*
		Capitella teleta (Ctel)	JGI
		Nematostella vectensis (Nvec)	NCBI
		Acropora digitifera (Adig)	*Acropora digitifera* Genome Project webpage *http://marinegenomics.oist.jp/*
		Hydra magnipapillata (Hmag)	NCBI
		Trichoplax adhaerens (Tadh)	NCBI
		Mnemiopsis leidyi (Mlei)	*M. leidyi* Genome Project webpage *http://research.nhgri.nih.gov/mnemiopsis/*
		Oscarella carmela (Ocar)	Courtesy of Scott Nichols
		Amphimedon queenslandica (Aque)	NCBI
**CHOANOFLAGELLATA**		*Monosiga brevicollis (Mbre)*	NCBI
		Salpingoeca rosetta (Sros)	Broad Institute *http://www.broadinstitute.org/annotation /genome/multicellularity_project/MultiHome.html*
**FILASTEREA**		*Capsaspora owczarzaki (Cowc)*	Broad Institute
		Ministeria vibrans (Mvib)	RNASeq data
**ICHTHYOSPOREA**		*Sphaeroforma arctica (Sarc)*	Broad Institute
		Creolimax fragrantissima (Cfra)	*Ab initio protein prediction*
		Abeoforma whisleri (Awhi)	RNASeq data
		Pirum gemmata (Pgem)	RNASeq data
		Amoebidium parasiticum (Apar)	RNASeq data
	Incertae sedis	*Corallochytrium limacisporum (Clim)*	RNASeq data
**FUNGI**	Ascomycota	*Aspergillus oryzae (Aory)*	NCBI
		Schizosaccharomyces pombe (Spom)	NCBI
		Tuber melanosporum (Tmel)	NCBI
		Neurospora crassa (Ncra)	Broad Institute *http://www.broadinstitute.org/scientific-community/science/projects/fungal-genome-initiative*
		Saccharomyces cerevisiae (Scer)	Broad Institute
	Basidiomycota	*Cryptococcus neoformans (Cneo)*	NCBI
		Coprinopsis cinerea (Ccin)	Broad Institute
		Ustilago maydis (Umay)	NCBI
	Incertae sedis	*Mortierella verticillata (Mver)*	Broad Institute
	Mucoromycotina	*Phyomyces blakesleeanus (Pbla)*	JGI
		Mucor circinelloides (Mcir)	JGI
		Rhizopus oryzae (Rory)	Broad Institute
	Blastocladio-mycota	*Allomyces macrogynus (Amac)*	Broad Institute
	Chytridiomycota	*Batrachochytrium dendrobatidis (Bden)*	JGI
		Spizellomyces punctatus (Spun)	Broad Institute
	Microsporidia	*Encephalitozoon cuniculi (Ecun)*	NCBI
		Nematocida parisii (Npar)	NCBI
**APUSOZOA**		*Thecamonas trahens (Ttra)*	Broad Institute

**AMOEBOZOA**	Mycetozoa	*Dictyostelium discoideum (Ddis)*	NCBI
		Polysphondylium pallidum (Ppal)	NCBI
	Archamoeba	*Entamoeba histolytica (Ehis)*	Wellcome Trust Sanger Institute *http://www.sanger.ac.uk/resources/downloads/protozoa/entamoeba.html*
	Centramoebida	*Acanthamoeba castellanii (Acas)*	*Ab initio protein prediction*
**VIRIDIPLANTAE**	Embryophyta	*Arabidopsis thaliana (Atha)*	NCBI
		Mimulus guttatus (Mgut)	Phytozome (JGI) *http://www.phytozome.net/*
		Aquilegia coerulea (Acoe)	Phytozome (JGI)
		Brachypodium distachyon (Bdis)	Phytozome (JGI)
		Sorghum bicolor (Sbic)	Phytozome (JGI)
		Selaginella moellendorffii (Smoe)	NCBI
		Physcomitrella patens (Ppat)	NCBI
	Chlorophyta	*Chlamydomonas reinhardtii (Crei)*	NCBI
		Volvox cartieri (Vcar)	NCBI
		Chlorella variabilis (Cvar)	NCBI
		Ostreococcus tauri (Otau)	NCBI
		Micromonas pusilla (Mpus)	JGI
	Rhodophyta	*Cyanidioschyzon merolae (Cmer)*	*C. merolae* Genome Project webpage *http://merolae.biol.s.u-tokyo.ac.jp/*
	Glaucophyta	*Cyanophora paradoxa (Cpar)*	*C. paradoxa* Genome Project webpage *http://cyanophora.rutgers.edu/cyanophora/*
**HETERO-KONTA**	Brown algae/ Phaeophyceae	*Ectocarpus siliculosus (Esil)*	EMBL
		Nannochloropsis gaditana (Ngad)	*N. gaditana* Genome Project webpage *http://nannochloropsis.genomeprojectsolutions-databases.com/*
		Aureococcus anophagefferens (Aano)	Uniprot
		Phaeodactylum tricornutum (Ptri)	NCBI
		Thalassiossira pseudonana (Tpse)	NCBI
	Oomycota	*Phytophthora infestans (Pinf)*	NCBI
**ALVEOLATA**	Apicomplexa	*Toxoplasma gondii (Tgon)*	NCBI
		Plasmodium falciparum (Pfal)	Uniprot
	Ciliophora	*Paramecium tetraurelia (Ptet)*	Uniprot
		Tetrahymena thermophila (Tthe)	NCBI
	Perkinsidae	*Perkinsus marinus (Pmar)*	NCBI
**RHIZARIA**		*Bigelowiella natans (Bnat)*	JGI
**HAPTOPHYTA**		*Emiliania huxleyi (Ehux)*	JGI
**CRYPTOPHYTA**		*Guillardia theta (Gthe)*	JGI
**EXCAVATA**	Heterolobosea	*Naegleria gruberi (Ngru)*	NCBI
	Kinetoplastida	*Trypanosoma cruzi (Tcru)*	NCBI
		Leishmania major (Lmaj)	NCBI
	Metamonada	*Trichomonas vaginalis (Tvag)*	NCBI
		Giardia lamblia (Glam)	NCBI
**ARCHAEA & BACTERIA**		-	Non-redundant database NCBI

Table S2. List of elements of the ubiquitin, SUMO and Ufm1 pathways sampled in our study, including their defining Pfam domain IDs. Examples of each element in the model systems *Homo sapiens, Saccharomyces cerevisiae* and *Arabidopsis thaliana* are also included.

	Type	Domain	Pfam ID	Human	Yeast	Arabidopsis
**General**	**E1**	ThiF	PF10585 *(See Material and Methods)*	UBA1 (Ub)… UBA2 (SUMO)… UBA5 (Ufm1)	UBA1 (Ub) UBA2 (SUMO/SMT3) - (Ufm1)	UBA1,2 (Ub)… SAE1/SAE2 (SUMO)… UBA5 (Ufm1)
	**E2**	UQ_con	PF00179	UBE2E1, UBE2E2, UBE2D1, UBE2D2, UBE2D3 (Ub)… UBE2I/UBC9 (SUMO) - (Ufm1)	UBC1-8 (Ub) UBC9 (SUMO) - (Ufm1)	UBC1-8, UBC10-37 (Ub)… UBC9, SCE1 (SUMO) - (Ufm1)
**Ubiquitin**	**Label**	ubiquitin	PF00240	Various	Various	Various
	**E3s**	HECT	PF00632	Trip12, Tom1, E6-AP, Nedd4...	Ufd4, Tom1...	UPL1-7
		U-box	PF04564	Prp19, CHIP...	Prp19, Ufd2P...	Prp19, CHIP...
		zf-C3HC4	PF00097	Various; see Deshaies and Joazeiro 2009	Various; see Deshaies and Joazeiro 2009	See Stone et al. 2005; Deshaies and Joazeiro 2009
		zf-C3HC4_2	PF13923			
		zf-C3HC4_3	PF13920			
		Sina	PF03145	Siah1 (Seven in absentia homolog 1)	-	SINAT5 (root development), SINAT1...
		zf-RING_2	PF13639	Various; see Deshaies and Joazeiro 2009	Various; see Deshaies and Joazeiro 2009	See Stone et al. 2005
		zf-RING_LisH	PF13445	Rmd5, Trim32	Rmd5/Gid2, -	LisH/CRA/RING-U-box domain-containing protein (not to be confused with Gid2/SLEEPY1, a F-box protein from the SCF complex), -
		RINGv	PF12906	MARCH1-9	Doa10/SSM4	MARCH6, Pitchoun 1/PIT1
		FANCL_C	PF11793	FANCL	-	unnamed NP_001032152
		IBR	PF01485	Park2, Ariadne/PARC, RNFs…	YKR017C, ITT1…	RING/U-box protein with C6HC-type zinc finger…
		zf-rbx1	PF12678	RBX1	Hrt1p	RING-box protein 1
		Cullin	PF00888	Cul1-7	Cullin	Cullin
		Skp1_POZ	PF03931	Skp1	Skp1p	Skp1-like protein
		MMS1_N	PF10433	DDB1	MSS1	DDB1
		F-box, F-box-like	PF00646, PF12937	F-box only protein X (Fbw1, Fbl1, Fbx1) See more examples in Cardozo et al. 2004	Mfb1p	F-box protein X (See more examples in Gagne et al. 2002)
		VHL	PF01847	Von Hippel-Lindau disease tumor suppressor	-	-
		SOCS_box	PF07525	Suppressor of cytokine signaling	-	-
		BTB	PF00651	Bcl6, ZBTB48…	YIL001Wp-like protein	NPR3…
		DCAF	PF00400 *(WD40; see Material and Methods)*	DCAF, RFWD2, COP1	DCAF	COP1, RFWD2, SPA1
	**DUBs**	Peptidase_C12	PF01088	UCHL1, BAP1	Yuh1p	Ubiquitin carboxyl-terminal hydrolase L5
		UCH, UCH_1	PF00443, PF13423	USP1	Ubp1p	Ubiquitin carboxyl-terminal hydrolase 1
		Josephin	PF02099	ATXN3, JOSD1	-	Ataxin-3
		JAB	PF01398	COP9 signalosome complex subunit 5; Histone H2A deubiquitinase MYSM1	Rpn11p, Rri1p	26S proteasome, non-ATPase regulatory subunit

		Peptidase_C65	PF10275	Otubains: OTUB1,2	-	Ubiquitin thioesterase otubain-like
		OTU	PF02338	OTUs: OTUD1; A20-like; A20, Cezanne, TRABID	Otu1p	OTU-like cysteine type protease
**SUMO**	**Label**	Rad60-SLD	PF11976	SUMO-1, SUMO-2, SUMO-3, SUMO-4	Smt3p	SUMO-1
	**E3**	zf-MIZ	PF02891 *(See Material and Methods)*	PIAS1, PIASxα, PIASxβ, PIASγ, and PIAS3	Siz1p, Nfi1p	SIZ1
		zf-Nse	PF11789	Nse1, Nse2	MMS21	MMS21
		IR1-M	PF12185	RanBP2	-	-
	**Pepti-dase**	Peptidase_C48	PF02902	SENP1, SENP2, SENP3,...,SENP7	Ulp1p, Ulp2p	Ubiquitin-like-specific protease 1,Ubiquitin-like-specific protease 2
		WLM	PF08325	-	Wss1p	no name (ex: NP_564704)
		DUF862	PF05903	DeSI-1 or PPPDE peptidase domain-containing protein	- (*S.pombe* has UPF0326 protein hag1)	PPPDE putative thiol peptidase family protein
**Ufm1**	**Label**	Ufm1	PF03671	Ufm1	-	Ufm1
	**E2**	UFC1	PF08694	UFC1	-	ubiquitin-fold modifier-conjugating enzyme 1
	**E3**	DUF2042	PF09743	E3 UFM1-protein ligase 1	-	E3 UFM1-protein ligase 1-like
	**Pepti-dase**	Peptidase_C78	PF07910	UfSP2	?	Probable Ufm1-specific protease
