# Supplementary material for: The Eukaryotic Ancestor Had a Complex Ubiquitin Signaling System of Archaeal Origin
Source: Mol Biol Evol. 2014 Dec 17;32(3):726–39. doi: 10.1093/molbev/msu334 (PMC4327156; doi:10.1093/molbev/msu334)
Supplement: Supplementary Data [file supp_msu334_Supplementary4_FileS2.pdf]

*******BTB***********
>Hsap_ENSP00000171111
MQPDPRPSGAGACCRFLPLQSQCPEGAGDAVMYASTECKAEVTPSQHGNRTFSYTLEDHTKQAFGIMNELRLSQQLCDVT
LQVKYQDAPAAQFMAHKVVLASSSPVFKAMFTNGLREQGMEVVSIEGIHPKVMERLIEFAYTASISMGEKCVLHVMNGAV
MYQIDSVVRACSDFLVQQLDPSNAIGIANFAEQIGCVELHQRAREYIYMHFGEVAKQEEFFNLSHCQLVTLISRDDLNVR
CESEVFHACINWVKYDCEQRRFYVQALLRAVRCHSLTPNFLQMQLQKCEILQSDSRCKDYLVKIFEELTLHKPTQVMPCR
APKVGRLIYTAGGYFRQSLSYLEAYNPSDGTWLRLADLQVPRSGLAGCVVGGLLYAVGGRNNSPDGNTDSSALDCYNPMT
NQWSPCAPMSVPRNRIGVGVIDGHIYAVGGSHGCIHHNSVERYEPERDEWHLVAPMLTRRIGVGVAVLNRLLYAVGGFDG
TNRLNSAECYYPERNEWRMITAMNTIRSGAGVCVLHNCIYAAGGYDGQDQLNSVERYDVETETWTFVAPMKHRRSALGIT
VHQGRIYVLGGYDGHTFLDSVECYDPDTDTWSEVTRMTSGRSGVGVAVTMEPCRKQIDQQNCTC
>Hsap_ENSP00000209884
MEGKPMRRCTNIRPGETGMDVTSRCTLGDPNKLPEGVPQPARMPYISDKHPRQTLEVINLLRKHRELCDVVLVVGAKKIY
AHRVILSACSPYFRAMFTGELAESRQTEVVIRDIDERAMELLIDFAYTSQITVEEGNVQTLLPAACLLQLAEIQEACCEF
LKRQLDPSNCLGIRAFADTHSCRELLRIADKFTQHNFQEVMESEEFMLLPANQLIDIISSDELNVRSEEQVFNAVMAWVK
YSIQERRPQLPQVLQHVRLPLLSPKFLVGTVGSDPLIKSDEECRDLVDEAKNYLLLPQERPLMQGPRTRPRKPIRCGEVL
FAVGGWCSGDAISSVERYDPQTNEWRMVASMSKRRCGVGVSVLDDLLYAVGGHDGSSYLNSVERYDPKTNQWSSDVAPTS
TCRTSVGVAVLGGFLYAVGGQDGVSCLNIVERYDPKENKWTRVASMSTRRLGVAVAVLGGFLYAVGGSDGTSPLNTVERY
NPQENRWHTIAPMGTRRKHLGCAVYQDMIYAVGGRDDTTELSSAERYNPRTNQWSPVVAMTSRRSGVGLAVVNGQLMAVG
GFDGTTYLKTIEVFDPDANTWRLYGGMNYRRLGGGVGVIKMTHCESHIW
>Hsap_ENSP00000215739
MAGPGSTGGQIGAAALAGGARSKVAPSVDFDHSCSDSVEYLTLNFGPFETVHRWRRLPPCDEFVGARRSKHTVVAYKDAI
YVFGGDNGKTMLNDLLRFDVKDCSWCRAFTTGTPPAPRYHHSAVVYGSSMFVFGGYTGDIYSNSNLKNKNDLFEYKFATG
QWTEWKIEGRLPVARSAHGATVYSDKLWIFAGYDGNARLNDMWTIGLQDRELTCWEEVAQSGEIPPSCCNFPVAVCRDKM
FVFSGQSGAKITNNLFQFEFKDKTWTRIPTEHLLRGSPPPPQRRYGHTMVAFDRHLYVFGGAADNTLPNELHCYDVDFQT
WEVVQPSSDSEVGGAEVPERACASEEVPTLTYEERVGFKKSRDVFGLDFGTTSAKQPTQPASELPSGRLFHAAAVISDAM
YIFGGTVDNNIRSGEMYRFQFSCYPKCTLHEDYGRLWESRQFCDVEFVLGEKEECVQGHVAIVTARSRWLRRKITQARER
LAQKLEQEAAPVPREAPGVAAGGARPPLLHVAIREAEARPFEVLMQFLYTDKIKYPRKGHVEDVLLIMDVYKLALSFQLC
RLEQLCRQYIEASVDLQNVLVVCESAARLQLSQLKEHCLNFVVKESHFNQVIMMKEFERLSSPLIVEIVRRKQQPPPRTP
LDQPVDIGTSLIQDMKAYLEGAGAEFCDITLLLDGHPRPAHKAILAARSSYFEAMFRSFMPEDGQVNISIGEMVPSRQAF
ESMLRYIYYGEVNMPPEDSLYLFAAPYYYGFYNNRLQAYCKQNLEMNVTVQNVLQILEAADKTQALDMKRHCLHIIVHQF
TKVSKLPTLRSLSQQLLLDIIDSLASHISDKQCAELGADI
>Hsap_ENSP00000230122
MAETSPEPSGQLVVHSDAHSDTVLASFEDQRKKGFLCDITLIVENVHFRAHKALLAASSEYFSMMFAEEGEIGQSIYMLE
GMVADTFGILLEFIYTGYLHASEKSTEQILATAQFLKVYDLVKAYTDFQNNHSSPKPTTLNTAGAPVVVISNKKNDPPKR
KRGRPKKVNTLQEEKSELAAEEEIQLRVNNSVQNRQNFVVKGDSGVLNEQIAAKEKEESEPTCEPSREEEMPVEKDENYD
PKTEDGQASQSRYSKRRIWRSVKLKDYKLVGDQEDHGSAKRICGRRKRPGGPEARCKDCGKVFKYNHFLAIHQRSHTGER
PFKCNECGKGFAQKHSLQVHTRMHTGERPYTCTVCSKALTTKHSLLEHMSLHSGQKSFTCDQCGKYFSQNRQLKSHYRVH
TGHSLPECKDCHRKFMDVSQLKKHLRTHTGEKPFTCEICGKSFTAKSSLQTHIRIHRGEKPYSCGICGKSFSDSSAKRRH
CILHTGKKPFSCPECNLQFARLDNLKAHLKIHSKEKHASDASSISGSSNTEEVRNILQLQPYQLSTSGEQEIQLLVTDSV
HNINFMPGPSQGISIVTAESSQNMTADQAANLTLLTQQPEQLQNLILSAQQEQTEHIQSLNMIESQMGPSQTEPVHVITL
SKETLEHLHAHQEQTEELHLATSTSDPAQHLQLTQEPGPPPPTHHVPQPTPLGQEQS
>Hsap_ENSP00000232014
MASPADSCIQFTRHASDVLLNLNRLRSRDILTDVVIVVSREQFRAHKTVLMACSGLFYSIFTDQLKCNLSVINLDPEINP
EGFCILLDFMYTSRLNLREGNIMAVMATAMYLQMEHVVDTCRKFIKASEAEMVSAIKPPREEFLNSRMLMPQDIMAYRGR
EVVENNLPLRSAPGCESRAFAPSLYSGLSTPPASYSMYSHLPVSSLLFSDEEFRDVRMPVANPFPKERALPCDSARPVPG
EYSRPTLEVSPNVCHSNIYSPKETIPEEARSDMHYSVAEGLKPAAPSARNAPYFPCDKASKEEERPSSEDEIALHFEPPN
APLNRKGLVSPQSPQKSDCQPNSPTESCSSKNACILQASGSPPAKSPTDPKACNWKKYKFIVLNSLNQNAKPEGPEQAEL
GRLSPRAYTAPPACQPPMEPENLDLQSPTKLSASGEDSTIPQASRLNNIVNRSMTGSPRSSSESHSPLYMHPPKCTSCGS
QSPQHAEMCLHTAGPTFPEEMGETQSEYSDSSCENGAFFCNECDCRFSEEASLKRHTLQTHSDKPYKCDRCQASFRYKGN
LASHKTVHTGEKPYRCNICGAQFNRPANLKTHTRIHSGEKPYKCETCGARFVQVAHLRAHVLIHTGEKPYPCEICGTRFR
HLQTLKSHLRIHTGEKPYHCEKCNLHFRHKSQLRLHLRQKHGAITNTKVQYRVSATDLPPELPKAC
>Hsap_ENSP00000232744
MDTSDLFASCRKGDVGRVRYLLEQRDVEVNVRDKWDSTPLYYACLCGHEELVLYLLANGARCEANTFDGERCLYGALSDP
IRRALRDYKQVTASCRRRDYYDDFLQRLLEQGIHSDVVFVVHGKPFRVHRCVLGARSAYFANMLDTKWKGKSVVVLRHPL
INPVAFGALLQYLYTGRLDIGVEHVSDCERLAKQCQLWDLLSDLEAKCEKVSEFVASKPGTCVKVLTIEPPPADPRLRED
MALLADCALPPELRGDLWELPFPCPDGFNSCPDICFRVAGCSFLCHKAFFCGRSDYFRALLDDHFRESEEPATSGGPPAV
TLHGISPDVFTHVLYYMYSDHTELSPEAAYDVLSVADMYLLPGLKRLCGRSLAQMLDEDTVVGVWRVAKLFRLARLEDQC
TEYMAKVIEKLVEREDFVEAVKEEAAAVAARQETDSIPLVDDIRFHVASTVQTYSAIEEAQQRLRALEDLLVSIGLDC
>Hsap_ENSP00000232766
MVEDGAEELEDLVHFSVSELPSRGYGVMEEIRRQGKLCDVTLKIGDHKFSAHRIVLAASIPYFHAMFTNDMMECKQDEIV
MQGMDPSALEALINFAYNGNLAIDQQNVQSLLMGASFLQLQSIKDACCTFLRERLHPKNCLGVRQFAETMMCAVLYDAAN
SFIHQHFVEVSMSEEFLALPLEDVLELVSRDELNVKSEEQVFEAALAWVRYDREQRGPYLPELLSNIRLPLCRPQFLSDR

VQQDDLVRCCHKCRDLVDEAKDYHLMPERRPHLPAFRTRPRCCTSIAGLIYAVGGLNSAGDSLNVVEVFDPIANCWERCR
PMTTARSRVGVAVVNGLLYAIGGYDGQLRLSTVEAYNPETDTWTRVGSMNSKRSAMGTVVLDGQIYVCGGYDGNSSLSSV
ETYSPETDKWTVVTSMSSNRSAAGVTVFEGRIYVSGGHDGLQIFSSVEHYNHHTATWHPAAGMLNKRCRHGAASLGSKMF
VCGGYDGSGFLSIAEMYSSVADQWCLIVPMHTRRSRVSLVASCGRLYAVGGYDGQSNLSSVEMYDPETDCWTFMAPMACH
EGGVGVGCIPLLTI
>Hsap_ENSP00000232974
MGRLNEQRLFQPDLCDVDLVLVPQRSVFPAHKGVLAAYSQFFHSLFTQNKQLQRVELSLEALAPGGLQQILNFIYTSKLL
VNAANVHEVLSAASLLQMADIAASCQELLDARSLGPPGPGTVALAQPAASCTPAAPPYYCDIKQEADTPGLPKIYAREGP
DPYSVRVEDGAGTAGGTVPATIGPAQPFFKEEKEGGVEEAGGPPASLCKLEGGEELEEELGGSGTYSRREQSQIIVEVNL
NNQTLHVSTGPEGKPGAGPSPATVVLGREDGLQRHSDEEEEDDEEEEEEEEEEEGGGSGREEEEEEEEGGSQGEEEEEEED
GHSEQEEEEEEEEEEGPSEQDQESSEEEEGEEGEAGGKQGPRGSRSSRADPPPHSHMATRSRENARRRGTPEPEEAGRRG
GKRPKPPPGVASASARGPPATDGLGAKVKLEEKQHHPCQKCPRVFNNRWYLEKHMNVTHSRMQICDQCGKRFLLESELLL
HRQTDCERNIQCVTCGKAFKKLWSLHEHNKIVHGYAEKKFSCEICEKKFYTMAHVRKHMVAHTKDMPFTCETCGKSFKRS
MSLKVHSLQHSGEKPFRCENCNERFQYKYQLRSHMSIHIGHKQFMCQWCGKDFNMKQYFDEHMKTHTGEKPYICEICGKS
FTSRPNMKRHRRTHTGEKPYPCDVCGQRFRFSNMLKAHKEKCFRVSHTLAGDGVPAAPGLPPTQPQAHALPLLPGLPQTL
PPPPHLPPPPPLFPTTASPGGRMNANN
>Hsap_ENSP00000240327
MSRVPSPPPPAEMSSGPVAESWCYTQIKVVKFSYMWTINNFSFCREEMGEVIKSSTFSSGANDKLKWCLRVNPKGLDEES
KDYLSLYLLLVSCPKSEVRAKFKFSILNAKGEETKAMESQRAYRFVQGKDWGFKKFIRRDFLLDEANGLLPDDKLTLFCE
VSVVQDSVNISGQNTMNMVKVPECRLADELGGLWENSRFTDCCLCVAGQEFQAHKAILAARSPVFSAMFEHEMEESKKNR
VEINDVEPEVFKEMMCFIYTGKAPNLDKMADDLLAAADKYALERLKVMCEDALCSNLSVENAAEILILADLHSADQLKTQ
AVDFINYHASDVLETSGWKSMVVSHPHLVAEAYRSLASAQCPFLGPPRKRLKQS
>Hsap_ENSP00000242810
MVLILGRRLNREDLGVRDSPATKRKVFEMDPKSLTGHEFFDFSSGSSHAENILQIFNEFRDSRLFTDVIICVEGKEFPCH
RAVLSACSSYFRAMFCNDHRESREMLVEINGILAEAMECFLQYVYTGKVKITTENVQYLFETSSLFQISVLRDACAKFLE
EQLDPCNCLGIQRFADTHSLKTLFTKCKNFALQTFEDVSQHEEFLELDKDELIDYICSDELVIGKEEMVFEAVMRWVYRA
VDLRRPLLHELLTHVRLPLLHPNYFVQTVEVDQLIQNSPECYQLLHEARRYHILGNEMMSPRTRPRRSTGYSEVIVVVGG
CERVGGFNLPYTECYDPVTGEWKSLAKLPEFTKSEYAVCALRNDILVSGGRINSRDVWIYNSQLNIWIRVASLNKGRWRH
KMAVLLGKVYVVGGYDGQNRLSSVECYDSFSNRWTEVAPLKEAVSSPAVTSCVGKLFVIGGGPDDNTCSDKVQSYDPETN
SWLLRAAIPIAKRCITAVSLNNLIYVAGGLTKAIYCYDPVEDYWMHVQNTFSRQENCGMSVCNGKIYILGGRRENGEATD
TILCYDPATSIITGVAAMPRPVSYHGCVTIHRYNEKCFKL
>Hsap_ENSP00000245663
MNNRKEDMEITSHYRHLLRELNEQRQHGVLCDVCVVVEGKVFKAHKNVLLGSSRYFKTLYCQVQKTSEQATVTHLDIVTA
QGFKAIIDFMYSAHLALTSRNVIEVMSAASFLQMTDIVQACHDFIKAALDISIKSDASDELAEFEIGASSSSSTEALISA
VMAGRSISPWLARRTSPANSSGDSAIASCHDGGSSYGKEDQEPKADGPDDVSSQPLWPGDVGYGPLRIKEEQVSPSQYGG
SELPSAKDGAVQNSFSEQSAGDAWQPTGRRKNRKNKETVRHITQQVEDDSRASSPVPSFLPTSGWPFSSRDSNADLSVTE
ASSSDSRGERAELYAQVEEGLLGGEASYLGPPLTPEKDDALHQATAVANLRAALMSKNSLLSLKADVLGDDGSLLFEYLP
RGAHSLSLNEFTVIRKKFKCPYCSFSAMHQCILKRHMRSHTGERPYPCEICGKKFTRREHMKRHTLVHSKDKKYVCKVCS
RVFMSAASVGIRHGSRRHGVCTDCAGRGMAGPLDHGGGGGEGSPEALFPGDGPYLEDPEDPRGEAEELGEDDEGLAPEDA
LLADDKDEEDSPRPRSPPGGPDKDFAWLS
>Hsap_ENSP00000248272
MAEGSAVSDPQHAARLLRALSSFREESRFCDAHLVLDGEEIPVQKNILAAASPYIRTKLNYNPPKDDGSTYKIELEGISV
MVMREILDYIFSGQIRLNEDTIQDVVQAADLLLLTDLKTLCCEFLEGCIAAENCIGIRDFALHYCLHHVHYLATEYLETH
FRDVSSTEEFLELSPQKLKEVISLEKLNVGNERYVFEAVIRWIAHDTEIRKVHMKDVMSALWVSGLDSSYLREQMLNEPL
VREIVKECSNIPLSQPQQGEAMLANFKPRGYSECIVTVGGEERVSRKPTAAMRCMCPLYDPNRQLWIELAPLSMPRINHG
VLSAEGFLFVFGGQDENKQTLSSGEKYDPDANTWTALPPMNEARHNFGIVEIDGMLYILGGEDGEKELISMECYDIYSKT
WTKQPDLTMVRKIGCYAAMKKKIYAMGGGSYGKLFESVECYDPRTQQWTAICPLKERRFGAVACGVAMELYVFGGVRSRE
DAQGSEMVTCKSEFYHDEFKRWIYLNDQNLCIPASSSFVYGAVPIGASIYVIGDLDTGTNYDYVREFKRSTGTWHHTKPL
LPSDLRRTGCAALRIANCKLFRLQLQQGLFRIRVHSP
>Hsap_ENSP00000255608
MAAGGSGGRASCPPGVGVGPGTGGSPGPSANAAATPAPGNAAAAAAAAAAAAAAPGPTPPAPPGPGTDAQAAGAERAEEA
AGPGAAALQREAAYNWQASKPTVQERFAFLFNNEVLCDVHFLVGKGLSSQRIPAHRFVLAVGSAVFDAMFNGGMATTSTE
IELPDVEPAAFLALLKFLYSDEVQIGPETVMTTLYTAKKYAVPALEAHCVEFLKKNLRADNAFMLLTQARLFDEPQLASL
CLENIDKNTADAITAEGFTDIDLDTLVAVLERDTLGIREVRLFNAVVRWSEAECQRQQLQVTPENRRKVLGKALGLIRFP
LMTIEEFAAGPAQSGILVDREVVSLFLHFTVNPKPRVEFIDRPRCCLRGKECSINRFQQVESRWGYSGTSDRIRFSVNKR
IFVVGFGLYGSIHGPTDYQVNIQIIHTDSNTVLGQNDTGFSCDGSASTFRVMFKEPVEVLPNVNYTACATLKGPDSHYGT
KGLRKVTHESPTTGAKTCFTFCYAAGNNNGTSVEDGQIPEVIFYT
>Hsap_ENSP00000257749
MSVDEKPDSPMYVYESTVHCTNILLGLNDQRKKDILCDVTLIVERKEFRAHRAVLAACSEYFWQALVGQTKNDLVVSLPE
EVTARGFGPLLQFAYTAKLLLSRENIREVIRCAEFLRMHNLEDSCFSFLQTQLLNSEDGLFVCRKDAACQRPHEDCENSA
GEEEDEEEETMDSETAKMACPRDQMLPEPISFEAAAIPVAEKEEALLPEPDVPTDTKESSEKDALTQYPRYKKYQLACTK
NVYNASSHSTSGFASTFREDNSSNSLKPGLARGQIKSEPPSEENEEESITLCLSGDEPDAKDRAGDVEMDRKQPSPAPTP

TAPAGAACLERSRSVASPSCLRSLFSITKSVELSGLPSTSQQHFARSPACPFDKGITQGDLKTDYTPFTGNYGQPHVGQK
EVSNFTMGSPLRGPGLEALCKQEGELDRRSVIFSSSACDQVSTSVHSYSGVSSLDKDLSEPVPKGLWVGAGQSLPSSQAY
SHGGLMADHLPGRMRPNTSCPVPIKVCPRSPPLETRTRTSSSCSSYSYAEDGSGGSPCSLPLCEFSSSPCSQGARFLATE
HQEPGLMGDGMYNQVRPQIKCEQSYGTNSSDESGSFSEADSESCPVQDRGQEVKLPFPVDQITDLPRNDFQMMIKMHKLT
SEQLEFIHDVRRRSKNRIAAQRCRKRKLDCIQNLECEIRKLVCEKEKLLSERNQLKACMGELLDNFSCLSQEVCRDIQSP
EQIQALHRYCPVLRPMDLPTASSINPAPLGAEQNIAASQCAVGENVPCCLEPGAAPPGPPWAPSNTSENCTSGRRLEGTD
PGTFSERGPPLEPRSQTVTVDFCQEMTDKCTTDEQPRKDYT
>Hsap_ENSP00000258646
MVDVGKWPIFTLLSPQEIASIRKACVFGTSASEALYVTDNDEVFVFGLNYSNCLGTGDNQSTLVPKKLEGLCGKKIKSLS
YGSGPHVLLSTEDGVVYAWGHNGYSQLGNGTTNQGIAPVQVCTNLLIKQVVEVACGSHHSMALAADGEVFAWGYNNCGQV
GSGSTANQPTPRKVTNCLHIKRVVGIACGQTSSMAVLDNGEVYGWGYNGNGQLGLGNNGNQLTPVRVAALHSVCVNQIVC
GYAHTLALTDEGLLYAWGANTYGQLGTGNKNNLLSPAHIMVEKERVVEIAACHSAHTSAAKTQGGHVYMWGQCRGQSVIL
PHLTHFSCTDDVFACFATPAVSWRLLSVEHEDFLTVAESLKKEFDSPETADLKFRIDGKYIHVHKAVLKIRCEHFRSMFQ
SYWNEDMKEVIEIDQFSYPVYRAFLQYLYTDTVDLPPEDAIGLLDLATSYCENRLKKLCQHIIKRGITVENAFSLFSAAV
RYDAEDLEEFCFKFCINHLTEVTQTAAFWQMDGPLLKEFIAKASKCGAFKN
>Hsap_ENSP00000261683
MDTASHSLVLLQQLNMQREFGFLCDCTVAIGDVYFKAHRAVLAAFSNYFKMIFIHQTSECIKIQPTDIQPDIFSYLLHIM
YTGKGPKQIVDHSRLEEGIRFLHADYLSHIATEMNQVFSPETVQSSNLYGIQISTTQKTVVKQGLEVKEAPSSNSGNRAA
VQGDHPQLQLSLAIGLDDGTADQQRACPATQALEEHQKPPVSIKQERCDPESVISQSHPSPSSEVTGPTFTENSVKIHLC
HYCGERFDSRSNLRQHLHTHVSGSLPFGVPASILESNDLGEVHPLNENSEALECRRLSSFIVKENEQQPDHTNRGTTEPL
QISQVSLISKDTEPVELNCNFSFSRKRKMSCTICGHKFPRKSQLLEHMYTHKGKSYRYNRCQRFGNALAQRFQPYCDSWS
DVSLKSSRLSQEHLDLPCALESELTQENVDTILVE
>Hsap_ENSP00000261721
MASLGPAAAGEQASGAEAEPGPAGPPPPPSPSSLGPLLPLQREPLYNWQATKASLKERFAFLFNSELLSDVRFVLGKGRG
AAAAGGPQRIPAHRFVLAAGSAVFDAMFNGGMATTSAEIELPDVEPAAFLALLRFLYSDEVQIGPETVMTTLYTAKKYAV
PALEAHCVEFLTKHLRADNAFMLLTQARLFDEPQLASLCLDTIDKSTMDAISAEGFTDIDIDTLCAVLERDTLSIRESRL
FGAVVRWAEAECQRQQLPVTFGNKQKVLGKALSLIRFPLMTIEEFAAGPAQSGILSDREVVNLFLHFTVNPKPRVEYIDR
PRCCLRGKECCINRFQQVESRWGYSGTSDRIRFTVNRRISIVGFGLYGSIHGPTDYQVNIQIIEYEKKQTLGQNDTGFSC
DGTANTFRVMFKEPIEILPNVCYTACATLKGPDSHYGTKGLKKVVHETPAASKTVFFFFSSPGNNNGTSIEDGQIPEIIF
YT
>Hsap_ENSP00000262630
MSLPPIRLPSPYGSDRLVQLAARLRPALCDTLITVGSQEFPAHSLVLAGVSQQLGRRGQWALGEGISPSTFAQLLNFVYG
ESVELQPGELRPLQEAARALGVQSLEEACWRARGDRAKKPDPGLKKHQEEPEKPSRNPERELGDPGEKQKPEQVSRTGGR
EQEMLHKHSPPRGRPEMAGATQEAQQEQTRSKEKRLQAPVGQRGADGKHGVLTWLRENPGGSEESLRKLPGPLPPAGSLQ
TSVTPRPSWAEAPWLVGGQPALWSILLMPPRYGIPFYHSTPTTGAWQEVWREQRIPLSLNAPKGLWSQNQLASSSPTPGS
LPQGPAQLSPGEMEESDQGHTGALATCAGHEDKAGCPPRPHPPPAPPARSRPYACSVCGKRFSLKHQMETHYRVHTGEKP
FSCSLCPQRSRDFSAMTKHLRTHGAAPYRCSLCGAGCPSLASMQAHMRGHSPSQLPPGWTIRSTFLYSSSRPSRPSTSPC
CPSSSTT
>Hsap_ENSP00000266269
MERVNDASCGPSGCYTYQVSRHSTEMLHNLNQQRKNGGRFCDVLLRVGDESFPAHRAVLAACSEYFESVFSAQLGDGGAA
DGGPADVGGATAAPGGGAGGSRELEMHTISSKVFGDILDFAYTSRIVVRLESFPELMTAAKFLLMRSVIEICQEVIKQSN
VQILVPPARADIMLFRPPGTSDLGFPLDMTNGAALAANSNGIAGSMQPEEEAARAAGAAIAGQASLPVLPGVDRLPMVAG
PLSPQLLTSPFPSVASSAPPLTGKRGRGRPRKANLLDSMFGSPGGLREAGILPCGLCGKVFTDANRLRQHEAQHGVTSLQ
LGYIDLPPPRLGENGLPISEDPDGPRKRSRTRKQVACEICGKIFRDVYHLNRHKLSHSGEKPYSCPVCGLRFKRKDRMSY
HVRSHDGSVGKPYICQSCGKGFSRPDHLNGHIKQVHTSERPHKCQTCNASFATRDRLRSHLACHEDKVPCQVCGKYLRAA
YMADHLKKHSEGPSNFCSICNRGFSSASYLKVHVKTHHGVPLPQVSRHQEPILNGGAAFHCARTYGNKEGQKCSHQDPIE
SSDSYGDLSDASDLKTPEKQSANGSFSCDMAVPKNKMESDGEKKYPCPECGSFFRSKSYLNKHIQKVHVRALGGPLGDLG
PALGSPFSPQQNMSLLESFGFQIVQSAFASSLVDPEVDQQPMGPEGK
>Hsap_ENSP00000272797
MALKGQEDYIYLFKDSTHPVDFLDAFRTFYLDGLFTDITLQCPSGIIFHCHRAVLAACSNYFKAMFTADMKEKFKNKIKL
SGIHHDILEGLVNYAYTSQIEITKRNVQSLLEAADLLQFLSVKKACERFLVRHLDIDNCIGMHSFAEFHVCPELEKESRR
ILCSKFKEVWQQEEFLEISLEKFLFILSRKNLSVWKEEAIIEPVIKWTAHDVENRIECLYNLLSYINIDIDPVYLKTALG
LQRSCLLTENKIRSLIYNALNPMHKEISQRSTATMYIIGGYYWHPLSEVHIWDPLTNVWIQGAEIPDYTRESYGVTCLGP
NIYVTGGYRTDNIEALDTVWIYNSESDEWTEGLPMLNARYYHCAVTLGGCVYALGGYRKGAPAEEAEFYDPLKEKWIPIA
NMIKGVGNATACVLHDVIYVIGGHCGYRGSCTYDKVQSYNSDINEWSLITSSPHPEYGLCSVPFENKLYLVGGQTTITEC
YDPEQNEWREIAPMMERRMECGAVIMNGCIYVTGGYSYSKGTYLQSIEKYDPDLNKWEIVGNLPSAMRSHGCVCVYNV
>Hsap_ENSP00000273963
MASDSMSSKQARNHITKGKRQQQHQQIKNRSSISDGDGEDSFIFEANEAWKDFHGSLLRFYENGELCDVTLKVGSKLISC
HKLVLACVIPYFRAMFLSEMAEAKQTLIEIRDFDGDAIEDLVKFVYSSRLTLTVDNVQPLLYAACILQVELVARACCEYM
KLHFHPSNCLAVRAFAESHNRIDLMDMADQYACDHFTEVVECEDFVSVSPQHLHKLLSSSDLNIENEKQVYNAAIKWLLA
NPQHHSKWLDETLAQVRLPLLPVDFLMGVVAKEQIVKQNLKCRDLLDEARNYHLHLSSRAVPDFEYSIRTTPRKHTAGVL
FCVGGRGGSGDPFRSIECYSINKNSWFFGPEMNSRRRHVGVISVEGKVYAVGGHDGNEHLGSMEMFDPLTNKWMMKASMN

TKRRGIALASLGGPIYAIGGLDDNTCFNDVERYDIESDQWSTVAPMNTPRGGVGSVALVNHVYAVGGNDGMASLSSVERY
DPHLDKWIEVKEMGQRRAGNGVSKLHGCLYVVGGFDDNSPLSSVERYDPRSNKWDYVAALTTPRGGVGIATVMGKIFAVG
GHNGNAYLNTVEAFDPVLNRWELVGSVSHCRAGAGVAVCSCLTSQIRDVGHGSNNVVDCM
>Hsap_ENSP00000277554
MSQMLHIEIPNFGNTVLGCLNEQRLLGLYCDVSIVVKGQAFKAHRAVLAASSLYFRDLFSGNSKSAFELPGSVPPACFQQ
ILSFCYTGRLTMTASEQLVVMYTAGFLQIQHIVERGTDLMFKVSSPHCDSQTAVIEDAGSEPQSPCNQLQPAAAAAAPYV
VSPSVPIPLLTRVKHEAMELPPAGPGLAPKRPLETGPRDGVAVAAGAAVAAGTAPLKLPRVSYYGVPSLATLIPGIQQMP
YPQGERTSPGASSLPTTDSPTSYHNEEDEEDDEAYDTMVEEQYGQMYIKASGSYAVQEKPEPVPLESRSCVLIRRDLVAL
PASLISQIGYRCHPKLYSEGDPGEKLELVAGSGVYITRGQLMNCHLCAGVKHKVLLRRLLATFFDRNTLANSCGTGIRSS
TSDPSRKPLDSRVLNAVKLYCQNFAPSFKESEMNVIAADMCTNARRVRKRWLPKIKSMLPEGVEMYRTVMGSAAASVPLD
PEFPPAAAQVFEQRIYAERRGDAATIVALRTDAVNVDLSAAANPAFDAGEEVDGAGSVIQEVAAPEPLPADGQSPPQPFE
QGGGGPSRPQTPAAAARRPEGTYAGTL
>Hsap_ENSP00000280098
MSREPTPPLPGDMSTGPIAESWCYTQVKVVKFSYMWTINNFSFCREEMGEVLKSSTFSSGPSDKMKWCLRVNPKGLDDES
KDYLSLYLLLVSCPKSEVRAKFKFSLLNAKREETKAMESQRAYRFVQGKDWGFKKFIRRDFLLDEANGLLPDDKLTLFCE
VSVVQDSVNISGHTNTNTLKVPECRLAEDLGNLWENTRFTDCSFFVRGQEFKAHKSVLAARSPVFNAMFEHEMEESKKNR
VEINDLDPEVFKEMMRFIYTGRAPNLDKMADNLLAAADKYALERLKVMCEEALCSNLSVENVADTLVLADLHSAEQLKAQ
AIDFINRCSVLRQLGCKDGKNWNSNQATDIMETSGWKSMIQSHPHLVAEAFRALASAQCPQFGIPRKRLKQS
>Hsap_ENSP00000280758
MARRGKKPVVRTLEDLTLDSGYGGAADSVRSSNLSLCCSDSHPASPYGGSCWPPLADSMHSRHNSFDTVNTALVEDSEGL
DCAGQHCSRLLPDLDEVPWTLQELEALLLRSRDPRAGPAVPGGLPKDALAKLSTLVSRALVRIAKEAQRLSLRFAKCTKY
EIQSAMEIVLSWGLAAHCTAAALAALSLYNMSSAGGDRLGRGKSARCGLTFSVGRVYRWMVDSRVALRIHEHAAIYLTAC
MESLFRDIYSRVVASGVPRSCSGPGSGSGSGPGPSSGPGAAPAADKEREAPGGGAASGGACSAASSASGGSSCCAPPAAA
AAAVPPAAAANHHHHHHHALHEAPKFTVETLEHTVNNDSEIWGLLQPYQHLICGKNASGVLCLPDSLNLHRDPQRSNKPG
ELPMFSQSELRTIEQSLLATRVGSIAELSDLVSRAMHHLQPLNAKHHGNGTPLHHKQGALYWEPEALYTLCYFMHCPQME
WENPNVEPSKVNLQVERPFLVLPPLMEWIRVAVAHAGHRRSFSMDSDDVRQAARLLLPGVDCEPRQLRADDCFCASRKLD
AVAIEAKFKQDLGFRMLNCGRTDLVKQAVSLLGPDGINTMSEQGMTPLMYACVRGDEAMVQMLLDAGADLNVEVVSTPHK
YPSVHPETRHWTALTFAVLHGHIPVVQLLLDAGAKVEGSVEHGEENYSETPLQLAAAVGNFELVSLLLERGADPLIGTMY
RNGISTTPQGDMNSFSQAAAHGHRNVFRKLLAQPEKEKSDILSLEEILAEGTDLAETAPPPLCASRNSKAKLRALREAMY
HSAEHGYVDVTIDIRSIGVPWTLHTWLESLRIAFQQHRRPLIQCLLKEFKTIQEEEYTEELVTQGLPLMFEILKASKNEV
ISQQLCVIFTHCYGPYPIPKLTEIKRKQTSRLDPHFLNNKEMSDVTFLVEGRPFYAHKVLLFTASPRFKALLSSKPTNDG
TCIEIGYVKYSIFQLVMQYLYYGGPESLLIKNNEIMELLSAAKFFQLEALQRHCEIICAKSINTDNCVDIYNHAKFLGVT
ELSAYCEGYFLKNMMVLIENEAFKQLLYDKNGEGTGQDVLQDLQRTLAIRIQSIHLSSSKGSVV
>Hsap_ENSP00000282570
MGSLSSRVLRQPRPALAQQAQGARAGGSARRPDTGDDAAGHGFCYCAGSHKRKRSSGSFCYCHPDSETDEDEEEGDEQQR
LLNTPRRKKLKSTSKYIYQTLFLNGENSDIKICALGEEWSLHKIYLCQSGYFSSMFSGSWKESSMNIIELEIPDQNIDVE
ALQVAFGSLYRDDVLIKPSRVVAILAAACLLQLDGLIQQCGETMKETVNVKTVCGYYTSAGTYGLDSVKKKCLEWLLNNL
MTHQNVELFKELSINVMKQLIGSSNLFVMQVEMDIYTALKKWMFLQLVPSWNGSLKQLLTETDVWFSKQRKDFEGMAFLE
TEQGKPFVSVFRHLRLQYIISDLASARIIEQDAVVPSEWLSSVYKQQWFAMLRAEQDSEVGPQEINKEELEGNSMRCGRK
LAKDGEYCWRWTGFNFGFDLLVTYTNRYIIFKRNTLNQPCSGSVSLQPRRSIAFRLRLASFDSSGKLICSRTTGYQILTL
EKDQEQVVMNLDSRLLIFPLYICCNFLYISPEKKN
>Hsap_ENSP00000284669
MDSQRELAEELRLYQSTLLQDGLKDLLDEKKFIDCTLKAGDKSLPCHRLILSACSPYFREYFLSEIDEAKKKEVVLDNVD
PAILDLIIKYLYSASIDLNDGNVQDIFALASRFQIPSVFTVCVSYLQKRLAPGNCLAILRLGLLLDCPRLAISAREFVSD
RFVQICKEEDFMQLSPQELISVISNDSLNVEKEEAVFEAVMKWVRTDKENRVKNLSEVFDCIRFRLMTEKYFKDHVEKDD
IIKSNPDLQKKIKVLKDAFAGKLPEPSKNAAKTGAGEVNGDVGDEDLLPGYLNDIPRHGMFVKDLILLVNDTAAVAYDPT
ENECYLTALAEQIPRNHSSIVTQQNQIYVVGGLYVDEENKDQPLQSYFFQLDSIASEWVGLPPLPSARCLFGLGEVDDKI
YVVAGKDLQTEASLDSVLCYDPVAAKWNEVKKLPIKVYGHNVISHKGMIYCLGGKTDDKKCTNRVFIFNPKKGDWKDLAP
MKIPRSMFGVAVHKGKIVIAGGVTEDGLSASVEAFDLTTNKWDVMTEFPQERSSISLVSLAGSLYAIGGFAMIQLESKEF
APTEVNDIWKYEDDKKEWAGMLKEIRYASGASCLATRLNLFKLSKL
>Hsap_ENSP00000286800
MSLSENSVFAYESSVHSTNVLLSLNDQRKKDVLCDVTIFVEGQRFRAHRSVLAACSSYFHSRIVGQADGELNITLPEEVT
VKGFEPLIQFAYTAKLILSKENVDEVCKCVEFLSVHNIEESCFQFLKFKFLDSTADQQECPRKKCFSSHCQKTDLKLSLL
DQRDLETDEVEEFLENKNVQTPQCKLRRYQGNAKASPPLQDSASQTYESMCLEKDAALALPSLCPKYRKFQKAFGTDRVR
TGESSVKDIHASVQPNERSENECLGGVPECRDLQVMLKCDESKLAMEPEETKKDPASQCPTEKSEVTPFPHNSSIDPHGL
YSLSLLHTYDQYGDLNFAGMQNTTVLTEKPLSGTDVQEKTFGESQDLPLKSDLGTREDSSVASSDRSSVEREVAEHLAKG
FWSDICSTDTPCQMQLSPAVAKDGSEQISQKRSECPWLGIRISESPEPGQRTFTTLSSVNCPFISTLSTEGCSSNLEIGN
DDYVSEPQQEPCPYACVISLGDDSETDTEGDSESCSAREQECEVKLPFNAQRIISLSRNDFQSLLKMHKLTPEQLDCIHD
IRRRSKNRIAAQRCRKRKLDCIQNLESEIEKLQSEKESLLKERDHILSTLGETKQNLTGLCQKVCKEAALSQEQIQILAK
YSAADCPLSFLISEKDKSTPDGELALPSIFSLSDRPPAVLPPCARGNSEPGYARGQESQQMSTATSEQAGPAEQCRQSGG
ISDFCQQMTDKCTTDE
>Hsap_ENSP00000287777

MALGLEQAEEQRLYQQTLLQDGLKDMLDHGKFLDCVVRAGEREFPCHRLVLAACSPYFRARFLAEPERAGELHLEEVSPD
VVAQVLHYLYTSEIALDEASVQDLFAAAHRFQIPSIFTICVSFLQKRLCLSNCLAVFRLGLLLDCARLAVAARDFICAHF
TLVARDADFLGLSADELIAIISSDGLNVEKEEAVFEAVMRWAGSGDAEAQAERQRALPTVFESVRCRLLPRAFLESRVER
HPLVRAQPELLRKVQMVKDAHEGRITTLRKKKKGKDGAGAKEADKGTSKAKAEEDEEAERILPGILNDTLRFGMFLQDLI
FMISEEGAVAYDPAANECYCASLSNQVPKNHVSLVTKENQVFVAGGLFYNEDNKEDPMSAYFLQFDHLDSEWLGMPPLPS
PRCLFGLGEALNSIYVVGGREIKDGERCLDSVMCYDRLSFKWGESDPLPYVVYGHTVLSHMDLVYVIGGKGSDRKCLNKM
CVYDPKKFEWKELAPMQTARSLFGATVHDGRIIVAAGVTDTGLTSSAEVYSITDNKWAPFEAFPQERSSLSLVSLVGTLY
AIGGFATLETESGELVPTELNDIWRYNEEEKKWEGVLREIAYAAGATFLPVRLNVLCLTKM
>Hsap_ENSP00000292431
MAQTLQMEIPNFGNSILECLNEQRLQGLYCDVSVVVKGHAFKAHRAVLAASSSYFRDLFNNSRSAVVELPAAVQPQSFQQ
ILSFCYTGRLSMNVGDQFLLMYTAGFLQIQEIMEKGTEFFLKVSSPSCDSQGLHAEEAPSSEPQSPVAQTSGWPACSTPL
PLVSRVKTEQQESDSVQCMPVAKRLWDSGQKEAGGGGNGSRKMAKFSTPDLAANRPHQPPPPQQAPVVAAAQPAVAAGAG
QPAGGVAAAGGVVSGPSTSERTSPGTSSAYTSDSPGSYHNEEDEEEDGGEEGMDEQYRQICNMYTMYSMMNVGQTAEKVE
ALPEQVAPESRNRIRVRQDLASLPAELINQIGNRCHPKLYDEGDPSEKLELVTGTNVYITRAQLMNCHVSAGTRHKVLLR
RLLASFFDRNTLANSCGTGIRSSTNDPRRKPLDSRVLHAVKYYCQNFAPNFKESEMNAIAADMCTNARRVVRKSWMPKVK
VLKAEDDAYTTFISETGKIEPDMMGVEHGFETASHEGEAGPSAEALQ
>Hsap_ENSP00000293303
MEMESAAASTRFHQPHMERKMSAMACEIFNELRLEGKLCDVVIKVNGFEFSAHKNILCSCSSYFRALFTSGWNNTEKKVY
NIPGISPDMMKLIIEYAYTRTVPITPDNVEKLLAAADQFNIMGIVRGCCEFLKSELCLDNCIGICKFTDYYYCPELRQKA
YMFILHNFEEMVKVSAEFLELSVTELKDIIEKDELNVKQEDAVFEAILKWISHDPQNRKQHISILLPKVRLALMHAEYFM
NNVKMNDYVKDSEECKPVIINALKAMYDLNMNGPSNSDFTNPLTRPRLPYAILFAIGGWSGGSPTNAIEAYDARADRWVN
VTCEEESPRAYHGAAYLKGYVYIIGGFDSVDYFNSVKRFDPVKKTWHQVAPMHSRRCYVSVTVLGNFIYAMGGFDGYVRL
NTAERYEPETNQWTLIAPMHEQRSDASATTLYGKVYICGGFNGNECLFTAEVYNTESNQWTVIAPMRSRRSGIGVIAYGE
HVYAVGGFDGANRLRSAEAYSPVANTWRTIPTMFNPRSNFGIEVVDDLLFVVGGFNGFTTTFNVECYDEKTDEWYDAHDM
SIYRSALSCCVVPGLANVEEYAARRDNFPGLALRDEVKYSASTSTLPV
>Hsap_ENSP00000293805
MGSPAAPEGALGYVREFTRHSSDVLGNLNELRLRGILTDVTLLVGGQPLRAHKAVLIACSGFFYSIFRGRAGVGVDVLSL
PGGPEARGFAPLLDFMYTSRLRLSPATAPAVLAAATYLQMEHVVQACHRFIQASYEPLGISLRPLEAEPPTPPTAPPPGS
PRRSEGHPDPPTESRSCSQGPPSPASPDPKACNWKKYKYIVLNSQASQAGSLVGERSSGQPCPQARLPSGDEASSSSSSS
SSSSEEGPIPGPQSRLSPTAATVQFKCGAPASTPYLLTSQAQDTSGSPSERARPLPGSEFFSCQNCEAVAGCSSGLDSLV
PGDEDKPYKCQLCRSSFRYKGNLASHRTVHTGEKPYHCSICGARFNRPANLKTHSRIHSGEKPYKCETCGSRFVQVAHLR
AHVLIHTGEKPYPCPTCGTRFRHLQTLKSHVRIHTGEKPYHCDPCGLHFRHKSQLRLHLRQKHGAATNTKVHYHILGGP
>Hsap_ENSP00000294008
MKLSVNEAQLGFYLGSLSHLSACPGIDPRSSEDQPESLKTGQMMDESDEDFKELCASFFQRVKKHGIKEVSGERKTQKAA
SNGTQIRSKLKRTKQTATKTKTLQGPAEKKPPSGSQAPRTKKQRVTKWQASEPAHSVNGEGGVLASAPDPPVLRETAQNT
QTGNQQEPSPNLSREKTRENVPNSDSQPPPSCLTTAVPSPSKPRTAQLVLQRMQQFKRADPERLRHASEECSLEAAREEN
VPKDPQEEMMAGNVYGLGPPAPESDAAVALTLQQEFARVGASAHDDSLEEKGLFFCQICQKNLSAMNVTRREQHVNRCLD
EAEKTLRPSVPQIPECPICGKPFLTLKSRTSHLKQCAVKMEVGPQLLLQAVRLQTAQPEGSSSPPMFSFSDHSRGLKRRG
PTSKKEPRKRRKVDEAPSEDLLVAMALSRSEMEPGAAVPALRLESAFSERIRPEAENKSRKKKPPVSPPLLLVQDSETTG
RQIEDRVALLLSEEVELSSTPPLPASRILKEGWERAGQCPPPPERKQSFLWEGSALTGAWAMEDFYTARLVPPLVPQRPA
QGLMQEPVPPLVPPEHSELSERRSPALHGTPTAGCGSRGPSPSASQREHQALQDLVDLAREGLSASPWPGSGGLAGSEGT
AGLDVVPGGLPLTGFVVPSQDKHPDRGGRTLLSLGLLVADFGAMVNNPHLSDVQFQTDSGEVLYAHKFVLYARCPLLIQY
VNNEGFSAVEDGVLTQRVLLGDVSTEAARTFLHYLYTADTGLPPGLSSELSSLAHRFGVSELVHLCEQVPIATDSEGKPW
EEKEAENCESRAENFQELLRSMWADEEEEAETLLKSKDHEEDQENVNEAEMEEIYEFAATQRKLLQEERAAGAGEDADWL
EGGSPVSGQLLAGVQVQKQWDKVEEMEPLEPGRDEAATTWEKMGQCALPPPQGQHSGARGAEAPEQEAPEEALGHSSCSS
PSRDCQAERKEGSLPHSDDAGDYEQLFSSTQGEISEPSQITSEPEEQSGAVRERGLEVSHRLAPWQASPPHPCRFLLGPP
QGGSPRGSHHTSGSSLSTPRSRGGTSQVGSPTLLSPAVPSKQKRDRSILTLSKEPGHQKGKERRSVLECRNKGVLMFPEK
SPSIDLTQSNPDHSSSRSQKSSSKLNEEDEVILLLDSDEELELEQTKMKSISSDPLEEKKALEISPRSCELFSIIDVDAD
QEPSQSPPRSEAVLQQEDEGALPENRGSLGRRGAPWLFCDRESSPSEASTTDTSWLVPATPLASRSRDCSSQTQISSLRS
GLAVQAVTQHTPRASVGNREGNEVAQKFSVIRPQTPPPQTPSSCLTPVSPGTSDGRRQGHRSPSRPHPGGHPHSSPLAPH
PISGDRAHFSRRFLKHSPPGPSFLNQTPAGEVVEVGDSDDEQEVASHQANRSPPLDSDPPIPIDDCCWHMEPLSPIPIDH
WNLERTGPLSTSSPSRRMNEAADSRDCRSPGLLDTTPIRGSCTTQRKLQEKSSGAGSLGNSRPSFLNSALWDVWDGEEQR
PPETPPPAQMPSAGGAQKPEGLETPKGANRKKNLPPKVPITPMPQYSIMETPVLKKELDRFGVRPLPKRQMVLKLKEIFQ
YTHQTLDSDSEDESQSSQPLLQAPHCQTLASQTYKPSRAGVHAQQEATTGPGAHRPKGPAKTKGPRHQRKHHESITPPSR
SPTKEAPPGLNDDAQIPASQESVATSVDGSDSSLSSQSSSSCEFGAAFESAGEEEGEGEVSASQAAVQAADTDEALRCYI
RSKPALYQKVLLYQPFELRELQAELRQNGLRVSSRRLLDFLDTHCITFTTAATRREKLQGRRRQPRGKKKVERN
>Hsap_ENSP00000300101
MGMRIKLQSTNHPNNLLKELNKCRLSETMCDVTIVVGSRSFPAHKAVLACAAGYFQNLFLNTGLDAARTYVVDFITPANF
EKVLSFVYTSELFTDLINVGVIYEVAERLGMEDLLQACHSTFPDLESTARAKPLTSTSESHSGTLSCPSAEPAHPLGELR
GGGDYLGADRNYVLPSDAGGSYKEEEKNVASDANHSLHLPQPPPPPPKTEDHDTPAPFTSIPSMMTQPLLGTVSTGIQTS
TSSCQPYKVQSNGDFSKNSFLTPDNAVDITTGTNSCLSNSEHSKDPGFGQMDELQLEDLGDDDLQFEDPAEDIGTTEEVI
ELSDDSEDELAFGENDNRENKAMPCQVCKKVLEPNIQLIRQHARDHVDLLTGNCKVCETHFQDRNSRVTHVLSHIGIFLF

SCDMCETKFFTQWQLTLHRRDGIFENNIIVHPNDPLPGKLGLFSGAASPELKCAACGKVLAKDFHVVRGHILDHLNLKGQ
ACSVCDQRHLNLCSLMWHTLSHLGISVFSCSVCANSFVDWHLLEKHMAVHQSLEDALFHCRLCSQSFKSEAAYRYHVSQH
KCNSGLDARPGFGLQHPALQKRKLPAEEFLGEELALQGQPGNSKYSCKVCGKRFAHTSEFNYHRRIHTGEKPYQCKVCHK
FFRGRSTIKCHLKTHSGALMYRCTVCGHYSSTLNLMSKHVGVHKGSLPPDFTIEQTFMYIIHSKEADKNPDS
>Hsap_ENSP00000300976
MAESGGSSGGAGGGGAFGAGPGPERPNSTADKNGALKCTFSAPSHSTSLLQGLATLRAQGQLLDVVLTINREAFPAHKVV
LAACSDYFRAMFTGGMREASQDVIELKGVSARGLRHIIDFAYSAEVTLDLDCVQDVLGAAVFLQMLPVVELCEEFLKAAM
SVETCLNIGQMATTFSLASLRESVDAFTFRHFLQIAEEEDFLRLPLERLVFFLQSNRLQSCAEIDLFRAAVRWLQHDPAR
RPRASHVLCHIRFPLMQSSELVDSVQTLDIMVEDVLCRQYLLEAFNYQVLPFRQHEMQSPRTAVRSDVPSLVTFGGTPYT
DSDRSVSSKVYQLPEPGARHFRELTEMEVGCSHTCVAVLDNFVYVAGGQHLQYRSGEGAVDACYRYDPHLNRWLRLQAMQ
ESRIQFQLNVLCGMVYATGGRNRAGSLASVERYCPRRNEWGYACSLKRRTWGHAGAASGGRLYISGGYGISVEDKKALHC
YDPVADQWEFKAPMSEPRVLHAMVGAGGRIYALGGRMDHVDRCFDVLAVEYYVPETDQWTSVSPMRAGQSEAGCCLLERK
IYIVGGYNWRLNNVTGIVQVYNTDTDEWERDLHFPESFAGIACAPVLLPRAGTRR
>Hsap_ENSP00000302586
MSTQDERQINTEYAVSLLEQLKLFYEQQLFTDIVLIVEGTEFPCHKMVLATCSSYFRAMFMSGLSESKQTHVHLRNVDAA
TLQIIITYAYTGNLAMNDSTVEQLYETACFLQVEDVLQRCREYLIKKINAENCVRLLSFADLFSCEELKQSAKRMVEHKF
TAVYHQDAFMQLSHDLLIDILSSDNLNVEKEETVREAAMLWLEYNTESRSQYLSSVLSQIRIDALSEVTQRAWFQGLPPN
DKSVVVQGLYKSMPKFFKPRLGMTKEEMMIFIEASSENPCSLYSSVCYSPQAEKVYKLCSPPADLHKVGTVVTPDNDIYI
AGGQVPLKNTKTNHSKTSKLQTAFRTVNCFYWFDAQQNTWFPKTPMLFVRIKPSLVCCEGYIYAIGGDSVGGELNRRTVE
RYDTEKDEWTMVSPLPCAWQWSAAVVVHDCIYVMTLNLMYCYFPRSDSWVEMAMRQTSRSFASAAAFGDKIFYIGGLHIA
TNSGIRLPSGTVDGSSVTVEIYDVNKNEWKMAANIPAKRYSDPCVRAVVISNSLCVFMRETHLNERAKYVTYQYDLELDR
WSLRQHISERVLWDLGRDFRCTVGKLYPSCLEESPWKPPTYLFSTDGTEEFELDGEMVALPPV
>Hsap_ENSP00000305721
MSSPMPDCTSKCRSLKHALDVLSVVTKGSENQIKAFLSSHCYNAATIKDVFGRNALHLVSSCGKKGVLDWLIQKGVDLLV
KDKESGWTALHRSIFYGHIDCVWSLLKHGVSLYIQDKEGLSALDLVMKDRPTHVVFKNTDPTDVYTWGDNTNFTLGHGSQ
NSKHHPELVDLFSRSGIYIKQVVLCKFHSVFLSQKGQVYTCGHGPGGRLGHGDEQTCLVPRLVEGLNGHNCSQVAAAKDH
TVVLTEDGCVYTFGLNIFHQLGIIPPPSSCNVPRQIQAKYLKGRTIIGVAAGRFHTVLWTREAVYTMGLNGGQLGCLLDP
NGEKCVTAPRQVSALHHKDIALSLVAASDGATVCVTTRGDIYLLADYQCKKMASKQLNLKKVLVSGGHMEYKVDPEHLKE
NGGQKICILAMDGAGRVFCWRSVNSSLKQCRWAYPRQVFISDIALNRNEILFVTQDGEGFRGRWFEEKRKSSEKKEILSN
LHNSSSDVSYVSDINSVYERIRLEKLTFAHRAVSVSTDPSGCNFAILQSDPKTSLYEIPAVSSSSFFEEFGKLLREADEM
DSIHDVTFQVGNRLFPAHKYILAVHSDFFQKLFLSDGNTSEFTDIYQKDEDSAGCHLFVVEKVHPDMFEYLLQFIYTDTC
DFLTHGFKPRIHLNKNPEEYQGTLNSHLNKVNFHEDDNQKSAFEVYKSNQAQTVSERQKSKPKSCKKGKNIREDDPVRML
QTVAKKFDFSNLSSRLDGVRFENEKINVIAKNTGNKLKLSQKKCSFLCDVTMKSVDGKEFPCHKCVLCARLEYFHSMLSS
SWIEASSCAALEMPIHSDILKVILDYLYTDEAVVIKESQNVDFICSVLVVADQLLITRLKEICEVALTEKLTLKNAAMLL
EFAAMYSAKQLKLSCLQFIGLNMAALLEARSLDVLSDGVLKDLSEFYRKMIPAMDRRVITPYQDGPDISYLEVEDGDIFL
KEEINMEQNHSETMFKKAKTKAKKKPRKRSDSSGGYNLSDIIQSPSSTGLLKSGKTNSVESLPELLTSDSEGSYAGVGSP
RDLQSPDFTTGFHSDKIEAKVKPYVNGTSPVYSREDLKPWEKSPILKISAPQPIPSNRIDTTSSASWVAGSFSPVSPPVV
DLRTIMEIEESRQKCGATPKSHLGKTVSHGVKLSQKQRKMIALTTKENNSGMNSMETVLFTPSKAPKPVNAWASSLHSVS
SKSFRDFLLEEKKSVTSHSSGDHVKKVSFKGIENSQAPKIVRCSTHGTPGPEGNHISDLPLLDSPNPWLSSSVTAPSMVA
PVTFASIVEEELQQEAALIRSREKPLALIQIEEHAIQDLLVFYEAFGNPEEFVIVERTPQGPLAVPMWNKHGC
>Hsap_ENSP00000306356
MSVSVHENRKSRASSGSINIYLFHKSSYADSVLTHLNLLRQQRLFTDVLLHAGNRTFPCHRAVLAACSRYFEAMFSGGLK
ESQDSEVNFDNSIHPEVLELLLDYAYSSRVIINEENAESLLEAGDMLEFQDIRDACAEFLEKNLHPTNCLGMLLLSDAHQ
CTKLYELSWRMCLSNFQTIRKNEDFLQLPQDMVVQLLSSEELETEDERLVYESAINWISYDLKKRYCYLPELLQTVRLAL
LPAIYLMENVAMEELITKQRKSKEIVEEAIRCKLKILQNDGVVTSLCARPRKTGHALFLLGGQTFMCDKLYLVDQKAKEI
IPKADIPSPRKEFSACAIGCKVYITGGRGSENGVSKDVWVYDTLHEEWSKAAPMLVARFGHGSAELKHCLYVVGGHTAAT
GCLPASPSVSLKQVEHYDPTINKWTMVAPLREGVSNAAVVSAKLKLFAFGGTSVSHDKLPKVQCYDQCENRWTVPATCPQ
PWRYTAAAVLGNQIFIMGGDTEFSACSAYKFNSETYQWTKVGDVTAKRMSCHAVASGNKLYVVGGYFGIQRCKTLDCYDP
TLDVWNSITTVPYSLIPTAFVSTWKHLPS
>Hsap_ENSP00000307604
MDFPGHFEQIFQQLNYQRLHGQLCDCVIVVGNRHFKAHRSVLAACSTHFRALFSVAEGDQTMNMIQLDSEVVTAEAFAAL
IDMMYTSTLMLGESNVMDVLLAASHLHLNSVVKACKHYLTTRTLPMSPPSERVQEQSARMQRSFMLQQLGLSIVSSALNS
SQNGEEQPAPMSSSMRSNLDQRTPFPMRRLHKRKQSAEERARQRLRPSIDESAISDVTPENGPSGVHSREEFFSPDSLKI
VDNPKADGMTDNQEDSAIMFDQSFGTQEDAQVPSQSDNSAGNMAQLSMASRATQVETSFDQEAAPEKSSFQCENPEVGLG
EKEHMRVVVKSEPLSSPEPQDEVSDVTSQAEGSESVEVEGVVVSAEKIDLSPESSDRSFSDPQSSTDRVGDIHILEVTNN
LEHKSTFSISNFLNKSRGNNFTANQNNDDNIPNTTSDCRLESEAPYLLSPEAGPAGGPSSAPGSHVENPFSEPADSHFVR
PMQEVMGLPCVQTSGYQGGEQFGMDFSRSGLGLHSSFSRVMIGSPRGGASNFPYYRRIAPKMPVVTSVRSSQIPENSTSS
QLMMNGATSSFENGHPSQPGPPQLTRASADVLSKCKKALSEHNVLVVEGARKYACKICCKTFLTLTDCKKHIRVHTGEKP
YACLKCGKRFSQSSHLYKHSKTTCLRWQSSNLPSTLL
>Hsap_ENSP00000307858
MPPPAEVTDPSHAPAVLRQLNEQRLRGLFCDVTLIAGDTKFPAHRSVLAASSPFFREALLTSAPLPLPPATGGAAPNPAT
TTAASSSSSSSSSSSSSSSSASSSSSSSSSSPPPASPPASSPPRVLELPGVPAAAFSDVLNFIYSARLALPGGGGDGAAV

AEIGALGRRLGISRLQGLGEGGDAWVPPTPAPMATSQPEEDSFGPGPRPAGEWEGDRAEAQAPDLQCSLPRRPLPCPQCG
KSFIHPKRLQTHEAQCRRGASTRGSTGLGAGGAGPGGPAGVDASALPPPVGFRGGPEHVVKVVGGHVLYVCAACERSYVT
LSSLKRHSNVHSWRRKYPCRYCEKVFALAEYRTKHEVWHTGERRYQCIFCWETFVTYYNLKTHQRAFHGISPGLLASEKT
PNGGYKPKLNTLKLYRLLPMRAAKRPYKTYSQGAPEAPLSPTLNTPAPVAMPASPPPGPPPAPEPGPPPSVITFAHPAPS
VIVHGGSSSGGGGSGTASTGGSQAASVITYTAPPRPPKKREYPPPPPEPAATPTSPATAVSPATAAGPAMATTTEEAKGR
NPRAGRTLTYTAKPVGGIGGGGGPPTGAGRGPSQLQAPPPLCQITVRIGEEAIVKRRISETDLRPGELSGEEMEESEEDE
EEEDEEEEEEDEEESKAGGEDQLWRPYYSYKPKRKAGAAGGASVGGSGLPRGRRPPRWRQKLERRSWEETPAAESPAGRA
RTERRHRCGDCAQTFTTLRKLRKHQEAHGGGSHSSRAGRRPSTRFTCPHCAKVCKTAAALSRHGQRHAAERPGGTPTPVI
AYSKGSAGTRPGDVKEEAPQEMQVSSSSGEAGGGSTAAEEASETASLQDPIISGGEEPPVVASGGSYVYPPVQEFPLALI
GGGREPGGGRGKSGSEGPVGAGEGDRMEGIGAAKVTFYPEPYPLVYGPQLLAAYPYNFSNLAALPVALNMVLPDEKGAGA
LPFLPGVFGYAVNPQAAPPAPPTPPPPTLPPPIPPKGEGERAGVERTQKGDVG
>Hsap_ENSP00000308759
MEGLLHYINPAHAISLLSALNEERLKGQLCDVLLIVGDQKFRAHKNVLAASSEYFQSLFTNKENESQTVFQLDFCEPDAF
DNVLNYIYSSSLFVEKSSLAAVQELGYSLGISFLTNIVSKTPQAPFPTCPNRKKVFVEDDENSSQKRSVIVCQSRNEAQG
KTVSQNQPDVSHTSRPSPSIAVKANTNKPHVPKPIEPLHNLSLTEKSWPKDSSVVYAKSLEHSGSLDDPNRISLVKRNAV
LPSKPLQDREAMDDKPGVSGQLPKGKALELALKRPRPPVLSVCSSSETPYLLKETNKGNGQGEDRNLLYYSKLGLVIPSS
GSGSGNQSIDRSGPLVKSLLRRSLSMDSQVPVYSPSIDLKSSQGSSSVSSDAPGNVLCALSQKSSLKDCSEKTALDDRPQ
VLQPHRLRSFSASQSTDREGASPVTEVRIKTEPSSPLSDPSDIIRVTVGDAATTAAASSSSVTRDLSLKTEDDQKDMSRL
PAKRRFQADRRLPFKKLKVNEHGSPVSEDNFEEGSSPTLLDADFPDSDLNKDEFGELEGTRPNKKFKCKHCLKIFRSTAG
LHRHVNMYHNPEKPYACDICHKRFHTNFKVWTHCQTQHGIVKNPSPASSSHAVLDEKFQRKLIDIVREREIKKALIIKLR
RGKPGFQGQSSSQAQQVIKRNLRSRAKGAYICTYCGKAYRFLSQFKQHIKMHPGEKPLGVNKVAKPKEHAPLASPVENKE
VYQCRLCNAKLSSLLEQGSHERLCRNAAVCPYCSLRFFSPELKQEHESKCEYKKLTCLECMRTFKSSFSIWRHQVEVHNQ
NNMAPTENFSLPVLDHNGDVTGSSRPQSQPEPNKVNHIVTTKDDNVFSDSSEQVNFDSEDSSCLPEDLSLSKQLKIQVKE
EPVEEAEEEAPEASTAPKEAGPSKEASLWPCEKCGKMFTVHKQLERHQELLCSVKPFICHVCNKAFRTNFRLWSHFQSHM
SQASEESAHKESEVCPVPTNSPSPPPLPPPPPLPKIQPLEPDSPTGLSENPTPATEKLFVPQESDTLFYHAPPLSAITFK
RQFMCKLCHRTFKTAFSLWSHEQTHN
>Hsap_ENSP00000312397
MEGESVKLSSQTLIQAGDDEKNQRTITVNPAHMGKAFKVMNELRSKQLLCDVMIVAEDVEIEAHRVVLAACSPYFCAMFT
GDMSESKAKKIEIKDVDGQTLSKLIDYIYTAEIEVTEENVQVLLPAASLLQLMDVRQNCCDFLQSQLHPTNCLGIRAFAD
VHTCTDLLQQANAYAEQHFPEVMLGEEFLSLSLDQVCSLISSDKLTVSSEEKVFEAVISWINYEKETRLEHMAKLMEHVR
LPLLPRDYLVQTVEEEALIKNNNTCKDFLIEAMKYHLLPLDQRLLIKNPRTKPRTPVSLPKVMIVVGGQAPKAIRSVECY
DFEEDRWDQIAELPSRRCRAGVVFMAGHVYAVGGFNGSLRVRTVDVYDGVKDQWTSIASMQERRSTLGAAVLNDLLYAVG
GFDGSTGLASVEAYSYKTNEWFFVAPMNTRRSSVGVGVVEGKLYAVGGYDGASRQCLSTVEQYNPATNEWIYVADMSTRR
SGAGVGVLSGQLYATGGHDGPLVRKSVEVYDPGTNTWKQVADMNMCRRNAGVCAVNGLLYVVGGDDGSCNLASVEYYNPV
TDKWTLLPTNMSTGRSYAGVAVIHKSL
>Hsap_ENSP00000314080
MTFPEADILLKSGECAGQTMLDTMEAPGHSRQLLLQLNNQRTKGFLCDVIIVVQNALFRAHKNVLAASSAYLKSLVVHDN
LLNLDHDMVSPAVFRLVLDFIYTGRLADGAEAAAAAAVAPGAEPSLGAVLAAASYLQIPDLVALCKKRLKRHGKYCHLRG
GGGGGGGYAPYGRPGRGLRAATPVIQACYPSPVGPPPPPAAEPPSGPEAAVNTHCAELYASGPGPAAALCASERRCSPLC
GLDLSKKSPPGSAAPERPLAERELPPRPDSPPSAGPAAYKEPPLALPSLPPLPFQKLEEAAPPSDPFRGGSGSPGPEPPG
RPDGPSLLYRWMKHEPGLGSYGDELGRERGSPSERCEERGGDAAVSPGGPPLGLAPPPRYPGSLDGPGAGGDGDDYKSSS
EETGSSEDPSPPGGHLEGYPCPHLAYGEPESFGDNLYVCIPCGKGFPSSEQLNAHVEAHVEEEEALYGRAEAAEVAAGAA
GLGPPFGGGGDKVAGAPGGLGELLRPYRCASCDKSYKDPATLRQHEKTHWLTRPYPCTICGKKFTQRGTMTRHMRSHLGL
KPFACDACGMRFTRQYRLTEHMRIHSGEKPYECQVCGGKFAQQRNLISHMKMHAVGGAAGAAGALAGLGGLPGVPGPDGK
GKLDFPEGVFAVARLTAEQLSLKQQDKAAAAELLAQTTHFLHDPKVALESLYPLAKFTAELGLSPDKAAEVLSQGAHLAA
GPDGRTIDRFSPT
>Hsap_ENSP00000314153
MESRKLISATDIQYSGSLLNSLNEQRGHGLFCDVTVIVEDRKFRAHKNILSASSTYFHQLFSVAGQVVELSFIRAEIFAE
ILNYIYSSKIVRVRSDLLDELIKSGQLLGVKFIAELGVPLSQVKSISGTAQDGNTEPLPPDSGDKNLVIQKSKDEAQDNG
ATIMPIITESFSLSAEDYEMKKIIVTDSDDDDDDVIFCSEILPTKETLPSNNTVAQVQSNPGPVAISDVAPSASNNSPPL
TNITPTQKLPTPVNQATLSQTQGSEKLLVSSAPTHLTPNIILLNQTPLSTPPNVSSSLPNHMPSSINLLVQNQQTPNSAI
LTGNKANEEEEEEIIDDDDDTISSSPDSAVSNTSLVPQADTSQNTSFDGSLIQKMQIPTLLQEPLSNSLKISDIITRNTN
DPGVGSKHLMEGQKIITLDTATEIEGLSTGCKVYANIGEDTYDIVIPVKDDPDEGEARLENEIPKTSGSEMANKRMKVKH
DDHYELIVDGRVYYICIVCKRSYVCLTSLRRHFNIHSWEKKYPCRYCEKVFPLAEYRTKHEIHHTGERRYQCLACGKSFI
NYQFMSSHIKSVHSQDPSGDSKLYRLHPCRSLQIRQYAYLSDRSSTIPAMKDDGIGYKVDTGKEPPVGTTTSTQNKPMTW
EDIFIQQENDSIFKQNVTDGSTEFEFIIPESY
>Hsap_ENSP00000314608
MAAAAVAAAAAAAAAAASLQVLEMESMETAAAGSAGLAAEVRGSGTVDFGPGPGISAMEASGGDPGPEAEDFECSSHCSEL
SWRQNEQRRQGLFCDITLCFGGAGGREFRAHRSVLAAATEYFTPLLSGQFSESRSGRVEMRKWSSEPGPEPDTVEAVIEY
MYTGRIRVSTGSVHEVLELADRFLLIRLKEFCGEFLKKKLHLSNCVAIHSLAHMYTLSQLALKAADMIRRNFHKVIQDEE
FYTLPFHLIRDWLSDLEITVDSEEVLFETVLKWVQRNAEERERYFEELFKLLRLSQMKPTYLTRHVKPERLVANNEVCVK
LVADAVERHALRAENIQSGTCQHPTSHVSLLPRYGQNMDVIMVIGGVSEGGDYLSECVGYFVDEDRWVNLPHIHNHLDGH

AVAVTESYVYVAGSMEPGFAKTVERYNPNLNTWEHVCSLMTRKHSFGLTEVKGKLYSIGGHGNFSPGFKDVTVYNPELDK
WHNLESAPKILRDVKALAIEDRFVYIAARTPVDRDTEDGLKAVITCYDTETRQWQDVESLPLIDNYCFFQMSVVNSNFYQ
TASCCPKSYCLENEEAVRKIASQVSDEILESLPPEVLSIEGAAICYYKDDVFIIGGWKNSDDIDKQYRKEAYRYCAERKR
WMLLPPMPQPRCRATACHVRIPYRYLHGTQRYPMPQNLMWQKDRIRQMQEIHRHALNMRRVPSSQIEC
>Hsap_ENSP00000317442
MMEGSRQTRVSRPYKISESSKVYRWADHSSTVLQRLNEQRLRGLFCDVVLVADEQRVPAHRNLLAVCSDYFNSMFTIGMR
EAFQKEVELIGASYIGLKAVVDFLYGGELVLDGGNIDYVLETAHLLQIWTVVDFCCEYLEQEVSEDNYLYLQELASIYSL
KRLDAFIDGFILNHFGTLSFTPDFLQNVSMQKLCVYLSSSEVQRECEHDLLQAALQWLTQQPEREAHARQVLENIHFPLI
PKNDLLHRVKPAVCSLLPKEANCEGFIEEAVRYHNNLAAQPVMQTKRTALRTNQERLLFVGGEVSERCLELSDDTCYLDA
KSEQWVKETPLPARRSHHCVAVLGGFIFIAGGSFSRDNGGDAASNLLYRYDPRCKQWIKVASMNQRRVDFYLASIEDMLV
AIGGRNENGALSSVETYSPKTDSWSYVAGLPRFTYGHAGTIYKDFVYISGGHDYQIGPYRKNLLCYDHRTDVWEERRPMT
TARGWHSMCSLGDSIYSIGGSDDNIESMERFDVLGVEAYSPQCNQWTRVAPLLHANSESGVAVWEGRIYILGGYSWENTA
FSKTVQVYDREADKWSRGVDLPKAIAGGSACVCALEPRPEDKKKKGKGKRHQDRGQ
>Hsap_ENSP00000317534
MSVEMDSSSFIQFDVPEYSSTVLSQLNELRLQGKLCDIIVHIQGQPFRAHKAVLAASSPYFRDHSALSTMSGLSISVIKN
PNVFEQLLSFCYTGRMSLQLKDVVSFLTAASFLQMQCVIDKCTQILESIHSKISVGDVDSVTVGAEENPESRNGVKDSSF
FANPVEISPPYCSQGRQPTASSDLRMETTPSKALRSRLQEEGHSDRGSSGSVSEYEIQIEGDHEQGDLLVRESQITEVKV
KMEKSDRPSCSDSSSLGDDGYHTEMVDGEQVVAVNVGSYGSVLQHAYSYSQAASQPTNVSEAFGSLSNSSPSRSMLSCFR
GGRARQKRALSVHLHSDLQGLVQGSDSEAMMNNPGYESSPRERSARGHWYPYNERLICIYCGKSFNQKGSLDRHMRLHMG
ITPFVCKFCGKKYTRKDQLEYHIRGHTDDKPFRCEICGKCFPFQGTLNQHLRKNHPGVAEVRSRIESPERTDVYVEQKLE
NDASASEMGLDSRMEIHTVSDAPD
>Hsap_ENSP00000321475
MDEESLDGLLFKDHDFSSDLLRQLNSLRQSRILTDVSICAGAREIPCHRNVLASSSPYFRAMFCSSFREKSEAKVQLKGI
DPPTLDQIVSYVYTGEAHIATDNVLPVMEAASMLQFPKLFEACSSYLQSQLAPSNCLGMIRLSEILSCETLKKKAREVAL
TSFPEVAASADLKELCALELRDYLGDDGLCGEEEKVFEALMVWIKHDLQARKRYMQELFKQVRLQYIHPAFFHHFIANDA
LLQSSPACQIILETAKRQMFSLCGTTVPDCKLLLHVPPRNSYQDFLILLGGRKDSQQTTRDVLLYSKQTGQWQSLAKLPT
RLYKASAITLHRSIYVLGGMAVSSGRSLVSHNVYIFSLKLNQWRLGEPMLVARYSHRSTAHKNFIFSIGGIGEGQELMGS
MERYDSICNVWESMASMPVGVLHPAVAVKDQRLYLFGGEDIMQNPVRLIQVYHISRNSWFKMETRMIKNVCAPAVVLGER
IVIVGGYTRRILAYDPQSNKFVKCADMKDRRMHHGATVMGNKLYVTGGRRLTTDCNIEDSASFDCYDPETDTWTSQGQLP
HKLFDHACLTLQCIPRTSGLP
>Hsap_ENSP00000321544
MEHAVAPCVLYPGTEPGAAGESESEGAASPAQTPCSLGASLCFSSGEESPPQSLASAAEGAATSPPSSGGPRVVERQWEA
GSAGAASPEELASPEERACPEEPAAPSPEPRVWLEDPASPEEPGEPAPVPPGFGAVYGEPDLVLEVSGRRLRAHKAVLAA
RSDYFRARASRDVLRVQGVSLTALRLLLADAYSGRMAGVRPDNVAEVVAGARRLQLPGAAQRATDAVGPQLSLANCYEVL
SAAKRQRLNELRDAAYCFMSDHYLEVLREPAVFGRLSGAERDLLLRRRLRAGRAHLLAAALGPAGERAGSRPQSPSGDAD
ARGDAAVYCFHAAAGEWRELTRLPEGAPARGCGLCVLYNYLFVAGGVAPAGPDGRARPSDQVFCYNPATDSWSAVRPLRQ
ARSQLRLLALDGHLYAVGGECLLSVERYDPRADRWAPVAPLPRGAFAVAHEATTCHGEIYVSGGSLFYRLLKYDPRRDEW
QECPCSSSRERSADMVALDGFIYRFDLSGSRGEAQAAGPSGVSVSRYHCLAKQWSPCVAPLRLPGGPTGLQPFRCAALDG
AIYCVSRAGTWRFQPAREGEAGGDAGQGGGFEALGAPLDVRGVLIPFALSLPEKPPRGEQGAP
>Hsap_ENSP00000323183
MDLANHGLILLQQLNAQREFGFLCDCTVAIGDVYFKAHKSVLASFSNYFKMLFVHQTSECVRLKPTDIQPDIFSYLLHLM
YTGKMAPQLIDPVRLEQGIKFLHAYPLIQEASLASQGAFSHPDQVFPLASSLYGIQIADHQLRQATKIASAPEKLGRDPR
PQTSRISQEQVPEASQLSQLTSNLAQVNRTNMTPSDPLQTSLSPELVSTPVPPPPPGEETNLEASSSDEQPASLTIAHVK
PSIMKRNGSFPKYYACHLCGRRFTLRSSLREHLQIHTGVPFTSSQQGESRVPLTLCSNAADLGKDAMEVPEAGMISDSEL
QHISDSPIIDGQQQSETPPPSDIADIDNLEQADQEREVKRRKYECTICGRKFIQKSHWREHMYIHTGKPFKCSTCDKSFC
RANQAARHVCLNQSIDTYTMVDKQTLELCTFEEGSQMDNMLVQTNKPYKCNLCDKTFSTPNEVVKHSCQNQNSDVFALDE
GRSILLGSGDSEVTEPDHPVLASIKKEQETVLLD
>Hsap_ENSP00000323670
MAGGVDGPIGIPFPDHSSDILSGLNEQRTQGLLCDVVILVEGREFPTHRSVLAACSQYFKKLFTSGAVVDQQNVYEIDFV
SAEALTALMDFAYTATLTVSTANVGDILSAARLLEIPAVSHVCADLLDRQILAADAGADAGQLDLVDQIDQRNLLRAKEY
LEFFQSNPMNSLPPAAAAAAAASFPWSAFGASDDDLDATKEAVAAAVAAVAAGDCNGLDFYGPGPPAERPPTGDGDEGDSN
PGLWPERDEDAPTGGLFPPPVAPPAATQNGHYGRGGEEEAASLSEAAPEPGDSPGFLSGAAEGEDGDGPDVDGLAASTLL
QQMMSSVGRAGAAAGDSDEESRADDKGVMDYYLKYFSGAHDGDVYPAWSQKVEKKIRAKAFQKCPICEKVIQGAGKLPRH
IRTHTGEKPYECNICKVRFTRQDKLKVHMRKHTGEKPYLCQQCGAAFAHNYDLKNHMRVHTGLRPYQCDSCCKTFVRSDH
LHRHLKKDGCNGVPSRRGRKPRVRGGAPDPSPGATATPGAPAQPSSPDARRNGQEKHFKDEDEDEDVASPDGLGRLNVAG
AGGGGDSGGGPGAATDGNFTAGLA
>Hsap_ENSP00000326200
MSSEESYRAILRYLTNEREPYAPGTEGNVKRKIRKAAACYVVRGGTLYYQRRQRHRKTFAELEVVLQPERRRDLIEAAHL
GPGGTHHTRHQTWHYLSKTYWWRGILKQVKDYIKQCSKCQEKLDRSRPISDVSEMLEELGLDLESGEESNESEDDLSNFT
SSPTTASKPAKKKPVSKHELVFVDTKGVVKRSSPKHCQAVLKQLNEQRLSNQFCDVTLLIEGEEYKAHKSVLSANSEYFR
DLFIEKGAVSSHEAVVDLSGFCKASFLPLLEFAYTSVLSFDFCSMADVAILARHLFMSEVLEICESVHKLMEEKQLTVYK
KGEVQTVASTQDLRVQNGGTAPPVASSEGTTTSLPTELGDCEIVLLVNGELPEAEQNGEVGRQPEPQVSSEAESALSSVG

CIADSHPEMESVDLITKNNQTELETSNNRENNTVSNIHPKLSKENVISSSPEDSGMGNDISAEDICAEDIPKHRQKVDQP
LKDQENLVASTAKTDFGPDDDTYRSRLRQRSVNEGAYIRLHKGMEKKLQKRKAVPKSAVQQVAQKLVQRGKKMKQPKRDA
KENTEEASHKCGECGMVFQRRYALIMHKLKHERARDYKCPLCKKQFQYSASLRAHLIRHTRKDAPSSSSSNSTSNEASGT
SSEKGRTKREFICSICGRTLPKLYSLRIHMLKHTGVKPHACQVCGKTFIYKHGLKLHQSLHQSQKQFQCELCVKSFVTKR
SLQEHMSIHTGESKYLCSVCGKSFHRGSGLSKHFKKHQPKPEVRGYHCTQCEKSFFEARDLRQHMNKHLGVKPFQCQFCD
KCYSWKKDWYSHVKSHSVTEPYRCNICGKEFYEKALFRRHVKKATHGKKGRAKQNLERVCEKCGRKFTQLREYRRHMNNH
EGVKPFECLTCGVAWADARSLKRHVRTHTGERPYVCPVCSEAYIDARTLRKHMTKFHRDYVPCKIMLEKDTLQFHNQGTQ
VAHAVSILTAGMQEQESSGPQELETVVVTGETMEALEAVAATEEYPSVSTLSDQSIMQVVNYVLAQQQGQKLSEVAEAIQ
TVKVEVAHISGGE
>Hsap_ENSP00000326240
MQYSHHCEHLLERLNKQREAGFLCDCTIVIGEFQFKAHRNVLASFSEYFGAIYRSTSENNVFLDQSQVKADGFQKLLEFI
YTGTLNLDSWNVKEIHQAADYLKVEEVVTKCKIKMEDFAFIANPSSTEISSITGNIELNQQTCLLTLRDYNNREKSEVST
DLIQANPKQGALAKKSSQTKKKKKAFNSPKTGQNKTVQYPSDILENASVELFLDANKLPTPVVEQVAQINDNSELELTSV
VENTFPAQDIVHTVTVKRKRGKSQPNCALKEHSMSNIASVKSPYEAENSGEELDQRYSKAKPMCNTCGKVFSEASSLRRH
MRIHKGVKPYVCHLCGKAFTQCNQLKTHVRTHTGEKPYKCELCDKGFAQKCQLVFHSRMHHGEEKPYKCDVCNLQFATSS
NLKIHARKHSGEKPYVCDRCGQRFAQASTLTYHVRRHTGEKPYVCDTCGKAFAVSSSLITHSRKHTGEKPYICGICGKSF
ISSGELNKHFRSHTGERPFICELCGNSYTDIKNLKKHKTKVHSGADKTLDSSAEDHTLSEQDSIQKSPLSETMDVKPSDM
TLPLALPLGTEDHHMLLPVTDTQSPTSDTLLRSTVNGYSEPQLIFLQQLY
>Hsap_ENSP00000328732
MANDIDELIGIPFPNHSSEVLCSLNEQRHDGLLCDVLLVVQEQEYRTHRSVLAACSKYFKKLFTAGTLASQPYVYEIDFV
QPEALAAILEFAYTSTLTITAGNVKHILNAARMLEIQCIVNVCLEIMEPGGDGGEEDDKEDDDDDEDDDDEEDEEEEEEE
EEDDDDDTEDFADQENLPDPQDISCHQSPSKTDHLTEKAYSDTPRDFPDSFQAGSPGHLGVIRDFSIESLLRENLYPKAN
IPDRRPSLSPFAPDFFPHLWPGDFGAFAQLPEQPMDSGPLDLVIKNRKIKEEEKEELPPPPPPPFPNDFFKDMFPDLPGG
PLGPIKAENDYGAYLNFLSATHLGGLFPPWPLVEERKLKPKASQQCPICHKVIMGAGKLPRHMRTHTGEKPYMCTICEVR
FTRQDKLKIHMRKHTGERPYLCIHCNAKFVHNYDLKNHMRIHTGVRPYQCEFCYKSFTRSDHLHRHIKRQSCRMARPRRG
RKPAAWRAASLLFGPGGPAPDKAAFVMPPALGEVGGHLGGAAVCLPGPSPAKHFLAAPKGALSLQELERQFEETQMKLFG
RAQLEAERNAGGLLAFALAENVAAARPYFPLPDPWAAGLAGLPGLAGLNHVASMSEANN
>Hsap_ENSP00000331682
MAEEQEFTQLCKLPAQPSHPHCVNNTYRSAQHSQALLRGLLALRDSGILFDVVLVVEGRHIEAHRILLAASCDYFRGMFA
GGLKEMEQEEVLIHGVSYNAMCQILHFIYTSELELSLSNVQETLVAACQLQIPEIIHFCCDFLMSWVDEENILDVYRLAE
LFDLSRLTEQLDTYILKNFVAFSRTDKYRQLPLEKVYSLLSSNRLEVSCETEVYEGALLYHYSLEQVQADQISLHEPPKL
LETVRFPLMEAEVLQRLHDKLDPSPLRDTVASALMYHRNESLQPSLQSPQTELRSDFQCVVGFGGIHSTPSTVLSDQAKY
LNPLLGEWKHFTASLAPRMSNQGIAVLNNFVYLIGGDNNVQGFRAESRCWRYDPRHNRWFQIQSLQQEHADLSVCVVGRY
IYAVAGRDYHNDLNAVERYDPATNSWAYVAPLKREVYAHAGATLEGKMYITCGRRGEDYLKETHCYDPGSNTWHTLADGP
VRRAWHGMATLLNKLYVIGGSNNDAGYRRDVHQVACYSCTSGQWSSVCPLPAGHGEPGIAVLDNRIYVLGGRSHNRGSRT
GYVHIYDVEKDCWEEGPQLDNSISGLAACVLTLPRSLLLEPPRGTPDRSQADPDFASEVMSVSDWEEFDNSSED
>Hsap_ENSP00000332791
MAGDVEGFCSSIHDTSVSAGFRALYEEGLLLDVTLVIEDHQFQAHKALLATQSDYFRIMFTADMRERDQDKIHLKGLTAT
GFSHVLQFMYYGTIELSMNTVHEILQAAMYVQLIEVVKFCCSFLLAKICLENCAEIMRLLDDFGVNIEGVREKLDTFLLD
NFVPLMSRPDFLSYLSFEKLMSYLDNDHLSRFPEIELYEAVQSWLRHDRRRWRHTDTIIQNIRFCLMTPTSVFEKVKTSE
FYRYSRQLRYEVDQALNYFQNVHQQPLLDMKSSRIRSAKPQTTVFRGMIGHSMVNSKILLLKKPRVWWELEGPQVPLRPD
CLAIVNNFVFLLGGEELGPDGEFHASSKVFRYDPRQNSWLQMADMSVPRSEFAVGVIGKFIYAVAGRTRDETFYSTERYD
ITNDKWEFVDPYPVNKYGHEGTVLNNKLFITGGITSSSTSKQVCVFDPSKEGTIEQRTRRTQVVTNCWENKSKMNYARCF
HKMISYNGKLYVFGGVCVILRASFESQGCPSTEVYNPETDQWTILASMPIGRSGHGVTVLDKQIMVLGGLCYNGHYSDSI
LTFDPDENKWKEDEYPRMPCKLDGLQVCNLHFPDYVLDEVRRCN
>Hsap_ENSP00000334996
MKLEFTEKNYNSFVLQNLNRQRKRKEYWDMALSVDNHVFFAHRNVLAAVSPLVRSLISSNDMKTADELFITIDTSYLSPV
TVDQLLDYFYSGKVVISEQNVEELLRGAQYFNTPRLRVHCNDFLIKSICRANCLRYLFLAELFELKEVSDVAYSGIRDNF
HYWASPEGSMHFMRCPPVIFGRLLRDENLHVLNEDQALSALINWVYFRKEDREKYFKKFFNYINLNAVSNKTLVFASNKL
VGMENTSSHTTLIESVLMDRKQERPCSLLVYQRKGALLDSVVILGGQKAHGQFNDGVFAYIIQENLWMKLSDMPYRAAAL
SATSAGRYIYISGGTTEQISGLKTAWRYDMDDNSWTKLPDLPIGLVFHTMVTCGGTVYSVGGSIAPRRYVSNIYRYDERK
EVWCLAGKMSIPMDGTAVITKGDRHLYIVTGRCLVKGYISRVGVVDCFDTSTGDVVQCITFPIEFNHRPLLSFQQDNILC
VHSHRQSVEINLQKVKASKTTTSVPVLPNSCPLDVSHAICSIGDSKVFVCGGVTTASDVQTKDYTINPNAFLLDQKTGKW
KTLAPPPEALDCPACCLAKLPCKILQRI
>Hsap_ENSP00000335615
MGANASNYPHSCSPRVGGNSQAQQTFIGTSSYSQQGYGCESKLYSLDHGHEKPQDKKKRTSGLATLKKKFIKRRKSNRSA
DHAKQMRELLSGWDVRDVNALVEEYEGTSALKELSLQASLARPEARTLQKDMADLYEYKYCTDVDLIFQETCFPVHRAIL
AARCPFFKTLLSSSPEYGAEIIMDINTAGIDMPMFSALLHYLYTGEFGMEDSRFQNVDILVQLSEEFGTPNSLDVDMRGL
FDYMCYYDVVLSFSSDSELVEAFGGNQNCLDEELKAHKAVISARSPFFRNLLQRRIRTGEEITDRTLRTPTRIILDESII
PKKYATVILHCMYTDVVDLSVLHCSPSVGSLSEVQALVAGKPNMTRAEEAMELYHIALFLEFNMLAQGCEDIIAESISLD
TLIAILKWSSHPYGSKWVHRQALHFLCEEFSQVMTSDVFYELSKDHLLTAIQSDYLQASEQDILKYLIKWGEHQLMKRIA
DREPNLLSGTAHSVNKRGVKRRDLDMEELREILSSLLPFVRIEHILPINSEVLSDAMKRGLISTPPSDMLPTTEGGKSNA

WLRQKNAGIYVRPRLFSPYVEEAKSVLDEMMVEQTDLVRLRMVRMSNVPDTLYMVNNAVPQCCHMISHQQISSNQSSPPS
VVANEIPVPRLLIMKDMVRRLQELRHTEQVQRAYALNCGEGATVSYEIQIRVLREFGLADAAAELLQNPHKFFPDERFGD
ESPLLTMRQPGRCRVNSTPPAETMFTDLDSFVAFHPPLPPPPPPYHPPATPIHNQLKAGWKQRPPSQHPSRSFSYPCNHS
LFHSRTAPKAGPPPVYLPSVKAAPPDCTSTAGLGRQTVAAAAATTTSTATAAAAAASEKQVRTQPVLNDLMPDIAVGVST
LSLKDRRLPELAVDTELSQSVSEAGPGPPQHLSCIPQRHTHTSRKKHTLEQKTDTRENPQEYPDFYDFSNAACRPSTPAL
SRRTPSPSQGGYFGPDLYSHNKASPSGLKSAYLPGQTSPKKQEEARREYPLSPDGHLHRQKNEPIHLDVVEQPPQRSDFP
LAAPENASTGPAHVRGRTAVETDLTFGLTPNRPSLSACSSEAPEERSGRRLADSESLGHGAQRNTDLEREDSISRGRRSP
SKPDFLYKKSAL
>Hsap_ENSP00000336800
MSVSVHETRKSRSSTGSMNVTLFHKASHPDCVLAHLNTLRKHCMFTDVTLWAGDRAFPCHRAVLAASSRYFEAMFSHGLR
ESRDDTVNFQDNLHPEVLELLLDFAYSSRIAINEENAESLLEAGDMLQFHDVRDAAAEFLEKNLFPSNCLGMMLLSDAHQ
CRRLYEFSWRMCLVHFETVRQSEDFNSLSKDTLLDLISSDELETEDERVVFEAILQWVKHDLEPRKVHLPELLRSVRLAL
LPSDCLQEAVSSEALLMADERTKLIMDEALRCKTRILQNDGVVTSPCARPRKAGHTLLILGGQTFMCDKIYQVDHKAKEI
IPKADLPSPRKEFSASAIGCKVYVTGGRGSENGVSKDVWVYDTVHEEWSKAAPMLIARFGHGSAELENCLYVVGGHTSLA
GVFPASPSVSLKQVEKYDPGANKWMMVAPLRDGVSNAAVVSAKLKLFVFGGTSIHRDMVSKVQCYDPSENRWTIKAECPQ
PWRYTAAAVLGSQIFIMGGDTEFTAASAYRFDCETNQWTRIGDMTAKRMSCHALASGNKLYVVGGYFGTQRCKTLDCYDP
TSDTWNCITTVPYSLIPTAFVSTWKHLPA
>Hsap_ENSP00000338157
MDLTKMGMIQLQNPSHPTGLLCKANQMRLAGTLCDVVIMVDSQEFHAHRTVLACTSKMFEILFHRNSQHYTLDFLSPKTF
QQILEYAYTATLQAKAEDLDDLLYAAEILEIEYLEEQCLKMLETIQASDDNDTEATMADGGAEEEEDRKARYLKNIFISK
HSSEESGYASVAGQSLPGPMVDQSPSVSTSFGLSAMSPTKAAVDSLMTIGQSLLQGTLQPPAGPEEPTLAGGGRHPGVAE
VKTEMMQVDEVPSQDSPGAAESSISGGMGDKVEERGKEGPGTPTRSSVITSARELHYGREESAEQVPPPAEAGQAPTGRP
EHPAPPPEKHLGIYSVLPNHKADAVLSMPSSVTSGLHVQPALAVSMDFSTYGGLLPQGFIQRELFSKLGELAVGMKSESR
TIGEQCSVCGVELPDNEAVEQHRKLHSGMKTYGCELCGKRFLDSLRLRMHLLAHSAGAKAFVCDQCGAQFSKEDALETHR
QTHTGTDMAVFCLLCGKRFQAQSALQQHMEVHAGVRSYICSECNRTFPSHTALKRHLRSHTGDHPYECEFCGSCFRDEST
LKSHKRIHTGEKPYECNGCGKKFSLKHQLETHYRVHTGEKPFECKLCHQRSRDYSAMIKHLRTHNGASPYQCTICTEYCP
SLSSMQKHMKGHKPEEIPPDWRIEKTYLYLCYV
>Hsap_ENSP00000338290
MESGAVLLESKSSPFNLLHEMHELRLLGHLCDVTVSVEYQGVRKDFMAHKAVLAATSKFFKEVFLNEKSVDGTRTNVYLN
EVQVADFASFLEFVYTAKVQVEEDRVQRMLEVAEKLKCLDLSETCFQLKKQMLESVLLELQNFSESQEVEVSSGSQVSAA
PAPRASVATDGPHPSGLTDSLDYPGERASNGMSSDLPPKKSKDKLDKKKEVVKPPYPKIRRASGRLAGRKVFVEIPKKKY
TRRLREQQKTAEGDVGDYRCPQDQSPDRVGTEMEQVSKNEGCQAGAELEELSKKAGPEEEEEEEEEDEEGEKKKSNFKCS
ICEKAFLYEKSFLKHSKHRHGVATEVVYRCDTCGQTFANRCNLKSHQRHVHSSERHFPCELCGKKFKRKKDVKRHVLQVH
EGGGERHRCGQCGKGLSSKTALRLHERTHTGDRPYGCTECGARFSQPSALKTHMRIHTGEKPFVCDECGARFTQNHMLIY
HKRCHTGERPFMCETCGKSFASKEYLKHHNRIHTGSKPFKCEVCFRTFAQRNSLYQHIKVHTGERPYCCDQCGKQFTQLN
ALQRHRRIHTGERPFMCNACGRTFTDKSTLRRHTSIHDKNTPWKSFLVIVDGSPKNDDGHKTEQPDEEYVSSKLSDKLLS
FAENGHFHNLAAVQDTVPTMQENSSADTACKADDSVVSQDTLLATTISELSELTPQTDSMPTQLHSLSNME
>Hsap_ENSP00000338671
MDADMDYERPNVETIKCVVVGDNAVGKTRLICARACNTTLTQYQLLATHVPTVWAIDQYRVCQEVLERSRDVVDEVSVSL
RLWDTFGDHHKDRRFAYGRSDVVVLCFSIANPNSLNHVKSMWYPEIKHFCPRTPVILVGCQLDLRYADLEAVNRARRPLA
RPIKRGDILPPEKGREVAKELGLPYYETSVFDQFGIKDVFDNAIRAALISRRHLQFWKSHLKKVQKPLLQAPFLPPKAPP
PVIKIPECPSMGTNEAACLLDNPLCADVLFILQDQEHIFAHRIYLATSSSKFYDLFLMECEESPNGSEGACEKEKQSRDF
QGRILSVDPEEEREEGPPRIPQADQWKSSNKSLVEALGLEAEGAVPETQTLTGWSKGFIGMHREMQVNPISKRMGPMTVV
RMDASVQPGPFRTLLQFLYTGQLDEKEKDLVGLAQIAEVLEMFDLRMMVENIMNKEAFMNQEITKAFHVRKANRIKECLS
KGTFSDVTFKLDDGAISAHKPLLICSCEWMAAMFGGSFVESANSEVYLPNINKISMQAVLDYLYTKQLSPNLDLDPLELI
ALANRFCLPHLVALAEQHAVQELTKAATSGVGIDGEVLSYLELAQFHNAHQLAAWCLHHICTNYNSVCSKFRKEIKSKSA
DNQEYFERHRWPPVWYLKEEDHYQRVKREREKEDIALNKHRSRRKWCFWNSSPAVA
>Hsap_ENSP00000338807
MDPVATHSCHLLQQLHEQRIQGLLCDCMLVVKGVCFKAHKNVLAAFSQYFRSLFQNSSSQKNDVFHLDVKNVSGIGQILD
FMYTSHLDLNQDNIQVMLDTAQCLQVQNVLSLCHTFLKSATVVQPPGMPCNSTLSLQSTLTPDATCVISENYPPHLLQEC
SADAQQNKTLDESHPHASPSVNRHHSAGEISKQAPDTSDGSCTELPFKQPNYYYKLRNFYSKQYHKHAAGPSQERVVEQP
FAFSTSTDLTTVESQPCAVSHSECILESPEHLPSNFLAQPVNDSAPHPESDATCQQPVKQMRLKKAIHLKKLNFLKSQKY
AEQVSEPKSDDGLTKRLESASKNTLEKASSQSAEEKESEEVVSCENFNCISETERPEDPAALEDQSQTLQSQRQYACELC
GKPFKHPSNLELHKRSHTGEKPFECNICGKHFSQAGNLQTHLRRHSGEKPYICEICGKRFAASGDVQRHIIIHSGEKPHL
CDICGRGFSNFSNLKEHKKTHTADKVFTCDECGKSFNMQRKLVKHRIRHTGERPYSCSACGKCFGGSGDLRRHVRTHTGE
KPYTCEICNKCFTRSAVLRRHKKMHCKAGDESPDVLEELSQAIETSDLEKSQSSDSFSQDTSVTLMPVSVKLPVHPVENS
VAEFDSHSGGSYCKLRSMIQPHGVSDQEKLSLDPGKLAKPQMQQTQPQAYAYSDVDTPAGGEPLQADGMAMIRSSLAALD
NHGGDPLGSRASSTTYRNSEGQFFSSMTLWGLAMKTLQNENELDQ
>Hsap_ENSP00000341342
MLMAGQRGAWTMGDVVEKSLEGPLAPSTDEPSQKTGDLVEILNGEKVKFDDAGLSLILQNGLETLRMENALTDVILCVDI
QEFSCHRVVLAAASNYFRAMFCNDLKEKYEKRIIIKGVDAETMHTLLDYTYTSKALITKQNVQRVLEAANLFQFLRMVDA
CASFLTEALNPENCVGILRLADTHSLDSLKKQVQSYIIQNFVQILNSEEFLDLPVDTLHHILKSDDLYVTEEAQVFETVM

SWVRHKPSERLCLLPYVLENVRLPLLDPWYFVETVEADPLIRQCPEVFPLLQEARMYHLSGNEIISERTKPRMHEFQSEV
FMIIGGCTKDERFVAEVTCLDPLRRSRLEVAKLPLTEHELESENKKWVEFACVTLKNEVYISGGKETQHDVWKYNSSINK
WIQIEYLNIGRWRHKMVVLGGKVYVIGGFDGLQRINNVETYDPFHNCWSEAAPLLVHVSSFAATSHKKKLYVIGGGPNGK
LATDKTQCYDPSTNKWSLKAAMPVEAKCINAVSFRDRIYVVGGAMRALYAYSPLEDSWCLVTQLSHERASCGIAPCNNRL
YITGGRDEKNEVIATVLCWDPEAQKLTEECVLPRGVSHHGSVTIRKSYTHIRRIVPGAVSV
>Hsap_ENSP00000343273
MAASGVEKSSKKKTEKKLAAREEAKLLAGFMGVMNNMRKQKTLCDVILMVQERKIPAHRVVLAAASHFFNLMFTTNMLES
KSFEVELKDAEPDIIEQLVEFAYTARISVNSNNVQSLLDAANQYQIEPVKKMCVDFLKEQVDASNCLGISVLAECLDCPE
LKATADDFIHQHFTEVYKTDEFLQLDVKRVTHLLNQDTLTVRAEDQVYDAAVRWLKYDEPNRQPFMVDILAKVRFPLISK
NFLSKTVQAEPLIQDNPECLKMVISGMRYHLLSPEDREELVDGTRPRRKKHDYRIALFGGSQPQSCRYFNPKDYSWTDIR
CPFEKRRDAACVFWDNVVYILGGSQLFPIKRMDCYNVVKDSWYSKLGPPTPRDSLAACAAEGKIYTSGGSEVGNSALYLF
ECYDTRTESWHTKPSMLTQRCSHGMVEANGLIYVCGGSLGNNVSGRVLNSCEVYDPATETWTELCPMIEARKNHGLVFVK
DKIFAVGGQNGLGGLDNVEYYDIKLNEWKMVSPMPWKGVTVKCAAVGSIVYVLAGFQGVGRLGHILEYNTETDKWVANSK
VRAFPVTSCLICVVDTCGANEETLET
>Hsap_ENSP00000343362
MAEEEVAKLEKHLMLLRQEYVKLQKKLAETEKRCALLAAQANKESSSESFISRLLAIVADLYEQEQYSDLKIKVGDRHIS
AHKFVLAARSDSWSLANLSSTKELDLSDANPEVTMTMLRWIYTDELEFREDDVFLTELMKLANRFQLQLLRERCEKGVMS
LVNVRNCIRFYQTAEELNASTLMNYCAEIIASHWDDLRKEDFSSMSAQLLYKMIKSKTEYPLHKAIKVEREDVVFLYLIE
MDSQLPGKLNEADHNGDLALDLALSRRLESIATTLVSHKADVDMVDKSGWSLLHKGIQRGDLFAATFLIKNGAFVNAATL
GAQETPLHLVALYSSKKHSADVMSEMAQIAEALLQAGANPNMQDSKGRTPLHVSIMAGNEYVFSQLLQCKQLDLELKDHE
GSTALWLAVQHITVSSDQSVNPFEDVPVVNGTSFDENSFAARLIQRGSHTDAPDTATGNCLLQRAAGAGNEAAALFLATN
GAHVNHRNKWGETPLHTACRHGLANLTAELLQQGANPNLQTEEALPLPKEAASLTSLADSVHLQTPLHMAIAYNHPDVVS
VILEQKANALHATNNLQIIPDFSLKDSRDQTVLGLALWTGMHTIAAQLLGSGAAINDTMSDGQTLLHMAIQRQDSKSALF
LLEHQADINVSRTQDGETALQLAIRNQLPLVVDAICTRGADMSVPDEKGNPPLWLALANNLEDIASTLVRHGCDATCWGP
GPGGCLQTLLHRAIDENNEPTACFLIRSGCDVNSPRQPGANGEGEEEARDGQTPLHLAASWGLEETVQCLLEFGANVNAQ
DAEGRTPIHVAISSQHGVIIQLLVSHPDIHLNVRDRQGLTPFACAMTFKNNKSAEAILKRESGAAEQVDNKGRNFLHVAV
QNSDIESVLFLISVHANVNSRVQDASKLTPLHLAVQAGSEIIVRNLLLAGAKVNELTKHRQTALHLAAQQDLPTICSVLL
ENGVDFAAVDENGNNALHLAVMHGRLNNIRVLLTECTVDAEAFNLRGQSPLHILGQYGKENAAAIFDLFLECMPGYPLDK
PDADGSTVLLLAYMKGNANLCRAIVRSGARLGVNNNQGVNIFNYQVATKQLLFRLLDMLSKEPPWCDGSYCYECTARFGV
TTRKHHCRHCGRLLCHKCSTKEIPIIKFDLNKPVRVCNICFDVLTLGGVS
>Hsap_ENSP00000343686
MARCGEGSAAPMVLLGSAGVCSKGLQRKGPCERRRLKATVSEQLSQDLLRLLREEFHTDVTFSVGCTLFKAHKAVLLARV
PDFYFHTIGQTSNSLTNQEPIAVENVEALEFRTFLQIIYSSNRNIKNYEEEILRKKIMEIGISQKQLDISFPKCENSSDC
SLQKHEIPEDISDRDDDFISNDNYDLEPASELGEDLLKLYVKPCCPDIDIFVDGKRFKAHRAILSARSSYFAAMLSGCWA
ESSQEYVTLQGISHVELNVMMHFIYGGTLDIPDKTNVGQILNMADMYGLEGLKEVAIYILRRDYCNFFQKPVPRTLTSIL
ECLIIAHSVGVESLFADCMKWIVKHFARFWSERSFANIPPEIQKSCLNMLIQSLVSIT
>Hsap_ENSP00000343930
MQPRSERPAGRTQSPEHGSPGPGPEAPPPPPPQPPAPEAERTRPRQARPAAPMEGAVQLLSREGHSVAHNSKRHYHDAFV
AMSRMRQRGLLCDIVLHVAAKEIRAHKVVLASCSPYFHAMFTNEMSESRQTHVTLHDIDPQALDQLVQFAYTAEIVVGEG
NVQTLLPAASLLQLNGVRDACCKFLLSQLDPSNCLGIRGFADAHSCSDLLKAAHRYVLQHFVDVAKTEEFMLLPLKQVLE
LVSSDSLNVPSEEEVYRAVLSWVKHDVDARRQHVPRLMKCVRLPLLSRDFLLGHVDAESLVRHHPDCKDLLIEALKFHLL
PEQRGVLGTSRTRPRRCEGAGPVLFAVGGGSLFAIHGDCEAYDTRTDRWHVVASMSTRRARVGVAAVGNRLYAVGGYDGT
SDLATVESYDPVTNTWQPEVSMGTRRSCLGVAALHGLLYSAGGYDGASCLNSAERYDPLTGTWTSVAAMSTRRRYVRVAT
LDGNLYAVGGYDSSSHLATVEKYEPQVNVWSPVASMLSRRSSAGVAVLEGALYVAGGNDGTSCLNSVERYSPKAGAWESV
APMNIRRSTHDLVAMDGWLYAVGGNDGSSSLNSIEKYNPRTNKWVAASCMFTRRSSVGVAVLELLNFPPPSSPTLSVSST
SL
>Hsap_ENSP00000346603
MAAAEAVHHIHLQNFSRSLLETLNGQRLGGHFCDVTVRIREASLRAHRCVLAAGSPFFQDKLLLGHSEIRVPPVVPAQTV
RQLVEFLYSGSLVVAQGEALQVLTAASVLRIQTVIDECTQIIARARAPGTSAPTPLPTPVPPPLAPAQLRHRLRHLLAAR
PPGHPGAAHSRKQRQPARLQLPAPPTPAKAEGPDADPSLSAAPDDRGDEDDEESDDETDGEDGEGGGPGEGQAPPSFPDC
AAGFLTAAADSACEEPPAPTGLADYSGAGRDFLRGAGSAEDVFPDSYVSTWHDEDGAVPEGCPTETPVQPDCILSGSRPP
GVKTPGPPVALFPFHLGAPGPPAPPPSAPSGPAPAPPPAFYPTLQPEAAPSTQLGEVPAPSAAPTTAPSGTPARTPGAEP
PTYECSHCRKTFSSRKNYTKHMFIHSGEKPHQCAVCWRSFSLRDYLLKHMVTHTGVRAFQCAVCAKRFTQKSSLNVHMRT
HRPERAPCPACGKVFSHRALLERHLAAHPAP
>Hsap_ENSP00000347193
MHFKCKYSYPALYQMDHTSPTYMLANLTHLHSEQLLQGLNLLRQHHELCDIILRVGDVKIHAHKVVLASVSPYFKAMFTG
NLSEKENSEVEFQCIDETALQAIVEYAYTGTVFISQDTVESLLPAANLLQIKLVLKECCAFLESQLDPGNCIGISRFAET
YGCRDLYLAATKYICQNFEAVCQTEEFFELTHADLDEIVSNDCLNVATEETVFYALESWIKYDVQERQKYLAQLLNSVRL
PLLSVKFLTRLYEANHLIRDDRTCKHLLNEALKYHFMPEHRLSHQTVLMTRPRCAPKVLCAVGGKSGLFACLDSVEMYFP
QNDSWIGLAPLNIPRYEFGICVLDQKVYVIGGIATNVRPGVTIRKHENSVECWNPDTNTWTSLERMNESRSTLGVVVLAG
ELYALGGYDGQSYLQSVEKYIPKIRKWQPVAPMTTTRSCFAAAVLDGMIYAIGGYGPAHMNSVERYDPSKDSWEMVASMA
DKRIHFGVGVMLGFIFVVGGHNGVSHLSSIERYDPHQNQWTVCRPMKEPRTGVGAAVIDNYLYVVGGHSGSSYLNTVQKY

DPISDTWLDSAGMIYCRCNFGLTAL
>Hsap_ENSP00000349503
MEFFISMSETIKYNDDDHKTLFLKTLNEQRLEGEFCDIAIVVEDVKFRAHRCVLAACSTYFKKLFKKLEVDSSSVIEIDF
LRSDIFEEVLNYMYTAKISVKKEDVNLMMSSGQILGIRFLDKLCSQKRDVSSPDENNGQSKSKYCLKINRPIGDAADTQD
DDVEEIGDQDDSPSDDTVEGTPPSQEDGKSPTTTLRVQEAILKELGSEEVRKVNCYGQEVESMETPESKDLGSQTPQALT
FNDGMSEVKDEQTPGWTTAASDMKFEYLLYGHHREQIACQACGKTFSDEGRLRKHEKLHTADRPFVCEMCTKGFTTQAHL
KEHLKIHTGYKPYSCEVCGKSFIRAPDLKKHERVHSNERPFACHMCDKAFKHKSHLKDHERRHRGEKPFVCGSCTKAFAK
ASDLKRHENNMHSERKQVTPSAIQSETEQLQAAAMAAEAEQQLETIACS
>Hsap_ENSP00000351539
MCPKGYEDSMEFPDHSRHLLQCLSEQRHQGFLCDCTVLVGDAQFRAHRAVLASCSMYFHLFYKDQLDKRDIVHLNSDIVT
APAFALLLEFMYEGKLQFKDLPIEDVLAAASYLHMYDIVKVCKKKLKEKATTEADSTKKEEDASSCSDKVESLSDGSSHI
AGDLPSDEDEGEDEKLNILPSKRDLAAEPGNMWMRLPSDSAGIPQAGGEAEPHATAAGKTVASPCSSTESLSQRSVTSVR
DSADVDCVLDLSVKSSLSGVENLNSSYFSSQDVLRSNLVQVKVEKEASCDESDVGTNDYDMEHSTVKESVSTNNRVQYEP
AHLAPLREDSVLRELDREDKASDDEMMTPESERVQVEGGMESSLLPYVSNILSPAGQIFMCPLCNKVFPSPHILQIHLST
HFREQDGIRSKPAADVNVPTCSLCGKTFSCMYTLKRHERTHSGEKPYTCTQCGKSFQYSHNLSRHAVVHTREKPHACKWC
ERRFTQSGDLYRHIRKFHCELVNSLSVKSEALSLPTVRDWTLEDSSQELWK
>Hsap_ENSP00000351933
MKVSLGNGEMGVSAHLQPCKAGTTRFFTSNTHSSVVLQGFDQLRIEGLLCDVTLVPGDGDEIFPVHRAMMASASDYFKAM
FTGGMKEQDLMCIKLHGVNKVGLKKIIDFIYTAKLSLNMDNLQDTLEAASFLQILPVLDFCKVFLISGVSLDNCVEVGRI
ANTYNLIEVDKYVNNFILKNFPALLSTGEFLKLPFERLAFVLSSNSLKHCTELELFKAACRWLRLEDPRMDYAAKLMKNI
RFPLMTPQDLINYVQTVDFMRTDNTCVNLLLEASNYQMMPYMQPVMQSDRTAIRSDSTHLVTLGGVLRQQLVVSKELRMY
DERAQEWRSLAPMDAPRYQHGIAVIGNFLYVVGGQSNYDTKGKTAVDTVFRFDPRYNKWMQVASLNEKRTFFHLSALKGH
LYAVGGRSAAGELATVECYNPRMNEWSYVAKMSEPHYGHAGTVYGGLMYISGGITHDTFQNELMCFDPDTDKWMQKAPMT
TVRGLHCMCTVGDKLYVIGGNHFRGTSDYDDVLSCEYYSPTLDQWTPIAAMLRGQSDVGVAVFENKIYVVGGYSWNNRCM
VEIVQKYDPEKDEWHKVFDLPESLGGIRACTLTVFPPEENPGSPSRESPLSAPSDHS
>Hsap_ENSP00000352314
MSRSGDRTSTFDPSHSDNLLHGLNLLWRKQLFCDVTLTAQGQQFHCHKAVLASCSQYFRSLFSSHPPLGGGVGGQDGLGA
PKDQQQPPQQQPSQQQQPPPQEEPGTPSSSPDDKLLTSPRAINNLVLQGCSSIGLRLVLEYLYTANVTLSLDTVEEVLSV
SKILHIPQVTKLCVQFLNDQISVQNYKQVCKIAALHGLEETKKLANKYLVEDVLLLNFEEMRALLDSLPPPVESELALFQ
MSVLWLEHDRETRMQYAPDLMKRLRFALIPAPELVERVQSVDFMRTDPVCQKLLLDAMNYHLMPFRQHCRQSLASRIRSN
KKMLLLVGGLPPGPDRLPSNLVQYYDDEKKTWKILTIMPYNSAHHCVVEVENFLFVLGGEDQWNPNGKHSTNFVSRYDPR
FNSWIQLPPMQERRASFYACRLDKHLYVIGGRNETGYLSSVECYNLETNEWRYVSSLPQPLAAHAGAVHNGKIYISGGVH
NGEYVPWLYCYDPVMDVWARKQDMNTKRAIHTLAVMNDRLYAIGGNHLKGFSHLDVMLVECYDPKGDQWNILQTPILEGR
SGPGCAVLDDSIYLVGGYSWSMGAYKSSTICYCPEKGTWTELEGDVAEPLAGPACVTVILPSCVPYNK
>Hsap_ENSP00000356375
MKKRRKVTSNLEKIHLGYHKDSSEGNVAVECDQVTYTHSAGRPTPEALHCYQELPPSPDQRKLLSSLQYNKNLLKYLNDD
RQKQPSFCDLLIIVEGKEFSAHKVVVAVGSSYFHACLSKNPSTDVVTLDHVTHSVFQHLLEFLYTSEFFVYKYEIPLVLE
AAKFLDIIDAVKLLNNENVAPFHSELTEKSSPEETLNELTGRLSNNHQCKFCSRHFCYKKSLENHLAKTHRSLLLGKKHG
LKMLERSFSARRSKRNRKCPVKFDDTSDDEQESGDGSDNLNQENFDKEKSDRNDSEDPGSEYNAEEDELEEEMSDEYSDI
EEQSEKDHNDAEEEPEAGDSVGNVHEGLTPVVIQNSNKKILQCPKCDKTFDRIGKYESHTRVHTGEKPFECDICHQRYST
KSNLTVHRKKHSNETEFHKKEHKCPYCNKLHASKKTLAKHVKRFHPENAQEFISIKKTKSESWKCDICKKSFTRRPHLEE
HMILHSQDKPFKCTYCEEHFKSRFARLKHQEKFHLGPFPCDICGRQFNDTGNLKRHIECTHGGKRKWTCFICGKSVRERT
TLKEHLRIHSGEKPHLCSICGQSFRHGSSYRLHLRVHHDDKRYECDECGKTFIRHDHLTKHKKIHSGEKAHQCEECGKCF
GRRDHLTVHYKSVHLGEKVWQKYKATFHQCDVCKKIFKGKSSLEMHFRTHSGEKPYKCQICNQSFRIKKTLTKHLVIHSD
ARPFNCQHCNATFKRKDKLKYHIDHVHEIKSPDDPLSTSEEKLVSLPVEYSSDDKIFQTETKQYMDQPKVYQSEAKTMLQ
NVSAEVCVPVTLVPVQMPDTPSDLVRHTTTLPPSSHEILSPQPQSTDYPRAADLAFLEKYTLTPQPANIVHPVRPEQMLD
PREQSYLGTLLGLDSTTGVQNISTNEHHS
>Hsap_ENSP00000356468
MIPNGYLMFEDENFIESSVAKLNALRKSGQFCDVRLQVCGHEMLAHRAVLACCSPYLFEIFNSDSDPHGISHVKFDDLNP
EAVEVLLNYAYTAQLKADKELVKDVYSAAKKLKMDRVKQVCGDYLLSRMDVTSCISYRNFASCMGDSRLLNKVDAYIQEH
LLQISEEEEFLKLPRLKLEVMLEDNVCLPSNGKLYTKVINWVQRSIWENGDSLEELMEEVQTLYYSADHKLLDGNLLDGQ
AEVFGSDDDHIQFVQKKPPRENGHKQISSSSTGCLSSPNATVQSPKHEWKIVASEKTSNNTYLCLAVLDGIFCVIFLHGR
NSPQSSPTSTPKLSKSLSFEMQQDELIEKPMSPMQYARSGLGTAEMNGKLIAAGGYNREECLRTVECYNPHTDHWSFLAP
MRTPRARFQMAVLMGQLYVVGGSNGHSDDLSCGEMYDSNIDDWIPVPELRTNRCNAGVCALNGKLYIVGGSDPYGQKGLK
NCDVFDPVTKLWTSCAPLNIRRHQSAVCELGGYLYIIGGAESWNCLNTVERYNPENNTWTLIAPMNVARRGAGVAVLNGK
LFVCGGFDGSHAISCVEMYDPTRNEWKMMGNMTSPRSNAGIATVGNTIYAVGGFDGNEFLNTVEVYNLESNEWSPYTKIF
QF
>Hsap_ENSP00000356674
MEKGGNIQLEIPDFSNSVLSHLNQLRMQGRLCDIVVNVQGQAFRAHKVVLAASSPYFRDHMSLNEMSTVSISVIKNPTVF
EQLLSFCYTGRICLQLADIISYLTAASFLQMQHIIDKCTQILEGIHFKINVAEVEAELSQTRTKHQERPPESHRVTPNLN
RSLSPRHNTPKGNRRGQVSAVLDIRELSPPEESTSPQIIEPSSDVESREPILRINRAGQWYVETGVADRGGRSDDEVRVL
GAVHIKTENLEEWLGPENQPSGEDGSSAEEVTAMVIDTTGHGSVGQENYTLGSSGAKVARPTSSEVDRFSPSGSVVPLTE

RHRARSESPGRMDEPKQPSSQVEESAMMGVSGYVEYLREQEVSERWFRYNPRLTCIYCAKSFNQKGSLDRHMRLHMGITP
FVCRMCGKKYTRKDQLEYHIRKHTGNKPFHCHVCGKSFPFQAILNQHFRKNHPGCIPLEGPHSISPETTVTSRGQAEEES
PSQEETVAPGEAVQGSVSTTGPD
>Hsap_ENSP00000358265
MPSERCLSIQEMLTGQRLCHSESHNDSVLAALNQQRSDGILCDITLIAEEQKFHAHKAVLAACSDYFRAMFSLCMVESGA
DEVNLHGVTSLGLKQALEFAYTGQILLEPGVIQDVLAAGSHLQLLELLNLCSHYLIQELNSFNYLDLYRLADLFNLTLLE
KAVIDFLVKHLSELLKSRPEEVLTLPYCLLQEVLKSDRLTSLSEEQIWQLAVRWLEHNCHYQYMDELLQYIRFGLMDVDT
LHTVALSHPLVQASETATALVNEALEYHQSIYAQPVWQTRRTKPRFQSDTLYIIGGKKREVCKVKELRYFNPVDQENALI
AAIANWSELAPMPVGRSHHCVAVMGDFLFVAGGEVEHASGRTCAVRTACRYDPRSNSWAEIAPMKNCREHFVLGAMEEYL
YAVGGRNELRQVLPTVERYCPKKNKWTFVQSFDRSLSCHAGYVADGLLWISGGVTNTAQYQNRLMVYEPNQNKWISRSPM
LQRRVYHSMAAVQRKLYVLGGNDLDYNNDRILVRHIDSYNIDTDQWTRCNFNLLTGQNESGVAVHNGRIYLVGGYSIWTN
EPLACIQVLDVSREGKEEVFYGPTLPFASNGIAACFLPAPYFTCPNLQTLQVPHHRIGTI
>Hsap_ENSP00000359942
MAPKKKIVKKNKGDINEMTIIVEDSPLNKLNALNGLLEGGNGLSCISSELTDASYGPNLLEGLSKMRQENFLCDLVIGTK
TKSFDVHKSVMASCSEYFYNILKKDPSIQRVDLNDISPLGLATVIAYAYTGKLTLSLYTIGSIISAAVYLQIHTLVKMCS
DFLIREMSVENCMYVVNIAETYSLKNAKAAAQKFIRDNFLEFAESDQFMKLTFEQINELLIDDDLQLPSEIVAFQIAMKW
LEFDQKRVKYAADLLSNIRFGTISAQDLVNYVQSVPRMMQDADCHRLLVDAMNYHLLPYHQNTLQSRRTRIRGGCRVLVT
VGGRPGLTEKSLSRDILYRDPENGWSKLTEMPAKSFNQCVAVMDGFLYVAGGEDQNDARNQAKHAVSNFCRYDPRFNTWI
HLASMNQKRTHFSLSVFNGLVYAAGGRNAEGSLASLECYVPSTNQWQPKTPLEVARCCHASAVADGRVLVTGGYIANAYS
RSVCAYDPASDSWQELPNLSTPRGWHCAVTLSDRVYVMGGSQLGPRGERVDVLTVECYSPATGQWSYAAPLQVGVSTAGV
SALHGRAYLVGGWNEGEKKYKKCIQCFSPELNEWTEDDELPEATVGVSCCTLSMPNNVTRESRASSVSSVPVSI
>Hsap_ENSP00000362206
MSVSGKKEFDVKQILRLRWRWFSHPFQGSTNTGSCLQQEGYEHRGTPVQGRLKSHSRDRNGLKKSNSPVHHNILAPVPGP
APAHQRAVQNLQQHNLIVHFQANEDTPKSVPEKNLFKEACEKRAQDLEMMADDNIEDSTARLDTQHSEDMNATRSEEQFH
VINHAEQTLRKMENYLKEKQLCDVLLIAGHLRIPAHRLVLSAVSDYFAAMFTNDVLEAKQEEVRMEGVDPNALNSLVQYA
YTGVLQLKEDTIESLLAAACLLQLTQVIDVCSNFLIKQLHPSNCLGIRSFGDAQGCTELLNVAHKYTMEHFIEVIKNQEF
LLLPANEISKLLCSDDINVPDEETIFHALMQWVGHDVQNRQGELGMLLSYIRLPLLPPQLLADLETSSMFTGDLECQKLL
MEAMKYHLLPERRSMMQSPRTKPRKSTVGALYAVGGMDAMKGTTTIEKYDLRTNSWLHIGTMNGRRLQFGVAVIDNKLYV
VGGRDGLKTLNTVECFNPVGKIWTVMPPMSTHRHGLGVATLEGPMYAVGGHDGWSYLNTVERWDPEGRQWNYVASMSTPR
STVGVVALNNKLYAIGGRDGSSCLKSMEYFDPHTNKWSLCAPMSKRRGGVGVATYNGFLYVVGGHDAPASNHCSRLSDCV
ERYDPKGDSWSTVAPLSVPRDAVAVCPLGDKLYVVGGYDGHTYLNTVESYDAQRNEWKESMQELLQNFYTTQKLKETLGH
>Hsap_ENSP00000362556
MEPGTNSFRVEFPDFSSTILQKLNQQRQQGQLCDVSIVVQGHIFRAHKAVLAASSPYFCDQVLLKNSRRIVLPDVMNPRV
FENILLSSYTGRLVMPAPEIVSYLTAASFLQMWHVVDKCTEVLEGNPTVLCQKLNHGSDHQSPSSSSYNGLVESFELGSG
GHTDFPKAQELRDGENEEESTKDELSSQLTEHEYLPSNSSTEHDRLSTEMASQDGEEGASDSAEFHYTRPMYSKPSIMAH
KRWIHVKPERLEQACEGMDVHATYDEHQVTESINTVQTEHTVQPSGVEEDFHIGEKKVEAEFDEQADESNYDEQVDFYGS
SMEEFSGERSDGNLIGHRQEAALAAGYSENIEMVTGIKEEASHLGFSATDKLYPCQCGKSFTHKSQRDRHMSMHLGLRPY
GCGVCGKKFKMKHHLVGHMKIHTGIKPYECNICAKRFMWRDSFHRHVTSCTKSYEAAKAEQNTTEAN
>Hsap_ENSP00000362609
MEISSHQSHLLQQLNEQRRQDVFCDCSILVEGKVFKAHRNVLFASSGYFKMLLSQNSKETSQPTTATFQAFSPDTFTVIL
DFVYSGKLSLTGQNVIEVMSAASFLQMTDVISVCKTFIKSSLDISEKEKDRYFSLSDKDANSNGVERSSFYSGGWQEGSS
SPRSHLSPEQGTGIISGKSWNKYNYHPASQKNTQQPLAKHEPRKESIKKTKHLRLSQPSEVTHYKSSKREVRTSDSSSHV
SQSEEQAQIDAEMDSTPVGYQYGQGSDVTSKSFPDDLPRMRFKCPYCTHVVKRKADLKRHLRCHTGERPYPCQACGKRFS
RLDHLSSHFRTIHQACKLICRKCKRHVTDLTGQVVQEGTRRYRLCNECLAEFGIDSLPIDLEAEQHLMSPSDGDKDSRWH
LSEDENRSYVEIVEDGSGDLVIQQVDDSEEEEEKEIKPNIR
>Hsap_ENSP00000362758
MSERSDLLHFKFENYGDSMLQKMNKLREENKFCDVTVLIDDIEVQGHKIVFAAGSPFLRDQFLLNDSREVKISILQSSEV
GRQLLLSCYSGVLEFPEMELVNYLTAASFLQMSHIVERCTQALWKFIKPKQPMDSKEGCEPQSASPQSKEQQGDARGSPK
QDSPCIHPSEDSMDMEDSDIQIVKVESIGDVSEVRSKKDQNQFISSEPTALHSSEPQHSLINSTVENRVSEIEQNHLHNY
ALSYTGSDNIIMASKDVFGPNIRGVDKGLQWHHQCPKCTRVFRHLENYANHLKMHKLFMCLLCGKTFTQKGNLHRHMRVH
AGIKPFQCKICGKTFSQKCSLQDHLNLHSGDKPHKCNYCDMVFAHKPVLRKHLKQLHGKNSFDNANERNVQDLTVDFDSF
ACTTVTDSKGCQPQPDATQVLDAGKLAQAVLNLRNDSTCVN
>Hsap_ENSP00000362763
MAAESDVLHFQFEQQGDVVLQKMNLLRQQNLFCDVSIYINDTEFQGHKVILAACSTFMRDQFLLTQSKHVRITILQSAEV
GRKLLLSCYTGALEVKRKELLKYLTAASYLQMVHIVEKCTEALSKYLEIDLSMKNNNQHTDLCQSSDPDVKNEDENSDKD
CEIIEISEDSPVNIDFHVKEEESNALQSTVESLTSERKEMKSPELSTVDIGFKDNEICILHVESISTAGVENGQFSQPCT
SSKASMYFSETQHSLINSTVESRVAEVPGNQDQGLFCENTEGSYGTVSEIQNLEEGYSLRHQCPRCPRGFLHVENYLRHL
KMHKLFLCLQCGKTFTQKKNLNRHIRGHMGIRPFQCTVCLKTFTAKSTLQDHLNIHSGDRPYKCHCCDMDFKHKSALKKH
LTSVHGRSSGEKLSRPDLKRQSLL
>Hsap_ENSP00000364515
MPRRGYSKPGSWGSFWAMLTLVGLVTHAAQRADVGGEAAGTSINHSQAVLQRLQELLRQGNASDVVLRVQAAGTDEVRVF
HAHRLLLGLHSELFLELLSNQSEAVLQEPQDCAAVFDKFIRYLYCGELTVLLTQAIPLHRLATKYGVSSLQRGVADYMRA

HLAGGAGPAVGWYHYAVGTGDEALRESCLQFLAWNLSAVAASTEWGAVSPELLWQLLQRSDLVLQDELELFHALEAWLGR
ARPPPAVAERALRAIRYPMIPPAQLFQLQARSAALARHGPAVADLLLQAYQFHAASPLHYAKFFDVNGSAFLPRNYLAPA
WGAPWVINNPARDDRSTSFQTQLGPSGHDAGRRVTWNVLFSPRWLPVSLRPVYADAAGTALPAARPEDGRPRLVVTPASS
GGDAAGVSFQKTVLVGARQQGRLLVRHAYSFHQSSEEAGDFLAHADLQRRNSEYLVENALHLHLIVKPVYHTLIRTPK
>Hsap_ENSP00000364675
MASGVEVLRFQLPGHEAATLRNMNQLRAEERFCDVTIVADSLKFRGHKVILAACSPFLRDQFLLNPSSELQVSLMHSARI
VADLLLSCYTGALEFAVRDIVNYLTAASYLQMEHVVEKCRNALSQFIEPKIGLKEDGVSEASLVSSISATKSLLPPARTP
KPAPKPPPPPPLPPPLLRPVKLEFPLDEDLELKAEEEDEDEDEDVSDICIVKVESALEVAHRLKPPGGLGGGLGIGGSVG
GHLGELAQSSVPPSTVAPPQGVVKACYSLSEDAEGEGLLLIPGGRASVGATSGLVEAAAVAMAARGAGGSLGAGGSRGPL
PGGFSGGNPLKNIKCTKCPEVFQGVEKLVFHMRAQHFIFMCPRCGKQFNHSSNLNRHMNVHRGVKSHSCGICGKCFTQKS
TLHDHLNLHSGARPYRCSYCDVRFAHKPAIRRHLKEQHGKTTAENVLEASVAEINVLIR
>Hsap_ENSP00000364798
MELPNYSRQLLQQLYTLCKEQQFCDCTISIGTIYFRAHKLVLAAASLLFKTLLDNTDTISIDASVVSPEEFALLLEMMYT
GKLPVGKHNFSKIISLADSLQMFDVAVSCKNLLTSLVNCSVQGQVVRDVSAPSSETFRKEPEKPQVEILSSEGAGEPHSS
PELAATPGGPVKAETEEAAHSVSQEMSVNSPTAQESQRNAETPAETPTTAEACSPSPAVQTFSEAKKTSTEPGCERKHYQ
LNFLLENEGVFSDALMVTQDVLKKLEMCSEIKGPQKEMIVKCFEGEGGHSAFQRILGKVREESLDVQTVVSLLRLYQYSN
PAVKTALLDRKPEDVDTVQPKGSTEEGKTLSVLLLEHKEDLIQCVTQLRPIMESLETAKEEFLTGTEKRVILNCCEGRTP
KETIENLLHRMTEEKTLTAEGLVKLLQAVKTTFPNLGLLLEKLQKSATLPSTTVQPSPDDYGTELLRRYHENLSEIFTDN
QILLKMISHMTSLAPGEREVMEKLVKRDSGSGGFNSLISAVLEKQTLSATAIWQLLLVVQETKTCPLDLLMEEIRREPGA
DAFFRAVTTPEHATLETILRHNQLILEAIQQKIEYKLFTSEEEHLAETVKEILSIPSETASPEASLRAVLSRAMEKSVPA
IEICHLLCSVHKSFPGLQPVMQELAYIGVLTKEDGEKETWKVSNKFHLEANNKEDEKAAKEDSQPGEQNDQGETGSLPGQ
QEKEASASPDPAKKSFICKACDKSFHFYCRLKVHMKRCRVAKSKQVQCKECSETKDSKKELDKHQLEAHGAGGEPDAPKK
KKKRLPVTCDLCGREFAHASGMQYHKLTEHFDEKPFSCEECGAKFAANSTLKNHLRLHTGDRPFMCKHCLMTFTQASALA
YHTKKKHSEGKMYACQYCDAVFAQSIELSRHVRTHTGDKPYVCRDCGKGFRQANGLSIHLHTFHNIEDPYDCKKCRMSFP
TLQDHRKHIHEVHSKEYHPCPTCGKIFSAPSMLERHVVTHVGGKPFSCGICNKAYQQLSGLWYHNRTHHPDVFAAQNHRS
SKFSSLQCSSCDKTFPNTIEHKKHIKAEHADMKFHECDQCKELFPTPALLQVHVKCQHSGSQPFRCLYCAATFRFPGALQ
HHVTTEHFKQSETTFPCELCGELFTSQAQLDSHLESEHPKVMSTETQAAASQMAQVIQTPEPVAPTEQVITLEETQLAGS
QVFVTLPDSQASQASSELVAVTVEDLLDGTVTLICGEAK
>Hsap_ENSP00000364885
MDFPQHSQHVLEQLNQQRQLGLLCDCTFVVDGVHFKAHKAVLAACSEYFKMLFVDQKDVVHLDISNAAGLGQVLEFMYTA
KLSLSPENVDDVLAVATFLQMQDIITACHALKSLAEPATSPGGNAEALATEGGDKRAKEEKVATSTLSRLEQAGRSTPIG
PSRDLKEERGGQAQSAASGAEQTEKADAPREPPPVELKPDPTSGMAAAEAEAALSESSEQEMEVEPARKGEEEQKEQEEQ
EEEGAGPAEVKEEGSQLENGEAPEENENEESAGTDSGQELGSEARGLRSGTYGDRTESKAYGSVIHKCEDCGKEFTHTGN
FKRHIRIHTGEKPFSCRECSKAFSDPAACKAHEKTHSPLKPYGCEECGKSYRLISLLNLHKKRHSGEARYRCEDCGKLFT
TSGNLKRHQLVHSGEKPYQCDYCGRSFSDPTSKMRHLETHDTDKEHKCPHCDKKFNQVGNLKAHLKIHIADGPLKCRECG
KQFTTSGNLKRHLRIHSGEKPYVCIHCQRQFADPGALQRHVRIHTGEKPCQCVMCGKAFTQASSLIAHVRQHTGEKPYVC
ERCGKRFVQSSQLANHIRHHDNIRPHKCSVCSKAFVNVGDLSKHIIIHTGEKPYLCDKCGRGFNRVDNLRSHVKTVHQGK
AGIKILEPEEGSEVSVVTVDDMVTLATEALAATAVTQLTGPATLPAVVPVGAAVTADETEVLKAEISKAVKQVQEEDPNT
HILYACDSCGDKFLDANSLAQHVRIHTAQALVMFQTDADFYQQYGPGGTWPAGQVLQAGELVFRPRDGAEGQPALAETSP
TAPECPPPAE
>Hsap_ENSP00000366886
MERPAPLAVLPFSDPAHALSLLRGLSQLRAERKFLDVTLEAAGGRDFPAHRAVLAAASPYFRAMFAGQLRESRAERVRLH
GVPPDMLQLLLDFSYTGRVAVSGDNAEPLLRAADLLQFPAVKEACGAFLQQQLDLANCLDMQDFAEAFSCSGLASAAQRF
ILRHVGELGAEQLERLPLARLLRYLRDDGLCVPKEEAAYQLALRWVRADPPRRAAHWPQLLEAVRLPFVRRFYLLAHVEA
EPLVARCPPCLRLLREARDFQAARYDRHDRGPCPRMRPRPSTGLAEILVLVGGCDQDCDELVTVDCYNPQTGQWRYLAEF
PDHLGGGYSIVALGNDIYVTGGSDGSRLYDCVWRYNSSVNEWAEVAPMLKAREYHSSSVLDGLLYVVAADSTERYDHTTD
SWEALQPMTYPMDNCSTTACRGRLYAIGSLAGKETMVMQCYDPDTDLWSLVDCGQLPPWSFAPKTATLNGLMYFVRDDSA
EVDVYNPTRNEWDKIPSMNQVHVGGSLAVLGGKLYVSGGYDNTFELSDVVEAYDPETRAWSVVGRLPEPTFWHGSVSIFR
QFMPQTFSGGRGFELDSGSDDMDPGRPRPPRDPDELH
>Hsap_ENSP00000366902
MDGSFVQHSVRVLQELNKQREKGQYCDATLDVGGLVFKAHWSVLACCSHFFQSLYGDGSGGSVVLPAGFAEIFGLLLDFF
YTGHLALTSGNRDQVLLAARELRVPEAVELCQSFKPKTSVGQAAGGQSGLGPPASQNVNSHVKEPAGLEEEEVSRTLGLV
PRDQEPRGSHSPQRPQLHSPAQSEGPSSLCGKLKQALKPCPLEDKKPEDCKVPPRPLEAEGAQLQGGSNEWEVVVQVEDD
GDGDYMSEPEAVLTRRKSNVIRKPCAAEPALSAGSLAAEPAENRKGTAVPVECPTCHKKFLSKYYLKVHNRKHTGEKPFE
CPKCGKCYFRKENLLEHEARNCMNRSEQVFTCSVCQETFRRRMELRVHMVSHTGEMPYKCSSCSQQFMQKKDLQSHMIKL
HGAPKPHACPTCAKCFLSRTELQLHEAFKHRGEKLFVCEECGHRASSRNGLQMHIKAKHRNERPHVCEFCSHAFTQKANL
NMHLRTHTGEKPFQCHLCGKTFRTQASLDKHNRTHTGERPFSCEFCEQRFTEKGPLLRHVASRHQEGRPHFCQICGKTFK
AVEQLRVHVRRHKGVRKFECTECGYKFTRQAHLRRHMEIHDRVENYNPRQRKLRNLIIEDEKMVVVALQPPAELEVGSAE
VIVESLAQGGLASQLPGQRLCAEESFTGPGVLEPSLIITAAVPEDCDT
>Hsap_ENSP00000367075
MSGSGRKDFDVKHILRLRWKLFSHPSPSTGGPAGGGCLQQDGSGSFEHWGPSQSRLLKSQERSGVSTFWKKPSSSSSSSS
SPSSSSSSFNPLNGTLLPVATRLQQGAPGQGTQQPARTLFYVESLEEEVVPGMDFPGPHEKGLVLQELKVEPDNSSQATG

EGCGHRLSSTGHSMTPQSDLDSSSSEEFYQAVHHAEQTFRKMESYLKQQQLCDVILIVGNRKIPAHRLVLSSVSDYFAAM
FTSDVCEAKQEEIKMEGIDPNALWDLVQFAYTGCLELKEDTIENLLAAACLLQLPQVVEVCCHFLMKLLHPSNCLGIRAF
ADAQGCIELMKVAHSYTMENIMEVIRNQEFLLLPAEELHKLLASDDVNVPDEETIFHALMMWVKYDMQSRCNDLSMLLAF
IRLPLLPPQILADLENHALFKNDLECQKLILEAMKYHLLPERRTLMQSPRTKPRKSTVGTLYAVGGMDNNKGATTIEKYD
LRTNLWIQAGMMNGRRLQFGVAVIDDKLFVIGGRDGLKTLNTVECYNPKTKTWTVLPPMSTHRHGLGVTVLEGPIYAVGG
HDGWSYLNTVERWDPQSQQWTFVASMSIARSTVGVAALNGKLYSVGGRDGSSCLSSMEYYDPHTNKWNMCAPMCKRRGGV
GVATCDGFLYAVGGHDAPASNHCSRLLDYVERYDPKTDTWTMVAPLSMPRDAVGVCLLGDRLYAVGGYDGQTYLNTMESY
DPQTNEWTQMASLNIGRAGACVVVIKQP
>Hsap_ENSP00000367471
MVDDKEKNMKCLTFFLMLPETVKNRSKKSSKKANTSSSSSNSSKLPPVCYEIITLKTKKKKMAADIFPRKKPANSSSTSV
QQYHQQNLSNNNLIPAPNWQGLYPTIRERNAMMFNNDLMADVHFVVGPPGGTQRLPGHKYVLAVGSSVFHAMFYGELAED
KDEIRIPDVEPAAFLAMLKYIYCDEIDLAADTVLATLYAAKKYIVPHLARACVNFLETSLSAKNACVLLSQSCLFEEPDL
TQRCWEVIDAQAELALKSEGFCDIDFQTLESILRRETLNAKEIVVFEAALNWAEVECQRQDLALSIENKRKVLGKALYLI
RIPTMALDDFANGAAQSGVLTLNETNDIFLWYTAAKKPELQFVSKARKGLVPQRCHRFQSCAYRSNQWRYRGRCDSIQFA
VDKRVFIAGFGLYGSSCGSAEYSAKIELKRQGVVLGQNLSKYFSDGSSNTFPVWFEYPVQIEPDTFYTASVILDGNELSY
FGQEGMTEVQCGKVTVQFQCSSDSTNGTGVQGGQIPELIFYA
>Hsap_ENSP00000368797
MQSREDVPRSRRLASPRGGRRPKRISKPSVSAFFTGPEELKDTAHSAALLAQLKSFYDARLLCDVTIEVVTPGSGPGTGR
LFSCNRNVLAAACPYFKSMFTGGMYESQQASVTMHDVDAESFEVLVDYCYTGRVSLSEANVQRLYAASDMLQLEYVREAC
ASFLARRLDLTNCTAILKFADAFDHHKLRSQAQSYIAHNFKQLSRMGSIREETLADLTLAQLLAVLRLDSLDIESERTVC
HVAVQWLEAAAKERGPSAAEVFKCVRWMHFTEEDQDYLEGLLTKPIVKKYCLDVIEGALQMRYGDLLYKSLVPVPNSSSS
SSSSNSLVSAAENPPQRLGMCAKEMVIFFGHPRDPFLCYDPYSGDIYTMPSPLTSFAHTKTVTSSAVCVSPDHDIYLAAQ
PRKDLWVYKPAQNSWQQLADRLLCREGMDVAYLNGYIYILGGRDPITGVKLKEVECYSVQRNQWALVAPVPHSFYSFELI
VVQNYLYAVNSKRMLCYDPSHNMWLNCASLKRSDFQEACVFNDEIYCICDIPVMKVYNPARGEWRRISNIPLDSETHNYQ
IVNHDQKLLLITSTTPQWKKNRVTVYEYDTREDQWINIGTMLGLLQFDSGFICLCARVYPSCLEPGQSFITEEDDARSES
STEWDLDGFSELDSESGSSSSFSDDEVWVQVAPQRNAQDQQGSL
>Hsap_ENSP00000368799
MQSREDAPRSRRLASPRGGKRPKKIHKPTVSAFFTGPEELKDTAHSAALLAQLKSFYDARLLCDVTIEVVTPGSGPGTGR
LFPCNRNVLAAACPYFKSMFTGGMYESQQASVTMHDVDAESFEVLVDYCYTGRVSLSEANVERLYAASDMLQLEYVREAC
ASFLARRLDLTNCTAILKFADAFGHRKLRSQAQSYIAQNFKQLSHMGSIREETLADLTLAQLLAVLRLDSLDVESEQTVC
HVAVQWLEAAPKERGPSAAEVFKCVRWMHFTEEDQDYLEGLLTKPIVKKYCLDVIEGALQMRYGDLLYKSLVPVPNSSSS
SSSSNSLVSAAENPPQRLGMCAKEMVIFFGHPRDPFLCCDPYSGDLYKVPSPLTCLAHTRTVTTLAVCISPDHDIYLAAQ
PRTDLWVYKPAQNSWQQLADRLLCREGMDVAYLNGYIYILGGRDPITGVKLKEVECYNVKRNQWALVAPLPHSFLSFDLM
VIRDYLYALNSKRMFCYDPSHNMWLKCVSLKRNDFQEACVFNEEIYCICDIPVMKVYNPVRAEWRQMNNIPLVSETNNYR
IIKHGQKLLLITSRTPQWKKNRVTVYEYDIRGDQWINIGTTLGLLQFDSNFFCLSARVYPSCLEPGQSFLTEEEEIPSES
STEWDLGGFSEPDSESGSSSSLSDDDFWVRVAPQ
>Hsap_ENSP00000368813
MSYFLSYCKAHGGALLTGYQALRAEGFLCDVTLETEGSEFPAHRSLLACSSDYFRALFKSHTQESRARVIHLHVPSAAGL
QRLLDFIYTAWLSLSMDTVEDTLEAASYLQVTEALGLCGRYLERQLAPENCCFAANVAARFGLAHTLDAAERCIVSHLQE
LLARGAGPAGLLELNPTSLRAVLGAPDVARVPEARLLGLALAWLRQEPTTERLAHCTELLERVRFGLVPADVLRRVYSGS
GLVLPARVKGLIIQALNYHTTPSRQPLMQGEQTSIRSPQTRILLVGGRRAREVVIEEVAAPQRAARGQVAAPEPEEEEEE
LEEEEEEEEWELTQNVVAFDVYNHRWRSLTQLPTPLLGHSVCTAGNFLFVLGGESPSGSASSPLADDSRVVTAQVHRYDP
RFHAWTEVPAMREARAHFWCGAVGERLLAVGGLGAGGEVLASVEMYDLRRDRWTAAGALPRALHGHAGAVGDRGVVYISG
GKAGRGEGGASSLRDLYVLGPEEQVWSKKAPMGTARFGHHMAVLRGAVFAFLGRYEPFSEIERYDPGADQWTRLRPLPYD
RFCYGLAVVEETALLLGGLKWRDSRQVPTRNVVGYDLDLDRWEDIGCALPWAWSGLRCAVLQLAEGGDDEREGEVGEALD
LVLG
>Hsap_ENSP00000369318
MSIHIVALGNEGDTFHQDNRPSGLIRTYLGRSPLVSGDESSLLLNAASTVARPVFTEYQASAFGNVKLVVHDCPVWDIFD
SDWYTSRNLIGGADIIVIKYNVNDKFSFHEVKDNYIPVIKRALNSVPVIIAAVGTRQNEELPCTCPLCTSDRGSCVSTTE
GIQLAKELGATYLELHSLDDFYIGKYFGGVLEYFMIQALNQKTSEKMKKRKMSNSFHGIRPPQLEQPEKMPVLKAEASHY
NSDLNNLLFCCQCVDVVFYNPNLKKVVEAHKIVLCAVSHVFMLLFNVKSPTDIQDSSIIRTTQDLFAINRDTAFPGASHE
SSGNPPLRVIVKDALFCSCLSDILRFIYSGAFQWEELEEDIRKKLKDSGDVSNVIEKVKCILKTPGKINCLRNCKTYQAR
KPLWFYNTSLKFFLNKPMLADVVFEIQGTTVPAHRAILVARCEVMAAMFNGNYMEAKSVLIPVYGVSKETFLSFLEYLYT
DSCCPAGIFQAMCLLICAEMYQVSRLQHICELFIITQLQSMPSRELASMNLDIVDLLKKAKFHHSDCLSTWLLHFIATNY
LIFSQKPEFQDLSVEERSFVEKHRWPSNMYLKQLAEYRKYIHSRKCRCLVM
>Hsap_ENSP00000372635
MMTVMSLSRDLKDDFHSDTVLSILNEQRIRGILCDVTIIVEDTKFKAHSNVLAASSLYFKNIFWSHTICISSHVLELDDL
KAEVFTEILNYIYSSTVVVKRQETVTDLAAAGKKLGISFLEDLTDRNFSNSPGPYVFCITEKGVVKEEKNEKRHEEPAIT
NGPRITNAFSIIETENSNNMFSPLDLRASFKKVSDSMRTASLCLERTDVCHEAEPVRTLAEHSYAVSSVAEAYRSQPVRE
HDGSSPGNTGKENCEALAAKPKTCRKPKTFSIPQDSDSATENIPPPPVSNLEVNQERSPQPAAVLTRSKSPNNEGDVHFS
REDENQSSDVPGPPAAEVPPLVYNCSCCSKAFDSSTLLSAHMQLHKPTQEPLVCKYCNKQFTTLNRLDRHEQICMRSSHM
PIPGGNQRFLENYPTIGQNGGSFTGPEPLLSENRIGEFSSTGSTLPDTDHMVKFVNGQMLYSCVVCKRSYVTLSSLRRHA

NVHSWRRTYPCHYCNKVFALAEYRTRHEIWHTGERRYQCIFCLETFMTYYILKNHQKSFHAIDHRLSISKKTANGGLKPS
VYPYKLYRLLPMKCKRAPYKSYRNSSYENARENSQMNESAPGTYVVQNPHSSELPTLNFQDTVNTLTNSPAIPLETSACQ
DIPTSANVQNAEGTKWGEEALKMDLDNNFYSTEVSVSSTENAVSSDLRAGDVPVLSLSNSSENAASVISYSGSAPSVIVH
SSQFSSVIMHSNAIAAMTSSNHRAFSDPAVSQSLKDDSKPEPDKVGRFASRPKSIKEKKKTTSHTRGEIPEESNYVADPG
GSLSKTTNIAEETSKIETYIAKPALPGTSTNSNVAPLCQITVKIGNEAIVKRHILGSKLFYKRGRRPKYQMQEEPLPQGN
DPEPSGDSPLGLCQSECMEMSEVFDDASDQDSTDKPWRPYYNYKPKKKSRQLKKMRKVNWRKEHGNRSPSHKCKYPAELD
CAVGKAPQDKPFEEEETKEMPKLQCELCDGDKAVGAGNQGRPHRHLTSRPYACELCAKQFQSPSTLKMHMRCHTGEKPYQ
CKTCGRCFSVQGNLQKHERIHLGLKEFVCQYCNKAFTLNETLKIHERIHTGEKRYHCQFCFQRFLYLSTKRNHEQRHIRE
HNGKGYACFQCPKICKTAAALGMHQKKHLFKSPSQQEKIGDVCHENSNPLENQHFIGSEDNDQKDNIQTGVENVVL
>Hsap_ENSP00000372683
MEPSPLSPSGAALPLPLSLAPPPLPLPAAAVVHVSFPEVTSALLESLNQQRLQGQLCDVSIRVQGREFRAHRAVLAASSP
YFHDQVLLKGMTSISLPSVMDPGAFETVLASAYTGRLSMAAADIVNFLTVGSVLQMWHIVDKCTELLREGRASATTTITT
AAATSVTVPGAGVPSGSGGTVAPATMGSARSHASSRASENQSPSSSNYFSPRESTDFSSSSQEAFAASAVGSGERRGGGP
VFPAPVVGSGGATSGKLLLEADELCDDGGDGRGAVVPGAGLRRPTYTPPSIMPQKHWVYVKRGGNCPAPAPLVPQDPDLE
EEEEEEDLVLTCEDDEDEELGGSSRVPVGGGPEATLSISDVRTLSEPPDKGEEQVNFCESSNDFGPYEGGGPVAGLDDSG
GPTPSSYAPSHPPRPLLPLDMQGNQILVFPSSSSSSSSQAPGQPPGNQAEHGAVTVGGTSVGSLGVPGSVGGVPGGTGSG
DGNKIFLCHCGKAFSHKSMRDRHVNMHLNLRPFDCPVCNKKFKMKHHLTEHMKTHTGLKPYECGVCAKKFMWRDSFMRHR
GHCERRHRLGGVGAVPGPGTPTGPSLPSKRESPGVGGGSGDEASAATPPSSRRVWSPPRVHKVEMGFGGGGGAN
>Hsap_ENSP00000376337
MAAELYAPASAAAADLANSNAGAAVGRKAGPRSPPSAPAPAPPPPAPAPPTLGNNHQESPGWRCCRPTLRERNALMFNNE
LMADVHFVVGPPGATRTVPAHKYVLAVGSSVFYAMFYGDLAEVKSEIHIPDVEPAAFLILLKYMYSDEIDLEADTVLATL
YAAKKYIVPALAKACVNFLETSLEAKNACVLLSQSRLFEEPELTQRCWEVIDAQAEMALRSEGFCEIDRQTLEIIVTREA
LNTKEAVVFEAVLNWAEAECKRQGLPITPRNKRHVLGRALYLVRIPTMTLEEFANGAAQSDILTLEETHSIFLWYTATNK
PRLDFPLTKRKGLAPQRCHRFQSSAYRSNQWRYRGRCDSIQFAVDRRVFIAGLGLYGSSSGKAEYSVKIELKRLGVVLAQ
NLTKFMSDGSSNTFPVWFEHPVQVEQDTFYTASAVLDGSELSYFGQEGMTEVQCGKVAFQFQCSSDSTNGTGVQGGQIPE
LIFYA
>Hsap_ENSP00000378201
MAKPSHSSYVLQQLNNQREWGFLCDCCIAIDDIYFQAHKAVLAACSSYFRMFFMNHQHSTAQLNLSNMKISAECFDLILQ
FMYLGKIMTAPSSFEQFKVAMNYLQLYNVPDCLEDIQDADCSSSKCSSSASSKQNSKMIFGVRMYEDTVARNGNEANRWC
AEPSSTVNTPHNREADEESLQLGNFPEPLFDVCKKSSVSKLSTPKERVSRRFGRSFTCDSCGFGFSCEKLLDEHVLTCTN
RHLYQNTRSYHRIVDIRDGKDSNIKAEFGEKDSSKTFSAQTDKYRGDTSQAADDSASTTGSRKSSTVESEIASEEKSRAA
ERKRIIIKMEPEDIPTDELKDFNIIKVTDKDCNESTDNDELEDEPEEPFYRYYVEEDVSIKKSGRKTLKPRMSVSADERG
GLENMRPPNNSSPVQEDAENASCELCGLTITEEDLSSHYLAKHIENICACGKCGQILVKGRQLQEHAQRCGEPQDLTMNG
LGNTEEKMDLEENPDEQSEIRDMFVEMLDDFRDNHYQINSIQKKQLFKHSACPFRCPNCGQRFETENLVVEHMSSCLDQD
MFKSAIMEENERDHRRKHFCNLCGKGFYQRCHLREHYTVHTKEKQFVCQTCGKQFLRERQLRLHNDMHKGMARYVCSICD
QGNFRKHDHVRHMISHLSAGETICQVCFQIFPNNEQLEQHMDVHLYTCGICGAKFNLRKDMRSHYNAKHLKRT
>Hsap_ENSP00000378286
MLREFSKWGVEASPGKAWERKRSLLRGAVGRYRGATGGDLFWAPFPSWGTMEFPEHSQQLLQSLREQRSQGFLCDCTVMV
GSTQFLAHRAVLASCSPFFQLFYKERELDKRDLVCIHNEIVTAPAFGLLLDFMYAGQLTLRGDTPVEDVLAAASYLHMND
IVKVCKRRLQARALAEADSTKKEEETNSQLPSLEFLSSTSRGTQPSLASAETSGHWGKGEWKGSAAPSPTVRPPDEPPMS
SGADTTQPGMEVDAPHLRAPHPPVADVSLASPSSSTETIPTNYFSSGISAVSLEPLPSLDVGPESLRVVEPKDPGGPLQG
FYPPASAPTSAPAPVSAPVPSQAPAPAEAELVQVKVEAIVISDEETDVSDEQPQGPERAFPSGGAVYGAQPSQPEAFEDP
GAAGLEEVGPSDHFLPTDPHLPYHLLPGAGQYHRGLVTSPLPAPASLHEPLYLSSEYEAAPGSFGVFTEDVPTCKTCGKT
FSCSYTLRRHATVHTRERPYECRYCLRSYTQSGDLYRHIRKAHNEDLAKRSKPDPEVGPLLGVQPLPGSPTADRQSSSGG
GPPKDFVLAPKTNI
>Hsap_ENSP00000378503
METPTPLPPVPASPTCNPAPRTIQIEFPQHSSSLLESLNRHRLEGKFCDVSLLVQGRELRAHKAVLAAASPYFHDKLLLG
DAPRLTLPSVIEADAFEGLLQLIYSGRLRLPLDALPAHLLVASGLQMWQVVDQCSEILRELETSGGGISARGGNSYHALL
STTSSTGGWCIRSSPFQTPVQSSASTESPASTESPVGGEGSELGEVLQIQVEEEEEEEEDDDDEDQGSATLSQTPQPQRV
SGVFPRPHGPHPLPMTATPRKLPEGESAPLELPAPPALPPKIFYIKQEPFEPKEEISGSGTQPGGAKEETKVFSGGDTEG
NGELGFLLPSGPGPTSGGGGPSWKPVDLHGNEILSGGGGPGGAGQAVHGPVKLGGTPPADGKRFGCLCGKRFAVKPKRDR
HIMLTFSLRPFGCGICNKRFKLKHHLTEHMKTHAGALHACPHCGRRFRVHACFLRHRDLCKGQGWATAHWTYK
>Hsap_ENSP00000379739
MANEDCPKAADSPFSSDKHAQLILAQINKMRNGQHFCDVQLQVGQESFKAHRLVLAASSPYFAALFTGGMKESSKDVVPI
LGIEAGIFQILLDFIYTGIVNIGVNNVQELIIAADMLQLTEVVHLCCEFLKGQIDPLNCIGIFQFSEQIACHDLLEFSEN
YIHVHFLEVHSGEEFLALTKDQLIKILRSEELSIEDEYQVFLAAMQWILKDLGKRRKHVVEVLDPIRFPLLPPQRLLKYI
EGVSDFNLRVALQTLLKEYCEVCKSPKENKFCSFLQTSKVRPRKKARKYLYAVGGYTRLQGGRWSDSRALSCVERFDTFS
QYWTTVSSLHQARSGLGVTVLGGMVYAIGGEKDSMIFDCTECYDPVTKQWTTVASMNHPRCGLGVCVCYGAIYALGGWVG
AEIGNTIERFDPDENKWEVVGNMAVSRYYFGCCEMQGLIYVIGGISNEGIELRSFEVYDPLSKRWSPLPPMGTRRAYLGV
AALNDCIYSVGGWNETQDALHTVEKYSFEEEKWVEVASMKVPRAGMCVVAVNGLLYVSGGRSSSHDFLAPGTLDSVEVYN
PHSDTWTEIGNMITSRCEGGVAVL
>Hsap_ENSP00000382450

MEAEETMECLQEFPEHHKMILDRLNEQREQDRFTDITLIVDGHHFKAHKAVLAACSKFFYKFFQEFTQEPLVEIEGVSKM
AFRHLIEFTYTAKLMIQGEEEANDVWKAAEFLQMLEAIKALEVRNKENSAPLEENTTGKNEAKKRKIAETSNVITESLPS
AESEPVEIEVEIAEGTIEVEDEGIETLEEVASAKQSVKYIQSTGSSDDSALALLADITSKYRQGDRKGQIKEDGCPSDPT
SKQVEGIEIVELQLSHVKDLFHCEKCNRSFKLFYHFKEHMKSHSTESFKCEICNKRYLRESAWKQHLNCYHLEEGGVSKK
QRTGKKIHVCQYCEKQFDHFGHFKEHLRKHTGEKPFECPNCHERFARNSTLKCHLTACQTGVGAKKGRKKLYECQVCNSV
FNSWDQFKDHLVIHTGDKPNHCTLCDLWFMQGNELRRHLSDAHNISERLVTEEVLSVETRVQTEPVTSMTIIEQVGKVHV
LPLLQVQVDSAQVTVEQVHPDLLQDSQVHDSHMSELPEQVQVSYLEVGRIQTEEGTEVHVEELHVERVNQMPVEVQTELL
EADLDHVTPEIMNQEERESSQADAAEAAREDHEDAEDLETKPTVDSEAEKAENEDRTALPVLE
>Hsap_ENSP00000384889
MVSGPLALRWCAWAGRGDMGPDMELPSHSKQLLLQLNQQRTKGFLCDVIIMVENSIFRAHKNVLAASSIYFKSLVLHDNL
INLDTDMVSSTVFQQILDFIYTGKLLPSDQPAEPNFSTLLTAASYLQLPELAALCRRKLKRAGKPFGSGRAGSTGMGRPP
RSQRLSTASVIQARYQGLVDGRKGAHAPQELPQAKGSDDELFLGGSNQDSVQGLGRAVCPAGGEAGLGGCSSSTNGSSGG
CEQELGLDLSKKSPPLPPATPGPHLTPDDAAQLSDSQHGSPPAASAPPVANSASYSELGGTPDEPMDLEGAEDNHLSLLE
APGGQPRKSLRHSTRKKEWGKKEPVAGSPFERREAGPKGPCPGEEGEGVGDRVPNGILASGAGPSGPYGEPPYPCKEEEE
NGKDASEDSAQSGSEGGSGHASAHYMYRQEGYETVSYGDNLYVCIPCAKGFPSSEQLNAHVETHTEEELFIKEEGAYETG
SGGAEEEAEDLSAPSAAYTAEPRPFKCSVCEKTYKDPATLRQHEKTHWLTRPFPCNICGKMFTQRGTMTRHMRSHLGLKP
FACDECGMRFTRQYRLTEHMRVHSGEKPYECQLCGGKFTQQRNLISHLRMHTSPS
>Hsap_ENSP00000385879
MECKIEGKEKYQHSLNLLNKIQNMKELAEMIDVVLTAEGEKFPCHRLVLAAFSPYFKAMFTCGLLECNQREVILYDITAE
SVSVLLNYMYNAALEINNANVQTVAMAAYFMQMEEVFSVCQKYMMDHMDASNCLGIYYFAKQIGAEDLSDRSKKYLYQHF
AEVSLHEEILEIEVHQFLTLIKSDDLNISREESILDLVLRWVNHNKELRTVHLVELLKQVRLELVNPSFLRQALRRNTML
LCDADCVDIIQNAFKAIKTPQQHSLNLRYGMETTSLLLCIGNNSSGIRSRHRSYGDASFCYDPVSRKTYFISSPKYGEGL
GTVCTGVVMENNTIIVAGEASASKLSRQKNKNVEIYRYHDRGNQFWEKLCTAEFRELYALGSIHNDLYVIGGQMKIKNQY
LITNCVDKYSVERDNWKRVSPLPLQLACHAVVTVNNKLYVIGGWTPQMDLPDEEPDRLSNKLLQYDPSQDQWSVRAPMKY
SKYRFSTAVVNSEIYVLGGIGCVGQDKGQVRKCLDVVEIYNPDGDFWREGPPMPSPLLSLRTNSTNAGAVDGKLYVCGGF
HGADRHEVISKEILELDPWENQWNVVAINVLMHDSYDVCLVARMNPRDLIPPPSDLVEEGNEH
>Hsap_ENSP00000386121
MSNSHPLRPFTAVGEIDHVHILSEHIGALLIGEEYGDVTFVVEKKRFPAHRVILAARCQYFRALLYGGMRESQPEAEIPL
QDTTAEAFTMLLKYIYTGRATLTDEKEEVLLDFLSLAHKYGFPELEDSTSEYLCTILNIQNVCMTFDVASLYSLPKLTCM
CCMFMDRNAQEVLSSEGFLSLSKTALLNIVLRDSFAAPEKDIFLALLNWCKHNSKENHAEIMQAVRLPLMSLTELLNVVR
PSGLLSPDAILDAIKVRSESRDMDLNYRGMLIPEENIATMKYGAQVVKGELKSALLDGDTQNYDLDHGFSRHPIDDDCRS
GIEIKLGQPSIINHIRILLWDRDSRSYSYFIEVSMDELDWVRVIDHSQYLCRSWQKLYFPARVCRYIRIVGTHNTVNKIF
HIVAFECMFTNKTFTLEKGLIVPMENVATIADCASVIEGVSRSRNALLNGDTKNYDWDSGYTCHQLGSGAIVVQLAQPYM
IGSIRLLLWDCDDRSYSYYVEVSTNQQQWTMVADRTKVSCKSWQSVTFERQPASFIRIVGTHNTANEVFHCVHFECPEQQ
SSQKEENSEESGTGDTSLAGQQLDSHALRAPSGSSLPSSPGSNSRSPNRQHQ
>Hsap_ENSP00000386389
MVRNVDDLDFHLPSHAQDMLDGLQRLRSQPKLADVTLLVGGRELPCHRGLLALSSPYFHAMFAGDFAESFSARVELRDVE
PAVVGQLVDFVYTGRLTITQGNVEALTRTAARLHFPSVQKVCGRYLQQQLDAANCLGICEFGEQQGLLGVAAKAWAFLRE
NFEAVAREDEFLQLPRERLVTCLAGDLLQVQPEQSRLEALMRWVRHDPQARAAHLPELLSLVHLDAVPRPCVQQLLASEP
LIQESEACRAALSQGHDGAPLALQQKLEEVLVVVGGQALEEEEAGEEPTPGLGNFAFYNSKAKRWMALPDFPDYHKWGFS
LAALNNNIYVTGGSRGTKTDTWSTTQAWCFPLKEASWKPVAPMLKPRTNHASAALNGEIYVIGGTTLDVVEVESYDPYTD
SWTPVSPALKYVSNFSAAGCRGRLYLVGSSACKYNALALQCYNPVTDAWSVIASPFLPKYLSSPRCAALHGELYLIGDNT
KKVYVYDPGANLWQKVQSQHSLHENGALVPLGDALYVTGGRWQGMEGDYHVEMEAYDTVRDTWTRHGALPRLWLYHGAST
VFLDVSKWTQPSGPTQEH
>Hsap_ENSP00000387462
MSFSEMNRRTLAFRGGGLVTASGGGSTNNNAGGEASAWPPQPQPRQPPPPAPPALQPPNGRGADEEVELEGLEPQDLEAS
AGPAAGAAEEAKELLLPQDAGGPTSLGGGAGGPLLAERNRRTLAFRGGGGGGLGNNGSSRGRPETSVWPLRHFNGRGPAT
VDLELDALEGKELMQDGASLSDSTEDEEEGASLGDGSGAEGGSCSSSRRSGGDGGDEVEGSGVGAGEGETVQHFPLARPK
SLMQKLQCSFQTSWLKDFPWLRYSKDTGLMSCGWCQKTPADGGSVDLPPVGHDELSRGTRNYKKTLLLRHHVSTEHKLHE
ANAQESEIPSEEGYCDFNSRPNENSYCYQLLRQLNEQRKKGILCDVSIVVSGKIFKAHKNILVAGSRFFKTLYCFSNKES
PNQNNTTHLDIAAVQGFSVILDFLYSGNLVLTSQNAIEVMTVASYLQMSEVVQTCRNFIKDALNISIKSEAPESVVVDYN
NRKPVNRDGLSSSRDQKIASFWATRNLTNLASNVKIENDGCNVDEGQIENYQMNDSSWVQDGSPEMAENESEGQTKVFIW
NNMGSQGIQETGKTRRKNQTTKRFIYNIPPNNETNLEDCSVMQPPVAYPEENTLLIKEEPDLDGALLSGPDGDRNVNANL
LAEAGTSQDGGDAGTSHDFKYGLMPGPSNDFKYGLIPGTSNDFKYGLIPGASNDFKYGLLPESWPKQETWENGESSLIMN
KLKCPHCSYVAKYRRTLKRHLLIHTGVRSFSCDICGKLFTRREHVKRHSLVHKKDKKYKCMVCKKIFMLAASVGIRHGSR
RYGVCVDCADKSQPGGQEGVDQGQDTEFPRDEEYEENEVGEADEELVDDGEDQNDPSRWDESGEVCMSLDD
>Hsap_ENSP00000388723
MARGPQTLVQVWVGGQLFQADRALLVEHCGFFRGLFRSGMRETRAAEVRLGVLSAGGFRATLQVLRGDRPALAAEDELLQ
AVECAAFLQAPALARFLEHNLTSDNCALLCDAAAAFGLRDVFHSAALFICDGERELAAELALPEARAYVAALRPSSYAAV
STHTPAPGFLEDASRTLCYLDEEEDAWRTLAALPLEASTLLAGVATLGNKLYIVGGVRGASKEVVELGFCYDPDGGTWHE
FPSPHQPRYDTALAGFDGRLYAIGGEFQRTPISSVERYDPAAGCWSFVADLPQPAAGVPCAQACGRLFVCLWRPADTTAV
VEYAVRTDAWLPVAELRRPQSYGHCMVAHRDSLYVVRNGPSDDFLHCAIDCLNLATGQWTALPGQFVNSKGALFTAVVRG

DTVYTVNRMFTLLYAIEGGTWRLLREKAGFPRPGSLQTFLLRLPPGAPGPVTSTTAEL
>Hsap_ENSP00000389910
MSALPRDLELSNPREKLTKPVQATLSSLKMLDVGKWPIFSLCSEEELQLIRQACVFGSAGNEVLYTTVNDEIFVLGTNCC
GCLGLGDVQSTIEPRRLDSLNGKKIACLSYGSGPHIVLATTEGEVFTWGHNAYSQLGNGTTNHGLVPCHISTNLSNKQVI
EVACGSYHSLVLTSDGEVFAWGYNNSGQVGSGSTVNQPIPRRVTGCLQNKVVVTIACGQMCCMAVVDTGEVYVWGYNGNG
QLGLGNSGNQPTPCRVAALQGIRVQRVACGYAHTLVLTDEGQVYAWGANSYGQLGTGNKSNQSYPTPVTVEKDRIIEIAA
CHSTHTSAAKTQGGHVYMWGQCRGQSVILPHLTHFSCTDDVFACFATPAVTWRLLSVEPDDHLTVAESLKREFDNPDTAD
LKFLVDGKYIYAHKVLLKIRCEHFRSSLEDNEDDIVEMSEFSYPVYRAFLEYLYTDSISLSPEEAVGLLDLATFYRENRL
KKLCQQTIKQGICEENAIALLSAAVKYDAQDLEEFCFRFCINHLTVVTQTSGFAEMDHDLLKNFISKASRVGAFKN
>Hsap_ENSP00000394472
MCSPASPKILYRNPRFLRLAFLQLHHQQQSDVFCDVLLQAEGEAVPAHCCILSACSPFFTERLERERPAQGGKVVLELGG
LKISTLRKLVDFLYTSEMEVSQEEAQDVLSAARQLRVSELESLQLEGGKLVKAPQGRRLNRECLQPTSAAPISARVVTPS
HHPHTPLPTNQTPCPLGAIRLKSLGKEEGPQENNRQNADNLSGTLLLKRKARACPTPQEKNSSPSSHSQEPRENKNDTAL
DPTVLSPPSLYPSVDKHLLPRKIRLSRSKPSPGICTSKPSSILSGSSSVPATPGRRLWRQRSVNKETPEDKPKPGRASPL
QSTPNPSGLGKTGGSRKRSPEVRAPNSDSAEEGQVGRVKLRKIVNGTCWEVVQETPLKNTQDSPQIPDPGGDFQEPSGTQ
PFSSNEQEMSPTRTELCQDSPMCTKLQDILVSASHSPDHPVVKSEFESSPELVEKEPMLAIDCREPYAFDTALLEQPCEA
EEYRITSAAATSELEEILDFMLCGSDIEPPIGSLESPGAEGCRTPTYHLTETGKNWIEGEEWCLPDMELWPRELTELEKE
PAGENRGPTELLSPLVMPSEVSEVLSVGGRWTPDLEITSSQPLDGQEDKLLHVSSLDTPQRSYGDLSPPCSNWVETGLEV
SLTTDELLYPSPKAGKEVSGHSELLGSLPASSEEEEIDVVDWTAEGRLVPTTVPSVWPDPSSESETEVDILT
>Hsap_ENSP00000395461
MEPLGLVVHGKAEPFSAALRSLVNNPRYSDVCFVVGQERQEVFAHRCLLACRCNFFQRLLGTEPGPGVPSPVVLSTVPTE
AFLAVLEFLYTNSVKLYRHSVLEVLTAAVEYGLEELRELCLQFVVKVLDVDLVCEALQVAVTFGLGQLQERCVAFIEAHS
QEALRTRGFLELSAAALLPLLRSDKLCVDEAELVRAARSWARVGAAVLERPVAEVAAPVVKELRLALLAPAELSALEEQN
RQEPLIPVEQIVEAWKCHALRRGDEARGAPCRRRRGTLPREHHRFLDLSFK
>Hsap_ENSP00000396292
MEPSPLSPSGAALPLPLSLAPPPLPLPAAAVVHVSFPEVTSALLESLNQQRLQGQLCDVSIRVQGREFRAHRAVLAASSP
YFHDQVLLKGMTSISLPSVMDPGAFETVLASAYTGRLSMAAADIVNFLTVGSVLQMWHIVDKCTELLREGRASATTTITT
AAATSVTVPGAGVPSGSGGTVAPATMGSARSHASSRASENQSPSSSNYFSPRESTDFSSSSQEAFAASAVGSGERRGGGP
VFPAPVVGSGGATSGKLLLEADELCDDGGDGRGAVVPGAGLRRPTYTPPSIMPQKHWVYVKRGGNCPAPTPLVPQDPDLE
EEEEEEDLVLTCEDDEDEELGGSSRVPVGGGPEATLSISDVRTLSEPPDKGEEQVNFCESSNDFGPYEGGGPVAGLDDSG
GPTPSSYAPSHPPRPLLPLDMQGNQILVFPSSSSSSSSQAPGQPPGNQAEHGAVTVGGTSVGSLGVPGSVGGVPGGTGSG
DGNKIFLCHCGKAFSHKSMRDRHVNMHLNLRPFDCPVCNKKFKMKHHLTEHMKTHTGLKPYECGVCAKKFMWRDSFMRHR
GHCERRHRLGGVGAVPGPGTPTGPSLPSKRESPGVGGGSGDEASAATPPSSRRVWSPPRVHKVEMGFGGGGGAN
>Hsap_ENSP00000400836
MEMQSYYAKLLGELNEQRKRDFFCDCSIIVEGRIFKAHRNILFANSGYFRALLIHYIQDSGRHSTASLDIVTSDAFSIIL
DFLYSGKLDLCGENVIEVMSAASYLQMNDVVNFCKTYIRSSLDICRKMEKEAAVAAAVAAAAAAAAAAAAAAAHQVDSES
PSSGREGTSCGTKSLVSSPAEGEKSVECLRESPCGDCGDCHPLELVVRDSLGGGSADSNLSTPPKRIEPKVEFDADEVEV
DVGEQLQQYAAPLNLAHVEEALPSGQAVDLAYSNYHVKQFLEALLRNSAAPSKDDADHHFSRSLEGRPEGAGVAMSSMMD
VQADWYGEDSGDVLVVPIKLHKCPFCPYTAKQKGILKRHIRSHTGERPYPCETCGKRFTRQEHLRSHALSVHRSNRPIIC
KGCRRTFTSHLSQGLRRFGLCDSCTCVTDTPDDDDDLMPINLSLVEASSESQEKSDTDNDWPIYVESGEENDPAGDDSDD
KPQIQPNLSDRETLT
>Hsap_ENSP00000401878
MAASADLSKSSPTPNGIPSSDPASDAMDPFHACSILKQLKTMYDEGQLTDIVVEVDHGKTFSCHRNVLAAISPYFRSMFT
SGLTESTQKEVRIVGVEAESMDLVLNYAYTSRVILTEANVQALFTAASIFQIPSIQDQCAKYMISHLDPQNSIGVFIFAD
HYGHQELGDRSKEYIRKKFLCVTKEQEFLQLTKDQLISILDSDDLNVDREEHVYESIIRWFEHEQNEREVHLPEIFAKCI
RFPLMEDTFIEKIPPQFAQAIAKSCVEKGPSNTNGCTQRLGMTASEMIICFDAAHKHSGKKQTVPCLDIVTGRVFKLCKP
PNDLREVGILVSPDNDIYIAGGYRPSSSEVSIDHKAENDFWMYDHSTNRWLSKPSLLRARIGCKLVYCCGKMYAIGGRVY
EGDGRNSLKSVECYDSRENCWTTVCAMPVAMEFHNAVEYKEKIYVLQGEFFLFYEPQKDYWGFLTPMTVPRIQGLAAVYK
DSIYYIAGTCGNHQRMFTVEAYDIELNKWTRKKDFPCDQSINPYLKLVLFQNKLHLFVRATQVTVEEHVFRTSRKNSLYQ
YDDIADQWMKVYETPDRLWDLGRHFECAVAKLYPQCLQKVL
>Hsap_ENSP00000406286
MLQPGPHPPSPQAAAPGEAWPGPSQAPWQSLEEKMGSPEDDLIGIPFPDHSSELLSCLNEQRQLGHLCDLTIRTQGLEYR
THRAVLAACSHYFKKLFTEGGGGAVMGAGGSGTATGGAGAGVCELDFVGPEALGALLEFAYTATLTTSSANMPAVLQAAR
LLEIPCVIAACMEILQGSGLEAPSPDEDDCERARQYLEAFATATASGVPNGEDSPPQVPLPPPPPPPPRPVARRSRKPRK
AFLQTKGARANHLVPEVPTVPAHPLTYEEEEVAGRVGSSGGSGPGDSYSPPTGTASPPEGPQSYEPYEGEEEEEELVYPP
AYGLAQGGGPPLSPEELGSDEDAIDPDLMAYLSSLHQDNLAPGLDSQDKLVRKRRSQMPQECPVCHKIIHGAGKLPRHMR
THTGEKPFACEVCGVRFTRNDKLKIHMRKHTGERPYSCPHCPARFLHSYDLKNHMHLHTGDRPYECHLCHKAFAKEDHLQ
RHLKGQNCLEVRTRRRRKDDAPPHYPPPSTAAASPAGLDLSNGHLDTFRLSLARFWEQSAPTGPPVSTPGPPDDDEEEGA
PTTPQAEGAMESS
>Hsap_ENSP00000409107
MEFPEHGGRLLGRLRQQRELGFLCDCTVLVGDARFPAHRAVLAACSVYFHLFYRDRPAGSRDTVRLNGDIVTAPAFGRLL
DFMYEGRLDLRSLPVEDVLAAASYLHMYDIVKVCKGRLQEKDRSLDPGNPAPGAEPAQPPCPWPVWTADLCPAARKAKLP

PFGVKAALPPRASGPPPCQVPEESDQALDLSLKSGPRQERVHPPCVLQTPLCSQRQPGAQPLVKDERDSLSEQEESSSSR
SPHSPPKPPPVPAAKGLVVGLQPLPLSGEGSRELELGAGRLASEDELGPGGPLCICPLCSKLFPSSHVLQLHLSAHFRER
DSTRARLSPDGVAPTCPLCGKTFSCTYTLKRHERTHSGEKPYTCVQCGKSFQYSHNLSRHTVVHTREKPHACRWCERRFT
QSGDLYRHVRKFHCGLVKSLLV
>Hsap_ENSP00000410157
MAGTYSSTLKTLEDLTLDSGYGAGDSCRSLSLSSSKSNSQALNSSAQQHRGAAWWCYSGSMNSRHNSWDTVNTVLPEDPE
VADLFSRCPRLPELEEFPWTEGDVARVLRKGAGGRRLPQFSAEAVRRLAGLLRRALIRVAREAQRLSVLHAKCTRFEVQS
AVRLVHSWALAESCALAAVKALSLYSMSAGDGLRRGKSARCGLTFSVGRFFRWMVDTRISVRIHEYAAISLTACMENLVE
EIRARVMASHSPDGGGAGGGEVSAEALEMVINNDAELWGVLQPYEHLICGKNANGVLSLPAYFSPYNGGSLGHDERADAY
AQLELRTLEQSLLATCVGSISELSDLVSRAMHHMQGRHPLCPGASPARQARQPPQPITWSPDALHTLYYFLRCPQMESME
NPNLDPPRMTLNNERPFMLLPPLMEWMRVAITYAEHRRSLTVDSGDIRQAARLLLPGLDCEPRQLKPEHCFSSFRRLDAR
AATEKFNQDLGFRMLNCGRTDLINQAIEALGPDGVNTMDDQGMTPLMYACAAGDEAMVQMLIDAGANLDIQVPSNSPRHP
SIHPDSRHWTSLTFAVLHGHISVVQLLLDAGAHVEGSAVNGGEDSYAETPLQLASAAGNYELVSLLLSRGADPLLSMLEA
HGMGSSLHEDMNCFSHSAAHGHRNVLRKLLTQPQQAKADVLSLEEILAEGVEESDASSQGSGSEGPVRLSRTRTKALQEA
MYYSAEHGYVDITMELRALGVPWKLHIWIESLRTSFSQSRYSVVQSLLRDFSSIREEEYNEELVTEGLQLMFDILKTSKN
DSVIQQLATIFTHCYGSSPIPSIPEIRKTLPARLDPHFLNNKEMSDVTFLVEGKLFYAHKVLLVTASNRFKTLMTNKSEQ
DGDSSKTIEISDMKYHIFQMMMQYLYYGGTESMEIPTTDILELLSAASLFQLDALQRHCEILCSQTLSMESAVNTYKYAK
IHNAPELALFCEGFFLKHMKALLEQDAFRQLIYGRSSKVQGLDPLQDLQNTLAERVHSVYITSRV
>Hsap_ENSP00000416886
MDVNKFEASVGFLDVKKFLSTWKLQNPRTHFVLSPHCFMGGIMAPKDIMTNTHAKSILNSMNSLRKSNTLCDVTLRVEQK
DFPAHRIVLAACSDYFCAMFTSELSEKGKPYVDIQGLTASTMEILLDFVYTETVHVTVENVQELLPAACLLQLKGVKQAC
CEFLESQLDPSNCLGIRDFAETHNCVDLMQAAEVFSQKHFPEVVQHEEFILLSQGEVEKLIKCDEIQVDSEEPVFEAVIN
WVKHAKKEREESLPNLLQYVRMPLLTPRYITDVIDAEPFIRCSLQCRDLVDEAKKFHLRPELRSQMQGPRTRARLGANEV
LLVVGGFGSQQSPIDVVEKYDPKTQEWSFLPSITRKRRYVASVSLHDRIYVIGGYDGRSRLSSVECLDYTADEDGVWYSV
APMNVRRGLAGATTLGDMIYVSGGFDGSRRHTSMERYDPNIDQWSMLGDMQTAREGAGLVVASGVIYCLGGYDGLNILNS
VEKYDPHTGHWTNVTPMATKRSGAGVALLNDHIYVVGGFDGTAHLSSVEAYNIRTDSWTTVTSMTTPRCYVGATVLRGRL
YAIAGYDGNSLLSSIECYDPIIDSWEVVTSMGTQRCDAGVCVLREK
>Hsap_ENSP00000419153
MLERKKPKTAENQKASEENEITQPGGSSAKPGLPCLNFEAVLSPDPALIHSTHSLTNSHAHTGSSDCDISCKGMTERIHS
INLHNFSNSVLETLNEQRNRGHFCDVTVRIHGSMLRAHRCVLAAGSPFFQDKLLLGYSDIEIPSVVSVQSVQKLIDFMYS
GVLRVSQSEALQILTAASILQIKTVIDECTRIVSQNVGDVFPGIQDSGQDTPRGTPESGTSGQSSDTESGYLQSHPQHSV
DRIYSALYACSMQNGSGERSFYSGAVVSHHETALGLPRDHHMEDPSWITRIHERSQQMERYLSTTPETTHCRKQPRPVRI
QTLVGNIHIKQEMEDDYDYYGQQRVQILERNESEECTEDTDQAEGTESEPKGESFDSGVSSSIGTEPDSVEQQFGPGAAR
DSQAEPTQPEQAAEAPAEGGPQTNQLETGASSPERSNEVEMDSTVITVSNSSDKSVLQQPSVNTSIGQPLPSTQLYLRQT
ETLTSNLRMPLTLTSNTQVIGTAGNTYLPALFTTQPAGSGPKPFLFSLPQPLAGQQTQFVTVSQPGLSTFTAQLPAPQPL
ASSAGHSTASGQGEKKPYECTLCNKTFTAKQNYVKHMFVHTGEKPHQCSICWRSFSLKDYLIKHMVTHTGVRAYQCSICN
KRFTQKSSLNVHMRLHRGEKSYECYICKKKFSHKTLLERHVALHSASNGTPPAGTPPGARAGPPGVVACTEGTTYVCSVC
PAKFDQIEQFNDHMRMHVSDG
>Hsap_ENSP00000420659
MSRHHSRFERDYRVGWDRREWSVNGTHGTTSICSVTSGAGGGTASSLSVRPGLLPLPVVPSRLPTPATAPAPCTTGSSEA
ITSLVASSASAVTTKAPGISKGDSQSQGLATSIRWGQTPINQSTPWDTDEPPSKQMRESDNPGTGPWVTTVAAGNQPTLI
AHSYGVAQPPTFSPAVNVQAPVIGVTPSLPPHVGPQLPLMPGHYSLPQPPSQPLSSVVVNMPAQALYASPQPLAVSTLPG
VGQVARPGPTAVGNGHMAGPLLPPPPPAQPSATLPSGAPATNGPPTTDSAHGLQMLRTIGVGKYEFTDPGHPREMLKELN
QQRRAKAFTDLKIVVEGREFEVHQNVLASCSLYFKDLIQRSVQDSGQGGREKLELVLSNLQADVLELLLEFVYTGSLVID
SANAKTLLEAASKFQFHTFCKVCVSFLEKQLTASNCLGVLAMAEAMQCSELYHMAKAFALQIFPEVAAQEEILSISKDDF
IAYVSNDSLNTKAEELVYETVIKWIKKDPATRTQYAAELLAVVRLPFIHPSYLLNVVDNEELIKSSEACRDLVNEAKRYH
MLPHARQEMQTPRTRPRLSAGVAEVIVLVGGRQMVGMTQRSLVAVTCWNPQNNKWYPLASLPFYDREFFSVVSAGDNIYL
SGGMESGVTLADVWCYMSLLDNWNLVSRMTVPRCRHNSLVYDGKIYTLGGLGVAGNVDHVERYDTITNQWEAVAPLPKAV
HSAAATVCGGKIYVFGGVNEAGRAAGVLQSYVPQTNTWSFIESPMIDNKYAPAVTLNGFVFILGGAYARATTIYDPEKGN
IKAGPNMNHSRQFCSAVVLDGKIYATGGIVSSEGPALGNMEAYEPTTNTWTLLPHMPCPVFRHGCVVIKKYIQSG
>Hsap_ENSP00000424198
MVWLEARPQILFVCTKQGHQKPLDSKDDNTEKHCPVTVNPWHMKKAFKVMNELRSQNLLCDVTIVAEDMEISAHRVVLAA
CSPYFHAMFTGEMSESRAKRVRIKEVDGWTLRMLIDYVYTAEIQVTEENVQVLLPAAGLLQLQDVKKTCCEFLESQLHPV
NCLGIRAFADMHACTDLLNKANTYAEQHFADVVLSEEFLNLGIEQVCSLISSDKLTISSEEKVFEAVIAWVNHDKDVRQE
FMARLMEHVRLPLLPREYLVQRVEEEALVKNSSACKDYLIEAMKYHLLPTEQRILMKSVRTRLRTPMNLPKLMVVVGGQA
PKAIRSVECYDFKEERWHQVAELPSRRCRAGMVYMAGLVFAVGGFNGSLRVRTVDSYDPVKDQWTSVANMRDRRSTLGAA
VLNGLLYAVGGFDGSTGLSSVEAYNIKSNEWFHVAPMNTRRSSVGVGVVGGLLYAVGGYDGASRQCLSTVECYNATTNEW
TYIAEMSTRRSGAGVGVLNNLLYAVGGHDGPLVRKSVEVYDPTTNAWRQVADMNMCRRNAGVCAVNGLLYVVGGDDGSCN
LASVEYYNPTTDKWTVVSSCMSTGRSYAGVTVIDKPL
>Hsap_ENSP00000427926
MQAWRKGPDGPQKTSSDSMSRLMDSDMDYERPNVETIKCVVVGDNAVGKTRLICARACNATLTQYQLLATHVPTVWAIDQ
YRVCQEVLERSRDVVDDVSVSLRLWDTFGDHHKDRRFAYGRSDVVVLCFSIANPNSLHHVKTMWYPEIKHFCPRAPVILV

GCQLDLRYADLEAVNRARRPLARPIKPNEILPPEKGREVAKELGIPYYETSVVAQFGIKDVFDNAIRAALISRRHLQFWK
SHLRNVQRPLLQAPFLPPKPPPPIIVVPDPPSSSEECPAHLLEDPLCADVILVLQERVRIFAHKIYLSTSSSKFYDLFLM
DLSEGELGGPSEPGGTHPEDHQGHSDQHHHHHHHHHGRDFLLRAASFDVCESVDEAGGSGPAGLRASTSDGILRGNGTGY
LPGRGRVLSSWSRAFVSIQEEMAEDPLTYKSRLMVVVKMDSSIQPGPFRAVLKYLYTGELDENERDLMHIAHIAELLEVF
DLRMMVANILNNEAFMNQEITKAFHVRRTNRVKECLAKGTFSDVTFILDDGTISAHKPLLISSCDWMAAMFGGPFVESST
REVVFPYTSKSCMRAVLEYLYTGMFTSSPDLDDMKLIILANRLCLPHLVALTEQYTVTGLMEATQMMVDIDGDVLVFLEL
AQFHCAYQLADWCLHHICTNYNNVCRKFPRDMKAMSPENQEYFEKHRWPPVWYLKEEDHYQRARKEREKEDYLHLKRQPK
RRWLFWNSPSSPSSSAASSSSPSSSSAVV
>Hsap_ENSP00000432163
MELAMDNSYAFNQRSTCNGIPSEKKNNFLVSEDHGQKILSVLQNFREQNVFYDFKIIMKDEIIPCHRCVLAACSDFFRAM
FEVNMKERDDGSVTITNLSSKAVKAFLDYAYTGKTKITDDNVEMFFQLSSFLQVSFLSKACSDFLIKSINLVNCLQLLSI
SDSYGSTSLFDHALHFVQHHFSLLFKSSDFLEMNFGVLQKCLESDELNVPEEEMVLKVVLSWTKHNLESRQKYLPHLIEK
VRLHQLSEETLQDCLFNEESLLKSTNCFDIIMDAIKCVQGSGGLFPDARPSTTEKYIFIHKTEENGENQYTFCYNIKSDS
WKILPQSHLIDLPGSSLSSYGEKIFLTGGCKGKCCRTVRLHIAESYHDATDQTWCYCPVKNDFFLVSTMKTPRTMHTSVM
ALDRLFVIGGKTRGSRDIKSLLDVESYNPLSKEWISVSPLPRGIYYPEASTCQNVIYVLGSEVEITDAFNPSLDCFFKYN
ATTDQWSELVAEFGQFFHATLIKAVPVNCTLYICDLSTYKVYSFCPDTCVWKGEGSFECAGFNAGAIGIEDKIYILGGDY
APDEITDEVQVYHSNRSEWEEVSPMPRALTEFYCQVIQFNKYRDPWFSNLCA
>Hsap_ENSP00000435749
MEMQSYYAKLLGELNEQRKRDFFCDCSIIVEGRIFKAHRNILFANSGYFRALLIHYIQDSGRHSTASLDIVTSDAFSIIL
DFLYSGKLDLCGENVIEVMSAASYLQMNDVVNFCKTYIRSSLDICRKMEKEAAVAAAVAAAAAAAAAAAAAAAHQVDSES
PSSGREGTSCGTKSLVSSPAEGEKSVECLRESPCGDCGDCHPLELVVRDSLGGGSADSNLSTPPKRIEPKVEFDADEVEV
DVGEQLQQYAAPLNLAHVEEALPSGQAVDLAYSNYHVKQFLEALLRNSAAPSKDDADHHFSRSLEGRPEGAGVAMSSMMD
VQADWYGEDSGDVLVVPIKLHKCPFCPYTAKQKGILKRHIRSHTGERPYPCETCGKRFTRQEHLRSHALSVHRSNRPIIC
KGCRRTFTSHLSQGLRRFGLCDSCTCVTDTPDDDDDLMPINLSLVEASSESQEKSDTDNDWPIYVESEIGSHYVAQASLE
LLALSQPPASASQSAGITDGVSLCCPGRSTVA
>Hsap_ENSP00000436187
XTFTHSSSSHSQEMLGKLNMLRNDGHFCDITIRVQDKIFRAHKVVLAACSDFFRTKLVGQAEDENKNVLDLHHVTVTGFI
PLLEYAYTATLSINTENIIDVLAAASYMQMFSVASTCSEFMKSSILWNTPNSQPEKGLDAGQENNSNCNFTSRDGSISPV
SSECSVVERTIPVCRESRRKRKSYIVMSPESPVKCGTQTSSPQVLNSSASYSENRNQPVDSSLAFPWTFPFGIDRRIQPE
KVKQAENTRTLELPGPSETGRRMADYVTCESTKTTLPLGTEEDVRVKVERLSDEEVHEEVSQPVSASQSSLSDQQTVPGS
EQVQEDLLISPQSSSIGSVDEGVSEGLPTLQSTSSTNAPPDDDDRTERPSPNGPDRPFQCPTCGVRFTRIQNLKQHMLIH
SGIKPFQCDRCGKKFTRAYSLKMHRLKHEGKRCFRCQICSATFTSFGEYKHHMRVSRHIIRKPRIYECKTCGAMFTNSGN
LIVHLRSLNHEASELANYFQSSDFLVPDYLNQEQEETLVQYDLGEHGFESNSSVQMPVISQVSSTQNCESTFPLGSLGGL
AEKEEEVPEQPKSSACAEATRDDPPKSELSSITIE
>Hsap_ENSP00000436713
MAVNSSGYSRWCCFADSWQREKLASMESPEEPGASMDENYFVNYTFKDRSHSGRVAQGIMKLCLEEELFADVTISVEGRE
FQLHRLVLSAQSCFFRSMFTSNLKEAHNRVIVLQDVSESVFQLLVDYIYHGTVKLRAEELQEIYEVSDMYQLTSLFEECS
RFLARTVQVGNCLQVMWLADRHSDPELYTAAKHCAKTHLAQLQNTEEFLHLPHRLLTDIISDGVPCSQNPTEAIEAWINF
NKEEREAFAESLRTSLKEIGENVHIYLIGKESSRTHSLAVSLHCAEDDSISVSGQNSLCHQITAACKHGGDLYVVGGSIP
RRMWKCNNATVDWEWCAPLPRDRLQHTLVSVPGKDAIYSLGGKTLQDTLSNAVIYYRVGDNVWTETTQLEVAVSGAAGAN
LNGIIYLLGGEENDLDFFTKPSRLIQCFDTETDKCHVKPYVLPFAGRMHAAVHKDLVFIVAEGDSLVCYNPLLDSFTRLC
LPEAWSSAPSLWKIASCNGSIYVFRDRYKKGDANTYKLDPATSAVTVTRGIKVLLTNLQFVLA
>Hsap_ENSP00000438526
MRQGHAPEESEPGCEAPCAGPCHAQRVLQALNAYRRSGTLTDVVLRAGGRDFPCHRAALSAGSAYFRSLFAAGRPERGPA
VVPVVPVAPEAPGTSPAGAAAALAVVLDYVYGAGVRLRAEDEAAAVLALAERLGVAGLREACVRFLEGRLRAANSLALRR
VAAAFSLAPLAERCGRVLRQAFAEVARHADFLELAPDEVVALLADPALGVAREEAVFEAAMRWVRHDAPARRGQLRRLLE
HVRLPLLAPAYFLEKVEADELLQACGECRPLLLEARACFILGREAGALRTRPRRFMDLAEVIVVIGGCDRKGLLKLPFAD
AYHPESQRWTPLPSLPGYTRSEFAACALRNDVYVSGGHINSHDVWMFSSHLHTWIKVASLHKGRWRHKMAVVQGQLFAVG
GFDGLRRLHSVERYDPFSNTWAAAAPLPEAVSSAAVASCAGKLFVIGGARQGGVNTDKVQCFDPKEDRWSLRSPAPFSQR
CLEAVSLEDTIYVMGGLMSKIFTYDPGTDVWGEAAVLPSPVESCGVTVCDGKVHILGGRDDRGESTDKVFTFDPSSGQVE
VQPSLQRCTSSHGCVTIIQSLGR
>Hsap_ENSP00000443191
MMRVQTLREKWAYWRRRQLSLKQADFKDIFKKSTSGSLVEEEDQHMKLSLGGSEMGLSSHLQSSKAGPTRIFTSNTHSSV
VLQGFDQLRLEGLLCDVTLMPGDTDDAFPVHRVMMASASDYFKAMFTGGMKEQDLMCIKLHGVSKVGLRKIIDFIYTAKL
SLNMDNLQDTLEAASFLQILPVLDFCKVFLISGVTLDNCVEVGRIANTYNLTEVDKYVNSFVLKNFPALLSTGEFLKLPF
ERLAFVLSSNSLKHCTELELFKATCRWLRLEEPRMDFAAKLMKNIRFPLMTPQELINYVQTVDFMRTDNTCVNLLLEASN
YQMMPYMQPVMQSDRTAIRSDTTHLVTLGGVLRQQLVVSKELRMYDEKAHEWKSLAPMDAPRYQHGIAVIGNFLYVVGGQ
SNYDTKGKTAVDTVFRFDPRYNKWMQVASLNEKRTFFHLSALKGYLYAVGGRNAAGELPTVECYNPRTNEWTYVAKMSEP
HYGHAGTVYGGVMYISGGITHDTFQKELMCFDPDTDKWIQKAPMTTVRGLHCMCTVGERLYVIGGNHFRGTSDYDDVLSC
EYYSPILDQWTPIAAMLRGQSDVGVAVFENKIYVVGGYSWNNRCMVEIVQKYDPDKDEWHKVFDLPESLGGIRACTLTVF
PPEETTPSPSRESPLSAP
>Hsap_ENSP00000446269

MRLPGQGLGAREGPSIPLSSPAPAPASPAPPAWPPRPAVTHGRLRSPTRRAYTSLVLVGCTNLCAVLFARCLDDHLVSLR
MSGSRKEFDVKQILKIRWRWFGHQASSPNSTVDSQQGEFWNRGQTGANGGRKFLDPCSLQLPLASIGYRRSSQLDFQNSP
SWPMASTSEVPAFEFTAEDCGGAHWLDRPEVDDGTSEEENESDSSSCRTSNSSQTLSSCHTMEPCTSDEFFQALNHAEQT
FKKMENYLRHKQLCDVILVAGDRRIPAHRLVLSSVSDYFAAMFTNDVREARQEEIKMEGVEPNSLWSLIQYAYTGRLELK
EDNIECLLSTACLLQLSQVVEACCKFLMKQLHPSNCLGIRSFADAQGCTDLHKVAHNYTMEHFMEVIRNQEFVLLPASEI
AKLLASDDMNIPNEETILNALLTWVRHDLEQRRKDLSKLLAYIRLPLLAPQFLADMENNVLFRDDIECQKLIMEAMKYHL
LPERRPMLQSPRTKPRKSTVGTLFAVGGMDSTKGATSIEKYDLRTNMWTPVANMNGRRLQFGVAVLDDKLYVVGGRDGLK
TLNTVECYNPKTKTWSVMPPMSTHRHGLGVAVLEGPMYAVGGHDGWSYLNTVERWDPQARQWNFVATMSTPRSTVGVAVL
SGKLYAVGGRDGSSCLKSVECFDPHTNKWTLCAQMSKRRGGVGVTTWNGLLYAIGGHDAPASNLTSRLSDCVERYDPKTD
MWTAVASMSISRDAVGVCLLGDKLYAVGGYDGQAYLNTVEAYDPQTNEWTQVAPLCLGRAGACVVTVKL
>Cint_ENSCINP00000000756
MIMDLEDKDDIKGCPRLCISYSDKESELVEDKTSKVMKSEEYPWMMLEQLNKQRMNNLYCDFTICIDDEEYPVHQGILAA
RSEYFNAMFTSKLKEGFEKRVEIKESSKQCVERILGFLYSGKIELSVGNIEETIRCAALMQIPDLLQVAADYVHSIFNAE
NCIHFRGLAKLHSMDDLVKKADKYVLVHFRDVCLDESWNELSVEDVEMFISSEDVVVESEEVVYECMVGWVKHDLPSRKQ
YFPTLFRNIRFQNLSTQFIRDVIRIEVLVYEFHDCCKVVCKHYFDNIRSTPTCNPRKGDFSDVNVTYVSDVRYGNFILNE
NKIEVKQYVAKRIQRFYHSIYCS
>Cint_ENSCINP00000000878
SRMSDTTENPNEETIKCVVVGDNNVGKTRLICSRAYNEEAPNNRCNISTSHVPTVWAIDQYRNNQKVLDNARCNIDGTMV
SLRLWDTFGDHHKNRRFAFGKADVIVVCFSVCDERSLRQVTKFWLPEVRKHNKNIPVLLVACKVDLRFAELREKIKCKGT
LTKAVKHADILFPEQCRYVAMETNCPYYESSVLLGYGINDVFENACRAALLYRRNLNFWNSSLKRIQRLQLQPPLQPPKP
SPPNFPHLDTKSPLLPNLFQMYKDNVLTDGEKIFAHKVVLCASSSVFAEVFTSPVLPATNDAFVVTIAKSGNTVESYKRN
YDITHHSIDDNQREVYESETPTEILKILVNFCYTGLLMLETPDVSILTQIIIAGQKFAIPELVNLVADLLNSMTFDPRPH
EGFMSRRAASMKSLFIETPTLADVVFHLDDGSIPGHKVLLASGCDVMMAMFTGSFIESGNKEVDLPDTTTTSLRCVLEFI
YTNNIPELDCCATVYDVIKLADRLCLPRLISRDERKYFEKNEKCLISKFCTDVIMMSQIADLHNAPELRQWCLYFLSIHY
NDACRSIPKIMKSLPPPTLSHLEEHRWPPVWFIKQQDVYDKSLYQRKEEEKGLRVYKKKVELCFENRRYEVF
>Cint_ENSCINP00000000883
MNFSFSSPDYPGQSHRKTEQEEKSGEYRFGQYLKNMLVRINQQRAEDNPLCDFTIVVGGRKFRAHSLILSTSSEYFHAMI
NSRMKEGIEKCVQIKNVSEEIMENILDFIYTGKIPLRVETIGQVIEAASFMQLPDLLISVVQYATKILDEENCIFFQKLG
ELYKLENLVQSSYKYLLDHFDEVSTTNDEFLSLTSSELNGILLDDITVSSETVVYKAVVCWVKHDLEERQQYFVKFFKLL
RLQVVSVDDLANIIVKEDMVTSNCECMAAVLDSYDARVRPRLNIEQPRKKPLSGDATSIREFNIPKPTPTFGTAPFGNLM
N
>Cint_ENSCINP00000001277
MSSSKFLSCDGHFDFIMDMLVKQRSNRRYCDVTLVAEGQKFPAHRAILAASGNYFDTMFSSQMKEEFERVIKIKEVTKVA
MEQVITFIYTNKILLSEENIDEVLHAASLMQVQGVLSLVIQFLSDEICLLNCLHYRQLGKLYSLNDLVLECDRCICENFD
QVSTQQKFFEMDEVEMEKLISSDELVIESEEVVYECMVGWVKHHLQTRKQYFPTLFKHLRLQYVRLDYLSRNIKKEGLVR
EFLDCRDLVEDAFYFHVKPDLFEKQKPRKSCYPCKELIFIPNKQQNITSYNMTENKWSSLQ
>Cint_ENSCINP00000001988
RMDNLYCDFTICIDDEEYPVHQGILAARSEYFNAMFTSKLKEGFEKRVEIKESSKQCIERILGFLYSGKIELSVENIEET
IRCAALMQIPDLLQVASEYVDSIFNAENCIHFRGLAKFLYMDDLVKKADKYLLDHFRDVCRDESWNELSAEDVEMFISSE
DVVVESEEVVYECIVGWVKHHLQSRKQYFPTLFKHIRLTSLQFNYIEQVIRKEVKNIILNLSLIHALINMFRIYFMSSVK
VVKLFANITLTTLDQQQPATLGMEIFQ
>Cint_ENSCINP00000003285
MDSNDKHVEQFKSCHEGRVLTKLNRLRESKVLTDVTFKLEDGEVSAQKTILAGYSDYYEKMFTTGFKEGFNEEVEVKEIK
TSILDTLFTFIYLQVIDISDKTVYQLYEASDYLQFDDVKGYCVSFFNKTLAIGNCVSYRSFAQRYQLLELINKCDKFIVD
NLESVSKGLNFKELSIDEAEALITLKQKKDFCQDSILKAILSWIKHDVAQRHQFIKRLFQVIDVKKLSLALLKEVVENPE
EWIQKTDFFIGVLNPEYITHIKTNQRVVSGEASKVTFEFMIVGGFEAKHNVHIYDVLEKHFRETKASFYERWGSISAKIK
NCVYVAGGGGADRWCASSAVLNGWFLIMLSIMLVDCYWRSSMAYHISREIIL
>Cint_ENSCINP00000005165
MATRNGKVYSSQRYSQLILVGFFQLRVHGSLCDVTLTAEGKSFKAHRGLLASASDYFRAMFTSEMKESFSEDIELHGVSS
TGLEQVLRFIYSGEIVLSLENIHDILAAASHLQVTAIMDFCNEFLISEVTLENCVDIGHIANAYNLEAVDHHVNVYMLQN
FNLVSDFNKVPFDRLTFLLSSNTLKGCSELQLFNAAKRWIEGDVDNRMKHAAALMENIRFPLVNPTDLITHIQTVDFMRT
DNACMRLLLEASNYQMVPYMQPILQTPRTSIRSDATHLVTLGGVLRHQLDVSKDLRVYDEKSKRWINLAPMENSRYQHGI
AVIGNFLFVVGGQSNYDTKGKTAVDTVFRYDPRYNKWIRVRSLNEKRTFFHLSAIEGKLYAVGGRNAAGELSTVECYNPQ
HNEWVFVEKMQEPHYGHAGTVYNGQMFISGGITHDSFQKKLLCYNPTTDKWEQRSPMSTVRGLHCMTTVRDRLYVIGGNH
FKGTTDYDDVLECEFYTTETDQWCCVAPMLTGQSDVGIAVYDGKVYVTGGYSWNNRCMVDIVQCYDPEENKWMKTFSLPA
ALGGIRACTLTVLPNFANSNAQSSSSSSMPKHEPDPSGDMMLSVDRPAPSARLPRRPQAQADADIGARPLQDR
>Cint_ENSCINP00000005691
FKSNSHNNAVVSGLNDLREDKILCDVTLIAQEQPFLAHKNVLASCSPYLRNLFNSEDGFKNEHEVVLKQGIGNNTIPDRI
YKVDVESAVQRSPHADCRCYLNTAENYLTGLFLREISVYNCVDIIHIADKYTLAGVEDKAYSFIAEHLNELVKTEEIQKL
TINNMSLLLDSYSLKNVSELELFEAARQWLLYKTERYQHVRTLMEKIHFPLIPPRDLLRYVNFVEFMRVECNYLLLEASN
YHMLPHSQPILQSVRTQVRSNDTRMVIIGGVDPHDRVSNQLFAINSDMSKSEFLPPMHEGLCSHCACVMNNFLYVVGGQN
MFDERGTTAVNSVIRYDPRFNIWMRIASMNDKRAGFTVSVVDNRMYALGGVNAAGRMSSMECYSLEDDRWRYVASVQTGL

CDHAQSVHGNLIYISGGFKEGRFNNQLLAYSPRHDTWHERTPMHFARGWHAMVAFGDSIFVTGGNAGLNKRVDIHETEIY
SAMSDQWTLVAPLPLPHSEGGCAYYDGKIFVVGGYSWTQQKCLSTIQSYDPNKDQWENPGNLPIELSGVRLANLTIPYTV
SPRSGLTQGSHVPLSWP
>Cint_ENSCINP00000006099
VAQDSIREMAQDSRFTDVIIEVEGHSFRCHRIILAASSQYFRAMFCASYRERDQSSVEIKGVTIDVMKVLIRYAYTSYLE
INTENAQTVLEAASLLQFTRVMEACANYFASQLSIENAPGIMELAQRHSLTELQQLATLECVTHFSNLRSSSDYLCMSSQ
LFEVILSRDDLNVANEEDVFEAVMFWCQHDTGKRKSELPNLLRHVKLPLINQNYFVRHVETNELIRCNEEVYDMMMEARR
YHTIGGVTDRKVIPRHNTTLHQVFVTVGGCNNRDSFIDEVCVLDFQHKTRASLCQLPNTKYMVQAGDAKWAEFACTAWRN
NLVISGGKETKKETWMYIMSLRRWVQMASLNTGRWRHRMVVCMGDIYVVGGYDGLLRVDNLEKYDERENLWIERRNMREA
VSSAAVCESEDRIYVVGGGPSVRISTEKVQVYDPNTDDWRLSTPMPDAAKCLSSIALRGKIYVVGGTLRYILCFDTREEI
WSKIGEDLSCARASCGVTLCNDKVSLT
>Cint_ENSCINP00000006415
LSGTSSVKEQTHNIDHASDLLPFANKSRQDGRFNDIIIRIGEKNIPANKMVLSCYSKYFDTMFNTEMEEKYKDVVELQAV
DAESVEKLVDFMYTGKININTNNVCDLLAVSDFLQMLSVKKLCIEYLLTTITQQNCLTIQALADRFTIPKLTEKFNNYVV
EKYQQVISDDHFKKLSKDDVIKLLQLTHDKVSSDLVYTAVMNWVKFDLASNESYLSELFKFVDISKVSPKMLEDVISVEP
LIRKLDDWGNLFVNALVQQKKETTEKSLISLGGIKSLNKVTKFDLQTMQWSPLPDLPVRRSSASAVVIDDILYHLAGDLN
TDGKETATNIVHRMKLKEKVLKWEKVASMNVERYVFGAAVINGVIFVFGGGDENNKRVSSGEYYVVPLNKWIQLKPMKIA
RWGHCLVAHNDQLYSLGGHDGQQVISSVERYDPSSDEWKDVASMQTPRRWFAAVVLNNAIYAIGGYDGKQTLKSVEKYNV
DDDTWVYVENMNIERSSHAACVAQNKIYVVGGLDSGKKIVKSIECYDDQTDKWSVV
>Cint_ENSCINP00000006826
MAHSSSFEPKKRTLDDLDRITRGRVISHFTEDATKIPVDTKQEVEPPVVNSKPQVKMQEVCVQTEEKINVDASVQNTLEC
KDESTQYRFRRKTKNFMSQYKVPTKTIGTYFKVKTKDSASQYEPPLPIQDTAADSTCLPLDDEPLVKDEQCRSKLSRKHH
ALMVLQSLSRQQDLGMLCDITLAAQGELFKAHKVILAACSDFFHTLFASEEIRQTPLSYIELQGITASALRLVLDYIYTS
EVSVGDSIKNTQEIITAAKRLKINSLLPACNALEQALLSDQDLSGLVISNTELVFPNSKINYGNSGSFMHSSDETSSLEF
VQKPHIKREHGIYDDTDSPVSPELKPIINETYEAPNSLKHKLSETKVKEKAVKRKERSQSCVEVSTTVKRRKSEDTVKPY
SCNSCHKTFWKEKSLHSHRAKSHPNLTLGQVTKALSTLANEVKPDANNPRCPHCKLYFKNSGALSTHIKWNHEDCQSSSS
KNSDDHSGSRSSPTQQKDKQYSDNDNKDLTRKPNRIGVHNPVHPKPKLEKTKSKSEKLKVEATNDKGRQDLKSLPVTTET
EDSTVRPRLYQCELCEEIFSVQCFFKAHMASHKRAAENGGER
>Cint_ENSCINP00000007379
MTYQAQLFQCCREGDLQKAQFLHEEKDVDLNLRDIWDSSPLYYACLCGHFELVEYLLQNGAKCVENSFDGERCLYGALTN
DIKNLIKNWKQVHTRGLRRERHYDMMRSLLDKGSFYDVIMNVHGRQFKVHRYILSARSEYFADAFSNKWKNVDEVNISHH
AVTADAFACLLQYLYTGRMDIQCDMVGQVKRLAKNCRLDLLHQEIIKVELRVDELVRLKPQMKKRVKVFSIEGEVCMSVF
RDDLRKLVDHVIPPSLHDWVEDGILPFTKMWRETLLFPDVCFNIDNHLFLGHKAIFCACSDYFTALFEDHFLENSFHSGT
RVLPMITLTHISPQIFKNVVVSMYSDEVEFTSMDTAYETMCTSHMLLFHGLTRRCGQILGQHLDESNLFPLLEVARNFDL
PKLEDMCAMYMAKTLDKMVDNEEFAELVENDAAKIKDREETDSIPIVDDIRYHLTSSVQNYSQMEEANFKLSALNALLSR
LGIDC
>Cint_ENSCINP00000008095
MYAERGVPMSMDITSVCGNRLQPVTGITVVNKSSSLRHTSDKHPHQILDVLNILRKRQELWDVAIVVGSLKLHAHRVVLA
AASPYFHAMFTVELAESHQTEIVIRDIEEKAMELLIDFAYTAQITVEESNVQCLLPAACLLQMVEIQEICSEFLKRQLDP
TNCLGIRAFADTHACRELLRVADKFTQANFQEVKESEEFRLLPVDQLIQIISSDDLNVRSEEHVYKAVTTWIKHDLKDRR
NLLPKVLQHVRLPLLTPKFLVGVVSVDPFIHGDEQCRDLVDEAKNYMLLPQERPLMQGPRTRPRRPITREVLFAVGGWCS
GDAINSVERYCPESREWRLVAPMSKRRCGVGVSVLDDLLYAVGGHDGTSYLNSVERYDPQTNQWSSDVQPTSTCRTSVGV
AVLDGYLYAVGGQDGMSCLDIVERYSPKQNRWNKVSSMNIKRLGVAVAVLGGYLYAVGGSDGQTPWNLVERYDPRENRWT
EMAPMSTRRKHLGCAVYRDMLYAVGGRDDTTELNSVERYNPLTDTWSTVVAMNSRRSGVGLAVVNGQLMAVGGFDGASYL
KTIEIYTPEANTWRMYDGMHYRRLGGGVGVIRLPQA
>Cint_ENSCINP00000008658
MDEIIEEINQLGRGNAENLASPSLIDSTQVLVRMADKPTRISGKATKCKAKKDSTSDWEFSKSTIRERNEKMFNNELLSD
IHFIVGPLGASKRIPAHKYVLAIGSSVFFAMFNGDLAENKEEIHIPDVEPKAFLALLRYLYCDEVCLEADTVLATLYASK
KYIVPYLAHACVEFLETSLSYRNACVLLSQSCLFEDEDLTQRCWEVIDAQTELSLKAEGFTDVDGKTLRSIVCRESLSVN
ETAVFDAANRWAEAECIRNNVEPTGKNKREALKDSLFLLRIPTMSLQEFADGPATSDLLTKEEIIQIFVWYSATTKPELT
FPTEHRRGLTPHCCHRFQSTAYRSNQWRYRGRCDSIRFMADKRVFIAGYGLYGSSTGAANYKAHIKLSQNGKVLSEETAG
FFSDGCSKTFSVWFKHSVQCEPGIFYTASAILDGNELSYFGQEGLSEVVCDGITIQFQCSSDSTNGTGVQGGQIPELIFY
YANT
>Cint_ENSCINP00000008842
MQPSSPRVSSATSSRSGSPAPPNDMLGTPVAENWCYTQVKVVKFSYMWTINNFSFCREEMGEVLRSSTFSSGANDKMKWC
LRVNPKGLDEESKDYLSLYLLLVSCHKSEVRAKFKFSILNSKREETKAMESQRAYRFVQGKDWGFKKFIRRDFLMDETNG
LLPDDKLTLFCEVSVVQDSVNISGQSNMMQLKVPECRLSKDFETLLDESHFTDCTFSVDGKEYKAHKAIVAARSPVFRAM
FEHEMEESKFNRVEISDIPHQVFKEMLNFLYTGKASKLDVMASQLLAAADKYALERLKVMCEESLCSNLSVENVAEILIL
ADLHNACQLKEMAIDFINNHALDVMETSGFNVMVRSHPHLVADAFRALAAIQSPPSGPPRKRLKSSS
>Cint_ENSCINP00000010031
MMDEVCTVKCNFGKCVDDLNHVITKATLHQVKEYIRLHCFNAVARYDSSGRTPLHVAASSGKSDIVRWICNGNVTIDSVD
VESNWSALHRALYYGRIASAMELLKNGANMWLKDKAGCNSLDMIIADISPWNNKISFKRNSDISLYTWGVNENMTLAQAV

QKSKKMPEMVKFFSKRNIYIKQVVMCKFHSIFVTRDGVAYSCGNGRGGRLGNGAEDVFIDPQLVDAGGSVITSASAAVDH
SVFVTADGQLLTCGLNDHRTLGINPPPQKSLLPQRVSGLRDHIIVGCCAGSCHTVVWTSQSALYTFGTNAGQLVFLLRCF
LGHGKGQPFIVQPRQVSSVHREGDKMTYVHACDAATVVSTAHGVVFLLHNYICRKIVTKQMDIIKLQVGGGILDRTKKEE
NLDSEKKDVKVLLLKKSGDLLSWNSGHSSLDRCQFDQKQLRWISDVSLSCNGVAALLVSADGTAYMAKFQAWIAGKPARR
GSALHKQEYLTSLDLMSSDAIQKFQLEQVPCAFRCHTAVLGPKGRNYSLLQSKPSNFLTQIPVVDAADITEEMRMLLESS
DASDLLHDIVIHVRRDGKPDVLFPTHKFILASRSLLLSKLILSRIPYSDEASLDLEKDKLILEGIHPKIFMNFLSIIYTG
SCEMLQPGSKIDDKYPDSGRVKIFWSDNATSAMEIPTKSNQVKVECFTMLKILSNLFGIRDLSNEVFSLHSIRDGVIFSR
KSGKKPHFAPSSLPQLTDCEIESDDGICFQAHKCILASRIPFFHGMLSSPWIETSLYESRLCAQLKVPVPGDVFKYILDF
AYMDSSSSIRGATSVDSLCEILAASDLLLMGRLKQIAEVQIAPLLTLKNVCMVAEVASAFNSNQLYESCLQFACHHLPAL
LHTPAFHYADEEFLDRMEVYYRSQIPDVYRRSSPPSMEGPDVSHIPEEDLSDDITEVDFSVDRHSSDLPTSSLRKRRTSE
AHSSRKGKNKKAPGIMLSDLL
>Cint_ENSCINP00000011706
MAHRDHNSPGSSRNIGPQNRARGLGAIPPNADGRMDFLEEPENNEEFPLQVVNRRHNQPRARAGGVRIVTYQSYRGYRLR
REDQQEQQGRFYNSPPVNQDQQNDIVPVLSSPDGFSSDSLAGEEHEWSDGMSSISDLDERLPPAAAPPAYPVPGAADSPP
PDFLGISMGSRGVGSSRGNKRTRTGSTSSYSSVESVCFNSRKQKISNTNSVQCISQRMALIDKLQKYRSNKEFTDVILTV
EDQEIACHRVVLAANSPYFHALLSSNMKEGREGRVHIQGHKPEIVHMLVRHAYAEAIKVTTENAQDVLEAADYFQMDMLK
SHCEKFLIRQVAESNCLGLMQFASLHSLDRLYNKAKKYAVKNFNKVSQQEEFLRLPLATLSKYLEDHGLVVQREEHVYDA
AMRWLEYDATRKEHVAEVLRCVRLFFVSSRFLFEVITKHPMFQGNPEVQNILSEACKYHALGSNEMKHGSLPHTKPRASS
GISEVLVVVGGICNNRRLLYTECYHPNKQDWISLSDISTSHATMHSYSVCSHKNDIYITGGHSNSGMTMDAVSVYFSNID
QWSLLAHMQHPRERHGSASVDGAVYVAGGLMASPKNRKKPGVLDNVERYRPDYNRWEKVKPLPKRCYSPGVLSYKEKLYV
IGGVSMTEEPSNSSKIILDCIQCYDPLKDSWSQLPLGRPLARLSCALYRDHFYMISNNASSIFRYDPNTCTLEEWRELHE
LNVEFAGLAAFNGKLVISGGQKGSTTLNHMLVIDFESGRVEEEVKMKRSRCMHGCVPIDKFG
>Cint_ENSCINP00000012687
MVKKSIVSLGKSHQAVVQYFKCYASVFEHDIAAISSLLQRKNIRKASYHSWPDANNQKVVWSLTLNDNSDIKTTIRELDD
FKEERRLVSRAAALELLLLESDIHFSDNNMVFTSTSSSPILTQLRKFRKEKRFCDFTINLDGKSFAVHKLLLVASSNYFC
EIIKDNQTSVDIGGIKAEIFETMLCYIYAEDVAVNESNIFELKENASLYELQGLVDMCMLFQDFVGAVTKIPEIKLDSLP
EAKSAIGNKKPGETESMVHVRKSERQAKREAYNTISKTINLVEEPDDISTDDRFLPVTEENHVLPQKENIENHNRTHSVE
VTKSQVEQIDNVSMLELQAASVPFVLKCEICHVELEDQKQRKAHMKKHFVQNQPNRFGCHLCMKAFRSASHLKRHMISHT
GIRKHCCTTCGKLFLKSDHLKRHERTHTSERPFTCEQCGKQFRDKDHLKRHNLTHTEERPYFCEVCGKQFKDKSTLLSHQ
KSHNANNSVVCGVCGRLFKTIPRLDTHMVKHMETKAHICEVCGKEFPFSGRLRKHMLSHTGARPFHCEICGKQFRDGHSL
RQHQKKHTGIGLHPCLECGKKFTNAFHLKRHVKIHSGVKPYSCDVCKKSFARPDNLDSHRRSCIVNMQIKQKKYLPNAPL
EAWETDNTDQAHESIPGCSNASFNVETTGHVSSVENLGDFSPQATTTTTHSQIPFTNIMETLQSQLNRSVQNDATSCSSS
FTSLLSTLTPTFEGEPAINDQTTDQ
>Cint_ENSCINP00000013326
EMMQRIRRQGKMCDVTLKVNECKFTAHKIVLAAVIPYFTSMFTTDMAEANGNEIEISAIDENALEHLINYAYTGKITVNV
ETVQSIMIGANFLQLQVVKEFCANFIKSHLHPNNCLGICQFAELLMCMPLLEKAKSYLNQHFVEVSKSDEFLDLSKEKVL
SILCRNELHVKSEEQLFYAASAWVKFKYTQRCEYMHELLQHIRLPLLRPQFLTDVIQMDAAIKSCLRCRDLVDKAKDYHL
MPDRRSDFPPNMVRPRYCTEIHEMIYAVGGLTSAGEALNTVEKYSGLVGRWELVASMKTCRSRVGVAVLAGQLYAVGGYD
GMNRLNTVEMYTPETDEWCDIKPMQEKRSALGCVAYEDQIFVCGGYDGVSSLSNCEVFRPHTQEWQKISPMNKSRSAAAV
GVFEGCVYILGGHDGLSIFNSVECYDQSIDKWCMKVPMLSKRCRHGVASLQGCMFVFGGYDGQKFLNTVEVFDRVTNQWS
FVAPMSMRRSRVGIAISGGKIFALGGYDGCTNLNSVEVYDPETNSWRKSDRMWAHEGGVGVGTIPLPLCVSFAW
>Cint_ENSCINP00000013720
KIHDHLKREQLCDVIIIAGSKRIPAHRLVLSSVSEYFRAMFNSEVREAVEEEVVMSDNDPDALENLIKYMYTGKLELKED
NVERTLSTANMLRLDAVVESGCCFLKQQLHPSNCLGIRSFADIQSCRGLYEEAHKYTMEHFTEVIRNQEFLMLPLPQVCE
LFGSDEVNVTSEINMFEAVLLWVKHDVGERKKLMAELLTRVRLTHMPKEYVADVVQSNPFIRDNLDCEHQVIEALIHHLL
PERRPCPVRPRKSTTGHLLYAVGGRDGSSCLRSVECFDPHTNKWTNCAPMSKRRGGVGVGVCGAHLYAIGGHDAPASAVS
IEQYDARIDQWSQVANMTGRRLQFGVAVLDDKLFVVGGRDGLKTLNSVECFNTRTKTWTRAWNYVAPMSVARSTVGVAVL
HDKLYAVGGRDGSSCLRSVECFDPHTNKWTNCAPMSKRRGGVGVGVCGAHLYAIGGHDAPASNQMSKLSETVERYDPKTD
QWSTVAPMSVPRDAVGICMVGGRLYACGGYDGQSYLATCEAYDPQLNEWRNIASLNTGRAGTVVVHVRH
>Cint_ENSCINP00000013854
MSAKLTSVDSLSVSSFQTSTDSFLQPAVRKGEYPETVYTSNSYNNILLSGFNALRFDNLLCDVTLIAGNTRFPVHRALLA
ACSPYFKSMFTKDSQNAQIGRNEGTKYTWFSESIELRGISSDGLKHIVEFMYTGKLPISMKSVQNILAVGRHMQIQPVLD
FCMEFLISAIDVNTCVDIIHIAEVFSMAQLEENAYKYMLERFPDFIKSNQLQKLTFENMSYLLDSNDLKVMSELDVFAAT
LKWIMYDHSRQAFIRKLMEKIRFPLMPPRDLMVHVNVVDFMRTKCNDLLLEASSYHMLPHSQPIVPSSRTKIRSSDTRLV
VLGGADNTDEVSNQLKVFNRELTAYTLLPSMDTGVHSHSVAVLNNFLYILGGQNHFEERGKTSVPTVTRYDPRFNTWMKI
ANMNEQRAGFHVSAIPAYNRLYAVGGVNSVGRLSSVECYCVEEDRWKYVASTQNALCDHNGSVHRDKLYVSGGFSDGHFS
DAMLCYNPKHDIWERRSPLQFPRGWHSQITVKDKIYIIGGNTGINKRVDVLDTEVYSPDFDQWTTVTPISMGQSEAGACL
LDNKIFVVGGYCWSARRCIKVIQTYDPEKDEWERVGNLPRGLAGLRLCTLTLPHHLVR
>Cint_ENSCINP00000014080
MPLRLRRPSSQMSVNPPVVKKAAMEAEEDLEPTEFSRASSEVYDFEDPKHPLEVLTCFNHLREKGLFTDVILSAGSREFP
CHRAILVAGSKYFRAMFCNDHRESREMLVQINGMQPQIMDLLLDYLYTSKAAINADNVQLLLEAANLFQILPLGEACTRF
LEAQLDPCNCIGMREFAEAHALSTLFTTASEMIMEKFADVVVHEEFLELPKSRVIDYISDDDIKVTREEIVYEAVKRWVG

HDAVTRSGDFAEMLSYVRLPLIHPSYFVNTIECDEQVFSNPACQSILQETRKYHILGNEINSERTRPRRSTGCSAAVVIV
GGCDRVGGYNMPYVEAFDPVTGQWNSLAKLPAFTKSEYAVASFKNSIIVSGGRIHSRDVWLYQSHLDNWVKIAPLCKGRW
RHRMLTMGGHVYCVGGYDGKNRLRSVERYDSFSNTWVEVAPLLHSVSSCGLATCHGKLYVVGGGDEENCFNKLHCYNPES
DQWILRSPMPSMQKTLSCATINGKIYIIAGSEKNIYSYDPLTDNWDTRQITEDKLESCGMTVCGGKLYITGGRGDGVEAS
DQVYGYDVRSGDMCHVSSMPRPSCFHGCVTIHRFGPLGEKSQNSGK
>Cint_ENSCINP00000014756
MLVCFWYERCNMIEVYGIELLRKLLEQRNRGSYCDIEIHIEDNVFYGHKCILAAASPYLESYIVDAESKYIDQTSLHRSK
IQLNGISHSGFERLFDFMYTGKLQINEETVNDVSVAALILDISVALDCCAEFKRKKFSVASLFKNNVLQISSTECSSSVS
HQQSKSSVENLMLQDAQPALVSPKNEIQINEVVSFSAMKPRSEEFTENRENFLTSPTDRIPSHSESDTESLGISLGRESY
YNTTSQGNDMISACANFIQSFEAMNNDPSALPTLLPLNGKDRIMPTTTTENAGSTSMLESPPNTFESLDERFNEVSFYAE
QTEKNGVPSWKCKVCKKVFRKLSYLKYVHVRLHNGNRPHICNLCGKPFATRGNLLQHHKVHLAKNGVLPFKCKFCKRAFS
LKGNCQKHEKTHTKPYRCEFCSKVFSVKNDCMQHLNKHHAEQSENPTIGLRCSKCLTLFNERFGDDYFTHKCS
>Cint_ENSCINP00000014893
MMLDIKTTTAHSSTFVKPNFSNEIVQGLRNLRTDGKFFDIVIHVADERFPCHRIILAAASNYFRSMFAGMMKESCTDKVT
LFGVEPEAAEILIEFCYTGTAVITENNVIELLKASDLLQFHSVREACCEFVGKRLDSSNCLSVRQFAEKLSCAKLADEAL
TYTAENIVTVACSQEFNSLNYQQIRPILAHHMLNIDKEEGAFEILMQWLKCDIELRKDDFCRLMEVIRLPFIRRIYLLSK
IETNPIVRASTLCKKLIKETRLFHTCMLDKNEKTSIRLQPRPSTGIAELLVTVGGCDENCDEATTVVSYNPVSGTWKALA
QVEDNLRGGYATVSIGNDIYVTGGSDGATVYNRVWRYKSQVNEWIKVCNMNYAHEYHGSVVLQSYIYVIGSEGCEKYDFI
SDSWTLIAKVPHRVNNCSVAACAGKIYTIGSLYGSEEILLQVYSPDRDKWDNIPINLPPMSFVPTISSLKGLLYFTSEDS
KEVLTFDPRRNEWINTTSPMLHVHLGGSTTVFGGKLIVSGGYDERYDLSGIIEAYDTDTNTWSVIGKMPQPIFWHGCVSI
YRYVAAPSGQTYCADPDLYKLSTYVDISQL
>Cint_ENSCINP00000015643
MADVIPTLKSPERTTIVQNLADLLARDAAVVSSSDDVSENEILKEDGVPEMHGASIYSERPRTDHQDRMEEDQPFDLSTR
RTSASDILPREGKPTTGPDPSETDVSNDEHYEANTKKGEFSRYLNGMMLSLNDLRLRCLLCDVIFKVQNETFHAHKAVMA
SCSEFCRKKFLPLLYSQDHRDSEILTLEIHNVSVLGFRLLLDFIYTGQLDINDVTASHIAQAAATMEMTEIADIVEKHAR
GSMNNYLSNSDGSPSKRSELHARSLPLQGTSYSQSLDLSVKPHCKKRTSWNGMDSESKENHCKPRRSSDSARSVRTQEIE
RVPAVDPRSPERGNPEIFNLRSGNGIINQAKQLSEQNEQTFSCDDSHTQHWKKKYHYLEHENQRNERKEHGSKGVYEQTR
YPTKEESQYQQSLLHYSKNNSPNALAPQMGSGRSPQQEYQFRLQFNRLFNWMPPLSGGENLLAQRVSAFMQPQLASKPTL
AQTQWANFLRKQPNNYQIPMSFRPDVLPFQPNFLFPRVDGTDNRNGWRVPFVPPPALSVINNQYQQSTATGTRLRSQSST
NTEEPMNVDTTLPPKLETKQSPIYINSESNESLPSLESMNTMEERRSSGSGEDKSDDTKSKPPCNARVRATGSSKPYKCE
FCDADFNRPANLKTHMRIHSGEKPFQCERCNARFVQVAHLRAHVLIHTGEKPYPCTVCGTKFRHLQTLKSHLRIHTGEKP
YSCQECRVHFRHKSQLRLHLRQKHGIQTNTKKTYKQVPGLCSADICAHIRRAQEIARSQTKNSNNAPFMEQSSSCSEAIK
QTEQHPKSFDNPGEKMRGLNTAFSH
>Cint_ENSCINP00000015848
MAQTLIAKRILSYINEMRMNKRFCDVEFITRCGKVFAAHSCVLTCVSPCFETWILNGNYDKETRVTKVLLPESVHASTFS
VLLDLIYTCNVKVRKLNNCNQNSLDELTLNVNVELEETASELSLSFDENVLKNINMSSDKTSHESKNTESRQRRSIRLSD
PSDDLLLESVEPDEVSLEFDVGSLHNEVTASASNEVQKLHNDGKQNVTAGKKVSCDICSRLFTNINGLERHKKCVHHGKT
SPIQCSECKKLFKSKTKLSKHQLTPCLGKQKSQCYICKKTFASKYIMGLHLKIHTGDELFKCHVCNKKFPYKSSLTNHLI
LHNHQNYFLCSVCGKSFSQKVRAKEHESKHIGVKSSLSCSECNSKFTQKSALARHVSSVHNQHRPYVCETCGRCFARKNK
LLEHTRTHTGEKPYVCSLCGSSYKANRSLKIHKQRRHGIKDLVVDQELSPKTGTVTILSSPPGVVRSELTSIATLDLMSP
QTLDVNKPCQTLKDNPHSLQGELNIGL
>Cint_ENSCINP00000017049
RIERFELKEYPNDSLRIINELRHSSLLCDVTIKLEYNGQKKKFSAHKLVLASCSPYFKAMFTGGCRERNMSEVNMREIHP
DVFSKLLDFAYTSRILISEQCVLYIMVGACMLQMNHVVQICCKFLENQLDPSNCLGLAAFAEDLGCIDLKRKIEKYVCAH
FEQVSQSEEFKTLSPCRLSKIIAADEIHVDCESQVYNAVLCWVQYNPKTRQQYLKMLLEAVRCHSLTPGFLERQLQNCPV
VNKEPRCQSISDNAVASGVSVPTSKATAANKKHSIINIHHFQCVFLNLPGAVVVVTPPGYNRIPKAPASLCTWLHNVIYV
AGGYLRHSLPYMECFHPEENMWLRLRDLPMPRSGIASCVVQGLFYAIGGRNNSPEGNYDSAACDRYNPMSDQWDHRSSMN
VPRNRSSVGVIDNMVYAVGGSQGPTHHNSVERYDPELDTWTMVCGMKTKRIGVGCAVVNRMLYAVGGFDGVNRLSSVERY
HPENDEWRDTQPMHTARSGAGVVALGNTIYAVGGYDGHEQLNSVEKYNVLDDTWQSVSRMKHRRSALAVTVHNGKIFALG
GYDGHDFLSSVEYYDPAKNEWKEVTNMSSGRSGCGSA
>Cint_ENSCINP00000017813
MNGSDTSMATRMYVCESVEHPSSLLSGLNEQRLKGILCDVTVRVQGHLFKAHVNVLASAMGYFRDLFCKPSKEKQVGIID
IPMIVNSVGFAKVMDFVYTSQLCLSQSTVMQVLCSACYLQIPYVVEKCQEFISTHGHVTSASSLAAVTAPLLQDLQRDGG
ITNGTNTQSASASLSLLSQLNKASEKSRARADALRHQSSPQPSNPENDEQHQECELINTPPSPASRSPTTGDRGITDSPV
VSAGSPSNLKIKQEQVDCRRNDEFPNETSSSGDDKQLVAVYSPQEERGANERSHPQPTSIKNIIFTTSRSRIEGNGNDTQ
QDREAIDHKSLAEYFSGDRDKMDTNGTYPFRFETRLEDPNSTEQSSFMLGNNQDRNNQVTTLTHYSLNVKQQKEKPKKEY
KCDYCGKMFGRQQHLKRHILTHTGERPYPCQYCEKRFRRSEHLKHHLVSHEQIMGIQNPPSRKRQRRMEREDAYSMSKMM
LDSYSQPEQTGMNMIMSAPVVLPSMYTPSDIQRTSPTLQITELHSDPQAPDSGMIDLNTGQRVSPAHSAASASPVSALNL
CRATVRDDMQPSPVSGFSDQMSNEDKIEQSNADVEHDFTPAALNGYEENPSSHDIIGMEGSEVLTSDLKRLIAAMDE
>Cint_ENSCINP00000017830
HEESVLKGLSDLRKCTSLCDVHLIVEGEKFSAHRSVLASCTVLDFIYSSTVYLTVQNMQDILSAANHLQILSVVKFCCEF
LQSEISAQNCVDISKIAKFFNLTDVDDCVRGFILANFTQFVRTDEFLRLSEADVCALLSSNNIRGHSELELFEAANRWLL

HDTSRFSSVFPVMQHIRFPLISPDDLDEKVQSVHYMQTDPDCMRLLFEATKYHMLPHKQPLVQSSRMQVRSIMPCLVAIG
GKESTNHVSKEVQLFNKEASQWLHLGEMDLPAYCHCVAVLNDFLYVVGGQEMFDNNGSTAMSNVYRYDPRFNRWLKMKNM
LETRTDFHVSAIDGFLYAIAGRNHTGPLSSAERYRVDRDKWEPIAELPHAVCAHSGAPCAGKMYIAGGFASDGFQRVVYC
YHPEENKWEPKPKLKTERGLHCMVDYKLRLYCIGGNNKTNGARRDVLSTEMYDLNSDQWTEVAPLFAGQSEAGAAVVDGK
IYVVGGHTWRERKDVRTVACYDPERDTWEKATDFPEPLTSVSCCALRIPATLLSE
>Cint_ENSCINP00000017959
PLYNWQATKGTLKDRFRFLFNNQLRSDVLFVVGRGVSSQRIPAHRFVLSVGSAVFDAMFNSILATQSNEVELPDVEPAAF
LTLLRFLYTDEVNIGPETVMTTLYTAKKYAVPALEGHCVDFLKKNLSSDNAFMLLSQARLFDEPQLATLCLDCIDQNTRE
ALAADGFTDIDHSTLVSVLERDTLGLREKVIFSAMERWAEAECSRQELEPTKDNCRKVLGEVIYLVRFPLMNVEEFAQGP
AQSGILSDREIVDLFLYFTLNPKPRVRFSDRPRCTLSGKEQSVCRFRDVAERYEVWGYSGTSDRIRFTVSRKIYIVGFGL
YGAVMGPADFQANIQVISVDNGIVLGQNDTGFSCDGTDKTFRVMFKQPIEIKANQSYVACATLNGHDSFYGTRGHRRVIK
DNPSDGQTVFIFTFAPGNNNGTSVEDGQIPELIYYT
>Cint_ENSCINP00000018116
MATASANTSVSEDELIGDKNVIVESISRFYNSTEMSDIVLKLGNQRFSSHRFVLVLLSDVFRAMCSKRWDNNKVNEIELN
ENEQCIPVFPRFLHFLYHGTVYVNTTTALPLLMLADKYNVQPLKQSCERYVHTQVHEGNVIGAIRWLPYLQLCGHRDLEN
SCIEVIVMEMEFVLKFDDFLLLNLDFLVLLLGRNDIVVSSEYSLYKGVVKWLEANNSGDNQNNFEVLIPQIRFSMMFPEQ
LIEIEKSEFYRLHQDVIGPFLSLAHRFRSLALEVSDDSFCDNIYRPRNYTNSVWCHYVTLDADSVNFHNKCVNLQNANDM
LMSAHQRDPNWQVQVLNSEKRDNNSSVLSSERGFRRQSSTLFANPSAATSTIPLYVMIRPLRPVRSNIVVDISMFQVKKE
KLSHHLDTKSLLPDSNLAENGAVGNGSQRPKIQINGPRFFYPATSTRDKMFQSVPIHFSLLPQTASEQKVILHHEFNRDS
LEHPPEIPCVSFGPHFTNPARPVHTVRLAIITKPRFRSNDDVAAEANNEEDCADEALPILG
>Cint_ENSCINP00000018854
MKKGQPVVTANHVADDDLNQCSESQCMIHAHYVLQILQIHRKSGMFCDILVRTNDQTFHAHRSLLAACSLYFSNLPTDPN
NQVYEVVLPQVTSVGFDVLLNYMYTGILGINSENIKDVISGAMALKMTNVVETCYQFVNSSQYMETDQMYEYEQNNHMST
DSDATMFHDQDKLSDQMHDEAPVDASSAPLDLTKPRSSDAGHASDNITI
>Cint_ENSCINP00000019558
MEYPIQQVPELLHNGAGDAPLKQAMIKQTIIKAEKNNEKSVENGDCSAHDCKNLNGNVIEKTADKNEIKKQSSEVLDQTS
RMCENDSSSDDKSYEGNVTLTMEPEHSEVPFKARSENGDAGLSKDDDLHRQSDVDADDSDIEEDMSKEFKTAYKKSHQPS
VLEWTDTQHHDNTIHRLDVFRKSKHFCDLVLQVGGKEIYCHRVVVGGAARSIFDELSRGEQEVGNLSRIPLDGRNGLLAD
AVEILIDYIYTAKLVIPSELLTAVYKACLQLGVERVTPLCRRYIIADLEPEHCVPMRRLASSVDDMELKQYIDNYIEENF
EAVAASNELLNLPRIKIEIVMNHGNGVISMLLTNLREGSIGHTALAWVRYIIEQRGGRYFEDLTEQVQRIPLDEINNTPS
SASGFHGNNYNRKLSHDEPRDYVYGKTNGAQQKIRLSRSSSIDSISSSDSDDSNSRALCCMDGEWSVIATTPAQGSDHVI
SLCSVGGVLCTLSIRLPNNGLLNGNETNGFATNATSSVSSASTTTSCSSSVASVSPLGQLAVARCAAGVAQLQGKLIVAG
GFNQSECLDTVECFDSSANRWSPMSRMSEKRARFAVAVLRGHLYAVGGSSGSHDHVTVEKFDPTTEQWCHVQSLKSRCSE
ASVAVLNNLLYCVGGVQKSTGVVAIKTCQVYDPSADEWSYKAQMHTGRSSLGVAALGSHLFAVGGSDGWTCLNTAEKYDP
VADRWTFTAALNVQRRGLGMAEHNGALYSVGGFDGTSFLSSVERYNPEEADVWVILPSPLGVPRNNAGLASLHGTLYIVG
GFSGRHFLSTVEALDDVNGDWTAHDIQSLATTESESD
>Cint_ENSCINP00000020045
MVMELPTEIDIETDEVLFESNHAEFLLQELNKDRVGGQELCDVRFCVGKMEIPAHKSVLGSFSNYYKKMFALEVREKYET
DIATFGITGATLQAIIDFVYTGRIQISRENVYDVFVASDYLSITPVRDFCIRFLKDSILVSNCVSMRQLGLTHNSNELVE
ATEHFMAVNFNAVTKQAEFKQLNVNDVTAILKLRVSSNEGFILTAVLNWVKHNTEARQQYFQQLFDLVNLKKLSTSFLNT
LVAEDLVKTSQSCLVKIVQQYSGRSMQQKTPTEFIVFGNKTKTKGVQTYNVQTQVWSDLPETRKERIGACGAFFDKKVYA
LGAAWSNSVECLSLKNLNLGWQSLSPMNKNRQFAASVVTDGNICVSGGHNNGGKLKSVELYSIKNDTWGMLPNMNFHRSG
HAMHCVDGILYSVGRVGLVSTERFDPRENKWTLVADMHSTRDGMASAVCQDGIFTLGGLRLRTVERFDPRNNKWLELASM
VHPRAYGAACNLDGNIYIVGGMGSEQSASTVEMYDSAKDAWKVSCTMPSAKACSVVLALG
>Cint_ENSCINP00000020104
MENSFYLKSIPLHVEGHGDKLLKALYDQQRNCQLCDVSLIVENNSFPAHKAVLASYSSYFQALFDRGKQSDDVVSKVCNV
TTIHLSSVSSKTFIPILHFLYTSKLHLFEENIRDVMKTAGKLQIHSIVQACERVIDDKIVLPSIHEIKTLNIPCTNVINE
NSTPSVLFVDLKSEDDDEKSCEAADSTNASFLPSSSDDTEKQKLCGNRTLKKKSNPKKIKFKENASTPENWKLTRSIDNI
STISPTSIFGRIGSQAGRRVGLSQQVSTSTSVDSANKTISSTSDFSIDPLIFEPTNESLGNIFDNDPALIVDDLASESCL
LSNNFTEQSSTSLATPKTTEALQVQSVYSISPKLTKKVKLFNERSPPEMNKNYLLPTSVDKNLVPSFSSSLFNERHTKPS
VYTCKECLKYFTNITDAFHHCYTEHQQSKKSLDISNRLLDTKSHSCQHCGRIFNNKNNLKRHILIKHTADKRFKCSHCGR
RYAIRQSLQYHIKSVHNKSINDITVDSPQTSSLMMENFSSLLAAPTVAHPTSRLKRE
>Cint_ENSCINP00000020666
MKNKPAFICQPLHKHKMATNDEHLIQYKGDHEGRVLTKLNRLRKRKVFTDVTFKLDDGEVSAQKSILAGYSDYYEKMFTS
GFKEGFNEDIEVKGMKASILDLLFTFIYLQVIDISDKTVCQLYEASDYLQFNDVKGYCVSFFNDALAVGNCLSFRSFAQR
YQLLELVEKCDKFIVDHLELVSKELKFMDLSLDEAESLIALKQKQ
>Cint_ENSCINP00000020667
RVNRVNCDVTFKLKDGEVFAHKGLLAEHSATFKGKFKHEEGNHEVVEVNGIKSSILDLLFTFIYLQVIDISDKTVCQLYE
ASDYLQFNDVKKHCVSFFNDALAVGNCLSFRSFAQRYQLLELVEKCDKFIVDNLELVSKELNFRDLNEVKTFIEIKNQQD
SCQDSIFRTILNWIKHDFKQRQQFIERLFQLIDVKKLSTAFLEEVVEKTEKWMKRSDLYLDILSPEYITRLKFQIPNTVA
AVTKATCMIISGDECEHLVNVYDVNEKSFKICKPTLYERVGSTSVKINNHVYTAGGVDSNIVECLNLNQVDGDWYKVASM
KEQRWRAASAVLNDQMCVTGGMDGGDNILSSVELYNPVVNTWTIIPSMKTKRWQHALVSYNGRLYSFGGENDPFSSNDLN

>Cint_ENSCINP00000020668
MASKEETVIQYKSNHEGRVLTKLNRLRKVKMFTDVTFKLEDGEVSAHRNVLAAYSDYYEKMFTSGFKEGFNKDIEIKEIK
ASILDLLFTFIYLQVIDISDKTVCQLYEASDYLQFNDVKGFCVSFFNDALAVGNCL
>Cint_ENSCINP00000020673
CDVTFKLKDGEVFAHKGLLAEHSATFKGKFKHEVIQGNHEVVEVNGIKSSILDLLFTFIYLQVIDISDKTVCQLYEASDY
LQFNDVKKHCVSFFNDALAVGNCLSFRSFAQRYQLLELVEKCDKFIVDNLELVSKELNFRDLNEVKTFIEIKNQQDSCQD
SIFRTILNWIKHDFKQRQQFIERLFQLIDVKKLSTAFLEEVVEKSETFILIFSVQNILLVSNFKFRTQWLRLQVSCMKCG
RKVAKQSTCMIIGGFGENKNSVHIYDVNEKSFKICKSTLYERNGSTSVKINNHVYTAGGRGSNFVECLNLNQVDGDWFEV
ASMKNQRWLAASAVLNDQMCVAGGIDDDYNKLSSVELYNPVANTWTNIASMKTERFQHALVSYNGRLYTFGGWNGLNYLN
SMESFDPREGKWESLKPMNNKRHGLCGVVYNDEIYAIGGRGLKSCERYNIRTNTWTKVSSLNHVRYCSCACVVNGKIYVI
GGFDWDDDESKSIEAYDATINEWKIETNTETPRYHASVVAL
>Cint_ENSCINP00000021014
KRKVFTDVTFKLDDGEVSAQKSILAGYSDYYEKMFTSGFKEGFNEDIEVKGMKASILDLLFTFIYLQVIDISDKTVCQLY
EASDYLQFNDVKGYCVSFFNDALAVGNCLSFRSFAQRYQLLELVEKCDKFIVDHLELVSKELKFMDLSLDEAESLIALKQ
KQDSGQDSIFRTILNWIKHDFKQRQHFIERLFQLIDVKKLSTAFLEEISANDVIFIHSQPCMKGLVQPFMFLISSEYCGI
RTNNFIIVICFVTDQMCVAGGCGGINASSSVELYNPLVNTWTNIVSMQTKRWEHALINNHVYTAGGDDSNFVECLSLIQV
DVGWNEVASMKEQRWHAASAVLNDQMCVAGGWDGYNALSLVELYNPVVNTWTNIAPMQTERWQHALVSYNGRLYSFGGRD
EGFGFNPLNSMESFDPREGKWKSLKSMNKKRSGFCGVVYNDEIYAIGSLFILYINSKLVCGDGLKSVERYNIRTNTWTNV
SSLNHVRYDSCACVVNGKIYVIGGYGDNESKTTIEAYDGTINEWKIETNMEISRRWASVVAL
>Cint_ENSCINP00000021363
MALVDKSISFNGTCSVEEKNHFVNHSYNLLKFANEARQDGRFNDITIHFEKKLIRANKMVLSCYSEYFNAMFNTELDEKY
KDDVEVHGVEPESFEKLVDFMYTGKININTNNVCELLAVCDYLQVLAVKELCIRYLSKMMSPQNCITIQVLADRFTIPQI
TEQFNKCFVTNYQQVVSSKHFKKLSKDDVIKLLQSTHGKVSSDLL
>Cint_ENSCINP00000022235
MKMSSSKFLSCDGHFDFIMDMLVKQRSNKRYCDVTLVAEGQKYPAHRAILAASSNYFDTMFSSQMKEEFERVIKIKEVTK
VAMEQVITFIYTNKVLLSEENIDEVLHAASLMQVQDLLALVIQFLSNEICPLNCLHYRQLGKLYSLNDLVLECDRCICEN
FDQVSTQQKFFEMDEVEMEKLISSDELVMESEEVVYECMVGWVKHHLQTRKQYFPTLFKHLRLQYVRLDYLSRNIRKEGL
VREFLDCRDLVEDAFYFHVKPDLFEKQKPRKSCYPCKELIFIPNKQQNIASYNMTEKKWSSLQ
>Cint_ENSCINP00000022638
MPGKVGLEHAARLAAHLSMQRELGKYCDVCICVAEKRFLAHKCVLSANSSKLDALILDAIGNNSNSQDIEIEIQGVSPVG
FEKLLEYFYVGEMNLEPHTVMPVWMTAAYLELPDVVSKCNDYCQRSVTCVSSNQPPTEETVKTSDMNNNSSGGMEQGMAT
LSPPNPCISPTAQVSASPKPGSTQHEQGPKYSQMNTVSATTKEDSPGVEQIGVQHSGSASQKVASHEENGIAWTPLQNAT
GETFSQNSGSSNNIQNYQQFHANTTQTWSQPQQTWVTTSYQDQPLQVQLASDSNQPAHTPNNLIVIKEGNGSTPEDGEIQ
IQANDGTFTVPSSGTSMVYTHPSNIQHASIIDVNKLSENNCIGDKGNCENIMLQVERLRIQHQQLQRLQQQQQAQQNADD
LQRTGQPPLLMNYLPQQQQHADEPPRKKSKKKKSSRSKSSDTDLPSDMEMQNYQQSTVTPLPMHTQQNQQMVVNNGLSLD
PTTGLRTVNPPPDFRIVTEAYRKSLYMKHSRIKEENPISATGTVAELLQRGKSRKRKSTEQNALNNTIRLILDMLEIVDS
SDPRVEDALFLIMDRFVSLRENINDPTVRRSLLVKVRHSIHCKKWRVKRQMLKEQEQGGAGVVVKLQHEEEEDNLHG
>Cint_ENSCINP00000022695
MAASSPIFRAILDRFSSSEIALTKVDDVAMLELVRFIYTGQCSIDRDNVVSVLRASRRFKIPFVAEQCTNYLDRHCGGFN
NFFSPANVLVAYKMAATYGCPKLREESRKFAISHFWEVAETKAFRELTSLELNRLISSESLKTARSRDPDLEHLFYNAIL
NWLVHAPTSRIPIMTSRVMKFVQFSNLKIGVLNNLVSK
>Cint_ENSCINP00000023099
MSGSDNDVPCGSSVEELVEDKTSKVMKSEEYPWMMLEQLNKQRMDNLYCDFTICIDDEEYPVHQGILAARSEYFNAMFTS
KLKEGFEKRVEIKESSKQCIERILGFLYSGKIELSVENIEETIRCAALMQIPDLLQVASEYVDSIFNTGNCLHFRGLAKL
HSMDDLVKKADKYLLDHFRDVCRDKSWNELSAEDVEMFISCEDVVVESEEVVYE
>Cint_ENSCINP00000023303
MGSPVSRVIQACPSVEKKRISQKFKPLPNPGNRLYSDNQLWVEILGNLNNMRHSGSMVDLVIVNGEHSYPCHSVVMCARS
KYFHDLVEASVATTNGTICFNSPVHLKVCEIKDNIAVKLLEYIYTGCISIPFQHSKEFVDASVKWGVNDYIEFHEKVSLS
QYYSRRSSSVLFHSVFSDEELRDLSINIQ
>Cint_ENSCINP00000023304
MFRVGDRSYMEATEDSVKLFGVDHDTLQVIVDYIYTGKVQFTPENINSIMKAAGWICPESELLKQGSLYLIERVTTQNCF
QTEIFAESYALTQLKEACSDFILKNLSQTKSTLSFTKLSSEYLRSVLSEDRLFAVSEKELFDAVLDWGVSHSAVEVLDLL
SKVIRTSLMEISELEASVEAVMNFGIPSHPTVDFFKDSLRFAHLAPMEERILETGQQHRNCS
>Cint_ENSCINP00000023494
MMKHFNDLRHHKKFVDVIVTSPSKKIRLDCHSLVLSSGSNFFEPILSLGGDAECEFEVTEVKEGVLEALVSFIYTGKVGV
NNHNVSKLMQGSDRMQLSEIIEGCAEYLETTTNAHNCVQHRSIAIDYKCNKLKETAMSCIAEHFSQVSRSTDFLLLSVGG
LIEVMTSNDLHLDAPLCEGEMSVFNAIRRWISHDEENRKTHVDEVMSVVRFPLMNQQKLIDHVISDLLVMESDALCGLVT
RVISQKPFHAFDEPVYMRGNEYIITLGGSSPVSDITSEERGYTAVAYNQHVTVYDATHDRFHELTTLPETRALHCAVACD
QYVFVVGGFDSYSCIVNNVHCFDLASGMWEDMPHLKIKRACFSLHVVSGFLYAVGGLTPNGYTSSVERLDRSRKTWELAA
ALPSTRYRHAGCVYKGEVLVTGGCEKLDSVLSYNPKVNEWKKCKPMNVGRDSHVMAVANDKAFVIGGDGINFSMEYYDGT
NWSLINTEMSCSVKKLYLPGVAVLNDTIYMLGGWKAESNAIGDVRYYDAVNGNFVESSETLPIPLGSASSCTVRFPKNYW
KRKFH

>Cint_ENSCINP00000023928
MRQKIEEIIGQRNIDIIHSAYVVHTLYQQQKLGKYCDLKVLVGDKQYFVHRCVLAAASAQLDSQINGLDTIQLVGVTYYG
FEGLLEILYSGKLKDIACLSDIRFQQIVSAADALNAETVMEYCTRERGDLMNLLMHQSANNAANLQKQNSPKNETEGNAV
ENKDSSYNNSNSNTETDNLNTCSPNTADDMNNKDIAEYRRTNSMSRVQ
>Cint_ENSCINP00000025128
MSADHDDTDSNGESDFEDDDRSDESFKVMKSDEYIGAMLEQLNSQRNDNIYCDFTICVGKKEYPVHQALLATRSEYFKTM
FNSKMKEGLDKRVEIKEISNQCMERILDFLYTGKMKFSLQTVEETIRCAALMQLYGLCLVSKVYNSYASAKIATDTVPIS
KPQYFYYFVKFELTSLNIFII
>Cint_ENSCINP00000025205
MQKSSDGCNKWRDCDSISHKHCVKNWRNLVRLWRAQKLCDVVITVKGEPFRCHAIVPASVSPFFRNAFLKKTAMSVPNGL
SCEKEVIVDFMTKEIFSQVLTFMYTADVLVTPATARPLILAAARLEMQGMVNAVLRRILDLEMAEIEDNKNNQPSNMDFR
LKSMIHGRQRYPFNQKVHNAMMDNKNQCPEIECCGELNDDDSQFPEAPGPQHGYKYP
>Cint_ENSCINP00000025251
NDVHTKDAFASMNALRKSNVLCDVILVAGDLEIPVHRLVMASGSPYFMAMFNGMMSESSADRVKLNGIDGKALLQLVDYV
YTAQIQVTEENVQSLLPAASLLELTFVRESCCCFLQSQLHPSNCLGIRQFADIHACSDLLTQARSFTEQHFTDVVRGEEF
HNLSCQQVCELINSDQLSVSSEEMVFEAVIGWVRSDLVSRSQYMPILLEHVRLPLMPSDYLVERVDQESLVKNNDLCKDF
LIEAMKYHLLSPEHRAMMKISRTRPRTPKGMPK
>Cint_ENSCINP00000025258
IVTSYYRSNVLCDVILVAGDLEIPVHRLVMASGSPYFMAMFNGMMSESSADRVKLNGIDGKALLQLVDYVYTAQIQVTEE
NVQSLLPAASLLELTFVRESCCCFLQSQLHPSNCLGIRQFADIHACSDLLTQARSFTEQHFTDVVRGEEFHNLSCQQVCE
LINSDQLSVSSEEMVFEAVIGWVRSDLVSRSQYMPILLEHVRLPLMPSDYLVERVDQESLVKNNDLCKDFLIEAMKYHLL
SPEHRAMMKISRTRPRTPKGMPKSMLVVGGQAPKAIRSVECYDFKEDRWRQVSEMNSRRCRAGVSVFQVGVSVFQVGVSV
FQLSGGCVCVSAFSGVTTIHYNAQGCVWAVGGFNGSLRVRTVDKYDPMTDCWTAGPSMEARRSTLGAAVLQNMLYAVGGF
DGSSGLNTAEVYDPKTNEWRSIAPMNTRRSSVGVAVLGGLLYAVGGYDGVSRNCLVSVECFSTSSGAWTFVAEMSTRRSG
AGVGVVDGLLYAVGGHDGPLVKKSAEVYNPATNTWSPISDMNMCRRNAGWWCVCNFCGLVVSYLHRNRRIPDTSVASVNG
LLYVVGGDDGSCNLVSVEYYNPRTDTWTLLPTTMSTGRSYAGV
>Cint_ENSCINP00000025741
MTDKQCIQMHQHSKNMVVQLNRQRLENTDYCDVIIKVEDVHFSAHRSVLAACSPFFDAMFTSKMVEQFKKEVEIKDVKKH
IFNDVINFIYTDQIVLSADNVRDVLHYSSFLQIKDLLDATVEYLTQSICPENWFVIRGLGQLYTLPTLVLQAEKCMLQNF
DESSLHESFLELTATDLDSIIKSEDLVVSSEFVLLLALISWVQKDVKNRQQNFLALLKYVRFQFIPIEYLTKTFRKMA
>Cint_ENSCINP00000025806
RNNREYCDVTLVAEGQKFPAHRAILAASSKYFHTMFSSQVKTMLQIEKEIRIKEVTGAMKQVIAFIYTNKVLLSEENIDE
VLHAASLMQVQDLLALVIQYLSDEISPMNCLHYRQLGILYSLNDLISECDRCIWENFDEVSAQEKFLDIDEVEIEKLISS
DELVVESEEVVYECLVGWVKHDLETRKQYFPTLFKHLRLQYVEIDYLSKYIRKEELVREFSDCRDLIENALLFCIKPELI
KKQKPRKRCYPCK
>Cint_ENSCINP00000025833
MGSSASKGRHSGTNVDQKIVSIPHQHSNEHINALVKGLSTLRKTSTLCDVTLSVNETEKGPLNKYNAHRCVLAASSVYFR
EKLGVSAAEFDGEQPPTPESKTTPRNDVTTDDVPIDFPITHKAMETVLQYIYSAVLESDPRDIPNVVEAAVKLGIDSLKR
RCIAEIQSGFQLEYFQRILKLSEQLDLSEL
>Cint_ENSCINP00000025878
THNIDHASDLLQFANESRQDGCFNDIIIRVGEKNIPANKMVLSCYSKYFNTMFKSEMKEKYKDAVDVHGVDAESVEKLVD
FMYTGKININTNNVCDLLAVSDFLQILSVRKLCIEYLFTTITQQNCFTIQALANCFTIPQLTEKFNNFIVENYQQVVSGD
HFKKLSKDDVIKLIQSTNNKVSSDLVYTAVINWVKFDLASNESYLSELFKFVDLGEMSPKMLEDVVSVEVLTCLLQYIIN
VRSSQQPALKCNLKNIYFSTNTFVFRILQKSLISLGGKTSLTKVTKFDLETKQWSQLPDLPVGRQVAAAVVIDDVLYHLA
GELQTDGDEIATKIVYRMELKEKVLKWEKIASMNVKRYMFGAAVINGVIFVFGGAEYSPISSGESYIVPLNKWIQLKPMK
IARCGHCLVAHNGFLYSLGGRDREQALCSVERYDPSLDEWKDVAPMQTPRSWFAAVVLNNVIFAIGGHDGKQPLKSVEKY
NVDDNTWVYVGNMNMERSSHAACVAQNKIYVVGGLDSNRKVVKSIECYDDQTDKWSVV
>Cint_ENSCINP00000025902
MACSKLLRSDGHFDAIMDMLSKQQDSGRYCDVTLVAEGHNFPAHRAILAASSKYFDTMFNSQMKEEFEKVIEIKEVTKSA
MEKVITFIYTNKFLFLYENINEVLYAASLMQVQDLLALVIQYLSDKISPMNCLHYRQLGKLYSLNDLVMECDRCICDNFD
QVSTQEKFFETDEVEMEKFVSSGELVMKSEEVVYECIVGWVKHDLETRKQYF
>Cint_ENSCINP00000026077
MPEQVYRNMTRVKHLRAKQRCSDIMKAMNEQRDNKLYCDITLVARGKLFHAHRNILAASSNYFKSKFQSQGSDPFEKMLE
IDGVSRLAMQHVLDFIYTNRVCVSINNVDEILYASWKMEILDLMKLVVQYIKDQLDPTTCLHFRDLGKTYSIDCIVKQAD
HCVRKHFEFVAQQDDFKALTISELNSVLQSHELVVTSEKVVYEALIRWVKYDLES
>Cint_ENSCINP00000026211
KYGDYYTSQPSELLVKLNKFREANVLCDVHFRVDGQQISCHKAVLAAFSKHFENKFTSGEYLDEEIELTGFSAATFLTII
HFVYNQTITISNSNVLQIYEACELMQVNCIRVFCEDFIIEELQVGNCLRFRFFALRYQNRTLAAKCDDFILNNFQFVTNE
NEFKELSVDDA
>Cint_ENSCINP00000026213
MENMNPGSLSLHSLNIVSRVAFNKFRKNGDYTNVVITVGSHDWKCHQCILAASCTSLHLLFQAAQSLDRVIPTVIMPDHF
NEQDIDLWLDVLYLNTLPNNLDSVKRVYTLAAYLKMPTLECICSSHLEKQMKKEEWIEWHSSTLKTIKSLCNLQVRHAGL

NKGTHHINILPPNDKLDSITHQPKTGELQNGQDNEFELEPSIPQTSATNERIKPSLLSCSHCGKLFKRKYDLVVHDRRMH
SNKPFRCPVCPKEYALNRELQIHLRIHKPGFRHQCKHCSMKFISTEGRRKHILQEHKTYACKNIGCKSAFVSQELLDQHP
CMKTKEKKHQCEVCGVTFRLAEYMRRHVSAVHVKEKPFKCTECERHFNRPEQLKLHQITHSKERRYKCDLCGKTFRQPAG
LWKHKRKHNGGYFVCEICGYSCRSNSVLSMHLKSHVSDTHNTCQQCKKTFKNMKSLNRHMKQHSKNT
>Cint_ENSCINP00000026502
LSGTSSVKEQTHNIDHASDLLQFANKSRQDGRFNDIIIRVGEKNIPANKMVLSCYSKYFDTMFNTEMEEKYKDVVELQAV
DAESVEKLVDFMYTGKININTNNVCDLLAVSDFLQMLSVKKLCIEYLLTTVTQQNCFTIQALADRFTIPKLTEKFNNFVV
EKYQEVTSGHQFKKLSKDGVIKLLQLTHGKVSPDLVYTAVMNWVKFDLASNESYLSQLFKFVNLSEVSPMMFKDEVSVEV
NNKHILSFITDNNKQIFIFLASCSKTGQSGLEKSLISLGGTESLYKVTKFDLQTKQWSQLPDLPVGRSTAAAVVIDDVLY
HLAGSLQTDGKETATNIVYRMKLKKKVLKWEKVASMNVKRFVFGAAVLNGVIFVFGGADENKIKVSSGEYYVVPLNKWIQ
LKPMKFARSSHCLVAHNDRLYSLGGHDGTQYLSSVERYDPSLDEWKDVASMQTPRRWFAAVVFNNAIYAIGGFLDSGNKV
VKSIECYDDQTDKWSVV
>Cint_ENSCINP00000026513
MDSVKFKFVQEVTQYGDKLLKRLDEFRRKNLYYDVRIKTIDDEFVAHKVVLAACGGVFRRNFVDPNTMMIIGDVTEVTFD
CCSIGLSPILNFAYSGILEITAENLIPVFVASGSTEMKEVQDICAGLTMERLGWIIDFDGKQSLCDVTIEESLAKLEKTL
RINLKSFGESPVGVEGEFPCLSHVEETWPELGVVYKLLVDCVKKAKVIDDGNKVNNKPDEVVTVDTTPTKKEFATQDTKQ
EPEKTEVKEPKLPATQESTSTENSKSTTEITKDETIKESTKEATKELEDPKTPAKEDPKTPAKEDPKTPANEDPKTPANE
DPKLPAKPPNINGNQPDGRPKLKPRTISIVDPEEPKTQQSPKVTKRKPTAPKPPIEASPVSNRTKRGSQHKPTQALPSPP
TRPPKPSPKVPPPRPKPPSIKKNKPKPTIYDDKLNPFSLDETDSNENDKDKKTSSTENNPFE
>Cint_ENSCINP00000026547
MEKSAVIVTLAQEQPTDYIPEHCDVQPQYVYESNIHSNNVLATLNEQRRSGLLCDMTVIVEGVELQAHKAVLAACSSYFN
GIITDPANVSHNIVLELSSISRLGMESLLEFAYTSKLTVSRGNINHVLAAARELDVKNLEYSCLNLL
>Cint_ENSCINP00000026571
LSFANKSRQDGRFNDIIIRVGEKNIRANKMVLSCYSKYFNTMFNTEMEEKYKDVVDVQAVDVESAEKLVDFMYTGKININ
TNNVCDLLAVSDFLHIPSVRKLCIEYLLTTITLQNCFTIQALADYFTIPQITEKFNNFVVENYQQVVSGDHFKKLSKDNV
MKLLQSTHDKVSPDLVYTAVMNWLKFDLASNESYLSELLKFVEFYEMTPKMLKEVSFEDCFKLALIKILFKVRHKCLFPN
INHSLEKSLISLGGTETLTKVTKFDLQTKQWSQLPSLPIGRDAAAAVVIDDVLYHLAGRLQTDGKNTTTNIVHRMKLKEK
VLKWEKVASMNVARLVFGAAVLNGVIFVFGGTDENNKSVSTGESYVVPLNKWIQLKPMKVVRSGHCLVAHNGYLYSLGGH
DGQQVISSVERYDPSSDEWKDVASMQTPRRWFAAVVLNNAIYSIGGHDGKQALKSVEKYNVDDDTWVYVENMNIERYGHA
ACVAQNKIYVVGGLDSGKKNVKSIECYDGQTDKWSVVG
>Cint_ENSCINP00000026681
QEKEVLGKMKMSGMSQMLNHREASHAQDSFARLNKLRREGILCDLTILVQGHKFKAHKTVMAACSDYFLNAVCGGKSTAS
VTVSPAGHMTIEMQYVTMRGFAPLLEYAYSSELRANASDVIDVLSAASYMQMFNVRFFLCYTNVLGQFQ
>Cint_ENSCINP00000027050
QRMNNLYCDFTICIDDEEYPVHQGILAARSEYFNAMFTSKLKEGFEKRVEIKESSKQCVERILGFLYSGKIELSVGNIEE
TIRCAALMQIPDLLQVAADYVHSIFNAENCIHFRGLAKLHSMDDLVKKADKYVLVHFRDVCLDGSWNELSAEDVEMFISS
EDVVVESEEVVYECMVGWVKHDLPSRKQYFPTLFRNIRFQNLSTQFI
>Cint_ENSCINP00000027152
MAMAAAETSFSINGTCSVEEQTHNIDHSSDLLQFANEARQDGCFNDIIVRVGEKNIPANKMVLSCYSKYFNTMFNTEMEE
KYKDVVEVHGVDAKSVEKLVDFMYTGKININTNNVCDLLAVSDFLQMPSVRKLCIEYLSTTVSLENCFTIQALADRYTIP
KLTEKFNNFVVENYQEVVSGDQFKKLSKNYVIKLLQSTHDKVSSDLVYTAVMNWVKFDLNSNESSLSEVI
>Cint_ENSCINP00000027212
MNLMREQSLLTDVTITAEDREFECHRVVLASSCPYFKAMFTYDLRESTERKVTLHGIEATSFAQILNFAYTGTCMLSKQN
AADIFVASDLFGILSLRNAAENFLKKCVDFSNCLQILRLSSMYSCPEVTKIAKEFARQNFIDVTQQNEFLQLTIDDVIDY
LNDDDINVAREDLVFDAAIRWLRHQPNPTNLTQRVLESVRLPFVRPEYLKIQSTMEVLLNDNAQCLQKISEAISYHHSDS
NCGTFLNKQRKPRKRTSREVVMVFGGETRAGSTHEASNDVSYYDPMTSRWDSISILPGAVSAEAFSVVSLGYEIYLTGGT
VNGKATNKVTVFYTYLNKWVKLSNMLVPRYHHTCTAIGDMIYAVGGTNGSRCLDDVERYRSDVDKWEKIEKLVHAIKCPA
VATHKEKLYVFGGFTDSYVISQSIQVYTIPDKTWTIISSSMIDYTCAHAAPINSKIFLLGGGSKIVKIYDTETDQVSRVS
DMLEKRDNCGVTVVGGKIFVTGGVSESSGPSLMTSECYCPLRNKWERIPEMPRPLHRHGSVSMRIPIYQRTDRVSPRTPR
TMTSSEEVTSPGIT
>Cint_ENSCINP00000027232
HSSDLLQFANEARQDGCFNDIIIRVGEKNIPANKMVLSCYSKYFNTMFNTEMEEKYKDVVEVHGVDAKSVEKLVDFMYTG
KININTNNVCDLLAVSDFLQMPSVRKLCIEYLSTTVSPQNCFTIQALADRYTISKLTEKFNNFVVENYQEVVSGDQFRKL
SKDDVIKLLQSTHGKVVSSDLVYTAVINWVKFDLSSNESYLSEVIKFVDFHELSPKMLEDVVSAEPLIKKHGDLGYSLFD
ALQHGFVPKNNLIYQLVVMTRQPLSSMMFCGDDGKQCLKSVEKYNADDDTWVYVGNMNTEISFHAACVAQNKIYVVGGYH
CSINSNGKVMESIECYDDQTDKWSKLASMNVKRWGLSAAVLNGHLYSLGGYESGYLCSMERYDPFLDEWNDVAPMQTPRY
GFAAVVLNNAIYAIGGYDGKQCLKSVEKYNVDDDTWVYVGNMNIERYCHAACVAQNKIFVVGGLGHSVVAHNGSLCSMER
YDPFLDEWKDVAPMRTPRYGFAAVVLNNAIYSIGGHDGKQCLKSVEKYNVDDDTWVYVGNMNIERYCHAACVAQ
>Cint_ENSCINP00000027294
SVEEKNHFVNHSYNLLKFANESRQDGRFNDITIHFKKKLIRANKMVLSCYSEYFNAMFNTELDEKYKDDVEVHGVEAESF
EKLVDFMYTGKININTNNVCELLAVSDYLQVLGESIFLILYLSKMMSPQNCITIQVLADRFTIPQITEQFNKCFVTNYQQ
VVSSKHFKKLSKDDVIKLLQSTHGKVSSDLLYKAVMNWVKLDLASNENYLSEIIKFVDFYQMSPKMLQNEVSVEPLIRKL

GDLSYPLFDAVLQQSKNTGTVNKSLISLSGTNILTKVTKFDLRTKQWSQLPDLPVGQDKAAAVVIDDVLYYLGGDAMRNG
RCTTNIVHRLKLKGKILKWEKVAPMNVKRWGFSAAVLNGTIFVFGGADDRLAKVLSGEYYVVSLNKWIKLKPMKIARHGH
SIVAHNGHLYSLGGHSGKQITSSVERYDPLLDEWKDVAPMQTPRGWFAAVVLNNAIYAIGGYDGNESLSSVEKYNLSNDT
WVYAKDMKIEKNRHSACVAQNKIYVVGGVNSNGKVVKSIEYYDDQTDKWSVV
>Cint_ENSCINP00000027315
INGTCSVEEQTHNIDHSSDLLQFANESRQDGCFNDIIVRVGEKNIPANKMVLSCYSKYFNTMFNTEMVEKYKDVVEVHGV
NAESVEKLVDFMYTGKININTNNVCDLLAVSDFLQMPSIRKLCIEYLLTTVSPQNCFTIQALADRFTIPKLTEKFNNFVL
ENYQQVVSGDQVKKLSKNYVIKLLQLTHDKVSSDLVYTTVMNWVKFDLSSNESYLSEVIKFVDFYKLSSNMLKDVVSVEP
LIRKVGDLVDSLFDALVQYSKKTAIFFNQSKVNTFYYLGIFLITLGGANSLTKVTKLDLQTKQWSQLPDLPVGRDNAAAV
VIDDVLYYLGGDLRTDGKATATNIVHRMKLKEKVLIWEKIASMNDKRLGFGAAVLNGTIFVFGGPVDNYRSLLSGECYVV
SLNKWIQLKPMKISRRGHSVVAHNGHLYSLGGRHGQQYLSSVACYDPISDEWKDVAPMQTQRGWFAAVVLNNAIYAIGGY
DGKRRLKSVEKYNVDDDTWVYVGNMNIERDLHAACVAQNKIYVVGGLDSKNALKSIECYDDQTDKWNVI
>Cint_ENSCINP00000027341
MAASIKLTSNKHFEFVMERLGRQRLNEQYCDVTLVVEDQNFPAHRAILAASSKYFD
>Cint_ENSCINP00000027404
HQSELHIDDINQEWLLMWGAKDLRDAGHLVDVTLIAEGKKLKPCHRVVLAASSPYFRAMFSNNMMEQNLDEVELQNVEFS
SINAIVDYLYTSKIVINDDNVQSIFTCCSMLQVLGLHGKCKRYIISRLSIYNCIDVYCFAKSLGSIELQIESFRFIASRF
CDFVKTKDFVELDKESLVDIISSDDLLVEKEETVYEAALRWLSHDVQNRSFMISEILSYIRMPLLCHDYMNECIVKEDAL
MNEPRLKEQVHATAMYKSIKTMWDQSEKRKLSTTKEDVKSNSNCTSEVRIPLTTRLRSGMKTETVLLFSNGKNTFLFDPI
KNKYYHGDSSSSDAAGLKSRSDRLMPVMKNAEIFATRNGTVYLLGGLEIDKEGSSSALRCKMFVFDLKRLQWSQRASYKS
PRCMMGIAELAGSLFVTGGKQLSIGCGVSTVSKYNYDDDVWTDFTPMPLQLYGHGCASLVDRIYIFGGKTTEKKLSTDVY
FLVNGSNAWCRAPSLIVPRFLAGCTTLISEHGGKQTETIYIVGGVGDVGLLKSIEVLRQYNTISWNMFGEFPSGRCAQTV
TIVGGKICVVGG
>Cint_ENSCINP00000027741
MSSSKHISCDGHFDFLMNMLVKQRNSGDYCDVTLVAEGQKFPAHRAILAASSKYFDTMFKSQMKEKFEKEIKIKEVAKIA
MEQVIAFIYTNKVLLSEENIDEVLHAALLMQVQDLLALVIKYLSDEISPMNCLHYHQLGKLYSLSDLVSECDPCIRNNFD
QVSSKEELLGIDEVEMEKLISSDEL
>Cint_ENSCINP00000028540
TPTTTTTTTTIHDKPPGRNIFMHNMWPLLSLLPPCLLEKINLVFTFGQSATEVIFTTLDEKVYSLGTNTSGCLGVGDNSS
SLEPRLLESFSGQKIISLSAGSGPHVLVLTQDGGVYSWGHNSYMQLGNGGTSPGTSPSLLSRNISNKVVQIACGSHHSMA
LTTEGEVYTWGYNNCGQIGSGSTANQGSPRKVTACIGNKRIIDITCGQTTSLCVTDSGEVYSWGYNGNGQLGVGNNVNQT
NPCRVAALQGVVIAQVVCGYAHALALTDNGVVFGWGANSYGQLGSGNKANTCTPLQVATRIGRVVCLAATHYSHSSAARN
QSGHIYMWGQLRQQATPEPKPTNFTDLNAVFAGFSSPPVSWRAMIVTSEVEPSVQQSVSRAFNDETSADLKILVEGKHIL
VHKSILKIRCEYFKRMFQSHWDENNQDVIEITGYNYPVVYSFLRWLYTDQVELPTEDIIGSMDKHQCQKLIKESIVVENA
ALLYAAAIKYNAKELQDFCFQFCLNHMTKVVQTESFKLLDKEVLYELVLEASKHGAFKR
>Cint_ENSCINP00000028655
MAKPNPRPMSDLFHKKTEKEYQQLCDNFYTELTASLTEYLEENMDKTDNVVGTIRSVTYDNVDVKSIIKSQETWESNESQ
SEILSNYSDVANHRSILADCLIQLLLNGFHYDLKIRVKSETFLAHKSVLYCRSSYFQAMLRNDHGGWVETESKVISLEGV
SVENFAILLLFIYGALVDLSEFTFDELISLASDADMYATMFVMNAVEFYVK
>Cint_ENSCINP00000028671
MEEKYKDVVEVHGVDGKSVEKLVDFMYTGKININTNNVCDLLAVSDFLQMPSVRKLCIEYLSTTVSPQNCFTIQALADRY
TISKLTEKFNNFVVENYQEVVSGDQFRKLSKDDVIKLLQSTHGKVVSSDLVYTAVINWVKFDLSSNESYLSEVIKFVDFH
ELSPEMLEDVVSVEPLIRKHGDLGYSLFDALVHHSKKTGKFPLISRLTLQMFIINVNCTIFVFDGYDNNYGSLSSGESYV
VSLNKWIKLKPMKIARLGHSVVAHNGHLYSLGGCWPEYLCSMERYDPFLDEWKDVAPMQTPRYGFAAVVLNNAIYSIEKS
LISLGGAKTLTKVTKFNIQTKQWIQLPDLPVGRDDAAAVVIDDVLYYLAGDLRTDGKATPTNIVHRMKLKERVLKWEKLA
SMNVKRLGLSAAVLNGTIFVFGGSDVNYESLSSGESYVVSLNKWVKLKPMKIARDGHCIVAHNGHLYSLGGCGSKYLCSM
ERYDPFLDEWNDVAPMQTPRYGFAAVVL
>Cint_ENSCINP00000028949
RNNREYCDVTLVAEGQKFPAHRAILAASSKYFHTMFSSQMKEKFEKEIRIKEVTKVAMKQVIAFIYTNKVLLSEENIDEV
LHAASLMQVQDLLALVIKYLSDEISPMNCLHYHQLGKLYSLSDLVSECDPCIRNNFDQVSSKEELLGIDEVEMEKLISSD
ELVVESEEVVYECLVGWVKHDLHTRKQYFPTLFKHLRLQYVGVDYLSKNIRKEELVREFLDCRDLVEDSFYFHIKPDM
>Cint_ENSCINP00000029056
MSGSDNVVPCESSGEELVEDKTSKVMKSEEYPWVMLEQLNKQRMNNLYCDFTICIDDEEYPVHQGILAARSEYFNAMFTS
KLKEGFEKRVEIKESSKQCIERILGFLYSGKIELSVENIEETIRCAALMQIPDLLQVASEYVDSIFNAENCIHFRGLAKL
LYMDDLVKKADKYLLVHFRDVCRDESWNELSAEDVEMFISSEDVVVESEEVVYE
>Cint_ENSCINP00000029057
MSGSDNVVPCGSSGEELVEDKTSKVMKSEEYPWMMLEQLNKQRMNNLYCDFTICIDDEEYPVHQGILAARSEYFNAMFTS
KLKEGFEKRVEIKETSKQCIERILGFLYSGKIELSVENIEETIRCAALMQIPDLLQVASEYVDSIFNAGNCIRFRGLGKL
LYMDDLVKKADKYLLVHFRDVCRDKSWNELSAEDVEMFISSEDVVVESEEVVYE
>Cint_ENSCINP00000029159
MANHGVNAVAPLEQAVPPLNVADLHPVVGAQPLYNWQATKGTLKDRFRFLFNNQLRSDVLFVVGRGVSSQRIPAHRFVLS
VGSAVFDAMFNSILATQSNEVELPDVEPAAFLTLLRFLYTDEVNIGPETVMTTLYTAKKYAVPALEGHCVDFLKKNLSSD

NAFMLLSQARLFDEPQLATLCLDCID
>Cint_ENSCINP00000029436
MKMSSSKFLSCDGHFDFIMDMLVKQRTNRRYCDVTLVAEGQKFPAHRAILAASSKYFDTMFSSQMKEEFERVIKIKEVTK
VAMEQVITFIYTNKILLSEENIDEVLHAASLMQVQGVLSLVIQFLSDEICLLNCLHYRQLGKLYSLNDLVLECDRCICEN
FDQVSTQQKFFEMDEVEMEKLISSDELVMESEEVVYECMVGWVKHHLQTRKQYFPTLFKHLRLQYVRLDYLSRNIRKEGL
VREYLDCRDLVEDAFHFHVKPDLFEKQKPRKSCYPCKELIFIPNKQQNIASYNMTEKRWSSLQ
>Cint_ENSCINP00000029472
MLNAVACKMALSKQDSSMSLMQKALYQQHLDRRFCDLIVKINDEVFHMHSCVIAAACPKLHDVLQERNNFHSEPLNQDLK
YFQQESFTPTCFSFLLTFMYTGNLQWENMKSEDIQDAFIAAQWCGFDKITDECRKLMTECDGTHKDAIELCENGESEEGI
GDTPTTTTTMVVLAKDDDEEMQQVVPYCSNDDIGAVGSVKKLAGMCYED
>Skow_XP_002742275
MAAADDNLWYSSSPNGSSFYDNSFIESQSKNVNVKKNHHAIELLAAMKDMKDESDLIDLNIQVGNRIVKCHKALLVAGSP
YYDDNISGEVNQETLGVVEKVLGSDIVHHIINYIYTGSLLITKDNASNLLQACYQHNIFRGKAVESAVSDFLARQTLNTC
KNIEESEYKFDEFHFASEILKILNQQRSDEMLTDIVIAVGDQEFPCHKFVLAATSAYFKAMFSSKMRETYEGTVSLEGLH
PSIMKILVNFTYTGKLTINCENVQEILRTACYLQHQSAMAACTEFVKDELHPSNCLGILQFAITVGCQQLCETAMAYSLH
HFVDLQKFEEFKETTFQIICQLIDDDDLNVPKEEDVYTAVMMWIEYDSENRQYFLSELLKRIRLTLVDASFMEQIENDPL
ILGCPCAIQIVNKIKNLRHALHKGEKVRDRRMKLRFSMKTEILVTVCGFTDNQQWVRNVRYYNPDNGLWNELAPFPGRNQ
RFQCVAVDNDIYVIGGQADHHAAHLSETLSDVWKYSSITDTWTQVTSLNKPRHGHGAAVLNGKIYVVGGKIGWSKKFNDV
ERYDPQLNIWTTIGRIKGHFVEKPTAVAHDGKLYVTGGSRLDAGINIPYVDCYNPEINSWIRIAVMPLPLCEHGCVTIHK
YIPNFHGD
>Skow_XP_002741594
MLSFRNQESDDRVEKKISVAACNVFRELRQNRQLCDIVIQVEGQEFPAHRNILSACSPYFRALFTNGMNETLLKTVKIPG
VSIRTMDQIIDYAYTREVVITESNVVDLVVAADQFHCLGIVEKCADFLADRLSSDNCIGIRRFAQSYYIPSLEESAKQYL
LKHFDTVSLNFDKDEFLELTCDELCEYISDDDLNVKLEETVFEAVIRWIDFDPENRIKHQYSLLSRIRLGLINPDYFISK
VKCHKYIQDNEECKPLIIDTMKYLYDLSVHDQHYQNPLARPRVPYDVLYAVGGWSGGNPTNVVESYDTRADRWRMVDSTD
NSPRAYHSVAVLNHFIYVIGGFDGNEYFNSCRCFDPVKRVWKEIAPMNTRRCYVSVTVCGRNIYAMGGFDGHTRTKSAER
YTQETNQWSLIPNMNHHRSDACATALLDKVYICGGFNGQECLNTAESFDPMTDTWTNIPNMRSRRSGVGVVAYNGCVYAV
GGFNGLSRLNTAERYSPMTNQWTTVQTMYVHRSNFGIAQLDEMIFVIGGFNGVTTIFNVECYDEKTNEWYDASDMTVFRS
ALSCAVVHGLPNRREYTWPRVSLDEDVASLSSHTVSDIGPATPRRMSAGTHRARSGNPRAAVVDQRMDSPEHSEADDEET
DTGSDSGMDEDADDEDENRGPESGNDSMDNNGFALVHMA
>Skow_XP_002741534
MNSHPGNNLVFDDDTQWASSMAQLNAMRKNKQFCDVLLSCDKQEIPAHRAILACCSSYLFELFNSEGDVGNTPILNVSVS
DNLTPTAVETLVNYAYTSRLDISPDQVFDVYAAARLFRMNRIKKVCTDYLVDHLNNYSCIGLYTFAARLKDTKLLRETEL
YIQKNIIDVALNQQFLLLPRLQVEIVCTDDFEMNDKQMFDKVLDWAKDCVISGCKLRDLTEETHALFLSSDNTLVDIHDL
DYDSDSEIIQEYRQQNNKRQTTPHKRKNTNSSNSNNNGSARQLIMTSGERNKKSQCEDWRVIAASQTSDNKTTYIALATV
NNKLVAISLHQKLPDSASKSPRLLRSLSIESQGSWTMLEPMKFARCALGAAELDGKLVAAGAAVLNDKLYLAGGSRGPIG
LKSCEVYTPETDSWSTISDLNVGRSQVGLCSLNDKVYAVGGAESWNVLNSVEVYDPTENEWSLIVPMNHCRRGAGVTKCK
GKLYAVGGSDGTSSLASVECYDPKVDRWNQIASMSTPRANVGIAVVDGHIYAVGGFDGKNFLNTIEFYDPENNKWQQFVP
RNGVNSRPDSPI
>Skow_XP_002741193
MAASSCSKGTCMSPQKNMFSNSHAKDILTTMNNLRKNGTLCDVVLSVEKTEFPAHRIVLAACSDYFCAMFTNEMSESQKQ
SIEIQGLTANTMEVLLDFVYTETVNVTVENVQELLPAACLLQLKGVKRACSEFLENQLDPTNCLGIKKFAETHSCADLLQ
AAENFSFKYFSDVVQQEEFMAISKEEVQALIESNDVQLVTEEPVFAAVLSWVKHDDEKRSQYLPELLGYVRLPLLSPKYL
TDVVDMEPLIKTSLECRDLVDEAKRFHLRPECRAEMKGPRTKHRTGSDERLVVVGGFGTQQSPVANVEEFNPKKQMWRFL
PNLTKKRRYVAVASLGDKLYIIGGFDGMSRLNTVEYLDYTMEDLGWSAIAPMNVRRGLAGVAVLGEMIYVAGGFDGIIRH
RSLERYDPHIDQWNVLAEMETGREGAGLVPANGMLYCIGGYDGVNILKSVEKFDPNTNQWVSAGSMSTRRSGAGVALLND
MIYVVGGYDGSSHLSSVECYNPRTDTWTLVTSMTIPRCYVGATVLKGKLYAVAGYDGNSLLNSVECYDPMLDVWEVMPPM
TVQRCDAGVTVMRKK
>Skow_XP_002741110
MEDNSDGECFRSVSHAEKTFKKMEDYLHNKQLTDVILVAGDMKIPAHRLVLSAVSDYFAAMFTNDVREAREEEIELHGVD
PEALRSLIKYSYTGRLDLKEETVESLLSTACILQLSEVVEACCGFLMKQLHPSNCLGIRSFADTQGCSNLLKVANNYTTE
HFVEVTQNQEFHILSVEEVCELFSSDDLNISSEEMMYYAMLTWVNHDLSSRKKDLAKLMQHVRLPLLTPQFLSDHVDTNV
LFKGDDQCQRLIVEALKYHLLPERRASLQCQRTKPRKSTVGSLYAVGGMDNTKGATSIEKYDLRTNSWTQVANMCGRRLQ
FGVAVLDDKLYVVGGRDGLKTLNTVECYNPKTKSWTMMPAMSTHRHGLGVGVLEGPMYAVGGHDGWSYLATVERWDPQSR
QWSFVSPMSMPRSTVGVTVMNGKLYAVGGRDGSSCLRSVESYDPHTNKWSTCAPMSKRRGGVGVTVCNGCLYAIGGHDAP
ASQQTSRQFDCVERYDPRSDTWTTVAAMNICRDAVGVAVLGDKLYAIGGYDGSTYLNAVECYDSQTNEWTMMAPLCTGRA
GACVVQVPDLTSHHHQVHI
>Skow_XP_002741092
MFALKSNLEKERLAAPIAAEEKEFDDDSLSSNEFTAQSMYGDSFSVLCELFEAQQLCDIHLRIGKKSIKCHRIILASASP
YFRAMLTLEMSESKQDVITIRDIDEQAMHALVKFMYTSKIKLTTENVQPLLYASSILQMETVARACCEFMKNHLHPSNVL
GVRNFAELHGRSALVKAADKYTYDNFIEVTECDEYLHISARHLGLLLTSSDLKVTNEEEVYNASIKWVSYIPEKRQKHLP
DLMSKIRLPMLSPEFLMNVVEKEDMIKKNHTCRDYVDEAKNYHLSVASVHPKTTPQHRFSLRSKPRKSTAGVLFTVGGRG

NLGDPFRSIECYDLREDKWFHITEMSSRRRHVGCTSVNGKVYAVGGHDGREHLNTMEMFDPVKNIWTILSPMKTYRRGCA
VTHLNGPIYAIGGLDEGGCYSDVERYDVTSDEWDFVAPMNCPRGGVGVVPLLNCIFAIGGNDGATSLNTCEKYDPHINKW
IEVAKMTTRRAGAGMATMNGLIYAVGGFDDNSPLDTVECYDPQSNTWSSVPRMASARGGVGVTALGGKIYAVGGHDGSSY
LNSVECFDPVSSRWETVSPISICRAGAGVVTCECSVSELKMMGQGINVVSCV
>Skow_XP_002741014
MAEAAEIEKLQRHLDLLRQEYVKLQNKLAQVEQKYSISAAASGNLEEDSFVSRLLKTVADLFDRELYSDLTVKLEGHVLK
AHKFVLAARSDAWGVPDLANVQSLDFTDIPYDVGVCLMKWVYTDQADMKSDDTFVLQLLKAASRYKLDGLRDRCEKALMS
SVNVRNCIKFYATADEIQANVLREYCSEIISNHWYKMFKAKTEYPLHTAIRHKREDVVFLYLMEYDSQLPAKLNIIDDHE
DLPLDLALSTKQESIAQELVKHKVDVDMTDNQGQCLLHKAVRRGDEFAACFLVKNGAKSNSASHREKETALHIVGSYNPG
IAEGDVIDGMARVAALLLQYGANPNMQDNNGRTPLHRAVSARNDAVFTLLLGYKQLDLELRSNEGDVVLWLALDNTTGDQ
QYDDNSFAARLITRGSSPDAIHAQTGDSLLHLASRARNEEAALFLVEHNAKVGHANLQGETPLHTAAHYGLAQLTSQLLM
KGANPNAQTKFDVTKPISRFDKDNTAAEASQQTPLHIAIVNKHTDVVAVFLEHRANAMHSNNSLQIIPDLNLVNSQEQTV
LGLALWMGLHEIAAQLLAGGANINHTMSDGMTLLHQAIMKQDAASALFLLEHQADINIKTKENETPLQVAIKRHLPVVVD
ALCVRGANMNVIDENGNPPLWIALESGQEDVASTLVRHGCDTNAWGRGPSNCQQTLLHRAIDENNEAVSCFLIRSLCDVN
TSRRPGPNGEGGEESRDGQGPLHLSCQWGLEQVVQSLIEMNADVNAKDNEGHTPIHIAISNQHDTLIRLLMSHPLLDLTL
RDKNGQTPFAAAMTSKNNKAAQLILDREPGAAEQLDNKGRNFLHIAVMNADIESVLFLISVHANVNSRVQDASRLSPLHM
AIQTGNEMIVRNLLLAGASITDLNNHKQTCLHIAAIHDHPAICSILIENHVDVDALDDNMNNALHVAVQHGNIHCIRVLL
TESQINAEAVNLRGQTPMHVLGQYGKDNAASIFDLFMECMHEYPVDRPDAEGNTVLLLAYQQGNANLCRAIVRSGACLGP
VNKQGVSIFNAQVATKQLLFRMLDMLSAEPRWSDDECCQECMTKFGIKTRKHHCRHCGRIVCAKCSAKMMPIIKYDLNKP
VRVCDMCFDVLTLGGSP
>Skow_XP_002740989
MAECLPPPQPCNGNKYKYQSTQDGAFIFRIEQHHSEAFQVMNELRYNKHLCDVTIQVEDKEFYAHKVVLASSSSYFRAMF
TSGFGECKKNKIEIKETHPCVFALLVDFAYTSTILVNEWNVLKLLTTAIMFQMNHVVNACCQFLEHQLDPSNCIGIAGFS
KEYSLVELHETSMAFICKNFREVSKTEEFMNLNLFQLVKLVSQDRLNVMCESEVYNACLQWIKHDECGRRTCFNKLLGGG
IRCSQLTPNFLKKQLQHCNILKCEPKCKDYLAKIFQDLTLHKKMGEKGRIPPSHCVIYSAGGYLRHSLSNMECYYPESNS
WIRLADLPEPRSGLSAVTIHGTFFAVGGRNNSPDGNMDSNSLDAYDPITNTWKICQPMNFPRNRVGVGVIDGLLYAVGGS
QGCRHHNTVERYDPKENTWTQVASMHTSRIGVGCAVANRLLYAIGGYDGTNRLKCVECYYPETDEWKCMASMNTTRSGAG
VAAIDNQIYAVGGYDGTSQLNSVERYDIENNTWCYVASMNSRRSALSVAVLYGKLFALGGYDGSDFLATVEVYDAAADSW
NILSQMSTGRSGAGVAVGMEPCLNR
>Skow_XP_002740966
MDDPNQAGGGEMFPDAGGSGNETKRTTVQDEVVSSTDMDQYRVLQLQRHAVSMLSFMHGLRERAELCDVTLSVKGKCFPA
HRIVLAACSPYFNAMFTSEVREKGETVIVLQDLEPNAVEAIINFAYTADVHVSEDNVQALLKASSLLQLQTVQTACCEFL
ESQLDPSNCLGIRKFAELHGCFDLQTAADTYTQQHFSKVAQNEEFLQLSCDDLIGLIHRDTLNVKSEEEVYSAVVLWIKH
EHESRLEELPTILEHVRLPLVTWEFLTTKVADDNLVTGSKICMKYYNEAKRFLASSFHPQLQGELNVRSLPRGETAPGRC
TISSVERYNPLIDRWTSVTPMRSIRRGVGLATLQNVLYAVGGSDGLNALNCVECFDPQTDCWRMVAPLLAHRSSVAVVTF
QNHLYAFGGYDGMTSVQTAESYNPSVNEWTLLPDMNTPRSMAGVVSLGNHIYVIGGYDGANDLITVEKYNPVVDINVIKS
NLTHKFTSHWKEEIHSDIREKSERKNKLRTYRTFKFNISMESYLINIKNIDLSLAKFRLSDHALKIETGRHNCTPLSNRS
KEWSSVSPMLTCRSMLGAAVLNNKIYVAGGCERALCLASVEVYDPTSDTWSLACPMNRPRNGVGVAAVGNRLYALGGYDG
MDYLNTVEVYNVVKDQWTEVASMETCRRRFGCCS
>Skow_XP_002740860
MFNFKCRNLPGPSSPIVLGLSSSSPLPELKSDELSPGLRTVAMRRQADFMLAMNAMRKNKQLCDVQLILEGKPISAHRVV
LSACSAFFHAMFTSKYTESKTAEVELKEVDLATMELLVEFAYTGDVDLTSENVQAIMSAANRFQITQVKNQCADFLRQEL
SPENCLGIRDFANFLNCTELYSEAVKFSYESFTDVSKCEEFLDLSMEQVTEYLSKDELVVRCEDEVFDAAVNWIKHRKDK
RMQLRTEIFAKIRFPLITRNYLSTVVEEDETMQDDTVCLKMLVNGMKHHLLDRTDRDKITATPVKPRAKKFDWQIAVFGG
SESDTCQFFNTHSRAWKSIPSMPEKRRDHAVACIDNIVYIVGGSYLFPLTRVDCYNVATKTYFSLAEKFESARDCLAACA
CKGLLYITGGSQSHGSTALKATDCYDPKLKTWTSRQPMFYARFDHAMVEYNGWLYVCGGGSGGGCNGRPLKSCEKFYSGP
NLLMNNRWAEIAPMQVPRRGLGLAVTNGLIYAIGGYDEHGTHDSVECYDPKLNSWKMVAKLPWKLRNIKCAVINNDVFII
AAHRDVGRLGQVLEYKTKEDRWIFHYNTRAFPVCNIGVTIVDACAVE
>Skow_XP_002740844
MDLTEDAHNANSVVCIGELELYSEHLLHGLNELRQREELCDVVLRIGDTKIRAHRAVLASCSPYFKAMFTGNMCEREKEE
IEFKSIDESAMKLLVDFAYTGKVHVSQVTVQSLLPAADLLQLKSVTKKCCDFLEGQLHPNNCIGIAKFAETHACCGLYRK
AYTYIFQHFEDVIETIEFCQLEGSEVAELLSSDDLNVKSEKSVFHALKTWIKYDINKRRCYLSRLLPSVRLPLLTVKFLT
QSYEADQLIRDDYTCQELVNKALKYHLVAEERLRVSPQMERSIRPRREPKVICAVGGKNGLFATLDSLEVYLPQNDSWTE
VAPLSCRRYECVCAVLDRKLYVIGGMKCIVRGGTSIRHHDNSVDRWNADSDTWTNIGGMIKCRSNLAVAVLEGELYALGG
YNGETYLRSVEKFCPRTMQWRLVAPMLKSRSCFAAAVLDGMIYAVGGYGPTYLNSVERYDPSHDRWEMVAPMVEKRINFG
VGVSRGFLYVVGGHNGVSHLSSVERYDPHRNEWVLVAPMDKPRTGLGVAVLDHKLYVVGGHSGSSYLNIVQCYNPISEKW
STVNSMSTCRCNFGLAAL
>Skow_XP_002740639
MDTNSHSGGSSGASFNSHYSSKSTRENSPHLAPLEWEHSRHGSFDTLNTSIPDEMEFIDKYYKIVELEKTPWTDVHVKNV
LQSGSKSRQLSRKVPYVVVQRISYLLQRPLVRIARETQRLCSTYGKCTKHEIHTAIKLILSRSLAESCLAASVKALSNFQ
MNPDHFRIGKKTRSGLIFPVGKFHRWLVDTHVSSRVNDHAAVYLTACMEGLAEEMFFRAFHGNIDELDEELTVEMLDFGI
SNDPDLWGISQTCEHLICGRNANGILSLTSSPLSGNSSTRSSSSGSDYSNQSRGYGRKCEIKTNGSLEVSKHLEQNLLAT

CVGSIAELDLKLGDGWMYVFSNRNLNQTEIMEECFVIVAWKIKIILNRYTPVRALLTDESICSSRHLDASACAASFQLDL
AFRMLACGRTDLVQQAIAMLGPAGINALNEQGMTPLMFACARGDEAMVQVLIDAHSDLDITVPSNPMYPCVHPAIRHWTA
LTFATANSHIPVVQLLLEAGAYVDGAVDNSFNAETPLQMAAAAGHYELVSLLLARGADPYLFTLDKSNANSGYLNSFALA
ASHGHRNVLRRLLSQPHVTKSSDFLSLEEILAEGTDTGYKAKKLDNTKTREAALQEAMYHSCEHGYLDVSMELRSLGVQW
SLHCWTETLSTAKQLRRKAIIQCLLRDFCSIQPNEYNEEFCDEALPLMFDIFRQCKNEVISQQLAAVFSLVYGYEPLPEI
KAINFTPPARIDPQYVNNPDMSDVTFIVEGRPFYAHKIILVTASKRFKALLSDKMNESTTPCVEINDFRYHVFKVMAAAN
YFYLDGLQRHCEILCSKLLLFENAVKIYKHAKLYNARALMEYCECYFLANMTELLDKSDHYKKLVFNSKLPTYDVIQGLQ
QTLTHRILLRQSTIV
>Skow_XP_002740428
MAGAVGSNHPLNATPQQQLQQHQQQRPNLAIAAAPSTGEQPIPYNWQATKTTVKERFTFMFNNEILSDVHFIVGKGIQTQ
RIPAHKFVLSVGSAVFYAMFNGGMATQSAEVELPDVEPAAFLALLRFLYSDEVQIGPETVMTTLYTAKKYAVPALESACV
EFLKRNLSSDNAFMLLTQARLFDEPQLAALCLESIDKNTSEALSAEGFTDIDLETLCVVLERDTLGIKECKLFTAVVRWA
NAECHRQQMSPTPENKRLVLGHALKLIRFPLMSVEEFASGAAQSGILTDREVVSLFLHFTVNPKPTVEFPDNPRCCLTGK
EQTIQRFLGIETRWGYSGTSDRIRFTVNRRIFVVGFGLFGSIHGPADYQVNIQIIHAEGGTILGQYDTGFSSDGSDSTFR
VMFKEPIEITPNISYIACATLKGPDSHYGAKGMRKVVHESPNGGKVVFQFTYAAGNNNGTSVEDGQIPEIIFFT
>Skow_XP_002740411
MTNRESQLSPSTINADDIETQTTAEVNKESGVITVFNDCQVLQVQMSSLFNDKDLSDVVINVGDKTYNVVKSILAMSSEY
FRKMFSSEWKESKTNEINLVGDSKECVKCFEAFLRYIYTYTVDVTLGNVLYFLMLADKYLVPQLTQSCLKHMCSNYNCNA
NDAGLTRVYSWLCYSQKAGHNNLEQLCVNLLSWNLKVRHLVEHGLGNDKDVSGKKRLDCSRRVCTTSVGCNED
>Skow_XP_002740260
MEVSKLFLSCKLGDLDSVKYLVETKEVELNVRDKWDSTPLYYACFCGHEELVDYLLQCGAKCEPNTFDGERCLYGALSDS
IRNVLKNYKAITAHCMRRDSYQEFLRRLLDEGSYEDICFAVRNSKFTAHRCILSVRSEYFAEMLKTRWRNKMSVNLKHPL
VSPYAFKAILRYIYTDRLEIHMEDIDDVIRLAKQCQLVGLISRIDDAVKKTDSFVCAKPGTRVTVISIDENCYQLHEDLG
VLAEQAIPNQIKNWVSHGELPFFTEEENIPYLGDICFSVQGYNFYCHKVFFCCRSDYFKALLSDHFSEVGTDSLQGIPVV
YLQDVNPDVFSQVVRYLYTDKPQISAALAYDVLCVADRYLLPGLKRLSANVISQNLDEFNVISVVKAARLFELHRLEDQC
AEYMAAILDKIIYTEEFAELIQEDASSVQERQETDSIPIIDDIRYHITSTILTYSDMAEANEKLAALDDLLIRLGVEC
>Skow_XP_002740064
MPNYYQQESSECLNSSRFENKCGLSEDLKFLASMPELCDVTFLVGENKEPVCGVKAILAARSRVFHKMLFMPSSSNTKKR
SGTADLKLSMRMFTKRSDSLPNLHLISNNTPKTIIIQEFEADVFRQLIEYIHTGCVTLQARTLLGLMNAADYYGLDDLRR
ACMGFMRCCINIDNVGALLSSAEKYIQYKATKSIVQRVLEYVDVNGDEVLNLPAFATLPEHVVRLVLSRDELCANELSKF
RAAISWATRNCQKNPHITLKESMAVFVEYISFYHIPTLALMREVRSCGAVPDQIIMNALAYQADPSSVDPAKFSSPCRQS
AALGNLAPREISPGTNFVETDAVSNKTCSLETLSSMGSDKTEESRGSEDTE
>Skow_XP_002739908
MSEVDFSETHGSLEEFVSAVGEYYNNPDLSDVVLKVGQQSFYAHKFVLAKSSDVLKTMLTNCNWKDSSQYEVPLEESQEC
IAVFSEFLKYMYFGQVTLSIHTAFPLLCLADKYNVLSLRSICSVYMGEKVLKQQNVSGALLWLPHSRRFGLKSMSDNCLD
VIVSQLSSVVESPEWLKLDIQLLCEILQSSDLVIKDEYVLYKAVEKWLLQSEYSTQIEKNIDRIMPLIRFSQLEPTKLYV
IECSELAKRFPILMKPLIGEAYRFYTLARSMKQEFQEEKYFPRCYSSALYCCREQNIKVSTIPVREHLCPHWYLDVFTND
GFFSFPENNSSEVQSCSKWLGSCHKRDCLSLSIKLYPGKEHAGKKYRVHVILESKDGQILSIYRKSGLVEERGEEQLHHG
LFQHHGPSASVGFSYTFGSGRSPFSQQASNVCGPMFGAKPENHQAQRGLFGCSALINVPQLPSVAPIVIETKCLELDMKI
NLSIGVVLLS
>Skow_XP_002739814
MSSILEDQILMKAEVEETPYNIVVSSQVNDLDYSSSRLDEMNVARKKSKLTDVVLDVGGTEFPCHKVVLATSCTYFKGMF
DSGMKECQQRKISIKAIEATTMGSLLDYFYTGKTTITSCNVQSVLRAANFFQVLTLRDKCAKFLITNMNAETCLDIWQMM
VSLSLSFSEQAKIYVLDHFKLVCKLQQFLELTQEELIELISDEHLAIDRENQAGSAVLDWFHYDEDSRRHYFVEILQHVR
LELITSLYLVKQIGKELSKVTPKTTLVSKLEGRLTSSDRTFENQKPRETNNVVCFIGAVLDGQKAGDINYFNPINNAWSH
LTTNTSSLKGSAVVYDNSIVTRVSKGIHTFNMDTREWNIEVDELIDGDRDELKMILVDERIYFIGGRFHAYDLNKKREVL
VEPMLKKVEGTCAVAYGGKIYVFGGRYMHYKETEMIQCYDPNSDTWMHVGDLPEEVPKKDTVAVVFDQFIYLFKDASTIY
VYDPKRYEWLRAIPGLETDTYHGRYTATVCNNKIYIISGIISVYDPIHFIYIYTLDPFEGVLELTTKIKVPSPYSSISLR
PDKHCVTVRLQS
>Skow_XP_002739771
MDLEISLEFDNPNHLTSLMSEMNEARVESKFTDVTLCVDGTIFPCHKLVLASSSEYFKGMFSSGMRESKEEKIHLRDVQS
HAVELMLDYIYTGKAVITGSNLQALLEAANMFQVLTLRDGCIKFMLENMDASNCLGVWQMAESFSLAAASEKALEYVINY
FKLVSTQGEFLELTKEVLIKLVSNDLLCVEDEDLVGAAVIRWYEHCKGDDRNNREEDFVDVLEYLKLGELSPNLYYDINC
QLSDMKGVTKKTIENRPRLKDRKNKGSPKRLVSVVCSVGGFRRGWRYCRNVEYFDAVKKTWHRITQTPVIKDKNWEVEYA
ATYDKYIITHVFRYPSMLNLETREWELFDDKIYLHLRECQSSKMIVLDNKLYFIGGEDRENSHYVAIYGGFTCCDLVTRR
EVQVPLLLKDGVFSSVVSFQGKIYVFGGSVRSTHGDTYDSISLIQCYNPNKKVWTCFDDIPPSVPREDTNAVVFDTLIYI
FQNASKLYVYDPKQKEWQPPIAGFPEARYYKDCSATVCNCKIYIMGGRIDRDDAHDDVYTNKMYCYDPIHSIWESNVGKL
KTPLSISRVSQYTAPPTGL
>Skow_XP_002739706
MANEQFMKGDISLFAREMRKLINHKDMSDVKFVVGENRRTIFAHRCVLSSRCEVFRAMFSDQAASGEDNTVPFILSDVDP
EIFLAVLEFIYTNCVTLSGKIAVDVLASSIEYGLDELKKLCVEYLIESLSVTNACYAMHAAVTYGQDELRESCLKFIEEN
TKSVFKTDGFHEMSDESLSVVLESDRLLLDEMDILSCVKQWALVNSVSYCFFPLSAKTIKAVVHKKIGIVAESVISHVRL

PLLSAEELKKTEQDNKKEPFVPVAMVSFAWKYHALRQPEWGNPQTKLRRGTTHRDTHDGLLRWDEQTSKGGKEGWSSLVS
>Skow_XP_002739684
MRQNNELTDFIIKAGDLEFKCHKALLMAASFNGHNKKTNEMWIRESTVTFHKVKSAAVGAVIDFIYTSNICIEEGNLKDV
TTIAKKIQLWSLEKACEQFYTEISTQQKHKIKTNTKKINFCDVNHARAILLSLNEMRHIPKLTDTVIEVAGKYFQCHRAV
LASGSDYFKAMFTNEMQEKKCNHVKLNEVNPRIFKILLDFLYTPQMLLNSSCLEEVLVTANYLQIQSEILTDHCAKLMLD
KLSLSNCLKVLNSSPALLCPAVDREAITLIITHFCSLYKSKEFLELSRHHLIEIISNDSLDVNEEDMVLDAVVRWINFDK
TRQSHIGEVMQHVRFPFINKNYLDSAISRQPSVMQSPIISQHIKQARKFPVTCPSQENNNQFKPRVSLTTPGILLFGGED
GENGYISAQCYMSQTGKWNNLSTLPSTLTCIKVVTLNNDVYVMGGEDFATFGDIMWKYNTRYNTWSKKSPMNMKRRNYAV
CSLSGLIYCIGGISSGKFCMDEVEVYDPETNMWQQGTPLLEGMGGPIAVAYKERNRLLVFNSDDSVDTMQAYLPAFGKWY
LISAYHPPPNIGHFRDAFLLNSEIYIVGGTKNTEDPYIWVFDLASLSWSRGIPLPHKQTCDNDILCITNVNRKIYLISKE
YTRTKLGIEVKQLHFDLFDCMTGQSTTLQSLTSTSNWPSCNMAAVNVYKHTAKEYSEMYTE
>Skow_XP_002739586
MAEELFRWDAHGNKKRMPWSVKVLSRLNEQRDKTMFCDMIVTVDGHEFNAHKCMLAACSPYFESLFRSNLMEARTGKVDL
QCTSVEGMQSILNYMYTGEIELSMDTVEDIISGADHLLLTRLKRICEKFIVQGTASSYRCLRALQLVEAYNLEKWVSNMA
NMVLARRFSEVLQDKQCQPLIMELSADRLCDVLSHDKMTVKKEEDLFEFIVKWTNHAPTERAQQFTKLLSKLHLIDVDKS
YLIDCIQEEPLVQQSMESRKYFTEALRYHLLPNRVDVKGKLKPQLKPRTTRLMDIIICVDKLGGCLYSYVLEENEWMSCG
TSSRTEQTPCYAGVKLGNLLLCAKQRRTCGGEKNMYDPLSNSFIDGAGTKHEYGQGSAVVSNGKIYTFGGGPHCQSVEMF
DPDNNGWIEKPSIPREPGYILHTAATKDGHIYVICYENNRFLTVWCLDRDDMWTCVLEYRGIEIKEVQPTSCIQPCISIE
SLNGIEVELPGKQKVVISNDPNHEVCIKDSTMPQIPFPRINAGICNVNGDIYVCGGLAKREHYTKFGDTNSVCMFNSSKQ
IWKNLPSMPRRAEIVGCALFQSPHEVYSP
>Skow_XP_002739550
MGCCQSGSKKKVVDPEPGNYIYPSITPSSEDGGHADIPMSNITSNGKVKDKDNCVKRPLNINLSNNALGSKTSLVSTERS
QSILALSDSPCSISQVKASCYSLRSISSYATLRSSFLSVYSAISLRSLTETEEEHRIDPSYAVKLLSDLNNFRKENRFTD
VLLCAGDVEIPGHRVVLAASSPYILSIFAIEALEDGRLYLDYIEPDILHLLVEYIYTSKLSVTKENVGALIKGTQTLQLN
SAEQACQEFVLNLENQDENGNGETASTKSVLYQPYHAIETLLGLNDMRREEIFTDVTFKAEDEEFVCHCVVMATTGDYFE
KLLEGSDDEHVKISIKNASASVMKRLVEYTYTSNLDIHHENAKDILALASILRHDAVLEKCTEFLKRRITPANCLGVQSL
ARKHGVEQLEKAATKVAVMNFKEVRLHPEFFEMCLNQIERLISEDTLDVKTESEVYSAVMQWVKHDVEKRKVYLPELMQS
VRLSYLSPKFLEEIPAAEPLIQESEACKKLIRDATIVHLVIRKGKFHRDPRPKARYSFGEVMVIVGGKNKNEQWIKDVQY
YHPNERHWYSLASMENDSVEYKVTTLNNDIYVVGGLNCNRVSGETWRYDSLYNDWSRASDLRVGRYLHSLGVANNRIYAI
GGQSSLDDNSQSLKSVEKFDRKLNTWQQVQEMYHGVLEPAVTSCGWKLYVISRKDDGDDCVVQYFDISNKTWSVVDSVNI
TGPVQIAGTIRGQVYIVGGKSKFAVQVYKPDTNELSSGTCEEHAEEHSRDLYSGTILNRRVYVTGGRWVIGKEDDDALTM
VTNNMESYDPVQDEWTTMKTVPKALTKHGCVTIRQYIGLPKSK
>Skow_XP_002739480
MAGGKSKSRRSRRGGRQRHIKVNENPIEFSSNGGHANHVLEQLNLQRLHGGLCDVALVVGGRKFDAHRNVLAACSQYFKS
MFENGRFRESKQKEVKIQSLDAEAIEILLEYMYTDSITITFSNVEGIIAASDLFLIQAVRDYCANYWTQTLCVDNVLSAY
KNADIYNLSQLRDEAEAFLSKHFTEIVESNEFVNYPYEVVVVMIKSDLIDVDEKDVFLAVKKWVMHAKERVSLLDDLMSR
IRLPLMPAADLIECVNVEPLVLKSEWCQALVQEAMNYQLLPNKQGELQTPRSRPRYVNLDEMIFAVGVTGTEGHIPDMLL
TKCYIPRHNAWYSLATLHAVSLRGLTFCNGLLYALCFNTENEQLDLYMYDIVSNAWSDGPSINKSNHVIRRSIQLSNTWV
QLIECRQKVYVMISVVCNHNQGVVSSVLCYDPSPKTWKSLPCFHTERLYYSAAATDIGIYLIGGKIPLGEGMSDVEFFDP
DTETWSVKSEMHLPRHSMNICFWSPERGILVSDYECRDPDVYYTQQDRWTSFSDEAELCDSLPVFFTDCPGGNEMLSTYS
PAMDTIYATCEASFDDTLATWYKFSFYEKRWEACASLPQLLMPYLCAVR
>Skow_XP_002739313
MAAMSDDSDSETSSSLAYYDIQNSTTILTGLYSLYTHCELTDVVLVVQNREFPCHRNVLACCSPYFKAMFTSGMRESSSH
KITIEDIEPLAVELMLKYVYTSHVDVDGDNVQALLHAASVFQMPMLARVCTAFLKSQLGRLDCFKILQLADVYSILYLKD
IANSFILHNFCDVSKSDDFLQLNRDALIGYLKDDDVIVENEEILCTSLLRWVEFNLEDREEAIIELMKLVKITVVSVSFI
RNLLQNNLIMKHSEVVNYLTQTLKVRRRYFGDVNETVVCEEENGSIPISEIDLHDPRYERGVVFTSTRNYNQCRLQLTYI
DPLNDQYEHHATEPDMGEPVNSVITMNETETYLLTGEHSMWYFGAVSKRWIKITKQIEHNGGTVINVNDKLYIIENTCSF
GKLLTENDGGLLEVISLVPDSRFDASVVVHAGKLYVIGGEDAQSRERSDNWCYDPSTDSWLRLASAPIPFQNCPSVSLNG
FIYVLGVKSSANLGKHTVLRYDPDIDIWCRVADLKCTGEVTGAQVFNGKMYCFILTDEEALSVHEKILRTYVYAFNSDSW
NEDQAYDIGNPEYGICFLKYRDFRAVKYFAKHHYIDYLGEGMKVTVVK
>Skow_XP_002739164
MTAKHVGGGAQMSDAFIEKTAKEKARLVHKLSNQLKTDMARLLEDTENADIILVCGGEHLLSHKIMLESRVPEFYNYFIG
DQCTAKKIMMHCVLLDDLKAFLRDIYTLNEIITWRDTIKEWIKKQQLANTHVAADTEDMDVTLLTNGRDADVQMISQEMD
AVPDDEVAMATTEDCNGNESGAFGEAVAMAVDVNSDVQLESGEKVASMEVGFQETEERFVNGKVATEIIVPAEYLEANMP
EATCDLARDLLRMFVKQKATDIGLRVQGRLIKAHRFVLCCRSVYFTAMLCGGWAESTAEEIYLHDVSCSTALAALYYVYG
GVMEHPNDANIGELVQMSDMYGLDGLKDNAAFILRRDKCHFFHKPCNICITGVPECLALSTAYGLHVISENCLRWITKHL
DKVWSTKTFAVQSEEVLQHSFGYATSSLGLDNAVAMAMQCDKLLTSLPHVKWVEPVRCLATNLHTTCLDYLVKNFWEVVD
HPTFAILLSASSWSHGLIEDLFNTIVQNLTVDNGCRMFVAANRLKGQSKFQDWNQDILNLVNSLHDGTRAYMVNNVNFLL
HTKDWQLIPESLQNNIKQDALFVDDTKKSTIPKPTLSSSLMGPGRTGIPGIKRPSRLPTTLRSIKDKTSKITIADRPKTA
PPAVRESRPQSPAVDKRTKEKRNLQQPRPSASSKVETKQDKKSTAMETRSLTRQASRPSSLPTKTMTVKPIEEKTKQTTQ
TARQQSRRPASASPTVQRSQRTTTTAIRSPLSPHMQRTTRDAFKTPLTLPVQKSARPSPVVKSPMLSPTEPLQNRIPSKT
ATWPRASTAKARSKNNSQNSKTDKLIKSNQKSMDTISTTKPKTPVSQRRSTDNTSLLISPQKERSFGFGFKASQEKERTQ

LSSPQKEHSFGFKATQEKEGKFKSPAVQYSSSGARPKIPSSPRNYGRKLAGLKSPNRVNEQPMEVEVSSQAVCEKTSYDM
SEDIMQSPWRDETEPEMQLSRESSVYSSVSEQSSQELPLRHVSPQRQLCSPGQSASSLDSSETPAKIKKSGSWRRYTTDS
EVSVTENITAWRKINQETEQKSVHGVSEVCESSNASSPGSTPGSPNRRLGARPKVRCGIPRPIKSPSPTLANKMYTSPAE
SNIPTKTFLRN
>Skow_XP_002739148
MALSKDGASMNFSQNDQGNRILEKLQQQRLDDKYCDMILQVENKVFKAHRNVLAAWSPYFDTMCYSGMEEGLRNMATIEC
TTADAMEEILNYIYTGEICISSDNVEPILRGADHFLIPALREYCCEFLSKKMDASNCLVVKQLADLYRFGELYNRSSEFV
RNQFMHVITDGNDDLMALTVDQLIDLITDEKLRVEKEESIYELVLKWTKFDLASRKEYFPELLSHVRLPQLNRQYLMSYV
EVEELIRENPKCMALFADAKHQTDTTRRSYCSDSPVLKPRKGRLTDVVVITGGVSTNGPLAETFGYIIEEDRWTALPGLP
NELRFHAASTLHSCMYVAGGSSNGLVSNKVHRYDPVTNSWTNTAFMQTPREYLALAACNGCLYAMGGIRWDNVLRSVEKY
DPELDKWTFVAPVSTPRYAMHLVTIDDRYIYAIGGRDGTRQPVTTVERYDTETDEWCTLQSFPVDGQPWEFPVVESQGDK
IFVTDLATKALCFNTERKCWTQEIHNFASLPERTCFTYCTLDRIVFVFGGWKSHVDNPYTSNSTLPTDACIYNSVLGLWS
SISSPPASTLASACCVLQIPYEFLMDAPPPSIHSY
>Skow_XP_002739105
IKMAYNLKVTVVGDGAVGKSCLLISYTTGSFPAEYVPTVFDNYCANVTVDGKPYNIAFFDTAGQEDYDRLRPLSYPGTDV
FLLCFSLASRASLENVTEKWWPEIQHHMPKTPVVLVGNKVDLRSSSSTSVITYDEGRILAKSLRIPYVETSALTQFGVNE
LFNKAVQHVNLSLSSKKPFKKGFGLFGFNRKNSKDDIPQPPYMPPAGKAPWIEIETSMFAEHMYKLLENPIYSDVTFIVE
DERQFQAHKCILCGASSYFRQVFGILPEKLNNQLKVEKPYSRVDLNDGKVLGISSIIDQIEGDVSKSIVSISGNISIKIF
KHVLEFLYAGLPHFAENLSRDTLQALKKAAEIFHLPRLSEICKNIENDEEFLNPSIGTYLNDCMGQQFKELFFNKKVLSD
IQFNVEGTTVYSHKAILIARCDVMSAMFSGRFAESSSNKIEILNSRPECFLALMEYLYSDHSPIEDNDSVGIVVLANQFS
QLRLINLCELYITKEIERSTAANIAEADIDVIGLLHSAQIHNADQLSDWCCHFISTNYMAFTQRTEFAELDGDNLDFITK
NRWPPIDYLNEVEE
>Skow_XP_002739001
MAAAAAAPLPSMKKNQTVTTVTTARSMSNPTTVGFISNKRDNPWHAKCLLDQFKAMFDSKELIDVVVCVENKQFPCHRAV
LSACSPYFKAMFTSGLTECKKHEITLHEVDAHSVSEIINYAYTSKLDLAMETVQNLFIAANMFQIQFVQEACAQFMSAHV
DLTNCVALFSFACSYNSTWLKRVTKELIEQNFQSLSQTDEFMELHGSELSLILASDNLNVQREEQVYDAARRWLEFDSRD
TTRVKFLLEIIQQVRFSLMVDTTVVQSLRQNELIRKSPESIQYLARLSENGLPRSVKPRLGMGESSMIVCFGSSPNFDPD
EVHRVPCFSIKSNEIFTMAAPPRGLWDYGVVVTKLNDLVVAGGCLVDDHSGSEDSYTSQDLGLRPQRDVYIYEHTHDRWH
KRAPMLTERFDLHLVCTDSHVYAIGGMGRGGQHLDVIEGYDLKNNQWKYVTSMPAGLSPLCAIVYGSCIYVIQGDTFLSF
DTEASMWSARLPSGGCLLVPSATVFQDELYFVAGYNLPKSGIPVQVYDPNAKRWHRITSIPIRSGDFLHFTCRPKVMVID
SKLHVLVKHEDDEEDDDDNAVFSLYQYSNSEDKWNLSHSFSFPYSVRVDVQFVVAGLNINYLYPAESDDSDSSEE
>Skow_XP_002738853
MTTEIKSPYVNRGDEYHHLSHAKKSLRIINELRNENWNTDVTLSAGDEYFRCHGVVLAAASLYLYDLIEQDREDVTSARE
VVITDLDMLGTIVDYIYTGHVCIGAQYVKALRKTSKLLKLKTLETVCEAFRDTIKYGREFKFDDEAHAQFLHLHLNILRE
ESCLTDVTFRVGDHVIQCHKTVLSASSSYFKAMFTHEMLENELDHITIKETDPYIFDSLVRFSYTGKVTITNENVQNLVL
AADRLNFTEVVDLCVHYMQQNLHSSNCLGVWEFAQVISHDTLSQTAWQFAVKNFSAVSEHEEFLCLASDKLETLMSAPLF
VLREEDVYESLMRWYHESEADRRAHLPRLLQHIRYPFMAPEYLQQIERELKDIVDTQMYMEEADDYRHMKYKERLKHHFN
LRMTSRVYIHVIIVVTSSGPILVFDPEDNRWFDLTIFPLVSREQIITASILGNDVLCTTNAGHSWVYSIPGNEWTRRAPP
LHMHERNVGHKSASIAGRVFIPGVARRCQTMEVYNPGNDKWSEAPSMPKAVCYHALTSCNGNLFVIGGMGNGGAITKAVQ
MYDVDENVWSLRAEIPVKCAHAAAAATESKIYVFGNQDECMDSVFQYDVIANVWSDVATLNAGRQRCTGVTCNGKVYAIG
GLREPLWKGVVDVVEVYDTGLNKWSVVSHLPYHVFVQASIAVPKYAIPRFS
>Skow_XP_002738834
MAAQTLCGESFGKISHSSDLLRRLEHYRESGELCDMTLVIEGLEFKAHRVVLAGSSPYFESLFHSNLQESQTGRVELICT
SPDGLECILKYMYSGRIEITADNVYDILKAADHLMITEVKGFCETFMEKQLTSSNCLQVRDIAELYDLMKLKASAEDAIE
CNFCDLLTQESFLNLSAERVCSMMSNENIKVKREEEIFEFVVRWTNKDINERKEHFPRLMSCVRLCQVDKKYLLDVVEKN
KLVEENTRCREYVREAIRYHMVPEEDVVCHNLRLQPRPCRLVDLLVMAGGTGYDINGSFATNTAYGYVFCEDRWVTLPPL
PEKISRSPAVAMDNKMIVIGGSLLLDNSNHVSMFDPLVSEWKTLPETRNPHRFGESVICNGRIYVLGGSLSPRSLEVFDP
DHNCWIEKASSMDDHAGFEMVVLNNRYIYAMGIREGGLIEYYDTHTDQWFRVSESESWCWKQMNWEPTCQIFPPLIMETP
TALEFSGLGRYKVAISKMTRRISLENSDINLIPDPPKRKGSVVRDIGDHGIFVYGGVQHLPDGSECLDNSAYLYKHSSKT
WKELAPARKPIMYPSCALVQIPYSYISSCQGSHGHCSSFSFTD
>Skow_XP_002738763
MNINTHSRVVFFRVYEHSLKFSYIYKLLEHIHQGCAPSKNRLKIMDNADCDKIDLSSRVNEGEFENRIHASILLEGLMEL
YENQQFLDVTLIVEDQLFSCHRNILAACSPYFKAMLTNDLLESRKTEITINDVDPRAMRPILNYVYTAKLNITKDNVQNL
LSAAHMFQMHAVVEACCQVMERHLDALNCIGVYHFADMYSCVDLKNAANTFLNDNFMSVCKGEEFLQLPPSELLEIISRE
DLNVIAEEDIYDAAMMWLNFEIESRIRVLHLVLNHVRLALIEPSFFHNIIACNQYVLSDENCKQLMENVRKFHLLTEKGR
HESLLNTPPRSGMWTNDMVVCVGLDCNTNKGRTHVQYHDPTTSRWHAIQSLPKPIGLPACTTAAADNTIYVAGGILYPWE
DSTDLCYSYDHRKNKWLQRQSMQVPRSYFTLETVDGQVYAVGGLNTLHDDQKSVVDTIECNDMNSDEWHIVTTLPEPVYG
HASVTHGGKIYIIGGVRTGTLISKKLMCYDPKANIWKELAPMKNPRALCSAAIKDGCLFVVGGLDRLSRWNTVESPAMCL
SSYEIYNFTTNEWTEHQHISGAGLFFPVIVSANSNLYAFQNGLGADGNDVMLLWDEDSSSWIYHDGLLPIEDCRYGVTVS
RFLKRGKASPT
>Skow_XP_002738762
MRRLLGGSGGGNSGDGDEMHHDMPPPSPKMSKRRTQAERDRAELQELLSGASHSPSPMVKRKSMPKVPNVNFLQDPDAED

NTTSGASNYSFGSGWSSRSERDLRELRDHMRGHLSVSSYTSNKYDTTQPSVVVTQSKPSLSTTITSSKASNECKPTAENV
FDFFPETYKYIDVQHLPKILSGLKTMYDSQDLVDVTLCVENESYPCHRIVLASCSPYFHAMFTHDLTESRQNKIRINGVE
AEAMRLIIEYAYTSELEITVDNVQAVMLASNMFQLLNLRDACATYMEKHVTLNNCINIYFFASAHECGKLQELARKLIFE
KFTEVCREEEFYNLSKDKIVDVISQDDINVDREEIVYEAVMTWVKRDLEGRKDDLLEVIKHVRFSLLSPYFIHDCMERER
LIYSSKPCQQIFEEALQYHLLKDRRPNLKLLSNMNVNTRRGMPFRDMIIILSRNEVSESAIGNDNYRIKTLPDLIEHPEC
VVMGENQIFAAGKQHLDFSARRMHRRGGGLFQYDLFEKKWLSRAPMAFSRTHFRLAVLDGLIYAVGGVGPDGVLSSVEVY
SPQTNSWRFVAPLPNTIKGHCVTATGGQLYVIGGECEETILDTVMCYNPRTDIWSNAANMILPRCSAGVAVFKQELYVVG
GTVSLMNDRQSESVLKSVEIYNPINNEWRFGPEIPDGRCSYQVVNYNGALYAMGGENGEEEPENRVWKLECEKKGWVDDQ
VNWPNITPPYNCVVARILRDA
>Skow_XP_002738648
MQKKEMAASRKRAEHLARTQGALQEFNKLRLEQSLTDIVVCSNDVQFHCHQAILAACSPVFATRLCNVPENSDRLTLNEI
DSSVLGQIIDFIYNGKITISDDNVEDLFAATSLFQILAIQNTCISYMDSQLDASNCLGIQAISDAFSCQSLSIKAGTYSL
ENYNEVCQQEEFLTLTKDRVVELIEKDLCVQREEQVYESIIRWVNYDLNSRKEDFQELFKHIRLEYVSSSYLQKKIMAST
LVLESLECSNRITQFLEGALSSMPERQYSEILVNVHGYNYKGCQGVELVPKIQCYDPRMDEWFELPSFVMEKGDKVASAC
NLKDDIFILTKHGKTWLFKSSKQEWVLCSCLFTTRRNHQTVGLDGKVYVLGGQDGANWCTNVNFYDPVTDTWTNKSDMLG
TRCIAATTYEGKIYAVTGRYNSLECYNPITDSWSVIATMPEPESAQAVALNGQIYILMHGSNFICYSLKENASKYVGRIV
PIQKSRRYACRLAVCNGKIYVTGGCDKKYQNVGTVDMYNFNSTKWDMVGTMPYATWGHCCLSIHRYPLRS
>Skow_XP_002738522
MATKSSIETPSELRPSSGGDRNQKMENLGLVIDINKQTGVLRRPDHAMEILQNLNDLRQSSEFTDIVICVEQDMYPCHRA
VLAASCLYFKAMFKNDLRERHQDHVTLTSMKSNIVKLLIDYSYTSTLTVTVDNAQDLLSAAQFLQYEPVVQACCEFLKTQ
IHPSNCLGIQFFADTHNCLDLVEEARTCALINLVDVVKQEEFFDLSVEQLISYVSNDELNVKSEEMVYYAVMQWVRHDVD
NRWSKLPVILEHVRLPLISPGFLMNHIENDPLVEHDKDCRNLVERAKTIQKQVWQGHDVRDPSLRPRPSMMTEVMVVVGG
IDSVRHWQRDITFYNPIEKKWDSLPDIPFSQTDYSVTSLDDNIYVTGGYLNDDAVADVWCFEPTSNFWMCVTPMQNARFN
HGSTSTNACIYVVGGENDEGDLQDIEGYHPFDDKWDSLGSIYPTESNLAVTGIKRNLFIVGWLTYEKTCAIQCYNLDTQD
CWVTNTVELNRHLFPIASLNGHVYILGGNRIKDTQIYDPEGDRTWQVADMLSKRNHPSAAVIDRKIYVSGGELRHYSDRV
EMYDSSLDSWTVVAAMPKPLCFHGFVGIRKYIGPPYI
>Skow_XP_002738426
MIQSRVYTINLHTSSAFRVMNELRQKHELCDIILRIEDHEFEAHKVVLAGCCPYLRAMFTNGMLETDKNYIEIQGVDPMA
MELLLEFMYTGKIEITVENVQGVLAGASMLNLASLRNVCSSFLQSQLDASNCLGIHSFADMYSCVDLENAARRYIYQHFL
EVIKTEEFLLISHNSLIDLLKSDKLQVTREEEVYEAAMKWIKIDYSNRKHLMFKLMQHIRLALLDMHYLKEVVYKTDCVL
QNAECRSLVEKALKIRNDNQALALIGNRAQPQSIYVVGGRNSVDCQLSSLERYDAIRDKWVPQECMTMARTAVGAATLNG
ILYAVGGECALADSEETMYLRCVECYDPLHKQWIQADDINVARSFITVAALGGYLYAIGGENRSCSFNTVERYDDRTNEW
SLISSMKRKRAGAGVAVCDGKIFVAGGYDKGYHTDRASVECFDPETQEWSFVAEMEKARSGLTLVAMDHFLYAFGGTLRH
TDQYFDVAERYNTQTHQWTCIQPMNRARAWPAVAIFDNCIYVIGGFDGSNRLRTAEKYDPHTNTWTYISNMNVSRAGCGA
AVI
>Skow_XP_002738422
MSETTAASEWVLKSSNHSAIILDKLNIMRENNIGTDLRLSNGEQEVSCHRVVFTAVSEFAKRTLYIDEPNSDAGTLQIDN
MDIKSLEQFVEYVYTGQIKVNDENANNLVNIAEMFEVETLRVGCMAFMKRSRKVPANAADSENTSQKVSSPQKCTVEVLK
KAGGGDEIQPSAMKKIAVTKLSSLTETRNTDESNCSTAKEDNTNKDSDDSEVEDGIPYGGDEDDDDDDWSPGKMKKITTG
KRKRFGKSLVANLTSGPSRGTRSSTQVSGKNNIKETKQASEQCTICEKYISTADGLVRHMRVHTGEKPYSCNVCGQTFTV
SSNLYKHCRTKHGKATPEMIGKRVRDKRKDVPACSLASNDVIVSNADDSDLFPKSEKIHTSPNEDENYANVAASRSLSPN
KNTQSRTKQNESLQCEICDKTLYGSTGLKRHMRIHTGERPYSCNLCGQLFTTSSNVYKHCRTKHGEVKPEMIGKCSGKYV
KKTKLEKYFTCNVCGSQFTTSSNVHKHCRTKHGEVKPEMISKPPRKPKKICERQEDDSLEYSNDELMSESSNDCEEESDS
EFSDMFSCPLCEETCADLSKLKNHLKEEHSSESNNPMDKQKDLKPIIIQIGSGKFIVDEKSTTVTDEAGTGTVSLDDCNS
TKVPHEEAETSIANVDKENSTTDTGRAEGTLTSTVDNENNTTVLNKDAATCTAAVDDENNTAAPDMEAETSIVVLHDENN
TAVLDKEAETSITALDDENNIAVPDKEAETSIAVLDDENNTAVADKEAETSITALDDENNTAAPDMEAETSIAVLHDENN
TAVADKEAETSINALDDENNTSVNDKLTGTDVTKDGLKSTEGGTEFNCPLCEFPCNNLLDFKLHVKQLHQSTQNSDQQTT
RAFHKCNVCNEVFKTRRALKRHMMSHRNDKKFICKVCDERLVSNVALYFHCRRKHPKLKVRRQTVQIDCTKVLNCPLCKI
PYTTRAGLNKHVREFHKEDSEALKLVDSLYIQPLEGLSHKIKKSGKMSYACSSCGKEFRAANTAKLHVRAVHLGERPHVC
GKCGKSFGYPQTLKLHNQTVHENLRPFKCTVCSKAFVRETGLKDHMKSHTKEKSYLCHLCGKSFGYIQSFNAHKKIHSDE
KPYECDMCGKRFKLKGTLTDHQNHVHKTGPTTECEVCKKVMNVNYLKKHMIAHSDYRPHECLTCGKTFKTERTLRNHEST
HNTTRPFVCTICRVTFKAQYQLLKHKATHKLPFRCGHCKQGFKTEESLRKHLKTHARKFSCSKCQRTFDRQRGLFNHQKF
CRFLPDADEVQPVVENATEVVDQIIYIQSDIDLTAEAVASLQEVVTTTHP
>Skow_XP_002738123
MGEQGCLDPQMQPARLVYNSDKHPRQTLEATNNLRKHRELCDVVIIVGQKRIYAHRVILSACSLYFHAMFTGELAESRQT
EVTIRDIDENAMELLIDFAYTSSITVEEGNVQTLLPAACLLQLQEIQDACCEFLKRQLDPSNCLGIRAFADTHACRDLLR
IADKFTQHNFQEVMESEEFMLLPVNQLIEIIGSDELNVRTEEQVYSAVMAWVKYIIGDRRCLLPQVLEHVRLPLLNPKFL
VGTVGSDLLIKSDESCRDLVDEAKNYLLLPQERPLMQGPRTRPRKPIRCGEVLFAVGGWCSGDAISSVERFDPQTHEWRM
VAPMSKRRCGVGVAVVDDLLYAVGGHDGSSYLNSIERYDPQTNQWSSDVAPTSTCRTSVGVAVLDNYMYAVGGQDGVSCL
NIVER
>Skow_XP_002738077
MNLNRGDSFKQVMLPTNEKSSCIKPKTPVTPPVTVLENWQAEKSTVCERNSYMFLNELMSDIHFLVGPKNSAQLMPSHKY

VLATGSSVFYAMFYGDLAENRSEIEIPDVEPQAFINLLRYLYCDEIDLVADTVLSTLYAAKKYIVPHLARACVSFLETSL
SARNACVFLSQSRLFEEPELTKRCWEVIDAQAEEALTSEEFCDIDCTTLESVLARETLNAKESVVFEAASRWSEAECVRQ
DIKINPDNKRKVLGGSLHLVRIPAMPIEEFANTVAQSGLLTLKETTDVFLHFTALQKPQLEFCSKPRIGLKPQRVHRFQS
SAYRSNQWRYRGRTDSIQFSVDKRIFIAGYGLYGSSSGSYDYKIKIELKRAGKVLGQRQTQFYTDGSSNTFPVLFDHPIQ
IEPDAYYTASVILDGTELSYFGQEGLPEVSCGDVIFQFQCSTESTNGTGVQGGQIPEMIFYA
>Skow_XP_002738039
MATSLSHRPPLQNLQSMYDEKTLCDITVVVCGREFQAHKVILAACSDYFKLMFTSEMQEQSLAKVELKCELLTSDSFDIL
LKYAYSGNLVLSSDNVFDVITAADHLQIVSAVETCCQFIIKSCLEIFDIKVGDCLAVATYADKYSQLEYLGKTLNKTLAK
NFVKVMAYPDTCEQLSYERLCSLLELDSLCASSELQILQTLLKWLRHNWEHRMQFAADLLGKVRFGLIDKYDLVELLEVD
DIQQIPECKELYYKAMVYHALPDRNREPCDLINQPRASCLALVSINTVVMQYYDRNTMRWVTLPKFVNNSTGRQITGFNA
PAVVAHGNFIYVAGGNIQGPTCNLSRYDIGRNAWDKLAPMKSCRFAFSLCIFDGYMYAVGGYSDSGYLAHNNAERFNFIT
GHWQYIGQLNTPRFHVAVTPYQGFLYAVGGQKDKFTALKSVQRFDPAKNTWETLNPTIHAHTQASLMEIDGKLYVSGGKT
NGPDRSSDLINSQTVEVYDGKTDCWRDVPQDLIPPGNIPAVNIGSDVYIVLNGYTYKTGIQTTPDQVYYVDLDDWANVVN
FIRGLSITCVPINVERLIDLNDVKDSKV
>Skow_XP_002737821
MELNVTMVTGNDGEDVVISTSRVCLSTGNLKLPDLNIRCRQVVFHVHSCLLRRRSVYFERRLSNPWSAVGSLNEVVFTTT
PPTLTGLKLTLRYLYGESLQVPNVKELVEFMEAADFLQVFLEWRYFVNCITRRNCFVLLDASYKFNHHSIVDCMYEYIAE
HFYFLAKTQSYRDTSHYHKTKIATKLKSERSRFKKSVIVCTSAYTTIDTNGVKRNLEYFYDDVTNQWYNLPLTYYITPCD
EKYSPFPHDDIICECYGKEYSYNVYKYIDKNGIVMFIARNNSRDKQWTEYFLPLPITTVSALSCLSVQHLKEIGGCIFMV
IENHQPRLQNVLKIQIQTQDDEHTFKCNYMLPWQLAAVHLLQEDPNVFLLTQSSDNSLRLYRNESGCGQIQEICETTLPA
TTTLTSDRITIANNKIYLLDSNNSEAVLYFNISSTPCWGCVKYPPSVEKAKYMTSLRQERIARDSVDYSRTARKYSFSEQ
HMDSKPALLKCLLRIVRLIIGQLAHSNL
>Skow_XP_002737797
MGKKGKTNRRQHSAESISYFGLKNNFCIRMENNTFFVDKDILTKHFEYFEVMFSRNYLETRNNEIVMKDVPTVTAMRTLM
EFVHSGILYVTPGNIIETLEGAEFMQMKSWVIDAFVYVMYLNICQDKCVKFLRVFSMKDILFNLQPNLKSIIFKWMSLHI
ELFLNAAAREKLYLKFAKYYECCTCVMSVNFKCFWNDLLKYGTNLQYDVMKYSTKYRHLRVACVIYGREQTPCYSRKPTE
TYLIKPLQNSLCDASTPEPYVMVIILCPRFIPYSATVPPNRRLCPNHNHEYYIRRLLKCTDRYSKFTRATGDLTEKQISR
LKETFTLFDKDGDGAITVGELKTILRTTGPLITEAELNNIIENRKDVAFPKSDTVQFVEFLTIMATRMTDREYSDKEIVK
SFRVFRKLESKYIKTSEIKHVLTTVGETLSHEEIDELIKDLDKDNEGVIKYEGVWF
>Skow_XP_002737737
MEDKLDKSSLDYLDKFFPSELLAGFEELRKHGTLTDVVLRVQDEEFHCHRTLLAVSSPYFRAMFTGGMLESSANNVTIHN
IESNTMKTVLDYIYSGRVSITMNTSQQLLDAASLFQFPRLIEACASFLQGELQPSNCINVYHLAELYGCSTLAEVARNFT
LRHFIDVTHYSEFLQLSVDQLRCFLQDDQLVYAGIDNLIRAIKTWIEYDETRSNYTKELLEMWKEPSQVLNEIQHSTNVN
KLSNYEVMLLFGDYWEIPGKSLSKCPVKCYDPVRKVFYALESVPNTVAARDYMNAAAVDDAVVVGMRTGKFMVYYPSSGK
WEMVAKYLVTPLFGNTIVAMGNNVYISGRDGHFFCYNMETKKLVSKADLLSPVRNCCMITLGGRVYVLGGNTSGDLYATG
VDLVQCYNPVNDKWQLCSPLPEKCTNCAAVVLNGLIYVIGANCREHSESTNRVYRYNQTTDSWCRIADFTIPRQCFGAVV
CNGKIYITGGVDVIGNQSQHEIPVQEVECYDPVTDEWSIVDTLSISIANHTCIALPWSKTNPELGLGSEE
>Skow_XP_002737544
MGNYVSGWGKTEGTTTPRKRNRDECEPDSDNDSDPEFLHTPKRKKLHTTSKYIYETLFVSGQNSDVTIQALGKEWHLHKV
YLCQSGYFSSMFSGSWMESSSSWITIDIPDDNIDIEALRVALGSLYRDDVMISPSKVVPILAAATLLQLDGLIQQCADIM
TETTSKKTVCTYHAASSAYGLSLVANSCVQWLERNLMLLQSVTVLRELSIELMETIIKSANLFVLQVEMDVYTMVKKWTF
LKITPSWQGTLKQLTADADSYFRSLEWLLPIYKHQWQAMLAVEIGTDRGPAEISLDKLGEHGMRCGRIINKEGDVSSTVM
LIVCKRNTVSHLVMNSVSLVPKRNVVFRVSVVSYDDMGQVKYSKTSGLFKVTLSKDEEVVVMSVDRHITFPLQISFNVAC
ITPRTNEPVTLAIPEEFNQLEAQNYGESADSHETDEES
>Skow_XP_002737158
MEVPHPAVGAVANGAVQEEPAQDVAVNESNPQAGSCKCIQPSTPCSSGSCAGSFGNVTGSFGSCTSEGVDCYGNERNALL
EQCKFFNNTLLSDIRLKVGTKIYHSHKLVLVRSSEVFERMFSSDWSDPNKGEVELVEDPECVHIFSHFLKFLYSCHIVLN
IDNTLPVLILADKYNVTDLRNVCIDYAATFIIPKLQLKDVFHVWFQYATKCYHKKLISSCVMALSAKADDIMLMPEWQPE
WLQLEKDQLIEIMKSSELTIRDEFSIFLALEKWLECSRYPERQQNKEALLRELLQYVRFPMMFPHQLCAVEKSKIAKELP
ALFVPHLMMSYKYHSLSLENRANTKEFNSSSFLLRNYTELRWDKRFVIQNYSQLSRCSEIMPRFSTRSSTFPSSSWDWEL
KIHPKGFSSSNDDFRAVLYSNLIMDQVRPIEFMLSIVDNSTILKTVTGRKNFSKTRYSADSEIDKKVSIDELLGDSALLV
NDNLVLQIIIRPMD
>Skow_XP_002737156
MDMLATHTPAGEWITNDRQILDSFGRLYNNPEISDVTLHVEDTTYYAHKVFLANASDVFKVMLTSRDWQDSSKPDIYLDE
PYATATVFGEFLRYIYTGQIYINTDVAVSLLMLADKYNMVDLKKACQSFMERNFVTIAAENHVLSWYQYSKRCGHKDLER
ICYMFIQNNMDIILESPDWHSMEKETLLMILRSSDIILTDEHKLYLGVEDWLTQPINENDLQQNLKDTLQHVRFPMMTPK
QLISIEKREFVKENWQHYQECMLNAYRFNSVPQCLRQNENFDEVEIYTHRNYTHAEYNICATIKITQYSQRKPFEYSCSF
DAAKTGSYADNCHKYKWELQFWPRGLQKPALTEFGNAYMIQTDEVTLAITPQGRNENAFKIVAAVIGYEDGLQYVKHVHR
ITHDFGTKMMFRIKHLIKLGDLMSDSSKYLNADCLQVYVIIRQV
>Skow_XP_002737154
MARSSSHIGDGRMEPSLSSDLAAIAQDSSAYGANQVANADEFLCQIGKYWNNPELSDIVLCVGDRRYYAHKLILVSSSDV
FRTMLTGNTWAESNTQEIDLEEAFQCEPVFPDFLKFLYTNRIFISDDTVIPLRTLADKYMVQRLSNLCIDYMCRELNCDN

VIGWDQYATKFSIKRLSFECLGFMQQNIDLLIDSKKFLNLDIDQFVKILKSSDVHVPDEYTLFKSVKDWAFAYDRSCSSD
DIKCNLSTLLPIVRYPMMSGEQLDSIEKDEAINACYPAMRDEINSSYKFHATAIPNPNWAMCKVTPRRYYQGYSCKFTLP
NYSKRHHDNNRYVKVFPAYSSNGTSRILNWDWFVSVAMGGRTDRLGSNNREPVLEISNPGPEREERSVKIMAVFYGKRNG
SIFKKMNYTTQLTVRDFDFHHIRLFGSSLAPEYIVNDTVTFHCIITPTDKIYVKC
>Skow_XP_002737132
MLRRLFGRRPNGQQLRVNNPYIQNIQIQQNQDIFLPPCNNTMDFDFDNVEQAEPDVIVHEADRRFGLKSLNLMNDLRKSE
TLCDVTICVGDKTFRAHKIVLASCSSYFWAMFTGDMAESQQDSIVMKEVDAHAMELLVEYAYTGRIEIRVETVQQILYAA
SLLQLPDVQVSCSGFLKRQLDPTNCLGIRNFADLHTCMDLVIASERYAQKHFSEVVKEEEFLLLPKQQLIDLISSEELNV
NCEEEVYNAIMRWAYHDKELRKDDIADLLQRIRLPLLSPNFLVDTVEAEELIKQDIKCRDLLDEAKNYHMLPDRRSKFMR
EKVKPRKSTVGLVYCIGGMDTTSYSLNCVERYDFSSGKVSIVASMNTPRSGVGVTVIDGKIYAVGGHDGTQYLSSVECYD
PATKRWRYVSSMTRPRRYVAVGTLNGMLYAVGGYTGTLVLDDVEMYNPKTNHWKFVPSMNCRRRHVGVGVVDGYLYAVGG
HDGNNYLKSVERFDPDTNTWTMMCSMGARRGGVGVAVLGNRLYAMGGYDGTSNLSTLERYYPDDDRWNFVAPMNQCRSGL
GVAVVGNLIYAIAGHDGAHYLNTVEIFDPHLGEWSSKGTIGSSRAVAGVAVLNDRV
>Skow_XP_002737036
MGPVAGLSVDSPSQGAGFASEIQALVEGLSVQIASLAERVLEEMSFLQISNIGLIEDKVPDEEAMETDNDVATPNVPSQR
DSYLDSTSKHDGSDVSMQNTSPNVDNAEPITDENTPKPRSSNKSGDISQTRSSTFEESDDITQAKTSVLQESDEISLAQP
SASKESDDTSEAKSMVLMESDEILQVKPSTSKDSDEISQVKPSISKDSDEISQVKPNTSKDSDEISQVKPNTSKDSDETS
QVKPNTSKDSDEISQVKPSTSKDSDEISQVKPNTSKDSDEISQVKPNTSKDSDEISQVKPSTSKESDDISQEKPSKSHKS
NKANQKSVDSSCAKSTNSDSDSDSSMMSDFSPRPPPGRYDNTPLSETFDMKTTDGEKVKSPYLFNDKKHALNLVEGLHGL
QKEGKLTDTIVSIDGKEFRCHAAHRVLLAACSPYFTAMFTSNMKESLERVVEVRNIDDSIMEKLIKYMYTGEIEMDENDS
QSMLETSCLLQILPILEATGRFLANHLDPINCFSMKQYGQIHCCQSLETAADIYILEHFDDIAQQSDFLKIGLDEFTTYL
KMNVCVEKEDIIYDCLMRYIRYDLENRKDSLSEMLKMIRWPFISQTFLNEKVKNDPLIVDCDELFDEAAKYHNSSIQERY
GIRQKHSWSHPRSYSQVVAVICGFRTPRNVRNVQSFDPDSNKWQPIVDLPDPTFDDIVSAVKIGTDIMLFTLKGNAWLYN
SSQRRWLQMKCPKNVNRFFYGAASLNDNVYLVGGKNLEGTVYKSVERYNPVTNDWDNIPEMPTGVHYPAVTSHAGELYVI
GGHTAKYHSPSGAVQIFNPDTNIWRRGADIPILTFIANALSIHEYVYVVSGEYGRMQRYSPRNDRWEEAATLDQRHYLGT
ITVCNNKIYAIGGYSDFSHSERNISNSIECYDPQKNTWKTVSEMPTPVPWHMSVALPRCVVECHNCV
>Skow_XP_002736900
MATASVVVEKKRESIGETIDYSEANHSVYLLKGLNELKSQQLFTDITLVVGRHEYPCHRAVLASCSPYFRAMFTNDMKER
NADKVQIKDVQVEVMNVIIQFIYTSKITISPHNVQAIFEGASLLQVHSVQDVCARYFERLLSPNNCLGILEYADTHSCVE
LQSKAQKMLLVNFVDVCKNEEFFLLSKDRLIEILATSDLYIDREEVVCDAICSWVNHDRPARESFLPDLLVHVRLPFLSP
TYLGKASNDPLLKHEHLAVAKDKMLERTRTYYAKSRDIAVTSLSATKPRNYTQVLLTIGGQNEDLQAVNCIYCYDPVTEN
WSYLYELPDELFDWDLAVASLGTDIFVAGKRPLISSLEPNFPAWIYKGVKQRWMKVLPPQGLETVEYALTSDDSGIVYVL
GGYDSNMRNQSNVRRYIVATNTWDEIAPMPMAASNLSATICEGKIYTIGGYTGTDDTSVIQCYDPKIDKWNFVKPLPITV
RDAVALTMNNCIYLLTGVAKSIVYRYDPCIDKWSQLNGMAKEVFMYGATVCNNSILAVGGYSGDYFDIEVNKTIQKYDTD
EDCWDVSETMPHGLHSLRCTTMYRYAALGT
>Skow_XP_002736828
MAAPGSHTSFPSLLHIEDVSPEEIANFLQSYDAHRFSSNYDLLNIVVRWVEKDLDTRSKNVSKLLRYIDLTQISAIDLVR
CVQSLRWLVNDIDSYNYVLHALNFHAAPRRQPVSPQLAILPDEDQTEVLVAVGGLQQHNGMMQLRVKSAMFLEPSQQQWT
ELSPLPEFVYSHSVAVLNGYLFVAGGSNHDVHHPVNQQISRRVHRYDTRSAKWVMSKPMLEKRAFFTLSALGDKLYAVGG
LNHSSPVYDRISIEEIAESNIKPQTILASPTAECYDPKSDKWELVEKLSEPSMLHSSIVHDGKLYMSGGCSSFDLQKVEG
DVAVLMTVMNSDFCREFSDSLSCYDPNIGVWEKREPMTTKRAGHKMCWLSGKIFVLGGKNNEFDMISSIECYSPDADQWN
KLSAVLPSPMVWCGTACLDENIFIVGGSKKLSELGEYQDVNLVQCFVTKESSWYTVAKLPVGYSGAECCSLKLPQNRPIR
QEFSPIEEDGFAPPNLKLDAIHESAEPPPDYFAANNGSSTVIVKYFYQLLSGLNELRISGSLADVVLVAEDKKFYSHKAV
LASCSDYFRAMFTGGMKESTTKQPEIQLHGVSAGGLATVLSFLYTTELNLSHSNFESVLTAASHFQMETALDFCVNYLQS
ELGVENFLDLSNAATMYSLLDRFVDDERTMDKFMLTATKDIDQIMELSVYQMNTFLKSDKLSLYPPLQVFSVFLKWVQYD
PENRQQGASLLVKHINFCRMSPEDVIDHVQSVDFMVDGVNCRTHLLEAMKFHALPYHIIDDDCLPCDEEVLLATSGVAGN
PYSCSQMYYFDGELSDWQVLTDMPMEHIAYSVTALDNFLYIAGGEDHDCYETDPMGCEYTSDAVYRYDPRFNRWSFVASM
HAKRTNFCLLACQGYLYAMGGRNALLRDGLVSYGTSLPRPMYGMGGCVYNGKIYISGGCTDGSLYSKNLLMYDPETLNWQ
QKAQLPNKRAWHTMAVVNDHIYVIGGRIVKSSMTIASEFSIECYDPKTDEWLTSVEIPTDELIQNRLSMTGHRSTVWNGY
ILTLHGQFAIGNLKTRRTLQCYNVEFDRWEPSLKLPSDGFQEVCTLRVPKYVLKTNRIAASGELGDYIASSAASSRRSSI
CLEDLDFLLPDAETSFSTSQHGAMVLGGLGELREYDMLCDVNLNISGTCYPVQGALLAATSGFFNSILCPKGNTKEEIDD
IKERKMPTVNGITATIRQSLHGGNKKEIELDGISATEIRRVLEVLYSTTLPIDGSVDLDCVKATAVRLELQPVITLCDDV
ETLKMG
>Skow_XP_002736827
MLKERALHMMQVLGNAVFVIGGRDSTGAPCLNIDIYLPATDSWSSVQIMLPVEKMWFGCSVHNNKIYLCGGLDGKEGCVA
SSMMFEHNRHVQCYDSTTESWQNVADIENLLIPLCCSIRIPKWRTTTGVQGFQLTSNLTLDIPTSVASNNSHGCDSVISE
MSEKKEQDYGDKFLAGLMQLYCSNDLSDVVIFINNQRFPAHRAILATCSDYFRAMFTGGMKESVDNEQEVELHDMSMEGL
KAVLEFMYTGKTTLNHRNSDTILAVANYLQVQPLVCLCISFLVDELVEENMMEVVALTTAYGLLEKFTRYEKVIDMFVAM
VTRQDALLELSDLTAEQLMAFLQSKWMETHSPLQRFKVLVSWLERQNVTVKEDVINRAINYMPLNEMSVDDLLTHVKPVQ
SLDTEHCQQLIIDALVHHTSDTPVVVSSDHSYSVETEQVIVAIGGFETTNEGKTIKNYICYYDCHTNHWHKLSDMPIERL
AYGAVLLNNHIYIVGGENYHCSDIDPSGCQFSTRTVYCFDLQLKQWRNVASMLESRTDFCVVTLHGYIYAIGGRNSRHPT
GINSVERYDPSTNSWSYVAPLVRALFASAGAVYKDQIYISGGCFYTDRLIYTNALLYYNPKTNQWGEASAMSAAFPIAWH

SMTSAGDKLYVLGGQQVELEEMDTKCSYSLKCYDPKLDRWSKCKDMPSHGSKDASCIINAYRSVYKDGKLYCIDGELTES
TFIVQKHVHCYDIAQDAWSDGPCLPQDGFQSLVIANLSTYMIKHELTKTKTNRYQSCDANKTVELYELSTSYLSILVHIL
YNGRLPVGYHGNGLDDLLEAAEKLGLQAAVDVLKAHCSQNDMAV
>Skow_XP_002736418
MRSLFSTSFSSTNWDQAPTVGGDVGETSLMDCMQRIDTSGVEKLVNAESAEISQAMKVLRTLKQQQTRGEGCDIVLQVSD
KEYKAHTAVLVASSPYFEALLRSGMKESVENRVELHGISNVAMEIVLQYMYSGCIDVDLFNALEVLVAADLFLMTSLKSM
NEHFLSNNIDTENCLFLRALSQRFHLAILLMNCNKFLQKRENLKEIVSGEEILDLGFDHFHDIITCDVVSVTVDNEEALF
ELIVKWTEFNRQSREDHFPALLSSIRLPLMEKEYFLNKVERQELVKKFPSCAEYVQETLRYWVSPGRKTSLDNSLLQHRL
GRMVDVILLCGGRDCRDRVRRDVVAYVIDYDAWAVLTPLPTFMSSHAAVTLDEKVYVGGALGTVTNSKLFCFDPVKNTWN
SLPPMHYLRDNFTLCACAGKLYAIGGKVRKNLLQSDECIAVATVERFDPITHGWDVVGSMGTPRSFAKAVSINDKYIYVS
GGEPSHNSTPFSVEYYMCNEDKWMPLKRINQQRNGLSPPPLLNLGDQLYIAYLNGENTKLSPYQDWKVDKTIPKLPIFGT
NGTVCRHESAVCSLDSGGIFVCGGWNDNLGRDVFNQKFAYIWNPASGSNKWQKLSPPPTSGVDSAIRLSDHNLEVELWRK
SIPKFEAKTTFLQTMQKM
>Skow_XP_002735621
MAEVENNYLTLHVPNLASRVLDILHDMRKHALCVDTVVCVQDEEFTCHRVVLSACSSFFKAMFSHELRETVEARVTLNDV
RPEIMKDLIEFAYTAKLHIHEENIQEMLTTSIYLQFDDVTTACCDFLTRHLTSSNCLEMWKLAENHACESLRANAQMFSL
WNFCEVKEQEEFLKLDKDFLIRYIRDDELQCDDEVDVCQAVIQWSLYEKSRFTELTSILEHVRLPLANLKAVMSHSPPEL
MADKQFKEFLDEAINCQFLIQQGYRVQGPRTRMRGIGKNSGYLVALGGQVRDGDGIMDATFNGNSKHCAVSSVGNKKGPW
QNLASIPNHRKRKYSLVNSGTDIYILGGYDSVKQRSTAEVWRYNFTNNYWTQMSNLTIARHSHGSTEYNGCIYVAGGKNS
LLSMRLNSVEKYNIETDEWVTVPSMPEAVSVPAAVASCGRVYVLGGSNVNNMPCHKIQCLSLNTLTWSVVRHITIQRKLL
SAAVLGDTLYIFGGKSARDVIIYNPKSESLRYELHACNEQRLYPGVLTWRGKIYLTGGKGTERVYNDADVYDTETNEWSR
IDAFLPKNMYMHGCFLIEEH
>Skow_XP_002735382
MTDSSRPAHTIDCQHSQKILHELKSFYETGILTDVTLVAGERSFACHRNVLAACSPYFRAMFTGSLREANMERITIKGVE
PQSLQYVLNYVYCSEVTLDAETIQGLVQAAHMFQIEPLINHCADYLERHIDAHNCVGVYNLAVLITHKQLETSAWEYFNL
HFVKVVEEEEFAQSPTEVIERVVKSSTLNVKNEETVYEALLKWYTFDKCNRKEVLWKCFLDLRLGFMKLTYLCNKIKCNQ
TIAESTECLKYIEETIHGHSDEAGSSEDFLFDGPRIERRLGMFARDILIITDKGWAVEDNRKLVCLDPSSAEHCSIPYPD
EMNECGAKFVVTSNNDIYAASSQYEDRKFYQYNHIRKVWMEREPMLSGREKFGFVSCNGCVYALGGENDWEILTSIEKYD
PLLGRWEYVAPMPREAMDMSAASVEKHIYVFSGSRTMSYNTETDTWNINLPPMVYPRFSSGITVHNNEIWLVGGEDDDSK
KVHDVEVYNPEIGEWWHALELFSPMKEYYPASLNNKLYVCVLSPGFNDGSSESVIEGRDDFSILKRYYSSIVYVGDYGSE
DDEEEVEYEAVKWFSRNTDCLAATYALRPEACFSARIFFEAEKDD
>Skow_XP_002735360
PIYEKDIISPYDCRQVAKDIGATYYETSVVTMFGIRELFDNVIRAALISRRHTRFWISSLRKVQSPLLQEPYLPPKPEPP
DLTLPPSSFCSDIGSLLDNPCCSDVVFLVKGILIPAHRVCLVAASPLFFDLFMMDLSAKIGDSTCKVEGVHPNMLDSIAG
NNGIKSRDYKEEMSTVVMTTPGTSSWTTFTDSTPDLTAKLNLPLEGAASNIPASEVVRTPSSEDSIELLGACAPKILPST
SLFNEPSTSHCSVHSTAKFLGYPGKILDHPALASIHLQPVDNPVNGKPSIQTVVTVQQHISPVAFQVCLEYLYTGTFDQE
RWHNINLSELITVADALKLTSLVTLVTGVMNHDAYVNEIILKAQYMLGRTSRLRELILRKGLFSDMLIQTDDGTVASHRA
LLMARCDMMYAMLGGNFKESTCYEVFMPGTKHCFQALQEYLYTDQCPPMKPAECLCLIEMANFLCLPRLVALGEENIIRE
FNLALEKERDIQEDVINMLNVAQMHNAHQLHSWCLTFLARHFSDLHQEYMKSLRNVASEDARYLQRHRWPPTWYIKEKDY
YDKEKREQDMSKRTTERKGKRKWCL
>Skow_XP_002735147
MSSPPVAENWCYTQVKVVKFSYMWTINNFSFCREEMGEVLKSSTFSAGANDKLKWCLRVNPKGLDEESKDYLSLYLLLVS
CNKSEVRAKFKFSILNAKREETKAMESQRAYRFVQGKDWGFKKFIRRDFLMDEANGLLPDDKLTLFCEVSVVADSVNISG
QSNQTQLKVPECRLADDLGILWEKSSFSDVTLSVSGREYQAHKAILAARSPVFNAMFEHEMEEKKLNRVEITDVDHEVLR
EMLRFIYTGKAPNLEKMADDLLSAADKYALERLKVMCEEALCTNLSVENVCDVLVLADLHSADQLKGVAIDFINSHAMDV
MDTSGWKSMVRSHPHLVADAFRALASAQSPLLGPPRKKSKQS
>Skow_XP_002734669
MEKSEYNDQLVQHASQLLDSLERLRQDQVLTDISICVGNTQIHCHKLILEICGFDFIKLSELGSRCDKKSGLCPQTVIKL
NNAEPAVIQEIIQYCYSGNLILTDENVKQMLELATLMDHEFLANECLKFLDSQIEEFQHHQPQPNSFIVCQPMHGINLLT
ELQCLRSQRRHTNIKLVCSDEVFACHSLVLAACSDYFRAMFSHDFTEKKAKKIDLLEPADIVEDLLTFMYTSMFDVECED
FLDLLESAAYFQVYFPPVTIESIKTIVQPDNCIKIFNVSNVFSTNLRNYTLRFILNSFPKLTNHEDFLTLSLSMMVQILS
DDRLNVMAEESVFLAASRWLAHDMESRAAYVDELMECVRFPLLHEKFLRKIMEKEPSVIKSQKCKEMVVQALCTKDGHAS
TQQAFSEKFHPRNSMNQDVIFVLAQATDEMSPPQCAFIGLRDLAVHDVCTKHNGLPLGLSRFTIICIDTDIFVIGGWDMT
SEVCCSTTWKYSITENLWSTCANLQLPRYDIGVCKMDQVVYAIGGQINSPDVCNPVNLVERYEVETDEWSEVAPLPSSLI
KPVVTVFKDRIYAMDSASHSKVIRTLCIYYSYTGMWAIVPAGVGLMNMRYIQNLFTVADYILCWGMYDAADYHQTFNAFQ
NFNAENFPPRRGGMLVRHPPIVSPRIQYPTKFEPVNLVGLALLNDEILVVSDAQARRGRYLLVHRYDPFENQWVEQLEIS
SRLSRGITSSLQCVAYRGYIASPWYEQKSTYN
>Skow_XP_002734502
MTTDLPNQTTPIIVNDRLQFSSKNHASALLSQLCKLRGRHEFCDVQFIVGKDVYHCHRVVMSACSPYFNVMFSGGLSETS
KSEVDIHEIHSSIFKVLLDFIYTGEVEVTSENVQELLSAADMLELPEVVQACSSFLQSQLHSSNCIGISRFAEIHFCTDL
KRAAEAFMSLHFLEVCQEEEYFDIKMDDLVTLLRSENLRIENEYQVFLAALGWILHDVGKRRKYLVQILDAVRFALISPR
QLFEYINSCEDLSLRVALGMLLAEYNPDRKPQQVKPTRSLSKKTLVQVRPRLSAMKHLYVIGGYCYPPGGRWSDSQSLSA

VEQFDSFNQVWCIKSSLIYPRSGHGVAVVEGSIYAIGGESDSLIYDSVERYEPGLNQWVEAPSLTVPRCGLGVCTIGQVI
YAFGGWIGSEMGKTIECYDPDIGKWCVIGNMKTLRISFGIAELDGNIYCVGGTSDLNTEMRLAEYFDPITQDWIKLPDMK
SRRTSVALGVLNDCLYAVGGWNDRKRALNTVERYSVTEKKWSTVAPLSTARAGASVASINGLLYVVGGRTNSKENTAPLM
LDSVECYDPHTDSWIHLGSMPTGRCETAIAVL
>Skow_XP_002734469
MAAAVSATTTTKMLDDIVVFQKNDLPSQGYPVMESIRRQGKLCDVTLKVGEQKFSAHRIVLAAAIPYFHAMFTHDMVESK
QDEIVMQGIEPSVLESFVNYAYNGRVELDVNNVQSILVGASFLQLQAIKEACCEFLKERLNPCNCLGIRTFADTMMCSNL
QEAANSYLHKRFIDVSKSDEFLNLSKEEIFEILSCDDLNVKGEDQVFEALVAWVKRDIANRQQYMPELLAKVRLPLIRPQ
ILTDRISTEELVKTCHQCRDLVDEAKDYHLMPERRAQLQSARTRPRCCNDITGMIYAVGGLTNSGESLSTVEVYDSICNN
WVPAKPMSTLRSRVGVTVLSGQLYAIGGYDGQSRLSTVEVYDPVVKEWWEVASMNSKRSALGAAAVDGRVYACGGYDGIS
SLNSVEVYDPENDKWHMVANMNKSRSAAGVAIFDGQVCAVGGHDGLSIFSSVESFNHFTGRWTMLPPMLTKRCRLGVAAL
NGKLYVCGGYDGSVFLNSVEIFDPVLQQWSFIAPMKSRRSRVALSANCGKLYAIGGYDGLTNLNTVEMYDPQMNTWTDVE
PMSGHEGGVGIGVIPLMPL
>Skow_XP_002734385
MADASSEKYETKKVVAVDYSSKILSGLCMLHSDALLHDVTLIAERKRFQAHRAVLAACSDYFKAMFTSGMRETDQKEVEL
KGISAKGLGDVLGFVYSGEMDLSMGNIHDILATTTHLQVTPCINVCSDFLESEVRIDNCLLIYQMAQTFSLNNVQAVAYN
FLMKHFKEVSRLEDFLQLPFMDLSTFLGSNDICGCTELDLFEIASAWIHHFEDDRLQFAKPLMEEIRFPLMKPFDLANQV
RSVDFMLEDPDCMKLLLEAFTYQSMPFHQHQQQSPRTMIRSAEQTLVTLGGYKGRPSEKMMAYCNSGKPVEIQPMAMGVY
YHNVTVMDNFLYCVGGQNVKDTAGKEAVSSAFRYNPRSNDWMEIASLQEPRCRFHLSALNGKLYAVGGKNARGQVNTVEC
YTPHENRWICVAPLDDPRNAHAGATYRNELFVSGGWSNGRYTDSLQSYSPANNTWQNRSCMHTRRGWHGMCSVNNRLYVM
GGNHLNSNGDRVDVLTVESYNPITDQWTDLCSLMTPHSETILSVVNMKIYIAGGYSWNAQGKTDRIERYDPETNSWEIIG
QLQQKMNGLACCSLVLPKELTDK
>Skow_XP_002734341
MNMMLGSLHGHYMGRRQPQQSEILQNVSHVKAAAVQKSTLQMFKKLPNGNDLEFLAPQHSVSLMDGLNDLRHEGQFTDIT
LQVRGQNFTCHRAVLASCSSYFRAMFTSEMQESKDSIITLNEVNPQAMETLLSYVYTARLVISDHNVQEILEAASLFQIF
PVRDACAAYLKRQLHPSNCLGFQRFAESHSCNFLRRAAEEIALMYLDEVSHHEEFILLNYEHLLEYVSHKDLIVTKEEIL
YEAIMKWIKHDSKRRIEHISQLLKQINFSKIKTDYFNSKILNEQLLLDLPNTQEVVSVSRQKPVRGKEVILLLSGKTGRP
NLPSQFNHDVLQYDPCSKSLETISCDPDREPNSFYDSIATAAKVNDQILFIRANNAWLFDIRTLQWNKVAPPLRGTYRSE
TICVELGGYVYVLGGHSIEFNREVAFVERYDAKRDVWEVVSPLIRATRGAAVAACDGKIYMFCGHTEVGNIDLTQCYDPA
TNMWQILAPLPIETSGATAITFNDSIYLLAVKNPSVNHNNPFPVYNHVFRYDVATDTWQQMANMNEGHRRCAAIVCQGKI
FAVGGTRRDFTSGNMQWATTVEMYDPNEDMWETCDQLAHPIFCHSGMKFIISDKEDIAEEDEKTDES
>Skow_XP_002733957
MGVNTSTSQEKEKEKEKEKEKQLARLQSSSSLTQYSDALAYPEYFLEPRGSSSSSGGAGISMSSSGKDKKKKSAGFATLRKK
FIRRRKSSKYDHARIIRDLVSGWSMRDLNALVEEYEATNVLKELTIHANLARPHASCLKEDLSDLYDYKYCTDIDLIFLG
TCFPVHRGILSLRCPYFRDLLRRHPQYGAQVHVDIRTKGMDISTFSALLRYLYTGDFNTQVSRLENLDVLVKLGDQFGTP
NLLEHDFRHLLETGEFCDAVLVFSSEPAAEVQGQEAASIGAIYEELFCHKSILAARSPFFRNLLHRRCRSGEELTERTLQ
QPTRIVLDESIIPKRYARVLLHAIYLDVVDLSFVIQDSPSMGSLGEVQAMVSGRGQLTRTEEAMEVYQIARFLDLTIMAQ
ACEDIIAESLCQDNLITILNWSSQAHGSQWVNRQAMHFLREEFLAIATSPVLCELNKGYLIEGLKSDYLQVRNKNTCWYS
KLHVMDCVLKWAEHQLIKRIEEREPNLLSNTQHSVSKKGIKRRDLDDSELREILSDILPYIRVDHIIPHSNEVLTNVIRR
GLISTPPSHMFGSDDESLVINPWIRCKNNPMYVKPRLFTPYMDEVKAILDEHMVAEMELVRLRMIRMSHIPDTLYMVDAK
SYQSRGYTQVDAVTDPSVIAGSLPAPDVHTTRAIMEREKELCRSNLTQRAFSLPCSDIGAVIHEIQKRVVREFGLPDEAV
EIFKSGGEDASTDPTNDYYSHSRRNVEQEWCGHPETPVLANPPDSPVAAAAAQVVPMLSEMMPDIAMATGPDITIATAPS
INQMLLREPELGDEHFVRDKNGQMGEL
>Skow_XP_002733689
MNKLRQDNRLNDVTLSVAGKCSPCHAVVLAASSSKLRDELLKNDDHVTSSSKRKRIELEQIDSDSLGHIVHYMYSGRCCK
ANKYKIIETAATVLDITTTRDDPGSTQQMPENSANTRQTSDEYINSFLTELGKLRQTESLTDVAIETDNHTIPCHRCVLA
ACSDFFLAMFSSELSESKAETVRLREIDSDIVKSLVEFAYTGHLELNGESVEGLLIVANQLQWREVEDVCVEFLEERLAT
FNCLGIFHLAESLGYSTLSKKAKEFGYANFSKVCREDEILEVDIEHLIDLLSDDVIIDREETVVNLIIRWIESNQSRYEE
IPYQQFAELIHVDALFAGRITNENATVVANLSRMESLQIVAPRQRRIYHQVLAFFGSYEMDKRQRNVEIYNPRTKKWRML
SVLPSSADAFISSVVPIGHDIWVSTLKRSTWIYSPVNDEWKEVRQQPGRVLNLYAAVYHCGHLYIIGGSNFHTNVVKATV
DRYDRVKDSWKAMAPLLSAVGTKAAVTWRSYIYVIGGWNGTVYTNQVQRYDSLRDTWEFVAHCPARDTCGIGAATVGDFI
YVVEGGDTFRYDPQEDEWSKKSSTNHPRSDFNLVTYNGYMCVLGGVHLGQHIRSIEQYDHRLDCWKILDTLEDFTIFSYP
RCVAIQQSIVHRDAYKYSLASNSNQTQGQRIVRKSSFKMPTDSSVSSLVLSLT
>Skow_XP_002733573
MNNIPRTKRFSGNSTALEFHDKSISHSILDGLLEFYRQNDLCDLVLTVSGYEYPCHRSVLSCVSPYFKAMFTSGMQECKT
TKVPLNDVDPVALRAILDYIYTSDITINQDNAQQLLHASSMFQMQTLFQVCCSFLKTQLNSNNCFGILKLADTYTCTNLN
NVTIDYILHNFCDICKNEEFMELSKDALIKYLSDRDLVVEDEELLCNALIRWIEYNKEERTKHVARLLRFVNVAKLERRY
IDRLLTEIDTVKNVERAQQILECTPKTFRGHDATDSRYSNVMILSSPGNVQGSLELFDYDAIKKTLIPYGVVKDITIEGH
MSSVALVDGYRTCLLNGQDSIWYVEGTKKGWVKTRQNVEHYGGTVVNIDEQLYVIENSATSADILTMKSSKCSHGDWQVV
TRIPDGCCDSGIVVLNGRIFIIGGNQGCVAYNQCYDLRARTWTKLKPAPRLFSECPAVALNGFIYILGIYSTEKKTNNEV
WRYDPDIDDWCQVADLSVPGPQIGAVACNGEILSFCTSKAEPEDTPISHVEDVPGPSLQTSTSITTSSGTVYATADDFYR
SIMGTSTEKNSKRQLHIQRYDFSHNIWYSDQCDTHCINAPDHGMCFLPYKHYKTVKVLMEKGLEHL

>Skow_XP_002733217
MASVPGFDFTHENHAANLLKGIQDMKFQRKFFDVVICVGEHEFPCHRVVVASCSTYFQALFSHDLKNQTRVSLHEVDPTS
LGLIIEFMYTSKLTLTDDNVQSLLETSDLIQVLEIKNACATYLERQIDFTNCLGIQQFAELHTCQALSKKAKMFIMENFS
EVSKEEELLALPKDSLLVYLSSDNVQVRREEDILEAVLAWSHQEEAREQILFKLLNFVRFMFIDEKYLEEKIQTHEALKR
WDGLNDMLKKVKQYRTMTATELSSIPCCQSRVFSEIVVILGGNVVGHECQVQVYDPKTRKWSSLPELPLHKDQVATAVSL
GNNIFVSSRRGKSLLYKWQQKKWLHKSSPLLKGRWSQRCVVLDGWVYALGGKDVNWASVTSVEKYNPAKNTWTETTPMIH
TARKPGVTVCHGKIYVIGGYSDVNPPPSQCYDPLLDQWSIVSSCPTLTAQQPACTAVTLNGAIYVICDVTGVLYCYDPLI
DSWQLKSPQIVGRTDPGMTVCNGRIYVFGGYYQSNLGAVETIETYDVVDDTWTTVGYMPVAVYRHGCVTIRRLGH
>Skow_XP_002733207
MDDFLRELQPFTAVFRLERELLEKRLGKARKSKRKQKEKHEQTSTTEDSGNRLELSLEKEEQEKWMKEKGNKELEQETDL
PGACAATSSPDECQMNDPEVDEFKMEALRTALLKFCAKLTTISPHFWQQKVFDDKSETSDKTVQPRRKKNVNKTVTLKSP
KHSVNLMQGMNALRRQNILCDLTLIVEERRFQVHRSVLAAFSGYFRDLLNGPPEDTDDSEIILEGLSSHGLELVINFIYT
AEIDLSIQNVPKVLEATTCLEMPSIIDICSDFLKGELLIENCVEILNIGTSYNVSDLRVFAEGFILANFKEITDKGVFQE
LNDRQMNDCLKSNKLAMYDETEVFQTVVRWLQLTPIRLPYSGKLMRNVRLSLIPPDDLVDKVQSHGFMMNDPACNSLLID
AFKYHATPHRQPCMQSMSSMLRSSNELFITVGGLRDNGATTNVECLNTFNGATYHLSQAPSRLVDHSVATLANFMYVVGG
QTSGSPGGEHSLATVYRYDPRINTWVTLPTMMERRSNFHLCVIANKLYAACGWKGRHERTRSVEYYDPQKNQWSFVAPYP
VSAVAPAGAVFDGILYISGGYGGQYSNAVNAYNPTTNTWEARMSMQTARAWHSMVTVRSKIYAIGGNCKDGNGKRIDVLN
TECYNPLSDQWHTLPSDLPSGCSVTNAVVMKGNIYVVGGYEWKTKQSKRSVLCYYVDDDRWGWKSDMVNTLNGMACCTLI
LPQDLKQTFLICPLHKHVLYISMSFT
>Skow_XP_002733124
MGECREMFYQDACQPSSVLATLDAFRSMGTLCDITIQVKEWQIPAHRLVLLSSSPYFRAMLTGQLAESNQHTVVMKDVDP
QAINDLINFIYTSKIKINEENVQNTMLCANLLQLTGVCNACGRFLEKQLHSSNCIGIWYFADAHCCNNLTKNAYQYILEH
FSEVAQEEEFLHMQFNELLKLLDSDFLGVPDENEVFKAVVSWVKFRLEVRAVHLYSLIEHVRLFQLSASFLEEQLKEEAV
VFNKDIDCSKMLFDIQRVLQEANNVHSVMLKYGKGKRICYGKRPKGLLAIGGESNGSTLNTVNCYFVDENSWTCNLPQSD
QNDTKDIQFGCMNTSRSEFGIASTFSNIYVVGGVNSMKVLGSVEKYNVASNKWEELEPMQSLRQGCTATLFTDQLLVCGG
RDNNQYLQSVEVFSSVARHWSYIEPMQVKRAFFGCVDLSGILYAVGGTGGNNGKDCDFLSSVERYDPNIKMWTNVAEMHE
RRAYLSVVQLDGYIYAIGGFNGSWINTVERYNPYTNQWIYVKSMKTKRSSASATVLNGCIYIIGGFDGFQCTNTVEKYDP
ATDRWSKICPMQSRRYGVGAATVILC
>Skow_XP_002733087
MTNKNMEPIAVFKYKDASHPAFVLRGLNELRHQKKFTDVVLCAGEEEDEIICHRAVLALCSPYFNAMFSNGMQESRVETI
MLKGIDPKSLGIIVEFLYTGCIDISEHNAQSILEAASLFQISSLESACSVFMKDHMDASNCLGILQFAYLHNLKELYEKA
EMYSTVIFSEVTKYEEFLQLPGELLLKYLSKDLYTDNEEVVYEAAMQWVKYDIENRSKLMFDILNVLRLPFLSPTYLVEA
GNNPLTSSEENLRLIEHAQKYQNMSFTELQKTSLFQCHPRNYKEAIIILGAESVDHMEIKHRTFMYDPQSKLKIQRQSPP
FQKYEKVQTAASLGSNVMVRTNLGNAWLFLPHENTWTEVASPSQPRWHGKLVSCSNHVYLVGGYGLNRTILDKMEVYRPE
SDSWEIITTLPTPVVDWHHSVIACREKIFIFGSSSSDCDGIPDIQCYDILANEWSLQGHMRVRTQKIRAEALNGCIYTIG
NMPYYNHIECFDVESSTWRTVKNLIESRHDFSTAVCNGKLYVFGGEKEGRYGSKCILNSVECYDPVEDRWNISDSSEHLL
NMHTITILHPEFQPSHLTGPKQH
>Skow_XP_002732994
MCDSHSLRPHTPVGEVEHVHHLSEQIGNLFLSEEYSDITFIVEDKRFYAHRVILAARCEYFRALLFGGMRESRPDCTEIE
LKDTTPVAFCALLKYIYTGRMNLQDLKDDALLDVLGLAHRYGFTDLEMSISDYLRATLNIHNVCLIYDVASLYQLRSLKD
TCCSFMDNNAAEVMSSEAFLALSESALREVISRDSFCAVEIDIFRAVLSWSEMNSDIDPTHIVSAVRLQLMSLPELLNIV
RPTNLVSPDSILDAIKLTSESKASDLPFRGFLSLEENVACMKHGGHVTHGEMPSALLDGDISNYDLDRGFTRHTIEEPCG
KNSIVVKLGKACIINTIKLLLWDRDTRSYSYYIEISMDEEDWVRIVDHSRYMCRSWQTLYFDPRVAKYIRIVGVHNTVNK
VFHLVSFECYYTARPFTLENGLIVPTENVATVHASACVIEGVSRSRNALLNGDTKNYDWDSGYTCHQLGSGAIIVQLAQP
FVISSCRLLLWDCDDRSYSYYIEISTDQHNWTRIADKTREPCKSWQTVFFEKKTVTFIKIVGTHNTANEVFHCVHFECPA
QLNGQGQDELSTSSAASAAATQTDGSSGSGLQGAVAGPPLPPFPNQLFPGPGMPPFHRYMPGAGDGMNRF
>Skow_XP_002732222
MEFKKYRVTGELSDITVIVNKQTFMLHKFPLYTKSDFFKALARSSMNDSNHVELNDFPGGAATFSSVADFCYNLDIQVSP
KNVAQLRCAAEYLQMTSPGNLSDQTDEYLHDVMYTAKMTSMESVVQILRGCCSVGPVAEQANITGKCIDALVEVWKMEDG
CKKAKDAISDSVLDKLTDIPLCWFLQLLIMSRDRGVSSTILALLARKYVWSVLKENQLDMYDTIDGILLELPAAAEALQA
VVNTEWVITVLKTATQKCKSQSIILELASNMLRKFTVRDLMQFPPNLLRELVQYASNSEKCIPDISCDVIDRYLHSLALD
QSLSTEVFMKLATCLPVDFRTSHDLIFQVVELLFKSGTEIPVDGRKAILELVDFRRLSEDTLRRAYETELVPLSYITKAS
LSLCTKLRAELETAKNIIKGQQDEFRQIYQCVNVTDGTWMKNEGNDFADVQPLSRDKMILQVSHVKERSPVAIEMTHEFI
EGKLAVCALAANTCKDQVLYLFNPFKSQLLEIQTTCDGKSESFKIENSYRKFETTGPYWPYSPRKK
>Skow_XP_002732022
MTTRKKPKRKTFTFQNHSCIVNDARGSLKFVGAKHVTKKTMTGLNEFRKQGIMCDVVLKVDNYEIPAHRTVLAVHSEYFR
AMFTTGMVESREKEIDIQGIENKSLETLIGYMYTGSLDVTKETVQDLLTAACMLQVEDVKDICSNSLLRFVDVSNCISVW
MFSDLHSLRELQIKSSQLLKSRFPAIIVQEEFLLLESNKLMEILAFDDLSLGIKNEDTVLEGIVRWLEHDPISRIQDISE
LLKLVRLHLVSMPYLQTVRQHSVVIDNHDAVSLIDNAIAIKEDKSPHNLKGARQPSSSIYVLGGYLQSRTGGNCPHTLSV
EKYDINKDKWTEVGKLPFNLECQTAVVLNGEMFFVHKKLIQPKLKIPTFTNVGTVYTLCTFSSLYNEWKLKEISQYSVWA
HLRDFKQCAIAVCKDENVVYVCGGKEPTRSLTAKSVLRLDIENDCWTELPSLPELRFNHSAIVVNRRLYVIGGNDISVHI
GNHRQEVKSSVFMYDPDEGCWSEKCEMNTHRLGLGLAEVGGFLYAVGGVSNVTRERSVEKYDPDTDCWNYVCDMNKPRSF

LSVVACHGLLYAIGGKSYSGDCGGARTVVRSGEVYNPATDTWTMIDSMKTPRCMMSACVL
>Skow_XP_002731856
MTSTQEDQVLKKTKYWEKPLNIMHSTQIHNPDQSVLLLEKINVARKESKQTDVILDVDGTEFPCHKVVLENSCSYFKSMF
DSGMQECHQRKITIKGVDSTTMASLLDYFFTGKIFITSANVQAILQAANLFQVLYVRDRCARFILNNLEAETCLGIWQMM
VSLSLAGSEQAKMYALNHFKLVCKVQEFLDLSKELLIEIISDEELVADSETEIGLAVLEWFLYKNGSRKEDFVEVIQHVK
LELITSVYLVERLCKELMLSGSQVGELEDRLISSQGGPEKEAQHRKNSVVCFIGGFVNGQPSDTINYFNPKNRTWSCLTK
ANTTYRGVSLSYEGGAVIYGNSIYTKVSDALALFNMDTNSWTNRASGLIDGEKWLKMELIDDRIFFLGGRFHCYDLNKKC
EALVAPILKK
>Skow_XP_002731630
MATGGCDINKAGQSDTDDVEDEVTYEHHDTNFPSELVTLLNNMRLTNDLTDMILVVGDREFPCHRAVLAASSPYFRAMFS
SDLRERHEERVTLYNVQADCMELLLDFSYTGKIVFTSRNAQDVLETADFLQHKKVVHACSDFLKSQLDVSNCLGIHQLAE
RHMCIGLVEAANLFILNNFSRITNQEEFFELSADTLVGYTSKDELYVRSEEDVYKVIMQWVMADVVERLPQLCRVMRNVR
MPLLSDEFFKNRVQDEIESLLRKSEECMELLAECQECRRGTREGPSEWSRPRPSTGIVNVMVVVGGARESQRNNKQSLNM
YCYEPKQQFWSNLCDLPYSLANVAMYSVLAYRNDIFVTGGYDGHRGGPIAQVWIYRTTEGSWDGCKSLKKARYQHASTTL
DGKIFVVGGYDGQHSLPDVEYYSTESNRWTLIQPMREAVSCPSVTAFHRSLFVIGGVQDNSTLCCPFTQCYNVDTRLWST
ISTLKIDKKGYQSVLLNDMIYVVGGSSRKTYVYDPNADKALEVAMTKEMHLCPGAVAIDGKIFLTGGDSVSDKSSKTVEC
YHPESDEWIVTNPSLPIPLYWHGCVTIMKNIKDLEKMTPQTALHSHPIFLYRGYGEADPTYDPR
>Skow_XP_002731536
MAEMENIHGSWKVADVKTLSAVADGLESSGLQGTDLTFDDSGEVTWKVPHNSETIPSFVTNFEIYEYISGDPPILKLVGT
TTGDVMEFKVSSRPDGMLVLNYENCFILSCEKLSPSDQIGDMPFSFLNAFEHGYFSDLTIEADNGKEYKVHKTILQVSCP
SMDWQRAPPPLTGLQTDVLHAVLYYLYAECLPKGLSEDTAKLCYRTASKLPGLEKLAELCETFLKNIALKQQIMGLISDM
HTCADKIIDIFNGQGGGGDDGISSGSQSFADPAKLCYSFKQALREGAVAGAKFLTLCDLFSRRKGEFSREERHEIIKYAR
TRLPVFLSQLSRLLEVCRQQFSQHLTPSQRQEIAIYLIPEIETSLDMLSKIVIDARTAIEQVITASNTLEKQKKHKGDAL
GRTIRNALHMKELVKLRKFHERTTTEFMNLLHKRDNFALLTQNEKVISVVRNMEQIIEEFPQYLKRIDLLASTIEEKLTW
KEWKYLFKFGSSRVAWVLNKMTQHKGTLQSVLAPVCDLVHRETFTTAIVQLGILDASVNKKTLSTKDGTQQQSTESSSPS
VSTGATSKTSKPTYQTNAVESLSIPPVPGKSKLAKSATELLKSGHLADMRFEIIHQNLPEEPDIVIDHTHGNAAPPMPPS
LAATTPHLNSCELDIDMEVYEIKAHRVIIAARCDWFRRALNSGMRESIDKKITVHDANPELFRLFLQFLYSGYLDTSGLS
VEQVADMMSLSDRYEVDTLKQLCESTLKRHLDGRYSILYVNISRPIQCKHITGAMNFIMENPSLAQSDVFDDLPDHLQTE
VEHLVAWSGNEMSPTHVCQYTTLESLSADTELQELEMKELACALEMSREATVAYSEEDSSSSEIDVAMTENSHELNTCIS
QLRDIVGDDVPREELVRVSLAADYDVNRALNFFFST
>Skow_XP_002731502
MPVTKILKWLNLQGVSSGEEITMNSLLGPLPLMPGEPTTALKPNIYEFTEESHRVSAFRELQQMRERQLFTDVILRVDNV
EFYCHRNVLAATSSYFMSMFSSDLKEKTENHITIQDIKSESMNLILGYAYTSKVVITEQNVQSLLEAANLFQIIPVRDAC
AEFLEKQLDMHNCLGIHHFADRYSCQELCEKAWHFSLENFPEVCGHSEIQLLQKDEILEYLSCDELHIEKEDSVFETMIR
WVQYDPKNRLKHLPELLEQLRINLLSSRYLLEVILRNEYVTTSEVFDKFMAVTKKYRSKKTNLITPPRMRARLQSNIVIV
GGVGIGNSKVMEVYSYEPAQQTWSLLTKLPKHSDSVYSVTSLGNDIYVTGLQGKVSMYSIKRNKWFESAPMNQPRHRHAS
TSLDGYVYVVGGYDGASRLSSTERFDPKNNNWEQVKSLLEAVSSPGIVTCDGKIYVLGGVTSNDIATDKVQCYDPKTDNW
TLVAPMPHCLACISVEVLRGCIYVVGCVSKIVHCYNPETDSWRQVECMNSQRASCAATVCNGKLYVTGGESQPNSPVDTM
ECYDPVTNVWTVLPTLPYSVKLQGCVTVSKKTTSLESSVATLLLPH
>Skow_XP_002731089
LSESTSIIGRTSQQDKMLLIRQDGKVFSVTGNGVMDHIVDLSGELSDTANWFVNKFTLGAVLSSKLIRFYSLSTGDVLFS
MNTSQYGDHIYPVITPDILTLFWTIHGAYHLQGTVNSELNVKDQVLSLLRGGPGNNNENYDRLEILMHHFPSEVTNTILD
ILPNTEDETATDSIDESTINWSGILTPETTFTSWKVTSHNNMPLFEVLCRLIYIGKPDKVVQFVEFANQMREKVIGVTAF
TRKKEKIQLYDRALKCLPRPENSDYENEAVKAQVCLVLGSGDDDCVMTSLKLLLQYKLWDDAIALVTKHADNTQQHCQLF
HLLAARLLKIYEHCLSAVAVMTEMNLDLKDFQLHFAEELICGLNSLHEDKILCDITLKVGTKEFKAHRVVLAALSEYFRA
MFTVQLKESKEDVIELKDISITPESFQCLLDYCYSAELDLTIENVFEILAVAHHLSITSAMECCCDFIKQQFDEQNFHFD
DFVKIAEMAETYSLVDLKEVADHYIAENFVDVSNSDDFLEYMSSKRLCEFLNRRDLAVPSELLVLKAVQRWLMYDKEKRL
MDANVLLRSVRLGFISAQNLVDQFNEDIKSVSECHKLFNGLMQHHAVGKPISGAFPLKNPELFAPRGCTKSLIAIGGRTS
MSCVRYYDVNAHDWVSLKNFAQPPYNTFTNHGITVVHHNLYIAGGQYYGSNKWQTRNYVYKYDASTNKWQSLAAMKVERS
YFGLVNLDGFIYAIGGLGKDGQPTDVVERYNIATNKWQIISALQSPRYDMAIAVFAGKIVIIGGQSSKTDSTEVLDVEVF
DPKRNQWEVKSKPLTCRNQGSTIVVDDTLYVAGGSQESSNDSATGKVELCNLVEEYNSEHDSWSTVSQSLIPKNEIGAFR
INSHVYFSICNHCYNSNVIIPQDEVYDVDLDDWIKLKQNTNGAAVTEFTFNVTKLK
>Skow_XP_002730985
MATRNRRNSENSDCSLETSIDSDEFLFRECSHSILLLMGLNNLRTEYAFCDVTLCVDGQEFLCHRVVLASFSPYFKAMFS
GELAESKQEKVSINGVEASMIEMLIKYAYTSEIWITKTNVQSLLSAANLLEILPVRDACCRFMEKHMDASNSIGIHCFAE
AHSCTELQEKSKVHVLENFTDVIKHEEFVKLSHSKLVEFISNDELNIKQEECVFDAIMAWFNANPENRRQYFEEVFTHIR
LPLVSAYFLHDFVEVQSAVRQSEVCRKLVDEAKNYQLLQDRRGQLYSPRTRPRRSTGTIEVIVAVGGEDDKVVLRSVESY
DPQKDQWKTLACLPFAVSKHGLVVSGNNFMYMSGGEFPDGSASKDVWRYDPSFDHWLEMAPMNVPRSELGLAIVDGSIFA
VGGWEGSARLESVEKYDTWTNIWMFVSPMKIAVTSPAVVAHEGLLYVTGGAVLEDGDGIDLVQCYNPKTDRWKELSAMLI
PRSGSAACVLNDHIYIIGGWHASTENTNKVERYDPKKNEWEIKAPMHERRYRPGVAVIDGKIYVLGGEEGWDRHHDTIEC
YDESKDCWEIVGEMPSSRSWLSCVAMQTRKDLPNKDAKVTSCIV
>Skow_XP_002730968

MSERLPNRANFQRDGRYCRRSNASLSNGKAEENVASQNGDADVNNTPKSNSPSDGVLRRMAEMWRKKQLNDVVLAVGKHR
FGAHKLVLSACSGYFHKIFADGTEENGDDQFVYNLHGISHRSIELLLESMYTTQLKIHVDNLEELLRASVYLEMPHAVQI
CCQHMMLNLTPKSCITILTLVSNFELNELQEAAGKMAARHFKTVSKSEDFRRIPPEQLKKFLCRDDIEVDSELEVFQSFV
SWILYDKERRICYSVMLLECIRLPMIKPVDLVDHVETVTFLMRIPECEALVKEALHYHCLPLRQSILQSTRTTPRSTIKL
ATIVVIGGHPRLSKDEVNSEVTYYNPQTKEWRLLTRMIHPLHHHAVAVLGGFLYVAGGRKTTNRSEIPTDTAYRYDPRTD
SWIQISSMKNKRESFQLGVLDGMLYAVGGRVDDDTSLADVERYNPLIDQWQAVAPLSDARRSVAVAAHGGRLYGMGGSGN
RRMSNKVERYNPKTNKWETKRPMATPRFFALLVSVKAKLYFVGGATVDSSGNLLCVPSVDQYDPMTDTWSNLTPMFEPRA
EAACTVNDGKIFIVGGYSWDYNTWLNSVECYNVSCDEWTYTESMPKAYTGMGCCTLTLHKLP
>Skow_XP_002730944
MSKHIIYIDSVMKIISRICILFRVSQGVTTEFLAVTTRKVYIRGNRPLRERLRYSDHSSLDQSAQSRSNVNVTMETKESA
ERCDDTDFVLYEPQYSNHILDSLNNFRKAGCLTDTVISTGGKEFPCHRAVLACSSPYFKAMFSAEMKETQEGRITLDNVR
AETLELLLEYAYTSKITINSANSQELLEMADLLQYKTIVQAASEYLERQLHPDNCLGIKYVAEMHACTRLYERASAYALD
RFPQVVKNEEFLQLPLFEVVAYLSHERINVRDEETVFDAAISWVKYELKKRLPKLLVVLQCIRLPYLTPAFLCSILKNPL
FKQQSSCFDVIQQAMHLQEYLQSNETNIYHVGHQPRWSTKTEVMVVVGGFDANHTWVSDVHYYNPVNKKWGKFAPFPADI
SGYKVVALDEDIYVTGGKSSRGVTGAAFCYHSAQDQWNQVPGLHIPRQYHGIVVLDSSIYVVGGEDGDGRCIGDVECYHP
SMENWGWVPQLPTAVGNPAVVSHSGKMYVIGGYCNGQLTYREMQCYNLDIDTWTVINTVTITTRHFPALVLNDSIYLLSG
AGKGGMQIYNPETNTCGRNSEMCLLERHLFAVCVMNGMIYVAGGMVNYKALGSVEMYDPSTNTWKIVGCMPKPLRAHGCC
ASIRRSLGSEDGSAAVFDCPCMPNNTMATPIKTFKESTARLIQYIFLKTDIFFKCNSTTCGPPSDLFIS
>Skow_XP_002730545
MMFVYRKLMARRRQDTNAAENGTEPVNDNKEAEESKVQSPKKVKKQKAKPVKVCDKNAEKAQVEEKGQHTQVETYISRIK
YNDTRFNNEPDFSLHTEDIEFRIHSSLLKEKSEYIQKALSAEAKCIDSVGKGHGIEFVKPPPTARALRILMDYVYTEKMI
VADDEKQEVYIAARVLGIQVDEEKLIGWNNPK
>Skow_XP_002730487
MRSDYYILCNDKKVALTTPITNPRCIYCAMDEPEGLSYEVPPYKNLQHTNKAFDILNIFRKQNQLCDVILEAEGVEIPAH
RVVLASCSPYFSAMFTGELAESRAERVTLQEIDGKALNLLVDYVYSAEVQVTEDNVQALLPAANLLQLPDVRDACCDFLQ
RQLHPTNCLGIRAFADVHACADLLNCAQNYTMQHFSDVIQGDEFLTLSAGQVCELISSDHLTVPTEEKVFEAVIAWVTHD
SPNRQEHTAILMEHVRLPLLSREFLVQTVEEESLVKSNNSCKDYLIEAMKYHLLSSDQRSMLKTPRTRPRTPIGLPKLMF
VVGGQAPKAIRSVECYDFQEERWYQVAEMTSRRCRAGVAVINGLIFAVGGFNGSLRVRTVDVYDPAKDQWSSVASMEARR
STLGVAVLNSYVYAVGGFDGSTGLASCERYDTKCNEWQPIATMSVRRSSVGVAVLGGYMYAVGGYDGASRHCLSSVERYD
PSINEWTQVAEMSCRRSGAGVGVVGGLLHAVGGHDGPLVRKSVEVYNPDLNQWTQVADMTLCRRNAGVCTVNGLLYVVGG
DDGSANLSSVECYNPRTDTWCLIPSCMTTGRSYSGVSVIDKPS
>Dmel_FBpp0070184
MLKSISGGVGVASTERGGGGGGCGGGGAGGSASGSRSTSGSGRASCGLGSSSISSGLSAVCGFVTGASGGGGFERDRDRD
RGLANISGGSTPAGILYCPACMAAANQQSPSQNSSPHHESFFLRSSFKSSKRNKARKSASTAGCLDAQHIRANLRMSMSS
SMRSSRGNGGGGAASSSHPPGSSCSSGPGGSGCSMAYDAASNASSGSGSGSGKATSPGSYSCNALNVDFTSYTATHQWTR
MLECAEFVGAKRSKHTVVAYKDAMFVFGGDNGKNMLNDLIRFGVKDKSWGRACATGTPPAPRYHHSAVVAGSSMFIFGGY
TGDIHSNSNLTNKNDLFEYKFQSAMWVEWKFSGRQPVPRSAHGAAVYDNKMWIYAGYDGNARLNDMWTLNLTGENQWEEV
DQLGDRPPTCCNFPVAVARDAMYVFSGQSGLQITNSLFEFHFKTRTWRRISNEPVLRGATSAPPSRRYGHTMVHHDRFLY
VFGGSADSTLPNDLHCYDLDSQVWSVIQPEQNSDVPSGRVFHASAVICDAMYIFGGTVDNSVRRGDTYRFQFSSYPKCTL
RDDFGKFFQDKQFCDIQFIVGAEEIRILAHIAFVAARSKYLRNKILAAREARQQQMEKVYGVGQVDALALNAGAGGDRGP
MLEVRLANASPEAFEIILNYIYTDRIDLKDTYSKNIIILITDIYQLAGLFTMPRLAHGCIQYLDYKINKLNVLEALYNAD
KSNIKIIKDHCMQFIIKEENFTDVVMSSEFSDLDKPLLVEIIRKRLYPSKLVIDTSYEGNIGTTLEIDLCAFLESTGKDF
CDISLVLEDHVIPAHKSVLSSRCTYFQGMFRSFMPPDNTVNIQIGEISPSLEAFHSLLRYIYYGETKMPPQDALYLFQAP
CFYGLANNRLHAFCKYSLEHNITFENVLQTLEASDITKIYDIKEYALKLIVKDFAKVARLPKIAGLSRELLLEIIRAVAD
SHGEFLTRININTDI
>Dmel_FBpp0070790
MATTQQYSLRWNNYLRHLTYSLDNHRLNDDFVDVSLCVDGRRIKAHKVVLSSCSSYFKEIFKENPHPHPVIIFKFIKFED
LNSIIEFMYQGEVNVQQEALQSFLQTAELLAVQGLTAEEKEKPQIPVAPLISEHKLIKTIPSTIRAVNEQQQQVSNVAQA
QTATQTIEIPAATLLQATNASQVPSQVVAAAAAAAAAQQLQVQHQQQQHHHVQTTQIQHIQVQSAPPPQQQQQQQQQQQQ
QQQQQQQQQQQQSQQQQTTPVQTQHLTITNAVALPAASSVKKRKITFSDDDDNMYTTETVDYAVKDEQTIVKTAEMTFLR
GAVKMDIPDYIVGEVDDRLSDGATPQTSQDATTAAAQNVAQYSSEYEILTESEMEEKYQADADNAEIAIEMTRLLGTASG
TPGAGSGQVMEDSGGGSATTGPSTTVSVTQVKSDSKASGTNAALKPGSVTVARSSKSGATASAPPAGSDEESVQYTGLAP
LNCSFCGRPLKSVNALRRHIASRHSEIQGKEHECFICMKSFKTKWSLSTHNSRFHREMGCSTKGFTKIEYSDH
>Dmel_FBpp0070823
MLPQQYCLRWKYHHSNLQTMFSQLLDRGCFCDVTLACEGQLIRAHRVVLCACSTFFDAVLSNYASERDPIIIMKDVTFAE
VKCLIEFMYKGEINVEHSSLPSLLKTADDLKIKGLAEVTWRDDEDGPPPPMAAAEFHSPPRSLAESYAQDLILQHQQQQQ
QGPAAPPLNLHSSAALLERDREREREREDRERLSPGMEGVLGRMPVMTPLTGASGSAGVGSVSGGTSLEGVAPVEHFLGPK
RKRGRPPLDDAYDVFNVRKLAQYAANLEPAQRAYLETARHFTEEPPLAAHAASMASPPAACPPKQRQRLRHQQQQQQQLL
QSESSDQEQPAAGQDWSNLEPNGSATPKLLSDGDANKQETEGGDESLPAVLTTASSSGTKKLEKIGERRERHGKLRHARR
LYEKSGEDEVDPPKEEPDELPQPVEEIENEHLVANRAESPRPTVVIPASFACKYEGGVTAAGGGSGSGTGSGSLGSSYLI
NEHGMLMAHEFAPNVASAAATAAVAVAAAAAAVANASAGNGNGGQDVSGSGTGAAADYPDIKLEDYDAAELRLTNEELSQ
WQDVIKMDDYLAKGRRPQFWEEPFTKRVLDAIKNKRLEMKKAARILGVSYGTLYGRYREVYGCLKHPYSGTAFRNSGSTS

TSQMGVQPRFDLGFRNGGVLPAQLVQTNPYLQSWAS
>Dmel_FBpp0071249
MSAVNQKESPSRDSTASTSGHISMLADNTLESLSRDYSLGSLNSAGSQDEFFRCRDHADNILKRMQLYVDSQQLCDVVLI
AGIDGKRVPAHRLVLSASSAYFSAMFTGSLRETKEQEVTLGEVHGDALHLLVQYCYTGFIEMREDTVETLLATACLLQLN
AVVTACCNFLARQLHPSNCLGFAFFAEQQSCTTLLRLAQAYTCQYFMQVCQNQEFFQLNADQLGKLLSSDDLNVPSEQDV
FHSLMSWVRHDSAAREQHIPELLALVRLPLLQPAFIMDHVENVCNANECQQLVMEAFKWHLMPERRSRIATERTTPRKST
VGRLLAVGGMDAHKGAISIESYCPRLDKWTPWKHMTGRRLQFGAAVMEDKLILVGGRDGLKTLNTVESLDLNTMAWAPLN
AMATPRHGLGVAVLEGPLYAVGGHDGWSYLNTVERWDPIARTWSYVAPMSSMRSTAGVAVLGGRLYAVGGRDGSVCHRSI
ECYDPHTNKWSLLAPMNRRRGGVGVTVANGFLYALGGHDCPASNPMVCRTETVERYDPATDTWTLICSLALGRDAIGCAL
LGDRLIVVGGYDGNHALKSVEEYDPVRNGWNELAPMAFARAGACVVAIPNVIPPAAQQPPPSATKKKKWKPVRLDPICYN
RRPRCVEFVDYYNM
>Dmel_FBpp0071426
MSSQGHHKMSGGGKKGAMEQDYTDVVDLGDRFSADMARLCMNEQYADVEFIVEEERIPAHRVILAARSEYFRALLYGGMA
ETTQRQIPLEVPLEAFKVLLRYIYSGTLLLSTLDEDSTIDVLGMANQYGFQDLEMAISNYLRQYLALDNVCMILDAARLY
NLEELTEVCLMFMDRNAGDLLLHNSFNTLSKESLEEVLRRDCFFAPEVQIFLAVWKWSRFNSNVDFKSVVSYVRLPLMNL
EHLLQVVRPSGILDPDKILDAIDERSTSKALPYRAALWPEENVAAETFLSRCIQGECRDALLDGDVTTYDMENGYTRHCI
TDSKDAGIVVELGTFCMINHIRMLLWDRDSRAYSYYVEVSGDQQHWDRVVDYSDYHCRSWQYLYFEARPVRFIRLVGTQN
TVNRVFHVVGLEAMHTAKVPRLVNHFVAPKTNVATVEMSAIVTDGVSRTRNALINGDYVRYDWDSGYTCHQLGSGEIVVR
LGQPYYLGSMRLLLWDCDDRTYSFYIEISTNRKEWQMVVDRRNDRTRSWQNFHFTPRPVVYIRIVGTRNTANEIFHCVHL
ECPTQDKNYLKKIADMEKEREKREKEKKTAKTDDDNIASTSGSSLASGHAESPSTSSSSSQSVLRSIHWPPQTREVAVAP
LTPPALSPPGTPALPAPLTPATSSPHNNHEQNQPSNISADASHHTSPSSRSNPSPSLSRSRSQSAELEPVPPLVELDTRE
TL
>Dmel_FBpp0071683
MSISAVDRKEPGSVSSGLGRRYGQLLRGAKYTDCVFHVCEEQVKCHKLILSSASPVFEAMFFGPMQNNEPEPEIEIHDIS
SAIFKVLVEYIYTGVVDYNGLELVACIELYYAAEKYLLEELIADTLMVITRKLRFANILPALELSVCMGLDGLLEVCMTF
FMRCCVNNGQYMSHLKEHYVHVSKECVKAIIAACKEPHKLLIWYVYEWTRQECEQLGLGPSDADLVVHDLGGKTSDWPAA
SGASDLESPALTPVVLVERCYYKACRPFTVDAETPLFRLRLKCSRFISLMGLVLNSRLTPNLLGHVQQLEYRESLRLDLC
EVPAESEGPATSPVWSHVVQSQNTKYNCNLHLGWRREEACVLNPELTYELQIRWDSSAYGAEYPCSLQSCQADGIRFTDE
DSFSGSLVKGLRYANLV
>Dmel_FBpp0072038
MKEFQRMLMLQYSKHGECILKEIGAAFRGEHPADLTIVCENKVKLHAHKLVLAASSPLIRNLLEDTHLSDCSTTVYFPDV
NATYFKFLLDFLYSGQTCITSRDVNYLHDLLLLLQIKSDSWKTTDSAYLSSKCGGLRDRADRRKQQYTSPQNLEPDQTLK
YEVDSVDESRNAADFSSAFNSNDNCESAAECERSGGHNNKEEDEDDCTHKDNKSDKDTDEIVNLSNAPPSGTSGSNSNIS
TSSNHQQQQHHHHHHHNHNNNNNNNNNNSSSSTINPVNLSLDLRTKSENSASRTLGSGSDHSGIDLAVTASESTKRKGLF
FDSHKDVMKPLSDGSDINSSPENYVVTPHRKRRPGFHNTQSDNQPFTSYPHSLLEELRLAKSTTSPISGFGSEKNMLAHL
EDGALNGDTLTPDRKHLLEAQRNRAQSPEIPMHLGPQFVYQWQSNQNAAMSAMPNLQSRLSSLSHISLNLDHPEGRSGSA
SGSGANLAGSNTHASSVREYRCEYCGKQFGMSWNLKTHLRVHTGEKPFACRLCVAMFKQKAHLLKHLCSVHRNVITTTNG
ADTENRYSCCFCSMCFESVQELVRHLSGHHNNLLLTKNLRE
>Dmel_FBpp0072358
MLKDAHQKLIKHRQSTKLSMTDFACQAAPVKQPLPLELPLPLSVQLQLLAKTPTSDHAPMHSTPPTTPPTPPPLPLNMSQ
SASAVTEAATPENSLPATPPSEGALAVPSAPQDHYSLRWNNHQNHILRAFDALLKTKTLVDVTLVCAETSIRAHKMVLSA
CSPFFQRVFAETPCKHPVIVLKDFRGWVVQAIVDFMYRGEISVPQQRLQTLIQAGESLQVRGLVESSVPEHTPTPAASPD
DFGMLDTSMLSSTFEDECPTMVRPSKGGKLLMPSARLFGNASSAIAALGLRRKREQESDRDLESDQELGGSSPMPRRKQA
RPRRRSGDVPHDFTLNKTDAESLQTVIKHELLERAERDQEEAPDQDNSQGEAEKISSSPAKTLVERAKEQKSMKEEGSDQ
PRSLNENHHQLELDDEDDDDQDHEEEEEQDIEELIHTTNELRRQAAAAAANAAAMSPNPSPCLSDGPEDLCTTKKGKELI
SGPSSSADCESNNNNSSKLQDNNQRIMLSLKDIRQLNANPNPTAIHTPTSCSGGNNGLLTFPPPGLRPPGLPDSPPCHME
ALEAQMHAAAAAAVAAAGSGEHPFHHMEHQMEMSLAAAAAAAAMHQREPRDPRDGRDHNAFASNLLGPMGMPPFGGHNGG
HPGNSGPGNGCPGQAAHERLEESMNRLSKELGKEFGKEFGKEFGKEFGPASPMSLQGPFNAPDGPPHPPSPLPFPGMSSA
MTLTPPHMFGLDSPLGLFPPGIDPGKLYNPLMEMSDPRDMPGGPPPFLKKKMPRPKGQHSAPRGGPPRSWTNTELTEALQ
HVWNKKMTTSQASRIFGIPYNSLLMYVRGKYGKSLKLEQLRKDCISGPPIEMLQMGIGGGSGGSTKNEKSKERKEKEKDK
NSMSSNGSGGSANSQGGAPTSGSGPMQHSGELGPMGQLDLDLGLPLGPPGGPRSNSSEPDLLSAPNALFNPFNPQGFYPD
FSGGFPGLPLSMLNLLPPAERHHAAAAMHHLGVSMDEDCKSVGSKQSSSVDEDYSGPGIPLSLEHRREISATGPPLTPSN
GGTGHD
>Dmel_FBpp0072535
MDMTKQIVDFEIKSELIGEIDQFEASDYTMAPPEEPKMVEESPQLGHLEDQNRKYSPEREVEPTLQDPSEVVDQMQKDTE
SVGEVKSPEKDVETELVKSKASPMNDQALTPPPRPLTSSEVVGLRDPEHTELRMCLEAKKSRSLPVSPQPQPNLKLAGSA
LFEFGQRSSPVETKIKTNPETKPPRRKIVPPSGEGQQFCLRWNNYQSNLTNVFDELLQSESFVDVTLSCEGHSIKAHKMV
LSACSPYFQALFYDNPCQHPIIIMRDVSWSDLKALVEFMYKGEINVCQDQINPLLKVAETLKIRGLAEVSAGRGEGGASA
LPMSAFDDEDEEEELASATAILQQDGDADPDEEMKAKRPRLLPEGVLDLNQRQRKRSRDGSYATPSPSLQGGESEISERG
SSGTPGQSQSQPLAMTTSTIVRNPFASPNPQTLEGRNSAMNAVANQRKSPAPTATGHSNGNSGAAMHSPPGGVAVQSALP
PHMAAIVPPPPSAMHHHAQQLAAQHQLAHSHAMASALAAAAAGAGAAGAGGAGSGSGSGASAPTGGTGVAGSGAGAAVGS
HHDDMEIKPEIAEMIREEERAKMIESGGHGGWMGAAAAATGAASVAADSYQYQLQSMWQKCWNTNQQNLVQQLRFRERGP

LKSWRPEAMAEAIFSVLKEGLSLSQAARKFDIPYPTFVLYANRVHNMLGPSLDGGADPRPKARGRPQRILLGMWPEELIR
SVIKAVVFRDYREIKEDMSAHQYANGQHGTYIGGGTTTNGYHSAAAAKLAAQNAALAPPDAGSPLSSMTETLRRQILSQ
QQQHQQHHQQQAHHQQQPSHHQQQSPHAQSMNMYKSPAYLQRSEIEDQVSAAAAVAAAAAKHQQQQGERRGSENLPDLSA
LGLMGLPGLNVMPSRGSGGGSGGAAPNSAASYARELSRERERDRERERERELSRQYGSQSRGSSSGSGSAKSLTASQRPG
AASPYSAAHYAKHQASAYNKRFLESLPAGIDLEAFANGLLQKSVNKSPRFEDFFPGPGQDMSELFANPDASAAAAAAAYA
PPGAIRESPLMKIKLEQQHATELPHED
>Dmel_FBpp0072538
MASAQAETNVGLASEQGPVAQRQRKGTGSGADSPKSNRSSPTQQEEKRIKSEDRTSPTGGAKDEDKESQGHAVAGGGGSS
PVSSPQGRSSSVASPSSSSQQFCLRWNNYQTNLTTIFDQLLQNECFVDVTLACDGRSMKAHKMVLSACSPYFQTLLAETP
CQHPIVIMRDVNWSDLKAIVEFMYRGEINVSQDQIGPLLRIAEMLKVRGLADVTHMEAATAAAAAASSERMPSSPKESTS
TSRTEHDREREAEELLAFMQPEKKLRTSDWDPAELRLSPLERQQGRNVRKRRWPSADTIFNPPAPPSPLSSLIAAERMEL
EQKERERQRDCSLMTPPPKPPMSSGSTVGATRRLETAIHALDMPSPAATPGPLSRSSRPHSQSPQQQQAQQQGQLPLPLP
LHPHHHASPAPHPSQTAGSAHHPASPAGDSRFPLGPAAAMAAARELSGLGPGPSAEPRLPPPPPHHHGGGGVGGGGVGGG
GAGGVGSGGGSSLADDLEIKPGIAEMIREEERAKMMENSHAWMGATGSTLAADSYQYQLQSMWQKCWNTNQNLMHHMRFR
ERGPLKSWRPETMAEAIFSVLKEGLSLSQAARKYDIPYPTFVLYANRVHNMLGPSIDGGPDLRPKGRGRPQRILLGIWPD
EHIKGVIKTVVFRDTKDIKDESLAAHMPPYGRHSPAFPLQDLPLSYPGASGALAGAPSSMACPNGSGPQTGVGVAGEQHM
SQETAAAVAAVAHNIRQQMQMAAVPPGLFNLPPHPGVGGGVGNVPGAAGGRASISPALSSGSGPRHAPSPCGPAGLLPNL
PPSMAVALHHQQQQQAAHHHMQQLHLQQQQAHLHHHQQQQQQQQQQHHQGGHQVAHKSGFGASSSSSASSSSMGQHHAPK
AKSSPLRSETPRLHSPLGDLGLDMASYKREFSPSRLFAEDLAELVGASVSSSSSSAAAATAPPERSAGAASAATGADAPS
SSSSGGIKVEPITTTSE
>Dmel_FBpp0072630
MDASPLSPEQAPILVTGRSLDQQVDASSPNHSNYSNLTNSSSSGSSASSPSACSSSSSSSSSAPQYSADIRAAALHPQQPQ
QQQRGSASGSRSRSGSTKTCELDVGEERDTFSPVSSPDCNGAAAVNVLPSSSGSVDNKIYEGGSKGKPPSKEEKQRQNEE
IVCMETSTVSTETGSWESMYPAASVAPQAEPEEMEATTVFKGISASTLLRDLEKRDQPLSTSGTSACFIDASSLRDEDEV
LSFPSLDGHGQEEEVGQEATGDEEPVDEPSPTDQLEQFHKSFARCKGVRLTQQQQQQQQREPEQEDPPDEPAEERHAPMS
VPYSFQHNAQHFSVLPLGSSPKFTHHQHQHNQSQQHHPDLSYTTDYSSSSPAPSLVWSEQGRNHHGASSSTPHNSMVHIE
PASAGSNNSSIHRDYTLSSSLSHHRDSGAYCSDATDSPLPLPRTPKRSSKFDEANPIVSGGASIEDFREKQCESPSIKRR
TDTCPIVSGGFVSLDFAPTRPLADDDDDEELGEDPEIAGVEMRVKTGVQRKPVPGIASWVVDMSDCRPERRRSTSSTTSS
FRERSDSNSSHKSGCGFYVSLDDMDRKPQEQQKAAAVAPPSQLSKSYTAPKSSGFFVDLSQSAKDEEVAPKSQEVAEGKE
ETKPEPKNIFSMFIDFGEQKTPRSNGRKEPLSLAQRLSSSLSSSASRRGDTEVMKSSASSTETLMAKSANLKDVSAGAGE
SKSLDYRCNLEPPLTVAALRRLSQQHRQQSDPSKRHSWGSNGQPEQASGSGTGNDCKRSISLTANGATDSGAGLMSIIDK
LPIISKASSLSIDSSVSPYDDFSGSNPGISSCSEEEQGQVQGRIKKRRRDVQINETYDKSSLASVTEGILSKDLSPASNT
DTDDLTFQQDEQLAKEQAELVSVPLQLTSSSNRTSSVMETIIEAKETASPKKQVTLSNGHTMESLHATIEKQKQLLETVN
EHGEQTTQQPSQIPSSFVKLSDMDKPVKHDLQSPDSMSKSVGNNHRSSQLGQRLYRDENRARPHSWAMTRSTGNTLQNFT
NSVDNIRSLSRLFPNFSKEFSNSLPNGMTLEQVQGQQVDYIQTDLSADSSLASSFSRSGMDESSLSCRQPRRLGEDLLKM
FLQEIATDMLVEVHGRRIRAHKCILRSRCQYFAAMLAGHGSQSVVSLQGYSYAAVHFALCHIYSGASHPPDGISLMELAA
LADLLGLEGLKEVTAHALKTNYCHNFHKPCSGCSDGILQVLPVALNHALDDLYRKCLRWTCRHYLKVWPTRQFAQLPPDI
LGRCRQQIVAYMTSDTVLDTVLDCDHLLAQLSAYRWGHVCEQLVREILEAAYAYVGDHFASLIASDSFLSLGHDRSRHIP
RLETLLLHTAAELTPEQACRSYQRVTRLNTVLQAKVIHMPASLGQSELARELQGLQEEQLDWQPEYIRLVGALVYAVEQC
LIRQCSRAMRVTAWQRMDLELRKKIQTLARLTEPLDMKRQNGKPVGKAFSFGGSTRSQDLVQIKLAIQAQTKRANAQETL
MYKATQTQDAAGGAGHVQTADRGVQANHESREGGSKLLKSNSRAYVPHRASSQVATERNSLNLHRRTQSEAPPVTHKKPI
TVAASTVESKSAQGKPNAAAAKLGEVRPRYLEPRKPRAMAPSATNGHGAGPVRSATSSSIPANGGRKAPAKNLHISSSDS
SRNSSPAARRVQNGTRLGNKSSNLSMDSLASPARRAKSSSRGPQERYSSDQNSLAESMKSSVITNKTISHESLSGSSKLA
RIQQINGHSKGAATGKIKATQRGSTVGNKSTRQLKQQHNAMASNGSSPSSVHTTKSAASRLSSVSSTLSTRELFKQRSIS
VPAGGTDGAPNKGRHSFLSAKSREILAKRAEKNKLQQQQQQQKQTEAPVEERGSVQLRSSAGPLRSSSHSAVSSMLQQHN
LRNSLPSNIPTRRPNTLHLKKTTAATNGVGGGPTKTTTNGMSNGTYKSAQAVKQKLDLLIDAQMESVVPRERVESKLERS
STFCKDSADLDISELQIVE
>Dmel_FBpp0072891
MNHLKWMGHSSTIMDIQRSLRNDNQHCEVVLASRDGVRVRAHLFVLSTCSELMRNLLVDVPRGQEATIMLPDIRGDLLEC
MLSFIYMGETSLPSASLPEFLEAINLLGIKSAISFECNPSASPPSVDVENHSLAVESAKSITGLQIAEAELLDDEEEPHP
MVSVPATTLAGSQHQPSHSSRSLEYIDVYEPPKITYSIEHMDGSSSGNQFILTENTGTFTITQSASSLSKIEADETGTSG
AEVDLGDDDVDADLAEDSEEHESQMIDEEFAQADPLMELEAGTEMDDDDDVHDQLVDEEIDDKPRKLGGGKTRIRRPISA
KAVKRLSDCKPTRIQQFAALKREVKDDINDALDLAADAVIIEGLSLQKAADRFDISKTVLWRRVRTNPAYMRNNRERPSL
QEAYERLKNGHSLKSISSELQIPMSTLHRHKVRLSAQGRLPNFVSCRKRDNTPKDELRDKLAKAVHACLNEGMSQNHAAN
LFEIPKSTLWRHLQRRTTSQNKKVKKELKANYGDDMLN
>Dmel_FBpp0073397
MNDNGNGGAAPVSGGVCGVGGAHGAAGGADGAGGAGGGGGCGAGGGGAGAGPGGGPGGGAAAADLAGEAAGGAAGGDAAI
NNQANANGTSESPAKGLNSSEDLDAKRRKCRETEEGQDEASTMIDANSVLNKIANLYAEQLMSDIVLLVDGKEFPAHRVI
LCASSDVFQVMLMNPEWNECSKHVIELHEEACCSAVFPQFIKYLYVGQIEVTLQTVMPMLALSDKYNIRDLIDLCVDYMN
KHVAKAATSGYLVSWLQYTLSFTPTHNDLTETLKRFLKWNLEMVAESRDFVEMDPAILILLLQQNDLVVTSEYKLFDILQ
TWLLHRREQMEATGSGGFMELIEQTVSHIRFGMMTPRQLSHLLMDPLVEYHKEFLVERIAIGMSYQSGHEDRVREVRATE
SGKLQFTPRLYWNDTWSVDIDVHNFTAIEDYKNYVTCFFSQRHIAETEEDDPCMTWEIEFFPRGVRYNKAKMVWGEDVPG

CSLNTVRLRVTCKHQNIGEERFKIAVLIVGMQNQIMHIRTVVERTEYFSDTSRVVNLDNLLPYEELEHTSLYLSPHLTGT
ARNTLTLHVVITPMGSHTCRDTPPFQF
>Dmel_FBpp0073518
MFTNWLTGSSSSSSSTPIIRSNAAGIVGAGNVTPSGVSATALVMSSPPDVILEVGPAPSNVRFVAHSLVLGMHSGYLRSA
IRLDETAAGTAASVSASASISITGATAASCNPSSSSSAASSSSSAVTTASGELLLYLSNVTADQFAPLLTYMYTGYLDLN
VDNIFAVLLATHVLHMPRALEICRSFLARAQTEGYLNGNPAAQLCPVPSIPAKIIRPIPSKATMPNFGFLPLPQAPPSVA
PSAQPTAHQPTTGIAPVASASILSSFGTNTQHMEHMEQPIAMLDNEVDEEDVEVFIDSNTVTDNEAEELEPDVDMDCNVS
VVSSLAASSAGSMNIERLDVKPPTPIPVSVATIISATTTKSQAQSLGQSKGQNSRRESNSKGNRTKSSASRSRKSSGLQV
AKAPVPAAQLDLTAAATPSITSSKFIIDVASCDGPVRFRRILNTAYGHKPEAMESNALAGPATAPAGGSSNSTISEQHRS
QQSVSLSFHQQMARAISSQQRQLSHQQQEESENSGDAIPATIGKQHATGATAASSAMSTSSTHAGSNRKSQATGTGTGSG
SATASSAGNQELYVCVYCKHTFKSQYCYQKHAKRHLNPLTLTTNDKKLLMESAASMANTIEMDNPSSVGQSGANNSSGSI
ATGNGATAVAATTALLRREVRPLDMNVQYYPCKTCGSKFPSYYFVHKHRKMCHADEIEATATSNTSNNSSSNNSSNNNSS
SSSGSSGSSSNHAKRDDQQQQQQS
>Dmel_FBpp0073904
MLMLILILILILILILFLTPNLCNLSKLINSQCPYVHFINVRTRERLCYLEQTFLNFANSIRTLKEDPTESEIVEIDHEV
PPEEYLVPYIQNYECADIIVTVHDRVFLCHNIILSIYSRRMLKILQEDVDHIVFKSNDLSPKGFSDAYLWMISSDGEVNP
CDMGEILRAAYFLDIPELLEACWANLDRITFFEISAFRLLFELRGATNLWDVFDKLTGRISISILPVASTREFLCLSETQ
ICYILKSNFLAINSEMEASSISIRRSSTYRVLSQIRFSFLPPLMLKKFRSEELHKMPEFSDILKEFSKSPDVITLLRDSV
FNSSLLLTVRNNPSVLDTHVEFTEVELMEPRHWVQDHTCDYHRKVTQLCPNMRFITLNEYQEYLKRLSIGPEFNTCFKHT
RDRESEGQEDMWKHM
>Dmel_FBpp0073924
MGANTSSSSYLTAASSGSGANSNASNSNGNLSAMPPGMGEIPGGGGGCSGAFGSSSSCGPSGNAGAAPGIVISGAHYHRD
QRRKRATGFATLKRKFIRRRRSSKACDHARVLRDFVSDWAPVELAALCEEFEALSALRDLSVQAELARPPAATFKQDLAA
IYEKNLCTDCDLVFRGTIFSVHRAILSARCVYFRDLLAGCPGFGARICLELPTSPIDVQLFSSLLRYLYTGDLCPHDPNI
DITLLRQLGKDFGTPNPLEHDLRYLLETGDYADAALVFTADGSNDYLRQDSGTSEYGFRPKIELPCHKAILSARSPFFRN
LIARRTRNMDEYVERSLHVPTRIVLDETVIPKRYARVLLQAIYLDSVDLSLILRGVGSGTSAGSLGEVHALTNTGRVRPT
TLEEAMELYQIGRFLELDILAQGCEDLILEWLSIETLPTVLKWGCQPHGSAWVFRQACQYLREEFAAVSSSPVLHQLDKS
QLIHILHSNFLQASELEVLQAVLKWGEQELIRRMEDREPNLLSHTAHSVARKGVKKRDLSDIELREILSELLPLVRMDHV
LPPHCEVLCQAIRRGLVSTPPSHMIGDDRENLRINAWIRGGKNQGLYVRPRLFMPYFEEVKALLEDRMSSSHHQVELMRM
RRCRHPPDIPDTLYMVSHMNSKANSDLSTVENRSTDGNVDILVGAAVIPPPDNQTLLAMRKREHKLRQSPMCQRALLLPL
SSKSEIDRQIRLRVVREFNLPDEVSDLLESALQTNPGRNESHAEETSASTSQKDDSLLEDEDQSPPPSPAATCSVSRHTT
VASSFLSPMGSSVASCGTDAAHPCYSRNLTFPRHHSNGLIGVANAFGSSSGPTLQCSYSRLSSSVHQRNDLPALITEGDF
RFPHGNGLGLDMGAEGGACGLDTANSHLSEMMPDVAMATASLGQLHLTNNGAGCAGNIGGRAVSSNNDMSESLQLDLGDG
PSPHIIGSAVGSMTLRNIQHQLPTNNYHHFMQRSNSPFEILRQGQPSPTSHPQHSGTYNSGPPRFL
>Dmel_FBpp0073935
MSGATQEFCVRWNSHLGSIGAAFPQLLAGQRFVDVTLACEGQQVHCHRLVLAACSTYFEAILAEHPCKHPVIILPREIKL
WEIQALVDFMYKGEVNVTQAGLGQLLRCAEQLQIRGLYGSEAPINYKKLQQASLEAAKDPAATTARSFKHVDSAETDDAA
TNSTTSTTMTTSSAPPLPVNHQQPTVLKRQNPDARQQQQQQQQQVQQRPQINGSNAQQPSKATSTNVLGSHSNAPTEDD
SGDEVPNNWNAYGEQFDDCNISAQAVDNKMNVHLSLQQARMQMQLQQQQYLDSNVEDDHNYVAAHDENNDSSFATGVPCG
SGLSLSLDTDLDTSANTSGGLSHSSIDGYTSYKRVRRSEASLAQAAKCVSKGETFQTVSNMFNIPVSTIRFYMARKGILP
KRKRGRGASHAGNIIITTNSGKLSTVPASITHPPAHPHIHTNPLPHTQSQQMSMDSLHLKAGIANTSGSNSPATATATSM
SPSFDMATTGDIVPFHLHSDAAAFKLNDQKLHMI
>Dmel_FBpp0075260
MGDLPGSGSTAQPRDAAVTGTGGNSTAGGGSSVGSTAVDRPPSPARLSHTSEKHPKVTLTELNMLRRHRELCDVVLNVGG
RKIFAHRVILSACSSYFCAMFTGELEESRQTEVTIRDIDENAMELLIDFCYTAHIIVEESNVQTLLPAACLLQLVEIQDI
CCEFLKRQLDPTNCLGIRAFADTHSCRELLRIADKFTQHNFQEVMESEEFLLLPVGQLVDIICSDELNVRSEEQVFNAVM
SWLKYNVAERRQHLAQVLQHVRLPLLSPKFLVGTVGSDLLVRSDEACRDLVDEAKNYLLLPQERPLMQGPRTRPRKPTRR
GEVLFAVGGWCSGDAIASVERFDPQTNDWKMVAPMSKRRCGVGVAVLNDLLYAVGGHDGQSYLNSIERYDPQTNQWSCDV
APTTSCRTSVGVAVLDGFLYAVGGQDGVQCLNHVERYDPKENKWSKVAPMTTRRLGVAVAVLGGFLYAIGGSDGQCPLNT
VERYDPRHNKWVAVSPMSTRRKHLGCAVFNNYIYAVGGRDDCMELSSAERYNPLTNTWSPIVAMTSRRSGVGLAVVNGQL
YAVGGFDGSAYLKTIEVYDPETNQWRLCGCMNYRRLGGGVGVMRAPQTENYMWCENSFKQPNS
>Dmel_FBpp0075887
MSIWPIRTLPNSKMRSCMMELGRTNRHTDCTFIIEDESGGSQSFPCHKLLFSCASDVFDRMLYGDYIESTSGVVRLNDVQ
PDIFEKFRDYVYGYECDKLQKYDFDTLIRLCEFANKYLVQSLEEDCVKDLLIRKNTFDMGELLRLFQCAHRMNRKSLINQ
IAWELKCTFKSTLDHSGVYEFNCEVFKHYIEVIASKISEADRFRLLEMYLKYNGIEELESAGQVDSQDATEANTTTITTT
NEVENQESELPSTSCVPLKTECSFAVPNKKASFVSDLLALIDFGKLSPKEFYDGPGKSNFLSLAEKYEHMYQIAKNCVTA
KDELQLKMALTTEQKPPLPSESRRIVHMGGSLMRDEPTASSVTSHYLSRPIRRYRAWSAHSDV
>Dmel_FBpp0076347
MAAENYHLKWDSHLTYLNSSIATLYKNEKFADVVLYSSYNSSGIPSDIPTVGISAHKFILSASSQFFATMFETAPITNPN
GVLYVVLPPDLSHRAIQILVQYMYSGEATVSNDILNEVLRGGEILKIRGLCRTSSSNGGGSGSVVTTTHHPHSLAGHLHP
REASTMYMSNGTRTALPPPPPPPQSTMSAQLQGDLYASKPGGSTRFALDHHHLSSHHSHLNHHNPQQQFRGLGASVMPKD
SPVIIKSPKLAAHTGLLTVASSSKLGISVNKEVAIDPEDKCCFAAPGQLESQSHPPPPTTTTTTVGGGGGSVGGSGAPPS

QPISICTEVGCSSCPLTAGPGADQAETTLRRTEYEEQALRERNEVGLVFERRLRRESACERSRDYYEAPHFEHVVSSRLA
ATPPPPPQQAPPSHPPTLRHPHNFLTIKQEPTDWSNNPPNASSASNNNNLEELPLASPKQPLDFKMSAVKLEVNRDRGTP
SEESEEHTLRDYNNFKQLLVCEICQKSFEDTKMLVRHLGTHAGDPTGGTERNLLPSSASGSTTSASSNLALRSVKTYVPK
KRRRVSQQENNMDHDHVTLLCDLCSTSFDTPAEWVRHMNSQHTEIELAMFNSKKDGEQKGQQPPPPPATSSSSTVSNSQM
QPKYPSSTATRSTHSLMLHKMSNSDAVATTTSPGASTSPNG
>Dmel_FBpp0077004
MAATPAPAVTPAATATSAAAKIPVNPVSPEEETLLRGLNKLRNEEKLTDVTLIVEEHRFKAHRVVLAASSDYFCAMFADC
AMIESRKDEINLYGIKARAMGSIIDYIYTSMLELNYDNIEEILAAATHVQVREVIDRCTIFLGAKIEMENCLAIAGMADI
YGIMDLSERAYRYMCAHFEEFATTSDFKEMKVDQLRFILSSNYPIDVAEQDLVRLVCSFAIEQQLNESALGDLLRLIKWQ
HISYKSVDELRNMNCSLDKGKQLPLPTSYYNRIKQEHMGHLLNGVVSLKDQSLPQGPTNMRGMELCLLKIGGFEWNGLTN
VIMCFSPKKMNWYELTAIPHIDQCNFGTAVLNNKLFIVGGAYDVFLKEYIHPFGFCYCPLRNTWMTIAPIQQDRCRFSLN
AVGTQHLYAVGGILDDDNEEALRMISNVERYDIAKNVWTYMPSLQENRSQHAGVVVGDKLYISGGVHLANILASMWVFDT
KTEVWQELASMPTPCCDHVLVAVDNRIYACGGWHESLTEWRVLVEHIYAYDIETNTWSVETKIPAPKFYTGVTAMGRTIF
FVGGLDSTESIDRASAETMAYDLDTKEWWHEKDSWDSPNDVWESTCVAIYVPNCDA
>Dmel_FBpp0077033
MDIDWQNGLTELKDRGQYLLHSEKWADCRFLVGSSPTQRLIAGHKLLLAMASPVFERMFYGNLPDKTDPIVIPDVQPEAF
EAMLEYIYTDRITIGSFDKACELCYVAKKYMLPHVVTRCTHFLWADLSPKNACRAYEFAKLFDEPRLMQSSMDLIAANTR
EVLSDPSFLDIEVSTLMAILDQNRLNIDSELDLFNCLLKFASERGILNESGQEETASGGQVLTKESPDNAAGHVLVEEIK
MEPDVAAMVQHMHQDDEADSFETDAGMASTSSAAAAAAAAPTAASPPLDVASGSDDLVIIDSDASADAAANMINIMDAQR
TILDGAMLRQAVKKIRFLTMTPQQFAEGPARSKLLQQHEALSILIKISSPSLNDCHMPEGFCVSRSTRNFYESGHRHAQR
ELSSSYRTGAAPSGVCFPGPGPAVRSGGPSGGGMMMGIAPRFGSVGTGFGFAGEELVIRPPEPVGVPPDAAPAPAPLRCF
GEGYESGAPVPAPANDDDGGDRGAPAPAGGAAAGGAGSAAGNSHNDMVRAYCMRSLTRQFDFRNTSVTDAGVTFQVDTNI
WILGVQVPTRVLCGELMTSAGFTERYTEVLYAHIQDMHGSRITYTHCTARVRYDSLLDITFDRPVYIYRNQIYKIYVVFN
KTGWYPMYSCVPDANCNRVKFMFNVGNPTESVRDGLIYAIIFSTPQDHGRHLVD
>Dmel_FBpp0077547
MDPQQQFCLKWNSFSSNLAITFSNLFKSDLLADVILSCDGVVFKAHKLILAACSKKFADLFENTPTNGQCVIILEATTPD
NMAALLEFMYKGEVHVSQEALNSFLKSAESLQVKGLSTETGRLAAQQAQQHMGDLSPLDSPTGRRSVRNSLSGGSSSIVP
GGVGIGLGGGATGANSMSGMGIGNGLSLAGMAAGGGMAAAANAAASSLSTLAASANIVDRCGSAGANIISGSAAGIGGSH
SGGAGNGSGTVGIGGNGVGSGGGNNGPISLGSGAGAAHHLGGSTGILKQECDSLMHPGGSSSSSGMGYTHVPPIYRPINY
EPPRKRAIVRSPYSEQEQRGSVLRDGSKSSECPSPINKPPYHRPSSSASSTAPTEADTMHSERASPQSSRYENHSPSTTA
GNGNATSSLERIVKSERNNGSANEANDDDRELMDESTDNGAEDLRVKLENLKYSPPPPPNSNTSSTTPNTLLENLKADGT
LSSNLAASIAPADMLNVWNATKMNNKNSVNTADGKKLKCLYCDRLYGYETNLRAHIRQRHQGIRVPCPFCERTFTRNNTE
AAATAAASVVMNANKEAAKQRKRKTLKMALEKCMQRRDAMVAETTTGAGGDGAEAGGESGEAVDGAGSEEGAGSEGTEPL
TYEQQQQRMHQELTQQIRAAMAAEQAAEAMDSTMNTTVNTTTTTTTTTNTTGATSDGCSDAEASDGSDRPMEVDEDQPPE
PQPGSELVVVEPKIEVLSESEDEEDRLHMSEAEPDIQAEAIYDHMPNTPTSPPHIIPPNDTPTLTSTPKVNVEQ
>Dmel_FBpp0077843
MPKMDNEQPHQELVKCVLVGDTAVGKTRLICARACNKHVSLSQLLSTHVPTVWAIDQYRIYKDVLERSWEVVDGVNVSLR
LWDTFGDHDKDRRFAYGRSDVVLLCFSIASPISLRNCKMMWYPEIRRFCPDVPVILVGCKNDLRYMYRDENYLSYFGEKG
TFVRAALKSDLVMPDEARAVAKELGVAYYETSVFTYFGVNEVFENAIRSALIARRQQRFWMTNLKKVQKPLLQAPFRPPK
PPPPEVTVMVGNYRQDIYNMFLSQAYTDLVLVGAGGTKFAVHRFMLAAASSIFQRLLSTELTDMGGRSSSESSMVSSTFG
EATIADFNDDTEALIRYESRTQRMWEHLKRRSSYQALPLMESKRSNDLYRELHHPVLQSIRLVHVENHRGVNGLQTIVTL
SKLISPQALHQCLRFIYTGTIDKDCDNIEEIREAADLLELPQLTQLLSRPQTVMENSSDEPNPHICLRIKESMERHCIGD
GCFSDVTFELDDGLMKAHRAVLVGRCDVMRAMLLGDFREAHSNVIVFPGVTIYTFHKLLCYLYTDQIPPISAVKCLNLLE
LANRLCLPRLLNLVECRVIEDLTLISQNETNETVDHCLKLLEPVKLHNAHQLAEWCMSYLCVNYNLICKFSLKGLKALHQ
DNQEYLREHRWPPVWYLKDYDYYQRCLNELNKELKLKTSRRESPSDDEGCLCFTGVFSCWLLGKSKRSADVSGTTDNSNA
DNQIFNSAGNSLNHIDLEADMDLNL
>Dmel_FBpp0078081
MPTRHNWDSERVGVAHLAGALSPLCITDANHEILRPKPRRPTGNLPPNFVQHQAPSQVTGLPPVPPPQMQSPHQVQSQLP
PSQTPLSSQKGLAVAMATPAPRVFKELAQQPNKGMPHKMVASSSSCSGGQANSSCKMPSPGTASKALIPQRAVAGPARLT
VQSTPVKSQPPQLSGSLSQSQVSPNRPILGIPTTAANPTPPRIHQHDQFRALQRVQECHSSSDENRSSGHASMSDTGGHS
SSPAGGMTLGPLPEDRLAAGVTNRSTRSRRHRGIMPGGKAPWSGTGLEDIKLAMLTLRSQTSSSTYSSLSAGSESSEPAR
RLGRYSSLETVVTSTSADEFVWVDSHNRLVELQHPPWSQQCILKVLRNGRCQQQAEHLAVEAVSRLGYLLQRALVRIARE
IQRFSAGVGLCSKQEVVGALRVVLSSSLADSCTKACLRSAAMFAVPGESALKQSKSARAGLQLSVGRFYRWMTDARLGKF
IHEYAAVYLCAGIENLLEEIALQCNGTTAAALDQSIASSGDVWGLLQPFAHLNAGRIASGALALPRWANSLDQVIASSGD
FRGHLATCVGSIRELKEKALRTQQEFQLAAALSGSALAALFYFMRCSQLEHTELLAASGCHAGQAQSAAPGGGTSSAGHH
VQELCYERAYVVLPPLAEWLRVAAAHAEHRNAMMIDKDDVMQAARLLLPGVDCPIRPVAHDEELPTKKTHFNTAPTVSST
GTGTGSSSCSSPVVSGIGIGIGIGEDTSELGRRATIGVAFKLLLTGRAELLAQAAQLLPPTTRYDTQNSAGLTALMIASIRN
DEVALHALLDAGCDPNVEVPPAGSAGYPAIQPDTQHWTALSFAASRANYVALRILLERGGKVEGGARSSEEKCTLTPLQL
AAGTGNLEIVALLLAHGANAFLSTQQKDSLCFAGSAQKGCFCAISVAAAHGHRSCLRKLLTHPVSPGTRDVLSLEEMLAE
GDVGGVRGSGGPTGAGGPNSASASGNNEDILPLLNKTQIKRLQEAMYHSAENNHLDITIELRKLGVPWTLHCWMHALSAA
HDLRLDAVIDQLLQDFLQVCPDDYSAQFVSECLPLLFNIFRNKNEGTTLLLADIFATCFGWETLPPVKEQPPMQPVQGSR
IDPKFVNNPELSDVTFRVEGKIFYGHKIVLVTASPRFQSMLSSKLSEGSSTPTVQINDIRYHIFQLVMQFLYCGGCSSLD

VAHGDVLELMAAASFFQLEGLLRYTEARCSEMVDVDNVVAMYIHAKVYNANRLLEFCQCFLLQNMVALLTYDDSVKRLLF
AKKIPNHDVLAGLLQTLQNRLKARKPNSGGGLVNSYRSPPVTPVKK
>Dmel_FBpp0079181
MSLPQLIEDLQRLSEDQDSADVVFICGRDERIYAHRLMLMARCKSFKTAKRGEVCRIPGCTVSPAAPGASTPTTIRLPHV
NPELFRQFILYVYTAKLVLQDSQVFQMMILAQDIGVVELRTACEDHVISTLSVDNACTFLTAVMDIHEKAGAKCAASFME
RCIIYIGENANECVKTNAFLTLTKDAIIKIISSDYFCLEEEEVWRCVLSWAKYQAGVTQPTAHWTEEERARVCQHLSGVM
GHVRLLLIDSQVFAEEVEPTGAVPMELSLERYRYAALHANKMMDNDKRLQPRLAVAVNMFPGSQILKNDNLALQTVLNNW
VGVGKQSWRLVFRASTHGYDSGAFHRYCDGVAPCMVIGLGSHGEISGGYTDVAWAKTSRKGGYVQSERAFLFALNPANGE
QPVKFDIVKKPYAICYHPDCGPIFGAGADLLISSNCNTNMDSYSNLPHSYDGTNAAHLTLFGDYNFTITDYEVYTLSSPA
GSSNSGANHSNNQNLNHSTNGQAPGVPTKAKYDRY
>Dmel_FBpp0079793
MTESTQLQTAENNNAGVVKMEPPPPATSSVSVSAAAAAHALSSLSSLTMAATGSALSPATPPPSLNLSHQQQQHQQHYAL
KWNDFQSSILSSFRHLRDEEDFVDVTLACDERSFTAHKVVLSACSPYFRRLLKANPCEHPIVILRDVRCDDVENLLSFMY
NGEVNVSHEQLPDFLKTAHLLQIRGLADVNGGYPYSKALSAALSHNSSNNNNNNSSSNNSLSNNNNNNNNNAESSNHNKI
SSYLSPNQTSAACNNSSNSNSNNHSSSHNNSSSNNISGSLNSSLNSPFSAPQIPPPVTASSAAAAAAAAASLTAAVAAAA
AATAASAGSSSSAASGQTSGTPAIQELKASSAASPVRNPNPNPSKASSSNHWDMGEMEGSRKSHLTPPPQKRIKSADLFR
AQHGISPERLLLDREFPVAGQHPLTRNRSGRDTSKDRERNLELRESLLGQALENSNGQQANPKHELGQSAGEDSNSSDTE
PSDRGDGQHDGTLDGIDNQRSHSFPNAFLGLQGIPGLLPGPSGINSDFVSRRSLEMRVRATDPRPCPKCGKIYRSAHTLR
THLEDKHTVCPGYRCVLCGTVAKSRNSLHSHMSRQHRGISTKDLPVLPMPSAFDPELASRLLAKAGVKISPAELRARASP
TGGSGSSGGGGGGGSSQAKLDLSNASGGPMDDAEDSDDDPEDLTTGNGLYGMGGSSSDLSRYHESLLSNFGHARMRNEAA
AVAATAAALGQPKDLGVQLPNSNAPGQSLLDTYLQFITENTFGMGMSQEQAAAAALRAKMAQLNAMGHSLDNLPPGLLPG
QFDLSKLAAGNPAFGQSGPGLTIEPIMRHEQAAGNLSPNRPLALNSGGRMMGHDEMAENDGDMRREGSEPMDLGLDNNQS
GSNHEVANSDAEENYSEDEGVHNT
>Dmel_FBpp0079889
MLHSQTMQSIPTLHQPLHSKVSNYLNNQRRTGQFCDLLLELDSDDSLSLSVHFCVLAAQSQFINSNQKQQQFSIHNPLKI
TIRNFSCTQCLHTIVDFFYEDLVSVSKEHELHFRELAQILAVTELLNLYQLQPLGEAKEATEIPAPGEAQPNPDPEKKAE
AVFENRQSYFKLKNPRAVKSSSKVNYCIGCDFKCYQVQKMIEHMGSCEPSHLICSLCEVGFLDWREYDTHLRRHSGDLRK
PFFCLQCGIRFNTRAALLVHQPKHSTETPHICPHCGKGFKWKQGLSNHILVHNPEKQMLCDVCGYSTTHMKALKSHKLLH
TGEFFACTVSGCKHRANRKENLKLHIETHKQGRDFICEVCGCKFSQSKNLKRHALKHTENGPNRYKCQLCGFSSHRSDKM
KEHVQRVHTEKPVQLELSETVDSSFPDDFELPVIETSPKKKPKSVKSKTIRNVNPDKRLKTLLPKETVE
>Dmel_FBpp0080063
MPPLKTYQIHEYNGNTSPNANANQNANQNPTGGGGDGDFFGQNLGSGATAASHRTNLPSYCNAQYPFKVLSNLNQLREQS
RFCDVEIIAGMATLSAHRAVLSAASAYFEAMFRPELGLNEVKQKSVVLHTIDGDILHILLDFIYTGRCEITQSNVQELLA
AADMLQLNEVVDGCCEFLCRELHASNALGILRFAEAHNCESLAKSALNFVHANFPAVTLEDEFLETPQTLLSQLLNSELL
RVDSESQVFQAALRWIKHDVTQRRCYVFDVLSHVRMALVPVKVIDKALKDDCRDMSVKIALRSICRDIASKRGQLVPLRV
CPRQLAKKNIYIIGGSHRDTPRTWNSADCIFETVAKFDIFRREWTETAPMEVGRILPGVSALNGKIYVVGGERGSQILAN
GEVYDPQNDVWQPIAPMIVPRCEFGLCTMGGNLFAVGGWIGDDIGGSMECYDPEQDLWKLMGSMPQPRFSMGVVSFEGLI
YIVGGCTTTTRHLPDLISFNPVTKEWNELARMQTARCQMGVAVLDRYLYVVGGSSISQDILSSVERYSFDEDKWTTVCAL
NVPRAIPAVVAADGLLYVAGGDQPCEVNFYRAQVTINAVECYDPLSDTWKNCPDLPVSRSEAGAVVV
>Dmel_FBpp0080596
MIALSALLTKYTIGIMSNLSNGNSNNNNQQQQQQQQGQNPQQPAQNEGGAGAEFVAPPPGLGAAVGVAAMQQRNRLLQQQ
QQQHHHHQNPAAEGSGLERGSCLLRYASQNSLDESSQKHVQRPNGKERGTVGQYSNEQHTARSFDAMNEMRKQKQLCDVI
LVADDVEIHAHRMVLASCSPYFYAMFTSFEESRQARITLQSVDARALELLIDYVYTATVEVNEDNVQVLLTAANLLQLTD
VRDACCDFLQTQLDASNCLGIREFADIHACVELLNYAETYIEQHFNEVIQFDEFLNLSHEQVISLIGNDRISVPNEERVY
ECVIAWLRYDVPMREQFTSLLMEHVRLPFLSKEYITQRVDKEILLEGNIVCKNLIIEALTYHLLPTETKSARTVPRKPVG
MPKILLVIGGQAPKAIRSVEWYDLREEKWYQAAEMPNRRCRSGLSVLGDKVYAVGGFNGSLRVRTVDVYDPATDQWANCS
NMEARRSTLGVAVLNGCIYAVGGFDGTTGLSSAEMYDPKTDIWRFIASMSTRRSSVGVGVVHGLLYAVGGYDGFTRQCLS
SVERYNPDTDTWVNVAEMSSRRSGAGVGVLNNILYAVGGHDGPMVRRSVEAYDCETNSWRSVADMSYCRRNAGVVAHDGL
LYVVGGDDGTSNLASVEVYCPDSDSWRILPALMTIGRSYAGVCMIDKPMMEEQGALARQAASLAIALLDDENSQAEGTME
GAIGGAIYGNLAPAGGAAAAAAPAAPAQAPQPNHPHYENIYAPIGQPSNNNNNSGSNSNQAAAIANANAPANAEEIQQQQ
QPAPTEPNANNNPQPPTAAAPAPSQQQQQQQAQPQQPQRILPMNNYRNDLYDRSAAGGVCSAYDVPRAVRSGLGYRRNFR
IDMQNGNRCGSGLRCTPLYTNSRSNCQRQRSFDDTESTDGYNLPYAGAGTMRYENIYEQIRDEPLYRTSAANRVPLYTRL
DVLGHGIGRIERHLSSSCGNIDHYNLGGHYAVLGHSHFGTVGHIRLNANGSGVAAPGVAGTGTCNVPNCQGYMTAAGSTV
PVEYANVKVPVKNSASSFFSCLHGENSQSMTNIYKTSGTAAAMAAHNSPLTPNVSMERASRSASAGAAGSAAAAVEEHSA
ADSIPSSSNINANRTTGAIPKVKTANKPAKESGGSSTAASPILDKTTSTGSGKSVTLAKKTSTAAARSSSSGDTNGNGTL
NRISKSSLQWLLVNKWLPLWIGQGPDCKVIDFNFMFSRDCVSCDTASVASQMSNPYGTPRLSGLPQDMVRFQSSCAGACA
AAGAASTIRRDANASARPLHSTLSRLRNGEKRNPNRVAGNYQYEDPSYENVHVQWQNGFEFGRSRDYDPNSTYHQQRPLL
QRARSESPTFSNQQRRLQRQGAQAQQQSQQPKPPGSPDPYKNYKLNADNNTFKPKPIAADELEGAVGGAVAEIALPEVDI
EVVDPVSLSDNETETTSSQNNLPSTTNSNNLNEHND
>Dmel_FBpp0082029
MDLSDNSQVEDQYFVYKHDKLFGESFPLFKEIRRMGKLCDVTLKVEDQTFSAHRVVLAATIPYFYAMFTNNMAESRIKEI
TMKESAIEPSALESLINYVYSGQVRIDNQNVQNLMVGASFLQLSNVRDACASFLISRFHPHNVLGIRTFADSMICRQLID

AADKYIDQNFAKVSQSEEFLALDCEQLLELMRRDELNVRTEEVIFEACMKWVKYAEDKRSELFPQVLAAVRLPLLSPQFL
ADRVAREELIRSSHQCRDLLDEAKDFHLMPERRGLLQSFRTRQRSGEFFTGQIYAVGGLASTGESVSTVEIYDPLTKKWK
MGEQMSMMRSRVGVAVLNGKLYAFGGFNGTERLSTVEVYDPRKNKWSQGCAMLCKRSAVGVAALDDCIYVCGGYDGVTSL
NTVEVYYPKSNTWKTVAQMMKYRSAGGVTQLNGYVYALGGHDGLSIFDSVERYDQAEDVWVKMSPMLNRRCRLGVATLNG
KIYVCGGYCGNSFLRSVECYDPQTDTWKLVTPMNCKRSRVALAANMGKLWAIGGYDGESNLSTVEVYDPETDKWTFMPPM
CAHSGGVGAGVIRIE
>Dmel_FBpp0082580
MGEVKSVKVDNWGVFFLQKLQNFFNKTDYCDLTLQFRDNSQLKVHRLVLSACTDYFNVLEQTCEIVDDALIMPNEFQADV
VVPIVNFMYTGTLEFELKMYGKLLRTAKEMNMTVLLKLLEAHRRTMENVNRQQRPPSPKGIRRRTVGQPSSGLPQQRVLG
PSPQSRNVATPIAQRANTQRGSTGNTMSRTSGGSNRSPYGDSSNVKQEPTSPFEQLRKGYNNNKRPAQTSLLSPPSKKPS
LEEVKEFAEQQRMRKQIAAEYGDNDPEYDGGMLYDDVHAGDDDDDDMPPQPSTSKQQSPQGTQTQLEHGSTTIILKQDSP
SQTPTIIVKDSSNAKLNHTKIIAEVLRQYPHIVKGHKNIKLKIMPNTPAAPTEKSAPATVKPPANQSSATTSPHKKLHVS
FKADKSTPLITAQQKAASSQQKSGTSQTTGNQGTGANPPANTAAAQKRRIDSKTMHALIAQGAENTTGPWLCLRCGVNGR
PISIPSYRGFRRHLINTHKETIDPALCEHCGWRSVNNRELHFHMYMEHQTKSLLYTFAECALCNQSYRTKGELEAHINEV
HTDDNKQQCIYCNKVFEQELQLYRHMKSYHKEQALEDGIIDETDEEFLGSQDEEEEAEGDEEQEPEQTGKVRILSDISLP
ATSAITVQQAQQEQLQEEDVEQVQQEVKFVGADGNEVELTDEQRKEILSQLNQQQAGATAGGVVMVLSEPEAEHVKQETD
EKSLAGTEEEYDDSQIYSELGAADSVESAKKNIADESKESIDNLEWAENLIAESEEQSNKEPKSDKPRDDISEKLKELTG
DWTEDENDDDVDDKPATAELASELANKDPEPTVHEEEDDIDLALQSLHKGPEEATEEKASEESVTSADDAVDAVPNINSQ
PEKMDVDSEAADEKASKAEVQIKKEAELENDQEEFIKEDSPIPHSDSVAELREAVTASEGEDDVHLEADNIRKELLDELI
AEAEKPDQEKDIVQSEENATTEALDRSVTDEDDLVPPTQVSTEQMEIDEPAAEKAAENNEDTRTADEKEAVEDKPNQTQD
VTTAEKPTLESAKAGDEATSGEAASVDKVKSLISEWGDDDEDEDENGVSAAAKEEL
>Dmel_FBpp0082864
MQTQMHSPMQYETHISDMPNHKGYPCRPEEAEMSNTSESCSISCGGRFGSAHSTLDDHDATDDDMTFCMSNYAKEALKMM
YMMRSHGMLTDVVLEVKKELFPAHKVVLSAASPYFKAMFTGGLKESEMSRVQLQGVCPTAMSRILYFMYTGQIRVTEVTV
CQLLPAATMFQVPNVIDACCAFLERQLDPTNAIGIAHFAEQHGCVELQKKANVFIERNFTQVCQEEEFLQLSAYQLIALI
RRDELNVQEEREVYNAVLKWVKYDEDNRHCKMEHILGAVRCQFLTPNFLKEQMKNCDVLRKVPACREYLAKIFKDLTLHK
CPGVKERTPNTTRMIFVAGGFFRHSLDILEAYNVDDMTWTTLANLRIPRSGLGAAFLKGKFYAVGGRNNNIGSSYDSDWV
DRYSAVTETWRPCAPMSVPRHRVGVAVMDELMYAVGGSAGMEYHNTVEYYDPDLDRWTLVQPMHAKRLGVGVVVVNRLLY
AIGGFDGNERLASVECYHPENNEWSFLPPLQTGRSGAGVAAINQYIYVVGGFDGTRQLATVERYDTENDTWDMVAPIQIA
RSALSLTPLDEKLYAIGGFDGNNFLSIVEVYDPRTNTWTTGTPLKSGRSGHASAVIYQPACSTTFMDYEDSDAPHPDGSN
NGERNAHTPQNTYGSGSPHYPGTASNMQFHTSYGMSGCNNCDSEMDIKPYIPAEHHSFQIPAIRSEELVNPNCPWSRMQE
RFRRTPPASFTDDERNANDMRHKLKKAQKECLFSTAAKVFHHHIEGRLRKLTSAAT
>Dmel_FBpp0082876
MASSSNNSSSTANNHGGHNPLNRLSLKSAGKRNQESMSHSQPNGGWINVETLNNGNGLLHSPPHNHQQQQQQQQRGGGGA
SPAGGGGGPSAAADHNIQITQPISAPSSPLASPGALNSSGSSSGGGAGSAGGISGSPTAFCLPSSSAAAAVAAISATPTS
GSGSYVCSAGTNWHFAAVAASNAIDTADPNWQASKATVLERNAAMFNNELMSDVKFIVGGEFDIDPIQTIPAHKYILATG
SSVFYAMFYGGLAENKQEIKVPDVEPTAFLTLLRYLYCDEIKLEPEHILATLYAAKKYIVPHLARACVNYLEVKLTAKNA
CLLLSQSRLFEEPELMQRCWEVIDAQAEMAVKSEDFVDIDLKTFESILSRETLNCKEIHLFEAALNWAMNACEKMSIDDT
PQNKRRLLGQALHLIRIPTMSLEEFANGVAQTGILSSQETIDMFLHFTAKMKPSLGFPTRSRAGLKTQVCHRFQSCAYRS
NQWRYRGRCDSIQFSVDRRIFIVGFGLYGSSTGAANYNVKIELKRLGRTLAENDTKFFSDGSSNTFHVFFENPIQIEPEC
YYTASVILDGNELSFFGQEGMSEVLMGNVTFQFQCSSESTNGTGVQGGQIPELIFYGPTTVTAMSSPTNSLCPTPIGGSS
SNAGATSTAAAGTAVPNSSNGSGNNANSSGEDGDGST
>Dmel_FBpp0083463
MADDEQFSLCWNNFNTNLSAGFHESLCRGDLVDVSLAAEGQIVKAHRLVLSVCSPFFRKMFTQMPSNTHAIVFLNNVSHS
ALKDLIQFMYCGEVNVKQDALPAFISTAESLQIKGLTDNDPAPQPPQESSPPPAAPHVQQQQIPAQRVQRQQPRASARYK
IETVDDGLGDEKQSTTQIVIQTTAAPQATIVQQQQPQQAAQQIQSQQLQTGTTTTATLVSTNKRSAQRSSLTPASSSAGV
KRSKTSTSANVMDPLDSTTETGATTTAQLVPQQITVQTSVVSAAEAKLHQQSPQQVRQEEAEYIDLPMELPTKSEPDYSE
DHGDAAGDAEGTYVEDDTYGDMRYDDSYFTENEDAGNQTAANTSGGGVTATTSKAVVKQQSQNYSESSFVDTSGDQGNTE
AQVKIKMEPSPTPGHSSDAAVAALAVTYLSDEESFRKPFTLPKLLDGKFYKNIQPNQKTPGAIQATCTTCHGLISGTTKS
TGNFLSHIKRRHKELLPLCQLYCQAKANGTVPAVKSSPPNPNHVLTSATPTPAMEMMTQVAQMPPTAAYATGPTHLGMPV
TVPVPVSMSLAMPISLPHVQTPQMMALMQQHQAHGAVFISKDY
>Dmel_FBpp0084527
MNCKLRRLDLLRNGQKSDCELHVSQPDKLKEGKRCPCIFRCHQVMLISASAEFERLIRDPEFQKNKRVISVDDASPTAYE
ALLLYIYTYEVCNAVTIDMCGDLMLLAERYEMLDFIDCYIDKLAHQEWPMEVVLQVFHLASEHHHPALMDLVAKKILPVA
TQVLMDNSFLKLSVKELKALMIILKKDGDLSDHELLTSLKNYQDVNNLRYEDMEQFQQLVEVTQLFGHVLFEVDGTIVVP
EEEKPSPEE
>Dmel_FBpp0084547
MSDSEKEAAGKAHLGSNNRWSDLEEEEGEANDQGGIQNLRPELVVPRTTAASDVEENLPLGSPSDGSKRATSRNRRLEY
FRSGADADCQVHVLSNCPQSDEPAVSVCYKKFGCHRIFLATASDKLEQDVYQNKDWNGVLQINGVSPESVEIFLEFIYTF
EVTSPLVQLKLVGDMFILSCAYNMPELLRSFAEKLKEQELPLDGIFPAFDLAFRHNIIDVERVCIEKIIEEGASLIHEPS
LMKLPVYALNYAIQHWLVADAVPPDELITILKQYQEINEITFANTQKFPHFTKIIKYFPNVLLDAEGFINQN
>Dmel_FBpp0084903

MFWTPEQVESMLEVLKTSTDRTVIYKCLVKLRTDLVKDKNGIVLFRTAGGVHIMVRLITKLNEKIMEVVLSILGNCCTDE
QSCIEAVECKVIHPLVTILKTIPNPLIQCRACRMLGNIAKSKKASQCLTVHYAAIAPAICHIIESTSAVQTRIMAFRVCR
FLLASSQFLKYFLMANGFQQLMRIFLAVMKNEEASKEPVPDIEMSTLIGLKRNQHREKYFEEVARNLEGVRSDIFDHQML
KNSTRNCDYALPKENEAAVELALEILKCLVLISAQQIGLKVWESISWNTSLASIVCFVKEDGDQRASALKILSNFCKDPF
AFYMLSTADAIVAACEMLMAVNMAKPLSESESRHCINIILTLSTDACSRSKIRRCGALRKLVAMIRDSNSQSERSSLFHI
LYNFQYDNLSMELMLYEGLMPMLVRELNDYLTSDDEHHKRRRDERLQGGKKRKLTDPTTPETLSKFSKTHETLGSDFESP
SSSPRSYRTTSPCSSRSMSPVCTDLLTNDDDDNYSPACSDDDEDEDDSTNNSNLDTRERRIAANRSQPSNVLDILKLIEE
GSEPIDDPLSDKEDLEELSSDVPPKLAPFRNTTCNTIDIIENLIYRITLMVNKRVELGKPETLDTLIRAIKLFGSNNNFP
NALTNILLESQFFAQIIKHGVVHQLYQFTKLKEIRKDGFAFLETLTNVGESNYGKEELVRLLRCDDVISQQRAAISVAYI
VKSHRLLYQFLYDGNALNMLFDMMLRKKTEDHYADEAADAVTSMARYTLGIVMPEAEPAESTAGICNQIANDTAPLHANC
DMRFLVGHGADSTSIGFNKQLLCDTSEVFNKMLNSDFREGHQGEIQLPDYTASGLRYFLHLIVCQERHLTEPDYPALLQA
YEMARVYIMPDMEAVLQQRLIQYLDSHNCLRLLEWALKNYHADLTETAISFYLCSALPAQEKLQLFRAAEDSTYASEWFQ
LLNDAVFERCRGYVF
>Dmel_FBpp0085186
MKMASQRFCLRWNNHQSNLLSVFDQLLHAETFTDVTLAVEGQHLKAHKMVLSACSPYFNTLFVSHPEKHPIVILKDVPYS
DMKSLLDFMYRGEVSVDQERLTAFLRVAESLRIKGLTEVNDDKPSPAAAAAGAGATGSESTATTPQLQRIQPYLVPQRNR
SQAGGLLASAANAGNTPTLPVQPSLLSSALMPKRKRGRPRKLSGSSNGTGNDYDDFDRENMMNDSSDLGNGKMCNESYSG
NDDGSDDNQPNAGHTDDLNESRDSLPSKRSKNSKDHRVVSHHEDNSTSVTPTKATPELSQRLFGSSSTTISATAPGGSST
GPSETISLLEISDERESAPVHLPTILGLKIRAINTTTPAQQGSPQTPTKSKPKIRQATGSNNSNSLLKQQLRGGAKDPEV
PPATRITGAVTPNAALNAEEQSKEMPKKNQDEVNACIGLHSLANAAEQQAAQVASTGNLHHQLLLHMAANNSMLNTTDYY
QQQQQESPSSAGQFMDDDLELLSLNDQQDKSDEPDHEMVTLADENAGLPGYQGNEAEATPAQEDSPAAETATAPPPAPRS
GKKGAKRPIQRRRVRRKAQSTLDDQAEHLTEMSVRGLDLFRYASVVEGVYRCTECAKENMQKTFKNKYSFQRHAFLYHEG
KHRKVFPCPVCSKEFSRPDKMKNHLKMTHENFTPPKDIGAFSPLKYLISAAAAGDMHATIYQQQQDHYHRQLAEQLEQQN
ASFDSRDSSLILPDVKMEHAEDQDAEQEAELSDGGYDASNPAAAAAAMLSLQQDVIIKDEIQISPSPSPTPPASCAVAEG
KSLALASTAQTAT
>Dmel_FBpp0085700
MGGPTAPVASSGEVGQTYCLRWNNHQTNLVQILHALHEVGSYVDCSLVVDDEQFQAHRVVLAANSPYFQHILKDVPQDHC
SIILPGVKGFEIAALLQYMYTGETTVTKSQEPEILRTAKELQVKGLYDNLMKFNHASVTPTSSSGAGGAKPQNGSASNHS
SSVISTSTHISPSAAISSSCSPPPPPQFGYQPGYSHYPQQQPMSASQIPAGEAPLTPTQATPHSAASGAGEAGGQWPLTP
SAAAAMLNSVYESAADMNPLKRKKLSAISSMLLSGNRDTPILRNVLAQANPADSSQPGPMNANGEKTPTHPHQNTQLPAG
LGVSGGERNHSFNGSDYGGDKEPLSPYTDRSFEEETGQSGGKKPEWKRYKQYTRADMMCAIQAVREGMSALQASRKYGLP
SRTLYDKVRKLNITTGRGTHRTPKRSPPGAESSQGFSYSAAAAAAAHNYGHGHGHHSEVKRDQKVDHVPAHHGMPPTIPP
SAAALLDHAFLQQALENRGGDMAGREALHAMALAAAAHAAANRLSSSPAEMDQRSNGHGMRSPSPQGRHYPHEQEAMDLE
MEKHEQNIIKRERDQDEREDADDEEDHEQEHVEDLSLARKERPPSPYSPQPTEAGAGVIMHASSASANGKDHVDYPSSPQ
FVPIGLKRELMEGEDAQARAD
>Dmel_FBpp0085813
MFLPVKSNHSGEASGNNEDVTTTSNNSTQTPSDLSPPGPNEEVLPQAHHQNPGHCIASFAAINQMRNNAQLCDVRLEVGG
DTINAHRVVLASVSPYFYAMFNDDMLERTQGLVRLHDVDSSALRQLIDYTYTGEITITEQNVQVLLPASGLLQMHSVRDA
CCKFLLRQLHPSNCLGIRSFADAHSCKELHTRSHKYALQNFQQVVGTEEFLLLPFEEVRELISNSQLNISSEERVFAAVI
NWVKHDLPTRRLHIAELMSNVRLPLVSRDFLMSCVETETLMRDDSECKELLLEAMKYHLLPEQRSIMGSQRTQERRPEGM
KPYVFAVGGGSLFAIHNECEVYNPRSNSWSPVAPMLWRRSRSGVTSLHKQLYVVGGYDGVSDLATAESYNPLTNKWSNIT
PMGTKRSCLGICSYDALIYVCGGYDGASCLSSMERYDPLTGIWSSCPAMSTRRRYCRLAVLENCIYSLGGFDSTNYQSSV
ERFDPRVGRWQPVPSMSARRSSCGVASTDGHLYCIGGNDGTMCMSSGERFSLRRNSWEPIAAMHSRRSTHEVVEVEGALF
ALGGNDGSSSLNSVERYDTRLNKWSVVNAMVARRSSVGAAVLECFHLERGLVQTTNL
>Dmel_FBpp0085955
MMSSDQQFFLKWNDFQTNMVTSFRHLRDEKSFTDVTLACEGQTCKAHKMVLSACSPYFKALLEENPSKHPIIILKDVSYI
HLQAILEFMYAGEVNVSQEQLPAFLKTADRLKVKGLAETPSSIKREG
>Dmel_FBpp0086255
MPEALILPGMADAEPDLSTFENKTGLAEDMKFLASMPELCDVTFLVGDTREPVCAVKAVLASRSRVFAKMLYAAPSPQRK
RETSTKENKLRLFLKRSSEPLLNLQNAAQQRTGYTQQLAPIPEPSGQQHQTLIIEEFEPDVFRQLIEYIHTGCVTLQPRT
LLGVMNAADYYGLEELRRACAGFVQCCINVDTVCALLASAERYIQYKCTKTLVQKVLEFVDEHGTEVLNLGSFTLLPQHV
VRLILAREELRADEFTKFQAALMWSKKYYDNNPNIDIKEILGTFCEYIQFHKIPANVLMREIHPLNLVPYAIIMNALAYQ
ADPESIDPGKLSPNSSRQHHRHRHHHQSLPKIRKAKSQSFRTRRSPSERRSPNNAPNLTLNTSLTSGNGEKKRSPLTPKS
PVMPVPESKSPGSSSQKTPTSLSRQGTLRASNRRKNSGQLSISLGTQGRRSPVGLNDRSPQGRRSPLFPSSGLRSPNDPM
TSPTVRSPTGEPRRSSPTFSVHTQERRSPLGAVAPTDFGCQFGVPGTRRSPTSTVHVQDMATEPEEAGFVGLKRPSISLF
TPIYFASEKRSPMGPIPPITVSNPAETYKSAEREREAAEAAAREKEKEKEKEAAQPQEKKSVMREILAFVRKPSKHLSSR
TNRFANAFTRAESGSSGGPLIRQSTFSASPAASSTAAKSAVQKQMSEVGFEPKISQKFTHYAKMSLRLRRSTKRDDEEKE
KQKQSSASGSKRPSADLSQTNADQQVGGSSDELPFELANVHFEKVGESYIKHERLRELQEPEVVKEEDTEKDAEAEVEVE
QTDAAMAQFVEEVTNSLKVVALNGEGGTAAVHHFTRRSESREPIEPRISEERESDSNDLMIPDDMRAELVEILKAYPPEP
VYVNLQALRRETEDADLAIAQAEAAALAAAEPAKKPLQCPTIEFEPPSRRSSFDPPRSPFLEQLRSPGVDTETDLINLQR
LDSGGDSFELVESKWSKSSRGESSFDCPYSSRDTSFDVSISRYQSTSYEDQTSSFEIVDTDEKGQGRRAVDLRKSSIELV
DAETFQRSGSSCGGRKSSLETHFDYTPTEGGGRSPSLPFPAMSKKQRTENFRQLHVSQFSAFSRARSPLSQQTSSNYSSR

DSYDSSGSYPHGYGYPPEPQRSPYGDPSRKHFPLTVRQKGEEEREVRTFLCTDQRCASIFEPRPSSVLTQQLSTGSMSTP
SGYTNGTPRIPGAAPVGPVPLPHPSAPLSSCGFSSGSEFEPPSPRRAASASPKHTFTFRIVMKKVDSSPEALCPERHRSR
IIDRYRRRDSRRKRIHDAGKSF
>Dmel_FBpp0086322
MSAARTQEYDCTAKCRLRQHGNAITAALTKRSIDNQKLASFIFKTCGNFANILDDLGRSAVHMSASTARYEILEWLLNHG
AYINGQDYESGSSPLHRALYYGSIDCAVLLLRYGASLELLDEDTRCPLQAICRKCDDDDLATDSQNDVLVWGSNKNYNLG
IGNEQNTNTPQAVDFFRKSNLWLEQVALGAYHSLFCDKKGHLYAVGHGKGGRLGIGLENSLPAPKRVKVSSKLSGDSIQC
ISVSRQHSLVLTHQSLVFACGLNTDHQLGVRDAAEQLTQFKEVVALRDKGASDLVRVIACDQHSIAYGSRCVYVWGANQG
QFGINSNTPSITVPTLIKLPAKTTIRFVEANNAATVIYNEEKIITLCYADKTRYIKTPNYEDLKSISVMGGNLKNSTKGS
AAALKLLMLTETNVVYLWYENTQQFYRCNFSPIRLHQIKKILYKCNQVMVLSEDGCVYRGKCNQIALPSSALQEKSRGSL
DNWQDNDQNKTEISREHVIRIELQRVPNIDRATYIFCDEGFSSFAVLQESHTKYFRKPSLPRREHSFKKLLHETSDCDAV
HDVVFHVDGEKFAAHKFIIYSRAPGLRDLIRCYLDKDIYLNFDHLTGKMFELILNHIYSSYWPTEDDIDCIQQSLGPANP
QQRTRTCEMFLPHLEKFQLVELTKYVQSYVRDHQFPLPNARKLFNRLYRSDHPELYDVRIVCKDGKVLGAHKCMLVARLE
YFEMMFMHLWAERSSVTMEGVPAEYMEPVLDYLYSLDNEAFCKQGYLETFLYNMITICDQYFIESLQNVCESLILDKISI
RKCGEMLEFAAMYNCKILQKGCMDFICQNLSRVLCYRSIEQCDGETLKCLNDHYRKMFKRVFDYRQITPFSEAIEDELLL
SFVDGCDVDLNYRMDAESKLKQAAKHKQKDLRKQDARHQYEQQAISSMMRSLSISESTQGTEVPSSPQDSARSEDKNWSR
VVDKKDQKRKLAETALKVNNTLKHEDPPTQELVPIERKPLKEQTPPPPSHETEPTTPLSKSYNLDFSSLTPQSQKLSQKQ
RKRLSSESKSWRTNNPPLVEQSSTPVAVPNAWGVTTTPSGSFNDSFTSPTTGSSTDPTSFANMMRGHATSSTTPTDQSQS
FSRILADEKRQRESYERMRNKSLVHTQIEERAIAELREFYNVDNIDDETITIARVSRPSDINFSIWLRQ
>Dmel_FBpp0087368
MAAVRGHQYFSLRWNNYQNTMTSVFQQLREDLSFVDVTLSCEHGSLKAHKVVLSACSTYFQKLLLENPCKHPTIILPADI
IFTDLKTIIDFVYRGEIDVTESELQGLLRTAEQLKIKGLCETAENADDLNDAATATITVSENIQQAVVGNIVNAAIVPGA
PSPSLEQQQQHHQQQQQQQAQEQHQQQQVHAQQQQQQQQQINAALLTQHGVSSGSVSLSGQLLSSSASGSSGSLAGGQQA
SQTPSGLQPTPRKSRLKRSKSPDLSSGGGAGSSGGSSSGSTQQQPQPAHHHHPQTILIQGQNPNSIVSLQQTADGNYIPV
SGSGDDSDAEDHEHEHNHGHGHGHGGHTHGHGHSHEHSHDHEHEHKPNKICKTEHSVASPASNSSSASNNAAGVSGVTST
GQAIVTQIVVARDGKDTKNMTSLGMGMNGGLLGVPMGFLDFTPEPPAPSATPVTVTEHVDLSCNPSTDTRDLSNTTEPLD
IDNHLAQQIHRLDQSPMHSISHHHTGDESNSNLVQHIKSEVIEAKHLAAQHHALSQAQQQHAHHQAHQQHQQHQQQQHQQ
QQQHLHAQQLLAQSQLQQQQQQQQQQQQHHQQQQQQAAAAAAAAGVHGQHGGHVTHADIGGATVMEIDPSQIKHEPGMII
TPEIVNMMSSGHMDMYNSDTSEDSMMIANGSPHDQKEPHYTNLDQQHGLGGSVCGPGPGGAGGGGGMSGGAGSGSGEKGQ
FNGPKAWTQDDMNSALDALKNQNMSLTKASAIYGIPSTTLWQRAHRMGIETPKKEGGTKSWNEDALQNALEALRSGQISA
NKASKAFGIPSSTLYKIARREGIRLAAPFNAAPTTWTPEDLERALEAIRAGNTSVQKASAEFGIPTGTLYGRCKREGIEL
SRSNPTPWSEDAMNEALNSVRVGQMSINQAAIHYNLPYSSLYGRFKRGKYDVVANTSGVALLNTSGNTTGSIEIIIEHSQE
NSLHMLQQQFPYSPSPHPPTPQHHSTPQHHSSAQQGPPTPQHMQQHVVHMQQQQQQQQQQSPHPGHHPQHMQQDVVTSSS
QVVHSQQQQQLQQIYQHHGTPERS
>Dmel_FBpp0087789
MGQIVGSMHMNVAEVFSNRRKRKRSTDSSLGKDDPAQLDTTQPKKKKLLTTTQYIYKALFKEEKNSDVAVMALDKVWHLH
KVYLSQSPYFYTMFNGTWREAQQNFIQITILDDRITVASLDAVFGSMYSDEIEIESADVISVLATATLFHLDGIIDKCAE
VMVDNISPETAIQYYEAACQYGVVGVKKSTFQWFQINLLSIYSKQPNLLRHISIELMSALTASPDLYVMQTEFSLYTLLR
TWMFLRLHPDYDPEDPVQRAEALKTQELLVNAGVETHAPSGDVVQWTYFTSRSEERSFLATPEGQPYVKVFQKLRTQYLT
NHYMDLKIIYNDNIIPKEWLYRHIHNHWDALLRIDHGQEDCSPQQLDDEQFFENCMRCGRMLLEPGYQKWRWTGFNFGMD
LILIMDSRRLNIRRHHRHEHERVLSLQTKRKFMVRTTVTSINAQRQAVFTQTSEICSLSLEKNEEVPLMVLDPKLVHPLL
ISINMLVVMPPNQSFKEIVPLSEEATTSLSIPISEIGANSDRPLSPSSADDSAVFIGDSEPSTPSSPAPRPRIAWSASET
GAICGQLAC
>Dmel_FBpp0087800
MPHTDDTFPEGSSCCPIDIVSSNKVILRGYASKANKDVEAVEELEEIQEMNDELRDLFINTTAINCSSLSELKIQTDAFN
TLKERTLFSLGYSTAGLLKKSKISKVLKGKVNLYQQIVSLVKQRTKTNSEITIGKTSHPVHLMVLQSFSRMFRDMGNDLS
VALPEKMITPRSFGLIYEWMIEDTPVLPRLGLLEVFHAAKFMEIPQLVRQCKYCLDHGFTEDSAAMLYFEAKILKLEVIH
LQYLERVSKFFLTLVASKEFLRLPLKSMLLLIQSDLIGVNTELEVFMAAARWLSHHWPQREENVTDVVSSIRFGLIPPWL
LIRLQKPDVTSVGVGRIVAQPIVRQAIHEGIAYTTTRMFYGKDREAFKHYLHKACVKPPVQRTWIYDRKCPYHHRMQCRN
TVDLTYDAFLEYLNYTQRQHRDYWKSLEPVDASNVCFSCQAKKSDLTKPNS
>Dmel_FBpp0088384
MDDDQQFCLRWNNHQSTLISVFDTLLENETLVDCTLAAEGKFLKAHKVVLSACSPYFATLLQEQYDKHPIFILKDVKYQE
LRAMMDYMYRGEVNISQDQLAALLKAAESLQIKGLSDNRTGGGVAPKPESSGHHRGGKLSGAYTLEQTKRARLATGGAMD
TSGDVSGSREGSSSPSRRRRKVRRRSMENDAHDNSNSSVLQAAASNQSILQQTGAGLAVSALVTTQLSSGPAAGTSSQAS
STQQQQPLTSTNVTKKTESAKLTSSTAAPASGASASAAVQQAHLHQQQAQTTSDAINTENVQAQSQGGAQGVQGDDEDID
EGSAVGGPNSATGPNPASASASAVHAGVVVKQLASVVDKSSSNHKHKIKDNSVSSVGSEMVIEPKAEYDDDAHDENVEDL
TLDEEDMTMEELDQTAGTSQGGEGSSQTYATWQHDRSQDELGLMAQDAQQRDPQASKQDKGEQTEGAQDEFELDDCLLES
NDIVITQNKDGFVLHVKKLGNITAAKLEENQAVAQQQGQAAVTVTGPAGQPTPTITELLNAAAASHSEPKPTLTTLTSTP
IKLPSSECELINIKKIIPATTTIATHHPHTSSTIIHPHHIIQHVSQEPHHQEHHQQHQTIHIEEVPQTSQQHHQQQHHHQ
LQTVQPTHTQVQSIITAHPGQTINLVGLRNVQLADSKPIASRIRYSRGKIIGPTVQNLQIVETHEPIQHQHHELSDGTKY
EISEIDLNNPNASAAIISDLVKYAEIDDIELPDGTKIGIGFAPSEITEHMQTSGGETHITTIEHEPQELQTVHQHEQTQQ
THHIHAGQLQTHHIQTVVQSSSGQQQHDQQQHHQHHSIELQDDDGVETITPEELGMHDSSKSYTILTTRPMKEESEHDPS

GMTYELSLSDSSLGPCDDPESRYVCRHCGKKYRWKSTLRRHENVECGGKEPCHPCPYCSYKAKQRGNLGVHVRKHHPEKP
QLESKRGRKV
>Dmel_FBpp0089415
MSLPMNSLYSLTWGDYGTSLVSAIQLLRCHGDLVDCTLAAGGRSFPAHKIVLCAASPFLLDLLKNTPCKHPVVMLAGVNA
NDLEALLEFVYRGEVSVDHAQLPSLLQAAQCLNIQGLAPQTVTKDDYTTHSIQLQHMIPQHHDQDQLIATIATAPQQTVH
AQVVEDIHHQGQILQATTQTNAAGQQQTIVTTDAAKHDQAVIQAFLPARKRKPRVKKMSPTAPKISKVEGMDTIMGTPTS
SHGSGSVQQVLGENGAEGQLLSSTPIIKSEGQKVETIVTMDPNNMIPVTSANAATGEITPAQGATGSSGGNTSGVLSTPK
AKRAKHPPGTEKPRSRSQSEQPATCPICYAVIRQSRNLRRHLELRHFAKPGVKKEKKTTSGKKSSSGSSGSGSGALSSSG
SVPQVQTVQSLHTLQGVQVKKDPDAQQQQQQQQQQQQQQQQQQAMTVSGATGGQVQQQVQQVQQQVQQQQQQQQQQQQLQ
HHQIIDSSGNITTATTSAQAAAAAQQQAAGQQQQLVAQSDGSESGAPLSIAQVQTLQGHQIIGNLNQVNITDFQQQQPQQ
QQQQQQQQQQQQQQQQQTQQTL
>Dmel_FBpp0099767
MFEPYVKIKKKRSVNEIYENENLEQQQHHLQQQQQQPATSDNCCCENGEPQNAPEVATATVAATSVAATSAAATVATGAA
ASSSSSGNCSRLQQLISTPPVLLRRSSLQQQQHQQQQHHPHTPAATATPPQQQQQQQAAPSVLQQHLGHLNYESGATAAA
AATAAAAAATVATSRSGSATLAQHLATPSNILQAAFGSSNLQHILTRSAPSPSSSAISSNNCSSACAGNTHYNGGNSNSG
SSSSNSNHHSNSIIASRLFGAASSSSSSSSSSASASSSSVAASSSSSSHHLHSHHSALTNSITNRINQSIRRHLNQQQHH
HPLSASSSSASASPSASTSSSSSSYQQSSVQQQHYNCAHPAQQQQHHHHHHSSSSSSSSSSSSHHHHNSSSSSNSNNQQQ
PQQSPLCLVLLVKCPNSKEFCNAAANFCDKRLPVNECQASQTARVTSNLHASSSTMAVSRVPSPPLPEVNTPVAENWCYT
QVKVVKFSYMWTINNFSFCREEMGEVLKSSTFSAGANDKLKWCLRVNPKGLDEESKDYLSLYLLLVSCNKSEVRAKFKFS
ILNAKREETKAMESQRAYRFVQGKDWGFKKFIRRDFLLDEANGLLPEDKLTIFCEVSVVADSVNISGQSNIVQFKVPECK
LSEDLGNLFDNEKFSDVTLSVGGREFQAHKAILAARSDVFAAMFEHEMEERKLNRVAITDVDHEVLKEMLRFIYTGKAPN
LEKMADDLLAAADKYALEKLKVMCEEALCVNLSVETAAETLILADLHSADQLKAQTIDFINTHATDVMETSGWQNMITTH
SHLIAEAFRALATQQIPPIGPPRKRVKMS
>Dmel_FBpp0111467
MGSEHYCLRWNNYQSNLLGVFSQLLQDGSLVDVTLVCSEGTSIRAHKVVLSACSSYFQSLFLEHPEGHLIVILKDVRFAE
LQTLVEFMYKGEVNVQYCQLSALLKTAESLKVKGLAEMTNQNTTLREPEREPDRLRPQGGGGSGGSAHKAADSPAAALDA
TAATQAATATATATAAAATSTSTSATSAAATAAATAATSASTTATAAAGSSNTTTTTAATTTATCATAATSTAATSSSSS
VTSAAAAAAAAASASGVAHSEEATSSSSSTGGQKREASDRSSPTPAKRSSRMHPADKVDVAEDREQEQNQADELLSEELL
GRNLKDEEDDDVDELDENEETKLRGRDEDDDDEDEEEDTPMPLDLYQRPNVEPKSAAAATAAQQGGSNSLSGSSNACHTN
SSSNSNNNNNNNNNNSSSNNNNTSSGKTSASSNGGTASSGGTADSVVAVDDDDDDDDGGHHHQRQVMDDRLEQDVDEEDL
DDDVVVVGPATAMARGIAQRLAHQNLQRLHHTHHHAQHQHSQHHHPHSQHHHTPHHQQHHTHSDDEDAMPVIAKSEILDD
DYDDEMDLEDDDEADNSSNDLGLNMKMGSGGAGGGGGVDLSTGSTLIPSPLITLPSSSAAAAAAAAAAMESQRSTPAMSA
AQAAGAGASDLSIGGCGGRPASRSSRDGGGNDGGRGGASSSSLLSPGTVANLLPVQSVPLSLKKEIDCSEDDNSSHSRHM
QQRSASAGGGLGGDLDYRASHESETSESPHQQQQPPPHAVGVGAGGGSAAAAAAAAAALLHQQQQQQPPHSPPPPPLHMF
PCIYCRSAFPNQSKLTRHLLTHSLKTLEYREPTTTALLHSHLLGDLNMAAAGLPSPFAFQMNAAAMAATSGSDTQEQVAA
LAAAFGIDMEAALSLASTGCTFQSLTASCLEASSGGGSGGGGQGSLSRSATSAGNGGGLMLGSGSGSGSGAAAATGGSGS
SGCGGGGGTATSGSLSPSTSAAAAAAAAAQSQSAALLGAASSDPNSVVMCDRPFKCEFCGKAFKLRHHMKDHCRVHTGER
PFRCNMCGKTFSRSTILKAHEKTHFPKYVRKFLSPSSPVDTKDELPQ
>Dmel_FBpp0111520
MDDEFKLCWKNFQDNIASGFQNLYDRGDLVDVTLACDGKLLHAHKIVLAICSPYFQEIFTTNPCKHPIIILKDVSFNIMM
ELLEFMYQGVVNVKHTELQSFMKIGQLLQIKGLATNSNSSPGSSVSEKSSSQPPAEESSNTNSTNHHNSSTNSNNNSKSE
TDHNESKGQSNSRTTSPGGASRASNDASHLYTSNKRPMPSDFGSDSLSIYSGKQLRRSLKDHGSGGSEGGGGDHADTAAG
LDNSMNSEEFFLPPIPQITMGEQRYDLGGLKRESDGHHHGPLSAGGGNSGSVGSLTSSASPSAPIRNPFAPNFMDSFNYY
KGGNSSGPSGSSNNSVGPGGVNNNGSVGLGSGAEYPNELYMPNDYSKSFANHMDIPSSGSNMVMLSTTSLLHGNCVFNRN
NTVATQQGMKTYWLCKSYRISMCRARCITHLGRIISATGVHNHTPHMRGGQGSSAPSTASVSGNPAPTPISSQAGYSLTD
GGGHLGPDLQHGNRVTNMFANQTPPPPPPPPPPLPVASTPHHPHHHHLGQPLPNLLVQHHPAPSHMEQHGSSASLLHNPN
LMHLQTPTHHHQQQHHQQHSPHNPPPPQQHNLELGGGSGSGAVLLSPAHLQQSPQSNRSSHSLQAQSPPPQSVEEQQPHQ
QQQQQQEGATTEVTTDPSTAHVINSITISPNTRHTFKMENM
>Dmel_FBpp0112152
MAANEIMTPTVNANCQYSTRYCWEAEKVKSLIRLRAELSPLFTGKRNASKYAWAVVERELNVPLPLSKIIKKWNNLLQEY
KAIKMSEEPKRREWPFFTLMDVYFSDQVNDPSLRLFSSTKTLKETLDDSVFEDDPIIASAIAAATSGAYMDIDELIRRQK
ERAALAAERKLAAAASGGTGDYKFDLKPMLSNSKFNSNSEMAKLSKSVDDLSMQQYKIKQELMRQREQEQQENMVKIKQH
VRQQQQQQHQQQQQQQHHQAQQQQQQRFLGHQQAMSQHPHRLSSSSTPPALQHALQQQQQHMLQQHQLQQQLQHQQQQQH
QQQLQQQQQQHHQLAHPHQPHTPRPSTPPTTAQQIHITATQHQAAQQQLHQQQQQPSHKQQQSYADPNTIDQYLLSWNNF
HGNMCRGFHSLQKDEKMVDVTIAAGGKIFKAHKLVLSVCSPYFQQIFLENPSSHPILLMADVEASHMAGLLDFMYSGQVN
VKYEDLPVFLKVAEAMKIKGLHTEKNLDSDASDISSPHESDGTECRYERGTHSVNITSGHAAGYGGAPSSQQSSPSPSLN
GPNRCPTPLSSGGSGNPNYAGFREHIKSKATLAETFMKNLNRNNNNNNSSNNYNHLGGSNGSLNLTEADSNSFAILQANS
KKMLDSARKQHKYLAKRKILMHYNTELGGEKRLKEMERDRYPSAEQHDDALNNNHSHAQDNIMEPIITTIVPQTPPPSTH
NNNFKIRLVESHHLTNNSNNNQNQSSNNNNSSSSPQSNDCNNDEEALNLVQIPNANGSRNREATPTCATPTPDIQLIKRE
VKTELSPAENLSNNNGHDDEMAGAGFDRKYDDNNNNSGLDMETDSNNNNSKPGGDNNNGLALALGKHKLLSAINRNIEQD
HPDHEHQDQQDREADHGGNNCNSDDNNNNNHSQHLDLSTIKNIATAEILCGLKSKVLKLEEEQREREREREREREACRSL
SEIKNAE

>Dmel_FBpp0112336
MEHVNSTNARQPQHHHFDQQLQHNDSQQQFCLRWHNHQTSLLSTLPILLDQSHLTDVTISAEGRQLRAHRVVLSACSSFF
MDIFRALEASNHPVIIIPGASFGAIVSLLTFMYSGEVNVYEEQIPMLLNLAETLGIKGLADVQNNNLPKTARSGGGSYMD
TTNEKSSEFERPTTPSPTPTPTLTPSHTPTPSHSLPLPQLPSAALNTPLLANKLGSVNSSGMGTTPLENLFKSLQFYPSL
LPQPLNFSQTALNKTTELLAKYQQQCQLYQSGMQEDQLETDCFGSKRLKGDSPPKELRRLEKSLLKNPKSSSTTNSSSSK
SPQECSNPNPIVATSPVTLAPPTMAHFSPQLPVVKCSSASYPSALGQGQLYSSKPPLYSAAVTPTAAQQAAQMHHHPQPG
PSPYISAEDHAKLQLHIEQYQREAAAAAAAAAGGMALVSAKSEPNLLSLSADRDKSLATAPIKPPSNSKLYATCFICHKQ
LSNQYNLRVHLETHQNVRYACNVCSHVSRSKDALRKHVSYRHPGAPSPCENEARRKRVSKLAATTVPTSTPMSMSASHTV
TSGDVGPAPATTLGCSGQEARNPYLFLPNQFQMAAAAAAVAVAESSPASGQPSLDLAHEAPPSIKSEREPPTASNGEATG
VEASASTT
>Dmel_FBpp0112408
MSRNQNESNLLDINEDLPFALALPPVSMDSLQLNADGNSPEDKTLGIGRKTILNAHTKSLAPIVSDEALNTLNELREQNL
LCDAQISVGEDVFNVHRAIMCSCSSYFRAQFTGFNADTPGCVDGSDAKKNNNFIHIPGVSSCIMNCVIQYAYLRQTNISE
SNVHELLICADYVGMVGLVKKCKDYLGRILTPENCVSIMGFARFRFLEDLHLKARNYTLRYFTEVAYRNIDILDMSAEDF
YSIISDDELNTREEDHVWKLCVKWIDRNPESRKRHVAHLMTGVRLGLMTPKCFMEEVKEHPYVLECEAAKPLIVDTFKFM
YDLDFLNPQADELTTPPLAMPRLPHEVIFAIGGWSGGTSKGCIETYDTRADRWVTINAEDPAGPRAYHGTAVLGFKIFSI
GGYDGVEYFNTCRVFDAVKKKWNEIAPMHCRRCYVSVTELNGMIYAIGGYDGHNRLNTVERYNPRTNQWSVIPPMNMQRS
DASACTLQERIYATGGFNGQECLDSAEYYDPVTNVWTRIPNMNHRRSGVSCVAFRNQLYVIGGFNGTARLSTGERFDPDT
QTWHFIREMNHSRSNFGLEIIDDMIFAIGGFNGVSTISHTECYVAETDEWMEATDMNIVRSALSANNIAGLPNKRDYIHK
ERDRLMEERRQRLMATAMARENVGHGNASQMLVEHNDGAMDDYESPDEVDETDEQEQSARISRTIPPSVGLPNPAVAAET
IRDQSQLVSTAQLNRVMEDRGMDNNRSNRVQLRNQRHGSHHEIRRRT
>Dmel_FBpp0112713
MKTGSNETFSVLKMEKILAVQSDEYTKLQRCYVELQRKYNENIASSENEESGLSSFLSRLSLTVASLFDKNTYADIYIRS
QTRVFPAHKIVLHARSEKWGNDLLSNIQQLDWSDLNEDVVLSLLRWIYTDLIDLQNDGLALDLLKAAHRFGLPSLLGICE
RALVTSVGVRSCIRFYCVAEEVGASTLLEYCSSIISTHWDDLTTDDFQHMSGPLLFEMLKTKTKHPLHAAVRLLREDVVS
LCIQKYGNSLVNTFSENGILPLEMALSSKNAKIAKSLVDNGMANINAVNMEGFSLLKSALKNGDAFSANFLLDQNCLLDL
PSKPSSDTALHIICNYGPDNTPEIMEVVKKILQRQLNINIQNIKGETPLHIAIARRNVEMVKLLLKVPNIDINLRTYDEK
CALELSLSMGDHEFLIASILLSMGARTDRTNSKTGDSLLQVFALDRHRGEKSAIFLADFADLDHINFRGLTALHIAALNN
MPNLVKKLIVNGASSNLKHIDCGLKSALHIAVEANAIDALEAFVELKNKSHDIDFNCQDINGDSPLSLCLSLKRTTLVPT
LIRGGSDVNGKNKNNLSPLHQSIKNEDSDISLFLLEQGADITALTENLDSALDLSIKHDLSEVVDALCRRGIALSINKNG
ESPLWSALEKGYEDVAKILVRHGIDTDCWDEGPEGCRQTLLHRAIDENKESVAIFLIQSQCDLDSSRQPGPNGEGGDEAQ
DKASPLHLCCHWGQTKVLQTLIDHGANVNLIDAESKSPLHVAIESQYDEIISILLCHPDIDLKLRDKSGNTPFATALDFR
NHNAAQRILDRFPTAAEQMDIRGRNFLHLAILKDDLESVLFLLAIQVDVNSRVHDANQSSPLHLAAASQNEMITRNLILA
GARMNERDAVQKLPLHIAIERGNLPAVSALIQNNADYDATDADGNNALHIAVRCAQFFIVRELLTESRVNAEATNLKGRN
PLHELCRVVEDSTAGLICELFLESMPKYPINIPDMDGNTPLLLSFMRGQSPLCKILVKAGACLGTENKDGINIFNFKLAT
DQLLHNLLDQLPQESPWAESDYCQHCTNRFTITMRKHHCRHCGRVLCSKCSCNDVPILKFGINKPVRVCTVCFNVLQCGN
GSLS
>Dmel_FBpp0271760
MSVQQFCLRWNNHQPNFISVCSSLLHNGTLVDVTLAAEGRQLQAHKIVLSACSSYFQALFTTNPCQHPIVILKDVQYDDL
KTMVDFMYYGEVNVSQEQLPHILKTAEMLKIKGLAEMPTDPANLTKSDSKSSTDGTEMVGGAGVGTGAGNSAGSLGAGSG
SSVGDSLWSSSEAQQFQQQQQQQAQQQAQQQHHHHQQQQQLQQQQQQAQQQQQQQQHHHHHHQQQQSGQAQAAQTHHHQM
RRTPSPLSAGTSPATRRKRLRKSSNNGSGDRNNAEEQHNSSLDAGSGAGNAGLSLAQMNQMTFGAGGGLAGHSLHAAKLL
KESASAELEQQPQDSDLDDGHGHLHMQIKPEVDIGGVNQTMPLDISGGTTPSEHDAPNSQSSHSGLQWTVVDSNYPRFSL
PACQSNLLGNGGGSGGGAGSQSSGANSAGGVNSDQRQQHEAQQQATQQQQHLQQLHYQQQQQQEQASASGQAYSSQIITV
NNLVGSYATAAQNLSPTSPNESNMVQSVYSQGPTPTQSPVHAGVGGASAAGGAAGNASAGNGGAPGAANQVVKRKRSVNP
QGDENFIRALEAVRTGGIGFCKAARLYGVNNRTLWLEYKKRGYPVSRPSIKARVVKQEPNLSPSPTPSTNQGDDNTNETL
GMQIPPQAETPTPSLMCTSHHTGLGSGAGSLPAGGNSHPAIGVMSLFDPRYMDSPGNVHSMTRQRYIEATGGGAGAGTGA
GTGTINNTISSQAGTATLLQAAAVMAASEPAESLSISAMPVSVPMGMAHSFLINNFVTVAAPPVATVTSTGLQYLDKS
>Dmel_FBpp0289659
MDDTQHFCLRWNNYQSSITSAFENLRDDEAFVDVTLACEGRSIKAHRVVLSACSPYFRELLKSTPCKHPVILLQDVNFMD
LHALVEFIYHGEVNVHQKSLQSFLKTAEVLRVSGLTQQQAEDTHSHLAQIQNLANSGGRTPLNTHTQSLPHPHHGSLHDD
GGSSTLFSRQGAGSPPPTAVPSLPSHINNQLLKRMAMMHRSSAAAAAEETSHAFKRLRGSDNSLPLSGAVGSGSNNNSPD
LPPLHARSASPQQTPADFSTIKHHNNNNTPPLKEEKRNGPTGNGNSGNGNGNGNGASNGNGISISDKLGSLTPSPLARAG
ADDVKSEPMDMVCSNNNANANDEHSNDSTGEHDANRSSSGDGGKGSLSSGNDEEIGDGLASHHAAPQFIMSPAENKMFHA
AAFNFPNIDPSALLGLNTQLQQSGDLAVSPQGGSTGSLLSGVIVPGGSGGTPSNSSSNNNNNNSNNQQQKVEQQSSPHQL
LQQQHHSTPHTNSPQLKQEQPKSGGGSCKSSDLHIAAGSERSLSRSSQGMPDAGGHSATPSPTAAYHKRERERERERERE
RERERERSLDHERDLERPGGTGSPPPPPPSHHSHFGQHPLSLLPSHHQLHATHHELSAAAAHHAHAHAHAAHAHALARAG
SPMEHHHLLHHRRASLSPSGAVSSASGAGGRGGGAGGPGGPGGSLLSSVRAQDVAQANRLLLPLPLNACHRCDVCGKLLS
TKLTLKRHKEQQHLQPLNNAVCNLCHKVFRTLNSLNNHKSIYHRRQKNHHSYFHHGAGVSQAGSPGSRLHQSLSSLSAAA
AAANNSVNVGGGSVGGAGGNAVAAAAAAAAAAAELLLSPIVGAAAVAGGTASSTLQLAAAHQQQQQQSSPGIQLFHVALN
NSAAAAAAAAAAAAAAGGSGGGPGGVGAGPESINGGGQEYGGVQGPSGTPTVDLTQLEHAAGGGGFSAAAALQHHLPHHLA
HQHPMSHQHPHQLTHQHPHPHPHQHPYERMNLLDQ

>Dmel_FBpp0290376
MALVHTFLGTWEIVCCTFEGKRELSGLEGIKFRLDDTSDITWYNDLVSPLPESKDCGNFCESASVLFSCETFDVHEINPR
LVFGAFAGHSIEFSTNTLTPTDTLVLSCENWYAVECKRLQDNQAAGQEKQLSFGEALQESYFSDVMVKSSSGLEYALHAS
ILRLNGFDCSMCVNSAPSSAAARPAARSCHSGPNTPNPIRISVAPASNDDGHEKSLPRLNLSPIYLQPPCDFQTMPSSGL
GAQRVHQFSSSFNCLSNGNAGDSESVVRTTGLLSLECGGGGGMYRLPPTSHSDSHLETSQSHCKNIFNFCQDLMLQPTPT
PMPPTTGLAICSIRRPPLSPYRARSPSPFPSSMDTPPSSPLTPVGVLCNLPNFLLGPILHWLYTESLLPDLDEDVCEKLI
SFAELQPSLTKLVEPTRKYLRLIRLKKFVVNVTMDLHGILNRVIQCINPGNITRDPAQLYATFQDALRECAIGCAKVLQF
CNIFITDAAHMTRYQKNEIVKYIRTRIPIFMSQIQQLLQNVLGVFVGLSVDEKAELVNYLVPEIESTLFVLTAVIEEIKN
SLEIMCKDLKCSHLDLPLQQQQAEDAIVMQLQIADGGEPSPRPRRGAPVGLTLYDNLSDKQRLSSAENDLKFVLYMYEVR
KMRDIYGRIVAALEIIKDKKTTFCEMDFLSKSSTINQNLEQLIMDIPAYIFIVENLSDRLDDKLGWKEFKFCFKLATSQI
NGVIAKLLEHKDALRDAISQICMLVRKQEFTQSVIELGLLDDTCLLSNNLKMADHENNLNQGLSEKDSMYLDRKQYYDYN
SIKLNLIRHLCEPPIAASSNLSKNALRLLHSTQLADMEFEVHTYAPTSGAAKSGGGTEAVPETAEPAAKVLQVHSFKAHR
VIVAARCEWFKKALMSGMQESINRKVIITDTSPVIFRRLLLYLYGAPIDRTVGAEQVCELMLLADRYSIDDLKELCENTL
YSLIDEDSVVCLLGIADRYMATALKSKCLSFLSQHAQLTKCEIFKELPQTLQLEVMDLIHWFGRVSEPWNDRGFKPRSSS
RHSLKSPSKPRSRSRKSSPSYM
>Dmel_FBpp0290499
MMATSQDYFGNPYALFRGPPTTLRPRESPLGVGHPHGHGHLHSHAHAHGHGHAHSHYAALDLQTPHKRNIETDVRAPPPP
LPPPPLPLPPASPSLQISRYNTDQGAMDQQFCLRWNNHPTNLTGVLTSLLQREALCDVTLACEGETVKAHQTILSACSPY
FETIFLQNQHPHPIIYLKDVRYSEMRSLLDFMYKGEVNVGQSSLPMFLKTAESLQVRGLTDNNNLNYRSDCDKLRDSAAS
SPTGRGPSNYTGGLGGAGGVADAMRESRDSLRSRCERDLRDELTQRSSSSMSERSSAAAAAAAAAAAVAAAGGNVNAAAV
ALGLTTPTGGERSPSVGSASAAAAAAAVAAAVAAAANRSASADGCSDRGSERGTLERTDSRDDLLQLDYSNKDNNNSNSS
STGGNNNNNNNNNNNSSSNNNNSSSNRERNNSGERERERERERERDRDRELSTTPVEQLSSSKRRRKNSSSNCDNSLSSS
HQDRHYPQDSQANFKSSPVPKTGGSTSESEDAGGRHDSPLSMTTSVHLGGGGGNVGAASALSGLSQSLSIKQELMDAQQQ
QQHREHHVALPPDYLPSAALKLHAEDMSTLLTQHALQAADARDEHNDAKQLQLDQTDNIDGRVKCFNIKHDRHPDRELDR
NHREHDDDPGVIEEVVVDHVREMEAGNEHDPEEMKEAAYHATPPKYRRAVVYAPPHPDEEAASGSGSDIYVDGGYNCEYK
CKELNMRAIRCSRQQHMMSHYSPHHPHHRSLIDCPAEAAYSPPVANNQAYLASNGAVQQLDLSTYHGHANHQLHQHPPSA
THPSHSQSSPHYPSASGAGAGAGSVSVSIAGSASGSATSAPASVATSAVSPQPSSSSTGSTSSAAAVAAAAAAAANRRDH
NIDYSTLFVQLSGTLPTLYRCVSCNKIVSNRWHHANIHRPQSHECPVCGQKFTRRDNMKAHCKIKHADIKDRFFSHYVHM
>Dmel_FBpp0291476
MECSDFMQQLRSFKPQSLGCAVARRKSSTTLIFPLPPQACSDEFCEEYPHNLMPTPGYWIECSRQKITMPTPHTIWDESD
SEHEDIRAGSASDAYLERECSDLYEDDEDSEATPSPRKKTTIEEQWDQQGAFELISVEQETYEKYFYGTEHWNYFTSDED
LGPVILSIKQETLNGRDQFRILVRAGSYTVHGLIPASCVFADRYNREEVVRSLGKEVNLNPPLTLGQLPDTPEELLKLDQ
VFIKSELKVGVIFVKEDQYTEEQILDNNENSPLFDEFLTLLGDRVRLRGFDKYKGGLDTVHDLTGLFSVYTNWRNIEIMF
HVSTLLPYEKHDPQKLQRKRHIGNDIVCVVFLEADNTRFSPACIKSHFLHTFILVRVSARIKHKPTRYEVSVVTRDEVGA
YKPYLWEQSVFEKGPMFREWLLTKIVNGERASYSAPKFARMQERTRSQMLEDLVMNLSNHAETGQIPKPYRRGSWRPIGE
DEYIKMLHARVLDNNTHHEQQQQLLLQQQHMQHTQHMQHLPSHNICCYCNNNATELHTAGAIHNNNDNNNTTTTTTFTHR
HNNNNNNYSEQQQQQLPQQQQHDSQQQQQLLQPQQHQLLHQLQLSTSSLTHTSCCAMLHLCARHSHMRPSSPLLDSVRDQ
FEDYDQLAKDFTRVFLNEEPSCLTNAHLFDVVFLVGQSKQKARFIGVRAILGVRSRVFQEMLYGIQTGFGSPQIPVAEIF
ARPAPSLVSPQNQKPKSNNYLTVPDSDSIRPKSVPSSPMVKRAFSRLGTITAGWGRSIRNKNTNQLNPDDKKKWISSTDY
RDSKDKDKDKPGNNQLAVPRLSVCADAQKVDRAKLAQTEFNIIEFDPDTFRVLLDYLHTGTCPLTCVSIPGLLCAAEHYD
LPELLQACFHHCKQFLRIEVVCPMLISLENYYWRYTSASELVNMILSFVESKAHSLFKCPEFLHLSESMVQMIMCRELQT
PEIRKFEAMLAWAQHKVGKLKNHPNKDTQFEFECIMERLTRDLNLCRISPSELLTVVLPSKSMKNERIMETLMVQVNLGT
YRMPELDAYRQQLRQQESAEATVQVHRAHQNHHNPPPIDARAYAMASLAAALEMQNSSNPLQSNAARDAADEEVELYASF
DNAPSTSRVAAQMQAAKEARRKRWAADGASGSSGDGGCASGSSGSGRRY
>Cele_B0047_1b
MVVANKEFKLSHVFTNVLKMKDGDELYSPQEMHFNIPWMLKIKRNADHLGAYCYCLKLPKDEDGVAWTIDGHFELTALDP
NGYRSLSGLPYLDCVFTNKRCSSGRAKFMSWNEMIEDYLINDSITIEVAAKITKMSGVIENLHRFFDKSMKQFSDVVIDV
KGTKFYVLRKLLAFHSTYFYTLLGDSTESKVTISDINADDFQTLLEVLYGESAINDSNVDGLLQFALKHEILLAIQKCIE
FLSEKSEKPMKDRVKIAKQHKLYNLKHALLMKIKTTDEIKAALSEDLLEMDTTVVAALLQKALKIK
>Cele_B0047_2
MFPKRSSPSIRFFFRKLFTSKFKESKMTEIPIKDVSYEDFGLVMSTIYPDTIFPNDETAEKLLEMADRFLMPVVTNIVEH
HLLYNSKLPNEKIIRLADQYRLEMLINKSTWKVDSLAKLRQLKNTPEYEKLSGDTKAKILDRLAGP
>Cele_B0047_3
MVAAHSVTRNAKNSFGTGNETSSWGWSPFINWNEMIDDYLIGDTITIEATVRITDMTGFSKKVLKSFDESNEEFSDVVVA
VGNEKFYVLKVYLASHSTYFKTLFLGKFVEADKSEVTLQDIDPTDFQCLMEVIHGESAIDDSTIDGVLQLAQMFDVSLAI
RKSEEFLVEKSKKSMRDLLKLAKQFQLENLKTVCLSKINTLDEIEASMAHNPAEMDPSVLAELLKKSTALVRMNEVLFAS
LP
>Cele_B0496_1
MNDITMKIPTASKLLKLPEDSKVGEEVEIPICNILGLHLTGFWTVLNTANGKFEIRCEKESLFRWKFLAQMDLQFTGEGK
VINIKNEIEFSPNVNTIMIKSIDRRYNQVKLKITRKSSFGIAIEDFIDFEKKSKWLDVLIRSEDGKKMIYANSGILALNS
PILKQKLENNKLAEIIMPFESFVSMKKLMNFLHPPFKMERENVEQVLDLASKWEMKSVITKYEQFLIKTRCHCQRSSIQD
VKLAEKFKMASLLEEIINDGETDVFALLDGNDYSSWTRAAMFEFIVNDEDENTIHEN

>Cele_B0496_2
MKTQTVTKLFKLPEDSEIGKKVQISICDILGLHWTGFWTISCTAEGRFEINCEEKNRSRWKFLAQVELQFTGEGQTPVIN
INKEIEFTPKLKIMVFDPIDQGCRYNKVNLKITRKSSVGVAVDKFIDFEKKNSWLDALIRSEDGKKMMYANSGILALNSP
ILKQKLQNDKKAHIIIPSESFAAMEKLMNFLHPPYKMEREIVEEVLDLASKWKMESVLTKYEQLLIRERCKCQDSSLKNV
KLADKFKMRSLLEDILYNDTCNVYDLLDGNDYSSWTRAAMFEFVLEMENDYADDDESENTLYGVLSMAELLYQNKIYKIN
KLFIFQDYFKFQNTSNYASSAQKKSTGSA
>Cele_C03H5_6
MKNSNFLPIAFCKAWCVECPDRELILANVQYEHVLEMLECIHPTYKAVDDQSVHILLPLAYDYQMEGLLHRCECFLISHN
LPFLEKVWIADRYKLNRLLVLCLREMRPNSKVDLNGSRYYALSDRVKVLLLERLHGAAAPEEILEPPLDLEPYQRQSDVN
FAAVRAKTGRLYYVNPFYMAAWSNVFEEKLCTTSSGIEEMFCPCTHEELKAFLMAIHPPQLRINETNIGPILMSACKMES
PALLRKCANLLLSPHTQLSVFVRLSLLDRCFLHEMLPQCLQMVLRPENLIQMTQQTTYDCLSTRAKAAMMDRLGVLLDNP
GLQTHHCSRCKVTNTCGAVTWMCPSCKTYSSDTTLMKNPNNVTTGTTTVTQGYGSGTAWKNLIFTNQTSQTNHTKIVTMT
SIPKAFNIRRTQNFDFENKIERFRFFSDPNKIDSEPGTEFSIKLTQLGLTINNFFTEKSEGFLIIIDNSTSTFGIFVFFL
LFPFLSLHNHNFVVKKNYFSQIVKQLEFNKVKFHIVRRHFLMKNYFFEMS
>Cele_C05C8_6
MSDNHAFGRPIQEDGRGIVETMRTESAAISNNFNAVNSVNKSVVQHLDELSQSFDEIFTSTDHSDVTLVLDDGTEFAAHR
LILAVRSSFFRAMLYTGFQESHQQLVTLQETNSVAFRAVLRYMYTSKIDFAGVELDILLEYLSLAHRYDLIQLMTAISEY
FKEILKNENLCSIFNAAYFFQFTDLIDYCMQYSDKHADQLLEDPSFNRLSGDSLKELLARDSFFALELKIFNAVRSWHQN
NPTMKEASKVLLELVRLPLITQTELLNCVRPTGLVSADTLLDAIEVQTQRPHEIPFRGCKSLDTNIIPHYPHAQPLSREL
SSRYTNDENISVFQIDLGKPFIINTIMMDFNWKNEVQAFTYQVHVSMDNRVDAHWNLVADYSEYDCRGTQRLFFTDSVVR
HILIRVNNSMSCKLEGSRIEAMFSTETMPVEPVHKVIVPHRNITTIENHARVVEGVSRCRNALINGDSSSYDWDTGYTCH
QIGSGLIMIQLAQPYIISSMRILLWNCDDRFYSYYVAVSKNQDEWVTIVDRTNEECHGWQELIFDPLPVVYIRLVGTRNS
INEVFHVVHLEAPSNVPIAIK
>Cele_C07D10_2b_1
MDNSLMQMRHAADSWSTSEVRSLVHKHMWTIRGFSQLECRYLETSAKIKDITKDESGLQVPLTHQPSSSGGLPELPDITF
RIRLHPQGNKESNKDFTFFQCFTNQATTTTTPTFRAKFKFTVFNQRTEETPTTVYSGTQQLHGYFEYIRREVLVGHVQPA
DDLILCLTITITFDTVTKAAQNTRSVPPELPMPSDVTKDLENLYRSGKHADFTLVVEERELKAHKAILAARSPVFAAMME
PHTAEAQNSRAILRDIDYEVVQAILYYIYTGTCTNMGGNALDILAAAERFALPGLKNIAEVAMRNGLATETVCKNLAFAE
MYGLVDFKKEAIKYICMNANAVINSEGFLRLSRQNPDLIVDIMTVLVNERHNSPSCFSLDGTLEPASKRARMTETNL
>Cele_C08C3_2
MVAASKEFVFHHTFKDVSQLEDGDFFKSPEEIHYNAEWFILVIRTKDQLEAYLHCDNEKDDNVAWTVDAEFSLKIASSSG
SYAMKFQKGCFDGFGLGWNDFVSWDSLTEDFLYDNSFTIEACVKITKMTGFSKDVLRSFDESEKRFSDVILVVGDEKFYV
LKLFLASHSSYFNALFLGKFKEADQSEVTLQNIDPTDFQSLLEVLYGEPAIDDGTIDGVLLLAHMLDVTLAIRKCEEFLI
EKSMKSMRELLKMANQYQLENLKTVCISKINTIDEIEAAMAYNPEEMDPIVLATLLKKSTALHRSAISDSV
>Cele_C08C3_4a
MSDNLSARWHRILDMTNRETSDDVVIARSSFDDFSQSTPWRKFEISCGNRSFFVNPGWLAELSPYFADELYVRKPNVQRY
VIDQATPDEVLEFLRCITFCPMRKPLTVKNVSLVLTFANRFEMRPVQARCENFIAQNATSLSRDKTKLFQFFPLQVTCAM
SQCDPNSSTMSVLVDKLAGIKDDELSRLHFAEMPGDVVAEVYAQKIQRTKDKKAQRQAGASDETAAGCCFMQWASSLFNR
RRQRNQADQSILPPSGDQQHHRSELHA
>Cele_C08E3_2
MAEIVYQHKSKSMAEIVYQHKSKSYNGNVQEVDTSNVAGIRCAFYYSPSDKNQRRWVFNWEELGKEGIINLPSDKNDAIL
LVEEQQLHVNKAFLSFHSDYFSALFSSNFKDGELDEIPLKDVTIEEFGLVLSTIHPETVLPVNDKTVEKLLELADRFIIP
AVLRLAETHLLTHSKIGNDAMLWMADKYRLLKLLDVCILRIDTVEKAKKLQLSPHFHEISDGTNAKLFKRLPKIA
>Cele_C08E3_3
MTKEFVMTRKVKNIAAMKEGDSQSTTSEQHFGLPWYFKVFCKDKHLQLCVYYGAQLIAEEQSIEVECDFKMITPTGKIFV
EKTGVRKYGGETQYIGIGFPKFMLWEKMMKEFVVEDTVTVEVCLKIIDTWGFEKKVKVRNFDEAMKEFSDVVLVVKETRF
HVAKLFLAAQSGYFKALPIGNFKEGNSSEVTLSSIDPNDFQNFLEVLYGHQEAITDESVEGVLLIADMYNAKLVTQNCEN
FLFRGAGSKMMLKKQLQLAYRYNLENLKRSCYSNLEI
>Cele_C15H9_2
MLINTNSTSDATTIIDLQNRYYHVNAALEKTSFDVDGFKWQIVFDCQQIIFTLISEMAEFCSIDLIIHCTDFKSSSTFTS
QFERSGESVVHEWLPGGRHVQLEIDQTIHESQFSGLDFSQPFDSSNVVLKVEDEEFHVHTFVLSMASQYFKVLFNGSFIE
SSDLTKPIELKGISSCSFRNLLNCIYGYPSVFRITRGNIVELLELSHFFDCKNVTMFCEYNLCNDTCYNIRSTQKMEWAI
RYNLNTLKNKLLRHEPYLVDLLSLT
>Cele_C17H12_12
MIKVSTTILTNVFDFEALQKKSMFHHKIRIFNSSDLRNPKVYTVQELTETIKRYDIQRIRGDFSQCNITLKFSSGAEIKV
HSLILQTFSKKFHDLRDGIIITRTVNMEPFDEGVVRDVIYWMYTGKLFLMDVPHYNLAEFWDMPELLVQITDELKNDVDL
TFRFINTSSTNIKDRPQAALRPHCTIEHDVTNVTILCVNLIADYESSPKLTMVQLMRLSTCSIYACMAVRMPLKKKIPLI
FMVCDWLIEKKPRLDTVIGIIQSLVFDRYTSHVFVERIRQHAYMFVTDLHQSFKFMIFFNGATRIDRDETKEYHPDPLDL
RVFNNDPNISRPNENPYKPRYSNDDWSFETGNQVELKKFRDFFDEEYERKLKAHQQKPPVPKPAIEVTWDASNFGKPPSE
SVKNCVLEERPKTPERPKFTLGITHLESDIRVEDRLMFRVRALNNPRHYAMAVEREVEAQKIKKMEMDQLYGDYEKSNYV
GHQNNPQPDVNRSQYCYTSRSVHHPADQYQHLDKRQRNLSYSAPPKNYVILSPHSNNNRRSVDSQNSNQSFDGLSANSQN
YYDQQFAGRYTHNPDTAGPSSSNSVYNRNMRDDGTSGANGNAEAGPSTSYASGGSSKKQPRETGSGSSEYIL

>Cele_C18D11_7
MSDVYRVGEDATSEKSLVMTPDTNPQHAYVANYLWFFFIKDRKFHIAAFFKETPEIEYQSSVKIWFKDEHFSYYERSEPF
PFEEKCEDTYDIEYLDWTSIEVTFKVTKLYGEEYIGPADKESENEDGLIVNVANQKIFLCMETIVSNSEVFNKLRDSSSG
IEITLLDVDPEVFLEMLRVLNPPHKRISRFYFTEFLLLACRFRMYRLLVLLDDFSRTDEYDKNWITRHSWKKFQLILQED
PSFSIQVLESFRVKRRMEKPVRRVKIRDPEIQSVHLLDDKKSWFGEACEYFAFNKSKLIFTILSIWFLEHVFIFTWKFAQ
IKKPS
>Cele_C25A11_2_1
MADDPHVLAFLARVHQGRRDRVEDHLHEHFIRERNLHQRPQFPPLQLVPVQAEHEYEHPFPLVDVFKFSLQGDSVKTASS
SWQGLQWKVKCSIYNLPGELLATRVSVDFFNLMDRVFKATVKYEMNEKFAAANEFAEVVIDSNHSTIVFPETGAFAPGRL
VDGMLPTEIAVKIKLEHAEIVDQFSYNNQSWVMGTRWVRYGDECSVRMNFDVLKNLNPCHDKFFDDLREQVLSPKEARAF
FDFASAIFNRSFMGLDLFHESVRNGLKFGFTTNWDSLNTVDSEFDFNSRTAFPGYQNVLYEQPSELTSISNRETAGMCQI
FKLNLDPSDVTTRFQVASMNNVKSHTSQFTFYLENSPNGYLLIAGGCFQMESRQLVNLKLTLFDSGVSCRNIQTFRILHK
EQSTIKVVIALLDDDKMEKMSRGKLLLNFQMHMNGTISNVVRNFEDQTKRIGDFAMPGLVTGYINCRDKKRIGVCKEHLA
NNSEHLNMLFFNTVFEDCGKNEITVNEDSDIVLDALDIIYHRSLAIFPAQINRVLDLAGYWLSATMHRFMEIAILHSTHI
SDSAKLELAAEHHFDIIKYVARHPRYASTYHLSLTDEEIMQVRIPADNEVDGGTGGRPRRVEDLMDLEEQQE
>Cele_C30G4_2
MIGPQEFDITLAVAKLNFNNVDYLGIEIKYNQCLPRGTSCEYSTEVTVMNRKFNLKHHQFDNDRLYLPTFMKWDDVVDLT
YEDESVRCCAKVVGFRHLENVERSFGDCSYHGTSDLRHVTTVNLTITVGRYKILVNKDYLMSMSGFFRTILENADKNSRH
DDLVLTESRDEAFVTMIDICHHKFWCNYQLKEDLFEVAKLYEFPFVINFYNQFFNSDRAFNADDELSLENTM
>Cele_C37C3_9
MRPYIINQTEQAQARFGINNDFPVNVILMQPTTSHETFLEGSDYVDFESTSKLRNWPIIVGNQKYFVDPAIFARNSDYFR
ILMENKFFLEGAIKQLNILDESPEDILKLITVISPNSLGLYPDVIDEQNVCTVLRLCDKYLMTNLKQNCVDYLKDYNPDS
REPTDIFHLFYHLCFSLNAEYEHDHSLADVALEAMFVCLVELFNSTNVTKFYRFLIELQRKSGEKDSENLKRVTDAVLHG
GSIFKHRISFSEEVQGLGCHQCSMRPPARQSRSKNRKSFVLYLCKSCNREVCVQCRRSLCSRLFEDWINELRS
>Cele_C47D12_7a
MLPNIYNAESDRLGPGLSHSNSRSPNFNKTSIYSNPTMLGEFYQNLNLMRCQQELCDVVLEAYPIQGTSSEDGMSDDVHQ
HIHAHRVILSASSSYFRAMFTGGLRESTQRIIPIKEVDVEVLSQLIDYMYTGRMRIDEQNVQTILATASLLQLTCVRDAC
ARFMLELLDMTNCVGMAEFARAHACHQLAHAAQLYTRQHFVEIIDNEELLSLDKDAFCELIQDDRITVPSEKPVMQAVLN
WVAHEEPSRRPFLAELMSNVRLPLLGDDYLFEKWRNDEMIKSDAACLNVIIEGMHQLKITKQDISSTEPHYPIQELNRKW
FVAREPMPESQHIMIVGGQAPKAITNVDLFDPDSQLWSSCASLPQRRCRSGVSMCNGYVYTTGGFNGAQRVRSVDFYDPR
TDTWRSANQMSARRSTHGITTCQQVLYAVGGFDGTTGLASAEYFDPHTGNWFPLPSMSTRRSSVGVAAFEEDIYAIGGFD
GVSKQCLNTVEIFDRRAHKWRPGPAMLNVRSGAGVTVYDKKIIAVGGHKGAEIHRSAEILIGEEWIELANMAIPRRNTAA
TALNGLLYAVGGDDGSSNLSTIECIQLSDRSDAQWKIVDAPMSQGRSYAGIALIPKEF
>Cele_C50C3_8_1
MSSRSSWSSTEQINRTISSRADDLPPQPRRLEVVSKQATRVTALSTKLEWKIEQFEKLMKLIKNGSNLISRMFSVPDAPT
VCWELHVYPNGKRDEDVNNVSFFLRQVGLARGEEPIMTEFQIYALDANNQRVSVCRDTKDFTNQQGRGKFQVSRDKMLGA
LRSDGTLFLICEVEYFPPGSKISVEPVVDEDVSTEEQEEMPEVIVRANNRSMWEDELFTDCVIHVGNKHIKAHRCILGQN
SPVFKSMFSSPNMIEAQKGEIHIEDAKYDSVRAMVEFMYTGATESLESQGNIDEILAIADKYEVLMLKDQCERLIAQTIN
LKNVTQIAMFSDTYTADYLKSAVIRFLTTHHRVVIKTQDWISLKKSRHELANELLEAVLSTDQDDDDVTSNIPISVSPPP
ARKRLRRSAK
>Cele_F10B5_3
MEEDNQSPSPSESSIKTDYDAKIRVLYSTKNNKSEEESVFAHRLVLSTFSPLFQQLLQDATSSSTSTTSSTSSDDVISIS
LDLTAFPNALAAFKVILEALYTGDIKMKSVAANEVLAVSRHCQISSLEPKIMSAVAPLPLNLANILNCSQSVPLNSNPFL
QYTSPPIQNYLSSMISLWSNPFFSALFGNTTGTPSLPFSPQSDTENSRTQSEGSSPRANSTSTPPTTDLEKIVPNDDKEG
WCRNKKYIEKVDGGFMCTVCRKIYGRYNSVSYHVTIYHRNPPIKCEENGCNFSTREARYIHFHKYYRHHIPLPENIDLGS
RKCPFCRHVSKSPAMLEKHIARHETDGTSGGTPGALKRAQSKKKIVPTMTTDVIDPNVSSIFAPTNPAGNIPIQCSLCPF
STHSTDLLFIHLAMQHAQELAQCSGLLADNSSETSTSSSSDGEPMITDIDLDVKPLIDQSLMLHLNTPLSN
>Cele_F12E12_4
MLVITKEFVSTYTIKAISNFKPDTTYCSEVKEYYYIPWKLHVNRRTDQLEVYLSCDQLGTIWSVQANVEVKLRSPNESPH
CKTATVNFNSEKPKENSWGWKQFADWEKVAKNFVIEDALHFKVHVQILNASGFKGRDPVLKFDNESMGELSDVVLVVDEQ
RFHVLRKHLALQCSYFKSLFFGSFGESKKSEVTLSGIDPEAFQAFLEVLYLEDSIDDNTVEGILQLADMYDSRAVIGKCE
KFLYKESEKSGKKKLELAIRYKLEKLEIHCCNTIKTIQDVRSIIAGNIDEMSPFLMKRVLKRVLEV
>Cele_F14D2_1
MSNSKKEFVLRFDFKKVWLMKKKDQYTSEEEDHFGIKWWLRVTRDDQFLYVDLEKSGTGSTNFHGNFNVRIESGHGVISE
NFKFSGSKSDAEIPSTSDRCESFKWNKLDYSKYLSIEGHVEIIDMTGYHDFQKRVDFGSDVTRRFADTVLIVRGQRFYVL
KQHLALHSPYFKNLLKECTLDSEIQLPDVDTDDFQKYLEILYGFSTIHDNFVEGILLLGKFFETERIITACENFLIDKSS
KSIRKLFELSNKFHLKKLNKNCVENIKTSTALRLMLPPDVLTIHHTAIVELFRRSMQFH
>Cele_F14D2_12
MSRSSVTGKEFVIRYVFNDVSNMNENDMRISTVEEYFNVPWIFQIQRNNGNLGIYLRCRKLKGQEMWSMFTELELKLISV
YSDTRDQKCKTDMCFGHDSGKFYSSYGWSEFISWNELETSFMDAGSITVEARVIINKSIGFYTPKLMNFDEKKKEYSDIV
VLVDGQRFYLLKKFLAFHSTFFESLFLGGFYEAKLTEVPLYDIDVDDFQKFLEVLYGFPVINDNTVEGILLLADMYQTPL
VSELCEDFLIQTTGKTYKKKLQMAIKFNLENLKQFCISMIKSPFDLRSMIPSDISSIDHSTLAAL FTKSLTLMK

>Cele_F14D2_15
MSNSKKEFVLRFNLKKTWLMKKTEHYTSEEEDHFGIKWWLRVTRDDQFLYVDLGKSGTGSTNFHGNFNVRVESDHGVISE
NFKFSGSKSDAEIPSTSNRCESFKWSNLDYSKYLSIEGHVEIIDMTGYHDFQKRVDFGSDVTRRFADTVLIVRGQRFYVL
KQHLALHSPYFKNLLKECTLDSEIQLPDVDTDDFQKYLEILYGFSTIHDNFVEGILLLGKFFETERIITACENFLIDKSS
KSIRKLFELSNKFHLKKLNKNCVENIKTSTALRLMLPPDVLTIHHTAIVELFRRSLQFH
>Cele_F14D2_4a
MSRSQVTGKEFVLRCVFNDVSNMNDNDMRFGTVEEYFNVPWIFQIQRNNGNLGIYLRCTNLKDQEMWSIFSELELKLVSV
YSDTRDQKCKIDMCFGHDSGKFYSSYGWSEFISWNELETSFMEAGSITVEARVLINKSIGFYTPKLMDFDEKMMEYSDIV
VLVNGQRFYLLKKFLAFHSTFFESLFLGGFYEAKLAEVPLYDIDVDDFQKFLEVLYGFPVINDNTVEGILLLADMYQTPL
VSELCEDFLIQTTGKTYRKKLQMAIKFNLENLKQFCISMIKSPFDLRSMIPSDISSIDHSTLAALFTKSLTLMK
>Cele_F22G12_4
MGSNKEPKIDYLDILKEQFNALQSEHLELRRKYELECSSSTPDSFPSRLLSLTSSLLEKPRFSDVTFKFAGNSLKSVPAH
KYVLAARTDFWKFENGGDEKSEIQISDDVDFEAFHVAIRWIYTDEIDLKMEDEKLLKVCETATSFRLEQLKNVCVQQLGA
RLHVDNCIKIYEFAEKQSLRQLSTVAGSMIAGAWPKLGPAHFAQMTAALLYRLIDGNTKNVLHSIVSIGREDVLFLYFMQ
NSAKIPKILNDLDPQGASALEIALCSEHEKSRQIAAQLVEKGADVNVKDAERGETILMRMCRKENLSAMDFLLGNGADGR
DSQSPGDYNVVHVASRIPSPALATWISENKEKLDLDKVDAEERTPLICAVMANNHIMCETLIRSGVNCDVTTSEGHTALS
TCLLMSDAPNRRIAELLMLNGAGVNFAICDSKTPFFNEIVSRKDVASVEALLAANVDCHVADGRGQTACHVAAEAGAPEI
LSKIVEARRGLKWPRDADDRTALDIAVEKRDLKSARICIKGGADVNARDANGQTLLVKAILANDDEMGVFLIEHDAKAKN
DERISSKTYVEAACERGLLNTVRSFISNGCKLNSRCSTGYSLLHSALSHQKLDVASLLVNFGCDVESRVALGAGGEVLDD
GDESWITKQTLLHRLIDDGDQQSAVFLIESGADVNARKEYRNPTDDDQFTPAHMAVSWAQNDVLRALRDHSANLCDVDSD
GRTPAHIGVREQNVDGVKILLDAENVEFIPIRDKFGQTILSQAMAMKDHQIASLIVARQPHAAVQTNGNGENLLHQAIRQ
NDIESVLFLLAVAKADPCRPITDGSLKTPLHLAAIAKDEMILRNLILVNDDVNVTSADGTTPLLEALKARNDKHAAILME
NGAEPNVKDEYGENAMLCAVRSGSLDCIRAVADSSRTNRYARNKIGYTSLHICALLTIDKLPKRTSSSDVIELVLIYAEE
EAQAQNEKQFAGFIDARDADGNTALMIAYSQGNAGVCRSLLKRRACMGQRNNGDVNVFTYETATKQLLLGLLESLEAEPR
WSDGDTCDCGARFSLTSRKHHCRHCGRHVCSKCSETTMPIAKYGEEKRVRVCDVCAHVISTGTAPRR
>Cele_F25E5_16a
MGRKKEKHNAKIGEANVKKTTIMRFLIDQLDGFTDKLFVCQAEKYNWTTHFYRGQFFDEDYLIVDIYCHSSIPSWSCDAR
TEVKFWNTTIQDIEHHYEMKRNCMSCPPFIKWKDLISRENNYVRNGRIVCEVLISEVPKKANKNQAVIVKEYPGIRNLEI
LVEKSKFHVNMELLAFTSKFFEKELARGRLNTADPYILEDITPIDFKLYMDLTYHPKQYFSAYHAKDILQIAKRFNNLEV
VTTCQNVILEDLNENKLSTKNKTKLAESYDLDTVRELFNPNTMTQPSARPRFMAPDKFREKQPTDAEMEGISELFAEPWD
NQCHCKSKGGKENFDEKLKIKVENQTEKEEDDEDYDISMPSTSGC
>Cele_F25H9_2
MTESITHRFVFQLPVLHDENGRGYVSMAQEAENVYWRCGLIGVEKGMLGIQLMCNSEDRSNFWYIDIEYEAAILYNDKEH
LNLKGKFHHAARKNLTLRFSLEHCKKAATKDKGMYMQFIVTIVKKIGIREKRIHDFTVPRDNLTNLILIVDGKNIYCNKH
ILAMNSEVLMELLFPDEDHDVNELQLSNDFEYEPTLRIVKVFYNDGDALTGDNVDETLEYATKLKSENARQRCERWISKT
QLLTAKKKMKLAEKYVLVELQNECVAMLKTSQQVQRLFENPHEFTDNEIVELMDKVTLSIVGHTYDGFSK
>Cele_F25H9_4
MSPGGVRKHCIEFDDNWWKTTFLVLIIVSFLSTRFKMSILQPLEDLSANIVTLVFNIYNFEHLDGSYTSDLKEHNGIYWC
VRIQSNKAAKSQKRRVSIYLVCNPNNSSPDWSVTTSFGFRIINSWGKSRNKISTLFNHTFTSNETSKGTSGYCTWDELTA
ANSGFLVEGRFQIEFDLNVNSSTGIRKEKISKEKYEKYIADGELITDGKTVKVCLALLADNSPILYNLFYVEKPGQTTFH
IFDFTYEAILGMVSILQLDSFEVSVYNYRDLLELGQRYQIPSVTDKCEEFLLKTRYVSIETKLKLSEIFELHYLQFRTLE
RVTCIHHIDNILDDHMDIGEKTYDALLEKMKHLKTQEDGQLCSCKRNHVR
>Cele_F27C8_5
MFFSNSLHANAAPQHFERIAKILQAKRRAGDFSTCDVKIQLKTGFDMVHSVVICAHSDVFSETFDNQRAPYQPFNMTDFD
PDSVRRVFDWMYSGEIDIPETTIADVLAVASYLRVTMLQRQIEQKILNHNGSPIMALNIASARAFSVMDHTMNDLVHGFT
EKMTGLGIDEVAKLTANSMIAVMAAVLPMKKKVPLVNMFISWIVCKQPERETINTIIQSLVISDITYDTLYAIRYSLKQY
LTNSEIASKSQLTISPSGTIEIKIVPKKESMVSEKSSSLHSVVELPPNQYYRTRSEISAIDKMPDPFVRNLPRTQSASSM
IARPRSSGGFPQYFTRSEVEDLQQMTDPFSKSERGMTPTRGPPMGFSSVQCTVKYPGWSKDVMETNKEMYKQCKMNGRYI
LSEAQTESNAPVFLRPAVGGKVSSSRGALSNLSNSEQNKKYGRGVTQSFSGHSLNDNDPMPKEHDPKVQPSVIISCLSLP
TTSPVSVVKPKMTGVKKTDSEIMEINALPSSFNSNSFYTAKTSNTSGHSNDQSAGKSEKSQRSQKSEKSQKLKKPIPQSQ
YLYPN
>Cele_F29C12_5
MAAAHKVFELSHVFKDVSKIGDDESVCSPNEDYFGVPWKIVLRHKDEKMGVFLSCEKPSNNDAHCIVNVVYSVKLMSSSG
VCYIKSDSKLFGEITEHGWVNFVNWNTMVDAFLIDDSIIIEVLVKTIFMSGLPKKSPKNFDESNKEFSDGIVVVKHQNFH
ILKKFLAFHCEYLEKLFFGDFKEAGKAEVTLKAISSTDFHYLLAVLYEDYAIDDDNVGGILRLASYLQVPIVIRKCEQFL
IEGSGKPMEIRLDIAEKECLQNLKKHCLSKVGTADQIEAARAILPSDIPKRALKKFDESNKEFSDVIFVVEHNKFYVLKQ
ILASHSSHFKKIFVENVDKREFTLADIDSNDFQNLVEVMYGVSFIDDLTVEGVLHLAHMYDRPFPMQKCVEFLIDLSEKS
PKEKLKIAKAYQLKNLEKAALARINLPESIRAALSCDMAQMEVEVLKALMQKALFHLSDTKVHTETFF
>Cele_F37A4_9_1
MEINNGAQPENAAVSIPSRSPSGKSEKRKSPSIVSGCRTVMNERSFTNYWSVERFTVQLELHNPAEFMLAPKFGDGDYEF
VMKLFPNGKDEETAGYLSLFLLINKCPNPRLRFRVSFTVETADGPRSCHLNKNLVTINRSGIVTASKFFSLDILRSAMNV
YIPNDILTIGCELTIFGECTTQISSLFKPYQRTHSASSTSRSLSSPNTKCLKIDESSAHDAFTEFLSTGEFSDFIIVASC

GREFPTHMCILAARSEYFKVLLRNHSTKEFMSKRLQFDDISARTLDVLLRHMYASAAGRVVKLEEDQLTEDLISAMDRLM
INSLRDEVARLIGSKVTVDNVLTRISMAAELRLDDTYDVLLDFFSNHKHECMKLTAWDELESAKPPLAIKILRDAFSNTD
SPSTTSMDSRIIDRITLT
>Cele_F38H4_7_1
MSSENGVEVSDPEEGPCNRRQSSGSCEPENSDSIFQGENQLYPSRRTAANHEHQQQQKSSSTLDLPANVLHEYASSSPPQ
SSSRHVHSSSTSSLGEVAALFGTRFPQDEGKQASNVEKAVNILPVSRNLAEHVSNLFAIGRKPVTIVQKKKPTNHPEENP
GARPLLGWQADKKTLRERIEHMYCNETLADVFFVVGIDDSRQRIPAHKFVLSIGSVVFDAMFNGGLTPKNTEEALEIELP
DVEPSAFLALLKFLYSDEVKIEAESVMTTLYTAKKYAVPAMEKECVRFLKQRLVPDNAFMMLSQAKLFDEPDLMQKCLEV
IDKNTLEALNGEGFTEIDLDTLCEVLTRDGLRIREIFLFQAVLRWAKFEAERRGMPANGDSRRAVLSRSIPLIRFPLMKI
DEFALHVEPSHILSDREMNKIFKYLAVSPPDRPVLVYSDRPRCQISSTEYVVSRFQRIENRWGFCGTSDRIKFMVDRRIF
VVGFGLYGAISGPHEYKTQIKIIHCGTNKTLAEHDTSFVCDGNSRPCRVCFKEPVEILPGITYIAAALIRGPDSYYGTKG
LRRVSTHDSDVTFQFTYAAMNNNGTSVEDGQIPEIIYYTAAE
>Cele_F39E9_11
MSQVPIPHERITYESKNVYIGGNIRFGAANVQSGPTVLDTSTTNGLKCTWSGEIVNNNSQINFTWKFEYDEAGNEDIDEI
AGTIDVKFRRNQVSGMFQTGQNQNFTTVHTLVSLTEHCQTLTKLVEVPNQPGAVEVMYVYALVPWINPLQFSFDEMFLSS
KKNDAVLVIGERKLHVNKAFLSYHSDYFRALFSSNFKEDKQDEIELKDVVYEDFGLLMTTIYPKTEFPSDRTAEKILEMA
DRFMVQSAIDHVEYHLLHNSRITNESMMWMADKYGMEKLMKKSILKMDSLEKAKKLKESPWFPSMSHDAKVIVLERLMNL
I
>Cele_F39E9_12
MFYKAIEYKSKITQTWLPRGPPPPAILDRSTTNVLKCTWCEDMENGLSQIHFTWKFDFASEDIEKFVGTIDVKFRQYQFQ
NYVTVQRAVTLTDRFKWATVIVENPTRREHVEVMFEYSLTPWLRPLPFSRDEIFLPSEKNDAVLVIGERKLHVNKDFLCY
HSDHFRELFFSNLKKDASVEIELRDVVYEDFGHLMSTIHPNPVFPNDRSVEKILVLADRFKVQSAIDHVEHHLLHNSRLA
NECMMWMADKYGMKKLLKISIQNIGSLENAKNLKNSTLFASLSHNAKVMVLEQLLKFQADMCEILIIVLTGLIILGSFLA
AVDFIFNNFYSIVIFTLPVFILIAVIFYPPLTTYFWFWIKDVAEVVFEYALIPWNFLFQPSYEDEFLPCEKHDAVLVIGK
RKIHVNKAFLSYHSDYFRALFSSSFKENEQNEIELKDVVYGDFELLMSTIYPKPVFPTDRTVKKLLVMADRFMVHSAIDH
VEHHLLHNSRIANESMIWMADKYGMKQLLKKSIKEIDSLPKVKILKDSPLFPSMSHDAKVMVLERLMNLI
>Cele_F39E9_2
MFYEAIEYESKKVPLRGPHMMSGQGGTTVLDTSTTNGLKCTWSGEIVNNNSQICFTWKFEYDEAGNEDIDKIAGAIDVKF
GQNQSQNFTTVRTLVSLTEPCQTLTKVVENPNRQGAVEVMYEYALVPWITPLQFSFDEMFLPSEKNDAFLVIGERKLHVN
KAFLSYHSDYFQALFSSNFKEDKQDEIELKDVVYEDFGLLMSTIYPKTVFPCDRTVEKILAMADRFIVQSAIDHVEYHLL
HNSRITNESMMWMADKYGMEKLLKKSILKMDSLEKAKKLRDSPVFASMSHDAKVMVLERLMDLI
>Cele_F40B1_1
MVLPKKEFVLSHTFTDVSKMEKDISVFSPGEEHFNVSWNMSISCEGDHLGVFLHCHKSRGKETMWTIDTEHQFTLKTSSG
KAYREDDNFNFQFSNAHSYGNGTTEFISWNKLIKDYLIDDVITVEAAVKIIKTTGISKKALKGFEKSNEEFSDVVLAVGD
KEFFVLKQFLAYHSSYFKSLLLGKFAESDQKEVTLQDIDPTDFQNLLEVLYGEPAIDDETIDGILHLAHMYDMSVPLRKC
EEFLINESEKSMKDQLKIAKQYQLEKLKKACLLKINTVDEIKAAVAGKLSDMDPTVVAALF
>Cele_F40G9_4
MADEVIVCEGALKHNCWEEYINGLKWGCYRNDNLLQLRFNWSDLKEQGVEGLNLTLIKSQFLTKSENSDAILLVEDTKLH
VNKAFLSYHSEFFRGLFEFESEEKQKNEFKISDVSADDMKAFLAIIHPGNDFPTDSNAKKLLELADRFMVPFVTSSVENH
LLKSTEIDHVTKIWLADKFQLEKLMNHAISTIETTEKARQVRTSDLFTEMSTEVTQKLLDRVLQLTTKS
>Cele_F40H3_1a
MVLSQTHHHHHRHEGNDRSLISILTDPIVNLFRSIPIEILDQNEELSPSQKDFLDDFLELQNDSDLEESEDFIQESQEVV
SSVDDEIKVILNEIINQVETETQLIPKIIIHSDDAEVPEESIEIAVQSFGCPLDSFSGLSDSGVDLTVDDVPDDEDDEIT
VQTFESTPKELPTVTKFTNCTFIRSQSSARCKAILPDTTYICTLPPVSATFSAPVVTPKKIVKRKKSTSAAAKMAAEVLQ
NEPHCFIAPSSSIDRPFKLLVQKETFLIDAESMSRISPIFNVMCFGKDFEKMDLSREIVDEKSNDIDCFLRAVHDTSMIN
SSNFALVLRLSNKYQVDPVIDACQNFIIRQHLDVLRADEILTMLIAAFEHHCKREVVQKLIKRLASEGNSVFTKLKISRY
LPAQVYGSVISTSMNLNQAKEHELMNGHVLKMERSKIRWRQSVCEECKSVAECANCEECKKTVCRNHVSKLKCSSDYGAK
LAADLKKKIVDFEWQD
>Cele_F45C12_11
MSSLTKQFVITQSVKNVSSIADGGEHVTPSEIHYGTEWYMRIKRDNGFMSFGLYCPMTGKTTNWSIVADIRVELMPLTGM
WKSVECECKIAKSPSLISEVLRSTIMPWGRMMTNYVVDDALMIEVFVTIKSMTGFERPYNLRSFDESMEEFSDVVLLVGE
KKFYVLRKYLAAQSKYFKALLLGSFEEGKSSEVTLTSIDPNDFQFFLEVLYGDSAINDLTVEGILHIADMYDTPMVVRKC
EEFLIKGTKLPAKKKLQMAFRYKMENFMVLFFGNIQRKVFNFFRETTTLN
>Cele_F45C12_12
MEEVINYRSDLFRLSDNLETLHIEEKFGIKCDLTCKIEENMTTFEFQFDLRDFKNNGFKQLTAEITIRPDPENPIFELIH
RKLDILDIDEHRVNFSVENKNTQSLAETRAILELILTPIREKFSYREMFMPSQKTDAILMIGGEKLHVNKAFLSFHSDFF
HELFSASSTESEIPEIPIRDVSAEDMGLLLSTIYPDLVFPTDKTAAKLLTLANRFQMRAVTAQVEHHLLWHSDIEIEKLI
LMANTFGMKDLLKECTCDGGNKTPGEVPISVQ
>Cele_F45C12_4
MSEEVIKFTSPRKQVKEEFKLIDTQKIADVRIQWFGKFTKNREKIMFCWKFFPGDKLQNEGIDFLVGHIKAYPHPTYNDF
EPFEFDFNVNGAEHKETREYEYLTKNDWCCATYECELTPFRLYGNMFGASTKHDGVLVVEKKKLNVSKAFLSYHSDFFSD
LFSREAHSKDIPIEGVTYFEFGLLISCIHPDHTVFPNDETVSKLLSLADRFSMRAVYRMVEHHLLHSSSNMGIREVIKTA

DQYGMKELMEKSISRITSTEKVMDLIQSAEFPELSAETKAKLMDCMAKMMR
>Cele_F45C12_6
MPQPAMETITFSSRPELYKRGYKHSNISISDEFQCELIDMRTSNEKQFQVGMDFYWNEEEIERITGTVTLCYSSGNGFGS
WVESETSVDLSKPGLTIGKVFDTNFHVMYTSYRYSLTIHRSHCLHRLYEKMFSPSEQSDAVLLVGDKKLHVNKAFLSCHS
TYFRSLFAKQADTGILIEGVTYDEFGLLMSTIYPEMVLSTDKTAELLLRLGDRFKMPAVMHHVETRLIEWTQLTDQKLMF
LADKFKLQKLLERTISWIDFLDDSRMLKESAEYSELSDSTKAKILDRLNSLPLSNENYTNYSSDGYFNRDVMRNILFILV
GVWLHNTACLIVSFCKSTKN
>Cele_F45C12_7
MDKTKNETLEFASDWMYATLPITFYSDCNGLKCNVSIFNPDDQFLSTVKFDWDQKVIKDLTGTFTWIFDNSGGSCNQQLE
SITLDKPGQCYAQKINWNVFDYNYLRCKYSLTPNCCQEKCSDNKVALTPKKSPGQKVPEHKPTDHEIGSDVSYEKLFAPS
NRHDAVLLIGEKKLHVSKAFLSYHSDYFSDLFADKINEEIRLEDVSYEDFGLLMSIIHPETGFVNDQNAEKLLHLGNRFK
ISCVIHQVEHHFTRGTNFSNERLIFLADKYELQKLLEKAIQEMNSLDKAKKFGGSDDYKKLSDFARGKISDRLMQLLIN
>Cele_F45D11_5
MWCLLLKIGSSMLLESSWPPIRPTSRIFCSETSQSPEVTLPGVNAADFQNFLEVLYLEPAIDENTVIGIHHLADMYDVEP
VVRKCEKFLIEKSQLTVNEKLHFAMQHRMENLKEYCLSKIKLWTISAQFYRLT
>Cele_F47B10_9
MVAIHESVLDGIREKISLFSTETVLRKFQIDTQSDTLYVNLSYLAELSEYFHILRTGSYTEKTSEKVTLDDVYTDELVVF
LSYVCPEGFEFDRTINQQNITPLVYFSDRLIFPWVKQEIKKYFKSDDFKNELYDAESLIQLCYLLHTQCYASGDIDPIFK
KIARLENISLVDKAVAAISDANMRSYFAERIIHFRPYSYQPRAQSFFDWNDTRARLFF
>Cele_F47C10_2
MFLPTNETDAILVIGDKMLNVNKAFLSFHSTYFSALFSSNYKEGQMSEIELKDVSYENFALLLGTIYPNTIFPNDKTVYK
ILDMADRFILPSAIWHVEYHLLNNTRIRNEMLMWLADRYGMTKLLEKSIRRMNSVESAKKLKASQEYSKLSNDSKAKVLD
RLMGLI
>Cele_F47D12_7
MDICDISASQLATSPPPRTAIDFNSSIEESPVKRVKFSPELTKSFEETLSPTVTLVLRNNEEVIFDRYLLSFYSNYFRVL
FSSKFRDSNSTTHRIRLISASDLHFLLTIPKAFEQGIKPNITLQKAIELLEPAAFLQMSIALDYISDIICKNLTHENIIK
IFRLALLYHTTLAVRVWRSMVRKFQTLFATNVYLSLKENELIGLLTDKHLNLKSEDETTVVVNWIKHNSPLQSDRLTQFA
QRNFSRRPQPDATKYEVIRTRQPMDAIVCFGGWASRGVAQKIEVCNTRSDRWQTCNFNYDIPNIHRAYHGIEVVEDKLIV
YGGFDGIKQFQTTVLFDLSTKEWRRGANMNDKRCYVTSARVNDSYGRPLVFACGGMNGVSRLKTAEMYDYRADQWTEVAN
MTQMRSDGAVVTIDNKIVAIGGFDGRNIHQGGEVYDPVLDLWHPLSSNMRTRRTGCTAVSIMNQVCMIIGGFNGNRRLDS
AEIYDMREGLWHPEPTLQTARSNFSACQMDTCYVYVAGGFDGQTTTKESERLDLRSKMWQALPDMAEAKSALRMVTLSDH
PFLDELFDIPDDTGIVTSW
>Cele_F52C6_10
MVAPGKKFTLRHIFKNVTKWKTGEIFYSPDEEHFNVPWQMCIHRENGNMCVILCCLKVEEDRVPWKINFEYQLKIVNISG
MSIVKAGNKICTEKRRFVRRQFISRNTLKSGYLVDDIITVEATVKITKVSKKQLRSFDKSNKKFSDVILAIEDEKFYVLK
KFLASHSTYFKTLLLGKFEEAERQEVKLQDIDSTDFQNLMEVLYGESAIDDGTIKGILELADMYDMPFPIRKCEEFLIDS
SEKSIKEKLKIAKQYKMENLKNSCLSKVNTVRDIKAAMTGDPTEMDLSVLAALLGKSLALH
>Cele_F52C6_11
MVAVKREFELSHVFKDVSKLEDGNTFYGPEEEHFNVQWRIVLIHQKEDVAVFLRYLHPKNEQAPWKIDSEFTLKLSNSSG
KSVTKTGSDIWNSYDALGFCDVFEWDSLLKDYLIDDVIIIEVAVKIKNMSGIPKKKLKSFERSNEEFSDVILAVGDKKFF
VLRKFLASHSTYFETLLLGNFAEADKSEVKLQDINSTDFQNLMEVRYGEYAIDEETVDGILHLAHLYDMPFPIKRCEEFL
VDSSEKSTKEKLKVAKKYQLENLKASCLAKINTLEEIKAALAGDLDEMDPSVVHALFRKSLALH
>Cele_F52C6_8
MVATTKEFQLHHSYKNVSKLEDEGTLLSPSEEHFNVQWYMAVSHKKEDMAVYLHCNILGKAEATWCIDAEFEFTLKNSCG
KRSTKKTAVKFTNSENIGYGWKKFISWEMLAKDYIIDDIIIAEATVKVTNMTGILKKKLKSFEKSEEQFSDVILAVEDET
FHVLKKFLASHSTYFNALLLGKFKEAEISEVALKDIDSTDFQNLLEVLYGESAIDEDTIEGILHLAHMYDMPFPTRKCEE
FLIDFSEKPIKEKLKVAKKYQLEDLKMSCLMKTNTIDEIKAALAGDSTEMDVTVLAALLEKTLTFH
>Cele_F52H3_3
MSEQYTTPERENPDDERAFDRVFDALSISCPCDNPSNIPNRLFASPPTQSAPEQIDNGAADVQPFDFQAYNQPRLARTEG
MLKLEIPNFSNLRSKVSTPFQYIGNLPWRLAAKTEKTKRTSDVKFFSVYIDCNPESESTLWSCDAVVEFRLVSRNRTIPP
FSRQFTNKFNYNSNNWGFPSFMAWEDVNNSNYVRNEMVTVTARVVVQKVLGVRNVPKYDFGAMQTNICDMTLVINKQKLF
VNKAYLALYSPVFYAMFFSNFQEREKTQVELEDVVLEEFRELLHVIYPCHKPITSDNVEYLLELGDKYEIQYVMDECERF
LVGSEDILQITKLMWADQYLLAKLQDSCLRNIKEVSDVKAIKLTEEFKNLSDATKAALLEKVLKIVN
>Cele_F55B11_7
MKENLRKPPEHFPLGSHSTILHVPKSSTNYSFRIRNIHNVKWKLNVETLNSVPTDKINLKLSKELNKNTDTNKIDSIQID
YGFTIKNHVNSNNSIVVKGTFTFGGNENELNVEMSENEKIIEDSNGFYDLKTQKLELECSFIVRSAELCDFVEYLDLYNF
NPVIHDTEANVLGVRMFLNKKIIALQSPTFLALIENQTVLSSEQLANCKFEDIHDFLQLIHGVDLSLDEDNIDTFLMLAK
RLDVPRVIDYCKRELIAGCGGISKKKVTEIAIKWAFWDVMPEIVASAHTLADLKKQNWDLNELPLNVFDMITRKCFE
>Cele_F56C3_9
MTQQKMHMFSTETALRKFQINTQSDTIYVNLNYLAELSEYFHILRTGFYSEMTAEKVNLNDVFAEDLVVFLSYVCPDGFE
FDRTINQHNITPLVYFSDRLVFPWVKREVHKYLNSEAFQNEIYDTELLVQLCYLLHSQNYSEIDVVFKKIALIDNPLVVD
RLVQEITDSDVQSFFTKKILQYRPYTEKPRPQMFFDWDHTPYSAYV

>Cele_F57C2_1
MADEVIEYKFTCSSFYYGRETSQVLNTSITKGLKCVWKTRYASIAEQAFFSWEFDWNELKRLGIDSLAGNITVKSASMSP
LNVQIDLRKPTEIVERAFYLRDDDYAYNSYINKTTFEYSFVPYCAQVDESLYEKMFLPSDENDAILVIGNKKLHVCKAFL
SYHSSFFRAIFSSAFKEGQMSEIPIKDVTYKDFALMLSTIYPDAVFPNDRTVEKLLEMGDRFIIQSVINHVEYHLLHNTS
IGNEKLMWMADRYGMPKLLVKSIQQMNSIEKAKALKSSPDYAKLSKEAKEKILDRLMALI
>Cele_F57C2_2
MADEVIEYKFICPNITIYRESLQHLYTSFAKGLKCVWKTRNTSGQTFFSWEFDLNELKNQGIDGLMGRISVNSDSIDLLN
VQIDLKNSTEIIERVVHLYHNNYQYGYNYIPSATFEYSFVPYYAQAEGSLYDQMFLPSHENDAILVIGKKKLHVCKAFLS
YHSSFFRAIFSSTFKEGQMSEIPIKDVTYKDFALMLSTIYPDAVFPNDRTAEKLLEMGDRFIIQSVINHVEYHLLHNTKI
GNEKLMWMADRYGMSKLLVKSIRQMDSIEKAKALKSSPDYAKLSKEAKEKILDRLMALI
>Cele_F59H6_1
MLNGIHDYKKEFVLSHTFKNVSKMKIDKSLISPVKKHANFDWYLSINHNGDDLGMYIHCKQSKNDGVIWFIEAEIEMTLD
GRFIKSTTKRGEFKFSTNAPFSHGFRKFISWNKCVYRIGDSFNAKVSVKIRKAIGNCKSVLRSFDKSNEIGLEVLIETYW
AEYYVMREVLTTQSAYFMDLLKDPGISKVKLDGDDLTEFQNLLEVLHGMPAIDENTVVGILHLATDYKMPLPIQKCEEFL
TNKSTKSTNRKLKIASEYRLDSLKVLATHTSYFKDRHVTLQNISAADFQNLLEVIYGESDVEDHTAHAILYLAHKLEMKL
IVQKCVDFLLQISESTIHAVIHLACRLQIHRLHQKCANFLSEKSENFNDELTNYQLENLVNNCIEKIDSIDNIKAKLAAG
WKDSILLADLLVISLEIY
>Cele_F59H6_10
MVATNKEFQLFHTYKNVSKLEDEGTLLSPSEEHFNVQWYMAVSHKKEDMAVYLHCNILGQTEETWCINAEFEFTLKNSCG
KRSTEKTTVKFTNLENIVYGWKKFISWEMLAKDYIIDDIIIAEATVKVTNMTGILKKKLRSFEKSEEEFSDVVLAVEDEK
FHVLKKFLASHSTYFNALLLGNFKEAEISEVALKDIDSTDFQNLLEVLYGEPAIDEDTIEGILHLAHMYDMPFPTRKCEE
FLIDFSEKPIKEKLKVAKKYQLENLKKSCLMKISTIDEIKAALAGDSTEMDVAVLAALLEKTLTFH
>Cele_F59H6_11
MVATEKKFTLRHIFKNVSQLEDGKYLYSPDEDHFNVDWNMSISHKKEKLGVYFYCYKSEESASAWSIDTEYDLALKNSSG
KRISIDFKYKFSTSTGFGTAELVSWDAMIKDYLIDDVIIMEATVKIINLMGIPKKTLRTFDESNEEFSDVILTVEDEKFY
VLKQFLASHSSHFKTLFLRKCEEEAEKSEYTLQDINSIDFQNLLEVLHGEPAIDEETVQGIRDLADKYDMPFPTRKCEEF
LIDLSEKSIKEKLKIAKQFQLEKLKMHCLSKIETVDHIKAAMTGDPIEMDLSVLAALLGKSLALH
>Cele_F59H6_12
MRAQKVFNRAPNEGTDAILVIGDKKLHVSKAFLSFHSNFFRKLFSSNFKESEMTEIPIKDVTYEDFCLMLGTIYPRTIFP
NDETSEKLLEMADRFLIPAVTNIVEQQLLYNSQMQNEKLIRLADQYQMKMLLNKSTWKVDSLEKVKELIKTLEYEKL
>Cele_F59H6_8
MVKKEFQLTHIFKNVSKLKVGKFAWTPMKEHFNVSWQIAITRTHFRNEEKMEIQVGCQKLTKDDVPAGVEGEFTIKLTNS
SGISTSKSSRLKLVKNGGLGFLKIVWKVMQDEYLIDDSIIIEATVKITKMTGISKKPLKCFDQSKEKFSDVILAVGDQKF
FVLRKFLASHSTFFESRFLGNFDEADKSEVTLRDIDSADFQNLLEVLYGEPAIDDDTVDGILHLAHMYDIPLPIRKCEEF
LIEVSEKSMKDQLVLAKQYQLEKLKVKKKT
>Cele_F59H6_9
MVKKEFHLTHTFKNVSKLEDGKTLYGPEEEHFNIPWQLSFCRKEEFVGVFLRCLRPTDGIVPWEINADCILKLTSSAGKC
LSHSSTYIFQSIGGFGRRKFVSWDTLVKDHLVNDSIIMEATVMITKMTGISKKKLRSFEKSEEQFSDVVLAVEDKKFFVL
RKFLASHSSYFKSLLLGTFAEADKSEIKHEAINSIDFQNFLEVLYGEPVIDDDDVEGILHLAHMYDVPVAIRKCEEFLIE
KSEKSMRDQLEIAKKYQLDNLKKSCLSKVNTVEEIKAALMCDLADMDPTVVAALFRKSLALL
>Cele_H31G24_3
MVDAPGTEVNDTRVIIGKGLSLDSIKFPDFHMFDTNWKMKLHAEWSEELETEVVSIKLRHRRRNVDDIWSMDATVKIHFE
QDWLKGEVQVDSQFSNQGQEIEILNCGTVVGKLNSSEIHLCVHIFMQINQSKGIQLRNYINFARPIPIFSDTIVLVDGVK
FHVNRMILSMASPIFLEAFVTNFDERSLKLCNISAPDFRRILNIIYPPHLPPKQWIKESDLVKQFEHIQRILRIGKSLQI
SIVLEVADKWLVKYGRHKLGDTLHLVQAFGLRKLMGSKLAKMRSLEEIKKCQDQIQVLSRKTKALILDRILSTL
>Cele_K02E7_9
MDNIVTQNIKFESEPNYYLRGYHLFDISPLNGLKCEITDEFETDKIHLKIKLDWDQQEIKKITGFIQLSYDGHQLKPIDS
NFDKRGGICIMTVPKYINAYRWSFSFDLKLHRYPAKPPQISHDLNPQVRSYDTMFEPSRKHDAVLLIGEKKLHVNKAFLS
YHSDYFCDLFAEKFDEKNGIPIENVTYENFGLLMSIIYPEAAFSNHQNAEVLLELGNRFKIPAVMRHVENQIIRDTKFPN
ERLIFLADKYELQKLLAMEIQKMESFDQAKKFKGSDDYSQLSDATKAKILDRLIELS
>Cele_K08E4_7
MDAENKSNTDNNYGEIQLVFKLVDKKYFFNPLFHRSEHSIAKIRNLPWTLSLVKKDQIKVTISCNEDSSSNVWKCEADIA
LEINYHDVRSDTPTKMETIFEGRKFDFNKRIMEETVEQWIDVFNGKIELKGAITVTAKVKVTKLYGYRKRKMLDFSTNHP
DYFDGVLLVENKKIYVQKRYLATKSTYFWNIFFGDFKEKEQEAVEIPGVKYDDVIQFLNFIHHTDSQINIETIENMMVLA
DLFLIDCLMCCCEKILIKADSVNLAKKLIWAERFALNELMDHCLDGFKKMQDFVEFRKNEFYAGVSDDIKSTLLDRVMKI
KGYRYL
>Cele_M01D1_3
MDNIVTESIKFESEPNYLQRGYHLFDISFLNGLTCVITNKIETDEIHLKIKLDWDERDIEKITGFIQLSYDGHQLKPIDS
NFDKRGGICTIIVPKFLYEYRWSFSFNLKLHRDPAKPPQISNDLKSQVSYDTMFEPSRKHDAVLLIGEKKLHVNKAFLSY
HSDYFSDLFAEKVDEKNGIPIENVTYENFGLLMSVIYPEAVFSNHQNAEVLLELGNRFKIPAVMRHVENQIVGDTTKFPN
KRLISLAEKYGLQKLLAMEIQKMKSFDQAKKFKGSDDYLQLSDATKAKIFDRLIELS
>Cele_R09A1_2

MKTAINYCFFFDYQLFNQKIFGNFLFVNEYSGEMKDSCKVSKSTTNHRIDMNKSLDITAAIHHFESVSQFLRDDYSGARE
LCDVTLRFDDESIPAHWVMLAAYSPAFAEKFRKPNKCFVLEMQPLDKTSVKRVLDYIYFGKVTFRLATLHEDLKAISYFE
IVTLQEDVEKRLVELAKGNCVDVLNLVTANLKTIPHSPSTILLISDESVRDIVSILHDTYSTNRLSYEEILSLSTNTVIT
CLSARIPDFKKVDIINMSLKWIYERHLNDHKACKILQGLTLGSMTYAELVNLRNSLIQTAIPVTIGRCVRLKRRENDTLD
IVFTYGESSGYQKPGIKPTITSSFTMNNDPTSGAGTPSTKTSRSPSNKFDDAISESWNVNLDDCNTAMSFNDEDLKRLGI
EKSKKMKKKEQEEM
>Cele_R09A8_3_1
MPLELNIDLTEERPKVPEIVAFAMDKPVLNKTNVLTLIPKRNRSAVSMRADETDNWGQEALDEKEAYTLMLRDPSIKNDL
LDRLNTFRRNRELCDAVLFVKEREIFVHKVVLAAVSPALLDMFNEENESSQDEKSPTSSSPTNGHTNGITNGNGEVAHEN
GAPVSAQKPQKSSTTFYEFSQTDFECFEALVNFAYTANLEISSRKVAELYKTAYALQMIPVIRACAAYLAENLQLSNCIG
IRRQANFHNDTFLMGKVDQFIVDNFDSIVNDSKEFTQLPVIQVRIIVPAEDGKVTNNANNNGGLAEMALFYFQNMPHDRA
EHSIDLLTCKTHILYMEENHLADCIEMDERSSVGSCDIIQDYKKSGKDRKDVAKAMTAQEPIINSVVQHRVNGAVPVRLN
ASRLPNMNASNESLESAGTDDSDPQDTIEARLISTHQTAPQYWVALVVLYRRLCVLSLQLTDNEELLRTKREPCPVDSQK
AALLSRLISCTGKQQKPLECMSAPRCSIGASFLNGKIFVCGGYDRGECLRSVEEYDVVNGKWRNVAKMKAERGRFDCTIH
GGKVFAVAGSNGNNDLKSCEIYDPKADVWAPLPNLATARCHNGCATIDNYVYCIGGSFDQKVLKDCERLDTTKLGTEEAE
WEPMASMEHPRYQAGVCTWKGLVVAAGGCDRWTCMDSVEAFDPKTNAWRQLPKLRQARRGGAVAVVRDTLYVIGGHDGTQ
SLDTVEILDSPSSQWRVGPTLTTPRANTHAVVTAGNVIFCIGGFNGVKFLDTIELLENEQIGWRNWQHCPQQILEEHEEE
DSSLMDDEATSTAPSSP
>Cele_R10D12_8
MKISRFFELFNKNGSLKSENQFQFFNFNWKLKLSFSEKHNVPSIDLKLECDEIPDGIFLSIWIHFHCLTQNTKYDFFQIF
DKSRKSFCYRRIVTGIRHVRKFDGKTIVSFPINRFLLDDETGEHYDSKKQTKWPFYNPFTWDKLKEGTINAIPCKSCYHQ
FGFRIDLKCVTTFNTLPVRYGLPKWPKATLYPIQVEFFEPAWPSIQKLLNDKKTSSMSSDEWWMLCDLLGAYYHEWNFTD
EKLSKLNLHATKLGFYGFMEGLGFDGNIHKNIKNRNSANIVQKPRNITLTKHRKATVEYQKFWTISRIPKLLEPPKLFKI
YLGHGLHCIFNFYQSKFREQSYLSTAIVLIGCFNTFHDCDIEIYLVHGKTSKTQKSCARRILNQVQNTICIPLLCPVEVL
EFGEEINIEVKLKTRQVDGNFKPDCPSFVEMPGMNNVILKFDENCAIRVNKYFLAHFAPFFVALFFNENFEEHKQNEIRI
ISASFNEMLSFLDVLYHKTKLFNVSEYYHVLRIADGWICDEVIRIIEKAVENTLCSSVNKEKLRMKFNLANSKQMPLAES
TLKRESKDKTSDWRSQADAKKSRVDKND
>Cele_R10E4_1_1
MAPNKAAAEEYPVDIVDLSDDFKSSKTLDLVLDEEHKVYRYWIHLKCMKWQIELRANSFECFAAYFKTPATWSAEVEFEI
RAHVTNSVRSEKFTGRYTGSNHVYFRPLKLQDHMVREKLRSMRVEITVNVIKTMNFGMLYDYFDVLPEEENALLKVEDRL
IRVNKRYLSMMSPFFKALFYGELGEDKEIYEMKEKFREIITFLRCIYPHRQPVTKKSLDYLLQLADRYQCQPIVDACEMF
MCAHYHEYQKKTEKCFSYVEKYKMQRVLRIIVEYVAVTQSPAQFRLQSYYRDLSEKTQLAVLDYMAKNQSRRGKELERRR
GVNSHRANIDPIDDQDPPNGRLLGIADPLRNADPRPPQPGYNVPRYGNFRPLVFGHH
>Cele_R11E3_6
MTLVASSENTGLCPMTFDDSSGQTSHMLFQRLQYQRKTGRFCDVEIVVQNKSFAAHRNILAAHSPYFDAIFKYCKITKEQ
LTINSKSPQVFELFLNYMYSGTVIIDRSSVSELLRFANNFLIVKLKVYCAAYLDRYLDAANCLSIRTLAQRYNLPGLVKS
ATDYFDSNLNRCLLESRDIIGYTYTQLTRLIGDPKYVDCITSDTYLKLIVRWVGEEVSKREEIFRLLLESCEFREVSADT
LEFLLDYSPLLSKSQKSRFLLLHVMETNNMLMEKYSTQLSSLRQKIVDLPILHDPSQFEDDIYDSSDSEEDPDDYIADGH
PTSAQVVEYGNDVNNRLKMKIAFQPGNEKKRLKRPKGMKKHHLHPIPPINATENESIPWIDEDEIDGVYATCSLEGCHTY
GPVDNLDPDDCEDEEDQPDENGPSTCIFCGLRTANEELLEKHKARHHNRNTYYMCHLCEFETNWSKQFYLHCAEHWTEIP
YRCETCPFTSNEIQEFLTHRLQHTDERFFKCGECAWKGRTRSQLFAHERMHSVLDDRSLHCEECGRGFQQHSTLDHHVAS
HNDPRPYICEDCGFATKTADHLSLHRRQHTGDNFSCHIAGCDYSSTKKSQLAAHLRTHMAVRAHLCKICGRGFIEKSHLV
RHERIHLEEKPFKCDQCEYASSRRDKLKEHIIKHHNSEVLTFQKTQRRKYKRQRMLAAASNIVNNPDALFRPITSDDAVS
NWQATNDFSTYSPNPNDYGGPSTYGNPSSPQRAFSVVHPSQHQHHAQSPGCMSMMNVPLRDNHHQMQRQDSPGDSVGGGQ
GGGGGGGPEGSDNTHGVLMTPNLVMRSPHNRSAVQPQFNNLSTNSDPQQQQQQQQLRPFSLPTYGQMDLAQAAQLQQLQQ
QLQAAGLPPTGTPNNQDNSNNAWNPWNVQ
>Cele_R12E2_1_1
MRGGQEISDPMDLPSTSHDSADMFESRKPQSKYKNSHFANSVLQQLGGLKNRDVLCDVTLICGWKRINAHRVVLSACSPY
FLSMFTSQMAECYMREINMEEIEPPTLEALIEFCYTGAIAIDDSNVQDILPAACLLQIHEVQTACCDYLKKQLDPSNCLG
IRAFADTHSCKELLSSADEFALKNFSRVIGKEEFQMLTVESLTTIIKSDKLNAASEELVFSAVIQWVRHDISKRKTHLSM
LLSHVRLPLCTPKFLVSVVSEEILVKSDPASRDLVDEAKNYLLLPVERPNMQGPRTKPRKPLQGSEVLYAVGGWCSGDAI
ASIERLDPMKGGTTWKCVAPMGKRRCGVGVAVLENLLYAVGGHDGQSYLNSIERYDPMTNQWSSDVAPTATCRTSVGVAA
FNGSLYAVGGQDGESCLDVVEKYDPRKNEWAKVASMGTRRLGVSVSVVNGCIYAVGGSNGPAPLNTVERYDPRVGKWEEV
RPMLTKRKHLGTAVYDGYIYAVGGRDTTTELNTAERYSVERDEWQPVVAMSNRRSGVGVAVVGEKLYAVGGFDGQTYLKS
VEIFDKDTNRWKTHSQMFYRRLGGGVGVVRVADAPETELHAVKRRDL
>Cele_R13A1_3
MSDISMKIPAVNKLLNLPEDSEIGKKVRISIRDIHGLYWTGFWTILNTIQGRFEIKCEEDSMCRWKFLAQVELQFTGEGQ
TPDVNINKEIEFSQKMKIFTFDPIDQGRRYNKVNLKITRKSSVGVDVVNFIDFEKKSNLLDALIRSEDGSKMVYANSGIL
ALNSPILKQKLEKDKLAEIIMPSELFVSIEKLMNFLHPPFKMERESVEEVLDLASKWEMKSVLAKYEQFLIRTRSYGQEA
SIKNVKLADKFKMKSLFEDVLYSEEIDVYTFLDGNDYSSWIRAAIIGSSLLFFHSYIENAVSLGIFCPLSFSFYFLSYAF
LQTSLIACSKCSIFNLLFQLLVICVFQSLSQICKSSMGDDSSQDKASAPPSAAAAGAAPAGNPQQQPASAIRNKVDVPPG
RASSWWRNLVMDDDKTAPVQSDTVISPENA

>Cele_R52_1
MVATEKKFVLTHIFKNVSKMKDSDFLYSPEENLFNVPWFMLIARKDDHFGVFLHCQKINEAGPTWSVDTQNTIRVKTLSA
SKVSSEIFKHKFEKNEGWGYFKFISWDTLTNDYLIDDCIIVEITVKITKMTGIPKKVLRSFDKSAEEFSDVVLEVGGKKF
YVLKKFLASHSTYFNALLLGKPEMSEVTIQDTSSVDFQNLLEVLYGEPAIDEDTIDGILTLAHKFTMPLPIQKCEEFLIE
KSEKSVKEKLKLVKKYQLKSLKTHCLSKINTPYGINALMPFDHNDMDPEVVMALLKKSLATDEFYLDCIMRFTVICLIFI
TVGLII
>Cele_T07H3_1
MPPKLRNFDKSMEKYTDVVLVVKQERFYVSRAILSEHCTYFQSLFCGDSNYQEHIISDVEPQDFQKFLEVVHGEPAIFEA
TVQKILPLAQKFGSKTVLQKCERFLITISTVRLSTKWNLAKQYGLDELQAHCKLEFKTAQIVRKNPQPEPKSLAYSDKFI
FDHSFEKVDGLAEGDMRRSQPITHLDFTWTIYITRKNQHLRVSLCCGKAHLTSDWTVRADFKMTLTAKGSPDLVSDTKGF
FHLDSEKNFYWELGQEVEWDEIRDKCLADGKLEVETRVRIVKIRELPVEEFKPPRIVTVNAVKSYEGVLPPTKVLFGDFP
FPKALAKEKPDYSDYIRVTPLTQQAKTSAPAETPDLQTPPRQQASAKPQASPAKSAGAPQLPQKVPLFPSGGAPSSPSAP
EAPSEPPPPKIYPTLGANAPPAPPVQGKKFILKNVFGNVEAMKCHTAMACQKAVAYDFEWNLIISQKNNRLHVSLCCSKS
NVTSEFRILADFELIIAGKNGGRPLKSKTNGCFNIEKTKNSSQELGQDVEWSTIVYDYMVDGRLTVEAHVNITRVTCLDA
PKFKKLRNFDATGNSWDVMLIVAGQRFFVLKEILISNSARFKNIISKINFTAAPSTEIKLPDSSADHFQNFLEACYGDPS
INDDTVHGILKIAQSYEAAGIVKRCEEYVIKYSKKTLEQKEKLAKHHNLKTLKAHCDYWKSRRKCC
>Cele_T07H3_2
MGPPPKLRNFDHQMEPYSDVVLVASQEWFHVSKAILCDHSPYFRCLLSNFPRIDEKPVHLLKGVNPADFQKFLEVLHGEP
AIFECTIYQILLLAVKFQTYTVIKKCEKFLIHTSMAKLETKLALAQKYDLTELQTHCNEEFQAAFPVREPEIQRKPAIYR
NKFTFKYTFEKFDEDCVGGYNVQLGFLWSLNIHRKNDHLRISLCCDKPEPTPYWEVRADFQLVLPGQDEAPVTKGYFRNI
DEELKCEELGREIEWEEIADKYPVDGYLTIEARVNVIWIKEVEKKPEIKVEKKSKLITAMKKLLTVGK
>Cele_T16H12_5a
MILLAKFRNLYSKSRANDTISEGDKPEKATGDLEAGRNRLVSMEVGMGNDEVVSSGSGNSAHGRSISPSPSSASHGDPLL
PVAENWCHTQVKVVKFNYMWTINNFSFCREEMGEVLKSSTFSAGCNDKLKWCLRINPKGLDEESRDYLSLYLLLVQCNKS
EVRAKFKFSILNAKREETKAMESQRAYRFVQGKDWGFKKFIRRDFLLDEANGLLPGDRLSIFCEVSVVAETVNVTGQTNV
SQLFKVPPCRLADDMYGLFDNKQFSDFTLVCKSDLGSPTQTFHIHKAILAARSRVFSAMFEHHMQESDTNMTTVDDIEPE
VMRELLVYMYTGQTKYIEQMAQSLIAAADKYQLDRLKVMCEQALCYQLTTDNASLTLMLADMYSASQLRAHSINFINVNA
NEVMDTEGWEDLVRDHPKLLEEVFRALATQQTPPVVLVQPPKKRPKHNCPY
>Cele_T16H12_6
MDISDISASQLATSPPPITTIDFNSSIEESPAKRVKFAPELTKSFEETLSPTVTLVLRNNEEVTFDRCLLSFYSNYFRVL
FSSKFRDSKSTTHRIRLISAPDLHLLLTIPKAFEQGIKPNISLQKAVELLEPSAFLQMSIPLDYITDVICANLTHENIIK
IFRLALLYHTTLAVRVWRAMVRKFQTLFATNVYLTLKENELIGLLTDKHLNLKSADEKTVVVNWIKHNSPLQSDRMAQFA
QRNFSRQPQPDATKYEVIRTRQPMDAIVCFGGWASRGVAQNIEVFNTRSDRWQTCNFNYDIPNIRRAYHGIEVVEDKLIV
YGGFNGTQQFQTTVLFDLSTKEWRSGANMNDKRCYVTSARINDSHGRPLIFACGGMNGVSRLKTAEMYDYRADQWSEVAN
MAQMRSDGAVVTIDNKIVVIGGFDGRNIHQGGEVYDPVLDLWHPLSSNMRTRRTGCTAVSIMNQVCMIIGGFNGNRRLDS
AEIYDMREGLWHPVPSLHTARSNFSACQMDTCSIYVAGGFDGQATTKESERLDLRSKMWQALPDMSEAKSALRMVTLSDH
PFLDELFDIPDDTGIITTW
>Cele_T25D10_5
MKIKCVEVNSDKYSIKSVLGKFCDTNCGGINVSLFVSETTSNKYRFEWELKWDELKKKGVKHLFGTITPETNDKLVPSID
FSVDEKRSTSNLETSKDWLYNIPFKYKIFYKESDYSGLTLEELFKPSERNDAVLVIGGTKMHVNKALLSFHSEYFRALFS
SNFKEGKLTEIPMYEVSYEDFSRMLAVLYGNEALLKDDNLEKMLELADQFQVPRVTAHIETHLCHFSKMKQDQLMRLADK
FNLRTIIEKIISEVDTTQKMKNLKNSAVFWNLSDETKVLFLYRLMQLI
>Cele_T27C10_4
MTNSTRKFSLKQKFTNVSCMVNHEKRYGPSQVHFDIPWRMKIQRSGSDLAAFLCCQKSENAENLMVHFEMKLISILGTEK
SRKTKFKICDLKPGNDGYGWDFMNWEDMENQFLIDDSIRMECHVELKYIERTSYRKFDESAKEFSDVILVAEKQKFYVSK
LFLSFQSSYFHALLLGNFIESTSSEIELKDVNSTYFQYFLELLHGESSITAENVEHILHLADMFDARTAIRRCEEFLNSL
CPNVKLKDKLRLAVQYRLGTLKELTNDLLARKHLTKTEEEIRKVFQDLDKDGDGFISVAELQAVMTNLQAWLTEEEIDDG
IRSADISGDGLVDLEEFIDVTMHSQQYSKRITNSNFSVPLKNFE
>Cele_T27E9_4b
MFASDVKHDKIAKIPLYTDDDLDFIKVDILDEMYKKSYSIFNELRSKCQLCDVALLVENRKLSAHKVILAATIPYFRGMF
TLDLMEANMKEINIEDSDMNYETVDALLSFAYTGELRITTSNVQSIMLGANFFQMLEVVQHCGNFLLTRLHPSNALSIRE
FCKMMCVEEKITEMTDDYIQKHFMAVSKDEDFKRLSLEDAIELLRNDHLYVDSEEQVYVAAMEWLNCDVIRHEQAAKILP
CVRLPLLSPTYLSSIVASNPIIKKDIPCRDLIDEAKDYHLLPDRRSLIKSFKCTPRLCQIVPGLIVAIGGLMHQSQSKSS
VEIYDPTSKKWSPIDGMVTLRTRVGVAVNQRQVYAIGGFNGQDRLDLVEKFDYDTLKWTTLSPLTRKRSALAAAFVTNRL
YVCGGYDGLHSLSSIEIYDINRNVWEAGPPMENMRSAAGVTVIDKHIYICGGHDGMQIFASVERLDTENQQWERIPSMIQ
QRCRFGAATFKGKIYVAGGYDGTSFLKSVEVYDPVEKKWSPVSPMNMRRSRVSLVSTNEGLFAVAGFDGENNLCSMEQYD
DVTDSWTIATALTCHEGGVGVGVIPMPPHML
>Cele_W02A11_5
MSAVNSEATTNLLAEFQKLRGETIGLHSIFSEKSTVDISNLNGNTTLKRIEDSCEELKNEIREHVQVSSDELKSIRTANS
GFLNKIEESLKTSQQEAREDLKAIEKKNESLLNKSLNELKFQKEQLKYVEATLISQQKLIEHMAKELTGKSISDMVKNNE
DDGTHFEFSHTFNSVATLEKGVERRSELKEGCGSLNWFMSIKRERPSRKLLVYLNLDCEKLPTNNWIIEADIQCTLNQYY
FNEWVRKFERKEDAIFKCENGEYTDKLVFNFGEYEEILRTFTKNGVWNIEFDVDIIKTVGLSKKKLRCFDESAAEVSDAV

MIVKDEKFHVSKMFLAAQSSNFKLLFKNSNNSEFTLDGINSEDFQCFLELLYGEPALTGRLLNN
>Cele_W02A11_8
MYNGYADVDDLHVVYVYCGNTPELRLQELVADVKITIQPENEKSCCIKKFLSLKDNKFESIAMSADFGDFDEMVYNYAIDG
KCTIEADITIIKEVGDARAKLRDFGEETRDVSDVVINVQNEKFHVSKMFLAAQSSHFKLLFKNSNDSEIALDGIIPEDFQ
NFLELLYGEPALTEDSVEGILQIAARFDVSTVIRKGEDFLLQKSEFSPEKKLQMSLKYNLENLKSKCLSEVKSVADIRSV
LPKDIKEMDHSVMAELLEKSLGLQKT
>Cele_W02G9_2_1
MPVYAKAIFGSFPSVQQVRVENGERCLIEAMDELNKNEKGLYRPVRLENGDQISTTDDVLNGKLARVSYTPFEEFELVSN
SKGSCMEYENQEQSSKIMEQMRILRQTEELCDVELLVAGSVIRAHRYILAAASPYFKAMFTNGMVEMKKLTIELQDIPEE
SVRIIVDYIYTDKIAITMNNVHQLIFTATVLQMDVIVVACQQFLATMITSHNCMSLYHFSDIYNCTNLISSIEDFASSQF
RCIRKSPEFNSISFHHLKSLLNRSDLNVSEEQDVFETIVQWVSSNPRDRQHHFVQLFKTLRLHLVGWNFLCEAVNSNSYV
KNSQECREIISAMVLDAMTPSKRKHPESNHENTSEYSASMACPSLTASSSSSTSTFRKSVAGAIFCAGGRGKAGGPFSSV
EAYDWRRNQWIEVPDMMSQRRHVGVVSANGNLYAIGGHDGTAHLATAEAFQPSIRQWKRIASMKTARRGIAVASIENVIY
AVGGLDDTTCYKTVERYDIEEDEWSTVADMDVQRGGVGVAVIGRYLFAIGGNDGTSSLETCERFDPMIDKWKRIASMKNR
RAGSGVCVLDGYLYAIGGFDDNAPLETCERYDPDADKWITLDKMSSPRGGVGVAALGGKVYAIGGHDGSDYLNTVECYDP
IANRWQPAAEIKECRAGAGVAWANVRMHQLSRTPEKCDSGCAPSGGSYCI
>Cele_W05G11_5
MVEDVIKFQSEIIKVQGGSGLAHEVKGLKWCWRSSFTPMTYDDYDDEDGFDYETNGQFMLTWNFDWSELKLAEVDRITGS
IVVKPVAGKSNISLMQIEVDMNNPKQSLVKNFNHPYKNLGLVAFEYVFCLHYNPRFLSFHSSYFRKLFSSNFKEAQLTEI
PIRDVSYEDFCLLLSTIYPNMIFPNDETADKLLELADRFLMPAVTNMVGFHLCHSDKLESMWGSKVHSANCPPNKARKTN
RLVCVSHSSVLSSLKQFKTELRAKVATEFIKNYDLLGAIEKADQLISSKKGVILNKNTVDEEGYKEYLELMKDNFVKLQR
LII
>Cele_W07A12_4
MVPKYKDIPNRLVIEENDPAGSDWFGDDRETLCDMAKFYNNAQFSDVNMKVGEESYPAHRLILSKSSDVFDRMMSQKWNG
DKFDLELVEDELCQKAFAPFLRFMYSNHVVLHKDNCLPLLVLADKYNVTTLKKVCLDFAQSEILPVIDLKELFSVWFSYA
TKAYHPSLIKSCMQAIALEFETLLTEEWEKDWQELHRDQMIEILKCNNLKVASEFKLWEALQKWIQAPNHSERRGNTAGP
LLAFLLPLIRFPFMNGDELNEVEKSAISEMHPKLFQPPLLLSYKFNALPLSSRMSCKDFTKKQFLLRQYEDNRWNQRMTV
AKTLLAYPCVDHAFTFTSRSSTVPMTEWKWTLKLTGLTFPNPTKVDVLRIILIAEGIDQYRSIEYMLQIVDEKKVLLSMS
GKKTFTKTRYYAELEMEKQISFDELLHDDSTFSCNGEFIFQLFLRVIE
>Cele_Y105E8B_4_1
MSDRHLYGSDHSYLSSKPSCSSCRRQSSTSQKNECMTNPGSIVLTQRWTVCNFESLLKLSRPGSCLRSTVFKDDAVPDAC
WQLCLYPGGKREENANNVSLFLKMSATSPSKEVVLKAEYRFYFLDDNDEPKFSNVNVGEFHAKPPKGGHSWGLRNIPTQK
VQNSIRQDKSLVISCHIELIPDASKVPCKRVPITPSIEMPFAQIPRAHVESELEMLASGDGTDMTIVAGPLDGEREQFRV
HAYKLRAHSDVFQMMLSHTEMRENQEKRIEILDFSPTSVRAMVEFIYAGVIKSDIDVYQAVDVMQIAEKYQILALKMTCE
QHLLDRLNVNNVLECITHAERYNTDVLYDACVDFAIHNRQHVMALTSWRNFISDEPVLASNLLEKMVKSNDNSSPPVKKP
RV
>Cele_Y108F1_5
MINGLELLYALGAIDETSQLTSPLGLQMAEFPLPPMHSKCLLKSAEFGCFTEMVTIVAMMQIQDVFITPYRQRHQADVIR
KKFAVEEGNHITMLNVFTKFVENGRSKKWCSDHFVNYRGLMRADNVRSQLVRLLKRFEIEKVSSRGHYTKVDIFHDKNDK
FEFLQFKTIDIFRNYRFLNRKIDNFWNFSFLAFHSTFFESLSLGGFYEAKLAEVPLYDIDVDDFQKFLEVLYGFPVINDN
TVEGILLLADMYQTPLVSELCEDFLIETTGKTYKKKLQMAITFNLENLKQFCISMIKSPFGLRSMIPSDISSIDHSTLAA
LFTKSLTLMK
>Cele_Y43E12A_3
MVEAPGTFVEVNDTRVIIGKGLSLDSIKFPDFHMFDTNWKMKLHAEWSEELGTDVVSIKLRHRRRNVDDIWSMDATVKIH
FEQDWLKGEVQVDSQFSNQGQEIEIPNCGTVVGKLNSSEIHLCVHIFMQINQSKGIQLRNYIDFARPIPIFSDTIVLVDG
VKFHVNRMILSMASPIFLEAFVTNFDERSLELCNISAPDFRRILNIIYPPHLPPKQWIKESDLVEQFEHIQRILRIGKSL
QISIVLEVADKWLVKYGRHKLGDTLHLAQAFGLRELMGSKLAKMRSLEEIKKCQDQIQVLSCKTKALILDRILSTL
>Cele_Y43F8C_3
MDDEEEIPVKYKLDGETEWQVKKEAVMKLGKTEWAIRLKLCKQGENHSYYSLHVAALTPNQVSRLQIKCTHQVEPPVVDW
EQEYTFGGAHTVLVNPCLLKIAEQKEKMTFVVELKLLNDSEKKPEKPFECEKEINFPGGDFILKSGNRKLKINSAYVKRR
CPLLGYLFENEETKEYDVGDVEFEDLVDIFGIFQNRWELEADRMIHLIPIARKFGFDIWGKSLNREFSQLMHRLDPVEIV
AEPNEEPVKFEFNTTLKWAIDDFELIGPSTLPKNQNLMVTWEEQNRNCIIYTYTQEILGEKYVSLGFQMSRPSTSATTSD
DDDDEEDVNVIDDESVYSEDQRVKYFDIDIHVINENGRRLHSDRIQRIRDDEYTIVGTPIFAYFDHLCQYLDEDGALLME
INWIENHDMVSDDEEDDDLDFSKLQKPRLKMPGLTNCFLRCEGQLIEVNREFLAHSSQYFSGLFSKNFREGYERTVNLTY
EHLPTLKLGLQVLYNRRLPLNDVEISRVLDFADRICAKSVMESLQRQLWSCKNMSFGDKKWLAEEFMLTDLKIKCTMEQN
EEMLRVTRVTRKRPRPVDFEDDVIEPPPQPRVPVISRRRLTRSSGSPRTNVL
>Cele_Y49F6C_3_1
MVKEIGLTHIFKNVSELEKGKYFHSPAEEHFNVRWNIKIARKNENIGVYLCCQRLKDIGEGAWSVDTEYRLTVKNSIGKR
LRNKARTKFVPNSNEWGWEFSWETLIKDYLIDDSIIVEAEVKIRKMTGIPRKLLSSFEQSDDVFSDVVLAVYDKKFFVLK
KFLATHSSYFKTLFLGKFDEPEKAEIALTDINAADFQCLLEVLYGEPAIEDENVEGILHLAHMYKMAVVIEKCVEFLSDK
SEKLGRDKFRIAKQYQLDNLKKSCLSKINTITEIKSAMAGDLSDMDPTVVAALLEKSVALH
>Cele_Y49F6C_4

MAKEFQLTHIFKKVSEFKDGECRCGPLEEHFNVEWLLYINRKDDELAVFLGCNSLQGTEETTLSIYVELKFTLKNSIGTR
VTKIAKCLLSNKSTESDFGLNKFIGWETLLKDYLIDDSIIIEAKIKITKMTGFPRKELRSFDESSEEYSDMIIAVGDRKF
YVLKQLLASNSTHFQSLIPLDFEEMDCEGKSEITLPDINPSDFQCLLEVLYGEPAMDDENVEGILHSAHMYKMTKVIRNC
EEFLSNKSEKPMRDKFKIAKQYQLESLKKSCLSKIQTIQDIKSAMAGDLSDMDATVVAALLEKSVTIEPPSKNGRPY
>Cele_Y49F6C_5
MNMIIWKIKTVFTYTHQCAATMIQMLNWKLSVHGKAAPYGLFNMKTPSGKRVTFNTSHCFESTNEINGYGWKMSWETMKK
DYLVDDSLFVKATVKITKMIGIPRKKLKSFDESVEEFSDVILAVEDEKFYVLKKFLASHSSYFKSLFFGSFAEAEKSEIT
LSEINSAGFQCLLEVLYGESAIDDENVDGILHLAHMNEMAFVIRKCEECLLDKSDRPSRCIFKIAKQYQLEFKGKDHNRS
WEI
>Cele_Y54F10AM_11
MAGMDFSMPKELVFDAIVVVEERKFHVGKQYLATHSRVFQSLFATPGDPEITIQDVSSEDFLKFLEFMYHNTDQITAQNV
ESLLTVSARFGVDFVMARCENWLSLDESMDFSLRLQLAETYKFWNLFDKLLETLQSEADCQQVMKMRFPPKLASHSMMAL
CRRAAALAKEAAKPEGAGAQITEEELDRTMKMLAPAEDDHSQMELTVPDDNASGSSGTLGDSEDTGAELVEPELSRILQR
TFVVPRRSRKLSVPEPEEYEEDGFSETLELFQEEARSCHDDTITLE
>Cele_Y57A10B_3
MSAMRRCTCFIICLLTSYTYGTTELVEYTRGDIEFDSSNGEKIIDISTTKGIRCEWKVSSDYYTACFKWKFIWDQVDKYE
VAGFSGQITVNYTTDSEGQKTRTVKVNLTDPGGEIWYNVSRSRYLSTYYASYNYTLEPQKRHMEAMELDKYFAPVDDRDA
VLIVEGKKLHVSSCFLSFHSTYFHDLFEYDNSTSLLNIEIPVEGVSYEDLGLLLSVVHSTATFPNDGNSKKLLELASQFQ
TPYVLGLVENHLLTNTFAWNETLMLLADKYGLMRLLGKSIRRIDSIEKARQLKDSVFLELSDSAKVKVAYHMIQLL
>Cele_Y59E9AL_8
MNSQKKMKEDINCGEIKLIIESVKDFKKISSSVGTETYTRKSDKLKINLSCNEDSANTLWNCQADIVMQLSYFDTKSEAR
KKLETIFEKKLFDSKNKKFEESMDQWAEIFNEKRELEGTIEVTANLKITKVQGYRKRKMFDYSTDHHRYFDGILLVENKK
IHVNKKIMSMSSTFFENIFFCNFKEKEAKFVEIPGVSYDEFMIFLDFIHPTGRQIESGFLENMMVLADLFMTDCVMNSCE
EFLIKTDKVDMAKKLILAERFSLCELMVSWKSFIKIVQAHFLRITVSIASKRFEISCRLQN
>Cele_Y62E10A_16_1
MGLAQSHSRTPSRDPLPNDHQVMDVYEESGPRRGRKRKAAKGSEIEMKKVAKLNTCAYVYQKLFLQGENSDIVLAACGRE
WRVHKLYLKQTKFFESMFDGLWTESNSGRVQMEITDPNIDADGLNSVLGSLYHNEIEIDLDKIEGTVAAASYIVLDSVTD
RCSEMMIEALSTKNAVRFYDVSTKYGLEKVREKSMEVLLHQFWKIMTDREKLNEVDRQLFVTLLNSPNLLIIEGEFDLYK
VVKSWIYMREVPDCCKDDSPETFTQNASKFFKESKTSMFIKYADIFASLRIEQFLTCSETIKTVKSDALIPLSIVDEMTS
ALWSSLLENEESPKTLEMDDDEFFKRCIRLGRSLDSFPKCWRWIGFNFGVDLLLHVNDYSVCIKRNCLNQKAPYSVNLKS
KHVLHYRLVICDSNGQILYDSGKTTWEMKPDEARTIYRMSEDLATQISVHFQYLIHQPICSTGHYVNKYLDDQNSTDDED
DEEEEDDDGDDEEDSS
>Cele_Y69H2_15
MASFLKKLVKDRSKSPQKRRNHHVPPENGHISRQNYVPPYAPYAPVCYLPLPPSPVPPHQKRSSSRSNESFGIHSWNTEP
REIDDTPPFFTQKSTDFQAPPTSPGPLMRTRSHSPRKLKRESNEERKLKSKSKSPQKRATSGTVISQLDYLNSQCHLEGC
RADIVVNSTRFMLCRHQLCHASVFFKDLLAGKETNKDYNVSRNRMSEPTPSTQFRWFVESCIPCPALKDISDETLETCMR
LSQRFQAKGLELRCMKFLIENAHLRQPIVVLCWLNWALKHNFDTQTHAALLPSVSRLSLNALQRHRHMITEHIYSDIITA
KLRSTYDKTVQVFLAIHKIDHFSADVDVCPRCKRSKDGMKIKIHCDPCRKQVGCDRCYHDGCEISSRAVEDLQAFFKCPH
GMTPINDATDDCQCQIPQMAQYLGNQPPVRYQENNDEYMVPTDRLVENGQKSRKHK
>Cele_Y75B12B_4
MSGKDNKGSIRFEIQNFSGLAKATEHKPVQIGHVEWILGAFTSTSDATNNAKHLGIHLKCNEELRSNLWSCDASIRFSLL
RLNSNEDDDAFSMEFQQKFDDLNKIVVVNNFKNWEEAICYDNRFVLDKHAVLEVQITVNKIAGIHERIIETFDQPKEHLT
DVVLVLEGKHVHVGKQVLATSSQFFQKMFYSNFAEKTQAEIKIDDVTHSEFIDLLNVIYPTHMLINSSNVSHLLKLSDRF
AVPRVLEKAETFLILDQKTHLIDKLKFAEVYRLSRLQNACLSQLETSEDVTALKHHENYKSLDHITYNALLQKLIDLLED
>Cele_Y97E10AR_1_1
MIRQEPKAYFLYENVDLSSFSELEPACKELDFAAFEDIHASCLLRITNTADLEVRLRRDGPPNDQWLAKGVMKVILWNEH
FEIDGSYPATWCNEKPFHLVDLMSRKSLKSEIGAEMFNVEISIKFDKWEGLKVLKLFQFDHQYLDSDLNINVQGENFYMR
SKTLFKEVPALQEYLEQNFEPGTKKYMMHELNGQRSIKAFRTLLQVSFRCSFITNIHEVGEVLTLAYRFEYPELKKKCEY
YLISEPESDWFQKLVFADLYELPLLRTHVLNNTIGLAQIWRNLHLHRFLSTETIRAITCKAIELKNQKNFGDGPSTGWAK
LDLHTITENRWERLKRVPICIAAPEPDVTARVEKFESTFMPKHFGVELITPEFSFVEKCLNLFTYKVQLGPDHRLLFKLD
WNNIQTDNFVWSATANVFIRLQYQGSQKFEISERVIFDYDHQSIFFDGLHIQNVFPGGVEHLLDGLGNNPTLPKIEIMVV
VDKIRGLELTPFDPSLDSDEEMDDEWILQPSRTSPIVCCGKYLYVDKEVLRKQSRVFSKIFEQTYEPITIPLTFDKFEAF
SSLLEFLHGEDIYFNYNNVFKIIELARAFDADDILEQAIDWIFNEPQNGKLEKLEFSERYAIPELQKVITDNILDLDELF
QIMKHPAKLSRRLLQILQDRMINLPIPADEEKEFE
>Cele_ZC204_11
MAEKIIEWKNQTCFSEKPISIIDSGITCNVKIVENRVHWRFAWVGAEPVYGLAGHTPLDEYETMFAKSNRTNAVLMVDGK
KLHVNKTLLSLHSTHFKKLFQSEGDQLPVDNISYEDFALLISTIQPNPIYPNHFTAEKILELAVRFRIPAATALVENQLV
NYSSFSHGKLLLIADKYGLERLLDQQVRKIYTKEVAEELKTCAEFPQLSDNAKAKVADFLLPLFT
>Cele_ZC204_3
MDWKGKLKFLQFCLLRNNKKKFHFSASYVDNDGQLEIFMDRLKKRLRFGWSCNCESRLNTHHAFYAFQDASRFPQGKYQR
HKCARYDLYLVALETPLAEYEKMFSESEDTDAVLVVEDKRLHVNKEFLSLHSTYFAELFKKSEITIEDLSYEDFGLLMSN
IYPKVIFPNDFTAEKILELAVRFKVPAAVALVENQLLHHSTLSHGKLLLLADKYEMHRLSGQQVRKIYTKEVAEKLRRST

EYSELSNRARARIGECLLQLF
>Cele_ZK858_4
MEPRIDGGVFIGGIGNSGNEMCSNGVPALGVSSQTEIKVEKVQHTWTVKNFSHCYQEYLENFVYLQRGDEQLTWSIKIYP
KGNGENNKDFVFLCLNRVINNNVKAGKIGFKSQFKLRTAENKDIEMRIHPNPSHSDYVSYIKRDVLFPQIMPRDMIIVNV
EIDVAVETITTTNEPIQFEPTNSEQQLIEDYQRLFSQELLCDFAINVNGKIIRAHKAVLAARSPVFNAMLTHQDTDEAKS
SMMYINDMDYDVIYEMVYYIYCGRCNKDITDMATALLIAADKYRLEELKSHCEKYLVENINIENACSLLIIGDLYSAPKL
RKRAVTYILARPKNVTGTPGWEDILKGHPNLITDIFSQIDRQSSTGATSSVSNLPGVPMDIPGITGNIVPPPSGL
>Ctel_214539
MGKSRSYCDGFRSHLKKDFQWDDWSPPTTPPRVTLILPDKSTLTCLESALTNASNFFKAEVTSNPGQEIKLNFSDSTTIR
TVVDYLNRHTIRISDENFEKLLVASDFINLTNLKGHLSSFVRDTIDSTNCIEFYKLARKHKAQYFYHESLEFILSNFIEV
CCSSDNIEELSEEEMIEVVSNHRLAVDDEDIVHDTVVRWANADIKHRESSFLKIAAFIRFPFCSKFGLTNTVKYGGALKS
PSIVDQLKELKQVRYTSPMILHWIHNHRFVPRPSYKKVKKLHLVRIYVSATNHFVVSQTTDCLNETNTEWTETFKTPEDY
FCGHSMSPKFFPTDDGICWAVKGDKPGLEFRKWLVGSQSVTTPESPGDLSSACTWTFLSPNLYCFVDRTVNSDFNSPFVV
EQRHVNMWHKQKFVHPRPVQSVNNPYVVHFKEKLFLLGGVDAVGQPSLVNQTYSLNARSERKKDMPGICQSGAAVSFNDD
IFVVGGTERVCYQYTPSTDTWVVLSRPTHVHKEFTAATVWEGNILLWGCYHAEQYNPKTDTWSPCDELVPKNAAPGFDRG
LYTYAK*
>Ctel_188992
MTTVKPKKSLNDDLVALWVSSVSGFSSQYNSTTWSANCVVGAPRVYPRYGDIHGAWAQSSCDADQYIQVNFEEALYVEKV
ELYETLNAGAVIRISALDDRGVWHTLWETDSPTHITSSRIFSPPFERTYFKSDTLKIDLNCTSCNSWCELDAIRLLGKRN
VFDLQKAQSLSIDLLTLVDNPSFSDIIFLVEGHSVHAHKAILSARSRYFEAMFTDGLKETNEKEPIKLENISHSGFIAMM
QYLYSDALHANPHPTQYNELIRIADQFSIDGMRIFAHYHLSKDLTDDNVIHTFQDASEQLPVLDDVRQTCLSYITSHMSA
VSKTKAFCQLPQPLMLEVIQDAAGKLKSKLIIANFWGHKN*
>Ctel_217135
METKKFLQTMSKSFCNMRAATELVDVILVFGDAKIPCHKVVLAGTCDFFRAMFVTEMKESRSAEVQMSEISADIGLTLVN
YLYSGNIEITKQNAQDLLAACNMLFLTDIKKDIAKFLSQAQEFLTKHWKELIDTEEMKELQEVDFVGLLKSLDSQEDRFR
YLQKWVRLGGSGRDDRFNDLVEHVTLPKCSKEFICNVVMDEERMNHPKGMKLIQKAMQALMLADQPTKSSHESLVVANRD
GNAWMCSDNKTWKSIEWHAHESTYFSGCSSPEGFIISGGHTNYNPMPQCSSYVALVKQWRLLPNMHIPRFAHASVYYQDK
LYIIGGMESRHGYVDSVERLDAKTMRREDLCDLPQGTSTPLVVIVKHRLFVLGGVTKVGHSKTVLEFNFKDDTWDERSAM
PKECRRGAAVEFNSFIYVVGGREQSCMRYDPRLDEWVSLQRTIFPHMFGPAMVWNDVIIVCGGEGTDVIEQYCPHSGTWS
TWQLKLPHSGHLRFLLKIKS*
>Ctel_144920
MTDVTLKLPDHSTIRCHKVILMAASPFFETMFQSGMKEGVDRVVRLKFSDSVTIKMLVDFIYSGEIDVDGDNVQTIVAAS
EFLLLKDLKTHCEKFLADKVNSSNCIQLIKFCQRFNLEKLNEIAQRCIRLNFQEVVALPEFKTLTEEDVTALVSNDQLKV
ANEDIVYHAVVAWVKADPKNRKGSFLRIAPLIRFAHCTQETLNGVVGREPLMWNPECMELLTEAQ
>Ctel_161127
MAASRMVPSPPPPEMSCPVAENWCYTQVRVVKFSYMWTISNFSFCREEMGEVLKSSTFSAGANDKLKWCLRVNPKGLDEE
SKDYLSLYLLLVSCNKSEVRAKFKFSILNAKREETKAMESQRAYRFVQGKDWGFKKFIRRDFLMDEANGLLPDDRLTIFC
EVSVVGETINMPGQSSCTPVKVPECRLSDDLGNLFDSSGFSDVTLCVGGREFQVHKAILAARSPVFNAMFEHEMEEKKQN
RVEINDVDHEVMKEMLRFFYTGRAPNLDKMADDLLAAADKYAVERLKVMCEEALCSNLTIENVSEVLVLADLHSAEQLKT
HAIDFINSHATDVMETQGWTHMLQSQPHLVADAFKALASQQSPPLGPPRKRAKQS*
>Ctel_172031
MAAKLTIEDGCHSNDVLTRLCTLRRNDQLCDVVLEVMGHNVPAHRSVLACSSKFFMELFAADKDCSQRHFKLDHIDYDSF
MVLIDFMYLSRLEVTGDRVRSVYKAARFLKLKKVVSACVNFLTENLTPFNCIGIRSFADKHSDEKFTACVDSFIQKHLSD
VLEKSPDAHQLPTIKVDIMGVEAFDLPSIEEKNLCHLVVEWLKGSLNNNLTISDLADNPSALYLTGNNDLQDCLDLEFEK
EEDDVVRRGIVEDYKKMHQKQNKRKLMIKDPSEVALDVPLSVCTTSPPTWEVLASKMVQEHKCVCVAALHSSLIIITMKL
HPRPSSPASTNGNESDTSAFNGHSPSLSPRLSTDKGCCLIPLAQMNNARCGLGVARLNDKLVAMGGYDRGECVDSVEVFD
VSTNSWSELPKLLTARGRFDATQIDDCLYACGGSNGASELNSAECFNSTLNKWLALPDMASNRSNAGVVALNGKVYAVGG
WNGSSLASCEVYDPDTDAWTSIASLKYGRSQAAVCAYGGCIYAVGGCDAWKCLASAERYNPETDEWTSIASAGTPRRGAG
VQVFNDKLYVVGGNDGQSCLSSVEIFDPVSHSWSFGPTLNVARANVGVSVIQDRLFAVGGFSGKLFLNSIEYLPIDGKQW
CVYVPNENKKLVAATSVHSLNGDFDDEALKSENGCHSECTVAALNGVEVE*
>Ctel_210697
MADVHFLVGKDAQLIPAHRFILCVGSAVFDAMFNGAMAATEARIELPDVEPSAFLALLKFLYSDEVAIGPDTVMTTLYTA
KKYAVPALEQACVEFLKKNLNSDNAFMLLTQARLFDEPQLAAICLETIDKSTTEALAAEGFTDIDLETLTAVLQRDTLGI
REVKLFKAVCRWAEAECVRKGLELTPANQREVLGDVLRLIRFPLMTVEEFAVEAAQSGLLGDREVVSLFLNFTVEPKPSV
SFSDVPRCCLTGKEQVVSRFVHIEHRWGYSGTSDRIRFAVNRRIFVVGFGLYGSIHNPSEYSVNMQILQTESGEVCGSND
GAFKADGSASTFRVMFKEPIEIQPNTNYTASATMKGPDSYYGSRGLRKVTHESVTSGKVTFQFNYAAGNNNGTSVEDGQI
PEIIFYT*
>Ctel_196313
MSTFKLRNSDNDNDVLFKDRCQARRAFLNVKQSMKDSLIAFFVLSESMDCVNEIGIPCDVELKTNDGGCFPVHRIFMVGQ
SPFFRAIFTNGCEESKQHEVQIGVVSERGLSNVIKYTYTRMLDVHDVMDTNHLGEIHFAADFLLVEGLRNVCELLLAMSP
RQRTRTAKEFFMNTE*
>Ctel_120430

MKEAGELLDVTLVFGECRIRCHKVILAGMCDFFRRMFTTNMAECGAQEIVMRDISAGTGTLLVGYLYTGQIEINTQNVQD
LLAASEMLMLGALKQNVEEFLRSHTESMNCISMINLARFYSLETLLAKAKDILHDHIKEIIDMDELRYLQERDFVEVLEE
RESQEESFLFIQKWMRSADGRTDRFNDLLQHIDLSLCSKEFIFSTVMDEELMHNSRGMKMLQLLFQSFGSTDAQPKSLAV
GKLEGEMWMCRDIHVQDWYPIQKPPSQHMYYSACASPGGFIVSGGSRNNVPQRDCFSYDSKSDSWSTLPSMSTARRSHSS
IYYNQHLYIVGGWDGQKKINIVETLNMRSLQWSHLPPLPHPVGLPYLAIISNKFLVFGVRNGSWNVDMHEFDFAQQTWQT
RSPMPEICDAGSAVTLGEEIYVIGGRSNSCMRFNPRADAWTFLQRPPMDHTNCSLLVWKGQILVFGADNHDSIEAYSPKT
NTWTKWALKTPARGGIRFAFEMKLPE*
>Ctel_128447
SHLKAFMASLHKLQENEVMTDVTLILPDQSAIQCHKVVLAAASPFFETMFQSGLKESAEQDIKLAFADSGTIRSLVKFFY
TGDIKLTAENIKAIVGGSEFLCCKHLKAHCEEFLMGNVGLSNCIDYYQFGKVFNLKVLVKTAFDFILSKFREFMEISDFD
KLTEDELAEVVSCDRLNAENEDVVFEAVVHWVNADPDAREEVFPRIAPLIRFPFCTQTMLIDKICCSSLVLKSECIQLAH
EAVWSQFHL
>Ctel_63634
SYLEAFMTSLQKSKESRSMTDVTLILSDQSTIDCHKLVLAMASPFFEAMFRSDLKENIQREVPLDFHDAGMIRKLVEYFY
SGEIDINSDNVGDILTECEFFCLADLKIHCGAFMTSQVDSSNCLAFYRCARQYKLGKLVPHCFEHMLSHFKNDFCSSESF
VDLSEEELIEVLSDDRLSVENEDIVLHSVVRWVEADLEQRRTAFVRIAPFIRFPFCTPELLGDIGPETLMTNNTCMELLR
EAAEVKSAKIGLHLKQSARCIPRQGYKTKKPQLFRVYGQYFEFTEELNGELTKWNTIPSKYAITLNHVLTYIQNKLFAFG
GKINEHLSTMVDSFDQQSFQWKPQKRSMLHAVCKPYTVHFGTKLYVLGGIGVDGPSTVNQEFDPIWDKWQLKQSMPGVCE
SGAAVSLNTSIFVVGGAERACFRYTPTTDTW
>Ctel_203209
MMQAVTSLPDIFLQMREAGEFVDINLVFGEQRVSCHKVILAGTCDYFHRMFLSDMLESTAKEVTMKDISASTGVSLVHFL
YTGRINITSQNAQDLLAASEMLLLGSLKQKVEEFLRSNTDSMNCLSTLSLARLYDMKTLMEDAKKFLHEHMREVTDSEEM
HLLQEDDLIETLEANASQEHKFCFIQKWIRSADARTDRFDDLVQHEILKEKCGSVLTIKFNIGNRCRSPQFSVGGGISKG
TASAVCYSYDVHDNKWNELPPMFTARNQHSSIFYNNHLYIVGGINSNTILDSAEELDMESLRWSRLPPLPHPVCQTCLAI
VSDNLQVFGGIQSKSTWIDDVWEFDLNERVWQQRAPMPEGCEGGAAVCLDDHVYIVGGKNRTCAQFNPSQDTWTSLQRPQ
FTHRFGPALVWNKNIIIFGGEENKTIEKYSPSTDTWSEWMLKMPTKGGMRFVVKI*
>Ctel_62981
IFLQMREAGEFVDINLVFGEQRVSCHKVILAGTCDYFHRMFLSDMFESTAKEVTMKDISASTGVSLVHFLYTGRIDITSQ
NAQDLLTASEMLLLGSLKQKVEEFLRSNTDSMNCLSTLSLARLYDMKTLMEDAKKFLHEHMREVTDSEEMHLLQEDDLIE
TLEANAPQEHKFCFIQKWIRSADARTDRFDDLVQHVSLSKCSREFICSTVMNEEIMQNKHGMKLIQEAMQSMTTDPPEQP
SLIVGDYEGEIEHSSIYHDHHLYVVGGYVAGNCLDSAEALDMKSLKWSHLPPLPHSALHSYLAVASNKLLVFGGFRASWT
TDVCEFDLNERVWQQRAPMPERCEGGAAVCLDDHVYIVGGKNRTCAQFNPSRDTWTSLQRPQFTHKFGPALVWNQNIIIF
GGQLNKTIEKYSPPTDTWSK
>Ctel_211281
MCDYFHLTGLLERGVKKVIMQEVSATTGLLLLGYLYTGQIEITTQNTQELLAAIEVEEFLCSQTESKNCVSIINLARIYD
TKALLAVAKRFEHVKDIFDAEEIHLLQKEDLFEILDADAFQYDNFCFIQNWDNKEFICSTVMGKKLIANLKGFKLIQKAV
QTE*
>Ctel_63514
LHKLQEKEVMTDVTLILPDQSVIPCHKVVLMAASPFFDTMFQSGLKEGAEQNIKLDFADSDIIKILVEFFYTGDIELTEG
NIKALVAGSEFLCCKHLKAHCEEYLVDTVGLSNCIDYYRYGQVFNLKLLIKTAFDFILSKFKEFKGIADFDKLTEDELVE
VVSCDQLNAENEDVVFEAVVYWVNADPDARKEVFPRIAPLIRFPFCSQRALTDSICCNTLMQKPGCIDFAHEALWSQFHF
NCRPPIHNARCIPRQAFHLRESMSSLYRLTKSRSLNQVSLCVASFNDGSGIKWETIMPCVAPTWQRALAFIDGKIFAFGD
DPASKSVNSINLQELNPEWKSEQEMLHAVNRPIIVQFANKVYAFGGWKTRVTQEFDPALNEWRMRSEMPGGCYSGAAVAL
GDKIYVVGGKEKVCYSYDPENDEWKVLSKPTH
>Ctel_176754
MESPKSTPCEEASVVNNVHVVKQEVAENDFFQDQQHCNRILQALKVQKDLGVMCDITIEAGDLTLPAHKCVLAANSAYFN
SLFASTSSDAPSTLNFALGNSDAFSNLLNFMYTGEILLQVDKIHSYLRLNRELKMPNIQQTCIQYLSKVISADNCKQASL
SDGLLMSRPSKRAKQAAVQEAVVTEIKEPEARAPVKRKRGRPRKIVTPVVEERLGEPLSCICRLHDLSINLFTATPEDDY
EDEFAAHDDDEEGDEVVDEEKEKVPPTKRKRGPYKKKTDGPSAGDYETYSHSLPPSGAVFQCTKCPKLFRSQSLLQRHMK
GHGSDQPLECDACDFSTLHRSSLDTHMQTHNGHPHVCSVCGKTFAGRKHLQRHERLHSGEQLKCEHCDWSTTRRDKLRDH
IRRHHPDWALESGFATEDEVNKWKLARGPRSKEKKETPPPKAQREFPKGKGHHHSCSVNS*
>Ctel_112974
MQTTAQKESICSGSSRCRHYEKLATNMMYLMTSNTFCDITLVVDDVSFPCHKVILSAASTYFSAMFQSGMKEANADKVQL
KFCEAETMKVLLEYFYTGSIAINEDNCQALIEAAGHLDLEDAKIDCETYMVSQTDSPNCIGFIRFAKAHDLKELHQTAQR
HLLENFEEVVMKSEEFVEMSEDELVDLISDDRLNTQDEDLVFISVQKWVEASNIQRREIFPRIAKHIRFSFCSKEFLSQL
VYGEPLLNNSDCNELLKWATYSAFLPQKQGLFAGWQPRLSYALKGTQSKLRSISQDSTQVALREYREESKQWPPLVQICG
SREKDACVLIKNSLIVFGSGGQSDSVVTIDLTNCNKWSTLAPMLKAPYQSLAVPFGQYIYVFDGGHGFNCCGVTQVYNTN
LKQWKMVSQMPCECSGGAAVVLNGNIYVFGGKIQRAMCYDPIQDKWDVLTPPTRTHDPASAAVWKGKILLNGSDSVEEYD
LQIDCWVERPELVAPGSVHIFTTTR*
>Ctel_136176
MREAGQFVDITLVFGEQRIACHKVILAGMCEYFHRMFLTNMLESESKEVVMEGISATIGCLLVDYIYTGCIKITTQNAQD
LLAASEMFFLGSLKLIVEEFLCTHTESTNCVSIINLARIYDMKTLLAGAKKYLYEHVNEVIDTEEVHLLQEEDLVGVLEA

NGSQEDNFCFLQKWMRSANGRTDRFEHLIKHISLSRCSKEFICSTVMGEELMHSTQGMKLIQQAMQSSGQAKPPDQQSLV
HWSTLNPPSFHKFSSVCGSPRGFIISGGSKVEIYKRDCYSYDAHTCQWNTLPPMSIARRSHSSIYHNGLLYIVSGWNEEH
VYLNSVETLNLQSLQWYHIAPLPNSRSHSYVVIASNTIFVLGGFKGEHWLGDVFEYDSHRGTWNQRSPMPQICEEGAAVS
FHDHIYVVGGRNRSCMQYNPSDDMWTFLQAPRFSHIHGPSLVWNETIVVCGGQDGDSIEAYSPSANEWSPWALRMPIKDR
MRFVLKIKSPP*
>Ctel_128118
MATALERECGPKCRSKQHALQMNASVTKGSFLQCQIFFSTCYNVTNNTDTQGRNVLHLAASCGQHVLMKWLIEGKKMDVN
ARDLEHGWTALHRSLFYGQLAAARLLIQHNANLTLRDKEGFTAFDVAMSDRLPHVTFEKSGLQEVYTWGDNANFTLGHEK
EQRKKHPERIEYFGRHGISIKQVLMCKYHSVYLSTAGHVYTCGHGRGGRLGHGNEQTILAPKLIEAVKQEECILVSASND
HTVMLMQSGKVFSCGLNAYSQLGHSTSGACLTPKMVRMKSNSSPLLKGHQIIGVIARKFHTVLYTQEAVFTFGLNAGQLG
KIYNVLGEEIVSQPRLVAAFNHPDIRIKLVSASDAATIVTTGKGDLYALHEYQCRRIASKLLDLVQLSVVGGNLDHRVAV
ELLRDSGGSDLLIVLRNLSGNVYCWRTSHPVLHRVVWSIKRPLSVKEVAVSVHQLVFVTTEGDAFSTSLSQLRSRSSPVK
AKVVAVQSHNSSESMSLTDLVNRENIVEAKVHRIPLVHRATAIAIDRNGVNLAVLQVHPNASITQIPMYGGSVMMQQMKT
LLNEADLTDSIHDCVLQVGTQRVPAHAYILAMKSEYFRRYFEKTPPSQNNPIIVKCSQNLLSQLVQYFYTDTCDLLTPGF
LWQPDDEEAEQEEEVVEESTTVLSAKSRKKAAFEVYKEMKEREPLVKPPQPVNAQGRLVQVATELGIGGLVHRLKFVKLQ
KGVVANIPGKLLPQPKIRWDHSKLPELCDVWLKCDDDQVVTCHKCILVARVEYFHSMLSTGWIESGSHEPLSLPIPSDIL
KVVLNYLYCDELVGNKVLNDAEMLCNVLVVADQMLIVRLKELCEVHLSALVNLKNACELLEFASTYTALQLRDTCQQFIA
INLATLLESGSLDLLSDDVIDELSDAYREMVPGVDRRRIESFDGYPSRAYLQ
>Ctel_128104
MGKERFLTAMSASFDQMRAAKELVDIVLVFGDLKLSCHKLVLASTSEYFRRMFQAGHKESQSEEIVMKKIDDKTGILLVE
YFYSGEININSENALCLLSASNMLLLDDLKGEVENFLCSHIGPQNCVAFLNMSHLHDLQDLIKEYREYITEEWKNIDEAA
KNQLREEDMVAILCNCEVQEENFLYLVDWVETDKRKVYKFMDLMVNVHLPACSKEFINNTVLGQKLMQNPKGIKLIQQAL
NTIYQRKHVVTVVGL*
>Ctel_228654
MGTIGRPDSCQLNRSSSLLSGLTQSSIDSSFDHFDPSIGQQKLVSDLEQLVNNKETADVVFIIGEQETPFYLHKLFLWTR
CLNFKKYGLQFWRSKPSSGMLTFKYRQYQPTVFACVLRYIYSGQFDPEESSVFSSLIIATELGLPDLQKRCEGFILSRLS
VDNACLFLAGASKLESDDLNDEPIAGAFTKTCMHYVMTNASQCLLSDAWCHLPKLAVKRIIQDDQLCLEEDEVWRAVLRW
TKFQAGIPAHLSPLHWSDEHRRLAAQPLSGLVEHVKLLLIDSKVFAEEVEPTGAVPMEVSLERYRIAALPPDVCNQSFDD
DRLKPRVHITLFHDTRILCNEHAHFQKLLNQWYNNNLRPWKLLFRASRDGYKAEDFHRCCDGFSPTFVIVKGETGNICGG
FSDVAWTSNTLPRGRFIPSNCAFLFTLINNQGIAASKFEVSNGRLATLHHPTSGPTFGAGADLCIADQCRTGMHSYSNFP
HSYHGDSSSSSVLMGDYYFNVADYEVFTLISSVDVRESTSSVHNNRGHSCHTTC*
>Ctel_207764
MANNEFLQEMSVTFRNMRHSDEMVDVVLVFEETRVKCHRLVLAASCEYFRRMFQTEMQERDAGEIPMKNVSSTTGLLLVE
YLYSGNIEMSTENAQELLAVSDRLLLTKLKKNVEEFLCEHVEATNCVSLKNFARLYGLESLLEVTHRFLTTHWKKLIDMQ
SEIDELTEDDLITLLTTYGSDEGSFLLLQKWVRSSHGRIGRFMDLLQNIELTLFSKEFVWRTIMSDELMQNPKGVKIIQE
AMEALLSADRNLSLVVGDFNRNIWISVDGKQWRVIQVPFSECGSYSVTSSSDGFIVSGGLANKTPKRECYAYVTHTRQWR
TLPPMSVGRYYHSSICDQDEFIVIGGRINEKSHLDSVECLNLKTLEWSQFPDLPQARCLSNLLHVQNQLFVVGGYLTKTT
TARDIYKFDSTERKWQTCSPLPEKCGSVGAVSFDNKIFVVGGQLRSCKQYDPCTDRWVQLPRPRFGHAFGPALTWKDKII
VCGGRGTDSIEEFDPQSDKWSTWGLKVPKQCDMCFALITNGHQ*
>Ctel_111659
MKEADEFVDITLVFGKQRIACHKVILAGMCDYFRRMFLTNMLERGSQEVVLDDISASTGVLLVNYLYSGNIDITQLNAQD
LLAASEMLLLGALKKKVEDFLLSHTDSVNCISIINLARLYDLKTLLADARSYLHEHVKEVVETEEMHLLPEADLIEVLEA
NYSQEDNFLFIQKWIRSADGRTDRFNDLMQHVSLSQCSKDFVCSTVMEEKLMHNTRGMKLIQEFMQSYRSADPPKQPSLT
VGNYYGEMWLCTDLNTPKWQSIQQPPFPIRRYSACASPGGFVVSGGEFQNYINQRECYSYHALNGQWNTLPPMPKVRSWH
SSIYHNHHLYVVGGLVNHLPLNSVEVLDMRNLQWNHLPPLPREVCLAYLAIVSDNLFVLGGCNSDWNWAADVHEFDSTQQ
TWRQRSPMPEICRGGAAVSFNDHVYVVGGEDRSCMRFNPRNNTWTSLRRPQFNHWEGPSLVLNGNIVVFGGEYSDSIEEY
SPLTDSWSTWTLKMPHKSQAWAFAARMDS*
>Ctel_63131
LILPDQSTISCHKVVLMATSIFFETMFQSGLKESAEKDVRLDFADSNTIKTTIEFFYTGFIKVTTENVKALSAASDFLWC
KHLKAHCDEFLKQKVNSSNYIDLYHFGKMFNMSALSSQAHQHILFNFKSFCEMPEFYSLTEDGLVEIVSEDELNVETEEI
VIEAVVSWLNVDPDVRKGAFARIASHIRFPFCSLNTLTSNECLNKLVLDSGCMQLLHEALRSPYHRNCHHAVHNARFPPR
QSSTLCFFGGKIFAFGGCTIGENTPLKFVASFNVEDANPDWRLEKEMLIAIEEPCVVHFADKMFVFGGRIGNTFSVVSQE
FDPISNEWRLLSEMPGPCHGVAAVALNNKIYVVGGLNRVCYSFDPVNDEWKSLSPPTQLCLDGSATVWKGKILLSAYLCT
SLVQEYDPATDRWSKQNQL
>Ctel_47985
MLENMKLTDLVLEVEGKEIPCHRAVMAAASPFFMKMLTSGMKECREGKVEINGASFNTISLIVHALYGGELKIDGDNVQD
ALVACNMYGINTLASECASYISERVDQDNCM
>Ctel_140542
PAGISLEDATPSSEDSSEIPSCCPLGKDILHMYLQGDDHDVSLKIDSTSFRAHKCILCARSGYFAAMLSGPWSESNSDEI
ELIGVAPEAMEIILLYMYGAVPNLDPSQCNITHVLAAADMYGLCGLMESVAYALRRYYCHFFHKPCTDCCNGVLFTLPLA
STYGLEELWRKALKWTAKHFARVWSNKQFSLLPQEIQKKCVDTCIEGIVREPFHLSFLFHSVNLTEFQNRC*
>Ctel_225601

MNEELTLLATPPRDEFWDCLYDLRLKGHLCDIDLGVKNADGNMFIIHAHKLVLVASSAYFKQALESGELLEFYNFDGIRA
SDLEIVLDYVYHQKAWQEVEREPAALTAANALGIDRHSTKREKPSKLYKDHLGKNFMVVSMEGKPRKKAAMKPNTEETRI
FIDDVNQVFEQSIEYSKSKANESAQDTGPQEQMLSIELTAEGETSLQSLLQPSEGSAQSQLSLLADASSSADTLSAIAGI
TGGHEMVQVITGDSGQTLEVIAGGGGRVVKEEEAAADEVEETVEETEEEVTVEAVDDPQGPDSSQATGSQVETAPQGSNR
YGTRGKKFNLPEALKPEKKIKLESEAEEEEEQEEHKCPTCGRGFKNAQGLSVHMGWAHNASGVSHMVKPVHCPECNADVK
GSSALSKHMRRYHNNADIHKCSQEGCEEKFSTIRSLRKHLGDVHSVSMFSCDICSISFKSPEGMKIHNSKFHTKGEASLT
CHICGKSFKLKHELARHIKYTHNAGFDYKCNLCERGFKTRGTLKRHISTYHSKTRSFICDVCESAFKTAGDLSKHRSLHF
RDVNPLNAGRKIQRSNPDGKELRPCKYCGKEFNRLATLTHHEYCVHEAKQEGITYVCSICNKSFAVQAYLQQHIQSKHRP
KKYKCGTCEASFTQRYLLHRHMPRCKCIPKHLKPFHCTTCGATFLNKRNLQVHHEQNKCEKELEKQKQDQEDVDYVLYMK
EEVEEEAGVEEGEVVVNDGVAHASAGEEGAEEAYQIVENEDGQFIQIGDKLVQVSVTMTSEEQEETTIIQHLDPSAEVAM
ETGLDNEQFVFRVIEDHALTEEVTQEILEIEPQGNQ*
>Ctel_114856
MAFSADFSISNADNSNVLEALEGQRSEKRLCDVTLKIGNDEFPAHRCVLAAASQYFSRMFEEYNFTESRNEEVELHSVTK
VALTTILDMIYTGRLHLDVDSVHQVLAGADHFLINEVKHACAEFMLMVLKSPSASDEALKVRQSAELYDLTDVEHEADRV
ISLNFAAVASSPAFCELSFGEVCSLLQSDYIQEGEEAVFWEAAIAWLKHDLAERTPQLEKLMASIRFPLIDSDALLLKIA
RDETMDKSNACYKFLDEAMRYQLLPKCQSSLQSPRTKPR
>Ctel_143807
LCDALLILADGSLVVHRAVLAKRSDFFKREFSNDVKQLNIRDISTRTMDIILDHAYTLEPVVHINNVRFLLPLACRFELS
DLVKECCNTLAQDLRVSNCLGILITASEFSCDDLVEETTQFVHKHFKAVSTQSNELFNVEPAILKSLMSSDHLNAADERL
VFDAICRWVSHAPDVRMKHVSELLSCVRMSLLKPEFLSCIINYENNEDDQCEQKVLQVLMHHMADNLQDPQLSMTGVHRV
PHELLLAVGGWSGRSPTSYIEVFDSRAEEWLPEADVGYQMGPVAYHGCIALGSDVYVVGGFNGSLFFNDARCFSLINRSW
KSVAPMNIARCYISLAALNGCLYALGGFDGHTRHNSCERFTPELNQWDFIAPMLRIRSDAGASSLDGKLYVTGGFDGQNC
LDTAEAYDPMVDQWTFVTPMLTPRSGLGVIALDDKLYAVGGFDGHRRLDNAEAFDPLAQKWTQTSAMTTGRSNFGIERVD
DGFLVAGGYNGTTTIHAVEMYSPAEDLWTENESMQLNRSALSVCRVEGVSIASRFLARHSGAEEER*
>Ctel_228722
MASISPRKRSSNALHTPEPEPTTSLLLDSPNYSGRILAGLNELRQTSMLCDYTLAAEDISFNVHRCVMVACSDYFHALLT
TDMRESRDNRVELKGVSSVGLKVVVEFAYTGSLNLNLDNLEDVLAAVSHLQVSEAMDLCCTFMKKALTIDNCVDMLHMSE
VYYLADSLDATRKFVLQHFEQLAETEQYQRLTSSQLASLLAENCLKVTSEYRLFELVLQWIDHKKEERQEGLPSLVSNLR
LPLLSGEELVDKVSKVDLMHSQRECSKLLMQAKDYHIVVSKQPLLQTDRTQVRSDKSSLVMCHAENLESYHFEAERHAFL
KDAMVPLYSPCVCVVDNFMYACGGKYDSNENNEIATARCFRYDPRFDTWFELPSMNEARKDFVMVALNKCLYAIAGQDEN
MVMCTMECFSIEKNDWELCQSLSHAVYTHAGTVCESKIYITGGQKFDGHCRMVKSYNSELDSWKEEPSLINSRSNHNMAA
VNGCLYVLGGNTEDPYGFPVPVTSIEKFNPVTGVWTVLKSVLNIREAGTCVLNSTVYIVGGINGQHYYSNLIQSFNPEDD
KVEFVTKFPTRIYGRACCVLTLAHFV*
>Ctel_220776
MEKNMTFLQTMSTSMNEMRTAEEFVDVVLVFEERRIPCHRVVLASTCEYFHRMFLTGMKESSSDEVTLNEIDGDTGDLLI
GYLYSGMIEITQENALNLLAASEMLLLCDLKAKIQAFLCCNIHAKNCVSFLNVSRLYKLQDLIKDSQRFLNTHCKDVTRK
GDLVELQEDDLVGILNFDPCEDSFRILRKWVQAVDGRDDRFMELVKNVDLSRFSKEFLCDDVMDDRLMQNVTGFKLIRDA
MKSILLCENRTEVTFRYTVHNISSLGPTKVYSPEYYMRNLRWSVYLRQRRVCLSDTKESKPESLCVGVQCKSDSSLAWSC
KVSHEQRLLKQKANGPPYTLSESQTLSGAFNW*
>Ctel_100271
MTFLQTMSASLNEMRTAEEFVDVVLVFKERRIPCHRVVLASTCEYFHRMFLTGMKESSSDEVTLNEIDGDTGDLLIGYLY
SGMIEITQENALNLLAASEMLLLCDLKAKIQAFLCCNIHAKNCVSFLNVSRLYKLQDLIKDSQKFLNTHCKDVTLEGDLV
ELQEDDLVGILNFDPCEDSFRILRKWVQAVDGRDDRFMELVKNVDLSRFSKEFLCGDVMDDRLMQNVTGMKLIQEAIKSI
LLCE*
>Ctel_108335
METSLPTAFSEMREAEQLVDVHLVFDGTRVPCHKVILAGTCEYFKRMFLTNMSESVATEVSMKGISSTTGPLVIDYLYSK
KTEITVDNAQGLLEASGILMLDTLKRNVEEFLLEEIEVENCL
>Ctel_108783
MTEDSSASVTFESQSVHREAFSVLLEFYAHQTLCDVEIVVGARSFRCHRLILASVSAYFRAMFTSQMTETLSSSVTIHDI
DPAAFELLLLYAYTAKISFSTENVQTLLYASSILQMESVASACGEFMKNHLHPSNCIGVRAFAEQHGRTDLVLKADDFIR
DQFRHVVAQEEFLSMTSPHLIKIIESNDLNVRSEQEVYEPIMKWVKHKADRHTHLPALLSRVKLALLPAKYLVEKVCTEE
FLKQNLECRDLLDEAKYYQLSLARVLPGMQLTEKILPRKSCAGVIFCVGGRGASGDPFKSIEVYDLRKNSWHQVTEMSSR
RRHVGVVSIGEKLCAVGGHDGQDHLNTGEIFDPATNTWSVISPMVSLRRGIGLACLGGPIYAVGGLDDSTCFSTVERYDP
ESNSWSAVQSMNFPRGGVAIATAKGFLYAMGGNDGATSLDSCERYDPHLNKWTMIASMKQRRAGAGAAEINGKIYMIGGF
DNNAPLDSVECYNTETDTWVCVAKMSCPRGGVGVAPLAGRIFAVGGHDGSSYLSSVEAYDPRSDKWSSVASISSNRAGAG
ISTVMCRFDSLIDISKVNLSPAGTVALHCRHLQATV*
>Ctel_189559
MSEYFRRMFLVDMVESGAQEVSMDAISLNTGELLVHYMYTGHIEITTQNAQDLLEASDMLLLSALKQEIEEFLRRHIDSG
NCISMMNLARLYGTEAVLRDARKFLSELAKEVIATEEIHLLQEEDLIETLQANSSQENNFCFLQNTVMDEKLMHNTSGVR
LIQQAMQSPRSTYPPQQQALAVGDRDGDMWLCSDTNNAHWQPIQKPPTECWHYSACASPGGIVVSGGLCNNIRQRDCYSY
DACDAQWNTLPPMSHARASHSSIHHNHHLYIVGGQDGQDSLNSVETLDMNTLQWSHLPPLPVGIQLCYLAIVSNSLFVLG
GCNRGWSADVHEFNITQQTWRSRSPMPERGDGGAAVQFDGHVFVVGGTTGTCMRCDPRSNQWQSLHRLKFNHIWRPSLVW

DGNIVICGGRNNDTIESYSPITDEWVALSLKMPMIGFMTFAVPPPPAPAYISIWLYNC*
>Ctel_227534
MATLKARLGAEFLDYAHNLYVTQSDCDVLLIADENCSLHVHQIVLAAFSECFRVQFSEDARREIYIPDMEFAVLKALINF
IYSGKLCINKQNAKSILTAALQLKISAAVDHIRRTHKKIKRLFDTNRDLEEIISANSDVLEEPTAAATDEISPGNVSDNQ
INEPLTVKRNCKQEGENEREEEEAMNVEETLQEELTHESEDVEQGENLHELKEMKHPSEEIPPEVKSKRKRKVFPCKECD
QVFVHGASLKLHFRRRHGIPATCWRKSWHPIDFVRSMALRQSVRKIKSRKQNKIGNNYICKKCLAYFRYRSEYQAHFHQH
LCPVSCSKCHKKFTKSELCRKHLQKHRDVRQCISHSCKHCGQSFKWISVLKRHKKEKHPSTNKTSLRCRHCKRTFKYVQA
LEAHLRHHGLSAREVELEFKDPGVLPNVGNVYSSKGTSRMRQQSLECKLCNRIFTTVYTLNLHSIRKHNHALFDQPPKKR
SLKWACTVCEFKSHTATEMSTHMKQAHPQVMLSCASCKYQTPVKGLLDNHITTRHDTSSKKGHQCTECNSKFQSAYHLRM
HLYKKHDIKHEALKVYTCPVADCSYTAFQRYIVDAHMAKHKVGKNVPCAQCQRKFKSQKNLESHIRTVHERIRPFICELC
GASYGHEAELRNHIARHEKSLLCVLCDFVTSDKSCLQSHMWSKHRVQLDSGMKIFTCEQCGYSTDRGPRFKEHMLKHGED
RPVPCPECAKTFKSAHNLRSHMLWVHKKKKVKCEHCDHVTATMTNLREHVRIKHTHCNVKPFKCPYCDQLFGTAGNCRKH
VACRHKDQPINVITVKDKYPNSRPLVGGQRNPHRANTIASFDYDIETLMNKEEEAPPAPPPQATPPSETSYVYALHFAPP
PQQPYEQLTTGDDMLPSSAQIEMLPSDFNLSQQPTSNYQQLYSVVPQHENAPQTL*
>Ctel_115500
MWTFGQLTDFTLCVNSVHDIRCHKVILAASSTYFSAYFESNKEQKVSHAFPFCDALGHEIVEQIIEYFYTGRININEGNV
RAFLDASEHLELDELKNECLKFMTSQLGSKNCVGLLRFARSLHLEGLQIAARDYILHNFEEVIAISEEIAELSEEELVLL
ISDDLLNVRCEDSVYCFVKQWMGSGSVVRNEAFERIAKHIRFPFCSRDLLGQLMFGESLM
>Ctel_115520
MTTLQSLKKQESAIDITLKLPDETTIPCHKLVLMVASPFFATMFQSGMKESKQPEVQLEFSDAATIRMLIDYFYSGDIDI
NSDNMMDLVAASEFLCLADLKQHLGSFMTNEIDSANCIDFYMISQKYSLTNLIPHCLEYIMSHFTEAFKESDDFLNLTEK
ELTTVLADDRLIAENEDFVFHSVVRWVNADLERRKGKFVQIAALVRFPFSTQGLLDHLSLEPLMLNPTC
>Ctel_34919
MREAGEFADVTLVFDQQRMPCHRVILAGMSEYFRRMFLVNMMESGAQEVSMDAISPNTGELLVDYLYTGRIEISTQNAQD
LLAASDMLLLGDLKQKIEEFLVSHTDSENCVSMLNLARFYDMELLLRDAQKFLHKLAKEAIDTEEMHLLQEKDLIETLQA
NSWQENNFCFLQKWVGSAEGRADRFDALIQHVSLSQCSKEFICSTVIDEKLMHNTRGVRLIQQAIQSPRSTNPPQQQALA
VGTDGGEMWRCSDVNNQHWQPIQKPPTECKWYSGCASPGGFIVSGGRRNGINQRDCYSYHAHDDQWNTLPPMSIARAEHS
SIYHNQHLYIVGGHDGIFNFDSVETLDMDTLQWSPLPPLPILVHRCYLVVASNGLFVLGGVKNLGVWSADVHEFDFRLHS
WRSRSPM
>Ctel_110519
MTQTTSHLPAAFLQMREAEEFTDVTLVFGMQRLQCHRVILAGMCEYFSRLFLVDMVESGAQEVSIDAISPNTGKLLVDYL
YTGRAEITTQNAQDLLAASDMLLLRDLKQKIEEFIDSHTDSGNCISMLNLARLYDMELVLRDARKFLHEHVKEVINTEEM
HLLQEKDLNEILKTNTSQEDNFCFLQKWVGSAEGRADRFDALIQHVSLSQCSKEFICSIVMDEKLMHNTRGVRLIQQAMQ
SPRSTNPPQQQALVPIQKPPSDCVVYSACASPGGFSVSGGMYRNGIPQCDCHSYDARNDQWNTLPPMSIARAFHSSIHHD
HHLYIIGGHGGHHHLNSVETLDLNTLQWRHLPPLPIPSCNCYLVVVSNSLFVLGGYFEGGLRACVHEFDFTQQTWRSRSP
MPEICAYGAAVQFDGHVFVVGGITRSCMRFDPSSDQWQSLHRPKFDHYWGPSLIWNDHILICGGSNNDTIESYFPITDEW
EAWSLKMPKKGNMSFAVRLNIL*
>Ctel_146989
MKENDVMTDVTLKLPDGSSVSSHKIVLMASSPFFDVMFQSEFKESTTQDIPLDFADAVTIKTLVEFFYSGEINVCDDNVQ
TLVAASEFLCMKDLKDHCDAFMATRVDTSNCIELSRFGKKFNLMRLISAARKFILLNFKDVVKCFSDFPTLTENELIEVV
SSDELNADNEDLVFSAVVRWVNHDADERKEAFSRVAPLIRFPFCTHETLNNAVSQEPLMWNSECMKLIREAQNFNMHNHH
HFYSPRTVPRRAFNGKRQRLFRASGTEAEHTASVRSFRLDDRTPKWKSEATMLHSLDSFSVVQFNNKIYVFAGRDADGLS
TVTQEFDPGLGEWRLRSPMPGVCENHAAVVALNDKIFVVGGKERSLFSYEPAKDTWTVLSSTQHAYEYCSATAWKGKILV
MGTEHAEEYDIEGDTWIPMDKLVPSDTYYRNADNLILRTFLI*
>Ctel_164360
MACSRSSPPSCFRPKPLTPDRPLDGSMHFSIMKHPKESFEVMNTLRVQGKLCDITLRAGSSSFCAHRIVLASTSPYFKAM
FCTSGMRECGMADIPLQGIRPEVLSAIIEYAYTSEIQVNEVNVCSLLPAATMFQILHIIEACCTFLEHQLDPSNCIGIAD
FSQAHGCTDLYNKAKLYIYENFADVSQSEEFMMLSPSQLVQVLKRDELNVRCESEVYNAVVRWVGFEQDKRCQKMESLLN
AVRCHFLTPCFLQQQLKKCPILGKNMKCKDYLKSICDDIMRHKRCQEKRRTPNAPHVVYTIGGYLRHSLGNVECYNPSTA
QWLKLANLPVPRSGVAVCVAHGLIYALGGRNNSPEGNVDIAAVDCFDPFTNAWHKCHDMTVARNRVGCGVIDGQVYAVGG
SSGGMHHQSVEKFDPSQDTWTEVAPMETKRIGVGVTVVNRLMYAIGGYDGTDRLSSVECFHPENNEWRFLAPMNCTRSGA
GVCGFEQHIYAIGGYDSTNQLSSVERYDIETNQWEVIRSMNRPRSALSVVLLNNKIFALGGYDGSDFLSSVECYDIENDD
WKEVTTMSCGRSGHGAASAIEPCLKG*
>Ctel_63511
LKRLRKKNSMTDVELKLPDRSTISCHKLILVAASPFFATMFESGMKESTQQVVQLDFSDAVTIKMIIDYFYSGEIDINVG
NVQDLIAASEFLCLDDLKGHLGAFMTDQINASNCFEFYRYARKFNLGKLIPHCLEHILANFAQAFRSIETIIHLSEEELI
SVVSDDRLKADNEDMVFHCVVRWTNADPKKRRDAFVKIASFIRFPFCTQELLDRVPREPLMMNSACLDILSEAMSVRSPT
QTPSWLPVHRSSITSGTLNLLVSSTGIYWIDEEGAHGKWRTESSNHSSFNWVPHWVTNYSSMFIQGKLFSFGGQKNGAYC
STVQSLDPIEREWKPEPPMLQPCSKPYIVHFGNKIYVLGGVDASGQTLFTQEFDPVWGTWQLKAEMPGVCQSGAAVSLNE
NIFVVGGAERVCFRFTPSSDTWTVLSRPTH
>Ctel_220698
MCDHHHLRPQTSSHASGDVEHVNFLSEDIGALFLQDNYSDITLVVEEKKFPVHKVILAARSEYFRALLFGGLCESKPGVH

EITLKDTAASSFQHLLKYIYTGRMLLTSLQEESLLDVLGLADRFGFVELKNSISQYLEAMLSIRNVCLIYDMASVYSLSS
LLQTCFEFMDQNAMDTLQSDSFMTLSASSMRAVLSRDSFCAPEIEIFKAVRRWAEQNPEVELSSVMCSVRLPLMSLGELL
NIVRESSLVSADLILDSINIKTTHRDTELSYRGSLVPEENIATLRHGAQVIRGEMKSALLDGDFNNYDLDRGFSRHPIDD
NNPQQGIVVQLGSASIINTIRLLLWDKDMRSYSYHIEVSMDEKDWVRVIDHTRFQCRSWQYLRFPQRVVKFVKITGTHNS
VNRVFHLVCFECMFSKKTVELEGGIIVPRENVATIRHSACVVEGVSRCRNALINGDIQNYDWDSGYTCHQLGSGAIVVQL
AQPYVLSSMRLLLWDCDGRSYSYYVEVSTDQQHWTMIADFTKKPCRSWQVFTFPSCAMTFIKIVGTHNTANEVFHCVHFE
CPAESSALSEFESRRSSEDPSPPTLRLLPPAGSNMALQGLNQAADAEDGYGGMLQDGERSASPTLSS*
>Ctel_206441
MQSTEDLNHETSKPATQGKEKKAGVLEVEYDPPGDIIIRHMNTDEILSSLTCKPESGATWRETSDKPHKIQNYLTNCWLQ
QNSCDVVLAMGDGEMQVHKAVFAAHSLGLAKDFLKFSPKKQLVIHLADYAPCHIFDLTEFLYTGALELTPKNAGWLYKCS
DELGIVSLFQKSRDFLRSYNNMSAVTILNACDAAGLLDMRRDVMSYISNHFHETTPSADFVNVSLGTLIELLSMDGLILR
SEMAVFCAVVRWVDQTRDQRMVHAHDLLKCVRFRLLDPELLARGVERITWIFEDSKCQALLGDAYKFHSLKACGSRLIDT
FPPLIYRQKSPK*
>Ctel_211783
METVSFLKAMSASFDEMRAANELVDMVLVFGEVRLACHKVVLASSSDYFRRMFSCGLRESQSDEVVMKEMDAETGRLLVQ
YLYTGKIDITEENAQNLLSASNMLLLTDLEIASEKFLCEHIQPSNCVSLLNLSHLYELKDLIKRSQHFVAEQWTKVSDES
IIDLKEEDFIEILEAHDSHEGKFIRLQRWVKANEEREQRFMDLMQLVDLPGCSKNFIRDIARDEELLNNTRGMQLIQKAL
KRVKITLHHEAIRIPSSPFRSESRSSPVRCHNLRWQLVVIRTSGWEDKLDVTIVCQSTETTRWSCRVNYEVRVWSHSPRR
ALLTKRGRYVFGPNQISLRLPQSVYGRTQEYGHGNEPFIVYRRTQMHGPIIVEVNIEEAG*
>Ctel_86160
FNEMRAANELVDMVLVFGELRLACHKVVLASSSDYFRRMFSCGLRESQSDEVGMKEIDAETGRLLVQYLYTGKIDITEEN
AHHLISASNMLLLSDLKAASEKFLCKHIQPSNCVSILNLSHFYELGELMKVSRQFIKNKWAEISN
>Ctel_140761
SDIRFVIGPNRKTIYAHRCILAARCEVFKVMFSEQAASKDNHADVPFVMSDTTAEVFLPLLEYIYTNCVTLTQKNCIEVL
GSSMEYGLEGLRQACINWLIARISDATACEILQAGVTYNQDQLKDAAVQYIASNTAKVFQSKGFNELSDTALSYILKCNK
LMMDEMEIVKHVTDWSTVNSVVLNKPVAQIAKRVNGLIRLPLLSPQELRNVEENNDNKLLAVDSFAYAWKCHALQSWDQH
NPLTTLRTGTLPRDFYKAMKSST*
>Ctel_38482
IVLVFGEVRLPCHKVVLASNCEYFKRMFLAGLKKSGSDDVIMEGIDSDTGVLLVKYLYSGNISITEENASNMLLLSDLTD
AIEKFLCEHIQPANCVPLLNLSHLLECQDLIKESRKF
>Ctel_158890
MALDESSLPPLERPSYKNQGHTQKAFEVLNVLRGNQLLCDVALSADGVELWAHRVVLAACSPYFYAMFTADLAEAHTERI
VLQEIDNRALTLLIDFMYTAEIQVTEDNVQSLLPAANILQLVEVRDAACEFLQSQLHPSNCLGIKAFADLHACQDLLQYA
QTYTEQHFSEVVLHDEFLNLSPPQVCKLISSDRLTVGSEEQVFEAVMHWTSHDMPSRLEHLAQLMEHVRLPLLTQEYLVQ
RVEEEPLVKNSSACKDFLIEAMKFHLLKPEQKIMYKTPRTRARTPIGLPKVLLVIGGQAPKAIRSVECFDFKEEKWCQLA
DMPSRRCRCGVTIISGMVYAVGGFNGSLRVRTVDMYDPIKDMWSPIASMEARRSTLGAAVLNNMIYAVGGFDGSSGLSSV
ECYDVRANEWKIVSPMNFRRSSVGVGVLKGLLYAIGGYDGASRHCLSSVESYNPETDLWTSVAEMSCRRSGAGVGMLDGH
LYAVGGHDGPLVRKSVEMFNPETNQWTQVADMHLCRRNAGVVANSGLLYVIGGDDGSSNLGSVEFYNPKQDTWTMLPSAM
TTGRSYAGVTVIDKPM*
>Ctel_22921
RDKKKKASKFATLRKKLIRVRRSSRSLDNSKVIRELTLGWSIHELSALVEEYEASAALKELASCADLARPHARTCQQDLS
VLYDCKYCTDVDIVYQGVVFPVHRSILSVRCPFFRDLLAHYPDQNSQVPLSIRTPGVDTHTFSALLRYLYTGEINSGGPL
DNRELLIQLSQEFGTPNPLEHDLRRLMESGVYSDAVLVFASDAHEHNSDSLLSVSSEARTLMRHELRCHRAVLAARSPFF
RNLILRRGCNSEQACIVLDESVIPRRYARVLLQALYMDSVNLACIVRSSVSVCSLSEVQAMVAGKGHVSLTEEAMEIFQI
AQFIDFPILSQGCEDIIVERLSAENLLCTLAWSEEAHGSAWVQHQALHFLREEFSSLSRSPLLYDLSRHYLLQAIKSDFL
QAGELDVLSAVIKWGEHELIKRLEEREPNLLSHTAHSISRKGVKKRDLNDVELRDILTDLLPFVRTDHVLPPHHDLLCNA
IRRGLVSKPPSHMIGGECSGQFSPASAWVRCGGKGMYTKPRLFLPYYEEAKALLEEQLAQVQENEVVRLRMIPSAIPDTL
YMVSEYACRSSPHLCRSQPTLDPSPQHSSLDVITGTIPVPDDDTLRKMQQRESELCQSPLVHRAYSLACADRREVAHCVR
VRVAREFGLPDSCADLL
>Ctel_199916
MSLDKRIHTTKRLDQHTRYDMTSALQRSPNKQSSDGGSSSTDEFLYKSSSLALVKHKHGMNKTEANTSSTHDRVDATMRY
LEASQKVLDFLRRMWLDQKSCDMVITLQNGEMLAHRVVFAAHSQILAEKFAHFGPDQIVTIQLSDFAVKPVFELTQFLYT
TDLCLTVDNVAEIYKCAAELGLNSVFEYAREFLSNYNVSNAILFYNICDQGNITDLRDEIFSFICEHFHEIIQHESFKRV
ALEDLMNLLKNNHLGVHSELDVFFAVCRWLEGDLRRRMPLSNRLLGCVRFQHIKPEDICTKVCSVEWLMEPKENVAIINQ
AYKFHSLKATGSHLVARFPVQVPRSRAPGLRRKSSTSKAPVQPTLPTE*
>Ctel_223523
MEKNMTFLQTMSASLNEMRTAEEFVDVVMVFEERRIPCHRVVLASTCEYFHRMFLAGMKESSSDEVTLNEIDGDTGDLLI
GYLYSGMIEITQENALNLLAASDMLLLRDLKAKIQAFLCCNIHAKNCVSFLNASRLYKLQDLIKDSQKFLNTHCKDVTRK
GDLVELQEDDLVGILNFDPSEDSFRILRKWVQAVDGRDGRFMELVKNVDLSRFSKEFLCYDVMGEPLMRNEAGMELIQEA
IKSILLCENRTEVTFRYTLHNISSLSPTNVYSPEYYMRNLRWSVFLGTDTKDSRPKSLAVFVQCKSDSSSTLSCEILYEL
RLLKQKADGAAYTKSQS*
>Ctel_227949

MVKKVGSDNEIWKYIPENGNTEERKKRMEWMSQGDDRDRSKQPTKLWLALWRDGNISLVSGKDASAAKRALEEAGESYNC
LIQQYDRHICLTFRPEMPEFKVNKSLSFETPGPPDLKEHFTEDMKRLMHDEASSDVQVHCGNRVFKCHSFILSLRCDFFR
AAFASGLSETREKVVRIDHLEEDVLQVILTFIYTGNLDFSSWDSFDGKVLSDADERMMKMIRLGEAADFLLLPHLRDAVH
HEISLLPEPWHHDCLKALVNIQRPHASLQQQIFLWVHWYLRNKRLDSDLQQDDLYSMLSEEFKSNLAEFVKFKLKTEPFY
GSRLCKLANWLSSESPLPGYEEFTSQVESVHDLHTVSALHKLCRKWDSEGDEQQNGCVLRLANYMVSEMGFDLGLRDERG
DLALHCAVEACAEPLVTLVMGANQDYVGIMNNELETPCDIAMKLRLREDVDSTIAKRIQRLMLSVAGDNLTSRMPDWLAE
DQEENFRELYENYANMKFEWLPSSGFNAHVMFADFFNFSFPEFNAALKRKNDALMHSHSMVTEEELISALKEDVYFRLAR
IARPQFDIFASCCWKQEEEAAEDRSGYSA*
>Ctel_34910
MKEAEELVDITLVFGERRVLCHRVILAGTCDQFHRMFLANMVESASDEVTMEGISASIGVLLVEYLYGGDVEITSQNAQD
LLEASEMLMLGTLKQNVEEFLCGQTDSANCISLLNLAQMYDLHSLLEDAREYLHNHVKEVLHSEEINLLREGDLVKVLTA
NDSPEDNFRLMQKWVKSDESRTGDFVKFIHHVQLSQCYKEFLHNTVMVEELMFNKQCMELLRQAIDTHPPEQQSLAKPPN
QNMQYSACAYPGGFIMSGGWSHNSVPQPDCCSFNVQNDQWVILPPMSTAREWHSSIYHKDHLYVVGGRGDTNKYLDTVES
LDIKSLQWSHLPCLPQKCEGGAAVSFDDHVYVVGG
>Ctel_159913
MASLHKLQEKEVMTDVTLTLPDQSAIPCHKVVLMAASPFFETMFQSGLKEGAEQNIKLDFADSDIIKTLVEFFYTGDIEL
TEGNIKEIVAGSDFLCCEHLKAHCEEYLADTVDCLNCIDYYRFGKVFNLKLLIKTAFDFILSKFKEFRGIADFDKLTEDE
LVEVVSSDQLNAENEDVVFEAVVHWVNADPDARKEVFPTIAPLIRFPFCSQKALTENICRNTLMQKSGCIDFAHEALWSQ
FHFNCGLSLDNVRCIPRHAFHSRTSLFQLIKSESPNEVSLCVASSNDGSGIEWETVLPCNTATFDSALAFIDGKIFAFGG
DSAPKSVKSINLKESNPEWKFEQEMLSGLHRPPIVQFANKVYAFGGWNMRLAQEFDPALNEWRMRSEMPGLFNDGAAVAF
GDKIYVVGGEERACYSYDPENDEWKVLSKPTHAHHSFPFATVWQGKILLGNMEHVEEYDPVEDRWSNRDELLPGGDINEM
ILYASCTL*
>Ctel_37139
LHKLQEKEVMTDVTLTLPDQSAIPCHKVVLMAASPFFETMFQSGLKEGAEQNIKLDFADSDIIKTLVEFFYTGDIELTEG
NIKEIVAGSDFLCCEHLEAHCEKYLIETVDLSNCISYYQLGKVFDLKLLINTAFDFILFKFQKFREISDFNILTEDELVE
VVSCDQLNAKNEDVVFEAVVHWVNADPDAREEVFPRIAPLIRFPFCSQKALTENICRNLLMQKTGCIDFAHEALWSQFH
>Ctel_48502
RFSILQEIRAERKLTDLVLVVQDERIHCHKALLGAASLFFQVMLVSGMKECEEGVVSLHGTDPVTVISIVDALYGCELKV
SGDNVQDLFVACHQYQLRDILQQCIDFMKQHLDSQNCL
>Ctel_146234
MVQSPTFGVETLAKLNEFRNGRSFTDAVLCVGHEEFPCHRNVLAVSSPYFNAMFSSDLKESREAKICLNEVSPWTLRRLI
DYAYTGKIEITVDNAQDLLAASSLFQYPVIVDGCCEFLMKHLHPSNCLGIEDFAHLHSCSKLEADAHRFALDNFSVVVEF
EEFLEMPLARLCSYLASDLIDVRSEEAVFEAGVRWLQYDIDKRRDSTLSVLEHVRLETMDMIFLEEVISRHSLIRCSEKC
LALVKQAQHRGQCRPSRVQQPRPSTVAKEVLVVLGGINNMNYIMQSVEMYDPFKDKWTPLPDMPTPASWCSASAIGNAIY
VTGGIVDGHIVSAVWKFESIKRVWSEVAPMLSPRARHTSTVLDGDLYVIGGVVMNGAKISAMETIERYHLLSDQWLLVGQ
APFPRVQSTMVPYQQTLVEVGGTQNGAQVQTMESYICKNGQVLYSGEQFILPECIQFSQILLIHETFYILWEDTKKMISL
SPKKRSFRRLPDMLHTHIKGGAAILENRIFVAGGLIDSKPSRVVECYDIATETWTPVKSMRQARACHATVSIQMS*
>Ctel_44894
EVVMNGIDCETGVLIVKYLYSGNIAVTEENAQDLLSASNMLLLGDLKDSIEKFLSKRIQPPNCVSLLNLSHLFELQDLIK
TSRKF
>Ctel_46405
LIVKYLYSGNIAVTEENAQDLLSASNMLLLGDLKDSIEKFLSKRIQPPNCVSLLKLSHLFELQDLIKTSRKFIADKWDDL
S
>Ctel_98406
MASLHKLQENEVMTDVTLILPDQSAIQCHKVVLMAASPFFETMFQSGLKEAAEHDIKLDFADSGTIRSLVEFFYTGDIKL
TAENVKTIVAASEFLCCKHLKAHCEEFLTGNVGLSNCIDYYQFGKVFNLKVLIKTAFNFILFKFREFMEISDFDKLTEDE
LSEVVSCDRLNAENEDVVFEAVVHWVNGDLDAREEVFPRIAPLIRFPFCTQTMLSDKICCSSLVLKSECIQLALEAVWSQ
FHLNCRPPIDNARCIPRQSFLLGGSTPSLILITESTSQKKVFLSVTSCIDGSQMKWETIMPCKTAKFDRSLAFIDGKIFA
FGGDPASKSVKSINLKESNPEWKSEQEMLHEVLNPLIVQFANKVYAFDSWITMVTQEFDPALKEWRMRSEIPGDCFGGSA
VALGDKIYVVGGYQRVCFSYDPKNDEWQVLSEQTHQYYRNVTATVWKGKILLGNTKHMEEYDPVEDRWSNRDELLPGGRI
NKICLHASCSL*
>Ctel_43430
SHMKTFMACLNKMSEDEVMTDISLKLQDESSISCHKIVLMASCPYFETMLQSGFKESTEQEILLDFTDADTIRMLVKFFY
SGEIDINEDNVQTLVVASEFLCLNDLKAHCDEFMSNLVDSANCVQLQRFGKKFSLMASTPVAEEFILKRFKDVVDAFSDF
KTLTEDELAELVSSDQLNVENEDIVFEAVSRWVNVDIHERKEAFSRIAPLIRFPFCTKTTLNEVINRAPMMWNPACMELI
REAQHYHLNYQHYLNSPRIIPRVGSVGKVKKPILVRTYGDTRGMMHCEHTDSIEDECVRWKEVICEQKYIRHLMTAPLGC
IGLNYEKSYKLGIFKRQMWEVSWKTNIKTHDFCQYFFFNGKVYAFGCQASAQTLTYSCLSINVDEFDPKWKTEAPMLHAL
EHPTVVQFADKIYVLCGRDVNGSARVTQEYDPMLNEWKLRSVMPGFSRGGAAVAFNDKIYVVGGQGRSCYRYEPATDAWK
VLSKPKLYYDVCSATAWKGRILVMG
>Ctel_38610
ETMSASFNEMRTANELVDVVLIFDELRLPCHKVVLAANSDYFRRMFLAGLRESQSDEITMNGIDADTGRLLVKYLYSGEI
PINEENAQDVLSASHMLLLHDLKAASETFLLHHMQPPNCVSLLNLSHLYELQDLTKQSRQFITNKWTELS

>Ctel_116561
MTTLQSLKKQESAIDITLKLPDETTIPCHKLVLMVASPFFATMFQSGMKESKQPEVQLEFSDAATIRMLIDYFYSGDIDI
NSDNMMDLVAASEFLCLADLKQHLGSFMTNEIDSANCIDFYMISQKYSLTNLIPHCLEYILSHFTEAFKESDDFLNLTEK
ELTTVLADDRLIAENEDFVFHSVVRWVNADLEKRKGKFVQIAALVRFPFSTQGLLDHLSLEPLMLNPTC
>Ctel_116580
MALYGDSDWSIVESNRFMLDTQCQCDVHFQLVPPATAEMIRVSAHRFMLMSRSSVFMAMLTGPLAENDDCIKVTDISSEC
FHALLKYIYTEEVDFSEDNAFGILYAAKKYLVGGLVKKSLSYLEANLTAVNVCSYLDNAFLVEEDCPELLVQCSALVQRE
TQVALHAESFYDMNRESLCRILEMPRLSMPESDIFSACDHWAKRKCNENQLAETPKNKRELLGRALYLIHLPVLEIEQFT
NVVKSGLLSTEEENAIFRYF
>Ctel_193275
MWERVRRGSGYVFIAMAQAWQSLPSSFHRMREVEHFVDVTLVFGHRRISCHRVILAAACEYFRRMFQTNMVERASSEVVM
TDISPSIGELVVNFVYGRKIELCMEILDDLLVACNMLQLGDLKYDVQNYLILRANGPDYIPNLKLARRYELDRVLCDSHE
YVLHHTSEIDTDQLALLDEEEIIETLESSDSQEASFRLLQVWVRSAVGRIDNFVRLLGFIRLDKCSNDFILHTVMKEELM
QNPQCSEIIDKAMQIDVNRVLVVGSYEGDMWMCRTFNQQWHLIPKSPSQNFAGSVCAVPGGIFVSGGLLNGYDQRDCYYY
DAHTDRWNTLPPMPTGRRCHSCISFNGCVHIIGGSSDGKALESVDSFNMSLLEWRDDIGPLPRPLALSYTAAFADSIFVL
GGYGEDAWIDLVYEYECKRGTWRERCPMPETCTTGSAVSFDEYIYVVGGTEKRCMRFHPDTNSWEEFMEPCVGHAHALTW
RENILLCGGAGDDSIIEYSPLSNKWSYWVLKVPGSMNMHFALRIDLCPPQ*
>Ctel_103627
MREAEEFVDVALVFGERRVKCHKVILAGACDYFHRMFLTDMAESASKEVSMKGISANTGALLVDYLYGGRIEVTAQNAQD
LLSASDMLLIDALKEDVEEFLCQNTGSSNCISLLHIARLYDLKALLKDAHTFLLGNIKEMIDSEELHLLQEGDLVDILIA
SYCQEDSFRLVQKWVRSSEARTDRFACLLEHVELSECSKEFICNTVMEEEFMMNKQGMKLIQKIMQSSTLADSSKKQALV
VGNRDGKMTWVCADGQLWERVCKPPSQNQYSSACASPGGFIVSGGSHSFEAQADCYSYDVLTNEWTALPPMPTARILHSS
VHHNHHLYIVGGSDNEDVYLDSVETLDMRSGQWSRLPPMPRPLREPQVVFVSNHLFVLGGFLNSGELSVGVHEYDAAHGT
WGARCSLPEECEGADAVCFGGDFFVVGGDDRCCMRFNTSSDSWTKLQGPPYDHYWGPSFVWRNKIILCGGEDRDSIEEYS
PGTDSCSAIWTLKMPNRKRMRFAFRIDVP*
>Ctel_106756
MDLHLRTFMRSLRRMRENEAMTDVTLKLPDESTIACHKLILMAASSFFESMFQSGLKESIDNYVKLEFTDSDTIRALVEY
IYSGEIEVNKENVQTLVAASEFLFLEDLKANCDDYLATLIQSSNHQDLRDFGQKFNLKKLLSSTHEFYLSHFQEMIETPS
FTNLTEEELVALVSDDRLNAKSEDMVFESVVQWVKEDPEQRKEAFPRIAPLIRFPLCTQKTLTKIVNCEQLMWN
>Ctel_228846
MYRMASDPEQMDSPLEDDDENQTLVPPNPAREAVLRDEANFIQNMSQFFNQEMLHDIILTVDGQQFYGHKFVLAKSSDVF
RKLLYENCWSEEKSKEITLSEAPECQAVFEPFLRYLYTAEVSISTDTAVGILCLADKYNVASLKDLCVGFMIDRARSPAV
SMALMWYPWAKVLHLPDLLHQCTQTIAWNFYEVLMSPGWMNMDLDFLTDLLSSSELVLPNEFVLWEGMERWLLQEGTLDN
LQENSALLLPMVRLPMMVINQLYAIESSELYENPLSKDTLHSLLSKAYRFRALCPSQAKVEPSLSFCDIFYQPREYTDLR
VHSVSMHNSMRLNQVDVRMFKGAVPSDVHEADWKITYRKNADNWNLQLYCHDTGLPMARVQPTLLFCCDEGKVIQVHREK
VLVIPRGTTMSMNAMAENPAITKHMALLLKPVAC*
>Ctel_64348
LQEKEVVTDVTLILPDQSAIPCHKVVLMAASPFFDTMFQSGLKEGAEQNIKLDFADSDIIKILVEFFYTGDIELNEENIK
ALVAGSEFLCCEHLKAHCEEYLADTVDLSNCIDYYRYGQVFNLKLLIKTAFDFILFKFREFKEISDFDKLTEDELVEVVS
CDRLNAKNEDVVFEAVVHWWKSEQEMLHAVDRPHIVQFANKVYALGRWKTTVTQEFDPALNEWRMRSQMPGGCYDGAAVA
LGDKIYVVGGSEKVCYSYDPENDEWKVLSKPTHK
>Ctel_183893
MDESPVDSAECHRSPEHADRMLRRMESFQAKGQLADVSLVCGSKRISAHRIVLSSASDYFAAMFTNDVREATQEEVRLKD
VDGEALATLVHYMYTGMIDLREDNVESLLSTACLLQLAEVKDACCSFLVKQLHPSNCIGIRQFADAQGCASLYTMANNYV
MEHFCDVIRNQEFLLLAPDDVAQLLASDDLNVPNEETIFHAFVLWAKHEATSAARKKHLARLLALIKLPLLQPQFLADHV
ERNVLFRDQRDCQELIMEAMKYHLLPERRLSLQSPRTKPRKATVGVLYAVGGMDSTKGATSIEKYEPRTDSWLQVANMNG
RRLQFGVAVVEDRLFVVGGRDGLKTLNTVECYDPRKKTWSLMPPMATHRHGLGVEVLEGPMYAVGGHDGWSYLNTVERWD
PQAKQWSYVAPMSTARSTVGVAVLSNKLYAVGGRDGSSCLRSVECFDPHTNKWSLCAPMTKRRGGVGVANCNGFLYAVGG
HDAPASNPTSSRFDCVERYDPKTDTWTLVAPISSPRDAVGVCLLGDKLYAVGGYGGQQSLNEVEAYDPQTNEWSKIASLG
TGRAGACIVHLRAS*
>Ctel_34906
DSHLKAFMASLHKFQENEVMTDVTLILPDQSVIPCHKIVLMAASPFFDTMFQSGLKEGAEQNIKLDFADSDIIKILVEFF
YTGDIELTEGNIKALVAGSEFLCCKHLKAHCEEYLVDTVDLSNCIDYYRYGQVFNLKLLIKTAFDFILFKFREFREISDF
DKLTEDELVEVVSCDRLNAENEDVVFEAVVHWVNADPDARKEVFPRIAPLIRFPFCTQTALADKICCGSLMLKSECIQLA
HEALWSQFHFNCRPPIHNGRCIPRLSFDLPLFRLTIFAFGGNPEFKSVKSFDVKGTNPEWKTEKEMLHALRQPYVVQHAS
KIYVFGESQLSGYGVFFTVSLVCQEFDPGSNEWRVRSEMPGSCTGGAAVALNDMIYVVGGTERVCFSYDPANDEWKVLSK
PTEVYNNRTTATVWKGKILLGNEKHVEEYDPVEDRW
>Ctel_54631
DSHLKAFMASLHKLQENEVMTDVTLILPDQSAIPCHKVVLMAASPFFETMFQSGLKEGAEQNIKLDFADSHTIKILVEFF
YTGDIELTEENIKALVAGSEFLCCKHLKAHCEEYLVDTVGLSNCIDYYRYGQVFNLKLLIKTAFDFILFKFREYKEMFSF
DKLTEDELVEVVSCDRLNAENEDVVFEAVVHWVNADPDAREEVFPRIAPLIRFPFCSQTAFTDNI
>Ctel_85628

LDDMRTASEFVDIVLVFGKVRLPCHKVVLASNCEYFKRMFLAGMKESQTDEVVMNGIDCKTGVLIVKYLYSGSITVTEEN
AQDLLSASNMFLLSDLKDSIEKFLSKRIQPPNCVSLLNLSHFFELQDLIKTSRKFIADKWDDLSDESIVELTEDDMEVIL
KEHECQENNFLRLQSWVKSVDGGEERFPRLLENVKL
>Ctel_151000
MADDVIVFHKQDLPLMAFPMFEEIRRRGKLCDVTLLIEEQRIHAHRIVLAATVPYFNAMFTHDMVESKQNEIAIKGIEAS
ALSDLVTFAYSGSVQIDSMNVQNLLVGASFLQMQVIKDACCDFMKSRLHPNNVLGIKSFADQFMCRSLEDACDKYIQIHF
MEVSKSEEFLSLDEDRVTAILARDQLHITGEEQVFEAAINWVKQQSSKRMHHLPNLVVHVRLPLLSPQFLSDRVGQEELI
KGSLKCRDLLDEARDFHLMPERRAQLQTFKTRPRCCTDVVGVIYAVGGLTSSGDSISTVEFYDPIIGKWQMAKPMSTLRS
RVGIAVLKGHLYAIGGYDGQERLNTVEVFDPVKLSWGLVAPMICKRSALGAGALGGELYVCGGYDGVTSLSSVEKYDPVS
NKWVMVSNMIRHRSAAGVCVLNGHIYALGGHDGLSIFDSAERFDGNGQWKAVASMLSKRCRLGVASLNGKLYAAGGYDGN
VFLKQVECYDPNTDSWCFVAPMNVKRSRVALVTNCGKLYAIGGYDGVSNLNSVEVYDPSFNTWNFSASMCAHEGGVGVGV
VPVDAARS*
>Ctel_198492
MTQEMSSLPASFLQMREAGELVDITLVFGDQHIPCHKVILAGRCDYFHRMFLTNMIEKDSTQVVMKEISARTGALLVGYL
YTGRLDISLDNAQDLLSASAMLLLGALKQDVEEFLSGHTGPNNCVSIMNLASLYEMKTLLANAKKFLHEHTKEVMGTDEI
RLLQEVDLLEVLGKNSCQEDNFRFVQKWVNSADGRTDRFYDLLQHVTLSKCSKEFICSTVMEERLMVSVNGMKLIQQAMQ
ASRQTDPIGQQLLVVGDVNGDMWLCPDINLQAWQSLQGPPSMSSYYSACASPGGFFVSGGYLNGIYKSDCSSYHAQDRQW
KALPPMPTARGYHSSIYHDECLYIVGGITGQNYLDSVEKLDMRSLQWSRLPSLPHATCYVYLAIVDNKLFVLGKSGGVWN
IHVHEFDLTQQTWRQRSAMPEKCVDGAAVSFNDHVYVVGGKDRSCLQFNPLQNTWISLERPQLTHHIGKSLVWNGNILVC
GGRNSDVIEAYSPGENKWSSWSLKMPTKKAENHFALKMQR*
>Ctel_204628
MIFLQRPIIENDGSRTVETLGSNPVRITNALSDKLNLESHLKAFMASLHKLQENEVMTDVTLILPDQSAIQCHKVVLMAA
SPFFETMFQSGLKEAAEKDIKLDFADSGTIRSLMKFFYTGDIKLTAENIKAIVAGSEFLYCQHLKAHCEEFLMGNVGFSN
CIDYYQFGKVFNLKVLIKTAFDFFLSKFREFREISNFDKLTEDELVEVVSCDRLNAENEDVVFEAVVHWVNADPDVREEV
FPRIAPLIRFPFCTQTMLSDKICCSSLVLKSECIQLALEAVWSQFHLNCRPPIDNARCIPRQSFHLRGSTPSLFLFVQSK
SRNNVSLNVASFVGESEMKWETLMPCIPATFDLLVTSPFGLYLIDNNDCYFVEPKLKRLTTLPWKPVNLDGSTLAFIDGK
IFAFGGGPPSKSVKSINLKESNPEWKSEQELLHAEDHPIIVQFANKVYAFGGWGTSVTQEFDPALNEWRMRSQMPRFFYC
GSAVALGDKIYVVGGNRRVCYSYDPENDEWKVLSEPTHQYYTNASTVWKGKIMLGNSEHVEEYDPVDDCWSNRNELLPGG
DINTMYLYASC*
>Ctel_177060
MSGLNLESHLKAFMASLHKLQENEVMTDVTLILPDQSAIQCHKVVLMAASPFFETMFQSGLKEAAEKDIKLDFADSGTIR
SLMKFFYTGDIKLTAENIKAIVAGSEFLYCQHLKAHCEEFLMGNVGLSNCIDYYQLGKIFNLKVLIKTAFDFILSKFREF
REISNFDKLTEDELAEVVSCDRLNAENEDVVFEAVVHWAEKNPLIVQFANKVYAFGGLVTRVTQEFDPALNEWRMRSRMP
GGCYGSAVALGDKIYVFGGSQRECYSYDPENDEWKVLSKPTHQYYLNVATVWKGKILVGDKEHVEEYDPVDDRWSNRDEL
LPGGDINQMFLYASCSL*
>Ctel_121014
MEPHFERPPSPARLPYTSDKHPRHTLEAINVLRKHRELCDVVLIVGQRKIFAHRVILSACSPYFHAMFTGELAESRQTEV
TIRDIDEAAMELLIDFCYTSMITVEESNVQTLLPAACLLQLAEIQDVCCEFLKRQLDPSNCLGIRAFADTHACRDLLRIA
DKFTQHNFQEVMESEEFLLLPVNQLMDIISSDELNVRSEEQVFNAVMGWVRYGVQERRLHLPTVLQHVRLPLMSPKFLVG
TVGSDILVKSDESCRDLLDEAKNYLLLPQERPLMQGPRTRPRKPIRCGEVLFAVGGWCSGDAISSVERYDPQTNEWRMVA
PMGKRRCGVGVAVLNDLLYAVGGHDGQSYLNSIERYDPQTNQWSSDVAPTSSCRTSVGVAVLDTFLYAVGGQDGVSCLNF
VERYDPQTNRWTKASSMSTRRLGVGVAVLAGYLYAIGGSDGTSPLNTVERYDPRNNRWTPVAPMGTRRKHLGVAVYSNMI
YAVGGRDDATELSSAERYNPQSNAWQPVVAMTSRRSGVGLAVVNGQLMAIGGFDGTTYLKTVEIYDPEQNCWKLFGGMNY
RRLGGGVGVVKMPQQESHLW*
>Ctel_45905
MLDSHVKTFMACLCNMKENDVMTDVTLKLPDGSSVSCHKIVLMASSPFFKTMFQSGLKESTTPVIPLDFTDAVTVKTLVE
FFYSGEIDVTEDNVQALAVASEFLCMKDLKDYCDAFMATRVDTSNCIELSRFGKNFNLVKLTSAALKLTLLNFKDVVKRF
SEFSTLTEDELIEVVSSDELNAGSENIVFSAVVRWVNHNAEERKEAFSRVAPLLRFPFCTQGTLNNVVSLEPLMCNPECM
ELIREAQNFNLNNQHRIHSPRTVPR
>Ctel_131628
MDVSGLSIQTSAADPTGSGANRDFQVIKCVVVGDTGVGKTRLICSRACGTKYTLQQLTTTHVPTVWAIDHYRKNRDILNR
SWCTVDGLNVSLHLWDTFGYHDKDRRFAYGRADVILVCFSAVRPRSLRSVYHHWFPEIQRLCPETPIILCATQVDLRYLY
QDPEFVSMDKGPFFKEIKETDILPPDRGRAVAQEINAHYYESSVLLDYGINDVFVNVIRAALIQKRDKHFWNQLGGLKRI
TRPLCQAPFQPPQPLCPKILIPPPSLDHDLLNLLDSQSYSDVVFMVQGIAIPAHKSCLAVACSVFEELFLSLDAGVSLRD
PKPLSAVLPSLSDSPLHTADTVNLLDDGDGRMTTAINRALQDLPLPVSQQFPFCSSIEQNSSWASLNLSGMNSTDDSSLT
VIHIVDCITADTFRQLLRYLYSEQTISSLETGLSVCDLNKLKKAAEIIGIQDLSFALINILKKEAFMNKALLLSVQFQRC
QRRRELLVKKNLFSDVLFQVEDGSVKAHRALLLAGCDVMAGMFSFNFRESSAKMIPLPGVTRESFVALQDYLYSGQCPVL
SNIDCVDLIALANCMCLPRLVALTEQYIVNELISAEDCGKSILEDVMLFIEPARFHNANQLAAWCQWCISTRYTQVYHSH
SKLLQTLPPENQVYLSKHRWPPLWYLKEVDYYEKCLLELERQRQKPRPYLRRGSEDFDSGCLGFCGRRNRKINREGASPT
TAVVN*
>Ctel_54628
SHLKAFMASLHTFQEDEVMTDVTLILPDQSAIQCHKVVVMAASPFFENMFQSGLKEGTEKDIKLDFADSGTIRSLVKFLY

TGDIKLTAENIKAIVDGSEFLCCKHLKAHCEEFLIGNVSLSNCIDYYRFGKVFNLKVLIKTAFDFILSKFREFREISDFD
KLTEGELAEVVSCDRLNVENEDVVFEAVVHWVNADPDVREEVFPIIAPLICFPLCTQTMLSDKICCSSLVLKSECIQLAH
EAVWSQFHL
>Ctel_228408
MNVVGGLSLHLSEQGDSVLRCLNQQRTHGRFCDVTLLVSGHSFAAHRNVLAACSPFFNSIFRERKSENESVTLQCKDHLL
FESLLEFLYTGQITVDQNSVQALTEQACELGLERLVAQCVACLESTLHAANCYQLRDFAIKCHLENFLKTIEAFLITNIP
SIIHQNEILEFPLCKLENLLVTRSAPLTEEDKLVLICRWTKYLLKDREKDFVKCLSHLNWGNLGFSQLNALIHQDELFVQ
SEWCLFSVLQALEENHLLFDIFSERLTSLRMKFISNDLPAVLVDTDISPAEELLSGHDSLVVDLPTSEDDEDSLPIEEYL
KKKLVKSRRTRSKAVKNIDQEEESPSSGKKRKLEPLSCDICPYSTLKGEERLRSHITKNHADDRTFSCPKCEFSCKWNRE
YNQHLKEHFKGPPFMCDIIGCTYKADRMRLLLEHRTVHTNERPFKCSECHLQFRTQANLYVHRKTHTGEKRFVCSVCSRR
FSSRGNLQQHSVIHSAARPFLCDLCGFSTKYQSHLKSHRRTHTGEVFSCQYSGCTYSTPKRSQLACHMRCHLKIRDHVCT
VCSQSFTEKSHLTRHMRIHSNDKPFACNQCEYKSTRLDKLKDHQEKYHNPEAKKEKKQRKGKKARTDDAVTPRDASTVSA
ASIVTQAMEVLGQNVVPPLMMNAVDCQQGALQPAAQDYATISSFINIF*
>Ctel_63547
LHKLQENEVMTDVTLILPDQSAIQCHKVVLMAASPFFETMFQSGLKEAVEQDIKLDFADSGTIRSLMKFFYTGDIKLTAE
NIKAIVAGSEFLCCKHLKAYCEEFLMGNVGLSNCIDYYQLGKIFNLKVLIKTAFNFILSKFREYREISNFDKLTEDELAE
VVSCDRLNAENEDVVFEAVVHWVNADPDVREEVFPRIAPLIRFPFCTQTMLTDKICCSSLVLKSECIQLAHEAVWSQFHL
NRRPPIDNARCIPRQSFHLRGSTPSLFLIVQSKSRNNVSLNRLTTLPWKPADKWSTLAFIDGKIFAFGGLPESKSVKSFN
LKKSNPEWKSEQEMLNAECNPLVVQFANKVYAFGAFGAFGGRWSTKVTQEFDPALNEWRMRRQMPGHCFGGSAVALCDKI
YVFGGSQRVCYSYDPENDEWKVLSKPTHE
>Ctel_121302
MSATFRNMRHSDEMVDVVLVFEKTRVKCHRLVLAASCEYFRRMFQTDMQERDAGEIPMKDVSSSTGLLLVEYLYSGNIEI
SVENAQELMAVSDRLLLTKLKKNVEEFLCEQVAADNCLSFNNLARLYSLKSLLEVCQNYLSDHWRKLLESDEIDQLTEAD
LFMVFGGKMDVESFLDSVECLNLKSLKWSQFPPLPLALSETKPVYVQNKLFIVGGWLSQKTYSRDVYEFDSAKRAWRSRS
AMPEECRAGGVVSFDDKLFVVGGDPKSCMHYDPRTDLWVQLQKPLFEHFYGPAAVWNDQIVVCGGAGTDSIEEYDPQSVK
WSTWDLKMPSMNDYCFALII*
>Ctel_140893
MTDVTLKLPDDSSMPCHKLLLIAASPFFETMFQSGMKESVDQVVELKFSDADTIRMLVEFIYSGEIDVNKDNVRTIIAAS
EFLLLKDLKAHCENFIVGTTNSSNCIQNMKFGRKFNLEKLISTANQFMVLHFEETLAAPEFNSLTEAELREVVSNDQLNV
EKEDVVFHAVVGWVNADLEQRKEAFPRIAPLIRFPFCSPETLNGVVGREPLMWNHECMKLLTEAQ
>Ctel_119696
MFQLLQRNSEMKELTDFVLKVEDQEIHCHKVIMAAASRYFEKMLTSDMKESTEGRAKLNETNFAIINLIVNAIYEGCLEL
DGDNVQDVFLACHHYDIELFMPLCTDYMFEHIDSDN
>Ctel_119690
MAGNEFLQEMSATLRDMRHSDEMVDVVLVFEETRVKCHRLVLAASSEYFRRMLQTDMQERDASEIAFKEVSSTIGLLLVE
YLYTGNIELSTENAQELLAVSDRLLLMKLKKTVEKFLCDQVSITNCVSLKQLARLYDLDTLLKTAHDYLNDHWKDLVAID
FESEIDQLTEDDLIELLTTHSSDEGSFLLLQKWVRSSEGRTHLFMDLLQNLELTQFSKSFVWRTIMSDELMQNPKGMRFI
QEAMEPFMTFDRDHVTSSSSDGFIVSGGLDRPVLNNKCYGYVAHTRQWRTLPPMNIGRYYHATICDQDQFMVIGGRISET
YFLDSVECLNLKTLRWSQFPDLPRALSLSNVVYVKGQLFVLGGWASKSVISRDVYAFDSTGGTWWFKGLMPEESRVGSAV
SLNDKIFVLGGEFRVCMQYDPCTDLWVELQRPLYQHAFGPAVVWQDKIVLCGGRQTNAIEEYDPQSDTWSTWDLKIPAYN
NIDICFALKIKRYT*
>Ctel_90025
MTDVTLILPDQSAILCHKVVLMAASPFFDTMFQSGLKEGAEQNIKLDFADSDIIKILVEFFYSGNIELTSGNIKEIVAGS
EFLCCKHLKAHCEEYLVDTVGLSNCIDYYKYGQVFNLKLLIKTAFDFILSKFREFREISDFDKLTEDELVEVVSSDRLNA
ENE
>Ctel_204925
MTQTISSLPAVFLQMKEADEFVDITLVFGKQRIACHKVILAGMCDYFRRMFLTNMLERGSQEVVLKDISASTGVLLVEYL
YSGNIDITQLNAQDLLAASEMLLLGALKKKVEDFLLSHTDSVNCISIINLARLYDLKTLLADARSYLQEHVKEVVESEEM
HLLQEGDLIEVLEANASQENFLFIQKWMRSDEGRTDRFNDLMQHVNLSKCSKDFVCRTVMAEKLMHNTRGVQLLQQFMES
YGSADPPKQPSLAVGNRNGEMWLCTDLNTPQWQPIQQPPFDIQDYSACASPGGFVVSGGLSQNSNQRECYSYAAQNGQWN
TLPPMLTARFSHSSIYHNHHLYVVGGWDGSWCHDGRPLNSVDTLDMRNLQWNHLPPLPREVRLAYLAIVSDNLFVLGGFC
DEWAADVHEFDSTQQTWRQRSPMPEICKRGAAVSFNDHVYVVGGENRSCMRFNPRNNTWTSLQRPQFNHYYGPSLVLNGN
IVVFGGTNDDSIEEYSPLTDSWSTWTLKMSQKSQDWAGWAFAVRMDS*
>Ctel_109893
MTDITLLLPDDSTISCHKLVLVASSSFFETMFHSGMKESIDKYIKLEFSDADTIRKLVEFIYSGEINVNEDNVQTLVAAS
EFLLMRDLKAYCEDFLTTLIRSSNHQELCTFGKKFNLKNLLSSAHDFYLSHFMEFVEKPAFEALTEEQLVEVISDDRLNA
ENEDIVFTSVVRWVNVDPEQRKEAFPRIAPFIRFPLCTQKTLSMNVIWEPLMWN
>Ctel_104519
MRAATELVDVILVFGEAKIPCHKVVLAGTCDFFRAMFVTEMKESRSAEVQISEISADIGLTLVNYLYSGNIEITKQNAQA
LLAACNMLLLTGVKKDIAKFLSSQICNSNCVSFVNLARFYELTSLRKAAHKILIKHWTELIDTKEIGELQEVDFVELIKS
LDSQEDRFRFLQNWVRLGGSDRDDRFIDLIEHVTLSKCSKEFICNVVMDEERMNHPKGMKLIQKALQALMLVQSLHESLV
VANRDGNAWMCSDNKTWKSIEWHAHESTYFSGCSSPEGFIISGGHTNYNPKSYCFSYVALVKQWRLLPNMHIPRFAHASV

YYQDKLYIIGGMESQHGYVDSVERLDAKIMRREDLCDLPQGTSTPLVVIVKDRLFVLGGVTKVGHSKMVLEYHFKNDDWE
ERSAMPEECRSGAAVEFDGFIFVVGGREKSCMRYDPRRDNWDLLQRTTFPHMYGPAMVWNDVIIVGGGGGTHAIEEYCPH
SDTWSTWQLKMPYSGHLRFILKIKS*
>Ctel_182588
MRAATELVDVVLVFGDCKIPCHRVVLAGTCGYFRKLFLADQVKENRASEIEMDEVSSDVAQTLVDYLYTGSVDITEQNVS
ALLDASNKLQLTSIKKDIGQFYIEQISSRNCISFLNLARRFGLKVLRKASQKFLIKHWKGLMVTDDIGHVQEVDLTALLK
AMKDSHEDGFLLLQKWVRLGEGRDESFLDLVEHVTLSKCSKEFICNVVMGEELMNHPKGMKLIQKALQDLMLADQPPKPS
SQPSHEAMMLADQSSCESLVVAGKSSTAWECSDQQTWKPIEWHAHKYTYYSGCSSPEGFIISGGHINHDIKPQCFSYVAR
AKQWRTLPDMDVPRYAHSSIYHKEKLYIVGGLISQHDDTDSVERLDTRSLEWQDLCALPHRVASPFIATVKDRLFVLGGV
QKRCTSHCQDVFEYDDVGNAWKECSPMPEECRRGAAVEFDGFIFVVGGREKSCMRYCPRRDQWVFLQRTTFPHMFGPAMV
WNDAIIVGGGEGTDAIEEYCPHTDTWSTLQLKMPNSGHLRLFSKMIS*
>Ctel_47361
SLDDMRTASEFVDIVLVFGEVRLPCHKVVLASNCEYFKRMFLAGMKESASDDVIMEGIDSDTGVLLVKYLYSGNITITEE
NAPKLLPASHMLLLCDLTGAIEKFLCKHIQPANCVPLLNLSHLFECQDLIEKSRKFIVD
>Ctel_199622
MSRSGLKERRKKEKRKKKKVHLDFTNSEMMRKLVDYFYSGEIDINSDNVDDKITEIGFLYLDDLKTHCGTFMTSQVDSSN
CLAFYRCTRLLGHIESETFMVNSTCIELFRKADPIKSVYNMVYIQSKVQDLFHAKGTKQPTNQEFDPIWDNWQLKQSVPG
VCESGAAVSLNTSIFVVGGAERACFRYTPTTDTWIVL*
>Ctel_180059
MSLDATKWPIFSLLEPSFIATIRMACVLGSAGNEAIVITNDDEVYSLGSNCSGCLGLGSDAMSGLEPRKVEQLCKKNIVS
FAYGSGPHVLATTNAGEVYSWGHNGYCQLGNGSTNQGPTPTPVTSSLLGKRITAIACGSHHSMALSSEGEIFAWGQNNCG
QVGSGTTSNQPTPRKVTACIGSKKCISIACGQTSSIAAFDTGEVFGWGYNGNGQLGLGNNINQPNPCRLVVLNNVFITQI
ACGYAHTLGLTDEGSLYAWGANSYGQLGTGNKANMISPVRIAVDLGRFVEIAATHYNHLSAALTQAGKVFMWGQCRGQSV
TSPLETKFGSMHDTFAYFGSPPVTYKPLEVGNMENSCVTESFRKAFDDPSTCDIKFSVEGRHIHAHKCVLKIRCEHFRMM
FQSEWLETDKEYLTTLTKYTEPSSNTYTRILLISSLKKPLGFWIWPIHTARVR*
>Ctel_161527
MASDTKKRRMRSQIYGSELIKSYAQMRENEELTDFVVSTEGQSFKVHKTLLAASSDYFRAMLQGVMRETKEERVDLKGLT
AASLCQTLDFLYSGEMDLDFDNLIEVLNAASHLQVNAALELCSDFMTTLLNFSNAEELLHVADTYSLDRVVKCYEAKVLK
HFEEFSNTEMFTRLTGTQLARFVADNQLRVRSEMVLFDIIARWYSGKKEDGVDGIITYVRFGLLSEQQLTQLHNHWLVRK
SATAALCIQRALQFHRDSARGHPKLSEQTQLRCTQNSLVMVHQDSLRPLEITAYDGKEGRFYQLLTDLSSCRDTRICSFD
NMMYMCRVVDCGGGTLMNSLLRFDLRHLRLQELTPCSRLRLDPAVVCCGKKLFVFGGVDDNYSILDSVEGYDIEANRWTN
FAPGLPQATHSLAAVEHNGCIYLCGGVSATDRQPTTSMYKFDPEARTYTQQSPMQCPRRLHEMSVLQDCIYVLGGIGSHT
FNQNTQIPIEAFCISSNQWSILTGTLAGRSVGHYMSYEGEIVSLGREHNGATEEDIWLYSLQSDTWRSFIKAPRRMTLAS
AACTKLDVNFFDEKIAQRIISDKR*
>Ctel_62970
EMKKNGVMTDVALVLSDDSSVQCHKVILMASSPFFEKMFQSEMKEGAEKDVKLDFTDADTIRTVVDFFYSGNIQVTEENV
KALVKASDFLCCEHLKAHCDAYIAGKVRSENYLDLYKFSKLYNLGILLAAAFHFILDHFADFIETSDFDQLTEEELVKII
SDDKLNAEKEDIVFEAVAQWVNVELGLRKEAFPRIAPLIRFPFCRMVCLANKICNSQLILDSGCIGFVSEALRMKLVSLS
VPWQPAKNGSSLAFLDGKIFAFGGKDGGSVESKSVQSFDLKCTNPTWKNVNEMMYAVCNHCVAQFDNKLFVFGGCSQGSA
SKVTQEFNLVTGKWRRGRDMPGSCSAGSALALNDEIYVIGGKERV
>Ctel_53735
FLQMREAEELTDVTLVFGDRRLPCHKVILAGMCEYFHRMFLTDMMECYSKEIVMKDINASTGVLLVDYFYTQKIDITTNN
AQDLLEASDMLLIDTLKHNVEEFLCNQADKSNCISLLNLAQFYDLKLLLKETESILHDLQVNDLIDTEEVHLLSEKHLHV
YVIGGNDGQSVLKSVDALDMWTLEWSSLPHMPQPLQCPYVVFVSNKLFVLGGLHGCMDPMEWSEDVYEYDSTSKTWHKRT
PMPEQCPDGAAVALGDQIYVVGGSNRSCMEYNPHMDSWLHLQ
>Ctel_99560
MSLRRSYTSDFHGNTVVDGFQRLWMKSLLCDTQLIVQGHTFEVHRCYLAACSPYFYSMFTEDFQERNQKRVELKGVTAVG
LRVLLEYAYTGTIIINDNNLQDVLEAADHLQFMEVLSSCAQYLREELTNDNCLHFLKMAEMYGLADCKQETKNYILENFV
PVAQNEDFKTISLELFYEFLSDDKLRAESELEVFQVALSWLESNSSNTDAGNPDLNKNVLSLIRYGLMDAEQMENVIRSN
LMLGESCRDVLQSALNYHMKLFEQPIINTPIARMRGYKESLVILGAGYLDNTLCTQMLAAGTKNGQLESFVQLNPTRDRR
YFAAVAAVNDFVFIVGGQTAMAGDGSNATSTAFRYNPRDDKWLQISSMSVSRTHFALIAADTCLVAVGGKHNRHALNTAE
KYDFTTNEWTPIANLPSILFSHAGCAHKNKVYISGGCPADDFTDETHVYDFNVNGWQFRAPMNQSRGYHAMVTHENFLFV
CAGNTNAGDRNDVRTTEYYDIELDQWTEVKASPHGQSEAPAVKCGSKIYILGGYSWDAHSFQDTIQSYDMDDNLWEIVPT
KLPEPMTGVVACHIKLPMKLYEDSQQNPANLNFPN*
>Ctel_177887
MAAAKSRWQERSRRENTLRFCHPLHANSLLQSLNETRNNPQLCDGVVVIGKEEIPVQKNVLAAASPYFRALYDYSLPMSS
TEDPSNRAALNEDVVTSATFHEILEYIYTSNISLGEENIQDTLQAADHLLMIDLKELCCEYLEQCITVANCLGIRSFSER
FSCPRVCHVATSYLDEHFLDVSYSEEFLQLSSTELQEVLSRDSLVIQTEDAIFDAVMRWVRHDVNQRKEFLQDIIPSCVH
MGLLDERYVQEHIIPDELIRSCHLDQTLLRLRGEDTHRKLRGYVNMVVVTGGEGRSHKWEDVRCAAFHETNESRASTWVD
LSPMLTARIGHGMVEAGGFIYVLGGRDNSGRILHTGEKYDPCSNEWSLIPSMHHGRVGFGLVTIDDKIYAIGGSNDMSDP
MTSVEEFNIYTSKWRRLPDMNLKRAWSAVAVCNKKIYVIAGGIMGKLYEAVECFDPRSETWVSVAPMKERRFDARAIGFG
DDIFVFGGCRRFECPSAMQTGSGMKFCGTEIYSSEHKQWTMMNRDPHMCTMNDLCHIDSVVRCKDEILIVGDIEVGGQQH

TVRALRPSTNTWRGVIQNHPSNQKGMQACVLKLPCALIQTLMCQQGKLSHKDAGSVFS*
>Ctel_174860
MRPSKDCPLKAFNFEASGHSASVLTGLNALREKDQLFDVTLVADGQSFGAHRVALAASSDYFRAMFTDAMLECKQTQIEL
NGVGALGLKHILDYAYTSKLTLTLGNIQDILRAASHLQLLPVVSACASYLHLQLDLDNCIDILTIAETYSLPELTRIVYC
YIGQNFDRLVQMPEFENMSSSQILHFLHSEFPISCREVEVLQALLLWLNKDPISRMDGAKELLSCVRFQSIRRNEFQDLK
KSCAYKEARGHSPHVANLLSGFMFGCRRSRVARYGTTEDTSCLINPRGFKKAIVNIGGFHCTRGLINDVTYFHPHSNQWK
RLTSIPHVEQCDFGVAVIDNELYVVGGCFNQSLQEHVHPFGFKYNPRSDKWTTILPMLQERCRFYLAAVARKLYAIGGMT
EQSEAMDPGSCECYNPETDTWHEISGLAVNRTQHAGAVQGHCVYISGGLDSGVVGDSVFDALLCYNVDEDAWMTKAPMLQ
SRADHAMLAHADKIYVAGGWHEDPSTGDRVITDTIDCYDPVADQWLCLTTVPTPRYHCSLTALGSTLYLIGGFGEGQFNR
ATKKIETFDVVQGKWEEHEDYPAELWEHCSSVLYVPTCRNDNNVTRGGDIGRRSRGHSSNPYAQ*
>Ctel_111094
MTSLQRLKKNESSIDISLKLPDETTIPCHKLVLKAASPFFETMLQSGMKESTQQVVQLDFSDAVTIKMIIDYFYSGEIDI
NADNVQDLVAASEFLCLDDLKEHLGAFMTEQIHATNCIEFYRYARKFSLGKLIPHCLEHILDNFGQAFGSSNKFTELSEE
ELINVVSDDRLEAKNENIVFHYVVLWVNADRNHRKEAFTRIAHFIRFPFCTENYINNMICEELMINPSCMEFLREATVTK
SSHIILHSMYSARHLPRLALFAVGGRDSNNGKHCSMVESFDENQNKWRPEPSLIHAVSKPYLVHFGTEVYALGGVDANGP
SLATQEFDPVVGTWQLKKDMPGVCQSGAAVSLNDKIFVVGGTERVCFAYTPSTDTWATLSRPNHVHNTLTTAATWKGKIL
LLSDQGERYNPETDTWSPRQALVPTDGNLTTYRKGLFTVYL*
>Ctel_145514
MASLHKMKKDDVMTDVTLKLPDGSAVSCHKIVLMASSPFFETMFQSGLKESTEQDVRLDFADADTIRMLLDFFYSGEIDI
NENNARIIASASEFLCMEDLKDECDSSLATLVNSSNYVELGRFGKKYNLAMLTAKSQDYILANFEEIVKTSCEFKTFTVN
ELAELLSNDKLMADNEDLVFESVIRWVNFEPDERKEAFTRIAPLIRFPFCSQATLNNIIPQEPLMWNSECMHLLSEAQNY
RMN
>Ctel_23415
MREAGELVDINLVFGDHIISCHKVILAGRCDYFHRMFLTNMLEGDSTEVVMKEINARTGILLVRYLYTGQIEITTDNAQD
LLSACEMLLLGALKQDVEEFLCIHIESNNCVSIMNLASLHDMKTLLGNAKKFLHEHMKEVVETYEIRLLQEADLLEVLGE
TLSQEDNFCFIQKWVKSADERAERFDDLLQHVSLYKCSKEFICDTVMEERLMISLNGMKLIQQAMRTPKQINPTGSQSLV
VGDVRFIVSGGLLNGVCKSDCHSYDAQSGQWTTLPPMSIARRAHSSIYYNKCLYIVGGHDDHNYLDSVEKLDMRNLQWSR
LLCLPHSAVYINLAIASNNIFAVGG
>Ctel_97326
MTQAISSLPAAFLQMREAEEFVDITLVFGSQRIACHKVVLAGMCDFFRRMFLTDMLERGSQEIAMKGISASIGLSLVDYM
YTGHIGITTQNAQDLLLASEMLLIDNLKQAVEQFLNSHTESTNCVSIINLARIYNMKTLQVDALRYLQEHASDVIDAGEM
HLLQEEDLIEVLEANYSQHENFRFIQKWIRSADERIDRFDHLMQHVSLSQCSKDFVCSTIMEEELMLSTQGMKHIQQFLQ
SYGSADPPEQQSLIVGNMTGDIWLNRNIHIHQWQPIQKPPFQTQNYSACARPGGFVVSGGRSQEHISQRECYSYDAQNGQ
WSTLSPMSTARRHHSLLYHNHHLYTVGGFDGNHLNSVEALDLRSLHWNQLPPLPLSVASSYLVIVSGNLFVLGGYNGVWL
TDVHEFDSKQPAWRHRPPMPEICVRGAAVSSNDHVYVVGGEDRSCMRFNPRNNVWTFLQRPQLNHGYGPSLMWKGNILVF
GGHSCESIEEYSPLTDSWSTWTLKTPKEGIGFVARMNCIGEH*
>Ctel_218689
MVESLALEVTIGGIDHHIGLIIVDYIYSGVVKITKDNAEDLLRASDQLLLFELKTLIEGFMLNYVEVSNCIGFLIFSKVF
HLQNLTKKSYDCLLDNFSDVTTSSEEIGQLQDDLLAELLMDDGLNVPNENCVFEFVTKWYRNVPGRSRTSFLDMVTHVRL
PFCTDEYFSSIVHDELVKCLRGSQLIQDATERHRVNQHIIQIRRPRRSYAQHSLVIGNDTRIWQRGSDVTDDWYEMEDIP
FNCRYYSACSTPDGFIVTGGCNESSIALDCKRGCYWYIAHENHWRILPPMSKGRSDHSSVYHNHTLYVVGGDEDHSFQSL
DTNSLKWSTLPSMPDPVYRAHMAVFKGRIFVLGGKSSYMNFRREVHEYSSAACSWQERKSMPEVCSYGAVVGYGEHIFVV
GGQERCCMRYDPEVDSWLRLQRPLHVHRWGPALEWKAKIVVCGGCNADSIEEYDPQLDMWSVWQLTVPYISGMVFALKV*
>Ctel_36834
QFVDSGRPAALLTGLGMLFEQKIFADITLVAGSQHFLCHRAILAVSSPYFLTMFNTELSEKNQSEIVLQEMEASTLELIL
EYVYTGQVSLCEESVQHLLSAANLFQLLALRNGCAAYMIKHVSVSNCIGVYFFARAHNCDQLASKAKELINSQFELLCRE
PEFLSLPSDKLVEIIQDDQLNVSKEETVYEACLSWLNSDLEERKPHLHTVMRHVRLANISSYYFCDNIACLPLLYEDGAM
AKALDQVKYYHMLRNRQ
>Ctel_93889
MSKIHVASFLLFSVHSISSSHSNHSDVSTTDCAPTNEVFVTNRRSSKTEFDHDQGQRINNHSQHIKKISALYQNEELSDI
QIIVGQNVFHLHKLILCCSSDVFKVMLTNPAWPESNKNRIVLKEEPECVSVFEDFLKYLYTGEINLTHYTVLPLLMLADK
YDVLDLRDVCVDYMCCHLVVTVQHNHAVSWLQYAALCNHRELARTCEDFIKWNFHKVSCTCDFFLMEREVLVHFLQLSDL
VIHDEYTLFQGAARWLCHQIRRNETSVTEDDLDVLSCIRFPMMSPQQLGQLNKTPIAEEFEEFFIDKVLHALTFHACSFH
EKQTIASISQESLDFQPRNYTNDQWSTSLTIDNFLSLPVHDVRPLLFSTPLSAAQADEQSCWEWNVDVYPKGIHFQKCIM
IGLWRNLEISGAVYNTVRLVLETRTPEKRNVEVAVLVTGVQDGVEFIKRVVQVHCLFDQKRRVRHINDVIPFEDLNSADS
AYLSGPEGNAFKITIIIKPAYGQVY*
>Ctel_93881
MREAGEFVDITLVFDEQRVSCHKVILAGTCDYFHRMFLSDMLESVSKEVTMNGISASTGVSLVHFLYTGRIDISTQNAQD
LLAASEMLFLGDLKLKVEEFFLSNTESINCISTINLARLYDMKTLMGDAKKFLREHLREVTDSEEMHLLLEEDLIETLRA
SASQEASFRFVQKWIRSADGRTDRFDDLIQHITLSNCSKKFICSTVMSEKLMHNTHGMELIQEAMQYSGKAEQESIIQHW
QPMQEPPAKFERNSACASPAGFIVSGGRCRGITVHDCYSYDAFGKKWNALPPMSIARNRHSSIYHNHQLYIAGGHDGKKN
LNSVEALDMKSLKWSPLPSLPYSVRHSYLAIVSNLMLVFGGFQTNTWVADVCELDLNERVWHHRAPMPYTCEGGAAVCLD

DHVYIVGGEKRTCVQFNPSKNTWTSLRRPQFTHDFGPALVWNQHIIIFGGKDHDFIEKYSPLTDTWKTLALKMPKKDLMR
FVVRINCPL*
>Ctel_93906
MREAGELVDVTLVFGDQHISCHKVILAGMCDYFHWMFLTGLLESGRQEVTMQEISAATGLLLIDYLYTGQIEITTLNAQD
LLAASDMLLLGDLKQEVEDFLCSHTEPKNCVSIINLARLYDTKTLLVDAKKFLEEHMKEVIDTEEMHLLQEGDLIEVLGA
NANQEDNFCFLQKWVRSAEGRTDRFDDLIQHVDLSQCTKKFVCGTVMDENLMASVKGLKLIQNAMQSSRQMQSPELESQV
LVVGKCDGDMWLCSDFDSQHWESVKKPPRQLDYYSACASTDGFIVSGGQLNDIPKSDCYSYNAQNGQWNTLPSMSTARRS
HSSIYHNHCLYVVGGSTNDSDPSNSVESLNMRSLRWSHLSPLSHPAYTPYLAVASDNLFALGSSDGKWSVDVHGFDFTQQ
TWRQRSPMPEACVDGAAVSFNDHVYVVGGENRSCMQFNPLQNTWISLQRPLLNHYGGASLVLNGSILVLWGEDNDVIEEY
SPREDKWSTWTLKTPNKRLMSFALTMQRSS*
>Ctel_142568
MKEADEFVDITLVFGKQRIACHKVILAGMCDYFRRMFLTNMLERGSQEVVLKDISASTGVLLVEYLYSGNIDITQLNAQD
LLAASEMLLLGALKKKVEDFLLSHTDSVNCISIINLARLYDLKTLLADARSYLQEHVKEVVESEEMHLLQEGDLIEVLEA
NASQENFLFIQKWMRSAEGRTDRFNDLMQHVNLSKCSKDFVCRTVMEEKLMHNTRGVQLLQQFMESYGSADPPKQPSLAV
GNDDAEMWLCTDLNTPQWQPIQQPPFQIRSYSACASPGGFVVSGGASQNGNQRECYSYDAQNGQWNTLPPMPKVRSWHSS
IYHNHHLYVVGGLVNHRPLNSVEVLDMRNLQWNHLPPLPREVCLAYLAIVSDNLFVLGGLCGDGFYDGWVADVHEFDSTQ
QTWRQRSPMPEICEWGAAVSFDDHVYVVGGRERSCMRFNPRNNTWTSLQRPQFIHNYGPSLVLNGNIVVFGGLHDDSIEE
YSPLTDSWSTWTLKMPQRSQGWVFAVRMDS*
>Ctel_219878
MERAVEQPIKRLSTHDHNWQQSCQSVRERNTMMYNNELMADCHFLVGRPPRVERIPAHKYVLATGSSVFFAMFYGSLADH
DENAKDVEIPEVEPVAFLNLLKYLYCDVVEPEPDSVLATLYVAKKYMVPHLARQCVQFLETSLTCKNACVLLSQSRLFEE
PELMNRCWEVIDAQAEEALASEGFTEMDYDSLEMVLGRETLNVKEISLFQAAVRWAEVECSRREIANTSGNKRKVLGKAL
QLIRIPSMKLDEFADSAAQSGILTLQETNDVFLYFTAQNKPDVPFVQNPRRGLQPQRCRRFLSSAYRSNQWRYRGRCDSI
QFSVDKRIFVAGFGLYGSSNGAAEYKATIELKRNGVQLGQTKTKFYADGSSSTFAVYFENPIQVDPEAFNTASVILDGME
LSYFGQEGIAELMVGNVNFQFQCSSDSTNGTGVQGGQIPELIFYC*
>Ctel_1815
MSSSSPPLNSLHDHPDISSDGSSTSGNGGVGGGGAQGASASSSASGGGGVRMEENPKHGMKVLQGLSKLQREKVLCDVCL
IAEGVKFDVHKVILVSCSDYFRSMFTSGMKESSQKEIELKGVTSKGLEKVLEVVYTSTTTLEADDIFDVIAAATHLQVTP
VIEFCEHNFLSGMTTSNFYDFIHTAKLYSMNNALRQIDTFIADNLLQIAKEGTLHLLTYDQMQSCLRCNSLRLREIDIFQ
VAWDWLRQDVGREQYAPSLMAHIRFPLISPADLVSRVQSVDLMMRLPELRDMVLRALNYHVVPHSQPVKQNSSTQMRCLV
ERLVSVGGREIHPHPGLHDEVFVFDDKITANSLLNRREVTTLPNALSHMQIVVFHNFLYILGGCTTQCAHGESAVNSVLR
YDPRFNQWHQVASMINKRAYFFAGVLHKKVFAIGGKFKEGSLATSECYDPERNVWEPIQAMPSAYHAHAGAVYGSHIFVS
GGYSNNHFTPDLQRYDPVGHQWEDMAPMLTPRGWHVMCVAQDKLLVFGGCNLNANQQALPVLQSECYDPSTDQWTIIAPL
SISHKEASCVLYHDHVYVLGGYNVQTKTGQKMVSRYDLYSGTWETLGALPQSKTGVGYCVLKLPSYSVEMSE*
>Ctel_106244
MKEADEFVDITLVFGKQRIACHKVILAGMCDYFRRMFLTNMLERGSQEVVLKDISASTGVLLVDYLYSGNIVINQHNAQD
LLAASEMLLLGALKKNVEDFLLSHTDSVNCISIINLARLYDLKKLLVDARNYLHEHVKEVIETEEMHLLQEADLIEALEA
NVSHEENFLFIQKWMRSADGRTDRFNDLMQHVSLSKCSKDFVCRTVMEEKLMLSTRGMKLLQQFMQSYGSADPPKQPSLA
VGNIEGEMWLCTDLNTPQWQPIQQPPFHMQDYSACASPGGFVVSGGRDCYSYDAQNGQWNTLPPMPTARREHSSIYHNHH
LYVVGGFVNLRPLKSVEALDMRSLQWNHLPALPRKVYSAYLAIVSDNLFVLGGCFFGFNWLADVHEFDSRQQTWRRRSPM
PERCVVGAAVSFNDHVYVVGGRDRSCMRFNPHNNTWTSLQRPRFSHCYGPSFVLNGNIVVFGGNDDSIEEYSPLTDSWST
CTLIMPQKTVTWKWTFAARIDS*
>Ctel_115045
MTQAISSLPAVFLQMKEADEFVDITLVFGKQRIACHKVILAGMCDYFRRMFLTNMLERGSQEVVLKDISASTGVLLVEYL
YSGNIDITQLNAQDLLAASDMLLLGALKKKVEDFLLSHTDSVNCISIINLARLYDLKTLLPDARNYLHEHVKEVVETEEM
HLLQEGDLIEVLEANVSQEENFLFIQKWMKSADGRTNRFDDLMQHVCLSKCSKDFVCSTVMEEKLMHNTRGMKLIQEFME
SYGSADPPKQPSLAVGNRSGEICACASPGGFVVSGGLSQNGIQRECYSYEAQNGHWNTLPPMPTARFSHSSIYHNHHLYV
VGGYDGSPLNSVDALDMRNLQWNHLPPLPREVYFAHLAIVSDNLFVLGGRCDEWVADVHEFDSTQQTWRQRSPMPEICVG
GAAVSFDDHVYVVGGNDRSCMRFNPRSNTWTSLQRPQFKHSCGPSLVLNGNIVVFGGFNDDSIEEYSPLTDSWSSWTLKM
PQKPLGWAFAVRMDS*
>Ctel_23567
CDVKLKVEKENFKAHRDVLSEASDYFSAMFSHNMKEKEQTVIELHEINPKGFIAMMDYFYHGHITVEPDNIEDVIEAARF
FHIEWLVHICCDFLIRFLSLENYHIVLHLVDKYYLGDLRPGIFSFVSQNFMTLAEQPKFVELSYDLLHLLLSEDHYIDAT
ESFIFNVVLKWLRHDEERAEHQSTLLPLIRYPLLEVETLENIPAEVMEVTSVKELVDDALLYNAHPTRQCLMSSDQASVR
GSSDVVALYSAVYDAHEVNYKIPNLDGFFTEAIDTGFMQNFFEFASVASLGNFLFVAGGYWRNSWCSSPAFYSYNPRNRL
WAQLSSMLIPRVSFSLCPAEKGLYAVAGIDHIVDEGRDQEIILNSVEFYDPEENLWSQVSDLPFGCFNCATAVIGDRLYA
SGGITDDPEDNVPVNYVHAYNPGERSWDVKARMNVER
>Ctel_213147
MAVQNREVDWQVNRSVLECNRYMWEKKIATDVEFEVGTTAEQIKNIRAHKYVLMSRSPVFEAMFNGGLSKNTGDLVQITD
VDSDAFNETLKYIYFEKAEINSQNVLATLYASKKYVLPLLTAQCKDFLAKNLHLESVCSILDQSIFYDEQELTQKCLHYM
APVIKEVFATEGFLKMSQPALKALMQDDYLFCESEVIVYDACLRWAEAQCNAKRQECTEGNIRAMLDDVIYHIRFPIMQD
VEFARVVGKSNILSASEKSDIYYYLLTQDGSSMKFGCTPRVSICARFPVLGTAQQWWSCNGPTDAFSFQGDKDLDVIGVT

VYGGKEKAQHTVCVEIRDSSERVLSKSGAVTFESDGSPSPIPIYLPTPAQVRANQTYTVMVTMRGPLTHYGSGGQEVLSC
AGINLKFFTSSKSTNGTNTKSGQIPQLVFIPRQHSPAVSRDSCSSRESTKVERAINPK*
>Ctel_109214
MKSLFDEGLLLDLTLCVQQRKFRCHRNVLACTSPYFKAMFTADLTERGQAEVELHEVDPESVYLIIEYAYTGHIEITRSN
AQNLLAASSLFQVTRVLKACATFMETQLDELNCVGVHYFAHIHNCTALRKKAQEFIEKNFVNVCHGEEFLSLPEDKLSEI
LSSSELNVEKEEIVLDALLNWTLHERDVRISSLSELLPQVRLALINSCYIQEVLGRNNLLSECTEFLKDLKTFEANPEKY
AGKHAISTVLRSGMIKPDICILLIGGVDHKKPSINCYNPLTREAYFMAGFNEELSTGLYAVEDPACIVTDKNQIFVAGGN
YIYHENLAECQSDDDSFEEYEEESVRKDFFMFDNDHDCWLSRAPMLFPKSNFALAQVGNTLFCFGGLTINQHPTEIIESY
DITLNKWSYVSMMPTTLVDLSAVVHDDLIYVLGGRTGVGAHNVVMRYNPKKSEWLSLAGMPTPRFNFGACVMADEIYVCG
GQIYSHTSHTINREALNSVEIYSIESNQWRQGPDLPEDMYNTGLFNLHGELYACGTTEYHRSAYRIYRFNVVYKLLSDQS
TWKQVESDLCGTLRDYACVAAHMHTRKLSQVFRPDVDT*
>Ctel_196617
MSCKSARAIKRSKNVIKKKDDSFLMETKNYASKFMQTLNETRSSKQLVDVTLVAEGGDISAHRLILSLSSEYFRCMFSAN
MKESSTSEVPMVGCDYDIVKIIVDFFYTGVFKAFNDQLPRLLQLCDMLQLDELKEICSQQLEQHVNPMNCLGIWHYRNRK
TFIYIFFQGLWQFAEQVRAETLQLQCERLIVSRFPTVSQSEEFLSLDLNPLLRVSHLKNLNCEREKINTEDEKSQLREIL
LSVQWNGVCEEVNTQVMQHDLVKDNQDLCNGIREAKEREFHERSVGTGIYVIGGYLQSRTGPLCPRVNTVEHLSLNTDGK
QEWISMKSLPDSASSIQAFNFHGQLLCLLTFPDSSNSRIPALTLWYTYHPLTNEWFNVTHTFGEEATKSLQTCVEKQGVL
CYDSKSSALYACTGKTCAKYSVSLNTDDDSLSIAKVKDLPSECAELLEGHAVVVCEDKLYLLGGQYQTTKVNNDNQPRIN
LSRCEDTHWTPLSGMNTPRAHFAAVAIDGYIIAMGGTTGLHRLDTCEQYSIFSENWFQIKSMERPRTHFSATTVNGEVYV
MGGKSYANRFGGARKVLKTCEIYDVKSRKWRSGPPLLGHRCWFGLTVL*
>Ctel_99318
MKEADEFVDITLVFGKQRITCHKVILAGMCDYFRRMFLTNMLERGSQEVVLKDISASTGVLLVEYLYSGNIDITQLNAQD
LLAASEMLLLGALKKKVEDFLLSHTDSVNCISIINLARLYDLKILLADARKYLQEHVKEVVESEEMHLLQEGDLIEVLEA
NASQEENFLFIQKWMKSAEGRTDRFEDLMQHVSLSQCSKDFVCRTVMAEKLMHNTRGMTLIQEFMESNGSADPPKQPSLA
VGNDVAEMCACASPGGFVVSGGQSENGNQRECYSYYAQNGHWNTLPPMPTARRQHSSIYHNYHLYVVGGWDDGSYLNSVE
ALDMRNLQWNHLPPLPREVFCVYLAIVSDNLFVLGGYNSDLNCVADVHEFDSTQQTWRQRSPMPEICDRGAAVSFNDHVY
VVGGEDRSCMRFNPRNNTW
>Ctel_136979
MAESESKVVEITSFKLDVIKPLLNYMYTAQIEINVDNVQSLVQACDQFDFERLKEACEQFLQDQIEPTNCIGLFRFAKLY
RLNSLMESARHCMLQNFKEVLESSTEFLELSPNELIEYIADDVLCVSNEDLVVEAVILWAKASMDERIIALDRVLERVRF
PFCSSTYLCHVAMKEPLLATSTKAHAMIHEAVTFHLLPDQQLTPRVRPRKSFRDDTRLVVVGGLLKDEQENKFCWYFREE
SASWELLAQLPRPTWKFYSACATKEGILVTGGYHCNVKKEVWFFDCMDKKWKSLAPMTTARCKHQSVLQGADVVVVGGED
DADQLLTSVEKFDSKHRQWSLQRPMQKALSDPLVVSFGHSLYVLSGIQDDDTSSSCTQEFDCTWNRWKFRSEMPEPCRLG
AAVTLGDAIYVVGGFTRSSMSYRPATDTWSVLSRPRGKHGNAPAVVWRGRVLVGGGDVLLSETVSTIEDYDPATNTWSFW
KVPLKEDLSCHYMLSVDLGGL*
>Ctel_145048
MTDVTLILADHSTIDCHKLVLAMASPFFETMFRSGFKESTQKEVHLDFTNSEIIRKLVDYFYSGEIDINSDNVDDIVTGS
EFFCLTDLKIHCGAFMTSQVDSSNCLAFYRCARQYSLGKLVPHCFEHMLSHFENEFCSSESFVDLTEKELIEVLCDDRLR
AENEDIVFHSVVRWVEADLEQRNTAFTRIAPFVRFPFCTSGLLNHFASETLIMNNTCVELFREALQ
>Ctel_145040
MSSLPASFLQMREAGELVDINLVFGDHIISCHKVILAGRCDYFHRMFLTNMLEKNSTEVVMKEINARTGILLVRYLYTGL
IEITTDNAQDLLSACEMLLLGALKQDVEEFLCSHTESNNCVSIMNLASLHDMKTLLGNAKKFLHEHMKEVVETDEIRLLQ
EADLLEVLDKTLSQEDNFCFVQKWVKSADERAERFDDLLQHVILSKCSKEFICDTVMEE
>Ctel_73018
SFLQMREAEEFVDVALVFGERRVKCHKVILAGACDYFHRMFLTDMAESASKEVSMKGISANTGALLVDYLYGGRIEVTAQ
NAQDLLSASDMLLIDALKEDVEEFLCQNTGSTNCISLLHIARLYDLKALLKDAHTFLLGNIKEMIDSEELHLLQEGDLVD
ILIASYCQEDSFHLVQKWVKSSEARTDRFACLLEHVELSECSKEFICNTVMEEEFMMNKQGMKLIQRIMQSSTLADSSNK
QYYSACASPVGFIISGGLLRSLRAVHSVVTDCFAYETQNNQWTTLPPMPSARYQHSSIHHKNHLYVIGGMDSRLTLNSVE
TLNTRSHQWDFLPAMPRPLQLCYAVFISNSLFVLGGSQTGFDASSDVHEYDPMSRTWLARCPMPECCSRGGVVCFADHLY
VIGGDERSCMRFNPRSDSWVFLRRPQLNHYSGPSLVWNEKIVICGGIVNDSIEEYSPLTNSW
>Ctel_73122
LQSLKKQESAIDITLKLPDDTTIPCHKLVLMAASPFFATMFQSDMKESKQPEVQLGFSDAATIKMLVDYFYSGNIVITSD
NVMELVAASEFLCLADLKKHLGSFMIKTIKAANCIKLYMLSHKYSLGNLIPPCLKYILSHFSEVFRESDDFLNLSEEQLV
TVLSDDGLVTQNEDFVFHSVDRWVKADLENRKGKFVQMAALIRFP
>Ctel_63636
HSDSVLRGIFAKFCSMREAGEMVDVALVFDGMRINCHRLVLSATCEYFSCMFKSNMQERFAKEIPIADVSSRAGLLLVEY
LYTGKIEITAENAHEIMAVSDRFILKRLKKNAENFLCEHVASTNFGSLMRMAYFYNLDSLMLSIAKNHWKKVVAMEDVDL
LREDHLVTLLRAHPSDEDSFRLLLRWARSSEERTKRFMVILHNVKLAHFSQEFIWDEVMTEELMQNARGIQMAMDSLRVM
KRHQSLSVRMPTPKCARYHMTSVCSSPQGFIVSGGQIEAGKTSNACYAYRTITCEWQSLPPMIVGRHSHASLYHCHEFMV
IGGRSSEDDLDSVERLDIKTREWSQFPPLPQPHPRPHSVIIQNKLFVLSGSSASGAASCAVYEFDSAARSWIPRCPMPEE
SRGGSAVAFDDRIFVVGGHSKSCMQYVSGTDSW
>Ctel_222192

MAELRETSFCDGANASALTSLTSALFQQKQAGVFCDVIMKVCDIEIYAHSNVLAACSPYFNTFLAQDLPRQFCQRSPQVI
EIQIDGVEPNSMYEEAVSHVVDYMYTGELVMSDQNVGQINEIGRIMQMDSVGQQCDLFLSGLLTSERKQVLRKKEQSRVS
RLKSNKYMSMVSVATQVFPKFLGARADLADAAVNTDSVARIVKAPTGKPRGRPRKIIQDEEAGTITQDVLSQALEEVREL
EEEQETVEIVERDLDEPDYEEEMEEGEAQEEPSSSIRRSSRRAKPKKFADYEEIEFIPRKKIKLESDDVIIMAEKEEMSA
EKSAEGCQTLFDLAAAAEEIQKETTDNGECGAADTSKETEVYEEVQLFEGEGEEESPQSSEKKPRAKSRIKVEREYKHKP
DGKFQCERCSFATNSVKAYGAHNKQHKIDNNQCYFCDAAFEDKESTLAHMATHAGPDKFLCRFCNANFKARTQLNIHLPK
HFNIKPFICEICSMGFKWKHALNAHMIVHSNRKDHVCDVCGYATAHKNQLTSHKRIHTGETLKCLYPGCDYQALKAQNMK
YHLMTHSGDKPHQCEVCGQAFSLKKNLRRHMVLHSTGNKRGCKMCDFTSPRADKLKEHLLRSHGVGTPPEKRMRVTDLVA
EAENQRVLSPNKLPDSTIIQPDDITDEQKPIERYIIEIETDGTKQQLEIAPNQIMVDPTGTEHVLSTHGITELGHLEIAG
STVEFATSTIQGEGMAGHEGFVEGVHYTEVMVSADEVIPELVEQVEQYEDSAQQD*
>Ctel_139164
MRTASEFVDIVLVFGEVRLPCHKVVLASNCEYFKRMFLAGLKESGPDNVIMEGIDSDTGVLLVKYLYSGNISITEENAEN
LLSASNMLLLSDLTGAIEKFLCKHIQPANCVPLLNLSHLLECQDLIKESRKFITDKWDDLSDAAIVEIQEDDMEAILKQH
ECQEENFLRLQSWVKSVDGGEERFLRMLENVKLSKCRKSFVHDTVMNEELLQNKKGVKLIQQALKDILISKFLLITLCFI
I*
>Ctel_192565
MAYSQSPKSTDSDWGDANSSYTSGENSPRHHGTTSDLSDMDLEFLDFTTPDDLSSLTLVIEGEKLYVHREVLAAWSPVFR
SMFTRDFKEKDQREIELPGKKVDDFVELLHCMYPPIKPITDSNFGQLLPLAEEYQIVQVKRKCEEYLLTKAGSIELLVTA
QTYGLQQLRLKCIEYARTKSFTELQKDPFYQLLEAENLIGILQLRVQDLESSDQQAKKQQGERDARLFGVINELASGYGN
FCTECKSRKVNDDCKNCLKMFRQKVRTKCDEAKNLRNQSQLL*
>Ctel_146149
MAQAISSLPPVFLQMREAEEFTDVTLVFGEQRTSCHRVILAGMSEYFRRMFLVDMVESGAQEVSMDAISPNTGELLVHYM
YTGHIEITTQNAQDLLEASDMLLLGALKQKIEEFLRRHTDSRNCISMMNLARLYGMEAVMRDAQRFLHELTKEVIATGEM
HLLQEEDLIEILQASSSQENNFCFLQKWVGSAEGRADCFDALIQHVSLLECSKEFISTTVMDEKLMHNTRGMQLIQQAMQ
SSRSTNPPQQRALAVDDMNGEMWLCSDINIQHWQHIQKLPSECWQYSACASPGGFVVSGGFRNNICQRDCYSYDACDAQW
SILQPMSIARASHSSIHHDHHLYIVGGQDGRDNLDSVETLDINTLQWSHLPPLPFHIQLCYLAIVSNSLFVLGGYNRRWS
ADVHEFDIAQQTWRSRSPMPEICDGGAAVQFDGHVFVVGGTTRSCMRFDPRSNQWQSLHRPKFDHYKGPSLVWKENILVC
GGHYDATIEFYSPITDEWENWNLKMPKKAGMSFAVRL*
>Ctel_217877
MTSALQRSPNKHSANGTSSSTDELLYKSSSLALVKHKHGLKTEEVSISSNHDRVDSTMRYLEASQKVLDFLRRMWLDQKS
CDMVITLQNGEMLAHRVVFAAHSQILAEKFAHFGPDQIVTIQLSDFAVKPVFELTQFLYTTDLCLTVDNVAEIYKCAAEL
GLNSVFEYAREFLSNYNVSNAILFYNICDQGNITDLRDEIFSFICEHFHEIIQHESFKRVALEDLMNLLKNNHLGVHSEL
DVFFAVCRWLEGDLRRRMPLSNKLLGCVRFQHIKPEDICTKVCSVEWLMETKENIAIINQAYKFHSLKATGSPLVSRFPV
QVHRSRAPGMRRKSSTSKAPVQPTLPAN*
>Ctel_204893
MKEEKVLTDVVFVFENKRIVGHKLVIEQFMPCIRESSSEIEIDNVNFEGLRHFLDLLYIPKITWGSKTAELVANALDFFE
NYALDPAQFESTVKFVSSLMTIENSLVVFRIAVGLDHKSLQKAAAHRLFCHFDSYQCTNPDMNSLLIDLRRSELIDILAI
EIKEAVCGTYAAFNMTLKWVECSPKRRKPHFLYVVKKLGLLNHVSKFQLKYLRCQSLEGHQMILEALDLSENATQRTPNK
PAMFHRKADDLNNLDVSEHLFHRLREMRTQKEGNLVLEIDGKEIYCHKEMMAAASPFFATMLTLDMKERREGRVEIKGTN
FNTMNLIVNAIYGGEFKLDGGNIQDALLACKLYEMEILISECVDTMIQYMDCDNCIDIYLLGKNYDIEQLNEGVADFLDK
NIISEKDIRHVSESDLKNFISKMNPVTQNFFLNLLNWVKTAGANNMECFRSIIENCQNEDIDTLRMTVDRTMTCRLKDIE
MLISDCVATIVKGMDDDHSINTKQLNEEVAHFLGKKLIPKTDLLRVSESDWITFVSETNLVTFGLCKDLLRWVATEGNNM
ESFCSFIENWRCRDERKLRRIANKIELFTHYRTFLDILEEKERKQTDFFDVDLYSDNYSDYSYHRSDSLSPYKSDFGGYY
DDFGHYKQKSNRQTS*
>Ctel_63624
GNEFLQEMSATFRDMRHSDEMVDVVLVFEETRVKCHRLVLAASCDYFRRMFQADMQERDAGEIPMKDVSSTTGLLLVEYL
YSGNIQISAENAQELLAVSDRLLLTKLKKCAEEFLCEQVASTNCVSLKNLARLYGLETLIKVIHSYLHDHWKKLIDMESE
IDQLTEDDLIALLTTHGSDEGSFLLLQKWVRSSQERTGRFMELLQNLELTLFSKEFVCRTIMSDELMQNAKGMKIIQEAM
ESLVIADRNQSLVVGDYKGNIWFCADDQQWQGIQVPFTECYNYSVTSSSDGFIVSAHTRQWRTLPPMSVGRLSHASICDQ
DEFILIGGCISPDAHLNSVECLNLKTLKWSQLPNLPLGQTFSNVVYVQSQLFVIGGFTSSTTVSRDVHLFDSTEREWQLR
SPMPEENEGHAVPFDSKIFILGGGSKCCMQYDPCTDMW
>Ctel_63247
FTQIKREGHFTDTKLEVEGHLISCHRIILAAASPYFYKMLTSGMAESRTQSIKIYGTSFKTMNMIVEYLYSNAIMLTADN
IQSIFIASDLFQMTQLKYGCEQFMSTLVDSLNCIGFLHFAGHFQTEILENAAKQCALDNFSDVIETPDFLDLSEKEMILL
FSSNELNIPCEETAIEALIRWVGNDPTRNLHNIIKHIRFAHCDPEFILRLQEVIGRVKTDDLALSAEEHDPRHSYDNRLL
VTVGDRHIGCIRAGNTDFTGWKFITDMPKHPDYYSACALPDGILISGGYRPDFSSNACWLFKTDTLTWKPLPRLKTARGF
HVSMFLCGSLFVVGGTTDQCYPVDSIEKLDMRKHSPWNEVTFLPDLLTRFCAVSLGQSIFVIGGSVRYPGTAEKVWNERR
TSSLITSEYDVIENIWCQRACLPEPCYSGGAVSFQDKIYVFGGKRNFCYSYDPAADLW
>Ctel_73060
MRHSDEMVDVVLVFEETRVKCHRLVLAASCDYFRRMFQADMQERDAGEIPMKDVSSNTGLLLVEYLYSGNIQISTENAQE
LLAVSDRLLLTKLKKCTEEFLCEQVASTNCVSLKNLARLYGLETLIEVTHSYLNDHWKKLNDMESEIDQLTEDDLIALLT
THGSDEDSFLLLQKWVRSSQERTGRFMDLLQNLELTLFSQEFVCRTIMSDELMQNPKGMKIIQEAMESLVIANRKESLVV

GDFKGNIWFFPFTKCKFYSVTSSSDGFIVSGGRNANNATRRECYAYVAHTRQWRTLPPMSVGRYGHASICDQDEFVVVGG
CKGLDDFLNSVECINLKTLKWSQLPNLPLAQMISNVVYVQSQLFVIGGFTSSTTVSRDVHLFDSTEREWQLRSPMPEENE
GHAVPFDSKIFILGGKSKCCMQYDPCTDMWIQLQRPLFKHAGASAVVWKDKIIVCGGNRTDSIEEYDPQSDKW
>Ctel_189418
MAHSVFPVRTTTIWYKSIRINIPLSDQRRKMRGLNLDSHLKAFMGSLRKLQEKEVMTDVTLILPDQSAVLCHKVILMAAS
PFFESIFKSELKEGAEQNIKLDFADSDTIKTLAHCEEYLADTVGLSNCIDYYRYGKLLNLKVLIKTAFDFALSKFQKFEE
ISDFDILTEDELVEVVSSDRLNVENEDVVFEAVVHWVNVDRDAREEVFPRIAPLIRFPFCTQSKLTDEICCSSLMLKSEC
IPLALEALWSQFHFNCHPATDNARCIPRQAFNSSASLFQLMESKSPNKVSLSVALFTASGIKWESVLPCITRTFDRIITS
PSGLYLINKNDCYFVEPKLKRVTPFPWKPVDQDSALAFIDGKIFAFGRTSAENSESGISVERPTSIFSTAKPASGFSFGS
SVSPKSTFGNGAGHSIERRLESKLVESLNLEESNPEWKSEQKMLHAVVRPHIVQFSNKVYAFGGSNKKVTQEFDPALNEW
RMRRQMPGHCYYGAVVALGDKIYVVGGSEKVCFSYDPENDEWKVLSKPTHGYDGIFPFATVWKGKILLGDKEHVEEYDPI
DDRWSNRDELLPEEDIRSMILHAPFTLYGNQ*
>Ctel_206095
MEAGLMRVSLNAHLISFIASLRKMKENEVMTDVTLILPDQSAISCHKVILTAGCSLFRSLKESAEQEIRLDFADSDTIRT
LIEFPYSGFVNMTDDHLMTLVAASTRLCHKNAANSLIGLHVMSFMTSLHEMKERQVMTDVTLILPDQSTISCHKVVLMAT
SIFFETMFQSGLKERLFLVGEDDQCVRFQADRDRKTLTSIPWKPPRQSSTLCFFGGKIFAFGGCTIGENTPLKFVASFNV
EDANPDWRLEKEMLIAIEEPCVVHFADKMFVFGGRIGNTFSVVSQEFDPISNEWRLLSEMPGPCHGVAAVALNNKIYVVG
GLNRVCYSFDPVNDEWKSLSPPTQLCLDGSATVWKGKILLSAYLCTSLVQEYDPATDRWSKQNQLTAHEPQQYFLFSLCY
*
>Ctel_142715
MSWSETFSNHAALMLEQMNVQRFYSAFCDVSVRVEDVDFRLHKSVLAAASPKLQTLMCGLRDENSNVMTMHDVSLTGFRC
ILEFIYTGLLKLDIMCVQEVLNTAQFLELRMVEKACLNYLKSVPPSNMSMGDSKSYRGVLPGPPPDDISTNGDDVDVEGE
DDASQPDTHLAHDAVSLSHNASVTAEEEITVDDFEMVPPPETPDIEVEATDSEEDEDDDDDDDGEEEGEEAEEEEEEECEY
HSHKLSNVERHASIHSERKPFICLQCGKCFAQSNGLRSHLHSCTEQRGYLCDLCGAKFNYLGALKSHRLLHAGKKPYTCA
DCGASFTDVRNYKRHRRIHDNAFPYPCPHCSKTYRHSNSLKKHLLAHHPDSKEAKESSLPEKETAKNTKKTVTAKKSTIE
KINKESSKVADADCDKSQNS*
>Ctel_3638
MVAAPTEEEEENTNCEDFLFIEASHALQVLSGLNELRQGSLFCDLTLCIERKKFPCHKIVLASFSPYFKGMFSSDLAESK
QDAVILNGVEADMIELLINYAYTSEICVTKSNVQSLLSAANLLEILPVRDACCRFMERHMDESNCLGIHCFAETHACIDL
QQKAKLFTLRHFADVCQQDEFVNLSQAKLTELISEDALCVENEEVVFNSVCRWLEHDPSCRIERFPQVMEHVRLSLLSPY
FLHDCVDDHPVIGKLAKCQELIKEANTYHLLKDRRHELRTPRSRPRLSTGTMEVIIAVGGEDDKVVLRSVESFDPITKHW
KTLACLPFAVSKHGLVVNASRSMWRYDPCFDCWQEMASMILPRSELGLAMLDGFIFAVGGWEGSSRLDSIERYDPTTNHW
SMVAPMKMAVTSPAVVAHEGLLYVTGGAVLEDGDGIDLVQCFNPRNNHWMELPRMKIARSGSAACALNSVIYVIGEMGCL
VLLIHKIYDYLMAF*
>Ctel_27529
SLQSMREAKVLTDLVLVAGDQRIECHRVILASFSPYIRRLVHSSPKKSLLEMNIQDIDFEILSILLDFVYAKECTITADN
AERIVDALDVFQIESTRKPSILRAMGIKEGDGLAVLRLSRNH
>Ctel_66377
TDVVIHVKDEQIHCHKAILAAASIFFKKMLTSDLKEKQEDEIHLHGTDFKTMSHIVDAIYGGELKLHGDIVQYILMSCHQ
YEMHHFMELCTHYIALHMDCDNCIDIYLL
>Ctel_210851
MADRFSDTSINIFERFQSLREDKVLTDLVLVIGDQRIDCHRVILASFSPYIRRQLMEDDSSELVIDDINFESLEVLVELI
YAGELFSVDADNANEILNAIQLLEVSALATADFIAAMMDIEDSLSILRMAESSKYDVVLKEAAMLKLCIVFLDVTVRYEL
DESNVEEFLKNMTESEIINLISREDLSVSNEIDVFRAVLKWTNADSNRKQYFMNIVEHVRFNLMEKSDLDIVKEEELMQN
AKGLRMIEEAEFKPRKVPEADIGLEEDSIASVEIMKFQFLERLNEIREEQQLVDLVLFAGNQQINCHKAIMAAASPYFEK
VLTSDTKQTEEGEVRLTGHTGDTIHLIVDAIYGKQLQLGDDNVEEVLVASHQFELTECEDECIDFICSHVTCNNCVDFYL
LAKTHDLDRLRCNVTDYLTKSKDKIENVHLLKAEDWIDILSAWDTNVTPDLLSTILEWAKTDARHMDSLASVIQSAYLDP
HPDYLPLIESNKELLSKAGAYDELRGMIEED*
>Ctel_133847
MVDVCLLFGGTRVSCHKLILVGTCEYFKRIFFTNMSEKHSKEVNIKGISGNIGILVVDFLYSRNIEITVDNAQALLEAGE
MLILDNLKQKVEQFLIGQLEAQNCISILNLGRLYELKTLVESTRQFLTD
>Ctel_160730
MTDVTLILPDQSTINCHKLVLASASPFFEAMFQSGFKESTQKEVYLDFTDSEMIRSFVDYFYSGEIDINSDNIGDIVTGS
EFLCLDDLKTHCGAFMTSQVDSSNCIAFYRCARQYSLGELASHCFEHMLSHFEYDFCSSESFVDLSKEELIEVLSDDRLI
VENEDIVLHSVVRWVEADLEERKTAFVRIAPFIRFPFCTSELLGYKAKTQTLLKMYGNSFHYTDELNGEHTEWKGFHNNF
SIKLNNVLAPNGLNWIYAKKHHIYDIDTKSEVQFGAQWMTPQRSDFSLTFIQNKLFAFGGKLNEQISSKVDSFDQQSRKW
KQQPKEMPHAVSKSYVVPFETKIYVLGGIATYGPTTVNQEFDPIWDKWQLKQSMPGVCESGAAVSLNTSIFVVGGAERAC
FRYTPTTDTWIVLSHPTHVHTLSTAITAWEGNILLCGGDHGEEYNPDTDDWSPCDTLVPDKHPSHSYKCILQI*
>Ctel_73011
RMREAEELVDVTLDFGGRHVKYHKVILAGKCDYFRHMFLTNMAEKASEEVAIEGIGANTGVLLIEYLYSGQIELSTHNAQ
DLLEASNMLLLGTLKKNVEDFLCRHTDATNCISLLNLSRMYDLEALLEGAQKYLHDHVKEVANTEEMHLLQEGDLVEILT
ANSPEEDKFRFLQKWVKSDERRTGDFVTLIQHVELSKCRKEFLNNTVMVERLMFNEQCMELLRQAMQTDPSQTQALAPPS

EKQHYSGCASPDGFIISGGLRNNIAQRDCFSYDVQTNRWKTLPPMSTARVEHSSMCHENQLYVIGGWDGNNILYSVEVLD
MNSLQWSYIPRLPFRVCKAGVVFFSNNLFVLGGMNDAGDWVTDVNEYDSLLRGWQRRSPMLHISKRASVVSFGDHIYIIG
GHNRSCMQFNPIDDFWISLKRPYFKHYKGASLIWNDKILIYGGRDEDSIEEYSPLTNQWETLA
>Ctel_64340
LKDEHVLTDITLKLVDRSTISCHKLVLMIASPFFEAMFKSAMKESTQQEVQLDFFDAVTTRMLVAYFYSGKIEINTENAE
RLVEASDFLCMSELKQDIGASLIPHVDSTNCMEIYQFACKYSLEQLISHCLELILANFNEVARSSDAFVQLSLEELIRLL
SFGGKIGEVYCAEVMSLDQGEWKPEPTNMIQPCSKPYIVQFKTKLYVLGGINEHGPSLYNQEFDCVCGTWRLKTDMPGVC
QSGAAVSLNDNIFVVGGTEQACFRYTPATDAWTVLSQ
>Ctel_223277
MASDGSKHPSASHADVVQKKKSPDLSLSKCLHNLQSSNSATLHIALTQIRCKYIKDNAACSWVVTKGILPKLFSLVSNPK
VTSKVLDLTLSILGNLATASEVRSKMISSDAVELIGAVLMQSSDPSIQNRACRSLANLAHDPQASCAIHLKTGLLKHIVS
LLKETESKENAQTLVRSLRVLANNRVHLLGVLQTSCVNVILNMMSSTSASTITSALNCLTHLTSIYNGDRQLHKDCDTRH
DSSGQAEIKEMTEDDKREATQEIEKKEPLDEAADALAKPNKISLSRCCDELTRQLVYCEAGLQLLIETAQRDNPALRKLA
RTILFTLTKQTALRTQLSNAGFIPFLIQSIKENRLQPTETDSMRLLNSLAVCSQEASCRAKIRQHGGLDLILTLLDQEAW
QKMHCRLICALVCYIYDSEGLETLLGLKLCQRLMPHLLASADLSQEETVVTNIPRNTDYKYSLDSPTYQEISSQQESVSY
DSDAPTNIWDSAALESDSSRAYSPMSAVSYHSPEWGGAGSSGSPSPARSISPALCISPLPGASSSPASPFSDFSPGASPV
HMHSPSYNSFSPSAMPDSPARSLSPLRDEAWVRQTTRKKFNVDPLPNKVPHRRTQHNILVLLSRISYLPDPTPHLICPQV
MRSLLRYIAHSRRPLSRATRIIQRLVSFPICFEALIATATPFAIATMLGGEEKLRESLLTDLSITMATAYGSGTMEHMLL
TAETKTNAAISLPFICRGDRDRDRPLLTTALDILFDLVFRSDHPQSLRTATEALAGLASCYAKHDMTQIPQDLTQLQAED
PTEIEFLLDDGSHVKVEREILSQKSEVFRAMLMGNYTESTQSTVRLPQVLTAPFKELMNLTCKSSPLTDSIIERYSRNAE
SARDFCLEMLALSNRYILTEEQQHIAAYVSQRFISGASSVSVSGFSSILLFALTHNSHSLVSACFTWLFTAAETEIRVDV
VQGWQSTGQCCSDEIEKSLREFLNTVMQAPRLT*
>Ctel_144257
MESTHSSAIRHTSPDHSKLLLSNLHNLYVAQDFCDIQVCVGDQVFHSHRNVLSAASSYFHAMFSGGLAEMNKEKVFIQGV
DGNIFKIILEYIYSGVVLIEDITVQDLLAAADMLGLEGIVGACCSFLRQQLHPSNCIGISLYAEKHACDQLKSAAQHFIE
NNFSKVSQEEEFCSLSKDLLVSFLHSESLVVENEYQVFTAAMQWMLSDASQRRKHVFEVLAAIRFPLISQRQLETYIESC
SDLSLKVALRKLTQDFRFDRRLPYELKTGRLKPYLFQPRKSARKNVYVIGGYTRDQGGRWSDSQSLCTVECFNTFHEQWR
CIPPLRHSRSGHGVVQLHGLIYVIGGESDSLIFDNMECLDPTTNKWTMLPSMMLPRCGLGACVFEDSILVFGGWVGSEIG
DTIEKYDPGLNVWSELGQMETVRYAMNVLEHQGLIYVVGGMSDMGTEMQAVESFNPVTRDWIPLASMQIKRAYAGIACLE
DCLYVVGGWNEHFGALCTVERYDIEKNSWSLVTPMSTARAGASVCAVNGFLYVIGGRTSSGEFTAPSTLNSVECYDPHMD
TWVMMGAMPSSRCEAGIAVM*
>Ctel_25374
SSPTSSEDVDNGGKCTARLSQLFNNPKFSDITLVVGRKRFFAHKLLLANASDVFEQMLTSEHWLDSVQRELVLQEPDDCI
PVFADFLRYIYSGQISLSSDTVLGLLVLADKYNVPDLKDCCCSYMGGHLVGPPDVTKAVTWYQYALACNSQQLQNACLNY
IVLNMDKVIQSPDWVYLDQENLICLLQRSDLVIESEYTLLQAVVCWLTEDLRLHQLTDNLRATLPFIRFPMILPEHLSEF
EESEFEKAYHKFFAPYLLAAYRYHALSVKGKKNHTASVPSHQFMYRNYTDDNYSIHVDV
>Ctel_101293
MKEADEFVDITLVFGKQRIACHKVILAGMCDYFRRMFLTNMLERGSQEVVLNDISASTGVLLVEYLYSGNIDITQQNAQD
LLAASEMLLLGALKKNVEDFLLSHTDSVNCISIINLARLYDLKILLADARSYLHEHNGQWNTLPPMPTARIEHSSIYHNH
HLYVVGGCNRNALNSVDTLDMRNLQWNHLPPLPREVCSAHLAIVSDNLFVLGGSCGGCVADVHEFDSTQQTWRQRSPMPE
ICEGGAAVSFNDHVYVVGGRNKRCMRFNPRNNTWTSLQRPQFSHDYRPSIVLNGKIVVFGGLNDDFTEEYSPLTDSWSTW
TLKMPQKSQGWAFAAIMDS*
>Ctel_35088
LTASLFEMREAGEMVDVHLEFDGLRVPCHKVILAGSCEYFERMFLSNMRESTAKEVSIKGISSTIGLLVVDYLYFRTVDI
TGDNAQGLLEASSMLLLDELKNIVEEFLVRRIEAQSCVSLLNLARLFELKALIEALLKFLTDNLDKAFDEIEMKELQEED
LVHVLKNHASQRVCFCALQKWVRCTGGDADQFMALLQHVDLSRCSKQFIQDIVKHEEMMNSPRGSQIIKMAMHNECVLYC
TREMEWSTLPPMTNARYAHSSIYYKDLLCLIGGRILSGQPTDSVEALDNRTMEWRPLASLPVRVYLCIVVCVSNRLFVLG
GAESKQVSSRNVYEYCASPEMWQRRAPMPEDCHGASGIELRDKIFVVGGI
>Ctel_58016
ESHLKAFVASLHKLQENEVMTDVTLILPDQSAIQCHKVVLMAASPFFEAMFQSGLEESAEKDINLDFADSDIIRSLVKFF
YTGDIKLTAENIKGIIAGNEFL
>Ctel_208069
MSQSDSSLQDGALPEGKAMFYDDFKEKFAPKIKLPGKHFDDILELMKVLHPPNKELDESNVGLLLPLAREYQMEHLLFRC
ERFLLTRPPSVQGLLLAEEFDLDCLKRQCMRYIKNNKLQDLKAEAEFEILKSQTKVQILLEKSMKYEALVYEVKEIVTGH
RPEQVRFRSGGTLCTFTCSDKEKSSGRLPHNAGTDMCCKRCALYMVSRIGKQIENLP*
>Ctel_43597
EFVDITLVFGMQRIACHKVILAGMCDYFRRMFLTNMLERGSQEVVLHDISASTGVLLVEYLYSGNIDITQLNAQDLLAAS
EMLLLGALKKKVEDFLLSHTDSVNCISIINLARLYDLKTLLTDARSYLHEHVKEVVETEEMHLLQEGDLIEVLEANASQE
ENFLFIQKWMRSVEGRTDRFEDLMQHVSLSQCSKDFFMRSSGSTDPPKQPSLAVGNYKGEMWLCTDLNTPQWQPIQQPPF
DIALYSACASPGGFVVSGGASQNISQRECYSYYAQTGYWNILPPMPTARRGHSSIYHNHHLYVVGGYDGIDLNSVEALDM
RNLQWNHLPPLPRKVSFAYLAIVSDNLFVLGGFCGEWIADVHEFDSTQQTWRQRSPMPELCEWGAAVSFNDHVYVVGGEE
RSCMRFNPRNNTWTSLQRPQFNHYRGPSLVLNGNIVVFGGFNDDSIEEYSPLTDSW

>Ctel_24807
QMKEADEFVDITLVFGMQRIACHKVILAGMCDYFRRMFLTNMLERGSQEVVLKDISASTGVLLVEYLYSGNIDITQQNAQ
DLLAASEMLLLGALKKKVEDFLLSHTDSVNCISIINLARLYDLKILLADARSYLQEHVKEVIETEEMHLLQEGDLIEVLE
ANVSQEENFLFIQKWMKSAEGRTDRFEDLMQHVSLSQCSKDFVCNTVMAEKLMHNTRGNICQRECYSYAAQNGQWNTLPP
MPTARRNIHRFITIIISTSSVAGNCSELNSVEALDMRNLQWNHLPPLPREVCLAYLAIVSDNLFVLGGYCGLNRVADVHE
FDLTQQTWRQRSPMPEICVAGAAVSFNDHVYVVGGRERSCMRFNPRNNTWTSLRRPRFIHTYGPSLVLNGNIVVFGGSND
DSIEEYSPLTDSWST
>Ctel_37076
RKSLEFCDISLKLNGVVHGAHKAVLAATSPYFRSMFRSKMKEQTSSLVDLSQSLVLDRDDSFKTILDFLYTGDIEITTLN
ADDMLRIADFFLLEDVKHYCQEFFLVHGNLNLKNCMYLSALADHHNMPEVAEASLKMVHSRFHDHLIHSDQILELPEDIL
AKFLRDPEAVQFAKPVHILRAVLKWLKHSFFERKSCLQQIFACIHFNISSLSDLDLKSIVDQEPLLSDEEICCKLSCSD
>Ctel_141439
MTRAISSLPVVFLQMKEADEFVDITLVFGKQRIACHKVILAGMCDYFRRMFLTNMLERGSQEVVLNDISASTGVVLVDYL
YSGNIVITQHNAQDLLAASEMLLLGALKKKVEEFLLSHTDSVNCISIINLARLYDLKILLADAQKYLHEHVKEVVETEEM
HLLQEGDLIEALEANASQEENFLFIQKWIRSAEGRTDRFDDLMQHVSLSQCSKDFVCSTVMAEKLMHNTRGMKLIKQFMR
SCGSADPPKQPSLAVGNERGEMCACASPGGFVVSGGRSQNSNQRECYSYHVQNGQWNTLPPMPTARRIHSSIYHNHHLYV
VGGYDGRFLNSVEALDMRNLQWNHLPPLPREVYSAYLAIVSDNLVVLGGCCGGWVADVHEFDSAQQTWRQRSPMPEICER
GAAVSFDDHVYVVGGSERSCMRFNPRNNTWTSLQRPRFIHSYGSSLVLNGNIVVIEGSNNDSIEEYSPLTASWSTWTLKM
PQKSPGWAFAAKMDS*
>Ctel_124196
MREAEELVDITLVFDERRVKCHKVILAGTCGFFHRMFLSECSSNEVVLKEISASTGVPLVDYLYTGHIELTTQNAQDLLA
ASEMLLLVTLKQNVEEFLCRHTEPTNCISHLNLARIYDLKTLLEDSRKYLHDHVTEVFNNKELHLLQEGDLVDIITANDQ
QEDNFRFLQKWAKSDERRTGDFVSLLQHVQLSQCCKAFLWDTVMMEELMFNKHCMKLLQEAIQADPPEQQTLAVGDAAGK
IWICTDNQKWHPIQKPPAQNMFYSGCASPGGFIVSGGCLNNIRQRDCYSYDVENDRWNTLPPMPTARSHHSSLFHKGQFY
VIGGRDDLNLDSVESLDTKSLEWQQHPPMPRHLYLAGVVSFSGNIFVLGGYEANYEVDVNEYDVTQRTWKQRSPMPEVCE
GGSATSFGDHVYFVGGRNRSCMQFDPREDTWCLLQRPLISHHFGFSATLNKKIWICGGNNEEAIEEYSPRTDKWTILPLK
MPPGRSIFMRFALRIDDFS*
>Ctel_33935
QMREAGELVDINLVFGDHIISCHKVILAGRCDYFHRMFLTNMLERNSTEVVMKEINARTGILLVRYLYTGLIEITTENAQ
DLLSACEMLLLGALKQDVEEFLCSHTESNNCVSIMNLASLHDMKTLLGNAKKFLHEHIKEVVETDEIRLLQEADLKEILG
ETLSQEDNFCFIQKWVKSADERAERFDDLLQHVTLSKCSNEFICDTVMEERLMISVNGMKLIQQAMRPHKQTDPTGLQSL
VVGDASGHMWLCSDINLQTWQSFQGPPTRNTNYSACASPGGFIVSGGWLNDVCKSDCYSYDAQSGQWTTLPPMSIARHAH
SSIYHNEGLYIVGGIDNHDYLDSMEKLDMRSLQWSRLTRLPRSVTYINLAIASNNLFAVGGSSGRGCCVVDEFDFTQQTW
RHRSAMPEKYANGAAVSFNDHVYVVGGKDRSCVQYNPLQNTW
>Ctel_142559
MTDVTLILPDQSAIQCHKVVLMAASPFFETMFQSGLKEGTEQDIKLDFADSGTIRSLVKFFYTGDIKLTAENIKAIVHGS
EFLCCKHLKAHCEEFLVGNVGLSNCIDYYQFGKVFNLEMLIKTTFDFILSKFREFMEISNFDKLTEDELVEIVSCDRLNA
ENED
>Ctel_215677
MTAAFQKMKEAEEMVDVELVFEETRVKCHRIMLAASSEYFLRLLQTDMQARDVKEITIKEVSSSTGRLLVDCLYSANTSL
SHLDCHPEALVYADDTMLYRTGKDPAHICPAIEEDLRIAAKWADIWGMRFNASKTVAMYIYQTTATPPPITFAGATLEYS
HEHRHFGFILDSALSFHDHKSVSCLLGVLHRGDSFLVVRGQDSLLDQFSIIPEKFIETSLSPHVGVGSRLECAMLCQRDK
CCYSALTKNDDYNVQCQMLNYVFPEEDLSDDEGGAYLIRIHEPDSQQWLVHYDAAIFGNNLFSINEITEEMCKAACVERV
DCLSIDFYTETGNCFLNYYTSKTVPLSEETGTIYMEKTCGL*
>Ctel_116986
MREAGELVDVTLVFGERRLACHKLILAGMCEYFHRMFLTDMVESYSKEIFMKDINASTGVLLIDYFYTQQIDIATNNAQD
LLKASDMLLIETLKRRVEEFLCEQIEESNCISLLNLARFYGLEMLQEDAQVTLYDMRADDIINMEDIHLLEEKDLIKILT
ANDALDESLCLVQKWILCTLRSNSFDSLLELLHSTHGLGLNDLLEEPSLVVGNFEGEMWQSFDNQTWQPIWKPPFLNEVY
SACAHPDGRIIISGGVFVDVVQSDCRSYDAHTAQWNDLPPMPTVRTQHSSIYHDHHVYVIGGNDGQTMLNSVDALDMRSL
EWSKTWHQRSPMPEQCAGGAAVALGDQVYVVGGWKRSCMKFNPQIDSWVSLQRPQFLHWYGPSLVLKGCVLIYGGSFVDF
IEKYSPLTDKWTTLTGNTPQKQEMRFALTLRIRHDLITL*
>Ctel_216344
MTQEITSLPAIFSQMREAGEFVDITLVFDEQRVLCHKVILAGTCDYFHRMFLSDMLESTAKEVTMKEISASTGVSLVHFL
YTGRIKITTQNAKDLLAASEMLLIGSLKPKVEEFLRSNTELVNCISSISLARQYDMKTLMEDAKKFLHERVREVTDSEEM
HLLLEEDLIEALNANASQENNFCLLQKWILSADGRTDRFDDLIQHISLSNCSKKFICSTVMSENLMHNTHGMELIQEAMQ
SPRTTDPLEQQSLVVGNLEGEMWLCTDAHVHHWQPMEKPPVQCLQYSACASPGGFVVSGGKCNGNACPNCYSYDAHENKW
NALPPMSTARVNHSSIYHDRHLYIVGGLSNEGHILDSIEALDMRSLKWSNLPPLPRAAYHSYLAVVSNKLLVFSGIQERF
WLADVNEFDLNERVWQQKAPMPELCVGGAAVCLDDHVYIVGGGNRTCSQFNPSRNIWTSLQRPQFARYFGPALVWNQSIV
IFGGKGKDSIEKYSPLTDTWTTWALKMPKEDCWRFVVRMNNL*
>Ctel_219008
MIARLATLDILDSLAARTLFTLDEVGDVSWKVAENAEYMPFFSCETYEVNLSGGEVLKFLGSFAVEFQVEISDDLMLLTY
DQCCILQCQRVLNSRPPNEVPFSFLPALEEGYFSDGIIRAENGAEFKVHKTILTLAMPEMDWNSDPMPLSGFREDVVHMI

LHYIYSECLIKGLSEETAHAALQAAEGVKGLEKFSELCLTFLKNTALKQQIISLVTDIHACADRMVDLLNGKGSAETGSG
SFGEPSLVLSPARLCYIIKQALREGAIAGAKLVILCDLFSRRKGDLSREERHEIIKYAKSRLPVFMNQLHRFFEVCKSHS
SSMTAQQRQEIAAYLVPEIEITLDAMSSFVVEMKSSLEDIISQSSSSEKNSEKGHVKDVLGKALRNALHTRELIKLKRFH
ERTTKSFHNLMRKKENFCLMTQEEKVRSVSKNLEHLMDEVPLFLVRIEDVMSALDEKVSWREWKYLFKLGTSKVSWVLSK
IQKHTDTVNPIFLKACEMVQRDQFTESISAIGLISKVTDGAGAAGGCVANKAASSAPKYAQLSSVESLCIPPYAQDSTLA
AKALILLQKNCGETDVTFEIISTSEPGDIVISHADDEEISHQSVPKVITEIPAHRVIIASRCDWFRRALLSGMRESIDKK
IPVHGTSPELFLMMLKYLYSGYLDTKSLSVDQLADLLSLSDGYEMDSLKCHCEYALNSSIDEESVLFLFSLADQYNARVL
KESCLAFITLHVHLLYSDVFNELPDTLQKELHDKCDISWPSNQALSPEGGALGRHPGSSSGDMDEVMKALELGDVQASPQ
STDSTSSSFLEDLHYVEDATNIEICLTELRGILGDEVPHEMLLRVASAADYQVNRAVNYYFSS*
>Ctel_53734
FSEMREAEELVDVYLVFNGKLVPCHKVILAGNCEYFKRMFLSKMNESSAREVNLKGINATHGELVVDYLYSKDIEITEDN
AQGLLEASSLLMLDKLKKKVEHFLIGQLEAENCVSILNVARFYQLETLLETSLKFLCDRLHKIIDTMELEELTKDDLVNV
LENHATKEACFILDMKTLEWGVLSPLHRPMCHSLVASVSNRLFVIGGTPNPTSISTGVHFIESGRLNWRPRTPAPEDCRG
ASAATLKNKVFVVGGASNSCMAYDTLVDAWAILERPQVLFTYKASLEWNGKILLLGGTEDD
>Ctel_221032
MLSSLPDFCYDLYKKGQLVDLTLTCANGERLLVHSVVLALKSKVIQEVLLHLGDLTQVTAVVHCGDVDVQLLRSLVEFAY
TAEFEEEAYNHRDTLLFVAKRLGLEAAVRELDNQKMALKRKSLDAEQMKAVNVSAQLKCKSCEKTFVSARQCKRHACEVK
VEVVNLSQMKHEEVQGHDDMVQIEQRVAEDNAAEEIMEEEETNQMPEAEEQQQEKSEEEKDEEVAEMEVEEEKDEMKMMK
EEPVKGTKHDEQFPEKKCKVWRRSHTQSLPFKCPHCTFSTSKAPYLSDHTRVRHPNAPETCPHCQEVFSRKEIGRHKKGC
EKRFSDAASVEALFHCDVCPFTAIRARYISDHTRKMHGGDSVLCVHCGQQFQRREIHRHQQRCKEKPAKVKVKVVYETDE
NALKCPVKDCEFVAEDREQKLLHAQTEHKVIVKKKAAVSEETFVCEHCGRTMNSKAAHRAHMAAVHSTVFHPCSMCEYQG
KSLLALRSHCQLIHSCNPDGSPYERRFACTVCDFKAIHRCTLKTHMKKHESKKAFTCDHCPYKTHRKDSLLKHKSTMHTT
SRRVKVCRTCGKAFVSETGFRMHNLRHLGLRPYHCTLCNLPFARLSTFELHNMRKHPTTCSQSPLSCTHCSYTCSAASTL
KQHYRTKHGISDVHIVGSRKGRRSGHSSFKNRFKEFNNVNQFVESNTIVGKVEDNSCVVDEEELQQQAAEEGEATLEMHE
ASEILAGIGTSSQVEVEGTVEVASTPTVYEVNGQTIAVYYV*
>Ctel_34879
DSHLKAFMASLHKLQEKEVMTDVTLILPDQSAIPCHKVVLMAASPFFETMFQSGLKEGAEQNIKLDFTDSAIIKILVEFF
YTGYIELTKGNIKAVVAGSEFLCCKHLKAHCEEYLVDTVGLSNCIDYYRYGQVFNLKLLIKAAFDFILSKFKEFKGIADF
DKLTEDELVEVVSSDQLNAENEDVVFEAVDYWVNADPDAREEVFPRIAPLIRFPFCSQTALTDNICCNTLMQKPGCIDFA
HEALWSQFHFNCRPPIHNARCIPRQAFHSRASTSSLFQLIKMKSPNKDSLFTTVTQEFDPASNEWRMRSQMPGYCYYGAA
VALGDKIYVVGGSEKVCYSYDPENDEWKVLSKPTHKYYNKAATVWRGRILIGNREHVEEYDPVADLWS
>Ctel_223819
MDGQDIGEFKKRGYLKGTKTIIIRNIVIGAGACIFLGSIAYFVNKIGESGPDSKLQKPIQLMRNQESERAENRLKVQIRR
MERARNVAEFASKALRIKSRGAYNKDPQIGMKNDEEKLCQTCKQLFGRSVYDDHVTKCLRAKFSRKNPGDFVICQICQED
ISNLRSLQQTVHVNKCIDKSKRQEVLLHNAKSQVADCPLCGQSRKSHLKKCSSQKGISTAELLDKVNEQEATRRSLLEAG
IVPPQNSCPPRKRLKKSERNEKKFAADMQRAIDLSVSEDANRAPVKDAFSVLMTPTKKIPARGKKRRPPVKPSRLMEQSA
EETRQKIAKKMAAIVVDEVKNNSSEVPNLKESQVRNRSEELQLWSKASSKDVCSGYYVPQLAAHLRKNPSIQRTAPVETL
NNTTLIASTQTALLLAELELDQTTLTQPERLANISRLNSSCNLLNQTGFCLEPDEETDPDIGASFEVMLGNQSLSDIVFI
CLDGQVPAHKFVLCSRLSQDVWRSVENERELNVDVNKDTLLSFLRYLYTGRVPRVLNVQFQSLARKFQVKFLEEACNFER
LKEDDLSESSESDESAIEEDSDQRELHEEEDEDILALLKDESPKVAEVASPQEVVYNSDADLFEDTAEIVDLTGDSSQNL
ITTPDMFPTEKVVRTKSRLNEDEDSSASQELLDSSPTFATQQAVDLTQNEASTPVFSEDELPDIATSPQATSPPTSPEAI
SPQATSPEATSPEAISPLTSPPTSPEAISPLTSPPTSPEAISPLTSPPTSPEAISLQATSPQATSPLSPLTSPPTSPQTT
SPQRVATQDVWQDFDDGGMDEILVEAEEEAMVAPPVYDPPTDIETDVSFMESAVTPVNKACEAKKKGKAPVVPSPFTPMP
SYKDMNTPALKREGQKYGVRPMPRKRMTQVLTNIYQETHQYETDSEVDESLLKEKASSDGLNLSSSDDVCLMPEESILIE
EKETLKLSRKDVAAKKELVWNFIRGNEEIYRQVLHYEPLEFDDFLLRIKSLGHVIGASIVIEALDDMCMGFTTRSKNDGN
RHIRKKRKTDKK*
>Ctel_223821
MACAPNDLLPLQDIITESAANGYGDQLLQRLGHFWRNKLFCDLTLRVESASYQVHRLVLAACSDYFEALFSHNMQERHSS
CLDLPSVEASGFEPLLHFAYTGNLPLTLLNVENILPAAAFLQITAALQAISDFLKSKMTFDNAKRLVNLGEHYGLIELRS
HLKEMILTDFTSFAETELFLELSPMELTGYLMEDCLNVPNELTLLKCVLRWYHADVTNREEHIADVLDKIRYTLDGWSAV
AFARKTEPFISNRQCREILSWCERYLQNPFGRVMLQSYRTRVRSLERSLVQVGGIRQEKNDPFPGFVLPDDDEVPEERTG
CMLNRFYHRTLQKWMPLGEMEATDAMSHCPLVEVNDFAVLVGGYLYTTDFNLGRYQHCSNEVRLIQAGCGFASWDLPCLL
GARAHHTAVFCQNYIYAIGGKNESGNLSSVERYSLEEEEWSYVEELPVATFDHASCIMQDQVFVCGGTQKGRVVNDVLCF
VPCAEEWTSRPPLLTARTGHAMAAIGDEILVCGGSTALGAHYRCLASVESFNVRSQQWTFIKPMRQDCCSMTAVVLDSKL
LLVGGENSVGDLLTQVQEFDPKSETWGTYCSLPTAMKSVGCCALTVKLTDAGKTAESEKELNCAESDDDYPDDDNDDDDD
DDVLRHLQHSAPFNASGFMERIGNVFF*
>Ctel_225321
MPPKMKVLRAHRSSKSCTDSCHKNPRVILRPLLADFSIQNRYRFNSPPPSASLYNKPELSDIKLKVGNDTYYGHKLILSA
ASEVFSRMLNSDWLESHTDVLKLEEEEECIKVFDLFLYYIYTGTIVLSESYVVPLYMLADKYHVTVLLDECVKIIRNGLK
IFITKSGSMSPAASEVQPLSGVGQSFAIGPSSHSSSSGDSASDDLTDSEASHDHSISLDECSRQSASRSSTPDNQPGPSQ
ALADPPQKTVLVASETFSLPLVMRLILTCHNESISNAALYNLEVRLANQISLGVFSTWNELDQVFLTRVLSDRHFYCKEF
YLFRAVISWLQFDAKRQTEDVITDVLQHVRFPLLKPCYLYQVEQDDLVSRSSATKQLIQEAIRYQLFKSCCHGDDRVRWT

GPQFEERLVRRP*
>Ctel_84268
LKAFMASLHKFQEKEVMTDVTLILPDQSAIPCHKVVLMAASPFFETMFQSGLKEGAEQNIKLDFADSDIIKALVEFFYSG
NIELTAGNIKEIVAGSEFLCCKHLKAHCEEYLVDTVGLSNCIDYYRCGQVFNLKLLIKTAFDFILFKFREFREISDFDKL
TEDELVEVVSCDRLNAENEDVVFEAVVHWVNADPDAREEVFPRIAPLIRFPFCSQKALTDKICRNPLLQKPGCI
>Ctel_137514
VSFLQMREANELVDVTLVFGDRRLACHKLILAGMCKYFHRMFLTDMVESYSKEIVMKDINASTGVLLIDYFYTQQIDITT
HNAQDLLEASDMLLIDTLKRSVEEFFCELTDKSNCISLLNLARFYDLKMLREKAQDTVCNIQVDGMDDIEDMHLLEENDL
VTILKASDVLDDSFCLVQKWILAVERSNAFDSLLQLLKKKMLHNKHGMELIQHLTQLYLVNDRPEKPSLMVGTFEGEMWQ
SIHSETWQPIQKPRNLNEVYSACAHPDGRLIISGGVFMNTIQSDCHYYDAHTAQWNHLPPMPTVRAQHSSIYHDHHVYVI
GGNDGRSFLHSVDALDMRSLEWSSLPHMPQPLQCSYVVSVSDTLFVLGGMRDGWVDWSVDVHQYDSTGKTWHQRSPMPEQ
CAGGAAVALGDQVYVVGGWNKSCMKFNPQIDSWVSLQRPQFSHWYGPALVWNGCVLVCGGSEEDSIEEYSPLTDKWRTWT
MTLPQKQELRFAFRILL*
>Ctel_137498
MSATFRNMRHSDEMVDVVLAFEETRVKCHRLVLAASCDYFRRMFQTDMQERDAGEISIKDVSSTTGLLLVEYLYSGNIQI
SAENAQELLAVSDRLLLTKLKKNVEKFLCGQVASTNCVSFKNLARLYGLDTLIKVTHGYLNNHWQKLIDMESEIDELTED
DLIALLTTYGSDEGSFLLLQKWAKQWRTLPPMNERRYYHSSICNQDEFMVIGGRINEDIYLDSVECLNLKTLKWSQFPDL
PEAQCLNNAVCIQKQLFVLGGWISITTVTRDVYKFDSAERAWQALSPLPETCEGVSAVSFASKIFVIGGQFRRCMQYDPC
TDIWVQLQRPQFEHIYGPALAWRDKIVLCGGKETDSIEEYDPQSNKWSTWNLKMPNEDYFCFALTIKRHK*
>Ctel_174903
MREAGELVDINLVFGDHIISCHKVILAGRCDYFHHMFLTNMLERDSTEIVMKEINTRTGILLVRYLYTGRIEITTDNAQD
LLSACEMLLLGALKQDIEAFLCSHTESNNCVSIMNLASLHDMKKLLGNAKKFLHEHMKEVVETDEIRLLQEADLIEVLGK
TLSQEDNFCFVQKWVKSADERAERFNDLLHHVTLSKCSDEFIRDTVMEERLMISENMWLCSDINLQTWQSLYAPPSQHRN
YSACASPGGFIISGGRLKGVCKSDCYSYDAQSGQWTTLPPMSIARRAHSSIYHNECLYIVGGRDGQNCLDSVEKLDMRSL
QWSRLPRLPRSACRLYLAIASNNLFALGGSSGGNWNVDVHEFDFTRQTWRQRSAMPEKCEFGAAVSFNDHVYIAGGKDRS
YMQFNPLQNTWILLQRPQLSHRFGKSLVWNESILVCGGLDSDVIEAYSLRENQWSTWTLKMPNKGEFIFALKM*
>Ctel_200473
MREAGEFVDITLVFDEQRVLCHKVILAGTCDYFHRMFLSDMLESTAKEVTMKEISGSTGMSLVHFLYTGRIKISTQNAKD
LLAASEMLILGSLKQKVEEFLCSNTESVNCISTISLARLYDMKTLMEDAKKFLHEHVREVTDSEEMHLLLEEDLIETLNA
NASQENNFCLLQKWILSADGRTDRFDDLIQHISLSNCSKKFICSTVMNEVLMANEYGLKLIQAAMQSPRTTDPLEQQSLV
VGNLEGEMWLCTDSHVHHWQPMEKPPVQCLQYSACASPGGFVVSGGKCNEIDSFNCYSYDAHENKWNALPLMSTARVDHS
SIYHDHHLYIVGGLSSQGHFLDSIEALDMRSFKWSNLPPLPRAASFPYLAVVSNKLLVFSGVQTGFWIADVYEFDLKERV
WHQRAPMPEACKNGAAVCLDDHVYIVGGLNRTCMQFNPSRNTWTSLQRPQFAHYFGPALVWNQSIVMFGGRDNDSIEKYS
PLTDTWTTWALKMPKKDLWRFVVRMNSLL*
>Ctel_36674
EASASPFFDFKDKRFPSQLLQGLSKQREHGIGLDLTIRVEERTITAHRNIVMASSTYFDALLGPNMKEGKESSVELQGIK
YAPLNQLIDFMYSADIAITSENVQDILETANFVGMISICGACCRFLAQHLDVSNCADILQLASALDQDHLKGDASDFFCT
HFFEIQGNEGFMSIDITLLTGLLAEDNLRVIDGTVQPTMENVETALLNIVMNYINFHMYDEPHRLAALLETVRLP
>Ctel_134985
MLESFCNMRAATELVDVILVFKNVKIPCHKVVLAGTCNYFRAMFVTEMKESRSAEVQMSDISADIGLTLVNYLYSGSIEI
TEQNAQALLAACNMLLLVDVKNNIAEFLSRQICNSNCVPLLNLARFYELESLRKAAHKFLTKHWKDLIDTNDLGELKEVD
FLYIVGGKESKQGYMDSVECLDIKSLKWGHLSDLPLCLSTPMLTTVKDRLFVLGGIKGGGHSRMVFEYHNEAWEERSPMP
EECRRGAAMEFDGFIFVVGGLEKSCLRYCQRRDDWNILQRTTFPHMFGPAMVWNDAIIVGGGEGTDANEEYCPHTDTWST
WQLKMPNSGQMQFILKI*
>Ctel_151100
MREAEELVDVTLVFEERRVKCHKVILAGTSDYFRGMFLTDMAESASREIVMEEISASIGALLINFMYGGLVEITTQNAQD
LLTASDMLLLDALKQQIEKFLCRNIESTNCISLLNLAQFHDLKLLMEDSRNFLLDHVDGIDVEEMNLLQEGTLIEIIQLS
ASSSPEDSFRLIQKWVRLVEGRSESFLNLMQHVSLSKCSKEFIRSTVMQEELMANEQGERLIEAAMQTDSHEQHLVVCGW
GGQLREEFGTSGRQNPVAQTDCFLYEALSNEWRTLPPMPTARYNHSSIHHKNNLFVVGGKKDGAALNSIETLDRRNGKWS
CLPPMPRGLEHALVVFVLNRLFVLGGSFRNVIIDQCVQVNEYDAILKEWVPRSPMPEECEGASAVSLEDHIYVVGGHDRR
CMRYNPRDDTWLDIALHRPHFVHIFSPALVWEGKIVICGGDMEASAHTEEYNHWVDRWARSWLTFDVLPMWQVRSAFAMD
IPS*
>Ctel_101775
NVSLVVEGRTFRVKKDFLMKQSSYFDAMFSVDMKEAREGIATINETRADIFEKIIQYYDGHSFHLTEDKLDEACSILITA
SMYQFRTVMEECINFLTEILAPRNCLKIMTFSFPHNFKNLANNSLQFALWEFDSICQQQQFLNLSQVELFDYLDNPNLNT
KCEINVLRAVMRWLEKQESLGVEKVEKILQVVRFQSITNADLDSVIHHPVWKDRGCTDVLGSFRSAGWQALYPKKVL*
>Ctel_214586
MTCFYFGKKTLQQLYTKCQHVLRLIQPMKMDFHKQKLLASLRKMKENDVMTDITLILPDDSTISCHKLVLVAASLFFETM
FDSGMKESIDKNIKLEFSDADTIRKLVEFIYSGEIDVNEDNVQTLVAASDFLLMGDLKAHCEDFLTTLIRSSNHQELCTF
ENEDIVFTSVVRWVNVDPEQRKEAFPRIAPFIRFPLCTQKTLSMNVIWEPLMWNPECFKLIAEAQHSQFHSRREPIENSR
TAHRSSKREYLVRLSADYNQVSIEYTDSIDEDEVEWECKLLDFTHSLNKNNCIPSESGVIWIINDETRMSLDLKSYKISS
HPWVKPRNEECSLFVGDAKIIAFGGKERLNPNSNPAAHVIYLLGNVYILGGYALCRERDYYGITMGNQEPRYRPQPSQST

QKFSIATQTWSLCTSMPDECTSAQAVALNDKIYVVGGENRICFCYDPEEDTWTVLSRPKFGNSDSATVWKGNILLSSSRG
HSTLQVYDPILDCWKCTENVFLDETKQHQTKLFCAMRF*
>Ctel_36636
PLSIMTQTISSLPAVFLQMKEADEFVDITLVFGKQRIACHKVILAGMCDYFRRMFLTNMLERGSQEVVLKDISASTGVLL
VEYLYSGNIDITQLNAQDLLAASEMLLLGALKKKVEDFLLSHTDSVSCISIINLARLYDLKTLLADARSYLHKHVKEVVE
FEEMHLLQEGDLIEVLEANASQEENFLFIQKWMRSAEGRTDRFEDLMQHVTDPPKQPSLAVGNIDGEMWLCTDLNTPQWQ
PIQQPPFQIKYYSACASPGGFVVSGGLSQNSNQRECYSYAAQNGHWNTLSPMPTARRSHSSIYHNHHLYVVGGYDGSYLN
SVDALDMRNLQWNHLPPLPQRCVGGAAVSFNDHVYVVGGRHRSCMRFNPRNNTWTSQQRPQFNHYCGPSLVLNGNIVVFG
GQGNDSIEEYSPLTDSWSTWT
>Ctel_47053
DSHLKAFMASLHKLQENEVMTDVTLILPDQSAIPCHKVVLMAASPFFETMFQSGLKEGAEQNIKLDFADSHTIKILVEFF
YTGDIELTEENIKALVAGSEFLCCKHLKAHCEEYLVDTVGLSNCIDYYRYGQVFNLKLLIKTAFDFILFKFREYKEISEF
DKLTEDELVEVVSCDRLNAENEDVVFEAVVHWVNADPDAREEVFPRIAPLIRFPFCSQTAFTDNI
>Ctel_63295
IPCHKLVLMAASPFFETMFQSGMKESTQREVQLDFSDAATIKMVIDYFYSGTIDINAGNVQELVAASEFLCLTDLKEHLG
AFMTDQIDATNCVEFYRYARKFSLGKLIPHCLEHILSNFSQAFGSSANFADLSEEELINVVSDDRLKTENENIVFHSVVR
WVNVDLEQRKEAFVRIAPFIRFPFCTLEECLNNIVREKLMINATCMDLLYEAITIKTSLIIQHSMHSSRHVPRLAYTTKK
PHLLRVYANVSHHYMVGQCTEDITQTNQISIVMGRTFPQRTDYQTIFNQDRLFAFGGCDSSNGQHCSTVESFDQKQNTWK
AEPAMLQAVSKPYIVHFGTKIYVLGGVDANGPSLATQEFDADWGIWQLKKEMPGLCQSGAAVSLNHRIFVVGGTERACFS
YTPSTDTW
>Ctel_111570
MTSLQRLKKKESSIDISLKLPDETTIPCHKLVLMAASPFFETMFQSGMKESTQREVQLDFSDAATIKMVIDYFYSGTIDI
NAGNVQELVAASEFLCLTDLKEHLGAFMTDQIDATNCVEFYRYARKFSLGKLIPHCLEHILSNFSQAFGSSSNFADLSEE
ELINVVSDDRLKTENENIVFHSVVRWVNVDLEQRKEAFVRIAAFIRFPFCTLEECHNNIVREKLMINATCMDLLYEAIST
KTSPIIQHSMHSSRHVPRLAYTTKKPHLLRVYANFSHLMVGQCTEDITQTNQVSGVGWTFPQRTDYQTIFNQDRLFAFGG
CDSSNGQHCSTVESFDQKQNTWKAEPAMLQAVSKPYIVHFGTKIYVLGGVDANGPSLATQEFDADWGIWQLKKEMPGVCQ
SGAAVSLNHRIFVVGGAERACFSYTPSTDTWAVLSQPTHVHNVFPAAAAWKGKILLLGNQGEEYDPETDAWSACQALIPT
DGNLSVPKGLFAVFM*
>Ctel_164103
MMERQIFTDSHAKSILTTMNNLRKANTLCDVMLKVDGQDFPAHRIVLAACSDYFCAMFTNGMSEAEKTEIVLHGISADVM
EVLLDFVYTETVDVSVENVQELLPAACLLQLTGVKEACSEFLEKQLDPANCLGIRMFAENHGCESLQAASGLYTHKHFEE
VVQHEEFRMLSEKDVHKLVASDEIQVGSEEAVFHAVMKWVQHGEPQAKEDDDLNAEDVQLTPLTNDREQYLSDLLQNVRL
PLLSAKFLTDTVDSQLLIRKSLECRDLVDEAKKYHLRPDLRPQMQSQRTRHRTGCDDVLVVLGGFGHLQSPVDVAEMYDP
KTKTWLPLPNITRKRRYVAAAAIKTKVYALGGYDGTCRLSTVNCLDLADEDPQWQTVAPMSQRRGLAGVCTYQDMVYVCG
GFDGIMRHTSMERYDPQIDQWSMLGNMSVGREGAGLVVANDMIYCIGGYDGVNLLNSVERYDPNTAQWTTVASMATSRSG
AGVAVINDAIYVCGGYDGSSHLASVECYHVRTGHWTSVAHMNVPRCYVGACVLKGQLYVVAGYDGNTLLSCIESYDPHAE
AWQLHDATMATHRCDAGVAVVRRG*
>Ctel_145181
MQNLKLVVVGDGGVGKSCLLISYATNSFPSEYVPTVFDNYSSNILVDGKAVNLGLWDTAGQEDYDRLRPLSYPLTDVFLL
CYSVDNRDSLENIANKYKAEVDHHCPGTPQILVACKRGELGKKDQSRFVSENEGRRMAKSLGIQFFETSALTQQGVAIAF
DHAVAMSFCPKKKKKGGFGLFKKSKKSEPQPPVMPPAGKAPWLEIETSTFADDWGKMLKQPEHTDMTFIVDGRHSVLAHR
IVLASASKFWARILTIPQEDNQLLKLDPQLTFENICDGKVKGILHIQLERAPDKRSMMTAVTLDPDYVNAKTFSYVLQFL
YTGKIANYDISKEDLDNVCNISRVFEMPELITICENITSGEEFLNPSIGTYLNDETGKKMKEMFLNQDRLTDVTFIVEGT
KIFAHKAVLSCRCDVMAAMVGGNFAEGNSETSEDTTLDEFLAFLEYLYSDHSPIEECDSVGILMLADRFCMSRIVNLCEL
YITKEVDRSVQKNIQKSNMDVIGLLLLAQAHNAKQLEGWCLHFISTNFSCFENRPEFPQLTGSNLEFVKEHQWPPLSYLK
EVEEYKQMSGGKCSVM*
>Ctel_35989
HKVVLMAASPFFETMFQSGLKEAAEKDIKLDFADSGTIRSLVKFFYTGDIKLTAENIKAIVAGSEFLCCKHLKAHCEEFL
MGNVGLSSCIDNYQFGKVFNLKVLIKTAFDFILSKFREYREISNFDKLTEDEIAEIVSCDRLNAENEDVVFEAVVHWVNA
DPDVRNEVFPRIAPLIRFPFCTQTMLTDKICCSSLV
>Ctel_76871
MTDITLILPDQSAIQCHKVVLMAASPFFETMFQSGLKEGAEKDIKLAFADSGTIRSLVKFFYTGDIKLTAENIKAIVAGS
EFLCCKHLKAHCEEFLMGNVGLSSCIDYYQFGKVFNLKVLIKTAFDFILSKFQQFMEISDYNKLTEDELAEIVSCDRLNA
ENED
>Ctel_32616
SHLKAFMASLRKLQDNEVMTDVTLILPDQSAIQCHKVVLMAVSPFFETMFQSGLKEAVEQDIKLDFADSGTIRSLVKFFY
TGDIKLTAENIKAIVAGSEFLYCQHLKAHCEEFLMGNVGLSNCIDYYQFGKVFNLEMLIKTTFDFILYKFGEFMEISNFD
KLTEDELADIVSCDRLNAENEDVVFEAVVHWVNADPDVREEVFPRIAPLIRFPFCTQTMLTDKICCSSLVLKSECIQLAQ
EAVWSQFHLNCEWKSEQEMLQAEKNPLIVQLADKVYAFGGWETRVTQEFDPALNEWRMRSRMPGGCCYGSAVALGDKIYV
VGGY
>Ctel_110271
MKEADEFVDITLVFGKQRIACHKVILAGMCDYFRRMFLTNMLERGSQEVVLNDISASTGVLLVEYLYSGNIDITQLNAQD

LLAASEMLLLGALKKKVEDFLLSHTDSVNCISIINLARLYDLKMLLTDARSYLQEHVKEVVESEEMHLLQEGDLIEVLEA
NASQEENFLFIQKWMKSAEGRTNRFDDLMQHVSLSKCSKDFVCSTVMEEKLMHNTRGITLIQEFMQSYGSTDPPKQPSLA
VGNLSGEMWLCTDLNTPQWQPIQQPPFQIKLYSACASPGGFVVSGGQSQNSNQRECYSYAAQNGQWNTLPPMLTARFSHS
SIYHNHHLYVVGGYDGSYLNSVDALDMRNLQWNHLPPLPRKVRFAYLAIVSDNLFVLGGLCGDGFYGWVADVHEFDSTQQ
TWRQRSPMPEICVGGAAVSFNDHVYVVGGRNRSCMRFNPRNNTWTSLQRPQFNHYFGPSLVLNGNIVVFGGFNDDSIEEY
SPLTDSWSTWTLKMPHKSQYWKFAVKMDS*
>Ctel_105762
MTQTISSLPAVFLQMKEADEFVDITLVFGKQRIACHKVILAGMCDYFRRMFLTNMLERGSQEVVLNDISASTGVLLVEYL
YSG
>Ctel_120168
MRHSDEMVDTVLVFGETRVKCHRLVLAATCEYFRRMLQADMQERGAGEISIKDVSSATGILLVDYLYTGNIQISVENAEA
ILAASDRFLLTKLKKIAEDFLCGQVGSTNCVSFRYFARLFSLNSLLEVTQKYLTDHWKEL
>Ctel_192804
MTDVTLTLPDQSAIPCHKVVLMTASPFFETMFQSGLKEGAEQNIKLDFADSYTIRSGKVCDLKVLLKTAFNFILLKFREF
REISYFDTLTEDELVEVVSCDRLNAENEDVVFEAVVHWVNADPDATEEIFPRIAPLIHFPFCSQKALTDKICRNPLMQKH
ALILPTKLSGRSFTSTASSNSQRQMHSSSRLSFAWIYAISLSIITESRMVLRLHSVTRSMWLGEKRKCATVMIPKTTNGK
SSQNQRMHEYVTNEATVWKGKILLGNAKYVEEYDSVKDRRSNRYEMLPDGGIRSMTLQASHTLRESITAKLVSS*
>Ctel_44497
FRRMREAGELIDIILVFGNKEVPCHKVILAGTCEYFKKIFLTSPNVSTRIPITGVNAQTGIAIVDYLYSGYIDITTDNAK
DLLQASKLFSLVGLIQIIETMLCDKLRPHFATQSFSSTDNCYSYDSRKKAWTTLPPMTVTRQQHGSTHHQGAILIAGGTD
GNGNGLYCKSVERFDLGTNQWSILPPLPHGVLVPSVVSLSNRLFVLGGHLGKSYSGAIYEFDFKRNVWLARASMPDESWC
ASAVAFDEYVYVINGFSYKNLRYDIHQDSWEEIQAPMFDHCVSPAVVWKGKIHTLGGHKEEEKNTMEVLDPQT
>Ctel_146749
MASLHKLQEKEVMTDVTLILPDQSAIPCHKVVLMAASPFFDTMFQSGLKEGAEQNIKLDFADSDIIKTLVEFFYTGDIEL
TEGNIKALV
>Ctel_63102
CHRLVLMAASPFFETMFRSELKESKQGEIDFDFLNAEIAGKVVDYFYSGEIDINSVNVSDLLEASEYLCLDDLKRHLCDF
MATKVDSTNCIELYRVSHKYGLDKLEPICFAYIVSAFHDVFSSPVSFEGLSEEEVVKVVSDDRLTAQNEDVVFHFVVRWV
NADLDKRKDIFPKVASFVRFPYCSQECLHQMTREPLMINSTCMDFLHEGLSLDVGSKRIELHSKQNARSVPRLGYDATVP
HLMQISVDIHSIRGQICIDESSHTWEWKHCFRAGNPSRQGELEVFFSPKAIHWREVNSCQSRNKSGQKSLSCWSMDTQQI
TKLPWVEPQIPDSTLMFVHEKIFSFGGTKNGQTCEMVESFDPTRRRWNAEPPMIHAVSKPIGVHFDARVYILGGTDADGP
TIFTQEFDPVLSTWRLRSSMPAVCQFGAAVSLNDKVFVVGGEERACFCYKPAMDTW
>Ctel_167175
MPIQEPFLFKDANRNACLLRGFKELHESSLLFDVTLIVDSKEFECHRSLLASSSDYFRCMFTSDLAERDQREVSISGVDA
TSMELVIKYMYSGEVKLQASTVQNLLSAANLFQLSDLKEGCAVFMGKKLDVDNCIGIHFFAQAHECGDLEFQAWDVITEH
FEEVAQCIEFLDLSAEKFVEIIQYDDLQASEEDVYEAALAWLQHSPEERTSNVYDIFRHVRFSLMDEHYFYDRVKNNTVL
QAEPRIAQMFDEVIRYKLLKNRWMEIDLRLEPRYGADFCRVAVYTAYLDDEKKQVLQLYGQRSDEVTRLHGNVLCDLPSD
IENPALLVTGNNDLYVAGGDQCSSAVYKWCSEDVATFDEIASMTIPRKRFCLVQLDKYIYALGGCNLEGNLSSVIRYSED
NDEWESVASMPKALRCFCAVAYCGKLYVFGGESEKVNKKVLKGRRELNNQTLCYDPRDDKWTTLAQMTIPRALSGCTVYK
NKIYVIGGFGSITESWIKDTSPDNVLDSVEVYDPVTNSWEVGVPLPIPLCAMGIFKYYGTIYVMGGENEEDVSNSVYYLS
DEGVWELKDEMVTGVTEITIHSSYAILNVDVDAD*
>Ctel_167525
MKHRSTVSLLSDAQGGSTRSKSSRSSSDSPSENSSSTTTCVADDQVQNPYDDHIYSEISSLSRTPLTFHSKTKRRLSWGD
AAAKHQPRRRDPSDAYASFLDQYPKLTNLDYVPWGEQEVLNILREGRAKSYSGHITVEMMQRLSVMLHRPLVRIAREAQR
FAAMFRRCTRHDLQSAMKVVLSRPLYDSCVQACMKAVALYNMKSGQYTKASKSQHCGLKFSVGRFHRWLLDAKISCRVQE
PAAIFLAACVENLLEEIVLRCLGSSVFSLEALEGGVSLNPDLWGLLQPCEHLVCGRISTGFSAPLSLPPPNNASPGTMSS
LATTAAAAYFEGIETTLLATCVGSVPELGNLVSQVMHHWQNVLAPSSTLRPALTWGADALHALFHYMKCSQMAPSSLQPK
LHQGDMKLCRERPYSLQPPLIEWVRTTLAHTEHRCALVVDGDDVTQAARVLLPSSDCPQRMFKPSVQCWRSTQQTSVQCQ
LQIQQDMAFSMLCIGRSDLLAYALSLLGPGGVDTFNDQGLTPLMCACLHGDEAMVQMLLDAGASVNMAVPSDGHKFPGLI
PDTKHWTALSFASLLSHVSIIQLLIEYGASVEGAGSDDDPLCETPLQIASSAGHYDVVMALLNYGADPYLTSTTSVLSSS
QSGHNAFTLAAMHGHRTIFKKLLANPTSKVDEALSLEEMLAEGQDCISDDYDCASQRRNPQDGCSPLRPFSKTQYKVLQE
AMYHAAEGGHLDIALELRSIGVNWSLHIWSQCLWAAHLLQRKTIIQCLLKDYLATGRHDEINIEFINECLPLMFHIFRNS
KNEVTSQQLANILSSLYGNQPVPPIPTLRGKTIARIDPFYVNNPEMSDIQFVVEGKVFHGHKILLVNASSHMRSLLEEAR
KHTKNSDPASVTKVEIHDIKFKTFEMMMMFLYHGTVDTQGVDTKELLDLLKASHTYKLDSLKRHSEILLSKHLNLDNSSA
VYKHSKVYHANELLAYSEGFFLQNLTHLLNQSDGFRKLLYSYKMLNYDLVNGLLATLCQSLEDKFSMQSKTSTLV*
>Ctel_219490
MGRKPKHSFKPFSRFLLDTLSCLRDEGLMFDIVLQCGETLFRAHRLLLAAYSPLFKQFLTSQVDNFSASKLNIIVPIQNV
GHMRLILDFLYTGTLDLSSNPDLQSLMLDVEKLQIPSLMFYIREIATEHGDGSQFVLKKSEIRANHSLVSEHFYRTDIEN
PVAKDKRAIAKKGWKRVEEEEEEEKEEAIEVVEESLPSAGRRTEMSLRRKQRKRSSNVLNCRYCSFTSMHQSSLSRHMHS
HERKVFRCPLCLKCFANKLNHAHLSCCRKNLKKKKGAHKLKVRPERLQFVDDFLSKRLECRLCCYEARSPVALLEHCITV
HTNFKMHLCVRCKTSFKAQSQREAHQKICGGTKVAPRDQFLFKRWSGCFQCSFCSFRSPRKGVVAQHLHTAHKTCMRCSS
CRKRFANEFDAQTHLKMHPGCHFCDCCGSMHRDRSSLWKHEMLTHGVRKQGFKVVKGLMSCSKCSKGYSSYERLRSHMDS

AHAPGINEEHICGICSYRAVKLTDLQLHLQETHANEGHLICRICKQRFPTQEEITEHVNSVHPGVEITSAESPLKRRKPA
SSSTEKKGLICSLCDEAFPSAELIKCHVEEAHPGVSYSLDPLPVPLRKKYEKRVLTEEYICGLCEQRFEKPEAITQHIQD
FHPGVQVDANTKKIISKQYNTLYQCCYCSYRHHIRSRLLRHEAFHTGNFPLKCKECGYGAFDHATMKRHFRRHNKPMDYM
CHECGKAYNTYSGYRLHVFAHRGEGVHKCPLCGKDFHSPSSKVAHMFDHEGKKAYGCHLCSFECKYPTPMRRHLLQEHNF
KAKKIFPRYHLLSFKARQRTKKFCPELVDQNSTDPIEETAMVEETAPSLEEGAMEGLNIDPVTMVLDMGGQEVVMVDEAT
LSGQSVIFIQDVAQ*
>Ctel_93209
MDFNDQTEFAKNVTHCRPHLSTDKRKIYEDSTRSGQVLSEMHRFYQDERLCDVNLIVDEEQFACHRLVLAASSLYFERMF
SNGMSEARAKDIRLRNLSPCALKHLIEFAYSSKLCVQKDTVLEIFEAADMLNFPAARMFCQDFLMDQIDINNCLSFITYA
DRFSCEPLYERAKSCAAANFQLICSTAEFLELPKNHLEELLRDDNIEMEYEEHIYEALKKWVLHDDSRLVNFADLFKCIR
LNFVSRWYLIEVISKDELIAQSAECSNIIQSA
>Ctel_85638
VSPFFESMFQSGLNERKSRDSPLGFADERTIRMLVKFFYSGEIDVNEENIQSSL
>Ctel_207879
MGTDITDPTLNNVVYLKAMLDSHMKTFMACLHKMKENDVMTDVTLKLPDGSSVSCHKIVLMASSPFFEVMFESEFKESTT
QDIPLDFADAVTIKTLVEFFYSGEIDVCDDNVQTLVAASEFLCMKDLKDHCDAFMATRVDTSNCIELRRFGKKFNLMRLS
SAAREFILLNFKDVVKCFSDFPTLTENELIEVVSSDELNAENEDLVFSAVVRWVNHDADERKEAFSRVAPLIXFPFCTHE
TLNDAIFVVGGKERSLFSYEPAKDTWTVLSPTQHAYEYCSATAWKGKILVMGRDHAEEYDVEGDTWIPMDKLVPGQTYSC
NADNFILRTFLI*
>Ctel_89939
MSLNFRNMRHADEMVDVVLVFGETRVKCHRLVLAAACEYFRLMFQTDMQEKAASEIAMKEVSSNTGLQLVEYLYSGNIEI
STENAQELLAASDRLLLVDLKKMVGVFLCGQADTNNCVSLLNLARLYRLESLGGISQQFLTDHWKELIETEEIYQLAEDD
LHLMVIGGMKSANFYLDLVECLDLETLEWSEFPHLPKALLLPSVVYVQKQLFVVGGEPSSTTTSGDVYEFDSAERAWQPR
CPMPQVYKGADAVSVDNKILVLGGVYRVCMQYDPCGDLWIQLQRSQFQHITGRAVVWKDKIVVCGGRDTDSIEEYDPCLD
KWSTWNLKMPFEDMCFALIIKGNV*
>Ctel_185452
MTLHWAKTTFEEPCIFCPPFRGFYHLPILVIQTQQSCHIQSDEKYFRDSLQKIKESNVMTDVTFVLPDDSSIQCHEVVLM
ASSPFFEKMFKSGLKEGSERNVNLDFAAANTIRTLVDFFYSGLILVTEENVKDLVETNEFLCCEHLMNSLQVLVLKMMGA
KLEWCPEKEIPHAVHDPCIVQFQNKVFVFWGIGINRPRMVTQEFDIAMDECRRKKKCLSMIQRTTNCLRKKAYQAVPQTF
PGFTQFVFNVTQVEAEWAFKFSDL*
>Ctel_213425
MNIKSPKLAQVRFHSGVDHFDIINFSRVRAVHKMISMKRSSMESCDVIPASFSQMREAEELTDVSLVFDGTRVPCHKVIL
AGTCEYFKRMFLANLSESTAAEISIGRMSSATGVFIVEYLYSRSREITVENAQEILEASSMLMLDRLKRKVEEFLIRKID
DQNCISLLNIGRLFELKALINTTRMYLTDTLEVAIDTMQMEILQEEDLVYILEHHTSPNVCFSVLQKWVRLAKGSAEQFM
ALLRHVDLNRCSKQFIQSTVMNEEMMKSTQGLLIAQNAAMHKSLIVGDFHQNIWIRDDRDVWKVISRHPMLACKYSACAS
PRGIVFSGGEVCGKPMSECYSFCTRERLWSRLPQMIKARHSHSSIYYKNYLYILGGKRMDQHSTASVEIHNLVTRHRRQL
TPLPESLHYCLVVSVSDRLFVLGGTHSQRGNSRNVYEYDSSQIKWVARASLPEDCNLASASAFEDKVMVVGGERMSCLMY
EPLADCWAMLNRPGLLHCLGASLEFRGQVVLLGGQCNDSIEGYCPGTDKWVKWKLKLPSAGCLCFALEM*
>Ctel_36920
MSNSLYQMRTEEEFVDVVLLFGERKFPCHKVVLASTCEYFHRMFLAGMQESSSAEIAINEIESDIGELIIGYLYSGTLDT
TEENAQRLLAASEMLLLGDLKEKLEEFLCDHIQAANCVSLLSLSRLYKLQELIKDAQNYLNSHCQEILNEGDLVELQEDD
LVGIL
>Ctel_63500
MREADEFVDITLVFGDRRISCHKVILAGTCDYFHCMFLTDMMESALKEIVMKDISASTGTLLVNYLYTGLIELTTHNAQD
LLVASEMLLLGALKQEIEEFLCRHIEGTNCISLLNFARLYGMKTLLEIARICLYDMDMKEFIDSEEMHLLQEEDFVELLL
LKPNDSQEDRFCLVQKWVRSGEGRAERFACLLEHVRLGECSKQFLRSTVMHEKLMHNTHGMTLIRQLMQTSLRAESRPRR
SLVRWQPMQQPPSQSAFYSVCSFPGGFVVSGGREESRNDIRCRGCYSYNINSNRWNTLPPMPVSRSSHASIYQNNHLYVV
GGMDDKSYMSSVNKLDLKSGEWSPLPSLPKPLQAPYVAFVSNRLFVLGGFNGSISLDVQEYDSRQNAWRGRRAMPERCVG
GAAVSFGDHIYVVGG
>Ctel_192349
MGTDKTDPTLNAMLDSHMKTFMACLHKMKENDVMTDVTLKLPDESSVSCHKIVLMASSPFFEAMFESEFKENTTQDIPLD
FADAVTIKTLVEFFYSGEIDVCDDNVQTLVAASEFLHTKDLKDHCDAFMATRVDTSNWIELSRFGKKFNLMRLISAAREF
ILLNFQDVVKCFSDFPTLTENELIEVVSSDELNAENEDLVFSAVVRWVNHDADERKEAFSRVAPLIRFPFCNHETLNYVV
SQEPLMGNSECMEFIMEAQNFNMHNHHYLYSPRTVPRRAFNGKRQRLVRFYRDYNGTQCEYADCIDGDSVKWKLLRGDHQ
NINQVIATPSGFIDLPVWGTCFIFTIHRDELIKVPSEMELNFNGQFIFFNGRIFAFSEDNFRASETEAEHTASVRSFRLD
DRTPKWKSEAPMLHALDSFSVVQFNNKIYVFAGRDADGLSTVTQEFDPGLGEWRLRSPMPGVCENHAAVVALNDKIFVVG
GKEQSLFSYEPAKDTWTVLSPTQHAYRYCSATAWKGKILVMGTEHAEEYDVEGDTWIPMDDLCCPTA*
>Ctel_101877
MLDSHMKTFMACLHKMRENDVMTDVTLKLPDGSSVSCHKIVLMASSPFFEVMFQSEFKESTTQDIPLDFADAVTIKTLVE
FFYSGEINVCDDNVQTLVAASEFLCMKDLKNHCDAFMATRVYTSNCIELSRFGKKFNLMRLISAARKFILLNFKDVVKRF
SDFPTLTENELIEVVSSDELNAGNEDLVFSAVVRWVNHDADERKEAFSRIAPLIRFPFCTHETLNDAVSQEPLMGNSECM
ELIREALYFNMHNHHYLYSPRTVPRRAFNGKRQRLVRFYKDYNGTHCEYADCIDGDSVQWKLLRGDHQNINQVIATPSGF

IDLPAGSTCFTFIIHREQLIKVPSEIKLNFQGQFFFFNGRIFAFHEDQFRAPVTTSAFGQFQAFATKWKSEAPMLHALNS
FSVVQFNNKIYVFAGRNADGLSTVTQEFDPGLGEWRLRSPMPGVCENRAAVVALNDKIFVVGGKERSLFSYEPAKDTWTV
LSPTQHAYEYCSATAWKGKILVMGTEHAEEYDIEGDTWIPMDKLMPGQTYRCNADNFILRTFLI*
>Ctel_89766
MWLEQKFCDTVLILDDGQMYAHKVVFAAHSQALAKDFQNFPPGKLLYIKLKDFDPSVVFDLTEYLYTGKLKLTEKNTAGL
YKCALELGITSLLQTVKQQFASYDVNNVFDHLLMCDQAGLHELRDDIMRYISQHFNEIVNTKGFLNLTLSYLLMLLSSDD
LCVMSELEVFYAVVKWVDFQRQDRMTHAEALLKCVRFRLLDPDILATKVETVDWLFRQRRCHEQLVEAYK*
>Ctel_199969
MTQTISSLPAVFLQMKNADEFVDITLVFGKQRIACHKVILAGMCDYFRRMFQTSMLERGSQELVLNDISASTGVLLVDYL
YSGNIVINQHNAQDLLAASEMLLLGALKKNVEDFLLSHTDSVNCISIINLARLYDLKKLLVDARNYLHEHVKEVIETEEM
HLLQEADLIEALEANASHEENFLFIQKWMRSADGRTDRFDDLMQHVSLSQCSKDFVCRTVMEEKLMLSTRGMKFLQQFMQ
SYGSADPPKQPSLAVGNEIGEMWLCTDLNTPQWQPIQQPELQNKYYSACASPGGFVVSGGQSQNGIQHECYSYDAQNGHW
NTLPPMPTARRWHSSIYHNHHLYVVAGCDRGDLNSVEALDMRSLQWNHLPPLPRKVRFAYLAIVSDNLFVLGGCYGDWVA
DVHEFDSTQQTWRQRSPMPEICVAGAAVSFNDHVYVVGGENRSCMRFNPRNNTWTSLQRPQFSHQYGPSLVLNGNIVVFG
GFNPISIEEYSPRTDSWSTCTLKMPPKSVTRSWAFAARMDS*
>Ctel_111686
MDTTTSYREDLMTSLQRLRKNHQMTDIDLKLPDRSTISCHKLVLMAASRFFETMFQSGMKESKQREVRVDFSDAVTIKMI
VDYFYSGEIDINVENVQDLVAASEFLCLVDLKRHLGAFMMDQIDAINCIDFYRYARKFSLDKLIKHSLEYIRSNFGQAFR
SSDAFIRLTQDELINVISDYRLEAESEDMVFHCVIRWVSADPKKRTDAFVKIASFVRFPFCTQELLDRLPREPLMMNSAC
LGILSEAMSVRLLGDDQSAYDPRYTGTQCIVRIQSTKCIFTQTPQWLDMHASSVMQSSVLCINCPVSGLFLSPSGIHWIN
EDGFHDKWLADSSKHSSFIWIPPNMTNYGLTFIQEKLFSFGGELNGVHCPIVQSLDQIESKWKPEPSMLQPCSNPYVVHF
ESKIYVLGGIDAYGASLFTQEFDPVWKTWQLKAEMPGVCSAGAAVSLNENIFVVGGTERVCFQFTPSTDTWAVLSRPIYA
HNETAAATVWKGHILLCGKYHGEDYNPKTDAWSACDALIQGNGYDPPSRMMLCTTFK*
>Ctel_73105
QMKDADEFVDITLVFGQQRIACHKVILAGMCDYFRRMFLTNMLEKGSQEVVLKDISASTGVLLVEYLYSGNIDITQLNAQ
DLLAASEMLLLGALKKKVEDFLLSHTDSVNCISIINLARLYDLKMLLADARSYLHEHVKEVVESEEMHLLQEGDLIEVLE
ANASQEENFLFIQKWMRSAEGRTDRFDDLMQHVSLSQCSKDFVCSTVMAEKLMHNTRGMTLIQEFIRLYRSADPPKQPAC
ASPGGFVVSGGASLNSNQRECYSYAAQNGQWNTLPPMLTARRWHSSIYHNYHLYVVGGYDGSPLNSVEALDMRNLQWNHL
PPLPREVRFAYLAIVSDNLFVLGGCYGDWVADVHEFDSTQQTWRQRSPMPEICEWGAAVSFDDHVYVVGGSERSCMRFNP
RNNTWTSLRRPQFSHKYGPSLVLNGNIVVFGGFNDDSIEEYSPRTDAW
>Ctel_205505
MAVLLTSEDLFELILISAPLCEKEVNVLKEMTEEVERCINFSEPWQYADITFLVEDKKIFASKMILCLWSPVFEAMFSHD
FKEKNASEITLPGKKFKDIEHLMKVLHPPNEEITDDNVSILLPLLQEYQIDAILKRAEDYLCSQCSSIKNFLLAQKFNLK
ELYESNLSYLKRAPIARLKSQPNFDEMDKEFLVELLMEKCLKFEQNLESLREVRMVLERKKPTTFPGMHLLCEDCTNARQ
QQVDCDGCMKSCCKKITEILRNLEK*
>Ctel_83392
KIPCHKVVLAGTCDYFRTMFVTELTESRSDEVQISEISADIGLTLVNYLYSGTIEITEQNAQALLAACNMLLLVDVKKDI
VKFLSRQICNSNCVSLMNLARFYELDSLRKAAHKFLTKHWTELIDTKEMGELQEDDFVELLKSLGSQEDRFRFLQNWIRL
GGSDKDDRFSDLIEHVTLSKCSKEFICNVVMEEERMNHPKGMKLIQKAMQALMLADQPTTSSLESLAQWRTLPNMNIPRF
GHSSIYHKDELYIVGGMESKHGYLDFVERLDAKSLKWEDLCDLPLGLSTPMLVIVKDKLFVLGGVKGGGHSKMVLVYHDD
AWEERNSMPEECRRGAAVEFDGFIFVVGGRNIGNRAKSQCFSYVAQVKQWRTLPNMRLPRYAHSSIYHKDMLYLVGGMVS
QNEYVNSVERLDTKLLNWVDLRDLPGVQGLSNALLVIVNDRLFVLGGLTRSCHNKMVMEYHFENDAWEERNSMPEECRRG
AAVEFDGFIFVVGGQEKSC
>Ctel_55892
LKQLNEMQKEQHLTDVVLYTGEKEIHCHRTVMAAASLFFKKMFTSGMKESREGEVHLQEIDFNALSLIVRASYGEKLKLN
GDNVQSILEASDRLEMREYIDDCIIYMCNHMDSDNCLDLYLISKKYSHDQLKRSVVEYLVFGDNFADCMTCSDS
>Ctel_227008
MDTRTSYKDAFMSSLRSLRDDGTCDVTMKLRNSHTIDCHRLVLMAASPFFKTMFRSGLRESTQREVDFDFPSSEIAVVII
DYFYSGEIDINSNNVQDLIAVSEYLCLDDLKKHLCAFMTTKVDSTNCMEFYRFSRKFNLGKLVTLCLEYILSHFDEAVTP
PKRFDELSEEELIEVVSDDRLTSKNEDIVYHSIVHWVNADLNTRKEVFPRLASLIRFPFCSAECLSSMTREALMINPTCM
EFLHEGLCFDVRSKRIELHFKQNARQLPREGYEATNPHLMQILMNESNLIGRTWTDVRGSTCVWDEFFNKRIQGKVEVFF
SPNAIHWKEARGSPTQSSREMLHTLHLETLRLNSTAWMEPHMSEYTLKFIRERIFAFGGTQYGQPCEMVEFLDQTGKWTP
ESPMLHPVSKPYVVHFDAKVYVLGGFGADGPSLLMQEFDPIWSTWQLKEPMPGACQSGAVLSLNDNIFVVGGSERACFIY
TPAADTWSVLSQPDHVHNISATAAALHGHIMLCGLSCAEMYNIKDDYWSPCNELLIPNTQRHHGGLALCTTRTTDLKPHA
SSSQSNVSHFFKTPRPEFVIIDSCSCLKDSNRDFVKMDTRTSYKDAFMSSLRSLRDDGTCDVTMKLRNSHTIDCHRLVLM
AASPFFKTMFRSGLRESTQREVDFDFPSSEIAVVIIDYFYSGEIDINSNNVQDLIAVSEYLCLDDLKKHLCAFMTTKVDS
TNCMEFYRFSRKFNLGKLVTLCLEYILSHFDEAVTPPKRFDELSEEELIEVVSDDRLTSKNEDIVYHSIVHWVNADLNTR
KEVFPRLASLIRFPFCSAECLSSMTREALMMNPTCMEFLHEGLCFDVRSKRIELHFKQNARQLPREGYEATNPHLMQILM
NESNLIGRTWTDVRGPTCGWDVRGSTCVWVECFNKPIQGKVEVFFSPNAIHWKEASGSRTLSSREMLHTLNLETLRLNST
AWMEPHMSEYTLKFIRERIFAFGGTQNGQPSEMVEFLDQTGKWTPESSMLHPVSKPYVVHFDAKVYVLGGFVADGPSLLM
QEFDPIWSTWQLKEPMPGACQSGAVLSLNDNIFVVGGSERACFMYTPAADTWSVLSQPAHVHNISATAAALHGHIMLCGL
SCAEMYNIKDDYWSPCNKLLIPNTQRHHGGLALCTTRTTDLKPHASSSQSNVSHFFKTPRPKSVIVDSCSCLKDPNPDDA

ECVIENNHVLPNLRAIQRKSIGHRPKKKTKKKTMKGCPSRLLDEN*
>Ctel_76876
MTDVTLILPDQSAIQCHKVVLMAASPFFETMFQSGLKEAAEKDIKLDFADSGTIRSLMKFFYTGDIKLTAENIKAIVAGS
EFLYCQHLKAHCEEFLMGNVGLSNCIDYYQFGKVFNLKVLIKTAFDFVLSKFREFREISNFDKLTEDELAEVVSCDRLNV
ENED
>Ctel_200443
MKESNSDEIEIKGIGDSTGLLVDYLYSETIQITDENAQALLAASDMLLLSDRKDHTKEYLCDHIEAKNCISLLKFSRLHE
LQDLIQVSMKFLTDYWQVVMLEGEIVNLQEDDLTDILSTCESEEDRFRLLQKWTKFNEERSKGFMGLVDLVKMSSFSKKI
INSTVLGRRANAELKRESNAFKRLYTQLIYRLKGDRASNDKKRKTTLLVIGKGTVFEFSVSQYLIVHSIFYYLHFFKSSV
VFTSQKHLDSENGYVKDDCIILEATIVADSPTDPCEVCPKSACIRTQMLGCGSV*
>Ctel_226967
MYIFSTRDPTRNKKARSCAERVSVSQKGTQVSDSSKAAISYGSADRYFLSVRRTTAEFNAGEKTRAVSDQLKTEMSGLNL
DSHVKAFMASLRKLQEKEVMTDVTLILPDQSAIPCHKVVLMAASPFFDTMFQSGLKEGAEQNIQLDFADSDIIKTLVEFF
YTGDIELTEGNIKEIVAGSDFLCCEHLEAHCEEYLIETVDLSNCISYYQLGKVFDLKLLINTAFDFIMFKFQKFREISDF
NILTEDELVEVVSCDRLNVENEDVVFEAVVYWVNADPDAREEVFPRIAPLIRFPFCSETALTENICCNTLMQTSGCIDFA
HEALWSQFHLNCRPPIHNARCIPRHAFYCLFQLIELKFSKQVALRVASYIDGSGIKWETVMPRIAQTCQKFITSPFGLYL
IDNNDCYFVEHKLRRVTPLPWKPVDLDSTLAFINGRIFAFGGWPASKSVKSINLKESNPEWKSEQDMLSGEYKPLIVQFA
NKVYAFGGWRTRVTQEFDPASNEWRMRSEMPWISYGTAVAFGNVAVALDDAAVALGDKIYIFGNHGYSYDPENDKWEVLS
KPTHAYSSLPSATVWNGKILLGNRDHAEEYDPVADRWSNRDELLPDWDINKMILHASCTLRGNE*
>Ctel_89870
MTQTISSLPAVFLQMKEADEFVDITLVFGMQRIACHKVILAGMCDYFRRMFLTNMLERGSQEVVLKDISANTGVLLVEYL
YSGNIDITQLNAQDLLAASEMLLLGALKKKVEDFLLSHTDSVNCISIINLARLYDLKILLADARSYLQEHVKEVVETEEM
HLLQEGDLIEVLEANASQEENFLFIQKWMRSVEGRTDRFEYLMQHVSLSQCSKDFVCNTVMAEKLMHNTRGMTLIQEFMR
SSGSADPPKQPSLAVGNDGGEIYSACASPGGFVVSGGRSQNGIQRECYSYAAQNGQWNTLPPMPSARRSHSSIYHNHHLY
VVGGWDDRSYLNSVEALDMRNLQWNHLPPLPREVYFAHLAIVSDNLFVLGGCCGLICFTDVHEFDSTQQTW
>Ctel_212216
MTQTISSLPAVFLQMKEADEFVDITLVFGKQRIACHKVILAGMCDYFRRMFLTNMLERGSQEVVLKDISASTGVLLVEYL
YSGNLDITQLNAQDLLAASEMLLLGALKKKVEDFLLSHTDSVSCISIIYLARLYDLKTLLADARSYLHKHVKEVVETEEM
HLLQEGDLIEVLEANASQEENFLFIQKWMKSAEGRTNRFEDLMQHVSLSQCSKDFVCRTVMAEKLMQNTRGVQLLQQFME
SYGSADPPKQSSLAVGNLSGEMWLCTDLNTPQWQPIQQPPFQIRSYSACASPGGFVVSGGASQNISQRECYSYYAQNGHW
NTLPPMPTARYNHSSIYHNHNLYVVGGFVNHRCLNSVDALDMRSLQWNHLPPLPREVSNAYLAIVSDNLFVLGGCNSDWN
CAADVHEIDSTQQRWRQRSLMPERCDGGAAVSFDDHVYVVGGRERSCMRFNPRNNTWTSLQRPQFKHIDGPSLVLNGNIV
VFGGQFNDSIEEYSPRTDSWSTWTLKMPQKYHDWAFAVRMDS*
>Ctel_89672
MREAEQLVDVHLVFDGTRVPCHKVILAGTCEYFKRMFLTNMSESVATEVSMRGISSTTGPLIIDYLYSKKTEITVDNAQG
LLEASGILMLDTLKQNVEKFLLEEIEVENCVPLLNLGRLYEMNLLLEATRKFLFDHLDEAIDTVEMKELREEDLVHVLRN
HASQETCFCMLQKWVRCTDGNADHFTELLQHVQLSECSKQFITSTVLGEEMMASAQGLQIIQRTLQAKDWKLIERCSRFT
SIYYSACASPEGIIFSGGDRDDKPRNQCFLFCAKEREWRTLPPMIKARYYHASIYHNSHLYIFGGRFTDTSYTASVEALN
MKTLEWSQLSSLPFALTDCHAVCASNKLFVLGGEDSSSTFSTEVHHYDSPLNTWRSCAPMPEDCYGGSAASFHDRVFVVG
SRNKSCMVYDPRVDSWTMLQRPQMEHMWGASLEWNEKIVILGGEVDSIEEYCPESDQWSKWDLKMPTQNTMCFALKI*
>Ctel_106678
MTSNTFCDITLVVDDVSFPCHKVILSAASTYFSAMFQSGMKEANADKVQLKFCEAETMKVLLEYFYTGSIAINEDNCQAL
IEAAGHLNLEDAKIDCETYMVSQIDSPNCIGFLRFAKAHDLKELLQTSQRHLLDNFEEVVMKSEEFVEMSEDELVDLISD
DRLNTQDEDLVFISVQKWVEASNIQRREIFPRIAKYIRFSFCSNDFLIRLMYQEGLMKNPECKDLLNRASYSVFVPEKK
>Ctel_196552
MESSLPAAFSEMREAEQLVDVHLVFDGTRVPCHKVILAGTCEYFKRMFLTEIRETEVSMKGISSTTGPLIVDYLYSKKTE
ITVDNAQGLLEASGILMLDKLKQNVEDFLLEKIEVENCVPLLNLGRLYELKLLLEASRKFLSDHLDEAIDTVEMKELREE
DLVHVLRNHASQETCFCMLQKWVRCTDGNAEQFTELLQNVQLSECSKQFITSTILGEKMMGSEQGLQIIQRTLQTGQQFH
QNKSLAIGDFEGKFWVRAESADKEDWKLIERCSLFTSIHYSACASPEEEYWINEDDDKEPDSDDDCDCDLELLRPPAVTT
LGLLSQATIKTWMIIEGTVSTGQCDTLKIEIAFSADGSNCLLRLIARQF
>Ctel_130853
MKEADEFVDITLVFGKQRIACHKVILAGMCDYFRRMFLTNMLERGFQEVVLKDISASTGVLLVEYLYSGNIDITQLNAQD
LLAASEMLLLGALKKKVEDFLLSHTDSVNCISIINLARLYDLKTLLADAQNYLHEHVKEVIETEEMHLLQEGDLIEILEA
NASQEENFLFIQKWMRSTDGRTERFDDLMQHVSLSKCSKDFVCRTVMAEKLMHNTRGMTLIQEFMESYRSADPPKQPSLA
VGNRSGKMWLCTDCNTPQWQPIQQPPFKIQDYSACASPGGFVVSGGLSQNDVQRECYSYDVQNGHWNTLPPMPTARIEHS
SIYHNHHLYVVGGCYRSWWHDGRPLNSVDALDMRNVQWNHLPPLPRKVSNAYLAIVSDNLFVLGGCNSDLNCVADVHEFD
STQQTWRQRSPMPEICAGGAAVSFNDHVYVVGGEDRSCMRFNPRNNTWTSLQRPQFKHYFGPSLVLNGNIVVIGGDGNDS
IEEYSPLTDSWSTWTLKMPKKGSIWRFAARMDS*
>Ctel_39183
SSHKIVLMASSPFFEAMFQSEFKESTTQDIPLDFADAVTIKTLVEFFYSGEIDVCDDNVQTLVAASEFLCMKDLKDHCDA
FMATRVDTSNCIELSRFGKKFNLMRLISAARKFILLNFKDVVKRFSDFPTLTENELIEVVSSDELNADNEDLVFSAVVRW
VNHDADERKEAFSRIAPLIRFPFCTHETLNDAVSQEPLMGNSECMELIREA

>Ctel_146450
MNVPHACYVVQEMNKQRLTNTTACDFALISNGQKFYAHKGVLVAVSPYFKTKFKDCYRDSSNSEEELPPVEGKHVQVFME
YVYTGSLFLDDAAIEDILTLADYLCVEDLKTKCGQYLLE
>Ctel_101946
MTQTISSLPAVFLQMKEADEFVDITLVFGKQRIACHKVILAGMCDYFRRMFLTNMLERGSQEVVLKDISASTGVLLVEYL
YSGNIDITQLNAQDLLAASEMLLLGALKKKVEDFLLSHTDSVNCISMINLARLYDLKTLLTHARNYLQEHVKEVVETEEM
HLLQEGDLIEVLEANVSQEENFLFIQKWMKSAEGRTDRFEDLMQHVSLSQCSKDFVCNTVMAEKLMHNTRGVQLLQQFMR
SSGSADPPKQPSLAVGNRSGEMWLCTDLNTPHWQPIQQPPFQIKYYSACASPGGFVVSGGASQNISQRECYSYEAHNGQW
NTLPPMPSARRSHSSIYHNHHLYVVGGYDGSYLNSVDALDMRNLQWNHLPPLPREVRLAYLAIVSDNLFVLGGFCGDWIA
DVHEFDSTQQTWRQRSPMPEICYGGAAVSFNDHVYVVGGRERSCMRFNPRNNTWTSLRRPQFNHRDGSSLVLNGNIVVFG
GQFNDSIEEYSPLTDSWSTWTVKMPKEGSVWRFAARMDL*
>Ctel_63864
MVDVVLVFEETRVKCHRLVLAASCEYFRRMFQTDMQERDAPEIPMKDVSSTTGLLLVEYIYSGNIEMSAENAQEFMAVSD
RLLLTKLKKNVEEFLCEHVEATNCVSLKNFARLYGLESLLEVTHRFLTNHWKKLIDMQSKIDELTEDDLITLLTTYGSDE
DSLLLLQKWVRSSHGRFDRFMDLLQSIERTLFSKEFIVTSSSDGFIVSGGLANKTPKRECYAYVTHTRQWRTLPPMSDGR
YYHSSICDQDEFIVIGGSIIEKYHFDSVQCLNLKTLEWGQFPDLPQARCLSNLLHVQNQLFVVGGYLTKTTTARDIYKFD
STEREWQTGSPLPEKCGSAGAVSFDNKIFVVGG
>Ctel_137808
METSLPAAFSEMREAEQLVDVHLVFDGTRVPCHKVILAGTCEYFKRMFLTNMSESVATEVSMKGISLTTGPLIIDYLYSK
KTEITVDNAQGLLEASGILMLDKLKQKVEKFLLEKIEVENCLPLLNLGRLYELKLLVEASRKFLSDHLDEAIDTVEMKEL
REEDLVHILRNHASQETCFCMLQKWVRCTDGKAEQFTELLHHVQLSECSKQFITSTVLGEEMMASAQGLQIIQRTLQTSQ
QFNQNKSLAIGDFEGNFWVRAESADKEDWKLIERCSRFTSDYYSACASPEGIIFSGRDREGKAKNQCFLFCAMEREWSIL
PPMIKARYYHASIYHNSHLYIFGGRFTETSYTASVEALNMKTLEWSQLSPLPLALTNCHAVCASNKLFVLGGTDSSCTSS
RGVHHYDSALNTWRSCTPTPEGCLDGSATSFHDRVFVVGGRSKSCMVYDPRVDSWTMLQRPQMEHMWGVSLEWNEKIVIL
GGEVDSIEEYCPESDQWSKWDLKMPTKNLMCFALKI*
>Ctel_89177
MASHPKTLLTSLQKIRDNGLMTDVTLKLPDDSTILCHKLILIAAIPYFEAMFQSGLKESQEQEVELKFSDADTIRMLVEY
IYSNEIEITEDNVETLVDASELLLLNDLKISCEDFFVEYTLSIEGESVKW*
>Ctel_38688
MRTANELVDVVLVFGELRLACHKVILASSSYYFRRMFSGGLRESQSDEVAIKGIDAKTGKLLVKYLYSGKIDITEENAQN
LLFASNMLLLDDLKAASEEFLCEHIQPSNCVSLLNLSHLYEIKDLVKRAQQFITDKWAEIS
>Ctel_38749
MREAGQFVDITLVFGEQRIACHKVILAGMCEYFHRMFLTNMLESESKEVVMEGISATIGCLLVDYIYTGCIKITTQNAQD
LLAASEMFFLGSLKLIVEEFLCTHTESTNCVSTINLARIYDMKTLLADAKKYLYEHVNEVIDTEEVHLLQEEDLVGVLEA
NGSQEDNFCFLQKWMR
>Ctel_214573
MTQTISSLPAVFFQMKEADEFVDITLVFGKQRIACHKVILAGMCDYFRRMFLTNMLERGSQEVVLNDISASTGVLLVNYL
YSGNIDITELNAQDLLAASEMLLLGALKKKVEEFLLSYIDSVNCISIINLARLYDLKILLADARNYLQEHVKEVVESEEM
HLLQEGDLIEVLEANVSQEENFLFIQKWMRSAEGRTDRFNDLMQHVSLSQCSKDFVYRTVMAEKLMHNTRGVQLLQQFMR
SYGSADPPKQPSLAVGNYEGEMWLCTDLNTPQWQPIQQSPFQIMRHSACVSPGGFVVSGGQSQNIIQRECYLYEAQNSQW
NTLPPMPTARRSHSSIYHNHHLYVVGGCDDRSELNSVDALDMRNLQWNHLPPLPRKVFFAYLAIVSDNLFVLGGCNDEWV
ADVHEFDSTQQTWRQRSPMPEICVVGAAVSFNDHVYVVGGENRSCMRFNPRSDTWTSLQRPQFNHWRGPSLVLNGNIVVF
GGFNDDSIEEYSPLTDSWSSWTLKMPQKPLGWAFAVRMDS*
>Ctel_130076
MFLTNMLERGSQEVVLNDISASTGVLLVEYLYSGNIDITQLNAQDLLAASDMLLLGALKKKVEDFLLSHTDSVNCI
>Ctel_140661
MTQTISSLPAVFLQMKEADEFVDITLVFGKQRIACHKVILAGMCDYFHRMFLTNMLERGSQEVVLKDISASTGVLLVEYL
YSGNIVINQHNAQDLLAASEMLLLGALKKKVEDFLLSHTDSVNCISIINLARFYDLKTLLTDARSYLHKHVKEVIETEEM
HLLQEGDLIEVLEANASQEENFLFFQKWMRSAEGRTDRFEDLMQHVSLSQCSKDFVCNTVMAEKLMHSTRGVTLIQEFMQ
SNGSADPSKQPLLAVGNRNGEMWLCTDLNTPQWQPIQQPPFKIQNYSACASPGGFVVSGGEFQNIIQRECYSYYAQNGQW
NTLPPMPTARTNHSSIYHNHHLYVVGGYDGSPLNSVDALDMRNLQWNHLPPLPREVYFAYLAIVSDNLFVLGGFCGDWVA
DVHEFDSTQQTWRQRSPMPEICEWGAAVSFNDHVYVVGGEDRRCMRFNPRNNTWTSLQRPQFDHGCGPSLVLNGNIVVFG
GFNDDFIEEYSPLTDSWSTWTLKMPKSWSIMRFAVRMDS*
>Ctel_132731
MKEADEFVDITLVFGKQRIACHKVILAGMCDYFRRMFLTNMLERGSQEVVLNDISASTGVLLVNYLYSGNIVITKLNAQD
LLAASEMLLLGALKKNVEDFLLSHTDSVNCISIINLARLYDLKTLLADARSYLHEHVKEVVETEEMHLLQEGDLIEVLEA
NASQEE
>Ctel_39952
MRQAEQLVDVHLVFDGTRVPCHKVILAGTCEYFNRMFLTKMSESAATEVSMKGISSTTGPLIIDYLYSKKTEITVDNAQG
LLEASGILMLDKLKQNVEKFLLGEIEVVNCVPLLNLGRLYELKLLLEASRKF
>Ctel_120272
MLLTPCLFCLQTPPLSIMTQTISSLPAVFLQMKEADEFVDITLVFGKQRIACHKVILAGMCDYFRRMFLTNMLERGSQEV

VLKDISASTGVLLVEYLYSGNIVITQLNAQDLLAASEMLLLGALKKNVEDFLLSHTDSVNCISIINLARLYDLKLLLADA
RSYLQEHVKEVIETEEMHLLQEGDLIEVLEANASQEENFLFIQKWMKSAEGRTDRFEDLMQHVSLSQCSKDFVCRTVMAE
KLMHNTRGMTLIQEFMESNGSADPPKQPSLAVGFVVSGGRSQNISQRECYSYDAQNGQWNTLPPMPTARRSHSSIYHNHH
LYVVGGYDGSYLNSVEALDMRNLQWNHLPPLPREVRFAYLAIVSDNLFVLGGF
>Ctel_141587
TYFRRMFLTNMLERGSQEVVLNDISASTGVLLVNYLYSGNIDITQLNAQDLLAASKMLLLGALKKKVEEFLLSNTDSVNC
ISIINLARLYDLKILLANARNYLHEHVKEVIETEEMHLLQERERERETSFISLEQVLHIQSDK*
>Ctel_48390
MKEADEFVDITLVFGKQRIACHKVILAGMCDYFRRMFLTNMLERGSQEVVLNDISASTGVLLVEYLYSGNIVITQLNAQD
MLAASEMLLLGALKKKVEEFLLSYIDSVNCISIINLARLYDLKILLADARNYLQEHMKEVVESEEMHLLQEGDLIEVLEA
NASQEENFLFIQKWMR
>Ctel_103978
MKEADEFVDITLVFGKQRIACHKVILAGMCDYFRRMFLTNMLERGSQEVVLKDISASTGVLLVEYLYSGNIDITQLNAQD
LLAASEMLLLGALKKKVEDFLLSHTDSVNCISIINLARLYDLKILLADARSYLQEHVKEVIETEEMHLLQEGDLIEVLEA
NVSQEENFLFIQKWMK
>Ctel_85541
ELVDIDLVFGDARFPCHKVVLASTSDYFRQQFSSNEVVMKGIDEETGKLIVNYLYTGEIDINDENVKNLLSASEMFQLND
LKAECEKFLCKHIQLSNCVSLLNLSHRYELKDLAEKSRQFVTENWTELPDDSIIELKKEDLESILIERLHENPENHFACL
QIWVKSDKERDEQFVELVQHVDLSRCSP
>Ctel_40445
IFCHKVILAGMCDYFHRMFLTGLFESGRQEVIMQEISAATGLLLTDYLYTGQIEITTLNVQDLLAASDMLLLGDLKQKVE
GFLCSHIEPKKCVSI
>Ctel_135854
MREAGELVDINLVFGDHIISCHKVILAGRCDYFHRMFLINMLEKNSTEVVMKEINARTGILLVRYLYTGLIEITTDNAQD
LLSACEMLLLGALKQDVEEFLCSHTESNNWYQL*
>Ctel_66668
DITLVFGKQRIACHKVILAGMCDYFRRMFLTNMLERGSQEVVLKDISASTGVLLVEYLYSGNIDITKLNAQDLLAASEIL
LLGALKKNVEDFLLSHTDSVNCISIINLARLYDLKTLLADARSYLHEHVKEVVETEEMHLLQEGDLIEVLEANASQEENF
LFIQKWMKSADARTDRFEDLMQHVSLSQCSKDFVCNTVMAEKLMHNTRGVQLLQQFMRSSGSADPPKQPSLAVGNDGGEM
WLCTDLNTPQWQPIQQPPFQIWCYSACASPGGFVVSGGESQNSNQRDCYSYHALNDQWNTLPPMPTARRSHSSIYHNHHL
YVVGGLDGCIIFNGRPLNSVEALDMRNLQWNHLPPLPREVCSAHLAIVSDNLFVLGGFFGGYCGDWVADVHEFDSTQQTW
RQRSPMPEICDGGAAVSFNDHVYVVGGSKRSCMRFNPRNNTWTSLQRSQFSHTCGPSLVLNGNIVVFGGSNHDSIEEYSP
LTDSWS
>Ctel_86950
IRMLVEYIYSNEIEITEDNVETLVDASELLLLEDLKISCEDFITKILNVSNCCQFCKLGRKYRIENLVANSLEFIASHFE
NVKSSNFNQLSEEDVVEVISDDRLTAKNEGVVFQVVARWVEADPEAR
>Ctel_203932
MYYKCDYFHRMFLTGLLESGRQEVIMQEISAATGLLLVDYLYTGQIEITTLNAQDLLAASDMLLLGDLKQKVEDFLCSHT
EPKNYAKKYFHEHLIDIFDEEEIHLLQEEDLFEVLDTNTSQEDNFCLT*
>Ctel_106365
IFCHKVILAGMCDYFHRMFLTGLFESGRQEVIMQEISAATGLLLTDYLYTGQIEITTLNAQDLLAASDMLLLGDLKQKVE
DFLCSHIEPKKCVSIINLARLYETKTLFADAKKYFHEHLIDIFDEKEIHVLQEEDLFEVLDTNTSQEDNFCLT*
>Ctel_111345
MASHLQSHQESFMNHLKLMKEDDIHTDVTLTLSDESSIKCHRLVLMASSRFFRAMFQPGSKEVAKKDVKLDFGDADSIRT
LVDAFYTGEIHATQENVKALVIACDFLSCDHLKAYCEEYLAAHLTSSNYIDLYNFGKLYNLQKVLSSAFGLILENFQEFT
KTPDFGQLSEDDLLEILTDDGLNAENEDVVFEAVLRWVHLDLDLRQRLFPKLAPHIRFPFCTTANLTHSICNDRLVLDSG
CIGLAVEALKSQ
>Ctel_131535
MKEADEFVDITLVFGKQRIACHKVILAGMCDYFRRMFLTNMLERGSQEVVLNDISASTGVLLVEYLYSGNIDITQLNAQD
LLAASEMLLLGALKKRVEDFLRSHTDSVNCISIINLARLYDLKTLL
>Ctel_118653
MTSYPKTLLTSLQKMRDNGLMTDVTLKLPDGATISCHKLILIAASQYFEGMFQSGFMESVEQEVELKFSDADTIRMLVEY
IYSNEIEITEDNVETLVDASELLLLEDLKINCGDFITKILNVSNCCQFCKLGRKYRVENLVANSLEFIASHFKKVESSNF
NQLAEEDVVEVISDDRLKAKNEEVVFEVVVGWVEADPEAR
>Ctel_41768
FVDITLVFGKQRIACHKVILAGMCDYFRRMFLTNMLERGSQEVVLNDISASTGVLLVNYLYSGNIVITQHNAQDLLAASE
MLLLGALKKKVEEFLLSHIDSVNCISIINLA
>Ctel_64361
QEKEVMTDVTLILPDQSAILCHKVVLMAASPFFETMFQSGLKEGAEQNIKLDFADSDIIKTLVEFFYTGDIELTEGNIKA
LVAGSEFLCCKHLKAHCEEYLVDTVDLSNCIDYYRYGQVFNLKLLIKTAFDFILFKFRDFREISDFDKLTEDELVEVVSC
DRLNAENEDVVFEAWKTEKEMLHALRQPYVVQHASKIYVFGGDQVLGYFSQASLVCQEFDPASNEWRLRSEMPGYCRDGA
AVALNDRIYVVGGRKRVCFSYDPANDEW

>Ctel_86006
LVEFFYTGNIELTEGNIKALVAGSDFLCCEHLKAHCEEYLVDTVGLSNCIDYYKYGRVFNLKLLIKTAFEFLLSKFREFK
EISDFDKLTEDELVEVVSCDRLNAENEDVVFEAVVHWVNADPDARK
>Ctel_187794
MSGLNLDSHLKAFVASLHKLQEKEVMTDVTLILPDQSAIPCHKVVLMAASPFFDTMFQSGLKEGAEQNIKLDFADSDIIK
ILVEFFYTGDIELTAENIKALVAGSEFLCCKHLRAHCEEYLVDTVDLSNCIDYYRYGQVFNLKLLIKTAFDFLLSKFREF
KEISDFNILTEDELVEVVSCDRLNAENEDVVFEAVVHWVNADPDARKEVFPRIAPLIRFPFCTQTALADKICCSSLMLKS
ECIQLAHEALLSQFHFNCGPPLDNARCIPRQAFHSTPSLYRLIQSNSPNIISLSVASFVDGSGIKWKTILPCITTTFARV
ITSPSGLYLINDNECYFVQPKLKRVTSFPWKPVNQYSALAFIDGKIFAFGDAPASKSVKSINLQELNPEWKSEQEMLHAV
DMPLIVQFANKVYAFGGYQTTVVTQEFDPALNEWRMRSQMPGPCYWGCAVALGDKIYVVGGREKIELLLSEKHLRSTDLP
KYRVMCQKMGLKYSAMQRFTPDSSWL*
>Ctel_218846
MAAAHANVRETDGAWSESSDCLTLDFGPFETVHRWKRMPECDVFVGARRSKHTVVAYKGALFVFGGDNGENMLNDLLRFD
VKDKSWGRAFTTGAPPAPRYHHSAVVHESSMFVFGGYTGDIHSNSNLTNKNDLFEYKFATGQWLEWKFEGRRPEPRAAHG
AAVYENELWIFAGYDGNARLNDMWTISLQGDAKRPRHWDEVCQIGDIPPTCCNFALTVARDSMFVFSGQSGAKITNDLFR
FHFKQKEWTRISTEHILRGAPPPPEKRYGHTMVSYDRHLFVFGGATGQTLPNELHCYDLDSQTWSIIEPSQDSQVPNGRL
FHASAVVQDAMYIFGGTVDSNVRSGENYRFQFSSYPKCTLHGDYGHLLDQKLFCDIDFIIGKEGEKIEAHIALVAARSAW
LRNRIRQALEQFKNQKVKSVDEEKEEKGSSLHPQVILPDADPRAFDLVLSYIYTDRIHPTKQGQDPNSNEVVLLMMDVYR
LAVQFQMSRLEHLCIQYLEAFIGHRNVLSALQNAAKLKLDYLKEYCLKFIVKDSNYNQIVMSKEFEFLEQPLMVQIIRRK
QLPPVRPLSDIHHIDLLSATLEKDLTHFLSSVGREFCDITLMLDNVPVPAHKAILAARCSYFEAMFRSFMPEDNTVKISI
GETVPSKQAFDSLLRYIYYGEVTMPPEDSLYLFSAPHFYGFTNHRLQAYCKHNLEMNVSFKNVVQILEAADRIQAHDMKK
HALSIIVHHYPKVAKLPQIRLLSRELLLDILDALAQDMALKESRYLSDISSLGDDSLSLNFHH*
>Ctel_74802
DITLVFGKQRIACHKVILAGMCDYFRRMFLTNMLERGSQEVVLKDTSASTGVLLVEYLYSGNIVITQQNAQDLLAASEML
LLGALKKKVEDFLLSHTDSVNCISIINLARLYDLKILLADARSYLHEHVKEVIETEEMHLLQEGDLIEVLEANASQEENF
LFIQKWMRSAEGRTNRFDDLMQHVSLSQCSKDFVCRTVMAEKLMHNTRGMTLIQEFMESSADLAVGNRSGEIWLCTDLNT
PQWQRIQQPPFQIKYYSACASPGGFVVSGGASQNIILRECYSYYAQNGQWNTLPPMPTARREHSSIYHNYHLYVVGGFVN
HRRLNSVEALDMRNLQWNHLPPLPREVDFASLAIVSDNLFVLGGCSGLNWVADVHEFDSTQQTWRQRSPMPEICGGGAAV
SFDDLVYVVGGRERSCMRFNPRNNTWTSLRRPQFSHKYGPSLVLNGNIVVFGGQFNDSIEEYSPLTDSW
>Ctel_96470
MTQAISSLPAVFLQMKEADEFVDITLVFGKQRIACHKVILAGMCDYFRRMFLTNMLERGSQEVVLKDISASTGVLLVEYL
YSGNIDITQLNAQDLLAASEMLLLGALKKNVEDFLLSHTDSVNCISIINLARLYDLKTLLADARSYLHEHVKEVVEAEEM
HLLQEGDLIEVLEANVSQEENFLFIQKWMKSAEGRTDRFDDLMQHVSLSQCSKDFVCNTVMAEKLMHNTRGVQLLQQFME
SYGSADPPKQPSLAVGNDDAEMWLCTDLNTPQWQLIQQPPFHIALYSACASPGGFVVSGGRSQNGIQRECYSYAAQNGQW
NTLPPMPTARFNHSSIYHNHHLYVVGGCDGYDIFDRSDLNSVEVLDMRNLQWNHLPPLPREVRLAYLAIVSDNLFVLGGF
CGLNWVADVHEFDSTQQTWRQRSPIPERCERGVAVSFNDLVYVVGGRNRSCMRFNPRNNTWTSLQRPQFSHWRGPSLVLN
GNIVVFGGQGNDSIEEYSPLTDSCPRGH*
>Ctel_151321
MIPVATRSLSFSNHAPRAFQVMHELLQRKELCDVTLQVGSREFRAHRIVLAGCCPYLRAMFTNGMLETGQNVVTLQDLDE
ELMETLLEYMYTGCVTISTTNVQSLLQGASLLHLTDLHRACCQFLQLHIDAANCLGIHAFADVYSCTELESVSRRYINQH
FSEVIHHEEFLNLPECRLVELLSSDHLQVSSEEQMFEAALMWVNWVPSERQALMCGLMKQVRLPLLSDEFLENVVLQTEI
VHGCPKCQSLVASAMHTKADPTSLSLISPRSQPQGIYVIGGRNSSDCQLKSMEKYDFLRDEWVTMGNMQIARTAVGAATL
NGLVMAVGGECALAEPQDETMYLRCVEAYNPRLKEWLPLADMKVARSFASVCVSGDYLYALGGEDRTSTFNVVEKYDYKA
NSWSFVSSMQRKRAGSGVAVCDGRIYMAGGYDKSFHTDRASVECYDPDTKEWTFVAEMEKARSGLTLVTLGHCIYALGGR
SRHNDMYYESVERYNTWTKQWSSVAPMNSPRAWPSVAVVDNRIYVIGGFDGSNRLRSVETYDPEHDSWTFIASLNMCRAG
SGATVL*
>Ctel_44314
SHLKAFMTSLHKLQENEVMTDVTLILPDQSAIQCHKVVLMAASPFFETMFQSGLKEAVEQDIKLDFADSGTIRSLVKFFY
TGDIKLTAENIKAIVAGSEFLYCQHLKAHCEEFLMGNVGLSNCIDYYQFGKVFNLEMLIKTTFDFILSKFGEFMEISNFD
KLTEDELAEIVSRDRLNAENEDVVFEAVVHWVNADPDVREEVFSRIAPLIRFPFCTQTMLTDKICCSSLVLKSECIQLAQ
EAVWSQFHLNCEWKSEQEMLQAEKNPLIVQLADKVYAFGGWETRVTQEFDPALNEWRMRSRMPGGCCYGSAVALGDKIYV
VGGY
>Ctel_90441
MREAGELVDINLVFGDHIISCHKVILAGRCDYFHHMFLTNMLERDSTEIVMREINTRTGILLVRYLYTGRIEITTDNAQD
LLSACEMLLLGALKQDIEAFLCSHTESNNCVSIMNLASLHDMKKMLGNAKKFLHEHMKEVVETDEIRLLQEADLIEVLGK
TLSQEDNFCFVQKWVKSADERAERFDDLLHHVTLSKCSDEFIRDTVMEERLMISENMWLCSDINLQTWQSLYAPPSQHRN
YSACASPGGFIISGGRLKGVCKSDCYSYDAQSGQWTTLPPMSIARRAHSSIYHNECLYIVGGRDGQNCLDSVEKLDMRSL
QWSRLPRLPRSACRLYLAIASNNLFALGGSSGGNWNVDVHEFDFTRQTWRQRSAMPEKCEFGAAVSFNDHVYIAGGKDRS
YMQFNPLQNTWILLQRPQLSHRFGKSLVWNESILVCGGLDTDVIEAYSLRENEWSTWTLKMPKKCEFIFALKM*
>Ctel_106809
MASYPKTLLTSLQKMRDNELMTDVTLKLPDDSTISCHKLILIAASPYFETMFQSGLKESQEQEVELKFSDADTIRMLVEY
IYSNEIEITEDNVETLVDASELLLLKDLKISCEDFFVKMLNSSNCSKLRLLGTKYDLRNLIASTFEFIALHFEDVVNSRD

FDQLAEEDVLEVISDDRLVAKNEGVVFEAVVRWVEADAEARTDVFTRMIAPRVRFPFCTESFLGRTV
>Ctel_220459
MAEQDTISAIRAAHAAITDIYQLPGGDEKLLEFYTDDALIIAPSFDTPVTKREHLKDFAKKRRTLGLVKATTEEYEIEEM
SDSCAYSATGVKFMNAQDCIMIQIKQCDDFHRMFLTDMTEKAPAEAVIKDINVSTGGMLLEYSYTSHIELTSQNAQDLLA
ASEMFLLGNLKQNIEEFLYGHTETTNCISHLNLARMYDLKTSQKTRGDI*
>Ctel_215644
MDTRTSYKDAFMSSLRSLRDDGTCDVTMKLRNSHTIDCHRLVLMAASPFFKTMFRSGLRESTQREVDFDFPSSEIAVVII
DYFYSGEIDINSNNVQDLIAVSEYLCLDDLKKHLCAFMTTKVDSTNCMEFYRFSRKFNLGKLVTLCLEYILSHFDEAVTP
PKRFDELSEEELIEVVSDDRLTSKNEDIVYHSIVHWVNADLNTRKEVFPRLASLIRFPFCSAECLSSMTREALMINPTCM
EFLHEDFDERVEPDWTNMDGRKGLYLCVG*
>Ctel_73812
DITLVFGKQRIACHKVILAGMCDYFRRMFLTNMLERGSQEVLLKDISASTGVLLVEYLYSGNIVITQQNAQDLLAASEML
LLGVLKKKVEEFLLNHTDSVNCISIINLARLYDLKTLLADARNYLQEHVKEVVESEEMHLLQEGDFTEALEANASQEENF
LFIQKWMRSADERTNRFDDLMQHVCLSKCSKDFVCSTVMEEKLMHNTRGMKLIQEFMQSYGSADPPKQPSLAVGNRSGEI
CVCASPGGFVVSGGQSQNAIQRECYSYDAQNGQWNTLPPMPTARIEHSSIYHNHNLYVVGGLVNHRPLNSVEVLDMRNLQ
WNHLPPLPREVCFAYLAIVSDNLFVLGGCNTDLNWVTDVHEFDSTQQTWRQRSPMPEICAGGAAVSFNDLVYIVGGNDRS
CMRFNPRNNTWTSLQRPQFSHYYGPSLVLNGNIVVFGGQFNDSIEEYSPLTDSW
>Ctel_46193
IPCHKLVLMAASPFFATMFQSDMKESKQPEVQLGFSDAATIKMLVDYFYSGNIVITSDNVMDLVAASEFLCLADLKKHLG
SFMIKTIKSANCIKLYMLSHKYSLGNVIPPCLKYILSHFSEVFRESDDFLNLSEEQLVTVLSDDGLVAQNEDFVFHSVDR
WVKENLENRKGKFVQMAALIRFPFFTQELLGHVES
>Ctel_92281
MKEADEFVDITLVFGKQRIACHKVILAGMCDYFRRMFLTNMLERGSQEFVLKDISASTGVLLVNYLYSGNIDITQLNAQD
LLAASEMLLLGALKKKVEDFLLSHTDSVNCISIINLARLYDLKTLLADARSYLQEHVKEVVETEEMHLLQEGDLIEILEA
NASQEENFLFIQMWMRSADGRTDRFNDLMQHVSLSQCSKDFVCRTVMAEKLMHNTRGMTLIQEFMESSRSTQTIVTGCG*
>Ctel_188904
MTQTISSLPAVFLQMKEADEFVDITLVFGKQRIACHKVILAGMCDYFRRMFLTNILERGSQEVVLNDISASTGVLLVEYL
YSGNIVITQHNAQDLLAASEMLLLGALKKKVEDGRTDRFDDLMQHVSLSKCSKDFVCSTVMAEKLMHNTRGVQLLQQFMR
SYRSADPPKQPSLAVGNYEGEVWLCTDLNTPQWQPIQQSPFQIMRHSACVSPGGFVVSGGQSQNIIQRECYLYEAQNSQW
NTLPPMPTARRSHSSIYHNHHLYVVGGCDDRSELNSVDALDMKNLQWNHLPPLPREVLNAYLAIVSGNLFVLGGCNSDWN
WVADVHEFESMQRTWCQRSPMPEICDGGAAVSFNDLVYVVGGSERSCMCFNPRDYTWTSLQRPQFNHWRGPSLVLNGNIV
VFGGFNDDSIEEYSPLTDSWSSWTLKMPQKPLGWAFAVRMDS*
>Ctel_93988
MKEADEFVDITLVFGKQRIACHKVILAGMCDYFRRMFLTNMLERGSQEVVLHDISASTGVLLVNYLYSGNIVITKLNAQD
LLAASEMLLLGALKKKVEDFLLSHTDSVSCISIINLARLYDLKTLLADARDYLHEHVKEVVEFEEMHLLQEGDLIEILEA
NTSQEENFLFIQKWMRSPEGRTNRFDDLMQYVNLSQCSKDFVCRTVMAEKLMHNTRGVQLLQQFMRSYGSADPPKQPSLA
VGNIDGEMWLCTDLNTPQWQPIQQSPFQIMRHSACASPGGFVVSGGQSQNGIQRECYSYAAQNGNWNTLSPMPTARYNHS
SIYHNHHLYVVGGYDGRRLNSVDALDMRNLQWNHLPPLPREVRFAYLAIVSDNLFVLGGCNTDLNWVADVHEFDSTQQTW
RQRSPMPEICKGGAAVSFNDHVYVVGG
>Ctel_63949
EFVDITLVFGKQRIACHKVILAGMCDYFRRMFLTNMLERGSQEVVLKDISASTGVLLVEYLYSGNIVITQLNAQDLLAAS
EMLLLGALKKNVEDFLLSHTDSVNCISIINLARLYDLKLLLADARSYLQEHVKEVIETEEMHLLQEGDLIEVLEANASQE
ENFLFIQKWMKSAEGRTDRFEDLMQHVSLSQCSKDFVCRTVMAEKLMHNTRGMTLIQEFMESNGSADPPKQPSLAVGNDV
AEMWLCTDLNTPQWQPTQQPPFQIKYYSACASPGGFVVSGGRSQNISQRECYSYDAQNGQWNTLPPMPTARRSHSSIYHN
HHLYVVGGYDGSYLNSVEALDMRNLQWNHLPPLPREVRFAYLAIVSDNLFVLGGFCDEWVADVHEFDSTQQTWRQRSPMP
EICRRGAAVSFDDHVYVVGGRERSCMRFNPRNNTWTFLQRPQFSHEYGPSLVLNGNIVVFRGSNDDSIEEYSPLTDSWS
>Ctel_99459
MTDVTLILPDQSAIPCHKVVLMAASPFFETMFQSGLKEGAEQNIKLDFADSDIIKILVEFIYSGDIELSAENIKALVAGS
EFLCCEHLKAHCEEYLVDTVGLSNCISFYQLSKVFNLKLLIKTAFDFILSKFKEFKGIADFDKLTEDELVEVVSCDRLNA
ENEDVVFEAVVHWVNADPDAREEVFPRIAPLIRFPFCSQTALTDNI
>Ctel_186627
MTQTISSLPAVFLQMKEADEFVDITLVFGKQRIACHKVILAGMCDYFRRMFLTNMLERGSQEVLLKDISASTGVLLVEYL
YSGNIDITKLNAQDLLAASEMLLLGALKKKVEEFLLSHTDSVNCISIINLARLYDLKILLADARSYLQEHVKEVVETEEM
HLLQEGDLIEVLEANASQEENFLFIQKWMRQHAALVWLRETAITALSSLVCHILQDCEAVN*
>Ctel_64557
LQSLKKQESAIDITLKLPDDTTIPCHKLVLMAASPFFATMFQSDMKESKQPKVQLGFSDAATIRMLVDYFYSGNIVITSD
NVMDLVAASEFLCLADLKKQLGSFMTETIESANCIKLYMLSHKYSLENLIPPCLKYILSHFSEVFRESDDFMNLSEEQLV
TVLSDDDLVAQNEDFVFHSVVRWVKADLENRKGKFVQMAALIRFPFSTQELLGHVSREPLTLNPTCQEIIREAVSAYTGR
KPHLVQAYAYQRTVQQQSPMPVYYGHQSQTQYISVTEQSVEIRSTVGINTQKTPWSNNLTASKTLSAVSKFFLSASGIHW
IDYNGSHNHWLADSGKNVIQFWVKPTRSDCTFSFIQERVYAFGGKVGGIDCTNVESLDQSKKQWKPESPMLQAVSTPYVV
HFDSKIYLLGGVDSKGPALFTQEFDPVWGKWQLKKEMPGICQLGAAISLNDDIFVLGGSENVCFKYTPATDTWLILSQ
>Ctel_47904

HKVVLMAASPFFEAMFQSGLEESAEKNINLDFADSDIIRSLVKFFYTGDIKLTAENIKGIIAGNEFLCCKHLKAHCEEFL
TGNVGLSNCIDYYRFGKVFNLKVLIKTAFDFILAKFRDYREISDFDKLTEDELVDVVSCDRLNAENEDVVFEAVVHWVNA
NLDVREEVFPRIAPLIRFPFCTQTMLSDKICCSSLV
>Ctel_167969
MAEENKASDLVLEVAGNKIHCHKVIMAAASPFFKVMLTSGLKESIEGHVKLPGARYDVIRKIVDAVYGQDFVLDSDNVQD
ALMACHQYGFTLIQERCVQQMLPLVNNENCLGVYILGKKYDIAELKAKAAPHFCDICAERSSNLSHLSAHDWDFIIKRTK
FSPSLFQLLIYWVHFDRYARLVALDSIIQQVAITKCDLLRDSLFREICEREKCLLLEANVL*
>Ctel_94441
MEAHDLFISCRKGDLTRVQFLVDEKEVEVNIRDKWDSTPLYYACLCGHTAVAEFLLAHGAKCEMNTFDGERCLYGALNDK
IRNLLRDYKAVTASSMRRGLYSEFLRNMLERGLYSDITFIIHGVSFSAHKCILASRSEYFADLFGTRWKDKRVVSLSHRM
LLPNAFESVLQYLYTARLESLIEDLEDVLRLAKHCHLEKLQHDLEAKMKAVLHFKSTKPGVNVTVLSLEPKANDFTLNDD
LGSLLLQSLPPAFCCQDAAELPFLPERTENFADIIFQVENHKFECHKSFFLLRSDYFKALVNDHFGECMEDETTSLPIIK
IQDFSPSTFVQIISYVYQDSCELTEKNIFDILYASDLYLLPGLKKQCSNLLAQYIDLTNIVQMLRTAKLFSLSRLEDQCA
EFIAENLDNVLADPNFADYVNEDANEVKDRQETDTISIIDEVRYYVSSFVQSYSEVDEANRKMLLIDWFLEEQGLDA*
>Ctel_189617
MYQEDCALPDTFLQMREAEVLVDVTLIFDHWHVKCHKVILAGMTKFFHSMFVVDMLERNSNEVVMSSIKASTGKLLIDYM
YGATIEFTPKNAQDLFIASDMLMLDNLKNRVVDYLCEKADYTNCVSILNLARFHKVDLLAERAKLVLLDYVEEVDEQELD
LLKEDDLIDILTTTANYYHEDSFRLVQRWAKSGEKRSNRFCPLMDYVRLKQCRKEFIENVIAADPRMLEGLCYRRLQEVR
NTVPPEQFVVGDDDGNMWMCSVGSEQWQLMRRPPARYELCSACIFPLGFIISGGLYDNVYQTHCHAYHAKYNRWDKLPPM
TNPRSNHASVYHNNCLYIIGGVDGEYAYVKSFEALDMRTLQWMDLAPTPIRLRDPIVVVESDHIFVLGGYLECDYTPCSR
DVCEYFDSKWEVKACSMPEDCDAGAAVCFDKHIYVVGGRNETCMRINPLLDQTWVDLDRPQFNHFYGPALVWDDKIIVCG
GDDVDAIEEYSPQDDSWSIWKLKMPKKGCMRFALRIALPL*
>Ctel_99345
MATLHKLQEKEVMTDVTLTLPDQSAIPCHKVVLMAASPFFETMFQSGLKEGAEQNIKLDFADSDTIKTLVKFMYTGDIKL
TAENAKEIVAGSEFLCCKHLNAHCEEYLADTVDCLNCIDYYRLGKVFNLKLLIKTAFDFILFKFKEFKGIANFDKLTEDE
LVEVVSCDQLNVENEDVFHFNCEWKSEQEMLCGVHRPHIVQFANKVYAFGGWKTRVTQEFDPALNEWRMRSQMPGRCCYG
AAVAFGDKIYVVGGEEKVCYSYDPENDEWKVLSKPTHAYHYSFPFATVWQGKILLGNKEHVEEYDPVEDRWSNRDELLPD
GDINEMILHASCTL*
>Ctel_97200
MREAEEFVDITLLFGEGHVKCHKVIFMILAGRSDYFHRMFLTSMVESALNEVPIEEISASTGVLLVDYLYTGRIKLTTRN
AQDLLVASEMLLLGALKQNIEEFLCEQIESSNCISLLNFARM*
>Ctel_90447
VVNYLYSGNIDVTQLNAQDLLAASEMLLLGALKKKVEEFLLSNTDSVNCISIINLARLYDLKTLLADARSYLQEHVKEVV
ETEEMHLLQEGDLIEVLEANDSQEDNFLFIQK*
>Ctel_86318
EFFYTGDIKLTAENIKAIVAGSEFLYCQHLKAHCEEFLMGNVGLSNCIDYYQLGKIFNLKVLIKTAFDFILSKFREFREI
SNFDKLTEDELAEVVSCDRLNAENEDVVFEAVVHWVNADPDVRE
>Ctel_221817
MDFSKFRDTGELSDINVVVDGKEFLLHKFPLFIKSDFFRALARSPKGESIKLDDFPGGPEVFAEVANYFYNVKLTLNKTN
VCQLRCAAEFLQMTSPGNLSSLADRVLQDTLTSAKLGHKFDIITDLIAHCRSLGAIAEQAGVVEKCINAIVDCWLISPKF
SRRYTLDGKFSNDDESIKLLSEFPLDWCKITCVTAREKSIRPTVLANLIQSYISHAVDSMFGDKSEKTSESVTPEDEAEK
EEVKGNDDQPKSNEDIKQETAKETDKTTESKTQIEDAQMPDNPDLDLGKVMDTLLFEVPLDSPVMVESFCHSWVIEMLKV
AQDIGCDSKKVLLILASRMMYRYSKDDLAGLPADILVQIIEEGCKDNRANPDDVCRVLYGYLMDQAAKDKLSLQTFQKLV
KAIPKNSRVSDDMLFTVLETLLKSKNISVDDKQKQELLETVDFSRLSECTLQCAYDSGLVPGAYVTKAALSLCTKLRSDL
DAAKVIIRDQELRLERYGITLGAGTSWLKSVEAGSAGRQKAKSTDFPDLC*
>Ctel_208218
MRPSKDCPLKAFNFEASGHSASVLTGLNALREKDQLFDVTLVADGQSFGAHRVALAASSDYFRAMFTDAMLECKQTQIEL
NGVGALGLKHILDYAYTSKLTLTLGNIQDILRAASHLQLLPVFKHFHLFLCMWRPYGGSIFKMPYFSIDVLGS*
>Ctel_125917
MASLHKLQEKEFMTDVTLILPDQSAIPCHKVVLMAASPFFDTMFQSGLKEGAEQNIKLDFADSDIIKTLVEFFYTGDIEL
TEGNIKALV
>Ctel_105049
TNMLERGSQEVVLNDISASTGVLLVDYLYSGNIDITQLNAQDLLAASEMLLLGALKKKSEDFLLSHTDSVNCISIINLAR
LYDLKILLADARNYSKTPLTRI*
>Ctel_81337
MLESGSQEVVLNDISASTGVLLVDYLYSGNIDITQFNAQDLLAASEMLLLGALKKKAEDFLLSHTDSVNCISIINLARLY
DLKILLADARNYLHEHVKEV
>Ctel_81395
SLQKMRDNGLMTDVTLKLPDDSTIPCHKLILIAASQYFEHMFQSGFKESVEQEVELKFSDADTIRMLVEYIYSNEIEITE
DNVETLVDASELLLLLEDLKISCEDFITKILNVSNCCQFCKLGRKYRIENLVANSLEFIASHFENVKSSNFNQLSEEDVVE
VISDDRLTAKNEGVVF
>Ctel_51488

EVMTDVTLILPDQSVIPCHKVVLMAASPFFDTMFQSGLKEGAEQNIKLDFADSDIIKILVEFFYTGDIELTEGNIKALVA
GSEFLCCKHLKAHCEEYLVDTVDLSNCI
>Ctel_51700
LMETKNYASKFMQTLNETRSSKQLVDVTLVAEGGDISAHRLILSLSSEYFRCMFSANMKESSTSEVPMVGCDYDTVKIIV
DFFYTG
>Ctel_177411
MREAELLVDVTLVFGHRRVPCHKVILAGTCEYFNCMFATGMVECASKEIVMEGINASTGVLIIDFLYGRCITITTQNAQD
LLAASDMLLLYALKQNIEEYLFCNTEISNCISLLNLARIFYLKRLLEVAQNYLQEHVCEFINTKEMHLLQEEDIIKMLAA
NRIQEDSFCLVQKWVRSVEGRIDLFSSLMEHIRLSQCSKEFIRSTVMKEELMTDELSLRLLHPPEPQSLVPIKKPPFLIA
TECHSACAFPGGFIISGGYTRDIIDCHECYSFKSHNSQWRKLPPMGTARRSHASVYHDHHLYVVGGRDTSEESTLKSVEM
LDMRSLQWSYLPPLPTSHADFLYAAFVSDRLYVFGGITDEYASDPLFREVLEYNPSQRTWRLMSPMPMDCSWGGAVSLDN
RIYVTGGYEGSCMQFNPRNDTWAVLQGLTLDYHSAPLVWRGSIVICGGCDDSSIHEYSPLNDEWTEWRLRSPRMHGSDIS
FALSLNIPS*
>Ctel_54206
LVDVRLVFGDRRVPCHRVVLAGTCEYFKRMFLTKMNEQSSEEVTIKEISSMSGVLVVDYLYSRTVNITVDNAQGLLEASS
MLLLDELKHKVEEFLVRQIEAQSCVSLLNLARLFELKALIEASLKFLTDNLDKAFDEIEMKELQEEDLVHVLKNHASQRV
CFCALQKWVRCTGGDTDQFMALLQHVDLSRCSKQFILTNVLSKELLNCALFCTKNREWRILPSITIGRYLHSSIYHKGHL
YIVGGRVSETSCSDSVESLDIESLQWRQLPPLPVHVYLCLMVSVSDRLFVLGGAASKQSSSRTVYEYSASQSSWRCCAPI
PGDCHLASATSFHDQVFVIGGYDKSCMVYEPRVDCWGMLQRPQRRHSLGVTLEWNGKIIVLGGEGDDSIEEYCPQTNQW
>Ctel_64689
LQENEVMTDVTLILPDQSAIQCHKVVLMAASSFFENMFQSGLKEGTEKDIKLDFADSSTIRSLVKFVYTGDIKLTAENIK
AIVAGSEFLCCKHLKAYCEEFLMGNVGLSNCIDYYQFGKVFNLKVLIKTAFDFILSKFREFREISDFDKLTEVELAEIFA
FGGDPASKSVKSINLKESNPEWKSEQEMLHELVDPLIVQFASKVYAFGRWNTMVTQEFDPALNKWRMRSQMPGCCCGSAV
ALGDNIYVVGGSQRVCFSYDP
>Ctel_197724
MDETKVSTTNNALMDNTSMLRVTAQKPPKKVAAGKADSAMTQAISSLSVVFLQMKEADEFVDITLIFGKQRITCHKVILA
GMCDYFHRMFLTNMLERGSQEVVLKDISASTGVLLVDYLEMLLLGALKKNVEDFLQSHTDSVNCISILNLARYYDLKTLV
AYVRNYLQAHVKEVMETKEMHLLQEIDLIEVLEANASQEENFLFIQKWTRSADGRTNRFDDLMQYFSLSKCNKDFVWSTV
LEEKQLKFSTRGKKLIQQFMQSYGSANLLKQPSLAVGDEDGEMWLCTDLNTLQWQPLQQPSFQIKYYSACASPGGFVVSG
GRSQNGFNQRECYSYDAKNDNLFVLGGCNDEWVADVHEFDLTQQSWRQQSPMPDICKGGAAVCFNDHVYVVGGRNRSCMR
FNPRTNTWPSLQRPPSRHNYGSSLVLNGNIVVFGDLNHEFIEEYSLPTDSWSKWTLKMPQKSKGWAFASRHYIVEGCV*
>Ctel_211658
MTQTISSLPAVFLQMKEADEFVDITLVFGKQRIACHKVILAGMCDYFRRMFLTNMLERGSQEVVLKDISASTGVLLVEYL
YSGNIDITQLNAQDLLAASEMLLLGALKKKVEDFLLSHTDSVNCISIINLARLYDLKMLLAEARSYLHEHVKEVVEFEEM
HLLQEEDLIEVLEANASQEENFLFIQKWMRSAEGRTDRFEDLMQHVNLSQCSKDFVCRTVMAEKLMHNTRGVQLLQQFME
SYGSADPPKQPSLAVGNDGGKMWLCTDLNTPQWQPIQQPPFQIKWYSACASPGGFVVSGGESQNSNQRECYSYAAQNGQW
NTLPPMPTARREHSSIYHNYHLYVIGGWDNFSDLNSVEALDMRNLQWNHLPPLPREVCFAYLAIVSDNLFVLGGYCCGWV
ADVHEFDSTQQTWRQRSPMPEICGGGAAVSFNDHVYVVGGRERSCMRFNPRNNTWTSLQRPQFSHEYGPSLILNGNIVVF
GGDDDSIEEYSPLTDSWSTWTLKMPKRGSFWTFAARMDS*
>Ctel_227326
MHVLMYTKRLLYVAIESMMHVHQKSCLSQLSARTTAAVSKSERIINGLSVEGKMIGLNLDSHLKAFMATLQKLQENKVMT
DVTLILPDQSAVPCHKVILMAASPFFETIFKSGLKEGAEQNIKLDFADSDTIKTLVKFIYSGDIKLTAENAKEIVAGSEF
LCCNHLKAHCEEYLVDTVDFSNCIDYYRYGKLFNLKLLIKTAFDFIPANFEEFRRIANFDKLTEDEFVDVLSCDCLNAKN
EDVVFEAVVHWVNADRDAREEVFPRIAPLIRFPFCTQTALTNKICRNTLMQTSGCIDFAHEALWSQFHFNCGPSLDNARC
IPRQAFNSSASLFQLIESKSPNKVSLSVSSFTDASGIKWELVLPYITRTFDRIITSPSGLYLINKNDCYFVEPKLKRVTP
FPWKPVDQDSALAFIDGKIFAFGRTSAENSESGISVERLASKFSFGSSVSPKSTFGSETCISIERRLESKLVESLNLEEL
NPEWKSEQKMLHAVVRPHIVQFSNKVYAFGGSNEKVTQEFDPALNEWRMRSEMPGRCYYGAVVALGDKIYVVGGSEKVCF
SYDPENDEWKVLSKPTHGYYGIFPFATVWKGKILLGDKEHVEEYDPVEDRWSNRDELLPDGDIDKMILHASCTF*
>Ctel_64582
VSCHKIVLMASSPFFEVMFQSEFKESTTQDIPLDFADAVTIKTLVEFFYSGEIDVCDDNVQTLVAASEFLHTKDLKDHCD
AFMATRVDTSNWIELSRFGKKFNLMRLISAAREFILLNFQDVVKCFSDFPTLTENELIEVVSSDELNADNEDLVFSAVVR
WVNHDADERKEAFSRVAPLIRFPFCTHETLNNVVSQEPLMGNSECMELIMEALYFNMHNHHYLYSPRTVPRRAFNGKRQP
FATKVAGIPVQSIRLDDRTPKWKSEAPMLHSLNSFSVVQFNNKIYVFAGRNADGLSTVTQEFDPGLGEWRLRSPMPGVCE
YRAAVVALNDKIFVVGGKERSLFSYEPAKDTW
>Ctel_64789
SHLKAFVASLHKLQENEVMTDVTLILPDQSAIQCHRVVLMAASPFFETMFQSGLKEGTEQDIKLDFADTGTIRSLVKFFY
TGDIKLTAENIKAIVAGSEFLCCEHLKAHCEEFLMGNVGLSSCIDNYQFGKVFNLKVLIKTAFDFILSKFREYREISNFD
KLTEDELAEVVSCDRLNVENEDVVFEAVVHWVNADPDVREEVFPRIAPLIRFPFCTKTMLSDKICCSSLVLKSECIQLAH
EAVWSQFHLNCRPPSDNARCIPRPSFHSNRSLFRIVETKSANKVSLSVRVTTLPWRPVDLDGSTLAFIDGKIFAFGGGPP
SKSVKSINLKESNPEWKSEQEMLQAEKNPLIVQFADKVYAFGGWGTKVTQEFDPALNEWRMRSQMPGRCYGGSTVALGDK
IYVVGGSQRVCYSYDPNNDEW
>Ctel_136065

MRWWTLCWCLRKHESSAIDLCWLQHANISVACSKLTCKREVQARLSIKDVSSATGILLVDYLYTGNIQMSIENAEAILAA
SDRFLLTKLKKIAEDFLCGQVGSTNCVSFRYLARLFSLNSLLEVTQKYLTDHSKELIDTESEMNQLTQDDLIALLGTHGS
DEASFLLLQKWSTSFKERTPRFMDLLRSLELRLFTKEFVLRTVMREKLMHNPKGMKIIQQALESLVIADPNQFLVVGDFN
RNIYEGRHFHASIGNQDEFMVIGGETSDNLYLKSAECLNMKTLEWSQMPDLPDALSLSQLVYVQNQLFVLYEQRYSVYVH
YYDHTGRAWRPRCQMPEVCVGGGAVSFDNKIFVVGGKSRSCMQYDPHTDAWVKLQRPQLESIFSPAVVWKGKILVCGGRQ
ADSIEKYDPQSDEWAVWDVKVPTKQDMCFALLIKEHC*
>Ctel_176531
MEPKSFMEAKNNVICEMRPANELVDIDLVFGDARFPCHKLLLASTSDYFRQQFSSNEVVMKGIDEETGKLIVNYLYTGEI
DINDENVKNLLSASEMLQLNNLKAECEKFLCKHIQLSNCVSLLNLSHRYELKDLAEKSRQFVTENWTELPDDSIIELKKE
DLESILIERLYENPENHFACLQIWVKSDKERDEQFVELVQHVDLSRCSPNFITSTVMDEKRMHNTKGMKLIQKAQEAEAK
RQAAQEKEAEAKRQAAQAREAEAERQAAQAWEAKAKRLAAQAREAEAKLQAAQRSGICITM*
>Ctel_116194
MSATFRNMRHSDEMVDVVLVFEETRVKCHRLVLAASCDYFRRMFQTDMQERDAGEISIKDVSSTTGLLLVEYLYSGNIQI
SAENAQELLAVSDRLLLTKLKKNVEKFLCGQVASTNCVSFKNLARLYGLDTLIKVTHGYLNNHWQKLIDMESEIDELTED
DLIALLTTYGSDEGSFLLLQKWAKQWRTLPPMNERRYYHSSICNQDEFMVIGGRINEDIYLDSVECLNLKTLKWSQFPDL
PEAQCLNNAVCIQKQLFVLGGWISITTVTRDVYKFDSAERAWQALSPLPETCEGVSAVSFASKIFVIGGQFRRCMQYDPC
TDIWVQLQRPQFEHIYGPALAWRDKIVLCGGKETDSIEEYDPQSNKWSTWNLKMPNEDYFCFALTIKRHK*
>Ctel_126136
MQKEQHLTDVVLYTGEKEIHCHRTVMAAASLFFKKMFTSGMKESREGEVHLQEIDFNALSLIVRASYGEKLKLNGDNVQS
IFEASDRLEMTEYVHDCINYLCNHMDSDNCLDL
>Ctel_110621
MSQVILAGMCDYFRRMFLTNVLERGSQEVVLNDISASTGVLLVNYLYSGNIDITQLNAQDLLAASKMLLLGALKKKVEDF
LLSHTDSVNCISIINLARLYDLKLLLADARNYLHEHVKEIIETEEMHLLQEGDLIEVLEANASQEENFLFIQKWMKSVEG
RTDRFEDWMQHVSLSQCSKDFVCSTVIAEKLMHNTRCLSSRVHAVKRISRSTQTTVTGCG*
>Ctel_119714
MRAATELVDVILVFGEAKIPCHKVVLAGTCDFFRAMFVTEMKESRSAEVQMSEISADIGLTLVNYLYSGTIEITEQNAQA
LLAACNMLLLTNVKKDIAKFLSHKIDESNCISLMNLARFYDLESLRKEAHKFITKHWKELIDTKEM
>Ctel_73742
VFLQMKEADEFVDITLVFGKQRIACHKVILAGMCDYFRRMFLTNMLERGSQEVVLNDISASTGVLLVEYLYSGNIDITQL
NAQDLLAASEMLLLGALKKKVEDFLLSHTDSVNCISIINLARLYDLKMLLADARNYLQEHVKEVIETEEMHLLQEGDLIE
VLEANASQEENFLFIQKWMRSAEGRTDRFEDLMQHVSLSQCSKDFVCRTVMAEKLMHNTRGMTLIQEFMQSYGSADPPKQ
PSLAVGNYEGEMWLCTDLNTPQWQPIQQPPFQIKYYSVCASPGGFVVSGGQSQNAIQRECYSYDAQNGQWNTLPPMLTAR
KWHSSIYHNHHLYVVGGCDSCDMFDCGFLNSVDALDMRSLQWNHLPPLPREVANAYLAIVSDNLFVLGGFCDNGLYDDWF
ADVHEFDSTQQTWRQRSPMPEICDGGAAVSFNDHVYVVGGRNRSCMRFNPRNNTWTSLRRPQFSHEYGPSLVLNGNIVVF
GGDGNDSIEEYSPLTDSWS
>Ctel_85072
DDSTISCHKLILIAASPYFETMFQSGLKESQEQEVELKFSDADTIRMLVEYIYSNEIEITEDNVETLVDASELLLLNDLK
ISCEDFFVKMLNSSNCSKLRLLGTEYDLRNLIAATFEFIALHFEDVVDSRDFAQLAEEDVLEVISDDRLVAKNEGVVFEA
VVRWVEADLEARTEVFTRKIAPRVRFPFCTESFLGRTVCCHHLMWNPDCMELVKEAQHSQFHTGKSPVQNLRTAPRHNGE
KLVRLYNGENNSICVEYTLSIEGESVKWKQLSSNLIDKGLLIFVNGQIFSICSCDGSVYSLHIADPNAEWYETVKFNYRN
TNWLDVINVSDKVYFVGNTMEMFDPVNNSKTTCKAMPGACTSGRAAALGGKLFVVGGTEKVCFRYDPATDEWTTLSRPTT
TSANYKATAWKGKIMLYDTEANKAEVYDPVFDRWTL
>Ctel_85897
LKAFMASLHKLQEKEVMTDVTLILPDQSAIPCHKVVLMAASPFFETMFQSGLKEGAEQNIKLDFADSDIIKMLVEFFYTG
YIELTDGNIKAVVAGSEFLFCKHLKAHCEEYLVDTVDLSNCIDYYRYGQVFNLKLLIKTAFDFLLSKFREFREISDFDKL
TEVELVEVVSCDQLNVENEDVVFEAVVHWVNADSDAREEVFPRIAPLIRFPFCSQTAFTDNICCNTLMQ
>Ctel_104961
MIAHLPHSQLLFRYYACLCGHTAVAEFLLAHGAKCEMNTFDGERCLYGALNDKIRNLLRDYKAVTASSMRRGLYSEFLRN
MLERGLYSDITFIIHGVSFSAHKCILASRSEYFADLFGTRWKDKRVVSLSHRMLLPNAFESVLQYLYTARL
>Ctel_226377
MEKNMTFLQTMSASLNEMRTAEEFVDVVMVFEERRIPCHRVVLASTCEYFHRMFLAGMKESSSDEVTLNEIDGDTGDLLI
GYLYSGMIEITQENALNLLAASDMLLLCDLKAKIQAFLCCNIHAKNCVSFLNASRLYKLQDLIKDSQKFLNTHCKDVTRK
GDLVELQEDDLVGILNFDPSEDSFRILRKWVQAVDGRDGRFMELVKNVDLSRFSKEFLCYDVMGERLMRNEAGMELIQEA
MKSILLCEYRTEVTFRYTVHNISSLSRNSVFSPEYYMRNLRWSVLLGTDTKESMPKSLAVFVQCKSDSSSALSCKVSFEL
RLLKQKADGAAYTKTTTQLFEHNGSKWGYSFFISWDRRIPCHRVVLASTCEYFHRMFLAGMKESSSDEVTLNEIDGDTGD
LLIGYLYSGVIEITQENALNLLAASDMLLLCDLKAKIQAFLCCNIHAKNCVSFLNASRLYKLQDLIKDSQRFLNTHCKDV
TLEGDLVELQEDDLVGILNFDPSEDSFRILRKWVHWAALQSHRRITIVIHNHNARKWVQAVDGRDDCFMELVKNVDLSRF
SKEFLCGDVMDDRLMQNVTGFKLILDAMKSILLCENRTEVTFRYTVHNISSLSPTFVSSPEYYMRNLRWSVYLRQRRVCL
SDTKESKPESLAVYVQCKSDSSSTLSCEVSFELGLLKQKADGAAYTKKNSWIPKIVMSKMIPS*
>Ctel_188501
MEKNMTFLQTMSASMNEMRTAEEFVDVVLVFEERRIPCHRVVLASTCKYFHRMFLTGMKESSSDEVTLNEIDGDTGELLI
GYLYSGTIEITQENALNLLAASDMLLLCDLKAKIQAFLCCNTHAKNCVSFLNVSRLYKLQDLIKDSQKFLNTHCKDVTLE

GDLVELQEDDLVGILNFDPSEDSFRILRKWVQAVDGRDDRFMELVKNVDLSRFSKEFLCDDVMGERLMRNEAGMELIQEV
KPFLILMPYITANSYNK*
>Ctel_192304
MSEYFRRMFLVDMVESGAQEVSMDAISLNTGELLVHYMYTGHIEITTQNAQDLLEASDMLLLSALKQEIEEFLFCHIDSG
NCISMMNLARLYGTEAVLRDARKFLSELAKEVIATEEIHLLQEEDLIETLQANSSQENNFCFLQNTVMDEKLMHNTSGVR
LIQQAMQSPRSTYPPQQQALAVGDRDGDMWLCSDTNNAHWQPIQKPPTECWHYSACASPGGIVVSGGLCNNIRQRDCYSY
DACDAQWNTLPPMSHARASHSSIHHNHHLYIVGGQDGQDSLNSVETLDMNTLQWSHLPPLPVGIQLCYLAIVSNSLFVLG
GCNRGWSADVHEFNITQQTWRSRSPMPERGDGGAAVQFDGHVFVVGGTTGTCMRCDPRSNQWQSLHRLKFNHIWRPSLVW
DGNIVICGGRNNDTIESYSPITDEWVALSLKMPMIGFMTFAVPPPPAPAYISIWLYNC*
>Ctel_194386
MYHPIHLCQGGSFIKIFSVYEAFKSMLKNRYVETPPLSIMTQAISSLPAVFLQMKEADEFVDITLVFGKQRIACHKVILA
GMCDYFRRMFLTNMLERGSQEVLLKDISASTGVLLVEYLYSGNIVITQXNAQDLLAASEMLLLGVLKKKSFMNRPTMFNG
ITRAYGL*
>Ctel_202593
MVLTPKESGPNLYLLVMQTLQMTAQVAQFWKQLDFTFSRRKCVHKIRPHFERPEANNQKHIFKRLQSMREEEVLTDVVFV
SERIVCHKIVIDPFFPCIKGILTDKSSSEMEIDIVNFEILSHFVDLLYTPKIAWSSKTAELGTNLPSLLDVRRSELIDIL
AMEINEAVCGVYAAFNMTLKWVECSPKWRKLHLLYVLKKLGLLNGVSKFQLKHLQCESLLDNEEGHQMILEALGVQSRQP
TRNMFQNKEDDLNNLEVFQHLFYRLQGMRTQNESNLVLEVDGKQIYCHKEIMAAASTFFTIMLTLDMKESREGRVEVKGT
NFNTMNFIVNAIYCDELNLDGENVHDALLACTLYEMEALISECVATMIQCMNCDNCIDIYLLGKNYNIEQLNDGVADFLG
KTRFMKKTYIMIVLESDLKNFICKINPVTQDLCSNLLNWVKTAGANNMECFRSIIENWRNKDIDTLRMAVRMAMEGTMTC
RPKDINILISECVSTIVMQMDDDDSINTKRLNEEVAHFLGKKLIPKTDLLRVSKSDWEIFVSETNRVTFGYSRMLDILEE
KKLTEKYSFDIDLYGDCSENIVYYSPGSYESDLGGYYDEFETKPDKETTDDIREKVIPNEIRVFTKVITTGFVCLRKAEY
KAKASKGIELQEMTGDYSNTWKATLMRPVTNDLNNPNASPPQQFYVLNQQNDTPIGTLR*
>Ctel_204662
TPPLSIMTRAISSLPAVFLQMKEADEFVDITLVFGKQRIACHKLNYLYSGNIDITQLNAQDLLAASEMLLLGALKKRVED
FLRSHTDSVNCISIINLARLYDLKTLLADARNYLYEHVKEVVETEEMHLLQEGDLIEVLEANASQEENFLFIQKWMRSSE
GRTDRFEDLMQHNTIKEEKAQAQVVITNAKEEDSDLQFMSSLCEIISHKSMPKNVSRMGTKLADRVRPMVVTFDFNFDAR
TFMSRFDYSKSDDDVPTPIKILPYRTREDQTMYKKKLEMARELNKKAKDESPNISFSLRNNGCISKYIKDADCKKWRWDL
EWSDLEDRDNTKPKESMSGNDYGTAQHNTHPLMEDDLKKRWHSAS*
>Ctel_208565
MGKSRSYHDGLSAYLKKDFQWDDWSHRTTPPRVTLILPDKSTLTCLESALTNASNFFKAEVTSNPGQEIKLNFSDSTTIR
TVVDYLNNNTIIISDENFEKLLVASDFINLTNLKGHLSSFWRDTIDSTNCIEFYKLARKHKAQYFYHKSLEFILSNFIEV
CCSSDNIEELSEEEMIEVVSDHRLAVDDEDIVHDTVV
>Lgig_63855
SNSNHGDMILAGLQILYESGELTDVTLQSPDSQLCCHRNVLAACSPYFRAMFTMKLRETVEQTVELHDIGPGILPNIVRF
IYTGQVSINHSNIQSLLIAANMLQLDSLSKLCIDYASSKLDIENCVDVYNLADFVNLSDLKALSKKFIYENFDKISETNA
LCNASSNFMIEMLSSNDIITEKEETVYNLLEKWIVCDHDKRSTFFPLLFQYVRLPLLDGSFISKTLACSELVTSNERCCE
LLNLATHAKGDGNSNQNAELRKGMCNRPLLIFSGGSISERSRSFTCFDTETHKSYIGVEQHPTFDAKFKIEHFQLIVGTD
NSILFLGGAWKEALQFEGIEALNDVLKYSQRDRKWIYCAPMTHGRCSFACCAYEDRIYVFGGKDRRHNGHVLDQSEFYAV
ESDHWQTIQSMPVGISHQAVTVWKNYIYTFGGIDDENAYLNTVLVYSVFRDTWSLVPTELSRPRADCQAFTFKDTIFVLG
GADKHENQTNIDVYDPEKNRWSEGQIFPDDRKIMAITKYKDAIYVC
>Lgig_73790
SILEHFNQCRSRNLYTDVRIKVQDQIFHCHKILLDASCPYFKSSFSFSEHKEEPNNLIEIGDFSAVAFNELLNYIYTAQL
TITADTVVEILQCADFLGFRSIVKSCSGFLLQHINSENCLKAFQMADCYSLHHLAEQTWNFILENFDAVSQSEDFCEITY
ACLLKILSSDFLKVFLEIDLLSVIDKWVSYHSERKKHLQDLIKCVRIEHSTLSSLDHKILSSVEFEKDVKIKPRLNKVIV
LVSRDTNPLHPRNSVKSDIIWYNQDYKCWDTLTSFPFDHRQLYGVAVVNNNIYITGGVGNEVPLFDTNKIVYNECWEFNV
KLSKWKEIFPMNFARFNHATSGFLNSVFVVGVRINNYEDAQYYDPEDNSWHELSALSRHIQS
>Lgig_82061
ECGPRCRSHQHALEINAAITKGGLREFKAYTSLCSNATNVNDKFGRNVLHMAASCGKLDIIEYLLEEKEYNLKAKDYESG
WTALHRALFYGQLAAARLLIQYKHDIRTRDFEGLSPLDIILIDRPSYISFSPREPNEVYTWGDNSNFNLGHSSQDRRIIP
ERVGVFKRISGFIIQVVMCKFHTVFLSQIGQVYSCGHGHGGRLGHSDQQTHVVPKLIEGIKKENCIQVAAAQDHTLFLTD
KGIVYGCGSNVNHQLGFTDPVLEVSFTPRMINQEVIKGYNIMGICAGRYHSVVYSRDAVFTFGLNAGQLGEVLLTLGDKY
QIHPRMISSFNESDINIVQVKCSDSTTVCLTSLGDIYSCSRYTCRKIGSRWLDIKTITVSGGTLDHLVTTDRDLLKEKGG
SELIVAMVTNKGMVFMWREKSPHFKRCNWMIRRELSICDVYLNTNNMGIITDKGEGFLAIWSAVSKDKKKQKMANKYKQV
NDEFPTVRLLDFILKNDVEDIQIERIPNIHRATNIVSDSKGRNFAALQVLPNGCLTEIPYIRESSLRKDMMCLFNQSSES
DVIHDVIIQCGNKRWAAHRYILASRSEKFDKMIKDTLTDNNDQTPIFDLSDIHPVIIEEILLFLYTDTCTALT
>Lgig_82889
LSSMETNDVANGSHRLEITSGISELWRKRLFTDLVLVVDGKYLRCHRFVLAAISPYFHGRLFNGQRESVTEKLFVNAASV
KIVHQVLDFIYTGSCKLTAKNSEELLKTASLFGIKSLRDKCDTFLSDSVGMNNCLKMMKLATETKALKLESKSRSAILNN
FITLYQTTAFKALSASDVISLIQDNDLRVPNEDVVCDAVLSWANSDPKLRKSALADIFKHIRLPHVQQHYMKYVLGNNEL
VKENVDCKDIIDEAKTIHQDPGRRHEVNLPRLEYRNDARMDDVIVILGGTASGGAKNHLRQNVSGFNMSLRKWFPLAPLP
YGYDSGTTACVCGNDIYISGGGGSRQGLTLYESCVNTWHICAPMLVGKRGHAMVGFGSSLFILGGVIGQSDDGHLRVTTS

VEEYDMKGNDWKKCGNLNEAVWGMSCAVHDGKIFLFGGYKDRHHSTQTIQIYDPATNSSINIGRLPI
>Lgig_92273
LSAKEKKKKASKFATLRKKLTRARRHSRSLDYGKSLKELLSTWGIRDISSLVHEYEALAAVKELAIAANLARPVANSLTQ
DLSNLYDYKFCTDVDLIYKGACFPAHRSILAIRSPYFRNILTRYPDYGCRVPIKLKTHGVDVQLFSAILRFLYTDELNTQ
ELSTESNEILSKLAEEFGTPNPLNHDMKNLLDNGDFSDAVLVFSSDNEHGDPLGTVDTAQSMHAGSSRLTKLEIPCHKAI
LAARSPFFRNLIIRRARSGEELTERALRAPSRIILDETVISRRYARVLLTAIYQDCVDLSCIVRGSSSMCSLSEIQAMVA
SGKYHMTLCDEAMEIFQIGQFLDFPVLSQGCEDMIVESINTENLVSILSWSSEAHGSMWVHRQALHYLTEEYLQIAHSPV
LFELSKNYLIEALSSDFLQAGELDVLSSILKWGEHQLIKRIEEREPNLLSHTAHSVSKKGVKKRDLNDVELREILANLLP
LIRMDHVIPYSNDILNGAIKRGLVSTPPSHMLGDDIPSNRMCAWVRGRNNGIFVRPRLFTPYYDEAKAILEEQLSQAQDI
DTGRVRMIHMSAIPDTLYMVEDRPFSLPYPTTPASPVDIIAGTIPVPDRSTMILMMHREQEFRKSKLAARAYSLPCADKR
MVTYNIQLRVVREFGLPDSTVEVLQ
>Lgig_93877
GGLTDLWNNAKHTDVRIYVEDRLFACHRVVLASMSAYFDAMFTSDMKETREGVVYLKNMKAHVFDMILKYIYKGETSEMF
PEFAVDVLYASSLLQIVSLQELCEKYFSDNLTLNSCLEIWRLSCTMHCINLKDNAWTFFMDNFVEVSHTDEFLEMSKDEL
LLAINDDNMNVENEELVCDAVLRWFEHDRKRVYYLLEIFHNLRLALMSYAYIDCITRRKAYIADNSECSELLEQMKHVSA
RAEIVYGQPHIFREHGKAACTLSNDIYVSGGTSSFGSAETRFVRFETKRNRWEYCLAMREPRCYHELVTVGHYIYTIGGF
APEDAVSNVERYDTIQKEWTRIGNLAIPVGGMSATALNGIIFVFGG
>Lgig_103262
MSRKPSNTDSFDTRPFKGDNVSHFVESYPQFLLKNLCQLWNDGLYCDVSIVVCEVTFNAHRHILAASSSFFKAMFMSGMT
EEKQREITLHGVDKEILQGLLSFIYSGIIEITLDNVQELMATSDMLGLTDVTEACGQFLQQHLNCDNCIGIYLFCEAHAC
YDIKRAAESFIRKHFLQVVHCEEFLQLPKETLIHFLSSENLRIENETQAFNAAIRWIQHDLIARRREVFDILVPIRFPII
SDKFLEKFIEECEDISLKIAIQKIIQDYKMSRKMCLELQLSKINPMMLQPRLSARKNIYIIGGYCRKRGDRWSDMHTLRS
VERFDTFHKQWQSLPCMRNPRSGLGAAVLDGKIYVAGGENDSLIHDSVEIFDPDEKKWTKLGTMTDPRCGLGLCAYRNNL
YAFGGWIGSVIGDTVEKYNADIDHWCIVGKLPTPRFGMAVIEHQGLIYVIGGLSEMGTELKFIDSFNPVTKEWMRLSNMP
SRKAYIGAAAIDDYIYVAGGWNEHEGPLNTVQKYSLSLDEWSVVPPLSMKRAGPSVIGVNGLLYVIGGRYYPDQYSAPIT
TDSMECYDPSSNKWVQMSNMPSSRCEAGAVVL*
>Lgig_109572
MNTLSSGLERLMSSHAHSDTTIDVEGRSFKCHRAILAAMSPYFDAMFSSGMRESITGDVSIHGISADIFGSIVNYIYSGY
DIVNEENALQVLHASALLQIECLMKRSEDFVCRKITDANCVEAYRKAMVLNCAQLKQKSKERILSNFVSLCESSEFYQMD
ADEMAMVVKEDNLECPSEEYVCDRVVDWVEYDLENREKDLKNIFTHLRLSLVKSEYIVDVLEKRDFIKKNADCVKIIDEA
KNFQLLPSRRHDFSSVYKLYRKTSTKEDVLVLISGGETPKPPYVRSKDVLAYSSQKHAWFHLAQLPYDPGIEFATCVYDN
DIFVSGGGARPRCFLRYRAKKNKWSQRGPELNFGRRRHAMVAMGSRLYVVGGFNSERPEGDKVLDVIEEYTLEDNAWREV
AKLAVPVSSVSAAALKDKIFIFGGETNTKSDTNSIQCYDTNLKCTTIIGELPMTCKLTRSVVCDDQIYIVLFDGQILRFC
DGENSELVSRIPKFERVHFGLLHLQGYLYIMGGQYVSGPDSRALCDLIGRFDTRTNTYCQLSERLPSARLLDSCAKISVE
KKFLIEEHLDE*
>Lgig_113379
MAGDRQIFTDSHAKSILTSMNNLRKINTLCDVTLIVQNQCFSAHRIVLAACSDYFCAMFTNKMKERDKSIIELHDISAPV
LEVLLDFVYTETVNVSVENVQELLPAACLLQLTGVKEACCTFLEKQLDSSNCLGIKIFSESHSCDSLCQAAESYSLRHFE
DVVTQDEFKSLSIDELERLISSDELQVNSEEPVFEAVITWVKFNEVERVPYLSNLLQFVRLPLLSARYITDYIDNEALVK
SCHKCRDLVDEAKKFHLRPDLRGQMNGPRMKTRIGTDDILVVVGGFGSHQNLVDTVEQYNPRTQSWSRLPDLTKRRRYVS
SASVNGCVYIFGGYDGQSRLNTVECLDISDEKPMWVTVTNMNHRRGLAGVCVYRDHIYVCGGFDGYSRHTSMERYNPATN
QWTMLSGMAKGREGAGLVEAGDMIYCIGGYDGIHLLHSAERYDPQTEQWSTISPMSTQRSGAGVSVVNDIIYVCGGYDGN
EHLSTVECYNTNTGHWTSLQHMIVPRCYVGACVLQGNLMVVAGYDGNLLLNSVELYNPLIETWEVLDGSMATPRCDAGVC
VVRVP*
>Lgig_114310
MSLSLRRIRFQASDNESSPGSGDESPSSSVVRTCQEKTKRDIGGPCDTNCHIDNTNPKNPTLTSKNHCCKLLQGLRSLRM
TDTLCDYSVIAEGQTIKVHRAVMAACSDYFRVMLTSDMKESREACVELKGVTASGLSFVVDFAYTGKLELTLENVEDVLS
AATHLQVADAVELCSRFIEIAITFENCVDVLNLAELYNLSATCMKARQYMLENFEIVAASEQYFKLTPIQLASMLAENKL
CVESEFKLFQLVLSWIKHDLNSRRENIALLMKNIRIPLLSGEELVEKVSREELMKTSQECVDLITEAKDYHIVVCKQPLL
QNTRTQVRSERRSLIMCHGHSVETYTFKTKKLGYLKESPVPLYNPAVVVVDNFMYVCGGKYDNNENNEIATARCFRYDPR
FDSWYETAPMNESRKDFVMVAMDGYLYSIAGQDENMVMCTVERYNIVTNDWDMRASINHSVYGHAGAVCNGLIFISGGQR
FSGCCKDVLCYTPSTDSWEVKAPLLNARCTHVMACVQDHLYVFGGNIEDNYGFPVPIIAIEIYHPETDQWTLCARTLNIR
EAGACVMDDRIYIVGGINGEHYYSDLLEEYDYRTDTLSAEEKFPTRIFGRACCILTLPQYVC*
>Lgig_114716
MLAGIRTLRDRCQLFDMILNVEDIALPVHRVVLASCSDYFRAMFTDGLKESKEENIKLNGVTAMGMRNLIDFAYTSKINI
DHENVYDVLAAANHVQILPVISACEDFLKSHLDLDNCIDIMNVAELYCLKNLYQVAQQFMCRNWSIFSLSNDFLRLNFKE
FQTLLSSKFPVDCDEKSILLSVLTWAEYDSQNRNSLFVELFKFIEFENIRREDIEEIQSTPVWKTVESNEVCLSRYLNQL
DKSVVSKDWLKCSGISNLRGFQHTLIVAGGFSNEGGLTNSVWYLDQSSGNLKHLTKIPHVDQCNFGMGVLNNKLYAIGGC
FNDQMQELIHPFGFCYDSQRDTWESIAPMSQERCNFYLGVLEGRFFAIGGDPHATAGAADMALCEAYDAETNTWVDIEPM
PGNRMQHTGASLGRRLYISGGLQEQDGDVFDDLLWYDLDTNLWQTGSSMRTPRADHSMFVYNDKLYVVGGWFYDTHTQQR
VIASAIDSYNPELDCWETVATLNECRLFATYTVYDGCIYVVGGWKGGNYRKKCNTIDVFDLERGEWTDKKCQSLQLWEHS
SCTAYLPRFVS*

>Lgig_117662
MVNQQQGQYEIVKCVVVGDSGVGKTCLIRALACNAKYDLAELVKTHVATVWAIDHYQNDLEVLDRSWCDVDGVRVSLRLW
DTFGYHDKQREFSYKGADVIVLCFSTVKPLSLKNVRRVWYPEIRRVMPTTPIILVGTRADLRYLYNDEKYLQTQKGLLYV
YDYVYIFRPIEEKDLITPEIGRDVAKDIGAPYYESSVLSHHGIEDVFINACRAALIERRKLKFWHAQLRHVQRPLIQSPM
RIPKPTIADVIIPPSTLNSDLLQLLHHQIEGDVIFTVQGTCIQAHKICLMLTCPIFEELFSLDILRQYPTGKKNRHSLRL
SLLGTGNSSTDMDKLIESDTESVSTDGELSSQRMSGFENLLNIDNANGNFPVITSYNKKAIDTVELKYCDDPFKPSESYL
QTVVTLNREITPRAFQVILEYLYTGMVKEVACINEIKRAAEIFEMTNLIIIISNLQKNESYLNKALEKEFHEKRQSKIHE
LALGLELLTDIRFKVDDGIVSAHKPLLMARCEMMQAMFSGDFRESVAEVVPFPGVSKYTFQALQEFLYTDEPPSMTGVDF
IDLIGLTNQMCLPRLIRITENFIINELSKSEKNGEDIIEEVMSLLEPCQLHRANQLAKWCLMYLCCHYKDLHQKYHKLYK
TLEKENIDYIEKHRWPPVW*
>Lgig_119223
MVLTDVGGGPPVSLKVVVLGDGAVGKSCLLISYTTNSFPADYIPTVFDNYSNSIMVDGRAVNVAFFDTAGQEDYDRLRPL
SYPDTDIFLLCYSVDNADSLENIKEKWIPEVDHYCPGVPKIMIACKAGMLPSRSERTIPVPKGVGEKMAKDLGLDHYTTS
ALTLQGVDVAMQESFRLGIADKKSHKHRKSKGIFGKLSKKKDTPVPPVMPPAGEAPRIEIQTSVFADQLYQMFVDAQFTD
VEFVVEEKHKIQAHKMSLCSASPHFAKILGVSQTINNIENYSLDDLNMGTTPGIAAAYYKEDETGKARLTVEISSDIKAS
TFLRVVEFLYTGKVTLNVQLSYLKELLRVAEIFKLGQLETVCLNYGNEEEFLNPSIGTFLNDQTASKIKTMFLNKPEGAD
VIFNVEGTKIYGHKSVLRSRSDVMNAMLCGHFTESAQALSEVGTTPEVFLALLEYLYTDHSPIDDVDSVGLLILANEYNV
MRLVNLCELYITKEVDKSCVKSIEKMDIDVIGLLMTAQIHNADQLSQWCLHFIASNHIAFNERDEYRLLDGENKEYIETN
QWPPLSYLAEVEEYERQMKKRGEKCVIM*
>Lgig_124272
MAIARPPGSDFDHNWPENAECLTLDFGPFETVHQWKRMPECDEFVGARRSKHTVVAYKEAVYVFGGDNGKNMLNDLLRFD
VKDKSWGRAFTTGKPPAPRYHHSAVVHDSSMFIFGGYTGDIHSNCNLTNKNDLFEYKFNTGQWVEWKFEMRTPVARAAHG
AAVHNGKMWIFAGYDGNARLNDLWTVTLDGVSKQWEEVNQTGEIPPTCCNFALALARDSMFVFSGQSGAKITNSLFQFSF
DNKQWTRISTDHILRGAPPPPEKRYGHTMVAFDRHLYVFGGATGQTLPNELHYFDLDSQTWSINEPSADSIIPAGRLFHA
AAVVGEAMYIFGGTVDNNVRSCEIFRFQFSSYPKCTLHEDYGRLLESKQFCDVSFIVGELKTPIPAHISLVCARSPWLRD
KIKKSKNQARIDAAEVSSDVKTSGMLNVYLPDAKPRAFEMVLSYIYTDRIHPTKTGESNDPHSNEVILIMMDVYRLSLQF
YMSRLEQLCIQYLETCIGNKNVLVALQNAAKLKLDFLKEYCLRFIIKESNYNQIVMSKEFESVDQTLMVEIIRRHQIPHP
RLLDTHNDLIQSNSLEKDMEAFLKTSGHEFCDITLMLDNIAIQGHKAILAARGSYFEAMFRSFMPDNNTVKITIGEMVPS
VQAFNSLLRYIYYGDVVMPPEDSLYLFPAPYFFGFTNNRLQAFCKHNLEMNVSFKNVIQILEAADKIQALDMKKHALNII
VHHFPKVARLPHIRKLTRDLLLDVIDALADDLSDSRLCQDMSSPSLNDSINIYKPYSMDNL*
>Lgig_124742
MDFSKFRESGELSDITITVDDVEFKLHKFPLYAKSDYFRNLSRQANSKEKSVTLENFPGGAEIFSLVADFCYNMVLPLTK
SNIVQIRCAASQLEMDGHGNLADIADKFLQDTITSAKMSRSIQSIVTLLINCGKTGDLAEEAGIVTLCVDAL
>Lgig_127223
MKSSKKTFSRNINDYIIRPLLRQRETGELCDVSIKLNGQTYQAHKAILALWSPYFLSMFTCDMREKVAGDVDLSESMLLD
SDSVFSSVLDYMYTGSINISTGNIEDIVRISDFLLLDDVKDYCKQFYLEL
>Lgig_131914
MYDYKCDIKLKCGNANFKAHREVLSAASDYFSAMFSHNMKEKEQDVIELKGISPAGISVILDYFYHGFVTIEPTNIEDIL
EAARFFQVEWLVEVCCKFLVHHLGLDNYTEVLTLSDKYFLGDLRWDIFRFLGNNLPELAKTPTFFENLSLELLSSFLKED
VYIDTTEDYLLYICHKWVKADLEKREAYLLPLLRLIRFPLM
>Lgig_132245
MFYDVVVNVKNQSFKAHKVILSCFSKYFADSFIVSSGGKTKHPLEIKLNGITSNAFKIFLEFVYSGVLEMKPEIIGELALA
AEFLEINCLKEKTMEFMNSITPDQALVVIETGSIPCTSEIFKTSYETVMEHFGECYDTNAFNYLSLDNVCAILRDDRLVI
FTEMDVFWAGLKWIAFHMNERSPFLNKVMECVRFPYLTQAEIFKCLETTDLLRTSDKCKEILIHANWEAFSCEVYDPIEP
EKEPEKTPDETEPKFVPSLYYIIYYQFFISADKQMQRYSAKAEQWRFFGQLPAPRHHHAVALLGGSIYLAGKRNMFMRNE
TATKAVFQYDPEQKSWKLCGPMNSCRMFFKLIALYGMLYAIAGEDDRHQILASAECYNPKTNGWIYVSSMHWPRKGFAAT
ACEGKIYVAGGHGESVGKKVALPILQSVESFDPRSNQ*
>Lgig_133026
MSLVKDEHNKCLRQSLKRFYEEEMLTDVVINVSGRKFKCHRIILASLSVYYETLFHSQFIDAQEKELTLQVDGSTFEEVI
NFMYCRTDTINTLNAENLFKIADFLQIESLKEVLEQQLLKDLSVENCLEWFRLADLYNCPRLLEKSLSMTAGNFGSVFDN
DQFLLLSSNLICNVLSKTNLSVPSEENIFEAVVKWYRADIKGRKSSLSFILEQIRFPQMSLGYLEKVGKESFMKPLSRLR
LIEEAKNYQSNPHLRYVKMSPSCMKHPLFLK*
>Lgig_135113
MQKKFSMQTCNVLAELRRTGDFCDAVIKVQSRSFPIHRNILSACSPYFRALFKYQHLSNGRIEVTIPGVSSEIMNELIEY
AYTRDIEIDSENVEELLPAADQFLVSGLVKSCCDFLANQIVPENCIGIRKFAKTYFCNSLERTALRYILQNFTTVHKTST
EFLQLGVEELNEILSSDDLNVKNEELVFDAVIHWTDHDPEKRRCHMAEILKTIRLGLLTTEYFIQKVKSHPYVKESAECK
PIIIETLKFLYDLDMDEEKEVDLNHPLAKPRVPHEILFVIGGWSGGSPTTMMESYDTRADRWIVCEYADKEARAYHGTVA
CEKRVYYVIGGFDGAEYFNSVRCFNPITKKWSEVSPMNCKRCYVSVAVLKSQIYAMGGYDGHFRQNTAEKYTRRKNQWSLI
QPMNVQRSDASATSLDGKNKLSVLGRRRKEATFIHRPILNP*
>Lgig_136271
QFQVHKALLAATSDYFRVMFGGVMAESKQNVVDLKGVTADGLNHVIDFIYSGEMALTLDNLTEIINTASHLQVSSAIDVC
SNFITSLMTFENAEDFLNIADTYSLDKVMSHWDSMIQDKFFEFTQTQAFLKLDAENLAKHLERNTLRSTSEYRLFLSLDK

WFRFNPARITTDAPNVLSHIRFALMSEGELASVRESDIIKRCPGAQQLVAKGFFYHNDCKKGHPSIEEVSNVRSSALCLV
LVHHGTPYMPFQVTAYNHHAGLFYSIFSDVDGNRDCRIASVDNFAYICRVVDYGGGSLMSSLFRFDPRHLVGQELRPMSR
PRLDFALVSFNRCLYVFGGSTEQFAILDSVECYNVLTDFWDTVLPLPTPVHSLAATTFRSKIYLSGGVSGQERQPTNSFL
CFDPTTQRYETKPGMFYARRLHDIVGYEQRIFVLGGIPRPGIPLHGQIPIECFDLRTNQWTMLSSTLSGRSVGHYINFEG
QILSLGHEHHNATEDEIWLYDADLDDWSKYAKAPQRTSLNSAMCTQLFINFDDEKISNKYMKEK*
>Lgig_137657
MDSQTLSLVLDYIYTGQVILTEETVQNLLSASNLFQLLSLRDGCAQFMMNHVTVSNCIGIYFFAKAHQCEVLASKAKEMI
NNQFSTLCHQQEFLLLPADKLVEIIKDSDINVSGEEIVYEACMEWLNHDMDSRYKHLVDVINCVRFANISSYYFCDRIEK
NTSLNDCPEIQKTLGVIRYYHMLRNRQQEMDLNLMPRKGMLYERGVMILANPYTEDALKKYNTMELLLPKTGEVITICRV
AVTGDNQIYLGGGAIRKINYRGFITMEGVCSGFYLFDQLEATWTCKTKMMVSRSQFTLTVVDGYIFAVGGHDENDILNSV
ERYDPHTNTWCMVAPLPQPLRLMTSVSYRGKLYVFGGETNSDFSNNAYRYDPATDKWTTLASMSTQRVLAGSVIYNEKIY
VIGGNSAISDKWKKEYLPEHCVSSVEIFDPVTDTWSPGPELPNALCGAGKWLNIYVCLILYTL*
>Lgig_143089
MSQETGEHNPGNPAFNLSSLSHDLPAAPFNYEMKTLFKDNFKTLYDFYESGDLCDVEINVNKRSFKCHKMVLACSSLYFR
AMFTSEMAESKQDTVTIHDIDESAMEKLIKFAYTSKIVLTVENVQPILFAASILQIETVAGACCDFMKTHLHSSNCIDVR
NFAEQHNRVELMRMADDYILDHFIEITDTEEFKNMSFKLLLHAVSSQDLHVDNESQVYESVVKWINVDLKTRKDHLAALL
TAIKFPLMSPTYLLDSVESNPMVKSNLECRDLLDEAKYYQMSLANLVPDFKVNQRTRPRKSYAGVLFCVGGRGSSGDPFK
SIECYDPRKNRWFPVAEMNTRRRHVGVCSANGLLYAVGGHDGNEHLNSGEVFDPKTNQWKQIAPMGTLRRGIALAGLGGP
IYAVGGLDDTACFNSVERYDPASDVWTFVASMNTRRGGVGVAALKGQIYALGGNDGNSSLDKCERYDPFVNKWTSLANMK
ERRAGAGVAVLDGNIFVVGGFDDNSPLSSMEKYDYSTDTWTLVAPMSTCRGGVGVGTLGGYLYAVGGHDGSNYLNSVEAY
DPINNSWEIVKSIQQFRAGAGVAFCECSAKHLRHIVRGHVEQLQCV*
>Lgig_143975
MEIKLKDDGYSDDLITTLNTFRKRGEFCDLTLKVGNEKVFASRVILAANSQYFRSMFSGGFCESSSPTTEIDLTGIATSI
SIVEHCIDSLYSG
>Lgig_177172
MSDIHFKVGKEPNEEVIPAHKFVLCVGSAVFDALFNGGMASSENMIEIPDVEPSAFLALLRFLYSDEVQIGPETVMTTLY
TAKKYAVPALERACVEFLKRNLSSDNAFLLLTQARLFDEPQLAALCLETIDKNTTEALVADGFTDIDIDTLCVVLERDSL
GIRECKLFHAVCQWAKAECMKKNLTPSPENQRQVLDRSLRLIRFPLMTVEEFAMGAAQSGILEDREVVELFLYFTVNPKP
NINFLDVPRCCLTGKEQVVSRFCQIESRWGYSGTSDRIRFMVNRRIFVVGFGLYGSIHGPTEYAVNIQIIHNDTGKVMGS
NDTSFPCDGTTSTFRVMFKEPVEILPNTSYIACTTLKGPDSYYGSKGLRKVTHESVSSGKVTFQFTYAAGNNNGTSVEDG
QIPEIIFYT*
>Lgig_177842
MAAQLNLPLERPVVNSPVSDDNSDQTVTVTPPSKTLTLALNSPEGIDCYGNERKALLDQGKFYNNPVLSDIKLVVGGQKY
YSHKLILVKCSDVFERMFSSDWSDENLKEVELKEDSICIGVFPRFLKFLYTNHIKLNTDNSLPILILADKYNIVDLKNVC
INFASTCIIPKLQLKDVFHIWFQYATKCYHQTLITCSVQTLSDKMEDITSTVEWETEWMNLDQDQLIEFLKSSELIIKDE
YALWKATIKWLNAPQNVEKIQENLKKVLCYIRFPMMVPEQLCEIESSAIVEQNTELFQHHLKSAYKYHSLPLTTRANMKE
FSSSAFLLRNYSDLRWDKRLIIPKYSTLPKGEEVSLRFATRASSFPAQTWEWELKISPKGFSPTCDDLRTILYSNLILDQ
PRPVEYLLSVVGSDQILQSVCGRKNFTKARYSIDTELEKKISVSDLMTSNSPLLVNDCLHLQIHLKPVE*
>Lgig_177864
MADDVVVFKREDLPSTAFPVIADIRRQGKLCDVGDQRFSAHRIILAASIPYFNAMFTHDMVESKQGEICMQGIDPSALES
LVNFAYDGRLQIDAGSVQSLLIAASFLHLQCVKEACCDFLQNRLHPQNCLGVRSFADQYMCTSLVEAANKFIQNNFKQVS
VTDEFMSLSKAEFVDILSRDELNVSNEEQVFEAALSWVKKDLDNRKDISLKFYKKYFVIENYLLDEARDYHLMPERRPLL
QTFKTKPRCCTDVPGMIYAVGGLTSSGESLSTVERYDPIVDRWKIAEAMSTMRSRVGVAVLEGKLYAIGGNDGAERLNTV
EVFEPEKKQWKKVAPMNCKRSAQGAAALNGQLYVCGGYDGVSSLKSVEIYDPKTDVWAMITDMQKLRSAAGVAVLQGEIY
ACGGHDGLSIYDSVELYNTALGSWQSVAPMQSKRCRLGVAALNGKLYAAGGYDGSVFLRTVECYDPAENKWKFVASMARK
RSRVSLVATYGRLYAIGGYDGITNLNSVEYYDPSTDTWKFAKIMSAHEGGVGVGVIPIEESV*
>Lgig_179800
MNELRLKEHLCDIVLRVEDCKYQAHKIVLAGCSPYLRAMFTNGMQETEQSLVEIHGMEAKTMNLLLEFMYTGLLEINIAN
VQDILQGASLLGLSALRTNCSQFLEGQLAASNCLGIHYFADMYGLTELASYARQFMFQNFLEIVRSDEFYELSQERLLNL
LNSDKLRVVSEHQVFEAACAWIQYDPVGRSHCACAILQNIRLALLDLTYLENIVIKSEFFNNCAKCQLVISKAIRTQQDN
VAMEMITPRAQPPCIYVVGGRNSTDCQLSSMERYDFLTDRWVANVNCNMNIARTAVGVTCQEGLLYAIGGECALLETQED
TLYLRCVECFDPVIKQWLPKPEMKVARSFVAVAAARGCIYAIGGEDRSCSYNIVEKYDPKAEKWSFTTNLKRKRAGAGVA
VCDGRIYVAGGFDKTLHMDRASVECYDPDTQDWTFVAEMEKGRSGLSLVTCDQFIYAIGGRFRHLDQYFDLVERFNTVTK
QWTTITPMNTSRAWPGVALFDGLIYVLGGFDGQSRLCSAEVYNPETDQWSTIKDMNIHRAGCGAGVV*
>Lgig_154271
MEFNTTSPLLSTEDLKFDLEFESDNDSVTSSGYDSGNPNAPEYWSDSDNEELFDDNDDLMIYENSKPIINTMEYILSMPS
LCDVIFIVGEEKTQQMGLKAALASRNRYLCKLIIEEENKMREEEMKMEIKKSKFSVFKKKTNKPTQQKLTINIEHYDAEV
FDKILNFLHLGQVCVTPETVIGLMCAAEEFDIPELQKACWDFSFRCLDNGNVATIVNSSKQYRSRLAAHMLTSKLIEMAH
GPRTTSV*
>Lgig_157498
MHFTIMKHPKEALEIMNILLRTNKLCDVTLAVGEEKFLVHKIVLAAASPYFRAMFTGGMREEEMSVIHLHGISACTLSSL
IEFAYTAEIRINELNVCYLLSAATMFQMTHVVEACSVFLEHQLDPSNCIGISNFASEHGCTELESKAKTFILKNFCEVIK

CDEFLMLSPCQMISLIKQDELNIKCESEVFQAVIRWVEYDPDKRLLKLENLLNAVRLHFLSPCFLESQLKGCKILQKMPQ
CLQTLSEKLKKLQLHEKCPERPRRFSPLVVFCAGGYLRQSLSNFECYNPQLNQWSRLPDLLVPRSGLSACHARGTLYVIG
GRNNSPDGNMDSASADMYDTLRNAWTPRTPMTVPRNRVGVAVIDNMIYAVGGSQGQQHHCSAERYDPDLDQWKAVASMST
KRIGVGCAVVNRLLFAVGGYDGQNRLRSVERYNPEKDEWNFVAPMNTMRSGAGVATMDSFIYAVGGYDSNCQLRTVERYC
VESNTWKFVASMNSPRSALSVAVIDDKLYALGGYDGEDFLSSVECYDADKDEWAECTNMTCGRSGHGVSVGAEPST*
>Lgig_157976
MESSTSSVNLTKYDQLETEDCFPDSDESHSSGYDSCDSSECGSVKVKVNETKCRKPASETMVFKSQESLCDSLDYILSMP
ELCDVVFLVGENKTPVYGVKAILATRSRVFYQMILQKQKEVKAKKVKGDKFKGKLVLEVMNYEEDIFRRFISFIHCGKIK
TDVNSVIGLFCASVEFSMKDLKQACWDYFLRCLDNQTETLMTSLEFYSHHRGIKHIMNKLYGVQQ*
>Lgig_157985
MESCTSSVNLTKYDQLETEDCFSDSDESHSSGYDSCDSSECGSVKVRVNETKCRKRASETMVFKSQESLCDSLDYILSMP
ELCDVVFLVGENKTPVYGVKAILATRSRVFYQMILQKQKEVKAKKVKGDKFKGKLVLEVMNYEEDIFRRFISFIHCGKIR
ADVTSVIGLFCAAVEYGMKDLKQACWDYFLRCLDNQTETLMTSLEFYSHHRGIKHIMNKIYNVV*
>Lgig_159792
MAEEQVPEEGMARNYEQELTELKEEFEKLQEHLSNVEVRYQTEIQETNKQKENDFAVQLMNVVTNLFGRSQYSDLKILLE
NGNELCVHKLVLLSRSNDWDVPDLDEVDVLDLKGIKYDVAYSMLKWVYTDQIEFEENKENLVEMLKLSTKFKLEPLQIRC
QNFMIEIVNVDNCNTFYQAASETSAELLKDYCQEFLSKHQEILDIHDDLEQTNLTFPEIYRLQNQVGELDEDKIESLITV
QEYNESVQLKKPKTTSKLPFACVICGLQFPNEAEFTSHGDVHTQSESFICKLCGHRFKTDAEVKLHLIQHSKKPVVKFVC
NICHKPFTEKRSFIKHMRIHEGVGIKCEICNRIFMSKADLDRHHTVHTQEKPFKCSFCEKRFRLKPQMKTHLVLHTGEKR
FECD
>Lgig_159829
MADENGTSSRDDHLQIPDFKALVEELAEVKTEYEELKSHIVELEKKYERMLSVKSGGGDGEINISARLQLLTADLFNNSM
FSDIVIMLQGGTQVKAHKLILAASGNDWGVEQFDTIDMLDLTDMRYEVCYAMIKWVYTDEFDFHHKINDDFLIELMQAAS
ICKLDSLFIRCQKALLAVLNSENYDKILKSSEDLSADILYEQCLKFTGVPHNKTGTELLVQKLKQHIGEQQIDLIIPDNF
TPTSAINYRIQSSDNKRLANEGNSITVYVSNSEELLNNVLSEDIAETYETLEETSEIPKSTSGTIDINSTETSENSHLNE
MLYSSSLTSISASHNQKVETVDYGPECVDIGPEHGDIGPECVDIDPEHDDIGPEHVDNGPVTSSQHCSNTTNSEVTNKIS
LKQGEIPSENHYFELVEDSVEDIPSEKFDLGTNSMHLTSDGALLAFENHSNASLQETYVDQTEDSLHIHKDKDSVIIT
>Lgig_159873
MFVLAEEINEELSDIKLEYENLRSQLCKLENLLCKENGGEFGLSTRLQLQASKLFNTDQFSDVQVILKGGNHIKAHKMIL
AASGDDWGVEQFDHVDMLDLTDLRYDVGYALMKWVYTDDFGGYSVQLDDNFFIEMMQAAKVCKLKALHIRCQKALSTMVN
NETCERFLKLSEEFDAKILKENCEKLLGKKTVINLPRMESQFERQVTKDTEQI*
>Lgig_160378
MQGSRLSPKNKEDETCERYIFKDHNHKTVNKLSKLFINKQLCDVTLKIEGKDLEAHKAILAINSDYFEAMFSHWYGESNE
HSVKLENLNYHAVCTIVKFMYTGTISLTTENVKDVFIASDLLQMLHIKSLCSGYMVNVLDASNCMGMLSLGEVYNCQYLY
DKSMKLLALQFPLVSSTSELYQISFHKLLDILASDDLNLGHEREALVLKVVVKWLDRNGSIDRDDLSLLLTYIRMKHIDK
TILQSLLDSSIYFHSQDKKEMVMKQLETCNECEKRNLNQSIYVFGGFLQSRTGPLCPRLKTCEKYNVEKDSWKNIEGLLK
TSSNAQIINIKGRILYFEPSSSEGTPQILEYNVVQNTWKTAVLNLSHDVQSLIHNYSRGGELILYDDQNDWLYLVHKSVR
KLILEFDEGEICCKQYETLSNTEQLDYEEYSCVVCNNELYLMGGVFKKSLTESEVKADVVCFSRNEWKRKADMLERRGMF
GAVYLDRYISQFYIIDGYISQIYIIDGYIYVLGGFNTRRVNTVERYNVHLNQWSYIANLIKERSHLKAVVCSQTIYVMGG
KSYSSNHCGARKVLNTCEVYDPVINSWRYIQSMKNSRCVFGAIAF*
>Lgig_162307
MEHPPSTARLAYTSDKHPRQTFESINLLRKNRELCDVVLIVGVKKIFAHRVILSACSPYFHAMFTCELAESRQTEVPIRD
IDENAMELLIDFCYTSNITVEESNVQTLLPAACLLQLVEIQDVCCEFLRRQLDPSNCLGIRAFADTHACRDLLRIADKFT
QNNFQDVMESEEFLLLPVNQLVEIISSDELNVRSEEQVYQAVMGWVKYSIQERRQHLPTVLQHVRLPRMSAKFLVGTVGS
DLLIRSDEACRDLVDEAKNYLLLPQERPLMQGPRTRPRKPIKCGEVLFAVGGWCSGDAISSVEKYDPQANEWRLVAPMSK
RRCGVGVAVLNDLLYAVGGHDGQSYLNSIERYDPHTNQWSSDVAPTSSCRTSVGVAVLGDYLYAVGGQDGVSCLNYVERY
DPQTNKWNKVTSMSTRRLGVGVAVLGGSLYAVGGSDGTCPLNTVERYDPRNNRWNAVASMGTRRKHLGVAVFNNMIYAVG
GRDDTTELSSAERYNPQTNMWQPVVAMTSRRSGVGLAVVNGQLMAIGGFDGTTYLKTVEVYDPETNCWRIVGGMNYRRLG
GGVGVVKMPSNDSHIW*
>Lgig_162329
MARPKENVILSEAFEQKCQREKEKLKVELKRNLKEDIIRLLTEEIHPDVNITDGVDVVPSHLLILKARAKRLFEIVKIDS
DKYDSINKVNIPWSNCRRLEDFVKQLYYTENVNSLIDEIEDSLNHKTTKKIDDLNHNSCYSNHKNNTLNPTPESNSQNTG
EIHLSDVHLTENVNTVENFNCNPVSICISVNEESRNGENVCEGKENISITDNNGSTSITKDNVSTSIAKDNVSTSIAKDN
VSTSIAKDNVSTSVAADNVSTSIAADNVSTSVTDDNVSTSVTDDNVSTSVTDDNVSTLVTDNTNLPDLIKLSYSLSLPHQ
CCSILGEDLLRAFLQEMCCDCSLVVAGQQFKAHKFVFYFIFQKKCILAARSEYFEAMLGGQWRESDSQTIELEGINPGAV
EQALLYLYGGVIDVIETCNVPDLLVVGDMYGMKGLKHISSFLIIKDYCHFFHKPCSSCLSLAGDALTLAVTYNLQDLQEK
CIKWINRYFTKFWSSRSFASQHEDILTLCCDQLISQISEQNVVDMIMDSHKLQGNVPHVKWAEPILHLTSQLMTATIQFI
SNNFISIMGSKQFLGWEKGAAWNLSVLEDIFGQVIESVSPSNGCKIYQALLRLDEYNASLEDSSGREDLHEFIKYLLKKS
ETFLRVNIHQVTLTEDWAVLPETIRNKILSCASYVCFDSGNNMTRKRPPKLPSSRKTNISGAVIQSLRDDHEFKKKATSL
NKNSTNSKKLISNSGSHTKSKQISNKTPVTSSASAANPSLPSTSRQNGHPKSGKSSKVIKKVGSIPKEEIGDSSKEVTHS
YLRLKAAPSYHRQISWSPESDRIPSPSNTSPRITTKIPIFRSSSLSPFQERSRPRSPDTVSVTRYADTNQSLLATQSSQV
KEADGLAEPVVLRKHKTLTDDMKERRWSSPAYTIISSDDSQLLKRSTSTMSSCLESRSIGRDSRTRLLPLFEVVFHQPSQ

*
>Lgig_162549
MESYTSSVDLTKYDQLEREDFDSDSDESQSSGYDSCDSSECSSVKVKVNETKCRQPSSETMVFKSQESLCVSLDYILSMP
ELCDVVFLVGQNKTPVYGVKAILATRSRVFYQMILQKQKQVKASKKVKDDKFKGQLVLEVMNYDEDVFRRLISFIHCGKV
KVDVLSVIGLFCAAVEYDMKDLKKACWDYFLRCLNNHKEVLLETVKNFNSHRGIKHILDKIYNCVTV*
>Lgig_162550
MESYTSSVDLTKYDQLEREDFDSDSDENHSSGYDSCDSSEFGSVKVKVNETKCRQPSSETMVFKSQESLCDSLDYILSMP
ELCDVVFLVGQNKTPVYGVKAILATRSRVFYQMILQKQKQAKASKKVKDDKFKGQLVLEVMNYDEDVFRRLISFIHCGKV
KVDVLSVIGLFCAAVEYDTKDLKKACWDFFLRCLGNQKETLMTSLELYSHHRGIKHIMNKLYDVQQ*
>Lgig_163705
MEVEYSYNIDLSNNGEYNNQLLKSLVQFRKDGTFCDVRLMVNGACVNAHRNVLAARSSYFEAMFSGNFQENVVDGVAVID
LSNVAEDVSHLEKIIDAIYEGSCTVTHSTTPGIFHLASFFMFEDLQARLVDNLVVRTYTSLRTILQGYILAVTYEIENLK
KTCLCVIDSRFHDYFIYQEEILALTDDQMKYLFSRKVHRYCSPLKIICFLLRWIKHNSDDKRVQYIEDSLEILKPILEPL
KHEKLLKTKLFLEKYKIDNISDEYLSVVKDLIIDIVSNLSSESNTVSKTIQPCDKTRCLIIVVPDKNLIQDVYKKEFEYT
LSKIDGKRVCLNPPRHNRQQVMKVAIYDMDLENWFEVGDFIPLAKFDNRNNWKAASLNDKIYFVSLHLRICYSLSLKTLM
CQEISIKDILDQFEGEIKSIMPLAVNNQLYALASNCRQIVAELKTEQKYFKLEEDLTWTFVAGIEHEHSNTPASLINAFN
KKVIAVKASIVVSLGKNLLEIKDGHVFDTVSSTIKKFSGPCYLSSPVRLLVKGDNLFIIDAEGWMRQYDWDIEEWMFPEK
IDLSCVRYCRSVASESEYPCLSHVSTNSGDSIWEIVNHGAFNSYLLKIDIDNDGELHTTKCSPPPFKYFTLCTAGTCSTE
LLQSLNDSQYLDPDI*
>Lgig_166873
MMKSIKNTPRRSPPPPPPPLPTSKVSEDHNWQATQTTVRERNAVMFNNPLMADVQFTVGVAPNQQQIPGHKYILATGSSV
FFAMFYGGLASETKEVDIPDVEPQAFLNLLRYLYCDEIHLEPDNVLATLYAAKKYIVPHLARICVRFLETSLSARNACVL
LSQGRLFEEVELMQRCWEVIDAQAEEAFKSEGFAEIDYKTLETILSRETLNAKELSIYKAVSRWADAECKRQGIDSNAEH
QRKVLGDCFKLLRLPTMTLSEFANGPAQSGLLTLEEANSIFLNYTARRKPQLTFNKNHRKGLQQYRVHRFQSSAYRSNQW
RYRGRCDSIQFSVDRRIFVAGFGLYGSSKGAAEYQVRIELKKNGLILGQNITKFKSDGSSNIFPVMFDNPIQVEAETFYT
ASAVLDGTELSYFGQEGLAEIQCGKVAFQFQCSSDSTNGTGVQGGQIPELIFYS*
>Lgig_167174
MEVGFVCLEMRLDDWRQSLFKRHIRKFYGRYSEKYYYLLVVEVYKILGRKRFKPYDMESEYDDGDMVKSKDEVCCCCLKS
SKGKNCLRLNGKKCSVAKTGEKIQKLFGLTDTGSRKKYLNLTLKNALICRGCLIKLDKFEEFTSSLRLSTDTFLGDSKQD
DAKRIRAAPKLARGNRDDNCTNTTCVNIKAKYLNLHSKVFELEDMVHTSQIKLSDEELLKSFFQITAKLFGKKLYSDIDV
YLEGGHVIHGHKIILSIHSLDWGIDGFDNSDELYLTDVKFDVGFAILKCIYTDKLDIDKLIDSDLLELYGACSRYKLSIL
FKRTEEVLRCKLKEDNVEMIYKYAVDKKFDKLINYCKAFKQTHDMNVEDDQDIKVEPDSDEESQSSVSITTNKSSVDHSS
IVDSNRQIRIVLNKIDSSHLEPGIDCKSTESELVDKDLEDSDVSIKQENESEVEGENNDDIDDVLENDDDDDPMNDIDDD
GSNTDKNDDEYSASGHVHTK
>Lgig_167228
MDAENARLRKKLNSLEEDYKRLQDHISHIELKYQAALIKEGGDIDEIDEDYRPSEDKLSTKLLRTVSDLFNNRLYSDIKM
KLEDGSIMYGHKVILASRSKHWGVYDLNEVNTLDFTNISYEVIHDIMKWVYTDDIDLFEYKEDFLTELLEVTLRFQLMSL
DIRCQKALISLIDKNNCQKFMTLSNELCADLLKKFCKEFMSQAQIEDISGVDELQFEEDSMSDEESLESDNTSPVSDNNS
LHTDNNSLHTDNNSLHTDNTLHTDNNSLHTDNNSLHTDNNSLHTDSTLHTDNNSLHTDNNSLQTDSTLHTDNNSLHTDNT
ATDKSPDGSNTSPDTVGADNTLPDIDKDNNLLDADSTLAETNDKIPDAGNTAFKSDNTSVEIENEANDNGVEAIEEVVEI
DGEISLEYLPQAFIDTCNTMNGIGTSKEALKPDSVSLLENGTGQEQKLEKQVETMKVSAEVKHEDV
>Lgig_167463
MASDPTDLMEVSLEPVGLVSIEDPVSLDGSAVIYIDNQMAVDQTEPMIVSQEDIDNSILAQDIDNSILAQDQLKSELLED
QVTMKLDDPATTSTLEGLVTEAPIEDLEKELNSVREEYVRLQDHLADIELKYQSALANEKEGRGSQFSTELQTVVANLFN
CEKYSDVQVLLEDDKIFYGHKLILAARSLYWGVTDLSLAPSIDFKHVNNDVAMAVLKWIYTDDIDIENVSVDFLMELLKV
SSKYHLETLFTRSQKILITRITTENSQKLYRLANELPSTTLKNHCLEYMTRGKDGVVIVTDDLSGNSPIKTSSTDQLHCD
VCSKSFTTPAKLKRHLLTHTGEKRFKCPVCGKGVMEKTTLKKHLRSQHPNEDLEVEYSCSICCRPFTDEDELQKHKNLHL
LDPIDHVCKFCFVKFPSHSQLQRHMIVHTNKKPFGCDECMKKFTSKYSLDDHTRKFHPDLAKTLPPRRQNSKTGLDKLKC
DLCDLMFRYEKRLVVHRRTHTGEKPLQCDVCNKCFTGKNSLNYHLKTHQERTKKCDVCGKCFYLEYQLKLHQRIHTGVKP
FQCQLCERTFRFRESLVVHMRIHRNEKPFVCEVCGNSYRTQWLIKRHKRTHTGEKPYVCEICKKGFTSSSILKRHKLNVH
KTSDASVNTSHIISNQSGGEFSSLNISTDQSVTEYTNVQIQDLDFHSAEINPDVEESIVEIPYGDQPVEIVKSENDEVSD
ESGRLSVSCDSSITEDQLTSLEKGESPSSEKLAKNQVKCDVCSKSFYSLRQLKKHVNTIHKVKFECKTCHTLFDTERKLN
LHQVSHEKEIICDECEKEFDTEQKLRRHILFHHKNDFSCNFCNVVFDTHQKLREHYVTHKDKVPFKCHLCIKIFSMEDDF
KNHLENVHKVEKPEYFCSICNKPCDDEVKLKDHEKLHDTDESDHICKYCGRDCQNHSKFLKHMLTHLGIQPFPCRHCSKS
FGTDSSLERHNRSYHAKLEGLEPTSSKRKPKQPLKCSYCEETFTAPKVLQEHERKHTGEKPFECDVCKQTFRLVDSYRSH
IKIHKGKPHMCDICGDCFVLKGQLTRHRRKHTGETPFKCELCPREFKYYNSIKIHMSSHTGHKPLVCETCGKGSFTQSDF
KRHLKVHTNERPFTCDICHKKFKSTNCVTRHKRLTHNKIVKGEKTDDFENGGQIIHLADDMNSNSLVTTLQVSNEDPTIP
HTIYVFHEKKCEMELNPPKYGKKPFQCETCQKVLSCFQKYKRHKLVHSKEKPFKCSECGKGLADNSSLIYHKRTYHSHDS
TKEVPTDYVCQVCDFKTSRKLDYCKHLKKSHPDLAANLPNQKKSGSEQHRETCSVCSLTFGNKRKLETHMRIHTGEKPFS
CDRCGQNFRREDSFKYHLLTHGEKQHQCE
>Lgig_170454
MSSMGSDYDAESVPEVANNCPIEEDDEFTKPTEFTDLTLVVEGKQLHTARYLLITASPVFKVMLTTTHFKEKNADKIDLP

CETFRDVQLLLKYISPDAGVLTADASERLLPIADKYQIEGLKSRCINCLCLDLKENPDGRLISLLSLTEQLGLDVLKEQC
IELLLDTHIDELTDYIHYDAPQKSTVSWHSVWRILRRHLEKLSTLTSMVNSMKALASKHAYHCRCATSNKRYCCKPKCSA
TTHQHDFVPCNWNEECLNNFARDLLTKGEIKNILKK*
>Lgig_171795
MAEADERSELMAIKEEYMKLQDHLADAELRYHTLLNIKEEENKVNIDYDRNLHKFVSKLFNKSNYSDIVILLEDGHLISA
HKVILASRSQDWGVEDFLLTDHIDLSGIISEI*
>Lgig_172763
MNSEESKIPLLQADYLNNDDIVDSVVTNSETDLLLISHFKTNFPEKRQVMVFKEKQPAVVTLLPELCDVIFLVGKNKMPV
FAVKSIIISQSRVLYQMLLDNKREKTGSKRPPNASKARVMLPDYHPNVIQKLIDFLHSGKLRLTTRNIVGLYCAAVEFDV
LEVRRVCSHYLNSLKPHQIPHVKLSLYSYGCKPGVKYILNILNWTKRCQISIQRAS*
>Lgig_173854
MSDMSDEISIDSEASFSSKDHLEFLIDNVSKFRMEGGYTDITIKIEDHKFDCHKIVLAAGSAYFRSMFSSGMEESRQNVV
ELKQLDGKVFEHVLQFIYTGSVNITGLMVQELFTQAHLFQITPLVELTVQFFQQNMNDNNCLAALTLADLHNHKPLYEFS
KKYACAHFSSVVLDEDFMKLSVDCIVDLLRDRRLNCKSEDIVFKAAVKWLEFDPDQRRKSRFKVLECVKYPLITESYLMD
VVIKMGHLSEEDKEKDLLDDAIMFHLVPSRRDMLNSYQITPRFSFPYFECAVLLGGRVMDGLSNEVECYRATTNDFISLK
TLPFKKRNEFAACTMGNEIYVSGGLRSSEFWKYDPNFETWLKGSNMMHARRRHAMASVDDCIYVLGGFDEEVVLCSIEKW
NKKSNTWEEAGSLNTAVENMGFVAFGKKIYLFGGKNSEELVTNTVQCFDTSSGQCEVLQNGLPANDMCLNACVLNSQIYI
VGLEGVFRYTPASDKWDILSDMSYPRDFVSLAILDAKLYAFGGRRRGAKDNLYSDVIEYYDPEANQWYITGTLNMNMYSY
GCVQKQYHIGAIYVLKYAHFVWFKLWQ*
>Lgig_175448
MEVGDSNEDGSMQKKFSMQACNVLAELRRTGDFCDAVIKVQSKRFPVHRNILSACSPYFRALFKNQEGNNKRIEVTIPGV
SPGIMNELIEYAYTRDIKIHPENVEELLPAAEQFHVTGLVKACCDFLTDQLAPENCIGIRAFAKAYFCHTLERTALKYIL
KNFCSLHQYSNEFLQLNVDEVSEILSCDNLNVKNEELVFDAVLRWIDYDPERRRCHIAKILKTIRLGLLTTQYFVEKVKS
HPYVKESSACKPIIIETLKFLYDLDMDEEKEVDLNHPLAKPRVPHEVLFVIGGWSGGSPTTMVETYDTRADRWIVCKTVD
KEARAYHGTVAYDMRIYVIGGFDGVEYFNSVRCFNPLTKIWSEVSPMNCKRCYVSVAVLEPYIYAMGGYNGHDRQYSAER
YSKQRNQWSLIQSMNIPRSDASATDLEGKIYICGGFNGQECLNSAEYYDPETNQWTLLRPMRNKRSGVGVIAYRGHLYAL
GGFNGVSRMNTGERFSLVTNSWQTIPEMDNSRSNFAIEIIDDMIFAIGGFNGVTTIFNVECYDSVTDEWYHATDMTVYRS
ALSACVIRGLPNIEDFIQQGRE*
>Lgig_186404
MNVNSGSSSVLDTEVDEQKKDGVIQKKFSMQACNVLAELRRAGDFCDAVIKVQSKCFPVHRNILSACSPYFRALFKNQEG
NNKRIEVTIPGVSPGIMNELIEYAYTRDIKINPENVEELLPAAEQFHVTGLVKACCGFLIDQLAPENCIGIRAFAKAYFC
HTLERTALKYILQNFSSLLQSSNEFLQLNVDEIAEILNSDNLNVKNEELVFDAVLRWIDYDPERRRCHIAKLLKSIRLGL
LRTQYFVEKVKAHPYVKESAACKPIIIETLKFLYDLDMDLEREVDLNHPLAKPRVPHEVLFVIGGWSGGSPTTMVETYDT
RADRWIVCKAVDKVARAYHGTVAYDMRIYVIGGFDGVDYFNSVRCFNPATQIWSEVSPMNCKRCYVSVAVLEPYIYAMGG
YNGHDRQYSAERYSKQRNQWSLIQSMNVPRSDASATDLEGKIYICGGFNGQECVNTAEYYDPESNQWTLLRPMRNKRSGV
GVIAYRGHLYALGGFNGVSRMNTGERFSLTTNSWQTIPEMYCPRSNFAIEIIDEMIFAIGGFNGVTTIFNVECYDSVADE
WYDATDMNVYRSGLSACVIRGLSNVQDFIQQGRE*
>Lgig_190348
MSNWHQKYLETISEGVKYLWNSGHFSDTTIVVQNKRFRCHRAVLSAMSPHFDATYSTGMRDNPDGVLTLLNIDVTTFDSI
LTFIYTGRDVVCDENAESLLRAATTLQVKGLIDRCAIYLSDNLTPSNCIRMWRLGRTHGCEELEERAWPLILDNYLELIK
SEDFKDVDVDELISIIKDDDLVVPNEELVCESVFKWIAMDPDSRNVHLSSIIEHLRFPLVSPEYLLTISQSKGLLKEDMI
CRMMMEEAKRYHMLPSRRQEFTSKRSSFRNSYDLEEVIVVITGGSTQMKPTEIICFSNRQKKWFYLAPLPYDPGVEFSAT
TYANNIYLSGGSRKWPKMLKYNSESNDWRECEQMAQGRSRHGMVAARDSIYVLGGYNRNLPQGNKVLNTIEKYNINTGKW
TMAGELPVPVEMVSAAVIGEKIFTFGGAQRNNRNTPAIQYFDTRIGQATLFADLPFACRLTRTVVCDGQVYIITTDGDVI
EFSEDAGCRTVGKIENFGRVHFGAVQLCGKAIIIGGKKRNDSECETMLSFDPLRAGAETLPDKLPFPNVIDACVKTVINS
NHLRTAFVLKTK*
>Lgig_190432
MDEMDLSDSSIDTNRFEGSDGDLDLNRALSRSSSSDSDAPNDQLGNKGKYTTITALNLQDNLAELFEENILTDFDVTVGD
TVFHCHKIILCAVCNYFRSMLQSGMAESSSGHTSLTSITPECFQSVLNYIYRGSQIFNLDIIEELFEASLLLQFDLLENF
CKEYMAESLAPSNCLVFYHLSKRHSCEDIADLAWNMCVTKFEDVMAATDLQELKEGTLTKLLTDKNLKFTSEDVLCTTII
EWVNAEPETRLDSLPELFNFICLPEVSGEFLYNNLYKNELIDKFPLITQAVDDAIKCHLIPTEKKNMVMRQRTIRDVKKY
RRVTVVLGGCITDCESLNNMSCWDDVSLSWYSLSSIPQHLGTDFASCVLENKIYVSGGSKGFDSVFCYDPADNTWSNLPK
LIVGREAHAMVAVGSTLYILGGRNDKR
>Lgig_194339
MFKKPNGFASKNTGSYRTILAKKLERQHKGSSQEGSRSSKTIFNFSDCLLNGIDALWRQKLMCDVLLIVEGKNFLAHKLV
LATCSDFFFEKFSDGKDKPGSNQIELEDVKASTIYSILDCMYTGKISLTPNTIRDVLSTATMLGFLGIHEACEEYLIEHL
TTENCFLYMDIASTYDLSMLSDKCLENAARNFQHATKGSEFRYVPVEQLILLLKRNDLNVKNEYDVFERMLAWIETDKQR
RIQYATELLANVRLPLMTPSEIIDKVESVRYLMDIPDCDEMVKDALHYHCMPCRQTLLQTSRTEPRIAYSDDCLVVVGGA
PRLKTDRISCDILVLDINNPDSRWELTACLPEPRHHHAVAVMNGFLYITGGEITNNRGFPSNSAFRYDPRHDTWLQIASM
KHRRESFQMVVLNDKLYAVGGRLDKKKSLSSVEVYDPSRDAWALMSPLSTPKQSFAATTHNGKIYVVGGSESKIISSRVE
RYCPSNDSWEIRKPINLPRFYAHLTSYNNNLYLIGGATVDIHDHLVCVKNIERYTPSTDTWVVLKGSNLPNPRSEFGCTI
INNKIYIAGGYDWDTKVRLAKIDCFDLITESWSTVQSFGKKYTGLALCSMRIYNFNFRK*

>Lgig_194758
MPRKLVSKRRKKRPRNMAEGQDITNGEIFRVEERPEHGMKLLQGLNKLKNDRILCDVTLIAESHKFEVHRVILVSCSDYF
RSMFTSGMRESKQKEIELKGITAKGLEKVIEVIYTSTTTLEGDDIFDVIAAATHLQVTPVIEFCERNFLSGMTTSNFYDF
INTAKLYSMNNALKQIDVFIAKNLMQIAKEGTLNLLTYEQMSNCLNSEKLFLREIDIFHITWEWLRQDANQEEHAWQLMK
LIRFPLINPSDLVQHVQRVETMMSNPDLRQMVLGALNYHVVPHSQPFTVSSSVQLRSYVERLISVGGREIHPNPCLHDGI
IVFDSKVTSNSLLNRQEKATLPSALSHMQVVVYNNFLYVLGGCITQCAHGESAVNSVLRYDPRFDSWYQVCPMLNKRAYF
FAGALNNKIYAVGGKFKDGSLATAECYDPAENTWELITSMPMSYHAHAGAVFERYIYISGGYSGNHFTPDMQRYDPQTNQ
WEDMAPMLTPRGWHVMCAAQDKLFVFGGCNLNVNQAAQPVMQSECYHPAMDQWTIIAPLSISHKEASCVVYNDQIYVLGG
YNVQTKTGQKLVSKYDIYTGIWETVGALPRSLTGVGYCTL
>Lgig_195500
MDDSLHYMSVNHADSLIGAINKLRCNRQLCDVIIIVKNYRIPVHRLVLSACSPYFQNILETNWDFGVKELTLRNVNGEAV
QMIIDFCYTSAISVNEENVWTILPVAFTFQLEELVNLCCDYITSLIRNETCLQTHTVAVQCQCSDLEQLSANFIQDNFES
LIEDSYFYDTTASSVLRYLKLLQQDVLTDDQVILGLQAWVKHSYNDRKYHTAKIIHQFPELKEKLLEFLPKELVEAPFNG
DDLK
>Lgig_197896
MEVAASPYLDSNSGLKEFRDQLNEIREQELFCDVKIRLNNGENCTLSVHRAILASCSPFFRSLFTSAMADSKKVEILLPE
ISKEEIETIIQFAYTNQVDLSPDTIESIFIAADRLHVLGLLKLCANYLEENVDPLNAVGVYRLAHHCHCRFLEVRVENYI
VTHFDQVWKESAEFLQLHINELLPILQHNRLNVPTENMVVQASMRWVHHYPKRKRHYYDLLATARLGRCDKFFIEDVILK
NKTIMAQKACRDLVYKAYAMSQFTLLQKTQNYLNAPCYMLMPRNPHGVIFILGGWTNDSVCGHMESYDNRVGRWYSLKTT
VEEKPLAYHALVTHGRFVYVIGGFTTATCLNTVKRWDPLEEQWEVIANMHDKRCYVGAAAIDNYVYACGGFDGETRHSSA
EKYCPQKNQWCYIAPMRSIRSDSGVCALDGLLYVCGGFDGGACLISTEVYSPKTNQWTMIEDMGIHRSGVAVASFEGYIY
VLGGFNGLQRLGSVERYHPATKKWSTVNGMSHSRSNFAITTLERKIYAIGGFNGTSTSNTVECYDIDKDEWTTVKNLVVG
RSATSACTVYGLLNVMDYTFFTKDLPYQQRSNAISTRV*
>Lgig_205955
MEDVVGDEFRSINHSEKMMRKMEGYVAKRQLCDVIIIAGQKRIPAHRLVLSAASDYFAAMFTSDVREKTMDEIKIKDVDP
DALTALINYTYTGKIELKEETVESLLSTACLIQLSEVVEACCSFLMKQLHPSNCIGIRQFADIQGCSNLFKVANNYVMEN
FVEVMNNQEFFILPFEEVCKLLASDDLNVPNEETIFEALVKWAKHDLPNRKKFLPKLLIHIKLPLMSPQFIADHVHNNPL
FQDDKECQTLIMEAFKYHLLPERRSSFQSPRIKPRKSTVGVLYAVGGMDCTKGATSIEKYELRTNTWTQVANMNGRRLQF
GVAVLDEKLYVVGGRDGLKTLNTVECYDPKKKTWTLMQPMSTHRHGLGVGVLEGPMYAVGGHDGWSYLSTVERWDPQAKQ
WSYVAPMSTMRSTVGVAALHGKLYAVGGRDGSSCLKTVECFDPHTNKWTNCTPMSKRRGGVGVANCNGFLYAVGGHETPA
TNPTCSRFDCAERYDPKTDQWTMIAPIGCPRDAVGLSPLGDKIFAVGGYDGQQYLNDVECYDPVTNEWTKVSSLCTGRAG
ACVVHVQSS*
>Lgig_208300
MESWENRIAVSVLDGFGKYFSAGTFTDIQINVEGEIFHCHKVFLCAISDYFQAMFLSGMKESLDGHVSIADISGSLFRDI
LAFYYYGKDSIVTMDNAEELLRAASILQMKCLLEKCEDFYLSQLHFDNAWDILKLAKCLNSQSLCQKTTEFIKENFEEVT
LEDEFLSADYEDLLTLIKCDELRIKNEDVVSQAVIKWMNCADERKSYAKSLLEFVRLKFVS
>Lgig_211219
MDVRPKIISGRERPAYKHPQHTQKAFDCLNQMRKCSLLCDVILVADVVEISAHKAVLSSCSQYFYAMFTGEMFESKAERI
TLQEIDAKALSLLVDFVYTSEIHVTEENVQTLLPAANILQLTEVRDACCEFLQAQLHPSNCLGIRDFADVHACSELFNYA
QTYSEQHFTEVVHYDEFLSLNENQMKKLISSDRLTVTSEEQVFEGVMSWIQHDIGSRKDIISQLIEHVRLPLMSQDYLVQ
RVEEEPLVKSNTICKDFLIEAMKFHLLKADQKVSFKTPRTQPRNPIGLPKVLLMIGGQAPKAIRSVECYDFQEERWYQLA
EMPTRRCRCGVAVLNGSVYAVGGFNGSLRVRTVDVYDPLKDTWTACPSMEARRSTLGVAVLNNLIYAVGGFDGSTGLDSA
EYFDVRTGEWKMMPSMSVRRSSVGVGVVGGFLYAVGGYDGASRQCLSSVECYNILADVWILVAEMSCRRSGAGVGVVDGL
LYAVGGHDGPLVRKSVEVYNPECNTWTPVADMHLCRRNAGVVANNGLLYVVGGDDGSSNLGSVEVYDSKSDTWTLLSSSM
MTGRSYAGVTVIDKPVL*
>Lgig_214601
METESTSSLSSSNQTHTSNSNTSRLVSSDSISDLQLWPIFSILDQETLNSIDKVCVFGSSGNEAIFITKGDDVYAIGSNC
SSCLGLGDTHSSFEPRKIEVLCKKKIIDIAFGSGPHVLAVSGLGEVYSWGHNGYCQLGNGGSSQGLIPVLVTSSLQSKRV
VQVACGSHHSMARTQDGDVKKFPLSHCFYLHFCFIAARPCIAIACGQTSSMALIENGEVYGWGYNGNGQLGLGNNVNQPA
PCKVTALASVIIHQIVCGYAHTMVLSDEGVMYSWGANSYGQLGTGNKANLVSPAKVNSGGERFISIAASHYSHISAATSQ
TGNVYMWGQCRGQSITSPLITRFVCTDDVFATFSTPPVSWRTYNVDQIKGNKLAQSIAKAFNDPITGDLKFIIESKEVYV
HKCILKIRCDHFRSMFQSCWDEDGKESIEITQYSYVVYKAFLQYLYTDNVDLRPEDAIGLLDLANAYCEPALKKMCELII
KQGITVDNVAMLYAAAIKFEAKSLEEFCFRFAMNHLTEVTQSEAFNNLDEYVLKELIRRAAQAGAFKY*
>Lgig_215797
METMSFENEAHMGRVLVEMKSMFDERQLIDVDICVQELCIPCHRNVLAATSPYFKAMFTSSMHESSQRHIILHEVDAKSV
AQIIDYAYTGELKITRANAQNLLAAASLFQIIPVQQASAKFMEQQLDAMNCVGIQTFAQIHNCEELKQKAREYTEKHFPD
VAKHEEFKALPLERVLEIVSSDELNVEKEDIVYESIITWINHDLKMRRKYIGQLLPHVRLVLLSMKYIKDHITQNYIIQQ
CDRCRALLNSLREFEENPELYTGEFNFALSLRSGMIQPEYCILLVGGLDQNKPSINCYNPLTREAYIMETFPETRERSGY
YCVEDPAVIVTEDNNIFTAGGNYIYHENYGETASDEDSFDDFEEETVRKDFYQYDNDHNKWILKAPMLFPKSNFSLAFHE
GKIYSLGGLTVNQHPTEIVECYDIAKNRWNYVGMMPTTLVDLSTCVYRSEIYLLGGRTGVGAHNTVMKFDPKKAEWCSLA
GMLTPRFNFGACVVDDELLVAGGQIYAQLTHTINREALRSVEIYNIADNQWRQGPDLPEEIYNVGLMIMNGTIYACGTTE
YQRSPFRIYRYNIVCRLDRVKNIWEQIESDLCDIRSYGCVAAKLHTRRLSQVFRPDVDT*

>Lgig_217137
MLSEDFLSCFSIINNRLLDLAGSSEFQRSTFAKMDEPKHPKREMGRKENHAVKIQDILYEQFVSESEFCDVTLLVGDEKV
KAHWCILVSCPFFQSLHDSGMKESFEVKIRLNYGTPWAMKEALKFLYLGTISVTYDKIKELLEAAEYLQITDLKKCCNDY
LCMTEINEENCVQLSLLSSLYDLDLYNSAFKFLRGHLPTVLKGEDALTLTKDSVMSLLTDETLTYVPQETFLEFITKWTE
QDEDFRSEFFKELFLALNLQNIPKSVLIDN
>Lgig_220364
MCDHRQLRSPAPLGVVDHVGSLSDSFGELVENSDYSDIDLIVDGGEFQGHKVILAARSEYFRALFYGGLKESQPGTAQIE
LHDVPATAFSFLFKYIYTGRLHLTEIKEESTILDILGLAHRFGFEELETAISDYLKSVLNSRNVCPIYDLANMYNLMSLC
LMCQEFIDRNANEILHSESFLSLSQNSVKELIARDSFYAHELDIFKAVCEWAEFNNGTDPTPILDSVRLPLMTMPELLNN
VRPTSLVPADSILDAIKTQTESRSMELKYRGLLLPEENIATALHGAHVYRGEMKTGLLDGDYHNYDLDRGFSRHPIDDNN
GQGIVLKLDRPYILNMFKLLLWDRDMRSYSYYIEVSMDDKDYVRVIDHTAYLCRSWQKLYFPIRVCKFIRIVGTHNTVNR
VFHLVAIEGYYTNKPFTLDRGLVVPAENVATIKNSAVVIEGVSRSRNALINGDVKNYDWDSGYTCHQLGSGAIVIQLAQP
YIIGSMRLLLWDCDDRSYSFYVEVSFDHQTWTIVADKRREHCKAWQLLTFDPLPIAFIRIVGTHNTANEVFHCVHFECPA
DLNGVNPATTHRAPGSPNHPVVSPRDPSAPQQPEIGVAGPPNNQCNQMPRVQGLDQYAVPDFQQDTPEADRIDAL*
>Lgig_221919
MGNSNTAPIDRKRKLEEVEGENSSDSSDSSPLHIPKKKRTKSTSKYIFQTLFINGENSDITIKAIGKDWNLHKHYLCQCG
YFNSMFNGKWLESELTEIEVDILDKNIDEKALKIAFGSLYTDDIMVKPVEVVGVLASATFLQLEGLIQYCHTLMKESIST
DIVVEYYEIAHQYGLMDIKKLSFDWLLHNLMSPTNFKLLKNISCELMIQLISHPDLFVLQVEMDVYSLLRKWIFLKNNPA
WDGTCKELLVEVDNFFKKFYKEKKLCYLECIEGKEYIPVFQHVRWQHVINENSALKAIKTECVLPEYWLDPFYKHQWFYR
LQSEQSIDKGPTAVDEELFYKECSRNGRVLLKRGEYCWRWVGYNYGVDLLLSYVNGMIIITRNCSNSASGSIGLQNTRNI
MLRLTAVNLDKNGCVEKSKSTELTLLSLSRDEECVLLRVDTDQIFPLVIILNVLVYTPLPDYPNPELSIPTLLSIDHSDV
AVQTIGLHESPSKSELQT*
>Lgig_225923
MALYEKVMRGDISNFAIQMKKMVNSKENSDIKFMIGPNKQPMYAHRCILASRCAVFKAMFSEQVGNNKHKDKDVPLVLTE
TSPEVFLAMLEFIYTNCVSMTSKIGLDVLETSIEYGLDELQKLTTDYLIEILNTSNACDIIQAAVKFSNNDLRDESVQFI
AAHTMDVFKSKGFQELSPDCLATVLTSDQLCIDELDLYKAVKEWANVNSTIEGKSIKEVGESFVHNLRLPLLTDDELSKL
EEENKTDNFIPAECFAFAWKIHALKKGEKGNPLTTKRKGTNHRDIHKYIDNSK*
>Lgig_227286
MDIKIPDLTSLLRDNIYRLYQEQRHTDIEIHINSVVFNCHKTVLAAMSPYFDAMFASGMRESKDNTVKLQGHVPKHFENI
LLFMYSGKCDINIDNAEDILKASSLFQMSCLALLCEQFLINHLGSENCIGMWKLARAHGCKSLETKCFMYLLEHFGDICK
SEDFECLDIDEVYSLITHDELKVLKEEIVCDAVFTWLSADVDARKEHAVTLMEVLRLPLVSPEYLVNEVEQRSIVNDNKR
CQEILKEAISYHMLPARRQEFTSPRLIYRKHSLYEEILVVLGGYSLNGEKVTDVLGYSIRNKIWYKLGPLSFKLGREFAC
CVYGNDVYVSGGSQRLSSLLRFRAEYNEWQKCPPMMQGRRRHTMVAVGESLFILGGYDDKISEESNRTLSEIREYQMATS
SWRTVADLLKPVRSMSSVVSKEKIFIFGGLLSDDTETACIQCFDTRSHTCTIVGELPFSSKLSRAIAFNRRLFVICTEGS
ILELNEEHECTVVKKIPFFNRRRFGLVAYQSGILIVGGECGEEIQKECLNFNPETGVVGVVNDLIIPSRANFGCAKIVIK
KQHLKHEYFED*
>Lgig_228420
MSSQEIGTALKYVSSSSEKHIYNGLVSIRKKLMKSKYAEKQLRNKGIISKLLHILHTQDLTQKENKLLDITLSILGNFCM
EEEARSDVLKIGYGITCIADCVLESPCSSIQNRAARTLANLALSPLSIKRIVQSEVLPQLVKLLLECKVKESQLVYCRTL
RLIGSNLYGKEKVLDENGLFAVAKLMSSDDDEVVKNSLRTVADLSVSGCSFKFARQILAAGIVNVMTDKVMNDNNTVSHN
ALSTLIRLAEYEYIRPVIGSAGGILLFINLLTDTRQHVDHIKILNVLCLCGKESVNRVKIRENGALEILLSALEGEKYAR
IHDRIISCFLSYLYDESSLNLLLDNKLINILLKHLQRCTEFKSRTITITFDEKMAVTEEMIMETKSDLILARPKQIPKRS
KTRGKRKCELEINKDKLLSSPVSSKSQNMEDSSKGFVSMPVPTAELISSTITMRPEEASFTVTASSEVDSMTDISDIPSS
SSISIEIPSTKDNRVYSIDSPTYCNHLWEPSPLSKGVTCKRSFSPVPSSSRGSSPTAVSSRSGSYSPLSNESYYSPGVSS
PSYSIPSPDSPNMPYSTVWSQPYQSDSSDELSSLGTGVAAASYWTFSSSEEEEASNEDSNGSAAVTAAEVELKSEGNDQL
LEETMVESDHSDEMSTFSDACSSLSLSEKKPESPCDTQVKRCGKHKRTSTSDNSDTNLETKRCKTTSKRLPASKITESNI
LILLSRVSVKNDPSPYLALPSVLCCMLDYIRDVPSAQSRCVRTISRILRDPMCLDSLLNIGAPVLIYQKLVLNGKGVDSS
DVRSLLSKSKVRLSIDSNSSWTSPFSSPRQTSSDFSPDPTGKPWNFPTDPGSPTSPGSSSDYSVASPHFKKEEKEEPPID
YEQAGLELLRDLSFTAESPFGQGAITHTLLRKSKAERLSCCLSLQFLCWSSSMQDHFLVKHFTLSSLLEVIKEKHNHTML
PYIVSGLNFLARISNMKRKLTKESPVKFPKLVTDGEEKCWYWNCNHDVFFQVCDVQLGGNREILVKQSPVFAAMLEGHYC
ESNQSEISIQDTSHDAFLFILHYLHGCGLDCPMLMKYYNDSPDVEFWENALSLCDRYLCQSLTNFLIWVLASRYLTFDLF
FHVFMYSAFHGFHPLTNECVRMLFSAEKPVILKVGIFKNIVQNKGADFLCTAIKSVFSEFIGKNFK*
>Lgig_228487
MALRDRTSHVIRPRQYIHERKFKHVVTRFKKTMDEAEADQAPPPQPEIDGDNILRDEDNFILNVSQFFNQEALSDILLKI
ADNTYFAHKFVLAKSSEVFRTMLYDQRYTQASSPEIELNESIECQQYFDRFLKFLYTAEININVDSAVGILCLADKYNVT
SLKLLCTKYMVKNTRSPKVKNALNWYSWAKALNLGDLIDQCSKTIAWNTDLILKMPEWLDMDFDFLKDLLSNSQLVVQEE
NCLFLALTSWLFCDQHKTNMEDHSKQLLPLIRYPQMQVSQLYEIEKLAKEDENYATFRELLLELISKAYRFRSLCPSQAE
LEVSFNQPFYLPRNYMSLTVDNVRMQNTLRFGIQVDVRTYVGPVPSENKEGEWKITYRKNQDSWTLQIYGHESAMINGEA
EIQSSVVIYNEYDHVIQVDAAPTQICSRNNNLSMTINVPKAEEAKTMAVLIKPMPK*
>Lgig_228743
MERQREKSTSPVRTVSLNDTPAKRSSPQRYPGRHNIFTKHHNVEDGQPLDRHHHMDDMTVAFKKKLSTKEITGPEERKKK
RKIKKTAEKEESSEEESWEEESSEDEEEEEEEEEEAEPGVIEEFLTGIWERQEMCDLIIKIGRHQVLAHKLPLAAYSTQF

AKQCLDEVPIGSCEIYLSDTNTDAVQEVMKYIYTNDIDLNSENIDAVISASLQLGVTDVIDSTKEYLLEYTKFNALDHMW
IAEKYEFLDVHSSTFNYICKYIVKISDTESFLSCPVDKVVHLLKSDNLNVKKELSVYNIATKWLDYNQKGRKKHSKEVLN
TIRFPLIVPDDVVKHVEPNMKNYSDECNKMLVDVFRYHALRYSGGGTDEFLPKQRHYYPIKYVVKETAGFSEEESEDEDE
DESGEEEESSEEEESSEEEESSEGESGEEEEESGEEEEESGEEEEESGEEEEESAEESAEESGEEEEESVEDQEELVEDE
EKLVDETEDKLKETP*
>Lgig_229085
MAESDLKSDKTAVETISDTSDFIQRFENLFDSSRLSDVVLCVSGERYRVHKFILVTASEVFCAMLSDQWQESKKTEIQLT
EDDACKDVFEQFLKYLYTGSIDLAIDTTLPILLLADKYSIKTLQKSCVQYMQRHIVATPDTNRTLSWYQYAKLTSQEELM
QECGKFILSNFSVILEASDWNTLSKNEVIEFLMSSDMIIESEYILWLYVEQWLLSTHNKDNIQENINEILPLIHFSMILP
KHLLMIESSNLCQDFNNLFKEKLNQAYRHHSLAVDCAEVGKSIEPYRNYCSDIYKICHPFSLHEYKDCQRIDSKITIRCQ
IPPRFISPNHPNCDKDSAMFHVYFYPKGFFSIITLYGSYVGRRNDGTTLKVVNRMPYEVPIKIHITLVLYSLLNSVKYVS
FTHNSHHLLTKQNNFEDTVDNVIDIKKLADDSQYLINGALEGKIFIKVEDMGEHLGPTPKRE*
>Lgig_229277
MADEESLYYGTWRIVECVSLAGVVETTGVEGTEFLLDENGDVSWKISEDAEQMPFFNCETYEVTPEKGSNRACLKFIGTF
AGHVIEFKIEVAGDLMLLTYERCCMLQCQKVMSHDPKDDAPFSFLGALEEGYFYDLVIKADNGKEFHVHSTILKLGAAEL
DWSCQPPPLTGLREDVLRTTLHYLYAECLPRDLTEDIVKNCIKSVGKLPGFSKFSELCETYLKNTALKQQIISLITDMHG
CADRIIELFTGKVTGVNSEGEPVLEDGLMSDPARLCCTVRLGLREASVACAKLLMLCDLFSRRKGELSREERHEIMKYAK
SRIPVFMNQLHRFLEVVRHHSSGLSSSRKVDIASYLVPELEEVIEKITHIATETKLALEQVINNSNHGDKTEKPEKSKKH
HVRDVLGKKLRNALHVKELKKLKDFHDRASTSFVHLMQKKENFNGMTQIEKIRSISKNLEQLIDECPMFLIRLEELESSA
EKLPWREWKYLFKLATSKTAWVLGKVLSNKATLQSLIDQLCDIVNRDQFTNSLATLGLTATESSTEGAAVGTKINQIPIS
KYAQLSTLESLCLPPLSHDSILASKSKQLFESQSNTDMVFQIVPESGPCNGDIVIDHTQVASPGQGAESIEKVEIPAHCV
IVSARCDWFRRALLSGMRESIDKKIVVHDTNPELFELFLKYIYTGELDSQDITAEQLADMLTLCDRYEMDSLKLLCEHCL
KHHIDEDNVLCLFSLADQLHAKNLREYTINYIVEHPNISNNEEFCELPDHLQAEIGDFITWNGLETRNASGLRDTNSSPS
SFSDTEDLTSNIDLGCSLPGDSSSSSEDLPFMEDATRLEFCLSALRDIIGDSVPRDELVRVSLAADYDVNRALNFFFSS*
>Lgig_229825
MATNVDSEKNPKNYSCQHHARNLLKALNESRKLPSLCDIIVLIDKTEIPAQKSVLSAASQYFRAYFSYEVKTESLKTERV
RVDLTPLEISIATFQDILDYIYTAEITLSTDNIQDLFQASDLLLMSDLKNLCCEYLEHCISPVNCVGIREFTARFASPWV
HFKATQYLDKNFREVSNGEEFLGLDVDSVREILIRDELNVRGESELPIVQCILQWYRYNRQERENDLTDLLINCVRASRL
TEDCFTTLRYEDCGQLNITEFLRNLQSHPRLDEPRGTTAVIVVCGGEGYVSRNEDTEVEVKPCLRCVKLYKNNSVKVGKW
IELTPMLTPRSSHGLVEVGGLIYAIGGRDNTCRLLNSGEKYNPKTNEWTVIAPMNHARVGFGLVAIDHNIYAIGGSNDMT
DPMTSMEVYNTFSNEWQSLPDITMKKVWSTYAAVDKKIYVIAGGIIGKFYEAVECFDSRTQTWTSVSPMRERRCDARAVA
VDDDIYVFGGFRRIECPSAMHSGHSLKFCGAEVYSTTNDYWVPISKRGLEPGMCIMHDKSHVEGTVYDGNEILAIGDLDI
GTGSDCIRGFDRYVNEWRTVVQNQPPNQRCYQCCLLNIPNSTFAKVLKQQASTPT*
>Lgig_232540
MAVCRTSTPPPPEMTCPIAENWCCTQVKVIKFSYIWTINNFSFCREEMGEVLKSSTFSAGANDKLKWCLRVNPKGLDEES
KEYLSLYLLLVSCNKSEVRAKFKFSILNAKREETKAMESQRAYRFVQGKDWGFKKFIRRDFLMDEANGLLPDDKLTIFCE
VSVVGDTINISGQSNCTPVKVPECKLSNDLGSLFERSLASDVILCVNGKEFQAHKAILSARSPVFQAMFEHEMEESKQNR
VEITDQDHEVMREMLRFTYTGKAPNLDKMADELLAAADKYALERLKVMCEDALYSSLTVENVCDILVLADLHSADQLKTH
AVDFINSHATDVMDTMGWKSMVSTHPHLIADAFRALASQQSPPLGPPRKRVKQS*
>Lgig_233079
MARRGSGPIYDPLGTPYFLYNQNYGIEILTKLNALRHEATFTDAILCVGHEEFPCHKNVLSVSSPYFRAMFTSELKESRE
TRICFNEVSPWTLKRIIDYAYSGRLEINQDNAQEMLSAGNLFEYPDIVEACCSFLKRQLHSSNCLGIEYFAHIHSCQKLE
NEAHEYALENFSIVIDSDEFLDLPLDRVIQYISNDHIDVRNEETVYHAIRRWLNYDSDERKPFLPQLLEQVRLPIMGLQN
LRLLESDQLLQGCHKGLQMIKEAQKNHQISNERPEHRRSSLPAIIHPRPSTVAKEVIIVISGINSYITPSVEMYDTQKDR
WFILPDLPRLVSWFSVCTVANSILIMGGICDGHVVDTVWKFDVVLRQWTEVARMPERRARHACAVLHDKVYVCGGVKGDG
NQLVESIDCYDIYNNSWSKAGQSENPRKQARIVPYKNSLLEIGGIQGEAKVNTIDTYICTGDKLKHSGEQYLLPSRIEFA
QILVLNGIFYIIWEDTKKMLALDPLKRTFQPLPDMNCSRIHCATTVLNGKLYVSGGLIDSKPTRLVESFDPVLKRWKKER
SMSEKRAYHGCLTLQMC*
>Lgig_237773
MEDLNMNGFHGEELEIIGENFHSNVLSTLNSLRQGQKFCDVTLVVERHDVPAHKCILVACSPFLFDLFTIDEENGVTRSS
CKLREVSHKGFQYLLDYMYTGRILIRSEDVKLVYVTARKLKMDEVALKCVAYLANNLTSINCLGIRSYAQDEEMKVKVDN
YIKSDINKVIVSPNFFSLPNLQIEIVGRKEDVTDSEQISHLLDLVLIWAQNNLQGKKNKLDRLTEQMNLLYLTRDNTLQD
CQEFEDEELKEDDGIQDYRKLKRKQNGERNLTENNNNNNKIISNSSLPFQKFSLIKDENIEREWKIIAYKQTADNTYMGI
AILNSCLVTISLRYRVAQQSPSTSVSDLSESPTNSTSTDPQYDRSRSLIPLNTMSTPRCGFGLRSVNGKLIACGGYDRGE
CLKSTECFNLESNTWTMLADMKTTRGRFSAARVGDTIFACGGSDGYHELRTVEVYDVEKDVWSFVAPMIKHRSSPGVAVL
GDQLFCIGGCMGQNSVAECEVYDAKTDSWSFIASLNTARYQASVRAYNGKLYVMGGTDGWNCLNSVEVYDPAINEWRYLE
SLNVCRRGAGVECFQGKLYVIGGNDGTHSLKSTEIFDVENQKWIFGPVLVSPRSNIGSAVLDGRLFAVGGFNGKKFLDTM
EYLDDANEWTFYTPFYKTRGINGGLSSSCDSLDNSNNNSALVMDGPLH*
>Nvec_XP_001642127
MASSDQNIELFIKNLDSSTHSTVKKALFSITKLTKRHNLVSTFIENGGLRKIIRFLQTTNSTIVDMSMSAVANCCMFDES
RKEVRNIDGIKPIVTVLKNLTSTSILNRTARALANLAEDEKNAIVIEELGAIEELTKLLTDASDTECQESVLRALLKICT
TPERKKIVYNTGGVQTMVKLLKSDKESLKHCCIRTICEFTKFCSKEVAQAVQEYDGIKHIVALSKSDKPVIQRAAVLSIA

NLSAHAQVRVCIGTEGGIQELFQLAKLDEPAHIKVKMLEGLCYCCREAVNRNKVFECGALELLMQQLLSSQHHVLHRKII
AAFCCFYYNDHCLEFLLNAGIIPALVTHLNKIISHCRSTDDHGDHSEHDFVYNHADFSSDFSSPPSAILAIDTSGDISLN
ESFLEEAENLALQVRDSTDSNRLLTFKRKPRTKSQSSTSPTSAKAIGLSPGMFSFCELGSCRVIESVRSSLTSVNTRTTA
LQSAPVGLVCSSVVDNRAKSQEIYDVSFQSSTCISLIDEDHVNVTTPMEMAEEKDSPSGAIVQSASKSSGFTERLQEQMG
SPVKGFEPPSPATPRKHTTQDGTQQNHGPGHTALGLLSRFSQMNDQTSVFSLLTKPCIKCLLDYVSFVSKPSPKCARLLS
RLTRDPQCFELLVTMGIPSDIHQQLLLGYVDPLHPDIEPFHDDDAESQDATGSSQTSLCKSVGLQLLENLSSLAQVPYGK
GILMHLLSKGSGTDWQNCVLSLPYLCRSKSLQQQVLLNRQGLQMLVSLLQDPPSNRVFVAAIYSLLELCSLTELSINVPD
THPCEPKVELDCTFTLSTETRKRKHVFKSGDRTAKKRKLEDNDHEKISTQVKCHQAEHNSDSDFDVKFELDSGHVIPAHR
KVLRQASDMFSAMLSSCYKEGTLEVIPLQDVTMTTLKCVIHYAYGCHLPCDERSTICPELKNIFSSVSNPESKFEFLFEL
LALADRFLMSELKANCEEHLQPLVTARNCLATCYYGLHYHSSSLCNFGMRYLLSQVSWPQGQCQMLQELLRSKEKQRLVE
TIYRLLLQQLRSVC
>Nvec_XP_001641792
MVKTFMRFEDFRKNSQLCDIKIVIGDKRIRAHKLVLASFSDYFSAMFTGDMAETSQNTVHLTDMDPAAVQALISYSYTSE
IEIRVDNVENLLSVACILQIDEVKNACSEFMRHQLHPSNCLGIRSFADGHGCAHLLKAADAFTKEHFVEVVKNQEFLLLS
AESVGELLSSDDLNVSSEADVFCALNIWLREDINSRKMHIYPLLSLVRLPLLSPKFLVDHVESSPLFRESVPCKELIIEA
MKYHLLPTRRFELQNARTKHRKSTVGKLYVVGGMDTSKGAINIEQYSLLTNEWTCVGPMASRRLQFGAAVLGNNLYIVGG
RDGLKTLSTVECYDPKTMQCMSVTSMNTHRHGLGVAALNGPLYAIGGHDGWSYLSTVERYDPDTKQWSFVAAMSTPRSTV
GVAVMDGKLYAVGGRDGSSCLNSVECYDPHTNKWKMVSPMLKRRGGVGVTVLGSFLYAMGGHDVPASQECSRQFESVERY
DPNTDQWTMVQPMINCRDAVGVACLGDRLYAVGGYNGSKYLSAVESYDPINNEWKEVASLNAGRAGACVVTVIDN
>Nvec_XP_001641691
MGDILIQEFTYEANDLPSQAFTILTQLLEQEKLCDVTIKAGERKIRCHRVVLASCSAYFHSMFTNSMLESSQEVITIQGL
SEKSVIQLINFMYTRKITITIDNIESLLTASAVFQLDPVVYACCEFMKRHLHVSNCLEMRAYAELHGCIEFIHFADKFAR
GCFTTLVSNDMFLKLSPKHLAEIVSGDDLNVKAEEIVFEAILKWVEHDPEKRSEHIADLISQVRLPLLPTDYLINRVEQN
SLLSSNMACRNFIDEAKNFKLCPVNVSSFRTQPRKSTAGTLFSVGGRGKTGEPFQCIECYDWFSDSWFMTARLSTPRRHV
AVASLNGRVYAIGGHDGIQHLNSVECFDPENNTWTDVAPMRTYRRGMSAGVLQGVIYVAGGLDEATCFETVERYDPETDE
WSIVSSMLHRRGGVGVAGLEGYLYAVGGNDGTVSLQSVERYNPHTGRWTRVASMNRRRAGVGVAVVGQYLYAIGGFDDSN
PLDSVERYDPKTNQWSYIASMSTCRGGVGAGSMGERIWAVGGHNGTQYLGSMESYNPAKDVWEASAQMSTPRAGSGVTGC
MCDVQALKALNWEMAVGIVFVRLNSENK
>Nvec_XP_001641588
MSSTPVAENWCYTQIKVIKFSYMWTINNFSFCREEMGETLKSSTFSAGANDKMKWCLRVNPRGLDEESKDYLSLYLLLLL
CNKSEVRAKFKFSILNANREETKAMESQRAYRFVQGKDWGFKKFIRRDFLLDEANGLLPNDTLTLFCEVSVEGDSVNVSG
SSHSAALKVPECRLSSHMGNLLDNATFSDTVLIAGGREFKAHKAILAARSPVFSAMFEHEMEESRKGRVEILDIDPDVFQ
EMLKFVYTGNTPQIQGMADDLLAAADKVGLFISKTLEVDVIKDHLTCDYDLERLKVMCEDVLCSNLTVENVCDVLILADM
HNATQLKSQALDFVNSHATDVMDSTGWKTLVCDHAHLIAEVFKAFLASTQTPMIGPPRKRQKTS
>Nvec_XP_001641437
MASEDGLLFCVPDFPTKVFSSLNELRSEEKLCDVTLVVKDRSLVSHRVVLAAASPYFNAMFTGDMLESRLQKVTLHGIEF
AALEELINFCYTGRTEIHVENVQSLMCASSLLQLSNVKQACVEFLHRALHPANCLGIRSFADTFCCGDLVQAADVFALKN
FSEVSQSEEFQALAPDEVVALISSEELNVRAEEEVFEAVMFWLKKDLDERRDFLSELLKHVRLPLVRPQFLCDRIATEDL
VRNSLACRDLIDEAKDFLLLPERRSKLQSSRTKPRKCSEAAGLIYAIGGLNSSGEALCSVETFDMLSCQWSPTSPMNTLR
TRVGVAVLDNRLYALGGFDGHKRLSTVEFYNPVLDKWIPAAPMNTRRSALGAATVNGKIYVVGGYDGHISLSTMECYSAT
ANSWSFLAPMSTLRSAAGVTELNGKLFVIGGHNGLSIFSSVEVYDPQTDKWGPGASLLMRRCRVGVATLNSCIYVCGGYD
GSSFLNTVECYDPQTQQWSFVAPMNTRRSRVAVVALGNCLYAIGGYDGLTNLNTVECFDPRANRWSFVSPMCKHQGGVGA
GVLPRGPFV
>Nvec_XP_001641173
MDFVDAEAPCFAFKQMNAFRCDGIFCDVVLMTEDGQEIDAHKLVLSASSEYFRAMFLTDMKESQQKFITIRAIDSQSMTT
LVEFAYTSNVRINSENVETLLYAASMLQFVRVEKACYEFLRKNINVPNCLTIWNLAELHGCAELTALAESYSRGNFVSLL
KHSEFKELSSKQLGSLLSHDKLNVPSESAVFEAVMSWIKHDVESRKKDLANLLEHIRFPLLTRKFLIDTVAKEDLIMNER
PCREFVLEAIDYHLIPERRACTRTTRTIPREKSSRAVYYVVGGEEQGTVLSTAECFDFNKKAWGTLAPMIIARKQVGAAVL
EGQLYAVGGVNREYADLVTVECYSPSTSQWTSVASLNKCKGALAVAILEGWLYAAGGSHNGSALKTVERFDPIRNDWTQV
ASMRLPRSQFGLAALQGRLYAVGGYNGISEIEHVECFDPMNNKWSDVNGMNKARMNHGIVTYGDRIYVIGGANSVGPLDS
IEKYNPDLNLWLIIRNTMDPRTGVCVVAVYGGSDGVQDLYIIANGSLYVCLTLMQALHWGTESLINIKGYVV
>Nvec_XP_001641034
MQAPKPSKKNTNTCTDLDPEAAHRSAWVKSTKDLFLNKALLSDITFVVKGVSVPAHRVVLITRSAVMAAMLDGKFRENDL
AMIELPDVPLAPFLILLEYIYTDSCNLKDTNAREVLVLADRFCLDGLAARCEQFIIDSMPGLGVICDNEEFVESILDVFM
FAKAFNSQYLTMWCLHVIATNYTIFEKTPEYNTILKENREYIEANRWLPRSYLKILNSYRARGTIVIPDRIQRHCPKHCG
CTIL
>Nvec_XP_001641015
MAGFTHEPVFTADNWQTKRRCIRERSEFLFNNKLLSDVSFRVGKEQGKYFIPGHKLILAISSPVFYAMFYGSMAEQKAEI
TVADSDADSFMELLRYAYFDEATINEENVLGVLYLAKKYILPFLADKCVAFLEQNIDHHNAFTLLSQARYYCEPKLEETC
WELIEKDTCRVISSDDFTEVDHSTLLAVLSRETLTVTEAELFKAIKRWAEAECVRRELSPDDPKNKRLVLSGALNLVRFP
VMSLQEFTDDAAVSGILTQEETINIFLYFGSKGATKVESFKSEPRRGTHRVFRCTRFMSDTSEWNCEGTNVDSIKFSVDR
DIRAVGVGLYGICNPADGHHTEPYCVDLELLDSHNDSLRAFEGTYFPEKPEKVHDVIFDEPVLLEAHESYTIRVLLKGLP

THRGTEGKRDVLCEDAGVRFRFDVDEASFNGTTTADGQIPEVIFKLP
>Nvec_XP_001640741
MCSLRERNKHMFNNELFSDVRFLVGRSAKQSIPAHKYIMSISSPVFSSMFFAFGALQAEQLTREQIEISECEPAAFLELL
RYLYYDEVQLTTITAPEVLYLARKYLIPHLADICTDFLVTNLTVDNSLTVLDQCCMLGVGKGLEKQCWDIVDKRAENIAD
DVTFVEIDHGTLTAFLCRDTLVAKETTLFNAAVRWAGKECQRLKLPLTAEARRQVLGDTFYSIRFPLMSMKDFTDQVARS
GYLTYEEVANMYIGFNSGFQKCHVKFPIRPR
>Nvec_XP_001640465
MEKKVEGFFTDKELYGTWKVTEAHVSKNDTEECIDNLEGIQFTLQEEDELVWELTSSAKKDPLPFFACETFEFIFSDHNT
VNELQFFGKTPECWIEFKVSGIPTKLLLTHGRFELLCTKVVTTFLDSAMEHFNLLGALREEFFSDLSLTSANGEEFHVHK
TVLSCTFFKHDWDTVPDCLLGLPSDVLGALLHFMYSGNLPKDITEKTLSKTLSVASSVPELEHLSELCSYFLEASAVKNK
INGLLKELDKSLATIVKEVHELCPSSSHSPLAGHDAFGLSRIYSTVKAVTMEMAIASLRFVLLCDVFTQRKSELNHEERH
ALFKDVKARHVEIIVWCTLPNNQMLTTFLEQNYNPNPMRKPNSNPSPNPSTWHTGFGLRAGCHGLGSEIESVWETITLLG
NKTRSSLDQILQSTDKEREERKANHSSTGKGNKLGRALRNAIHIKELTILKKLQKKATETFTFIIDIREDFITFDQDHKI
RLIVKSINKISAEIPYYIRLLERFPNSKKLSFKEWKYFFKVGTSKASWMLGRAKQNTPALQPLVQTSRDLVNQDMFNELL
SQLGLVRDGGEQYIPGGTTQETSSLSTNKTCPPTTPPPGFPVPLAQAVTRLLESGEHADMTFLVSDSSLQDTDSTLDSRA
NQKAAVSCVEQMKNLNVEDERETSSQACCVLSEGTGNGYVELRAHRVILAARCDWFRRALTSGMKESIDKKISIPFTNPA
LFKCFLCSLYRGNMETSTMTTEQLVDMLTITDRYEVDSLKQACEAALSSRMTEETALFLFGLADQYNAFCLKVSRSEEFD
RAFPPPGQEAQETRMEACIRALTDILGEVPRRELIRVTLAADFDVNRALNFYFS
>Nvec_XP_001640335
RIRQECKLCDVTLVVEGKEFPAHRIVLAASSKYFYGLFTSEMIEKNAPSVKLQELRASVMNHILTYLYTGEITVTELNAE
DLIASANYLLIPRLKGIACKYLERHMTSSNCIYFYKFGEKYECKDLKFYASQLIKKNFAAVGRSKDFMRLGAKQIEDLIA
SEDLVIGNEEEVFETVVEWIKYDPEKREDYFADLFRHIRLTSITVSYLFATLVPNPLVKSNPECMAKILEAIKQQHTADA
PLLAQRPRKCLEHHLDALLTCGGLGPDGSVRDLTYCYIPSEGSWYELSRMTVKRCRHGLSASCGFIYAIGGKDECFHQSV
ERFDPKTNTWSLVAPMKRRVKLVGTTSLNGNLYVIGGIEFGSESSRRRCDTVQKYDPTTNVWSLVAPISSRRSSVCAVSD
NRYVYSIGGLGDNDFQDVLERYDPKLNTWTKLAPMTEKRGCACGAHFGGKIYVFGGTVDAFSRQAMRSCEVYDIAVDQWH
EIAPMRVPRYHAGASLLRDRIYVLGGTGSDSLDSEMRQMVECYNVEKNKW
>Nvec_XP_001640178
MDYCRELLHTLNEFRLENHLCDVELIVGNNRFAAHKNVLCASSIFFNGLFSSSMRERQENTVNLKQFPVNIMEDLLTYLY
TGKLEVTEATAQDFLAAADFLLLPKLKTILESELEQYINPFNCIPLLHAAKAYSCQRLASVCIQCMKVSFSEVSKLEEFS
SLNFSQVFELISSRDIIVQSEEEVCEAVICWVESNPNTKDYFTKLFQQIRLFCLQSDYIRGLLSNELLDERCLRAIELSK
HHTPTSPYLMKPRTCLDDYIDVVLLCNRAFDPAVVCYAPCLEEWYEFQPIAGGISWQCLAYCNGCVYAVSGNGWSGCINP
ASHVHCYDPSTNTWAAKAPLLHDADEFGMAAIGKFIYVVGGHNRHGVRTELLQRYDTCTDTWELMSPLHPARSGTCVVSH
NNQLYVFGGSTNDKCLTSSMIYDQLSDSWSQVAPMSYSRSSACGIVVSEKVFIISGHSDEAYGTNSINDIKPITACEVYD
IRTNSWSSIPDITLQRSNAGVVHIDGKIYVFGGDDMISFAQQGESMEYFDIESCKWSSPVEFSDLHVTIARPRACALRLP
KSFVQALNNAI
>Nvec_XP_001640060
MSINQRPKNSRKLCEIDQTQAVIQQLDKLRRSGLLCDVTIVVNGREFPAHRAVLAASGGFFAGLFASNMQENSQNIVNLG
EIDPEVTEDVLTFIYTGQIELTTENAVAILKAADFLLLETLKSPVDSFLASMCQHISANPLRSYGIALDYKSPLFKTTAL
DLVCADFLDVIKSNEFLEISTETIQEIFSSDDLLVNSEEEVFHALLKWVKHNPGDREPLFPTLFPLIRSLSEEFLKSVVF
SEQLVFAQPKCLGHVLDCITNEPRPRDCLDREDAVSSN
>Nvec_XP_001639711
MACHHLLCALQDAFNGVLSSTMCITGCIQWRVIIFYVHYGMHSMACHLLLCALRDAFNGVSSSTMCITGCYSMELPFCTM
FITGCIQWRVIIFYLHYRMPSMACHHLLCALQDAFNGVLSSTMCITGCIQWRVIIFYVHYGMHSMACHFVLCALRDAFKA
CHHLLFALRDSFNGVPSSTMFITGCIQRRVHLLFALRDAFNGVSSSTMCITGCIQRRVSSTMCITGCIQWRVIIYYVHYG
MYSTACHHLLCALRDKFNGVSSSSMRITGCIEWRVIIYYVHYGMHSMACHHPLCALRDAFNAVSSSIMCITGCIQCRVII
YYVHYGMHTTACHRLQCALRDAFINVPILSVIARIWVKYLESTPPPSKNKQNKDIHKLTSCEIYTPISKSYPKATVSLRG
IHATYRRYLPREPRGVMDEVPDFSSPWHFSDVVLVIEGTKFHVHRSTLSIWSPVFERMFTSGFTEKTAEEIPLPGKRAAE
IEVLLRVMYTFGKAQPITEGNYTFLLELAEEYQMDRVKELCAEFLNYNLQESNCLKFYRVAEHFKLKEVMARSLEESRYQ
SLANLQDCEDFEKLGPEIQLKVYGEKIKELERTLQEYAKTCSALIDNIYRTVAARVQESGTDCDNIEVHRTGVREPFKMS
CKCCRRRVQNTDCKLEYSAFRGLLKKLFDLEHYNRTARRAKNSGD
>Nvec_XP_001639233
MKHISDRHPKQTLDSLEALRQCEELCDVVIKVGSSTIHAHRVVLAACSPYFRAMFTREMAESRQAEITIRDVDESAMNLL
ITFAYTASITIEETNVQTLLPAACLLQLTEIQEICCEFLKRQLDPSNCLGIRAFADTHSCRDLLRVADLFTQHNFQEVME
SEEFLLLPFAQLVDILSSDELNVRSEEQVFYAVMNWMKYNVTERRAYLSHILQHVRLPLLGPKFLVGVVGTDGLIKSDET
CRDLVDEAKNYLLLPEQRLLMQGPRTRPRKPTKCTEVIFAVGGWCSGDAISSVERYDPQTSEWKMVATMMKRRCGVGVTV
LDNLLYAVGGHDGSSYLNSVERYDPKTNQWSSEVAPTSTCRTSVGVAVLDGFMYAVGGQDGVSCLNIVEKYDPSENRWAR
VAPMSTRRLGVGVAVVDSFLYAIGGSDGTSPLNTVERYDPSCNKWVSVASMGTRRKHLGAAVFQDKLYVVGGRDDATELS
SAERYDPKTNQWSPVVAMNSRRSGVGLAVVNGQLLAVGGFDGTTYLKTIEVFDTLTNQWKMSGGMNYRRLGGGVGVVRLP
LSDTIGC
>Nvec_XP_001639182
MKSRSKTLAKRGTSNRSAAKADPQGNQDTSESEKDSFKTKTSQKKISPVFGNARLKCFIPKDSAKGSEEESCKSSSSYYA
FDYDSESGPTVETCTGTKRKRGTGIDEGNDGASNPHYRRAKQALVEVKSSISSNKGNASKKRKTSCKESAKKKDHLVVES

ADSENELDFVDKPQTSIPQHVLGEEHKLERVKSKRDALKRKTTRQAASSGNENKSASERKVCEQPEASSSVEMPNIECKV
VDDKLDNSESKCPVCSKVFPATDSEEEMNAHALKCLENTSTVDQVIESDPTDPRSSHSLLRDQIEAFQYKRSQPKDDPEF
KEELFFCHICQKDLSKLNSQRRQQHINRCCDKIESCKPKEEKKETNTRSSKISCPICGLVFKTTKSRNSHVKKCAQKNNI
PANQVIEIMHRVQQDEQAEELEKKDGSVPTSSTATNPIHIGKPVPSKKRKPKESAKVMDEQTQIALALSASMAVPSVVED
DGLTTTRSKKRKKKGNEAIPALLLRSKEETDQVIAARAAEVVCRQIYKKKYLTKSIPKTKGYNWTSFLSFLCVDGEEDDG
IKICKKSGPEEIKEKENLAPPSALENAKVLPKSSLWALSSLVNSQLPLNTYYVTDLLPPVSPAKCTSTKSPSKQQRKSYH
LDKTLPILTGDLPESVTPKPEENNYFEKNLAIQKSQKEDLGKSLSESISDTQAVNVDILMGLANEDEDVRNNEVDVSMAT
SKLSSCTDEGDDDVFPSSGFCLPFGQPQDYKAIDSSTSPYLTTLMRDLSSMVLNPSLSDVVMITRDTHEIPAHKFMLAAR
SSVLAKMIEDASSQVVNGKVSLKFENTESAVLGEVLKFIYTATASVPPPLIDGVVKLAESLELEELVQTLQDQNRHPSGD
GTNVDDATGECHSTDPEDLLNTIWHESNDEDTETTHKQNTQETDYEFNEEEIEEIRIFQTQAASRTKFCRYDLEENGSDQ
QGYKEVNTDQTECTAMAEDMDMPMNELDKNDVVAEDYINIAVETERENDEVLESHIVGGMKTTFRELNECSPTTKSNESN
LTNVGSLRERLRAKIGIDKDQFESQKQDHTDESQVKADPATDIKKYGEHNLHVKSKPSEDDGMLSSVLAVDDIPWDDEPT
IAYDLERSPSSGADRMGISQQTRSFRNTRANLSQAFQESSVTGFDRQEYVTVGYPLTDAIDSRVKCAKKNQKYLRGNNDK
EEVVGAQNIEPRKDNVKDQITSELSNKRKLIVDEENVGNCDKDESVNCDGVNVTLNESISLISDKEDNDNSQVKQRGDFD
KNGGESGEDTAAADDDNKDSDVDSVGTANEDIDEVMQKGSGGDNCGKPEVDTKNDSIFRDANWADTVAGDANCDDAEFFI
PETPNSKKTGRSLISLSDKRPAPMNITSRLSKISPLRALDSNAECAVSSDSDSELEDEKKPLASFCSQVKMHKTKEQQCN
VEKTLPEESAQKRTFVKNRTDMASSLNIRRRHLEDSKTLEKSKSFSSGLDVPVVDNAADQTQGITPFNLASPEDGNALWS
DASEPGEDPCPPCSPGDEDGYSSGSSLTQMIPPANPSPSYFEFQSFSPPESPPEKASEEDEDMPSPHSVDLTRDGTPPTS
PSPEATNPLSDPKITTFKVPESPTSSIEATTAPTSADSPTVATSPIDSPTVPVVEQSYGCPKTSVALSDKDDEELKGFCK
DEDHSDGYLSPTISVSTSSRKILQRSDAENTSEDDNNDDGLSAGLKTGEKSSGILTKTSLFQRLKARKLGIEICHRVESI
SDNDHVDKERASTPIDNSDGKPNSQSQGRTSNQPTLNQTEYFDDGGFNCDVDCFAECPGADDSLLAAVDIEQLEQELPSK
GTPSPGRETKVHSRATHSQQSLSQGSITQRTVTQQVVTPGSVTQGAFTTPSATWRAQSSVGQSPITPMPHYSDMDTPQLK
NKVKEYGVRAMPKKRMVKKLKEIYQYTHPGAPPQEEQRPDKKRSNRGRKQAPRGKGKGKRAPASQRQLSSESESEDDQEAIK
KQFSLKQTSETQLNKISRVKSTVSTQKPAMSGSEEEMIGVRDASNKDDVETVSQSSEESDKEDEEFGEVSVLMENEHATA
SQQVNSDDLKQTLLKYMTGDRDLHFKILTYQPMQVVHLHEQIKSDGIKCPISKLVDFLDEQGFGIMERDLKTLRRSRLMN
YSEFRTMATALERFHKDVNVAMMITGKSTGGDKKEMDMRPYLYDEGDSYERPFYTTLLKMLYEVATSTFYEKQYEDLVKI
YKWYCANKKLITSPPKIPIPSPKSCTPEPEVTDTPDIEQDGSSDDLEITDKYIQVPKLHIRPKTAPPVEHRQGARYVTWN
DAQKDNGPKRPSSGKPTGFQYQHNKADYVKEQLAKSLRVRQCGPYKEVPYIPDKSYRHHLARPVTAPPPNRTLQVRMEDP
AGLKSFVLKPFSTPVPKSSPINSPLTSPRDSPVPFSRAKSPQLSVDFAPITESSVKLNVRLPQTSDSSHQNAGQALTGNG
NANGAKENGRHQNGHDSGSGSPPENQAPDDLPAKLPNQSPLPNNASASEIQRILENQRREEELLEEQHSLLQKRLLHEYD
RYARDCSKLDRERTASVEMVSGELAVNLNGRVVHPAPVGTYDLSSPQIIPSYNRVYSHKHDTTNNRSAQGPAKQSGYSPA
RYLKHAGMERPDEVIRIDAVLGMATPIGMQMEYDRERASGYTGFRQPVAYIPHSVPNTQASPVAVPTERIQLTTSSSAST
SSQDSDSSRLAGPRVASIPSPLPLLPSPHPFTHT
>Nvec_XP_001638917
MVNESDGLGIEFLHSKQLLIGLNQLRQRKELCDVELCVGNVQISAHRVVLSACSAYFDAMFTGNLLESKKQVIYIKGIDE
TALQLLVDFAYTGKAEITQENVQLLLPAANMLQLYKVKDACCQFLSDQLDYSNCLGISKFAEAFTCQTLVKKAKKFIQDN
FRSVVEQEEFLLLPYSHVTELLEDDNLCVDSESVVFEAAVSWIKYRIVERGPLLSNLLQYVRLCLLNIRFLSQCLDTNEL
VKADRQCQNIIKDALKKRLLPARKSHKSLITQRRAPTRIFAVGGKNGLFATLNSVEFYEPRTEKWTEVVPMHLRRFEFGA
SFLEGKLYAVGGLVCGTGTNLGRAPFRYCDNGVECYDLVRDGEWTRVPPMHQCRSNHSVLTLGGYLYALGGYDGNSYLNT
VERYCPKTKEWTMVSPMIFSRSCFAAEVADGYIYAFGGYGPSYLSTVERYDPSMDAWEMMPAMSMVRINSGVGVVAGCLY
IVGGHNGVSHLQSVERFDPITNEWSMVAPMGRPRTGLSVAVLDGLLYAIGGHDGSGYLNLTQCYDTISNTWHSVKPMNSS
RCSFGIATV
>Nvec_XP_001638837
AHRYALAVGSPVFKTNFEDRWSAEKLKLTDSSKLLIPIANYTAEGFSEMLRYVYYGEVELSESNVMEIMYLSEQYDLPGL
QVNCSQYLNEMLQANDVLHCLLNALEKGDEKLQRQCWEVICKKTADVVASDSFLNVTQQLLYNILRRNDLRIRELALFKA
VDRWAEHQAGRQGLLIEADKVGHLKRRVLGDQAIRLIRFPLIPQQQFIDEVLTRDVLLKDEILDICKHFS
>Nvec_XP_001638605
MDPSIESPNEEKVVVPPYKNNEHCNKAFQVMNSLRQQNMLCDVVLKAGSIEIPAHRVVLASSSPYFFAMFTGELSESRQT
VVTLKEIDSLALELLIEFVYIAEIEVTEDNVQVLLPAANLLQLNEVRDACCDFLKAQLHPSNCLGIRAFADMHSCPDLLR
TGHIFAEQHFSEVVQGDEFLGLPFMQVAELISSDQLTVPSEEKVFEAVVRWVGHDPEHRHECLALLMEHVRLPLLSREYL
VSRVETELMIKNSVACKDFLIEALKYHLLTSEQRSLLQTPRTRPRTPIGMPKVMFVVGGQAPKAIRSVECYDFKGERWYP
VAEMNSRRCRAGVSVLDGLVYAVGGFNGSLRVRTVDCYDPIKDQWRPAASMEARRSTLGAAVLNGLLYAIGGFDGTTGLN
TCEVYDPKLNEWRPIAPMSTRRSSVGVGVLNGLLYAVGGYDGGSRHCLSSVECYSPANNEWTLVPEMSTRRSGAGVGVAY
GVLYAIGGHDGPHVRKSVECFNVDLNTWKPVAEMSMCRRNAGVASVNGLLFVVGGDDGSTNLASVEVYNPRTDQWGLLPS
CMSIGRSYAGVAVIDRPNNT
>Nvec_XP_001638484
MRYHSILTDVVLIVDGHEFPAHKNILAASSDYFMAMFSGHMATVDRTVVVQEITSTAMEVLLAFIYQGKLLITEENVEDI
FCGSCLLLLDTVTHACCKFIQERLTLANCWGIRSLADRYNCREMLKKVTSFIEENFVDATATEEFLSLPEEEVCQLLSDN
DINVPSEEVVFQTLLKWVQHDQSTRMKYFSKLLLKVRLPQIRTSYFEETVAQCELVLESPEAIEIISDARKMAQSFEFYE
EGKATSNTSMETLNNMIYIVGGSKEWKRLNRAECYNPETNEWKLLNPMAMSRSNIGLVSLNGLLYAVGGYNGRSPVSDVE
CYSPERDEWQFVSQMNNCRDGACVVSDGRLLYAISGYDGSNYLSSVEYYDPSTDKWTVGEISPIIERREDAMAACVDGKI
YVIGGYHGSTFLSSCEELDLDQNEWDFKSSLSSPCYQAGIAVLGKAIYICGGWSGNGLASSRVERYDILTDTWSQMASLN

VPAAARTVPCRFPRKLIEQLKRERYCGKQAKEGSKSSASS
>Nvec_XP_001638047
MVQWQTNLKTLKERGAHMYNNRTFSDVEFSVMNADGEKVSIPAHKYVLATSSPVFEAKFFGKLAETGFTIALPDCSAEGM
LEMLRFIYTEEIRFTINIAVEVLYLAKKYILPCLEERCHEYFNEAMGPDDVLAVLPHAVRQADQELQKICWNLVEDRSQE
VLDSDAFLGISHELVKETLDAELFAVTELSVFLALNRWAEHQAKKQGKSSDGKTKRSLLGDEVIKTICFPAMTVQEFVGE
VLPTGILKKSEIVELFTYFNLPEKSSIECKSSARSGKQIEPNIVCKRFPSPQLLNIGQGKKFDAISFTVSKPVYLCAVRL
MGYNGSNYMVKLSVCCKETVCSQVAEKTYQAASIEGISYRFDVKLDKPLFIEPGVHYTIKAESNAPG
>Nvec_XP_001637692
TLQVLNSMRTHAELCDVTLLAGDRKIPAHRAILAASSPYFRAMFLSGFVEAKEESVTVKEVAFDALESAIDYFYTAKLRL
DCDNVEDILKVCVVLRLDGLVDHCEMFLRRNMTPSNCLGLQSLARLFCLDGLSEHANRFALWHFQSVYQEEEFNRVPYQQ
LKSLVQDDTLKVQSEEQVFQAVWKWLNHDFHGRSMFVADIMQYVRFPLMNLHFLSDNELVKRLTREHTICRELIREARIY
QSSVESGALVQVDNFRVRPRMASEEIYVIGGWSNGQKISSVQCFNVDTLEWTVVAGMSVAHVNREDYFRVVAVRDELYTV
SRYKVGKYDPISNSWIQAATGPDVQCKWAGVCEWDGYIYVVGGHSSMSAKRFDTESLSWETLPLMTYARYYPGVAVLQGH
VYAVGGLDHLWAPLNTGERYDPIKNQWTEISSMTTARWSLGVAVLGEKLYAIGGSDNRESHSNSVEVYDPSTNEWSEAVA
NMNNGRRCLGVAVVNDLIYVVGGRVANSIEYYDKDQNEWTVVGAVNTRCNFGCVALRLM
>Nvec_XP_001637613
MGSTSYNWQAEKTKVGDRISFLYNNEILSDVYFLVGKGPQRRRIPAHKFVLSIGSAVFDAMFNGGIATQSDEVELPDVEP
SAFMALLRFLYTDEVQIGPETVMTTLYTAKKYAIPTLEKACVDFLKKNLAPDNAFMLLSQARLFDEPQLAALCLECIDKN
TVEAVNAEGFPDIDYDTLYVVLQRDTLAIRESQLFTAVARWAEHECLRRRIPSNAENKRTALAKALYQIRFPLMTVEEFA
SCAAQSNILTDKEVVSLFLHFTVNPKPQVHFLDKPRCCLTGKEKVINRFAKVESRWGYSGTSDRIRFSCSRRIFVVGLGL
YGSMYGPSDYQVTVQLIQTDTNEILAHNETAFNSDGNDSVFRVMFKEPVETKPNVSYTASATLRGPDSHYGTSGQKKVVY
DLDGKEKLTFNFSYATGNNNGTSVEDGQIPEIIFYT
>Nvec_XP_001637448
LDRLNSLRKENVLCDAVIQIGGKTHPVHKNVLCAASPFFRGLFTNDMQEKNQEHIELQVISGDVGEEVISFMYTGAIKVE
KENAQDIVMAGDFLLIPSLKSAAARFLEATLNAENCVFLRDFAIRYNCSALGEAANCLIDRSFVDISRASYFRELSYETV
VDLVSRNEINVSKEEEVLTAIFRWIKHEPIARKQYIHSLIGKVRMFSLSKYTIQECLYEENLIFEDPICTQMFIEGLQIH
LFPDSMLNKLIRPRDSLWDTAFMIILIGGENRLNEASNVLSIVQEYDPKVNKWRYLAKMKCGRAAHIAALIGDHIYVAGG
FGDKSVHGCGEKYSIETNEWVGIADMQSRRRFAAGCAINGKLFVAGGYHAEDWIAYSSCEIYNPTSDLWCMVSSMPEPRA
ACGVVKIEGSVFLFGGEKGGRPNDKLGSVVQYSVDNDSWELQRPMPEVRS
>Nvec_XP_001637410
MDVLDLFHFCRIGDVENTRNLVEEEDVDVNVRDCWDSTPLYYACLCGHEALVAYLLSIGARCEPNTFDGERCLYAALTDR
IRKILQNYKAITSTCMRRNPYYEFLRRTLESGDFADVCFVIHGQRFCAHRAILTTRSSYFASMFETKWKDKHVITLKNSL
VKPWAFKALLNFIYMDRLDVDVSQTMDVLLLAKQCKLHVLKQLLEMRLRDIEMMSHSKPGTHEVKVISVEPDASLRTMQG
EYGQMAEIAIPQPYRQQFFFPFSLSLPNDDRIPGDLPSYTDICINVQGNKFFCHKMFLCGRSDYFRALLIDHFAEVSTEP
NSIPELALHDVTPEVFAAVVSFVYRDNAELTDEILYNVLCAADIYLLHGLKRLCEHKISGLLDRANVLTVLRTARLFSLD
RLEMNCCDFLAGNIAEFIDSQDFHELILEDAAAIRNREETDSIPIVDDLRFHLYKNILPNDGSGSLCLEGGDAEVNLLIL
NNILEKLGLDC
>Nvec_XP_001636833
MAGSAEVILDFSQPWRMSDVVLLVEEQRMHVHQSILAMWSTVFEEMFNELRKKNSRALHLEGVKAHEMRELLLFIYPTIS
RPINEDNCYCLLELAKRFKMGKVTEDCEEYLMRTTKKGSDAINMLVLASKYGLKRLRDDCLHTVKKLNWSTIRSHEAYEE
IALPDYRTIAEQIIKNLQNELSEQKYKTEELEDEVESLLPRGKPVSTGRVSPMARQQSPERLRPVSPNPCTQDSIVKDFG
MDTPHPGPPTHDYHSPHSTETSRRDFEASPKMQRKFSPPTPTHKTSVKSTEGLANPKLIHPNFYSTSKVEKKSSCQRAGQ
PSPQTSKKSSVTTEESSESREKFSSFSTTHETSTKKSGFGASFSGDRYQPTISPDAPPKITLPAEDMLSSISGPAISPIQ
SKYSTPAQADEALSTMPSEPRASPRMQRKYSPTSGPSSYKQKKHLQVTLSSAANTTKGASLPATKSGPPVPKRTHPVKPQ
PIIKEF
>Nvec_XP_001636804
MASQVAGIDFEKWQLKVTTVRERNEFMFNNELLSDVHFLVGSKKARIPAHRYVLAISSPVFFAMFFGRMADDTKEIPVVD
TEEECFTELLRYVYTDKCLLTLDNALGVLYLGKKYLLSVLVNKCAAFVEKHLNPDNALTVLCHSRYLGEKCLEEKCWDII
DVHTAKVLQSEYFAELDMETLLLLVQRSSLSVKEISLFHAVKSWAEAECYRKDIEPSPQNQRSVAAEALKYIRYPVMSPV
EFADEVARCGLLTSEETTSVFLFFLSSFETKISFTNAPRVPRPLAAQKTYRCSRFKSCCGNDWFCDTRKHDGIKFMTDRR
MYIKGVGLYGASKTEGDYKTTVELKEQDRILAQTKATYSSFTHDLVCDILFDESIAIEKNIVYTITVLLKGPCSLSGTNG
QREVLVEGAKFTFLEYTSPNGTSLSAGQIPEIIFNF
>Nvec_XP_001636664
MADSSKSVAEENVLHPLEFLQTLDKMRVSGEGCDVTLKVTNQSFPGHKLVLAANSTYFRAMFGEGFVESSKNDVVLHDLD
PKGVNAVISYFYNSKIEINADNFGAVFAVANMWDVKFVLTACENYLRVHLSASNCLGFQILVHQSSAFSENFRTLLDDFV
NKNFMVICRSKLAILL
>Nvec_XP_001636663
MRVSGEGCDVTLKVTDQSFPGHKLVLAANSTYFRAMFGLREGFVESSKNDVVLHDLDPKGVNAVISYFYNSKIEINADNF
EAVFAVANMWDVKFVLTACENYLRVHLSASNCLGFQILVHQSSAFSENFRTLLDDFVNKNFMVICQHEEMLIIPVEHLEK
ILKGDELCVKSEEIVCQAVLRWLRHDVTGRKQNAARLLLAVRLGLLSDSFITLVLFQNDVIQCSDECKNLLRQVLHHSES
LPAYLWQARHLSALYLFAVCTKNFIYIIGERVMRYSIATKEWQDLNVEMPLRNYIIHCGLVAHGETIYAIGGLCNAIKLS
SDVTQQSLTWEGLPQTVVDHYRPGVCLLGDNVFVVGGCDRAHSDAHTVVEQLDISDPHAGWAGVVSLTTPRWGLGAVVLN

KKIFAVGGFDTDNRAPIGTVEVYSPRNDTWTQIKPLNRARRRFGIAEVDGTIFVAGGFSEDSSTNSTSLKTTNSEKSSTG
TQSKSVIAA
>Nvec_XP_001636233
MEDAAEVIDTLSLDFGPFEKSNRWKKMPDCDEFVGAKRSKHTIVAWSDALYVFGGDNGKRMLNDMLRFDIKECSWSRAVT
KGAPPAPRYHHSAVIYGQSMLIFGGYTGDLYSNSNLQNKNDLFEYRFNAGQWIEWNVEGRSVTRKATQGKHDNIYPVIPL
MWKGEEGKFRLFRGVVQQYGDRPPTCCNFPVAVARDSMFVFSGQSGAKITNDMFQFSFKENRWSLIPAAHLLRGSAPPPQ
RRYGHTMVAYDRHLYVYGGVADNALPADLHRFDLDDQTWDIVQPSSDNQLPTGRLFHAGVRIDDAFFIFGGTIDNNVRSG
ELYRFQLVSYPRCTLRDDFGRLLGSQQFCDMVFVVGEDDKIFPAHTALVAARSPWLRRHLLHLKETIQGSGEEEGQIEIR
LHDAHPRAFEITLHYMYTDSIYSLVKGMMNFLMKFLLSDVNGSEAISLMMDVYGLAVKLEIGSFEHLCVQYIEASITQDN
VLVALERAARLQLESLKEFCLRFIVREANYSSIIMSKEFESVPRDLMVDIIRRRQAHPQEYSIPLQFTITRTLEQAMTGF
LCSGQDFHDITLKVDGNPVGAHKAILAARCSYFEAMFRSFMPENNIVTITIGETVPSQKAFDSLLKYIYFGNVTMPPEDS
LYLLSAPFFFGFTNNRLQAFCKENLEKNVSFQNVVEILEAASTIGAVDMKKHALDIIVHNFPKIARSSTLRSLNRETLLE
ILDAIADYMNEMSFGQNSL
>Nvec_XP_001636169
RNDEVLCDVTLRHNERRIPCHKVVLAARSPYFRHLFINSESGQYVKNVELPSYFSILAVDEVINYLYSGKLHFTPTNTVD
IFKCALHIELDALVKECAFVLERFLTPVTCLTYRALAVTYGLRQLKHRCWRFLCQRFSDVCSTQEFLELSEEELAEVLVS
DAIMPCGEIQVFDSLLRWTETDRQNRGLHFPKLLHLVRLPFLDNDTLLRRVEPEPLVQRSPTAKDLVMEAMRYNMADEAL
RHTLQSPRTAPRTYQRLRKVIAFLGGRLGRERMGTLCYEPTSNSWSTLAAAPVSLTNHSVVESDGVVYTCGGSIEGKASL
VLQDQVLSFDTHDNTWIPLAPMQIARAQCVTLAYQGLVYAIGGMTDNPTETAEVYNPIRNTWSFVPVPRIPRRNHAAVAT
ETHLYVIGGDSDGIEPTVSVERFDPAASAWNFVLPMSCGRSGACAVVRDNKLHVIGGWNGYQTIVQCETLDLTTQRWSKM
ADLNECRQHAQAVMIDRTIYVIGGHDGNKHVDSIEYYDSSLCKWQLLRTMPLSRRGYKCASFQVLDKYLIPVDIPSNGGL
>Nvec_XP_001636151
MSSNAWQPAFKTIKERGKHMWKNPVLSDVEFVVCTSAGEKISIPAHRYVLSVSSPVFEAMFHGAMAESSREISLPDCYAE
ALSEMLRYAYYDEVKLTGSNAMAVMYLAEKYNFPGLKEKCSHYLQENLEPKDVLFVLPEAMKIQDENLQSHCWELIGEKT
EEVVTSDAFLSVTRELLCYILDRDKLRIKEIELFKAVDRWAEHQTSSQGLGTDGESKRRVLGEEAIRRIRFPVISQEQFA
KLVLPKDILIKEEIIDIFAHYALPKVSDLFSSKARVTSLSSVTRFMSKEDCGWSYSGGEDSLCIKVSNQVRLVAVSLFGS
EGNTYTVELAVEKEGQQVYQQEKRTFSSSELMNPTNGSYYNGFVVSFDHPVTILPNTKYCLRAWINGPNSYYGNNGQSVV
KCEDVEFTFMGSATSSS
>Nvec_XP_001636025
DVKFIVGPNRKVVYANRCILAARCEVFRAMLGSDPDREGRQKSAEPDIPLVLADVSPEVFSSILEFLYTNTCTLNSNSVS
VMDIMGSAMEYGLLQLQKICEQYIAETLAVNTASGAMQIAVTYNQEELQARCTEFIELNTPEVFKTKGFHELSEEALEIL
LLSDKLNIDELDLISSIREWATVNAVVGSTSVAETAAKVVRHIRLGLLTPEELRQIEIDNEKDNLIPVLMISEAWKFHAL
RKSPTLSAATIPPKPRAGTRPRDD
>Nvec_XP_001635994
MDWQTSRKTIRERTQYMFNNALHSDIEFAVVKSNGELDKIPAHKFILAIGSPVFESQFHGPMAEKDCRTINYNGTVEGLL
EFLRYVYYDEVQLNQNVVMEVLHLAKFYIVPSLVSMCSHFLEENLGPKDVFIVLPEAIKYEEVKLQGICWKCVEFDTELA
VTSEAFLNVTQEILCDILERDSLRIREIDLFKAVYRWAEHKMQRQGSDGEARTMRDIIGDKAFHLIRFPTMSKEEIGEVL
SQASDLLTKKELFELFLYF
>Nvec_XP_001635857
MTESVFCRSKAFREKEKCAREILRAKNEHELIGDLARLFEEKSFCDVTFVVNDQEFPAHLSILRTRVPRFCQRFVPHLLS
PNAVQSCICIQQLNQDEFSAFLRSVYLDNNVSDYNPDEYPWQDKAVWIFPCNKTPGKTDHPLDVTDAIHDSIEESIFINT
DINQSSSESDLPDSDEKMKTSSANEHTENDDDVILENGVGHDDSNIQLSENEGKENIGLKACCKLGSDLLSLRDDSLSSD
ITFVIENSSNIKAHRAILCARSNYFSAMLYGGWSEGQGSKIVLHGVDPLAFKTVLNFLYGGITHIDSMTTSLCDLLQLSD
MYCLEGLKNVIQAQLRIEKCHLFHKPCLDCSVGVMETVEIAHIFNFIELKQTCIRWITKYFDRVMGLKSFAAINKQLQDE
ILAELQGSLTIMICIDHLKRVDRLKFNTPHIKWAEPVHIVLTAFMDSCLTFIAQNFPVISQREEFLDMLQGVGWSAPLLD
EIFAALGTKLSIENCCEMYRAANKLRVSLIQDSQPNEQSQKDESECTTFGYVQYADQLYSRCKEFITSNLHRVSRTESWA
NLSPEMQSELQQSQDNLMFFEVGAIDRSKEALNSSSKPQTTRGLNRTGQPVGPASASRVHDRPGAAVRAPSSTSRAHNQS
GATARPSSSTSSTSRAHNQSGATARPSSSTSRAHNQSGATARPSSSTSRAHNQSGATARPSSSTSRAHNQSGLQLGHLVQ
PQGPIISQGLQLGHLVHFIYFKGTKSLRGYS
>Nvec_XP_001635779
MLSKEDSREFHPEESHRDILFRSMDNFRRQEILCDVVLIADGTRFPAHKNVLAAGSSYFLGLFTTDMKEQNETEVNFEDF
KSSTMDELLCYIYTGEVNLTETNAKDLVFAADYLLVGGLKRKGSEFLEDTLTPGNCLSVRSFAETFDCPELQTKAECYIL
DNFVEVSRCEEFLSLDPVQLERLISCDGLVVDEETQVFEALLQWTKNDEEKRRASFPALLRCIRLPFISKYYIVENMETE
PLIRDSLDCMRLVYQVMKHFAFRSQLSEDATTYDPATNKWQEVAPLSVSRGGPCVIAHKYVYAIGGKTEHGSEAEIGVYM
KTAERFDARANTWTEIPAMQTRRAYAGGVAMNDRLYVIGGTQDDLLSAHNTCEVFDCLSETWGFIASLVIPRALGGVAYL
NGRIYVLGGKKNCRERTDKMEMYDLELNEWTVMGTVPNCIGGIQCCAVSLNRDLVNSLQSITRNVP
>Nvec_XP_001635692
MEARGADGTPDKGSKLDASRKHDNPAGLARDLSQLSSMPQLYDVYFTVGKAGTCIGGVKAILAMRSRVFFKMFYPNPDHA
GRTASTNQSAQSKTQVIKHAFMLLKKEVSPKRDQKTDASTFHVGDLEPLAFSRLMEFLHGGSCDLDARTAVGLLAATDYF
AIDDLQAAVLSFLETRITIELACYLLSEAEKYIQFKKIKALLSRLLEFTAKNACEILRLPAFCSLSRSVVRLILSRGDFN
ASEWDKFKAAQAWGVAYCRKNKETSLKEALEPLVECIAFNEVPAVLLMQNVRKLDVVPPHVIMEALAYQADPTSVASTPE
RGTNIRQTTDALDTREERSYKG

>Nvec_XP_001635055
MADHHSTSTPKHDDDVSLDKKKLIYIGARRYKSSGCAMRTLNRFRKEGGSGFCDVILQVEGRHYPVHRCVLAANSPFFYT
MFNSGMKESMQQTLQLQSIKAKAMESILEFFYTQEIVLEEDELLDLLDAASFLLLPALTDAIIELLSSILSVDNCFSIRM
IASKYNAKDLLSNADGFIKGNFVYLSLWSNEFIYLPCEEICNVISGDDIHIEDEIQALSAVLRWLEHDLSERVQHLSKLL
KEVRLHVISGAAVNELVTNRPKLTENPSAKSMVKKNIARALSEQEEATADKSTQIPRPSTVLHNTLVGVGIGECRKVFCY
DVEDKETYVLPDLPQVQAFPCLVSMDTDVYILGGESQKNINYDPVNSMRIFSFQVMKNSTERRVGWRKMASFREVRRDTA
VTTHDKKIYVLGGWMSGPTGTVECYDLLSANWISVASLNVPRSQLAAVASDSRIFAVGGSVDQVVGTNTVEYLDPVINSW
SFLPPMKTRRIFANVSFLRNKLYVLGGYANVFERISSCEMFDLDKNKWFDIASLPDNLPTNQSTVTLNGEIIAVVGAQDS
CCSAAWYNPHSDVWEKVQNFDVYGNIAGYRLCCLQVKKYYLQHLPHADSESRDWPIKQDLYYEDNSFVSVNAGDLGLSLS
GDDQETNSEEEYFFL
>Nvec_XP_001634764
MAKFSYKSSANSHRVLQGLFSQIGLKELCDVTLETEDGLSIAVHRNVLAAVSPYFRAMFTGNLLESGKDRILLKGIAGVA
LQALLDYVYTSSIEIFDDNVEEVLNAACAFQIPEIINVCSEFLKEQLHPSNCLGILALADRFSCEELANEAHKYTVKHFG
RVSECDEFKALPFNEIKLLLNDENICVRSEEEVFEAALAWVNASEDRCKELAEILTCVRLPILSPSYLTEHVLTNETLLA
DDECKLMIDEAMLYASSPSSVKRQQVYHSRMQPRMPTGFADALVAVGGLYTGNSVASAERYNMYTDEWTEFPSLLTPRYG
FAITQLCGNIYCLGGYHNGEFLKAVEVFDAEQNIWISKPPMLTARKYFGADCLYGKVYAVGGSDGQHRIASVDCYDTFTK
EWTATAPMLEPRMYHGVVALGGLLYAVGGHSGTVRLSSVECYDPQTDSWTKVAAMSKPRSVAGIAALNGRIYVVGGFDGH
DYLKDVECYDPQTDTWLSVAPLNRARSAVSVAIMKGRLFALGGFNGQFLDSVEMFDPQENIWATVASMSIPRVHFGVTVI
>Nvec_XP_001634696
MGSVLSNEVNKGKRSWSEANDDDEEESEDTDQVIEIPKSKRPRRTSGYIYQTLFKDGEGSDITIAALGKQWKLHKIYLKQ
SGYFQGMLSGSWKESTEDFITIQVPDDNVTVEALDVAFGSLYNDDVMITPATVISLLAAASLLQLDSLNMQCCDAMLETI
NASTVCAYYAAAQVYGELAVEKRCMQWFERRICSSWTVQLLQNMSIDLMEQVIKSPNLFVMQIEVDLYTLLVMWTYIKLH
PSWEGQRKDLRGVATGFLQQRNQLTDTSLLESDEGKPFVPAFRAVRLEHVINDARSVQLLENDNVLPQDWLHPLYKAQWT
RMLTVYQNLDTGPSTEMTVDKFNLIAQRYGRILERNTRYCWRWTGFLYGLDIIILYNNVTGKISLRRNTYIHPTHGVVCV
QQQHSFVIRVDVAAFPPSQSKAEGPQSRAGEGQAESYFGSSGQQYVTLGLDEEVQILQVDLTKASYPLHISTHIALVRDQ
IPQL
>Nvec_XP_001634445
MQPFRTTAKQEEKPEFISHFCPTHTEEAFFSMKQMRCNSELCDVVLLVGDRRIYAHRLVLAACSQYFHAMFTSELLESRQ
KEISLQGLQPDAMELLVEFAYTARIQVSEDNVQALLPAASLLQLESVKDACCEFLKNQLHPSNCLGIRSFADVHVCTDLH
ESSYRFALQHFVEVSQTEEFMLLKVNEVLDLISSNDLNVKNEEQVFNGVISWVKHDADSRRKYIARLLHHVRLCLLSREC
LMMRVETEEMIKGSEECKDLLIEAMKYHLLPEKRMMLECTRSECRRPSGQVPILFAIGGGSLFAIHSECECYDPRIDRWC
MITPMSTKRARVGVGVVNGCIYAVGGYDGSVDLATVEVYCPQDNQWSTVTPMGTRRSCLGVAVISGLIYAVGGYDGASCL
NSIERYDPLTAQWTSVAAMSTKRRYVRVGVVGGIIYAVGGYDGSSHLNTVECFDPVTNTWKSVANMASRRSSAGVVVLNN
MLYVVGGNDGASCLNTMERYNPETNTWTSLAAMSVRRSTHDIAIIESCLYAVGGNDGSSSLNSIEKYDPATNMWSTVVPM
STRRSSVGVTVAGVLVV
>Nvec_XP_001634253
MPKSIPPVQESFYTNFSYDRVNYIVTYWDTCTLARTEELDLSKLRPLEYRDTDVFIICFDITNPTSFQNVSTVWVPELKH
YMPETPVLVVGNKKDMRTNTSFLPCMVSPSKGEELAARLGTVYRESSGYTGEGIREVVGTAVRLAVQHRLQTQTNKKRFR
MFRRSPSKLCNHHPEPPLLPVPVPAPEIKVEDSKFAEDMKCMLDRSTCYDVSFRFGSNLPLLQAHRFVLSIGSEWFRDFF
ENQDHLHKNQTHRTIDIHSSTHETQEISESLRLHTSPIYAQPRSFTHLIRFLYLGQFDVFDKGLVSEVLSTLNCVHIAEL
KEICTNILNNEDFMNKDVIESFIEKKSKIAKEVFLNRPLMSDVVFRVEGLQVHAHKGLIMARSEVMAAMFGGSFAESSNE
EIKLKDTPLKAFIGLLEYLYTDKLSPHESLDEDKQLLTLADRFCLNRMRSLCERRISEHLIARVKKQMSVDTETCEEVID
AYLLSQALNADQLHRSCAYIMKTNYADFEFKEVLSQLKPADIQHLTEHRWPPLSFFRQLEEYKKKTGRH
>Nvec_XP_001634098
MAGKDATFCVNAMQSMDDFRKERFLCDVTLNVNEVLFHAHRNILAASSPYFRALFTSEMRENQGNEIKLNNVDVEIMEDI
LAYLYSGSVVVEESKAIPLTVAADYMLLPKLKQLGIDFLSHNLTSANCLSILAIAEKFNFQTLKDNAQRYTLEQFGTVSQ
AEEFERLNVEQLLAIISCDDLVAKEQEVFEAVVRWVDFSLEERRKHFEILFRHLRLVYLPRCYLEDVVKGEELVRESDAC
KLLVVRAEEYFSLSYDERKRRNLFPQPRTCQNGIFIAGGWLETNRITNEAHLFVPNLSECYDLAPMITARKNHGMTLHDG
GIFVVGGASMEETALRSAELYDPRSNSWSTVMSMRKEVAALGVASFGNYIYAIGGHDNTGRPLNVVQRYDSKDNSWSFVA
SLNESRTRLCVVATEDQIYAFGGFSSEEYMPLKSCETYKVDNDAWYEIPPMRHRRSGASATYLHQQIYIIGGEDLLEVPF
VLDACEVYTPVSNSWSVIAPLCVPRSHGCSGAIKDKVYALCGINKGRELHSIECYDTYKRQWEVVDRLKVAIEGLACCIT
TVPGELLASIM
>Nvec_XP_001634017
MEQQELNTCTEFASVAFQQFNDFRNSKELCDVLLCVDDEEIPSHKLVLAASSPYFRAMFTSNLLECTQRTITLYDIDVGA
LQQIVEYFYTGKITIDEDNVQFLLHASCLLQVDRVRDFCSEFLAKQICVTNCLGIRALTDSFSCKEIYEAAQKFAVENFS
KVMHCEEFLLLQPYESLLSLIELDFLDLNNEAELLVGVMEWLRYSADTRGHFAYPLLRRLHLMRVESQILENLLQDPVIKP
NRDCVELILETLEALASPTISAALEFPMFQSRRYRENKKVLLAIGGESIGVNLKSIECFDPETQQWSWEYPGDDSDDRLL
PQLNDARIYTCVCSTGHHVYTIGGKSSWKPLELVERYEWPENCWKPCTPLNHSRLGAGSALLDGQLLVIGGCGQTGYLSS
VESYDPLVDEWTVTKSMLLHRSYHGVASLGGNVYVVGGFGGTIDADNCWLGNAECFIPSCDLWAPIAPVREPRAYVSLAS
HEGSIYVLGGYNSSWLNSVERFDPREGIWYSSPPMKTPRTSMGATVLGNRIYVAGGFNGVKNINTMECYDVTAGKWLSLS
PMRMIRYAMSLTTLQL
>Nvec_XP_001633639

MDVKVDDSFTFYDDKYSKAILHRINQLRHHGAMCDVVIKAEDTEFLAHRNILSASSDYFFAMFNGNMKESSQDVVTITGV
TPDSMRSILNFIYTGEIVLDWDNVELILQGANLMLVQSVKDACCRFLESRLNVSNCLGIQSFSETYACHELWHIASRYVY
QNFVHVSESDEFLAMSPKNVAILFSSDEISVENEEKVYEALVRWVNHDLSQRRDLFPELLELIRLPLVSPYYLVDVVEKE
ELMTVSPRCVELLLEAQHFHMLPDRRSSLDNARTKPRNYERFHEMLIAIGGNGDFTNFTTVIRYDVREKQWHPVPSMNTR
RSKHGMVEVDGSIYVIGGFDGTTTVNTVESYSVQTNRWKVRAPMPTRRRCVCAVAHGKFIYVIGGHDGSSILNTVERYDT
TRDVWSSTDVQPMRDRRSFPCAVVCDDSMYVMGGYDGNDTLRSVEMYNFSSNQWTPLPSMFVPRSNAGAAVFNKKIYLVA
GWDGISLNSVENFDITTQEWQRLPSLPRPTTGATVVGGQMVVLGGGGGGVKTLDYSVYAISDNENKRSI
>Nvec_XP_001633531
LSIENPKHGGEIVDGLYKQFKARRFCDVILLFEKTEVKAHKVVLASASRYFDAMFSDHFSDCEQEKVELPALDDKTGPAL
IEFAYTGKIDITQDNVLGLLAGSNFLQGFQFVEESCCNFLRLGLKETNAVDIMRIADTYSLQELKKDTKKYILRNFIKIL
DTEHFIGVPVTVLAELISEDSLLVVQE
>Nvec_XP_001632305
CDVNLEVEGQVFAAHRCVLAANSQFFYTMFTSGMRDSNDSRIKLCSLTSGALSSILDFFYTREINISRDNVVDILEAASF
LLLTPVKSACAQIILSALRINNCISTRRVADKYALEELKIKADSFIKKNFQQVRSEEEFLDLSIGELCELISSDDLNVET
EEEVFEAVLKWVKHESQNRAMHLPTALPCLRFGSLSSDFVERQIEGESLLQQCTFCQNFIKMYQRRQRATRAKKGGMSQE
ERPSAKVHNVMVGVGRGNLRTSFCYDTDSDTMYQLSEPYSPSFVLGTNLVAMEGQLYIVGGCNGGASGRVQCYNPGNNVW
TTVASCLTPRCQVGAVAFNSHLYIIGGTTEHLSQPVNLVERYNPAADSWTLVCPMKSKRRAPQVIATSTSIFAVGGATQP
GHDTVTCEVYKPAFDQWIIIAPL
>Nvec_XP_001632261
MALNSSSLLGNEAQRNIQQQLNAIFGQLSSAGLTVQVLQNKAVITQAANSETFRIVNGNEALKFVNNSRFADKILCIGNQ
RFFCHSDYLALRSEYFRALFNNEFRENSMDEIAIELPAPCDFEPILRYLYTGVANETDSIFHEKNIFPVIQNTNFLGVDD
LLSKAVTVFAYRWRVLTKSPMFRRSVIDYKFLSSVLEFGARNDLFKNGDKLKIVVSWDEEDSEEFPECRQLLREHKCLEE
AAVSDIEWGIQVKPKLFTRLDSLDFRSIFNRALHDSERAQEENRRSEEKIRGLTQCIRVLTKQLEDVRCLRCFTMVPRAA
LKTRTCVTTQHTGSYAQNRGWSCCGELQKRSKGCKPVCLSRHRINSH
>Nvec_XP_001631936
MADEGQVAKLERHLGLLREEYVKLQNKLVEMEHKYSIAKASAGQGEENSFVSRLLKTVADLYDKDLYSDITVSFGGQKIK
AHKFVLAARSDHWCSRDLNEVTELELSDVSVDVGLTLMKWVYTDKAQIPKEESFLINLVHASNKYRLKDLRLRCEKILMS
SVTVSNCIKFYQTAEDIGAKELQKYCAEIISNHWHDLKTEDFVGISAPLLYEMFKSKSTYPLHLTIRHHREDVLFLFLIE
FNSQLPGKLNEVDPTTGEVPLHIALSEKLDSIARTLVGHGCDVDMVDSSGNCLLHKAISHGDEFSSTFLIKNGARVSAAN
HAEKVTPLHLTASYRPWMAGSASSPAGANHMTSAEGMARIASLLIDKGANTDAQDLAGRTPLHRAIEAKNDKVMNILLNR
SKLNLELRDKQGRVPLWLALSCDSFDSMDEESLAAKLVKHGASADAVNTVTGDSLLH
>Nvec_XP_001631436
MDSSSWQTEKTSIKERVHYMYSNSLFSDIEFLITKADGTEVRFPAHKFVLSVSSPVFEAMFFGNLAESGPTVRLPDCTVD
GFQELLRYLYCDQVVFTGKNVLDVLYLSKKYIISHLIEKCWDYIERVIGYDDVFHVLPKAITMGEERVQGICWELVDIDT
AKCLASDAFLEVSKELLCDLLKRESLSIPEVKLFRAVNRWAEKKVEEKGMLGNPKAKREVLGEDLIYLIRVPLMTAMEFV
EVLSNNILNEVEIQELSNHHVKKEALTRFNAEPRPGLEPIDELPLGKANSSDVSGLLDGEVIRFTVNSELYLTGIKLFGE
KILDNFNINLMILNGNNEVVSQCNFGKTFLSWVAGRDLDEIALKLPEPVRVTPGLSYCVKAHIQGP
>Nvec_XP_001631232
MAGRTVLKSLPPPKDFCLDVMLALNKQRLEGVLCDVTLIVEETSFHAHKSVLAAKSPYFHKLFTSEMAEKAQEMIKFELS
SLGLSKSVMGDMLCYLYTGNLNVLASQAQDFLIAADYFLLSELKDAAINVLESMINQENCCILLDLGERFSSDHLANSAR
MFIENVFSDFRHTTEFQELTLRQFQEIISSDNLVVTDEKEVFEAILAWVSHCEDKRNIYFEQLFSKIRLNSLDNEFLQFR
VLGDKFVAKSTKCVSRIKKVFRARLEAKPYDIPLEIPRKCLQPPVDIVLTCGGMTPRRPAHAHMLRCYVPGGQWYDLPPM
PCKRNWHGVASYSNAFYVIGGEKDGEPSASCDRFDLPSRTWREMSPLLRGVVIPAVAVLKEFIYVIGGVRNYRARLQIFS
TKTNMWAYGESLSVAREITCAVAKAPYLYAIGGLRSDLGAYMCSVEKYDPARDSWSGVSDMLHARAGAVGLALNDKLYVL
GGESEVRVALTSCERYDIDLDEWSSIARMGVPRYFAGVAAIGDGFYVFGGVGGSNVDCDEKDVVEFYDVSEKKWTRTLRM
PWGERFFRICREQACNLATGGR
>Nvec_XP_001631191
MGDNQDFQLAKIDHVELLSSQSGTLYQSRELTDVTFIVEKTKFTAHRVILAARSEYFRALLFGGMREANPGIEIEVADAS
SIAFDALLRYIYTGKMFLAEYREEIVMELLGLAHKYGFLALESAIQGYLKAILDVKNVCLIFDMASLYQLKDLYETCLEF
LDANAIEVIASESFSMLSRTSLIDIIKRDSFCAPEVQIFRAVSDWIEVNKDSKEEDIESVLQCVRLTQISLHELFHTVRP
TKLYSADAILDSIKIKTESRVSEMNFRGHLVKDENIATSSYGTDVIEGEKRDGLLDNESLNYDLDRGFSRHLIADGDKGI
CIALGRPSIINTIRMLLWDRDLRSYSYYIEVSIDNQDWVTVIDYSKYLCRSWQILHFAPRVVRYIRIIGTYNSVNKVFHL
VHFECMYSTGSFKLSDNGIIVPSDNVAVPTASASVIEGVSRSWNALLNGETKCYDWNSGYTCHQVGSGSIVVQLAQPYFI
GSMRLLLWDCDDRCYSYYIEVSTDRQHWTKVVDKSNEQCRAWQHLEFKPLPMTFIRIVGTHNTANEVFHCVHFECPSTIS
NTENCNEITEKLEIGRGGRYLINIDYFNCYPFLPSSPL
>Nvec_XP_001630614
MSSNAWQPAFKTIKERGKHMWKNPVLSDVEFLVCTSAGVKISIPAHRYVLAVSSPVFEAMFHGAMAESSRKVSLPDCTAE
ALSEMLRYAYFDEVELTGSNVMSVMYLAEKYILPGLNEKCTQFLQENLEAKDVLFVLPEAMEIQDENLQRHCWELVDETT
EEVVMSDAFLSVTRELLCDILDRDELMIKELELFKAVDRWAEQQTSSEGLGVDGESKRNVLGEEAIRRLRFPLISQEQFV
KHVLPKDILNREEIVDLFAHYVLPKEKGSVLFRATSRGLKIRGVSSSVTRFTSVSLTPSYRTYNEHSICFEVNTEIELSA
VILFGSLGNTYKVTFDQRVVIHPNTTYTLRAWINGPKSLCWQTGRRVVRNGDVEITFMDSPSSENRTRVEGVQFPVFHFS
Y

>Nvec_XP_001630590
MGVDNIGLIIHLVLTYHSERHEEIESQVSKAFKEMSLRDIKDLPETKEMTMIANPLVEKIGELENWIRYVLPLILDMMSM
YGQAHSSFEETFEVTCDLLEDLNDYDRARHIVTCEECWNNMSAYPDDVKPKVQHPFSEPWDESDVILVVEEQEFHVHKFI
LKMASPVFKAMFEHVKDSKPIQLPGKKFNQVLDLMNHIYPTSNNSSITMDNVEHLSALAAEYQIKAVTNSCFDYLKGLEM
GVDNISLIIHLVLTYHSERHEEIESQVSKAFNEMSLTDIKNLPKAKEIMIIANPLVKRMEKLEDWVRYVLPLIIDMMGMY
RLDQEEFKIHGSVGGRCYYSNRLDGYDRARHVLITCDECRNYMSAHPGVSRCLKKYGNTSLLRRMEDIVEYIR
>Nvec_XP_001630504
MKPKEEQKATMNSDIYCGEMLQRMNGYRVAHSLCDVDLMVEGLTFSAHRLVLAAGSPFFHGLFTTEMKEKQENKIVLKQV
KASVMENVLEYLYTGKTSLNPENAEDLVVSASYFLIEGLKEKASNFIRSSLNVDNCIQVREFAEKHDCKSLSLAARVFIG
QHFGEIINGEEFLGLEFEKIEEFIASENTVVPKEERIYEAVTKWVRKAPAIRKKYFKKLFHHVRLSLLSRKFLNVTILQD
ELVTSNVACMQFVLQSMSNVQSLPPIQKPRKCLDSCVDAVVISGGFDDDNLPTASTICFIPEMNNTWYELTPLSIAKYGS
AAAYCDGFLYSVGGWEVQASQGPIEEDVPTTSVERYDPTTNTWLSVAPLKHAEKFVSVISLDGHLYCLGRSKSMYRYTPA
KNTWEYITGAPEELHGACVVADASNIYVIGGLDQNNMVTSSCWKYDPEVERWSLLSSMSTKRYLAAGTVKGREIFIVGGL
NEYGSTQALNSVEVYYIDTDEWSCGFSYLQFPRYSAGVTCIWDKVLVFGGEHEGETQNSIEWYNKIERRWELCPKMPCRS
NFCCNWLRIPKNLLEVVEELEF
>Nvec_XP_001630128
MNDQRKESTKLCDVVLRIDEQSYAGHRAVLASCSAYFYAMFNGELAESKQKIITMKDILPDYMQVLVEFAYTGRVEITVE
NVQNLLATASLLQFHEVKDLCCQFLESQLDPSNCLGIRKFTESHGCCKFLEVIDKYVLENFKQVMKSEEYALLPSELLVK
VISSDDLNIIEEEEVFEAVMNWIRHDLNTRVAKLPSLIRYVRMPLISKHYLLNRIDTDSLIRSNLTCRDLLDEAKNYHLL
PDQRARFRSDRMRPRKSMMGTMFAIGGKEAGETISNTTECYSLQTNTWQSSAPLIVPRQQLGVGNIGNRLYAVGGSNGYT
RLSTVEMFTPESNKWTYCKSLNTSRSGVGLGVLGETLYALGGYDGRTCLKTVERYDPQVDCWSSVASTTVTRSFPGVAEL
GGRLFVIGGNDGASFLNSVECYDPLSNKWTTLPSMCRPRAGIGAGAIDGLLFAIGGFDGMLRLDIVEMFESRMNTWTQVS
PLKSCRDGVCVAAYGCWIYAVGGIDGPSYLNTVEAYDPKTDLWETMPSMSRCRAAAGVVVLPPI
>Nvec_XP_001629915
MEMPSIVQSQSRLTFSRENHEKRLLELLNEQRKATKFCDVLLEVGDSEIAAHRAILSASIPVLSERLSDRKEGLKIKLEG
LQHNAVKAIVDYLYTSCLDVPADSVLNVYLAARSLGMNELVSDLEDFIRKEILPTEWLAIRVFAVRNDVQELLAMVEEFI
ANNIEEIFQKQAFLKLPRLQVELTARHSEDIFRPCFEAYRYSVLQVDLNHIPCSSSKLFSGIVLLSLIRCWQQSPYTSSS
RCHAMILCPASRLVMMNGRCSVGAAEVNGKLYAVGGYDRGQCLETVAYYDIQTNEWMPVTSLRRRRGRLQVAILGGKMYA
VGGSDGHSELNSCECYDEASDSWHIVAPMNYCRSNFGMATINNRIYVVGGYQGSHNLKTAEVYNPDSNKWVMVTPMSSGR
DNLSAVALDGKMYVLGGYNGWAYFNTVECYTPETDSWSFVTPMKFARRGAGAAAVGGYLYVIGGYDGTSFLTSCERYDPS
TNEWTTIAEMNTPRHNVGVAVVNGLIFAVGGFNGSAFLKTMEYYDPKTNKWSSFVSSFT
>Nvec_XP_001629817
MAEESKEPEVLIYKDSFHACNVLETLRTLFQGRKMCDVTVVVGKMEIPSHRLILAANSSYFYSMFTSGMSETAQNRINLK
EVDATVVRQLIEYCYTSTIEINENNVQNLLSIGNLLQFTTVVETCSDFLKKQLHATNCLGIGNFADHHGCGKLKLAAQSY
AEKHFLDVAKSDEFLLATYEQISSMLKSDSLNVISEKDVFEAVLAWIRHDLPKRQNYLPELLKHIRLTLLPPKILVDCIE
SEKLVQGDDMCIRIISEAKNFHLLPERRDKMQMLTKIRQKDHGTRMFVIGG
>Nvec_XP_001629193
MNELDFDEEHQRELIKCVVVGNSGVGKTRLICAEALGVGMKTQEANPRHVRHVHYPTVFAIDQYHVSAEIRDRANFTIDG
VSVSLRLWDTFGDHSMNRRFAYQNAHVIVLCFAINTPSSFKDVNAVWYPEIKKYCPRVPIILVGTKSDWRSQLNVSVVCQ
NTSLSKRMRECFLVSPDMGRQVAKEIDAVYYEASVVDMWGYHDVFENAIRAALNSRRQTRFWSSHLKRVSQPRLQPPNLP
PKPTLPNIIVPSSLFRENLKTIFHSSLCSDVVFLIGNQSIPAHAMLLAAMSPTFAHLLLGHELFGELIEEEKLQKFLVMG
DKSVTSFQEGDVMGEKAWLPRGFTSINLSGMQCVPTPGNKKHQLLVKLDTFISHSAFLLVLDFLYSGNLSVSACTKELQD
AALYLQLANLASYVENVINNMAYMNTEVYADINMRVTSCARAFVGKECMSDVAFDLGVVVVHAHKVMLVSQSEMLAAMFM
EGHFLEGGRQSVRLRDTNHDHFLALLEFLYTGRCPNLFLDDALGVIALANFFCLPRLVASCEQLVVKELQASMHSDELAV
SESVIVSMIEAELHNASQLQQWCRTYISCNYNALCRKHPKELKALNPDTQKHLEQHRWPPVWYILENDWYEKEKREQESR
ANIFRKYSLNVRYCCYNGQNIDCGLYGLFEF
>Nvec_XP_001629068
ERKRRFPKLINLRRRLLRSRGPYRTLDHGKVLKDLIENWSTQEITELVDEYEALIAQKDSITQSSICRQPVSSLKKDFQE
LLENAYCSDVVLLYSGSRFHANKAILSARCSYFKDMFSDESNQPLTYSVDIPVEEISTGMFASLLQYLYTGDFLSQNSGL
DSLELLIQLGDDFGTPNVLEQDLKCLLYNADHSDTLLVFQGFNLSELPVDSSVTETCFEVPCHKAVLCARSPYFRSLLLK
KDSSFPVLNESGVLKLLLDERVFPRQYARVVLQCMYTDSFDLSAVVKWTSEEERGHGLETHKLLTTAEVAMEVFEVARFI
DFDLLAQGCEDIIVEELSPASLLVTLEWSAKPHGSQWVAKQAEQYLLEEFTTIMRSEVFTKIPKLYLQRAIQSDFIQACE
QDILAAVIRWGESQVAQRMTALQGTCWVVFLISYSVIMDGKRSVKIKDIDNKALKRELAGLVEHVRVQHIIPPQCEVLEN
ALARGLIKQGMPLDIGQGSFYPRCPWMRRKNDKDFTRPRLFLPYYQEAKSILQERQDAELDLIQQRMLHMGKSIPDTLYM
VSNSKR
>Nvec_XP_001628616
MNYQNVKLVVVGDGGVGKSAFLITATTNAFPGEYIPTVFDNYSSNIMLDGKAYNVGLWDTAGQEDYDRLRPLSYPMTDVF
LVCFDIGSRTSFENIESKWLPELNHHMPETPKILVGNKRDLRSAQQNTSRDPITYEEGKALAKRLGMQYLENSSLTQDGV
KDVMSAAIRVGAGHVQANTFTCRCQGSRFCIHKVWRKSGQSKKADLGPQPPIMPPAGQAPWIEVMTSTFADDWKKMVLGN
DDSADVKFTFSGNAPTLHAHQTILSAASPIFRELFLGSKKVNAVPRKYQAIFSDISWSIAGNNFDSGKAKNSTELVEFQL
CSDISCESFEKILQFLYTGLPGFSEIEDSDFVLDVKKTAAKFHLSWLEQVCENKFGEDVYLNPSIGTYLNDETGAKLKSL
FFNKSQLCDFRFRVDGQVVFAHKVVLMARCDVMAAMLGGRFKEGKTENETPILDASLAAFLAVLEYIYTDHAPLETVDPL

EVLVLADRFGLPRLVTLCELYITKRVDRMIQKKVADSTSDVINLLNMAQVYNADQLAQWCRHFIASNYSVFESTGDLHLL
QGDDVDFVQENRWPPLSYIRELEEYEAKMNEMGKGDKCSVM
>Nvec_XP_001628243
PDGPPPDNKVYLFTDVAHSSNILCSLNSLRQQEDLCDMVLVVGGSTISAHKVVLASGSPYFRAMFTGGMSESRQDTVTLQ
ELDEKAMQNMIDFFYSGKIEISELNVQEVLPIACLLQVQSVQEACCEFLKRQLSPENCLGICAFADSHSCTELVKFSDAF
ARLHFVDVVQSEEFMDVPLKQLSRILVEDDLNVHSEERVYEAVMAWIKYDQDLRQEYAPEVLKYVRLPLLSAEFLMDRVA
TEDIIRNNRLCRCVSFL
>Nvec_XP_001628153
MLRRVDSEQEGSLEKFAQLRKEHILCDVEVIVSDGVSKAHGVIISIGSEYFRDRLTAIACRENKRIELKYKITKGTLENV
LDFLYSGNIKITESNACALLEAAVYLRISSLKFHCEEFTISRLSLNNCFQIMDLSKKFKSGKLDRASMEYICKNFLHLSK
NAEFLCLEPEYVEKILCCKFLTVKKEEDVFVALSSWVQHDISRKQTLPHFFKKLDFFAMSKFFIADHVANDQLIVSSPKC
TAHLIRVISSMLLPGREDVDLFLADRVTHSCNVDCVILAGGTGRKSRSIMCYVPSRKAWRLLRDTRIDREGHVMAISENL
SYIFGGRASESKNFLSNLNTLQLDPVLKTSQPIRGPEQDRYFAAAAILDGIIYIFGGKTSDRFYNVTETVVSYDPSTNTW
HSEPSLTYPRYAHCAVALHGNIYVMGGQSGSGNILRSVEKYSPRTGEWTCAPSMTIARMFASAVVLKNQIFITGGKNGWE
NSEILSSTEIFDACTNVWTVLGAQLCVALFACGIARVGSRVYVFGGADGLGRGQGSVECYDAGRDEWTITDKMPSKRAFL
QAGVISL
>Nvec_XP_001628138
MPSFSEPWGYSDLVLVVEEERFHVHRAILAMCSPVFRAMFQSKFKEASLEEIPLPGKKADRIYDFLCMLYPFPIQIAEHH
DVSSLLELAREYQIPKLTARCEERLLQKQSSVDLILLAQEFNLTKLLDKCLVSLSRMQLHDLKEKADNVGMRHHMNHHTF
LKTASICEHVPTGQPKFDDIDSSNLIVLLNNNIRWLKEQQARECRQLKDQHNREKATLLETVNELNECWGYNKLPIRGCA
CPSYTKSCDTCNTVLEKYVKTKCAELIEKLSADS
>Nvec_XP_001627035
MQCLNDFRKHNVLCEVTIVVNGKPFYAHRNVLAAASPYFRAMFSSHFREQNESKPVILENITADVMEELLNFIYAGTIKI
TPFNVKDLVSASNYLLMNSLKDACVSFMKSMINPSNCLGIETAANQFDCEALRKTANQYILDNFATVSQTDEFKSLPADK
LEEFLSSDETKVDREEQIFEALETWVSHNEDERKPLFPRLIQHVRFPLMSPYYLADFVETKELVLKTPECTPLLLEAKNY
HMLPDRRHLIKGSRTKPRKSMGFVTVIFSAGAAQSSLSSVERYDPRTNTWTMVAPMNVRRSLLNVAVLDGRLYAVGGCDE
NNFRLNSVEHYNPFTDTWHYSAPMATCRSSPCVLATGRALYVVGGVNYVGMSLNTGECFDPLANTWSPIAPMIEKRASAC
GAVCNGKAYVIGGWDGQKHLNTGEMYEPEIDQWTVIPQASTARWDAGIAVESDRIFVVGGCDRNALCTLETECYDPEKKK
WSKVASLPVATHGLKCSTIQVILESFVQHVNALVHWGQGLHATCDENKAFKE
>Nvec_XP_001626972
MADRRRQESANLAKEHAIGILNRLQHLRRLNTMCDAVLKVEEKQFPIHKIVVSASSPYFEVLFSGGLRESYLDTVTIQGI
DSETFSALLDFIYTGVINVNEENVQQLLPAAKMLQLDAVEKCCCDFLMRQLEHANCLGIYLFAEAHSCADLSTNALEFIH
RNFVLVCESEEYLELDRWHLLQLLDSEDLKIESECQVFEAAMRWVMFDMRSRREALGKILERIRLPLISPKYFEKFLSEC
QNESLHRMLGNILEGYRSYQSLAHSAVKGIVQPRRASRKCFYIIGGYSRSAGGRWSDTASLANVEKFDTFTQTCYGSTPT
SMSYPRSGHCVASVSGLVYAIGGENDSLIYDITECYDPTLNSWSVVAPLTAPRVACGACVVDGFIYVLGGWVGSEIADSI
ERYDPKENLWEIVGKVETKRLHLGVTELHGNIYVIGGISELGEQLKCTESFNPLKDKWTKLADMSTRRSYLGVASLGDAI
YAVGGENEHEGVLRSVERYDYATDEWVLFAMLETPRAGLSLATNNNMLYVIGGRTAGGEFSAPITMATIEVYNQQKNKWT
HASDLSLSRCEFGVGIV
>Nvec_XP_001626607
MGFFKNSVEAPKSLSKNTHPTLMEGLLLKSLNDQRQSKILCDVVLLVENEEFSAHKGILAANSHYFMAMFTTDMIEKEQE
RVILKKLKPSVVKEILDFLYTGRIEIDNKNVRDLLEASSFLIISSVTDKCWQFLEEYLNVETCLIVLSIADEFYNLDLNK
KALKFLCREFLSVAKTREFLELSTAEIKELLSSDDIYLDDEIQLLEILIKWVNFDRSNRRKYIPELMKLVRFHFIKPGIL
DSQLFKDLATDLEIVSQTEQLSSLTSGGEAPRKSYSNIEVIIIAGGVDETRILDTVCCYIPSANKWCELASMNTPRWRSQ
MVLLNNSVLAIGGLKEVSTTNPEIPFLETYQPRKDSWQAIHTLDLPAAPMNQPRFGASAASIDNKVYVIGGCHGRITHQS
GEVYDPSTERWTFLAPMATARVHCATAVHDGKLWVFGGLSEPRGSAVRDVECYDPDTNNWTSVAPMPGARA
>Nvec_XP_001626365
PLLPRIDDVPWCLNDVSKVLRLGRAREHFRAISPQTIERISFMLQRPLLRIVREARRLALRHERLSKAEIQTSVRLVLSL
SMSRSCAVLASKALSMYSMCGEPFLKSKRARSGLVFPVGRIFRWLLDMKVACRVYDAAAIYLAATLEYIAEETIYRAVTT
REVIDHVVPETVEKCVNMDPDLWSLYQPYCHLLSGRTCYGIVETLEPYAVTKVKRTLSPSLERSPVAPKTHKPLEPCFVT
TCVTSISELKELIAHVQKRLGSESLLTKDNRALSSLAWLPVALQTLLYFMTCSHQVDVDITGILANTKRSHSHLPPLVEW
IRVASIHAEHRASGVVDDDDVRQAARLLMPFTDCEPRLLSSQCWSPGLSPSASIAACQFDMGLRMLLCGKRELAPQALTT
LGPEKINAIDQQGMTPLMHACAQGDVDLVKLLLDNHAYLDSQVPHRDAQRYPQVAAAVQGWTAICYAVMIGHVYICQMLL
DAGANPNASQGTGADAALQLAVAAGHYDLVSLLCARGADPHVTWALGPGKTGFGSTFATAAACGHRKVLRKLLAEPPPAR
DSDMMSLEEILSEGSPVSKGGNSPKLNRKRMKVLQEAMYHSSEHGFVEIAMELRSLGVPWSLHCWTQTLYHAYEQGQTAH
ARCLFRDFQSIPVNEYTVEFCEDGLAVLFHIFRECEDLSLMKDLASVFSACFGDEPLPTIEELPSTAPKIRIGADYVNNQ
EMSDVTFVVEGEPFYGHKIILATASPRFKQMLTIKPSENSEGHVPCIEITDIKYDIFTHVIRYVYSGKTQDPQEHWRVLE
LMHAAHYFMLAGLRRHCERLTADRMNVTNAIPAYKCAKCYEAKELLEYCECFMLANLETMIGDKTLRDLVLGEAGRTVSK
GGRGN
>Nvec_XP_001625907
MCDITLKTTSEDIFPAHKIVLAAKSDYFKALFTTEMAEKNCQEISLDISTRTLKAILKYVYCGDVSLNVSNARSVFVAAD
YLMMDHLKKRAAAILKRTITNDLYFEIRNLAETYNSSILDRSTMRYICRHFSLLSQRGGFNNLNFETVERVIRSDEVIVK
QEEEVFEAVQRWVGADMRRREHYFEQLFRHVRLCSISEDYLRLMIDDMLVRNSLACMKQILKSLQRRVTKEDNEDIDEPR

TCLRKHSRAVILFGGMSGGRTITDTVSWIPGANTWSKLSDMRTPRERHAVVTCQGYVYVIGGYNNSCAVDRYDPRTNTWC
SVAQVPDKTIASAAVAFEGKIYVLGGSDGFNAMSTVQRYNPHTNTWQLLAPLTQKRKAHCAVVLHGNLYAISGCTYDNDS
LASVERLDPNVDHQPWQRVASLRHHRKYACAVASTNRIIVAGGYQDTSSTALRSCEAYDVMTDEWQGMAPLLLPRAAAGM
VRMRGHVFVLGGRSDLQSVRSVERYYEGEGEW
>Nvec_XP_001625887
MMNELRKKRELCDVLLHVGGREFRGHRIVLAGASSYLRAMFTNGMLESGMRDIKLQGIDPAVMEILLDFVYTGIVDISVE
NVQALLSGATLLNLQTLSQACCSFLQTHLDASNCLGIRAFADLYSCTELENAAFKYICQHFLDVIKCDEFLQLPYRDLKS
LLESDLIQVRTEDQVFEAMESWVYHDFKQRKDYCSDLLARIRLPLLSLEFLEERVFPSKLIKSSTDCQLLLARVLNETSR
NLPPYMTTQRAQPLSIYVIGGRNALDCQLATLERYDVLADEWVSMENMKHARTAVGACSLNGLLYVVGGECAVNSPHDDT
MYVRYMECYDPIVRQWILLADIAIQRSFVSVVAANGYLYAVGGEDRTCSYNYVERYDPKSNHWITVQSMRRKRSGAGVAV
CDGKIYVAGGYDRGVHSDRASVECYDPENDSWSFVTELEKARSGLVLAEYNGCLYAFGGRNRSTDHYFDLVEKYNPQTHQ
WTPVAPMLTPRAWPSAAVHDGKIYLLGGFDGASRLASAEVYDPELDTWSYIRDMHVSRAGCGAAVL
>Nvec_XP_001625690
MSCNKLEDSHVYGSHLVSDDRFRRFVFRELNEMRKREFLCDVIILADNGCRRFPAHRAVLAASSRYFHKLYNGTTLARYS
RETILSGICDRELETVVHYIYTSEVCIDGDNVRALLHAAYNLGLDLLTLACEKYLVENVHHENSIELMVLAQMYSCDKLL
KTTGQCIGEHFLRLSNTQPFFRMSPRQLQQIIADDDLNISREEEVYNAVIKWIRFDYERRRDDFPNLINHIRLQFISRRF
LLNTVDKEPLVTSSEFCRGLLADALALKAVDRKKICIYGHLHHKKRWRTHLMKVIMIVGGITEDPGSSSHCYNPLTNTWY
SVARIKQDRLDFGMSRVGYWLFVVGGSSPRESGSVLASVERFDPKYNKWEKMESLIQARSKFELAEVEGKLYSIGGTIGG
EPLTRDNAVECYDQVNNTWSSRAAPHQLRHFCSTAVLHCRIYAIGGISRCGTVLSTVERYEPQYDRWMTAAALNTARGGA
CAVVLNGHIYVMGGSSERSALSSCEVYNPSMNKWTYFSDMSIKRDRAGAAVFDDKIYVFGGSYGNVVIDTVECYDPAVGR
WETVAHLPNARHGFKCACALVNKELVREVKVKAKSEKLI
>Nvec_XP_001625538
MEETDEGVCQNFVDSLHPGCVLSRLQELRKNEELCDITLLVENKRITAHKAVLAATSRYFNAMFTGQMRESSTDLVEIHG
VDSVSAELLINFAYTSRLRITDANVQNIFLASDLLQFKSAKDACIDFIMRQIETSNCLRILALAERHGLKILKEAAFSVI
MDNFTEVVETDDFKNLSTSQVIDILLRDDLKVTEERQVFKATVTWVDHNREERKQHMSELLKVVRLSFIPLRVLCTEIGE
NPLVQSNVFCQDIIEEAKTCHCECACVTMRRSYAKTLFVLGGETSFMKEVKSVERFDHERTEWLMSKPMSEARASFAVAV
YDNKLYVIGGYRRGRKLNSMERYDPVEDTWTGLPSTSKCQGDMRAAVLGDYIYVAGGSSDRLLTCNYVERYSPRSERWQT
VATMHRQRRRFALAVLDSRMYAVGGFDDNTGDLSHVEHYNPETNSWVEDPEMLICRYDFGAQALSGYLYAVGGANGRKGS
LNTVERYDPKAQTWTRVASMKHCRGGVSIAVHCGKIFAINGMNEYMSIVDSVECYDEVENRW
>Nvec_XP_001624475
DWQLTKTSLNSRVAHTFNSPLMSDVAFVVEDPAKQDVVSIPAHKYILGVSSPVFFAMFYGQMSTSISKVELPDCDSVGLI
EFLRYLYCNRVKFTMESAIQALYLSKKYLVPSLAKHCVTYIRDNVTPQIVFDVLPYALKLAEKDILDRCFELVDFHAVTL
LGSQSFLTVTEDVLVALLQRDSLRVTEVDLFRAVQRWLAHNVEQECSVTGELNFLHTDKHVQRLMALIRFPLMKQSEFIN
QVVPSGFLSPDDVTQIMLGYVTPQKACNFSSQPRSGPIKRCARFQSTERWFLYSGKRAETVAFTVSASVRLHGVRLYGDE
HACYRVNLELFFTMSPEHKHTKNGCYNSELIQTDSYHGYDVKFDTPVMLKAEIEYQLSALIVGPPSCYG
>Nvec_XP_001623399
MSTAATLPLTPPPLLRSWQPTPDGPPPDNKVYLFTDVAHSSNILCSLNSLRQQEDLCDMVLVVGGSTISAHKVVLASGSP
YFRAMFTGGMSESRQDTVTLQELDEKAMQNMIDFFYSGKIEISELNVQEVLPIACLLQVQSVQEACCEFLKRQLSPENCL
GICAFADSHSCTELVKFSDAFARLHFVDVVQSEEFMDVPLKQLSRILVEDDLNVHSEERVYEAVMAWIKYDQDLRQEYAP
EVLKYVRLPLLSAEFLMDRVATEDIIRNNRLCSDLLLEATELLLLPRRRLQSQTTRVQPRKAVSSHQVLYAIGGMSRRDA
SKSGERFDPRQGKWKPIGDMKICRWGAAVGAIGPFLYICGGSDDASRLETVERFDPFTNVWVPSVSMDASRNGVGVAAGH
GRIYAIGGFDGSMPLNTAEFFDPKVGRWIEVSRMNHCRFGVGCAVLDTCVYAVGGSDGTNLKTVEKFDPETNTWTVVAPM
NTARKQVGVAALGGYLYAIGGCDHGTRYDTVERYDPDKDRWTYVCPMSTPRSGCGVGVLDGFIYVVGGYDGTTYLQTVER
YDPLSNKWHP
>Nvec_XP_001622928
MAAAEDDNTKALRSRNVLGSHENEAFSVFKELRDDGELLDVTLHVQGEEIKAHRVVLAACSPYFRAMLTTGFAETFMSTI
PLHECDPVGVQSIVEYFYSKRLTITKENIEGLLSAASLFEIPSIVHECGEFIQGEIQIDNCLGIQSLAGQYSLSSLKSKV
DDFVCWNFMKVACEDEYDMVPPDHLRDILSQDTLYVNGEEDVFEAVMAWFEYDQERVHKYPDIMSLVRFPVMDHKYLEEI
YSDEDIMAVPTWNKIISDAVSNPLRRKAGKSRQSPKVLYLIGELYNEPIQMFNLETYSCTTVSRMKQNAGTAVVADGSLY
MVVGGGLMVDRFNPRLNDWVRVADGSKESEFAACGGVGRMYLVGGGRRAKFLDLRTKEWTNLPLMSIRRNYHAITCVENK
VFVTGGVTPPGEQAIADAECFNTELNKWESIPPMNHVRFRHELASAGNHVYAIGGCDKVGSAHKTVEVYDLKSCTWQLIG
ELNHPRRDFAVGVIQGMIFVFGGGGTTSIEQYNIDMDKWTIVGSLNKRWSNFRCVLYPFINFKL
>Nvec_XP_001622433
MVTPKKSSKCDSSSYLTSGHAKDILTSVNKLRKDGKLCDVVLQVEKKEFPAHRIVLASCSDYFYAMFTNDMLESQKGVIE
LQGLASDTMEVLLDFVYTETVKVSVENVQALLPAACLLQLTGVQKACSEFLQHQLDPTNCLGIRAFADTHCCDELKAAAE
NFIHKHFTEIVKSEEFMMLNSDDVMCLSKSDNLTVPSEEAVFDAILKWIKHDQVQRKDYLPVMLENVRLPLLSPRFLTDV
VDNEALIRESLACRDLVDEAKRYHLRPECRHLLQSPRTKARYGLAEMMYVLGGFGNMQSPVDIVEKYDPRTKQWTEVQPM
SKKRRYLCAVALGNRLFALGGYDSSSRLNTVECYNPIVSQWNTVTPMLQRRGLAGAVTLDGKIYVSGGFDGTVRHTSVEC
YDPNIDRWSMASRMLSPREGAGLSNMDGILYSVGGYDGTNILNTVERFDPRTGQWTAVAPMGTRRSGKDILNHFILEDSA
KIICYILGAGVTVLDGQLYAIGGYDGNHHLATVECYSPCTDQWRPVASMQSKRCYVGGSILGGKLCAVGGYDGTALQDTI
EIYDVVSNAWSILSSMSTSRCDMGVCVLADPYG
>Nvec_XP_001622304

MSASTTSTSDSVKSDTVYYVSKEHGHAAFQVLNEMRTNSEHCDITLKAGNLVLSAHRVVLAALSPYFRELLLPTNNEKAEK
EYVMPDSLKPGAVVAMLEFFYSGTLQINLKSIEDLLAAASTMKVSHTLREKRMILLSFSVAITFLYEDFDLKSLRKSHFI
TSMGMHFLAGDHLYNCTAFDYGLTQIHSKYYRISHV
>Nvec_XP_001622152
MAANMTCIRSYVINPMDSASQHIMTVISYSIAILAAASNAFIIYLMLRAKKPRVLKAFYRSHASLFTLSLSFSDVLTGTI
SIPLQNAARIMNIHSSIVCQTHGILILLFPTITVLNLMVISIEKYLAIFYPFYLPSTRAVRGLIASMWLLGAAITTAYNI
NTRNIFPVIVNEDSYTLVCAPDSSLPTYSATVFAFLILVFILPIVAQIIFALKIRRHLKQRHRWRPSRQALTLNKFLWLS
VVAFTVPCIPWLLYLMINIIFQLKLDYEHDTLARYCTFLSLLSNALINPVIYFFHSTRQFRQVLCNKRSPRCAVIPRGNG
GAFAFQAAGGDVAAVNVGGFGDRVIGCTGDVAAICIGGIGDGAIGGGGDGAIGGGGDGAIGSAIGDGVNSDDGAIGCDGG
GGANVGGDIGGNNGAIGGDNDDGAIGVSCTMQASPGVGLPHEQEEDVPDYGKVFKESFKVFKEQGPKDSAPQTCNLDFSK
QWKRSDAVLTVDNNQKFHVHTATLARVSPVFEELLTPENLAKYPNKELYLPGKNADEFREFLKVLYSAEPVREYKIHFLL
PLAHEFKTLVVLKRCEESLMPKVKNETLAFKYLSIAQMYGLEMLRRCCVKILKYKSLPDLRANRMYREIAPKSGWDITEG
RVEILESELGQVQTKLSTICEQLITELFGELYTKQSGGRPPPMNATMANKLCFVKDSGIKSPEIRIISEALEKLEKLYS
>Nvec_XP_001622151
MQKKGSEPDFSVPWHLSDAVLVVEEKHFNVHKSTLSMWSPVFEKMFRERNDQEICLPGKKSKEIKEMLLVIYPTSKKVTE
ENCYYLLTLAQEYQMEQLTERCEKYLIQREKTPFQAIDFLVLAHGFKMSELCKQCIEIAKHISLNELRKHEKYSLVEDGN
GKQLAERRVELLEGKVATLEQKPNSIRKEIKETCEWAIREISRVVFHKKHQGDGKHSPRALGDCLRVLEEEALQSPEMNL
VVQLLQQRLRKIYEPQQRM
>Nvec_XP_001621571
MASHRRFQIPNHSNEALQMMNELRKKRELCDVLLHVGGREFRGHRIVLAGASSYLRAMFTNGMLESGMRDIKLQGIDPAV
MEILLDFVYTGI
>Nvec_XP_001621522
MDFDRALHGGERADETPSDVVKYEAGSKHGIKALMVMDDLRGRKQLCDVTLCVDERQIVAHRLVLASFSSYFQAMFTGGL
VESFEDSVTLRDVDSGAVELLVDFAYTGKLDITTENVQSIMYASSLFQLNAIQKACSEFLERQLHPSNCLGIRSFADAHS
CVGLLHASEKFINEYFSDVVKNEEFLLLPQEELAVLLSSEDLNVDCEEEVFVALIAWTKHDVDERKDLLAELLQNIRLPL
ISPHFLIDQVEKEELISHDIKCRNLLDQAKNFHLLPERAPKKISVVHPRRSLMGALYSVCGMDSTGHSVKIVEQYDFHGG
KVKVISPTHVARSGVGIGVLDNKLYAVGGHDGTNYLNSVESYCMVTKQWRFVAPMCNPRRYVAVGVLGGLLYAVGGYDGT
TVLDSVEVYDPKSDQWKFVSSMKNKRRHVAVGVLNQLDLCLGYLYAVGGHDGVNYLKTVERYDPETNEWSYVASMGARRG
GVGVATLHGCLYATGGYDGTSNLSTSERYYPSDDRWAFVAPMSVCRSGHGVGVAGGRLYALGGHDGVSYRNTVEYFDPKV
GEWRMVGSMGMCKAVAGVAVIKGRH
>Nvec_XP_001621258
QLPTGRLFHAGVRIDDAFFIFGGTIDNNVRSGELYRFQLVSYPRCTLRDDFGRLLGSQQFCDMVFVVGEDDKIFPAHTAL
VAARSPWLRRHLLHLKETIQVIQPRYFPKAHPNVGFQGSGEEEGQIEIRLHDAHPRAFEITLHYMYTDSIYSLVKDVNGS
EAISLMMDVYGLAVKLEIGSFEHLCVQYIEASITQDNVLVALERAARLQLESLKEFCLRFIVREANYSSIIMSKEFESVP
RDLMVDIIRRRQAHPQVRTLEQAMTGFLCSGQDFHDITLKVDGNPVGAHKAILAARCSYFEAMFRSFMPENNTVTITIGE
TVPSQKAFDSLLKYIYFGNVTMPPEDSLYLLSAPFFFGFTNNRLQVRFHSTK
>Nvec_XP_001619954
WQTSMCSLRERNKHMFNNELFSDVRFLVGRSAKQSIPAHKYIMSISSPVFSSMFFAFGALQAEQLTREQIEISECEPAAF
LELLRYLYYDEVQLTTITAPEVLYLARKYLIPHLADICTDFLVTNLTVDNSLTVLDQCCMLGVGKGLEKQCWDIVDKRAE
NIADDVTFVEIDHGTLTAFLCRDTLVAKETTLFNAAVRWAGKECQRLKLPLTAEARRQVLGDTFYSIRFPLMSMKDFTDQ
VARSGYLTYEEVANMYIGFNSGFQKCHVKF
>Nvec_XP_001617883
MADSSKSVAEENVLHPLEFLQTLDKMRVSGEGCDVTLKVTDQSFPGHKLVLAANSTYFRAMFGLREGFVESSKNDVVLHD
LDPKGVNAVISYFYNSKIEINADNFEAVFAVANMWDVKFVLTACENYLRVHLSASNCLGFQILVHQSSAFSENFRTLLDD
FVNKNFMVICQHEEMLIIPVEHLEKILKGDELCVKSEEIVCQAVLRWLRHDVTGRKQNAARLLLAVRLGLLSDSFITLVL
FQNDVIQCRGLRVY
>Nvec_XP_001624184
MDCTPSCRLPKHAELIQSAATWENLPGFLSVARRICANPTNFTDRFGRHVLHVAATCGKGDLLEWLVKECKVDVGLRDVE
SAWTALHRSVYYGRLDCAVRLIRCGADLYACDKEDLSPLDILMLDSPYKMSSADDHETQLYTWGSNTNFTLGHRDENMRQ
HPELVAMCKFHSAFLTGDGKVLTCGHGRGGRLGHGDEKTQLAPCMLSGIGDDKCISIAAAQDHTLLVMESGVVFSFGLNN
MHQLGHSPPSMQSLVPKMVQARVWKGKQVIGVAAGKYHSAFYTHTELFTCGLNIGQLGHLKGEKYQTIPRQVSALVQKDL
RLASVMCSDNATVCATDRGDIYLLQHYTCQRVINRFPELTRLQVCGGNFDVTSTPDVNKAHEKLTLTLLNAKGEVYQWRS
SNGGLRKALWSLKRELFVSDIAVGKNLLIITDKGEAFMCTQSDDVITDVKRTPSSSSLTQLGLLMGDWNRKDFIGYHAER
IPMVHRGMRIFTDAKSKGFAVLQSHPSAGLGKYPKVSASRMCEDFKQLLDESTIEDSIHDVTLMVESHTFPAHSFILASR
SAVLRDLLQAD
>Nvec_XP_001623193
MADRILVVTVRKQGWEQLRAAKLAKNICQSKKSSTETGKAEDEALSDEDSDIEIDKTAEIKGKNQSEETEAGGVTPGTLV
DITNEALGNKGPEPSPAFDVDRGKVSDGEKKPKCATKRKKNKSDDSFTMPDDIYKFTFDSIPQSTNGTDLEEIPGKENDK
SQCNTSIAHNNCRETLPQVEPERELCDGTETCKIDDIHDLDVPESVVREYEIHVHSFWLALNSSYFRGLFFSSGMKETRN
KKVSINVTEDMSAMFMILIESLYRPDVVINKSVDDLLILMRLSLIYDADSTLKCCQRLLSTAELSIPICDKALNMQEHER
LSEMDEFIKRCEQHLVEAFSPLDYQWTREEFFSLSATALKSVLGSNELCVSSENTVFLALMRWAEMNEVLDEGLEPLLNH
VCFKAMTINYLHDVVTSAHPIASTVEKFPLLFEEAVFFHAFSKERQAEEGDYEPRKAFEEPILFNWHVENYEDTEFEKKK

KIVKSPHFWVSGYEMFLELKLRSSGFQGLYLTICTQQFNHNSKGFVKLKYSLSSNFLKATHVFNEVDIFDKEGSGWGYEE
FFPLTWPEFRDQLKRSPLIITAQVRLLE
>Nvec_XP_001621835
RYYACLCGHEALVAYLLSIGARCEPNTFDGERCLYAALTDRIRKILQNYKAITSTCMRRNPYYEFLRRTLESGDFADVCF
VIHGQRFCAHRAILTTRSSYFASMFETKWKDKHVITLKNSLVKPWAFKALLNFIYMGMLI
>Nvec_XP_001620300
YFRAMFGLREGFVESSKNDVVLHDLHPKGVNAVISYFYNSKIEINADNFEAVFAVANMWDVKFVLTACENYLRVHLSANN
CLGFQILVHQSSAFSENFRTLLDDFVNKNFMLICQHEEMLIIPVEHLEKILKGDELCVKSEEIVCQAVLRWLRHDVTGRK
QNAARLLLAVRLGLLSDSFITLVLFQNDVIQCSDECKNLLRQRVMRYSIATKEWQDLNVEMPLRNYIIHCGLVAHGETIY
AIGGLCNAIKLSSDVTQQSLTWEGLPQTVVDHYRPGVCLLGDNVFIVGGCDRAHSDAHTVVEQLDISDPHAGWAGVASLT
TPRWGLGAVVLNKKIFAVGGFDTDNRAPISTVEVYSPRNDTWTQIKPLNRARRRFGIAEVDGTIFVAGGFSEDSVEYYNP
LSNQW
>Adig_212v118523
MATVQENWQTKCTSISQRTKYIFNTALLSDVKFIVSVSNGESESKVIPAHKLVLAIGSPVFFAMFCGQMADTRDSIELPD
CEYEGVLELFRFIYSDEVKLTGSNVMHVLYLAKKYVVPLLAEKCAEFLRKNVDASSAFSILSHSQKFEDKDLENRCWKVV
EVKTEEALASDDFVVIERSLVESVVKSEKLNVKEVELFKAVNRWAEKNIEKQGIASDGNAKRAIIGEEILKEIRFPLMSQ
EEFARFVIDSNILNVQEVGDMIKHYSQVLTSPLPYLQSPRNEVLRRIGRFKEYCSGCWGYGGSLDSLILSVDKDG
>Adig_213v118522
MATVQENWQINCTSIRQRTKYIFNTALLSDVKFIVPVSNGESESREIPAHKLVLAISSSVFFAMFYGQMADTRDSIELPD
CEYEGVLELFRFIYSDEVELTGSNVMHVLYLAKKYLMPSLAKRCAEFLRKNLDASNVFTILAHAQKFEDKELEDRCWKVI
EMHTEEAVTSDDFVVAERSLVESVVKREKLNVKEVMLFKAVNRWAEKKIESQGIASDGNAKRGIIGEEILKEIRFPLMSQ
KEFARFVIDSNILNVQEVGDMIKHYSKVRTSPLPYLQSPRTARVCRFNEYCPAGVESCWVYGGNLDSLILSVDKDISLHG
VQHFGHEGCVYTVSMEIKVTTSNLSLAKKSGIYSSKKDLDHIYYGFDVLFDTPVILKSGKRYEVSSMISGPRSWYGKHGQ
THVNFEGINFTFSRSDSVRNGTDEKRGQFPAFFFTRSG*
>Adig_1270v119354
MFYGQMADTRDSIELLDCDYESLLELFRFIYSDEVELNGSNVMRVLYLAKKYLVPSLAAKCAEFLRKNLDASNVFSILPH
AQKFDDKDLANRCWEVIEVHTEEALTSDDFVATERSLVESVVKRQKLNVKEVELFKAVDRWAEKKIEEQGIVTDGNAKRR
IIGEEILKEIRFPLMLQKEFASFVIDSNILNMQEVGVMIKHYNQVLTSPLPYLQSPRILVLKRACRFKNFKQHPAEGESG
RGRWNYNGKPDKLVINQDKDVRLHGVQHFGREGCEYTVSMEIKDTATNLSMVKKSGTYSSKKDFYHAYYGFDVLFDTPVI
LESGKRYEISSMISGPKSWYGEKGETPVNFEGINFAFSALDAPGNGTTEKVGQFPVLLFTHSG*
>Adig_1368v119439
MDEIDYDQELPRELIKCVVIGDTGVGKSRLICAEALGVGILPPQPSQKQHVHFPTVFAIDQYHVSQEIRKRGNFKVDGVD
VALRLWDTFGDHEFNRKFAYQHAHVVVLCFAINCPNTFKNVNAVWYSEIRRYCPRTPIILVGTQSDWRMHFKNSSLVNVS
RSLSKRMQDCSLVTPDMGRQVAREIGASYYEASVVTMWGIHDVFENAVRAALITRRQTRFWSSHLKGVSKPQLQPPYLPD
KPDAPGINVEVSTYKENLLALLHSGMCADVEFLVQGQPIQAHRAVLCAAAPVFAELFSANELVINSGIRTPRRLVFQDAL
VFGKAGSLPRGFVAITSYNKTWVSDECSFEVQVIIDNFVSHKTFMYILEFLYSGVLPVEACTDELEEAAQDLQICNLVDY
VTNVLNNVGYMNTEVHSNFIRGMASCSKKLVNTQMFSDIVFKIEGRIILAHKSYLVAQCEMMAAMFMAGHFKESGVQVVE
LKDTNFDAFLKVLEFLYTGECPCLALDDTLAVMALANFFCLPRLVAFCEQVIVKELQVSMASDEMAVAEDVIGYLVEAQV
HNASQLEAWCIHFISTHYNAICRKCPKLVKNLDTSTLKNIEEKRWPPSWYIVEADWYAKAAEQLNNEKIAEKLRKSHKQA
RRKQKCTCLRVSA*
>Adig_1383v103255
MGDSPDIRFGNGHIAAGEIDHGELLSSQTGALYRSQEFTDVIFVVEGSKFYAHRVILAARSEYFRALLYGGMRETDPDTE
IEVKDTTAPAFDALLKYVYTGKMFLGECREDTILELLSLAHKYGFLALESALQGYLKATLSIRNVCIVFDAAVLYQMRDL
CSTCLNFLDRNAVEVLSTDGFSCLTKSSLVEIIKRDSFCAPENQIFRAVKEWVEAAEEISDESIRDILDKVRLPLISLHD
LFNIVRPSQLFSADAILDVIRIKTESRVNEMNFRGHLVPDENVATSRHGAEVIEGEMPEFLLDGDTTNFDLDRGFTRHLI
LEGDKGICIALGRPSIINTIKLLLWNKDSRSYSYYIDVSMDNEDWIRVIDYSKFLCRLLLWDLDERRYSYYIEVSCDQQT
WVRVVDKTNEACRSWQHLSLDPRPVVFVRIVGVQNTANEVFHCVHFECPASSKEPSVCGSDSSDHSGTTETTISSIM*
>Adig_1599v119618
MFTSELSETGQAVVNLPDLDPQAVEALVQYAYTSHIEIRVDNVENLLSVSCILQIDEVKDACSEFMKHQLHPSNCLGIRA
FAEGHGCNELYKIADAFTKERFVDVVKNQEFVLLNSECIAQLLSSEDLNVTSESQIFEALCAWTEHDERNRKRFMPKLLA
LVRLPLLAPEFLVDKVEMSPLFRDMSPCRELIIEAMKYQLLPKRRFQLQNPRATPRKATVGKLYVIGGMDSSKGAIQIEH
YDPRKNQWSLVAPMVTRRLQFDVAMVGGDLYVVGGRDGLKTLNTVECYNPITKQWSPVQSMNTHRHGLGVAMLGGPLYAV
GGHDGWSYLSSVGVAVLDNFLYAMGGHDAPASQDCSRQFDSVERYNPNTDQWTMVAPMINCRDAVGATCLGDRLYAVGGYD
GSKYLSAVESYDPEKNKWEEVASLNYGRAAACVVSVPINPAEKGI*
>Adig_1739v119736
MQGPMDHNPDFSRPWHFSDVVLSVEGTKFHVHRSILSMWSPVFEKMFTADFSEKDAKEIKLPGKKAEEIGVLLNIIYSCE
TAGVSEENYEFLLALAEEYQIARVKKLCIQYLSRNVNATNCEHIYAVAEEYDLYTVMESSLEESKYRTLRSFERADDFEN
LTHKAKVVFLKIRVQELEGTLSKYVTTCSSLVKRVYEMVAEEGKESVCDNYEAHSGWSRDARTFKPSCRCCVRRVQNSSC
QIYYGTLREDLKTLFDLRKNNQTVQRLQEEQRKGKWGWRH*
>Adig_1759v119756
VKGRTIPAHGFVLASRSQYCHQLICENKEKRQGEADERTKTIPLNENVDYIAFLSWLRRLYSGGDICDEEVFVLKDVVVK
GKNGYHNLNEGGSKETNKTVNEMGKELDEEEMLSSRVLADLLFLAKTDHDVFPAPTCEEDEGFVQDSEDDVSEDDCTCLE

TRPLKKIEETMTKRD
>Adig_1845v103367
MSLHEVFKPVESVPFTDDSHNTYILKRISRMRLHSVLTDVVLVVEGKEFPAHRNVLAANSDYFMAMFSGHMATPNDIVEV
KEITAKAMEVLLDFIYQGEILITEENVEEVFCGSCLLLLESVSQACCKFIQERITLSTCWGIRALSDKFNSSDLLNRVDT
FIEENFMEAVNCDEFLELPPSHLGDLLMDDEIVISREEDLYELLLKWVDHNRGERIQHFPSLLEKIRLPQVHPDYFEEKI
ATDELVTEHPEVQEILAKARRQLYPIYPMMVERDDSKWQIARRTVECYDPQANGWKFVSPMNNQRDGACVVTEGRYIYAI
SGYDGHSYLSSVEMYDPSSDAWASGAVVPIIERRQDAMVAVVENKIYVVGGYHGNTFLPTCEALDLDKNEWNFMASLSSP
RYQAGVAVLNRKIYVCGGWSENGVVTASVECYDVATDCWAMVMPLPTPAAARTTFVNFPRKMIEKLWLKDIKKTGSEEQL
AQPTKKDSLGADHNLHGATY*
>Adig_2016v103420
MFGAQPDKSGVDKTCPDEWQTTKATIKDRCSFLFNNEFLSDIRFTVPALGESESKKSKHETFAHKFLLSISSPVFYTMFY
GAVPETRDIVELPDCDYEVLLEFLRYIYCDELTLNESNVSGVLYLSKKYMMPPLADKCWNFLMSNMYPSNVYSVLSVAEF
HEEEKVANLCWKMIDGQTEAVLRQSIERPQLEAMVTRDTLKIGELNLFIAVDKWATSECGKKGLTPDGKTKRTVLGERII
KAIRFPLMGEYDFRYYVIPSGILSDEEVGHIQKYFDKTLDGPVGFPEHRRTSLDHLDRRKRFRYSGGSWSYHRGLRDALV
FEVGAIELYGLRLFGRLDSNYSVNLELINEESGEKVLSQTGNFTSVPLKTYGGSEYDGFEFLFDQPVNIDTVTKYRVEAL
ISGPSSAKAWNCYRNTDWFSFSASDNENNGTNAREGQFPEFVFLRCQKR*
>Adig_2585v120404
MMAMAENWQREIPNIKGRIEYVFNNDCLSDIEFVASLPNSEYKGGKKAKMAIPAHKFVLAIGSVVFHRMFYGPLAEKGKT
IELQDCDYESLLELFRYMYTDEVRLNSSNVMQVSYLASKYMVPSLVDKCSEYLMANVNTSNVFSILMHAQKFDIKDVEEL
CWEIVKSEAKDVVPSDEFLTLPRSLVEDVVKMKKLYIKEVELFKAVDRWASAEIEKQGLIPDGKTKRQLLGDEIVKSIRF
SSMSQKEFAAHVPELNILTMEEVSSIFQFFSGVSPKAELKPSPESSSRYGHFLRCCRFTKVHPPIDDSSIGEDVYSLGVT
VSEPIFLHGIQLFGSEGNSYEVYTKVSEERTDIRYSALFEQSGSYLSEKKADDKRFYVFDVFFNEPFLMERAKEYKIMAT
VYGHRAWYGTGEKAFANQDDVSFSFNNKSQACTNCNAVNPCNDIPALIFTIR*
>Adig_2616v120430
MAAHSEILLSKCAHFREQGEFIDVRLKVGEYEFPAHRIVLAANSDYFHAMLARGMKESYQEVIELKDENISAAATKKLMD
FMYSGELNVNDENVFEVLIAADHLQVTSVVQQCCKYLETKLVELGFDVQFYGRIIMVADRRGLKDLKEAMESKMASLYKE
ICEKEEFLSYMNADLLSALLCRDDLRAPSENFVFKSVMQWIKYRKEERMDLAAQVIGGVRLGLVDIKDVIDELDTEEMQV
IPEMKTLLHEAFMYNHRPSRSSAFALANGKPRSKSMVLVAIAPKHVACFDVQYKTWELVTSMQKLVRQGQCYCAEVIGNY
LYVAETASSICRYDIVHDIWSNLPPISRSLGIQKICSLCHFEDHLYVIFKSSAPYRYNIATNQWQAVASSKAVCNLSHET
FSNKAAVVYKSCIYVLYGQGEFKQVENDHWGSGTFSPEVSALYCFHPKKNVWQKKASTKTPHFGSSLLVVNNNLCVAGGR
GSLSYSRECVPAAVEVYNDQKNAWSVVKQTHIPPNKLGAVEIEGRVYFIINSFPVDSGIRIPPGEVYPVVLEGWENLRKV
NRDAVLCYVPLKTEDLITEHISSLGSGLELEYTLGLSS*
>Adig_2617v120431
MVAHSEILLSKCAHFREQGEFIDVRLKVGEYEFPAHRIVLAANSDYFHAMLARGMKESYQEVIELKDENISAAAIQKLMD
FMYSGEISVNNESVFEVLIAADHLQVTSVVQQCCKYLETELVELGFDVQFYCRVIMFADRRGLKELKEAMESKMASVYKE
ICEKQEFLSHMNADQLSALLCRDDLRAPSEKFVLKSVIQWIKYRKEERMDVAAQVIGGVRLGLVDIKDVIDELDIEEMQA
IPEMKALLHEALMYNHRPSRSSAFALANGKPRSTSMVLVAIASTRVICFDVQSKTWKQLTSMKKLVEEGEHKQKRLWEEE
CYCAEVVGNYLYVATTGSRIHCYDIVHDIWSSLPPIPRSSGFERIGSLCHFEDHLYVINKSSVPYRYNIASNQWQAVASS
KAVCNLGQETFCNKAAVVYKSCIYVLYGQVKQVENEYGGSCTYADVSALYCFHPKKNVWQKKASTKTPHFGSSLLVVNNN
LCVAGGSGSLALLRRGHYVPAAVDGPSGDPAAIEVYSDQKNAWSVVKQTRIPPNNLHAVEIEGRVYFIINSFPVDSGIRI
PPGEVYPVVLEGWGAFERVERNAVLCYVPLKTENLTTEVRGLLF*
>Adig_3170v103671
MATVQENWRTNCTSICQRTKCIFNTALLSDVKFVVPVSNGESESREIPAHKFVLAIKSTVFLAMFYGQGADPSIRSIEVP
DCDYESLLELFRFIYSDEVELTGSNVIRVLYLAKKYLVLSLAEKCAGFLRKNLDASNVFSILPCAQTFDDKDLEDRCWEV
VDVHTKEALTSDDFVVAERSLVESVVKRQTLTVKEVELFKAVDRWAEKKTEEQGIVADGNAKRRIIGEEILNEIRFPLMS
KNEFVSFVADSKILDMQEVDDMIKLYNQVLTSPLPYLQSPRISVLKRVCRFKDFKQHSSVKDLWNYRHGYPENLVLNVNK
DVRLGGIQHFGREGCEYKVSMEIKDATTDLSLVKKSATYSSEKDLDHIYYGFDVLFNTPVILESGKRYKISSIITGPQSW
YGEQGQTCVNFEELNVTFSDLNSKSNGTCYLSGQFPVFLFIHYG*
>Adig_3451v121128
MSSPETWQTERSTIRERTKFMLNNDLFSDVKFVVRKSDSEGESESKKMIPAHKFVLSIGSPVFEAMFYGELAETRDSIEL
PDCDYESLLELFRYLYSDEVNLSGSNVMQVLYLAKKYIVPSLVDKCSQYLQDHLQPENVFNILTMAEKYEEKGLLDQCWE
IIDNQSEEAVKSDGFTTIELSLLEAVVSRDTLAINEIDLFKAVDLWATKKCEKQGLEANGEVKRRVLDETVVKAIRFPVM
KLRDFSDVVLNSKILSLDEVTRLVQFFTSSRATEVGFPATSRTGVFTKQIGTWCRFGKFNFNGWNYSGSSDFLVFSVNKE
ISLHGLCFYGSDNNFYSVDVKIMNPVEMSTLVKETGLFTSKLIHYGESSCYGFEINFDKPVRVKKNSRYEIEAKLSGSPS
FSGSRGLRVVETSGVKFTFYDSTHLPTSNGTSVSNGQFPQLLFSF*
>Adig_3862v121466
MIKSYCGILRKGVTHPHEMTASGNNSSGVLKRSLSDTTEALLFTDENRGLTILKVLFAFYKDGKYTDISLKIGPEQTLRA
HRAVLASFIPYFEALLGDNWKEGNKEEIEILVLGLDESAVSDLIEFAYTGKINIDKDNVLTLLEAANYLGIEPVKISCGN
YLEQRSRVDAKSCLDILQLADMFPLGELSSVAKEYALKHFTDVCKEDEFLCLPVNLLSDLLSEETLVVVVEDLIPCVEER
EKMVLESVLQYVEHDVENRKTHLAHLLGLVRLPTLSAEYLAEVSRNKLLESEFGNMLEKAKNLKLYPPEKGTLDETWAAP
RKFGKVAYTWGRCFPYRRQKKYDVECLAAEDMLVGLEDDSYVTGMELWIGRVLHGVKVYFKDRKVMVFGQDCAGTYAETA
WDEQVHHEFHLDENERIVKVEVKFRRISDCLTFYTNKKDKDGKPKCYGPYGRNGGSIITESAPESFGYLCGMSGIVVQSQ

GKACITTLRFLWRSYCIHGYDGHYDNMTIN*
>Adig_4027v121590
MDQIDDCLRLAKHCKLEYLENQLQMRLNSIQMFGTSKKRSITVVSIEPESQSTSLQDAFGRLAEIALPCPFTAQQFPKPF
PFEISLDLFEVVPSSEDPPPFFDVCFVVDGYRFHGHKLFFCGRSDYFRALLEYKNSEASSSGQERLSESVVEVFLNDVSP
DVFAAVLAYIYQDDALVRHSTNVTFELITSRVDSVIIENKEFHSLILEDAASVRERQETDSIPVVDDIRFHLMRGVLPYN
VSERDDEEFVDCEGSLQMLDNLLLYLGIEC*
>Adig_4094v121642
MVAHSEILLSKCAQFREQGEFIDVRLKVGEDEFPAHRIVLAANSDYFHAMFANGMKESNQEVIELKDKNISSSAFKIMMD
SMYSGEISLNDENVFEVLLAADHLQVSSVVQQCCEYLIQMKFDVQTYCRVITNADRHGLKDLKEVMESKMASMYKEICEM
DEFLSDMNGDALSALLSREDLSAPSENFVFKSVMRWIKYRKEERLDVAAKVIGAVRLGLVDIKDVIEELNTEEMQVIPEI
ILLLQRTLIYNQMPSSSSGFALEKAKPRSMSSEGASHRTNMVGEKVLLFLSFTLHENLVHGTSLIIFPNSYEPFDLSACH
ATM*
>Adig_4172v103910
MSGSNLQDSDAASNEVSADFSKPWQFSDVVLVVGGETFHVHRSMLAMWSPVFSSMFTAQFKEQKADKIPLPGKKAAEFKE
MLQVIYPICDKEVHTTNCFSLLELAKEYMMTKLTNKCERFLLGELEELKFDCLSLLSAAEAYGMKELEKACIEQAKLMSF
DQLKRNALYRKIKHSNFQTIVEAQMTKMESDILDKDFKIQALKNNVEFLKDCGGEALDELEKLTDATLIDLGNTDRVNAR
MDSKLRWLNRNRGSLFVPFDRLHEKLKEISDYDNV*
>Adig_4175v103911
MLFSEEEAAAASREDLPDFSEPWLLSDVVLVVEEEKIHVHRSMLAVWSPVFAKMFTAQFKEQTADEIPLPEKKASEIKEM
LQVIYPTIGKEVHEGNYLSLLNLAKEYMMTKLTKKCEIFLVGKLQNSHCLEYLDVAQAYELKELEEACIKKAKRESFDVL
KRHRVYEEINYSNYRALAEAKLTKMENTLAKMKKENERLSDQVESLKDEGEKALEHLEYLISNITINMGYTNISETIEKK
LYRIRTAFDQEKKSCTARWVICGHHSNDSNK*
>Adig_4974v122322
MSNGESERKAIPAHKLVLAIGSPVFYAMFYGEMADTRDSIELPDSDYESLLELFRFIYSDEVKLTGSNVMHVLYSSKKYL
VPSLAEKCAEFLRKNLDASNAFSILSHAQKFKEKDLENRCWEVVEVQTEEALASDDFVVIERSLVESVVKREKLNVKEVE
LFKAVDRWAEKKIENQGIGSDGNAKRRIIGEEILKEIRFPLMSQKEFVSFVVDSNILNMQEIVNMIKHYSEVLTSPLPYL
QSPRTGVLRRICRFKEYCRANWGYGGVLDSLILSVDKDVRLCGVQHFGREGCEYTVSMEIKDATSKLSLAKKSGTYCSEK
DLDHRYYGFDILFDSPVILESVKRYEISSMIRGPPSYYGGKGQTSVNFEGINFTFSRLTSPYSGTNEKGGQFPAFLFTHP
G*
>Adig_4975v122323
MLIQFSYTSQLTITDSSVQNLFLAADLLQFKWAKDSCVQFIMQQIDIANCLGVRALAERHSCKVMYEAATLYIMENFTKV
TQTEDFHILSSGEVIELISHNEVKVCSEIEVFDACITWIKYNLEKRKCHLSEFLKVIRLPFIPMKQLQMDVAEHPLVLND
IYSRDIVEDAITFHFDHASVQMRDCYAEMIYLLGGETSFMKEVKSVERYNCETMQWEMVKSMTEARASFTVATLQDKLYV
IGGYRRGRKLNSMECYDPVQNAWLSLKPMSKCQGDMRAAVLDGFIYVAGGSSEGLLTCSFVERYSPITECWQVVSSMHRQ
RRRFALAVLDSQIYAVGGFDDNRGDLKHVEYYSAGTNSWTE
>Adig_5005v104163
MFNNDLFSDVKFLVRKRIGDSYIKSKQVISAHKFVLSIGSPVFEAMFYSELADSSDSIELPDGEYESVLELFRYLYSDEV
KLNGSNVMGVLYLAKKYIVPSLADKCTEYLAENLDPSNVFSVLQCALKYHESMLAEACWRMVDEQTEEALGSEGFCSIDR
SLLEAIAIRDTLFIDEVDLFRSFDSWATSNCQKQGLAVDGEMKRKILGEQVVKSTRFPIMTQEEFAVVVLDTKILTQEEI
VRFFQFFSSTLTAPLGFPETKRPGIIYRCGRFERLQYDCWFYNIDGNGFTDYIDFVVDRDIVLYGIYLFGGENSSYNVSL
KVNYFHKNSCLTSRTGTFTSKQLKYSTTGEEYFGYEIRLEPPVHLIKDTWYQIEAYISGPESGYGLSGLDTLLWHGNWQT
KRPSIRERTKFMLNNDLFSDVKFVAYKSSGDSESKEVISAHKFVLSIGSPVFQAMFYGELAETSDCIELPDCEFESLLEF
FRFLYSDEVNLIQSNVMGVLYLAKKYMVPTLAGKCTEYLKNCVEPSNVFNILSMAEKYGEKNLVSQCWEVVDGHTKVALR
ADGFATIEKSLLEAVVIRDTLNVKEVDLFRAVNSWATKKCQKQGLPVDGKVKRKILGERIVKSIRFPVMKQEEFAAVVLD
SKILTTEEVITFFKFYSSALTSSVGFPQIIRSGVIHRCERLGSLRLDRWLFGSGYTDPLSLLLS*
>Adig_5246v122505
MFYGQMSDSRDSIELPDCEYEGVLELFRFIYSDEVKLTGRNVMDVLHLAKKYLVPSLAEKCAEFLRTNLDASNVFTILPH
AQRFEDRELEDRCWKVIEMHTEEAVTSDDFVVIERSLVESVVKREKLNVKEVELFKAVNRWAEKKIENQGITSDGNAKRG
IIGDEILKEIRFPLMSQEEFASFVIDSNILNMQEVGDMIKHYSQVLTSPLLYLQYPRIGVLKRACRFKNFKQHPAPGSSE
SGKRYKISSKISGASSWYGEEGQTLVHFAGINFTISTSDGATNGTSEGRGQFPVFLFTC*
>Adig_5419v122638
MATVQENWQTNCTSISQRTKYIFPVSNGESESKVIPAHKVVLAIGSPVFFAMFCGEMADTRDSIELPDCEYEGVLELFRF
IYSDEVELTGSNVMHVLYLAKKYLVPSLAERCAEFLRKNLDASNVFTILPHAQKHEDKDLEDRCWKVIAMHTEEAVTSDD
FVVAERSLLESVVKREKLNVKEVELFKAVDRWAEKKIEEQGIASDGNAKRGIIGEEILKEIRFPLMSEKEFVSFVVDSNI
LNMQEIVNMKKHYSQVLTSPLPYLQSPRTGLLRRICRFKAYCPTAWCFDGASDSLILSVDKDVRLCGVQHFGREGCEYTV
SMEINDAVSNLSLAKKSGTYCSEKDLDHTYYGFDVLIDTPVILESGRRYKISSMISGPRSWSGYQGQTRVNFEGLNFTFS
RSDSWCPHSPNFGTGNGTNELSGQFPAFLFTHSG*
>Adig_5634v122832
CAQFREQGEFIDVRLKVGEEEFVAHRMVLAANSDYFHAMFAHGMKESNQDVIELKHENISVTAMKIMMDSMYSGEIYVND
ENVFEVLIAADHLQVASVVEQCCKYLTQLRFDVQTYCRVIMVADQHTLKDLKEATERKMASMYKDICEKEEFLSDMNANI
LTAILCRDDLSAPSENFVFKSVIQWIKYRKEERMNVAAQVIGAVRLGLVDIKDTIEELDTEEMQVIPEINLLLQRALIYS
HSPSRSSAFALEKGKPRSMTTVLVAIHHKTDICYFDVQSKSWKLLSSMEKLTNVYGCYSAELVGNYLYVTVKFPTWEYGI

CCYDIVHDIWSTLPPIPHSSGIQIGFLCHVEDHLYAIYESSAPYRYHIATNQWQSVASSKALCNHSKIKFCNKAATVYKS
YLYVLHGQGQILFCFDPKKNVWEQRASTKTPHFGSFSLLVVNNKLCVAGGHCSLKSSSSEPGDKAAIEVYNDQENVWSVV
QQTHIPRNDLGALEIDGRVYFIINAFPVDSGITIPPGEVYPVILDEWENLKKIRKDAVLCYAPIKTENLTTENE*
>Adig_5635v122833
MVAHSEILLSKCAQFREQGEFIDVRLKVGETEFGAHRIVLAANSDYFHIMFARGMKESNQEVIELKDENISVAAVKKLMD
FMYSGEINVNDENVFEVLIAADHLQVTSVVQQCCKYLEAGVVKLRFDLPTYCYVIMVADGLGLKDLKETTQSKMASIYKE
ICEKEDFLSDINADILSALLCRDDLIAPSENFVFKSVMQWIKYKKEERMDVAAQVIGAVRLGLVDISDVIEELDTEEMQA
IPEIHMLVYGILMHHYRPSSSSKFALEKAKPRSMSLHAVKINTEDKHRETCKFTSIVLNKREPQV*
>Adig_5778v104374
MMETVSHSMSADPSQHHEELIERINALRTKQRFCDVTVVVKGEEFQAHKLVLAATSPFFLSLLESQMKERTEDVIKIELQ
EATAPVMEQVLAYVYTGNVSLTTENGHGLIATADYLLLPGLKTIAESFLKLHLTIENSVFNYYFAEKYQCSGLKEESCKM
INENFRKVMETDDFLSLDENQAANWVSSDDVIVEAEQDIFEGILQWVLHNKSEREKNFPKLLQQVRLSSVSHEYLVDKLL
NEELIKTNLDCVNFVMECVKMIFNCRAESGIKPARKCLERNENVIFVCGGSKALCYLPAENKWHELMNTTLEHKDHCIIQ
YRDRVYVFSPQKVGDNQSHLVEYYMPSTNSWGAIQTKFKYGSENFTGLFVLDDNKGWWILTESEFCDSYIYEYNPDKNIW
VGSFRCLSRSGACFVTDGHCIFIIGGTFSSHKNITATTEVKKIIPAEATENGEVEFKEVAPMNEARHDAFGAAMNGKIYV
AGGKQRDATLPIGERVLASCEVYDPSTDEWQVMPSLNVPRYSASMVCFKGALYVVGGLKNTARVSLERGRDPGNEVVKLS
PPPLKFLLLTMETASHLMSADPSQHHEELLERINALRTKQRFCDVTVVVKGEEFQAHKLVLAAASPFFLSLLESQMKERT
EDVIKIELQEATAPVMEQVLAYVYTGNVSWTTEIVYGLIVTADYLLLPGLKTMGESFLKLHLTIENSVFNYYFAEKYQCG
GLKEESCKMINENFRKVMETADFLSLDENQVVNWVSSDDVIVKAEQDIFEGILQWVLHNRSEREENFPKLLQQVRLSSVS
HEYLFNELLNEELVKTNLDCVNFVMEYMKLIFNCRGETSIKPARKCLERNENVIFVCGGSKALCYLPSENKWHELMNTTL
EHKDHCIIQCRDRVYVFSSQGMVGDKQSHVVEYYMPSTNSWGTIQTKFEYDNEEFVALFVLDDNKGLWIITNNESDHDSY
ICEYNPDKNIWVGSYRELGRWGACFVTDGHCIFIIGGTFTENEKITAKTEVQKIIPCDETEDGKLEFKEVAPMNEARHDA
FGAAMNGKIYVAGGKQRDENLLSERVLASCEVYDPSTDEWQVMPSLNVPRDSASMVCFKGALYVVGGLKSSNCGALSVEM
FDSVADQWQEKSNIPVKGKKETICFNACFATVHKNCLDV*
>Adig_5781v122946
METASHSMSADPSQHHEELIERINALRTKQRFCDVTVVVKGEEFQAHKLVLAAASPFFLSLLESQMKEKTEDFIKIELQE
ATAPVMEQVLAYVYTGNVSLTTENGHGLIATADYLLLPGLKAMAESFLKLHLTIENSVFNYYFAEKYQCCGLKEESCKMV
HENFRKVMETADFLSLDENQVANWVSSDDVIVEAEQDIFEGILQWVLHNRSEREENFPKLLQQVRLSSVSHEYLVDKLLN
EELIKTNLDCVNFVMECVKMIFNCRAESGIKPARKCLERNENVIFVCGGSKALCYLPTKNKWHELMNTTLEHKDHCIIQY
RDRVYVFSPQKVGDTQSHLVEYYMSSTNSWGAIQTKFDFGDEKFTGLFALDDNKGWWILTESEHDSYIYEYNPDKNIWVG
FFICLRRFGTCFVTDGHYIFLIGGTFSGSPLSQTIKKITATTEVKKIKPADRTENGKVEFKEVAPMNEARHDAFGAAMNG
KIYVAGGKQRDVTLPIGERVLESCEVYDPSTDEWQVMPSLNVPRYSASMVCFKGALYVVGGLKNTARSKCGELSVEMFDS
DVNEWHKKSDIPVPTGDQNRVKLKTNKETIRFNACFATVHKNCLDDSNNSSQLL
>Adig_6304v123344
MEAVSHSMSANPSQHREELIERINALRTNQCFSDVTVVVKGEEFQAHKIVLAAASPFFLSLLESQMKETTENMIKIELEE
ATASVMEEVLAYVYTGNASLTEQNGRNLLATADFLLLPGLKAMAENFLKRHLTVENCVSIYYFAEMYQCCGLKEESCKMI
NENFRKVMETADFLGLDENQVMYWVSSNDVIVEAEEDIFEGIIQWVLHNRSEREKNFPKLLRQVRVPSLSHEYLVNRLLN
EELIKTNPECFNFVMECVKLIFNCTAESSIKPARKCLKGNEDVIFVCSLRKALCYLPDENKWHKLMSTTLEHEDHSIIQY
RDRVYVFSSQAPLENQSHVVEYYMPATNSWGTIQTNFKHAGAEVTGLFVLDDNRSFWILAKYGDNDYIGEYNPDKNIWVG
SFRELWRWGACFVSDGHCIFAIGGILFFSESEAVTADVDKVFPGEKTENGGVEFKGVAPLNEVRFDAFGAAMNGKIYVAG
GKQEFMNENSDLASCEVYDPSTDQWQVMPSLNVPRYSASMVCFKGALYVVGGMKSVSECGELSVEMFDSGVNEWHKKSSI
PNGDENEAEFDNRNIHYNFKACFATVKLG*
>Adig_6310v123352
MVAHSEILLSKCAQFREQGEFIDVRLKVGEVEFAAHRIVLAANSDYFHAMFVHGMKETNQEVIEIKDENISVAAMKIVMD
SMYSEEINVNDGNVFEVLTAADHLQVASVVEQCCKYLETELVELGFDVQFYCRVIMVADRCGLTDLKEAMESKMAAIYRE
MCEKEEFLSDINADALSALLHRDDLIVPSETFVFKSVMQWINYRKEERMDVAAQVIGAVRLGLVDIKDVIDELDAEEMQV
IPKINMLLQRTLIHNHRPSRSSSFALKRGRPRSMNPVLVAILPESVISYFDVQSKTWKELSSMQQLTGVQECFCAELIGN
YLYVAAKCKREFVICCYDIIYNTWSTLPPIPSSLGISSLCHIDDHLYVIYESFAPCRYNIATNQWQSVASSKAVCNLGLE
TSSSKAAAVYKSCLYVLYHQGLNGASCAHLYCFNPKENVWEQKASTKTPHLGSSLLVVKNNLYVAGGHCSFFCRNPHEET
AAIEVYNDQENSWSVVQPTHIPPNDLGAVEIDGRVYFIINSFPVDSGITTPPGDAFPAILIEWKKLGNVEKNAVLCYNKG
VATNVVIQ*
>Adig_6311v123350
MVTHAEILLSKCAQFREHGEFIDVRLKVGGDDFAAHRIVLAANSDYFHAMFAHGMKESNQEVIELKDENISVGAFKIVID
SIYSGQVNVSVENVFEVLLAADHLQVSSVVQQCCKYLETKVVELSFDMQTYCRVIMVADRHGLRDLKETSQRKMASLYKE
ICGTEDFLSHVNADILSPLLCRDDLSAPSENFVFKSVIRWIKYRKEERMDVAGKVIGAVRLGLVDIKDVLEELDTEEMQT
IPEILMMVYKTLRHNCRPSSSSAYALEKAKPRSMSPVGESLFLFLLFSLHKNLVC*
>Adig_6365v123393
MVAHSEILLSKCAQFREQGEFIDVRLKVGEDEFEAHRIVLAASSDYFHAMFAHGMKESNQEVIELKDENISVAAIKIVID
SMYSGEINVNDENVFEVLIAADRLQVASVVEQCCKYLIQLRFDVQTYCRVIMLADQHSLKDLKEATERKMASMYKDICEQ
EEFLSDMNVDVLSALLCREDLKTPSENFVFKSVMQWIKYRKGERLDVGAKAIGAVRLGLVDSKDVIEELNTEEMKAIPEI
NMLLQEALIRNSRPSRSSALALEKGKPRSMNSVGEEVFVCTKTPFVLRSVIIFSQSFEQSYLCLSRNPNQSPPFIDLLKM
RLLASTRRRL*

>Adig_6372v123394
MMEAVSHSMSANPSQHREELIERINALRTNQRFSDVTVVVKGEEFQAHKIVLAAASPFFLSLLESQMKETTENMINIELE
EATASVMEEVLAYVYTGNASLTEENGHNLIATADFLLLPGLEAMAEDFLKRHLTIENCVFNYYFAEKYQCCGLKEESCKM
INDNFRKVMETADFLGLDENQVMYWVSSDDVIVEAEEDIFEGIIQWVLHKRSEREENFPKLLRQVRVASISHEYLVNRLL
NEELIKTNLECFNFVMECVKLIFNCTAESSIKPARKCLKGNEDVIFVCDSREALCYLPDENKWHKLTSTTMEHKGHSIIQ
YRDRVYVFSSQEVEENQLHVLEYYMPSTNSWGSIQTKFDYNSWEQFTGLFVLDDNRGLWILTNYGDNDYIYEYNPNKNIW
LGSYRKLWRSGACYVSDGHCIFTIGGTSSENETVTTEVDKIFPGDKTENGKVEFEEVAPLNEARYDAFGAAMNGKIYVAG
GKQDLHVDNSDLASCEVYDPSTDEWQVMPSLNVPRYSASMVCFKGALYVVGGMKSVSECGELSVEMFDSGVNEWHKKSAI
PTGDVNEGDFDNRNIHYKACFATVRSGLVRL*
>Adig_6771v100408
MSSPETWQTKRSTIRERTKFMLNNDLFSDVKFVVRKSDSESESESKKVIPVHKFVLSIGSPVFEAMFYGDLAETRDSIEL
PDCEYDSLMELFRYLYSDEVNLSGSNVMGVLYLAKKYIVPSLVDKCSQYLQDHLQPENVFNILPIAEKYEEKGLIDQCWE
VIDNQSEEAVKSDGFAAIELSLLEAVVSRDTLSINEIDLFKAVDLWATKKCEKQGLEANGEVKRRVLDETVVKAIRFPVM
ELRDFSAVVLNAKILTFDEVTRLVQFFTSSLATEVGFPATSRTGVPKKQFGIWCRFEKVDFHTLGYSGLSDFLVFSVNKD
ISLHGLCLFGSYNNFYSVDLKIVNLVEMSTLVLKTGKFKSELLYYGESSYYGFEINFHKPVRVKKNSRYEIEAKLFGPSS
CSGSSGLREVETSGVKFTFYDSTHLPASNGTSVSNGQFPQLLFSF*
>Adig_7004v123874
MGKSKGFAYVEFKNEGFGYIEYELETGASSAIVALDGTRVGDRQILVALSNPPSRKPRQPGAEQQQRPKEQPPGSRGRSR
TQLQLLPRSLQKPVSLAQASSANGSISPSDGKTEAKNGVEPKESLSNDYFRNLMQKKNNCLMSETENFVSAMNWQTMKPS
IKERIKFMLNHDLFSDVKFLVFDEVNKSKQAIPAHRFVLSISSPVFEAMFYGQLAETSGSIELPDCDYESLLELFRYLYS
DEVNLKDSNVMGVLYVAKKYAVTTLADKCIKYLQNMVDPSNVLRILSSAIWYQEKNLVDWCWRIIEEQTKDVVKADNVFA
TIDKSLLNAIVHRDTLNIKEIDLFRAVDFWAKRNCESQGLIASGKEKRKILGERIVKSIRFPTMEQVDFAIATLDSKILS
GGEIVRVIHHMNSVLSSPVGFPAVKRSGFFGDIKQCCRFGSVSFRRRWKNDFSKDCINISVDRDLRLHGVRLFGGENNSC
WVVLEIRNAENRAILTSKAGWFSSQTLHGTLGQYCGFEVLFDSAVSLKRCISYSIEVSITGSDSCSGRNGYKSVKCHAAT
ITFVNSKYCENGTSVKCGQIPEVLFSISILPCVISFETFARKYLCAFPRVANEIFEGLLGIMPVKESQKSKVLLLLLLNF
VCVLGKDAAFCFKQETHYNTTLKGGLDAGEYTPWEPRGVDFMTVDLCTKHCCKSPDTDAVFLLNSHCFCVKCYSVRSCSL
QKITPPPGYIPVTVIVMESISKFSTTSTPHTHPKTFREIPKTETSSQNKQLSFNRKEEVGNFPKVFFGKTFSDEHIYNPP
PSDSTKNKQSKGQNDTAMSRQHPPRRRVMVDPDEELGKTRKLFRLFSRKHSPQTALLARYAVCFFLGGLVGLVPYLVFAI
EVCKANLESVRNVNFPVPNGYYQTKDSPKRETESPFSGITIQSVTTANIEVNITSPNISATGATTPRPTRKVTKQKKGIL
NYRLSFKLSLYKQPGKQM*
>Adig_7344v124135
MTTPGMKLNMAASGNKFSGVLKRSLSDTAEALLFAGENHRSTILKGLFFALYEDGKYPDISLKIGPERTLRAHRAVLASF
SPYFEALLGDNWKEGNKEEIEILGFDEGAVSDLIEFAYTGKIKIDKDNVLTLLEAANYLGIESVKKSCGNFLEQGVDTKC
CLGILQLADMFALDELSSFAKKYALRHFTDVCKEDEFLCLPVNFLSDLLSEENLVVVVEDLIPCVEEREKTVLESVLQYV
EHDVENRKTHLANLLGLVRLPTLSVEHLAEVSRNNLLESEFGEILEKAKKFKLHPPEQGNPDEKWAAPRKFAKHVFKWSG
CYPAGGRALPETAFCTDKMFEDLENDCYVTGMELWIRKWDIWDVLGGLKVYYNNGKVMVFDQDCGGTYAETARDDHVHHE
FHLEENERIVKAEVRFGFTINCLTFYTNKKDKDGNPKCYGPYGGYGVSFDAVSAPESFGYLCGVSGAVVKSQGQTGITHL
QFVWRLYVLPGGHVPSKHLCIADVYLPFTSCFSRCKYHLLL*
>Adig_7367v124160
MGANASSEQPSPDSTLVRVPRYTLEEKRKKTSKLNTLKKKLTKSRSTFKSQDHGKMLRDLTQTWTRQEINALVEEYETMM
NVKDLKNQTNLVRPSASCLKKDLQKLYEVETCADCTLVFEGTSFNVHRAILSSRCPYFRELFTQHMGEHPIIQMEFKTEG
MTKEIFSALLCYLYTGDFTPKKSGLEYIDVLINLGDEFGTPNVLEQDFKLLLNCSEYADVALVFPAQASMQERYSTSDGG
IISDHNYEILCHKAVLCARSQYFRSLFLKDCLHSLEQSSKVQRLVIDESVMTRQYARIALQCMYTDSVDLSSIVKWSSDD
RGYCAFGTHKLLTLAEIAMEVFEVASFIDLPVLIQGCEDIILEELSPSTLLCTLEWSSCAHGSDWVYRQAMQFLQDEFVN
IAQSEVFPFLPKKYLVETLKSDFLQEKFSSFY*
>Adig_7375v104839
MEELSQPLSSEPSTHVKHLLERVETQRRNDHFCDVTVLVKDKYFKAHKVLLTASSPFFRKLLSSGMKESNEDVIKIELDE
ATEDVMEDVLSYIYTGDEKACEVVTSNFTVVMETEGFLRLQAKEVSDWISRDDIIIGAEEEVLNGIVKWVSYDRGEREAH
FTELLRHIYLQSIPADFLLKEIVQEDLFSQNAEFALKFVLDAMKLKSQNSTNGQVPQNYRKCLETYKDGIFVCGGKKALA
YFPIEDKWCKLRDAAVDYQSHCLVQWKDKIHIFHEDCNTEGESNAFREYYKPNIDSWGSIQSDHIKSINRCITFKGDLYV
LRFEMFSEQKIYSYDAKTNGWNEVDPPSAVQNNPCVVASDQHLYLIGGKNKVHQCSARSTRFDPTHNTWEDIGNINEARH
SAFGAAMNGQVYIAGGLKHFGNDIVSSCEMYSPMSNEWQLIGCLKKARYNASMVCFEGKLYVLGGEGSSRGSFSRPSRVL
TIEELDSESRAWTVKSVVPVDNFETSEENKNKREYKACFATFSKRLIADLQPLN*
>Adig_7417v104858
MSNGESESKAIPAHKLVLAIGSPVFFAMFYGQMADTRDSIELPDCEYEVVLELFRFIYSDEVQLTGRNVMHVLYLAKKYL
VPSLVEKCAEFMRKNLDASNVFSILSHAQKFEDKDLENRCWKVVEVQTEEALASDDFVVIERSLVESVVKSEKLNVKEVE
LFKAVNRWAEKKIENQGIASDGNAKREIIGEEILKEIRFPLMSQKEFATFVVDSNILSVQEIVNMIKHYSQVLTSPLPYL
QSLRTGALRRVCRFKEYRAAGIERLWNYFDGSWNAIILSIDKNVRLYGVQHFGCEGCEYSVSMKINDAASYLSLAKKSGT
YSSEKDSDGIYYGFDVLIDSPVILESGRRYEIRSMISGPQSWHGAKGQTSVNFEGINFIFSRSNDPLCCSNVEQGQFPVL
LCTHSG*
>Adig_7769v124461
MLNNDLFSDVKFVVRKSDSEGESESKKMIPAHKFVLSIGSPVFEAMFYGELAETRDSIELPDCEYESLLVLFRYLYSDEV

NLSGSNVMGVLYLAKKYIVPSLVDKCSQYLQDHLQPENVFNILPIAEKYEEKGLIDQCWEVIDNQSEEAVKSDGFAAIEH
SLLEAVVSRDTLAINEIDLFKAVDLWATKKCEKQGLEANGEVKRRVLDETVVKAIRFPVMELRDFSDVVLNSKILTLDEV
TRLVQFFTSSLATEVGFPARSRTSVSTKQIGTWCRFEKLDFNAWDCNGLSNFLVFSVNKEISLHGLCLFGSDNNFYSVDL
KIMDPMEMSILMRKTGKFKSKLIYYKETSYYGFEVNFDKPVRVKKNSNYVIEAKLSGPPSCRGLSELHEVETSGVKFTFN
DSLHFLPSNRTSISICQFPQLLFSF*
>Adig_7881v104993
MQTKGSQPDFSVPWHLSDVVLVVEDKKFYVHKGTLSMWSPVFEKMFLSEFKEKTASEIPLPGKKAAEIKEMLLVIYPTSK
VVSEENCYYLLSLAREYQMEQLTERCERYLLQREKTPHQAVDFLVLANEFLMEELCKQCVEIAKHISITELRRHEKYTLV
APENGRQLAERRVELLENKVVAGEQKVNAVRKEVKDACEWALREIARSLYRKKYPEGRLCPRALSDCVKLLEETAVESSE
INVILQLLQQRLRCIYEPFRTV*
>Adig_7887v104996
MATVQENWQINCTSINQRTKYIFNTGFLSDVKFIVPVSNGKSESNVIPAHKLVLAIGSPVFFAMFYGQMADTRDSIELPD
CDCESLLELFRFIYSDEVELTESNVMHVLYLAKKYFVPSLAEKCAEFLRKNLDASNVFTILPQAQKFEDKDLENRCWKVI
EMHTEEAVTSDDFVVIDRSLVESVVKREKLNVKEVELFKAVNRWVEKKIENQGIASDGNAKRRIIGEEILKEIRFPLMSQ
KEFATFVVDSNILNVQEIVYMIKHYNEVLTSPLPYLQSPRTGVLRRVCRFNEYRAAGMAGCWHYGGYWNALILSVDKNVR
LYGVQHFGCEGCEYSVSMEINDAASNLSLAKKSGTYCSEKDSDGIYYGFDVLIDSPVILESGRKYEIRSMISGPQSWYGA
KGETSVNFEGINFTFSRSDSPGIDTIVGRGQFPVFLCTHSG*
>Adig_8137v124743
MSTPEKWQTTRPTIRERTKFLLNNDRFSDVKFVVRDSNSESESGSKKVIPAHKFVLSIGSPKFEAMFYVGLAQTRDSIEL
TDCDYESLLELFRYLYSDEVNINASNITKVLCLAKKYEVPSLVDKCIQYLRDVLSSSNVFEILPTAEKLEKKELIDECWE
VIDNQSEAAMKSDAFTTIKHSLLEAVVSRDTLTINEVGLFKAVNLWATKKCEDQGLTANGEEKRRILGDKVVKAIRFPVM
KRHEFASVVADAKILTPEEVINFFKFFSSAAAAPKGFLNKKRSGLGKSYKLRKPRS*
>Adig_8482v105388
MEAVSHSMSANPSQHREELIERINALRTSQCFSDVTVVVKGEEFQAHKIVLAAASPFFLSLLESQMKETTENMIKIELEE
ATASVMEEVLAYVYTGNASLTEENGRNLLATADFLLLPGLKAMAENFLKRHLTVENCVSNYYFAEKYQCCGLKEESCKMI
NENFRKVMETADFLGLDENQVMYWVSSDDVIVEAEEDIFEGIVQWVLHNRSEREEKFPELLRQVRVPSISHEYLVNRLLN
EELIKTNLKCFNFVMECVKLIFNSTAESRIKPARKCLKGNDVIFVCGLREALCYLPGENKWHKLMGTTLEHKDHSIIQYR
DRVYVFSSQEVEKNQSHVVEYYMPSTNSWGTIQTKFNYSSEQFNGLFVLDDNSGLWILTTVTETFESSYGDYIYEYNLDK
NIWVGSYRRLRRSGACFVSDGHCIFTIGGTSSIDINTVTADVDKFFPGHKTENGGVEFEEVAPLNEARYDAFGAAMNGKI
YVAGGKQELHVGNSDLASCEVYDPTTDQWQVMPSLNVPRYSASMVCFKGALYVVGGMKSVSECGELSVEMFDSGVNEWHK
KSAIPTGDENEGEFDNRNIHYKACFATVRLGLVRL*
>Adig_8561v105466
MREQMENKPIILENITADIMEELLNFIYTGSIKITPFNVKDFVSASNYLLMTSLKETCVSFMKSMLNPSNCLGIEAAAFK
FDCIDLRNSANQYVYDNFVAVSQTDEFKSLSAERLTEYISSDEIRVEREEQVFEALMEWIKHDLDSRKGHFKELSQHVRF
PLISPYYLADHVETEDIVLTSPECTTLLLEAKNYHMLPDRRHLIKGSRTKPRRSMGVISVIFAAGGIQGSSVMRDTFGFF
SATNSWSPLAHMITARCRHGLAVSGDMVYAVGGQSREGAAQSSLSSVERYDPRTNTWTMVAPMNMRRSLLNVAVLDGNMY
AVGGCDENNFRLNSVERYSPATNSWSFTAPMTTCRSSPCVLTCRALYVIGGVSYVGMSLNSGEYLDPVTNTWRAIAPMHD
KRASASGAATNGKIYVIETEKIASTKTVPTAHKRNALTALNVSIRKPHKAVPIPHPTPMIRLFTTPAIKNDDTGLCKCLI
FKEDVSQPTTISCQEGPMLLQAIISHTPTVFSSQPENPLTDCYQNTQAFSPVGYGHVANHHRVSPSKVRSPLYAGSPGSA
QPQSPYQQALQHSPSDHVNRVHQMASSQHQVAIQSRSPVACSSYSGRMSTAQQNTGNPLAENLSIVNGSSPNIHLMQPCI
NLSPTQTLSPLKTRKMRQGLPSTEPQLKTKQDKPMDRGSMTAPHHNKSQRKLDLSPSVTRNLNDQEFYQHVAQAQVQSSA
ADVYNQVEYNSMSALEALASQNGFNFGASVLGQASQQLAALTENQRNASLKPAASPKTLHYQEIDSNRSCGSSSKPKKKR
NQEAMNEKCTQHQRPSKFGWRSVEDQNTNTDLKATEVMNEGLPLAPMSAMDLNIQNNRTPDRTDKPMSTKDGSGENIKSL
SPTLKLSPALKGIPLYVPKLVITKIRQRRGSKEIETHQVREVSPGSDSTTLQRLKKRRRSSEEKSHHNIYSDEAPPKKKS
KAKKRRKQESCESDALVLQLIQCSVKIPDKADDISEATGAKDGGDENKSQVDRNLLIITSYENALRTAQNFLSMFLNKCI
SVGKDEEDYRPLFENFLQDLLVTLNRPDWPAAEVLLTLLGKLLIVTFNNKGNDRELQRF*
>Adig_8963v105869
MENTSNVFYDFTDEQGASDALGKMNDFRKERTFCDVVLSTEDGEEFNAHRLVLASVSAYFRAMFLTDMRESTQTNITLKG
VESQALRDLINFAYTSTLRITFSNVHSLLSAASLLQFLTVENACYDFLRNSINVCNSLQIWNLADLHGCTELRNLSENFI
RANFVAVLKLSDFHSLSSKQLAALLSHDKLNVPCESVVLFGVLDWVKYDLDNRLVNLEELMEHIRFPLMTRKFLMDTAAR
EELIMGSATCREFVLEAIDYHLNPERRAKNRISSTIPRERLSRLLYVVGGEEKHQVLNNVEYFDFNEKKWRIITPMIIPR
KFAGSAILDGLLYAVGGINKEYADLVTVESYNPCVGQWSSVASLNKCKGALAVSVLEGWLYAAGGSHNGSALKSVERYDT
VKNEWTQVANMRLPRSNFGMASLHRRLYAVGGHCGFSEIEQVECFDPMTNKWCDVSNMNKSRMNHAVVTFSDRIYVIGGS
NSVGVLDSIEKYNPDLNLWLIIRNTMDPRSGAAAAVVLGDEEGAQELFIIGGHDNHHRDTNTVKKLNLKFADYSTAIAPC
MNHPRVYASVACA*
>Adig_9048v105954
MESFQSEGKTWLNVEGLLKDLEKLIDNRESADVVFLVGRDEVAIYAHRLILVTRCKYFRNRQRELWSSKSAAHTQLTVRK
PELRPDVFREVITFIYTGKVQLDSGTAFEMLRIAEDLEVTDLQQACEQYFMDNLSVDNASEFLADAMSDSRAKGSEGVSS
LVDKCIAFMEENAEEIVKTEGFLLLPKSSIIRLVSSNQFAVSEEELWRAVLSWAKRKCHVNKPINQWTEEDNSKIREALS
GVIEHIKYP*
>Adig_9061v105962
MVSHSEILLSKCAQFREQGEFTDVRLKVGEDEFAAHRIVLAANSDYFHAMFTHGMKESNQEVIELKDKDISVAAMKIVMD

SIYSGEINVNDENVFEVLIAADHLQVASVVQECCKYLTQLRFNMQKYCRVIMVADQHSLKDLKEAAESKIASMYKEVCEK
EEFFSDMNANMLSALLCRDDLCAPSENFVFRSVMRWIKYRKEERMDVAAKVIGAVRLGLVDISDVIEELDTEEMQVIPEI
NMLLQRTLIHNHRPSRSSAFAVEKGKPRSTSMVLVAIPPKSDMCYFDVQSKTWKLSSVQKLPDVLECYCAELIGNYLYVA
VCFSLFCSGICCYDIVHDIWSTLPPIPGTSLGFCSLCHIEDHLYVICTSRTSYRYHVATNQWQAVASLKDVCDLSQGTFC
NKAAAVYKSCLYVLYSQGKNMGKGSKKWYNFHSLVSVLYCFDPKKNVWEQKASTKTPHFGSSLLVVNNKLYVLGGCCSLQ
TSSSEPHGSPAGGQAAIEVYNDQENAWSLVKQTHIPPNNLGAVEINGRVYFIVNSFSVDSGITIPPGEVYPAVLDGWENL
GMIDKDAVLCYVPLKTENLTTEEMFVQKKI*
>Adig_9062v105961
MVAHAEILLSKCAQFREHGEFIDVRLKVGGDEFAAHRIVLAANSDYFHAMFAHGMKESNQEVIELKDENISVGAFKIVMD
SIYSGEINVSDENVFEVLLAADHLQVTSVVQQCCKYLETEVVELSFDMQTYCRVIMVADRHGLKDLKETSQRKMASLYKE
ICEKEDFLSHMNADILSALLCRNDLSAPSENFVFKSVMQWIKYRKEERMDVAAQVIAGVRLGLVDIKDVIDELDTEEMQM
IPEIHTLVYKTLRHNCMPSTSSAFALEKAKPRSMSPVLVAIVSRGEICGFDLQSKAWRKLSSVQQLTKAGVCYCAEPVGN
YLYVADSSTVFCYDIVRNIWSTLPPIRSSSYFKFGSMCHIEDHLYVICKSSESYRYSIATNQWQSVASSKVSCKLGQKTF
FNKAAAVYKSCLYVLYAQAIEEYENNSFISKPYNSLLCCFDSKKNVWEQKASTKTPHFGSSLLVVNNNLYVAGGKCSFSR
PNLEPCGSSAAIEVYNDRENAWSVVQQTHIPQNNLGAVEIDGRVYFIINSFPVDSGITIPPGEVYPVILDGWENLGKVEK
NAVLCYMPVKNSLT*
>Adig_9063v105965
MVTRSEMLLSKCAQFRQQGEFIDVRLKVGEDEFAAHRIVLAANSDYFHAMFARGMKESNQEVIELKDENISAAAMKIVMD
SIYSGEINVNDENVFEVLTAADRLQVTSVVQQCCKYLETEVVEMKFDVQTYCRVILVANLLGLEDLKEATQRKMASVYRE
ICEKEEFLSDMNADSLSALLCRDDLRAPSENFVFKSVVQWIKYRKEERMDVAAQVIGAVRLGLVDIKDVIEELDTEEMQA
IPEIHMLLHRTVVHNCRPSSSSAFALEKARPRSMSPVLVAIFHNGVINYFDVQSKTWKELSTMQPLTPVQECYCAELIEK
NVWEQKASTKTPHFGSSLVVVNHNLYVVGGDCSFIPQSFQLSGSPAAVEVYNDQENVWSVVKQMHIPPNNLRAVEIDGRV
YFIINSFVIDSGITIPPGEVYPAVLDGWENLGMVVRNAVLCYVPVKTENHTIEIE*
>Adig_9353v106248
MAENPAEEPSESSYCSKMMQNLDKSRKENSLSDVTLDVGGQMFPAHRCVLSASSMFFQGLFSNNMAEKSASVVKIEGIPP
SVMDQLLSYLYKGEIQVSEANAEDLIVSANYLLLPRLKSIACKFLEQRMSASNSIINYLFAEKYECKDLRSYAGELIKQN
FGVVGRSKEFLLLSFDLVKDLISRDDIVISGEEDIFEIILEWIDENFEEREQYFAELFSYLRLSTVSQHYLYTKVLFNEL
VHGSEHCRQLLMTELYRRALFAGQDISSQRTRTCLQTHEDSIITCGGLSPDGQIRNATLCYVPSNKTWYQLAPMLSRRCR
HGFSACQGFIYAIGGKGEDSFHSSVERYDPRTNTWSYVAPLAKKVKLVGAATLHGYLYIVGGIEFTTEQGRRRCGTTQKY
DPATNSWTPAAPLNSHRSSVCLVSDANYLYSIGGLADDDFLSCNERYDPKLNMWTKMASMKEKRGCACGVYLNNKIYIFG
GTVDAFSRHASVSGEVYDIALDEWHAIASMCVPRFHASAILLQDRIFVFGGIGSESVQHHNSQMVECYDVKTNQWMTPHS
MPYEETYFRGCSVSMFKGLLDSLEKVSQN*
>Adig_9362v106264
MACFFNMVKHCHVLLYSDTRAPTREFNMEIEVRRANYCYNIKAYHERKGEEQFYDFIIKDKNGDIMKSHKFILASQSEYF
AGLFRECPTASQTTFRNFSIEVIKICIEYLYTQELKLAGNNVRDVLVFADNINLVDVVDICTNHIIRSIDESNFTLVMDL
GNKRGIDSLIEAAVFYAIRNQKHKAFDDLTVKTIMKVARWQQKRPTIMTHQQWMISQLLYKFVSNPAEPDMVFETRCSSV
YMDAGNYGPQLAVNGKVSYENGGFFHSLEEYYPWLEVKLPSPILIMSVTVINRQDMCYERLRNLEIRVGMKPVPDDFTTH
PQGREGKKQLDCNSRCGFFAGPAEHPQVEPVFNFQKPILAQYLTLQIIDKGILQINGLKINGGTILNSNVMQPE*
>Adig_9826v106703
MYPAKFSLSLLFGICCLTSVSQSVDQASALSSSVRSKGREVRPHEDKTRTANSSPEFSLGKRSYGCPQVYGFEYARDFFE
DTAAGSQVYRYHPERVDTVTCHSSCGTSCREVRVRMWVTRIHKYECFNSQTRSKEVPVKCEIDGIPSFEKSQLKATTVRE
RNELMFNNEILSDVHFLVGKDKARIPGHKYVLAISSPVFFAMFYGRMAEPASDVVVPDSEVECFMELLRFIYTDKAILTL
DNALGILYLAEKYMLSVLASDCINFIEASLNHDNALTVLCHARYLGKEDLQERCWDMIDTYTSQILESEAFLELDIETLS
LLVQRNTISVKEIQMFKAVNCWSENECRRRSLEVSTENKRALVSKAIKYIRFPLMSPLEFADEVARSGLLSYEETTNVFL
YFFSSHETKLHFSKIPRSPRETLIKKTLRCSRFKSCCGNDWFCDTRKSDAIKFMIDKPIYIKGIAVYGASKMEGEYTTSI
ELLKQNKVVSERKMSFSSYTSSTLHDILFERPIFIEKNEVYTINLLLQGPSSLSGRDGFSVVETEGIKFTFIDHTSPNGT
SVTAGQIPEIIFHL*
>Adig_9970v106846
MSSTNQPAGGTQSDFNKLRKDQTYLFVDRSFQDSIIEYFQFLRREAILCDVTLRIDSQKFFCHRSILGVASPYFRSLFID
SESGRFIKNVDLPPEIDGVTMEALLGYMYGGKIFIDTTNVLDIFKGASFFKLHSLLEECSFLLVKFLTPENCLEVREVSL
VYGQEKLHQRCQRFLCDHFPAIANSGAIFGASTR*
>Adig_10187v107035
MAQADLPANSHFLSLAASTENHVSPRNEGQLMVIDTDWDEWLPMLPSIDDLPWSLKDIAKVLRLGRTREKFRAITPQAVE
RVSFLLQRPLLRIVREAKRLSFLYSKCSKQEMQTSIRLVLSLSLAKSCLSLASKALSLYQTSNDRFHRSKRARCGLILPV
GKMFRWLVNLKVATRIHDAGVIYLSACLEFIAEELVYRAVTKQGEILKVTPEVLEEWISTDADFWGIFQPYYHLLSGRTA
FGVTDSIDVYAVPKKVSRNPKTGSPRQRGLDKSLAITCVSSLNELTDLISQAQRQFIDVYQSKDNKFISQVDWSPSALNS
LFYFIKCADPTDENGPDILAGTRRSHSDLPPLVEWLKASSLHTEHRASGMVDDDDVRQAARLMLPFHDCGPRSLSSSVLL
CLSKALSSAVTISSFQQDLCLRMLSCGRADIIPRALEVLGPEKINAINVQGMTMLMYACADGNEDLVRTLLEHGVLVDTQ
VPNNQQIYPLLNLEFKSWTALCFAATKEHVNICQLLLDNGASPNGAIGYGPENQVDTPLQLASATGNLELVSLLLRNGAD
PDQSVNVTSFSPVSRGFGSDYINSADMSDVTFLVEGRPFYAHKIILATASKKFKALLSEMPSESEDGNDPCIEISDIKYD
IFTVRKEAAMVVMLVPSQLQLPEKSEKYMHAYV*
>Adig_10374v107204

MASVQENWQPKCTSISQRTKHIFNTALLSDVKLIIPVSNGESESKVIPAHKLVLAIGSPVFYAMFYGQMSDTRDSIELPD
CEYDGLLELLRFIYSDEVKLTGSNIMHVLYLAKKYLVPSLAERCGEFLRKNLDASNVFSILPHAQKFEDKDLENRCWEVV
EVQTEEALASDDFVVIERSLVESVMKSEKLNVKEVELFKAVNRWAEKKIENQGIASDGNAKRGIIGEEILKEIRFPLMSQ
KEFVSFVVDSNILNMQEIVNMIKHYSQVLTSPLPYLQSPRTGTLRRVCRFNQYTPACWVYSGSLDSLIFSVDKDVRLCGV
QHFGCEGCEYTVSMEISDAASNLSLAKKSGTYFSAKDLDHIYYGFDVLIDTPVILESGKRYKISSMISGPNSCSGNQGQT
RVNFEGINFTFSRSDSSSNGTNEKGGQFPVFLFTRSG*
>Adig_10375v107203
MALLSDVKFIVPVSNGESESKMIPAHKLVLAIGSPVFYAMFYGQMSDTRDSIELPDCDYEGVLELFRFIYSDEVKLTGSN
VMHVLYLAKKYLVPSLAERCAEFLRKNLDASNVFSILPHAQKFEDKDLENRCWEVVEVQTEEALASDDFVVIERSLVESV
VKSEKLNVKEVELFKAVNRWAEKKIENQGITSDGNAKRGIIGEEILKEIRFPLMSQKEFARFVIDSNILNVKEVGDMIKH
YSQELTSPLPYLQSPRIEVLRRIGRFKEYYSACWGYDGSLDSLILSVDKDVRLCGVQHFGCEGCKYTVSMEIKGATSKLY
LAKTSGTYCSEKDLDHLYYGFDILFDSPVILESGKKYEISSMIRGPPSWCGEKGQTSVNFEGINFTFSGSASRNSCTNEE
YGQFPAFIFTHSR*
>Adig_10376v107202
MATVQENWQINCTSINQRTKYIFNTALLSDVKFIVPESNGESERKVIPAHKLVLAIGSPVFFTMFYGQMADTRDSIELPD
CDYESLLELFRFIYSDEVVLTGSNVMHVLYLAKKYLVPSLAEKCAEFLRKNLDASNVFTILPHAQKFEDKDLENRCWKVI
EMHTEEAVTSDDFVMIDRSLVESVVKREKLNVKELELFKAVNGWAEKKIENQGIASDGNAKRAMIGEEIVKEIRFPLMSQ
TEFVSFVVDSNILNMQEIVSMIKQYSKVLTSPLPYLQSPRAGVLRRVCRFNRYCSGVCFGDCDSLVFSVDKDVRLCGVQH
FGREGCEYTVSMDIKDATSNLSLAKKSGTYCSEKDSDGIYYGFDVLIDTPVILKSGKRYEIRSMISGPPSWYGEKGQTRV
NFEGINFTFSRSASSVYGTSETEGQFPVFLFTHPG*
>Adig_10583v107400
MYTGDLYSNSNLQNKNDLFEYKFNTGQWTEWHIEGRLPPARSAHGAAIYKNNLWIFAGYDGNKRLDDLWTICLTDLNPRW
QELASYPRCTLRTDFGRLLDSNQFCDMVFLIGEEGTCFPAHAAFVSARSPWLRTQLLRAREKCQV*
>Adig_10710v107515
MDFDSVSPDPSVQQLDNETFKFEAGSKHGIKALMVMDDLRKRRQLCDVTICVGSKKIYAHRLVLSSFSSYFLAMFTNGMM
ESVEETITLKDVDGNVVELLIDFAYSGKLDITIDTVQSLLYTSSLFQLTAIQKACSDFLQRQLHPSNCLGIRSFAGTHSC
TVLLQASEKFIHEHFNDVVQNEEFLLLPREELATLLSCEEINVNSEEDVYNALIAWARQDVDERQSFLAELLEQIRLPTL
SPHFLVDEVEKEELIRQDIACRNLLDEAKNFHMLPDRRSKLGSQKSRPRESTFSKLFSVCGMDSTGHPLRTVEQYNFHSG
KVKYIPPLNTPRSGLGVAVLDGKLYAVGGHDGTSYLNRLRCTCNSDWSYVIVYRQKVKIISNSRFSVTFCLSGSLFEQTV
ALLIMEKEAEDDMSKGNFNKAREKFEMLLQQDKSKTKFWLLGKADCLACEGRLHESLPVYCEAFKYGKVNPDRLHRFVKA
LSKLIEKDSCATEQQTPVEDSNGLQCGVCTGLLYDPVTLPCGHSFCRLCLKKQESEKCVRCGNQFSYSRLKTNVILQHVL
EKSFKVELEATGLRLQGNQLHRKKNSLEAIEKYEEAEKLSK*
>Adig_11018v107792
MDSTSIVIMESNLSTDIMAVLDNMRRYNELSDVTLVVDNREIVAHRAILAACSPYFRAMFLSGFSEATESKIVLKEVDGN
AVQLIIDYFYTSELEVNVYNVEEILRICVLFRLNSLIDHCETFLRRNMTPANCLGLLFVADLFCLEDLSQHANRFALWHF
ANVYKEDEFKKMPYKQLKGLVMDDSLKVQSEEMVFEAVWQWIVHDIDARKIHVADIMQYVRFPLMNLQYLNENSSIQKLK
REFRICKELIREAICYQTALESKSSTAIDNFRIRPRMASEEIFVIGGWSNGQKICSVQCFNVDTLEWTTVAGMSVAHVSR
EDYFRVVAVGDELYTVSRYKVGKYDPIANSWLQIATGPDVQCKWAGVCEYEGCIYVVGGHSSMCAKQFDTETLTWKSLPS
MMYARYYPGVAVVRGKIYAVGGLDHLWAPLNTAERYDPQNNQWLAIPSMSTCRWSLGVAVLDDKLYAIGGSDNRECESNS
VEVFDPETNAWNQSVAAMNAGRRCLGVAVVNDTIYVVGGRVTNGIEYYDQENNKWVIVGSVNTRCNFGCVALRIL*
>Adig_11139v107906
MFGVHQDQSVVSKTCPEDWQTTKDTIKDRCSFLFNNELLSDRDIKFAVPVPGESESKKSKHEIYAHKFLLSISSPVFYTM
SYGKMKEIRETFELPECDNEILVEFLRYIYCDELTMNGNNVSGLLNLSKKYMMPPLADKCSEFLVSNMYPSNVLSVLKVA
ELHKEEKIVNLCWKMIDGQTEAVLRERIERPHLETIVTRDTLKMDEVKLFAAVDKWATSECEEKGLTPGPSCSKPD*
>Adig_11140v107907
MSNLLNTEVKFSTICKSICKMFGGQLDESDVNKTCPDDWQTTKVTVKDRCSFLFNNELLSDIKFTVPVLGESESEKSKHE
IFAHKFLLSISSPVFYTMFYGAVPETREIVELPDCDYKVLVDFLRYIYCDELTLNESNVSGVLYLSKKYMMPPLADKCWD
FLVSNMYPSNVYSVLSVAELHEEEKVVNLCWKMIDEQTEAVLRQPIERPLLEAVVTRDTLKIEEVKLFTAFDEWATRECE
KSGLTPDGEKKRAVLGEQIVKAIRFPLMGEYDFSSVVTSSGILSAEEVSQLQKYFDKTLDGLVAFPEHPRLWQPLVRRQR
FRYCGGSWAYERGRKDTLVFEVDRPIELYGLRLFGKLNRNYSVTLELIDEESEEKVLSQTGNFTSIPLITYEECEYAGFE
FLFDQPVDIDTLVKYRVEALISGPSSAKAKSCYRNXXXXXXXXXXXXXXXXNARQGQFSEFVFLRCYDQ*
>Adig_11141v107905
MFGVQPNQSVFHTIYSEAWQTTKLTIKDRCSFLFRNELMSDITFTVPVPGESESKKCKHVIYAHKFLLSISSPVFYTMFY
GAMPETRETVELPDCNYEVLVEFLRYIYCDEFTPNERNVSDMLYMSKKYMIPPLADKCWEFLVSNMYPSNVLSVLSVAEF
HEEEKVVNLCWKMIDGQTEAVLQQPIERPLLEAVVTRDTLKIKEVKLFTAVDEWATRECEKSGLTPDGEKKRAVLGEQII
KAIRFPLMGQYDFSSVVTSSGILSAEEVSQLQKYFEKTLDGPVGFPEQSRTSLDHLDRRQRFRYSGGSWSYQRGLRDALV
FEVDGHIELYGLRLFGRLNSNYSVNLELINEESGEKVLSQTGNFTSVPLKTYGGSVYYGFEFLFDQPVNIDTVTKYRVEA
LISGPSSEKAKNCYRNTDWFTFSAYDNENNGTNAREGQFPEFVFRRHRL*
>Adig_11644v108366
MQWTFHVLYLNNIHDHDHAGHEGGKLNMADSGNKFSGVLKRSLSDTAEALLFTDENHGSTILKGLFASYKDGKYVDISLK
IGPERTLRAHRAVLASFSPYFEALLGDNWKEGNKEEIEIPGLDEGAVSDLIEFAYTGKIKIDRDNVLTLLEAANYLAIES
VKKSCGNFLEQGVDTKCCLGILQLADMFALDELSSFAKKYALRHFTDVCKEDEFLCLPVNFLSDLLSEENLVVVVEDLIP

CVEEREKTVLESVLQYVEHDVENRKTHLAHLLGLVRLPTLSTEYLAEVSRNKLLECEFGDILEKAKNSKLHPPEEGNPDE
KWAAPRKFAKHVFMWGRCYANGGQVQPEIACCTNEMFEDLENDCYVTGMELWIRQWDGRPVLGCLKVYYKDGEAMVFGQD
CGGTKAENAQDEHVHHEFYLEENERIVKAEIQCGWMIDRLTFYTNKKDKDGKPKRYGPYGGNGGSFSAESAPESFGYLCG
VSGAVVQSQGEAGITRLQFVWRLYVLPGGHVPFKHRCIVAHAQKGDYEDEYDDPYDDIYDEFDDYEDDDDYGQYDDFEDI
FEFEMGAQPYMFEPLA*
>Adig_11645v108367
MAASGNKFPSVLKRSLSDIAEVVLFTDENHGSTILKGLFALYKDGKYADITLKIGPEQTLRAHRAVLASFSPYFEALLGD
NWKERNKEDIEILGLDERAVSHLIEFAYTGKINIDNDNVQSLLEAANYLRIEFVKKSCGDFLAQGVDAKSCLGILQLADM
FAMDELSSVAKKYALRHFTDVCKEDEFFCLPVNLLSDLLLDENLCVVVEDLIPCVEEREKMVLESVLQYVEHDVENRKTH
LSNLLGLVRLPTLSAEYLAEVSRNKLLESEFGDILEKAKNFKLHPSEKGTPNEKWTMSRKFAEHTYTWGCAYANRGQEQA
EVEYCTDKMFEDLENDCYVTGMELWIREWYERYVLGGLKVYYNNGKAIVFGQDCGGTYAETARDDHEHHEFHLEENERIV
KAEIKCGCMIDRLTFYTNKKDMDGKPKCYGPYGGNGGYFSAESAPESFGYLCGVSGAVVQSHGEAGITRLQFVWRLYVLP
GGNIPLKHHCIVAHAHKGDYEDEYDDSYDDINDEFDDYEDDDDFGQYDEFEEIFEFEMGAQPNMVEPRA*
>Adig_11750v108465
MGAHSEILFSKCAEFREQGEFIDVRLKVGEDEFAAHRIILAASSDYFHAMFAHGMKESNQEVIELKDENISAAAMKIVMD
FIYSGEINVNDENVFEVLTAADHLQVATVVEQCCKYLIQLRFDVQTYCRVIMFADQHSLKGLKEATENKMASMYKEICDK
EEFLSDMNADVLSALLRRDDLSAPSENFVFQSVMQWIKYRKEERMDVAAKVIGAVRLGLVDVKDVIEELDTEEMQVISEI
NMLLQRTLIYNHRPSRNSAFALEKGKPRSMSLVLVGISPNGDICYFDIQSKTWKQLSSMQKLAKAGACYCAELIGNYLYV
AAKINSDHVICCYDIVSDIWSTLPPIPGSSNIRIGSLCHIEDHLYVIYKSSAPYRYNIATNQWQSIAGSKAVCNLTQKTF
CNKAAVVYKSCIYVLYGQGNIRRQDCVYGDPIFYSEVSLSVLYCFDLKKNVWEQKASTKTPHFASTLLVVNNNLYVAGGR
SLLQSSSSQQYGHLPAGGPAAIEVYNDQENAWSVVKQTHIPPNNLGAVEIDGRVYFIINSFPVDSGVILPPGEAYPAILD
EWKNLRNIGENAVLCYVTIKKENLTTEKTT*
>Adig_12079v108770
MENKQKKKLSQLQKPVKKKKSLRQHLLDKNQQNRKRKRAKAVTSPEACEQKEKSQTSDSKTRKRYILFLGNLPFTVKEED
VKSHFRCTGKDTIKQVRMLTKKTGESKGCAFLEFNNKFSYWKALNLHHSIIAGRKINVEVTCGGGGNGEQRQKRLRERKS
KFQESLLLCVPEFSTQVFSGLHSLRKEEKLCDIELSVGDSNLRSHRVVLAAASSYFNAMFTGDLRESRQDKVILFDVEFS
ALEDLVNFCYTGRIEINVDNVQNLLSASSLLQLASVKQACVEFLHRVLHPTNCLGIRSFADTYSCEDLVEAADVFAVNNF
SDVARSEEFLALAPEKVVEMISREEINVRKEEEVFEAVSTWVRRDEEERKDFLPELLKNVRLALISPQYLCEKVSSDELI
KSNLSCRDLVDEAKDYHLMPERRYKFQSIRTKPRRCTEAAGLIYAIGGLTSAGEALSTVETYDTLTGQWVPGLPMSTLRT
RVGVTVLDDKLYALGGFDGHKRLSTVECYDPQLRVWKAVAPMNTRRSALGAVALKGKIYVVGGYDGHISLNTVECYTPTT
NTWSFLTPMGTLRSAAGVTELNGKIYAIGGHNGLSIFNTVEVYDSHSDKWSSSPPMGERRCRVGVATLNGRIYVCGGYNG
SAFLNTVECFDPETGQWSFTAPMNTRRSRVAVVTLGGRLFAVGGYDGLCNLNTVECFDVLANQWTPVASMGMHQGGVGVA
VLPRGPYA*
>Adig_12487v109151
MRSKGELCDVNLVVGESQRRISAHRVVLASCSPYFQAMFTGELIESRQRDITLQGVDPEAIQLLVDFAYTARIQVSEDNV
QSLLPASSLLQLTSVKDACCEFLKNQLHPSNCLGIRAFADAHTCMDLLESSHRFALQRFRDVSQTEEFMLLSVKQVLDLI
SNDDVNVESEELVYNAVINWIKFDVENRKVFVRDLLPFVRLPLLSRECLMTRVETENLIRSTPDCKDLLIEAMKYHLLPE
QRSILQSPRTACRKPAGQVPFLFAIGGGSLFAIHSECECYDPRIDRWCMVTPMSTKRARVGVGAIDRKIYAVGGYDGSND
LATVEAYCPQSNAWEVMPSMGTRRSCLGVAVFNGLIFAIGGYDGASCLNSVERFDPLTAQWTSVAAMNTRRYL*
>Adig_12755v109413
MNQFATPEELEKHHVRDVYDKIAPHFAGYSHKAWPRVEEFLLSLPAGSLIADVGCGTGKYLGLALDSFVVGSDSCVEFAE
IAMQRGHSVVACDNQSLPFRESCFDAVISVGVIHHFASGKRRSKALEELYRILRPGGRMLVYVWAFEQEQRKFDGQDVLV
PYKHFQRNKVLARRSLSAPVRPRQQAQFHNGSTPNISSSSWRSAHSSHNCFGGHVYKRQQTQTSKREDSSKNDTGLGRVQ
VNDTSKYAVLRKIRNQEPVSSLRLEKSEEDNPMTKISKFLNSVLNKVFNNADDDGGEPEKLPSASANEKTHQRNRSSENA
SSEFSPKFSALMTYFSSEKRETILKTDVLSQLARKLFAWKDDLFLDNDFVKTLDNCDEIDKEQHEKSVVVIGLKKENVGV
DGTCGHQAPSATTSVSLNVMARLVDSLVLPSDVYNDSSSSSDALSSDDSSSAEGWNQVEPSHPTHSKKTFQHTTISCQEP
AEPVCAADKDSQYSSSSSFFSTTASSSSQSSPLLSSSSSSPRTETSSKLATRKLQRYYHVFCEGELPKLVQDGIPSAVIL
KEYYDHGNWAAGERKLKCHRVVLSSCSPYFRGMFTNEMMECTQDTVVIQGLSEDAVIQLINFIYTRKITINIDNIEALLT
AAAVFQLDSVVHACCEFMKRHLHPSNCLEMRAYAELHGCLDFIEAADTYARGCFLTLVNTEALLKTSFKHFRSIISGDDL
FVKREEQVFEALMAWVRYDLKTREKNLGELLSHVRLPLIGVENLIQRVEQDATVRANLTCRDLIDEAKNMMLCPSLMKVN
IRTQPRKSASGTLFSIGGRGKSGNPLQSIECYDWFRNCWFRGADVSTPRRHVAVASVAGKVYAIGGHDGSYHLNSVECFD
PEINQWNTVSPMDISRRGMSAGVLEGQIYVAGGLDETT*
>Adig_12987v109617
MERKTTFDLGMLSQLSKPRMRHFSDRHPKQLLDTLETLRQCKELCDVVIKAGNKTLFAHRVILAGCSPYFRAMFTGEMTE
SRQMEVTIQGVDEAAMELLLDFAYTASLEVEESNVQALLPAACLLQMAEIQEICCEFLKRQLDPSNCLGIRAFADTHSCR
DLLRFADIFTHNNFQEVMESEEFLLLPVTQLLDILSSDELNIRSEEEVFTAVINWVKYSVSERRGHLPEILQHVRLPLLA
PKFLVGVVSSDPLVKSDESCRDLVDEAKNYLLLPEQRLVMQGPRTRPRKPTKCTEVLFAVGGWCSGDAISSVERYEPQTN
DWKMVATMSKRRCGVGVAVLDNLLYAVGGHDGSSYLNSVERYDPKTNQWSSDVAPTCTCRTSVGVAVLDGYMYAVGGQDG
VSCLYIVEKYDPQENRWCKVASMSSRRLGVGVAVLDGCLYAVGGSDGTSPLNTVERYDPKTNRWSPVAPMGTRRKHLGAA
LFQDKLYVVGGRDDATELSSAECYDPKTNQWSPVVAMNSRRSGVGLAVVNGQLLAVGGFDGTTYLKTIEVFDPAANQWKL
YVGMNYRRLGGGVGVVKLTLNDAFGC*
>Adig_13021v109650

MQFFANFRAVLLNTCSKYCSWIGPKLHSSSSCYSSLPGAGQTVERVVKWMGQHAPVFAVKGNEITVLHEPEQFFNSLKDN
VSNAKERIILASLYLGTGNKEQQLVDCIQEAVRQSSLAKQSLNVEILLEHTRGSRGDHNSRTMLLPLVQNFSDTVKVSLY
HSPSLRGLLKLLLPERFNETVALTHLKVYLTDNTLIMSGANLSADYFTNRQDRYIEIKNCAALADYFHELVSTVRSFSLQ
LQPDNSTVMSKYFPLHPTKGFDHGKKFKVEVGKTMLKYLSQQKEQNSYINDVSERTDDQCDTWIVPLVQMFPFGIRQDEF
ITSELLRSANHSSVICLASGYFNLTRKYIDIMLKSSAKFKILTAHPTVNGFYEAKGVAGGIPHAYTLLLYDFFKLILKHK
LDHITVHEYACSGWTFHVKGLWYYLGNKQLPNLTLIGSPNFGHRSVYRDLEAQIALVTKNQDLSEALHEEKSRLYHKAKC
VSKETFELTELKVLIGNMFKLNARVPSEDEILGCWKIVSCKLSDGNYPLVEGVKFKLDEAGDLIWMQPATFEQETDLPFF
SCETFVVDQDQGLENLSCHPYTLIPALDEGFFTDITFVSSSGKKFPAHKTILSCLFSDVLAWDKIPPFLSNLSSDVLQSL
LHYCYNCSLPSNISEDTAKELLRISQQHSSSLGNLGELCTEFLDATAVKNRIKSLMSELCNILENILQMTESVAAAFPYN
RDILQLLI*
>Adig_13023v109651
MFLSQKQFDFTSLSEEQKQRCVMKSMDKMLVEIPQQVRTLHKFPRIFEKKMPWREWKHSFKVWTSIVFLEYLYSGILDTR
SVSMDEVPEMLALSDKYEVDSLKESCEAILIKDIDDETVLIYLGIAEQFTVKRLKEGCLRYVTAHPDVMDSDVFEELPDN
LKQEIADKVHQSM*
>Adig_13226v109833
MPPRRRSLHKSLNTLKRKLTMDDSFVFSDEKYSRDVLMKLSGLQKQGIMCDVTIKIEDEEFFAHRNILSASSDYFFAMFN
GNMKERHAREVAISGVDKDSMKTIMNFIYTGEITLDWNNVEPILQSANLMLVQSVKDACSRFLESRLNVKNCIGIQGLAE
TYACHKLYGVTTTFIYQNFSNVAENEEFLNLDQKQVELLLQSDDISVENEEKVYEALLGWINYDLENRREYFVELMEYIR
LPLVSPYYLVDVVEHEELIISSSRCKELLQEAQHYHLLPDRRQLLDTARTTPRNYERFHEILIAMGGNGDFHNQSTGK*
>Adig_13460v110048
MEVCSGSLSREVPSPFALTHSVRVLEIMNKHRRDCLKLCDVVLKIGDEHIPTHRAVLAACSPYFYAMFTSDLVESRQKVV
TMKDVDAGIVQQLVEFAYTGKFDVNVENVQSILATASLVQFHEVRELCCQFLETQLDPSNCLGIRKFTEAHGCVNFLEVV
DNFVLENFREVMKSEEYALLPCELLIKVISSDDLNIFAEEEVYEAVMSWVKHDLNNRVKQLPNIIRFVRMPLVSKHYLLN
RIDVDNLIRSNMNCRDFLDEAKNYHLLPEQRAQLRTDRMKPRKSMMGTLYAIGGKEAGETISNTVESYSLQSNVWQPAPL
LNVPRQQLGTGSLGGWLYAVGGSDGYSRLSTVERYSPDSNRWMYVKSLNTSRSGVGVGVLGETMYAMGGYDGRSCLKTVE
RYDPLVDVWSHVAPTNVTRSFPGVAELGDRIFVIGGNDGVTFLNSTECYDPHTNRWTQLAPMSLARAGIGAAALDGRIYA
IGGFDGAQRLDVVEMYEPRMNSWHDAASLNSCRDGVCVVTYGCWVYAVGGIDGPSYLNTVEAYDPRTNQWEYVVSMARCR
AAAGVAVLKKSS*
>Adig_13478v100953
MSQDTIDDENYRNKLLSKLFRQQREGVLCDVVLIIKDREFSAHKSVLAASSEYFLALFTTTMIERNCSSVRLDLCPDVVQ
DLLEYLYTGKVTFTKSNVKGLIITADYLLVSNLKQEAEKYLRSVISEVNCTSMYVFAVHSNCDKLKADATSIIGGNFASM
SKTNEFLNLDFNLLLPLVSYDAIIVATEEEVFEAVLRWGIVDVPQVGYVRFGFILGWD*
>Adig_13481v110073
MEELCDVVIRTDDDASISAHRNVLSVCSPYFRAMFTGKLRESRNEIVALKGVTAEALRCIVRFMYTSTIDINEANVDDIL
KRFGSFSDEREALAAGFSCEELENEARKYSLKHFTEVLKSEEFKELPLEAAKKLLSDENLCVRSEEDVYYAAMEWIFGSA
DRLKHSAEVLSCVRLPVLSPSFLNSEVLSDKWLSNDFECRQMVEDALRYASSPTSEKRKHCLTTRMRPRIPFGYGDVLVV
IGGLHLGNPVPSGERYNMYTDQWTSIEDMPTSRYGIATSHLHGQVYCLGGFDNGKYLTSVEAYDPEENVWTIQAQMLVAR
KYFSATSLGGKLYALGGSNIQRRLKSVECYDPQDDKWTFVSSMISPRMYLAVGTLGGLIYAVGGHDGMLRLDTVECYDPQ
KNEWSYVASMGKPRSVAGVATLCGLLYVIGGFDGTNYLSDVDVYDPLTNCWTSVSPLNEARSAASIAVMKGRIFALGGFN
GQFLSSVEVYDPAINQWSYVQDMSIPRVHFGATVV*
>Adig_13958v110530
MWGGFSIICKMFAGQPDQSVVDRTCPEHWQTTKITIKDRCSFLFRNELMSDIRFTVPVLGESESKKSKHEIFAHKFLLSI
SSPVFYTMFYGAMPETRENVELPDCDYKAFVEFLRYIYCDELTLSESDVSEVLYMSKKYMMPPLEDKCWQFLVSNMYPSN
VYSVLSVAEFHEEEKVVNLCWKMIDGQTQALLRQPIERPQLEAIVIRDTLKIEEVKLFTAVDKWATSECEKNGLTPDGKT
KRTILGEQIVKAIRFPVMERHEFSRVVTPIGILSSEEVSLLKKYFQKTLDGPIGFPADRRLPNDLVRHERFRYCGGTHLY
HNGLEDALAFEVIYPIELYAVCLYGNKNSNYSVTLELIDEESAESWLDMRLVKTHSPLCSLLVFTISHLMHTHGTITLLL
NNCPLAISPISINSHTLSMAAIFG*
>Adig_13960v110529
MFAGQADQSVVDKTCPEHWQTTKITIKDRCSFLFNNELLSDIRFTVPVLGESESKKSKHEIFAHKFLLSISSPVFYTMFY
GAMPETRETVELPDCDYKVFVEFLRYIYCDELTLSESNVSEVLYMSKKYMMPPLADKCWQFLVSNMYPSNVFSVLSVAEF
HEEEKVVNLCWKMIDGQTQAVLRQPIERPQLEAIVIRDTLKIEEEKLFTAVDKWATSECEKNGLTPDGKTKRTVLGEQII
KAIRFPVMERHEFSEVVTPSGILSSEEVSLLKKYFQKTLDGPIGFPADRRLPNDLVRHERFRYCGGTHLYYYGLKDALAF
RVIYPIELYGVRLYGNKNSNYSVNLQLIKEERRETVLLSQTGNFTSVPFPLEGDAEYYGFEFLFDRPVYIHQNTKYCVEA
LISGPGSVNGTKCYPNTDWFIFSKSDHDRNGTNEYRGQFSGFIFQRKREG*
>Adig_13964v110536
MDAKLSCMVTMEAVTQTQPEEEKIAVPPYRSTESHNKSFQVMNSLREQNQLCDVLLKAAGNEIPAHRVVLASSSPYFLAM
FTGELSESRQTVVTMREIDSHALELLVQYIYTAEIEVTEDNVQVLLPAANLLQLTEVRDACCDFLKGQLHSTNCLGIRAF
ADVHSCADLMAAGHSFAEKHFSEVVQADEFLSLPSQQVEELICSDQLTVPSEEKVFEAVVTWVNHDPEHRQDCLAQLMEH
VRLPLLSREYLVSRVETEMLVKNNNMCKDFLIEAMKYHLLTNEQRSSVQSPRTRPRTPIGLPKVMYVVGGQAPKAIRSVE
CYDFKGEKWFSVAEMNSRRCRAGVAVLDGLVYAVGGFNGSLRVRTVDCYDPIKDQWRPVASMEARRSTLGAAVMNGLLYA
IGGFDGTTGLNTCEVYDPKLNEWRSICPMSTRRSSVGVGVLNNLLFAVGGYDGASRHCLSSVECYNPIINEWSLMTEMST
RRSGAGVGVAEGLLYAIGGHDGPHVRKSVECFNTDVNQWRPVADMSMCRRNAGVASVNGLLFVVGGDDGSSNLFSVEVYN

PRTDQWNLLQTHMTIGRSYAGVAVIDRPNT*
>Adig_13976v110546
MVAHSEILLSKCAQFREQGEFIDVRLKVGEDEFPAHRIVLAANSDYFHAMFAHGMKESNQEVIELKDENISVSAMKIVMD
SIYSREINVNDENVFEVLTAADHLQVTSVVQQCCKYLETELVELGFDVKFYCRVIMVADRRGLKDLKEASESKMASVYRE
ICEKEEFLSDMNADSLSALLCRDDLRVPSENFVFKSLMQWIKYSKEERMAVAGKLIGAVRLGLVDIKDVIEELDTEEMQL
IPEINMLLQRALIHNCRPSSSSAFALERARPRSKNPVLIAILPERVISYFDVQSKTWKELSLMEPLTEIQECYCVEVIGN
YLYVAAKSNHTFVIFCYDIICDTWSTLQPIPGSSGIRIGCLCNIEEHLYVIYKSSAPFRYNITTNQWQSISSSKAVCDLG
QNTFCNKAAAVYKSCLYVLYGQAVIGRSYYDSFEYFSAQLYCFDPKKNVWEQKASTETPHFGSSLLVVKNSLYVAGGRCS
IVSTTRQLGDDPAAIEVYSDQENEWSVVQQTHIPPNNLGAVEIEGRIYFIINSFPVDSGITIPPGQANPVDLTEWENLQY
TKKNAVLCYAGVSTSFLTSDW*
>Adig_14008v110579
MGEASHLNDDVQSAYDGDEGTEQPNIIRDWAEQPYDVTLVVKHGEQFKAHGNILRGTCCFFEKILDGNWKENQDGVIRLE
VLSEEIMEAMLRFIYTGTLHVNDVERAMGLIETADYLLFPKLKDVPEHFLEQNLSTSNCVLLNQFAGKYRCEKLLATSRK
FIQSNFTSVANTGDFCELSVDEVEEWISSDVINISAEDDVFRIILRWIDYEQEERKEKFPSLFRHVRLGFVSLSCLLNEV
VTNDYVKKDELSLNNILEWTSGSPVCCELFMQSPRKSLQEDALVALGGEKTAWCFVPSKNEWFQLPDIPSSVDNPEWLLS
FQNSLIAVGLNSTWLEEFPFYDPFVNEWTTSFLPTPQALLEQGFEPEIATVMVVGEDICAIVEDILPTDDDQGQYSYQRT
LFKYSPGSEWWTMVPSFHLEPNLKAKEYYYNAIIKCLKMSAFSYPEPDNIVFMAQFIKKVYRKKDSIKQTDKEGRCPK*
>Adig_14081v110648
MKTLPAVMNSSKAWQPIGKTIGERCAFALTNELLSDVKFVAGERESTTDNERRVIAAHKFVLSISSPVFLAMFYGEMAET
SEVIQLPDTDYDSLLEFFRFLYSDEVKLTGDNVMQLLYLANKYMVPSLADRCTEYIQNNLDQTNVLCVLSNAQKFEEETL
VQRCWEFIDKKTEDIVKADEFFELDRCLVESIVNRDGLCIKEVELFKAVDRWVSVESERQGFHQNSGGNVKRKILGETIV
KSIRFPLMSQKDFMSHVPDSNILTSDEIIDLMKHFNGVPLQNPLPFLQTPRKRTIRDSQKTRRLGTYIPPAVSWFSHTRC
KPPESTRC*
>Adig_14421v101055
MKEKQKLKVKLEGFKPVVMNDLLSYIYTGQAEITDENAKELVFAGDYLLIESLKKKGTFYLEDTLSPSNCLSVRAFSEKY
VCDELKRKSESFILDNFVAVSKSEEFLCLGSSEIEKLISMDDLIVETEEQVYEAVISWVKHDIQKRKEDFARLLSKLRLG
SMSKYYLAEYVENEELVSGNLECTKLLYKAMKSFAIFATQGKLTGDICKIRRCLDSNIKAIVTIWGPGDELRSSTQCYVP
SVNQWFNLAPMLIPRFSHGAVACEGFIYTVGGVSLNGHLSSMERYDYRTNTWAGVAPMAKEVSGLGVAELNGCLYTVGGW
HRGRPLNSVLRYYPSSNIWEAVAPMTATRGGPCVVSDKYLYAIGGKTENDNANDPFKYLNTVESN*
>Adig_14852v111356
MSSPETWQTERSTIRERTKFMLNNDLFSDVKFVVRKSDSEGESESKKVIPAHKFVLSIGSPVFEAMFYGELAETRDSIEL
PDCEYESLLELFRYLYSDEVNLSGSNVMGVLYLAKKYIVPSLVDKCSQYLQDHLQPENVFNILTIAEKYEEKGLIDQCWE
VIDNQSEEAVKSDGFTAIEHSLLEAVVSRDTLAINEIDLFKAVDLWATKKCEKQGVEANGEVKRRVLEETVVKAIRFPVM
ELRDFSDVVLNSKILTLDEVTRLVQFFTSSSLATEVGFPATSRTGVSTKQIDTLCRFGTLDFNSWGYGGSSDFLVFSVNK
EISLHGLCLYGSNNNRYSVDLKIMNPVEMTTLVQMTGTFKSKFIYHGKSPYYGFEINFDKPVRVKKNSKCEIEAKLSGPP
SCSGTSGLLEVETSGVKFTFYDSTHFPSSNGTSVLIGQFPELLFSF*
>Adig_15226v101156
MDQIDDCLRLAKHCKLEYLENQLQMRLNSIQMFGTSKKRSITVVSIEPESQSTSLQDAFGRLAEIALPCPFTAQQFPKPF
PFEISLDLFEVVPSSEDPPPFFDVCFVVEGYRFHGHKLFFCGRSDYFRALLEYKNSEASTSGQERLSESVVEVFLNDVSP
DVFAAVVAYIYQDDALIIENKEFHSLILEDAASVRERQETDSIPVVDDIRFHLMRGVLPYNVSEGDDEEFVDCEGSLQML
DNLLLYLGIEC*
>Adig_15434v111864
MALSAEMSESIPTLPTLEIVGYPRNEESESKRRDDEAGSEQQEKESVEAAKHPFCEPWEDSDLIFVVEEERFHVHRQIMS
IHSPVFRAMLNSVGFKEATATEIPLPGKKANEFLDFLEFLYMKKMDEIQLNQVEHLLKLADEYQARGIVDVCVKMLKSEA
RCKDNAVKILHLATCTPTVRDDERLILVREQCYELMKNVELTETQGKESYKNLERDALERVLVKRIQRLETFVKEIYPQF
IGLVQCCLWYVLENAKVISTCPQHFLNKKPTENLLKLMKDCPVCRQMIKQLVNYVRPPRVSHLTPVPQLQRATPVPVIPQ
AAQLQRVIPRQVLNAPSLVSSPTSSVFGGVAPTTYECKYGGNCHFDEEVITIIKDFEEIIRV*
>Adig_15437v111863
MASSTSGETIEEPTVKHEQVEDAKHPFCEPWEDSDLIFVVEEEKFHVHRQIMSIHSPVFKAMFTSVGFKEATATEILLPG
KKANEFLDFLELLYMKKIDEVQLNQVEHLLKLADEYQAGGIEDVCVKMLKSEPKSKDNAVKILYLATSTATARDDQKLFQ
VREECYELIKDMELTEAQRSESYNDMEKNTLTRVLMKRNERLETFVKDIYPQFMEPAAKQEQVEFSKHPFSEPWEESDLI
FVVEGEKFHVHRQMMSIHSPVFKAMLNSVGFKEARATEIPLPGKKADEFLDFLELLYVKKMDEVHLNQVEHLLELADEYQ
AGGVVDVCVKILKSEPKSRDNAVKILHLASSTATARDDQRLFEVREQCYQLTEDMELTESYENGKSLEKEIWERVLVKRI
QRLETFVREIYPPFVDFVEFSLSESLREKTISSCPRHFSNGKANEHLLKRMKDCAVCRQMIKLLIFNLKKQSQQPFQFGA
STSNLFASCSASAFESDPLKDEKTMMVIMDFEKIMRV*
>Adig_15438v111862
MASKKGFTFRPPSSDSILFQDHEESSRRNSIATSESDDESEVDNFDDTPNLFKGLERDLPISEQQEKESVEGVAKNPFSE
PWEDSDLIFVVEEEKFHVHRQIMSIHSPVFKAMFTSVGFKEATATEIPLPGKKAHEFLPFLKLLYVTKYDEVQCK*
>Adig_15454v111882
MNLSFNIMNDLRKRKELCDVVLCVGDLEIYAHKVVIASFSPYFYAMFTNDVVESRQNSITLKSMDSKSVELLIEFAYTAR
IRITEENVQYLLPVSCILQISPVKKACCDFLQSQLDPSNCIGIKDFTEMYGCVDVAKAAEKFIFRHFLEVSQSEEFENLT
REQLIDLIRRDELYVKSEDEVLAAMLRWVTHDLNNRTQELKELLNFIRVPFVSSSYLKSLIQCCEQGMRTEFKVLLIEAF

TETKRVSSVTAIQKRRPPGPTVIFFAGGYLRRSLDVLECYDPLTLTWSILPPLSVPKSGLGAAYFDNMLYIIGGRNNTSS
GNIDSASVDRYDPLRDEWKSMKSLSVPRNRLGVAVIDGYIYAVGGGNGNSLHTSVERYDPNHDEWTTVMPLNYRRIGVAV
AVLGRKLYAIGGLDGANRLRSVECYDADNDVWTLVCPLMERRSGAGAASLHNHIYAIGGYNGDAQLNSVERYNPSSNTWT
VVSPLIHRRSALSAVVYCQRLFVLGGYDGEGFLSTIEEYIEEKDCWRLASSMDLGRSGAGVAVGWKPAT*
>Adig_16311v101294
MSDLKQQKMISPVFSRNDVDFKRKIKQSGMRGSCRSTISEASETGSLSHRLRHAQSVLTALENLRKREELCDVILKISET
EIKAHRVILAAVSPYFNVMFTGDLAESKKNVVELNNLDASAVSIVVEFAYVAQANITTENVESLLTVASFLQVQSLVEKC
CRFLDRKLQPSNCLGIRRFALMNGCFTLSKTAYQYAMRHFIQTTQLEEFLQCSLEEVLELLSEDALYVRHEEEVYEAAVR
WLRYNPGERSEKIPTLVEIIRFPLMAWEFLVTRVVDDGIITSNSCTSHLFEEARTFHLNSPLNNEVRYGSRLRPRKLYGQ
AEWIYVVGGETSPGRQTIKSVTKINPSSSMWVEVCPMQTPRRGLGVAILDGILYAIGGSDGIAALNEVESYDPSSDSWTT
TTSMIEHRSSLGVCILDGFLYALGGYNGQTTLNSVEMFNPLTREWSMVTAMNTRRSMLGVASLNGRLYAVGGYDGISDLS
SVECFISQPDIWQQVASMKTPRSMVGVAVLNRRLFVVGGCNKHSLESVETYNPDTDSWTIVASMKQPRSGAGTCVVDGLL
YVVGGSNGMDYLNSIEVFHSQTERWTSANFTIARRRFGCCS*
>Adig_16461v112821
MSSPTKKTKMSAPENWQTTRPTIRERTKFLLNNDRFSDVKFVVRDSNSEGKSESKKVIPAHKFVLSIGSPVFEAMFYGEI
AETRDSIELPDCDYDSLLELFRYLYSDEENLSGSNVMKVLYLAKKYMVPSLADKCSRYLRNHLDATNVFSILPMAQKYEE
KELTDRCWEVIDNQSQAAMRSDGFTTIEQSLLEAVVSRDTLTINEIELFKAVDLWATNKCGNQGLEANAHLCCLQRIVAF
IIWTATDCFTAQESQEKRATIHRHRRQMFAPQSWQTERPSVRERIKFMLSNDLFSDVKFVVCKSDHTVESKQVIPAHKFV
LAIGSPVFGAMFYGGLAETRDSIELPDCEYESLLELFRYLYSDEVKLSGSNVMGVFYLAKKYMVPSLVDKCSEFLLENLD
ASNVFNILPTAEKWDEKELIDQCWEVIDNQSEEAVKSDEFTTIEHSLLEAVVSRDTLTINEIDLFKAVDLWATKTCEKQG
IESNGEVKRRILGEKVIKEIRFPIMEQKEFASVVPDTKILTPVELKVIAMGFYDRKRSGIWVCDRFGSIYNGCKTYDGLK
DYLLFEVDNDIILHRLRFCGSENCKYSVKLKIGNVDSCPFEQCVCKTGTFVSKRLQCKNRNEYFGFEVKLGHSMALKKNE
RYLIKAEISGQPSWIGNFGCSVVKVAGVNFNFITSLKKGNGTCSTFGQFPQLVFSLCHHSSADAAGIDRLRLAKNTATIS
AYIHIFSVGTQHRRLRRRRFTVLPSLLPSRSKDRQNRLGGRAAGAPEGFLQTNRSGKWTVYGGLRSPKAKQSPHGYNNYR
RDGCDVKFVVRKSNGEGESESKKVIPAHKFVLSIGSPVFEAMFYGKIAETRASIELPDGDYDSLLELFRYLYSDEVNLNG
RNVVWVLYLAKKYMVPSLADKCCNFLRDVLSTFNVFFILPTAEKYQEKELIAQCWEVIDNKSEEAVKSDVFTTIEHSLLE
AVVSRDTLTINEIELFKAVNLWATKKCEGQGLEATGELKRRILGETVVKAIRFPVMKLQEFATVVPDAKILTPDEVINFF
KFSVQQWPPQKDF*
>Adig_16464v112822
MFAPQSWQTERPSIGERIKFMLSNDLFSDVKFVVHKSDHAVESKQVIPAHKFVLSIGSPVFGAMFYGDFAETRDSIELPD
CEYEILLELFRYLYSDEVNLSGSNVMGVLYLAKKYMVPSLADKCSEFLLDNLDASNVFNILPMAEKCNEEGLIDQC*
>Adig_16467v112823
MSTPENWQTTRPTIQERTKFLLNNDRLSDVKFVVRKFNGKTQRGSIAFVKKVIPAHKFVLSIGSPVFEAMFYGELAETQA
SIELPDCEYESLLELFRYLYSDEINLSGSNVMKVLYLAKKYMVPSVADKCSKYLRDHLDASNVFNILPMAQKYEEKELTD
RCWEVIDNRSEEAVKSDEFTTIEHTLLEAVVSRDTLSINEIDLFEAVDLWATKKCRNQGLKANGEEKRRILGEKVVKAIR
FPVMKQQEFASVVPDTNILTPHEVINFFKFFNSAAGAPEGFSQMNRSGKRTGLFFVLYAGFVVNLIEIRFGDVKFVVRKS
NSKGKSESKKVIPAHKFVLSISSPVFEAMFYGELAEAQASIELPDCEYESLLELFRYLYSDEVNLTGSNVMEVLYLAKKY
MVPTLADKCSKYLRDHLDASNVFNIMPMAQKYEEKELTDRCWEVIDDQSQAAMNSDEFTTIEHSLLEAVVSRETLTINEM
GLFQAVNLWATKKCRNQGLEANGEEKRRILGERVVKAIRFPVMKQQEFASVVPDTKILTPDEVINFFKFFSSAAAVPEGF
SKIRRPGSRGMSGVRKNPRVPPPASYFRRDENWFCPDCATALRGNFCPTCGQAIYMW*
>Adig_16476v101324
MSSPPYNWQSERTSVQDSITFLFNNEILSDVHFMVGKGTQRRRIPAHKFVLAVRSAVFDAMFNGGISAQSDEVELPDVEY
SAFQALLCFLYTDKVEIGPETVMTTLYTAKKYAVPALENACVDFLKKNLSPDNAFMLLSQARLFDEPQLAALCLECIDKH
TSEAHAAEGFTDIDFETFCVVLQRDTLAIRENQLFAAACRWAEHECQRRSLPSTPENKSDYQVTIQVIATDTGKICGHND
TAFSSDGTVSTFRVMFKEPVEILPGVNYTASATLKGPDSHYGTSGGKKIVYETDQKEKIIFQFSYAAGNNNGTSVEDGQL
PEIIFYT*
>Adig_16513v112863
MEAVSHSMSANPSQHREELIERINALRTNQRFSDVTVVVKGEEFQAHKIVLAAASPFFLSLLESQMKETTENMINIELEE
TTASVMEEVLAYVYTGNASLTKENGHNLIATADFLLLPGLEAIAENFLKGHLTVENCVFNYYFAEKYQCCGLKEESCKMI
NENFRKVMETADFLGLDENQVMYWVSSDDVVVEAEEVIFEGIIQWVLHNRSEREEKFPKLLRQVRVPSISHEYLVNRLLN
EELIKTSLECFNFVTECVKLIFNCTTESSIKPARKCLKGNEDVIFVCGLRKALCYLPDENKWHKLTSTTLEHKGHSIIQY
RDRVYVFSSQEVEENQLHVVEYYVPATNSWGAIQTKFDYSREQFTGLFVLNDNSGLWILTNETEGSDYIYEYNLDKNIWV
GSSRKLWRSGACYVSDGHCIFTIGGALVEDERVRVTRVVDKFFPANKTEDGKVEFEEVAPLNEARYDAFGAAMNGKIYVA
GGKQELFVDNSDFTSCEVYDPSTDEWQVMPSLNVPRYSASMVCFKGTLYVVGGRSSVSEYGELSVEMFDSGVNEWHKKSA
IPTGDENEGKFYNRNIRYYKACFATVRLGAS*
>Adig_16539v112886
MEDSRASSCSPSSIAKEHSMKLLNKVQELRMQGLMCDATLTVEGKHFPVHRVILSASSSYFQVMFTSGMAESFMDHVEIH
GMSPEIFQTILDFIYTGIVEVNESNVQQLLPASKMLQVQDIEVICCDFLKHEIDSTNCIGIYQFAEDHSCSSLSKSALDF
IHRNFVEVSNQEEFLQLTRTVLLQLLESEELKIESEEQVFEAAMRWVLFDLTRGREALGKILVRIRLPLISPKFLENYLV
ICKSASIQRMLSGILDGYRNYQSLSHGQQKVHTKPRRASTKCFFVIGGYNRRVGGRWSDTSSLYTVEKFDTFLQTCDSFS
TAPMSFARSGHSVASIGGLIYAVGGENDSLIYDTVECYDPALNTWSLVAPLIAPRVACGVCVIEDFMFVLGGWIGSEIAE
DIERYDPHLDRWEVVGKVETKRFHLGVTEMDGLIYAVGGLSELGEELKICECYNPVSDTWLRLPDMNYRRAHLGVTSLGG

SLYAVGGSNELDGTLSSVERFDWQTNQWTEQKVQHKDRRSGNEYMTTKMAASMSEYENAMFLEVMDDDGLFVSAKGLNIE
RVISKFLSIHCVTSDPSSISLVLVINTTSQEEDFILDELRSSDVPLPKVITTEFSSSERESVYLRGGVLFVTSRILVVDM
LTKRLPIHLVTGIVVYRAHKIIESCQEAFILRLYRENNKTGFIKAFSDNAQAFTNGFFKVEKVMKNLFVRRLYLWPRFHA
TISSFLEKHKPEVIELHLHLTPSMLAIQTAILDLINTCLKELKRANPTLDPEDLTVENGISKSFDKALMSQLNPIWHQLG
NKTKQLVSDLRVLRLILLYLTQYDCVTFYNFLETLRASQRDVKNQSLWMFLDAADTLFVHAKARVIECTERKHKKKKGEK
PEEEEKDSEGLVLEESPKWKLLAEVLEEIEQDDTKNESKSKYLLKLMN*
>Adig_16772v113098
MATLQENWQTKCTSISQRTNYIFNTELLSDVKFIVPMSNGESESKVIPAHKLVLAIGSPVFFAMFCGQMADTRDSIELPD
CEYEGVLELFRFIYSDDVKLTGSNVMHVLYLAKKYLVPSLAEKCAEFLRKNLDASNAFSILSHAQKFEDKDLENRCWKVV
EVQTEEALASDDFVVIERSLVESVVKREKLNVKEVELFKAVNRWAEKKIENQGIGSDGNAKRAIIGEEILKEIRFPLMSE
KEFVSFVVDSNILNMQEIVNIIKHYNEVVTSPLPYLQSPRTGAFRRVCRFNEYCSAVVGSRWSCGGSSESLILSVDKDVR
LCGVQHFGCEGCEYTVFMQIKDTTSNLSLVKKSGIYFSEKDLNHIYYGFDVLFDTPVILESGKRYQISSMIIGGPSWYGE
QGQTPVNFEGINFTITSSGPNNGTSESRGQFPVFLFTC*
>Adig_17109v101423
MDTNSSHYWTNALKIVLVGDTRVGKTWLLARAHELYATLRDDGCSFSHKPFDPTLTYSGTSFVTCITNVDTADLGNYQTN
IWDTGGSGLFNFLRPLAYICADVFMLCFDLTSRESFDNLTERWLPELRKYQPKTPILMVGTKYDLLDGDVSNSSEHSIPS
CKVKAVADREGCLYQETSALSDHLVADAFEKAVKLGHKKREGEGKRSERETFVTILEFIYTGLAGISKDTEQEELKQLLF
AAEKLQIHSLVEICKYFSKLAEPKKACSSHEKIQQQNNSENQLQPPSTPMHTIQQGFFLDKEEAVFSDVTFLVEDTLVYA
HKAVLVARSPVFAALLSDNFKERFCNQVHLQGIDCISFLAVLEYLYTDACPSLETISTENVLIVADQLLLSRLVQICEDH
LHKSLQQVVPASSILDALAFSKLFNARQLTKWCLFYMALNYTRFRGITKEMDSFVKDDQSFFEEHLWPPREFLRVLEGYK
LKTEKAARLKGTERPISFIHWTPFGRS*
>Adig_17228v101445
MKQALAGTNYDLTGAISLGHITANQLKAFRDFLYKPEAILDEEIVADLEQFAKKYRIHSLAKLCGQRKYQREGAEGMAVW
AKHYEDLLAQLYEMFKKMEFTKAILKGDEESCEIPVHGPLIGAASPVFRDMFCNELFCQKGQKYRLRGLSSNTLNCLLRY
IYTGEVTVDGATVQDLLQKSCTYKIPAIISACCDWLSSTMSPYNAIGIWHLARETESDHTNSLEREAKAFIVENFTKVCE
EDDEYYDLEYEDLKEIIQDDDLGVDCEEDVFNAVNAWVGADKEARSQYFCDLFKCVRLANTSVKFLRDLETHPLVQDYAT
CLQAVRRALAEIVAQRFRVNKSPPQSGSKEKTTLNPQETEVPLKKNGTPDRRFKVNRNTLKSRSQGNTVEEKEQLHKRRT
FL*
>Adig_17260v113519
MSAHRRFNIPLHSNDALKTMNDLRRKGELCDVVLHVDGRQFPAHRIVLAGASSYLRAMFTNGMLESGMKDIKLQGVEASV
MESLLDFAYTGNIDVTVENVQALLSSASMLNLQSLRHVCCGFLQTHLDSSNCLGIRAFADLYSCSDLEETAYRFVRQHFL
DVVKGDEFLQLPKQELRNLLREDLLQVRSESQVFEAVETWILHDFSRRKDHAVELLSNVRLPLLTLEFLEARVFPSKVIK
NNADCQLLLAKVLNESARNLPLYMTIPRAQPQAVYVIGGRNGVDCQLASLERYDTLTDEWTVLQSMKYPRTAVGACSLNG
LLYVVGGECAVNIPHDDTMYVRHVECYDPAVNQWMSLADIAIQRSFVAVTALNGYLYALGGEDRTCSYNYVERYDPKTDQ
WSTVQCMRRKRSGAGVAVCDGLYSKSMYFSVVRMNFVLPSMYAPY*
>Adig_17327v113556
MAASGNKFSGVLKRSLSDTAEALLFTGENHRSTILKGLFFALYEDGKYPDISLKIGPAERTLRAHRAVLASFSPYFEALL
GDNWKEGNKEEIEILGFDEGAVSDLIEFAYTGKINIDKDNALTLLEAANYLGIQTVKESCGNFLKQVVDAKSCLGILRLA
DMFAMDELSSVAKRYALRYFTDVCKEDEFLCLPVNLLSDLLSEENLCVVVEDLIPSVEEREKMVVESVLQYVEHDVENRK
TRLAHLLGLVRLPTLSVEYLAEVSRNNLLESEFGEILEKAKKFKLHPPEQGNPDEKWAAPRKFAKHVFKWGRCYANGGQV
QPEIACCTDKMFEDLENDCYVTGMELWIRKWDGQDVLGGLMVYYNNGKVMVFGQGCGGTYAETTLDDHVHHEFHLEENER
IVKAEVQSCWIIDRLTFYTNKKDKDGKPKRYGPYGGNGGTFRGESAPESFGYLCGVSGAVAKSQAGETGITHLQFVWRLY
VLPGGHLPFKHYCRVAIALEGEYDEFENISVFKNGADRYLSVSNSFSRCRQGWARVRVGQQQSFSGLQSPK*
>Adig_18105v114204
MVNASMETVPPNSLSSRHIAIKIVEIIVVCVIAFLSLGGNILVCITVYRKKILRTITNMFIIAVCVTDILLAAVAMPLTI
GALIIDYWPFGSLICQIQGFSIHLLVFQSLHIITLTAVNRNLNVVRPWLYKRIFTKWTTLFLILAVSFLTAVILAALVIS
LSSRYEFHPSKAICVMIFPNRGASLQFTIVFSLLFVAFPTVIIFACYARVLQTIRTHRREYDATRGEHDSQSVLSRKEMK
LTKLIFIIILGFSSCWVPCVAIDIVDTTQTKWFPRQLYIFYTCLGYTSAVLNPWIYGFMNKLVRKEMVFLLTST*NASSL
GNSKTHSNSKLKVKARESFLMASKEENEGWQTTRKTLTERVEFLLNNSLMSDVSFVVRNASGVEESIAAHKFVLAVSSPV
FYAMFYGDLAESKNKIVLPDCDSESFLEFLRFLYSDKVTLSGGSVMQVSYLAKKYIVPELETKCTNFLKKNLSPENVFDI
LPLAKKFDEEELVIRCWEVVDRSTQVALQSETFPSITKELLAEVVKRDDLTIHEIELFQAVDRWATAECERKDLEPVKDS
KRIVTGEEILSNIRFPLMSQDQFAEKVPCSGLLSKDEIIDLFMCLNGVQVSTLKFIKRPRKLIPPRAISRCKRFPKVSYG
WNYSDDKNDVLVFSVDSPVLIRGVRLFGSKGLAYDVSLGLWSLEDGLDVELCSVDGFFTTDNEERNGYYGFDVVFDHLIP
LHKDVRYEIFAKISGPSSWYGSSGLGMVDCNGIHFRFINQTSPNGTNESEGQFAEILFHKY*
>Adig_18605v114634
MESLDPITQADRAPFCVEMLRRLNVQREQDYLCDVTIVAKEGNEFRAHRNVLSAASQVFEKILQTEMKEKEERTVRFEEM
SELILVKVLEFIYTGTVEIEDAQNAEDLIMAADYLLLVGLKTAAGRVMEREMTNCNCISTFYFAEKYRCEELQGKSRMFI
YDNFIEVSKSEEFLCLECEKVESWISDDEICIEDEDDVFAIIQKWIGDEDSDRKAKFSELFAHLRLAYISREFLLDLMKD
EQVKANPCCQKKVLDTLLSGEDEVIQSPRRRMKTHAIVFRGGKFTFCYLPEVDVWKPLAVGPSKLKHYKRKSTIAFLNQW
YIEHSSNQTERYDPEFDSWSVLNDFELSNNQVVVGRQRYRIYRSVTFRDDEDEDEDDEDEDEDEEDVMVDKYNMGSWKNV
HSSGTRGSSNACILAVDNSIYVLGGGSAHVDRFDTLQNKWEELADMKKGKRSPFGVASQGKIFVADNNYPSSCEVYSIDT
NEWQLIASLNFRRFRRTMVCVNGNVYAVGGQNCIVESYDPTMNQWIEKTSIPKEMIPEEEQGSTFFKCCALKLSRKALRG

IWRKSGFKLK*
>Adig_18713v114738
MTSESAIAHSPPRNSPLRKEKHVGHAQSHCGSSHLSLLVEEESKKDVLRFHLERIRHVHRGVRVFTDSKSRGFALLQVSP
KEGLNTAPNVRPTLVWRHLTQLLEETTCEDDIHDVEFLVKGRTIPAHGFVLASRSQYCHQLICENKENRQGEADERTKTI
PLHENVDYIAFLSWLRRLYSGGDVCDEEVFVLKDVVGKGKNGYHNLNEGGSKETNKTVNEMGKESDEEEMLSSRVLADLL
FLAKTDLDVFPAPTCEEDEGFVQDSEDDVSEDHCTCLETRPLKKIEETMTKRDRENKGDALSVRGEANAFSRLNW*
>Adig_18767v114783
MALTQHSCEMFETLNKMRESQLHTDVVLRVKGKEFHAHRVILAASSRYFDAMFSSGMKESLQTEVQLTELFLTERVFEIL
LHFIYTSFLPLTEANVLDILEAADHLQISGAIKNCSLYILNNLREEKFTIETKLKICRVAENHNLPELHEEIVHAVALQF
GEICSDKAFMENVTSDELLLLLTRNDLSVPSETFLFQTVISWIKADKEERLQYTAQLLDKVRLPLVDIMVLLDELESEEF
NSKSECFYLVHMCLLQQVCPSLSSPFVQDKGEHRVASKALISLSASTGAKFFNSETKSWEPFPEFKIPVLQEKPATLYTG
NHLFLFDDNAVHQYQIDKNTWTSLPHMKNNHHVFQTCIFEDFLYVIGGSEVDHMVVSERFSFAKKTWQFISFHEEHNLFG
TAVTVFKDNIYSIGGAWAEGSAGREVYRFDPAKNAWTKLEPTKHSHAQACAFVVNGKLYVAGGKTDPPSKRHRGLVPSKF
VEMYDEENNRWYDVPQSHMPPNNYGAVEVQNRVYFILGCFAYNSGVAIDENEVYQIDLEDWKSISSHDQDAAFVYLPLNR
VQLSNEYSKEQEPRGHEMQREINSEGGQKRSLSVPMNGELKKKFKKNLGERNSQPVGEKDNQPVVDQQKEHENDDTRESM
KQICEKLVAYRLIKLIGRGGFGHVLAGTQKSDNLPVAIKFVRRKYVKEFVETGGEQMPSEAYFMKKIQHPNVIKLYQYLS
VNDFCIYIMERPASCQDLQRVLRKRRLTEKEARRYFKQIIEATICCEENGVVHRDLKPENILLDKESDEIKIIDFGLASR
IQYEPYNKFRGTRAYIPPEFLRSGWYDSCQSTVWTLGMILVDLISSSMAFSNPEEALLWPPRLPVYLTQDSVDDHEVSPS
PVCSISLNLEDLASDERQTNCSSLLNIERLFVSASSMAIDKSYATSLSENTAMIGHSHQRIQDGQLHISPDMSNRNESTD
KKSFDLERTHNGKSCVLRKRTLDSKYGGFDVNVCDRAVKHKKMFCDIRRNVKESNGRFDELWMLPKWLGKKVRWKIWTKF
TKKNDQERRDWKRKNFILKLERRRSGEMKQRRTIFNYGKTVVRYKIEKKIPPFTT*
>Adig_18824v101757
MATSSKYSEVLMCQQPLKQDEAFTTMESHREPSDITVVVQNGIKFRAHQDVLSEASPFFEKLLNSGMKESREGLIRLETY
TEATMKALLQFIYTGNVHICDLETAIDLFTISDYLLLPNLKSLAENFLLSAEILSTFNCISTYYFAEKYKVSELLSKAES
FIYANFVTIAKSQDFLKLSNKEVEMWISKDEISVDDEEDVFQIILAWVYYDTAERKKYFANLFRHVRLLYVSRDSLYGEV
MTNELVLCSEGCLELVRRVLKPFPCRNNDEFSVRPRRSLEVPVIVVSAEMFVLCYFPRENKWCELANNVSTHLRECEMAS
CGGQLYFLHLIDFVIDSTISIYEMSMLRYDSIANSWKAMPYKEERYLKQIFVRNDTEMYALVTDACRECENRSCICCRAI
RGEPPNSTRKKLMSYITKYIPESNSWTEIASFDFGLREGICIVTKGNFIYFIGGGALMQRMDRSLRDTERYDISTNAWDK
VADIQEDRMFACGATCHDRIFVVGGINRQGASVTKTCEVYNEHTNEWHFIASLISRPAMLSSMVCVDDKLYIVGGCCDLE
GNQTGKLQCYDPEKNEWREDIEIPVGRKGSKNSLKYYSNACSMRIFRGFVDNLDKSSSKRRRSIRERLKCLIL*
>Adig_19094v115039
MASKEENEGWQTTRKTLTDRVEFLLNNSLMSDVSFVVRNASGEEESLAAHKFVLAVSSPVFYAMFYGELAESKNTIVLPD
CDSESFLEFLRFLYSDKVQLSGRCVMQVSYLAKKYIVPELETECTNFLKENLSPENVFDILPLAKAFDEEELVVRCWEVV
DRSTQVALQSEAFASISKELLQEVVKRDDLMIHEIELFQAVDRWATAECERKDLEPVKGSKRIVTGEEILSNIRFPLMSQ
DQFAEKVSYSGLLSKDEIIDMFRCFSGVPVSGLKFIKRPRKLIPPPAFSRCKRFPQVSCGWSYSDDKNDVLVFSVDSPVF
IRGVRLFGSKGLAYDVFLELRSLGEYVQLCSVDSSFMTDNEERNGYYGFDVVFDHPIPLHKNVRYEICAKISGPSSWYGS
SGLGMVDCNGIHFKFSRRTSPNGTNESEGQFAEILFQKY*
>Adig_19256v115177
MVAHSEILLSKCAHFREQGEFIDVRLKVGEYEFAAHRIVLAANSDYFHAMLARGMKESYQEVIELKDENISAAAMKKLMD
FMYSGEINMNDENVFEVLIAADHLQVTSVVQQCSKYLETKLVELGFDVRFYGRIIMVADRRGLKDLKEAMESRMASMYKE
ICEKQEFLSHMNADLLSALLCRDDLRAPSEKFVLKSVTQWINYKKEERMDVAAQVIGGVRLGLVDIKDVIDELDTEEMQV
IPEMKTLLHEAFMYNHRPSRSSVFALAKGKPRTTSMVLVAIVPKGFMWCFDVQSKKWKLLTSMQKLAEEGESMHKLVGEG
ECYCAEVVGNYLYVAARGSRIHCYDIVHDIWSKLPPYPSYTQIGALCHFEDHLYAIFKSSAPYRYNIATNQWQSVASSKA
VCNLSEETFCNKAAVVYKSCIYVLYGQAQVQQYG*
>Adig_19257v115176
MFANGMKESNQEVIELKDKNISNSAFKIVMDSMYSGEINVNDETVFEVLLAADHLQVSSVVQQCCEYLIQLNFDVQTFCR
VITSADRHGLKDLKEVMESKMALMYKEICEKEEFCSDMNADVLSALLCRDDLSAPSENFVFKSVIQWIKYRKEERMDVAG
KVIGAVRLGLVDISVVIEELDTEEMQVIPEVNMLLQRTLIYNQKPSSSSAFAMEKGKPRSMSSVLVAIHPKSDICYFDIE
SKTWKQLSSMQQLSEAKECYCAELIGNNLYVAAKGFRGVVIYCYDTVRNIWSTLPIPGLSDNQIDCLCHVKEHLYVMYKN
SSAPHRYNIATNQWESVAGWKAVCDLKGQEIFLLVEAAVQYKSCLYVLYAQGIRESLKIYPLGTDEAIVCGKKCRPCNSV
LFCFDPKNNVWEQKASTKTPHFGSSLLVVNKNLYVAGGRCSFHLSSNKPDGSPAAIEVYNDQENAWSVVPQTCIPPNNLG
AEEIEGKVYFIINSFPVDSGITTPPGEVYPAVLNDWKNLGNVDKNVVLCSVPVKTENLEKKKNNAQGELKS*
>Adig_19588v101920
MSSSSPVAENWCYTQIKVIKFSYMWTINNFSFCREEMGETLKSSTFSAGANDKMKWEETKAMESQRAYRFVQGKDWGFKK
FIRRDFLMDEANGLLPNDTLTLFCEVSVEGDSVNLSGSSHTAALKVPECRLSKHMGSLLDSGTFSDVTLCTRGREFKAHK
AVLAARSPVFGAMFEHEMEESRRGRVEISDIDPDVFHEMLKFIYTGSTPQLQGMADDLLAAADKVGFCVVCY*
>Adig_19813v115611
MESLEPITQADRAPFCVEMLRRLNVQREQDYLCDVTVVAKEGNEFRAHRNVLSAASHFFETVLQTEMKEKEERIVRFQEI
SASLLGKVLEFIYTGTVEIEDVQNAQDLIMAADYLLLVGLKTAAGRVMEREMTNSNCISTFYFAEKYRCEELQGKSRTFI
YDNFVEVAKSDEFLHLECEKVESWISDDEICIEDEDDVFGIIQKWIGDEDSDRKSKFRELFAHLRLAYISREFLLDLMKD
EQIKANPCCQKKVLDSILSSEDEVIQSPRRRIKTHAIVFRRGEYTFCYLPEVDVWKPLAVGPTKIFKKAQPYYIRTPMIT
FNNQWYITNSSNQTERYDPEFDSWSVLKDFALSNSQVVVGRQLYRIYSGYDTAGNVMVYKYNMGSWKAIHSSGTKWNSSA

CMLAVDNSIYVLGGGSSNYSSGSVHVDRFDTLQNKWEKLADMKEKKRHPFGVASHGKIFVAENFSRSCEVYSIDTNEWQL
IANLNDASYWRTMVCVNGTLYTVGGKNCVVESYDPTMNRWIKKTSIPKKMIPEEEQGCTFFKCCALKISRKVLSGIQGIR
*
>Adig_19830v115621
MPFVNGCALSLVPFSSNMIAHSEILLSKCAQFREQGEFIDVRLKVGEDEFAAHRIVLAANSDYFHAMFARGMKESNQEVI
ELKDENISVAAMEIVIDSMYSGEVNVNDENVFEVLTAADHLQVTSVVQQCCKYLETELVELGFDVQFYCRVVMVADRRGL
KDLKETTQRKMASMYQDICEKEEFLCDMNAEILSALLCRDDLRAPSENFVFKSVMQWIKYRKEERMDVAAQVIGAVRLGL
VDIKDVIEELNTEEMQSISEIHLLVYETLLHNCCPTSVSEFALVKVEPRSMSL
>Adig_19836v102009
VEIKGVDFYSVQMLIQFSYTSQLTITDSSVQNLFLAADLLQFKWAKDSCVQFIMQQIDIANCLGVRALAERHSCKVMYEA
ATLYIMENFTKVTQTEDFHILSSGEVIELISHNEVKVCSEIEVFDACITWIKYNLEKRKCHLSEFLKVIRLPFIPMKQLQ
MDVAEHPLVLNDIYSRDIVEDAITFHFDHASVQMRHCYAEMIYLLGGETSFMKEVKSVERYNCETMQWEMVKSMTEARAS
FTVATLQDKLYVIGGYRRGRKLNSMECYDPVQNAWLSLKPMSKCQGDMRAAVLDGFIYVAGGSSEGLLTCSFVERYSPIT
ECWQVVSSMHRQRRRFALAVLDSQIYAVGGFDDNRGDLKHVEYYSAGTNSWTEVKPMQ*KQCSFYQINSVTLQMRYS*
>Adig_19874v115656
MEAVFESMSADRPSKHHEELIERINALRTKQRFCDVTVVIRGEEFQAHKLVLAAASPFFLSLLESQMKERTEDLIKIELQ
EATAPVMEEVLAYVYTGNVSLTTDNVHGLIATADYLLLPGLKTMAEDFLKLHLTIENSVSNYYFAEKYRCCGLKEECCKM
INESFRKVMETADFLSLDENQVVNWVSGDDVIVEAEQDIFEGVVRWVLHNRSEREENFPKLLQQVRLSSVSHEYLFNELL
KEELVKTNPDCVNFVMEYVRLNFNCRAESSIKPARKCLERNENVIFVCGGSKALCYLPAENKWHELMNTTLEHKDHCIIQ
YRDRVYVFSPQMVGGNKSHLLEYYMPSTNSWGAIQTKFEYGEQKFTGLFVLDDNKGWWILTTYKFCSYIYQYNPDKNIWV
GSFKELGRFGACFATDGHRIFIIGGTLSGNEKITATTGVQKIVPSDQTEDGEVEFEEVAPMNEARHDAFGAAMNGKIYVA
GGKQLDENLVRERVLASCEVYDPLTDEWQVMPNLNVPRYSASMVCFKGALYVVGGLKRTCMLRSGELSVEMFDSGVNEWH
KKSNIPVKVKKRATICYNACFATVHKNCL*
>Adig_19927v102036
MTSQELMFCANVMKTLNDFRKDETLCDVVLTTQGMCFPAHRNILSANSEFFKALFSSEMKEKAEKVVHMDEFQPEILEQL
LNYMYGGGINIGKENALDVTISADFLMLSELKEKACEFLISDLKSENCLFSLYTAEKYSFRELRDSAQKHILENYVAVFT
SSAFVTLKIEQLLAIISSDDLVAREEKVFESVLKWTKHDKEKRRRHFASLFSHVRLIFLSRCYLITQVEQEDLVKNDETC
FHLVNAAKEYFSFPKGAISSDLCLQPRACQTAILLVGGWQENCDVTASAKVFMPSIKQVFELSPMAVPRNNHGIAVHEGT
IYVVGGTTKDGSVTRAAELFDPRTNSWSTVMSMRKEVAGAGVAMLGDFLYATGGRDAEGWPQSIVQRYNTKLNKWEFVSS
MTSSRTRHCVVCCCSHLYAFGGYCSDDDLPLNTAECYDHVKDCWSAITSMNQRRSDASVVSLHGKIFVMCGEDVSSSPVK
LSFCEVYDPQSDTWAVIPPSLQPRSHSGAAVIKNHIFLLGGVNKNNRELKSVECYDTNGCAWTIVANLPMGIEGFACCTI
TVPGELMPVLV*
>Adig_20016v115756
MALNTEISESLPTLENVSYPRDEESDSKRRDDEAGSEQQEKESVEAAKHPFSEPWEESDLILAVEEETFHVHRQIMSIHS
PVFKAMLKSVGIKEDMATEIPLPGKKADEFLDFLKILYLKEKEGIECK*
>Adig_20017v115757
MALSAEISESLPPLRTLEIVDDYPEDEESETKGRDDEAGSEQQEKESVEAAKHPFCEPWQDSDLIFVVEEERFHVHRQIM
SIHSPVFRAMLNSVGFKEATATEIPLPGKNANEFLDFLEFLYMKKMDEVQLNQVEHLLKLADEYQARGIVDVCVKMLKSE
ARCKDNAVKILHLATCTPTVRDDERLILVREQCCELMKNMELKETQGKESYKNLERDALERVLVKRIQRLETFVKEIHPQ
FIGLVQCCLCNLPSALTMMFIKRHMVNVKRRHAYINAIETVFSLKVFATSSNTAAESQNLGKFTHRSHIMASSTSGETIE
EPTVKHEQVEVAKHPFCEPWEDSDLIFVVEEEKFHVHRQIMSIHSPVFKAMLNSVGFKEVTATEIPLPGKKPNEFLDFLE
LLYMKKIDEVQLNQVEHLLKLADEYQAGGIEDVCVKMLKSEPKSKDNVVKILYLATSTATARDDQRLFHVREECYELIKD
MELTETQRSESYNDMEKNTMLFLAAFANNSSPRIYEFTHRSHIMASSASGETIEESTEEHEQVKVAKHPFYEPWEDSDLI
FVVEEEEFHVHRQIMSIHSPVFKAMLNSVGFKEATATEIPLPGKKPNEFLDFLELLYMKKIDEVQLNQVEHLLKLADEYE
ARGIVDVCVKLMKSEAQSKDNAVKILHLATCTPTVRDDERLILVREQCYELMKNMELTETQGKEGYKNLERDAMERVLVE
RIQRLETFVKKIYPQFIGLVEGSLWCVFENAKQPCFLLKLFATSCNIAAESQNLGEFTHRSHIMASSTSGETIEEPTVKH
EQVEVAKHPFCEPWEDSDLIFVVEEERFHVHRQIMSIHSPVFKAMLHSVGFKEATATEIPLPGKKANEFLDFLELLYMKK
IDEVQCK*
>Adig_20037v115774
MFYGQMSDTRDSIELPDCEYEGVLELFRFIYSDEVKLTGSNVMHVLYLAKKYLVPSLAEKCAEFLRKNLDGSNVFAILPH
AQKFEDKDLEDRCWKVIEMRTDEALTSDGFVVAERSLVQSVVKREGLHVKELELFKAVNRWAEKEMEKQGIVSDGKAKRR
IVGEEILKEIRFPLISLKEFASFVIDTNILNVQEVGDMIKHYCQVLTSPLPYLQSPRISVVKGVYRFKRVLEVVSSPPCV
RSSLILSVDKAIHLHGVGHFGHSKRECTVSMEIKEKRSNSILVKKSGTYSSETPLHYSYCCFDVLFDTSVILEPGMKYEI
SSTISGPSSVGRTIGSKLGPPTYSWSGVDGQKLVNFEGVHFTFSASDFSNSETIVQCDQFPFFLVTHSG*
>Adig_20124v102105
MGSTVSYLGSSVGGKRKYSECDTGDDGGCQTDEDAVVDVVKRKRLKNTCDYIYQTLFLDGEGSDVTIRALGKDWKLHRIY
MCQSPYFQSMFHGSWKESNEEVITLEIPDNNIDIEALNTAFGSLYCDEVTVSPVQVIPLLAAANLLQLIGLSDKCAEVMM
ETINRATVCTYCLAATQYCEATVKSKCVEWLERRLTSETSISLLKDISIGLMSEIVNSQELFVMQIEKGKVMFVSLKVMK
VERLFRPSDLSGSIML*
>Adig_20125v102095
MDLSSDIQTDDWQSTRETVRERNKYMFLNPMMSDVSFLVQDSTNKESVKVSLPAHKYVLAISSPVFFAMFHGVVAEQSRQ
IELPDCDSECLTEFLRYVYYDELQITANSVLGVMYLAKKYLVLPLMRRCGQFLEERIDPNNVFETLTRARQFREEELEKR

CWFIVDLNTKQCLESSSFLELDCAVIKSLLRQETLRIEEGELFKHCMLWAEHQCKKEGVEASGCNLRTILGDSLFCLRFP
AMTQRQFAEQVVPRGILSDREALNVFLRFSSIKSDVSFPCVPRNGRPMRRCCRYSEAPSPHHWYRPPSSGTGRREEVLSF
TAISASPVYIAGARLFTRTLGLTDADNRQDKVQLWIKDESQDKVIGFTEGEYSPDNSGAYSGDGIDIKFNTAILVRNGVR
YSLRCVISFSQSVTSNRNTPQKDVVFDQTKFTFENLYYGSHFIEVLYYTPWSK*
>Adig_20154v102124
MRLVGSLSPYYKPRIYVLAESDRMSAEKIVNFEKIKTYRSSSEFKILRIPRSREVHQSWLSTILTTLNAVFFSFPLVFGE
KPDLLLCNGPGTCIPICLAVFVLKVSGLKDVRMVFVESICRVKTLSLSGKILYHFADHFFVQWKQLQRKVPQHIISRKTY
SEPLQRGSPMAGPHKYSPVSFAQISPNRADEDSCPSPQYYFTDVAHSKDLVNGLKFLRQDEDLCDVVLRVGNISISAHKV
VLAASSPYFKAMFAGSLSESQQDTVTLQELDEHAMETVVDFFYSGEIEISENNVQDLLPVTCLLQVKSLQQACCEFLKRQ
LSSENCLGIGAFADRHNCGDLVTMSESFARHHFIEVVQGEEFMEISAQQLTRLLSDDDLNIQSEERVFEAALAWIKFDSD
VRQEFATEILSYVRFPFLSAEFLMDRVATEEIVRNNRACCDFLLEATECHLLPKRKSQLHSQRGAPRKPTTVNHAMYAIG
GMSRREASKSGEKYDPRERKWKTIGGFDGSMPLNTAEYYDPKVGKWTEIARMNQCRFGVGCAVLHNMVYAVGGSDGTNLK
TVERYDPDRNTWKMVAPMSTARKQVGVATLGGFLYAIGGCDHGHRYDTVERYDPCKDEWVNVAPMSTPRSGCGVGVLDGF
IYVVGGYDGTSYLSSVERYDPLSNEWHAAPPLSQPRDCVAVCVASCRRTFGNKRLFETSPNRMPPV*
>Adig_20371v116014
MSTLTNKTKMSTPKNWQTTRPTIRERTTFLLNNHRFSDAKFMVRESKGKGGTESKKVISAHKFVLSIGSPVFEDMFYGEV
AETRAVIELPDCDYESLLELFRYLYSDEVNLSVSNVMGVLYLAKKYMVPSLVDKCSKYLRHNLNASNVFNILPMAQKYEE
EELIDRCWKVIDNQWKEAKKSDGFKTIEHSLLEAVVSRDTLTIEEIELFKAVDLWATKKCKMQGLNANGEEKRRILGEKV
VKAIRFPVMEEEEFAAVVPDTNILTPDEVINLFKFFNSAVDAPRGFSKTKRSGSRATSRVRGSQSAKLSLSRSFFRDDL*
>Adig_20372v116015
MSAPKDWQTTRTTIRERTTFMLNNHRFSDAKFAVRESKGKGGSESKKVIPAHKFVLSIGSPVFEAMFYGEVAETRAVIEL
PDCDYESLLELFRFLYSDEVNLSGSNVMGVLYLAKKYMIPSLADKCSQFLLDNLDASNVLNILPMAQKYEVQDLNDRCWK
VIDSQSEDVTKSDGFKTIEHSLLETVVSRDTLTIKEIELFKAVDLWATNRCKMQGLKANGEVKRRILGENVVKAIRFPLM
QQEEFASVVLDTNILTPDEAINFFKFFNSAVAAPMGFSKTRRSGSQAISRVRGSQSAKQLPTGYLKDDCQQQPSPGPAMR
LVRGIIRPSSAPKPASASYGDQQWVVTSPAVLGQRRQPHIWLQQQNAQAGQQHIWECPVCTALLPPHIDKDQHVNGHFG*
>Adig_20815v116383
MDRVKLVIVGDGGVGKSSFLISATTNHFPSDYVPTVFDNYSSNVIVKGKPISVSLWDTAGQEDYDRLRPLSYPQTDVFVV
SFDVRSRISFQNVRAKWIPEVSHFAPNVPILLLATKTDLRDGYNPSLSDRQIVKSKEGKSLADSLGVTYAECSSLLNDGV
QQALHTAVELAVNNIHAPRCFKPSYRGFKMSKKKTSNFDNENKLLPPVLPPAGLAPHVEVLTSSFASEWHTMMSDTGSAD
VRFLFFDGQHIDAHKTVLITASTIFENIFLNKVTLACKSYADILDNIYWVCDDKDCCPNRIHNEEICIGKGKTVITLHKS
LSKNIFMEVLTFLYTGSPDLAGNEDVNFVKEVQSVSIKFQLNWLETYCTNILKGESFLNPSIGTWINDSTGLAAKKLFLN
KELQADVKFCVKGSIVFGHKVILKARSEVMAAMLSGAFREGDLNTEINIQDASLESFLALLEYLYTDHAPIEEGNSVEIM
VLADRFCLPRLVTLCELYITKKVDRAVQKKVADGTSDVIDLLFTSQAHNAKQLSGWCLHFIATNFSVFENCEEFHLVQGE
NRDYIDEHRWPPLSYIKAQEEYEMKVKSDRDQSKCSIM*
>Adig_20908v102318
MLEKCAYQRKPKFQSKIPEEAASAKKEMADLQSEAKVALMQERQNEREMEKERESEPSDVSNELDSGIFEDKKKVQSQQD
LALFTDKSISKSQICPVCSMELLTTESMNVINDHINNCLDKQMNNNSYIMLENGFSEPPGESMGEDVFFCLLCQKDLSKM
NSQRRGQHISRCCDQSSKGERIPPSNDSQSQRLNSLQCPICGKGFKLSKTRQSHLKKCAQDYGVNTNQLLEIMHREKQQE
DLTIGTTVLTLPPIKPHQASRVKRKRVGGGKKEDLDEETQIALALSASIAPFPLHSDPCTKKTVKVKGGKKRGDNELPPL
LLRDNESAQRAVALRAAQVLCTKDNGDDDEDEISCTPSLAPSRFVQSNLACPAVLSVGNLENNFDSSRASDEKEDVDNDP
KEAVKIDEGGNCTNETCESSFTVDPEFSKSFLNSLYSAEIEEDSDVITKVPRPSPLWSLAGLMEPPSFTYVVPGLLPPVS
PQKGKNLLEQSTGPESRGINENASIKCGNAKTNQGGNLEETKELSKSAVQHQTITTVTEKCAEITSSQVADMEILLELAQ
DDEKSNISLDSQHVIPTSGFCIDRQVKVEEVDIVPSGVPELLQDLSSMINNSFLSDVMIKLQDGQEMVAHSFILALRSEV
LAQLLTEQRAVCEGTTCNGSWTGKFCLLFEDVNKDVFEPVLRHIYTGDVSMLNTNDLDEIAKLAERLRLPCLLKTCKATR
EKLRSSFGSDQDDGMADFATTQLDKLEENLWRDKNINELSDERQASANKLDEGEFLNDDDIEEIMIFQTQSLRRKGSLEW
GNVEQRQSCSHEKNFDNGELFAPGVNGNRGLQKEAEYDNLEVKNESSMKENALVLPNLIEGLNSSRDTVLINGAQRGNIS
SKLDASVSLSHNIATVNEKVDAVENTNNICGRNERKEMIVGLSTVHGKEYIEEDRVIAANKLRGEMSPRAKDISRMSDFE
NTQYEFHGRHRMFTGDDGQLSPDISKSVIDIEALPCNSTISNDKAISTGSRLETKAKMECFSFNEVPSHRNDNAGIGNEP
QNEPIVINVPIDLSSENVQGVNGDRLLEADEKREAFSDFTSLANQEHSSQVWLSVLPEEGCVEYTNNNGSPEEATLLEDD
EVGESAVDGSITLISDNDDEEAVDDSSPVKKNLTIVPETPRISRSLPQSKSSSTDTYSEENDVDGDNEVTYTGSSPSKTQ
KKLRDEAIEFLSMLKERVGSGKGKKSRNKKSSRRESVKRVSPAKLSSQCTEENQERKSPLSPREFRSQGSSQLGHFSRMG
DISPLEELPCDFGSSPIWENDDKPDCFTVDKSPPRSPVHVQPNLVLAASEPESNKNCSVSTPCNPTFGRNNSGYRGCVST
RLTTADLQNCVDHEVLDVCDEKTEGNFDAAATISTFQDVMSPLVPSPDLLRPRSPTLPISPLPPRVVLSPDLTVTNCKSP
LPGGCPQSDSPMSPSVCSFSSQQTALGLQSEKRECAKKLDLKQGSNSTESVKPSISETFDLNIPLMERIKAARNGKKMCL
LTNVEEDSSDNSIDSDSIGDENDSKDKKSSIDLRLQDAGNAASGEINEMRDVTTVTDFDFYDDGGYNFDIDEFDIVDNCL
ENINTEANLESGEGTYLKVTEVLPGNKGGKEFVGKKKISASYRSNDDIERASVPKKRGKKMDSKSHQTPAPTVNEPVTPM
PNYHTMDTPTLKETKNDPHAVGVLSSKTAGSCDVDEVSGSGTASSDESGVEGDTEFGDCTVMIEDEAPMELVSLHKNLKQ
DGISCGLGKLVDFLDEQCITFTTAGSNARTTKRRRRKKKPEN*
>Adig_20934v116456
MVNETESYPGTLEVVHSNQLLKGLNQLRQRRELCDVELCAGDNRVSAHRVVLSACSPYFNAMFTGKLLESQKQVIHLKEV
DENALHVLVDFAYTGKARVTQENVQLLLPAANMLQLSRVKEICCHFLIQQLHPSNCLGVTKFAETYCCQTLQTKSSKYIQ
DHFQEVIKHEEFFLLPPSRLIELLQDDDLNVISEKVVFDALLSWTRYQLPERIPLLGNLLNYVRLPLLTVRFLTQIYETN

ELIRGDRTSQVLLSEALKCKLVLEKRVACEKTVYPRRAPKRIFALGGKNGLFATLSSVEYYEPEREKWIEVASMNFRRFE
FGAAVLDGKLYAVGGLVCGIGVHSGAPFRYCDNGSECFDPETGHWTRVTSMNQSRSNHTVQPLDGLLYSLGGYDGNSYLN
TVECYNSQTKQWSHVAPMHYSRSCFATAVCDGYLYALGGYGPSYLRTVERYDPSTNSWEMMPAMSTYRINFGGDVLNGCL
YVVGGHNGVFHLRSVERFDPVASEWAMVTPMNRPRTGLSVTVLNGLLYAVGGHDGAGYLNLVQSYDPQTNKWQNMKNMCS
SRCSFGIAAL*
>Adig_21410v116812
MAEERNRKTLVVSNKEYKEVLLKTADQLRKEGVLCDVRILVDGHGFLAHRSVLAAGSAYFRGLFTNDMKEKELMECKLDG
LPSSIMETLLSYIYTGNVTLTEENTESLVSASDYLIIEGLKNIGCQFLESLLSPSRCFGLRDFAEKYSCEDLKLAATSYI
VKHFTEACDTEAFKEIEFKVYSDIIARDDLEVSREEDVYETLITWVKHDLASRREHFDELFGKIRLSTMSKRYVIDQVEK
EELVSSSFHSTKLVLQALNSFVLHSYDVQEPARKNLERHLDVIAVCGGNLSKEATCYSPERDKWMPLAYMLRSRDEHCTS
VHDNVLYAFGSTQPENGQSVEQYNAHTNTWLAVANMPQMRCAATAVTCGDHIFIIGGRIPNGGSTKEVLRYDADINKWFV
ETPMSYKRSGLCSAACGGFVYAIGGLSDRNEFLRTVERYNSRKKVWSDVSPMQVARYFACAVEMNGKIFVIGGQSSTGAY
LSSCETFDPVTGDWASLPNFSVPRQAAGVTKLRNRIFLFGGYNNSGSLDSVECYDERKRSWETLTKMPFKRSWVQCGVLR
VAKDLFPLNS*
>Adig_21746v117061
MRGRRTTASKPYVSRLFGDTTLSDVFFIVKNVEFQGHTVILRARAPNFCQRYLPSHSSSVSVKTFIQIHGLEATEFETFL
RSVYIDDNFKDYDESSHPWKKQSTSSDSEEFVDGFCPKSGSLCSDLQSFEWSNVEKTSMIIQSLSQQEDKASIESDYTSD
TSSLEETTISRNTVIDCNENSLCPQSHEFEDISKLSQLDSWECFSGAKITDVGCRNSASLNDALLVNDSLIMNSASLDQD
TSHFDSCGLVDSLDKMTVEESPGKYVSIGTQTITDIGANGVSVLLSDNAAHEEKDVNDKPSLSAENNHPVAKLKEEKTIK
ISVKDLEVSLKV*
>Adig_21823v100187
MATVQENWQVNCTSISQRIKYIFNTALLSDVKFIVPVSNGESESKVIPAHKLVLAISSSVFFAMFYGQMADTRDSIELPD
CDYESLLELFRFIYSDEVVLTGSNVMNVLYLAKKYLVPLLAEKCAEFLRKNLDASNVFIILPHAQKFEDKELESRCWKVI
EMHTEEAVTTDDFVVAERSLVESVVKREKLNVNEVDLFKAVDRWAEKKIEKQGIASDGNAKRRIIGEEILKEIRFPLMSL
KEFASFVIDSNILNMQEVGDMIKHYSQVLTSPLPYFQFPRTGVLRRICRFKECFAHDWGYVGNLDLLILSVDKDVHLCGV
QHFGREGFEYAVSMEINDAASNLSLVKKSGTYCSTKDLDHFYYGFDVLIDTPVILESGRKYEIRSMITGPPSWYGEKGQI
SVNFEGINFTFSSDEVELTGSNVMNVLYLAKKYLVPSLAEKCAEFLRKNLDASNVFTILPHAKKFEDKDLEDRCWKVIEM
HTEEAVTSDDFVVIDRSLVESVVKREKLNVKEVELFKAVNRWAEKKVENQGIASDRNAKRAIIGEEILKEIRFPLMSQKE
FVSFVVDSNILNMQEIVNMIKHYSEVLTSPLPYLQSPRTEVLRRVCRFHIFYPPLWGFVGDFDSLVISVDKDVRLCGIQH
FGSKGCEYTVSLEIKDAISKLSLAKKSGTYCSEKDSDGISYGFDVLIDRPVILESGKRYEISSIIRGPLSWHGANGQTSV
NFEGINFTFSRSASRNSGTNVEVGQFPAFIFTHPG*
>Adig_21986v117268
MLVDKKIDAEMNRLEMATVDENWQTNCTSINQRTKYIFNTALLSDVKFIVPVSNGKSESKVIPAHKLVLAIGSPVFFVMF
YGQMADTRDSIELPDCDYESLLELFRFIYSDEVELTGSNVMRVLYLAKKYLVSSLVEKCGEFLRKNLDASNVFAILPHAQ
KFEDKDFENRCWEVIEVHTEEALTSDDFVVIERSLMESVVKREKLNVKEVELFKAVDRWAEKKIEEQGIVTDGNAKRRIM
GEEILKEIRFPLMLEKEFASFVIDSNILNVQEVGVIMKHYNQVLTSPLPYLQSPRIVALKRVCRFNNFKQHTADGESGSW
NYGSGKPDNLVLILDKDVRLHGVQHFGREGCEYTVFMEIKDATSKLSLAKKSGRYCSEKDSACIYYGFDVLFDTPVILES
GEKYEISSMIRGPQSWSGRQGQTCVNFEGINFSFSRSASPSNGTNEQCGQFPVFLFTGSG*
>Adig_22245v117472
MVAHSEILLSKCAQFREQREFIDVRLKVGADEFPAHRIVLAANSDYFHDKFAHGMKESNQEVIDLKDEFENISAAAMKIV
MDSIYSGEINVNDENVFEVLTAADHLQVATVVEQCCKYLIQLRSDVQTYCRVIMLADQHRLKELKEATESKMASVYKEIC
EKEEFLSDMNSDVLSPLLCRDDLSAPSENFVFQSVMQWIKYRKEERLDVAAKVIGAVRLGLVDVKDVIEELDTEEMQAIS
EVNMLLQRTLIYNQWPLRNPAFAMKKGKPRSMSSELVAILPKSEVCYFDVQSKTWKQLSSMQNLAAEARECYCAELIGNY
LYVAAKINSDHVIGCYDIVRDTWSTLPPIPGLSGIQIGSLCHIEDHLYAIYKSSAPYRYNILSATNEWQSVAGSDAVCNL
TQKTFCNKAAVVYKSCIYVLYGQANIMQGDVDVFYSEVSSSVLYCFDLKEDAWEQKASTQTPHFASTLLVVNNNLYVGWG
EMLTPIIKFSTIWPSSSW*
>Adig_22586v117776
SNTNLQMASTLENVAYPRDEESESKRRDDEAGSEQQEKESVEAAKHPFSEPWEESDLILAVEEETFHVHRQIMSIHSPVF
KAMLKSVGIKEDMATEIPLPGKKADEFLDFLKILYLKEKEGIELNKMGHILRLADEFKVEGVHDFCSKMLRDLPKSKENA
VKVFFLSTQTVMAREDDRLESVRLQCKAIVRDMDLVDIQGSGVFKKLNKEACESLLVERIGRLETHITEVYPQFVGLAEY
CLHLGLKCSLPGLVKCPEHFINPKVNTTLPERIVSCSHCKKMIDKLVQFSLESEGHGHVYGGDHHFNSDLISIVKNFTTV
MRTLQRKTSLSVFQPDFSFGTGPEPPRPVEAVQPATRKRICGSGSGQPFGTGYETSSVSFASLAAASSKIPDHFKSQTND
IH*
>Adig_22587v117777
MASNTEISESLTTSESVGYPRDEESESKRNNYEAGSEQQEKESIEAAKHPFSEPWEESDLMLVVEEEMFHVHRQIMSIHS
PVFKAMLKSVGIKEDMATEIPLPGKKADEFLDFLKILYLKEKEGIELNKMGHILRLADEYKVEGVHDFCSKMLRDLPKSK
ENAVKVFFLSTQTVMAREDDRLESVRLQCKTIVKDMDLVDIQGSGVFKKLNKEACESLLVERIGRLETHITEVYPQFVGL
AEYCLHLGLKCSPSGLVKCPEHFINPKVNVGLPERIVSCSHCKKMIDQLVQLSLESEGDEHVYGGDHHFNTDLISIVKDF
TTVMRSLQRRTSLGYAENLGIAGTAPFEATSHAATALCESKATTETVKSGGVGVNFGKSAYDFSKALGSVAPAPFEGKTH
CGTSIFGGAGKQTSGKSSRSIFNGAGLVQETNSAPKFVFGVQKMK*
>Adig_22739v102776
MDNMLNILYTGKCKTDQNNIVAMLSLADTLGIQDLFSKFAENIEGNKLSSSKRRVFNCQYQDALLTKLSSFQMGGLFCDL

TLTMSSGKVVPVHKNVLASTSCYFKGLFRSEMREVHERNVYFGVIDEAIVEDLLKFVYLGEINITFDNLRSLLQASDYLL
IEPLKRQIIDFIKENSTSSNFWKVYSLVRSFDCLDEVYQEMVNFVCSHFWSIATSSEFLHITDQDVRWFLSNDDILSSEA
QMLESLIRWYKHSEEDRKESFRRLLNLIHMSNIPDQYLKNLAMEQGIDELNRFSGYKLKDNVAMEDLLKTSAFHNVVLFG
LTHRLHYTHSYNLCYWLPFAGPWSLIARIPHNKSMLANGSPLVYTNSGLYVQVPWNNPKLAFHRNPLTARHLMPNSGVAP
TSEIEPSAPMTFDCAAVTMGKYIYLIGGMSMQERTEVKTVQRYDVEKQSWSSVADMREPRYELTAVNYKNKYIFVFGGID
GSSHRECLKTVERYDPLVDRWSYVTSMHQPRSSAFAFVHNERIYVIGGERGEDVTPTDCELYDPLTDEWQVMRIQVNTLY
TPRGQTDQGSFLYHPATENKEPSVGKSCAGGLSVLSNISAQHDQPIFNEAKITRPYVTCINGSIVILDFSTCADGQRKAN
FYFLDPESGNFRILQSLFTWPTDISYGALMPLSRKDMIKALKD*
>Adig_22765v102787
MVAHSEILLSKCAQFREQGEFIDVRLKVGEDEFAAHRIVLAANSDYFHAMFAHGMKESYQDVIELKDENISVAAMKIVMD
SIYSGEINVNDENVFEVLLAADHLQVTSVVQQCCKYVHTEVVGLRFDVQLYCRVIMVAERHGLKDLKEATESKMASLYRE
ICEEEEFLSDMNADVLSALLSRDDLRAPSENFVFKSVMQWIKYRKEERLDVAAQVIGAVRLGLVDTSDVIEELHTEEMQV
IPGINMLLLKTLIHNYRPSSSSTFALERGRPRLMNPFGFTTHHPQSGVLHILTTESKFIKRKVLVAILPEGIISYFDVQS
KTWKELSSMQQLTKVQECFCAELIGNYLYVAAKSNKNFVICCYDVVCDTWSTLPPIPHSSGIQIGSLCHIEDHLYVIYKS
SSPYRYSIAKNQWQSIASSKAVCNLDQEAFCNKAAIVFKSCLYVLYGQGQVLWEISAYGCSSHPDVSSSVLYCFDPKKND
WEQKASTKTPHYGSSLLVVNNNLYVAGGHCSLQSSRCGGTSFPAGDPAAIEVYNDQGNLWSVVKQTHIPKNKLGAVKIDG
RVYFIINSFPIDSGITIPPGEVYPVVLDGWVNLGMVNRKAVLCYVPVKTENHTIEDE*
>Adig_22767v102786
MVAHSDILLSKCGQFREQGEFIDVRLKVGEDEFAAHRIVLAANSDYFHAMFSHGMKESNQEVIELKDENISVAAMKIVMD
SMYSGEVNVNDENVFEVLTAADHLQVTGVVEQCCKYLETELVELGFDIQFYCRVIMVADRLGLKDLKETTQRKMASMYKD
ICEKEEFLTDMNADIFSALLCRDDLRTPSENFVFKSVMQWIKYRKEERMDVAAQVIGAVRLGLVDIKDVIEELNTEEMQS
IPEIHMLVYETLLHNCCPTSVSEFASVKVHMAPRSMSLVLSAICPKRVISYFDVQSKAWKELSSMQQLTEIEEYYCVELI
GNYLYVAAKINTGVVYHYVVYCYDVVCNIWSTLPPIPGSSGIQISCLCHIENHLYVIYKSSTSYRYNIATNQWQSITRSK
AVSELGQKTFCNKAAAAYKSCLYVLYDQQERQWINNKWLSYTCDSMLYCFDAKRNVWEQKASTQTPHYGSSLLVVNNNLY
VAGGSCSFSLASYEPSGSSAAVEVYNDQENAWSVVQQTHVPPNNLGAVEIDGRVYFIINSFPVDSGITIPQGKACPVVLD
GWKNLGKVDKKAVLFYLPIKTETLKTENEQGELKS*
>Adig_22936v118070
MSIHSPVFKAMLNSVGFKEARATEIPLPGKKADEFLDFLELLYVKKMDEVHLDQVEHLLKLADEYQAGGIADVCVKILKS
EPKSKDNAVKILHLATSTATARDDQRLFLVREQCYELIKNMKLTETCKNGKSLEKEIWEKVLVKRIQRLETFVQKIYPQF
EDLVELSLSDSLREKKISPCPQHFSNGKANEHLLRRMKDCPVCRQMICSLKKSQEASAFSSLFASANSANYFASTAINRN
EDLGEKAMKTILDFEKIIGV*
>Adig_23241v118315
KSQHVRLLLRNLAEMQAEGVQTDLIVQTKSKDLEEPFHSELLAACSPSMKQALAGTNYDLTSAISLGHITANQLKAFRDF
LYKPETILDEEIVADLEQFAKKYRVHSLAQLCGQRKHQREGAEGMTVWAKHYEDLLAQLYEMFQKRELTKAILKGDEESC
EIPVHGPLIGAASPVFRDMFCNELFCQEGQKYRLRGISSKTLNCLLRYIYTGEVTVDGATVQDLLQNSCTYKIPAIISAC
CDWLSPSMSAYNANFWDMLRETESEDTNNLDREAKAFIVENFTKICEEDDEYYDLEYEDLKEILQDDNLGVDCEEAVFNA
VDTWVGADKEVRSRYFCEVPLKKDGTPDRRFKVNRNTLKSRSQGNTVEEIATSDMRENGIPRKKDGTPDMRFKVNKTAPG
SSSSSCSSPRPSSGESIGPLKSDGTP
>Hmag_XP_002161530
MENQEGPHNLNRQQHVNPPQNGRTSPVGAAFPNVNFEAYLDTLEDPSANGTYNWQSSRPSIKERIAFLFNNETLSDIYFI
LGKGTAVQRRIPAHKFVLSISSVVFDAMFNGGFASKTSNEIEIPDIEPASFLLLLKFLYTDDITICPDTVMSTLYAAKKY
AVPILEQKSVEYLKSKLGPDNALTLLSQARLFDEPQLAEMCLNCIDKYTVASLNAEGFTDIDADTLKIVLLRDSLSAREN
QIFDAVVRWSEVECRRRNIEKTNENKRIVLGDLLYLIRFPLMTLEEFANNAAQSGMLTDREIVDLFLHFTVNPKPNVKFP
DRPRRCLTGKEKSVIRFAKTEARWGYSGTSDRIKFMVNRRIYVVGLGLYGSINVPTDYDVNIQIINSETTAIVGYTDTKF
SCDGTDSLNRVMFKEPIEILPNISYTACGTLRGLDSYYGSKGMKKVIIELPNHEKVEFKFSYAAGNNNGTSVQDGQIPEI
IFYT
>Hmag_XP_002161103
MKKSMLIWKEHAIAVNLDPVPSENAAGLEVPPKVPAPVAENWCYTHVKVIKFSYMWTINNFSYCREEMGETLKSSTFSAG
ANDKLKWCLRINPKGLDEESKDYLSLYLLLLACNKSEVRAKFKFSILNAKREETKAMVKLLRLNQVSVVADSVNVSGQSL
MSNLKVPDSNLAANMNTLLEQGQFSDMILKIADTSIPVHKAILAARSPVFAAMFGHDLEENKKGVVTITDLDIDVLKEML
KFIYTGKVTQLETMADTLLAAADKYALERLKVMCEEALASNLSVENVCDVLILADLHNAQQLKSVAIDFTNSHAQEVMEL
ASWQRLVKNHSNIVAEAFRALASVQSFSCSPPRKRQKTARVEPCV
>Hmag_XP_002160795
MDVFIEKENMRLHHKCVLLCLAPTLYEFLFLSENVFSKIFDFPENVPQIERNSLRIVYKQNEQDVICTSHEVDITSILNS
AVHSDVAFKINNDILHCHRCILSVRCEYFEIMFSGHWREKDQNVIPINGMSMSTLCTVIEFLYNPKIKLSEDMNLLEIAS
AADMYMLLGLKDYNIKKKTMEINTRTFVVIFLILLFDLLAFTIILPLFPSIIDHYDTINKDGESSDAWLEYIHELLEWSR
KTLKMPDERRYNNVLIGGILGSLFSFLQFLSSPIVGACSDIFGRKKVLLFTMFGTTLSYIIWWKAVTFKLFILSRIIGGL
AKGSVLLCTTMMSDITTKENRGKGMVFIGLSFSLAFISGPLIGAYFASQHRDKLAESFEKPAIVSMGFQLVSIFITVFFL
KDTLLSKEILELQGRIAVRGSAAAKNYHILFGEAHELPNVYLPTHKQESALAERRLKRKSVLRSTNKISNRCVRVAEASD
SDNDTVQTTSDSETYSPYRFGTSDRAVAALVSATLVDFSLEHMGPVDRNKIRRERKLLRSAYKNTDCKISGLYFDDPGSR
YIDHSSPVSGEEWSIYLDPAKSIAESILDTVDCNNIVCIGSDSTNINTGLNNGVIRRIEIELGRPFHWSICLLYLNELPL
RHLFSSFDGATTGPHSFNGPIGKSISKLLQPPIVNFAVIAGEPINCDKDLLGSDQLYFFKTINAVKTGILPQGFESRLPG

RLNHARWLTLASCVLHLYISNVAPSNELTLLYRIVVQQVLKRNNNPAHIESIILAMLTDNRQVIRKLALKRILHAWEDDM
PNRKFQIPEVRLQATSYEELIDWQTTSRLEPVLTKHISTAQILAWLQSDEGIVVDIEPFPCYNQSVERLIKIATESASKL
SISDGLKKVMKLVNPFSLFNFSSSKECGYVRRIKVNKEKNAIVQVSQDIRGF
>Hmag_XP_002154764
MDKNIWEFNKKFDITFLVEGEELYVHRYVLSSSSPVFKVMLESDNFVEKNKRVISLPNKKKENIQEMLNYIYPFGHQISD
ETNIDSLLILSREYQINKIHNLCEEFLLKKEPTFELLITAQEFQLKNIENKCIEYFSKKALISLENYPKYNELSEDNKLQ
LAERQNMTLQAACGKARKCQLHPMQMLWMISSQLVKYIVYQLQTPYVTAMTKHSQ
>Hmag_XP_002157627
MAGPLPSGWEARYDPNYKRYFYIDHSTKKTQWEPPVVPTSGFGLTIPKAASQSQTQQYQQMPQYPQMQQQYQRVQQNYPQ
QQQIQQQHVQPQTLLRQPVQPFTSGGSLSQQYPQMQQQYQKVQQNYPQQQQIQQQHVQAQTLLGQPVQPFTSGGLPLQEV
SKTQNQYKPTQMVQSCCTVACPATVTQVTVAGASSSSGISTSTGYRSNASNAPKPGMSSSKDVNSDISDAQQNELLRQLK
NQLDLYGVLDAAIIETLAAGNYDLALTISIFEGMGFTKKGEQKTSSSASRFEEEEKKAKLEAEKRRKEKEEVKKKEEEKK
EKEIRKKEQEKLQKDVETEARVKEEEARKKEEEIRNKEVERQASSSYSTVSSATNSNLLNKLVYSGNANYFNNSAVTSKD
DATYQLQKKITAVGSDPTLLSENRIQTKGGDISLVAGPLQRLDNGKVKSLAKGSLVEPVGSGMIAKGIKKTDFVKIMFVE
VAGGDRVCLREPSYSKNVMSSMAQFRKDGTLCDVTLNVDGKKFFVHKNVLASCSEYFKVMFASSLIEGTKSEICINTVTS
EVFEELLDYFYEGTLKMDMHSIVDLLHAASLFLLNDVISECFRHLNRQMNATNCLRIKEREILKTSLQELKSLLLSDDIV
CQSEDSVALIVEKWLRERIHELEESQIRILFKTIRFPFLSENGRIKLMKFMEELDVMYPDAGYASILSFEQRHQARHFRR
CSLTCRASQRVETVIFCVNKRGETFCYNILNHKFCRLERFPEYRLPGYLNVNNNVLYVTGGSNGLTTVFSRDVYSYDVTE
IKWNKLMPMVNARDQHCATFFQDNLYVIGGRNLQNFVENLSKKVECYDVAKNVWSTVADTLHGRVNSCATSSHKYMYVIG
GNQNVDGIDASETVEKYCIRKHSWELLPNMCQKRISCSSVFYNDKLYVFGGSNGFFDLPNFEFYEEKSNKWTLVSVGVPG
PINVALAMDNEILTMGVSAWNDDSVYRYSAVAKRNCDVWTEIKEFIHPSHRVKHFKYAVLRISNYYFNKKLDGLCDCCAY
DPESSFDEDELLTSSNEDSDGDEWALHWGLWI
>Hmag_XP_002155857
MGHAQSAAFNDKNQLKSSDLILPHDDSSKLVEKEKKKQSKKLLKQGSIRKKLSRTLRPIKSSLYSKQIRDLVQSWSITEI
QLLLKKYETLEAVRELKFLSDSARSINGSLQNDFCQLFYSKHQADTIIEYKDTLFHVHKIVLISRCQYFRSILIDINHSH
VKIDCDVLDVNISDFIDLISYIYCGYTNNNEILKTISVLEEKFGLLNTLENDMQKLFNSKERTDLVISYRNGQKNMFCEY
SEVLKPFDPTLKVDCHLSIVCSRSPFLKRLFETKYCNQTELTIPLLLEINDHIVPQPFLHVVMECIYFDQVSFNSIFNEK
FHESCNNNNLVYVEIAMKVFEIGQFLEIPSLMRGCEDIIVSNLSATSLIKILEWSSIDSKYVYRQAIHFLREEFIPLCKS
SFFLANLSKIHLLKVLESDFLQADEETILDSIITWCEWEIAKSGNPTIILTKSLPRHPCPKRYNITNDALREMVSSLVGC
VRLSHILTQNSVILSNACQQGLLQMPFTACDVVSSEKKLSSTVYWIEEMHHSDYLIYQERLTENKNTCDVNNLPSTLVEI
PLSITTCATSDERQSMPLVDIFKLSEDVIKNMVTRQKELMSSEQVLKYLTLAPDNVEIIAEIQLRVVREFDYPDELANEI
FQQYPDLVENFTNIPTKRSTLSMHRAAGNDCFSPIL
>Hmag_XP_002162072
MDPKLKHTKTGTLVNSNQINKMKDDFFKTLKSLFKKTDFSDKVLCIIEEQLDKTKEDSNVHLKDPIVNNGSFLPKQISIP
IHSLVFCINSRFFRNLFYDSNMLETKENKVVLHVTPGDIQYVELVINSFYDPDIINNINTIKLLKVLEYADRYLCDSLLK
HGLSIVKNISDGEFLFSENSVVTCVLNWLEANETHQTEENIKALLSECRKWSGYLNWYINAVSYPSMQNNAAATSKRTNR
QKKITSSSTEFRNMVNHKKATKNNELCHKYELKMKYIVDTKRWISDKNMLYRVRITEPNIRSKFSALGNLLARFFNQTDI
LPKQGNGVIGKKLVVHCKDLQELINEVLLSRKVFSKPLIKLGIDGGGGFLKVTLGAIEKMPCEDDPPPCKKNLTTNSSKD
SGCNRQLIIAIAEDLFETYLNVQNVLSLIKPNPKDFVVSCDLKLANILCGLQSHSSPHPCTWCDVPSNNLAQSGNLRTFG
SLKSSCQAFDAAGRDMKKAKEFGNVVHPPLLALDDEQLVLDLIPPMELHLLLGVVNHLFKILMSM
>Hmag_XP_002157473
MPPTVHKLLLHSSSISNKLPLPIGVYSEDALESLNKQLAEVKSKNERTNPSILENNITETMADKIAELEDRSRRNNLRIN
GIDESGNKSWNDCENKVKELLLTKLEIKDNFVIVIKRAHRVGKKDESENNKKHRTTVVKFLNYKDKTAILDKYIKMKFWN
DRLFINEDFCERTSRLRKKLFTDAKDLRQKASNLMSVMFTDDTNLFLSDNSITTLFRDMNMELTKISDWMSTECHETCFD
ISEQHHKLVFLKLPLLKKDKFLCDTTLVVGRKKIYAHKILLACSVPYFYSMFNHDVVESRQNVITLKDLDPESVESIIDF
VYTSKIVITQRNVQAILQVATMFQINLIQEKCCDFLESQLHPSNGLGIYLFAELYGCSKLKSRAKTYCNWHFSDVVREDE
FLNLSLEQVKWFLNQDELCVRSETEVFNAAIRWVSQNSQQRKKDLQYLLPLVRFNFLPKSFLSLQLETNDLIMDDEFSLK
LLFDALNDIEVKQLVATKRKPIGNNVVFYMGGYNRKSFASYGYFNPETETWFKLGDMCSPRSGAGCVSLGGLIYMVGGRT
NSLQGKSDSNSVECYDPYSQEWKNIASLNSSRHRLGVAAVDGVIYAFGGSDGMVHLNTVEKYDSEKNLWEPAPSMNTPRI
GVGGTVLNGVIYAVGGFDSENRLQTVESYMVGESSWKFLASLNTPRSGAGVTSMNGHVYAVGGYNGVAQLNSVERYCPYE
NRWTNISSMNERRSALSVAVVRNKLFAFGGYDGERFLDSVEVYDPDNGEWQLLNPMPDARSGAGVAVCIAPII
>Hmag_XP_002161665
MDCEKLEEYSNHIQELCTLNSSKGEINHIQLLSSQMDSLFISKKFSDVTFIVDNQKFFCHRLILAARCEYFRALFYGGMR
ESNSTSDIVICDTSSTSFQMLLNYIYSGLVVLKTLKDHEVIDLLNAANKYDLLALQNAVGSYLESIISIENVTIIYDAAC
LYSLTSLKQKCLIFIDHNAIDVLASENFVSLSETSLLAIISRDSFYAPEINIFNAIVDWIKNNEPMLDKNSILKYVRLPL
ISLHDLFHVVRKSKLFNSDAILDAVQMKTELGVSDMPLRGYLDSETNLAQNCFGATVLSGEFCDTLFDGEFACYDLERGF
TRHLISDEKQGIVVALSRPSIINMIKLLLWDKDQRSYSYIIEGSLDNQNWIQIVDYSIYMCRSWQKICFPQRVIRYIRVL
GTNNSVNRYFHLVTFQCFYTSGLTHVKNGIIVPSCNVASVTASAQVIEGVSRNPNALIDGKTEAYDWEYGYTCHHIGSGC
IIVQLSQPYLISSCRMLLWDCDDRDYSFYIEVSLDCKNWKMVVDKRDVLCKRWQNMIFEPEVISFIKIVGTKNSVNEVFH
VVHFECPSSIECFNLTKS
>Hmag_XP_002164975
MDIDSWPASSNSFDAYDNKPSEEFIKCVVVGDTAVGKTRLICSYIFDQPPTSIQSLHLKPHIPTVFAIDQYVGNSDIRKR

GKTVIDGVNVELRIWDTFGDHEKDRKYAYENAHVVVVCFSIGMQSSLINVNAKWVPEIKKYCPRAAIVLVGTQLDRRYTN
PEMYKNALQVTTLTNILNTGGRFKLHSGFSMSDIIYAETARQISKEIKAACYIEVSVVSKHGVNAVFENSIRAALILRRN
SKLILKSHLKNIKTPHIQEPYLPPCPEAPDINILAEGEMFDFSCLHSVSSFCDIEFIVENEHLHAHSVVLISADEVFQSL
LMHPFILDLLGFPKGKNIQNFESFSLEYAPINGVLCYGIPRGFSSISVVTDFNLKRKVTVNVSQVTAVSFRNILEFVYCG
SISNIKDSAQLLTNATYLQLSMLCNYLTSSIEESSQLKNTFYQMLFKKRKKILLNFVFNKSLYSDISFSMEGALIPSHKP
LLVVQCNVMAGMFRKKTFKESITHVVDFSNCDKESFMTVLEYLYTCDLCNHGDLHNVLQLANFLCLPRLITLCELELTKK
ITEVNSNQCDEACALVLDTFIFAKFHNAVQLSDWCLYFMASNYNKMCQYQGREMKALDETTKNYLEKNRWPPVWYLKDQD
LYERCMQKIEKENTFKISKERKESRCFSFFKNL
>Hmag_XP_002164510
MFLLYFIFYLLVFTKSYDILNFIFIFVLVPFVKDKWKLYQAANISKDWYSDSDTKNSKRIGYYWTKVFKSDNEYGKTKYL
YLSKVIKSRLTIQNGNARIERSLSDNKNKLTSERVKLNDETLMALRISNARSCNQAYNVDALFKDVVQSMSLSGCVYDGN
ENQMSLSGCVYENENQFQMQFGFNESFESVVTHQPEVIYDGKQNAFRSSFSIPLRLSQSTFMKPYFFYSSQTYPKSLLTM
LDNLLKCNELCDIDIRVGSRRFRAHKVVLAACSSYFRAMFTREMAEQRQEEVLIQDIDEKAMELLIDFAYTGNIKIDEAN
VQIVLPAACLLQITEIQEACCEFLKKQLDPTNCIGIKLFADTHSCRDLFHIAHMYTLRNFQDVILNEEFLLLNVEQVCDI
IQSDELNVISEEDVFRAVLKWVHFDLIDRRSKLKDVLQHVRLPILNAKFLVSVVSTDMLIKNDAGCRELVDEAKNYLLLP
EQRAVMHGPRFKSRRQNKREFLFAVGGWCTGDAINSVERYDSQTCEWHMMCSMNKRRCGVGVAVLDDFLYAVGGHDGSSY
LNSVERYDPKVNQWSSAVSPTSTCRTSVGVAVLDGYLYAVGGQDGVSCLNIVERYDSKANTWSRIAPMNCRRLGVAVAVL
DGLLYAIGGSDGTSPLASVERFNPSTNTWTFVHQMSTKRKHLGSAVFQNFIYAVGGRDDTTELSSVEKFDPKTNKWAPVV
ALNSRRSGVGLGVMNGSLIAVGGFDGTSYLKSIEIYDPTVNQWKLHPGMNDRRLGGGVGVLTLHYSDWINLCES
>Hmag_XP_002162610
RSKHSLVAWGDLLYVFGGDNGKRMLNDLLRFDIKDSSWGRVVTSGTSPAPRYHHSAVVFANSMFVFGGYTGDIYSNSNLR
NKNDLFEYRFNTGQWIEWQVNGNIPVARSAHGAVVYKNSMWLFAGYDGNARHIQQIGKRPPTCCNFPIAVARDSMYVFSG
QSGAKITNDLFQFHFLEKKWTRITTEHLLKGTPPPPSRRYGHTMVTHDRHLYVFGGAADNTLPNDLYWYDLETETWDVIQ
TEGELPNGRLFHDADVIGDRLYVFGGTVDNNVRSGELFYFTFSSYPRCTLQEDFGRLLESRQFCDMTFSLNGEEEKIGSH
ISIVAARSPVLREQLRHLLNKENNSIKESVERPSDDSASSIRQLNRSSNFKEKICIDVGDVNKLAFKVVLWFMYTDQIYP
YIKDEHGATTDVMKLMMDVYNFAVRFNLMRLRLLCEQYIEASISTKNVLAALECSQALHLEFIKEFCMRFIIREVHYNQI
IHGTDFEDLDKKLMIEIIRRKQLQQHNSIPDTPSQTVLHAPTLKDDLKALLTNDFGEYFSDFVLCLKGKEIKVHKVVLAA
RSSYFEAMFRSFNPVECKATVKIGDMVPSPQSIETLLRYIYYGDIKMPPEDSLYLFSAPFYFGFTNSQLQAYCKHNLEMN
VTHQNVIHIFEASHGIGAKDMKRHALSIIVQQFPSVARHPDIRRLKRELLLEIIDAIADSMLPESNTEVVSVMAFDPYK
>Hmag_XP_002167170
MPGMKINSEQANSQDIKMERDDNTKKVIDKFHIDHILKQMYNDIFKGGNFADIILSFHNKEIRVHRIILAASSEVFSTML
QTNMKEKIDGKVDMELSGLLPRFADDFIKYLYTGEIELTVENVESLFSCANFFIIDGLRDACSSFLKDILSPMNCLSVST
IANRYCCFDLEQKALKMLHDNFSLVANGTEFLNLSLEDLIEILSSDEIQVSSEDVVFSALMKWIYFDSSVRLLHFEDLFK
CIRIPYLSQKFISTTKFQNSYYEKYLSRAISFSKTVSPRKCLDTATVIMTTGGYDGNNCLLASFAFNTITQKWGSLAPMK
VARHDHGTVLIWNKVFVIGGLNSHRGPISNVECFNPSQNQWSEVCEMNSKRKSAGVCVYNDKIFVSGGLDGSYNALDTVE
FYDHELNTWNMFMPMNEARYCHGLVGNETCLFAVGGWKSASLERYYNSQWCLLSPMSIPRAGATYLIFNNKIYVFGGYSE
RYCVSSLEIYDITLDNWVIQSSSQISRWRAGSTLVNNKLFIIGGRDCSWKYLDIVESYDLERDEWSIDLPFPFQVMGLEC
STLNLPKHFIRFSK
>Hmag_XP_002160995
MKKDEFESLLVTDNFHFTSAFQVLNEMRVNEEMCDVILCTDDSLRVPAHRVVLSACSPYFRAMFNTNFLEAHQSVIHLKD
LEGNTLEDIISYFYTGKLRINYANVEGVIKLAKVFYINNLINKCEIFLRRNMSPKNSLGLEAFALHYSLSNLKDHATRYS
CWYFDFIKDEEEFCLLPFQSLKCLIQNDSLKATSEEFVLEAVIKWLFFEYQKRKNYVPELLPFIRFPLMSMQYLENSQTI
KLLVDNFSLAQVYINEALQYKNFGISKNLDIGKVCSPRAASEDIYVLGGWSNGQKLKSVQCFNVDTLKWTPVQNMSVAHL
TKEDYFRVIVSNDELYTICVGKVMKYDPVDGMWCKVASGPEMPCKWSGICECNGIIYVIGGNNMKESKKFNTDTCQWSTL
PEMHQGASPKILGIVSGEHLNWTNLITI
>Hmag_XP_002154264
MVLKTLKRYREDAIKLCDLVLQVEEQVFPVHRSVLAACSQYFYAMFAGELKESSQSTVVLNDVKANILKQLLDFAYDGEI
EIHSQNVEDILRLATQLQFIQVCDICCNFLEQQLDTMNCINIRSFATLYNCISFASKIDIFMENNFKDVINSDEFKNLPF
PVLKSLISSHKLNVVLEQDVYNAVIKWVKFDLNQRRNQFFALLNEVKLPLLSKKFIMQHIINEELIKSEISCRDLLDEAK
NYHLYPKLRAIFRSKRTIPRYSTVGLLFAIGGKETGEQITNKVEFYSMFDNSWKSLTSLWSPRQQLGVCVLKSKIYAIAG
SDGDNRLNSVEVFDWNTNSWNHSTPLQVIICSMFDNSWKSLTSLWSPRQQLGVCVLKSKIYAIAGSDGDNRLNSVEVFDW
NTNSWNHSTPLQTCRSGVGVGALRGSIYALGGYDGHHCLSSVERFNPIDNKWHFIASMNFARSFPGVASLNDLLYVIGGN
DGSTFLDTCECYDPHTDKWCTINSMNNGRAGVGCAVLDGCLYVAGGYDGIKRLNLVEKYDPNTDTWVCLSPMTSCRDGVS
LASYGGYIFAIGGIDGPSYLNSVEYYDPSNDTWMPSQEMITSRAACGVAVLGNKDIINKR
>Hmag_XP_002163870
MRTYSLFELLSSTGREEARNFISKDPKYWEDDESYQKLAERVQRMKVVNDSAERGIALIGQYNESITKDEEQKQRMLEYK
CAGINIPRKQQHDGQQCQLILPINCTVWSCNEKDSSHCRRQSVEMTHASFKLMKAFYFTRRRSGLPRVTQLLFNTILSLS
AAHSKLMLHPKVEIDDAVAAIYIVEQLDTLMHGESLSGIRPVIHFPKEEMFKRIGPEVHATHQLSGSFYLIYKFKFQGMK
RFVHGLKNYFKEQQLSDRILSIVEIDEEFISYKKRKFECETEGTGFKKIKFHEISTTDYQALHDSSLNATSIESVLEMTD
RKGCDLNSLSTGSCQSEISEGTFKKTASDESVYVTFPIHSLVLCISSDFFKKLLVDSGMKENSMNSVTLKVNKGEGKHLE
MLIEAFYSSDVLNNISLTDLLCTLNVAARFSCFDFIQQGLEHLNHSEIKSVTECDIILSHISQILALFNNNERYENIKNC
CTKFLAKKFFPLEMQFEQQKVFNSLSHFTVNLLMMSDHLLVVSENNALNLIYQWLLNNEEYQTPEIIESLLKTIRYQNLH

VDQFCDTLSVCDKILNKWSSYSSWYVDVLKYHALPQSARSIRGIKELPANHAFPIAINNLAAKRHFKLEKENLNLIDEQS
FKIIWGGCCLVPLLSLKEANGNFIVKMKIKLINVFQPEINKNAVHQIIDIYYCLLPGYVDFQESLMHTSKFVQKYKKMSS
IIIEQYLEDLKGYFKKEELSDKILHIMEAETTQVVLKTQKIRRKRDQTRDEIEKKKSEEIIFNENLKDSINTVESEDINI
HKCEEKEVDLRASSSEENFYDALTEVEQLSEKLIPFDRTTSQLITDKPSNQSTYEKSKTDAQKKNLKWFCDSICDKLIFT
NPLTRQEECKNVGELDGKPLAGFVISFDQYEDQQRETLSALAVSLGACVQDEVFSINKDHRVIASTHLILKRCEGIQFLT
ASHWQIPCITEEWLWKCLDIQGVASTATYEISEKQSSKFTSIHCFFPVHGLILCISSSYFKFLICDQNKASNNKDIIVYV
NLGDSQYVELLIHSLYNFDVLKNVGTLKLLKVLEYADRFQCDALLKQGLPLINNIVIDNIEECNLLLEYICVFQSLEHII
PRDFLTKVKETCSYFLANLITPLECCIFQESFGLINLSGLLILLKSNYKFLFNENSLIPFLLDWLEFDSSRQTEDNIRIL
LSECRYEFMSVEFLKDCLSPTDAIFSKWPGFFDWYIDAVTYPAVPLNSKELYLCKKRENRSAVNFSEGASLKHERNIKLI
WINDQFVQPFKCRQRIIYKGYLLTPVVKICKENNKHKVLLQMYVGKKITKREMRNFYFRVDFHFAILPGDLECAEELTYR
KCKKSLDIRIASCMIFSYDSNRVEFQNSNLAEISFGTIDGGLVQRQLEDRQRRNNLRIEEVNENDNENWDDTEVKIINLL
ENNLNVKDVIIERAHRTGIIDNKKPRTIVKNTVEYQYLAI
>Hmag_XP_002154942
MDFINDEVDFDENYEDMSDDYQIVPKDYSYKHFLDSKKKNTVEIDKHAINILNFYNECFQKGNFCDITIKTNSRSFNAHR
VVLAMFSDYFRAYLQHDGKDKDVITLMDVNGDIFYQILLYAYTGHVERHDDTIEELVICADYLGVKPLKDICENILMELV
SSNNCFHYWQFSDIYTMKSLFEVCLEYFRRNFITLTKHEGFFKLSSKYLMLILEDCELRLYNSVNDVDPICSKQREEILL
KAILKYIGNSLSKNNNELTEELLKLIESVKLPEIDLVFLEKQVEKHSLKKYPAINNLLNSAMMFKSRKNETEWDGRLVVG
GIELQFNDGFVVGGGEKQLNSVEFHEIYLKPDEVITKVTISSGWLIDRLMFTTNIGNTYGPYGGDGGRTAVEEESGCFLY
SISFSSVRTQALLAPIELQLHWIKLV
>Hmag_XP_002164351
MKYALLLLKMYVDNSHSVFLLKRLSLLRDHEELCDIELKVGDNGTCFSAHKVVLAAVSDYFKAMFSTKMEESFVKTIILH
KTDEKSVSILIDFAYTGHISLSSINVQPLLVTANMLGIYQVVTACCNFLESQMHASNCLGFIEFAEIHSLHELKSSSLKF
SVHNFLDLMNYDEYLHTNGNTLKLIISHDSLNVSCEEDIFYFIEQWVLYDKDSRIEVFYELFNEIRLDQLSTNFLSYLLF
HSLFSTKAGYLSKLKINMFYNLKSSPSRMLSNELCLAKSKRNSSDCVFLSGGKDGLLNNLVSCTLYNLKKDTWIDCSPLP
TPRINAAAVANNGVVYVVGGYIPYSTSFLPTSSMIKYSIYTHTWKYSLEMTEKRSGHVAVNSQGFIYVIGGYDGQSYLSS
VERYNTISGQWELMPAMSCKRINFGAAQVNGYIFVVGGHDGTNYLRSMERFDPISNEWAVVSSMSSPRTGIGVSVLYKKL
YVMGGHNGSRYLDTCCSYDPFTDKWEDICSMNTPRCYMSYTDVCLNFSS
>Hmag_XP_002165897
MCDVVLFSPPKEYFAHRNVLCSASPFFLEIFTSNPNSQPTLGSRVDISNISSEVIEDILNFIYLGEICVSEQNVIQLIAA
SDILRMSNLKEIVCRFYERRLCPSNCLSIASLAEKFNCESLREAADKFIFFHFMDVCKYQEFKTLTFKQVLRILSSDEIA
VKCEEDVYFSAMEWFFYDVKERAQYLSELLQSVRLLQTSKYFVSDVIENNEYIVCDNALKHEPRKASDLTSIIVAFSGNQ
DRISTNEVLCYVPEKDFWYPLVPLLFNRYQSSSVVLNNEVYCIGGQNDNGAINSVERFSFSTDKWFECSKTHQPIFNHAA
CVFNGEIHLIGGEIDGASVSQVLRYSNNLGKWIKLADLKVPRKALAVATHKKIYAIGGYGPSNEALASVECYDPYMNEWS
KIFSLSSPRAGASAACVGNKIFVFGGEYAMWSYYRSAEVFDINTDEWRNIADLYLPRAYMGIATYNESILLVGGMVSVEG
ADQYGSGRDDDDDFVDVTESKLVECYNVANNSYKRVCSLPIATAAINCSVISVSKTILQERCVQVKTRLVTLIKPENSLL
KKKHIKTRALLGISAQAITDELVLVHGDQAPKYSTVAKWATLFKDGRESLEDDPRSGYFQTTYTAENIERLRAIIEKNPH
VSRDIIETLTSIDRFTINKIIHNAL
>Hmag_XP_002156939
NETLQLINALRKEQQLCDVILKVEKNEFHVHKVVLAGTSPYLRAMFTNGMLESGQKVITIHGIKSKTMEMLIEFSYTGVI
EINIKNVEELFCGSSLLNIESLQSACVRFLCHQLDSSNCIGIRDFANIYSCTQLERYANRYIHQHFLDVSNTEEFLNLNV
DDLCNLLKNDSLQVRNEEDVYNSVEKWLLHDYTKRQKLFPEVLKYIRIPLLSLEFLESKVFPASFIKANSTCQLILAKII
NERPEHLPDYLCIQRALPQSIYAIGGRNSMHCHLSSVERYDIYTDSWFIEKNLSIARTAIASVCLNGCLYAVGGECAINN
PQDETLYLPNVERFDPKTKYWYRVADLSISRSFVSAVVCNGKLYAIGGEDRISSFNLVEQYSQKHNCWKIKRPMQKRRAG
AGATSHDGMIYVAGGYDRTMHCDRASVECYCPIKNEWKFVAELEKARSGLNLVSMDSFIYAIGGRNRSSDTYFDICERFD
LSTMQWTLISNMLSPRAWSGVAILKKKIFVIGGFDGINRLSSIEVYDFEKDCWVHKRNMNFARAGCGAAVL
>Hmag_XP_002156314
MKEAIMERALALTNQEVCENSVILEDKKILKAQRDIDMIPKQSIFMLSDRFMQNNNCKLWELASMPKNKNDATYYIPLLQ
TNLSTKEKTLSIEDNLHKKQCNEAITDTQHEKLSILVELADETCQSNGFIRESKQNGNLSHLEMLSNDMKLLLEESLFAD
FIFHCDSNIQVKAHRNILAARSEEFKKRIMATSSRSKNPLWNHFKVSDEDVGKAICILCKKILSRGSKDAHNMSTTNLQN
HLKKVHYNIHSMILSQKKIINHESLPNPNEKSIKQLCTTRTICLDRTSSAESVQIITSTATDETQGSSRVSNNTIAFFQK
ASTQVSLREILQRKELWQLDNPKAQAITKLIGEMICLDLQPYCIVEDKGFIRLLKNLAPNYTIPSRKYFSTKVIPLMYET
IKAKICAVAKLKEIQSQLKLPEHNIIQDVSTRWNLTYYMLERFIEQKKAITLYCITRNQANAKNPTENQWQLAEMLVCIL
KHFESTTKDMSKETACISEIIPFIYAMEKFLDYACDTATEIKTDNVDLKIAMMLDPRFKLKFVEDKNHNMFKEILHLEFY
SFCSKSHLEQVKEIEFGVARGSGSDSEESLSSFSESKIHESDSDFDGHGSPLKKKN
>Hmag_XP_002161289
MKEETVSPLSYNHHSFLSANLLKFRQCGVLCDVTLLCEDALFQVHKVVLAASSAYFQAMFTIGMCEEKKRQIPLNDVDKK
ALSVLVDYAYTGIISIDDRNVQVLLKTADLLQFSTAKALCCKFIMDQMNSENCLDLLHLADVYHCSDISALSQLMFNENW
EKIIKTESFVLLKLDILIKLLSSDNLHVNKEADVFYIIAQWVESDIESRKKYLFGLLEYVRWAFISPKDLNKMRQHPLML
VDKESLSKIDSYLILNAFQIENFHMRDSYIGWMYVLGGEQSFLMEMKTCEFYNNQLKEWNYGFSLNGPRTSFAAITLKNR
LYVIGGMRFGEKLKLVECYDQYLGKWKRLASMKKCQGDVEAAVINDTIYVAGGSSGGKPACRYVEKYDICNNQWSAVASM
KSRRRRFGLCEYNSRLYVFGGFQDSLGELSVCESFCPITNMWESIAPMKTNRCDLGVTVLSDFIYVTGGVNSYAGSISTV
EKYDPALNTWSMCQPLIHARGGHTMVTYFGRAVAIGGMNSYMQTCNDAEWYNELSLQKKRLSYGEHNCEK

>Hmag_XP_002156126
MADIEVTKLQKHLTLLRQEYIKLQSKCVDIEKKLSLANAATGEIQEDSYISQLLRISHNLFERETYSDITVKYANKSVRA
HKLILAARSQCWCADLNDVDELLLDGVSCEVGNMLIKWVYTDHADVRNDESVIIELVKAANRYKLKALRTRCEQMLMSAV
SVANCIRFYQTAEDIGAESLRTYCGEIIATHWDDLETKDFLGMSAPLLYEMFKTKSNFPLHFAVRHHREDVVFLYLIEHN
LQIPAKLNEIEPHSGLLPLQIALKERQESIAKTLVLHNCDVNMANNNGETLLHQFIREGDTYASIFLIENGADVKASTKH
LGETPLHLAASYKINLESRDNNGNVVLWTALLSYKESLDSNDKTSYASMLIKNGSNPNAVNPLTGDSLLHLAATFKNEHA
GLFLIDHGAHLNHTNKLGESPLHIASRNGLLLLVEKLLKSGANPNYTTTATDALKAKEAARKIEFQLMCDESRRRIIMLE
KSKAMKRVKEIRELLQEEEKLKLQKKKSEIKSYQPESTNPFSEDDNSNAYDGNPFLDQDEEEGLQLNQLDRESSFLNEMQ
AASSAAMALTSSSMFGYQNVNKGGVKQSKRLITSFDGRIDVQLIQSPTDVEKFDFDAMQITQLDIEDGLQQDVITDCWNC
TPLHFAVIEKQEEIVKCFIKYSENASLSGGRLPIVPDFNVLDSAGQTPLLIALLTKQYKIGKLLVNMDVDLNICGEDHQT
LLHKMIMHRNVEACLFLLENGADVNKRSKENMSPLQMAIHLRLTPVVEALCIRGADVNSSDEDGNTALWVALKSHQLDIA
SSLVKHGADTDFWSTGINNCVWTLLHRAIFERDEETACFLIRNKCDLHSPRRPGLDGGSASEGIDGLTPLHLACASSQDK
VVQCLIEHNADINMQDADGRAPIHIAIAAKNPIITRILLSHPDMNFYIKDTQGQTPFIVAMNCRDNDASTAILAREPYAA
EQFDNKGRNYLHKAIMNKDVDVILFLISIEVNVNSVIMDGSQRTPLHLAVPTGNEIVVRHLILAGADVNAVDKNRQTPLH
CAAIEDKPSIISVLLQNGSNPDLVDISLNNALHLACQHGNLASVRTLLTESSINAEVYNIRSQNSLHVLAAHSSDNAAAI
FDLFRQTMPNYPFDALDGEENTALFIAYSNGAVGLCCALLKAGARLATMNKHGVSVFNAPVATKKLLFKLLDLLNAEPSW
SDGPNCHECGDKFGITTRKHH
>Hmag_XP_002167734
MKQMEFFRKQGIMCDGTIVVDDMELPVHKNILSATSPFFKNIFSQLTNPDDSKITLRNLTGQIMDDILHFAYTGEICIHD
GNVRQLVATANFLQLQSLKDMTVNYLEQKLSPASAAEILILADKHNCKTLIQSAEKLIMDNFVIVSKTSGFKNLSFEIVH
QLVKSEDIRVIKEEEVLEAVLTWVKSDKGNEKEKESHLANLLHEIRFLLISPSYLSETEKDEFVSKSSLCLNIIKEGINY
HLSNSKHSIIDPKLAHPRKFMGVVCGIVCVGGWQGDKPTKDVFAYITSNYKWFPLAPLPKARYGHSVVSCKEFLYVIGGR
NESTLLLSSVLKFDPTYNKWEEVAPLPYQVTSLGVCVFEDQLYCVGGLSSVGSIDIVLRYSSCNNVWQRVASLNCPRGAL
SVVASEKYMFAIGGIRKSGSGVNTQWEYLNTMEIYQRESNLWKYSTELLTKRAHASAAYLNHFIYLVGGQSELLGVNRGM
DLYNTVAKEWSSVQYSGTPRTMSGIAVSETRFYVVGGMSKEGECLNTSEVYDTTKDRWTKISPLPVSTSSMQCCAINLRL
AVLQGMTASFSE
>Hmag_XP_002156698
MAMDGYEFREEKSNRASFSPSDNHFSSLRSIDDEGLSESEIFSLSSRPDILRDIMKSFLKLKQNDCLCDVVLVAEDNEIK
AHKIVLAATTPYFSAMFTNKMIESSSPKIYIHGVDSKSLQALVDFIYGENLYVRIDNVHNLLSAASLMQINCVKDACINY
LMKKLHPENCLTVRNLADAFLCEKLLKAANSFLEKNFVEVSQSDEFMQINIDNLIEIIKKDDLNVRSEEQIFEAVVSWVK
VDIAKREEYLPRLLAHVRLPLLSPQYLSDRVLTEEIIHNNIYCRDLIDEAKDYKLMPERRKELNSERTLPRRCNESCGMI
YVVGGLTSSGESLSIVEKYDSVSGKWNHVLPMSVQRSRVGVAIHDGKLYAIGGFDGTVRLNDVERYDPALGCWKKVCPMN
IRRSAVGAAVLGNKIFVVGGYDGNSSLNSVECYDAELNQWRFVASMSTLRSAAGVSTLNGKLYCAGGHDGLTIFASGEMY
DSTLRQWRAIAPMTTRRCRLGLTVLNGRVYACGGYDGTSFLSSVEFYDPCNNQWTNVASMTQRRSRVSTVTLGGKIFAIG
GYNGAANLSSIETYDPWTNAWTLTTEMSMHDGGVGVGVLPRIP
>Hmag_XP_002158300
MFTIIMFLTANKKEIPATHVEENHLKDVLKRVDSLRHHGQLGFCDVILRLDGHEFSAHKIILASCSDYFYAMFNGNMKES
KEKIIEINSVSLDVMKLVLNFIYTGSIQLSNDNVEDVLQAANLMLIKSLKEVCCRFLETLLTVNNCLGMQKFAESYACEN
LFNITTNFIHENFGYVMDCDEFLQMQAAQLEPILASDELRVLNEEHVYEALIRWIKYDIKIRKLYFLNLLSLIRLPLVSP
DYLVDRVETEPLINEFPKCKELLLEAHHYMLLPNRKIGSLNSRTRPRKYENGNEILVACGGNGEPSSNSTVKKYDMEQAI
WVSVASLHARRSKMSVIECAGNIYAIGGFDGAQTLNSVECYCPITDRWKFVSPMITHRRCTAVSCGNFIFVVGGHDGAQ
ILNTIETYDVERDVWSNTEIAPMTDRRSFPCAVNINDEIYVMGGYDGHDTLRSCEFFSISGNEWVSIEPMSVARSNAGAI
FMNRKIYVVGGWDGVSLNSVEYYDLVTHEWVRISSLPRPATGTRFCYYYA
>Hmag_XP_002159777
DVKFAIGNEKVLFHAHRCILALRCQIFADMFKEMPITDEPFLFPDLQSDAFMLVLEFIYTNKCNVLKSDVIFVLSTALEF
GVMELVKVCEKYFEENVTLETACQAMQAAVTFGLDSFKRSLLPFFRQNTAEIFNTSSFNELSEETLAYILQDDELNMDEY
DIIKAVENWTLVNSVALDKPASEVSRSVVCNVRLPLLSSDELAEIECLNVKNHFIPIEQISLAWKHIALKTPLNASFETT
PRTGTRIR
>Hmag_XP_002163342
MEERKRAFQTIAQPPLFLTPDLKRSRSCSSSNSSSISPRRPSSIPIKQVTLSEKMSPPVSDTSIVKIEEDDSPKSHTPSD
VSHSNTESLTYCCDMMKLMDDFRKANILCDVTLCVDGTVFFAHKPLLAAASPYFKAIFSSDLNKCSENTKPIILTEIGSE
HMETLLDYIYTGHINLTKENINQVLSAANFLLLSSLKSRCTSFLEKILSPANCLVIESTAEKFDCEALKNTATHYVHDNF
ITIANSNEFLHLEVDRLIEIVSSDDTKVEQEEQVFEAIMNWVKFDIINRRRYFKDLVTHVRFPLISPYYLMDHVESEDLV
CQTPECISLLLEAKNYHMLPDRRWQLKSKRTTPRISMGIVNGIIAVGGIQGPYIGEVWKYGNSKEKLPNTSVVASTSCYL
LCANQWFVLAKMQTPRCRHGLAVTGEFVYAVGGHSGAAQSSLNNVERYDPKTNNWSVVASMLTRRSLLNVVALEVLQTGL
ALPPYRFSINVMWM
>Hmag_XP_002170423
MAFLSHKYTGSCSGFSLPIFKNPLHLTKSFDILYAMWKQNLLCDVTICVGNKSIEAHRAILASSSPYFYAMFTGEMSESK
QNCIVLKEVDAYALELLIGYCYSGEVQVTEENVQVKKSAIMDFVVVLLIE
>Hmag_XP_002169891
MKQMEFFRKQGIMCDGTIVVDDMELPVHKNILSATSPFFKNIFSQLTNPDDSKITLRNLTGQIMDDILHFAYTGEICIHD
GNVRQLVATANFLQLQSLKDMTVNYLEQKLSPASAAEILILADKHNCKTLIQSAEKLIMDNFVIVSKTSGFKNLSFEIVH

QLVKSEDIRVIKEEEVLEAVLTWVKSDKGNEKEKESHLANLLHEIRFLLISPSYLSEIEKDEFVSKSSLCLNIIKEGINY
HLSNSKHSIIDPKLAHPRKFMGVVCGIVCVGGWQGDKPTKDVFAYITSNYKWFPLAPLPKARYGHSVASCKEFLYVIGGR
NESTLLLSSVLKFDPTYNKWEEVAPLPYQVTSLGVCVFEDQLYCVGGLSSVGSIDIVLRYSSCNNVWQRVASLNCPRGAL
SVVASEKYMFAIGGIRKSGSGVNTQWEYLNTMEIYQRESNLWKYSTELLTKRAHASAAYLNHFIYLVGGQSELLGVNRGM
DLYNTVAKEWSSVQYSGTPRTMSGIAVSETHFYVVGGMSKEGECLNTSEVYDTTKDRWTKISPLPVSTSSMQCCAINLRL
AVLQGMTASFSE
>Hmag_XP_002160151
MKNETSGNNVADNFAKKEIVPQWQTLKANRLERNEFMYCNELISDIKFYIGKTKQVLIPAHKYVLGTSSPVFFAMCCSEF
RKDDVININDCEPEPFLELLRFIYYDQVHLNKTNALDILYLADKYIIPSLSKECVNFLLDNVSTENVLEVLSASICLNEK
RLEKHCWSILSRKTKEILQSDSFLEIDPSFLIKIIKKDCLDILEIDVFEAVKRWVERECFRNSIEPTSNNKKVFLTEILP
LIRFPVMSAKEFALSPAQSDLLSLEDIKNIFIYLTSGVGGSNLNYPLNPRHFKPHVCNRYIKPMKNYLWRYDEKDIDAIR
FKVDQEILLAGIGLFGSPCGGEYSTEISISINDEEIFKTKGAFVSPPDPKFNNIDNPKIHEVMFDEPIHLKEGIFYDIAV
LLDGPPSFAGDDGKQEVIEEGVNFVFKNSSGSTNGTSYDEGQIPSLLFYF
>Hmag_XP_002168622
MDNNTTKSATAFQSNSTHGIKFSDVAYPNEFLSAFKSMRYHDELCDVYLLVGSTKITAHKAVLAASSPYFHAMFTGSLLE
SQMKEVVIHNESENEFSQVIDYFYSSKIYISDSNVESLLQIAGLLQLPRLQHACCEVIKRKVTVNNCLGIAAFAESHNCL
QLVESAHNYAVNHFIDVIQGDEFMKIDIAHICKLISEDGLNVSSEERIFEVAVAWLRYDYENRK
>Hmag_XP_002170243
MDNNTTKSATAFQSNSTHGIKFSDVAYPNEFLSAFKSMRYHDELCDVHLLVGSTKITAHKAVLAASSPYFHAMFTGSLLE
SQMKEVVIHNESENEFSQVIDYFYSSKIYISDSNVESLLQIAGLLQLPRLQHACCEVIKRKVTVNNCLGIAAFAESHNCL
QLVESAHNYAVNHFIDVIQGDEFMKIDIAHICKLISEDGLNVSSEERIFEVAVAWLRYDYENRK
>Hmag_XP_002160977
MEESVDRLVYECESYYSLAFKKMAEFRNREEFTDIILVAGCKKIPAHKVIISSLCSYFSAMFRNELSESQQQVVTINNVD
PDALEALINYAYTSKIEIRVDNVENILASACLLQVTDVRDACCTFMKSQLHPSNCLGIRAFADAHACEQLFDIANSYVKD
NFGDVARNQEFLLLNSEQLVEILQSDELNVLKEDDVFHALSLWVNHNPDSRKNQLHKVLPAVRLALLSPQFLIDEIEPMI
QCDEKCKDLFIETMKHHLLPDRSSRNSMLSVKARKGTIGVIYAVGGIDEVKGAATGIEEYNPRKNVWSLAASMETKRLQF
GVAVVSNKLYVIGGRDGLMTLNNVERFDPKSNKWETMTSMLTHRHGLGVAVLCGPLYAVGGHDGWSYLNTVERFDPQTSK
WCFVKEMNTPRSTVGVAVLDNKLYAVGGRDGSSCLNSVEVYDPHTDKWKIAAPMVKRRGVLGVGVAVLRGFLYAAGGHDA
PASCESSKQFSSVERYDPRSDQWSLIASMNNCRDAVGMTALGDHLYSVGGYDGQAYLDAVESYDPDSNKWVDVGKLAHPR
AGACVVALKFN
>Hmag_XP_002168260
SLYFKSMFKGDWSEKDQTVIKLTFPDSFITSKALDIVFGSMYRYEVDVEVLIANLIPVIAAARMFDLGGLLKHCEDTVLE
TINAENVCIYYDASQRYGLTEVSKKCFSWLERNMLLCKTLTLAKYLTKELLINLLQSPNLAVMQVEMDIYNFLKIWLYFQ
MCNEHSEVSCPKSLITDTQKYFQCMQKKTKTCFLSSEEGLQYADVFKCLRFENMLGDAKCILLLEKDAIVPQNWLMPVYQ
SQWKSMLSVYNGEDTGPKQIVEDLFYKQACRCGRILETDTRYCWRWTGLFSFGIDIILIYNNRTKILSVKRNTYSQPCNF
AVCLQSQRAVVLRLKAFTIKTNGEELKALDTGILLWNFHTDEELPLLTLDDTWTFPVKAHLKSKHRIDWSQTEKIPPEQV
VAGKKPKTMLDFVQRKSLNEIVSGMATDRISIRAITRNQFICDAIKKEGYENGVMKLINADFHNKKNQMVSEIVSKIGKE
RKFSISVDEFTTVRRRRFFRRNLQ
>Hmag_XP_002170055
MRRIRTKLAVVETTDELFYDDSFSKKLSQRTNHLRKQNILTDVVLLVDDQMFPAHRIILATSGDYFFSMFSGSLATNEKH
IRIHGVSANTMESILSYIYCGSIEISEENVQDILEASALMLLESLKERCGKFLRDRIDEENCLRIFSLSKQFSLSTLQTK
TFELITSHFSELCSHPDFQLLDASALIDILSCEEISVPNEDTIFNAALTWFNYDSHQRSDDFKNILKVIHLPFVSIELLK
KCKESVSVDNSNIFSLLDSAIELILSRSFNDDCFPENIGGVFPEPWMSARKCEQFVTSIVALGGPTLNIYNFEVDHWTEL
GKCTPRHCPGLAVLGSSIYVVGGSSEWKRKSSGECFSIESCTWSTISDMNTPRSNFGLVELRGKLYAIGGYDGNLPLXXX
XDMNSPRDGACDVSDGECLYSIGGYDGSRYLTSTDFYDPETKLWNDEKIPQLQEKRQNAMCAYYDAKIYVIGGYYDDHFH
KSVEVLDLDQMKWLSVAPLPKPRHYGGASAINGRLFVCGGWSVQFPVSTVDVYSIDEDIWSTISDLPTPTMVIAGFCLVF
LRQCFGDVLNDLWCVY
>Hmag_XP_002157659
MLHSNWKESDKREIEVAQFSYNVYEAFIRYLYTDFVDLPPHDAIALLDLADAYCELRLKKQCENMIKKGISIENAAMLLE
TSIKYSAKSLEDYCFRFALNHLTAVTQTEAFRNLDGETVKAFIIKAGISGAFKR
>Tadh_XP_002118193
MASQDSQQDSSSQAKRKLKLRKSTTMASSHTTIQTNSAIHLTTTPTNHAAIQPNSIIHPTTTTPSSDTVIQPNSAIHPTT
TPSSNIVIQPSSATTAPTVNPIQAVEASSSGRVCPVCLEMISLGQEDGMELFNLHVNQCLDHHRSSQMSSSGKKTADTCD
ADSIVAAQILQDEKENEEKSMQILSEFCPVCGRLLCNLSMEAKQRHVDQCCQSQTNKQEKPLAPAATKSIACPVCGKDLS
RIKNHLIHIKRCGQAHGITTEQLIRCCQQKGFTDLGQNLNEPKAKPAKRKTLSKVDEETQLALAMSASMHDKSKREKDNT
FALPGDLTSVGNKKRRKKGFKEMPQLLLCSEEEAKENIARNACRLLQENEENDIQLTQSMSSSKLSNLYTKPQESIPSPD
KPKWTPEVSSTSTTTTTCPTSSIWSLSANLTPNKNVDNFYVNQLLPDQKSISQTSKSKLQSTHLKNEPSIPDVKNENNEP
NAILTDIRSQLLQDFTLMLRNDNFCDVTIVTKDEEKVMAHACILAARSPYLYKEFHSYGNDCKYEVILNDFSAAVVKALL
LYMYTGSVNFTSEILIDVAKLSKKLKLRHLCRYVKSLFDSSSNVIDNTKETTVNQEMNDNNSSQSIADEDISLDIIDLTQ
ITPHKPVTTESTDSSCVGHLMEQSQRKESNSHLSQEEVLVVSSKSDHEDSIAISDDILVLSQDNLRKIADGSPQSMHENP
AENSDDNEDFQFIDDGGYNADLDFFLLRDESPTVVEDPKADIDHFSASQDRNLEFETSLSATLTAKGSPHYPSSNTTNQR
LVTDYSTPELKNQLQKYGVKPLPKRQMIAKLEQINQYLNDAATQSDKSDCDANSQNSASYGSCKSSLVADDDENSETDDI

LSTQEIRESNIADRISDFIKSNRAVYMSVLKYEPLELRDLHRLLKASSIKCPRQKLPAILDQLGVMFSMQRNTATGNKKD
NNLSSKESEG
>Tadh_XP_002117687
MEKKNSIVEEHKETIKAAISVSPKYLELYHDSNFTDIKLIIGDRSFLCHRIVLAACSEYFCAMFTSDFKEKNEVDVNLVE
IDPVIASLVIKYIYGHELELTLMELSRVQAIYALALQWTIRELQKQCHDYFTAIMNAENCCQLASFADYYHAAKLCDEIL
VYIRENFFECIKVKSIKDLSYDLLIQLIQCDYLNVTDEDSLLESISIWIDSDISQKKRYLISLLSNLRLPYISPESLIAI
EERYIDVIENSLEYLNMLRRVKNYSLIPENYFDKSAKPRMYIKNVDYCGVTSIHKSDAKKRQDCFNVYGIINNKRYRLQR
LDSSTLVDASNINQIERVRLLTYAAVCVGHKIIVFGGVNEDKQALDSIVAFDVRKTKWTCPTWAFELPEPLAYLAACIYK
DDIYIMGGLNKNNQCSNKVWRLCRQNKTWEAVAPLNTARKCCQVVVVENFIYIGGGLDDNEDLITTFERYNPLLNTWNIV
YELKTIHHSIRLIYARSKLFIYHSESTYDCYMQSADGKWYKASEREISECSREIHGIDYITDGYIAIDQKMNGYGFFIPR
CK
>Tadh_XP_002117675
ITDIKLIIGDRSFLCHRVILAAHSEYFRTMFTSDFKEKDKAEINLIEIDCSIASIVIKYIYGYVLKLTLMKLSQVQAVYA
LALQWAIKELQEQCHCYFITILNARNCCQLATFADYHAPDLHDEILAYITDNFFRCIKTKSLQDLSYNLLVKLIQCDYLN
IINEDQILESLNIWIDSDTSSRKKLIVSLLCHLRLAYISPES
>Tadh_XP_002117674
ELYHESSFTDIKLIIGDRSFLCHRIVLAACSEYFCTMFTSDFKEKNEAEVNLVEIDPVIASLVIKYIYGHELELTLMELS
RVQAIYTLALQWTIRELQKQCHDCFIAIMNAKNCCQLATFADYYHAPNLRDEILVYIRENFSGCIKAKSIKDLSYNLLIK
LIQCDYLNVTDEDSLLESISIWIDSDRSQKKRYIISLLSSLRLPY
>Tadh_XP_002116816
MAGNVQVDNWCCTKMTVDRYTYLWKINNFSYCREETGETLKSSTFTTGPDKLEWCMRINPRGLDEESKDYLSMYLLLLYS
NKKEIRAKFKFSILSRNEEEVRAMESQRAYRFVQGKDWGFKKFVRRDMLMDTSYGLLIDDHLTLFCEINVVSDPVTLDGR
FTAEEAEVPKCRLAQDLDQLFKTKKFADITFNIGKDQLKAHKAILAARSPVFDAMFKHCMEEQRQGTVDVSDIESDVFEE
MIKFIYTGEEPERIDDLAAEILAAADKYDLQRLKSLCENSISNNLTVENAAKVLIIADMHNSEVLRQNVLEFINSHALEI
VETEGYQHLLKSYSHLITDAFRTLARSCNSKTDVTSTRKKVRLS
>Tadh_XP_002116805
MIPQMAGKVKIDKWCYTEMKVDRYTYVWKIENFTYCPLKTGEFLKSSTFVTASSDKLQWCMKINPRGLDEDCKEYLSIYL
VLLSCNKKEVNAKFKFSILDSNEMEKRLMESQRAYSFIQGKDWGFKKFVRRDMLMDKTSGFLTDNRLTLCCEINIVSDPI
THDGRFTAQETNVPTCTLSQDMDQLFKTKKFADVTFNIGKDHLKAHKAILSARSAVFDAMFKHSMEEQHQARLDIPDIAA
DVFEEMIKYIYTGKEPSRMDDLALEMLAAADKYDLQRLKSLCENSISNNLIVDNAAEVLVIADMHNAEILKKNILKFINS
YALEIVETEGYKNLLKNHSHLITDAFRTLARSTDSNINSVSRKKRRI
>Tadh_XP_002116765
MEVNRHDQESVKCVVVGDAGTGKSRLVCARAYGHTYSKKQLAARHVPTVFAIDEYRNNRIVQERSNCMVDGVAVKLLIWD
TFGDHDKDRRYAYENANAVILCYSTHSRQSYDNCRNKWKPEIRKYCPNVPLVLNATQVDLRYAEQDEVWKSINELNIGIR
GHFSNSLNADELLLPEDGRQLAKEIDAAGYYETSMINNYGVNQLFENAVRMALVQRRKLRFWNSQLRQVLKPQAQKPYLL
PKRSLPNVSAETSTYWSDIKSTVGNPDYADIQFIVRGTVIYAHNIILCAAWPRFHDLVKAVGIINKYNRTDSDQISLENT
ITGLKFLYYKKNCQQNSYIRKNLSADHIVFTVDQRITLRAFWKMLEYLYSCQLEKNLERDLLIELHIAADILSLDGLQVI
LANLADGKPYKNAVITDDARQKFIARMKMLYTSDHFQASIAPDATFEAGDTIIPVNRVLVTCRCDVLAAMFSGSFLESGE
QHAKLPGICKDTLLSFLEYLYTDECELKSDFALEVLVLANQFCLSRLVSICESFMAEELREIDNDYDLRNWAVNKNVIHL
LRIAEMHNAFNLHACCVAYISNNYLEIQRQHSKFFRESIDYLTQKKIEEERWPPSWYSAELQMYEKEYGKRNQDIDKQSV
KKRRQKRRLFNVRH
>Tadh_XP_002116532
MDKEEQHWIIRSSTSYNESFGVFSSLRQSGVLCDVIIVVENSKIPAHRLVLASYSPYFYSMFTSNMIEATQQEITIGNID
ATAAEKLIEFAYGGQIYLNKNNVQSLFTASDLLQIDLVKEACRNFIIKQMDASNCLGIRAFADTYSSEELVLSANQFINR
SFAEVSHSDEYLSLSVKQVQELLLRDQLNVASEELIFEALIAWTRYRLAERKQYFSYLLKFVRLPLLSPQYLAEKVANVD
LIRKNIECRDLVDEAKDAYLIPSKRLSIESYKIKPRCFTEELGYMYAVGGLAANGNSVNAVEYYDNVRDEWFPAPSLQSM
RSRLGVTALCNCIYAIGGVDGTERLSTVERLDISHQQASWEYQTSMRVHRSALGAVNIQGSIYAVGGYDGTASLNSVERY
EFGKDTWNYVAPMTTCRSAAGVASLGGRIYALGGHDGLSIFNTVEFFDLREAYWRHMVPMATKRCRHGVATLENKIYVCG
GYDGRSFLNTVECFDPIADKWTFVAPMSIRRSRVAMVALGGVLFVVGGYNGFCNLRSVECYDPKTNKWSYVSDMSQHEGG
VGIVATPRFH
>Tadh_XP_002116242
MCEISTGSVSEICHIRSLADDLKNLMDSGEFTDITIVVEEHRFSCHKAILACRSNYFKALFFNGMKESQSSSEIRLHGIK
SQAFDRLLTYTYSGGLDLVLFSQDEIIDLLAVAHQYCFELLQEAICKYLASILNGKNACDIFEIAGLYEIPSLRQQCLQF
ADANAEDVLKSDGFLTLSKSSLVDLLSRDSFFTQEIVIFNSVAQWLNANPEIDKEDVIPCIRLHLINRRDLLLVVRETKL
ISPEIILDTIQDIDVNNYLESTPRGYSVQDNNVAAKSLGATILAENQRDPSEISISYDWQNGIGYHDIDLKDDHGGVLIT
LGREYIVNMIRMLLMDKESPSNSYYIKVSTDNINWREIIDYRGYYCRSWQHLMFRPTVVRYIRVIGSRNIINRRFCVMSF
ECYLNKLSWPIREYMNGLMVPNTNIAVHDKGAALIQGVNRSNNKFLSGAFSEDYDWDCGYVCHQINGCPCGIVIQLGQPY
LINSLRFLLWDLDDRWYSYYVEISVNQEDWIRIKDQKGTQCRSWQELQFDTKPVSFIKIVGTKNSVNDVFHCTYFECPSQ
ASNNNITNAD
>Tadh_XP_002115044
MTMIMALRSIKQCIQYYERKLCDVTIYSGNKKIEAHRIILAACSPYFCALFTSNLIESGQASVTLHEIDADALEVLIHYA
YTAEVEISEANVQALLPAASLLLLHDVRENCCEFLLSKLHPSNCLGFRRFADVHSCHDLKVKAHNFALGTFTEVVEFEEF

LNLPLSEICELVSDDGVRVACEEQIFEAVLKWVQHDIAHRSVHMAELFRYIRLPLLPKEYLVNLVESELLNGSNLECKDF
LIEAMKFHLLPNHRRVSYRSPRTRPRKFFSSTTVMYVIGGQAPKALKGVERFDRESNSWTDVAPMTSRRCRAGVAVVDGL
IYAVGGFNGSLRVRTVDSYDPIKDLWQPVASMELRRSTLGVAELNGSIYAIGGFDGATGLNSAECFNVITNCWKNISPMN
TRRSSVGVASLNRYIYAVGGYDGSSRQCLNSVEQYDPALDEWRFVREMKVRRSGAGVAVLDGLLYAVGGHDGPDVRKSVE
FYDPATNEWTEAAEMNLCRRNAAVTTVEGLLYVFGGDDGSKNLNSVEFYDPFCNKWTLSEESLGTGRSYAGAATLQLPRH
LSLPGAFSSDCESSL
>Tadh_XP_002114331
MTENNTNSQKGEGLYGNPIGFAAEMKKLVNKSEYSDIKFIVGDTKQMIYAHRSILSARCDVFDTMFHDNSVAIENNDVPY
ILSDVQPDSFLAVLEFIYTNCCSLTDKNVVDVLASAIEYGLRDLAKICVNFMSDSITIDNACETMQSAVTYGQAELQKKC
LSFIESNTKAVFKTKGFHEMSDEALAVALASNNLNIDEVDIIKAVREWATVNSVVLDKKVSEVAFGIIKHIRLPLLTPEE
LTVVEADNKADRLIPIEHISYAWRYHALKKKEAGNPLTTKRSGTKPRPNYS
>Tadh_XP_002113342
MDVLIHHGIHHTNQAFSAIGQMKSRDELCDVELIVDGNRLKAHKLILASFSPYFHAMFTSQLAESQSNTVTLHDIDFEAL
KTLVDYTYTSVIDINQHNVQVLLSTASMLQMNCVRDSCCKFLKTQLHPSNCLGIIAFADTHSCNELHQLSHEFALGHFKE
VIQSEEYVLLSYNQVESLISSDVMNVDSEECVFQAVMSWIDHDRANRQKYVVNLIQHVRLPLVSRDFLLLHVETNELIRN
CNECKDLLIEAMRYQLWPEKRSLYQNFRTQYRRLCGTSKVAISIGGGSLFSIHSECEIYDINRDSWIPVASMAERRARLG
VAVINNTVYAIGGYDGGSDLNSVECYLPQTNTWTLIQSLGTRRSGLGVAVTSNLIFAIGGYDGALCLNSVERYDPLTNQW
SCVADLNSRRRYVRGATLNDCIYAIGGFDGGIHLASVECYDLNLNQWKQSASMLARRSSAGVTVVDNILYVCGGNDGSNC
LRSFEKYDPEKDEWISLPPMNSKRSTHDVIAVDGWIYAIGGNDGSASLSSVEKYSIAANKWYPSSAMNMRRSSVGVAFCE
VLLHE
>Tadh_XP_002113136
MPSSLSLTEICVRLNSEVIHYLDLKRVPLVHHAKQVAMDDKGSNFAVLQWDAYQGMNNRASKSESKLNADMAKLLQTASL
NDHIHDIIIKVDGLVIPCHRFVLCSRSIAFAEILGKGAVDEATMGIYQIDDMSYDVAINLLQFIYTRDCILLQRNTAYSR
QKMQDLETSNSKLPLTDIELSRKKMEHEQASTTANYEKTKYGYIPVNVKELRRAAKHYKIPGLRESQESSDFTLESNDGE
TFCCHKCMFIHRSESNDAEFLSNIVVAADYLLLSDLKQICEWRLSKLITLKTVITFTDFSLTYNAKLLNDACIEFICINL
GYLLEGRIFDNVTLSVLENVSDMYKTKITGILSRTVSFQSSHIDYTSASNDGASFVTLESNSNVEPLKDDLPKSTVAAKS
LHTSTPTKDIHKTLPSSSGACSKIDDNTHSSASVEDSIMSDILAYDLSDILDTSDTLLLPAINQQATNEEASFTTTISKN
NENTNSNSVSQSNKKKKKRSQKMRKAELLKVGTAATVTAEPPKNTPPLSKSQPTVNTNASSRVKKWNSSQSANSNSLLSI
MAEEEEVARKQQACYHFQWIRLAVLYLNLSANNTTYNEAIKWGVAQDVKKKSNRQRNISTSSNISLSSNETEMLASSSSK
CPWGNVKQSASFSLGDVMKQEEVARLDGRGSNNRKVQSSSSSSKTMPSKGTSRFLDIVSSQAEENYISFRRGKKSLSSIQ
LEEKAMSELLHEYGGRYNPEEYIIVEYERPEVSLNPSWFRK
>Tadh_XP_002113089
MESDSVLNYVRSQFPIETLHQLYTNGELCDIQVKIGHQTYHCHRIVLASNSTYFHAMFTGNLTESKQNSVEIKEADREAV
AILIDYFYRGKIEINTNNVQSVAVTSSMLGVDEIVHVCCQFISDLLAPSNCLEIRYFAQFHHFEALGEAADNYIANHFSE
IVETDSFVEMSYEHLINILPMPHLKMIEEISVFNAVMKWIRSDLKARNEQLPKLLNCVRLALIPVETLIAKVESEDLIVH
EFQCQMYLHRAKNYHFMPEHESIYLLGGILNENHQVAPNGNTTSISDVYAYHNGIWSKKASMLTARANFGAVVHEGRLYC
AGGRTEGQLTCVALTNTVEIYDPKDDKWVEGKPMNEAREGCGLISLAGQLYALGGYNGNNVLCSVEKYDENKGKWIQVCA
MKVSRSHANYIAYKGEIWAIGGGKFFMYDYICQYINAKYRDLQMKNYN
>Tadh_XP_002111701
MAKMEDRIAHDGFSLDLLKHQFEALRQMRCKQELCDVTVIVNHTHTIYAHRVLLAAYSSYFFAMFNTDMVESRLGVLHID
DIQPNLMDQLIDLAYGIDLHIDDDNVADMLSACHRLQISTGINRCCDYLILSLHPSNCLGILKFARINQCQIVIDAASKY
CWHHFTAICDEEEFTLLSLDEVQEYFSNDHLHVKEEAEVLRSAVIWLLHDWQQRHPSLSAVMDCIRFSYISKEEIVSIAR
KNCPADIFQCIADYLSDKSNRKKRKPGQRHYSSAVFIIGSTSLANCCRRYNICSRTWVKMPSLVEKKLNTLVGDIDGKLY
AISSTPASDEITEIGQNLECYDPNQESWTIISALPSCRWQFSMACIDDRICFIGGYNFKNGFSHLATVDCYHPKLNQWTK
LPDLPEKRRGSCTAFLQGILYVIGGHDGTRILSSCECYDMTQQCWLHINDMKVPRESHKAIVINKKIYVIGGISKSGYTK
CVECYDPVTNSWQCCANAPVIYPRQQVVSLNNRLYLLNSDTESPVLCYNTMEDKWYKSRWPKFAEGYNGGTAISCASVDG
L
>Tadh_XP_002110875
MGSPKYCTLEPIEELPQLEESQAGLTNLITDLSHLIDDRQSTDLIIYVGENKTPFYTHRLIIWARCKGFRNQHPSILSQS
HGCQSPAIINKPYLNAQYFQQVVDYLYIGKINLDSSKVFPILAIADDFGIASLQSACVDQINSQRTIDNAISYYMVIRDI
ILSNDSEWPAVNEVKKSCLTFIDENAEDVTNTESFLHLSNESLIELVGRDDFALSEEEIWRSVLRWAKNKADVTGQIKSW
NDSEKMAVSHHLSKVIHCIKLLLIDSEVYAQEIEPTGAVPMKLSLERYRFAAVPAAFNTATDNRLQPRKMKTLFSGSVLL
SGDNLYLQSILNGWYGNELQQWKLIFRASRDGFSARQFHVYCDNIDPTFVIVRGPDDNLCGGFSDQPWSRKGASGSYTRS
SKAFIFTLTNQQDMPPSRFDITQEKYALVYDEEYGPTFGAGADLCIASNCDSNDQSYSNFPYSFEGLNASVNLLMGSYNF
YVKDYEVFTIK
>Tadh_XP_002110405
MENCHHYYQSFAKNSFTVIRQLLSDGYLCDVELHIGQWQFPCHRLILASFSSYFRSMFTSNMIETTLHHITIHEVDQQAA
SMLVQYAYNGELAITKDNVQALLETAVLFDVPDVMEACSQFMSNHIDIQNCISIYNFAGFHHLTTLSNYARNYILENFTD
VFQSNEIQYLSEDYLIQLLKHSKLNVQNEMQVYSAVKKWINYDYDERKLAAFKLLNHVRFAMIPRQQLILLTKNDDELIN
LDQHCQHLINQALRFQLYPKKVQNIETKPRESYSGLLYVFSYADVYSHYNTVECFSYKHQKWIIVHDAPKKLMSEDFAVT
ASEQNLYTLSGNRNHIISNQMSCYDVITKSWSAFPSVQTPRLEASICYLNQLLFVIGGYKIDYNRYTRSSRIAESYDTDT
GLWTALSPMNEKRFGAGIATMDGLIYIVGGLSYDSSSTINFLNSGEYYNPKDNTWTPIRNMNYHRYKLGLVALNGYLFAI

GGKSYDFECGNSFVTNSVERYDPEADEWIEITAMSEKRFAAGVATMGGRIWAIGGKCDEDNALDSAENYDPVSGEWESIP
PMCNKGYNLCAISIQHKIFIS
>Tadh_XP_002110225
MTNRHEPSYTDSLWSSNNCSDVIFQIAGNSIRAHTPILKARTPLFYDCYVKAATDKDYPIPIDNITVEQFNHFLRQVYLN
DEACDIDQLEGEWLTGKVLTDKSDHENLVSNNDNCSNLIIDGYNITDAATSRLGKDLLNLFQDNLSDVTLIIDDVELKCH
KCILCARSEYFNAMLSGSWTESNNNTIELHRVHPSAITALLYYIYGGIVELPSATDLTFLAPIADMYNLDGLKDVVSYTF
MRDWCKFFDKVNNELTDNISRCLEIADTFGFEYLRESCMGWIIKNFNRVWCSKSFAKLPTEIQQDALKHMQELLALDSVT
DILRGCDYLLVNMADGIKWTENIREITTTLQQSCLDFISARFRNLIHQLNFLSLFRGMGKSDKIVKNIFDNLAMQLTPDN
VCSHFKAIEELRHQTKLEEWEEMDSITLIEDFHANLKRFINSNAGRVRTSPGWNSLSQKQQEELKQGLVIFENGGSRTSK
IAIGKNNLAGNRSGNKSSIRSKNIAVTQQNHRLGQRGNNLNLMRPQRGTNHSNQGSSRLPVTATTSTAVNSIAGRSNNTM
SQSRRVSNPCGWNSRSTRSTVDAKRDHYSSRTKATATVTVTCDASCLPKRNASKFNPTLQTRSGSIKLKQASLDSSKLQN
LDVGHLDSKLNKNASKSANGTSRIPIRNKQTINYIGNVDNQRQGQVSRIPRNVALSRLSPSKCLLSRYPLFPSDSVKISS
TIINHSNGGISASTVKMKTSSNPELAVTTYRPNMVSPDSMNEAKDPINSASVSTAVKNKLEMNISDAAMKLQITKNSEES
STENKQFFSEELSNSSMTIQPPVKAACSEENSYEDHTSLDISSDLCLELVADLNVRDFETPAQSLPDEIEKEITTTCTHS
DEDVDINAMESSYDRASQELLDAFSEGNEQDPESRVYQNLQ
>Tadh_XP_002110137
ELYAEGTFSDVTIHTNDTVLHCHRFILAAFSDYFRAMFTNGMAESQQRKIELRHVDGKTMTSIIDYFYGGSLKITSENVQ
NIAITSSMFNVSEIVDYCCTFLADSLSYTNCVQILIFAELYNFHPLTQIANNYILDYFEKCTGYDTFYEIPYPLLNKILP
SERLAVKNELTVLNTLKKWVELDPSIRKDHLVGLLSHVRLAMIPVEQLIDFEEKETLLKESLPCFQMLLEAKNFHYLP
>Tadh_XP_002110136
MPELTIKGRLPQSSFQLFQDKIFSDVIIKINDAQFHCHRFVLASFSSYFQAMFVNEMAESQQNIIELKELNEDVFADIIH
YFYGVDLKITEKNVQILAITASMLNIMDVVDKCYSFLSEKSPNRSSYGALAIGHKILVFGGITKDNVDILRSIIAFDTCK
VRWSNADLAPLPKPIALLSPCLCDNRLYIIGGSHENSSTATNEVAYLESGSDHWVYVSPMNVARNDSVVAVLDNLIYVGG
GQNNKWKVRSFERYNPSTDTWELLNWSIPFSEFPDSHVLTVYDGCLNYIWNSCRRRHCHTYNPIQNEWTEASESASAILR
FKYRAEEDQFNYDECLNKLFIIRVTNQLRDKDNVK
>Tadh_XP_002109948
MATENLHFNDNRHLINVVHSLDKLRIQQELCDIQLVADDGKVNAHRILLAVSSGYIKQMLRDEDTADLSSYRVEGVDKLS
LETIIDFIYSGTINITPEQVLPIFRASNKLQISSISEACYNYLQSNVDLNNCLDIRTLASSYSCSNLIVAADKFISNNIE
QVARLEAFLNLPQLRFEIYIDEDAATSGPSGEQYAAETVKWLHKNANLLNRQRNRLGESIQYLFLIENGELTDIDNLSSK
QLSSLDESVRQKALQSRYRNDGLSSRPSSPTAGEKHTPEKCVNSAIAVRQLILSPQDNSIFSWHVVAVGSMQRGRCSFGV
TVLNNEVYAVGGYDRLGCLNVVEKYNLASNTWLTCQPVNQRHGRIAVAASSDHVYALGGSHASHDLKSCERYDPITDSWK
PFKSMRIARSCLGATVIDDQIFAIGGQNDKGTLSNVEICNIASNSWNIAKPMIAARSHLAGATTYEGLLYVCGGSDGSQP
LNSVECFDPRANKWILLKEHMNHARVNVGIVNVDGWIYAIGGFDGSTFLNNIERFNPSTNRWTSIVTDNDDGMIAL
>Tadh_XP_002109926
EALNRLRDAKELCDVEIATDNSTIHAHRLVLASASSYFRAMFLSNLVESQQKIVHIEGISYSILSNLVNFCYTGNLVISI
HNVAKTLEAADRFQLLDAKATCCLYLSRILQPYNCLGILKLAQIYDCPGIVSKASNISCRKFVHVVQSEEFLEMSFQDLV
KLLSKDCLHMTSERDAYSAVSRWIKFDKTDRIAHTHDLMMLIRWKYIDEDFMQSSVYEDDLLPQSVFPLVSVDSFQSSLS
YYYHNQFPRMYTEYLVIIGGMRSSKSCKCYNIHRQKWMQLPSMLMGQRRSENAAVVVVNGKVYCFGASSDSKLEGGSECF
NPSKNSWKLIASLNTCHRFMFSCVNLNGIIYALGGYDTFSQAVHCAVESYNPKYNIWEYAPPMLARRYAFTAQVIDSKIY
AIGGYNSTECLSSCECYDTTTEQWRYVANMPQPRARHVTANLHGLIYVISGVITFKGDFVDSVLAYDPIKDQWQCRAPLP
FIPNNPQLVAFHDRLYLIPVSHNQNILQYNPRADEWRIV
>Tadh_XP_002109389
MNSSSQNLNLPFALNERPRGGSRSSSTASADISSNGIKHRSRKHAAKVLHYFNKLRRKGQLCDVTLVVESKRFYCHRGIL
AACSPYFRAVFNENSSINPEEEICIQDADEESVEAILDYMYTANIIINENNAHILFPIASLMQLDEILDTCSQFLRDQLT
LSNCLSVRTYADDHSSQDLYQTATAYIQENFTEIVLQTEFLALSITQLVAILRSGNLNVPNEEFIYQAIIDWARYDIKQR
RDQLHQALQHVRLSSLNPRYLVTVVSQEPLIRCDEKCRDLVDQAKNILLLPEAIANRANTTTVRIHQSKQPPVTNMNGAS
TTMIYVVGGWSHGKADNTVEVCHACYGNWKVVSKMNKPRYGVGVAVLEDSIFAIGGHDGKNYLDTVEQFNPKTKYWSLDI
ATTRTCHTSHGVATVNNCIYAIGGQDGVSSLNLVERYDPHHNEWYSVVPMKSRRLGLATAVVNNCIYAAGGFDGTAILST
VERLDPRENQWVAISPMNKRRKHHGATVINGILHVAGGRDDSKELKTVEYYDSRNNTWIPTTSMTTLRSGMILTAFNDQL
AVIGGFDGVDYLKSVEILDRELEEWKYCSGMNHSRLGAGVAVLESP
>Tadh_XP_002109220
MATKNVQHTVPTYKTLNQDQEALFRSCRTGDISKIQYLFEYCDIEVNLRDAWDATPLYYACLCGHKEIVQILLANGAKCN
ATTFDGERCLYAALYDEIRNLLRSRRAITSHTMRRDAYHEFLRRYCILNNVFNAYISNVNLKSTTHCLKVNAAGAMRRHR
MLILNLGYVDVLLEEASSFSELAKQCGLQELRNRLISESQLAIKFSLSKSHSQVNMITIMEDEGQISVQNHMKILVNRLL
PKEFRSDLDELFDNFEPLNSILRPDLIFCVGGYKFYGHKVFFCERSDYFKALLLGNFAESIVSNCSEEIPVINLNDCTPD
IFSIVMVFIYAGDVKIPCDLTQEILYAADKYLIPGLKRHCCKVIIQNLQTDNVIQLLETARLLTMPKLELECTRFISKHL
LEMVERDDFAKIIIDDATSIIQRQETDSIPIIDEVRYHLGEESLQQSSEKYNEILLKNNALDKLLMNLNIEC
>Tadh_XP_002118162
MTIYEEKLVIEDISSDIFSLLIEYIYKAAVNLNPITALYLYCAAEKYPVSGLMEYCLQSLSNENSVEDLLKAYVEYESVK
SSLSSKLEDRFEKALVDQLSSKFRLVTKSESFQKLDKDLLVSLVRKIANQIIIT
>Tadh_XP_002116777
MEALISSPTFPLPGLSKNNISKSPPLKIEGEKMNGLNSSSNEEKKSPRAHLLKPVRVRPRTQVGTTNRWRFPESSGGDLL

GTSQAVKSVPAIDDTPFIPIINYQTYSEFEQQNIPVDHEEVKPLPGIDGKFASGEETRIIKPTKSTYFIPKEKKTKLSPE
AFHYHSRSTTSPYEPDVILSCLGTLWELHKPFLQRSNKLTALLEKAGDRSAATYTNRIDHNHDRQITSKYRAGLISGSEM
NISSELPDHKRQQHDTVTKADDQLIKIKLSANDPNIDKEAFAIAICNLYRDDMIVDETHVIGVLAAAHQLQFSKLENKCA
QVMLKSIELSNVAQYHQMATKCKQSTVAVACERWLEINLMPLMSKRINLRQLSLDLLHKIIQSPRFFTYNEYDVFKLVCV
WIYLRENSSEMLMPTYATVVQYFESIPKNICFLLGAEGKKYTSIFQIIRYNGIIDVRHLDEIEKMNIFPQSHFYSLLHQH
YHSLQNGGDMLLDPRIELNGIRIGFILQEEPKFHSELIALHGFYFEAKAIRQSHPNSYSFYMQRIRPTDNVLSYKECERQ
AFSVRPERKTMYIVRVQSTQGGNYKVHKTDMLTHNFSMTTHGRKSQSYKPETNKFVITHFSYPAIILVTILRENFGAIRN
NLRRIYEDKQLAGWKIGLYDIKSINNQTRGAKIGKSKFDS
>Tadh_XP_002115262
MAKNLKRILAYTKCTNDVQTIYNRGGITVLLDTLYQSSEKNLNVVQFALIVIGNCLKYKRDLPQAYTKADLVWEELCEFL
VKTLKAAEDSNYISTHCLRILDKLAREECMREYIYKLVDGFQLLLQRLTLDTLRYEENVYILDIMVKLLHQKNSSVTFVS
QNGLLRLTKLMRSTDEVDLFRRCADIVLSLAKRRDRQCYFQRLKDDGCFKTLVTVGMDKKKSSNIRKKTVNLLFNLCEIK
PYSRFLVLADIHTLLIRLIETAPMGHTKSCAVRSLSLLCCDVANRSTLNKCGAIEMLISLLKDNNVKKLHHVIVDGLHWF
LYDQEALQVMVKEYDLVSALSGHLNRLIDGLANCVRHTNSTDDNYCYDNTIGVEREQSQSCVNPVLSSSPLINSMDNCEW
NGSLSPVSLMSISCSSESSSIYLNDSKINENNSISDMKNSDRLCIATILNLLKAISRLAHSYHINVVKFCMKGLLGCIVK
DSENRADAYKVMSLIMENKCCLKVMLRRCVPWLIYQYLMNDHETNVSSGNFTYFATHYLHDESLKEHWNTLTNDEEVTTS
ATDQLSSLKDSVNLTSSFITAVTDTLQADDYIATTSSSPKEMAVSLLQLIGKISSSDYGKGEIKNILSTGNTSWKRACAF
SLPYLLSYERSMYKVLLRNNGISTILEPLLLENVDDFTTTITCHCISSLVERFQSCKQVYRRKRARSNDGTQHFTKKVQK
LEMSQDSSQSYFFIDDSRYTYYQQQSFMTADAILVTENGSELPVNREIISQQSVYFSAMYSGYFIEAKLEKSYIKEIDDT
AMIAILLYLYGFNWRIQPSIMQLKTILSANSINEVKFTVKLFDRLDEEDNIEFYWNVLISADKLLLEELKEACEPYIIDL
LKPHNRSETLFLSCVYSCEGVFMYCIDQFFRYWTSAEQCNCFKQFPEPYKEDFIKRVCNFVNKIFFCCVDNI
>Tadh_XP_002108906
MGNYFFRKELTEDSNYDHSVQFKRPQNKRKRTINQNEDTEDATDSLQACTKRQRNDEAIYHQYTLSKLFFNDENGDTTIE
ALGYKWKMHKICLIRAGYFKSMFSGFWQESKATYVKIEMIDKNITVNAINTAFASLYDDDTEIKSSEAISIVAVATMLQI
DNLIKRCTDVMIATISEENERTFREAASAYGLLEVHGLACTATADYQTIRLDAAAVYRCKSNGKNCKLSIFACAAGGNGC
ICDVKEGSTNFLVTEEGKVFSLAFRQLKFNHIITDTKIARQIEKDRIISQDWLLPIYRQEWRTLLAVEEGDDFGSPKRIS
LKRNISEHDVAMSLSLENSRCIMTK
>Mlei_ML00107a
MVADSPSEHIHANVVDSTNSPPIAESWCLTQVKVIKFSYMWTINNFSYCREEMGETLKSSTFSAGPNDNMKWCLRVNPKG
LDEESKDYLSLYLLLISCDKSEVHAKFKFSILNHKREETKGMESQRAYRFVQGKDWGFKKFIRRDFLMDDHTNGLLPDDK
LTLFCEVNVVADSVNIFGQSNHRSLNVPKCNLSADLASLLSSGSFADVTLEIESQEFQAHRAILAARSPVFSAMFQHAME
EQKNGRVEIKDLSPEIFGEMLEYIYTGRSPNLERLANSLLSAADKYQLERLKLMCEEALCSNLTVENAADTLILADLHSA
EHLKEMCINFINSHVSEVMDTPGWTVLCSHSHLLAEAFKALAAIQAPHMSMLMPPNKKRKKLG
>Mlei_ML00112a
MDFGIENLALPNHLNVSLITANRDVVKANSLVLSYNSTVFKRIFEEQELRHSVDLIEYTRFVVDEFVKILYSGKVDFEKL
PFMGQICIWKLADRFHVEWLLKQFDQYLAEYATLKRKEQAPTEEYPTPLNELNNSTPELHYQDEPDFQESFEKRRRMLLQ
GQDCFEEIRPYFRDEEIATSSSTSARSIHDRTVEHRAIVKHRRSIYKRTVQVTSVSVVDRPIIGHSLSSSGHFRDDAVEE
NVIRTHLIPNLLFSLPYDLDTFLPSFNYLAKFLDHLEGSSLSFPNMFMFIEFVFVLKRMPCTNSYRVVDVSDLKEAFVEL
TQRIVDTKKRFGWRKVPKSFILDTCNLPDISRENRGLLKFMCNNEELTTETEAVKIVSYRGSESMNRSRRYSDYDTIRME
EFMSGAIKKYKFFWKHPSTHYCTNTGDCGFILCVVPISKSFDIRLSTRPEDYSEDIHCHPELLDAKNMHLVIRRDFSTKG
KSSLNSPTDLIHISWQSKPVVHNNQVFWAGQSIPDKAYIALVVYYTFSCSVAGILKY
>Mlei_ML005223a
MVANIRVPAKFSKKYLNSPVHGNLILNLKNGQEIRTNSVIMSLNSPVIDRLTSELHQSSLEMDDFTAGAVNCFVDSIYTG
DVGTLQKQIFEDVNKMSHVFEVSWLTKWCFKFYKRDVLNFTNNSYQEVLFACEIASRAHYNLKQTRYVTCFVKNLMSRDI
SRKMFLQRYMADFAELSKRQLEMSLSMARNDFSLIMVPLTSHLTMNLKCKNLDKNAMYLLEACDVQKFRQDHPDNFDDAA
NLLLEISELSECDEVKEVVTKFVRPNSGTEASSSENVVDLVETTEECEDDDEDDSDDEDFPEVSTQTDVEPERGWIQAVD
CNTYPLISDNTLQFITTDNEVQRNIFMCVIILYLKKEDVLGDGLQ
>Mlei_ML005314a
MAPKIRVPADFGREHINSPVHGNLVLKLRDGQEIKTNSMIMSLNSPVIDNLTTNLTQSSLEMDDFTKEAVDCFVESLYTG
EVESLEKQIIEDVNKMAHVFEVSWLTKRCLRFYKSVVLNYENNSYHEVLFACEIASRAHYNLKQSCYVRCFLKNMMSSNL
SRAMFLARYLANFSERSLRQIDMSLAVAGNNFDMLIIPLMSHLSSNLKCESLDKNSLYFLQKLNLQRFCKDFPILFKNTA
DLLIEISEVSDSHEVSEVIKNFTKNKGDFSCSNYIDALEITEECDSEYSDDEKSSEVSCNCRQPDLEPERGWVQVISHKK
YPLIFGDTVHLITTDNTFMQRIYIHYEFILDDGQPSPNTYFAMRSENPTGNRWSYIIRGCTANEYKERYLSDVPLDKVKH
WKITRKPSHLIIMCNKVTVLNFNFANNCDTDKKGDCKVWTRNSNSEIELRYI
>Mlei_ML005353a
MYKIPKVQVPMDFGVQYLGLPSHSNLRFVLGNGEELLANSMIMSFNSPVIEDMTTNLFQHTIHVPEFTKNAVQCFLAASY
SGGIADRISKSNFREVNKIAHMFEVGWLIEGCFGYFKTLTFGMETDNFSEQLYVFNEAMFVMKNLKKRNYIEIVIKGFST
RLSETRYFISNYLNTAPSHCSAAHLDVIFEIAESKEYILLEALVEQLDKSKYSISSNGRYILNKVCSIERLRFTHKSLYQ
NLLKKLEGIENPSGEDFMLILKIRQESQKKSKIQASIPYATIPNLFHDYEKLKSIGDIDSLFKFLVESPEVTNCYIFYDA
VAIWLNQKGYSSYAQFIPEGLVERFAEHKHSRGWKPLSLHYIGADTANSFNRKIFENMNPSLITYNRYHRIESTRGYTPD
ELFARNHDMKFKFREESIKECTKKGDCGFILRVTAGSSKKDNNFDIGLLTETKLYPDDIHFHDESLSMIDFIHFTLDVTE
NDGPTKDPTFPTVSWDSLKVTWYGKPRKDAANKYWSWGAHRFHKKGSGRPHPGDRYRRGQYFGDPSAIRLRPAVYYFH

>Mlei_ML00672a
MGDIFTACKQGDLQRIKLLVEEMMCDVNVTDKWNSTPLYYACLSGYDDIVVYLLQAGAKYDQCNFSGERCMNCALTDRTR
RILRNPPKRKTFQSTHFRETMAFMFREGYQNYSDVCLVVGSENYRIYAHKLILYRQSPYFAHSLLGPNRGKDFLYLKHFK
ESSVEAWIQVLKYMYTQQLKIGKYRHIYEDILHISRRLGLQDLTTQLRAVCKKLAKKNADTILIETETCTYPDIADIAIS
DFNSEGRKERQYFADLCFLVETHRFYCHKGFVVAQSPYFSALLDSEQFLEVTTSNRGKLTFITLSNVKADTFALLLKFIY
TGNTEITPETLQDLLFYSDHYMIHGLKVGCGDLLASQLDTANAVQTLSLARMLMMSKLQHAALKFIAYNFENILDKEVLA
KILVLDEVSNRSNRLVRISESSVDSDLHKERDEEQYEENSIDYKDAGVDVQLDLRLYFREITNKQRHTAEAQEREEKCVR
ELQTMLCNHGNTTSY
>Mlei_ML006914a
MDIPTQDSSTPGFCFEERFLALFSFQKDGWLCDVEIVCDDGNVQAHRVILAATSSYFKHMFSSAMMECSSDTIKMKGMDV
ESLSCIVRYLYTNELVISKDNALNVLNAANLLCLPIVEEQCCKVLLGPMAKETALDYAILAEQYGMNSLLETSVEYLVKH
LKYFLSNGLNKLDNRAFQMVVASDDLDVKDEMMVFDTVVQWIEHDLPNRKKYFKSLLEHVRLPLLPPKYLVDVVEKSCYV
KTDLQCRDLVDEAKNRHLLPNRTQGYLSERMTPRKSTVGYIFLVGGLTVKERLLSSVERYDLPLQQSFKMPDMTLARSGL
GCAIVDKVLYVIGGHDGTKALKIGECMDFGKGSWTWVPQMDLPRRNLGVCTDNTRIFAVGGQSDSNTTLNSWESYDPREG
IWMKHTSTMNRARKYASVCFLNNGLYAVGGNDGTHYLKCCERYDLRADKWENIAPLPHAFGANGLAVWDNCLYTVGGFDS
RQCLEVLYLYWPPSNTWSELPHPMGHRRTGLGAVTAGHSLYAFGGHNGETYLDAVEMYDSDARGWVRLPPLHYKRGAMAV
SVLENCYMTNTDPL
>Mlei_ML00784a
MSTVATLRSQSRPSHLSKRRYIESDTEQSSEHLLSAFKHLGRLRRNSLFCDVILEAEDGQISAHKVILASRCSYFRAMFM
CGTRESWATEVKLTGISLRTLDIVVGFIYNEKIRILGEDVQGVLSASEMMDLGDLKRMALKYMTSHVSSSNCLAVWQVSE
LYSCNIAAAEARSYALKNFVKVIDTPDFLQLEPQKLMMLLSDDLLKVPEERLVLEAVTKWFKHDKEARREFMPELLNSVR
FHLFGTTDFVKAITTTKCVEAVPHLESALETALLNHFEPAKRQVCTVPGPPRNRERRKIVEELAVIIGGQQENSIWSKTW
GWDLSSTSQQDKEVSSSDSSWYSLPLPLTQRRDHVAIGRGCDVIIAGGSNGTDHINNVERYDAYIGQWQELPSMLHTRNH
LCGAALHGNVYVGAGTNDSNSYLSSMEMLDTETNRWLEVTGLTTPRRSCAATSNANQIVVAGGAGRNCMDLATVEMYDPR
EGSWRTGPSLRQGRRGFGLVSIDNTIYALGGFTTSPSLGYTSSVEQFDVRTNKWTVFTDLPIKTGYFGCAVTKSKKVLIC
GGMSHSQLLVWDPSSLDLATFSGPPLPYSGTITACGAIS
>Mlei_ML007811a
MAPKVCVPADFGKHHIGAPSHGNLTFTLQEGVKIKANSIILSLNSPVIEELTTNLHQTSLEAEDFSKEAVDCFIEASYTG
EVEALSRENFREVNKMSAVFKVNWLVARCKEYFVSCLSDINNGSSYSDVVFVVEEAIYVMSALKSKEFYNLVVKKIISLD
YLAKDAFISDYLSDLSASTYKQIDFCIEIVAAEVHILVELLTNHLEQEDGACFNQSSRYLFKNLDIGICYENSPEIYKKL
LKVLEDLDDIEKDDYILVLEKLEKATAHSKCVTLGATKPGTSTSTQCDMYDLKTFTLASFSQYLVPARLDDVIEMLAATG
VVDNLYQLIDGVWLRLCQCRKDVVNGSVIQKIVGIKRIKGWKPINYDYLDKLLVPQNATSCLNFKYLLKSSDSLVIKNTA
ISWGSTNQLYGGDFVEDIYKSDKDFLFKINNPALSGVRFILSITSMKNEDPDSFSIKWTIFSKTNAAYPTDLQLHFALER
WNGFSWTLFPISWSGKPTCDQTKRHWAWGYIYSKSYPDRLIKLMKEKDRLLLSESKAYICHEYRYRFVAFVV
>Mlei_ML008026a
MAPKVVVPADFGVENLHCEFHGNVVLKLQGGEDMRANSLILSYHSSVFVRLFLELHQSVLEMDDFSKASVKSFLEALYSG
EIQLDRQLFRDVNKMCHVFEVDWLSRRCGDYFAGLVGDVSTSTDYQTLFFLFEEARFCSKTSKSNVLLDLVVQKMCGLEN
RAEIFVQPYMKNYTQLETSQLDIMLQITRENPVSLLTIVKQNIENGALFHDNDAYLVENIDLCKCLINDEALVEETFDVI
LDSSDLSRENERFVNKLYRQVYKDFKASNIEEEIEHSVSEDSLTSEYIELDSLFVSFQSFSNLGLQAFYDQSMVSPLMSN
LYMFIEGLWHNINRLPPQPAMGFDWSTMILNINRLLSERNWKRISPKYLYDQTEDYKLVLQTLVRRAPHIISKTDDTYRL
SGKVIDIVGSTEKKLRLSDLFARRVRIAYYLPQSATTKLCCSRSGSCGFILETEPVLKDGNPHKFKASISYDISKYPDDL
HPHPEIIDVKETHLMLSAYFKNNFTRMKMMPCCLIWSGDGVRIEESSSGETWHLDPYILDPKEPVEIVVFM
>Mlei_ML01342a
MAKVRAPMDFGVENMDCPVHSNIDLRASNNETVRANSLILSYNSPVFHEIFFKKLQTSVDVTAFTKNSVYQFAQCLYSGK
VNLSRQSFRELIELADKFVVEWLLEQCDLFYMKLSRSIKFGCKNQIELETVRFLFDEAIYAPRRSRQKYTVSVKEKISTM
GKFAKQEFIHRYATNYMYLSVDQLSALLELARGHDGILLKIVQENLAKQKYYFDEKSFHILNGIDFEGCLQSKVERSAFN
QLFANTFVVKTQQELDNLEKILNLGTNAVEKVVAQTPSLLSDVVSIKDNSSIHIPNLKTVLKLKTVANAFRTDTVQGTGC
PVPQRSLICDFNIVDELHDPASIPNLFSSLDQLQLKTLTYKDMKHRSLLFDRGVRFSVEDILAVSSSSKIFRNLYMVMEL
IIMHGMIIKPESDTTKSYLESIVKRLVEIKNNRGWGRVDSLFFGHRYLNMSFLEEENVKKNPEVALRILLSHCQELVSVR
DSSAIVCQRGKIQSHASLRYSVLPDTIRMGEFLSTAQGTKRIYKFYWRHPDTTDCLNPKQCGFIINVTAMTENSSEAFNI
GLCTDVNDYPGDIHFHPEIISAQKMHFVLRRDYNVKSSKRPSEMVYISWDGKPVYHSNHVHWGGQSIPPNGYIALVVFYD
VSDVDLDALKKSDINLSRALRLEDLKTKSSF
>Mlei_ML014420a
MFGNNMMECHKKEIRIHDVDEASFQTILSSFYTGFLSVCSQCVIRILKTGPAANLFETEDFLNLTPDRMSMLLDNSLTNC
SETELATAVLRWAARTWEWYDILSPSEHVSSPNSSTKRVSSPNSITLSPGVSTGCLDSSSDTSLNDSGIVLTEEYFVEFP
GEMPDIPSPPPSLPSSSSSYDVDDFTDSPSRSGRATRTYKRRSRSSPRNRKEPETSSKRKRKREEAPAEVETIRGLAQVR
QGQREIGEVLNKIEFNNMEVETLRGFLKFDIVGRIPELYHHIRRLAGSVRYRCLPERGSKISVSLCYHRCYRSNTENKDL
TFRSTETPTRTTSLYYLNNNRWHTLDTSFNINLISVKSLVLNNFLYLFGMQKVPGQHAVPRAFRFNQFTGIWREIAVPPH
SFNSNVRCIAFKDSILMIGATTSLGSGLVTFQQRRVKILRYFPQTDSWIELEQELPITNHQLQRYVSSIICHDGLLYFVS
RNALCSFDIEAEEPRMKEVAVISQMFTDISNYWLFAYQDQIILLGGLCSMQGGGNWQNREILIWNLTSESWTILQNNKPS
PMYEMLTGMVYQDQIYTVSHAAAGGESIAKIELFDLVRHRWKKGTNLRYESPVHCVEYGLTVGCMLVKPLQETD
>Mlei_ML01862a

MASHVEEKRQSGRLFPFIAITETWLKSYYSDAQLPIPGYSVTRCDRSKRNGGGVLLYSLSSLPVSVTESYDDGICQGLCN
IYPSAKLLVAVIYRPPDASLSSFSKLLDQVGHAIETHADNDYDLFITGDFNLPHIDWETLEVRSGGTSESNLSAQRLLNF
MSTYLLSQMVNVPTRGNNILDMVLCNNNRLVLDVMTEPTDLSDHDMVNVLLSFDPGVLEDAQASYLDASNFRSLDFNRAD
FSELNKVFSGIDGKELRDSGTFEEFPAKFIKKLPNHFDAFEIYFEICTYQVDKELLSRYSDYFDAMFSSPFSEQSSDTIT
LKHIHFKSVKVIVGEWIHKLSVQHGLKKTEKSAVKTIMKCFEQIYEDHEYLDFTGNLLFALLSDCDLNISDEMNAFNAID
RWIHHDPLTRKLYKDELLRCVMVELLTNQDLHKVITSDLVKNSSAVKQHYKELLSSNDVTGKRRNIKGGKGLGKERLGMI
SGRVYIIGGLTNKKTPGDVEVFIPSYKEQQRDCNTYIRRSFTQKLSADDVITQSDDVTKMEYCSKYVTQSYGGSAAVIDG
QVYVVGGRIDGDISDRLYKVELHTQSYIQLGKVPVWCDHATVTCSDKKLFVIGGYQLSDSGSTKFPSDLVQKYRKKTKAS
LLFGSTECLHCGLLAYVIEQCLDIPDRVRIDSGGGLASRVLVLGGYGKNKLNSVELITDGKTVEVMPPMIKPRHAFCAAM
VNGAIIVVGGWNERSVEKYDFRNKQWSLLNNFIKHRDCIQGAVCEEVLYVFGGVKKNEYVPTIEKYDPEKDSWKTVGQFI
VPRLYSAVAVT
>Mlei_ML019411a
MAPRVKVPMDFGRQLRESPSHNNLKLNTNEGKEVLASSVILSFNSPVIDHMTTTLHMTSVDMLEFSEAAVQMFVDSAYSG
TAEGINREIFRDMNKMANVFEVAWLVSKCSDYFTELTNSVTSGSYAELFFLFEEAAFVHENLKMKDFLNTTVKKIESLKW
KKQFLDKYLENAERFSTKKLDTVIELAGSDVDIVVQTLVNQLSEMFRIQGPSLPVYCKYLLDNSELHICQENNGALFNEL
FDVLRELPNEQLRWTLDLHRQSTAKMLSQQQTATGKVVSRPSCSYRSTEENPPQSSSATIVKQDNFTNASRCDIIPNLYH
YIDITMDFEQFKEWLTNSKSITSLLMAIEACWTWCNCSPNYSYPHFERLHSSINSLMKRRGWPPLPMIMNNFDWSFHSSP
EEMLYNLNWTRFCFQQTELDRTHVIITGVDPDRKHLITFLSQSSKLIFYFQHPSVNSCTLPGKCGFILKTIPNDAPCVLW
KLKLCTDDKDYHGEPVHFHDQFRAENMHLLYNDIDFSDALDLFPLSWLAWINSSTQRQCFVDYFF
>Mlei_ML019412a
MDFGRRLRESPSHNNLKLNTNDGGEVWASSVILSFNSHVIDHMTTTLHMTSVDMLEFSEAAVQMFVDSAYSGTAEGINRE
IFRDINKMANIFEVAWLVSKCSEYFTGLVNSVTSGSYTELLFLFEEAGFVYENLKMKDFLNTSVKKIESLKWKQQFLDKY
LENAERFSTKKLDTVIELAGSDVNIVVQTLVNQLSEMFRIQGPSLPVYCKYLLDNSELHLCQKANNRDLFNELFDILKEL
PSSESKWTLGLYRKCTEKTLSGYDNTTEVTSFTGKGSGLEVSRNCITTATHSNMVPNLYHDFAVGMTFKELLNWLSESEH
VTSLLLAIEAACVWCYYRAHCVSEKKLILPDTDDLLQKLTDMMASRRWSKLPAEFRRFRWRSYNFDLSQFCLQPSQSSKQ
YVIINSVRTYNLELDVLSKSSKLTFHFKHPSVASCNLPGQCGFILKTVPSDAVLWRLELCTKKEDYLNEPVHFHDSVRAE
NMHVFFVGTHLSFSDNTLVFFPLTWLAWIRNENMIKNWRSAPLRRDSRFKILYKLTNDDT
>Mlei_ML02161a
MAPKICVPADFGKHHIGSPSHGNLRFNLQEGSKVYANSIILSLNSPIIDDLTTNLQLTSLDAEDFSREAVDCFIEATYTG
EIEALNLRNFRNVNKMSRVFNVSWLIAKCEKYFLSYLGNLDSESSYSEILFAVEEAVYLMSAMKSRHFLDLLVDRMSFVS
VYNRANFIKRYLNLTDIPHLTKYRIDACIAVVKSDVHVLIDMLILHLEKQGNISLDENTRYLLKNVNLSSCYYREPELHT
KLYSVLGNLQCVGKEDFHLLLSISKQLTIQSEARIDARKSNTALQSNTSSFVPVCFIESNGSFNSENLIKKVATNDDVQN
IYTFFDGLWFGLFQADHLPLNSIVSKIVSFFKGKQGWAKLDSEYIDCLYRTEKTSEFVCKLRLCDKLFRSNKLSSKIVFF
DFVGQESFVEAIFLQNSCFQFQIPDPEFADQQFVLKTTAMIGENPDTFNLNQCLDRTVDLPDVATIVGGSNVVTDYAPAS
AVSSDEVTDPDSTSNGGSDGLIDSASGSLSGSNDRTEPATGSTASIDSGKLNKLNEVFGFNLLETTDDQIDNLKKHPLAL
AVIQNLVKRAGPQGNRKQMDRIVKATFTKFSLRHSVTAGFDFLHLLGGPSKSTIKNNFKPKVPVLNYIDKDKIRDHIKVA
IAASHFTHDSINSYHFRSIMASKVIVPADFGKYRSTQPGHGNIYFIFKRGKQIKANSIIMSLNSPVIKDRIKNQQLNSFS
TKFGRKSVRYFIDAVYTGGVGVVHVNHIINVMKIAQEFQVSWLVAKCEYLVPFIDKLDCETSRFAIKHAKSSRAPKVSIP
IDFGRHLIGAPEHGNLPFVLDDGTKIKANSIILALNSPVINDLISRRSTESITAENLSKEAVGCFIEATYTGRIDALNLE
NFRHVNKMSRMFKVSWLSAKCEEYFVSFSEMLNNESSYSDMLFAVEEAVFMSAVNNGYFLNLVSEQMATLSEFKRKNFIQ
KYTFNLNYLSSHKIDNCIAVVKSEVHLLAELLTAYWKLTENKGLNKNSRHLLRTLDMAECYQVRPDLYAEIFHILESLSE
VSRNDFKLFVSLQKQNTEHSIPITPRQVILLETPQHWNQSFNIDEFFEELSAREDITSLYSLIDGIWMKLYGMKKVNVSA
YYRVIKMTTNLAKKHGWQPVNKDYTSCSCFKSHTTTEILIGKLNSFGNIVSKSSDVKAITVLEYSQHDFVEELFHRDNMF
KFHIPGDHYAGQTFVLLSSAVKYGIPDSFDLRWCLYNPPGGHSPFTLPKLHFALVVEREELRGDAVCGGSVTKYAHISSS
DTVKMVAFEIRSAMSSKFILPADFGRHHIGAPSHNNLTINLGKGDKIGANSVIMALNSPVIADMTTTGQLTSIEASDFSR
EAVNCFVEALYTGNLKAVNGSNATEVIEMGRVFQVSWLVAKFEKCFGLIADKMESLCFHNDEMLALRLKKAPKLCIPTNF
GRHFIGLPSHSNLTFHLGNGNKIKANSIILSLNSPVIDNLTTNLHQSSLEADDFCKEAVECFIESSYTGDIEALNLENFR
DFNKMSRVFEVNWLVAKCKQYFVSYLDNLRNGSSYLDMMFAVEEAVYLKSAMKNESFLNLVLKKMLSFPVSKRRDFIKNY
LSDLNKSSAYKIDACIAMVKSNVHILAELLIAHLRQSGNISLDRNSRHLLRNLDMTMCFFKKRELHAEIFGVLENLTEVT
NEDVKLFVHLHKQSTSKACVPFVPVIQRGSFERFLKECCSIDDILDELATRKDVTDLYSLIDGLWLRLYNVKEKSIKSYY
EFVEKITNVAKQRGWSTIDRKYAEALIHHSYTQDFLHAILACDDLVSKDSNAQFVKVFEYSRSRFGSTLFLQDNIFKFQI
PGEFYLGLTFVLLTSSVESSPDCFDLRWGLFDAPPGCNVTCLPMLHFFLVELDDEGEWKCILPITWCGQPTCDDTDGLWN
WGYVKLHQKNKGNMIKLVGNAKGYVDWRCYHRVGVNSRLRMLFLDMSDDSEPGEIKSPDLSSLSRQKRSTDDKRSYKAEK
KIKLEKEFSTKLGREDYKDYRKEDYRREERSNYDRDRRDYDREDRRDYHEDRKDYERDRSDKRDSDYYRRDETGGKRSAV
RDDYKEEKLAKKRPDEERVRLSREDELREKIRTINEEINKDKKREARSPSSSSDKKEKKEKKDPSEKKKKKEAKKNKEKQ
PPSTQEKYEQLLKEKLTAPSAQSPEEVTISHTTVPTVAAKKKSLLVDSSESSDNDMSDEEMAEAESSPVPLRPTPSPSPP
PDPLAGLPPYYPALQGCRNVDHFEYLNRIEEGTYGVVFRARDKRTNEHVALKKLKMEKEKQGFPITSLREINTLLKGQHE
NVVHVREIVVGSNMDKIYIVMDYVEHDLKSLMENMKEPFLIGEVKTIMRQLMSGLAFLHDNWIVHRDIKSSNLLFSHGGI
LKIGDFGLAREYGSPLKQYTEVVVTLWYRAPELLLGSKLYSVPIDVWSAGCVFGELLSQKPLFPGKSEIDQINKIFKELG
TPNEKIWPGYSDLPAKQSVSFVNHPYNKIGNRFPPSMLSKQGLALLNKLLCYDPSKRITAADSVDHPWFHEGPLPVDPSM
FPTWPAKSEMNANPKKKIKSPTPPEGGGAADKLENSGFKFLNALQGSSAKGPGFSLKF
>Mlei_ML02196a

MEVSKVSLPADFGIENLDCPVHGNIEITLRGEEKMRANSMILSLHSSVFQNMFFNHGRTAVDLVDFDTDIARQFLGGLYN
GKVDIEQKNFREFHKLASIFKIGWLKIRCQLAFFKIIKANLAKYGDGAEEKEISDEIMKETKWILDEAIYGKVVLKDPKL
FDVSVQVFSIRVGDGRKSRFIRYYLSGFSRLLTEQLDICIKISGENVKVLLEILKDSMMDRTGMGLGKNTTYLLEHIDLV
KCRELEPDLYKDVFNSIVQMKNSLVSKTTELKKQVDGAV
>Mlei_ML02809a
MSNIPLPADFGLNKLRSRHHGNVLISLSGGRKIRANSVILALNSPVVESMFVETDQDPQLDLREYNSAAVIKFIEAVYTG
QLKLDTVLFRDVFKLAVVFEVAWLQERCEGYLERMISQAVQDDYDSMYYLLEETRHTAELTHDKTLLNIVIDKIFNSNKS
NFITRYMADYRALSKSQLDLMIDVSRDQPTLLVKIVIKHIESNGLILDEATKYILTNLNLLGCLVADKILYDCLFDLLFE
KVVDLSFDDLKLIVTLHKTTFEAYLAALDKDDDSSDKETGNLSHIGRQSTPSILKSITIDSLPIISRLKSFDNDTVTISS
NNSSLTEKSDSSLPALHTPISSRSRENSTKDNRKTASLPCISSETLELQSRSVPNLFHSLESLRGQSFHGMVALNRLLHY
DKVANLFMVIEALTYLPNLPTYQAIEALISARKKYNWARIPPKFLESFSRQPHTHFIQQIKKTLGLCSVRDTVQLESKNF
TTLHDLVRKGCRVHFQIPARLGGGDFTLNVLPASTKNMNNFDIKFQETNYHDQYLNMSVDKLHLVVKEWDRYHGNRVLTR
HVTWCGRPGVTWSGRNRERRMSWGEISEFRRVKLVVYCDIRQFSVR
>Mlei_ML034615a
MYYFRMRINWLQRSTTGLLEGIDFGVKYKSDTCHGNIIIRLKHEKTLKANSLILSLNSPVFDHLITDLGQGEIFMELFEE
PAATRFFRALYCGEIGKLTRLSQLQDMTALAHIYNIPWLTNKCLNYFLDYPHSRVTAEFIRKIEVSATWNKMTEENFKKA
AEEAAAKMPPPVKLEPLPPRPLSFMSNGKVHRVENPPKTSDSQNTLLDKVTKN
>Mlei_ML03611a
PLLAMFRSRPYNSGDAPPPLHPPTPVPAESPPPAAPGDEILLESLLITAERTGNSAAAAANEMNLERWPIFSLLDNDFTG
TIKMVCVFGNNGNEAIVVDHNDDVYAIGSNSCCCLGFGYQTSTLSPRKVEALCKKDIKTFAYGSGPHVLCITGSGELFSW
GHNGYSQLGNGTTNQGVSPTPVTSLGNKKIVQIACGSHHSLARTDEGEVFAWGYNSCGQIGSGTTNNQVTPRKVGGVLNG
LKITDIACGQTSSMALSSQGDIYAWGYNGNGQLGIGNTTNQPTPRISLLTGQVVVVKITCGYAHALALTDGGEIYVWGAN
SYGQLGTSTKCNTLTPVKIAEEYGRFVDIAACHYNHISAAVNHSGKVYMWGQCRNQSIQTPTETKFAHVVDVFACFATPS
VTPYPMSVEHSKLGSLNQSIESAFDDRSTADITFQVNGVAIYAHKAILKIRSQYFQGMFRSPFTESSQDVIEVSQFSYDV
YRAFLEFLYTDQVSMEPEDAIHLMDLANAYCEPKLKYLCERIIKQGITTGNAAHLYSAALKYKNQDLQEFCFRFALNHMT
AVVQSEAFIKLDNDLMKSFILKASEQGAFKT
>Mlei_ML03635a
MGSTTGEKDGDRVEYPTFEETWEFSDLVLVTKQRKFHVHKAVISMWSSTLKCKILDLDNSLELEIEDGESLPSDDVLQLL
QTIYPPNKPINEDNCEKLVKLANRFSIFELLRRCEDFLIKNKTPTL
>Mlei_ML03784a
MLTADTPVKLPSKKSSKKKSARPKESEIVQIPGQNSFRALDPDRGRPDDVTKSNSSGPAKQSAKQSKDGFQKGTPPTSDP
IPSEIKCTQRKPSNPLSNVPSTPSSTEKQGPCQIRNTETKPGQESLTRNNRTCHGKPIQLAASQSQTLKTTTSQTSQQAT
SETITPSPRTACPPVQCHEAWKLQIVTSFRRLFNDENSSDVVVFLGNDRIYVHRLILDVNSKLLRNQTESLHNIEGRLTL
DLLDYTEDQEVVRDIIKSFYTGVLSINRLAIKLRCEKLKELCIKKLDTPLFCTLMDSGDMWTLEPELVKALLAADDLCIE
ESDKFLQLCFHTCQLIISVKNQVFYYVSRWYEFDMEKRGEYMTELLMNVRYHLLSQEKLIGPVYDWISNVKGLEDATKLQ
LFIKINKYMKVPGLVSLKSIPEVEHVTQSTSSTRKLSPDDESLLKLIVHSVQNNNSAKCADAQQLIKVLRESFPRANPTT
RQCVIEYISDNQLVDPLNITSLLQTYSVTKHLNILKLCVQYIMSSITTNIHHVNWEQLSGEVVRDLLLEIHAGKYEFLLV
ECAMRWSEHHPSDQTTIRSLLQTICYHNIPESYTSRVLLPHVQHLLPDVRALTCTRHKTHNITTRPMTIHHTAPPEIYLL
KPFKLFTKDHIISLDKKFADQPISVKTKMRAIKNRKGRPLPGYVVLYDANRILDRYPVSAKLTLGALRQILEKAGNLQFI
FLC
>Mlei_ML03788a
MPIQMEGRKVKKTPPLQRSSRPPTIDLSVTRDKPPTIDLSVTRDKVLDDPFPLVFEKSVLKSEPPGLTEPRAAPNNDISY
MAQDYAKQILEIMNDLRKIKKFCNVVLIVNDAEILAHKSVLAASSTFFRAMFESKQAENQSASVTISGISEVIVKELIEF
AYTGNIAVGRDNVKDLLSAASSLKFLPVRDACCDFLHRHFSASNTVGIRYFALLEDCTELVQKAEDFIARNFTDVAKSEE
FFMLSFSQVCEIINLQKYPFRNEQVVAIALFCWIHHDVDARRHFFKDLFKLIKLDMLPAPFIMTSLTHNEMVNDCPETKE
AVLLTVAKLKLLSEDVSKVITLQRMFLRICLQEKKRPIKIPMTVGEVTSAVTTSESIRSNENISTHRESSGVTKRDHITK
RDHVTKKSELITVDVDQKKKGEFVRGCGNAMFVVGGACNVNMTDAINKVEYYNPTLDKWYEAGPLKKSRSLVGVTVLRGT
LYAVGGFDGISPIDTVECYNQSTNTWSSMGRMISARRGAGVTNLNDKIYAVGGYDGTKLLSQCEVYHDNTNSWEPIATLQ
TCRFALGAAALDNKIYAVGGFDGVSHLSTVEMFDPYTNTWKNVASMSMSRSVLSLRLNTASSNMSLPGEYNFTPDFTLSS
THLEDFNSLGYILLRNFLCPAEVAKALRVFEETDIIEKHGFGIPDVSGRMPRMVLWNTPGDDVTGMIC
>Mlei_ML03807a
MTEGSYEGMPLIVNDPGHASLALQAMDELRKKRCFCDVLIAVEECDISAHRLVLAASSSYFYAMFTNDLHESTSKKIEIK
NVDSQTMQNLVNYAYTSQIEIRKDNVQVLLTAAGLLQFDKVQEICTDFIKRELTPGNCIGIYNYADRYGFVDLLRAADDF
AKRNFVEMCLSDEFLILDKGRLLQLVSSNELNVLVESKVYEGVMSWVHHNTEERAEHLPELLAAVRLPLISSKYLVDHIE
KEELISENESCRVLVEAAKNHHILPEHSSGSNQVVPRHAYDSEGLYVFGGYHRTVLRTGEMYDANTDVWRELPMMECQRY
GPGSARLEGLLYVAGGHDSCFHLASVECFNPRSMEWTKAAPMLDQRFGVALGELNGWLYAVGGRDSKERHSSVECYDATL
DSWKYVNKMSFPRSGVGIGVLNGALHSVGGYDGHSDLASVERYDPRIGEWHCIYPMTHARTLCGVAVLNSALYVVGGRCG
NTKLATVECYEPRTNTWSLVEPLTVPRDGHGLGALNGRLYAVGGKTDTTKLEVVECYDPVLDCWSMVTPMFDKRNIVTLA
VI
>Mlei_ML04284a
MGRRKKTSSRSLSESEHRASKIVPECSTQATENLAVLTADQAKYLLGNPRVFTEIFQDNSSGFTILHQICKEGKAALLTD
ILQLISLAQVSTSHLEHQDLESGYTALHDAISYGNIHCAVKLLQQGASLSTPAVDGFTPLDLAVWNQFKNGFVPESKNLN

VLTWGTNCNYNLGHNHDGNLKVPEKVKRFSRDKFTSSICNVNFGKYHTLFLDKNGNVYSCGYGKGGRLGLGSESTHLEPT
LIPGLKNIVKISCSNDHSLALSADGKVYSWGINTNMVLGHPEQKNLLKPQVIKATFTFEKILTVKAAKYHSILVAADQIY
TFGFNGGQIGHMYSEEEYQIVPRTVTRLRHKPGDNLGKIAHVHTSAAATVVGFENGSIFVCNQYDIKKVATLNRSDQVMS
LNIRQGHSYSSVKTKKIRVTGGSLARQATTENTNKKSKPLLITILDTNGLVFCCIPDMFKNVLLYHWDVYNEKFLVSDVA
LGSNSLLLLTTNGEVFSCDPYPTLKSEKEIRRDHSHKTWSSASNRKKESLKMLRPIKLLKKAGLHNGTKIVCNGTGYVNA
CIVQDRISTLDFLPSVESSTFTSEISALFDNDDIYETDITVYCQSGRKVSAHKALLANASEKLSNLVQNAEESEQSYIEL
SETDESVTSMLETIYMINLPPTAKRQTKHPLLCNGMIDVNRCVKIDRNAIEELSDVVLVSEEGEKFKCHRVVLAARVEYF
MTHFLVNSRWGAVSLNQEIPVDANKRTLSDFIYYVYTDTFPSNYTFESLKLLLKLSDQYLVSRLKSFCEVSLVAFLTEEN
LIHVLRTANDYNSHNLRAVCLHIVAMNLPFYLEHRLLDHLEEELLEQLDIIYKEVVLFHLTRSPNLDYSVMEYLKYIADF
TTAVEEKAVVVKTPIKGLPDELDEYFSEEDDVTVSSVPEQSTETSAVSYDVLPSSSSGGSLTLPEDKTEDVSLESAIVEV
NINKKQKNRKLKVGAAPPVYNKQVVSGPDALTAQSSSAVVPTSSHENSITTKSWGTPTTGAVSLNRSLSEIIEEEERQKK
LNQKTKESKKAQSRLSAQNIVTQNLAKNEGGTRTSGTPWGNMGGHARCFTPRHNVNGEFWYKLQTKTFPFG
>Mlei_ML051328a
MAPKVTVPMDFGRHLLRSPTHSNLTLKTKAGGEVSASSVILSYNSPVIDHMTTTLHMTTVDMLEFSEAAVRLFVDSAYSG
TADEITRENFRDFNKISSVFKVGWLVEKCSNYFSELSESVKVANYSDLLYLFEEAAYVYENLKTKDLLKISLDKIKSFSW
KRQFFEEYLINAARLSYRKLDLVIELAGNDVEFVVQILTKQLSDLVLKQGLPVYFEYLLEHSDLSLCRQSNKILFDELFD
ILDGLPDDKMRWTYGLLKKNSKNDTRNRDETTPPNFTISAAPLLLSVEGGVIPNLYRSFNMDMSFEEVYQWLLISDEVDS
IILAVEAISTWNWYQCFKKSSPTVKFDVDLEVLQGMLEGLRSIRGWCKLPNSFSSYQCLGGGSLSYLCSTSIEDTTYHYL
TPELSLTSETNGLDALKSSTKFMFRFKHPAVASCNLSGHCGFILETVPSDLALWKIRLCTETEAYAPGRIHFHNEIRAEN
MHVFVRGVQVGLTTSIIPVIYPLTWLGFLLEEEDTLEKFISWTRSNSFRSFGVFYKV
>Mlei_ML051329a
MAPKVTVPMDFGRHLLRSPTHSNLTLKTKAGGEVSASSVILSYNSPVIDHMTTTLHMTTVDMLEFSEAAVRLFVDSAYSG
TADEITRENFRDFNKISSVFEVEWLVEKCSHYFSELAKSIKEPNYADLLYLFEEGAFQHENMERKDFLNISFHKIDSFNW
KSQFFDEYLVNASRLSTKKLDLVIELAGNDVESVVQILTRQLSGLVLKQGLPVYFEYLLEHSDLSLCRQSNNLLFDELFD
ILEDLPDDKMRWTYGLLKKNSKMVKIKSGEPSSFPSLSPIPLELDYVQPNMIPNLYRSLNMDMSFEEVYQWLLISDEVDS
IILAVEAISIWNCYRCFKNGQRNLIFNVDDDILTDLLDGLRSLKGWSRLPFPFKDYECLGGVPLTSLCLQSNEDPTYYYL
SSEIPRYIASKQWIGLLRKPTKFMFRFKHPAVTSCNLPGQCGFILETVPSDLALWKLRLCTEKEEYTSTLLHFHSEIRAE
NMHVYYGVHKYWTSKKKHYSKSILCPLTWLGFLQNNLDVVFEKFISWTESNGIGFFNNINLGVMYKI
>Mlei_ML05698a
MEFREEIYNTRGFSSFLQYRQDRRFCDIALKCQDKTLHAHKIVIASQSPYFHEKLAAGDDDAVAVVDIPKHISSSTVESV
LDFMYTGSIAIDSFNYRDLMIVQKLFEIPDLSEMLYRTLRKFIIPQNIRHFIKSYPDFTNITSLKEHFDHVFLHSFEDIS
KDLLSVGPELLNVISLLGRAELNLKEEESVHAFLDSWLSLNSLEVRNFSILQK
>Mlei_ML059210a
MASYSNKERFGSEEEAATKLEMICSPSPSHIKSEGIILISNEGTRFQCHRAILAARVPYFKQYFTFNSKWGRESLNKETS
IDVNEKTLTSFINYVYTDEFPGQQTINDMVQLLIFSDQYLVTGLKSFCEAGLAKRIYDDNLFYILQIANVYNADALRAFC
LNIIAINLPFYIYHRLLDDLDEDIVRQIEKVYKEVVLFDRNTSSNEDSDSLLYLATMVEIFNLVEHEERNESVMGKKPDY
SKSNSPHNIAVNENPGAWMLHADEMFQEGEQIQQTSGSVASPPLENHSNFTPEVQFNSTSRSWRVFNDCSKKMSMPLKHI
IEEDERIRLSEKKKNKHQVASHSNSMTEPRGWMSLDNGPTTSTRSFQEIMQEEEEARKSLAKSKIRTSRL
>Mlei_ML059817a
MSQQRPTSPPYKIREPVMYDWRGTFAQFYKLQQSGTLCDLTVQTRDGDLPAHSLVMFTRVPYFRALLEFVSTSDRQHPNQ
TDLMNYDLETVECVIKFVYTGVVQIESLSLLNKVFCLADFLQISNLRDQCEDVIFSTLTLDNCVELANFSSLYSTIVSHH
HKTKLLEFLLSRWNYVIRTDNWTELKYDIFIKLVISDEFNVKNEEQVLSAVLEWVSAEPSRLSLLTTLLQDVRLHAVSEK
VLEKLEQHPLVASTPGCSRLIEEGRFFASNVKCDTAPIRLKPRSYMDRSVCVFCIYKTYPPEVLYPRPDFSNVACPHNRE
TLSRQYDARYNAAYCNKQVNNCNVLVYSKIHDHWEQPMDRVTRSWAQDTAMATRGYSVFLLGNRQWEFGGASPSPSDEVY
DGTVSFDRLSQTWEEQGNLPFPVMFNSCGSVCGRLFSCGGLNLARSVGAYCIPTFLLSTEDGHRWEFMNDMLCARRHHGA
ASLQERVFVVGGKTKGEDLNPCFFSESWDPVVNEWVKIPNPWNNRSKLSLCSSSDRLFVLGGFNLNPEHLCYDYTWEWYD
PRDGKWSTPLPLPGKYFDFRTAYLDDEVYMIGGCTSFDPYGDEVWSRDVNVLDLRAMKWRPGAPLPLRAPPELSELDEIL
NELSLEDSPNIPIPTGTAVLS
>Mlei_ML063312a
MAPKVCVPADFGRHHIGAPSHGNLTFNLEEGVKIRANSIILSLNSPVLDELTTNLHQTSLEADDLSREAVDCFIESTYTG
EIEAVNLDNFRDVNKMSRVFQVSWLVARCEEYFVSYLDKLDSESSYTDMLFVVEEAVYLMSAMKERDFLDLVIAKIDNIS
VAFRNDFVQNYMSDLYNSSKIMIDACVAIVKSDSHVLVELLIAHLEKQENKHLDVSTRYLLKSIDLTTCCRNDPDIHKRL
FDELANVKCEQEDFHLLLTITKQVTSQGHFQDMGAAREYYQVCYPNVSAKSLSDKNWANINFFPYSNDF
>Mlei_ML063314a
MIVKCSSLCSQLMFMNNLIIIVVNASNLVGSQTMAPKVCVPADFGRHHIGAPSHGNLTFNLGEGVKIRANSIILSLNSPV
IDELTTNLHQTSLEADDFSREAVDCFIESTYTGEIEAVNLGNFRDVNKMSRVFKVSWLIARCKKYFKSYVNKLDSKSSYS
DMLFVVEEAVYLMSAMKKRFFLNLTVKKIAYISVTRRDSFVKEYLSDFGNASVHKIDACIALVQSDVHVLVQLLIKHLKQ
SGNKSLDEACRHLLRNLDLASCFERMPEVHDEIFRILENLTNVSKDDSLLFLSMFKQLTSKKPCSSGTLSPVLHLRSFKS
YLDTSVNINNVLSEFSAREDIANLYTLIDGLWLRLYCINETNVPFDSNFIQKISDVVEQRGWALICAKYVMSLISDSYTN
QFLEKIKSCDKLVLGPDGSSYATIFEYSCSEFITEVFCKDNFFKFQIPGPYYTDDEFVLLTTAMRHGNPDSFDLRWGMLD
TQNGHIFTSSGLPELHFALVVMKDDHSNEWKYVLPISWCGKPTCDKTEGYWNWGYIKFNDKTG
>Mlei_ML064928a

MRQLQPISRKRARLDGKLLLEDRNHNSVCMIAMNDFRKRNLLCDFVIHAVDLDGDVVENLIDFTYTGTIELTLVEVQAML
VAAHQLKFITVIRACIDFLKRHMSAMTCISLRQFSKTIQCDELKLSADVYILQHFREVSNTDDFCKLAYSQVWELISSDN
LQAESEDLVFEAVMTWVKYDVQNRRSYLQSLFSAIRLPLIPIRYLLQKGGNIMTWVLGELSNSRISQVETEELITSDVNC
NSLLHKAKNYHLLPGFEQSSALSSVERYDIYNNRWSYLPPMIKERSCAGATVLDNCLYVAGGNSSSTTLNSVERFDPRDN
GWSAVGNMQFPRNGVTVCQCKNMVYAIGGHDGHQELKCVERYNIRMNKWESVCSMHSHRSYAVAGVVGGEIIVVGGYNGS
INLATVEKYNPVNDSWSMLSMLGSPRSYAGCGVVDDNLFVVGGCDVTQCLNTVEMYDSASNKWSPSERINVPRACCGVSG
LYFSS
>Mlei_ML064956a
MELASPEPQKIPEVATSSRGSTPHSSSNSGPRSIVRSSSSTSSSTSGQDKYFYRDKLVFDPDVYINNPSYSDFIFKVGSS
EIGLSGIVLAANSSFMHYKIHSENCKRLDVTTLELDVDQVKEMVRPLYNGKLSLSSRNLLCFLTFSTLFEFQWIFDCCLV
FFTEVFFQTYA
>Mlei_ML06977a
MFTNGMKESKSKEIHLKGIAGSTWANIMIYMYTRDIFINKSNVLDLVGASSMLQIEDLKQACSKFLAQQIEPNNVINIRS
FAHRMFLEELKEIATNFLTDNFEKIIENEEFLDMDVFDLIDILKFDFLRVRSEYVPLFAALKWLQRDLKHRIDHSHEILT
NIRLLHFNNTSYLQRLIDKLPQQLESPEDCEKFIANIGKRCSKRRLLGALTEDSLSYPRFLLQ
>Mlei_ML073023a
MHELQLVALKLFNSFLMFFSILITIHCILTKAEKHLLTITSLKAMAPKVVVPADFGVENLHCEFHGNVVIKLQGGEDMRA
NSLILSYHSSVFVRLFLELHQSVVEMDDFSKESVKSFLEALYSGAIQLDRQLFRDVNKMCHVFKVGWLSRRCGEYFADLV
GDVRPSTDYQTLLFLFEEARFSLKVMQCDKMLDMVVQKICGLENRAEIFVEPYMINYRELSIGQLDLMLKITKSNPVSLL
KIIKKNIKLKEMLFDDMSRYLLENIDLFKCISDDEPFFEDLFDFLEGAKFSSVSDTLLVNKLYRTVTKALKNRIGAKTST
VPENQLPNVFCSFESYYGLSRSEFFEKMRISPLVQNLYMFIEGLLPVFYKIGFSPIIVHEIEVTRRERSWSRISPEFFTS
CHSVFEAALVPMLENSCDLVSVEESFKRVGKVVGPNNGISKKRSCKIEDLFFSPMSFAFHYKHPTTEICNLTGKCGFLLK
FQPETKSGQPYKFKVNLCLDQKEYPVGLHLHAGLIRADKIHLVPMFQNFGLGELKYNHATITGNGNGVSKIFTDEDSLTW
WKIGVSKVQPCSKVKIMAFVTI
>Mlei_ML073246a
MSRVNVPADFGIENLESPVHGNVTLLLSDGLHRKANSIILALNSPVFRRIFIEQEQSTVDFKMFDAGSVRLFTRFLYSGK
CRFHHVSVEAVREVREVSEAMEVDWLREKCERHLAEHESTLTPSIPVIERPSSRSSFRSESRSSRDKSNKHDLTPEDNIA
DFNYNRSVTDLQMDVSEMDLKLLHTPSSEEVPSTSDETRTTCSDESYRSRDKLRKEASRPPEYKRIRRNVSCGSGMDSEG
DSDKRSKRYRQSDSDNKSSGSRSSRSCSEFSDRGHTLQSQIITPTKQKDSRSKKLLFRDINPDLSNMFHQYHDTLEHLVM
NKTDLASVLGVLKSCREIRSLYMLFEALDLVVYNNTDISVTELDLGLISELRKRRGWGKVSPHFLENCSFYNQHFVDSET
LASERKELTSRSESVKVPDSLPPFSVRLEDLLRSVRVIEFQFKHPNIETCSKRGKCGFLLQLTGETQRNRATQRDRSTLL
HVRLLRSSRFYTNSKVHYHDEVMCPDSMHLVVEEHFMTRSATGRNSRTHFVSWRGRPFLERHGKSEMVSWGELQIPAQRH
VRLVVYYDVGGGEDKGRRKKS
>Mlei_ML07447a
MASSLPETLNVSRFFNRKDFSNTVEIIVNDTKFICSGVVLAHQSPVFEKLFSTGASFVLLQDFFYSGSEIFVEECLNLLY
GGKILVTLQNIEALTRFSLIYDVKFMYELAIGWIRDNITYLNVFELFNIGNLPEVKSRKQDVYNHCLEIMKRCDVEVSQE
MSSRLKSGKVVAGDFIRDMIRMTNCPALMGFLVSFCGQSEQNTNFVLDLRENVDFENLFLDQKELFNSLMCSMKKHTESP
FRLKQLLEIQMEVLKL
>Mlei_ML078928a
MSVTSFFGRRSSRASVNSNVSVSSSNLTSITEDRKKSIINGTNKKYLLKKRSWSILVHDTHVYVWANCSKYRRYINPFLL
HDLNRKQNFQSVPELDSSSDGAFADDDVQPGAEKKQGSPNLGRGSHYGVRSPSKPPRSPDTRRGSPDVAEVSSYEPLDDP
VDLAADKEVEKITGPPVRPEDDHDESRSQSISLNPKIDFDDVTDSDITLRSVVTADGLLREYNSERHARLLLDGIGDIFQ
ARQFTDVVVKIGETEIKAHKCILSACSKYFRAMFTTDLKEGQTDVVELKDMTEDSVTKVINFMYTGRIQIVTDGVEDLLK
VADMFDIQEVREACTDTLIRNLCPENCIKSILAVILLRLIRCLVMNHDRSMLSDQYPRMITKHIIFLCTLISTLALQELK
DDTFISFITKSTKFCIMFYSPSCGTCRASLPIFRNAAMKIEAENITNFQIAQIDVSEFTASGDRYDVWHYPTFIIDGVDL
PYHQIYKGSPTSAEELAKWIRQLGSPDYLPPVSALWVLDEDSIDDCVEERICLIQFTDSRCVQCLGRAYQIHELANHIDN
ELHGEGVKVAQVDLAKYPNLGKKYSVELRGAEVRKVFRIGKMYEYNGPDDWEGAVNFIKARLESPLKEISTMAEYASEFL
SGKVSCSAALLLPSNGNITATKDHFLTWAESTRDFCSHFLVTNNVVLESMAVESPKFVIKHSAKTLISSEPPYAEIDVVA
EALPIWFAEHFKKHQFSLVNEIDGRRITTELQNLDQYVGIYTENPSWDNVDFKEEIAKIAGKFQDSDIPFFIGDVADVSV
TFEEADTINADVPFLFLIRDKNDRIYTPNYKTLEKFGHKPKNQYLTYVTTYISQYLEGKIAPRITSDKDHGVEDGVLDIV
GTSVDRVFNRTDNDILLLMTSSNQCRSCPRFEDVLLKFGEKYENSETLTVGLINVETNRLPRKLVMRSSPPHLVLLPAAR
SGPFDGLVYFYDEVTENKLLKFVTDHGKHDVIEKKYQKQIPRNGQRGNSEFFDKEDVTSEEDPGSFQSMRISVTEEKKDE
TVNHDNLMVENPEFDKLKEKTEKMLKERFGSSNYMHGSNINPSEHKYDEIFDMEVEWDPEETESMFGRSDSSLGVEQSSI
EDRKSADDFGDSSGGSVLHDEL
>Mlei_ML08445a
MDLAGSTVVMYSEAHGNMMLKHLQQLWDDELYCDVVLKVGEKLIKSHRVVLCAGSRYFSTMLTSGLRESNQDDPIEIQTV
DPRAAELLIKYIYCGEIQLDDQTVEHVLYAADLFDIPEAVSLCCKFLLRSLTPYNSLYTLRLGDMHNNQELYNAALTIVS
QQFTEVVKSVDFLSAPPNQAIDLLGSDDLNVKTEITVIQGALAWLDHCDLLRDYKSTQRDDTDEMGETAEHQSETEAVKP
LSASEKRIRRQMLDTIRFPLLPRDYLEGLTDDIATRVCKQELKAALKERKSIPELRKPVTLRRGMHYDKIYVAGGAGSSL
AELSHPKLKMEMFDPVHNEWVKQAEPDFMTTSPHVGSLACTGDYLYVIGGSDNSGRPRNLVHKLNILTGVWEEVCPLVTS
TTKPGVCVVEGRRIYVGGGWDGSHPQSTFERYEESTGIWVKMRSMNRARMEFPMVTCGGKIYSICGNTSDYSNVAQSLEV
YDPSDNGWTELQPTIRPRWCPGAAVIRDFIFICGLPGKDRAGLREADKMYTVERFSPATGQWNIMPALSNPVGGACLVNL

QDTLYVLGGSDGTAPLASCSTAQRYDAEEQAWKSLPPMYLARVGASVAVTTRP
>Mlei_ML085025a
MNEDDPLSPMIKSSVGSIKKEIGVEVPSHAQRILRKLNEMRANCYLCDTELICKDQSFYVHRSVLSGGSAYFSNYFDRWF
PNADQKKVELQSVDPSALSVLLDFLYSGVLNITTSNMFDIFVAADTLQVEEARHVCITKLHVHLNPDSCVDMLNFAMHFF
LHPLAEAAINLVCSELLVYNTESRTSLTDASWSEIQTWIKRNRMVINPYTVTYNLLQIRPGSSSKLREIFTDMIDERTEG
LLTAQVLPTALSILSAARANVETKGEVTSNTPTCSSSETPSSDIPEIPQENKATSQTTLDLEPHEDVERAPEEVVRVDRE
ITSIRIPPLPDSWTEVAGPTTSQYFRPSRLWTISCNHPGGYGIDERAAKKRRVAIEDGTPRWVTKIVLLGGCNSTTSTVH
TIFHNIQPFSVVTGVTVVRDNLCHDILPDPGKNHEQEWEECGTLSQLCYGCAATTAGTKAYVFGGKDANSQYLAEFQEFD
MCTNTSRRLARGPQPKEDAVAVRIGEDIYIVGGRNSTVQNSQYLVDKYNIPSGKWEPVSPCFKIGYQGAAAACNGLLYTF
CGQRPHGKATDTVMCYMPHSDSWSPQPNAPTTMNSCQAATLQRKIFLLATEYKNLILYHFQPDQQLWTKINKSEPSINEP
QNFFIMNSAVVAHEGHLYVLLFMCEDVVRVIRVQRFNIGTGEWEIVSTKPVPVQNSGVVLNSFITNIKPLS
>Mlei_ML085027a
MTFYEEPAPKLSVPADFGVELLGDPNHCNVSFNLRDNQQIHANSVIVSLNSPVIQRLISDLSFKTIDVFDFQNDAVQCFL
EGCYSGRLAKINKVIFREVNKMAHVFEVNWMEKRCVSYYRYLTDNIPDKYNFLDLYYLFDEACFAARQLKTEASLNDITS
KLSSSASYRKCFVPKYLEKKAGKLDDIELKGIIKVSQRQPELLVAFVIDQLKVDKPSLSKEAKYFLQNINLLRCFRKNRD
LQKSLIDQLDRIESPSAEDYKLVLSLYKSCNVVVEKYCSLNNIFNSFETINKFTDFTELHEFLNSSPDVKSFNMFFEAVF
CWLFNHANPATKLPADLSDSILKMMSQKGWRNIASHLVKMLIVDGERLSVFKSFENAIRKCQAVSADGYDYITSSREYGW
EDLLFNENYVEFVTNELKLNPIEGPCSESGDCGFIIHITPSNEEDNTVFNIQLLQDPAAYPRHIHFHEDVISAENMHFLL
EMTFSGKKRLFPVSWYGKPNCNVNGSIWKWGAVYFIDKGETPLGFSDENIDWRWGLGRQAKISPAFLIQKN
>Mlei_ML086627a
MNSCEKCGVDLSEYFNNQIFSDVRLYTKSRTFFTSSLVLSIHSPVLKKLIVEENEKDIFLDSFLDEEEEMEMIISSFYNR
SNLQVTAETLFTVAKFGVTYEVPWIWQAAIEYVKSNISRDNCISLSRAGYRLKENFNFPPVSDICCAFLRSTKFLEEIVV
DLENIEEDELFLSLPASFFKNLLQNSFRRDESRMVNLVKRWLGRDSNVIQAMEILPLLQLSQLFLVNNTLHSEFFEFLAT
SDKLTTEDKKTILELSARSIEKSKNCMITALGFSKEVPKEMKGSQSIREFLLSPTWLKCSFDDILMVRQFRGANQFIHIE
IFLVWMETNPITESQVKAVLNSVDLSRVTKEYVDDLIAHLKKFAYPVLPDNFHTLFDAKIAAHNFEEQTIREYAAQNLNV
DNVKSLKMKKHTLSKRPCKLSRCYERTLGSVEIKIKRDCFPFYKTGRQNIGPTMLEHSHNSQVHHVYFVVESRIVSCYGH
PWERLEEMLRPDCKGCVTIRVVCQGG
>Mlei_ML08884a
MAPKVCVPADFGRHHIGAPSHGNLTFNLGEGVKIRANSIILSLNSPVIDELTTNLHQTSLEADDFSREAVDCFIESTYTG
EIEAVNLGNFRDVNKMSRVFQVSWLVARCEEYFVSYLDKLDSESSYTDMLFVVEEAVYLMSAMKKRDLLDLVTPKMNNIS
VACWSKFVQNYLSDLSNSSSVKIDACLTITDSHPFVMLEVLISHLEKQENKALDKSTRYLLKNVDFTTSFENNLDIHTRL
FDVLENIHCLEKEDFQLILSLTRQLSQPTTPAHQQISKEKQHSESMSESKAVSVELLPFSNDLFKATHQMKVRFDFCQSE
IPNLYTLVDALWTWFYEENYGYQHEYQHKHQHVYQDEYHDDSQDESQDDFFENELENFPDLESVFIKVNKHHDWDMLDYK
YVDKLYRDTYTDILLASLRRCEMLTPKNEELGKAIISHYYDAHNFVMEFFLQDNSFNFYLVGDPIFENQKFVLKTTAMKD
SCSDTFTMKYDFADSNGKLDLPKLHFCLELSAGESDTVCNLEPYLLPLSWCGNPTCDVTKTYWNWGYIQFHDKDMEDIEM
ITVNEDMKNGGCYHNVASKLCRLVAFVVS
>Mlei_ML08886a
MAPKICVPADFGRHHIGAPSHGNLTFNLGEGVNIRANSIILSLNSPVIDELTTNLHQTSLEADDFSREAVDCFIESTYTG
EIEAVNLGNFRDVNKMSRVFQVRWLVARCEEYFVSYLDKLDSESSYTDMLFVVEEAVYLISAMKNRDLLDLVTPKMNNIS
VACRSKFVQNYLSDLSHSSSVKIDACLTITDSHPFIMLEVLISHLEKQENKALDQSTRYLLKNVDFTTCFENNLDIHTRL
FDVLENIHCVKKEDFQLILSLTKQSTKPTKPTTPAHQQISKKNKHSESTSESKIVTVEFSHCMN
>Mlei_ML094328a
MASKIIVPADFGIENLNSPVHSNITIEMSEFPELLVNSLILSYNSPVFLDIFRDTDMRVPVLDMKKHNAAAVRKFIEALY
TGDVRLEKSLLKDFSELSSKYKVAWLILRCHNYFYDLLETVSSSIEDMHLLFEEACQIKLLIGDSVYLNQVTKKILSNGH
KISDFIELYLNKDYLGLTKPQIKLILSLNPLNYQVFLNVVYKHVFESQSFDETSRAILLRVDLVRAIEADVQGYQTLFNL
LLVKLQDVSRQDRRLIHRLHMDALKRYKANHPEDRIIKSEAVSCCAKAFKTLKKMGSKTELKLLPSKYHLFQCYKIFEDM
ELSDFVDVLALSSEVKNLFMLLEALDMFAAWEKFNSATLHKVINCIHERSWSKVPPNFLRSLYWLNNCPDKLDMLQGCTQ
LISHNTHSVIISQQTTTPRRFLLEGGFYKFPFHRPRTTNCVVLSTQCGVLVKTEPLSHDGEKKFALQVVKEQGLYSAHDM
HFHAQLLLPERMHFVVEMYNEGEEDGIDMEIAWARGKPEFREEQIYWGGWNWGDAHLARMVLYFDNS
>Mlei_ML095317a
MSEITESFPSCDVDNEESTYSYQTYKTEPPLGEEILTKFENRSALAQDLNLLSGLPELCDIIFLVGEDKQPVCAVRAILA
ARSRVFRKQLYSHVPRRCSLKDTSPRSIKSKSKPPNTIVVPEFEPSVFKQLIEYLHTGVCTLQPRTLFGLVNAADHYDVE
ELKQACINFVGQSISTDTVCHMLSNADRYIQYKSTKVLIQKVMEFIDRNGDEVLSLPSFSILPKHVVQLIISRESLAVSE
VNKFVAVYNWSKSQYEKEPCLAINCDEKVVKLSDIVEGFLDYIQFHKIPIHYLMNEVRQSGAVPDTIIMMAVAYQMDPAS
IDVSKFSRSYSH
>Mlei_ML110512a
MSQHYYTNNLKDRLPLEAEKSWQGNLSVSLLPFFEYTDEYADICIVVGGEKIFCHRLILSLNSTIMKQCEPWKDSDQNEV
YSITLISEFEEYPQILRKIIRSFYCGEISLSYENIKVVYKFAKCYQVKWLTSYAQSMFGEILEAENFVDLFAFAEKFGKQ
NNKLILVCLGHLDSQLLKKLYLLESVRSVFQFNFYCLKSITSSDKTLLSPIEIFRLITNWAEFELENRRCHVEILLFDIK
YHRIAKSDLVDFVYPWVMHTKGIEDTLRTSLMKLITKRIYESGQREDFFQKGNLVSDITTMKVDKLLGDIIKEMETKVTS
GDYYPLPKSTVSAEEVYFVLKLRYPTANPYVKRCIIEYINDNKIIDNSIVTDFIKLYEENREDDLRYILLKFLNGKSSIS
ALTSVQWEVFPFEVVHDFLKDVALEEREYVKVECIMRWSGGVHFEEEKLDKLLSTLVCYYRLPEEYTSFVLRPYLKKILP

NYNLSLVCGVHQADSMDHCEETTIQCVVKPLSKEFSLLLDAVQQLQECKKWLQLKINASSPLQLLTKPKTVNVDSDDYVM
FGEENELYENCCSDYLLFDLKEPLNSYPIHSDVSPSLAELRELFATHKNLHILVMWIPNDAKEGGETRAPPLPERLSVPP
LPPRRITA
>Mlei_ML11323a
MTFKEYKNLNKYKLKIHEKMSIRQSLKFMKENGNLYSWWKVLDLDGMSVVPIVYTDTSDQFGIVENLLTWISTSKPTYND
ENLVLKMISEAFVKMSKNLTNNSLVNYANAVTGSLQSFDIEKKFPVQRTPSHFDVSGLQRIGWFVCGDGLVSPNESCGSQ
CTSVEHITLTGYANVSRFRSASSSVFARNRKCNTSEVHVEFKMAYTVKLDFNTDFTEFLAKDDYNDLICVVTESETIKCS
SVLLAQHSSVLKEYLKEEKELFLTDNKHVRECLSILYGGSVELTEENFQDILKFMVSFDIPSARDQVLNWMSQRKWNLDN
VDLLIGASMTAVKNYSGNANSESIREKVYCPSRLFFQNSLDDIISSGNDDERYRSLDSAMRYILSCVTDKKEFLVMLMHQ
DLIPRFIPWIQILIEQPHYPVFLNSLQKAEILNKMSLCTRTQFDGLFDKIEEFDSMTLREYKHLSKFKLKINEKMGIYQS
LRFMKENGNLYSCWKCLDVDGLSVIPTTFTETSDQFGIIENLVAWLFANNGCSESGREQVIKMICNAFKRLSENSNNDSF
TAYALILAEEFKKILNINSTLLYSYTKPKPVQTNIYGSCHYQMSYPLSITQWTIGDKNSVLARGRSFNQAYNPAVVPKDQ
ILFSGSMRQYSKLNVTVKIFQDRIPEVIFKNDNEIEFNVISNRKFHAYVYACKLDNARIDWNNSHRWETLRVVNVPTHLP
LYTNPAEAFQTLKMYPYLCNYRIWKTQQQQQYQQQYQQRFNESAIQFGILLV
>Mlei_ML11571a
MLITLSPLGNLSRYIRRHDKVSVHETALLVVEGSSVSCSGAILAARSPVLERVLRRDYEVVLEGFQGMIPQLNQCLEVLY
GGAVFLTHENIIPFLKFGITFQMDELVNLCCVWLQETGLYDEMGLLLEDNIEKRLKLLEKLAPRVRQHSAGSAGSSHSSH
SENLDHSSPSTSPDSSSPPSFSPLRLPDHEKRWSKAPEVQNYSSSEARPKVSGDVSLVPLRSKMRSDNEREWKPASVKKR
IGARHYVAHDETRFEEGTTPAAIVGGDSLNNLDSINNSTSSQKGWKMAFSKKNKPPSSKPSPVPVSRPCQTSPNLSKWRS
YTLTDVERLCSSMCVYPEFVKLEVVVSWVTQTQNITPNSRQELLTRLLGDMDPLLLSDRYIGMVQEDLNCSHGLSLPDNF
SNNFGDVFSDEKSGSGCKFLALQWSVSTAEDLTKLRSKVTVSGKSLSYLSFFGDCGLCSKFGKQEMVLKMVDGTPCYDLK
TNNFRKKYPSSGVSGNHYHPDTVFHWFIRTKISKKSCSMVSLITTEYSRVLDILNMNNMFKVVCLQLNKKLR
>Mlei_ML11573a
MLISLSPFGNLSRFVKQGQRVHETVLLLLDGDSDSIPCSGTILAARSPVFEKLVRENYEIHLYGFTGFAEQLGQCLELMY
GGEVELDSTNIAIFLKFSVQFDINELLNLCAVWMENNNLFRESEQILNEQDKSKCLDIISCINFDKSTFDRNSYQNGFSS
EEEYIDLEEICSSFSETTIKTEQTNGHTATIVSSRGRLAAELSALATPTHPTEPAKYPNSQQYTNEHSQNNQQYQSEYTN
GHLASNQYKTNGHSKRTSPSLEKTNQAGEWTKVPTKTKLSVLNKTETETISKNPVTSFKPNLGKWSQFSSEDLLMLGSPL
CPYPDFAKLEVALAWLANSELTSHQVQVLLDQFAENIDPLNLSDRYISLVSNYLASWDVSFPSHFKPHIRSSYITDDPGS
KSKYFMHLWTLQRGDLAELRELNYVSFFAKCPLCDQGTGESVLKLVKETPCYDLKVDNYRRKRHKHGIEYPVHYHPNTVF
HWLITTGSGSKPILHSLITTSLQGLQNILKKHDRLIITCLQLKSR
>Mlei_ML11637a
MDMSGTSSVQPTLRTLVSYSSTTSVTEIKHEMYAEHLLNNLHGDRSMETCDVIIKTKENTSIFAHSAILRPTNMYFRKNL
DFRLCKAGRYTIDLSHLSATALQDIIDYLYTGQFNIVNKKFLSMVLTLKELEMEEIIDQCMEKLKTSLSDKNVFDILEQS
TGDIGVLNSVAMTYIEDNFKTLYKNDRILRLNWDSFHKILSSDRLNVENEDKVYQLALAWVEKDDIGRRSHVNEICSKIR
IPFMTDYGLQNMVCNKNILTSNEEFYNNVETLLGNPSALQIPYYQRVNDKMGRIFVFGGYLNRSHITTTMEVYNRRTGAW
ETIEEMNMTNTPPSIYSATMTSVAVDNKIYIAGGQSDSGTRLDTTFVFDGILSIWASIKEMPTVRVKSAAARFGNYLYVS
GGQKDRHTMNEVIRLNIHRDVWETVSPMIHARHSHGMVTAGQKIFVAGGDSGTGPVATAEVFDKGTGQWSEIASMSNARF
GCQLVSIKSQVYVTGGWNGRRALHECEKYNEENDSWGYISPMATRRYGHTSVSFGSKIYVLGGQQVQHEDAIASVECYDS
VENTWTRAPDMGNARAWAAIGAVM
>Mlei_ML11854a
MSDHDPPVTPNKTNITPCNFGIKYLNNAEHSNIRLVLKNNADILANSVILSLNSPVFDRLTTQLGLRTVQMDDFEEAAVW
LFMESLYTGKMEEIKEEHFRDLNKMSRVFEVMWLSAVCVDYFEHRLRSLTDKDFDGMVFLFGEVCYMEEFLKDSSMTHLV
IRRLQYLDTGDCYVLRREEGTIDDLRTSMTIYANSMKEFCERRQNIHSVASRTIPNLFRRSWKEIRAFMNPISIACQDFL
SIAQLINQIKHEADDMYMLISITLFIFTSFFKIYRVSSQDLHKKSINELVDKVIGQAIQQLIEIRQEKGWGRICPEYLEI
NIDKDDYCSENSTENIKIIVNKFVNKLKESEISSGGTEGVMLIGSRTNDVYFEEFLTIRRKPGKCGFILEVNPVLPGNPD
SFNIELCTLPEEYKTDVHFHDKITADKMHFALISKVGESAETYMITSYLGRPSWNGDNISWGSYRDLGTRSEAKLVTPVV
FYDLT
>Mlei_ML124210a
MNMEISPLVKGGLAPLKLPKSLIKFFRDHEIPQIVILELGSVNEEKQSHSRFICNSVILALHSDKFERVISAGSQVIFLE
GFNDETGVDTVRQCIEFMHGESLAVDKCGDLLGMLRFAENWEIPSLRNACFNKMKSKMVNLQLIPDSEFFDEQTFLKFKS
VLLDEHGGSKNYVTQSNVDHRTSSDKIPKHLPGQSQEKRINIKCDDNASLLQRAIGGSSNSSSSDSSSGNKSSKPQNGFK
HGLQKKLIADSLKLVIQCSYAVKDYPKKIQPYSLYSGLDIIAQMVSTKTNLSSKNLVKLLSPFFKNSSVAACILEDIRDI
AIKKCSDQSNSLQKVLSTAMDTGKRVETYSVSKHFHSVSEFVEKLTADKRINFHLDHGILCQICHGGTNSICGNSASKIA
RKQINCVLKNLDIDFSAEEKIDFISDKEFHGEHIIHCYLIGMSTKNKPISYVSLLQLRVAEIVGKIKDKVSRVKVVVVFS
RKEPASSSPFRGKLISKCSG
>Mlei_ML124211a
MEYFEFPKDLSIFLSDPDIHQVVSLNVSDKHNEIISRIYANSIVLACHSEKFRNIILDGEEDIFLGDLKPSTFDKRAVKR
SIDFMYGKRSAVSNANHDSLVKMYQFAKIWSIPSLQKACSDCVKKHLSCDPTLVFTYYKRLRKLDWFNEEIWELVSELVE
NNSDFLIEHILKGNEKKAKKMDDLLKKELIKSSRSASCGKFLMFLLECKTDNKFVLDNLQSIPKTAFLNYSEFLLFKKKF
SPESESKLNEMATFYENHRADPPSFSCKDDNKENIVDIVLQSNFHNVTCYDKAKDSRNITNNLSNQIGEDMPSLQFESNR
DFDRLNLSKEMGIKESWEDDSLEEYELQSNNDSAESPSKYVQIASDNQPTEYTSNEKVIPDLRNCTEGERDLLLMCSQ
>Mlei_ML13025a

MGTEHNRLHQLKLLLTNAKLKEPPGLFTKGDPYAEVTVDGQPPHRTDPAKGTWEPCWNHPFDLLVSKSSEIEIKVHIKYR
FKNDLYLGSNTVNLAQLLRQNASSLQHSNGPVCNIDKHLTLFDKSHNNVGAVFLTFTIAPEADTLNSLLSSLQVATTSES
SSASGAHSQENGGTEEEEPLPQGWELKFDQNGRRYYVDHNSRTTSWSRPKPLPAGWEQRLDPRGRPYYVDHNTRTTTWQR
PTTQNVAEFQSWRRQLSDNQEANRQNLENRYANSDAEPSSGFTGTGSSNSEEPLPSGWEKRVMDNGRVYYVNHETRTTQW
EDPRVQIKKASNLPLPPGWEERYTGDGTKYFVDHNTRSTTFQDPRVAISATSSGIHYERSYRWKFGQFRHLCSRASLPTG
HIKISVSRSSVFEDSFNQILNIPPQDLKRRLFISFKGEEGLDYGGLAREWFFLLSHEMLNPMYCLFEYASKNNYSLQINP
ASPINPHHLTYFKFVGRIIALALFHNKYIDKGFTLPFYKRILDKPLTMLDLKSIDEDFYNSLKWVKENDLEECGLELYFA
HEFEVLGKVEIHELKPGGEDILVTEENKEEYVNLMVSWRFTRGVEEQFKSFLEGFNEIVPIQWLQYFDERELELILVGIQ
DFDINDWQEHTIYRNYRRSDKQIQWFWQFVRDISDEQRSRSASSGKRSRRRRSASFGSSSTHHHSSSIDYHSDSEASKML
DEMQYFREKKFFCDITIVVGDKEIEAHRIVLSSCSPYFRAMFTSDLAESKQDKVSLYDVDPDAIEALIDYAYSNKVRICE
ENVQELLPAARMLQLDYVVDQCGVFLEQHLSIQNCLGFYLFADKHHCEQLLATARRFIQRNFTEVYMKGEEFVQLPAESI
IEFLSMDKLNVSSERVLFEAVMKWYNTDRTNRVSHLADVLSYVKLSLLPSSILLGEVLDSIGDVVDVKIKDLLMQAMRYQ
MQPRYRSSMQHFGTIARKKPDHIATMMAIGGGSQFTTHCECECYSITDDTWYRVTQMSTPRARAGVLELDGFVYSTGGYD
GACHLLSMEKYNPSVNEWTAAPSMTIRRSCVGITELDGIMYALGGYDGYTCLDYMERYDPYMEVWMAAPVMTTKRRYVRA
VTSDKQIFAVGGFDGTSHLSTVEMFHPDREKWYTMVQMHSQRSGAGVIALDSCIYVVGGNDGCTSLSSVERYDTREGKWR
WLDPMHTSRSAVDIAVVDGALYAVGGNDGCSSLNTVEKYDLVAGQWISVASMSMRRSSVGVCIVDIPIRILDSVKVRRSE
I
>Mlei_ML132514a
MASNVVVPANFGKHHLKSRTHNNLELLCAEGEVQRANSLILSLNSSVIEDLIARLEITSLDMQDFAAPAVNCFVESMYSG
ELENCDRTNFRDLNKMGHVFDVTWFVERCLGYFTELVDKVTGRCYEDVLFVAEEAWFVEENLKKCELMDLIIGKLSKLGN
KKSIFLKQYAADLEKISINQLKMVVKIAEQDVGILASVLANHLEKCGASTMSDTTRFLFENMDFVQSFSNRRYFDITHNI
ITRILNDSHNTEFKSAVKYISKLSPPEKFRGINSICGIFRYPVPLDIAIEDFLKLDWYSLCVFLSQSDKIRNLYHFFDAI
YMWLFIARGDLDEKVPRLDFSDHNYVMEISDIAYDRCWGKVSRVYLELLSSSDVLGTDRLIKSFSNSDELSCENGTIFES
KQGYNFNMLLSEENLEIQLLLRGEVKVPGCTEDSKCGILLKCSLLNVQETVKSFQLEVVRGLVNEKNTHIHYHNVSGSTQ
TVNLLINVCHRESEDITPIPFNTPPLSYGDGSWHWGFHYYPVDNQVSSPLPCRYRFKYQSDVLIKFIACLSANNY
>Mlei_ML132517a
MSQIDLNQTKPNVNISKGHGLGDCSIFTQNSDSESSKNQSQSCPKEEYEEVSNSSIEKCGRTEKSEKSQSQRTNNDRGRN
RGGGRRGDGSRRGQEKNKKHSVSSSGCADESESPKNETCSSQFRNDNSKQARDNHRGGGYNRRRDGQWKAHREKSVPANR
PVRQNSSTMSMEYPFYYSPVLSNNNLSPRMHQNMPPHMSQNMPPQMLQNMPPHMSQNMPQRMQTNMPPHMSQNMPPQMPT
HMSQNMPQRMQNNMRAHMQQKMPQNMSQRNMPHYMQQNMPQRMQNNIRPHMQQNMHQRMQNNMPPHNKPQQAVHHSSHKE
KKANFISSKDPTRKKVSKSKVNPDSVNDKKERPLPSRENAGKDHQKVTDSQTTSENDHAGTTKIDQKEKENKKDSPPASP
ASPASPASPASPVIKSNQGLDSATTDTTAKIQSALKMEELGPSQGDQCVSFNSELSLDEICHELKIKDDRERLSVPLQRT
LSLTMQHEPKLATDMVPMNFGIELLDSAVHNNIDLLADGGAVIRANSVILSCNSPVLHRMTTSLELQEIDMKEFSEKSVR
IFVEAAYTGKLPDIPSDIFRDLHKISHAFQVGWLSKSCFAMFTEFSLKIDEFLPPFIEQVFMFEEAAYVMSKIKLTHFRD
LAIAKIQSYEFGRKIFILNYLKDLSSLSACHLDMIVLLAGSDIDLIVDPMKDLLVKQSVQKGTVLSDECKYLLNKCDLSP
CRRYNRKLFEQLFDVLQDLAGDSSEDMKWVLELHRKSAYTCEETLKEEEDAREDKNSSSGNDGQTIKKSTWFWRKKHVKK
RFTTFFLSLKNAHFSTTKQYRNVLMKISLLGHLFDLRSDTCLKCGVSYKICNNHACVNNIGGQSNAFLAENTDEPQPRSV
YERRGRGDRGWRGNNLKSSIFKTPDLSVASELPKNRASLPQSSHENMNREKVNRRGRGRGPRQDDQCQVNRRHDHDKSMK
AFDPSLYYTPVFQVPTPDYSVIRSPPSSNVSVLQGLSKLCPDGNDIFNPFVHPPSVELLNHQQELSHTNQIYQGTTFNYP
DKSKQDTVPKILSAMQIPIAENGQAPIYYRLQPSDSGETADEVNGGQSTVVQGKAGGVTPVIKSKKKGKNKKKQVLVVPT
NEELNPVTLAMEIDLENKQEEIRPEHHLRKPEVHQGNLFQSIPVQKETVISEVKCERKSKKGEEPVLPASTNKILNSDWV
DIKPLRSPCPSLKLEPDENIDHPLQENDESVSSKTLRGLTLKENQRRLSVSLHKMLTLTSQHKPILVTATDMVPMNFGIE
LLDSAVHNNIDLLADGGVVIRANSVILSCNSPVLHRMTTSLELQEIDMKEFSEKSVRVFVEAAYTGKLPDIPSDIFRDLH
KISHAFQVGWLSESCFAMFTEFSHQIDEIVPLYLEQVYLFEEAAYVLSKLKLVHFRDLAIVRIQSSESGKKVFIKKYLKG
LTTLSACHLDMIVLLASSDIDMIVDPLKELLVQQLGVKGTAISDECKYLLNKCDLSPCRRYNSKLFEQLFDVLQDLAVDS
SEDMKWVLELHRKSAKNSEEVEKVVMDQEKTAKEGNEAKISTKNKWFWQK
>Mlei_ML13533a
MAGAGWSKGNINHSREMSVQLGNLLKDMELEGDITITCGSRTFSCHRAILGARSKYFKALLYGGMKESRPGAVIEIKDSD
PDTFEILLEYLYSSNVKLSRIPTTQVLQLLELAHRYTLSLLEKDICSYLRTQINANNAAEICQLATFYNLVQLQEHCLTL
IDMNPSDSLTNIEDLSYHTLHAIICRDSYPLPEMEIFNIVRSWLKEPGSSVSETEKQFLLNDVRLSLISPEDLFLKIIPT
ELFSSEKILDALRDQTLMNHVDLPHRGISSTSGELVSSSVMGVVSGENGEVLFTQFECKADRPHCRHMIGDESGITIKFY
YPHIINKISFLLPCNKEFNRLYSYRVAVSINGKSWKVVANYEEYHCHGWQHVILPSPKSVVYIRIQGLWCSTSKTFCIEQ
MSVKLDRYSESTSVDCNGVIEPADNVATIERGAIVVEGVSRHRSTLLNGNTTQYDWEDGYTCHQVGSGAIVVLLNQPYLV
SSISLLLWDCDTRCYSYIVSVSKVQTDITYFITFRLSNCIQEAKGFYKEKFETK
>Mlei_ML13872a
MSFVFSKSCVHAKDVLSSLHELKTQKSFCDVILVVGKLEIYAHRVVLATTSTYFKALFDDGFLEKDTFRIELHDVDGEAV
NTLIDFIYTGQFEITMRNAKSLLAVSDMLHLLDAKEACCEFMTHNLATDNCLEIMLFSDRHSCLSLKTAAVRWGEEHFSE
LVESKRYLMLPADALVSFLSSDRLNAVCESQVYECVAQWADDKVDENGTLSSLMLQYLDTLLDCIRFPCLPVPFLVETAS
NNKVLLQSPKFRSLLDKTKTLHLMINCCPGYDTVKYTSPQKPRVGCYTNIVYIIGGMKNIRNWRCLQSQELTDLPSCKMA
AAVAKLGRYIYVIGGRNKDGLVLSCVSKFCTYNNTWTTACKINECRANGAAAVLDESLYVCGGYNGYITLSSVEVLAKDG
LSWKQLPPMNHRRSDFGLLPIKNRLWAVGGYDECNGAPHCVESYSVSSKQWSVVSPLFTKRAGISCCVLDHLIYVVSHNT
LERYNTQQQDTMVEITDDEAALYDRQIRLWGVDAQRSLRAANVFVKRCGPLGAEVCKNIVLSGVNSLTILDIRMVEERHS

ATNFLIRTATGKSFAETCVPNLQILNPLVKVEYVSEELNEDTIKQYSVVCITDGSKTEYETTNELCRKHGVTFIATNTWG
MAGFMFQDCGDAYKYVAEKPKIARGKTSEKPDTKRRKVEEDSSEYEEKIANFSQFGKLAPLSSMKHSSPALLIADGMLNE
VADLSEHCKTMCKKLGGKEVPEELIRNVSGELVPICAIVGGIVSQEIIKLVSKKDDPIVNSLFYDGLSGAGITENLK
>Mlei_ML14119a
MDYKKPRHGETLIEAFYAYKQHELFCDATLVVDEEQIGVHRAILAQGSPYFQSLFDSRFAYDHIAQVVLHNVESTAVRTA
IDFIYTGKITIKESNVENLYCVVDLFQLSDLRELCVDVLEKSLDHNNCIDRLRFSEQQDIERLYNAAIRVSKSRFKEVIE
EEAFRLMPFEKVYSLISADDLNVDEERVVYEAVMKWVNHNQERRKKHLAELLTHVRFPLIPPIYLLEIVAREKILLEDPV
CRQMLDDAKDFHILKGSTGAVETSGPSPRQGVQSGMVFIFGGEGTEHTVDCYNPRTNNWSKRGKMRAPRREASAVCIKNK
IYILGGENDKRGILNSTIIYFIDSDKWVASTKMRYKRSGHGSCVINGHIYVTGGHNGETILNTTEKFEPETELWMTLSPM
NMERCWLGVASTGGHMFAAGGYDGDCVLDSVEVYLPDNDKWSYVAPMLLPRERLGLCAVRGTLYAVGGRRGRAIADCECY
DSRSNSWRKISPLLAPRDGMGVVVNDKFIYAIGGFSVKSHALTVERFVQKKNLWERVCPMTRLHDLAAVVAT
>Mlei_ML141120a
MASQFWRDNCGDRLSMFINMEKFCDACIQFGDGSSSFTLNCHYVVLSLHSDVFRDNIEEYPMKGSKRCISFSKYSQVEQT
VIADIILSFYNGILAIDEQNFKTVYKFSISYKVSWIQEQALVEIHTFLKDTNVLSFLLLSEETRCKKLRGELIRFIVQTR
LSDYRRIMSSADLHSLSPTTLKTFLKATKQLDDRPELETLQGVISWMGDSVVRLAYINKFFDLVCWERLAGSESLVEALT
TRLEEELFPKLSEEQLMYVKKCLQDAANFAACSGTMKEAMAVFSASRWTEFPLELAEFVLSNDFINATNEYVIAEAALVW
LDDQERSSLQAADIISIMSTIRVDRLHGTYILKTLQPHVMKYEISPNDWIQSCSRSHVPRKNTDNTASGSVVSLTVPTDK
KGKELLALKNGIYNLMAVCKCSYHCTGGPRECLTVTITVNNKASPPVSVKKSQCDFCNRMVLHVYCENGNLSKYPFLTCG
SSTRIKQVLKQSGLSFYAVLSEAEDRSGGVRRSSWYNTPVRTVVRTLDNNNVSSLTEYELLEAALDWLDFRMPDKTYHEV
VLSCIRLNLLHESYLKDVVIRYLKTNYFIKAELLKSLLKSREQERRGCSISPCYKKIIVCRVPLPVVELKHGDHVIYEEC
LCEVSSFQGEIYSGTLTVKLSVRVKQIPALTITAKCSKCNISLRHAYLTSTSRLTSFPGLATASVYRLREVFTACLQRNE
SSESDVSTPSEVSYSAGGTGSREIPGLIPLDKLALNLILGP
>Mlei_ML141728a
MIAVIRRATHWNRKSQEKELNYYQWQCISGIQTGTGNGIETDTGIGIKTGTCDGVETDTGTGIGTGTGTGIETGYGTGTG
MGTGIETDTGQGVGKKLRGRVSVARCSLTSVSNNKPPQFGAGSPLCRDEENVFVPRGSVSNQPAEQEVRKFPSAALQNRT
SQVNKEWFNNCPLSTHCTLNHHSAGGGGGGVTDLMVTSGQAELTIPCRTDDSSEARMERQVRCREKTAQTSKVFKAWCCT
FIVLIAVLILIPIIATEILNRLLNSEELTDVKIACGGETFSSSQLLLAANSEYFKAMFLNEYEEDAGSSFANFTAIIAGQ
SLASPFGESRERSVSIKEVDPQVLKTLIDFCSTSRITITQDNVYDIFNAANRWLDYPLMLVQTVLGSDYLTIYNESQLFE
GLMSWVKHDPGSRSSHLTELFACIRLEEISLKYLYHVISEEKMLLENEVCRNLINLVKDRRTLPLSLASVDRESKHTLKR
TPHIHYEDFLDLVCTEDRKCLMRLRSSSHRFNCETGRYVTDKELATSNALKSWYKRCEFCVSDDTKWLTHLPFCNIIEED
EHHILISCPKFHQLRTELHEETKSLLLRNEDHHYLYNQPHIYRFGGYVRKIFNQRFPKTKKKKDTK
>Mlei_ML14391a
MNTDRGCGGVPKGVGERWPFEMEVSGEDDSGEEECYFVKNHELFNGVSDYFNNAKLSDVTLIVGNKRFSAHKFLLSCVSP
VFKQMLDNTSNWEESKESTITLSELPECVDTFELFLQFMYSRRVSMNSNTVKPLFLLADKYAISSLQELCSNFFKRHLSA
STVLDVISFANKLHNQDLSDRCLKYIAENAEEVMYTAGWIDLDQSILIVIVQRDDLQLWNEIELFSAVLRWVLADPKRDA
LVPQIFHLLRFPLICYELLTHLVEPNDIFQRCPEIQYLLTEAYKYQLLPEDQREKYFANNIRCRPRQYNFVELGRYAYCV
KNYSKIKNLTRLKTDTFTTLACMGGQPVPAPPLRPDGTQPEYPRWSWTLWVEPKGDADIDTGCNLAEDETHKFGIFLMPV
NIKNTEAEQREIDCVFVVFGRDADGQPMGHSVRDSYVYMSNNCIGFGEPDLFQKDDNNSQSYIHNDEIDILVLIGYRPKR
VESPQVASRSSSMVQ
>Mlei_ML14893a
MADLKDAVPHNFGEKYFNSKLHSNVEVTLKDGKKIKLNSMILALNSPYLEAKLEETMEDGKKIKLNSKILALNSPYLEAK
LEETMEEKLEEKLEETAGRNLKTIELEEFDAEICREFLKSLYTGKFETIDRENFRQVSKLSYTLQVGWMKENCLQFFNGL
MNTAPDNTAVDTDKFLLEEAVEAKQFDKDRQYLDALAQKKSSNYCIQDVTITEKMLEDLTNLSIDQLDFFIRLANKCNRK
VANYQQRCNIHGNEYHNKEYSTIIVQKVTEHLVKLEKIDSRTRYILETLDFSGIGQKFNNQGFKSSGYQDQFKMAASKLF
QGLLNLKEISNDDMKMTLKHYMSLGN
>Mlei_ML14965a
MMGISEQNVQYDCKKTVASSSITLPLQKNIIKFFTSFDLPHVVCLRVNNSRFFVNSVLLVLHSSVLEEIIYSGIEEIILD
ESLHLPGIDEIVQLCLQFIYGDTVNISLEQVPSVHQFANVYKIESLLKECEKSFLEQVCNVSEYVKVLPKWCRPKRESWY
KTLVLKYSCEIVQYVTADESVPVMLPLIRDESSSHDSLIPLMLPSHEMVCMFYAKMLKKVSEPLDAVQLNLIKNKYARVS
KLPQAASCIDNISCSKIDDDTDTNLHKPLSKKGFVDKQSKVCKSYDSLQGVKNQDFLKLATEDRKNLVCLTFKEVVFLTK
SVINNSSYKYLIIDIALTWVFLKNYKLSSACYDTLFTSFESGLVCREFLCDVNEVLCNMGNVSPNYYDDNGLKDNVFTIS
SGVLNQDFVVNKLKNNGCIYLDGQDCKLNSCKVVGHKLSLFVLLKKEPNDSYRVVPQPSSCDIHSNKILHVYLITLDINM
YVDSYISIKVLTKEEIVKICKQYPYFVLKVIFQNDPTSVRYEKINLQNEETPYPLQNTNEGYHEVFASRNWQNFSFSKLT
KFIRTGYCKEKMDEFKKYNLLTSKTDFIYLCIDLFFTWIILGNSSFQEIFYQPLICAFEHSHISAHFIKDLKSIFKSALP
ISVPVVSKSGNKDSILVSVKLSKFALQNSIRNNGMISSGEFACQIPTCQLGDHTLCMQILLNKSSNDLTVSLQSTKKYPE
IHKRKVQHFYLLALNANDEATQLISLKLLRKSEILQSVKKFTKFRLCIIFTK
>Mlei_ML149644a
MPSNSASTDKNKTLLKSSDEWQKNLVARLSSLQVDDTTSDVDIIVGAHKIATHKTVLLLNSDLLTAGEISHNSRSQKMEI
ELLPEFSEMFEIVKAVVESFYTGKISVTHKNAKGVYKFGICYSVEWLTEQTFPILKEMINPQNFAELFDLAQNVGCKSLT
TACLQKFDEKLAAAMAPTPEWLEQDYYRVKLISLSNDVQIPEIKMFEIVCTWLDHKPLERSSLSLSLLSNIHFELIDHTN
LVNQVFRWINGSKSIDDTVRLSLFKIVSSATQKNKLGYPAGFSRSKFSTKESESATSSPSPMQFLNSNPMSTPFSFGKPK
PFNSSFTQF

>Mlei_ML149652a
MDEGDLNGGDISDSPTISDARTLSDSEADPGRASDFFDYTPSQSFTAPESYLEECHLVMNDLRRDRDLCDVTLIVKNEVF
HAHKVILVSRSPYFLGMFKSNMAESQQEEITLTGLDANSFKLILEFMYLNKIKVTESNVTHLLPAASLLQIMPIVEACAE
FLKDKLQPSNCLGFQSFADLHHCEDLKKQALQYATEHFWEVCRQEEFLRITAEQLKCLVKSDVLNVRSEEKVYEALLKWV
KHDVDARHQYIPDLMQCVRWSLLPQSYIAKEVVNEPVLKSYLAVKDMIINALIYHLDNHPDTGKQNRRFFPRHPSSLVVV
GGKNPTVRDTVESYSAKNNSWRPLAKLTVKRSSVGVAFLNGYLYAVGGFSVTSRLSSVERYDPEKNEWTLVAPMSVCRST
TGVGVIGGKLYAVGGRDGTSCLATAECYDPETGTWTPIPPMTMKRGGVAVAVIGDLLYAIGGYEVTCSSCTNTVESYNPE
LEVWTRCAPMTCMRSYLGVGVVNNFLYAVGGRNGAQYFNSVERYDPLQDIWTAVVSMNTRRGFVGVGVLDGCLYAVGGCD
GKTNQMSVEKYDAKEDCWTEVAIMKSRCSSLGVATLG
>Mlei_ML15421a
MEVKQVRGPMDFGIENLALPNHLNVSLITANSGVVKANSLVLSYNSTVFKRIFEEQELRHSVDLIDYTRFVIDKFVKILY
SGKVDFERLPFMSQIWKLADRFHVEWLLKQFDQYGEEYATLERTEPAPTEEYPTPLNELNNSIPEVHYHEMPDFQESFEK
RRRMSLQGPDYFKEIRPYHRDEEIATSSSTSARSTYNRTVEHMAIVEHPRSIYKRTVQVTSVSVVERPIFGHSLSSSGRF
RDDAVEENVIRTHP
>Mlei_ML156511a
MAARLRNNPDNLSLGSFGRLCRSGLGVLTSSSQGSLGCPDHVPHASSLLSNMEDLRQQEKLCDIWLEVEGEKLPAHRVVL
SACSPYFCAMFTNDMKESVETCVTIRELEFSALKALVDFVYTGYLDVNVSNVQSLFQASNLLQIHSAMKVCCSFLESQLH
PTNALGILNFAEIHSCQDLKLAALKFVDAHFMDVVEGEEFPLLPSNQVSDILCRDEIIIKSEEQVFEAVQRWVSYVSSDS
DITPAERQVACPRLLNHVRLPQLSPEYIMSKVEQTPFIRKNLACRDLVDEAKNYHLCPKMASHVISQERFQPRLSTCGQL
YVCGGIDSSWDAAAVESVEVYDLRANSWDLSTPMTSKRGRVGVATLDQHLYAIGGYNGTSYLNTGERYDTILNSWVAIAP
LNTPRRSLALGVLGGPMYALGGYDGASSLKSVERYDPQSNCWTKWSKVAPMAVSRVGVGVATLNTNLYAVGGHDGRSYLN
TVEMYDPNVDVWKEIKPMRQARGGVGVCTLGARIFAVGGHDGSAALRTVECYDPRAGVWSEMASMDTHRFSAGVGVIRDI
>Mlei_ML17371a
MANRKACYGSNKAFAADMKKMVNNPLLSDVQFVVGEERKQVFGHKSILSPRCEVFKAMFAQASDSKPKLENGAHVFVLSD
TQPHIFLAVIEFIYTNRCYLDACNVTDVLTAAVEYGIEDLKKLCIDFFKSTLTVDTVCEALQAAISYNNSDLKKTCLGFV
ERNTLVVFKSRTFVEMSEESLSYILQSDKLKADESEVLNAVKEWCTVNAVITGQSMKDVARKVIGHVRFSLLDPEYLSLV
EKENELKPFIPIRLLSEAWRSHALQDKDSNNPQLRPRAGTHPHDSLARKLARSQGCRTAED
>Mlei_ML184422a
MGEQTNLEPLQSLSLAGSIWMPDASILYKEPNSSQSLLDTLSDFKENDLLCDVVVVAGNTKFACHKVILAARSRYFNSMF
LSGMTECNQEEIEIKGVSPEIMAILIDYTYTGQVTIDVCNVQDLVLAADLLSFINLRTACFNFMLNHIDVNNCMGLLNIA
ERLDCKETITSSLNYISRNFEEIYTTDEFLEIPLQKIKAILSSDELNVQSELVAFDGMLKWVKHDLDSRADSLAELLHLV
RLNLLTVEDLIEKIECEPLIMSNADCNRILHNVKNHHLMPHKRRFVKLHGVPTNSDRVPRKRIRPHALFVCGGIDSKRIF
LKSVECYDVEREEWFPCADMLTKRGSHGIALMNGKIYAVGGHDGNEFLDTVEMYDPVTDVWTSIPNMTRARNGAGAAVIG
DNLYAIGGRDKQSRHKTCERFSHERGSWQQISDMNHGRAGAAVAALNNRIYAVGGRSNDKTYLRSMEYYDPETNQWNIQK
PMESCRYFFSMAVVNEIMYVIGGNIYHENDDVEHRPVPVELFDPQKEAWEMLDIKSDGITLRSVCVLENKVILAGGIKNG
AMLKTVHCFNTSSDEFTELPSMKTDRGGCGMIAFTPSDMVSSPEIPASTSSHTTL
>Mlei_ML185518a
MKSEILQMVIDFSYSGEISMTQSNVEDVLRAASFFQIYSLRNACCKYLTQLLATDNAIGIYCFAEKFSCTQLQEAAFRYI
LHNFGLVSIGEEFLSLDAETLIKIISEDKLKSSSEEQVYTGVVRWARHDLQNRKDELHRVMSFVRFGLMSRDKLAELSQG
EESLLLIINQLSSKSYPLRHPHNQPRDPQRLSRRIFIAGGIAAGVHSALSEVQSYNPDTDTWENLTSLTFPRFGAGLVSR
LGSLYMLGGHTGSSYISSVEKYDFEGNCWVTLPGIYGPSRRYFSTVGVDGKIMIMGGCDTASYMDTAAVLDIPSKQLIFA
GKLNEQRVYCGSCVLNDIVYVVGGYGMCRQFSRLNTAERYDQLTGCWEPIASLKEPRSGLGVVAIGRKMYALGGFDGKNY
LNSVEEYDPKTDVWKTISPMKHARRAHGTAAINGYIYVYGGFDGTNFLKSVERYDPCLKIWESMKDMPLAKAYFGSTVL
>Mlei_ML190426a
MMAPNVSVPMNFGLKLLDSPTHNTLELKASQGAVVRASSVILSFNSPVIDHMTTSLHLTSVDMEEFSEEAVRYFVDAAYS
GLSPPISKELFRNVNKLANIYEVSWLVSKCVEHFIEFADSIKQISYEDFVYLFDEANFVLNWLNSRDLIKIAHTTIRSLD
GQKSFIGKYLENLTAMSSQQLDLIIELAGNSVDFIVGPLTEQLTATLENGEAIVPVNCKYLLENCELHLCQINCTPIFVK
LFDSLEKICEESLNDTKWMFNLYRKATEKRERFSEGLTTINSDSVDSVVSEDYLELQPRICDSLTDPVIPNLFHTLDFSL
KFEQVVDWLGESELVTSLLMFFEGIWAWMFVNKNVKVKCTSHVLENIQRIQKKRKWNFIYSRWLNFIKSTNGSSERFFTV
IRNDLEKKILSSSWEGNKWGVIMDNQQASHSIWSIFEKETKLVFLYRHPSVKECLEPGECGFILRTIPANDGANVVLCTD
SSDYFQGLHFHEETKAKDMHLSITTERNGCKIPLSWLGKPELKNGQVRWNMLSSLRESRLECWFLVIFDLEK
>Mlei_ML19911a
MADLIRLAVDPIGLRRTQADFWKECFQDVPPKTNCITSNHLNTNKLCECDEKFWIEIPAQYDLTPIEVTSGTREHFRILV
RGYCHIAHGLLQPTAITANRYERDEVVRAVGKEMGFTNPFVTVSHHRAKRELLKLDEAFFKRQFKVGVVYLKEFQSNEEE
IFSNQPDSPRFEDFLRLLGKRKRVHVSCINSLTILLRGSATDGPTVRFRPSCIKSHFLHVFIVVRPVSGGGYNVALVAKN
GVQLFGPQCSPDYTFQRDNTFRDFLLCKVVNGQHAAYNASKFKIMIERTRHQMLDAVFERYENANKLGKDLENEYISSTM
TELCDVVIVAGKKKSLLFGIRAIMAVTVEEFNCKVMSVVLQYIHTGSCYIVTSHLPSLIAAADRFDLPELREACLKILPS
AISFDTIIDMLNAMEYFIQYKSVKSMLQKLLEFIDQNIRDVLILPSFCNLSEHVLQLVFARDLDIDEIAKFNAAIRWGKN
FCAKQEGRDLKTVLQQLVNKIQFHLIKPRELMQVVYPTGVVPDHKLIVALAFQADPRSVEGIKGAPVLEHTV
>Mlei_ML200244a
MKRRRHDIVELSSDDSLDDDFVPAKLKSKRVSIVLSKNPVSLTKLKTRRNASANNCKNSEEVQYSTLAGSIKTKRGRGRP
KRVDISKITSLPRHVETSEKSENQNVKLKPQFSDLDDSLLIESLMPGSSKINPNQGTLSEDTSDSLKQSKLSISDTLNST

ENATVDNAELQSLKTQNNSYTPENETETRNVSSLFQSNTDTDTQSQPGPSYEPQTDMRALNKKSKEVTSTRQSQADSLGY
SFCQICQKNISGYSTERRRSHVNKCCDQLERANNFGGSRSSVNSQGGRSVPSRVNCPLCPKNYSDKNGLKKHLKSCCIDH
GKCFETVCEVFLSAGKNTVEIVDGKKQTTKKSKSKEKKPTALQLENRSAINAAQNKILDIIAEQDTQEIPATPALKISHL
TTYRSKRNSIWNVAGSATTTVSNVLAHDKSLINTPPPQVSLLESVPPLTASVPDDPICLEKSSLPSQADITHLTQFLTEL
GKDDSVKERLTSLMLSPSKTSQKSVETSQESVKSSQKSVETCQESMENSGVAENKTRGHGSLEKKVIQVQNSLKSKPLSS
PSITKKPCKISSVNESRKPLLDPSENQNIDVEESDQTTENHSFDNEPEPKMENENVADISKSPHVTLVTDRSPNPSEISA
VDNSTDVRTPVRGTSEMVSSIRDAESSLPDQSSVQEDSNLDLFGEEAGPGTRREVYDRSPDRSLLNVSPITDSGINEVDV
QTGPNGKLKDKKRQISPIYRPIVCSSTTRDSVAITVPVALSNKNIQVGKETKSLAVGTDLRTRAKASNTNMVECRSVSCN
TATLDKAAVQCQADGDNRLTAMMQRLSQHTPSTDITLLCSDWKEKRAHRVILTVSCPALLDCCDPQSSEDNPVVKLDIGS
EAADMFLEYVYTQKIESEVVGTVVLSELFDIAERLDMTELTNHLHTKRTETIELEFEQESGVRILLNLSAQDVVNLSQQI
ELVSSQNKSIVSLENTFQQDIPSFQDLFNRTESGVHILDQDLFNGTGNPTSPVKGGVSDDKEQEENNENGADGSDCDDLP
ASGITFGAENLYVIDPQKYRLFVIVLVKSMWLLKRPFQMTNLAPGIVLDEKEEPTAMNPSPPSPEMTEEDRRASQLIQDV
EFIDLVKKLDVPVVDSPLSFIDDLSPLNLTTDKDISLINLDCQTPGNPSGKPTGTSGIYQSTPDISKNRRNVRRSLLDTP
SNLELSEYNKTPNFKGMDTPKLKAEVGKYGLKPLSKKRAVAKLEEIHRFTNKKFLKCPLDTDTEPTSSAAKSNKQKERTS
RSSSSKQSTKERKRRNSSSSSSSSEKNCPVGDISVSEKLPKSASREVSEQVRSVIRSREDLHCQVLTYTPIDIIEIKGLLK
RRTSVGLIAQVLDDLGITYFNSETKAKGFRKGNKKPKK
>Mlei_ML216319a
MEIIFGNTSVITTIKSQNAMCWIITTMVLCSLMDLYFKEAMRIVVSPIKDETATKIRKPSHVYFETSLFILPSDFGIENL
DNPGVCDVTLYLQENQLLRVSSTVLSYNGSEFRRLFADLLLRSLQMDDFPVVVVRSFVEAMYCGSINVTRSHFRDVHKMS
HIFGVEWILKRCDDFFENFCENLQEGSYEDFLYAVGEAVYAEQFTKRVKLRAIVITCCAKFSASQRNEFLKRYLENYVNL
TDLQLRLMCDIENSGTLAADPIIALLLLDKLSSPDAVMDCKSMFLIRSLDLETYCSSQNEQKRSVFQSIFEVVVKVDGCS
IDDLQYLLRLHQKSFSLYVNEVDLSVNKALIAPNLFRSWEPYMHDTPGLDALINGLLNSPSVCSLYMLIEGIVVGFLSRP
YDFNEDSGKELIQRVAATMIERHWTELPGNFLKSLPYSGNRNISKLCEILRLSDLVIDRSSMAHSAVCVAVSDSTPTMWD
FLTIPNTYNFHFEAVEFKKGTVLSSPPFQVTVIPVEVDEADEARFDIQLKFEGISHTHLHKEGTPLSGSIHLVLEEWCTF
ADQEWMNIGLLSWYGRPRYTHVKKNAYILFSGAGFFNPTYRAKMSRPVRLVAYYTVPFRTQECLLLEY
>Mlei_ML22139a
MNDRESSAIVNCRVKEQTRNESLDGITLDSYVQSLNCWTKLPESSELNVGLSRTHHTAVAWKDSIFIFGGCFSNDSTSEL
AGNGNGLNDMISFNITERVWKLVSPQTRPPAPRYNHTAVVYENSMMIFGGFSNKINKNDVHQFRFDRQVWTPWVHISGEQ
PNPRSANCAFVYDAHMWIFGGYDGRNKLNDMWMLALDDDSPTWKKVEQHGQIHPPVLHAVCAVIDDKLYTFSGLCGVSTS
NDILAFNLTTKTWSIVLPDLINNCCAAPDRRRGHTMVSHGKCLYIFGGQNTNSRSPNDLFCFDTEASAWHIVKSRNKPSK
QVPSNRSYHAATIIDNIMFISGGVITGNKDKSDRGSDNFYCFIFASFPKSTLREDLSGLLDSRMLCDLEFVVGEGPRKES
LYAHISIVAARSPILRMKIMDQLNELCDSKNHQNRDHILSVLDLDNSERVVLPFPKVMPAAFRIVLTFIYTDRIPHSVRE
DDNSIVTLTDVYGIANYFQLSRLEKICITYIQSYISIENALSALKVAKDRNLTELVGVLESFIIRDQNFKAIISREEFEL
LEKSSIIELIRLKTSTVTAIRSPHILSDTGQQSLEEDLQALLKDKKLSDITLTLTKDGIARRAHRAILIARCKYFRGMYR
SLPPPQDTVGIKIGELEPTPDAFNSLMNYIYFNDTEIPPEDSLYLFTADKFYGFSNNQLQAYCKKSLETNIKTSNVIEIL
KAADQVHSEDMKCHALKMIAEHFTLVQPLPAFRTLEKSLLLEVTDYVATHMKGTRVTPHSTPKHGSKV
>Mlei_ML26321a
MSMNEEFEETVLIKIRTGEHIEVPKHRLIKDSAKFRELFVTLNFEEHEIDDFSPEAVEIFLTLLDTKVLEHLELNMFREL
HKLAVVFEVNWLTESCRVWLKGLINTASTREEKTYIFEECWYIADRLNNEVMMDVLICVLAHKDNRALLSDYLSDISVLE
T
>Mlei_ML267921a
MENIPKVVVPMDFGIEYLGSSSHGNLRFLLDREELTANSVIMSFNSPIIKKMTIDEGQKTIDVEEFCKDAVQCFLEASYS
GNLEKISKSNFRQVNKLAHKFEVDWLIDRCFKYFQSLTDTTEEDNFDDQLYVFEEAMYVLEKLKKRDFVADVIRMFTSKK
GCTQHFLNIYLEDLGSCLFENLDVILEMMSDEEHVLAEIVVKDLENNKSSLNQNSRRILEKINFSACPPDHLPVYEKLLE
KLENMENPSPDDYRLIFKILKQLNQALHISKQAMSFVELPNLFHKFDHLKKLADLDGLLTFLTESPIVSNSYIFYDAVYS
WLVEKQKSEGAPFVAIPETFMQKFADLMSERKWKPLATEYVKHNLFRNLGGFTEKILKNPKLNTQNYSRIPATSEYIAAE
LFARNHDIKFRFKAPGSVQNCSSDGHCGFILRVTTSANAGTSTFNIQLITDPSLYPEDIHFHTESLLTNKNIHLVIDITT
SENLKKYARPITWYDKPWRGTSRLWRWGAHYFLSKNHFSEVTSPASSSSVAPPKLAHAVIGRHGSHAKIRPVIFYFGDR
>Mlei_ML270529a
MYAWSESLISIEHRVFEVNLQFVQLTQFRLTTYIQFAIVKLLSISISPKRSRHVTDFPGSDWSTLGRVTSSCSMIGIVLH
RTPVFAGSARPVLLLLCLMEGCLLSRVSESYGASSAADQLQSLLDNKSLPLMFPTTPTLKMNDVISSLLQYKADLCSGTV
KMEPHSPLYSPDSKAPKQTSSPEKAGVTEPGTLSISTAGILPNSSSLNTTSTPMLSSSSSFLPSSSSTGLFSSNSSLLPP
NTSLLPPNTSLLPSSTALFSSSCSSSQLPRPSLLQNNTILTGTPLIPTSSLLPSNGLVPNPSLVPTSSLLPNCSNILSNS
TANILQGPNSLFTGSNSLLNSSSLLNANQIIANKMCDMGKKSDKTMASPSTSNPTVNNGVKIFQTKQGYTLLICNGYRDL
ELWRIIRYHVKNLEHEVFVPSSHQHPTLTTRTSVRTTTCDLLTELNALRLTHHLCDVALCVNGTLIYAHRVVLAAASQYF
RLLFLANTKLLSYIELRGVDPESVELLVNFAYTGTLNINYVRVTEMLTAANLLHFTPVVEKCCDYLRRKITPQNCIRMRE
FVTLQGLTKLQAILDKYICNNWNRVIEHEEVYDARPETLLFFLGNDDLVVQNEADVYQVIIRWVRRSPLYRQFYVVELLR
KVRLVQLNAQFLLDNVESEELIRQHRECETLLHQAKNHFLLPERDHGLEDYHFRKRKRKQNVCLLGTENSRTIAYEYDTT
RNTAWEASGASDDSGFFVIGGKGPNGVLSDKVEHISLTSHAVTTLPPLPHGVAGAAAFSTGDSILVFGGIAAGGASDYVF
VYNKSIKLWKAVASMPEKVESPSIFRNSDEMIYILTGSNKQFRILSSKLYDEYNSTQPFNFECKKTSHETITKKDMPSTK
KKIVTLGQVEDTIVKKIEQIKAKKNKSGPVVLSDCKSSRVNKESFKNKKKGPLKKVEASNKNNEAGDTDSDSDIKAVKPS
AKLMLNDVIDLGGTQDDYDLLQNLSDNEVELEIDSSVDPNFNVGELITFMKSNGFKTFKKVKEKNVDKSAEMNDKKPISK

TCPTAPHSPTPHEKLLFKSNEPWYTNIRVNKTTVPFIGIDKWKLYAEQLWKNEVDLSAKLRDKYQSSDQQWMKTVLSSGT
INDKVSAHCLLLQESPVHHIKSLDALLGMAKKKSKRMAIQSIDTLKEMFLSTILPDSRKLFTFEQHDFSILTGENVNLIA
LGWLVEDKLKSCYSDYLEIIKAHLNDNEVAIRTKLCNLTFQLLNEKPEKEQELLAILVNKIGDPDNKLASKIIYILTQLL
QNHPHMKKVVASEVERVLFRPNISERAQYYSICFLNQLQLSTEDSVFAAHLIEIYFNFFKESIENTNVDSRMLGGLLTGV
NRAYPYTASQDAITKDNIDTLFKLVYSDNFNVSVQALMLLYQVSGGKTEICNRYYQVRKKHVVMFLL
>Mlei_ML271524a
MNLDAKAHHPPDSFFRTLDSSVKKNSVFVKKLSSLTEQNRTKLVADLNSLNLTKYIEEVVNAICSAKLKMQDMNCAIHLC
SLLHRRYSDFSQLLLKTLQSALSNLNKEDKVTRVRIELRLLGQMLVLGIITDQKEGFTLLHTTLCKIVESDKDRLEFLPA
MVSFVKHCGEDILGVQSTKNRQLIEQHNITVPAMKILAKDKQQIFAGLLKEYSSAVILTLVKLHNKLMKCLKHNKKTLLV
KGEVSAEKIELSEKLSAQHSKLLMNATTLAELLDITIPDLPSLQQLEDEEMDCNSPTNANADMSEVSLWEDEDQKTFYRD
ILDLTGIVPQILYKKDKAKADQKAKEEESKKKEEKSEKDGKDDKEEEITEESIEEEFKKLNIEEEDDDDDDEEEIVVTET
TQNSNNKENPDDFGTSTITISNKAMMESFISKLSNISNRDLVNEAAQEFCLKLNTKSNRKKLVRALYGVSRNRLDLLPFH
ARLVRTLSPILPDIPTDLFTLLKKEFIYHLRKKDQNNLETKVKNIRFIGELCKFEMCEKTDALWCLRVLLVKSSKMAVPQ
NFGSEYFDSEHHSNIEIILKDGSRVRLNSVVLSMNSPYFKSIIEEKGIKKIEMDGVDAKVCRRFLKSLYTGVSGVLNRRN
FRQVSKLSFTCEVAWMQLECLVYFMKVLGCNDDNNKFLLEEAIAAKDYDKNCQYLEALVQQKTQSVFDQDIQAMGLMLDD
LNSLPVDHLDFFIRLANKCNRRQANNGNPNYGNYFYGELPGAYGETYGEECPSEECQSVVVHRPDTVGSYSSQAVIPRDR
LLFLPKITSLTFQKVRPPLHEYIRKLLIKDLNKNNIEKILRQFRKLPWNDKEVAMYAVKCLCRVWNVKYNNIYCVASLLA
GLAQFQETVGILVVDNVLEAIRIGMEINQPHYNQRRIAEAKLLGELYNYRMVESNIIFNTLYLVISFGALPDSTNPIDLP
QDMIRTRIVLTILETCGQYFEKGSLKKKCDIFLLFFQKYLFTKVQPLPIDVEYAIQDTLESIRPNITMYTTVAAVDMAIA
EISPQLKNLLGNQEEAEETEEDEDTETEDQGEETPDEKEEEVKVNKKLEVVKCEEDDEFIAELERTMTSELTGRKMDSTV
NKVNSSDLALPMNRGFKGHKIDGNSVQFSVMMKKGSKQQFKKMDIPVESEFASNLFDKQKAVVREREEMKRLVLSQEMRQ
EEEELMAEQMDQHKSSGVGYRAGERKQKPRKAPQLTTDMFNFSSGK
>Mlei_ML279816a
MITPKRSDNATTANRSEPVIHPLRQWPWRHKHIENTLKNPNFTFCRTDINLIVEKDAVEYIGEILKHPLKKLSHELKHLS
VRFLRTSKAEVQTALRLIFHDNLSHLALASGVHALSLFVMSMDKYKFSKSVRCLLSFSVGHFFRWLVDHNISKRVTDCSV
VYLAAAFQCLTEELLTRTIQTKVTADSSKGKQDFVLTREVLQSVIQHDADLARVFGVEFARGGEVGDDGPLPGIPQQAAS
DSPNLEDSMGALDMTEYFEDCGCQHCTDIKSPLDLCCVHSLSVLRELVSKQQQLQHHQLQTYRRTHGNVTQPLSWSNNAF
HSLLYYLNQHNESRTCNKITIREWIRVAGIYAAHHLSELIDEDDISQAAKFLLGIDYPPRRYGLEDPLCLSHKLNYASSF
NSLPFKLLTCGRNDSIRQALSAIGPDGINSHIYQGMTPLMCAANAGDYNLVHLLLDADAHPNIAVPVPLDNTQIIPMENR
RWTALSFAVNQGHTDIVKLLLSRGAAVEGSMNNPADITTETPLQLAAAAGDSSIVELLLEKGADPFLSSSIHSSGSTPRN
RGGSNAIALAVAHGHVEVRHKLLQHQPSVKSNDMLSLQEILAEGYLFQNNRAHGIRLSRLRKLQEALYLACEHGYLNIAL
ELRSIGVMWNLNFWTRCLETAYEEENLEIVRYLLNDYDCLRVDEFSNAFCKDGIVLLFEIFQNSKEEDIVMAACKMLQHC
YGVFQWKPISNFTQHAAPEPRIDLNYVNNKEFSDVTFCVEDKMFYAHKIVIVTGSERFNAMLSKHFVEGSSAVVEIPDIK
YSIFEIIIKYLYAGSVNCVDIKSSIVLDVLSAAHFFMLDELQFYCESLAAGSITKDNVITVYQKAKLYNAHDLVHSCLHF
FLGFLPELIKKKISCFCISIEGHLKDSSSFNGLLIQPSITIHSFLVICLSQPKKIAEVKPNEILQSSTAVYEVLEFIGRG
TFGQVVKCLRKDSNELVAVKILKNHPHYARQGQVEIQILTHLSSESSDKHNFVKALECFKHHNHTCLVFEILEKNLFDYL
KMNEFSPMPLNHIRPILQQVLTALIKLSELDVIHADIKPENIMLVNANLEPFRVKLIDFGSATYTSRALDNTYLQSRYYR
APEVILGVPFSEKIDIWSLGCVIAELFLGCPIYPGGCEFDQIRFITSTEGLPPKEMLDVGKKSRQYFVPPNSFSPNWRLK
TEEEYSLETGTRPKESRKYKFKRLQDLMFFSATDAVTDQDKFIEELDRFQFVELLRQMLSLDPNLRCAPTWCKTHEFVTM
NHLQDYVQCKRVRQGIEVMQRACRREKPINRMLLAQQQQIKYLQALNQQVQQREHIQQQLKAQQQHDLNWSLHTGPSTSL
QYFISDNLQTISDRQPGIYPTPDWSPASADWQQPEYGSKSHPIVIPDSPNTRPVTSEQLLQEHLYKAQLLRNALKQSTQH
SAPSLDLSSHFMNPVMNPVTLPNSLNLTGLTNALTNRYL
>Mlei_ML30558a
MTISLPLHFGLGLLKSSTHNNLTLEASEGETVLASSVVLALNSPVIDHMTTELNLTSVDVTEFSRDAVQMFVEAMYVGDV
QKIARAQFRDINKMAHVFKVSWLVSICERDFLRLAKEIVVPSWPDLSFLFEEAAYIRCNRNCDKLKEIALDRIKDLEWER
NFIKWYDEYNSNIPQLKLDMLIELAGTQVDCIVLPINKYLALVETGKIPKEVLPEKYRYMLDKCDLSYYKLGREKLFEKF
MNRLREVPAESDDYFKWLFDLNKKACSTSKVSLQVARKPRSRQASKISLGDRSVPRTF
>Mlei_ML32434a
MAPEVIVPMDFGRRLLSSPIHNTLKLNTSKGGMVYASSVILSLNSPVIDHMTTTLHMTSVDMLEFSETAVKFFVDSAYSG
TADELNRDIFRDVNKIANIFKMSWLVEKCFEYFNKVADSVQTASYPDLVYLFEEAGFVLEHLKSKHFAQIAIKVIEKVGF
KQQFIEKYLERADRLSKHMLNMVIELAGFETNCVVKAIAYQLKEQTSVQGTSNILVSHRYLLDHCDLNHCKNSDRVSFDL
LFDMLGYLADKVDDMKWTFQLYRKSSNNSRRKMQSESVDTICLANSPQEMRTEPVLAKGVDSVLPNLFHNLNMELSFDEL
LVWISQSELIKNLLMAIEAVWTWSWYRRLYTKKVYSVNHSKLTSELLELREIRRWKFLPSDFMKHHVCLSDQLYFDREPF
CLTPQDTISSVIIDCIETCSEPLTLLSKEAKLTFHFQPPSISQCNLPGECGFILKTVPDETALWKLALCTDDEDYDNESV
HFHNEFMAEDMHIYFHRNAYFYPLSWLNWLFSSDAAQQRIDWESKFRIHDAHSRFKVLYDLVVPSECSLVRDPDSKSRFR
EIVSKLRWDTSLDEAK
>Mlei_ML398335a
MTKLNFIKMRRRPTNKQQEINDLQTEMSDVTSDTPTHSRSLTPDDITIISDISDISTTSSTSTQHTQFNLETSPQHTQDK
QQTPAQHWSSQLLTSLRSLRLNLPPDCHVIVNTHHIPVHRTVLYMNSSLGETASFNQESGVYEVRVEGDQVRLVTDILGS
FYTGELEIEHQNLESVYRFAKSYSVGWLQKIVWEKFAGYITAEPGCARFVEIFKFAHTVWCTQLVDLCISTLDNTRVMSL
LAKPDLILQLDFFSMRSITTSSELRVEEKWVFKLVNRWFKEVKQRDQLDNCHLGYLLTSVQYHLIDKPYLHEVVFDLVLD
STSLEDGVRRMLLKKIRDAMKYTDENQTARNQLSGDTSNKDDDLSSTVSKAVPKNQRPIRPDNLIRKIVGIMAGNIPGGD

RSVSANEIDLLLSEKYQNLEPNIQDLVVDYLATRDRISGSNCTRCLQLYGSTRNRKLILLVVNYLTRDPGDYNEIYGIGW
GEVSFETIRDMIQDTGIAEIFHQHDSPEEDYWYPNYRRFPNCTQVELIMSWSVAHPNQTSNIASLLNTLCYRSIPSKYRK
LVLLPFVRLLLSDKTELKCGRHKHKKTLSSHNYIGAKGPRELYDDDEEREESVELVRIGDCRYNLDFSEGMLKLTSRLSG
EDNRDQDAAQHKFILFDALAPTETTQYPLFTCNLSLARVRETVGKSSDVFVMFVKRRGMAT
>Ocar_g52_t1
MELRSLGVPWNLHCWLHTVAAADRARKLELIVMLLRDFKSINHDEMGEEFMQDGISLLFSLFRKYNEEALYRELADTICG
CYGDLPPPRPPELPPDANAFKMPFPRIGPEFVNNPEMSDVTFIVEGKPFYAHRIILVAASSKFKXXXXXXXXXXX
>Ocar_g98_t1
MVSNGGLGFAADCDGKLHYTSSNKIYTLFPNGEKKFVCGSGRNKSIDGQGLHASLASPRCLRYDARSGCLFFTESKAIRK
VRVGSPKFQPYSDPKLPLDLARLIDSPDMPATDQAVFLVEERRIHVVKSILCIRSEYFHTMLRSGWRESQNEQSAPIPIG
DASYDAFYAVISFIITGVFDVEKHRSILQEIAVLADRYLLDDFRKLCLQHLKRGVSKDTVIEYLCLSERYGFGELKEACM
VFAVKDFRVLSQKEEFKSLSKETLVEILRRIR
>Ocar_g160_t1
MGDSQKRATEASSTASGRSLGKIDHVKKLSNDLGSLLDASRRSAISDVTFIVENRRFPCHRVVLAARCPYFEALLYGGMK
ESLPGTEIPIHDTTANAFAQLLSYVYTGKVDLLNGSDVTQVMIHDLLSLAHKYQFVDLQMSIADYLEVGLTTETVCYTFD
VASLYRLDGLRRTCLTFLDQNAVKVMQTDSFLDLSLSAVEEMLQRVSFFAAEIEVFRGVQRWVERNAVEVGPEGVASIVG
CVRLALVSLEDLLIEVRQSELVTADDVLDAIQKQTIKAESPLDYRGLLGEPWHLYSRYKHCCTAY
>Ocar_g248_t1
MQASSSSSSVPISIEDASYEAFHALITFLITGDPIMKDVLVLADRFLVTNLREFCMEYLLKEIALKTVLKYLNLSDQHGL
VSTVARNASFSDLWGIVTLPNGFILAADQQKHCIWKVNPTDGTVSSFSGMPGTHGYRDGPADAALFNGPAGLTVHGPRVF
VADYRNYRIREVCEGVVSTVAGDGLCQEQDGYCLQASFYQPWGIAVKSDKKIFTSDINGETVRLINVGGRVQTVAGLPGQ
YGHQDGPCKAVAGLPWEYGIGNLGAIYNQATFNLPDQIAVGPEGSLYVVERGNGSIRRIKDGQVQTILDGLWCPNGIAVD
DDGNVYFSDFTNKIYKFKIGGGTEILCGTGKRKSVDGQGEMASLNDPRHLHYNSKSSCLYFTERRAIRKVIIHRKPFSDP
QLSLDLAKLIDSSDAPPNGQAMFVVEEKQIK
>Ocar_g521_t1
MDDYHDPDHPENCFIQMDKLRHKGQLCDVSFSVRSHLFSAHKLVLVSCSAYFATIFLKHDQQYAMPIHLDDMEPEAFGEL
LKFAYTSRVRINEANVHSLFHAADLLRFNGVKEACFRFLKSRLGPDNCVSMWTFAQSHRCVDLTRAAFSTIETHFVETSR
GLDFLELECDNVVEILSSDELCVTGEQQVYEPVMAWVHHAPQRRAKDLWTVMQHIRFTMLDYEFLLRVVDKDDLIKEDEN
CLDCLIEALDLMAEAAEEEGNHRLSKVCSYTWNARRPTQAIDEIFLRKNGRALGFRIMGGVDRPSHVFRHGDKPGVFILK
ILPGGAAWCSSLRVGDRLLAVNGNDVQLVTHNDAVKCLSQAEDPLTLTVRHEPPPRGLKVGRLTVEMGTFFTFWTAMQEV
NLVAKPGQGFGLSIRGGVNGYAGNPTDPTDEGIFISQARANIHVTNWIIQ
>Ocar_g854_t1
MSLPPSPKRSKLSETRIPIYALAVLRQMNEFRRKRHLVDVDLRIGTHRIPAHRLVLASVSPYFTAMFTGSMKESNERVIE
INHVEYHAVELLIDHAYTCEIELNAVNIQPVFEAASFLQFAEVKTKCLSYLERNLSASNCFGIHRLAFQHGCRNLEKNSW
LFALENFETVVAEDEFLSLEGSDVIQLLRDDSLNVQKEEIVFTAALRWVHHNVSERSKELANLLKSIRLFQLPWQFLVQN
VFTNDLVANEENCKNIVEDLKKFRLSQKTDEKHLKPRSSFSSFELIYIIGGISPSSRQPTNTVEAFNPVTKMWKSSVPME
TSRRGCAVAVDRDNIYVIGGSDGHKALQSMEIFNVSQSKWNRGPEMKSPRSSVACSLVGSNIYVTGGYDGETQCLATGER
YSVDLKRWEEFASMTTARSMHGSVDISGLVFAVGGYDGHRDLRLCECYHPEENVWKAIPDMPLARSMACVCAASSKVIVA
GGMEGNQFYNSVDVFNVNTSAWYQLKSMLSVRSRAGAVEWGGRVFVFGGAAKGREVEVDAIYVNEDEEDADWCSVAKMDT
VKERFACCLV
>Ocar_g872_t1
MVKHGGTVVFVVEEKQIKVHCNILCIRSEYFSGLFLSGFKESQASSSSSSVPISIEDASYEAFHALITFLITGVIDVEKC
HPIMQDVLVLADRFLVTNLRKFCMPYLIKEITLKTVLKYLNLSDHHGFEYLKDACLNFAVENIQLLRRDAEFESLNKEVI
EELFKRAKPEGHSNSYSITTGTVAMIV
>Ocar_g1164_t1
MFTSCLRESHESTVKLHDVDPAAAKALVPHSYTGHVEIDDGNYLAFFKAAHMLQFSDVLQHCCKWMESHADKSNGLKWDK
ESGPSCAAKERVGNFGVVLAVGGIKEECHHNGYESFIEDDQIESPPISSVEFYDPAFGQWKELPPLTTPRSDPGVVSISG
NVYVLGGNKRGANWDNCGSCDAEWYDSQLKEWVADVEPMRSPRLQRHFFAFDGFIYGLGSCDRMCSSACERYDPVKNTWD
ELEPIPTEISNGKVLRRAFVVDGSIHVAIGDDEVDFLEYDPVMDAWSETGMCLSFMYGGENRREQYEWSYSTVAMDKHLL
VYKCEVQEGGNVEIKEFEVGEVVTAVAEMAESGDDYVRGNRIAGGNKKFYIVAGYQGCKKIPGSDYVNDYFYPGRNFCWL
DICTREWEKGPNLTQARGCCGVAVVDREDILG
>Ocar_g1239_t1
MAESTQAVVTMRDVDPTILEQLVDFIYSGRLEVAVENVQDLLSASSLVQLEAVQNLCCDFLKEQLEPANCLGIRNFAESN
GCQSLAEAVDKFAEERFLEVAAHEEFLAHPCERLATLLASDDLNVHSEEEIYEAVIRWVEHDPKERAASLPVLLQHVRLP
LLNTDFLMENVDANELIKANMECRDLLDEAKRYHLVDKCRRSMDLPKSAQVRKSCVGTLYAVGGKESSDSISRSVECYSL
RENEWSRDKEMIVRRQQLGVGFLRKKIYAVGGSDGTTRLSSVEAFDADTCNWAFVAPMNTCRSGVGVGILGENAMYAAGG
YDGRNCLNTMERFDLDLNMWNYVAPMTTRRSFPGQAQLRATSVTVLDDKLFVVGGNDGSAFLSVAECYHPHLDRWAPLPS
LNMSRAGIGGRLFVGGGNDGSSRLESVECYDPRVGHWEMKESPFSASSPVEVFIHFKLDMEEQLSMFARRGWKRKGSFPG
GDDVDRKVFCDDGVGVAFSASATLPSSITVSSSDGNMAKFRVKREMEFPQIRNVFESSNLLSVSFDELKSKVREKRYQSV
IRRAADFRDKIRHSGILGRGSGGGQESVDGEQPNKDEMWVKKHGVNRILLSWLKLWDGAVFGLSEASKKFSIEHADLQPR
FPSKPNDKSVNFPQNVDASRRPKQKVSFVLADFVCLALRQVALLCGPPGLGKTTLAHVLCDHAGYSPIEINASDDRSPEM
MKSVLENVTKTRSLSWANEKPSCLIIDEIDGALPADHPVEMEVIESMCIGQKDMKKSLFSFWQDVFILSRKKQQPVRMAD

GREHAQSQFDSVILGMRSCGDHDKAIQGLYENYLKAKVLDRSSDSFSLALEWLLFADQIHCSISHHQTYEMLPYFPYVGG
IFFLLFASAAFPRVTYPSSFYEHSVQKEKIRKTANSLTCDFPVGVNQSTSVSSVITSVAPRLLQILSPTFRPVDVKLFGQ
SEKEQLKATINIMVTHGINYCQQRSLEGQYAYEFEPNIELITKFSESGQHQLSYATRQLISHEIEREKMRKSEANYGINP
LKTWK
>Ocar_g1355_t1
MDAESRYEGEERAADCFSGGFEFVLKDFPQRGWSDFTIRTIDDQSHRVHRLFLAFHSDFFRQTIFSHENNESVRCEFKMS
HEVDPTNVTPLVIDFLYSGKIRLTPTTAVAVWKVADFLQIPGLASVVETYVTDRLDKTNGWRIWTDCVDLCVDRFRDSAL
DHVVKDMEATKARDFSDIPEADFLALLSHKDQDGKDVFVLYSKYVQDLSFDLSDEFLQKASSVLESAVSTITSKFCRVVA
PADVAFVPALHLGSVLSLDDLDVDEELAVYDFIKELVQVRGKTSSEEVDTLLRAVRFSQLTKRQLFDVFQARKVRSDLLL
AHVLRRLDTFEFGQCCCLSDSDSDVSFGPRSSYDGPHLVSISILLSQCSSVDIEQFRIGLALTEATSLTEWETDSIRVVH
GPVDVPVSSVQSSQWLEFKMKRPFKWRKRRNLVVEMSRLNCSMTCDLGSLFCFHKNGSHTVEGTSDSLLAEHGPAHYPLS
LSRCPNYIPALRLFTVEGDEYQIRRLATYSLTRGPFNGQCTSSWMQSLYRWGDFYVVD
>Ocar_g1961_t1
MIADEGSIRRKRQSSSTFLRSNSKRMKRVSLGTGDGGNRRLFRTPDYASDVLQSLNRLRNAGERCDVELSVGSRVFRAHK
LVLVSTIPYLNAMLASGMKESRQSTIELKSIDSHAFETILDYAYTGQVEVSAENVQELLPAAGMLQMESLTEACCSFLEG
EMDAGNCIGIRTFANLHCCVELEKKANRFVCTRFADVAKGEEFADLEWSEVTTILKSDELQVK
>Ocar_g2028_t1
MENSPRHLHYDSTSSCLYFTEYHAIRKVIIQRKPFSDPQLSLDLAKLIDRSDALPTGQAVFVVEEKQIKVHCKSNLCIRS
EYFNRLFLSNLKESQASSSSSSVSIAIEDASYEAFML
>Ocar_g2124_t1
MAAMAAQLEPVNFPDVYGPSTNPSLRHMDQKHSSELLEVMAFLRGRNNLCDVALMVGDREIVAHKLVLAAASPYFQAMFT
GELSESRQSKVTIRDVNPEAMELLVKFIYTATIEVNEDNVQALLTAASLLQLNRVVTVCCEFLIDQLDASNCLGFSMFAE
RYNCFDLQKEADRFALQHFRQVAESEEFAILPADHLARLLASDELNAESEEQVWHTVLRWVKHDEAGRQNQIGSVMQHVR
FSHLSPKFLVDVVSAESLMRGDDLCRDLVDEAKNCHLLPDYDPRLETTVRRLRTPVRQGQVMFAVGGWCSGDAVSTVERY
HPADNTWTVVASMTKRRCGVGVAMVGDDLYAVGGHDGSSYLNTVERYEPKLDQWFTDVPATNSCRTSVGVAVLSGLIYAV
GGQDGVSCLNVVERYDPALKEWEQMKAMNARRLGVAVTVMGDNLFAVGGSDGASPLSSVERYDAATNQWANVSLMMTRRK
HLGVAALDNSIFAVGGRDEISELNTVERFDPRTGLWSNVATMTSRRSGVGLGITNGKLYAVGGFDGTSYLNSVEWLDLPA
NQWRQVAPMNFRRLGGGVGVVKEALVMNKENK
>Ocar_g2161_t1
MIEFDCASGYSAAMKELYSSGVYSDVDFLVGSHGKKLTAHRMILAAHSDVMARMFFCGMKESTLKEITLPDANTAAFEAL
LLYFYTGRAEVEDEKVVMELIKVCDYYNVSCLKRKCCEWIAAYLDASNVCSFLDFASAFDEDLYGKCMDVIVLETSEVLK
SSSFGSCLSKKQVQIIAENTSLEASEFELFEGIVRWADHQKEQRETVSHILTCIDYTKLTPGELLGPVQKCGFVEDKVLL
SALSVVFLPNGSHPANVQRSVCFDQGLTRGFRLLHGDTVADDSNLGCKRITGVLIDDVLSCLQKQGSKIRLELKLDKPMG
LDQEDAGSSRKSKKTKPAASTASVIVGFQMPAIAGIKVSFSSFGFGTLSTADGRLFGNLSGCKSLILDVSLRKKEILFDA
PGTLVHLNHFTVCQEPMKLKERRAHLCVSVSNAVHGFHLSASLQSLSS
>Ocar_g2329_t1
MFTSGMLESRERRVEIKDAIDPSSLRSLVDFIYTGKLAIDTENAQALLSASAFLHVTAARXXXXXXXXXXXXXXXXXXXX
XEHACQELVRKCDEFFWANFSAVVKEDEYLLLPVDALMKLLASDELNVASEKEVFDAVVRWMKYDGTSEKRDDCMANLLS
QVRLQELPIDYLTTVVEKDSIIRQNLECRDLVDRAKNHYLLSKMRRTSVSVLDADTEQPRPRKSTVGNVYAIGGRGKLNE
PYKTVEMYDFFRDKWSEVVALNSRXXXXXXXXXXXXXXXXXXTSPQFRRMLPPRNRKVDGNRTNADKTPWSSCRIIGRTSL
RSWYV
>Ocar_g2555_t1
MLALQRSNAHYDVIFSVGLGKARRTAHRAILATHSDVMERMLYGDMKESSNDQITLPDVDEKAFDELLSYFYTGQTQIES
SDHLLAVAKLCDYYNVTGLKNECTKEMNRTTDVTNVCAFADFGGQFGVPTLKRNCCAWILDNMKFDNMCYFIDLWKDRTT
NYLLINS
>Ocar_g2805_t1
MFTSPKQIAVGPEGTIYVTNDLNHSIRRIKDRQVQTNGFDYPFCIAVDYSENVYVSAACKIYKLKIGGEKEVLCGTEKGE
SVDGQGGVASLNYPCHLHYDSKSGCLYFTEERAIRKVIIQETSFSDPQFSLDLAKLIDSSDTLPNGQVVFVVEEKQIKVH
CNILCIRSEYFSGLFLSGFKESQASSSSSSVPISIEDASYEAFHALITFLITGVFDVEKCRHVMQDILVLADRFLVTNLR
KF
>Ocar_g3076_t1
MAEGLVSTVARNPLFSCLSGVVTLPSGFILAVDAGNNCIWKVDIADGTVYPFAGVPGRRGYQDGPADSALFSSPRGIAVH
GSRVLVADYFNRRIREVCEGVVSTVAGNGLREEPDEDCLQPSFISPFGIAVTSNQKMWTSDYYGHRVHLLDVGGRVETVA
GRPVWKGKQDGPCNQAKFSYPCQIAIGPEGSPYVADQFNGCIRRIKSGQVQTILDGIGSPCGIAVDYNSNVYVSDLFNSK
IYKSKIGGEKEVLCGTRKCESVDGQGRMASIFHPRHLHYDSMSGCLYFTEHDAIRKVNVHQRTFSDPQLSLDLAKLINSS
DALPTGQAVFVVEEKQIKVHCKSVLCIRSEYFNRLFLSQAGSSSSVPISIEDASYEAFHALITFLMSGVIDVEKCRHIAQ
DILLLADRFLVTNLRKFLMEYLVSLETVLKYLNWSDQHGFSVLTDACLNFAAKNIHLVRIDAEFESLSKELNVELLKRAK
CE
>Ocar_g3129_t1
MDSLRLSSELCDVVLSVGSLQIPAHRVVLAALSPYFRAMFTHDLKENRESAVTLYGVDPAAAISLVSHSYTGHVEIDDDN
YMALFEAAHMLQFSDVLRHCCKWIANHMDESNGLNWGILAHNYGFVDCDFQKTADCWSALSLKSYADRGADEFFSLPCDY
ICHLLGMDQLQVDSEEEARELAWMWIRHDEENRLNHADKLLACVRSPSISDETNPSDTESSSSQSVESDHTAKERVGNHG

VVLAVGGTNEESQPYHNESTRYKEFDFDEIDCSPISSVEYYDPAFGLWKELPSLNTPRSDPGVISVGGNLYVLGGNKKRY
TYGSCDGERYDRQLRKWVADVELMRSPRVQKHFVAFDGFIYGLGSCDENIYAACERYDPVKNRWDELKPISFIKQETPIG
RAVVLDGSIHDGSIHVAIGDHE
>Ocar_g3667_t1
MDSLRLSSDLCDVVLSVGSLQIPAHRVVLAASSPYFRAMFTSDLKESHESTVTLHSVDPAAVKALVSH
>Ocar_g3762_t1
MDNLRASEDLCDVVLSVGKLKIPAHRVVLAALSPYFRAMFSSGLRESHQSSVTLHEVDPEAAKALVDHSYSGNVEIDEKN
VISLFKAAHLLQFSDVLCHCCDWLSGHVDESNCLEWGLISWRYNLSDSEIEWRTDRWVALRLSQMAKCNDDSFSSLPFEY
LNRLLAMDELKVDSEEEVEGMALKWVRHDEENRQRYTDDLLACVRKSLLPCSEAESDITESNEEESTDDDSQKSKSDIPA
QRRTGNLGVVLAVGGFTFVDWNKQTRTSLVEYYDPVFEEWVELPSLNTPRYAPAVVSVADSVYVLGGNEGICDAERYDRK
NKTWISEVEPMKNPRHRNQLVAFDGFIYGLGSSECGEKRCQCERYDPFVNKWHPIKAVPLKKSQYVIGAVVFDGWIHVAC
GGESSSYFEFLRYDHMANVWFETSIRYRHHNILHKRPHIFSALGNDRLLIYDGSGHRLIELSEGKATVKQLSQLDNCPLQ
EGAVAAGNGRLYFVGGVKQKKKGLCESHNFCWLDTAEPESKVEKGTLLIHRRSLCGAAVVDRSAITM
>Ocar_g4119_t1
MATSEDFGLRRLFRPPVGHLSVKEQLKLCSTRDLGKSALYRTAAKIWGAPNRTRVLIARSTDENPENEAKEVVERQAEAA
SAEKSTEEDEMVDYHRWMTERRQLRGDLDSLGLNAAWLRSKRDKTDIEGRVLERMERPVFPLPLNKPEPKKVSPKRKVKS
RGRGVRPKLDLPLPDALTIIEEFVQKNRLRLVDLFARFDKDKNWRVSKAEFRRVFKLAAIPVSDRQLDSLILALDKDDNN
ELDYKELVRGRQSLKKLLRDAKRDISSRTSMSSSPLLGGTPTILEEGEERKSPSMLLPIPKVSTSEKVKMVSREAKMTER
KRVRKMKRKQEKTAAVVHKEISVQLERHCAVSTIGCGGGGAVGEAVNAYREMVRREYEAALHLCRMNGVDLTKEILEKGE
CQAKLRQPGLDLLSRKDLEIPDSSPKKSSRLRSSHAGFGAPSTRLKRVKLSTGWAKVGPKTDCWLTYEEYEALTRQLQPK
FDYKRKSRSVDSNAFWPGSLLDRLRVYLPSDNETFGFLSSVIVLSNGTILATDRKNCCVWKVDPRQTTLSTFAGVSGKMG
YKDGPANEALFDNPAGMDTDGSSIFIADSGNNRIRKVSEGVVSTVAGNGACRVRDEDYRQASFRCPFSIVVKPDKTILVT
DMRGHVVRLLRADQVLTVAGVPKSSGRKDGPCDEAKFFGPIQIALSSKGGVYVADQNNQTVRTIRNGQVQTVLGGFNSPR
GVAVLGDESIYVSDSSNRIYKCKPTGEKEILCGSGEKKSVDGQGVAASLDFPQQLFLDARTSFLYFTELQAIRKVRIACS
YQDPLLPQDLARLIDNSSLSSSDLATFVVEGKRIEVKSKTLLCLRSEYFNRMFASGWKEVQTCKLSGSVTIPIEDANYDA
FHALITFLVTGFLDMVKCRRFMMEIAVLADRFLVPDLKNLSTDFLSKGVTMANVLEYLTSSEVHGFEDLKEACVNFAVRN
MRALKREGMLHFLSKESLVRILELVQLDQ
>Ocar_g4295_t1
MNDGSIYVTDSGNKRLKKIANGKVETILQNCCSLHGVAINNDGSLFICDFGTNEIFKWTPTDDELHVLCGSRKKESQDGL
GVSASLNGPCFPYYDAKSGYLFFTENKAIRKVKVIHASAEAANFLVEGRIVRVAKTTLCIRSEYFQRMLQSDWKESQTEE
SGNVITVRETTYDAFHAVITFLVAGVFDEEKYRSIMIDILLLADKYLVEDLGEICMRQLTKQLSPTTVVECLLLSTKYGF
DVLMEASLTFAAENIKHLRHQDGFDSLSKGIMSVRL
>Ocar_g4641_t1
MEAVLPWDAKVEAKVEAQVEAKDESCLPADPDAVVADATMHCIWRVDPQGRLSVFAGVKGRKGYSDGIADQALFNSPQGL
CTDGSCVFVADYFNSKIRCICRGVVSTIVGKWSEHSKESEARFSHPFGMASVPGGNIVIAEKLRVSLIGNEGHVTNVAGK
ITSSVLSSLQNSSSSTSYECSCRDANFSHLRQIAVGKDGSIYVTDYNAKALLKIANGKVQTILGDLSSPYGVAIDTDENI
FVSEFRRNQIITFTPTGEKKVLCGSGRKASKDGNGLLASLHCPRYLYCDSMSGFLFFTEENAIRKVRFAEKRPSHLDHKL
SRDLMKLVDNSDIPSTDAVEFLVEGRIIQVAKTSLCIRSEYFQKMLQSGWKESQSEDSEKAIPIEEATYDAFYAVIAFLV
AGVFKADTSFAIMTEILVLADQYLVDDLKEMCMRELATNLSVETVIECLLLSEKHGFEMVLEASLRYAAENVKEIRVCEG
FDSLTRDILSKIILNLDICGCGSAINK
>Ocar_g4957_t1
MVRYPWQLVRIHQAMSXXXXXXXXXXXXXXXXXXYTKKGDQSATLTRRMQDKKVTSLEVHPSAGSKYGLAISHSKQMLFVS
TEKNIAVVPLEDSDFPSKEKVQFAFCPTIGGNVADGKYGTXXXXXXXXXXXXXXXXXXXXXXXXXXXXXXXXXXXKGNLSF
LIGSGTPRDVVFVAESEEFQSIRAALSARCQPLHKLSVNPILYNTKITIENTSAKAFRAFHDYLFTNEFPLEQCSDFLDE
VFVLSHRFEVRELKDFCERHLIAKLSKQNALHLLQLSFENKGPEMKKSATKFIIHHLSSFQKDGSLSKLRPDLLLDIVMQ
M
>Ocar_g5475_t1
MAMAQHTKLNCFSGFFESLGELYESRANSDVTFLVGPDKARQRAHRVILATHSDVMERMVFGDMSESKKDEIPLPEVNKT
AFDALLQYCYTGKVDVGSSDEMIELAMLCDFFNVSKLKSRCALWMAKSVREDNIRDFMKFASERGEPSLQHKCLQWLISN
VKATNVCALMTIGNRLKEDNNDFAEKFLDKSLAIISLNATKVLASDEFAHLPRDEALAVVQCQALQTDEINIFRAIARWI
KCQDESDSHNDFPSSSILYDLLTPDQLVGEVQNSGLVANDVILSALTKLRRPGEDSLGLPSCRRRFVMAFEGSSDDCPLT
NNGVQVEPSSQLEFSTFIPKTIVAVLKNTELKISLQIRRVSCWSFGSCDWFTFPPRSILNILFNVGSGKFGIRLQKGKNG
CSANATAATVTAITPNRTYALTENRLKSEFVVEVSSTKDGDIIFEIPSVNCKCCSNHFGVSAKPISERGHLYLEGSGCLT
ISCECSLDRHN
>Ocar_g5529_t1
MQNIKVVVVGDGAVGKSCLLISYTTNAFPSEYVPTVFDNYAANVMVDGKPVCMGFWDTAGQEDYDRLRPLSYPQTDVFVL
CFSITSRSSFENCAAKWIPELRHFCPKTPVVLVGNKWCAGYYENSALTQEGLKCVFDNVIRAVFNGPSAAERHGWMLWNR
RKGKKGQVEEPVPQPPILPEFERHAPWINIETSSYAEDWGTMVDNLTSSDVEIHVENQTFYAHKVVLCSASQLFRNIFHV
KSDRAVDVSSAGGVASKTKSKANQAIPVTDIIAGKIPGFQFLKEKKINSGMNTTALYLSDDITTKMFKHVLQFIYTGIVD
LEGESDEAKDSLQKAAETFDMPDLVKMCENAKNGMEFLNPSIGTWMNDRCGAAAKQLFFNESLLADVSFKVDGTNIPAHS
AVLTARCEVMAAMFGGSFAESQSKGVEITSTSLECFLALLEYLYTDHAPVEETDSVGLLVLANRFCLPRLVTLCELYITK
EVDRATANRIAEADIDVIGLLFTCQMHNARQLSDWCLHFIATNYTAVKRRPEFKLLDGDNLQYVEENQYPPPSYFKELEE

YERMMASREKGKKKSQSNTSCCIM
>Ocar_g6020_t1
MSGEQAGFGDPKVLAVEMSKLLNARDHSDIKFLVGEDRKVFFAHKCVLAARCEVFRAMFADQSATVSSGQPGDPYVLSDM
KPQIFYALLEFIYTNKCTITTDTSIDTLGAAIEYGLEDMARVCIDFIRDSLSVANVCEAVQAALTYCQDDLARDCLTLID
SDPESVFRTRGFTEMGEEALIYTLQSDKLMADEGEILKAVKEWATVVSGESMGDLCRNVIEHIRFPLLDPEELSFIEKEN
SRKKYIPLHLISHAWKFHALKEFDPNDIQTRLRAGTLPKESLRVVGLNLTDGCNIAQAYAGTAPASKASATTHASSERQH
TSGRETTGRGSTDSAVVCRPPWCKEMMVRSLFKPFKEYTSPTEVLSTSNVLGRLTGGLKDLYRQIGTKETPRPHSETRRD
GPLHIVDSVDHSVAGKCTADNGRFTIPHRAARLAQRYQMLDVIGKGTFAVFIEARIDPYRVEFEVQSLWYRAPEVLLGVE
VGLPIDVWSLGCILIELYVGRPVFEARQKQDLLDLMVRLLGPVPDHIYNTRKFKDLVASHCQLTPEVCRLKASENIYKLF
KKMKRQGRQEREFADFLSCLLCYSARARPSQALLHPFLRHEFPLRFCIEEPYNYGVTGKGGDFEVDQLPSISLPSNLEHL
SPLLSSTASSSLSKSCRLIAEETGSASGTGSEVAVEPGSNSEEMDSCLDDCMFDKETEPSIPFSLGADLEFKIIYVGSAS
SKDHDQTLDSILVGPVPAGRHVFVFEADSPKPELIPQQDAVGVTVVLLTASYLCQEFTRVGYYVNNEYADSEMKENPPAK
VEFDKLQRNILATNPRITRFKIDWEGTGKDEYAEQLQREAHEVENEADTAGCLPEQAIGHSSE
>Ocar_g6241_t1
MADIQSQSLASEDTSGPSSAPYSSFDAPENFSDFKLQVGDRIFHVHKAVLSMSSPVFRTMFTSDFKEKNAEVISMKDKDP
DQFLEMLQVIYPPQKPVTALTAPYLLSLADEYMMLAVTDRCEDALLSSSFTFDTLILAERYRLEKLLSAVTSGIAKMPLR
KITDRDGFQKLDLSTRLSLVEKRLNFVTEQGSRVREIFKKWNKLYYPYLGKIVDGRDVALIKNPKSNFTRNYRWETIKDK
IKKRLGITGLTSSMSQVMEEHVCDLEILKAASDKLSY
>Ocar_g6594_t1
MSASSVLSTAKKSVLDSGPRSSHFRTEIERQKADFLRLFESGNWSDLTLVAEGRHFHVHSAILKARLGSAFGSCPVSSQL
TLCDIKSSDTEQTLRDIYSSDFVSNADVLNELARLSLPQSDYEKLSKPIGATRTLNQCLSILFESKELSDLTIIVEDKSI
KAHNAMLSGSWIESSSKEITLKGCEVTHSSVQLALRYIYCCAVDWSPEMKISDLFIVATMYGLEGMMEDICFLLQEKYCC
KFHRPCKNCTENVPLCLKLSSDFPLDKLKENCLNWLCKYFDRIWCTKAFASLPVDLHQLVLETKKSSLSYGNVIGVLRGC
DCLGGAMSSYCKWAEPVQELNQKLLKYCVHFVSCNLYNIVHSRAFRDLFEGIGSSSTLVNLIFQKLLSDMSAENCCLNLR
CSSVLMDNGFADDIQALVTDFHLSCKKFALCHLAQVRRMALWKTLPKLLCDELEAAAVTSFVQFGPAKNVKPKSRAAPKR
FNSKKKS
>Ocar_g6998_t1
MAEALDPADCSSTPSTEQQKPQSSLPRSLYKPTLWKPTSSSTKAPLTFPSSAKSTNAPTSQTKTSSSEASQSSSVDSHQT
TAKAEEPDNSDVASESAHPDPPAPPCPNKEVADTGNSGSPTHENFEEPAPPLEESVEVPEGYPDDDVGLPLPPPPPPLPI
LGSPHDDGDVQAENDEVAVNPLAQVMADIRKGASLRKVPEEEKKRTEPGGMKVVDVQSELRKKFMKEKTEKVKIMKSQID
LHQDRIEKVKRWILDAQHGYLAIFQMKKLLGEIKEVQQLVQVTPELMALRISWPTDLIDAVEAIVDVLTALDQAVFKLEG
VLKNAESAGTEERPSSVHLNVELAKNTINTIILKVKSMGMAESRYSQAAVVFTVSSSDVGGIIARARDAAAGILNLLFMK
GFHTAEVRSVYEPGLKKFDQNFRISNAHRLLLMGCQINDSVIVHLDGVKHHDYSEARLKLTELEQINSKAVNVSRRSGEW
DTAEQVVLKSSGSQTMSRPGSSSLEKIVQAVKAKQIDEAKQLLSSVTTKEFLAIEKSLILAALGSEAVSVVLQQRNSLGL
DPLSSSKQFEFINEAMAKGEKESVKQLVELHGFNVNCKSSDAGNTDVTIVSEEGSGQKKFPAHRIVLCAQSDVFKAMLDS
PHWGISRQHEVVLPGLRAAVVERLLEYLYTGLCVFPKDDLNLGVELLAVADQYLLTPMRSQCESHLTDMIEKEIVIPLYY
AAYRFSTLSLPVQYTTLH
>Ocar_g7064_t1
MSYEVGYALMRWVYTDKFEIKTNTDFVLSLMKAAEKYQLMDLKRKCENSLVSAVNVKNCVKFFQTATDVKAVELRKFCLE
MIRTNWTQLDSTAFADMRAELLFDMFKEKSEYPLNLAIRYEREDIVFLYLVEHSSQLPAKLNEADENGDLPIHLALLGRN
EGIATKLVENGCGLEMPDGDGRCLLHKAIARGDDFAACFLIEHGADVHRAIASTKDTALHLASASKAAEKMKKVVILLLE
KGANPNEQDSTGSTCIQRSVMSHNAIAFDLLLHHGHQSRYSSHSRIDRRCGATAATSLVVVSEAAKKDGLVH
>Ocar_g7150_t1
MAFSALRESGEYSDISVEIDGETLHLHKLPLLAKSKYFKALMNAKVDSANSVELKDFPGGLPVMNLLADFCYGVDISEKI
ACDNVGPLICGASYLQMTVDSDNFLEKAQQKFHCLTEESVLNCLRILVGCLAITFHAESEALVSLCFDSLVDCWSSSSLQ
LNETERRTLQSLPLKWMLALIDRLKTKIPPEFASVQQLSSVFDEKVMDDCRQGLVDPGQFCELMNSTLSNRKSTASFDRS
FETLEVFLQSCPTLSQDETTAVSQAVDYAQLSEDCLERAASNGRIPKEIVLLCAVKLCRKLNSEKKQLVDKNTELTTLSG
QILAEKEEFEQRIAEKVEMKLRDWELRYPFLQ
>Ocar_g7841_t1
MTDLRVFPRRRPIGKPSLSHRLVSAVFHGALEEVKRTCALAGGRLARMTDEWGRSPLHVAASLRNGVETLNWLLENAPFD
KEGKDPESGWTALHRAIYYGNLQNALSLLKRGGSLQSCDRELLTPLDLVNKNRLLASAGRVSGHTNSELAVQSEFLAWGL
NNNMTLGHADASSKSIPEKVAGLALCNVSEVVLCKFHTVFLCADGRLFTCGVGQGGRLGHGDEQTHVIPSYVKSLHGVRI
VSVAAARNHTVILSDSGAVSACGINDYCQVGSQDSSAEILSPRKINVLKGKRVTLVAAGRYHSAVCTKSELFTWGKNEGQ
LGYCKADTIQAQPKQVTLIPERSDIVMVCASDAATACLVQLKGGGGGSDDVYVMHEYGYRKISLGLLGLPVKMALMASLG
QLQGVEQDGACVFVGLFKDGRVWKWSPGLRAFRPCQFANKCDIEGVHIVDMALGKHVFLTTDEGQVYRATFPSPPPPSPH
RKEVSPLGRHTRIPKGRKSFDRQSSKSREWEADSGRVLELTRVPHLHRVQRIFCDYKGQNIMAVALNPIFGISGFPLLSP
SQLRSDLERLLAEADLFDPIHDYHLQLGARKLPAHRVILADASRRFRRVFQNGANDDQINFDSFDVQTVNIVLFYIYTGQ
CKPLEAWKGSRCRRRRPQLSSAASETDNNEMNFVLDLDEVDGLREIDWSVEEVHFESVLAGPEDAAGRMSGQRRGERKPI
LSPNSTSAFARFQTSVVSDDDDGGGADADAETTSLLRRDSVPADEICKEKEFMKTFQALPEILQKVYKAAEYLEVFSLVQ
TIERDCGLREHPVNDTCFGSLDREAHLHLCDISLVANDGTTFLCHKCILSARLDYFYSMLNFGWREASSDLPSVNLPFSS
KVLKVVLDFLYTDTATVVEESTNLELIGQILIAADQMLVTQLKQICELSLAKNLNLRNVVDIVDFADTYGASQLKKTTLD
YICVNLAALLEGGFLLNMDGRLAEELAMSYQTMVRKIDMQ

>Ocar_g8274_t1
MFTGELAESRQSHITLHEISPIALELLVEFAYTSEIAVCESNVQVLLPAANVLQLTAVRDFCGSFLQSQLHPSNCLGIQA
FGDLHACADLKSAAHVFAQHHFVDVVRGDEFLALDAGDVAKLIESDDLGVQSEEQVFEAVLAWVKHDPLTRERDLASLIG
LVRLPLLSKEYLVNSVETEAITRRNNVCKDYIIEAMKFHLMSAEQRSQFQTPRTQPRKSIGLPKIIVVIGGQAPKAIRSV
EKFDFLVQKWSPAAELNSRRCRCGVAVSGGLVYAVGGFDGSSRVR
>Ocar_g8536_t1
LSLDLKAAYLNRDLCDVIFLLGKQEIPVYGIRAILSARSSVLKNLLCSASASVHRKSSFTKSLNQRRERNYVKKLQLSAI
PQEVADESPTSPIPVAIKKEKKKKGMREKLKMKFQRNSLKSALKEPKKESEQETTATSADVRIVAGKCLVS
>Ocar_g8610_t1
MWSGWKDERRMDERRMWSFPAKLRCSDAFSKSLEQLYQSRANSDVTFLVGPNKARERAHRATLAAHSDVMERMLFGDMSE
SKKDEISLPETNEKGFDSLLKYFYTGGVEIENSVELLELAALCDFFNVPELKRECSNWMKRNLNAESACPFMKYASAHNE
PLLEEKCIEWFLHNVNAENMCAFMDFVSTFRDSKRDFAEKFLDKTRAIVFLDATSVLASESFAKSLSRKQALAIVQRPHL
RTDEINVFKAVIRWIESQGGDGNYEDLLSSIHYDLLAPGELIGPVQRSGLVAEKVILSALTKLHVSDEFQLRLPSMRRRL
VMGLMVFDKNLSQNCNLSDDGATAQSSNKLAFSACILNSLVMVAKKKGLKVRLEIQQVSYTLPLKSMVQIYFTQPYFGIN
VYCEVRQKNITLEKIEEITSTGVIGKSFPVSGSLAEFFVDVSGNEIIFEIPSVNFKLSSTPAPLSKGDLQLHTSYASVYS
FHCSCC
>Ocar_g8707_t1
MQTSAVQTSAVQTSTVALMSTSQMPTSISPVITQSPTEGSSGLSSSEWAAVAIGIVIAALVLFVLVALLAKYVYERRNKR
SYKFKSKAQEKYEGEEQSGNRVIPQDPDDGSPGRKGANHSSSLVTHHLVPSCTLRNRLRSSFPRPPCTLSPAIFDANIMF
AARRDINIYEAGDHADALLRRLNALRNQKKYCDVILCTQSGKIEAHRNVLAAISQEMNTMMESETATVDLTQFDPQSIRL
LVDYAYTGRIDGTAMNLPSLARVAKFLKAGDVFDACVKAIGDTLNGQSCLSVRRLGSELGIWKLVGGVDDFIAGANDEER
EEIMESSDFVNLPRVRIDVIGDRKLGDSKKLFERVVDRMEGAYAGKIVLLQETGCRFQFDSDDETDDLSHDVTPSGTPDR
PKGSGVNRSSVKTPARKLLLPHACQRDDLLDWKVLALFPVTEDHVSTGLVVLDSQLAVLSIIRHDHIRASLPRNSTGSMS
SCSQELIGSLHTARSGMGACSNSGKIYAVGGYNRSKCLSSVESYSIETNEWALEASMSAERARCGAVSVGGNLYAFGGSD
GQNVLTSAERYNAVTAQWTPVASLKCGRSRLAGVALGSSLYAIGGLNDKSSSALKVVEKYDMQRDVWTVAPSLLRERVEA
GAAVLDGKIYVVGGSNGWSCLSSVECYDPYVGSWQYVAPLQVGRRGAGVAAFDGKLYVAGGHDSVRSLSSVEVFDPVEDK
WVFGASMSCPRSSGPLAELDGALYAMGGFNGSTFLNTVEYYLPDEDRWHQLVTY
>Ocar_g9195_t1
MASGIYFGQCPIREKKGPQQPIYRYESPEHAAGTLLRMNEMRKSDEMCDIDLHFTSGHVIRAHRVVLAGFSPYFHTMFNG
HLVEKDMTQVTLREMDREAVESLIDFAYTSTLTITEDNVLRLLAVADFFQIQNVKDACSDFLAHHLDASNCLGIWSFAER
HHCDMLEKKSFIFVKLVELRLEMYRLEPYQTIYKALL
>Ocar_g9422_t1
MAGIGVERSGIQSESGRRPKPGKTSKIKVSDQLNESDDFISTENLSCHVCKKDLSSLNTSRRQQHVNHCSNPLSNENSKV
KKVSCPFCDQALSGLMQGKRHVKTCAERLGVDESVLGEVVENLKECFETKYAFEKGKRTKKRLQPKKGMDDDTLLALGMS
ASLVDTKRKKKFRQRLGDVDQIPELLLRSKEQNEEMMAARVEEIISTEEESDIEPTPALAPSKMAEKYQPYKKSDCEVED
VCHSQFASKGNVTELSYLDKPRADFWDLTTKSTKKASLSHFFVHGLIDVPSPAKVEAKESSSTTEEIKTDPIEEQDDGAV
NVSILPSQGFFDTEVNREESERKVESSNDSIETLLSDLGLLVNSPLSSDVTILAANRKPLFVHKAILSARSTALADMIEK
AQGGSLVCDDVDHDDILIALRFIYTAKCLANQENASRIYKLAERWALSGLMAACLEIDLSAEVANQQQSSMSQSSRGSPI
LIPGSSSLIEASPVCLTIPDSPYPVQDVPDSGSFSDECEVANTDFGNSPIISLSPTAASVHHVNDSDSDDSLPPFQCASK
WRPKKMSETPEKVHDAFPLSDADDDLLFGCSGNMAKEKGSPSFSQEKETSMTAAVAAENNEDDEEKEDAVPLVGFATPSE
PIQKRKRRLKLGKTPDYNSMDTPALKGLGKRFGLKRMAKGKVKKVLVNIHRKM
>Ocar_g9721_t1
METVSLDFEPREVVHRWRRMPSCDEFVGARLPPARSAHGAAVYDDKLWIFAGYDGNARLNDMWSIPLNSKTPKWEELEQT
GDCPPTCCNFPVTTARDSMFVFSGQSGAKITNKLYQFQFKYKRWTRITPTNLPTGGTAPPQRRYGHSMVAFGRHLYVFGG
AADNKLPHQLHCFDLDKLEWSVIQPASNIESPSGRLFHSAAIKDDAMYVFGGTIGDNARSGEIFRFQLSTYPKCTLQDDF
GRLFKSQQFCDTAFLVGKAGKVKGSPEFQISFPNMEPEAFQVAMVFMYTDAIHTCFDVQIDQLTTEDLKLIMEVYGLSKE
EYCLRFIVKDVNYHSIVMSPAFENLEKSLMVDIVRRKVQPVSHQLSTGSPEPIFTLTSGVGKTFEMDMKAFLTEGVGEPF
YDITLLLNRSPVYAHKAVLAARCSYFEAMFRSFMPEKNEVNIAIGETVPSPSAFESLLRYIYYGDVNMPPEDSLYLFAAP
QFYGFTNNQLQVFCKLNLEVNVSPRNVVEILEAADRIEAHDMKRHALSIIVQHFPQVARTSNIRNLPKELLLDVLDALXX
XXPASDSLK
>Ocar_g10884_t1
MAGNLRRKVPSHFGHAFDLMASLKDRGDLCDVTICVEERAIPVHRIVLASCSPYFHAMFTTSQMAESHQERVHLHGVDAP
SVSELIHYAYTAEIDVSEDNVQRLLPAASLFQLQEVMDVCCEFLALQLDHSNCLGIKSFCESHGCHRLVETAQKFAMNHF
NAVRKTEEYLSLTADEIEQLISNENLKVSSEKEVYEAVMAWIMHDSEHREQFAHQLLQHVRLPHLPRAFLTSEVVENPIM
KSTGQEFLIEALAYQLAPERRSVMTSPRAQARTSPKLSDAIFAVGGGSLFAIHGNCESFNLTTETWTTVATMNARRARLG
VASTSRMLYVAGGWDGSTDLNLVEAYDPELNTWDYVHPMGVRRSCLGLSSLHGLLFAMGGFDGASCLNSVERYDPLTDLW
TVARPMNVKRRYMRSAACEGVVYACGGFDGIAHLSLVEAYDPRCDSWMNLTPMNCRRSSCSVAALNGSIYVIGGNDGSLC
LNSVERYDVRNQVWETCASMIIRRSTHDSIVVNGRVYVAGGNDGSSSLSSVEVLSDGDWHLATAMNVRRSSIGLAVVKC
>Aque_XP_003391072
MDTSSSPTAPAILIPASPGEGAEEKDNDPSMTLLSTSPTPQGELALAGELGTTDKLIFEAKKHPSDTLRSMQELRDEEKL
CDVTLLVQGVEIKAHKIVLAASSHYFRSMFAGDMIESRSSSVELKDVEPDAISLLVEYSYTSQLEITSNNVQSVMAAASI
FDFPFVLEATAKFLATHLHPSNCLGMRAFGRTYGSEALVNAASRHFRNHFTDAIKSEEFLQLPPEVFSELLDSDEVNVRT

EEDIFRSLELWLNYEPESRKDSLPKLLNHIRLPLLPLNFLKYRVESNPYIKRSLDCRDLIDEAKNYHLFPDDYSQIKGTQ
FHPRKSTVGVLFAIGGRGAVGEPFCSVECY
>Aque_XP_003390903
MASGVSDCTVNISTADGGTELDFSLKEPPHSWKQLQSCEEFVGAKRSKHTMVAWDDKVYVFGGDNGKRMLNDFLVSHVND
SSWARVVITGGQAPPPRYHHSAVVFRNSMFIFGGYTTGDINSNSNLRNKNDLYEYNFTTSQWIDWADKVTGPLPPARSAH
GAVIYDNRLWIFAGYDGNTRLNDMWSIDLTSATPTWERIDQSGDSPPTCCNFPVAVVGRSMYMFSGQSGAKITNNMYEFK
FNERLWVRIPTEHLLKGDTAPPQRRYGHSMVAYAGQLYVFGGAADGILDNEVHCFNVETRNWSIIKPYDGSQVPSARVFH
TAAVSRDCMYVFGGTVDSIASRSGELFRFKFSSFPPCTLIKDLTKLLHDLPLCDVHFIVGDKERNIIKAHAAIAAARSPY
LRRKILEAYEKPSGEEELPPFIPSSPVEVTLGGVSTSTFSHSMYYMYTDRIHPELEAPPQGDQVLLMMELYKFSLLLEAR
RLELLCMQYLEASVNVDNVLLVLENASELNLQRLKEYCMKFIVRENNYRKIIMMQSFERLDHPLMVQIVRRQQYRSRPTS
PSPESDDQPLPSTLQDDLRLFLLSDYGRPLSDIVLKVGDQKVLAHKPLLAARCSYFEAFFRSFMPSDNQIEISFGRTVPS
QQAFQSLLKYVYYGDTDVPPEDALYIFSAPNFFGFSNNCLHIHCKAILEQNISISNVVSLLEASDLISLAEMKEHCLKLI
SEHFPSLVHQPEFRDLNKSLLLDVLEVIAKKIA
>Aque_XP_003389395
MATLEKLRVYKQVCLKIRSSLVSGRDDLVSPTLVTRNFVEGFVASLNDPILSKHTLERKIREEEEERERSEITGYRSSSS
YGNGASANCGGGDRKRGGEGSSPECPFIEPAEGWSTEYYETRCQVCDSESLCACRKDISSNEFELRPLEEAAYRAHFNGV
EHWNYFTDEPDIGPIVLSLKQEPCGEKFRILVRSSDHYLHGLIPVGELDVTQLSREEVVNAISRAMNLQTSFKPAGLPNM
VEELLKIDETFVKNKHKVGLLRVKAGQTNEEEIFSNQHEPGPFEEFLGLMGDRVKLQGFKKFLGGLDGDRNFTGEESVFM
EYKNMEVMFHVATLMPFSANDPQQVNKKRHIGNDIVCVVFLENSEAVFNPSWIRSHFIHSYVVIGVTNRSSVPTFSPDLQ
FIPTMPHCKPLQHILLSKIINAERASYHAPKFLKLTNRSRAQLLIELTACLTEFEMATKNCRATRKISRRGTWLPIGISR
PPSPLLDPVREKYTNDEKLVEDLKNALAGPELADVMFIVGENRVPLFGVKSILACRSRVFKSMFKDGVSQTRIGSPSGRR
RLNSSGARVNTTVSSPQLPRKANGFQPSPQKAKLMSAVIVKKKKTSNSSDLLDRSMSSSPEPRTPFLEEPKQIIQEQYTI
PEFNDAVFSQVLKYLITGSCSIQPSTIVGITCAAEHFEIPELKQACFDQLSNCLMVKSVCKILTQLESYLLYRCAKTMVV
RTLEFVDSNAEDILVSEDFLKLSENMVHLILRRDIEVQEILKVNAALDWTTKNIKPNQDFKKLVRPLVKHVRLHLIDAGD
LMKIVVPSGIFEMEKVMTALAYQVDPSSVETNSYSMFKPKIKSSVLSSR
>Aque_XP_003389388
MDTYETNVGEEEDLKSRPHFVDNTLPREALQTMDQLRRNSELTDVELVADSQVFRAHRVVLAASSMYFRAMFTRQMAESG
QRRIVIQGVEPSALSSLIKFAYTCVLEINEENAQSLLAASNFLQMLHAGEACCQFLKDRLDSVNCLDVADFAEFQSCQDL
LDAALTYCRRHFTDVSRSERFVLISYNRMKHFLSSNDLLLSEGEESVFNALVRWVKHNTQERSGHFYELLDHIKLPLLGQ
EFFAVEVAANSLVSSNEQCMKLIRDAARALYNDSYPNNLSHLSRLPTQIPMKWWPRDTLRAGEVIHILGGVSEHETLGNV
ECYDPETNRWVVDLIPQMNYRRSGVGVAVLQGLLFAIGGYLEGKTSTDAVECYNPRTMRWTQVSSMLTARMNLGVGAIKD
MRDAVTGATFSAIYAIGGYSGKSILGTAEKYDMQTDTWSEISPMKTPRRNVGVAVIDNLLYAVGGSNRDDGTRSNLNSME
RYNPDRDEWEEMPPMHRSRGAASVTALGGCLYAVGGYDSGQWLCEVERFDPQMNQWSMIAPMHHSRTGVAVTALKGEVYA
IGGYNGVKTVDVVEKFDPEEGTWKEVAPLTYGRSVPGIAVAYLWPTFSLYNFLLLLISAWAFAFAGVACFPGLWRETAYL
AEVDKEMAGVSIRFLLIVGLVSYCAASEEIIEDKGDIIHGVYLEPFSSLILRDSDQSLRLSIYYDDSITNGIPSSVRDRI
QNSIVPKLKTFFESLLMVRPTQAPILLQRSCTGGSYYPTNINNTIRCAYTCQSSTTCGQTTVPSDHLKACIQCNGNSNTC
TKTGSDGSGISNADMILYVSAVVAGSCGGGSGSTSTIAFATTCQMESALDRPIAGSVNFCPSAITDKVTDDFIIATAKHE
IIHALAFSPSLYPFWRDQNGKPRTDRDSNGYPPRGSGYYNYMWSDSTIKQVTYNDWQVYKGSVSHTVNLVVTPTVVAEAA
RYFDCSSLVGVELENQGGQGTQLSHWEKRILGNEVMTGIIDSNPTLSNITLALLEDSGWYKVNYVYGEYLAWGYKKGCSL
ATKSCYQYMNERSKSGESLTPFCNQFSSSNQGLRYGCTADREYKAECNLAKYTTSLPQEYQYFSDPTVGGGNQVGDFCPY
QNSFSYTSGRDTQCSNPSNQPPDNQDIAAETYGPGSKCFNHANQWTLGTNIGPLMDSASVEKYNLELLIFLIIRRF
>Aque_XP_003389213
MATRVYGEVYKGLSLCPSSSPSSEELEKLMLLSNGFLNELIELVYRNRVNDDAFLTDVLTEKRSHWGLIVSEVNIPSILS
KHSSPFADRVSSQLSCLNQAICEEHWDGAKILFECKCFQAMIRALLDTWRPGLDMSDRVLVYSDTDDTAGLDCTTDIEKD
SLYNDGNLKVNINFSMHSRLQYAHLLKTKENFDVIFLINNQTIKAHRIILAAQSNYFDRLLFGEMSEGSSSSTVPIVLKD
VRVSSFQLLMCFAYTGFVELKSAQLEGVLELLEVADRFCFVKLKDALSKKLTSVITVSSVLPIFVTADHLSLNEVLSLCV
CVLVWVCVCTVKYKQDELEGIEIFGV
>Aque_XP_003388085
MAEMESAIQGRDAVCYGDPEAFASEMIQMINKKENGDVRFAVGEEKQVVYGHRCILAARCEVFRAMLSVPPGNEASASLV
LSDIRPKIFLAVFEFIYSNCCSLSTHMVIDVMAAAIEYGLDGLTKLCVRFMRDSIQCSTVCEFIQAALTYQQTTLQEECL
LFIEINTEEVFNSPGFNEMSEDALSFILKSDKLTMDEEDILMKVKEWAHVNSVVTGSTLSEVAKTVIQHIRLPLLDTEKL
SEIEQQNAKDHFIPVTLIAASWRFHALKRPDPADPHCRPRAGTLPRESLKIVGRGP
>Aque_XP_003387682
MPRNRRGGNIFKVNDEILKELVGRWKITATEIASSSGTDLHTDDNLVEFTLDDGYDIDWNVPVAMETNQLFQTSSFQIDD
RKITFSGTTDGTTFVFNVTFLDDCIVQLKSLDDTISIYIKKSIQVFDLLNALKENYFSDLTIRSSEGIKFEVHRCVLNSS
VQGVKYRDWEVFLSKMSSDASRAALHYIYSSILPSGIAAEPVKELLELSSSNPKLSHLHSLCDEYIDSMKLNKRLIHLVD
SCLHSLEVVKELIVSATMDDPAKVVYTIKQIFKEVVAGVSRFILLSHLYTVNKKGMSKREKREILEYIQGRLVVFIDGGR
ELLQLSISKWETLTDDQQTEISLYIIPNINQIWLDINEYTKELNVSLTNEPTDGPAAPEVPPALTLQQKLAAVVQLNTSK
TEMKRIKKMKKEFKKFVEVGDYRRSQFIGLSEEEKVEKIKRVIEEMTHDGQSIIGRLEDLKKKILIANALDWGSYKLGVS
ISSSYISWGLNRLCFYKDQLVPLLKKLSRLISSDEFNQTLIELRLADPQTLFDETPPTSIPTPLIPTGKKCNAVIPLVGG
CLELLKIQEMSDMILIVQELALPSDDPSHKPQNDEEGVVIHAHRVILSSRSEWFRSALQSGMMEDRNKKIIIKDIDVTLF
RTFIEYLYGKPLEYETMDNNEIIELLAVADRYETSSLHVSCEKELIGRIDQSSVFSFLVVADQLSAARQLRSSTLQFILD

NPDVLIQQVDAYTDLPIELKSEVRQILSGTQQMRYRNETIHEFRRGPPKRDDDDDTHQVMYSSELEELIDTTDTGYTGNT
GVGEIESLLSEMRLVVGDEPTDDRLKSLLMAADMDINRAVNFFFGVG
>Aque_XP_003387455
MASNQHDIAQGIQPDHLTTSRLFKMKEHCSASFTVMNQLRRDNQLLDIRFRIGDHAFSAHRVVLASCSPYLRAMFTCGMK
ESTQDEIMLRDIEPQAMELLIDFAYTGEIEVTTENVQDLLPAAGILQLRDLKTACCEFLSDHMDVTNCLGIKQFADMHSC
PDLVKKANRFIVRKFADVVKTDEFLDVPHHILCELLENDHLHVENELQVFTAFLRWIDYDIDGRAPFAYDLFGCIRIPLL
PKQHWENVFTNHRLFQRSRECQAYMRGYLMGLDFTSLSLKPRSPIATIYTIGGRNSQKCLNTAERYVTEDDRWEELPCMK
QVRTAVSAGSIDGRLYAVGGECETKFSHEGTLYLSSVEYYDPIQNTWSNVAEMRYARSFAAVAVLNDKLYAIGGETTQYC
YKSVEEYDPVANTWSIVPDMHTARSGAGAAALDGRLYVLGGQDRAVHYSSMECYDPNEKRWYMCPSMKHPRSGVATAVLG
RYLYAIGGRDRHRQAYYDIVERFNVDTNTWESFPRLTHSRAWPAATVFKNEVYVIGGYDGQLRLKSVERFDEKEQKWKRS
GDMVEFRAGCGSAVL
>Aque_XP_003387285
MSGLSLGACDGRDFCRSSIRDSTIGINFDALGEVEHYEHVSGDLFDLLKSGEHTDVTFVVGGVRFPVHRVILASRCTFFS
VLLFGDMREAQPGNNEITLQDATPESFRALLEYIYSGKVCLGDLPEQVILDLLGLSNKYDFSHLQSSILAYLKATLSVHN
ACIIYNVANFYQLSDLCLACASYIDINAQAVMRSEAFLSISHQSLVELVSRTSFYCPELEVYFGIRRWLDNNEVSKDEEK
NILKIVRLELIPMSSLLGEVRESSLFEANDILDAIAMINKKNMIELNQRGLLLPEENVATVQHDATVSDNQTILLSGDTE
SYTGDHGYAIHTITRNGPSKGIIVGLNQPYIINCIKMLLWDRDNRSYSYYIEISLDGSQWLTIIDRRDHLCRSWQELFFE
DKVVKYIRIIGTFNSMNRSFHLVSFSCLHTSRRFSLSKDGLIIPDENVASISTSATVLEGVSRSRNALLNGNTRDYDWDS
GYTCHQLGSGYIIIQLAQPYVISTMRLLLWDCDDRSYSYYIEVSTDQKSWTKVIDRSQESCRSWQHIQFDPLPVTFVKIV
GTHNTANEVFHCVHFECPAAPLSSSPNNHFN
>Aque_XP_003387060
MASGVSDCTVNISTADGGTELDFSLKEPPHSWKQLQSCEEFVGAKRSKHTMVAWDDKVYVFGGDNGKRMLNDFLVSHVND
SSWARVVITGGQAPPPRYHHSAVVFRNSMFIFGGYTTGDINSNSNLRNKNDLYEYNFTTSQWIDWADKVTGPLPPARSAH
GAVIYDNRLWIFAGYDGNTRLNDMWSIDLTSATPTWERIDQSGDSPPTCCNFPVAVVGWSMYMFSGQSGAKITNNMYEFK
FNERLWVRIPTEHLLKGDTAPPQRRYGHSMVAYAGQLYVFGGAADGILDNEVHCFNVETRNWSIIKPYDGSQVPSARVFH
TAAVSRDCMYVFGGTVDSIASRSGELFRFKFSSFPPCTLIKDLTKLLHDLPLCDVHFIVGDKERNIIKAHAAIAAARSPY
LRRKILEAYEKPSGEEDLPPFVPSSPVEVTLGGVSTSTFSHSMYYMYTDRIHPELEAPPQGDQVLLMMELYKFSLLLEAR
RLELLCMQYLEASVNVDNVLLVLENASELNLQRLKEYCMKFIVRENNYRKIIMMQSFERLDHPLMVQIVRRQQYRSRPTS
PSPESDDQPLPSTLQDDLRLFLLSDYGRPLSDIVLKVGHQKVLAHKPLLAARCSYFEAFFRSFMPSDNQIEISFGRTVPS
QQAFQSLLKYVYYGDTDVPPEDALYIFSAPNFFGFSNNCLHIHCKAILEQNISISNVVSLLEASDLISLAEMKEHCLKLI
SEHFPSLVHQPEFRDLNKSLLLDVLEVIAKKIA
>Aque_XP_003386880
MASLPNSGGTTASPFISSSSSISARSESDQQQMRSAAGLTRSLSNVSSQLSRTSVPSSTTSPSVVPTTIGSGKVERSCAE
SEQQEPSAPIKKVYSSKKIIEKAFHVLDEMRKGGHLCDVTIRIGTQDFSVHRVVLAATSPYFLAMFTGELSESKQDVVTL
KEVEPTAISLLIQFMYTGSIEVGEDNVQSLLPPANLLQLNEVRDACCDFLKDQLHPTNCLGIKAFADIHSCHDLLSDAQA
FAQKHFSKVMESEEFYCLSHTDVIELVSSTELGILLEEDVFEAVISWTKYNTKERATYLPELLKHVRFLFLRREYLVHRV
CEEELIQSNPACKDFLIDALKYHLLPPNDRTSLAGSNCPPRKRIGMPQSILTVGGQAPKAIRNVEIFDVNSHSCHNGPEL
LSRRCRCGVTILNNSVYAVGGFDGTSRVRSVERLDLDTERWSHVEPMLSRRSTLGVAVLKGEMYAIGGFDGNNGLDTVEK
YNPETKQWIAVASMNTRRSSVGVAVMNDLLYAVGGYDGFARQCLNSVEVYDPNTNEWSTIEPMIQRRSGAAVAVIDNILY
AIGGHDGPDIRKSVECYDPQSNKWSRIPDMFTCRRNAAAAVVYNLLYVVGGDDGVTNLPNIEIYDPIFKTWKVAQGTLSL
GRSYAGVAVVDKPGDGPTATAISQS
>Aque_XP_003386475
MSLSPEPLIDLSDTTSIGSSSSGSSGGGRRRPPPPPKPSLIRSKTIGEVSRPAPTNNTVGAGVIKKPLPPLPPPKNKPLP
AVPIPTESSPTTNRPLPPPPAKQKLTRTVSTPPSTRRPIVNKDEVSSPPSRSPPPPPPRKPTASYHGDSAQSIGQTTPPT
SKRQLNEKLSESLPPAQPNETEADSPVDNGKLVDIDSPPLSPPLSPPVPERDRRPVSLTSQSSFRRTQPVCLPNPVSREL
VYEEKDHPRNSFKRLAQLRKKGELCDCVLSVGDTEIKAHRIVLASCSGYFESMFIGEFSEPEGVPVVIEEVDEHALATLI
DFAYTSCITLTQGNVYHLFEAADTLEFQGVKNACFRFFRSQMNKTNCIRTWLFAESHNCTELLDASLKYIEVNFLNIVRG
REFMEVETETVCRILSLENIAITCEEQVYEAVIGWLNYDIENRRASAYQVLQTVRLPSINREYLMHINDNEAIIHDDADC
LQLIINAIQSHMTNVRGTLMKKMNKENTKVLPRAAAMAVEEVELHKSNCSLGIAVMGGSDRPQHIFRQGDKPGIFIRDVL
PGGAAAKSGQIRIGDRILSVNGVDVSNSTHEGAIKLLVTSGDPLKVLIRHEPPPDGLKELTLVTKPGEGFGFSIAGGVNG
DPANPFDETDEGIFVSEVVPGGAAARDGRLAVGIRILQINSVSLIGKTHQECLKVLQGILDRMTLLVCNGYDPSSLISFS
NSD
>Aque_XP_003386216
MGVGSSRSSSVTSLSQEQEETQSRRRGRRRKCKEAVDDIPSDDEPSLSPKRQKIQHTSTYIYDTFFLKGTGSDVTIKALG
REWKLHKTYLKQSHYFSSMFSGHWMESELPVIQIDIPDANITTDALDIAFSSLYKDSVHLSDSIVMSTVAAAAMLQMDGL
IEHCKQFMKETIGSDNVVPYLRTASEYGLLEIKNDCQFWLQTHLLTEYTVTMLLHLDAEMLTEFIISPDLFVIQVEMDVY
SLAKKWLFLHSHPQWDECISKLSSAADNFFRQREGEAPFLNTERGSPYSQVFRSIRLQHILADPVSIGVLEKDNIIPQEW
LMPLFRQQWLKMIAVDQRTDPGPSVMTSEEFNETSIRCGRILKCSDEYCWRWTGFSFGFDIILSFNNKIISVKRNTRSQP
CLSAVSLQNVRPLSLRLRIASFSEGEGGISSSTQDTGIIQLSLTPDETAYILTVDSEAQFPLYISFDFSLTSHNTSSLVS
S
>Aque_XP_003386107
MEQRVINKRKEYRPTSIALNLRDGQIEIPEVSVESEEEKEPTFSFDNRAGLAHDLSFLAGLPELCDVTFLVGEDRQPVCG

VRAILAARSRVFYRLLYGDAFQPKKPKKKALYRPSSDGYVDKTDFRGQPTVTVLDFEPEVFKQLMEYIHTGAVMLQSRTL
LGLMNAADHYGLDELKMACVQFLDRAINTDTVCALLSSAEKYIQYKSTKILVQKMLEFVDNHAEVVLNLGSFATLPQHVV
RIILGRDELLASEQTKFESAFRWCLRYIEDHPELDLKTAFEPFASKIAFHKIAATHLMKKIKPAQIVDDSVIVTALAYQA
DPSSVQFNPLQLKHCKTASAIPSLDGSPAHIRRVNSSGNSLKLSDEDDGIEEGYLRGGSVPLSNDARPDLHPSRVFDVRA
DSQASELSYSSTGSSLSSACGTVTPQSPVIDRSASHSGFLHISPDPPSGCGGPGKMARKPSTRSESFITLSSTSVEV
>Aque_XP_003386072
MAASNEAAKLQQHLELLKEQYVRLQQRHSDLEQKYTQAVASSGSVGPDHFVTKLVGMVTGLYEKQLYSDITIQLADRSFP
GHRFVLATRSSSWSPSDETLGNSDSLDLSHLSPHVAVLLLRWVYTDAVFLPADQTAVIELLSATNQYQLGQLKEKCEQSL
ISSVTVRNCIRLYQTSEEHSASELKRYCLQIISNHWNDLDTADFADLSAPLLYDMFKAHSKYPLHLAIQHQREDVVFMFI
IEHNSQLPRKLDELDDRGTIPLNLALINKNEGIANTLVSNRCNLDVTDKDGNTLLHLAIARGDIFAATFLIKNGASTILC
KREGQATPLHLVAQYNRAESERYILTRLIKGAGAPADDLVEIGSLLLQYHANLDAQDSQGYTPLHRAIEKGNTPIFEIFL
EHQVINLELRNNSGSTALWLALTQLNSTRGESDRYAARLIERGSTPNAIDSLTGNSLLHQAALESNERAAIFLVCHGANV
DHVNHQGESPMHVAAAIGLHNLIQVLLQYGADPNLQTNLKKAPPSISIPVKVVPAQTPPISHNSSDLISPTSTLGALSVL
SNIASTSPSLGGGRETDSFASQQLLQLNSLSTAASCDTSPVIGRAAASAVNSNPFGDDSDEETAPPTRTHGYGFVPSGHN
SPRMSPVPPARQAPPPALTTDPGTEFIEEPEPFDINMDPGQRTALHLAIAHKHHEVVKVMLQYKESGSTSGKLEISLNVG
LKDGGDETPLSLSLWTNQLEVALNLLDAGADIECQRRSDNMTLLYQAIVREQPSASLFLLDHGSNFRKRTSNNETLLLVA
IKHQLEPVADRLCDLGAEVGVADATGNSPLWVALRSRQESIAAKLITHGCDPNAWHTAPDGTSLSLLHRAIILQDPLTAC
FLVKSGADVNSSTRPSESSSGSTGFSPPLHMACQRGLDSIVQCLVDHHADPNAKDSQGCTPLHVAITTRHPSCAQILLQQ
PRLDLSVKDNRGSTPFAAALATKDNETGLAILKREPKAAEQMDNKGQNFLHLAVLNQDVESVIFLLSVAVDVNSKVQNPT
QNTPLHYAIKTGSEILVRHLLLAMANVKAVDNHNRSVLHLAVEGDFSSILSVLLEHGANPDHADEEGNNALHVAMQLGHE
NCVKVLLTDSDINLTAANMRGRNCLHMLSASPKHNASSLFHLLLSAAAKFPVNTADEDGYTPLLLAYVAGSSSLCEGLIA
AGAHPGMCSKQGLSIFNAPVATKQLLFRILDQIQSEPSWLEGQYCMNCSMKFNISHRKHHCRHCGRLLCNKCTSQQMPIV
KFELNKSVRVCEVCGDSFKISSMR
>Aque_XP_003385938
MPTDQSREDPDLEEGGEGDSRPSKNSHSRFTGQQQHQRGGGGGGVRAVGSAKSTSESSKSKIKVDTSLVEKSESSQVSKF
GSISYASALKNRPMVSHAVEPVRVPGQTSSDPVPLSIPPVIEDVPISPTIPPEVPDINNSPLSWKSETVPLSPLPPMGVA
PEVLSSPVVCVPPVDISEAIPLQTTPIPEPIPLPVPPQSTIVPVDPSVPSSSYPFDLRGGEGPLGAEPEGVEILATVEPH
SDVKSNLNITANEFVPYHRSVPENSNAFSSSSSTGLTPSTTPLVTTVLGKPTTTSSSSSFIPSSSSSCYTELSTCKEPVH
PSYIERADGIHGSMAPPIHPVLSHGLSHQGIASVVPTTLQAPPTMYPSSHLPHQLATPSHVFMSPALPAGTGGLPVTFQQ
INPSLSRVPISTPTHIQSIETKIHSTVRPPLLPTPPNFNLLVSATPTRPVQTTMATPAVFAPPPAAVWTGMRLGTSLNGS
GHAPPPQHIVSPEGHPMALPHQVVSTSGPNGTECSDTNTFRHLAIMNIVAGGISVLFHPLIVSNVSENTGYCKFIGLLGV
YSEHLQLFIVTWIVVDMFVMSSFQFYARRVFEVLEVLTTLIVPLLYVWIPLPLGMYGPTGALCEITYVNISECKEEKNGV
ILITFLCFVPQFLALFLVIVFYIASHVNLHREKQKYTGIYDPQEKPRIEGLMKKLRVLQAYPFIYFGFELIVIIYIIVDI
NIVQILHPYVQSIPIIALNIQGLALSLAYIFDSKTCSRIALSFRYKVAAMRSNRSDSIITYNASGMYVEFQESLVKKGSL
PSYLKGTNSSHAFQCMNGLHSLRTDGKLIDVRIEAGGATFNAHRAILSSCSPYFSAMFTGEMKESENPSVTIHDIPSHIM
EAILNYCYTATISITEDNVQELLPAACLLQLAWVRDVCCEFLKSQLCSSNCLGVRSFADAHSCPDLRDAAHSYALKHYLE
VVESEEFLDLSSEELVCLIQSEELNVQNEELVYESVMKWVQHKVPERKSELPNVLEHVRFALLEREYLVSRVSSEPLIRQ
NETCRDLVDEAKDYLLLPEKRSQMGGTRTRPRKPMGSNEMMFAVGGWCSGDAINMVERYDSVNNKWKQVASMNKKRCGVG
IAVLDNFIYAVGGHDGVSYLNTIERYDHMTDYWSSNIAPTSVCRTSVGVAVLDKKIYAIGGQDGISCLDFVECYDTGTNS
WSSVRSMNSQRLGVAIGVLDGCLYAVGGSDGVSPLSTVERYDPKSDKWANVSPMQVKRKHLGVAVIDNVLYAVGGRDDTF
ELSSVERYDPRNDRWCSVVAMNERRSGLGMCVLNNKLYAVGGFNGNSYLKTVEWLDTVEHQWKNACAMNHKRLGCGVGVV
NLPN
>Aque_XP_003385509
MDGRGESYYEDLEIEGPIEESFLPSRPSYTPYLSTRDREAPQFIYEPEDFTSAAFSSLWSMREEGLLCDVIIKVGCHTYR
AHKVVLASCSLYFRAMFSGDMKESVTGEVTIKEFDPDAVKSLLDFCYSSVLTINLSNVLHLLQAASLFQMNGVQEACCNF
LIAQLHPSNCLGFCKFADTHSCYRLWKKCNVFVQQRFPEVALHEEFLELTLEELINITSDSHLNVRGEEQIYEAVITWIK
HDLDNRKDHVGILLKNVRMPLMSAAFLSREVQSEPLVMDSFDGRGLLIEAMDFHLQKNYMRDTRSAKNRSVNTTPRQCPG
LEYLFSIGGSGPPVLDDPYLDICECYDAERNEWRQVASLNQRRSGLRVATCGGYLYAVGGFSATDTKALSSVDRYDPMTD
SWRSMAPMNQPRRGFALAVLHGCMYAIGGINGGIYYDSVEKYCPKKNQWRFVQPMTVERRAVYAAALDNYIYAAGGHDGD
CLLDTMERYDPSSDIWVVIANMMSPCCLGALVSLKGCLYAIGGYDGTTILQHVQKFNPETCEWTSVASLPIKRGGFGAAV
MDGLLYVVGGCDSLTKVNSVDRYDPEKDKWTSVAKMSIRRSGMGVAVLETTLYNQWMKQFHQCQHILLHPDFCPSEIAIK
PGLYSSMLLHLVEPNIFNLPTTSSDHWTMLNGISYSTVNTIWG
>Aque_XP_003384548
MADHMSVPGDLDGPCSTLTTTQPESPKIPFHKKILLKVHQLRSNRRLCDVVLRAGDTEIPSHRSILAAVSSYFTAMFTHE
LMESRQEVIEIKDMPGHVLSSLIDFAYTGDIELNVDNVQEILSASSLLQIHEVQDLCCSFLMKQLDVSNCLGIKTFVEAN
GCPQKITSDIDRFACRHFQQVAMGTEFLSSSSENVSSLVSSLDLKVSNEEEVYTAVIEWIKQDPEERKTHLPSLLSHVRL
PMLSVSYLMEKVDTEPLIRDQPECRDLLDEAKRHHLLPHQRDIRSPIPRFHPRKSTVGILYAVGGKESSESITRSVEIYS
LLDDSWTEATGMIVRRQQLGVGVLDGKVYAVGGSDGSLRLSSVECFDPATNFWSFVAPMGTCRSGVGVGVLGGAMCAAGG
YDGRSCLNTVERFDPDKNLWSNIAHMSTRRSFPGVAVYDGQLYVFGGNDGTSFLSIVERYDPHINRWLTIPSLNKPRAGI
GVAVLGSQIFVAGGNDGTSRLDSVEFLDIRTNAWQTVAPMRSARDGVSLCALGNQLIAVGGINGPSYLRSAELYDPVSNS
WEDLKSMQTCRAAAGVAVIKC
>Aque_XP_003384303

MEGEAREESEEGSDCSLYKETDHSQRCFKEMAQLWYEKQYCDVHLMIVDSFDSPTSAILAHRVVLAAVIPYFKAMFESHM
REKEEKEITLHNLTPTAVKLIVEYAYTGQVRVSPHTAQDLLVTADKFGLTEIVSFTARYISRQLCSSNCLGIREFALQQS
LVELHKDAAQFVIQNFTAVSKEDEFLQLSLEKIVELVRSDDIRVESEEDVYHAVTLWIYHNSEERGEHADVLYNHIRFPL
TPQRFLDNVASKNPYLQSAKGQVYLKDAYEYYKNPGVTIFSNPKKTQPRHSVQGIICVVGGVDDGGSSLSDVTLYNPHNK
EWTDGPKMRFRRSRLAVALFQGELYAVGGHDLGYSLTTCEKYSPSDRMWKPVCDLETARRSLALVPVGNRLFAMGGYTGS
IYLKSVEVYNPTVDEWSPGPPMLEARSELAAVLLDNRIYAIGGSNSSGDLKSVEVYDLLNKKWELIATMEMPRTGGAACL
LGEEIFFSGGMGGKEIYNVASSYDPETDMWEQIACRMNDCRVGLAAVSLGNIVYVLGGSNGSRYLETVEYYDPSRLTWLQ
GTSLPFPRFAAAAVTLSRNELMDS
>Aque_XP_003384184
MASYDSPRLKMVVVGDGAVGKSCFLISWSSGSFPSEYVPTVFDNYAMDKFVEGHGNVILAVFDTAGQEDYDRLRPLAYPE
TDVILMCYDISNRSSFENIKSKWLPEVRQYIPDAHVVLLGLKSDLKDTAHSIVTYDEGYQLAREEVLSGFHEVSSLRHQG
LEEAMHTAIINGLSDKKKKKRFNLFKRKKSSKSSEPAVPPPPVMPENTRAPWINITSSTLASDWSKLIDHTQFSDVVIHL
SNKRYHGHRYILCTASDVMRRLLGVPLSQFKPSESLSEYECPQWSLKRLTELTFDTINNGQEEGFVSIVAENQSSSNSLP
TVHVTLDEELFTETALKRCLEFLYTGSITVDKISEELDETIKAAGLLNLPELIIICKNAQSDQEFLNPSIGTWLNDRNSS
IAKDLFFNKTFLSDVEFLVEGVTVPAHKIVLTTRCDVLAAMLTGGFSETNTHQIEIPEVSSETFLAMLEYLYTDHSPIEE
TDSVGIMILANQYVQPRLSSLCQLYVTKQVDKACAKSIAEADINIIDLLLLSQLHNADQLSGWCLHFISSNYLVFENKDQ
FKQLETENKEYIEKHRWPPVSYLEAMEEYQRQYGSDEEISGGKKKREKCSVIFTPFTENHLNLADSFTPFTAGSPPIKQN
KRRHD
>Aque_XP_003384001
MAYVVHSPKTHFSDAFKALKSLYYQKDLCDVILKTEDAQFHAHKVVLVACCRYFAAMFRSGMEESQKSEVILHDVDPVAL
KCVLELFYTGHVTIKSDNVQSLLSAASLFQIDHLVSACLSFLKQQLSPSNCLGIKSFAGLHSCQFLVEAAERYSLARFTE
VTCENEFLELTFEEVSSLTSNNKLNINSEDEVFDAIKNWILHDKSSRIIHLTSLLSNVRMSQLSPSVLVDKILRNDLIQN
DIQCRNLIDDILIYTHVLTDRKHLLPSSQLQKRMSISEDGVIYVVGGLGCTENSVYSVERFDIHDGAWYISEPMDIQRSR
VGVAELEGKIYVFGGYDGTINRLSVVECYDIQTEKWSSCSPMLTCRSAMGVAVLGDQIFIIGGYDGIHSLNSVEVYSVPD
DKWTMAPPLLTNRSAPGAAVVNGCIYVMGGHDGLSIFSSVERYDPELQQWVFVANMNSQRCRLGVTAAVGKIFSIGGYDG
HQCLDSVECYDPATNVWQLLPKMIYHRSRVAAVTVGNQIYAIGGYNGVSNMSSIEVYDIQREEWSVGPPMRKHYGAVGVA
VIT
>Aque_XP_003383757
MSLSKWHILSLSKAEFLDDIKWVTVFGPSGNEAILVTQSNELYAIGTNGNCCLGLGTPITPGFTPKRIESLKGLNISSIA
YGSDPHVLALTEDGTIYAWGANGYGQLGRGSTSVHSNLPKPVNHALGGVIVSKVACGGFHSLALSNKGDVYSWGFNNCGQ
LGCGDTTSRYNPNRVTGLLDGKTCIHVTCNHTSSFALTGEGEILSWGQNGSGQLGIGSTLQQLQPVNVQINDAFIDQIVT
GYSHALALSECGSIYAWGANTHGQLGNASKQCVSSPTIINNIGRASEVAAIHLSHISAATISGKVYIWGECCHQNVCAPL
VTDFNSIDEVFACLSSPSCSWRPFMNILPKKDLSSCIENRFNDKETSDMVFIVEGNHIYVHRVILRMRCAYFYSMLQNGK
WKESEMDTFEINDYPYHIFKAFVSYFYTDNVDLPNEDIPDLLELSDCYCETELKKKCELLLKRTISVENAIALYTIALRY
QAKELQECSFRYITRHCSAVILSKDFIGMEESISKKLLVSLAEAGVFKS
>Aque_XP_003383580
MQELRDDEKLCDVTLLVQGVEIKAHKIVLAASSHYFRSMFAGDMIESRSSSVELKDVEPDAISLLVEYSYTSQLEITSNN
VQSVMAAASIFDFPFVLEATAKFLATHLHPSNCLGMRAFGRTYGSEALVNAASRHFRNHFTDAIKSEEFLQLPPEVFSEL
LDSDEVNVRTEEDIFRSLELWLNYEPESRKDSLPKLLNHIRLPLLPLNFLKYRVESNPYIKRSLDCRDLIDEAKNYHLFP
DDYSQIKGTQFHPRKSTVGVLFAIGGRGAVGEPFCSVECYNFRTNQWYEGPELRSRRRHVGVACLNGKLYAVGGHDGNQH
LNTVECYDPKVGRWEYVQPMKTLRRGIAVGVLEGPMYAVXXXXXXAALNGFLYAVGGFDDASPLETIERYDPVTNTWQFV
QSMNVCRGGVGLTTLGQYLCAVGGHDGKVYLNSAEMYDPKRDKWEIISSMNTSRAGAGLVTLDASTFSLPGCISIPESLY
SL
>Aque_XP_003383224
MAVTTNGSSTISASPFYFQSVNHPGHILQSLQDLYHERRFCDVTLVINEQEFPAHRIVLAAGSPYFSAMFKNDLKESTQT
RISLKDVDPLPLESLISFLYSSSLVITESNVQALLHAASLLGIMSVVEACSDFLLARLDPENCLGMYQFADIHGCSSLAE
ASWKYALEHFREVMESEEFLSLSSIHLQTLIQSGDIQIQTEVDILDSVLLWYGHDRPERVRHLPSLIQYIKLPLISQSIL
QEKVLNGVFPPNDPLHSLIASQLNGFLSSQTTDFDPYHPRQSTRHSLIYMVGGETFPRTTVNTVEEYDPLKNTWRELASV
HIARRGVGLGIIDNLIYVMGGSDGRDALRLAERYDPNLDKWTRVGDLNQERSSVSGAVVNGVLYAVGGYNGYSSCLKSVE
KYNPESDSWSYVSEMNISRSMSATAVLNDKLYIFGGYDGASDLSSCEVYDPLTDKWTLIAEMGSPRCMSSAGVLGETLYV
VGGCYCSRSLAMVDSYDPNTNKWTSVNRMIDARSGVGVAVVGNKMYALGGYTGTEYCVTVEEFSQSLNQWTVVSQMSKGR
RRFGCCS
>Mbre_XP_001750581
MGGTDDDIPPRPHPTQAAPTPDRDADSNQPPTPAPPVASLPAALEGPARPNQTAVASPHSPPHSVLLSAPHLVLKPMTAT
LVLEAAGDPISPSPRPKAADPSRGQPTTIMAPTMPLAPPDRDRFQACHHSRHGLPALHEPQTPLPSPSSSPTPSPGHKLH
SDLSELLLVPAPAKSCYAGATFQVQVLHTRHTPQLIWRSSIYHPLLRAQAPVCPCLLRGPPASSTLGPHAKADLLCHLSA
VLAAPELLRPCASCIVNQTAYLEARTDLSFFRRKANAHTPGTDPLPDSELRAAPAEPGLDLSALLTTGTYSDLKILAANS
KIFRAHRTVLAAVSPRLALQFRVRADGGKLVVPYHPVVVARCLQFIYSRALPPPNFPPQHWSALAQLAAEWGLDSLHRFA
CAAEQLLVHRASPLPSPPAVPAQTPLAPCGQTGLDCQCMMCDL
>Mbre_XP_001750553
MFSFCSDSSATATRTKPTPDRGSQPRPPPSTQSADMATVFGDAGEFAQEMKQVINENQYSDITFIVGDTREKIHAHRIIL
AARCEVFRAMFAEQRAQAKSGKKDNVPLVLPDVRPSATNAPKEKKQSSHVNGNQLFLAVLEYIYTNSCTLKPSTVVDVLA

SAIEYGLDGLAACCANYITDNLTVDTACGAIQAAIAYDQIELRDRCMTFIEEHTAEVFHSKHFVEISAETFAHILESDKL
RVPESTVLEVAKNWASVNAVVTGQSLKDTIAPVIEHVRLPLLDAATLQQIEADNEKDPIVPERLISKAWKYQATRKAPAD
DKHFVPRAGS
>Mbre_XP_001749188
MTDARGLPLPKRRLAARQLQTAPATNLPSPSVQAALSLAGSLPSSPARASASLPKPRRQSHAADPPAQLSAPAAPAAATV
EPMPSAPMPSAPLLHDRDPRPADPPSGNLTGLKEPAPATVLEEQVAPVVASPQTTPGSETNVSPDTSVAAPSVADPQATA
HTDRAPSRPPVPSRSPSQQLRTSPTPAQDPAPAETPATAPASSPVRPKPPNRPMPPNRPPSASKPAPPQRPPAPALNDPP
APVAAPRRPPRPTGPSPGPKVTPTPASRPSRPPQPTASAPASISKPAPPSADVPDTRQPEVAPETLPSPRRPVQVPSRPQ
PSPKRRTPPPRPTGPSRTPSSTSVTSPVVLVEHQAPDQPDLVELDDDAFEDFVGDFGAESDSDTNEYHPDQDIVSAQKDD
SGNQPQTPGDGGAPSTKVNELSSGLSPTPSEPTDCTSLRADEYLEIDGADAHSPASESTATGVLAEPTLMPGTAPTLQVA
SPPSPPPLPVPLTLASATPEDRVPSAVASQAAAATALSPPATSVCSSDQNAIPVSPPLPPAPAIASASEMVESDGPHLPQ
ARAQLLQEIAQGSGRLRRVVPSESAEQARPKTFDDDLRAAMASVRKKASLERLPLPADALSASIIAPFKDTLNMIMPIAW
QKLSEGMHALDNVFVNKFDRDAMVEIAFNRTQAFLLLCQLLEQRLNDPTWLGSDAYTEAQEHLARLREQHGQVIAVRQRE
GAWDQQESPSLRQAEPQPDRRDPNLQAAVNALQNFQGNTDDLRAVLAPLATRQASGQLRKLMRAALESGKPLALETLLTS
QAWPSVDVHPLRDMHVLAEVVAIGADFAVPILVQHGVDVNAASHDTATTAMHLAARNNSRRLLARHFRVAQTNGLAQADI
QASDVTVRGQGEGPGLPAHRIVLCARSQYFRGMLDSQVFLEARQPEIVLHDIPTPVLKLILKYIYTGDMAFNTDQIQLAV
DCLMACREFQLPQFFNAIQKKLVGSVDLQTCISLVEAAILCHAPVLLASAMRTFLEHYDQLQAGQDPDGAFLQQFLLGIT
DEHLKVPDTHRDENVNGFFDSGH
>Mbre_XP_001747447
MASNHSFNEHLWHRFDPCQEFVGGARTKHTAVAIDDGIFVFGGHLGTRMSNDVIRFDTRDCSSGAPAAPHPYPCVSLDLR
LSWSVCMTTKGIRPSPRTHHSCDVLERSLYVSRPPDLLARPHLWATPPPDLALAFAWVGRAQLHQVFGGYTGNIAANSNF
RNCNDLYEYRADKGSWRLVHPGSVSPPPDSELGGALGAASVATATAAAPATHGGSGVSNPISVNSRLNLHRSFRETRAAR
LLTARRVAGEDDASAPGHRSPPWGSNPTQAVPLGTSPPWHPDLASAATALSLAALPAPVPAYQAAAPPQAEDEVPCGRAG
HSSCIHNGGGEGTNQSRISYEAASHFYGSVPRSTPSPRSDYLYIFGGFNGTRLLNDMWRICLSDLAPRWECVQQHGTLPA
AFCNSPAACVGDFCYMFSGMSGKDMSNRLFRCHLPTATWSELTGLPRLVGGHEPPPARFGHILHYDGHARLWVFGGVADG
RPCNTVHCYHIEANVWAQVARQVGNDAPSPRSFAAGAVLDRSLFVFGGTIDLKADEISNEMFRFDLTITERSTLATDLLA
LWHSATLADIDVVAADTVVPAHAVVLAARSSVLAQRIAALPEPNPSKPGQALPRRRLVLRELSNGDTLQSILHLCYSDRL
PPSFLHLPPRAALKHVVLLYACARWYKLHRFMADAVEYLTAVARTDLLLALLALLHEHRLQDLLALFAAEACQPRRFQKM
VQSDEFATLDRDLIVFLVRRHESSRRGSTSRGGPVEGTTYTASDDPATAPPLERALRSGSHSLRFHALELDLYRVVEWCL
RQGEAVEASTPSVGTATSADVGSTQDGTSQGSSRVSDLLPADEAIDVVGGLADCQLVAAGQFHVPLHRAILAARCQYFHG
VFCSGLQNVDSRHTEAILGDAILPPPVLTSLLRYIYADVTTMPVEQICRRQMQVSFDENDAVNILLAAHRLNDVVMIQRA
LAVGPGLLYSTVFGCPLLNFGGPEES
>Mbre_XP_001743876
MPWRTKSTTSIKTKTKTSPGTRTRTTSSNHPHSPDDSKETPNVSSPSPQTRANSPALASHTVPPTTDEVPTKRGQQDDLE
PLHTSSTVVVVAANSPENAHLTSALPHHQTIQAAALASVSASSASTGSQPDHRTLHQQQSPSSLVAPSPFASSPSLTATV
PGSSRLKSTRASTTAASDTAAASVPPPHDTPGKPISTSAPTDMAATATATASPHLPTPPQDIEWDVLRKALAGTSFQVNE
HQGAQGLFAALFDSNHQHQKQASPRASTVTSPITDVTTTSTIASHATKAATVQNSYVSVDQLQEKAAPRPASESGPTKPV
AMPATTTELASASASASLLPRRDVGSMGSLPSAQQMMPASPRPADAHAAAPQSAPIKFREGEYVLHSQLMQAADAISRMQKS
AAGVPLRPDPNSPAQSGRNGPRKGSPTPTAFRTLDHRSLPTPNAPSPFADRAMSVRSNSDARPRSRSRKRVLAALARRRS
AVTSPADLVGPHSHTSDGMVTHVNAHGSRASPPAVRGMGRPLDTHNLLVAPATSNYSTMTSRANKGGVESHHSPHVLART
MLDMLEQRHDLCNIRFLVGPQQESIFALSAILAMHSSTFEAMFKPYFAASRDIPQVTVALPSVDANAFAAVITWCHTGEL
RLQGTTVRAVLLMAETYGLAHLEVLCNSILTQNIDAESALDVLVRSLREENLFLAEQALQHVGSHARELLQPSRLVQADV
RPEELAIIMQLDAIQGMSELELYRTVVAWAKQMSFRCDLSIEELVAEPLACLRLNLIDLDSLLSVVRADGLIESSRILDA
IASHSNPHNHRHISHRTQEPPTTPLQPLSWDASLSEAPFGVLNDMRIFLPHTSNRLERMAHAFSVPVGMPCTLVVNVSEL
DDANWVAIGCTATTNTATSILSSPFVAAISSVGAVHGNLLEGAAFNFEEGDELILTLVPEEGTLTITRASDDGTDFVVMD
LPSGVLRLFAVASGGPVGIDVAQQPS
>Sros_PTSG_02617
MPSKASRRKRNNSGNSGSNGDKAVQQTRLRGFHLAQRLHQAIVNDDDDALRSLLKQGKNVNLCDKYGRTLLHVAAACGAV
RCLKHLIQREEVDITITDDENAWTPLHRAAFHGQVHAAVLLMESKTPTSRSVDTSGCTPIDLFTSDLDALTNAPTLLTRK
EELDIARSMLNPSAALSRHRQPLRSHRHVGGQPLHAGNASHGRSYHAHDANGDDDDDEEEDDEDEDEDEDDDDDDLLLLG
GFGFGIGSGGRNSGGSGGGGGNQWAALANLLSSAHPSEVAPMEQTQAVSSDVYGHVYTWGSNINFTLGHADDANRSHPQR
ITAEKHLRSSHDGFVAVSTNKFHTVFVASDGRVYACGHNHLGRFGTGRGSKSHLEPVHIPVSFTCDSAVAGRDHTLFVSR
KGTAYACGSNDVGQLGIISVDVAPTPVPIRFGAGVKVLRVAASKCHSVFLTTHGLYMCGQQNGQLGLPSSVVARDAHTKS
PQLVALTSDLTPVDIAASEAVTACATTSGKLVVFSHFKKRVFSPEWRSAGLEYQLVLESYTAVRASKRDFVIAITRLGRV
HLHHRGVIYPVVFRTPKPITVIDAAIGVHEGRIVLVVATADGHVSTCSFTVDDVKAKDKLGLRGVTLTTVSTLFQVTAVA
CSPSGYHFAAVRQQYRNPLSIHPSALFPEEYAAPVQEIHPSLRQAEDSEDSDEEPEDHAAPNLERDYRRQRRQRREAER
AEQQQRQQQEEEEQRRRGFAVGAGVSVAPATVAGRRHRGGGGGGGGGADEDEVNFWQPYQEPAGSDWQTTAQLHHDWHWA
ALTGTVHTAAPNAVASTRQLYTMCCVLADTAGSWQTHARANRVEALAKRTPGGIAARALAWIERTLVAAGRCGVNTDGTA
NVGQRQASAPSASSSSSAAAVSSSSSSPSSLYSSVAPGAGGAVLACMDTAFLAHPDVFSFFSLHSPECSDSGSMHRTMSA
LTSLRADIIILAPHQPLPAKSRNAAAAGAGVEKGRDAVAGGGEACDMMGGQHGKNEKVFAASVAHSSILSSRPEATAILD
TLADTAAQFDVAGRTVTLFVVNAFTHTHVRSVLRLLYTGRIDEVLSSVRELTQSGLNAAFRTRPSRPASHTCSFAFFSSS
TTAAPEHTTTTTTTTTTTTTRGGDGDAGGVAHETHGDGGETKTSSSATIAKTTMREKNSAAGEANTAADRTPHGDTGVH

EGGDDDVDDDAASVDTTIASISDADDHDDDVGADGDLDGSQDGSQDGSQDGSQDGSTPPAVMSALDARIQAMLDNPEEAL
ADALTPTPSGVAPAPGDGGSGDGSGDATTTAAQRVVVQEEGDGVDEFELMYGDDDDDDDDDDDENEDEDENEDAEGVAKV
GSGAAQEQTQPDEDHTKTEDEQQQRDEQQQQQQQQQHEGASATDATTHEAAEPSTTATEATAAGALRARVLLPRAFAATH
DISLPELVDVLCNVGQFEMAEEIIARARAYACDGLRGQLATKATWFVNAMLARVKAHRQQYRRVYRHVPKRNELKRLLET
HAHHDDDDDNNNNNNNNNNNNNNNNNKNNNSGTGNNDAAGSRGGEDDGDDHSSDEARQRALERHAVLFDAAVHAVLCSERN
TVTADDDDDDDESNGGMETRAVTSASYGSKGKKGRKQGKGSKKQHQQHQRQKQHQRQRKKHDRHGSREYAAAADDDEEEG
DEMTEVVTFLLSGNEFIHTHAVGNCDVAVCCGNTRFMLPSSVLAASSPYFGAMFSHRWNDDPVQRAAIDEAQHVATSLAA
RGGGGTADHSGGDGGGGDHHRRGKGRRRNHSSSSSKSRSRTASCRGADAMPNGGGGGGHNTTSAGSHGDGGDDTKEGETK
ARRAQQERDVGVWGVGEWCNDADADVVDAAFVEEVMEVEVELSPVLWEQCHRSIFHQQLSVDDLRPNQLKDLIVFADYVM
LEDVHAAAQEALASQIASHNVVSFIEFADTFNATHLYYACAHVIVRNLELLLCQRALSKLAEHIVTDVTRVYTASLPRRP
LVLASPTPATDYDADLLHLIPYRTVDPRTGATDDKGEDEAPPIWRADVSNGTSGGDGGGGGGGRENGEQAMPTLSREQLA
ALPRREQVLVQLMGMGFSRAAANWAIERHGLTEAGVLRAVQTLVTRPEVEEMFGGRRGSEAEGKKAKKKKKKKKKLSQQP
QQQQQHEGEEAAAAATQPDQQQQQVGSGDGEQCVEGSKQQRDAGAFPSLSESSLVTAVSKRHKRKAKQQQQQQQQQQQQ
QHTWVSTSASAVKRTQTSPATLQPPSKQTPAMPPPPTHAAATTPTTVDSNQPSKAASASSKPASLLDIMAEEEKHVREKE
KKKKAEQRRRQQEAEKRRKEQVVTRPLSLADKLAGRRMATNAASSSSSSSSSPPPSSSSSSSSLPLTAGAVSRSSSAPSAWT
AAKAPDTRTRAQRRKAAATHATTPPPHFSGTKPQWASTPTPTSASTVLASAQRVGPPSPSPSSSSSAWAEAIAARPTPHV
PLHSSFHDLELEATLEREYAQPSAGGKGKLKGKAKTKQKSRGGRQRTVSGDASNLFTGKSAASSTAVSSSLSSSSSPPPP
SSLPSRWSSGGASHHAPVTSPATATATATVGVGLGSSPLSAMASAPKATPPSSSSSPSVKAGALSSGVSSGGLGGGVKSA
WKSTTEKSLQKAMQAAVEGGQTPGQFLRELEAERARVQRRRQRAMQCIAIEEAAMRDLEDMYNDGNDYDELVTISRVAKL
V*
>Sros_PTSG_05304
MDGRKEGEGGQKPAVARKPVPPPKPRSLSFHVSRTTSGSSGNGGSGSSAWSVDASPPATGQPGAQRRATMSPAASFRSKH
QSPHHVQPAQLAAVPPQLMSGGPLLYTSLDTGVRRSKKTAAKLSSSSSSSGRNRANGKDADDADAADDDGDGDDNINDIN
APHPSLSLRLQGGGGGSGDELPLDESDELHLSERQQQGVVVGGGGRGGIQNASEVEDEAEGGARRTHASDGSHSMHAAEA
AAQQPEEQAQPQQQHDGRQAVLSPMDHRVSTSSLSDMNPPPTPPPPPQFADAAHEAMPLPPVHVLSLDDRERITTIDDND
GDDDDGDDGDHDGDGDDDDAGGSSHVSMLQAIRAGVQLRQTQPREEQQQEDEEEQLRASQPTHVMDELAWRLRRRQRREE
ERIADAAGDAWRQRAHLFLRNLPQAQDVPPVSTLRQLLTDALEIEDSVGGRDAAKRLFPSWPHEYVDDLEAAITAYNTFI
SAHEALRETCHSSKPVTDAHLQGAIDRAVSAHAALFVRTNSVVYFACRRVHLDDSQREPCTHVLQETLRLALGRLWQRAE
QVLASLHDPGLRTFDRRDRAAVCYRLHHTFMGYCRQFAQTLDMHDLLTSTEYSRAEENSFRLFEMHRDAIVVRERVGTWD
KAEHVRLRGQQATSSTQAPPETEPQSLVHRVLSAIADHEPTQLRAALNDLREVSPARVQAKYQSFVQAAAGHNHLECLAV
LLSWTHVPTRETLPARDMAWISAAVNKHNMPAVKMMLDHGLWINDINPNSHATALHMMAHLIRNGADIFIANRHSRTPLQ
VASNVFLPFAAPPPLPGQHGHIHSGQRYTSHTHHHHTSQQGDQPPSPTSAHHQHQHHQQHHHLDLVRYLETGVFSDVTLV
ADDGTAFQAHRIVLCAQSEYFRAMLESEWAEASQREISMPGLTPATLRIALHYLYSGHADYPLDDDELCLDLLETAFILL
IEPMQRHLQVRLQQKLSIHNALEYYRTAVRLGSSMLVAHCCVFILNNYADLQDQDEGREVLYHLLEDLKPTDYQHNTTAI
PRPYAI*
>Sros_PTSG_08954
MCPEHAAVFLTVLEFIYTNRCQLTQNTVVDVLASAIEYGLDGLAQCCSDFIKAHLTVDTACSAMQAAIAYNQTDLRDTCM
RFVEDNTRAVFKSRHFVEMSADTFAFILQSDNLQIDEVDVLASVKEWGTVNSVVTDQTMAEVLRGVVDHVRFPLLDAATL
QKIEEENDKTSIIPVRLIAKAWKFQATKRSDPTDPHFRPRAGTHTA*
>Sros_PTSG_10143
MQRAAIQTASNDSIHTSVNSTFPHSLAVSRAACFPFSNSAMFVVPTAHDKTKKTHPQQQQQQQQQQQQQQQAWNSMDVAD
DINICQQKQQQQQVQEPAQSAALTSTETAAAKTTLPFLPATSNTKATAAQVAAVTTALLASSLATPKPLASSGSSSPARS
AAGSSPACSPPVSVRSSSFALTSSPSSPGSPPPAIGMEHSQPQQHQMQPQLQSNHNAQPADNSSNTVLTLEAPVDSPYSG
AAFNLVCDTAANTLTWASPIYHPAFEQGSRVCACVTGSTRAVADAEVQRRIRALLSNPDLFRPCSNCQHVNTTAYTEAQA
DPEFFRRKARAHTPAAAPPSDDDLRAVTTPKMTSFDFSMLLRTGIYSDVRIKAGSAKTVFKCHRTILAAGSPVFAAHFAT
QADHGVVTLDVAPRTLAHVLHYIYSQRLPRMASMDVLDCIHLLQLSLKLRVPALVKFTVAALRAACVGQEGPLVVCELQR
CGLDKLVLHMRSSAAAKATAQAGSGDEEVGLGGDDHMGGAENNNQQQEQQQQQEQQQQYGSDDGHVAATTGQVDQVDTSE
MQEQQGGRRQQEQTTETGIEVGGGHYAGQHNDPQQIQNDRMVTATDDNDDQQQQQQQQQQQQQQQQQQQQQRVPVSRYEQR
RPCGSFPRANRMRKRRPSPYSLGLCMH*
>Sros_PTSG_10974
MADRTPARHALHQAHHAQPSQQWFPASTTHAVMPLTPSSPSASTSHPGSEAAPLAGFASTACPSQQRQQQRQPAVVLQAV
ASDMQPFCPQTDADAGRPLSDTALVGHRDVSTAPPVRQPPQTYVQHWQQQRQQHWQPHHEQQQHLALKLSPGTCPAPRGL
THERVVQELVWRLHALSHSTHTRTGDHAPHQPCEPEHCAACARLVQKMTGVSTDDQYEKKEKRHSSHAARRSASADTSAP
GRGSQRATSPIERRRTTLLQRLRRRVLGSRPSLSASSHSTSSHTATSDPITNHTTESGRPGSRGSMGRHVSAVAGHADAR
DRQATARDGSSIAPYQSTIDYRATQYPKPSSQQRDGDSGHHHRHHQHGDQHRASTVGRSLHGMDDYVPSGLLQGHALFSS
THSSLPPVQPATIPQPTHVQGALHSPLGEADACRQREQMIQQLWVRTQQQQHHQQQHQHQHHHHQQQQQQQRQQHHHHHQ
QQQQQQQHSLGSPPSHSSDVPPMIIDHRQQISISAVLTSAAPVLERTAATCDVEFHLAPGHPPLYGMSAVLCHQSRVLAA
MIDAALPVSHSTAALASNVTAPLPPTPAATLPSRRAVRIDKWSPSVFDAFLAYMHTGRVAVSSTDMLPQLAACAGAFNVP
RLAAHCQQLMPSGMSTPRNPSSKGISRIPGASTSAPRTASPSLSTYTRLSDMSQSRTYDSSTV*
>Sros_PTSG_12431
MDERALLRWHAWRQLPGVAPFVGSSRSKHAAVTVGDGLFIHGGDSGSTMLGDMLRFDVHDHSWSSCQTVKGDSPSPRYHH
SAVVYKHSIYCFGGYTGDIFSNSNLENRNDLYQHFPDKGMWRPIILADGSNTPPARSAQCCAMYGSWMYVFAGFDGSDRL

NDMWRIDLSANDKRWERVNQQGDVPPPCCNMPCATSTTTMFIFSGMSGKFSSNQLHAFDFGSHTWRRIEFLSQFHGGGEP
PSRRFGHSMVHHNNNLWIFGGCADNMLTNSIYRFDLAKRVWCRIVPEESAFTPSGRCFHSACVAADTMYVFGGTLDVSHS
TRSGHMFCLSLVEPEQPSLMKDIGSLHVTGMLADVYLHPRHRTPGDDGCGGGDDGDGDMKGRVDCGEGGGARRQAAQGKE
NHDDDDDDDENDDGGGDDGGENAGESISCGRGAGGCGVQCHAALMAVRSDVLRTRIREALAKQQQQQQQQQQQRRRQRRG
VGVGVGVGDGARGGGGATASGEGREGSDGGEGSARSDGRGEATEAGSCIEATDAADEPVHTTHEADQQQQQQQQQEQQEH
QQEQQQQQQQQQQQPQLSAFKLRPRPSAPLHLVVAEAASTSTLQALVHFCYTDTLPEWLDVTLATNSTIANLLDLCSLAD
HMHVARLKALCVACVRINTRVDNVLTILQAVTRRNMMDVRQHLLDFVLRPCNYRHVVMSPEFEHLPRDVIVELVRQHELT
HGAATLRTAAAMSPPNTGGTTATTTTATMGGSARVSGSGSGSTMGRGSGIATSPSPLSRQSSSTRILLELTGDAGRSGSG
GSGGISGGGGADHIQRALFRLFDRVLESFVLTHAQGPSRTTSESKRDALLDPDTLVEFDGGVCRVHACIVAARCGYFASL
FRSGMVEARSNCVRLLLGDRLLSPSAAKHLLCYVYLGEVEAVSPQDAVVLASCANYFSFSTAHLHDTCHQRIQATLSTSN
AVPVLKAAALVHNDDAKEQAMAIIVKNLPQLSQQSDFDTLDKQLLLDVLKRVAEAPRVPACTTASTTAHETTV*
>Cowc_CAOG_00261
MSQLYASGAPPSAPSSAQTSPLPVATPMLSVVKEGMLRKQGGRVQSWKDRWFVLTTNDIAYFKTRNSEEPIKRIQVSAIK
SCDEVQAQPDSRGARPDHFYFTLNTEGRLFTICSRTAQERSDWVAAIRQYLPAPAVVASPIEISAEQAEAERAEAETREK
AAAELEAANAAAAAAAVIVPKPFTPATRAPTSDVPLERTFEIEQFVGQMKSIVRSEKFSDITFEVGESNFYAHKAIIAAR
CPKLAGLFQAHTARVPLVGIEPAHFSIVLDFIYADEAALTPANAVDLYHAAATYGLSRLQQLTETYVRDNISVDNVLTLL
KAANRREARELERLCLGFVSDVQRYQSVIRQKEATEIAQVNKDLYVRLSQVLAEAPEAFAHSLGDVIVGPTTLTSDLLGL
LSSVAHADVHIKAKDSEIFAHRILLVTRCESLLHEGGAGNANLAQSGFVTLRLPTLEGQVIYTLLQYLYSNSVSVDRLHD
TYGLIALADSVQQDPIVSVCNEMMRQNLNASTALKILMVSSKYQLLTLKKATLALIVRESGAICTRKDLKPVLNEFPNNL
IDICRALVKGEGGAFSDDEDDDDDEDEDTSDFERI*
>Cowc_CAOG_01182
MSTSWFSSLSPSSSSSSSSSSSSSSAAADVNADSSSRVARSQDGSAKEAPHRTLRSAFESEPSSTSASASTSASTSSASS
AAATTTLLRNSTLSRGLSCSDNDVAVLLNEITALLTPTSELDLGSNYEALPEQWSQQWVQDVPEWSSEYNTTTWAARCII
GAPRVYPAMGDIQGAWAQGTVGMETEFVTLQFHKPVVIKAVNIYETNSPGAVVRILALQRTDLPGGRVAAPALAPAATPT
TEMARVPQGNWVVLWSGAPTVGPHHVTSRIFTPPLERLDVASNYIRLELNCRGATTWCEIDSVQLVGDPGYDETTRKEVM
NSLRQQPVNTGSKLSQDLGALLSSGEFADVTFVVQGVSIPAHRNILSARCPVFRPMFAHDTLERASSQVVIPDIEPRAFR
KLLEFMYTGTVQVSPDVIAWLYHTADKYDVAEVRRICVDEFRFALTVDNVVPIFERVAEFYPELKEVCLRFLLRNAATIA
GGRALETLPQPLLLELTRTFAGGLSATRPAV*
>Cowc_CAOG_01769
MALPPFRARTESAGGGADLSLDFRAEPMYRWKRMAGCTMAPSRSKHTMVAHGNSLFAFGGDDGHKMLNDLLRFDIEKGVW
SKMSVEGKSPSPRYHHSAVVFGNSMFVFGGYTGDLYSNTNLENRNDLFEYRFDLNSWQEWSTVGARPVARSAHSACVYGD
YLYIFAGYDGKARLNDMWRIRLSPEESTTPATPGSAAPTHEWQEVSQSGNHPPTCCNFPMAVASDSMFVFSGHSGAKTSN
DLFEFEFSTGRWTKLTHRHMIDGGDPPPPRRFGHTMVAFGNHLYIFGGSADNALPGDVMAYDLQADIWRAVEPHGNSQIP
TGRCFHACAVVDSGMFVFGGTVDFSQNIKSTDLFRFQLAVIPSCSLHSDYARFYQSGELTDLVFKIGPEQKSIRAHVAMV
AARSKYLRELILRKRGPTSLYNSVVHATKGATATTASDSSSSSSGSSGGGAAPTQGYQTLYPTVAQDVEQTKGARSDAHA
PSRQLAALDTLVDESPPTDYATAPVLELHFPNANFAAFELVIAFIYSDTISHVEYNSNLGAINIEMITHAYELAIQFNLA
RLEKLCELLILAAVTVKNVLSVLRIVSALKLGNLKSDCLNFIVREGNYSEIVGSPELEQLEQGLLVEIVRRQQMPQQREQ
KLSPVDSPEGSLDLDMSNFFQGMTGAYFFDVSFVVHGEVFFAHKSILAARAEYFRAMFRSGMQESKTTRLSAAIGDVMPS
RSAFASLMWYIYCGDVGMPPEDAMYLFSAPHFYGFTNDRLQAFCKYNLEENVSPRNVLQILEAADRIQAHDMKRHALHVT
VECFSKIAQSAKLKSLKKELLLEILNALGEKMTNCQCSHSAGSASHSGERLRAYESKKEKEKEKEKEKEREKDKDKDSKD
SKDRDTAERLVERTTREPVRRDKHDRRFRQYLLEQAENNFPPSDRAAESGVSRHPNSVWRVSEGSSRGRGRQTTSASAAA
SSVGSGALDDADSPLGTPNSASSGAAAGSTSASSTLGTGGSSGSGFAVALAALQSTTGSAPASVGASGSTVAAAQPTALS
SSASAVGSTPVERARSPTRRIMSFV*
>Cowc_CAOG_03206
MLRSLSIEPFERQSCPVEQLEVQVEAILPTTAPATVDRTIPIMMTTTTTTMNDSDDDEDEDDQSDEDEFDDIEGNDNLDT
NHSTANAANSADAAAGSSNQAAGSNDGQSMLEISSASLLLLEGSILTFEADGDLRIVVPPESRDALRPLKLHRFVNYECN
KDDSLLTLLSSDQRAMVFEVQQGDQVLLLDTVRMHWVLVREPGRQHSTFSLERALKDASFCDISFQASDGVVLQAHSAIL
QLRCPELVGMMKDPAKDSIRLATFDAGVLRALLFFLYTDTAPPELVKVARPLSQLAKALHLRRLEAQCVQLFTDDAVLAE
VMTYTHGIEAILQQILVTARRPEVSLDRPEIVLGVAKQHGADLCVAGLLFIRGADVLGRRRASLSREQQKVLYTNLQEWA
LRCMAILNRINLEVGHKLATALRQQNVSSVALPTSLQPYVVQALQSISSFAKDLGVGLRTLASVIKQSEAQNRKRKHAEA
SSAAGRSERSGVLRSMSVFAKYRSYAKLKSAFKSLRKYLKAIPKVCDKVLKADPSKQRERITGALLYMRDSVLQDDEEEF
TRNLERSLRGSRLKKLISATTQGVSMGLNQFIEHPEAVQPLIKRLRDQVQSKEFDMQLVALNLLSASEVDSMDALAASTS
ADSTGRMVEEELVDDVGISFLSSMQQLYTERDATGDMSFVLSDGSAVPAHRVVVASASEWIHAALTNGMQEALNRQISMP
DIPPESFMLFLNYLYTGDLDLSTSETAAVDLLELAERLAMPLVRETCTQYLLKSIDEDNAIALVTVADRFSIPKLQAAAM
DFIVSHGDVIMQEDIQELDPHCAAELQRRVVQHQQHLEAALQRQKERDEQIKQAELLGNLVVPSFGTEPPELEFPEETIS
VDDGVAVSSLVAEMQAIVGEDVPAARLEHHLRAANFDVNRAVNFFFSE*
>Cowc_CAOG_03675
MEDDVTAMLAHVPMGRLTWREDPASTFADWTVVVQDAALSGCQYGDVPALLESSAAENAAAETTTVTRGDTASPPKPGTS
SPPPPPPLQAQQPGLLQQQQQGQSSLAGVIPGANGAAAVGEPLQVAPVALVTTPTTTPQPAAAAVQPPRPAAPTPLRPNR
NAQVRGARAGAGPVQGALPAATPTRVIIASAHPARQQQQQQQQHATHNPHHHHTHHQNHHHQNHHHHHQQQNQQPNPRSP
AIRLPFRGLRHEQQQQPQSQQQQQHQQSQQQGQSQTDRSDAQQHPPPMPLALTQPGEATYHVHACILAVGSRGSGFFRTL
MQTPLESTERQSRTTILYLPQACLPVFPLVLDYMYDGSWDAVTSSTAVPMLRVAHQLCMADLHARLMRFIDETMSTWTAP

VYLADSLVLALRQLYDRALAVCASDFLQYAADQFNVLPVGTLIELLQHPSMHAPPANISHCICSYLLANETLPLCHSEDT
APSISDANPASTPATSSNGTATAVPVVGKSCVFVVGMSQPPTASASQCGSDALVRSADAVPAPNSADAVPANNSAGATPA
LTMSPVLDQKHPSTSTDDALSDEEFDSLTAVLSDVARDSAVPILLSACAHRNQRVIALASKSIAESFFLRQVGELDDVPC
EVLYNILVHEALAAHTEDVVYDVIQEYLQNSSHRWHMTDADVSRLWSACRFTWLSMERLTLAAMQPSMPPALIAQATLAR
CWRLERAPAGVHAQAGAVFSSTAAPERVRTELAQRKTYLQTPILRWHVGPLRNTDATRGGSSGASTSSGAFSFSDIPSSA
SNVFSAMAVAPPVIWVGAHAAAFSSPAPNAATTTTTTTATAASNQSVPSSTNHRPGFAAPTVSSTAAAQATFNSGMPTTT
IFSSGIPSSSSSSAPTFSVSGTATQSASGSIVNNNVRYTAEQRQFTVLTPEHRLVRSVETFSLSECSRVYVEVLINKLHC
CGGIGIADLGLLNTTPSHQQPATWSYSMEGVISSRGVRVTAISPTAWGTNDAIGMLIDLAQRQVQFYRNSVFQGRCEDIA
TSNICVIADLDGNGSSASFSATTLDVSAMLQQQQRM*
>Cowc_CAOG_05579
MPVISQTEGELRRIADASAADELVARLSALIPHDDDGHDADDSHRATQPDQQQQRGQRRSSIVRLVAQPRQAVPVASSVR
FHAAVLAARASQAFIDAFTAPNEVRVQSLLLASSPSSPSTTRPESVETQILRQIRTFLYTDRLLEPKTASKHQERPSSND
LHAVRQVGIHYSMPDLVSAADSLLLSSQAPEPQRCPELVLQVPTWTRQSDASQSFSLIRHIPVQLSPCSTLAASIARLLE
ARESGAQAPAVPEGNVALALDDGTHILAHQVVLQGAAPYFYSILAGAWSTLPSDSAALRTLALPGIAPEIAVQLVVSMYA
GNCSVSAENALALAAAADTFGLHTTVRRVEDFLSKHCHNFHVPCTDQCVSEVINVCNATSEIPACSEMADRCMDWLGLHC
DRVWMLRSFMNLKEPLREIVYNKPFMLLAVNSQQDLGDSDFAIDLDKSVAARQGGVSPRVVVSELLRATRGIAANPQVQW
AKPSLKALEAVRSACLKRLEDSFCDWVLADQDRHMAMPCLCQLVLDSTQDERSPFVLFAERFMLSTTAKTACRRLLAIHT
LAQRLNATQNGLALPEALQRLLAWHIQFAQRNSGWVRRSAEFALIPASLMETIQAGESFIDLDDSASPATPKRNNSQPSA
TTGRSGSRWEASSGAHPATPIRHSAQVRATSDSHPSPISSRAALTPDHHAASRVSRSTAAADHLSGSHAAPATAPVRRNI
PSMAQRVHGGAGLSSAASSPARPSQARDSVQTSLAANQLKSGMRVRLRDARLGTVRYVGNIHVSPGTFVGLELDEPGPKY
HDGCINDVRYFDAPKQHGVLVKLANVETVLSES*
>Cowc_CAOG_05755
MAASPSSTPSPQHHHHGRAHHAPVAHPMGSDASPLSAADAAFQVFLLAAQLGRRDDLAELHDRLASHPSQLQGSSAGTSA
THGQRGGGGSASSSEVKSRPRLLNRAPNASSSTLQVHGTTGQGRGVSDIVNRTSARPSGSGITALMLAATGDHIHAATWL
LEQGAQVNAVDAESNWSSLHRALYMGQIQTAILLIAAGADVHLKDAEGMTPLDLIARDREWPFRVRPTPVAPLALPLRRS
SPVVGDAAFASSPAASDQHESASLSSAASADEPAPSSSSSVFDKPSSALAFYDMLISTEQLLEGADVFSWGLDRNYTLGH
VGAEDRNHPDRVDAFVRMVPRARIVSAVTSSFHSAFLTDAGEVYTCGFGLGGRLGHDCDDTVIVPRRVGGLLDGKACAAV
SVSRDNSAVVTSKGEVFTFGSNEHGQLGVAGLPVGPHANCPKPQLVKALRERKVPIIGVACGRTHSAFYSEQELYTCGQN
FGQLGHAQRDPRILAPKAVPAVAPTSGRISQVVASDTFTACLTSVGDIFVCHSYAVHRLRLPTTIFRPNIVVFRQRKPVP
FRIVAGHGSSLLCVATDGSLFIWLPWLGTQFVHLAFGSQRALNVIDVAMGVNQMAVVSAEGNVYVGNSPDLDRMLAKAKA
LATSHNLVPAPFTLHPSLVDALTVPTLNVYRTRNLHRTVSVSCNPQGSHFLAVTSATMYARGPDRVKVLPSQARTDFGAL
LTSHSSFTSDMHLVSSDAEIYGTIPAHTAILASRAPQWFGALEKDEALTQELAAAAADPFALPQTSYELIAPTPSSAHEE
GESPTKSVMEAFLVPELPQAALLALLEYLYTGTCDLLELPLESKQRPTTSQSRQASGKEGGGKKKGNKARSSSQDHFTAP
PSDADSEHQALTSHEIARTLCDFSLKHGMTELHTLLSSKLETRYMGRRSLRGAIPTFRVTLGTPDVDLLSAEDEHIPVHK
AILVARCEYFAAMFGSPFWSETGAARPALGLPSPTPLRALTVLVDYMYTDSVDLTARDPVSGAFAVNVPLAFEVLVVANV
LLLERLKSLCEGALATCVDIKTVVSFLDMAAVYQCTQLKEACLSFILHNLEAVLEARQLESLLGGSKPANLTPVESGLSS
DSPYANDAFFADLPELAEDASEKILDELGRRYRSTLPPTRRPTQVDLMSFHSAAAAAIPQRSAEKMPAGSMRSSANGKRA
AGASTSAQHNDSDEEAPPAPVAHDGDLARQLQEQEDAEFAAALAASFVELDDDERQQRADQAQRQQQKSRKKAAKLSDRQ
KQQAAQLQEEVAKRKQIQQQSLDVEEPAGPVAKPKKGGRGIRVDLSNPDEALRKIAGGGDSPPLPTVTSDLPTPAPWAAT
SAPAAPATASTVAGSKAGRGAAASSLRSIMSEQQQPKQAQKQQPTVHTAPVSQPAKAAQEKPVPDAKSKKASPAATTAAL
PGAAVASKSGTTPPLLPSSVPLSPASPQTAASILFAPKSPAAAPSAQAALETWAAKGSDRKLSQRERKRSQSRDVAEHTE
PAVPESRTATAGASVAWKSASPAAVQQTLSTNHFPSLGRAPAVAPSPASPAKQVSLTQIHQEQRQLAYNADAVKRKALDL
IQLEEKAIAAIEAFYAHLSLEEETDEMIVIERVVG*
>Cowc_CAOG_06560
MSVYTWGASYNPEVKEQLVPQVEDSLRGRRIVQVACGEQHTLALDDLGNVFAKGRRREGQLGIGDGANSLPAATSFHPIV
GLQHEKVLSVACGAHHSLAVTASGAVYFWGLAHERIEHAAPELITVTARPLTERQRKMVEKSHYQYFSATSSRTAAAGSG
AVLSQAERDQTRSATNPPTAVQTSDTPREQSNPAANVASQTPPATVEHPMNAAGRAALARQNGTSATTTTTTTSSVPPPP
QQPTVSESRDEFHAATDSSLGTGEGESDSVSSSGFRRRIYSRPVRLRLPSARNAHLGSFVAINEHALAERADQDTSSSAD
NLEDIPSEQHVTAVAGGYCFSLVVDTRGRCYSIGFNDMGQLGLGHRFMQGQLQRIPVFDALFPHRQIKQVAAGVQAAAAL
AYSGDVWVWGCGVFGLGLGDTEDQLKPRRITNIWNVTLPDEPPLPVDLVMKAPIVQLVAGSHHFVCQDEGGAVYSFGHGE
YGQQGTGDEGESGATARHGHALPHLVNFTPSATHTSTAKQAAVATQVAMIGSGSIHTFLVTVDRQLYSFGWNSAGVLGHG
DRRFRVLPTHVMALEGERVLSVAGGWKHSIAVVESESATFAFDFQHLCNPPHARLRPSLSGISEFVPALNINQPDLAFVV
ENKPIFVHRAFVASRCRRIAAMIAMTEKYGGKDVRMEPDTAESNPEEFVRAVASTRNGDPTESVQLLQGSIEALAPLVIQ
VSGPRFVVFAGLCAYLYTDHVRIPTFFLRELAQLAQQYGLPRLVLLCKRQSMQHHTPAEGESAPMSTFAADMEGLVASPR
FADVVIDIGKASIPAHRMILSARCPYFRALFEGALAVDSGTRVIRIAADADDGLVNDAEPEETFLNPSTVHDLLMFIYTG
NRDVIGPENAADLLAASDYFMMHDLKQVCEAELIRCLAPDNVQVLQQYADAYRAMRLRHECRKVEV*
>Cowc_CAOG_07172
MATPQSPSPMAASSGGDSEVGKLQKHLSLLREQYVKLQARLAEVEKAHAAASTAAGQIGVDHFVPRLLRTVEELFDKDTY
SDIKINLAGRIIPGHRLVLAARGGNWGVTDLAQVTELTLADLPHEVGWALLKWVYTDRVDIRPDINFTIEVMRAAHRFRL
EPLKIRCEHALMALANVSNCTSLFRYADEAGAQDLRRTCLQLILTYWTQIDPAQFDTMPAPLLFEVFKQITAFTLHLAVS
RGREDVVFLFLIENDTNVVEKINEVDEKGDVALHLALLGRHESIAETLVDHGCDVDSPQATTNKTLLHRALERKDETTAA
FLVSHGASVNAVTSAATGSTTPLHIASKLNLPVTAKLLINSAANVNAQDASGATALHVSIGAGFAEIVNALLAAPAIDLS

LKDNLERTTLWLALVQRNEQLASTLVAAGCDTNAPDNLRNTLLHHAIQCQYTAAALFLISNGASVDAANAAGTTPLHLAA
LFAYAELATTLLNAGAKVNVRDSKGRTPLHNAIRGDNGSQPLGFPATPVHVSDAPRRATGPTNPASEDVARRLIRHAGVD
LNARDEEDNTPLGLALLLQAKTIATALVEAGADKECVSAQGGYTLLHQAILRKDAGAARFLLEAGSSINSKTGSNQSPLM
LAITNEIGPVVEVLCSMGADPNGFDNAGNTALWTALTLRQADVASTLVKYRADVDAMNAQGEALLHSAIRKRDSFIATFL
IGNGAKVNARSSKGQCTPLHLAAEQGLEDVCVFLLQNGADINAQESQGLTPLHVAVESRHPTIVQCLLHQNGVDLTIVDG
LGATPFATALNTKDLDISALILSVDASVAEQADDRGRNFLHQAIAENDVDRVLFLIELKVDVNSRVQDAEHLTPLFLAVR
LGAEAICKHLIQAGADVNARDGHDSDALHVACSLNLATVVNLLVEAGADLTAVDSDRRTALHLAVANQHINVVRRLVKES
AVDLNARDAKSQTVLHVLAAGAAQNAIALLDVLLQFAGGSLDINAVDIDGNTPLFYAFGVGHELLCVALVKAGAQIGLQN
KAGKSVMDEANKGRIQRKIIDAVPAVLTWLDGKACQNCNDKFSVSKRKHHCRHCARVLCSKCCPSDKEVLLAKFDEKKPA
RVCDACHEVLSAPF*
>Cowc_CAOG_07578
MQNIKCTIVGDGSVGKSCLAISYTTNAFPGEYVPTVFDNYSANVMCDGRPVNLSLWDTAGQEDYDRLRPLSYPQTDVFLV
CFSVISRSSFENIAAKWVPELNHHAPGTILVLVGTKGDLRDDTDTRARLAARGTSVVSIAEAQAMARSVGANAYVEVSAL
TQANVKQLFDQVIRATLSPTRSKRKNVKTIVGRSGERIEIPLPPPLPKGVPAPWINVSTSTIAEDMRKLLNSPYAADVSI
QCGATTFHAHRIVLASASRFFRRLFHIAHTDTDPRNFLDHARVNSRQISGLEGIEVSGMPPAYDESDAKPGFAPARPAPA
PPAGTSRGMTLAEANAASAALSPSAVQAFNASSSINALGTHVTIRVAASAVSAPVMLKVLEFLYTGLASFDDKNDHVRET
STASSLFDLGELSTICQNVLEDDQELNPSIGTWLNDQTGQVMNQLFLNNKLLADAQFQFADGRTMYVHKAMLAARCPLLD
VLGHDSNPTALQTIRLYEQEEVLDVLRPFMEFLYSDHATIDTRTANPATLLIIADRYEVKRLVTYCELHVSKAIERETAS
RVAKAEIDVIGLMGVARKCDAEQLVAFCKHFICTNYSAMCKRPEYRLLDAGTRTYLYNQQWPPLSYLAQVEEYEARVAEL
TKMSKTKAARASKAPSSSFSVAPEGIAVGADEGVAGSRCCMM*
>Cowc_CAOG_07856
MGNTASSASAGSSLSAGQHPQHQLEQQQQQHQQQQQHWQFGSHSKTAIFPLSSSQQLPQQPPHQTSSWPPFAASFTSSLF
PADLELSRGPALDPQSDTYSPLASLSSPARRTFSQRESPPSTRAAHLTYESASLPVSHATADAASESAGPTHAAMSDLAG
STRPFDPNPSSVSKSTVDASGLIRRAPMHASKRPRRSASLYIVETLFEDGEGSDITIRALGQEWRLHKVYLKQAEYFCCM
FDSCWAESTSPTIDIEILDSNIDAQALTVAFESLYHDEYTLDEAIVVPVLAAATMLQIPHLVAHCASVMSRTYSATTLPI
YYAATHQYGQTQLLPSLADWMDHFLFVLPPVDLVTLLRQMPLELLRQTIASPKLCVVFVELDVYVICRIWLFLQLQPKVA
DEFIEEQTKYQHELDELKAKRRTTASLSTPASMVKRSGKFFRARGQDVQAYLDTKEGKGVAALFAVLRFQHMLTTMSNVQ
ELRMDNIIPRSWLLPYYEALWLSSLQGGYCDAVPLKAFAESIHTPGVASLSSTTSGSTPNRPAASPLFGNLSPATSAADA
LPEFPEETQQSETESQDDDGASADDADSEGVFEEIEVQTTQSAADQAASLSPAASDSSPDAELPTSPPTPAQHNIASARH
TLTPGASSPLALAAPPPTKPETPSLLRELECFASESIRCGRFIETDVDVTWRWTGFAYGFDLLVTYVASRRTLSLTRNCA
FRTPRSHESALTCINTVEHQPLRYQMSVFSVASFQEYSQASTGVTDMTLAQDTDRVVMTLDPSLKLPLVVSLRLLFPVPS
ATFRSRVVARRLL*
>Mvib_comp15094_c0_seq1_fr4
LCCLIYSYSGALAALPSGHVLA*VLYYCIEIVDWT*HPKKLNCELSNFSRWWKEREARMSNSREQLVLHDLNSFAASREN
LVELRKSSKLCDVTLIVGDEDFRCHRIILAAASPYFRNLFLNEGFLESQQESVVIEQLEAEAFQQVLDFVYSGQCTVSVN
NVGAVLYAADMLMIERLRVLCDAFLRERLDLDNCLTVWTIAESTNMEGLRDACKSLALRQFDTLRQSSAFLSIEEERLAE
LLEDDVLRATEWSVFEGIVAWAQAEPGEERREALQRLFKFVRLLRMTSEELEKVTAEPLVAEHHTLVNRVLHQRYLQLRA
LQQPDEADTFDTEDTTAPSTPHALSSDPSPSSRRRLSTTINLAAAAVASAAAIATTAPFATTTACTSATPTTPTAVSSAF
SIMTSHAKSALRLGGRASSGRATSVPCSPARSAPVTPQSGQHFVTAIGGRSSLGGRVGGDDVGDRSLSIEGVHEGGTASD
AAAPDDELAKRIAKLTVRGRVEMECMFALGGKSGRGHALNTIERYVPRENRWEVIGPRLITSRYRCAAVTVGDCIYVIGG
RAGFTRESVVTVEEFHTTRGTVREVAPLIAPRSCCAAAALAGKVYVLGGLHLDVRLDTMEVYDPSRARGQWERAPSMHVG
RTMCGAGVLHGHIYAVGGHSGMKQLRSVERFVPTREQWEPVAPMFTKRDSCRCAVLNGLLYAIGGYGSGRYLDSVERYDP
ARDCWEMVASMNVQRAGCGVAAIDGRIFVWGGRSRENDVLDSVEAYDPLSERWTIMASLCCGRRGSAEATGLC*ERLVER
GESNRVIERESIFQFL*KQHLLFIFC*KKKKK
>Mvib_comp15094_c0_seq2_fr4
LCCLIYSYSGALAALPSGHVLA*VLYYCIEIVDWT*HPKKLNCELSNFSRWWKEREARMSNSREQLVLHDLNSFAASREN
LVELRKSSKLCDVTLIVGDEDFRCHRIILAAASPYFRNLFLNEGFLESQQESVVIEQLEAEAFQQVLDFVYSGQCTVSVN
NVGAVLYAADMLMIERLRVLCDAFLRERLDLDNCLTVWTIAESTNMEGLRDACKSLALRQFDTLRQSSGRYMVR*VNV*H
*FFFFLGG
>Mvib_comp142230_c0_seq1_fr4
RCVHACDMIDDVLYIFGGTISFYHMMKSSELFSFTFTPIPQPTLSRDLERALSNGIAFDVEFVLPLANARVSAHRSIVAA
RSPALSRHICAALDRQQETKTCPLEVVLDTISDLRAFRTAIHFMYTDELDQEARPTRTATTLSLNNSNSATMTTSPGPPR
RHLPADSSISSFSPASTPSTPPA
>Mvib_comp6440_c0_seq1_fr5
GAA*VTLDIHVVKLLCKHSR*GGFSWTGEMSSRPNCAPNLPQPPVSPNELVMRNTRLWRMRECGQLCDVTLVVGGGEFPC
HRAVLAAAGEYFSILFTGPYADAQRDAVPLEGVDPSAFQQVLSFAYTGAYTFDDSTVTSVLAAADLFSLDALRELCGEYL
LHRLDVDSCLVVWRIAEATSMTRLCDECLSVALREFPTLAFTSAFQDLEPDRLAELIGSDLLRARTERDGFEAGLRRA
>Mvib_comp9598_c0_seq1_fr5
YLRGQHCACTSGGVAPCSGPPQRLLGAVYRAIIQQPEFQLLDKALIVELVREQQDPSRETQWIAALGEWTSTLAADLGAF
LISDVGVRLADFTLTCNGEKLSVHKPILIARSEYYAGFFRSNMSDSRQNSCEITIGDVVPSPRAARALVEYLYTDTVRVG
PADAVYLFVAHDYLAFPNARLQVLCKQSLEEDTSHVCILQIFEAAHCVHAHDLKQHALDMIMDNFLMCAPMEQLQLLKKE
LLLEILQALTQRMGTCSCGILSPATPSSAAATSSRSLSFLLEKPV*L*NTSTTR

>Mvib_comp15778_c0_seq1_fr5
KNQKSNFSMFPESRGRKRRRCDEADSETIEQDSFSASFSNHQTLTAPLSALRVANTILNELRKQEHLCDIYVNCQGRQFP
CHCVVLIAHSSYFRGMFTGSFAEARQWLPAPAVASVFDQHHPQPHYARRQPAQVTGPPPLVSTTTAEAVASAPSGAPASL
PVDSVLSESVSMDIDAKSTTNDNHDDSRDDNHIDNYDKIATLVRPTPTPVKGVKRQRSTPTVPMRRSRRTTRALTSDKST
ATESMGAANESQGALAAAANAVESAALRDSASAKPPTTATLTTTTATTNATGKDNESVSSQSSCGSPAGAATDDEADGDN
GPSSRRKATKHHHHEVLTTTTSPPAPSFPRPPPVFDLPDIDPEAFEQVLAYIYEGECVVSPDNIAFVLMAADRLVIEPLC
QLCVGYLMQHLDTGNCLDVWQLGLDSSRNDLTERALRKTLNYFKQVVQSPAWLGIDLERLIQILKSENLPRDCEGTAFAA
VVNWVRCGLSTEAIERGDTGDVDPAELVQRQAQLDILLPLLRFPLMDPDFLENVVDADPFMKEHPMGKDLLLEAWKFRSR
GKPIGDRSRERAGRWDRVILLGGRRGVESRNMSLSWAPDDREWEVFDPQLPQGRKNCCATTLGNYIYIVGGKMGTTAQNM
RQCPSIRRPIFPANSPWEPFDVQREFSHRSRCQLVASPANDSLYVVGGLNAHEAQHSMYRYNARTNHTSECQHMGVRRGG
CGATLLGHHIYVCGGYDSNHHLQSAERYSIEEDVWEPIRDMSTKRRSCCAVAWSGKIWVIGGHNGQMTLKTCEVYDPNTN
TWSDFASMHTARGGFGVAIIDDRIYAIGGRNSADGFISSVERYDAANDKWVFVTPLPMPCSHFGLVVIKRTSTVINTAAS
PCAND*ILITTIIIIKKIN*ESRAQCQNKSNRNASAGDKDRMRKLRQKYKAHMRLS
>Mvib_comp15778_c0_seq2_fr5
KNQKSNFSMFPESRGRKRRRCDEADSETIEQDSFSASFSNHQTLTAPLSALRVANTILNELRKQEHLCDIYVNCQGRQFP
CHCVVLIAHSSYFRGMFTGSFAEARQWLPAPAVASVFDQHHPQPHYARRQPAQVTGPPPLVSTTTAEAVASAPSGAPASL
PVDSVLSESVSMDIDAKSTTNDNHDDSRDDNHIDNYDKIATLVRPTPTPVKGVKRQRSTPTVPMRRSRRTTRALTSDKST
ATESMGAANESQGALAAAANAVESAALRDSASAKPPTTATLTTTTATTNATGKDNESVSSQSSCGSPAGAATDDEADGDN
GPSSRRKATKHHHHEVLTTTTSPPAPSFPRPPPVFDLPDIDPEAFEQVLAYIYEGECVVSPDNIAFVLMAADRLVIEPLC
QLCVGYLMQHLDTGNCLDVWQLGLDSSRNDLTERALRKTLNYFKQVVQSPAWLGIDLERLIQILKSENLPRDCEGTAFAA
VVNWVRCGLSTEAIERGDTGDVDPAELVQRQAQLDILLPLLRFPLMDPDFLENVVDADPFMKEHPMGKDLLLEAWKFRSR
GKPIGDRSRERAGRWDRVILLGGRRGVESRNMSLSWAPDDREWEVFDPQLPQGRKNCCATTLGNYIYIVGGKMGTTAQNM
RQCPSIRRPIFPANSPWEPFDVQREFSHRSRCQLVASPANDSLYVVGGLNAHEAQHSMYRYNARTNHTSECQHMGVRRGG
CGATLLGHHIYVCGGYDSNHHLQSAERYSIEEDVWEPIRDMSTKRRSCCAVAWSGKIWVIGGHNGQMTLKTCEVYDPNTN
TWSDFASMHTARGGFGVAIIDDRIYAIGTRTYFDSFRFPPDYYYDSHFSFFSMFAA
>Mvib_comp15778_c0_seq3_fr5
KNQKSNFSMFPESRGRKRRRCDEADSETIEQDSFSASFSNHQTLTAPLSALRVANTILNELRKQEHLCDIYVNCQGRQFP
CHCVVLIAHSSYFRGMFTGSFAEARQWLPAPAVASVFDQHHPQPHYARRQPAQVTGPPPLVSTTTAEAVASAPSGAPASL
PVDSVLSESVSMDIDAKSTTNDNHDDSRDDNHIDNYDKIATLVRPTPTPVKGVKRQRSTPTVPMRRSRRTTRALTSDKST
ATESMGAANESQGALAAAANAVESAALRDSASAKPPTTATLTTTTATTNATGKDNESVSSQSSCGSPAGAATDDEADGDN
GPSSRRKATKHHHHEVLTTTTSPPAPSFPRPPPVFDLPDIDPEAFEQVLAYIYEGECVVSPDNIAFVLMAADRLVIEPLC
QLCVGYLMQHLDTGNCLDVWQLGLDSSRNDLTERALRKTLNYFKQVVQSPAWLGIDLERLIQILKSENLPRDCEGTAFAA
VVNWVRCGLSTEAIERGDTGDVDPAELVQRQAQLDILLPLLRFPLMDPDFLENVVDADPFMKEHPMGKDLLLEAWKFRSR
GKPIGDRSRERAGRWDRVILLGGRRGVESRNMSLSWAPDDREWEVFDPQLPQGRKNCCATTLGNYIYIVGGKMGTTAQNM
RQCPSIRRPIFPANSPWEPFDVQREFSHRSRCQLVASPANDSLYVVGGLNAHEAQHSMYRYNARTNHTSECQHMGVRRGG
CGATLGRLRC
>Mvib_comp343_c0_seq1_fr6
GSHSLGFIAFLNDPALCDCAFLTKDAPAPLYASRMMLARSSPFFRTMFSGDWAESANSKDPIKFTSWHAPAVALTFIHIY
SGWLPGTPPPTGGRT
>Mvib_comp3659_c0_seq1_fr6
DGHDQLRPLRVRALGATVVVGLSAGAHHIACVAMDGTVFVFGHGEYGQLATGDLDGEGGGARGGYNKLLPMQLDLTHAVR
QISCGHLHTLALTDSGEVYSFGWGSAGALGVGDRKHRVRPTLIESLQGERVTMIGAGAQHSVALVDDGNASFAFDFAALF
GKAKAIANETVVFVVEGKDIRVHRSIVRARSAVLSAMVAFTERFSRNNPAEETRIPISRKTARFVVFMGLCSYLYSDRVR
LAAFFARELAELAHNWRMDRLEALARKAAQLNGGNGHDMTLSTFSRDLSVLLECGWHSDSDIVIDNQCYSAHSYILAVRC
PYFRTLLHGPLQPVPSPETGRYVFSLDNAPFPAHCLPLVLAYVYANTFDPPSDQVCDLLAAADFFLISELKQLCEAELVA
ALDADNMLTLKEYAEYYNANRLCARINELMARGNNSSFA*TSYRIIIIQTQHNIRFIA*
>Mvib_comp12596_c0_seq1_fr6
F*QVSKKEEMSTRKRRRLGSRATRAYSATCGGEAEAQEELADAEWMPDRGSLQFRLREKGQLCDVTVVVEGQNFLCHRIV
LAGASEYFQKSFTGDFEDSQKTSLELNDLTAATFHSLLRFMYTGACDVNSETVAEVLAAADRYVVLPLRDLCGQYLARHL
CIANCNTVWSIAESTGMTSLVKMCQEKALELFGDLIVHESFFYMDAERLDSLLRSDQLDADEECVFLALVAWARFHDTTA
SGEPIPSAAALAVSAVSNVNALHQAAGPDLNVERLLEAPIFDRPAFMTPTKPALLTRSSALNTHTSFSPSTASTSTAALL
ASAAEPASVEADDDVPVAGSNACKNNTQQDQLQQQQQQQ
>Mvib_comp13352_c1_seq1_fr6
VRQANAIEQQTGIEGEMLERRFQWPAQQLSDLKQIAVVTTELSTSIDCANSFMNTHRDIVVAAPGKVSGTQVTQMVSDII
KRLLKLNAEWFVTIVMSYRVNLASLKERSVAAVRALMEVLIVKALAAHSDLVGLEHEPSAPMGMFNINAAAHKAYDLLTS
SLVLSQRALNSLGLHITDNERLLHVNRLRNEVIESYPAQITVRERVGEWDMEQLVKLKSAFKPQHQQQRSASMSSTGAST
TDTGGTSAPSGSRRAASMSGPSSAPAPVAITIAQLKQSAMEALQRNDPTALSAVVEEFRESNEDRLPENVMVHLAREVFK
FGSPDCMEAMLRVSEPIHRTRWLAECVQAVSMVSPSPQEDCLLRMIHYEGCSLNDQAGESLQTPLHVLGRRHNAAALAIC
IRNGGDPTIRNAFQQSPLQLFSINLPWPFPTLHARGNHTSFMDLSSFCFDPRFSDVQLRLDNNEVFPAHRLILAAQSPVF
DAMLDRSRGWGESSASEIHLQDLQTAPFKVLMRYLYTGECLFPRDDIATGIDIMEIAR*VIKKERREHANSVFVCTADSS
*ATCLHNANRFCRSA*ATTRP*SCTTTPSATKPTGCGTPPPRSSSSTTLSLMTRTTRPFCISLSLLPAPLPPPPPSVKIV
NFQSCSSNNIARLQGFSY*YIIIND**MGCLTFSKKKKKK

```
>Mvib_comp13352_c1_seq2_fr6
VRQANAIEQQTGIEGEMLERRFQWPAQQLSDLKQIAVVTTELSTSIDCANSFMNTHRDIVVAAPGKVSGTQVTQMVSDII
KRLLKLNAEWFVTIVMSYRVNLASLKERSVAAVRALMEVLIVKALAAHSDLVGLEHEPSAPMGMFNINAAAHKAYDLLTS
SLVLSQRALNSLGLHITDNERLLHVNRLRNEVIESYPAQITVRERVGEWDMEQLVKLKSAFKPQHQQQRSASMSSTGAST
TDTGGTSAPSGSRRAASMSGPSSAPAPVAITIAQLKQSAMEALQRNDPTALSAVVEEFRESNEDRLPENVMVHLAREVFK
FGSPDCMEAMLRVSEPIHRTRWLAECVQAVSMVSPSPQEDCLLRMIHYEGCSLNDQAGESLQTPLHVLGRRHNAAALAIC
IRNGGDPTIRNAFQQSPLQLFSINLPWPFPTLHARGNHTSFMDLSSFCFDPRFSDVQLRLDNNEVFPAHRLILAAQSPVF
DAMLDRSRGWGESSASEIHLQDLQTAPFKVLMRYLYTGECLFPRDDIATGIDIMEIASRFLLSDMLAQCEQILSERLSND
T
>Mvib_comp13977_c0_seq1_fr6
FVSLSLISTFVCVTPQVTSMSGAEEIAKLQRHLALLQGEYTKLQQRCVELENQLAAQASLNGTGDPAATDHFVTRILKRI
ETLFDRDELSDVAVVVGGRRIKCHKFVLAARGDSWLADGHNLASVDEFEITDMRYEVVYTMLKFVYTDKVEIKEADQFAF
DLLTASNRFGLPGLKERTEKALMLHANERNCTELLVIADTANAPTLRSYCAGLIVSCWKSIPQEKFDVMPARLMYELMKR
VTNTPLHMSIKQGREDIVFLFIVEFDRQLKTKLDERDSENSQCALDIALQGRRESIAMTLISHGCDPNLTDTSTGETLLH
LALQRGDEYSAAFLIRNGASVNATTSLKRLTALHIATRQGMRTLVELLMQNNVDLNAQDAEGSTPLHVAISNTHDACVEL
LLAGPTIALNLRDNKERTALWLALMAGKEALAKTLVDRGVDCDAPDANKETLLHLAVRNPSMETAALFLIENGASIDVQN
KAGETPLHLACAQGKMIILQRLLAKGADVNVADEAKCVPLHVALDGKHIDAVFALLDSNKCDLAVRNANDDSPLGLALIK
GLTTAATRMVANGADVEARSTKGLTLLHQAILRGDAKGVTFLLENRCSINTMTDANETPLILAINHAMTSCVDALCNRGA
DVNAADANGIVPLWHALSRQEEAVAITLVRYKCDVDRRDNEGASLLHRAIRDGDEFSGLFLIGNGADVNAVCDGMTPLHM
AAQCGMNEIIKLLLQKGATVNTVTSEGDSALHIAIAENNPDVVDTIIQHPGVDLSLRNNKDQTPFAVTVVTRNPGLAYRV
LEQDPQCSEHFDKRGYNFLHCAVDSGDESCVQFLINLNVNVESRVRDPTNVTSLYLAVQRGLENITKHLLNAGADVNTTD
RNGVPVLHQAAALDQPNVVSMLLERGVNVAACDREGNSALHVAVENRNVNCCRRLLQDSVIDVNLKNHRSQSALHAMALG
AGPNSCAVFDLLLQYGTNVDINLLDSEGNSPLLLAFGTGFEPLCAAMVRQGAHVGVENHRGQSLMSNENRGRLQRQLLEL
IPRELPWLAETKLCQGCKDKFSVTKRKHHCRHCGRQLCSKCSCKEISIPKFDLRKAQRVCEPCYESLAMPSTGGGRLT*S
RTRTHTPIHTHTHTH
>Sarc_SARC_02238
MMTASGTNDLVTKRARESSPPPAPPLRTQTHIQSNNLPLVPQPSAMKRVRVEDTSRDQRVWSNNPSRDVFASMCRRSGIM
DDNEHEKDFSIMCTSKAKDDETQEAKRFYIHKLVLRLVSRELNDEIKATTDKKLYLECSEPAGQIFVNYCYLGENVYNEL
KPSNVFEVFEIAKKYAVRPLIKLCENYLHNNRKQRCLALLAYAPEESVAVYLETAKQNFCSLVGAPADVVHLPKQIFKNV
MTDERLMVDSERFVYETVMQWIKANSDGPECAAELLTDCVKLERLLVEEGPKKAEIYTAEMMQVAPEVTQGLWLAAMQNF
FGLQNQLWTEELVNIMRPRCPYGFLQLRITDFSSKPGTEWKGPLTTIVDTQFRLRVSINQTGGYFSSGPVKIRAKVEMMF
DRNGADRYHPLWPYSVDYRIHDLQTPPKSMKVLRSTTPDWVTLTNISQLVNQHHVNNGFMVVSARIARA*
>Sarc_SARC_10925
MGQYMSRTRRTRLSQLPRTTAYYATPTTMGAHTPATTHVLIAGDARAPPVTLKRSRTQDDGSEDEEGDKAQIKKRRVENT
YYQVVGDLKHTSEYMFTQLFENGVGSDVCITALGHSWHLHKVLLRQSKFFDCMFSGRWADDSDSNVTLTIDDPNVDVAAL
STCFASMYGDSIVITVDGLISLVASATLLQLDNVLDRCLATAKGSLDSSNVLTLYEAARMYGMTGIETLTVDYLSSRILL
YAEDTHTPPINWLLGLDAALMQRLVVSSTLWVFTVELDVYSLCCMWMYAQIERDTIEEYIDKTSSDDCSKLLKGWSEEFF
SSWAKENKDTAFLNSEIGQRYIPVFRALRLEHVINAIEHLDKVETDAIIPRDWVHAAVYNWW
>Sarc_SARC_12103
MHSSVITFVVNHRAPTAELSGANMGTTFAWDNHGADIPIPVPTSTLDGSVPSSPEAGVGHEGSKIWETHATRIVMPKPHS
TVDNPRVSPSHSWDTHTAHVLIPAPHSMTAGSESSPQPDSATSRETTTSEDVPFAQTPTLAPSSTTGKSVSSLLPGAATG
HRLSGDGEGNRGVCTRTKRNGDGLDTNSEGRVDPDKETSGTDQKETQPSLFSVDKDNLCAVSTYFRSMLNGRFCEGDQNE
VQITSCGARAFQGLLAYLLPRPYGVSQAKSKLGGARPVGKLRKGGELIKKEGAVFSVIGDGEELIDTAGALSESVGEGIK
GATSGTDKREPQVKTEVDGPSRRESGEWKIWARLKPRRDASKVPMSEDCAVKNPMEAASSGQLKRKWTDSDGRKTDSDDD
VVNVDSDASDLHRGKKAKTEPNPSTGIDPQLHDQQTVEEIRVAQSAFAVDSDHNEDLIEDTRWRDDADTTEVRDQSECGD
SSELLDGDVDTDDEYDMEGISRDVVGNYTNAKTTTGGTAPLIRRRNEMDIHNAGWRDVGGSGASNETQSADSSVHVRGPA
ATASIHTHGGITVRIEKTIGPITNACCEKYFYDNLVPEDALALIPVAQQYMLTELRALCIHKTLENLKQFPEDAYQIGLD
TNIEILQFAGIGTVIEMLPQLCEDRRWVPNHRILNGIKSYLADQALHIS*
>Cfra_g190
MMAREGSSTTDGKRPFTGCEAVQAIDFSAFKRSKTYDLVDENKQWSADPSKDLLTKMIKESNIMQDNELDKDFIVSCQLP
QSTNVKQEEFMFHCHKLILRLVSSRLSAEIRACEGKGMIKLNCSQSAGRIFVDFCYLGRDIFDEIKPSNVFEVLAIARRF
NIRSLQDMCQQYLRNNCLSRCLALLAFSPSSEAEMFMIKAKQSFRYLVKAPEDASNLPVDTFINVMTDCQLNTDSEDMVF
RTVVTWLKKNNDDESLAEKMLEDCVRFERLLVEGNSSQVSFYKNQMKDICPHSVERLWNVAYSNLLALQREPWNMEVMEP
MVPRIPYGVIQMTITDFPDEPTVTVEWTGEPQVALGRKWRIVAKCEVNKGFFSSKPAEIQASAQLLHETEMDRYSHDDSS
WPYFVDYRIHDLQDPAGTIRMDCTSTPGMSVVANVSKLKDHRHINNGHLVIAATIRTA
>Cfra_g2309
MGQANSSVSDIPHNSLRPRIGHTSLSPISSLTPTSRTIPPTNANKRKRDNGSESDDTGAGKREEKRLRNDVSYHDLEPEL
KHASDYMFNDLFQQGLGSDVCVTALGHSWNLHKLFLRQSKFFACWFSGRWQNEDDKYVELTITDPNIDVAALTTCFASMY
GDNYTISPDGLVSLIAGATLLQLDNLLDRCFETAKEYLDIDSVITLYEAGQAYGHTQLEEYTYKWIEGRLFMRAAWHTHT
HTNTLPVNILMEVSIELMEKLVASPALVVMTMEMDVYALCCIWMYIQQNRSNVTYMLSSKANQEVENEIPTLSSMYYETY
KLNPDNEGVRFLHSSEGLPYSSVFKFIRLGSIVNEPQRLRLMEEDGIVPEEWINTAVRQSWLSLLKLGQVSPMLVEKEMH
AMFDKMAFRLGRCLLNDRTDQWRWTGCYAGLDLVMKYWDASREISITRNNTLHESDSVKLVRCATTLRRVLLRLKLMTFD
```

ENGHLAWHHTTELIDMALGKDAVVH
>Contig55830_Abeoforma_whisleri_fr1
LFYFNYFFYFCVSEQNLRLSLLIFT*FLHWKTFSRCSRLRLCLS*SAFFSNELLRMNNNTSSSSRLATDSNSTIGPTGQQ
VGNSIVDLEKSKKTKKNSRAANQKKINLNLKMGTSSPAREFYKWRSLPNHIRGRAKHTLVSHGRSIYAFGGDDSSQMVND
IIAYDIVTQVWSSVKPSRGVPPVPRYHHTAVVRNGRMYVFGGYTGDTQSNSNFENKNDLFSFDFKTKEWIEIVTAGPRPA
PRSAHGSFVWKDYLYVFAGYDGTDRLNDMWRVKLSETVKRWEIVQQQNTPPTNNNFPCALAGDKLYVFSGHSGAQSSNYL
FEFHVATGKWFRLRQLHLLNGGDYPPGRRYGHVMVYHNNWLHVFGGTNDGVLPNQLSSYNLLKHQWVLHSPTGETNSPSG
RCFLSATMLNNTMYLFGGHDLIQVKSRAMHSISFSPFSPPTCDQDYLEMYEQHVMCDLIAEVGENRREFPLHSTILAARS
EYMHKRIIEESKDDINDVVIPNGALLKDKTSQLVQKRGAMFAQGKEKRIRLDDVDGDAFSIAMNFVYSDRIIFNNKDHFD
MDLRSETTVLSASTREKEKMCSNNKTLLVLVNVYTLAARLGLHKLKYMIEGLFCSTVSAQNVLFALGVLTLDKRKEAVTQ
DLKKQCMNIIIDKNNYPSIICTQEFAELEKDLLVDIVRMHQKPFRPIISPAAEPTASWVSPDLMSESENAGPSIEASNFI
RQARKRNNNVHHSTGENSSTFLELTDTTESSLCNDLRNFYANSTGKLHHDIILNLSGVYIYAHKFILAARSKYFRGMFES
NMIENSTSIVQLSIGDIIPTEDSIHSLLRYMYCGDLIMPPEHSMYLFSTPFFFGFTTDRLQSYCKQNLEQHMSPVNVLEI
LEASQRIQATEMKNHALEIIVRNFSEVSKLPKLKDLSKATLLDILEAVARNADNCSCGSF*QMFTLLEKK*NKVIYDAYF
NVWRLKGRRGKNNDFYDKRFGDL*KPLAPMECWIYIFCFTTNENRYYDIWKTAAIRTR*TGDSAEGRVE
>Contig67656_Abeoforma_whisleri_fr3
LRFRFFFSTATSYSVALSLNPFPSPLLPSSSILHSFYPSALAWRLLPRASCLTSTSLTLIS*LMLLLLFHRLLPLLPLLL
LLQSSPISNLHSYFPAVLSLKDPASTLILTSLSTSTLSPISFLFLSYFFPNSPVIKQNIVNLIKFTLKKKKKEKEK*KKE
KPPSSFF*GVAFAFLFSIFSL*KMSLLISKPVLKVCTLDNGKEITTYEIIISNLSSSPAHEGMQYSDSFAAGGFHWVLGI
RDKRGLFLHYHESNSDDVYIEFTLTTLNRKGKMIKYYFKKESSDNGYGYPKLLQAWETKDIIKASVEFHKVEKIEKVIEK
YQQFVKASSLLPFDNPLFSDVCFRCSTDSTDDQRLIHCSKLLLAGRSPYFTTLLNGSFKEKSLGSTEDPIIVKDTSYDAL
YYVLKYIHVLDESASSTLFSSISFLKEAYTLTEMYELPDLQAILKTKFVPTAVNFGEMFEYADTHALTSVVERILIFAQG
PGRKDLFDSEGFEYINGDGKRELRKKIFKLLIFPNDEKEEGTEKKRKVQDN**LLG*DTLAVLPIA*IVNEP*GKKVRNR
>Contig13442_Abeoforma_whisleri_fr4
FDVIFTIQDDNDELVRSNLLLLKARCPKLASIVTNTLQSEEVDPLTGLIHVKISGVSSENFRFMLQFLYSAHTSIESAKD
QEGLLALANRMGLSRLVALTELYITKSIERKIEKSISKCGIDFFDLLEFSQENNALQLKAFCLHFIASNYSVFENLKKCK
LLEGDNKDYIKANRWPPVSYLQKVKEYEKKNGTKKTK**KN*S*L*NEGP
>Contig13443_Abeoforma_whisleri_fr4
FDVIFTIQDDNDELVRSNLLLLKARCPKLASIVTNTLQSEEVDPLTGLIHVKISGVSSENFRFMLQFLYSAHTSIESAKD
QEGLLALANRMGLSRLVALTG*LIPYFYFNPYPR*
>Contig19114_Abeoforma_whisleri_fr4
PIFKFASKCGIYLTPTIMKRVFCHNYSSSAISCFKNGNIFHFHISEHPVVFTKLDFHHFDRKFHLQIYPKNDFVRPGGVY
LHLIEGEPMSIQFRLSALDYQTKTNPLRKDVICTGRARKESHIGNKSFAKKIKEKGYVLSLTFGTEDSPLKNTSPIPALK
TGLFNHARLSDVEILCSLDLQHRPIYCSKNTLASQSSYFQSMFESNFREASKTRGYESKRFKVNAVEEDPEIEKEAKKQI
KEQTKEQTKGKTKKIKKDKTDEEKKNEEKTKKDEKEQTDEETVFEDKTKGVRKDKVLSKSSLASSSSLSSFAEERHVLEI
RDICYEACFLVLQYFHTGKLKIKEATSTLEGFHLVRDMYTLVEMYDREDLKTYLEFYFDPSLENFGPMVIFARLHGITSV
LQKIKGFVILEMNENQEHFFKSESYNIIYQDRNLSDWFFGFMFKK*SIYKPKKQNY
>Contig63705_Abeoforma_whisleri_fr6
LSDSGVNERTLGDHCY*IMSFLTVGNSASAPVISSPDPSLLDSEGGVDASEVETITKEWSGDLLKKIFEQEICTDLTIVC
GDVRIKCHRAILSGTSDYFRAMLSGGMLESFSEEIKIPKCEVNGEALQSILRYLYTGKIVLENSNVTQVLQCSQLMQLTS
IIEICENFLILKLSDENCCAILTHCRVYNMERLEKKAMTHLRKRFSHISNSVGWLENDISTLCSIVEDNDLECEEEEVFC
SIIKWVEPRFSPTSSWDFLYNSDNRIKKIDYNIYFNVVDDHEIISMKASQSTSTSEKSSCEFYNGTTNSNGNSNRYCARP
DIDSSCNFCNDQSSNTINATTISDTNLLYWKDKVDQNNNRNSSSNSNKKINNIVNNNNSSSNSSSSSSISSSSSNHNNGS
NSLSNNNNKTNNHNANGNDNLNNNDNNESNSNANNKNGNGNDSFNSYTNKNNNIINNSNNS
>Contig68074_Abeoforma_whisleri_fr6
KKTVA*ILP*PWK*QRLCSLVYIICTFPII*KPCGKSNIAMGNSLSSVSQDESETELGFKRKRGAEDITENETFTYWCGT
PRKKSQSTAKYIYETLFVEGKDSDIKVEALGKEWNLHKLYVQQAGYFNSMFSGRWEKIPETKFLLEIVDNNINIEGLNSV
FASFYSDTYEIPVDNMMGVVAAASFLQVDGILDRAAYSMAENISDQTAIEFCKCAEFYGHAEMRRVCEEWVSQCICTGLV
EDVMAKLPLALVTRQINAPQLVVIHVELDVYAACRKYLYLQLFPEKINASKNLASEISAYFRAQPKDISFLETEKGIPYQ
ALFAGIRLNTILNDRRNIDSITNDNIIPKARIENVMALCWKSVLTSGLDPEEYEFEKYAFRWGRIIDHNRTLTWRWMGFS
YGFDLLVTYHKATKELSIKRNTSHQRPTSYHNANTMVCMHPRRSIIAKLMVSNKEEFGRQSYRQMTDVLHLSFSTNEEHR
VMKLDENIKFPICVSLNVLFESVES*FLYAPLRSTTITW*QNERNI*KSAET
>Contig68373_Abeoforma_whisleri_fr6
DWQTLDDQDMH*FQKTKPIMKADYKKMEDEGFEVLSVSDLSEYSKTGNFISNLTTKKVTKTTEKRSQLASKVLAVSSQYN
TTTWGAHQCLGPARVYPQYGDIGQTWAQKKTRSSFEYIVLQFDESFAIEGLELEFWETYGCGAITKISCLPEEKNNFEFT
NFGFDTESNYDFYFNNTESKYDFQCNNDFLDKKSEAVEPTAFVREAYVDFEEVWSSPEPHLISKARIFKPELELEFVKSP
KLFTKTILVEINCSKAESWYQLDSVRMTGQTVDDYTEHTSQLDCTRLGSIASDVSKMYLDESFSDFSFVIQGQKINVHKF
IVASRCPDLASLIHRKHQLIIDNFSHDVFSKFVEFLYKDLDCFETGHVALIPLLELSLFMKVEKLTTFCLERLSSNLTAD
NVLYCLINLENLDVVKELCLDFIASNHKKIVPQDQFTTLNSELMLEAIRRISDFASDIS*ASFAIRRISDF
>Contig9696_Pirum_gemmata_fr1
LLMVLLAMFQ*IVVGAR*NVTVLF*VLRANTLLRCLLGLKSVLWFLYTGELSLNNENVSYILQCAQLMQIRTAINFCDDF
LSKSLSNENCCAILSYCQVFVMAELERKCLHHISKHFIEIYKTDDWLNSSLECIIKILSEDNLEGEELEIFKAGMLYIKY

QTDG
>Contig9696_Pirum_gemmata_fr3
VDGIASDVSINCGGRSIKCHRAILSASSEYFAAMFAWVKECVVVFVHRRVKFK**KCFLYFTMRAINANSHRN*FL**FF
IKKFK*RKLLRNFVILSSVCHGRIRAEVFTSY**AFY*NL*NG*LVK**S*VYYQNIIRR*LRR*RTRNIQSRYAIH*IS
NRWD
>Contig9697_Pirum_gemmata_fr3
VDGIASDVSINCGGRSIKCHRAILSASSEYFAAMFASNMKEAQSGVINIPYREIPYDVR*KQNNNINNTNN
>Contig39303_Pirum_gemmata_fr3
NDDVNDKRVYIFKVQMELLTKQSEYFKAMLNRSGMIESKLRSITYNDIDPTVFSWFISYINQNNLEEIKNQVEP
>Contig36317_Pirum_gemmata_fr5
ENENENQDGNKKLEVMEIDLQRRSSGKSLESNDGEIDLKRKSSGKNIENNGFKRKRSKEEVKLCRDSDIWGFAKENLKHL
RDVQIRTTDTDKKIMAHAFVLSCQSRIFLEHLKNNEGICYTIGIGYDYETVQIMVEFFYTGNTGSYALLTPRNVFKVKKL
SIDYEICDLTKMCNQYAELNRNTQCLSLLANCPEVDECQPTMQYAARSFMNVANPTQIVQLSFERLSQLLKHPDLEITKE
SFLLNMLISWINHHKFSIETSTIETIFKEHISFGRILVESSADEISKLSDNPVFLGLEKLWVNGYQQRDRVKQDLWRMEI
LVPLLPRPHSRKIQIVIDGLHLNKKVDLNLSWVIAKKINNRQWELTLELTQTKSFWKAPVKFLTATLKDVDADQSQYSEK
YISYTATYSIIDVVDQTHSSIRLPRCECPHIQKELIETSVLAARQNSLHNNGFMSPHGN
>Contig30798_Pirum_gemmata_fr6
TNNNNNNSNDNNNNNSNNVRNCTWAMAAHKAILDARCSVIRRSLRPDIKTAAFYGFSPEDFKINDDNCQSDLDTLGQDLD
KEKCKITLPSDDTNVIEAFVEFLYTGHCLLSMAKGVDAMALLVMAYRYRLPRLVALCELYLTAAISEATTEGIANSNISI
VNTLLVAIACGAKQLEDWCRHFISTNFDAVAKTIDWEILIDSENQQNKLLTQELKQDRWPPLSYLEDLEQFEVRQAKREA
MKKWPLIGKYIIYKDNKYRKILENEKKTNKELDDGSNIQKRRIQREDTGVYGIGGVQAPLAMEIPKLCVRN*NILVTFYC
GTFFFNF*FVSLFFYNKKK*S
>Contig59437_Pirum_gemmata_fr6
TQTQTSTHTHTTKKRMGSSVSSITGYGTNVENPINSSNSNHNNSNNDESNPTESSSENENTGSHATSRGNDADNLNEGDE
LVYRLMNRKRAAENIDCPSPSKEGDVYWCGTPKKKLKTTAKYIFETLFVEGKDSDIEIRALGHEWKLHKLYLQQAGYFGS
MFSGRWDQKSGSAIDLIVVDENINVEALNSIFGSFYSDDYEIPTNNILGLVAAASFLQVDGLLDRCAKSMAENICEETAA
EFCKTAECYNHPIMKKVCEAWISQSLCAGLSESVISALPLELITR
>Apar_comp15821_c1_seq1_fr4
GCGRTNFFVKDGTKTRRAALQRLPPPKMSIKRCVVCSVCRSGVEL*QSCQD*ISLYHIVLVLVLSLSSRMPM**KVKAS*
SEHSQNQTIQLERMAPFTYDSRAPEDPFQQRMLHMYENASLFHSDVTFKVGGRLFQAHKCIVSAASEPLRAMLEGGNSSG
GYKLASSQKGGFMESTQREVEIGGMSAAAFESILRYMYTGSLCFASWHDAVDVLLAANMYLLDHAATMCTQYLVEHMLPH
NVFVLLEVADYICDETLLKAAQAYITEHAHTLCALMASGDMPGRHVTILIGWPEIHVDEDTLMNSLSSWLKIKEEKRLAK
MAKKDGEARKRSWEELKCWLDQGSNKSSCSINLQTGPKIPCLAQWDKQVSNHPSLTFNQSGDTVRATESNGLTRWVGAVG
KPTRMFPSTKIEFTVNEVGVLGGIGFGVIAGEHDEPIRWRDDGWMLCYWRIAGAPTLCHGDEPVWQ
>Apar_comp15821_c1_seq2_fr4
GCGRTNFFVKDGTKTRRAALQRLPPPKMSIKRLFQAHKCIVSAASEPLRAMLEGGNSSGGYKLASSQKGGFMESTQREVE
IGGMSAAAFESILRYMYTGSLCFASWHDAVDVLLAANMYLLDHAATMCTQYLVEHMLPHNVFVLLEVADYICDETLLKAA
QAYITEHAHTLCALMASGDMPGRHVTILIGWPEIHVDEDTLMNSLSSWLKIKEEKRLAKMAKKDGEARKRSWEELKCWLD
QGSNKSSCSINLQTGPKIPCLAQWDKQVSNHPSLTFNQSGDTVRATESNGLTRWVGAVGKPTRMFPSTKIEFTVNEVGVL
GGIGFGVIAGEHDEPIRWRDDGWMLCYWRIAGAPTLCHGDEPVWQ
>Apar_comp18382_c0_seq1_fr4
SLLG*NSHPRHVISSIHSGKLSYFSW*MNK*KL*G*INVY*CCWVLPNVEPSVLVVCRVYGLTVTLKALLACSRCFRDSF
TSFAHDLFDTKMREINGRISGSAELQSRLGFRQKHEEEERCTGGAVTAIDNVEVVRRMTAGLFDQGDLSDVVLLVGDLRL
SCHRIILAASSEYFRVLLTGPLRESKQKEIRLHDLRPDSFLAAIRYIYTGRIDLDVHNACNLLMVADRLQIDSL
>Apar_comp18599_c2_seq1_fr4
HVVCTVAAHRVMLAARCDFFAALLRSGMRDATDRTIHLHTPCPAEPPVLTCILEYLYTGTIIDWPRSLTQLVHTITLARS
YGIVDLIGSLERRAK
>Apar_comp18599_c3_seq1_fr4
VHGMGCHTGKLFARLLEGQDTTLTDVVFVFPDGVRIAAHSSILAARCATLAGQLAGLENGGEKKVKCPSRTFRHLLRYLY
TGTVGPLTPQETSHLLVLTKQCRLVELQRHIARRPCVPARVTQDIGALWLKTRNARSDTISQAPFTDCTVVLHCTSQPGN
DHVVCTVATH
>Apar_comp19707_c3_seq1_fr4
EEAHCHAHQVILSARSAVFRQQLARLRREGCNMRLDLPDMRPAILRHLLRFLYTASVEIDMDVACELFKAADTYGLEELK
LSCEEFLKQNATIDNITSLLLSAERWKVGPVRSFCLDFVLWHFDAVSVTQQFARDIVPKHDLMRDILLARRPFLEKYANS
VGTDAHGGGLSQGRQRRDCIAVSFAAPLLADLTSLLKVSQDDKFVDVILNVGEGNEQQAFPAHKAILSTRCEVFAALFRS
DMKETKDHVIQVPNIRPGVFVSVMQYLYTGQAAISTLNVFELYRAADQYVLDALKHACEEFVIQEITIDNVASFLVEAER
WQVSSVRAHCLGLAVEAFNEVSITASFLDSVVPHHALMLEILQARIPGTLH*GGHSRGIPHFMLPRYCRTPPCPHARDTP
GQDPGHTALGGTLMRCPSLHASSIVSYPTIRSSPRYSRPVNISNFRMYV*RASSA*LFILIPWHDIHPFHRI*KAQDLYR
MFLIYTMGWKGTKEKRYYFFRATQLI*PDGIKEAG*LTNRVPSSALALG*ICPSRCLQGTTKTFLLGTQSLFANRKNALY
VLQITFE*CVTSIVALRPTFDQVAVIYTYIIDYDHEHIGCYAVDQ*ELAKKVVDFYCVPFQPIAHFH*AFWCTKEPGAFV
HDYLTVIRSTVVATIHTEVLT*S*LNASDTSYWTS*IYRRCSTSNSPRSMYLLLAIINRHNIGLV*QA*QIYV*MQRSVP
TRLFTYATRSLRVGHTPVKHKL

>Apar_comp7266_c0_seq1_fr5
SAHNGGGNLVTGYGTNGDLPIMAPNGQPPRSQEEISSLLSLLRFDPTSSQAIQAVGQCLTPPGSESMLVDGNGIIGGGRC
NANIAHNNNNNGGGNINSQTNAFSMQSMKRHPIATSASSHPSNWVANQNSQESGEEKLGWGAVVSEVEGGFNTVQANFKR
DMLLLARATFYGEDIGLPDRTDCTVVYDDVTFRAHKAVLASTSGFFRRAFYPTMPTQESTASTVRVHRNVCDSAEGLRSL
LAYCYVGDAAVFSLNSLVSIGPSLSYFSLEEDMWLTSYFEKVYIYYCEDMEEEGEVECSNITAQPMDLTVSGHGAM*LAA
FSNHPPITDRFKCTQLPNTPIHALR*KRIEFRGFLLAVGFGF*LSTVASIWSEGVFGPRFVIW*VVMQ*GGGGFST*PPC
PPGIVLLTEVRSDEGKND*VRSVMITRWFGVN*VAARIHPVYC*VFQRRL**DEICEGMGWSFVWEVLVSYYYKVCVGQI
YRVKSGAHTLM*HA*HQRTLKDNMYITRFATCTQTQKDTSTDTRSRTHIQTHAG*VDFARWNRMVWGE*RIP***MPFRT
CMGLI*TV*SCKIISFLT*RGWFVLLLFVFPPCVHADWRLREVVPLLVPG*LLV*HVP*LLSTIPVHVRTPMNFPTSPFS
PSPCSVCAL*HRKWF*HILSVVLYFSV*DTAD*LVR*NKVKL*ARDLCVG
>Apar_comp9153_c0_seq1_fr5
SVAKNIR**V*LWFSSLSFHFPVPSASTPASAQHTLSSLPSSSAHFNNMAAAGLPPFAFLPPGPNNPFPFSDDDDDSDDD
SMDGWNDDRSLGQLWKRLHTVVWPDTTVGFCLAPNEYDMNECLDMVADPGKARIKKLDPKELEKGECRGTIYVEFSSSVP
LVKWKNKVKEFFANTKQEPTKGGEALRGWINNSQFADVSFVMFSTGEKIYGHKVILAARSEKFAAMFRFHDLATPGQVPS
LRIDCDESVVFLALLEFLYTETISDDTNRALLKLPSKHGYQQLLVLADEYGCPDLVSLCEGPVVKELSVEDLGENPRGMY
GNDNEEINQARDRMMIKNMVQAINLLIFADRHAAARLRSYAMYILLMNTHFTKLVLGDKGTVARVFGAEDGGGNGAVLGE
LRRLVSLVPDDVKAGAWKTTPFGQCHRIHPPEELYITHPEFSPVCVDENEAEKIARNIANGDSARKTLRAILQLDANTGF
GHLQEAKELDHSMRAEVIPETWEVLKTTARYLDEDDFDGMEEDEEENREEDDEEKRRKTEEKVKDVKTKVIAALVRDIAF
TKTTEAEKEMEKMAMMGHPLALIAMRAGLTAPTSYLSVSRLSDVMRDEEGMNGDGER
>Apar_comp10452_c1_seq1_fr5
RASPDPVRGVVAVATALGAVGLLHGLRNSGRIRRGGPQEAAFYRALSGEADHRAQTLQRGDGSGPYAHLYDATVEIGGGA
VTLAAHKCLLVARSQYFQAMVLGGFREAGTPHL
>Apar_comp15715_c0_seq1_fr5
SRLFLPTLDDYFPAPLKLNPARVESAGVILPAATNMLGTHELQLMQFVKRDNQTKRPQRLLTYSSVVRDGEVFFAFTGPI
RLTESNSTVSVFVKLFDPSRKTPCAQLSYKGILQCSVKDTLSSLKPRLCELARIPCNTVIRLFECVHEHMVNALNEDDTV
ESAELITGDIIVIQPANDEWGTFAECYRHFPAQVEHTVPQPTPPVDQEPSLGEHLRDALADPMFSDVTLKVGNELIQAHR
VIICRLPFFMKLLSLDMIEARAAGVEPITLKELDVHAFKHVLNYVYSGTFYQADMLPTHTLLKIITTADY
>Apar_comp16488_c0_seq1_fr5
VFHVHHT*ELRACDAWCGAFFAAYEELFWHIGASTFFQRVGQDFTERFGDHWVTGAHRESHSNQ*LFKMAVYSWGYGRSG
ALGTGADKDVLEPAIVDRELRGKRIVQVSCGEQHTACIDDLGNVYCWGRQREGQCGFGRSIKPLLEPSMVPGLEHEVIVR
VECGHEHTLALTDGGVLYMWGLMHVQGPQVKTQYFGLTDLNEGQRRDMIEKSYYEYLSNGGVSDDIHQSMETVAGFRRHV
VTQPRQVVIPNCPHKVVAMAAGYCFTLIVVSNGDVYAFGFNDKGQLGNGTRFMTEVPTLVPELSHECVVSVAAGTQHSVA
VTRDGSLFTWGGGIFGLGHPVPKDLLTPKRVMALGPDIGRRVLSVSCGAYHTVCLVEGGAVYTFGHGEYGQQGVGDFGER
GARQRSFALPNLLTSLPAPALKVACGHLHTLVETTKGLYSFGWGCNGVLGVGDTKYRLLPSLVTTLRGDKAVALAGGWKH
SVVAVQGGDTTYAFDFAHLVTQTERAWRWADVVFVVEGKELPGHMAIVAARCPLLAARLLMAVRFCGADPHKICLPLPGT
RYIVFQAFLSYLYTDHLRCSAFLARDLQTLAATYQVPRLLTLRPRLVIVHR
>Apar_comp17282_c1_seq1_fr5
PGRCQSVPATYQLPRLVTLSRRLMNVHQNIEIPPSTMGEDMETALYSSSNFDVVLQAVDGSVQAHRAIITARSPFFYTII
EEARFGDATSGKIDLEHTGISKDTLQDVLVFMYTGKRTIIDGGNVLPLMAAADFPVLPAVKAPLG
>Apar_comp19714_c0_seq1_fr5
IGGKGGEKEKSRGSALLLLRLSLLGTSVHTLMPHTYCVADSFIDPVPPSRRIPHSWFLNSSPHTRRKQQHTSHCFSARCL
NVQSINI*DQAASFPELLSKAIVEKQSNMQLKEGLFASTAGRTVGEIEKRELFLDLVEACRKGDVERVQYLVEFEEVPIN
VSDDFDATPLYYACLCGRTSVVKYLLDKGARCAENTFEGERCFYGALTMEIRNLLKAAKIVSGLTLLATNSYRQFLLGVQ
ETGLYSDITFSVHGVDFTAHRAILAARCPYLSDMLGSKWAKKTMVTHNKAEIDPRAFEVLLRYLYTDRVDMPSSMAIPFV
RLLRQCKLHELIGHVAEAEKQNKLQLVIEPLRGSLVFQQQMSSLHRLSLPPELRVSLEDGVIEVADVSDMEVEVDGHVFR
CCKVFLCGRSEFFRTISDGFFVQHQATQRQAQGEMENQGPIKVGHIPAEIFKLVLEYIYTDQVAVLPHSMALPLLRAADY
LMLPGLKTVCGTVIGQTLTTENVLGIFGVARTYDLLRLEDRAVMFMAEHLHKVIQDPSFVDLVLEEANSIKNREEIDSIP
IIDEIRTYLDQLYGFADVDSEDEERVSAEPLEFTDPKLQIKQEKLLAIDRLLERLNLVA*FRGHPP
>Apar_comp19789_c2_seq1_fr5
S*LNRLEVGS*HGATNQSLEATTPRSHSQSTFPAMSFFASANLFCKGTRYGRYYEEGRLCDVTLQAVARDGTSRDFNVHR
IIMAGAGDVWEALLGGRGFLEEGQKVIQIHETDAETLGLIVRSVYGLEGPVSAEDCVHMINAADKFGMENLRTALSARFI
KLKPIFADNRDLFLRILLGTAAQTEDSSSDGESDSDSNSHETAAKRGKFDHTEQPEQPGDDATLKPFYCVDEAVREYCEE
QVVAEFGILVADEELLSQMTDTIMVRLLEANNLNMPEIEIFRAAMKWSELTDIPLCRPEAVEDCIRYPMMNGAEIAEVRK
SELVCDSTVLLAYRARTEGHIDETDQEWPFKWLCPRFSQEKMVKLPRFSKDIVGMEYSSKKFIICGNFWRLILEYREAAG
QATHAAQQADGQAAKSVGLWVFLCWDGASGDGEDDSAGLVVSYGVRCRSLPGTPQVWDHIHYKARFSWGQHLFMLDLDDL
PPVLAAGGTLRFDLGLCVRSMRSPWGDN*CSRML*SMHRDIIYPPQYLFNNPAIYLPRYFSSYFSSCHR*LLEVCEHTPG
*YTARAQ*GGGWV*SDRQ*SLLRYVMILSVFTGAYREGKLALYNRD
>Apar_comp19789_c2_seq2_fr5
S*LNRLEVGS*HGATNQSLEATTPRSHSQSTFPAMSFFASANLFCKGTRYGRYYEEGRLCDVTLQAVARDGTSRDFNVHR
IIMAGAGDVWEALLGGRGFLEEGQKVIQIHETDAETLGLIVRSVYGLEGPVSAEDCVHMINAADKFGMEHLRAALSARFL
KLRPKFADNCHLFLHILLGAAQMQGPDPENASDWNSNNETAAKQGKYDQPDDRALMPFYCVDETVREYCEAQVAAGFRIL
VGDEELLQQMTDTAMKRLLQLNNLNIPEIEIFRGTLKWSKLTGIPLCRPGAGIELEDCIRYPMMTGTDIAEVRKSGLVCD

KAVLLAYRARTEGHIDGIDEEWPADWLCPRFSQEKRVKLPPFTLALSHQTNFGKTFAFCGNDWRLMLQYEYDDPDEPGET
DGQPPKKLTLWVYLFWNGASADEEDDPAGLVVSFGVRCKSLPGKPQAWGHKQYTNSASDWGQCMFKLDVDDMPLLLAAGG
TLVFDFGLCARSLRSPWSAL*P*RRYNGYLVAIYII*SDDA*YVFFLFS*HAFSIFRAKFERVLKHVVIHYDL
>Apar_comp20054_c0_seq1_fr5
VEKQQRHVVSRSFR*LKPNLISFELFCFFLPPFFFCLPLSSLCSVLPIPIMSEGVRNDSSRARPASMYKSGQMADVTLVA
VAPDGTKQTFQVNRAIMARNSDYWEKLLGTADFLEKRTTTVTIPDIQPRILDMVLQVCYDGALENIDPESHILLISAADR
FCMEQFESRLVKELFDSRLTYTDNQDLLIRIFLGSLQAEEKTGEEENESENEENEEKSEKEKGTAKEPEKRKRGRKTVES
TPKRAKREKENEKGDKGPTGHVPLFCSNAKVRNYCTDLVLANASFVLEDKEILHRLPAGAMKLLLSHDYADAKEIVFFRA
AMEWCKSTGEKFQEVAECVRYPLMSSEEFAEVCKADVLDKKGYRMLVRVLQRTRTESAAKEPIECPKSWMVPRLTNRLEA
KITFPETFSGQKTDRMEFSSCGFDWLVYCEFAKARDTDNFVVHVRPKYRVVDSAHSTYVCAVGMQWKGMPFKQRPQFLHH
SDYFRSMSESCRFVFAPTKEAVGKLKEKTNGEITLYVGVYVKLAG*EDCAGGW*WWFDGGF
>Apar_comp39010_c0_seq1_fr5
GAHKRYIRKLWKGSSILISSETIIRRARLQS*NRFWSLCVCEASF*P*FRIAFSNRLVASAITGQVLCSVAWSCLMPTGG
HVQFGFHGINQVEIIVVL*ILHILFSSINTWKSPRLGTPTKMSKTRPSKRLARHQATAPYSTPSKITQEEGDVKRNSSFL
VNQVGRLFLSKDLSDCTLLARTGKGKGSYKRFPAHRQILGACSDYWRGLLCGQFREAHQSEIKIEDVSADVLEEVLRFVY
CGHVQLTADILVDVIMAAKQFQLDELRDYCLEQVRQQMTPEFCCTLLESTDDLDLDDLKAECRRYLGTSADMVFETPELA
KKLSKETLLWLLKNSDMTVDEYVVFQGVCMWCEAHANDPNDKEELRALYEPFELLVRYPLIHPRELAEEVRLTGVVKESL
LLDAWEHLHGINTSRQPQWMLPRSSHLRWRIPWDKFKTEEKYVDSHPIHINGNTFRMRIFPKGTDGTSTHLSLFVAINKD
AATEKTWRTAAKFSISLVNQVNEAKTEKKNYEAKFKHDTLDWGHRQFVKLSDVVAENSGFIVNNTMVLDTFFEEVWVPAD
TPAESEAEDAEA*FIVFLCVCVCV
>Apar_comp166944_c0_seq1_fr5
FRLSPSSRTS*LHKNNHIAEGGSRTPVHVQI*VLAFFLTQSDLALKKGNCKKPTHPSKIHKQAHTSFSKKMAEQGNPTPP
SLFVTSLDGQQERFARLYSERRLCDLVLTAAEEDGSGRKEFHVHRCFLATTSDYWEGLLGREGFVEAGQARVGLEELSAH
TLGLLLNCVYGIPCHVGPRDCIDLINAADKYVMDGL
>Apar_comp221596_c0_seq1_fr5
IHKAIVASASEYWSNLVATESFVEKE*SVVQIWEVEPATVDLVLRHIYGLKAPMDAASSVAVINAANAFLMSPLQDAVTK
ELLNSNLSFASNRRLFAEIAYFHNEQPEEYVMSGPTGDQESDSEN*NGAMDVDATIETEPNKCFCSYWLFG*RSGSWWLS
IVSESGQW*PKMLGKVGFGQGTTSAGLKNVKLLLPAGSSTQIWSAFVSQCVMYW*CALQCFAVV*EYIRAFLDYKNV*TL
SRVLPY*RLY*P*KKRVGDTISVIALQRFAGLYASLRIPVL*AQHYVCVVLHCVMITLWGAGI*RCIIVCILHI*TPVKN
TWGNNMSTVLRCSTTCFSAPHKQLIFQADQTNK*TVRYFYP*AKSIQICEL*NK
>Apar_comp12887_c0_seq1_fr6
FFDVKIFLVGMSTASKRFKWKTYVHTNSQTSLVILTSPFG*ATNLKGV*RVADNVPCFLLVPLNIFSACRFPHTSSPAGT
*FHH*NIPITYSIVLAP*RRIRHK*PFLGARPQMWNLLNVFTGSGQNPPIPPLFAQGGELGVQEQNKNMKDSDAGDRGLK
RSRSDSSDEEEEVVETAGRPPKKPKRADDFIYQTMFLNGEGSDVTIKALGTEWKLHRVFLKQCGYFEAMFNGPWEDANKS
THDLIVSDCNINVEALDTVFASLYFDRCKIDVRNLVAVVAAATWLQLEGLQDRCARMIEKHLCDDTVMDFCIIAHEYSNR
EMEDRCLSYLELRLFLVTERQLSRLPVHMLKRLVRSAKIFLIHIELDLYVLCRTWMYMQIAPTKLADTATPPKKDQMSKE
SQAYFYGRKSTVPFLETEEGKEYREIFEPLRYHTIMNDPRNINMLQRDNVIPTAWYTNTYKRCWLNILRGGMELTSTLEK
DGTRCGRRLERDNQIMWRWTGFAYGFDLLVNYCHVQRKFTLKRNNNLRPSTFHSGNNLACMHERRKITFQMAVASFSEAG
ENTYHDCTDVMEHEFRQNEDMT
>Apar_comp16488_c0_seq2_fr6
ELE*GYHLDLEHLLTGALDRELRGKRIVQVSCGEQHTACIDDLGNVYCWGRQREGQCGFGRSIKPLLEPSMVPGLEHEVI
VRVECGHEHTLALTDGGVLYMWGLMHVQGPQVKTQYFGLTDLNEGQRRDMIEKSYYEYLSNGGVSDDIHQSMETVAGFRR
HVVTQPRQVVIPNCPHKVVAMAAGYCFTLIVVSNGDVYAFGFNDKGQLGNGTRFMTEVPTLVPELSHECVVSVAAGTQHS
VAVTRDGSLFTWGGGIFGLGHPVPKDLLTPKRVMALGPDIGRRVLSVSCGAYHTVCLVEGGAVYTFGHGEYGQQGVGDFG
ERGARQRSFALPNLLTSLPAPALKVACGHLHTLVETTKGLYSFGWGCNGVLGVGDTKYRLLPSLVTTLRGDKAVALAGGW
KHSVVAVQGGDTTYAFDFAHLVTQTERAWRWADVVFVVEGKELPGHMAIVAARCPLLAARLLMAVRFCGADPHKICLPLP
GTRYIVFQAFLSYLYTDHLRCSAFLARDLQTLAATYQVPRLLTLRPRLVIVHR
>Apar_comp17269_c1_seq1_fr6
FFFRLIST*L*LYEPRISFICEHQLISVKHVRTMEDQITLEPFGHPSNFAQLYEAGKLCDLTLRAVGREGITQDFNVHRV
ILAGASTYWENLLASEWPVEAGGAVVCVQTDPRGVAGLARASYGPG
>Apar_comp20104_c2_seq1_fr6
EPPLGVEIYEGDMHIELRPNLPYRLKDHPQALHPTKEAFPSLEEDIGSMRNDPEWADLEFSVEGINICAHTAIVCKRCPY
LSAMVRHASRAKAMSMPTTAG
>Contig1926_Corallo_2_fr1
DTTKSKVYGQGSSKLGGKYNYSWKPYA*NLEGPWEQKQRDISK*PSRGVNIFIDTMSEHEQQQQQQHLDEQDRAEESTPL
RAENRSSSDEDDDMEGLPQQARRMHFDTQFIRLMSAFISVGASTEIHTNQELLEQLRMRRALHSTRVQNAMSRLSRGDFV
PEDQKGEAFYDCPIHLPRYNFNISAPHMYATVLEELNIEYGDRVLDVGSGCGYFTALGSWLACYEYDADSDIVSSRKGRG
ICVGIDIQQDIVSFGRRNVRATLGKLLPTARWSIGLHNVLLPTFDFADDGMFDKIHVSAAITDEMIGPIMILLAEGGTAV
FPCGSELLKCTRKDGTLQFSSPWGHKARKGSNGLALKRSETYSTASIASVGSPPQGGDDMAECSTGFRKRKLKVPSPSSN
GDDCENVNRMRGISVSSTDVEDNWEEDDAVRHPREQKRREEFIAWWNRMRTGLPSHSYETKVISNVRYGDLVPPDSAMTM
HARFELERLYFSRIHVTPPLTALEQKDLTDASRELEDCEIFLAGGEMPLAKCHRSILRRRCPMMRARFDSGMMDANDSKI
VLDYPGADAMLARAFIDFMYKDSMPVGLDADKMILLLGMSNYYAVNRLTEMLEERLSKEINADTVVELLVAADMHGAYQL

QNACISFILDNYATMRHDGRLNELPPSLMLAITDEAARQLSEIRSLNGGLQICIHDEW*VSTALDSKSSQAKMCPRGFPP
*Y*LFSMVFILFSVSRHCKNICCIMNVMQPQRSDYVNGQGPSSCLL*ALLLSNQSRIAKRVPSVTCCVLAREL*RLTPPN
KFPV*NLALTCRAEGGGSTRYPLQDRLEVLLFWITS*MHPASSQAAASVSLGDSFRAIFLRFCDSFTW**TTKWCLEGRP
SHSFTGYKDSQTPSIVGLMERSWAKSMPVSSSTSRSMASTRVSPLSTWPPGGTQAWGKARMFLARRVRRMAPSALSTIQQ
TARLTGRRRYSAGRVRPCAQVRSAGQLRL*PRGILPLELRLRVTSP*PS*ISQRSVP*VIPRGVATTCSINIHRLKLICV
CTTLSI*TMMGLSSR*ALLEGRGTSAYCSIPAWLSL*GSLGSYFVPVTT*GGIFCVEGFRGRSLFIKLDILRGIGGGD*S
PDWSSSTIVASSLSELALDIPGSISFSLSSLCE*IGESSRA*GGRIGCAAKIACSCKEIAWAIVYFG*EYWFK
>Contig3889_Corallo_2_fr2
GG*EPGGCTFQTLICSEVQ*WPRRYYLEFRSDHVHQGNCARCCESTPTHLYGLVLQILESVAMTGSQSQNGRSDELTSQD
FDHDHRTIYRNALGLNDEKSTKALKEFYGIEFTSTTGTNVSDTENEQTNIAEAKAEEVPNVDDLQSHAQQFSAVQTFQRR
KGYYQPGVSPFDLGTEMIKGRPALKDDDADIEYAPEIDYDEDEGFEYFEDDAFDNQDLRLVTAIKDSDDAEEDTDDMYMA
CRKGKVDRVAYLVEVKHHGVNSRDRWDSTPLYYACLCGHLDVINYLLSKGARCEEGTFDGERCLYGALNQGIVKVLKRYK
VATALDAFHEALRKAFEDPQNTAAETKDISFHFSLSIKNDPHNSIVTTETLSAHRCVLAAQSQYFLDKLQGRWRAKAHVH
LKSELLEPKAFNALLRFFYTGRFECFRRDFGAVRLILKQAGLLLLRDELEAIDENLKPEEEMLVVQKRTEYGPLFAACLP
EEFLGKYADSVLPALKNFARTGTGTVVDDDTADDTRQSPITDQNVPGAGADTNDKPGTAVESLLAFTSLIDGFIAFADVV
FTVEGHEFPCHKVFWISRSPYFEAMLGNEGFMEGRAVERQGGDSDGEREGGLVTIALHDISPTIFARVAEHVYCDYVSDV
SIDELMPLMKASDLFLMSSLKNFCATIMIQHVTKQNVFRMIEISRVYDVPRMEDHCAKVLAQHLDELAHTKLFHGLVLAD
ATAIEGRQETDSIPIVDDIRFHIDRLYGTAHESAYFGASLRKMQRREELLAALEKALNALGLDV*QGTLSNNHTNSKWAR
ALVVVVVNKTAYQTM
>Contig3001_Corallo_2_fr3
RDYRKGYCPLVLTVFSAGRIVCRQHLGTWGAVVLLPT*GRVPMQVLSTL*RRIR*DLSYSHAMFCSFVLNGTVQDRRYPV
AGQINEKSGGSIIFCATARANIKIYFYSSASPSEAAPYEILLGGWKNTKSAIRKNDAEVVIVDSAFDADAKLDSEFGDGT
WFWVQYWRGCISVGKGELGAHIIMEWRDVTPIQGIDSVAFGVWKEDIRLDSVCLAGSVPTTLYLNETSHLPAFGNFSGYD
STELSTAFLLGSRDFADLSIKVIDSSAKGTWTDDIPLHSAILYPYIDELIDVNKAEDFVTKWIDLVHEKIQLYYEAKDID
RGRRMGHKRRQSGSNTYAKIPQLPVKKNIIGHVLAYIYGDQRMDLSQEEIEPCMECAQMFGMTSLAGGLKELLAVSRQGA
ARNGVVSHDATALRSFKSWRFPMSTPDRPTFNITTVYGGGNMRRYRLSTNWKSLDRHLKVMEVFPTDMVITAHNECIGAH
RAVFANESSVVHAMMSGDMIEAKEQEMNVSEATGDVIEVFLRILYSSLDVKVVNNIPDIETALSCLRLTDKYLMMRMRGL
VSEYIATKLLNTKTVTGVLAWAKRLRCPVLYSRAMLYLKNNFQDVSLEEAFKELGYKELLNVIKYDDLAVVDENALYDSV
MLWLKAQNPDFERDESDKITALLSCIRFPSMSMTKLEKHLLDRNIYEVEREFILEAQGVIAERAAARARGQLSSSPTALA
VSPISLAVMDTHGNAVQAMQAAPSSTPNTMNTSDEDLLSEVRGTRPPTFTGQRIELKPPAAGGHMRSRLRTFARQDRPAA
NDGKTIVRAPPAPGTKITTNDTLNGIVYALATNLNTSDWIDPHNHGRVTVKLTAPEGPFVKAANFVSKNARVRNALPGRT
AAVTLDLGPKCSANVTHYALRHDSENAGFLRHWCFEASNDGQEWTLMREHRDDESVNYAGQTVVFRTHATLAMKAPSSGG
SPGSPLSLGATQPAAYFRMFRVRLLGPNNDGSFAIHCSTIELFGTYRSEVMIKS*SFMYS*KLILVFKEPYLELFTCKLV
RARP*PAFRKASNAVSKPLDRRSTL*TECVVPYRPCPHSHPRPYLTSCQILLIDKRSCCPVCAASSFQ*SSADSLRLS*N
QPKQSRPVHLPCRGSPNSICRTVSGPGCVYLLP*W*AVLPGARSDGTMTGDSAALELLPSFRYFLSRPLCMQHFHILQ*N
QASPNFICLSY*RNPNTKIPLMTLIPLGITALINLTL
>Contig5630_Corallo_2_fr3
TCSFLVAPLMSM*RERDTSKRPSTS*ELGIMDDDVTADEPLGHIVPLSVAMDHSRQPGYNDLFVEQLRRFREEGLFVDLT
LKVVYDGPPNLDSFVDEYMSASITTPVGTNTGPIASTSPLQLSASELFRVHRCVVAAYSPVLRQMLSGPWSENESDEIVL
RGIHPRAFRNFVNFCYGQRNILASPHSAVEDVVDQLGRTSLDTDTAGGQRTAERDPSPSVTMTAESLWELRVIETAQFAD
QYHVPGLSMECEEYVRQAIDSDNATVLLNAALAHNLQNLADIVVAYLKDHLHEVQHLRKIPQQVMLDLLRSSDLNFDEFY
IFIKLAAYCLVPLEPEDGDEEEDGTQSKRLQEPDDDQQNRRKSNDEDEEDAPVSSTQLQQPSTDGDPELWRSIEQMSLIG
ELIEDMGRDPPWSYMNKAPTSQKDSYTEAANAVQRGWKAVDLEFFEQAIEEIDFDVMTVDMVTVASDIMRLFGSTVGKDA
IIRTLLKRSSPQINAPPLQVFTRQRHFEVTLKVDLDMTVSYNKTDLPKGGFWNNGTRWFLRVFPCGNRNQRALSVYLLYD
KGVNGHSQGQSGAGGVPYGAMPSEQGSDMAPSGGGGAGQRGGATGAHDMRTK
>Contig6577_Corallo_2_fr3
SSAQI*LQNGNITMTVTTGSGPSKMTLREQNSQNYVAHGSSTKKKKAKRKIKNTCQDILGAETLQYVEKFTHGSIRVLRM
LSLRTDMRRLWAASAKEQISQPLLDSEVDLYFRFGHSNYVGCHRAMVLALCGMLSDQDRNGIVKETRQDQEPQIVDVEHL
LETQRHASLTAATYIHVFRKLLRNLYMGDFKLLEHREVEMSSHDLNVSMEGLHKVPSDDHTVPDASHPRLSDTTMDNVSQ
PQDSDDDMDGEIDWNAAWVQDALHRHLALEQQRSDELSKGTQRGNDKVKQASGTVPNKSVGRSWGSSEKEQRVYTAFTSD
HALSLQNFFLQALAAAIVDMPEEYTAVDSVVQKYKESVAGTDEEDHMSQVEIVSTAIFKFLGSDEDIEGQTSVQSVGAVA
QPPVQAQLRSLYKHPVFSDVCIRVRGGDVIHVHRVVLMACCPYFRMLLGGPWMEASTGVIDLTAHEPDVIHAIISFLYCY
PFAFMVDPADGQQLTDAVEADLSTTEPDRILPIADLFGLGALKKCIEYYVMNVTCHGVSNPCPICLPEWLHWLGTVHNCN
CSWLENKFLKEIAANFSKIMSNRAYMEICPKHIRAEIEAEIRKSLNLNTLLDHMAAVDILLAAVGHTGRWAQPVVNFAKA
YQQEINDYCIRQLDRMTSADFEKILKSDRTGHMISDTFNTLMESLSPQTACSTLQTLHRVSGIVSEVQNEELHLGMMLDR
VIEYVAGNVELICRHEALKKSKKTKQTGRRPNLVPLDECLGFNDELVRRVTSSPGYLPILAEDVRHLSAQHPLQSPAPAS
ASSLTTIECQLIEDHRPLSRSGLKPTVPASAKQQGLQGRSPVNALDTISASQPPSSSMQRHRFVTHRSQGPDTDETFVVG
DRIKTTTSGGRTGTVVFVGTVHFAKGTFLGLRLDDESGKHDGTIDGVSYFSTEPNRGIFVRAASASRQPVE*EY*YKIG*
EFA
>Contig858_Corallo_2_fr6
CWRWRCTKVSTTSPIQRF*SYPRHRI*VNNVRNMVSPVTIAVLAVIVAIVYWKVFRRDPRENSVFKGGKSLQSIPGPEGK
PILGVIGDFEGDCFHRYVYDLHVKYGPVVRIALGPSTWLSVADPSWVSEILHDKRKAFTNSDVFRNVFGFYFPNSVICVE

GDEWKVHRRIVHSAITAMKPAKWLPVVKKVAERRLDPVLESAATDGHAVDVEDAVGSTLFESFLRFGYGIAEPDTDQGKE
EEMQQYENAKVISGSLSIRSMLPVRALWYLPTEYNRRVDASYQGMKEYVTRVVNARKEYLDRHPADASDNLLDGLIIASQ
SERVSDAAVADNVMTLFFAAYDTTSNTFAAMMSHLALNPTCQERLRNDLRAWDRNTAGDVTLLADEVPYLDWCIYESQRL
HAAAFAFARTVTEPVQVAGINMPTGTYCLINNMFMGRHAKQFELKDSGLIKADSSALIRPSNVPDGDDAHGFRPERWADD
NLRKRVNLEYAFNPFGAGGRMCPGSKLALNEMRVMAANMLMQYCFKPDPENPIDYTCRLAMGPKDGGWVLVQRVTPADGA
*LVGTFFPRACANAIRIVIIIRSRERIDRLVTFCFSTPPP*LSLNKPI*FYCDTTITEHKIV*GDILLHLSLPATTTYQV
RAVEVLTLSGLH*KLA*MALFFPARRPFPRCCCSIVRLE*MDDESCCIGCSWKSTMDTPRLAWTR*PCCACSCWWSATGM
PRLG*ARWLWYCACCWWWSATDTPRPW*ARWPRTTGGPSVCRRMASLFE*YEFSVPTDPAGDDGTAWACPTALAGRRRMA
ARLE*CELSAVDDFDSKVSRLA*WRRIASSVVIVPCMLSVPRLSAKCTELGTEAALVKLWFSGSCPYCWGCCCCCCCCTV
*RSSSMFSYGLSSSFSLSSSGAAAVVLRLRVVGTT*PRSSDDFFFVCSCPLPGTALRSSPLLLPSLLPLLSLIPFGAVVW
LPLRPPVNVEYGLGAIAPCC*SWYSPNSRRSTASYSLTGLKAVPS*VCAAS**PPLAVQAGLGSECVLSEGVETWRL**L
RLWLLVEPSIEESKER*EYTSDVRNLLLPTRDI*TTLRFGSKSARRFRRRCERMYKVSPIAATTTAPPTEAPMIIYSSVL
LLEVEPPPSGFTGPFWSSVCILINSNSEIPTSVASVPSEIVDEYS*LYEFTWLASTLTTVTS*LSDAVWRRFLLL*TFER
SCTVTKFSSTS*VAAVLILRLVVSVPSSG*DALIIIDTRTNFVAAVEVLEVVVVVFDDVGVVEVVVDVV*VLELVCELDE
VEVVV*VEVVLDVEVVVDVLDVDVVVLVGGVDVVVDVETIWSLDKPLQDMFFRMIMP*TDALNEAAETAVMSAGTVDSIL
RHLSSILDETSKSTRGNVTPRAAKAQVAGIFRNFTRTLYKKYVNSIPSKGRHSITAQDIQGISSSLHEQGWNRRLKQTFD
LVLGPVQGTDQLPAVDKAQYNDEATATLLAGLEDLVCDRDLFEPLFTTENLQIILLQLTKPLSVGTDPTGKAALHILSLM
LTYFRIGTEIGAEKRRIMLLRPVLRELLRAVITTIEDYSKKLLHERAYRVVAQVLPHMDSDVKREIWDNRDFLRVWQHAL
EMYLDPATDDLHVEIFASLSSQVEVRGLEDQWLKSVHLLLQRVVNVADRGRRLRGLDATLNGIHVLDSNGQGVRKPLIES
LTSKVSSTSGGAGTNEQLLAIQCLSHAPRALKQFRGESVVLKETNSIVTSMIVNATREIVNNGWALRPLHILDHCCTYWM
TLYENNIVSTRDFGLRNATTLKEQVFGPELMITLLRFIRSTYPLSATQGVIKLMFLLFHIDSYVAQMKVPVHEVFLVVVD
FMKTIKNRHDNAGHAILWAPEDPTFSVLLHGAILGAKLTSMMESIVMEQQEVRNAIHQLRALGAHSTEFEGQKGFMLFQT
YIALAMAHMGIYGIEDSIGRDLSHFIDNPLLHDTKIVGGDGLPLYAHKAVLSARCAIFANRLEEVSAATQDGVVTLSMTS
MPRRSLLHLLTYIYQGYVENLTAGDQENLLTVAKKAKMKALRDLLSLKRPVLTDLRTVSADMQEMYLADRLCDVCITGFR
DMEGRDIEVRCHRIMLSLGAQYFSAMFHSEGVDDAWQEANTGNVSLGDFNVAVIRACVDILYNMPMEAALHGDVALSLKT
ELLLQWVMPTAPTILPQIQDYTAHLCEDLIQDGNVLEYLWILSSGTRNDEVLDVYLNCVIRNYSKVQDTAAFQDLDNRTR
QMIHQFYLESAKGT*ITGLRIDRRFTNYTHSCNRL*LFRRFYA*FGASFSVPCGLCGF*CDAACSCSSVKWVRRIVGGKR
TVNVYMENASVMMRPVCRSTLVGRSRTRGSKLTWKPRSVSTSITISTNPSSSNAGLSLRTRQIPAKQCLRSFGTKSDSIK
RTALRNFVGVFCW*PSRRPTRSALALRLICDAKGTTCRASKVSSLSSMLLWYSNCC*MLRWTVSSEPWALMR*KGKCFRI
SSRVS**SLKIW*RLRIRRSS*SVARSSFSCPCPESRTRLLCSLIWTPRFS*RSSDSFSVT*STTTRCCGRGNSSRRAVP
K*LLRSCSIRSFRRCRHRTCTFVRGNPSTNAPFWYSSRSKVRNSSSTTSASPIISPRSMNFWKSAFLSRSLMTIASDAMP
RAFSRKPVLAPLPAPGAPFSQIISRGKFIFSCPNSFSRRPQT*SKMTLASLISRRPLRFSRFSLRRSSGSLSASTAAFTP
FE*KRRVAKTAPIVEICPTEEAGRMS*RCDIDDDANRGRACLRLALSTFMVPLYIQHAQNPVLLLQHAINPQRNLSSMLR
SHSYRSNC
>Contig3275_Corallo_2_fr6
*SSG*QAQEVTCPIAMAHALERITRWQQDELTSDIVAAAVHGRLDVIEKIVSHVNHAVGKEVLTNVQSMSKISQKVKEKQ
RVGLKAVDNRGLTALHVAASCDQPHLVEWLVENGMGGHEKDTENGWTPLHRAVYHASIRSAVNLIASKCPMYATDREGYA
SLDLIALDHKADGLQLKAPGNAFSWGLNSNFNLGHAEADGHAYPEMITALKGTQTRINDIVVAKYHTLFLTEGGQVLSCG
FGRGGRLGHGDERAYLEPTLIKSLSGMKVVAVAASKSHSAVVNSSGRLFVFGLNSEAQLGIPPTQEQKILMPQAVKSLKG
KQVIGLAVSNIHTICFTCNEVYSFGSNKGQLGHARGDDRITQPRIVSYMHMKYPSIVAATASEDMSALMTENLDVVIMRN
YNCFKLSLPFSRFPQEIKISRREKFTVSRLVAAPNDTLFVLNMAGDVWFWKEIEGVGPVMQSIDWAIPRRLRITDIAASH
DKLMLVTDLGHVYWAKFGDVLKAYRKRVPMQRVEMIDHVFKISTDTSGGRFGALRSIIAGRNADLNLLIPQSNVRKDLAH
LLETAEPGDGVTDIALVVGEGKELRFAHKFVLASRSRRFREILTEKKGEDSWYSFTVESPIDLSEALEESVADWVLYYLY
TGQLKDEAFNAKGKDRAALLKAADALQLPALSSLVNGRMRDDPTLSFDLVGLEDFCDIEVRIGDQDTVQLHKCILVARSQ
YAHAMLFGSPWSESHTAEGATIGLPTHTDRKTFEIVRDFFYTDSLDLSEYDLQQLFNIMAIANEMMCEDLKNLCEFNIAL
MLDVRNVSAVIDAADVFAAENLIDQCLRYIYANLEYLLSHRSLEQLPMELLDRISYEYRHTGNKILRGGIGVNTEVIEVL
DEIEPKRRRRRSSRLVSQGESSESSRTVTPSASPLLSATMAKQAMATPLDTFSLMSVEDHAQGQGQSNIQCSPKTKQQSI
GGGSTVPSIKSSEDWDLVSATGRKNKNRVKHKTRTSLESVTENLLSPDRGGAPSPALKARTIDSHQNPLQVTPVKSGSAR
GKSTPSSVPWGGSGSRTGVRKTSLREVMNEEVSHRQISPGSSMSVKTTPMKDVRSPSYAGMPSWRRLAAPSAQEDAHRVA
SRVMENAWGTNNTASPQGGGSSNKPGSMAAATLGGPVPISREFMESHVEPRRSKMIDISPLVREKKKSQREREQDAREQA
EAAARAAASAVKPRGWGSANRTTPETSPTPTGITCVTGTHIHSSLSDSSVETTPQKVYSLREIQEQEMHAQEQLRLRGYS
SNVTRGPVKTAWASPTLKVTSQSLAGRGRRSLDVVQMEDNAIKQLAVLYKTDGSEDDYIEIQRVSETPLAQF*ERSLQ*K
AVTCNLDNAQNC*RAMSIPDRRK*KSRIAVHYRLP
>Spom_NP_593682_1
MASQDQNTGTTHVELQGGENSKKLFSKYDLWSKAMDEKKLSSSLFTVNDTQEFLELCEACRRGDLEVVKSLVENYNTPIN
QVDQFDYSPLVLASLCGHEPVVKFLLENGALCERDTFQGERCLYGALNDNIRRMLLSYDITKAIDESQPYASHITSLLSN
SALHFTTDIVFAGQYGRVFAHKFYLAARSSYFKSKFSKLGPSEHEIEVKHFAKEFESILRYLYLDTNAVFTKQYNNALLS
IGKKFQLNDFIALYEKDREQLHSRDWKKIQLAKTQNDLGEFLDYIISNYKVPIESLNQPSDQYSFHDAYLQSYTHRYPVH
RAIMCRCEYFLDMLAGPFLESNQELPVLSLPFSSSVVEIVLKFLYTDKTDIAPELALDVVYVADMLSLDKDRSLKSLASI
VITKQEEPIDSIYDILRTAWDTSTPRLEQYASEYMANHLEHLIDDPEFCELVKESADRILQRQETDTIELIDDIRYFLSK
RFGIYHEDLCIDGVVDTLTPYESEYNQKMEMIDDLLDKLELQA
>Spom_NP_596074_1

MSMNSSTEISQEPSIMKPVSSISSFVFNAGFLQGTFSDTTLIIKGETYHLHALFLGRSPVLLQKLIENNPKGDVHYTIEV
ETEDPYVTKESCLFVLSTLYCDSPRIPAEVNVCSVLAVSDLLGLDTLAFEASSLIEKSIRPETMESVIRFLDPNFEGLER
LKMGMYPRFTSGLFNKAIQVMYNTLVSNWNTEYARLLCNLPFEVIKDLLESDKLTVGASSMARYKLANEIVKMRASFRKQ
NHIEGKGDESVVLAFDEGSRGIQLVHIAPGAESRRKIWKATSVR
>Spom_NP_587711_1
MSHLLFAYYLCNDIRSFQNLLKQDDSKVKEPRKGFSEKSGQKLRINQKDRYGRTVLHIAVSENKNSFVRSLLQHKGIDVF
VQDEESGYTALHRAIYVGNLEAASLLLSKDPSFRSLRIKDKEGLSPFQFLSRVLSSTIHPVLDLPIIGNELYGFGTNVNN
TLGIANGKEPSSPERVFLLKNQTESPTSGQLFSRDKILDVQASKFHSVVLTDEPSQNVYVCGIGAGGRIGFNTDVQYNFI
PIPGIIHKVIQISVSHTHSLALTKFGSIYSWGKNGSGELGLSNDELKKDDPIQITPRRISAFKDYQIIGMAAGKSYSVAW
TDTDIYSWGLNNGQLGISDHISVVSTPRRVAGLLSPVIHAVCTTRATICLLQNNSIIAFCNYNQVKLPFNVDFGSSLKVT
KHPLSLTRFNVRKLLASENKLAVLTELGEVYEFDMKLLLDRDSTSSKNSTRTSFKFTPLWIFESSDLAALDIAWTADNSL
ILCTRNGTCWKRELRSKKREKSSSSPYSRGPYKYNRIENLQMVVGVRASASGSFFAIRNDYLPPPIYKPSNMLIDLLRSL
LPYDHLLHVRQPRLIPPEDEDGVPIFDEDRAASSNEMQLLFEGSIPILTSYENYKQSFSDVTIYCGTSMFHSHKFILCAR
SSFLRKLLLQKKKSSVSNIIYIEEITQSHSTIRVEDIPPLAVAILLHYLYTDTLLSPWHLDSRFSPLKENLSKLANLLEL
PHLAEVLPFSVSRQPLLSLTNDILQLYNNFYVLCEETMDTVIKLKDGELKAHGLFLSLRSEYFSSYFQFVSMESNSFDIP
ITVNLSHLTVEHMSIVLRHVYSDLKVELFDDLKESDFHNWLETMFEILSIADELLFLELKSIAQQSLLRFLNLKTLPTLM
DLSLSYHAEELYSRCIDYACHNIEFFLEANRISEWDGFHLKKVAQRLTELLSDQRVHLPSSKIANRLLIRDPVLMEKRNY
ELKVLREYLFSQESSQLWDDSPYRSIFEDRRCSTSAVILESGIVPSSNQSDSLNKEDAEEKSPKPNVVNVTSITKTAGAS
VEIQNNIESASSGGDKTQLNGPGADQPVTATITFDKTSPWRNRENLSHNNNTTRASLRELLEQEKADASTTTVLSDSRFM
KAPTKKSQREKKKELSKQVPISKTNVGHIDIELGKSNHSNPWSVATHQRGSFSSSTGVKKSFNGILREAAREEGSSQVIY
QESKKRISNGSPTSWNLLTKPSPRSASLPKNSQPLSISEIMTEQKEEIESQKRRSSFRKTIEEIQQEEEFQKWWEEESLR
VQKELGILKTERDTSTNRKQGQASKQPQRRHRKEKDSKVSESTAEFKSLPIDIPRTTHKKGKARAVK
>Tmel_XP_002841253
MALQSDRKTPISEEKNGPLEGLKWNNWIFKEGIVPLYLLFLRHLMKLHGTGGYRFWPAPPSELKNPDHASLTISTEFWKK
VGKSLYDLYPQAPPPLLTPRKSQSQATENLSIKRVIFDFLSPGQSKFIVPLLMQLGITNIVTLPPTIMKGLTGATTDEVS
LLSVTPAYVHDILRNPTRCKLLQDHWKKDPGNLARNTSMLLSFLLQDTSEDCLTGCSLLPLDGGSWGTFSRKSTSQIQYF
TSRTPLERSILEITDGRLVSKDLECNVINGLLEKMNISLLSFDHIPSLCRLVESKDPEYRKTWLANVWEYFTFCVEKDPA
YMDYYLKSIESLPVYCGSVVGKPGSLRFLTPLSFASGSPPAIIDPNIPPSEKGKSALFQALNGLILVNRSTFPKTGLIAE
SLSWPQGVRRLIKSIGSLPSTIHTAHSLSQIFSGMPAEGVEALSNLILPHISLLLNEDYSLVGTLKQLPIWPVLSSSFQS
AEKLKLAPHTSLTLTRMIDQTTFLRPDLASKHRDELQKLGVPQLSYLDFLNNEVGLARGYLPDKKIGEYRKFTEIVYRVK
PGIFQSYNLAVNGDLCFCLPITLYDSSVHLFQAAFRDQEGSRFLHKELVGSAIWKAFLIQNISGPTYIECARSIERRNNR
TLPDDQLESDSCTVFNHLCWDYQEMHSWPVSAWESLSNIRFAPAQEAITSSGHSQLRSNQGKIFRQRNRLIALSEAVDPK
FEGISWSQKPVLPVRMGPLALGKITSNKPMITPTTVLGHLEFLASHREIITKAELPPRISEIKKAYEYLEEKVPAYAIPE
SVPIWLNVENEDLVGMTPETFRNSWSCTRDLCLNSNYDSGEIKRVRSFLGRFHQLLVHARVRRIIPPPSIPEPGKTQSPI
LEGLLNLREQKALFDVTITAPPQTFKAHKVVLASVSEYWKGMFTSNCRESSTAEISLQDDPSTVKVLLDYIYTNKFIKPP
REDDVTRQLENLLDQLEKSEKWLLPSFQHSMENYLSDPHWIRPETVKPILRSAKMYKADRLADVCRKYIENNRVIVERES
PNEE
>Tmel_XP_002839204
MPDETLGDFCFDTEDAAEPRAANAAGCPRQSPNMRSSSLQKSRLKVSSQVLCLVSPVFRAMLGPSSMFKEACELRASTSN
YRLQLEDDDPEAFVAILYALHCQTEKIPSSISFEDLFHMAIICDKYDCALAVAVWVEIWTAGWREFAFETTYSEWMFISW
TFCLPSIFEPLSRKIVMEGRYEDELGQLLWGGRSLDEVMVPDPVISAILQLRSDAIKAILDDTVSYLDLYSNAEGTMCSY
GIPECDVMILGSIVREFRKRGLHNREKFLLMSLNDVVASIKGISIYFYTNYSGTVFHGDTCSFLPDLFAKVDKICEGIGE
LKLERFTKGVVWCRYKDDFGGKLEPLEYELV
>Tmel_XP_002839163
MSNYLWRYYLENDVGKFQRLLAGSASSSYSRGASGSSATGLTTNSPALSKQHQPSSDGKPRPGSPPTNIVITKQSLKELD
TFGRTILHLACSTSDSGGLPFVEALLTHPLTDPCIQDLESGWTALHRALYHGNISAARAILSNNLSTVKVKDHAGDSPFE
VFEASVHGIEKLFLRKSKSVPRSDNIEGEDEILPGRQGREASHSDDEGGDEIFAFGSNKNLTLGFPDGDDRSYPERVPIQ
RSEKQLLQLVKDRFHNTDGDIKELLGSTTLFAPLRILDVQLSKLHTAVLTSDLSSNLYVCGFGRGGRLGFGDEQTQFTLR
PLASPLFPKRRVIGVALGLDHTVVVFDEGDVWTWGSNTWGQLGYIIQRKTPQDEPIQTTPRQVFGPLKRERVIGCAASRI
HTALVTDNSLFTFGKNDGQLGILDSLDTRTLASQTTPRKVAATFLTVSSKICDVAAIDKATAVLLNNEEVWILANFGYYK
LKLPFQRGLYRSPVSQFSKHSGGHSLSKITGGGDTLCVLSVSGDIFTLDAGSFVRESAGKGPRTGKITTVRAWSLKKEHM
AARDVDVGHDGSIILCTKNGSVWRRVERVKRKENRALGIEGSRYKFERVPGLTKIVVIRSNNFGAYAAIRRDPNVMKTGL
LVDDHMLWKDLGPLLPFTGAFGNRKQPDDSKDKIQTRTPPRTPEGYVTAVKWFMSSSLKSELREYLSYPELSGSEDCDVL
VSTSNCPEINIPAHKVFLAARSPVLRNLFVEGAQCASDVGEFTPSTTPEEKARLVFKDIKLQTLLILLYYVYADSLVDIW
NQNNIHHSRMQHYRVIRSELSSISSLIGLDHLAKALEVAPHPSCSMNRDFSNALLGPSRSKFEESADMMIELANGESMLA
HSVIMKARCPFFKALYGSSGRWLIQRREQGIVKVDMKHISKPVMDMVVNWLYSDWGVEGFDGVKVGKESGKVEEFIDFVL
EVLSVANELILGRLCQVCQKILGRYVNTRNAAYMLTALAGCSENGFKDMCMQYICLNMETMLENHLLDELDADLMMDLDG
VVKSVQRQLFPHSRTGVQEELLRGIYPDMHEIAAKEKEALIASYLPEGNDTATISTSFKNSRVGSYAGTLTPFGSPHAQK
AKIKASKESIRIGASPTTEPHAGGEMIFDMDGEGSGIGFALEVAKAEETGLRNAWASPGTPSKATRTGWYPSPASSIPRA
SGGWVDVKGKSIGTPSSNPDLQLLAGGSPRSHELPTPTKIPTTPSKPWGASPILLGDKKLDMKEIMKQASSSRKSNLSTG
LSSEGKLAPAKLSQKERKRQAALVAAASSELAVSTEIETPSTSSAKPAWATASTSARVSLKEVLDSEISAPAPPNMPTPQ
MLKQRTGDGGGRAFNRMMSTTTPSAVPPTTPTAQTTRSSPPIPGTYRPRLKTATPTTPPQPALLRRSVSTTPIDPTPKLS

LADIISQEEAHKEVFREHTAKRSLQEIQEEQEFMRWWDAECERVRLETAVAPEGPVRRGSMKSNGRGGGGGDPGDGQRGG
SVGGIAGEGTGGGHSNGSARGKGRRGRSGGRTGGGGSAEVTKVGGPSKRMCS
>Tmel_XP_002837073
MAFVPPTTPAHHQHQHHQVQQNQHQGIYASGGASSNTSFNTPPSQIMSGAGVIQQLDKSGLLLGRKRALDGGSIEDTEGA
AYRPAAYRTEGARLACLVNASVTVCGEDAIYAFGGFDQYTDEVYNHVLRLDLRTYTWTLVDNYGDIPSVRMGHTTVYWRD
NKLIIFGGENEHRMFLSDVYIFDIPTATWSQPVLSGRPPCGRSRHAVVLHDDKLFILGGIHNRSAIPSDFDPEEDNEYNP
QEVLDDICYLDLKTMTWSRSWTWIARFDHQAWIRDGKLWVFGGMGQGMDRTGELISLDLNHPAFARTEIKQTGYVTRQPG
LGGDAATVTGGQSRILRSHRPYGPNSPRPIASSRRNTPPPKKQAVPAATASVSFLWGRDIPSEAHGTHFHHLCGNTLLDF
VTSANTIRPSETGLCALNLDDMTWRKIADGMDIVGGGLWRWHYLAISPDSTMAYLLGCPTDTDEDVGEGEEYLSDVLPID
LSRFGMNVDQKEGDNPKAGTLGMDLANFFDKPDFGSDFVVRAIKDEAAEGDEWDMTGVWGRRPPIRVHKLILLSRWPHFA
TLYSARMVEFHKNRMYLPEPYTVVRAFLYYLYTDSVATSPYCPNLSTVAGLLVMANLYDLKRLRALALNRLFSELDVESA
AVIWERAGVAGEEGLRKKAAGFVLTYWGRIVRTMGFRRLSRRSLMELCEEVDVEGRIMGSEELGVVGAVDGGVVGLYQGD
IGGFMTEEARMRTKRRRYVDDPEELETGEAEEDEEMS
>Tmel_XP_002836473
MTAGDNGNTANSCGSGNNGSSSSSGCGSGSNSSGNVGGHRDGLRNLVEGLTLSPVTSPISPTSPSSAPPFNHPSQSIFNN
PGIGSSAPSLLAVPYSNAGARLHSFTSRPLSYGGGSGSALTVASSNVKATAVAGGSSGSNGHGAGSDPAVKEVDDPTTPV
SHLDKAAALNTFSRLLATPASQTAFSRSGRNCSYRGSDSVKLEDFLYTRGFLEGACSDVTIITFGSRYCLHRLILDRSPF
FSSCFNGGPWLESTSSEISLSPSDPNITQHAFELALGRLYGHVDRVEEDRHALPLLAAASYLDLQDLAESCASSLLRNLG
TSNISTVVRFVTNSYYGPLTDRLMESAKAMLYRDGWEMSFEEWEGISGEVAAEIIGYDGFYVPSEWLRYCFAKDMIDWRI
CHSLSYNRATHEGCSLDDEDYDADGASAFDDEETDVKPLRELLENGIYYIHMSFEELQKIEMDRDVLGRRTVAQDTIREA
LWNQMQLRQKVMNVPLNSPDLGITETETVRQPTPLGRKSSQRLPTPRFGEKLGEPGSGRATPTRQDDGDDGPDGPDTPTT
IRGRQTKRKYYIPTEDTTTVIGDCQDTPQHLAVSHPVHRASASRASECQAGAERTGGGEERDSSIPFEELRYSEFPPFRF
SAEFKSVRSLKEKKRVYSKTVFYAGSHWNIYIQKVRSKNLQLGVYLHRAKDRETAGGSHGSSSASRDSENIGQVITVDEK
IGTLELHTRNTSHPTEGLGDVTVTTDGDITTSAFTDGNVSGRPGANTSATRSAMPQPSPPEVGVPAIPYYIDSRPMIQTY
FKIFSPSRKGKVLSMFSSGPDSFNFSQSWGWKSSSLILEEGMIGGDVDKDARLRFMVVLGNV
>Tmel_XP_002836343
MLTRPEDLSNPPKTGFFSHIFQDRSEIRVKNQLDESEPFQKLCEACRRGDLKTVQDLIVSPGGVNLNAVDRFDNLPLTLA
SLCGHYEVVELLLESGAVCERDTFQGERILYNALNSRIRNLLLQYDYSQSIDPAQPWAAHITTLLSREPLDTTDIVVTAT
TTTGLETAFHLHRFMLSAHSPYFRRKLSLPLPTSTNEQNPKKTIRLSQNFSSRAFEAAVKYIYLGEVSAAPSSDKELMAN
IERLSKHLEIPELWRLLLANTPKLRRQLRSEGLKRAQERMEEWMRENVLKKRVVVRKSQVGQIKVFPGNDSFADVLIRSD
EPDWRDADADREAEEEEEEEEDYLVHIYPTHKSLLRSEFFTAMFTSPFLEGLPTAPTSPLPIIPLPLPASTLPYLLLHLYF
ETPTPPPLQHSLSLLYTSDHLFLDRLKSKCAQVIATAPNDLPFHIHDVIRAAWATRAPRLEAFGAKYIAERLEDFLPDPE
FAELVAESAGRIKGRQETDTIELVDDIRYYLNERFRMRVEDNLGFCGNGEEVEVEVGEVDRSLETEKGHVYEELLGRIDA
LLEGLKLDA
>Tmel_XP_002835379
MAMRKKGRKNASGLYHWSGSARRTGSTLQADTTSALQATSALQAAITPPETPQATSHWQATGAFEASNVPEGSSHPPKIA
DDTPEQSDATPASQMIPEASKQSPPIPFDKFASSDIFTIRAGTELQAFHVHKDLLAGLSDEMRNHVYNDMREGKANVMDL
KHISPETMHRFMEFCYSGDYLFPPGAERKDPLKYKEATEALPILLTHARLYVFAEMFNIVSLKRLSADKIIAITPLFGRL
EERAHGLAVISLMEFVLDNIPATTETTDRLVKFLARYSGYFVEQIGGYPEFHALLASSAREDFFRVFCKWVRAGGDREPW
TDIE
>Tmel_XP_002835276
MAASIVHIPLSPMTLEINSVSSEYVTWVYRWRNVDLSADFETYSQNFGHPDGWVWRMLFQQNVDSAGNRVYGIFLYVVMT
DNELQERTWTRDIRYLQFVILNSEGRQLKDKSTVAVYRENQLSWGFPGLLDNSILLRDPVVNTRRTVDIVCRLRYHPDGE
KMRRPLSPTMTGNLLGGTMISANHAAARAASAPASYWDQKTSGVAVVDPVGKNLRSLINSRKYSDLELIVGPDRHIVHGH
KAILAARSEWFEAALRPGFREAREGRIILPDEDPKIFEALLEFLYTGEYPLASNMNTNGYIPGSPSVVVAAKKSTPDGMH
LSPRSPPNIPPRGMDDAVSTIAGSFAESQQPPEEPTYDYLNTYENTVEMMDEEEELDEQKYALFLMADKFGISQLKSLIR
IHLMNELLDLASPNPPSQRLLRLIRFAFEDLPESTSTRKRDSGVGSSLSGSGMMPREGSPSAGYTHGYVYGGGSGSGYPL
NDPMVASSVMENREGAASAFGQAAEGSDVGLAMTTERRSTSRISNSSSMTGTGNGNGGYRGGVYGKDNETALLRESITTY
VAKMFHAVRQLEGFDELLREGGEMGGFARKVMDSMFRP
>Ncra_2344NCU02617
MAHPSTPAGHSPSIFGGPTPVMSGAGQPSIPSSSMTTSTLTPASAASSTNTYIMPNSPFKNRGDGYRPKVTRTLGQRPAC
LVNASVTYCGNNQIYAFGGFDQYTDEVYNHVLRLDLVSHQWSLVDNYGDIPGVRMGHTATLYKGDKLLVFGGENEHRTYL
SDLIIFDLKTAHWTQPQVTGPIPKGRARHAAVLHQDKLFIIGGITGHDNYVLDDICYLDLKTFTWSRSWRFVGRFDHSAY
IWGERVWVFGGLSEEMDKVSDIWWLDLKGSPAFDSPPQIGSMDRSGMARSAISPRPSYPAAQSPIVGSSGYAANSRTAQV
NPPSFHLKTYAPPAPGAVSALKFVNGPNVPSQGQGIHFHVYSSGTLLDFVTPAATISSKECSLSALDLGSLRWQKLADGR
EIFKPGYRWHYCTMNEDGTKAWLLGCPTEPNVLDHGGTGLEEYLSDIMEIDLRRYGFLGNNFAPEPRADFTRPSSRARHI
EQPSKGLGCDLAKLFDQPPETGSGTDFIVTALSREYDEDEIIGSALVHTSDSMDGDQTWLSPDAPTSPPIHVHRLILQAR
WPHFARLWSSQMAEFHTKKMHIPEPYSVVKAFLYYLYTDRIDPGSEEDESITADLSDVAGLLVMSNIYNIPHLRLLCVNR
LAKELDVDHACIIWYCAGMANEEWLRKRAATFCMTHWGRVVRTLGFQRLPRAALVELSQEVDMEGRVVGGDELDLMVGID
GRYHDGGSGSSRRKGSISSNHTQVLESELDEDEGMEMS
>Ncra_2850NCU03186
MVLRKDQLELKLKDDEQLIRQGVLRNEHPFDDSPEFHEFIHACRRGDLKHCQELISGGVNINGKDKYDYTPLIIASLCGH

YELVQLLLESGALADPDSFERERAVYNALNNKIRNLLLSYDYTKTADPLQAWSSHITSLLVREIPKTTDISLLTASESFH
LHKFILSSRSPYFRRKFADAPETSTWKLSHSIPVEAFRVVIRYLYLGELPRDLVGPRSTATEEEVFKGIDKLCKQLEIAH
LWEAVLSTTDRRLARQRHQDEVQRAQAQVTAYFRDTVLKHKFTVDTCRVGDVKWPRDNAISANCLLRADEFDEGEGEDIA
EPAEEALISRNGIPIGPTAALNSTNGASNGAKKPKRSVLFPVHKAFLIRSPYFETMFSSEFMEAKETEHLHVIKVDCLPE
VLEIILTFLYTERSDCPLELALDLLYAADMLLLDKLKTKAAVAISTLGSGTSNALVDRTHGGAAEELQSSPLATSTSTPS
SGQQQTPTGSSPEADGQSVEVEPINVYDVIHAAWDLKVQRLEDFAARYLASRLEDYIDEEEFAELIQESASRLKRREETD
TIELLDDIRYYLGERFRFRFEGDGIEEMLNEEGEIDAAQVEDLALEAEAGEIRLEQPSDGQREVGGEEVGKCTERDGEEE
GGGGLRTLDGKLVEDEFVSDAVNYQILLEKIDKMLDRLKLGA
>Ncra_4040NCU04521
MFKRSFRSRDAGNSQREPTQISTSTDSQHRVDKSARLMKKSKRDLQKLSSLKQPLVGENSSRTQPQSTQAKPAEKPLAIT
PSPIITITVGGEGRLFAAHEDVLCQAPLFERMLAQSKRILLEDEEPEVFSAILEYLYKGDYYPRLLHNQQQDSWELEDSL
SIPANRAAPLTPPTGHNSPQLGGGHAGQFPIEPTVFLSSLNQYLLRDTVVYCAAERYGLEELKRLALRKQGLQTGIDVGI
ILRSAQYCYAHTPDSDSRLRAHYLALIIRCRKTFKRSGTMQAEMEKCGSSNVYGQGSGKLFFDLFVAMCNHLDDIIDASN
ANATRTPKTI
>Ncra_4308NCU04838
MTQTHLLWKYYWEDDVEKFRRLLSLAAHGPQPAGRSSNITQGQAGGAGSLGTSPRPSGKSRKQPNGALSRIDVNSRDHVG
LTLLLRAASSTTKNAVSFAEALIEHPAIDIHAQDPESGWNALHRALYAGNISIARMLLAKERRVLAGPTASFLRVGQLIK
TKDHEGNSPFDLYNATIGERDLIALRELGGAQDEEDGSDNEAEVAQESVSRKPRHGALGEDLYTFGSNRNLTLGLGDQDD
RQFPERVYLERPAHLLQRFYNEYLESAGLEGSTAQNLRDIPALVLNRPIVIEDVVMSKLHSAVLTTDPVSNLYVSGVGRG
GRLGLGDENTRFKFTPVQGPLGDKKIIQVALGQNHTMALDESRALWTWGSNAHSQLGYTLPEPTKKDEDPISTTPRQVFS
SLKKESIQGIAASSIHSVAHTGISLYCWGKNVGQLALMDSDSRSLEYQQTPRKVAASLFSSPIVMVSAIDKATTVLLQNY
TVCVFTGYGYNIVKFPFASMDTIGHLSMSNRYEPGRSQISYITSGGETIAALTKRGDLFTMHLDHKMETNPPATSTTNPS
KIKGAVTQPQCIWSARKDGVRSVGVGEYGSVIISTHSGAVWRRIKRAKVKDAYSWSSESKRNDFKFQRVPHITKVAAVRA
SPFGAFAAVRRDLDVLKEQLVVSEQSLWKDVAPLNPLADFRASEPKPRVRNKEKVKFWGNETLKSNLGSVAYEVLKSSDL
EEDLESYLVEWNHLHQPLDALVCSSSLPDIRIPVHAWLLSARSSVIRHALAQWRRVSSDVQHEAFTISNVDGKPLLVFQG
LDVITLLNFVHFMYEDKVIPAWNFTRQAPPLAYRYRQVRQEVMRLATRLNMHNLEAAARLQVDPKKCMDEDFRLAIKDRT
FFDDGDILLELDGREVAVHSSFLCQRCPWFQVLFHGRSRGIWLATRREEAERENERIGIDLKHMEPGAFAYVLRYLYGDY
GAELFDPTMCDTFADFLDLVMDVMSMANYLMLDRLSQICQTIMGRFANIRNIANFLNAISPCSITEFRDAGLEYICLQLE
SMLENHLLDELDDDLLLELDAIVRDNQAAQSPFVRSGRFEMLLHEAHPELAQDIDEERQIRVKEIAFRASREDDRKLSTA
FKGKGGSGVDDASASTPAADKTRRASKAARNEPFGHSPELKPKTSQADLIFDMEEEDGPATLSTPLGAKAPKVPSSAVDK
DQFPTLGSSWKGKAPIRSGQSPAMASRSSLQAGTSPATKAGGVPWANAPMPASKVGLRDIIQLETTTTPVAAGRSALSEG
LAAQQQQQTGKEGTSAPKVAQPKLSQKERKKQQQEQAAAQAALHFAKASLANKPAWGDTESKAPPPWKMVTANKAKPAPP
HVNPPVSSPPVAKPLVAAEATPVVRRTASPDTRFAGQRTSSNNSVHTANPNPKSKSTPTLSSSSAQQFPSLGDSSSKPIV
PHSKVYIAPARKDEHLLGFSMEEIILQQKLEQERIKGAMSKRSLQEIQEEQAFQEWWDAESKRLQEEEEERKRKATEKEE
RKEKGDKARGKGRGRDTVNGAESSKAGGDATAKGTGKPGGGEAKTGERKGEGRGGRGKGGPSGKKDGPPPGEGKGAVVAS
AASTSATPAIPTGPKGHGGAAAPQHHSRKADGGEGSSNPRRRDRGRDRGKGGRDATQVAGAVSTPTGPAIPAAGR
>Ncra_4474NCU05044
MSNPDFTTYFNSPLFSDATLSYQTHTGDKRHLPVHRLILSLHSPFFASAFTLPFRESSDPDHLLTLHDDDPDALVSAIRW
CYGHSLYQLDGDDELETQLAYKDLITMKFTAEEEQQIAGLLLHLMRVYVVADKYDLGRLREEVVVHYERLAPLVLLHKQG
MFEEMVEVVYGLGKGDKLRRRFLGDVAREVYPVAAGKDFKNDKMLKKGNDWELEEVEKVVRASIFKVRTVGMGREWKKEF
MRDMKREVRREVKKSQAQSYTMDKRFLSSDEKLLESGLFSDVVVKCGDRTWNLHKNILCSRSIWFEKALTGHFEEAKTGV
VNIENFEPAAIDILIRYIYTGICDIPALSPHTKTNFVSCFEVYTVGDYFALASLTRIALDTLNAEFDAKIAPLQLHYDSG
SSASDGNGVSPDWLPELFEAIRLVYNEVPLTDTSPPTPGIRATFLSFIHAARFYFLQNEHFNAFLDQVPSFALDVFRAMR
NTGDFIAHLPDAHCSLCKSKPTRAEKGYYTHLATEKLKLKASCSTCAGKRDLSAGWTDWSGKLSKATTTATGTTAAGSGS
G
>Ncra_5191NCU05921
MDSEPAVIETAPVTDLVLVVGPSKERIPVELAILKAASPVFASMFSPPWLESKSKEIPLPEDDPDAMRFITLTLSYHNEH
ELVTRPQTPQEILQIAIAVDKYDLRTALKFVLEFLFREASKTCSVAKNEDGSQTAMAGKDIVYLCAAAYVLNRCDYFSNF
GLHLMCQYASRISTLMDDELISSILPAKFFIILGEALASIKLGLSRAVHECLKTNSPYRRCQCASAKSIIQRLEQFTVTY
NKAKNGSVLLQEMKTMQKILDEDEPFLRGICHGCNRPHIYSFGAYDSLSPKVRTAYEELDILCVRELKNKDACDAEHARL
TVAGPMN
>Ncra_6536NCU07410
MNQHNSPGSGGVFATAEPSSSSSSSMRANDTINGERSSNSRREIEEIPNEYGEGSNSNDDSSGNGNRNEDLSASIVENED
QSQQYANPRSQTLGDVMREPGSRAPNVMVLEAMPEETSGRKRETTLSSKEDLWLSTADGRYNSTIIPIRVGEAPQAQIFY
VHKHILLKVEYFEKALCGNFRESETQSIELPEEDPAIFHYLIAFLYEGKFEPIKPAASVLVPDEQKGKGKDAMETNGGGA
GEIDSENNNNSDDSELDTDLSAAAALRRRQQRERQREWDRANGKHPGRHRPNCGCPRCVVEPGPPCWSCFAPRSPLPPPG
IGTSYTPTGTRPPFPHPPGSEYRHRPYPERLLQGDGLGAGANRRRPGGRARLAAPPPPPPPPPPLVPPATTQTLPPAPPA
QVSSFFSSSSNQPPLSLTNSTSSPTEGNTRLSTPADLRTWLLCYEHTLSVYILANKFLLDDFKAEIARYAIDMLESAGTD
AAVPQVLFLCKRLWEGLPESDPLLKMVFARVGFLQPWRSRSSGYVYGSGNRDGVHGGNGERINENGKRRRSGHGDEEGMT
TRKETDEEIGDWLHQNPEIVTAVLVEMAARREEDFYQTQYQSSGLQGPPPGMGGYMSGMGLGMGRMDAGGRSLPSMERPW
AAAGRQPGTTGGLGDMGGGGPLPWMNHWEPIRPNTLRPGHTAHHHHWQVGGR
>Ncra_6575NCU07450

MSTRRSSRSRLGSEMVQVLVGKEQRDFRMHKKLLCRTSVFFRNHIEAAQQESKTTKSESEDSVLWLPEESPEVFELFIVW
LYDRRAFQAYVDSAVEKASRNRHSPTSSPSRISPARHSPSRMGTTRHAETTDSERRALRWNLVRLHLFAEVIDLPALQDD
SMDALQDLYLRCDWDMSPGFITFLYGDCSLDNAVRLRKWAVAMLAWTLHGGDGTSSEFEKLFAVFPKLKADYGMHFNKMA
ASKADLRIKNPQLRLPMNNLRSEERLFGFRQCSFHSHRSAVGEGVCPHLVTGQEDSDDMETGSTLMGEDIISEEEDEEKL
EGEAGSSDSEVIITPVCDMETSFLDLS
>Ncra_7438NCU08387
MSSRCLDIPHSKSGSRPPKLVRSNSSLSFAKEAGISKRRHDLRHRRLNVATVPGTVARADGNLNPISPIVTLLIGQESRI
FAAHENVLSASPFFKNVLDTQFSEGETKRILLPNEEPEIFSSVLEYLYKGDYHPRLVHDRRRDTWELEESAAAGRNGNGG
REAPTIYHHSVDGEVLKDTVIYCAGEKYGLEELKRIALRKQGLQCGRVASTILTSARYAYANTPDTDSKLRAHYLALIIR
GRNTFKRSGTMQLEMFHGGSDLFFDLFVALCNHVDDISVASSTQQNTQQSSCNEDGPQP
>Ncra_7785NCU08809
MDMSASPTACQDLARSVLENISGINRALNVTSDDVNGVDSDAKINETHDDHNDKAMTEADNEAAGTVEAVKQEAPRSPVE
SFHELPFSWLQPHALFVIIMVGPEGMPFGIQKDFLCAKSSYYRRYFREKAANNESVEDVVELRDASVDVFGYTQHYLYTG
QVYPDTPPDYEVLIGIWKLGHSLGIDGLCDATIDAMIEYRRITNSIPATALLVQVWEETPEGSSIRKLLLSWAADYMRAS
EDRAEFAKALPQEVLSELVVAMSSLDATPAPAATPSSPVSQGVVNSAHQGWHDAVANCDGEQARPAKRARYSDAGPTNGA
KATPNGRKGPGRPSTTGPKTGQKRKYNVANGEVELTTSKKLAFCDDLLTRMLSGPGFWTRLVGPFRDPVNPESDGVPDYF
EKVAKPMDLQTMRAKMDRNEYADENEFLADMNQIFNNCYTYWAKKDPMWQACERLQKTFEDKYSQMSKWIAKMEGQEGSN
>Ncra_9794NCU12146
MDPSRPLGIKSEPVESSHMTTIPQAIRQAAPAIQSAFTAATLPATDSITVATTSAATTAPTTAAKAPPAKKATAAARKPA
AAKKEAAVKTPAVPKTASAAKATPKTTPKAPPKATPKATPKTAAAKATPTTNTQAEAEVTPPTTTGTGTARPRGRPKKNA
NATDAATTAAAANDNTNITDTAKASISSPPPAAPSSPIIVATANRPTTATATANRLIPSPSPSPGPGPAPATATTTPAGR
PTTTTRGRGRGTGRGTGRGRGRGRGRGGASTSRASSSTTTPARKRTASAASIADADADTDPDDDDDVDADAYGRASKQRK
VASAEGIAATLRAFEASRAGSASALASGAGSGSGSKQVVRAAASGGAGGSGGNKGAAGPGTSVGKKGWSVDGQEINRRHK
ENDLHLLETGEFSDATIHCLKKKWKVHKMRLSTRSAWFREAFAGSDGTGQISEINLHEQNAEDIETMLRFMYADSLDDNL
TRPSSLASPITTYVTLYNLGHTFRIPLLCRDSITLLGQYLDTKLLLLCTYSVPASGRSGVIDHSDPLLSPQSYANDLFSA
IFQAYSGVSESTKLHSLLASFLWAGRDRLFRLPGLRQVVDQCPMLGTDVFKLQLGDNSSSFIPPTTIKLTNVPGGGGGGG
GGDVEELKAKHFGPTCSRLDHTRKTQHPDRCEACDEVFDEERWAKRVYNPFKVVVRPAAWCRRCVEGKTNPNAPGGGKGG
KGGIGGRFDLGVGGPVVVDGDGEGEGAEGGNGERHRRKGVLEDVVPMWRLRVGGDDE
>Scer_SCRT_05180
MADKLMDKNFEELCYSCRTGDMDNVDRLISTGVNVNSVDKFDNSPLFLASLCGHEAVVKLLLQRGAVCDRDRYEGARCIY
GALTDTIRDTLLSYDISKAVDVKQPFATHISSMYNDEGFLKRDITFRVSNGKLFTAHKFLLCARSEILAEKMVNEWAKHE
IVSLEVRPDIFDIFLKFLYLIPILHQIEPGQYEELIELSSKFDIELLPEFLDKARHTADPTEKSRLMSDYQYKFTEVARS
QLLIFVNNCIFRSTVDLANSERRVFSLMNCPAYPDVQLMVKNRNGAIRIYPCHLAVLSRAEYFKVMFTNNFKEKVTYIKA
KHVTGKYNSIIPQLTLPNCEFEVAEIILRYLYADNTDIPWMYAVDVLLLADILLEDRLKTIASTIITQSKEFIQQYNVFD
VLYLSWEIGVERLEQFAAKFIAIHLQELYKDPEIKRAIMLSSQRISLRQETDTIELVDDIRYYLLRKYSFEPDDVELFEN
QDDLEYLKQVGYLEYRKDMGMLDNILADLELDV*
>Cneo_XP_569325
MSFDIRTGTGNFALGTGDQSDKAFPDHINLLTQAQAAGRSDPSQKFSHVGVKDVVMSKLHTGVITSESRGNLSLCGFGAN
GRLGKSIHSQLALVPMTDLQHSIVSIALGQDHTLALTSGGYILSWGYNRFSQLGYVIEAPEKPAGMGKEGDDLIQVSPKR
IVGPLKKEFVRGVAAGRMASACWTADAVWTWGTNAGHLGYDKASNPVQTVPRRVTSITQPVVDIAFSDYAMICLLDTSEV
ICFHGDTNFKISFSIPRLLPEAFPFRPPQTTLKPMITKVASSASSFAALSSIGDVFTFTLPNPMEDVYKDGRHVVVKPQI
VWALRKNFTAVKDVAIGSDGTVIVCTHSGHVFVRQRLKSGSGQLKFRRIPYLQRVIKVATNESGAFGAIRVDARPTPIKL
VGKTLEEDLFLLQPHFHRLEHQMTAEDFEKLNANSKKTDDDEEEDDESTNSVAKDTSIALKMCTILSRWRTGEGDSLFAW
SDPLLSSDCHLIIQGLAIPVHSAILSMRAVKIAQLLAGEYTSNVLSLGSYGTSPAIKVEACHPLVGLLLIQYLYSDDVAA
IWDARVARIIQDKYAAEKIPVGDIKSELKALADELDLSPLSSVLSYAAKQPLPHHTLSSDMQSFFNTTFTVPAGPESPCD
VTIFLSDKQVSCNSVILRARCPFFEAMFEDRDWTLNRKSEGAVVVRMTHLKWTPMKLVFRWMYEGVEDDLFDYLHTLDEF
LDFVFEVMAAATELLLDRLVLVCSRAIIRHCNAFNAAALASEASFYQANTLKLSVFDYIISCLETMLESGLLDEMEQSVL
QDLSSVIAEKQKKRSPVAREGVLVKELMAKHREWLLVQDIPAPIVRQPFKYRARSAMSPVDLSSAKPVAPRRKPPPSPMV
NPSAEVSDKSSAADGIFQMDDDLPTPTTSASEVRTPNRGSRSMTPLSLGATPFQPAASTSGSSTPAKTPIWKSKTVETGK
ADLRSIMAETAAAKTPLKPVSMPVVASAVGNGSAARGGFPLPASGAAPSKGMLARSPPSAGPSTGGPWRATEVRKTSFTA
LQGQQATNGLGTGSPGNTSTSTSTSANANANANTNTLNRSAGSQASPAPQRSGSAKVITPVRLQAPATQPRKSSTPTAAW
STPSTFIPPPPISVSPVAQGLSLLAIQQQEREAAEALARRPAKSLREIQEEEQEAERARVQEEEFMRWWHEEEARVAKET
RQGAVGGNQQGQSGRGRGRGGRGKARGGGGGGRGRGGGQGGQNGRGEDKQGGAAPTGTHGQPSQGRGKHDGGRGDGSGPL
FVYL
>Cneo_XP_566864
MTTGASNINGISGPAVLSNDVAESSKMAAEIDPVFHAVNLARGIFNVGYLNQEWSDIQLTFFASGLKSHRLILARSPYLA
HLMSAAAPGTTIHLSFPDSNVTEESVHIALQHLYNPSMNLVYPQNARAVLATAFLFGGMPELIHHAYLVIRDSLDAKNVV
DLVYWLEAPSDSSAVYDGQSSLRNHREDSVKAWLSDPYPRYGEWTVRLRHDAVNYLLHSLPEQVIQENKFNTLDDPLSVT
YAHLPYELFKALVESPELPVVSHQDRFTFVKKVLVQRKKIAPSGGPQMEENVVLAFRGGEGMEVHVTRRPKKGRTFFKVE
G
>Ccin_CC1G_00139
MSGSSKKPMRRAVVPVSKTLPTDPQDQHSSSEEVDPIFFTPSPSPPSNTPTPDNASALPCDHATGEPHRHPNPTRHPDFW

FHDGSIILQVESKLFKIHQTILANHSEVFAGLFDVPPPLPTGTTEGDDMVLEGCRIVVLHGDTEKDFESLLRAVYNPSYF
DDTLPPSADVETLLDFISGILHLSTKYLIRNLRQRCINLFTSKFPATFDEYTAKSTPQPSYSSTRSSSHKQERYRSDTLM
RALHLAHSCNITTSLPYLYYCTARLSLHRLLKPRPTDVDWQTKSVVLAGRERLRYATLAISYSFLLVFERAPTCAEMRCG
FASGPGREWRSIVLGGESGIGSAPTPPTAATSITNAGGVNGISASATAERKSKSSSNRAPNPLRLYTRWPELGVCDRCVE
WNKGVHERGRREVWERLPGMFELEGWDELRKAQGS*
>Ccin_CC1G_00185
MSTPVSQSNWDPRASSSGPSASGNVPMAPLTTWRANYTPRAGAHAIGSNGPLRQEAVETTRQWSLTGFEWAVKDIAKLRN
FVEGSPTGTDQPQLSADDFEILKESPTLADSKFKLEIGMSVTIAFESRDNTFLSSDHPWRRAWKGTPVSVYYFSVLRLRV
GRLGNIGHDDGGHQMPGRQNRSKRCSPRMECPLPSLSDLLRNERIRETDSFVICLQIHSPSGPSIPQQPATCYVPRDLLD
GLEASLDNPITDTGDVKFICLERLVEGDEPPLSASTSSMSRQTSSSLGSPRHPFSSQATARKRVIYAHSDILVRRSEYFA
TMLSSSFSETSSTVPGERKIHTIVVEEADFETIYWLLKFCYANWLLFKQNDDPRAAVEGVGAGWSAKWLTSPENEWEWKT
FYKSAAEDNVFDARSATSGDSIPASGISRSTSKKSDIPEDVPPQTSGQSVSTRPAGTSSKAPPTVARPATSGARRSTTNT
STLGPGASMSGTSRPKTVPIATSGFATSRAYPVSPRAARPLPPPVPDPHPHPTPPPPPASALAMYQVAHRYAMNGLATLA
LEHIMSTITPQSCFPLLLASYTWDELYGLVEWDEVAGSDEFEQCCKEIAAGEWGSDGGQTLMSVFRRLRSPSTLTN*
>Ccin_CC1G_00753
MSSTLASEPCTPRLAAHPFDHPEADLILRSSDPRPTDYHVFKLLLTLSSPFFCTLFTLPQPSASKDGLGIHEVPVIQMAE
DKQVLDVLLGFCYPLSAHALPRFTSLTTLQKVANAAIKFDMEGIVTHTRSELVAPRFVDSQPFRVYALASQYGWVHEMSI
AARCCLRRPMPGPYASEVKDMPPDVYKQLKLHHRTCGDIARSQVLRNLRVIPESRTGWVWWTCRKCPPLKTEDIADKGSS
PISTLSLSMKPRKWWAEWLDDVAAQVGSKPLSGSVRSKRLLARALEKAARCSTCSRKSREDLECFSQTLDDLVRKDLLSV
GSVGIIF*
>Ccin_CC1G_00928
MVLRITPAASDSTGMDPDNSDPTTPTGTSSISTSSSSMLTPLSSLRAARSNSTLASLVSQKSQIRSHRSMSTLNPPETRF
SSLRYGDKDDLGKATICDAETGLRLHRHPSLWLEDGSVICRVEDTLFCVHMSVLARHSICFRDMFAIPQPESNLRDSLVL
EGVGMRSGAQRIPVITLFDKAEDVANLIEALYDIGTNFGNNDEKDFRAVAGILRLATKYIIDELREMALNHLSIAWPTTL
KAWDLREDLARSYEIDATIPSARLYPHPFEVINLAREVEAPSLLPAAFYDLSRYSFSQIFEPHEDDALYRPPEQPPPPLS
PADLQRLCVGKEHTQQMITALIQAMGNGQHIRQSQQHPPHSLSRRTSSSGAVCVSAAACRKDFTELVELATQHYLFDRER
GCHDPLYVAEELGQLKSAEISECKACAKSLEGWAAREREKIWKLIPVWFRLDMPVDASAKFH*
>Ccin_CC1G_02055
MQTTANSSSDALMGTPIDESNACFHPDFSDPDGDVVLASRNCKMMFRTHSYTLKTTSGFFRSMFSLPGRKCSEVIYTNEE
TDVLEHLLRMVCGLPVLPLDPTLVEPILHAAETYDMPGPMSIVRLGVKSSPLSDDPLRMYVICSRYGWESEMKYFSTRTL
TYNLHDPTHFETLQSLSSTALLKLFALHRGRRESLRKRLNDPPFVSGGAAVCIQCQGRIEYQTWRELKYRIILEIDERPL
GDTILPGLAEWPEANACWQAVCPNITCHRRLYDKAESFRVIRDCIERLPDTV*
>Ccin_CC1G_02709
MRKPRVRFVPCLCDSRITSLTGFFQNLQDFVLDCRSHPSERLCKAEFADLQGPALLAFNNATFQPDDWVAVKSISRSSKK
ADTSKIGKFGIGIRSCYHSLSSDGLVLQLDGDVTETIDHFRPFGHFMGEDWRGGAFDGTVIRLPFRRYVGEIQDRVVTPQ
EIEDLMLDFINDEFNISMLFLQHLRSIALYKIDDSGTITELGSCNITPKPIADKVFKKTVAITKSGMDVEEEWLVILSEF
SEKEAFEALLSRVKTELDPRILQKHKLRPDVGIAYPLHKTTNSIGYLFTYLRLPLATGFPAHVHAFFALTPSRQNLRNPG
DNGIVKGSNDHLLVEWNKVLFEVFIPRAWTRFLQALIANEPKHDVFKAWPASQELSRCGESVYWNNFPQAVLHSIIDADA
AVWPVFGGAHVHKRLRDIIIASSDVPSKVTQAFSNLGLSITLPPQDVVKLLRSRDRSPSHTPYAKAARILSPESAAAELR
KSCLDILRQPEVTATIDLNVITDYLLSTNNLGNIVDIPIIPTVKNERIALHASRNTKGHHTLLEKEDIDLFGGCDAHAIA
LNVFSDRVADLLRREGPKKLDVKRLDADIVVTYLNRLPKERGIEFSAVKIDRPAVHLLTAFWMWLGGSRLKKDLIPKLQP
MYLLPCANGVRQASATVFEVGNTPERLVALLQRLGVPFLHPDFLEKARKVVRGLGTVKSVDDIPALLNCLADHQGQFPVG
MADSDAAMLLKHFSDNINRHTVLSASQTTTLKKLPIFPSVSAAPDSARRITQWVPVNDSKIYGVSSKAILPSIPGIAYLT
TTHVQSDMLRLLEAASLKSLSEVEVLQLALKEQALPLQSPVILEDLMKYLVEIEEKLPESLRKPLKREKFVLAANGRFAA
PENLIDPSNPLRPLYSQDRSRIPATSTPTQKGIVEAASALKLLDYQLSPVNVKDRIEWISSNSRDRAFFQHSIDLLKILD
RANFDYSTLGDTNDARQNWLPTVCNKLVSPSECRPAPGQSDELALFDRVLKRLHPKAAISTSFARAAGWDKPVPTPVILQ
QIGLVLKETPDPHYRVMVILKELGRRSLRQSEIEQLKRTLEGTRWVPTMGYRLVQPNCAVFQQAIPEAEIEEVHPILTRE
QGPYSLLKQLGCTDRPSFDSVLALLKNMSIRPPSDDTTRIAMSLLKFVTRNSPSEEQLSQLLIPDDRDTLRPRSEVIFND
IPHMVDLVPSDDYHMASPSIDEPLAQALKLERLGLKFGDLQQSTEDMGASPVTLVQETIKQYTERQFLSEFLANAEDAQA
TTFSVVLSEDLAAMTTGRPRLLSSDMEKFYKCPYVFVYNDSFFSEKDFQGILRIHVGGKREDSHSIGQFGLGALTMFHFS
ELVVILSGDQALFLDPSKRHLPMGRASWRIPLKQLAQRYPDSLLPLKGLPWEIQDSRRFPGTLFMLPLRAGSHCSEQGIS
SKVWEASAFKQLLQEFRSTAAQSLLFIKLRRIEAFSCLYSGKASPLWIVSASRTDTARDDSNFIVTNTSIEEEGIGGQLT
PARWLVVSATISPDNIPENLSTNLKRVKHVRVGLAAPFSRRAQQCRFFSTLPLAITTELPVHVNASFALSSDRRGIRHDT
YNSAETAFNQWLLSEPLVQVYLAMLEEIARTQDNAQWWPAAFKLDRIEPPVGASQGADLTKSLVESFYRVHLPSTERQVC
WPFFKNSNNFASMTPGNALLFPPFLPRPVLTFLSRSQPANVTELRIPQSYQDEAKLKSITPEHVKDWVLALPAAAQTSYK
DEEFQELLSHLASSDVTNLIGLPLLRLQSKQWTVFRQRDNMPPKYLWRPGPVAAINLFPPSSFVDHDTLDRLLEQKILDG
GLNIAPFDATGLKALTQTELTSLRTNELPSWINKFWTAYPSFETQGIRESIEDLPLVPSTAGEYISLSDCKSGGALIAAD
GELADDDVMRLIQEFEITVVAKERCPPALANILNMEEYLKSSEGNFITRLLRSLKSDIQAVKQRIDSWPDHERETLSHWF
TSQVQSRIPDEVIRTARELPIWRGHKQHQVAWKSASGVRLLPIGVDFEAQAFLNVFVVRGLPPTYFNVHRLTINELETAL
RFPPRLEAPDEPGYRSILNFLLDGIHNTYDELEIPDGTRAFVPARSLYTRENLFRQAFGDRPERFLLESLRNPFEQLLRA
KRLIIREEDLNMDIFVTCARALDEDVLDNNLNPQEVVERARVVFRAYCEQLPLRISSQAVQQWRRVENLRFIPRNMATSR
QYAGGTLEIPERVRNLPMVVPPNDLKAIFAEQPDNRVVLANPKLGEPPVSVVIDHLRELKTMRQGPDVLRYLKSTYAWLD

KNLADAREVVAALKDEALFLNVNDPAVDAWEWISPSRMAFENHDVANIQRVRGFLLSYKDLLKEAGVADVNYPDFDPSGA
LGGKEHELLLRSRASYARQREKKQLTDVILRSKEEDHLEDGERNKFYAHRSFLASYGGYFESMFTGEFAEGQEASPSNPV
EVPLDYSVFAIKTVLDFLYDGDSAIRDESAKWEDLLQVMELANYLDLSNLFENVQVVIIRRKLVDPLNLDYVKAFAERVG
ANRLKEWCVQYEAKNKDYVDRIKASIEQQGG*
>Ccin_CC1G_02725
MADTFVERVSPVAVIKGILSNYPFSVGILRELLQNSDDAKAKLQTFVLDCRTHGTNPLQGPALLAFNDAQFSKDDWVALQ
NISESSKREDTSKIGKFGIGIRSCYHVTDHLQILSGDTLVTFDPTKSLFKEGASVVTLSSEVDETATERLRPFSRFVGAN
WTGGPFDGTVVRLPLRTTPSELQTKVATTEEIGQLMKDFVRDELKICMLFLQHLRSINLIMIDDQGETELGSSTIESNQI
YDNVDEKLVTLIDGTSVTKRRWIVQASHFADEDAIALLAARTGGLSDTLLKRHKLRPHVGIAFPLDGDDAREVGQLFTYL
RLPLSTGFPAHVHAFFALTPSRQNLKNQGDPTLMKGSDDHLLVEWNKLLFDIFIPRAWGHLLSSLVQTQCGTDVFKAWPL
AQSISWCGESIYWNNFPHNVLSSAIEQQAQVWPVHGSSPLSHKILQGVFVATTATPTEVLDILSSLGFSITRPPAYITQM
LQSQDYSNRGVKFLTPDTAATELKTRKPDLAEVNHKVILEYLLSTGNLSNIVGIEIIPTIANRLVALENRGSTTISHTLL
EEFDAQLFRECDDQAIPLHEFSTAVSSLLRRLGPNSLNVKLLDVGRVVEYLSLHPTRVGRDLASSQPDSMAVEFLSDFWV
WFEGWGMKNQLRPMLSTLYLLPCTHGLREANSPVFSTVGRDPVLVKLLEGLGVPFLSSTFKQASEKVLRNCHLVVDIDNI
HALLNTLSHCRAPTLSESDAKLILGHITTCHARSVALRGSLTNSQRDALRRLPIFPVLHVDETTARKSTVWQSLESYNSI
QGISSNCIVPVIPKTAFLPTDIISPSALSLAGATTTTLSPLGVLDLTLQSNRLQHQSPAVLEDIMAYLATRRIWINDNTR
RALMREKIVYNASGTLCAPEDLIDPSNPLYTLYSSDPVRIPSTSDETRAKICKSASTLGLFDAELSSANVMDRVNWIAAN
ADNPSFVSHSLKLLEILEARGFNCCHLNPSIRTYAWLPTQDGCLVPSTQCRPATDDPQLFDQVLKTFHPNAALPSSLIRH
LRWNQELPVNVLTDQMRILLQGSPSCQQIVTLLKALGQRTLLSTEQGKIREVLSGRLWVPTSHYELVEPSQVVFNHSIPE
AGIFVIAPALSKDKGASRLLRLLGCSESPSFDMILAALNRLSNQPPSQATASAAISLLKFVTANGDLSDTQRSQLLVPDD
GNRLRSKGEVVFNDIGQQAILVQRDGFDLAHPLIDNSLAITLRLDRLGLKYIDLQEEFEDMGATAVTLVQKAIAQYTEKQ
FLSEFLANAEDAGATRFSVVLNEGSGPAFTSKKLLSTEMEKFYNCPSVLVYNDGVFSDNDFRGLCKTHIGGKKDNPSSIG
QFGLGALTMFHFAEIAVVLSGKYVLFLDPSKSSLPVPRASIKLPLKDLVRWYPDHLLPFDGCGLPDIEKIKSSGEYPGTI
FFLPLRSGSHCSSRGISQKTCSALKFHNLLSEFRETAAQNLLFIRLAHIEVSSRHVGSPRSLWKVTATRTPYDHDGFTFI
DTVIDGDGKRATTKWLIVSSSRSLNEVPEEVRYEGMKHIRTGLAACLSSKHPPPSKFFSTLALAIGTTLPVHVNASFFLS
PDRRQIRHDAYANKETRFNQWLLSDPLPQAYLVLLEEIAKSRKNDRWWPENYANYASSAVDDVEPDDILTSNQDDALTST
LIRSFYSLLPSTERRILTPRYAHPSRSALKVNEAVFQPTGLPDGVKRVLDITEPLYLTKPAIRSRVWREARFSCLTPEHV
KDLILGGAVAAIQSTFSNDERRGLLSYLCSSDPLNLRGLPLLRLQSKEYTTFESSGQDATKFIWESSAIVESPFPLSSFV
AQGALPSDVILSARKSGLNIAVFDLSAVENLTRQELSRRSPLERKNWITVFWKAYRTLPGGRDLHLAIESLPLVPSISDN
FVSLRQCKDDNVLVASALDQEDRLELIQDFGVTVVSRDRCPSALAEILRMSEYHKSGPFFHRALRCFQSNLSGVISCLSA
WPDSRRKEFSKWFRSQLYTKPPKGLLPTARQLPVWPARRLASKDWKAASEIRCLPSDLDVSAAELLDVWVCKKPAAQHLG
VETLSVEELKLHLRLPHILSQPDESVYLPILSKILYLRKPGAVKVPNMNRVMVSSKVLYSREALFEAAFKQDPTKFLLRS
YSALEGQLRQKQLINRTSELNMSMFTICAQALHDSDDDEGDKVERARVLFRVYGEDLPLKVSAITFTNQWDQLDHLRFIP
RSMSTDKTHGGWTLPVPSRVAGLPEVVSPRELVRREFEDIAWTQLASFAQQPNQRVLLANDNLGRPSLSNVLAHLRALCK
MRPGPDVLHYLKSTYAWLNAIASENTTAIEQLNQESIFLNIDNPNAFDTWRWTPAYHMAFENHDLPTIHRVRPFLLDYKD
LLKAAGVQDVVYPDVDTSPAKENTDEELLKRMRTGFAKMRDDLQFTDVVFTSEEDESTEKPQQFFAHRNFLSVFSGYFRD
MFTGSFAEAQQASVSAPHAISLPYSNFAIQTVLDYLYTGSVINPKQPDLPFEDLLQALELSHYLDIKSLFDNIQLEIIKR
RLIDPLTLENVKGRADTVQATRLSKYCDDYARSNRDYIRRIQQDAN*
>Ccin_CC1G_03050
MFSPPAQLARLGYGLHPELKNRTQAAIVCLQPNPSSYSGAHRHIFFIYTQRPSLEEQALFTLTTSVLLFTARLRRNLPDV
DAKMLALKWSCDLICSRSDVPTRSDLNPLHPRIHSFDVYGLDMSEQTGSSSLSLLAKRKRVEGPDGASPDSEPIQRAKTW
WFDDGNVIIQAEKVQFRVHRGVLSYHSAILSTMFSLPQPSTNDTSTETVEGCPVVPVSDSAMDWDAILRVFYSSVLFLNG
GASPPLEVVFAMIRLGHKYDMPHCRNEGIRLIRSACLSTPNFSLSPSLSISDLVQFSFEMRLETVQARLFLYTVENPGFP
EILYRGTTSATGKTTQISPSLHPTLTLGREKLWAEMQTHLFSYLFKNLDSAGCHDTEECSKAKLRITCKIFTPVPQLGYI
LTAWDPDLFGKDLCKHCLAEAKATVTEGQYTMMKLLPKAFGLPPWECQKDLNF*
>Ccin_CC1G_03051
MSGTSQRASPAPPSAKRRRTNLSDSNAEEQVVNSTEYWFSDGNIILQVENTRFRVHKSVLAIHSPILHDMFSVPQPPEGE
EFVEGCPVVQLWDRAEDWELLLWISYRGVQYRLQENTRPHLNTVAALLRLGHKYEMADYRDHGRRGPYGFHHAAAQADRH
VMWATFGDTSSFSDLFNLVMELRLETVQPQLFLSTMCRPDFPEMLWTGTTAADGNVSKISTSMHGALIMGKEKLCQALRD
DIFDCILHWEPDHSACTSPARCEPEYRKLIVNLFTPVVQLQKAFSSKAMNSFSQAVCQNCSITAKQSVTEGQEAVWERLP
EIFGLPNWDELKDFNFD*
>Ccin_CC1G_03060
MPTSTLNNTSHQPPLKRRRTEDDSAMDASAAAVWGGGGCPHCAHCAALRSQASSSLPSSSQPSPSQAAIPQPASQSSTSE
PSPSTSQLTQPSQRPSEPTHSHPTPSQPTGTSPPDRPTPPPSYTLTPPPTRSPDVWFDDGSVVLQAQNVQFRVHRTMLSR
HSEVFRDMFEIGRLGSSINASNTPQAADPTPLVDGCPLIILHDDPTDLTHLLLSLYDKPHTTTDALPFATLAALLRLGRK
YHFPSLYAEALRRLQSEFPASLEEWEKLPQTYTYIVEEEGLLFDIVNLALEMEVHEVLPSAYYLCVQDLNDLLYGIPRPN
SPNGPAMLPPHIKNLCIQARERLLALQYDMTFAWIDLLGNWNQEDHGVNTDCLKPDHCGRIAGQLVREVFLPLPEVNRTL
EKWTDFTTYTPLVNQLCAVCLEDARVCHEAGRRKVWEGLSGVFGLG*
>Ccin_CC1G_03065
MPETRGIKRARPGDESPDSSPLSTIFVSESDLESTLSDLEDFRIKRSEAVWYDDGNVILQVGDTRFRVHKSILSAQSRVF
RERFLEKPEAKKTKGKPGGGRPRRPRFVDGVEVVRLEGDSVQDWTYVLQIVYDGHRTYRSDTSYPFSVVRAMLTLGDKYG
FEYLRSQAVYLYEKTFPSDFSTFSGIYYSPSTNMKMTEKWIRMDARTTLVDVADMANLFGLIACLPAIYYLVVSQKDFMS

CIATGFAREDGTRAIFSRDFIQILLSGREKLITAAARKMFNWLWSIPEIIPAAGSCKDKISCRNSLREIHAKYWWPSFPP
SKILGQWNTRYAQDLCPACAKVAQAEFCKGQKEVRKDLPGFFGLSAWEELQNRGRA*
>Ccin_CC1G_03075
MDTQVPRISQVEDTKEDSELKEEVETCDIDMEMSTKRSDVWYDDGNIILQVEKTLFKVHKSVLSEQSYYFEELFSKPQPF
PTPENTGEGGCPILNVSDDELDYTVQWTKCLRTIYNTRRDLNPNTTYPFALVHAMLVLGDKYGFHYLHKEAVNLYERTFP
SDYDSFCDLGTTSEFESDYFVLNEQWTIHDIVKTAVEFGLERCLPAIFYLTLTHRDSENFLFGPITPAIGEPTRFDNDIL
EILVKGREKIYHSMARNNFAWLWSNPHSVPAGTSKCQKYKQCKLALERLLKKYWLPVPAIENALCPWRDSFGKGLCDSCY
KAAQRQFESGQKALWKELPTLFGYPPWDELKSSRVEKEEDEDDSPDGEVAGGSDAIMAITKRYLR*
>Ccin_CC1G_03092
MSEPGSSRKRPRTNDDAPVSDNSIQIIGHTTRNQKLWFQDGNVVLQVEGVQFRVHRGILERHSNVLHGMFFVPQPEPVAA
AQETTIEGCPVVTLWDSSRDWEELLQYIYDGFLKRRTHLTFPLIRAMIVLGHKYEFDTLKEEGLTYLSQVYPPSYTALNS
TERKDKLKLFYTDDTTFIDAVNLALEHNHKRFLPALFLAVLTHTQYPEILTNGLADKTGRIVKIPAETAVKLAIGHEKLY
EGMLKHPYGHLTGKTTSPRGCFRALGPQSTDSSSSSSSDSDSDSTDSDSSDSSDSSSSSTDSSSSDSDSSSSEGNSGDDF
GQALDQAMDQAIAQRVEQSIRARLRISPCESVRLEMLKDLVAPPVTVSKVFSSSIARGRHAKLCSNCLEYGAARVREGQR
KLWEELPTYFGLPPWKQLEQEQEQA*
>Ccin_CC1G_03319
MAAADDFSTNQNQASSTVPGVEDPSLKLGFSSLQRHPLSFEDGNLALISEDHYFLVHRGFLCRHSAALKDIILKSEDQRR
LFNRPVVTIDEKWADLGCFLSSLYDGVTHLSSSIEDFAVVAAILRLATKYQVEHIRIEILRKLASTWPRNLLAWDIRESN
ATSLGLYKPRAVYPHPIMVIPLFYEVKAHELLPSAYYDLSRCVASDIVSGWSYPADPSTSWHLDNDSILTVLRGKEQASR
FLSTFIVNELEGREPSTGCIYKTDPDPLKRRICPASFEAVTFEIVRDCNGVVCHRVTDPLFAILDSYLMQKRDNPIGRGK
ITFRACDSCREDFAHAVEAARNNLWQKLPSWFGVKLDLWPQTAGY*
>Ccin_CC1G_04002
MEPGIFQYVMTLQRRPPRISLYSKHVGAKENAHAYYQDILLTLSWVSCHCERAFASRRKARFALASPPGWWVAQRMTQRA
QMTHMSFYTVCMTLAHDYFNTRNLQQFQRLLDGGLNDRKQQAGNGASAQPSTSGAKSWNKPSPLATMTPPVDVNARDWLG
RTVLHLACSAVENVEYVRALLKHPNINVNVPDTESHWTPLHRALYHANLPAAQMLLQRSDIDISLKDWEGYTAFDLYNST
INGTNPDSRQLNAELFTWGANRNAALGLGDGNDRSHPDQVIIPGKGNPEDLKDNKLTERFIPISIRQIQMSKLHTAIVTS
EESGNLRVCGFGSGGRLGPGQHTQFSLKPLPQFSYTITQVALGQDHTLALTKNGEVLSWGLNHFSQLGYPLELTGGKLEE
PIQPTPRKIPGPLKKETVIGIAASKKASACWTLDEVFTWGTNAGQLGYDKNAQPVQVIPRKVTKFSQPVIALALSDNAMA
GLLQTQQVECIWNDRHYRINFPIHAFPSEIQPFRPRQAIKDFHIAKVTCCGDLFAALSSNGEVFTFSPPTDGGDGVDGYH
HKGSPFKPQRVWALRKKFSSVRDVAMGAEGSIIICTESGHTYVRIRTGKGGGKNFKFQRIPHLQRVTHVCANSTGAFGAL
RVDFRPKKIDIQGPSIAEDLSRVQPYRLPFPAEAVPSGHRFHPTVVDALDEAGRSKTSINVPALSLDDDNYIDEDGDDAD
IEKDIRTLQGFMNIIQREERKVKAAKGSLKYGGVALPHGADTLISIGTIHVPAHSVILAARSTVLDDILSKRTTATSSSI
SIVVTRRLPGPGNGVSNLTSLAFSGIHPLSLLIILHYLYSDEVLAIWDRRVYSSLTDHALSTLKVDVAEIKSDLRTLAAT
LELPHLSQVLQAPVKRPPAPSLSTDLSALFEKAQGSKQRRSLPCSPDVVLELRDKEVWTYSVILRARSEFFSCLLDEDTW
TRKRWGQDGVLRVDLKHMRWHVMEFVLKFLLCGKDKELFDVLPFTESVDDVLEFMFDVMAAASELLLDRLILICSSVILT
FLDIHNCCYILSEASYYHAYQLINSVQEYMAANMESLLEMGMLDDLPYSLVKQLSKFIKERQLEKSSFSRTDVYLDGLLA
KHWDWLEGQDIPVPYVRSGKQHPSRELAKTRLSPPVPPKEVPSTPSKQLKIPTSAVQRRPSGDDLFAMDLGEPATQPSQS
STPWKVPDAPRVDMRALMAEAQESNRSIAPPISASPSRGQTPRTTSGPSTINGPGWKTPGPSTTPLTPSRSSASQGPSWR
QHASPQLPPPASATPPSATPGAGPLAPKAAPPSPSPLRSKASKPTTPLAPLPGMGPTFTPTKQAVSKSSGIRNVSGGKAW
TAPPPEPVVVTTNSKAVSFAAIQEAQQTQHAPVKDRRSLKEIQEEEEALQAEADFLKWWEAEENRVKEEMAALEKINQGG
LTKGERGSGGGRGGRGKGRGKSEHHRPRSGNKPPATGGSGGAPADAGEGGPSRGEGETQGKGKPTKSSNPDDGQPRRHRR
PPKKQAQGQQAGQVPSS*
>Ccin_CC1G_04507
MDILDELAKPEIPFQTLLDKRNYSLCMRHPQFYFSDGNVTFLVESVLVRVHRYFFQRDSPVFASMFSLPPPPGEGPEGAS
DEGPIVLEGVKLGDFERFLSVLYPKDFHSGNTLATEGWISTLKLASLWQFTSITKLSLSKLTKLTSTSPIDRIHIARQLS
PCIPELSTWFMTAYLDLCTRDEPLNLEEGEKLGMKDVIRIGVVRHHIRFVKGSDLDRSKDMVLLLVAQTWGKDLYH*
>Ccin_CC1G_04513
MTSTNTNASSASPSYYPPNILQAPYLGPPRMSREDLQGRWGDGMFAPPVPSRRTSGRSKRRSYLPAPHPGERLTGLGNPS
QGDVSNTHPATGSGRSQNQGVPTPAQVPAVEPTAHATGNTPGAPKEVTLHLTMPITFPSGIGGPAQVKVPTAVDTSSAVP
GPLGPTVIVPPSSDGSGRSNKDWVSVDSVPSAKLSGSNARSVSRSRSSSPATTVSPRSRSPSTPETCDVCPEAAINTVLI
NSRYDFMGAPNNNLEALANNFGRMSVKDKDNKNVRFSRRRSRNPLPDLPIPQPLFPQSIKGDTRDTNTGPIPVSAPYIPS
LASPTQSPWSSNRSLPSPTAVPAPFPRFSPSPPPLIMPPFAPAGYCSKCSVPLAHTGSTVCDACRPWDAMNGYIYPQGRP
IAPSGYPPFAPPVAPTGVFAPPAPSLYSYPTRGPSLCSLSGEVIYPYRVFYPGQSGVVHPVYYFSDASLAFVVQGYEFRV
HRYLFENGSAVFKKALASFPIERDNIVTISLYTATADAFEAFLKALYARPGENIFENTGDRAEAGSSMTSLQRAQAIFSL
ATRWEFHTLCKEVMAHYKDVGSLVDRFIVAKKYVKLNPAKEPVVLSTWGGPYSPYIPPANMATPWDPLKDAKKVDDEKPD
WEAYLLDVLKGLCLGGNPVTLEDARRLGLETAMKIWHAQDQIRTHFPDVMRPTYRACCGLAKPQNSASDYPHVDTHAMKA
IETYIKKKFGIKTCE*
>Ccin_CC1G_04664
MLTATHTEAPQASRRNKDFYFDIVTFKVENEFFRVPSLYFMKESDFFKGMFQLPVAEGAVADGHSEDQPLLLEGVLAKDF
NSLLRVMFPSSVGQEETLNAEEWEGVLKLADMWQMEYFKTRAISKLREILFRDPATRIVHSRKYNIDEWLLPAYEQLVTR
SDPITIEEANLIGLETTLKLCAIRESYVLKVGDDYGYGRWVLQTRRGNIDTPTAALPLRVRMKKDFGMDLELDESDLLLQ
TSTPVRVRSSTFTVDSP*

>Ccin_CC1G_04665
MYLNPGLAFKLSSLVSLNLHVHNRAINRLIHYRWNDDLYSTLRWPAIMIGTPSPDAPSNTSTIISSISTAKTAAQTSEQY
FFRDIIFRADDELFKVPALYFLTESQFFRDMFDLPNPEGSIVDSTCKDKPLVLEGVRASELSALLRVMYPPTFREADLLS
KEEWAAVLKLADMWQMKPIKALAVEHLVQDLSEDPVTRVVLGKKHHVPEWIIPAYKQLIKREEPISPKDLEGLGVEIALK
ICAMRESCSRVEYPGGKGRAPQTVWSLGKSRPVIPSSEDLRGLDERLQKELIDN*
>Ccin_CC1G_04672
MSSIPNCVKVQDESASGRTTLTPERSLTDQPTENKFYFFQNLTFRAEDELFQVPALYFLGESHFFREMFELPPPEGQPLE
GSSKECPLVLEGIRAMEFQALLKVMFPRTFGKAEILSKDEWEGVLKLSDMWNMQTIKELAITNLTPILLEDPIARIVFAR
KYNVGRWLTPAYVDLVRRQEHIGVEDLEAIENELFQVPDLYFVKGSQFFRDMFELPTPSGQPADGSSEDRPLVLEGIRAA
EFLSLLKVMYPQNFGEQERISESEWTSILKLPDMWQMSAFKKFAVESLALLLSEDPITRMALARRYGVEEWLYPSYAELV
RRAQPICREDVDAIGIETALKICSLRERCVEETTKTITIYGANQGRLFANIPTTNTVQTLVLNPQRKQVGSDQITDMEQH
IRDIFSPERR*
>Ccin_CC1G_04675
MDTSAPDAGLGQDPPPTKNALYFLRIVVFKAENELFQVPVLYFMSESQFFRDMFKLPPPEGAPVDGSSEDHPIVLEGIRA
LEFQALLKVMFTQKKEELGTEEWEGVLKLSDMWEMHRFKSLAVKNLGSWVSAQDPVSRILLGRKYNVDDWQGGAYAELVR
REEPISLDDLSALGADAALKICSLRERCHPVKTITKVIQEYVYHPNRPPTSETETEACEWTLQARRQEVPGKELIDLEEQ
VGVIFGIRMPLHRGPYICAGYTVNV*
>Ccin_CC1G_05070
MAKETRSAKRKRGESTTAEALTATTSEPASVNIQRSQQVWYDDGNVILQVENRQFRVHKSILSKHSPVLRDMFSMPQPDG
QLKEDCPVVVLEHDLAKEWEQVLALIYDGFREVKYGVKLWFSEIRSILSLNHKYDLPSLREEALSRLKLEYPLGPGFEEE
NAVKEFGERDKSMELDNHTSMWDVVNMATKYRLERWLPAIYLQALMQRDDDEALTTLIFDGTVAKDGTTTTQLEPAIQRL
LLKARDRFRKVMKDEVFNYLSNERVIPGNACPQPKRCALAQAGLLRETWGQGPTVSSVFALSEPDVGKLCPACSKAAKCT
FWKARVELVKKLPAMFDLPLWEQLKEYEGQVAEDEEGNNQNNQ*
>Ccin_CC1G_09209
MTEDTQAKATGRGKYFYRDLVTFQVESTTYRLPREGFERQSEVFKSMFELPQPEGPILEGAGEDNPIKLEGVSTVQFESL
VEILYPSQVPYTPLNKDQLLAALGIAQRWEMNEVIHYIESALLRLKLSDLEKTALGKKHGLNRLLVEGYLPLVTGSRALG
LEDAAVIGWDCVGQLLYLRRLVDAKFARERRCGWEDLACIKCGGCPRENRAWVFSDCNCAKENKATLDVVRVKVVTEEPW
SGPTLEKIREFCRETMAKELGIESGA*
>Ccin_CC1G_09212
MTGRVYLSRTKPVFASMAVNQFLLARRRAYASIADRYLPIQWTARTSIKLDSSLSATLDVLVRKLKPMNKASRSDKYYYN
SQFAILQVDDTLYNVPQHALERESSFFRTLFTVSRGDVEKNSDDNPIHIPQVEKEEFEAIIEILYPSEIRHEWTRDQLVA
ALRLARLWEMDAVAHFIEESLRNLKLPLSEVDKIILGKKHSLAKLLADGYLSFMTSDTPLSVELANQIGWDSIGHIAFVR
QEFRQDWRFKPKLVCSISGAQCSQPGCGGYLTKDNPRGRSSRIEDLGCQSCKTMSSMVVLSDVEKELPTEADPEDVRKAL
EKALSRVFQSSGESKGSD*
>Ccin_CC1G_09695
MDVDGKTTEGLPTEAVLNRSATIWYPDGNIVLQAENTQFRVHQSILARHSEFFSNMFSMPYPRDPMGPTVEGCNVVSLAY
DSASHWQALLELLYDGGTKFLKMDSSWPLKLVEAVLTLGDKYTFDNQRTEAETRFLNTFPDEFGIMTPKDPVFSERWIYL
DKDRSFCDFVNLAFEFEALHKALPYYCSGLWSRGYQNSG*
>Ccin_CC1G_10014
MSSSRSPSTNPTPPTHTTTTTLSSTLLTSTSPPTLQESIKNSTKQWQEDLASLFKHAKDRFPDVVWETSNEGLLEDDGLG
LSDGFSVSYVNGYGDEEERRRTVLSRVRVEEVWGHKAIVYARAPPSFQNRYFSFNRPHGATSPLGYNQSDDYHGTDDDHH
SHFQHQQSLYPAESSVSLGLDLRTPSPYRSGTPSSMVHPTTNLLRISTTINPALFSNELEYLYTGQGFGEAFEFLFDSSE
HAALGGGLVTGVDGEDGEGGGGGGVVDAEALRIDKLRKDLVFMWRSRLYSDVRIALTGTFASSSGTHENTTAIFSSHRFM
LVSRSPYFHSALLGGFNSKRRIGGVGGGIGGGDDGDPPTLTLPSPPFTPASLHFTLGFIYTGTLIFSHRTYDLSTAFAIL
RASLYLSLTTLHDEIQARITTEMLHGLFHAFLPFAEYEKLTQGKWGVGGCRCRQCARRAPRVLEFSLEPDVGNQHLERGA
RRALVGLFGEGWCTTEFASLPPKLRESLLKGLAKRTTPQNVWALLFAAEHALLKLGPVIDAWADTVRENIFAARKVVDEV
LCRESEKCFGMEEWVVGVVGEDGEGVGFEDMEKVEWAMAAVLRGVRERWAAGVYQTLVSSILLRPHPTEENAPLLSATSP
IRQHVEQTRLELLKWIGKRWIGIRNEHGFDELEGWALKEISDYIEVPIEDLLNPIPAEKSSPRAGGGHAQSASAASRTKT
GSAFLRPHSQHPHTSRIDADSDTASSMRVSVLTRNTSTVSSSNRYGERSYTASLTPSRRRGGGNESVASSVRSSARSTAT
FESGASGSTVGASGRRRVTGGGSSVVSSSVASTRREASSTVSSSRAGGARDGEDSPAKRIAASVRSRMKADEVRERERER
PDSKLTPVEADVLRAGGVESLYGDDEDLDDNNDDDDEDEGEMRTAPEGRSTVHEDDDDGALTETEAGDGEDRVTDLEDGE
EEPQEGDSDKSVDGRSTSTAGGRRQARQVKRTPTSASSSTTSSSVAARSTANRTPTRTNTMSSRRTTLSNASSMSASPAV
KKMVITRASLAGSGVSLSTRGSVSSMRSAASGVGGRKVSGGSVVSGVSGVSGTTGLSRSTGVSGSTGVSGRPSSRGSTRT
GTGTTLSSRSTASRSTLSSITTSTTSTRSSTSRPASRLSSVSTSTAGGGRPVSTVSTASSTTEGTWRTASQGGGLATSPL
SPTAPSSARTRKTSAASTVSTASVKTTTTATTASAARARKVSTGSVASRKEPPAGASASARARKVSTGSVSSVASSRSPV
ARRGGGKAPPVPAIDPTRLAPAVGGRRSAGVSPTASSFKGTSPTTSTSKPGSSPSNSTQRPGAAGGRTSKGTPSLRSSAS
SASLASTAGGGKKGVGAGGTVKKVASSPAAGGGSAKGGEKEDDRSKRSPEKKSDSLPTTPALSTKSLGSSTSVSSDATSV
AGGGEHKKTSSSASNSSVATLTMKKRDSVDTITGRTRAASPPLPALPQQEEPEPYDPADYVPKGATLDIGIPCIISCKRK
RFKAYARYIGEVAGELGPWVGVEVPMPSWESANYGYSSISSSSTSASSHPTSEIDDRQWNDGSWGGIRYFEISNHGGDGW
GGEDRASRRRRVDAGSVGGAVWGHQSSSSYSGSSSMFGGSVFGRGTLKREGDTLSINADRMKKMRSVSPTMSESSSMVES
RGLFVRPQQVLYVVDAIGPD*
>Ccin_CC1G_10647

MSAARDQSPLFRFPDADVVLLSGGSDATTEFHVHRRVLSLASPFFRDMFSLPQDPSKPNDIPRIPVAEPAPVLEKLLQLV
YPMADPVLTSLEELKDVLGVAVKYDFEGAVSMLRAWLITPQFLHSAPVRVYAIACRYELEDEAKVASRYTLGVNLLDVPL
SDELKWISAYHYHQLLVLHKRRAQAALGYLKDVKGDLKCMQCNGSNFHLQLPTPPKWWTEYEARAREELSLRPTTDVIFR
LDFLYKAAAKGGCPRCPGSVLESWKFLMDLKNAIDDLPSTI*
>Ccin_CC1G_11551
MSTNPSHILIARNSRGHNPSASLSSPTETHAAASGIFRSSTSPPWHFEECETDDIVSDLSNEDGENNNEEAIQLDETFTR
NSKFPGTVKIIVEATTFWAHKEVLFFASPFFEAALSGNWSETGGRPQSISSVITISQPPSDPANKEQHDIPTEMTFAPMD
SEGEEGLDGEGYPDPDDDIEILTDPENLKSDPESSSDTESAKQRARGDSLAKLEASSTAAKSPTPSPSKSTKERDPSATR
KKKLSPALATIRRRMKNGPDAVIVLKEEKATTFHDFLKFVYPHLECTITWNNVEGLMNISHKLCVPTLQRECLSFLLTHA
AGRPIKAMRIAELFEEEELYREASRFVLDNPGGWSEHELNTLSQETLLKLEKRRNWFLERVLKLGLTMIAKEYQCCPTCP
DPATCARLLDEKWRQAYHAVFRFGPPQPSMVFRYLRMLEGVSPPLSLTQTACQASAKAFTATLFDRMFSLGLRGSGTETT
PIGARVAVAAVAGAASGPRRHFLYCALKNESPQKNKRTRSDQT*
>Ccin_CC1G_12438
MSSSDSQAPEPHQPIHSSHFWYKDGNLILQAENTQFRVHKSILAAQSPVFRDMFSLPQATIENPSDPPVVVLEHDSSVDW
ELFLSSVYNGFKFIDPGKVPPFAMISSAFRLGKKYQCRDIFESALGRLRSVYPPGEWESLTAEFDEKIIALAPHASLWDV
VNLAVEYGLQKMLSKIYLQALMQGAKPGDLVTLILEGMKNQDGAITRLSSETQRVLLKGRDRLHNAIISKPLRYIWNSHI
PVYKCTNSVTCTQERDKLLRSHLLPELKIDKAFALPVDLDQFCYRCAGIAEDSFVKGQKVLIASLPEMFGLPPWDEMKDY
DWQDDE*
>Ccin_CC1G_13560
MGSFSPLPGWENLIRSDFWFLDGNIVLLAGNAAFKVHRGQLERQSEVFRDLFSIPQPASNQDLIDGCSWVELHDRPADVF
YFLSALYDGLYFKIPRTNDFACVSAVLRLSTKYLVEALRTRCLARLELDWPTTLSGWDIREREALDDKGRYLPREVCCHP
ILLMELAIELGAQHLLPSAFYDLSRYGPSKIMAGTTYHAVALDVAASKTPIEASRERPATLNKDMLVRTFKGREAAQRYV
SDFVAKELENRPPAPDCATRNEKENPSRNCLESFYFIMLNILRSVGGIACGRDADPLFTLVQATEMLSRTDFSDGQRQTG
LRICHACKVDFATSVGKAREEVWGLLPVWFGLKEEGVPLKVA*
>Umay_XP_762572
MACHSPNAAATNTRAGTIPAELEHISPSVSESASASASTSTSTSTSTSTSTSTSAVAPCTAAQTSASQLASCVSLSTSST
ATNTTDDLHNHTSDAAAPPSTLDAPATSDVDPELRAPTLSAHNADPSDSTDQAAHNPAPSSTASLLPRASTTSTISSASS
GSATPTNRSISTRPSQPHVAHARSHLLAASANAATLGPARRSISRLGVQPSNNRERIARNNVAASALSSSYTSNAVPSLF
GFYSHDANPTAYANSAASSTNSTTSNVAPHVWQPPPRDAVYHNRTASNSSAPTRSSHPAFASPIATPRVGPLSFYPSTTP
RQHARVSSNLGNAYRSTETQRTGAFHSASTTNSIDSTLSASSLPAEQPTFEETTTISFTWTIRDLHLLREEVEHSPPPSE
GGRSVSAGAGKSDIWTNQPIFGDNKWKLELVRTTRPLPHHAQPPAFSPHGAVDNADDPSVSPTASALDRVPVETDDSNPT
RTTITILSVYLTALVLDYTHANVEIPASIMVALRPIRAAVVRRGSQTGGYLWQRFYDYTFQKEADLFTCHDLPSVSDMLQ
DTNVARDDAIALTIQLGLGPGHTRALFSQPDHPAALHAPVEVAGHHIVPRAILNSLHGLLDDANTGDVRILVRERGILLP
DLSDDASISDHGHHDGTVADSLGRIVPFPIGSTCPAAPASTESSEAATMTGIDDDQDRIFVRDRVLWAHASVLKNRSDYF
DIMLHSDFSEGISRNYGSVDTSFGPARNVRTLQISDADFVTAYWFLRYLYTDDIHFADKQDVRSAVLDEEWTKGADLGYL
TTGSTSGDAAPAVDLRKSMLVDWTPISQLKHYDDFNVDGMGYAMAAGTATGARSELSGSFVLQGPPTSKTAATSGSLRQR
TSAAGLGDGSSASASVLAGGGNGVGARTLSAPNSPSGTTVSGAGQHRSADEASRVAGRAVCNVSAPLDQHDHPCAEPGPA
SALSIFKLAHRYNMYELMRLASLHMVATLTPQSAFPMLLATCMYTELHARIKTYVYQHWHLVSHTHEFEQCCDEVCMGMW
GADAGKTMRAFVTSLVSPLRAAAS
>Umay_XP_760428
MTSSSSNIAFNSNNPVPALIARRTTVLAPPSLSTVNHLDGAIAATASSSSSSTPASAANPRPSINSVSTRRHRPDLQQAV
SQPTAAVPVSTHENISTSKSWFSGLHLGSNAGSSASTVAQEQRRSSGGAGGGDDAQGFAVHPTFRSDAESRGDVLVRVDG
VEFWVHKDVLLFSSPFFKSVLEGGWSESRLSRLVNKGEADAVTLAHTLPMQQEGETHDAAINHLTGAHHVSERSTRSLDR
EAQAQAQPHQQDLYRPDDRVDVTEESASEHAVNHTVELESGLLGVAESQRSSLLRASYHTALWSQDDESRGSQMHFHDTT
EDSLSVDSDANDVGSASEDGSASEGALASEADQAVPQQKHTPDGDRDAVSAISAHQLRQRRSKTKHLATLRKLEAAQREK
LIPTRSTSRSLDTACKNEALVETGAQQRLESLATPPLSRRASGYDDALGVPVTAPEPNTEAAAESMQCATSTVNSSSEDR
HRAAALPAHYQGIISVIDLTEETASTFHDFLFHIYPHLDLFVTWYNCGPLLRFSDKFQVPFLRRSCIAFLRAALAGRPIE
AMRLAELHGIDDLYRQASRHVLDNFAAWLPDELAVLSHETLLKLERKRTWFLERLLKLGLANPARDYECHAACPDPQACA
RALHDKWQTAYKAAFRFSPPQPSVIFRYLRELDSIGVGINASSGVSSAIGSVTGLAGHREGLGARSVRPYV
>Umay_XP_759930
MSRSPARRRSGELSVPVDKNNPMETHSITFEWPLHDLKQHFDSSNSDAKSKVIKSVPFGQGRWTVLFYAQSGLQQFCSLY
LNAEPTPEERNHSKSPWDSYLASNSNTRSQASLADKLAGATDEGWTRQGLFRFTFKILTLNKGIVLATKEAFNHAFSHKT
SNWGWAQFASRDTVFYTNSEVRETDGFLISVTITASAERPRPLTLGVTVPPALIQAMGSLLDDPDHSDVVFVIRRKRRHH
KTGAIVTRERRIYAIKKILASRCEYFRDMFDSGFAEGDVTAGSDDETDDEVSLAHANSRGVGQDGLSLIGSNCFPSSVEE
DDDELGLEDSDEEMDVDEVYEMGMSNLTDQQRNSEAGLARNLASDTATTPVKAAATSSESSAARRMRETSSDVAQLSPFS
STQTPSIPSNSAQASDYVSLQQTTPPQSRNRQSNDQDAGTDSGEDSMRVQSMSAFQTPQHSPRSLRPDVSAFVSPSKSSS
ASMSLQLRSTRPLQAQAATQTSHNGRTLSQLRRSVDLGASNVHSGKKRRKVVVRDAAYATYKALLYYLYTDTIAFAPLHS
RFYSHATSSDDKSDVLGGELDLADGASATYAASDAEASPVRSSTNTAPGSSFAKDRRRAQHLRQYFIDEYCAVHTNVPAP
CSAKSMYRLADKLQIPELKKRSQQELANNLTVDNIVWEAFSGFNSQFPTIRKMETDFLLKHWNEVKSTEVMKNIVSRPSA
HPGLADIWPHLLSQLEFRGSNSENKSEGSA
>Umay_XP_759382
MSIPLTSSGTQTAGPSAYADSARRGRVRKARPSTDLQSEAGPSKAPEPRATRHQHDDPVLSAQEEATRIWKADMRRLLEQ

AEHRFADICWTTASAETLEQHDLDSKLDHSAYAHSETAGQSSSTAAHYALISTKHASLPNPTAPSDTLIWAHKAILYARA
TNTFQTRFLNLRAPAAQLRHIGSTASFISLPLHQSESQLSLASNFSGSTQTGTVPTPTTNSAVTRPGVPAKPRSSVRGAA
PPSSFSMRKPSLRKPVSRSSISRPLRKGSIDEANSIALGFATSDSEGEGAPTTSLRASRLINTGSTAAASHTTSAISSAE
RSEPGTGGPERKSSFASVTSTADSRLMDTRSVVSDGSTLRAPISLGGVSPAFFEATLQYLYTGEEDMVNTFDFLFEDRLT
LDLHASPEEKLDKLRSDLTFMWRSKLFSDVKLVLGDDGGEDKSDMEGVASNNNGRHRRRAPTNLMDIPDANMSVLSLAQT
IDTDRIEDSDTEDDELTSFSTHRMILASRSQYFASLLLSPYADARVPVLHLPSPPFTSASLHFTLGFLYTGTLFFSNRTF
DLSTAFSLWRAGAYLQIETLQALVTALIDREFCHGFACSPPCRRCAKRVPRTLAFATSPDVCEQALQEKAIAAVSGAHFG
IYWAKDVGNLDPMLQDRIVDSISDRLTQDPTLVVSVLRQLSIVGQRIDTERSNRWVEALRSMAETVENRVMPVLHANLDK
IVQSAAWSDLLDGVGFLGDVLEKSLVMLIDGLTEARAAQVYQTLVGQVLLREQGFEVIASREAVETARASILRYLKKRWI
NVRALSGFNKLEKWCLKEIADELEITTTDLYLAEVPPSVKSTALPGRLMAARRNSSLGGGAVAASAVASGTATTRGSARQ
SSSLGGAVDSSCSSIISSAPAASPTGTTASPARKLIRDDDERGDLSIHMRAAVLNRNAARTSVANGHRSTSCASASSTSF
SAARSSPTPNGAMSTIARAKAAESPPSNIAKPTMTALSTAKTSGVSASVARSVTPSAAASSKQQRLRTTSGISVASVAST
RSTNSKAAITPTASPKRIPDTVAAARSSPTTINQEAIPPRHPTKLPASDRDKTPTKAIRPVRSATALPHSRPSTGTSDPK
PCANSIDSATPTKSAKPPVLAHKSSFLRATPGPGGTFSLSSTSSGADLRARTISNASNRKEDAISGEEAPRARSTTPTRS
MVTRTSACAQSSIGSSGRASADRATAEAVGEAMPADAVQNVQRARQASSTALPRRDSTKTIVYCPDPPSEHERAHESQVE
QNEVKGVVTSVEGGVSLHVAIACIVALPLGGSASDAGAAGASRPSAIETRTKSFATRPSASLAAGGGKRSTIATSSSASH
PKPLPSVTSRASPTATPAGAGAGAAQGVGRKLRAEIRYLGPVLGTRGVWVGITLRVACSELAALGAKSILRLERGVYKGV
RYFHPDVNDQDVTQHMSGVGAKEAPRSMERRKQLCTVVSPGRPFAAALAVGQRQIESADWETTGKQKAELVELFVRPHAV
VWVVQ
>Umay_XP_758781
MLAPTLLDCWHVGDLQRFRSLLKGSPPDKSASTQPSAIPSYGGHSAAFSKSPHSYKDPSTPCPPAEVNRRDHLGRTVLHH
IASSSDPSCLSYLNAILAHPSVNVNLQDAESGYTPLHRALYLANLHTAILLLRRSDLDLRIKDFEGLTAFDLYNSTVAGT
NPPPDALSFEPNRHHHSRTLTQLFTWGSNRNYNLGLGDGDDRQLPDKISLRAPSTSNINTSHSIHHSFHTSLPAGKKFDR
IGIKDVAMGRLHTVILTDEKVANVWLCGLGSNGRLGRSPQTQTSFEPLKDFTDTARSLAVAQDHTLIITASGAVYSFGLN
RFAQLGYTIEHGLGTVASTTPKFGAGAGVTFGQTSASSAQNLELDIQISPRRIAGPLKKEVVLGAACSKLHSVVFTADAL
YTWGTNTGQLGYDKSATAVQVQPRKVTALAAGAGIQQVAASDFGTACLMHGGEVFVWHNDTHFRINFPLARFSSSISVFR
PRQVQPKTLISKLSSSGNTFAALSDMGDLFTFNLEHPSEYGNTATPPRAAPSTSSTPKSSAVQLAPKPQLLWSVRRKFTA
VRDVAIGADGSVILCTESGHCFHRPRRSDAAGAKPGGGKNFKFHQIPYLQRVVKVATSESGALAAIKADPSLPDLRVKGR
SVEEALCDLLPHLKVQTLSQAIDQEKGITSKVVEISESPSRLDSDTDSEDERPDPALSTDTAKAAKRWVAGRMVSGPSSA
QTLHDLTQTQTAPTTTRRQAFYGPHNLTPPFGCDAFLIADGHYLPVHKVILGARIPSLRTVLQDSSAKDSLPPGIKLSAV
PAAAGTNKSVSILTLSNCSLISVLFLLHYIYSDDLPPIWVPSVGLVIDKELKAAKVDRIRIHDELQLLSQRLGLSALTTS
VQASMPRSPEPTLRANMSTLFESFVSMHVDKTQETKTFNDVLLRLEDRTVPAHSVILRRSPFFSALFQPHWTAARWDSSN
IITLDMSHLRWEVARIVLMHLYTDNGETLFTGCDADRSLEQYMDFLVEVMAAANELMLDKLKLIVSSLLRKRILAHTVGA
VMTEADFYNADQLKDITMDYCTKSMEALLEGGRLNELDHGMIQALTVYVRHKQDEKLHRSLAQDRLAALVEKHRDFYDNL
DIPPPSLHLIVNKVRPKKGTGLAAAKSPPLLAQDAGRRHSSARFGVASATSPGALRPVTAGIMQRSMADDTSEVFSMDDD
DFTTPRKYPSARPHGSPWAVLPQVAHLSLDDSMDKSAPSRPWKSRTVEAERKQAGVNPSMSPALRPADAPTDLRSIIARE
QKRQQQQSVVPHISNATTPSGSGTCTPVHKSATPGFELSPISIANARLSQKERKRQQQEAGLAAAAAAQAQPIVARRGSV
SSPWKLAAAGSSPPDKTTASSPWSRKAAATTAMALESSHEGTPPLTTTFSSRRPAALPLQSGSGTATHATFSETGASPAN
GLGPTYTPMRMGSKCTPKRMGSNEPAWSTPAVAPPLGMYGTSPSPAPPLGASRAARPEAQRGASSNLSSSLRNADAQSIA
RSSSEVAHIDQAGSLACTAQLTFAQIQEQQRMAVEAEQETIRLAAAARKSFIDIQEEERQAEERRKEDQKQAAEFERWFA
QESKRMQKEKKANNAGAKGKAHKKQVKQAEQSRSQSQSQSGSKASQDTGLASGANGNSSKSKKSQQAGKKSGNEGRQLNG
ATSSKGAQSKGGAKQTSGAANHGENDKLSHRSNTKSHMGSADATTSNAPPHASSVQLPSKHPPPARSALLSAAAPTFMPR
QE
>Mver_MVEG_00006
MTAQNTFTTTRRFKYAFLVEEHLKNNLWTAYQSNHGTFRIAALKVNNYYIFFAAPNPSYAQSYWVKSLDGARLSSGKLDP
GRADTECVEIARIHVEALPVKNGSCEVEICLSGDDPLERSPSRSHPEVMMTKLYYDTKYQDFSFAFSEESTDDEGEPSQV
IELGAHKHVLAQWPYFRAMFEGGFSESDDDAKQVKIKDIKPTTFKRLLRHMYLGDLEKEDQPTTAFMDELNDEEAVSWEE
IFIAAHRYDLEDLCQLAKTKTVIGLTPMNAVSFLFRSAYLYDELRVPVVKYAATSCASTIASKDFRLKYLDHPEFGALLH
ELFIEK*
>Mver_MVEG_00012
MSLILGFPSNVSRKTCIKSLEFSFDWSVTESTPPPQNYDGGDGFFTLSIVLAGPFNNSGITFRLSPRTTFAQSFTIKTPS
GSCLASGPLVPGVRTPGFGMTFETQTLAERIPRSRLEILNGRYQVDVCLSSRQYSAPAPKPFVVAPKPVASDPATSLLNR
LYRDVDNRDVSFILSPADLAASNETLVRKAHKVVLNQWPYFKRMFNSDFMEGGKGEKEIKVTNVKPKVFQLLLRFMYTGI
IPQEERPFVAFTDAIANPQEASWEDVFLAAHRYELEELCDLAHKNILDKLTPQTAIPFLFRTGYLFETLRAPVIKYTAKT
SASQAASKSFRDTYQDHPEFGALVFEIFEAYHGSK*
>Mver_MVEG_00114
MTSSIPEFILRVQYPILSENPPGDSTDISLLEPCNKNRWQVTLTRVYDLLKIDVRGLELNGMNIDSEYMHIIPMNGSKPI
IAKVCPQTLRLYNTVSVLVRTEIVQKGDRYEFYIVQAKEKEIPWFFHASDGIKSPPLPAYEEERPSAITNDTASALLCDI
YSVDTQIVFGLSRTTDETCIWAHRTILSKYHTFDILLKQLSFDNRSALTGPIKLVVTKVSFATFATLLRFIYTGEIHRTS
HPEYFAISKFLKGNNYFVAGGIKDLHQWHFMDLHRPLSDEPVTWQELLDAANIYKMDALRAQCEAALKSIAKVSMARSTD
AT*
>Mver_MVEG_00137

MFASYEKNMDTEREQLTFTCRWPKDSAKEDVVHFNFNSTLGSFRVCISEEASDPELSITVEKTDNDRSTLQRFTKCSISC
SQGSRTILSPFVYQFFGDYSLEENIPLSLLEPQDDFFELDFFLTTDASPRLVREPIYEHALLAKAFSDPSTYDIYFMPCE
NNFNVDSNGSEAYIDPAQPLETEKPGSEKPQACKERAQPTKPEKSKWTFFFMQAASSTTTTDSLKSSTKQTDESKDMASK
EQMNNEETDNSRDQEAVEDAPKKIGAHKMILWQWPYFRTLLESAPAHKPFGPVTIRLRSTDADTLKIMIRFMYIQMLPTR
LSTFQDEEDDDTTWTSTSNWEKVFIAAERFNVDELQRIACSKVLDGLSEATAIPFLFRTAYLYSDFRGTVVEYVAQNLHH
VVTKRPFRDAYGDHSDFRELHGEIFQAFAENSEGRRRRIV*
>Mver_MVEG_00138
MKNRLFVSADTGARTLGFFCAWRDSTSLGCFTHTFLPGWGSFRIHVHRNEDNYVCSIRIDSSDDTKKYTEFKSCSLRVHE
GNWLVSNTIQLQPIHTIDQHLTAVQKSLLVPANGKFIIEFRLSTALVSAPPLKSVPPPRTIAETILDKIYRESAHGDVVF
IFDQPATSLSATQEPDITQDSVADTDMDFGTSLDYQGDQDEQSQWTQTQEKQNKIHQSQDCNKSTPAVARTQASAVSLRA
HKLVLCQWPYFKAMFEGGFAESGPGEKRIPIKDVKQSAFQLMLKFLYVGKLPRDINSMVVCADELVDEKKSSLEDLFLVG
HRYDVQELCSQVAEKILSRLDASNCIPFLYRSAYMFHDLRGPVIKIVAEKCGDQIAKRSIRDSYKDHPDFVDILGELFEQ
HHALHDK*
>Mver_MVEG_00139
MPEAYTFNKKTGVHRLQFGLNWPDNHDWATGYYSFSDGWGKFAINGSNNGCTIGYSVKLDEVNEAKYGVFKSCSVSVKGG
KWLAYNAIEILPNVVDNQFLATILKSQLPGKNTKFILEFILTSNTVTVPPSEPVAPAPTLGDVILEKLYNDDVHEDVFFT
FDPPSDVITTAEETDVASDKVSGSNMDARESTKHTGEVPSESGVDLSKSDNVSNSPNPYVACARRSTASTIGAHKLVLCQ
WPYFKAMLDGGFAEGGTGNKTIRIKDASPKAFHMLLRFMYTGKLPDDAQPTSVYIDPLNKYTDASWESLYLLAHRYDIQE
LVDITRENILSNLDPKESVPFLFRTAYLFADLREPVIKYAAESCRSTFASKSARAKYLDHPEIVELFGELLEQMCAIEE*
>Mver_MVEG_00140
MFDSGFAEGGTGNKTIRIKDASPKAFHMLLRFMCTGKLPDNAKPKAVFMDPLNKHTDARWESLYLLAHRYDIQELVDSTR
ENILSKLDPEGTVPFLFRTAYIFADLREPVVKYAAKSCGSTLASKSTRAKYLDHPKSAELFGDMLGQICTINK*
>Mver_MVEG_00144
MFEGGFTESSPGEKRIVINDVQADAFKLLLRFMYTGKLPLDAQPKNVYASFMTKQHLSWEQIFAAAHRYNILELCQLAQE
KILAGLSRSMSVDFLFRTGYLYDELRGPVIKYIAKNCGSQVATKMSRDKYKNHPDIIDILEMGSISMTLKRNCGRADFLI
KADGSNGSYLTTFKSCTARMPDKNYLYFGAVNLQNSSKLQFVMTIPLSYLNHYQDGNYYLDINISSQEVSVPTPPPLVSP
PAPLPSPKDVYSEKLYTDSTYHDVFFVFDGSSTSSDTQQGVTTTSTVGAHKLILAQWPYFSMMFESGFSEGGPGEKRIRV
KDTKAPTFKLLLRYMYTEKLSEFSQPTVAYRDAITNMEEVSWEDLYLAADRYEVDALRQSALGHLLPGSNSEGVIPFLFR
TAYLFEELRAPVIKHIAMICSSEIADKTTQQIYADHPQCRELFGEIIAQLNARALNIGK*
>Mver_MVEG_00145
MPWTQWTEESTGIEKASFGINWKEKTSLTYYWNFKTFNFTATRAEDKYTFSINSSDKRYSVLSRYKRCQIRTKDHKDLGT
FPFIYDFFGKFRIDAEIPTSEFMRVDHNHSVWFYFSSEAPVAAPGKSLAAVPTMADFVMERLHADEASQDILFVIGEEPI
DADSGSEVSESEDTLQQAPIKVADENLQRASRKKTTTRIRTTKWTLRTRKIRTIKKRTKNKEDSDDKETSSAVSNGDAKT
AEEDAREKSKDAEGKGSVDDEDKKGDEEESKEEEEKEDPVPERIVGAHKMVLSHWPFFKKMIDAKGGGPDKTRIYVRDVD
WIAFRVLIRFLYTGRLPPSLEPTTIFADSDTKDGEISWEDLFLVADHCEVDELRQVALTTILSKLTLEGAIPFLFRVAHL
HEDLRTPVVKYVATTCGAEISKKSVQQEYADHDECVAIFGEIITELHAHIESLE*
>Mver_MVEG_00157
MTDGPTLTSSSVVQNGLDKMQDARTMDIAFVFTHSPGVRVALWAHQLMLAMSSNRFRNLYAVDRFNNLHRIATNHNESLS
SARVYEICTYSLKELSIEAYCALVRYLYTSVLEFKVDMREFVLTHYPVDSPDQYLKRPECSREMNKLMKELLDQPVRQAG
WACLFTLADRVTSELNMSSPVQYTLTDMKDMATLDFAFVFKQPTIHSQVAIWTHQDVLASSIQLANYFMTPNKVYGRSGP
RRYSISFVRVSYCLLESYCAMIRFLYSRQLALEIDLREFVLTSTAILSPDKQRLVRSAVVEGILVKPLRETTWDDVYKLA
MGFEMKSLKARCLEKV*
>Mver_MVEG_00186
MEIPRANLKATNQKYELEFRLISAKNSTTAPVSSEPMYTSVLMDRYYRNKESHDVFFSCEVGSFVNSDNSQNNKGQNDND
QNHIAKSNIDLENEAVKPVEASTLGAHRFVLSQWPYFKAMFESEFEEGDSGAKTIRVKGVRMRTFQIMIQFMYMGTLKSD
IAALYEDNATDQASWEGLYIAADRYRIDDMRKLALATIERKLDSIAAIDFLFRSAYLYSELRDLVIKYIAKEHHAEISKR
EVRQAHKDHPEFSELLGELYEAFHELMTYNGLLNSRSNLKYRP*
>Mver_MVEG_00187
MTAKGTLILHRSRAPGKASESSDPGQLNLKENEGCPTSDIGNSEHEKTCTGQDDTTVSRTLEISARNIVLSRWPYFKMMF
SSEFVEGSSEKIKICIRDVNFTTFRVMVFFMYTGRVEIDQPVIYQSQDGPKAKSTSYEQLYLAARRYRIDDLRKLVLGWI
LKCLGKADAVDFLFRTGYLFKELRGPVIRHIAKECHAEVAKKEILKEHKDHPEFCELLSELLDAYHELRK*
>Mver_MVEG_00189
MDNGKQEAIYCRKTSTDPESLSFIVHLATTPGNQEFEFTSQLCSFVVRAKETSDNKYKFIIHTPESNSLTLTRFRKYSVT
NPKNTINGNFIYSYFGEYKLSELIERDKLIPNAGCFELRFCFTEGSGSIENLPNHSTLQPSFPRIIVERLFYEPIGVDVT
FVCDDVLKEDCPSKADLAIIMEQASILSEVETVAVVETSNQSQAIIEKRGSAGSSSTNKVDPRGSITSTTSTFGAHKVVL
AQWPYFKSMFSSEFIEGGSGMAPIRIKDVNAKTFREMIYFMYVGEVESDSVELLKSHDPIQAAKVSWEALYLAADRYQID
DLRKLALNKITGNLKKMDTIEFLFRSAYLFEELRTPVIAYIAKVHHSEVAKKKTREKYMDHPEFCELLGELYDAYHNLAN
K*
>Mver_MVEG_00190
MEDDQTQAVYIADGPDIDESLFFTLRFSTTATSDQRFKFKSEIGEFAVIVCVHDDKYSFMLDATESDYLILNQFKQCSIS
PLLGVESFVSPFLYRFIGEYEIVEHIAKTRIDAEEDYAELQFSFSVEQLPNHTSYERPFTFVMMHRLFHDPSGEDVTFVC
DEVVTDKEQIRWRASPTTQQPSPLDESANVGDSSYDALRRLSLDQSNYAMVPVIRRFGARKIILSQWPYFKAMFSSEFAE

GGSGRIEIRVKDINAKTFLETLRYLYEGQLHNPPNAENIFEGDDPAKATWEALYLAADRYRIDDLRKDALDKILGGLSQI
SAVAFLFRMAFLFEELREPTVKFVARVCSTEMADMKEIYNKYKDHPQIAELVLEVFQAYQGAREV*
>Mver_MVEG_00191
MSGLENFPDTSGASRMKTLKDEITVAKIKEDESKAIMAALLRNVYSVQTQFVFTQDKPCSDFGLWAHRSILSKYPVFEQH
FKQSEEDQSNSHDDGVVIRPLTLTVDKVSFATFCALLMYIYTGEIKTIIDPSQFAIGKVSIVLPSPLSDSPGTSDRQQGT
PCWHPLVPNSPWDLNPVQWSDLLFAADLYKIDDLRSQCQKHLIDTLDDSKVVDTLIHVGPRFPEVKEAAVNYIAKNMKVM
YAGGNDPFAPFVQHPSVMTQDCQKQKK*
>Mver_MVEG_00640
MPIYSNPDILGADFLRLVNSPVGSDVRLIVGEEKVVMHAHSLILQARSTYFTRALSSDWKEASQGVIHKPNIRPAVFERV
LQYIYGGRLMLDDAIVMDLIKAADELCMEDLLHGCERHALTTICRDNVLDLVHLASQHNLSQLKAECLDFIASNIDHLKK
GKPILTLDADILKDILSMDQLDLEEIEIWKIAVRWAYYQQGLDWAQCPVLDFPRGSGCVLIQTFTDEEPSGYHIGDDGDE
DDYDELDDARVPLGGTNIHISGLGDDEDGFRPPAQSVFQRATSHNIVKMSATVHEDLRRQLAPVLSAIRFMRIPSLEFLR
LIEGTGLLPVHLCAKVYRYHSVPSMADPYQTSLRRRMAFSTVLNKDQKEVVLEWLQMSIVDSQTNATSSSSTTSSWHRRS
YVYSPPIGNASPASNGSYYFPATTAIPSVGSSSMSPPGFSSRPNAPSPSPSLSRLAPPVVTTPNGQRPSRSSSSLSAAQP
LLTLSQRDSSLPLRSLSLSHQQHQQEQTPTHAVQSQLPLPKLTLQYRATRDGFDAGNFHMACDQRGPTLTVVRADNGAVF
GGYNSSSWSSHPSGVYSTSKTNFLFTLKSREHPTRENMRFDVKGDGNAAAYNKADFGPTFGVGHDLYLASNCNLTCQSSS
TMPSSYNGPGASTAALAGSYYFRVSEVEVFLVQSASAASP*
>Mver_MVEG_02408
MDNFDSFEVVEPLTRRVAGILQDYPDGTQIARELLQNSEDACSTVQWYLLDHCHHQTDPERLFDPGMQAYQGPALLAGND
SLFEQRDFQSLKNLAESGKKDDERKIGQMGIGFNSVYHMTDTPSFISGDKMVILEPHMRIFNGIPYRGGVVGSFTNQGQE
MRDFSGQLELFAAKEKIDFSRPYNGTIFRFPLRTQAQARVSELSKSAYPAEKVRDMLMNLKNEAIKCLLFLKYIERMVIY
ERRESDTTVHKLFEIEIVNNTEVRASRQAFMAQLKSHVYASDNDDPSKTLQYAIRPKFRLTQEDGTVTEEEWAVVSYIGN
VPEARRIMKEEYQADISNQRMIPWVGLAAPVDPTVSVGTSNLFCFLPLGIHTPHPVHINGHFAVKQSRREIWTNYTDDIS
SQSAAGIKSNWNEFLFKRILPVAYARFLDSIGTSHGTNYDLWPRFSESGLGMESLWKNLLYQCIEEVTTNNGIKVFFVNS
ADGETSAVSYQVATIADPAMDEYPLLLDLLKGMVNLVCGVPEIISDLLRQVVDATGVQDNILTPQKVRDLLRKHKHSWQH
NTSDDARIQILNYCISDDQIMDLEGLPLLPMADGSWVEFKRDLANSRYVVDSLAYRVLTYANSDIVETQHNGLPMTEHNV
HDFYQFMSALRNEDLVSKIRNSFDSELYDGEDPPQGAIAQPPTGLFPSLDWIVDVWVLLRESRLTEASLNTLLGCHLVPI
EMNQLAPLDRNRQVIHRSGILNYTLDSDQVLQLLGTHLGCYVVRRDLQDCFSWLSQCFVSITDVEAILTLLADTSDTTLT
DLTSPQRQLLCDFVSATLSTHRALRGSQRLSLERLPIYQGYVSSELLSLQDLGGVMTSLVCQGFSSEDRPWELTSITLLK
EDQPMSAHLLHVLKMRTIQESEYWYRLISEMAESNSDNRSQWNPIMAAFLPSFYRHSRDRNFKALIADIPFVDVKSNSLM
DEDDLSMKLSPKMVVSHDLAQFFFQDETVFPAGVYCQSFNSSVLAELGMTTRFDTEFATSRLRKISFISADYLNNNTDSL
RVITDFFARLNVEVDPSYLDDSAFVKCVRNLSWVPARAHDDDPLCLYRPGQCRPQSESLVIGSQRPISSLECTSKHLSSL
MGWSNPPPLEAVLANLLDLSEQSSAGHHPENFEESIQAIYRVLNGRIRDPQAVSIMRNSLGHHRCILINRSLHPIDRVAI
KIQSECKLDPHFIQVPDSDFVELFRAFGVREEVTPKDMEGILITINSGYNHGDRLSDDDSGLVVRILGTIASVDHSSTYS
PDMLILTEDSQLRKLSDVVYNDMADSSRLQEYSADHVFSSSRISKKVASKLNIQMLSERIWSGCNDDFFQNWEQEISVVD
SISKILNDYGPENIFTEYLQNAADAGATQFSIMFDNQSHSCEGILNKDMSVGQGPALLIWNNAEFTQQDFEGLRKMAMGS
KRHDPEKIGRHGLGFNTAYHLTDLPSVVSGRHIVIFDPRHAYLPKRQTMSGMAAEGAVRIDFIASQLAQKFPGQVAPYQG
RFGCDMTTQFRGTLFRLPLRQAQFSQSDNTSAFGGIWDMDNTKALIERWAEDAKVSMLFLNRINTINIINENDKWAIVKE
SWPVRGLFEDSDTNSSFSKVRVRTYGLGDQPSADSTWLVGLDDAYPTAISTIREVAQNNRWIPHRGIALPIEQPKGESSP
AFKGKVFSYLPTPISTDLPFHVHGVFALLSNRKGLMTSPAHNSHAASWNEYVLSTLLPPLIVQTMSRLLRLRFDCLDKRQ
SKTGIDSAFDAILVTYFKLWPLKSTGDMTSMVQRFWELAYISPIFPIRPLQKEADTSMVQGSTGKDVCFPMRKIPQDVLP
KLKQLLRENNVPLCECRPDVSASAIYHWGKASLALTQTDATLLRKILRGNKDFMLKIRSQEGREWLLELLLGVLLDKEQD
GIDHLNGLALLPLEDDSWVSLSPNPAYYTANSTARSLIKNKTLVKESAFTTPKLGKILRALEKDPQYGVMPLPPAMFTEY
MCLENSTGIPPSNLPRIWEYLRGYNDLSGFGDLPILTTIWGSMVPLRGCHGALRVTESDRLDSGSMLQKLGLVLKRASVV
VFRMEDNDKNKYLEAKSGPCSNASVISAFVKSGGSLSSMTFNDIEASTLRAVITGSSNEFTHAVMSDLGLLRIWPSFGVA
HGESGLICAHGNQMIEGGCDIRNLGDSHDVIQGAFSKTFQRMGARSAHLTDFTRERIIPRLQSQVFTPLSNRNQMAAYIN
LLKSLMRIATTKSGQKIDAQRLLQSGSLILARDGSFHSSSVLFDPDDELIKTIYGRGSPARFPDNETWSALNSEKPTVTK
FRQSNQDDVLVECANTVLEEIEARPHAPATKAKAKALVTKIYSKPSSANWMQSRWKIVPVSTSLTEPYASSAPYLPAYMS
FSDLIYEEHRDIVWTHCGFFPSDLEPPPTCRKVWTDVGQPGIHTIVDHLHTLAKDLAPKWTSDSDQLKLQLALINTYTAL
NKYANKSMTNSKTLSTLLDNLTVPYLYNSFEKEMSDPEAWLRPNQLILDVENPTVKERLVHSKLQQFRAFLAAAGVGEMR
SVVGKVIVAKEPNKSEFVNMLCTLFETQDRSNGFMDVCFRFQPDGQDIYANKIVLAHWSVFFKTRFLGAWAEHVTRDPAE
PMVDVIDMSALANVDEDFYAAFWGVLYYLYSHKLTPWNGPPTLVQQPGTEQARKVDDIGERVHYLLSLLARADEYEIMRL
KDLIAEKLVGSSQMIVQGNVFEVREHAKLARCTSIVDHCENYVKENAGTLKKFVAGEIAVLEAELQQSGHARGGRELAEI
KEELAERKKHREVIDKIAPK*
>Mver_MVEG_02410
MTVDIKRNFVSFRQREPLTRRLAGILKEYPDGTQIARELLQNSEDACSTVQWYLLDHRHHHADPERLFDPSMQAYQGPAL
LAGNDSVFKEGDFESLENLAASGKKDDESKIGQMGIGFNSVYHMTDIPSFISGDQMTILEPHERVLGGGVRGSFINKDRD
CSGQLEPFAAKEKIDFSKPYDGTIFRFPLRTQAQAEVSELSNSAYPAEKVRDMLMNLKNEAIKCLLFLKYIEKMVIYERR
ESDPTVHKLFEIEIVNNTEVRASRQAFMAQLKSHVYASDNDDPSKTLQYAIRPKFRLTDEDGTVTEEEWAVVSYIGNVPE
ARRIMKEEYQADISNQRMIPWVGLAAPVDPTVSVEMSNLFCFLPLGIHTPHPVHINGHFAVKVSRREIWTNFTGDISKDD
FQSNWNEFLFMCILPVAYARFLDSIGTSHGTNYDLWPRFSESGLGMESLWKNLLYQCIEEVTTNNGLKVFFANSADGETS
AVSYQVATIADPAMDEYPLLLDLLKGMVNLVCGVPEIISDLLRQVVDATGVQDNILTPQKVRDLLRKHKHSWQHNTSDDA

RIQILNYCISDDQIMDLEGLPLLPMADGSWVEFKRDLANSRYVVDSLAYRVLAYANSDIVETQHNGLPMTEHNVHDFYQF
MSALGNEDLVSKIRNSFDSELYDGEDPPQGAIAQPPTGIFPSLNWIVDVWALLRKSRITEAILDPLLGYHLVPIETNQLA
PLDRNRQVIHRSGILSYTSDADQVLQLLRTHLGCYVVQRDLQDCFSWLSQYFVSITDVGAILTLLADTPDTTLTDLTSPQ
RQLLCDFVSATLSTRRVLGEPQRLSLERLPIYQGYVSSELLSLQALRGVKTSLVCQGFSSKDRPWKLSSVTLLKEDQAMS
AHLLHVLKMGTIQESEYWHKLISEMAESNSDNRSQWNRIMAVFLPSFYRHSRDHNFKALLADIPFVDVESNSPMDEDDLS
MKLSPKIVVSHDLAQFFFQDETVFPAGVYCQSFNSSVLAELGMTTRFDTEFATSRLRKISSISADYLNNNTDSLRVITDF
FARLNVEVDPSYLDDSAFVKCVGDLAWVPARAHDDDPLCLYRPGQCRPQSESLVIGSQRPISSLECTSKHLSSLMGWSNP
PPLEAVLANLLDLSEQSSAGHHPENFEESIQAIYRVLNGRIRDPQAVSIMRNSLGHHRCILINRSLHPIDRVAIKIQSEC
KLDPHFIQVPDSDFVGLFRAFGVREEVTPKDMEGILITINSGYNHGDRLSDDDSGLVVRILGTIASVNHSSTYSPDMLIL
TEDSQLRKLSDVVYNDMADSSRLQEYSADHVFSSSRISKKVASKLNIQMLSERIWSGCNDDFFQNWEQEISVVDSISKIL
NDYGPENIFTEYLQNAADAGATQFSIMFDNQSHSCEGILNKDMSVGQGPALLIWNNAEFTQQDFEGLRKMAMGSKRHDPE
KIGRHGLGFNTAYHLTDLPSVVSGRHIVIFDPRHAYLPKRQTMSGIAAEGAVRIDFIASQLAQKFPGQVAPYQGRFGCDM
TTHFHGTLFRLPLRRFSTIQNRGWCTFGKNWDYAKVTELIERWTEDAKVSLLFLEQVKSLSLINQTSKWTILKESESLPG
TYDATGSSSVCKVHIQASGPRDQPSVTSTWLVATDKAYIEVTSDIREVARNNRWIPHRGIALPIGQPKGESSSAFEGKIF
SYLPTPISTDLPFHVHGVFALLSNRQGFVTSPAHSSWNEYVLSTLLPPLIIQTMTCVLQVKFHMNALQSKLGIDTAFLAA
LRAYFELWPLKYTGDMTSMVQRFWELAYTSPIFPVRPLRQDVDAPVVQGFAGKDVCFPLSEYPEKAQTIVPKLMQLLREN
DIPLCECRPDISDSAINHWSKTSLTMILTDATLLREVLRGNKDFMLKKISSQDEREWLLELLLGVLLDKEQDGIDHLNGL
ALLPLENDSWVSLCPNLAYYTANPMARSLIKNKTLVKESAFTSPKLRRILQALEKDPRYGVMPLPPAMFTEYMCLENSTG
IPPSKLPRVWEYLRGYKDLSGFGDLPILTTIWKSVVPLKRCHGALRVTESDRLDSGSMLQKLGAVLQRAGIVVFRMEDNY
KNEYLEAKSGPCSNASVISAFVKSGGSLKSMTFDDNEASTLRDVILGDANDFNPAVMSDLGFLRIWLSHGVAHGESGLIC
AHGNQVIEGGYDIHNLGNSHDVIQGTFSEAFQRMGARSAHLTDFTRERIIPRLQSQVITPLSNRNQMAAYINLLRNLVLI
ATTQSVQMSEAQRLLQSGNLILSRDGSVHRSCEMFDPRDDLLTTIYEQSSPARFPDNETWLALNSGRPTVTKFRQSNQDD
VLVECANTVLEEIEARPHAPATKAKAKALVTKIYSKPSSANWMQSRWKIVPVSTSLTEPYASSAPCLPAYMSFSELIYEE
HRDIVWTQCRFFPSDLEPPPTCREAWPEVGQPRIHKIVDHLHTLAKDLKFRWITDSEPLQLQLALMNTYTALNKYAAVSD
AHVTILSNLLNNLTVPYLYNSFEKEMSDPEAWFRPNRLILDVENPTATQQLVHSKLQQFRAFLTAADVCEMLSVAGNVNV
APAPNMSEFMTMLCTLFETQDRSNGFMDVCFRFQPGVQDIYAHKLVLVHWSDYFKTRFLGSWAGNVTRDPTEPMVDVIDM
SKVDKGDPSNVDKGDENSYDTFWGILYYLYSHNLTPWNGPPRFSQHSDAASAIKEEEDDPSERVKYLLSLLARADEYQIT
RLKDLIASELVVGKSKILHGNVFDVCKHAELAQCTPVVEHCEKYIETNAGSLKSFVAGEIAVLEAELQQGGHFRGGRELA
EMKKELAGHKEHLEVIDKIVHK*
>Mver_MVEG_03286
MEFSSFLPSFPPSRSNSRRVSALTISSPTSHSPVLTPEPTPVPMPHLSASRVLFEEGLYSDITVILQVPGSTKKILRLHS
RILAKESEFFATLLAGKKHGDKAIVLLDEKDSHGSRVIEIQCVDTPELYAFESSLRLLYTGSWCSDCDFQTSSLDARLRR
GLDVMSQAARLSLKGSTTALLQEEITTMCQNQPELLEKVSRTLRPEDREDDQAILKFDRDALQVMCRVAVTKVEIRDHCE
LAIARNLYGPKKFRDLRMSEVQLAELRPGYIFPPGGQESIYLLKGAPSLPLHPCRASNVEALRHVVDYYTAYNSQQPLRT
REFYAVPDEEAQKGTDLQKATYYRISFTSGFQWLLDQVIKVRAPFELIILLLRDAVDENADRRNDVYFGEFASTTDGDVY
LQGVNYIADGLLHAFQDPTLESFRNPGTSGSLLNMISYGINSLTDLPPLRARPETKTGDVHQDYPGLSTLVMEFFWQVLE
LALKRPHHLEFVQLLLDSITRIAPLSPFDVVDRIEERLAELLEETRKRIMEQRGDAGEAGDDAEEGNEDEDEDEEEAKDK
LEEEIRIGRERTYSNAVHSDVSNTVYEDFDDRTSMVSSVLLASEPIASKAAVINTRAKDEETASLLQEEPEPEQEPEVET
PWMRPEYLQPLCLQLLTKFPMNIQSSLLWAIHDLQLL*
>Mver_MVEG_03799
MGFMEETLTDADSGPIPHRKIGSQVACFEVEVVLSNENYFAEHPHAFVTGSLELAHRMSQMDTGTSESINLQETFSVNNA
TLGAHKHVLAQWLYIRTMIEGGFLKSGPSEKRVIIRDTKPTTFRLLLRFIYLVYVTHHQDRHRTTVFADTFEDKNQSSWE
ELSVSADRYDLQNLRKEAMHMTVNSLNTSQADHEPLGLLP*
>Mver_MVEG_05064
MENTYSINPVLEAISSSTTLSGDFPPVTYLANKIASQLLQSGEGAQMFDRRCDADCVLNVGQRRYYVHVQMLASRSPTFR
RIFDDMIENKAWGDLTDRSTSSQGQSQVGDDMDYSFEGSIEDEDQGSEMDRDLQQRSSPVSPLERSIQLCSIDQLSQSIQ
HSLHLSSNPIPMSAEDDEEEIDPDIYDDDDDENSDPDSEEDKYLPELTINLADPEASHFDELLYYLYTGDGPRWQVFFRP
DNYIYILQNIAHLNMLTRHVLNICLAFEQTTPPEMHLHGLALQLLHKSTLLTVPMPGGPGPGLGSGILCQDVDPEQ*
>Mver_MVEG_05498
MPAPAPGLQSISDLTTTSRITTGDIPPPLVGATMTIVDDKLYLFGGRLIQTRQMTNSLYILHLSEFRWENVTEVVNVVPT
KPSIGDPGAEDEMEVSAPPAERYFHSANAYKNSIVYFGGMGPSATSSSASDAQDLIVFDDIKVLDLELLQWRQVRIPTSA
HSPKPRYAHLSSISGSKLVVVGGQDIANQYIEEINVLDLDSWQWVASMPFEKHCGSYRSIAVSGSGQGSTIPQIGSEVDQ
ASSGQLSPTSSDGFPTSYLRKASSNSSLHYTPSLRKSSSNLALKKGGPMSRLSGSSSTFGSSPPSENNRSSIYRLSHLMS
PSPVQQQEHPIYLYSNFNFTDVKRELQVLNPPQFSTQDHSHSMSGSILPPGLRFPTGAIAGQHLLLSGTYLTNAQQTFSI
WSLNLSSLVWTRIDTGTVFNSGSWNRGLLMEDSNAYLVFGNKSRNLADDYQNRQTNFEHVSIVDLEAFGIYQQPPTTMTQ
TAQELGLGLLNDPAMADFEILTNDNQRIPVNSAILSARWPYFGALLREQEEQDANDAALGVTAEGSDYDPTLFSKRTLTL
PEPYTVVLAFLQYLYTDHLVTAQQHHSQVLSQLLLLADMYGLDRLRDLATHALHQMLNMSTAAMVYETAALSQQTGLQIR
SLKVMIAAKKMLQQQQLKLQQQQAQSQSVSANQAWRSTEPIYGSDPQAYASNPYLSQQQHINGLFQPSPPPPGSPNGSEY
RRMSITNNGSQPGLPHPMVKRGSLLGSMPPQHWNTEPLTPPPVSPPPPAVVPPQPTPAKTSFFSFPGSKSKAAAAAAAAS
SNASISSSSASIRSGSANIPAFAGMGVAPMPFGMQSDTSSIRSGMSGGSDEAKRAQDEKQRQDAMRRVMDGQFGGY*
>Mver_MVEG_05548
MTTISTAAGSLAQDMLLAFRDQHLSDITVVAFNQTFHLHRLILLRSGYFRSLILGQGEWIEKTRQAVNIQFGDPYVTLES

FQRIIHWLYGIDFHYSEGSAPINFRNLTKTAVTATGLGRGAALMRSASASQYPSSTNVESGRHGLGLQHAATSFDSHQAT
SNTHADKAPSNGRNDNIMRDPSDKVDELLISMYAAASYLNITALMDEICSDIAHRIREGRGLIHYMGYAWKLNNGKAWDI
ILAQCFYQFFWSTYRSSILQDMLVSPEMPLAGLVKCLVSESLWVPNEYERYQLVKTIMLRRLNLDTDKLMNWVYDYHDQD
WFMVLKDLDDNGTWSYELSTDDFLNNLQDWESTSTEQYEDAMDTDHDWASTTKELRRRSSVASATSFYGIDRNATPTIAS
SSSEPTLALAQGTTSSSSSSQGSLIQKPPQDLANLEDIEETQEPEEEKIDKIENPTPITTSTSAPEKMTTPQSPILDSPI
STPDMSVISISNETLPRHQVRDLLIMMYILHHSIHYTHMSFDHLHQILTDGLVLPARVKDAFWRQHLLRRIAVSTSSTLP
YLTAPSPLHGRHHGADTHRERRTPRAPMISRSKMASASSRPSPSTSLCQPCNSHGLLPIRFSTSLYISRRDLMTDGKHFT
SSTFYGGSWWSIQVGNNLNSKGIVGVFLSAAPVPRVVPNSRGTGAGGATLGGGSRSNLSFVGGGTSPGSILGGPPPRARG
SENAGGDCSSVYSRQRELTCSYRIYMTSNVVEPPEYFAATAASRLDEIRGSDYNYDHQNTSNEFPSISRTGLGASSSSVP
NSPEMFSGAFDSNTTGTRSQQHRIASEDEMICHDSFKLNEGWGYSKSNYLTRVARHIRDQPPRSNPSLYSHDPHGAPPPP
RAPPQQQQQQHAPGQQQQPGHPGQNMPAFPQGHAHPHPQGNVTQQNAINNNNNINNMTQPPTTSSSGVNSLHPSLNEELE
DDGLWIHFVVKVGWSDLKPEGETA*
>Mver_MVEG_06760
MRTAELINLLRNPERNAAAREHLSTLHHTVLVHLLGELANWLTVEIVKYQERQTPTFSSSTPTSPQPVSRSASRAKSGEG
DNSPRDSSGALETFQQYQIRKQIRLTLSAIQTVLGSVQVATPASSNSIDATDQTNRGKKAIALDDAKQEQILDDLLGPIM
TGCAPLGPRADSGEIQLMCAKILKFFTNKRAENGLFPTEDVIKRILALSDTASRSNGMHAQEPPKKKPRLAVDLAATLTA
LLSSPLVKLQDCGIFLLANHRSIRQQDAAWASISPLKSTLESLRSNLDDIGLGNLETDDDDAGRQTVDKELDATLSHQHR
ALTLLNLFIQDVVHKGSTDVGASHLRALKTAGEIGLFVDLWTGILRIIVLDQVKFTVRDQLVAMTTNVVYGTCYVFKDQA
IGHLMGEGSETLMAWYAYFIVPHDRSIEGGKSEEESYKSRAQMLQCCARLGEIMVTTERYQPVSMAENALVGRIFMRRSI
EFLETILEATLPITEMEPQPLDLDVTTTVPYSIKIIRQRPEVLEAMLGVLVGCLGYLKDGYMAVAKGNLVFVLVALLVDL
KSLFGQISISASSQQRIHSHVLSLLVRILSWEVDTGLQDIPLNLWTLGCVALVDLIMLPLERVKDATGQLEASSSSMDSQ
GVKAVHIFIQFWKQYPKGRSLLAELYGPRFYQPYMVRILLDDSKNDLQQSSRAAITSWGRDRVSNLLELMIYLGVESSVR
IHMRETWSTLIFMVALLSASLKRLQDHRFRLSDKLSHLVAWKCFQALACFWFDQTALVQLIHMELDSESFDLFGSLGKLM
LPDSITAGIPGGNPATLSIVPVLLGILEPPCRKWTVDTMLGTESLRDDKHRVMRRKMHHPLLERDDALVLEAAMILSKLT
QFQEGQLAVISKPGAVWTLSRILIERAIVQEKRRLVPPEQGDQEQGSLDKGSQSATSPLELALDGNQGSFNSVYRRPPII
TSMQRDPLNRALLEILTRTVSSTNLTKALVVNNTITELFSSILAIDHSLYFHRDVTVFDQGELGGAGNSETSTQDNSTLP
THDILPSACQHSVYCVFFAYFRQAMEPLFPQLQRLHGFAAGGASAIAGDAEEISDILFGLQEQSAVVFLYLSRPESFSIL
DSSNLLASESHIGDVFRMLTLEMDYTADSEVNEIDIDQVAVEGAHREAAALRKFMAGLAIQPWTWRYVDTWRERHEELMH
TYNDVLTTEREDHVEKLRRQGISTPLELAVPIRFLVQGQVISFADRNLLACASPHFSSLLTGEFLESHQDQVTLHDVDPV
DIQMLLECLQESLTMPSHYLLPEDLPFDMVLRLMACAERYSIQFIKRLAELWILTTLQKREQRWHDSAGRGQDDAEAKKR
DPESPLSGHENKKAKVEDGDLKIDQVYPDDEELSEAKDEEEEESIQECLLMVYEQCSDPRHGDIYTPQHPFYGLVWDALA
RLCLRLGSVAILPRFQRILDQGGEEKIQEFLKAVYELMVNQAP*
>Mver_MVEG_09048
MSSERQFMSATLQDHVRMGSVRSSRAELNDTANDGFLIPSAHIATSSLSNTNDGPKVRTLPSVPISSTENSCANSLSISL
PPSATPLSTTASSISSIGHSSDSNMSDACKGVASIASDPISAAPDTASPPHRSGQESHPPPAVNETRPTRPKSPLVPPPW
SKPLALISTPEIVPGMVNPATVLHRLFNNPLYSDLALTVNETTFHVHRGILAEQCSYFRRVFEGARSRDPTTEIKKIDCS
YTHVLYTHAPKAERDLIETHGEEVDENGIGQPAIAAHESKDTKRATSLQTGEFQSAGNGVDDNEKQPDPNTEDEAPHHHC
SRKKPYLEPFPDLRSNTRGAILAAGYTAQHFALFLQILYGIQPFSTLKDTDLMPVFRIAHLYELSWLVSLLGKQIFQRLT
LSVETWLPILRFAERYQLDTIRRATIDHASQHQVLWTLAVETLSLEDFKAFLRGIHQKDTVSSVKDELLTMFLLVHYQDT
TSFSAAAMSSLNTAGSSSSTVHVQQPGRQAALLRRLSRSHSRRKRAVSGSSSAVLKIRQQLHGTAVGSAQGADSLAQHEP
NDHLGLVQNTQERRGEDGSIDQSSDDKAEKAKLWMRRFKTECGWEGRISVLD*
>Mver_MVEG_09344
MECVEQNYGSTLVFSIGATEFDESDNASSRAQVNVRTFEVESPNLSTHGHWKCDFTETMQQGTRIVGLKLALKWIRRGGP
GSKKTLKSDDLISQIESIKVRSDSTLSELFTDKVDGRSILKGDSIQVKLEAGRVLQCGRYKFRVSLHTQDISRARFLDKP
IFTSFLDLNTSFDPVRCKKDTPTDVCFRFPVSRHCAYPTAIHAHSSELTGSTYLLNKMAELNLEKVKEKIKREKVTTEED
VEHSLECTVNEFSPAVFRVMLEHMYTGKLLLQPYNYTQKGPDHNRTESCHFEDLYRIAERYEVQSLKETALKAIRYTLNM
SIAISILTKITLDDFEEGDVGHAEKAYHQSAKTMVMDTFKEYIMFFGTEIFLLQASKHPEELSIRERQDLISHLGEYIVT
NLDRIWSV*
>Mver_MVEG_09375
MLPTGVPNTSAPDIQVHLPSGTKTLCFIFAWSTIINGSTPFASPYMTALGDFNFLVFKTESNFRFAITRKADPECFFAKS
YLIKSLDRRTLASGKLVFAQKPVAGEVHSPTLIAEKIPMAMIEPRDGHYMIEVCLSNQEVTDALRPLRPTIADMIMERLY
NDTEHHDVTFGFYHDEAATNHTLGMLHAMFARKLIVISAHKHVLRQWPYFRRMFGSEFAEGGEGEKQIQIRDVHPKTFQT
LVRFMYAGYLPQDEQPKRVSDCNQASESETSWEAVFLAAHRYELEELCQAAQRHIVAKVTPDKAIPLLFRSGYLYKDLRA
ALVKLIANTSASVVAGKHFREQYRDHPEFGLLLHEIYQEGRQPHEGFYMEPAAVVPDVRANAVARTRVRR*
>Mver_MVEG_09644
MPNSYTIDIKVPVLSGTDDPTEASLDSTDTTAIVTDVATPAGAWECIIQQSFESGEPALTIMLKWVPDTAGLSDPDEQAK
VYQFLAHFTHVQVVQGLDNVMSMHLRRPAMLLRGVQVHVDPTMVRCRNLYDFSIHVVTLMQFRQRQTYSNYSTAQGPGQY
HALNPLDAHRRRPQFDDYAADDPSRKLAAFLGRIETADMRLSCMIPGTTNNNEPEEHYSVIPVHSCILKAADTPARRLMR
DTASSQNERTIADYFQRLLSQGRDLGRERLLAQAPSLPGTSMPLEHPRRTGIVKEIRFVDAIPQAVEAVTHFMYTGQRPT
PEPYSHYTVKDLMELAAYFDLPDLQDHCIALALGWGQPWSGAPVVYRQDMENQYVYASAIAELLQDGQGSFDQVEQPRQQ
PPFSVDPSFFQPALPLLQGVCASGSGPSRMSQLSPESLLQTLFDWGHRFPRMRWALVQALCRDHGHVFTHADGFSRYRDH
PAYSEILTEMIGEQARMVSG*

>Mver_MVEG_10039
MTTPRAPAALLNDPYSHRGHGGSTYQQQPQQYTGLGLHRDQPQIEQPQPQQPSTKTLAVTSRVTKGVQPPPLMGSAIVLV
NDSALHCFGGRLENRDLSNCHYVLDVETGFWDTIQSVPDDPEVSPQSSSSKLYSLSLTTDPVNSNSGSLSEALSIPPKPR
YFHTLNAFGTSLILFGGMGQMPVLDQDSDNEGSAESRVRMEALDDLHVYDIITQRWQQKYPTRNQHTPRARWAHLATILD
HYLVVIGGTDTVKAYVEDACVLDLHTWEWVASIQNIGQCGSYRTVAATGPTAQGSSSAFTPSSPSFPSAEASCPQTSSNF
SWPSTDSGPIDAMASISMVLSGRISAPSSATSSESIHLSKDTKEAKGRKTSERLMSSELTPAFKAGSEMPCIYLYSNYNF
LNLQRDLKIVTPHYTHSGENDASSPSFSILDKTQTLTKTGVELPPGLRFPQGHVYQNQLILTGTLIVPGKAPTLAIYAFN
LSQFKWERLSTDTILETGSWNRTLLHPASGTLLIFGHREGNADADYSNRIQRHDHLMMINLQVYGLYEQPVPSFFSAAQE
LGSDLLKDPSLNDMHVASVTGALFEANSTILAARWPEFSTLLLSPPYITPLILTLPVPDDVVPLFLHYLYTGALPQSTIA
PGMADYLLILARRYQLNGLHALVMDVLHQTVTQSPLRIYSSALMAGELGLQARAVELTILTLPAKPQHVPRPADTPPPPP
PAGERRRPSLAGFDPSGLSRNYSTRVPPPSSRPPFPPGRQHSRTASHHSRDSVSTEGTIVGDSLMMQYTNAKSGIHKPQS
IHESDNVLYLSSPEPGAAPARQRKMRSPPVPPSQQPHPMSFMSTSSGGSNYTGPASPRTPQGAHQAQYSYPSPLSPNYQQ
NHKFSPPPSEQGYMSRAGSEGAPSIYSYDGQDDGSSIYSGHHNHQLQQQQQQQVDLRAQQAKKRLQMQMQHDMDQQRQQQ
QLHLQQEQQLMREQQQLREQEQLMIEQHRLQQQQFLQKQQRLKQQRMELEAQAAAAAANNKAGAMMGALDYHFGGSQSSK
SHYAPSQMTVESTAETVSSQKSGSSKASTKSSKSDKDKKGILSKMKPPKPKASGAELMKSAGF*
>Mver_MVEG_10127
MPAPAPGLQSISDLTTTSRITTGDIPPPLVGATMTIVDDKLYLFGGRLIQNRLMTNSLYILNLSEFHWRNVSEIVNVAPV
ESAIGDPGAEDENEMAAPPTERYFHSANAYKNSIVFFGGMGPTPNAPIPETQGQPQDLVVFDDIKVLDLERLQWRQVRIP
SSPHAPKPRYAHLSSISGSKLVIIGGQDIANQYIEEISVLDLDSWQWVNSMLFEKHCGSYRSIAVSGSGQGSSIPIIGSE
LEPTSPTQLSPTSITSDGFPTSYLHKTPSNTSLHYTPSLRKSSSNLALKKGGAMSRLSGGPNTFGTSPPADHRSSIYRLS
HVPTPVQQEEHPIYLYSNFNFTDVKRELQCLTPPQFSTQDHSQSMSGSYLPPGLRFPTGAIAGQHMLLSGTYLTSAQQTF
SIWSLNLSSLVWTRIDTGTVFNTGSWNRGLLYENSNAYLVFGNKSRNLADDYQNRQTNFDHVSIVDLEAFGIYQHPPTTM
TQTAQELGLSLLNDPSMADFEILTQDNQRIPVNSAILSARWPYFGALVREQEEQEIARCKQDGVEYDPTLSPKRTLSLPE
PYTVVLAFLQYLYTDHLVTAQQHQSTVLSQLLLLADMYGLDRLRDLATHALHQMLNMSTAAMVYETAALSQQTGLQIRSL
KVMIAAKKMLQQQQLKLQQQQAQSQSISADHVWRSNNESHHGEDPHHPPYASQPFNPYPNNPYLSQQQTIHGLFQPSPPP
PLSPTSINGPPSPDYNRLSIHGAQGSTALPHPMVRNRSSLMGAMPPQHWNTEPLTPPPVSPPPLTSAPPSTAPPTATKSS
FFSFPGSKNKQAATSTTNLSSSASTKSGSSGKAKVPAFASMSVMPIIAQSDAASVKSGKSGVSSGSSDAVDKKKDEQHKK
EVMQKLLDSNYSMTTFSMR*
>Mver_MVEG_10147
MPTHHHGAALYNQQQAHAGGPFQFHHSGHSSIDTSGTIADPRLSTGDRMTCIVNASVTIVAENQIPQNLTKLQRNGGIGG
NGPTMGSALSNFSDSSSPHQPYHQSRSRASSSQSLHRHSSQQFRHEDSPLSKERERERDRGTERLRLYVFGGFDQYTDVV
NNDLYRLDIETMAWEKLIYTKGEPPSKRNDHSASLWGDDRIVIFGGSDETNQYCNDVHILNIRTMTWEAPEVDGFIPAGR
VKHAATIVDNKLYVSGGCMEGEEKDGVSQELLCLDLLTWEWDEPIDFVPRYNHTLFAYREKLFAYGGYNEEMDRTGAITY
MDLQSAQVTKINVSNDIAPNVAAQQYSQVCGHYLVVVISQGLRSELQKFTGGIWTLDLDCFQWTKHDDWKILGENHTWHY
FSMERDGDQFYLIGDVNDSDEYFKQILTVDLREYGSFQEPTVNIGNELGRLLQDQISLADFRIICIDTSQPEGHDSSFSN
LSDLDHDMVVDSQPVVPSMKRSARPSATSSLAGSTSNSRAHTPQQDPILCHKLILMARWPHFANMVQSGMVESQTNTLTM
PEPYETVRAFVEYLYTDSIQEMNTDTIADLLVLGNMYVMDRLVQLCSSALHQQIDIETVSKIYHRAGLSGQIGLRQRALK
FMFRNYGPVSKTIGFRSLPKEALFDFWDSTPEGAVINISM*
>Mver_MVEG_10537
MSCRSTKRAFLPNRMASKGKNITYSIRSSFGPVLVTTSRTLASRSQPQREQPLDTPIHSSSNDPCREYDTNTHPRVLNPP
CTLGHVISSLSPISVDASFWSSFMTGLLPPTPPLPTTITSIPSIASDNPHHGDNSQTQLSESASIMLLQQMASLARHIAF
QGLMQGTGSDISLCAFGKTYHLHRLILAQSPFFDSMINGPWKESGQTLIELTLDDPNITLEGFEIAIGRMYGIWTVEEDD
PAYEPHQQQPVTVVFAGSQAVYASRLTPDNALSVLATATYLGFDSLCAQCTAYIVRTLAVQRVVAYVRFSHNHSYHPWSS
QIADACHTFLCRNGFDDPRIECFEVFESLPAKWLPHVIGSDAFWVPSEWDRYLFCRRIVDRRRRRRRRMHKKKSFRHKGG
GDKRGKSRWEQEQDLLDKQNRACDEGYDDDPEGDEEEEEEDEDEETIYEKLFSTSIVYMHMTLEQLQLILNDHDPWTERP
FTVSHLVHESFWRQTEFRTLIEGADRKNPELGLTTVVSRLALATASFGSEYIRDDPIPVKDKTAVASAILPFDDPKGLPG
EHGTNVPSQLYSTCSPFRFSVQFEDVSQLEVGFPLSSNSFYYAGSHWNVYVKKLPYRETGTQLGIHLHRLSAATPLPSTS
RLRRPVFAPSHYGRGTSSSRPRMPSPPSIPSMGLHLSGESDDDSDRAEVSEVDELCDLADTISIAESFSCFPDRRESTRT
WFRVYAASVGPLHQVTHFEAAPEDFPMSHSWGWTSPKLYTTCYLPELLIQPPSGDLEACRGSFYDQKTRTRTKTQGQTGH
QSAVYDMQNNEETCDCEAQAKYGPHHHHHDHPAQLKLSFVMGHI*
>Mver_MVEG_10895
MTSNINGFKSASEDVNAKDANGATQLHLAVRDNNIGRVLALLEHPKIDINAKDLESGWTALHRALYLGHLRIAVLLVQHK
DINLMIEDKDRNTALDVCLSTLPNSLKQGPCPADALYTWGTNSNLTLGHNDGDDRKMPELVRFPYQSASFTFPRAKSAKP
TLYHLSMNKFHTAISTSESGLSAKVWGFGAGGRLGSDNKMQLCPAPVHGIQGIVATTALGRDHTVLVTARGEVFTMGNNK
YGQLGYPLDEPKNGQDPFQLVPKRVIIAISKLVVKGAAASRWHTVVHTENELFTFGFNYGQLGYERKGDIQMGPRKVATI
PSGKILQVAASESATACLMASFDVVVFHKYAYHKISFPLSPYPEAFVQSIVKTRGWNSPCKISCSENKFGAMTALGDVFV
WSYPEVDSGITLGSLPANHSIPFSTIPTPEKPRRVWAYGGDRTQAIDFALGHNGSVILLTKGGHVYIGANKGNSLGRNIK
WRRIPHLDRVVQVNANPSGAWAALRSESSLTPVPIRNGCLGADLEQSLSQFHLYQMNEKNQGGTLNKIVASINDDDDSEE
DDDQKAEPNPWRINAQGWVNIDQSWDFDLVPLLDQEASFTSALGNSVSGSHLFDVEVQAGKRCLGAHRIILAARSPAFRQ
AFVENSSSRSKVGSLVMVEPLSDLSQGRLYTVHLKVELATAVLLLQFLYSDRIDPFWDAFDLPKPNKPSSLKIRQELYHL
ALELGLPTLQAALQYTFTHTCSPSLSVNLAQVLDNGTLFNGLSDVKLMLKDGFSMNAHQVILGHRSPFFNAMFVRTNEWI
RDRQGQRRLPPSKGASDVEDSHMLSVDMRHMSFESIQLVLRYMYSDCGPEMFDGIEKDEMDDLVQVVLNVLQISDEWLID

RLKDICEQVLGDQVRAKTVVSFLEFALMYGAKSLEATCIDYLCHNIESALDQRWLEGVEDDILILVENAIKEKQHSMSPW
VRSGGHLPDPVAVEKMRDKLQTHGPVYYVEQGGGECHDVLSTFKPLDRSRSMSRENITESAAPHAGSSTPPQPVTALEAF
APPTSASSVTETWPSGLVESPSLDRSSAEPPTRNTTMEWPSLSDPPLPERRDTPALPQRKASWGQVPSENVGQNRQSSRD
DNSDKVSVTLKPSLRDILEHEQRQQREHGGQLEHGGGIKITGSMAAPKSSKLSQKERRKLLQQQPSLEDTPPQSTVPSNA
WSRVLPGEDTQSLVRRDSHAAGLVGSSASLSASLPSSSPLFLDIQQSELALFRSQPKEVPKVAIATSKPVKETMRPSSER
LSSEAPWRLDAIPEPLRAVSRHRSQMYPTSGSPTSRLYAGKKSSSNVSLSSQAQSSPLSDAALLGPEVLIGNMDAAHSGP
SVLSSFAIIQNQQLRDRNTLLKARHHKKSMYQIQIEEQALSQIRLMHLEQAKSKSSEGTGEWFTCEISV*
>Mver_MVEG_11243
MIKSVKPCGTTGQNLVGLWRPSFTRVDQKLYLYGGGGHVTNDLHVLDLVTMNWDCVLAQDGVPPTKRYGHTAVLWDQYII
IFGGSNEYLEYRDDVIVFSLKTKVWSRPDIKGEVPARYLHSATVYKNKMYVYGGFAKDSKRTYVLEEMRMLNLETWTWSE
AYQVPARYNHSASLIGNKLWIYAGKDEAGQTVSDLHSIDLDTLKVLPHIGITGKVVLLKSQHFSESIGSQLIVFGKYLNE
FTGTSLYGLWIMDLEQLEWRKLDIDHCLEDGVWNYFTVVSKHQRSIEYGTDDCFIDTSDDRDDGGSAPSLIFLGNTEKER
PQPYDHFRDVLSINIEFLGVFQIPPSTLQSHMSLMLNNEEFSDFCIISKGSQKIHVHRMVLSSRWPHFRNMHASGMIEST
RGSIVLPEPYPVVFAFLKFLYTDSVDTKLDYSIVCEVMIMANMYLLERLKKLCASILHKHHLHIDTCVRIFQAACVSQEH
GLKKLSQEFIFRHCGAVMKTEDWLLMWSQSESGAQRAALEEFIDGIPDEASLELSPNQRPYAEPAPFEPSLRKEL*
>Pbla_Phybl2_137950
MSFLKSNQPLQTTGSNLVGLWRPSFVKVDQRLFVFGGGGNVTNDLHVLHLTDMRWETIQNIKGTSPCKRYGHTATLWNNC
ILVFGGCNEFQEYCNDLHIFDLEKLTWFQPKTTGVIPARYLHSAVAYDDKLFIYGGFAKNADCTFVLDELNVIDLKSMNW
SKHGTVPPRYNHSATIIGNKMYIYAGKDEQGCTVSDLFSIHLECPPYVPHLVLNGSHSNSTNQMVLLKSQHFCEAVCGKL
LVFGRYLAGGQPDTPSDATYGLWMLNLDRLEWERQECNANFQVGGWNYFTVLSENMQSSETGETDVKINNLFFLGNTDPF
RPQGYDHFRDALIINGESLGLYDIPALACMNEFGHLLNCPELSDFVIVPAGGQALHVHQVILMTRWPHFRNIYKSGMVEV
QQGKMEMPEPPEVVLAFLTYLYSDNLDPDTSCQIVCEVLILANMYLLHRLKKICCERLYRQHLTVEHCGLIFEKAILSEE
HGLRLLVLDFIFRNYGAVLKSNVLLEMPLMIRQEFLQAVPDEAVLEVTRSRFFSHASPSKTALTSVAAAMTSHTGNISIT
PLSHPYPINYSFGSNTFSGTNNSPSANVARMRHNRTNVVSTSILSSNTTPTSGMSVGV*
>Pbla_Phybl2_139010
MNTCKFYDLNIRSLTDSYTYKISYYFEGINDFLSEDLIVSFEVISGEPLCLLPFVDFSSYHDSSEFFDVAVKVINTQNEH
DVVAAATSPWIKTLFTAEMRDYNKEDVIIRGIYLEIFEKILIFIHSSVYDVKDIYEVSEITKAVDQLELSIICNIMFRYL
RSNIDITNMWPVWDLAVKYECKETEIAYQKITVIKVLQIDHLSPPVPEQDIYNSILSWRQNAISDTKSRIPKTVNPNTFT
DSAIVNQNNSKETKKSLKSLEKIEKAFIEMIQLIRFSQMSLEYLLRDVKTNEILTKTDICMSSISQAISSIICNNLCADS
RNA*
>Pbla_Phybl2_162075
MNAELVDISHYQLDLRLPRFGYRAPSVSTAVKHNVFNGYRCQGHTMRITLQKMKMRNEDRMVPDHIKATCIFRINQEEEK
MSNVSEKGNSDGNVYYLWRFSSKAPISFHLEPVIRILVGFEDTTGLPLRPTSLIALPTYVNSSEFFDVTVNKNLNVEILN
ATVILSAASPWFKTLFTSGMGDSNSKRVIIRGIHPRIFSKILSYIYSSSYKVQDVDEALETIKAADRIELRSLCNTMFDY
LKLNINITNVWSIWELTVDYDSKDTDFLCQEFIRKNLSTLVKNKEWLDLGPGTTIRVLRLRRVLSHALEEDLYNHIVLWR
ENAKNKVAPKSPNATNPNTANVLATTSQSNLSGIQTPLLTLECIEDFFAEMLELICFDQMSMDFLLNTVETNPAVMHINS
CRELIQRAVYLKVP*
>Pbla_Phybl2_162078
MTQKGFIEKRQCAAVFTVKRLEEVFPNDSTTGSIYFLRGYRCQGVIRQDTTRLLKRVLELEKPEESKKAISIKVTLQKIS
GSEKKLRLPNAIKANLKLETTINYTKENLNRMWRTKYTELCRNNGQTYPLEYSIGPEIKVPVSFEDLINQPVALLTYPPL
SSYLDCPSFSDIRVTVYENEVMVGEQSVFNCHKVILAAASPLFDKFFTEKMDGSEKKGKIIHGVHPEIFKKILVFIYSSD
YDVKDVNEASKIIEAAHRFELPDLSCYHCTDTQGYCQKFIRKNVRKLLSSEAWIHLDPESVIRTLNIDHISPPVKENLFY
EAVLDWREQALYNIRSNSEDHDDPGSSHTLVDDTKKSLEKGPGYPQTEEDVEEAFEEMISLIRFNQMDTKYLFEKVEGDF
EIMKSDKCKKLRLFLLLIISSMLHLPMAVPPETLFVFKTLAAIYPNKIPPPYDEREKYHSRLQLLLSTKQFFPYLRAINT
VCCNKNAWLLHLFHDALQHSNVKIKHATIDFDMYTSNVKNLNTWIKHYSESVNILCIYHGNDYSSIPAARKLHLKYMTLN
EIKYHCSDFDETGQALVDMHFSPLKVLITYDTSFKYFDSRSSQPPKLIVIEQKGNDNIDQGQAPQSSWYHLCADRTNRKS
RMLVWELGRRDIEFSQRHLKNFKRRKGRENI*
>Pbla_Phybl2_139045
MSSDTLNTFDFEIILQKTSTKENSRQLASSIRAFYTIIDSANKVISLEAARKDKVKNNEHTGIDLYLWEFKHSIFHVSSN
LSATVLIRFEDKIRQLLCPTPSIDLSSYADSPDFSDVTIKVIKSESNDDSDSRKRIQEPNKYMYQTLNICIEKLKSRDFR
CHKVVLAGASPWFKALFKIKMSDSNSKDVTIRGISPEMFEKILVLIYSSEYNVKDLDEASKILKVAERIEISTLCNLIFA
YLKSNINNKTNYRRKDIETACKNFIKHNAATFVRSKEIIAFDSESVIKILETNNLSPPVNEEKLYDAVLSWR*
>Pbla_Phybl2_162891
MNRRPTSPPAQQSPEASVQDEVLLPLPTPAPTPCRILGESLASLVGASLTVVGDSVYVFGGFDRFTDEVFKSLYRLERHA
GQSRWTRIIYTKGAAPSELSGHSVTLWNDTKLVVFGGSSEEDNTFFNQIAILDLSTMVWSHPTTTGHIPEGRVNHSATIY
QNKLYIAGGNTANPSSFADTLLILDLETLEWQPPVSFVKRQQHATFVYDHRLYLYGGFREDMGRSNHLSFINLDTYAITH
LDFESASAPPLTGQRFGQICGDQLVVVVVTAPSTAATVRATRETHQPQIHATGVWTLDLTSMQWQQREMGSRYGVCNWHC
FAMGENDTAFYLFGTEEDEPYDYYAMALRVDLQELGIVVVPPPQLGHDLVNLLASHTGDFSISSSLNDEKGEVRVHRLVL
MARWPHFAALLQSGMEETVQSTLMMPYGLESLQALVIYLYSDSIDQIRQRGVIADLLVMAQVYELPRLSTLCIRRLHHPM
TIDSVSKVYHCAGLAGQPGLQHSALAYMFQHFGAVSHTHAFRSLPREVLFQIWDAMPDNATIVGYTSRSPSVEQDAGRQS
DSEMSGVDS*
>Pbla_Phybl2_185572

MFHTPLKTLKQSLHTVLNQSQEYLADVSLVYPTQKIWAHRALLVTRVPNEFRIRYMPELQAESTSLTSLATIVPQELMMY
LLRFCLDSLSIHDSRSSDEIRKQITDLEETLSMRLLPECETGKTDDEQWINDLTRMRADQVHSDVMINIFKAPVEIKKEP
QSNQTPSQPSPAGLRIMSKSGGVPSILTSFSKTTDHQPVQTSPPVTITFPAHRFMLAAQSPYFRTMFCTEFKEAANATIH
LPADLFSAAILDLILHYLYTDTLLIPPMPANTQSSNAVQQRLTIKKHSLRILQKVYRAADYLGHSDTICVAVLCELGNIC
HDFKCSCSDCAVLLPSMLSFSSQHAGLVVSEMRRKLITFYSDPVHSLSLWSQKPFAILIQSTLPTTPDGPKPTTQNATLI
DDIVQQTFANITRHNAIHVLHSLHLCLSHIRSSDLSTTWSAPTLDILHVFLDHTVQMVSSNFDFYCVEYPILLSCVDGIG
AGFSVDFLGFVLKHVLEEGIEDNNATVLYQGIVRDLVGRQEMVKNVAVDSVLLDARQSCASYIRRRSAAIKAIDGFNSLD
KDVLRQLSDDIGIPVRTLTKPVESDFSAIFGFHPKAKAAFRSRTAESDEKLKTVCATASGGGNGGSGGTIDSRSSGRRLS
FSGLMTHRYSVPTKTPAPAEDASASAIRPVPRARSHSSDAVPIQLGITSASAVSTSSASSVVSVGTDEKPAPRRRFHPPV
SQGSSSSLTDVLLPIDSVASVVAVKGETPRPRKLKFELPATPLRTKLTPRKQISAQKRAQSPRRSRWGLGGSTTSDNSDE
DESATIVIGTKIELLRRPLPTLGTIKYIGNVEFGKGTWVGVELESRVGNSDGAVDGKRYFQTDAQRGVFIKPDDFKVVHL
PTSKA*
>Pbla_Phybl2_87911
MAPSPSTQSITDLTTRFHKAKGDIPPLLIGASVTIVGENLYLFGGRHKASRQISSKLYVLSLETYFWKSVIPKNTPPTAR
YFHSADCYENHILLFGGMASDKSSPNGLCALGDIVLLNLQDENGDLAWEYPDTQSTTLPQPRYAHLSSISGSELMIMGGQ
DIENEYIPEVNVFDCKQKTWFNPIPIEPHYGAYRSAAVSIPTSSFASLNMPRIVDSANTSFASPFDENNAYNHSRQRNKT
STVHVYSSSNIDGPVRQFHALTVSTKGEFIDLTDRSNLLAGTALPPPLRFPVAFACGQYMVIAGSCITPTSSQYHIWALN
FAAMAWTRLDAGPALSKGSWLRGALYKNSNRFYVFGHPDRSMGDDYKNRIVSFEYLVSIDTEAFGIYQPPTPSYDAEAQG
LGLCILNDMTLADLTIITTDQQSVPVNSAILAQRWPSIRALLSPLLSPENPDPSVQTTHKLTFPDTHSVLVAFLQFIYTD
QLLTAQQHQPHILSRLLLIADLFGIARLKELAVHALHQMLNMSTASVIYETAALSNSISLQIRALRVLIN
>Pbla_Phybl2_142117
MTLHDYGDTSYYTYYWRVPRFSSIPLKTEVRSAWFRLDKYSMMIQFYKGTEDEPNALQVDLVIPRVHERLSDEEMIEPLN
AFNPSIVEISDETKYECKRFISLDQLSTIITMDEIRFSVCPENKSSSGYNMSPLKSLSPINIKSFRESEIFHDVLIRVIN
DNSVQDVSVELYVNHDDQTASETSSITGTPLEQPEEESFFVHRLVLSAASDLIFEKLMEEKHEGFKREIVIRGVDPRIFE
RILDFVYGLSPGPNSVQEAIDLIPVTYQLKLDGIRQYLFQYLQLNISNQNMWGVWNCAEKFMCPDTQDICERKMKTKPLD
TLQSVYWFSSDPKVVLNALKVDYLQKPVDEVVFYEAALIWRDAQIKKAKKWLYLDNKKVALSDVFAQIIACIRFPQMDAS
FLSEVVEKNEAVMNTTGVQELIFEAYRFQACGKKKITSVRCYPRKRF*
>Pbla_Phybl2_142118
MLQISKGTKEDKNGFEVFFYLKNVNPENKAAQDVYVNATISVGLPESHYNSYVGSVYSRGELKASHGFRWTHFASLDEIK
QRLVGDTLTISVRVGRDLCAKSNRPVDPIKTKILNLEKYTDSKMFHDVLIHINNDRNNRKKGDNPPKINYCGEEDDHDFL
YYNASGNRTFYSHKVILCGASGWFKKMLEPKNGATQKVELTLNNVDPEVFETLLCCVYSSELLVDDFVGAIDLLIPAKEF
GFKTLCSQIMRFFRQDINHQNIWLVWSMANRTACTQTETACRKYLEENPVLSMKNACWLAVKSSLAINFMSMETFTKPVD
ESIFFEAAVAWRTQNMEWTLNGGGPIHEENAVAIERGFSEMIRCVRFPQMDPHYLTNVVEKEVVFKNMKGSKDLILEAYR
FHAGASEPSSFRCYPRYSPSESIASKALVKKTS*
>Pbla_Phybl2_109978
MQMQSISGIITQVRLTQGEVPPPLVGASVTVVNDQVFVFAGRLVSSRKMTNHLYILDLNTLLWSRHFPAPDSDSPPQARY
FHSASVYGNSLVFFGGMGHSLETEQIAVLNDVSLFDLSTMSWKKPPLQSSTHAPSARYAHLSSVTDDRLVIVGGQDISNK
YISEINVLSLKRWEWVQAKTFDKSIGAYRSVAITTPTGTRLPVLLREPMSDNEDHPQSQQDKDQPVVDYNHRASLVRQYG
QSLQSTREPSPIYLYSNHSFSDVRRELQLIFLPNATASNIEDCSSFMSGTMMPPGLRFPTGHTLGHHLILAGTYLSPQSQ
AYTIWSLNLGTLTWARIETGAMFSTGSWHRGVLHENTNRFLVFGNRTRSFLEDYTHRQMNFDHLATVDLEAFGVYRLPRT
TCSSLAQEMGLSLLNEPAVADFHIITREQQTIPVNSAVLAQRWPYFADLIQVSTEGLDVVDLPETSGTKSRGMAFPYPYA
VVIALLQFIYTDNLLTAQQYQPHILSQLLLLSDMYNLPRLRELSTHALHQMLNMSTAPLIFETAALSHQTSLQIRALKMM
IA
>Pbla_Phybl2_76118
MSKAVRLWRKTQSVLRRNKILRLSDHKRDTKKETSRHSKIKGQVLSAFALLFTFLLFHFSLLWSMFEKAPQTLKSSLHTV
VDNAKEYLADVCFRFAHGDIWAHRAILLARVPLKVRQQLMPTLDHNVNETCLMINLSKEISPLLFQSLLRFWYTSELYSP
SSFEYNSSTTSISSTPATSATTSTASSLSILSSLEDIPGIITNESEMLKERKVLEERLAVGLLPVHDDKETDYDQLVKDL
TNMSKTELGSDVTLNLFPASVYALHSPGDRKGILRDTSEGGLGSSLAAHRFILAARSSYFYAMFCTDFRESSSSTIHLTD
DIFSPWILHVLVHYFYTDSIVVPPLPSTALLASPVQQRLSHKKHSLRVLQKTFYAADYLGHASCAGQAVLHEMSVICHDF
KCACADCSAILPSMLLFAHKNKASVPIMLPGLMSLYTDPVRLLAPLWAAKPFSILVGSMAPTAVDLVEPESTIFSKAPAT
DDQPTFIKDMSELTIANITKHNSIHVLHSLHLCLSLIRGHDPLSHWSAPAYDLLQPVVRHTVAMISQNFDYYCVEYPILL
SCVDGIGFGFSVDFLEFLLTRVLEDGIQDANAGIVYQGIIRDLVGRQEVVKNVAVDDVLISARQRCAAYIARRWTSIKAQ
RGFRKLDKETLQKMSADIGVPYRALSRPFDSDFSAIFSFKPKAAKAALKSKMSEVDNSSTLFKTLSHTPGSNRRLSLGNL
RPQRSNGALNAKASLDASRMPTRPRSHSTESVKINSINGHYVNQTSMDAMSSQPLIHLLSLETKARHQAQEGSFESPTQH
RRNSVRSLADELLPMDVVPIVVSPPGPSAASVPRVTRLTFEIPTTPLRAKSPVTSYLSTPQGSRRRARSPRRHRWNIGSE
SEDEDLRVTPVIGAKVELLRRPLPTLGKIKYIGPVSFSKGTWVGVELESRLGNMDGSVDGIRYFQTDAQRGVFVKPDDYK
ILSMPV*
>Pbla_Phybl2_72015
MNNTDQQILGNTSHYKYPIHEWSNIPLFSNIYSPHFYTQDYTCLKKGSKEAPGSLIVATRLALSDNSAKPYEIMITFSIK
KKSDTSKSTRRNGLVLESNILEGLHAPITLKALEPYIYDDCLNVVVSIDKLPRLLGMFGKYPPGHGFLLVVYDDLRPSPN
VIIRVSKDEKETENVTSEAQIYYSNKHILIKNSAWFSNMFLSGMRESEDSRITIRGVDPQIFGRILHFIYTQEIPIESMK
DSIDIIKVADRLQFDKVNTKAFNVLKDGISRHNVLETWRCKYLRVQKRAYTPIDQYEYTEIQDLCYKYMRRYCGDILKSP

EWLDTDGQQATKILKIDCLDIPVDETIFFEAVINWRKANITRANRLYEDNVSNLCSEPSADNKVVYNCENEFRGADSKLQ
EKFHKKKATLLKEIDYYFAEMVRSVRFTQIKKEYLAETVQQECSVRDVYGLHFLLRMAYKNLAFSKGIKLEKNYGPR*
>Pbla_Phybl2_61272
MNGRTPFSTVGHVDAAGYYWTIPEFTMIAHKKRIISPCFNINGCTWFISSLEIFVCLKEANISRSSSDQKRVVQATVSIE
HANLHSCVYDPKITKEMDEIMDSFDERGWVSFIALKDITPYLIKSQLVISIRIKKETENLLTISPDIPRNLVPKLEHFAG
SPEFHDVIIRVINCPKKESDEPSTSTVISKKHKHKIDSKGKDTTPIERLFYGHKVVLASASVWFKNLFTSGMSESSGHEI
TIRETDPGVFEKILMLIYTSNFEAQDVFEALSISKAADEFQLHDICNQAFSYLQTQVTRENLWLIWNHAELLENEDVQNF
CQNYLRRNCIACLQSSAWEDLEALWAIRTLQIDKLTSNVKEVDFYRAALRWRDNKQHYLAAKNESGDRSGLRLRKRAFQQ
LRDCEDERQINDDFSKMIHCIRFPQMDMQYLVTNVETDVPVMNVSGINELASIYYFFSSYDSLIIN*
>Pbla_Phybl2_145183
MSALKSGKPICTTGKNLAGLWRPSFVKVNHQLFVFGGGGNVTDNLHALDLYTMRWDAVQALKGKAPSKRYGHTATRWNDS
IVIFGGCNEFQEYCNDVYIFNLHTLTWTSPEVSNIVPARYLHSAMAYDDKLFVYGGFAKSMECTYVLDELNVLDLQTMIW
TKYQDVPPRYNHSATLIGQKMYIYGGKDEHGSTVSDLYMINLLVPPYTPHLVLSGEQSSTSSKMVLLKSQHFCDAVCGKL
LVFGRYLSKNQETNHSSSGISGSSNSSNSSSSSSSSSSGSGSSSSGSSSSGNGIPEASYSLWMLDLDTLEWERQECNIHF
EVGGWNYFTAFKEQMMPGTEDDDQSVATVNSLLFLGNTDTYRPQGYDHFRDALIINSETLGLYDIGPPCCLNQLSRLLTN
PELSDFVIVVSGGQEIQVHQVILITRWPHFRNMYNSGMTEALERRLEIPEPYEVVMVLLKYLYSDRLDETESWDVLCQVL
VVANMYLLSRLKKMCCEQLYRRHMSVESCGAIFEKAILTDEVGLKMLTLDFIFRNYGSVLKSSTLMHMPLLVHQEFLEAI
PDEAVLDIGQMRASNTTKPIGGKASGLSKTYSTPSLAYSSSDNIYHTQTYTSVNIVRVSTDNPVGATDVIIGI*
>Pbla_Phybl2_145288
MGIDNSLGTLKSFKNIKPASCEWEFPSVLPSSTIQDSINNDSLNISVHIKQDPANPKEHFLTAYIPESVVKETNYLPPHD
ATVYVFENIEETDLSEYQSFNCHKSKLTEASGWFKQVFNDAEGPTTNEAIIRGMDPEIFKRLLQYSYTLTCDIMNIEDAL
TLIEAAERLEFNNLIKQGFQYLQTNINSSAIWNIWKVADVYDCKKTIEVCKDYLKRNIVSILKDTSWLRTDVDYALRALN
VDGLPEAVNEAIFLDATLIWREWHIQEIERKTIYIDSWQYERQEQQEREEIDHKFASMVRCIRFSQMVKEDLRYISNNIQ
YVMNVEGIPRMIARAFRNIQFDSKETIPKEYQKRHIH*
>Pbla_Phybl2_168423
MSVPLTITKVNESKNFSFGSSCNIEDWNSVANGTIYESNNMNCGNIGWHTEKTLVKKGVFRHRITADNRGFKIPKFCLLN
KIAPGKISSRCIIDIHIIPELEDDLIESTKNLLVPAEHKLKNYIGSNKLHDIVIHVTESKDSKKSFESDLKKDFYGHKVI
LAAESLFFEDMFTCNIGPKTKKEITIHEMNPDIFEQILGYIYSHKLTIADIDNAKELVLRGDYLQIPAIIEQAKEYIYDV
LSVNILWDTWKWSEINHCNEINEVCKMYFSNNIEASFLDPAWENAYYPTTLKALSVKNIQGTFSEASFYHAAVKWRKEIS
TADMDEAETKYLNEHFSLMLQNIRFGQLDFVFLADTVQNEPLVMSSNIIDKLLLEAYRYKASKSESKTLWSRCHSRAQP*
>Pbla_Phybl2_65765
MNKYPDSDTRDSIQKIKVSISIIKSVIQPINKGVSYEPEYRGESSNGRVYFEGTTLQNLQNYITDDQLALSVSLTIGHKN
KNPVDEPRSYPKPRPLKFYDQDLKNFQDVTIHVLKNNKDEPKEIESNLNNNSTASITISKNKKDESTQEFVSDKKTTLHG
HKFILAAASPWFRDIFLSGIKESTKNEVKICGVDPIISQLIFDFSYGKDIYIKDSTHCINILKVADRLQFKNIKVYAFSY
LRNQVKKSNIFGIWEASDLYDCDETRALCEKYRKSGYPDIFMSPGWLSANDKCAISVIKIDGLQGIIDETRFYKAVLARR
EFAIQTVINLRKAKEKESEEKIAKAPHVTPTPSNVNIYEEIQKEAKDIKEDDKDNSCNSDTQTKTKTERLKRLENIRWEN
ILKRNWKLYKSILKL*
>Pbla_Phybl2_65771
MSKVHENVGLFNECNHKWTIPEWSKIPLKAILNSQGFNVGNHEWTVQHIGIFVMSMGKYSTSDKGDSTEKIKVSLSIIKS
GMKSIDKEGGSSYGREYQKVTTLQNLQKYLTNDRLTLSIGLAIEQKNKNSLDGPRPYPKPQPLKFYDQDFKDFQDVTIHV
FKDEIDESEEIESTEGLTSDEKATIHSHKFILAAESPWFRDIFLSGMKESTENEVKIRGVDPIIFQLIFDFSYGRNIYIN
DSTHCINILKVADRFQSKRIKDYAFSCLRAQINKSNIFDIWEASDLYDCDETRALCEENMKSSYPDIFMSPGWLSANDKC
AISAIKIDGLQGIIDETRFYKAVLARREFAIQAVINLRKAKEKESEEKIAKAPHVTPTPSNVNIYEEIQKEAKDIKEDDK
DNSCNSDTQTKTETERLERLENIRWEKYIKEELEAIQKHFEIMIRNIRFHQMETEFLANTVRKERAVMQIPGIKDIIFEA
YRDKTFLGKKELSKKYKSRSIESK*
>Pbla_Phybl2_187045
MKSNKKSTLHGHKFILAAASPWFRDTFLSGIKESTENEVKIRGVDPNIFKLIFDFSYGKDIYIKDSTHSVNILKVADRLQ
FEDIKVYTFSCLRGQLDESNIFDIWQASDLYSCGETRISCEEFLKDCYADIFEPPGWPAASNDYALKAITVDGLKGRIDE
RIFYKAALARRDISVQKMINLLKTKEKKCKKKVAKALPGTSTLEGAKNTKEKGRKKNEYKAETATATETETETETETETE
TEPEPDTDTETEIEAEIEAEAGSLEGLEYIRWEEYVKKKLDDIHKHFEVMISTIRFHQMDIKYLADTVEKDDDVMQIPGI
KDIIFEAYRHKAFLGKRQVSKKYQSRSIKSN*
>Pbla_Phybl2_168637
MGLFEECNHKWTIPEWSRLPLKSVLKSQRFNVGNHEWIVELYKGSSKHPEHLGIFFEPIGTHLKSHKENYIKTFKVSLSI
IKPGIQPIDKEGGASDGWGYYEATALQNLQNYLTDDQLTLYFGLNIEQKTKNSLCGPYLHPKQPLRFSDQDLKYFQDVTI
HVFKDENDESNENENDFNKDSIASISRNKVKKAKSNGGTTSNEKITIHGHKFILATASPWFRDIFFSGMKESTQNEVTIC
GIDPRLFKWIFDFSYGKDIYIKDSTHGINIIKVADRLQFKYIKEYAFSCLRTQINMSNILDIWEASDLYDCTETRTSCEI
YMKSNYTDIFISPEWLSVNDNCALNAIKIDGLKGIVDETVFYKAVLARREAATQKVIDLQKLKEEESGDEVTKALPGTPT
PSNVNLYEKIQKEIKDIKGAKGAKDTKGESKDNDYTTDTQTETETEKLERLENMRWERYIKEELEAIQKHFEVMICHIRF
PQMDIEFIADTVEKEDAVMQIPGIKDILFESYRHKAFLGKRELSKKYQSRSIESK*
>Pbla_Phybl2_145692
MGTYPTSDKAYFTERIKASISMINPEWLPIVNEGKHSSRFGYDKFTILQNVQNYYLTDDQLTISISVAIEQTTKNLLNGP
RSYPQLLALTFHDQDIKNLQDVTIHVFKNEKDEPDEKKSNLNHGSTGSTNENENKKVEDTEGTISNEEATLHGHNFILAA

ASPWFRDIFLSGMKESTENEVKIHGVDPKIFKLIFDFSYGNDIYIKDSTHGISIIKVADRLQFKRIKVYAFSCLRAQIKN
SNMFDIWQASDLYDCDETRKLCEKYMRSNYADIFVSPEWLATSDVYALKAIKIDGLKGIVDETVFYKAVLARREAATQKV
IDLQKLKEEESGDEVTKALPGTPTPSNVNIYEKIQKEIKDIKGAKGAKDTKGESKDNDYTTDTQTETETEKLERLENIRW
ERYIKEELEAIQKHFEVMICHIRFPQMDIEFIADTVEKEDAVMQIPGIKDILFESYRHKAFLGKRELSKKYQSRSIESS*
>Pbla_Phybl2_181494
MDTDNVEEKIANGRGYDQFITLQDVQNYLTDDQLTLSINLTINQTHKNSLRGPRSYPKPQPLKFYAEDLENFQDVIIHVF
KDENNESDKNESNLNHGSTDSINKNENKKVEDTEGTISNEKTTLHGHKFILATASPWFRDIFLSGMKESTDNEVKIHGVD
PKIFKLIFDFSYGNDIYIKDSIHGISILKVADRLQFKRIKKYAFSCLRAQIKNSNIFDIWEASDLYDCDKTRKLCEKYMR
SNYADIFVSPEWLATSDVYALKAINIDGLKGRMDETVFYKAVLSRREAATQEVINLRKAKEKELEDNMLKAQLGNPTPSG
DNESEVVKGLDEGAKDPREGAKDSEADDKNSEDASREDTGEKGKEDSKNNGNEKMDTNVKATDNGWEDLPENVKPVVETE
EEKLERLENIRWEEYIKEELEAIQKHFETMIRYIRFPQMDIEFLADIVEKEDAVMQLPGIKDILIESYRHKAFLGKREVS
KKYQSRTIESS*
>Pbla_Phybl2_65929
MLLIVMIKYLFLLHLHLLSDSEPTSQANLEFDTALSVTQIYNGINLRKGTRDRPEHLGVCVVPSPHTLVVDKGDSIEKIK
VSISLIKAGMQPIVREFSRDFEFKGVLSGGRGYNEFITLQHLQNYLTDDQLTLSIVLAIEQKIKNLLGGPSLHPKSQPFN
FSNQDLKNFQDVTIHVFNEKNDGSDEMETNFNHDSTASIRSNKRKRVESTEDIKSNKKITLHSHKFILASASPWFRDTFL
SGMKESTENEVKIRGVDPNIFKWIFDFSYGKDIKIKNSTHGINIIKVADRLQFKIIKDYTFLCLQNHINKFNIFDIWETS
DLCDCVETRRFCMQYMRSNYVDIFLSPGWLSANDEYALKAIKIDGLKGVMDETIFYRTVMTRRRAAVMELAKLRITKEKE
WKEELLKAPSGSSTPSGEDESEIVKEVKEEAKVSEGDVKVSEGDAKVSDDNSREDTGGKVKEDSKDNGKEKMDTNVKVTD
SGGEDSLGNVKAVVETEAEKLKRLEKIRWEEYIKKELESTHKHFETMILHIRFPQMGIEFLANTVEKDDCVMQIPGIKDV
HMFSDNLYGVCWFNNNLYAICFLTGISIDYRNLFALRLMSKFSRFGYVARLLPH*
>Pbla_Phybl2_65416
MDQIIHGIYVCTLFIEKRLSSECDDQPVAIALRKMDVKDTRRLPSSIRGHIKLYDIVSKPTEIKSVKKAGNYIWQFTHNF
SLAGTSEPTLIIVKFEGETDQPFCLSPFLNFSNHTRFSEFSDMIVKIVKSENEDDTESGHETRIFRCHKIILATASSWFK
NLLTNGSYDSKDWTVTIQGINPDIFKKVLDFIYSSNCDVNDVDEAFEIIKAARLIELDSLCEIMFGYLRPKIDFDTFWPI
FSLAVDYDCIPTIMAGKEFVKTNAKEFYSCAFSFERDAADILKSQSQDTSSSDISNLASSVIQLGLDETKQKKDKDTLEK
LTKNAHDLFEFTIACHTEMTYWKTSK*
>Pbla_Phybl2_159680
MVVWPSSYGPPNISVVLGNPADTQNADTGLVSRHLSLSLFRIPVLQAHRRLMSSVPQLGTLDAQKDGSVLPCHRSVLVAH
SSYFAALLEGDFQESQMSTVYLPHELSDAPSIKTTLEFMYDHKLRLSCSPAESLKLLQTTYLVADYLDIPLLKQALLDEW
NKILHNWTCNCPGCSSAVPLVLQFARQYPHDEHLEAIHKKIYLLLTHDPDKALAAFWTEKSLATLLLFDKHFAQSLHMDI
ISRIGQHNAIESLFACFSAAKQLATKDPLFNWSEPLHITLAAAEARATKIIATHFDFYCSSYPALLSCIDGVTYSFDFLE
YLLNQILEDEMDCTNAGVLYQGIVRDLMGRQEVQSHERVSSILQGAKYQILCFVADQFDQVKQTNHLNNLDSDILQQLAK
ELTVPPRSIVPTWHASNHSKQSFLSFFSASGDLRFALHPRRRPFQPSPQPLSSCPEPFVSRLASYIKRLWAKEPPLKGRA
TYSRSTSTFSGQSRSTFSSRYNEKLRKRSFEVRHVHEQST*
>Pbla_Phybl2_149292
MPSFSGLCVDLLGIERNIIICFQFCQSFFGAPFGFYKFFDLSTVDQYINDDSLCISIDIKILSISSYYRPPGFVIPKHLT
DSNGFYNSNKEPTACASEVEVALDRYMSFMEFHDVIIKVTENERLGNEDNKDNCFRIFYGHRLTLSAASDFFSALFTSDF
GDGTKKEVTIFSVDPYVFGRLLYYIYVDMIEFKCVDDAKELFLAADYLQLPKVVSKAQLYLQMKVSPLNIWSSWKWAVAC
GCDKMDEYYKECFCLNAVLLLSQPEWLLADFSIVSRVLSIDNLSESVDEKIFYQSLINWRKHQLEEEEAGRRNKDNDGDQ
QFGLLLKKVRFPMIKPISFFNVVEEEPLVIKAAGIHELVYQKVKSLFDVKPDP*
>Pbla_Phybl2_149462
MPSVSIFKAIKDNNIDLVKFLIGLGTKSNQSANVTKEDQQWLRNHLKTTGQKQYNLNKRSSKGKTALHYAVQWNRISIAQ
CLIECPMVNINLQDLESGWTALHRQDWEGLSPFELYNLTVKDSSYPNISTLKVETPASKLTQLDDLLNLNSHYQLQHQYQ
YQYQQQNQNQNQNQNQHVVAGGTDLYTWGINTNYVLGLPDSDNKSRPERVRLDLESQRIPEITSRPPYLVRTVVMSKYHT
AILTSEPRNNLLLCGFGRGGRLGTGKVVDTQLTPIAVQWPEYIVAVALGRDHTIALSKSGNVITFGNNRFGQLGYETDAG
KDETPMQLVPRKIQAANLKRLPIIGVAASSVHSVVYTSSDIYTFGYNQGQLGYTYLGDDICQTTPKKVTFSEKIIQVVVT
DNATAVLFSTNQAVLLFNYGQQKLILPWKRFPSKMQVHKPARNLISKLITSGGGDYMGAISNMGEVFIWTCKSNAATTRR
QNEGSGTGTGTGTGTGTGTSAGIGTGTGTSTTGSGTNAGTGTGIDTSASTGTGTGTGIDFGARSRSTIITTMSSPRIVWA
PRLAHLEARQASIGQNGQVIVCTAMGEVYIGAPSGPNENGEYRFNKIPFLQRCIYVSANPSGAFAAIRAEYKASKQQAII
PHVSDHLVSALPHKAVAKMLDEQIKKFDAQKQSALEELRNNQLAGEYGHDKNIKNNHSHNHNHSNTNTNTKTKTKIKINN
NHNKHNKNNKNNNINEDEDEDEDEVKEMKQKIHTRYNFQIETAVSDAWKSVENLSRDDATLDLVFVVQNRRIYLHKFFLS
SRLRFLIGGGSSNNRSGGGDDTYSGDLVEPKIPHSSSIFHQLLAVINNPERSEIHLATTHLESFLLLVDYLYSDNYAHPM
STLYKSPLLCRASDIPTATAAAAGTATAAPFLVVRPLDVQKGLINLAEELDLAILLDSAQSTFNHTPQSSLLTDLELLLT
NKVTQDVQLNLKDGEVLFCHELIVRQRCPFFEALFCPGSVWMEERREANRTNAAVDGNNGTPAGNKQSYLPVDLGHVPVE
VMASVLEYLYTGHNEVELFGKVAKNTVEEMVEFLMEVLCVADELLLPDLKALCERSLIDFISLRTAVVLLERADACLANL
LKQECLGFIGANLSTFITCGMLEYTDNRLVGYLEEHVQSWQAQERKFSGIQRDLAQVDSKDREMDDPEFSTSLYALSLED
MPIISSQRDILVTVYPKKPAVPDHNEPSRASLAIKDKKPKAAHVSSQKKGGESRKRGVRLDLGATENEIEAIARSLTKTS
HSLTSRQGWATPVVGIQAESLKPSLREILEQEPQAIDLSSSGHSPPTQAKLVQPHLKKVSQKERRRLQQQQEMAGSGSG
SGLKTGVWGKITTEKPELIKATVPNNVPGLSLNPAINLAQKYVPEELVNAETSAIRRPVENDIKIGSNSDKVVSDTFEAG
GLVDGFVLTPIRRRNAYGDLMALCSDDVAETGSFEKILRQQALDKMRKDRRPKKSLVRIQTEERAVEGLREFYVQTIEVE
SGEWFEVNRVGQ*

>Pbla_Phybl2_149691
MSLIEPNQTIFWLLCDKDNVCYCDWRLYLKDCVLQDELRHILLGGAIVSTIVAVVGQSVWEYSPTTNFIRPKPIDSFLLM
LGLYNGLRAIQTYIVLGDVLQNVIFRSFFHELPWQFAVASMTCYFFGIIHTIAENHKTLSIRWIRRPVLIDRICTVLIIC
PFITNNIFSICSGVYAQNGNLKMAYIFSRVLHITWGAYCGFALLIIFLSGKRLVRLLQEHRKMRIKNNSDIGAINVGIIK
VRVVALAASAALVMFTFLSFSYGAARNEIMMRKGLSLTICVFWMFNAPVAYSVCEVAIILTPKLTTALGFSDHSTHEEED
DIARPSSKIFNHIRSSSLPQNQVRFPKRTLSKIDTQQKNGNGNGNEIDYVFSKDVTNSDTINNGYGLNKEGLGVGAGHYS
ANTSPVRYEFNKVSSEHPADFNGFVTALDAPRHHTSREKNREFSTKATDTIPLQQISSSDDGFPPARSNCRLSLELEGTY
PVGNTYGIDATVHGTFSIEKPEVKQTKLANMPHYLPKNKTSRTPDWISCVSLSTIQQHVINDRLTISVYIDNDSPCFGQK
RQSCPEPRILKVDNEKFSKFHDITMHVFENEDEDEDEGDNGDGDKDGDREGEHDGSNKTFRGHKVILATASPWFENLLLS
GMEPRLNEVTIRGVDPEIFKRFLHYSYKQEIYIRSVSDALKLIKVADMLKFDDIRKDAFYYLQMRINSGNFWSIWKAADM
YSCKTTQKLCQVFIRENCPNVLSSPGWLTADFEYTLKALKIDCLPVPVEESTFYEAVIDWREAAIERISRNKRRMTENAQ
AAAATAAVPRSPDSVSSDDMTKHTTHDIGRTENKSISDSKRKKMTTEDALLLTGDEKTKIELDIIEQQFSTMVHCIRFTQ
MKKEYLADVVETEKAVMKVNGIERLLISAYQYIAFGGKRPVPKELMPRYISSA*
>Pbla_Phybl2_149712
MQQKPVIESELKFAKGSKTFPENCGISLELVGTYPAGDITEISMTVDTILTIFKENALKFSPSTRHQFKLENGSTNLCEY
SFYPTKRGIGDYIDNNQLSMKIDFLNLKQSGIDIPIKNLEERRFVEPKSVPEFCDTIIHVIETEEDSDFSDMHSGDYETP
RSRFTFKSRKAILSKASPWFHNLFFLGMKESRDSEVSIRGIHPDTFQRLLNFIYGFELEINGINDAVKLIKAADRIHLTK
ALEFIFVYLNMRVNNKTVLRIWKAGDDFECKETMEYCKSYTKKHWESILADPSWLETDGRDTIQILSIDSLPLAADESIF
YQAVLNWREAKIKQAVDVPEMLQSNVLQIFNHLRNNRFTKDCKLWSNEIGNELEDVENHFVQMIPLIRFNQLTAKYLMDN
VETSGIVIDIPGMKKKVLEAFRAIAIRTEIPETVNTDKRHI*
>Pbla_Phybl2_183000
MTERFVFEDAPPNVIVAMGPDHRSSTAAKTDIQLESGERTRWSCHKAVLAKHSPYFSSMFESEFRESRATIVFLPRGIFT
VSALDGILHYMYTAELVANTPPLLGDIYSAADYLGMQLLCSLVADRIRALGHQFNCYCESCQTNVPQILEISRAQVQYQE
DDQQMIQIAQAAIKVMTHDPDKTLQTFYTSPELADQLKNAPEVHDYFNQQILERINKGNAIESLYGCFVGLQALQKNDPN
VSWSQPLHTTLQAAHSIVTRTIASHFYFYCSEYPSLLSCVDGISYTPEFLSYLLTKTLSNEMNENNTAVFYTGIVRQLMC
RHAVQHGERVKEILQVAKDTILAFMASHVDQLRTHHILDQLDKGLLETLAQDLSVLPKVLVHPEPEPPKNHHFFARSTQP
PPPPPPPVRRRVIAVQEVESSKKPIGKVSWFLKHVFVVPFSSKTAKQESKRTFVEKTKEMEKKEAAFSAQNSLRREDICQ
*
>Pbla_Phybl2_152168
MDIKPNTVHCIVNNLHTLSSQAKVSNVQIVLESYKCTITLSKDSNPESDQLCVEIILQTVGNKDNIHKLPDSLKANIVIE
IVTTYTDKNLKEKHRTEYDKLSKNNGQDVYKWKLSNLHKFPETSCIDTSHISVHVRLEDIYSQPLNLISETTSLYPNPSD
TYDIRIRVIEDENENENEEKEDAQTVSKSRMFYCHKYLLTDISDWFKDMLLSRSDSTGDLKITIRGVHPDIFEKLFTFMY
TSRYDAKSVYEASQVIKASTRFQFGSIRHQAFQYLRSNINNETLWIIWDLSVSYGCEQAVCACEDYARKECRTLLSVYGW
QDLDQESITKMICVGGLPISITKTLLHTTLLAWRSSTLNRIMSESSKANSSSSAGSDKAGCNTQRYFKELQRYPKSEEEV
EKVFFEITQLICSSEISIHRVRKQTKRVTRATIPPSSRKLVFKPMYFNNSGPKKHCKSRDS*
>Pbla_Phybl2_71105
MSPALLKNRDKARQMQRTSKPASILERTFEPKEERLLIANSVDYAETFNEQHPSWGYSKFILLNSVKNYLEDDRLHICVK
IREKIRWKIKYSSCHPEQVPFYVNNPSYTLMIPDITVHVSSEDTDSSNINEKTNTITIPAHKEILAASSVYLKTLIIVLE
PFNRDKLFIRGVDPGLFVRVLRHCYIADAEIVDIKDAIKLADIAYRFQLNNLMIDALYYLRMRVNVDNIWDIWYISAFVR
HNCRSVLESSSWVHADKKLVLETLKIDKLPEAVDEVIFFQAVLAWRTTAITKFCHEFIDSSNEYSGQLKKDFTEYILNDS
KEELEYSEKIEEKDSETNEYSNIEKTFGTTNTERLRDIEISFSEMLHCIRFSRMELVFISDIVENIDSVMNVEGIEELLS
NAYRCLAFKGKKYVPEESRQLHIN*
>Mcir_Mucci2_105336
MSKERPHRQDISLTSIYRAAQQPTASSSVRFFNQHLNKASTKEIDYTLRKLLSYVKHNNEQWPQKRLVRCLDCIYLHAIK
PHVQERVSLPSFKACVRWTVAMLADTADMNNNNNDDPMSLQIQCATKFICGLLEDLAAAVATQPSPDAEAATTTIMAEIR
HHHGVKSLLARTHDCPLPIRIYSVRALTSQIRCFAKDMIALNAYETLSQSLSNIPHLLHVLSLKPPVTDAAQQRSELDQC
VMVCRLLESLFKYEVPSRPSPARQQFAASEAFHQLLSVWQAACHHDYASLVDDATPVGSSSSSSIKRQRVLVYSLTSIVQ
KCALTASGLIAEETSRAKWQPLLALCMQRWIRGTCCTMNKAIFTSAMLDQDKSIMKKLVQISLDILPVLKQHNYWLDYVD
QAVCDLTNFFMAYVSQPILPEQASLVGTQSTLLHTNLCIDVEERGYQIDGAMLNQHQDLFVLLLESFIQHVQLATPSFVK
MISGRVAWCLKSILTILLNANQLETSALRSRILKLIVFFLHYDDAIHSFATSSTNIASLVWGPTIDTAKQGLLTAVSLSK
SQALTAEQSVAIYKAKRAFVSLAMISKHPRACERLVDCQVLQLVDVSLIPAGDIIEKTASLLSVYALFGQFVAALSRRTA
FVRTKLRDECNLIPMIMKLLQEAVQLKEAKRDMTCTVSKDNNQDAIAKGWNHVIASCLMVISTFQYDEPSMRMWLSWDNT
AATRPSTTHDDVSTKRDDYDMKMEEATVAIKQESMDPSDMAVLMEEYTVAANAPKRLSILPILLSVLFPWRNHTDSKVNF
AEINTFLKADLQVILLASQLLDQLSVIPICGRQMIVDPTALYNLCSLMICLTAAYCPDQPECMFKLMTTTISSDANDTPT
LLTMQDGDMVEKLAPLSTAEPVQEDAEMADTAILDNVHLDLDGQQEAVHRPQEPPTLCCAEHLRRAAVRILITHDNIQFT
MLSDAFTSFFQPVLQHPVRGTSHEYWRRLVCDDLFEKKMGDFLSLFRFTETADNAIKLHEMAAIAVGYAAMGAPTELEWN
QSLGLVSLDGCIVGSTQPFGIFCQMLVYELEYEEEAEEEEKDTPRADALLSMITPFRRHAAAQVLEALALEFEVVWKVET
ESIREHITLPQHMPLDQPAEMVTFVTDDATSTTPTISGNRQLLRARSPIFDALLSTDYAESVLAAIPLHDVTFHSLELFI
SVIHQLNDKAEMTTGGHLLVDKVLAPSTSWNDIVDLLLISDRFGSTVVKSLCEHWILEQVKHISSDAQSRFVCLEGMVGL
YRQCCDPMERDGGIVSDTWPFATILREVLKTILQYMSESCQTASLIQMIKDKHVEELDVFCNGLACLLQK*
>Mcir_Mucci2_159482
MTMLMKPGQPIKTIGQNLAGLWRPSFVKVEQRLYVFGGGGNVTDDLHYLDLKSMRWETIQNTSGIPPCKRYGHTATKWRD

SIIVFGGCNEYQEYCGDVHIFNLKTSTWHQPSITGSVSSRYLHSATVFEDKLIVYGGFAKSSDCTYVLDELCVLDLGTMV
WTRYHDMPPRYNHSATLVGNKMYIYAGKDEQGNTVSDLFMVNLQKLPYTPHLVLSGNQQQTQTQQTQQTQQGCSNRMVLL
KSQHFCDTACGKLIVFGRYLNSNKQQNSPESTYSLWMLDLDTLEWEKQECDGHFEVGGWNYFTIINETSGAQTEAQSQIT
INNLLFLGNTDPYRPQGYDHFRDALMIHGESLGLYDIAEPRFTSEFVQLLNSPELSDFSIIAANGEEIYVHQVILLTRWP
HFKNIHKSGMSEAIERKMTIPEPVQVIMAFLKFLYSDRLDESEPWQVVCDLLVMANMYLLHRLKKLCCERLYRRHMAIES
CGLIFEKAIMAEETGLKLLALGFMFQNYGYLLKSNMLMELPPSVRDEFLEAVPQEAVLEVGRSRYIQATAAAATAPVAQQ
SQNVIFTSNSAHHIYVSNAQSATTTTSSTSSTNYHHHHHHPRHHVQHHHHRHPQNANYVTTDVILSQRSTQTPNELIPIT
NTNSNTAAASTDMTVEV*
>Mcir_Mucci2_79248
MESWSMFGKCATSTFGFYAHKRVKYSHCSNIEDQPDQCAFVKSSCHGFNALYLELYYCSTFWKPIAIAMLVTWLLVLFGA
ISVVASDFFCPNLQTISTKLQLSESMPLSAMDSGAGSMAIGELIGAAFFIVAIVSGCMGIIRPFQSQKITFTRDASFLTG
AVAIMTWIVYHQQIHWHHSVILIGYYLCYVTAVVFGAYSSSSSSSNDPLQENKLVVHLDESTHLLSQHAKPPRLSIPDRG
FSSSVQSNLSELNHLGHVIRPTSLNSIYQSVLPRSASTNGSIMSTATRRTYRRPMTPRIGIRTSVFGAIEFQEQVNHMRR
SSHNSSQQHIMSPIHQRRHRQQSMPPPMWQTLQLPSSHDLSRRQRASTMTDQAFPTVTPSIHVVSPTSQQQQQQEQDYFT
FISTQHDQPLFHIQPSSPPPPPSHSHVPEIRLDVPSSTLLTPDASRSIHTPSILSDYDLFVSARQSFELSPSSATPSFSK
NLADHLMTAAGHNNYPLLLESPSLLQTLFPTLQDWRQKSFFSKWSSLAVLPLVLVFTLTLPVAEIDQIKVDDIQVVEEEH
DQDENDDDDDEESTVNYLSVPAAKSAAATMSDHDISAKITVDNIDTKQGWNKQLLEIQCIISTAFILGVFAANNVIPSYA
ISIGMLVGGLLAIYIKKTTLSHEPPSWFWILSFVGFFVALNWIFLLANEVVGLLQAIGQIFSISDAIMGLTVFALGNSIG
DFVANTAIAKMGFPTMAISACYAGPLLNMVLGVGISSLYQFWKTGASYKLDIAPTIIISSVGLLTVLSMVGHIKSSHTCH
WRVPQWSQLRFGIEYSSPPLLFDDLKWSIKMLSIYVQIHETSPGSLRGIRKNVQLSIKINNTNPSSYYNDFGVGKRLQPV
WFSEKNTKWGEDSIGTLSSIIPFLSRDQLSLSVNITILKSEMNNKAAIPTITPPFSRYIDSPEFSDVSFRVIEQDEEDLE
YANKRDSSRLQDAPPPQEEDPPAIKVRVFHGHKNILAAISPWFHMLFTNGMRESLQNEITISGVKHDIFHRLLKYCYTFK
MDIDDVTDAYEMLRASDRFQIVNIREEALCYLRQELDEDNIWDIWECADMYGCEKTVNTCVSYASVEMKTLVKHPSWVYA
KARVIKMALDIEVGDLPEETELYEAVLVWAQQRGANVTMTLAQQEPVEKRDTVNMSMTRTQLLSSVVNVEPLAISAAVIE
DEDDEEEEDQPPAIDYSHRVVAIPDPKELQADLASILQCIRFPMMPPDYLANRVEIDAYIMSIDGMRDMLYEAYKYHAVL
GTSSSFRCQPRRAQIESDEL*
>Mcir_Mucci2_155704
MQDQSESIAVGTIAPPALRFPAAFMCGQQFILAGPHFTPTSQQFQIWALDTNTFVWTKIEAGPALSRGSWLKGMLCQDSN
RFLVFGHPERSMQMDYKDRVNCFEHMACVDIEIFGIYRPPRSTYSGFGQGLGLTLLKDPVLADLNIVTIDQQHIAVNSAV
LAQRWPIIRSLLSPVLTPQADNALVLDFEKRELQFPDTYVVLIAFLQFIYTDHLVTAQQHQPHILARLLFIADLFELPRL
KALATHALHQMLNIQTATMIYESASLSNAVSLQIRALRVMINAKKMMQRQKMLEMNNRQQQQLPKKQPELPQAERPFSPP
PTPASANSIFSMPQQQQQQSANSSRFLQKYDLNRQQQRQQQSLPSTPMSAMSSFSSSSQASSHHHRSPSQRSLSTESHSA
PAIPTTSTFSNTTIPTSSKQAIKLPNFRMRQASSTPQSSDGEYSPLSSATSSPKITPSTSFTVNHKPPSASRSATSFWRQ
TTTSSGTTSLKKKSTTPSKSEGTNRMPAFNFIK*
>Mcir_Mucci2_80924
MSVAILLTRAPRQFLERYLPELLEEDHIADIDLPIKLDISDIISQDVFTPLLRFWYTASYYNLVSPQSSISDLSVHTPIN
GSPRLTGKDALLQQPAIQSPPPGFQSSDIQQELEQLGIELGTALLPPHCDSIPDFVQLVSDLKRMRDQEIATDVTIRLIS
SSASNDVSHSSSSNPQLTTLPSLLSTSPDNHTHPVDITVAEFKAHRFLLAAQSSYFYSLFCTQFQEASSTMVHLTDDLFN
EAIIEVVLNFFYTDKIIVKPLPTNDSKLSSPLQRRLQHNKHALRVIQKAFYSADYLGHSDTLGGAILHEMERLCHQFKCV
CAECAVLLPSMLAWSDKHVEVVPKLRRTLILLYSDPVHSLAPLWSQRPFAMLITSMVPSAASLGEDTLAAVIKQEPLFRA
RTPKTLIHDIVERTFYNVTKHNAIHVLHSLHLCLSQIRSADPNPSWSRPALDLVNPILHYTVGMVSQFFDFYCVEYPILL
SCVDGIGAGFSVDFLDFLLRHVLNEGIKETNAGVIYQGILRDLVGRQEVVQNMAIDDVLIKGRSRCAAYIARNWTKVKSQ
GGFRTLDKATMRQLAEDINVPYRQLTKPFDTDFSNIFSFKPRKSKKYHQNAQDDKNATLTEVYTRRLSLGHLMTRSSTTQ
ASESSRRVQRPRALSTESVLVSSLRRYHTDQTIPPSDNQPLINMLAYETLERGRQLEEEDNRSEPLTTTMSVEEHEIQTA
TQNNTIANTLSEFTRPLLRTSSSFTSLTDQLLPVDAVLQTHVASSSQTVPASLTTRPSRLKFELPTTPSRSKSPSAPSLP
SSSLYTQPNSNNSNNSSSTNISAAGFLSPPVNQKHNRNRRGSKTRRSRWSIGSNSDVSDDEESTLPPAVGDKIELLRRPL
PTMGTIKYIGPVQFAEGPYIGVELESRLGKSDGSVDGVRYFYTDPQRALFVKPDDFKILSNHHR*
>Mcir_Mucci2_190266
MNNSRPNSPRTAAPSRDRTPSSNGVPVVHYSQPVRTTGERLASLVGASLTTVNNSIYVFGGFDQYSDEIYNKLYRLDYDD
ICQWKQVIYTKGEPPAKRNDHSATLWNGDKLVIFGGNSEEDSYFNDIAVLDLTTMTWWHPEVHGFIPEGRIRHSATIYQD
KLYIAGGNKRGGESNVAFAETLLTLDLHTWEWDQPIPFVKRSQHMSFVYNARLYLFGGLREDMSRSNHLAFIDLNRTEDV
TQLDISSPSAPSLSGQRSAQICGDQLVVLVTLPFREALVEVPVTGLWSLDLPSMQWQCREVGTRYEACSWHCFSMTEHDT
SFYLLGTTEDDPDEFYSMVLKVELEELGIVPVPPPQLGTDLIGLLLNQQQLEKQQTDFSIYSSTEPHAPPIHVHRLVLLA
RWPHFVHLMDSGMQESISNKLTMSEPISTLESFVRYLYTDTLDDPHFTTALIADLMVMAHIYSLPRLLALCVRHLFSKMD
IETASKIYHTASLSEQRGLQQASLHFIFQHFGAISHTEGFRQLPRNILFQIWDDMPSNAAIVHQQQELIQQQQQQQQQQQ
QLNGHDDGMIEDEEMDA*
>Mcir_Mucci2_115574
MSFLKTGQPIQTTGNNLVGLWRPSFVKVDHRLFVYGGGGNVTNDLHVLNLFDMKWETIQTVKGLAPCKRYGHTATLWGNY
IIVFGGCNEFQEYCNDVHVFDIEKLTWFQPELNGSVPARYLHSASVYDDKLFIYGGFAKNSDRTYVLDELSVLDLKSFTW
SKFHGVPPRYNHSATLVGHKMYIYAGKDEHGNTISDLFVVNLNTPPYTPHLVLSGSTNSQMVLLKSQHFCEAVCGKLLVF
GRYLTHPNNTALMSTSNSNSSNSSNHSSSSANPDSVYGLWMLDLDTLTWERQDCNANFKIGGWNYFTIISESNEQATDQQ
VTSNSLFFLGNTDPFRPQGYDHFRDALVVNGETLGLYDIPPPSISSEFSRLLNNPELSDFVIVPSNGQELHVHQVILITR

WPHFRNIYKSGMMESRQRRMEIPEPYEVVLAFLKYLYNDQLDDNESCDIICQVLVLANMYLLYRLKKICCQRLYHHHLTI
ETCGLIFEKAIAAEEAGLKSLTLDFMFRNLGYILKANVLLQMSPLIRMEFLDAVPEDAVLEVHHRSARHPTCSTSSTFPA
SKSTSYTTTTNTTAYNTTNHHHHLHHLHQQQGVIAVGASGNNDLIPVARNSANNNLNNPSSAATVGV*
>Rory_2238
MVSLTNSGQLKQSITDLTSETKNTKGDVPPALVGASTTVIGDHLYVFGGRVAATRQMTNHLYILHLPTLVWIRHIAPPDS
ASPPSSRYFHSATAYKDRYIVVFGGMSNVSPITRRKQPQNEEKLYATDDISMFDIETMSWKEIHVAPSIFTPQARYAHLA
TIWDDDKFLVMGGQDVSNQHIHEMNVFDLTQSSWIHGEPVQGTFDAYRAVAFGPTMRNISSYYGKMAKPFWKQAKEEQSP
VCVYSNYNFNDITRELQSFWPLQSSSSAGVEFHSHEPTGSFLPPGLRFPTGQLIGQYFLVSGTLLSNAQCGYHVWALNLT
NLTWTHVDAGKVMAQGSWNRGVLSGQKYYVLGHRQRDLKDDYSYRRLNFEHITIIDLEAFGIFELPRATCSLAAQELGLI
LMNESGMSDLTIWTSDKQSIAANSTVLAERWPDFAQLLQEKQDSQDKQLLYFPETYTVTVAFLQFLYTDHLMTAQQHQPH
ILSRLLILADMYSLGRLKQLATFALHQLLTISTASMVYETATLTFQNALQIRALRVMLNTKRMLLQQKEQQLQQQLLTPP
LPHTSSYEDWSPSGVPVSAPMTPHQNNYHVSGDESYPASPPSSSYSFNGLRRLAAQPSLPPTPSDSRFGPYTPPLKSNPM
PKKKMPDYGFPVNVL*
>Rory_3433
MHSNMQMQSVSGIITQVQSTLGDVPPPLVGASVNVIKDKVYVFGGRLASTRKMTNYLYILDLNTLQWIRHIPPPDSDEAP
QARYFHSASDYNDQLIIFGGMGYCQFSPGDLSVMDDVALLDVHTLCWKKVTIQPSLYSPQPRYAHLSTVIEDRLIILGGQ
DLENNYLDEMNILNLKTWEWEAIKALEKHVGAYRSMAVTVPFIVKPEIEKNEEGMRTMVQEKGGCSTLGVYLYTNSNFGD
VQRELQVVGSDFSIEDYTGYMTGSSLPPGIRFPTGYALGDHLILSGIYLVPGVQTYSIWSMDLSKLTWTRIETGSVFQTG
SWNRGVLYEDRNQFLVFGSPSRNLLEDYGHRQHNFDHIALVDLEAFGVYRLPKITCSSLAQEMGLRLLNEPAVCDFYIMT
LDNQTISVNSAVLCQRWPYFAKLIESSEPKGRYLSFPYPQTVVTALLQFIYTDHLLTAQQYQPQILSQLLLLSDMYQLPR
LGELATHALHQMLNMSTAPLIFETAALSHHTSLQIRALKMMIAAKKMIQQQQNHTRPRTLSTPGPSSPSLQLPMTSSISS
DQRSSLLDYHSNTASPTLQSFRARKQSLGNSSLKKSGFMQSEVDMIPESGTVTPNDKPLKEKKKKKTFLEAFGKISSSFQ
*
>Rory_6179
MPQLLTSNSLDIASTIPYPTFKRLLRFWYTADFDAPLDLSSSSPSGLQTQVVDITIRQEIKQLEAQLGCCLLPEGEDMSD
KEQWIADLKRMIDDKLCTDVVLNIFEANQQQQQRSSEQNQVFPAHRFILASQSTYFHSVFCTEFREASTSSVHLPSDLFS
PDALQVILYYLYTDKLILPRLDSSNAQKKHWGLNEKKYELRILQKVFVAADYLGHANTICKAVLVRMQTICHQFNCVCQE
CAALLPFMLFFADREEKKGLTKLKEVIIRLYSDPLESLAPLWSQRPFAILVQRSSATYIQTIAERTLKYPILLSCVDGIG
QGFSVDFLEFVLTKVLNEGINDTNAAPLYQGIVRDLGGRQEIVKNVAVDGVLNINIPYRQLTKPLENDFSTIFSFKPKKP
QATAKAEPLSLKRLSIQEVEENKPRESRARSLSSTTTTTISILKKHAESDVTEDNTPFQKARRQEETSLTEALLPIDVKQ
ESIAKPPRQSRLRFALPDTPSRPHRPTMQQQQKKRTKSPKSRSRWSLNMSDSSDEEGPASIVPEIGQKVELLRRPLPTLG
TIKYIGSVEFSKGTWVGVELESRVGNNDGSVNGKRYFQTFPQRGVFVKLDDFKIISTPEKK*
>Rory_7767
MSYMPKMIINNVCVAQQEEANTFWKTMQKYSVDFTDKIPPNVILALAPKLSSDSSLHTMIGDLNPTISKPLSATTIPIVS
KKTQWSCHKDVLRSSSNYFKSIFNSQLHEVEASIVFLPNGIFSASVLDIMLYYMYNKSIFIEDETDIEVLQSLYLASDYL
GLESLCATIEQHITINLAHSFNCYCENCITIVPFLLSFAGSNQQNNPSLSKMAQSIVKLLIQDPEKTLSTFWTSQSMVTL
FTQNPEMELLHEYLENNLLKHVNKSNAIESLYGCFLAEEVLIKKGKEFQTKLLEGTIQKVRESGSSILANKFDFYCTKYP
KLLSCVDEIGLN*
>Rory_9000
MVWKIAFPNNTPPIPRYFHSANAQNDTHIIFYGGMTVKQRNILNIKTFTWEYPNFNDQTLPPSRYAHISTLVNDRLIVVG
GQDIHNNYLSDINIFDCKKRVWCEPISSQQFQYGVYRSSIVGVIPTQLTPPFAPNVDLLSDPFDESAVMPKDTDIDIHIY
SNYSTSETTRQLHSWKLNSRNECIEASNQEEQVGTSTIAPPPLRFPSAFMCGQQFILAGPYINGPVQQFQIWALDMTSFV
WSKIESGPGLSRGSWLRGMLCETSNTFLVFGHPGRIMQDDYKNRVHCFEYLACVDIEVFGIYRPPQPSFSTIGQSLGLSM
LKDSLLADVKIVTTDNQTISANSAVLAQRWPLVRSLLGPTATLQEQSKMLNTDTRTLSFPDTHIVLIAFLQFIYTDHLVT
AQQHQPQILSRLLFLGDLFKITRLKELAIHALHQMLNMSTAAMIYESASLSNATSLQIRALRVLINAKKIMQRKQKMQQV
AENPMSQSDTTQNTHVHHTRLLSNVQNEPINPYSTSKPLQKQDIHASSVPTVSQTAVARNSNDNTRYLNNGSPFSPPQSV
VSFSQRSLSDRSMTTDSQSSYSGAPLTPVSSRSLKLTTFLGRQPSQLQNVYSDQETSPILTKAPSPKLSHTQQQQYTENL
IKNSNNSKTHFWRHNTVSTKKKTKLETRTFGFSLNK*
>Rory_9099
MVSLTKSGQIKQSISDFTAETKSTKGDVPPALVGASTTVIGDHLYVFGGRVASTRQMTNHLYILHLPTLVWVRHIAPPDS
ACPPSPRYFHSATAYKDQFIVVFGGMSNVFPNSRRKQSQTEDTLYAVDDISMFDIETMSWVEFHISPSIFTPQARYAHLA
TIWNDDKLIVMGGQDVTNQHVQEMNIFDLTQSAWIHGSPIQGTFDAYRAVAFAPGTISSYYGKMSSPFWKPSKDEQPPVC
VYANYNFNDLTRDLQSFCPLQSSHHVELHPHAMAGPFLPPGLRFPTGQLIGQYLIVSGTLLNPTQRGYHIWALNLTNLSW
THINTGQVMSEGSWNRGLLSGRKYYVLGHRQRDLKEDYSFRRLNFDHIGIIDLEAFGIFELPKATCSLAAQELGLAMMHE
RSLSDVTIWTADRQSIIANSTILAKRWPNFANLLRQENDEGKRILYFPETYPVALAFLQYLYTDHLLTAQQHQPQVLARL
LILADIHNLERLRELTTFAMHQLLTISTASMVYETAMLTSQTALQIRALRILLNTKRMLLQYKDSQHLPPPPLPSTPLED
WNQAPVLPSYVSPPHSANASYYSFNNDSHLPSPPLSSYSFSGSLRRLAGHSPEPRNIYSSPSKSTPTSSIAKKKLVDHNF
SFNVL*
>Rory_10298
MFDEPISTIHNSLNKLLSKPKEYLADICFCFPSSEIWAHRAIILARAPKLFVESYLPELLYESHSPLKLDISQIICQELF
QHLLRFWYTASFVSLESTDATIAGDTANQLEKLSAQLGVPLLPLCSDSVPDSVQLIDDLKCMLHENIATDVTIHIIPSSD
DKKKPDTVMILSTCPDNNTQQVNIPTPSFKAHKFILAAQSSYFYSLFCTQFQEASSSVVHLTDDLFNVAIVDTLLTFFYT

DRIIVSSLPKDKTKSRSHHDLQQSKHDLRVMQKTFYAADYLGHSETLGKALLYKMQQLCHGFKCFCSRCAVILPSMLAWS
DKHVELLPELRRTLILLYCDPVQSLAPFWSQRPFAFLISSLVSSSSNLGKNVLQTVLKQEPLIDPQIKFKSVIHEIESLT
FSNVTKHNAIHVLSSLHMCLSRIRSADPTPTWSQPALNLINPILHYTVSMVSQFFDFYCVEYPILLSCVDGIGAGFSVDF
LDFLLTHVLTEGIQETNAGVIYQGIVRDLVGRQEVVKNMAIDDVLLVARARCAAYINQRWTKVKNQGGFMKLEKNTLRQL
AEDIGVPYRAICKPSDTDFSHIFSFKPRKSKKNFNYSRRFSVDPIIEVVRPKSRPRALSAESPFVVESIRETEAKRLSTS
SSLLEQQRPMLRTSASFTSLTDQLLPVEPLAVTPRLEEKPSRLKFELPTRPLRSNSPIKSSSSNAYLTPKKTKRGQNGKL
RKPRWSMGGSDVSDEEDVDNVPLIGDKVELLRRPLPTLGTIKYIGPVQFAEGPYIGVELESRLGKSDGSIDGIRYFYTDP
QRALFVKPDDFKILYHQR*
>Rory_11005
MAAVDASLGQYGEIVICTVSGHVFHGRPENSGYKFKEMPYIQRCIRVCANSSGAFAAIRAEYLLHPTEVGPSTLKYDISQ
SLPHMKISKELQMKLNDLNKQMEEAIVNIPVEDEENQSKHQMLIRQRYEGLTNMAVESAWELIEEKSRKDEALDTVFVIR
NRHVYCHSSILACRSQIFKRIAKMADKSIGSFNVRLTKRQDNRIEVHIDNCELSSLLLMIDYIYTDEYEHPMKVFFCSPA
LSDEADVRKIQKDLVTLAELFHLPHLLASAQSSFNHQPTPSFLSSLDGLVGQHHPNVVLKVKDKEILCHEVILRQRCVFF
KHLLSPQSTWVAQRREQQNEMIKVDLEHMSSEIAETIIQYFYLDKSSSSLFDHIEKGTEEAMLHFLLDLLCEADALLLNR
LKLIVENAIVQFIKLRTASTILEHADVYWANSLKHTCLSFVRANLSAFLSSRMLDHLPVSLILELEEHVRQSQSKEIPLV
PRGNFSSTNVVDTAEIEDDEFSTSLYALSRGDGSSAGAFTELLVTLYPEKSSSAIELEIKKKKEPDELTDKSLAKTKKSV
RIPLHELVESAAQPVTPSYNWVSSTGNILG*
>Rory_12244
MAAGTPTQSIAELITEFNKTTGDVPPTLIGASSVLVNNHVYVFGGRLQSNRQISNRLYILSLNTMTWKLSISQNTPPIPR
YFHSANAQNDTHIIIYGGMTVKQYQNKRTSSADELVTLDDLVLLDLRTLTWEYPDFHNQSLPSPRYAHISTLVNDRLVIM
GGQDINNNYLSDINIFDCKKRIWCKSLSSQQYQYGAYRSATVGVTPIQLTPPFAPNMDSLADPFDDSNTTNKDKDITIHV
YSNHSASDAPRQLHSWKLNTGNECVDILNQTEQIGANNTAPPPIRFPSAFICGQQFILAGPYISGSSHSFQIWALDMTSF
VWTKIEPGPGLTRGSWLRGMLCESMNKFLIFGHPGRTMKDDYKNRVHCFEYVALVDTEVFGIYRPPQPSFSTVGQSLGLS
ILKDPILADLKVVTTDDKHALVNSAVLAQRWPLIRSLLNPLTSPQSKMSEILEHDKRELLFPDTYIVLIAFLQFIYTDHL
VTAQQHQPQILSRLLFIADLFQITRLKELATHALHQMLNMSTAAMIYESASLSNAVSLQIRALRVLINAKKMMQRQQKLG
QTVERPLSPPPVFGQSPLVRSNQIAIGSQSEPLNHYTTSKLLQKQDTTVPSTPTSLASMAQGATRLQNNNSVPFSTSQSA
TTFAHQRSGSDRSITLDYNPSIPGAAPPTPQQNSFLET*
>Rory_12451
MPSTMQMQSISGIITQTHNTLGEVPPPLVGASINVINDKVYVFAGRLASSRKMTNHLYVLDLCNLRWTRHIPPPDSDKPP
QARYFHSASVHDSQLIIFGGMGYSRQSTGELCVMDDVSVLDIETMCWKKIKIQPSLYSPQSRYAHLATVVDDRLVVLGGQ
DLESNYLDEMNVLDLKTWEWEMIKALDKHVGAYRSMATAVSLISKVEQEKSKEESVKAIEEKKAMSGLYLYTNSNFSDVN
RELQLILSNLSVEDYSSHMTGSLMPPGLRFPSGHALGNHLILAGTYLCPQSQSYTIWSLDLSKLTWSRIETGSIFQSGSW
NRGVLYKDKNRFLVFGHTERNLLEDYNHRQINFDHMASVDLEAFGVYDLPKTTCSSLAQEMGLRLLNEPAVCDFYIMTLD
NQTIPVNSAVLCQRWPYFAKLIESATSRYLSFPYPQTVVVALLQFIYTDHLLTAQQYQPHILSQLLLLSDMYHLPRLCAL
ATHALHQMLNMTTAPLIFETAALSHQTSLQIRALKMMIAAKKMIQQQQERTRTVSSPMPSSPQPSLTPSLSSEPRSSLFE
YQANSASPTLNSFRSRKYSVGATPAKLGKSSFMQSEPDMSEQQQPKEKKKKKAFLEAFV*
>Rory_13321
MDSTQPTHLSLTVIYHSLKDPNSPNASQCRVNLASASVKVMDHTLRKWHHYAEGDHKKWSITRLIRCLDYMLTNGIKPSQ
HREAILTLDSFQSCLKWILHRLPEQDKDPVQLHSQVKFLSNLLEATDREPVVKLFRDFEGIEHLLSLACSDNSTSAIRFY
CIRGLTAIQQFTETLLALHAPERLTSLLNRLISSEETEQAVMISRLLDQITIPKNNDYYYADKDDSWRELVYLWITLGDR
YRDCVVHKRDALFKSQASLVYSLTNIIYKSASASSQARMELTEPSIQHKWFNLLVFLSQRWCHQRIFIEPDSSTMLPQEE
RTMLKLLNITLIVVNVLPQRQWYTIIVKNTVNLLTCFFMAFLCQGVLAERDVFLQWLDYQLCIEVDERGSAVDLETLDKH
QRLLVFLLDIYIQYIQLFPNEGRRNLAPSLSGIITWCLKSFLLLLQSHGRLEESPLLPRLKKLVIFYLHNDKAIEVLASS
PKTISQYIWLPTIDLAREGLVMIAGLTEEELSEINVKESTTLYKADRAFVSLELVAKVNARACERLVECNVLDLITFITA
GLQWAPSLLTLYARFVRLMATLAGRSAFIRVRLRDESALFSKIKGLLNTAVLYRQQERDTAGPYSNMEGCYKLIKGCLLV
ITSFQYDEPSMRQWLSSNDGEVSAVSLILPIVFPWMERPHIRIADMQAIYSYLRTDEDLMALASYLLEHASSHPLCVRQV
IEHELALPNLCKVTIDLLPRCSESQCIQDEKPDILMDMNDDNDDLKESEEAKSVPGEPSSSEKPLLMSHYALSIQGTSLR
ILEPRNNIRVPILSNAYTLYFQAVIQRPRLRGDHHKSICIHLYKHRMEDFQKLYRFAQTEDDSALKLHEMTAVAIGYAAI
GASSAAEWNASLGLTGDDDNTNDELSRHAFGALCQMLVYNLEYEHDQENTNRLDTVITPLRRNAAAQVLEILALEFASLW
SAQTVMAKDQGSFVFQPMNCIEEDMVMFVTDDGQKVIGNRALLRSVSPIFSALLSKDYVESEMSLIPLHDVTFHSLQGLI
EIIHRMNAGEVDILDRWGYHFKYLAIFNCIERKSQDGNTISYRDMSNKRVYRNGARKFFMLIKQQHIGSIRTIMLTSISL
ISSVYGHPLSSYTNTPEANTEEPEIDYGSPEFYEKLVLIMGLVLLGGVFAGLTIGLMGMDETNLQVLIQTGSPNERKNAK
KVLQLLERGKHWVLVTLLLSNVIVNETLPIILDGVLGGGWKAVVISTALIVIFGEVIPQSICVRYGLAIGAKTSGMVLCL
MYVMYPIAYPTALLLDYFLGESHGTIYKKAGLKTLVSLHQSVNPSDVDALTEDEVTIIGAVLDLRSKPVSQIMTPIADVF
TLSTDDILDETLINKILTAGYSRIPVHTPGDRVNFVGMLLTKKLITYDPEDAHPVKNFQISTLPETGPDTSCLDILNFFQ
EGKSHMALITSDPGGQSGALGVITLEDVIEELIGEEIIDETDVYVDGK*
>Rory_13953
MKWETIQNVKGIPPCKRYGHTSTFWNNCIVIFGGCDEFQNYYNDIHLFDIKSSTWIQPQIEKSVPARYMHSASVYNNKLF
IYGGFAKNPECTYVLDDLSVLDLNTFTWAQFHGIPPRYNHSATPIGPKMYIYAGKDQQGNTVTDLFVLHLDTPPYTPRLV
LTGQMVLLKSHHYCEAVCGKLVVFGLYNHDAYGLWVLDLDQLEWQRQDCYGSFDIGGWNYFTVMTDTADRVKTKKFLFLG
NTDPNRPQGYDHFRDAMVIHSETFGLYDIPQPEHTLEFSSLLNNQEFSDFVIIPSNGQALYVHQVILIARWPHFRNMYKS
GMLESEQKRLVVPERYEVMLAFLKYLYSDTLDPEESCAVICDLLVLANMYLMTRLKKLCCLTFYRHHLSLQNCAMIFETA

MMVEEVGLKSLVLDFMFKHYGQLLKSDVVSSMSSFCKTAILGGCTKRSKTRNQPSPSSISSYAIHVNNFLFYLCFPMMYL
FFLWYYCAVITVFYQVIGFFIFIVIFTRTDITVIITPCWWWKVCLKSDSIQ*
>Rory_14675
MRNFSAGSINTTPPNVILALTPEPDNSDLYSVSNSSSPTTSEESPAASTFNEKIRWSCHKDILKAKSNYFKSIFNSQFQE
AEASIVFLPHGIFSASVLDAVLYYMYTNSLSIEDDEDINNLELLQSLYLAADYLGMESLCTTIEQHITIHLTHGFNCYCE
NCTMIVPLLLSFTGPNQQDDPRLSKMTRAILKLLIQDPERSLPTFWSSRSMVILLTQNPEIDSSLHDYLENGLLGHVNKH
NAIESLYGCFLAEKQINKNRQEFQTDLLEMTLKKVVEAGSSLLADDFIFYCTKYPKLLSCVDGVTYSFDFLNYVISIVID
HQMNETNACSLYKGIMKHLMCRDTVQHTPLVKAMLQSAKNRILQYISTNIVAVKKLNLIDKNIIDLLAHDLSVAPSALTD
QEDLKLGQRVQLLNRPVMTTGTIAFAGKIGMKEDNKKQTEYRLGIQLDRTVGTNDGSINGKRYFTTTSNRGVFVKPSDVI
LL*
>Rory_15320
MSQSIADLTTEIRPTSGDIPPPLVGASTTVVGNQLFLFGGRLVSSRQLTNHLYILNLDTFVWHHHIAQPNSPPPPRPRYF
HSTDFYKRYLIVFGGMANSSRSSLSALDDMCLFDVKLMVWKHVELRPSLYKPQARYAHLSVRSADKLVIMGGQNMSNQYI
NEINVFCLSSLTWIAGASLQRQYGAYRSVAFCPTDTDTSFMPSAPFWEKNDNSSHEDDTSRICVYSNYNFIDVTRELQSF
SSMPSLDFRDHSGDMSGVSLPPGLRFPSGHLLGHHMILTGIYLTPTVKAFHIWALNLANLVWSRIDAGSMFAQGSWNKSI
LYEERQQLVVFGDRSRDLLHDYNNRQVNYNQVAIVELEAFGIYTFPQEVSAPMAQELGLSLLDEPSMADMEIITEDKVSI
PVNSSIIRLRWPYFATLLKKKDQKTFKRLVFPETHRVTLAFLQFLYTDHLITAQQHHPQVLSRLLLLSDMYCLPRLGDLV
THALHQVLTISTSSLVYEISALTGRIALQVRALRVMINTKKIIQQQQQQQASNPLVTNTPLALSINRPTVPSQPFSPVSQ
RTSTDSERFPTGIQSPTSPTMPRWAAVSKPPSNDSIFTPALKSSRSSAVHQRIPPGVLERTRKASVPPFPLPHLHTGLIS
PTYEGSNKAFRF*
>Rory_15730
MYIYAGKDEQGNTVSDLFVLNLNTPPYTPHLVLSGSRQVVLLKSQHFCEAVCDKLVVFGRYLADHSKTDNVYGLWMLDLD
SLEWQRQDCHSMFDIGGWNYFTVITDHTPDGVRLNKLLFLGNTDQHRPQGYDHFRDAWVIHSESMGLYDIPQSQFSLEFS
KLLNNPELSDFVIVPSNGQEFYVHQVILITRWPHFRNMYKSGMIESQQRRMEVPESREVVLAFLKYLYNDALDPEESCAV
ICDLLVLANMYLLNRLKKMCCQTLYHHHLTVSNCGLIFEKAITAEETGLKLLVLDFMFKHHGQVLKANILIHMSPFVRQQ
FLDAVPDEAILEVNHRSISTHTISKSTTLSLSTMKKHLFPKNALFNNKKFKNIVPRILLFSIEDD*
>Rory_17060
MSSKESDEQAVDETIFQELCTAAKEGDIEKVESLVNHFNAPINIVDKWQCSPLYWACLCGHYAVVKFLLENGAQCDRNTF
QGNLLLSYKLTKAIDENQPYLLFLSNLLENHNHHDLTFSILLGDKQHEFHVHKFILAARSSFFAKNLLNRWRGQSCVKFQ
KNMIHPISLLSILRYIYTGYVDHDLDKDIVENMIFATKHLELTHLHQLLLNQEDDNALRSHTKQEITILRNDFEQFYNDL
IKISIIAQPNDDHTSWRMLSQWLTDKSEMKQPDALFTDIALRLHNNIIFPCHKAYLCRSEFFNTMLNGPFGEQEAGCVTI
RYPDQDLELPLIEVFDVEADIFGHYVLQFLYTDKCTIPPEDAYDVLIVADMLLIDRLKAIAAIAITNQEEPVVDIYELIQ
TAIELEVERVEQYCIKYFADHLDECIKQPEFLNLIKMSAQSIKRREETDSIPFVDDLRYFLSKKYFIADEDLNESGRVNE
EYQDTWTEMESLYNQKLEILDQILESLGLEA*
>Amac_AMAG_05019
MADRAPSPSLHHDASSAGPLQPASATAPPSPTLTTFCFPNALSASASNATASAAPPQASLSTASDTGSSSSVRRRHSHEH
HLAASSSSSARPATAASTSNSARSSSHYTPAPLHHATASSSRYPLAHGVHASSTPLQVQDHTLHYMASPDTSSSTADPVL
ISPRPCHAAPLYLLHHAADAAPMPGPDTDDASAAVSSTATDPAASSALNFYGNSPAMPFPSGHSSATTSTYDAMMTPTPH
AAPPPLPFPHSARASRAHSRHASAQAQVVASSPAASSSTMTPLLPALAQGIAYAHQHAASPAMYGIADAIAHMPLPPDSV
DVAESRENVPQSTSASAASLPTTAYAPGTNSATDVTAGASGEASSSSTRSASIAHGGEGNAQLEQQQLHGTPVYPWALI
PHHPPAISSSRAADDNPYHHHAHPAASSSVHGHQRGATVALATPAPAPVPLNTYTPRSNAAATPPRPGPHLASPYHHADP
VLDGMRTHLSSHPFSPRSIKRKSKARSHAPGSPFPTAYPLGYSPGYPPSTQLRPFSAIRGTVVPEVTDDDELDSDVSGAG
PLAGDETAFRVVGVLPSASGTSNRVAIFLSPVGATAMPATTPATATAPGPASIPSSPGPYAYNSPPVSAAATAASMSPYH
HGALLHAAHFPTTVAAGSQSVHHIPRSTYRGSSSVMASPASVTAASPATGTISSLASPALSSVAPPPAAGRETLKEIDLT
EFNTDRILVRFSTSEPNPNEKLVRGEWKGRARNNELYSSPRMGPRIEVTRPGGITGDKSGFVVFNTLCKAAKMQSLYLAS
ELRDVGLFQGAQLHTLYFKVSDVPDRALENVRIACAWTYDESLTEFVDPADMLVVHGPKTLLQSDLVHGDWLGFPLDAPV
EWNGRTNLVVELSKDETHASFSWPATGGLYFKSTTHIRTVAHKDNNDSSGQYPFTNWTKPARFRRVPMLAVGAACPPAYK
LVAEVPLATFHGALVLAEVALDGARFSKYGACFVHHDPEPIAMSRVLTDLARLLPAPNRGVEGDDESMVNAFHDVTFLVG
AEMVAFHAHQAILASRSPVFKGMFTVPPPAPLGAMDTPMHAGELLDQSATGAALPPTHRHIPVPDLSPAIFASILTFIYT
ASVEIALDDACEVLKAADRYALLDLKRSCEEFLKQNTSIENVTALLLAAEKFKVPSVRSFACDFVLWHFDQVSREPSFAR
DVLPRPDLVRELLLARRRGVAQHVGHLRPALPAGTVGGAVGGPAMAGMMVGGAPTTRAAAAAAGTAGANDPAAAAPGAGN
PHHGVVPAAAPHMAMTVAFPAGLFADLKTLLHAGPLGFPDVQLLVGTPNPAGDTPPVGTLDLRMSQLSLVSSSTTGSSPS
FQVFYAHKVILCTRCEVLEAMFRSQMRESAAPHHVAIPNIRPAVFASLLAFLYVGRCDLTYGNVFELYRAADQYVLHDLK
NACEDFLMQEISIDNVGAVMLDSDDHQVGPVRSFAIDLAVKEFDDVSVTQKFLSDVVPRQDVLLEILQARGSLL*
>Amac_AMAG_05173
MRSGCDVATREEFQLSSRVTAFSAASCVAHDILHDYNVNMLSISSVSVTTDGISRTHRFLSPQSANISMNTTHASSPVRS
VIPSGIDFAVVIRPAAIVLEPGTQGTGTRVDVGVVAKIPVHLRGKTLTVQGSLSYVPIVALPRFGSTNNAVPFELRILPS
TDAMPVAVYTTLKDFVSSDVMDLAIAVTWTVSEHTVVTRTSMVPEQLGFLRFLGDEQVSDCAFLAKGSDKPIYASRLMLA
RASVFFRQMFLGPWAESSTTEPIPFDSWHAAGVVLVLIHIYSGWLPGQSLPLNTPAQLVADFACDPAKLEYAKWRNFLEL
AQMLELKPLTLAINRHLVTLLNDQYRELSATPDADPDVNMPPTIGSLVRVGAGTSALAPFGVSASSATTTTAPVASVPIN
SPFAPVAATLTSSGPSGPSASSASTASTLPTTATAAVTGRFAFGGTGTAASATGYAGFAFGPPASSSTTSAAPATASANA
PASAFSSFGTAPALSFATPSTTTGSSASAFGVTGFGAPVTASTAPATTAASAPARFGFGAFGSTSAAPAPVLARPTASSS

PSPALSAGTFSPQPSTGSSSSGRVSSAGSDQ*
>Amac_AMAG_05182
MRPSELQPTQDQLEKRPHLRFVEVEVMAWFSNALSNKVFDVAGTILYLDSGLAPDRLSYRFSMQLEAPPECPVISSDPPV
PRPSVAATVVVAVDVSDPEQKVMDMELKFELAESEPTTLPHTPVALPSLGVLQYLHDTKASDCAFRIRGTETLLYASRIM
LMRGSQFFRSMFSGNWTETQSKDPISFTSWDAPAVALAFVHIYSGWTPDQPTLPEDVPPNLVDDFGCDPDSFDEDAWRHL
FKLARFLGLKPLARAVNRKLVALLEEQFQELSEVTDVLPAKATELAPARKRRRIETKTDDVARAS*
>Amac_AMAG_05194
MEFGLVLFWEGKTVPKVLDGKALAISCAFGYTWSASCIYGGKRTESYPFSMNVDPLHSGVCVISPLLEKTISANSYFSNM
SLAVTINLTAQASLASGVDHSMLLPHSLGFLAFLNDPTLCDCYFLAKDAPGPLYASLMMLARASPFFRTMFSGDWAESAP
KTEDPIKFTSWHAAAVALTFIHIYSGWLPGTPLPKGALKRVRSFSCDPTKLEYPTWRNMFELAQMLELKALTLAINRQLV
ALLEAQFQELGATKATCGVRDEDFESNDGLTGL*
>Amac_AMAG_06692
MQGPARIGAPFLTTTPLPNPGDGSSAARTEPPGPHPDGLEYIYRWRVENWSQLTPATEYRSPAFYADELPWTIKVYKGRQ
RNPQAFSVYLGCDQPMVAIKKQLRIAFHVEHPAPSAGSAGQAGNAPASAPAEFVKYERPVWFSSRHSTWGEESLLTLPEV
AASYVKDDVLIVGCRFAVLSSVHDPPILPTSPLPDLFQSEMFSDVTVLVHWHAADTPTAFHAHRLVLASQSTYFATLLYS
PHFAEAHQPTVTLTHTDPLAFQRLLAFLYGRADTTRATLDDLEVCLATADRFGADHWKPALMRDARQWIADATVWRVWAL
ALAYGCATTAGQCAAYVAAHFARLARLVMAPETVVHQTTEAGVLGDISVDLMRAVLHADALNTDGEEQVFEFVAAWTKYH
FGDVSTPTGENLVAAAAEADACADTIDDHDLDPPFVDAVTITTGTLVLDSASARCASAASTIAPSMVAAAAAEDAAAAAI
MTSLLPWVRFPTMHRQYLVDQVEETAHMMRWSVMKDLLLHAYKHHLFGGQTNRSGASGADADNVASAQEPRGRRMWETCS
TVPRARVMMSEELAGDGE*
>Amac_AMAG_08450
MCPARHQAHRAHQHHGQHHHHHHHHHHHHHHHHHLHHRHDTSEHAAAPIEPDTTLHVTTTALESASIDCHRALLDPARLH
ALVDRDLANDVANADLATHLDLQVNVLHQNLAHLRGVIESFYAGSGQSESDPLQTTLQHLLDAHPWTDCTLVLEDATGQV
MADAAVHKFIVAARVPYFETLLYAGFAEAGRSTIKLPLDVFGSPTVFENVVSFIYLARLKHAPDSALQLFDLFRCANFLG
LDSLEAHTIAEIRLLLHGFQCHCTHCCSTLPSALLFADRMSVTALYDALVDAVSAAPLVFWTSLEFATSPTKIRDDVLLA
YVARAEHDGTTLLQGIQTLDEVLETLLGRARSAAYLSLGTAVARARDVLVKQLPPNVPAILATIDRPPAMDVNARFETLA
RYLVPQLTDRNAVPLALALFTFVETHRNDDPNAAYRPTATDLAATAVDFVVKRWLNLSFAPPRGVYPGSDAWRAFSRPRR
ARAA*
>Amac_AMAG_08701
MPSTPTTIAPATPAAANFDAIQAVGKDLVRALREPIAPDTLLEVTADTQLDPDEVLCHRAMLDAARLDPFLATPLDAQLA
SASTVDPHDGLFHVHVHARTSRGTADLTELYRTLEDLYYGASDPATLANRMQSLLDYGDDADCTLVVEDTTSAPIYLVTA
HRFVLAARSPYFEAIFGPAGLGGLARTTTAHRFRLPLAVFGSPALFDNVLTFIYTGAFRTPPASALQAFELFHCAQFLGM
PALQAAVVVGLRVLLHGFKCSCTSCCATLPSVLLFAEHMGATDLCSELVPAFSRAPAKLWTSLQFLNFPPPIKESIFRTF
LLEAEHNGAALLDGIMNLDAALTAIQGRAGFHELTRVVTHMCDTLVPQLLRNLPYILGTIDRAPVTDMPRRIATLRAHLV
PQFDDGNIVLLAFNLFTLCQSRVSTPKNASESDTDDHEDDEDDDDENDVNAGEIHAAQHHRPGTLGDLADDACIFLASRW
PQIALTPPRGVYATSGAWRAFLEHMAARCHVPVREFLREYGGMGGRSANAAGLIAPRPVSLVGLAQEDLQRLVDKDDDLV
TPAVRKPRVRSMYAEVTASSASIGSNKSTGSGKAVASTAASNTAARRRTVAVATAPAAPSPKRQPAALASAAAPKPNTSK
PVPPVPASKTTTTTTAAATPRKPPASTSAAPTAHDRTLARRSMVATTVAFGRSTPVPASTQPTPQTRTPSRLAPGTPSAR
RAVSTSPNPQEPATSPKPGTLGRSKSLSRAPVRARTDSGISGPRSSSAARAPAAVVATTPPKAAASEKPAVTSSLTRRPS
AKSTTASQPSPVVTRRLPAAVARETSPTSSLTRRGSVRAPTTNGSTPKPAPKPVAPATAKPVSSSSKPASSSSVSKPSSS
SSTAKPTSSSVVAPVFRRVSTSTAKRNPSVVSIASTGSAVSSRTPTAGSRVQLTESPTGSSSSMHSSSSSSLIRPPPGKA
ARDRSPLRIHIGPDSPIAPPAVAVLDEAANPIPADEAVSLDSINDDGGVPRISTSPLPMEPTPPVPPVPPLSSFPYIVEL
ARELGTPPPDGGEYVAKDGGEYVPADGAELVATHGAECVSAHGAECVSAHGAECVSAHGAECVSAHGAECVSPPFATLPP
GSSLLGPSAAPAVLSPPVPPKDGRRFSTLLPPPTSRPASGMASPRPASLLMAPLPQPPPPASRPASAATSASSLGAVIDG
TKDRPTATAAELVPIKPKKKHKSFLARIGLGSLADPARGGSKGGVGVVTATGMARGKKTKE*
>Amac_AMAG_09363
MTAIAPPPFPTFPMFYDWRDTQTLGRYAWHYGADVHLAQTPWSDPVLVDLSAVVQPGHALPPTLAQHPAFSLYATVRPST
IYRSLPTVGDSTIGGLTYLDVVLGAVFPPSAGGELVDIAGLVSYTVPPVGVEAAAAQLHRARLAFRVQLAAPVAQTVHPI
YLVAPVAADTPLPAHGRVHDLVVDVTATHIGSVVPAPDWSLVPARLGLAGFIGDAHVADCTLQARDAPYALHVSRVMLAR
ASPYFRDLFSTSTDRDITLTDWSAPAVALAVIHMYAGWVPPHALPDGSPSAVVRDFACDPAGVKYATWRHLFDLAHWLGL
KQLALAANRQCAAVLDAEFREVEMEEEPQVVGTVAEAEVGKEETEAVRMEQAGTGSSSGGTGMETRASRAGLVTPASPTA
AGEG*
>Amac_AMAG_10132
MPGTPTTVAPATPAAANFDAIQAVGRDLVRALREPIAPDTLLEVTADTQLDPDEVLCHRAMLDAARLDPFLATPLAGQLA
SASTVDPHDGLFHVHVHARTSRGAVDLTEFYRTLEDLYYGASDSAALAARMQSLLDYGDDADCTLVVEDTTGAPIYLVTA
HRFVLAARSPYFEAIFGPAGLGGLARTTTAHRFRLPLAVFGSPALFDNVLTFIYTGAFRTPPASALQAFELFHCSQFLAM
PALQAAVIAGLRILLHGFTCSCASCCATLPSVLLFAEHMGATDLCAELVPAFSRAPAKLWTSLEFLNFPPPIKESIFRTF
LLEAEHNGAALLDGIMNLDAVLVAIQGRAGFHELTMVVAHMCDALVPQLLRNLPYILGTIDRAPVADMPHRIETLRAHLV
PQFHDGNIVLLAFNLFTLCQSRAGTPKNASESDTDGHDDDDDENDENDGPAGEIHAARHHRPGTLGDLADDACTFLASRW
PRIALTPPRGVYATSGAWRAFLEHMATRCHVPVREFLREYGGMGGGSANAAGLIAPRPVSLVGLGQEELQRLVDKDDDLV
TPAVRKPRVRSMYAEVTSSSASIGSNKSTGSGKATAANARAAVHGGNDCCVRSIHARAGNDAADATDTDPLSARARYPFV
APVRVHVPEPTGARHVPETTRDARPVQVAFARTRALTHRLGDFGAAVVVGGPGACGSSECTTKVRNDREAGGDVFAGAPP

IGQEYYREPAVAGRRTPLAGRRCKRDEPHVVPNAARVRGVPAPRVGACANDERVNTQAGTETCCATHVEAGLILQAGVII
ERLQAVDLVKRRQTSSIICRCARFPPCLDVNDQAEPVSRFHCVDKLHVLVTQPTRRISRAAHRVPHRI*
>Amac_AMAG_11130
MGRRARTAKLLHTLLKTVGRRRSSSTASAGAASAAEPSSSPRESPLATSSTMSDQQPAPASPATPQTPQQQQTRMWNGRL
ERCKHCSPVYASLLDYRHANRFTDITVLLPKGHPQLQTIADSLGNFEDIVKARDDGRVAVRAHKLVLAIASGTLSHYFEN
VIDEAAEGDVVELDLDKVLPADPQHVAGAVLDWMYARQVHVHAGNVLGMLRAAEDLDVQDLLQLAKTILARFLAVDPALV
LPSAVQFARHDLVDTIVRALLQPPPPSPSQSNRAPRITDPRTWPAVAHLPVPQFTALLDQTVSVLTPSDQFRLARSYVDT
NMPCAHRVCLMHPDLTDLGDRAIMARQAAEAIAAAVKRPDDDAGDEHTEDDHELNAPLARAQLCTCYLATPLTNDQVAAI
FGRVEFRALDADLLTEAFHAPHVPKELVFYALMAKLASLQQPAPAPVSAPAPAPAQAPAPAAAPAPAPAPSASLQPSDAA
AGVTQDQLHDMIASTLSRVKAAEEAAPRPAATNDTAPAAEGGAAPALQQQQAPPQPPQPPQPQPIPSPPATPAPQSAPTP
APTQAPLAAPPTNPAAVEESVSPIAKTLPPPIPVSAPPNAHHAHGAPVPVGLPPQQQQQPVPVPSPQPVPYGAPLPVAHP
HGAPVPVPTVDTPAQAMAIPPRSVAGVRPRGASAAPPSARSSMYSDTSSPAAPMLPQPQGQLRPVSYAGPAPQMQPRAVA
YPARPPPANGGQYGGGPHVAQLMGAVGGDRHSHEGSHASFDSSNGGNKRSSGIGKAFGKIFG*
>Amac_AMAG_12850
MVAIKKQLRIAFHVEQPAPSASSAGSGNAPGRASAEFVKYERPVWFSSRHSTWGEESLLTLPEVAASYVKDDVLIVGCRF
AILSNVHDPPVLPTSPLPDLFQSETFSDVTVLVHWHSHDTPTAFHVHRLVLASQSTYFATLLYSPHFAEAHQPTVTLAHT
DPLAFQRLLAFLYGRADTTRATLDDLEVCLATADRFGTDHWKPALMRDARQWIADATVWRVWALAVAYGSGVTAGQCAAY
VAAHFARLARLVMASETVVVPRTVEVASPPPVVTADDDAMQDLTPAAQSVVPEPVANEAAAVSAPTVVARRRARTLTQDG
GAGGVPHVQHHVAVQHQTEPGVLGDISVDLMRAVLHADALNTDGEEQVFEFVAAWTKYHFGDVPTPAAGENTVLAAAAEA
DNACTDVGRDEHDMDPPFVDAVTITTGALALDSSSARCASAASTIAPSMVTATAAADDAAAAAIMSSLLPWVRFPTMHRQ
YLVDQVEQTAHMMRWGVMKDLLLHAYKHHLFGGQTGRSGATADAQDVSGAVQETRGGRRTWEACSTVPRARVMMSEELAG
DSE*
>Amac_AMAG_15184
MTRRTSKRFILSVRAQVNNSRHDIGQYSRFLPGLLSRRRVHGLTAAQQLSTTTVVKSEDQKLLKYRLNPETRYAKFPCMN
QTIISTAQVTPFGFSFIVFLQRMAASSTSDAAQYDYEDGNVVAERVTVEIGVIVSWDMNGVREHDGKNVHIYGSMSYKRI
VGESGPTRSETKRYSMIVKASPAFPNPSRSATAIHTFTNVLTPLNHQVSAFSLWVNIVHVGPTATSIDRSMTVPPALGFV
VLLNDPTVCDCSFLAKDASVPLYASRTMLTRSSPFFRAMFSGEWAETCTKGEPINVISWDVAAVVLVFVHIYSGWLPGSM
LPKGAEKVVRDFKCDPETIEFASWHNMLELARMLELKALTLGINRHLVTLLDVQFKELAAAEPTCGVH*
>Amac_AMAG_16062
MPSVLEGKNVTVSGTLGYAWSMSTGYNNVTPLNNVASSIDNTVSSVSLEFAITLTPTDPPASCIDQTLVVPHSLGFIAFL
NDPALCDCAFLTKDAPAPLYASRMMLARSSPFFRTMFSGDWAESANSKDPIKFTSWHAPAVALTFIHIYSGWLPGTPLPK
GARKRVRSFACDPDTLEYPTWRNMFELAQMLELKALTLAINRQLVASLEAQFQDLKATKATYGVRDEDFESDDEVDGGLD
KTLAAA*
>Amac_AMAG_16436
MPPMDKCKGNDLLKSLNDLAISDCAFSTKNTTTLLYASRAILAAKSSFFRTMFKGTWAESARGATLEPIQFDSWEGAAVA
LVLVHLYSGWLPGEPLPKFTRKLVRDFACDPNALDFATWRNMFDLARMLELKELMLAINRQLVAHLEEEFVEIKNMAEAA
EEERDDDEHENVDENEKTCPDAAE*
>Amac_AMAG_17052
MSPARHHAHRTHPHHGPHHHSYHHHDASEHDAVPIDPDTTLLVTTTALESATIDCHRALLDPARLEALVDSDLASDVANA
DPTAHLDLQVNVLHQDLAHLRGVIESFYVGNGRSENDPRLATLQHLLDAHPWADCTLVLDDATGQVMADAAVHKFILAAR
VPYFETLIYAGFAEAGRPTIKLPLDVFGSPTVFDNIVSFIYLARLKHKPDSALQLFDLFRCANFLGLDSLETHTIAEIRL
MLHGFQCHCTHCCSTLPSALLFADRMSVTELYDSLVDAVSAAPLVFWTSLEFATSPTKIRDDVLLAFVASAEHDGTTLLQ
GILTLDEVLETLLGRARSAAYQSLGTAVARARDVLVKQLPPNVPTILSTIDRPPAMDVHARFETLSKYLVPLLNDRNAVP
LALALFTFVETHKDDDPNAAYRQTATDLAATAVEFVVKRWLNLSFAPPRGVYPGSDTWRGFLDHVATQCRVKVTDLPGQQ
VPAVAVTGRRTTARPASMAVPGELVASTRRMAPRVRSVIVDTPAPSPPVAPTPAPRPRSTYGPSRAGTTPAVASRTRASS
SQNATTATSAALPSATRAPTRSGTATRAAPPRAAARSPIRSATTSRASSLAHRPSFATTTSSTSSGTASSATPRSRAPSP
LAGRMSRRPILTASSSSPSTTPASAPSPSPTPATMPRQRTSTVAPRPASLHLGTTARSPRVCTPPGSLPTSPTAPTVSSS
SSSMTPVPSSSSRRPSAFPSSPVGLAAILDALPSSRAGFHVPSASPVLFRTSPILATTELRPTTPLGGAGFSSSGGAGLP
ATPSTPPRARSPLAPLARLSTLVSALGFGAGGSSGSAAAVPEPEQVQRSVVPNEMVAVTADTGAAGGSGGGLLGRFGLGS
FMDWRAGASSSGNEGADAGTAPAAEDMVGLTTPDESDGDDEERTEVPLTRRIVLDAPPTTSLPASAPAPGLGPGPGPGLA
PTRNMPSRLPRPRVRVPQLQATARTNWRP*
>Bden_Batde5_86125
MDCVTHSAHITWTITNFHNRTGLLVSPPFGPSDCQWELNLYPQGNGFSRGSHIGLFLKVIKSSLELQSDSSLQKWSRPIL
YFHLAICDGNSGRNAIIKERNAQGSQGFGFGFSHPVSWGWDTMLPVTALDRLLTAEGEMVVAASVSWKQKTDSMMTGHST
LGSDSSVHDCYQLLFNDWLSDLQLVIDYCDPVLQPSDTGSKRSAGTLRSTTDSEKDGSLELPQSSHAESNPTLHTVIPAH
RAILASRSTYFCTMLQSGFAESSQSNDSGDLSVIHITQFTPQEIRAMLEFIYTGNLITPPTDFAQRTRLLHTADYYQLAN
LYVYTSRLIVDLDINLNSIFTVLEIAHKYSSVSVYLKSCCMTYIHEHLKDLKKLQSFKEWIQTTDSRDLIVELFSVL
>Bden_Batde5_23286
MSVIEQTTGNNNCVVNASCACVGDKIYSFGGFHFVSDVVYNDLHVLNLADNVWKNINHIRGVWPVKRTDHTMTRWGDDRL
VIFGGSDTNENFLNDVFVLSLSQLTWEKLVCSGDIPTGRTKHSAVIHENKLIISGGCQRKDEDISNQVNILDLNSKTWDA
PRNFIHRHAHNSWVYQDRLYIYGGFDDNMDRVKCLSFMDLLTGSRAIISIDSPNAPCHMEQQHVQCFGKFVVVVGVPVHW
RGADDVSQMQASGVWALDLDALRWQRLSNGHILQPFVWHYHAIDPVKPMIYLLGENPANDEDGFMSNILIINLEYYGISS

KLDSTMSAEFGQLLQQTHLADFRIISSTSPGLPEVPVHRLILMTRWPYFLNLVSSGMSETTTETLSLKDTIDSIRLFVTY
LYTDTLQMATSRTYADVMSMANRYCLPRLQRLCSAKLMIAEPDTDVVWVYLCAMRCEESVLCQRTLEFILEHFGMVSKTK
AFRGLSGSDVDDLWNSLPDSARLIY
>Bden_Batde5_35225
MASRVDSIQGLSRQDSIITLQINELETEYNGQSSSNSTFESNDSPTNRLDQMSHLKKLTQALYARGFLDGMFSDITVEAL
GHKFKLHRIVLVSNSYFAGMLSNNFWIEQSQSSISIQFDDPNITVEALTVVFSRIYGQTKMSITAENAKSLLAAGIFFDD
LDICQQSMQFIISTLDESNIIEYLIFCDDFSYGTYSDSVLNTIVTQLCHRGYTEFSQLMEKLPLHWLARIVGSDCFMCPD
EFSRYEFLRDVLCRRSANDTEWSKVQLRSDTACSNHTLVESNALTDLDANVPKGFSDDDSDDILCDASMDSSKSTTKDKG
EACNEHNRDHLYVFANAIRFCHLSFTELKAIKKERHVQNTVLERAIWNQLELEDKIVGSSAEDLEIGSVTLADKEPIPHD
DTDRIDGQFLVNVIKGPMFPYKNSTSPPFRFGIKFSNLQHLATGSKLVSKRAYYAGSYWQVYLQQISKNGLPKLGIYLQR
ISPNSEGWVQDESKDESESSIKPIASAVQSGRYEDKRIQVRTWFQLYCFFPNKCYTLESKPDIFKTNQSWGWRSHKLYRD
AMDSGQKASFECAVVLCHV
>Bden_Batde5_91182
MQGNQQRRRSSHKESIKDDLVNERDVFGNTPLMVAVVEIASNCVSSDSLTIPDYESKRASILALAESRKLNINLQDLESG
YTVLHKLRPDANLGIKDKEGCTCLDLLNITMKQPRLFADLDSPSGYLHQINKENQTGPYLHHNGSTATYAVEDTTSHDSD
GVDTFLDDQSFPNSSTLLNPATSVWSWGSNKNFTLGFPNTQDRAIPEQIDLVHSLHTTGNVPVSYATLSEYDSQVEQVVM
SKYHVCIRTKDRLYTHGFGGGGRLGLGHEETTMRPTEVTELTNNIGFVAAGPDHTIAITLNGDVWTWGSNKYAQLGYYSE
ASGDSLPRECHPQMVQLKKTVCVGGAGSKYHSVVYTTSGVIYTWGTNNGQLGYQQPVQVVPRKVTSFPVQMILSVASTNC
STAVLVGSFDVYVFAENKISKVVFPFSKPLKSFTRPQSSNASLLMSGSRPSQPAKHGSGSSHTGSRSVVKIVSGNHQYAA
LMSTGDVYMWSPPESRFQDSWQQVNFPQQRPKLVWSVRKKFMAAHDVDIGVDSSIIIGTISGHVFVGTRRVQAQSTQPLA
SSERSVYVSPAVAAAKMLNESSVPGASSSGIASTLSLASYFSQGGLSSMHPKDSNVFFKYTMIPNLQHIVMVVASSGGAF
AAVRSDCRPSLPPLPDNQLSSDLYRALYPLPLHLEQFQQQLTIDHYKSLETPSSDQSANFPVDAAISHIEAPLFPVFEPP
EIFPNSSFDIEFEFEGCVKLYAHRAILAARSPVFWSALSSINCADSPTEYIAKSPKKSKSPETRHIGYASSLKSLLDTMT
FPEKDVPLVRFRFPHIHSASGKSFLYFVYTGCIYTEFLLSDSVFTSENDFNDQSFGSTDKQNNHRNSNLFNRAQIEFTKL
ARCFELDETEILYANIKAGLNPVSPTLAPITLLPRAKSDRICYTNERFQKSISALYHRHRDRFTDVVIHLADNHRIEAHS
VILSARSTFFNAIVGPGSLWNCHAETDSCAPLLKKQNIDLTHISHAVFLVVIEWIYTNADASTLFESLNHSNPAEFIEFV
TSVMAAADELLLSDSTYLKDCCSDILVHLLDIKTAVSFLKIADRYSSARLKNICLDFICWNLETFLECSRWFRDLDEFMI
FGIESRLRVLQTQKSPCMRGSDGFYAQLRARTIKAQEASNEKKQAVYEIRKQAGLNHNTINESATSVGTTDLPFIYASSN
VDYGLLKENVISSSSFGSRCAIGSQSTINSGFSASSTYGSASCPGSSLKPVCRFSKTQNASITSPLLSAVSPSLSAISPG
ESGTVGDHLDEQVFQLELDTDLPDQQETVSKSRANDKLPLVATSRHRKHGTSSQADPNTSTYSSVKSKKSTFVKLDVFEA
SSDSSKTTGAFPAGSSLPETTPVLKGWSPVMQPVNTGPKMSLREIMDETRKSETGSGSGPSLSIPSPVKLGPSVSFLIPP
SPSSLKPIARSIENAPHSPFNLGEPMEPLKLVKSKPSQKQRKKEAAKLAAQTNIVELLPSSSPAWKTIPICADVLTNSLS
DTRDLDVESINYATSSSLGSVTPWPNTGKSVPTKFSTSASSVVFTPVVKSTRKHSGSIAKTQQGPTTDSCNQPRQQQQKP
LWPVQSFADIQNEQVVEAQTLARTLQKSLLRIQMEEEAVRALTEYYTMTQANGTGEWFHIRRGL
>Bden_Batde5_36268
MVGEPNTNTAASQPTETWEKSRHPFAQVFVSGKYADAVLRVRNLVETTYVFDVPVHRVILASGSPYMDNLFSQPPEDQTT
LHGVGKSRQPQICGVWSVAVHSDSAMHVFLQWMYFGTAIHTKESCWGVLALSRHMYVPDLEEHVGKWISDNLLARTLGEP
RPTSEWYEYILGALHARVTQNVVDNLIEAAVIDDQILSPTPFGIPQTFHRYTILRNLVRAAEIKGTPLTSKQVRYMFKTI
DYKKWPRDQLQQAYMDGLLPRDKICTALSTSEPNPSEFTSKVADITPTDLQVPTVSATNLPKPDVEQSHVLDHESDMQTI
KPSTVKLARKSIINFHSDVKDLQKSTLPTEPAPDSIQKTVDNVASIDTVPSLLPGAYPNPTCNHSSKMDSVRADTELPSH
STRAIMNTFDEQKENAEFTGLNLNKSSLSVESASTYNQHTSPSNIEISNQVNTDSLAYSESLIRETPEQCLNDIRLQLDI
MFANEPTISTDSYSSDISNQAVVPINGTLIKSPLVQKSVQRTWGSKILRNITANPLSFSTGFDSHTNASTSPIPLAIHDV
RSLHPLTVLAAEVKALHNTVQNTLVSPHNSITKQKDGGLNATMHLVKTDPFGHGLSKPRDRSKTDPSDFVSNQANHNSAI
GDSKHFLKKPHFTRALGENEHSVAKSDLSASIRQTEQILKKHGLMLEPRRLDIGNGNFKTWKDKESRMGVLSSTDTLVNE
EDTETPTRAREVKAGQHTTGIVSPLNSRQLSSAYPFPLPPSINVGNNSTLRGISARQGRGALPNPPKPTDVQSEQSKRRS
LNPTFLMESATDSLTMPHGLSNKQLDSNALFLPRRSMFGMKHPKQLQNDASSSRSKSVDVRFSLPPPEASSLFMDNSLDD
IQAHESKRQDYQHNGTFKMLKLKHASFVPPGLPVPDSQVMPPKTLKEHMDDEANPSMSSDDSYQLAFPLSKSATMTKQPS
QMDPPHRGLKKQWAKSTTFLKQDIRKKQSLMNMLKNFV
>Spun_SPPG_01422
MNDPKARKFQANITHEKAVAEGFTSEYYLIFSACDFSGKAVDAEKPIVISDRHNFPDADVRVVVDSTAFHIHTELLKIHS
DYFRALFNWPWMESHRVDGFGRSIREITLKDIADEESFKLFLDCIYVRIVEFNGEIALKLLNTCDPLGLVFKNDIELYFV
NHPDLITWSTIEDLVRAATPLGALQQSCQEFLSKVTAEHWIRCLYLAMTHGMHDLLSDAYQEKWPLLPLLDPFFYRLSVE
VQNKLLIHTVNRLLRCPGCGGTSQGLRGSGVWYCALCGCWFDAPYVLPKRTEELG*
>Spun_SPPG_01517
MGTSNNVAITPLKLTENRRTLELAIRNFSKAEHGKTTFSYGATPFLVCNRKWGCNVYPFGLRPASKDYGQRIDIYLHIWA
QNANETEEQFLSWIRANNIDFQITITEPEPAALHVVRSSQSTWCDMEDAAGWWLISLRSSPRLMELTDDTLIIRISFTTG
EAGLRQKPDVINGKDVITLPIGPVQAAGALPAGLFDDARISDLTVIAGGKPIYVQRSVLASRLPGCELPEDGSPLVIDDI
PYEVVRATIAYAYTGALHVENTAKNHAYLQVLAERFGMEQLRRTSRRMVYSCLTVTDALDVYMALGGLSPSLRELICVYI
IQSFAEIRSSDAFRRLSTHPTRRSTASLLAILRRMEVIAPHRPPIFEPTESAGNLDWRRSMAFRSLLANPAVADVHFVVE
GKILTAQKSVLSSLSDYFLAMFTRGFQESTTERDITVVEIPDFPYSTVRNMLLYLYVREIDPPSSISEIGYLYVIADKYQ
VHDLMSKAKSLLQTHIQPDTVTEFLFSFAYKYEPLRRLVLQYVLADFNAVRKSDGFARVVRHPHLYPEYVTLMDEILEHL
VVKDFEQTEASRWENETEVPEYEQMLGEVDAIANVDELDREANDDEEEIFRDAPAFVGSDAYLDTVQRQEFQGKRHAVDD

DTITRPVEKDDNETGPQSPRRATGVESTGKFDVSNEQVDIESSS*
>Spun_SPPG_01731
MFAAVRNRDVSTLRSLLLGSDTSSSGHTGGPGSGSFGSLGKHSVLKQRKGTVDPNERDGEGNTVLHIAVASGNVEAVNII
LQCPKLNVNVQDTESGYTALHKALYDGNLSLALHILRQREDCDLTIRDREGNTCLGLLVSTIESATSISEPLPPISDPPA
ERSYDSDEDSPSQEKRTDDRPDAAASLWTWGSNTNYLLGHQNSDDRAFPERVDITATYRKSDRPTVTVQTFSEHDPDVKS
IVLSKYHSAILTSDRLFTNGVGSGGRLGLGDEDTMLRPMVVRGVKGKVASLAVGPEHTVAVTTTGDIWTWGSNRYGQLGY
VSETVGKDTPCELKPREVGGTLKRVRILGAAASRHHTAAFSDAGTIYTWGISVGQLGYQQPLNTIHNYPKKVTDFPQQNI
LQLSATKSATAVLTESYEVFVFAEFKYSRVTFPFQQFPKHVQVHHPRTEKPPYITKIVSGNHQFAALTSAGDVFLWSPPE
KQFAETWQHLTFPQRRPKKVWSVRKKHLAARDVAVGIDSSIIIRTDSGHVFIGIRRKDAKVRNVSETDEAKDVVYFKYHK
IPYLQHVQHVVASASGAYGAIRLDRRPRTVTVSPGTLREDIHKAFSADPDPDKPDFHGVGIGPDVEGVGDVVFVTRDKRT
LHAHRVILACRSPFFKRLFCDGNLLPGTKKTVKDVTVEFKLNTMSNVVEISVPTCHSRTLSLLLDYIYSGGFRKAWDHTI
FMREDTTITPHGSLQPSTIYKEFNQFAKTFQLGDTDVIQLTDSKGRAYHQHIFSILNDSKDTYDQFSDVLLRLSDRDVRA
HRVILTTRCPFFNAMLGSGSRWSFAEEGGLALVQLDHFRFEVMEVVLRYIYGDGSADEVFAGIEKRGISEFVEFTIEVLA
AANELLLERLKEICAVILERAMDIRNMVAMLVVADTYEATKLKDACLNFVCRNMETAVECRMLEDVAEELITDIENRLRD
MQSRKYPFTRGENSIYQRIRAIAVNEEEERKRRRRSAYDERKRVEREMGLVCATSSYEEASAGSKHFPLRESTEGDSGDD
QKDVPRERGFDGEEEDLFEMEMEAPMKEGDKKTGSSSQEACTPTPGAKATSRGKKDKRAWRKLDLNGMTAVSVSPVAATT
TAPPVAPAKVESPSPVKTWGAVIAKDQKASFRDIMDETRATSSTPVSNLSIAMASRKKSFLDIMAEAEAKPPTSPDFSVG
VQKRTMSPSPAKKPPSATSAQRPPAPISTPTKPAPWTSSGSPATPSATHPCTPPPVSHFPITIKVTSPSRTKMSQKERRK
SAAAAKASLPSPPLGPSPSPWANTVPAWGLNSKPNGTGSMGKTLKQIQDEERAGLSGGVELAGRSSSNGSNGSISSNNPA
SHWLGGPGFASAAASAVSSSSATVSSFTSIQAEQLHYQQARHKMLKKPLSRIQAEERAVKDLIEFYAMTKGVGSGEWVVV
TRGEKS*
>Spun_SPPG_02542
MSLSNSAALMPVLPRKTTGAPAHACLVNASCTLVDNKVYVFGGFHMYTDEVYNDILVFNVNDSHWKRLEHVKGNWPVPRN
SHTTTYWKDGKLVIFGGQDVNDEFLNDVYILHLRTLTWEKPEVSGDIPSGRAKHSAVIHNDKLYIAGGCQGKENDVSRDL
HVLDLNTFTWEPHVPFVRRYSHFSCIYRNRLYIYGGMYASLDCATDIAFIDLDDMNVSQVVVKCEDSPPKLGQHFAQLCG
NNLVVVVTQCLKYDENAIATTTGIWTLGLDSLRWRRHENSDMDQRHASWHYYVMNPDDRRFILFGTNDRDPDESNLTLVL
TLDLETYGIVYVPPGAAGRDYSILFEKPEFADFTIMSSEKDMPPILVHRLILSTRWPHFRNICTSGMSEAASATLTLEDP
YHSVRAFLRYLYTDSLEGMSVETVANLLVMGNRYCLDRLQKLCCADLHARMDVESVATVYARACMVQDEGLCQRALFKIM
DDFGRVTKTAGFRELTHEQLIALWDAIPREAFITA*
>Spun_SPPG_07109
MPTTLPHEQLARQQQYVWRPIVSSPLPCYSAVPIPDTYIIDKLRGFGWRYFGNTQLADCCLWIYSPETTSPIDQDKAAAP
IAIIPAHSLFLKTMSPRLRDLLSTAAKAMSDPYQPYATISTTPLLIHPPSPEAFHAVLQWMYTADVGAIREFFESHENAL
ASVIANAEWLGLSDRELLHVCEGWQGDLKAGRVQVRVQEASDEDGDFEQDEHDDVDGDYEDEEEEEEEESEVEEADYLNI
PHMY*
>Spun_SPPG_08118
MSTSRTDVLSIVMDEVEDAIDVATRDWVKEYHGTLPIPKCSPTTTNPTLSQTLEPLKKLSESLFRKGFLSGLFSDFTVQV
LGMTYNLHRIVLLNNPYFAGMLDGGPWKEHESRQVQIAIDDPNVTVEGITTVLGRMYGNTQLHLTSTSAPSVLAAALFFG
DKELCDMTVNFIVEDLSPDTVLDYLLFSDGYCYGTHSETIVEACLTYLCREGYARKELRPVFERMPVRWLERMVTSDCFW
VPSEEQRWNFIVKIIEERRRTELGKRDDEAIELGKRFEQLASLEGTIETCTKEDAAACAAESEGHVWTTIKDQPSDDEIS
ADDDSDTIYDNILARGIGYIHIPFDSLLRIHSYTELQDIIDLPDLLESSAWTQKELQHMITSATEDTMEFNIDEPVVPRS
DTRHIDGLRLTEVMDEDNLYVHQGMRFCPMRFGVEFSDLRRIASGEKVASRSVFYAGSMWQIYVQTVTVDETPKLGIYLQ
RMPVPVPKFSTYPTYAQLPSTTPYIDPRKTTLTWFQLYCFFGDKCYVLESKPDKFQLTQSWGWRINKLYRDAFDAGRSLR
CCVVLGHTSQK*
>Spun_SPPG_08682
MRRDSRTPVVVKEREPSGQEGFGKGFSHPRSWGWAALFPLNRLAEAATADGTLVVHAEVVWQQRAELVGLLDAQAKIVPS
SGGLLFNWRLADVKFVVLQQQEREDDGDYNEVDEGKEEVEDTYENMKPAVKTEEESSANMDEDDESATESPETPYPEEIV
PSPTDDSRTELEEQAVKLKLTMHRPSSTTSSTASLPTLPSASHPQTVIPAHRAILASRSDYFAAMFSSGLREAGGSDFSQ
PSVVEIRDFSAFAVRAMLEFLYTGRLVSPPTDLTARSELIRLADRYQLPGLHNYVGAIILEKDMNLDVALEVLELADVYS
SASGELKLACLGYVRENIGKLKGRDSFKEWVRSTERRDLLVELFSLM*
>Ttra_AMSG_00215
MAKKYDDLLFGDLDAAAATSATSPGSLTDIANTLESVTANATGAPKAAPAAASETDARATELAADVPVVSEARRKKRRRA
RTLTKAETELKEAQARGTLLAPIDASLLYDIGPASASEAANAQGLSASLFSMAQAGVLCDATLVVSGVDELEAATPETPV
VELTVHRAVLAAVSTAFRSAFDSDAGGADCRLPVAGFDVELFAAMIEFVYLGCVRVFVEARSDGKRPESVLIDLLRIGMR
FRIVQLVAAVATETIKRVCVSNCVELLLAARSMLLGDDEVPGARYLCALANVCLHTIRINLDAFAGKQLLPLCLEDLVAI
LGSSDVSVSSEAVVVDVAFAWMKATKKRFKHKDVVLNLIRYYALTEDELLATRGMLNKAMNASIIDVQLAAVAGGDMAAP
PRRHYVHAKTCMPTHELRGSGAPLFCVSLADSRIFVSGAADGTVSVWSVKGWKRLKVMEGHKGPVRGAVVCGTTLYSVSH
DRSIRGWDVVAWEPTTSLFSAHQAPIWSVAEYDLMLVTGDASGVIKVWQEDDGVDIFNGGVTGTGLSQIATLKDHTGGIV
AMATSDASLYSASDDATVRIWNIMNQTCLKVLSIGSTPLTTIAASPLMVAAAYVDGSVHVWRKNKAGPGTAKDDPAGTGG
AASMRSFDIAASDSGRTVRALLFIDDLETDTPSRYVLTGATDGVIKVWDVCPDSGHQAAPSFSDAEQHVDADVDASRSSA
STTADPNDADSSNGPVDAVLVRILDVHAQSVNGLVGSGSGFFVSASSDGTARVWE*
>Ttra_AMSG_00345
MDKRSKKRKSLALGFSRGRGTVSSSSAAAAAAPVTQNYPYSPFSSSLRTQGLFVEAKQSNSWAQVMVVGSHMPSGRYGHS

SALYAGHMFVFGGFNSEVGTLNDMHAYSLEACKWTPITYASRSVPSPRFYHASLVDGESMYIFGGLHVADSSASAPEFCD
DLWRFSFRTLKWKRIKATGHLPHGRHNATLVAYEWPRGSMELLAPLDLETPDVALATAVAAAAASKSAMRSVSSAVDIPG
MAARVAREVPEPGPGAASFDRATPPSPVVSGAALGESDSPASPRTGSPASAADEPVSRTRSRAASGSSVSSAQGASVSGA
GVSSKFLYLFGGYGQRSALMYRFEVASCEWRIVLTKGPFWLFGGRDGKMIVNDVYCFDLATSTWSKRDVVGDVPTRRFGH
STLVQGGYMYVFGGVDKDGTCSDLFRFSFDVTKTGQHEWARLICRGHTPSGRYYHTAVMTSSQSMLVFGGYSDHAVDKKL
AFLNDAYAIWIDPIVVPPSSLAFDLTAMLLSGAWADVILRAGGRSFPAHRVVLGARSPVFSYLFELAEASSTAAVAKLQA
KLRAVVHQLARGVAEASVDDPMAAYRDAWAPPSARSLLSATSISSSSDALDDAMWEPRPPGLTKAKSSKAGANAAEVTLD
DDGGRGEWVSAESSAELGASAIGFGASSGSSVEFVSGTGTTGTDGGGGGGGWRRPLFDGERAGLGSLGTVPEHASLFDPV
FAAHVHTPAPLLSSSVGADAADEEEEALEAPIMEFLESVETASAAETESGKTEVLFTTMAPNAFAVMLQFMYLDTLQHGL
GLDVMVSLLEFADLCLLPRLKFMCMAELRQRIELANVTRILLAADARSAHALKAACLAFIAGHLDQVRSHASFAELIQNP
DLLFEVTHMVADRVTCSPTRQRFGSASMAGPSE*
>Ttra_AMSG_00480
MVCVFGGDNSRAAEDYMEAEPDHKPSSRYFHTASYVADARVIIVFGGFAAGANLNDVYAFDVETREWIDASVEDDPIAGR
PDSRSGHAATVVGGNAVVVFGGFSGTTRLADTWRLAVARTEGPGSDVRLVWKKLLDNGGGSGPPPTCNTDVAYDPESDVV
YLFSGHAGVASSATLYALDGASRDAPAWRRVATCGMEVERRTGHTLHWYNGAVYVFGGESSAQLCATLLRCDVAEGRWEV
VDTTPVNSRCFHAADVDSSTGTLLAMGGTMVDGLDQGLVRVHLPPLPHKLSLADDLDTAFAALAPEARNETPFGVPMALS
GGVTPASASGWPARCSLVGTILAEYIAVGASKTLHADDGRVSVIDAARVYSSALDAGLDAFANHVAVTCLLDPPGAWDEL
LASSELGSLGPTALAGLMRAHASRPPVFGQQALRIDRMRQQLEHAAETGDYDLVANDGTRIRVHTFVLCARSPMCAALIG
GAGLLMAEGASDDWQVRLGEAAEPLTATALQALVEFIYTGSVDLVLADATVAASLVQAHAYLGFRSEVLPSAVQASMDGI
GSGLSALVAAIDAAKRAGGPQIVDGLLAQIAAVVTGSADPRSLRPELERLDAEELVSLVYAVADMADGNTDE*
>Ttra_AMSG_00759
MAGEAAARSSRRSASVTAAAAAAAAAAAAAVAAAASSIGPASAEGAAGRGGGGGEGSIFDSGFGSFLDSGLYADVVVHD
AAADVVFRAHKLILCNASDQFAAALTAHPDEAHAFGPRRTGSASSRGMVAMHAAATARRRRRQRTLSRGRRASKGKASES
ESGSSSSSSDSTSASSGEASSASSSSSATPASLGHVRSPKGAMPPLWKRPRALLTPPTRTSSPVAVLDPASAAAVTAAHS
GDLEGIVLDSVVVVRVSAPDPQRLLGQLLSFMYTGKARVTPDNYIALMALADAYGVDELRNVLTTYVRRAIKADNAIDAL
STAISLGCESVASRAIDTIARNFHRLRKADYSSLPLHLAAKLFFHPRLSAKTEFAVYTAVNAYCAAMLADDPPRSLPASA
AFDRPRLGSSSSSTSNPPAASDRVAALFEAVRFTFMTVAQLEEVATNKLVPRELLLEAALARLRRVELGEDPMQVSSAAR
LQPRNAYGKHFEYQYDFDENGILFYIGTGGGAHEFRNPAIPVAGAEFQSVKVTASSIQKGTPLLVLARDGQAPFWTANVP
ASWFCIDFGDERRVRPTYYTLRHGGNYKADSLRNWELQGSVDGVSWEDLKRHRNDTALNGKFASASWPVDGAPRAYRYLR
ILQSGRNSSNHNFLALSGFDVYGDLITTARFNAL*
>Ttra_AMSG_01388
MADGSMPSIYELNMLALMTAAELADVTLLVDTDPFAEPDADGAALLDEFLAASKSEAEPATRIDGGNGGECDHAGGDNAI
DELEASPTETATATTTADVVEIDAHAALLAAHSPVLRAIIVDERRAGVTTKILVPISGLAEAAVRAALTYMYTGRVTEMD
AGWVVDFIEFGIRYQVPLLVELGELFVAEEVTVETAAVMWTAGLRFGNATVVERMAQFALDPSFSCAFLGHPSFASLPYQ
VVAALAADTALVVDELTVFSALAAWIAANLPGYPATLDTEAATQAAGLLEAVRYPLLSPDALADTVEPSGLVPAWLLAEA
YRFHATGRSPSSSDSGSTLRFTPRAEPPPASPLTASPRM*
>Ttra_AMSG_02031
MALCSGSLELVEAALVHPARPMPLALAGAWSGDDGSASGGADWPVRVASAGYGPAFLHASGSDSTQWALRGVAALRHAGS
IAQVAASSRMAAAVSDSGQLFVWGRGSRGELGLGKGVTSALTPTRALPAIGAVAKVALGAAHMLVLTEAGSVYAVGSNAQ
GQLGQPSDEVDVLLEPLPVTLPDGLAVADIAAGSAVSVILTTDGQVLAFGANDYGQIADRSAGVVASHVPTALMLGSHSA
AVAVVTSAFHIALLDDGGTLVIFGLNDRRRRLSLSRGAREALFRAETSSAGAADDADDFTLASLPALTFIELAVAPFATA
VITSSGSLAIFVHTGAFALLTAAAKKSKRRRQGASDDSATRHRAVTSSAQLTRNFAVGGSRHRTYVYPWPACFGAPLRSV
GLSATGTVAVVSESGLLFEWSLLSDKQPPLYGVGKLPRLLNSRLDLTSPIRVARIRHASAVAVSDSAVLALHTPLAPVSI
DSAEGEAQGALQADLASMAVDRPEFPDLNLDTADGVLPACSWLVALRAPALASKPRALATVPKATARQLLAFVYGAVLPE
SIEPEAMMALRTMATRLDLHSLRSTLISVYPNGITCNEDSPAIAAASRSEAGATLQRQLAAAWHRSPEGNTRLVLADGET
LSVDASVLAARSVPFRAMLTLPMMEGLRREVDMSFHQPDTVRAFVEYLWTGRVKSLASHKLELGEVLDLYAIAHEYMAQP
LLALCEAAAVWWLDLDNAIAVLNTIAPFVSPLDTAAPLGSYVLVDAALMVLVDSLEALAWSRKAFDALDQVYQARWEQAW
RLTVARPQLSARRDALLKIHEEAEADRKAALAAAAAREAAAAAEAAAAAADARDWDEYVFVSWDESGPERDAELVVSNIH
LELGELEREIAIVRTLDERARSGARISHSDLERTSNLRALRKRRKELRARIPSAPVAVGIQESSTASAWGSPSASSPPAA
STPPALDLRASLRASEAASIAAAVSPPRGWSAASPSSPHIDIRASLAAADTSQASSPVPIRGSRRKPKWGGLAASPPTTS
PSRSLAEIQAEQAAEKAGGGGFATSAWGMVKSSSPLSLAQILEDEEIQREVALVDAAVAAAEAAAAEASTATPSTGASRG
KGKGKGKGKGKGTRTRAWAKSRARARARAWARARK*
>Ttra_AMSG_02890
MAKHLREFSQQEALTTRLANILEEYPLSVGSTVAEWAQNADDAGASVFGLVVDGRTARNDLPLTPCGGEALPDMIEMARR
PALYVYNSAQFSDADLENIISLGNSMKRESGASIGKYGLGANVMYSFTDTPMFISRSSLVVFDPHGHRLPGGMLGTITDY
MACGLAESDPVLASAFDLELAGVFSASLSAHFDGTLFRLPLRTPEDAAASAISSHALSIDDVTNLLSDFAPQASDMLLFL
KSVSTVFAHVLTDDGLTTLITTEIVGLGPELAAKRALLLDVVAGERQALHASFELRFTESSDILASRPAPRWWVAVGTLD
TLDADAEALAKAVGYVPWAGTALPLHQLPPNWHGRAYTFLPLPITTGFPFHINGAFALSANRRQLWRGDAELSDAAATKG
RWNSFLLNTVLPSIVIRAFVDSQATQLALDPLAHLPVLASTPPPFDDFAHALYTAVTSLGPDAPALFPDPRSPGSFAPLH
TFTLMDPEFAATASSHMFTALIAADVALLALPTHVQDALDALDVPLPTTSPAMLASALRRVVAVDPALLTRDVAVAALNY
VLSGASEESALARLACLDGLAIVPLRGGGLGTLRVTNAPHALAAAALPLDEKRRESKAERAARRRAARKAEKAERKAKAK
AAKRRARGQELEARLECGLEPAPELKGSMPSFMPDTIVLPPHPQFLGLIPDDAACLDMDCRAAAVLAAPAILAVTNIVPL

GLEYLTRFALPRFLDDAFLGMGMNDALVLSGFDAGLRQPFGPRWRSLRRAEPHAAATGREILNLAWSLLASLQLSTDAIR
RACAGLPMVVTGQGVAMTLDAAVAHFVLAPDELDLAGESAALARDFGVLSLAAEWAAEAALAAQLLPAHWYDDGLLALAV
CVPRGACIDVVSDADAAALCLAARDTIANWANADASVLHPQLRPEGLDRLPLFESLDGSSMLASPRADPMRPLPLPRGEG
WAIARKSLAALAGLRIIKTDGSARTVLGLVGAGTADFTTFMRRVLLPRLRKLPRAVIEEHMSVELAAAIFDGLVPVMVLP
WQGDDAVMSMVLGLRGVPLLPSSSGWRTASEVHDPDSELLARIERAAAASTDGSIELVFPSHAFSTPGVLTVLRALGMTD
LTAPECMFAAARAVAELRDAELAREFVASVVQHARQWPVHQLRELGAIAFVPAYDLRGAEMVPTHLLPSLPFNAVTSLET
QACLGFQAQKYRVDDLVSAQVSRRQPSGAHTVRFDDALARAHEQLRMVTGSTSASEIPSAVVTFVAPNEASILAMDASVG
FRKALVLPAELDRAPLRMLSAWGVPHPPPASVVASNLTDCVAMWAENGVRMLASHNRLAARNAEAYVHGTVLLHLAYLAR
YLAAMTDMNWVAALAPVPSDKFGVIRKGSSGKRWLISQLRALPVVFVYSDLEHDLADDVRALPSSYLPVQGLLEELGVHN
LDHEPLILPRVTAEAPNTLLGPIVAAAFNDARLADVVVRSRSGREWFAHRLVLGTSSAYFASAFESGMCANEGGVIALTT
PHWASDSAYDAMMQHMYIGDGWLSALKETLEDVMTVTEVLRLSDVYMLPRLKQVCELWLASGEAVAILNLCTLYALADAT
NAVQLQTAIAYHMRQVLPALIKTDDWGELSDEQQSRIMH*
>Ttra_AMSG_04224
MSVPGAAVHDPFVWRIVSPAEPQTPVLRYGHTAIEFDGCMYVFGGFNPEVGTLADLWVFHFDSNVWTQLEPGGDLPAPRF
YHSAVRCGRAMYVFGGHSGHGGTYYDDVLKFDFDECEWVPVQVSGLVPAGRRSHTAVVWPPAGQTVGDGGVEPERAMYVF
GGFCGTHHSSQIHRLSLVTFEWSRVETVAETVPPVLQLSPAVSPRAVSAVESPVLRTRPAGQQPTRHRRRPSGADVMGAD
GAPQALKGHTAVVHEGLMYVFGGQTGALSASSILFQYQFATRTWRVLEFDGEPPELRSGHVAVTHYGDMYVFGGKHGERG
VVWRVALRPQANGRFEWRRIECRGIQPRARSFASAVVCNDRIVVFGGYANDSIKKRKHFRNDLLELALEPIVVPPPSLDS
DLRAFLNCPELADVTFRVGASRVAVPAHKLILCARSETFRLMFRNPMRESLESVVELPEVEPTVFAPLLEFMYTDHVQLR
GQFELAAQLIPLAEQYALPRLKALCIDTLRTAISVPNVVFLLMLADLYHSDGLKRLCLDFMVAHLPVVRQQPSFAELKHN
PELLFEVMQRFGVAARTRS*
>Ttra_AMSG_04895
MAAAPWLEHHGCALCGAAVARKGAKCADCLANVRFELGGRETYAPLQSENHAGTYAHHPGPSSLSASMPYPRAAVRATPV
PAWAARSAAGPPSSMSAAAAAAAAATDAADRRRVALATPPPLSMQYETHAWHSSPLQSSPPRPASPLLSPPRMPPGLGPD
PPTSPPTSPPTSPPASGPPPPTSPPVPPTSPPAPVLSPLGTNVASGQAPPGQGPSREEMFAAQLAQQVQAAYATRKKTVG
KRSFKSSQVRVVAPDQVMMLVKPPVPNSPLTRLQLYMRQMLMSPQYADVFFSVGSETVHGHRAVLANHSPVFRELFAKYP
EHRAVIPVTDTRPIVFRKALKFLYMEMTEITLELAMELVPLCQWLQLNYLLLICAMRLAQNISVANAAWVYEGATKFGLA
PLRRVALDFIIAEFDSVSLTRKFQDLDREPLVAIISAP*
>Ttra_AMSG_05038
MASQQVYNIPALKGRIVWVDEPYMSLAEGTGAVVFEAKAKADLNVALATHPAGVLPSQDGRGDGAPEFELVIGGWLNTKT
ALRVDGKVAAAKTLSEDPAAAIAHCGFDHYWLVVSQSFLAYGKGPRLGMAPLLVLPRPQHHPHGHRLFMGFTTWGWPVTI
RSLNVYPHYEPDLVLAAALARLSPSLSPSPDSLDLPLYANAPQNDARAGAVADLADLVVVWPDGAELKVHSLVLAALSPL
LADAIAESDGSGLTFLQRGEPRPASPPALVSHITRLIVADAAALPPQPLVELLSVAYGVLPPDRVSTAAHDVAAVLGIEQ
ARFAQPLASSHSVWRPSLLMWPQVWRTRWSACLADMPHSDVVLVLDSGDVLAAHRLVLAAWSEYFAVMWASGLREAQEAR
VVLPDVGAGEMTALLNYCYFGQFVVDGELAAGDASAPPLPAELAPGVLALADRFGMHKIKALVATALYRCLEVDNVLDAA
YLAEVLGCRELLRGCLVFLAKLLPELLQPESELADDPTELGTDLAFLTPDAMLALVSAARLGALAPHLGPALVALVVAWH
AERTAVARLDPDEREARSRSRSRSRSRSRSRSRPMSRQVSRPMSRSSSPTRERRRSLAAEPTGRSVSDSDDSGLRIMSMPRS
LSSASLASMVSLALSDLDDELASQVSRASSPTPLPIARRDDSGPETAFAALAAVLDTLAGASPPSDYLAFRKHVLSSLTS
ASGCASPKSRASDSAGASGESVPAQAAEYVADVGEVLASPAGEPDGELLLYSFDGDTNGVVYHYGTAFGTSRTFRSPHLN
GVLRVLSDSPESRYTQRKRLVDRKAVSNNSTTAWWMLDLGPGARLAANYYTVRHTHTGGNYLRSWILEATNDEAALGPAT
ARGDVLWTRLVVHNDDHSITYAGHSQTMRSGSSASG*
>Ttra_AMSG_05420
MDPHTLRNGRHHQPAHGTPPPRSSAGSDYPYPSSYSARYGVAQAESGSQARLASSYTLAHNPPLPHSASSASAHRAQPQS
PAVASWSALPGYVSPYATRTTPGLHIGGSYRPGEIQGPTAAGAGAGAGDSYRVRGTAPGSQQPLHPSPLKETMPKPSP
YASYLARNYGGGQSLTASQIGGEPMRDATADQAPSSPVAKAPQHTPFYTSHAGEWSSAPPAKPQQPRASDAMLRPTRNYG
ESNTAMPLSASALGGPALAVRDSDDSPAPASLDKIQAESRARAATASPGLVASPADVDHTRRERSPSPIRAEPTSPGSPG
VDASSVVDGDGRQSNLPPLFGASFLDADMYGHPEKVIPGVPKPAKKRGMLARLLKPKPKQLLAPDEKILVKAQPDHAEFP
DFTPLHRDMRELLLDPIYADVTFFVQGRPIRAHRAVLAARSAKFREMFAETGATEFELDMSLEAFTALLAFVYIDRTEIS
LLQAMELLTAVNLFELADLKAMCESVLLNNICVENAAWMLQGAVEHECALLHEASLSFIVAHFDAVSQTEAFQELERGPI
VHVIRARASRGK*
>Ttra_AMSG_06508
MESEVDVSVRVFRRGFGQVLRTGEHADAEVVVGRRRYKVHRLILAKGSEFFATSFASSFTEAQTQDNAMALLAMADLLLI
EPLRAKVLLHLAKAIDVDSVLAFLKQAILHAQSSILPLCLRTVSKNFHILADADLSFLPPDLFLDIMEDPWLAVKSEKTV
FDAIGAYISARDAAGVPVPQDAVVALYETIRYPFMPYELLVEAQANPLVPQALLVEGLMARLRRHEGAPRSGPASTAGTD
GTNGGADDADASDAGDAAASGDDGSVSDVLAGLPATADAKCRLRHTRRPPYSITLRYKADFDGGGVIHWIATDRGRGPWT
NPALPQLPGVRPRIQITVSSLEKGEVAAFVALDPVQTWTKDVPASWITWDLGHDYSVVPTHYSLRHGGNYKADSLRNWDL
QGSTDGVTWSVLRRHVNDSSLNGPFDTATWPIDDVSTPYRYFRILQSGHNSSRRNFLLLCGFELYGDLYIRNEAYA*
>Ttra_AMSG_09592
MASPGPSVATSSTASTATTAAGAAPVRYNDDGMAVYVANGEETTTAPGGGEEAGERATKRAKLNSGSSSKAVARSSGRTR
RSSKKAESAALQKKRSSGKGKKQVSAKGKRRGKEEVSDDGGGSGSGSEPAPMDVVGGSTRRRRGSGLVAPKLPPPSGSVV
LTSSMRRMVPSINEPPLMPSRKTALKWETSVPLVDRINFDELEVETIAGKTGRHGKRNGPAPEAEFFRCYGMVENAARTH
LFVCDNGNGLVRVLHDGEVSTFAGADKSSGVVELETPRNGETTPQPLAVRFNGPTGLCIDENDTMYLVECYGNRVRSVAA

DGTVKVVAGPTADDKGYKNGRGSVARFNGPRGICLASDGHLYIADCDNHCIRKVEIATGKVSTFAGKGRQSGYRDGAATH
ARFSWPAQVIHAREPGTGESVLYVSDNHNRCVRRISLATARVTTIAGSQSQQVRDGEGTNASFESPWGMLVDAHGDLYVM
EHNVNCIRKVMLSESLKNPKVVTISGSPLNKLVTLDGRGKTVSYKHPICMLDDGRGNMYISEYAGHCIRVMKGVLPVVGV
EPPPPSRLLDDLASLELDEATHDVAFCVSVPHHTEISAHRSVLMARCKYFKAMFSNEFSEAQASQDAARAAVAKRAAAAD
RDAAAGGTSAGGSLPRAVINFPEATASALYSVIHYLYTDVLDVHEDDVLDTMKLGEFLLLERVVALCRVYLTDALSVKTA
IPWLIWHSEYAMSDSVDDGDNRFIFPRARRTAFEFVVLNMIKRDEAATGTFAALQAYPKLLAELLTSAFAEDLEEAANKP
RTVIASFGTTAGALAALISFLQSGELPLDTDTACEVLGLANRFSISSAIDRASRYMIDTLTPATAIERLTWVSQQLQSPK
PRSRKPAYMFPNLRKTCLDFVAKNFTEISVKYFAQVQEFVQGHASLMVEVLDRQRKSATPVKSKSKSSKSNA*
>Ttra_AMSG_09812
MVKLGTARARRDEADGAVDVLVSHLAAGLASAAFADMELSSRLGDIGDVLTPLFVPSIAPIRPTATIATTATTASTGTTA
AATNAAVAAVSGDVNQESAAAAARAVMLARAIPVHAVILAMRSPVVAQALKGRRLRMRITGVPPDVLRAVVEYVYTGECE
LENEGYSELQRLGQLAHAAQLLGMDHLLGECLRDLAVAQLTVADAICLHGLIVTLPAKLVDAISFHWKSFMRRHYEDFIR
MAANGEIHTGYEPERADMHVRIRTGDVIALLSALPQDGGTAAEQVVRTACHLYIETNAYAVLMSPHFLRSSVDSLRVVLT
SRTLQVKEKLLFQAVLQWILIDPASREAECTELMTHINLDKVPSRLLLEAKRQGFEFPDKAYKAALVKHHRSRHVRDVAY
RYNPRLTAINMHSTKRPGKDLRALRGGVRSHRNARDVISASSSSSSLEELARFQQTVLNSLKPSEARANRATTAGAGDDG
ESSSAAPLSLSRSNSFDNTSIASSTASQQPSPNHPLLRGAGSSLSRAWRQNSYVSLVKRVRKQNHVRARGTRPSGLGGGG
GGGSGETTVRHTPQPPPGRSLGRSVSARRVRTRAPPS*
>Ttra_AMSG_10025
MQAIKCVVVGDGAVGKSCMLIAYTTNAFPGEYVPTVFDNYSANVMVDGKPISLGLWDTAGQEDYDRLRPLSYPQTDVFML
CFSVVSRASFQNVSSKWMPEILHHCPNTPIILVGTKADLRAGDVDAERLVSRDEAQALANGIGAVSYAETSALVGTGLKA
AFDTAIMAVINSAGSRRKSRFSLFKSSGSGAGSSSSAVPDYDYEYEYDSDDGVEDHNAGIPAPPALPEVVDLHAPWINIE
TATFGADLHTLYNNSLHADLRFVVPSLGSAPETSFFAHSPIVLAASTLLARLLLPALSANEADEVARIALPRGVSWIARV
DNVLYRVEAPDGSASSSSAALLDLGVDARLTELEYVDEDIDDELLCSICTLPFLEPVAVPGCEHIFCATCLKDWLFQHAN
CPACRGTVAASAVAPASRIIRNLVDKLEAHCPEDGCEHVAARSTLVAHLDAEHGSPEEVSSQSAGEASSSLAAPPNNELG
WSLVVVLDAAEVTADGFGALLEFLYSGATDLGARGSERLGLVRAAAELFGAEHLLTSCNNVAGSLDELNPSITTYKNDLV
GERLKELFLGRPLRADAWLEVGKARVPVHRALLAARCAWLHSLVRELPCGGSVVVEDARLGAADLGACLEFVYTAHCESV
SECTVETVLHGESHVFSIAAAAAHCNLFRLLQFTELYGSKILERATTASVGQSGIDVVALLHASQAQGASQLAAFLLHFL
ASNVVPLAPHITPGALGPDNLAWVRSHQWPPADYLDELDAWEGQVAEAKETGAVPDDWQWRRRERVRMRIANDGTRRPVR
SGKKNCIVM*
>Ddis_XP_637561
MQAIKLVVVGDGAVGKSCLLIAYTTNAFPGEYVPTVFDNYSANVMIDGKPFNLGLWDTAGQEDYDRLRPLSYPQTDVFLI
CFSIVSRASFENIRAKWYPEILHHAPNIPIVLVGTKNDLRGHHDLKRPEVSAAEANNLVRELGFSGYVETSALLQTNLRE
LFSLAIRTATSPKSASAKKKGGFFSSSSSSSSSSSSKSSEKSVPIPPVMPPAGKAPWIDIITSSYDKEMRETLNSESFSD
VKFTFFEERPVYAHRVILCSASEFFRRVFGYTLSSDQAEFKLDDLNEGKVKNIRSVTIKPNEKNDDGTKSSGEFVEISID
SSINRRVFNRFIEFLYTGVLNYLDKNDQLQETIALAESVGFKYLASACKNVLGGNEDLNPSIGTFLNDQLGETSKELFFT
KKILSDIQFIVEGRSIFAHKALIFSRSNVVNALIGRNFIEREGKVEMSDDVTFDSFMAILEYLYTAHAPIEEGDSVGILV
LGNRFDLRRLVSLCELYISKEIERATTIGIFRSELDIIGLLICAQRHNAPQLEKFCLHFISSNYQPMRRRKEFALLDDKT
KKHIEDNQWPPIHYLRAVEDYEKEMEKLKSSSKGWFKK
>Ddis_XP_640165
MSLFYTKDAISDFEKILNGLNTFDSFPPLRILSLATYCRKEEVKDFFSFIDSNGDTCLTFIIQSTVENNVTQQYRSIVDK
FKLLEENIELLNLPNSYGETPLHVSSISNNIEFMKLLVLQGVDGSLKDNRNMTALDIAVMMDHKTIIKELKKLKTSMMTM
EQTSIYQWGQILTSSLTLKSCAKATNMLFNDEISAPIQTEFGQNYNLILTDTGAVYSSGLCSYGKTGHKKKRDVLIPTQI
LELSNKRIKSIACGTHHSLAMDDLGQVYSWGNGASGRLGLGNDFSIVPTPTLISVINRFGSNEIATAIAAGCDNSMILGS
NGDVYSFGSNLHYKLGYKECDGALGFQSTPKKIPSILQMKSIACASWHSVFLDLKGRVYTCGSSTNGRLGRAISSYSSFY
PERVSITNSKTNGTLPQEEVIKQIACGTNFTTVLSESGSLYSWGVNTKSQLGLINLVGISEYYSQPKLVTALNPYTIHSF
AVGDQHVVALSDQGEVFCYGDNSTLQCGFGEVLNFEINLTKVKIETTSSDAPIIGGGGVFKVSAGGSNTMISLSSDHNIF
GEELLKSLNLQMFTDCRLIINDNKEQFIQAHQVVLAARSNKFNKLLELQHNSTLVGVDQQQQQQQQCKIPLTQTTFLKSF
IESIGTFDIDGNENRVLYISFPSISSISILSLLLKYIYSDHTILSQSTNQELGELAHSLEIERLAYLCNFSHGKSLDKLP
TSTLLEDFRILKDPKFSSIYSDIKFKCINNGGGGGDGNMVDEAYLEKNKQQIETVESYKLFAYTGSSYFKTMLSGSFIES
NKKEIELFETSASSLRLLLNYCYSEEIPTDSNDCIDCIIICDVHQISRAKDSCAAKIRSQIDIQYICYIYHISLIYNVKL
LKTWCQANFNSIADIKALPYFNQLPIEIQSELNSIKKKY
>Ddis_XP_643384
MDEIINVKDQIICNSNDLKNYWNKAYACGRVPAPRHAHSSVVIGDSLFMYGGYGVKGTHLDDFFRFDLKLNQWFRILKNE
KKKFQSSSSSSSSSSSSSSSSSSSSSSSSLYSSSIDDKKNIITARHSYSMVAYGECIYIFGGIGIGLNESEPNVFNDLYE
YNTCTDEWKLISPPRESTFVPEKRWGHSAVVYNDNMVIFGGMGQSITFYPTILFYNFKSKLWSRVTVKNTIPSQVPRGRQ
YHTCSIINDKLYVLGGYDGAVCPTDMFEFDLITNLWSIVPCNNNNGNHVIVRCGTSAVFDNKIYLFGGRNKVVSNSLHEF
NPSTLTWREIKLVDRPSPRQYSTCFIYSNSFYIFGGQSDYNENDLFYYQLTQNNNNINNNSIFNQLFENRTSFSDIKFLV
ENRIINAHKCILVSRNEGFRALITNGMIESTLDIIKINNCSYNCFLAIIQFLYTGNLIFDDLNILELLLISDQYMINEIR
NECSRYISNLVLNEPISDIYHQIINQSLSEKNLL
>Ddis_XP_642619
MSVLSVGQSKNGQLGHDSIKECLEPTVIEGLLGKRIIQTVSGEAHSLAVSRFGDVYSWGRSKEGQLGIGQGYGGSDKVMF
VAKPTLIKSLQHERIIKVACGNFHSLALTDMGKVYEWGQLHRLDESQSSLDIQTSNGLIEMPRLSSQRIIEASVSQYLSG

EKKAYDANGGTKPKDDDEDKEASNSAQSKSKHLGKIIDLNQTIPMQIQDIPEEVVDVSGGWAFSAAVTKSGRLYTWGFNE
KGQLGLGNRWFHSTPQLVKTLIDVNIVSVVCGRQHICAITDQGEVYSWGLGVFGQLGHGNVKSYLHPKKIQQFVELNERI
AQVACGSNFTMVRSVQGLLYAFGHGEYGQLGSTEETQHLDFGGRDNHFKYSIPIVVKSLETKKIKNVACGHLHTIVVTDE
NEVYQWGWGSSGALGLGNKRFQLVPQLITSLSGEEIASITAGEKHTIVVRCSDVTSFAYDYKSLINEKKYADLIFKVQDK
KIYSHSLFIKSRCQKLYSIILFSSRFINQYSNNKQVVVAKEEKEEEKEEEKEFNENGEEIKIIDLGNSVKYQIFIGFLQY
LYTDHLVIAPHLRIELGKYAEKMGIERLSAICKRYTYRLRLTEKIPPSEFSKQLIQAVDELTKADISFKIKESDTSSIYA
HKFILTQRNLYFKTMFECSFKEKDQMEFIVSGKDENDISSKETFKLLLQYIYGNNEEIINDDNAIDILCLADRFMIEDLK
YLCEYHLEQMVLSNNEILLKNVPKTTNNIDGNQQKLKKEDILNFEETLTSFDNICILMQVADGFMVKRLKRICMDTISQI
DFDLFKILNTKNETLKKIRINSPLLIRELDNYARNFGKFQQNSLIELIR
>Ddis_XP_641620
MLPAKRKEYNYPTECSEAKRTMRVSNNSTQNNIVDDDMSSPISSPNNGTMSYMEIEPTGEQEYIVFCIDLDEEISTVFKQ
SCPRLDHVKKALSLFIRSKLRTDPTTKFALCTLRVSSIWTLDFTNDYLDVKGKLDKLTHGGKFTAFDMTTLFDMIEIKYN
EIVESKKTQLINFPFSSEVKRDWSLRVVFIYSRSNTIPQYLGGENSMESLFKESNFYFEPVYMHHMNNYHPDHTKIVESM
TKSKNKLIYKVSDQSSIRKFYYCFLGLLFVPPSQRPPAPKYQRFPVGLINENIHRLGLWIHQKYHSNTFPYSMNGKIFGG
PMHGAGGQIQNMFQNPAQYLNINQLLQQHPHFAPPAHFFQIQHHHQQILQQAQQQAQQQAQHQQQQLQLQHQHQHQQQQQ
HQQIHAQHQLAQQQAQQAQQIIHHLQQQAQQQAQQAQQAQIHIQQAQQQVQQAQQLVVQAQQNINNNNNNNNNVNNNNNN
NNNNNNLNNNINNNNNNNVNNNNNNNNNNNNNNNNNNNNNINNNANVNNNVNNNNNVNNNTNNNTQVQNTQTNQVAQNIDDVQ
PVAPEAIPPTPIPTPVPATVSIPLNNNNNNNNNNNNNNNNNNNNNNNNNNNNNNNNNNNNNTNNNNQPNNNQQNNDNPQPNNTPN
NTNIQPNNTNIQPNNPQVNNQPQNEAPHNNINNNNNNNNNNNINNNNNNNNNLNNNLNNNNNNLNNNNNNNNNNNNNNNNN
NNNNNNINNINNNNNINNINNNNINNNNNNNNNNNNNNNNNNNNNNNNNINNNNNNNNNINNNNNINNNNNNNNNNNNNNNHNN
NNNNNNNNNLQINNNPQQQHHHNHNNHPHVTYFPQFLHIFPNAVTPHLIYHPAIAENGFDPLTVPFQFAGYPILGTQQTA
YLQHMAGVQQPNLQNPSNLNAMAAKHHSLKSSTSNSSDSQTPYFPSIGLDMKHYLNNEQFSDVIFVIGKCEDSPGEKIFG
HRVILSARSKYFKALFTNGMRESGSRGGEIVVPGVSREVFLALLCYLYTDDLPSNSISTSTDGNSCNPSTWLTELLILAN
QYMLDGLKVKCCELIGETLSYSNLVSLMTLASIHEATELKKACIYYFYTNLPLFLRDCCFEPDPTNDGPEQLGYLIVDYF
TKVAKLGLTEKQPVNQTTTTTTSTNDNENSNNNNNNNNNNNNNNNNNNNNNNNNNNNNNNNNNNNNNNNTNNNNNNNNNTNNN
NNNNNNNNNNGNNNENEVNSASDPTLAQKEFDELVSLF
>Ddis_XP_641246
MSNSASSSPSSSGYMNNSNSNSNSNSNSNNNNNANSINLINNSNNINNNNNNNSNITIGFSIPNNNNNNSSSNSNINNINNN
SNNNNNNNNNNNNNNNNNNNNNNNNNNNNNNNNNNNNNNNNINNINNNNTNLNNINNSTNVNITGSTLNNSNNGNNSNSNNNN
NNNNNNNSNNNNGGVNNQQTSTSKIIEAARKGNTELINKLLKKDWKKINKRDEKGDGCLHKAAFFGHADSIEAILKYASN
NGLLANNQVNVNMKNHEGYTPLMYAVQNNWKDCLKPLISYGADINEKLPSNPGLPLTYSISNGTGIYNWKIENFSKIKDR
KIQSNTFLVSGFSWKLVAYPRGSKDDDNLSLYLEVANYESLSEGWSHMANFTFTITNQFDQSKKIIREVLAHRFHRNHTD
LGFSQILKKEMLKDKKSGWLLNDCLLVEFKIEVLHNSSYQNDETSIYTWKINNVSAMKERATSPIFKVGNCRWTIALYPK
GKNGGNNLSVYLKVADKSILPPDWFFLVSFKFSLIDQKNGTKFTRQVEGKRFKENVEDWGFPQFMKLSSLYDSNGSGFLK
VVDDSIIIELQMEIVNDFTKKKHRETNSDWGLSPLHLSSYRGFYDTVRLLLMLGANPNITDSRGRTPIDWCAYNGDLPTA
EVLITIGCADINCKDYEGYTPLHKAVMNGHLEVARLLVKHGALVNAKTNSKVTPLELAIRVNRMWCVETLIQWGADLNLA
DKQGRSPLHWAVFLGDPKLLSLLVKYGCSDKNVDKKPCPIISFNNRTKRNSNELLNANNNNNSNNNSNSNSNNNNNNNNNN
NNNNNNNNNNNNNNNNNNNNNNNNNNNNNNNLEYVSSENNNDYTGNDHIEQNNSQSTTTTPSSSSLPIITTTSSSSSAVLS
SSPSSSLNSNNNNSNKKSITPLKINIVDQDGFTPLHKAIIRGKGSFVKLLLEKGADPTIFTKDNRNALHLSVSANEYYIT
QLILDYCDKTTELFSLDNQHQTPLHKSVVNGNLKMQELLVSRGADVNDGYFNSTPAIHEAVKKNDILCIIYLLQNNAEIH
KSPKLLMNLLSKKIRALKNINSNNNNNNNNTNNNNSSNNSNSNNNSNDNQIPMCSLGRDMSYLVNNPLYKDITFIVEDKP
IYAWRGILCARSDYFRAMFETPLKESLESEIKIESVPHSTFLLVMQYLYTGEIESLSLNNLDIENNNSNYSNNSSDANNF
DTNNNNNNNINNNNSNNNDSTLTNSNGIENDGDNNNININSNNQFSNNDNDNINNNNNNNNNNNNNNNNNNNNNNNNNNNI
NIEEYNRNNYNNNNNNNNNNIQSNGLGFEDYIQLFIAANRFMITRLKMICETLIIKKYSIDNIYSIFKLTEIYESSFLLDE
AVRFFSENFQIIDEANFILKMPSFRKSLLDIFKRSLMSNNKNACNISAFNFESDIVTSNNSSFIKNSTSNINNHSNFDNN
SDGDNDNNNDNNTNNNNNNSENNNNNINNNDENDNQNHDD
>Ddis_XP_640746
MEKTKINSNELKVLINNFGTVPLFKIDENENQDVTLKKLNSSRPVGYHQFCFDSKLNKYKSIDKGDLIENQSEELACNPY
LYSKNIKFVHERDMEMCYICRDRDAPSVCLWSWDFGKKLEISKLILNMLFMAFEENSGIDWYLSTEISNDMVLALRNKNH
EKVMKTIQDEFFKLPLELVSPVYFDKKLNLTELVGGSKRFYLSCIMSNSGPYKTQLFRSKFLTKFTQEDDDYLHLHTPPK
FNYPFNIYFEVSLSSNNSTTTTTSTTTKKENQQENLDINGKQNNNNYYFVEIDENIDTVQDNSTFKTKNWDSGRVGDWLF
DMGFYDLIERFSFQNVNGETLLTRGLSILPNSFLQNSTKRNKYIEAINRLLSLNKMDSILSPQQQQQQQQQQQQQQQQQQ
QQQQQQQQQQLQLQQQPIEQPIASADDQQMLVPTANASPLYSNQYSEKSILFLNYLDETKKKQLNNFKKLPDESSKNQIN
LDDNNESKLILNDLNSSNYKLIIKNNVIHVHKSILSQNSDYFNALFTSSFFENLNSTCTFESDDEKLTIESLELLMEFIY
LQTQEEALNFLKKQDFKQLKVLLHLSDRFLFKQKELIEKFIVWSIKSVNLKIIDQEFNNNNLISLFTKYYLNN
>Ddis_XP_628970
MDIEQKQFFTEISDLFKSNLLIKAIRLINKKLTTITDNNEFKYKLILILGKLCLKGRNVDTIIPMIENELKTNEIKGSIK
FNLMSLLYRCYYLKSSSGSKSVTNNYQYKSKTTTNHPETMQIMNQSDNISDMVLSRNRSVSVQFENETLEQRESKRIKFT
TTNTDDESCDDQKGVNGDEKNSVYNFYDDLNFQYYLFSSDLFVRDRIFKFKTPIKSISCGRYFSSILDSNGMVWNWGLFF
EVCSNGQASPTHPYPMKKPIKKIASGHHHCIALATDGSVYGWGANTFNSIGIVTSNNGNIKTPTPIEFINYNGEINNNNN
SNNYYINDYTRNNNENDSEILKISAGGFNSAFITKNGNLFVCGRNDRFQLGIEETTASPPPLFTIEPDKKYLPSILVASS
SITRDYSYLINNKQFSDLHITFGKDKNTEEIAYCHLILLTILENDELLNQLIQLKLKSINKIVKIDIIEFLNLVIKTKNY

NGKGDDDDDFNKESNKKIILDQLIEENQLFSNSFIDFISFIYTNTTSKRNMKILFQYSKLFNCKQLTNYLNETSFNIIQE
ESDLSIPSKLLKRFNNDDDNLSDCTIISNDGKIKIKAHKSILISRLNYFKNLLLVDQIDNSNDSNDSNEIKLNDKTIPDN
HLILIIAYLYCDRIKFDLLPQEIVTGDFVLSLCSTSDQLKLTRFKDGLLKLISNSITKENAISLLLWSKNNSYSLERFIK
CFISLHVEFINSEHYDPTFLPFPLNQSPANQQTPTHVAKHEKFVDVAVGAKLMLALTVDNKLYQLGIGSDRSRKPIQQIT
LPFPDENTIIKKISCGSSHWIAVTENNKIYGMGCADLNQLVEGSRKFCYNPTLISDSNKYNNAKAGFYASMYWNDLSNFP
LHPNGLNNNNNDNNNNNSLKVDKPITIEVYNSSSSSLWFFKNYDFIDSLLKNKLSGSDDEKQQEQENSNDEFTKSIISTT
SACLPPYLYELISNKENGNDNTIQLFGFSKELIEKSFLKTTKDLNINSGDEILDLLFLTTSFGNYKLTNELFGILKKSID
LKQACILFTLIHKSKYSLQLAFLFNYLLGYIGKNRLKILQDTELFNSIPISLLDLISSK
>Ddis_XP_628948
MSNKTLPYCCEHGLKDEVIALLAKGEDVNQKDGSNRYPLHYAAIGGYIEIVAMLLERGALVNCSTPRGATPLHYASRGGR
IECIQLLIDNKADVNCRDGAGSTPLHTALQCNETKCAIALIETFGADVNLKTAEGSTALHLASARGLVPVIVALLENGAR
ADVRDSKENSPFQSLPINLTENEKKEKIENSISQDISKLFNINKNREFSGVGKKEDDDNNMKIDKQESEQQSTSDKLYSD
ITFLIENQKVYAHKCILQARCPNFMSSINNKIEQQQSQKPIEIKDYSFKLFLSFIEWIYTGSIEKFESTSKSIDLSFQFS
ILLMGEKFDCKGLVSYCREFIKESIGDSNVGSIWSIIKKLPTSTRSNLGNLVRDCLITMVRSWNIFTMTKTFSTDINKSD
IIEIIKSLAPFITETPISEIKQTRTANANASNSNQSKTPAKRTSTTNTNNNIPQQNITSSNNTPQQNTSSSSSSSTTSST
PSKSSSSTPSKSTSTSSSSSSSSSSSSSSSSSNYSDSMNEKNLTFCKGLINGMFKKKTSLAFQRPVDPLAEGIPDYFDVIK
HPMDLGTIKGKLDNNGYSTIKDFAADVRLMFENALTYNADSSPVWKHAKTLLNAFDQKFLQNFPNEKPPTYKPPPPTPTP
IPTQQQQQQSTSSTSTPTSEKKRKHDEHVKVKEDTNSAQPTSSSSNHTNGENASSSSSSSSSSKQSNNNNNNNNNNNSNST
TNSSSSSSSTTTTQKKYSDEERRSLMERINELAPDDVQEVLNIIDPNAIKQADESLEIDMYQIDDKNLSQVESFINECFK
KQKQDE
>Ddis_XP_001733000
MEFFSNLYGKYLSKQTNVNNHYSNNNSISSNIDFESMNLGSINRIIGFEFDEDFFKKIDSNGDTALTFFISEIIKFQKSQ
SFNTDLKLFNNSKYRYLLDIQNDFGETPLHVAGFVKANANAGIKDYRNLTPLDMAIMLEYNDIIKILETSETSKQSNVQS
NIYNWGEILTSDFLLKSECKPIKILFNDELCSPISIEYGQNFSLILTDTGAVYSSGLCSYGKTGHKMKRDISIPTQIKSL
ENKRIKSISCGSHHSLALDDLGLVYSWGNGSNGRLGLELHILGEKKIVSTPTLISTIASNTLDPVVVQISAGFDNSFLIT
SNGSVYSFGSAISEKLGYDGTVLGYQALPRKIESIPPMKQVSASNLHTVLLDTCGNVYAFGSPTNGRLGRPSNGPRNDYK
PTMVSFGSGGGDGTVIKKIATGTNFTIVLSDSGELFGWGGNSNGQLGVEPICNVSDSFDSPTLIKCLSNLNCGKITIHDF
AVGDQHVVILSDVGEVFCFGEDRKAQCGIGISYPGYNDISLTQIQSNNFNGRNIFKVYAGANNTMVSLSPDHHIFGEELL
KLLNSNLFTDIKLKVTESDQIINAHKIVLAARCKKFHSLISLQLNNPTKPQSIKYNDYDNNSTTKYITSEIIDGILYITF
PISFKVLNLFLKYLYSDHVNLTQSITDELGELSHCMGVGRLSYLCNFKNNLSLQKLPISTLLEDLKKIQNQEYIDIYSNV
QFNCVSNADPLENGVIKSYKLFSNLGSFYFKKMLGGSYIESEQNEIHLNQTSIHGLRSLLKYCYCEEIPEDINECIELII
LSDIHSMHRAKDITSAKIRSLMDNDSICYIYHISKLYNVKPLSIWCLAKLNLISTTQENLKKLPYFDQLPNELKIEILNS
LKK
>Ddis_XP_644898
MMAETAEDSDPISEAVGFISNILSSDTELQYKGLKGLMICPTSLVVELFKNPAYFYHIKKHPNHKFNNHNNHNDNNNNNN
NNNNPSPTSSTTSSNGSSPNNGNSPNHGSSSPNNNNNNNNNNNNNNNNVSSNTSSSSSSSSPHSSSSPTNCPSTLPHGVDP
CNQCSPNGSPKYSDIDFDKITKKRKSNIKSLIELCLSENRKVQSSALRLLWHLSAQADLKVLLFEEGVLDKLKSLMFSDF
EKYNINNNNNNNSKYPNNNNNNNNNNNNNNNNNNNNNNNNNNNNNSGSTSPTDIANERQINVHRTDVQLASSAILQNITEFRF
EKGEINPNQVKIVNEGIVDLILIPRSKSTDKRVQFLTTLTIANLSMNDENHTALTKSKAFDVVEAFVTSNSLHMDLVCHW
ITLQPHIPLLHSKYHQVQLFALYCLYNLIRNGMNYLKQYKVEVWKGLSVNNGVQSIFILLHSMHPKVVELAKKIADQLQI
EEPSVTVNTTKIGTDLKKLFNNQEYSDVIFTCEGKKLYAHKAICASRCEQLRAMFTWGKESKEQDINLPHTPYCAMYGVL
EYIYCGQATITWENACDLLQWADFFSLAGLKSSCEFYLWHYIDLENAPIILTVADRYRCTQLRNVAANFVLRNWDKIKDF
DVWINQVTVDVKNYISERIYSLITCSCGCICSCVDDAKNNNANSPSQIVDEN
>Ddis_XP_645290
MFLETKNNNNNNLNNKLIRNKIKKDKMVSQGKNKLIQKYSSDFSYYFNNQELSDIIIQVSENGYLDEDVLTMKPIPINIK
NDKKPTFEEIIELINQRNNCLNNSNKIKNDETNRIDYFYTHKFVLSARSPVFMKMLSCQMMESNSVIITSHFPRSIFYIL
LKFLYSGKVEMNATNVLEVLAIADYYQLEDLKDFCGKYSLQTCIDGELDICRVLYASEQYGLEKIKQLCLDYISQHSMDI
LLSSPNWHLLSEENLIEILEQDNLRVPEVEIFDSLVRWAEKQYSILLDLDPSLGDSINKPDFLKKKLSKPLKSIRFASMF
PYQLSSHIEKTNLVDKEILYEAYRYCAAQIPTSINSSSCELRQGVKEFTFQFNGDTNGVLYYFGTCEDMEDYESPVTRKV
VTVTSSTMSIGYPHPFVGRQSTNMYTDKEENSFFCVDLGVKHVLCPTYYSLRYGGDSQGSIYAAPKNWQLLGSLDGQEWT
LLRDHSNDLSLKDGYGIAGWDVKSIHSYRYLKILQTGPNQRDGNELCLCCFEVYGRLVSLEDDNNNTTCSSLTDGESDDD
EMKNTTSHIDDDDSDNEDVNDNNNGDDSDETNNENDDDMDDN
>Ddis_XP_644257
MARTNNENNNNNNEESESGSSTPPNIELDQAPIVNWKIIATANTPTPRFDNTLVYYNKFLYSYGGSENSINGGFQQFVKL
DLSTFQWQTLPAPARTRIVHTSVIHGDCMYVFGGGVYEDVRTSFLGGTTKVFKTINELWKFEFLQNQWTQILSNAIQQTT
VPSQSMINESLNNPEPRDGHCAVVVGDNMYIIGGSQTISTNNSISSVFFTNTKIYNITTNTWRSVISPEFGRLGSAVVYK
HLDGTKCIYFFGGYGLDSNPTNKLYKFDIESEEWVEVIPKKSTSPSTSTSTSTVDDIVIIPESRYGHSANVFQDKMLVYG
GYSKKKGFLRDFYSFDFDKEEWKQILTEGPPRRRRHSTVLIENFDGSNKPKLFLYGGSYQSCLYNDLWEYSFPNPVQIPS
DNLIEDFSTLFKNAKQYFSDICFSLESTELVGDENDNNNNSNNNSKPTIVTKEIYAHKNILSVRSQYFYSLFNSGLKESF
EKVIKINERYEDFKIFIEFLYSGNEDLVLLENCIGILYLSDLYCVPRLKTICEAKATEGIDCETVVQIFKEADSFKLAKL
RKLCLNFIARNHKELVKQGSLNQLSPSVLIEIMDYLSSSSTSTSTSTSTSTTN
>Ddis_XP_646157

MEVIKNDLEYKVKINNKDIVPLYMLEIDIDNLTAKEIEFKRPISYHEFSYDTKSNQYKSIKNKKKEKEEKEKEEHDEKAI
IIIKNQYLYSSNLKYKYETGWEMCYIGRDNHSRGIAIWSWEFDPRLEITNLFINMLFMTFDPNSIINWYISTKSNEAILK
ESFKRMDSHEVYSIITDQLNFTLLPLNLPTTPIHFDKKLDLTDYLKLNNKNNNNNNVTRFFLACVMENNKNDRTQLFRCK
SSNYFKKFGTSNTNPNQHPDPIYDYPFTIHFNLSQKSTITNQLIGDEEEEGEEEEEEEQDNIYNNNNNNNNNHSEDCRQN
NNNKFNQLNNFYYFNEEDESIELIRKIKLSKIHKWDNERVLQWLFDIQVYSLIRLFKELRIDGIKLLTFQIELLPISFTE
NNIEKLKFIKELSKLLLISGQRNEILLKSIEEEEEEQQQQQRFTKFQHQYNQSKSTILSLKSNLIQNKKLIRKIKIRNKN
KDNNNKEEEIKLLNDENTSNCQLIIQGRIFKAHKEILIKSSEYFKTILTCESFEESTNGVINFECDIDLTSESLRIILEL
IYMQTNDIQLEFLKNLGLNQLKSTLILSDRFLLVDSVCKLSQLIENFIIWKINSENIKLINNLFDFNQKIFFFTNFILQY
NK
>Ddis_XP_637721
MEQKMDFLNVKPIELELQGLSIATVKNNEQWDLDGFNQYKFVDESEYEDQPSFQKNLSHDLTFLLPTLDQLQDGADSSGH
VDQSHLRGRIYAHKEKVALKSEYFSLLLTNGMKESKQQEISIPNISVDIFFEILMFLYTSELSVNQENLTSMFLVCDQFL
LEDGKQICRDFIKTLDNLILERFLLEDNGLCSELTDLYIEQVCSNSSFFLNIERVSKLGKDVLIKVLNRDDIPMKEIDIF
RSTIEWVSCNTVLLSSSSTSTTTTTTTNNSNSILIEQQDKNLVSKVFHLIRFPSIDYEDMVSVVEPYSHLIPSNLLLESY
RFLSKPKLIPMNENDLLNPRLTPRKKPNQTTLVPGCDVISFQCPDHKKCYSVSWPIHNFSAIKSQKHVSNCFEMYGLTWK
MWAYPAGEAKHSDSFSVYLEAVRVKEKESYEFLRNTTFFFGLVNQKNKTLCRHYPSSPNVLFNYEKSVWGNGLIELKLLY
DKSLGYMDNDCVTIQLHILECIALDG
>Ddis_XP_636804
MLLTMENQQETNNIINNNNNNNNNNNNNNNNNNNNTNNNSNSIKSDGINNIDNIINNMGSINGEHTKSDIIIKHDTGGGG
SGSGGGGGDEESSSNFFTHNLLKDDSAITTNKNDINSSSNTNTLDNSGNSTTNNSDNSNDEPLENCIQEINLSQQPQPQP
QQQQQIQQDNNNTTNNTNNKKDKHSTSMPNCTSPILEAVNNIEDNNENDGSFKIRSTSFQDSLFTCSRIMNKEDDEENNN
NNNNEKELSCKNNNRELIENNEEIIENTNNNSNNNNNENNENKEILIENNSNKINGNFKGINESGESIPIVMITSSSSPD
GIPPPSLASSSSSSQSHPQSPRSVSVPLIRTRVNRQQQSTRQRPPGAFERPLLHAVLLQEEIKNLISYDDVNSRDFDGNS
ILHRVSIIGNSVAIKHIILRGGRVNAVNNLGLTPLHYAACANVPSVEILLYYGSQANAKDNNCETPLHYAADRGQLSIVS
ILLSRGCKASLANKSMGRNPLHLASIKGYSQILRSLLEYKININSVDKSGCTPLHLSVFFSPIINNNNNSSNSIGGGNNG
PSPLYSTQEINSCVDQYLTCCEILLQRGCNIYSQDVDGSTALHLASQHGKKNFIEVILFYANKLKSPSLSSISNGKSISK
SDCLKSELVLYIPDNKGRLPLHVAAISGHTKSLPILVGTNRKAAFIRDEMGMTALCLASLFGHFDCVQVLSREKRPYNFK
FKYFDNDNDNNSNSNNNNNNNSNNEVFDIKTPFDPLGNDFKLMLDGQYCDCVFIVEGREIYAHTVLLRQFKVFREMINLF
KLPYGHSNSYINNNSSSNNNNNNTYSTSSNGASSYCYTSVIQVPPTSSSNVLSIQQQQTTPQPQQHYQQYLSSFPANSLV
SDYYNNENITTSSSFNDDDCSEQSSQIGIGGGVRNIDKSGGGAGGLLTAVKSRSRVGSVMNSHTLTRIPIEKSVSYDMLK
AVLHFTYTSEIPRELLSNPNKFSEFIDLCTIYNIDAISEVNQNRPRYGTLDMASYLYRQVGSSIAADIIFICESGYAINA
HSVVLAARCQYFKNHFEIHHQTPSKSTIPIFPTTTSTTTATTSTSTTTTTAVANTTTTTTTTTTTTTTMNGNSSLQSNGG
NVNGGVGGGGSGGGGGFSLFNVENTPRKLSCASSPGFGSDIGLNYVWTIDVSDVNHKVLTALLEFLYCGSIVYANPYQPP
STAVSNTLGSGVFTPSEVFKFRNSGNDQLDVLDILTLARLANTLSLKVLQQYCGVLLYHIIDCSKVITIIKTIFELNTLQ
MKSQWLSTNTTNNNNCGGIGGIEEIWSVCLAWLSRKQNWLDLTKNEIYCKQFSPKFTKELKKERDDKKKRLEKQEKQRKK
RETSDLKKQQQQQSNQTLNQTNQHQLNHNNYGGSSLNLSYSGNSQQQLLQQQQYQQQQQQQQQQQQQQQQQQQQQQNSPP
INSVKNNMNSMTTNHTFNRSATTPQLNTPPPSSPSSPVINSSSRLTVSNCSNGGASPMFSNSISNSQPMASPYPTNKQQG
DEADDDGDGDDNDDDESKKNIQKQTGEGYEDDDDDDDNIYGNDDDDDEINNNNNNNSKNIVNSNIDDNSSSSNNNNNNNT
HDMDFNSNNVNNDNFTQLNLQKQKKSFWSFTKKSLKANK
>Ddis_XP_635359
MSTPFNISNSININYPNNLNSHTIDNNNNNNNNEDLNSLYPPNNHHHYTISTSPSTSSFFPITYTTSNSTNSNTNNNSNS
NNNQNGSSINISISNGGQILSGSSPNNHNNININNNNINNNINNNNNNTSLNGGYFSSRLNNNLDFKSYVGQRIQTIQNN
NLSSSSHLTPTRNNNNENENASNSINGVISSHDNHQGSLLYQSNIFRDGFGTFLKSSYFSDVTIIANERKYFLHKVILAH
SSQFFSDHFDQQEPIEINNNSNDTPSSSSPPINNKNNNNNNNNNNNNNNNNNNNNNNNNNNNNNNILTTSTNNNNSNINT
SNNNNGYFNYLGNNTYELKGGLFLEFFELVVIYMYEGKVNLTPKNALPILWLSNTYSIRGLKKVTTFYLTSSITKENALS
MLNKAINIDSEDLVSKCITVICKHFNQIILPNSFYKLYLNQQQNNNNNNNNSNQLILPNFGSGHSRNNSYNVDENNNNNN
NSNNNDTQHTSNSDESFVHSVDDNNNSNNNDELSTTTTTTTTTTTTTTSTNTNTSSIPILITPSTDKQINYQILKDSEK
SLDPNTINTIPELLSLPLNIMIRLMKQNNLSVSNESIVYKTVLKYIQYNRSNLLEKDICSLFEVVRFPLFSYQQLEELGD
TSMVPSFLITEALLQRLKIHEGPKEKDLKDPTKDSKDLKESCISNVGSGGGGGGNSGSNSGSSLSVSTTTTTTTTIITT
SSSSSLNLNNQSINNLNIQDCEEGGNSLTQMRKIPRTAYATSLEYSFDFDTFGVFYYIGTNGGKEEWSNPALRGRVRVAC
SSQEKGSVIDCFNRTSSEFWSMDVPASWISVNLGSSRTMVPTCYTLRHGGNSKADCLRNWTLQGSMDSKNWTVLVRHSND
ASLNGNFSTCSWPIPNCTQAYRHFRILQTGRNSSNHNFLSISGIEFYGDLYETRVSQN
>Ddis_XP_629606
MSFNLNTIGLNTITTSNTAALNSTINNVNNNNNNNINNINNNNNSNNNLNMLSTTISTPTSNPPTLQPQQAQQRQSGDNG
GITLTGQLLAIQQPQPPQPQPQQTQLQQSANNTPPPSVENITIYILPVGIQTTSLPPFLMKKHTLFKRLFGHISQRFSIE
ESQLIFLFQDRIDPEQCPNDIGLNSGETIVVRKLKNKLRANNLHSIFVNNIGSLFNNPVYSDIAFKLLDGSLLLSHKNIL
SSRCQKFQGMFQNDMKESQLKEIEIVNYEPAVFRKMIEYLYSDSLNEDNIDMVLQLIIIADEYLLDTLKHRCELKLITEI
NSNNVALFLLKSDIYNCKFLKKSSMEFILGNVKKLFQNKEFNKVLSESPSLLLEVIQEMAPLYEDNVNGSRKSYFTINGN
>Ddis_XP_646199
MTSNYQYPNPKSYTNNNKNFDQHYVWGWNEGRQLGIGHYQNGMEIVKEPYSLGGSHNFDTKSISIGMAHSLAIDSRGQLY
SCGKTAYGRLGLGDYLSQNQQNIKEFTQIKLNKDDNDDHDNSYSYSYTSSRINNKINNKINNSINNYIKVNKISCGSAHS
AFITENFQLFTFGKNDQGQLGQGLINELHSPKRVIYIQYSFNPNEIDQSKAITEKSTNYHDNVDLVACGLNHTVFSLKDG

CVYSMGSNKFSQLGYQSTINDKVSPIVQPDGIVQPSPPLPLLEHRPRLIESLNLPPQHLEQRFTNIKSISCGSNFTVCIK
QGNLYTWGTNNLGQCGFNPKENDDIITTTSSSSSSTTTNNNNNIIINKPKLLIIYGDKSTLSTDSNNGNGKRVFNFTQVS
CGVDHCLALTNNGQVFGWGDNKYGQIGQLTFSSVIKIDTTTPIRIGESLTNEFITQISSGAYHSMALTKSSIIYAWGLND
KCQIGEIGKNQQEIIQTPCRVRFTNAKENCAPILQISAGGRSSMAIKYSPSKVDIRPSTYFDDILRILENKSFQDFNLNI
TDNEGKIKEMFKLNKLFWNRSLVLQSLKSKELTSTSSTNVAIEKSEFIKTFNDYSIKEIIQDLYIDAPSNQSFITTSISE
YLFSMINQSSFSDVKIKVICRNDQDDKEVTKIFNAHKCLLVNRSEKFKTLLESNYFKESIDGVITIDDSPSFQVYETFLT
YLYTDVLNLNNENVIDLFLLSHRESQNRMKDLIEEYLFNSLDIDNVSNIFELALQLNDNAISIDSKGINNFLIGQCIFFI
AKNLEQVSKTESFINLSDNSKIILSSNSINLNNSKSNKSLNNNNNNNNNTNTNDDNNDNDLNNGNNGKKCIIC
>Ddis_XP_639138
MATINIINNNNNQNNNNQNNNNNINHTEYFNNGFKSLLGEFKKYTDIKIKCGGQIFPTHRIILANSSEFFGRLLLSDFKE
SSQSVIELKQPDPFNVFPMVLEFMYDGIIYLSPENVIPLLAMSDHYLIRDLQHLCLLFLDQNLHRENVLTILKYSIDFHF
EPITERCITIISKNFTFVCENLYKQTKINQNNNNNCINIENNKKHLNTSSASSSLNSSTSSASSSSSSSNNNYYTTTTTT
TTTTTASSSNFNKNLTNCKVTGQQQQQQQQQQQQQQQQQQQQQQQQPIYDFSFLPASIFYQILDHDYLVVNSEYTLYRIV
LNFIEKFFGENFTEITYNTNITDQLKVSDLMSQIRYILMPYDQLTLITKDPLIPKEILFETLLERLKRFEGKTSSPSSSS
TSSSPSNTSNSPTKQTSPSKYQQQQQQQQQQQQQQQQQQPQTIHQMIQQSQLTIHQIVQQNIQQQQQQQQQQQQQQQQQQ
QQQQQQQQQQQQQQQQQQQQPQQQPQQQPQQPQPQIIQQQPQQPQIIQQQPQQLQQLQQPQQPQTIQQMVQQQQNIQQQQ
QQQQQQQNIQQQQTQNMQQQLQQQLLQQIQQQQQQQQQPQIIKQFTPSSSPLSSSFISLNASQSKLSINNATNTTNNNNN
NSNNVTNTNNNNIINNNNNNNNNKQTQSNNNDNKILKRYQPRPPQSILFEYSFDFDFKGIIFWISTDGGNEKWSNPHSTSK
IKITSSSIDKGNLYDIVELTPNAFWTKDVPASWVMIDLGPNRTVVPMYYTIRHGLSYKSDSLRTWDFQGSTNGEQWTVLK
RHTNDPSLNYKYATHSWPVTGCETAFRYFRILQTGKNSNNRNFLVIGGLEIYGELCETNPNPN
>Ddis_XP_629612
MEIEPVVRISFNGNNNQNNNNNNNNNTNNNSNNNNNNNNSSNINSTNKPSVKKPINQKMISNINNQKSPNPLNSSVDDNN
NTNNNNNSNSNGTDEDIIKLARDLTNTFGILLGDSDQPSQFSDVIFKVGDRKFFASKIMLVARSEYFKAMFTGSMKESSI
KEITLEGVDPDVFFTVLKYICIGILDLDYDHRMVSSIYQYCDLLGLQRGKELSLATMGKIAQMYLEKPDVESALFLWDSL
NQSAIPVESVHPHLRAFVLQYASISLHCDAFYSISLTTLVQMLRNDGLRMEELDILDCVIDWCRENSQASIQQQQQQQQ
QLQSANGASGKSHGKRSSSSHLKKHDGDGGSDGSCSSRCSSRRNSLSLKDHHRQHIGNGGGGGHYNNNDCHSDTEETYS
DIDDEEHRNGGGGVGDDFENDSEDGDDDDEDDDEDDDFTDDDDKDDSANDDYEYSTNKDDLDIIEDWDTTIDKNLLDEVL
PLIRWENMNIGEAFERLDNLKIIPDKEISNLLRHILKSNRGESFSFLGYHYRRPRNNRKRPYPIEFLLGITQPFDSNVTN
EIIPLNSFSSEFGQSTNQFLYRIKKDIKLPANLERFSFKDREFFVQLKEREKGQLAVYLAFGSKLTKPLAISVLASVIGF
RFQDSIEFSFKKMFDEGFDSAWGWPKFISLDTLYKQDKYSHYSDSFCLLIELTSQWG
>Ppal_EFA86792_1
MSFLDGGNGLLDDSITIGSDKLNECWESVMVYGRIPVPRHAHSCTLIGDTIWIFGGYGLGGVHLGDLNRFDILTGQWHRI
KTSAKINARHSHTAVEYGGCLWVFGGIGKGSDDNVYNDLWMFSPATDQWTQITHRGVESSAGSRQPDLAGSAWTPVPRNG
GDRQQADHSRWLRRTGLSERHVRAESRHDGLAWNHGGQRQTRHSSWRLGGQSDYNENDIYRYQLDRKKNNQPSSSSSLSS
TKQLSLNFETLVDNPIFSDIKFLVEDKIINAHKCILYSRNQHFKAMITSGMKESTEDIITISDVSYEAFKAIIHYIYTGQ
LHFHQVDILELLSLSDRYLIDDVKHQCTKYLINHINSNQTNLASLKSIAETFNLSDLLKKILIIEDNKIKE
>Ppal_EFA85705_1
MESINEYLVVGDNIDENNNNNNESTTQQQQDGNVHFNLTSNISDLDINVNNQTNSPSSTSNVSFVTDSNQSITSTSTVPT
TTTSTTSTTTTPPTTTTSESVVVESTEDKIKPIKWNIVPTSNNPTPRFDNTLVQYQGYLYLFGGSEVGSEMKSAFSHFQR
LYLDTMAWQSLPCTTVTSRVLHTAIVHENAMYIYGGGIYEDEKSSYFWSSNTKKLFKTIGSLLKFDFLTNEWTTLGEHQP
RDGHSSVVVGDNIYVMGGSQTVYQKGSIFSVFFNTTEVYNITTNKWRQINSPQFGRLGAAASYGLSEDKKFIYYFGGYDQ
SGRSNNDLWRLNLETETWRLSEQFAEFQSMLRYIYSGDETIVTLDNCIPLLHLSDLYMIDRLKTICEAKAIEGIEFDTVV
ALYKQADFYKLSSLRQTCVTYIAKNHKDVLATGTLNDVDSSFLLEIMYCLDPSLNQKSNDNQESSKIVK
>Ppal_EFA84542_1
MLPSKRKDTYSFTECSSKRRISNRDITMEDDDMSSPIPSPTSSNHSFMEIEQSIINEQEYIVFCIDLDEEINSTFKQTVP
RLDHIKKALSLFVRSKLRLDPNTKFAICILRVSAIWFMDFTSEFNEIKSKLNSLKHGGKFNAFDMTTLFDTLENTYRQIV
ESKQSVRLLQTSQSSGPSTPVPMSWNLRVIFIYSRSSIIPQYLGGTNSLDSLKKNPNFSFESIYMYHIYHTDHGKIVESI
GQATKNISKISDQSSIRKFYYCFLGILFVPPSQRPLTPKYQRFPVGLINENLDRLGPCGKNIITGKANQPPAFLSHHLIQ
LPHIPGHQLHTPLIFQIQTHHHHHQNQQQAQPQQPNQQAPPQPPQNQVPALVVPILPQQQAAPPPQPPQNQQVLNNINQQ
PLNNIQPNPPQQQQNQTQVEQPAQQPQQPVVEPPAQQIELAAEQENNENIEILHAENENTNEQQQQQNNNNLNNNNNNNP
DNNNLQQPAAQQPAAQPPQQPDPVATIQQQQQQQNPPIQIALPQPVPPHNNIFPLVYYPFFIQNPNANPAQPENLIIDPL
QMYNPYSFVVQGQNPGVINPQIQQQLQQQAQQQAQQQAQQFIASKTANKPNQNLRSSSDGEDSAALPSLGSMLKPYINSP
QFSDVVFVVGKSEDSDGELIYGHKVILSARSKYFKALFTNGMRESGTKGEIVVPDVSKDVFLALLCYLYTDDLTPMDGSP
SMSTTGSSSSSSKSSKPPTSTTTTNSNNNSVGTPPSSSTPPAHGFSSTLLTELLILANQYMLDGLKIKCCELIGETLSVNN
LVSLMNLASIHEATELKNSCILYFYSNLPIILRDCLFENDSNNEGSEALGFLIVDYFSKVSKLSSTDGKSDDSNNNPELS
AKNFEELAMKDVEFTDSPTFTVGTISGQDAPVSSDGGSDGRHHHLHHAGSSSSSDGNSGTGITGTGSSGSGFVY
>Ppal_EFA83284_1
MSVISFGVGKQGQLGLDVNKDQLDPAVIQLLKGKRIKQIACGEAHSLAVTEFGDVYSWGRGKEGELGHPQKAMTAPPALI
KALEHERIVKVACGNYHSLALTDTGKVYQWGQLHEIDATKSGVKSQGGLVEMTGIKSFASKVADHSLSMYLNGEKAAYDE
ESASSSVLQDNPTANGLVTDDQEEEEEFLPEAIVDTQQEQQPEKSGSKVGEIVDKNQMTPVLVDFGSKSIKIVDISAGWA
YSAAVTSTGNVYTWGFNEKGQLGLGDRWYHGNPRVIRSLSNKSIVNIACGRQHMAAISKSGDLYTWGLGVFGQLGHGKLK
SLLHPKKVLYFEQQQKKIVQVATGANFTMAVSENGELFSFGHGEYGQLGATEDTQYMDWNNNGDRDDHLKYSLPKQVKAL

ESVKVRKVACGHLHTIAVTTDNDVYTWGWGSSGCLGFGDRKFQLVPQRVPSLSGEEISSVSGGEKHTLIVRSSDTTTFAF
DYKQLVNDQKTGDIAFLVQNKYVYAHKLFIRSRCPKLYGSILFSDRFCKNKLEYIDEDGHMEQEGAVKKAVVEIAKVKYQ
IWVSFMTYLYTDHLVIAPHLRKELSDVAAAWGVSRLVSLCHRYNYKLRLTDKIPPSSFSTDITEHIDSFDFSDIHFEVAN
KEFIPSHKLILGARNHYFKTMFECGFREKDQLNFNIGNDIEKESFKLMIEYIYGNNEAVVNQENAVELLCLSDRFMVEDL
KLVAESFLESMVRESIQPILTTIKPSSPIQKDQGNSSEQTSPDSAKENISEDAIISFENICLLLQVSDKFLAKRLKRFCM
ETIAHLISTKQMTFFFDILKKVRYESPQLIRELDHFASLNTTLSANQLISLIR
>Ppal_EFA82827_1
MNGTINHIDSSFITSMRNESPVLSFIENVDRILNLTKSQYPQLQHTGMFSLSELTFQRFKYMVTVFAKFRSFTPILSLLA
SKEKKTRDIVISLLWNFSAEESLKIKMFDEGIFDYIFDNMDKLGEDQTLAISAIIQNLSEYRFERGHFQPILDFINEYHE
DYQFPATFNWVTLQPHVPLLESKDVEVQSFVLYCINSFSSGSRYDRSLWKGLMENKGVLSLMNLTKSNNQFVSSLAMKIS
KSLGLGESSILVEDPEVEMKENIQNLYIQSLFTDFTIQCGSESFKIHKSICLARCPKLKDILKEQDKNIKIANDSEGMAS
EYLEVQDYALFEILREIFRFLYGFNPVMNDNTAKVIIRYASRLELEELKQYCEYYLWHYIDCDNAGEYLDIAIESGAQQL
KKVASEFIIRNIGFIYNGNFAYYVPTNSPPTTSLNINNNNNNNSNNNNNNNNNNDINNGMNLENCKIESDEEGEDEPTSS
NINNNNNNSNNIKKQTKWRGHWNDTTKVHVYENYRKFLEKQKKEIFQLNLEYQ
>Ppal_EFA82299_1
MIILTPLISPIVVDRLPAMMQRSLLLLKTRSYPHLYNKHHTKMIYLKGVNPHMNCCDEQLIKCPTENTQPTQSSKYEDDR
FYYAQPSFEKNFSHDIVFFLSPPSPTSTDNIIILNNNNNYNQNNIRYNINDNGDSFININDYITLNESFEKEVRIYSHKT
MILNGSDYFKKLLNEVSVDTTEIEIAIPNVSVNVFFQVLMFLYTSTLTIEIDTLNEIFLVCDEFQLENAKEICIDFVRSI
ENKVLENFLFDQKKTICPELYSYIVDQAVENSTYFLQVNRVHKLSKDVLFLILKSDGVYLKEIEIFNSVLVWIRANIEGV
KIERIVDSDIYGTSENNNNNYNNNNKIVRTQLEEIFQFIRFPLMSYEDLISGVEPYHIIPDRLLLEAYRYISMKAHSKLP
RSSIIISNHRLKNRNITTLQPGIDVFSIVCRSHKCCSSVLWPINNFKSIRTHKHVSNTFTMFGLQWKLWAYPAGETKHPG
SISVYLEAVRVCGKESFDFLRKITFFFSLVNQHNISYSKHYPSSPNILFNNHRSVWGIGLIDLNSLYNPELGYLDNGTVC
IELHILKCYSIKR
>Ppal_EFA82284_1
MSIYTYAENGNKKELAQLINDGVDINKPGTSNRSALHHAAMNGHTEIVAMLLGKGATVNCQTNRGATPLHYASRSDRIEC
VSLLLDHGADVNQVDSTNSTPLHSAIVYKSDKTALALINNYGADINAQNNDGSTPLHLAAQRGYREIIVALLEKGAKVEV
RDHNGDTPFSLIPLTDTIDIISSSAKNTLANDISSLMYNSSSSSSSSSGSNTSTTETTTTTTTATETAEATATTTTTTA
EPNGESKMNIDQVKSEDETAVSNSGLFSDVTFIVESNKIPAHRCILSVRSEHFKRLLLKHKSEKELEIKDISFKVFQSIL
DWVYNESVSTFKVKEIVDLSFATQLLIAAIKYNIKALVQLCEQYLIETVTSTSISSIWQDLRSTEIKSNCPELAKRCAHL
LCKNWNVVGIIKAIADMPRVDMIEMVNLLPIQPLQEPAAAAAAPAANATAASSSSTSSSSSSSTSNASSTPKGKTTSRTR
APTTPSSTATPTKSSTTTPTRSSTSSSKVPSSPPTGDSMDKKNLEICKNINSQIHRKKMAEIFHFAVDPIALNIPHYFDV
IKHPMDLSLIQSKLDGGLYKSIKDFAADMRLMFDNAMLFNEEGSLIYRNTKKLMAEFNKIYWDHFPFEKSATPAAAAAAP
TTSASTSSTPAKAPAPSPAEATESSDKKRKSMEPSKAAPSTSSNTPSGESSSKSSTSSSSASANNESASSKTNASSNNND
VKMTDTSPSLKKYTDKERKNLMEMINDLNEDQLTKILEIIEPKAIKKSDEGVEIDMYEVGDANLAQLEEFIFSCLKRQKV
>Ppal_EFA81791_1
MELESAMRISFNGSKSNIKKTNLKSSNEFQLSRSPITFSSGTEDSGSTDEDIFKLAKDLTNTFGTLLGDTDQPPPFSDVI
FRVKGRRFFASKIMLVSRSEYFKAMFTGSMKESSVKEITLEGVEPDVFYIVLRYICIGILDLDFDIRIIGSIYNYCDLLG
LNRGKELALGIMTKSANAYMAKPDIDGALQLWDSANRNMISIESIHPHLRAFIIQYASMILYSDLFMVMSEKTVCDMLRN
DGLRMEELDILECVVDWCRVNSPATATSSQAKDNNTLSNSGKRNSMSYSDRSRRSHDDGESTGSCSSSRCSSRRGSIHEK
ETGDMNRTDSHSETGESFSGSDANDESDYDDDDFTDDDDKEDSANDDYYFAEQDEQVDEYDETIDRNLLNKLLPLIRWEN
MNIGEAFERLDNLHIISEKDITNLLRGIIRANRGEAFQFLGYHYRRPRNLRKRAYPLEFLMGISQPFDAPQVTNEVIALN
PFTQEFGQTSNQFLYRIKKDIKLPANLERFTFKDREFFVQLKEREKGQLAVYLAFGQKITKPLAISVSASIIGFRFQDSI
EFNYKKMFEEGFDSAWGWPRFISLEALQKDKYTHYGDSFVMLVDLTRIGVG
>Ppal_EFA81627_1
MKINVKRDFLQIMVAGVGNNRLRKRFMSDFSYYFNNPELSDIMVQVVGCSDDYIECSSSDKERELQSSGGITGNNGTDTF
YAHKFVLSARSPVLMRMLSSSMMEAASSVISMQFSKDTVQCILRYLYSGQIDLVPALVLDVLSCADYYSLEELREYCGWY
SMRICMEGDPEMDICKVLYAAERYRLDKIKTLCFEFITQHSMDILLSSPNWHLLTEDSVIAILKQDSLRVPEVEIFDSLV
RWAKKQYSILKDISCEEIVEIDFLKKKLQKPLECIRFASMSSHQLSKHIEASGLVDRDIMYEAYRYLATKETPKQLDMAV
VRQGVTDFTFSTPGDTKGVFYYFGTVEDTDDYESPSIRKTVKVSSSSMSIGYPTPFVSRTPTNMYTDKLPNSYYSVDIGS
KYVLCPNYYTMRYAGDSQGSIYAAPKNWQLLGSLDGEQWDILTTHHNDTSLKEGYSISGWPIDSIHSYRHLKIQMTGPNQ
RDGDELCVCCFEVYGRLVKLEEDSNLITEENDSSE
>Ppal_EFA81459_1
MDNYLQFFNHFVETNYSSNEELIEKLKSKYSIDNLEVIDALITIPRGDFIPEELTEQAYHDSPIRCSRLGFNISAPHIYI
NCLAELDIKPGNKFLDIGSGCGHFTCLAGYLVGPYGQSHGLEISEEILNFGRDNQIRFSNKSGIDLSNVEFKLRNCFLPD
RDDILYDRIYVGSACPKKMMARILDRLAPNGILIIPISDELRKYTKDEKGEYTKTTLLGVRYSELQIPTDQEIENANFLV
ERERKLEIKIPSENLSKSFGNLVNSELDSDIIFMVMADEKQRKINAHKLFLRVRAPKYFESIEEQSVIELPIELDYESVM
IVMNYMYCGEEPMEVINQKAVLAFRERLSSVVLVCQYFEQLELLAYCQELLELYKEVQSDSDKLTMVTPKTKHHFEQLRC
TAKRTLQTQMLELSTSSLNSDITLSVGGSKIKCHKLILKSRSKFFKSFFTSGMKETFSDMIEIHGPFDLSSFIEYIRFVY
SGDKSIINSDNVIDLMCVSDYFNDNCLKSLCEEFLVGVLDSSNVSSFLQASSNYNALQLKAVCMEKIFEEFDTVSKMESF
KQLDKDLIVNCLSECCQYFKKVVNKQPVGTGTGTSVPTSPLF
>Ppal_EFA81116_1
MSLSRIFGLIPSYDQLYLPDPEQQFVWGWNNEGQLGVTMSAASMNSGGDVVDFKTMSKNFVTKDVAIGLSHTLLLSRNGE

VYAVGKGKYGRLGNDSERSLRSLFRIPISIRVLKIAAGSFHSAFITEKHQLFTFGRGDSGQLGHNDYIDQFSPKQVSCSD
PIDLVACGSDHTVFVTFSCGTYHTNVLTAEGLIYSWGTNDYGQVSPDTRGIINVPQRLRTPNSKGGSPIMNVYAGGRSSM
AIRFYPTLIRVRESTYKNDIENIFTNNTNTDCEVILKDKSGDEEKTNIILSHKIFLTRSSLFSDQLDSNTIDVGDEFESF
AQLNSFITKLYKDDENEIDLVSNHLFLYINNPLYSDVKICVKEKAFYAHKCILVARSNKLKVQLESFFQEGINNTIFIDN
VNPDVYYAFIHYLYTDKLNIDAENAIELLFLAHEENLERMLELVQEYLINGIDDDNVSPLYDISTRLDLKSLGDQCLYYI
AKNIDNVSKTETFSELPEELKKKLISSSVPFSMKKDKEKNCILINF
>Ppal_EFA80319_1
MVLKKCTSDFDREKKIIDLTVGDMIESYFKNGFKEYCENNKFSDVKLRCGDQVYNVHRIILAYSSEYFATLLSSEFRESS
QPVIELRQPDPHNVFREVLMFMYDGRVLISPDNVIPLLSMAKYYIISALTTLCLQYLESHLHRENVITILKSSISFHFDD
IVDRCLYIVAKNFPYICESTNINNINNNNNNNNNNNNNSNSNNNGSYNCKDNYFKFLPPPIFLNLLKQPYLVVKNEYSLY
QIVTNFINYHSNICNSNNNQNNSNNNLNLSNSSVDNSFNSSNNSSNCGYNYKFEQSDIVELMSQIRYIWMSYDQLLEISS
NPLVPKEILIETFMERLKKYEPHDLSNEPPISHSRFLPRPPQSILFEYTFDFDNKGIIYWIATNGLKDKWINPHLSSKIK
ITSSSVDKGNHYDMVELSPCAFWTKDVPASWVTIDLGPNRSVIPMYYTIRHGFFKSDSLRTWDFQGSVHGEQWTVLKRHT
NDTSLNLKYATHTWQIQGVTTAYRYFRILQTGKNSNNRNFLLLGGFEIYVPIMNINTSSKLLLLLYKESIFES
>Ppal_EFA79484_1
MWESNKQYHSNTMETIAAMLEAEQLDWQEVINFHHPNFMYFLRNSSISIVMAVLTRYDRHQNTIFHTAAFCPPRDDFIND
IRILLRKLDYNISADLQNLLTQRNINDETPLHLAAMHQNIQYIRSLVGLQVDGSLVDNRNLTALDIAVWLDKKECINLLT
SLRSSKMTVSNGTGGSVPIYQWGQMLCPSLSLIATGTPTGVLFNQKEHSPTTIKFGQNFAVMLTDTGGVYSWGLCSYGKL
GHPTKNDISIPSMISAFKNKQITQIACGKDHSLALDEIGVVYAWGNGSGGRLGLGNSEVVSIPKIIPHFEDEKITSIACG
QEFSLALSDSGKVFSWGKGLHGNLGYDVSTMLSQSTPKVIPMLPPASQISCGNWHSMVLSKSGDVYTFGSNIDCRLGHSG
NGSTPTMVQIGSKIKKIGAGANFNAVLSESNAIYTWGSNIHCQLGVLLPDHRNYTGEPQVVKTLLPYPISTIEVGYEHVV
VLTDTGEVFTFGDNSMMQCGVGEAIKEVCNFTRVKIPDNSTVFKIAAGGNSSLISLSSGHNIYGSELASLLTDESLVDLR
FIVNGDTAQYVNCHRAVVACRVPKLLPLISSEIKNPNSDNSHEISITSKITASTINKIIYIDFKQISFESLKLFIKFIYT
DHVPLIQHYVDEIGMLSTCLGVERLAFLCNLVLGKTKSLESIPSSNLSEDFKKLSDPTFAESLYDITFKVHEPSGNIAEV
QSFKVMLCLRSKYFKMMLSGSFIEGSMNSINIHDVSISSFKDLLHYFYCNEVPSDPNDCFELVILADFHQIARAKELCSA
VIRPSIDNTSLLFMFQFSKQYDLKSLTVWCESRISQVPNAELLEGFDRLTEENQKFVKENCAKAPPKVEGFRSSTPDSAS
QEQRHYRRKPKNQEKPKKSWFFG
>Ppal_EFA79126_1
MFNAGFQQYLNTGLYSDLTLIVNHTKEYQLHRIIVGYNSEYFSNILQAINQHGTEIFNQPASPALSSSSSSSSSLNNSP
NVVSGSPTLSLIRTLPTSSPTVLASHSNSPFLILSPLTPGSVYSEHSNANGKFRLGSNRLKQQNNNNNNNNNSNNNANI
NININPLSLSSPNISNTNSNVSASSSWVKSSPAQIQQQHQQQQLLNQPNQQTSQPSQLSHSIQNINSQIHHGLQPPRRTT
SFSRSISMLLSSVSSKSKLSPPNTSSSSLINNPSTSTSPSSSFINSQTNNSTNNISLSSSTSNLTPSKSQPNLLININEN
NNNNNSNKPLPPTPVLPAHLSSSSSSSSSSGRKDSNSSPSFVVSLSQIKQQFNNHQPKPNISWNTSRNCLTIEIEINDPD
NTFDSIITYMYRGKLDISEKNCINILYFANHFLIHSLKKMVSDYIVEHITSMNVLEWLNKSIEFNLTELIPRCIFVIARN
FSQILEEHKNRINQNITSGENGKLKIQLPKQPFCNLPFDLFLQILDHPSLSVFTEFSVYLAICGYIDANRGHLEIKQIEQ
LFMKVRFPFLTYNEYLQVIRNPLVPQELLTDGLMIRLGNFECPGSEQLKKRLSEPRFMKRKTPGRIFEYLYDYDNRGVLY
FLGSYGLPDWINPALYPSADNPRVKTGHNSTSHNFLSLSGIEFYGELIIQQHQPITTITTSILSSNNNNNSNTTSNQSSN
SSLTTTSSSSSQASTQPTITPSQLTFQQQQQQQFQQLVTLLQQQQQTNQQQQNNITNTSTTTTTTTNINRNQIQSNNINT
TT
>Ppal_EFA79079_1
MSSMRTSSGISNNNNNNNSNNNNTVSNVIVNNGVTISSSPNSYSGFLNVGSNNVNSITNSNSNSSNSINGSNNTNSSSSNN
NNFHNSSKIIDAARKGNAELVQKLLKKDLKKLNKRDEKGDTCLHKAALFGHADCIENTSYFDEESSSVYTWKLQKVSTLR
ERAISPVFKVGQCKWMIAVYPKGKSGGDHLSIYLKVAETVTLNNIPEWFFLVNFKFSVINQRDGSKFTRQVEGKKFKANV
EDWGFPQFFKLSILYDAKNGFINYTDDSILIELQMEIINDFSRKKFKDNISDWGLTLLHWVAYKGSYDGVRFLLSNGANP
NVTDYKGRTPLDWCAYNGDLPTSEILISTGMADVNCKDHEGFTPLHKAVMNGHLEVARLLVRYGAQVNAKNTCLTTPLSL
AIRVSRLWCVESLIKWGADINIPDKQNRTPLHWAICLSESRLLGLLIKYGCSNQSNNNNNNNNNSTPNNQNNNGIELNIE
DIAETSDNFDTSENNETQNQSEGQENLEDTDSDDDQNGGRIALSGSPNNVNPIIIHQNNVNNTKDDRSLSIPNTTITTTT
NNNNNYNNNNTIIYLNNNEYHFQNNHYEEFIASVVPLQMNVRDQDGYTPLHKAIWGKGKISFVKILLERGADPKICTKDG
RNAVHLAVAGNEYTIAQLLIEHSPQCLHQFDEKGHTPLHKAVINGNLVMMEMLIQHGADVNLCSPTHPDVIPLADALRES
DISCIIFLLQKNADPKRCQKSLLNLLYKRVKVYKNKEIDPTIPKCTLNSDMKYLLNNINYKDITFIVENKPIYAWKGLLC
ARSDYFRAMFEQPLKESLENEVRIESVDHITFLHVMEYIYTGELSSKLTLEESMPLLIAANRFMLPRLKLLCESLITKEF
NTDNIYNIFKLADMHETTLLLDECVRYLAENHLYIPETDDIVKIPSFRKSVLEFLHDSLNIH
>Ppal_EFA77059_1
MHFDTLNEDTEWLLSFFFVDCEEGLHYVSYTVMHYQSWVEYLHKLFFPMMIKRNFYQYSSIMISTSPGNQSSFLIPNNSN
SSGNNATLNSGSFINGANVRSGSVIGISNSNISNNNNSNTFKGHSRTNHSNGGNNTIGNYFEAPLFQEGFGSFLKSGEFS
DLTIHSEGKQYHLHRVILAHSSKYFSDYFDLDNNNSNNNNNNSNNKMTTIKEITTTNYNNNSNSNNNDIPVTTYFTKIDN
NNYELKGPFIEHFDMVVNYMYEGRVNLTPKNALPLLSLSNCFQIKGLKKYATFYLTNSITKENAFFMLNKAIHINSDDLT
TKCITVICKHFNQMIQPHFSHIFTQPQPFIMQPNYFTNNNNNNNNNNNNTSTANYSDELDSIATVDQFTSNLESSLNNNTT
SEPTSTNSDSDDSDLDSFSSSTTATTTTTITNNNNTHSNSSHHHQSSIHIPQTSNPIGISSKYGSPSPSNSILSSSMAND
KSLNISTIHTIPELLSLPISIMIRLMRQNNLSVSNEAVVYKTIVKYIQANKSSLSEQDIESLFECVRFPLFNYQQLEEVQ
DNTLIPKSLITEALMLRLRLHEGPKDVNSASNLSLSPSNNLLQSNSKSPTSSSINVQYSGGNNINSNTTSTSTTATSSSI
QITPSVIVTSSSLPSSSLLTSTLTSALSSTLNINNINNSNSSNNNTGSILTSLSNNSSNSSIGDSLVASTESANDSSALI

RRTPRAPFAISLEYNNEMKGVFYYIGTNGLKDEWSNPANRGRVRVTFSSIEKGNVTDVVGRQPTECWTMDVPASWIAINL
GSSRTLVPTFYTLRHGGNSKADCLRNWTLQGSMDSKNWTILSRHSNDSSLNGNYSTWTCPIENCTQAYRHFRILQTGRNS
TNHNFLSLSGIEFYGDLYETRKDD
>Ppal_EFA76882_1
MQNIKVVVVGDGAVGKSCLLISYTTNAFPGEYVPTVFDNYSANIMVEGKPFNVALFDTAGQEDYDRLRPLSYPQTDVFFV
CFCVISRASFENVKYKWNAELDHHCPSTPKILVGTKSDLRGSGRPEISTKEAHDLAKELGFIGYVETSALVQTNLTQLFN
QAIKSAVNAPAAKKKKGEKPVPIPPAMPPAGKAPWINIVTSTYEKELKSTLEHQGFADVKFTFGNDKPIFAHRVILCSAS
QVFRRLFSVTLNTDHSPFAPEDIEAGRVKGIKSMTATPMKHYEDGSISNGEMIEIEVDPKFTRRIFYRYIEFIYTGLLNC
VDRTDQIKETIEIADVFDCKYLASASKNLSNGSEDLNPSIGTFLNDEMGDKCKELFFGKKIFSDIQFRVEGKTIYAHKAF
IYSRSAVVNAYVHATKDYAESQDATVELQDVSHDNFKAVLEYLYTAHSPIEEGDPVGIMVLANRFGLTRLITLCELYISK
EIEKATTVGIFKSELDIIGLLLCAKSHNAEQLTKFCLHFISTNYQPMKKRPEFTKLDKDTQKYLEENQWPPVWYIQACGE
YEDQMAKFNGKKDNCSIIYLFRYAVCYA
>Ppal_EFA75696_1
MSINTSMSNNSLHQALASSAIINSGTTSSNSNSSGNSVINSSALLFSSGLSAMSSPNNSIVPSSSTTSSNTSQNNSNNST
ISNTNNVSNNNTNSNSNTTATGEDITITIYPIGIKTYGCLPPFLMRKHTLFKRLFVHVSNRFCIDENELLFFFQDKLLPE
QCPSDIGLGNGDIIVVRRVPPKPKIANESKQASSLVLEIGSLYDNPSFSDISFILDDGSTLYAHKNILSARCDKFKAMFQ
GSMRESTENEIKVNEHSSIVFKKMVEYLYTDTLAEEGGIDMVLKLIVIADEYLLHSLKNLCEQKLITEINIGNAPLLFSH
SDTYSCALLKKHCLSFILTSIKKLAASEEFDRALFQFPNLLLEIIKEMAPSFDNTPNNTKRRKDFEI
>Ppal_EFA75556_1
MMMDKNSNSEFEKNSTTTLPQQQQPPGLPLSPCLATMTTKTTTTTTTTTITEHNFLVPELLVISPRLETPLNTTTTMSSS
FTQPIILVPEQDSNSNNNTSSSPPNNNTIDQISNNICSTNNTSSSDYIMDDLNKSQPQQVQQPSNSTPTPQPQQSQTQPQ
QSSSAAMDSPVIFKTTSSTSTTTTSHTVTTPALENRAVSTSPPSTPMLDDCQIELGARNNTGLSKSPPLQSSSNSTNMEN
DKLSPEFINAASISKQDQTLLNNINNNNNNNGNNVGNSTVPNSPTTPQIQIQLAPSSPLSTSSSSNSNGNGSGSGRKSH
RSTSVPLIRNRIQRPIRQKSPSSMGERPLLHAVLVQEEIKTLINQEDINMRDSNGNSILHRVSIIGSMLAIRHMVLRGAR
VNSTNNSGMTPLHYAACANPQSVEVLLYYGSLPNSKDNNRETPLFYAVDRGQFSIVSLLLSRGAKVTTVNKAMSRNSIHV
AALKGYSNILRLLLDYKSNINAPDVNGSTPFHLCVYHSPPAQNLMNPINYQSVNNIDAYMSCCELLLQRGATLTLQDTQG
YTPIHIASRQGKKNIIDVILFYCKQQKNKLPQSILSSIPNASKFKKELKSEIVLYIDDMGMTPLCLASMFGHFDCVEYLY
NLDPKLNQQCRFYTSSSIDCSKTPIDPLGNDFRFALDGDYCDLVFIIDGRDIFAHSIMLRQYKFFRDMISSCKSLASLHP
HSLNNATGHGSHQQLHSHQYGGIYGSQYSYNNSYISRSHDDSSIHYHSSSIMDGGYYDVGDDSNYYNHSSSNSMDYISAN
ISVGNGVATSRARTRSRMNSVVNPGTLTRIPVEKGISYEIFRAILQFAYTSEIPREIINTTGKLSELLDFANQFDIESLS
NLCVDTLSQHSTGRMGTLEMSKYLLKQINSKHSADVTIIYDGGQVVLAHSVILANRSTFFKLHFESYPVLSRSAIQLFPN
NVNNISSSMSSSYGNSSLSNIAGTSTPHRKLSCPSSPALSDIPTMGWVIDIGEASQKAVFVMLEYLYAGCIPSGNSLMQP
SLYPLLNQKLPSTASEMDVIDMLLLSKLAYALNLPVLQQYSTVLLYSALDPLKLVTIIKTIFSFDGNVGWGRTSSLELMW
GVCLEWLTKKCNWEEVNRCEAFISLGLKFIKDVKKERKDKKRKQKMNERQTSKSSQHNNHTSNNNSNKQSLSSSTTTTTT
NNNNNNGQDGNNNTKKEEKFSTWSKMHPKEILRSVSSGFLINSNNNNNSNNNQSSFSVQTPNSPPTNQQQETPAYTSKNN
NLNNGADGQQQNKTDSPKLTKQNSISNFLSKKKVFSTFARKPMSNRSKSFVI
>Ppal_EFA74882_1
MSEGDDGSDPISEAVGFIENILQPNIDLQYQGLKGLMLCSTTHVVELFKNPSFFYHIKRHPNHKLNHISSHGHHHQSSPI
PTGNAANPGSNNSSPSSSPSSSPTNSLIEPCNQSPSKYSDIDYDKYSKKRKSTLKSLIELCLSETKKVQSSALRLLWHLS
AQADLKVLLYEEGVLDKLKYLISSDSQAPKDDIESRQNGIHRTEVQLASSAILQNITEYRFERGEINPNQVKIVNEGIVE
MILLPRSKSTDRRVQFLTTLTIANLSMNEENHQILQSHKAFDIVESFVTNNSLHMDLVYQYKVEVWKGLSVNNGVQSIFL
LLHSPHPKVVELAKKIAEQLQIEEPSVTVNTTKIGTDLKKLFNNSDFSDICFVCEGKKIYAHKAICASRCEQLRAMFTWG
KESQENEIHLPHIPYASMYGVLDIGTDNIDSIGTEIIGSVKHFSYYVLNFIFLV
>Ehis_130_m00126
MFGLAEDIQQGKISISNLHLRDVEDITMNEETVNTFFKELYESGDGDYSFEGDKEMKVYKFILSTRCSYFYQYFSLEKQP
TIELNKETLKGFITWCYTNKIEMEMKECLELLHFIQKNWINEPISSIFYCIIKRIKNDFEANIYDNWALVEEFFLNEAHR
RYYEDYFKSLSQLCLKEIINKKDYSFVGKILPYVLLSMNKHIEIIKEKKNIEKKKKTKKNITKPIETDLTVTLLHQEKKP
KKLNTKELLETNVHKEKKLSKKEISNGIEDELLISSNRLDTQFPLNSQNTSLINKLLSSLLRNKASFAFKEPVDPQLTGA
INYFDIIKHPMDLGTIKNKLKDKTYKNVNEVLSDIDLVWNNAFKYNAPNSEVWNLAKTMSDAYEKKLSTMKFSI
>Ehis_217_m00087
MNNLIECPICGQMVPQNQIEQHANLCLDAMNEGRSPPIVILPNEINSLNNGRSVLTIVSPLSSHSSANLNGRDGSQRREN
SKELNNTNQNYNNLADLLNSITTNENTETKEIPEEIENNKVNSQSNSNELISSPPIEEHIVNIQKPVDVTINITELSSTG
IYQMIQKQYFNNPCKDFKGDNQKNIKEQQKLIELPIELENPVDPLIYMTNQQQKTKKDIHSEDIPIGWGFKGNIYNEIMN
KDNRYFDTILYFPNEPGKYIRAHSFILAARSPLFQIELEEMTRIEGMFLYEVKQYKYDDFLYFIKVLYGVSPKITVTNDN
KQLKDIDGMYSPSSENIINDILNLQNPKFFDGYFIITKEGNEVERIGFSRILFYLFSDYFKELFDKKQINYINDGIKRNL
SSISSLGFDVTQITPTGDSQPFVFKIKGDNEEICYIHQAITEFIKCIYLNKNYSLKEDKIDEITIQRVIPILLHFGSLFK
ENLLVFNCERILGHNLIIKPTNCNALFYSMRASSNQTLPGFTQIDVIEQWRKEIVDMIYELTKELTGYVFINKENDDQLP
LEITCINLRDIQKLASIALVSFVNQNSDKSLLISRLCSKKFSSLTQRQDVIDFLCYKGFRDILPVLMACFVGSMKKIQVQ
ELLKVIVHDIIVTICHSFSILSTLRGYIPITEEFFNIVNTILQSFFQNNEICCVCGDQLGFFSKKCELCQINMCKKCMVK
GMFINFKTNNLVQKNICYRCHRIWQFLSSETYYQINK
>Acas_g137
MEMDDEAEANEVRSRRSVSTPQHQQAAASQSPPTKNQQSNRLRGASSGSSGSFLIDALKKTKMGKKHKKQLKQLLVDSLD

QAAAATPTTSSSSSAPQVPPHLSTTTTSTTPSTASTRPQPHQVLSISQGTDQSWSASSTTPPAPFSLSDVSDMERKFLNT
SFGTDRSSFSAYDRRSSAGVSGASTPSSPSFSPGTSPSFSCSPLLAGVFAGATADRRDSTDTTGGGVGGRRSFSEEAAAG
SGTTTTTTSAGGAKEKKKKKKKKEVKRKGKREHRKKGSESSGSRLLMSTSPPSSTLPMLTSSPTVSPPMTVTTPMTPTTV
AGGGYGGYDRQPMNIDLDSLKKKVKPKRCEADIFRDGFRHFLQSGDYSDLTLECEGKEYRVHRLLLAYSSEYFSRLLLSN
FQEGRQTYIHLRFPDPAHVFPLVLQFIYQGHVTIAEDNVIPLLAMADHYLVQELQQMCSDFVNQTLSRDNALARLKRAID
FNSEEVVLKCLFITARNFSHASLGKADYTFLPVDIFMLLLHHRYLAVKSEQHLFNTVCTYVEAHRDDLTEEQINELMEAV
RFRWLSYEQLEEAARNALVPRHLLVEALMAILRDQKYPDLKPLHEDNPRLQERVSYGIEFEYQSGVENNDIFWWIGTNSG
TDSWQNPGLCGRIKDVPASWVQLDLGPNRAVVPTFYTVRHGGTFKADSLRTWDFQGSMDGTGWSLLRRHVNDEVL
>Acas_g263
MLGDDIYHLIQRGDYEDDLNRVLAVGFPVDKPDYTGWTALHYAAHMGRTGCLSTLLECGADVNALTSREGQTALFLAARG
GHLPCVQLLHAYHRWAVAEATTTQLGRAAAVEDAEQLLPAAAGLDTSGTAEEEFGRWTPLHIAVHCDQVSCAHYLMAHSL
VDINARDYYDRTALQLLIEKRLNPALTVWMVLRGARVLSKPSAPPMWDSTKLDLDHMVDTFDPTVKQWSSRANEKVAMAQ
PQHDACFVVEGKRLYAERAVLCARSTFFAGMFGSSMKEAVEGEVELESTYNAVFAMLEWMYTGCISTDNFDDILQLWQSS
HMYDMEALQNYCQKRLLKLFEARSKQLGDHSAQETWNYALALFKATEGGSSFDSALKAISGIYLLGHLEAVLQRYRGDPI
AFVGRLFAAVERKAYDPASYPTLPEIEAEIAFFRRYKRLPKGQREIINVQEMEERKKGKCLLWFEEATSEGVEGWRETLA
QLRMLRQLEEDCFFFDGRKPHRCFDLIAGTGMGGVAAICLGRAQMAVPAVEALVCELVAAAGDLGGGGSWLSYVGGMMDW
GGETTRAHLAQRVREIVERHLAASHQSQQSLSLVGLTDDYDHLRLCEMQDLDEETHPRDDRVKVLVIAKRAGASKDVSRA
DNRRELFLKLYGDRVVARSGSSEAGVNLTDVIVAALAAASPGLLNPVKLADVVVGGDGAQGASSGLGGRGRDTLLAEMQK
LWPRWELKLLLSLGSDQAPASAQSSSSWSSSWSSPSLLPWAWSVLADHMSSFAGYVSRDRGKNVWDKAHERCLDRTNADD
AGYTVGDKVATVLAVEGWRPLRSCDYDDDDRQGHDQGQAVHAPDGAGRSQRRRRRDEGEDEGGEPGEEGHEPINGEEGGR
PPPSGSGQDEEEESWVLV
>Acas_g324
MNFKRLYSQALHDHSKLCKVSFNEQLAGIMDNPESADVEFLVGDDGTPVYASKVFLVMRSSVFKALLCGNFREALPPSSS
TSVSTSTSTSDTSSSLPPVFWHRVEIRDMEPRTMRHLLHWCYTTRLHPDFFREDDSSRSPTLQESDLVSLYVAADRYLLDSC
KESVKCKLTLMLGKNNRNALSVFDQAFEFAPQLASLAQSCITTDATKFLGGKRKETEAQWLAMSPAAAAQLVEGDLEKFE
EVDLFRAVVRRYEHHRAEAYDASADSDDHDNRRSAAAAAAREKVACLLEGIRTILMSVEELIDVVEPSGLFTDAQLGAAY
KQAARNHRFIVPELRSRRLFSSDDRVRHGKIVWHLAVRRGWNEEWVLVKVAALRKKDETRPKAVSAEGVSVSFFHNRRCL
LHYVAPAATSVSVPGELCVPVPVDSAALVRATLIVHVPATAFIE
>Acas_g326
MVDNPLCADVQLLVGQEATPVYASKVILMARSPVFEALLGGNFREGQPPPSSSSSASSSLAPVFWHRAEIRDVEPRTMRH
LLHSLYTDQLHPDFLSPAPYSDDGNNDSTAPTQQQQDLVALYMAADCYLVDKVRALVRRELGRSFDDDDANVLPVFDMVL
AVAPELAMHVCQPIIETHAWSLLGNQHAYLEALWLGLSDTAAARVVRMNLRGIGEIELFGAIQRRYEHHLAEAMELVEDD
EDDDCEKGQENGDDKKMMKKREAAIAEAKNKVAGLIEGIKTSLMSVDELCSVVEPSGLFTDAQLLAAYRHAALNHRYVIH
DLWCGYSLFNGQVEDGNIRWRLAVCRTPTSRTPTSWTVDFRIRTDRADPHSTDSLSAEGVTITIYCDRRCVKSFWAPATT
VVTMPDRLSVPFTMQPHTGRVTIFVHAPPTAFTQAGPI
>Acas_g425
MEKEDDLAFALSMANLGSGRVSQEEVQDESTVTLAQEIKRLAAKYGPERFTETSLGFKLAFVSAEVNVVVEVELDTMMYL
EDDPPQFKVQSNGAHVGGADCRELRSLLEEYAEDWMVEEMPILERLMDAVRDWLFWKTEDAQFAPPAPAPRATSPGKAGA
SAAAGAGFSDHKEKKAKEEQKKQKAAAKPKPTPKPKPAPQRVAYTPVYQPPVYRPPPVYQTPWNYVAPRNPPPAPDMTAV
RTRFEGLYGRPLSADFLETWNLPSYGAGAPPRDHNRDKFIDRPETECDEETLIKIEEAYVNENRILSITNKGVPALPIEL
MGRLTTLTELNLHNNKLSALPVTICQLSSLTKLYASFNQITEFPAELTVLPQLLNLDLSHNKLITMPPQVDQMTTLQILE
LNHNQIRGLPPQMGEMPSLRKLYLNNNKIMFVPTELGYLPKLTLLRLSHNPIKNLPVDEYQQGTAKTLEFLRTAGPGVET
GRQLKNFAFVKEFSDVEFIVGSVVFPAHKVVLCARSPVLRVLLHSMSESESASGVVELRPPAELSHIPVDVFREFIGYLY
RDTVEKDKLKEIGNDLGVVANYYGVERLEEMCKRLKFGYVVVRESTFAQDFGVLMEEPMRYSDVAFLVEGVPIKAHKVFL
AASSEYFKGMFTSGLKEAQQDEIELPHVREPIFRAIREYCYTGDVEEITGDTAVDLLGASCAYTLPRLKMIVESLLGYSL
DVDNVACLYPVAVMYEAQVLERACEFFMAQFLAQVKATEAWADLPPELKAKVEEQTIKWGLNKNKKW
>Acas_g526
MGKKIIIIGGMGAHFVRDDICIIDTENLTWREVPSECSGEMMPRSGLSASLIDTRIYVIGGHHDFTIHNNVFLLKTERPI
ERKEVYAQGSFLQDMSFLWENRQFCDLTLVLEGQEVQVHRALLWVRCAYFRSMFSSGMTETKKDRIELAGVPLQYFMYLL
EFIYTSQCSPLSTMSECDDDDLDTVKGILVLANEFMMEDLVAQCENKLIDIMDCNNVVPLLELASFYFASTLRSACVNFI
SNNYDIVSKTMEFDDLPSETKDEVKRLKAL
>Acas_g794
MASPTALGYKQLQLDLWSLLSGADSSPYADVVFRAVDPNQKPADASSGSGGQDRPAAQSFHAHKVIIYHTAPMLFASLLS
VEDHTASADNAAAPPPPATATIEIEPHIFKLCLEFLYTGAVTTPGVTREDKVRLLSFAEHYNIPGLMTAKAWKESSDSCW
ILPEPHQDSVTDKNPPPPKPILAARHSPLFADVTLLVDGQRFHAHKVHTTTTATAATATTLTTDNFQAILAARSAYFARM
FGSGLAESFQSEIAITQASAASFGLFLDYLYSATLRDWSLLTPDAAVELVPVCHQYGATSLVNPLQSAGPSDLHLKAICE
QLLSDVLDGDNVCLIYDIALFHNATGAKPLMTIVDSALVGSAEQDPVFYANCGALQCDSIKTGKKSPSVISQDRALKVHL
DTLMRLLPADRVTITPVSRAFPSPTPDQPKARGGGRKQG
>Acas_g977
MKLLRRTSLLGSDGSRDVHLLRGTAEVVLVEGRNLAIRDSCGTSDPYVILRLGDKKYSSTIKYKTLNPVWKEKFTFQIHA
DEALHCDVWDKDKFLRDDPLGNVVLHLGKYASTQVVDVWVPLENVECGELHFQILYRTFADDLQKSFQAIEMLPLQIASL
VATPIMWTEAGKCKQALADHLASLVNNEEWADVEFVLDADGDKRIFWVPPTSPVISRARATHSRAARRVQLNRHSAILAS

RCPVFQSSLPKSAARKRSASRRASSADVQLSGEFGFSRNASPKLETITGMSSSRLKEKRRARVLIKEHKADVFLAMLHFL
YTGAVPSVPCFGYTLRKQEEKPEQGAAKAEEPPKPPVRWTWFADPDWADFEPEVNDELEKAYQQAVTANDGNSIRVRVDS
ERFVEVRVGSSLYQKRYDDESRQRSVKREDPSVGSSEQSSSSSSSTPTDRLSQFNAQQLMAKQDLDVRLAKIAIEYSLSD
AFVLQCENAIRFGEYSLTELPVNLVDDLKKAIETTDYAQVTVVVADDADESVHHEVLAHPCFLALSPFFRAILQSGMKEA
QEKRIELTEVTRDCFLLVLEFLHTGLFAKFPTEYALEMLRISDQYDISPHLRQMIEQFIATHLLESHNAIAIYHYAVLNN
AANLEATALAAIKRNAAYPAWTKRNREALATLNPTAHQTVFGS
>Acas_g1063
MESQASSSSSTASSSTYDAASLPPVDLFVERLHKAAASGDLAALNLLLDALRETAGRERELAAFVSSSLSSLLDSPGLEA
ASAQRGRRQRDVRIHLGIGRDDEGRGDCYDLFKGPDLLGRTALHQACYHGHSDCVKALLEFDVALRELMFGAEQPSGSKK
AAQQPQPQPPPPIRRSTSSVGAGGGQRNALRKSMARSVAGQPTLAASTGAGVAGSVAAGAAAAEQTSVVPADAETTEAGH
VDEGEREQEGEGMERRRLVDTGDLRGLLPIMVAIYRGNKDCVQVLLGHNPTLDALQTQVFAYEQRHPYRAMEKRSVQSLY
AVTLNPNDLYRVNLSLDSLEVCHIVGCELDRSNDQISFYLNGQPMGVAFTKIGDDIKLVDATERCRLNFGGRGGEGFRHL
PDGCKSLCDLIPEAQARLTHWKLRALHGTFVSEPAMKLPCMNLGQQRLKLPLAWATVYGGAGAEMFDLLLREASPQAVAG
QLRLLAADGAEQGLMPVPHLASLCGNEAVLRWCVEHSKILGAKGRAQAKPQQQQQLLFDLANDRDRVGATALHYAALGGH
LACVRLLVEEAHVPPDTTIKKSTARTLNVIDGHTGVSSVITARPDATSPIGASGITPLHFACQAGAQDVVDYIYQRWLDG
QHRGASKKGQRPTALPVDEHGATPLAYAIQGGHTDLVRHLIAPTAPTSTDASSTDGVDGNAEAAATQNEPGLGWAISMAK
HSGSRRATLLSLAMSAGWVDIARLLIAEVKRRDKRKLKTFVTQRAADGTQLLHSACYFGSVECVDLLLSLRDDVTGEPLI
DADGPDSDGLLEEGAPPTVNSGKTVLHMCANTGNVPAVEMLLQGWKENKWGLRDKLDLNAEDPNAAGSTPLHAAIFSTSA
LAHKNVAALLDAGVDPKKRLHNDDGSLLLHAARSGTAKAVEELLLRNFDVEEADSFGSTPLTAALARQYPTLATSGQQVL
LRLLEAGAKVKWGATPLVDVECYINTGSPEENMKSGHTATYYDAGRSAYQVVVGGHFNGVHPPGIYFLSTHYRAWDATYF
DNNVFVNRRGHTCTLVGDTIVLFGGLRYAPLPRTHFNDVYFISAVNGRVTKPLILGEAPLARESHTATLVGRKIYFMYGC
SATAFMDDIVVLDMDSLEWSRPSVTSLKRPSMRFGHTATLVNDHEIWLYGGINRVVNVAGRTHLVSTDPDWHVFDTHTLQ
WRTINASVAVPASGKGSPAKLPTPRANHTATLVGEEIYVFGGDASQEINELWIFNTRTHVWRQQEVIKDFSGTGLTGHTC
ELVDGNKLIIYGGETGVIVRDNVACIETGVLSPASADMAKVWSEREETGDVEFVLPSGQTVAAHRVVLASLSHYFRDLFA
RQPPSALRKGSGEKLQVRLGNEVRREHLEIVLRFIYTDVIDVDNIPGFPPSGTHCAAPPFVRDLIDAAKLYAAERLGQFV
ARLLRQSMPFHRDTADYEPRSLLAFLNNIESDESEDDVEELVITDTSHDILQVILRYLYELADENPVGEGAFVTEETAVE
VLITAEKYGLDTLKLWCEKLIVEVMEEQNIPYFLQLGDTYHAPHLRRYCLSALSAQYDHIDAVLRAAPGSAGGVEEQIES
DELRAEVLGIVHERRRKRNALLADYEDVNDKVYFNTVLPTLADSRQPLGRASAVFGVMVGVAAAWAFLGGI
>Acas_g1173
MEHKPASAKRKSHQQAPAATTEDAQVAQVAEALSLAGQAPAAKVVCSSYHAWFLLAVEDQRVRQVACAGSIAVLLMESGK
VAWCKVGSDSSHPPLIELTLVGEKNPTFPPVKEVACTEGYVLLLCEEIAGSVANFAALTAGGDLYTWEPSTEPTKVEGLE
NKGIKSIAAGSYHMAAITNDGQLYTWGGNSMHQLGLGGGEKKTAIPKRVAALYGKPVVSVSCGSSHTACLTEMGRVYTWG
AGTSGALGHGQTADQITPRLVEELRDMTVSHIACGWYHTIAVVSVGADPNKTKLAKDISSLLAFDGDNFSDATVYGRGSA
RGLACHKMVLGRLPAWKRIIEENDAKAGLEAEPVLHVDVEDRLLPFVVRAVYGHPLAPDLATATYLQLAAVAKRLKFAAF
FEEVVATFGSGINADNVCRVWRELAHLQKRIPGIAGNDLGPNDDHGLAPLVQGDLARFHSPCFVMVIFNFEQKWSSDEEF
RAVAYEASQHSHKIESMIKEKTRHQTSRSVREQRHQDQADKTVGTSDEGAPTTMPEEVAQADLAGASAATGATTETTALP
DHEPETSNTTGSSMFEEYESTATAEEEESLSSTLAAFVDNERYADVKLVVEGRDIQAHKAILCARSSHFRAMFTLGMREA
TTNVIEVGDISYEVFATILRYLYAAEVELQEETVVELMISANQYVLLPLQEQCEAFIEQGLSAENAGYFLEMANRFQAQH
LKALALEYMVQHRAEFESNEEALEDLSPELLHEYRQKSTGPASPSV
>Acas_g1175
MATARPNVQNERVKQVACAGSTAVLLLEKGSVKWCRIISEDAPTQRLSELPAQTIASMGKIKAVACTDAHVVLLTETGAV
YQFSSVVTRRTPKPVLVIPSGVTQLAGSVYICAALTSANEIYTWQPAGGVAKPEKVNLELASGIKSIACGSYHMAAITHD
GQLYTWGTIMHQLGPEKDQLVPRRVMELDGRAVASVSCGATHTACVTEMGRLYTWGVGEWGALCHGDVEDQPAPRLVEAL
DGVTVAQVACGWHHTIVAVSVGTDPNKTRLAKDISSLLDPNGTENFSDVTLVGAEGAREFRCHKIVLHRLPVLRRLIKES
SAGGDDGALRLKLDGVPDHLLEHVVEWLYGQPLSAELSLSVLVQLATVAKRQLGSAQLQQACESTFGMGLNCVSVCQRLE
DLASFDVDEREQGRQQAAGDGVDADLSHLQLTATDLRGFYERCFTLIARNWDLLKKDRQPELRSLEALACKHSPRVAALL
KGDTRAAAAAVAEAKQSDAQAEAEAEAEQEAATDQEDNEEDGLADSLLKFVDNPLYSDVKLVVEGHPEVHAHRAILCARS
SYFRAMLTLGMREATDGVIQIRDLSRETLMAVLRYIYAAEPELDEETVVQLLMAAHLYGLLSLQERLEAFIEQGLTMENA
GYFLAIGHRYEIQYLQVLVSNYMIQNRGEFASEEGKQALAAHLTPEQIEEFRRKIACADKCIVHGLHRNHAALWLVCRFT
IEARNEADQVPTCGGERFEVKLFPIKLEKPTSEDASLLSPDAPLFDSAIASRLEKHGFLKGRVAEQKDKDGHPTGEGIYE
VTYTVPYRGIYSVEVTLHGQHLRGSPFTVQTHAELTADKPATTAGQHKAAGKKPAARRGRGVTAAAADTQSEHEGFALLP
PPSASSDASPAASLTTAFGGLSVSATPPMFTASTTPFAPSSSTTLESPPAAFVFGSPSPSTTSSSSSSSSPSSSGFSFSF
GQ
>Acas_g1260
MNVVGGAQEEVSAEKMGRCVRRVLFNQFVVQLPVSFLLQPLLEARGIAYSAELPSAVEVVLHLLIFLAVEEVLFYYSHRA
LHLWNYQRIHKIHHEFRAPISIASEYAHPVEYVVSNMLPLLAGPLLMGSHLATVWVWTAIAVTGTSNHHCGYALPWLRGL
SSPRFHDHHHLSFNTNFGLVGLLDHLHGTRHKPLIAHRRVDAEAELLLALAKLKQIKATEWKQGTTAETKQKAVQVLSGA
VANEEYRLLLQAHAAAALAPIMEQGGSTGASALVLSEAVIEHLLRRIADVTPQPDLPFAQKKFLAVKATCDHVANKGGIE
VVAGFLRRTPLAESKRQRLRWATSCLWIMATDLTRILALASCDLIPVIRDIFQDYIDTYARSNFSSRFLSEALMSMAQHE
VTLMEVIREECLPTVWRYLLYHVRCKTEFPKRPDVLKVYHPRVTKLRARGLVCSVKSLVCCSVRGKDGHGFKPLLALTFM
FMDKAEKRQFFIDADGLSLFFKELQENKDSALTILTAFNCLVFGVEQIYSGTAVVPPAIPEPQPDTTAISTTTAHLPEYL
APIRRTVVQLGSFAVWRDDKSLHDVTFSFPYEDNQTVTAHKLLLAGRSAVFKAMFTLGMREAQRTSEALLTIPIPNMSPA

TFNLLLDFVYGVHLEAAAPPAQPGTTPAAAGSASGPSTNGHQSAPAGGGQPGQRPLFANSSALDAAVAWLERSGVGQDGA
SALMAAAGQYEILDLEDACAIFLGGKVVASNVLNLLREGLLHRSPVLIFRCFEFLLDNMWSLFMHTATATAATTTASTPQ
PEAAATTSSSSSSLSSSSSSSSGDGKQDKEEAAVADAPKLPPVTRAFSPLVRGILMEADCLQWLRGFVVRGMLGLPHVEFE
VVASAGPQSKVPRPDGAKACCLQ
>Acas_g1316
MEDQRTLGEDEEQLHMTDEVEEDNQESIQQQQQQQPTDEAGEGEGDDEAEAEHLDEAAQQSSMVSAAAPPPPPPGGPPPL
PDWLQRSIAGLPPSASADRSDEAAPSSAAPGPAGPADLAAMLSQRQLRATVVVEKQVVETPQDELRRALTRRMSSKAMAK
KDEIADLVAKLNEGRFRTFDDLARLVQHAVRVQNEVGNIELIKVARWPLDLLNALKEVVAIIHTIRRNIKRTEDALREQA
NKGLVANEQAVSNIRDCVRLLRETGPSLKRATDLVVMREVSVPLHQEAALAKTNALTESCVNVVFELALSKYKQPIPRNS
FNLRYRLNHTITLLQTAITLCHEAGVEAKDKRQMANQAILDMQHKYADVLQINAISSGEAFEQAPPDESSDVDTLGTPGS
TASSADSPKPAPAAGASSRSTGLLHSSSGASSRFPLHSYISEVAAYVSGLKESEGAEKVVAELAREDNRARTPMEVAILQ
ADVRAVRELLSLCPLSASVASFFASYRNGEGNSYAHLAASLPPMASATAASAQPSLSTSSSSSSSSAAASILSALVDALS
AETGFLSANLNDRGETPLHCAAKIGNADAVRFLVHQGVPLLDKDKFGRGALSLLALKCIERVTVAPPRPANDRACGLPGL
SQYLLNASLADVVFNVRGTLYPAHRIILCAQSPNFKSMLENKEWKEAQNVEVRLEDMSPVAFKHLLEHLYCGDSAFVTTN
LDLALDILHAADRFLVDGMKDKCQYVLFKMLTLENAFLLYTRASLHSARMLREATAHFILTHYDDIAFGDEDPGWQLSVD
PYGRPVYVPVLAPPATSAVGGLRGSHHHPIIATAPPASLTQPLTLTQPPATSSAALLSSLPLPPGWEEGKTATGKSYFID
HNTQTTSWEDPRRRLLSAAPLLPSSGANAPSAAGAVFPAVLSPSVAQTQPSAAAVSSPIASSSASPSPASSAQQQHASYS
SPPVSSSPPLSMLLGSPGPTTAHAHTHAPPPSLSSGAAAAAPTGRPAGETRPRAGSSGAGGGAQLARGKPVWDTAKEQPN
CSRCGAEFSFFKRRHQCRCCYHQLCDTCCSLRQRVRAFGHDEPVRVCEFCHNHLARGDAACIPRVVPCLQEDDLQLRAIG
ELYDLVRTDKAHVEDALSMGLPITILPLLLSENAPLQQACMRLIAKMAEDGRAAAALIGEQRTMPAIVHALSTGEDDTRA
YTASLLAQLARHEGGAELVLPAMAPLVACVQSSRSQSMQEAGLSALRALADQAGAEPHLLRAGAVPCVVGLLSASPAFTV
LEAAADTLQLWCSRGTSRSSCVRXSMRGPVIEAEGARVLVGLLAGHPSIQSYALVALSGLASEPSGSLAIACEGGIAALL
HFLPAAEEWAQERIARLLITLAANREAADIIAPQVSEGLPSVVALLDGRNARLAQVGLSFLSTVVELPGDLREKCAPSGL
IEAAVGLLGTKEERLRMQALSFIAKMAGHESNGMVLINCGGVREMVSLLACPELPVRTAAIGALAALSATGSERVAAAVA
AVGGIGALVPLLASSHPPQGQAYAACGLANLCAHLICREGFHLSNGIQHVPGLLASEAGEVRVQALRLLVAVAGEPKVRA
RAGPCVPPLIALLADTEWMASLGPELTEQAALALLHLCEDSPANRALLLVHKGLPCAVRLLQSSRPAVAQAAAHLLALLS
QDEQARGGMSGTRLVELLLGSEEAGAAALEEDVCCVLSRLPLQEPEAARLLGAGGIPTLTARLSAALRPVTRRYALVALT
NLSLFPATREAILQAPALPHVLPYLAGDEAGPREQAVSALLLLSDDPAFALSLHDANGFEVVVNLLLGLGPADGRLREEV
IVVLLHVCEQDPPAWGTFVEKGGVRGLLLLAASPNSTAQRRAALELRDMASRPHLRAGLVEDGAGEALVSLLCASADPHA
REAACQGLAHLTPVDPRVCGFLLDFGASAALILMLQSPDPVTREAALVSLAHLTAYPHSAATLRAEGLLPALLAVLAALA
DEQRETETCGGAALGHASGALRNFLAADPAEHIADFLALSASPAPVALLIDLLALVTTSSPADAAAGGDGTPANEESREA
AAAVEVALAECLDRVCAHGAGRAAVLDLPEAQQLPRVLVALLGSRETGAQSSALSAAEYLSREAPFRFAFHSSPGVAEAL
VGHAMTDLRPSSPPGAVEVFVALLARDDDASPSSLAEEAQELVARLLASIAADALHRAPLCTAAGVPALLRCLGVTSRPP
TVMLHSATALAHLALSREGSEAIGAAGGVEVVLGLLREPCPDDAACALLTALSHLLPHDEREKEFVEAGGPAVLVSLLRT
AGRGEPLARALDLLVDITACAGDKAVGQSLRREGAVAALAALLEADHHQPGDRSSRLAAAADDDEAQLMAPALHALYHLA
LSDDGREEVEQAAGRHLHALLGAQQPQQQRTRGRRGGEEAASGPPPPAPAPAGGGGAAVVRRAALRLAYALGLSLSVHTY
TPADLGS
>Acas_g2076
MSSSFRYASSGPFNGETEDGEEEDEEAEEERCRIPRGKDRLTWLIENLAYHFEATKRVYGFVKDACTPKSCPSMTAGPQY
EYLWMDEVGYSRPTRLAACKYISRLFHHWCDLILLDHTFGPPQGCEELLGTVFRRLFRVYGHVYYSHLRKIVARRKEAEL
NRYFKRLVLFAWHHKLIPPSEMEPLQSLINTLLCIKPYCYLATGTGLVAAHAYRRATDGTYTVTYLPTEPGRFSFHFSSL
TKTKVVAEGFRVQVFPSLASDLIKVLTEEQFVDVTFKAGDTRNAIKAHKMIINARLKGFEILNIIDEIQLSSSVRETTFR
EFIKYIYTGYVDTRNMKNYMIAELLLMAKNYGLEVLEKALERMENGGHEEEGKNNDEDDDSDDDDDDDEQEEEEELDDD
EDDDDDGDDSEYGVKKKEKGIEGLGYGSEGEGMEGIQTGYDGDEGYNDHSVHDDKENESDSDSDYPVNRKAKKKKEKRRK
GQQYHHQRSMSKTMKASTTAAAAKKTARSAINDFSYRDHGADFEKCINSWKFSDFIFSVYLATEKDGGRNVPVRNIYTHK
LFLFARSGFFRDLFNVYRDLRKTNIIIREDRAVVGPGVPTSEEADRSRSSGSGSGNGSANSNELVMSEEKKNFIYVPSYF
VFLVFLKWVYTDAVDLDLADSDHMAKLLDNPHVPRAEPEEEERTMSDLLFLARKFKAVGLIAILIQHKNQKEKKKREMQR
ASELAAARGGQQQQPHLTKKLSGGGVALDRAHKGGGDPTKSSQAHHRLKKKSGSKLKSGSGSRTEKLKKFIASSPSNLSS
GGVPTRYPYGEMTSSSTTQGTAAAASGSSAKVTYPYGEMAAPAPALSASLSPGHQPLRLAYDSDDDDSAIGLKKANAAAA
ARKSGKSGGRGGQSASGTASSARPRAETNSCEYRTGIMRLVASSNSPAPASPPLPQESPYVGLAPHTRPTSTSLLAPPPV
AFHPPTASSSLLRPPPTSFATSPYAPPPGTAAVSSSSDIRASADARPAAGQPSAATTPLFSLPAYTPAPFSIPATTIQQG
SAKVKAKAGPSSPRRSSSPPPSSSSTSPPYSLPHPRAVQAAQPPASPYAPPSSGGAYSPLLSSMPHAQQRLSPPLRGRAF
SIPTLIIEDASSPSSSSSSSATNNAGSSSTPLLFSLPQLPSASRVSAAQTSPRPNGHDRLAIPRRLQHQGRPAQPVAPPP
SSSSAGYSDLEEAPPPMIFTSSSPFPPPAHFNPHHHHHHQQQPPSAITHTTAAHALGAKRPTSPPLASEPPVASHRAQPS
ALLRSSPASTPSSEPPPSPRLVMSSSPRDTTASTAAKPKRSPSPAPSSYAPSATSVPSSATTAGLGSSPRFTSSLKSSHS
RNHSAPGLSSLDSGAGSGAAQHQDEAQVSFLKNGRVTLLTSLERKKTTTTKAKAKTKDGLKTSKKDKKASKDKAATADAS
LVADVKTKKTKAKTKTKARADVGSSSSSYSSGSTSKEGKATSMAEKKGKQLQEPTDKQQHQLSKSGARVNGEDDDDDVEE
EEAEVEIEASDESEDDGFDGVLHQRKTSCIVKEEDVRPGQESHLMKNNLKHNWRDSL
>Acas_g2207
MGDIEPASYAFILFFLYCILASIATFQIARIVCYGRSHPFLFHFSMLLFRIPINLQFSTFSLLILFYAHIYYKTEKQWES
RRLPFTLIFAITNLAYLVVTIVAVSLVATEGTQGDWENKMWNIVSAIGFFMLVFVLAYYGLRVASLMREGKAQISFQPKK
SSNVQVVSVTAVIFLLFTTRAIFNVLSAVDTIGFTVESGLELNDLGMFALFFVWEIVPTSLVIILFWRIPSPRRQQALLP

NAQAVPFLVSAVNEKDRAAYSTDTTVRTSLFDNPQRYDSDEDSPNYSASPRFYLPANSPYSPYDTNPTPPSFFISSDINL
PVAIKIVSFHNVVVPPPKSPLEDLFDFLEEPQLLRETDAQEVYLTLQLLGNNGKPLIPETTVPRIHLREWQRLPIKYRDL
PLSAQVVLKIWSCVAPRKAFCIAGVSYGLFNKHQQLRKGKHKVQLWPIKENDESLDYVAPGKISVHDDELWRIEKLVQEY
KRGQVPRVDWLDQITFKQIAERINAQSVMDHKAHMYIEFPAFEHTVVFHERDNPGCANQANLNSETPFIEDRSNRKPHLS
DAASAAIGLAANVVSTGAELKFTVVHDPEALRKDNPVENKYLKLMRYNRGLLDKDLKPDQKERNQLKRILNYPTTKQLTA
EEKEFLWKFRFYLKNNAAALTKFARCVDWSHRKERKQAMKLISEWEAIEVDHALELLSASFAGINPLRRYAVKRLEEAKD
EELLSYLLQLVQALRYEKGFLSDEQILASQTEQQIIEPGLPTKKVSRTRDLPATLRRKKTSAGNVAAKRSLTTALSNIRK
EEEGAAGSINGGVMIYTEMPRLAQFLIDRAMKNPKLANFLYWYFTVEATQDQKPTAYGLSYRRTLDHFIAQLGEVEDGQQ
QLLYFSRQKKLIGELIELYNQLKASGKNVKRKVERLREWLEGYRSFDPFPHPLNPDIIVTGLIPEKASVFGSAMAPFKLV
FRTDTGGEYSIIFKCGDDPRQDQLVIQLISLMDSLLKKVKFDLSLTPYGVLAMSCNEGMVEFIPSEPLSKVLAQYGRNIQ
NFLRQHHAEDTGPFGIERETLDIFIKSLAGYCVITYILGIGDRHNDNLMLRSEGHLFHIDFGFILGADPKIFPPPFKLTY
QMVEAMGGEASEEYEQFKSYCCSCFIILRKNANLILNLFSLMVDGDIPGIADLGGEKSILKVQEKFMLDLTDEQAIKEFQ
DLMKSAVENMWAPLFDKGHDIVQKCGSNFQSFERDNGAVYTYGSGRLGQLGHGNKKDVEVPHLVEALRQHHITTVACGRD
HTVAATDTGEVFFWGNRVDFVGSGSVADAEVLPVRIESLAGVVIRQIACGAYHTAVLAENGDLYVWGEYQATKSCAQCNH
RDPFLVSKAIEGQIVSIACGPHQTIAVTKDHRVWAWGGGVLKNPTNGHADLHQFHRPTPVEAFDGYKVAQVACGDFNIVG
LTVHGEVVIAHDLDYFKKSRKNTASRGYGWGGFYALGDYRDTPLEVDDSKPHRIVATNVKPLFGIRMLQVTVYRNTAMGV
TAFGEAYTWTLPDPWQSFSNAVTSVKVDALNGIVVAQVACGGEQTALLADESTFLATQLRHSLDAPQFADLRIVIDGHQI
LAHKCSAVCSILAARCKHWQTLLSLPAAQLGGFHLDRDSPDGIDTLTFPSGHPDDGREPASVDAQHGKAHKQVFSYSIVL
ALLQYLYTDKLIVPKQFEKLATDEKKRTKAKQTEKHKKVEEDEEAEADETENQEDEEEAKKERLAWYRQLERAAKYFKIA
RLRRLCQRATRNRGFAVQVPSSSLGLDLRWLVEPQLQAFGVRDPHCDDDDDDEDDNGNDQAASFNSDHHTSKAPTDATEN
QKAVTSNLRFAEMQAAMKKIEEEYAQSLERAHDRYLGQMQQAVELYRSELSIEAVVLRTNHQAWARERARADASASAQRA
AEAAELEAKERKAREKEEKRQAQIRREQEEQAKKNDDDRKRAKQEELARHREEKAKEQVGLGGGDESDLERQMVQERGAD
VKFLLGGECLWAHKLVITSSCEHFRLMFASGMRESELGEVDLGEGVEAGVFVHMLAFLYTDSLAGVDANTAIDLIAIADQ
FMLPRMKSICEIRIQKDIDPDNVAYVFQGADMYQATRLRSFCLGFMAHHFDEVQHTETFAALPPHLAKEVRDKAKTVQRA
APNKPAPVPQAPPPPAAKIGGGGPAMKAGGRPLPPPAPGGGGPPPPPPPTTAQTLAAQQAIAAARAEFDEAAVEQAIEQG
VYTSAFNSLRKAAAAWVHVLSFVDANSIANRVGLVSHAFHALATEPQLWRRLVLRDFPASSSSADSARRNLYEGEPDGWR
KTFYAHRWLERSKRPRFCLLGHVDCGKSTLLGRMLLGLGQVTQQEVNRLRDAAFEWGKGSFMYAWCSDRTDAERQRGITI
EWHAKECTLSSGREIEIIDVPGHRDFNKNASIGASMADVAMLVVGAGHGEFEAGVSDHGWFLGGTNHHLLLAKSLSMSKV
IVVVNKMDDRSVNYSQTRFNEIKDEVLRIAGGVGYKPEDLEVVPISAWTGENVVTAPSSMPWYNGPMLFEVMEQVARAVT
TSAPGEVVAKRPVFGPTRVSILQRVNKRCTTGGGTDIAQKQEEVVKALVQWGTPTTSVATFTVRSIQRFQTPRSRAAAGE
SDVFAFGRNSSRAIPLLRRGMVLLAAAERAGAVAGGNALTGRVESLTVRLDHSTPVSLRCGSEPYFIIHSSAAPYRVDKI
LRCWDKDGQEVKVDDASDGKATVITVKVGMSTLLLVKPLGDVCVEPFVNNAAMGTVLMFDRRRLVASGRIETVQRLGSEV
ID
>Acas_g2502
METFQVYSFGSNSDGQLGYATTAKNIGIPALVESLQGRRRVKQIACGASSTYQLLENGECYAWGVLPGALVHSTTSRQIP
QLTGKVAKHISCSSSPAMYSTVVLDNGECLLSQLGSDFQPSDKWGSARQVSLLMADLGRICEAEELKGGHTLDEEECFYD
VKLRCAREPLKPALRAHKAVLAARCPAWHALLPQQELKTVDAADEEAPSSSSSSSPAAAADMWELVVDGMEHAVMVEFVR
YLYTDRVSPDLEDRLLVLLCVVAERFHLSRLQAKCQAYWEQGVTPANVEAKMALLLDEEQLALAEAAERVKEESEASTLK
NSDAPYHLVVFTRLRIACVEFLQQRLLEMEAMTEASGPANPHADAQREQQAVIRQNLDRLRESTGTLEHAQLKSLATDGV
DNSAPTTTTATTPPSDKADDVAHHVNDDDDDDDDEFIQEMNEPAAEDDELVDYLDEEDYAEVLRQRKAREEKRVRVEKRE
AQKLRAKRESEDRQEEEATRANHSLGADLGAYVGSARYADIVFEVEDVRIPAHKALLCARSSHFRAMLTSGMREAQSGRI
VVPEISSSAFSTVLKYLYTSEADVNEENVIELLIVCNLYSIQQLQEQCENYIESGIQLDNVTELLQMAHQFQTHHLRSVA
MNYLVGRLKGDFSKLEGFSELSQDIQDEFKRGIPCPGESLIKAKSGGFQGKRFYFVVISRDFFKKRVLKGEEKWDIVVQF
EPHPTDSAATTTTATADQAASNAGSAAGGGDDADKGDDKADDVADKAEAGEGHHDAGAASSSSSSSSGSSGSSSDAMAKL
FDFGLHSGTQRGRRPAGRRSHAGGIPLRGRSDKHRTASSPLLPFSAPLGAAAAPAAPATTPTSPASPVSFDTSPASVAVF
ALIALVFVFVLYHRHHRVSSGVALAVIVALAAECRHRDDQQPTKEPAPSTTGAGFIRGRRRVKKSVSAEVGDAGHAQASA
ASYFAATATTPATNEEEEDIAAAAAIARRSAGDEEDRPSVLLTSNSDGTYTASFMPYKLGQYSISVSLNGTPIKNSPFRV
RFEKPIFSRNCRAAFKGDSGSGVITAGKEATLIIQSRDINNRPITVGGAEPGFRVCVYLPSAKESTVMSSNPSFYFNPAR
QGLSDKGLTDVYVRDNKDGTYTATFALRRSGDFLVDVVRHEISDKMRLPIFGSPYPIACFPAGAHGPKCEAEGTLSLHPC
DGLVSAVMGKPASFHIRTFDRYANRRDVGGDTFVVRLIRRGAVPLVRNEEEEREKEEERKREENSKKQNEEKCSPGGMMS
LSSSTSSSTSSLSTPLTPEQQQQFEDENIDAAEQRRGDERKNKKQELAERKRKQAEEWRQLDALLQQHELVRGVVTDEGD
GTYHATYTVPPDIHDIALRNRAALEAAAAAATTPDATSSSAEATSSGAEATSVAAEAAAEGDGEAWPQEYQLVVTLLDGG
MVRDSPFNLLLLHEGFVFDDDHAGVGSAHASLLGAAATIGASTPRGLASAENAEPTTPRIAIQRVAGGDDNDAFLRGLKS
DESENDDVEDELKSDDSENDDADEKNENGKNKDDESEDDDNDDVQSKRVVRKVMTKKTGPRPRPRTLSSFAKPTTITTPP
SFSPPTSPRAAPTSTASAPSSPFRLPSSSSSSAPSLLLPATVTTANPAASAPPVPLLPMPALAPAQPSFVPASPGSPFST
EIFSTMFTAHMELMQPDAATFSFSPSGASASFLPPAAALPPPSFSFSSSPAPSSGSSTTTTTATSPFTFAFASADVSSST
SAALRMPSTPPAAFSFGPAPTTAAAALLPFFGPPAGGLDGTPADESDGEDSELSSGSKKSRPGSD
>Acas_g2571
MRMATFFSAGFGQYLESGEFGDVALVAAEGGERFVAHRLVLAYSSAHFARLLLPQPPSPSASAPHSSCPSSPSVSSPSSS
RAPSSSRCGSPLASPTSPPARAANRSVSSDGALLGGSSAALVDGDDINWPKATKKKKKKKKDKDKEKEKRKEQRRAKKGK
ERALSSGNDSTEGSTRDSGSSLTVAVAAPTVPVATTSGGHKVVGEGAADKGSGTASGETAAEGGAAQPQIPAPPPPPVRL
ARRATGGSTSSGSVVLQRPSAQTLKRSNDTHHHHHHSHHANGIAISRSLSGSSNFFGSVRRGGGGGGGMKTASGSVMTLS

SFGTPRSYSSSLSGEGAALAGSDLTTHTMDEVIYPSSSPTPPGTAGHERQAAVADELPVIQLDFPDPNRIFPAVLRFIYE
GVIEILPENAIPLLAMADHYCIEILKKLCSDYVVANIDRENALWMLERAMLYNAEEVITTCVNVIARNFSYIYNANYNIL
PYRLLVSILEHPCLAVKALGEWPLFQIVWDYIKEHKDLTSQEQYRLFGTVRYRWMTTDELAQTQAANIVPSDFIIEALMA
RLKEAERDKAEEVGMCAEQDPTSRRLLPRPSFGILFDYAYDYDQKGIIFWIATNRGRGDVPASWFAIDLGPSRTLVPNYY
TLRHGGNYKADSLAHMGSTSAGSSDGKTWIVLKRHTNDTSLSGPFATHSWPIPSVTEAYRHFRILQTGHNSSNHNFLVLS
GVEFYGELYES
>Acas_g2583
MNGAPPHNPHHPPQALLDDVEEEVTITVEDVIAGLADADKARRLDMFRLLHEAVSGDTRKAVSVGRTDAAWHAIHQAALF
RPSWDGDQPGVDDLICLEALSCVWAISAHEENKPLVVLYGGVDAVMLTFNKIKYEKGHPNWADFLQTMSAIIQNVCESHY
DSGKKNDLHGVLVNKGVIEFLHFMVGERVFRRREDASNELECIKFLLCLTIGNLAYNRINRALLEEIGAFTFLRNCIRDT
KILIKNSWRTLQPFVPLLGLVRDIRQGHNDPLWAVITLAILCLEQFSSKFGERARHFLWRTFALNDSPETLRVLLLSPVI
RRTENEHLLEALLSLCKNMNLNVDQLTHNKDAVAAKEHMEKDLEDFGMLFNRREMFPDLVLVAPRHEPSSSTTTLVGEVS
AVRRPRNVTAAAHNDSSGGGDGEGLIYCHRAILAARCKVFAALFLRWEASDDEEGNDKESGEEKDNGSTASTAASGGGLE
LDDDEEEPKKRKKKEAKATKKKATGAKDLKKKRKRDGPPEHKHIRRKKDSQSNGKGDLQRLVVQDVDFDTMHKIVEFIYK
GRVELDESCVVDVLRAADIYGLDPLKSKCEAFLGDSLDLDNIAKLLEIANIYSAWHLEKRCQEYIRFTYNGHFARVVESA
LFVDLFERMPHYRESLAEVCNVVSFVPSVTTSLVAPARNATAC
>Acas_g2896
MATKDYKIAIGGPGGVGKSAIVLQFVTGNYVSEYDPTIEDSYRKQFALGDKVVMADMLDTAGMEEFSAMRDQWMRTHNGF
IIVFGLDSRSSDDVDHRQVESAEAIAMARRWNVPYVEATAKESVDHIFAHLLYDMEAARVALATAKEKEEPATKKTEANK
NSTWQQDMLALVNEPSSADIRIFIDHQGAVSGPIFGHSMIIGRQSPKLRELFAQAKQSAGACGVGQAADELPAVTLSHVS
PAVLQFLLGYFYSSTLALPSFLEPQGQAVIAELYRSIGGTSDAPKAPEAADSSSKSEGMRRAWKLVRRVYKSKRPYSIIL
DPVDVASTATVGPESVAAIMPLLQRVMAQGVSTAGAAHLCALLQHMHAFAAAYEISALQEFLDQRLAALNLLSECGKAMD
QVMSVVAPFFEEWEAVKASSATPASAYFARFLELLARHGVRFVFEMVRFVKLLADVRGLMGADVMRMLTTLIVDRADAVF
LRLSLALVDLHLARTAHEGETEQASAEYVTRLLVQWELLLDPLAALLDARQSGGAVTAAADGDGDAGATASEGDEAPAAA
EKKTKNLEHTLNLRKKEKKRRAQSPDEQAKYFRSYMKTGLSSLKEFLPELREELIKHATGKPTTLTRKHLPTFFNDLADN
LCKWSKANKHYNEAMHHPNLVTAAVEQLVAALAYGREAEKHIGRDIHAVAQVVLSDELREILIETGVIKREYASEADEQN
KPGLLANGKEKATATPKTEEPEIETSKAKEVAKADESEHGATTETSKEQEGGDRAVAEAQPDRTKAFLPRLGIEAESDLA
FVVEGQRIFCYRAVLMGRSQHFRISFTGYMERYTKEIPVPDHSCAAFAAFLHYLYYDQLPHLDASDNATLVGWCDGQFRV
ASPLSAGDVLLEVVAISDEYHHEHLKELVLGQLLPFVDVDNVVFLHMMAQLYNATDLRNKCVAVMSDNFEKIHDEHFPAA
LKVLQGVVPQEATASEAEAESSSGHKEDRIELDEATAQQLIKGWEERRKQGLPVSGRKAELVQRVIDHLDSDSDSGSPNK
EDDSENEDEEDEEGDEREKEEQKKKAKIKQPANAKRSSKKQQESEDEASDTDGTVEASGDDDEEDEESDGEGAAPLLTLG
PSTTESEEEEEEADQESSEEEYDFEPYKVNPGFQKLLMRIGGVCESVADPLPMRLIDQFEQQLRTNLGKHVRLPNDLRQL
YRFCNGLQNEEDNFTVCPLVDVGDGRGGPMTHADIFGPGHCGWLDMAEDPAWIPFATGSMEEEHEIFMVNANLDSESYGH
VMLLIRDCFDDGERVEARSIYSMMSKRWRNVC
>Acas_g3311
MEAPGPGDSNWTGGGGEKSSKKKDKKKDTEKKYFTVKNSKAKSSPAASKKDKEREKGERDERITAKNRGGSRDEAEAELE
PDESSSASTTSHTVHDELDLTLDDLSTGSDVEVIEVPIRAYEDQDHWAVVACTGSPPPSRNKHTAVHYNGSLYFFGGDDG
NSRLNDLYQFIIAKKEWRKVATSGNPPKPRYYHSAVVEGPCMYVFGGYTDHNHNDTFQYHFGTREWTQLECAGEVPSQRS
GHNAVMHNGAMYVFGGYDGSKRLNDLFKLDISKREWEAVESTGKAPTSRCNASAVVIEGSMLVFAGHSGVATNADLYDYN
FASRTWSQIECSGDAPSKRLGHTSVCNQDHMYMFGGTAANNFCNDLYRLNIQKKEWVLIRTTGPTPFSRCYHTAVVVDTV
MYIFGGAPPDAHPLDLYSFTFAAVDPPCTLSDDMGAFLEKGELADVTFLVDKEPITAHSLILHVRSSVSLDNVIAVLKLV
QSDKGGVVSAVVKEFSIMFVLRHYQDVIVQQAFETVGPALLVEIMRLYQQFTATKSSKYQAAAAARPVEIPPSKLADDLA
RLRRDETTWDFTFYVRGKPLGAHLVMLLARTGFFQGALRSTMKGGDLHTRQMAVVIGSVQPTPEAFASFLDYIYTGRVDT
PPDEAVYLLTASGFYGLTNDRLKAIASKQITQNLHRNNILRVLEAASDVGARQLKHFVLEQVVKDFAHLMRTQADQVRQL
SKALLIDNMTSLALTMPPSSSSSLSSSLSLSPSASSSSSPSSSSSSSSASPSVPPAQSGLGSDSSGTRP
>Acas_g3432
MKQQLPYAKELDDPAHGDVQLRLNGDSCFYVHRFLLKASSPVFAAMLTNGMRETGSLTIDITEEPDHAPAFGQLLSFLYP
YSHIDITDNNVASLLFFSDKYQLAPKLKQECELYLITQRQSWDLLLMAQQYGLHRLLDQHVEWAAFRTTGLFRKEPQRLD
QLKKSTLKKILLQRG
>Acas_g3433
MKQLPYAEQLKDPAHGDLTIRLNNQTNFHVHRFLLKLSSPVFEAMLTNGMRETSSATVQIVEEDSNHSHAFGQMLSYFYP
FCRVVVDDDNALALLHFADKYQLPPKLKEDCECHLTRQPRSWELLLTAQQYGLDRLLELHVSQAVQWNKLQ
>Acas_g3527
MEAEVAQSRWVSVTHASMTARIGSEEAGAGGAVWQWLKPEVVSKKAAPGGGGGTTRAQELDAWRSTRRADAGVKGTPPSV
RMAHTASALGASGSEVLVFGGLSDTHRTLRDAFLLNTKTWEWTSVEPASEQCPAARAYHTASVISGNRVVMIGGESMEVV
ARYYHANRTVDLELNAVDVFDLTTKTWTSVATTVSPRKQPDSRAAEATPQADDFPQRRSEKPWRWEAIDVGTHHTLVSVN
DTSVLLFGGKDYKYTFDDVRLISPDFKEMDFLEPAADSFPVPSLFGHSAQQVGSQMVVIGGKAFNSSSTEIYVFDLHHRT
WKRQATSANPENYLPRYYHACCVFGGNKILMFGGYDLDWGCQNDMAILETGIVTSGLVTDLSRLLKQGTLSDMTIRVRSS
SPAQDHQIAAHRALVAARCPSLFELASSGPHDERGCVVDVEGDPEAWTHFVHFLYTGLFADRSGDDAAFAEECGRSQVKK
SLTTDDTEVVAQLLDINARHPVVGLADLLVESKGGAPRRVLPEREVIHSETLPPLVNALHDMLKQERQQKQQKAKLQDIT
LKLQQNGGDAPARSAHRFMLAARSEYFRAMLRFDGHESNEEVRLLELSENVSPQALDVILAFLYTDRVDVGHLEVDEALE
LVGVANEYLLGELKLFVERFVISSKAVDEENVFFVLDTSEHFDAHELRYHCVGLLAQSTNTSAEFNQQLAALPPHLQAAI

RERQD
>Acas_g3679
MASLEELRWQAYAMPCSGSASTTEQLEEPVASLQNAMKTLLADPTFADVTFEVDGEEVRAHRSILSARSPTFKAMFTSGL
SESAEGAVIKLDCNKRIFEDVLKYIYTEQVTLTDVDTAIEMLGRAEEYFLPGLKKECESILLKQYLTEDTATSLMVVADM
YRTPALKKACVDFILRNSRSVNVAELDKAMLAELLVTACSITAPGPHRPPHDRPLCCVGHHDLGHHAHSSRVRLKPPTAP
RRRRRRRAWQEM
>Acas_g3922
MRRYWSERKARGYARGGVDDDGAGRRFTGPQVGVWQVFVRTLQGQLLTIRAGPNDTVEALKFKVWALEGIPADQQRMIFA
GKRLEDGRTCADYGMSDQCTLHLVLRLRGGGPGQESEEEQRRSQLWPAPDGAEPTTTNPQPNSFGSMAAEAQARKGDSMY
AGRTFMFDIKFGVLDEVDWHRLKGTSLSSSSGRPSATLELQLRSGTVNLKTLTITTRDMTIGELKDTISARDHIERSAIE
LHDYGQAGLWDDDKKIRMVTPFFHPQVSHDGLLCLSRAHRERVWQLSDCQTDPSEFTLRTLHPANHTPDPTSAEGIELPK
SRLINHLSRLVNDLDALPDVEFVLNNGEDKVYAHKSILVGRSPVFRAMFSGHDPSTTGLTQVPIDLPEHATAATFASFVR
AVYTLAYDEPTASPDVAHEGVVSLAQEFGVAVPTDPAARQSELEVAQLKLMTDDQFKDVTLVVGGDKLRAHKYLLMARSK
YLHTMFTSGMKEGREGVAEVELHDIAFPVLQHILEFIYTDKINELPENLAELLMAADLFQLARLKALCGKALAEALEVSN
ASWLYEIASRFDAEPLRQAALHFIMRHWTDVAATLDVAAEPAHVLNCCVFLAGGLVYAVALWRSLPCALVYITGIYAFFL
GSCFVLSFELAQAVQRSDRTVVAVFTSLLSFLFAFTMPRCYKAERDFVGELLEERRQLRMWEEVSVMVPITAALLRKFDL
HLLAANKTLGGARGLHRDALELLGQYEKEEQQRAGTAHVGLRSLRVSAIHPRPQGMQGLLAPFHQLRSLSLHVFIKPNDL
RCLRSMPQLRSLDLSRSSLCDQGMTHLAIRSLEELSIAECTMATDAGLLALTQKKGVLTRLRVLDIARTKFTPAALITVS
DLDDPAFLRWTQVLRLTHLNLHATRVTGASFRHIIGMKHLRHLDLSGIFSGNQCSHHEVLAAPRLEYANLSYTLPRSGLW
RRGHIRMLSRHLRVVVDTKGINERLLSPNTHRLNPRATPRKTSGVCQALAKGVRDGDDDLVDEAVRVLNRSLAPSVNLYD
DSQFEPRFADKTERYQHFQYKAAVGRQLLEHFLRVYDPPAFRPVGRATCLPQRLASELHLFQRAHLDYFRLRRQGRLVCD
GPTQLAHHVLSADLCNLSPADVEELTKNVMVCLAESFTRLRADDGSGSKAKASASLDLKNLVQRREVGSVERAVRLLARL
VALANASAHARLIEIIAVDSGGLRLVVDVMWHLGLAGDDTALCLVCLQIVAGLVAYETPPAHLLPRAWLGQVAARQGSDA
DETTRAARVQEQLAANRLWQSRLCKAALLFGATEPGISEHVLRAFPIKLESLSVSGLGHLVISNLAHYVRGRPAPCPLKS
WLVPFDSSYSLRPLIKTLLESTSHDTVRPDAPLAFHALGTENALRLVLAAALRSARAAAGFGRAAWARERQIEEAHAASV
AQAWAKLALNDHHKPEPNFWASACTPLAAVCTTDDDWVAPSTNGVNAAVAEVCDQFVASFVEAGGVALLVEVVERECAES
QRTTRCDGRRIALTLARVEELYEARYRYRGRGVGRRSAPNWSWYELQLRWERQRKIALLAAALLRTLVAAKRDDDNDMEI
GRHVDGSTIAQLTSIEPGPGMDRLLASARSLHALGHTGAVAAAWEDDVGLRPYLDLRRAKQANEAHNRLRRLKKKQERKQ
RNHRPA
>Acas_g3930
MREALGMIRFALFTTPQLVEVQKSALAVRLLPEGYFLRVYAHKLELQQFPTTTPSTATASAEFMPRTPPPAGPHDSNKTV
VIGQVITGAMDQSFLWRIHQVRETLGLDDLLSTATRTFRSPSFALDGSKRQFVLSYNAMERSLRLYVARSRAVSDGIRGD
AQLPELVDDSDFPSGRRTTTHYVAVVHPQSSHDSITQEVTPQSAALRVALVDLTSQGFLGRASEDSSSDLLLVRLLSVSL
PSRSAPASHLTNAINEEGEDVAVDTNQHAGDQLVAQKEENGQGESMIDFMHFFNSGKLADVTLRLVDENDGENEDENENE
GKSESEGKVQGRVVVKEVKAHRLLLCCGSDYFDSMFGSPWSEALTNEVCIPLASGADDSCNPTGVQLLEKGEVGMFTEMI
ASFYGKTITPDESNVLPLLKISLRFLAHALSESCLAWLKEAADVEQLFSLLLRANHLNLPQAQDCLMNRLASEFGGMVHR
GQMHLFEQLPYEQIVELTQRDQLYTLSEYQVWLVVSHYINTHGQELHESEVAYLLRMVRYPLLTSVEILQAALAFRLAHH
EPLPSSDVAYGQGATRPRSRDGLWCFTIPGIERLLDAEKRRKIQRKEHKRLEEKARNPALINVYYSTPTSSSNISTNSPS
SRQSKAKRSAGGANSVQSSEAFVDREGCSWTLRLVPEFRQGQQFVSLEIEFYPPASTAHHLLSRHRTGAQPSGRIEEAQL
SASSLAARFGMVFAGRVILQFITQSPFQPIKREFPFAFRYPNERVAYTQLFTIGDLRSLRLIDDAVAYPGRDALTIQFEV
RLDPRRSQPSSFERLVTARFRHQPPRRSDDGAGSQYQHEPAAAEEAEFLCEVVDKTQLPAVLAGYGVLPDGRGLVVVVGG
RERRDAHVDSLQIRGAKAEVDVVLVDSRKRVVAIEHRLVFATISANHACYVLVVNTRDRATLLPHLAPGDTGDVDC
>Acas_g3967
MQNIKAVVVGDGAVGKSCLLISYTTNSFPGEYVPTVFDNYSANVMVDGRPVNLGLWDTAGQEDYDRLRPLSYPQTDVFVL
CFSVASPTSLENVKSKWIAEISHHCPEVPVVLCANKVDLRDDDSIISRLRDRGQHPITYDEGAAMAKQIGAAHYVETSAL
TQAGLKNAFDVIMRVAMGGERASSYKGKKSKGRGKGGKSAYDAKPALIPPVLPKPTHAPWTNIQTSTFADDFKGLLESSA
EADVKFVVEERAVKAHRVVLAANSLLFRRIFGADGAQSAEATTAAAAATVKAKAKQSQSQSNKAASKSKSSTSAKKKGKG
ADDKQQQQKKKTKKNKKEKKKEEEEEAKVEVADTKGEEESKEADGGEESEKDPVLTCDGHTYERTSIAEWLQTHSTAPIT
GAELENKALIPNHLSESESEEEEEETETQHRADADPSVPPLHRQINAGQIPGFSSIHRGRTSGGGEEGVAYTIGISKRVS
HAVFARVLEFFYTGIATIRDKLDGVQDLLAAAELFGLEHLATICRNVMDDSEFLNPSIGTWLNDEAGAVAKQLFLNRPLF
SDVAFEVEGKRIHAHKAILMGRCKVLAAMFSGLFVEDSLETIPIGDTSLESFLAFLEFLYTDHSPTLREWRLRRHSHPRQ
PLRRSRPATSDGIEKADIDVVGLLHLAEQHNADQLVGFCRHFIASNYQPMSKRPEFSTLEGENLEYIEANQWPPKSYLAE
LAVYEKAIKETDKDAKYSTRAQDRDQR
>Acas_g4071
MQVQESHAAQRAKEDGVMWAWGSNAYGQLGWAPTPTVRQQAKPSPIVLECVGARQVVQVACGLYHTVCLTADGLVFSWGR
NVELQLGHAGPKSSGAAAAAAVQVDAAQVLEGLEDMPVQQIACGRYHTAVLTTAGAIMEWGWGEQKAPRLVEGLLGLPIR
QLACGVYTTIALTSDGQVYTWEARKLGQAGYAPQLVEALQDKGVVKLACGLWDVMALTEDGEVYTWTDHSWSPFNSNKVI
PSLVTALSGQGITDIVCGKAFSVALTDGGDVWSWPKGREDEAKPMEALRDKGVRMLACGDTHTGGPCHDECTVFTSGLND
YNQLGSVKDRTTDEPSAVDDLENMSFCQVACGGYHTVALTGPWMGLSSDLVDDLAAILHSGQMADYEFVVDGRSFPVHKI
ILAARCSHLALAETDTTTRVEMNDVTAEIFGVFLEFVYKDRCRVSRANGKALLSLAERFGVPRLARLCHNALSSTTSRGD
TASPLERLGNELLQVLDRPQLSDITFLAEGHRIQAHRAIVAARCPYFKALWHAGMRESEERELSVGEIEAGVFTDVMKYI
YSGQVEFNPDTVVELMAAANQYQLVQLKRRCEKYIIQAIDEDNVEVRANHDL

>Acas_g4117
MEDANVIKIKVIAAEDLYVDESKEALRVVHGQSVEFRIPKTIRLLKLKKRYCEEYNLHLDGANTSTRSGVQFKFGNRTID
DNDTVCPSSLPRPFTLFFMHRDADERSFVCGTGGGQCGSLCLTTNDVIKVHHTPEFHCYLNSTSVPGKTLQSDMRELYLE
AMRSGGHGSTDVVFNVGKEKVHAHKLILTARCEKLRAMFSSSFAEYNEQKTGEVAIEGHDPSTFRLMLEWIYCDNVQMPT
TAEGCLQLLALAEEMLLFPLKTTCEVVLMSHISKQNAVTLLICADRYHVDFLKQKCKQYIMDHYDEMLREQPEFEKELCK
VPELLVELTRSALSREEPRRYVVLETSIGSPSTGRGEKRRRGN
>Acas_g4129
MEQGTGAALADGWSSFSVDNVANPALFYTHAADHLWSRDEPPPTAEFITNALNSLHTRLHYAVEIEDVEMVKHTLQTEPD
LLKFSFKWRGTPLHVAAVKGNVEIMRLLLNAAEANEPSALRQQLTSPASFGRTPIMIAAANGKSECLRLLLEEAKKAKCE
NALKLTDDLGMSPLHAAASTGSVECVTVLLEYGDDVNERHDDMSALHLAAFYGHVDMLKLLISKGCQVSWCSRRSGETPL
HRAAVRGHTECVRVLLDSGAVIDAKDSMGNTPLHQASFFGRIDCAGLLVEAGADVNAKNSIGAIPFSRTNEDLKLMWRTP
RITGLHPGPRHGHSATKVGAKLFIIGGSSEKEERVDVVVLDTDAMMWYRPTVKGDAPASRSFHSATLVGSKLYLFGGSND
SHYFNDLFIFDALTMQWSAVEAKGDIPEPLSGHSATLFGSQIFVFGGYDGQTYHDQLYVFDTQTLEWRKQNPSGDIPPAR
AWHTGNQVRTKIFIFGGTGASAYNDLHILDPGVMRFYKQSVVGQPRACSGHASALVGNKLFYLAGGMFDSGLDDLNILDT
ENFTWSALKIGGQRSKWTMANFSGHNLTLVGSSLYCYGGYFFEKYNTTVRVMETGTRTTLESDMKNAFITNDFADVTFRF
PNEDNALIKAHKIVLASRSQKFRDLLQGRDEEGLTIDINDIPRELFQVLMELCYTDHLTSCPHRAQQLLSLVKAYIPQSY
KRTLACLIQKGKVASSLGDDLRQNALGSPLFSDVIFEVEGRDVPCHKVVITSRCPQFQAMFLSGMRESTAEKIPLDLHYP
IFLMFLEFLYTDDVDFTKVSPDDVIELLGVANQYTLDQLTDRCDRELQKFIDFENVVVLFQAASLYHAERLRSSCVKFIL
RSYDKLEKEGVLEQLSEDVVEELNLLKENMDTDKYSSSLQGPVLPRAV
>Acas_g4592
MAEGAVLGLSDSVDQQILQAVVQGDVQELAILLRNFGKDPSSIIGKKGRTPLHFGCLNGNAACVTLLLTIVQTVDSEDAK
HKTPLALAAKRGFNKVIELLLARGANLNTRDENGNTPIHQASYHGQKATVELLVKHGAQPNVPNDIESAPLHFAAILIKA
SADVNSVDPYNQIPLHLAARTGHYDCLQVMLEDGAEVNAQDMEGNTPFDWADDYGHGECAVLLWRRGAQIKTQTGKQTMQ
WRRPWGGRERPTMRYDHAMTALGSSIYMFGGYGISPQQEGYEITPQEGDEPMALHMSDLYVLKAEGELQSLGTIATAEDR
RDHSYALDLRRIGENLVVAEDGLTVEYVVDASVLSPSKPAVVKTSTPIRRIGDETLFYFEITVLDQGLKGYIAVGLTDVN
YPLNKQPGWVKHSYGYHGDDGMAYHNQGSGIPFGPRFGTGDTVGCGLIYETGEVFFTKNGKFLGVGYSNAQGKELFPSVG
LHSSAEKVVFNLGKKPFKFNFEAEVVEWTALNPRGQEPEGRSASHLLPFGSKLLLFGGYSPVDFFDDVHVLDTDTMVWSE
MATKGPLPRYRDVSVSICMNKLVVFGGRSASRCVNFVYFLDLATWEWENVTAKIEGDGPRARMAHASAVVGDKVYIFGGV
TAEKQLLNDIHILDVGRMQWSSPQVYGRPPTARQNATVTPVDKRLYVFGGWDGRRQHNDMHVFDTERLAWYKPHVSGTVP
RPRNHHTAAALNVDGRQQLYFFAGWNGRGYMEDLDCLDLQSEESEELLSACNQPDFHDITFWVAGKPIYAHKVILASRSS
YLRSMLTTGEDAMACEEEQRGAWEDKSEVVLTDVTDYSVFLAFIHFLYTDIADATTEMIGALLAVTEKYGKEHIERMSEY
LILTRLKTPSSLYKNLRWAFNNPRFSDVRFLVRERTRSEREEEEELFGGHLKGKERDADSPATAPTAQRVYGHKVVVCGR
SPYFKALLLGGLKETSQEEIVINPSDDEGLGDDMAVEGEFARGRGSFDAVDAVTSSAIRYAIFTSLMKYLYTDEMELEND
EDRLDLLVLANLYSVESLKKLIEDDLMQSLDFENVSCLFALADTANALNLRRKCLHFITENENTLQGIKLTWSYMDLSGE
LKEEIARFWATRQAQ
>Acas_g4714
MKPMTNAFGPGTTGSSLFSGFGQPAATAATTTSGGVFDFGAATSGGFGQPVTTTSGGGFGGFGSATSGGFGQPAAAATTT
SGGGGLFGSTTTTGVFGQPVATAAASGGGFGFGAATSGGFGQPAATTPTSGGCFGTFGAPSTTTPAFGAPAATAAGHFSS
FTPTPERVNVARLFDRRDLFPDVILQYQGQDIYAHRAILALGSPKLQKMFDDHSTQPPMMQENKPVYLLDDDIKGLQLTD
VSRIIKVPMQPNPPLSSTTYHTSHRSATRAVFDADLPLARLVQIALVAEEFKMTSLSSICQTLCLHKAMAETSLEPILET
LETAHQNGLVELKKGIMTFLKEASSNLGATVDEFMRLDKDLLAEFLLHVTGMLNPSTPSGAFDFGSSV
>Acas_g4928
MSSWLSSSSSSSLMFTRVETVVVSARVTGQRVELSHRDFSGGQAGGGVLSPEVVRDVLAKNAHTPVVVLVLRSCKLAALP
RELGQLTFLHELDVSDNLLTDLPIELVALTNLAKLTLSGNKFKELPVFIGHLPGLLHLQVLGMPTLTTPHNLLRHGTEAI
CAYLRSSFVTLEEKQGELELHVRETFNVAKHADICFVVDGEPIHAHRLIIGLRIASPIFESFLASPNKEHKIEGVGAETF
VRLLRFAYGNGLYDSLKSPLENRAFLAMLDTLQITTFGRNHPPPETQQGTTKRRGKQKRETRAEREMRVFAEAQARRAAT
ERQRELEAQAERDKAERQARVRSEQAQADELAKKAQVERERQAAAATAETAAAAARAASGLLPPGTLLNEAFLRRHGWFA
DLRVRCASDVRGGGGGESEEDEEEEAQVENETGHGVFVCHKFLLERLRSVVEKFFGDNIDHDNVLSLLHLADHYQAYHLK
VFCTSYIFKHQDAITSCVVGNS
>Acas_g5029
MMGDQHDRGDHEGHNYIHAIDDTNFMRFDSLAVRALGAGLSWRDEDAIEDDIFQCAVMGRHERVRYLVEVKRVQVNQRDK
WDSTPLYYACLAGHEPLVRYLLEQGARCEENTFDGERCFHAALTNDIRRMLREYKAVSRTHDPFSDFLRNSFYYHMDDLS
ERPDDERPPHSDVHFIVNGVHTWAHRVVLASRSAYFKQMFRTRWQGKRRVLLAHPKLDADAFKAVVHYLYNERLQVPRIL
LDETEAMVKQCKLWDLHGVLQREIKKQNVGLPLPERVVIELADIYRSTETQRTGPTPLQSAFGQVAMRSIRNAFNESGDC
DDDDDDDDMFEDIVFKVEGKRFKCHKFFFCGRSEYFQALVMGNFREGEGNAAGDNTPGSDREDDADVSVDHDSPATRVTR
ELELSDVSADAFAFIVDFMYTDNVTNALPFVQELRRRRATTTMTEKNEDEGNEEENESESEKEWHLVYEVLQLSDMYMLP
VLRNLCSMLLSQYIDLGNLFDLADLAQLMGLDRLEEPIIQFMALNLEELVRQRKDTFSEMIRESAYSIANRQATDSIPIV
DDIRSALDRMFPTDDLEAAQTYEEDDDWEDWEEDNPDGDDDRDAGDDGEEEEEVSMKSRVKARVRRNDRMVQEEEGDEGE
DGERSAGKVPDERSNAAWTRYQYVVAAKNRKLELLDRLIEELGLSG
>Acas_g5239
MESRRQRAGKNFYFEFMERPSYYFVWGEGAWGKLGFGGSHDYCHPPDQPEPRQGLDQQLRGKQIVRVACQKRHTFILTEE
GQVFSFGSNPPGLDILGTGSRMALTQDEPKPTHQQSRLVAKAIAASSNHALILAHSXGGDVLVWGAGEDGQLGQGDYSSV

STPTFCDALRGKRICQVACGSGLTAALSVDGRVWAWGQLLQWHLTPTLVMDADDRVSQIACGANNMAAWQGPLSPFMESV
RSQVANVADPSTLLSTALRSIIDKEEGSDCCFLVGDTEGDGQGRRIHAHAIVLAVRCAQLGEVIEAEWAKLRKSESESAG
GGKARERLSIRLPPDVSQKSAVHFLEYLYCDELRLAPKHYAAYAPRQAKEKNEKRNDEAEDEAGEEDDESGEEAKVVEDL
LQLSRHFGTKALEKEISRRVASPQLSASLATGPISATLSTDLARAFDNPLFSDLTVQLTGDENSDEKNEGEDQEKNESEG
QATTTATSIRAHKCILAARSRYFNAMLLSGLKESYMGEMVVSHIRPSVFVALARYLYTGGDATITPDSVVELFLAANEYS
IEGLVKQCERMIENALELEIVADLWAMASLHSSEELRQVCINYVLKEIDDHNKVAADALHHLSSEVIEVVCSRLQSQLAG
K
>Acas_g5391
MALTGSSSPHFYLARSRSTSLPPLSSSSSSSSSKSSSVSPFAAFCSALTWPNHVLPSLALAFDEPSFFADLRVDINPIIT
PTSSTPSSPSPSSSPSSSSSSSSTQQGEGEECQHSTNTEESSPRLGQDCVWVHRMVVAVHSPVFRSLLRSGSEAGGSRES
REGVIDMCGPQHPPGRVVRCLLKMMYASPGLAIDELGHEPKLSDEWEFLLQLFKLAHLYQVEVVLDACTRRLKHILEQRK
DTTATTPAARQATVQHHRRGSKAAAAAAANAAAVADVDLNGGGGGELSDEEEERTRQTCYALELLAFLLHIKEAETSDHP
NSDPPSPTNTTTTGRRSQSAPDVSLSSSTQPRDVDSSGDDVSTAPGKKVVEVGVEAEEAAAAVAPVGEQREEMLVRMLDH
HLYPQLAMIVQHIRHAKLYSA
>Acas_g5603
MDVAKEPVERAVSFVAKAFGGHLQKGLRQLLAARMFTDVTLNVLQENQAPVSVPAHKFVLSMASQEFKLLLSMDEEDACE
VKEVDVDIGNIPLRIFNYMLDFIYTGTTSFAESDLLLLIELASKYRVVSLREKCGEMLYDDNKDVASLMEIAAKYGCVKL
GQLCAQYFARNWTRASKDGTLMRLNPGMWEELLRRDEIEAYEEDIFAAVIRYSQDQIYERADRVLEQLLPLIRFPLLETR
FLLEDVGKNPILEILPITHELLHEAFRYKLCPDPHLTQAGRCKPRKGALRWSATQKSGDIVVSDNRMTASYRASSAANRW
NTVLAETYVRAPGKHTFYIRVDACNSGWMFIGVATKDWNGWRSSGGAGGRGRVHSATTYIGNVAPSWSIASSAGWGKTDA
GGSNVPYGNGFSTGDVVKLVIDSDEKTVSYAVNGLDLGVAFTGLPHELRVGVTLYGPGDRVTIVQGDKKTSD
>Acas_g5770
MSLWRAASEGNAKQLNHMLRSGKAAGFNITNTLGQTPLHVAVIAGQVECVSVLLADPSLDVNRRDADLGWTPLHYAIFKK
FPRIAVMLAARGAEIDVLDHEGFSPVDLAFVDTYPPPQRSDFDLHQLNGQSAAASADSDEWLEDGGSQKRPLPTSKPDEV
FGELFTWGEGSNYCLGHGNPNSVAQPKRVTSLQEDSVVQVSTSDFSTGVVTRLGRLFTFGVGTGGRLGHGDTEIRLVPKR
VVGPLLAKRIVGVSMGFTHTAAVADDGSVFTWGSGAFGQLGHGTTDDSLAPRKIRGGFEGQKPIAVAAGRHHTAVLTSKG
GVWTFGCGENGELGYLTDALAKKAYCHCTPRQVVMSIEAPVVAIAVGKAHTACTTQAGDIYLLGFGSPQARKLFLPTILP
SAFAGYQQAAGASSSSSPSRVLPTPVYSISPSQTSFSVLSGDSLSAPPTAAASRGHRLLTSLVASGNMTLAVFNRRDVFL
SDLYHVDGLHTRIPKPIYIPAIPGNNTIASAALNTNKEIVVVTDAGHLWTCGENKEKAREWVWRRISVGKRFSQVACSRN
HYAAIAHKEALVKETIDISPWSLSAHLLKELENDDFTDVEFVMDDGRRVPAHKIVLAMRGHHFATLFAAEDEDGGDSEAP
VWGESDYYVPDMVTNHLRPNKKRREVKVDQTAYPLFHKFIRLVYTDPSDPRAPKPTESDRGQVAPLLKRFKMHSISSSSS
RDKARAKGKERVVETDADENAAHATTPSPSPAASAAVGSASSLSHFETELRAAFFDERLTDVDLISSEEDRIPCHQVIIC
SQSEVFRTMMTIGMLESHKSEIFIQNASTDTLKKVKEFLYLGNFAELHGEGGKKVGGAREVIPFGTVMELLNISNQFMLQ
RMKETLEHLLLHQYLDYDNLVSLLITAENYSCHNLKRQLFEFARRHFITLLQGNVARDLLDHPDYAYLLDDLDYFVQSGG
KLRDVDGEDGRRRRRRRWRDGGGGGAAPGRGRRAADVLLRGGDPEPEGDGGLLGARAADREGAREAEGQRAATPRGQARQ
GRRQGASGQGRPHGQVSVDKSVDKSQSAKGKEKVMDNDGAADDDNDDVEGSTSTTTSVPPEEDPRRVQLRALVERANKAL
GKDYVGANEEATRVVKRLRAAKKKLSQIDSLLDKQAKGEELDEGQLAKLDQEYPLADEIAALFAQLAEATRPPPPPQLQP
TPVAPVVVPTTPAKPAPTGSVAYQKKRDNDDFFSGKAAANRNRRRTNSTKTAAPTPAATAAAAVTATAHTTPAPSLAPWA
ANKPVAAASEKPSGSSATATSAAAAASPQKAKSLLQIQEEELRRRKETVSRPKPVATTAPRQERTIKRLDGSVEKEAIGP
SRPQQQQQVVGNADNESSVSSPTKNYAISLAIGGKLAASPVRVTVPGAPPQHQPNTTNSAGKPMPALRSSTNGVGKPSAP
AAATSTSRAAQQPQPVGGVARSVALFPPLGALPRAPSLADIQREERERVEAERKKREVVKPTKKPLKQIQEEQEEARKVQ
ALFGDELKQIYGHDIDLESIW
>Acas_g5832
MTATALRTCTHPQLQDPLHGDITVIVHSSDESTTTTNINNTNTTTTYYVHRLLLSLWSPVFRTMLTNGMRESTCDVVHLR
EHPLHAPRFGLVLARLYPNAPPMAPATVHSVLYFVDKYDLPAALRLECEAVLLRQPRSWALLVTAQTYGLASLLARHAEW
ASANLDSVPDAPPSAVEGALLLGEIEVETWVKIMRHTMARLTNTKQLLRKMLLASNCNMCGGAQGSAAVAVCGRSLYCSH
HVQFMCLLASLTDTSP
>Acas_g6990
MKRVEDSKTQLEDDELQLRTPSPAQATQGEMLTLSAAQHQDSDDFEANPFVFSMEQPSYVYLWGSGGWGALGMGGEDDDD
DYYDEDGAADSARPTLLRNPALDGLQLRQIVCTKRHTLFLTGCGRVFACGSNVGVELAHDEGRRVVARPEEVKLPQPAVG
RAFELASGNGYNHRFLLCSSASSSPSSGSSEAGDTQVYCWGESTQGQLFFNARCVEQPRLVESLQGLDVVQVATGDRHSL
VLTSQGQVFSSGKNGDGQLGLGDSVLHGPGPHPVIGLDGFKVVQIACGESHSVALTDAGTVYTWGNGLSGQLGHCNESSV
NRPQLVQACLGMKVLYIACGSYHTVALIDTGDVVVWGSNEESQLGIEGESFLSAPTSNLHLSGQGIIAVACGTGLTVALS
ADGRVFACGTLLERIPIPRVVLPAGKGVTQIACGAQYIAAWRGPLSPSLAGLRARISRAVDPNSWLALSALIDRDECSDL
LLRLGDGSAIHAHRVVLAMRSDRLSAELVDLEAPQGARHCRRPGGAHHSLPAALGDSPRGSGVPALAVGDDSDSDVEDVV
GQLLHLARLYATKPLEDLLEKMVAFPRLAAAAALDAPSPVSNTLASDLLAALEQPLFSDLSFIVTPSAATADDGAGYDEV
AKNESGDKEMQKNEDAHADEKIESEEAQANEHPVAIEIVAHKCLLAPRSDYFRAMFLTGLKEATQRRVPIAGLRPEVFRQ
VVRYHYSGFVGEIDPEQVVELFVVGNEFGMDALVRPCERMIERGLDLDNVNDLWGMATTFASEQLKQLCIAFLLKQLRPK
KIGTGRVAALSLELLEAVCARMIATPDSNVMNCGSCQESITRLDEREMCPHCVLCGRVLCQRWKKRGCIHKLLRHDGPPQ
LLRLFSPHGKSGFVPGVVQPLCHPCRAILSAHGT
>Acas_g7254
MITDNDEDCDVAPMAAVVSPSVALRFKEYRDVVVIPYNNSEDYRQLSVAAWVYCEKDASCSSDPCYLLAKKGSFALGLRS

DEIAMAFHNKTWTHVVVVYDSTYEQACTYANGRLVSTQQVKGKLKGSTQPLLIGVELKPDKGTRTRFKGLISYVTLWKHT
IAPDTINQHLSGAITGTERGIIGHWPMTEGVGEKIFDASPNKLDGDINFALWWMNSDSVGHVDVPKSTLVEDMRKMFNSP
LGSDLKLIASDGRELHAHKIILAMRSEAFRALLFGGMRESTQAEIQFPDIKYEVLALVVEFLYTDTANITGDIVVGLFMA
ADQYQLGRLRALCEDFILQNISIENVCTIFQTADQLQAHKLRGFCFNWIINNFGEVLTCDAYPQLPAELQREINYAAAKM
HFGKKRKLDEATSEP
>Acas_g7310
MDQRQSQAPIVVVGTAGLDGQVAAAIEHAARRLSSEEEREQVLGIKDLWSLSCDDPQFAIAIGQCGPLLDRLLELCSLAS
CPSYTPSPSLPPGPSWKLVILALHCLWRHLKEAKALDLVASFIEPENWAEATEKTSIWNSWLGLQPFVPLLSLTSGNGDN
VDRDMLSAVGFAALCLEKFSGAKRYRKKLWHDLALNGGIQPLKSLLRSSLLSGRLERVRTAVAAVFRNLDIDTSDVGVAI
DPSSYETDLSHIFDRKDSFADCVIVFPQVGTGSRATPEREIYCHRAVLAARCKVFAVQLSRWDRSGQADEQPREQNSSGP
STGAPPAGPSRIIVSEFDYPTMKRVLEYIYTDRPALDWLCAVDVLRAADIYGLDRLKQMCEDLIVQGLDLENVGYLLELA
DFHNASQLGRACVEFLCRRHAGNFAAIEASPQFEALLQNYPAWREQIEGACRTFTSARHPGDKPSSAAAITTACAGPDPM
RIET
>Acas_g7401
MDRNLTDEGTSPSKRVEPSNRLKKPPGSDEEISSSGIFYKMMGEGALTSLGYSRSSPGREGRGPAMFPPHDGEDSQPSSA
SASPQPKKKRSWIDRFMQKDGRHEIKKEIAETEALRKSRERQQHQQQQQYQQAPSSSPPQQQSRWPPKEERPESSFCVNW
RAREELYRKKIEELTEAEIRANIQFYETSDLLVLVKSVRGRNDHAAFRILQRHLEELYLLKHILYERGFYLNSTDPPLGH
RGVVGDDGTASSHQTDANAPDQFPRFLNSSALTLLVRDQNRIADVVLQVPAPDIESARKIYAEVNYHNDVLELYQRCIDV
LEFADIESRLDSSHSHQYDAVPSPSTVSSTSTVGLRDQLLSKLFLDFGEYFLWCGDYVESAQALVRALVETAGVLAKLPP
NIPPDGDKDIISLFNHVLNDHSGPYSLHIRFRVLAWVIARLRYYKENKVNIEGVYGLLRILVRLFNDDNKHVRQLAKGAV
SDFGDRVLRIVDNATKDLFKEYHVFSAVLKERSEFFQRMLESEMMEGQPEEPITIQVDNPRVFHAAIEYLYTQQIILNEE
NAVDVLMCANQYSFTDLLHLVEDFLCVHLQSSSLEETEHLYHVTEGMVCPRLQGILRQRLRYLFEKTIVNSDHEKFVSMA
HDYPILKRQLFFTDHYLCLSLYTGNKNIAMSMLSWGVGIEHKEKEKKEKKKGSSKSSKGSSKSKPKSRVRTKGKDKQKSS
KGGTKDSKDKEREREREQPTSEEDEERDQRGSYSSSSSASSTSFTPSNTPAAPAAATPDEFLPLVPPWYSHTSLPAFIAP
QSEFGSPSPPSSPRLHGSQLGVPPSPIALSGSPHLLSPLASAGPARSPPVTRRDMRDSKDKEAQQQRDPMLGCVTIHELK
KKEKKMWKGWEAALLIAIRRGYPDIVKILLDGDADTNVFTHSGKTPLELAVKRGKSGPGSDIVGMLVEAGSDVNRRRTKK
AQEQPVFNEDMYVVHFEREKYNAIFDIKNTLRKDTTPLMVAAQYGHMQTIEWLIAGGADVNVATENMNTPLMVASAFGHL
ECANKLIEAGAQVNKCNIFGWKALHFAVHGGYADIVIALFKAGARDSLAIHSTNPDNTNPKSKTAQEMAVDVGRDDVIRV
FDIYLTKIPAYEREIKKKTILEKKNSFHHSKG
>Acas_g7637
MWTFGSNLKGQLGRTLRSRRLGGGPLLPGPVTDFAWGDGPGEVEVVAPSLVAVAAGDWHSAAVAADGRLFCWGNNSDGEC
VSGGPKVLQPRLVGGDLKDRVFVDVACAGHFTLALTQNGEVVVWGKINDRLIQPTFILFQEGSQCPIVQIKCGWAHAACV
DSEGRMFVFGAGAFGRLGLGNNEDVMYYQPKLCPASLKLKVDKVALGMSFSLVLLQTGELYACGDNSRGQLGIGKFGGMR
STPVKVKVLSQDRVVQVVAGADHAAAVTVNGRALTWGDNKYGTLGTGDRENGHTPTAVQALASLKIASIGCGSAHTIFIT
GTRHHSSCCVCCVSCHSGKVYLCGGGSALMDGMNQGTVITTPQELEGVQHITHAVAGVEHTILFAGPAKSSSPLGVDWLG
AYNQPGGYADLRFTTTSTGDKTPFHAHRCVLAGSAFFRGLIAERWTEADGGEVGERPVLEVELPAGVSGSFFGRLLRSFY
GGPQLTRLQLIAAQADLAALQQMAPTIAATFDAATATDDDAIDTAADVIDDTAATDESEPTTTTEPTPPASWRRKLRGWL
WQRGEAADRPPTEAESLPQQRHGRPQPPPPQDHQPLPPLVAWALACLLPYGADAGGEEDQEERQKKRPKKKETEEEAAEG
GGGAVRDPLEAHMRAMVDNPLFSDVAFVVGGRTIRAHKAVLALRCSYFEAMFSAGMRESGQREIDMDASDCTYAVFRRII
AYLYTNTLDPVDLDRRADAYDDNDDGQEKEKEEEESTLDDYVQLMNAAESFNLPHLKHLAEEQTIAAIDASNVAQLLDAA
VVLRASSLAKSCFVFLVQNWKLLLRDRPDVVAQLDDATRQKIENHLRRTHRL
>Acas_g8019
MEKPQSKVLLLGDPGVGKTSLAIQLAHNAFLEIESPDGKYEYHRMVDGQYRVVHLFDNVQTEDWGWSDQIIRECTAFLLV
YSITSRSSFDSLAAWVDRIVRGKNKADREDLREVSYAEADLLAQTYDALFFEASARTRFNVEQCVLAATSQEAPERRQEW
HQKKVARLKAKGKKIPPIPPMSPVSSTYGPDFWSLVGEHKCSDIEFEVAGDIVPAHSVVLIAHSPRFMKSFIREGRRRKA
VLGVDEETFRAVLDWIYAGIFPKPESTDMRAYIRLYNAAVKLGVDSLAAVAKAFLQGQNYIEAHRQYAPKGALGLFFNSK
MLSDITVIIDGREIRAHKAVLSARSVQWHDIIADYEVNQAAAASYWDGSQPTTPRTNKNQSLTLVEQSAAAGGYHAPAII
FITLLHSPCLQSTDIFPNSGMTRSASSPTLSPHHADFEAGGKKKTKKKDKAAGDLQLYDLYHLMLSYMYTDEVEIAESYA
LELFLLALDYEVTHLANLAAQVVLDSLTVQSVAGVYEKLVGIHKSSSVLGELRRKIELWISFRFKSVAKTKAFKKFGTRY
PQIKAELKRNKKAGFSSSVKTPMSPPESTTAAAAAPRSPQREGGGNSGGLRKSSGNSSNSGSSSGDLLSTATTTTASSTP
NGKRLPRFLNSSMPTVPTNRSSFFTSPRATAPVDEPDDELIDLYESYQEYQYAKKIAEARLPVIDQRRKWAKHIGDYLAS
LQVSGEQFGSLNMANGFPRKVMFVPDEITEISDTLRKLKLDNNLLGNDPIVVYDRQGREQEKIEPLSFAGMRSLKMLTDL
SLSQNNLHTVPRSLCHLRSLTSLNLSLCNITAVPSYICYLHALERLTLIGNKLTRLPASLCLMRNLKKLDLASNELSDHS
TKPGYFPHSLTSLNLTNNQWNSFPLGIVIKADKEEERQRTKEEIDLKSSAEFFRFLSLGADIHLPSVLSGEAAPVMMSDR
PVALAGRVTTEEAERARSGTGELYKALGKGADKFIREQKIQQELLRFAKDAMKREREVEKSRKRASGADDDKALDEREDV
IFQRLLKVESGDLDIEVRPLENLVQLNLDGNNISSLPEEIHQLSSLRCLRLCDNYALRSLPDGLARMPDLQDLVIPLDLP
LTVREDQIPIYQHKVSPSVWRTLMDFIEVPIPHPLTPVTGPMRLIPEELQLRILSYISQRELADTVSLVCRHWHVLANDA
SLWHSFPWKLLFSARQEQITLERRIRRLCVMHPDIGELTLRNTSAFPERAIEHLLNRCKKVKTIDLDNSMNYYKSHLGSF
LDIITQNLSMLRHIACQNCYLPYPALACVLQAPRLSRLLLVDCEFSSSVSGCHGRSLAELPDTLPLTHLSFERSRIDPVA
LELVVTRIPNVVDLNLSACEIVPAVLNCIGERCRALLHLNLSHTAITATDVANILNQRKASLQTINIRDIPNVISSFYSL
ASNLLVDPEDVVSPSSSPRGNPTVKIQEFAQHFAHIRTLTISTTTENEAAGINHFIEGLARTNSSLVSLAIRSTSLSAEH
LANLALARSTTLRKIDLSCCNKLDAKAVITITANCKRIEALNLNFLPQLNDSALMEISRKHSGSLRVLSLYQCSSITDEG

VLAVIFRCARLKFLSLTKTSVTARSVRAVSELPELQVLCLSYADVELLKKIAQCSSLRHLVLDPFAVGQTGMGEGQGLFP
HTAINEYSEQKLLQMHWQLLGH
>Acas_g8179
MMDPVHVRHIVDASAQNVLPLRPMMGSYVWLVDDQFHLPATGRAFVTFEAQATNDIHVSFNKDTSRVEGRDGLADQSPNY
EVVIGGWMNRKTAVRKRGQQQAAARKEENPNAVITSPHFTQYWFLLDNVTGTIPVSCLSLSRLSAHSTFRQGESSLFLSD
LFADVHFHVGSAVFPAHCCILSCRCPALLEQLLDASAPTEGPRTTPFIVTLAEEETRADEFRELLRFIYTGEVKLRTTDR
ESFDGLLALAQRWSPQLAAHLTAATAAAGGLVALPWFGELVGSERWHDVVLTFPGDSSSSSASSSASSSSSAAPPPPTGR
LPAHRAILATASEPLWALLGGAHGWRERTEGEVALHDVDEAAFRILMECVYKRTCRRFTSLVEEVLPLLLAADRFDLLNT
PKLYPPPAADSTSDVASPGEPVPLADHCLAYAMAHFDLSTCCPTFHIADVLALPALRLAAQHFIQDNFDRSYVAEHILPH
PYAKLPGVEEAVKLNSNPLKLSGGSAAVQAGKVVLRQSLADILNRPRTPRSLQGWKELPYSGTPGDASGLFHWLGTSGGA
GGGAKERGWVNPCRAGLVKVVCSAPPLALLQPGSSRRPRLPHHVKEKAFVMKGYAIRQDGSTVFLRSWSMQGSNDGQSWV
DLSTHVNDCGLASPSRWVFWPVSSVVPYAQFRLIMTGPSASPVSPNTLALSNIEFYGFFN
>Acas_g8199
MMEAPVAGEAGEWQAVAVRGVVPGARHVHTAVEYEGAMWVFGGYGSSSTYYNDTHRFSLEEHKWECVLAAGRSCSPVGRH
SHVAVVHADSMYVFGGISRAKGNAELNDLHRFAFRDKWWTKVAALAERSGSLPSPRWGHTGVCHEGALYVFGGFAGTYSA
ELWRFTFTTVYEGRMYVGGGYSRVFHGDLFEYDFGEGDELPPARHFHSAVLYKGLWYLFGGITALNSNDVVRYCFEKVEG
RVLASRRVAGDFRMLFNSELFSDISFLVHGHKFFAHKCVLYARCDRFRAMLGCGMREAAQEEVILDDPHLTPELFLLLLE
YIYCVHVAVRLFVVADYYMLDGLKATCEMVIGRDVMEENNVLPLLELAILHHGSALLRYAVDWIADRQDMIEVPPTATTT
NAALHTHHGRGGTSAPVSEEGADPLKPVAPSSGAAAKNTLEDIVTNTFRTFAQVIIQARSQRLGGHDPAPSSSPHHRPDV
YSPEASTWLNYYLDCLWAVDEDLAHFWRDWKRNERRSAIDLTLDIFAQLPSSDPPSRSRGTSQQPPPPQQHQQQLRRDLA
TSQPFLLERWVLSFNAKPNGEARHNTINVKKTAMVLRSVGSVVRALPAHKLVRLRRKQPTSFSIDYALYPSACASLSMPS
LASPSRCEWAGTAGKQSLPFSSVVSRTGTFAVSVEYATNLPSFKFQALKIEPFVISDYAPSPSEQSAARGEPSTVGAPGV
SPTRRKEESATSSPRGATDISDRSARDRKSLERATSLPVFLPSSSPGMQPPITSLGGHATPPVMAPALPPAGRANLPSSA
SIPIRGAGASGQPRGGRGGGDDDHLDIGALAMLGTSTSPPKMMSPPVFSGGGLVSPSTSAGGGGGGGLSTRGTAAVGSAG
KGNANFFNFSTPPFTRDFFLQQQQQQQHFPRSSPGFVPQAITPFVEDSGSSPWEGAGSSASRSLETSGSYSESIFPTLLP
PSSSAEDVGEIPGLLYTKLPASQPAFSVATQEIEVSSFLESCAHPPDLKMFSQSKNMAQGTEDLETLSRSLRRLSIDLGD
ARKRAPGNKQSSRETTEDDPLFFDM
>Acas_g8435
MLKRSISTGQLTTLIQTEVDALEGELDQQRKLAAEPNAPPSRANDLLTKSFEEVLRDQYSFIENDEDYAAQILGVADDGD
RVEEGGRHATPTAATRGRAEKTVQEAKETKKEREAVAELLRVLKAGEGSHEVGDKKAKEVKVEHSYFVGWLQKARIFCRL
DRAVQQQPQANIDIFDDLLAAETLDYGKEALGYYQKCISLLPHLAGLTAPDLKQAKVYLFSELGQYYQGKGMYQKALQVA
VTLLSENRQSQEMQKMLRDSVTVLRETADANQAAFIISTISKLKSPINACVDFISATYMQLEPLLQRSSDPTFIQARISI
VNWVTLTLCVDLVDLLQLMMDLLLGYREHDHSPEVSEAAKLAIEQVAEFTLDIIEEGYEHVQAGTEEAEGHLISIQVHME
SLVQRCSYFQTMLSLNMVEKQTQRMRVVTSQPYVFLDMIDYLYRSEVEFESFDHALAVYMIANQYGVAELKELASNFLST
SAHTYAAEIESDWAKFKEVYYLADTMNLPNLKAALMQHLTTNQRWLNNLDSLPPFGSDMQHLRDIESHFPEVFARVSNDG
LIKKIQSAAKHDQLAIAEFLVDLLTKRKRGSSVPWYSSTSYEPAAGSQEESVAATVDAQGMCSECRIVMGHLTTCSMFKN
FRNIAGRARPAVPVAPPPPPASAAASASQYTTTGGGFVPEPLLLSLLEKANSVAWNDLVLKLLRSGFCAITPSVLAKPAE
ANDVALLGQLIDILAAVYTEEDAETPGEADGDDVSKGKGKEKLRDHEEAAAAAAKEEVLRRREGKWQWEGDSSDWHDYDE
TANQLIEAAYASGAVMTTLTHGYFAGGSGYTIMLDRQKQRNNATNFERNIRRHDLLPGFPSAAAAPLVLPQEPKRHLAML
MNAPTHNGKTLLITFIEKRIPLSVLKRFLALGKDVLDVNVRASLESGESVTALALAIQSVADAWNEADELISLLLALGAD
PNLEFTVASLLGMKPSTPLLQAIKQNNFELVQRLVSAGADVSYNVPTARPPLNSIGADRGPVATLYSPEEDLLASLNSHA
DRNPLFAACFGREESEDSARIVRLLLEKGAAGGPPDHQATSAAAGPHSTGSARSRALAPPPVLVLPNAASATTSQALDAS
VVRGNTPLMAAAFYGRADLVQLLLNVGSADQTAAAKPKKAKGKEKESDDEPSPRPRSARPPVSRACDVGARNWWHWSPLH
FAASAGHEEVVRLLCQAPGAIDSACVVSSNGIVNTARQMAADRQFNRVLSLLCALPEDPVVPSLASSQGSGAGGENRHSS
SGTFQHSAELAPLQTFTSVSALGGGATTTAVSTLSGSGTAKSKRRLWDRLKFL
>Acas_g8733
MHLSGRNSINLNGVQNGCTPLYLAAQNNKPAMIRLLAKESVNPNIHCQGYSPLGIAAMNGFTECVKELLELRANPNSTDL
PLHAAAYSGFASVAEVLLQNGASLDKKDDEGRTPLMLALIHGHEKVAELILQKGLKQGMPSVNDKTNLPPTSVYNTQDYA
YSNPNCGRAALHFAAEFEGGRHLVELLLSPSLAVDVNVTDRFGRTPLHLALATNHGTEIAKRLLKEPAIDVVSALVDLAP
HLINAKDEHGATPLHRAINNGHDNIALHLIKRNADIKARNVRDQTPFDGLPKGDRLQMLEECYQSFQEGLLNTLNDEKLS
DVAIHLEGGSRVILCSRLVLFARSAYWRKVLLEKEAEAEAAAAAANTSEGGRLEIVVNEGRYEVFHAMLYYLYTDNIQFP
QLAALAAAPHPGLKPADAEARRAQERNNFLSDVLVMAERYGETRLHELCSQAIDSDTLRSLERRRKRVDAARRLLVSHFS
SDMKKMLNNKRYSDVIFSIEGKIVYAHKVILCSRNEFFKAMLLGPWAKEKCTEEDPILITDTPYHIFYSVVEFSYTGDCP
QIEPDTVVDLYQASHQYMLLELRKRCENIIEEAVGVDNAVPIYELGHIYEDVKMKEKALAFITQDADTFQLISANSAFSD
LPEHLLVDIYRELLVRIGGFSASTTDALVTQENKQGDTPLHLACSIPGMVVAVRWLLQNGANTVRALLDQPGLLVNIADP
ATGETALHIACGAGHENIAFLLIERNGDVNAHDKRRRTPLELIAETNHTFATDFKEHRELSKIKFGQALNDPAFSDIVIT
NGDARYYCHQAIEDSSRSEDITLAGGHVRKVPVVEIEGVQEQTIAAFLRYVYTDEVEIDLDNVYEMMGLSMMYPVTGLLA
LCERANDPTTAPPPEASTMAVDFVSLFNQEKFADVRFIFDPPTFVDTGGGDDPDGDLDEEWKQHMPSFTPAELREEPKID
DDHDGEDEVVVIHAHRAIIATRSPLLLTHFPELKSETTQRTKDGKNEAVVEIDMRRWTMPQQRGKSVVGKNEASTKNGKS
DGGSSSDEQHNYYASFFVFLRFLYSGVMSLPATVAAGDRSPDAATTVRSNDEEAELYREILGLGELLGVKALVRQVQPKV
DEEERRRKKAHLLTSAF
>Acas_g8927

MQVAISQLFATKELSDVVLVLPGSRIPAHKIVLASGSPVLAAALNSQGEAGAKELALDDEADEAAFHTILAYLYTGKASV
ASDRVYNLLIAAHRYGVDALKEKCGVYLFQRHLESARRRKKMAAAAKKGKEKSEEEEKEEDHVDNVDGDSDDMQVDDADE
EGQEGEEAQGVGHLLRLAQTYKCHELEKLCAEDLARNFSTLCESGKLNTLPLSTFIELIKSEKVAAAEEEIFDAVVAYVK
FQRDTQGLDEAALLEQLLLFVRFPFIQSQRLVEDVEGNPLIAGLKLTGELLFEAYRYNFYSQLYNQNILSPKSQQPASLS
HADGEESLYLDYATKSSTAKGPKFAFTSPLPHYELRCSPRSGAVKWNSTKKSRDIAISNSMLTASHNGSSSVWMSVVADV
PFSDHLTSIYVHIDRNASNWIFIGVAEEQWMDYAASSGNYLGVGAGTWSHGSAGGYGKVHNGVAGAYGVSFATGDTVGVH
LNPETGVLSFSCNGRNYGPAYLSLPVSKRLFLAVSLYHHSDQVTLARGRPKKFKAALNSIHPAAALPTDEEEDPNLFGLF
G
>Acas_g8932
MATRTSGSAIYSTTGALLAASPREAAKRREERGKLRDHARHLAQCRIEEEQHKRKWWDEQIKERDWTMQLMMALLDPTLS
CAQKNDQIQKLREQFIEQHKRKWWDEQIKERDWTMQLMMALLDPTLSCAQKNDQIQKLREQFIVAVRQGVSKIFNSKKSH
SAFKFNGYAVGPGEDVYATGGIRFQQVSSSAQASRFFRVRQEVLDCKINGVLIPLSDEDKLTGRILQLAKALNLRCDFKK
LVTVNDSNVILRAQPWLLKGYKAPAKDMFVLDNLQQLFPDDVRPELLTIHPRPFSTPLLCSQAKDPIGRRKYLRTVRALL
RTKVVDLALALDSACRGKDGDETFALAPDTIADHLHRAGLNLRYLGQVYARLGEELARREVMREMVQRAIKVIVRGHITN
GVDAAAKEILNAIFSRLGDRMWRSDGVIQRKIEDKFFLAWPGARFMSHEDEAGEALRLTRTSSSMIDESDDDSESEGMSV
ESETGRMMSTTTTSSTSSSSHELFVNPSNAAASTTLVPSAWPVLSPVDATLWQTLHSRQKRKLGENGGEQTLQCGGGGDA
ATPSDSGKRKAKRRRFELAALEEALNQVEEERSDVDKNRSVKGKEKADQSAAPEDDQNDDDGDAVGDQERFNLTYSRAKV
FGQIFVNGQSHTMKAVTASDWASHFARTGTPFSNEEITLKPVVSRCTTYAIPAEATHSALNLGCALNNSDYSDVQFVFPD
EGVTCYGHKVILASRSEYFARLFSCGLKESREEKPVVEIKELSSHTFLAMLKYVYINDVDFADRSVDLVELLLAAERFQL
GGLKKLCEERLASSFSLPTAIRLLTAFNISEDLRNQCISFVLAHWGELEKTDEFRDLMLYYPDLLQEIYGSLAIEEADSF
EPQDVNAIQDNLNPLMWTAKVRSLMGEEIDEGLSAFTLLFNDDYPNDPPLVRFKCNRSHPNVFSDGFLHHGVLSLGWNCT
KTVKDILQKVVRVIRQPMAEYPANLHCPQPVGTSTVSSQPVDSAGHQQQEQEEEESSMQEALEL
>Acas_g9031
MLRVSVSKGKGKEKDEEETLKVHLPSAKRQFNAVLYGDMVQKGFAHLYGDTKISDVTLVLGDEKVAVHRLVLSVWSDTFR
TLLEGAKAEVVIPLEKEDMEDFKLMLKHMYTGETDFINGQNVLSLLSLASYYNIHSLKETCGELLGDLVTDDNLFFFLDV
VDKYEVKALEAACGSHLAEHFQDLVNNGKLDDLEPSTWAEIVKTDDIRVRSEEELFDAVIRYTKQFKAKDVREQALTTIL
PYIRFPFMSPRFLVTKVEGNESIRDLPVVQDLLHEAYRYRVNPSSVKNIRSTPRTGHQFNPEQSHASITLSDDKLTATLS
ASIGWANASCTTPLTRDNPYIEFKVGNGTPGQLMIGVVSGTCSTTGYAGQYSNGWTYYSSGQVYNSSTTPSTGQTYTTGD
TVGVFVEFDKNIVHFFKNGKSSTTVSNVSTSNGQLFPNVSLNSMGNNVSVIPYAPIPEELKAKMKNAPSTRKKRAKLTAS
SLGRTYGDENDGTPGIEPSEEEAAEAIDGTTFIKRFLRFKF
>Acas_g9067
MREGQQQSSIDGEARKAEVELPEIEPRVFGHLQRFLYTGEVSVEPDDLLPLFRVAHTYAVQPLAEACSGMLQSGVDQRNV
MCLLQVACLYDDHALREQCLSVIGPSAATLLDSDEVLAQSRPIIAAILRSSKLCIGEVELFKLLLKWRAIDPESRHLDAL
ALVPLVRLPLISPEYLCSLVKPSGLVPLEPLFEAMTFHANPDCLPDTTLTRFQPRDLRELSKGQQIARQVMNKRAAARKL
LSASMSEPLPPARSGQDFEAMSA
>Acas_g9272
MSVLSWGTGKVGELGAGRDNKEALEPILLERFRGKRIRSVACGEQHSMAVGETGDVYVWGRAKEGQLGNGERKAANALKP
VRVEGLRHERVVAGVCGYNHCLAVTVGGKLYQWGMLHKHVEGSANKEYFGMAIGLSGLNSDRMKRMVGRSHSTYYAAGAT
GEELEELSRVQNFGSFTPYTQPSPEVVTGLQHVKVVEVAAGYSFSLAVTSQGEVYSWGFNEKCQLGLGHRYNQSVPQLVP
HLQGVHIVSATCGQQHSVLLSAAGHFGQLGHGVSSDERFPRRVEALVPAEEGVESAGERIVQIACGSHHTLALGVSGTVW
TWGSAEYGQQGGTNAYEDWATGQRAEKSAAMICATPRSLSGCFDGKHIAHVSCGHLHNLATARDGSVYSWGWGSYGVLGH
GNHRFQLVPKLIRRIGAGSSTLFAFDFKVALNNPRYSDVSFRVDGKTFAAHRAIVFARCPYLGAYTLLAHRFRPTDADSS
PDPNPVVQLPQMRQQVFTAVLHYLYTDHLKAASHWLGEIKRVAAKFRLPRLEALCTAQLHSQSHSRQYKPPKSTFAAELA
QVVDGPEWSDVVFAVADEEEGEAEGQVRHVHAHKVLLTSRSDYFKRIFGSSFAEGEQPKSKPVAIQGTQPRVFMAMLEYI
YTGRLPAEETVSSNGTSASGSIEEAAADDPSLPNINPDTVVELLLAADRFLLDDLKQLCEAALEQSLESSNATWMLELSD
RASAPRLKKATLEFLAALLYRAQRPRAGTPVEDDEEEESESKEEGHDDVPAGPTSVEELGGQLSPHLLEELRAFLAAYAP
ATVPPPTKDTPSSSSSSSSSTATLPATTNQVSAIDSLPPLVD
>Acas_g9404
MQARGNNAISQFVAVTGADEDTAKYYLEANGYDLEKATVLFFDSGGAAAVQSGGAGAAPKKDAEDTGAKNVGLEFDAQAY
GEQLQRTFAVLLDESDPHAHAFSDVTLVVHGEKVPAHRVILSAWSPVLRDYLLANADKREVELQLPHQELSVPVFKQVLR
FIYTGCTELSKEIAIPLLELSNQLQVQSLKDKTSEFIFVAEQKKDIGHLLELARKYESKHLEKRCAAHLADRFEDCLRDD
SLNSLPVSTWSELLLRDDIRVSNEEDVFKAVIKYAQTNPVTKDDTLKALLPLVRFELLPNRFLVEEVDLNPELKELAFVH
ALVHEAYRQKLYQQTGGAPPTARRAGRGKGRGVAGGVWSSALMSPGIEVLEGGKTVRHTGAGKGTTQSWQSVALDSWLSS
GTHVYTFKIDKNASNWLFIGVAARSWEGYTDTSNGYVGHYHDSWGFGSYTGWGRAHASKNSAFGPSFKTGDVVQMTVNMD
LKTVAYSVNDENLGVCHKNIADEVCPAVTLYKKGDQITLQA
>Acas_g9628
MEVAQVFSFGNTVGSHGAPCPTPKLVETQFGKRQVKQVSAGLYQTWATSANGECHLWKEGTSMSADLRALATKEKCVQVS
CGQTYSALLFESGVGKVYNSGHLTSLPPVLETEDESSVVIKQVACADDHILALLEDGRVCMCSSMPGARESDWRLLDFGG
KSVASIAVGQMYSAAVTTDGGLWTFGIGPLGHAISVKQDPEQVEALRGKKVVAVACGSFHMGCVTESGELYTWGSSVWHQ
LGHGERKNVVEPRRVTALEGKRVVEVSCGAYHTAVRTDAGRVYTFGAGSLGALGHGDLVDQEIPRLVDSLVNRCVQHVSC
GWYHTTVVVGMDPNISLLRDEMGKLLHEQSDFFDIDVYCGQEPAKPPIRAHKVVLAARCPKLAEVLAADPGKQELYISGM
DSATLAEFLHFLYTDTTRQGLDVDMLLNLLSVGVAYNSALLQKRCVELFQDELSLDNVLAHWRKVLDKPEPWVLDYRQML

HNAVVGFILREDNLQLLATKDVEALQAMMPHDPRLTSAAEAQLVRRPDGQLLVDRPLSSSRGALRASQQRIRPDEVIEEE
VATEEELEEEKKKEEKEGEAKEAKKESEESEAAENKEEQQHEVVESLKTTDTQEEVVEAKAEATEAEPRARSQCETEEIA
RWEAETANEEKVAQETSTTADDATERDTETKEEEETTTTETEQERNTGSSGSEASTSAPPSVEKSNQEEIKVEVVVVEEE
EPEEPTTYTFHTDLQRFVKCSQFADVTFAVEGELIPAHKAILCGRSEHFRAMFTSGMRESQAEVIDVHDITLPAFNALLN
YLYSGVVEITEDNVVELLMISNQYTLTHLQEQCECYVEKGIYKDNAAYILEMAHRYQTHHLRTIAMNYMLQQRDHVMRTE
GFQELSDELLQEFKSNVACAKNCKATGTGASKGYEGETAFFKIEAFDEFGNKCARGGENFKVQILEETPAERAVRKRREA
EAKRKVAEAQKTTATTCTVTPDTAAPGDTTTTAAVEETSDTAAAQAGEYKSGAEEKEATKEKLEAEEEKEKKNEGEEEEV
EEEGVDSEKEEEDYRNRKPIVDAEAGEEEEHQHETQWLAYHDDEFWVCEECTLLNSKVSYVALRSGRYSLHIKLGDEPIA
GSPYFLRVEPAYAASQCTADGEGLRRVEIDNETTFTIQARDMFGRPIRAGSKWFVVRLSHQEESANSHMDWKADKGMVTD
NNDGTYTARYTQRQVGKFYVSVLLGHMHISGSPFTVRAVSTVAHAANCVAFGDGLERAVIGRPAAFTIQAKDRQDNPLDK
GGDKFVIVITRTTDQGSADDVNGDNDNDDDVRSLLASLPVGGPPPAPAKARPTRAVAVQDSSAFAGGLGQLRHKLADDEQ
AAPMVDDGAGDAAAGATATPASAQLPRTFRRAQRRNRDALSPSPQPADEGGDGSTSPEENEKENEKEKELEEHPQAKTKR
ESKESEEDSEEGSEEEGEEEKEGRRKRNLLDVDSNATTVASTRRPLPPPAVIQSRISKAAEASQREFNITVEDQGVGVYL
VHYTVKEASDSYRIHILHNNQHILGSPFALANVTEAPQPTSQQLGQTEPQSTSFLAFIEAEAGGGRPASRKGRGPRRPGS
KSSTTTTTTATPLAADLDTPAAAADASGEEKGDSGWDVTPHRSEGEEVKTEAEAETEAPVVDEGQVAAPLGFTVCWESTA
VSTFETTDTSSPFFRPSFTFTPAASDPASTDATPTTTEGTALFSPSPSASPSPTPASPFGSPFSSPASTSPFTGFSPFSA
ASTSSLTPSLTTTTATTSPSSGLSPVAKARAAGKRSAATRTKAKPAAASPPSRIEF
>Acas_g9789
MDEELAARAAEVAELRALYGPRKEWTELDAETILIKRTANYGRVELRVLLRLPASYPQARPSIRLKAVKGLSAEDCVVLQ
SLLTSQAIALKGGVMISTLIKSADDWLEDKILSATFGDSSSSPSPMQSTASSTTSSSSPTSSTASTAVTSEPESYAEVDD
ETLQDFKCLTALEIISFIGKEVQYVQELLKVPQTTARILLSHYMWNTERLVTDFLESPSRVFFSVRAAGGQNSGEAASAK
GKEKVGDDFDEDNEGADETKSGGDAEETFACPGCFDDSPNYTKLSCGHAFCNDCYKEYLSIKIKDGQADKIGCPAYKCKQ
TVPESVIKDLVDEDVFKKYNSFLANSYVENNPYVMWCPAPGCQYAVQATIKLNCTVTCQCGNSFCFKCKQEGHFPCTCGM
VEQWAKHSKAEKETFQYISKFTRDCPNCETAIEKNGGCNKMVCSYCNFMFCWVCMQDWSTHNFFYSCNRFDPKAKKKAAS
SKPSSSLLAAANASELFSHHYERFTAHDRARHFAAQIKAATQEKLRQLQEAASRLAASQGTQRDRHSENLLFASVNDAAA
LENDFVQSVNVLMQCRRLLKYSYVLSFVMADEVKANKKLTKKQGASTHSVAAAEPQTAKRQMPSKELFEFLQEDLEIATE
KLSEVLEGDLVAASSAGESDSETTVGADAAQQFFAKVSRKRADIMSAMKIARQRMRGLTDLIDREFVPEEDEEDTRGRKG
RKGVVGGLHPSSAANNVICIGEWSYPIGTPMALPAPTTGTAASAAKASEAELQKWRDQGFTVLDPRENTDDATEEKGYGA
EDDDEEEGDFEYIPEKILKKRVRKDDFGRPQPQYLVKWKGVPASEASWECIKLVNAFEKEKLEKAKANVARAKGQAAADA
EEKPAPKKKKVKKEKEDLDKKSKKGKEAELERLLEEQIKFISKQNMTSLHLLSEASLKNKSEEFIAEYYKNIKEQTGLIL
SWGANSDGVLGHTGGIEQLWPLAIESLSSVQVKMVAAGEFHSLCLTENGEVTSFGLNDQGQCGQPITKIAESSDLAAVAR
PRPIVLPDDLRKKTVVSIAAGGYHSALVTADGELYTFGRGGEFQLGHDTVMALGEGGNALHYRSNSAQTSYFQPKPTRLS
ALNGQVVKQVACGRFHSACVTEEGALYTWGRGANGQLGHGDMTDQATPKRVEAGIKDKIMVKVACGENHSAAVDSAGVIY
TTGGDESGQLGKGDEWICATHGEGCTGSGWSCQERIPTFLSILSVRHVQFVDIACGHGFCAAITNRGLLYTCSIDGVVSV
ACGDIHAAALTDKGQLYMWGSSEAGRLGDGRSTEGVTRTPKLVPAFADIHQARCLGVSIGPRHALAISSTSSSLPKTIGA
ALDSPDFADVSFMLEDRYTVRAHQAILTARSQHFRSLFAQRKAKQQRNTEIKVKKVTLETFKRLLEFVYRDHIAADMTKG
VATQLAQVARMFGMERLQAMCESFLEHEVAEVPKSHFVENMRSIFNDKESSDIAFSFPEEENGEDESMLIHAHRSILGMR
SEYFGAMWRSGMRETTQATIPITYADRKSFYFTMEFIYTDSITEQAGADVDINTGVSLLSLADLLELPRLRELAEKKTLC
KARVENCATLYHIAMLHRAELLKRYCMEIVFQQFAAVKRTSGFDELPHDVQDTLTKAHASWWKNRTSTLS
>Acas_g10197
MLATRKKPVDGYLLKQSVPKPSSSSSSSKKAKHQEQAGLFRKLFFSLDRDAGVLLYYTSQQEHANASSSAASSSSSGGPE
GSIPLLDIEFVEELAKERPARELLLQAEPTKKDKKDKSGEHGAASEEPLPRNAWQWPFVIHTTSLGTHLILAPTAEVRDN
WVVNLEEAIAETKLRARKSKSKSQRLDDPNGRIRSESEVQADFLDILVFDSYVHELRKKEKAPEEAAESVSPRNATYHSA
LPLSPLNLKPPPKVPSPTSSRSPRYGDINSPRPPSEAAQAQRPEAERLTQSQSSHPRALARPSAPPATKESHGEGGGETG
SEIGSPGAKRKGSRSQTVWRKEERKGNAGALGGGTDTPPKRRIGVRERVDSNNSSSVDAETSGTRPIRKTTSEETVMTAK
SSLAAQHQEETTKATPTPTPTPTTVEPKSENEGESDAETAKDRAQVPKRLSAGKRMYHTVRGPRFRGKEEHAEGGGGSGPRP
PKLPQLPKNENEIQKKNEDEMTLKGEAKNNTEKSEESPPQQQQRVEDEPVQLRDNVARRLAHAKAKLSREAADDDSSESS
ESESAAADSEFVEQMHFMQRRLSVIYKRMNDMEGDFFAQQQPPHTTEPHTEPLNESSSPDRPRKTRKKQLASNNTATATL
LRKEDQQAVRKDQEMLQAVVRREAVCRRLHKLVDNPMDHDVQFLISRDTSTRRIIYGHRAIIRTRSSFFDKLFTQTSASG
MDPKGPYVIPETTREAFIEVLSYLYSGNMSLTVDNVQDVYKAAATLRMKEMKAACRQFASRNAVTLPPRPTSSPYHVAKR
QSVMDLFKNVNFEEARAAALASCPQHEPQGVSAVASTPAPAPAANTTDDGAGGEDSPKSGGKSRSKRMSQGLARVKSVLS
IVSPRSSQLVASQGDDQPSTPTSSVEDEREGKNENESESEGGSGEEDKLRTSGGLKGSFFSRSGNHSKTKRTAKDS
>Acas_g10396
MDSEMEIEKRFIKNLDKGKDKIDAQSFVPFLFPSGANAAGAESGDRITELEVLFDARTFGEQLQQGMKDLFFNSHLSDIT
LIVGRDADRIPAHKFILYAWSPKFQAQLKELGEEGRELHVAVEGDDAAQLFKMMLQYMYTGTSDQKINERNVMPLLQLCH
EYGVDPLKEECGNFLFSNSEQVNVAYLLDVADKHGIKNLEKRCAEYLAANFSDFIESDDRMLMQLKTSTWLELVQSDEIE
VTREEEVFNAVLKFADVNGKTAAETRDILNQLLPHIRFTLCSPKFLIEEVETNPELMQVPVLKSLLFETFRYKANPKTPF
LFTPLNTKRRKANLNVMLQWDKDQPFTHGVYTLENEELTAHKSSMPGTIYVLRTKTKLEGSGSFYWEIQIDQTAGNQELH
IGIAEEHFNFHTAYLRANGTHYIERSGGIMQGHTQVVQGPTLNTNDNVGFALNLDDATLQIFHNGTPVHTFTGVTGPVWP
CVAARDVGTKLTLVGKKPKIYQFPSLPK
>Acas_g10432
MEGRGGRRSEEASKEGDEQEEQEQEELQSDEEELWVETSLDFAPFLTVAGLRRSQQKVGASLDLKAKKSPHNNKSKKKDG

FEELKRRQNEEEEGEWPGERYGHTAVVEERGGGVLLVWGGSDPHGTFLRDLWHYNFESKKWGRIEAEGGPPGRHFHSAVM
YEGCMYVFGGTSNGYYNDLHRFDLNNGQWSVISPANRAPSPRYGHSAVVHRYYMYVFGGYDKDGFECNDLYEFNFLNRQW
RKVKTKGIIPKDRYLTSTFFQSGRWQRMATSEGISERAHHSLVAYGDHLYSFAGCNIFHFCFNDLFVYNLVDGHGGAPNT
LVDDFKHLLDDPSLSDIAFVFPNEGDKKIRAHKNILSARCAPMQALLRSGMREAFSGVVVIDSIEHATFYALLEFLYTGQ
VFMQADGLVDLLQIADQYQLERLKYLCERKIIGYVDAENVVNLHLLSEQVQAPDLAKHTLEYLAKNWAALREQLQGVSSL
GKEQTERIERVWRKRYSGDDGKAQSSAKAGSVEATVLNEP
>Acas_g10726
MEAPHEEEAKKVPPKDTMEEEQEAQEAGQLLKIDAKGRPFDAALFGETVVKGFEKMYNNPLLSDISLVVGLEKLPAHRMV
LCAWSDTFRSMLENKDWAESSLHDLPIHLDDPEDQLHFKNMHVLRYMYTGSIDFVSGDNIIPLIRLSDYYGILSLKEICG
DLLGEQINEDNLFFLLDIVEQFDCRRLNAHCGEFLAEHFGDMWTELPERLMGLKVDTWAEMLKSNELHIRSEEELYEAVI
SYANQFKAEPQMRDKALTTLLPLIRFPYLRPRFLVQQVETDKSVMHLPIVHSLLYEAYRFRMYPAAHTTFRTEARRGFQR
FDRDHCSSAMQLSDDELKATLSNSSGWNNVRCVCPLSPSYNYVEFKADNGPNMMIGIVDGDCGRNAYAGQFSNGWTYYSQ
GGLYHAGGTPATGARYSPGDTIGVEVDFASSDGKINFYRNGQLSVSLTGIPKESTNLYPVVCCSAMGDAVSIVVGAVSPA
DEKAGKKSEVLGAWNHDAALELAREEGKPDAWWGRARLARGVECRYKYFIREGAAIAKWESWRENRRLVPLGVELFVDDG
RFGVEKTSPSSSPSPSSPSPPSSSSSSSSDVWEDHGWLVSDIQLRITLGAKSLEHHSKPRTPPVELRDSQLRPHKLVFSSK
DPNIVAKSVFLDDDARDELKEHTFHTGGFSSLHLYVDFFVAGSTTKVGRAIITPALLRDLIGTAAIPIVSPELEEVGIFA
FEYLIVKPFEHENLSDVLARTYWKSTKFVDVGHRGCGANRAVSKSGKKTVVSENTLLSFVRAGQLGRGAVEFDVQLSKDG
VPVIFHDFLVPVDERGHYKVPVYSLTLQKFKELAKCSHSWSKDASGAVPYPARKGGQHSSSKAKQVEPSEEPTLFPSAAE
KHKLMRKRNSLGHVYHEGDENRPHTPLQDSFATLEEVFDIVPLSTGFNIEIKYPTIEQLAKGKVALYPERNQIVDAVLKV
VFDHAKRDRSIIYFSSFDADVCTMLSLKQPRYPVFFLTQGGSKLCLDHRRNSVPQAIRFAKSVNLLGLVVEVQPLLNAPE
LIKLVKSSGLLLATYGSPNNEQEYVRLQEEMGVDAVISDHLIVPRHKDASE
>Acas_g10917
MSIHEAAEDGREEELQKLIQRNIQAVNEEDEYGETPLHLACMAGHPNCVKLLLHNGAQVDCQNSNGTTPLHYAARYGRQA
CVTLLLRNGANVARKDNAHWTPLHYAAINAQTKCAALLIEKGADANALTSDHNSPLHLAASAGVLDCVLLLVKHGAKLNA
KNKEGNTPLSLLTIKAKAAVDKEKNSLQRLSEDLRGLVNDKTYADVAFIIEGEDRRTLYGHKNIICLRCPRLKEIIQQHE
RHKGSNGSTSEPEQIGNLTIVHVQGMSASVFENLLHFVCTGEVPALVSLAAFLSSASSETDKVRELAQLVELLRAAEIYQ
YSHLADYCEEILSANLNVESAIEFLSATDYRKKGATGLREWGTLPGMNNDDVSRQCLARVRKTFGEYLIAHFNEAAESKG
FRKIKKEMIVDLLPELSKHKPAEYVRRVPKKKVEKVDAMKGNILLECKKIMTSLISDTDAWPFNQPVDPVALGIPTYFDI
IKEPMDFGTINQKLKNGKYEVLAQFERDVHLVFANALLFNEPDSDIGYWAKKLRGLFERRLVRVHNMLKEEKERYASAVA
AAPQQPAAAAGPPAAQKEPAASTSTSTTSSSSSSSSSSSSGASLTGAKKKPSRPVAAATAAKAAPATVISPEELEVARAR
LSQNVNRLSPHQLMEVLTMIQQASSMPTGEEGDSNGDIEIDLNELPARRADPGDEEHEEMTLEEEEADEPVAAAPATALK
TVSAPRKRQRSEFDFDATPEELGGGLDPKNENGVARQQRREKGRERRKDKADRGSGSGSDAGGGDKKRKRHHHHHHSSSA
SSSSTPSGGYASPGNAGTPGVSGSSASAAAAAAQQRKERKRIKEERDETYSQGGGGSGGRRNESYAPTSFEHIEFGHS
>Acas_g11396
MQASLPSTPGLLPSTTSLCQPTERPSPAPADVSDIYVWGSGQVGQLGLDPESEFELEDQYVSHPQRLRHEALLHKRVLQV
CCGRDWTACLTDAGLYTWGSNAYNQLGHHHQRELFTLMRELKQSYPSSPDAKWGYWRVDEPVESEHESESEGQAAERSPV
ERSQGLEKSRELTGCRLASSTTWLCATPKLVGGPLLNKTIVHVACGAQHMACVTDEGDVFTWGYGGRGALGHPNTRITGE
PMLVEYFRSPGGDAATRIFIKSVACGANGHAYGWGEHAFGQLGLGPIPETPMQHSAFYPCPTLISSLAEANQVVVSVACG
ASHTLFLTDVGHVYSCGSNVLGQLGNGTDHSLPVCIESLKSTKVTSISCGELFSAAVTDRGELWMWGAGSGQFGCKEGRH
AEYPGQVGFVGNVRQVACRSRSVMTLLGPPREASRPDYQKMLFARDFSFAVTLLLPDVNEAVFRKLLEFIYTGDFALLAS
AGLNDELYRLALELNFTGLVRKCAMLLGRPVPDAHQAAGGSAVRHIDDLREDLLRLLREPQFSDCTLRVEQQPGLEAKTF
ECHRAVLSARCPFFEAMLSADRGYKESQQAVISLSMPFDNDDVDAGSTVAELLKWIYAERFDPAPDTSQALLVLADEYRL
PLLAQMCETLIQEGVDETNVCDVLQFAETFRANKLRSYCIDHMAAPTFPSHLIDAAQLKPELLEQIQLRKGSWSEHSRSR
RKVKEREARYQQYEEERQTQVQRWAARDSPLFALGQALRESLERFDQAVAQADPAAKAPCWPRWMWRMSRQERRERLERL
HAERKQRKEAKRLRRLEEQEQAAAAQRERDEAQRRKRERMNRLDKGKEKAVDSDNEEEIDDFEAPLDLEQQQGREEEAPN
YDKATLTLMDTTQQATAEFGQISPKGALQLEVRALRREFNSIEEEARSLAPQVSVALDSSALSQEVVALRAELDALEREA
EQLVLERASQSLPPSLLVLPKEVRAIRAEFDAIEREAQSLAAECRAHSIVDPLLPKEVSAIRAEFDAIKRQAQQLAAWRE
AAVKEVSALRDEFAAIELAAQALQITLREADAHEEVVKREEVTVRSEEEERGYESEEEEEVKQNEEEEEEEEMKVRGMRDS
AESPDGREDKHTTDEEKEKDRKMEDGEDDDNEELNRAIRLVEQAFALEREDPAKADHLFTQSSIGFLQAMRIADAQGKSD
VRRIVDGYIARIDARNLRRSAQMIKRKESESLHSAPEEEFMATSGMELESCDYGQLVADSSEEVATVSSLEEVVPQDSYL
TAVLSDELPRRATNNTPLRSPVVDAAAVATSAAPSTADTTGESESESEVSAELLASAEMEPEDGAYMQAVVEEEEEADTPQR
SRASTVELRRDAAEAAAEVLNTITAGGSQAQTTGVSAAARWKPADNNGARKHDALANTRVTAPALRDINRRSEGIVFVVP
TSTQSSSSSTSTAPSPSPSSSSSSPTSTSTTPSPSTARVIFSSNRVPTTAATRLEPAARPKRPASTSFVSGRVSSSLASR
LALFESPSSSSTSPPQHPNSTTQTTPQLGPSSPVAVRATSFGQTRPAALSTPQPPGALGLRRRSSITTFQSPATATVAQD
AIERYQQQQQQSAEVSSGQRPAATAAASNGHETRTLSYVDLKDNRSSLPDWVDQAQLEVYLSDEDFGVVFQMSRQEFNAL
KSWRRNQFKKAAGLF
>Acas_g11645
MPKRAAHPTWWSGGVASVGSYVFAFGGIDPHTSLPTNSTHVLDTGSGEVSRLDCSGSAPAPRWGHTLSPVGNRLYMFGGA
LSPTICTNDLFLFETGTTLWRELRVKGDCPARRTGHAAAVVGTKVYIFGGKDQQGRLLNDLYILHTSGPARWEQVTGRGV
APAGRMDHALSAVGTRLFVHGGVVINDPATRASDLHVFDIVTQTWREVDVQKELGIALGPLGRYAAAVWKSAIVAKSEQG
TSIIDTRKILFSDPPADRLRDDLAGLLDGGQASDITFFVQGQLIPAHRCLLSVRMTEAGLAALLGRTPSTTEETPELESD
PSAVIEVADCTVDAFKAVLGLLYSDRARVLSPDSDAFLRLAERYQLSERVAPIAFYPLIVDQQQPLLPADQQQQQLVNAD

ADSLAVDLRKLFNLSLTTDQTSPQTERERQVLGLRDIAFVVGQRRVTTYKCVLVARSEYFRALLSANFGGEARSEIVISS
DGDGDGVVVDYESLVGVLEFVFTGTIDRFQAEARAHEAEQRRRLYCGRRPARMRQKQKKKNKLRRKRGLGGDSDSSSDSF
VGRVVALLAASTQFALFDLGSLCERTLIAELARRTPPVRQQPQKERRRPDNEEKVKATEKEIEDEREADDDNDEEGEEED
AADLALPLLIVADRFACAQLRSACLNVAALQADAFIWSGVGGGVIVLLALFAALTAYLCARHNHRRRPGGGSPYLPLYDE
EAAMPRGRKGKGGVMSAEASLREYLEDNVATPPETQQHRIREEEALMNARYYLRSSMAAMDTSLPGIGHRKRKFSFLVRH
PQRGLHLMTMVHRGINCVLSFGSDSGKATFCNLLQSLRHPYVCPTLDADFLAEKEMAVIFRAFQPKGSLKDLIYRGLAFF
QSKGFMYGHLHSGNVILDNKNVPPHRWRRGRELTAHDQVNEDTLASQPQVLDILKRILGLAVGQEEALRTTRIDSKGLDM
LKAAHRTSIIGKDDFLALEGEHELGRSLRRSIRGERRGRRSGASSPRSLAASTSSTPRGGGGPATQQNPLSPPLSPLLPL
SASAPAHQLAPLLLYSPPPGLRHHHLPTHRHQPPVAAVDSADHQQHQQQNQQHDGPAGQDEEETTTTMTDTNINDIDNRD
KKDETDPPLERMSPPAPTTLSTPLPPLSPSSPSTSTTPSAGVSPSSP
>Acas_g11659
MQNTNNNVNYFSFVRQEKEKEKERGKWHEFNEDWGETFPGKTNPRPHVVDLATGKVRALRVPAHISFGQLRWLPDGRRLL
AVGWETTERRLGIVYCTNRRSAVYLIGVDGDDDGKLTRVSADGGEDVTTPWSVPTPDGKGAVYASSPSLAPHNACFALRY
VDLSAEATGSAPRTVVPIVDIPSADAVPPAFPGLYDTCGSDDYWLDDRTLVTPSLWGSRQVLLAINVDTGAIHDLSTGKL
SGAKGGGEVEGERVSWDLCDVRKGAAILAIVSSPSLPPTPCFGALDTTSSGPRFVWSLFPATTSPRLDDMQQQFATIEHA
IHSVPVSQGEYERFEVVAIHQSGGEDVAKRRVLVLPHGGPHSTFPSGFQPLYLFFALQGYVVLLVNYRGSLGYGEKPLES
LMGRCGSQDVADVMQAVDYVTGTLKKGDPTKLYVCGGSHVMEQLPFADEFKDPAHGDLVVRLNQGQAAFHVHRAVLALAS
PVFSALLTNGMRESAQAVVDLVEEHLDHARAVGQLLAGLYPYHRTTIDMANVRALLHLGDKYQLRPRLREECEAFLLTLP
AAWDLMLLAQRYAGLDALLDRQAQWVAANVRLFRLRPELLVQLEAATVQRVVAHIAKLVHGLDPHPGRVLSHTGLDVDSA
QRVRAVFPFH
>Acas_g12068
MGDEADAVIKAEALSVLSAAVLSPRYSAEVRAHAAGVVGLVVNHDKRTTLGEQLAECLVAMVASDHRLAQKKALKAIHHS
QYASLQNRNLLVDKGVIEALARFIGNTPFLATNWMRFRWACGALGHGFTHNVEYLHRIAACDNLLHTVVELLKVRSPNYG
YAKCSVSLYFQELARSGEGALVMVEHKLPHAITTFINSALQSLFSSRFQFTALMNLFKQDVALHTLIHEQSLATAWRYLL
YHAKHSPAKKERVAMLKLYVEAIVAQRSKVKLLARARVGATAGDAQSGLDALLAFVFMSPDREEKTQMLLDRCGLDALFK
ALANESSALNVLTAFNCLTLGDAQIYEQSGVHPPTFSLPRPSAAAIPVVATATAPRAAALVAVAPPAAAAVAAPDPRIPR
PVRRTITQSGCFAQWRADQALHDVAFRFPSEGGVTVTAHKLLLAGKSAVFKAMFTSGMREARRGGADDDDNNNDADGLPV
VAIPNIASRTFQLLLDFCYGVHLLEPPSPVATGTTATTTAPDPQRAILNTLDAMAAWLSRAGASLDEVAALLAAAGQYEI
LDLEDGCAHFLGARLSGLNVMDMLRLGLMHRSPILVFRCFEFVLADPSVVLLKRAVTTKSAKVVTSRKSPSRAAATPSTQ
PTTDDEQAKKNEGKGEGDNEGEDGGGGEDDDEDEGEMEIDGTSHGNTTDEPGAAAPAEEPVTIQRSRWAWRPKARRVLLG
DPECRAMLRDFVLRGMADVPQLEFVGGGDASAPPAGPGVGLHAKDSKNCLLQ
>Acas_g12197
MMMDEQGGEQRLDAVSLASLTSLGAATKAAHDRDKKRALRERNLLDLSTRLATLADDPGAWFSDVVLHLGPQREPVPAHR
AVLATASPILHALLLSSASSSQAGAVAELELPDVDPQTFKMLLRFVYSGALHAGSGLSTDAGLQLLELAERFDVPGLAGV
CLDRYVRKVISHENAFDLFAETYTKHKQQISIECLYFIEEHFSKLLKHNRAGFRQLPEAVLAEVLKSDRLSVGDEYMLFL
ALVDWGRAALAREQPDLVPSSSPSSSSSSSSSLSSSSSSSSSSSSSLSLPSATSPALTDALRRRLASLMAHIRFPMMSPQG
LEEAEKTGLVPAELLYEAFRHHVLLRDGVVADGESPDVGDAALSAAASFSSSSSSSLLVEEDQKNPRLRRRKYHTYFLWD
PSWHGQNIQIEDQGRTARKVKAGWYSLAIGSTGFSTGKHYWEIKVNGQEFSVGVVLKSLPSTQLDNYLAASTDAWAYEAV
AQRKRGGVRTAYGEVYGVGDHIGVLLDLEQGHLSFFKNGKSMGPAYTDLPTGTPIFPAIGWRDGTATIVPAWCCSMDL
>Acas_g12391
MQLSASESGSKRSKGGGGLQLASMVDNRTCADVEFLVGKEATPVYASKVILIARSPVFEALLNGSFREGLPPTSSVSCST
SASPSLPPVFWHSASVPDLEPRTMRHLLHWCYTNTLHPELVHEDGDRDGDDDDDDDDDEAPSQHERDLVQLYAAADRYLL
KDCRTVAAEELSKVFENDSSRVLATFDLALGIAPELARSVCLKVIRDDASSQLGGPEIVQLQVEQQWLHLSLAAAEELLE
ADLEEIEEVDLFRAIERRYKHLRQFPDDEEQTGPQVASESAVEKVASLVWLLETHLMTTEEIKEVVEPSGLFSTKALMKA
YRRAASSHRFVVPDLHSTGTVEFDDRVAQGKVTWRISVFPGDTPTTYKIRFIIAPRKNIDISGKVSCKGISLTVFVNRSC
IKKVTMRGSCAVPGRVATTIHSRLFARLTILVRVPATALREIGE
>Acas_g12724
MELHEMMEDSVPAADEDALPMDEDEELIPGVGRQTCPWRRLRLRLPVEEDDDATDEDHEEEDEEDDGGAWPAGEGHSATL
VGHACSAEMLVLDMTTGRWRFSTDGLVARAFHSATLVGSLIYIYGGTSGDESLAAVHCFDTETETWSEVEVVDGGGDGDG
DNSTVPPPAFFFHSTTLVDNRLLVWGGTGGHLHYNNHLYSFDLATHRWSQCPVAGDPPSPRANHTATLVGSEVYFIGGFE
LSVRGPGDRQYYKKKYYDDVFILHTDRSPMAWEKARVGGAAISRRSGHIAEYFPEAFGEKEAIVVFGGRTKAAGQTFALN
DTWSLCLRPRLQWVEQKFAADESLASPQSLALPALPAAALLPLPRRNHAGCRFGTKLVVIGGWSGRENYGQSLADVFLLE
TNYVQEVPSTYAADMRQAMQAFFDAPQFSDLVFIVGDQRIHAHRAVVAARCPKLAAQLRCGLRESTSGEIAIGHHAYPVF
RKLVEFLYTDRVEVEPDDALDLLQAADEYMLERLKALCEEVVAAAVAEDNVAQLLEAAERYGARRLRSACVSLVAARPQL
LTSAPFLALPPTLLHDLRLFVPSS
>Acas_g13349
MGRWIREQWVSFASTNKLLCFRIEYAKPVELLSIRAAGNAWMSKRPGHQLTLYDENMAIIAETCVHSDDDINMYMEVVLE
PQDKTAAHGKVFYLEEKHDLCILRNYNVVARPFRPAIPKGAIMDDVHSLFNNKELSDLTVVVEGKPIYCNRAMLAVRCPV
FKAMLYGPMKEGQQKTISIPEARHEVFLRLIEYLYTDDIRSELEADAYVELLMLGEQYNLPRLKAMCGVKLKQSISVNKV
STLLMTAHRHNSQQLKDLCIQYICEHHNET
>Acas_g13707
MNTGGGDDQHEEEHEEEREEDEEEAARNNAFLHHLLAAMVTRQRTNDTNDELIASLKRSGALRSPEVEAALRAVPRGPFV

PADLADQAYVDTPLRLAAFGFNISAPHIEVVLHDTDYVPFMACLEFIYSGQVKIPDPDFAIELIGEANKLQLVRLKALCE
DLISKNIDIENAAYVYQVGSYHAVPRLRSIALDFVVTNFDQEEEHEEEREEDEEEAARNNAFLHHLLAAMVTRQRTNDTN
DELIASLKRSGALRSPEVEAALRAVPRGPFVPADLADQAYVDTPLRLAAFGFNISAPHMYAMCLEALGLELGHTFLDIGS
GCGHMTALGGQLVGKAGRADGIDLLPEYKRFAEDNLQRLKVETGLELPNLHFEVRNCFLPDLEERTYDRIHVGACCPTPR
LQSLVDLLNPGGILVTPNEDSLVKVTKSADGQSTSIDKLASVRYGDLKVPSDAEIKEAQLALERKKATTIEIPPDTFVQD
FARLLNNPDLSDVTFVVEGRKVPAHRFILQVRSEHFRAMFSK
>Acas_g14417
MCTYGSDFQPSDKWGSARQVVATGTHFLALLEDGWVVQRDHRLGGLPSTEHQVLPPHHSIIAIAANELYSAALSDTGELY
TWGIGPLGHKGASNPIKPKPEKVLGALENKRVISVACGREHMACVTDAGELYTCSGDGEVGAKLAEPQRVQALAGKKIVA
CSCGDYFSACVSDTGVLYTWGDGSTGALGHGEMTNEPSPRLVEAFKNKTVKSVVCGACHTVCIVLAGINQNQSLLMADLG
RICEAEELKGGHTLDEEECFYDVKLRCAREPLKPALRAHKAVLAARCPAWHALLPQQELKTVDAADEEAPSSSSSSSSPA
AAADMWELVVDGMEHAVMVEFVRYLYTDRVSPDLEDRLL
>Acas_g14556
YHRMVDGQYRVVHLFDNVQTEDWGWSDQIIRECTAFLLVYSITSRSSFDSLAAWVDRIVRGKNKADREDLREVSYAEADL
LAQTYDALFFEASARTRFNVEQCVLAATSQEAPERRQEWHQKKVARLKAKGKKIPPIPPMSPVSSTYGPDFWSLVGEHKC
SDIEFEVAGDIVPAHSVVLIAHSPRFMKSFVREGRRRKAVLGVDEETFRAVLDWIYAGIFPKPESTDMRAYIRLYNAAVK
LGVDSL
>Acas_g14888
MFMSQWRESDLGEIEIIDVEPDVFLHLLKFLYTHRLDADQELNLQLAVKLLLVADQFLLPSLQIAVQKYLAQNIHKNQDS
FVMLWEIAEQFEFKEVKKACLKFILDDLGHQRRRALEQQQHDTDQEQQQRKQQQQEEEKKRKLDVDAAAMEIEGEQQPQQ
LATDADTATATATATAAKKRRRTDSPEEEEGEGEGEAVAATIDGGMEVEKKEKGKEKAGDEEEEKEEKGDEYREVCRKLI
FEVLEKKMDLPRKYRSIHWKKDEEVASASSSSSAAAPASSP
>Acas_g15036
YHHTAVVHGGSMYVFGGKKSFNEIVEYRFSTETWSLVQSEGSGPRPRWGHGACVWRGGMWIFAGRDNVFSFKDLLVFHFE
SGRWQRMATSEGISERAHHSLVAYGDHLYSFAGCNIFHFCFNDLFVYNLVDGHGGAPNTLVDDFKHLLGDPSLSDITFVF
PNEGDKKIRAHKNILSARCAPMQALLRSGTRESCFSSSPRHGHFM
>Acas_g16742
MMTGLGARCPPLYELAAQEQQRPSAEGGSSIGGGGGGGKKQKRQGKAKAKAKKDESSTSSSPPSSSSFPASSSAAFFAPV
EVELSDVPSAQAFREILYFIYTDEVRFSPKMTLSQLFELLALATRFRLDRFVRLCERYI
>Acas_g18389
MWDSTKLDLDHMVDTFDPTVKQPQHDACFVVEGKRLYAERAVLCARSTFFAGMFGSSMKEAVEGEVELESTYNAVFAMLE
WMYTGCISTDNFDDILQLWQSSHMYDMEALQNYCQKRLLKLFEARSKQL
>Acas_g18470
MFMSQWRESDLGEIEIIDVEPDVFLHLLKFLYTHRLDADQELNLQLAVKLLLVADQFLLPSLQIAVHE
>Atha_AT2G39760
MSTVGGIEQLIPDSVSTSFIETVNGSHQFTIQGYSLAKGMSPGKFIQSDIFSVGGYDWAIYFYPDGKNPEDQSSYISLFI
ALASDSNDIRALFELTLMDQSGKGKHKVHSHFDRALEGGPYTLKYKGSMWGYKRFFKRSALETSDYLKDDCLVINCTVGV
VRARLEGPKQYGIVLPLSNMGQGLKDLLDSEVGCDIAFQVGDETYKAHKLILAARSPVFRAQFFGPIGNNNVDRIVIDDI
EPSIFKAMLSFIYTDVLPNVHEITGSTSASSFTNMIQHLLAAADLYDLARLKILCEVLLCEKLDVDNVATTLALAEQHQF
LQLKAFCLEFVASPANLGAVMKSEGFKHLKQSCPTLLSELLNTVAAADKSSTSGQSNKKRSASSVLGCDTTNVRQLRRRT
RKEVRAVS*
>Atha_AT2G05330
MATQTNQDHFSGDLRRSWQKIGKLTYGSRQWIVMMASRSAVLKKMLESDEFKTSAKQVGTITLLEMKQEELEAFVEFLYS
DGSMLSSKVKQHARALYRAADKYEILRLRELCRSELISSLNSTNSLNLLELAQIPFDKVLNDAALSYIKTNELMFPSFDE
FKLFVDNYPNLAVEVMMASLTRTPSTSCSRCGLITYHNQTGTSCCSCGFDYPRRS*
>Atha_AT2G04740
MSPIENSSSWTLESDLEDLDLDLQDYKPSVPLKKVPNGDIFEASRAGDVDRLRYLVETGVNVNARDRWDSVALYYACLAG
HIDSARLLLENGAICSEHTFDGDRCHYASLNLRIRKLLKAFEARPPPLAPLQASLRDTFLGCCHNRDYLQQEEANLDVSD
TLSEFGSSNYFPPDVMFYVQGRPIEAHRVILSARSPFFKQKFENEWKDRREVRFSKEKLSYPALCSLIHFFYSDRLEISV
DDMEDLVRICKVCKCESLQKIIEKELIHQRYAEYKTHRDLDNSMKRFILQGISLPEEDRLPASLHRILRVSLAKSFVGDV
IDSSVGDTRVGDSVESLADVCVRVDKRNFYCHQVILASRSEYFRARLSRVNDFHEGKNGLPGDTLPFLEEHDLSAEAFEK
MIEYMYTDGLKEINPNQAEEIFDVASRYLLFPLKRAVADALLPHLETATPAELCQWLVLSDMYGVLKIREYCLDLVACNF
EAFVETHEFRAMLLTLPPPSGDSSLRTTVPSAPGAMMTTDQGNLLDDLREKWLEAEALELDMRDESALIFDKRLAMLVEI
AEREKSESEAEDYKDTSA*
>Atha_AT2G23050
MKFMKLGTKPDSFLSKGDNVRYVTNELETEIIIIIGNVKFYLHKFPLLSKSGFLQKHIATSKNEEEKKNQIDEIDISEIP
GGSVAFEICVKFCYGITVTLNAYNVVAVRCAAEFLEMNETFEKSNLVYKIDVFLNSTIFRSWKDSIIVLQTTKDLLSDDS
EELVKRCLGSIASTASIDTSKVKWSYTYNRKKKLEKVRKPEDGVPKDWWVEDLCELHIDLYKQAIKAIKNRGKVPSNVIG
EALHAYAIRRIAGFSKESMQLIDRSLINTIIELLPDEKGNISSSFLTKLHRASIFLGCEETVKEKLKKRVSEQLEETTVN
DILMYDLDMVQSLVKEFMNRDPKTHSKVSVAKLIDGYLAEKSRDPNLPLQNFLSLAETLSSFPRHSHDVLYRAIDMFLKE
HSGISKSEKKRVCGLMDCRKLSAEACEHAVQNERLPMRVIVQVLFFEQIRANGSSTGYSTPELTTTTLNTEDDEWDHEKE
F*

>Atha_AT2G30520
MATEGKNPINMNSMSSSLARTGQWVFSQDIPTDVVVEVGEANFSLHKFMLVAKSNYIRKLIMESKDSDVTRINLSDIPGG
PEIFEKAAKFCYGVNFEITVQNVAALHCAAEFLQMTDKYCDNNLAGRTQDFLSQVALSSLSGAIVVLKSCEILLPISRDL
GIVRRCVDVVGAKACNEAMFPCRTPPNWWTEELCILDVDFFSDVVSSMKQRGVKPSSLASAIITYTEKSLRDLVRDHSGR
GVKYSDPGDNESDERSQQRDLVQSIVSLLPSDKGLFPVNFLCSLLRCAVFLDTSLTCKNELEKRISVVLEHVSVDDLLIP
SFTYDGERLLDLDSVRRIISAFVEKEKNVGVFNGGDFNRGVCSVSLQRVAKTVDSYLAEIATYGDLTISKFNAIANLVPK
SARKSDDDLYRAIDIFLKAHPNLDEIEREKVCSSMDPLKLSYDARLHASQNKRLPVNIVLHALYYDQLKLRSGVAEQEER
AVVVLPEALKTRSQLQADTTLAKENEALRSELMKMKMYVSDMQKNKNGAGASSSNSSSLVSSKKSKHTFFSSVSKKLGKL
NPFKNGSKDTSHIDEDLGGVDITKPRRRRFSIS*
>Atha_AT2G36370
MSEILSYGSVKVRAHRTRLIQESSYFHGLLSGSFSESGLDHISVEWNLESFLNLLMCLYGYDIEITSSSFLPLFESALYF
GVEKLLSICKNWLSVLASSNDNALPKVELSDLIQIWSFGLEHAGEFVPDLCVAYLAKNFMLVKSDKYFGNVPYELLMWCV
KHPHLTVHSEMDLVDGLLIWLDAGGRLSDLPESSQDNTINLMEQVRFSLLPLWFIAGRSKSHGFSKFADQSIELVTKLMK
MPSTCLVDSLTDGPPTDVRVRLTEYSEILDLSGCPQLNEASLLLSILPNSYFANLRWRKSLESFLKNPDDDERHQEQISH
RTLPILSFESVKEIDISKCQRLDYKVVIKCFSKSFPSLRKLRAAYLLNIKVSTLLELLLNFRELTEVDLTVDVSPIIPVQ
ASVFYSGQGHCLLSSITRLTLEGRSDICDMELRSISRVCESLCYLNIKGCALLSDACIASVIQRCKKLCSLIVCYTSFSE
NSILALCATISMTNEHMDINSVASNLQTLHMSKCEGISETSLLNLITHSQKMKSLCLRDTKVSDSVLCEFPGSTLEALDI
SNTTISWMALARVISRNPNLKTLKARGCKNLLQLEVDGRTDNFSPLVSGQEVFKCLSKGSGLEELEIGWGFSYFSFESLR
PAASFLRVISVGLGASLGEDVLKLLPSTCPLLESIVLHFQEISDSALTSVLTSLKHLQELALSYCFGEISLQSFKFSMPN
LRKLRLERVTRWMTNDDLLVLTQSCPNLTELSLVGCLHLTSDCQPIISAGWPGMISLHLEECGSITENGVASLYGCIALE
DLFLRHNGSGIQKSFLLDATLKFPMLRLVSLDMCDAKEGGFDVPEEKEEGRSLSIVKISRCKSDRCSLGRRAAPMHRETL
VMLWNGQTLTKTLLKQRL*
>Atha_AT2G30600
MVAAKENKFLTVAPFECAWSDDLKFREAGRGCVAFDAFAHNDVTVVFRENVGTQHYHYKKDNSPHYIVIIGSNRNRRLKI
QVDGKSVVDEEASDLCRCSLEFQSYWISIYDGLISIGKGRYPFQNLVFKWQDPKPNCNVQYVGLSSWDKHVGYRNVSVFP
VTHNHILLWKQVDCREVRGDESGDEKVVEEGTGYDYEQWGLGNFLESWQLSDTVFLVGEEEMDVPAHKVILQASGNFPLR
SSDGDVIQLRGVSYPILHALLQYIYTGRTQILESELAPLRDLSSKFEVMSLVRQCEESIDHFKLSKTAFDSCRKVKLLCP
ISHPLSGFMFPSAFPVDVGKLVKLYSTGEYSDIKIYLSDHSLTFQSHKVILSLWSVAFAKMFTNGMSESHSSTIYLTDVS
PEAFKAMMNFMYSGELNMEDTVNFGTELIHLLFLADRFGVVPLHQECCKMLLECLSEWVAVSSLLKLKEKRNEIPHSNYR
VKRILILCHVYITCRDGVPFWKKDSVCSVLQVVSSISSCKLIEEMCKRKFSMHFDYCTTASLDFVLLDQTTFSDILESAD
LTVTSEEKILNAVLMWCMKAEESHSWGVIDEMMNYADPKSLFKERLQSLDDLLPHVRFSLLPYELLKRLENSNLSKEIPV
FNRLLKEAASFLTSGLISPGNEPISRFQHRRSSFKELQYIRDGDSNGVLHFVGTSYGSHQWVNPVLAKKINITSSSPTSR
FTDPKALASKAYAGTSFAGPRMEDGHISSWWVVDLGEEHQLMCNYYTFRQDGSRAFTRFWKFQGSMDGKTWTDLRVHEDD
QTMCKAGQFASWPITAANALLPFRFFRLVLTGPTADTSTPWNFCICYLELYGYFR*
>Atha_AT2G40450
MATQSNKEAFLGGFKKLLNEQWQADVRLKAGDSDETTSIFAHKLVLVARSEVFKKILESDEFKASSKQMETVTLSEMKHE
ELEAFVEFIYRVDGSICSASLKKHARSLFHAADKYEIPHLRDLCRNELISSLNSSNALSILELAQIPFDKALNDPAFTTI
ITNISTIASGDEFKLFVANHPNLAVDIMKASITRLSTSRKICGYCNRYY*
>Atha_AT2G41370
MSNLEESLRSLSLDFLNLLINGQAFSDVTFSVEGRLVHAHRCILAARSLFFRKFFCGTDSPQPVTGIDPTQHGSVPASPT
RGSTAPAGIIPVNSVGYEVFLLLLQFLYSGQVSIVPQKHEPRPNCGERGCWHTHCSAAVDLALDTLAASRYFGVEQLALL
TQKQLASMVEKASIEDVMKVLIASRKQDMHQLWTTCSHLVAKSGLPPEILAKHLPIDVVTKIEELRLKSSIARRSLMPHN
HHHDLSVAQDLEDQKIRRMRRALDSSDVELVKLMVMGEGLNLDESLALHYAVESCSREVVKALLELGAADVNYPAGPAGK
TPLHIAAEMVSPDMVAVLLDHHADPNVRTVGGITPLDILRTLTSDFLFKGAVPGLTHIEPNKLRLCLELVQSAAMVISRE
EGNNSNNQNNDNNTGIYPHMNEEHNSGSSGGSNNNLDSRLVYLNLGAGTGQMGPGRDQGDDHNSQREGMSRHHHHHQDPS
TMYHHHHQHHF*
>Atha_AT2G40440
MATETNKELFVGGFAKILKEQRQVDVRLKAGDSGDEGASTSAHKLVLSARSEVFKKMLESDEIKASAQLETITLCEMKHE
ELEAFIEFIYSDGSMLSAKEKQHKMANKIEFIDSFAKYWKEKVGVDVLLKAGDQTDPIPAHKIILAASSEVLKQMIDSTS
SASDPITFSEMTHDELYIPLLSSCTKVTFRIQNC*
>Atha_AT2G46260
MRGSNNTDLFDPKTEMDSNFSRHGSSSEGDFGFAFNDSNFSDRLLRIEILGGPSDSRSDAEGCTSIADWARHRKRRREDN
KKDNGVAISDIVACAEEQILTDNNQPDMDDAPGGDNLDDEGEAMVEEALSGDDDASSEPNWGIDCSTVVRVKELHISSPI
LAAKSPFFYKLFSNGMRESEQRHVTLRISAQEEGALMELLNFMYSNSLSVTTAPALLDVLMAADKFEVASCMRYCSRLLR
NMPMTPDSALLYLELPSSVLMAEAVQPLTDAAKQFLASRYKDITKFHDEVMALPLAGIEAILSSDDLQIASEDAVYDFVL
KWARGQYSSLEDRREILGSRLALYIRFPYMTCRKLKKVLTCSDFEHEVASKQVLEALFFKAEAPHRQRILAAEGSDSMNR
RFIERAYKYRPVKVVEFELPRPQCVVYLDLKREECAGLFPSGRVYSQAFHLGGQGFFLSAHCNMDQQSSFHCFGLFLGMQ
EKGAVSFGVDYEFAARDKSTKEEYVSKYKGNYTFTGGKAVGYRNLFGIPWTSFIAEDSQHFINGILHLRAELTIKRSSDL
H*
>Atha_AT4G31820
MKFMKLGSKPDTFESDGKFVKYAVSDLDSDVTIHVGEVTFHLHKFPLLSKSNRMQRLVFEASEEKTDEITILDMPGGYKA
FEICAKFCYGMTVTLNAYNITAVRCAAEYLEMTEDADRGNLIYKIEVFLNSGIFRSWKDSIIVLQTTRSLLPWSEDLKLV

GRCIDSVSAKILVNPETITWSYTFNRKLSGPDKIVEYHREKREENVIPKDWWVEDVCELEIDMFKRVISVVKSSGRMNNG
VIAEALRYYVARWLPESMESLTSEASSNKDLVETVVFLLPKVNRAMSYSSCSFLLKLLKVSILVGADETVREDLVENVSL
KLHEASVKDLLIHEVELVHRIVDQFMADEKRVSEDDRYKEFVLGNGILLSVGRLIDAYLALNSELTLSSFVELSELVPES
ARPIHDGLYKAIDTFMKEHPELTKSEKKRLCGLMDVRKLTNEASTHAAQNERLPLRVVVQVLYFEQLRANHSPVASVAAS
SHSPVEKTEENKGEEATKKVELSKKSRGSKSTRSGGGAQLMPSRSRRIFEKIWPGKGEISNKSSEVSSGSSQSPPAKSSS
SSSRRRRHSIS*
>Atha_AT4G37610
MENMDDFSPENVLAPPPPPPPMKKSTDLFMQRSNSFVSKATRDSWDRMFDEAHGADVLIHTDDNGLIYAHSNVIGMASDV
IRGMMKQHKRKSHRKSISILGVPHHALRVFIRFLYSSCYEKQDMEDFAIHLLVLSHVYVVPHLKRVCESEFESSLLNKEN
VIDVFQLALLCDAPRLGLLCHRMILNNFEEVSTSEGWQAMKESHPRLQKELLRSVAYELNSLKQRNRKQKEIQTYTQLYE
AMEAFVHICRDGCREIGPTKTETPHMSCGFQACNGLEQLLKHLAGCKLRSIPGGCSRCKRMWQLLELHSRICVDSEQCKV
PLCSSLKERMKTQSRKDEKRWKLLVRNVLSTKRIGGSPFFLQAIDVTL*
>Atha_AT4G37590
MKFMKLGSKPDSFQSEGDNVRYVSSELATDVIVIIGDVKFYLHKFPLLSKSARLQKLITTSTSSSNEENQIHHHHHEDEI
EIAEIPGGPASFEICAKFCYGMTVTLNAYNVVAARCAAEFLEMYETVEKGNLVYKIEVFLNSSILQSWKDSIIVLQTTRA
LSPYSEELKLTGRCLDSIASRASIDTSKVEWSYTYSKKKNLDNGLRKPQAVPRDWWVEDLCDLHIDLYKRALATIEARGN
VSADVIGEALHAYAIKRIPGFSKSSSVQVTDFAKYRALADSIIELIPDEKRSVSSSFLTKLLRASIFLGCDEVAGLKNRV
GERLDEANLGDVLLYDVELMQSLVEVFLKSRDPREDDVTAKASVAKLVDGYLAEKSRDSDNLPLQKFLSLAEMVSSFPRQ
SHDGVYRAIDMFLKEHPEMNKSEKKRICRLMDCRKLSAEACAHAVQNERLPMRVVVQVLFFEQVRANNNGSSSTGNSTPE
VIPASRSTNTTDQEDTECWDTEDIKALRGELANLRLAKNQQESHNQGKLMKGGGLGVSRVFSKLWSGKERSGDMISSSGT
SSPGSVNDDSKSSSSTSKKH*
>Atha_AT4G04090
MGTQTNKGFFYDEFLKVLKEQQVDVRLMARDSEKDAAISAHKRILSARSEVFEEMFESDKYKASSKLETITLSEMKHEVL
EAFVDFTYSDGSMLSEKAKQHAMSLYSAAKDYEIPRLWCLCRKELIASLNMSNALRVLQLAQIPYDESLSVAAFTTIKNS
KLKLSSSTKVKVFVVNHPNGPLEITRAIFPRN*
>Atha_AT4G08455
MRCISCREEYLPRNAGTCKECYVEAGETEEELKREIDDLKAKVAFLRLSSSLDHGTSSTSRSFTDVVLIASEDNAGSPPI
PAHKSVLVSRSPVFKAMLENEMEESLSGTIKISDVSYDALRTFVYYLYTAEACLDEQMACDLLVMSEKYQVKHLKSYCER
FLVTKLSPDNSLMTYAFAHQHNAKHVLDAALSQIVENMDKLTKREEYMELVEKDPRLIVEIYEAYLSKQVNTAAGGTSTS
KTS*
>Atha_AT4G15840
MDQEYRNSRTTVTHHQHHHQMRMTVPPPPSPQPSPPVLPHAESDQSTSELRSLDCNLTSLCDHIQSEGFGSGAFSDVVVK
AMGSSYHLHRLILSRSSYFRTMLQGPWKEASSPVVNLLVDDKNVNGDAISVALAYLYGYHPKLDDDNAFRVLAAASFLDL
QDLCAICTDFIISELRTSNFLAYQVFAESQDYGIHGERVRNACWGYLCQSGAVELREMLPKLSAQTLCALLTSDELWVPS
EEKRFELALYAFIANSALSNSDHSNQDSRFERETSFPLDSAISKGKNVMDDFSFRSLDCKLGHLDIEADLRDASDDVIVP
LTEGVIDFQRGVSGSNLVFQQSSNSQTNFGRPCTSVVDKTEGSVVAIEGPSEEAYHLSNDSWLSGADSRNSPTLGSSSDG
FVVNEWGNCGVSALTWGGRVVGTRQGTGSVKGKYGFTEEEYSAFVNTFEGGSLLYCNMSFEILLNARKELEELGFPCKAV
NDGLWLQMLLSQRVQEVAANTCKRCCLISIACACKQGFGVSHGATFNNYYCQENVQNNMMGDMDNVYVPESSQGDGIGIF
KPVRISVRGQHIDGLAGIGCEATFVPPPAWPPTPFVYSRVPINRNGQQSIASDGSEGRIDQSGEISKDGLTALVGLSQGT
SGVGNNPRGEQTEGGRGSGATVGMSEPKEPSVGTEWENASCTISLDTKTPLCHFPPFRFGVEFEDVHRLANGHVEHSPEF
FYAGSLWKISIQAFNDEDPQGRRTIGLFLHRRKAEIVDSVRKVHVYIDPRDKVTARYQLICPSKREVMLFGSFKQRGTLL
PKAPKGWGWRTALLFDELSELLQNGALRVAAVVQLV*
>Atha_AT4G26120
MATTTTTTTARFSDSYEFSNTSGNSFFAAESSLDYPTEFLTPPEVSALKLLSNCLESVFDSPETFYSDAKLVLAGGREVS
FHRCILSARIPVFKSALATVKEQKSSTTVKLQLKEIARDYEVGFDSVVAVLAYVYSGRVRSPPKGASACVDDDCCHVACR
SKVDFMVEVLYLSFVFQIQELVTLYERQFLEIVDKVVVEDILVIFKLDTLCGTTYKKLLDRCIEIIVKSDIELVSLEKSL
PQHIFKQIIDIREALCLEPPKLERHVKNIYKALDSDDVELVKMLLLEGHTNLDEAYALHFAIAHCAVKTAYDLLELELAD
VNLRNPRGYTVLHVAAMRKEPKLIISLLMKGANILDTTLDGRTALVIVKRLTKADDYKTSTEDGTPSLKGGLCIEVLEHE
QKLEYLSPIEASLSLPVTPEELRMRLLYYENRVALARLLFPVETETVQGIAKLEETCEFTASSLEPDHHIGEKRTSLDLN
MAPFQIHEKHLSRLRALCKTVELGKRYFKRCSLDHFMDTEDLNHLASVEEDTPEKRLQKKQRYMELQETLMKTFSEDKEE
CGKSSTPKPTSAVRSNRKLSHRRLKVDKRDFLKRPYGNGD*
>Atha_AT4G19660
MAATAIEPSSSISFTSSHLSNPSPVVTTYHSAANLEELSSNLEQLLTNPDCDYTDAEIIIEEEANPVSVHRCVLAARSKF
FLDLFKKDKDSSEKKPKYQMKDLLPYGNVGREAFLHFLSYIYTGRLKPFPIEVSTCVDSVCAHDSCKPAIDFAVELMYAS
FVFQIPDLVSSFQRKLRNYVEKSLVENVLPILLVAFHCDLTQLLDQCIERVARSDLDRFCIEKELPLEVLEKIKQLRVKS
VNIPEVEDKSIERTGKVLKALDSDDVELVKLLLTESDITLDQANGLHYAVAYSDPKVVTQVLDLDMADVNFRNSRGYTVL
HIAAMRREPTIIIPLIQKGANASDFTFDGRSAVNICRRLTRPKDYHTKTSRKEPSKYRLCIDILEREIRRNPLVSGDTPT
CSHSMPEDLQMRLLYLEKRVGLAQLFFPAEANVAMDVANVEGTSECTGLLTPPPSNDTTENLGKVDLNETPYVQTKRMLT
RMKALMKTVETGRRYFPSCYEVLDKYMDQYMDEEIPDMSYPEKGTVKERRQKRMRYNELKNDVKKAYSKDKVARSCLSSS
SPASSLREALENPT*
>Atha_AT4G01160
MDLSLSGGDFRFAFNNVNFSDRLLRIEITQSSGEGEVICSSIVDWARDRKRRREDIVNENSNNKTQVLVTAAEQEPKSGG

EDENERLTNNNTSVLSVQELHISSAILAAKSPFFYKLFSNGMLESEQKQMTLKIDASEETAVMELLKFMYSNSLSVTASS
ALLDVLMVADKFEVASCMKYCSQLLLKMPMTLESSLLLLDLPSSLLMADSVKPLTNAARQFIASRYKNMSKITMEELMAL
PLVGIEAILASDGLEIQSEDVVYEVVLKWVKSHYSVLEARQEVLGSHLARYIRFPHMTTDRLKKILTSNDFRPSVASKLV
VEALFFKTESLAHQHVLLAHEQPASTSRRFAKRAYVHRPIKIVEFAVPRPQCIIYLDLKRKECESIYPSSRISSQQFTLG
GQGFFLSAQCNMDHLCLIHCFGLFIGMQENGSASASVTVDYDFSVRSKPTMEFVGKFKGIYTFTRGKAVGCRNLLGIPWD
IFTAKNCPYFINDVLHLRADLSIRL*
>Atha_AT1G04390
MASSKGGNTTAHINTLHHRLYHALNLGFRVCDEKEKKWKCTDIEIQRHVVKSISAFLDCFSRATANNRLIKDSISDIAGA
LVFILGSKNRAVVGLAANVVIRLIRIVPPSILHSYSLDLVESLSPLLCCQQFDVSLPCAVALNAILVNVRETKEKEVWKI
LEDEKTVVSVVGNLQIFSEGSMSVEWFQEMALLLSTIMLKWPQSRYSVWNNPALMGVLESVSQKPDMGLTVATLKLYSSL
ALCGHGANELLDNGKPMLDMMISCMEESSSQNARIEGLKLAQRLAATVRTMGKWFLSSGKLELDQMSLLVEACKLALITR
WEGQHHIYFWKYRISEALLSLVVENFHSQSLDGYVSLEEEVLVAEKRVSIKSSANDDMFSLQIHIIEGPSYPVIYPRRRC
WYQRNWENIGASSFAPSSQSITEKRICCWVCTEDWDNKDAFLLYALLALAELVNHSFFGQNHAEELSMKSGNLKDRLCTT
LKEIRDGTYGSGPRWYAAHILSYFGYYGFEHKLGKRLMCAYEDEEYSDMRLLFASGNSASVNKVIIAVRCPMLLPPKEGA
HSSSTISTEKSQRTVQEIRMSANVDILALVKLLEFAYSGYVEVESTTLKKLKPLAKHCKAKVLLQMLCRRRPKWGSSIPE
IDIPLALTPKLIHFSDVILVPKETNVACFNCRMCSLTSPHAHSHRVILSSGCEYLRALFRSGMQESHLDRLNVPVSWLGL
TKLVSWFYSDELPKPPSGCKWNNMDTEAKLDELQAYVEIYSLSEWWIMEELQNDCAHVILSCLESARELSIKTIELAASF
SMWKLVEAAANHAAPIYHQLRDSGELDELDDELVNLIRTAAVQFSQQGG*
>Atha_AT1G64280
MDTTIDGFADSYEISSTSFVATDNTDSSIVYLAAEQVLTGPDVSALQLLSNSFESVFDSPDDFYSDAKLVLSDGREVSFH
RCVLSARSSFFKSALAAAKKEKDSNNTAAVKLELKEIAKDYEVGFDSVVTVLAYVYSSRVRPPPKGVSECADENCCHVAC
RPAVDFMLEVLYLAFIFKIPELITLYQRHLLDVVDKVVIEDTLVILKLANICGKACMKLLDRCKEIIVKSNVDMVSLEKS
LPEELVKEIIDRRKELGLEVPKVKKHVSNVHKALDSDDIELVKLLLKEDHTNLDDACALHFAVAYCNVKTATDLLKLDLA
DVNHRNPRGYTVLHVAAMRKEPQLILSLLEKGASASEATLEGRTALMIAKQATMAVECNNIPEQCKHSLKGRLCVEILEQ
EDKREQIPRDVPPSFAVAADELKMTLLDLENRVALAQRLFPTEAQAAMEIAEMKGTCEFIVTSLEPDRLTGTKRTSPGVK
IAPFRILEEHQSRLKALSKTVELGKRFFPRCSAVLDQIMNCEDLTQLACGEDDTAEKRLQKKQRYMEIQETLKKAFSEDN
LELGNSSLTDSTSSTSKSTGGKRSNRKLSHRRR*
>Atha_AT1G21780
MGDTKVETISRLAQWRIENFGPCSFKKSDPFKVGIWNWHLSIERNRYLSVRLFPELSRVSKEQPPVAKFVLRVSNVGPNR
RFYISPVYEKLLRTTDDCVWHVDSSFHGRFTIDVEFLDLKICPVNGGEASPVWPTDATMQSISTQTTLKCLSRMLEESIL
TDVIIHTADGTLSAHKAILSASSTVFKSMFHHDLMEKESSTIHIDDMSRESCMALLSYLYGNITQEEFWKHRLALLGAAN
KYDITDLKAACEESLMEDINSSNVLERLQEAWLYQLEKLKKGCLMYLFDFGKIYDVREEISSFFRQADRELMLEMFQEVL
SVWKPV*
>Atha_AT1G30440
MACMKLGSKSDAFQRQGQAWFCTTGLPSDIVVEVGEMSFHLHKFPLLSRSGVMERRIAEASKEGDDKCLIEISDLPGGDK
TFELVAKFCYGVKLELTASNVVYLRCAAEHLEMTEEHGEGNLISQTETFFNQVVLKSWKDSIKALHSCDEVLEYADELNI
TKKCIESLAMRASTDPNLFGWPVVEHGGPMQSPGGSVLWNGISTGARPKHTSSDWWYEDASMLSFPLFKRLITVMESRGI
REDIIAGSLTYYTRKHLPGLKRRRGGPESSGRFSTPLGSGNVLSEEEQKNLLEEIQELLRMQKGLVPTKFFVDMLRIAKI
LKASPDCIANLEKRIGMQLDQAALEDLVMPSFSHTMETLYDVDSVQRILDHFLGTDQIMPGGVGSPCSSVDDGNLIGSPQ
SITPMTAVAKLIDGYLAEVAPDVNLKLPKFQALAASIPEYARLLDDGLYRAIDIYLKHHPWLAETERENLCRLLDCQKLS
LEACTHAAQNERLPLRIIVQVLFFEQLQLRTSVAGCFLVSDNLDGGSRQLRSGGYVGGPNEGGGGGGGWATAVRENQVLK
VGMDSMRMRVCELEKECSNMRQEIEKLGKTTKGGGSASNGVGSKTWENVSKKLGFGFKLKSHQMCSAQEGSVSKSNNENV
KIEKLKDVKERRGKHKKASSISSER*
>Atha_AT1G01640
MTEAEQKAAFLGGLVVSFKEQMHTDVLVKPGEEAPPIPTHKAVLAARSKVFRNMLDSDECKTSPEESITLPDLSHDELKS
LLEFLYSGNLKAPYNQYRSLYLAADKYDISYLQDVCRNHFIASLSSRNVLDILELASIPCDTILKDAAINHIVKHMEEVV
VPMKYETFVQRNPDLSVEITRAYLRETKAKAKDHGAPLNGNTRPRIW*
>Atha_AT1G55760
MTDSAYRVDTISRLAQWRIHNLSSSTYRKSDPFKMGLWNWHLSVEKSKMLLNVKLYPEVSNLTRENPPVASFALRVVSST
GERKALSHPEVIDKRIKTNEDFIWTIEVPLTGKIIIDVEFLDLKVLSQDSGELYSIWANGSTENQSQVTAVTSLGRMLTE
SIYTDITINASDGSIGAHRAVLAARSPVFRSMFLHDLKEKELSEINVLDMPLDACQAFLSYIYGNIQNEDFLIHRLALLQ
AAEKYDIADLKEACHLSLLDDIDTKNVLERLQNAYLYQLPELKASCMRYLVKFGKIFEIRDEFNIFMQCADRDLISEIFH
EVLSTWKGF*
>Atha_AT1G05690
MSSSTKNIPKPPPLPCITYQRFQSSTRKPSSLMRLVPKEALETWDKLFKEGSGADTYVETDNKSHFPAHSSVLAAASPVI
ATLLNQSRDKNGNTYLKIHGVPCEAVYMFIRFLYSSCYEEEEMKKFVLHLLVLSHCYSVPSLKRLCVEILDQGWINKENV
IDVLQLARNCDVTRICFVCLSMVIKDFKSVSSTEGWKVMKRSNPLLEQELIEAVIESDSRKQERRRKLEEREVYLQLYEA
MEALVHICREGCGTIGPRDKALKGSHTVCKFPACKGLEGALRHFLGCKSRASCSHCKRMWQLLQLHSCICDDSNSCKVSL
CWNFKEKMKKLSKKEQSKWRLLVENIIRARNSLGPFSSRSSGLI*
>Atha_AT3G48360
MEAVLVAMSVPATTEDDGFSLITDKLSYNLTPTSDVEIVTSDNRRIPAHSGVLASASPVLMNIMKKPMRRYRGCGSKRVI
KILGVPCDAVSVFIKFLYSSSLTEDEMERYGIHLLALSHVYMVTQLKQRCSKGVVQRLTTENVVDVLQLARLCDAPDVCL

RSMRLIHSQFKTVEQTEGWKFIQEHDPFLELDILQFIDDAESRKKRRRRHRKEQDLYMQLSEAMECIEHICTQGCTLVGP
SNVVDNNKKSMTAEKSEPCKAFSTCYGLQLLIRHFAVCKRRNNDKGCLRCKRMLQLFRLHSLICDQPDSCRVPLCRQFRK
RGEQDKKMGEDTKWKLLVTRVVSAKAMTSLCQSKKNKCEQAQGV*
>Atha_AT3G03740
MKSVIFTEEKNLQRQNPLQKSEQQRRNFEMPSPPTTTSLSVTQTINGSHSFTIKGYSLAKGIGIGKHIASDTFTVGGYQW
AIYFYPDGKNPEDNSAYVSVFIALASDGTDVRALFELSLLDQSGKGKHKVHSHFDRALESGPYTLKYRGSMWGYKRFFRR
LMLETSDFLKDDCLKINCTVGVVVSEIDCPRLHSIHVPASDIGSHFGMLLENEDGSDITFNVSGEKFRAHRLVLAARSPV
FESEFLDVTGEEDRDIEVTDMEPKVFKALLHYIYKDALIEDAESSSSSGSSVGPSASDTLAAKLLGAADKYKLPRLSLMC
ESVLCKDISVDSVANILALADRYNASALKSVCLKFAAENLIAVMRSDGFDYLREHCPSLQSELLKTVAGCEEELSGGGGK
TRSVWGQFSDGGAETNGRQAQTWGDINGGAERSQSVWVEVVNANGSGRNNNDNNNSDDPMAELED*
>Atha_AT3G50840
MANSKKQAWFFYTTGLPSDIEIEVDDITFHLHKFPLMSKSKKLHQLITEQEQSKVYSHIKLENFPGGSEIFEMVIKISYG
FKVDISVSTAVPLRCAAEYLEMTEEYSPENLISKTEKFLSEFVFTNVQESIKALKACESVSSLAESLCITEQCIDSIVFQ
ASSTDPSSFYGWPINNGGIFTVDRKKQSKDSKTELWFEDLTELSFPIFRRVILSMKSSVLSPEIVERSLLTYAKKHIPGI
SRSSSASSSSSSSSTTIASENQQRELLETITSDLPLTATTTRSLFGLLRAAIILNASENCRKFLEKKIGSNLEKATLDDL
LIPSYSYLNETLYDIDLVERLLRRFLENVAVSSSSLTVVGRLIDGVLGEIASDANLKPEQFYNLAVLLPVQARVYDDGLY
RAVDIYFKTHSWILEEEKEKICSVMDCRKLTVEGCTHAAQNERLPLRAVVQVLFLEQLQLRQVITGTLLTEEDGDKTVVD
LGRWKEAVKENQVLRLDMDTMRTRVNQLEKECLYLKKVIAKIDKESLLKAKHGAGKWSIGKKFGCKFSAQVCDSQEATMV
DRRSRRFLS*
>Atha_AT3G61600
MRGTTENTDLFDPKTQMDPDFTRHGSSSDGDFGFAFNDSNFSDRLLRIEIMGGPSDSRSEVEGCTSIADWARHRKRRRED
IKKESGVTISDIVACPEEQILTDEQPDMDGCPGGENPDDEGGEAMVEEALSGDEEETSSEPNWGMDCSTVVRVKELHISS
PILAAKSPFFYKLFSNGMRESEQRHVTLRINASEEAALMELLNFMYSNAVSVTTAPALLDVLMAADKFEVASCMRYCSRL
LRNMPMTPESALLYLELPSSVLMAKAVQPLTDAAKQFLAARYKDITKFHEEVMSLPLAGIEAILSSDELQIASEDAVYDF
ILKWARAQYPCLEERREILGSRLALSIRFPFMTCRKLKKVLTCSDFEHEIASKLVLEALFFKAEAPHRQRSLASEESASL
NRRLIERAYKYRPVKVVEFELPRPQCVVYLDLKREECGGLFPSGRVYSQAFHLGGQGFFLSAHCNMDQQSSFHCFGLFLG
MQEKGSVSFGVDYEFSARSKPAEDFISKYKGNYTFTGGKAVGYRNLFGVPWTSFIAEDSQYFINGILHLRAELTIKRSTD
P*
>Atha_AT3G08660
MGSDSTLSLPSSSPPCNNRSSVPPTFTTRIFSDVAGDIIVVVDGESFLLHKFPLVARSGKMRKMVRDLKDSSSMIELRDF
PGGPSTFELTMKFCYGINFDITAFNVVSLRCAAGYLEMTEDYKEQNLIFRAENYLDQIVFRSFHESVLVLCSCETQEIAE
TYEIPDRCVEAIAMNACRKQLVSGLSEELKGRDCLEMWTEELSALGIDYYVQVVSAMARLSVRSESIVASLVHYAKTSLK
GIIDRNCQEQRKIVEAMVNLLPNDEKGSYSLSIIPLGFLFGMLKVGTIIDIEISCRLELERRIGHQLETASLDDLLIPSV
QNEDSMYDVDTVHRILTFFLERIEEEDDECGYDSDSTGQHSSLLKVGRIMDAYLVEIAPDPYLSLHKFTAIIETLPEHSR
IVDDGIYRAIDMYLKAHPLLTEEERKKLCNFIDCKKLSQEASNHVAQNDRLPVQMVVRVLYTEQLRLKKALSGDSEEGSW
VLPSGVQSRAVSPRDTYAALRRENRELKLEISRMRVRVSELEKEHNLMKHEMMEKSGNNGGTFLTSLSKGIGIRIATFGGE
TRQKVNRKSRSVSERKSSRSGR*
>Atha_AT3G29740
MASQTNKELFVGGLARILKEQRQVDVRLKAGDSDQKGVSISAHKLVLSARSEVFKMILETEEIKATTTLDTITLSELKHT
ELVALVE*
>Atha_AT3G57130
MSNTFEESLKSMSLDYLNLLINGQAFSDVTFSVEGRLVHAHRCILAARSLFFRKFFCESDPSQPGAEPANQTGSGARAAA
VGGVIPVNSVGYEVFLLLLQFLYSGQVSIVPHKHEPRSNCGDRGCWHTHCTAAVDLSLDILAAARYFGVEQLALLTQKHL
TSMVEKASIEDVMKVLIASRKQDMHQLWTTCSYLIAKSGLPQEILAKHLPIELVAKIEELRLKSSMPLRSLMPHHHDLTS
TLDLEDQKIRRMRRALDSSDVELVKLMVMGEGLNLDESLALIYAVENCSREVVKALLELGAADVNYPAGPTGKTALHIAA
EMVSPDMVAVLLDHHADPNVQTVDGITPLDILRTLTSDFLFKGAIPGLTHIEPNKLRLCLELVQSAALVISREEGNNNSN
DNNTMIYPRMKDEHTSGSSLDSRLVYLNLGATNRDIGDDNSNQREGMNLHHHHHDPSTMYHHHHHHF*
>Atha_AT3G06190
MDTIRVSKEVPGSSKSTAQSLTESTSRTETINGSHEFKISGYSLVKGMGIGKYVASDTFMVGGYSWAIYFYPDGKSPEDN
SVYVSLFIALASEGADVRALFELTLVDQSGNERHKVHSHFGRTLESGPYTLKYRGSMWGYKRFFKRSLLESSDYLKDNGL
LVRCCVGVVKSRTEGPRCYNIPVPVSGLGQQFGKLLESGKGADVTFEVDGETFPAHKLVLAARSAVFRAQLFGPLRSENT
NCIIIEDVQAPIFKMLLHFIYWDEMPDMQDLIGTDLKWASTLVAQHLLAAADRYALERLRTICESKLCEGISINTVATTL
ALAEQHHCFQLKAACLKFIALPENLKAVMETDGFDYLKESCPSLLSELLEYVARLSEHSLTSSGHRKELFADGCDLNGRR
VKQRLH*
>Atha_AT3G56230
MDCSICTTMPSILRPPRNTICGSCYEGARTTIALLKKLEGSKEDRHDKSNHNSTINNGSSISSSPLFSCEPQPLEKVIKW
MKNMKETEEEQKKRIVFLSSFVSGFKEQLHADILLKPGDDGPPIPAHRALLASKSEIFKNILDSDGCKTAPEYAITLQEL
NSEQLQALLEFLYTGTLASDKLEKNVYALFIAADKYMIHYLQELCEQYMLSSLDISSVLNVLDVSDLGSSKTLKEACVGF
VVRNMDDVVFSDKYEPFSQKNQHLCVEITRAFLMETRSKRRD*
>Atha_AT3G44820
MSPVAKVSEFHREGNDWFCKTGLSSDITVVVDDVKFHLHKFPLVSKCGKLARMYEDSKSTDKQSLWTTVLEEFPGGADNF
LIVARFCYGARVDITSKNLVSIHCAAEYLEMTNEYGEDNLISQVETFLHKHVLRNWKDCILALQSSSPVLKSAEKLQMIP

KLMNAVSTMVCTDPSLFGWPMMMYGTLQSPGGSILWNGINTGARMRSSGSDWWYEDISYLSVDLFKRLIKTMETKGIRAE
SLAGAMMYYARKYLPGLGRWQSGTSDSSKSRRRVVSFNLAKASSPSSMPPLDQIALLETILSLLPEKRGRSFCKFLLGLL
RVAFILGVDGNCVKKLEKRIGMQLELATLDNLLILNYSDSETLYNVDCVERIVRHFVSSLSSSSSQLPEFSPPSLDPVTS
PSPAPLKKVANLVDSYMAEVASDVNLKPDKMRSLAAALPESSRPLYDGLYRAFDIYFKEHPWLSDRDKEQLCNIMDYQRL
SIDACAHASHNDRLPLRVVLQVLFFEQMHLRTALAGGLNVANTETAHAVTIPGGRTGQEIVQRDGWVTVVRQNQVLKVDM
QKMRSRVGELEEEFQSIKQEMKKRVSKSSSSMSSPRLVKLGCKFLLPRASDAKNDTVQNSVSSTPRSATADHTLPRSSRH
SKHRKSFSFFG*
>Atha_AT3G43700
MSKLMTRTSGSSSPNTIPDQIESPTSSRSVTQTTNGSHQFVIQGYSLAKGIGVGKHIASDNFSVGGYQWTIFVYPDGKNP
EDNSSYVSVFIVLASECTEVRALFELSLVDQSGKGKHKVHSHFNRSLDGGPYTLKYRGSMWGYKRFFRRSLLETSDYLKD
DCLKINCTVGVVVSEMHCPRLLSIHVPDSELGSHFGKLLDTLQGSDVTFDVAGEKFQAHKLVLAARSQFFRSMFYNTLAE
NNSDVVISDLEPKVFKALLHFMYKDSLPGDVEPLTAHSFDLLRPSEIDDTLIVKLLAAAEMYNLSRLRLLCESHICKGIS
ISSVSKILALSDKYNASELKSVSLKFTAENLAAVLQTKAYEDLKDDCPNLQSELLKAVAGYDDTSSSGGGKSQSVWAQLS
NGGETSSRRVRQRTT*
>Atha_AT3G19850
MSLCCDLQINLNNQFTFFVNQDLISEYSGFLRKMIKQSNKKKKNHKNSRIIIEVEDFPGGSDGFDLVLRFCYGGGISIDV
SNVSILHCSSVFLEMTEKLCSSNLLLRTEKFLEGMFYWSWNDIVLCLKSCEQVFLHADSYGLVDKLVFGVLAKIPQNSDV
SHVFSSSSPSSSASASASSQSPETAMIRSYSDKRSTSRSFSCRTSNEWWFDDMSILGPKIIEKLINTLGAHDKNNDSLVL
TKFLLHYLKTKVPNKSTNKLEYSGLADTAVQGVVFAAKTAFSCRKMFWVLRVLSGFSISKESRIGLERVIGEMLDQATLD
DLLIPAGGKGEKGVYDVDLVIRLLKVFVRIGNTEEGDQNLRMRRIGKLIDKYLREISPDQNLKVSKFLEVAESLPDSARD
WFDGLYRAINIYLESHPKLSSEDRTKLCRCLNYKKLTLDTCKQLAKNPKIPPNIAVQALKSQQLSNETRPHSREDKNKVN
KIWNSRKYLEEKPILVCLKGFDMSEKFEDDLMMNLERKQWNNSEKVSKEKKSEVMSRSVRHGHTHSSSSFPRLC*
>Atha_AT3G05675
MEPRDNQAEASYTFGDRSSSDIVVRLRNEEGRDDWIYCHSKILSEKSQYFADRLSDKWPTCKILDSRYCVEVICQESDYD
HHINLLRLLYVVSDDVHEDNLCHNVKSALGILSVAKELSCPQIVTACVNYLEAVPWEEGEEEEILRIVPRIGSEAEPILA
RLQPVDQSAVLEIFVSAFRFATSSPPLLLGDIKSSAQEQIEYMITEDDDAPLLIADEEVKLEVKQCVKSLFVRFFQCLEE
ITLKPVESEVINKKGSFRMVLSDMCWVFQILTKMEVVRDFVITWADISEKLVKVVEQLETTVVEAVEIRVKVIEVTAKVI
EAIGYGTVILPTAKRLQMVKLWLPFVRNTKPLVDSPVREDEENDTVRYKIDGEIWQALESSFVSIILALPSADQAEILTE
WLSKNGLYPDLTEAFEVWCYRSKVAKRRLGLVGGEEENGMS*
>Atha_AT3G08570
MGIISDNAQSHSSSSAPAPSIFSSSFATRIFSDVAGDITIVVDGESFLLHKFPLVARCGKIRKMVAEMKESSSNLSHTEL
RDFPGGSKTFELAMKFCYGINFEITISNVVAIRCAAGYLEMTEDFKEENLIARTETYLEQVAFRSLEKSVEVLCSCETLY
PQDIAETAHIPDRCVEAIAVNACREQLVLGLSRLNRGTESGELKRGDSPEWWIEDLSALRIDYYARVVSAMARTGLRSES
IITSLMHYAQESLKGIRNCKERTKLDSGTFENEQRNVLEAIVSLFPNDNVPLSFLFGMLRVGITINVAISCRLELERRIA
QQLETVSLDDLLIPVVRDGDSMYDVDTVHRILVCFLKKIEEEEEYDEDCCYENETENLTGSMCHSSLLKVGRIMDAYLAE
IAPDPCLSLHKFMALIEILPDYARVMDDGLYRAIDMFLKGHPSLNEQECKSLCKFIDTQKLSQEACNHVAQNDRLPMQMV
VRVLYSEQLRMKNVMSGESGEGLLLSSQKHSSENPSRAVSPRDTYASLRRENRELKLEISRVRVRLTELEKEQILMKQGM
MEKSGHGGTLLTSLSKGIGRISIFGGGPTEGKLRNANRKSKSRLERKTVRSRPESMF*
>Atha_AT3G49970
MLEKLSFLLHKFPLVSKCGFIKKLASESSNDSNIIRIPDFPGGAEGFELVIKFCYDISFEINTENIAMLLCAAEYLEMTE
EHSVENLVETIEVYLNEVILKSLSKSVKVLQKSQDLLPIAERVRLVDRCIDSIAYAICQESQSNEDIVDWWADDLAVLKI
DMFRRVLVAMIARGFKRYSLGPVLKLYAEKALRGLVRFLNFLTEQCDIFGKEAKKMEAEQEHEKRLILETIVSLLPRERN
SVSVSFLSILLRAAIYLETTVACRLDLEKRMGLQLRQAVIDDLLIPYYSFNGDNTMLDVDTVQRILMNYLEFEVEGNSAD
FASDIGELMETYLAEIASDRNINFAKFIGFAECIPKQSRMYRAIDIFLKTHPNISEVEKKKVCSLMDCKKLSRDVYAHAA
QNDRFQENLSNSDSPAPATAEKTLSPPELSSYKNELSKLNRENQYLKLELLKVKMKFKELEKEKAFEVMSGSDCSSSVST
ASVAKPRLPRKSFINSVSQKLGKLINPFGLKQGQTKQPKSRRHSIS*
>Atha_AT5G48510
MATPTNKEHFSGGLAKVLAEKWQVDVMLKAKNSDEGSAISAHKLILASRSEVFKNMFELDEFKTSTKHVETITLSEMKHE
ELEAFVEFICSDGSMLSANVKQHARSLYLAADKYEILHLRDLCRTELISSLSLLNSLDLLVLAQIPFDKVLNDESFSYIK
NNISTIASSDEFKLFVAGNPNLAVEIMKVSLITLTLRCSSCGSNHSLQGMYCCNCCRYGTLRLI*
>Atha_AT5G58550
MRNLKLFERFKSTQVHAFTTQDSPSTSSNGSPRMMKFLGHPKSKSRSLLPHGFPTTDLLEPPLDSYLKPIDLVESLSNLY
RRIESSSESEASMLYLEQYAVLRSLGDAKLLRRCLLNARRHAIDVPCKVVFSAWLRFFRREHELVGVESMDCNGLASECP
KTSLTHGCDLNVDDEGCECSTVCEDEFGSDDVKISKADEFSGLDEVSDISFCVGSEKAKCVRSRIAALSRPFEAMLYGSF
VESTTSEIDFSENGISIEAMLALNIYSRIKRVDLFRVETVFELLQLASKFCCDDLKSECEARLAASVTDLDKALTFVEYA
LEERTTLLLSACLQVFLRELPQSLHNPKVMRFFCSSEAKEQLAFLGSECVFLLYYFLSQVGMEEKLTTDTMLILLERTRE
FARTNWQKALSLHQMGCVLFERKDYKAAQFHFRLASSLGHVYSLAGVSRTEYKQGKRYSAYRLMNFLISNHKPHGWMYQE
RSLYNVGVEKLKDLATATELDPTLSFPYKYRAVMKFEQKQIKEAFQEIDRLIQFKLSPECLELRAWLYLATGDRESCLRD
LRAVLSLEPNYVVFGGKMRDDLVEALTAQCIEVESEADCWVRLFDRWSAVDDVASLAVVHQMLQNDPSKNFLRFRQSLLL
LRLNCQGAAMRCLRMAWNLATSEAERLVYEGWLLYDMGYVEETLTKAEEAISIQRSFEAFFLKAYALADKNLDADEISCV
VQVLEEALKCPSDGLRKGQALNNLGSIYINLGMLDQAETAYKNAIEIKHTRARQGLARVYFLKNQRKEACEEMTKLIEKS
CSKAAYEKRSEYCEREKAKEDLDMATTLDPLRTYPYRYRAAVLMDDQRETEAVEELSKAIAFRPELQTLHLRAAFHEAT

GNLSLATQDCEAALCLDPNHTETLHLYSRSKDQASSIDNTIFGLD*
>Atha_AT5G21010
MSESVIQGSNPDRVLSPTSSKSVTQTVNGSHQFVIQGYSLAKGMGIGKHIASDNFSVGGYQWGIFFYPDGKNPEDNSSYV
SVFIALASEGTEVRALFELALVDQSGKGKHKVHSHFERSLDGGPYTLKYRGSMWGYKRFFRRSILETSDYLKDDCLIINC
TVGVVVSEILCPQLHSVHVPDSELGSHFGVLLDSMEGSDITFNIAGEKFLAHKLVLAARSPFFKSKFFSEFEANNTEVTI
NDLEPKVFKALLQFMYKDSLPEDVEPATAHTFERLKLSEIYETLIVKVLAAADKYDLIRLRLLCESHICKGVSVKSVAKI
LALADRYNAKELKGVCLKFTAENLAAVLETDAYQQMKDECVTLQSELLKAVAGHEEGSNSTGGAKSQSVWAQLSDGGGDT
TSRHVRQRTT*
>Atha_AT5G45110
MATLTEPSSSLSFTSSHFSYGSIGSNHFSSSSASNPEVVSLTKLSSNLEQLLSNSDCDYSDAEIIVDGVPVGVHRCILAA
RSKFFQDLFKKEKKISKTEKPKYQLREMLPYGAVAHEAFLYFLSYIYTGRLKPFPLEVSTCVDPVCSHDCCRPAIDFVVQ
LMYASSVLQVPELVSSFQRRLCNFVEKTLVENVLPILMVAFNCKLTQLLDQCIERVARSDLYRFCIEKEVPPEVAEKIKQ
LRLISPQDEETSPKISEKLLERIGKILKALDSDDVELVKLLLTESDITLDQANGLHYSVVYSDPKVVAEILALDMGDVNY
RNSRGYTVLHFAAMRREPSIIISLIDKGANASEFTSDGRSAVNILRRLTNPKDYHTKTAKGRESSKARLCIDILEREIRK
NPMVLDTPMCSISMPEDLQMRLLYLEKRVGLAQLFFPTEAKVAMDIGNVEGTSEFTGLSPPSSGLTGNLSQVDLNETPHM
QTQRLLTRMVALMKTVETGRRFFPYGSEVLDKYMAEYIDDDILDDFHFEKGSTHERRLKRMRYRELKDDVQKAYSKDKES
KIARSCLSASSSPSSSSIRDDLHNTT*
>Atha_AT5G03250
MAFMRLGSKSEAFHREGQTWLCTTGLVSDVTIEVGDMKFHLHKFPLLSRSGLLERLIEESSTDDGSGCVLSLDEIPGGGK
TFELVTKFCYGVKIELTAFNVVSLRCAAEYLEMTDNYGEGNLVGMTETFLNEVFGNWTDSIKALQTCEEVIDYAEDLHII
SRCVDSLAVKACADPSLFNWPVGGGKNATSGQNTEDESHLWNGISASGKMLQHTGEDWWFDDASFLSLPLFKRLITAIEA
RGMKLENIAMAVMYYTRKHVPLMNRQVNMDEQVIETPNPSEEDQKTCLEEIVGLLPSKKGVNPTKFLLRLLQTAMVLHAS
QSSRENLERRIGNQLDQAALVDLLIPNMGYSETLYDVECVLRMIEQFVSSTEQAGIVPSPCIIEEGHLVKDGADLLTPTT
LVATLVDGYLAEVAPDVNLKLAKFEAIAAAIPDYARPLDDGVYHAIDVYLKAHPWITDSEREHICRLMNCQKLSLEASTH
AAQNERLPLRVIVQVLFFEQLRLRTSVSGWFFVSENLDNPDNQHGANGGLLKPRGENVRERVSELEKECMNMKQELHKLV
RTKRSWKNFTRKLNFKKKSECCKPKDQATPAI*
>Atha_AT5G13600
MASLKLGSKSEVFHLSGHTWLCKTGLKPDVMIQVVDESFHLHKFPLLSRSGYLETLFSKASETTCVAQLHDIPGGPETFL
LVAKFCYGVRIEVTPENAVSLRCAAEYLQMSENYGDANLIYLTESFLNDHVFVNWEDSIKALEKSCEPKVLPLAEELHIV
SRCIGSLAMKACAEDNTSFFNWPISLPEGTTTTTIYWNGIQTKATSENWWFNDVSSFLDLPMYKRFIKTVESRGVNAGII
AASVTHYAKRNLPLLGCSRKSGSPSEEGTNYGDDMYYSHEEQRSLLEEIVELLPGKKCVTSTKFLLRLLRTSMVLHASQV
TQETLEKRIGMQLDEAALEDLLIPNMKYSGETLYDTDSVQRILDHFMLTFDSSIVEEKQMMGDSHPLKSITKVASLIDGY
LAEVASDENLKLSKFQALGALIPEDVRPMDDGIYRAIDIYIKAHPWLTESEREQLCLLMNCQKLSLEACTHAAQNERLPL
RVIVQVLFFEQMRLRTSIAGWLFGSEENNDTSGALEGNKNTNANMVMHGMRERVFELEKECMSMKQDLDKLVKTKEGRNF
FSKIFGSRSKTKTSPCGKGGEDALVIPETKN*
>Atha_AT5G67480
MQGREDKLNKRMVTGCVDLHQSFKSADSSSVPIPPPLPSKSDGLKKKLGHSSVSTATRDMWDRLFNDGYKADVVIYTDNG
SIIYAHANILGTASTVIKGMLKQAKRHGKWHTISIRGVPHDAVRVFIRFLYSSCYEKEEMNEFIMHLLLLSHAYVVPQLK
RVCEWHLEHGLLTTENVVDVFQLALLCDFPRLSLISHRMIMKHFNELSATEAWTAMKKSHPFLEKEVRDSVIIEANTRKE
RMRKRNDQRIYSQLYEAMEALVHICRDGCKTIGPHDKDFKPNHATCNYEACKGLESLIRHFAGCKLRVPGGCVHCKRMWQ
LLELHSRVCAGSDQCRVPLCRNLKEKMEKQSKKDESRWKLLVKNVLGSKKIGGSPFFLPVTNC*
>Atha_AT5G48130
METFSLKDCSSVASSPISSPNISTLLKIKVLSWSKETGLPASVHVRVCNKSFNLHKSLLCAKSGYFKEREDQLSEIEIPQ
EFPGGAETFEKIMLFIYGCPTLIHPFNIAGLRCAAQFLEMTEQHSTGNLCERFDLYLNQVVLQNWDDTLVVLKKCQDLVP
WSEDLLIVSRCIESLAFTACMEILDPERRREKPVIMLEGMVNQPWEYTNIERIINQDTWIKDLTDLPFEFFKKIIGSLRR
QGMKERYVSPLVALYASKSVIPEGQTNTDILQRALDLLLTRDKAYRFVPVGFYFACLAHNLKHDTVLKLQDQIVSLLHTA
QPENFIYPKAGNRQVAFSQELLTMESLFSVYVSTESERHLTSSSSNVRVGKLWDIFLSRLPYDQEMKTTRFIELIETVPM
SFRESHDQLYLAVNAFLQVHTNISQEEKGSICSYLNCQKLSQEASLELVKNEKMPLRLVVQALFIQQLNTHQAFKDCSDS
FRFTNSADFSGSVVPSSRPLTSQQSPCTDDETGPRNRPLCFLMQKDATLDEFESTSFRIHNLEEQLVSLKKSLHSHDNLK
KPNCLGKRSASRNKNTFGQVTTACIGSVSFTSQRKYANRLLQILRRVNLFGSRKTNRSKAGESER*
>Atha_AT5G48800
MDKHHHQHLLLQHHQHLHHHQKLSLAKSSRQSCSEWIFRDVPSDITIEVNGGNFALHKFPLVSRSGRIRRIVAEHRDSDI
SKVELLNLPGGAETFELAAKFCYGINFEITSSNVAQLFCVSDYLEMTEEYSKDNLASRTEEYLESIVCKNLEMCVQVLKQ
SEILLPLADELNIIGRCIDAIASKACAEQIASSFSRLEYSSSGRLHMSRQVKSSGDGGDWWIEDLSVLRIDLYQRVMNAM
KCRGVRPESIGASLVSYAERELTKRSEHEQTIVETIVTLLPVENLVVPISFLFGLLRRAVILDTSVSCRLDLERRLGSQL
DMATLDDLLIPSFRHAGDTLFDIDTVHRILVNFSQQGGDDSEDEESVFECDSPHSPSQTAMFKVAKLVDSYLAEIAPDAN
LDLSKFLLIAEALPPHARTLHDGLYRAIDLYLKAHQGLSDSDKKKLSKLIDFQKLSQEAGAHAAQNERLPLQSIVQVLYF
EQLKLRSSLCSSYSDEEPKPKQQQQSWRINSGALSATMSPKDNYASLRRENRELKLELARLRMRLNDLEKEHICMKRDM
QRSHSRKFMSSFSKKMGKLSFFGHSSSRGSSSPSKQSFRTDSKVLMERTCASTD*
>Atha_AT5G17580
MSASYVSDLHINVKGVPFHLCKEMLAKRSSKVSSLLERNEIDELRLILRDLEVDPETFELVARFCNGSEFKFTSDTIVSV
LCIAYYLGMNEEQSSNNLLGKASEFLEHRVFPSWSETINALRSGDKSFDKLADVGLVDVFFDSLIEKASYDPRLLGELIK

NRAETDDYRPNPRRRLFVIDWKSEDLITIPLRLYEPFMIRAIKSRSIPVEYIVLSVCKYAKKWVFDTEESLSGQKREAIE
VVERLLPYQRGLISCELLFESLKHSIWLEASSECQNGFMIRICKQLDMAKSTDLKILSRGYGEKAEGFENIELVKTVVKS
FYTYYANEDSETVSHFVKVAKLSEEFLFLAASEASLKLEAFVELAEMTVAVSQGILSYSDGIYRAIDVFLESHRYLTESE
KMEVCKVLECGKLSQEGFERAAKNQKLPLRIVVNVLCVSQLQIRDTVAKEIKGMEEKVDEEEEEEIEVSSDEDEMEKMSN
KLLGLEIENDECVVHRRKNMKKKKKKISVWGQVKRKFGCLNSSSSSYSVDACTCDVKKKKKKIHHHYE*
>Atha_AT5G67440
MKFMKLGSKPDSFQSDEDCVRYVATELATDVVVIVGDVKFHLHKFPLLSKSARLQKLIATTTTDEQSDDDEIRIPDIPGG
PPAFEICAKFCYGMAVTLNAYNVVAVRCAAEYLEMYESIENGNLVYKMEVFLNSSVLRSWKDSIIVLQTTRSFYPWSEDV
KLDVRCLESIALKAAMDPARVDWSYTYNRRKLLPPEMNNNSVPRDWWVEDLAELSIDLFKRVVSTIRRKGGVLPEVIGEA
LEVYAAKRIPGFMIQNDDNDDEEDVMEQRSLLETLVSMLPSEKQSVSCGFLIKLLKSSVSFECGEEERKELSRRIGEKLE
EANVGDLLIRAPEGGETVYDIDIVETLIDEFVTQTEKRDELDCSDDINDSSKANVAKLIDGYLAEISRIETNLSTTKFIT
IAEKVSTFPRQSHDGVYRAIDMFLKQHPGITKSEKKSSSKLMDCRKLSPEACAHAVQNERLPLRVVVQILFFEQVRATTK
PSLPPSGSHGSSRTTTEEECESVTATEETTTTTRDKTSSSEKTKAKGVVMSRIFSKLWTGKDKDGVGDVSSSDTSESPGS
VTTVGDKSTPSTRRRRSSS*
>Atha_AT5G13060
MIISKSFKAPLKFSVKSSTAPVISNHPPMENHPKRQRTTRLAARNLKRKLSHNTDGAPIVTQLIDIDDEPIDLVVAIRRH
VEVLNSSFSDPDFDHEAVKEAAADIADLAKIDENVEIIVENGAIPALVRYLESPLVVCGNVPKSCEHKLEKDCALALGLI
AAIQPGYQQLIVDAGAIVPTVKLLKRRGECGECMFANAVIRRAADIITNIAHDNPRIKTNIRVEGGIAPLVELLNFPDVK
VQRAAAGALRTVSFRNDENKSQIVELNALPTLVLMLQSQDSTVHGEAIGAIGNLVHSSPDIKKEVIRAGALQPVIGLLSS
TCLETQREAALLIGQFAAPDSDCKVHIAQRGAITPLIKMLESSDEQVVEMSAFALGRLAQDAHNQAGIAHRGGIISLLNL
LDVKTGSVQHNAAFALYGLADNEENVADFIKAGGIQKLQDDNFTVQPTRDCVVRTLKRLQNKIHGPVLNQLLYLMRTAEK
TVQIRIALALAHLCDPKDGKLIFIDNNGVEFLLELLYFSSNKQQRYSSSALYELAKKATSFAPEDSAPCSPTQQVFLGEK
FVNNPTMSDVTFLIDGKQFYAHKIGLVASSDIFRAMFDGLYKERNAQNVEIPNIRWEVFELMMKFIYSGRINIAKHLAKD
LLVAADQYLLEGLKRQCEYTIAQEICLDNIPEMYELADTFNASALRRACTLFVLEHFTKLSSQLWFAKFVKQIIPEIRSY
MTDILTRPVEASPPTVV*
>Atha_AT5G63160
MAITATQNDGVSLNANKISYDLVETDVEIITSGRRSIPAHSGILASVSPVLTNIIEKPRKIHGGSSKKVIKILGVPCDAV
SVFVRFLYSPSVTENEMEKYGIHLLALSHVYMVTQLKQRCTKGVGERVTAENVVDILQLARLCDAPDLCLKCMRFIHYKF
KTVEQTEGWKFLQEHDPFLELDILQFIDDAESRKKRRRRHRREQNLYLQLSEAMECIEHICTEGCTLVGPSSNLDNKSTC
QAKPGPCSAFSTCYGLQLLIRHFAVCKKRVDGKGCVRCKRMIQLLRLHSSICDQSESCRVPLCRQYKNRGEKDKKMVEDT
KWKVLVRRVASAKAMSSLSQSKKKKSEVLFKEEAEDLIRIRNKLM*
>Atha_AT5G19330
MDQQPERREGRSFPERKGQKRKLEEGAAAVEDREISAVSTDGGQALLSEVAAQVSVLNSAFSWQESDRAAAKRATQVLAE
LAKNEDLVNVIVDGGAVPALMTHLQAPPYNDGDLAEKPYEHEVEKGSAFALGLLAIKPEYQKLIVDKGALPHLVNLLKRN
KDGSSSRAVNSVIRRAADAITNLAHENSSIKTRVRVEGGIPPLVELLEFSDSKVQRAAAGALRTLAFKNDDNKNQIVECN
ALPTLILMLGSEDAAIHYEAVGVIGNLVHSSPHIKKEVLTAGALQPVIGLLSSCCPESQREAALLLGQFASTDSDCKVHI
VQRGAVRPLIEMLQSPDVQLKEMSAFALGRLAQDAHNQAGIAHSGGLGPLLKLLDSRNGSLQHNAAFALYGLADNEDNVS
DFIRVGGIQKLQDGEFIVQATKDCVSKTLKRLEEKIHGRVLRHLLYLMRISEKSIQRRVALALAHLCSPEDQRTIFIDDN
GLELLLGLLGSLNTKQQLDGAAALYKLANKSMALSPVDAAPPSPTQRVYLGEQYVNNATLSDVTFLVEGRTFYAHRICLL
ASSDAFRAMFDGGYREKDARDIEIPNIKWEVFELMMRFIYTGSVDITNEISKDLLRAADQYLLEGLKRLCEYTIAQDITL
ESIGDMYELSEAFHAMSLRQACIMFILEHFDKLSSMPWQNELVQRTIPEIREYFCRALTKSTTNLQSLRL*
>Atha_AT5G47800
MKFMKIGTKPDTFYTQEASRILITDTPNDLVIRINNTTYHLHRSCLVPKCGLLRRLCTDLEESDTVTIELNDIPGGADAF
ELCAKFCYDITINLSAHNLVNALCASKFLRMSDSVDKGNLLPKLEAFFHSCILQGWKDSIVTLQSTTKLPEWCENLGIVR
KCIDSIVEKILTPTSEVSWSHTYTRPGYAKRQHHSVPRDWWTEDISDLDLDLFRCVITAARSTFTLPPQLIGEALHVYTC
RWLPYFKSNSHSGFSVKENEAALERHRRLVNTVVNMIPADKGSVSEGFLLRLVSIASYVRASLTTKTELIRKSSLQLEEA
TLEDLLLPSHSSSHLHRYDTDLVATVLESFLMLWRRQSSAHLSSNNTQLLHSIRKVAKLIDSYLQAVAQDVHMPVSKFVS
LSEAVPDIARQSHDRLYKAINIFLKVHPEISKEEKKRLCRSLDCQKLSAQVRAHAVKNERMPLRTVVQALFFDQESGSKG
ASSRSESQELFTRGKETPTDEHSMMHKLHLGPASTGKAKNVLEEGCKRGEEKTRSSTDPKKIVWKGTGSEHKHHISRDR*
>Atha_AT5G19000
MGTTRVCSEVSSGSSKSLSQSLTVSTSTTETVNGFHEFKICGYSLAKGVGVGKYVASDTFMVGGYSWAIYFYPDGKSPED
NSSYVSLFIALASEGADVRALFELTLVDQSGNGKHKVHSHFGRALDSGPYTLKYRGSMWGYKRFFRRSSLESSDYLKENS
LLVRCRVGVVKSVTEGPRYYNIPVPVSNLGQQLGNLLESGKGCDVVFQVDGETFNAHKLVLATRSPVFNAQLFGPLGDRN
TKCITIEDMEAPIFKVLPLTLLLIVYSRMYHPGSSPGALLLFSSLLTRDKVLLHFIYWDELPDMQELIGTDSTLASTLVA
QHLLAAADRYALERLKAICESKLCEGVAINTVATTLALAEQHHCLQLKAVCLKFVALPENLKAVMQTDGFDYLKESCPSL
LTELLQYVARLSEHSVIVSGHRKEIFADGCDASGRRVKPRLH*
>Mgut_mgv1a003050m
MKFMKLGSKPDAFQTDGNSVRYISSELATDVIVNVGDVKFHLHKFPLLSKSNKLQKIVSKSNGETPEELSLADFPGGPKA
FEICAKFCYGITVTLNPYNVVAARCAAEYLEMTEEIERGNLVLKIEIFLNSSILRNWKDSIIVLQTAKSLLPWSENLKIV
GRCIDSIASKTSVDPTLITWSYTYNRKLASLDKNILPNGVKCPKDWWVEDICELDIDLYKRVMVAIKSKGRMDGAVIGEA
LKTYAVRWLPDSVVALLSEVHVLRNKCLVETIICLLPSDKGISCACSFLLKLLKVAIFIGADDSSREDLIRSIGLKLDEA
SVDDLLIPARPPQVGIYDIDLVQLLLDRFVENEKSGVDLKISDKNCKNDGDFVLGSGPWMKVGKVIDGYLAAIANDPNLS

SSSFIKLSQSIPDSARPIHDGLYTAIDVYLKEHKSITKSEKKSLCGLIDVKKLTTDACMHAAQNEHLPLRMVVQVLFFEQ
VKAAAAGTHQTLKNDNDVAVPSRNSEANWGKTVPENRKSLRKQMDEMKIKEDEKIGKLSKRGHKNRGSGPQLLPSRSRRI
FDKLWIVGKGFGINGENKSSETSGSSQSPSSMFRGEIKSSGSSSRNRRHSIS*
>Mgut_mgv1a002099m
MENQNQNSGRRHREQSATSSRRSLKRKLDEDSGEDRGRIGGESPSDSQQDLVTEIRTQVEILDSVASSSAEQDRASVKRA
IHVLSELAKNEDIVNAIVDCAAVPVLVKHLKAPPLVTEIESGPRLYEHEVEKGSAFTLGLLAIKPEHQQLIVDAGALPPL
VDLLKRHSAVPNSRVVNGVIRRAADAITNLAHENSSIKTRVRIEGGIPPLVELLEFADAKVQRAAAGALRTLAFKNDENK
NQIVECNALPTLILMLRSEDAAIHYEAVGVIGNLVHSSPNIKKEVLAAGALQPVIGLLRSCCSESQREAALLLGQFAATD
SDCKVHIVQRGAVPPLIEMLQSSDAQLREMSAFALGRLAQDPHNQAGIAHSGGVVPLLKLLESKNGSLQHNAAFALYGLA
DNEDNVADLIRVGGIQKLQDGEFIVQPTRDCVAKTLKRLEEKIHGRVLSHLLFMMREGEKNVKRRVALALAHLCSPDDQR
TTFIDNNGLALLLELLESTNSKHQRDSSMALCRLANKASSVSPIDAAPPSPIPQVYLGEQYVNSPTLSDVTFLVEGKRFY
AHRICLLASSDAFRAMFDGGYRERDAKDIEIPNIRWDVFELMMRYIYTGSVDVNADIAQDLLSAADQYLLEGLKRLCEYA
IAQDISVENVSLMFELSEAFNALSLRHASILFILEKYDKLCIMPWYTQLIQRILPETRNYFARILTRPVQGEIRR*
>Mgut_mgv1a025266m
MKCLSCKEEYGDRNAGTCRECYEEAGETEEELKRQIVELDSKVNFLSLIAISPKFVPTANPRCFSDVVLVASGPPESDNP
HCNSPSIPAHRAVLTSRSPVFRAMLENEMEESLTGTIMIRDVSYDTLRAFVNYMYTAEACLGENMACDLLVLSEKYQVKH
LKNYCEKYLMSTLNWENSIPRYAFAHQHGAKSLLDSSLSLILDNMDKLGKREEYVKLVERDPRMVVEIYEAYLSKQVNTG
LSKDRFVKA*
>Mgut_mgv1a003178m
MKFMKLGSKPDQFQTDGDSIRYVATELATDMVVSVGDVKFYVHKFPLLSKSSHLQKLVATSSDDISEEIDIHDIPGGPAA
FEICAKFCYGMVVTLNAYNVVAARCAAEYLEMYETLEKGNLVYKIDVFITSSIFRSTWKDSIIVLQTTKSLLPWSEELKI
VSHCLDSIATRASVEPLRVDWSYSYNNRKTENGSDPHWKTGGVKKVPRDWWVEDLCELQIDLYKRVITTIRSKERVSEGV
IGESLKAYALKRLPIFGNIQGGDILKYKYLLDTIIWLLPKEKNCVSCSFLLKLLQASVVLESGETGRRELMRKIAEQLDE
ATAGDLLIRCPNGESTVCDVDLVRDLIERFVMMEHSDDDDFASEASKAKVARLVDGYLAEAARDSSLPLSKFVDLAEMVS
GFPRSSHDGIYRAIDMYLKEHAGLSKSDKKRICRLMDCRKLSPEACSHAVQNERLPLRVVVQVLFFEQSRGAHDLPGPVR
ALLPRGSYGSSRSTTTNSDDDWEGNQTSEELKDLKGELASLRLRNNNVGGGGNNNNNINNNGDDIIKSKKVKKVFSRLWS
NKERQGENSSSDTTSESFASTSAEEKNEVAVKVKVLKPVQRK*
>Mgut_mgv11b023775m
MTTEDSLRSLSLDYLNLLINGQAFSDVTFSVEGRLVHAHRCILAARSLFFRKFFCGPDPQPGSDPLRMGPGGVGGGGGAS
SSSPRQQQQQQVIPVNSVGYEVFLLMLQFLYSGQVSIVPQKHEPRPNCGERACWHTHCTSAVDLALDTLAAARSFGVEQL
ALLTQKQLSSMVEKASIEDVMKVLIASRKQDMHQLWTTCSHLVAKSGLPPEVLAKHLPIEVVAKIEELRLKSTLARRSLM
PPHHHHSHHHHHDMGGAAADLEDQKIRRMRRALDSSDVELVKLMVMGEGLNLDEALALHYAVENCSREVVKALLELGAAD
VNYPAGPAGKSPLHVAAEMVSPDMVAVLLDHHADPNVRTVDGITPLDVLRTLTSDFLFKGAVPGLTHIEPNKLRLCLELV
QSAAMVISREEGNAGTPPSAAIYPPMNDQDHGGAAASIGAAGNLNLDSRMVYLNLGAQMGGGGKNMSGEEDSGGGGHRDQ
VCNCPYNFQ*
>Mgut_mgv1a001528m
MEKKQKKFLTVAPFECAWRDDLRFREAGRGCVAFDAFAHNDVTVVFREKTGSQHYHYKRDNSPHYTVIIGSHRNKRLNIE
VDGKTVVDVTGVDLYCSSTFQSYWISIYDGLISIGKGRYPFQNLVFQWLDSNPNCSVQYVGLSSWDRHVGYRNINVLPLT
QNHVSLWKHIDCGESNGTEDADEEMEEDIGEYENWGLKNFLESWELSDVCFVVGSEERAVPAHKVILAASGNFGFGQSGK
DFIHLEDDCYPILHALLEYIYTGTTKVPELHLSSLKSLSLQFEVNTLAKQCEEMMERFKLNKKLFDSGKSVEISYPSARL
NCCTVFLNKLPIDVKRLNSFRLTGDYSDVDIYIEGHGQIAKSHRIILGIWSAPFTKMFTNGMTESIASKVCLKDVCFEAF
NIMLDFMYCGEVNNTMDIDTLLLQLLLLADQFGVSLLHRECCKRLLEHLSEDSVCQILLVISSIPSCKLIEETCERKFSM
HFDYCTTASIDFVTLDETTFGNILQHPDLTVTSEERVLNAILLWCCKAQELFGWDRVDEILLSSPPELVFGERLNSLKEF
LSFVRFPLLPYPVLQKLERSNLSMCIPTFCQLVKEAIGFLEFGSSAHEKDLNKFQHRRSSFKELQYICDGDSNGVLYFAG
TSYGEHQWVNPVLSKKVIITASSPFSRFTDPKVLVSRSYLGTSFAGPRMENGRNTAWWMVDIGHSHQLMCNHYTLRQDGS
RAFMRNWNFQGSMDGNNWTNLRVHENDETMSKPGQFASWPVVGPTALLPFRFFRVVLVAPTTDATNPWSLCICFLELYGY
FR*
>Mgut_mgv1a002789m
MPPAGKVSGFHRQENDWFCNANLPSDILVVVDEVNFHLHKFPLISRCGKIATIVEESQTTSDKILTVTLQEFPGGADTFL
TAVKFCYGARIELTPKNIVILYCAADYLEMTDNYGEDNLLSKSENYFHKHILRHWKDCILTLQSCKPVIITRADKLQIIN
KCTNALSVMICTDPSLFGWPMMMYGSLQSPGGTILWNGINTGAKIQSNESDWWFEDISYLCVDLFERLVLTMEAKGVRKE
NIARAIMYYCKKYLPGLDRRQGVVVQGGASRAVASFSMTPANVDQSALLKRVVKLLPEKKSKSFCRFLLGLLRVALILGV
NDTCQDYLERRIGAQLELASLDSLLIPSYSDSDTLYNSDCVERMIGHFVSSEPNVDSVLSPSSFDLGASPSFERLKRVAK
LVDNYLAEVASDVNLKPGKIRSLAEALPESSRLLHDGLYRSLDIYFKEHSWLSDKDREQLCSIIDFRKLSIDACAHASQN
ERLPLRVLLQVLFFEQIQLKTALASCLNVFDAENAPAPPLSANPHETGGEIVQRDRWVHVVRENQGLKIDMENMRSRVGE
LEQEFVQIKEEMKRVSKSHSYISSSPRFIAKGLGCKLLPRSSNVDILESSSPTPRASVERSRPSTCHHRHGKSFSLV*
>Mgut_mgv1a003926m
MRDFGLDLFDPRTAVMDSDSNSSPTGGRDPDFGFAFNDSNFSDRVLRIEIVADPPDAPLETEGFRTLADWAVRRSKRRRG
DAVKKKNALDITSSSREQVLCCQQPAVEEGIGDENYDEEESLKMEDSPSGDEAENSDDSNWSEDDSRILRVETLHISSPI
LAAKSPFFYKLFSNGMMESEQRHVTLRISVSEEAALMELLNFMYTSTLNTTTAPALLDVLMAADKFEVASCMRYCSRQLR
NLPMTPESALLYLDLPSSILIADAVQPLTDAAKQFLAARYKDISKYQEEVMKLPLSGIEAIFSSDDLQVASEDAVYDFML
RWTRTHFQNPDERRDILCSRLSPHIRFPYMTCRKLKKVLTCNDFDGSFANKNVLEALFFKAESPHRQRTYATEDSTSTSR

RFVERAYKYRPVRVVEFEQPRKQCVVYLDLKRDECANLFPSGRVYSQAFHLGGQGFFLSAHCNMDQQSSFHCFGLFLGMQ
EKGSVSFVVDYEFAARSKPTEDYISKYKGNYTFTGGKAVGYRNLFSLQWTNFMAEDSLYFINGVLHLRAELTIKQ*
>Mgut_mgv1a003106m
MAYMKLGSKSEAFHRDGQTWQCTSGLQSDVAIEIGEMSFHLHKFPLLSRSGLLEKLIGELTTTDGSQCTLQLSQLPGGAK
AFELVAKFCYGVRIELTSMNVVSLRCAAEYLQMTEEYGEGNLVTQTESFLNEVFGNWTDTIKALETCEEVLPHAEELHIV
SRCINSLAIKASADPTLFNWPVSDSGRGPNTTNPAFWNGICTSTSSNSKSKPAHHAGDDWWYEDVSFLSLPLYKRLIRAV
EAGGMRAENVAGALVFYAKKYIPQMNRQSSFKNLSSGSTISIPSEADQGGLLEEIVELLPIQKGVIHTRFLLRLLRTAMV
LQTSPSCRENLERRVGMQLDQAALEDLLVPNMGYSVETLYDIDCFQRILEHFMAMDQASTAASPCVIVEESQLVEGTHSL
TSLTMVANLVDVYLAEVAPDVNLKFPKFQSLAATVPDYARPLSDGIYRAIDIYLKAHPWLTDSEREQICRLMNFQKLSLE
ASTHAAQNERLPLRVVVQVLFFEQLRLRTSISGWFFVSDNLDSSQNPNSVLSLTKNQHHHHQREASLETGPGVDDMRERV
FELEKECQSMREEFQKIVKAKRSWNIFCRRKSHVSNPKSPKNSNVKNH*
>Mgut_mgv1a003636m
MSELDGIELSLDDFNSSLPLKKVPYGDVFEASRAGDVDRLRYLLDSGVNVNARDQWDSVALYYACLAGHLDAARMLLESG
AICSEHTFDGDRCHYAALNLKVRKLLKSFEARPPPLQPLQASLRDTFFACNCKSNHPQFYANNHHHLINPLSQGVGNTSS
NGGESSSSYSPPDIVFYVHGRPLEAHRLILAARSPFFKQKFETDWRERKEVRFSRERLSYPSLFSLFHFFYSDRLEVAVE
DMEDLARICRVCKCHSLQILVEKEIVHQRHAEYKSLRDIENSQKRFILQGISLPEEDRLPAALSRSLQISLSNSSRGNNL
DIVSLVGSLQVQTSDLEDDLADVCVRVEDKIFRCHQVILASRSEYFRARLSRMKDFLQGREDSLLPDPTLPCLEEHDLSK
EAFQKMIEYMYTDGLTDIDPDQAEEMFDAASRYLLFPLKRAVADALLPQLEMVSLAELCHWLLLSDMYGVSKIREYCLDA
MACNFETFAETPQFRAMLLALPPPSGDSSLRTTAPSAPGAEANTTEGNVLDDLREKWLEAEGDELDKRDESALLFDKRLE
MLMLMAEQEKIS*
>Mgut_mgv1a005124m
MEELGMSGWNQLGVVGTIYEEENEDSYSSSPSTIPSPEFTPSPTTQLSGAAAQPWSSERRHDPDVVIHVQDSSFRLHKGA
LTAKSTYLKGQLTEFPEITLSPPLNITAQTFSLVTDFCYGSHVAVTPFNVAAVRTAAELLGMTEDGGGPPGEENLQQKTE
AYFRQAIYVNKEYACIVLRSCFPLLPEAETAAALVSRCVEALALTAESDGDMSCLEGFKSVAFDDLQVILESENIETVSE
DEKIQMCNHIDCSILTPRLLMHAVQNPKMPLRFVVQAMFLQQLNTRHSIISAATSSPDHHHPHPRRIHQEHSKKDPVTLG
AILERDAALRQVSQLKSIMTATNSRIQTLEKELHGMRQLLNESESVKNRAGSGRSASFRFSSEPTSRNYNKIERGQMGSI
SASSFRSLAERGRRTEISSSSEESYSDGSSGGEKSFGQRLMSGLKSAFRLQKKKCSSESKVPVKINGENLREDGNKDNIV
IIKKDQAFHRRTRSRS*
>Mgut_mgv1a003409m
MDSRTAFCDSNDVSGGSSSTCCAATATDSSLSPSPVQLPADVAAIKRLSDTLESVFLSPSDSDFFADARLVAASGKEIPV
HRCILSARTPFFKNLFASRERNAKLKLKELMKEYDVGYDALVTVLAYLYSGKIRASPKDVCVCVDDECSHLACRPAVAFM
VEVLYASFIFQISELVARFQNSLMGILDKTTTDDVLMVLSVANMCGTVCGSLLARAIEIVVKSDLDVITLEKSLPQHIIK
QINESRIALGLLSPENSTCPDKHVKRIHRALESDDVELVRMLLKEERTNLDDAYALHYAVAYCDSKTTAELLDLAIADIN
QRNPRGYTVLHVAAMRKDPKIIVSLLTKGARPSDLTSDGRKALQISKRLTRAIDYYGSTEEGKSTPKDRLCIEILEQAER
RDPLPGEASMSLALAGDDLRTKLLYLENRVALAKLLFPMEAKVAMDIAQVDGTSEFHLSGIHKNQAGSQRTEVDLNEAPF
KIKEEHLNRLRALSKTVELGKRFFPRCSAVLNKIMDRDDYPDIAQMGNGSPEECPLKRQRRMEIEEVLSKAFTEDKEEID
RTINLSTSSSSSSVGIMRPNGMLPFKN*
>Mgut_mgv1a022041m
MSMTWMPNIGFLSFEEIFTVSKFIHSDIFTVGGYKWAIAVYPDGISHDKTLGGDKYEQGHVYFDLFLSPMSTDCDGVSFC
VKMTLLDQSGNGIHRTEFDDVPRVISKESRRGFVCFIDREVLEKSSYLKDDCLKIECTVYVVLTSSSSSDIIDDVSKRDF
DDDDGGVGGDFLAVFSSAEEDSDVVFSIGGEKFHAHKDILSARSSVFESMFAVSDQREMVITDVEPRVFKALLHFIYSDT
LPENERRSLVGGYAFGPVVSNTIGAKLLAAADKYDVKRLKVICESHLWRSISLNRFAEILSLAVNYNATELKHLCFKYAA
DNYDVLVKLDSFSLLEKKWPSLMNE
>Mgut_mgv1a003483m
MENSGEPSSSISFTSPSHISNESTVNKVHTSVGSEAVLDLETISLSKLSSSLGQLLCESGSDFTDADIVVEETSVGVHRC
ILAARSKFFDELLRKDKGSVGKEGKPKYCMVDLLPYGKVGYEAFVIFLNYLYTGKLRPSPLEVSTCVNCVCPHDSCRPAI
DFVVELMYASTIFQVSELVSLFQRRLLNLVGKAIVEDVVPIIKVAFDCQLSQLLNECVHRVARSDFEAISIEKELPPIVA
QDIASLCLKSQSEQENKTGPVSPLHEKRVKRIHKALDSEDVELVRLLLTESDITLDEAYALHYAVAYCDPKTVTEVLSLG
LADVNLRNNRGYTVLHVAARRKEPSIIVSLLTKGACAADPTFEGRNAVTICRSLTRPKDYQMQREKGQEANKDRICIDVL
EREMRRNPISMDVSISSIAMVDDLHMKSLYLEDRVAFARMFFPTEAKLAMEIAHAEATSSLAGLLSSSGNLMEVDLNESP
ITQNKRLLARIETLSRTVELGRRYFPNCSQVLDKFMEDDLPDAFYLEKGTLEEQRMKRVRFMELKGEVHKAFNKDKAKLH
RSGFSSSPSSFKDHVGHKIRKL*
>Mgut_mgv1a009657m
MPEPDVHVVTSAGILIPAHSKILASASPVLESIIEATQNRHSSGRKIPILGVPHDAVSVFMQFIYSSRCSEDQMEKYGIH
LLALSHVYIVPQLKQRCTKGLAQQLTVENVVDVLQLSRLFDAPDLYLNCMKVLSNNFKIVKKTEGWKFLQNHDHYLKLEI
LHFIDEAQSRKKRTRRRKEEQNLYIELSEAMDCLVHICTEGCGSVGPCDMDPGKDKGPCSKYSTCYGLQLLIKHFGSCKN
RVNGGGCWRCKRMWQLFRLHSSICDRSDDCRVPLCRHFMTIEDRRGSDVRWSLLVKKVASVKAMLTIPKRKREDELRMDV
DHQIMRSSKLQNVAP*
>Mgut_mgv1a003257m
MNAPISDVSSDLTIEVGSASFALHKFPLVSRSGRIRKLLMESKDSKISRLNLSGVPGGPHTFEQAAKFCYGVNIDISLSN
IATLRCAAHFLEMNEEYSEKNLESRTEAYLKDTILPSIPNSITVLHHCQNLIPISEDINLVNRIINGIANNACKEQLTSG
LSKLEHNFPPKPVETEINSDWWGKSLALLNLDFFQRVLSAVKTKGLRQDIISRILINYAQNALVPVKGSTISDLDMQKKQ

RVTVETIVSLLPTQSRKSSVPIAFLSSLLKSTIAAGGSTSCRCDLERRIGLQLDQAILEDILIPVNPHGNNHSPLYDTDS
VVRIFSMFLNLDEDDVDDDEEDNRRMRNDEGEMVYDFDSPGSPKQSSIIKVSKLLDNYLAEVALDSNLSPSKFTALSELL
PDHARVVQDGLYRAVDIFLKVHPNMKDSERYRLCKTIDCQKLTQEACSHAAQNERLPVQMAVQVLYFEQIRLRNAMNGVG
GHNQFFFGSINGPNRAGSGAGSGCISPRDNYASVRRENRELKLEVARMRMRLTDLEKDHVTMKQDLVRSHPANRLFKSFT
KKLSKLNLLFRFKDGGSKANSDSKLLFQKRRRHSVS*
>Mgut_mgv1a003057m
MDKHQHQQQQPPLAKSGRQKCSEWVFRDVPSDIIIEVDGATFSLHKFPLVSRSGRMRKLVAEHRESDISRIELLSLPGG
AASFELAAQFCYGINFEISTSNVAHLCCVSDYLEMTEEYSNNNLSSRAEEYLNTIVCKNLEMCVQVLHHCQTLLPLSDNL
KIVTRCIDGIASKACVEQIASSFSRLEYSSSGRLHMSKQIKTEGDWWIEDLSILRIDLYERVITAIKCRGVRPESIGASL
VNYAEKKLIARNLLSGDQSAVIIIEAIVSLLPIEKNAVPITFLFGMLRSAVALDCSVSCRHDLEMRIGSVLDIATLDDLL
IPSFCQAGDTLFDVETVCRILVNFCRRDGGGDSDGDDVDGDDVDGDSGGFECGSRDYSDSSLPSQSALFKVCKLVDSYMA
EIAPDANLELDLFVAIAESLPAHARTVHDGLYRAIDVYLKAHQALSEPEKRKLCKLIDFQKLSQEAGAHAAQNERLPLQS
IVQVLYNEQLRLKNSLFSSYPEDDEDPKKPPMHHHQSWRINSGGVRSAAMSPRDNYASLRRENRDLKLELARMRMRLNDL
EKDHVCMKRNLERSGSRKFMSSFSKKIVRLNVFARSYSRGSVSPSKLSQRI*
>Mgut_mgv1a001244m
MTKEPSNSDNSDGGAAGERRGGGEMRRAAVDCNLASLCDHIQLEGFNNGLFSDVVLNAMGSTYYLHRLVLSRSSYFRNML
QGPWKEANAPVLTLHVDDKNVNAEAMEIALAYLYGHHPKLNDTNAFRVLAAASFLDLQDLCAICTDFIVAELWSSNFLTY
QVFAENQDYGIHGERVRNACWGYLCQSGAQELREVLPKLSSQTLLALLTSDELWVPSEEKRFELALHTLLAKGTLCKAEH
HEQRTPSCEVEASTYPDSSRVIRKHLADESGNNLPEIERGCTKPKDEIEGRNTARNILVELADSVVDSHSDVDNVDQAQT
AHSGSNLDSRYDCYDERPSASNTFYSDGIIPSCSYLNIHNAVGMSGSAGNVLALEGPSDEDSCYQLNSSWPSGDQMHCMS
MNSSCNVMIPNEWERCNMSSLTWGGRIVGRREVKTCLKAQCGMSIEDHDSFVNIFEGGSLLYCNMSFEALLNVRKHLEEM
GFPCKAVNDGLWLQMLLSQRLQEIGADTCKNCCRMSMACACRQPFGYSPGVTAPGYYVQDNDHNNLPPNDIGHVYINSSA
QGERNGIFRPVRVHDRGHIDGLAGIGRGTTFVPAAAWPPTRYVFSRVPFGIGNRSNQQPHANDDPENRGDNNGELAVDGL
TALVGLSQGSSDVTHVHEVQMGREYETGSVNPGSSTSGVPVQMTESPEHAAGIEWENTSSAISLDLKTPLTHFPPFRFAV
EFQDVHRLVDGQVKHSPEAFYAGSLWKISVQAFSDEDPQGRRTLGLFLHRRKAEIYDPLRKVHMYVDSREKVTARYQLIC
PSKREVMVFGSYKQTGTLLPKAPKGWGWRTALLFNELGDLLQNGALRVAAVVQLI*
>Mgut_mgv1a009133m
MVQLKKKLSAQRRVKSIPPIAGAVEMPETDILVITSDGVRIPAHSKILASATPVLENIIETPQKHRSTAAKKISIPGVPY
DAVAAFIQFLYSSKCTEEDMEKYGIHLLALSHVYSVPQLKQKCTTGLAKRVNVENVVDVLQLARLCDAPDLSLKCMKLLS
NDFKTVEKTEGWKFVQNHDPYLELEILQFIDEAEKRKKRAMRHRKEQSLYMHLSEAMDCLEHICSEGCTSVGPYDMDVTK
HKGPCGKFSVCQGIQHLIKHFGACKKRVNGGCSRCNRMWQLFKLHSSMCDQPDECRVPLCRQFKLKAQQNRRGNDTRWKL
LVRKVVSAKAMSSLSLTKREPRIAMDNRGVKL*
>Mgut_mgv1a003527m
MEENCPPQRCSEWVFRDVPSDITIEVDGTTFLLHKFPLVSRSGRIRKLVADHRDSDISRIELVSLQGGADSFELAAQFCY
GVNFEITPTNVAHLYCISDYLEMTEDYSKNNLFTRAEEYLEIIVSKSLEMCVKVLQQCEKLLPLADELKIVQICIDSIAS
KACVEQIASSFSRLEYSNSGRLHMSKNQTNCESDWWIEDLSILGIDFYQRVVKAMKCRGVRPESIGSSLMNYAKKELLKK
SKTDSISGHERVVVEAIISLLPVEKFAVPINFLFGLLRNAVIFDCAISCRLDLERRIGSQLDTASLDDLLIPSFRQAGDT
LFDVDTVERILVNFSRSEDSEEDMDDFSLFESDNYPLSPSQTALFKVSKLVDNYLCEIAADANLKLNKFADVAEALPAHA
RTVHDGLYRAVDVYLKAHQHLTDSDRKKLCKLIDFQKLSQEAGAHAATNERLPLQSIVQVLYYEQTRLRNALFCSYQDED
PKNVNMQQSWRISSGALSAAISPRDNYASLRRENRELKLELARLRMRLNDLEKEHVSMKMNIERSGSRRFFDLFSKAVGR
LFAHRNETKVTERTCDKHVS*
>Mgut_mgv1a027075m
MSDKKKELLSTAMKRTSEWIFSQEIPSDVTVNAGGTSFSLHKFPLVSKCGYIRKLVTESTDADLAVVEIPDFPGGPEGFE
LAANFCYGINFEITTENIATLRCAAEYLEMTEEYSVGNLVGRTESYVNEVALKSLAGAVSILHSAENLLPIAEKVKLVSR
CIDAIALVACKDSQFAAPVRAGGTGLNDSKPVVDWWAEDLTVLRIDLFQRVLIAMMARGFKQYALGPILMLYAQKSLRGL
DAFGKGRKKIDPRQEHEKRVVLETIVSLLPREKNTMSVSFLSMLLRAAIYLETTVACRLDLERRMALQLGQAVLDDLLIP
SYSGCGVHADDQDHVSPSAIEVEKVAKLMENFIAEIASDRNLSVSKFISIAELTPEQYRMTEDGMYRAIDIYLKAHPALS
DMERKKVCSVMDCQKLSREACAHAAQNDRLPVQTVVQVLYYEQQRLREAVDGSSSLNDTSAIPAKPGPYSGDVHIVTDEV
SSLRKENQDLKLELVKMKMRLNEIEKPSPTAFGSPSPATTVSRPGSADKPPLPPLPKKSFISSVSKKLGRFIRADGLSAQ
GSRGRTKPAKDRRHSIS*
>Mgut_mgv1a009872m
MSESAYRVETTSRLAQWRIENLASCTYRKSDPFKIGKWNWHLSMEKNRALFIKLYPEISNLTRENPPIASFVIRVVSSVG
DRKCLVHPEITDKQLKSNEDFVWAIEVPLSGKFIIDVEFLDLKIGSPDGTELCSIWSEGFTQRQSNETALTSLGRMLSES
IHTDIAINASDGSISAHRAVLASRSPVFRSMFSHDLKEKELSAINISDMSIEPCQAFLNYIYGNIQNEEFLTHRLALLRA
ADKYDISDLKEACHESLLEDIDAKNVLERLQNASLYQLPKLKSSCMKYLVKFGKIFDIRDDFNAFVLCADRELISEVLNE
ILAAWKGF*
>Mgut_mgv1a004464m
MAACMKLGSKSDAFQRQGQAWFCTTGLPSDVIVEVGEMSFHLHKFPLLSRSGVMERLIADASEQEEGCLIQLPQIPGGSK
TFELVAKFCYGVKLELTSANVVHLRCAAEHLEMTEDYGEANLISQTEIFLNQVVLRNWKDSLKALQTCDDVISEAEELQI
PKRCIDSLAAKACTDPNLFGWPVMEHGGPMQSPGGSVLWNGISTGARPKHSSSDWWYEDASSLSLPLYKRLISAMESRGV
KREIVAGSLNCYAKKYLPGINRRQGASKASNNRVVAPLSEEDQKILLEEIDQLLPLQKGLCATKFLFGLLRTAKILKANP
TCVSNLEKRIGVQLDQATLEDLLMTNSSDSMETLYDVDCVQRILEHFLTMDGGGGGGGGESPESVEDGGSQLMGSPSLT

PITMVAKLIDGFLAEVASDVILKLPKFQSLAAAVPEYARPLDDGLYRAIDIYLKSHPWLAESDREQLCRLMDCQKLSLEA
CTHAAQNERLPLRVIVQVLFFEQLQLRTSIAGCFLVSDNLDVVRGS*
>Mgut_mgv1a021791m
MDCSICSAMPYILRPPRNTICGACYEGARTIIALTNKLDTTIDKGSDKPSTNPVSSSNSAKGFANALKWVKEMKEVEEDL
NERINYLSGFADVFADQIHTDIQVQPGNNQPSIPAHRALLAARSCIFRNILDSDECKAPPNQTITLSELSHDELESLLEF
LYSGNLAKEKVEKHVYALSIAADKYEIPFLQKFCEQHMLGSLSSSNALDILEISDTCSNQSLKETALNYIVRNMEDVVFS
ARFDAFALKNPHLTVQITRASFMDIRNRRAAGV*
>Mgut_mgv1a004127m
MESDTSPDTGSPVSSSDFGFAFNDVNFSDRLLRIEILPDLPGSKPDADDSLADWARNRKRRRDDQLTNAVELADQREEQI
LNGNMPDLEDGVTYENQEEVVAMVEELPVGGEIATDQSGHEAAQRNEASSSMDYLTVQVKTIHISSPILAAKSPFFYKLF
SNGMRESEQRHVTLRIQASEEAALLDLLNFMYSNTLPRTTPTALLDVLMAADKFEVASCMRYCSRLLRNLPMTCESALLY
LDLPSTILMADTVQPLTDAAKQFLAARFKDISKFQEEVLNLPLAGIEAVLYSDDLQVASEDAVYDFVLKWARVHYPKLEE
RKEILSNRLLRFVRFPCMTCRKLKKVLTCTDIDQELASKVVMDALFFKAEAPYRQRSLVAEETKASFRRFVERAYKYRPV
KVVEFDSPRQQCVVYLDLKREECVQLFPAGRVYSQAFHLGGQGFFLSGHCNMDQQSSFHCFGMFLGMQEKGSVAFTVDYE
FASRTSPSEDFVSKYKGNYTFTGGKAVGYRNLFGTPWTAFIADDSPYFINGVLHLRAELTIKK*
>Mgut_mgv1a010667m
MRRHHHPRRRQLSSDSDIDFRGDDDDDDEDEDEEEVEEGSSAARRRRRIKMKCLSCKEDYGDNDAGTCRECYEEAGETEE
ELKREIDDLKSKVNFLRLLSVGPGPVGPNCITRPNTPCFSDVVLVASGSKDSDGSQCISPSIPAHRAVLTSRSPVFRAML
ETEMEETLSGTIKIGDVSYDALRAFVNYMYTAEACLDEHMACDLLVLAERYQVKHLKTYCEKYLMSKLNWENSLPYYAFA
HQHGAKSLLEEAALSLIMDNMDKLSKQEEYMELVEKDPRVVVEIYEAYLSKQVNTAAASKDPSAKA*
>Mgut_mgv1a007354m
MVGMGRVYQETAKPSSSSAAPSTPAVTTSISISETVNGSHDFKITGYSLSKGIGIGKYIASDTFMVGGYAWAIYFYPDGK
SMEDNATYVSLFIALASEGTDVRALFELTLLDQSGRERHKVHSHFGRALESGPYTLKYRGSMWGYKRFFKRTALETSDYL
KDDCLQVHCCVGVVKSHTEGPKIYSIPLPPSDIGQHFGQLLESGKGSDVNFDVNGENFSAHKLVLAARSPVFRAQLYGPM
KDQNTQCINIEDMEPPVFKALLHFMYWDTLPDLEELTGMNLPWAATMMSQHLLAAADRYGLDRLRLLCEAKLCEDVAINT
VANTLALAEQHHCFQLKSVCLKFVALPENLRAVMQTDGFEYLKESCPHVLTELLEYVARTNEHSIAMGKQGIEAILDGSD
VNGRRVKQRL*
>Mgut_mgv1a018386m
MEEEDFASESASATVRGMEKGSHLFVVKNYSLHEGIGVGGAIESEEFIVGGYGWTIRFILNQTTIAVGCLYSAHFMDQSG
KGNDKGLSFFDNSKIMYFERDRVIGRNFFIRRSSLNCPDYLKDDCLKIRVTIEVFTCRIHNLPLIKVPESNIGADFGKLL
RSKQRADVFFRVKNKSFCAHKRILAARCPQFLSQVSDSNYEIEVTYMEPNIFKALLWFLYTGTLSDEEHDDSDIGHSMLE
SFIGKMLAAADRFELKRLKNICELSISKRISGDSIAYILHLAERCHAKELKDACLGFGADNGADVMSCEGRRYLKERCPL
LFLELAYNIR
>Mgut_mgv1a007664m
MGSPGADISWPSSVIESFDRSFDINIEEGNSSDISYFLEDKMPSPNNHHNIPKPPPLPRKKLNRGLPKHCFVPKETRDVW
EKLFKEGYGADVHVITENGSIIPAHLSILSVASPVIGNLLQQSKVKNGVRFIRILGVPYDAVCTFIRFLYSSCYEEEEMN
KFVLHLLVLSHSYTVPSLKRICEYILEQGWLNNENVVDVLQLAKNCDTPRLALFCLRIIMNDLKSISKTEAWKFMKRDNP
SLEQELLESVVEADSRKQERMKKIEERKVYLQLHEAMEALLHICREGCRTIGPRDKVLKGSSIPCGFPACKGLETLIRHF
YVCKTKVPGGCVHCKRMWQLLELHSRMCNEPETCKVPLCRHFKVKMEHLSKKEEVKWKLLVRKVVAVKNAQKLFSPRRL*
>Mgut_mgv1a002655m
MAAATTTHLKFHNQPIKIKRRRRRETTITSTPTPVDSPLSSSSSAATNTTRSNHYHSRKIDTPITLSPDNSGCRAASEPP
PEAADRTSALVVSSLPSSSSSSDLKTRSSTSTMGSYTTPTQTSAAAAAAAAAAQDNFPSSLSKFNSALTAGLLNPMSPPPL
LDKTRSSPTLFEMMANEPDSYSVAATLAENGAVSAQNQIQNHQNPKISANAPPPPPDKQALMQQRLTDILASRSPGNQFN
EAASSDVKLTLSSKDGVSISISVHREILVVHSRFFAQTFNEKWVKQQRNLGPHIVEIADCDDIEVYIETLRLMYCKDLRK
KLMKEDVSRVLGILKVSAAIGFDAGVLSCLEYLEAAPWAEDEEEKVASLLSELRLQGIGAREVLKRVSLDATEGAEEAND
NEQVLVKLLQVVLEGKDDKARREMKGLVTKMLRENSSQNDLRKESLYFACNGCLKLLRVSFLQAAQGDMQDVGQIARQAD
NLHWLLDILIDRLIAEDFLNIWASENLLSESHSKVAPIHRYEVSRVTARLFVGIGKRQLLASKEARYMLLQTWLVPFYED
FGWMKRASRGLDRHLIEDGLSNTISTLPMEWQKEILLSWFERFLNGGDDCPNIQRGFEMWWRRAFWRRNNGEVERPRQIR
VVSDAVEKS*
>Mgut_mgv1a020824m
MELSDNESKEPLKIGDRSTSDVVVRIRTHEGRDEWIYCHSHIIVKKSKYFADRLSENWPTCQILDSRNCVEVHCEERDFD
YQITVLRFFYAQSDVTVTDLCNVANAIGILRVAFELGCPQIISTCVGYLEAVPWEESEEEEILKTIPKMGSQAEQVLARL
QPVNRGTVVKIFVSAIRFATSSPECEMKDLKTSAQEQIEYMLTEDDDAPLLTADDEVKSEVRDCVNGLLARFVNVLESVD
EEMKMGLFEAHLSDLSWAFQIVAKLEIWGDFVDTWVEASAKIVRVAEVAFLLTGVAKLKVLEVTAKVLETVAYGSVVLPS
VKRLHLVKVWLAFVRLVKSSVDDSVAVSDDSVVVSDDDNGGGGGDVAVKSDDEVWQTLEGAFVLMILALPSSDQAEILTE
WLENKQIRYPDLTEAFEVWCYRSKVAKRRLGLLDGDSRKTNGV*
>Mgut_mgv1a003084m
MMKLSVQPQSPASDSDEIYQADDQRIAIPARFNATTDSFEKNEHSCRKIMSQVPTDLSIQVQDVTFYVHKYPLISRCGYF
SRVEFQFPKSNLNCDIELDNLPGGPETFETILKFCYDIPINLKVNTVAALRCASEYLEMTEALEDGNLISKTEAFFTFVV
LSSWGGSITVLKSCESLSPWAENLQIVRRCCDSIAWKISQEASEVINGEKNWWFEEMSTLRIDYFTRIVTGLRARGSKPE
IIGSCIVHYGERWLPEGRGNELQWNITGGAIREEDSRPIREQRIIVESLVSLLPSEKEAVSCKFLLKMLKMAILYSASPA
LVAELEKRTGLVLETADVNDLLIPSYTAGNKIKLISSTEENIMHNVDVVQRILEYFLLYEQQQQQQLLPGPLAVSKLLDN

YLAEIAKDPNLPVTKFQFLAQSLPENARKSDDGLYRAIDTYLKTHPSLSDHDRRRLCKVMNCGKLSVDACAHAAQNDRLP
LRTMIQVLFAEQMKIRATVQGKSQTEIADNSDQESSWLSTNKEVKALKLELEKVKSQMAEMQRDHSDLQQERQKQNIKQG
NSSGWTIRWQRIRKSALFNLKTDGEETDEVNKRPNNIGKGSFRRRQSLS*
>Mgut_mgv1a002133m
MENQNSGGRHRDREQSSSTSSSSRRSLKRKLDEEFDGRIDDQSPLDLHRDLVREVRAQVEILDSSFSSAEKDRASAKHAI
GVLSELAKSEDIANVIVDCAAVPSLIRHLQAPPVSEGDSGPRLYEHEVEKGCAFTLGLLAVKPEYQQLIVDAGALPHLVD
LLKRHKLNSRAANGVIKRAADAITNLAHENSSIKIRVRVEGGIPPLVELLDFADPKVQRAAAGALRTLAFKNDENKDQIV
ECNGLPTLILMLRSEDAAIHYEAVGVIGNLVHSSPSIKKQVLAAGALQPVIRLLSSYCSESKREAALLLGQFAATDSDCK
VHIVQRGALQPLIQMLQSPDTQLKEMSAFALGRLAQDTHNQAGIAHSGAIEPLLKLLESKHEFLQHNSAFVLYGLVENED
TIADLVRFGGVQKLQDGEFIGQPTRDCVARTLKKLEDKIHGSVLSYLLRMMRVGEKKVQRRVALALAHLCNPDDQKTIFI
DNDGLTLLLELLESTNSKHQRDSSIALCRLANKASSLSLMDAAPPSPIPQVYLGEQYVNNPTLADVTFLVEGKRFYAHRI
CLLASSDAFRAMFDGGYRERDAKDIEIPNIRWDIFELMMRYIYTGSIDVKLDIAQDLLRAADQYLLDGLKRLCEYAIAQD
ISVENVSLMFELSEAFNALSLRHACVLFILKNFDKLSVMPWYSQLIQRILPETRNYFVRALTRPDQMVI*
>Mgut_mgv1a003749m
MRGSGLDLFDPTTAVMDSDDNSSPGGSRDPDFGFAFNDSNFSDRLLRIEIVADPSESQPDSDACQTLAGWAARKTKRRRA
DIAKKENDMYHSFAALDINNCPEEQILDCESDADDGLGDADHDEEEIPMIEDSPSGDEAANSDDSNWSGDGSRVQRVKTL
HISSPILAAKSPFFYKLFSNGMMESEQRHVTLRINASEEAALMELLNFMYSNTLSTNTAAGILDVLMAADKFEVASCMRH
CSRVLRNMPMTPESALLYLDLPSSVLMADAVQPLTDAAKQFLAARYKDISKYQDEVINLPLAGIEAILSSDDLQVASEDA
VYDFAVKWTRTHYQRLEDRKDILCTRLGSHIRFPYMTSRKLRKVLACQDFDQDFSKQAVLEALFFKAEAPHRQRTQAIDD
SSTSTSRRFVERAYKYRPVKVIEFDLPRQQCVVYLDLKREECSNLYPSGRVYSQAFHLGGQGFFLSAHCNMDQQSSFHCF
GLFLGMQEKGSVTFAVDYEFAARSKPLEEYISKYKGNYTFTGGKAVGYRNLFNIPWTSFVAEDSLYFINGVLHLRAELTI
KREITT*
>Mgut_mgv1a002858m
MKFMKLGSKPDTFQIDGNNIRYVASELATDVVVQVGDVKFYLHKFPLLSKSARLQKLVSSSNEGNSDEINIHDIPGGPTA
FEICAKFCYGMTVTLNAYNVIATRCAAEYLQMHEAIEKGNLIYKIDVFLNSSLFRSWKDSIIVLQTSKSLLPLSEELKLI
THCIEAVASRASVDVSKVDWSYTYNRKKLVEENLTDPSWHGVRTREVPSDWWVEDLCELEIDMYKRVMTAIKNKGIVSNE
VVGEALKAYAYGKLPGSGKNVVPQNDVSKFRSILDTIVWLLPQEKGCVSCSFLLKLLKAAISANSAESVKMELVKRIGQQ
LEEASVSDLLIRACDAGVEEGMMMYDVNVVKKILEEFTMADKNEIEENGGEIKEIRRPGILSEASKLMVAKIVDGYLVEI
AKDPNLPLSLFIGISEMVSGFSRPAHDGLYRAIDTYLKEHPGIGKSDRKKICKLMDCKKLSADACMHAVQNERLPLRVVV
QVLFFEQVRAAASSGSSTPDLPKAIKDLNSGSYGSSRSATTTNTNTNTDEDWDAVASAEELRALRGELAALRLGNEVVGG
GESRSGNSERGAAVSKMKGLIMSRRIFSKIWSNKAPAVENSGSDSSESLGSTNQDEAKCTPTRRGRHSVS*
>Mgut_mgv1a003347m
MENGADLSSSLSFASSSYLSNGSSGHEMPSTAGFEVGANLELLSLSRLSSSLEKLVIGGAEYDYSDAEIEVEGIAVGVSR
CILAARSHFFHEIFKNNNAESRSSTKEGKKPKYILSELVPHGKIGYEAFTVVLNYLYTGKVKASPAEVATCVDESCAHDA
CGPAINYAVEMMYASATFQIKELVMVVQRRLLNFVDKAFVEDVIPILIVASTCNLNELLSHCLQRIARSDLDNTIIEKEL
PYQTQNEVKSLRLKSNNQEEEHDSIQADPTNEKRIKRIHRALDSNDVELVKMLLEESGISLDSALALHYAAAYCNPKIVA
EVLNLGFADVSLRDPRGYTVLHVAARRKDPSIVVGLLTKGASVSDRTRDGVTAVTICRRLTRPKDFNEASVHGKETNTDR
LCIDVLEREIRRNPLAGNMSVSSMMVADDLHMRLLLLENRVAMARTLFPLEARVAMQIAHAESTFEFAGLSASKGSYGNF
REVDLNEIPSEQVKKLHLRLQALQKTVETGRRFFPNCSEVLDLLLEDDTLGDLLLEKGSPEEQRTKRMRYIELKEDVIKA
FNKDIAEHNKLSGLSSSSSRSNSPRVKVRRR*
>Mgut_mgv1a008185m
MDRRNEESPSRMEKSLPHPPPMPPHFNTRFHHKGLIITGSKIRGHSFLPSIAVNSSWDRLFDEGYRADVTILTDDGGVIY
AHSSILGVVSPIFKAMLNKIKTRGQSNRRSISIRGVPSEAVRVFIRFLYSSCYEEESMKEYALPLLVLSHAYVVPQLKRL
CECWLEHKLMNTENAVDIFQLALLCDVPRLSLISHRFILSNFKSVSSSDGWKDMKQSHPVLEKELANSVRHEDFRKKEIT
RKMNERKVYMQLYEAMEALVHICRDGCRTIGPHDKVPPKDDNTPCPYSACKGLEALVRHFAECKMRRPGGCVHCKRMWQI
LELHSRLCANSDDCRVPLCRKFRQRRRKQSKKEEMTWRILVKKIVRSKSISGGPYFSLESTT*
>Mgut_mgv11b024042m
MISNGKVILQDCIWFEGFGFNGAGYLVLVLLTDLSTQEDVETKSASQCLTRTVKGIHLFTLENYSLCKGVGYGNPICSNT
FTVAGYQWVVFFFPDRTPSEMVELSLKEKKCMRGDMGPIVLNTGQQIGAPHLIICDLLEKSPYVRVPQHNEPGFHFLGML
ETGEGSDVTFIVGGMKFRAHRVIVQHAPLYLNAYFLATWLLMVITDIKKLDTFADLEKNCPLLHKELVDYLSKERAVLHP
KQIEEVLTLAVAD*
>Mgut_mgv1a009909m
MTDSKVETIARLAQWKIDSFGPTTPYRRSDPFKIGIWNWHLSVEKSRSVYIRLFPEPSRVSKEQPPVARFVIRVTTAGSN
RRPYISPIHERLLRTSDDFACPIDINCQGRFIIDVEFLDLKICPLNGGEGSSIWPSDETMQNLATQSTFKCLSRMLNESI
HADITINTTDGKLKAHKAILSASSPVFDSMFTHDLKEKESSTIDIEDMTCESCMALLSYLYGTMRSEDFWKHRLALLGAA
NKYAIVDLKDACEESLLEDINTGNVLERLQEAWIFQLGKLKKGCLVYLLDFGKIYDVREEMNDFFRKADRELVVEMFQEL
LAVWKPT*
>Mgut_mgv1a024013m
MADTKVETIARLAQWKIDSFGPTTPFKRSDPFKSAYGNWHLSVEMSRSMYPPIARFVLRVTTSASNRRPYVSPIHERLLR
TSDDFACAVDITFQGRITIDVEFLDLKICPINGGEGSSIWPTDTTMQTLSTQSTLKCLSRMLHESIHADVTINASDGSLK
AHKAVLSASSPVFHSMFEHNLKEKESSIIEIEDMTSESCTALLYYLYGTINQEDFWKHRLALLGAANKYVIVDLKDACEE
SLLEDISSGNVLERLQEAWLYQLEKLKKGCLVYLLDFGKIYDVREEMNEFFRQADRELVVEIFQELLAVWKPT*

>Mgut_mgv1a021528m
SETNVVDLQCEGDDVGVDFLTMLTSGEGSGVVLNVGGEKFHAHKVILSACSSVFESMFASHLTISDQHAIVISDVEPQMF
KRLSLAGKYNASELKHLCFEYAFENYAVLVELDSFRYLEKNCPLVLDEMNG*
>Mgut_mgv1a003487m
MANRHNLAMERTGQWVFSQEVPTDVIVEVGEASFSLHKFMLVAKSNLIRKLILDSKEADLTRINLSGIPGGAEIFEKSAK
FCYGVNFEITVHNVAALRCAAEYLQMTDVYCDNNLAGRTEDFLSQVALTSLSGALVVLKSCEDLLPMAEDLRIVQKCVEI
ASAKACVEANFPSRSPPNWWTEELTILEINFFSKIITSMKTRGAKGLTLASAIITYTERSLRDLVRDHSGGNGIGIRSTE
YSDTDLRSRQRGLLESIVSLLPPEKSAFPVNFLCCLLRTAIFLKADAKCKTELEKRVSAILEHATVDDLLVLSFTYDGER
LFDLESVRKIISGFAEKEKNVAVFNAGDLKEVCSTAMQRIAKTVDAYLGEIATYGELGISKFNGIANLVPKAARKVDDDL
YRAIDIYLKAHPNLDEIEREKVCSVMDPLKLSYEARVHASQNKRLPVQIVLHALYYDQLMLRSGADEDDHKTPDAGSTRN
QLQADVSLAKENEALRTELLKMKMYIQDIQRPQGTTSKAVSTSTSTSASASASTSRKPKFFSSMSKTLGKLNPFKHGSKD
TLNLDEGMPDLTKPRRRRFSIS*
>Mgut_mgv1a022299m
MTFKNIVEGVHGFTLEKYSLCKGFGVGECFQGSNNFTVGGRTWAIYVFPEGTISDKTHGGDRYEQGHVYFGLHIRLVSTK
SDKVLLRFKMTILDQSGNGDHWTQSSLHLSPVSIAPLQDSNFPCFIDREILESNNSSYLRDDCIKIECTIGVVGPPDMRL
ISIPKYCNDVGVDFLAMFKAGEGCDVVLNVRGHKFRAHRVILSARSSVFKSMFAPSKKEIIIGNVEPRVFKALLHFIYSD
TFPEDEKCLVDGYAFGAASVSSTMAAKLFAAANQYDMKRLKSICESHLWRSISLKRFGEILSLAELCNAPDLKLLCFKYA
AEYYD
>Mgut_mgv1a007421m
MIVNCSEPNNGLDSSSRSVNETVNGSHHFTIRGYSLAKGMGPGKYIASDTFSIGGYEWAIYFYPDGKNPEDSSMYVSVFI
ALASEGTDVRALFELTLSHQSGKGKHPLHSHFDRALESGPYTLKYRGSMWGYKRFFRRTSLETSDFLKDDCLSMHCTVGV
VRTRVEGPKSYSVIVPPSDMGQSLKYLLDSETGCDITFQVGEESFRAHKLILAARSPVFRAQFFGLVGNPNSDKVEIEDV
EPFIFKALLQFIYSDELPSFDEIMDSTTTPSAIMMQHLLAAADRFGLDRLKQLCEVKLCEEVNAETVATTLSLAEQHRCP
QLKAICLKFAATNLGVVMQSEGFRHLEESCPSLLSELLETVASVDEKPNSVSGNNKKRSGSSIFGLDLAADGVAESVNPN
GRRLRRRA*
>Mgut_mgv1a009478m
MIVNCSEPNNGLDSSSRSVNETVNGSHHFTIRGYSLAKGMGPGKYIASDTFSIGGYEWAIYFYPDGKNPEDSSMYVSVFI
ALASEGTDVRALFELTLSHQSGKGKHPLHSHFDRALESGPYTLKYRGSMWGYKRFFRRTSLETSDFLKDDCLSMHCTVGV
VRTRVEGPKSYSVIVPPSDMGQSLKYLLDSETGCDITFQVGEESFRAHKLILAARSPVFRAQFFGLVGNPNSDKVEIEDV
EPFIFKALLQFIYSDELPSFDEIMDSTTTPSAIMMQHLLAAADRFGLDRLKQLCEVKLCEEVNAETVATTLSLAEQHRCP
QLKAICLKFAATNLGGACSP*
>Mgut_mgv1a003173m
MVSEHSLSTPHHHHPPPTKFSTSFANRIFSDVAGDITIIVDGESFLLHKFPLVARSGKIRKLVADAKISNSNLSKLELNN
LPGGPETFELAAKFCYGMNFAITSANVAPLRCAAEYMEMTEDYRDENLIERTQTYLIEVVSHSLQKSVEVLSSCENLLPL
ADEIGISDRCVCAITTTACKEQLVSGLSRLDCHSGSLEPKGRCHEWWIEELSVLGVDFYHRVISAMGQSGVRTDSIVASL
MHYAHTSLKGIGKPHLWNPARTNNPGFVENAQKTIVESLVTLLPTEKSSSVIPLTFLFGMTRVAIMVDATLACRIELERR
IAYRLEMAILDDLLIPCVQTGESLFDVDTIHRILGHFLQRIEEEEENEEFGYESDGHGSLLKVGRLIDSYLAEIAPDPYL
SLAKFISMIEVLPDYARVIDDGLYRAIDIYLKAHQTLNENECKKLCKFIDCQKLSEEACNHAAQNDRLPVHLVVRVLYFE
QLRLKNALSGSCGDGLLSQKISSGVASAAMSPRDTYASLRRENRELKLEISRMRVRLSDVEKEQLVMKQGMMDKSSQGGK
TLLTSISKGIGRIGIFKAQNQDQHNKRGKKLKSAGRGKRHSVS*
>Mgut_mgv1a007584m
MSETPEKTPNPNPLISPTSSRSVTETVNGSHRFVIQGYSLAKGMGIGKHIASDDFTVGGYKWAIYFYPDGKNPEDNSTYV
SVFIALASEGTDVRALFELTLVDQSGKGKHKVHSHFDRSLESGPYTLKYRGSMWGYKRFFRRAMLETSDYLKDDCLKINC
TVGVVVSAIDCSRLHSIQVPDSDIGADFGTLLENMDGADISFDVAGEKFQAHKLVLAARSPIFRSEFFDVMDSDEQEIVV
SDMEPKVFKAMLHFIYRDALVEDELIASSSCSSPLITDTLTAKLLAAADRYDLGRLRRMCESHLCKDISVNSVAHTLALA
DRFHAAELKAICLRFAAENLAAVMRSDGFEYLKENCPALQSELLKTVAGCEEDCSSGGGKSRSVWAQLSDGGDTTGRRVR
QRT*
>Acoe_002_01212
MELGQDEKLHKGSQKIEIKDYTKLKGSGIGTMIKIYYFTFGGHGWMIYFYPNGVTVDDREFVSFFIDMIDDRKKQVKALI
ELKLVDQSGKGNDVSFKNSTSFSMKGRGTSWGFKRFYKRANLENSDFLKDDSLVIFVTIGIVVSKISNSWRVYVPESDMG
RYIGKLLNNRLGCDVVFNVAGENFHAHKVVLAACSPYFRDQFFKANKADMVEEGEIVVIKDMKPEVFKAMLHFIYTGTLL
EDGMLVEFISSSEIYDETLLGEAELHGLEGFKLKFYSYLWANISANNAAKIFTFANRHDDAMELKSACLSFLVENLAAVV
QSNGLKCLKEDFSLLHSEIFQMVPGAEEKYSRGEKIRNSWTRLSDFRYDLGVLLEDPVGCDVVFNVAGKKFDAHKVVLAA
RSPVFQDQFFENGQAKAMDESEIGVSNVEPEVFKAMLHFIYRDSLLVDNELNRPTASSSKSSALDTFVAKLFVAAYRYRL
TRLACMCQSHLCKDLSVISVGETHALAVSHGATDLKFACLHFALENLTAILRVNGLNYLKANFPSLLFDVRMEECERELA
MMYTKGG
>Acoe_003_00750
MKVVIPSSSSDDNDGSNNVGEGRELRGGGGGGGTTIESSGVALDSSNLVSLCDHIQLEGFNSGSFSDVVVQVMNSTYHLH
RLILSRSSYFRNMLHGPWKEAHAPVLNLHVDDDNVNGDAIAIALAYLYGHHPKLNDSNAFRVLAAASFLDLQDLCAICTD
FIISELWTSNFLAYQIFAEGQDYGIHGERVRNACWGYLCQSGDMELKEVLPKLSSQTLQALLTSDELWVPTEEKRFELAL
YTLLAKAALLQAEQPEHGSPSFEMGRSAHCDSSGEKAENVIDNNRDEKLIEAENISLKDDCDGHKTAHNILVQLADCVVD
FHSGISDSKPLGQEVAGSQLNQETRYPDQKDQAASMLPSLSHPDGSRTSYSYVEMPTSVEASRMRGSGVAMEGPSEESTC

YHVNTNVWLPRDQTRQSSSASSSCNNNMPNEWGRCGMPPPSWGGRVVGRRQVKNHAKGYSAMLGEEYDAFINIFEGGSLL
YCNMSFEALLNVRKQLEEMGFPCKAANDGLWLQMLLSHKVQEIGADTCKNCCLTSMACACRQYGFPQGTTTGYYMQDHDR
NNLSGSMGNIYVTDAAQGEGNGFSRPVRVHVRGPIDGLAGIGRGTTFVPATSWQPTRFVFSRVPFGARADYNGEFSGDGL
TALVGLSQGGGNAVPVHGEQVVRIYEPDVQSRYSGATVSGPSNSGIPMQMLESSDHDGGLEWENGDGSSISLDTKTPLRH
FPPFRFGVEFEDVHRLNDGQVKHSTEVFYAGSLWKVSVQAFNDEDPQGRRTLGLFLHRRKAEVSDSLRKVSPFTGKRKIM
MI
>Acoe_003_00831
MGVSFGELKPSISGKRSFRPSLSARHTTEWPVSDVSSDITVDVGTTNFSLHKFPLISKSGKIRKMLSEAKDSKVSRINLQ
AAPGGSEAFELVAKYCYGMNIEITLANVAMLRCSAHYLQLTEEYAENNLEMRTEVYLREMVLINISNSIAVLHCCESLLP
VAEEINLVNRIINGIASNACKEQLVSGLSKLENNFPVKPITTVEPETLLDWWGRTLTVLNLNFFQRVVSAVKSKGLSQDV
ITRILIIFAQNSLQGLVVRDPQMIQENFLDVELHKKQRVVVEAIVSLLPTQSRKSTVPMAFLSSLLKIAIASSATSTCRS
DIERRIGLQLDQAILEDILIPVNTQGNNHHPLYETDAIFKIFSMFLNVDEDDSEESHTRDENGLGYDFDNPGSPKQSSIL
KVSKLLDSYLAEIALDSNLSPSKFIALAELLPDHARAGSDGLYRAVDIFLKVHPSIKDTERYRLCKTIDCQKLSQEACSH
AAQNERLPVQTAVQVLYFEQIRLRNAMNGGHNQLFYSSTNSHFPQRSSSCGGSGAISPRDNYASVRRENRELKLEVARMR
MRLNDLEKDHVSMKQDFVRTNPANKILKSLSRKLRNLNALFRIHNIKPMGVKTNSENRFVFQKRRHHSLS
>Acoe_005_00177
MDCSICGSMPIALRTPRNLICLTCYEGAKSLITFVSNNVENVNSPVSQSNSSAKGFANAMKLVKEMKEREEMLNEKLGFL
SGLLVAFRDELHSDIQVKPGNGPSISAHRSLLAARSIIFKNILEESDGCKAPPTETISLPELDHEELQSLLEFLYSGSLP
SEKISKHVYALYTAADKYDIPFLQKFCEHWMLGSLNLSNALNILETSDVCPTTTLKDSAMNFIVNHMEDIVFSTAYDAFA
LKNPHLNVQLTRALLIETKRKNHDSPV
>Acoe_007_00103
MGTEKPSSKGQAWFCTTGLPSDIVIEVDEMCFHLHKFPLMSKSRKLHQLITEQETSSPASSTEQKDDEQDENEEQIELHI
TLPDFPGSSETFEIAAKFCYGVKIEISSSNVVSLRCAAEYLEMTEEYSEDNLISKTESFLTQSVLRSIKESIKTLISCES
IMDLAESLNIPKRCIDSIAARACSADPSFFGWPMNEATIEVRNSNQILWNGIDTGARKKASRSINAESWFEDLAFLSLPL
YKRIISAMKDRDLSSEIIESLLISYAKKSIPGISRSTRKQTANSIATEAEQRVLLETLITNLPLEKSSRSSTAVRHLFGL
LRTANILNVSEASRTILEKKIAWKLEQASLDDLLIPSYSYLIETLYDVDCIERILKYFLGNLHEKAANEMDGGDVEDGEA
IAGSPVMLVGKLIDGYLSEIASDANLKPQKFIDLAVALPDQARSFDDGLYRAVDVYLKAHSTVTEEEREKISGVMDCQKL
TLEACTHAAQNERLPLRAVVQVLFFEQLQLRHAIAGSLLAATSDHQDSPLPCVLQLEHQESVEHLHENHNNTWKAAIREN
QVLRLDMDSMRSRVHELERECSTMKKVIHQIDKNVAVGSSGGWRNTISKKFGCKFKTQVCDSRERAVVETRKGSHQRQTS
L
>Acoe_007_00874
MDGKKQKKYLTVAPFECAWRKELRFREAGRGCVAFEAFAHNDVTVVFREHVGSQNYHYKMDNSPHYTVIIGSHRNRRLKI
EVDGKTVVDEAGIGLCCSSAFKSYWISICDGLISIGKGQFPFQNLVFQWLDSNPNCSVQYVGLSSWDKHVGYRNVNVLPL
KQNHISLWKHLNHIENEQDENDEDEPENNSESYDDWGLKNFLESWDLSDMFFVVGEEEKVVPAHRIILSLSGSFPFSSSS
EDVIQIPEVAYPILHALLQYIYTGRTEIVEPQLGFLKDLGQQFVVISLVKQCEEVIDRVKLNKKLFDLGKTVEISHPCSR
PQRCAVFPFELPINVQKLKESISVGSYTDVDIYIDGHDLVARCHKLILSLWSAPFMKMCTNGMCESKSSQIILRDVSFEA
FTTMLEFMYSGELNVEDTVDAGRLLIQLLLLADQFGVAPLHQECCKIILECLSVDSICPILQVVSPISSCELLKETCERK
FSRHFDYCTTASSNFVLLDEATLGGILQHPSLTVTSEERILDAILSWGILTNELYGWEAVDELLISSRPELLFGDRLKSI
EALLPLVRFPLMSLSLLKKLAKSNLSVRIPEFGKLVKEAIRYNDSGMTQPGDNQNARFHHRQSSFKELQYICDGDKNGVI
YFAGTSYGIHQWVNPVLSKKITVTASSPASRFTDPKALVSRTYQGTSFAGPRVEDGRNCSWWMVDVGQDHQLMCNYYTLR
QDGSTAFIRSWTLQGSLDGQSWTNLRVHENDQTMCKPAQFASWPVNDPTALLPLRFFRVLMTGPTTSDSTPWNLCVCFIE
LYGHFHMDGKKQKKYLTVAPFECAWRKELRFREAGRGCVAFEAFAHNDVTVVFREHVGSQNYHYKMDNSPHYTVIIGSHR
NRRLKIEVDGKTVVDEAGIGLCCSSAFKSYWISICDGLISIGKGQFPFQNLVFQWLDSNPNCSVQYVGLSSWDKHVGYRN
VNVLPLKQNHISLWKHLNHIENEQDENDEDEPENNSESYDDWGLKNFLESWDLSDMFFVVGEEEKVVPAHRIILSLSGSF
PFSSSSEDVIQIPEVAYPILHALLQYIYTGRTEIVEPQLGFLKDLGQQFVVISLVKQCEEVIDRVKLNKKLFDLGKTVEI
SHPCSRPQRCAVFPFELPINVQKLKESISVGSYTDVDIYIDGHDLVARCHKLILSLWSAPFMKMCTNGMCESKSSQIILR
DVSFEAFTTMLEFMYSGELNVEDTVDAGRLLIQLLLLADQFGVAPLHQECCKIILECLSVDSICPILQVVSPISSCELLK
ETCERKFSRHFDYCTTASSNFVLLDEATLGGILQHPSLTVTSEERILDAILSWGILTNELYGWEAVDELLISSRPELLFG
DRLKSIEALLPLVRFPLMSLSLLKKLAKSNLSVRIPEFGKLVKEAIRYNDSGMTQPGDNQNARFHHRQSSFKELQYICDG
DKNGVIYFAGTSYGIHQWVNPVLSKKITVTASSPASRFTDPKALVSRTYQVCWRYTSCNVIMDGKKQKKYLTVAPFECAW
RKELRFREAGRGCVAFEAFAHNDVTVVFREHVGSQNYHYKMDNSPHYTVIIGSHRNRRLKIEVDGKTVVDEAGIGLCCSS
AFKSYWISICDGLISIGKGQFPFQNLVFQWLDSNPNCSVQYVGLSSWDKHVGYRNVNVLPLKQNHISLWKHLNHIENEQD
ENDEDEPENNSESYDDWGLKNFLESWDLSDMFFVVGEEEKVVPAHRIILSLSGSFPFSSSSEDVIQIPEVAYPILHALLQ
YIYTGRTEIVEPQLGFLKDLGQQFVVISLVKQCEEVIDRVKLNKKLFDLGKTVEISHPCSRPQRCAVFPFELPINVQKLK
ESISVGSYTDVDIYIDGHDLVARCHKLILSLWSAPFMKMCTNGMCESKSSQIILRDVSFEAFTTMLEFMYSGELNVEDTV
DAGRLLIQLLLLADQFGVAPLHQECCKIILECLSVDSICPILQVVSPISSCELLKETCERKFSRHFDYCTTASSNFVLLD
EATLGGILQHPSLTVTSEERILDAILSWGILTNELYGWEAVDELLISSRPELLFGDRLKSIEALLPLVRFPLMSLSLLKK
LAKSNLSVRIPEFGKLVKEAIRYNDSGMTQPGDNQNARFHHRQSSFKELQYICDGDKNGVIYFAGTSYGIHQWVNPVLSK
VNFMDGKKQKKYLTVAPFECAWRKELRFREAGRGCVAFEAFAHNDVTVVFREHVGSQNYHYKMDNSPHYTVIIGSHRNRR
LKIEVDGKTVVDEAGIGLCCSSAFKSYWISICDGLISIGKGQFPFQNLVFQWLDSNPNCSVQYVGLSSWDKHVGYRNVNV
LPLKQNHISLWKHLNHIENEQDENDEDEPENNSESYDDWGLKNFLESWDLSDMFFVVGEEEKVVPAHRIILSLSGSFPFS
SSSEDVIQIPEVAYPILHALLQYIYTGRTEIVEPQLGFLKDLGQQFVVISLVKQCEEVIDRVKLNKKLFDLGKTVEISHP

CSRPQRCAVFPFELPINVQKLKESISVGSYTDVDIYIDGHDLVARCHKLILSLWSAPFMKMCTNGMCESKSSQIILRDVS
FEAFTTMLEFMYSGELNVEDTVDAGRLLIQLLLLADQFGVAPLHQECCKIILECLSVDSICPILQVVSPISSCELLKETC
ERKFSRHFDYCTTASSNFVLLDEATLGGILQHPSLTVTSEERILDAILSWGILTNELYGWEAVDELLISSRPELLFGDRL
KSIEALLPLVRFPLMSLSLLKKLAKSNLSVRIPEFGKLVKEAIRYNDSGMTQPGDNQKMDGKKQKKYLTVAPFECAWRKE
LRFREAGRGCVAFEAFAHNDVTVVFREHVGSQNYHYKMDNSPHYTVIIGSHRNRRLKIEVDGKTVVDEAGIGLCCSSAFK
SYWISICDGLISIGKGQFPFQNLVFQWLDSNPNCSVQYVGLSSWDKHVGYRNVNVLPLKQNHISLWKHLNHIENEQDEND
EDEPENNSESYDDWGLKNFLESWDLSDMFFVVGEEEKVVPAHRIILSLSGSFPFSSSSEDVIQIPEVAYPILHALLQYIY
TGRTEIVEPQLGFLKDLGQQFVVISLVKQCEEVIDRVKLNKKLFDLGKTVEISHPCSRPQRCAVFPFELPINVQKLKESI
SVGSYTDVDIYIDGHDLVARCHKLILSLWSAPFMKMCTNGMCESKSSQIILRDVSFEAFTTMLEFMYSGELNVEDTVDAG
RLLIQLLLLADQFGVAPLHQECCKIILECLSVDSICPILQVVSPISSCELLKETCERKFSRHFDYCTTASSNFVLLDEAT
LGGILQHPSLTVTSEERILDAILSWGILTNELYGWEAVDELLISSRPELLFGDRLKSIEALLPLVRFPLMSLSLLKKLAK
SNLSVRIPEFGKLVSMLWIRE
>Acoe_009_00556
MMRESGTLEKSGGLISPTSSRSVTETVNGSHKFVIQGYSLAKGMGIGKHIASENFTVGGYQWAIYFYPDGKNPEDNSTYV
SVFIALASEGTDVRALFELTLVDQSGKDKHKIHSHFDRSLESGPYTLKYRGSMWGYKRFFRRAMLETSDFLKDDCLKINC
TVGVVVSTIDCSRLHSIQVPDSDIGNHFGMLLENQEGSDVLFNVAGEKFHAHKLVLAARSPVFRSEFFEGMDEDRNEIVI
TDLEPKVFKAMLHFIYRDTLMEDEELVSSSSSCASSTSDTLAANLLAAADKYGLDRLRLMCESYLCKDISVNSVAKTLAL
ADRHHAMELKAVCLKFAAENLAAVMRSNGFEYLKENCPSLQSELLKTVAGCEEECSSGGGKTRSVWAQLSDGGETNGRRV
RQRTMMRESGTLEKSGGLISPTSSRSVTETVNGSHKFVIQGYSLAKGMGIGKHIASENFTVGGYQWAIYFYPDGKNPEDN
STYVSVFIALASEGTDVRALFELTLVDQSGKDKHKIHSHFDRSLESGPYTLKYRGSMWGYKRFFRRAMLETSDFLKDDCL
KINCTVGVVVSTIDCSRLHSIQVPDSDIGNHFGMLLENQEGSDVLFNVAGEKFHAHKLVLAARSPVFRSEFFEGMDEDRN
EIVITDLEPKVFKAMLHFIYRDTLMEDEELVSSSSSCASSTSDTLAANLLAAADKYGLDRLRLMCESYLCKDISVNSVAK
TLALADRHHAMELKAVCLKFAAENLAGDFFVQLLFPFFTQKQCNELMVNKWQVFIFNKISFILVREWRRKSKMKLSLISM
MRESGTLEKSGGLISPTSSRSVTETVNGSHKFVIQGYSLAKGMGIGKHIASENFTVGGYQWAIYFYPDGKNPEDNSTYVS
VFIALASEGTDVRALFELTLVDQSGKDKHKIHSHFDRSLESGPYTLKYRGSMWGYKRFFRRAMLETSDFLKDDCLKINCT
VGVVVSTIDCSRLHSIQVPDSDIGNHFGMLLENQEGSDVLFNVAGEKFHAHKLVLAARSPVFRSEFFEGMDEDRNEIVIT
DLEPKVFKAMLHFIYRDTLMEDEELVSSSSSCASSTSDTLAANLLAAADKYGLDRLRLMCESYLCKDISVNSVAKTLALA
DRHHAMELKAVCLKFAAENLAGTPQVID
>Acoe_009_00695
MEIHANKETITVCNVGDKSTSDVVVRIRTQEGRDEWFYCHSHILVEKSKYFADRLSEKWPTCQILDSRHCVEVYCQESEF
NHHVSLLRLLYVITNGSTTNTWHGVKNALGILQVAAELECEQIVHECVEYLEAMPWEEAEEEEILRITPRLGAQAELILA
RLQPVNPTAITGIFLSAIRFATSSPSSSMNDLKSSAQEQLEYMLTEDDDAPLLAANDEIKSEVRECSQNLLKRFYGLLEP
FVCNPMGLVLDADKLLELHSLLSDLAWASQILTKLDALREFVQSWVEASEKIVKAVDVKTTDPDMLEILVKVIEVASKIL
EAIAYGTVILPTSKRLHTLKLWLPFVRRKKPLVDSVTADSVTANDTDTPSFKIDGELWQSLESAFVSMVLALPSGDQAEI
LTDWLRNEYIRYPDLTEAFEVWCYRSKVAKRRLEGIPCVTNSSVTKSSCQAPSLSS
>Acoe_009_00883
MGKSDTKSTENKKRQSIADSSQNSCSKSINETVNGSHEYTIKGYSLAKGMGAGKYIQSDIFTVGGYDWSVYFYPDGKNPD
DNSGYVSVFIALASEGTDVRALFELTLLDQSGKGKHKVHSHFDRALESGPYTLKYRGSMWGYKRFFKRTMLEASDFLKDD
CLSMHCTVGVVRTRTEAPKLYTVPMPPSDMGLNFKEMLESGIGSDIMFEVGEETFRAHKLILAARSPVFKAQFFGLIGNP
NMDKVVVEDVEPAVFKGMLLFMYSDELPDLLDLVSSDPECSSAILVQHLLAAADRFGMDRLKLLCEAKLCEEVTTETVAT
TLALAEQHHCSQLKTVCLKFAAVPGNLGEMMKSEGYRYLEESCPSLLSELLETIAVGDEDSSQQSSKKRTSSSNIGLNID
HLIDQNLRRLRRRV
>Acoe_010_00212
MKKSLQANTSQCVAIVEKFEKKERSWFPTSQIPTDLIIHVQDINFYVHKLPLISKSECLSDLLKSRPSQSNSSCDIKLDN
FPGGAETFELIIKFCYGFSVDLTASNVAPVRCAAEFLQMTEEYEEGNLISKTESFIAFIVLSSWRDSITVMKSCECLSTW
AENLQILRKCTDAVALKIIRRTSMCNDTDYDGERWWFDDISILCIDHFVRIITAIRAKGSKPEIIGASIMHYAEKWLPGM
EELQGQKGFGHRKNELHMTILSGKWEEGNVGQEKEQRRMIEVLVSILPPQREAVPCKFLLSMLKMALVYTATTALVSELE
KRVGMVLGDATVYDLLIPIPSYNNGTEGKLGSPSEERRTMHDVDTIQRIVEYFLMHEQQLQQQQSNVGKLLDGYLTEIAR
DPNLSIAKFQALAEVLPQDARTCHDGLYRAIDTYLKTHPTLMEHDRRRLCKPMDCEKLSLDGCMHAAQNDRLPLRTVMQV
TFSYKVLYSEQIKMRAAMNGKGPMQHDIMCEQDSTWSTKKEIKNIISELQKIRTSIEELQHDYSQLQQEFEKANKQKIVS
GWSSGWKKFKTSTLFHGKMNAMDTENVQLGQTTVSSKNSRRRRKSISMKKSLQANTSQCVAIVEKFEKKERSWFPTSQIP
TDLIIHVQDINFYVHKLPLISKSECLSDLLKSRPSQSNSSCDIKLDNFPGGAETFELIIKFCYGFSVDLTASNVAPVRCA
AEFLQMTEEYEEGNLISKTESFIAFIVLSSWRDSITVMKSCECLSTWAENLQILRKCTDAVALKIIRRTSMCNDTDYDGE
RWWFDDISILCIDHFVRIITAIRAKGSKPEIIGASIMHYAEKWLPGMEELQGQKGFGHRKNELHMTILSGKWEEGNVGQE
KEQRRMIEVLVSILPPQREAVPCKFLLSMLKMALVYTATTALVSELEKRVGMVLGDATVYDLLIPIPSYNNGTEGKLGSP
SEERRTMHDVDTIQRIVEYFLMHEQQLQQQQSNVGKLLDGYLTEIARDPNLSIAKFQALAEVLPQDARTCHDGLYRAIDT
YLKTHPTLMEHDRRRLCKPMDCEKLSLDGCMHAAQNDRLPLRTVMQVLYSEQIKMRAAMNGKGPMQHDIMCEQDSTWSTK
KEIKNIISELQKIRTSIEELQHDYSQLQQEFEKANKQKIVSGWSSGWKKFKTSTLFHGKMNAMDTENVQLGQTTVSSKNS
RRRRKSIS
>Acoe_011_00389
MKTTHTQDTNETMTISPASFSDEMYGLRPLSIGVHKSSIEAPEADVRIVTSGGLSIPAHSSVLASASRVMEKILDQPRKY
RNSNKIIQILGVTNEAVVTFVRFLYSSRCSEEDIEMFGMHLLALSHVYSVSKLKQRCTKGLAGRLTIENVIDVLQLARLC

DAPDLYIKCMKYVSKQFKAVEMTEGWKFLHNHDPWLELEILQYLDEVDLREKRRRKNKKEQSLYLQLSEAMDCLEHICTE
GCTNVGPYDMDPIKSKTKKPCSKFSTCQGLQLLIRHFATCTKRVNGGCSRCKRMWQLLRLHSSMCDEPDHCRVPLCRQFK
LKMGQDRKGDDARWRLLVRKVVSAKVLSSLSMAKRMRCPSWPRHK
>Acoe_013_00045
MDKAGASKTYILGDRSSSDIVVRLRNREKRPEWFYCHSSILIEKSKYFADRLALELTNCIEVYCSEYDYDYHVKLLKLLY
VCTDLLIDSWDSIRTALGVLQAAVSLHCEEITQSCIQYLEAVPWEDKEEEEILKAVSGLGSLAMPILARIQPVDLNATKN
VFLSAIRIATSRTGAYPPFADELKTSAQEQIEYMLGEDEDTPLITADEEVKSEVRMGLSNMLSVFTNELSSLLLDSNISS
EAAENEVLQSLLNLDWMCNILPKMDLLRDFVFSWVENSNHVLAIVQDKKFDSIIWGVKMKLIEVTGKVLEAVGYGNVILP
APIRVQLLKIWLPFIRKMKPLLDSKGTEEINFPFKMDDDLCQSIEGAIISLVLALPSNDQADILSEWMQTEELRYPDLSE
AFEVWCYRTKSAKRRLVVGFDSAGNATVSL
>Acoe_013_00174
MKKGVLDLFDPKTVMDSDSSEGSKNGFLSDRDFGFAFNDSNFSDRTLRIEIVDGMAECKSDGEGCNSISDWARNRKRRRE
EIKKDNATELSVYRDERILDLNCTQFDAEDENQDDEAVAMIGDSQSGNEAVQDNSSPTSSMDCSMDAVVRVRTIHISSPI
LAARSPFFYKLFSNGMRESEQRRVTLRINASEEAALMDLLSFMYSNTLSANTAPALLDILMAADKYEVASCMRSCSRKLR
NLPMTPESALLYLELPSSILMAEAVQPLTDAAKQFLAARFKDITKCQDEVLNLPLAGIEAIFSSDDLQMASEDAVYDFLL
KWARIHFPKLEDRREILGRRLIRLIRFPFMTCRKLRKVLTCIDFDHDLASKAVLDALFFKAEAPHRQRSLAAEESAAISR
CFVERAYKYRPVKVVEFEAPRQQCVVFLDLKREECANLFPQGRVYSQAFHLGGQGFFLSAHCNMDQQNNFNCFGLFLGMQ
EKGTVSFSVEYEFSARAKPTEEYMSKYKGNYTFTGGKAVGYRNLFALSWQSFMAEDSLYFINDILHLRAELTIKH
>Acoe_013_00243
MDFSNSSLTSAGYGSYPSSYKNFNFALNAGLLNPSSPPPSILDKTRSSPTLFEMMASEQDCNPRTQISSINNINNIPIVN
GKTPISVQDKQLLLHQRLADILGNCSPGNQFNDPTTSDVKLTLSSKDGLSVSMNVHRQILVAHSRFFATKLSDQWSKQQR
SLPHIVEISDCDDVEVYVEALRLMYCKDLRRKLIKEDVSKVLGILKVSAAIMFDAGVLSCLEYLEAVPWAEDEEEKVAVL
LSQLHLPGAGAGEVLKRVSAEVMVEPEEGSNNEEVLVKLLQVVLEGKDEKARREMKGLVLKMLRENSSQNDLSKESLYSA
CDGCLQLLRLHFLQAAATSSDLNDIHQIKRQADNLHWLLDIMIDRQIADDFLKTWADQSELSEVHSRIPAIHRYEVSRVT
ARLFVGIGKGQLLASKEVRCMLLQTWLEPFYDDFGWMRRASNGLDRHLIEDGLSNTILTLPLAWQQEILLTWFDRFLNSG
EDCPNIQRGFQVWWRRAFWKRFGESEQHHL
>Acoe_014_00231
MTSSSSSSSGVGDSKVETISRLAQWRIDSFGPCSYRRSDPFKVGIWNWHLSVEKNRYLYVRLFPEPSKLCKDQPPIARFV
LRVSNIGTNRRPYISPIHERLLRTSEDFNWPVDCAFHGRFIIDVEFLDLKICPVNGVEASSIWPSEGFVNTLSSQGSLRC
LSRMLDEGIHADVTINAADGTLRAHKAILSASSSVFHSMFLHDLKEKESSTIDIDDMSMESCTALLSYIYGTIKQIDFWK
HRLALLGAANKYDITLLKDACEESLLEDINSGNVLERLQEAWLYQLEGLKKGCLMYLFDFGKIYDVRDEINNFFRNADRE
LMVEMFQEVLTVWKPA
>Acoe_014_00660
MSLEDSFKSLSLDYLNLLINGQAFSDVTFSVEGRLVHAHKCILAARSLFFRKFFCGPDPPPAGLDPAGGSRMNSSSSPRA
GNSQVIPVNSVGYEVFLLMLQFLYSGQVSILPQKHEPRPNCGERACWHTHCTSAVDLALDTLAAARSFGVEQLALLTQKQ
LASMVEKASIEDVMKVLLASRKQDMHQLWTTCSHLVAKSGLPPEVLAKHLPIDVVAKIEELRLKSSLTRRSLMPHHHHHH
HHHDLSVAADLEDQKIRRMRRALDSSDVELVKLMVMGEGLNLDEALALHYAVENCSREVVKALLELGAADVNYPAGPAGK
TPLHVAAEMVCPDMVAVLLDHHADPSVRTVDGVSPHDILRTLTSDFLFKGAVPGLSHIEPNKLRLCLELVQSAALVLSRE
EGNASNPPNNSIYPPMSNDHSNSNLVSLSLDSRLVYHNLTAAQLGCKMDGDDESSHSNQRDHQVMNRHGSQGGCDPSMYH
SHDYMSLEDSFKSLSLDYLNLLINGQAFSDVTFSVEGRLVHAHKCILAARSLFFRKFFCGPDPPPAGLDPAGGSRMNSSS
SPRAGNSQVIPVNSVGYEVFLLMLQFLYSGQVSILPQKHEPRPNCGERACWHTHCTSAVDLALDTLAAARSFGVEQLALL
TQQLASMVEKASIEDVMKVLLASRKQDMHQLWTTCSHLVAKSGLPPEVLAKHLPIDVVAKIEELRLKSSLTRRSLMPHHH
HHHHHHDLSVAADLEDQKIRRMRRALDSSDVELVKLMVMGEGLNLDEALALHYAVENCSREVVKALLELGAADVNYPAGP
AGKTPLHVAAEMVCPDMVAVLLDHHADPSVRTVDGVSPHDILRTLTSDFLFKGAVPGLSHIEPNKLRLCLELVQSAALVL
SREEGNASNPPNNSIYPPMSNDHSNSNLVSLSLDSRLVYHNLTAAQLGCKMDGDDESSHSNQRDHQVMNRHGSQGGCDPS
MYHSHDY
>Acoe_016_00240
MKFMKLGSKPDAFQADGNCIRYVASELATDIVVNVGDVKFYLHKFPLLSKSNHLQKLVSKANDDNTNEIDMINFPGGSKA
FEICAKFCYGMTVTLNAYNVVAARCAAEYLEMKEDVDRGNLIFKIEVFLTSSIFRSWKDSIIVLQTTKALLPWSENLKVV
GRCIDSIASKTSMDPSNVNWSYTFNRKLTVSDQITESTDRFQQKFNPVPKDWWVEDICELEIDLYKRVMFAIKAKGRMSG
NVIGEALDCYAVRWLPDSVTGLVSEEHIRQNKILVETIIWLLPSSKDVCCSCSFLLKLLKFAILVGAGDGPREELMKRIA
FQLDEACAKDLLIPSTSDETTIYDLELVQSLVNRFVLHHAQNTDYEKNEKGTDDLVLGRVLWLKIGNLIDAYLAEIARDP
NVTVSSFVSLSQAIPPSARPIHDGLYRAIDIYLKEHPSLTKTDKKTLCGLMDVKKFTTDACMHAAQNERLPLRVVVQVLF
FEQVRAASVGMNPPQNISCPSSRSTANTEDWGKTVPEDYKSLDKQMKSMKIADEDCRKNGKKYNKKSSKNKGGGALLLPS
RSRRIFDKLFVSSKGHGENKSSETSGSSMSPTSLNPGETKSSGSSSRNRRHSIS
>Acoe_016_00420
MTSTTKNNNTSRLSTAMERTGQWIFSQDIPSDVVVEVGNTNFSLHKFMLVAKSGYIRKMIIESNEPDLTRIDLSDIPGGA
AIFEKTAKFCYGVNFEITVHNVAPLICAAEFLEMTDEYCEGNLAGRTEEFLTQVALMSFSASLVVLKTCEDLLPMAHELN
IVQRCVDVVSSKACNEANFPSRSPTNWWTEELTILDVDSFKKVISSMKSRGAKVHTLSSALITYTEKSLRDLVRDHSGNN
LKSSESNDVGVKKQQRNILEVIVSLLPSEKATLPVNFVCCLLRTAIFLNASTSCKNDLEKRISAVLEHITVDDLLVVSFN
YDGEKLFDLESVRRIISGFVEKEKSVAVFSGGDLKEVSSTAMQRVAKTVDAYLSEIATYSELSISKFNGIANLIPKAARK
VDDDLYRAIDIYLKAHPDLDEIEREKVCSVMDALNLSYEARVHASQNKRLPVQIVLHALYYDQLKLRSGDDDRSMPDAVT

TRNQLQTDVSLVKENEVLRTELLKMKMYVSDLQKNQASSSKVVVAPKKPKFFSSVSKTLGKLNPFKHGSKDTLNIDDGEV
EVTKPRRRRFSIS
>Acoe_017_00056
MRGWKDLELVETIYEEEDHDETYSSNSSSLSPTLTHSPPTPLQSIAKTWSLATGIETDVVIHVQDKSFHLHKDSLIAKSG
YLKRRLKDSNDITISPPLNITAETFSSIVDYCYSYQIVITPVNVAALKVAVELLDITDDNGNGVNNLRNKTESYFDHAVS
VNREYATIVLRSCLTLLPEAESASMVSRCIEALTLTHHGGGGGGDNVNGWVDDIMSLTTEDFQLIADSMHEQFSDNHDLL
YRIADLYLQEHTGKITEEEKTRICSSVDCNKLSPHLIMHAVQNPRMPLRFIVRAMLVEQLNTRCTIISTTTSAVNNHHPR
HYPQNSNENNQSMTLGAMLQRDAAFRQVAQLKATMEATSSKIETLEKDLLRMKKVLDSSKKKRVSSDSGRSASFRFSSER
KIEKEERGSRSTSLRFSDKRVKGDVSLSSSSSEISMNESVSSKREKSFGWKLMNGLKTALRMSSPTSKQDSKSSVSNKVD
GNGLQDVEEDGSIIVYREERPSHLRSRSLV
>Acoe_017_00245
MGVATFAELKPVIPGKFRSYRPSSSIRHVVEWPISDVSSDLIVEVGARSFSLHKFPLISRSGRLRKMLSEASDSKVSRIT
LTNVPGGSEGFELAAKFCYGVSIEITLSNVAMLKCVSHYLEMVEDFAEKNLDSQAEIYMKNLVLPKISNSISVLHCCESL
LPIAEEINIVSRIINGIACNASKEQLVSGLSKLEKFAVIPPNIEPDTPPDWWGKSLTMLSLDFLQRVLSALKTKGLKQDT
ITKILINYAQKSLHGLVARDPQIIKGSFSDAELRKKQKVIVEAIVGLLPNQSRKCSVPISFLSGLLKTGILASASIVCKA
DLERRIGLHLDEAILEDILIPANSHNNNHHTLYDTDTISRIFTIYVNVEEEDNDENHMTIENEMDYDFDIPLSPKQGSIL
KVSKLLDSYLAEVALDSNLILSKFIALAELLPDHARVVEDNLYRAIDIYLKAHPNVKDSERYRLCKTIDCQKLSQEACSH
AAQNERLPMQMAVQVLYIEQMRLRNAMNGGHNQFFGSIHGQFPQRSNSAAGSGAISPRDNYASVRRENRELKLEVARMRM
RLTELEKDHVSMKQELVRSNPTNTLLRSFTKKLKKLNTLFHVKDDIKPISSSSSDTWFLFQKKRRHSLS
>Acoe_017_00398
MVMGRVCSETSKPSSPITTSTSITETTNGSHQFKITGYSLSKGLGIGKYIASDTFMVGGYNWAIYFYPDGKSNEDNGTYV
SLFIALASEGTDVRALFELTLLDQSGKERHKVHSHFGRALESGPYTLKYRGSMWGYKRFFKRASLETSDYLKDDCLSVHC
SVGVVKSQTEGPKIYSIAVPPSNIGQHFGKLLESGKGTDVNFEVDEEIFSAHKLVLAARSPVFRAQLFGPMKDRNTECIK
VEDMEAPVFKALLHFMYWDALPDMEELTGLNSKWASTMMNQHLLAAADRYGLERLRLLCEAKLCEDVAINTVATTLALSE
QHHCFQLKAVCLKFVALRENLKAVMQTDGFDYLKVSCPAVLTELLEYVARVGEHSVMVSRHGNEAILDGSDINGRRVKQR
I
>Acoe_017_00539
MRNMFLSESCKETQVHALNPQSWLQVERGKVAKSSSHSPSSIETLIKVPQPPILPFFKPVDYVDVLAQIHEELESCPSYE
RSNLYLLQFQVFRGLEEVKLLRRSLLLAWQKASTVHEKIIFGAWLKYEKQGEELISDLLASCGKCTQEFGPIDIASQLLT
DSNCYLPETFEMIEKHVSSSVFFRIEHEKIPCDRHKIAALSAPFNAMLNGGFTESLQEEIDISKNGITPSGMRAVREFSE
TGSLGDHCPDVLLEVLIFANRFCCERLKDACDRKLASLVSTRQEAVALMEYALEEHSPVLAASCLQVFLYEIPNCLNDDR
VIKIFRDASKYHISIMVGSAAFSLYCLLSEVTMARDPCSDITAYFLERLVESAVSNRQKQLSFHQLACVRLLRKEFNEAE
QLFEAAVSAGHVYSVAGLARLDFIKGQKFRSYEKMSSVISSFSPLGWMYQERSLYCDGEKKWENLEKATELEPTLNYPYM
YRAASLMRKENVQAALAEVNRILGFKLALDCLELRFCFYLALEDYPAALCDVQAILTLAPEYRMFEGRVAATQLCTLVRE
HVENWTTADCWLQLYDRWSSVDDIGSLSVIYQMLESDAAKGVLYFRQSLLLLRLSCPEAAMRSLQLARQNAATEHERLVY
EGWILYDTGHCEEGLHKAEESIQLKRSFEAFFLKAYALADSSLDSSCSSTVVSLLEDALKCPSDRLRKGQALNNLGSVYV
DCGKLDLAADCYISALKIRHTRAHQGLARVHFLRGDRNAAYEEMTRLIEKAQNNASAYEKRSEYCDRELTKADLEMVTQL
DPLRVYPYRYRAAVSMDSHKEKEAIAELSRAIAFKADLHLLHLRAAFHEHIGDVSGALRDCRAALSVDPNHQEMLELHNR
VNSQEPMRNMFLSESCKETQVHALNPQSWLQVERGKVAKSSSHSPSSMGLEEVKLLRRSLLLAWQKASTVHEKIIFGAWL
KYEKQGEELISDLLASCGKCTQEFGPIDIASQLLTDSNCYLPETFEMIEKHVSSSVFFRIEHEKIPCDRHKIAALSAPFN
AMLNGGFTESLQEEIDISKNGITPSGMRAVREFSETGSLGDHCPDVLLEVLIFANRFCCERLKDACDRKLASLVSTRQEA
VALMEYALEEHSPVLAASCLQVFLYEIPNCLNDDRVIKIFRDASKYHISIMVGSAAFSLYCLLSEVTMARDPCSDITAYF
LERLVESAVSNRQKQLSFHQLACVRLLRKEFNEAEQLFEAAVSAGHVYSVAGLARLDFIKGQKFRSYEKMSSVISSFSPL
GWMYQERSLYCDGEKKWENLEKATELEPTLNYPYMYRAASLMRKENVQAALAEVNRILGFKLALDCLELRFCFYLALEDY
PAALCDVQAILTLAPEYRMFEGRVAATQLCTLVREHVENWTTADCWLQLYDRWSSVDDIGSLSVIYQMLESDAAKGVLYF
RQSLLLLRLSCPEAAMRSLQLARQNAATEHERLVYEGWILYDTGHCEEGLHKAEESIQLKRSFEAFFLKAYALADSSLDS
SCSSTVVSLLEDALKCPSDRLRKGQALNNLGSVYVDCGKLDLAADCYISALKIRHTRAHQGLARVHFLRGDRNAAYEEMT
RLIEKAQNNASAYEKRSEYCDRELTKADLEMVTQLDPLRVYPYRYRAAVSMDSHKEKEAIAELSRAIAFKADLHLLHLRA
AFHEHIGDVSGALRDCRAALSVDPNHQEMLELHNRVNSQEPMIEKHVSSSVFFRIEHEKIPCDRHKIAALSAPFNAMLNG
GFTESLQEEIDISKNGITPSGMRAVREFSETGSLGDHCPDVLLEVLIFANRFCCERLKDACDRKLASLVSTRQEAVALME
YALEEHSPVLAASCLQVFLYEIPNCLNDDRVIKIFRDASKYHISIMVGSAAFSLYCLLSEVTMARDPCSDITAYFLERLV
ESAVSNRQKQLSFHQLACVRLLRKEFNEAEQLFEAAVSAGHVYSVAGLARLDFIKGQKFRSYEKMSSVISSFSPLGWMYQ
ERSLYCDGEKKWENLEKATELEPTLNYPYMYRAASLMRKENVQAALAEVNRILGFKLALDCLELRFCFYLALEDYPAALC
DVQAILTLAPEYRMFEGRVAATQLCTLVREHVENWTTADCWLQLYDRWSSVDDIGSLSVIYQMLESDAAKGVLYFRQSLL
LLRLSCPEAAMRSLQLARQNAATEHERLVYEGWILYDTGHCEEGLHKAEESIQLKRSFEAFFLKAYALADSSLDSSCSST
VVSLLEDALKCPSDRLRKGQVIFFISIFVE
>Acoe_020_00445
MDSRTTVVSDSNDNNSGSSSISNETLSPEISAPDIAALKRLSENLESIFESPNSDFYSDAKIIVSGSCDIPIHRCILSSR
SSYFKTLFSSCSSNSSKREKHFKFDLKELMKDFNVGYDSVVLVLGYLYGGKVKTLSRNVCVCVDDDCEHLGCKPAVDSMV
EVLYASFVFQIPELIALYQRRLLDILDKVSVDDILMILSVAKICSKACERLLLECIEIVVKSDIDIITLEKALPHDILKQ
VMESRSELAIGDPECNTFPNKHVKRIHRALDSDDVELVRWLLKEGHTTLDDAYALHYAVAYCDAKTTTEVLDLGLADVNH
RNARGYTVLHVAAMRKEPNIIVSLLTKGARPSDLTSDGKKALQISKRLTRSIDYNRSTEEGKSSPKDRLCIEILEQAERR

DPLLGEASASLAMAGDDLRMKLLYLENRVGLAKLLFPMEAKVAMDIAQVDGTQEFPFAANSRRSSGGQLTPMDLNESPFK
IEEEHLNRLKALSKTVELGKRFFPRCSDVLNKIMDADDLSQLAYLGNDTPEERIVKKRRYVELQDVLVKAFSEDKEELDK
SVALSSPSSSSSLALLRSKNMLNSDMKMDSRTTVVSDSNDNNSGSSSISNETLSPEISAPDIAALKRLSENLESIFESPN
SDFYSDAKIIVSGSCDIPIHRCILSSRSSYFKTLFSSCSSNSSKREKHFKFDLKELMKDFNVGYDSVVLVLGYLYGGKVK
TLSRNVCVCVDDDCEHLGCKPAVDSMVEVLYASFVFQIPELIALYQRRLLDILDKVSVDDILMILSVAKICSKACERLLL
ECIEIVVKSDIDIITLEKALPHDILKQVMESRSELAIGDPECNTFPNKHVKRIHRALDSDDVELVRWLLKEGHTTLDDAY
ALHYAVAYCDAKTTTEVLDLGLADVNHRNARGYTVLHVAAMRKEPNIIVSLLTKGARPSDLTSDGKKALQISKRLTRSID
YNRSTEEGKSSPKDRLCIEILEQAERRDPLLGEASASLAMAGDDLRMKLLYLENRDWLSCVWSGKTSISHGSESCNGHCP
SGWYSGIPICCKFQKVKWWAVNTDGFERVTF
>Acoe_021_00015
NSCSKSINETVNGSHEYTIKGYSLAKGMGAGKYIQSDIFTVGGYDWSVYFYPDGKNPDDNSGYVSVFIALASEGTDVRAL
FELTLLDQSGKGKHKYRGSMWGYKRFFKRTMLEASDFLKDDCLSMHCTVGVVRTRTEAPKLYTVPMPPSDMGLNFKEMLE
SGIGSDIMFEVGEETFRAHKLILAARSPVFKAQFFGLIGNPNMDKVVVEDVEPAVFKVTLLETHVVLSNSCTM
>Acoe_021_00028
MSSTRLVDTTSTTTTTKKKMKCISCKEEYGSSDAGTCKECYEEASETEEELKQEIEELKSKVAFLRFPSPVDYNRSSSSS
SSSCFTDVFLVASDDHDRDKGAPFVPAHKTVLASRSPVFKAMLENEMEESRSGTIRISDVSYDALRNFVQYLYTAEVSLD
EQMACDLLVLAEKYEVKYLKSHCEKFLMSKLNAENAFMCFAFSSQHYAKNLHETSLSLIVENMDKLTKQEEYQELVEKDP
RLIVGIYEAYLAKQVNTAAAKD
>Acoe_022_00252
MACMKLGSKADAFQRQGQAWFCTTGLPSDVIVEVGEMSFHLHKFPLLSRSGVLERLIAETSDNGEEGCVIHLSNTPGGSK
TFELVAKFCYGVKLELTSSNVVFLRCAAEHLEMTEEYGDDNLIAQTELFLNQVVLRNWKDSLKALQTCEDILPQADDLQI
VRRCIDSLATKASTDPNLFGWPMMEHSRPMQSPGGSVLWNGISTGARPRNLKSDWWYEDISLLSLPLYKRLLSVMESRGI
NQEILSGSLTSYAKRYLPGLNRRQIVSESNNRLAPVAMGAALSEEEQKLLLEEIDQLLPMQKGITSTKFLFGMLRTAMIL
RASTSCISNLERRIGMQLDQATLEDLLMPNFSHSMETLYNVECVQRILEHFLAMDQATGGASPCCIDDDQLMGSPSLTPI
TMVAKLIDGYLAEVAPDINLKLPKFLSLAEAVPDYARPLDDGLYRGIDIYLKAHPWITDSEKEQLCRLMDCQKLSLEACT
HAAQNERLPLRVVVQVLFFEQLQLRTSIAGCFLVSENLDGSRQLRSGFAGSNEGGGWNAAVRENQVLKVGMDSMRMRVSE
LEKECTNMKQEIEKLGKGKTGGTWGNVSKKFGFKMKSQMCSAEEGSVSKQLKDKHLKVEKVRKHKHLIKDEMACMKLGSK
ADAFQRQGQAWFCTTGLPSDVIVEVGEMSFHLHKFPLLSRSGVLERLIAETSDNGEEGCVIHLSNTPGGSKTFELVAKFC
YGVKLELTSSNVVFLRCAAEHLEMTEEYGDDNLIAQTELFLNQVVLRNWKDSLKALQTCEDILPQADDLQIVRRCIDSLA
TKASTDPNLFGWPMMEHSRPMQSPGGSVLWNGISTGARPRNLKSDWWYEDISLLSLPLYKRLLSVMESRGINQEILSGSL
TSYAKRYLPGLNRRQIVSESNNRLAPVAMGAALSEEEQKLLLEEIDQLLPMQKGITSTKFLFGMLRTAMILRASTSCISN
LERRIGMQLDQATLEDLLMPNFSHSMETLYNVECVQRILEHFLAMDQATGGASPCCIDDDQLMGSPSLTPITMVAKLIDG
YLAEVAPDINLKLPKFLSLAEAVPDYARPLDDGLYRGIDIYLKVKHHNFHNSLSGFNSNVVCHKHVFSEQIMIKPMPCDI
RQFLVYNNLAQVHVHHCFYSLNNLPIEVGIGLL
>Acoe_030_00142
MASMRLGSKSEAFQREGQTWVCTTGLSSDVIVQVGGMSFHLHKFPLLSRSGVLEKLIEDFSSEDGTKCVLQLHDIPGGAM
SFELVAKFCYGVKIELNAMNVVSLRCAAEHLQMDETYGEGNLIKQTEDFLNEVFGNWVDSIKALEACEEVLPHAEETHID
SRCINSLAMKAFYWCRWWYEDVSFLSLPLYKRLIMAVEARGMNPETIAGSLMYYAKRYLPGLSKQFSFKESRNHVNTGST
ISSPSERDQRHFLEEIVGLLPSKKGVTSTKFLIKLLHTAMMLHASSSCRENLEKRVGAQLDQAALEDLLIPNMGFTVETL
YDIDCVQRILDQFILADQAAAGSSSPCIVDEEQLVDGNPPLTPITMVAKLVDGYLAEVAPDVNLKLAKFQSLAAVIPDYA
RPLDDGIYRAIDIYLKAHPWLTDSEREQLCRLMNCQKLSLEACTHAAQNERLPLRVVVQVLFFEQLRLRTSIAGWFFVSD
NLDNSQGQNGNLVLPKIEDATEANPGHAETEDHFMNINDMRSRVNELEKECLSMKQELEKLGKTKKRWNILSRLGFGKTQ
PSNDTKAAAEPSSSEPHENGEQNHEGS
>Acoe_032_00035
AFQREGQTWVCTTGLSSDVIVEVGGMSFHLHKFPLLSRSGVLEKLIEDFSSEDGTKCVLQLHDIPGGAMSFELVAKFCYG
VKIELNAMNVVSLRCAAEHLQMDETYGEGNLIKQTEDFLNEVFGNWVDSIKALEACEEVLPHAEETHIVSRCINSLAMKA
CADPTLFNWPVSGRNSIQNPSAPVLWNGIYTGTKPRSIGVEWWYEDVSFLSLPLYKRLIMAVEARGMNPETIAGSLMYYA
KRYLPGLSKQFSFKESRNHVNTGSTISSPSERDQRHFLEEIVGLLPSKKGVTSTKFLLKLLHTAMMLHASSSCRENLEKR
VGAQLDQAALEDLLIPNMGFTVETLYDIDCVQRVLDQFILADQAAAGSSSPCIVDEEQLVDGNPPLTPITMVAKLVDGYL
AEVAPDVNLKLAKFQSLAAVIPDYARPLDDGIYRAIDIYLKAHPWLTDSEREQLCRLMNCQKLSLEACTHAAQNERLPLR
VVVQVLFFEQLRLRTSIAGWFFVSDNLDNSQGQNGNLVLPKNEDATEANPAHAETEDHFMNINDMRSRVNELEKECLSMK
QELEKLGKTKKRWNILSRLGFGKTQPSSDTKAAAAAPSSSEPHENGEQNHEGSDVLTHTLLKVCTTGLSSDVIVEVGGMS
FHLHKFPLLSRSGVLEKLIEDFSSEDGTKCVLQLHDIPGGAMSFELVAKFCYGVKIELNAMNVVSLRCAAEHLQMDETYG
EGNLIKQTEDFLNEVFGNWVDSIKALEACEEVLPHAEETHIVSRCINSLAMKACADPTLFNWPVSGRNSIQNPSAPVLWN
GIYTGTKPRSIGVEWWYEDVSFLSLPLYKRLIMAVEARGMNPETIAGSLMYYAKRYLPGLSKQFSFKESRNHVNTGSTIS
SPSERDQRHFLEEIVGLLPSKKGVTSTKFLLKLLHTAMMLHASSSCRENLEKRVGAQLDQAALEDLLIPNMGFTVETLYD
IDCVQRVLDQFILADQAAAGSSSPCIVDEEQLVDGNPPLTPITMVAKLVDGYLAEVAPDVNLKLAKFQSLAAVIPDYARP
LDDGIYRAIDIYLKAHPWLTDSEREQLCRLMNCQKLSLEACTHAAQNERLPLRVVVQVLFFEQLRLRTSIAGWFFVSDNL
DNSQGQNGNLVLPKNEDATEANPAHAETEDHFMNINDMRSRVNELEKECLSMKQELEKLGKTKKRWNILSRLGFGKTQPS
SDTKAAAAAPSSSEPHENGEQNHEGS
>Acoe_032_00118
MSNRQPWTIDPDLDGIDLDQSDFNTSFPLKKVPNGDVFEASRAGDVDRLGYLLEKGSINVNARDKWDSVALYYACLAGHL

DAARMLLENGAICSEHTFDGDRCHYAALNLKVRKLLKTFEARPPPLGPLQAALRDTFLGCCANRSHTSNFQLFGSSSNDD
SNSSYFPPDVTFFAQSRPIEAHRIILSARSPYFKKKFEMDWKDRKEVRFSRERLSYPAFYSLIHFFYSDRLEVAVDDMED
LVRICKVCKCKALEKLLEKELFHQTYAEYKSLRDIENSQKRFILQGLSLPEEDRLPAALYRILQACLANCKEGPDNFDAL
TSGVKGIQLTEHEEDLADICVKVDKKIFRCHQIVLASRSEYFRARLSRMKDFLEGRNEFYGYTLPILEEHDLSTEAFEKM
IEYMYTDELKDVDPDQAEELFDVASRYLLFPLKRAVGDVLLPHFDMLPPAELCQWLILSDIYGVIKIREFCLDVIAYNFE
TFANTPEFRAMLLTLPPPSGDSSDRTTLPSAPGSGVNSDQGNILDDLREKWLQTEAGELDKRDESAIQFDKRLEILMVMA
KQESSDNHDDAMSNRQPWTIDPDLDGIDLDQSDFNTSFPLKKVPNGDVFEASRAGDVDRLGYLLEKGSINVNARDKWDSV
ALYYACLAGHLDAARMLLENGAICSEHTFDGDRCHYAALNLKVRKLLKTFEARPPPLGPLQAALRDTFLGCCANRSHTSN
FQLFGSSSNDDSNSSYFPPDVTFFAQSRPIEAHRIILSARSPYFKKKFEMDWKDRKEVRFSRERLSYPAFYSLIHFFYSD
RLEVAVDDMEDLVRICKVCKCKALEKLLEKELFHQTYAEYKSLRDIENSQKRFILQGLSLPEEDRLPAALYRILQACLAN
CKEGPDNFDALTSGVKGIQLTEHEEDLADICVKVDKKIFRCHQIVLASRSEYFRARLSRMKDFLEGRNEFYGYTLPILEE
HDLSTEAFEKMIEYMYTDELKDVDPDQAEELFDVASRYLLFPLKRAVGDVLLPHFDMLPPAELCQWLILSDMYDLFLLFL
VLLILLDNIVASISVGMSNRQPWTIDPDLDGIDLDQSDFNTSFPLKKVPNGDVFEASRAGDVDRLGYLLEKGSINVNARD
KWDSVALYYACLAGHLDAARMLLENGAICSEHTFDGDRCHYAALNLKVRKLLKTFEARPPPLGPLQAALRDTFLGCCANR
SHTSNFQLFGSSSNDDSNSSYFPPDVTFFAQSRPIEAHRIILSARSPYFKKKFEMDWKDRKEVRFSRERLSYPAFYSLIH
FFYSDRLEVAVDDMEDLVRICKVCKCKALEKLLEKELFHQTYAEYKSLRDIENSQKRFILQGLSLPEEDRLPAALYRILQ
ACLANCKEGPDNFDALTSGVKGIQLTEHEEDLADICVKVDKKIFRCHQIVLASRSEYFRARLSRMKDFLEGRNEFYGYTL
PILEEHDLSTEAFEKMIEYMYTDELKDVDPDQVSLVAIPTTMTTSCFLIFFFL
>Acoe_034_00123
MDLHNKQLQLSMSKSTRQRCNNEWMYSDVPSDITIEVDGRTFALHKFPLVSRSGRIRKLVAEHRGSDSSRIELPNLPGGS
ESFEWAAKFCYGVNFEITSLNVAKLCCVSDYLEMGEEYAKDNLGSRAETYLDTVVCKSLEMCVDVLQQCENLLPLADELK
IVSRCTDAIASKTCAEQIASSFSRLEYSSSGRLQMNKQAKCEGDWWIDDLSVLRVDLYQRVMAAMKCRGVRPESVGASLQ
NYAQKVLTKKPSLWNASAHTKADLVVNLIENEQRLVVEALVSLLPVEKLSVPISFLFGLLRRAVMLDCTIACRLDLERRI
GAQLEMATLDDILIPLFRHTADTVFDVDTVHRILVNFSQQEDSEDDMEDISAYESDSPCSPTQSALLKVAKLVDSYLAEV
AADVNLKLSKFMVIAEILPAHARTTHDGLYRAVDIFLKAHQGLSDLERKKICKLIDFQKLSQEAGAHAAQNERLPLQSIV
QVLYFEQVRLRNALCCSYMDTEHKPMHQSWRISSGALSAAMSPRDNYASLRRENRELKLELTRLRMRLNDLEKDHVCMKK
DMEKSSSSKFMKSFSWKISKFSFFTRSTSRGSSSPSKYSPRTDSKVIGRTMDLHNKQLQLSMSKSTRQRCNNEWMYSDVPS
DITIEVDGRTFALHKFPLVSRSGRIRKLVAEHRGSDSSRIELPNLPGGSESFEWAAKFCYGVNFEITSLNVAKLCCVSDY
LEMGEEYAKDNLGSRAETYLDTVVCKSLEMCVDVLQQCENLLPLADELKIVSRCTDAIASKTCAEQIASSFSRLEYSSSG
RLQMNKQAKCEGDWWIDDLSVLRVDLYQRVMAAMKCRGVRPESVGASLQNYAQKVLTKKPSLWNASAHTKADLVVNLIEN
EQRLVVEALVSLLPVEKLSVPISFLFGLLRRAVMLDCTIACRLDLERRIGAQLEMATLDDILIPLFRHTADTVFDVDTVH
RILVNFSQQEDSEDDMEDISAYESDSPCSPTQSALLKVAKLVDSYLAEVAADVNLKLSKFMVIAEILPAHARTTHDGLYR
AVDIFLKVRISSIFLMICLNSHYFIIILF
>Acoe_037_00025
MKESNNTNLDLFDPRTVMESDFGPGGEGNSEKKIENSGKVSLSSDGDFGFAFNDSNFSDRILRIEILSECPDDKSDVDAG
SISLADWARVRKRRREDIKKDNPLESVYEEPDTEDVLVYENQDEEAVAMIEESPPGEEAAENSDSSWSMDCSVVLRVRSL
HISSPILAAKSPFFYKLFSNGMRESEQRHVTLRVNASEEAALMELLNFIYSNTLSTTTPTALLDVLMAADKFEVSSCMRY
CIRMLQNLPMTPEAALLYLELPSNVLMAEAVQPLTDTAKLYLADRFKDITKFQDDVLHLPLPGIEAVLLSDELQVASEDA
VYDFVLKWARIHYPKLEDRREIMGSRLCRLIRFPFMTCRKLKKVLTCVDFDQDLASKVVLEALFFKAEAPHRQRVLAAEE
SAPSNRRFVERAYKYRPVKVVEFELPRQQCVVYLDLKRDECVNLHPTGRVYSQAFHLGGQGFFLSAHCNMDQQNLFACFG
LFLGMQEKGSASFAVDYEFAARSKPSEDYVSKYKGNYTFTGGKAVGYRNLFGTHWETFVSEESPYFINGILHLRAELTIK
R
>Acoe_038_00192
MDYQTTLVSDSNSIGSNNICKYEKTHSPEITIIPNIVALNHLSLNLESILFDTQNSNFSCYSDAKIIVSDTIDIPVHRCI
LSARSSYFKKLFSSSEEEEKIEEYTMKEIMKDFEVGFESMVLVIRYLYSGKIKDLPMNVCICVDDDCQHLGCKPAIDSLV
ELLYAYFVFQIPDMVALYQCRLLDILDKVLWDDNILVILSVAIICSTACERLLSECIEIVVKSDINIVTLEKALPPDILK
QIMDSRSDLAIGDPECNTFPNKHVKRIHMALDSDDVELVRWLLKEGHTTLDDAYALHYAVAYCDVKTTAEILDLGLADVN
HRNARGYTVLHVAAMRKEPNIIVSLLTKGSRPSDLTPDGKKAIQISKRLARFRDYNRSTEEGKSSPKDCLCIDILEQAER
RDPLLGEASSSLTMAGDDLRMKLLYLENRVGLAKLLFPVEAKVAMDIAQVHGTVEFSFVSNSTRFVVGQLAPMNLNTIPF
KIQEEHINRMKALSKTVELGKRFFPRCSDVLNKIMDADDLSELAYLRNGTSEERVAKKRRYAELQDVLIKAFSEDKKELN
QFVAMSLPSSSSSLAKVLLRSKSILHTKMDYQTTLVSDSNSIGSNNICKYEKTHSPEITIIPNIVALNHLSLNLESILFD
TQNSNFSCYSDAKIIVSDTIDIPVHRCILSARSSYFKKLFSSSEEEEKIEEYTMKEIMKDFEVGFESMVLVIRYLYSGKI
KDLPMNVCICVDDDCQHLGCKPAIDSLVELLYAYFVFQIPDMVALYQCRLLDILDKVLWDDNILVILSVAIICSTACERL
LSECIEIVVKSDINIVTLEKALPPDILKQIMDSRSDLAIGDPECNTFPNKHVKRIHMALDSDDVELVRWLLKEGHTTLDD
AYALHYAVAYCDVKTTAEILDLGLADVNHRNARGYTVLHVAAMRKEPNIIVSLLTKGSRPSDLTPDGKKAIQISKRLARF
RDYNRSTEEGKSSPKDCLCIDILEQAERRDPLLGEASSSLTMAGDDLRMKLLYLENRGAMCIIHQHGRLIYCVRMDYQTT
LVSDSNSIGSNNICKYEKTHSPEITIIPNIVALNHLSLNLESILFDTQNSNFSCYSDAKIIVSDTIDIPVHRCILSARSS
YFKKLFSSSEEEEKIEEYTMKEIMKDFEVGFESMVLVIRYLYSGKIKDLPMNVCICVDDDCQHLGCKPAIDSLVELLYAY
FVFQIPDMVALYQCRLLDILDKVLWDDNILVILSVAIICSTACERLLSECIEIVVKSDINIVTLEKALPPDILKQIMDSR
SDLAIGDPECNTFPNKHVKRIHMALDSDDVELVRWLLKEGHTTLDDAYALHYAVAYCDVKTTAEILDLGLADVNHRNARG
YTVLHVAAMRKEPNIIVSLLTKGSRPSDLTPDGKKAIQISKRLARFRDYNRSTEEGKSSPKDCLCIDILEQAERRDPLLG
EASSSLTMAGDDLRMKLLYLENRGKILYFFFYF

>Acoe_042_00127
MVEPDSEETASTSVPISSKKKQLLTSAMKRTSEWIFSQEIPSDITVQAGGASFTLHKFPLVSKSGYIRKLVSGASDADLS
VLELPDLPGGPEAFELAAKFCYGINFEISTDNIAMLRCAAEYLEMTEDYAVGNLVSRTEAYLDEVALTSLSGAVSVLHLS
ESLHPMAEKVKLMSRCIDSIAYIICKESQLCLPGRTETNNDIVHSSLVSNAKTIVDWWAEDLTVLRIDIFQRVVIAMIAR
GFKQYALGPVLMLYAQKSLRGLEIFGRGRKKIDPKQEHEKRVVLETIVSLLPREKYAMSVSFLSMLLRAAIYLETTVACR
LDLEKRMGLQLGQAVLDDLLIPSYSFTGDTLFDVDTVQRILTNYMECEIDGTRLSYSADDDFVSPPPSDMERVGKLLESY
LAEISSDRNLSVSRFISLGELIPEQARITEDGMYRAIDIYMKAHPALSDMERKKVCSLMDCQKLSREACAHAAQNDRLPV
QTVVQVLYYEQQRLRDVMNGSLSGGESPVITQKVDLYSEDNHLVPNELSKLRRENEDLKIELARARMRIKELEKLAVNTP
SVGTPTSSSGKPPLPRKSLINSVSKKLGRLYPFVRADGVKPSDSKGRTKASKDRRHSISMVFQLFNRIFSQEIPSDITVQ
AGGASFTLHKFPLVSKSGYIRKLVSGASDADLSVLELPDLPGGPEAFELAAKFCYGINFEISTDNIAMLRCAAEYLEMTE
DYAVGNLVSRTEAYLDEVALTSLSGAVSVLHLSESLHPMAEKVKLMSRCIDSIAYIICKESQLCLPGRTETNNDIVHSSL
VSNAKTIVDWWAEDLTVLRIDIFQRVVIAMIARGFKQYALGPVLMLYAQKSLRGLEIFGRGRKKIDPKQEHEKRVVLETI
VSLLPREKYAMSVSFLSMLLRAAIYLETTVACRLDLEKRMGLQLGQAVLDDLLIPSYSFTGDTLFDVDTVQRILTNYMEC
EIDGTRLSYSADDDFVSPPPSDMERVGKLLESYLAEISSDRNLSVSRFISLGELIPEQARITEDGMYRAIDIYMKAHPAL
SDMERKKVCSLMDCQKLSREACAHAAQNDRLPVQTVVQVLYYEQQRLRDVMNGSLSGGESPVITQKVDLYSEDNHLVPNE
LSKLRRENEDLKIELARARMRIKELEKLAVNTPSVGTPTSSSGKPPLPRKSLINSVSKKLGRLYPFVRADGVKPSDSKGR
TKASKDRRHSISMVEPDSEETASTSVPISSKKKQLLTSAMKRTSEWIFSQEIPSDITVQAGGASFTLHKFPLVSKSGYIR
KLVSGASDADLSVLELPDLPGGPEAFELAAKFCYGINFEISTDNIAMLRCAAEYLEMTEDYAVGNLVSRTEAYLDEVALT
SLSGAVSVLHLSESLHPMAEKVKLMSRCIDSIAYIICKESQLCLPGRTETNNDIVHSSLVSNAKTIVDWWAEDLTVLRID
IFQRVVIAMIARGFKQYALGPVLMLYAQKSLRGLEIFGRGRKKIDPKQEHEKRVVLETIVSLLPREKYAMSVSFLSMLLR
AAIYLETTVACRLDLEKRMGLQLGQAVLDDLLIPSYSFTGDTLFDVDTVQRILTNYMECEIDGTRLSYSADDDFVSPPPS
DMERVGKLLESYLAEISSDRNLSVSRFISLGELIPEQARITEDGMYRAIDIYMKV
>Acoe_042_00147
MKFMKLGSKPDSFQTDGNNVRYVASELATDITINVGDVKFYLHKFPLLSKSARLQKLVAAANDEIDDEVYIPDIPGGPAA
FEICAKFCYGMTVTLNAYNVVAARCAAEYLEMQESVEKGNIIYKIEVFLNSSIFCSWKDSIIVLQTTKSLQPWCEELKVV
SRCIDSIASKASMDTSKVEWSYTYNRKKLPSENKTDPQWNGVRKQQIVPKDWWVEDLCELEIDFYKRVIMSIKTKGRVSG
DIVGEALKAYALRRIPGFSRGVIQSCDVAKCRSLVESMIWLLPIEKGSISCSFLLKLLKAAMLLDSGEMGRRELVKRIGR
ELDEASVTDLLIPAPAGETMMYDVDVVLSIVEEFVMQEQNLEIEPPPTEEMQEITSIGFISDTSKLTVAKLVDGYLAEIA
RDPNLPLSKFIDLAEMVSGFSRPVHDGLYRAIDMFLKEHPGITKSERKKICKLMDCKKLTADACMHAVQNERLPLRVVVQ
VLFFEQVRAATSPSGSSTPEIPGSLRALIPQENGGSHGSSRTTTTNTEEDWDAVPTTEELKALKGELASLRLGNGRSEKN
EGDSRSIEKAAATKVKGLLMSKKLFSKIWSSKWSQDENSSSDTSESPGSVNPEETKSTPSRNRRYSVSMKFMKLGSKPDS
FQTDGNNVRYVASELATDITINVGDVKFYLHKFPLLSKSARLQKLVAAANDEIDDEVYIPDIPGGPAAFEICAKFCYGMT
VTLNAYNVVAARCAAEYLEMQESVEKGNIIYKIEVFLNSSIFCSWKDSIIVLQTTKSLQPWCEELKVVSRCIDSIASKAS
MDTSKVEWSYTYNRKKLPSENKTDPQWNGVRKQQIVPKDWWVEDLCELEIDFYKRVIMSIKTKGRVSGDIVGEALKAYAL
RRIPGFSRGVIQSCDVAKCRSLVESMIWLLPIEKGSISCSFLLKLLKAAMLLDSGEMGRRELVKRIGRELDEASVTDLLI
PAPAGETMMYDVDVVLSIVEEFVMQEQNLEIEPPPTEEMQEITSIGFISDTSKLTVAKLVDGYLAEIARDPNLPLSKFID
LAEMVSGFSRPVHDGLYRAIDMFLKVCS
>Acoe_042_00179
MGAPKAPPLPESVNARYGLKNSGVDRTSRRGYNYVSTAAKDMWERLFDESYRADVSIITSDCSSIPAHASILGMASPVFK
SMLKKSRKRGRRHTISVRGVPHQAVRVFIRFLYSSCYEQEEMNENVLPLLVLSHVFMVPSLKRECEQQLENQLLNDDNVV
DVFQISLLCDAPRLSIFCHRLILKNFTTISATEGWRVMKKSHPMLEKELVESAIEADSRKRERLRKMEERKIYHQLYEAM
EALVHICRDGCRTIGPHDKVLKGNEAPCDFSACKGLELLVRHFAGCKLRVPGGCVHCKRMWQLLELHSRLCSEPNECKVP
LCRNFKEKIQQQTKKDEMKWKILVKKILRAKNIAGAPFFSSALAIGAS
>Acoe_054_00095
MEPNHIHQEEQIHSSERKGQKRKLEEESVELEEDREYSLSSPDARQTIVSEVNKQVEILGITFSWKEFDRSTAKRATHVL
AELAKNEDVVNIIVEGGAVPVLVKHLEAPPVLSSDSNQKPYEHEVEKGSAFALGLLAVKPEHQQLIVDAGALPHLVDLLN
RHKDGSNSRAVNSVIRRAADAITNLAHENSSIKTRVRVEGGIPPLVELLEFADTKVQRAAAGALRTLAFKNDENKNQIVE
CNALPTLILMLRSEDAAIHYEAVGVIGNLVHSSPNIKKEVLQAGALQPVIGLLRSCCSESQREAALLLGQFAATDSDCKV
HIVQRGAVSPLIEMLQSSDMQLREMSAFALGRLAQDTHNQAGVVHNGGLVPLLKLLGAKNGSLQHNAAFALYGLADNEDN
VSDFIKVGGVQKLQDGEFIVQATKDCVAKTLKRLEEKIHGRILSHLLYMMRVAEESVQQRVALALAHLCSPDDQRTVFID
HNGLELLLGLLDPARVEQQIEVLNKLKQQLDVLNKQKQPQEYSLLLNKIKQQQAVILNMPKEQHDSAVALYKLSNKASTL
SPMDAAPPSPEPQVYLGEKFVNNATLSDVTFLVEGRRFYAHRIALLASSDAFRAMFDGGYREKDARDIEIPNIRWEVFEL
MMRFIYTGAVPVTLDIAQDLLRAADQYLLEGLKRLCEYTIAEGISLDNVSSMYELSEAFHALSLRHRCIKFILEQFDKLS
DKPGFSSLIQGIIPEIRNYFSKALTKANQHSIFGYSH
>Acoe_054_00138
FIRTNATSDLTVCVGETRFHLHKLPLVSRSGYFSRLVFHRRKNGETNSTLNIDNFPGGYHIFHLVVKFCYGLAVDLTATN
VAPLYSAAHFLEMTEDFQHGNLISKAEAFLSFVILSAWKDTLLILKSCEDISLREDELGIVKQCSDSIAWKACLDPQEIG
CSVDEEVSLNSLSNNAKNSKYKYGSDGKIVEESWWFADISILKIDHFVEVINAIKAKGMKPKLVGGSIVYWISKWLQKIK
HGKDCDHKEVRGDTFECLIRILPSERNSVCCNFLLHLLKVGLMLNIDCDLTMELEIRVATMLESHCAQDLLVKNFKDEDT
MNDVSLVIRVVDAFVTYISKDPKARMDLVAGVVDDYLALVARDGNLSVECFQLLAEVLPEDARSCNDKLYRAMDMYLKAH
PTLTEEERRTVCRSLKYHKLSQDARNHAMKNDRLPVNLTLGLILWEQVNLTKLTLSNNMKYKRSKPQEVVGRTKEVDKRW
FKPHKELQTMKTDVEKMKVQLSNLQVFSLKLQRQVGILLEITEIGN

>Acoe_056_00054
MDTIAITPTSFPINMKVVCSGNAVIDSISSESPGEADLLVVTSGGLRIPAHSSVLVPVSTVFKKMLEQPCQNLSSKNIIP
ILGVPCKAVIAFVQFLYSNSCTEEEMDRYGIHLLALSHVYAVHHLKLKCVKGLGEQLLLENVVDVLQLTRLCDAPDLYLR
CMKMVSNNYIEVQKTEGWKFMQDHDPWLELEILQFLDELESWKIQRKKNREERSLYMQLSEAMECLEHICTEGCTSVGPY
DRAPNQQKKPCDKFPICRALQLSIKHFATCQKRVKGGCCACKRVWQLLLLHSAICDQTGICKIPLCRQFKLKMQLEKREE
NGKWRLLAKKVVCAKVMSLLSSHKTESCSSY
>Acoe_058_00077
MKRRTSKKRVPSAHMVTLHQRLYQALNLGIKSRDNGGWKWQCTDIEIQRLTIRYISAFVGCISPDMLQHPLVKDSVADIV
MAIIGVTQSQSAAVLGLAAEVTITFVGVLGNSIPFHQLSKLVGSFSRLLSFHQSSVAVSCANALNHILAKLEPRRLKSHN
EVWEILKENKTVDCIVCSLQDYIDGRKSYENFQEMVSLLKTILWRWPPSRYPVWSNVKLMEGFRAICKNPDSSVEVVVLQ
LYSAIALCGGGVKKLLQNGETLVPMILSCMDNSKPQSVRVEAFKLTQSLVRLEQGWSAIARTCYESIVKAIIDAMGGWNL
SSGKVATDQVPLLSEACKLALITRWDGEHHSYFWKFGVDRVLLNLLVDNFYKNRPSQSYSSLEELIAEARKGLDANYLEV
LRPFIWASLGWLVTHCPEDFTPDTRGNNCLDVLLTCGCLTLMDSLRKGRQSFLNAIMTSKLQPASRAVLQMIHSPCKYIA
SKARYILSELSTPNVDVYLDYVLSTSIEVGNVGNKDQTIIDLIIFSCLSTLPKYRNTIIQRDGIMHLSTFIMWCLRNNAG
GGPCMQSTFKSCRCCMPDMDNWEGDDTLLFFSLWGLAELMQVQHSRLGRNHGELIFNQDLSGLEFGGSDVQTLIGKLQEI
SGSTSTSPGLRWCATYILSYFGFYGFPSRFGERIGKAFHTKELADIHLKLSSGECLRVHGVIVMVRCPSLIPSRESPSNE
RLSDESSMGQSVEQQGRILRREVCLSARVDDKTLMKLLKFVYMGFVQVDDDLVRQLKMLSRGCGFHSLTQLLSKKRPKWG
TKVPCLEFSPALGPSGQRFSDIILQAKADELELNCTYCTLLLPHVHVHKIILWLSCDYFKALFQSGMQESHSQTIEVPVS
WGALSKLVSWFYSDDLPKPSSGCIWYNMDVDSQLQEVHPYVELCWLAEFWLLDDLREDTLKVVISCLRSSRNLSLKIIQI
AANLSQWEIVEVAANCLAPSFPHLRISGDLEHLNEELVDMVRVAYVRLSQTGDCDYSD
>Acoe_088_00007
MSESAYRVETQSRLAQWRIDSLSSCTYRKSDPFKIGNWNWHLSVEKNRGVYIKLFPVVSNLTRENPPIASFIIRVLCSHG
NRHALLHPGVTDKQIKTNDDFIWAIETPLTGKFIIDIEFLDLKAVSQNGGEPCSIWSEGLVQRRSNATALATLGRMLSES
IHTDITINASNGSIGAHRAILSSRSPVFQSMFSHNLREKELSTINISDMSIEACQVFLNYVYGSIQHGEFLTHRIALLGA
ADKYDISDLKEACHESLLEDIDTKNVLERLQTAYLYQLPRLKGSCMRYLIKFGKIYDIRDDFNAFLQSADRDIVAEIFQE
VLAAWKGF
>Bdis_1g02830
MDAGEQSATAAGMELDFSHSSVVPSFEFPFNSANFSDRLLRIEIVASDDAGGGSAADCARHREEKGDKGQSIDSSPTTVC
TAVLREKTIYINSAILAARSPFFLKLFSNGMKESDQTHTILRIVDLEENALMELLSFMYSGKVTATVPTLLLGILMAADK
FQVVSCMRQCSQLLTSLPMTTESALLYLDFPCSISMAGEVQALRDAAKEFLANKYKDLAKFQDEVMNLPLAGIEAIFASS
DLQATCEVNEVDNQEYSFGFYLVVQKKPRNARCLMVDLEFAARISWSGKFGYGLESDYTLTEGTVGYNNFFDTLWPSFLA
DDSLFINGVLHLRADLRVVEEQPELET*
>Bdis_1g02840
MAERMAADGESSVAAAAEAEMKMECFDFAFNSERFSDRLLRIEVVASDDIAEGFLPDCTRHRKDKDTKLYVTKLKVKRST
HGVPYYPSHPYFSSSYSDKRQRIASSPTMADTPVLRVKTLHINSAILAARSSFFLKLFSNGMKESDQTQTTIRISDSEEN
AFMELLSFMYSGKLMTTEPSLLLDILMSADKFEVPSCMRHCSQLLISLPMTTESALLYLEHRCSISMTAEVQLVIGAAKQ
FLANKFRDFDEFYDEAMKIPLAGIEVIFSNNDLHVHSEDDVYNFLLRWARAQYPESEERRKILSSRLLPLVRFSHMPGLA
LQEILMCTDTDIDHDQITKLVTEVLLQKGYPAQLEGALGAPAMVAERAYASKPMKMVAFDQPCRQVIVYWDLTFQECSRL
FPSGEIYSHRFYLAGQEFCLVAACESDEENESHGFCIYLGISNKPEGSPDMTVDFEFSASKRSPRKFDDGSCSQHTFTDD
PECAFKGLFCTPWLAFIADDSLFIDGVLHMRSDLAVVEQPQLQT*
>Bdis_1g02880
MKESDQTHLTIRIADSEENALMELLSFMYSGKLTTTEPSLLLDILMSADKFEVPSCMRHCSQLLTSLPMTTESALLYLDH
GCSSLLAAEAHSVIGAAKQFLAEKFRDFDKFCDEAMDISLAGVEAIFSSTDIHVESEDDIYNFLLRWARARYLESNERRK
ILSSRLLPLVRFNHMTGSALQEILTSTDTDIDHELVTKRVTEVLLQKGYAAQLEGALAADATTAERAYIRKPMKVVAFDQ
PCRQVIVYWDLTLQECSRLFPSGEILSHPFHLAGHWFYIVTICEMVEQNKLHRFGLILGILGDSTCKDRVVREVCQPL*
>Bdis_1g02890
MAGRGESAMPEAAAEVETKLECFDLAFNSEFSDRLLRIEVVPGDDVAEGSLTDCARHRKVQEENAFMELLSFMYIGKLTT
TESTLLLDILMAADKFEVPSCMRHCSQLLISLPMTIESALLYLEHGCSISQAAEVQCVIGAAKQFLAKEYTDFDKFCDEA
MNISLAGIEAIFSSTDIHVISEEHVFKFLLHWARTRYLEPEERRKIWSSHLLPLVRFSHMTGTTLQAILACTDTVIDLDH
EELTKRVTEVLLRKGYRAQLEGSLAAVTTTAERAYVIKPMKVVAFDQPCQQVVVYWDLTRQECSRIFPSGEIISHPFHLA
GQRFSLMVVCKMEEQDEIHSFAVLLGIHGNPKGSTCMTVDHEFAARTGLSGKFVSHFGRKHTFTDDPASECEVLFRAPWS
SFIADNSHFIDGVLHLRADITVVEQPAVQS*
>Bdis_1g05397
MATLSVSRCTAETEKGAHTFEINGYSLHRGHGVGRFLRSANFTVGGHLWSVVFYPDGCREDCKDFVCVGLQLMSWSIGMV
HTSFILSFLHPTNIQPSSSSAPVVFNFYQACSSFVCHRFMKRSMLEKAGYIVEDNLKLHCTLTVSKLPRVGETMHLETIL
PPPSDITEHLMKLLETQECTDVIFVVQGEEFPAHKLVMAMRSPVFKAQLYGQMMEKDMNRIIVPEMQPFVFRVLLHFIYT
DALPSLDDLDGDSMKDMIKHLLLAADRYLMERLKLVCESILCKELDVKSLANMLALADQHSCTGLKDACIEFVSSSSIAN
EVAETQAYKELKRTHPSVVMDMWEKIIKRPRT*
>Bdis_1g06070
MDDDDAGGGGDASPPHAGAVVEPGAAPRDVAASPTSSRSVTETVNGSHRFVIQGYSLAKGMGVGKHIASETFTVGGYQWA
IYFYPDGKNPEDNSAYVSVFIALASEGTDVRALFELTLQDQSGKGKHKVHSHFDRSLESGPYTLKYRGSMWGYKRFFRRT
ALETSDFLKDDCLKINCTVGVVISTMDYSRPHSIEVPESDIGYHFGTLLDTEEGADVIFSVAGEKFHAHKLVLAARSSFF

RSEFFDSESDEEKNEADISNEIKEIVIDDMEAKVFKAVLHFMYRDNLVSDDELSASSSGCSIFDTLAGKLLAAADKYELQ
RLRVLCESYLCKQISVNSVATTLALADQHHATELKSVCLKFAAENLSAVIRTDGFDYLKDHCSALQSEILRTVAGCENEC
SSGGKSQSVWGQISDGGDTSGRRVRPRV*
>Bdis_1g12870
METSTVTSSSSPPPSPSPSPSPQAPQAPPANLDAVSLGRLSANLERLLDPAFLNCADAEVVLADGGDEATVPVHRCILAA
RSNFFLDHFSSLSSPAAGGGKPRLELAELVPGGRHVGHEALVAVLGYLYTGRLKPPPQEAAICVDDRCRHQACRPAIDFV
VESTYAASGFQISELVSLFQRRLSDFVNEALAEDILPIIHVASTCQLPDLLNQCIQRVADSSVDRHYLEKELPGEAFSRV
KEIRRYSLHDETDESTLDPEHAKRVRNIHKALDSDDVALVGMLLKESAITLDDAHAIHYAAAYCEPKVLAGMLNLDSANV
NLKNDSGYTPLHIACMRREPDIIVSLIEKGASVLERTRDGRDALTICKRLTREKDCRKKLEKCKERSKAYLCIDILEQVI
KTKSSISEERLCEEVQIATPLLADNFHMRLLNLENRVSFARIFFPSEAKLVMRIAQADSTEEFTGLTLANFAKLKDDLND
LKLRERFDALTKTVELGRGYFPHCSEVLDKFLNEESTELFFLETGTPEDQRIKRMRFSELKEDVLKAFSKDKAVAAIASS
TSSSSSPRYDGKVRHGNKRAKLLR*
>Bdis_1g13890
MAASVKLGSKPDAFRRQGQAWFCTTGLPSDVTVEVGDMSFHLHKFPLLSKSAVLEQLIEESSDQEECTITLSDIPGGTKS
FELVARFCYGVKIELSSANVVYLRCASEHLQMTEEISEDNLIAQSEMFLNQVVLRNWKDSLKALETCDDLLPHAEDLQIV
KRCIESLAAKATTDPNLFGWPIREHGIMQSPGGSVLWNGISTGARPRNFSSDWWYDDASALSFPMYKRLISAMESRGVRP
ETIAGSLAYYARKYLPGLNRRNSTGIAPPTATLSEVEQKNLLEEINTLLPVQKGLASTKLLLGLLRIAMIQRASSTCISD
LEKRVGMQLDQASLEDLLLPNFSYTMETLYNVECVHRILEHFLAMDQANGGASPCMDDVMASPSMIPITAVAKLIDGYLA
EVASDVNLKPPKFQALASAVPEYARPLDDGLYRAIDIYLKAHSWLSEAEREQLCRLMDCQKLSLEACTHAAQNERLPLRV
VVQVLFFEQLQLRTSIAGCLLVSDNLERSRPLRSGIATSGEAGGWTTAVRENQVLKVGMDNMRIRLAELEKECSDMRQEI
LKLGRGKSGGWTSHVPKKFNLRIKSQMCSAQEGSVSEQQKSMSAKIDKLQAKLSKQKKQLSADA*
>Bdis_1g14770
MKHMKLGSKADVFQTEGSNIRFVATELVTDIIISIGDVKFYLHKFPLLSKSSRLQRLVASSNEENNDEVDISDIPGGPSA
FEICAKFCYGMIVTLNAYNVLSARCAAEYLEMFETIDKGNLIYKIDVFLTSSVFHAWKDSIVVLQSTKSLQPWTENSKVI
NHCIDSIASKASIDSSEVEWSYTYNRKKLLSENGVGSHWNGVRMQQIVPKDWWVEDLCELEIDLYKRVILTIKAKGRTPA
IVFGEALRAYAFRWLLGSLEDAVSNGIDCAKRRAALESIVFLLPTEKGSVSCGFLLKLLRAACLLGSGESYRDNLVKRIG
MQLDGASVAELLMPATSGENTVYNVDLIMVIVEEFMSYHRENGKMTFQDDDEEIMEVKNFASVSSMSMLAVAKLIDEYLG
EIAKDPSLPLSKFIALAEMVSASSRQMHDELYHAIDLYLKEHPSLSKSEKKRLCGLMDCKKLSQDACMHAVQNERLPLRV
VVQVLFFEQVRASAASARGGSAAELSSAVQSLLPRENGNSYGSSRSAATTTTDEEGSGIPTSSDINSFRSMRLANNCGGS
DSSLSSDINKNGEDKSTAGKAKAMLMPKKILSKLWSGKTNVGENSSSDTSESPGSANPEELKSTPSRNMRRSMP*
>Bdis_1g19390
MGAGRASRGGPSTSAAPALAPANGLPAAGRLFPPIAPPPPAPSYPSPSSSSPAAAAPPAETASTSVTKTVNGSHHFKIAG
YSLAKGIGVGKYIASESFTVGGFEWAIYFYPDGKSAEDGAAYVSLFIALASEGTDVRALFELTLVDQSGKGQDKVHTHFG
RSLEGGPYTLKYRGSMWGYKRFFKRSGLETSDYLKDDCLLVNCTVGVVQSHTEGPKIYRIPVPQSNMSQHIGHLLTSGKR
TDITFEVDGEMFPAHKVVLAARSPVFRAQLFGPMKDKNMKCIKIEDMEAQVFKALLHFMYWDELPDIEELTGLNTTWVST
LMAQHLLAAADRYALERLKLLSELKLCEVVAINTVANTLALAEQHHCYQLKTVCLKFVALPENLKAVMQTDGFDYLQQSC
PSLLTELIEYVAKVGEHIVSPCLYSNEVLDGGDANGRRVKPRT*
>Bdis_1g30767
MAAASSQSTRRTTAAATTTTTSTCAPETAKGARVFRIEGYNMYKGLGVGRFIQSAPFAVGGYDWRICYYPDGDVESSKDC
VSVHLELMTEGADVRALYSLTLIRQATAAGSSAYFMWANPTEPVVFSSAHGTSARGFSRFAKRSVLEASTYIVGDTILIS
CELTVIRLKEAQAPQPEARFVVRVPFLGLMDDLKNLLETGEEADVSFKVRDEVFHAHKLILAMRSPVFKATLYGPMGDNC
GESITIEDMHPVAFRGLLHFIYTDEFPELDDIDDDDDEDEEEDMIQHLLVAADRYGMERMKLMCERIFSECLDSKTVATT
LAFADQYNCSQLKDACIGFINSLDTMDPVLSSTGYEHLKRACPNIFVDIWEKAAKARKN*
>Bdis_1g32230
MANACAKLTGVASSVRQLRIDGFSLASSTMESQEYIKSRCDVDGYEWEIRCYPATSSFGKWVELNLVLLSEALAPSSIRP
MHSSSRVSLVDRSYLPVPASRADDYLAVQCTITVLKELPDDAATASSVPVPASDMHRHFGEPLRTVTGAHVTFLVSGESF
MAHKNILAARSPVFMAEFFGNMKEACLRRVEIEDMEAAAFRAMLHFIYTDSVPELDQELGAVATMAQHLLAAADRYGLDR
LKLICEGKLAGGIAVDTAATTLALAEQHNCLHLKAKCVEFIVSTPAILDGVLATDGYKHLEASCPSVLTGLLKSVRGRKN
*
>Bdis_1g32247
MATESSSRSISLTGAASLVQQLKIEGFSATASMALHQFIASRWTVDGYECEVRIYPATTCIVIRGHRTTWVALELIFVSE
SRTGGSRVSMGCRLVDPSGKLQPSEEHKVSVSKKFCHPNESSGLLFLQNRNDLAASGYLVGDCLALECTITVLKELPVPT
IPATKEVVEPPSTNLHQHLGELLQNGTGADVTFLVSGESFAAHKNILAARSPVFMAEFFGHMKEKSSRRVEIENMEAPVF
KALLHFIYTDKVPEELDQQKPDIVGACTMAQHLLAAADRYGLDRLKLLCEIKLSAGITVDTAATTLALAEQHDCEQLKAK
CMEFIVSTPAVLDAVLATEGYKHLEMSCPSVLTGILKSTRGRKN*
>Bdis_1g32260
MEKDCKSITNVARWVKLLKIDGYCAAKTMGNEDCIKSSCSVGGYDWEICIYPAMVPPDRRARDRTPWVVVKLVFLSETCP
SIVRANLSCRLVDPRGVLGPSPEKSVSCIFNRSWISRCLEMCSPFVNEDCSLPVCLKSTGELAASGYLRNDSFTVQCTIT
VLKEDVPAARIPVKEVSVSSPSLQHHLAELLHNKTGTDVTFLVSGKSFAAHKLILAARSPVLMAEFFGHMKETSSQHVEI
NEIEAVVFKALLYFIYTDSVLEFGLQHEAVTMLAQHLLAAADRYGLDRLKEICQGKLSDGISVDTAATTLALAEQHNCPQ
LKAKCVEFIVSTPAILDAVLATDGYKHLEASCPTVLPDLLKSARGRNS*
>Bdis_1g32690

MRHTASKATGREKGGRKGGGGEQLLTGRVLSLRTRLHDALALGLTSCPKSKIRGLTNSLSAWDFVKMCESISDILIVLEG
VLQTKNVLVLIQAADVSLKLVSSIGNSIRQYQFLEILTSFSCQLSADQIPLAISCAGALNCILNNLVTATVSTHTEIWKA
LERTNTVASIISALRSYAHDVHPLNYLAEMISLLRTMMWIWPSSRYHVWSNCNLMGKLAHHYLGTETTVTTKVLRLYAAL
ALCGNGAMILLKDEVLMAKICELMGASHISITRIEAFKLCQILLRSSKGCNQLMTSHCQPIVEAIIDAMSRSDDKLLVTE
GCRTALLVLRYAGNHHQCFWSNAIDKVLYNILTSFHISSHQTHHILCHDELFNMVSKNFTDIHPYVWDILGYLAVHCQNE
HLSVRKRKDHILDTLISCACSLAAGVMQSSPLKLSKDVQEPALRAVLMMLLSPCGYILSEASSKLSEVVICLGDDYLNIV
LSSLESTTTRSVTTSFDSFQIMINLMNLVCLVLSQPYHNLLNKRNCVDVLSTIIKECLHNHIHITRDHFTEGFLRVLQRA
LSQSLSSGPKLYIAHTLSLFGLCGSPSKLGRKMRSALDDNELADLELLLSDGESLNAHTSIISVRCPKLLPSVKYALGSN
GKINDEWGKSMYRVQMSDRVDSRALKKILEYTYSGFVMVDDDIVKAIRTLAKYCHLKSLQDMLQKEQPRWNSDCPSYDLT
VALGPVDHSFSDIILEAQSNEQMKCQHGSCQLSTPHVHSHKIVLSMSCDYLRALFRSGMHESFSEVIRVPLGWQALDKLI
RWFYSGELPNVSPDCQWKNLGSDAQLSHLSSYVELSSLAEFWFLEGVKEKSLEIVTACLNSSTDAAVDVIAFAASLGQWE
MVEASTRSVAHLFPRLRDSGQLEELDDDVIDLLRTEYVRHSQHGGRSD*
>Bdis_1g39260
MRGPWRGSSVGEASSRVLDWRKRARMDPDFSRASGGPSYEFAFNSVNFSDRVLRIEIVAGDDTAGAKGATGEGCSSIADW
ARHRKRRREELRREKESGKHMLDLTNVKVKAEEPDTYEEINEEPVAMIEESPPDIGQDGEDGGSSDSSWNMECNSVLRVK
SIYISSAILAAKSPFFYKLFSNGMKESDQRHATLRITASEENALMELLSFMYSGKLTTNQPTLLLDILMISDKFEVVSCM
RHCSQLLRSLPMTTESALLYLDLPSSISMAAAVQPLTDAAKEFLSNKYKDLTKFQDEAMNIPLAGIEAILWSNDLQVASE
DAIYDFVIKWARAQYPKLEERREILGTRLLPLVRFCHMTCRKLRKVLACNDLDHEQATKSVTDALLYKADAPHRQRTLAA
DVLTCRKYCERAYKYRPLKVVEFDRPYPQCIAYLDLKREECTRLFPSGRIYSQAFHLAGQGFFLSAHCNMDQQSAFHCFG
LFLGMQEKGSTSVTVDYEFAARTRPSGEFVSKYKGYYTFTGGKAVGYRNLFAIPWPSFMADDSLFFINGVLHLRAELTIK
QP*
>Bdis_1g42730
MVLDINLDPEDLQPSVPLKKVPTGDLFEAARAGDCDRLALLLEGGANVNARDRWDSVALYYACLAGHADAARMLLEAGAV
CAERTFDGDRCHYAALTLDLRRLLKSFEARPPPLAPLPAALRTTFLACPANRTTFLEILQGSSSGSEAAALAQTAGFGLT
DDASTASLFPPDITFYVDGKPIEAHRLILCARSCFFEKKFKTDWKNRREVRFSNQKLSFGALYNLIHFFYSDRLEASVDD
MECLLRTSKVCACEGLHKLVHKEVMHHRFAPYKSTMESELENSQKRFVLHGQSLPLEDQLPSALQRILEKCLANSREKDY
FNNEPNEMCRNLKDDDLADLIVKVDDRVFRCHQLILASRSEYFKARLSRTVDFLEGYNGQQESLDLPFLQEHDLSTEAFE
KMLEYMYTDKLEHMDPIQAEELFDIASRYLLFPLKRAVADLLLPNLEHVSPAELCHWLMLSDIYGVVKIREYCLDIIACN
FEMFADTREFRALLLTLPPPSADDALRTTRPSEPGAAGNTDQGNVLDDLREKWLEAEAAELDKRDQSAALFDKRLEMLMH
VAEREANDYNAGLHGELEFQGHGTFLV*
>Bdis_1g49730
METVKEAVHGCKFADETTSDVRVCFKRADEQPEWFPCHSSVLSGSSKYFADLLGQGDIPSSIEVECPRAEYGSYVKVLKL
LYLPSESILESFGSVKSAVGVLRASTTLRCEHITRLCIEYLESASWDEKEEEEILEAARSLGSEGVPLLARLQAPSTDTV
KNVFVSAMRFATSLESPFPPFLGDLTTSAQEQIDFMLHEGDDPALVTMDEDVRSVVREGLTKLLSTLRAGLDLLASEFDE
LPEQAEQRIMRSLVDIDWMATVLTKIEMMNEFVSGWSEISCLVISVVQDKKYSSGLWAVKAKLIEVTGKALDAVGYGSVI
IPSTSRVHLLKTWIPYIRTTKHLLDGKTEDEAFPQMDADFCQNIESAMVSMVLALPSSDQSDILSEWMMNADQFRYPDLT
EAFEMWCYRSKTAIRRLKGGGLNKARNPTISL*
>Bdis_1g52450
MEAAAEEPKKRSLTVAPFECAWDEQFRFTEAGRGCVAFEASAQNDVTLVFREQLGSQHYHYKMDTTRHYTVILGSHRNKR
LKIEVDGRTVVDQAGVGLCRSSAFQSYWISIYDGLISIGEGRHPNNNLLFHWLDPDPNRNVQYVGLSSWDKHVGYRNISV
LPSAPRNSILWSQIQYACAERHGDGSVHTKHQPEPKDGCQQRVLADFLECWDFSDAVFLVGAERKAVPAHKIVLAASGDF
SFTFMDGSAIELPSVSYPVLHSLLEYIYTGSTQVAEYRLNSLLELGLQFKVQPLVKRCEEILDCLTKMDNELSASSKNLE
VSSSGPQTRRFDHFPFKAPVNAQKIKHFHESGEHSDINIYTSGQGLVAQAHKLVLSLWSMPFAKMFTNGMKESRASNVFF
EDVSAEAFSLLLQFMYCGELKVDNRYITSELVQLLLLSDQFGITILQFECCKRIMEFLSEDTVCSVLQAVSSIPSCKLLE
EMCKRNFAKHFDYCTTACTDFVLLDEATFKDILQHGDMTVTSEERVLDAILTWCMEACDTFCWNSVDKLLSTSTPEQLFG
ERLTAINALLPSVRFPLMQPSVLQLMEKSNLAKHIQLFRQSVAEAVEFSNAGPWMPTTIKCERFLHRRSSYRELQYISDG
DNNGVIYYAGTSFGKHQWINPVLAKNITVMASSPNSRYTNPKALVSKNYQGTCFAGPRDENGKKCSWWMVDIGEDHQLMC
NYYTVRQDGSTTFMRSWVFQGSMDGENWTSLGVHEEEQTICQPGQFASWPITGPSALLPFRFFRLALTGPATGMSNTWNL
CICFLELYGYFR*
>Bdis_1g53140
MFCQSCKDVYEEEEAGTCKECYEEASETEEELKREIDDLRSRLLFLRLPSPTLDASSTAHSDLLLHAIPASSSSSPSPSG
DADAGRFDTPAIPAHRVILASKSPVFRAMLDNEMEESRSGIIKIYDVSYDVLRAFVHYMYTAEALLDEQMACDLLVLAEK
YEVKHLKSYCEKFITSKVNNDNAITHYAFAHRHSAKQLLEASMSAIMESMPTLAEREEYKELVVKDPRILVEIYETYISR
QDNTAADKDSDCSCRK*
>Bdis_1g56500
MDDDAGDASPPPGPPPAASAPSSRDMAASPTSSRSVTETVNGSHRFVIQGYSLAKGMGVGKHIASETFTVGGYQWAIYFY
PDGKNPEDNSAYVSVFIALASEGTDVRALFELTLQDQSGKGKHKVHSHFDRSLESGPYTLKYRGSMWGYKRFFRRTALET
SDFLKDDCLKINCTVGVVVSTIDYSRPHSIQVPDSDIGYDFGLLLDSNEGIDIILNVRGEKFHAHQLVLAARSSVFRSRL
FDDESAGDKDKIDENDDLKVVVIDDMEPKVFKAMLHFIYRDTLVDDYELDASSSEGSIFDTLAAKLLAAADKYDLERLKL
LCESYLCKGISVASVASTLALADDHHAMELKAVCLKFAAENLSAVIRTSGFDYLKDNCPALQSEILRTVAGCEEPCISGG
KSQSVWGQLSDGGDTSGRRVRPRI*
>Bdis_1g59760

MAAVPRPSWSRSVSETVRGSHQYTVKGFSLAKGIGPGRHLASDTFAVGGYDWAVYLYPDGKNPEDNASYVSVFVALASEG
TDVRALFELTLLDQSGRARHKVHSHFDRSMQAGPYTLKYRGSMWGYKRFYRRSQLETSDFLKNDCLVMNCTVGVVKTRLE
TPKNIQINVPPSDIGRCFKELLRLRIGCDITFEVGDEKVQAHKWILAARSPVFKAQFFGPIGKADLDRVVVEDVEPIVFK
AMVNFIYSDELPSIHELAGSFSMWTSTAVIQHLLAAADRYGLDRLRILCEAQLCDGLTAETVATTLALAEQHHCAQLKSA
CLKFTAVRENLGVVMETDGFNYLEETCPSLLSDLLATVAVVDDDPTSVNRKRGVCINEDVNPVESVEASDRRIRRRV*
>Bdis_1g70760
MQTETQFSSAAMKRTSDWIRSQEFPSDITIQVGDGTFNLHKLPLASKCGYIRKLVSGANGSRVTHLEIPGLPGGAKAFEL
VIKFCYGANFEITADNVAMLRCAAEHLEMTEECKPGNLIGRAEAYLEEVALASLAGSVAALRASEELLPASERVQLVGRC
VDAIALIACDGGDGYESGVVSVPAKAAVDDWWADELTALRIDTFQRVMIAMKARGFKGIALGTLIMLYAQKSLRRLDVNG
RERKKMDPRQEHEKRVVLETIVSLLPREKNTMSVSFLSMLLRAAIYLDTSLACRLDLEARMAAQLGQAVLDDLLIPSSAS
PDADGGAAAFDVDAVQRILVGYLEHHEGGGEATAAMAMAPRGLDYNTDDDFVSVASPPPTDVALVGRLMESYLAEISSDA
NLPVDKFVALATLIPERARFNEDGMYRAIDIYLKVRNSGIALLRNVILSFN*
>Bdis_1g70830
MKFMKLGSNPDTFQDDGSEVSIVESELVSDITVRIGSTKFYLHKFPLLSKCARFQKLIPTTGDENIEVPIHDIPGGPKAF
EICAKFCYGIVVTLNAYNVIASRCAAEYLEMNENVDKGNLIYKIEVFLSSSIFRSWKDSIIVLGTTKAHLPWSEDLKLVS
HCIDSIASKASADTSKVEWSYTYNRKKLPTENDLDLQWNGVKKQQSVPKDWWVEDLTDLDIDSYKQIITAIKTKGMVPKD
VIGEAIKAYTYKKLPSLSKVSMIHGDAKVRAMLVTITCLLPSEKGSVSCSFLLKLLRAANLLKCGEMCRKELVKRIGRQL
DEATVSDLLIPTVDGETTVYDIDMILSIVEEYVRQDSKNAQKHNAAEVSGHIQAPSASMNTVTKVIDGYLTEVAKDPNTP
VLKFINLAEAVSGNSRLIHDGLYRAIDMYLKEHPNVSKSDKKKLCSLMDCKKLSPDACAHAVQNERLPLRTVVQVLYHEQ
TRASAAATIRAESICIGSYESSRSGATTNTEDEWDGVMAVEDLSLSKTTKVHTAGTNAEKSHGSNRSTSSKVKGAATPKK
ALGKMLSSKGLTGERSSSDSSDSAILQRQEHPKRTPSRSTKPAAA*
>Bdis_1g77537
MASNLASSDKPISESSSRCLTECLSTAHNFEIIRFSLLEGMGAGKFISSSKFRVGGYDWKIRIYPDGWKEEDKAAYMSVF
LCFCSRPARDAKVKFTLSLLAKDGKVRSVHSTTHTFQETGQQKEGNYWGWREFIEKPKLQELLSVNDDCFTIRCVLTVIK
EPHTEDVSTVVVPVPESNLHAHFANMLKDGKGVDVKFSVGGQLFSAHRCVLAARSPVFKAKLYGQMKETTMKCVKIDDME
PSVFKALLHFIYTDSLPSKNRDVDENTALQHQLVAADRYGLDRLKAMCEGKLCQSIDVQTVAITLALAEQHNSVQLKNAC
LGYLCLQDVLRVVKETDGFKHLITSCPWIMMDILEKVGPPSRV*
>Bdis_2g05870
MEAPLTSHVTTAFSDCDSAPMEMEDDAAAAAADAADVEALRRLSDNLAAAFRSPDRFAFLTDARLVACPGAPELRVHRCV
LSARSPFLRAFFARRAAAEGGVGDRVELRELLGDEVEVGHEALVLVLEYLYSGRVREPPKSAFFCVDEDGCAHVGCRPAV
SFMAQVLFAASVFQVAELANLFQRHLLDVLDKVEVDNLPLILSVASLCSKSCMKLLERCLEIVVQSNLDMITLEKTVPQD
VMKQIIDSRLSLGLVSPEDNGFPNKHVRRIHRALDSDDVELVRMLLKEGQTNLDDAFALHYAVEHCDSKITTELLDIALA
DVNHRNPRGYTVLHIAARRRDPKIVVSLLTKGARPSDVTSDGRKAVQISKRLTKHGDYFGVTEEGKPSPKDRLCIEILEQ
AERRDPQLGEASVSLAMAGDCLRGKLLYLENRVALARILFPIEARVAMDIAQVDGTLEFTLGSSANQLPEIPRATVDLNE
TPFKMKDEHLARMTALSKTVELGKRFFPRCSKVLDTIMDDETEATELASLGRDNTSTERKRRFHDLQDVLQKAFSEDKEE
FARSALSSSSSSATSIGGVRPRR*
>Bdis_2g26076
MKCAILPEHEIIPIALGLEPGTHTLCFRPLLRLDLRQSLARPRRRSPQMEAEQQKPQRPRRKAQKRRLDDEAAASAAAAA
SAAAAAAAAAAASAAASSPLGSADAEDDNEEDEGSAGPEICCRHSQAAVAREVRTQVDALNLSWRHADRAAAKRATHVLA
ELAKNEEVVNVIVEGGAVPALVCHLKVPPAVAAVQEEQQPRPFEYDVEKGAALALGLLAVKPEHQQLIVDAGALPLLVNL
LKRHKNATNSRAVNSVIRRAADAITNLAHENSNIKTCIRIEGGIPPLVELLESQDVKVQRAAAGALRTLAFKNDENKTQI
VDCNALPTLILMLRSEDAAIHYEAVGVIGNLVHSSPNIKKEVLNAGALQPVIGLLSSRCTESQREAALLLGQFASADSEC
KVHIVQRGAVRPLIEMLQSADFQLREMSAFALGRLAQDTHNQAGIAYNGGLAPLLKLLDSKNGSLQHNAAFALYGVADNE
DYISDFVKVGGVQKLQDGEFIVQATKDCVAKTLKRLEEKINGRVLKHLVYLMRVGEKSVQRRVALALAHLCAPEDQRTIF
IDNNGLDLLLDLLVSVSLKHQQDGSVALYKLANKAAMLSTMDAAPPSPTPQIYLGEQYVNSSTLSDVTFLVEGKRFYAHR
IALLASSDAFRAMFDGGYREKDARDIEIPNIRWDVFELMMRFIYTGSVEVTNELAQDLLRAADQYLLEGLKRLCEYTIAQ
DVNLDNVSDMYDLSEAFHAMSLRHTCVLFILEQFDKICIRPGFSQLIQRVIPELRNFFVKALRPSHRTAQP*
>Bdis_2g51030
MEPSSSITFASSSSYLSNGSSPYSAALPPPGATQAAPLTPSEGYGGGGGGGGSSSLEVVSLNRLSNNLERLLLESDLDCS
DADVDMADGGPLVPVHRCILAARSPFFHEFFAARGRGNSGDGPPSASAAGVGGGGEGTGRPRYKMEELVPGGRVGREAFL
GFMRYLYTGKLRPAPPDVVSCVDPVCPHDSCPPAIRFAVELMYAASTFNIPELISLFQRRLLNFVDKTLVEDVLPILQVA
YDSDLGQVLEKCVQRIVRSDLDNISLDKEVCPEVADKIKKIRQKSPPDDGDTVILDPVHEKRVRRIHRALDSDDVELVKL
LLNESEITLDDANALHYAAAYCDSKVVSELLDLGLANLNLKNNRGYTALHLAAMRREPTIIMCLLNKGAVASQLTCDGRL
ASSICRRLTRAKDYNTKMEQGQESNKDKMCIDMLEREMRRNPMPVEDSVTSPLLADDLHMKLNYLEIRVAFARLFFPAEA
KVAMQIAQADITPEVTGVSAASTSGKLKEVDLNETPVTQNKRLRSRVDALVKTVELGRRYFPSCSEVLDKYLEDDLPDGL
DIFHQQSGTPDEQKVKKMRFCEVKEDVRKAFSKDTADKSAFSALSSNSSSSPPPPPQKIAQK*
>Bdis_2g51777
MATMKLGSKPEIFVLEGLTWRCMTELESDVVVEVGEVSFYLHKFPLLSRSGVLQRLISEYHPLSDGAGVAGMCTLQLDDI
PGGAKAFELAARFCYDVKIELSAQNVVCLRCAAEYLGMTEDYAEGNLIAQAESFLSRDVLASWKDAIRALETCEGAVLPA
AEDLRIASRCITALATKACATDASAASASACAWAPKKSASLDCGRVDPALWNGIGSGDHTPRAASAGNSVDWWYEDVSFL
SLPMFKRLIQAMEAKSMRPESIAGAIMFYASRFLPGLRPNTSSGFSNAAAAAEECISITTPRAAGANGLSEGEQRQFLEE
IVALLPARKGVASTRFLLGLLRTAMLLHASPLCRENLERRIGAQLEDASLDDLLVPNLGYTVDTLYDIDCVQRILDYFMS

STDGVGTGYTTPALAEEGSLLGVPQACTPSTQSPIAMVAKLMDGYLAEVAPDTNLKLPKFQALAAVVPDYARTVDDGIYR
AMDIYLKSHAWLSESEREQLCRLMNCQKLSLEACTHAAQNERLPLRVVVQVLFFEQLRLRTSIAGWFFVADNDGGAGAHP
QHPGNNAVAIVPKGGSVAESGQADPVASEGKGSEAMSDVKARVSELEKECMIMRQEIRRLGKPRRSWNILTRKCGFGAKV
QQTHPAMRGK*
>Bdis_2g57476
MASLESGSSQFLFNSNVIDNTFDIYECNSLAGTEVPDYRRYSQSRRSSVPDPPPLPGTNFGVSRTCRNTKACISVPEEIQ
DSWNKLFLEGYQADVRVSADDGSDILSHSCILGIRSPVLRTMLEDARVQHGLRHILISGVPSEAVHIFIRFLYSSSFEQE
EMKKYALHVLVLSHVFSVPSLKVVCINQLERFFLAPENVVDMLQLARLCDAPRLSVICTRMITEDFRTISLSEGWKVMRQ
VNPSLEQELLEFLVEVDTRRQERAKRMEDKKVYLQLHEAMEALVHICLDGCRTIGPRDQNLKGTQAACKFPACKGIEFLV
RHFSACKMRVPGGCTNCKRMWQLLELHSCMCSTPDKCRVPLCRHFKEKMKQLSRKEEIKWNLLVCKVLESQGTISSIPQR
RKFSSLKPSS*MASLESGTEVPDYRRYSQSRRSSVPDPPPLPGTNFGVSRTCRNTKACISVPEEIQDSWNKLFLEGYQAD
VRVSADDGSDILSHSCILGIRSPVLRTMLEDARVQHGLRHILISGVPSEAVHIFIRFLYSSSFEQEEMKKYALHVLVLSH
VFSVPSLKVVCINQLERFFLAPENVVDMLQLARLCDAPRLSVICTRMITEDFRTISLSEGWKVMRQVNPSLEQELLEFLV
EVDTRRQERAKRMEDKKVYLQLHEAMEALVHICLDGCRTIGPRDQNLKGTQAACKFPACKGIEFLVRHFSACKMRVPGGC
TNCKRMWQLLELHSCMCSTPDKCRVPLCRHFKEKMKQLSRKEEIKWNLLVCKVLESQGTISSIPQRRKFSSLKPSS*
>Bdis_2g58152
MPEAPRADGAAAVPDVDVVTAGGRRRIPAHSSVLGSASPVLERILQRRMQKIRDSGKPGRAVVRIRGVTDDVAAAFVRLL
YAGRDGGNEESEMEEELESHAEQALVLAHAYGVPWLKRRCQEAIGSRLTPATVVDALQLAALCDAPELHLRCMRLLAKEF
RAVERTEAWRFLRDNDPWQELEILRHLHDADMRRRRWRRKRAEQGTYMELSDAMDCLQHICTEGCTEVGPVGQAPAKSPC
PGYATTCRGIQLLIRHFSRCHARATCPRCQRMWQLLRLHAALCGSLPDGHCNTPLCTQFKRKEQQKEAEKAGDGDGDGRW
GLLVKKVKAVRVMASLGKRRSPTQC*
>Bdis_2g59770
MSAAAAAQPHPPAEGSAPAAPLSTQPPSAAAPRPYELAVAAAELRPVDCNLAALCDHIQAEGFGAGSFSDIVVEAMGATY
RLHRFILSRSTYFMNMLHGPWREAGAPTVVLHIDDANVDSEAISIALAYLYGQAPKLNDNNAFRVLAAASFLDLQGLCTI
CTDFIISELWTSNFLQYQLFAESQDYGRHGERVRNACWGYLCQSATLELREVLPKLSSQTLHALLTSDVLWVPNEEKRFE
LALFALLAKITVSDVEVSGNENLDLMSSSPTVDRSIGKGKSPMNETGEEQLMESELQKLKLHDNMRSNTAHNIIDVPNII
IPQDSIPHSVPQNAEASKRMENDISTGGPSSESTSYQFNENIWLSSEQTKNYYSRTSSSNGLVPAEWGKPTAPIWGGRVV
GRRQVRCISGNSSLSADDYNAFMNIFERGSLLYCNMSFDALLSVRKQLEEFEFPCKAINDGLWLQMLLCHRVQAIVADTC
RNCCLTSNACACKQAYVSTHSHYRQEHDRSSASGTAGSIYLAEAQGDGNGMFGPVRVNVRGAVDGLAGIGRGNSNVPGAA
WAPTRYVFSRVPYGLGSRNGQQSFANDESEPRVDHNGDIPGDGLTALVNLSQESSASHHQAESIFETGMQTGYSSAASVS
TPGGSSLQMQESKEHELGSNWESADNTTISLDMRTPLSHFPPFRFGVEFEDVHRLGDGQVKHSAEVFYAGSLWKVSVQAF
NDEDPHGRRTLGLFLHRRKAELLLEPTRKAHMYVDPREKVTARYQLICPSKREVMIFGSLKQAGTLLPKAPKGWGWRTAI
LFDELADLLQGGALRIAAVVQLV*
>Bdis_2g60710
MDDTLKSLSMDYLNLLINGQAFSDVSFSVEGRLVHAHRCILAARSLFFRKFFCGQQQQASADQPLMDHLSPRSPSGAGAS
SPRGGSTAAAAAAAGPGAVIPVSSVSYEVFLLLLQFLYSGQVSLVPQKGEPRPGCGERGCWHTHCAAAVDLALDTLAAAR
SFGVEELALLTQKQLAGMVEKASIEDVMKVLMASRKQDLHQLWTTCSHLVAKSGLPPEVLAKHLPLDMVAKIDDLRLKSS
MSRRSSPFLAHHHGHHDTQQQQQQPSSDQQQQHLLDDAHHKIRRMRRALDSSDVELVKLMVMGEGLNLDEALALHYAVEN
CSREVVKALLELGAADVNHPAGPAGKTPLHVAAEMVCPDMVAVLLDHHADPNVRTLADGVTPLDILRTLTSDFLFKGAVP
GLTHIEPNKLRLCLELVQSAAMVMSREDNNSNNPAAVSMAYGGDQHQHQHPGGAAVYGGGSGVTSTGMNLSLDNRMVYLN
LGMDVMSHGGGGAGDRDDGGSRGQGGPSSLFSPHGFP*
>Bdis_2g61167
MSDYSCLVPFKLRYAETKNLSQGDAVSSEVVSAGGHLWRIRCYPCGIDDDDEDEEEEAAGYLAIYLELMSKPSARGVKAV
FEAFLMNKKGEPSSSHADRIMEVFSPKNLRCWGWPRFAKRRDLESRYVVDGWVTVVWGVMVVRSDDDDPLVVPCSDIGAH
LGRLLDDGNSDVSFVVGGDTFPAHRAVLAARSPVFKAELFGSMAESTMSSITLADIAPATFEVFLRFIYTDALPDTPIET
CKHLLAVADRYAMERLKIMCAKKLWDGVSVDTVADTLSCAETYSCAELKTK*
>Bdis_3g09770
MAGRDASTVAGAAAEAEMDPDFSSGEGGPSFEFAFNSVNFSDRVLRIEVVGTDDAPGSRGDVAGGSLSDWARHRKRRREE
LRKEKECATHMSDQTNCNDVEAEECDAYEENQEEPVAMIEESPPSIGLDGDDGRSIDSWTVVSTPILRVNTIYISSAILA
AKSQFFFKLFSNGMKESDQRHATLRITDSEENALMELLSFMYSGKLTTTEPTLLLDILMAADKFEVVSCMRHCSQLLTSL
PMTPESALLYLDLPCSISMATAVQPLTDAAKEFLANRYKDLTKFQDEVMSIPLAGIEAILSSNDLQVASEDAIYDFLLRW
ARGQYPKSEERREILSSRLLPLVRFSHMTCRKLRKVLTCTDIDHEQATKCVTEALLYKADAPHRQRALAADTMTCRKFAE
RAYKYRPLKVVEFDRPYPQCIAYLDLKRDECSRLFPSGRIYSQAFHLAGQGFFLSAHCNMEQQSTFYCFGLFLGMQEKGS
MSVTVDYEFAARTRPSGEFVSKYKGNYTFTGGKAVGYRNLFAIPWSTFMADDSLFFIDGMLHLRAELTIKQP*
>Bdis_3g12700
MREARMLYVVIKDMQPTVFKALQNFIYTDSLPDFGDLVDIIGHLLVAADWYAIDRLKLIYQDILYKNLDVEYGAITFALA
DQHSCEKLKDACIQFITSADRMDARLCKSRRSQSSVLVDVFEKTRRFEQSIDSQF*
>Bdis_3g13110
MSGGGSLSGPDSRVETISRLAQWRIDTFGPCSYRRSDPFKLGIWNWYLSVEKSRSVYVRLFPEPGRLAKEQPPFARFLLR
VSWAGPPRRSSVSPVIEQLLRSSDDFVWQVDAMSHGRFTIDVEFLDLRIATNNGTESSSSIWPNEGMVQKVASKSTLGSL
SRMLTESIHADVTINTTDGVLKAHKSVLAACSPVFESMFLHDLKEKESSTIDISDMTVESCSALLGFVYGTIDQEQFWKH
RVSLLGAANKYGIAEVKACCEESLLEDICSANVLERLHVAWLYQLEKLKKGCLAYLFVFGKIYDVRDEIDGFFHHADREL

MLEMFQEVLSVWKPI*
>Bdis_3g14350
MACLKLGSRADVFRKQGQEWYCTTGLPSDVTVVVGEQSFHLHKFPLLLKSGLLECRIREKIETGEDSCVIDLSDIPGGAK
AFELAARFCYGVKFEMTSSNVVHLRCAADYLEMTEEIAEGNLIAQTESFLTQTVLKSWKDSIKALHTCDNVIDIAEKLQV
VKRCIDSVATKSCTDPDLFGWPVVQYGGPMQSPGGSFLWNGISTGARPRNSSSDWWYDDVSCLSLPLYKKVISAMEYRGI
DQDIIVGSLNHYAKRRLPGLNRRKSISDVSSCLSMTTLTAMPSEEEQKYLLEEIDRLLPFQRGVTSCKLLFGLLRTSIFL
KASSSCVSNLERRIGLQLDKATLEDILIPNMSESVETLYDVDCIHRILDHFLTMDQETGGASPGLGDDGQILASPSLMPV
TMVAKLIDGYLAEVAPDVNLKLQKFRSLAAAIPDYARPIDDGLYRAVDIYLKAHPHLPESEKEELCRVMDCQKLSLEACT
HAAQNERLPLRVIVQVLFFEQLQLRSSIAECLMISENLEGGSRQLGLPISAEQHRGVGWPLATRENQALREGMDGMKQRV
AELEKECSTMREEIERLGRSRNASKSWLSSLGLGGTKPQICSTKDAAPTTASDDNKLAVVKADATPRLKLSRHKKNLSID
A*
>Bdis_3g18250
MKEPIVFNSRDVHLGRSRTRAVGCVPRSDLETSKYLDDDRLVVRCLVIVIKESLLTKTRVVCSEIDVPPSDITEHLAKLL
EAKEQTDVTFCVGGETFEAHKILLAMRSPVFKAELYGQMKETNMQAVTIEDMQPAVFKALLHFIYTDSLPGLDDLEGDDK
LEMIRHLLVAADRYAMDRLKLVCQSILGKNLDVENVSTTLALADQHNCDKLKDVCIEFIDSSDKMDAVVATQDYANLKRS
CPSVLVDVLEKTSRFRKA*
>Bdis_3g18350
MAANWQNWQALHGVRSPPRSTPTLIPFIPPAPRMGLELTDTASRCTTGKAVGSHEFEIDGYSLKKGMGVGKFVRSATFTV
GGYNWAIRFYPDGFTEDAKDHVAICLEFMSSNAKVRAFHDMGLVKHATGLMGAGFVRSETMVFTSTAAIDKTTIPRVKLE
GSKYIQDDRLIIKCVLTVLKESQVFQTKGSSEIEVPPSNITEDLGKLLGAKEGADVTFSVGGETFQAHKIVLAMRSPVFR
AEFYGPMLETRMQCVAIEDMQPAVFKALLHFIYTDSLPNLDDLVEDGDANCEMMKHLLVAADRYAIDRLKLICQNVLAKN
LDVENVSTTLALADQFNCDRLKDVCFDFIVSSNEKEAVVATNGYANLKRTCPSVLVDLFEKTSRLRKA*
>Bdis_3g18357
MGSQSKRTLSRYSTVTEHGSHTFEISGYSLKKGIGVGEFIQSSTFTVGGYDWVIRVYPDGSCDAVKDYVSVYLEIMSRNT
EARACCSLRLINQDTGKPVIMWSEETPKVFRSCDSSRFGPQNGQFVLRSVLEEESLGYIKDDFFQIECDITVIKDSYVYE
SSVWSEITVPPSDLSQHLGKLLSDKKDTDVTFSVGGENFVAHKIVLAMRSPVFKAQLYGQMKERRARRITVEDMQPAIFR
ALLHFIYNDSLSADMDDLNDDEYSETIRHLLVAADRYAMDRLKLMCQSILCDYINVETVAATLALADQHNCDKLKAVCVE
FLEYTASSEGMDAVVATQGYADLKRTCPSVLVDVFEKTSRSRKA*
>Bdis_3g18370
MGSVSKKTISRHTTHTEQGSHAFEISGYSLNKGIGVGQYIQSCTFTVGGYDWAIRLYPDGVVEAFRDYVTIYLELVSQDA
EVRALYDLSLVKQETGLPVSMWCKSTPREFRSRDSSRFAPQSGGFIPRSTLEMDDIGYVLDDYLTIECAVTVVKEAHLYQ
TSAEYEIALPPSDLSDHFGKLLLEEEGADVTFSVGGETFAAHKIVLATRSPVFKAELYGQMKERTAQSVTIEDMQPAVFR
AFLHFIYTDSLAQMEDLDHDDYSEMIRHLLVAADRYAMDRLKLICQNVLCQYIDVDTVAATLALADQHNCESLKNVCIDY
MTTSDEIDAVAATQGYANLKRSCPSVLIDVLEKRSRIQKE*
>Bdis_3g18410
MATEMTTQSGSKKTASRCITEKAEGTHVFEIVGYSLKKGMGVGKAVRSGTFTVGGFDWGILFYPDGTSRTGHQCVAADLE
LRSKGKQARACCDLRLVNEDTRLAYSFESISPKAIPTSYNDCSHYSRENARVVEKSHFEEEGSGYIKDDSLTIECVLTVM
KQSLVAKITGDSEMQVPPSNLSSHFGKLLLEENGADLTFSVGGESFAAHKIILAARWPVFEAELYGQMKEREDQCIIVED
MQPGVFKALLHFIYTDSLPCVDDLGDDDYSEMIRHLLVAADRYAMDRMKLMCQNILSENLALETVAATLALADQYNCERL
KDVCIKFIASTDEMDTLMATQGYMDLKRTCPFVFVDVFEKSKRLRGA*
>Bdis_3g18440
MPNQMLPTRTVSSCTPRTVQGIHVFSISGYSQHRGIGYTEYNKSGAFSVGGHEWEIRFYPDGQHKQDFIAVYLELLSKGA
NVHASCDLRLVDQTTGLSSSVHKTAPRMFNPNNDLRFAPQDGNFMSRTEFEASAYLRNDHLAIECVVTVLKDAIVSQVKP
RSRIEVPPSDITAQLGKLLEAQEEVDVTFSVGGETFAAHKIILAMRSPVFKAEFYGPMRENAAALIIPIQDMQPDVFKAM
LHFIYTDALPAMDDLEGDDRSEIIRHLLVAADRYALDRLKLVCQSILCDNIDVQTVATTLALADQHHCDMLKDACVEFIS
SSAMDDIVATKGFVDLTRNCPSVLIDVFVRMSRSCKS*
>Bdis_3g18640
MAAPQMAGTKTVSRSVLDTARATHAFEIIGYSHHRGLGNGKSVRSAAFAAGGYHWCIRYYPDGDNTEDSNDHVSAFLVFL
SKDAKVRAGFDLRLINPVTTDFIYRVQPLVFDDANRTWGHRRFMKRSDLEASPYLRDDRLLIECDVVVLNEPRIELTLLD
FEVQVPPSDLSDDLGKLLEAKEEADVIFKVKKDVFPAHKIVLAMRSPVFKAELYGPMRGNKTRRKYITVEDMQPAVFKAL
LHFIYTDSLPSMDEIIGNDKKELIKHLLVAADRYAMDRLRLICEGILCKSLDVDTVAATLGLADQHNCSKLKGACVEFIL
SSNRMDVVVESQGYVHLKRSCPALIFDIFERATKFHKI*
>Bdis_3g27067
MSAAEAPISRVYRSRSRSASAIVAGTVTGHHLLNIEGYSHTKELPTGQYIKSRPFMVGGRLWRIKYYPNGDRPAKADYVS
IYLIPGESFAQPVKARVRFGLVDLARKPVPSQTLTTELHSFTANGFGFADFMERKELDMSEHLVNDCFTIRCDVIIYKEV
RTEDRSSPLVMVPPSDLHVHFGDLLATKDGADVTFQVAGEMITAHRCVLAARSPVFKAQFFGSMKEGAKSHCIQIEDMMP
EVFKNLMHFIYTDSLPEMKEQEEAVMVQHMLEAADRYDMQRLKLICEDKLCTLINVSTAATTLALAEQHSCDVLKQACMD
FLKCPVALDAVMETDGFDHLSKICPAVLKELMSKIVAR*
>Bdis_3g27074
MSAVASASAIVADTASGFHHLKIAGYSSLKGLPNGHRLSSCPFTVGGHHWRIDYYPNGDRQESAGYVFVFLVLDENMIDG
VKAQFEFGFWPKKRGLFFRKRTKSGLRLRYLFSQSTPSWGYGEFMKWEALDRSEHLKDDSFTIRCDIVVLNKVCIQGRAK
KAAPKFVSVPPSDLNKHLGSLLVSEKGADVLFEVGGETFAAHRCMLAARSLVFSAELFGSMKEGDINDLVRIDDMEAQVF

KALLCFVYTGSLPEMKEEDDEEAMYHHLLVTADKYNMERLKLICEEKLCKYIDVGTVVNILALAEPHHCHGLKSAWFDFL
SSPTNLRTAMASDGFEHLAITCPSVVKDLIAMCSAT*
>Bdis_3g27081
MSAAACKPSRSASTIVAATASGYHLLKINGYSRTKGVPHGQKLQSRPFSLGGHQWYIGYYPNGFNSDHTGCISPFLFLDD
SVTTAVKAQHEFCFIDEVKDQALSLASSAVSEFASKQGWGTSKLIKRADFEKSKHLKDDSFIVRCDVIVINDYRAEDLPE
NTPPTFVSVAPSSLHQHLGDLLKTEKGADVVFEVGRETFAAHRCMLAARSTVFGAELFGVTKEGDTACVIRVDDIEAQVF
KALLCFAYTDSLSEARNEEEDVMCQHLLVAADMYNMDRLKTICEDKLCKYINVGTVATILALAEQHHCDGLETACFHFLR
TPAKQI*
>Bdis_3g39210
MTGGRVEAAPRLAQWRVDALPCYTYRKSDPFRVGLWNWYLSVERNNKQTCVKLFAELSNSAKNTVPAPIASFVTKLLISF
PPNEKIIVHPGIFDKHLKHDGFVWAIDSTVTGRFVIEIEFLDLKVPDPSGGEPASVWASHQIKQSSDNTALSSLTRMLQE
CILTDITINATDGSIRAHRAILAARSPVFRSMFSHDLKEKGLSTVDISDMSLDACRGFLNYVYGDLRNEEFLTHRLALLG
AADKYDIADLREACHESLLEDIDTGNVIERLQVAHLYRLPRLKEGCLRFLVDFRKVYEMHDDFNAFLQTADRDLVAEVFQ
GVLAAWSGR*
>Bdis_3g40527
MVDSGSVQFKIDYRTIQTFASDDRYILEESICVGEHRFSASCYPQECSEDGEFEYLSIDLSLRSTSEVTSPVFEALVVGK
DGAPSPALAKRKRGTASDFNGLIDGVWKSWCWLDFSKGGDDDMESLWAANGRMITISCGFSVPLENPITVPASDIGDHLR
GLLDCADGSDVSFSVGGETFRAHRAVLAARSPVFKAELFGAMAEARMPCITLHDVEPATFKALLCFIYTDALPRDSGELV
GDPVDVFQHLLAAADRYALDRLKLLCAHKLWDNVTTDRVADILACAETYNCRELKDCCMDFFVTEQNFKHATLTEGFVRL
GQKCPTIIAELRKKARI*
>Bdis_3g40720
MVDSCFVEFKLDRQSYSEKLANGSDGFMELVTIAGDDAFIVNYYLPQCRQYGKKSEYLRLGLLLLSKYKKTNPVFEAFVV
GKDGAPSPSIANRATGAVFPGWTDEHWSFLYSWDWDHFLKPSDLESLWAANGGVVTIICGVVVRRDNPITVPASELFGAM
AEARMPCITVHGVEPATFKALLCFIYTDALPMDSGDLIGDPVDVFQNLLAAADRYAMDRLKLFCAQKLWDNVSADTVADV
LACAETYSCRELKDSCMDFFVAEKNFKQAMLTEGFVRLGQKFPSILTELRKRAGI*
>Bdis_3g40730
MVDSGFIEFKLDYQKTRKLALGHKLSKSIRAGEHTLTVDCYPRGLFGGNYPGDDLPLHLFLAIKSKNINLIFEAFVVGKD
GAPSPSHAKRTVAFESIVGGGTRHATLFSFLKQSDLESLCAANGGVVTVVCGVILRHDENPITVPAPNIGVHLRDMMGCT
DGSDVSFSVGRETFHAHRAMLAARSPVFKAELFGSMAESKLPCVTVCGIEPSIFKALPHFIYT*
>Bdis_3g40740
GDGPSSCKELLWRKNMLQREASQLYAPSWSSMNTPYLCRLQISAPTLAACSITPTGQMWHSSSTARHSMLTGLCLQPARR
SSERSSLDPWPRLQMPSITLHEIAPATSKAMLRSMYTDALPREKEIGDSSVEMFQNLLAAADRYALDRMKLTCAQKL*
>Bdis_3g40745
MSDVVSVGGHLWRIQCYPRGYHGHGDDKGEYLSIFLAHMSSTTTSAKAILDVFLMDKDGKPSVKDEIRFLHTFKCSQVRQ
SSGWPQFVKWTTPEKDYLAEGHITIVCAIMVTDDSPIPVPPSDIGTHLGSLLDCADGTDVTFIIDGETFHAHRALLAARS
PVFRAELFGSMAEATMPCITLHEIAPATFKVMLRFMYNDALPGEEELGDSSIEMFENLLAAADRYALDRLKLICAQKLWD
KVSVDTVSTVLACAETNNCPELKNKCIDFFVVEENFKEAMFTDSYALLVLKFPSVIA*
>Bdis_3g40750
MRTTIVCSSVLKLTIDYEKAKQLPIGKYVESDVVSVGGHLWRIDCYPRGHSEDDKGEYLSLFLAPVNMSNTRSANAILDV
FLMDKDGKPSATDARRLLHAFEVNSDGYSWGWPKFVKGTNLEKDYVAEGHITIVCTIMVTDERPIPVPPSDIGTHLGSLL
DHNDRADVTFIVEDQTFHAHRAFLAARSRVFKEELLAPWLRLQCLPLSGTTLHLLHSNDLLAAADRYALDRLKLMCAQKL
WEHLSIDTVATTLACAETYNCPELKNKCFNFFAVEKNFKKAVFTDGFATLLQKFPSITDELKSKVET*
>Bdis_3g40760
LWRTSTKTTIFALIKRQCHLGRLLDRQGRQAIHHHCRKVSPRLRCVSWGWPKIVKGDTLEKDYVSDGHITIVCAVMVIDD
SPLPMPRSDIGNHLGRLLDGAAGTDVSFVIDGEIFLAHRAVLAARSPVLKAELLGSMCDAAMPSITRHDIAPATFKVMLH
VHGCLARRG*
>Bdis_3g40767
MSKRAKTTALEAQTTMVGSAAVQLRVDDELASQAPIGYAVYPDIISVGEHLWRIECLPRGDVEDGSGEYISIFLRHMSKS
TSVTAIFEVFLIDNYGIPSMTETRRILRTFRIATGRGRGSGDSWGWRHFVKRTILEDSYVDEESHFTFVCAIMIIDDCPI
CVPPSDITTHLGRLLDDTDGTDVSFVIDGETFTAHRAVLAARSSVFRAELFGSMAEATMSSITLKDITPAAFKAMLRFMY
TDALPGESELGESPVEMFQDLLAAADRYALD*
>Bdis_3g40777
MCDSCFLPFKLHYAEMKKLATGDFVQSDVISAGGHLWSIRCYPRGKGEMDDDDSDSDDEDEVAADYLAIYLRHMTNARGI
KVIFEAFVMDKNGEPSTSHARRVVHFFSSKVSKSLGWSRFLKRRNLESRYVVDGWVTIVCGVIVVPDGDHLTVPPSDIGS
QLGRLLDCADDGSDVSFIVDRTTFAAHRAVLAARSPVFKVELFGPMAEASMSHIPLEDIAPATFKVFLRFIYTDILPEDD
KLEFGDDGDSPVEMYKHLLAVADRYAVDRLKLMCAKKLWDNVSADTVAETLSYAETYSCAELKTKCIAFFAEDKNFREAV
LTDGFVALVQKFPGIIAELRKKAKD*
>Bdis_3g41280
MSDSCFMPFKLHYAETKKNLSQGDAVNSEVVSAGGHLWRITCYPQGNEESGFIDDDDEEEDEEPAADYLAIYLELTSESK
GVKAIFEAFPMEKNGAPSSSHAKRLMDVFSSEEDLQSNWGWLHFVKRIDLGNRYVVDEATMSSITLEDITPATFEVFLRF
IYTDALPGDDELGEDGDSPIEMYKHLLAVADRYAMDRLKLMCAKKLWDDVSVDTVAETLSYAETYSCAELKTKCIAFFAE
EKNFRKAVLTDGFVRLVHKFPAIVAELREKPKK*

>Bdis_3g41290
MVGSVVQLKVNYEKAKNGKAIHSDVVSVGGHLWRIDCHPRGVSEDCKSEFISILLQHMSKSGSVTAIFESFLMDRDGQPS
SKYQLRSLPRSYQSDSWGWGQFIGRTTLEKDYLEDGHFSILCTIMITDDSSIPVPPSDIGTHLGSLLDRADRTDVAFIVD
GETFHAHRAVLAARSPVFRAELFGSMAEATMPAITLHEIVPRTFEVMLWFIYTDALPGDKELSDSSIEMFQNLLGAADRY
ALDRLKFICAQKLWEKVSVDTVSTILACAETYECPELKNRCIDLFVAE*
>Bdis_3g54372
MSSSGAHQAGGQSSRSASPITADWSSGHLHLKIHGYSGTTNLPTGQFSSDYFSLGGHRWRVDYYPNGVRADVADYISLCL
VLAQDAPPPVKVQCELSLVSESGEEHVVPPVAWAWVDTFFSPYGRLCCSRFVRRAVLEASKHLGPDGSLTIRCDVIKKFA
PEEIAAFVPVHPSDLARNLGELLETGKGADVVFEVGGKTFAAHRCVLAALSSVFAAELFGPMKESNTAPGVVRIPDMDPE
VFKALLHFAYTGSLPEIPKERESMAFQHLLVAADRYKMERLKLICEQKLCEHIDRDTVASIFAVAGQHHCAGLKKACFRF
VDCPENLVALLGSDSFKHLIRSCPDLVSELVELVAKAMKP*
>Bdis_4g01780
MGSVKLGSNSNSKPDAFTRRGQAWFCTTGLPSDIIVEVGDMSFHLHKFPLLSKSDILGRLIEESPDQDECIIKLSDIPGG
AKSFELVARFCYGVKVELSPANVVHLRCAAEYLQMTEETAEDNLINQTETFFNQVVLRSWKDSLEALKACDALLPHAENL
HIAKRCVESLAAKASIDPDLFGWPVSEHGVMHIPGGSILWNGISTGAKLGNCSSDWWYDDASSLSFPTYKRLISTMESRG
TKEEIIAGSLTYYARKCLPGLNRRQSTGLVPLTTAASLSEEEQRHLLEDIDGMLPLQRGLVSTNVLLWLLRTSMILKVNR
ACISNLEKRVGMQLDKATLEDLLLPNFSYTMDTLYNVECVRRILDHFLAMEQSMGGGSPCLDDVMDSPSLAPITAVAKLI
DGYLAEIAPDINLRPPKFQSLAAALPEYARPLDDGLYRAIDVYLKAHSCLPESEREQLCRLIDCQKLSLEACTHAAQNER
LPLRVVVQVLFFEQLQLRTSIAGCLMVSDNLEGSSLPLRCSGATTTSGEAGAGWVTGAAVRENQALRAGMDSMRQRLAEL
ERECSGMRQDIRKLGCAAPGKDGWAARVQRMFNLKMKLQMCSTEEGKMSERHRSVSAKLEKLQAKVAKHKEHLSIDA*
>Bdis_4g02767
MEESRPRRAPARIRAVPIAVTPGGVLDAPAVEAAAADGREEPAGGGEAQLQQHEICVGFGQPETSDLTVMLYGKEGIAVR
MNVHRDVLCGSSAFFAAKLSGGGNGGPPVPSVEIHDCDDAEIYVETVGLMYCDEAKHKLLKQNVSRVLRIMKVAESLGFE
ACVKSCLDYLEAVPWVGEEEDNVVSSIRHLQSKAYGVSPLLKRVASDSLNSPADTLSHIMEMVLKSNDDRGRREMKALVL
NLLKDSNRCTDGTSDICSELFHSSCQGCIDRLKLLFAEASEAGFPSQVTRQITLETDNLLWLVEILVNQRICDDFVVMWA
SQSELASLHAKLSVASRHAVSCITARLFVGIGRGEMLPSKNTRLLLLQVWLQALIDDYSWLQCSCRSFDRKLVEEGIGQT
ILTLPLEDQRSILLSWLGRFLKLGDNCPNLQRAFEVWWRRTFVRPYVNQAR*
>Bdis_4g03397
MKYMKLGSKPDVFQTEGNIRFVATELATDIVITVGDVKFYLHKFPLLSKSSRLQTLVASTNDEGNDEVDISDIPGGPSGF
EICAKFCYGMTVTLNAYNVLAARCAAEFLEMFETIDKGNLIYKIDVFLSASIFRTWKDSIIVLQTTKSLLPWSENLKVIN
HCVDSIASKASIDPSEVEWSYTYNRKKLPSESGSDSHWNGVRKQPMVPRDWWVEDICDLEMGLYKKVILAIKAKGRIAAE
VVGEALRAYAYRRLFSSLDNAVNNGLDCTRHCAALETIISLLPAERDPPWRPGQEDRLAARRSFGLGSSDTCELRRNAMY
NVDLVLAILEEFMAQRHGGGDEELQDDDDEEEVTMDGENHRHLVSSVSGDSDLALARLIDGYLAEIAKDPSLPLPKFIAI
AETAPLAARPTHDALYRAIDMYLKEHPGLSKSEKKRLCALMDCKKLSAEASAHAVQNERLPLRVVVQVLFFEQLRQSSAA
AAELPSSALRSLLPRESGNSYGSSRSAATTATTAEDDQWGTGAGDAGSFRSAGGGAMATNKSGGGGDGGSSKNGGKAGKG
MMPRKMLSKLWSGKASSGGEHSGGSDTSESPGSAAHLDAMAAGDAKSTHSRSTRHSVS*
>Bdis_4g12820
MSISELLSALRDAGRQHLSASTVGTRQATGSHLLQIENYAQIKKQLSVGEYVKSSTFTVGGHDWRVELYPKGCVREAHGH
VSLFLRRVCDAKTGGRDDSATAKIEFSLLDGATGEPSLTKSSPPRTFKPGDEDWGWREFVNKDDGLEDEKTCLGQDGCLR
VLCDVSVDLGIRADDATPEPETTGLFPAAPSFELRGELAGVIWNARRPDVRVEVGGEALAAHRWMLAPRSPVFAAELSRT
FQKNPAVLRVEDMDADVCRALLEFIYTGALHDMDRKLESSMAEKLLVAADRYGVEQLKELCEKAVCGRVGMSSVAASLAL
AKRHGCPLLRDACLEFLSAPGNLEAVVEMDGFEELMQEGCPSGLTKLIVKQVRRHEQWSPFSG*
>Bdis_4g12910
MSASAIVAKAVSGSHILKIDGYSRTKGLGNGKFIKSEKFAIGGHRWRMLYYPDGDVVSEKAADWISIYLAFDRANANEVK
AQFGFSLLDQDMQPVPSYSRKSKKTRTFSSKDTAWGFRKFIRRKELEESSYLKDDVFSVRCDVTLTTEIVTQSQPAVQVP
PSDMAQHLGQLLSASDGADVTFHVGGESFPAHRYMLAARSSVFKAELLGAMKEKTAAHVRIDGVEAKVFKALLHFIYTDS
LPAETDDDGGDTAAMAQHLLEAADRYNIERLKLICGDKLCNLINRSTVATTLALAEQHGCGALKNACFKFLTSPGNLKAV
MASEDYEHLKRSCPSLLEELVAKLAL*
>Bdis_4g12915
MSASCPSSSVHSASPLSASAIVAHADSGSHILKIDGNSRTKGLGNGKFIKFGKFDEGGHRWCVMYYPDGNVSDTTDCISI
YLRLEHGDDANEVKAQYRLSLLGQDMQPVPAYSFQSNQIRTFSSKDRSWGYTKFIKWKDLEESLHLRDDVFSIRCDVTMP
KEIITEPTAPAIAVPLSDITKHLGQVLLSGDAADVTFDVGRETFAAHRCILAARSPVFKEELLGSMKEKEADCVKIEDVE
AKVFKALLHFSYTDSLPEMDKDETAAVAQHLLVAADKYNMDRLKLICEDKLCSRICSSTVGTTLALAEQHGCGALKEACL
NFLVAPGNLKAARACQGFDHLTRSCPCVLEELAATP*
>Bdis_4g13857
MSSSPLSAVLPTTASAVVTKAVAGSHVLKVKGYSLIKGLGVGKFIEFGKFSVGGSSWTVRFYPDGGPGSDYCADWVSIAL
FLLDPNPTTDVRANFKFNLLDQAQGKHVELNPQPGMRSFSNAKTGFGQDRFIKRMELDESTYLKDDCLEIRCDVTFALMK
QISKVAVEAAKTQFVAVPPSDMHRHLAALLSGASGGGEGSVDVRFEVGGETFAAHRCLLAARSSVFSALLFGPMKEKTAT
CVKIEDMEPRVFKAMLHFIYTDSLPEMDKGETTVMAQHLLVAADMYNLPRLKLICEHELCKHVDGSTVDTMLALADQHGC
RGLKEACLSTINFTRNLKETTASDGSEDSASAFEILAGVLIIVFISYWVFRIFSLI*
>Bdis_4g13864
MSTSSVAAALAAGGGGMHSASVVVAEAVSGSHVLKVQGYSLIKGLGVGKFIGSVKFNAGGRSWCIRCYPDGWGSECTDWI

SVALFLLNPDATEVKAKYRFSLLDQAERTHVPLHTEAVSTFSAKASGKGHDKFIKRQKLEQSAYLKDDCLEISCDVTVLK
EICLAKDVAVQFVVVPQSDIHQHLGALLSDAGSEGADVSFEVGGETFAAHRCVLAARSSVFKSGLFGPMKEKTATSVKID
DMDPRVFKAMLHFIYTDSLPQMDSADATMMAQHLLAAADRYSLARLKLICEHELCSNIDKSTVTTTLALADQHGCHGLKE
ACFSFLKSPGNLKEITASDDLEHLMKSCPSVLKELVAQLAP*
>Bdis_4g15130
MRKLFFSELTSCKETKLQSATHSWLPLEKGKLCKFTGHSSSSSSIESLMKMPEPAVLPHFKPADYVDILAQIHEQLEYCA
PDEKSCLYLLQFQVFRGLGEAKLSRRSLQAAWEKASTIHEKLIFGAWLKYEKKGEEPISDLLGSCGKCSQEFKLLDFVSQ
ISAESHGLSYDDESDEFQGSPVVHFRIKDDMIAGDRRKLAALSTPLYAMLNGGFRESHLEVIDMSRNGISPIGMRAISKF
SLSGRLPYLSAEAILEMLDFANKFCCKGLKDACERKLASFVCTRQDAVDFMECAIELGCSILAASCLQVLLNELPECLND
EQVVRIFSSANKQQRSTMAGNASFSLYCLLSEVSMSISATSDVTISFLEKLVESASDSRQKQLALHQLACMRLLRKDHTE
AERLFNAAFNAGHHYSVVGLARLASLRSNKHFSLKLLDSVMSSRWPLGWMYQERALYLDGDSKLENLNKATELDPTLTYP
YMFRAASLMKRQSVEAALMEINRILGFKLVLECLELRFCCYLALEDYRAALCDVQAILTLAPDYRMIGGRVAAKQLRMLV
MENVEQWTTADCWMQLYDRWSSVDDIGSLSVIYQMLESDAAKGVLYFRQSLLLLRLNCPEAAMRSLQLAREHAASQHERL
VYEGWILYDTGHCEEGLQKAEASIAIQRSFEAFFLKAYALADSSLEPSTSATVVSLLEDALRCPSDRLRKGQALNNLGSV
YVDCGKLDLAAECYINALKIGHTRAHQGLARVHFLRNNRTGAFEEMTKLIEKARSNASAYEKRSEYCDRDLTKADLQMVT
KLDPLRVYPYRYRAAVLMDNHKEKDAISELTKAIAFKADLNLLHLRAAFHEHVGDISGALRDCRAALSVDPNHQEMLELH
HRVNSQEP*
>Bdis_4g23730
MAERSNISIAVVAEKRSLVIKIDGYSATKRLLEIGQSVNSSPFSVGGHQWFVAYYPNGRTASGCISFFLVLDSADAKDEV
RAKVRFNLLHKDGRTPVPPLSRSTVERVFRASDQVLFLNAIRHRDLEGSGHLIDNCFSIRCKINVFKDPTQKCHQFVEVP
PSNLHQHLGDLLNSTDGADVTFHVGGETFSAHRSVLAVRSSVFKAELLGGMKENLGDPIKIDDMEAWVFKSLLSFIYTDS
LPLMTREGTDEGATPADVVTASHLLVAADRYNIERLKLICEEKLCSCIDSAMVATSLVLAEQHSCHGLKEACLQFLASPS
NLKAMVASDGYEHLKRSCPAVLRELIARLLPVELKVAKDIVMSI*
>Bdis_4g23737
MAEPPVVTATIVAGEERRTHLIKIDGYSRTKELLETGKFTTSIPFSVGGHSWAVKYFPNGCKGATNYIPGYISLYLVPDL
ADANDVKAKFSFSLLDKDGVPVPSYSQTSEHTFTSKVPDWGFTKFIKHKELEGSAHLRGDSFRIRCDVTVVMQIRSEQTL
RNQYVEIPPTNLHQHLGDLLKSMDGADVRFQVGGEIFSAHRSVLAARSSVFKAELLGAMKEKTGDPIQIDDMESDVFESF
LYFIYTDSLPVTDAVMAGHLLVAADRYNIERLKLICEDKLCNHIDVNMVATNLALAEQHSCHGLKVACFDFLASPSNLEA
MKASDGYEHLKNSCPSVLKELLARLLPVELQAAKDIMMDI*
>Bdis_4g23747
MAERGIVTTSALVAGEDRRTHVIKIDGYSRFKELLRTGKYTTSVPFSVGGHNWAMKYFPNGSKAAAGYIPGHISVYLVLD
SDDAKDVKAQFSFNIVDKDGVPVPSYSRTTTEHIFPRKGSDWGFSNFIKHEDLEGSAHLRGDSFRIMCDVTVGMKIRCEE
TKCNPLEVPASNLHQHLGDLLKGMDGADVTFQVGGHKFTAHRYVLAARSSVFKAELFGAMKEKTDSPIQIDNMESDVFES
LLLFIYTDSLPVTDTVMAGHLLVAADRYNIERLKLICEDKLCNHIGSDMVATSLALAEQHSCHGLKEACFEFLASPPNLE
AMVASDGYQHLKRSCPTIITELLARLLRVELQVVKDIGMDI*
>Bdis_4g23767
MEAADVTFLVGGQKFTAHRIVLAARSSVFKAELFGAMKENSGDTIEINDMEADVFKSLLHFIYTDSVSVLEMDSNMGEAH
EDVFMAGHLLVVADRYNIERLKLICEHKLCGNIDANMVATSLALAEQHSCHGLKQACFEFLASPSNLEAMMVSHGYDHLR
ISCPSVLKELVARIIPVEFKAAKDIIMSI*
>Bdis_4g23820
MPLPMIYDFSSSVVPESEGRSYVFTVYGYKWTTKYVPAPLVCKVFCVGGHDWVVKYYPNGARKDYADFISVYLVLHSANA
KDVEVVFTFTLLDKAGELVPSYSRTMAGHTFAKKGSSGGYHDFIKKVDLEGSLYLRDDDSFSFRCDVNVLKHNQQKCDQP
VVIPPSNLCRHLKDLLESMDGADVIFHVGGEKFSAHRVMLAACSSVFKAEFFGAMKEKASSLIEVCDLEADVFKSLLHFI
YTGSLPETNNGDALGDVVMACHLLVAADRYNIERLKLICEYKLCKHVDSNTVATSLALAEQHSCHALKEACLQFLDSPSN
VEAMMTGDGYEHLKSSCPTVLKELIARFLPAELKAVLMSI*
>Bdis_4g23830
MAELRYNISAAVVAETEGSSYVFKVDGYSSAKWKLRNGEPLVSKLFSVGGYDWAVEYYPNGGRYEYSDYISVYLVLHSDS
AKDVNAIFTFSVLDRNGHPVSKYSRTIIGHTFSKKGSSGGYHDFIDRGDLERALYVRDDNFSIRCDVSVVVKDNDQQKCN
QSVVVVVPPSDLLRHLEGLLKNMDGADVIFRVSGEEFSAHRAVLAARSTVFKAELFGAMKEKECGLVEVCDMEADIFKSL
LHYLYTDSLPDDRTCGDGTIGDVVMAGHLLVAADRYNIERLKLICEDILCRHVDSKMVATSLALAEQHSCYGLKEACLQF
LASPSNVEAMMASDGYEHLKSSCPSVLKELVARFLPAQLKAVLMSI*
>Bdis_4g23840
MAKRCKISPSIVAESEQRSYVLKVEGYTMAKKQFETGKPVVSAPFNVGGYSWVVKWHPNGGRTEYAEFISVYLALHSAHA
KHVKVNFWFSVLDKAGEPVPLRCRPVVGHIFSSKGSNWGHHDFIKKADLQGSNYLRVDSVSIKCDVTVLKHIQKGSKFVV
VAPSDLHIHLQDLLNSMDEADVTFHVGGERFSAHRTVLAARSSVFKAELFGAMKEKARGPIEIDDMEADVFKSLLRFI*
>Bdis_4g25540
MDCCICGPMAAIYRPPRNTICAPCYEGAKAIVASLDDADDDNEQLAAGAHAGVVFKPARGPLINRNNNGSTKGMREVWEH
VKDMRSMGEEANERARFLSLGFGPNAAHTDIVLRPGSGPPIPAHKAILAARSEVFRHMLCDEERVKAGPGAGGCVTLPEL
AHDELELLLGFLYTGTLDLDSTETETGERRLRALLVAADKYDVPLLARVCEARLAVEVGLANALRTLEVADRVGGGGGGK
LKERAMAVVVEHAGTVVFSEEYEAFAARNTVLAVEVTRALLLAADNAGAKA*
>Bdis_4g33580
MDQDLIRRGGVVPSFAFAFNSPSFSDRFLRLEITAIDSLPGSSGVDDAGSLADSARHRKRSRLELREDKDSARHLLDTPN

PNGNEVETKDCHLSEEDQNELGSLTEEHLPSIGEDGDEGHLDTSPSMMGTPALRVKSIPVSSVILAASSPFFRKLFTNGM
QESNQKSVTIRIMETEEEAMLELLSFMYSGELKTNDPILLLDILMVADKFEVTTCITHCTELLSRLPMSKDYALLYLDHP
CSHSVAVALEPVKAAAKEFLANKYKHFLRFQDEVMRLPLSGIEAIFSSSDLQVPSEDHVYNFLLRWAIAQYPDAKECRKI
LNTSLFPLLRFSHLSYLKLQKVLAFMGLDRNEQASNVISSLLYKADASYRQNCLAADGVAPRKYEERSYSCRPLKVIVFD
RPFLQCMAYLDLKIDECFQLFPSGCALSQEFCFAAHGFFLKACCMMEQQSMTHRFGLYLGLLNRGPMPVTLDCEFAARER
PSGGFVVKSKYTHTFTDCGHSFGSRDLLNSLDGVDC*
>Bdis_4g37750
MRRGTRPRMGDRATSDVVVRLRTPEGRDEWLYCHSAVLAAGSKYFADRLSDAWPTCQILDSRYCVEVHCAEPDLSSHVTA
LRLLYADEPVSRFGVRGALALLQAAEHLGCPRIAAACADYIESAPWDEADEEEILAAAPFLAPQHGRLLARLRPIDPAPA
AGIFLSAFRHATSAAAPRELKSAAQEQLEYMLTEDDDAPLLAFDDDSVKSQVKDCVTGLLNRFSDFVSSALTKQKEAPCS
GGDDGEPQQELHSFISDISWVCQVLSKLEMMKYLVIYWLGASSHVVEVVEAACPGFDCLKTRLKVIEISAKVLEAAAFSN
IVLPAEKRCHAVNVWIRFAGRTKALVDQADVGDGDAETPKIGLDGEVWQGLESAIVSIVLTLPSNTQAEILSEWLQSKHA
CFPDLTEAFDTWCYRSKVARTRLSFLNNINEAP*
>Bdis_4g38300
MSALEAVISCDEERVKLEKDAEVLAAQDDGGGEALERVYERLEAIDVSTGEKRAAEILFGLGFNKQMQAKKTRDFSGGWR
MRIALARALFMNPTILLLDEPTNHLDLEACVWLEETLKKFERILVVISHSQDFLNGVCTNIIHMQNRKLKLYTGNFDQYV
QTRAELEENQMKQFRWEQEQIASTKEYMARFGHGSAKLARQAPRRILTFRFTDVGNLPPPVLQFVEVTFGYTEDNLIHKK
LDFGVDLDSRVALVGPNGAGKSTLLKLMTGELVPLDGMVRRHNHLRIAQFHQHLAEKLDLDLSALQYMMNEYPAGNGEER
MKAAIGRFGLSGKAQVMPMRNLSDGQRSREIFAWREPQQLLLDEPTNHLDIETIDSLGEALNEWDAVNESPHDDSLGTLP
PIVRLKKIHVSSVILAGSSDYFKKLFTNGMLESTQKEVTLRIREAEEMPLQHLLSFMYGEEILTTDPAHIIGILMVADKY
QVLSCVTHCSELLTTCPISTEVALLYLNLDCSIPTALEPAKDAAKKFLCNKYQDFLRFEHEIMDIGPSGLAAILSSSDLK
VPSEDYLFDFIVNWGRIQYPDRGERRTVFSSLLPLIRYSHLSCGKLSKIMKCQDIDLNVARLPLVRALFFKSDPVSRQHL
IDGPEPWSYEQRAYLYRPLEVMQLHRPVKQCMVYMDLNTDDCSKMFPTGSILSEAFHFAKSDFFLTAGCIVQQPGPLHSF
GLFLGVTGDCPLPVTVKYEFSARAKPSGDFIVKWSYTHTFTQADVRMGRRNLFSMPWAAFVDYDNNPWFIGGVMYLRAIL
TLVIE*
>Bdis_4g40865
MAVDHRDLSSSIVAEAEKVSHVIKIDGYSITKGLRENGSCATSLPFKVAGHSWVVRYYPNGYRSEDAGYVSVYLFLQPPY
ASTTTKVILGFSLLDKDLTPVPSYSPKGPVPARTFSKQSPSWGHGRFIRKENLERSAHVRGDCFSIRCDVTVLKDIRVRE
NKVEFVVVPRSNLGRHLAGLLESMDGADVTFLVAGQRFSAHRCVLAARSSVFKAELFGAMREKEAGGVVEIGDMEADVFG
SLLHFIYTDSLPEVTIGNEESAVDDVAMAQHLLVAADRYDVERLKLMCEDRLCRNIDSTVVATSLVLAEQHGCPGLKEAC
VEFLDSASNFEAMLTSDGFNHLKNSCPSLLSELIRRFMPAELNAANKDIVMAI*
>Bdis_4g43150
MSSEDSLKSLSLDYLNLLINGQAFSDVAFSVEGRLVHAHRCVLAARSLFFRKLFCGLDPAHQPPPPPPLNWPNTASSAAG
AGGGGSRGGGGAPGAAPELVIPVSSIRYEVLVLVLQFLYSGQASVAAPKSGPLPGCGARGCWHTRCGAAVDLALDTLAAA
RSFGVEQLALLVQKQLESMVKEASVDDVMKVLMASRKFEMQELWATCSHLVARSGLSADLLAKHLPIDVVAKIEEIRSKS
PPIISGLSSSSPTAAGPRSPFQLTHSYLPMTTPNPADRDHKIRRMRRALDAADIELVKLMVMGEGLDLDDSLAVHYAVSH
CNRDVVKALLELGAADVNSRAGPTGKTPLHLAAEMVSPDMVSVLLDHHADPNARTLDAGVTPLDVLRGLTSEFLFKGAVP
GLTHIEPNKLRLCLELVQSAVMVTTRDDGGGGGGEGGTSDGGNNGFQRSDVADDSLVSLTMNSTLMYQGQEMAGDQGRNK
ASSGGNNGGRGSPSTLYFPNGFP*
>Bdis_5g09870
MATQEKRRRKAFRTKPKGLPPPSPRPPPRGQGTREEEESKLRSREKSAKNPTCDTDLKHSSSLDTTVQATMTKTAVEKKR
KNSLRSGWKIRREKEGQQFSSPFLVHMSSYSGSVRGDGRPARQSASAIIADAVAEPGLVDGDKAAQNESAAIVADSEPGS
VHDCGDMLPQQAASTTITEAVSGPAIGQGHTSAIVVDSVPGPFHADADAGDEMPPAQHGASTITNATMLAGCDSDASAQH
TASAIVAKTVSGSHVLKIKSYSHTKGLGLGGILSDTFRVGGHCCRIVYYPDGCRSDCADWVSIGLLFVETHASHINARIK
FSLLDHGGKPVPLYTTETHTVTTLSTRQEPLVSARFIERKTLESSYLKGDCFTVRCDVTITSGIRTEDTTKQSVVVPLSD
IHQHLGQLLLTGKGTDVTFEVGRETFAAHRCVLAARSPVFMAELFGPMKENTATSIRIDDLEPSVFQAFLYFVYNDSLPD
IDKGEEILMAQHLLVAADRYDLKRLKLICEDILCNSIDTETAAATLALAEQHGCCGLKDECFKFLTTPGKNLKLVMTSDN
FQHLWSVSPSVLKELLAKLDAINGLQVI*
>Bdis_5g13663
MTFCADFDARRASPADLRVVTSDGQSIAAHSYVLATASPVLERMIDRARRGWGAAECTIRVLGVPTDAVRAFLHFLYSSK
VAPGEEDLVGAHGAQLLALAHAYRVGWLKRAAEASVSARLAPERAVDMLKLAGLCDAPRLYLRCARLAAKDFAAVEQSDG
WRFARRHDAALELQLLQLLEDADQRRERWAREKAAQEACRQLGEAMASLEHVFFGDGAGAGTCAGAECTCQGLRLLMRHF
ATCAKKIAPGGCARCKRMLQLFRLHASVCGRTDQTCRVPLCSHFKAKAQTEKTDKTWRLLVKKVTRAKAMSCLTNRKVPE
VVAMSWARYNSRVAKLR*
>Bdis_5g22350
MDLTAPSFSLGIDFDDPPPPAAAAGSDRRGQARGYEAPDAPSFSLGIDDDDWGVREPQLPAGSRREEQAPRYEAPDAPSF
SLGIDDDDGGGGGPHLPAVSRREEQARRYEAPDPPSFSLGLDDDEDDFLAGGQRHEQARPQVATGDPSWPDDEDDGFLLA
GGKRPDPARGGTLERDSMPLQPGTTRLKRLRRGPAPPPCAPTPPPPRAPAPAAMGATPDFNGKAALGAILSLEDDIEDLT
TDDERPPRGMPPSVGSCRTSSNSKSSLLNNSVLTSQSTSKSKTKKFTQMSNSVASKPLEESYTKKLLPKITVSPLRKIHL
LDSDTDSDDNKNQKKAKKPVSPIKRRQESIHNYVQKKPTIQHNDKSEGSTIVQKSNNVMNDSWATPALDEFCSEYFKSVT
DSRLPQQKEFNGSSGSKVSRPYNSLGELGEHFQQQSIPSGGVLEEDLTDSQPPAMHYFFHHDLMVQKLVRERLPHFIPIG
TQASQGNEYSGVENLNYRNQFGRSGDANDRWVTPNRRTSVPTDVGKRRVHASGSQSGSGRWFTGEDGRKVYVSKNGQELT

GRGAYLQFKKYLTASRAVPFIGHVYLTRCATERVTGTHDMVVANYSLLSGHMGAGAAVSSAPFTVGGYEWVLKFYPDGAT
LDDTGCASAYLYLNANRNALSGARIKASFTLSLLVGQGGGKLKASSCFVGRRRRKSTRDFLAGPGWGWGASGFGHFFWKP
LLRLSRGSCLLSGNAKFFDDQGTLSWGYPKFVEKSKMEDTAVLNSDSWIIRCLLAARSPVFKAELFGPMKDKSTQLVKID
GIEPPIFEALLHFVYTDSIHDDEHNKEGSTAELQHLLVAADRYGLDRLRMLCESELCESIEVETVATTLVLAAQHHCKDL
QEACLEFMAPRNVLGAVMATDGFKHLVETCPLLMKEILEKICPPGPDPCSWPSPKRLSWADACDDEENGEFVPDSIPLPD
CLRPQPQPLPMQIVPGCPRIYLPPQRRSSAVTPTSSPADLARDPIRVAPHFPEDYFVNLSEQTVCEAAVDKEQFHSHGRE
FLILPWSAERHSEWTAMPFHVHLCVENLPGRLSRLIDVVEDLTELTPAREAEPARPRGRQVIKGYTWSFGSLDSGRRDDA
RRPPPPETYSCQEGRHSSHRDDRDHDDERDGHRDRSGRRKETLPSIKARPAPYPQRRGVPAHASLHAGGRRHAASVGPVH
HQGGASSLLCLPTRRASSTSAPDPSLPFTDLDDCITPEPQTPSSFDSFVASITKELPPAILDLAPPPSPPVQTAVADSVP
FVRRSARLAGRPPGLSSTLSAQALLARRLGSLLPSAPFDGTAQAAYIKLFDGPLSDDAVAAIDGLVKEAKKAKKPPAVLG
AGRGRGHGPGHGVDRGRGIPAV*
>Bdis_5g22356
MANKSTSVVTDGQCQSETSSSCFTESFTTTHNFEVTGYSLLDGIGVGKSVSSSTFSVGGHDWNIRFYPDGNSKISADSTS
VFLYHLGPAKDVRTKFTLNMSYKEGKEVANFGALEHTFSPESDNWGYPDFVKKVKLKPSPDINSGCLMIRCVLIVIVIRE
PHKRVRLEAPDSNLKDHLRQMWKDGQGADVTFSVGGQMFNAHRCLLAARSPVFKAELFGPMKEKELQCIQIDDIEPAIFE
ALLHFVYTDSMPDDERSKEDMTSKLQHLLVVADRYGLDKLRVLCESKLCENMEVETVATTLVLAEQHHCKDLQEACLEFM
APLNVLRAVMATDRFKHLLASCPLVMKDILDKVCLSEESLSDDK*
>Bdis_5g22362
MGNSSTSAANHGQPSLPKTSSRCVPESFTGAHDFELTNYPLLDDGVGVGKFVSSSTFSVGGYDWAIRFYPNGWKEGFCAG
NVSAYIYCVDLAEGASVTTKFTLNMLEKEGKAKVTNYGRTGEHTFSLPINMCGYGNFAEKSKLESLSRTANNGNFIIRCV
LTVIKEPRTERSDLIVLVPQTNLAGHLGRMWKDEQGADVTFSVGGQLFSAHRFLLAARSPVFKAELFGPMKEKSAQLIKI
DDIEPPIFEALLHFVYTDSMPDDEHCKEGRTEKLQHLLVAADRYGLDRLKVLCESELSKSIDAKTVATTLVLAEQHHCKV
LKEACLEFMVPPNVLRAVMATDGFRHLLASCPLVTMEILDKMCRSE*
>Bdis_5g22370
MASNANSAVNPAGPKTSSRCVTESSTGTHDFELPNYRLLDGMGVGNYVSSATFGIGGYNWAIIFYPDGGNNDCAGYASAH
IKCLNPTDEVRTKSTINMLHQDGEKQVQLTDCDEGAGDVYSPAKLRWGYTKFVEKSKMKSVTQSGSGSLIIRCVLTVIVK
HSTEVTRKTNLVVVPGPDLQGHLRQMWKDEQGADVTFSVGGQLFSAHRCLLAARSPVFKAELFGPMKEKSIQPVKIDDIE
PLVFEALLHFVYTDSMQDDEHNKESSTAELQHLLVAADRYGLDRLRVLCESKLCEGIDVKTVATTLALAERHHYKDLKEA
CLEFMVPRNVLRAVMATDGFKHLLASCPWVMIEILDKTCRSFIRHVFPWHNPALYVCVVRCIFMA*
>Bdis_5g22375
MGVGWFARSSTFRAGGHRWHIQLYPDGCSKHCAGSTSAFLVCVLGLGERHAPEVRTRFSLNVLPQKEQEQEGRPEARSDK
LTDFPVKDGIFSPTADNKEFDYGYPVFFHNSKLVPSKHLDDDGCLTVRCVLTVVKNKKTTRVRVKKSAVVAPPPKPDLQE
QLARMLKDGEGADVTFSVGGRLFPAHKCLLAARSPVFRAKLFGPTKEDSSTRCVQIDDVEPAIFEALLHFLYTDCMTEEY
KEGETENLQHLLVAADRFGVDRLRVLCEGRLCNSINKRSVATTLVLAEQHGCKALRKACIKFMARSRATLRAVSETEGYS
HLVASCPNLVLEILSVVGKLPVSETAVVIKPASRSVDAFSRRLR*
>Bdis_5g22400
MGDHHRDSILGAPGHCLPKTSSMSMTDSVTAVHDFRVTGYSLVDGMGVGRYVSSSTFTVGGLDWAVRFYPDGSSTTCLGN
ASAFLYYFSREKDVRARFTLNLLEKDGRLSPVTNSHMKHTFSPASDNWGFIKFVEKSKLQGSPFVENDCLTIRCLLTVVK
ESRTEDVEMNSIVVPPSNLHLDFGNMLTDGEGADVTFSVCGQLFRAHRCVLAFRSPVFRAELFGPMKENNATQCIKIDDM
EPLIFEALLHFIYTDTLSDTCKDGKAAAMQHLLVAADRYGVDRLRLLCEKKLSDSIDVETVATTLALAEQHHCSQLRQAC
IRFMASPNMLGPVIETDGFKHLVASCPLIMKDILGKVSLIWSDKSCEK*
>Bdis_5g23307
MATAKAFIGSKSPDCFQLRDPNSWVCLTELASDVVVEVGEVSFHLHKFPLINRSSTLQKLITSSPTSDDDNKQPCTVNLD
DLPGGPATFQLVAKFCYDVRIELTAANVVPLRCAAEHLGMTSAGSASAANLAELTESFLRDAVLPNWDASVRALQTCSGN
ASALLSRYAEELGVVSRCVESLASKACADPTLFGSPMLAYYTAKEDSESASGSAAVMWNGIATAGKPRAPGAGWWYKQAT
SLHFPFYKRLISEMKSKGMSPHSVGGSLMHYATRHISGLNTRKLLGAAASSEPETTTTVVTDVDGERALLEEIVALMPPE
RGAATARFLLAALRTAAVLHAGEKCRSALETMAGAQLEEAALEELLIPNVGYASETLYDVDSVQRMLERFFVMAAEYEGE
LSPEIVMADGGDGGREVSGGELAAACGAVAKLVDGYLAEVGSDAGLKLSKFQTVAALVPDHARPLDDGLYRAIDIYLKAH
PGLTNTEREQVCRLMNCQKLSLEACTHAAQNERLPLRVVVQVLFFEQLRLRTTVAGWFFVSDNAVAAADPQRPGSSNSSS
RPRKSFAGGLGDIESAAGSEEEDDDEAEIEDHDCRSSSEQASVEEIRQRVVELEEECSSMRHEIHRLGKPKGALGRLFRK
LGLANAGGGKSSSSRQLLKSSSEGKRSRFLDLGC*
>Bdis_5g24790
MGAGQSVPADAAAAAATTASVSQIEKVTSIHEFTISEYSRTRGMGIGKSVLSQCFDVDGRRWYVRFYPDGYCTADAAWVA
FYAQTLYKPQLRAVRAEFSFALLNADGDPVYTRRSDRACKYDTLCNSWGIRRFIGRNQLEAAALGAVHGDDSITVRCIVT
VHKDRRRSLKSRRLFNRHGLPEAPPSCHAANFMRFLATGKAPFDVRFDLGGEVFEAHRMVVAAQSPWFESLLYGHGSESR
SNTVEINADTDDDDAITPAAFAGVLYYIYHDELPDEASAVVACQS*
>Sbic_01g005590
MEDEDAGPGGGGEASPPHHAAAASGDRARDMAASPTRSRSVTQTVNGSHRFVIQGYSLAKGMGVGKHIASETFTVGGYQW
AIYFYPDGKNPEDNSNYVSVFIALASDGTDVRALFELTLLDQSGKGKHKVHSHFDRSLESGPYTLKYRGSMWGYKRFFRR
NALELSDFLKDDCLKINCTVGVVVSTMDYSRPHSIEVPESDIGYHFGTLLDTQEGVDVIFSVAGEKFHAHKLVLAARSPF
FRSEFFDHESGEEKNETDTKDEIKEIVIDDMDPVVFKAVLHFIYRDNLVDDDELSASSSDCSIFDSLAGKLMAAADKYEL
PRLRLLCESYLCKHISVNSVATTLALADRHHAVELKSVCLKFAAENLSAVIRTEGFDYLKDNCISLQSEILRTVAGCEEP

CSSGVKSQSVWAQLSDGGDTSGRRVRPRV*
>Sbic_01g006980
MQGRRKNTREMAREQPQPQPPPAAAAASSKGQAWFCTTGLPSDVVIEVGDMTFHLHKFPLMSRSKKLHDLITNKESREAS
RRGRGAEQEEDAGEIREEELDVVLEEDEEADVHRIRLPEFPGGAEAFELAAKFCYGVKLDLTPATAAPLRCAAERLGMSD
DHSEDNLVSRADRFISQTVLRNPRDAIRALKSCEGLLPLADDLGVVSRCVDAVAAKAAASTPTALFGWPIADDARAGDRQ
RRKNGAAAGATWFDDLAGLSLATFTRVIAAMKEHGVGPEVIEGALIAYAKRSIPGLSRTGRHVGGGGGAAAAPPSSDGEQ
KALLETVIANLPEETIKSSAHTGTAVGATTARVLFGLLRTANILHASEASRDMLERRIAARLPDAAVDDLLIPSYSYLVE
TLYDVDCVERIVRYFLEGRDVADEGNEEEGSEAETPGREASRRAMLAVGRLVDAYLGEIATDANLKPDKFCDLAWALPDG
ARVYDDGLYRAVDIYLKAHPGLSEEEKEKVSGVVDGRKLTLEACTHAAQNERLPLRTVVQVLFFEQLQLRRAIAQTIMAN
EGGAAGSGEEGGESDGGGTWRVAARGNQMLRLDMDSMRNRVHELERECTSMRKAIDKMDRRGGAPVDRGAPSVPADGRWG
AIVTKRFGCKFPAQVCQSQQRTVVARTRRPRIEQSP*
>Sbic_01g013680
MASVKLGSKPDAFKRQGQAWFCTTGLPSDVTVEVGDMSFHLHKFPLLSKSAFLERLIEENSDQEECIVKLNEIPGGAKSF
ELVARFCYGVKIELSPANVVYLRCASEHLEMTEAVAEENLIAQSEMFLNQVVLRNWKDSLTALETCDDLLPHAEDLQIVK
RCIESLASKATTDPNLFGWPIREHGMMQSPGGSVLWNGISTGARPRNFNSDWWYGDASSLSFPMYKRLISTMESRGIRPE
IIAGSLTYYAKKYLPGLNRRHSHSMGAVATTSTLSEVEQKNLLEEIDRLLPVHKGVASTKVLLGLLRTAMILKASPTCIS
NLEKRIGMQLDQATLEDLLLPNFSYTMETLYNVECVHRILDHFLAMDQANGGESPCLDDVMASPSLAPITSVAKLIDGYL
AEIAPDINLKPPKFQALASAIPEYARPLDDGLYRAIDIYLKAHSWLSEGEREQLCRLLDCQKLSLEACTHAAQNERLPLR
VVVQVLFFEQLQLRTSIAGCLLVSDNLEGSRPLRSGITTSGEAGGWATAVRENQVLKVGMDNMRMRLAELEKECSSMRQE
IEKLGGRAGKGGGGGGWASRVVPRRLGLKVRSQMCSAQEGSVSEQQRSMSAKLDKLQAKVSKQKKQLAVDA*
>Sbic_01g013780
MPGSSPSPAAGYSGSGDGSHSASAIVAGAVTGSHVLKIVSYSRTKEVPNGQHIDSRHFYLGGHTWYVEYHPNGSAADNVD
FISLFLAIHGAVPGKAAKAQVTISLLDQGGKPVPCYSKVAGFVDFAVKGSWGFPKFIERKALEKSEHLKDDSFTVRFDVT
VMRDIQTVGTPSVVVPPSDMHRHYRDLLLSKEGADVKFRVGGKTFSAHRLVLSTRSPVFKAELFGPMKESTSTKAIRIED
MEPEVFDTLLTFIYTDTLPETKEGEECAMAQHLLVAADRYNLERLKLICEDKLCKYIDTDSAATILALAEKHNCHGLKDA
CFAFLSSAKNLDAVMETDGFDYLTVSCPSVLKQLLSKFVPR*
>Sbic_01g014760
MKYMKLGSKPDVFQTEGSNIRFVATELATDIVISIGDVKFYLHKFPLLSKSSRLQRLVASSNEEKNDEVDISDIPGGPSA
FEICAKFCYGMIVTLNAYNVLAARCAAEYLEMFETIEKGNLIYKIDVFLTSSVFRTWKDSIIVLQSTKSLLPWCENLKVI
NHCIDSIASKASIDPSEVEWSYTYNRKKLPSENGIDSHWNGVRKQPMVPSDWWVEDLCELEVDLYKRVIMTVKAKGSTPA
VVIGEALRAYAYRRLLGSLEDAVSNGVDCTKRRAALDAILFLLPAEEGSVSCGFLLKLLRAACLLGSGESHRINLIKRIG
TQLDGASVSDLLIPPNTDENNTYNIDLIMPIVEEFMLQNSDSGKEKFQDDEEIVEIENVTSVSSTSKLAVAKLIDGYLAE
IAKDPNLPLPKLIALAEMVSSLPRPTHDGLYRAIDMYLKEHPGLSKSEKKKLCGLMDCKQLSQDACMHAVQNERLPLRVV
VQVLFFEQVRASVASARSDPSSELPSVVRSLLPRENGNSIGSSRSAATTTTEEECGVPTSSDINSLRSMRLANNSGGSER
SSGSSDINKNSDDKSTAGKAKGMLMPKKILSKLWSGKTNAGENSSSDTSESPGSVNPEEVKSTQSRITRRSVS*
>Sbic_01g015810
MAGGGEPSRSASTIVANTASGYHILRIDGYSRTLATPTGEYIASLPFTVGGLRWHIRYYPNGNKSEDKDGISLFLYLHDS
VAKPVKAQFGFRFVGDVAEQPLTLGGMHIYDNQSAWGRPQFIKREVFEASKHLLDDSFAIRCDVVVTTEFRTEEAPEATT
PADSISVPPSDLHQHLGGLLLNEKGADVVFDVAGETFAAHRCVLAARSPVFSAELFGAMKESNAGGGVVHIEDMEPRTAK
EVEEADVVDDDDDEEGVFSQHLLTREVEEGGIDDDDDEDVLSQHLLVAADKYNLERLKLLCESKLCEYIDVGTVATILAL
AEQHHCHELKKACFHFLSSPANLRAVVASDGFKHLSRSCPSVMEELVAMLGNLVP*
>Sbic_01g015820
MAGGGEPSRSASTIVANTASGYHILRIDGYSRTLATPTGEYIASLPFTVGGLRWHIRYYPNGNKSEDKDGISLFLYLHDS
VAKPVKAQFGFRFVGDVAEQPLTLGGMHIYDNQSAWGRPQFIKREVFEASKHLLDDSFAIRCDVVVTTEFRTEEAPEATT
PADSISVPPSDLHQHLGGLLLNEKGADVVFDVAGEMFAAHRCVLAARSPVFSAELFGAMKESNTGGGVVHIEDMEPRVFK
ALLYFVTQTYANGKENKSQRQR*
>Sbic_01g018710
MPTFDTASAIVSAKSTIGRHLLHIEAYSAAEVYRGQPSSISASFSIGGRDWCIWYYPHGLPEGPSDYISVFLVLEDDIPE
PADAEATFHLLDLHPVHGLQKVLAKREVLEASEYLVDDCFMIRCDVTVKRRCYTENRGHVPPSDLHRHLGDLLASKEGAD
VTFQVAAETFSAHRSILAARSRVLKAKLLGRTTTGTGACCVIRISDMLPRVFEAFLHFVYTDSLPAEVMMTGWQDDEDDG
SMAYHLLEAADRYDMQRLKLICEEKLCWQVEINTAATMLMFAQQHRCHRLREACVDFLRCPHALNAVMATEGFQHLVQSC
PALLKDLISKLARVD*
>Sbic_01g018720
MTTTTSDSASAIIEGGSHTGSHLLHVEGYSRTKELPNQQSIIHSESFNVGGRTWRIQYSPRGQGGIHSKHIWLKLVLMDS
IDHPYPPRARAAFVFLDQAGNEVPAYTRRVEWNWYAHGGYRFIVLIEREAVEAASPDLVVNDCLKIRCDVAVYIRYRRET
RGPSTEGADVTFQVAGEAFRAHRCILAARSPVFKAELFGAMKESTSTGACIRIGDMLPQMGAGQEVVLMAQHLLEAADRY
AMQRLKLISCHGNQRI*
>Sbic_01g021370
MAVSKVADCIYLALALDEEDIAEAVKVQFQFNLVDKAEKQLPSNIHETEVTNFSKEILCWGSAFMKRNELEKSKLLKDDS
LTIKCDIMVTKDVDINRTGANTAPFVVVTASDMIQHLSSLLQSTEGSDVTFDIGGEIFAAHRCVLACRSMVFKGLLLGSM
NEGTTAARSAVFRAELFGPMKEGTTTGVIHINDMEARVFKLMLTFIYNDSMPNINEEEDMEEDEEKDDDVDDIEVMWQHL
LVAADRYDLQRLRLMCENKLYGYINATKVASILELAEQHHCRGLKEACLDFLNFPLN*

>Sbic_01g021380
MSFAGVSFIGSGGLSGRSTPATAASLVTDSGYHLLVIDGYSRTRETTPNGDCIKSSPFRVGGHSWCIQYYPNGRVSEVAD
CIHLALVLDEHNIEEAVKVQFDFSLVDKAEKQLPSNIHETEVINFSNETPCYGCAFMNRNELEKSELLKDDSLTIKCDIM
VIKDVDIDRTGANTAPFVVVPASDMIQHLSSLLQSTEGSDVTFEIGREIFAAHRCVLACRSTVFKGLLGSMNEGTTAGVV
RIDDMEARVFKLLLGFIYRDSVPEKEAEDDDVMWQHLLVAADRYDLLRLRLICEQKLCTYINTTTAATILALAERHHGRG
LKDACLDFLTRAHANLQEVTVLGGLDHLASTCPLVLKELIVKLVSPNKKARIDVNTAFVTVPASDMHQHFTHLLQSGEDT
DVVFEVSGEKLHAHRCILAARSAVFRAELFGPMKEGTTTGVIRINDMEARVFKLMLTFIYSDSMPDINEEEDMEEDEEKD
DDVDDIEVTWQHLLVAADRYDLQRLRLMCENKLCGYINTTKVASILELAEQHHCRGLKEACLDFLNFPPNLQQVMAAGGL
SHLSSSCPSVLIDLIAKLAYLKPSDEL*
>Sbic_01g021390
MSFCGVSFVGDSCLPPSTSSTPLLVGGFHLLVVNGYSRTKQDTPNGKCIRSNYFKLGGHRWIIEYYPNGYEQENTDYISF
YLVLDEHNIVEPVAAQYVFSFIGQVQSPESSLLVGERGAHFFSSTDAYSLACVMKRQSFEKSKHLRNDSFTIRCDLSMTK
NVNIGEAGAAVWFVNVPPPDIQHHLGNLLLSQEGTDVTFQVGDDKFMAHRCVFAARSAVFKAELFGAMKEGATDTVVHID
DMDAKVFRQLLGFMYTDFVPDFEEDEEEEYMWQDLLVAADRYDVPRLRLICERMLCPYINTDTVATMLELADKHHCNGLK
DACLDFLNSPANLQEVTAAGGLNDLTISCPAVLIELIAKLTSSLKLDK*
>Sbic_01g021410
MASMSFAGVSVVGEVRPPQPAAATTAVTYSGYHLLVVNGYSSLVKDIPNGDCIESRHFRVGDYRWTLQCYPNGYGAEQDG
FMSFYLFLDQGNVVDPVMVQYEFSFVVDQVLQNQNYCPALLDGAKQTLRFFSRYPYSFSLPYLTKREIFEKSKYLKNDSF
TIRCDIVITKDVNINEAGGTVPVSNVLPPPPDIRQDLGDLLQSGAGADVTFQVGGGTFRAHRCVLAARSAVFKAQLFGPM
KEGTTTGVVHVRDMEEKVFKLLLGFIYSDSVPEVDDIEKDEIMDSGAGALPPVFDVPESDLHQHLSSLLLSEERTDVTFR
VGGETFRAHRCLLAARSAVFRAELLGPMKDTASDVIRIDDMEARVFKLLLTFIYSDSVPETKEDEKEEVSENDDHADANV
MWQQLLVAAEGYGIQKLRLMCETKLGRYISTKTVATILALAEQHHCRRLKEECLDFLDCPAHLKQVMAVGGLDHLRSMCP
SVLIDLIAKLAAL*
>Sbic_01g021440
MSAPQPQSAALVVNEPRSASAIVGDVEGYSRTKDELPTGKSIRSRPFSVGGRQWSIRYYPNGTDSECADFVSFVLFLAKS
AGGPMKARVRFHLLDRAGNPLPSKSRTTGPCEFSPGGSGYGFSIIKREFLEKSEQLLMNDSFKISCDIIIPMELRTEDRI
VSLLDAVPPPDLNQHLSDLLMSKEGADVTFQVAGEAFSAHRFLLAARSRVFKAQLCGAMKESTATGDCIRIDDMLPQVFK
ALLQFIYTDSLPQMEEQQEAVMAQHLLEAADRYDMQRLKLICEEKLYRHIDVSTAATTLVLAEQHCCRGLKKACIEFLKS
PDALEAVMETDGFEHLTKSCPALVKELMSELYVFKQCKRRKLKA*
>Sbic_01g021460
MPTHLPPPCATSDARSAIIGGEVTGHHILDIEGYSHIKEQLRHGDSTTSLPFTVGGCSWYISYYPNGCRHSADGFIGIFL
CLERSRGAAAACVKARVKFSLLDRAGKPVPSHSHTTVLIFDDKIFVSADYPCCFGFAEFMRRADLEKSEHLKDDSFTIRC
DITIANKLCKERRRAAYGLTLVKVPPSDLHHHLGELLASKQGADVTFRVAGETFRAHRYILASRSPVFQAELLGPMRESH
AASSVVEVQDMEAQVFQALLEFVYTDALPQDMTREEEAVICQHLLVAADRYSMERLKLVCEDRLCRHIKVASVATTLALA
EQHRCRGLKEACLQFLESPAVLNAVAADEGFDYLANTCPSVLKDLILKLTDCHLDLWGKQVS*
>Sbic_01g021770
MAPTVSPSAPIASDDRFASAIVAGSTSGFHVLRIEGYSGTKFTIPNGQHVESHPFRIAGHTWTISWCSASSTKWISKGRL
TSVVSRQTGSSVTTASFMELEQSDRLRNDDCFTVRCDIIVLSEPRAEAARLAAGNSFVKIPPPDWSEHFRELLRGGKGAD
VRFLVGGETFAAHRCVLAARSPVFGAMLLGPMKEDTTTQNQIQSCIPVTIDGMVPQVFRSLLHFIYTDSLPAETEDQGNN
IYRGWHQRHNVAAFA*
>Sbic_01g021780
MAPVIPSYRRIDRDDRVPLVSASAIVGGADSGYHVLRIRGYSSIKVAFPNGSHFDSHPFRVAGHTWVIRYYPNGDRPETA
DHISFYLRFMDQVGPGEEVMAQFVFSFIDQVEMQKPAFVGNLEARRFGTNGSWGNKEFIKKESLEQSNRLKDDCFSIRCD
IIVAGLPRAEAAAAFVVAVPPLELPQHLGALLLAGKGADLRFLVGDKTFAAHRCVLGARSPVFDAMLFGQMKEGTATENC
IRIDDMAPQVFQTLLHFIYTDSLPETIEQDDSGATMAQHLLEAADRYDLQRLKLICEDRLCQQIDVSTVATTLALAEQHH
CQALKEACFEFLKSPKTLDEVSATDGFQHLVKTSPSVLFQLMSKLAER*
>Sbic_01g021790
MARASSSSSSIVANSASGSHVLKIDGYTRTKGLATGIHLRSCSFRVGGHSWHLAYLPNGDTEQNADFISFFLVLEDPPAN
GAPVLAQFCVALLDRAGKPVPSQTQAHPVTRFTATAAHWGFNMFIRRQMLERSRYLNPKDDSFCVRCEVSVVTGFHAEDA
ASVAAAAAAAKAAAAACVRVPPSDLSRNFGELLRSELGADVRFRVEGEDFAAHRYVLAVRSPVFMAQLFGAMKEAKGDCW
VEIDDMRADVFRNFLHFIYTDELPKPDEPNNNEEALMAQHLLVAADRYGMERLKLMCEDILCKHIDVSTAATTLALADQH
RCQGLKEACFQFLKKSPGSLNAVMATDGFDHLANSCPAIIKELMSRLAATHSN*
>Sbic_01g021810
MPSPGDIGGTLSASAIAGGTMTGHHLLHIDCYSRSKAELQNGEGIKSCPFSAGGRSWFIMYFPNGVRSIVEEFISLYLRL
DQIAADQPVKARVRFSLLDQAGEPVPSHSHITPLHGFCAAYPDFGFGDFIKRSRLEESEHLKDDRFTIRMPAVAVPPSNL
HLNFGDLLSSKQGADVTFLVAGETFEAHRCVLAARSPVFKAEVFGRMKESTRGAVIRVEDMDAHVFRALLDFVYTDTLPD
FQSSMKKQEEAALAQHLLVAADRYNLERLKLICEDRLCGHIDTASAATILALAEQHYCRGLKEACFRFLSTPWTLNAVMA
TDGFDHLTRSRPSILKELLSNIAARLPVHVNKAR*
>Sbic_01g021830
MASGYHDLQIDSRLLDATSVPHGESVLSCAFTVGGHTWRMMCYPNGSRSAAGGYVSMFLRLDEEVAKPVPAQVEFSVMAE
KRALFLVRSKKKKVPSTKKEFVTTSDFASRRHNVGYPNFAKREDLLKMVRRQDDPLTIRCHIVVFNGHRAVQQFPKQQAA
ASAVHVPPSNLHTHLGDLLSSKRGADVVFEVGGETYPAHRCVLAARSPVFAVELFGSMRESDAAAGGVVHIDDMEPQVFQ

ALLHFAYTDTLPEMRTPEEGVLCQHLLVAADRYDMERLKLICEDKLCRYIDVGTAAIVLTLAEQHRCHCLKKACIDFISA
PANLKAVVASDGFEHLSSSCPSITKDLLAMHAS*
>Sbic_01g021850
MARTGCGCNSELSRSASSIAADTARGYHILKIDDYSLTKGTPTGEYLKSHPFTVGGHHWHILYYPNGWKSEYADFITIFL
KHDGSVANLVKAQFHLRLVDDVGEEPVTLFAATSFASNVGCGYPQFIKREELEKSKHLKDDSFSVQCDIVVVNEFRAKVE
MAVESPPRFVSVPPSDLHQYLGDLLRSEKGADVVFDVGGQTFAAHRCVLAARSPVFSAELFGTMKESNTTGVVRVDDMEA
QVFKALLYFVYTDTLPKTEEAGQGEERDEGKEEDDDIMSQHLLVAADRYNLERLKLICEEKLCGYIAVGTVATILTLAEQ
HNCHGLKKACFHFLSDPANLRTAMAMATDDFKHLNRSCPAVMVELIGMLVT*
>Sbic_01g021860
MSSASGRSKPSRSASASTVFADNAGGYHDLKIDGYSRIKGIPAGVPIKSSPFSVGGHRWRISFFPNGDRTANSGCIALFL
FLDEDVAEPLTARFEFAFMGEERASFFRSRKRTEEKVRSLPAVVTSFGSRTGRWSSGLLSTNALDSYVSKHGSLTVRCSI
VVFKEFLAEEPVTPTTFVSVPPSDLHRHLGDLLQTEKGSDVVFQVGRETFAAHRCVLAARSPVFSAELFGAMKESRTQAD
VVQVDDIEAPVFKALLCFLYTDSLPEMRQEDEDVMYQHLLVAADRYDMERLKLMCEHKLCEYIDVGNAATILTLAEQHHC
HGLKKACFSFLSNPANLRATIASDGFKHLSISCPSIKKDLMSMLES*
>Sbic_01g038420
MARVTFSLLDQEGNPVPAYTFTTQMCNFSVNKSQGYENFIRRVDLERSGHLTDDSFSVGCYVVVTKEAAASSTAVAPPPP
SDMHLHYGHLLSSKQYTDVEFLVGGETFLAHRLVLAARSPVFMAELFPLMNEVTAAEEVVQIDDMEAQAFKALLTFIYTD
TLPEFNQHEDDDEMTMIQRLLVAADKYKLQRLKLTCENKLCRHIDIGSVATILVLADKYHCEGLREACFEFLSSSATLSV
VVETDGFLYLTQTCPAVLKDLISNIVAHQLERTDPFSVNK*
>Sbic_01g038430
MTTTLASSDGDVPPPWSIIVARGDDDAPPPRSASTIVAARANRVLKIDGYSSTLKARTLRSFSFSAGGHTWHINYRCTGS
SDNSSTDDDDYISFYLALEDEVDEPVMGQVTFSLLDQNGKPVPSRTRTTRLFSFSLSDSFGFQNFIRRKDLEQSGYLKDD
RFAISVHLVITKGSPSVKVPPSNLHCHYGDLLSSKQGTDVEFIVSGGERFTAHRLVLAARSPVFKAELFGLMKEGTTTDA
IQIDDMDAQVFEALLIFIYTDTLPKIDDEEDEAAMAQHLLVASDMYGLQRLMLICEDRLCNHINADSVATMLVLAEKHHC
VRLKEVCFEFLSASTALVEFTESSDFLYFIQSCPSVFKDLIYNVVAAHGK*
>Sbic_01g038440
MATTLASNTGDGPAPSSASTAVTTRANHVLKIDGAGGHTWHINYRRRGSSDNSTDYISFYLVLEDVVDETVSAAQITFSL
LDQDGKPVPYHTHTTKAFNFSPGSSSFGFDNFILRKDLEESKHLKDDCFAIGVHVIIIVTKQEASSSSSSVKLVPLPSDI
HRHYGDLLSSKLGSDVKFIVGGETFAAHRLVLAARSPIFKIKLFRTSTCKDEGTTPDAITINDMDAQVFKAMLSFIYTDS
WPEIDQENEAAIAQHLLVAAERYGLDRLKLMCEDRLCHGIIDMGSVTTNLVLAENHYCHSLKKACLELLGSSTALAEFME
TDDFRYLTKSCPGILNDLICNVVARERERTKILLGNKGRQCDSIDIRLQPWKNSAAAQKDQVKIMLSNTSFYSGTSFV*
>Sbic_01g043470
MEERRLDTPAMRRISDWIMSQEFPSDITIQVGEATFNLHKLPLASRCDYIRKQVSGINGSRVTHIDLTGMPGGAAAFELV
IKFCYGENFEITEDNVAMLRCAAEHLEMTDDSMGGNLAARTEAYLEAVALTSLAGAVTVLRRSEDLLPVSEEVDLVGRCV
DAIAYIACNDSQFSMPMGSNTNGNAGMDVSSFKAIDDWLADELTSLRIDTFQRVLIAMKAKGFKGIALATLIMLYAQKSL
RRLDTHGREKEKMDPRQEHEKRVVLETIVSLLPKEKNSLSVSFLSMLLRAALCLDTTLACRLDLEKRMAAQLGQAVLDDL
LIPSSSPDAGTTFDVDAVQRILVGYLEHEAEAARLDYNTDDDFISTASPPPNDVGMVGRLLESYLAEIASDVDLPIDKFT
GLAEMIPERARFNEDGMYRAIDIYLKAHPHLSEAGRKKVCRVMDCQKLSREACAHAAQNDRLPVQTVVQVLYHEQRRLRE
APKAPSPPYRPAPTPSLQGRHARTVLDEVSRLQRENEELKMELLRMKMHMREPPAALPQPSGAPPPGRRPPPKKAAGGSG
GFMNNVSKKLGRLNPFLRLDAVGTEPPKDRRRRRSIEW*
>Sbic_01g043540
MKFMKLGSNPDTFQDDGNEVSIAASELVSDITVRIGTTKFYLHKFPLLSKCARFQKLIPTTGDENIEIHIHDIPGGAKAF
EICAKFCYGMIVTLNAYNVIAARCAAEYLEMNETVDKGNLIYKIDIFLSSSIFRSWKDSIIVLGTTKAHLPWAEDLKLVS
HCIDSVASKASIDVSKVEWSYTYNRKKLPTENGHDSPWNGVKQQQFVPKDWWVEDLTDLDIDAYKQVITAIKTKGMVPKD
VIGEAIKAYTYKKLPSLSKVSMIHGDAKVRAMLVTITCLLPSEKGSVSCSFLLKLLKATNLLKCGEMCRKELMKRIARQL
EEASVSDLLIPTVDGDTTVYDIDLILSIVEEFVRQDSKNAQRHNGGEVNDHVSAPSASMITVAKIVDGYLAEVAKDPNIP
VYKFFSLAETVSANSRPVHDGLYRAIDMYLKEHPGLGKSDKKRLCALMDCKKLSPDACAHAVQNERLPLRIVVQVLYHEQ
TRASAAATIRADSIGIGSYESSRSGATTNTEDEWDGVMAVEDLSLSKTTKLDKCHTASTIVEKNHGGNKGANGRVKGGAT
SKKALGKIMSSKGQTGERSSSDSSDSAILPSQEHPKRTPARSTTKSAAA*
>Sbic_02g000320
MAIPPRTPPPPPSWSRYVTETVKGSHQFTVRGFSLAKGMGPGRHLASDIFAVGGYHWAVYFYPDGKNAEDNSNYVSVFVA
LASDGIDVRALFDLTLLDQSGRGRHKIHSHFGRKLDSGPYTLKYRGSMWGYKRFYKRSLLEASDFLKNDCLVMNCTVGVV
KNRMETPKDIQIHVPRSDMGHCFKELLSRGIGCDITFEVRDEKVRAHKWILAARSPVFKAQFFGPIGKPDLHTVVVEDVE
PVVFKAMVNFMYTDELPSISELAGSASTWTSTVVVQHLLAAADRYGLDRLRILCESKLCDELTPETVATTLALAEQHHCA
ELKSACLRFAAVRENLGAVMGTEGFDYLEETCPSLLSDLLATVAEVDDDPASLDRKRGVCGNQVLAPVESVEATERRTRR
RL*
>Sbic_02g002490
MPVSTTKPPAPTSATKPPVRRRPAPPRQISGGGSRSPPPAPPVLRALPWLRSMSDLPAALARLLETTTTTTTDGAVGADV
EFEVGGKVFTAHKLVLAARSPVFREQFFGAAKEEATGYVRIWDMHPDTFEALLHYVYTDSLPPPAVLVPVTRGAGRGGAR
REEEAAAAAAVLVQDLLRAADRYGLRGLKSLAETQLCRRNNVVGVDTVLPMLALAERHQCWELKNRCLEFIASGENARAV
MAATDDVEHLARSCPSLVKELVEILEAKEKAAATTSRRRPLMDSADALFFLFLALGFILACILPLRMWGFFEQK*
>Sbic_02g002510

MEYFKNHMTMKIGSCAEANKLGNGSYVKSDSSKAGGHSWRIAFFPNGRLAGTTASMSLFLLLDDDDDNDNAKGARTATAA
AADGDVVHVKFRFMMHEVYDDRSPVFMSGTVATAAFSRRSNAHGFERFVSRDDFEKTALFKRDRFAIQCRLTVFPAGSKR
AAVQMSRSVRPDPSVVQAPPPSPASPKPSVVRAPPPTPTVPPPAPAASPKPPVVTAAPPSPESASPPAPSAAMSSAPPPP
SVRPEQVVAGVSGSPASFPARQRPRAPPLSGLPADLGRLLATKEGADVELEVQGKVFAAHKSVLAARSPVFMEELFGPAK
EEDTSYVRIMPDMSPEAFEALLHYVYTDTLPPEMAMAMASLEEGVVLAEGLLAAADRYELKDLKLLTEQKMCNHVGVSTV
LPLLALAEHYQCCKLKKMCLGFISSCGNTRLVVMATNDLEILARSSPSVIKDVITEILDAREERRRRLINFCIFAFSFIL
PFLLFAIFS*
>Sbic_02g003880
MDEDAGGASPPPVPLAQAPPPQHPIAVAAAATATAAAPSQRDMAASPTSSRSVTETVNGSHRFVIQGYSLAKGMGVGKHI
ASETFTVGGYQWAVYFYPDGKNPEDNSVYVSVFIALASDGTDVRALFELTLLDQSGKGKHKVHSHFDRSLESGPYTLKYR
GSMWGYKRFFRRTALEASDFLKDDCLKINCTVGVVVSTIDYSRPHSIHVPDSDIGYDFGSLLDSQEGVDVILNVGGERFH
AHKLVLAARSHVFRSQLFDDESDGEKSEVNETDELKEFSIDDMEPTVFKAMLHFIYRDTLVDDNELGASSSDGSIFDTLA
AKLLAAADKYHLPRLRLLCESYLCKGISVASVASTLALADRHRAMELKAVCLKFSAENLSAVMRTDGFIYLKDNCPSLQS
EILKTVAGCEDQCSSVGKSQSVCGQLSDGGDSSVRRVRQRT*MDEDAGGASPPPVPLAQAPPPQHPIAVAAAATATAAAP
SQRDMAASPTSSRSVTETVNGSHRFVIQGYSLAKGMGVGKHIASETFTVGGYQWAVYFYPDGKNPEDNSVYVSVFIALAS
DGTDVRALFELTLLDQSGKGKHKVHSHFDRSLESGPYTLKYRGSMWGYKRFFRRTALEASDFLKDDCLKINCTVGVVVST
IDYSRPHSIHVPDSDIGYDFGSLLDSQEGVDVILNVGGERFHAHKLVLAARSHVFRSQLFDDESDGEKSEVNETDELKEF
SIDDMEPTVFKAMLHFIYRDTLVDDNELGASSSDGSIFDTLAAKLLAAADKYHLPRLRLLCESYLCKGISVASVASTLAL
ADRHRAMELKAVCLKFSAENLSGT*
>Sbic_02g007170
MATSAAGTGGKRKRSGSSSAIFAATASGYHILKIHGYAGTKGTPTGEYLKSHPFTAGGHCWTIRYYPNGYSSQSSDHISF
FLHLDESIAKAVKAQYQIRFVDQEEKNLLTSEPVTSFANQTSSGYAKFIKREEFEKSEHLKDDGSSFAVRCDIVVIGDVR
VEATAAPVYYVAVPASDLHQHLGDLLRAEKGADVVFDVGGRTFAAHRCVLAARSPVFRAELFGAMKESDAASGGVIRVDDM
EARVFSGLLHFVYTDMFPETSKEVEGEEEEEEDVVAQHLLVAADRYGMERLKLMCEDKLCKYIDVGTVAAILTLADQHHC
HGLKKACFEFLSSAANLRAVAATDGLKHLTSSCPSVLEEFLVTLGNLVP*
>Sbic_02g007900
MWCHSCKEGYDEEDAGTCKECYDEASETEEELKREIEELRSRLHFLRLPSPTLDAASAPNCDLLLHAIPSCPSSSGAGGD
GDAARTPDTPAVPAHRVILASRSPVFRAMLENEMEESRSGVIKIYDVSYDVLRAFVNYMYTAEALLDEQMASDLLVLAEK
YEVKHLKAYCERFIISKVNNENAITHYAFAHRHSAKQLLEASLSVLMGSMSTLADREEYKELVEKDPRLVVEIYEAYLNR
QVNTAAGKDTDCSSRK*
>Sbic_02g010170
MQVQNLTKVVRSKDLLLKVEGHSVTTAMSDGELIKSQKWSVGGHDWQIDWRPKNHWAGRHRPVTLNLVLLSEARGGDVKA
KLTCRLVDPTGRLKPSKEKSLSHKFYKSGDYSNPLEATSREDLEASGYLVDDSYTVQCSITVLKGAPAAAAPSNTAATAM
DRGRRPDDAALPSPDLHRHLGELLRKGTGADVTFVVSGKKFAAHKAILASRSPVFMAELFGGMKEVACQRVEVKEMEPAA
FKALLGFIYTDTAPDLLGQNQKGEDDATAMAQHLLAGADRYGLDRLKLICEGRLADRITVDTAATTLALAEQHGCSQLKA
SCVEFIAGYLDAVLETEGYKHLEASCPSVLTDIIRATRRSTN*
>Sbic_02g022400
MAEQTKACTVFHARESKSFNLRVNNSATKHLSGDECIHIDVSIPAAAGFTCTAKYWPNWQSSDSIKISVVITRTDDQQHH
YQQRQHKLLAAHIDLPARTGLTPPPVTVRREITVVTTDDDDCNCNQQGTAVRREQPAAAAVVTSTDCKKEGMVVNVKQEA
VTVVTTADCKEEGISLIAKRSDVEASCVVDDHFTAICMVAVSKVWPPLPLPTLSRLEHDILMTSDLTDVSFQVDGETFRA
HRLVLAARSPVFRAELFGRQMAEESSQQEQASSIAVQDMKASTFKSMLHYMYHGLLPAVAAPPVAAAAETEADCRVSEFQ
NLYVAAGKYGLDTLKQTCEEILCASVTTDTVLSSLQFAEEHACLKLKSRCLDFLAIGEKFKAVAITNEYIDLMKKVPSLL
DHVQNRFKRPRLS*
>Sbic_02g022410
MAGEPSRACDIYRTRDVQGFHFRVSHSATKNLAAGECVEKVLALVNGFRVTARCWPHCWEGIIKLSVVITSMLSQQRLQA
PKVAAASIDLRNRDGRPVRTGRQQPETVVAGDDCFGGVAFYASRDEVEKDCLVDDHFTAICTVTILRDWPPLDLPVPYNL
GSVIFSMPDLSDVSFQVGGHTLKAHRLVLAACSPVFRAMLFGEMVESRASSIAIEDDTRPNTFGCMLYYMYHNVLPPPPA
AAAAGTMMGDDDNAAEMMEFQHLFVAADKYGLDKLKETCEEMMFAGIKTSTVLSIMEFAEECARPKLKAKCLDFLAVPGN
FQEVAATQEYLDLMTKLPSLLAQVRDRCRRPRLVV*
>Sbic_02g024890
MKFMKLGSKPDAFQSDGADVRYVVSDLATDVVVHVGQVKFYLHKFPLLSKSSKLQRLVLKAAEEVTDEVRIDDLPGGAKS
FEICAKFCYGMVVTLSPHNVVAARCAAEYLEMTEDMEKGNLIFKIEVFINSSILRSWKDSIIVLQSTKALLPWSEELKVV
GRCIDAIASKTSVDPANVTWSYSHNSKKGGTCAEIVESAGKPSIAPKDWWVEDLCELDVDLYKRVMVAVKSKGRMSPELI
GEALKAYAVRWLPDSYDALVAEDYMRRNQCLVETIIWLLPSDKSSGCSCRFLLKLLKVAILVGGGEHVKEELIRRISFQL
HKATVKDLLLPSTSPNDGTYDVRLVHNLVQRFVARTALSHNGGFIEKSDEKMIELNFEQESTLSVGELVDGYLSEVASDQ
DLSLSMFVELATAVPEVARPVHDNLYYAVDAYLKEHPDINKSEKKKICSLVDVKKLSTDASMHATQNDRLPLRLVVQVLF
FQQLRAGSSKELALIDNGEQACTRPMEDQCDPCERRIPKHPNSLNKQVTSLSARESQHHRISEHRGGRNSFKDQIGGILL
QSRSRRIFDKLWSSKGQGEHGGKGSETSGSSQSPPLSAKPAEVKPSPLPPLRNRRYSVS*
>Sbic_02g036820
MPRHMRTTTVVSTCTPVTEEVRHVFEIVGYSKHRGMGHDGFIRSGTFSVGGHEWAIRFHPDGYCSSSGNVDTDFISVYLE
LLDKGAKVRASCDLSLVVQNTGLSTSVHKTGPRIFHQGQSSAFAPQTNLFMKRNDLVASGYLQNDRLTIECVVIVMMKPQ
VATTGFLNNIGLPPSNIIENLGSLLETEEGADVRFSVGGETFAAHRLVLAMRSPVFKAELYGTMMEGRSELVTIQDMQPT

VFRALLHFIYTDSLPDMVDHRGDAITEMIRHLIVAADRYAVDGLKLICQSILCERLNVKDVAITLALAYQHNCDRLKDAC
HEFLTASNSNVLDMVIATPGYKNLKTTCPSVLADAFEKAMKRRKT*
>Sbic_02g041470
MGAGRVCRGGPSSAHPAAGAGAGRPFPPLAASSSAFPSSAPSETASTSVTKTVNGSHHFKIAGYSLSKGIGVGKFIASES
FNVGGFDWAIYFYPDGKSGEDGAAYVSLFIALASEGTDVRALFELTLVDQSGKGQDKVHTHFGRSLESGPYTLKYRGSMW
GYKRFFKRTALETSDYLKDDCLLVNCTVGVVQSHTEGPKIYTIPVPRSNMALHIGQLLTSGKRTDIAFEVDGEMFPAHKV
ILSARSPVFRAQLFGPMKDKNMKCIKIEDMEAPVFKALLHFMYWDELPDIEELTGVNTTWVSTLMAQHLLAAADRYALER
LKLLCELKLCEDVAINTVANTLALAEQHHCYQLKTVCLKFVALPENLKAVMQTDGFDYLQQSCPSLLTELLEYVAKVGDH
SVSPCLYSTEVLDGGDANGRRVKPRI*
>Sbic_03g002980
MEPMESQLTAVALSDSDSASVEGAAAADAADADLQALRRLSDNLAAAFRSPDDYGFLSDARIAVPGAPDLRVHRCVLCAR
SPFLRDVFARRAASDGEEKDKGRDRGKVELRDLLGDEVEVGYEALRLVVDYLYSGRVAALPKAACLCVDEDGCAHVGCRP
AVAFMAQVLFAASTFDVAELTNLFQRRLLDVLDKVEVDNLPLILSVANLCNKSCVKLLERCLDVVVRSNLDMITLEKALP
PDVVKAIVDARLSLGLVLPEDKGFPNIHVRRVHRALDSDDVELVRMLLKEGKTNLDDAYALHYAVEHCDSKITTELLDFA
LADVNHRNPRGYTVLHIAAMRREPKIIVSLLTKGARPSDLTFDHRKAVQISKRLTNHGDYFGPTEDGKPSPKDRLCIEIL
EQAERRDPHLGEASVSLAIEGDSARGRLLYLENRVALARILFPMEARVAMDIAQVDGTLEFTLGSNANLPTEIQRTVDLN
DTPFIMKEEHLARMRALAKTVELGKRFFPRCSKVLDKIMDDETELASLGRDTSTEKKRRFHDLQDLVHKAFSEDKEENDR
SAARSSSSSSTTSIGIGAVRPRR*
>Sbic_03g035720
MEPSSSITFASSSSYLSNGSSPCSVAMPPPGPPQAPPLAGGEGWGGGVATAGSGSSVEAVSLNRLSKNLERLLLDRDLDC
SDADVEVPDGGPPVPIHRCILAARSDFFYDLFAARGRGGALRGDATAGAGGAAEGAASGRPRYKMEELVPGGRVGREAFQ
AFLGYMYTGKLRPSPVDVVSCADPVCPHDSCPPAIRSAVELMYAACTFKIPELTSLFQRRLLNFVDKTLVEDVIPILKVA
SHSGLTQVIDKCIQRIARSDLDDISLDKELPPEAVEEIKNLRKKSQTADGDGDAFISDPVHEKRVRRIHRALDSDDVELV
KLLLNESDITLDDANALHYAASYCDNKVVSELLDLALANLNLKNSRGYTALHLAAMRREPAIIMCLLNKGANVSQLTADG
RSAIGICRRLTRLKDYNTKMEQGQESNKDRLCIDILEREMMRNPMAVEDAVTSPLLADDLHMKLLYLENRVAFARLFFPA
EAKVAMQIAQADTTEEFGGIVAASTSGKLREVDLNETPVTQNKRLRSRVDALMKTVELGRRYFPNCSQVLDKFLEDDLPD
GLDQFYLQRGTADEQKVKRMRFCELKEDVLKAFSKDKADSSMLSGLSSSSSCSPPQKSAKR*
>Sbic_03g036420
MATMKLGSKPEIFVLEDLTWRSTTELESDVVVEVGEMSFYLHKFPLLSRSGVLQRMISEYQPPAGGGGGGMCTLQLDDIP
GGAKAFELAAKFCYDVKIELNALNVVCLRCAAEYLRMTDDYAEGNLITQAESFLADVLANWKDSIKALETCEGVLPTAED
LHLVSRCITALASRACNSDAAAPLVRNAGVVVDKDALWNGIRSGDTASAAAAAASGMDWWYEDVSFLSLPMFKRLIQAME
AKGMRAESIAGAIMFYAGRFLPGLKRDTSFSNALASYGADGAGGGGAGGMSSRNITPRAASVSAPSEGDQRYFLEEIVAL
LPTKKGVASTKFLLGMLRKAMLLHASPLCRENLERRIGAQLEDASLDDLLVPNLGYHVETLYDIDCVQRILDYFMSSTDG
IGTGYTSPALAEDGGGSLGIPQGGTPSSSLSPITMVAKLMDGFLAEVAPDTNLKLPKFQALAAVVPDYARPVDDGIYRAT
DIYLKSHPWLSESEREQLCRLLNCQKLSLEACTHAAQNERLPLRVVVQVLFFEQLRLRTSIAGWFFVSDNAAGGDGARPH
SGGGIVPKGAAAIAGSAQAEADDSDVEGDAPEGKETMTDVKARVSELEKECKSMKQEIRRLGKPRRSWSLLPKKCGFGAK
VQQAQTAMSGK*
>Sbic_03g042420
MECLEFDCSDLFLDGDAIDFPLEIHLESNSSAGAKAAPDHRRHAQLHRSKAPDPPPLPGTSYDAWRISRKAKACRRVPEG
VVDSWDRLFLQGYQSDLRVWTDDDTEILSHSCVLGVKSPVLRAMLEEAKLKDGFRCIRISGAPSEAVCVFIRFLYSSRFE
QEQMKKHVLHLLVLSHVFSVPSLKTVCIDQLERNFLAPDNVVDMLQLARLCDAPRLSLVCTRMIIGDFKTISLTDGWKVM
RRVNPTMEQELLESLVETDTKRQERAKRMEEKKVYLQLHEAMEALVHICRDGCRTIGPRDQALKGSLAAACKFPACKGIE
LLVRHFSTCRVRVPGGCANCKRMWQLLELHSRMCFIPDTCKVPLCRHFKEKMRNLGRKEETKWNLLVCKVLESRGTMAFM
PERRKKLSILTPAGSCHVAPLVGPI*
>Sbic_03g043250
MCEAPRLGYRAAGAGDADAADVDVVTTGGRRRIPAHSSVLASASPVLGSILERRLQKDRESGKPGRSVVRIRGVTDDAAA
AFVRLLYAGRCGDGGEEEEEDDAMEKHAVQVLVLAHAYQVPWLKRACEGAIGARLTADSVVDVLQLADLCDAPRLHLRCT
RLLAKEFAAVERTEAWRFLQENDPWQELHVLQRLHEADMRRRKWRRKRAEQRVYVELSEAMDCLDHICTEGCTEVGPARR
APAPAPCARYATCRGLQLLIRHFSQCHRKSCARCQRMWQLLRLHSALCDRPDRCNTPLCMRFKQKEQEKVAAKAGDDDDK
WGLLVKKVKAARVFSSLANRKQTSTSTQC*
>Sbic_03g044910
MAAPPQPHPPAAVEGAAPAAPLTAAAPGPRPYDVAVAAAELRPVDCNLASLCDHVQAEGFGSGAFSDLVVEAMGATYRLH
RLILSRSAYFRNMLHGPWREAGAPTVVLHIDDANVDSEAIAIALAYLYGQPPKLNDNNASRVLAAASFLDLQDLCTICTD
FIISELWTSNFLQYQLFAESQDYGSHGERVRNACWGYLCQSATLELREVLPKLSSQTLHALLTSDELWVPNEEKRFELAL
FTLLAKVAMCEIQVSGNETNLPSADRSLRKGKTSMDESGEQLLMDSELQNLKLHNNLGNEIPHNIIAISDMNGEAPKRME
NDCSTGGPSGESTSYQFNENNWLSSEQTANNYFSRTSSSGVVPTEWGRPNAPLWGGRVVGRRQGTCIRGISCLSTDEYNA
FMNIFERGSLLYCNMSFDALLSVRKQLEEFGFPCKAVNDGLWLQMLLCHRVQAIVADTCRNCCLTNNSCACKQAYVSSHT
HYRQEHDRSSASGSIGNVYLADGQGDGNGVLGPVRVNVRGAVDGLAGIGRGNSNVPGAAWAPTRYVFSRVPYGLGSRNGQ
QFANDESEPRIDRNGDISADGLTALVNLSQENSAAHLQAESLFETGMQTRYRSVASVSTPGGSSVQMQESKEHELGSDWE
TAEDATISLDMKTPLSHFPPFRFGVEFEDVHRLADGQVKHSTEVFYAGSLWKVSVQAFNDEDPHGRRTLGLFLHRRKAEL
LDPLRKANLYIDPREKVTARYQLICPSKREVMIFGSLKQAGTLLPKAPKGWGWRTAILFDELADLLQGGALRIAAVVQLV
*

>Sbic_03g047153
MAFPGDGEHAAANTAGDAVVLEITDTSSVSAARAPSSPHPLPVSISDLALIESSSYFRALLGGSFSESGREHVHVSCNLE
AAVQVLRYLFEPSESFNITHDNFLPLLEGALFLAVESLLVECERWFSTIASQTSAIVSVPLDFIIEVWDFAQEHGVTFVQ
DICREFLAQNFVENMKTCEQSNPNSVDGHLFLLSKVKICLLPLGFAAGTKRHWFEFGNNIVCTILDLLKDSLKTLLDAIA
DDNLERYCIRITEYSKNIVLSGCPQVTTAFLYISVLPTDLDVSFKRRIVSSYTQVDHRSFILYDELEKAAKTLSFKNVHM
VDISKCPNVHFGAAIDWLKLFFPELRIFRVSHCLSFQFDDLLYLLLRCPWIDEIDMTIDTSTVTPQHSVVSSSSEVLSKV
KPNQKRYGIHCPPYDMQLNSVFLNISRLTLEGRSDIDDVGLLEISVLKSSLCYINIRNCFLLTDDVIIVFAFHYLGMLVD
IGICQYVGLDSAALSQLMSIISITKFLSLRETSLTDGALCNFVGSSLEYLDVSETVISMVSLAPVIRRNCKLNCLKTAGC
HSLLLECGNVEHISGNKYGDFLQEVGITCCLEDVEMGWGFCPIRIEDLVPSFSKVRNMTIGLGTALAENVLCAVPMICPF
LESLILRFQVISDRVVRNLLESATNLQVLCLHYCLGSLTSFSFQTKAPALRVLRLHWVTPWLTNDDLTILTENCDLAELS
LSGCKLLDSSSQDIISSGWPKLALLHLEECGKVTVEGVSSFFNCKALEDILLRHT
>Sbic_04g009720
MDPDFSPGGGGPSFEFAFNEVNFSDRELRIEVVAGDDDAPGSSGGGAGGGGLADWARHRKRRREELLKEKESTTHMSDQT
SCNGVEAEECDAYEENQEEPVAMIEESPPDVGQDGDDGQGIDSSWTVVGTPVLRVKTIYISSAILAAKSPFFFKLFSNGM
KESDQRHATLRITDSEETALMELLSFMYSGKLTTTEPTLLLDILMAADKFEVVSCMRYCSQLLTNLPMTTESALLYLDLP
CSISMAAAVQPLTDAAKDFLAVKYKDLTKFQDEVMNIPLAGIEAILSSNDLQVASEDTIYDFLLRWARAQYPKSEERREI
LSSRLLPLVRFSHMTCRKLRKVLTCTDIDHEQATKCVTEALLYKADAPHRQRALAADTVTCRKFAERAYKYRPLKVVEFD
RPYPQCIAYLDLKREECSRLFPAGRIYSQAFHLAGQGFFLSAHCNMEQQSTFYCFGLFLGMQEKGSMSVTVDYEFAARTR
PSGEFVSKYKGNYTFTGGKAVGYRNLFAIPWQTFMADDSLFFIDGMLHLRAELTIKQP*
>Sbic_04g024950
MAFCADFAALRPSPADVRVVTSDGSTIRAHSSVLAAASPVLERMIVGARGRGWDAADCTTVRVLGAPSDAVVAFLRFLYS
NPRAAAGAGEWAEEEDAHGPALLALAHAYRVPWLKRRAEAAVAARGLTAERAVDALKLAALCDAPRLYLACARLAAKDLD
AVERSEGWRFAGRHDAALRLNLLHLLHDADQRKERWERERASQHVYGQLSDAMAFLDRIFAGTEEASPSPCEDEDEDGVR
WGLEQLMRHFAACGGRTRKPAACPRCTRALQLLRLHASVCDRAGDGDGDGGEPCKVALCSNLKAKMQEEGVDTTWKLLVK
KVTRARVMSALASREVPEVVKKSWAKYSSRRAARIR*
>Sbic_05g000930
MDCCICSPMAAVYRLPRNAICAPCHEGAKAIIGFLNKDDEHQHEEEGSLSHGSVLKSRGSVKPNSPTKGMRDAWEQVKEM
RGREEEAHQRAAFLAMAWKEGVHTDIVVKPGTGPPIPAHKAILTKPSQPIIISYLASDLMLMQAARSEVFRHMLAADERC
KAPAGDAISLPELTHGELDLFLAFLYTGALENNKDDDGDHQLHALLVAADKYDVPFLRRACEARLAAAVDASNVLRTLEV
ADLSSSAALRERAMDTLVHHAEQVVFSPEYDDFAVRNAGLCVEITRALLGKMKTAPL*
>Sbic_05g002500
MSSEDSLKSLSLDYLNLLINGQAFSDVAFSVEGRLVHAHRCVLAARSLFFRKLFCGLDPNHQPPPPPPGSSAGRAAPPDL
VIPVSSIRYEVLVLVLQFLYSGQASVAAPKSGPLPGCGARGCWHTRCGAAVDLALDTLAAARSFGVEQLALLVQKQLEAM
VKEASVDDVMKVLMASRKFEMQELWATCSHLVARSGLSADLLAKHLPIDVVAKIEEIRAKSPAVVSPAGSGGGPRSPFLT
HHYLPINAASSAADRDHRIRRMRRALDAADIELVKLMVMGEGLDLDDALAVHYAVQHCGRDVVKALLELGAADVNSRAGP
AGKTALHLAAEMVSPDMVSVLLDHHADPNARTLDGVTPLDVLRSLTSEFLFKGAVPGLTHIEPNKLRLCLELVQSAVMVA
TRDDGAGTGTGGGDTGGGGSDGGGSFPRGDAADDSLVSLTMNSTLMYQGQDLAAAMAGGEARKGSGGGGRGGSPSNLYFP
NGFP*
>Sbic_05g022780
MRKLFFSESACKETKLHSAPHSWLPLERGKLSKFSGHAAASSSIDSLMKMPEPAVLPHFKPADYVDILAQIHEELESCSP
DEKSCLYLLQFQVFRGLGEAKLSRRSLQSAWEKASTIHEKLIFGAWLKYEKKGEEAIADLLSSCGKCSQEFRLLDFVSQV
STGSHVMSYDDDDDESDEFQGSAVVHFRIRDDMIACDRRKLAALSTPLYAMLNGGFRESYLEVIDMSRNGISPIGMRAIS
KFSLSGRLPYLSADAILEMLDFANKFCCKGLKDACERKLASFVSSRQDAIDFMECALELGCSILAASCLQVLLNELPECL
NDEQVVRIFSSANKAQRLTMVGNASFSLYCLLSEVSMSTNPTSDVTVSFLEKLVESASDSRQKQLALHQLACTRFLRKDY
PESERLFNAAFSAGHLYSLVGLARLAALRGNKHFALKFLDSVMSSRWPLGWMYQERALYLEGDNKLENLNKATELDPTLT
YPYMFRAASLMKRQSVEAALMEINRILGFKLVLECLELRFCCYLALEDYRAALCDVQAILTLAPDYRMIGGRVAAKQLRM
LVLENVEQWTAADCWMQLYDRWSSVDDIGSLSVIYQMLESDNAKGVLYFRQSLLLLRLNCPEAAMRSLQLAREHASSDHE
RLVYEGWILYDTGHCEEGLQKAEASIAIQRSFEAFFLKAYALADSSLDPSTSATVVSLLEDALRCPSDRLRKGQALNNLG
SVYVDCGKLDLAAECYINALKIGHTRAHQGLARVHFLRNNRAGAYDEMTKLIEKARNNASAYEKRSEYCDRELTKTDLQM
VTKLDPLRVYPYRYRAAVLMDNHKEKEAIAELTKAIAFKADLNLLHLRAAFHEHIGDISSALRDCRAALSVDPNHQEMLE
LHHRVNSQEP*
>Sbic_05g024640
MPTFSSMPIAGTGGGTSAGVDTRSSEIHVMKVSASHVLTISGYTHSKSAVAPSEHIGSSFTAAGHRWFVLYYPRGHGHGL
EDDADYISLFLSCTDTDVVKARLSLCLLDKDGKVAYTCKCRSYIPNPPMSSLHGASRFITRKDLENSGHVLDDRFRIGID
MTVFKVVRKEHAGAAVAKLVDVPPSDISQHLGDLLSSGEGADVMFEVDGESFGAHRAILAARSPVFKAELFSPMEEGTAT
CVWVEDMEPRVFKALLHFVYNDVLPEIDEAEAMVMAQHLLVAADRYCLERMKLICEEKLCSYIDTGSVGTMLALADQHGC
LGLQKACLNFLMSGGNMMAAVVAGGLERVMSNCPSVKELLLKSCSRS*
>Sbic_05g024650
MSTNAASAVDIIGSDGEALTTTSAIVAEAVTGSHVLQIKGYSRTKGVGSGNFINSSTFCLGGHQWFIRYYPDGDCVENAD
WVSLFLHHNNTNDVEFKAGLKFSVLDHTGKPVPMFSKKTSTTFSPNRRSWGFDKFVGRKELELRESSYLKDDCLKVSCDV
IISKGISTEPTVQFVAVPPSNMHRHFGSLLSGAVGADVSFEVAGEMFAAHRSVLAARSSVFMAELFGHMKEKAMKCIQIQ
DMEARVFKAMLHFIYTDTMPEIDKEDVFVVTQQLLVAADRYDLERLKLICEDRLCMCIDTSTVATTLAFAERHGCHGLNN

ACYQLLKSPSILETVMATESFDHLMTTCPSLIKELLAKIATCP*
>Sbic_05g024660
MSSTAESAVGSGHGEAVTTSAIVAEAVTGSHVLQIKGYSLTKGLGIGKFIKSSTFCVCGHRWYIRYYPDGDCLDSADWIS
IYLQHDHTDAVDVKARFKFSVLDDIGEPVPTFSQKSCMRTFSSSKGGSWGFNKFVARKALEESSSYLKDDCLKVRCDVTV
SKEISTEATTTTQCVMVPPSNMHLHFGCLLSGAVGADVTFDVAGEMFAAHRCVLAARSSVFKAELFGPMKEKAMNSIRIQ
EMEARVFKAMLHFIYTDALPFIEKGDVFLITQHLLVAADRYDLERLKLICEVKLCKCIDTSTVAATLVLAERHGCQGLKK
ACFELLKSPVHLKTVMATEGFDHLLTTCPSLIKELLLAKDAATCP*
>Sbic_05g024670
MSSSSTSVVGAGGVAHSASAIVAQAVKGSHVLKIDGYSRTKGLGKGNFINSEPFDIGGHSWCIRYFPDGNCTEHSGWISF
FLCLSYIHATGVEASITLTLLDDVGEPVPSSYCLTDGIVYTFKFCNGLGFPKFIERKALEESSYDCFRVRMDVTVSMEIR
TEGTSQFVMVPPSDMNKHIGRLLSSGVEADVTFQVGDETFAAHRLVLGARSSVFMAELFGPMKEKGTMFRDMLHFIYTDE
FPKFDNGDTIAMAQHLLVAADRYDLERLKLMCEHKLCECINTSTVTTTLVLAEQHGCKGLKEACFKFLKSPGNLKTIMDA
DDGGFQHLTNSCPCILNELLANVAS*
>Sbic_05g024680
MSRSASVDGSGDANSASVIVATAVKGSHVLKIDGYSRTKGLGNGNFLRSVPFDIGGHTWFIRYYPDGATKERIGWIELYL
HLDRNNATVAEASYTFSLLDDVGETVPSYCFSDSVIRTFNYSTVRCDVTVSKKIHTEGSSSQFVTVPPSDMNRHIGRLLS
SGLEEDVTFQVGDETFAAHRLVLGARLLELLGPMKERNGRVVRIDDMEPRVFRAMLHFIYTDEFPKLDKRDTIAMAQHLL
VAADRYDLERLKLMCEHKLCQCISTSMATTTLVLAERHGCKGLKEACLNFFKSSGNHGW*
>Sbic_05g024710
MSTSSAKMVTGWHVLKIEGYPRTKGLPGRRSIQSSTFIVGGHSWYIGYFPGGMGSLGLGNTDYVSVQLNLHHPAASAEVF
TQFKFSLLDHAGESVYSGTNSIFTFSSKTISHGISGFIKKTELESLYVKDDSFKIRSSVFMAQLFGPMKEKTANHVRIDD
MDARVFKAMLRFMYTDMLPDMEEDEKTVMSQHLLVAADRYNMERLKLICEDMLRKRINTDIVATTLVLAEQHGCCGLKDV
CFKFLISPGNLKTVMANDSFQYLKNSCPSLLDELLANVAP*
>Sbic_05g024750
MSSWSRRRRTMLRNDDDDDDDGRASTTMLTEIATGWHVLKVERYSKINGLGVARRMKSCPFVVGGHTWCIAYFPDRRDAV
KVRTTFSLLDVAAGGPVPSLTVSCGVWTFSRIGQSCCDARFMERKEFESCYVKDDEFCIRCDVTVLSAAAAPPLDLRRQL
GDLLAGGDGADVTLDVGGESFRAHKNVLAARSPVFMAEFFGGPHKEQVAGRVSIHGIEPSVFKALLHFIYTDSLPDIDGG
DMTAMAQHLLVAADRYGIQRLKSICEYLLRTFVDRSAVVTTLVLAERHGCHQLKEACFKVMKSSANYKELVSGEDFQYLA
STCPSLVQELEAA*
>Sbic_05g024760
MAGQLQPAVGHAGTSTSWHQLVSTTDSDTYGSSSTLSAETTTGSYVFTVADYSRDKELHAAGSALPSNTFTAGGYSWRIT
YSHDTDDWIRFSLCLEPPVAGGGAGTGGGRDLGDAAIMVRTRYILLDKAGLPLPSSIRKDSERALSTMRSMRATQFMRRK
RFESYYLKDNRFSVRCDVTLVRRTMCGRETGAMPPPDMRRHLGELLAGGVGADVVLEVGAEAFRAHRSILAARSPVFKAE
LLGGPTDDKDAPPPTTSTTSRRLRIHDIEPSVFRAVLHFIYTDSLPPEIGAGDEMVMAQQLLVAAQRYKLEGLKWICEDV
ISTLCHRRAANESSCPCLLYQLHAYAKTILSYSRHCRLAESQARSPCSPKTQLLRVSSPWLKKTTTSLVLVYWAPTYRRS
KLIARLGGPARSLRGTS*
>Sbic_05g024790
MSTSASSSATGDDDDDSGSTSTITAETTTGRHELKGESYTRTKGGVGKFIDSVTFRVGGHSWYIRYYPDGNRDESADWIS
VYLYLDGAGSEDDGGGVKARYKFSLFRDAAAAGDGEAPPVLIHTRTASNYSFWSADQHRRGYCRFIKATDMEEATKGGGF
CIRCDVAVMKETCVETTMADSVTVPASELNQHLGALLDSKVGGDVTFDVGGEQFTAHRYVLAARSSVLMAELFGPMKENT
MSSIQIDDVEPRVFRALLHFIYTDSLPEIDGEDEPGMAQHLLVAADKYGMQRLKLMCEAMLLKHIDTSVLATTLTLAEQH
DCQGLKDGCFKFLRSPGNTKAVVESDGFQHLRSSCPSLIEEMLVKVAP*
>Sbic_05g024820
MATSPAASSGTATGTIGETIVGSHLWKIDRFSATKDLGVGKSIKSSTFNVGGHTWYIECYPCGDNEYSAGLVTLFLCLSA
HKEQVEAKCRFTLMDDDRDYYQDGYNHTYNAEDSRGLGIDLIKRADLKKHLKDDCFRIRCDVYIVREFPAVPPPDLHRPL
SDLVASGMPGGDRTFQVGGEVFTVHRGLPDPSAHIAASPTPAGTSSSTAGSISSVSHVLTIDGYSKSKKMFRTDDYVESE
WFRVVGGHSWYIKLYPSREKEDDDSGCLAVFLNNNWWLLDEDHIEDKVLKVRVELTLLTFAREIISSAQFCYAFTSRYDK
CRCYFPFNTEMEPLIGDSFHVKCDVTVVKEITPILRPPDLNLYFGHLLASQVATDVTFEVGSDLFSAHKLVLATRSRAFM
AEFFGPAGKGSSTTVTRVQIDDMEPRVFKALLHFIYTDTLPELHEDDDKIVMAQDLLVAADRYDMGRLKLICADMLCNYV
NDARAAFAMLELADKHCCRRLKVKCMKLLKGHFAKVAP*
>Sbic_05g026450
MAASMVPAETRKITWRCSPDVDRCSHMFEIHGYTLQAEGLGVGKTVVRSATFAVGGYDWSISYYPDGYYGQESSGYASVY
IDFMSNKTCPAGTKVSGHFRFRLFNPATGEFRIMLGGYLPFKHTVNPNGSHAWGSAKFIDRRWLQTSPYLCHDRLTIVCD
LSVLVGSRVSGPESTETCENEIIIEVPPSNLSENLEKLLDMKEGADVTFKVKGVVFHAHKVVLAARSPVFKAELYGLIGE
RKTCRHGHIAIQDMEPGVFKALLHFIYTDSLPAANGDCDLDDEGVVKHLLVAADRYAMDRMRLMCESMLCKRLDVDNVAA
ILALADQHYCCKLKDACVSFMSSPKRIKDVMATQGYDHLKKACPSIVMNLLEESVKSSVC*
>Sbic_06g000720
MSSTAGRSKPSGSASAIVADVASGYHILKVSGYSRTKGTPTGELIKSHPFTVGGHRWCIQYYPNGDSSECADYISLYLCL
DESVTDAAVKAQFKFHFIDDVEEEDQTQALTTVSVRSFESNQSWGHRRFIKREDLEKSKHLKDDSFVVRCDIAIANELRT
EEVAMAEAPTTTFVSVPSSDLHQHLGDLLQTEKGADVVFEVSGETFKAHRCVLAARSPVFSAELLGQMKESDTTTAGVVV
GIDEMEAHVFKALLSFIYTDSLPAEMFMEDQTAMSQHLLVAADRYGLERLKLICEHKLCSCIEATTLATILVLAEQHHCH
GLKKACFSFLSSPTNLAAVLASDGFEHLNTSCPAVIKELIAKLGT*

>Sbic_06g000740
MSSSTAASKRRKLARSASTIIATASKGYHILKIDGYSHTKAKPTGESIESNTFTVGDHTWYIGYYPNGDDSECSAYISLF
LFLNETVPKPLEVQYDFRFIDEVVEEAPPSSLASADIVTFECRNDCSGYPKFIKREDLERSRHLKDDSFIVRCDIAVINK
FRVEELANAPTTTSVSLPPPSDLHRHFGYLLQTEKGADVVFQVSGETFKAHRCVLAARSPVFSAELFGQMKESDATAGGV
IHIDDMEAHVFKAMLFFVYTDSLPEMGKEDQTAMFQHMLVAADRYHLERLKLICEHKLCRCIKAATLATILPLAEQHHCQ
GLKKACFSFLSSPANLAAVLASDGFEHLNASCPSVIKELIAKLGT*
>Sbic_06g020420
MALLYADLDALRASAADVRIVTSDGQTIAAHSYVLASASPVLERMIDRARCRGWGAAADCTIAVLGVPSDAVLAFLHLLY
SSDAAALARSRRLVAPGAEDLVAAHGPQLLALAHAYRVGWLKRAAEAAVSARWLTADRAVDMLKLARLCDAPRLYARCAR
LAAKDFAAVEASDGWRFARRHDPALELELLQLLEDADTRRARWARARASQEAYRQLGHAMGSLDRIFAAAGGGTDDDASS
AGTCQDGDDPPCQGLRLLMRHFATCAPKVAPXXXXXXXXXXXLFRLHASVCDRPEQDQPCRVPLCSHFTAKMQTEKADKT
WRLLVKKVTRARAMAGLANRQVPEVVAMSWAKYSTSSKWARN*
>Sbic_06g026230
MEPPPASSSATSSYATPPQPSYAPSSYPSSYTKFNSALNAGLLNPMSPPPLPLDKTRSSPTLFDMMANEQDYHPRSAAAA
AGVHSIPAPPHQPHHQLQPVRSMDRQMLLQDRIADLIGSCSPGNQFNDADSSDVRLTLSSKDGLSVTLCVHRHILVAHSR
FFAAKLSDRWSKQQRTLPHIVEISDCDDVEVYAETLRLMYCKDLRRRLMREDVNKVLGILKVSAAIVFDAGVLSCLEYLE
AAPWAEDDDEKVAALLTQLHLENSGAGEVLKRVSLELAPSVVVEEAEVGGSCNTGNSSGGEEVLLRLLQVVLEGKDEKAR
REMKGLVSKMLRENSTSRGGAIGGDLRKESLYSACNGCLSLLCEQFVRASEGDHSEVAQIARQADNLHWMLDILVERQIA
EEFLRTWAMQTELAAMHRKVPAIHRYEVSRVTARLFVGVGKGQILVSKEARCQLLSTWLEPFYEDFGWMRRACKGLDRHL
IEDGLANTILTLPLATQQEILLAWFNRFLNSGEDCPNIQRGFEVWWRRAFWKRNSEPELPSRLRITAICENS*
>Sbic_06g029110
MARVPADARGEPSKTSSWRVTERATATHDFVVIEYSKLKMLGVGQHVSSDAFRAGGHDWKVMFYPKLAQAEGRIADYCPP
FFPGGASLHLCDADKDTELMVRFTLSVLHQDGRVSPMASRTRTEIFGAPPRQRHGFNRLVHGSMLTWRCIHNDALTIRCT
LTVVGTRDPVPPPELAAHLARLLGNGMGADVTFQVGARSFPAHRIMLAARSPIFSAEFFGDMTEKDTPCIKIVDMRPEIF
ELLLRFIYTEALPGDGEGCDAATMQHLLVAADRYGLDRLKQICELKLHASVDAETVDSMLALAERHSCPRLRDACVACMS
SLEQ*
>Sbic_06g029130
MPVRPPCRARTRPPYRERRRTRPRCENGGGPSLAGTFPRRRTEVDRAELTRTTTATNDERGLGWAAIGAKTWAGRQPGLG
GDWHAARTTYCLSQTSSRCVTGTVTASHNFEVTNFPMLDGMGIGKFVSSSTFTVGGCDWRIDFYPDGNDAANQGAYVSVF
LYFVRGTGGASVTLSFSLLLGNSSEQVTETSARRTFESAGGDWGFNKLIEKSSLRESLRRLNNHGCFTVRCILAARSMVF
RAELFGAMKEKDAQRIVIDDMEPAVLIRDSLPDDADNNNNVAMQHLLVAADRYGLDRLRLLCEAKLCRDIDVQTVATTLA
LAEQHRCTQLRGACIGFIASRDVLGAAMETDGFEHLATSCPTIMNEILDKVAAVWSNK*
>Sbic_06g029140
MANNSTSPVNHGQCLSDTWSRCVTGTVTAAHTFELTNFSLLEGSMGIGKYVDSSTFSVGGHDWNIRVYPDGWKEDDDDYV
SVFLNLERGAVGVRVKYSMSSLDKHGHVSKVRDDIHTFEWTNGFRGWSKYMDKCKLQPLLDLNNDCFTIRCDLTVNQDPR
TEAVASTIVVPQPNLQQHFEHMLKDGRGTDVTFSVAGELFRAHRCVLAARSLVFQAELFGPVKEEATQPIRIDDMEPTIF
EALLHFIYTDRCNVGENVAMQHLLVAADRYGLDRLKAICEDKLCHAIDVETVATTITLAEQHQSVQLKDGCLRFIIASRD
VLGAVMKTEGRMQRIEPAIFEALLQFLYTDSIPPPGDDDDDCGGGAHQEDNVTSQHLLTAADQHGVDMLRLTCEVRLCRS
IDLLTVGTTLALAERHHCIQLKDACLEFIASQDMVGAIMETDGFKHLLTNLPFHHERDLRQDCRRKSRSTLKV*
>Sbic_06g029160
MAGDTNSSSSANPSLLPATSSRCLTHDYKVTNFSLLVGIGQVVASSTFSAGGCDWSIRFFPDGDNETPEEAASFTAVYLC
LVAGPAGTRVRYKFTLLDDRGRRVSTKTGKRRRRKKGKETAADLTRTSTSTYPYVGAMWGTNRFFDRSVLEELLRDSNDS
FTIRCAVAVIRTRTEDDVVIEVPESVLRHDLARMLRDGDGADVTFIVGNRFFRAHRYVLAARSMVFKAQLFGAMTKEAQQ
QRGVDGARCIIKVDDMDPAAFAGLLHYIYTDSLADDCTAGRVVVAQHLLVAADRYGLDRLRVLCEARLCGWIDVQSVATT
LALAERHQCAKLREACLRFLSWPDMLRAAVKTEGFGHLIASYPSVASDILDKVVSARVDDH*
>Sbic_06g029170
MDARSVPGPPQRPTPSAPLPAGCSCLPKTSSTSLTKSFTAVHDFRVSDYSLLEGMGFGRYVTSSTFSVGGHDWAVRFYPD
GATAGLLGDVSAFLYYYSRDLDAPGVRTRFTLNLLERDGKMPQVTNPYMKHTFSPVSDNWGFTKFMEKSRLQQGSPYLDR
DCLTIRCVLTVVIESRTMANETNSIAVPPSSLHQDFGEMLSDGEGADVTFTVDGQLFRAHRCVLAFRSPVFRAELFGPMK
EKAENRIRIDDMEPAIFEALLQFIYTDRLPDSCSDGRNPAITHLLVAADRYGVERLRLLCESKLSEAIDVETVATTLALA
EQHNCSQLRRACIGFMASPNMLGPIMETDGFNHLIASCPLVMKEILDKVSCIWSGK*
>Sbic_06g030040
MAAATRVIGSKPSDCFQFQDPSTWTCITELASDVVVQVGDTSFHLHKTQLISHSGTLKKLVNESTGDDDSKPCTVRLDDV
PGGAEAFLLAVRFCYDVETELNAGNVVPLRCAAEHLAMTEDYGEGNLVQQAESFLSEMLAGWDDTVRALNACDDAVLPAA
EDLLIVPRCIDSLADKACADPTLTGWPMLQYFTAKSLEETAIWDGIGAAGRPPSPGPDWWYEQASSFGLPVYKRLIAAVR
SRGMSPENVAGSLMHYARRHLSGVRRRGDNSDGSSRGRAGASGTAAVFSAGDQRTLLEEIVALLPVEKSVTPTRFLLGLL
RVATVLHAAAACRDALERRAGNQLEEAALEDLLIPNTGYSAETLYDVDSVQRMLEQFMVTTPPAFAASPETTDEGQLVDA
PPAELRPVCSVAKLVDGYLAEVGTDANLKPSKFQTIAALVPDYARAIDDGLYRAIDIYLKAHPWLTDSEREQLCRLMNCQ
KLSLEACTHAAQNERLPLRVVVQVLFFEQLRLRTTVSGWFFASDNADQGSSSDNCVLQRRRSDNDLDFVAAGSLSEVTTE
EDGFAAARHDEPSSPAMSVAEIHRPGKGKPKTKSALSRLLGRLGLCGRSSSSRQQLQPPPLPGASGKRRKSFDFGC*
>Sbic_06g031210
MACDPSVRGYHILKVDGYSRTRSISKYSVAPGFESGPFRVGGHTWIIGYYPRGRSYSGPTYKGFISFCLYLVDPVADQSV

KVRATFSFLDHDMKLKLYSRPPTTFTFSEQLGWHGYQTLVAKTALANKCLKHDCFALRVDLLIIKEEGEPSSSIALPPSD
MHLHLGDLLSSKEHTDVEFHVGEETFAAHRLLLGARSAIFKAELSAGDRKTTRVIQVDDMDPQVFRAMLTFIYTDTWPKL
QKDSAMAQQLLVAADRYGLQRLKSMCEYKLCKRIDLDSVEDILLLAEKHQCAALKEACFDFIGSTATLLSARESMLTLRR
SQELRFNRLVSLCPTITKDKIFNVLDHEPDRNSVIRGINITLDIE*
>Sbic_06g031500
MLSTKTFDCPFLKYSKAAARLSAGGVLKSKPFEFHGRSWRFKVYPAGLSPDTGDFVAVFVKCRTEAFGRFELTVAILDGT
GRYTVFDDATARSTLAVREHGHSKGYVEFARRREIEASSCLHTDTLTVQCTLSVDATVVPKPSAFPWGFMARNRKAGGGV
AMDRSAPYMDTGASSSTSTRAPEIVSESHTLTIASFSAKKASLQCGECVRSAQFGVGGSNWYIKVYPNGHDDGSRDSVSC
FLARGKSGEPETTAEFTFELVNAAGNDNKSAKVRVTFANDASNAEHLGLQRAAADLQQQMRRRDRDDDRLVVRCRLGVFR
GKPPSPLLAEQPTITVPPDDLAAAFLWLLNSKECSDITYEVGGTPFRAHSCVLAARSRVFRQQVRELNDRQDNLWRNIQN
EDEEMTANAFEALLHVVYTDQLPDMTYVEPTDETVAAMLFAAERYDVERLKLRCEEWLCTFVTPLTVADILCMAVRYGGQ
LLQDACVRYAAPEYIWELVKQTEGFLQLRASCPHIVREIESKHTGANIS*
>Sbic_06g031510
MGSCLSAAGDGGQAASSASSVSGTHQFTIRGYNATKGMGVGKSILTRYFTVDGRTWFARYYPDGYNMETSDFIALYVQTL
YKPLCRPVRARFTFELLRPDGSVAYARRSDRPCNFDMRCSSWGIRAFVARGDLEGAELGVLHQDAIKVRCTVEVVNSQRK
RGGGRPRAPVAPASDYATNAIKFLSSGTAPPFDVKFSVGGVTFEAHALVVAAQSEWFAAALYGHGAGERWVEAGLPCISI
SGTTPEAFQGVLHYVYHDALPEELIKAKGEAVMMPQLFEAADMFLIGRMKAICAIRLRRFINNDTVRSIMELAQAHSCEE
LQQACQNHLARRRRL*
>Sbic_07g000460
MNGGGSGGASGPDSRVETISRLAQWRIDTFGPCSYRRSDPFKLGIWNWYLSVEKSRSIFVRLFPEPGRVAKEQPPLARFL
LRVSWAGPPRRTCVSPVHEHLLRTSDDFVWQVDAMSHGRFTIDVEFLDLRIANNNATESLSVWPTEGMVQKIASKSTLGC
LSRMLTDSIHTDVTINTTDGVLKAHKAVLAACSPVFESMFMHDLREKESSTINISDMCLESCSALLGFIYGTIEQGQFWK
HRLPLLAAANKYGIGDIKDCCEESLLEDITSANVLERLHVAWLYQLERLKKGCLTYLFVFGKIYDVRDEMHSFFHHADRE
LMLEMFQEVLSVWKPM*
>Sbic_07g002130
MERVKFSTRHNAACKKRSAEAANAVISTPQTVVPMPIIGGGRGEREPSRSASAIVAASTESGQHLLKIDGYSGTKDVPTG
SHIKSRSFRVGGHRWHICYYPNGCNSTCSDFISIFLKLDLDDHNVNVAHGHGVRARFTFSLLDYGEKKPVPHYTFSAEHT
FRDDDRGFRTFIRRDELEKSEHLKHDRFTIRCDLTVAATGEIQTMDLSSQGCPTPTLRLIAVDAAVVVVPPPSDLHRHLG
GLLATGDGADVTFMVDNRTFAAHRCVLMARSPVFHAELSLSTCPTTTTTNDSGGAAVVVQIEDMEAQDFEAFLHYVYTDS
LPPESKGSAAAAMLPDLVAAANRYQMERLRLVCEEKLCEFVDVTTVAVILAFAVDHHCLGLKEACLRFLEDPENLREVVK
TDGLEHLSKSCPSVLKDMIAKLAAAQ*
>Sbic_07g002400
MAPSPCTVRGTHQFEIVGYSLIKGFAAGEYVRSGSFAVGGYRWSVRFYPGGFSAPHRAYVSAFVKLTSKNARAWARYDLR
LVDRCTGLSRSVHHAAEPVVFVYSAPPPPGKCKPKRGARKFMPRSELEASAYLRDDRLTIECVVDVVADEARVPEAVALC
KLRLPPSDLAKHLGELLLNQQHGDQAADVTLAVQGEIFRAHRVVLAARSPVFMAELFGDMKEKAMDRIAVDDMQPVVFRA
LLHFIYTDVLLLPGDLDGDDDYREMVRHLLEAADRYGMERLKLICESILCRSVDGATVETTLALADRHYCEALTDVCLQF
KSLELDG*
>Sbic_07g002410
MALPANTKSTNTTETVRGEHRFDIDGYSGKLRAGRVVTSETFAVGGLDWAIRYHPAAAEVGDEEYVSVFVKLVTPNARAW
ALYDLRLVDRATGLPRSVRRRREPVAFDASKARKRERGSRLFMTVSELAASPYLRDDRLTVECVLDVVETFLSETTASPG
TAEPPPPDLSRHLGALLLQTPVGADVSFAVQGETFRAHRVVLAARSPVLKAELSGSAPAKEDDDDDASEVVAVDGMAPPV
FKTLLHFVYTDTLPEDLLGDLGREEQQEFVRDLLAGADRYGMDRLKKICELVLRKPLDAKTVAAALDQHRHCQCQALGDG
NVQFMPSA*
>Sbic_07g002590
MACLKLGSRADVFRKQGQEWYCTSGLPSDITVVVGEQSFHLHKFPLLSKSGLLEKRIREKIDKGDDSCTIDLSDIPGGAK
AFELAAKFCYGVKFEMTASNVVHLRCAADYLEMTEEISEENLIAQTEYFLTQTVLRSWKDSVKALQTCDNVLEVAERLQI
VKRCVDSIATRSCSDPDLFGWPVAQYGGPMQSPGGSLLWNGISTGARPRNSSPDWWYDDVSCLSLPLYKKLISAMEYRGI
SQEIIVGSLNHYAKRRLPGLNRRKSIGDVSNCLSMTSLTSIPSEDDQKYLLEEIDRLLPFQRGVTSCKLLFGLLRTAIFL
KASPSCVSNLERRIGMQLDKASLEDLLIPNVSDSVETLYDVDCAQRILDHFLAMDQETGGASPGIGEDGQMLASPSLMPI
MMVAKLIDGYLAEVAPDMNLKLPKFRSLAAAIPDYARPIDDGLYRAIDIYLKAHPHLSESEKEELCRVMDCQKLSLEACT
HAAQNERLPLRIIVQVLFFEQLQLRSSIAECLMISEPLDGGGGVGPSRQLGGLPVSGEHHRGAGWPLAARENQTLREGMD
SMKQRVAELEKECSAMREDIERLGRSRSASKSRFPFALTAKPVCSTKDKEAAPESSKSKAKAAATGSEDKLAVVKGGAAT
DGAAQPKHRKHKMNLSAC*
>Sbic_07g003210
MASQARVRASASICSSVTDSELHTLEISDYSQHSDLGDGGYISSTRFTVGGHDWRIRYYPDGIGNGEDCKDYVGVQLELM
SEFTEVRLAYEFRLADDDDDVAAIKSTTVLTAETSSTCGTDKFKKKSDLPRYLRNDSLKIQCFVTVFKESQLKEAAVSSN
PVVDVQVPPSNLSSHLGKLLSPERKDGDIMFKVKTEEFRAHKLILDIRCPALKTTAATHTQPHFDGQTIMRDRRTTSGTD
NSSTVVVIQDMEPKDFKALLHFIYTDSLPKEHRNNEEMATNLLAAADKYDLTRMKLYCGNILSKRPTVDSVTTTIAQAHK
HHCTELKDACIKFINSSERTRDDVLSSEGYKELKATCPEVALEILENSDISKFVFEFVFSMFTYWMWEKSIQEGSRKATR
RP*
>Sbic_07g006430
MSQTPRRTASSHNIKAHTSTHVFEIDDYSQKKETNVGEFIRSSTFTVGGFDWSIRFYPNGIDENSKDDIIVFLELMSSDV

KLRAHYNIQFIGGDDGAKKDSMISWYSTGGMHLFKSKGGEYRPRHMGYLMRDNLEEEFVKNNRLTIQCDLTVIRLYSLSE
NRVPITEIPVPPSDIKVHFGKLLEDKKGADVTFVVGCQTFDAHKAILAARSPVFMEELYGLTTRDIGAGPMIVENMNPLV
FRDLLHFIYTDTLAFMCDPQGVDYCVRIIYLLVAANRYAMDRLKLMCEDILAKMLNKEAAGTTLDIAQSHSLHKLKEDCI
EFMASQCERDVVQACTLLKRSCPSFVTAVLTRKEKLELVNKDAVIILK*
>Sbic_07g006440
MSLGHRSTESTFTVEADTGTHTFEIDDYNLTKNLISVGNFTRSTTFNVGGVNWAIRFYPNSTCLLFRNSVAVCLELMSSN
AEVSAHYNIQFMGSRYPKMDSQLSWLSSEASCLFSSSDLSDGSYLPIRHMGYLLRDLLEQWFVHNDHVTIHCDLTVIRKP
TLFQNMAQTEISIPPSDFTMHFGKLLEDKKGADVTFVVEGQNFDAHKAVLAARSPVFMEKFYGSMTRETETGSVIVEDMK
PLVFKSLLHFIYTDMLLETFDPLGHDYIEMIKNLLVAADTYAMDRLKLMCESILAKKLSIENVDEFMDIAQRHSGHKLKE
ACIEFMASLSERDFTRTCGKIKSSDSSFLIAVFDRIKRIKKN*
>Sbic_07g006450
MRMSAVPKKTSSRCITVAETGSHTFEIVGYSLQKGTGVGKFVRSGTFTVGGYDWSIRFYPDGSSTDSEEYVTICLELMTS
NATARASYHLRLASQQSPLCWWGCKSGPRLFKSCDVTRFGPRNWDFILRCDLEEEESCYLVDDSIRIECEVTVIREPQLS
ETRVESEIEVPPCDMMAHFGKLLEQKKGTDVSFIVGGDSFEAHKIVLVARSPVFMAEFYGRMRESGMCCVTVQDMQPDVF
RALLHFIYTDSLPDMDDLDRDEYSEMIQHLLVASDRYAMERLKLMCQCILRKNLDVETVATTLALADQQNCDELKAACIE
FISSSRDVSALVATQGYSSLKRSCPSVLVDLYEKTNT*
>Sbic_07g006460
MGSEPKRTESSHTTEEETGTHLFKIIGYTLTKGRGVGKSIRSGIFTVGGYDWAIRFYPDGSTEATKDSVVVSLVLMSENA
EVRASYDLSLVNQTTGLPENVFRESTFRVFNSCKGNWFTPRIIISKRNKLEFKSADYIVDDCLKIECTITVLKESWVETT
GDFEIEVPDTDLPEHFGKLLSEKEGADVTFSVGGETFPAHKIAQLYGQMKESRARKITVDDMQPDVFKALLNFIYTDSLP
ADWDDLDHYEYCEIAKLLLIAADRYAMDRLKLLCASILVEYLDVESVATTLALADQHNCNCLKEVCVEFMASLDEIDALV
ETEGYATLKRTCPSILVDALEKRSKYRKTGCQLN*
>Sbic_07g006500
MPTSRTVSTIAAQTEQGTHVVRILGYSQLRRMKEVINSDKFSVGGHEWFVKLVLDSCSTATAFSHPDCIIAVLLLKSHAQ
TKEVQASFDLNLLDLASGSSLSVHKVAPSFFHPGYGDTTVACCQIKNDLFETSSYLQDDCLTIEFSITVFKGPLSSETKS
LPRIHVPPSDIAADFGNLMETMEGADVTFSVGGQEFTAHRIVLATRSPVFRAQLYGPIREAGTGTAPIVIEDVQADAFRA
LLRFVYTDSLPPSDDLQSDDYAEMIRHLLVAADRYAMERLKLMCQSILCKNLTVQDVPTTLALADQHNCDLLRDACVEFI
GCLSATDAVAATQGYKDLKRTCPSIVTDFEEASSQVNYYWSELITGLSSSFLVE*
>Sbic_07g006510
MDRPIGICFGRGRIARCGGAPVTPQPSPSALPPPMPKTASRCVVEASRGAHVFEVADYSLHKGIGVGRSIRSATFDVGGY
EWSVLFYPDGKAENAEDCVAVGLELMTADAGVVRASYQFGLISQTTGEPWFVAESRTVEFHTHTIWSRRDLMKRNQLEAS
QYLRDDRLAIQCAVTVVKETRVLEARAVPEIDVPPSDLVADMGRLLDQGDGADITFEVQGESFPAHRTVLASRSQVFRAE
LHGQMKERSVDRIVISDMQPAVFRALLRFIYTDALPPMDDLSKDDSLEITRHLLVAADRYAMERLKLICAQILSKSLDVE
SVTTTLALADRHNCSGLKDACIEFIISSNKMDDVTKTQGFANLKRSCPCVLVEVLEKAGAGRG*
>Sbic_07g006520
MAGRTPTSSTRTVATRSTCTPVIQHGSHVFDIMGYSVHKAMGSGLSISSGIFSVGGHDWSIVFFPNNPIYSVDDGGGAVQ
PCISVYLALMSKGAKVLASCDLGLVDHTTGLPSSFHRTPEPREFRYGDGSMYFPQTTAAAFMDSAQLEASAYLHDDRLTI
HCDIAVIKKPSVFTGNTFAGRLEAPPPPNITEHLAKLLETGEGADVTFVVGGETFVAHKFLLAARSPVLRAELCGPMREA
ATQHVTVDDVQPDVFRALLHFIYTDSLPPDRGDILLLQGRLNKLH*
>Sbic_07g006560
MASPKWQVCFMNYKVVSVRSPVFKAKFYGPEMEENLGCIAVEDVRPAVLKAMLHFIYTDSLPDMDALGDVDKQEMIRHLL
VIADQYEMKRLKLMCEDSLCNSLSVDSV*
>Sbic_07g006570
MRSLSLAITSHFVEHMPLTAWADPIAGDARQQGAHMLEVTRYSHLLHRRRRGVGTGDRNRCVVVESVGFTVGGYQWAILF
YPDGDDDAPGSSGYVSVSLALVTRPARVRASFNLGLVNRATGLPSFQPEDTTAVFDNAACCCCRVHPYPYPIPTCRHERR
RRSSHGVPRLMRRCELEASSSAYLRDDAVTVACVVTGVTGTALTSSSSSSRGAPEIGCRVPERDLHLHIGDLLVKAPMTF
DVDGEAFAAHRIILAARSPVFEAELYGSSSTTETADDAHTVVVVVTVRDMRPETFRALLHFIYTDSLPAMSHLGMDEMRE
LVQDLLAAADRYAVDRLKVMCEHILARTLHVENAARTLVLADRHGCDRLKHASIQFMESSSDKSEMGLMASSSNKVEMGD
DVAIEALEKTM*
>Sbic_07g006580
MTTQAKTASTCPPPETAQGTHVFDILGYSKHRGMGADYFIRSGVFNVAGHQWVIFFYPDGYGEELAGGFDFVSAYLRLLS
TSGKVRASCDLRLVNPGTGVSTSVHPTLVITRELDPEKDKVVCHCMCIGRGELEGTYVKNDRLTMECVVTVRKEPKVSKS
RTFPSIRVPPSNLKRQLAELLESREGADVTFAVAGETFAAHRLVLAMRSPVFKAELCGPMREVGMGTRPVVVEDMQPDVF
RAMLYFVYTDSMDHNEDLSRDYHSKNCDMVRHLLVAADRYAIERLKLICQSILCNNLDVQNVATTLALADQHHCDKLKHA
CVEFMCCSNNAQDVVATQGYMDLAKTSPSVLADAMGRMSKISKKVMTRALQAQDGSSIS*
>Sbic_07g006590
MPPLRRFCQFLPRSTPSMARHVPTPTTASTCTPETAEGRHLFEVTGYSKHRGMGHDKFIRSGNFSVGGHQWSIRFYPDGF
VKGKDYISVYLELMGGSKVRASCDLKLVDLTTGLSASVHRTEPRMFNSGDVSRYAPQNSSFMERSGFEASVYLRDDRLVI
ECIVTVMAEPKVSATKLRAKIEMPPSDIGNHLGKLLEEEVGADVTFSVGGETFTAHKLVLAMRSPVFKAEFYGPMRERST
QIVTIEDIQPAVFRVLLQFIYTDSLPDMEDLVEDVDCEMIRHLLVAADRYALDRLKLICQSILAENLNVMTVATTLSLAY
QHNCSMLQDVCLEYMTSSNVMDAVVATEGYKYLKTTCPYALADAFEKTMKLGRT*
>Sbic_07g006600

MPTAVCTCTAKTDQCMFVFEIFDYSQHRSMRTGEFIRSSTFSVGGYDWAICFYPSREQNDWGYITVYLELLSVNTKVWAS
CDLRLVDQTTGLSTSLHKTELRVFNSSNASRFAPQTGSFMNRSQFDASLYLKDDHLTIQCITTVKQLQVSAPELLNEIEV
PLSNSAVLLGKLLETGEGADVTFSVGGQTFTAHKIVLAMRSPVFKAELFGQIKEANEQLVTIQDMKPDVFRALLYFIYTD
SLRGMDGQDGEGKRGMIRHLLVAADRYAVERMKLICNNGFEWSVFIDFTSLATVRKEKGISKPGWKIVDVTLMPYC*
>Sbic_07g006610
MRTAVSTFAAKTEQCMLVFEISDYRLHKGMGIGEFIRSGTFTVGGYDWAICFYPDGDVNSAGYISVYLELLSKDTKVWAS
CDLRLVDQITRLSTSVNRTELRVFKSGDTISRRAPYNGTFICRSQFEASSYLKDDHFTIHCITTVRQPHVSGPELLNKIQ
VPPSRITELLGKLLDEGEGADVTFRVGGETFAAHKIVLAMRSPVFKAELFGQMKEAREQLVTIQDMQPDVFRALLYFIYT
DSLPADMDVRDGEGYREMIRHLLVAADRYAVDRMKLVCASILCKNLHPKTVSATLALAYHHNCDRLKDVCLEFITSSSDV
MNSVMATQGYQNLKATCPSALVDAFEKSSKKFQKT*
>Sbic_07g006620
MSTPTKVSTCTADTEQGKHVFKIFDYSQHKGMGQKMLIRSGTFTVGGHDWSVLFYPDGYSLASIDCISVYVELLGKERMV
WASCDLRLVNHTTGLPSFVHKTDLRMFNSADFSRFAPQTGMFMNRSKFESSAYLQNDSLTIECNINVKKAPRVSATRLLN
KIEVPPSNITVQLGKLLDAEEGKDVTFIVGGETFAAHKILLAMRSPVFKAQLYGPMSEAKAEHVTIQDMQPAVFRTLLHF
IYTDSLPLDYHGGGTYNCEMIWHLLEAADRYAVDRLSLVCQSILCKNLDVENVSTTLVLACQHGCNRLKDDCLEFITSKS
VMDAVVATQSYKNLKIAYPSVLLEVLEKKMKSCKT*
>Sbic_07g006630
MAAPSNMTSVTVSSFTPEILYSAQVEFKIQNYNERINIGYATFLRSPAFTVGGYEWTLNYYPDGRSEQTEGHVSVALELM
GTETLWAILSLTFADQVDRSAWAGWATAAFLNSNNNFTFYWPELRSEVFEQMGLVQNDCLEIQCQISVCRPNQLLKAGLL
PEVEVPASDMLQNLGRFMADHVAADVMFKVEQETFQAHRIMLAACSPVFDKQLNGQMREKDMGCILVHDMQPAVFQALLH
FVYTDSLIDMSDKVVGDQIELIRHLLVAADRYCMDRLKNICEGILCKCVDMESLLTTVGLADQYHCMKLLAACVDFLKYS
VQRDIVESQWYQVLRDNHPDVAAQICKGVEDVRKLIN*
>Sbic_07g006850
MAASQGPRRKTVSRCIPETDQCTQVFDISGYSLLKGLGAGKFVESAAFVAGGRDWCIRFFPDGHAGEDLKDYVAVYLALV
TNSAEARALFEFRLVNPATGGSSSVYTCKTPMSFKAGGNQGAWGCRKLKKRSELEESVYLQNDRLVIQCDVTVIVGPPVI
TSEADIQVPPSDLTDTLGKYLESGKRSDVTFKVKGEAFHAHKFVLAFRSPVFEAEFYGPMRGKSRRQNITVEDMEPDVFK
ALLHFIYTDSLPPLDDLDEDESQEMDKHLLVAADRYAMERMKLMCESILTKRIDVHSVATTLALADQHHCEKLKEACIGF
ISSSDKMDLVASNGYEHLKRACPAIFMDIWENEAKSRRMM*
>Sbic_07g006860
MNLVTGSATVAYYGLYIYHPIGGYPHVVLPPSTIESNVFAAGPHYWSARFVCQTYGGHPSLSMSLQLCSRGVSVKASWEL
SVIDPRCRSLLPTKLYGTPPLYFEESVNMSWDLDYVFLSDQCFWYVDGDSGRLVLLITVTVFPDDDDPETTTIGKQIDDV
PPSDMLDQFGEIFETREGADVTFSVDGELFPAHKIVLAMRSRVFKAQLYGEMKESVAGAAQPIEISAMTADTFRALLRYI
YTDASPASSIVDQQDEEEDGSHGNTTKVWELLTAADRYSVERLKLICERILCKRIDVDNVADTLGLADRHHCGTLKDACI
DFMTTSSRMDQVKKTQGFLQLKHSHPLLLVEVLEKSSKF*
>Sbic_07g007550
MASESPRTWSASVSAVETAHGTHSFRIARYSLHKKASVNKCFYSAVFTVGGHDWRIRYCPDGAEPPMTMIVNSSYDSDSV
FLELTAGDGGKLVEVRATYGFRMVNPVTGISYSRNDSGLQVFHSNRRSHGFTMFVRKVEQHKYLQDDCLVIECDVMVVKE
LQVKEATCVTSGFDIQVPPSDLSHDLRKLLESEEEADVKFKVKEEVFHAHKIVLAMRSPVFKAELYGPMRDKGMVESITI
QGMDPAVFKGLLHFIYTDRFPRMDDLVEDEEQQDMVKHLLVAADRYGMERLKSMCEGILCSNLDVESVAYTMALAGQHHC
SKLKSACIEFIINSNLMDVVVASQGYEHLKRACPDVAVELWENSAKSPITRSSCIGGQLMNYPILN*
>Sbic_07g007730
MSAALKRPGTRTASMYVAAETARVTHSFKIVGNGLHKNFGVGRCIRSAPFSVGGHNWCILYYPDGRTEDCKDYVSIFLEL
MSENTEATALREFRLVNQTTGISTSISCTCQAVYNAENAARGSRKFMKKGDLESLGYLKDGCLEIECDLTVIKGDDIDLP
PSDLQDNLGKLLESEEGVDVTFKVKDELFRAHKIVLKMRSPVFEAELPRDKRKRIIIVEDMEPPVFKALLRFIYTDSLPS
MGDLDGDENDEMVRHLLVAANRYGLVRMKLMCESILCNRLAVQNVAATLAVADQCRCYKLKDACIQFISSSNRLDDVAAS
QGFEDLKKACPALIAAIYEKAAVPHKI*
>Sbic_07g007740
MSAAAERPSTRIASMCAAAETARVTHSFKVVGKSVHKGFAVGMSIRSATFSAGGHSWCIKYYPSGNADNCKNYASVFLEL
VSKSTEATVLYDFRLVNQATGLSSSLFSSKAVFNDEKPTWGPRRFIIKSDLEASGYLKDDCLEIECDLTVIKVDEIDVPP
PNLQDDLQKLLESEEGVDVTFKVKDEVFQAHRIVLAMRSPVFKAELYGPMRNNKKGSIAVEDIEPPVFKALLHFIYTDSL
PIMNDLDAKDHHEMVKHLLVAADRYGLERMKLMCENILCKKLAIDSIATTIILADQYHCSKLKDACFQFINSSNRMDDVV
ASQGYNDLKRECPALTIEIWEKAAKSRKI*
>Sbic_07g026660
MGSERGVARPRRSGRAKSFTHQFKLNFLETEKVAIGHLVSSEDISAGGHLWRIKCYPRGDRKENYGQYLAIYLQHQSKSK
DAEAIFEAFVMNKDGTASSSQRKRQVRVFTSKSSAKESWGWTRFVERSVLESLYVTNSGSFIIMCGVKILHEDSLEVPPS
DMGNHFGLLLDCAEGSDVSFIVDGEKFLAHRGCACRSLACLQGSMADSKMPSITLHDIAPATFKVMLRFIYTDAYPEEDE
LGDSYDMVRHLLAAADRFALDRLKIFCAADLWDNVLVDTVATTLICAETYNCPQLKKKCIDFIAEDNNFKKVVLKDGFVQ
LAQQFPAILDELREKVGV*
>Sbic_07g026670
MLDSCSFTHQFKLNFLETEKVAIGQSVTSEDIFAGGHLWTLLCYPRGSRTEDNGVYLSMALQHQTKSESVKAIFEAIVMD
KDGTLSSSHKNKFVKVLAPGSNWGWPRFVERSVLESLYVTNGSFIIVCGVKVVVGDDPIEVPPSDIGSHLGLLLDCAEGS
DVSFVVDGKKFPAHRNVLAARSPVFKAQLMGSMADANMSSITLHDIVPATFKVMLRFMYTDACPEESELGDYPDEMFRHL

LAAADRFALDRLKLLCASKLWDNISVDTVAATLICAETYNCPQLKKKCIDFFGEGKDFKTKALLTEGFAQLVQNFPSILD
ELRQKLGG*
>Sbic_07g026680
MLDSCSFTHQFKLNFLETEKVAIGHLVSSDDISAGGHVWRILCYPPRGDRKEYNGKYLSIYLKHESESESVKAMLEATVM
DKDGNLSSSHRNCTMTLFTPKGSASANDSWGWDRFVERSVLESLYVTNGSFIIMCGAKVLLDNDPLKVPPSDMGSHLGLL
LDCAEGSDVSFLVDGDKFPAHRAVLAARSPVFKAQLLGSMAEANMSSITLHDIAPATFKLMLRFMYTDACPADAELGDSP
DEMFRLLLAAADRFALDRLKLLCASKLWDNVSVHTVADTLICAETYNCPGLKKKCIDFVGEEKNFKGAVLTDGFAQLVLK
FPSILDELRAKVGL*
>Sbic_07g026690
MSASVSSIFDSCFMYPFKLNFVETEKVAIGHSVRSMDFSAGGHVWSIDCYPRGDEENESGEYVSIFLQHESKSEDAKAIL
EAFVMDKDVKVVHDDPLDVPPSDIGNHLGLLLDCTDGSDVSFVIDGENFPAHRAVLAARSPVFKAQLLGSMADASMSSIT
LHDIAPATFKVMLRFMYTDACPADDELGDSSDEMFRHLLAAADRFAMDRLKLLCASKLWDNVSVDTVAATLIYAEKYNCP
QLKKKCIGFIAEEKNFKKVVLTDGFFQLAHQFPSIIDELKLKVGA*
>Sbic_07g026700
MLDYSYFTHQFKLSYSETENVAVGHKVCSEDILAGGHLWRIECYPRGRREEDNGKWLSILLRHVSEFRDAKAIFEALVMD
KDGALSSSHRYRCVRDYGPHRGAAESWGWKHFVQRRLLKSLYMTNSGSFTIACGIKVIQKDPLDVPPSDIGSHLGRLLLD
CTDGGSDVSFVVDGEKFPAHRAVLAARSPVFKAQLLGSMADANMPSITLHDITPATFKVMLCFMYTDACPADAELGDSPY
EMLQHLLIAADRFALDRLKLLCASKLWDNVSVDTVATTLMCAEIYNCPELKKKCIGFFGEGKDFKTKALLTDGFVQLAQQ
FPSILDELRDKVGA*
>Sbic_07g026710
MFDTRFLEFKLDFSQTKNLAAGEFISSEDISVGGQVWRINCYPPGSRPQKYGDHLSIYLHLVSKPSKSVKAIFQVFVLDR
DGEPSFNHTRRFLKVYPLPKGMQEWGWHRFATGSDLKLLYMVRGVVTLECGVTVAAASKYETLPVPPPDLVSHLGSLLDC
ATGSDISFVVDGKTFPAHRAVLAARSPVFKAQLFGFLADARMSSITLHDMDPAAFKVMLRFMYTDCLPGDDELGDAPAPS
AMLQDLLAMADRYALDRLKLFCAKKLWEYVSTDTVGVILHCAEMYNCPELKRNCITFVADEENVKKTLLSDGFLQLVQTI
PAIVAELRKKLGV*
>Sbic_07g026720
MSSITLHDIAPATFKLMLRFMYTDTFPGDAELGGCPSEMIRHLLAAADRYALDRLKLFCAQKLWDIVSVETVGATLACAE
MYNCSELKIKCIDFFALEKNFRQAVLTDGFVQLVQQFPSIIAELRERPVA*
>Sbic_07g026730
MTMLEQRTIDSASREFRLNYEQTKRFAIDKAVYCDPFSICGHMWRIVCYPRGDIGSDSGEYLTLYLELLSTSTSGKAICD
ILLLNKNEENFLTEGHITWVCSIMVSRDVSIPMPASDIGKHFGMLLDSMDGTDISFTIDNETFHAHRAVLAARSPVFKSE
LLGSMAESTMPNITLCDIAPATFRVMLRFMYTDALPRDDELGDSPLEMMRHLLAAADRYALDRLKLICAQRLLDGLSVDT
VGTTLACAEMYSCLELKSKCIDFFTAGENFKKAFLTKGFIQLVQHFPSIIDELRERMGL*
>Sbic_07g026735
MWRMNCYPSGVRGRNKGEYVFFFLELLNKSSCVDAIFVAWLKGNDQLNSTSVPRALACVFDEEEDEQGWRTSSFHKLTYD
MVNNYVEDGHIKFVCTIVVLQSNSVIPVPLSNIGKHMGTLLDSMDGMDVSFVIGGETFHAHRFVLAARSPVFKAELFGSM
VEATTMTPITLHEIAPATFKLMLQFMYTDALPGDEELGNTPIKTLWDLLAAADRYALDRLKLLCAQKLWEDVSVDTVAST
LACAKMYNCPELKDMCIDFFAEANNFKAAVLTDGFAKLVQQFPSIISELRERI
>Sbic_07g026740
MADATMSTVTVQDIEPAAFKVMLRFMYTDSFPADKELGNSPTDMLQHLLAAADRYALDRLKLICSWKLIGHISVDTVGSI
LVLAETYNCPELKNKCLDFFGVEKNFKEAAFSDGFAILLQKFPSLAAELRRRVTGQS*
>Sbic_07g026750
MAVGDLVCSDDISAGGHLWKINCYPRGDKEDEKGEYVSLYLELISDSKGVRTIFHAFCLGREDGASDSNFSLYKRFVKVY
SCECPEYPEGPVTWGWRQFVKRSVLESVCVANGRVTFLCNIIVLGADTIPVPPSNIGSDLGCLFDSNVGTDVSFILKGET
VQAHRAILAARSPVFRAELFGSMDEATASSITLQDMEPAIFKAMLAYMYTDEMPEDDELGGSPTEMVRHLLAAADRYALD
RLKLMCAQKLWGIISVDTFASTLVCAETHSCAELKSKCIDFFAAEKNFKRIVFTADFMWLVQEFPDLAAKLKQRVEI*
>Sbic_07g026752
PARRSPVFGSTADARTRCITLHDVKPATFQILLRFIYTDELPRDE
>Sbic_07g026755
MATTSSQSGFIEFKLDFAGTKTLAIGDIVPSDDFSAGGHVWRVNCYPHGDKAHSSGVVYLSLFLKLVSGSKNVKAIFDAF
LLGRDGQPSSSHGNRCVKVYPPEGFGSWGFPQFVERSVLESDYVKDGSVTFMLGVIVLQRDNPIPAPSSDIASHLGYLLD
HMDGSDDVTFSVGVEKFRAHRAVLAARSPVFKAQLFGSMADAKMRCITLHDIKPATFQILLRFIYTDELPRDEEIQSSSS
NIELFQNLLAAADMYQLAAPASYG
>Sbic_07g026757
MGRDGEPSSEHSVRTSVLVYPPDGYSANRGWGLPQFVRRSDLERDYLTTDGFVTFMLGVIVLQQRDNDPIPVPASDIAGH
LGRLLDCADGSDVVFSVSGEKFSAHKAVLAACSPVFKAQLFGSMAEAKMRCITLHDIKPKTFQVLLRFMYTDELPRDDEI
QSSSNNIDLFQNLLVAADMYHLYRLKLMCARKLWDCVSAETVAEVLSCAEMYNCSELKSTCLDYFVLEENFKIAVLTDGY
FQLMQSFPSVIDEIRARRRFDRQT*
>Sbic_07g027390
MSSPLVATCGRSSSLSVGKTSLARLSESGNVYAMFETFLLHRNGQPRHVNSGTCSIRPLTGLRLFKSDCPEWIPWKHNIL
RSDLERETSDIGKQFGSLLDSSDGTDVSSVIGTETFHAHRAVLAARSLVFKAELLGLLGPMVESTMSSITLHDIAPTTFR
AMLWFVYTDAFPGADKLGDSPSEMVQHLLATADRCLELKNKCIDFFASEKNFKKAVLTKGFVQLGQEFPSIIDELRQRIG

L*
>Sbic_07g028630
MTDGARVEAAPRLAQWRVEALPCYTYRKSLPFRIGLWNWYLSVERNNKQTCVKLFAENPNSPKNGPSAPIASFITKLLIS
LPPNQQTIVHPGIFDKQLKHDGFVWAIDSTVTGRFVIEIEFLDLKVADPSGGEPASIWASRQIKQCSDSTALSSLARMLH
EDILTDITINAADGSVRAHRAILATRSPVFRSMFSHDLREKELSTVDISDMSLDACRAFLSYIYGDVSGEEFLANRLALL
RAADKYDIDDLREACHESLLEDINMGNVLERLQTAHLYQLPRLKGGCLRFLVDFRKVYEMHDDLNAFLQTADRDLVAEVF
HGVLAAWSGR*
>Sbic_08g001180
MSSEDSLKSLSLDYLNLLINGQAFSDVAFSVEGRLVHAHRCVLAARSLFFRKLFCGLDPNHPPPPPPPPQPPTGGGGGAR
TPDLVIPVSSIRYEVLVLVLQFLYSGQASVAAPKSGPLPGCGARGCWHTRCGAAVDLALDTLAAARSFGVEQLALLVQKQ
LESMVKEASVDDVMKVLMASRKFEMQELWATCSHLVARSGLSADLLAKHLPIDVVAKIEEIRAKSPAGAAVSSATTPRSP
FLTHHYLPINAASSAADRDHKIRRMRRALDAADIELVKLMVMGEGLDLDDALAVHYAVQHCGRDVVKALLELGAADVNSR
AGPTGKTALHLAAEMVSPDMVSVLLDHHADPSARTLDGVTPLDVLRGLTSEFLFKGAVPGLTHIEPNKLRLCLELVQSAV
MVTTRDEGAGDAGGSDGGGNFPRSDADDSLVSLTMNSTLMYQGQEMAAAAVAAGEARKGNGGGGGRGSPSNLYFPNGGFP
*
>Sbic_08g015510
MDLTLTSTHLSYVARSTHLLNISNYSLPSTHSKVYCKSSVEVDGFEWEIRFYPMCSPHYQLELQLVFLGEAGADKLTAAL
SCRMVDPSGTFEPTAEKTSETTSFQHHSDSSQPLVIVSIFEAIDLPYRYRPYFDSNNESLTVECTVTVFRDPEAIPVPSS
NNLQQHLGELLASKAGSDVTFTVSGESFPAHKNVEKSSGRVEIKEMEAPVFGAMLRFIYTDVVPELDDVKPAEAATATAT
TATTLAQHLLVAADRYGLDRLKVMCERRLAFAVDAAGSVAATLAIAEQHGCSRLKAKCVEFIAGGSRENLDAVLRTDGFK
DLEVNHPALLTELLVAAHGRKKD*
>Sbic_08g017890
MAATSAAVLSAAARLISRSAAAAASSGGCAHLLSAAKRMMSSRSASSIITREVTGHHNLTISGYAATRKAPVEWIASSQA
FDAAGYRWRIKYDPNGNSWNESNECISIFVELAHDNRGPRQEVKDPVQFKLSLLDRAGNPSPKYCRSNDASFFVDGQARF
QDFIRWKDLEESGCLKDDSFIVRCDITVIRKWAEGDDGSVGVGGVDGASSSRVVVPPSDLHKDLYDMLWKKQGVDVAIDV
AGETFEAHGWLLAARSPAFEAELLAAPKEKEKAAASGGAVRRRVEIRGMEPQVFKALLHFMYTDALPASMMEAAQDKQRA
VGMARDLIAAAHRYKLERLKLLCEEMLCRRIDVDTVAGTMAVAEQHGCGALEAACAEFLARPGNLKAVMETEGYEKIKAN
PTMMDLIMKQLV*
>Sbic_08g017910
MASAARACMSAAHRVTRSSIVTREVTGQHNLTISGYAATRKVPADWTATSPAFDAAGYTWRIKYDPNGNSWNDPISMSLF
LELADADDAGDGRQQETEPVQFGFSLLDQAGNPVPEYTRWFWEVGYFNGASRSKGFQRFISWRDLEESGCVKDDSFTVRC
DIAVVKKFATEAEGAAAVAPRVAVPPSDLHEDLGDLLWKKKQGMDVVIDVGGETFEAHGWLLAARSPVFEAELLAAPKEK
ASGGVVRRRVEVGGMEPKVFKALLHFMYTDALPPSKTEEEEQEQGGVDVAMAQGLLAAAHRYKLERLKLICEEMLCQSID
LDTVAGILAVAEQLGCGALKAACVEFLSLPGNLKAAMETQGYEKIKANCPIMIELILKQLLV*
>Sbic_08g017920
MAVATPLLSAAARRCTRSASTIGRREVMDCHSLTIDGCTTFKMIPNTWFSTSESFMAAGHVWRIRFYPKGARECFLRRGY
MSIYLELDSAAGGGGGYYRTTDPVDFRFTLLDLAGNPVPQYSRAMAARVFSAESRRMGFNDFIRWKDLERSGCLKDDRFT
VRCDITVFQDYWTADDNDGDGAVAPVQQQVTVAVPPSNLHEHLGDLLGKKQGADVKVDVAGEALDAHGWLLAARSPVFEA
ELLAAAKEKAPSSGGGAARRRVVVEGMDPRVFKAMLHFMYTDVLPEMDQEEETVAMSQGLLAAAHRYELERLKLICEETL
CRRIDVDTAASTLAVAEQHGCRALKAACLEFMARPGNLKAVLETKGFEKIKANCPAVMLDLIMKQVAARMAIQQP*
>Sbic_08g019560
MKYMKLGSKPDAFQTEGNSIRFVTTELATDIVITVGDVKFYLHKFPLLSKSSRLQTLVASTSEESNDEVDISDIPGGPAA
FEICAKFCYGMVVTLNAYNVLAARCAAEYLEMFETVDKGNLIYKIDVFLSASIFRTWKDSIIVLQSTKSLHQWTENLKVV
NHCVDAIATRASMDPSEVDWSYTYNRRKLPSENGGGGGDAHTWNGVRKQQAVPRDWWVEDLCDLEVCLYRKVILAIKAKG
RTSSDVVGEALRAYACRRLLASLDHAVSNGLDCTKHRAALDVVADLLPAEKGSVSCGLLLKLLRASCLLESGEAYRGELI
RRIGAQLDRASVSDLLMPAESGDDAMYNVDMVAAILEEFLLQHKIDGSDQAKMQEDDDTMDVAADDVISSSKLAAVAKLV
DGYLFEIAKDPCLPLNKFIALAESMPPMSRPVHDALYRAIDVYLKVCANADESDDHGVDKKLTNLCCAHACIQEHPGMSK
GEKKRLCALMDCRKLTADASAHAVQNERLPLRVVVQVLFFEQVRQSAAAAACSAAGPARSLVLPRDDGASYGGSSRSATT
TATEDEQWGGGAAVGGAPTTSASGGDASSLRSVSQLSANKSGGRSGSGDRKKAKGGAVAPAPARRVLGKLWSGKASSVEN
SGSDDTSESPAGSLNLEETKSTPSRITRHSVS*
>Sbic_09g019610
MEAEQQKQPQRPRRKGQKRKLEDEASAAAAAAAAAAVAAAASSLGSAGADDDNEEEDGSAGTPEICCRHSHAALAREVRV
QVDVLVRCASSWRHADRAAAKRATHVLAELAKNEEVVNVIVEGGAVAALVCHLEEPAVAAPTQEEQQLRPFEHEVEKGAA
FALGLLAVKPEHQQLIVDAGALPPLVKLLKRQKNTTNSRVVNSVIKRAADAITNLAHENSNIKTSVRMEGGIPPLVELLE
SQDLKVQRAAAGALRTLAFKNDENKTQIVQCNALPTLILMLRSEDAAIHYEAVGVIGNLVHSSPNIKKEVLNAGALQPVI
GLLSSCCTESQREAALLLGQFASADSDCKVHIVQRGAVRPLIEMLQSADVQLREMSAFALGRLAQDTHNQAGIAYNGGLA
PLLKLLDSKNGSLQHNAAFALYGVADNEDYVSDFIKVGGVQKLQDGEFIVQATKDCVAKTLKRLEEKINGRVLKHLLYLM
RVGEKSVQRRVALALAHLCAPEDQRTIFIDNNGLDLLLDLLISMSSKHQQDGSAALYKLANKAAALSPMDAAPPSPTPQV
YLGEQYVNSSTLSDVTFLVEGKRFYAHRIALLASSDAFRAMFDGGYREKDARDIEIPNIKWEVFELMMRFIYTGSVQVTS
EIAQDLLRAADQYLLEGLKRLCEYTIAKDVNLDNVSDMYDLSEAFHAVSLRHTCILYILEHFNKICTRAGSPQLIQRVIP
ELRNFLTKALSSSRSPSPSDRNSQI*
>Sbic_10g000240

MRSVEETKRSLTVAPFECAWAEELRFGEPGRGCIAFEASAQNDVTLVFRHLLGSQHYHYKTDTSRHYTVILGSHRNKRLK
IEVDGITAVDVTAVGLCCSSSFQSYWISIYDGCISIGQGKHPSNNLLFQWLDPDPNPHVQYVGLSSWDKHVGYRNISLLP
SAPPNSIFWTQIEYANVHWVADAGDYVRKQLSRDDCHQRVLADFLENWDFSDAVFVVGTERKVVPAHKLVLCASGDFPFE
MGDEATIVLPSASYPVLHSLLEYIYTGSTQIAEWLLTSVLELSSQFKVEPLVKCCKEIIDCLEVDKKLSDSGKILIKFPK
SGLQDHRFDSSPLKAPLNVQKVGEFLANGEHSDINIYVNGHGLVSKGHKLILSLWSAPLAKMFTNGMRESSATDIFFGDV
PPEPFFLLLQFMYYGKLKLDTRDTTSVLVQLLLADQFAITVLQFECCKRIMECLSEDTVCSVLQAVSSISSCKPLEEVC
KRNFAMHFDYCTTACTDFVLLDEAPFKDILEHGDMTVTSEERVLDVILTWCMGACETFHWTSVDELLRSSTPEQLFGERL
SGINTLLPLVRFPLMQLSILKRMERSTLANDIQAFRQLVAEAIEFSHAGQLMLTSYECERFQHRRSSYKELQYISDGDNN
GVIYYAGTSFGKHQWMNPVLTKNITVVASSPNSRHTDPKALVSKNYQGTCFAGPCIENGKKLSWWMVDIGQDYQLMCNYY
TVRQDGSTTFMRSWALQGSMDGRNWTSLRVHDDDPTICHPGQFASWPIVGPAALLPFRFFRVALTGPAAGCAWNLCICFL
ELYGYFR*
>Sbic_10g001810
MEPVKLVEGYNFDDHTTSDVRVCFKLIDEQPEWFSCHSSVLSQNSKYFADRLGQNDIRSNSCIEIECPRAEYDHYVKMLK
LMYLPGESVVDSFDSVKSALGVLRASNSLGCELVTKGCIAYIEAASWDENEEEEILEVARSLGSDAVSLLARLHAPSADA
VKNVFISAIRFATCMETPFPPFLDDLKTSAQEQIDFMIHEDDETALVTMDEDVRSVVQEGTTKLLSGLRTGLDLLATEFD
QSPDLAEKGILSSLADIDWVANLLAKIEMMHAFVSGWSEISDHILSVVHDKKYSSGLWAVKAKLIEVTGKALDAVGYGTV
VLPASSRVHLLKTWLPYIQMTKPLLDENSKDETSLQMDSDMCQNIESAIVSMVLALPSDDQADILSEWMKNAEQFRYPDL
TEAFEVWCYRTKTAKRRLVGGLNGSSNPTVSL*
>Sbic_10g003110
MPASKTVSTSAAETDLGTHVFHVVGYSHQKAICAHKDTSILSGRFSVGGHEWALVLVADIYNDDHPDCVAISLLLDPFAA
PGVLASFELRLVNQSTGLPFTVHKEAPMVFDDNNDMAATCWIKRSLLESPTYLQNDRLTVECIVTVIKERLVSKTKSLPR
IEVPPPNMAENFGSLLETDLGADVTFSVGDETFKAHKIVLATRSPVFKAELYGPMKEEGMKPITIKDMQPDVFRALLRFI
YTDSLPPLDDLEADDHSEMIRHLLVAADRYAIERLKLICQSFLCENLNVQTVATTLALADQHNCDILKDACIDFITCSNE
MDGLLSSQGYKNLKRTCPAVVIDVFEKTSKFRKA*
>Sbic_10g005710
MKFMKLGSKPDAFQTDGSDARYVLSDLPSDIVIHVEDARFYLHKFPLLCKSSLLQRLIIEASQNGTDEVVIQDIPGGVKT
FEICAKFCYGMVVTLNAYNVVAARCAAEYLGMTEDVEKSNLIFKIEVFLNSGIFRSWKDSIIALQTTDALLPWSEELKLV
GRCIDSIATKATVNPSNVMWSYTYNRKSASSDEIVEARKSSQAVPKDWWVEDLCELDVDLYRRVMVAVKSRGRIPSDVVG
EALKAYAARWLPECCDMLAEDAYSEAYKHLLETIVWLLPSDKGSSGISCCFFLKLLKVTVLIGAGELLKEELVDRIVLQL
HKASVNDLLIPSKPPAQTVYDVQLVQTLISRYMSHAGVAQDGIFLNNLDQEMFETNVDSESLLALCKLVDRYIAEVASDP
NLSVSSFVDLVTSMPETARPTHDGLYTAIDVFLKLHPGLPKMEKRKISSLMDVKKLSKGACIHAAQNDRLPLRVVVQVLF
FEQLRAAGAAAAATPNVSVARCMARLAEEEEEDDHEHWKEGRALPEPPTPGALKRQLGSLKLGGDGDDGRRLVARSSSVA
NQSSRLSLSSRSRRIFDRLWVGGSAKLPGEAAATAAVGKGSSDTSGSSQSPRSSAKPLESKSSSSSSRNRRYSVS*
>Sbic_10g012120
MDSEPDFELRGLDIDVELDPEDLQPSVPLKKVPGGDLFEAARAGDCDRLALLLEAGANVNARDRWDSVALYYACLAGHAE
AARMLLEAGAVCAERTFDGDRCHYAALNLRLRWLLKSFEARPPPLAPLPAALRATFLACPANRTAFLEMLQGSAGAESAA
LAAAAGFGPKDDPSSACLFPPDITFYVDRKPVEAHRVILCARSPFFEKKFKTDWKDRKEVRFSNQKLYYGALYSLIHFFY
SDRLEVAVDDMENLARACKVCKCEELQKILDKEVVHQKYAEYKSAKELDLDNSQKRFILQAQSLPKEDRLPSALQRILQT
CLANSREGDYSEESNEMIRNSEDDDLADLYIKVGDKVFHCHQVILASRSEYFRARLSRTVDFLDGNSGFQAAQNVPLLEE
HDLSAEAFEKMLEYMYTDKLEHLDPDQAEELFDVASRYLLFPLKRVVADMLLPYLEHVSPAELCHWLMLSDIYGVMKIRE
YILDIIACNFEMFADTREFRALLLTLPPPSGDDSLRTTRPSAPGTGGNTDQGNILDDLREKWLEAEGAELDERDESAALF
DRRLEMLMLVAEKEADDVDA*MDSEPDFELRGLDIDVELDPEDLQPSVPLKKVPGGDLFEAARAGDCDRLALLLEAGANV
NARDRWDSVALYYACLAGHAEAARMLLEAGAVCAERTFDGDRCHYAALNLRLRWLLKSFEARPPPLAPLPAALRATFLAC
PANRTAFLEMLQGSAGAESAALAAAAGFGPKDDPSSACLFPPDITFYVDRKPVEAHRVILCARSPFFEKKFKTDWKDRKE
VRFSNQKLYYGALYSLIHFFYSDRLEVAVDDMENLARACKVCKCEELQKILDKEVVHQKYAEYKSAKELDLDNSQKRFIL
QAQSLPKEDRLPSALQRILQTCLANSREGDYSEESNEMIRNSEDDDLADLYIKVGDKVFHCHQVILASRSEYFRARLSRT
VDFLDGNSGFQAAQNVPLLEEHDLSAEAFEKMLEYMYTDKLEHLDPDQAEELFDVASRYGVMKIREYILDIIACNFEMFA
DTREFRALLLTLPPPSGDDSLRTTRPSAPGTGGNTDQGNILDDLREKWLEAEGAELDERDESAALFDRRLEMLMLVAEKE
ADDVDA*
>Sbic_10g020060
MDPDFSRASGGPSFEFAFNSVNFSDRVLRIEIVAGDDALGAKGAAGEGCSSLADWARHRKRRREELRRGKESGKYMPDPG
NCKVEAEECDAYEEGNEPVAMIEESPPDIEPDGEDGESSDSSWSMECTQVLRVKSIYISSAILAAKSPFFYKLFSNGMKE
SDQRHATLRITASEENALMELLSFMYSGKLTTNQPTALLDILMVADKFEVVSCMRHCSQLLRSLPMTTESALLYLDLPSS
ISMAAAVQPLTDTAKEFLANKYKDLTKFQDEAMNIPLAGIEAILWSNDLQVASEDAIYDFVIKWARAQYPKLEERREILG
TRLLPLVRFCHMTCRKLRKVLACSDLDHEQATKCVTEALLYKADAPHRQRALAADVMTCRKYAERAYKYRPLKVVEFDRP
YPQCIAYLDLKREECSRLFPSGRIYSQAFHLAGQGFFLSAHCNMDQQSAFYCFGLFLGMQEKGSTSVTVDYEFAARTRPS
GEFVSKYKGYYTFTGGKAVGYRNLFAIPWPSFMADDSLFFIDGVLHLRAELTIKQP*
>Sbic_10g026720
MTDPRHPYSGGDWIMSRVRLINNGAREVAALFSCRLVDPRSPADSLEKTTSSVFYENKSRDVFLIRRSELWESSRRYLKD
GCIIVQCAITTLPGHPKDAVAYAAPSVPSSDLHKQFGELLLSQVGADITFVISGEWITAHRCVLAARSLVFMAKLFGNMK
EKASSCFEIDDMEVEVFRAMLHFIYTDTVPKLDQLKGEEATVIAQHLLEAADRYGLERLKRICAEKMCMNIGVDTVATTL
ALAEQHGCSKLKSKCMEFISACPKNLQAVAATEGYKHLEASCPLVLTELLSSRLSLKGNKHEVVGHSTVGDSK*

>Sbic_10g026730
MRMQNYAACINLTNSARSVQLFKIDAFTATREKGQGSIASTRTSVGGYDWVINYHPRVWFSDQYWIMFRITLDSNGTGVV
ASFACRLVDVVDQTGGGQHLGSSSSPVPAAITSATLSKGQANDVYITTRSKLASSGYLKDDSFLVECVITVLIENRSPTQ
DAAAAAAANLAPTTTSAAAAPPSDLGRHFGELWRNQKGTDVTFLVSGEPIAAHKCVLAARSPFFMAELFGDMKEKASQHVE
IEDMRPEVFRALMQFIYTDTSPPELQVEGKEEEEEEDARMMAQHLLVAADRYGMERLKIICEEKMCADISVDTVSTALAL
AEQHGCSELKARCIKFIVATPANLRAVVKTEGYAHLIASCPSVVHDLLLAIMADRHK*
>Sbic_10g026740
MPSSLQQQYSITPSMGVSFSNLTDAACVVHQFKINGYSATKSMARTDSLPSKRLAVGGYEWEVHYTPSLVVDGGYWIAFR
LVLLSAPRRNDVKAAFRCRLLYTSSNSLQQRGACVRDAIGNVEGQISHAFKRAKESSGWIPLRKRNDVEAAGVIENDSFT
AECTITVVTELPPEPDTAKTNVVRPPMPPLSGLHSLHHDLGELLSKATGSDVVLVVSGETFAAHKAILASRSPVFMAQFF
GPMKETRSERVEIMDMEAAVFGAMLRFMYTDMVQELERQEDGAIIAQHLLAAADRYGLDRLKSMCEDKLCDGTRVETAAT
TLALAEQHCCPKLKARCVEFIAANLDDVMATEGYKHLMTSSPLVLNDLLRAVRGRKN*
>Sbic_10g026750
MSDGDALKSRWKVGGYDWEVQLFPNSIVVLHLYLRSNIVSPDSSVKVMFSARVVDPSGKLKRHQGILSVAHNFKRSGYRC
YGGPLMSRSELQTSDDLVPSSGLNHHLGELLHKGTGADVTLVAAGESFAAHKAILASRSPVFMAEFFGHMKEKRSPRVEI
KDMDAAILGAMLRFIYTDSVPEPEDGDDGEVAAMHTAQHLLAAADRYGIDRLKVACEDRLFDGVTVDTARGDDSGLGGAA
RLCVELIAANLEAVMATDGYKHLTASCPSVMDDLLMAVHRRKN*
>Sbic_10g026760
MKPATAITKITEAARSVQLLQIDGYSATETMSKSDYIGSTWNVDGYEWEVRVDPEYDYSRASWVALRLIILSKPRAVEVR
AHFTGRLVDPTGRLQPSEDDDSISHVFHHRREQTKVLLVEKRDDIASAGYLDNDSLTVECSITVLRELPEVVFPPAATKE
EVPSSPPPTPSSDLHKHLGELLESKKGADVTFLLASSAGGGERFPAHKTVLAARSRVFMAEFFGGMEERTSRVVEVQDVD
PAAFEAMLRFVYTDVAPPELDDDDDEEPAAAATMAQHLLAAADRYGLDRLKAICEAKLAGCVDVGTAATTLALAEQHGCA
MLKAKCVDFVTGSPETLDAVLATEGYAHLAASCPLVLAELLKSARGKKI*
>Sbic_10g026770
MEHDGKNLTVTEVVRSVQLLKIDGYSATSTMVGSSEFIKPRSRWNIDGHEWEVHFYPDHCQFLEDEDVIPPFDDFIQWVA
LKLILVSEPQRDKLRVNLSCRLVCPSQRHLGPSEEKSVSHVFHRGSKCSPEVLLMTKYDVPSSGYLVNDSLTVECTITVL
GGDEEQPLPVPPPSDMHKHFGELLQSQEGVDVMFHVSGESFAAHKVILAARSPVFKAKFFGGLEETSSESVVIEDMEPVV
FRSMLHFIYTDMAPELDGDEDPQAAAIMAQHLLAAADRYGLDRLKLICECKLSGGIGIGTAATTLVLAEQHHCSLLRAKC
IEFITKSPETLDAVLATDGYAHLVASCPLVLAELLRAVAHGRKI*
>Sbic_10g026780
MAEFFGGMNERSSQTVRIEDMDDDVFKAMLHFIYTDRAPELDEEPDVAVTMAQHLLVAADRYGLDRLKLICGSKLAGGIS
IDTVATTLAVAEQHSCSLLKAKCVDFIARSPETLDAVLATEGYNHLVESHPLVLTELLRAAHGRRI*
>Sbic_10g026790
MEHGRTVLTEAVRKVQLLKIDGYCATSVMKHSDYIKSKWNVDGHEWEVRIHPNYYNNNNHWDPWMAVKLILHGEPRRNKL
KVNLSCRLVDTSHRLDPSAEKSLSHVFPSCDGCTVAVMSRDIIPSSGYVVNDSLTVQCTITVLKELADIEVPANTEAPPL
PPSDLERHFGELWQGQRGADVTFVLDTRERFLAHKAILAARSPVFMAEFFGGMNEKSSQSVRIEDMEASVFKAPELNEEL
EVAMAMAQHLLAAADRYGLDRLKLMCETKLAGGISIDTVATTLALAEQHNCSLLKTKCVDFIARSPETLDAVLATEGYNH
LVESCPLVLTELLRAAHGRRI*
>Osat_LOC_Os01g09800
MEPPTSHVTNAFSDSDSASVEEGGADADADVEALRRLSDNLAAAFRSPEDFAFLADARIAVPGGGGGGGDLLVHRCVLSA
RSPFLRGVFARRAAAAAGGGGEDGGERLELRELLGGGGEEVEVGYEALRLVLDYLYSGRVGDLPKAACLCVDEDCAHVGC
HPAVAFMAQVLFAASTFQVAELTNLFQRRLLDVLDKVEVDNLLLILSVANLCNKSCMKLLERCLDMVVRSNLDMITLEKS
LPPDVIKQIIDARLSLGLISPENKGFPNKHVRRIHRALDSDDVELVRMLLTEGQTNLDDAFALHYAVEHCDSKITTELLD
LALADVNHRNPRGYTVLHIAARRREPKIIVSLLTKGARPADVTFDGRKAVQISKRLTKQGDYFGVTEEGKPSPKDRLCIE
ILEQAERRDPQLGEASVSLAMAGESLRGRLLYLENRVALARIMFPMEARVAMDIAQVDGTLEFNLGSGANPPPERQRTTV
DLNESPFIMKEEHLARMTALSKTVELGKRFFPRCSNVLDKIMDDETDPVSLGRDTSAEKRKRFHDLQDVLQKAFHEDKEE
NDRSGLSSSSSSTSIGAIRPRR*
>Osat_LOC_Os01g56200
MPARSAVVVIAMEPSSSITIASSSSYLSNGSSPCSVSLAPPGAGAVAAQAAPVAAGEGGGGGGGGGGGGSSSVEVVSLNR
LSANLERLLLDSDLDCSDADVDVADGGPPVPVHRCILAARSTFFYNLFAARGRGGDGAAGGGGGGGGGGGERTGGRPRYK
MEELVPGGRVGRDAFLSLLGYLYTGKLRPAPDDVVSCADPMCPHDSCPPAIRFNVEQMYAAWAFKITELISLFQRRLLNF
VDKTLVEDVLPILQVAFHSELTPVLEKCIRRIARSNLDNVSLDKELPPEVAVQIKEIRQKSQPNEGDTVISDPVHEKRVR
RIHRALDSDDVELVKLLLNESEITLDDANALHYAAAYCDSKVVSELLDLRLANLNLKNSRGYTALHLAAMRREPAIIMCL
LNKGAAVSQLTADGQSAMSICRRLTRMKDYNTKMEQGQESNKDRLCIDILDREMIRKPMAVEDSVTSPLLADDLHMKLLY
LENRVAFARLFFPAEAKVAMQIAQADTTPEFGIVPAASTSGKLKEVDLNETPVTQNKRLRSRVDALMKTVELGRRYFPNC
SQVLDKFLEDDLPDSPDALDLQNGTSDEQNVKRMRFCELKEDVRKAFSKDRADNSMFSILSSSSSSSPPPKVAKK*MPAR
SAVVVIAMEPSSSITIASSSSYLSNGSSPCSVSLAPPGAGAVAAQAAPVAAGEGGGGGGGGGGGGSSSVEVVSLNRLSAN
LERLLLDSDLDCSDADVDVADGGPPVPVHRCILAARSTFFYNLFAARGRGGDGAAGGGGGGGGGGGERTGGRPRYKMEEL
VPGGRVGRDAFLSLLGYLYTGKLRPAPDDVVSCADPMCPHDSCPPAIRFNVEQMYAAWAFKITELISLFQLHLQDYFFLQ
KQRLQCKLHKQTPHQNLALFLQLALLEN*
>Osat_LOC_Os01g57230
MATMKLGSKPEIFVLEGLTWRCMTELESDVVVEVGEMSFYLHKFPLLSRSGVLQRMISEYQAPQEDGGGGGGMCTLQLDD

IPGGAKAFELAARFCYDVKIELNAHNVVCLRCAAEYLRMTDDYAEGNLITQAESFLADVLANWKDSIKALETCEGVLPTA
EDLHIVSRCITALASKACASDAAACSTGHAAASASAVAVAAKNASYDALWNGIGSGGTPRGGGGAAGAAAGCSGMDWWYE
DVSFLSLPMFKRLIQAMEGKGMRPESIAGAIMFYAGRFLPGLKRNTSFSNASFGGDCGAGSRSITPRAANVSAPSEGDQR
YFLEEIVALLPAKKGVASTRFLLGMLRTAMLLHASPLCRENLERRIGAQLEDACLDDLLVPNLGYTVETLYDIDCVQRIL
DYFMSSTDGLGTGYTSPAVVEEGSQLGALHAGSPSSLSPITMVAKLMDGYLAEVAPDTNLKLPKFQALAAVVPDYARPVD
DGIYRAMDIYLKSHPWLSESEREQLCRLMNCQKLSLEACTHAAQNERLPLRVVVQVLFFEQLRLRTSIAGWFFVSGNAAA
GGDGAPQPHPGGSAIVPKGAAAAGQSDADAEADEGKGKELPAEAITDVKARVSELEKECMSMKQEIRRLGKPRRSWSLLT
RKCRFGTKVQQAQPTMSSK*
>Osat_LOC_Os01g66890
MACLELDSSQFLLNGDGSVIGSPFDIQLECNSFTGSKAVQDHSRYTLPSQCTNAPDPPPLPGTSYGTHRTSRNAKACRCV
PEEIQDFWDRMFFEAYQYDLRVLTEDGNEIMSHSCVVGIKSPVLRAMLEEAKVQGGIRHILIPGVPSEAVHVFIRFLYSS
RFEQYQMKRYVLHLLVLSHVFSVPSLKRVCINQLETSLLSPENVVDILQLARLCDAPRLSLVCTRMIIGDFKAITQTEGW
RVMRQANPSLEQELLESLVEEDTKRQERARRLEENKVYLQLHEAMEALIHICRDGCRTIGPRDQTLKSSQAVCRFPACKG
IELLLRHFSACKMRVPGGCANCKRIWQLLELHSRMCSALETCHVPLCRHFKEKMQHLSRKEEAKWNLLVSKVLESKATTS
SISERRKFPSLKT*MACLELDSSQFLLNGDGSVIGSPFDIQLECNSFTGSKAVQDHSRYTLPSQCTNAPDPPPLPGTSYG
THRTSRNAKACRCVPEEIQDFWDRMFFEAYQYDLRVLTEDGNEIMSHSCVVGIKSPVLRAMLEEAKVQGGIRHILIPGVP
SEAVHVFIRFLYSSRFEQYQMKRYVLHLLVLSHVFSVPSLKRVCINQLETSLLSPENVVDILQLARLCDAPRLSLVCTRM
IIGDFKAITQTEGWRVMRQANPSLEQELLESLVEEDTVSLSAAT*
>Osat_LOC_Os01g68020
MCEGVRAAGDAAAAADVDVITSSGRRRIPAHSTVLASASPVLESILQRRLKKERDAAAGGGKVRRAVVLIRGVTDDAAAA
FVRLLYAGSSGDEEEIDEKSAAQMLVLAHAYRVPWLKRRCEGAIGSRLTAESVVDTMQLAALCDAPQLHLRCTRLLAKEF
KAVEKTEAWRFLQENDPWLELDILQRLHDADLRRRKWRRKRAEQGVYVELSEAMDCLSHICTEGCTEVGPVGRAPAAAPC
PAYATACRGLQLLIRHFSRCHRTSCPRCQRMWQLLRLHAALCDLPDGHCNTPLCMQFRRKEEEKAAAKAKAKAGDDDDKW
GLLVKKVRVARAMSSLGKRRQMSCSQC*
>Osat_LOC_Os01g70670
MAAPPVSGDGAAAAAPVVALGAAGAVGGPRPYEVAVAAAELRPVDCNLAALCDHVQAEGFGSGAFSDVVVEAMGATYRLH
RLIISRSAYFRNMLHGPWREAGAPTVVLHIDDPNIDSEAIAIALAYLYGQPPKLNDNNAFRVLAAASFLDLQDLCTICTD
FIISELWTSNFLQYQLFAESQDYGSHGERVRNACWGYLCQSATLELREVLPKLSSQTLHALLTSDELWVPNEEKRFELAL
YALLAKVTLSDVEVSGNENLNLTSSSANSDHSMRKGKSPMNEAGEEQLMGSELQNLKLHDNTETISAHNTSDIPDMVIPQ
DSTAYSIEQNAEASRRKVNDFSTGGPSGESTSYQFNEDIWLSSDQTRNYLSRTSSSNGLVPTEWGKPNAPLWGGRVVGRR
QVRCVRGSSSLSADEYNAFMNIFERGSLLYCNMSFDALLSVRKQLEEFGFPCKAVNDGLWLQMLLCHRVQAIVADTCTNC
CLTGNSCACKQAHVSSHHHYRQEHDRSSASGTVGNIYLTDAHGEGNAVFGPVRVNVRGAVDGLAGIGRGNSNVPGAAWAP
TRYVFSRVPYGLGSRNGQQPFANDESEPRVDYNGDISGDGLTALVNLSQESSASHHQTESIFETGIQVRYSGAASVSTPG
GSSLQMQESKEHELGSNLETTENTTISLDMKTPLSHFPPFRFGVEFEDVHRLADSQVKHSTEVFYAGSLWKVSVQAFNDE
DPHGRRTLGLFLHRRKAEPLDPLRKANMYVDHREKVTARYQLICPSKREVMIFGSLKQAGTLLPKAPKGWGWRTAILFDE
LGDLLQQGGSLRIAAVVQLV*MAAPPVSGDGAAAAAPVVALGAAGAVGGPRPYEVAVAAAELRPVDCNLAALCDHVQAEGF
GSGAFSDVVVEAMGATYRLHRLIISRSAYFRNMLHGPWREAGAPTVVLHIDDPNIDSEAIAIALAYLYGQPPKLNDNNAF
RVLAAASFLDLQDLCTICTDFIISELWTSNFLQYQLFAESQDYGSHGERVRNACWGYLCQSATLELREVLPKLSSQTLHA
LLTSDELWVPNEEKRFELALYALLAKVTLSDVEVSGNENLNLTSSSANSDHSMRKGKSPMNEAGEEQLMGSELQNLKLHD
NTETISAHNTSDIPDMNAEASRRKVNDFSTGGPSGESTSYQFNEDIWLSSDQTRNYLSRTSSSNGLVPTEWGKPNAPLWG
GRVVGRRQVRCVRGSSSLSADEYNAFMNIFERGSLLYCNMSFDALLSVRKQLEEFGFPCKAVNDGLWLQMLLCHRVQAIV
ADTCTNCCLTGNSCACKQAHVSSHHHYRQEHDRSSASGTVGNIYLTDAHGEGNAVFGPVRVNVRGAVDGLAGIGRGNSNV
PGAAWAPTRYVFSRVPYGLGSRNGQQPFANDESEPRVDYNGDISGDGLTALVNLSQESSASHHQTESIFETGIQVRYSGA
ASVSTPGGSSLQMQESKEHELGSNLETTENTTISLDMKTPLSHFPPFRFGVEFEDVHRLADSQVKHSTEVFYAGSLWKVS
VQAFNDEDPHGRRTLGLFLHRRKAEPLDPLRKANMYVDHREKVTARYQLICPSKREVMIFGSLKQAGTLLPKAPKGWGWR
TAILFDELGDLLQQGGSLRIAAVVQLV*
>Osat_LOC_Os01g72020
MEETLKSLSMDYLNLLINGQAFSDVTFSVEGRLVHAHRCILAARSLFFRKFFCGAAADQAAAPPGALLLDHLSPRSPSGG
ASASSPRGAGGSAAAAAAATPGAVIPVSSVSYEVFLLLLQFLYSGQVSLVPQKGEPRPGCGERGCWHTHCAAAVDLALDT
LAAARSFGVEELALLTQKQLAGMVEKASIEDVMKVLMASRKQDLHQLWTTCSHLVAKSGLPPEVLAKHLPIDVVAKIDEL
RLKSMSRRSPFLSHHHHHPHAAAAGIEASSAAELDDHHKIRRMRRALDSSDVELVKLMVMGEGLNLDDALALHYAVENCS
REVVKALLELGAADVNHPAGPAGKTPLHVAAEMVCPDMVAVLLDHHADPNVRTVDGVTPLDILRTLTSDFLFKGAVPGLA
HIEPNKLRLCLELVQSAAMVMSREDAQTAAVNAAPIYGESPGGGGGGGVYNASGTSSSMVNLSLDNRMVYLNLGMDAQFG
KMNDGGDGDDGGSRGPSSLFSPHGFP*MEETLKSLSMDYLNLLINGQAFSDVTFSVEGRLVHAHRCILAARSLFFRKFFC
GAAADQAAAPPGALLLDHLSPRSPSGGASASSPRGAGGSAAAAAAATPGAVIPVSSVSYEVFLLLLQFLYSGQVSLVPQK
GEPRPGCGERGCWHTHCAAAVDLALDTLAAARSFGVEELALLTQQLAGMVEKASIEDVMKVLMASRKQDLHQLWTTCSHL
VAKSGLPPEVLAKHLPIDVVAKIDELRLKSMSRRSPFLSHHHHHPHAAAAGIEASSAAELDDHHKIRRMRRALDSSDVEL
VKLMVMGEGLNLDDALALHYAVENCSREVVKALLELGAADVNHPAGPAGKTPLHVAAEMVCPDMVAVLLDHHADPNVRTV
DGVTPLDILRTLTSDFLFKGAVPGLAHIEPNKLRLCLELVQSAAMVMSREDAQTAAVNAAPIYGESPGGGGGGGVYNASG
TSSSMVNLSLDNRMVYLNLGMDAQFGKMNDGGDGDDGGSRGPSSLFSPHGFP*
>Osat_LOC_Os02g16000
MAGSEAAAAQEAEMDPDFSGGGGGGPSFEFAFNSVNFSDRVLRIEVVAGDDDDDDDHAPGSSRDGGAGSLSDWARHRKRR

REELLKEKESEAVMPDQINCKVEPEECDAYEENQEEPVAMMDDSPPSVGPDGDDGPSMDSPWSGGVSTPVLRVKNIYISS
AILAAKSPFFFKLFSNGMKESDERQATLRITDSEENALMELLSFMYSGKLTSTDPTLLLDILMAADKFEVISCMRYCSQL
LTSLTMTTESALLYLDLPCSISMAAAVQPLTDAAKEYLSNKYKDLTKFQDEVMNIPLAGIEAILSSNDLQVASEDAIYDF
LIRWARAQYPKSEERREILSSRLLPLVRFSHMTCRKLRKVLICTDLDHEQATKCVTEALLYKADAPHRQRALAADVTTCQ
KFAERAYKYRPLKVVEFDRPYPQCIAYLDLKREECSRLFPSGRMYSQAFHLAGQGFFLSAHCNMEQQSTFYCFGLFLGMQ
EKGSMSVTVDYEFAARTRPSGEFVSKYKGNYTFTGGKAVGYRNLFAIPWSTFMADDSLFFLDGVLHLRAELTIKQPTV*
>Osat_LOC_Os02g20590
MANHCNNTSSVTVAEVARGSHVIKIDGYLRTKELMENGKYVSSIPFSVGGHSWFITYFPNGVNTESKDYLSVFLTIDFAC
AGGVKATFSFALLDKNGRSVQLYSKLYPLHTFTEKGSDWGHSKFMKKTDLERSVHLSNDSFSIMCDLTVMKDICSKETTQ
KQFVVVPPSDLHQHLGDLLLKNMDSTDVTFNVGQDIFSAHKCILAARSSVFRAEFFGAMSAKARRTIKIEDIEAGVFRAL
LHFIYTDSLPETAQNIVMAQHLVVAADRYNVGRLKLICEEKLSKHIDSNMVATTLALAEQHSCYGLKEACFEFLASRSNL
ERMMASDDYEHLKISCPSVLMELVARFLPQQEK*
>Osat_LOC_Os02g20600
MDGTDVTFDIGQDIFSAHKCILAARSSVFKAEFFGAMSAKAHRTIKIEDMEAGVFRSLLHFIYTDALPETALDVVMTQHL
LVAADRYNVERLKLICEEKLSKHIDSNMVATTLALAEQHSCHGLKEACFKFLSSDANLERMKASEGYEHLKVSCPFVLKE
LIARFISPEMEAAREITMDLQNLKIWVETFDSLCYNFENYKKFWRSNKDTRKGKEMVFCTHGRIYSRKAGVRRHR*
>Osat_LOC_Os02g20620
MANDCNTISSAIVAEAVSGSHVMKIDGYSKTKALIKNEECLSSTPFSVAGYTWTIRYYPNGQSTECREYLSLYLFLDSFA
RDVKAIYSFKLLDKNGRPLLLNSIASPVKTFKLRGTGWGYPMFIKSKDLKASESLRDDSFSIRCDVTVMKPICSKETPAM
PKPSVEVPPGDLHQHLGDLLKNMDGADVTFDVGQERFSAHKCVLAARSSVFEAMFFGATRAKPRRSNIKIEDMEAGVFRS
FLHFVYTDLLPDTSQDVVMAQ*
>Osat_LOC_Os02g20690
MANDTNNTSSSVIATETTSASHVIKIDGYTVTKDLMENGEFVSSIPFSVGDFLWNVRYYPNGNCSKNADYLSFSVFLESH
WAEDVKAKFSFKLLDTNNKPVRSRNFISNTHNFSRRGSNWGYSRFIKKRDLEQSEHLIDDSFTIRCDLTVMKGFSSKGSH
CKPSVEVPAGRLDLHLGNLLSNKKMNGKDVTIYVGKERFRAHKCILAARSSVFRALFFGAMIAETPRTIEIEDMEAGVFR
LLLHFMYNDSLPETWSQDAMMAQHLLVAADWYNVGRLKLICEEKLAKHIDCNMVATTLALAEQHSCQGLKEACLEFLAST
NQS*
>Osat_LOC_Os02g20720
MLHPSPESHPTFRMTDNCNTFSTIVAEAVSGSHVIKIAGYSRIKVLLRNGESLTSIPFSVAGHSWTIRFYPNGDSAESQD
YLSFYLILDSANSYDVKVIFSFELLGKNGRSVSSYSFTTDLRTFSYKGSLWGYNKFIHQTVLEESSAHLRDDSFSIRCDI
KVFKEIYSQETKGVHSKFVEVPPSNLHQHLGNLLDSMDGSDVVFEVGEERFSAHRCVLAARSSVFKAELLGTMKEKADGA
IQVDDMEPGVFKSLLHFIYTDSLDTMAQEDQSRDEASEEEDLVMAQHLLVAADRYNVERLKLICEEKLCESIDSSMVATS
LALAEQHNCNGLKEACFEFLASPSNLLEMMASDGYDHLKTSCPAVLKELTTRFLPPETKASEEITIGLYN*
>Osat_LOC_Os02g38320
MALSTAFSALPTSPADVRVVTADGSGIRAHSSVLASASPVLERMIEQAPRGGVVPIAGASTGAVVVFLRFLYAASVRGAA
AAAAAAEWEEAALAEHGAALMALAHAYRVAGPLKRRAEEAVAARVAAEGAVDAMKLAALCDAPRLYLWCARLAGRELAAV
RESEGWRFAARHDAALRADLLQLIRDADQRKERWGRERGSQGVYLQLSDAMAALERVFARAAHGSPPPLPPPRTGQCCRM
ASPCAHRRGLLQLARHFFAGCGRRVAGGCTPCRRFFLLLRLHSSVCDKSDDDSCGVPLCSNFKTNMEKGKVDKTWKLLVK
KVMRARVMSAWAKRPVPAPEIVQKSWAKYNSSSRSRAARFR*
>Osat_LOC_Os02g52316
MVAAAEFPSYVSVPPSDLHRHLGKLLTSGDGTDVTLEAGGETYKAHRSVLAARSSVLKAELLGPMAQPRSTAAATPTRIN
DIEAPVFRAMLHFIYTDHLSSTMATDGFEHLTTSCPAILKELMSKLVVH*
>Osat_LOC_Os03g10800
MKFMKLGSNPDTFQDDGNEVSIVATELVSDVTVRIGTTKFYLHKFPLLSKCARFQKMIPTTGDENIEIHIHDIPGGAKAF
EICAKFCYGMIVTLNAYNVIAARCAAEYLEMHETVDKGNLIYKIEVFLSSSIFRSWKDSIIVLGTTKAHLPWSEDLKLVS
HCIDSIASKASTDTSKVEWSYSYNRKKLPTENGLDLEWNGVKKQQFVPHDWWVEDLADLDIDSYKQVITAIKTKGMVPKD
VIGEAIKAYTYKKLPSLSKVSMVHGDAKVRAMLVTITCLLPSEKGSVSCSFLLKLLKATNLLKCGELCRKELMKRIGRQL
DEASVSDLLIPTVDGETTVYDIDMILSIVEEFVRQDSKNAQKHNGGEVDSHVQAPSASMIKVAKVVDGYLAEVAKDPNTP
ILKFIHLAETMSINSRPVHDGLYRAIDMYLKEHPSLGKNEKKKLCSLMDCKKLSPDACAHAVQNERLPLRTVVQVLYHEQ
TRASAAVTIRADSICVGSYESSRSGATTNTEDEWDGVMVVEDLSLSTKTTTKLDGAANSHCSNGKAKKGGASTPKKAAHR
RTTTVPTGKGQSGERSSSDSSDSAILQKLELPKRTPSRSTKPAAV*MKFMKLGSNPDTFQDDGNEVSIVATELVSDVTVR
IGTTKFYLHKFPLLSKCARFQKMIPTTGDENIEIHIHDIPGGAKAFEICAKFCYGMIVTLNAYNVIAARCAAEYLEMHET
VDKGNLIYKIEVFLSSSIFRSWKDSIIVLGTTKAHLPWSEDLKLVSHCIDSIASKASTDTSKVEWSYSYNRKKLPTENGL
DLEWNGVKKQQFVPHDWWVEDLADLDIDSYKQVITAIKTKGMVPKDVIGEAIKAYTYKKLPSLSKVSMVHGDAKVRAMLV
TITCLLPSEKGSVSCSFLLKLLKATNLLKCGELCRKELMKRIGRQLDEASVSDLLIPTVDGETTVYDIDMILSIVEEFVR
QDSKNAQKHNGGEVDSHVQAPSASMIKVAKVVDGYLAEVAKDPNTPILKFIHLAETMSINSRPVHDGLYRAIDMYLKVLL
QISTSNQKCTTHRTCSQAH*
>Osat_LOC_Os03g10880
MQAEARFVSPAMKRTSDWILSQELPSDITIKVDDAAFNLHKLPLASRCGYIKKQVSGVGGNKATHLEIAGMPGGSKSFEL
VVKFCYGVNFEITVDNVAMLRCAGEHLEMTEECRPGNLVGRTEAYLEEVALASLEGAVAVLRKAEELLPASEEKARLVAR
CIDAVASIVCGGDGQFSMSLGTPGGGGGGGYNGVGAAASREVDDWCADELTALRIDTFQRVMIAMKARGFKGIAMGTLIM
LYAQKSLRRLDMHGRDRKKMGARQEHEKRVVLETIVSLLPRERNTLSVSFLSMLLRAAIHLDTTLACRLDLEKRMAAQLG

QAVLDDLLIPSSSPDAAAATTAYDIDAVQRILAGYLDLDYTTDPPPRLDYTTDDDFSSAASPPHSDVAQVGRLMDSYLAE
IASDENLSVDKFTALAELIPERARFNEDGMYRAIDIYLKAHPSLAEGERKKVCRAMDCQKLSREACAHAAQNDRLPVQTV
VQVLYHEQRRLRAPSQPPSAAPSYAGGESPALSYRPTPSFNGRHRPGVPDEVSRLQRENDELRMELLRMKMRLRDPSVAF
SAGGVPPSGRPPLPKKPGGGGGGSSGGFMNSMSKKLGRLNPFLRSDVLGGGRVRTKPPKDRRHSIS*
>Osat_LOC_Os03g43990
MASSVKLGSKPDAFRRQGQAWFCTTGLPSDVTVEVGDMSFHLHKFPLLSKSAFLERLIEETSDQDECVIILNDIPGGAKS
FELVARFCYGVKIELSSENVVYLRCASEHLQMTEEIAEDNLISQSEIFLNQVIIRNWKDSLKALETCEDLLPHAENLQIV
KRCIESLASKATTDPNLFGWPIREHGIMQSPGGSVLWNGISTGARPRNFSSNWWYEDASALSFHMYKRLISTMESRGIRP
EIIAGSLTYYAKKYLPGLNRRHSMGAVPLTATLSEVEQKNLLEEIDRLLPVQKGLASTRVLLGLLRTAMILKASPTCISN
LEKRIGMQLDHATLEDLLLPNFSYTMETLYNVECVQRILDHFLAMDQANGAASPCLDDVMASPSLAPITTVAKLIDGYLA
EIAPDINLKLPKFQALASAVPEYARPLDDGLYRAIDIYLKAHSWLSEAEREQLCRLMDCQKLSLEACTHAAQNERLPLRV
VVQVLFFEQLQLRTSIAGCLLVSDNLEGSRPLRSSITTSGEAGGWATAVRENQVLKVGMDNMRMRLSELEKECSTMRQEI
QKLGRGKSGGWASRVPKKFNLKLKSQMCSAQEGSVSEQHKSMSAKLDKLQAKVSRQKKQLAGDA*MMTLLPSCKSPRFCT
TGLPSDVTVEVGDMSFHLHKFPLLSKSAFLERLIEETSDQDECVIILNDIPGGAKSFELVARFCYGVKIELSSENVVYLR
CASEHLQMTEEIAEDNLISQSEIFLNQVIIRNWKDSLKALETCEDLLPHAENLQIVKRCIESLASKATTDPNLFGWPIRE
HGIMQSPGGSVLWNGISTGARPRNFSSNWWYEDASALSFHMYKRLISTMESRGIRPEIIAGSLTYYAKKYLPGLNRRHSM
GAVPLTATLSEVEQKNLLEEIDRLLPVQKGLASTRVLLGLLRTAMILKASPTCISNLEKRIGMQLDHATLEDLLLPNFSY
TMETLYNVECVQRILDHFLAMDQANGAASPCLDDVMASPSLAPITTVAKLIDGYLAEIAPDINLKLPKFQALASAVPEYA
RPLDDGLYRAIDIYLKAHSWLSEAEREQLCRLMDCQKLSLEACTHAAQNERLPLRVVVQVLFFEQLQLRTSIAGCLLVSD
NLEGSRPLRSSITTSGEAGGWATAVRENQVLKVGMDNMRMRLSELEKECSTMRQEIQKLGRGKSGGWASRVPKKFNLKLK
SQMCSAQEGSVSEQHKSMSAKLDKLQAKVSRQKKQLAGDA*
>Osat_LOC_Os03g46440
METSTISFSSSSPPSPPPPQPAPGDIDAVSLGRLSRNLENLLDPAFLNCADAEIVLASGGGDPGGGAVVGVHRCILAARS
RFFYDHFSSAPAPAPATAGDKPQLDLDGLVPGGRHIGRDALVAVLSYLYTGRLRSAPPEAAACLDDGCSHDACRPAIDFV
VESTYAASGFQISELVSLFQRRLSDFVNKALAEDILPILVVASTCHLPELLNQCIQRVANSNLDNRYLEKRLPDDLYAKL
KEFRVPDEPHSGILDPEHEKRVRNIHKALDSDDVDLVGMLLKESPVTLDDAFAIHYAAAYCEPKVLAELLKLESANVNLK
NSSGYTPLHMACMRREPDIIVSLIEKGASVLERTQDGRDALTICKRLTREKDRNEKSEKCKERSKAYLCIGVLQQEIKRR
PQILEDQMSAEESIATPLLVDNFHMRLLNLENRVAFARIFFPSEAKLVMRIAQADSTQEFAGLTSANFSKLKEVDLNETP
TMQNRRLRERLDALTKTVELGRRYFPHCSEVLDKFLNEESTDLILLESGTAEDQQTKRMRFSELREDVRKAFTKDKAAGA
AISSSTSASSSPRYETKLRPGNKKGKLSR*METSTISFSSSSPPSPPPPQPAPGDIDAVSLGRLSRNLENLLDPAFLNCA
DAEIVLASGGGDPGGGAVVGVHRCILAARSRFFYDHFSSAPAPAPATAGDKPQLDLDGLVPGGRHIGRDALVAVLSYLYT
GRLRSAPPEAAACLDDGCSHDACRPAIDFVVESTYAASGFQISELVSLFQRRLSDFVNKALAEDILPILVVASTCHLPEL
LNQCIQRVANSNLDNRYLEKRLPDDLYAKLKEFRVPDEPHSGILDPEHEKRVRNIHKALDSDDVDLVGMLLKESPVTLDD
AFAIHYAAAYCEPKVLAELLKLESANVNLKNSSGYTPLHMACMRREPDIIVSLIEKGASVLERTQDGRDALTICKRLTRE
KDRNEKSEKCKERSKAYLCIGVLQQEIKRRPQILEDQMSAEESIATPLLVDNFHMRLLNLENRVAFARIFFPSEAKLVMR
IAQADSTQEFAGLTSANFSKLKEVDLNETPTMQNRRLRERLDALTKTVELGRRYFPHCSEVLDKFLNEESTDLILLESGT
AEDQQTKRMRFSELREDVRKAFTKDKAAGAAISSSTSASSSPRRERRGRSRRA*METSTISFSSSSPPSPPPPQPAPGDI
DAVSLGRLSRNLENLLDPAFLNCADAEIVLASGGGDPGGGAVVGVHRCILAARSRFFYDHFSSAPAPAPATAGDKPQLDL
DGLVPGGRHIGRDALVAVLSYLYTGRLRSAPPEAAACLDDGCSHDACRPAIDFVVESTYAASGFQISELVSLFQRRLSDF
VNKALAEDILPILVVASTCHLPELLNQCIQRVANSNLDNRYLEKRLPDDLYAKLKEFRVPDEPHSGILDPEHEKRVRNIH
KALDSDDVDLVGMLLKESPVTLDDAFAIHYAAAYCEPKVLAELLKLESANVNLKNSSGYTPLHMACMRREPDIIVSLIEK
GASVLERTQDGRDALTICKRLTREKDRNEKSEKCKERSKAYLCIGVLQQEIKRRPQILEDQMSAEESIATPLLVDNFHMR
LLNLENRVAFARIFFPSEAKLVMRIAQADSTQEFAGLTSANFSKLKEVDLNETPTMQNRRLRERLDALTKTVELGRRYFP
HCSEVLDKFLNEESTDLILLESGTAEDQQTKRMRFSELREDVRKAFTKDKAAGAAISSSTSASSSPS*
>Osat_LOC_Os03g48120
MNISLRTLFPPIFTFLKYILYAGDDDGTIPIPRSNLNGQLDDIADRADGSDVLFSVGSETFHAHRAVLAARSPVFKMELL
GSMAESTMPCVTLHNIDPATFKALLHFVYMDALPSPTEAVRIGEVEADVCTEAVGECVGGDGGDDSRLRAYAERYHCPEL
KSKWLSFLMAEINFKKVAVTDGYFHLRRDFPLIIEEIKKRIES*
>Osat_LOC_Os03g55830
MQGRKMKSAREMAREEKNHHHHHHQQQQQQQSSSKGQAWFCTTGLPSDVVIEVGDMTFHLHKFPLMSRSKKLHDLITNRE
SRAAGGGEQDEEEEEDAGEIREEEVVLEVDEDSDVHRIRLPDFPGGAEAFEQAAKFCYGVKLDLTPATAAPLRCAAERLG
MSDDHSDDNLISRADRFMSHTVLRNPRDAIRALRSCEGLLPLADDLGLVSRCVDAIAAKAAASTPTALFGWPINDAAAAA
AAAAGDRPRRKNNAGAGATWFDDLAGLSLATFTRVIAAMKERNVGPEIIEGALIAYAKRSIPGLSRSGRHVGGATAAAAA
APSSDGEQKALLETVIANLPEETIKSNAHTGTAVGATTARVLFGLLRTTSILQASESARDMLERRIAARLPDAALDDLLI
PSYSYLVETLYDVECVERIVRYFLEGRDADDGNDDGSELETPGREASRRAMLAVGRLMDAYLGEIAADANLKPDKFCDLA
WALPDGARVYDDGLYRAVDIYLKAHPGLREEEKEKVSGVVDGRKLTLEACTHAAQNERLPLRTVVQVLFFEQLQLRRAIA
RTIMANEGGAAGQGEEEGDSDAGGTWRVATRGNQMLRLDMDSMRNRVQELERECTSMRKAIEKMDRRGGGSTPGERGATP
AMEGRWGSMVTKRFGCKFPAQVCQSQQRTVVARPRRPRIEQSP*
>Osat_LOC_Os03g57854
MEDDDAGGGGGGGEASPPHAGSAAAMAGAGRDIAASPTSSRSVTQTVNGSHRFVIQGYSLAKGMGVGKHIASETFTVGGY
QWAIYFYPDGKNPEDNSAYVSVFIALASEGTDVRALFELTLLDQSGKAKHKVHSHFDRSLESGPYTLKYRGSMWGYKRFF
RRTALETSDFLKDDCLKINCTVGVVVSTMDYSKPHSIHVPESDIGYHFGTLLDNQEGVDVICNVAGEKFHAHQLVLAARS

SFFRSELFEHESDEEKNEVDTSNEIKEIVIDDMEPKVFKAVLHFMYRDNLVGDDELSASSSDCSIFDTLAGKLLAAADRY
ELPRLRLLCESYLCKHISVNSVATTLALADRHHAMELKSVCLKFAAENLSAVIRTDGFDYLKDNCPALQSEILRTVAGCE
EECSSGGKSQSVWGQLSDGGDTSGRRVRPRV*MEDDDAGGGGGGGEASPPHAGSAAAMAGAGRDIAASPTSSRSVTQTVN
GSHRFVIQGYSLAKGMGVGKHIASETFTVGGYQWAIYFYPDGKNPEDNSAYVSVFIALASEGTDVRALFELTLLDQSGKA
KHKVHSHFDRSLESGPYTLKYRGSMWGYKRFFRRTALETSDFLKDDCLKINCTVGVVVSTMDYSKPHSIHVPESDIGYHF
GTLLDNQEGVDVICNVAGEKFHAHQLVLAARSSFFRSELFEHESDEEKNEVDTSNEIKEIVIDDMEPKVFKAVLHFMYRD
NLVGDDELSASSSDCSIFDTLAGKLLAAADRYELPRLRLLCESYLCKHISVNSVATTLALADRHHAMELKSVCLKFAAEN
LSDALMYLQL*
>Osat_LOC_Os04g35310
MPTATGSRTPVRSASAVIAGTESGQHHLKIDGYSRIKDELPTGSDIKSRSFRAGGHSWHLRYYPNGFNSDCAECISIFLQ
LDYNVMKGVKAQYKFSLLDRARKPSYSRSSGKADVFLNTGWGYRTYIERGLLESSEYLRDDCLTIVCDFTVFKDLRTEDI
DVDDAMPPPQSPPTVVVPPSDLHRHLGGLLATGEGADVTFEVEGKTFAAHRWVLAARSPVFRVALFGATTGGADDVVRVN
IDAMKVQDFEALLHYMYTDSLPEMKGGEAAAMLPDLVAAANRYKMERLRLVCEHKLCEYVNGRTVVAMLAFAGEHQCNGL
KEKCLRFLDDPVKLRLIVQAEGVENLSKSYPSILKDVIAKFVATPVDS*
>Osat_LOC_Os04g35370
MPMATAPTVAGGKLLRSASAIVGGTESGQHLLEINGYSSIKDAVSIGNCVQSRHFRVGGHDWYIRYYPNGFNSNVSDCIS
IYLVLDGHEAHDYYYGRSIVRAELTLSLLDQEREPVTSYIYSHGLQIFDGYGRYRGSLRFIQKAVLERSEYLRDNRFTIR
CDITVMKNPEAKDTGGRRVTLPPSDLARHLGGLLATGVGADVTFEVDGKTFLAHRNVLAARSPVFHQELFSLTEKGNAAT
GGAGVIIRVDDMEAQDFEALLHFIYTDSLPEMKGGDAVAMLPDLVAAANRYKMERLRLVCEDKLCEYVTVRTVAAMLAFA
GEHQCPELEKKCLQLLEDPANLRNIVETEGLEHLTKSYPFVLKDLIAMFATKP*
>Osat_LOC_Os04g40630
MTQWADLDALRPAVAADVQVVTSDGKSIAAHSFVLGTASPVLERMIERARRGWNAECTIRVLGVSSDAVFAFLQLLYASR
VTPEDEEVVTAHGPQLLALSHAYRIGWLKRAAEASVTARLTPEHAVDMLKLARLCDAPRLYLRCARLAAKDFAAVERSEG
WRFARRHDAALELEILQLLEDADQRRERWARERASREAYRQLGEAMDSLEHIFSDDGCSCADADADADTDAPPCRGLRLL
MRHYATCGARKAAPGGGCTRCKRMVQLFRLHASVCDRAAPHDDGDRPCRVPLCSHFKGKMRAEKADKTWRLLVKKVTRAR
AMSRLAAGREREVVPEVVAASWARYSSSGGAARLR*MDFWFFSVQGTASPVLERMIERARRGWNAECTIRVLGVSSDAVF
AFLQLLYASRVTPEDEEVVTAHGPQLLALSHAYRIGWLKRAAEASVTARLTPEHAVDMLKLARLCDAPRLYLRCARLAAK
DFAAVERSEGWRFARRHDAALELEILQLLEDADQRRERWARERASREAYRQLGEAMDSLEHIFSDDGCSCADADADADTD
APPCRGLRLLMRHYATCGARKAAPGGGCTRCKRMVQLFRLHASVCDRAAPHDDGDRPCRVPLCSHFKGKMRAEKADKTWR
LLVKKVTRARAMSRLAAGREREVVPEVVAASWARYSSSGGAARLR*
>Osat_LOC_Os04g53390
MASSSTSSRCLTASVTGTHNLEVTSYSLLEGMGVGKFVSSTTFSVAGYDWNLRFYPDGITDNDRKEGYGAVWMLASVYQI
SIAKEHAIRRIRFTGNDSFKIECSLTVISESRAEDVSTIPVPPSNLHQHLAGMLHGVEIADVEFSVGGEPFRAHACVLAA
RSPVFRAELLGPAAARSIKIDDDDDMEPATFKALLHFIYTDHLPNDSGFGKDAAMQRRLLVAADRYGVDRLRAMCGVKLY
KSVSVGTVVDSLEFAEKHHCAQLKDACLGFMASPNVLGVVRKTDGFKRLVEGCPWVLKEILDKVPRF*
>Osat_LOC_Os04g53400
MGNSLFSMASSTASPSDGRSPRLPETLSRCVTASVAAAHNFEVTRYSLLAGVGAGEFVTSGTFSIDGHNWNIQVYPDRWK
QEMNAGYVSVFLCLCGGATGVRAKYTLSLSENGGESVQRSLTHRFDTVGAFWGFPRFMERPRLRQWLLRRGPGGGDDCVT
FRCSLTVIREPRTEGVAAVAVPPSDMRRHMANMLRGGDGADVVVLVRDQPFRAHRCVLAARSPVFRAELFGGGHMRERRT
SCVVVDDMEPSIFSAFLHFIYTDSLPENPDTPGDDQDCMAMQHLMVAADRYGLDRLVLICEEKLCRGIDVQTVATTLALA
EQHQRVALKDACLGFIVSRGVLGAVARTDGFKHLLTTCPSIMVDILDKVASVMSK*
>Osat_LOC_Os04g53410
MGDHRDPAFPAAAGGCRLPKTSSVSVTESVTAVHDFKVTGYSLIEGLGIGRYVSSSTFTVGGVDWAVRFYPDGSTVTCLG
NASAFLYYCGREKEVRTRFTLNLLGKDGKLSQVTNSYMKHTFSPASDNWGFIKFAEKSKLQSSPFLHNDCLTIRCLLTVV
RESHTKDVEVNSVVVPPSNLHTDFENMLQDGEGSDVTFTVGGQEFRAHKCVLAFRSPVFKAELFGPMKENGTQCIKIDDM
EPEVFEALLHFIYTDRLPDSCRDGKAAAMQHLLVAADRYGVDRLRLICERRLSETIDVETVATTLVLAEQHHCSQLRQAC
IGFVASPNMLGPVIESDGFKHLVESCPLIMKEILSKVSHIWIDKSC*
>Osat_LOC_Os04g53430
MLTSSAARTSSRSVWEGITGTHDFEVVGYSLMDGFGAGRHVCSGDFSVAGHDWYVAFYPDGLDQDSAGYASACLAYRGKE
RLVRAKYSLSLVARDGRASPLAGDTLRSHYFTPTSRSADVLKFVEKSNLSSSPSSSSYSCLDDDTLTIRCVVTVVTGPRV
VSVAPAKERGPRVTVPPPSLHEHLARMLRDGRGSDVAFRVGGRVLRAHRCVLAARSPVFDAELLGPMMETTAPCIEIHGV
EPAAFEALLRFVYTDSWPLAGVDVAATVRLLSAADRYGLERLRLMCEEKLHEGIDVDNAADVLAMAELHHCSQLRDACVA
FIASPSTLGPVLASSGFEDLIMATGASVTKEILHKVSESWSGPGNRNNSSKRK*
>Osat_LOC_Os04g54400
MAITKVLGSKPADCFQFQDPNSWTCMTELVSDVVVEVGDFSFHLHKFPLMSRSGTLQKLISEAAAGADDGEPCSVKLHDV
PGGAAAFELAARFCYDVRAELDAGNVVALRCAAEHLGMTEDHGGEGNLVEQAEAFLRDVLGSWDDALRALRSCDGALLPL
AEELLVVPRCIDALASKACADPTLFGWPMVEYYTARGLEETVMWNGIATAGKPRSPGPDWWYKQASSLKLPVYKRLITAM
RSKGMSPENIAGSLTHYAKRHLSGLTRHSGYVGGGGASGTVLSDVEQRALLEEIVALLPVERGVATTRFLLGLLRTATIL
NAGAACRDALERMAGNQLEEAALEDLLIPNTGYAVETLYDVDCVQRMLEQFVAANTSAFAASPEITDEAQLVDGPSGELM
PISTVAKLVDGYLAEVATDTNVKLSKFQSIAELVPDYARAIDDGIYRAIDIYLKAHSWLTASEREQLCRLMNCQKLSLEA
CTHAAQNERLPLRVVVQVLFFEQLRLRTTVAGWFFVADNVDQGSPIAAGRYAPERSGELDFGAGPPEEEDGDDDDDEARN
NVRSSSSATMSVDDIRQRVVELEEECSSMREEIHRIGKPKGALSRLFRKLGLGGRSAARRQQQPPPPPTSSGDERRKSM

SLEC*
>Osat_LOC_Os04g56460
MGCCFSVIDDDGYEHDDAVSGSVNEVPRVTGVHQFTIRQYSGVKGKGVGNSVLSRNFTVAGREWFVRFYPDGYNSDTSDH
VAFFLQSLYRPSCGSVYHVEFSFALLGGGGGGGAVLHDVRIDRPCRFDNRNNSWGIRKYIAREQLEGAALGAIHDDALTV
RCTMHVIQRRRGAGRPVVTGAGGPVRVPVPPSCHARNAMEFLLSGDAPFDLEIHVGDATFGAHRLALAGQSLYFRKLLYG
QVGNEASSPAIRLREMSPEAFGAVLHYVYHDSLPPEANKGRNAAAMAREVFEAADMYAMERLKLMCASNLCRFIGDDTAS
GIMELAKAHSCDPLKKACENYMKRRRIPFNPDS*
>Osat_LOC_Os05g27880
MKVEEKKRSITVAPFECAWDEEFRFRETGRGCITFEASAHNDVTLVFREQPGSQHYHYKMDNSRHYIVILGSHRNKRLKI
EVDGKTVVDVAGIGLCCSSSFQSYWISIYDGLISIGQGRHPNNNILFQWLDPDPNRNVQYVGLSSWDKHVGYRNISLMPS
APQNSILWSQIECAYVEPDGAGGHTRKQESKDGLDQRALANFLENWDFSDSIFVVGSERKVVPAHKVVLGSCGDFPFNLM
MSRPAIELPSVSYPVLHSLLEYIYTGSTQISEWQLVSLLELSSQFKVKPLVMYCEEIIGCLKMSDAVSESSKKIQLSSGG
SQAHQFYYFPFKAPLNTQKIEQFLVNGEHSDVNIYVNGHGLVTHAHKLILSLWSMTFDKMFTNGMKESSASNVFFEDVPV
EAFFLLIQFMYSGELKVDIEEITPVLVELLLLSDQFGITALQFECCKRIMEFLSKDTVCSVLRAVSSIPSCKLLEEMCKR
NFAEHFDYCTTACTDFVLLDEATFKDILQHGHMTVTSEERVLDAILTWCMEACDCFNWTSVHELLSTSRPEKLFGGRLTA
INTLLPFVRFPLVQPSVLHLMEKSNLAKNIEAFRQLVAEAIEFSNAGLRMATNTCERFHHRRSSYKELQYISDGDNNGVI
YYAGTSFGKHQWINPVLAKNITVTASSPNSRYTDPKALVSKNYQATCFAGPRLEDGKMCSWWMVDIGPDHQLMCNYYTVR
QDGSATFMRSWVLQGSMDGRSWTSLHVHEDDQTICQPGQFASWPITGQTALLPFRFFRVMLTAPATGVSNTWNLCICFLE
LYGYFR*MKVEEKKRSITVAPFECAWDEEFRFRETGRGCITFEASAHNDVTLVFREQPGSQHYHYKMDNSRHYIVILGSH
RNKRLKIEVDGKTVVDVAGIGLCCSSSFQSYWISIYDGLISIGQGRHPNNNILFQWLDPDPNRNVQYVGLSSWDKHVGYR
NISLMPSAPQNSILWSQIECAYVEPDGAGGHTRKQESKDGLDQRALANFLENWDFSDSIFVVGSERKVVPAHKVVLGSCG
DFPFNLMMSRPAIELPSVSYPVLHSLLEYIYTGSTQISEWQLVSLLELSSQFKVKPLVMYCEEIIGCLKMSDAVSESSKK
IQLSSGGSQAHQFYYFPFKAPLNTQKIEQFLVNGEHSDVNIYVNGHGLVTHAHKLILSLWSMTFDKMFTNGMKESSASNV
FFEDVPVEAFFLLIQFMYSGELKVDIEEITPVLVELLLLSDQFGITALQFECCKRIMEFLSKDTVCSVLRAVSSIPSCKL
LEEMCKRNFAEHFDYCTTACTDFVLLDEATFKDILQHGHMTVTSEERVLDAILTWCMEACDCFNWTSVHELLSTSRPEKL
FGGRLTAINTLLPFVRFPLVQPSVLHLMEKSNLAKNIEAFRQLVAEAIEFSNAGLRMATNTCERFHHRRSSYKELQYISD
GDNNGVIYYAGTSFGKHQWINPVLAKVHGSKGFGFKKLPGDLFCWASS*
>Osat_LOC_Os05g33050
MEAEQQQQPPHRPRRKGQKRKLEDEAAASASAAAAAAAAAAAAAATATPSSLGSAGADDDNEEEEDGSAGPEICCRHSQAA
LAREVRTQVDALHRCFSWRHADRAAAKRATHVLAELAKNEEVVNVIVEGGAVPALVCHLKEPPAVAVLQEEQQPRPFEHE
VEKGAAFALGLLAVKPEHQQLIVDAGALPLLVNLLKRHKNATNLRAVNSVIRRAADAITNLAHENSNIKTCVRIEGGIPP
LVELLESQDLKVQRAAAGALRTLAFKNDENKSQIVDCNALPTLILMLRSEDAAIHYEAVGVIGNLVHSSPNIKKEVLNAG
ALQPVIGLLSSCCTESQREAALLLGQFASADSDCKVHIVQRGAVRPLIEMLQSADVQLREMSAFALGRLAQDTHNQAGIA
YNGGLVPLLKLLDSKNGSLQHNAAFALYGVADNEDYVSDFIKVGGVQKLQDGEFIVQATKDCVAKTLKRLEEKINGRVLK
HLLYMMRVGEKSVQRRVALALAHLCAPEDQRTIFIDNNGLELLLDLLVSVSLKHQLDGSVALYKLANKAAALSPMDAAPP
SPTPQVYLGEQYVNSSTLSDVTFLVEGKRFYAHRIALLASSDAFRAMFDGGYREKDARDIEIPNIRWNVFELMMRFIYTG
SVEVTSDISQDLLRAADQYLLEGLKRLCEYTIAQDVNVDNVSDMYDLSEAFHAMSLRHTCVLFILEQFEKICVKSGSSQL
IQRVIPELRNFFAKALRPSHRNAQP*
>Osat_LOC_Os05g44530
MSGISDPVPPPCPSTCLKNYADRSGGVAASSSRGKSWRSRGKDADVAFEVGGGGGVRFAAHRCVLAARSKVFKAELFGAM
KEGDAACVVRIDDMEPQVFRTLLFFVYTDSLPEMRKEEEEAICQRLLVAAEVYGMERLKLICESKLCKYIDVGTVASIMA
LVDQYHCHGPWPHKGLLRFCQLSRQSNGGVSH*
>Osat_LOC_Os05g44540
MASRSASIVQANVTSGYNLLTIDGYMATTPIPTGVYMTSSAFAIGGHQWRISYYPNGKNSGCADYISFDLILDENVAAPL
HERERGEGPSRFVKRRILERSRRRHLKNDSFIVRCDVVVTEFRPAEVAPGSVDVDGPPSDLHRHLGDLLRGETGADVVFE
VGGERFAAHRCVLDARSSVFDLELFGATTAKEVVVGQSTGIVRVDGMEARVFKALLFFAYTDSLPEMMTTTKKKMEEQAA
GDGDDRYDIDAFTVGKVIALAEQHDCRVLRKLASISSCVVLDFRRMYCWAPATAAPSAAPFLLDKSCLKASSKSGS*
>Osat_LOC_Os06g03840
MEPSDLVDGYKFGDQTTSDVRVCFKRADDQAEWFCCHSSVLSGNSKYFADWLSRNDIGSNNCIGVDCISADYEHYVKVLK
LIYLPAESIIDSFESVRSAVGVLRASTLLKCELITRSCIEYLEAASWDEKEEEEILEVAQSLGSEEAVALLARLQAPNVS
AVKNVFISAIRFATSMESPSPPFLDDLKTSAQEQIDFMLHEDDDTALVTMDEDVRSVVREGLKKLFSTLKIGLDLLTSEY
EQLPEQAEQRVLCSLADIDWMANVLTKIEMMNEFVSGWSEISGYVLSVVQDKKYSSGLWLVKAKLIEVTGKAFDAVGYGS
VVFPASSRVHFLRMWLPFMQTTKRLLDEKSKDDAIPQMDADLFQNIEGAIVSLVLALPSGDQADILGEWMKNAEQFRYPD
LTEAFEVWCYRSKTAKRRLVGGLNGSGNPTVSL*
>Osat_LOC_Os06g08550
MKFMKLGSKPDAFQTDGGGDSRYVLSELPSDIVIHVEEARFYLHKFPLLSKSSLLQRLIIEASQNGTDEVYIHDIPGGVK
IFEICAKFCYGMVVTLNAYNVVAARCAAELLGMTEDVDKSNLVFKIEVFLNSGIFRSWKDSIIALQTTDALLPWSEQLKL
AARCIDSIASKATSNPCNVVWSYTYNRKSASSDEIVEARKNSQPVPKDWWVEDLCELDVDLYKRVMVAVKSRGRITSDVV
GEALKAYASRWLPECFDTAAIDDDAYSMAYNHLLETIVWLLPSDKGSSCCSCRFFLKLLKVAVLIGSGEMLKEELMDRVI
LQLHKASVCDLLIPARPPALTTYDIQLVLTLVGRFMRRAGVTEDGIFLNNLDQEMFETDVDDESLLALSKIVDGYLAEVA
SDPNLSVSSFVAVATSMPDAARATHDGLYTAIDVFLKLHPNLPKAEKRKISSLMDVKKLSKEACIHAAQNDRLPLRVVVQ
VLFFEQLRAAAGGNNPAAAAAAASGGIARLLVEEEDDDDDVGGGGGGDWSKSRALPTPTPSLLKKQLGSLKLAAAGDEG

GGGDDGRQLARVSSVANQSSRLSLSSRSRRMFDRLWAGGKPPGGEVVSKSSDTSGSSQSPRSSAKPPASKSSSSSSRNRR
YSVS*
>Osat_LOC_Os06g14060
METPTNLTNTISAVHLLKINGYSVTRALGCSEYISSRRLAAGGYDWEVLYYPRYYEHGVYWIALRLMFMSKECKHEVKAA
LKCQLVHEAQIYLPSGSKSVSSKYTGQRDCGPALLLVKQDDLPGSNYFIGDSFVVECTITVLREPQEAVTNVSPNVSNPC
CDLQMHLGELLLSEKGADVTFVVAGESFLAHKIILAARSPVFMAEFFGPMKESSSQCVEIKDIEASVFKAMLHFIYTGTS
PELDQQHVVSDSEQDITTMTQHLLVAADRYGLDRLKLICQDRLHDDINVETVATTLAFAEQHSCTQLKDRCIEFIISSRA
NLDAVMATEGYKLVIASCPSVLSTLLRAAVGR*
>Osat_LOC_Os06g21330
MDRPRQQHEPEHLGGGGIDVEVELDPEDLQPSVPLKKVPAGDLFEAARAGDCARLALLLGGGANVNERDRWDSVALYYAC
LAGHADAARMLLEAGAVCAERTFDGDRCHYAALNLRLRRLLKAFEARPPPLPPLPAALRATFLACPANRAAFLEMLQWTA
GSEAAALAAAAGFGPTDNPSSTSLFPPDITFYVDGKPIEAHRVILCARSSFFRRKFNTDWKDRKEVRFSSQKLSFGALYS
LVHFFYSDRLEVDVDDMENLARACKVCKCEGLQKILIKEATLQRYAEHKSPRDLDSSQKRFILHGQSLPEEDRLPSALRH
IFEECLANSREQECYNDESNEMSRDSGVDAAADLYIKVCDKVFHCHQVILASRSEYFKARLSRNMDFLEVKSGLQSTQSL
PFLEEHDMSTEAFEKVLEYMYTDNLEHMDPNQAEELFDIASRYLLFPLKRVVADILLPYLEHVSPAELCHWLMLSDIYDV
VKIREYCLDIIACNFEMFADTREFRALLLTLPPPSGDDSLRTTRPSEPGTAGNTDQGNLLDDLREKWLEAEAAELDERDE
SAKLFDNRLEMLMLVAEQEANDGNV*
>Osat_LOC_Os06g31100
MDPDFSRASRGPSFAFAFNSVNFSDRVLRIEIVAGDDAAGAKGAAGEGCSSLADWAHQRKRRREELRREKESGKYTDLET
CKVEAEECDTYEENNEEPVAMIEESPPDIGQDGEDGDSCDSSWSMECTQVLRVKSIYISSAILAAESPFFYKLFSNGMKE
SDQRHATLRITASEENALMELLSFMYSGKLTTNQPTLLLDILMIADKFEVVSCMRHCSQLLRSLPMTTESALLYLDLPSS
ISMAAAVQPLTDTAKAFLANKYKDLTKLQDEAMNIPLAGIEAILWSNDLQVASEDAIYDFVIKWARSQYPKLEERREILG
TRLLPLVRFCHMTCRKLRKVLACNDLDHEQATKCVTEALLYKADAPHRQRTLAADVLTCRKYAERAYKYRPLKVVEFDRP
YPQCIAYLDLKREECSRLFPSGRIYSQAFHLAGQGFFLSAHCNMDQQSAFHCFGLFLGMQEKGSTSVTVDYEFAARTRPS
GEFVSKYKGYYTFTGGKAVGYRNLFAIPWSSFMADDSLFFIEGVLHLRAELTIKQP*
>Osat_LOC_Os06g45720
MENAAINLTQAARRVQNLVTSCTIAVGGYDWLIEVFPAAYYHGTSSRNSGPYIKLRFTLSSDGERTVSATFRCRLVDDHQ
INQTAASASSSFKEVIVTSIFSNGQPKDMFLVSRSYASEYRYVQPDDSLLIECAITVLLEAPVNAAASVPAPLSDLQKHL
GEMLTSKNGADITFLVSGEPVAAHRCVLAARSPVFMAELFGDMKEKDSQSIEIKDMEAEVFRTLLHFIYTDTLPEQDDDD
VEAETMAYGLLEAADRYGVERLMLICAEKVHAGISVDTAAMALALAERHGCTKLKARCIEFILASQENFHAVAATEGYKL
LMDSCPSALNDLLVAVFLRYKLTVL*
>Osat_LOC_Os06g45730
MGKLTDVTRSNDIQLKIVGHSLTMAMDDGEFFSSRRYCVGGHDWEIRLRPKDPWVGRRDRPLTLKLVLRGAPRTGSGSVK
AQLSCCLVDPTQKLRPSEMKTVSHKFHKPGDYSPRAVFMARDELEASGYLTDDSYVVQCAITVLREQPEIAAAAAAAGDS
ANAAVAPSSELHAYLGALLESKTGADVTFVVSGESFAAHKAILASRSPVFMAELFGAMKVKASERVEVKDMEAPVFKAIL
HFVYTDTVPELDHRDGEETEAASTATAMAQHLLAGADRYGLERLKLICESKLAERIDVDTVSTTLALAEQHDCSHLKAKC
VEFIAAGTAENLDAVLATDGFKHLEASCPSVLTDLVKVARGRKN*
>Osat_LOC_Os06g45770
MSNSGKEQRRMDRFIVIPFSTCRNGSSVDVVDGGGKSGKKPQGGGGEGGGAADHHRQHVAHIGWDGSTNTTTSLRSWNRA
APPSSSSSTTTASTSSALAAPKTRYNGSLNYQNQSGSLSGFGLCGVYYVAGVAQLLPGAPVDHALPCFRGEERQSHAFGSA
NESSPWVLLVKRRELEASGFITGDSFAVRCTITVLSKNTINSAEPSPDLHLQLGELLRSGRFADVEFIVSGVSIAAHRCV
LAARSPSLAAAVLKGGTRKKDGSVRVEVKDDMRAGVFRALLHFIYTDTLMELDWREDGSDPLLPRTMVMSLNEAAGRYGL
ERLKQICENMLGFDDACSADCAVM*
>Osat_LOC_Os06g46240
MRAAAAASKAAGKEKSRRKGGGGGAGGGGGEQLLTDQVLSLRARLHLALALGLAKSDGGPKKWQSTDAGIQSHVLKAASA
FLGCLTNEMLRLPPIKESISDILIALEGILQSKNVSVLIQATDVSLKLVSSVGNLARQYPVLEIVTCLASQLSANQITIA
VSSASTLNCILNTLATARSSIHAEIWEALEKTDAVTSVIGALQNYSPDVHPLNYLMEMMSLLRIILWIWPSSRYHVWSNC
NLMGKLAQYCVASEMDVAVRVLKLYAALALCGNGAMVLLNNEDLMAKVGALLGKSNPSIARIEALKFYQILLRSSKGCDL
LMAAHYQHIIEGTINAMSRDDERLLTIEGCRTALLVLRYAGDHHRLFWSHAIDDVLYKILTGGCTSSHKANQILCHDKLF
NMVSENFMDIHSYVWDILGNLAVHCKNEYLSVRKGQDSALQALIHCICSLAADAMQKSNTMKLSKDVHEPALRAVLMMLL
SPSGYILSEASSKLLHVLPLGDDCLNILFTSLESNTTRSITASFDNVKIMSNLMSLAGMSINFVCIHCKRNLDVGIVCND
CRDHYSEGLIRVLQNASCQNLSPGPKLYISRILSLFGLCGFPSKLGGKMRRALDDNELADLELLLSNGESLKAHTAIISV
RCPKLLPSAKSLGSDGKITDEWGRSFYHVRMSDRVDSCGLKKILEYTYTNSVMVDDDNIKPVRTLAKYCHLKSLQEMLQK
EQPRWNSDCPRYDLTAALEPVKCSFSFSEVINVPLGWQALNKLIHWFYSGELPKIDPDCRWRNLNSEEQLSQLRPYAELS
SLSEFWFLEGVKEESLSVVTSCLSSTSTAASVEFVVFAAQLGQWEMVEAAVGSVAHLYPKLRDSGQLEQLDDDVLNMLRT
EYVRRTQRTGVGSAAAQAGARVVTAVYRRGQRADYWQSGGFGDNWNFQMVILNASEEHCRESKFDTIGVCKARFLYGKVS
RGFRLRTSGINKEGGPRGGTVIYSRSSGGLPPWCGAGSHDALAAVRWPSLPGLESHQTAQVIRRGAGRRGEGRDVNVTKQ
SNAPMRPPETMQREQPQSRARANGRKWPPPRRWRSGIREEQGVPSAKAWQEKRKRTQQQRCALPAAIAASRLQL*
>Osat_LOC_Os07g01140
MTAAASWSRSVTETVRGSHQYTVKGFSMAKGVGAGRYVSSDTFAVGGYHWAVYLYPDGKNPEDNANYVSVFVALASDGAD
VRALFELTLLDQSGRGRHKVHSHFDRSLQAGPYTLKYRGSMWGYKRFYRRSLLESSDFLKDDCLVMNCTVGVVKNRLETP
KNIHINIPPSDMGRCFNNLLNLRIGCDVSFEVGDERVQAHKWILAARSPVFKAQFFGPIGNPDLHTVIVEDVEPLVFKAM

VNFIYSDELPSIHELAGSVSTWTSTVVVQHLLAAADRYGLDRLRLLCEEKLCDELTAETVATTLALAEQHHCTQLKSACL
KFTAVRENLGAVMETEGFNYLEETCPSLLSDLLATVAVVDDDAASFNRKRGVGGNEGANPVESVEASDRRIRRRV*
>Osat_LOC_Os07g03150
MEEFRFGRLDGQPAKIRNVPIAVTPEGFWCCPSQAILQKSMKNQNQHARPKGGASPSVSKASSVQRTPTISLEKRTHSTP
TRSRTNSDEQVLPPADDAIPDQPKVSPVPDKRHNKQHKISVGFGQLHTSDLKVMLYGREGVAVKMIVHKNILAENSTFFA
DKLSRQSPVSCIEVSDCEDVEIFVETVGLMYCKDVKQRLIKQAVARVLRILKVAESLGFPTCIMSCLNYLEAVPWVGDEE
ENVLSSIRQLHCENYGVSPLLKRVASDLTNPPSDTLAHIIELVLKSSDDRGRREMKSLVLKLLKENNIWTNGSSDSCVVT
FYSSCRNCLESLSNLFRQASEPEFSEQSSDSKEVIFRQITLEADNLLWLAEILADRNAADELTSIWASQGELAKLHCRIP
VMHRHLISCVTARLFVAVGKGEALPSKETRQLLLDVWLQPLMDDYNWLQHGCRWFDRKVIEEGIGQTILTLPLEDQQSIL
LTWLGRFLKVGDSCPNLQRAFEVWWRRTFVRPYTDQQASSSSQSRWS*
>Osat_LOC_Os07g07270
MDDDAGDASPPPAAAGVGATAAQSRDMAASPTSSRSVTETVNGSHRFVIQGYSLAKGMGVGKHIASETFSVGGYQWAVYF
YPDGKNPEDNSAYVSVFIALASEGTDVRALFELTLLDQSGKGKHKVHSHFDRSLESGPYTLKYRGSMWGYKRFFRRTALE
TSDFLKDDCLKINCTVGVVVSTIDYSRPHSILVPDSDIGYHFGTLLDNHEGVDVVLSVGGERFHAHKLVLAARSTVFRSK
FFDDEDGEKNEPGENDDVQEIVIDDMEPKVFKAMLHFIYRDTLVDDNELGGSSSEGSIFDTLAAKLLAAADKYDLARLRL
LCESYLCKAISVATVASTLALADRHHAMELKAVCLKFAAENLSAVIRTEGFDYLKDNCPSLQSEILRTLAGCEEECSSGG
KSQSVWGQLSDGGDTSGRRVRPRT*MDDDAGDASPPPAAAGVGATAAQSRDMAASPTSSRSVTETVNGSHRFVIQGYSLA
KGMGVGKHIASETFSVGGYQWAVYFYPDGKNPEDNSAYVSVFIALASEGTDVRALFELTLLDQSGKGKHKVHSHFDRSLE
SGPYTLKYRGSMWGYKRFFRRTALETSDFLKDDCLKINCTVGVVVSTIDYSRPHSILVPDSDIGYHFGTLLDNHEGVDVV
LSVGGERFHAHKLVLAARSTVFRSKFFDDEDGEKNEPGENDDVQEIVIDDMEPKVFKAMLHFIYRDTLVDDNELGGSSSE
GSIFDTLAAKLLAAADKYDLARLRLLCESYLCKAISVATVASTLALADRHHAMELKAVCLKFAAENLSDTLMSLQL*
>Osat_LOC_Os07g15490
MWCQSCKEEYEEEDAGTCKECYEEASETEEELKREIDDLRSRLHFLRLPSPSLDASSLSHSDLLLHAIPADAPARPDTPA
VPAHRVILASRSPVFRAMLENEMEESRSGIIKIYDVSYDVLRAFVHYMYTAEALLDEQMASDLLVLAEKYEVKNLKAYCE
KFLTSKVSNDNAITHYAFAHRHSAKQLLETSLAAIMDNMSTLADREEYKELVEKDPRLVVEIYEAYLNRQVNTAAGKETD
SSSRKG*
>Osat_LOC_Os07g46160
MGAGRACRGGPSSSSPAAAAAAVGRPFPPIAASCPFSSSSAAAAAPAAPSETASTSVTKTVNGSHHFKIAGYPLAKGIGV
GKYIASECFTVGGYDWAIYFYPDGKSPEDGAAYVSLFIALASEGTDVRALFELTLVDQSGKGQDKVHTHFGRSLEGGPYT
LKYRGSMWGYKRFFKRSALETSDYLKDDCLLVNCTVGVVQSHTEGPKIYTIPVPPSNMSQHIGQLLTDGKRTDITFEVDG
EVFPAHKVVLAARSPVFRAQLFGPMKDKNMKRITIEDMEASVFKALLHFMYWDELPDIEELTGLNTTWVSTLMAQHLLAA
ADRYALERLKLLCELKLCEDVAINTVANTLALAEQHHCYQLKTVCLRFVALPENLKAVMQTDGFDYLQQSCPSLLTELLE
YVAKVGEHSVSPCLYSNEVLDGGDANGRRVKPRI*
>Osat_LOC_Os07g47260
MSGVRRAAGRPKIGDLATSDVVVRLRTPEGRDEWLYCHSGVLAAGSRYFADRLSDDWPTCQILGSRYCVEVHCQELDLSP
HVTALRLLYAAEPCSRFGVRGALGVLQAAAHLACPRVAAACVDYLESAPWDEADEEEILRTIPCLGPQYECVLARLRPID
PAPVAGILLSAFRHATSTRSPPQELKSAAQEQLEYMLTEDDDAPLLAFDDDIVRSQVKDCVAALLSRFSGFTSSILMEQG
EAPLGHGDAEVQQELHSLVSDISWVCQILSKLEMMKCVVVYWIGVSSDVVEAVDKACGGISCLKTRLKVIEVSAKVLEAI
AFGNIVLPTEKRCDAVNVWIGFARRTKPLVGHPEHDDDDGDAEAPKINLDSEVWQSLESAIVSIVLTLPSNSQADILSDW
LQSKHAKYPDLTEAFEVWCYRSKAAKRRLSFLSHANRVS*
>Osat_LOC_Os08g01320
MLPGVITSRVRGDGAAAAAAAGWRGRTGWRPSPGWPSGASTPSAPPPTAAPIPSRSASGTGSLPNPNPPLLRDHRFALSN
QIIRQFDPNPCRYLSVEKARYVYVRLFPEPGRVAKERPPLARFLLRACWSGPPRRSCVSPVHEHLLRSSEDFFWQLDTMY
AGRFTIDVEFLDLKIASNNAAESSTSIWPNESIAVQNIASKSSLGCLSRMLTESIHADVTINTTDGVLKAHKAILASCSP
VFESMFLHDLKEKESSTININDMCLESCSALIGFIYGTIKLDQFWKHRLSLLAAANKYSISDIKDCCEESLLEDINSSNV
LERLHVAWLYQLQKLKKGCLTYLFVFGKIYDVREEINSFFQHADRELMLEMFQEVITAWKPI*MERENRVETISRLAQWR
IDTFGPSSYRRSDSFKIGIWNWYLSVEKARYVYVRLFPEPGRVAKERPPLARFLLRACWSGPPRRSCVSPVHEHLLRSSE
DFFWQLDTMYAGRFTIDVEFLDLKIASNNAAESSTSIWPNESIAVQNIASKSSLGCLSRMLTESIHADVTINTTDGVLKA
HKAILASCSPVFESMFLHDLKEKESSTININDMCLESCSALIGFIYGTIKLDQFWKHRLSLLAAANKYSISDIKDCCEES
LLEDINSSNVLERLHVAWLYQLQKLKKGCLTYLFVFGKIYDVREEINSFFQHADRELMLEMFQEVITAWKPI*
>Osat_LOC_Os08g03470
MASRSISSCTTETAKGIHRFEIFSYSMMNTEAEDDSIRSGVFNVGGFDWALLYYPDGIDDDSKGYIGVYLELISKNGEPW
ALVDVNLINQLQPGQPRQLFTKTDVPTPFRSSSFQESTLGSLKCMKRSDIESTPGFIVNDCIVIECNVTVIYEPKVSKTR
ALCDAETSSALREIEVPPMEISSDFAKMLKDGVGADVTFRVGEDTFRAHRAVLAARSPVFHAQLCGPMKEKKETQMQEIT
IQDMQPSVFEAFLYFIYTDCVPRMDDLGNGEKMHLMMHLLKAGDRYGLERLRIMCERFLATNLDTEHVSAILGLADLLDL
KKLKEACMEFMVPSERMDAVAASQGYQQLKRAFPSLAFEVWERRSRVRRN*
>Osat_LOC_Os08g03480
METAPSSSMSPASGTYVLDVHGFSGLRRQHCGGGGCIVSPTFTVAGLEWAIRYHPEGDADEVTDDVAVFVVLVTKDATAW
AHVEFRLLDQAAGEMVTFFGEKDPILFDSGSEDLSTWGTGELAARSFLDGSPYVAGDCLKIECAVDVCRDRLTFHHDTPP
SGEPFRQYPADDEPADVTFKIAGETFPAHVSVLAARAPGLLNNTTSQAATITIDDDDDDTPAAAAAFGALLHFAYTDTLP
VASGLDGAGHTALLGRLLVAAGRYGMARLGAICERAMCRSLDAGTAADTLAMADGTASTR*
>Osat_LOC_Os08g03490

MASSRPISESTITRSTCATRTARATHQFEIVGYSLKRCLAAGEFVRSSAFAACGYRWSVRVYPGGFGPAHREFVSVFVKM
MTNRGKAAARFDLRLIDRATGLPRSVFRAAQPVVFDYSVKHKKCKGKRGTRAFMRRRDLESSAFVRDDRLIVECVIDVVV
ANGGDDDTAAAASPLAGVPAPDLSRHLGELLERADGVGADVTFDVRGQPFAAHRIVLAMRSPVFMASLYGSMREHRAPRI
AVDDMEPEVFDALLRFVYSDTLALPGDLGEGEYKEMVRQLLEAADRYAMDRLRVICELILSRSLDAKTVAATLAMADQHS
TTATRSRMFVFSSWPLGWMTTIND*
>Osat_LOC_Os08g03500
MPMETTTTTATESMSKMETVRGTHRFTFHGYSLCKGGGAGRCIRSGTFTVGGYDWCICFYPEGQGGGGGDREHVSVKLRL
VTRCATATAFYELRLLDQDTGRAAAVARASGAPRVFASSNPGTACFGRRAFMERSKLEASPACLRGDSVVIDCAVRVVVH
DPVVAAVRRREAPDDVPPSNILRQLVAQVESEGADVTFAVQGETFTAHRLMLAARSPVFKAELYGAMKEKDADHVIAIVD
VQPAVFKALLHFIYTDDMPPDLGLAAADDDDTDRIDMARHLLVAADRYAVERLRVICERVLRRSLGVETVIDTMALAEQH
SCGELKEACLEFIDSHSKRIVESDGYKNLKRACPLLVADMWERIVLSRLE*
>Osat_LOC_Os08g03510
MSSAAPAGHHAGEEPPPPSTTRSTMSTCTMGTVEGHHRFAIRGYSRLRALLAAGEYVRSAAFTVGGYDWAVVFYPRGATH
ADRDHAAVYVQLLTDRAAAAATFDLRFVRADSGRPLSVHPPLAAPRTFSTVLRSSSAAMYGVKVEAIQALQANYVRRDRL
TIDCAVRVVGKPRVSAAAPLTAADVPPPDLAAHLGRLLDLKSHADVTFDVRGVQFAAHRVVLAMRSAVFAAELFGPMRNN
AGGAIKVGDMQPAVFKVLLGFIYTDTLAAMDDLDADEDDRRELARHLLVAADRYDMGRLKLICADMLARSLTAQTVASTL
ALADRHGCRGLREACVEFVIAMGMNDEVVISRHPDQLSCISLFKYFFYQIGSLLKIH*
>Osat_LOC_Os08g03530
MATECTTSTSTTATVEGRHRFRVAGYGATKGAAPGHRVASGTFTVGGFDWAVVFYPEGVTAADMDFVSVYLELKNAAAAA
GGGGGGAVARAFYDLRLIHPATGEPRSVRWPMDGSTSRVFSQAFPAWGHLRFMRRRELEEMGFVRDDRLTIECVVNVVLD
PVVTAGDAPELDHPPSNILGHLAGLLGDKGTADVTLVVRGEEFAAHRAVLAMRSPVFKAALYGPMKESTDANAGRVAIDS
VEPAVFRALLHFIYTDTTAAMDDLDDDDDDKAQMIMHLLEAADRYDVERLKLICELMLCKSIAVDTVAATLAMADQHHCQ
KLKEACIEFLATSKKMEGVMESQGYEKMKLSCPSFMVDLWEIIGRKMTSYSVIPNIYARDKD*
>Osat_LOC_Os08g03650
MACLKLGSRADVFRKQGQDWYCTTGLPSDITVTVGEQSFHLHKFPLLSKSGLLERCIREKIENGDNSCVIDLSDIPGGAK
AFELTAKFCYGVKFEMTASNVVHLRCAAEYLEMTEEIAEGNLIAQTENFLTQTVLRSWKDSIKALHTCDDIIDLAEKLQI
VKRCIDSVATRSCTDPDLFGWPVVQYGGPMQSPGGSVLWNGISTGARPRHSSPDWWYDDVSCLSLPLYKKVISAMEYRGI
NQDIIVGSLNHYAKRRLPGLNRRKSISDVSSCLSISSLTSIPSEEEQKYLLEEIDRLLPFQRGVTSCKLLFGLLRTAIIL
KASPSCVSNLERRIGMQLDKATLEDLLIPNISESVETLYDVDCVHKILDHFLAMDQETGGASPGLGEDAQMLASPSLMPI
TMVAKLIDGYLAEVAPDVNLKLPKFRSLAAAIPDYARPIDDGLYRAIDIYLKAHPHLSESEKEELCRVMDCQKLSLEACT
HAAQNERLPLRVIVQVLFFEQLQLRSSIAECLMVSENLEGGSRQLIPTISGEQYRPGWPLASRENQALREGMDNMKQRVA
DLEKECSTMRDEIERLGRSRSTGKGRFSLNMKPQICSTKEAIPTTATTTATASEEKMAVVKGDTTPRLKLSRHKKKLSIE
A*
>Osat_LOC_Os08g09830
MASLFGDMKEKSSRSVEIRDIEPQVFGAMLGFIYTDSVPELDQQDGVVVAQHLLAAADMCGLDGLKIMCEEKLIAGATVE
TAATTLALAEQHGCPRLKARCVEVVAANLDAVMATEGYKHLMASSPLVMNDLLRAVRGRKS*
>Osat_LOC_Os08g12960
MVSKKKKTVSRHTTESEEGRHSFEIVGYSLKKGIGVDEFVESATFAVGGYDWCIRFYPDGKGDGAKDYISVYLELLTKDC
AVRAAYDLRLVNLATGLPKSVYSETTHRMFNSEDSSKFAPHYATFMHRSQLEMEASGYIKDDRLTIECFVTVVVQESMAS
NTVKAHELIKVPPSDILENFGELLEKGEGADVTFVVGGEKIAAHKIVLAARSSVFKAELYGQMKEKRARRVTVEDMYAMD
RMKLQCESILGEHLDVQTVATTLALADQHNCNGLKDVCIEFIATQNKMDDVVATEGYAEDLPFCLS*
>Osat_LOC_Os08g13000
MGTGSKKKKTVSWCTTEVSEGTHAFKIVGYSLNKGIGVGTFIRSGTFAVGGHDWAIRLYPDGVTEDSMDYVSVYLELMTE
NAKAMAFYTLGLVDPVTGGIRCNWSRSSPRLFDSSDSSRFGPRSPLFIPRSDLEMEESGYIVNDRLTVECEVTVTKGPQV
SRTIGCSEIGVPPSELSEHFGKLLEEEEDVGRDVVFSVEGESFAAHKLVLAARSPVFKAEFYGEMIERGTFSIDIKDMQP
SVFRALLHFIYTDVLPADIGDLEGDDYVEFIRHLVVAADRYAMDRLKLMCQSILGKYVDVKNVATTLALADQHNCDKLKD
VCIQYICSLDEVDAMVRTKGYANLKRSCPSVLADLFEKTSKFRAS*
>Osat_LOC_Os08g13020
MGSKKRMSRYAKKHEEATLKFDVVGYSLNEGMAAGEFIRSPAFAVGATTGQSASTPTAPRACSPVFKAELYGGMKEREAR
SVTVDDTQPDVFRALPHFMYTDSLPDMDGVEDADYVEMIRLLLVAADRYAMDRMKLLCESVLDDLLDAETVGTTLALADQ
HSCNNLKDVCVKFMATSKGMDAVMATEGYDNLKRNCPYVLIDVLEKLNRGKAIHTTLALLVVTNIFSSSNMFSLFEHED*
>Osat_LOC_Os08g13030
MVSKKKNTTASRHTTESEEGTHSFEIVGYSLQKGIGVDEFIESATFAVGGYDWCIRFYPHGKGDGAKDYISVYLELLTKN
CAVRAAYDLRLVKHATGLPMSVYSETTHRMFNSDDSSKFAPPYATFMNRSNLEMEASGYIKDDRLTIECFLTVIVKESMA
SNTVKAHELINVPPSDLSENFGELLEKGEGSDVTFVVGGEKIAAHKIILAARSSVFKAELYGQMKEKRARRVTVEDMQPD
VFRGLLHFIYTDSLPDMDDLSDDDYYEMIRLLLVAADRYAMDRMKLQCESILGEHLDVQTVATTLALADQHNCNGLKDVC
IEFITNQNKMDDVVATEGYADLKRTCPSVLVDVFEKASKFRRI*
>Osat_LOC_Os08g13060
MPSSSSAAAAACGPPEFTPGWKTVSTCAPDVVGEGVHVFDIFGYSDHKGMGAHEPIRSGAFSVAGLDWVACLYADGYGVA
GIDDVSAYLRLLGDAPTPRVWVSCEVKLVDQRTGVASTPQPFLRYALAFGDKCKALHCMMIPRGQIEVEPYLVDDRLTME
FHVVVRRDPRVSRTARFPRILVPPPDIKRQFANLLQSKEGADVTFDVAGEPFSVHKLVLAMRSPVFKAELCGLLREPGTQ
PITIVDMQPAVFRALLQFIYTDQFPVTRGFERRDNCEMIRHLLVAADRYAVDRLKLLCQGILCKNLNVHNVATTLALADQ

HQCDKLKDACIEFMSCSKKMKGVVASKGYEDLQRMAPSVLADAVAQMSKLNKMSRGSVPQDESKSC*
>Osat_LOC_Os08g13070
MKRHQMESVPRTVSTVVPNTAEATHVFDIVGYSQMKGRGREEHVTSGTFVVGGLHWAILLFPDTHVILLDDEEDNVTAFL
ELQSQGGSKVRACCDVRLVDQTTGLASSAAPAQPDAKTFKVFNADESNRVSCLKMKRTEFEAPPYLVDDRITLECVVTVK
KEPRVSRARPVPRIKVPPSNMMQQLGDLLESKEGADVVFDVAGETFPAHKLVLAMRSPVFKAELCGPMRESGTEPISIVD
MQPVVFKALLQFIYTDWLPSIRDLEGDDNSEMIRHLLVAADRYAVDRLKLLCQSILCKNLRVGNVATTLALADQHHCGML
KDACIEFMSCPNMLDDVVASQGFVDLENTAPSLVA*
>Osat_LOC_Os08g13090
MARAKTVSKCITETAEGSHVFSITEYSQKRGMGVGKGVEVHASSDLRLLDHTTGLSTSVTKTLPKASAYLRDDRLDIECV
VTVMKEPRVSQTKSSPKVAVPPSDIAAHLGKLLESKEAADVTFYVGEDTFAAHKVVLAMRSPVFKAELFGPMREAGAQVL
PIKDIQPDVFKALLHFIYTDSLSIIDDLVGDDRGEMIRHLLVAADRYAMERLKLICESDLCENLNVQTVAATLALADQHH
CASLRDACIEFMSSSSMDDIVATQGFVDLKTNCPSVLVDAFVNMSMLNKR*
>Osat_LOC_Os08g13180
MVASASRLRPSSTTRTASSCKPETARGTHVFTIHGYSLQAVDGAGSNKASFIRSAAFDVGGFDWCLRYYHNGNIESDDDY
ISVFLELMTKDAEVRTIFDIRMLDQYTDDSSCVLVSTTNNTRRVFGTTNFKSKCLVWGSKNFIRRSELEGSVYLRDDRLM
IECNLTVIKTPLVKTEERAAMPGDIIHFQVPPTNLSRDLGKLLEDNVGADLSFEVGGDVFPAHSVVLAARSPVFMAELYG
PMRAKRGERIAIQDMQPVVFKALLHFMYTDSFSPAINDDLSRDERQELAKHLLVAADRYAVEGLKTICEKALCMSLSVDN
VATIVALADQHNCGRLKEACVKFIASSNRLDDVVETEGYGRLKTSCPSILLDVIEMATKSRKIN*
>Osat_LOC_Os08g13250
MDYFTGTGTIVVSDDSLYAAFGYGDPFGFAPRARPPIRSLPFAAGGYQWCLWFHPTTFAGFFGFGVELLTAGAKARASFE
FGPVDAASHNVIVRMPPFLFDHPHHPMVIMVWPKAMLAEEATLFVRDHAVVFRVDVTVVPDEPLPPDAGVGDDDVLPPSD
MLAQLGNVYDTKEGADVTFSVDGELFAAHRVILAMRSPVFRAAVYGEMRESGRGGGPIAIDDMRPDVFDALLRYIYTDAL
PAAADDDDMEATWSDLLVAADRYGVERLKLICERALRGRLDAGNVADMLALADRQHCETLKDACIKFMATSGKMEEVKAS
QGYIQMVADRETGAKDQIGVYLELITKGTKARASYKLRLLNHVTACPCLWYTCPLKIYDSIDDNKSFTWGTKKFADRSKL
EVASEYLQDVRLVIECDVMPLVRIEAIAEIIRSPSSPWSQLSEDIGKLLEMETGRSRRELQGQRSTLNTIDRVASTIRRA
NEREEKYLGEEERKELIKHLLITVDRYAIEGLKTMLEDGLCKVLSLGNVKEMFALTDQHDCSILKDVCFEFITSSSVHGL
GDVALSEGYESLRESCLAAIVDALQRVQ*
>Osat_LOC_Os08g25240
MAAPSPSSGDRAPTRETASTQRLTITPYSSFSGLSSGKPVISGLFSLGGHLWDILFFPGGYYSGSPYAAVFLRLVSSDHR
EQVRVLVDFTLVYRRGGMTGGGEDDGSYTRCGYHVFGPGPATVGRGCFGFPEFILQHDLAASGVLLRGDRLVVECAVLLA
ADADEVLRRGPRPLDDELRRGLRRMLEDGTGADVTFVVRGERFRAHRCVLAARSPVLLAELHGPAARAMGETQDTDDATT
TITIDDMEPDAFAAMLRFAYDDTLPELPGNSERDATGVHMAQHLLAAADLYRMDALSQACQDRLARCVTPATAADTYALA
DRLGLRLLKAAVVRDVAATGARGIEAVKNSEGFRRLAAADAATAEEMVRKVMAAASAHVKREEVSTVSSMEMILGSLGVA
VFAGIALAALNFGPFRPIK*
>Osat_LOC_Os08g31420
MAAASNVPPSSSTSSSSPHNTTSTHSTELVKGTHRFTVAGFSLQKRNGAGHFAKSGSFDVGGYSWAVMFYAAGEKEEDQG
HVSVFLELQSTGVEKVTVKYTFNISGSSLLSAGWGDFKPSSKCRLGFNKFMEIETVEDVYLMNDCVTIHCAVEVVREKKA
RATVSRRIAVPPPAICRHLEQLLESKKGSDLTVQVGESKYDVHRAVLAARSPVFRAQFFGPMAAANRRASGGGGRCVRVH
DMRPAAFEAVLHFVYTDTLPPVKEEEGFLTNNSASRRHLVNLRDAAAGCSKGEVRVMVREWLAAADRFGLERMRLLCEDA
LCESIGVANAAATLRLADRHHCALLRALCMEYIASPGMLAAVMATKGFKELKVACPSLLIEILEKVGCCRSE*
>Osat_LOC_Os08g31430
MHILSLPMAAAAASTVPQSSSTSSTPQNTISTHSTELVRGSHEFTVAGYSLQKRKGAGHSIRSGSFEVGGYSWAIRFYPA
GSTKEEERHVSVYLELRSTVVEKVTARFSFHVHGASASSLHMRGSFDDYTPTSKSWGYPKFMEIETVESEYLINDCLTLL
CDVEVVKTVKTGATISCFITVPPPAICRDLELLVGSKEGSDVTLQLEQSEYDAHRAVLAARSPVFSAQFFGPMADEDAAA
AGSRRNVRIHDIKPAVFEAVLHFVYTDTLPPATTSWSASHRDKRPKLSDVAAASCSEEEVRVMIGERLAAADRFDLERMR
LLCEDALWETIDVANAAATLRLADRHHCPQLKELCMEYIASAGVLAAVMTTEGFRELKLDCPSLLIEILENFGKRSEADE
E*
>Osat_LOC_Os08g31450
MATAASTASSTTSPPHTTTMSTHSTELVKGSHEFTVAGYSLQKRNGTGHFVRSGSFEVGGYSWAIRFYPAGSTKEEERHV
SVFLELGSTVVEKVTARFRFRVNGATASSWGQFNDFTLSSKTWGYQKFMEIETVESEYLINDCLTMHCDVEVVKELKTGA
TMSRFITVPPPAICCHLEQLLESKEGCDVTFQVERSDYDAHRVVLSARSPVFRAQFFGPMADTGGGDRYVRILDMKPTVF
EAVLRFIYTDRLPPVEDGEAAASSCWREDVREMARS*
>Osat_LOC_Os08g38700
MSDGARVEAAPRLAQWRVDVLPCYTYRKSEPFRIGLWNWYLSVERNNKQTCVKVFAELSNSAKNTTPAPIASFITKLLIS
FPPNQKTIVHPGIFDKPLKHEGFVWAIDSTVTGRFVIEIEFLDLKIADPSGGEPASIWASHQIKKSSDNTALSSLARMLQ
EGILTDITINATDGSIMAHRAILASRSPVFRSMFSHDLKEKELSTVDISDMSLEACQAFLNYIYGDLRSEEFLTHRLALL
RAADKYDISDLKEACHESLLEDIDTKNVLERLQMAHLYRLQKLKDGCLRFLVDFRKVYEMHDDFKVFLQTADRDIIAEIF
QGILTTWSGR*
>Osat_LOC_Os08g40460
MEHRGTRGEHSPVRMKHLAANREGDVRAIGAAHHDAELVPQVGRWHCNRFVVVAQNDEATYECNHIARVKFTQLDEAPPG
IKAEALGCLAYQQTPLGMLHGWYSTLCSMKLETRSITHGWVGTPKAGVGDREMRIVTGQRRRLGMLLVGERRSRGGGQPC
EPPKTREERWGGEISLEMKKRSSGGGGGWGVRMASSARCRRRARGLGDGAAAIHMCRVDHLNGSDVFFFVGGETFHAHRA

LLAVCSPVFKALLLSSTAEAAACSITLNDIKPAMFEALLHFMYTGDFLPAGAHSSSPDSSDTNTDTLHRLLAAAHEYKLD
RLKLMCARKLEESLSVETVARTLGYAKMCGCSELKSKCLDLLLLGEEEHQSIGHTWLLLALA*
>Osat_LOC_Os08g40490
MLASGFVECKLDYLESEKIAIDDSLPETKVSAGEHHARIRLYPRGIEGGHGEHVSIFMFIDDVDDDDPRIDAAVFEVFLT
DKHGAPSPQHARRSTEPCRPPTWPRISPAWLGCPDGSDVSFSVGGETLIHAHRAVLAARSPVFRAELLGSMAEATMPCVT
LHDIEPATFRALLHLVYTDALPASSTSSSTAAAAVEFFRGLLAAADRYALDRLKLIYTCSWMLKSLKAASTCIMVMGEEY
PSSSGCSSVYEQFSDADLVVADPRSRAKKNDRLFWRFVGWYRFVKRSAVDSLHAIDGVVTFVFGLVILRDDRHHPIAVPP
PNLGGHLAAMVGSADGSDVSFSVGGETLIRAHRAVLAARSPVFSAELLGSMAEGTMPCVTLHDIEPATFRALLHFVYTDA
LPPRDILSPSFFKKLFAAADRFALDRLKLMCAQKLWESVTVETVAETLACAEMHSCPELKSRCLDFFVEENNFRKVVVTG
GYLRLMQGFPSVIDEIKARLEI*
>Osat_LOC_Os08g41120
MMISSSFAELKLEYPGTTTTTNSFAVGDGEQSWVIRCYPRGYREEDNGEYVSLRIGVPARSNTVRAIFHTFLMRRDGGVG
APSIICSDRAFPMSVPGHPRGYGGAFRHLVRRSDLEPLYAVDGVVTIVCGVVVFADAGGGGGDDDDGGPIPVPRSNLGGQ
LGGIVDRADCSDVSFSVGGETFHAHRAVLAARSPVFKAELLGSMAEAAMPCVTLHDIDPATFKALLHFVYTDALPSPSTS
SSSSSTTTGFFESLLVAADRYALERLKLMCAQKLWESVSVETVATTLGYAETYHCPELKSKCLNFLMAESNFKKVAVTNG
YFHLRQDFPLIIEEIKKRIES*
>Osat_LOC_Os08g41150
MEYTAASMLASSFIEYKLDYLETQKLAIGECLPGIRISAGEHNATILLFPRGCEGRNGEYIAVFLLLTEIDPKINVIFEV
FLMNKDGKPSSLCAKNSSIDVIRGTSSGFRFLGWHRFIGWHRSIGWHRFITRSDLESIYVIDGMATFICGLVILGDGGAI
AVPPSNLGGQLGAMVGSADGSDVSFSVGGETFHAHRAVLAARSPVFRVELLGSTAEATMPCVTLHDIEPTTFRALLHFVY
TDVLQIIEGSSSSTTASTSDHLLHHQRLLAAADRYALDGLKLMCAQKLWESVSVETVATTLGCAEMHGCPELKSKCLDFF
MAESNFKKVVVTDGYFLLIQNFPLVIEEIKEGKIRAMPL*
>Osat_LOC_Os08g41170
MLSSGFIEYKFDYLEASWMTVIGEWVPERRISAGEHNATIVFHFYSHAGCKYISVFLLLDENEIDPKISIIFEVFLMDKD
GKPSSHHAKRTMGPNMETASGLRFTGWRHFVKQSDLQPDHVSDKGTVTFVCGFMVVRNDVHGNPIPVPPPNLGAQLGAMG
GSADGSDASFAVGGETFHAHRAVLAARSPVFRAQLLGSMAEATMPCVTLHDIEPATFKALLHFVYTDVLPPLLGTSDLLE
PLLAAANRDGCNDSDLCRVYNCPELKSRCFNFLTADSNFKKVVVTEGYFHLGQSFPLVIEDIKARIETCDFFEKYIKGPI
*
>Osat_LOC_Os08g41180
MVSSGFIEYKFDYQQIHKLAIGERLPATTISTGEHNAKIMCYPHGFGYGNGEYISLFFVMLKQIDPKIKVIFEAFLIGKD
GTPSSFHAKRTMQCWASQDGYDWFGWHRFVMRSDLESLDGMVTFICGLVVLRNDDDGDDHVAVPPSNLGSQLAAMVGSAV
GGETFHAHRAVLAARSPVFRAELLGSMAEATMPCVTLRDIEPATFRALLHFVYTDVLQIEGSSSTSTTDLLQRLLAAADR
FALERLKLMCAQKLWESVSVETVIATLCCAEMHSCPELKNRCIDLVVTKDNFMEVAVTKDYFHLGQSFPSVIEEIKPRLK
K*
>Osat_LOC_Os08g41190
MAPASGFVELRLDYSATNASAIGDPINSDLFTAGGLTWRVNCYPRGDKADNNGDYISLYLELISKSKNIKAIFDAFMVDE
HGNPSDGSNRLVQVYPPAGYPAWGWPRFVKRSNLSSVFVVDGKVRIMCVVVVLRDDDGDGDGNRVPLPSPGVTGGHLDGG
LLPLPPPNIGVHLGGLLDSEDGADVTFVVVGGGGERFAAHRAVLAARSPVFRTELFGCKSESTSPSSSCITLQGIEPAIF
RALLRFIYTDELPADAGKLHQGSSSTNVFFKHLLAMADRYALDRLKIMCGQRLLDNMTPDSVAAILVCAEMYNCPELKNK
CIDFFAVEENFRKAVFTDGFALLMQKFPVIVAELKKRVEKL*
>Osat_LOC_Os08g41220
MEDKVLPEWYSKEDNGEYLSLFLELVRESTSNNVRAIFDAFLVEKDDEPSSTHADRGVHVHPTNGYTAWGWPQFVKRSDL
ESSSSSYVVDGKVRIMCVVIVIRDNTVPVPPSDIGAHLGGLLDRGEGTDVSFLVDGETFPAHRAVLAARSPVFRAELLGS
MAESKMSSITLHDIEPLTFRALLRFIYTDKLPADDGGDQLKMAAMATDELFQKLLAAADRYDLSRLKLMCAQKLWEAVSV
DTVATTLIHAEMHGCPELKSSCLDFFVQDKNFKETVLTEGYVQLVQRFPSIKDEIRGRTERKKGGGCLLAGRRARRGNHR
VGKLASMRGEREEAGRPMNGTGRPARGRGGRRVGWGGRRACEEREKAGKRAEGDSHRVGRPMNGTGRSACARGEGEGGEK
GGGRQPSLHGGGGGGEALSASILVASTPRVFTRDAAGSLHPDNSDAAGVHPTASATPLIMGARKLCVALVFGLLAADVMV
TSILKGSFVLMLVPKSFESDHARVRGQQQPKVCQGLMAGNTATGDCQTGDGERRVRLREAWAFRRWRLEDRGGPGVPVAS
RPLGQAPGDLAPSTRRAGEETRRGRGVRESLVEAES*
>Osat_LOC_Os08g41230
MTNKENYCYSKLEEFDHTVLITLISVYNQDYGSWILFESIDRYISDSIPLAIAFWFAAPYAGMPDSGSLELILDYEATNH
CAILVDGETFPAHRAVLAARSPVFRAELLGSMAEAKMSCITLHDIEPVTFRALLRFVYTDELPADDGGELNTTAMATERK
KGGGCLLMGRRAGSGNHQAGKLASVRGEGGGGEADEQDGEPTSGTGRGGRRAGWGGRRAREEREKVGKGAEGDSHRAGRP
MSLRGEGGGGEALSPSISLSWWP*
>Osat_LOC_Os08g41240
MLDGGFIELKLRYEDLAAGDFVRSDDIYAGGHTWRVLCYPRGGGAMNSNGQRIGGEYLSIKLDLVTRSTNVRAIFAAFLV
HLDGHPSPVHAKSFVAVYPLGAGGRGGGKAAAGWMYFASRSELEKKFVSGDGWVTVVCGVLIPSDSPQALSPPPPPPPSS
GGGGGHIGRLLYGAADDTADVALVVGGETFRAHRAVLAARSPVFKAALFGSMAEATAPSVALRDMDPAAFRAVLHFIYTD
ALPDDIDELAGFSPVDMFQHLLAAAERYELGGLKLLSTKKLLDNVTPENVAGIIVCAETYGCPELKKKCLDYLAREDEHF
RKAATTQGYLRLLQDFPSLMDEIRAVIER*
>Osat_LOC_Os09g06890
MPYISREKIIFLTAKVSGATVEIMGTPYPYGTVIRLQVYADTLGWSEHNGRTIGLYLMAFSFKMLNEEGQGDFVLLFAID

WPSVDISEFMYSTGTKKRIAGLEDGIRQGCVNNILLLPFKFPPIFTLLKYILYAGDDDGTIPIPWLNLNGQLGDIVDRAD
GSDVPFSVGGEMFHAHHAVLAARSPVFKTELLGSMAESAMPCVTLHNIDPATFKALLHFVYMDALLSPTRGGASSTSTTT
GFFKSLLMAVDMYALKRLKLMCAQKLWGSVSVETVATTLGYAETYHCPELKSKCLSFLMAESSFKKVAFTDGYFHLRQDF
PLIIEEIKKRIES*
>Osat_LOC_Os09g16850
MAAASTTDQGVAVINDSPLCKLLSSGDGRHVTFRVDYAAARRPAVAGRTLRTTCAIGDSGAYRCEASFRLLIGRRKLAAF
AVVVSGPRYRDVHKVVVDLVLVDNARSVALQPPTRSMAIQAAAGSNQGGCGLLVSKDYLEENCVQDGVLVAVCSVLFLPE
LPPCLWLDSLGHRLAAMSNKKDSTLTDVCFDVDGERFNAHRLVMAAQSEVFRSLLFGSDDAETKTETAVVTIDGISATTF
KHMLHYIYCNQLPPPATGDGDDDDGEADHVTRIAELQRLLVAADAYGVEALRQACEDTLCAGINMDTVASTLALTEKGSY
PKLRGSCLEFLSNTQIYSVATNDECYEVVQSYPDVLTEIRDRFKKPRLTPKFPSTDTKDQNNP*
>Osat_LOC_Os09g16870
MASSSSPCKLVSCGDNRHFMFRVDGAAIRRPVGRIVKETCEIGNGYTCEASLFVPSLLGERKVMMILAVTVNSDRAQSLA
RAHKVFIDMALLDDTKSPVLPPFARSVMSPSAAAGSSPKLPPPVAGSNLAASCKLVASRDHLLANCVKGGILAALCWVVF
VPSSPRSPYSLLAHCLATMSNGRDLTDVCFDVDGKSFHAHRLIMARQSEVFRAELLGSMAESKMECITISDMSASTFKHM
LHYIYCNDLPTCVKDTDDQSSWIFELQHLLVTADRYGVDTLKDLCEDTLCADITTDTVTSTLELAETRSYPKLRTSCLVF
LSNTQNFAEVATTKEYYNLIQSYPSVLSEIRNRFKRPRPSLMLAPSTVTENQNKRPRLSPKLTPSADTKDENNP*
>Osat_LOC_Os10g28760
MPASAAATTGSSDGGRPPHYSSASAIVGGTVTGHHILQIDGYSYTKEKLPSGKFIQSRSFKVGDHQWRLSYFPNVKGSDY
ADYISVYLCLVEGQPVKARATFSLLDRAGQPAPASASYYTRDMPMGRFAVSDIGFGYHQFIKRELLEKSGHVRDDGFAIR
CDVTVVTELRTEDRTPPLVEVPPPDLRRHLGGLLESGDGADVTFHVAGEEVRAHRYILAARSPVFKAELFGQMKESSSSN
TVVNVDDMEAEVFRALLVFIYTNALPETKTKANQEDELVIAQHLLVAADRYGMERLKLLCEEKLVEYIDRGSAVMLMALA
EQHHCHGLKEVCFRFLESKETLSAVMATDGFLHLMQSCPSLVKELLFRVVDHSLEITHVVRL*
>Osat_LOC_Os10g28770
MPSSAATGGSELLRPPPPYSSASAIVGGTVTGHHILQIDGYSYTKEKLPNGKYILSSSFKVGDHQWQLSYFPNGVNRYGD
ADFVSVFLYLVEGQPVKARATFSLLDRAGKPVPSYTRDTGMRDFAVGGSGFGPGDFIKRKLLEKSGHVRDDGFAIRCDVT
VVMELRTEDRTPPLVEVTPPDLHRHLGGLLESGDGADVTFRVAGEDVRAHRYILAARSPVFKAELFGQMKESSSSSSNTVM
NVDDMEAEVFRALLAFIYTDALPETKTKAKQEDELVIAQHLLVVADRYGMERLKLLCEEKVVEFIDRGSVATLMALAEQH
HCHGLKGACFRFLESKETLNAVMATDGFLHLMRSCPSLVKDLVFRVADSHFQ*
>Osat_LOC_Os10g28780
MPSSAATGGSELVWPPPPYSSASAIVGGTVTGHHILQIDGYSYTKEKLPHGKLKYSRMFNVGDHQWHLRYYPNGQGSANA
DYISVYLGLDAAAAGHAKEQPMKARATFSLLDRAGKPVPSYTLDAGMHDFAVGGSGFGYHQTGPWHQFVKRELLEKSGHV
RDDGFAIRCDVTVVVELRTEDRTPPLVEVPPPDLHRHLGGLLESGDGADVTFHVAGEEVPAHRYILAARSPVFKAELFGQ
MKESSSSNTIVKVDDMEAEVFRALLAFIYTDALPETKTKANQEDELVIAQHLLVAADRYGMERLKLLCEEKVVEYIDRGS
VATLMALAEQHHCQALKEACFRFLESKETLNAVMATDGFLHLMQSCPSLVKDLVFRVADRHFQ*
>Osat_LOC_Os10g28790
MATGGSDDGGGARPPPYSSSSAIVGGTVKGHHILKIEGYSYIKEKLPAGKFIKSRTFKVGDHLWCLLFYHNGSRASPPGF
VAVYLKLVVAGGKQPVRARATFGLLDRLGKPMMSCKLDAGMHGFTVSETGFGYHEFIGAEVLEKLGYVRDDSFTIRCDVA
VVGALRVEDRTAPVVAVEVPPPELRRHLGGLLESMEGADVTFHVAGEEVPAHRSVLAARSPVFRAELFGAMKESVSGGSN
AVVEVDDMEADVFRALLAFVYTDELPETETKQQVVMAQHLLVAADRYGMQRLMRLCEEKLCGRVELGSAATLMALAEQHH
CRGLKEACLRFIDSTATMVAVMASDGFEHLIKSCPSLVKELIVRGSQLLSGAK*
>Osat_LOC_Os10g28810
MPTPEPSTSASAIVSGTVTGHHVLTIDGYSRTKAKLPTGRFTASRPFTVGGHSWSIHYYPSGDRSDTAGFISVFLELNPA
ADAAAAAGSGGSEPVDARVTFSLLDQAGRSVPSHTMATDLHDFAATGFGFGRFIERSYLEQSEHLKNDRFAIRCDVVVFS
DELRAEARTADAAALSVAVPPSDLSQHLGGLLAAKELGADVTFLVAGETFTAHRCVLAARSPVFRAELFGPMKESAATAV
ITVDDIEPDVFRNLLTFMYTDTLPETNPQELEEEEEDDDDDYEDDQAQAAAMVEHLLIAADRYNLERLKLICEDRLCKHI
DGESVATILALAEQHSCDGLKEACFQFLSSRSALNSLVATDGIEHLARWCPSVLNQLMSKVAALVPVDFVVRETR*
>Osat_LOC_Os10g28820
MAGDTTKPTESAIVGSTVTGHHLLHIDGYSHTKDRLPNGCYMDSRPFTVGGHLWRIGYYPNGDVADASAYMAVYPSIDEN
VIVAVKAFAKFSLFFNGEPTPPAFVHTTEPFVFSRKGIGYGFSKYAERELMEGSIVDDKFTIRCDVGVSTELRAEDRPPS
DFAAVVPPSDLHRHLGDLLDSKHGADVTFQVGGEAFRAHRYVLAARSPVFRAELFGAMREATAAAAASSSDSEAIRVDDM
EAPVFSALLRFVYTDALPAPGGADDGQAAGGGSYSEEAAMAQHLLVAADRYDLKRLKLLYEDKLRRHIEAASAASMLALV
EQHHCRGLKEACLVFLSSPANLHAAMGSDGFEHLSRSCPGVIKELISKLVPRCD*
>Osat_LOC_Os10g28840
MGSTSGPACSAPPAALGSSTTTPTATPPRAQFTVSLLDELGRPGWSYRSSSCTCKCSGSSGEEYSMWGGERFIRRDQLEQ
SEYVRDDRLAIRFDVAVMDKLRTTEEIAGGGGGAVPPSEMSRQFADLLASGDGADVEFRVGGETVAAHRAVLAARSRVFR
AELFGPMKEGVAANGTIQVDDMDAEVFRSLLHFVYTDSLPPETGTPREGAAMAQHLIVAADRYDLERLKAEANLRGEAVR
AHWCGHGGDNFRARRAAPLPWAQAGVHGVPQLADESEGRHGNRWV*
>Osat_LOC_Os10g28860
MSPTMPMTMTADEPTTASAIVAGVKTGHHVLRIDGYSRTKNVVPNGQFITSRSFRAAGHSWHVFYYPNGFDDESIEYISL
YLLLEDAATATTATTTTVQFTVTLLDKDGRQVPSQKANSGVFTYSSEIQKYGFTQFISRDELEQSEHLDGDRFALRFDIT
VVGKFRAEEIAGPVGAPYVAVPPSDMRRHFGDLLASGDGADVEFRVRGAGGEEETVAAHRVVLAARSPVFKAELLAGVPA
KDGGGAVIQIDDMDAEVFRSLLHYMYTDSLPPEKGTTREEAAMAQNMIVAADRYSMETLKLMCEDRLRKHIGASSVATML

TFADRHHCHGLRAACTEFLSSPTNLKAAMATDGFGQLSCPTVLKELMAKALF*
>Osat_LOC_Os10g28870
MSSSSSTSSTGNNNGGIPSRSSSSSSAIVVSKVSGCHFLNIDGYSHTKEMLSHGHCSRSCTFRVGTHSWYLEYYPNGRSF
LHNASDHMAICLVRDDDGDAGDGGAYEQMTARFHLLDHHAGKPVPGHTRGVTSPLLSGKIWECSNLVTRKELEEHVLDGD
CLAVRCDITIVTVPRRAAPAPAVVVDVPAAAPDLQSQMGALLLSKEGADVTLQVGGGETTTFAARLLVFRSELFSATATS
KAGSGGRVHVVDDGIDARAFEALLRFIYTDAPPELDEEDDDFSSMAWLLVAADRYKVERLKMICENELCKRIDGNNFEAT
LALAEQHHCSCPWFNLSFSAFVSEGADIPKFQKFRKFRFEFSKILNKFWSNLNKILPNAQKNEKN*
>Osat_LOC_Os10g28970
MSSSSTSSASNNGIPSRSSSSAIVVSGCHVLKIDGYSHTKEMLSQGDCSRSCTFRVGTHSWYLEYYPNGRSLHNASDHIA
ICLVRDDDDGGDLGYGGAAREQMTARFHLLDHHAGKPVPGHTRGVTSPLLSGKVWACSNLVTRKELEEHVLDGDCFAVRC
DITIVKVPRRAAPAPAVVVDVPPAAAAAAPDLPSQMGALLLSMEGADVTLQVGGGEVETTTFAAHRCVLAARSSVFRSEL
FGATATSKAGSGGLVHVVDDGIGARAFEALLRFIYTDAPPELDEEDDASSMARLLLGAADRYNVERLKMICENELCKRI
DVNTVATTLALAEQHHCSSLKKACMDLVDANPRAVEAAGGFEYLSNKCPSILRELIARLADFDLKNDG*
>Osat_LOC_Os10g28975
MKEPSAVADPPAVVDVAVPLPELNRDMEALLQSEEGADVTFEVGGESFAAHRCVLAARSSVFRAELFGAMKESTAGGGKA
RVDGVEARAFKALLHFIYTDAAPELDGKDQDETSSMAQHLLVAADRYNLERLKLICEDKLCKRIDVSSAATTLALAEQHR
CPSLKKACMDFLYSPGNLKAVEATDGFEHLATSCPVILRELIAKLVAL*
>Osat_LOC_Os10g28990
MRRPIHRRTTRASAAAAAATGSTSTIFAGAMRYELKIVEYSRTKAVPNGCSMKYPAFTAAGHTWHVGYFPNGVIGAEEAE
ADYVAFFLYLNDNDAAEEAVKAQAIFSLLVIEGNPVSSYTFTTVLVNFSEKKYWGYKNFIKRESLENPLYLKDDCFSIRI
DLAVTPPLTVVVPPSDMHRHYGRLLISKEAADVEFQVGKKVFDAHRLVLAARSPVFKAELYGRMKESTTKSAIAIDDMEE
EVFEAMLTFIYTDSLPKMKRRDEAAMAQHLLVAADRYNLERLKLICEDKLSKNIDTGSIANILLLAEKHSCHALKEACFE
FLRTSRSLNAVMETDEFEYLIDTCPGVIKELMSKLIVNLGQSNEWRRDGALVIPIAKLILRYYQVQ*
>Osat_LOC_Os10g29020
MPASSRASASASDGASSSAIVAGTVNGYHVLKIVGYSLTKAVPNGKSIKSRPFRAGGHTWHVAYYPNGQNAEKAEYMAFF
LCLDDTASKGVEAKAIFSLLDMEGNSVSSHSFTTRVVNFSEERSWGYSEFMKRGSLEKSEYLKDDCFKIRIDVSVIADFH
EEETPLIVVPPSDMHRQFGDLLLSKQGVDVEFQVEVFAAMLTFIYTDALPEMKQQEEAAMAQHLLVAADRYNLERMKLIC
EDKLSKHIDAGSVANILALAEQHSCHTLKEACLEFLRSSRSLKAVVETDGFRYLIGSCPGLIKDIFSKLSPR*
>Osat_LOC_Os10g29050
MTASSLAAAASDGASSSSGSASAIVAGTVNGHHVLKIVGYSFTKAVPSGKSIRSRPFRAGGHTWHVLYYPNGNRAEKADF
VAFYLCLDDAEACSEAVEAKAIFSLLDMEGNPVSSYRFTTRVVNFMEHKKGWGFDFMKRESLEESEYLKDDCFKIRIDVV
VITDFHTEEETPLIVAPPSDMRRQFGDLLLSKQGADVKFQVGKKKFDAHRSVLAARSPVFKAQLYGRMRESTTRGAIRID
DMEEEVFRAMLTFVYTDDLPEMKQQDEAAMAQHLLVAADRYNLERMKLICEHNLSKHIDTDSVVNILVLAEQHSCHMLKE
ACLKFLRSSRSLKAVMETNGFGHLISSCPGLIKDIMSKLSPC*MTASSLAAAASDGASSSSGSASAIVAGTVNGHHVLKI
VGYSFTKAVPSGKSIRSRPFRAGGHTWHVLYYPNGNRAEKADFVAFYLCLDDAEACSEAVEAKAIFSLLDMEGNPVSSYR
FTTRVVNFMEHKKDFHTEEETPLIVAPPSDMRRQFGDLLLSKQGADVKFQVGKKKFDAHRSVLAARSPVFKAQLYGRMRE
STTRGAIRIDDMEEEVFRAMLTFVYTDDLPEMKQQDEAAMAQHLLVAADRYNLERMKLICEHNLSKHIDTDSVVNILVLA
EQHSCHMLKEACLKFLRSSRSLKAVMETNGFGHLISSCPGLIKDIMSKLSPC*
>Osat_LOC_Os10g29100
MSHGALSPTTAAAVSASAIVANTSRGYHYLKIDGYSHTKATPTGEALFSCQFAVGGHRWRICYYPNGNVLEAADYISMFL
VLDEIVVRNVKAQFQICFAGQVEKQAPSLAWKTVRAFNKQTSSSSSWGYPKFIRREDLEKSEYLRDDSFTIRCDIIVVDN
YRAEDASSGAAGFVSVPPSNLHSHLGDLLKNEKGTDVVFEVAGQRFTAHRCVLAARSPVFNAELFGMMMESDTTTNDAIQ
IGDMAAPVFKALLHFVYTDSLPETMEEREDTMCEHLLVAADRYNLERLKLICEERLCKYIGIGTVMDILALADQHHCKGL
KKACFDFLRSPANLSAVTGSESFEHLSRSFPSLMKELVDILGTSHNYAW*
>Osat_LOC_Os10g29110
MGQCAMSPARSGTTAGSGGGPTPSSSSSRSASTIVAGTASGYHLLKIDDYSRTRDLFPTSTALKSRAFTIGGHRWRIQYY
PNGNTPNCGDYISLFLHLDEEVTREVYAQLQFRLLDDELGDKLPPPPPPPSLDANKFFSHASWGQPKFIKKEELEKSRHL
KGNSFTVRCDVVVITEFVAKDMPEAATATAARRRTPARGTGSFVSVPPSDLHRHLGELLLGEKGADVVFKVGGKTFTAHR
CVLAARSPVFGAELLGSMKESRRKAVVRVVDMEAQVFKALLRFAYTDSLPEMKEKDEGAMCQHLLVAADRYAMERLKLVC
EEKLCERIDVSSVATVLALAEQHHCDGLRNACFDFLSSPENLKAAMAGDGFEHLSRSCPSLMTELVAMLGNCHDPQPTHR
SMPNTGSNR*
>Osat_LOC_Os10g29120
MATAVSRGTASSIVADAVTGYHLLKIDGYSRTKGTPNGAALTSDQFVVGGHRWRIRYYPNGDIAMFADYISFHLMLDENA
TSTKGVKVKAQFQICFADQVRREILILSRRRRDPHREDDGDRHRNLRHRASASDLNQKLGKLLDTEKGADVVFGVGGETF
AAHRCVLAAQSPVFSAELFGPMKDSDRAGVVRIDDMEAQVFKALLRFMYTDSLPEMEEEEDTMCQHLLVAADRYNLERLK
LICEDRLCKHVGVGTVVNILTLAGQHHCDGLKKACLHFLGSPANLSAVLAGDGFEQLSRSCPSLVNELVAMLAPPIPPSS
HA*
>Osat_LOC_Os10g29150
MSHTRSTTAGAAGGKPPIAPPPTSSASAIVADTASGYHLFKINDYSRTRDIFPTGSALKSRAFTIGGHQWRIHYYPNGNT
EECGEYISLFLHLDEIVTDKNVYAQHGFRLFDEFAGDNDDDDELQPSSIADLGQVSTFGGNNIGLGRLRFIKREELEKSK
YLKNDSFTVRCDVVVTKRIRSEETPLVVRTSPKPKVARLVTVPPSDLHRHLQDLLCAEKGADVVFEAGGETFTAHRCVLA
ARSPVFSAELFGSMKESDTTVVIRIDDMEAQVFRALLFFVYTDSLPETKKEDEYAMCQHLLVAADRYNMERLKLMCEERL

CSYIGVGTVTTILELAEQHNCDGLKKACFDFLSSQENLKAVTAGEGLEHLSRNCPSLVNELIGTLGNLIQ*
>Osat_LOC_Os10g29180
MATAACGESPSRLGSASAIVADTETRYHLLKIGCYSRTKATTPTGSFLSSGQFTVGGHRWRINYYPNGESADSADYISLY
LLLDDKATNSSVKVQAQFKFQISSTDQVKNTPSLASTNVNTYGEDSSWSWGHRKFIKREDFEKSNDLRDDSFTIRCDVAV
IGEIRTEKTTEIPSATTFVTVPPSDLNQQLVDLLETEKGADVVFEVSGETFAAHRCLLAARSPVFSAELYGLMKEGDTAG
VVRIEDMEAQVFKLLLRFMYTDSLPKMEEEDVMWQHLLVAADRHDLQRLKLICEDRLCNYIGVSTVLNILVLADQHHCDG
LKKACFSFLGSLENLSAVVTGDGLEHLSRSYPSLMKELFVVMALPPNHGRYVIFF*
>Osat_LOC_Os10g29190
MSCSAIVADGVTGYHLLKIDGYSLTKGTPTGTAIKSNPFTVGGYRCSSKPRISRSPTTSGTTRSPSGVTWSLSARSVPRR
GRLTHLAETFVSVPPSDMNRQLGDLLETEKGADVVFEVAGERFAAHRCVLAARSPVFGAELYGLMKEGDAAVVVCIEDME
ARVFKLLLRFMYTDSLPEMKNKDAGVMCQHLLVAADRYNLERLKLICEEKLCKHISMGTVSNMLVLADQHHCAGLKKTCC
NFLGSSANLSPISRGCLSVMKELVLGCWCIH*
>Osat_LOC_Os10g29220
MSSSAAGNSSRSASTSTIVADTETGYHLLKIDGYSRTKGTPNGTAIASSQFIVGGHRWRIYYYPNGDHTDNADYMSFYLL
LDEKKNTKTKSVKVWTLFQICFADQAKALPTLTSKTVRTFGDGSSWSWGYSKFIKREDFEKSKDLRDDSFTIRCDIAIVR
EFLVETTEVLPPKSFVSVPPPDMNLQLGELLETEKGADVVFEVAGERFAAHRCVLAARSPVFGAELYGLMKEGNAAVVVR
VEDMEARVFKLLLRFVYTDSLPEMKKKDEGIMCQHLLVAADRYNLERLKLICEEKLCKHISTGTVSNMLLLADQHHCSGL
QKACCNFLGSSANLSPVSRGWLFVMKQLVLGCWCLH*
>Osat_LOC_Os10g29230
MSPAVGRGNPPSRSASSSTIVAETATGYHLLKINGYSLTKATTPTGSFLPSSPFTVGGHRWNIKYYPNGDDVKTADYISF
FLVLEEEETNMGLTVQAKFKFSFANQVKKQPSLKYRPIKTFNLEDSCGWGYVEFIKRVDLEKSDDLRDDSFTIRCDIVVV
REIRTEETTEILPVESFVPVPPSDMDQQFGDLLETEKGADVVFEVGGQTFAAHRCVLAARSPVFRAALYGSMKEGDTDGV
VHIEDMEAQVFKLLLRFVYTDSLPEMETEEDVVCQHLLVTADRYDLHRLKLMCENRLCKYIGVSTVSNILALADQHHCDG
LKKACFSFLGSPANLSAVVASDGFKHLSRSCPSLMEELVVMLALPPSHA*
>Osat_LOC_Os10g29260
MARSLSPASSPSAATAGASDEKVKVQAKSDFQISYTGQVDEPPWLATMKADTFDGAGFRSFGYEKFVRRCDFEKLIRDDS
FTIRCDIVVIDEIRAEESTEITTTTAAVVTVPPSDLNQQLGDLLESEKGADVVFEVGGQTLAAHRCVLAARSPVFKAELY
GLMKEGGTAAGAVHIEDIEPRVFKVLLRFMYTDSLPEMEEEDVMCQHLLVAADRYNLERLKLICEEKLCRHISVGTVWNI
LPLADQHHCDGLKKACFDFLGSLANLSAVVASDGFKHLCRSCPSLMEELVVTLALPGSHA*
>Osat_LOC_Os10g29290
MSPAAACRGNPPSRSAIVADTATGYHLLSIHGYSRTKGTPTGSPLKSTRFTVAGHRWRIHYYPNADRADSADYISMYLFL
DEKSNATRSVKALFQIRFADQVKAQPSLALHAVRTFGDGSWSWGYAKFVRREVLEKSKDLRDDSFTIRCDIVVVREFVAE
EATEILPAGSFVSVPPSEMNRHFGDLLETEKGADVVFEVAGESFAAHRCVLAARSPVFGAELYGLMKEGDTAGVVRIEDM
EAQVFKMLLRFVYTDSLPEMEAEEDVMCQHLIVAADRYDLQRLKLICEEKLCKYIGVCTVSNILALADQHHCDGLKKACF
SFLGSPANLSAFVADDGLDHLSRSCPSLMKELVVELALPPSHA*
>Osat_LOC_Os10g29310
MSPAARRGNPSRSASAIVADTATGYHLLKVDGYSLTKATPTGSSLTSTQFTVGGHRWRIKYYPNGDSADSADYISIYLLL
DEKASLDLKVEAKYLISFADQVKTQPSLKYRTVRTFHRQGSWTWGYGKFIKREDFEKSDHLRDDSFTIRCDILVVHKIHT
KETAEILPVETFVSVPPSDMNQQFGDLLETEKGADVVLEVGGQTFAAHRCVLAARSPVFRAELYGLMKEGDTAGVVCIEE
MEAQVFKVLLRFLYTDSLPEMKEEEDVMCQHLLVAADRYNLERLKLICEEKLCKYISVGTVSNILALADQHHCDGLKKAC
FNFLGSPANLSAVVAGDGFKHLSKICPSLMEELVVVLALPGSS*
>Osat_LOC_Os10g29320
MSPPVAEESEGGGGRMPESMDHPPVVEESEARRQQRRPGDGKLHQHGQIDGAVDVEWRADEKSNADFSVQAKYQISFANQ
VKMQPSLKYIMVRTFIRGCSWTWGYKKFIKREDFEKSDDLRDDSFTIRCDILILRKIRAEETTEILPAAESFVSVPPSDM
NQQFGDLLETEKGADVVFEVGGQTFAAHRCVLAARSPVFRAELYGLMKEGDTAGVVRVEEMEAQVFKVLLRFLYTDSLPE
MKEEDVMCQHLLVAADRYNLERLKLICEEKLCKYISVGTVSNILALADQHRCDGLKKACFNFLGSPANLSAVVAGDGFKH
LSKICPSLMEELVVKLALPATQA*
>Osat_LOC_Os10g29330
MSPAACRGNPSRSASASAVVVDTATGYHLLKIEGYSLTKGIPTSLSLKSSQFTVGGYRWRIDYFSNGDCADSADYISLFL
SLDERANKDVKVRASWRFQIGYTGNVDKPPSLSTAKACTTFGVGPDGSWSWGYDRFIRREDFEKSDNLRDDSFTIRCDIA
VVRRFRAEETTEILPVEAFVSVPPSDMNQQFGDLLETEKGADVVFESPVFRAELYSSMKEGDTAGVVRIEDMEAQVFKLL
LRFVYTDSLPEMGNDDEDVMCQHLLVAADRYNLERLKLIYEEKLCSYISVDAVSNILALADQHHCDGLKKACFHFLASPG
NLNAVITSDGLKHLSRSFPSLMEELVAMLAPLLSHALVI*
>Osat_LOC_Os10g29340
MPSSANGHGNSSTRSTSAIVVDRVTGHHLFKIDGYSFTKETPTGTPIASGEFTVGGYRWRIEYYPNGRGKKSADYIPLYL
SLDKNTSGEVKVKYQIELADRVKKKKKQPSLISKPFMRRRKFEKSKYLRDDCFTIRCDIVVMREIRTEEATFVSVPPSDL
KQQLGDLLETGKGADVVFEVGGGETFAAHRFFGSMKESDAAAGGVVRIEEMEAQVFKLLLRFVYTDSLPKMKEEDVMCQH
LLVAADRYNLKRLKLICEKKLCKYIGVGTVASILALADQHYCDGLKKACFNFLGSSENPPVASII*
>Osat_LOC_Os10g29360
MNQQLGDLLETGKGADVVFEVAGQTFAARSPVFSVELYGPMKESEPTGVVCVQDMEAQVFKALLRFMYTDSLPKMEEEEG
VMCQHLLVAADRYNLERLKLICEEKLCKGKLF*
>Osat_LOC_Os10g29380

MSFAGVSLVRDGRLQSPSSSAITSGATSGYYLLVVEGYSRTKDTVPNGDFIRSRPFRVGGYRWVIDYYPNGESSDDADSI
SVSLQLDQDSERPFMAHYEFSFIDETERQKSTHICSEALFDFSDDNRWGYTNFIRREELEKSKHLKDDCFTIRCDIILKK
DGSNTTGDDVAAPLVAVPPSDMHRQFTDLLLTKVGADVTFQVGGETFAAHRCVLAARSTVFMVELFGPMKEGATTASVHI
SEMVPEAFKAMLAFIYNDTPPPETEEDEDGKVAMWQHLLVAADRYDLPRLKLICEEKLCGHIGVGTATTILLLADKHHCR
GLKEACLEFLSSPANLEEVMEHGGLEDVVGTCPSVLVELIAKLALLRTQV*
>Osat_LOC_Os10g29410
MSFAGVSFICDGVHVCSSPANGAAGSAAYGYHLLVINNYTRTKQAIPNGFRIKSGKFKLGGHTWHIKYCPNGDRSTISGF
VSFHLVLDCDGGDGAVAAEPVNAKFEFSFADQVAKHQATRLRATKVCEFSRDCSAWHVGRFVRREALERSRYLVDDCFTV
RCDIMVVHAGAGANGVAAATAAPSMAGAVESFGRLLDTKLGADVAFEVGGETFAAHRCVLAARSKVFDAELFGPMKEGTA
ASVVRIDDMDADLFRGLLSFIYTDELPEREDHGGEKETSSDDDDDDDDDDNGAQSDQKHKQFTWLQQLIVAADRYDLQRL
KLLCEEEMYDHIGEKTVETMLILADHHHCRVLKDACLGFLGSHGNLQKMMAADGLDRVIKNFPSLTKEIIGRFAVVMANK
S*
>Osat_LOC_Os10g29440
MGASASSSAGRSKPPPPPPCSSSGVAVSTSRGYHLFEIRNHSRIKSMTPLGRGETAEAADYAAVYLDYVIDSYPPKSVTA
YFLLRLVDKVSNDLMDPPPDDDVRAAAVHEFSAGQNSWGYYAFWKKDELESSGRIVDDVLTIRCDVGVVGRYATAKTDAA
AAAASVASSVHVPPADLGHQLGALRSRAVGADVTFQVGAGSGARRFGAHRCVLAARSPVFEAELYGPMVERDAGRVIRID
DMDPQVFDALLDFMYTDALPGMRKRDAVAMSQQLLVAADRYDLKRLRLLCEHELCKHVNKGTVASMLALVEQQRPSCQGL
KKACFEYLRKTPKVLREIMATEAFDHLVNELLLSNKLAIRE*
>Osat_LOC_Os10g29495
MSSPSSQQPASAIKAPTTSGYHRLRIDYYRSLGSPTGWALSSRDFVVGGRQWRISYYPNGNRPENAEFISVFLCLDSSSP
KPAMLQVTITFDDEAKKQSQLRKAPVITIAPGACWGYHRFVKRDDLARSKRIRPDGFFTIRCDVSLIDHFTAQEDEPVFV
SVPPSELRRDLGGLLDTGSGGDVVFQVGGEAFTAHRGLLAARSPVLAAALYGPMMEGGGLQGGVAIKIDDMDPLVFKALL
RYAYTDSLPPQMQQGELEEEGRAMAQHLLAAADRYGMERLRLLCEAQLCKHIEVASVASILILADQHGCSGLKNACFEFL
KSPGKFAAAMATQEYDYLKTNHCALADEVVKGNQAKWMLATVETFMRHRLNSKSILETATREIALHASTSNETPPVFLDL
EESLRAKGDMTLREEATLKVLKVVATLGKAISTGAIAAVGYYVPGIVGAKFGEPSLPRLPRFAMAAVTSWFAGKVMYYAI
LQGSTEFILKHGEERMKMELSNIILNKHSDDKTLVEAVKTQFFAEHLFSDQYQDRTLFRWHLRRTYVDSAFMERVKEIEV
KSSNDGPGLISGQRIISTRPFGDLMEDPLACILGSPDSNMESNKSAEHTGTIVKRREIACPPAVLVEPIRLQPNVPICSL
LARSRTGSNLEVNPVILDLEESLLAKGNMTLEEEVTIRVSKMTASVFKVFATAAFGASSYYALGLGELSLHARARLDL*
>Osat_LOC_Os10g29740
MATTTTMTLAASAGDGSPSSRSTVTTIAAQAYHVLKINGYSNTLEAGRHHALSSCPFSAGGHTWHVSYYPNGGRDSNKNC
ISIFLVLKDIVTEEDVMAKVTFSLLDRYGNPVPSYTYHTQLRNFSTSPSRAKGFENFIRRDELERSEYLNDDCFAVAVHV
IVPKEKPSIVVPPSNMHLHFVDLLVSKEGTDVKFLVGGEMFAAHRLVLAARSPVFKAELFGPTKKGTIDVIQIDNMEARV
FKALLDFIYTDIWPEIGHGEDNVAMAQRLLAAADMYGLQRLKFVYEDKLCNHIDTGSASTMLVLAEKHHCCKLKEACFTF
LSSMSPPIVEDLNSSIFGSESEKTVSSSDNHGSQINRIDIRIQPWQNADV*
>Osat_LOC_Os10g29750
MTTTTTAAAAALTSSVGDGPPPSRSTTTITAGRTYHILKIEGYSSTLKAGRGQALRSSPFSAGGRTWYISYYPNGGRETN
KHCISFFIHLDDDTVNDDVMAQVTFSLLDRHRNPVRSHTVTTTLYNFSVASSNALGFENFIRRDDLQRSEYLNDDCFAIA
VRLVITEESPSFTVPPSNMHMDYGDLLSSKEGTDVEFVVGGETFAAHRLVLAARSPVFKAELFKPMEEGTTDVIKIDNMD
AQVFKALLVFIYTDTWPEIGQDETTMVQQLLVAANKYSLSRLKIMCEDKLCSYIDTSSVVTMLMLADKYQCHGLKKVCFN
FLASSRALSLVMKADNFWCLIQSHPTMLKDLIYNIVTHQLEIKLSV*
>Osat_LOC_Os10g29790
MSTATPTPNVVDGDCGGTPPSRSASTIIAARTLHVLTIHGYSDTLKSNVDPSQHLLLSSPFSAGGHTWCIRYCPIGCTEE
SKDFISIYLVLEDTITDVVSAQVTFSLLDQQGNPMPSHTLTTPLLKFSLQGTLPKALGYNSFIRRDDLERSGHLKDDCFA
IGVHVVVTKEAEPSSITVPPSDMHLHYGDLLSSEERYATDVEFLVGGETFTAHRLVLAARSPVFMVELFGPMKEGTTVNK
IHIFDMEAQVFRALLKFIYTDMLPEMDQEDETAMVQHLLVAADKYGLHRLKMICVEILSNHIDAYSVATILVLAEKHYCY
GLKEACFEFLNSSAILSAIVNTSDFLYLIQSCPDVLEDISFNIVARQLERAIFLSENQEGQINSVEIRINSVMTN*
>Osat_LOC_Os10g29810
MATASIASNVAGGGCPTPSRSAAASTVVTTQAYHVLKIDGYSRTLQVHCYRSLSSFPFNAGDRTWYICYYPHGKNDISKD
FISIYLVLYDAIAEAVMVQATFSLLDQHGKPVPSHTHATRLLSTSNQDDMANNLGFETFIAKGDLEKSGHVQDDCFAIGV
HVVITKETPPPIIAVPPSSDMHLHYGDLLSSKRCADVEFLVGGETFAAHRLVLAVRSPVFVAEHFGPMKEGVNVNDVIEI
NDMDAQVFKALLNFIYTDTLLEMDQEEDATMAQHLLVAADKYGLERLKVKCEERLSNHIDADSVATLLVLTDKHNCRGLN
KACIEFFSSPTALAKIIETDEFQYLTQSHPNILEDIISNIVASQLEKAIFSPENEGGKINKVDIRIQPWQNSNARCG*
>Osat_LOC_Os10g29840
MTTTTITSSVCAGPPPSRSTTTITAERTYHIIKIPGYSSTLKVGHGQALRTSPFSAGGRTWYISYYPNGGRETNKHCISF
FIHLDDDTVNDDVMAQVTFSLLDRHRNPVRSHTITTTLYNFSVPNSSALGFENFIRRDELQRSEYLNDDCFAIAVRLVIT
EEPSSFTVPPSNMHLDYGDLLSSKEGTDIEFVVRGETFAAHRLVLAARSLVFKAELFRPMEGGTTDVIKIDNMDAQVFKA
LLVFIYTDTWPEIDQDETTMVQLLVAANKYSLSRLKIMCEDRLCSYIDTSSVVTMLMLADKYQCHGLKKVCFNFLASSRA
LSLAMKADNFRCLIQGCPTMLKDLIYNIVTHQLEIKLSV*
>Osat_LOC_Os10g29850
MATTTTSTVTTIAAQAYHVLKINGYSNTLKAGRHHPLSSCPFSAGGHTWHVSYYPHGCRDSNKDCISIFLVLEDIVTDED
VMAKATFSLLDRYGNPVPSYTYHTKLRNFSTSSGRARGFENFIRRDELERSEYLNDDYFAVAAHVIIPKKKPSVVVPPSN
MHLYFGDLLVSKEGTDVKFLVGGEMFAAHRLVLAARSPVFKAELFGPTKKGTIDAIQIDNMEARVFKALLEFIYTDIWSE

IGHGKDNVAMAQQLLAAADRYGLQRLKFVYEDKLCNHIDTCSVSTMLVLAEKHHCCKLKEACSTFLSFMSPPIVEDLNSS
IFGSELEKTVSSSENHGSQINRTDIRIQPWQNADV*
>Osat_LOC_Os10g29920
MATSSIASKVAGGGCPTPLRSAAVSTVVTMQAYHVLKIDGFSGTLQVHRYRSLSSFPFKVGGRSWYICYHPHGKNNISKD
FISIYLVLQDDIAEAAIVQATFSLLDQHGKPDDLEKSGHVQNNCFAIGVHVVITKEVPPPPPPIVVVPPSSDMHLHYGDL
LSSKRCADVEFLVGGETFAAHRLVLAVRSPVFVAEHFGPMKEGVNVNDVIEINDMDAQVFKALLNFIYTDTLLEMDQEED
ATMAQHLLVAADKYGLERLKVKCEERLSNHIDADSVATLLVLTDKHNCRGLNKACIEFFSSPTALAKIIETDEFQYLTQS
CPNILEDIISNIVACQLEKAIFSPENEGGKINKVDIRIWPEPWQNSNARCG*
>Osat_LOC_Os10g29950
MATTAPNVVDSDCGGTPPSRAGGHTWCIHYCPIGSTEESKDFISIYLVLEDTTADVVSAHVTFSLLDQQGNPVPSHTLTT
PLLKFSLQGTLPKGLGYNSFIRRDDLERSGHLKDDCFAIGVHVVVTKEAIPSSITVPPSDMHLYYGDLLSSEERYATDVE
FLVGGETFAAHRLVLAARSPVFMVELFGPMKESTTVNKIQIFDMEAQVFRVLLKFIYIDMLPEMDQEDEAAMAQHLLVAA
DKYGLHRLKMICVEILSNHIDANSVATILVLADKHHCYGLREACFDFLNSSAILSAIVNTSDFQYLIQSCPDILEDISFN
IVARQLERAIFLSENQAGQINSVEIGIRS*
>Osat_LOC_Os10g30040
MATTTATTLAASAGDGSSPPSRSTVMTIAAQAYHVLKINGARGFKNFIRRDELERSEYLNDDYFAIAAHVIIPKEKPSVV
VPPSNMHLHYGDLLVSKEGTDVKFLVGGEMFAAHRLVLAARSLVFKAELFGPTKKGTIDAIQIDNMKARVFKALLGFIYT
DIWPEIGHGKDNVAMAQQLLAAADRYGLQRLKFVYEDKLCNHIDTYSVSTMLVLVEKHHCCKLKEACSTFLSSMSPPIVE
DPNSSIFGSELKKTVSSSENHGSQINRTDIRIQLWQNADV*
>Osat_LOC_Os10g30350
MATTLASGAGDAPGLEWYIPGLEIELCSRGGCSEWLGLYNRGLSGPPVIFSSGCDTWHINYRRLGSSDSSRDYISFYLVL
EDQVVEDDTVTAQITFILLDQDRKKPFILRKDLAESEHLKDDCFAIGVDVVIVTKDEVSSVKVPPPSDMHRHYGDLLSSK
LGTDVEFVVGGETFTAHRLVLAARSPVFKIELFRTTEERTTPNAIPINDTDAQVFRAMLSFIYTDTWPKIDQENEAAMAQ
HLLIAAERYGLDRLKLMCEDRLCNGIIDMGSVTTNLVLAENHYCHSLKKACLELLGPWTTLAEFMATDDFRYLTKSCPGI
LNDLICIVAARERERTIFFLGE*
>Osat_LOC_Os10g30360
MKQISPPHHDFNPVSATGCKDYISFYLFLEDAVDEPVMGQVTFSLLDQDGKPVLSRTHTTRMFSFSLNSSFGFHKFIRRE
DMEQSKHLKDDCFAVSVHLVITKGAPSVKVPPSNLHSHYGDLLSSKQGADVEFMMFEALLIFIYTDMLPKMDQEDEVAMA
QHLLVASDTYGLQRLMLICEDRLCNHINTDSLAIMLVLAEKHHCIRLKEVCFEFLSSSTALVEFMESSDFLYFIRSCPTV
LKDLIYNVAAHGK*
>Osat_LOC_Os11g02070
MDCCVCSPMASMYRLPRNAICAACYEGAKAIIAFFNDDDDEHADADQGSVKPSRLTKLNSTIKGLRDAWEEVKQMRCREE
ETKQRASFLQEGFAAAWKDGIHTDIAIRPGTGPPIQAHKAILATRSEVFRHILAGDDDCKAPAGDSLSLPELTHDELSHL
LAFLYTGSLATCAEERHLHALLVAGDKYDVPFLRRACEARLAAGVEAGNVLRTLEVAELSSSAALKERAMGTVVEHAEEV
VFSPEYEEFAVRNAALCVQITRTLLANKSLPAKTP*
>Osat_LOC_Os11g04600
MSSEDSLKSLSLDYLSLLINGQAFSDVAFSVEGRLVHAHRCVLAARSLFFRKLFCGLDPNHQPPPPPPPLNWPMAGGGGG
GSGGGGRGGAGGGGGAPATPELVIPVSSIRYEVLVLVLQFLYSGQASVAAPKSGPLPGCGARGCWHTRCGAAVDLALDTL
AAARSFGVEQLALLVQKQLESMVKEASVDDVMKVLMASRKFEMQELWATCSHLVARSGLSADLLAKHLPIDVVAKIEEIR
AKSPLAAVAAPRSPFLTHHYLPMNPASSAADRDNKIRRMRRALDAADIELVKLMVMGEGLDLDDALAVHYAVQHCNRDVV
KALLELGAADVNSRAGPTGKTALHLAAEMVSPDMVSVLLDHHADPNSRTLDGVTPLDVLRSLTSEFLFKGAVPGLTHIEP
NKLRLCLELVQSAVMVTTRDDGAPVTGGAEAGGSDGGNFPRSDADDSLVSLTMNSTLMYQGQEMAAAVAAGEGRKSNNGR
GSPPPAMYFPNGFA*
>Osat_LOC_Os11g24550
MYSITAAPATVSHLVRIDGYSRTKNLRRGRFIEAMNFTVGGHRWFIRFYPNGHGPRDVGVVSVYVGIAGAYRRGGGDAKP
VIADARFSLVDRDGRPAPPSFVQGMPAVDFSGNDFGMNIKRAELETSGFLKDDGFLVRCELGFVNSAGDGDGRRGVQIKE
GIKVPPPNLHRHLADLLWKNQSSGDVFIEVQGKTFTAHRWMLAARSPVMAAELSSSDSDDAAYTDADATKNTMMTLRVDD
DMDAETFRALLHFIYTDALPPPPQPRARDTKEKEDEAAAAEAARRLHAAAARYGMERLQLMCEDALCRSLSVLTVASTLV
FAEKHGCRVLKAACLDFLSCKRKLRQVTRLDDDFRLLTTTCPSVIKELFAQVLK*
>Osat_LOC_Os11g37520
MRKLFFSELTSCKETKLHSAPHSWLPLEKGKLSKFAGHSTSSIESLMKMPEPVVLPHFKPADYVDILAQIHEELESCPPD
EKSCLYLLQFQVFRGLGEVKLSRRSLQSAWEKASTIHEKLIFGAWLKYEKKGEEPISDLLSSCGKCSQEFKLLDFVSQIS
TGSHEISYDDESDVFWGSPVVHFRIRDDMIACDRRKLAALSTPLYAMLNGGFRESHLEVIDMSRNGISSIGMRAISKFSL
TGRLPYLSADAILEMLDFANKFCCNGLKDACERKLASFICSRQDAIDFMECALELGCSILAAACLQVLLNELPECLNDEQ
VVRIFSCASKQQRSTMAGNASFSLYCLLSEVSMSINATSDVTVTFLEKLVDSASDSRQKQLALHQLACTRLLRKDYAEAE
RLFNAAFTAGHLYSVVGLARLASMRGNKHFSLKLLDSVMSSRWPLGWMYQERALYLEGDNKLENLNKATELDPTLTYPYM
FRAASLMKRQSVEAALMEINRILGFKLVLECLELRFCCYLALEDHRAALCDVQAILTLAPDYRMIGGRVSAKQLRMLVME
NVEQWTTADCWMQLYDRWSSVDDIGSLSVIYQMLESDAAKGVLYFRQSLLLLRLNCPEAAMRSLQLAREHAASQHEQLVY
EGWILYDTGHCEEGLQKAEASIAIQRSFEAFFLKAYALADSSLDPSTSATVVSLLEDALRCPSDRLRKGQALNNLGSVYV
DCGKLDLAAECYINALKIGHTRAHQGLARVHFLRNSRTGAYEEMTKLIEKARSNASAYEKRSEYCDRELTKSDLQMVTKL
DPLRVYPYRYRAAVLMDNHKEKEAIAELTKAIAFKADLNLLHLRAAFHEHVGDISSALRDCRAALSVDPNHQEMLELHHR
VNSQEP*

>Osat_LOC_Os11g40220
MMTRRQDAYYRYPTTSTIVASAPMPTGHHVLRIDGYSLTKAKFAAGEECDESCSFVVGGHAWRIRYYPNGAVVSGSGGFV
SLMLSLDHQPGAALPAAVVKARFAFSLLDMDGEPVPSRTYASDGVVSFSANSSSTFGAERFIGHGELEASGHLTGDRLAF
RCDVTVVKRDGPPTMSTLCPVEHDMFRCLLDTGDDADVAFRAAGGETIAAHRRVLERRAPELAKLCRRRGGASCFMGLVG
RHIDVGDMDAAAFKALLHFVYTDTLPETMAPREVPAMAPQLIAAARKYGLDWERLRLLCEDKAWGWRVDDTSMDTTAAAA
PATGDDPKRSQRRLPWFIRKCF*
>Osat_LOC_Os11g40490
MSGSSASAGDAPPPTTSVTASTIVAEVVSGSHVLKIDGFSQTIGASDGGSYVKSGRFVVGGHGWRVGYRANGDGDDDGAD
AGWISIALHLDDPNVDGVRNGVRARFKISLLAAAHDGSHPPPPRSDQSTATRSFPRFITAKALEESGYLVGDSFSLRCDV
AVVKDIRTEDDATTVKKLVGVPLPSDIGAHLGRLLAAGHGADVAIHVGGETFAAHRCVLAARSPVFMAELFGPMAMSRHN
NEETIRVHDMEPRVFEAMLHFIYNDSLPEVDDDDDGEVAAMAQHLLVAADRYDMGRLKLICEDTLCSHVDASTAATALTL
AEQHHCERLKEACFKFMENPSNLKAVMASDDFLHLTRSCSSLLKKLAKLAA*
>Osat_LOC_Os11g40680
MASNSPATSDAATGDVPEPSRSSSVVKAMSGYHVLKMEGYAAGVKGLGVGKFIDSGSFDVGGHRWCIRYYPKRSPASPGD
GDWISIYLNLCSTAAAIGDANASFTISLLDQDDDEHQPVAAHSRSCSSTVTFSSAATKAWGFPRFVERKTLEESPYLRDD
SFVLRCDVTVFKETIIEPAAPTPLVAVPPPDMHRHLGSLLSGGHGADVTLQVGDETFAAHRCVLAARSPVFMAELFGPMA
TSRHNDRETIRVHDMEPRVFEAMLHFIYNDSLPKVDDDEVVAMAQHLLVAADRYGMERLKLMCEDTLCSHVDASTAATAL
TLAEQHHCEGLKDACFKFMADPDNLKVVMESDGYLHLTRSCSYVLKKLAT*
>Osat_LOC_Os11g41260
MTTMSATDAAIVWLGDCEGDISGRRGQRTSTSYLEPVASRTYGGNVHRFPSPRGSSSWGFHKFIRHDELERSGHLTGDRF
AVRCDVTVMRATELRVEPACLAVPEPDLRAHLRRLLSTGDGADVTFRVGGGETFAAHRCVLAARSPVFKAELCGRGGAAA
GRCVDVDDMGAGEFGALLHFVYTDTLLEMASRDVPAMARRLIAAAGKYQVERLKLVCEDMLRRRVDTSMAMATTTDSAAT
TDDQLSQRPWLTQLFFKFASRMVGGMVVDAFTPEPPTAAEKSGDASPSLSPSPSCSTVAVSEASGHHVLRIEGYKRTKMM
MATGEHLNSGEFHVGGYTWRLRYYPNRYDQEFSSSISFALVRTGRDDDDVVVRARVKISLLDVAGEPVTRYSHSDNKCTF
YEGHDLWAIKSFIRRVDLEDSGHLDDGGGGGDSFAVRCDLTFNVPDIRVDVDDAAAVTVPAVPPPLLHRHLGDLLASEAA
ADVRFNVDGEAFAAHRCILAARSPVFRAELFGSMRERAARAIVRVDDMDADAFAAFLHFVYTDELPEMDDDGEEAAAVMA
KHLLVAADRYGMERLKKVCEDVLFRHVVVATAATSLALAEQHDCPELKDAILRFVTSPARLKADMASDGYEHLITSFPSI
ATEILAMLAAQLST*
>Osat_LOC_Os11g41290
MARRRPSSRRRSPPAITSSRSTATPGLRPWSPPATASTPAASTSATTRGASGTTPMAPTAATRIPTPYPSCSSCRTPPPP
PAATAPPSRRSSCSACSTRRRTYRSSVHSFPSSDGFKNWGFLRFITHGDLDKSEHLVDDGFAVRCDVTVMGGIELRVEPA
SLLAVPEPDLHRHLGRLLSTGDGADVTFRVAGGEAFAAHRCVLAARSPVFRAELYSRGGFLRPAAAGRPETRVVDVDDMD
AGAFRALLHFVYTDTLPEMA*
>Osat_LOC_Os11g41300
MSTKKSGAADVQPSPSSCSTIVVTEASGHHVLKIDGYTRTTMMVATGEHLDSGEFHVGGYAWHLRYYPNGYDQEFSSSIS
FALVRTAGAGDNVRLHARAKISLLDLAGEPVARYSQPVDKCSTSKASDPWVCKSFIERDELEKSGHVVGDRLAVRCDLTF
NVQDRLVRELVAVPPPLLRRHIGELLGDARTSDVRFKVGGETFPAHRCVLAARSPVFRAELLGPMREHAATTIRVDDMDA
SVFAALLRFVYTDELPELDGGSAAAMAQHLLVAADRYDMERLKKVCEDRMVRHLDVGTAATSLALAEQHDCPELKKAILR
FMASPARLKAVMASDGYEHLVTSFPSIATEILAMFAAH*
>Osat_LOC_Os11g41310
MAASSSAAASTSCAVAAEANGSTSTIVATTKPTGHHILKIDGYSRTKAMVAAGDSIDSSRFHAGDHAWRIRYYPNGTDRS
NQNPDAISVMLELQDAAAGRNNGAAAAAAVKAKFVFRLLNKDGEPVPSRTYRSSVHSFPSSDGFKNWGFLRFITHGDLEK
SGHLADDGFAVRCDVTVMGGIELRVEPASSLAVPEPDMHRHLGRLLSAGDGADVTFRVAGGEAFTAHRCVLAARSPVFKA
ELYSRGGFLRPAAAGRPGTRVIDVDDMDAGAFGALLHFVYTDTLPEMASADVPAMARQLIAAADKYKVERLKLVCEDKLS
RRVVADDTSMTPTPMSTPTTMTDSTTTGDDPQRRQRRARFLGKFVKFGACFSDRYFLPLI*
>Osat_LOC_Os11g41350
MSAAAADDDSPPLLTATPAAADDYCGSADSPAACTIVGKVERVCYNVRVDGYSKTKETTKNGSYIASTEFVAGGEPWRIR
YYPNGYSQSTAGHVSVFVYRVGGVDVGLHADVQIDLVARHGDATAPPETEVAGRFRCTFWPDSSFGFQRFISTEKLDMSP
WCVRDDGFTIRCDITVEGPPFVVAVKPSSSPLGWHLGDLLGDTDTADVAVVVGGDVGDGEETTFAAHRYVLAARSLVFKA
QLFGPMKKAAEGNGGAAMISVDDMRADVFRAFLHFVYTDELPPGELDVAGDGDADTAAIMAQHLLVAADKYDLPRLKLVC
ERKLSESLGAGTVATTLALAEQHGCHDLKEVVLRFIRLPANMEAVKCSDGFKHLLESCPSLHQDLKSRHIIS*
>Osat_LOC_Os11g45560
MGQTTSKSAGDGKPKPKTTSAAAAAAAVVSETATGSMTMRIAGYSQTKGIGVGNSINSSKFHAGGHTWYIAYYPDGDREE
YSDWVSVYLCLARPAAGAAADDVVEAKFTLSLLSGTYGAVVEEKICTAKKFSFANGYWPSWGHTRFIKRKKMDSRLWSCL
HLDGQSFYIRCNITMDIIRCEAATTAVAVPPPDLHRHLAALLGSGVGADVRIRVGGKLFAAHKNVLAARSPVFMAELFGN
NGGKDQKEAKAAAAATGNGVIRIDDMDLRVFRAMLQFIYTDTLPKIDKGDTAFMAQNLLVAAHRYGIERLKSISVDMIRK
AARTMNLIAMIIRNNDEGIATQLRTRGRLGLHLPDAPEVNRAQIFDTGRG*
>Osat_LOC_Os12g02030
MDCCVCSPMASMYRLPRNAICAACYEGAKAIIAFFNDDDDEHADADQGSVKPSRLTKLNSTTKGLRDAWEEVKQMRCREE
ETKQRASFLQQGFAAAWKDGIHTDIAVRPGTGPPIQAHKAILATRSEVFRHILAGDDDCKAPAGDSLSLPELTHDELSHL
LAFLYTGSLATCTEERHLHALLVAGDKYDVPFLRRACEARLAAGVEAGNVLRTLEVAELSSSAALKERAMGAVVEHAEEV
VFSPEYEEFAVRNAALCVQITRALLANKAFPAKTP*

>Osat_LOC_Os12g04410
MSSEDSLKSLSLDYLNLLINGQAFSDVAFSVEGRLVHAHRCVLAARSLFFRKLFCGLDPNHQPPPPPPPPLNWPTAGGGG
GGSGGGGRGGAGGGGGAPATPELVIPVSSIRYEVLVLVLQFLYSGQASVAAPKSGPLPGCGARGCWHTRCGAAVDLALDT
LAAARSFGVEQLALLVQKQLESMVKEASVDDVMKVLMASRKFEMQELWATCSHLVARSGLSADLLAKHLPIDVVAKIEEI
RAKSPLAAAAAPRSPFLTHHYLPMNPASSAADRDNKIRRMRRALDAADIELVKLMVMGEGLDLDDALAVHYAVQHCNRDV
VKALLELGAADVNSRAGPTGKTALHLAAEMVSPDMVSVLLDHHADPNSRTLDGVTPLDVLRSLTSEFLFKGAVPGLTHIE
PNKLRLCLELVQSAVMVTTRDDGAPVTGGEAGGSDGGNFPRSDADDSLVSLTMNSTLMYQGQEMAAAVAAGEGRKSNNGR
GSPPPAMYFPNGFA*
>Osat_LOC_Os12g39380
MKYMKLGSKPDVFQTEGNNIRFVATELATDIVIIVGEVKFYLHKFPLLSKSSRLQTLVASTNEESNDEIDISDIPGGPAA
FEICAKFCYGMIVTLNAYNVLAARCAAEYLEMFETIDKGNLIYKIDVFLSSSIFRTWKDSIIVLQTTKSLLPWSENLKVI
NHCVDSIATKASIDPSEVEWSYTYNRRKLPSENGLDSHWNGVRKQQMVPRDWWVEDLCDLEMCLYKKVIMAIKAKGRISS
EVIGEALRAYAHRRLFSSLESAVSNGLDCTRHSAALETIISLLPSEEGSVPCSFLLKLLRASCLLGSDEACRDNLTKRIG
AKLDEASVSDLLIPANSDEAAMYNVDMISAMLEEFMAQHREDDDGAKLQEDDDQEAMDGDDDNLNGVSRSSKLAIAKLVD
GYLAEIAKDPNLPLSKFIALTEMVPLATRPVHDGLYRAIDMYLKEHPGLTKGEKKRLCGLMDCKKLSPEASMHAVQNERL
PLRVVVQVLFFEQVRAASSASAAAAADMPPAARSLLPREQDGNSYGSSRSTATEDDQWAPPPTTTSVDVTSFRSMSLANN
KNGGVSGGGGDQAACKKPPPSSASAKGSGGGGLMPKKILSKLWSGKASSGENSSSDTSESPGEETRSTPSRNTRHSVS*
>Osat_LOC_Os12g41910
MGSMKLSSKPDAFTRRGQAWYCTTGLPSDVLVEVGEMSFHLHKFPLLSKSAILGRLIEENSDSDECVIILSDIPGGAKSF
ELVARFCYGLKIELSSENVVYLRCAAEHLEMTEETSGDNLINQTESFFNQVVLRSWKDSLEALRTCDGLLPHAEDLHIVK
RCIESLAGKASIDPDLFGWPVSEHSTMQSPGGSVLWNGISTGAKVRNCSSDWWYDDASSLSFPTYKRLISSMESRGIKQE
IIAGSLTYYAKKFLPGLNRRQSTGPMPLPAATLSDEEQRRLLEEIDRMLPLQRGLISTNVLLWLLRTAMILKVDRACISN
LEKRVGMQLDEATLEDLLLPNFSYTMDTLYNVECVHRILDHFLAMDQTMGGGGASPCLDDVMASPSLAPITAVAKLIDGY
LAEIAPDINLKPLKFQSLAAVLPEYARPLDDGLYRAIDVYLKSHPTLPEAEREQLCRLIDCQKLSLEACTHAAQNERLPL
RVVVQVLFFEQLQLRTSVAGCLLVSDNLEASSRTLRGGGAVAASGEAGCWATAAVRENQALRVGMDSMRLRLMELERECS
DMRQDIRKLGRGRRRLRGGGGIVAGGKDGGGWAARVQRMLTPRMMKLQMCSAQHDAAEQQRMNNEHKKVEKVAKNKKQLS
MDDGDDEEEA*
>Smol_XP_002993488
ERREVDKGVLTVHDMNPDIFAPFLDFLYTAQVSNKVLEKNASDLLAAAHMYSIPSLKSICEGFICQQMSRDNAVAMVELA
CKYDAVSIREAAYDAVTMTYRDILACPNFKKLLEREPRIAADMLKEILLRTTA
>Smol_XP_002990974
MFLPKLADPPINPNFKPVDYVSTLAEIHKELGVATSNQEKSRLYLEQSFVFRGLGELKLLRRSLRLARQHATTNHHKLVI
AAWLKFERRGEELDENPGESASHRAAATSTRLADHIPCLALDYCDEDEQQQQQQHERWHFSDVVFHVEGDRIYCNRQKM
AALSLPFDAMLNGCFTESRRTNIEFSRNGISAMGMRAVDKFARTGTIGRPSPTVVIEVMTFANKFFCDKLKEACDQRLAT
CVHTLQDAITFLDCALDENAQSLVGACLQVFLRELPKSLYIAPVSKLFSTQDGRKRLSAVGHSSFSLYCLLGQAAMDDDF
SSDMTVKLLHFAKDCAVSSRQRALALHQLGCSMLARKQYKEAHEFFEAAADEGHIYSLAGVARVKYMRGHRMAAYSEAAS
IIACYKNSGWMFEEKSLYCLGHDKLSDLNTATELDPTLTYPYKYRAAVLMDEKKVHEAITEISRVLRFCITKDCLELRVY
FSLALLDYEAAVRDLRALLTFDPGYRMYSGRVCASQLLDLLKQHVVQWTKADCWMKLYDHWSSVDDISSLAVVHQMLETE
AESSKGLLFFRQSLLLLRLSCPKAALRSLRLAREHADNNQEKLVYEGWLLYDTGHRQEALRKAEESISLQRSFEAFFLKA
YALADTSLDPTASTKVIGLLEEALKCPSDGLRKGQALNNLGSVYVDCGKLEQALDCYVNALKIRHTRAHQGLARVYFLQG
DRKSAFDEMTKLIEKSMNNASAYEKRAEYCDRDLVMADLSMVTQIDPLRTYPYRYRAAVAMDSQRDREAIAELSKAIAFK
ADLQLLHLRAAFHECSGEISDALRDCRAALSIDPTHSDTLELYGRVQHLS
>Smol_XP_002988207
MKLGSKVDGFERQGQAWFVATELQSDLVVQVDDLKFHLHKFPLLSRSGRLNRLVFESRDTEKDHIDLTGIPGGPDSFELA
AKFCYGMAIDLTAANVAGLRCAAEYLEMTEDLEEGNLISKTEAFLSFMVLGSWKDSLTVLKCCEKLSPWSENLQLVRRCS
ESIAWKACTDTRGINWSTIGKESSSRKSDSPESNEMRSSEDSKSVPADWWFEDLLTLSVDHFLKVIAAIKVKGMRSDLLG
AVVTSYVLKWVPGLSRDTGYFNAENNIHMTASTGQDDFTVLQNKTQKLLENIVGILPLQQDTVSCSFLLRLLRIANMHGT
STACRAELEKRVGMQLENASLADLLVPSFLHTCETLYDVDLMQRLLQYFLLQEQVTQASPSTLEQQLGFDGAMRSAKVKV
AKLLDGYLSEVARDQNLSLAKFQALVEAFPDSVRTSDDGLYKAIDTYLKVHPGLTELERKRLCRGMDCQRLSSDACLHAS
QNERLPLRVVVQVLFSEQVKLKNALSPVFGKDMDGWDNPQQGDGFHPLVSDSWAQELKAVKADLEQVKEKLAEFQSEYSL
VRQQLQKLLGPKEKNQHSSSSSSWSMGWKKLRRASNLFHRDGMDDPEQRPSYVVQKDAIDNVAKPRLRRNSLS
>Smol_XP_002987220
KSPVFKAMFSAQMKEAQSGVVRIDDFSHDVLEAFVRFFYTATVCPEVLKKHAASLFCAAEKYGVKLLKAVCEEFLVSNVS
RDNAINLLDLARKYDSETVKDAVLRTASMDMQVLPTFGDYSMYAEKDPKLLVELYEGLVKRMSRKRSRIVAGSSTLTLVS
NF
>Smol_XP_002986952
MNLEEPLKQLSSDLLNLLANGQAFSDVTFTVEGRPVYAHKCVLAARSQFFRMIFCSSEASQDIPGRPPIPVGIVGYDVFM
LMLQFLYSGQLSLVPPHPTGCKEGACWHVYCRSAVDFALEALHAAQVFSIEQLSILVQRELAGIAEKASIEDVMRILAAA
RKQDLLHLWSVCSKLVAKSGLSSEVLRKHLPGEVVAEVEAIRQKCGYGFEAHSSDALDDQRTRRMQKALDSSDVELVRLM
VMGEGLDLDKTLAIHYAVANCSRKVVKNLLELGAANVNMPGPDGRTPLHIAGELADPEMIAVLLDHHADPHSTTPTGATA
LNILQNLASEALAVGALTGVTADHNKLRLCLDLLESAAAVPASCSRDEDDSSSSMLTEQQQQRERRLPMMIGQPVTTPSS
TQQSDPPAILMHQNYYPVITAKGTCQNSQQTSHQP

>Smol_XP_002986835
MDCCVCSSVVVGTIAYRPPRNTICAGCLEGAQSILSVSSRIEQNWKLGEDDRQLSSKIMLLHASKRIEHLELMEREASRK
LRFLESLAVGFREGIHTDVELVANDGHSIRAHRIVLATKSSFFKVLFEVDACKSEHSGAVMVPELLNHSQLENFIEFLYS
ARISDDDLSKNSASLLVAADKYDIPALARTCEEYLTSSVTSSNVLEMLELATLCSAEALKESAVKTVIDDYQTILFSKEY
EEFALRSSMVALEISRAVISHLSSLREEIDKL
>Smol_XP_002985255
MARAPAVALQQEGVVSSTPSRSRSVTETKNGSHHFTINGYSLAKGMGVGKYIASNTFTVGGYQWAIYFYPDGKNAEDNSL
YVSVFIALASDGTDVRALFELTLVDQSGKGKHKVHSHFDRSLEGGPYTLKYRGSMWGYKRFFRREALEMSDYLNNDSLDI
TCTVGVVMSWTQVTKIYSIQPPEQDIGKHFGALFESGEGADLTFDADGETFKAHKLVLAARSPVFKAQLMGPLRERTSDV
LRVEDILAPVFKAMLQFMYTDALPDTAELSGVSSSSSPTLLYQHLLAAADRYGLDRLRLLCEAKLCETVSVDTVATTLAL
AELHHASQLKGVCLKYAADNLSAVMQSEGYDYLRESCPVLLFDLLKTVAGVGKDLFVGSGGKGGYIWGQLSDGGDVNGRR
VRQKT
>Smol_XP_002983725
MAVTSSKRVGRNSFSMRRTNDWVMSTDVPTDITVDICGTSFALHKFPLVSRSGRLRKLVAEARDSDLSRVEFNDIPGGPE
GFEMAAKFCYGINFEITASNVAILRCVAEYLEMTDDFGQANLASRSEAFIEDVVTHSFGSSITVLHSCEKLLPMAEQLNI
VNRCVQAAGAKASHEALSLAARPEYRNAKTPPQLDWWAEDLSILSLDFYQRVLAVMRSHGMPQENLWGSLMHYAQQALKG
FNSTSSKSPGRSVTGLEHEQRMLVETIVGLLPPDKRSASCGFLFGLLRTSIILDTTVACRLELERRISLQLDEATLDDLL
IPSFSFAADTLFDTDLVHRLLSSFLQHHLQDESKASQSSLAKVAKLMDSYLAEVAPDANLKVSKFIALAEVLPEHSRMVE
DGLYRAVDIFLKAHPLLVEADRKRICKLMDPQRLSQEACSHAAQNERLPAHFIIQVLYFEQLRIKTALANYAIEMPKGDH
GPTPIAAALRSSTAAANAAAAAAADANPQSALRRENKELKIELTRMKIRLSDLEKEQSPGVSRASEEKRLSSPTSSAASS
PGSGRFFYTVSRRLGKLNPFGKSSLKPPATPELQKPRRRRHSIS
>Smol_XP_002983257
MDTDTCSGGTSPSFAFAFNDSNFSDRVLHIEIVAGASGEAKSSGEPCSTVGAWARQKKRRRGADAKDKGTLFSCLFFCLS
FEEQIMTGTQPEPDDAEEEGDVMIEESPTSMAGNQDQMQSTSSWNSESSTVLRVKTIHISSAILAAKSRFFYKLFSNGMR
ESEQKAVTLRITDSEEVPVMDMLQFMYTGGLQANTAPALLDVLVAADKFEVATCMRHCSRLLRELQMTPESALVYLDLPS
SVLLPDAVQSLTDAAKAFLADRYKDIGRFHEDVMHLPLAGLEAVLSSDDLQVASEDAVYDFVLKWARAHYAKIEERREIF
RSKLVWLIRFPMMSSRKLRKVLVCNDIEHDLASKLVMEALSFKAEPPHRQKHIMVEEIVHKRFSERAYKYRPVKVVEFDA
PYQQCLVFLDLRKEECLTLWPQGRVYSQAFHLGGQCFFLSAHCSVDQQTAAHCFGLFLGMQEKGSVSFAVEYEFAARSKQ
EEWKFAPRVKGNYVFTGGKAVGYRNLFSCPWQELMKDDSDYFHQNVLHLRAELTIKPQGQGM
>Smol_XP_002983215
MTSDVPTDISVEVAGTSFALHKFPLVSRSGRIRKLVVETRDLDPSQIKLLEIPGGAEVFELVAKFCYGINFEITTANVAV
LRCAAHYLEMTDDYGEGNLVVRTETYLNEVVIQDFSVSLAVLHNCENLVPLAEELKIVSRCVDAVAAIATASLPSREQLS
SSMEYNGSSGWIDSQVQSCKTDHWWSEDLAILRIDFYQRTLAAMMSRGLLHESIGGALIIYAQRYLKGLNMKPEQRALVE
TLTSMLPCEKNSISCSFLFGLLRSAIILDASISCRIELEQRIGMQMDQAALDDLLIPSMSFTCDTLFDVDVVQRLVINFL
QHKEKEDSQAGGYESESTSPPSQSAAIKVAKLIDNYLAEIAPDTNLKLLKFYALAELVPDYARVVNDGLYRAIDIYLKAH
SNLSEGERKKICKLMDVQKLSQEACSHAAQNERLPVQVVVQVLYFEQVRLRAAMAASMMEGDNLGHFHHKLGSGALSAAI
SPRDHYASIRRENRELKLEVARMRMRLTELEKEHVCMKQEMEKTSSTAHNFLHSVSRRLSRLNPFGSRNQSHVAAPQTPD
SRVSSRRRRHSIS
>Smol_XP_002982871
MGICKDIHILHVASSEKPAPTAASKPVHQKDSSQSMFLPKLADPPINPNFKPVDYVSTLAEIHKELGVATSNQEKSRLYL
EQSFVFRGLGELKLLRRSLRLARQHATTNHHKLVIAAWLKFERRGEELDENPGESASHRAAATSTRLADHIPCLALDYCD
EDEQQQQQQHERWHFSDVVFHVEGDRIYCNRQKMAALSLPFDAMLNGCFTESRRTNIEFSRNGISAMGMRAVDKFARTGT
IGRPSPTVVIEVMTFANKFFCDKLKEACDQRLATCVHTLQDAITFLDCALDENAQSLVGACLQVFLRELPKSLYSAPVSK
LFSTQDGRKRLSAVGHSSFSLYCLLGQAAMDDDFSSDMTVKLLHFAKDCAVSSKQRALALHQLGCSMLARKQYKEAHEFF
EAAADEGHIYSLAGVARVKYMRGHRMAAYSEAANIIACYKNSGWMFEERSLYCLGHDKLSDLNTATELDPTLTYPYKYRA
AVLMDEKKVHEAITEISRVLRFCITKDCLELRVYFSLALLDYDAAVRDLRALLTFDPGYRMYSGRVCASQLLDLLKQHVV
QWTKADCWMKLYDHWSSVDDISSLAVVHQMLETEAESSKGLLFFRQSLLLLRLSCPKAALRSLRLAREHADNNQEKLVYE
GWLLYDTGHRQEALRKAEESISLQRSFEAFFLKAYALADTSLDPTASTKVIGLLEEALKCPSDGLRKGQALNNLGSVYVD
CGKLEQALDCYVNALKIRHTRAHQGLARVYFLQGDRKSAFDEMTKLIEKSMNNASAYEKRAEYCDRDLVMADLSMVTQID
PLRTYPYRYRAAGPVAMDSQRDREAIAELSKAIAFKADLQLLHLRAAFHECSGEISDALRDCRAALSIDPTHSDTLELYG
RVQHLS
>Smol_XP_002980693
MARDPEEGATAAANPSIAASSRSRSVTETVNGSHHFTINGYSLAKGMGVGKYISSNTFSVGNYQWAVYFYPDGKNTEDSS
LYVSVFIALASEGTDVRALFELTLLDQSGKDKHKVHSHFDRSLESGPYTLKYRGSMWGYKRFFRRAVLETSDYLKDDSLS
ITCTVGVVVSSMQALRQQSLPVPESDIGRHFGALLESGEGSDITFEVDGEVFHAHKMVLAARSPVFRAQLNGPLSDSNVK
LLQLEDIKAPVFEAMLYFIYRDALPDASEVLSSSSYSSTSLASTMMAQHLLAAADRYGLDRLRIVCEAKLCEDVSIDTVA
TTLALAEQHHATQLKRVCLKFAASNLAAVIQSDGFDYLKESCPSLQSELLRTVAGVEGGDDDELRPGSKRRSVWTHLADG
TDGTGRRVRVKQP
>Smol_XP_002978524
FVATELQSDLVVEVDDLKFHLHKFPLLSRSGRLNRLVFESRDTETDEIKLVGVPGGPEAFELAARFCYGMPIDLNASNVA
ALRCAAEYLEMVEELEEGNLIAKTEAFLALVVLASWKDTLTVLLSCEKLHPWAEDLQIVRRCSESIAWKACTDPKGLNNW
PTITGGKKNVVCPSSYIAKDGRNLALKQVPQDWWFEDVSMLCIDHFTKVLTAIKAKGMKSELVGAVIMCYALKWIPTLSN

TCHESSKTGRAVATSSPAIVTKEDFTWLQARSKLVVESMVGLLPGQKNSVSCGFLLQMLRIASMLNASGPCKVELEKRIA
IQLEQATLGDLLIPSAPHVSDTRYDVGLVQRLVEYFLAHEEETSPDRSPDAETPSSIDKAKLSFDNKSSSISVLNEHSSG
SKMKVAKLLDSYLAEVAHDPQLPLTKFQALADALPVYSRACHDGLYRAIDMYLKAHPTATEQDRKRLCRIMDCQRLSSEA
RLHASQNERLPLRTVVQVLFSEHLKLKNIYGYGSFGGGGTQVTPPYSEATWETASQDIRSLKKDIEKIKEQFLELRTGYN
HIKQDLQKLLKPSKRSSASSSSWGCGLKRLGSTLASPRFFHYSSRDDGPSTPDIFKSRRWR
>Smol_XP_002977926
MNLEEPLKQLSSDLLNLLANGQAFSDVTFTVEGRPVYAHKCVLAARSQFFRMIFCSSEASQDIPGRPPIPVGIVGYDVFM
LMLQFLYSGQLSLVPPHPTGCKEGACWHVYCRSAVDFALEALHAAQVFSIEQLSILVQRELAGIAEKASIEDVMRILAAA
RKQDLLHLWSVCSKLVAKSGLSSEVLRKHLPGEVVAEVEAIRQKCGYGFEAHSSDALDDQRTRRMQKALDSSDVELVRLM
VMGEGLDLDKTLAIHYAVANCSRKVVKNLLELGAANVNMPGPDGRTPLHIAGELADPEMIAVLLDHHADPHSTTPTGATA
LNILQNLASEALAVGALTGVTADHNKLRLCLDLLESAAAVPASCSRDEDDSSSSMLTEQQQRERRLPMMIGQPVSTPSS
TQQSDPPTILMHQNYYPVISAKGTCQNSQQTSHQP
>Smol_XP_002976378
KSPVFKAMFSAQMKEAQSGVVRIDDFSHDVLEAFVRFFYTATVCPEVLKKHAASLFCAAEKYGVKLLKAVCEEFLVSNVS
RDNAINLLDLARKYDSETVKDAVLRTASMDMQVLPTFGDYSMYVEKDPKLLVELYEGLVKR
>Smol_XP_002973958
VATELQSDIVVEVKDVRFHLHKFPLLSRSGLLNRLVFESRDTQKNRIRLDEVPGGPEAFELAAKFCYGMPVDMDASNVAA
LWCAAVHLEMTEDLEDGNLVSKAEAFFNTVVLGSWKDSVTVLLSCGKIHAWAERLMIVQRCTESIAWKACTDPRGVRSSH
GSRMGSRAQSPSRRSMDLSSSNKQELMKEWWFDDLLKLPVDYFKKVMAAVRVKGMKLELIAGAVALYALSWLPRRSEIEY
DQHSFRGKGRLLLEQVLSMLPRGKDLVPSRFLMHLIRMAHLLEADLPCKIDLEQRAGTQLDSAALSDLLVPALDASSCCG
LKVDVDLVQRLVEHFVRQDEIVTSNYGSGNSVVLDHRVRVATLVDNYLAEISQDRNLSVARFKALAEALPDSYRASHDYL
YTAINIFLKVHPELKDEERKDLCQLLDCKRLSLHSSMHAAQNERLPLRTVMQILFFEHLKLKNS
>Smol_XP_002971221
MARTSVVLQDDSGQVVGSPTSTATPSRSRCITETVNGSHHFTIHGYSLAKGMGVGKYIASDTFTVGGYQWAIYFYPDGKN
TEDNSLYVSVFIALASEGTDVRALFELTLLDQSGKNKHKIHSHFDRSLESGPYTLKYRGSMWGYKRFFRRAVLETSDFLK
DDSLSITCTVGVVVSSMQALKQHSLLVPESDIGQHFLSLLESGEGTDVNFNVKGEAFSAHKLLLAARSPVFKAQLFGPMK
DENGDVIEIDDMEPPVFKAMLHFIYKDSLPDTNEMTGSSSQSTATMMAQHLLAAADRFCLDRLRLLCESRLCEQITVDTV
ATTLALADQHHASQLKNVCLKFAASNLAVVMQSDGFEYLRESCPSLQSELLKTVAGVEEEAKAGTKNRTVWTHVADGGDG
LGRRVRQKI
>Smol_XP_002971080
MASVDPSESKAVVVGLERKGLKRKLAETSLAQEPQDGPGTRLLSDAEVLLETLNTSTTWRENDRFAARQAAHALAELAKH
EDHVDLIVDKGVVQALVPFLSAPVLEEGEGPIAYEHEVEKDAAFALGLLAVRPEHQRLIADAGALPSLVSLLKRRVTGQN
ARVVNGLVRRAADAITNLAHENGSIKTRVRAEGGIPPLVELLESNDPKVQRAVAGALRTLAFKNEANKNQIVEYNALPTL
IFMLRSEDVGIHYEAVGVIGNLVHSSSNIKKEVLAAGALQPVIGLLSSSRCQESQREAALLLGQFATADPDCKVHIVQRG
AVRPLIRMLEAADPQLREMAGFALGRLAQNTHNQAGIVHDGGLRPLLDLLDSKNGSLQHNAAFALYGLADNEDNVSDIVK
EGGVQSLQDGELIVQASKECVAKTLKRLEEKLHGRVLKHLLYLMRQPDKLVQKRVALTLAHLCTPEDQRLIFHENNGMNI
LLEMLGSFSSPKQQRDGALALTTLAKKATGLSPVDSAPAPETPQVYLGGKYVNNSTLSDVTFLVEGRRFYAHRIALLASS
DAFRAMFDGGYKEKEAKDIEIPNISWKVFEMMMRFIYEGQAEIGSDIAQDLLRAADQYLLENLKQKCEESIAQDLTVENV
ANVFELGEAFHAVSLRHTCVLFILEQHSQLCTLPGYQNLIRRITPEILEYMHRILRPRPTTS
>Smol_XP_002970915
MADERLEPVPRLLEGDQALPIADPQELPPKLESLESVDLSGSLGKESLLSAGAAASSPRSSASAASPRLDQVKPSVEVVG
LSDDAGHDLRLDQATFFRCVEDGNVTALEGLLKNRKLDVNAYNDDGMTALHLAVYKYEQSRNLEMVDLLIKHGASVCAKA
AGTPATHRISIIRHDVKHTGVLETRKVDFHQKPPLLVVLELKSSLYLKGWEYRHWDSMLKLLAEATIRHYVANNVKEPTV
HENAIPEIIQKNWKSVFHSGKHETIELWAEGRHIEALKLLLSGASKILKLNIESNSSRIDMKDASFNIVKAMVEFLYTGH
LDASLIEQRGIDLFVAAHKYGVDVLKLRSEAAIVATPDNWIKLLSVATECNSDVLAWKCATSIKNLMDTRQDRGHTLRHS
FSESHEAPQQLFQPSA
>Smol_XP_002970451
MKKLRFLLVTTASRGKAKRGNFFCSSSLIPFRASELFSFVVFFPSPRAHKCRFRFVATELQSDLVVEVDDLKFHLHKFPL
LSRSGRLNRLVFESRDTETDEIKLVGVPGGPEAFELAARFCYGMPIDLNASNVAALRCAAEYLEMVEELEEGNLIAKTEA
FLALVVLASWKDTLTVLLSCEKLHPWAEDLQIVRRCSESIAWKACTDPKGLNNWPTITGGKKNFVSPSSYIAKDGRNLAL
KQVPQDWWFEDVCMLCIDHFTKVLTAIKAKGMKSELVGAVIMCYALKWIPTLSNGCHESSKTGRAVATSSPAIVTKEDFT
WLQARSKLVVESMVGLLPGQKNSVSCGFLLQMLRIASMLNASGPCKVELEKRIAIQLEQATLGDLLIPSAPHVSDTRYDV
GLVQRLVEYFLAHEEETSPDRSPDAETPSSHSSGSKMKVAKLLDSYLAEVAHDPQLPLTKFQALADALPVYSRACHDGLY
RAIDMYLKAHPTATEQDRKRLCRIMDCQRLSSEARLHASQNERLPLRTVVQVLFSEHLKLKNIYGYGSFGGGGTQVTPPY
SEATWETASQDIRSLKKDIEKIKEQFLELRTGYNHIKQDLQKLLKPSKRSSASSSSWGCGLKRLGSTLASPRFFHYSSRD
DGPSTPDIFKSRRWR
>Smol_XP_002969653
MADERLEPVPRLLEGDQALPIADPQELPPKLESLESVDLSGSLGKESLLSAGAAASSPRLSASAASPRLDQVKPSVEVVG
LSDDAGHDLRLDQATFFRCVEDGNVTALEGLLKNRKLDVNAYNDDGMTALHLAVYKYEQSRNLEMVDLLIKHGASVCAKA
AGTPATHRISIIRHDVKHTGWEYRHWDSMLKLLAEATIRHYVANNVKEPTVHENAIPEIIQKNWKSVFHSGKHETIELWA
EGRHIEALKLLLSGASKILKLNIESNSSRIDMKDASFNIVKAMVEFLYTGHLDASLIEQRGIDLFVAAHKYGVDVLKLRS
EAAIVATPDNWIKLLSVATECNSDVLAWKCATSIKNLMDTRQDRGHTLRHSFSESHEAPQQLFQPSA

>Smol_XP_002969487
MSLEDSEPLKQLAEDFSNLLDNGQAFSDVAFAIDGRHVYAHKCVLAARSRFFRMVLTSNGPTQAPLVMPVSAVGHDAFML
TLRFLYSGQLCLLPSNSQPDRGCKESSCWHSQCRAAVDFALEALHAAQMFGIDELSVLVQKELAAMAEKASIEDAMRILV
TAREQDLLQLWSVCSKLIAKSGLSTEALEKHVPVEIAAEIEAIRHKCGYYNASRADCSDSLDEQRTRRMQKALDSSDVEL
VKLLVMEEGLSLDKTFALHYAVAHCSRKVVSILLQLGAADVNAVDVEGRTPLHIAGELADPEMIAVLLDHHASPHVRSPA
GTTALDMVQSHVFQALTLASEGGAPADHSKLKLCLELLQTAEPVSSQERIVQDEGNCLLMAPGRNNDFYPAPKGTQ
>Smol_XP_002969279
MDCCVCSSVVVGTIAYRPPRNTICAGCLEGAQSILSVSSRIEQNWKLGEDDRQLSSKIMLLHASKRIEHLELMEREASRK
LRFLESLAVGFREGIHTDVELVANDGHSIRAHRIVLATKSSFFKVLFEVDACKSEHSGAVMVPELLNHSQLENFIEFLYS
ARISDDDLSKNSASLLVAADKYDIPALARTCEEYLTSSVTSSNALEMLELATLCSAEALKESAVKTVIDDYQTILFSKEY
EEFALRSSMVALEISRAVISHLSSLRKEIDKL
>Smol_XP_002969106
MSTIKLGANANLSEAKGRSWFVALRFSFVDGLDFSGFRCVATELQSDIVVEVKDVRFHLHKFPLLSRSGLLNRLVFESRD
TQKNRIRLDEVPGGPEAFELAAKFCYGMPVDMDASNVAALWCAAVHLEMTEDLEDGNLVSKAEAFFNTVVLGSWKDSVTV
LLSCGKIHAWAERLMIVQRCTESIAWKACTDPRGVRSSHGSRMGSRAQSPSRRSMDLSSSNKQELMKEWWFDDLLKLPVD
YFKKVMAAIRVKGMKLELIAGAVALYALSWLPRRSEIEYDQHSFRGKGRLLLEQVLSMLPRGKDLVPSRFLMHLIRMAHL
LEADLPCKIDLEQRAGTQLDSAALSDLLVPALDASSCCGLKVDVDLVQRLVEHFVRQDEIVTSNYGSGNSVVLDHRVRVA
TLVDNYLAEISQDRNLSVARFKALAEALPDSYRASHDYLYTAINIFLKVHPELKDEERKDLCQLLDCKRLSLHSSMHAAQ
NERLPLRTVMQILFFEHLKLKNSSQANQKLPMEVPKIEGGGTAALQQEIRTIRCELEQMKADFSEFQNHFRSLKLEKQNS
DVRSKLKKLGVRIY
>Smol_XP_002964957
MVAAAEDDLDKQHADEGYDELDSYIRRKKKVPCGDVYEAARAGDIDRLKILLESGINVNARDEWDSVALYYACLAGHEDA
ARILLEGGAICSEHTFDGDRCHYAALNLRVRRLLKLFEARPPPLAPLPDSLRALFFRSGQNHRFLDPTLGDTGNFDADEL
GDPVGPDIVFYAQGQPIAAHRAILSARCRFFQRKFEKEWKSRSEIRFSRNRLSFHALFRLISFFYCDVLDVAVDDMEDLL
RICKVCGCSGLQRVLEKELVHQKFADYKSLNTDDDSLKRFIMQGSSLPDEERLTWAMDRLFSLLKHKSFGEEKVDGGQDF
ADICFLVEDTSFRCHRAIAEELLDVASRYLLFPLRRAVADAVYPHLENASPAELCKWLLVADMYGVTRIREFCLDTIAVN
FESFAATSEFKALLQRLPPPSGDTSTRTTIPNAPGGVKDDSQGNLLDDLRERWLEAEGAELDQRDESARDFDKVLELLRS
TAENDDTESLELV
>Smol_XP_002963496
MKLGSKVDGFERQGQAWFVATELQSDLVVQVDDLKFHLHKFPLLSRSGRLNRLVFESRDTEKDHIDLTGIPGGPDSFELA
AKFCYGMAIDLTAANVAGLRCAAEYLEMTEDLEEGNLISKTEAFLSFMVLGSWKDSLTVLKCCEKLSPWSENLQLVRRCS
ESIAWKACTDTRGINWSTIGKESSSRKSDSPESNEMRSSEDSKSVPADWWFEDLLTLSVDHFLKVIAAIKVKGMRSDLLG
AVVTSYVLKWVPGLSRDTGYFNAENNIHMTASTGQDDFTVLQNKTQKLLENIVGILPLQQDTVSCSFLLRLLRIANMHGT
STACRAELEKRVGMQLENASLADLLVPSFLHTCETLYDVDLMQRLLQYFLLQEQVTQASPSTLEQQLGFDGAMRSAKVKV
AKLLDGYLSEVARDQNLPLAKFQALVEAFPDSVRTSDDGLYKAIDTYLKVHPGLTELERKRLCRGMDCQRLSSDACLHAS
QNERLPLRVVVQVLFSEQVKLKNALSPVFGKDMDGWDNPQQGDGFHPLVSDSWAQELKAVKADLEQVKEKLAEFQSEYSL
VRQQLQKLLGPKEKNQHSSSSSSWSMGWKKLRRASNLFHKDGMDDPEQRPSYVVQKDAIDNVAKPRLRRNSLS
>Smol_XP_002963329
MDLADGLGGEVIPCAADEEVSVCYQCGQLCENNFTYCHSCFEQRSQSDFRNEQRWRNTESSLQSAELVLQRERANIRRLE
ATVSAFRAMLLQAIHPDVTIETGDGDTTPAHRAVLASRSPVFNAMFEHELKEKTCAVIRVADVPTPAMRALLLYLYTGEH
DTKVMKDHGMALLAAAHKYDIPDLKKICETAVSVHASNALEVLQNARLYDANHLKRVCIECIARNIKQLAFADEFRQLVF
KNEDPEAVLDIIQTLAHSC
>Smol_XP_002962973
MQSQPSQIHLTFSLNVEILSRLAQWRIDSLTPNLCWKSNNFKMGVWTWRLQIKKCGPLHVWLFKEHSDHPSDQVPTVAFV
LRVLHGPGLRQVVQSQAVNDKTFLTNEYHAWSVETAIHGKFVVEAEFIDMKCGSQAGDPFYFTLGKSIQPAAHQAALSCL
SYMLDSGVHTDVTITTPSGSIGAHLAILATRSRVFQVMFSHDLRERKTFTIEMEDMSLESLKALLAYLYGSINRPAFHKH
RMALLAASDKYDIEDLKQACEESLMEDINSDNVLERLHNAWMFHLPNLKKDCMRYLFEFGKIHDIRANFDQFLHSTDKEL
LIEMFQEGLNAVHF
>Smol_XP_002962812
MKLVENNVLVVPPFDCAWLFEEELRFPEAGRGCVAFEARAENDVTVIFKELAGCKHYRTDSDPNYTVIIGSNLNKRLKIA
ANGRTVVDVAGPVVSPLVFERYWISVDEGSIVVGKGELGQNVVYQWVDPEPNCKVQFVGLSSWDKHVGYRNISVAALPPC
LYRTTSAIDAQWSTGLANFLESGDLSDFEFVLFPENRRVCAHRAVLVTGSSSKECPFLTTTELELHGVDYLVLHAALRYV
YTGKAEIGKEHLVQLRGLAELIGFQNLAAQCKQHIREVSYDSSGDLVLLTMHCYDTNSPVHVNPVAFTRMLSTGDLSDLE
VYLEDYDRCLQLHRLVLSVWSAPFAKMFTIGMKESNRTRVTIKDVHLDAFMTTIRFMYTGRLDLEGKDNAGSLLLPLLIM
ADRYGVRVLHQECLDHLLACVNEDSSCAILQVASSMARCETLRQACEESCAKHFDYCVAASATEFTELDGQSVMRILQHP
DLLVTSEEKVLDAVFMWGSKMEGVQCWEEADTKYKAGLQVICPDREEDLKVLLPLIRFPLMSMELLQQLDSSALCENLPL
LRQMVGEACDYLKQESFVSDDEKDLIRSSNQGRFISLPKNGLWFTRRLSSFKELNYLCDGDGNGVLYYAGTSYGAHEWMN
PMVTKQITLSASSPHSRYTDPKVLASRNYQATSFAGPCIERGETVSWWRVDLGPDHKLMCNYYSVRQDGSTNFARNWTFQ
GSGDGETWTDLRKHEKDHSIYRPAQYASWPVHGSKSLIPFRFFRVLLQQPIAAVAAPWNLSICYLELYGYLH
>Smol_XP_002962007
MASVDPSESKAVVVGLERKGLKRKLAETSLAQEPQDGPGTRLLSDAEVLLETLNTSTTWRENDRFAARQAAHALAELAKH
EDHVDLIVDKGVVQALVPFLSAPVLEEGEGPIAYEHEVEKDAAFALGLLAVRPEHQRLIADAGALPSLVSLLKRRVTGQN

ARVVNGLVRRAADAITNLAHENGSIKTRVRAEGGIPPLVELLESNDPKVQRAVAGALRTLAFKNEANKNQIVEYNALPTL
IFMLRSEDVGIHYEAVGVIGNLVHSSSNIKKEVLAAGALQPVIGLLSSRCQESQREAALLLGQFATADPDCKVHIVQRGA
VRPLIRMLEAADPQLREMAGFALGRLAQNTHNQAGIVHDGGLRPLLDLLDSKNGSLQHNAAFALYGLADNEDNVSDIVKE
GGVQSLQDGELIVQASKECVAKTLKRLEEKLHGRVLKHLLYLMRQPDKLVQKRVALTLAHLCTPEDQRLIFHENNGMNIL
LEMLGSFSSPKQQRDGALALTTLAKKATGLSPVDSAPAPETPQVYLGGKYVNNSTLSDVTFLVEGRRFYAHRIALLASSD
AFRAMFDGGYKEKEAKDIEIPNISWKVFEMMMRFIYEGQAEIGSDIAQDLLRAADQYLLENLKQKCEESIAQDLTVENVA
NVFELGEAFHAVSLRHTCVLFILEQHSQLCTLPGYQNLIRRITPEILEYMHRILRPRPTTS
>Smol_XP_002961253
MSLEDSEPLKQLAEDFSNLLDNGQAFSDVAFAIDGRHVYAHKCVLAARSRFFRMVLTSNGPTQAPLVMPVSAVGHDAFML
TLRFLYSGQLCLLPSNSQPDRGCKESSCWHSQCRAAVDFALEALHAAQMFGIDELSVLVQKELAAMAEKASIEDAMRILV
TARKQDLLQLWSVCSKLIAKSGLSTEALEKHVPVEIAAEIEAIRHKCGYYNASRADCSDSLDEQRTRRMQKALDSSDVEL
VKLLVMEEGLSLDKTFALHYAVAHCSRKVVSILLQLGAADVNAVDVEGRTPLHIAGELADPEMIAVLLDHHASPHVRSPA
GTTALDMVQSHVFQALTLASEGGAPADHSKLKLCLELLQTAEPVSSQERIVQDEGNCLLMAPERNNDFYPAPKGTQ
>Smol_XP_002993946
MQSQPSQIHLTFSLNVEILSRLAQWRIDSLTPNLCWKSNNFKMGVWTWRLQIKKCGPLHVWLFKEHSDHPSEQVPTVAFV
LRVLHGPGLRQVVQSQAVNDKTFLTNEYHAWSVETAIHGKFVVEVEFIDMKCGSQAGDPFYFTLGKSIQPAAHQAALSCL
SYMLDSGVHTDVTITTPSGSIGAHLAILATRSRVFQVMFSHDLRERKTFTIEMEDMSLESLKALLAYLYGSINRPAFHKH
RMALLAASDKYDIEDLKQACEESLMEDINSDNVLERLHNAWMFHLPNLKKDCMRYLFEFGKIHDIRANFDQFLHSTDKEL
LIEMFQEGLNAVHF
>Smol_XP_002992598
MQSPSIAASSCTSAGGGACGGVLAATPNDGSGSGGLEEDQLQAWVGLNKLGQDLEGLVTSAEDCHFYSDAEIIVAEGVTV
PVHRCILAARSPFLRRIFAEKQREQQRVDLAELAGGTGKIGRQALMIVLGYFYGGKFQRIEEECSGVTCMDSQCPHVACW
PVIEFLLELLFVGSLFQVSDLKSMAQDRLLRLLPKTPAENVLQIAAAAAAQQGCEALQEMCLPILARSNTPALAIEKSLL
AHAPALVRDIAQLRHRLGIHPVDAAEDKRWRRVYKALDSDDVELMGMLLSESNSSVDSVYALHYAASYCDRKTLTELLDL
GLGDVNLRDRYGYTVLHAATLRRVPEVVGLLLGKGASPLDTTPEGYTALQVSRRIARNIEPLESAEAREDWLRDRICVEI
LEQADRANPCPVFPVPMGERELLMRLLYLENRVAFARLLWPRECKVVLGLSQLDTTKEFSMEDTSSLMDLNKEPTRSDTD
INSSLLQRVNALQRAIEVGHRFFPRCTAILNSYMDDGSFEHECIQNRRIPEDQLRLLAKLKRVTDDKQLSEAYQRDVEEH
HNLEHHNFGDNACLYSN
>Smol_XP_002989054
MAVTSSKRVGRNSFSMRRTNDWVMSTDVPTDITVDICGTSFALHKFPLVSRSGRLRKLVAEARDSDLSRVEFNDIPGGPE
GFEMAAKFCYGINFEITASNVAILRCVAEYLEMTDDFGQANLASRSEAFIEDVVMHSFGSSITVLHSCEKLLPMAEQLNI
VNRCVQAAGAKASHEALSLAARPEYRNAKTPPQLDWWAEDLSILSLDFYQRVLAVMRSQGMPQENLWGSLMHYAQQALKG
FNSTSSKSPGRSVTGLEHEQRMLVETIVGLLPPDKRSASCGFLFGLLRTSIILDTTVACRLELERRISLQLDEATLDDLL
IPSFSFAADTLFDTDLVHRLLSSFLQHHLQDESKASQSSLAKVAKLMDSYLAEVAPDANLKVSKFIALAELLPEHSRMVE
DGLYRAVDIFLKAHPLLVEADRKRICKLMDPQRLSQEACSHAAQNERLPAHFIIQVLYFEQLRIKTALANYAIEMPKGDH
GPTPIAAALRSSTAAANAAAAAAAAAADANPQSALRRENKELKIELTRMKIRLSDLEKEQSPGVSRASEEKRLSSPTSSA
ASSPGSGRFFHTVSRRLGKLNPFGKSSLKPPATPELQKPRRRRHSIS
>Smol_XP_002986018
MAVASSSSFEGMVVSVAKSRFCCDELKLAAASDLFRKIFTSVRHTGARNLFVELKNFPGGAPAFKKILKFVEDQFQADHL
DLHTSSFPTLCAAAKYLGMPLLASSCARFWSSQVVTTTSSIFEVLAIVTQDHGVALSAEERSEVISKCFSATLQALGRSS
LHEFFVNMVTILPVGGISVLLKFLERRVDFFSMQSKVWDKARYVVLASELIFVACPTGVKARDVLHRAGILARFVLGLFR
EGLDYAKDQRKHWLVPSFDDHHDLHESVALLNLLALCDFLLEQDSITADTSTSDRIKAQEKKLFALLAGSDAHSKASTIL
LSPGSAMLLAFNFGKHHEDKKQLAVEFIEHYLQQISLTQLSREETLEICKFISAHSLSHDNLFNHVWRVVTRSTTLSRKR
LQELMDTISSSKLSTEKLMEKLQERAHIRLCVEPDVDHGRGTADVKLLLSHGLEQPQGREHRAEAQGQDGDAQGGRVPTH
VQ
>Smol_XP_002977615
DNWDKLFCEGLHTDVCIVTEDGGMTAAHSFVLAASSPVFLRLLQEQQPSGKHHRRSIHILGVPYDAARVFTRFVYSSRYE
EKEMEKYVLHLLALSHSYSIPLLKKKCTEALERGLLTTENVVDVLQLARLCDASRLNLLCLRLIVASFKTVVKSEGWRVM
RESDQTLEQELVGHVIEADARKQEKLRKSEEDKVYKQLHDAMEALVHICRDGCRTIGPHERPPDGINRQNSFSSSSSSSS
SCGFPACKGLESLVRHFAGCRTKVSGGCLHCKRMWELLELHSRMCGDAERCKVPLCRHFKDRIGQQTKKEESRWNLLVAK
VKAAKGASTA
>Smol_XP_002977036
MVAAAEDDLDKQHADEGYDELDSYIRRKKKVPCGDVYEAARAGDIDRLKILLESGINVNARDEWDSVALYYACLAGHEDA
ARILLEGGAICSEHTFDGDRCHYAALNLRVRRLLKLFEARPPPLAPLPDSLRALFFRSGQNHRFLDPTLGDTGNFDADEL
GDPVGPDIVFYAQGQPIAAHRAILSARCRFFQRKFEKEWKSRSEIRFSRNRLSFHALFRLISFFYCDVLDVAVDDMEDLL
RICKVCGCSGLQRVLEKELVHQKFADYNFAATSEFKALLQRLPPPSGDTSTRTTIPNAPGGVKDDSQGNLLDDLRERWLE
AEGAELDQRDESARDFDKVLELLRSTAENDDTESLELV
>Smol_XP_002975208
DNWDKLFCEGLHTDVCIVTEDGGMTAAHSFVLAASSPVFLRLLQEQQPSGKHHRRSIHILGVPYDAARVFTRFVYSSRYE
EKEMEKYVLHLLALSHSYSIPLLKKKCTEALERGLLTTENVVDVLQLARLCDASRLNLLCLRLIVASFKTVVKSEGWRVM
RESDQTLEQELVGHVIEADARKQEKLRKSEEDKVYKQLHDAMEALVHICRDGCRTIGPHERPPDGINRQSSFSSSSSSSS
SCGFPACKGLESLVRHFAGCRTKVSGGCLHCKRMWELLELHSRMCGDAERCKVPLCRHFKDRIGQQTKKEESRWNLLVAK

VKAAKGASTA
>Smol_XP_002970328
MEVESPAHLSAACERLLSSGLYSDITFQTGDDSTPAHRCILASLSPVFSTMLSAGLQEQETATIAITDMTTPQLRAFLLF
LYTGRLEQQKVAESGLAVLAACHKYCLPGALMNACVAGLSRSITCENCLGLLELADLYGQETLAAKCRSFVTSHLSE
>Smol_XP_002968092
MEVESPAHLSAACERLLSSGLYSDITFQTGDDSTPAHRCILASLSPVFSTMLSAGLQEQETATIAITDMTTPQLRAFLLF
LYTGRLEQQKVAESGLAVLAACHKYCLPGPLMNACVAGLCRSITCENCLGLLELADLYGQEALAAKCQSFVTSHLSE
>Smol_XP_002968043
MQSPSIAASSCTSAGGGACGGVLAATPNDGSGSGGGLEEDQLQAWVGLNKLGQDLEGLVTSAPLEDCHFYSDAEIIVAEG
VTVPVHRCILAARSPFLRRIFAEKQREQQRVDLAELAGGTGKIGRQALMIVLGYFYGGKFQRIEEECSGVTCMDSQCPHV
ACWPVIEFLLELLFVGSLFQVSDLKSMAQDRLLRLLPKTPAENVLQIAAAAAAQQGCEALQEMCLPILARSNTPALAIEK
SLLAHAPALVRDIAQLRHRLGIHPVDAAEDKRWRRVYKALDSDDVELMGMLLSESNSRVDSVYALHYAASYCDRKTLTEL
LDLGLGDVNLRDRYGYTVLHAATLRRVPEVVGLLLGKGASPLDTTPEGYTALQVSRRIARNIESLESAEAREDWLRDRIC
VEILEQADRANPCPVFPVPMGERELLMRLLYLENRVAFARLLWPRECKVVLGLSQLDTTKEFSMEDTSSLMDLNKEPTRS
DTDINSSLLQRVNALQRAIEVGHRFFPRCTAILNSYMDDGSFEHECIQNRRIPEDQLRLLAKLKRVTDDKQLSEAYQRDV
EEHHNLEHHNFGDNACLYSN
>Smol_XP_002965955
MMKLVENNVLVVPPFDCAWLFEEELRFPEAGRGCVAFEARAENDVTVIFKELAGCKHYRTDSDPNYTVIIGSNLNKRLKI
AANGRTVVDVAGPVVSPLVFERYWISVDEGSIVVGKGELGQNVVYQWVDPEPNCKVQFVGLSSWDKHVGYRNISVAALPP
CLYRTTSAIDSQWSTGLANFLESGDLSDFEFVLFPENRRVCAHRAVLVTGSSSKECPFLTTTELVLHGVDYLVLHAALRY
VYTGKAEIGKEHLVQLRGLAELIGFQNLAAQCKQHIREVSYDSSGDLVLLTMHCYDTNSPVHVNPAAFTRMLLTGDLSDL
EVYLEDYDLCLQLHRLVLSVWSAPFAKMFTIGMKESSRTRVTIKDVHLDAFMTMIRFMYTGRLDLEGKDNVGSLLLPLLI
MADRYGVRVLHQECLDHLLACVNEDSSCAILQVASSMARCETLRQACEESCAKHFDYCVASSATEFTELDGQSVMRILQL
PDLLVTSEEKVLDAVFMWGSKMEGVQCWEDADTKYKVGLQVICPDREEDLKVLLPLVRFPLMSMELLQQLDSSSLCEKLP
LLRQMVSLVSRFIYDFLPKNGLWFTRRFSSYKELNYLCDGDGNGVLYYAGTSYGAHEWMNPMVTKQITLSASSPPSRYTD
PKVLASRNYQATSFAGPCIERGETVSWWRVDLGPDHKLMCNYYSVRQDGSTNFARNWTFQGSGDGETWTDLRKHEKDHSI
YRPAQYASWPVHGSKSLIPFRFFRVLLQQPIAAVAAPWNLSICYLELYGYLH
>Smol_XP_002961299
MDTDTCSGGTSPSFAFAFNDSNFSDRVLHIEIVAGASGEAKSSGEPCSTVGAWARQKKRRRGADAKDKGTLFSCLSFYLS
FEEQIMTGTQPEPDDAEEEGDVMIEESPTSMAGNQDQMQSTSSWNSESSTVLRVKTIHISSAILAAKSRFFYKLFSNGMR
ESEQKAVTLRITDSEEVPVMDMLQFMYTGGLQANTAPALLDVLVAADKFEVATCMRHCSRLLRELQMTPESALVYLDLPS
SVLLPDAVQSLTDAAKAFLADRYKDIGRFHEDVMHLPLAGLEAVLSSDDLQVASEDAVYDFVLKWARAHYAKIEERREIF
RSKLVWLIRFPMMSSRKLRKVLVCNDIEHDLASKLVMEALSFKAEPPHRQKHIMVEEIVHKRFSERAYKYRPVKVVEFDA
PYQQCLVFLDLRKEECLTLWPQGRVYSQAFHLGGQCFFLSAHCSVDQQTAAHCFGLFLGMQEKGSVSFAVEYEFAARSKQ
EEWKFAPRVKGNYVFTGGKAVGYRNLFSCPWQELMKDDSDYFHQNVLHLRAELTIKPQGQGM
>Php_XP_001784949
MGRLLQCEESYSEAFLESADEQCEDEFVLNLNPLHSVKPPSEVYSFDPSVLSPNISPALQRNVRAWRDATGLPTTIVIRC
KDRVFHLHKFPVVSRSGYLKKLLKDAKDVTIPSHVPGGPDILDLAFNFCYGSNILMDPSNIAEVCCVAEYLQMTEDYGQA
NLFERSMLYLTQVTLQNWDDTLVVLLHCENLAPFAEQLGIVRRCLDAMAFMACMEFFDPVVKKAFPTKAEDPHRWTTNMR
ETSSLHWWIQDVVAIPSNLFVKLVLALRREGMQENHVGQVIMAFADRWIFGSRAGSLLVQKGNDKCWVLESQRHVVPIRF
LFGLLRRGLRCALHDDCRIQLETRIALQFEHATLHDVLQPCKEEKDDIYIFRNEIDSMERILQLFLTRFRGYDEGRLPDM
AILSAVEKLWDEYLTDIACHSSLSPSRFAELIDRVPAYMRVVHDHVYCAIHAYLKAHPNITHEERLRVCRILNCQKLSQE
MCTHAVQNDLMPLPLLVQAMFMQQLQT
>Php_XP_001784176
MSPEDQSLKTLSADFLALLDKGQAFSDVTFKVEDRHVFAHRCVLAARSPFFRMVFCDDQQLNSAQPRPGIPNVISVGVVG
YDVFMLLLQFLYSGNYSNFFSPQNCGRQCKDKSCWHTHCSSAVEFGLDTMKAALFFGLDQLSTLTQKHLAAMAEKASVED
VMRILTTAHTQENKHLWNVCSKLVAKSGPFSEILQKHLPANIVCELEDIRRKSGFGFEAAMSSNTTSEQKTKRMQKALDS
SDVELVQLMINGEGLNLDKAFALHYAVSKCSRKVVKTLLDLGKANVNLRGPDGLTPLHIAAKLGDPEKIVMLLNHEADPH
VQSASGATAMGIVQFGMTEIVSAGGYNSKGDQNRLRLCMELLERAIL
>Php_XP_001783726
NLTALCEHVQVEGWKSKAFSDVILKAMGQTFHLHRFLLSRSSYFRKMLQGPWKEAGAPEITLHIDDDNVNADALETALAY
LYGHYPKLDDSNAFRVLAAGSFLDLQDLCSLCTDFIISELWTDNFLAYQAFAENQDYGLHGERVRKACWGYLCRGGAVEL
REILPKLSAQTLQKLLASDELWVLSEEKRFELAFYVLIARATKSDVSFECDDSAVTNSSAGLSSPTTSLSLDSRNSVRAV
QCSGVSTPTHDHDFETPSGKPLKEVDSPQLFNEMPKNKEASTCGDIRVVPDQLSYSGVESSSYVLVCSTRSTCPTPSWGG
RVVDRRVRKAFRNGRHWSIAEDWEAFLGVFAGGGVLYCHMAFDHLLHMRGRLEKLGFPCKSVIDSLWLQTLLRQQVLTIA
ADTCRNCCLVYENCNCRQFNASYEHERLPGLHYRDDSQSSGSTSTSIFPGAEGIGGNSESVGRIPFRNTMDGLAGIGRGT
TFRTPGSWPAPKFLYVPSLGAYGARGNPQQGEDGKSQMDPRLEIFGRDLPAAHCTLDGPAGGTGLTQGGTAPSGVGASGQ
ADLNPRNHEDSSAKPPNTDSTSFKLNITKNLTEDIDTRESEEGEGGSILLDLNTPLRNFPSFRFGVEFEDVHRLLDGQAK
HSPETFYAGSLWKVSVQAFNDEDPRGRRTLGLFLHRRKAEDVGPHRKVFFYTDTREKVTARYQLICLAKRGVISLGSLTQ
AGMLLPKAPKGWGWRTAFLFDKLPELLQQGGSLRVAAVVQLV
>Php_XP_001783439
MDTQHSILKQTFQVAVLETTLICTSVGGTKSVHLRKRLRAPGSPPNSIGPPFSPPPVPPPFPSSTSRKPWQALSATRCRG

SRSCGGSFISNHALNTWDQVFFEATNADLTVCTDDGSQIHVHSMVLMASSPVFKFYLQKEVRKSRFPAIDIFGVPFHAVR
CFLRYLYSSRCERSDMEECALHLVVLAHTYMVPALKQTCTVHFEQGLLNTDNVVDVLQVARLCDAPRLYLLCLKRTTSDF
KAVVRTDGWRAMKHSDPALEQELIEAVIRASMRKQEKTTRLEEDQVYGQLHDAMEALTHICRDGCRSIGRSDKSLNRGST
NCSFPACKRLQSLVRHFADCKLKVAGGCVHCKRMWQLLELHSRMCTTSDSCKVPLCGHFKDVAKQQASQKVELRWKMLVQ
RVKLAKRVVDTLSVAAIAQQLERPPI
>Php_XP_001783005
MARTPMACTVAGNSNTRSRCVTETVNGSHHFTINGYSLAKGMGVGKYIASESFSVGGYQWAIYFYPDGKNAEDNSLYVSV
FIALASEGTDVRALFELTLLDQSGKGKHKVHSHFDRSLESGPYTLKYRGSMWGYKRFFRRAVLETSDFLKNDSLAITCTV
GVVVSSTQGPRLYSIPVPEPDLGHHFGALLDSGEGADVKFEVDGELYHAHKLVLAARSPVFKAQLFGPMRDRNSDHIEIK
DIEPPVFKALLHFIYRDSLPDTKELIGAPSTSTLLAQHLLAAADRYGLDRLRLLCESKLCENVSVDTVATTLALAEHHHA
AELKAVCLKFAASNLAAVMHSEGFEYLRDSCPTLQSELLKTVAGVYEEGVGTGVTSNRNRSVWGQLSDGADAGGRRVRPR
T
>Php_XP_001782716
MGTLTINCGTSFTLSNSDSLRLVLTPEAVHIRSSPCNPHKHPHLLTLTHERVSNSEEPEHNDAAMEALVDINSQGTGNLD
DCDVSFDGDESDDSQNGFQDKRRSKRVESWARARFDAWRALHKRPTAEAIEDLCDHDRKELEELVELFLTQVCKQNGKEY
PHQTIGSLLRALGRVTRAHQEARIAETQVPEAPLNIMSDVRFKKAGQAVSDNVASAGRLGLGKRRKCISILTLLDEAAML
ALSTYKCTSAKGCSMRFAYFCTQKFFIRGTAELHELTDMDFTLGKDQHGDYEKRLKSWSSDCPLSHPEKFSLPMTCYIED
VSTHKIMIQHKPKWAEYEPPPRPIILTDVKNPTSRAVMSWRFHVSVLFIRFCVKNIRCTVNKKTKERRVHLERRLHSKEF
EGAGDPDRPPPPYSDIVLETSDGWKAYKHEAVVASQSEDFKAMFSMPMVEEYPLHMETSHTALDTIIFFFYRATVNQGVL
RNHLVELLQAADKYVMELYDGVLKRLKRKRPRQDTLSTVQGNLSIQLLDPKLHVCANARITKEIRSSVSIVEDVSKEIVD
DVFLERFSSMLPDITVAFVSKAEYFGPLYLCLKRSMIRTVHFYYKV
>Php_XP_001782701
MVKFLPSLCTSIELNDVQSQLQKIMEKQNFITTWDPETMLPPHTDVILEANDGRLVHAHKSTLMGKSTAFKTLFSATRDV
VNPQTVKMDMDHASLEAFIHFFYTGFVKDDLMDSFADKLLRASDTYGISLLHNLCQEKMMTNIHPERIFQYFLLGSKCHA
EQLVHAIISFVANNYSDIAEINGYDDFLKDDPTLVAKLGNGIVKKLQAKLKNHPKIM
>Php_XP_001782309
MDCCLCSSLGNSYRPPRNSLCLACYECAKSIIDLCQKLEEQSRDDVEDGDKARQVPSHGLQYAYNQIKQLEQMEENVSEK
LEFLESLVEAFQKGMHTDVELITKDGVSIHAHRVVMATKSSVFRAMFESDELKEPHKGAVYIQELTHDELRYLLEFLYCA
EIPHDAMAKHGRALLIAADKYDIPVLSKVCEAFICLTVSSSNVLEVLELATLTHATSLKETALNVILESYEDVVFSKEYE
DFAMQNALLSVEITKALIKDLKK
>Php_XP_001782174
MAKGEITEVEPPSNVFSYSCKLGERATSDVIVRLRTVHGRDDWFYSHSEILCAKSKYFAVRLSDDWPTYHLLDSRNCVEV
ICIESDIDHHVNLLRMLYSNLEEPVGDMWHSVKNALGMLKVATKLGCDAIASKCALYLEAVPWEESEEEEILKALPAIMS
STHKLVAGLGAKVEPILARLQPVDTASIRNVFLSAVHMATLSESGSNNLEKLPSDLKVAAQEQVEYMLEEDDDAPLLIAD
DNLKAELKKHFLKLYGEFKDGLSALVTNSIPENLEVAEKAILEKISDLVWAWRMLPQVGLISDFVELWVEASSDVTLALN
SDKLRDAFWNTKLHVVEITATVLKAVGYGNVILTANKRTQLVKVWLPFICETKPILDTLFAGEKIAMEMDMDLCQSIENA
LVSLVLSLPSVDQAEILADWLRWDHARFPDLSEAFEVWCFRTKAAKRRLSIETPKSANAISSGGIS
>Php_XP_001781926
VLKSTIDTWDRAFFEGINTDVTVCTNDGHQLGAHSTVLMSSSSVLKCLLLEQFQLSRHAVISIRGVPFQAVRCFLRFLYS
SRCEQSDVEEFALHLIVLAHAYRIPALKRICTDSFEQGLLNLENVVDVLQISRLCDEPRLYLLCLRRIVSDFKDVARSDG
WRAMKESEPGLEQDLLEAVIEFDSKKRDRLRKREEDKVYTQLHDAMEALVHICRDGCRSIGPHDKVLDERKGDCAYPACK
GLESLVRHFAACKLKVSGGCVHCKRMWQLLELHSRMCTVQESCKVPLCRHFRERVGQQPSRKEEMRWKMLVRKVQFAKAA
SGPFSLAAVSARLAQV
>Php_XP_001780118
MRHDRTWGSSKMVTFDFAYNNPVFSDRVLHMDVAPVGNEFYEAVGKSEEQHIEKLYVSSVILAAHSDYFMRMFSNGMSES
SSENAVVHVNEEEKFGLQQLIQYMYTGRLSEPLDIEATVMLLRLADRFAISSCMEPLAKILKLFPNTLSDCLLVLSLPES
LKADRSDISSKKGDFLTLSIEGVKVILDSDALMVSYEEEVFQILLDWVDSNCRTAEEKQRAAEEVAGVIRFPWMTGDFLI
DVVSTNPQMQTAACQALLMEALRFKSFTHARQQQMLWKKTNHNRYRPRNNTILENFWGNSKTFQVKQPDGSCQVLFEFPL
ELVICIGQSFQSRTFRLCDKYEFYLEARHGQVKTSYNQQLTCFVNLVLPPRNDSVSSCEEVEDLKFVDYTIAMKRDYSQN
YDTKTSGSFCLTGVESAGACIPFTDFFSGWGIERGFNVPRWNLTINGPVFLKLDLKLRVEAFATDLDGQLQRRDESSSLA
TSHFEFPPLGPGTTDAIPF
>Php_XP_001780098
MFFEEINTDVSVYTSGGHRLGAHSIVLMSRSPVLKFQLQEQFKTSRLAEISILGVPFQAVRCFLRFLYSSRCEQEDMEEF
SLHLLVLAHSYRLPALKRVCTDSYDQGLLNTENVVDVLQVARLCDEPRLYLLCLRRILSDFKDIARSDGWRTMKESDPAL
EQEIVEAVIDSDIKKRERSRCFDEDRVYSQLNDTMDALIHICRDGCKSIGPHDKAWDGRKGDCSFPACKGLESLVRHFAA
CKLKVSGGCVHCKRMWQLLELHSRMCTLQESCKVPLCRHFKVRVGQEPNRKEEMRWKMLVCKVQSAK
>Php_XP_001778891
MNDAMEMSSVGGMGGDGGHGSPRGELRPLHARGADAARVVVAVAVRVVGVVALASRAIAPGATGRAMWRRMRPSVAGQHL
QPRVWVNLHVIEELLALFPQHRQGVRELVADGRLLRRLEAELAGCHRVHPADVMSEALLGRSLQELTQPLLYRPLSCSHR
NVPTVTAAGEQISSCFGSLCDVEDEFERDTLLKWLSGEMRRSRVALDNHLTDYRVAPAKGVHARTNARESYSSDHGCGTN
ANLAAVKTLLYLKTHSNQRYRTKPLASAGALSGVSPNSLQITSAVISVLLGFSASCHRVFHEENMEAFASQSQGHRICTR
KTVVASSSKLGTAQPAVNDNLTFPKTKGYITGSAVSQWDQLCPKCQTIVKEQGYETELKSGPSFQVQVVRLEMEKKLKLV

EDRLHEREESERALQEQIIFLSTWDPVVMSPGYTDIILQSGGHIVNAHRAVLVMIPTFSSILSSLYALELMYRRMHRVSP
RLDYLTLNCVSTQKNIEADTEHDQFDTQAAKSDVILKLMKGNLDQLGTVSMELHIFPEALDAFVRYFYTGHISHRDLKRY
ATKLLTAAKRYGVRSLQLVAERYIAKHVSKETALSTWELGTDHNSEIIKESVLQILVKDVQDIPKLGEYRMYRCQKDPEL
LVGLFENLLGRSIPFQGLHDGCAKDPFRHSKTNWVNIRIGSSKGRYTQVTAHTAFNNESERSSAPLLTAWVKS
>Php_XP_001778807
MGEGDGHGCPTNQGILDGSSFVGVWVTSGEGVDSNPRQHRSDATTVVEEADAEGHSGEQFSQGFTPGIVLVFRSLPREPF
VVESGILSFRIIRVGYDSTMADSMGELTINGGTSFSLSNSGPLPLDVTAEALHVRPPPFDPLVHPHSLTLAHELVSNGEE
SEHNDAAMEAVVDMNSQGPGNLDDDDVPSDGEESDGNDLIGDGKAESFEQNANAVSGEDSQNALRDERRSKRAESWARAR
FDAWRGVNKKPTTESIEDLCDRDMKELGELVGLFLTQVRKQNGKEYPPETIGSLLRALGRVIRAHQEVRIAETQVPEVPF
NIMSDVRFKKARQAVSDNVASAGRLGLGKRRKRIDVLTLHDEAAMLALSTYKATTAKGCSMRFAYFCTRNFFIRGPAELH
ELTDMDFSLGKDHHGEFLRYEKRPKSWSSDSPQSQPEKFSRPVTCYIEDVVSTYKIMVEHKPKWAEDEPPPRPFFLTDIK
NPTSRTVWYTRSPVGVRTLASWLRMMTKETGLGGEYYTNKTGRTTGLGRLQYAGVPIDQDMLATGPRVAGTYARDDPEFE
GIRERAMQRIVSGEVDKSTGRLIDWSKALALEMELSKKPQSAGTGIQTMAIQSYSRDLCANLCCQDVGSEGSELTMYKEY
IGCDTCKQDTHSKDAYEQYRGLVEELEKTKERLVHLERRLQGKEFDGTGDLDHIPPPYSDVVFESLDGRKVYGHRAVLAS
QSDVFKAMFSMPMAEGTPLHMEMNYPALDAFIFFFYRATVNQAVLRNHLVELLQAADKYGIEALKSLCEQEILQSLNPTK
VLVTYVVGWRHNSDVVKEGVIDYAAKEVDDVSALEGYDTFSKKCPEAVMELYDGVVKRLKRKHPRQEPLSTLQDETCTAD
DTESEHSVKRLKVQ
>Php_XP_001778796
MAKKVGLNGWLQRVRETGEHSDVVVRVTGEEFRLHMLPLRNESGYFRDLPSSSNGIEQVDEKTGCKLVNIHHLPGGVEGF
AIAVNMCYLIKPSFTVKNVALVCAAAEFLAMEDVTESARKFMHSNIFSHWRYCVNYLQNYTRIGSPVDEYVEFRCQKVLA
TACVKCFTEIKHVSAPSAYISGPLTAVPPKQSSSACQTLTELLVQVCSLPDIYAAEIINTLVDSDVNLNLKCRQGRNVRR
WLESVIDDECQSDKARCYVLLCLSRMLLKNAPTKRPWMELSSQYWCSLLEHADQLLPLLGDPLKERLIGVKAMLEEKIGE
SLNEMDEYLRDYKFGPDTLMSLVEYYISMGEYTEQSLEEIAGDVDRFLWFYANECSISVDAFISLVKAFPPSARDCHDTI
YGAIEKLISTAPNCSAEEQQQLWSLIDHFKLSPSVNERALNNPVFLSQPEILESVLLHHSQELAKVDDTDGRHLRHIMQK
VINASLKLLEENSRRSREILELQRQYGELLGCRSQMLNPEGIVDLLGKQHHSNLSQNSSLQRESETEESEISDSPSLATV
SSTGWIQTCDWGQLIQKPAKVLKSLRNSYAPSAEDNLIQHETKINLLNHQAFPVQHFKAGGTIAAKRHITNASIVANSKA
SIYTRSIIDAIV
>Php_XP_001778510
MGGVVSPTEDRVDGRNLTPFLVVGSDADSHRVGDPELGILGESSEGSIRFIACWIRCCRGSLPWDITLVSWVCEGAAEML
DNWNRELNYRKLMDGGRGAGAGDGAVPAAAAAEAVTVDLSMCNQCGNLIENTVTYCHACYDLRSESDFRNERRWRELEGD
FMVVEADLHKERAATKRLEGALQSFRHMLLQGIHADVTIETGEFDRTPAHRAVLASRSPVFRAMFEHELKEKTCAHIHVA
DVSTPAMRALLLYLYTGDHDTKVMKEHGMALLTAAHKYDIPDLKRVCETAVANSVKPSNVIETLQQARLYDATWVKRACV
DCIAQNLEKVAFTEEFRNLIFRNDDPEAILDTIQSIAALQ
>Php_XP_001778211
LQKLGSDLETLIRIPDQEYSDLTIILDGKQVPIHRCILAARCPGIRKVFSEMGVTGGNRKLELEFSTIVEDGKIGYDAFM
AVMSYVYSGKMELWLTGIACYDSTCVHITCRPIIDHVLEVLQLSLLLNLPEVTTVAEQHLIDHLENFQVDDMLHVYRSTA
ISECSELKSMYLTALASSSLDNLTAEKEFSGAALQQVRRFPKELRPGMLHLSASQEKQQSFLNRALDSDDIELVQLLLDE
GKLDLNEACGLHYAAAYCHPRTLAHLLELDIADVNVRNERGMTVLHVAAWRRDPLAIAKLVEKGAQLQALTLDNQTALDI
SKRLTRKFNLVGEENFKDSLCVSILQQAERSVAVPNAAAAMLEQPCTEKDLMSKLLYLENRVALARLLYPREADIVMGIS
HLDSYATSTEILRNSSGSEITSRKRKSSVELNEEPTKRLAGLYPCLLTMLRVMYAAEVARKYFPCYSAVIDKYVLDDDYV
EPDEDCSVEDQLVKKKHFAELKGILQDCFSKGKVEE
>Php_XP_001776409
MACTVAGNSNTRSRCVTETVNGSHHFTINGYSLAKGMGVGKYIASESFSVGGYQWAIYFYPDGKNAEDNSLYVSVFIALA
SEGTDVRALFELTLLDQSGKGKHKVHSHFDRSLESGPYTLKYRGSMWGYKRFFRRAVMETSDFLKNDSLAITCTVGVVVS
STQGPRLYSIPVPEPDLGQHFGALLDSGEGADVTFEVDGEVYQAHKMVLAARSPVFKAQLFGPMRDRNSGNIEIEDVEPP
VFKALLHFIYRDSLPDTKELIRAPSTSILLAQHLLAAADRYGLDRLRLLCESKLCENVSVDTVATTLALAEQHHAAELKA
VCLKFAASNLAAVMHSEGFEYLRDSCPTLQSELLKTVAGVYEEGVGTGVISNRNRSVWGQMSDGADAGGRRVRPRT
>Php_XP_001774594
QRRGQAWLCASGLQSDIVVDVEQMSFHLHKFPLVSRCRRIASLVERASREDEDTCDIRLENFPGGTLAFELAAKFCYGMK
VEFTPCNIVALRCAAEYLEMTEDLANQNLIERSESFLEEVVLRNWSDSIAALQSCPNFYPMAEELGLVDRLINSVCAKAN
MNDSSRRVWPMRGQRALQSPGGSLLWNGISTGARARVPGDGDNWWYEDISVLSLTLFEKVVLGMEAAGVGGESIWGAVVH
YAKKALPGLHRRHSGREPAHSHRKAIVPANPIVQENEQRILLETIESLLPLLKVGSSTRFLFGLLRLAIILNASSQCKSS
LEKRIGMQLDQATLDELLVPNYSHVEETLYDIDLVQRILDHYLMLEQNIPPESDEEGLLLGSPSLCPIMMVAKLIDAYLA
EIAPDVNLKPTKFIALAEALPDYSRFLDDGLYRAIDVYLKAHPWLSESEREMVCKVMSCQKLSLEACTHAAQNERLPLRV
VVQVLFFEQLQLRNAIAGSFLVADTVGASGRQLGQQHHAGPGGARQGESWDDSLEVGQKLKDDMGKILARVSQLEMECGT
MRQEIENLHRAKKPVNPLSKAFGCKMFSTV
>Php_XP_001773928
MRNIDSNLGTLCEHVQVEGWKSKAFSDIVVKAMGRTFLLHRLLLSRSSFFRNMLQGPWREAGAPEVTLQIDDENVNADAV
ETALAYLYGHYPKLDDTNAFRVLAAGSFLDLQDLCSLCTNFIISELYTENFLAYQAFAESQDYGVHGERVRKACWGYLCR
GGAVELREILPKLSTQTLRKLLTSDELWVLSEEKRFELASYVLAAPVTNFSAGISSPSTSLNLNSCDSAQGNQGLGAPVC
MCDHGLEALNGKPLKEVDPSQAFIEIRKDGEACTSGDTCSTPGQLNFSGAESGFCGRFGFEEPWNPPRTGEHVGHDLCSP
SPETTSSPSSKVPQDQCACPTPSWGGRVVERRMKKAFPNNGNCGIAEDWEAFLGVFEGGGVLYCHMTFDDLLYMRGRLEE

LGFPCKAVADSLWLQTHLRQQVLTIAADTCRSCSLVYANCSCRQFNPSYAHSRISGLLYRDDGQSTDPIPTGIFPGAEGI
GGTSGSGIGVSSQSAGRVHVRNTIDGLAGVGRGTTFGAPGSWPAPRFLYIPTLGAYGGRGNPQQSGGGNNQADVRLDLIG
RDLAVAHYAPEGPTQGGTASASVPSPVQPDLTSRTEGSSVAGKPASIDTSLSNVSIDKDLATKEYEAGGGGSIQLDLNTP
LRSFPPFRFGVEFEDVNRLQDGQSKHSPEAFYGGSLWKVSVQAFNDEDPRGRRTLGLFLHRRKAEDVGPHRKVSFYTDTR
EKVTARYQLICPAKRGVIPLGSLTQAGTLLPKAPKGWGWRTAFLFDELPELLQGGSLRVAAVVQLV
>Php_XP_001772248
MARTPMACTAAAGNSNTRSRCVTETVNGSHHFTINGYSLAKGMGVGKYIASESFFVGGYQWAIYFYPDGKNAEDNSLYVS
VFIALASEGTDVRALFELTLLDQSGKGKHKVHSHFDRSLESGPYTLKYRGSMWGYKRFFRRAVLETSDFLKNDSLAITCT
VGVVVSSTQGPRMYSIPVPELDLGEHFGALLDSGEGTDVTLEVDGELYHAHKLVLAARSPVFQAQLFGPMRDRSSGNIEI
KDIEPPVFKALLHFIYRDSLPDMKELAGAPSTSILLAQHLLAAADRYGLDRLRLLCESKLCENVSVDTVATTLALAEQHH
AAELKAVCLKFAAANLAAVMQSEGFEYLRDSCPTLQSELLKTVAGVCEESVVTGVISNRNRSVWGQLSDGTDASGRRVRP
RT
>Php_XP_001772109
MAMKRGVISVGPLESSIVKRRDNLKVRARAAAGVVASLRAQIKELQQQRDFLVTWKADMFTGADAEGSLVDVELQGVDGD
RVHAHKAILSVGWVLVDPSLCSELGQVLLSTIHESVRRRCPYVIAEKKSVASKSIEFEAMFRADTDRKVVWNLEMPYVAL
KAFVTFFYTGRVSSSVLIEYLTTLLDAAHKYNVQFLEVVCEDSLVKNMTSDNVISIFDVAKKHCSSGCKEAVLKKARSLG
ELSSFTEYKFFTQSNPGLLLELYELLTDRSDQSPTKKMKLSRGNESEQANGDKAPLSDEDNMKSETDEDESVDDEEVKTA
TDSMPDRWIPHAAQWYIPVWNTNLLAWNTDARSPIPSSWIRECMLSTSSRGFLRGALTHSPGQLEAPILEEVVAGKKKGI
PLRKTTNSEIVSYKARVVSEGEDPKKPKVTPWQQKEKRCLLRGVKTTHNKNKKFIATITVKGQQIGLGKCCSEEEAGRLY
DRAAFVIGRKTNNELPDDMKEELKGISWDDFIASLQEVTALESGSFHHQIIFVPFYSNHANVSKNYVSFDYRSNVRISSN
MNSPDDCNGYVDAVVVLQRFIMGSWIMEIVTRLIVIKSTKKNSKNRNMSKHLSNYNSKSPVDQFEAKRHGHNSFTMDYTG
LTGISLQVTSFRHYLARQRERTAGIRTNDEGQVHLDRSNTACNHNKNWRLYLKLVA
>Php_XP_001771946
MARKAAPLSCRGWHLSVAPQLHHLYSSRNFGAGQNGSEAGRAQRRAFLFWEDAAGKFLWQHIMFVGSFHYYANGVLVFCD
PGHKMEEDFSNSVVLVITEGDHDSRKRSLSREIDGQEMGCEEWQSLVADSFTLNEIRIRVDKELLSQHSTYFKGYLNFSE
RNCESLNVNWSLPIFCDLLRCIHGFEPCLSSSMIASLIEGAIYFGVEDVITKCKAWVASDTFCVKWKIEDDLVSELLSIW
LSATVNGTGDASGNEMDLCKALLLWYESQKRNSRNGAVVDDPILLFKKIQLNLLPIDFILRELVGKYPLNIFHVSVAATE
SDVDVLNSIIDFKGELAAKPVKDYLNFKPVIRLTDHLKELDLSGCHQITCHHLISAAAQNGMKPKRIAQKTLVTIEQETW
PELAEFINLETLILSHCWRVEQRNLVTWLNLVCPNLRELRAPHCPQLLQVILEIGSGLPNLSVLDLSVSEELVEIEGVVT
RTKSTISQRLPLRWSFGKLHETNKLTDLSLRGHSEITDEILLFIARSCPLLRVVDLGGCLELSDVGFAAFLNFQENLAFQ
KLSAAGTRFGPVSCKALISQANISVNVLDTLNLNKCLGIVSVTSSWDIVGRSLGCGHLSNLLQVLPSLVSLELAFTRLDD
MALSAFSGTSLRHLNIRETKVSGGVLYRILARNFDLKDLNIRGCTQLALNSFDHLPSEKTFERMWENVGIGWGLSDSTIS
SFGFASCTLQNFAVGVGGTISEETLLCIAEQCHELKRLSLCFQFVADEGLIQAVSQLKSLHTLELQNMACAPRNLLTEIA
SSLPNLRNLKLERVTPLLSDDDLLLFSQSCTGLQSLSLLGCHLLTSISLSTMAHSWRGLQDLRLEECKYIITEENVRLLL
NSFPALKLLHLRHIGKVLPEYFILEAIRRLPLLQKLAIDLCDAANEKFSFSEGVERSALKSVYINRCKSVRDAFYGVRDL
TSEGISPYDSRTHKDTIVLEWNSVRLTESVVNERLTFE
>Php_XP_001771610
MVPLEKIGFLKNAKRKNTCSQNLASCENDSPIHRPLMTLTSTPAVNMLKPLHHIGLHEVPPMNLEALSGIRNCPKNRRRR
DEHNLGWWAMRGMEGLSLCVNAKSASQWASPHLCEKRQPNLASEYGLSGAQNWNRFAHHVQKLPDRVCWVDALEKQRLQV
AVALRSPCLTAGSSLRDLGGLGRWSLPEMAAEKSHAVDEVTAEVEAVGLAEDPGRNLKLDQATFFRCVENGNLSALTALL
KNRKVDINAYNDEGVTCLHVALYKYEESRSLDMVKFLLAHGANISLKAAVMPSAHKISIIRHGHGGSVANPLETKKVVFD
QKTPLLVALELKSALYLKGWEYRHWDEMLRILADATIEHHAKSPELKGERLPYIVIQKNWGNVFASGKHETIEVWAEDKS
ITVLKLLLTGASKILKLNIENPDVQYSNRLDIKEASFNITKAMMKFLYTGKVEAEFLEHRGLDLLSAAHKYGIKSLMWVC
EDSIRATPDNWIKLLSTAMECNSDMLALKCAQSIKEVMDERHEKHTSLKKSYSNMHNVPNQLFTSFP
>Php_XP_001771535
MDHEGCSSGGMSAPTTYTFAFNDSNFSDRVLRIEVVAVSEKNDAGSASSRQKKRRRAHRHSEAGSGVSKLLGGPVEAQLG
EGQEEQVMYVGEDVAPQEADEEAVAMIEEPHGVNSMFSALGGTWNMDTSVVLRSKTIHISSAILAAKSPYFYKLFSNGMR
ESEQRDVTLRITQAEESPLMDLLQFMYSGRVQANTPATVLDVLMAADKYEVATCMRYCSRLLKNMPMTSESALLYLELPS
SIILAEAVQPLTDAARTYLANCYRDITRFIDDVMGLPLAGVEAVLASDDLQVASEDSVYDFAVHWARHHYPKLEERREVL
GSRLVWLIRFPMMSSRKLRKVLTCTDFEHELASKLVLEALFFKAEPSHRQRQLAMEETMHKRYCERAYKYRPVKVVDFDT
SSQCLVYLDLKLDECRALYPQGRVYTQAFHLGGQGFFLSAHCNLDQQGQYKCFGLFLGMQEKGSVSFAVDYEFAARMKPT
WEFTPKSKGSYVFTGGKAVGYRNLFGMQWQDFIAEGSPYFRNNIVHLRAELTIKKSPSG
>Php_XP_001771155
MAKREGGFSSWLQRVRQTGEYSDVTVQVEGEEFRLHLLPLLNASTYFRNLISSPNWSSNASDQRSSRIISLSGLPGGAEG
FRVAVDYCYLIKPNYTVQNVAQIRAAAEYLGMSPVLESTKKFLYTNIFAHWRASVGYLHQYQPLGSPVDEYIETRCLKVL
VAALARAFGETKHLSAPSPLTTGNLQSSTCVALAEILVRVASLPDTYASEALDALVEADVNLNVKCRQGRNVRGWLGSVV
DDECTTVRARCWIVLCLSRMLERGAPDTRPWLELSSQYWCSLLEHVDHLVSAVADNEVMQVRLIDMKMILERRIGASLDE
LDEYLHVYKFGPDTLMAMVKHYLNEGHHDPDSDSIEEIAGEVDKFLWMYAEAGSINVETFVSLFKAFPSSTRLSHDTIFG
AIEKLLTTMTDATPEEKQILWRLVDPAKLSPAVNEKALNNPGFLSQPHVLEMVLQKHSEDLAKVPDTDAQNMRHIMQKVI
NASLKLLEENSRRSQEIVELQQQYSALLGGKICHLQDSCESSPSTCWRPSLTITHKSMVHETETEGSEFSETPSVATLSS
IGKTFRLAFPAGNRLFIGALAEEKRSENYF
>Php_XP_001771144

MTECETAEVESPLNVVSNGYEVGERATSDVIVRLRTVHGRDDWFYTHSKILCAKSQYFALRLSDDWPTYHLLDSRYCVEV
LCIENDFDYHVNLLRMMYSNLDEPVGDMWHSVKNALGMLKVATKLGCSAVASKCARYLEAVPWEESEEEEILKALPAIVG
SSHKLVAELAAEVQPILARLQPVEAVSVRNVFLCAVHAATAPQSRSKYLEKSPSDLKVAAQEQVEYMLEEDDDAPLLIPD
DDLKTDLKKHFLKLFKGFKAELSAHLTDSTKDLKLSEKTIFEKVCDLVWASRVLPHVGLNPDFVEFWVEASSEITQTLNT
AKLRETSWNTKLLVVEITATVLKAVGYGDVILTTNDRIQLVKVWLPFFRETKPVLDSLFEGEKMELEMDLDLSESIENSL
VSLVLSLPSDDQAEIFADWLRWNHSWFPDLSEAFEVWCCRSKAAKRRLNIEVPNGANAISSVGA
>Php_XP_001770455
MAKSCGETHLCLLASTFLIVSHTWCRVLTTDVPSDIVVEAGGMNFSLHKFPLVARSGRIRKLVANLADSENPNLLQLTDV
PGGAEAFDLAAKFCYGINFEITTSNVAVLRCAAEYLDMTESYGENNLVARTEAFLSEVVLQSLADSIAVLHNCENLLPLA
EDLGIVSRCIESAATKACREQNSNANFKLSSSSNLSHASKVPMADWWAEDLAVLRIDFYQRVLAAMRMKGLRVESIGGAL
MHFAQRSLKGLNRKQNGRSDLKPPKIKHEQRILVETIVSLLPPEKNTASCSFLFGLLRTAIILDTTLACRLDLEKRIGMQ
LEQATLDDLLIPSFSYTGDTLFDNDIVHRIVVNFLQQDDSEDSQVAHPMYDSDGGGSPSQSAMMKVAKLVDSYLAEIAPD
ANLKLSKFIALAEILPDYARVVDDGLYRAIDIYLKAHPALSEVDRKKICKLMDCQKLSQEACTHAAQNERLPVQVVVQVL
YFEQVRMRNALAAGAFVDAVDSGGGHYQHRLGSENLSASHSPKENYSVIRRENRELKLELARMRMRLTDLEKDHALMKQD
IHEKPIKSSKSTVGRKLSRLNPFARRDSKDLSSSNGSGKPVHTPDPSSRSGFGRRPRRHSIS
>Php_XP_001769458
MASSGAKSETSQDGTSQRRGQAWLCTSGLQSDIVVDVEQMTFHLHKFPLVSRCRRIARLVEEVSREDEDTCEIRLENFPG
GTWSFELAAKFCYGVKIEFTSSNIVALRCAAEYLEMTEDLANENLIVRSEAFLQEVVLRSLSDSIAALQSCSNLFPMTEE
LGLVDRLIDSVCAKANVNDSSRGGWLMQDHRSLQSPGGSLLWNGISTGARARILEVSDNWWYEDISTLSLPFFEKVVLGM
KAAGVRGENIWGAVVHYAKKALPGLHRRHSGREAAHSHRKALVSSNPIVQEHDQRILLETIESLLPPLKVGSSARFLFGL
LRLAIILNASPQCKSNLEKRIGMQLEQATLDELLVPNYSHVEETLYDIDLVQRILDHYLMLEQNGSPESDEEGLLLGSPS
LCPIMMVAKLVDAYLAEIAPDVNLKPSKFQALAEALPDYSRPSDDGLYRAIDVYLKAHSWLSELEREMVCKIMNCQKLSL
EACTHAAQNERLPLRVVVQVLFFEQLQLRNAIAGSFLVADTVGGSGKQQEQHHIPPGVVRQGESWEKSLGGSKTLKDDMS
KILARVSQLEMECGTMRQEIENLHRAKKPVNSLSNAFGCKLEREWVRLVG
>Php_XP_001769287
MQPATSTDNDGGPNRRGMDSNMGALCEHVQLEGWKSKAFSDIIVKAMGRTFELHRLLLSRSSFFRNMLQGPWREAGAPEV
TLQIDDKNVNGEAVETALAYLYGHYPKLDGSNAFRVLAAGSFLDLQDLCSLCTNFIISELWTENFLAYQAFAESQDYGVH
GERVRKACWGYLCRGAAVELREILPKLSTQTLRKLLTSDELWVLNEEKRYAVHLSITVFHISMIGRAGNSCLDSTNSIRG
DQREGASVCMYDHGLGALNGKPLKEVDPAQPFIEIRKDGEACTSGDTCATPDQLSFSGIEPGFCGRFCYEEPWHPPRTGE
QVGLGPCSPSPETTSSSPSSKVIEDQWPRDNACPIFSWGGRVVESRVKKAFPNSGNWGSAEDWEAFLGVFEGGGVLYCHM
AFDDLLYMRGRLEELGFPCKAVADSLWLQTLLRQQVLTIAADTCRNCSLVYGNCSCRQVGSSYAHGRTPSLHHRDDGQSA
GPVPTGIFPGAERIGGNSGSGVGANSQSAGRVHVRNTMDGLAGIGRGTTFGPPGSWPAPRFLYIPSLGAYGARGNPLQSG
VGNNQIDPRLDLVGRELPPGAHSTLDGRTQGSTASASVLGQGQPDPTSRAEDSPAASKPADIDAMLNNMDTNKALATRKW
EAGEGGSILLDLNTPLRSFPPFRFGVEFEDVNRLQDGQSKHSTEAFYAGSLWKISVQAFNDEDPRGRRTLGLFLHRRKAE
DMGPHRKVSFYTDTREKVTARYQLKCPAKRGVISLGSLTQAGTLLPKAPKGWGWRTAFLFDELPDVLQGGSLRVAAVVQL
V
>Php_XP_001769248
VSTDAPADIVVEVGGQSFALHKFPLVARSGKIRRLVASEKADENGILHLQLPDLPGGSEAFDLAAKFCYGINYDITTYNV
ALLRCAADYLEMNEQWGDNNLVERTEKFLNEFVLQNLAESIAVLHNCESLLPLAEDLKLVNRCIQAAAIKAVRDQAGDYE
GSSHLDNVKVSHNDTPTAPVVEWWAEDLAVLRFDFFQKVLAQMRTRGMRCESLGGAVMHYAHRALKAQRSTHEQRILVEA
IVSIMPPERNAISCSFLFGLLRAAVVLECTLACRLDLERRIGLQLEQATLDDLLIPSVNHSGETLFDVDAVQRILNCFMQ
QDGDGEVDHTDPMYEAEGVGSPTQSALIKVAKLLDTYLAEIAPDANLTISKFMALAEVLPEHARRVDDGLYRAVDVFLKA
HPAIIEAERKTLCKIIDCQKLSQDACTHAAQNERLPVQVVVQVLYFEQLRLRNAMSTINTRDPVLRLQLSQRMTVGNVAT
GQPPNENYESVRRENRDLKLEIARMRMRMTELERDQNNLKVDIVKNGGGSRFIDSMSKKLSKLNPFQRRESRDFKNSGEL
GTNSSGTPLTPDTRSAKPRRRRHSVS
>Php_XP_001767403
MVISPLVEEDMDIGLYGDEPEEPALPRKKVPMGDIYEASRAGDVDRLKLLLDEGVNVNARDNWDSVALYYACLAGHLDAA
RILLEKGAICSENTFDGDRCHYASLNLQVRRLLKAFEARPPPLDPLPRTFRELFVSFEANCRYLEGAPVNYNDNSIYGMK
QDGRSHTETAAHEKILRDAEDCLGPDVIFYISGRPFGAHRALLAARSPFFKEQFQGKWKHRQVVRLARPRLTFAALFSLV
HFFYTDRLDVAVEDMEDLVLICKNCKCLALQKVLENELAHQKYADYKSIKRVDDSQKRFIFQGSSLPESERLSSALYSLF
TLSLSNSAQRKKSDSKGDEDRSKPSRTSVDSSKSFSANNRDIIVDRKDTSEGGDIEKLEVEMNECSISKQDEHCEEDHAD
VCFLVGNEKFRCHRFVLGARSEYFKARFSRTTGFREGVAGLATNSGADSLLILQENDLSASAFEKVLEYIYTDSVKTVDL
DEAEELFDAASRYLLFPLKRAVTDALLPQLETASPADLCGWLLLADKYGVWKLREHCLDAMAANFEFFSGIPEFRQMLLC
LPPPSGDLAERTTAPKAPGVEDGPQAGENVLDDLREKWLNEEGSELDKRDESALEFDKRLEQLIAIAEEEYVQNES
>Php_XP_001766772
MESKSYPESERKGHKRKLADAYVRFPAVDEPNNAFATSVRDQVEILRTCVSWKENDRIAARRAAHSLAELAKHEEHVDTI
VEEGAVDALVAHLCAPSLRESEGPIACEHEVEKDAAFALGLLAVKPEYHRRIADAGALPLLVALLSRRGGTSNARVANGV
VRRAADAITNLAHENALIKTRVRTEGGIPPLVQLLESTDAKVQRAAAGALRTLAFKNEANKNQIVEGNALPNLILMLRSE
DVGIHYEAVGVIGNLVHSSINIKKEVLAAGALQPVIGLLSSRCQESQREAALLLGQFATTDPDCKVHIVQRGAVRPLIRM
LEATDTQLREMAAFALGRLAQNTHNQAGIVHDGGLKPLLELLDSKNGSLQHNAAFALYGLAENEDNVSDIVSEGGVQRLY
DGYFIVQASKDCVQKTLKRLEEKIHGRVLKHLLYLLRTADKVVQRRVAITLAHFCCPDDQRLIFIENNGMDVLLEMLNVF
SNPKLQRDGALALCILARKANALSPIDAAPLPPTPQVYLGEQYVNSSTLSDVTFLVEGRRFYAHRIALLASSDAFRAMFD

GGYREKEALDIEIPNISWKVFELMMRFIYTGNVDMATDNAQDLLRAADQYLLEGLKRLCEYSMAQNLTLETLMNVYDLAE
AYHALSLRDTCVLFILKHHEQMCSITGYPALLHRITPEIREYLRRILRPQPAALQ
>Php_XP_001766377
MADCISKVETTTRLVQLRVDSLGTGCYRRSDPFKIGLWNWHILIEKKASLIVRLYPEHCRKEPSPIATFTVRILVRGSDG
QRVESKVSDKLLRSSSDDFMWPLEYSVAYGRFLVEIEFHDLKVASSHSGDPHSIWTGEEYLQSRAEVGIARCLSRMLEEG
LHTDVTICSATGCVGAHRAILAARSPVFDSMFSHNLMEKQSATVRINDMCLESCRALLSFLYGNLKYPDFRKHRVALVRA
AHKYDIVDLKEACESSLVDDIDGSNVLVRLHDAWLYQLKDLKRECLRYLLEFGKVHDLRNEFDAFLQDVDKDLVVELVQE
QIKLLKIHGL
>Php_XP_001765920
METKSYPESERKGHKRKLADAYLRFPAADEPNNAFSIGVRDQVEILRTCVSWKENDRIAARRAAHSLAELAKREDHVDAI
VEEGAVDALVAHLCPPSLGEGEGPVACEHEVEKDAAFALGLLAVKPEYQRRIADAGALPLLVALLLRQGGGNSGRVVNGV
VRRAADAITNLAHENAHIKTRVRTEGGIPPLVKLLESNDAKVQRAAAGALRTLAFKNEANKNQIVEGNALPTLILMLRSE
DVGIHYEAVGVIGNLVHSSVNIKKEVLAAGALQPVIGLLSSRCQESQREAALLLGQFATTDPDCKVHIVQRGAVRPLIRM
LEATDTQLREMAAFALGRLAQNTHNQAGIVHDGGLRPLLELLDSKNGSLQHNAAFALYGLADNEDNVSDIVSEGGVQRLY
DGYFIVQASKDCVQKTLKRLEEKIHGRVLKHLLYLLRTADKVVQRRVATTLAHFCCPDDQRLIFIENNGMDVLLEMLNGF
ATPKLQRDGALALCTLARKANALAPIDAAPLPPTPQVYLGEQYVNSSTLSDVTFLVEGRRFYAHRIALLASSDAFRAMFD
GGYKEKEALDIEIPNISWKVFELMMRQSGFIYTGNVEVASDNAQDLLRAADQYLLEGLKRLCEYSIAQNLTLETVMNVFD
LAEAYHALSLRDTCVLFILKHHEQMCGMTGYPALLHRISSEIREYLRRILRPQPATIQ
>Php_XP_001764279
VSSDAPTDIVVDVGGQSFAVHKCALVARSGKIRRLVACERANEDGIIHLQLADLPGGSEAFDLAAKFCYGINYDITTYNV
ALLRCAADYLEMNEQWGDNNLVDRTEKFLNEYVLQNLAESIAVLHNCESLLPLAEDLKLVNRCVQAAAVKAVREQIGGGL
NRSEFDGSLRLDNAKPAFNDSPAAPVVEWWAEDLAVLRFDFFQRVLTAMRTKGLRCESLGGAVMHYAHRALKGIHKRQTM
KSPKPQHEQRILVEAIVSILPPEKTAISCSFLFGLLRAAVVLETTLACRLDLERRIGLQLEQATLDDLLIPSANHNVDTL
FDVDAVQRILNCFMQQDGAEDVDDTHPMYDVEGAGSPTHSALVKVAKLLDTYLAEIAPDANLTMNKFITLAEVLPEHART
VDDGLYRAVDVFLKAHPAISDAERKTLCKLIDCKKLSQDACTHAAQNERLPVQVVVQVLYFEQLRLRNAMSSINSRDPAL
RHQLSQRMTVGNLTGSQSPNENYESVRRENRDLKLEIGRMRMRLTELERDQTNMKMDIVKSGGGTKFIDSVSKRISKLNP
F
>Php_XP_001763910
MSTIAQTSRHCVRGSSCRAVANERCCWLYMRVKLERPEMLENWKRKVKYRRMMDGGRGAGDEDGGGGGGGAGVALATDLS
MCFQCGNLIENTVTYCHTCYDLRSESDFRNEQRWRNLESNLLAVEADLQKERAATKRLEGTLQSFRHMLLQGIHADVTIE
TGEFDKTPAHRAVLASRSPVFRAMFEHELKEKTCACIHVADVPTPAMRALLLYLYTGDHDAKVMKEHGMALLTAAHKYDI
PDLKRVCETAVANAVQPSNVLEILHQARLYDATWVKKACIECIAQNLKQVAFTKEFRNLVYRNDDPEAILDIIQSIAALQ
>Php_XP_001762013
PVDKIKASPTLFEMMTHEQELGPKAVHSISLSQQLTFQEKMKSILSGTSPGNQFNDTTTSDLKLSLNNREGCSVTVNVHR
HILVTHSRFFAAKLSDRWLKQQRANPNLIEITDIDDVEIYLETLRLMYCEDVKKSLLKENVNRVLGILKLSAHIMFEGGV
FACLEYLEAVPWADDEEEKVTALMGQLQLESVGVASDVMKRCSSLESTNNEDILPRLLCAVTKGTDDKARREMKSLVSRM
LRENMSQNKNTVDISKESLYRACHGCLDSLLHLFTQSKSGEDRNMLVEISRQADNLHWLVDILVDRRIADDFVRMWAHQG
ELATLHAQVPVMFRYEVSRLTARLLWVRLDKQVLSPKDVRFLLLQTWLQPLVDDFAWMQRCCRNLDKKVVEEGISQTILT
LPLKQQQCILLSWFDRFSSSGDDCPNLQKAFEVWWRRTF
>Php_XP_001761592
MAKGEIAVVEPPPIVFSYSFKLGERATSDVIVRLRTVHGRDDWFYAHSEILCAKSKYFASRLSDEWPTYHLLDSRNCVEV
ICIESDIDHHVKLLRMLYSNLEEPVGDMWHSVKNALGMLKVATKLGCDAIASKCACYLEAVPWEENEEEEILKTLPAILG
AEIQPVLARLQPVDAVSIRNVFISAVHMATSSKSKTSDTLSTLPSDLKAAAQEQVEYMLEEDDDAPLLIADDDLKSALRV
KFLNLFGSFKTELIVLTDSTRQFSEATILEKVSDLLWASRVLPRVGLNPEFVDFWVGISSDVTGALNGEKVREVLWDTKL
RVVEITATVLKAVGYGNVILSAGERVQLVKVWLPFIRETKLVVEEMFAVEKVSMEMDVDLCQSIENALVSLVLSLPSGDQ
AEILADWLRWDHARFPDLSEAFEVWCFRTKAAKRRLGVENRKVGSAIRSGCAS
>Php_XP_001761402
MKKTIMRGSLRGGDDAVVHGLIPMIITTGKNVPPECARERNHGDDGDENIVVVVVGQSEEEQFGWYEEEDGEEKRLGFAG
EERGRGEEWRWRKRNLKVPRVSGEPTLKSLQVQVKRLEQQREFLSLLRPVLASDVPDVGAAYADVELQACDGSPVYAHKA
VLAVKSKEFHEAFRANADVKVIEVSEMTPQELKVFVTFLYTGTISNNISLECMPTFFRAAEKYKVQFLAEVCEDTLVSNL
SRQNAISTFDIVKKHCSSAVRESVLVKAMKMGEISTYDEYKHYTQKDPGLLLELYELLSERIGPLLAKRRRISTGLSGEA
SLYIQADLDACSDVIFGFHCPGIDIGGAFGRDSRDEREMKPENEIASGKFILMTRFSILLVSKYFDLKNDSASLVHSTSS
LGAVMLLNGSSRDGGNGCSSAGGGVFWQGGQPIPPQHGRTYSAGYDRAPGPRPTYDGQMMSPAYVVPPYLSPMHMGGSLP
RGGFVGAGAQGALGMMERPVGGDELMVQDTRGKKLPVEAQTKIEGRRGQNWTNCEVEALIALRGEMHEEFERNKQKQGVN
TWNKLHARMLGVCKGFRKSANACKKKYSILYNEYKKDRECAKDINNDAFVAGGLPRSKKCAFFDQMDMWYQNRPRLKIDA
NQGEHLESPCTSADDVDLKEE
>Php_XP_001760430
MDHEGCSSGGTSAPTTYTFAFNDSNFSDRVLRIEVVAASEKNDAGSTSARQKKRRRADRNTEAGLGVSKLLGGASETQLG
EGQEEQVMYVAEDVAPQEADEEAVAMIEETYGVTNFAGEDGGTSSGSWNMDTAVVLRSKTVHISSAILAAKSPYFYKLFS
NGMRESEQRDVTLRITQAEETPLMDLLQFMYSGRVQANTPSTVLEVLMAADKYEVATCMRYCSRLLKNMPMTSESALLYL
DLPSSILLAEAVQPLTEAARTYLANHYKDITRFIDDVMGLPLAGVEAVLASDDLQVASEDAVYDFAVRWARHHYNKLEDR
REVLGSRLVWLIRFPMMSSRKLRKVLTCADFEHELASRLVLEALFFKAEPSHRQRQLAMEETMHKRYCERAYKYRPVKVV

DFDTSSQCLVYLDLKIDECRALYPQGRVYSQAFHLGGQGFFLSAHCNLDQQGQCKCFGLFLGMQEKGSVSFAVDYEFAAR
MKPTWEFMPKSKGSYVFTGGKAVGYRNLFGMQWQDFIAEESPYFRDSIVHLRAELTIKKPPS
>Php_XP_001759240
MSPEDQLLKSLSADFLDLLDRGQAFSDVTFNVEDRHVYAHRCILAARSPFFRTLFCGDTQLMNSAQPRSSLPSVIRVGIV
SYDVFMLLLQFLYSGNCNGFFSPQISGRQCKVNSCWHSSCSSAVKFGLELLDAVSFFGLEQLSIIIQTHLGAIAEKASTE
DLMRMLIAARYQMENHLWKLCSKVVAKSGLTPEILHKYLPAEIVGELESIRQRSGYALEASSSGNDMLENKTKLMQKALN
SSDVELVRLMVMEEGLILDKAFALHYAVNNCSRKVVETLLKVGAANVNLQDQDGETPLHMAAKLGDPEMIALLLDHEANP
LMQSVTGATAMDIVQSGAAGVQSAGGYNSKSDQVRFRLCVELLQSAALSYSPPLQVKATRVEIGSPVSIRTEHGSSTLLP
DECDNDYHGSMSRMVGGSTQGIHFGNRDVYSSFTHGQGKT
>Php_XP_001758201
SRNISPTLQRSVQSWCDATGLPTTITMRCAGRVYHLHKFPLVSRSGYFKRVLKDAKDVTIPDDVPGGPDVFELALNFCYG
SSILMEPSNIAEICCVAEYLQMTEDYGRANLCERSELYLNQVALQSWEDSLVVLLHCENLAPHAEKMGIFRRCLDAIAFM
AGLEILDPVARKVAPIKNRDLHCWSTNSRECSHLHWCIQDLVALPPNLFVKLVLALRREGMQESNVGQVITAFADRWIFD
SGVDTVLVQRGNDKCWAVVRVLPLEHYVVPTGFLFCLLRRGLSCALHDDCRIQLETRIALQFEHATLQDLLLPTKKEKEN
GCVFMNEVDSMERILKLFLTRFRGYDDARLADMSMLTAIGKLWDDYLSEIAFDSNIAPSRFAELIERIPAYMRVMHDHVY
RAIHAYLKAHPNCTQEERMVVCRTLNCQKLSLEACTHAVQNDLMPLRLIVQAMFMQQLQTGSVLASHIESASQSFRTEPH
SFTRSILPDT
>Php_XP_001757508
MAPGDTDSLRTLSLDFLSLLGEGQVFSDVAFEVEGRHVYAHRCVLAARSPFFRTIFWSDVQMMSNTQPKPSIPQVISVGI
VGYDVFMTLLQFLYSGSFQFSAQSSGRLCQDKSCWHTHCSAAVKFGLDILNAAVFFGVEQLSAITQNHLASMAEKASIED
VMRMLVIARKQNDLHLWHLCSKLVAKSGLSPKMLLKYLPGDLVQELQSIRQKTGYNSDTTASGSATLEQKIKRMQKALDS
SDVELVKLMVMGEGLNLDEVFGLHYAVSSCSRKVVKNLLELGAANVNLQDLDGRTPLHIAAQLGDPEKIAMLLDHHAEPH
TRTATCATAMDIVQSGAAEIQSAGRYNTKADHNRLRACMELLERAALASTRLTHTRSAAVVDTGSPASILTENGSSTWFP
EESDNFQGSMSRMKSAGTPGLLQSGNDIFSSLWNGLPGKGMKRPPTELVPISRNLAEKVSWPW
>Php_XP_001757364
WCDATGLPTTITIHFQDRVFHLHKFPLVSRSGYLKRALKEAKEITLPSDVPGGPAVFELLCNFCYGSTILMEPSNIAELC
CIANYLQMTEDYGRANLCQRSELYLTQVALQSWEDTLVVLQHCVGLAPHAEQLGIARRCLDALAFMACMELLDPVARKLP
IPGRKGDYQTSYVHWWIQDLVAVPPSLFVNLILALRREGMQENYVGQVITAFADRWIVGLQGAVDSTVSAHKGSAKKSWF
KEPPTTNLEPSVLIESVVGLLPLERNVVPMGFLFTLLRRGLSCALNDSCRIQLETRIALQFENATLQDVLIPVKKEKESG
YIFMSELGSMERILKLFLTRFRGLEEVKSTHMAMLSATAKLWDGYLAEVAFHTSLTPGRFGELVERIPPYMRPEHDHVYK
AIHSYIKAHPCTTQEERLAICRTLNCEKLSQKACSHAVQNDLMPLRMIVQAMIVQQLQTRS
>Php_XP_001756524
MDCCLCSSVANSYRPPRNTLCKNCYEGAKSIIDLCRKLEQQWRDDEDVERGRQAAPYGLQYAFNQIKQLDQMEENVSEKL
EFLGSLIEAFQTGMHTDVELVTNDGLSIHAHRIVMATKSSVFRAIFESDALKEPHTGVVHIPELSHYELRYLLEFMYCAE
IPADAMAEHGHALLMAADKYDIPVLSKVCEAFICATVSPSNVLDVLELATLTHATSLKETAINVILDSYEEVVFSKGYED
FASKNALLSVEITKALIKVLKK
>Php_XP_001755146
MMVIGFGALTQQMEPLEQRSVRMNDLSRLNTENCSVELGRISVKLSKIKLKEVNATTEGRWIDLEVPALQGMLRALPGES
EAKREGGFSTWLQQVRRSGEYSDVTVRVEGEDFHVHLLPLLNASAYFRNVTSHPHWSSGWSSDASHQGSRVISLPNLPGG
AEGFAGAVDYCYLIKPNYTIQNVARIRAAAEYLGMNDVLESTKKFLYQNIFAHWRPSVGCLLQYHPVGSPVDEYIETRCL
KVLVAALARAFGETKHLSAPMPLRAGNLQTSPCQVLSEILVRTASLPDAYAAEALDALVEADVNLNLKCRQGRTVRSWLD
SVIDDQCTTDRARCSVVLCLARMLLRGAPVCRPWLELSSQYWCSLLEHVDHLVSLVGDDEDMQEKLLDVKRVIERRVGVS
LDELDDYLHSYKLGPDTLMAMVGYWLDEGHPDPASFEEIAGEVDGFLWTYAEAGAIDPETFVALFKAFPNSSRQSHDTIF
GAVEKLLATRPDASPEEKQSLWRLIDPAKLSPAVNERALNNPGFLSQPHILEIVLQKHSEELAKMPDTDAQNMRHIMQKV
INASLKLLEENSRRSQEIMELQQQYSALMGGKICHLQDSCDNSPSPCWRPSLTITHRTTQSETEGSEYSETPSLATSGFE
IVELLDDEEEKSVHYLSPKQTHSFQESLASSTVDPSSFRVLLTNCKFAALNTWSLNRIAKILSKVGRHLIYELE
>Php_XP_001753499
MCYFPSAFNCYHNVDFNNLAGGEEADFTTLKAENDADMAEWMRPPPKQTPAEEMGYNIHVHKLISPTHYNLDRQDVLRNN
YVTMLRDGVNSDITLRSKRGSVRAHRSVLAEASPVFEAMFKHDMQERRSSSVRLPDMTIGGLRIFLTLLYVTSGSHAPAI
DNLANLFEDAILTHFDELADACMKYQVTRVMNVLQLKLRRKLTPANCWTYIHKFLADSETVTSKISYKSCISFAVKHVVQ
LIDSDNFLEEMSKNPKIVHEILMHVFKL
>Php_XP_001752591
VEVGDRGFNLHKFPLLSRSGRLNRLAYESRNTERCQIDLSDIPGGARTFEHVVRLLYGVKLELSPHNVATLRCAAEYLEM
TEDIRNENLVAKTENYLTKVVMSSWRDSISVLKTCSELNPMADDLEIVSKCIESIARKCNADLYAVRWSFSSRAVAKEVQ
RDWWFDEVCSLSISAFKKVMNAVSKKGLNPVDFGAAVEYYAHKWLQLAKELDFSDQQMVPQDATKTLHRHRAVLQGIVNL
LPFQLSATSVKFLLKLLRVACLGNASSFCKTELAKRISSQLEKAEIDDLLIPVPGESNCDLDIVQKIVEFYLQKLESKFY
VNEGPSRKTQLPPTTTNCKVAQLLETYLEEVGKDSNVSPAKFITLAELITEFPRDSDDSIYRAIDAFLRAHPSLTELERK
KLCSFIDTGALSLGACVHAASNEHLPLRVIVQVLFHEQIKLRNAIT
>Php_XP_001751401
LVVPPFDCAWLMGDEQSFLTPGRGCVAFEARAANDVTIVFKEHPGCKHYRTDLGPNYTIILGSHGNRRLKMLVDGKTVVD
VADIVLCPSTFERYWISVLDGRIIVGKGEPGQSIVHEWSDPSPNFKIKYVGLSSWDKHVGYRNVRVLPPAPISGKYMPNL
RDLQGIGGGLAQYLESSEFSDLQFVIGPNRRVVRAHRVLLASSCTGFSHFSGDVCRLASVNYSTLHAFLQYIYTGRTVVT

RSELSGLQDLSEKFGLDCLALQCRQLKIVASEEVNYEGANVSQENTKFELVYQSPVAIFEERSTHALNIPVNAAVMVQHL
DSGEFSDVEISIEDYGTVARAHRLILSAWSEPFAKMFTNGMSESSVGQVLIRDTEPQVFMAMLWFMYRGHLDLEERSDYW
ALLLPLLVLADQFAIQPLQQECCLRLLDCLAEDSACAILQVAASMPNCKALRDACQDCCARHFDYCIIPPAAVFQALEAS
CISQILQHSALSVISEEKVLDAIMMWGANKDDIHGWEDANQRAMAEGAELFQDRLEDVKLLIPLVRFPLMSLPVLQMLQV
SELCRLIPAIEELIFQSLDMRTQGRLVKFPRSTARFSQRLSTFKELLYISDGDCNGVFYYLGTSYGSHPWMNPALTKKLV
VSASSPPSRFTDAKALVSRNYQGTSFAGPCNVGGQMSAWWKVDLGADQQLLCNYYTVRQDSSSSFMRNWSLQGSVDGQRW
TQLRTHHNDQTIGHGGQYASWPVFGANALLPFRFFRVILLGPTTSVSTPWNLSLCYLELYGYLR
>Php_XP_001751363
PVDKIKASPTLFEMMTHEQELGPKAVHTISLSQQLTFQEKMKSILSGTSPGNQFNATNTSDLKLILNNREGYSVTVNVHR
HILVTHSRFFAAKLSDRWSKQQRTNSNLIEITDIDDVEIYLETLRLMYCEDVKKSLMKENVSRVLGILKLSAHIMFEGGV
FACLEYLEAVPWADEEEEKVTALMGQLQLESVGVAADVMKRCSALESTNSEDVLVRLLQAVTKGTDDKARREMKSLVSRM
LRENMSQNKNTVDVSKESLYRACHACLDSLLHLFMQSKSGDDRGMLIEISRQADNLHWLVDILIDRRIADDFVRMWAHQA
ELANLHAKVPVMFRYEVSRLTARLCIAIGKGQARQSNLDVRFLLLQTWLQPLVDDFAWMQRCCRSLDKKVVEEGISQTIL
TLPLKQQQSILLSWFDRFSSSGDDCPNLQKAFEVWWRRTF
>Php_XP_001754601
MPNQHRGSGGARGGHDEERLQQLIGRMRTALQLGLRPSSRKDGELKWGNTDAQVQGQAVRAIAAFVAVIPPRSFSSSQLQ
VVTSLMLLQIPVDYTCEPPVESFKYFSRSSLEVLPIYVLIFLESFISSRLRFLSSLSKCIVFSISRGDIVFFSQVHLEEA
VAALEYLLSVEHNTIYRQAAVVSAGLGRATNGRYMLRLLSPLSCLLAPAKATEAVVSAATAIQSILATVRPQISFDGTMG
DDPVWRVLEDSDALKHILRRLEDPVATTSEIAECTEVLAMILERWSGARYRVGQVGAVRSSLLNHCMSSDGEVAVASLQA
CQALGVAVLFLQGGDLLWETLAHCLYQSRPLLVNREALRLLSLLGVGRLDCLSPGRIFPSYVNRLSSFTTFFKGPHIQVI
LESCIRNINEGNRWRLTVVEQGVVVEAAHTTSVILRWAGEHHNIVLGLGIFKAIFLQLTVVETKSATNAKFWDGTPEKVD
VEWKSRGPGKVTSSLRSLLWEIIGCLAAHSNSMTTKEVLSGRLPAGPGLGGITVFACATLLRALHRSGDHTPVTTVPESG
SGEQRISSQELLQICKTMYLLLSSISQDVSAPTKACMEIAMKFHDQTWLARLVESLAFGHTGPSQVKPEAVQTATNLFAL
ACFTFFPECRELLPQERTLDVLMAIVKLYGNKALAKKQQASSCSTKWSLVHQRAGIAAKTCCEEITEDWEGADTVLFASL
CAFTKLMQGSYRARVIAKSLPRGRLSEANCAEALYGDTVGLLWKLAESTNVATGVRWWAAGGLACFNIYGFPSLLGRDIC
KILDESMFPDIIFLFKDGRRLLAHGVILTIQCPSVLPKGLILKEKKVVGDYVGHEIQLSKMVSFNSFKALLEYVYSGVVH
LNLEEVDEMKVLSRGCGLDTLTNLLHGRAPVWGQPPASCNLASALDSGGYPFADIILRGKSQDDTIGEAIPCRFEEGCIE
TYTHVHGHRIILSSRCDYYRGLFCSGMRESSAKAIDINVSQQSLRTLSLYWYTGKLTRLTETSGWCAWNNLEADKQIRYL
QNLIELAQFSGQLLMTVLQEQCNLLVLQHMVGCNQHLGPSVITHAAICQQWELVNMCANSMASAYPHLRDTGALEHLDET
FRDLLRAAHIQFMRGLD
>Crei_XP_001703701
MSGPSQAAALLEVLRTGLAPPSYAPPDKASLRKASHALAELCKQDEFIDDVVAAGAIEVVVPLLNAGSSSAREQLVDDAA
GTSGVSLQEELDKELCFILGLLAVKPEYQTRIAHSGALSGLVRLLKEHKLTSITKPQPGSGGVARRAADAITNLAHENVD
IKNMVREQDGIPPLVGLLEAMDVKVQRAACGALRTLAFKNEPNKNVIVEQGALPTLIQLLRSEDSGVHYEAVGVLGNLVH
SSQHVKLRVLEEGALQPVINLLNSDCPDSQRESALLLGQFATADTDTKAKIVQRGAVPALVRMLGMPDVSLKEMAAFALG
RLAQNVDNQAGIVQLGGLPPLLELLESKHYNLQHNAAFALYGLAENEDNIPDLIREGALQRLEDCKEKLQASKDCVQKTI
NRLEQVGARRALTSMVFLLRSSTKCVQQRAAMSLARLAPEEQLKSIFIDKRGIDVLLDMLMDPAVSHRSHREAAAALLQV
RGSSVCVCVTWAGQPGRTERSVYLGSEYVNNPTLADISFLVEGRRFYAHRIALLASSEAFRAMFSGGYREKDADCVDIPN
ITWAVFEAMMRFVYTGQLDVTPDIAFELLQASDQYLLEGLKRLCENAIAGSLTADNVLATHEYSEQFSAPALGRRCLLFV
L
>Crei_XP_001703456
MRWEVPDYTLVHSLAYDTAGDVLYVLTPRSVYRAAAPADPSSRLELVAGSEEASGCVDGGYGRAALFGSICDAAVDGAGR
LLVLDMEGPGGGAVVRVVDPRAGYAVSSVQPALGDLSGHAGRLDLLPSGELLILLARCEYLRFQINQQKAQGVTGKFSIP
IDDADAEAFWLLLRHIYTGRVDFPPLMVEPVLQLAERMRMQRSADIIRTLLHPSSSTADGSQQSQQQPQQAQQQAASGYG
QGAAASTPSPYGGEYGAYAAPAAAPAAAQQSPYGYSATTSAPPYGGPSASPYAAAAAGAPSPYGPPPAAAASQSPYGPPS
AQTPYGAPPAASAQTPYGAPPAASGQTPYGQTPYGAPPAAGAQTPYGQTPYGAPPATSAQTPYGQTPYGAPLGASPYAYG
APPQQQQQQPAGYSPYGGGYYPGQQR
>Crei_XP_001703289
MSGRPYTWDEDSDREAFLYAVPCPSNSGVVYFSTHVTLMRLNPDNSVQVVAGSWDRPGERDGHGSEATVNAPLCLALTGT
LASTTRGGAGAGAAGVAGVEGAAASGVAGGGATGAAGQEPGTPSATAAAPGYLFFGDGGARTHAEVVYVIRDPLGPSPSM
VPVAGHDWTDEEREEVGEFGMGPAGDGRRDGRGVDAKFYCICDIMLDSRGQLYVTDLGMQTAHSLPCVRRVDPAADYAVT
TLPIALGAATSASLTQLPGSWLCVYEWGGSRLQLVHIPGLEPCVLPPPAPPAPPPDLAGLSADWGTLLSDPVGTGSDIVV
HVGERSFAAHRLVLAARCEYFRRQFAGGCRDSNAAEVHLHDADPEAFAQLLRHLYTGDLDFPAARLRPLLELADRLLLPR
VAERAQQQLLAAASPGGVVEDMLWAERMGFDQLLAGLKAYYLQYEGRVLSTAGDAVRGMMVSHPDLFFELHVASVVRRAG
GGAGACPLRYAAELG
>Crei_XP_001701338
MQYLQNVAGCKLPLGNNLDDVFLRLDPSNEQRVQALVCDAVLRPLLGTDARWGFSLGDPIDLFWAGGSGAGPRRCFNSER
RDFNDFCWDPWSAALYFVCGKAVLKLLGDEVELVAGSILEEGEADGEGPAARFYWPHTLVSDGAGSLLVVDGGNLRRIRR
LQLPASWQWAGITAAATGGRVETLVTGLPERASCYAVLPNGFLALGEGSLILLDLGLQPLLPQMPVTAAAAAAVAAPPPP
RSLPADLGALLDAQPDGTADLTVRVGERRFHCHRGILSARCDYFRQRLVGDGFADARAAELELPDTDADAFALLLRWLYT
GTATIAVDQACGVAELADRLLLPELLGVALAVVAESVSAATVIDKLLWAAGCCETRGEGFGGLLAQLKAWYVQHHEQVAA
EAGAGRKRLVSEAPELMIELMDAVAVWCTDAGAKRARRS

>Crei_XP_001700338
MLRAKEHVAMCTLAASVKNILVRPDPVDDGETQVLATCDQGLCPLRGVSGTARSGFFLGEPLRLYRAPVGAEEGSQLFRG
DIAVPIWDSWSASVYFKSGCAVFRLVGDQVTLVAGNPTTAGEEDGPGPEARFEAGHLPGQPGQLASDGAGSIIYAGGETL
RRIRLPGAWRAVAPVVAAGGAAAGGADGAAAAADGAPDDVALVATLGDLALYHVDTQSVAYVPAPQPPQPPPQQQQAGGG
QDMMVGREDGRGDRAAFTGFVGITADASGFVYLLDKQPTEGRLRRVSPDGTVTTLVQGLPACLCTPTILPNGYLALGTGT
ELVLLDLGLKPLLPQLQPPPAAGAAALVAAAPPPRSLPSDLGALLEAASDGADTAVVVGGRRFHCHRLILSARCDYFRQR
LAGDGFADARAAELELPDADADAFALLLRWLYTGGADIPAEQARGVAELADRLLLPELCDAALEVVASAAAADNVVDRLL
WAAACCDARGSGGGGGGGASSFARLLARLKGWYVEHHGQVAAEAGASRKRLAEAAPGLSVELTDALIAWEREGARERAKR
PRC
>Crei_XP_001700099
MTIISKASFKTGMKMFTAGSYEWAIPEFERLTAADKQVSPTFVIGGSSWRMLCFPRQNATPHQNVSVFLEYPEASFTPNH
LSPTASFKLIIKNFKDPSKNFEKSADNTFKSHQEDWGFSQMLPLQDLNKESGYLREDGAMVVRVEISVPAPLPGGSYPVK
LSTATPGLSTDLLSLLDNPGTTSDLTITATGSELPGATGCDAGADARPQSRSFAVHRAILAARCPYFRTLFESGLADSGA
RELNLPDTDPDALRLLVRFMYGDGLAVTSREQAYSCLSLADRLLLTQAVELLREHLLSSVSAATFAADLLCAAGLAGEQT
LLRSLVARFADLGDDVPEADLKRVEAERPQLLLELLLSCRAAKRARTAMAGAPSLGRWRVGEGSRRRGAEMVVVEAEDKE
GSGQGGPPIMCGRAAAGT
>Crei_XP_001700012
DFEDPLSIAVDGDGNIFVLDRAADDDDDENIDCCIKSPPDATSDLTLHVGGRKFAVHRAILAARCDYFRQRLAADTFADG
AAAELELPDADADAFALLLRWLYTGAVELPAAAGQLQFIAELADRLLLPDLSAAATHKLLAAVTPATVVDAMLWAERQGE
AGAALLAQLKEWYLEHDEQVVATARDSLKRLAVASPDLMVELHVAKKKRPRAA
>Crei_XP_001699805
MIRDPTWCAWSQQVYLMDGQAVLSLNGTVLSLVAGCLRGGALDDDEAEAADGVGATARFSCARFVTSDHAGCLYLADCAR
LCRVTLPRSWRAADAPLGSARAADLGTQRPPAGLQPTRADGGPPRSLASDLGSLLDSPPDATSDLTLRVGGRHFAVHRAI
LAARCDYFRQRLAADTFADGAAAELELPDADADAFALLLRWLYTGSAPLPTEPAAMHALAELADRLLLPELCHEVQVHIL
ATLSPETVVEHLAWAEHLAGGGAPPTPDGPKDEPPPADLAAAAAGTGDTDEDERQAGSHGPNSIHAGRPQPPQLRHPNLQ
PQSIDATPFSQLLARLRSWYVARHLQIMEAAPDADVSAQPTDHPIAEEEPMPPAPSLHGHRTSSHFATGVVREDEVAEEH
EALGGLASTLAGTLHLGGPFAAAAGNGGGREEPDGADADGHGGGMGMDMGMGPRAHRDRKHRSEKPKKAPLLSSNWLKID
FKGNDTIIRVDRHKLMHKLGVQARDLRLLDLTSATPPAILDRDKAIIVNLWHIKAVITLDYCLVVSPDEGGELAAAFVAE
LKAKLASDNNPSTHGGGGAPSGRTYMGLFGTSSQVTASSGYSSLQLPFELKVLEVCLDMTAAHLDAATKALESDAYPTLD
ALTHKVTAFNLEKARRIKNRLVRLTTNVESVREVLERFLNDDGDMHRLHLTGAELSRQVSMKPGDLSRLSSGLLRNMSGG
APGGPPGAGGDASDSSSDSSSDSSIDEAETAAVEMLLEAYFMQVDHTFNRLQTVHEYIKDTEDLVTIKLDQHRNQLITID
LVLTSLNAALALMTAVAGYFGMNLDSKLQMRPHLFKAVVLTTTLGALALFVLFLFFTWRAKLLYFN
>Crei_XP_001698726
MGSNNQLKLLDLGLQPLLPQLLPAPASAAAAAAAAAAGPPPRSLPADLGALLEDAQQPGGGGTADLTLRVGERRFHCHRA
ILSARCDYFKQRLAASGGFADARAAELELPDADADTFALLLRWLYTGDTDVPLEQARGVAELADRLLLPELCARALDVVA
ASVDAAAIVDSLLWAAGCCEARGGGGGFGGLLARLKEWYVSHHKAVAAEAGVSRARLAAEAPLLMVELMDAVLSQPDGGD
RKRQRA
>Crei_XP_001698681
MDVSGAILLVDREDEQGRLARPAILPNGYLALGSHYGDTLLLLDMGLTPPDDTSDLVIRVGERRFHCHRLILSTRCDYFK
QRLAAGGGFADARAAELELPDADADAFALLLRWLYTGGAAIPPALALAVAELADRLLLPELCLAAQGRLMSGVQPSTIAT
ALLWADARAPASVRLRVELKAWYVAHYTEVRRVAPEGIMRIMMASPALALELMDATHEAARQQHK
>Crei_XP_001698675
MDCYLSARLSPLIACSTAPASVASGIWAGRAKTLVVRLDVENTESVDTVLGVSSRGEAIRVGPSLRLAKATNPEEHYGNL
DIHSMTWDAFTSSFVALGGERSPQVLIMTPSGDDTLLVASLPSDLPAESGDGEGPRMRYDRVPDTLTSDGRGALYYLAWC
EVPNQSGVPFKQYAIRKLQLPQSMRRQPAPQQQAQTQGDAGGSTAGGAQAQAEGGPAAGAPARAAGGGITDRSWQLPEDW
CQAVLKDRGGIWGMLLAGFEEGTGGAAVDGPGGEAQFVGVMSNLAVDGHGHVLLLDKGWEPAPGAQGPTMGAVRRIRPGN
GAVTTVARLKCPAAHAVICLTGTGFLCASWADACDGTVFDLGLAPPAHLQQPQPAASLMRPLSANLADVSAADLTICVGE
RRFPCHRTILAARCDYFKHRLASDAFADARAAELELPDADPDAFELLLRWLYTGGAAAAIPAEHARSVAELADRLLLPEL
CAAALASVAGSVTAATVLDAALLGYRGNGIAVVAPLGNPTAARSAESAAVGASCVLVANQGNKTACIAAQQSGGSD
>Crei_XP_001698661
MFSLSTTNISDVPLFWETVNLVYDSFTESFIVVTGACIQQLIPALHGEDDEPLVLAAVAGAILPVRVQANGRGNVAQFGK
PTHIATDGKGTLYVLDQANIRKLQLPAAARYQPHQQRQRINSMQVEVTTLSQQLPPDMTASGMVYVPAGESPGGSECLIL
AGTKGIYRLPLCNNDAAIEAGGKAGMQGSGSGAVAGGTGGAAEATTATGSLHRLAGNSDTAGSWGIRFDAFGAQAKMLAI
SSGLALTGDGRVVFLDYSATQRDTAVRCIRMSDGRVSTLYEGLDGQWQWPCLLPSGCLAMTSGKDLFIIDLALPPPRPPP
PPPSTGPPPRSLASDLGALLDGAAGAASSDLTILVGGRAFKAHRVILAARCEYFAKRLEEGAYADGAKQELELPEAEPAA
FEVLLRWLYTGAADVPAELAQEVAVLADRLVLPELCDAAQAVVLESVTPGSVAAALVWAASCVPGRGSSFEQVLRRLKKW
YVAHYDKVRSEARASVVALMASNPELAMELQEEVLGATERRVSKKQRV
>Crei_XP_001698217
MVGVHAALRAALYDAWSASVYVSLGNAILRLVESQQCEVVHVAGNASAGESADGTGDVARLECRFQTQLASDNAGRLFFI
DSALRIRKPDGTSDVAIRVGERRFCCHRAILCARCDYFQQRLAGGSFRDGLQGDIDLPDAEPEAFAQLLRWLYTGAADVP
PEHARAVAELADRLLLPQLCDVALEVLLASVSAEGVVEVLLWAAGAAESRGAGGCFGVLLGALKRWCVCHHGELSASVAR
>Crei_XP_001698083

MYPHQDKEPRTHISLFLEAVDVAAGAQPPTLAFKLYVKHWKDSNKDSICESKEPKTFNVRWGFSAFFPRAQLTTDSGFIR
RRDGALLLAAEIELPAGLAAAAGAAAGGSCRSSSSSAYPASITDGAARQDVSGDLLALLEKPGSTSDLTIVAIAGSDSGA
DTGGSGNGEAPAATWLKRKLVTDKGRKGGCVGSPDTRRRFDVHRAILAARCPYFATHFASGMGDSAARELDMPDTDPGAL
AALLRFIYGGELVVASRAQARAGLALADRLLLPKAVALLRAQLLASLCPSAIAADLMWAAGCGDQAGLLVELLDFAAEAA
DEVPQSDLQQLAAAHPGLTAQLFAASVRAAKRSKS
>Crei_XP_001697641
MVFVPPHLAAVLQAGGGGAGQQGGSGQGLQGRRAANQGCLVFCTPLAVYRLPLPAAGPSSRAHHAQRRQPSQPQLQAQLL
AGVEWARGRTDGPPTVARFNNIRGLAVDAEANLYVTDECDDDTDCVRRVSFADGTVTTLVTELPGPFWWPTVLPNGYLAV
SSHEDGLLIDLGLQPLLPPWPPVVEAAAGPPPRSLPGDLGALLEDAQQPGGGLMSDLVVRVGERRFPCHRLILAARCDYF
KHRLLAPDAGGGGGDSGGGSGSGCGSSCGGFVDAREAEVELPDADADVFALLLRWLYTGAADIPPQQARALAVLADRLLL
HDLCAASQAVVASSVSAATAVDCLLWAAGAAEARGGADGCFGWLLEELKGWYVEHHEEVKAEADESRERLAIEAPLLALR
LADAVLERVGWERSAAAADRKRRRA
>Crei_XP_001697572
MVCCPGWRSQRERERDKARAREPPGHYSEHVGVYEWAIVDFAAQFNAAEEKAVLRSETFRLGSYDWQLQCTDDLKDDDSV
DEHEEVNEHIVRDPLFHLERIITEPQEQLRPTAAGGGSRLPPASAWCRVAICNHDDPVKDFRIYAEHRFGRGHSRRHCIK
YKRKHALLLATITKPGSGFLRPDDGALVVRLELWLEPPPPCAYPATLPEDLCPVHRKAAAGAAVASCYCGEAAAGLSADL
LWLLQHAESTSDATITAFTTRPPRESSGGADAAGAQGPGGGPAAATAVGLVASGSGQRGLEAEIACSSAAGSGGAVSGSD
LTVPATSVTPAPAAEAPASNAAALEVSRRTFPVHRGILAARCPYFRALFDSGMADSGTRDLTLPDTHPAALEALLGYLYG
GRLELPSRELARSCLQLADVLLLQPAADMLTRHLVATASPDSFVADLLLASGLGGEGEGGQTQLLTGLLGWRSQRERERE
KARAREPPGHYSEHVGVYEWAIVDFAAQFNAAEEKAVLRSETFRLGSYDWQLQCSDDLESDDSDDEHEAVNEHIVRDPLF
HLERIITKPQEQLRPTAAGGCSRLPPASAWCRVAICNHDDPVKDFRIYAEHRFGRGHSRRHCVESYGPEHALLLATITKP
GSGFLRPDDGALVVRLELWLEPPPPCAYPATLPEDLCPVHRKAAAGAAVASCYCGEAAAGLSADLLWLLQHAESTSDATI
TAFTTRPPWESSGGADAAGAQGPGGGPAAATAVGLVASGSGQRGSEAEIACSSAAGSGGAVSGSDLTVPATSVTPAPAAE
APASNAAALGVSRRTFPVHRGILAARCPYFRALFDSGMADSGTRDLTLPDTHPAALEALLGYLYGGRLELPSRELARSCL
QLADVLLLQPAADMLTRHLTYYWPRGSAAKAKAARHSS
>Crei_XP_001697321
MSIDTGFLRCDGSLLLRLELQMPEKKQWSDDDDDSKYDSDEEEAYPAVLKEGSGGGSSIGSDFLSLLADPGPTTDLTITA
TAAVAGGVTGAGKEGGSKKRKADTASSNGGSTGASSSRFPVHRAILAARCPYFATHFASGLGDSNTRELHMPDTDPDALA
ALLRFVYGGELRVASREQASRCLALADRLLLPKAAGLLRAHLLATLSPATVMADLTWAAGLAEGQGQAELLTGLVDYAAE
QEADIAEEQVEQLAAAQPALMAKLFTARVQAAKRCRVWKAC
>Crei_XP_001695493
MNQANRTMSQHAGGLPASDGRTDVMGSSSERLPAGSGSCLHPGCSGLCCLAKAPVSDTIVVSTAAPSAGCDLKLVCCDGA
LMASRCVLCRASSVLRSTLELELPEAGELRLPADKAESWRMALSLLSLEAYPLSLVTSDNVVDLLLLADKYDIPIVRGAC
AHFLHLNARQLSLVPPLSSASNLLTAASLVIKFVQPYPGLQQYGSTVQARLDDELAMLRMPPDGGCDLGAAGGAQYTDLH
TRGCRLCNAPMLPTHARFCNSCAYRKHKKS
>Crei_XP_001694726
MDITSPRDMAVASASGRQASEAPGTGPPPFSHSRLRSYASKVEGLLDPKMVLSSSVALAGSGLADPFPSASSSSASQQLL
TPKSARFAGTGGGSNGIAAARPGGHNQPSAAALGSNGQHSHQQGSQPQQPQQQPASAEELAAALNQIQTLKAEIAGVETV
LGYLKGNEAATTTMGRKAHKLPSQQQLRQRADALAGALDEALPPGSITAKYGTGAGAAVTGGLEPAFWSGGEARRDFDML
DMVQREVARQCEAARQVAASCSERGRIIDKLADRHAELYGAVAAAARSATEAQRGLSERLAGVTTRASEQATEIARLRSE
LAARGAEAEDLRQQLAETRTQHEHYVWDCEQELETLQGQSDILAAEVEHLHVRLAEAVADKDAALSRGLARLEADLEAVT
DERDDLSQRIRFLERQLHKLRTEMNTAVVTAVAEVQTDPVNWAAEEPEDVPDDVSDTPSLAEIRARYAAHGYGALSPEER
LRLGLPAHMDGEGGGKSGKGKKRKRALGHFQWTVNAIATMYYDKMKADARSDLEGNPRQRLADFAVDWHMTRFGLRNLAE
LNLLDLIASVRQHYKTAVRVRWFGQFTGLVEVGDQVDTTPHVSFYLHLLACLAAPNSLVSLFPDTAASGGAASEEAGGGT
PPVAVKAAVLPEAIKAIYRYLNEPESATAFLARACDPLTDPDSQTVPLDPLVALLMAEYQKRWERNAAHLRALFRAGDYD
GDGLIGYDEFVAIVRQLLPDSGDRAFTKMYGEAMRKLPAGQNLLDVETFLGVARAFGCDRWRIDAGPTASAYGGGPYGIV
TSPSGAAAGGGGLNGPSRSGSVLGSAPSMGALRARATSAASNLRRTESVVGLSEPDRALLRVLDGAVEGLEPPLEEQLTS
LLDKLRTSAAATAQRAEALTAATAALPGFELGGGPNAKELATAAAEDNAMVSVAWGGISKLEAQYSHFKTTYEDRTDPAA
AWLAFRMLLASLAAAAAGARVKPNPSNARSRPGSAKSQAGGDRHWAGAKALARLTVPAVPFRAGSSAAASRPGTNGSLAA
SGPSGLPGSPNELITSLEKVIWHSRRILKSGNFQEHHVAHTKLEHANLTQASDLTSWARAATAWLGAAMRLQAGDAAAAT
QQGGGIFGGVAARVSHARDSHRRRRRHLSSSENVFSTREDDGAEQLTLLVPVMRRLLLAQLLKAWLRDMRQQLPAEPQAA
QALAVLIRKLFEVHVGSVVQRAALEYFHISKAGGTSWTVAAKSNKCPRPAARGCRVKEFDDSCRWLNRTALLDVGVPQGR
HHKAPWGRYGHVDRSTEYATCAQRYEYVRRRGFVYTTNEYTLHRDLAPERSLDERAEVLSAEQVVERLQSPEGQDAWHSA
HLCPQFVNILTVREPRQRLLSNINFMLPFLQSQLFPIRKRNDTQSAAQVADFQAIFCNAPSETWETVVPPVADNYNVRTL
LGELGFHLPLWSVGPEHVAAAESQLLRFDMVMDLNAGSGAADLVQKQGLGWHVTLAEVHALDSSQRQDRVPCGLGNLTKL
LERQGPDQQWYSFARVEPPCHLDLSKLIQAGTKSFFNNPLLSDIVLVCPDGRALHCHSVILAAASRRLAAALKQGNWTPG
QQMPVKGVDSDALETLVYAFYTAECPLELARVPALYDAAIKLEVPSLAPALEQYTASALQPHNCCAVLELCLGAGVGPLA
ELVLEWIRSRVADVVPGPEFRACRLETAALICKDLAGRSQLVGLQVAVAWLTASSARGSLVGVFAEATGLAVDAIQAVAN
SGGALGPIGGLGAAGDLSSALMDGPLRLGGGGPGRDLDLPSLSMGGKGMCHVQGCNRSLAGLRDYYQRYKICEHHLKVSQ
VLKDGVPHRFCQQCGRFHPLTEFDGDRRSCRTMLQRHCHRRAKQKQELAEVLAQRYEEKQVAASMTAVMAAMARGTPAGV
PALPPMLQLQYLAGLPAAAAAAAGRDDGGAKDEDMDGRGGGGRSLPAGLHSPPSRAALTEAVQQRARKQQATVLSQSGLL
GLAGSGEGLPEGQLAAAMAAAAAGLTLGLDNLPAVGQLPGSGGVAERKFGDQELLTGGG

>Crei_XP_001694074
MGVGHIPSPPVAYKQPGGGAPPPSGRKQHGLLQTLNKLEQESEDRAFELIRLRTLVLKLEKEKEDAATVCQDMKRENAEL
QQELTKTSSELQRLKREHNELRCRQWLDNAQGLSHLLTPAEGDPGPPSPRSDTLPVDAPLGHQPITQSLSAIEESRDEET
ETSTTQGAPVASASETALTPRSKSVTTALSPLASGPVPPTESPAAEASSGLGRTGNSSSSTTAAMIARSQSAKARTATAT
ALGPGALAAAVSGISVTATPPMSPAVGTRGAAATTAAAGGTPLTPTAAHATAVSIAAAAAAGGVSGATPGTPVALPSQQV
QQARAAAAAHALLQDEIKMLTLQLADFKDRIMYLQQELTEEKARHDATIASRRQLEERLSEITSAVSGAAHGGGGSRQGW
RNSSGGGLGGAGAGGAGSRDLHRAQSQHAHSPMRPGGDQTASGHGDAPPTPLRDRTSGLSAVTEHSLSALQDLIAKLRDD
KDSGLAAKQKEADGLKQKLEAAQKEVETLKGQLGDAQRVIVEKLARAPSEAAVEALKLQIDKLKVAALAQHDTAIAELHR
GMRDMQERLDATSADRDAARREAAERGGELSVARAEAEALRAELETRSAELAEKATKLAAALEQVAAAEPSLAAMRSDLA
AATARGEQLSGQYAASSARADSLAAQLADATSRVAELSERLAAAEARLKDLGTTLAAREQEASSLRATVDAQEADVADLG
ARNGRMQGLIKDMAGQMEVLQGSLAKQTALVAKLEAALEAAGMQAAEIEDISRTQAIAYKERIEDTERKLSSTRLKLSEL
NRGGGGASSTAAMLASSRAELAGLFKKLKAVSQAYRLTLPTVLSKQLRGDGANLTELQLFQMLHAVLAALEAGGGVGASA
ASNASSSIGPPPGSAGPLPPPPLPLPANYDVRAALAGLGHPPAYDGSAGGAGPGPHMHRSPSFVGASSGGGARPASHQQA
LALAASGAAGSGGGAGDPGSPPSFGHGVGMGRSGGGSGNYANILQVSHDGSMAGGGPNSGGGGGNMRISGAGGGGGHAHQ
GQRGGGRDRQVTDTGSLKTQRGEHSRGASMADSVMEEVVAAVANDPYDSDDSLPPGLTSLLRRRRGGAAPDSEDGSTTPH
SGAAAAVGCVTASSGGPASRPATVSTLQLTLAAAPVGVAVDFQGFAYVADTGHCRILRVRLDTGEAVVLAGGGGYGHRDG
PGRKAKFACPMYLAVDARDSSLVVSDQHCLRRVASDGFVTTIAGSSMPGHLDGPAATARFYNLRGVAVDGEGNCYCSDSS
NHCVRLLHAADGMVSTFAGSPGQAGFRDGAGTEARFRNPCGIAINLQDGSLAVADAENNRLRRIDRERLVACLAGCGVAG
PPQPWPLEEMALSCHLNHPQGIAVTHRGEVLVVECAANRVRVISARLHSPHTYLAATKSTFVGDMCGLLAGGDGADVTFM
VSGEEMRAHRWLLAARCECFGRMLASDYLEGRTAVVEVRDCSAAAFREFLRYLYTDVLEFKGDVVLDVLLLGRKYMVGRV
FQHCCSQVRRGLCGQNALALLSWADEHRVEEIRPHVYRYVLQHLRHILRKYPATLSYLEARPDLLMQLLVA
>Crei_XP_001693593
MSHIAAGAKAGALSHLWSCKCPDMATVTESVGLQFPEWGVSAVCLHPDNSVQVFFDFKERYNVEDYEPPKKPFSMALVGL
DSAAPSKGPPVPLALPSLTPRTYDPPYPHQVVHDPCTDLTYLLGPELGLLSLSRAGAVVQVLAPSVLLGMGVRTANGMVP
DGKGCLYIAGDWWTGKTDAAVDSTFPPGRAALVRVFLPGVCGREVASAALQALLPPEAGITAGLALHYPSGTLYLAADAG
VYEWVEDAPGGPGFRLLVRGEGRIDGWHEEDINGAMGLDGEWDGESGDLLVVHTILPVPDIEMADDVYDDGTRLDRGWLA
RVRRLPPQGGGHSGSSSGSSNRVSVTTLIRLKSWAELQQQPRGWSPQLRILPHGWLAITEPQADCREDFPLHRAVLAARS
SFFADLFGAVGSAASGGVVPLPEPYTAPVFRHVVAYMYTGAAEDWDAHTSMAVADLAHLLDLPELRAAAQRHLLEVQQQQ
QAGAAAGTGPAASGFNPAKVDAKVYLANERTLLTWMRQAAVETGGSGGHGVGAAAACSSLAAAGKVLVMEVVGAAARVSP
QELLSRLGGMALMLLGAGVAAAACRNYYLRVDMIRSGVGSDSSRWDSKVLPRLLTGCLAAVMLAAGGEVSLTNVAVAAAC
RGAGLGRRITVELLRRLGSGQGPTFLEVRVDNAAAQALYARLGFEHLATPPSGGQHLHTLSYHSDYVTCLAAAPARSLVV
SAGLRAEIFLLNIKVGRDVGRKITKLRGHQDTVRALLVNADGSTIISGASDGTIKLWDVGMQRCVQTYNLHSDSVWSLLS
PDDSFATIYSAGRDGALYRTRTQHRTSELLAVEDRPVTALALDPEGEPLGPGVSSRTHGLSSHMRNRMSLEANRQPLVRS
PAAVIAGVPALEAHEVLADRRHVLTRDSRGHVELWDVLTGAKVQSYGKRCGSAACPSLHVDMDAKRRELWEARSVPAWFS
ADTRLGCLCISLEPGSAFAAEEYAQQLEYSGVPDDCKLNSNNMNALVLTRHRGAVFADRIRMLGVSDSIVEGNQAGGSGG
FVAAGTLIDTVSFARARVSRNVAEQGPGGVLSLSIPPPGSQLLGHLFPLPGPDGSLPPPAPGTSAYYPDIWPNSSSLRPR
SPPRSFASVMCSRHDGQRWRRLINAFDGGEAEPGEVPTWVGDVVLRGANVTPKEAKCAFVLLPMEGSNLPSLMQSKLNAP
RILQVHKVAHYCCSKLQELNIALEVRPIYLRRPPAAAPDLADEPGVPPRPVLELTCNGVLVPFEMSLATVKKFIWKKSDD
LIFNYKISGPGAPRAHAPARTQQLR
>Crei_XP_001693015
MTVDAAGSIYVVDDDRRLHRVTPDGRAATLVKDLRAKNCRKPVILPNGFLALAAGETFYDDSAAAIVLVGLGLQPLLPQM
PAAAAATAVAAPPPRSLPADLGALLDANGGDGSSDLTIRVGKRRFHVHRAILSARCDYFKQRLAGGAFADARAAELDLPD
VDASAFAVFLRWVYTGAADITAKRARGVAELADRLLLSELCGAAAAVVAASVSPTTVVDCLLWAAGRCEVHGREDGGGAS
SGFGALLAQLKAWYAQHHEEVAAVAGDSRKRLALQAPDVMVELLDEVAAWQREAGAKHARLALLGKACS
>Crei_XP_001692281
MAAHLACGLRQTCRAARSLTDVCWRRLLDHPAQLGADGLAALFSTLRAQSSLGPLRLACRATRAFVDANTHCLDLRLHGT
QPLRPGGWAALLARCGGAAEVRVRADPSGYCRQRKAAVREPGCMLGVAFGEFPTPATARVRTLEVDIHRGRYVESGGWTT
AGAAAGGGGDGDVNGGGGGGRVWWNPATALYESASSGEADPGLNTRRRRPLPRPAAAAVAAAAAGAGGRPTVSSSKASHV
QYTSLLAFPHLVHVALCGEWACPPEEPAARAVGAVLGSLPHLRSLRLGRFSAAMLQQLAAGAAGIDIDSSGGERGSGGSG
SAHHCRVDCVELRLMPPPSAAAAAGELAAAAAAAAAHAAATTEAIGQVAALLAAPRALHISAPLVASPGQILEPLLALDR
GVAEAVRELVMTHAPALSPALVPQLMAALPGLEALVVGRGCRQLWHNSHCYELVAGLAVAAAATEATEAAAKRPVGLVTG
RGAELLVWSAGPDAEQQPVSDGRGGGGVPAHLHSLPGAALAAVTAAAAAATDDPCAVQRACEAAAAAAEAIPWVAGTPEL
WVFGPQTWSALAFDTDREVLYGATRTEVYCAAAAGGRSSVSRFDSPARRTSLLGAAFIGSSGGGSHGIAAVQQAAASPGQ
GPFTGIRCLLPLPGGAGLLVGDGPHLRRIDPDGAVRTLLPHALRSTAAADGSGCGSGGGPCPLTSLAFVPGGGGAGGGTV
VAASAGCPRLLMCSAEELSLSYGHLEAVAALQRQAATAVAYSGRLMSALRQQASNNDVTAAADSTAGAVAAAAQNAAAVV
TIRVCAAGSNRCEEADCGGGSGGGSGGGGGGDGAPSAAACFVAHRAVLSSHSDFFKQLLALEPPSRGGGGGGGSSGGGGD
GSSFAEGGAAQVELREADPDVFARLLAYMYTGKQCALGEDLDLLQPAQLKAVAELAGRLLLPGAITLLQPRLLAAAATAP
ETAVAELVWAASHSLTGLVEELSGFVVAHAVSVLPATPQEALAALAAAQPELMAAIMQAIAVAVAAPV
>Crei_XP_001692264
MVVHYFLKTAVPLNEPTGVTARLDASGEVQTLLTTTAGEFYPILGADNRAGLELGPSLQLFEASDSIEQPRRPFGGLSKW
VGSPTWDAFSHSVFFIHGGAILRLVGDEVSLVAGDVEERGEQDGVGPDALFSGPEFLVSDGVGSLYCADERSIRRIILPA
CWAAAAPPYPAPSDALASATGVGSGSGADKRPSVAEAGIALLRVAPDGAVTTLARGLARMYARPFVLPNGYLVLSCQSDC

QLLPADLGVLLEDAQQPGGGGTADLVIRVGERRFHCHRAILSARCDYFKQRLAGDGFADAYAAELELPDADADAFALLLR
WLYTGAADVPSAQACAVAELADRLLLPELSTAAQAVALTCITPANAVDCLLWAWGCCDEGRGGSFAHLLADLKRWYVRHH
EEELMAKMMQPHSTPPLQCCTTTPPVSSTARLFRAIYYGSCWESLARFMMHRLGRVVFAVLYPAQVETLLRTGVQVSAAA
DAFAVVVADVMLGKPTCQPF
>Crei_XP_001692162
RSSGISHERKVASYRCEAEFYKQHAPALIKASGLGVPRPLVVESTAHGWEFVLTDLSMDFPESSGCLDDSQVRAALDWLA
AYHAYFWEQPSAPAGLQEQGSYWYLDTRRDEFAQIGRSWSDLKAVADKIDARLKTSNNEFMTCIHGDMKSENLLFTSDGR
RCAAYDFQYTGRSYGVRDIAYLFASSVDSSDLDSRADDYLAHYHSALAARLAEVKGQAAAAEAIARYHPAVMRRHFDLAL
LDYVRFMAGWGMWALQVCSHRGQGGLGANDTAAAASAPLLERVAALAAAGVSCFDLDVVSTSDGKLVVGYGPHIAAALHQ
AGRPVKAGQQVESVPWSEYVQAGLTERYPQLAEVLEAFEKVVVQTGHKAAAAATTGSSSGSGGGAADAAGEAATGAKGKG
AGDAAAGGALGTDGAATTATAADTAGTAGSSSTGAAQGATGTGTNRDSAAGAKVTGTAAAGVAGANAAATGTAAAGTGAV
AGGDGAAGAGSAAAGAAAMDGGRHHHHRRRRRRQLHQDDDAGSSTATATTTGSSSTSSSGSGSSSKARPLLLVEFKDGA
MSERSVAQLHNLTGGLGVEGFVGVWLVLRPEPHPATKEVDAYLRRPGVGLVKVLGLPDSSRLPNGTLAPLPDAEAALLQV
ADPFPAAAYDAYGPSARHSDAFLQRVAALASGTDGGDGAGAGAGGGGGGGGGAAAGAERVRKPVVWWVVDSEAEATRAARL
GASAVVSNDPGAIGIATVWANGRLTTLLSNDQGQLFELMGTTSHQGLELAPPMALVAGPSTRHRHSLLYAATNGFYGAAF
DEFSLCLWVVDNCALLRVEMGGGEGGGPHAGTTTIHAVAGHVDQEAVRDGAGAAARFDHPNFLACDGHGRVFVEAGTSIR
LVRQPPSWRAYGSGGGYGYGGFDHYGMAGGATGSGWGAAADSRRASGAASADPGSAAAAAGAAGGGGAGAAAGAGAAGRG
EDDGADPNLDPEMEEVLVSTLPFEAPSTIKGLAYVCQAGGGGPAGGGGGAGAGGGGGGGGGGGAAGGPLGWQHANGLGGG
GAHERRASLDAVAGAAAAGAAAAGGTPPYHQRGGQQRQQPQPQQASLVFATRTALYRLPLPDSLTGELNVAMAGRGGGGA
GGGGGGGAVGVRGDGAGAAAAAAVAVVDGDGAGGGGGGEAAPALPLPILLFQRLAPLPPQQQQPQQRWGLGAVLLAGHEHE
QGAVDGPGSAARFHDIWGIAADAAGNIYIVDRDEDAGSSAVRVYNPREGLVSTLVVGLDGRHGRPAILPNGYLALCGGEM
VQVLDLGIAPPRPRSAGAEGGERGGGGYGRAGGGGWGGHLQPPPRSLPADLGALLDAQPDGTADLTIRVGERRFHCHRAI
LSARCDYFKQRLADGAFADAAASELELPDADADAFALLLRWLYTGGADVPSDQARGVAELADRLLLPELCAAAQAVVAAS
VTAGTVVDCLLWAWGCCESRGPGGFGQLLGDLKGWYLEHHDEVLRVADASLERLSAESPRLMGRYVTIIRLDGKLACIDA
ICFHAGGPLGIGDIEDVNGRKCIVCPWHFYKIDVESGDKYYQTMEFKEGKMQAGEWKSYVQLSTYERRPSHADYVESDGY
AFDEKCGDRCLNSPPSANKLKVHSAGADGRKPPSGQVLRGQYRTSPAALALRRLTLTNVAQASQGTTSPRGGPPGTSTAP
GGASRVGASASVAASASGGTPVPNSPRSPRGVGGAYTPILAGSGFISPAAAAATAAAAAAAAAGAPVAAVSPAVPAAAAV
SPVRPALATAAGTGASAGASSGAGTMGSGAGMGPLAAPGFAMRMTQSAVMSPRAGTAGGSGAAAAAAAAAAGGGGAAGPE
ASGGSSAAAATAAAVLVARRSLGGSFAVGLDLLGGATAAGAGAGGFGAGTGACGAGGTSGASSGVTASTRSGIGSGIGSG
IGSCSGSGLTGNGVELAGVGAGGAGLAWGAAASGGGERISPARRAAVGGNAGGSGASGGGVAATAFAMAAAAVTAAAAAA
GLPMTHSGTLAGPVHAAAAAPVAAAALPSRSRSSVLASSVAALASGSAVFTGAGASSGAGGRAGGSSNGSGTPSRGSTAA
AAAVATAAGQAQGQSQSYSQIQGRGLGEGPARGAAVGVGAGDVESRFRGTGSRRRSLELQISRLRRSSIDDGCGGGAGGM
GAFAAAAGALDDDGPSSGCPSSLASPTGSFVGLPLGRPLPPWGSHGHGHQGHQGQGAAAAVAAAAQEDEGDGVDALVDED
GVRVGPPTSARSSSKGLGSWRTLPAAATPPMSWSQQQQSQPQSYQPQSQSQQQPQHTVSGLLAQRAQLLEELQAAHRREL
SLAEQLGRARADALEAQQQNQNQHQHQQQNLYQHQQGSNPFQHSSQPADTAAAAVAAAGRRADELAATVESLRAELASAK
ARAAEEVRRWRGRAVAAEEQLATALAQLGCLRAAAVGPAGGATVGGGGGSGGAGGNGLKSAAAAGDDWVQQQHGRQSGAH
SPPLLVSRSGSLGGTGASAGAGAGEAGRGGAGAGDGAGGGANGETATWWGAAASAAALDAAAPLAAGGASARAEVVVVSA
VSPCGSPRASGGRLVEAGEVRAGAAVAVSGGPAHPASSMRRSFSDLAPPPRHHHHPSEHPDGAGSLVAANATAAAAAAAT
GGPYAHGSASRTSYSHLRSPQLAESSSSPPDFSPSQAQTQRGTAGYKSSTVYGTATHGTPPYSPRLAPDSPAWPPRGLST
GSGMEPHAAATASASATAATAAAAAVVYARSSLYAPSASAAPGAPALAGGSAAAAAAAAAAAAAAVAADAAASAAAAFS
DDTGAAPAWSRRATQSYATQRAPSAAGAAAAATAVRAPAAAAYAEAPGAATADLHGGGGVNGRREAEGGAAAAAWAPGLV
ETSAASWTAGSRAGSSAGGSAGGAAGGGSSLRLGVGDLASAGNDGWSIGEAEGGGGRGVKAGGQAGTGQVVGEGVPGGGS
ASVTSVAAEVERLKQAGNRAYTSQQFPLARHCYSCALDLMLQQPQQQQQPGSSSTTTGTTTTVSGSSRGSSNGSSTTSGR
AVAVLYCNRAAALLAVGQYADALADCCAAAAHDGAYARPWKRRADALAAVNAFSAAAAALERCVVLAETAAVATATTAAE
SGSSSGGSAGAGAGAGGDVAAAELPELRRRLQQYQALARAAGAAPGVGSGSASATALLLAAVSGAASSTPPPPPPPLVDH
YAVLGLQPQASAADVRTAYRRLALRYHPDKAAAAAATVTVATAGDTAAAVSSSASVSVSAPAAPPPPGGGVVAAAAAAEA
AVVVFPLVAEAHAVLSNAVSRRAYDLRRARAALGSSRNRQA
>Crei_XP_001692154
MAWDSYSGSVLAIDGESIVRLIPPAEGGLDAECAWTACVLAGDRDDGGTPQDGRGKAARFAEARHLTCDQRGVAYVADGS
LIRKQPGGGGAVADLVIRVGERRFHCHRAILSARCDYFKHRLAGDAFEDARAAELELPDADPDTFALLLRWLYTGGADIL
PKQARGVAELADRLLLPELCARALDVLFASVDAGSIVDSLLWAAGCCEAHGGGGAFDQLLLRLKRWYVERAAEVRAAARD
SLRALMTQQPDLMLELMEASEQRAVKRARTK
>Crei_XP_001691885
MHLTYVKQPTKASQQIPAMDGPRRQAAPSASSLPSNVEMYSSMLSSANVELVLDGGQKRLRVHSAILELTSDVFRRMLSE
ESCVLGEQSGETIRHPQQLQLDDDSRDWEVVLPLLYPRMSPPRLDWTELRRVVAITKKYEIRLLRHACERFLEEAQLSAH
SADPNYVLTWLLVSEQLELGALFSKCLQFLDSHLAPRAPGFCDYCSTRSRSASAGSGAGSSSNGDPNSRIGCTCACAVCG
GSTLRRDCRCGRDVESMLPAGSGSSSTGGHGAAFGHGSTTAATYIGPTAGGSGSGSGSGRNYPGMPAPLAAAYEATARSR
YAAAPGPAAASGARRDLQLLYRRDGAAAPTAAPAAALRIQVHAPTAAALVQRYASPMPTRVHISVRSNGSGGSASSTGRG
VLTSPISGGSAAHARPLVPPPAPVLPRVVELVRGDRKHLVKLSVPTLVELLGML
>Crei_XP_001691135
MPVTAAAPAVAAPPPPRSLPADLGALLDAQPDGTADLTVRVSERRFHCHRGILSARCDYFRQRLVGDGFADARAAELELP
DADADAFALLLRWLYTGSATVAVDQACGVAELADRLLLPELLGVALAVVAESVSAATVIDKLLWAAGCCLAHGRARPAIT

RRCVQVRADANGNGNGNYGNGSNEPNNPLANGVSPNYRVPYNTNVNAAPRVLAPFLTNPAAQPSSAITPAIEGNFKATGR
TVVITGGSQ
>Crei_XP_001690225
MDDPGPTCDLTIIATAAAAAGAAEGDSGGGGSGAGTQGETTGGKATGSEGQKKRKRGALTAAATVTAAGAGAAAAAGGAG
CTRSFAVHRAILAARCPYFATHFASGLGDSNTCELHMPDTDPDALAALLRFMYGGELRVASREQARSCLELADRLLLPTA
VGLLRDHLLSTLSTAAVVADLVWAACLAEGQGQEELLTGLVDYAAEQEADMPEEQVAQLAAAHPVLMAQLFRARVRAAKR
LCTASS
>Crei_XP_001689973
MASLVYSHEWLISNFLKVEAQSVDSPSFKLGPHAWKLQLYPSQDKTHLSVYLRSVEPKAPRAVNFKFVLRNWQDPKDDFK
SADASYTYTDACVAGYGFPSFIPREKLSIASGFLRPTSPTNGGALLLRIELEYNTLPAASSAAADGSSGGDGGGVYPAT
VCDGAVSAGSGDIATDLLSLWKRPGPTSDLIIIATAPAGAAAAVAANPTAEVLGTGAGAAATIKPTTATAAADGGGSSCG
PSNTGMRRFDVHRAIVAARCPYFATLFDSGMRDSSARELPLPDTDPAALEPLLHFMYGGGLTVTTRQQARSSLELADRLL
LPKVAALLRTHLLSTVTVASVVQEVLWAADAAQTELLTGLLDFAAEAEADLPERDLQQLAAQQPALMAQLFTAARRAAKR
SCT
>Crei_XP_001689841
MPSSPRSPPTRREGSYANLARADAGEQMISTGGRIVRAKRYGGRFGNGFLDFLKTSELADCTVVGPNGQEYRLHSLLLAY
HSEFFRRALGSEFAEGAARRIVLGFEDSGDTWPLLIEYFYTEEITLTDANVLALFAASRELLIPAIQDFCADFAQNSLEP
SNALSFLRQAVQYNCDAFRTSCVTLVATSFNTCCGKSTDGLPVEVIQEILDHPQLFVQTEEQVLLFVQNYVRRHQLDDAT
VRQLFRGVRLPFLSNDRLAALAAETPGHGPPIEFICIPMELVWEELATNLTVCMSGDTVEGDADSILRTSSSDSDTGAGS
WFTVSSEDPSRPVWVEIAFPPCMRVVELSKYTFSHGMNMIAHMFPYGISDSSWFQMKGMSTHVSPGDEEPFTPLRTQDSC
QQHTEYVVTVDPAHSKQVVWRRLRILAGDENAQQQVRLSIRKLKLYGRVEDRDGRFVAVLQPRQAAPRLTRLWEVVLEEL
ACGNRALRYNGGGDGGDRAAAFSAAFDAIAPVLKCTGGDAWPVLMAHGSLPAAAALLVASSGDFDAQEVVIDFFAAWWSA
AQELQLLQPAAAELFGQRGTALAAAAAAALVVRLWGHEELAPSGRGRLKNSVTLRLNKVLIPTVFVDMVVNWERPALSDA
ELAKLEKAAQSNGLYLLRVKADKSELLSSVPASCLLGDKLFEKAEVAVDRGGQLLSFNYAITDCAKRAAGTATGASAPSP
SEEGVGTRPPSLPVRVVLPRPGPALLVPEFVGDFVGAKPRAPVQGQAARKGAPPPKDERTWLQKNWMFVLAGAMMVFNMV
MKANAPEAVVAARPFIGLVSRLVWPPSNGPPMRQTLRLPRELPVRPVVQVDALPGAGDGSGIDDNRQRMLKRVMQMAPPA
HDASRLCFGSGAGAAASGAAERWVPRAAPAADGAAAAPGSGAAAGAVAAPPLERALRNPAAWPPPNPSARHEGEQPPQLN
DLVHAVFQGSCAVPALLAHGDLADAASLVATLGAAARTVLCPRSQQQQQQLDALFMGETAVTAGILIESVVEQLCAALDM
QQWLPVLIFLAGHCVAADVLMMSHQAPAVGHNDPSTQAAGVVALAPAAPGVPGRPQCVDPAGRRQAVQQVQGGPVADTGA
A
>Vcar_XP_002959617
MSSAKAGVLQLAPQTGEQAQCYIDLSRLLVTGTRSFYNNPTFSDITLVCPDGRQLRCHQVILAASSKRLASVLKQGGFTG
SKLPVQGVDSDALDTLIDSFYTGECPLTLATAASIYDAAVKLEVNGLPNAVEQFVSASLTPNNACLLLERAMGTKTGRLA
EVCLQFIRSRLADVVPTLEFSSANMDVVGQICTDVANNRHPPQLALQVAIAWLRGPPSGPSRTHLLPALADQIGISSDIL
QTYMRNSGNSPTAGGGGNGGGGGNGGGGSAAAVSLLGEPPSLDGSLSLSSLPLLQLLPAGQLQQLGMGGGAAGAAAAQLL
LGQGKGGAGTAALRSLLEEDGGGAAGGGGGAGVGSMEGAALAQRLGNLAGLRRDLEEGAAAAGGFAGALGSMSASGGGRG
KRARTEDSRRNSGWEQEPLTSSLGRGPSGDQLDMLLSPSGPGAGGGGGFGGAGGLGALLQGPQAAAYTADPPPRDRRTPQ
VKGVCHVEGCYADLTGLRDYHLRYKI
>Vcar_XP_002955801
MSSAGPGRRSRLSGFTSLLRRRGGGGGGAGGGGGGHGAESGDDSTPRSSDRSSVYSTGCGSVGGGGPSSGPLTARLPAVS
TLHIYMAADGVMGSSRRSNGSSSSSSSAANPGLYQQQQQQPHSAGPISAATGETAGGGRGSGGGGNGPSGVDGSGGSPPA
AAAVAAPGDSGLQAPVGVVVDFGGNAYIADTGHCRILRIRLDSGEAVVLAGGGGYGHKDGPGRKAKFSCPMYLALDHRDG
SLVVSDQHCLRRVASDGFVTTIAGGTTPGHADGPSGSARFYNLRGVAVDGDGNIFVADSSNHCVRQLTAADSIVSTLVGS
PGNAGFRDGTGTDARFRNPCGAAVNLQDGTLVVADAGNNRLRKVDRDRLTTTIAGNGTAGVAIDGDGNYLLSDLDNRCVR
LVSAGGIITTLAAQGIAVTHRGDVLVVECSANRVRVIAAKLHSPHTYLIATKSTFVTDMMGLLSRGDGADVTFVIDGEEL
RAHRWLLAARCECFGRMLSSDYVEGRTAVVEVRDCSAAAFREFLRYLYTDQLEFKGDVVLDVLLLGRKYMVGRVFQHCCS
QVRRGLCGQNALALLCWTDEHRVEELRPVVLRYVLQHLRHILRKYPSSLSRLEGRPDLLMQLLMAQAAADGGSRAAAAAA
AAAAAAAAPPLAAHSPPWRQRWEIRAVPVAAPAERVTWVNASHLIHVLSPHFDWRWGTGFAHYQEAMDAQSVLVEMEAAR
NAPTTTTGFSFGTTPAAAPAAPPAFGSAFGAPAAKPQTFGAAPSAPAFGSQPAPTFGAQTAGAFSFPTAAPAASAPSLFG
GTSAPAFGAPSSSPSLFGMSTAPAASAPFGAAGTTTGGLFGATNTAISVFGQPQQQQQQQQQQQANMFGAAGQAQLAGL
VDPSGLTQDDAVRALTRLAAAYNPGSPDFRFQTLFVSVVERAEQRVKPPGYVDENRWRAALAAIKGPNNPQKLWPVVANG
FADLNTRSRMQADAVAQNEGRLRELQDRAAALSRRATGEFRSRMSELQREHTALSHKLLHVMRCMDALEGRLALSAGYNA
QQSRHQVAELGRHMTRLEEALAPAAAAAGLQRRLEVKLDERSQAQLFTVLRDHAEAVRQLQAVLKQDELDVEVLKRLTSG
GGGGGGMADGGSGVAASDLSSGTLGGMGL
>Vcar_XP_002954685
VRMPPPCLGADLAGLLQTGEGADVTLVADGEEFKTHKYLLQARSKYFHSLLNSSMREGRAGRAVVQDIRAPVFRALLHYV
YTDSLPEGMEDGNLETEMAQHLLAAADQMQLERLRSICERRLCETVEVDTVSYTLALADRNHSEDLKKVCLDFVARNLAA
VMKTEGYTHMVDACPGLQSDILIAVA
>Vcar_XP_002951988
MLVGQTAGTLLETLRSGLSNSSYNAPDKASLRKAAHSLAELCKQDEYIDEVVFEGAIEVVVPLLNAGAGGVRDQQLDEGL
GATGPSMQEELDKELCFILGLLAVKPEYQTRIAQSGALTGLVRLLKEHKLTSITKPQPGSGGVARRAADAITNLAHENVE
IKNMVREQDGIPPLVGLLEAMDVKVQRAACGALRTLAFKNEQNKNVIVEQGALPTLIQLLRSEDSGVHYEAVGVIGNLVH

SSQHVKLRVLEEGALQPVINLLNSDCPDSQRESALLLGQFATADPDTKAKIVQRGAVPALVRMLSMPDVSLREMAAFALG
RLAQNVDNQAGIVQLGGLPPLLELLESKHYNLQHNAAFALYGLAENEDNIPDLIREGALQRLEDCKEKLQASKDCVQKTI
NRLEQKLRPDNTAAPNLAAAKRALQSMVFLLRSNTKTVQQRAAMSLARLAPDEQLKAIFIDKRGIDVLLDMLMDPNVSHR
SHREAAAALLQLTKKLDAHLPVVDQLPQQPGRAERSVYLGSEYVNNPTLADITFNVEGRKFYAHRIALLASSEAFRAMFS
GGYREKDADSVDIPNISWHVFEAMMRFVYTGQLDVTPDIAFELLQASDQYLLEGLKRLCENSIAQSLTVESVMSTFEYSE
QFSAPQLGRRCLLFILEMYDDVSKVYDNTKDRAFYFDCLRRMVPCLKDSLYCGAGPIWDNSALGQAAGASSGGGAMQTGT
QVQVGRGLQAEGGG
>Vcar_XP_002951740
MAASAIEKQPSGLITLLTEDGSELLVDKELLCSSSKFFAGMLTTCDTTRLHVEENREVMESVLTVMRGSPPANTFDTRST
ACWAIFKKILVVADKYDMSLVKERCEKWLVSRCLRGRMRSQSFKFQDVGEGFEWLAICQKFDLKDLGWYCAYDLGTQLAQ
GAWSRPLHECLEGVTCADMLKTILEAVQAAVLCSGPLGGVSSGATMGADPGMPGLGPE
>Vcar_XP_002951732
MADPDADREARGPLDADSDNEDGPEAPLQGQPRRERLERIALFDFLRLVAGNRDLQPGFRTVEELVNHLKANDTITSDAV
SRAMLACPRDLFVPPAHRGEALADRPIRVAEAGFNISAPHVQAVALQALALDQGQRVLDVGCGCGIVTAYAAYLTGSSGD
VVGIDIRDSAINLATSNLQRLIAHNIEFSEVSAPIRIERHNVFIPLQRHRGRYDAVHVGGAMPASRLGAILDLLGPKFPD
GTVRQRVLSQVSFSELEIPRDVEVVRALLEEQAEAARRVEVPASTFASDLAGLGAPGVDLQQAAQKAAAAAAPCSGPSSS
SSGAAAAVGGGTAPVSESSRRGATNGVRPRSQSSLEGDHTGPATLPPAHRASSKGSQLTGEAETAAVGAGAATSSATAAG
GGVGGHVGTGLASFVQRAGVGGWGWQWERPAQARTKAKGQDDEATRRSDVEMDEEAGEEKSQRAAASASAEAPAGPVQME
KAEEEAEVEALERRGGGSGDGDGDGGGLPYLAGVAEVLSLGPTDCELEGDGWRLPAHKAVLQARCELLRAQLSSGMRDSD
TTTYRVPEAVERRDAVEAFLNYVYKDCLPDDVDMELVPQLLHAGIYFGCHRRVGGGEEAERAQDTRALRSWNAAGREEGL
VRLCESLLARELVAAASSSSSQDAAAGAGADPELLEAAVAAAGPLLALADEGGLDQLRRVAMQFVLDHFPAVSVSEGYRQ
LPRPLVDEVAREALARYNSLLQQLAKLGDTTQGLEDADD
>Vcar_XP_002951420
MSVSSRLLSLAASEELQAKGRPPRTKRYGAKFATGFLEFYRQSEMADCTVVGPDGREYQLHGLLLAYHSEFFRRALSSEF
KEGQARRIVLRFEDSGNTWSALIEYFYTEEILLTDANVLALFAASRELMIPAIQDFCADFAQSSLDSSNAIQFLSQAVQY
NCDQFRSSCVALVAQSFNTCCFKPTDGLPVEVMLEILDHPQLYVQTEEQVLLFVQNYVRRHQLDADTIRALYRTVRLPFL
SNERLAALTLGSVCGDGGIRGLGKGGDGTDGRVDASGVVVAVAAGGPMGQGPPKDLLLAAMAQRLCWIDHPERLPPAAGG
GVGGGGLGGGSAPGDSSDSGGGGSGNGTGTGTGASRMLGGLLGFANGASDINGDMAGVTAPRKTYCCSIEYGLPGGSEYV
SIPMEAVWDDLAANLTVRVSGETEGDPDSILRTLDDPASWFNVTGDDPLRPVWVEVVFPPHMRVVELTKYTFSHGMNMSA
HMFPYGISDSSWFQMKGMCTQVVWRRLRIVAGEDGSQQVRLSIRRLKLYGRVEVSLLREPDGRDVVAAWRRRKEADTELV
WQRPALTPDELALLEKSASSDGLYSIRVKTDRGTLLSSVPAKCLLLDNLKEQVELQAGHDGFIESFNYGVESCSSAGAPG
AAASGGGGGGIPDGSGTGVGPRPDRLPVQVKVPAQGPTLLMPEVLGEFSDVYVDATEAGSVGAAAYDPNAKPRTLPSGGA
GGQAGARRGGPPPKDERTWLQKNWLYVVAIGMMVLNVVAKANVPPPQQGGPGAAPGGGGGGAARRS
>Vcar_XP_002950531
MPECTSVDDVQQERGPDGELKPYCCQVALHRAVYDPYSKAVYFVEGRALMRLGPDNVVSPLAGHREQCGTTDGPGPGARF
QLPQTLVCDGTGNLAARGSGVSAAAATVVAAATLGAGFRTCGGEEVKRLREILGDEKIAVGGKMAIVRADWYTGPGSFVD
VAYDIINRRLYAATIHAVFHIPEDGRAPIRIAGSERPRTGTGSGFGSPQAAGPPGFAHSASNSGSSSSSSGSGPLATAAA
AAAATAAAAAGAAAAATGGGGGATAAGGFFTCISGIVADLDGGLLIADRHQVFRLEAAGGSFEAVICLGKSDTSWGGVGA
YPVIMPGQGGWLALCQYHHKRVLLLQLGLRYSRALFGGGTVQLPYGRSYPWQLHHEGVAGLVGDLASLLAFPGGSEDVEV
AVGGRSFLCHRLILAARCLYFRRLFAGGFADSGARQVVLQDADADAFELLLRYMYTGDMGFPPHMLRPVAELADRLLLSQ
VVHHVHRRLLAAAQPGSVVDDMLWAHQHGFEDLLVVLKEWYLSHQAEVLAAAGDSVRQLVVAAPSLMYDLHCATVRQVGR
RR
>Vcar_XP_002950348
MAAVAGDDDVVAARGDGAAVVRAAVAAEQDVGEVEDLPAHRVVLAATCEYFRALLTGAGANMSDMATAAAAGQAAAAVAA
AAAAAPLATAMETAAVVVSLPGLDGWALRLAVGALYERRVEITADNLEGLISAASYLGAAALLDACTAFIRSSVGLSTCL
PLLLLSWRYQLSPLRDELLGEMSYVCRRFSVLVRMPLSNDSSSGGDVATEYEYGSAGSDPDSVSEVLRLSSLPLELLLEV
LRSEELLTECESDVLQCALEWLVEQELPDGPQAPYTDIGTAVGGAAGGGSSSLGDSVKPGSSNGCSNPQPPCIDSCKPCD
GTSRISDTASAARELVRLVNWDCLPDPPHLHMTRGSLAAQTRALALLLAAYAEEHLRAPASEALSTTAAAVVTAAAVIAA
AFAPAEGAGGSAVPPPAVLRLRRLLAELQAAATAVDLAPHGPATESTAAAAAAPSPPSAGAVAAASLAGAVASNGGGGIN
GALRRIGAVVSSDVESRAYGGIGGGSGRRRRHLASCLLAAGGHDASWRAVKAVEVYDPRTDQWMTGPSLLQGLSFTGAAM
VPGSGFGFEFGSGIGSTRGSCGAVYLVGGTPLCSSVWRLPWGGDGPMGRGWEPCPPLLMPRAHAGVVAVAGTLTVLGGRS
QVQQRGPDMVLPRSAHACAVLGGRIFALGGAAGGGARGGGGGGGGGTHRSGEFLDLGSGRWHLLPCDMSTERKYTAAAAH
GGRLYVCGGVKDSRTRLPSLEALDPREGRWATLEPMTVPRSSHSMAALGGHLYVAGGQCSSVDIRPPATFTSAAVGIGAG
GTPAATSAAAAAWGWGPARRTLSPPAFPGGNFSALGSSPPSLPYGHVSAGGSGVYGSAPGPGGARTAAGLLGLGLGGGGA
AAAGAIGVAVGSGGGGGCEDMTVLSAVECFDVAAGRWRVAAPLSHGGRAGLALSATLGETNCELTNDGRRQLRPWTVGSQ
LLFTAPPGTAKYLSRPT
>Vcar_XP_002949882
MDRPPAPLQVSGRLIGGPPYGRAGAPVSFDPSAAVPGPLTTAKAPHAKDYGSCFSEGFLQFLKSGELADVVVDVVADDTT
VIAATSSCPTSCSTPVLAASTSLPPPASALPTAFPTSLSSSLSSLASSPFLASAATCGNATTAAAPAPALAASSSSSVSS
PIYGLGEYRLHGLLLARHSAFFAAAFGQDHFADSAARRIRVTLDAAATPAWPALVEYFYTDRVRLDDSNALPLLALARQL
LVSELDSYCMDYVYGRLCVANCLVYMRAAVRFAFHDLHAECVALAAQGFPLLYGSDLSGLPPGSLLEILTHPELQVHCEL
QVVEAVTRYLATTAVSTESQRALCSQIRFPYLDNATITRLAVQPGLVLAAMTGGQQPSQDVSTAAGTTTVATTAAAVADA

PPTRSEHSVGIGDFEASSGPDAPTPIISMSMPTPTPPADAETGPGNRRGLDASESMPMAAADAVGRRSSDGAAAGSSSSA
VAVEGDIAAGEGTAAEDEVYGDGGSGPLAPHAAATDGDSGGLLSASSSAASSPRSSSSGGGDAAAAAEGHSLPYNTSRSD
PSSYNCRHEQDLRRQQQRCGADGQAVAPPAAVQQQEEQQQHHHHHHHHHHQQQPLLPRDLTLEGAMARLAAMEYHNSLPQ
IQNQFQYYPYGSGTTAGGTPATAAITSVAAATGSGRRPLAVFRMSGDPQQPTAAPPGGSGRPHPHALAHGLYYSHPAGGG
GGGGAQMPHHPAHVAASRGATSARGPGNGIGGVANQLAPGSGGGGGGGGGAAAGGVTLDAAAAFAAFCLPPPRASYCCN
VVHGLPGGCAWVDVALEVLWERLVPYIHVQVSGCVEGNPRNVITSDPDCFFETNDSSDPLPWVEVHLPHNVHVLQLHRYT
FLHGHRRAGYYRARNFKTQIAVRTANDIRSPAARSSSAGGGGGAGVGAAGAANPAAAGAAGGGQRPGQVQVFFHGSGAAG
PHVGAPLPPASRYVDLTTRMADQFEVGVLAGAASPPGPWRVMRLQATGPQEDGVHRLCVRGLKQQQQLPAAAAAGPGLLA
AAALQGTDTALAAALPATAAAAAAAASFTLGWPGTKHTEVLFRGI
>Vcar_XP_002949057
MEFFVRFYDSYLFRVTELQEVKHTRCNQRQQMEIRGKACQQSVCGCSPRTRDVGFPPCSMNRIGRSADNKLSKSTFWSRG
QSRCEQTTPSPHQQHQQSEHQLQILIFLRDEIRELQPGNAVGDGQPPLQLGPKLPLAGGSGPYNAGLAVFEPISGCVYYL
EGSSAVMKLDCNDAVHLVAGRDGCRKGRWRAPYCWSLLARRRRRRRRRRRQRPPWGEDDGPGVAAHFSQVSALAADGKG
AVYIAEPDRIRRLDVESGTVMTLPNSAPPAMHWTALSYNADDDMLVAATPYAICHVALPYNEGSCGLDGGEDNESSGSGA
GGGALPPRLVAGNWGSYGRADGAGTAAQFYNINAILSGAGRNLYIADATSLRVMDCCGYVRTVLSRGCLPYCCSFMTALP
NGDLAVSGFSYTNPAIIFISDGSFAPAADALGGGLQSLEGGQPPLRHCTGRKATAGRATGAAAELFELLSTPLTPSMIAP
GGGEVYVGLGRHDGGQTLTILVGAERKSFVAHRAVLSARTGFFRELLSPRRLCAMAIASGDGASGSNGAAQAGADDAAGV
FVLPDADPVAFGWLLVYMYCGELAVPDELLDTAADLAERLRMPAVCLAKLQERRLARLPKPASMVSEVASTRLIPSLGSC
VMLCGIWEK
>Vcar_XP_002948986
MSNTTSGTDDGALQFGWLWLNYQEGDEDSEGALSSGLTAPSPIKRACTPAVPPPAAVAAVAPPVHDPDGRTAAAAPVADR
LQWGEDDDVDANAAAAAAAAGAGRGGGGGGSGESRNMRITSLQSRRVAPAAAAVAGAACGSRSGRVSGVMAGHHNGNTTV
MMPPRAGGDGGGDGGLQRGANGHVHNVSFATISPQTSQQSSGPPAAAASLSAPTEAAATRDGVGRRDGPTGRRPPSSGSR
SVSGSSAGTATARNSAAVAAAPSGPGLLSAGSATGEQLQEGPSSASAAAAAPTRAAVAAAAAAEAASTDEVSPQRPVTSD
DLFHGGSDSGSGGRHATGHAVDTLPSRDQRSPQPPPPTAPPPPTAPAAPLTPGSDERTSPAGGAGVGAGGEDQGGRRGRR
APAGGADAAGYCGTAAGRRRSGAVDRALWPARFSGDGVGGAPAGPTGHAGGDGDGGGDGGERRHGEMASPLVTAAAASGA
AAAAAEPDAAGTESEMAAGEVTAASTAATAALSSTSAMSVAATERESVAGGGGDGGEGISYSSSPQWGQLPAAAARRHAA
GCGSASSARSRCTEARGDGEAAGEARLTRAHSAPAAAQHQDIAAVLYDPIMHLELDDDGGDGSAGGGGGGGGSAAAAAAS
GREAADALEPINRWPADDDTLPCGEAESTAAARELVPYSGSGAGAASRWDSWTSGAGDGLDGGAVCWSGGGAAPTTRNLC
RSAAATRAAAGAGAAATARPSPLPPQRPRPVRVYPVHAAVLAATSDYFRTLLQNWTDSVAAGDGGGGGGGGRRGVLKMFV
DADHVDIAEMMLKFMYMGRLPPGLSPSQLLSLLVLADQYAVGRLLAAVDTAFAGLSLSGLYLEQLLRLYELPEVLLAGGA
LNSALSKLVEKLVSELGDLELVMDCPARRAQLLGLPYEAIRLLLQSSETRVWNENTAVAALVEWVAGPEGSTASLEQRRQ
LAALLRLQHCTHSYLSYILPMRVTFDWSIDFATLQQLYADSRATRSLQVVTSEPAFFGGFHWWLRYDLDASQARALYTSA
PPICTPAVCGFGRQPPEYREHMGLACNVKLAGKKHSLPCVKVKATVCARQDAGVLGEGSYSLRKSFSYHVMNACPVGLWG
FFGLTPDRDEAAWRRFVFEGRVWLRCSIEECQ
>Vcar_XP_002948739
MTCPLSWVGGLRVARLFPDVVLKAFGKAYPAHRVILASQSAYFATLWSWVSWQPASGRGCYAEEEWSSLPSHDLVLGEDV
TQKGFEWILEYFYASPTVIITSENAFDVLAAAHYLGVIAVQTACVEFVAQHLDPHNLAACLLWATRADHGEASDMLASSC
RRLLALRLPQNLSAWAPALARIEPRILLDIMSSDHLAVPTEYDRYELVKEVAKICAAAAAAAPSGALAAAAATTAAEETA
GGGAEGGADGAIGAGGDGHSGGGASGTLEVQRMLTSALRLSDEGLAGHLELARSCSSAGLPCPGLPPTVAAAKEPATAAA
SASAAVDCTLPRRCLSSSASLGSVAGDADSDASLVGSASPAASALGTSSSECTSVDVASVLTSSQASSRSTLRLCELAVP
TTPEEHRIEDAVTDTAGKAPCKNATVVVATAAVSSPPLRSHKTQGSNDSSDVVDVPQRAISMVTAGSSVAATTAAAAVAA
CARMLLHEAVRYEHLTVPQLMAVMGEGLVPANVLHTALWERTRLDVEVHARSSQAAAATGERAGTHSIAGGAGGGSRSGR
VDAAEAVPVGALVRPLRFRAFRMCWRLPCGALRKLQPKDVLQSDLHQHAGSLWHLRVCCSDRSRDHLGLFVGRHLAGAAV
GNATGLLRLWQGARLSPARQMEPAGREAGGQQPHRLRPGRRGRLQPPQEHPLIDPVPPPPMQQWQQRQHAALSRPFFAYT
DQNASLRGRCRLSVFQSDNTALQGRLCLGDTGDFTADFAAGGYFGQGQLIARADLARLPDEQEMLVFMSLQVPVDTAE
>Vcar_XP_002947391
PQLQHGNSYHNHYHAQQQQQQQYQILVVCNGGICPLLAACDSVDASSSPKLGSPLTVTGAMTSHISRVLDATFEPRSGMV
VLAAPKALISLDARNEASRLSGQLKGWFANSCDGVGDAARYTSLQRIVASAPGYLHIMDDQGQGKTYLRQVHLLGGSCAV
STLMPGDGVFTYDRSTGSVLYVTDYRTINRVTAQGLLEPVVGHEGCSYDGGVVACRASHRVDGPIGLARFVSVIDITADE
YSSSGTAGSAAMEPLRIAAESPPDVVIIAGDGGTSFPAHRAVLSARCLYFRKLFSASFGGGGGGGAASSGSDDIVEVPLR
GTDPDALAVVLRHIYTGSFEHLTATAAAAAAASTSEPLRAVAALADRLLLPQLCAHAQELLLRDLAPDTVVSEMLWAERAG
LQTLLAQLEQYFVVHAAEVAQQAPQSVDRLLEGGNTENPG
>Vcar_XP_002946947
MTNMEQITLSTERVTAVFVRKLPGGAQQALVCTERGIQPLLGLEDDAAGATEIRLGPPLALMERTADGRSLPYTWSPPPA
AGGAGDNDRADGAARAPVYAVGDVGPVGDGCLCFVSTYDTIVRLDADGHVELVAGQPGCPGRRDGPGGAALVRSPLVMAP
DGTGGVFFSDGSRDCVRRARLAAPPPRSAAPPSPVLPVTVATLPFQIPDWSLVHALSYDYAAQVLYVCTPRMVYHAVGVG
TASGTAAPGLQPVAGGEEGGGSGCVDGVGRAALFGSICDAVVDSRGSLYVIDMEGPGGGAVVRRVDPAAGGVVQTLPSAS
LGYLDRAVRLAILPGGWLCLYEWGGNKLWMLQLGLEPSRPGVRAPRLAAAAAAPAVGPDLGGLADDFGALLTDPWHIADV
IILAGDNLFPAHSQVLIARCEYFRRRLLQTGNPQDHVPVAAATAAAAAAAAAGSGGDGSGADVAAAAAVRLREVPLADAD
PEAVAQLLRYIYTGSVDFPDLLLRPVLELANRLLLPRLAAAVQQQLHQKHPPMQPPPAAVYDTAAGTEAEAPVPPAPNSA
VHVTSGGDAAGLQGDAPGALHGAQVTSPWGSVGPTAPTAAMAVPLPLAEEKEEKEEEEKGDDDDGNAPSAPPAEAGQPLQ

PLQTQQRPSQPLSQPQLQPYTSYCYTHRPVAVTTEGTRPTAPSAPAAPPTFLAPPTAAAYGATQQTYNTVPGQMYGTAPP
LPSAAFSGGPSLEAGGYGFVQPTIYGNTELSHPYGAHGLSYTPPPVSSSLGSLYGSATTAPLPPPPNAGYGMPLPLAPAC
QPSSSYPMPGYPPQMYGDASHPHPHPQPPPPPGGGYYYPPFR
>Vcar_XP_002946300
MPGTSHTQYGMCTEKLFAPVTACPLSPGYSQGTLASSCLVSACLASVGLGTDRRLQYCLTLDSIIGPGERTTPMSTAASE
LKAKVNLVAGDWQATGNTDGPETKARFTRITAILAGVHRQLYILDGDKFRIMSASGYVSTALTGLPTDQCSIAALPSGEL
AVGAKGSRLRIIRGPQLGPSSPTSGGIKASHSAPRQRPSAALSKLHRTLLLCATGADVNPATVNIHTRDGTVFVAHRSAL
VSHSQYFEKLLVPGGGFADSGAEDVKLPDADPAAFQWLLAYMYTGELYMPDKLLRPAVLLADRLLMPQYCVGQLQKWLLA
VVTPQSVLSDLLWAEQHGMTSLVPQLKLRLLRQKKSVVLGEEDLRQLIVRCPCLVADLIREP
>Vcar_XP_002946298
MELTDVRYPVYEPTSDCVLFVKGQAVCSLDSNNVVDLVAGNMLARGFLDPWDPYTEQETDCSGDGYRGEARFSTITALAP
DGKGSVYIAEAERIRKVDLLSGQVTTLPGSAPPTGYTWTGLSHDDDADVLFAAMASAICRIDLITLTTTAASAHVSDANG
EECTTPMSTAAGELKAKVNLVAGDWQATGNTDGPGTIARFTHIIAILAGVHQQLYILDHDKFRIMSASGYVNTALTGLPT
DQCSIAALPSGELAMGVKGSSYSLLRIVRGPQLGPSSPTSGGIKASHSMPRQRPSAALSKLHQTLLLCVAGADVNPATVN
IHTRDGTVFVAHRSALVSHSQYFEKLLVPGGGFTDSGAEDVKLPDADPAAFQWLLAYMYTGELYMPDELLRTAVLLADRL
LMPQDCVGQLQKWLLAVVTPQSVLSDLLWAEQHGMTSLVPQLKLWLVRQRKSVVLGEEDLLGEEDLQQLIVCCPCLVADL
IRMP
>Vcar_XP_002946297
MILRCLCRYTPQETVCISDGYRREARFSAITALAPDGKGSVYIAEAERIRKVDLLSGQVTTLPGSVPRTGSWTGLSYDYD
ADELFATTASAICRIDVITLTTTAASAHVSGANGGERTTPMDTAAGELEAKVNLVAGDWQAAGNTDGPVTIARFTRITAI
LAGVHRQLYILDHDKFRIMSVNGYVSTALTGLPTDQCSIAALPSGELAVGAKGSRLTIVRGPQLGPSSPTSGGIKASHSA
PRQRPSAALSKLHRTLLLCATGADVNPATVNIHTRDGTVFVAHRSALVSHSQYFEKLLVPGGGFADSGAEDVKLPDADPA
AFQWLLAYMYTGELYMPDELLRPAVLLADWLLMPQDCVGQLQKWLLAVVTPQSVLSDLLWAEQHGMTSLVPQLKLWLVQQ
RKSVVLGEEDLRQLIVRCPCLVADLIRMP
>Vcar_XP_002946295
MALTNMRYPVYEPTSDCVLFVKGYAVCSLDSNNVVDVVAGNIYTPQETVCISDGYRREACFSAITALAPDGKGSVYVAEA
QRIRKVDLLSGQVTTLPGSVPRTGSWTGLSYDDDADALFATTASVICRIDLITLTTTAASAHVSGANGGERTTPMDTAAG
ELEAKVNLVAGDWQAAGNTDGPGTIARFTHIIAILAGVHRQLYILDHDKFRIMSASGYVNTALPGLPTDQCSIAALPSGE
LAVGAKGSRLTIVRGPQLGPSSPMPGGIKASHSAPRQRPSAALSKLHRTLLLCAAGADVNPATVNIHTRDGTVFVAHRSA
LVSHSQYFEKLLVPGGGFADSGAEDVKLPDADPAAFQWLLAYMYTGELYMPDKLLRPAVLLADRLLMPQDCVGQLQKWLL
AKVTPQSVLSDLVWAEQHGMTSLVPQLKLRLLRQRKSVVLGEEDLRQLIVHCPCLAADLIREPVAFRDTCLASAGLGTDR
RLQYCAALDNIIGPVSMLGRIRHKEVV
>Vcar_XP_002946294
MELTYVRYPVYEPTSNCVLFVKGYAVCSLDSNNVVDLVAGNIYTQETICSGDGSLEDARFSTITALAPDGKGSVYVAEAE
RIRKVDILSGQVTTLPGSTPPTGSWTGLSYDDDTDELFATTASAICRIDLITLTTTAASAHVSDSNGGERTTPMDTAAGE
LEAKVNLVAGDWQATGNTDGPGTKARFTRITAILAGVHRQLYILDHDKFRIMSARGYVSTALTGLPTDQCSIAALPSGEL
AVGAKGSRLTIIRGAQLGPSSPTSGGIKASHSAPRQRPSAALSKLHRTLLLCAAGADVNPATVNIHTCDGTVFVAYRSAL
VSHSQYFEKLLVPGGGFADSGAEDVKLPDADPAAFQWLLAYMYTGELYMPDELLRTAVLLADRLLMPQDCMGQLQKWLLA
KVTPGSVLSDLLWAEQHGMTSLLPQLKLRLLWQRKSVVLGEEDLRQLIVRCPCLVADLIRELVVMP
>Vcar_XP_002946292
MLTCQIIYLYTHARQFPDDAVLTRNALLCYNPPLPLRRYTPDEIVCSSNGYRGEAWFSAITALAPDGKGSVYVAEAEQIR
KVDILSGQVTTLPGSVPRTGSWTGLSYDDDDDVLFATTARAICRIDVITLTTTAASAHVSGANGGERTTPMDTAVSELEA
NVNLVAGDWQAAGNTDGPGTKARFTHITAILAGVHRQLYILDHDKFRIMSVNGYVNTALTGLTIYQCSIAALPSGELAVG
IKDPRDSPLRIVRGPQLGPSSPTSGGIKASHSVPTQRPSAALSKLHRTLLLCAAGADENPATVNIHTRDGTVFVAHRSAL
VSHSQYFEKLLVPGGGFADSGAEDVKLPDADPAAFQWLLAYMYTGELYMPDELLRPAVLLADRLLMPQDCVGQLQKWLLA
KVTPQSVLSDLLWAEQHGMTSLVPQLKLRLLRQRKSVVLGEEDLRQLIVRRPCLAADLIRELVVMP
>Vcar_XP_002945940
MYAVSSTVANPYSHYSSNQWPGAVVRPSNAWATQTQTFVFNDRGVHELQHCSPESSGSRGGGSGWRYTSSSDVKLSTALT
DVRYPVYEPTSNCVLFVKGHAVCSLDSNNVVDVVAGNMYTQQETVCSGDGYRGEARFSAITALAPDGKGSVYVAEAQRIR
KVDILSGQVTTLTGSTPPTGSWTGLSCDDDADVLFATTASAICRIDVITLTTTAASAHVSDANGGECTTPMDTAAGELEA
KVNLVAGDWQATGNTDGPGTKARFTRITAILAGVHQQLYILDHDKFRIMSASGYISTALPGLPTDHCSIAALPSGELALC
TKYSSLRIIRGPQLGPSSPTSGGIKASHSAPRQRPSAALSKLHRTLLLCAAGADVNPATVNIHTRDGTVFVAHRSALVSH
SQYFEKLLVPGGGFADSGAEDVKLPDADPAAFQWLLAYMYTGELYMPDELLRPAVLLADRLLMPQDCVGQLQKWLLAKVT
PQSVLSDLVWAEQHSMTSLVPQLKLRLLRKRKSVVLGEEDLRQLIVHCPCLVADLIREPVAFRAIVSSLWRRGANSDAVA
AHPAAGGGVCTHRRCWHEVETEDTTTTRGLIVAHPYGRPVVFYPFRTRTSTTCTSSRSYDCLQITQLTGKWMDWVNCIDR
FGRRQPAAAAAAAVTAQQNIKGVVGRGMLPSGRARDSLLGLDVPQNAEGFATVGRLKTRCMLVWNARRTLLRGLSTNVNS
RFKGATCA
>Cvar_EFN60076
MVTALPHFRGKSKTFIKRADVILEVEGVGFLAHTDVLLMQCGTVFEGLCREFLQEGADGEAGPSNGGRKRQRGTGTSARA
QLPTLVLSSKQPGTRATARSKAPGPSIITPKSFQLFLNHLYDWDAEIASEEDAIDLLAVADFFGADALKIACDQTLSKMA
VYRQPGPFYSRGAPPKVEHVELASRYRLPQLMAKVATSLAAWMHHQDKIQAMDSTVRDVMLGRLCEFCSLLPESDFATAV
AEDLISKKWHQG

>Cvar_EFN59376
MSSSESPEAPQLWRLFDEAPEEEVYPPQALSRPEAAQLWRLFDEAPEEVVYPPQALCDFTLTTKDGGTFRVHTAILAGSC
KVFDRLLQDVPGTKAMSVVETAHELQVGLLDPLYRRPDMLSPPVLTNMADVRLNLELARKYQCEALTALCDRFLYQCELS
ICPE
>Cvar_EFN51298
MNQMNHEGAAAPGQALEQPQQQEAPAGQANGQPGEAAVPAAAAAAAGAPPSQQQPAAQQQQQQQQGQRPAGTAAVEPATG
GDANGADGEHGPALKKRRLTPPPDFSYEPSAAVDLVLKTADGKRLGVHRLIVLRSCGFFRALLDACEEAEVAVPEGFEEV
KAHLLDIIYSRSVLIKPEIAMKVFQLADKYEAEDIMQACRPVFMSEAFKMRIATPAPEPAFPFGMGASAAAAAAAHAEWL
DVLALASKHRYEDLFDKATDFLVEESKDAECLPVEAATVLMRGKEQLAQLHQFHLLKLVATFALCTHNMHKKMNKEVEET
NSKLKDSQGLVAKLEKKASDRLARTVAERQKTYMKLLAKFNRLVNRCEKEGLTWHRELDPKEPGGKKKKKAAAAAAAPAA
AGAAAAVGPAQ
>Cvar_EFN55399
MLVRTSSGNTSVGHLSLSPLGFSALHRPDFTFQCQALLFPVHSVLLSASSKFFTEFFTYIRGQKAPEVISLDAVKCEALA
AVSLGYYLEFVYSGGVLPAFDTVPDAVQALRLSGFFGSASLAAQLGASLAARLPGLGEEELLEVFRAAVDLKLRRLLTEC
LVQLLPQFGRCTADTTVAAMIEHMDATCAKAVLAQLALHSFPVPGAAALAAVPPASCGPLAAAAAASASDGASGQQPPGP
GPAGPVRGITTPGRQDSGEFAAAAAAAATGPAAALDLEQVKQQFVRVLEAKGWGQ
>Cvar_EFN55097
MIRRYAGRNDVHLTGVPAQTCRRTSDAITNLAHENNGIKNRVRQEGGIPPLVSLLHSVDPKVQRAVAGSLRTLAFKNDEN
KNIIVDLGSLPLLIQMLRAEDTTIHYEAVGVIGNLVHSSAVIKKRVLEEGALQPVINLLSSSCTDSQREAALLLGQFATA
EGDYKHKIVQRGAVPPLIEMLSNDDNQLREMAAFALGRLAQNSDNQAGIVAQGGLPPLLDLLETCQSNLQHNAAFALYGL
SDNEDNLLEFVREGAVQRIHECELVVQASKDCVNKLTKRLQDKLSTRILGQIMYVMQSSQAAGKQRIAVALSQLTSKEQP
SGAQLRLIFLEKKALDVLLDMVQDPHMPVDMQRSAAKSLYRLAESCGAADRASVDDIMPKEPTVGGGRGEQTLLGVAYVN
NPKSSDVTFVVEGRPFHAHRAGLLGSSEIFRTMFDGHYREKDASTIPIPNIRWEVFEKMMVCIYTGKVEVTPDLAQELLE
VADQYMLETLKHLCEQAITDQLAPDNVSAAYDLADNYNAPELSKQCALYCLREQPEMVKGGSKTTPASYAIVMQKMAPRL
REAVTDAINEKSNAMVS
>Cvar_EFN54989
MEAAAACSSAGGSESGHASPPDTPCPLRLTSEELGPPDCVLEGGGWTIPAHRSVLRQRCDHFRARCGAGWADASADRVRV
PDHFSKQAVAAFLHYVYNDSMEGCSDPQEAIGVLHVALYYSCPRLVHL
>Otau_XP_003083629
MHAGLVKRTNDEEAFMRWTRAMDDAVEALNAKAPDTGTSLPEHLVHASEYERKITLALEEKSALIGTSGGHIRALGGTSA
TRSMDTMRLAAKLLKACAKAFDESRLVRKRIWLAMAATPESWRLCWKTREDELIRSWVKLLMTSGVRLSMQKDRDVVSEC
ESYVSAMVKEITKREANGDVPDEFGRRASDFRTIIQIFKTIHASFEYCAEAVCDVVFKPLKAFTTASITNENFDSGVHFL
SWAIRAVQIASDLSCLKEAYHHRITHFIGEDFLTWSRTLLEVFYKNLSSMRAGSISETLGAWYGSHQNVHSDEATKRACY
EELCKLIESLAAVSFFFPLKGQRYPRGHRTNDERFSDKLQRKLTRYVLAVVSCCVKELYEEIKAIGSDLTRLPKWTNGLM
STIERNKSNSTKEMARDILLKLDIGYVFADHACMSACESDHEAFTADISSLFTLLTVNPSKTRSADIENLVDPVFSIFFC
YLRARRGKVGSSSDIHDYEAEWKLLGICACFALSSDEQEDETMDGEILATRCEMMTKKVQAAVLDNFLDVESALLSVLGL
ASIDGSRTALQSDAKTKDKVIPAALAALAMFVRKSPELRAQFYNSGTIINTLLEIREYKGMNAGRAISACILAFEEHHKL
VTNLYKPSVNIDTKGERVMEISNWDTDKLRISLRALAEVVKESPSSAVGFDTAVLEVICVDRRLQIMMSKFTSSLASKID
ISDHIDKFHDESYESWVFKMLASSGRVLEEAADEMNERGKPIPSGLTCPLVEHLQKTYGVSGPEYPLFEEEAIRPKTDGV
RDGRTFWRSIHEWYEGVITHMRTVKSGLKADDELSDVCCILMDGDTDSACSHVGIIVPILMLLHKCRILQEHNLVEELEV
SGVGDVEKEACFVIGLLATKVANQNRIADSFQVNSKNGIEQLIPLLKRYQPCGTGPANVANASITRRAADAITNLAHENN
RIKHMVRDAGGIPPLVALLDSQEKKVQRAVASTLRTLAFKNSENKNQIVECGALPKLIFMARLEDVQLHKEAIGVIGNLV
HSSPHIKRRALDEGALQPVIELLKSPCSESQREAALLLGQFAARLEPPAQGDPDYRTKIVQRGAVQSLIKMLSRHREPGL
REMAAFALGRLAQHGDNQVGICHSDGLQPLLNLLESDVDEISHVLRLNNVTGKSDQELQADAKRFVNNLQHNAAFALYGL
SDHYDNVANMLKENAFMRLNFSNLEVEQSKQCLTKTINRLKDRILRKDVFNYLAFLISNGKPFEQQRVTLALSWLLMDKN
PDELYTVFITKGGLKVLSDMLLGTPAEMIDFSEAQTGLSGGRRIVNVVMEALRQVKNNICPKNATELHTMPPPSTPTAEE
HMPANFKDPELSDVTFIGRENGERYEFGAHQIAFTHASDAFLSVLDSGKRLPDGTLLVDLEDVSRSALEAMMDFIYSGTI
SPMSSVCHPSFLQERCEEILSVATRFDLLGLKHLTEKLFIENVQLSNLSLDRTCNVYRSAVKHEAAAIQGYLLNHVLDKY
DEPWIEDEGSTLALAEYKVRVKAIVECFGKGFVTHIRRLFKEQHQERQEQRAATRARVEHDAQE
>Otau_XP_003083136
MSERRGHGGLELTTSHDVRARPDAGSGQPSGVSAASRTSTTTVIGRHEHTISGYSLLKGIGDGEPIASDRFMVGGHEWVL
LFYPDGKRSMSDGGAPPTQQDDPYAALFVALIGEGPRPLGVVQSGQGRVVRAFHRFTLVDQSGNGAVKISCARQDPQARN
CHGYRKFVRRSVLEASGSGYLVDDVVVIRYEIELVVTNGGALNKNAKLLPAASVNVPSYPTIGKHLVGLLYDRNDETFDC
TFDVEGEKFRAHKLILSSRSSVFAGMLRTGAAMKEGREGVLKLNDVKADVFRLLLHFVYADEIPRGGGAEASLYEHTARL
GAEEAPGAAGDELTTRNGRASDELALDVPMTQHLLVAADKFDLSRLRAMCEARLCDSVDMSTVANTLLLAELNHAYALKR
ACMGFIAANLSDVMSTEGYEAMNTACPHLASEILSAVAEMRPLRPQGAPLALPQDTGTAIQSAAAANPATAAAFATAAAA
AARPDRDIAQGGVLAVPPTVAHDLTNLSFPAMSTLSNFLSRAVQPPTQALDHPPPSTPHAATLVNNAGEEAQREVAAAMA
ARHAQRGGDIANVVDDSLLLFASPQAQRVPSEAPAPVREGDEIAGGNDGQEGRRVRPRR
>Mpus_Micpu40407
MDAASKMLALSGQSKTQTVTVTGEHEHVISGYSLLKGIGDGEPIASDRFTVGGHEWVLLFYPDGKKGTDAAAQARGDEDP
YAALFVALIGEGPRPQGVVTSSSGQRVVRAFHRFTLVDQKRVNGGGTDITKGRQRDQGAVKISCAKQDPNARNCHGYRKF
VRRSVLEKPENGYLVDDTIVIRYEIELVVTSGGALNRPCKTSALNSIDVRRFPTLGDQISTLLHDELDSADCVFEVEGER

FPAHSLVMSARSAAFRAMLRTGAEMREGSEGVVKLGDIRAPVFKLLLNFIYSDRFPGSGPGEYQHGGRDGSLDDEDGSAA
AHHALDGGGPGSGAPGRSPDGGVNQFARIVGGAHVGAGGHAIGAPEAPAIGTASAARAAAASALAASAAAARGGADADAA
GGATGTGTGTGPDLGGAGAASSSSSCHEVDVAMTQHLLVAADRFDLTRLRAMCESRLCDMVDVDTAATTLALAEQNHAHA
LKHACLEFVAGHLAEVMNTDGYKHMERSCPQLASELLRTVAALNAQQAAAANVASVNAVVVGGGAGHQGPGHQAQGLQHA
PPPHPHHGAGGGGGHHHHASYPPLPHPAAQQQQQQHAGHGGVGGLAALPPPAGHGAAQAQAAAQQGVPPSHDAAFDLHA
AAAAEVGGGHGPGADQIASAMPVTTPRTLGTAAQAYAAASPGYAPNLSWEPVAPPDDALDGGTFTDERLERVRGELEEML
RADPIQLKRYEEALRDIRAAAAAKARATAAGGATKKTSGGASAAPDHLVAELARVWRDDAWIAARDAWVKALPPKRKLDI
AAMHLDAEKLALVVWSAEGDATKVRAKARRALLEAAGAVLAKFRV*
>Mpus_Micpu52522
MSDPGAQSASHDADVAKDDADGNLDALLSGAYASPRRATSAATSSSSSSLTTRRAALADASVGLAAHVQSLGWEEREFSD
VVVSALGKDYPLHRLVLSRSPYFAALMRGPWLDASSPTLTLRFDDVSDLVDVDAVETTLGFLYDKPPTLTTDAAPRVLAA
GSFLDLRDLCDICADFIVADVDERTFLRYQNLCEQSAFGMGDPGERGAFYLISHWSPYDPVGVVNAELRGTLHHLSPTTL
LKLLRSNDLWVANERARFELVADVTSRLLEESCDGDGDGDGGPDPAAVALRAEALSACVNYQHLPFADLLAIRAELELED
GDFKAACLGSMVAALGEAALRGEAGVGAADAVNAVSAAADAAATSPTRAVVDGLWAQTLLKAHVAAAQGRDGSERGAGAS
DADAPSRARAPPSRVDESLSRIEIEDGGGGARGESDEDDEDDEDDDDDDDARAGVSNRAAGAAGSSASPMKKRADGGAGT
RCVLYTGPHTTALSW*
>Mpus_Micpu173459
MSTEIGEKCTPNIGDIEKEACYAIGLLASKEDNQNRIAAAGALPGLVALLKRYPPQLSGSIPPSVARRAADAVTNLAHEN
NDIKNQVRTEGGIPPLVSLLETRDPKVQRAAASALRTLAFKNDENKNQIVECGALPMLIFMVRSEDQTIHYEAIGVIGNL
VHSSSHIKRRVLDEGALQPVISLLSSECPESQREAALLIGQFATTEPAFKVKIVQRGAVQPLIQMLNNTDPQLREMAAFA
LGRLAQNEDNQVGICHADGLRPLLDLLDSNAGNLQHNAAFALYGLAENPDNIPDIIMQGTVQRLNDGELIVQASKDCVAK
TLKRLEEKMTGRTLRYLIYMMRTTDKEHSARIAVALAHLCGGADKEQEKGGLETLSDIFMDHGGLEILLEMIAPKHSSTL
PSHLRLSPKPRDQKDAAAALYKIAEKITRLAPEEAAPLPATPETHLEEHFDNPELADLVFERAEKRTFYAHRIAFSRASD
AFHDMIAQGKRQDEATTTGSDADAKREGACRVDIKHITVEAFEALLKYVYTGQIPPSNDPELGFVPKLACTMLKLGEKYQ
MLGLKRQCETALAEDVKNAGVSFDAVKLIKFYDLAIDHRADKLARACVLHALEHHASIIKSIGPDSYSKLLQCMVPTIRE
HLHGIFYRVGSALDAANAR*
>Cmer_CMK275C
MQRAKGAHRSNAPSPPSTAGSSEGNVSGSEDSSSRASSCLVTDARTLNRLNLGQSHDPVAYESRHGNVAWNAGGCEHSPE
SWVKRSRHIGVGVGVGFSSQTVGTLDAPDRTEKSAAGCTHTTECPEEPTFAAPGRVQPRAVPLASVDADVQRSASVGDHT
DAAGSQPQHRQVVERAAAAPQQGPAGTAVDRTCEGNTFGAENGKDSMDSSGSGSRYATRMRFQSIPVSGNLRYLSAGRAG
HAMASVDNRVYIFGGHRSATGTPDPDTLPPPGLYRVTTDGQKRYTVHFCDLIELDGETRLWRLMSNAADEQPAPRRHATM
VSFDGSLYVYGGFDAENHVLGDLWRFDLHSERRWVLVSCQGRARAPGALLENVGHAEHRATETASDAALGAATSISLARV
PTAAAAATTTTGTSNVRGSEVRFRAVERGQSRSGRRESFRAPHPSIGAGLSATPEHATSVQMIPLPEGGPTSGRAEHSAV
TYGHLMIVFGGYDGRRKVNTTAVCDLRSGKWFCPRALQETMATGRPGIDYPHRRCKHSAVVYDDTMYVFGGFQYRNKLNY
GCSDLFVLNLCTWSWHCPVLMHGGGPQAVHGHCAVAGAGAMYVFGGKVRLDATQRAAQPVLAERPEHSLPPHALTSAVSA
DTSPTPSELRSSDLSRDIWKYHFHLNMWQRIALDELTEASLGQASAERVRVELHPRQLASAAVVAPRDPLRCSMYLFGGT
NRAKDRFFGDMYVFYGLGTDRGDVLQPCRFCTELGKALHEPTLADVIFEVPESLDALPEEDQPAGPAVATAHTASPVDAR
CDRIDASNACVQGATSARTRHVSAAMNAGQCAAGFPREATQQRQRQQRASRCRLFYAHRVILAARSEYFADMFHSGLRES
LHGEWPRVIHLPDVSAPVFEAVLRYLYTGELPVHDARIAARASGRACHDNAMDASAMLSVASTSERDGRFLLECLRTADM
LRLDELRNFCVALVERAIRPENVAFVLEIAAQREDGMGGLKAYCLAYITHHFAEVIETAAFQDLLRRDPAGLGKHVLQAY
HESMTGPDAQRRPHSR
>Cmer_CMP151C
MSRENGNNGQNGNTTDEEAFLVDERTMTVDGLPLHIWCRTPGNLAKVRALVERGADVNAVDEHDATPVYYCALTGNAPVL
RFLLEAGAVLDPKTFVGERVLYAALTPEIKRLLLQYREQTLPSYTDALTEHLRAVLEGNENVGGHVDDDADVGFRLEGNR
CIWAHRVVLAARSPVFEKILEKRTSPGKVLRLKHVSTDAFEAFLRYLYTSTVSVRVRHAEEFMTIAQRFHLDELAQEVDR
QRQWLLRKREHKYEVVTHQRLEDLHINLFGASRVHRLEQDLEHLLSRTDNMPFVDVYVQVTCPGPTYEECSLLVYPAHWL
FLRRAEYFRALRDFYRDRSSACAIVRIPSNISPFAFECVLHHMYHSSLPRNWREKALDCARLRREPCNHHAVDVDMNEFL
VEIIAAADLLGYPHVKQSAASAMEINRINVFGLIYAAACYQSRALRAACVRILAEMLLDAVQPLLVPSVTAISCSATRVP
EPGSIAEALVPHVDSQLRLPPADSRTRDSSIATSQESNHASQCSSIAAVSATDTCTMPSWQVLNQCPTHVVQVDRHATVF
QRARDFIALRQVLSAVEFSEIMEDVREEALRLGLVHKFSSAVRDALGETLDCIVFEITDGLVLAEGSAERTTRHHA
>Cpar_1374Contig12142
MEEDETARPANEFPEFSAHLKKAWCNERTSDVVLQLLRLRLHRIILECGSEYFRSLFSENWNRDVQRCEFMHDVRVDVPE
LAAHAVEISLNSA*
>Cpar_4246Contig25730
MRTEYFKNGLHLAEAAAREVEVGFSADAALLLLCFVYTGTLDTELVGSDREIPTPELLVELLQAADYYGVPALCARAADL
LARPGVVPVREVAFERVRRGRGALLASAEYRRLAEGALEPEGAADSRRLLADIVAALAQG*
>Cpar_8662Contig41164
MKKAHHQQKPDQSYPIGIEKKNSKLLQPNSNGALASLQELLRSGEHSDFTIVVVPSRAIAEQPDDVAAGSRAPALALAIR
ILHMRGEYFKRMMSSGMKETRSAEMRIKGRHPDAIRALVHYLYTDEVKELEPELCIKKMR*
>Cpar_10158Contig53050
MQPAPESDLAPAFPLLSFLESSEFADVTVCCGDRAWRLHRVVLAAGSEYFRSLFTSSSAFVENREACVRLDEAPENEFFE
DVLRVLYAAPGGRPPLDLLPDDFGRCVGLATLADRLRVTSLLPHLAALVESHLNVATAPALLRDAVLCGNAPLASLCVRY

IAAN
>Cpar_10636Contig53532
MELDPKAAAMSEAGPSQRRSARDSVIVPICDDEQLRHRIDEDFEPSCHQGTLACWSALARFLPPAPDKPAVLEKKPLPDI
ELVMSGRSFPAHRAVLAKSFYFRAMLQGPWKEAKEARVEITVDDPNISVDSMALAIDYLYGKDVAIPPHLALQLLATACM
LQLDDLTALCIKAITDTLSPHTFIDSVEFAIFYSQRNLLEVCLRWLRCHLVSSGAGVGRKRLNLLPREGGACCPNECIEE
QSRSNFGSAWLRRSPPLHERRSCTDCDIVLDGKHISYHNRRLSLDVFRELIASEDARRERFERHCYIRRVKDAELKEERR
YYLYAQATDELLLVARKRSGGRTSEFEISFDPFDLSRFGPRFVGAMRSNFLGTQYVVYDKGFDPEEVDPALFPLPERSEL
AMVFY
>Cpar_12672Contig6652
MPSPSPNCAVPDRNPSECPPADVTLRVHPPPLDRNLPNTAQAQGDYFRASLSSGMLESQSRVIDVHGFSADTFRSFLWFC
YTGGLRVPYSKTWRDNLRARASLI*
>Cpar_13087Contig6998
MASRKRRKVEPGSSVASVSADDDNPDSYSNTTAITGLSGLLEDTALLDVTLVFEDGEKLRAHRAVLAAISSVFRAMFTAG
FRESKQDQVQIHDCNAVTFKKFMKFCYSSISLSVEDVGEIATVADQFGVIALRDLCLRFIKDKISMETACKFMRE*
>Cpar_14394Contig8225
MRTCSNAALALRYFMESGYSDAKLRLDNGRVFSVHIILGAQSKTLDKIVSGARHGSPVEVNLGAPSLDDAFPLLLNYLYG
IPIEIEDSNVPLYLLSLDLEIQSLKDSTLEAVRAILEASPPKAVLLRQIARTRPPHAHHASPLHRDDALVAACVAAACRR
EGSEVGEAKLRRALFAIAGDSDLCFLEHLRTIEEHLQSGSRTRPTPLCETVRRKVGKTASRGRDPKRLRRGEGVLFTLEE
EGDDEAELELKTDVDLDSADEEDARLLDDFARHMKIGPSRAVAIPRRARPADFNFEI*
>Cpar_15196Contig9155
MAFQPAAPAFGSADFESGVHWAQLHCTADVTLTLVSTTASASARASSRNFAFSPQTETPQASNELLFNELVPLEELRSRR
GGFLEDDTLTIRVSLKTPPVPSKPRPSLVLIFLDTRTQPQTIDLTLDTLIASPELWATVDNLHDSVPLNRLKTLAKHIPF
TPHDFRIVVGGQSLGAHREVLARGSSYFRSGALEHMREGQEGVLQLEEGSHGQPVTFAAAEAALRYMYTHTVTVANVVET
FLWAHSQGAADGPEGEEAVWRLLQARCRDIVSWNLAQVANWTPAASWEAIDEQIVLEFLGGPNAKPGLESVALLEAWARA
RHLIGPADHLATLLPSLGELPPEPVSGQVQRPAAAPVVPFGAAAVAHFTFGAATPAAPPVFGAIGPQQQQPAAAAPASSS
EAPAAAGSPTLLQRLCLLGLEMGLDAVAAAAEREMAESAEGPGGGPALRLLLWIDAPRLASVIRSAAAARAWIRARPNCP
AEAAEQISLAEYPVYLQDPVLKGYACLDRLLAEAQYERMVPPDQHGKRPPVSDGSLAEAESAWKRVRTGE*
>Cpar_15924Contig10110
MAEQRLVESAATADEGSWKDAHDAIEGFATQASAITVVAAGAVMFIVMPADASGRSVATRSAATRAERTPAPPGCISSTR
AAADAAPDNSEPSTPANSRTRSYPLAPLLSTPGLSTPPQAQPARLRTTARKRRITKRASPFSGFHGGRYDLEVFTAADGG
SLPAHRHVLASRSPAFRAILNENHAGRIVVEEITSDVMEIVLIYLYHGTLADGRTLARLRFP*
>Cpar_16324Contig10675
MPFQAELYTSGEFSDVLLCIRGAGYELRRNLHRLVLSRSPWFRAMFLRWDRRTEVELDFTSEPSMTREALLTCLRRLYGP
VPLPPDFSGVSALLGPALYLQVEDLIEEIVADVAGRLERARPAECGSTLAAVAELAARYDVDALTQARAGLS*
>Cpar_16458Contig10870
MASKSLEPSVPETIQLVVDAQGSAEGEEGVLVSIGAAWDVVEKASNRASATVIVREGTYEEELPALPSPAADSKNECAAC
EARVQLGARSITLAGEGAVKLSLGPPCRACRRGDYSWTTVQQCFASCGGTLVLRNLVVASALKLSGEGARWRLERCELRA
GLHVSGGTALELSHCYLDGEMLDVEGVATFALEACRLSRTDIRVAAGVRTEVAGCLFERRGCLRLELPSSGRLAGNLFDC
EVYPVLSRKGKEARWERVRGACPPPGHVVEGNLGLDHETLVPLSDASLAIPAAGPDSELLEPALEGYGQLLDACLGLPAP
PGLPEPDVTFTFAAEEAPGGEKRRRVEPRTPVRAHSFVLRAQSEHFRALFASGMAEAASRSVEVRGFSPETLRAVLRYLY
TGQIAAGAHSTAELLELHRAGDYYALPALARRAALRVDLAPEQHEEALACLRAAEATGSAELRRGALEH
>Cpar_24202Contig39927
MRESESGEIDAANFKKRTMFELLRFVYTSEIQGGLTALELVDLIRAAHFYQLPRLANGCIERLALCENLSAEEALNIFVA
AHELGSPVTKTTKSANTPAPASASAIDVEIDFKKMWKVAMKTLRAKRHDVTKSPAFGALIAAGAEDVVKILFDLLLK*
>Cpar_24220Contig39955
MRQMEFDCPFEDCEEGLSVSELQDHLLDEHPDVFPRHTECEKQLKSLKSLKSDLVADFGALFERCCVRGDAASCHLPPDV
VITCQAEAPVTRPAKRSRACAAKGNAAPSAPAPPPRIHAHSFILKARSEFFRAMLAPREGPRLREAETGEIDMSAYPRET
VLALLRFLYTGDLPDHDQDHGRLVGLLRPAHYYQLPRLISKIEERVLGLELDDPATALELLHVADELRAEALEYVEANKV
AVAASPAYRALLSRGGCEDLLVDLAMALAKPAARKGDDGEEESDDEDSEEERPVVARPYASR*
>Cpar_24651Contig40874
MDITVTVCIPGSDSRAYALHKLVLMQPFFRAMLRGCWKESEASQLELQIDDPNITREAVTMALESLYHEGAVELTPWNQY
QMAAAEYLQIQHLQERCLDFIVRNVGVATCRTELLKGCSAWLKRHLPALARTEQFCRIPVDVFAELIGADDLWVRDEFER
FRLAR
>Cpar_26321Contig53058
MKRDTIFSAARAGSIARLRRLLELPEDGDGFESAPKNALELLHDRNSGANALHIAASCGHADVAEFLLAVEAACVQARKK
ERPQLMRVADWESSWTALHRAAYEGHVSVMSVLCARGNWEALYVKDRDGLTPLDLLNGFPMRPLAKGDARRGEVFTFGSA
ANYQLGHSDQNALCAPKRVEAVLPHRVVSVSASTYHSVAVTDAGLVYTWGFGTGRLGHGDEATRIQPTLVELRGKRVIQV
AAARDHSVALTDTGKLYAGDNSSAQLGYDTTPQGGFSPSTPPASSFLARYGGGSPLAAGAQPSGSPLAANASLGPRKVGA
IADLSVVHVACGRSHTVAVTKEGEVWSWGRGAEGQLGHGRPEPQPMPKRVEALAGKARAVMAACSSSHTLVLSDAGDVYE
FGHGRANKQGLIVPRRVKLEPPAAVRGIRLHRAASLRVVHVSAASGRSVAVTESGDVYQWQAPLATATGDYIFPAPQRVD
GFRGRRVVSVAAARDHTLAVTDAGELFSWGSNTAGALGLGSAPSASATTAPSRVYGVARVLAAAAGESHSVVLASPVPVD
VPAVSPSTFDRDVEGLLLPEDGDADDSDRVVLVPREACADGASSCAAAAAADGEDEDDGPIVASRALLAARCPFFAAMFG

AAFADSASREVAMPEFGRAALRALVRYSQTDDTDPRT*
>Cpar_27031Contig53791
MTVNASEKLDFGHKQLLEWPSSAIAGVRGGPAAIHTLNLSGNHLSSIPDRLAQLVNLEEMNISHNQLKSLPPEIGCLTKL
RYLRIGNLKCLQHLFLENNSLTSLPVEIGLIPDLSSFYVVSCGDIRGEGQQVWPVNVLQNLPPNNTAILTHLRTLAQPSP
PSDLIETTANDALAALCSLHDACLRGGGASFEGLPAPDVALAVEHGARESLPAHSFLLQLHSAHFRRVLGAGMRESRTRE
IPVTEYGPGTVRALLRYLYTGERDVDVGGAGDGDPSPALLELLRAAHYYELPRLAQHVEGRLCLPGFLGAAAALRALVAA
DQLGRPRLREAALAAVWERGAGRTAEYRELAATGQADLLVEVGEALARR*
>Cpar_28774Contig6636
MHQSGMREIPVAGCERDAFLDLLRFLYTGATPDLGRKKFAVFRVADRYAVEELRSKALSRIFGGVSAKSVIPVLRFATHF
DRDDTRRLK
>Cpar_29033Contig6824
MRRSGCELSEANASALLETLKSYKNRDDLSDVTLVVGPSRKYRCHRFVLSMRSEFFRNLLTGPMREGKEGETVVLPGFTE
ELFEVFLAYCYGDRIELTLENVLFVADAYCLGELKGLCGRLLWSRVDEESALEALERAELLPESLAAPEVDVILA
>Cpar_30865Contig8537
MEINEDAEVALLVGEGRERLAARAAVLRRSPLFAAMLDAPMRERATGEIVKPNTPDAARAVLEFLEHERTSIPPALLSDV
FAAADEAQAGELLGALRSPGLLLQLRTLDELAAYARFADACGLASEPDRASRKLEIEIDRLSAAGRDGLLTLESECAALR
ASTDCETAYRIHIVESPVAQCEECEGSATSMCLSSNSKSSPDCSTKLVDILVPKDAAAQALEAGADLRSELATAARRGSS
YFVEFSVFSFEFFLEFSFFVTARRDFDGPNSQY*
>Cpar_30882Contig8552
MKEAATGTVTVPGCDPATFLTLLRFLYTSELKVDATNSKSGEGGELEGAALLELARAADYYLLPRLRARATARLRELAIA
TGGASSSSSAEARAALAALMISVADE*
>Cpar_31392Contig9155
MRFDTFVSLEELRGGKGAFLEDDKLTIRVSIKAAIAADPSKPAKKPQPQQTVHLTIDTLVASPELWATADNLHRTRGARA
TGAVPLSGSSGVPLNRLAELVGSVPFTPHDFRIVVDGQSLGAHREVLARGSAYFRSGALERMREGQEGVLRLDDEGPHGQ
PATFAAAEAALRYMYTHVSPAVPAAELRPLLALACYGVRGLPSLCVEAIRQTLTPATVVETFLWAHSQSGLEGPEDEEAV
WRTLVNHCCAFVKENAEEVAGETGAETWAEIDEQLMPEMLAKTITFTRPDALAYSGAAPSRIYAILGQLTVAPGSETLLR
TWALARRRIGAEVPVGSLLPSFGPLLDLPAAPEQPQPQPSPQRSPQQQPGAPAAAAPAGEPLLEIPIGLRLQLACLAAVR
DELGAVAAASEREFAARPRPPRLPKPEDSPTVRMLQFLEPHIFAGVLQAAAAAKRPAASSAAAARAWIQAHPGYAPEAAE
QVLSSVPWAQISLAEYPDPVLKGYACLDRLLAEAQYERMGLLEESARVWKRFRTGE*
>Cpar_31619Contig9436
MTGSAERGCSAGGPCSLGELLWTTGKYSDVVVVVEDDEKTNSKKLQLHRLILEAATYWKTLFERWAPGATSFEARLAIEG
ESAEAIGAALRLMYVRDFGSALRDFAAAASLYRVSSFLVWEEGLRESTRFAISLVARETMGTMREAAEALAWEELLAYCT
AFTHATESNIFTEGNLAKLLDMALQQNLREAQ
>Esil_CBN80052
MSAVWGWSKVESTPDGGAPCQRSLHAAAVLNDSIYVFGGYDGSNRVNDFYEFKISTGSWSKVQASGTSPSPRDRHTGVVH
GNSFFVLAGFDGNQRVNDFFEFNFDTMQWSPVVAATGSPPSPRHSHASVVHGNSMFVFGGYDGSYRSDFHEYNFASSSWL
QVNAAGRVPRARYRATCVVHSGCMYLFGGHDGTRHLNDVHVFDFDGTGTGGNERGGGEKGWSRFWMTLNTEGTAPIPRDS
HVSVIHGQSMYVFGGSTGSAMNDFHELRLDKCRWQQVQALGNAPNQRFCHVACVNKDSMFVFGGYDGTSRLNDLVEFRFS
IDLMSCDIPESTLIGNLRDMVNSELLSDVTFIVEGKPVHAHKVLCMRCTYFRAMLTGEMKESRAKTITLPDVRRPIFLAL
LEYLYTDELDVELEVAMELFQAADQFGVDRLKRMCESTMLASIEVENAASIFHAADERNAKICPAPHQHHRRCGKSA
>Esil_CBN79063
MHQEVAGLVYSWGRGEDGQLGLGDTNDQDRPVLVDALKSKGVVQVACGSGHTVVLSEDGAVYTWGRGDDGRLGHGDNGWK
YVPRIVEALEGQRIVQATCGSYHTAAVTDKGELYTWGGGMYGKLGHGNESGHPTPCMVKHLRDEGARVTQIACGSRHTVV
LTDLGKVYTWGDKENGVVGHTNADGHQYLPRMLESLQAKTVTQVAACGFHTAALTAAGEIFTWGEGKFGRLGHGMERNQM
VPRMVEALMGKRVGHVACGGFHSAAILDSGELYTWGGGEHGQVYTFGNGDHGKLGHGDVRKVSVPTLVAGLVKMHVVKVA
SYNEHTAALAGASSSTPALSSMSSSFMTDIRSLLDNQDLSDVTFVVEGSPVYAHKALLAARCQHFRAMFTSGMRESHEQE
VVIPHVRLPIFKVLLEYIYADSVDVSLEDAVELFIAADQYTLDRLKGLCELAVQKGITADNSASLLHTSDDLRATRLREI
CMRFVVRHFDTVSKSEGFKVLSRELIFDVLSSR
>Esil_CBN74994
MHGHGGAAGFGSGSFAGVHADAASGEHIFGGGGGGGGLMPPGRSIPRCGGGGGGSSTSGPPFPGSRPSRKRGDSEEIFEG
SSRGSTPVGGKRRRRDGSDIAGHWHCVFKLTPDENTSFMDNIRELYEKEQLCDITLSVEGEEFRAHKAVLACSKSFLGTL
MMSNMRESTQDVIELEDISSRQFKILLDYMYNKPISVPSSQMMDLLSLANRYQVEGLKDQMCDALSTHLTHDNACSVFAL
ADSHQCHTLKTEAFSKIVQHFALATRSDGWTALTKDQLSEVLSSDQVLDCDESVVFDAASRWLLLDSSPSRRRFAPEVLG
MVRFPLMDAGLLSDVIKDHEATKGPECQKLIAEAWEHHALKAVGREGLTGTPRTKQRRRSCSFKQHTLLKEHQDAVSALA
VVNGKLVSGGSWDTSIKVWDPQSWTTERTLSDHTGPVRCFAQCAGRLLSGSDDSCIKVWNTDTWSLVRSLDDHTDAVNAAT
DCNGRLASGSDDGTIKLWNTENWQCEVTIHQHQADHTCGVLALATCGDYLVSGSDGGIKVWNTHNWTCHKEVLGHGDEIW
SLAVVGDKLISGSIDSTIRVWETQTWGCEKQVEDHAGPVYALTVLEGKLVSASSDHTIRVWGPDWVCCRTLECSGVWSLN
VFNDRLVSGSLDNAVKVWGA
>Esil_CBN74438
MDPDVADVDGLSAEAAEDISSETRQPEAVALATGYLRQVHDGHGVGSAVDPEESSTGQRKPDKTIAGEGTARSAAETADD
DVVAEKLPPDDNAPEGGYHVQAGETPAVDATGASTSPDAAQLPTLNSGAASLQDSPAPASTTSVSSQGGGGETDEEERSS
TEGNQHGQAEPHSSPDEVDNSNDLPSKLHRLWGTSPPDRAEGLAVLRNMLGYRKDLDGILSRHVPTKGPRYTRPFHGNDY

RASSPFSNPRPFTVATLAGRFAPTVTNPSELRERVGTRVRVVTAAGLRVGLARRVFAREPLLPSDVEAEEERQSRQQPDA
SPAPVDVEDLRALVVPVRASASSAPEMSPEGGPAPYPSMAEVSSVMWCEYDEDDEEGGGDGGRRRTSAESSPGKKRLLRV
SVQTLDGDVCDDEDEDSESDDGDSETDVETDIETDFGETEEGSFFDTASPQQGRESTGARGTAAGRSRGGDLSEGGRGNA
EGARRTSGMRNSKAREEELDEVEYQRKEEVASVLRRLRVAIRRMDGTWTEEKVRSMAKRVVRMAVMTASKSELVTTTAAR
GALRGLGDRTRLRRQQAQRGGAGSGDVGWVTVYVGVGRWGPNFFGPGFAVETQEKYRLGWLSLSSQPAAGMMGGGGASGN
APASEQRRVGGAGVRRGAWAGRRGFFGLGRRRNSQRREDDDEAGIEGWVGSLTCNYCEGHPLGDVPSAGACGEQQGIAFK
TADSGIRNSPVEQHATSTAVPLNISGEEEGDEVFSEARGTPVTVAESDNDDDRTSQENAAALGSMRTPPNSTLPREASGG
SLRVGSGNAVEEKEEQEEWCTGHVCLRLRPDGLRERRRGSTSGAGMGLGGDAEEGSEDESGSDTDPASGSDGENLEDLDG
IDVEGVSGSTMREPPSEGNGRTNRGSRGVPLSSITTSLFEGERRNSGDPCGVSSEKSSKSLEVFAWGDNAGGCLGLPADT
SAGLLPRVLQPFGLLPGERVVAVCCSERHSLLVTGMGSVYSAGDGTDGALGLGGRESSDEFRLVEWFAEQMPPPKVCQAS
AGSDLIGCHSAALDADGRLFTWGVGAATGHASLKPVLLPREMETLGGFGTGSEARDADGRPAGLRSRAKGVSCGGGFTLA
VTNGGKVFAWGSWARGRLGLGRPPERTIGRKKKVPRFQGTPRKVSGLGRSPAVQVAAGAWHGMALTEDGEVFTWGHNASG
QLGFLVTPSPASQHRQHQHQPATTNTPTAATSTTSLSEMLRASWTPVKLPAFGKTSEPREKLQRAPVADEVLPGVGREES
LLRKIVHIACGPEHSIAIDSGGAAWTWGAEGRACLGHGEAGFGAPGGGTAVAAEAKAKAMGLAGEGGGTDNRQTQTFIPL
SREREVSRARARRALAGGWAVPREIASLASPPPPPKTTASNTRDTREGTGRVKVVAAACGFSHTALVTEDGRMYLFGEGA
ALAGGDLQALEGENGGVLEGTGLREAGGLVRRRCSEGGEDHGGLDAVAALSAVAGPTPVPRETCSSWFPTLAARRVAAVA
CGGQHAFALLAGDRIGYTLGMDLFRAAMGTERHLPGGPASSERGVLRSGEGSVDEDGEGAVGWARGGVDCELLVAGSYLY
AHRVVLARRSPVLRDMIAQEERPAISGDDGDDDGLTPPLQLLLPDIRFDVARALLEFLYTGELRRSLDLDSPLPYDLRAA
AKSYGVSRLEVLCTEAISLGADPGEGGPDAGWGEVVPPPSLAGDLGEALGTMEHADVKFVAGGRPIYAHRAVLACQSEYF
AAMFRFRDQMRRGAADTSSGRDEHGGHSTAMAEIVVPDSYAGFVRLLLFLYTGVLPDANPESVLEDLVCADRYRLLGMKS
VSQSMVRLAAGSCLQVLLSRLHATAANTSAVSSAAARQKRLYARSREQRVEADGHGKSNGLVEDGGDPSFRADSPFPWGA
ALVAVACGTIYYKVSNVVAVGMFVPVVNGVFLVCLAAFAFHKIRDKGLFEGGREKRIHRRRQLQQGRGRVVRKRGGWRER
RREAAGGSGRGGSTYLFG
>Esil_CBJ28052
MQSVVMRSSAGREWESSRSVSPKNRWAVITPPLAPSCSLSPLGSGHRSAAGGKRRFFGLSETAEAEAEAAAVKAELEGGR
RRGGAVADKAPAPPAGEGGGAKDSIDAHRALLSLRSDPMRAMLRSGMRESFTMDVLVKDIRPAVFTALLTYLYTDTLQLQ
RPEDITELLVVANQYTLTDLLSLCEGFLTEGWMHLPPDLRARANAFRSRHVHLFMLGQRDHVTWKGGESEMMADWVLDRI
KSDGSSWMDDVTEEEEALAMAGEEGWQTASEGEGDGAHSTATATAAFFSPESQSPATSQSPTPASRRRARAFLSP
>Esil_CBJ26957
MDVDIDRFQERELYGFGQNACGELGLGDTLERHTPTLSQTSRGKDIVGVTAGNELTAVLTNTGEVYSCGYNSEGQCGIGE
IDETVPEMVPSLTKVTLREPSFRPAGRVSLVHSGNGSEHLVVVTEEGELQTCGRNNSGQLGHGEVGKGYSLPRPVAGFAG
YRVTKVACSYSHTVVVTDDDRTWTFGGNGYGQLGHGDHSSRSAPAALACFQGLGVLSVACGVYHTVVSVASGGLYSFGKN
DHGQLGLEGGESRFTPVQHRACTSVVEQLACGYYHTVALSRAGEVVAFGRNDYGQLGLGHRESTWQGGQVPDLAGLRIIQ
VACGSYHTISLDDEGRVYPFGRNNHGQLGTGTREDACRPCFVEGLSQSFVCQVAAGFYHTLCLTGPPLERKAGHGSAFVV
RDSKSLSSDLYRLLNNPSQSDVTFTVEGKVVYGHRCILGARCEPLGRMLDGPMREACSGGGSIPMPNHRHAVFLAFLEYI
YTDKVIALGADSLDLEFCLDLMDLSDQYLMGSLKRLCEDSVLRNITVKPR
>Esil_CBJ48840
MATANANAAPGATRPVDQHVEFVVKPAEDLILEGSERSVIRVRAGTPYSCLMLKNQRFSSLFRHYAKHHGLRKDDLAYYF
TEELQNEDTPETVFLLVRDEIVVRRRKSKSDSVSDDEAEVDPDSLCPDDMFIEQMGSLLDDDEHCDVTFFVGPSRTEVKA
HKTHLVARSEYFRSMFRKGAMRESETSEVTVMEHDEATLRQMLEYIYTNRVKDMPGLSADEVIKLLALANEFLLEELKVL
CEHSARKIVSVANVAKMLLAAERYQAETLKSSCLAYVQKNMPEVCRYPAFEAEVSANPKLSMFILQGMADAREEASGGGV
KKATAMPRAKRRRVGSSGGMGARGRDGAEVPSGRL
>Esil_CBJ33079
MPPGGSRSRSWRQVKANALPPGVSVGDQVPGPRSGAASVVVGNKLFMFGGYGGSGRLDDFWEFDFESRIWKEVHCQGPSP
GVRENNGVVEYKGSLYLFGGYNGSQWLNDFHGFHIETRTWRKVEPAGAPPVSRFGYVAVVHSHYFCLFGGYDGTTWLNDM
HRFNFDTSLWEEVNTSGQIPSIRSCPSWCKDGDNVYVFGGYDGVQRMNDFYRCDLETMTWAQIPGIGDVPTPRYFHSCAV
HNGSMYVFGGYNGSDRLCDFFEHNFDTGTWTELEPHGDLPTGRSSLVAQVHGNSLFIFGGYNGQVVLNDFYEWRFQPLLV
PPPTLQEDMRKLVNNRELSDVTFIVDGFPVYASRVHLALRSEHFRAMLYGGMRESEKGAEIEIKDVSHAVFLKLLEYLYT
DTLSDVTANQAVHLLVASEQYLLARLKTLCEEAIRTSITVDTVCTIFLLAHKHNAEGLKEIALDFVLDNMEGVKDTAGFL
ELKQEPDLLMEIIFRQAS
>Esil_CBJ26291
MGSRRERSLVWAVGGNSKGQLGNGTPTQSSFLPCRVAFQLAPTQRIAEVFCGYEHTFALTSDGDVLGFGNGVKGQLGIGN
TDSHSNPQRVHLLSGKGVVKMAAGLEHSLALTESGDLYAFGDNLSGQLGLGHNFTAKEQTPQKVPPLGRAKITQLACGQD
HTAVLTSSGELFMFGNNRDGALGVAGHANEGPPIVASAPQLVTGLKGKHVVQIGCGLQHTVALTAAGGVYCWGENGRGQL
GTGDTQNRYTPSLVCKPLSAFKCIHVACGGQHTIAVTDCDDVYGFGSDRHGQLGLGRRGVIVPVPTRIVRLGGRRVKNCV
GGAWHTIIGTVTGEQYIMGCILPSANSPPVAGGGAGGAAGATCSSDPFALSCGDFRRLLLPRGAEACSKGHSLLYGQGLG
APPSALREVSKSLGRLKCDFSRMVVRQKPDVLFTPHAHHDSDSLHDDDSRIRSVGADSLSSIGGAAGREGEGQGNCSSCD
NPVGAHLSVLKARCPPLYEHAATVVSRSRSRRATDADQPGERETDSDGRMAAWRVCAPAGVPLDALQLVICYLYQDAGVF
DKPKPETAETDRDYKYEDASFSGNTGDAAQSEPNRARDRRDRLFSIRSKAGVADEDDEQLKHGTAEMSMNPWDEGDEGEH
ETAAGFGSFGGAYGGNGKYDGAGLDRLVRLMAAARTLKLTRLQGLCEKRAEAFIDCRTVHKVLRRAAEGGFPYLKRRCLE
FCLVHLGEVLADVLSLSAASEGAKHAAASEESFEGDGAIPCIEALRGEAACVVPAEDYQGDFLALFEEIAAESVAAEGRE
EISPPRPTQAGHAAKAHSDEKKKRQPTPGPVTSFAQGNDKGELPRASGDGKGEGSVLVPGEKRRAAGYKDVGKRPAEAEG

DFKIRAGEGMPAVTVHKSILAARSGFFRALLEGRMADAGQDELTLKFEPVVTICALLEFIRFLYTGRSGNDIEPLIALEV
LFLTKGDEGSGGFFQVAGTDELRRFCTLRIFDGMDSDNALSILVRAKAFGDERVLEAAIDYILGHFDSCMSSLKPPDGFG
GEAEDGGGTLEEDPMTTLIPACLEDDESPAVDLLLALAKRCTVQIKPGT
>Esil_CBJ30427
MVDGDTCDVTFVVTSVASAPSSSEGLIPQPAGTPERARPRTETLEQGSHRGDNCSGKNVSGAGIADSDTAGSCENRERHR
GEGQEALAGGAGGGTGGGELESDREGADKLTQGTTDVGTAAVTTSGSVSSTKLSSCSPAHDTSGNTSSSLGTTSREFRCH
RVLFASCSAYFRALLYGGMSESETRRVELRDVTPEGFEAIMRYVYTGKVSVDAANVMDIFSLAHRFGMGELLKACAEVLD
ECMNCDDVCRVLEAAEYYGHDELAAKCWDLIKDNTPRVLKSESFLELRCQQVLSLVREGELQVNEAELFKAVQAWVSRDT
AERRRHVDELSRHFRLPLMSLKELMSVVRPTNLIAADAILDAVTYHADPTRWTGDCLTARPRGKRLAWDASSLAAGAWTA
DDNRTGLGFIGGPSERTRSAEPRSGALGQPSSESPDRIRRALEVVAGHSTLRNAFSCSTGCPSTARPYLPVTGFIAGTGS
SKLGGNTAVDEYSSTSITDGDVVTVMYDADGGVVTFFVNGTSLGQAYSGLSGPNVAAIAVNAAGAQWECVDC
>Esil_CBJ25968
MLLPAPAADVVRLALQRRNGGDCLRILVVLACLASCRRGRAALSLAVCKVTSPVSFLLYCIGLSRRRDRNNGTLLCGNDN
GDTNAGQRPRGRIGGWRGAFRRGIPSCESGAGGELFDQQGGGALASRSALREATLGARGRRAAFERFGELRRVGACCDVT
LAVGGKHFKAHKCVLAAQSSPLRAMFEGSFKEGSEDVIALLDVEPSSFSLLLDFFYDRRVEVTDENVEALLDLSARYGVS
LLRRHCCAFLAGSASPATACSLLSVADRYDCHRLRRVILAYTLERFPTTCRDSSSDGFRHLSAALVVEVLKDDRLAAGQE
GELAVFWAAISWLEAEQSRRRNADEVLSHVRFGLVSAKALADAVEAHPLMQSAAGKAFVHGAYRYQALPPESRDDLASSM
AARARPRTAAGGDGGGGGGYRTGRSSSAALKPDHGDGVGDFSGWLRADGFGDDQGDSSGCSSCEGQGGGGGENESEGEGD
PPVPGGEGSWHGNSNGAAAAAAAAADGGIGDGMRSAPVSGSAEEIGAGREESASGESRVGSESRPRKLHMTDGSPIRRLWG
AGTRMYV
>Esil_CBJ28734
MLSMLTDPALSDVCLVVEGIDVPCHKAVLAAWSRPFRAMFTLPMLESRHQCRVVIEDAEHKHLMVVLHYLYSGRIGLNDQ
NVMPVSALANRYEVLPLMAQCEGYMKWRLHPSNVVAHLLSAEEHRVPKAAAFALEYLAVHFDEVHHDSSGDFNSLGGDTV
LRVLQHDGLTVDEETVFLAVDGWAKAWEHRRSIRGATRNYAVGRSPPPAVAEQDASPGARGWGGRESNGGAGEERSTMDV
GDEPPKGLAEGEAGRVLQRRASGPAGGEGKQGGYAGEVLQRAASFSHPPPPPEPPVAGVRKTAAVGAPESSSPIEGMTDE
RKGTLETMAPIPEEEQAFVDELLMEVRFPLVSTSFLCQVVEKSPVITGSRVAQRLLHEAYRFQAIRPDGVDVDELFPGNH
RVKYRGHERVLFGEDEGCVGHVEDYSSSLSEVHLVENVKQRDEKYWLPGQPEEAYFCVRV
>Ngad_00464
MLIVEPKKETLIEHFKLLAEEVVTRFPEDADVVLFTGDGKSFALHRVILAARSPYFRTMLRSGFKEGQQAGRLIVPHILP
TMEVSAETRIFSSEINYKGSKRVMGEKKDLEKKKPSNQDQVEEKGANACCDASEPQALPSIHLSLSGEALRRVIEFMYCD
EPLSPLEVPLVFDVLDAATLFMLPGLRNYCVSALMEHVDKIDIFSLLEVARLYDLPRLENFVVERMAINLEKVVEADKFR
LLVEESAATVKAREERDSIPLIDDIRYFVCRLHRFDQDVQEMKIALIESLLERLGLKT
>Ngad_03402
MLRWIRQDRIMDMPTLKNHTATLVGNRIFVFGGYDGQRNHSTLHVFDHTTLRWVCEKVSGKAPPGRNGHTATLAENKLYI
IGGWLGSGPLAASDVHYIDLAEMKWVEPPILGEAPGPCNMHTSDYLPHRRQILVFRGGDGREYLNDLHGLHVDTGRWERL
RCSGTIPEARANHCSAVLNRHLFIFGGWNGQTRLNDVHVLDTDTMVWSRLEPRGVAPPPRAGMTLTRIRDRLYLFGGSGP
AAKCFDDLQILDLKEMCWTEITSRSKSGGGGSSGTALSSGSGVGEERAIPGAHLASAAENSGLERSRGGGWGGNQANPND
EHVTESVSIWGEGPGPRAGHSATVLDRRLVIFGGSHGSRYLGEMFVLDTDPVPEPLVIAPSCAQLIGSHLRHYLDREDFS
DVTLIVEKRPVRAHRLVLSIASDRFKAMFSSGFRESSEREVEVEGCSFEVFKLMLGYIYTGEPPSALLVGSQSDPILAAE
LLALADQYMLDHLKQLCEARLTEEVREDTVDSIMDLAERSNSWQLRAVCRHFVRNRDERHASFASSF
>Ngad_03902
GDRGRARCSWMVRQQFLRRAYCDVSFVVEGKTFPAHRCWVAAFSLPLCAMFNSGMRESQESCILLRDTDAGAFELMLNFI
YGGPIELNQDNIESVLELSVCRRHAYGAML
>Ngad_05984_01
MIMTPSWGFLFASFLHIVPGSLSWLKKHPSKLLVGSSAGVIQLCDIEREAWETENAIVLTYEEFDRLTSLHGNCDDTCVL
VSGYSHTVQLYDTQTGAIVRRFEGIHSDHINISRFANHSPHLFASSSFDKSIKLWDLRCAGTRPLYTARSEQGHVMVCFS
PEDVHLLASRVDNDIVQLEAVDGRIALHLKGLPKTGLADNYTRAYYTASGRAILSGSSEEQDVRVHCAQTGDALASFEAY
PGRRHHSLYVQSLRGDPLCDHRFSILVNYRDASFPLEIISIDMLGDGHSHTLQAATSALFPFPSPRPLPSIPEGKLDIVP
MSSSLAFRGPDGNGGHAELSSCESLSRGREGQKETMDVEQEDTMEKEKEEKETMDISSFFPSARLRDFLHDLYREDVKGQ
GHGPPMEEQELAWLGIVKDESDGGGEGKRGEMMYFRVHLPIVCARSLFIKEAAARVLVAPSFSPRPSASSSPFSPAPRVI
IPLPLRYSARGSEDQEIATSESVSGHLPSLHVVFETVLYYLYTDELPSLHIEAKDSDAVLDMLPFLEALLMIAQEMEISR
LFTLVEARLKEMVAVPTCLPLYALARRVKARQLETFLAYFAACHQELLAVHFCSSPLLPSLLKSVEIMMEKEGGYQFVAS
TDSSEPETESGDFDRQEGREAAEEARLDLHVEALDGERRTNQEGGHVHEAEKEEEEVEKQEMEQSGNETEYGKGPGQAHP
RSPPVGASIPTHMGHTATFLHLYPSDSFAASSPCKDSIGTAPFMSPSSLPACPPASPLPLMVVLGVGSRRSCLSFCSLLL
YNTRKKRWNRVQASGPLKSIPPSLVGHTTVPLDDPFSRQDPRRRLLVFGGAHYTRHEGKVQEEERGREGELEEKSEEDAL
GLMEPFYLLSIPSLSWARYHPHDEVGKVVVDASLRRVAFHRGLSVAARNRHTMVARHFPRKDDQREQLKTEMWVFGGLSI
SHRAALQDTLLLSYFEGGRGCSGQEGRFEWSLPEVEGTPPQPRWSHGAVLLPGKEEEGGDRMLILGGVSTEVIFNDLHCA
RLETISPASFPSSAPSSSSPLSCSRLRRKIVWEKVDVVNGSEQGARYGHTMTLLDRRHIAVLGGVSEIGVNVPISDMLKI
LVIDSLSLTEGLVCHWKTYPTIYRPLPATAADALPSTLDAPALRRLAIPPPRSQHSVVWCPPEGPKAGGRGHIGPQGRLL
VFGGVRDTHDNFAAGKDSDVYELEFLCGHAALWAAEADQRERGGWSLERRSREARFFGREGQAGSETMHIRDFPHVFSPV
GGLTEERALQKYQVVIASRPRASIVALPKEPPVFLLPSTLALDLLQLLGFGEGRQWGRDGGRVCDVYRRTVGVTGEEAGS
VGEGGREGVLEGREAGQTTAEVVWAHKSFLRRRCPYFQAMLSSGMREASEPIISLGDVSPPVLRALLLYLYTDTLQVTAE

IQTVLLPLLLLAHRYGLHPLMQMCSAYVRRALTDRAPCADVLEWSDVFHLVDIKRMCMSVQVMRGWNEGGGKEGSCEGEK
EEGLSERLKRQLQEFKRGLSWEKGEEGNGEGAEEEEEDIGVEEMEEVLEEEEEEEEEG
>Ngad_06508
RTGAMSDKKSGIPRRKDATSSAESAGGEEEGRAAATMNISLAVETGTDGLEEEANDNMVSSPMNSGVSSPTSAEPNLSSS
LCRKRRRPGGRIDLKTGTGGNTGGAAGGESSSSSLPSSSLSRSRSLPVLADLRQRSHSHTQEHELSPAVDADDMPVAGEY
ASNMLQTMNSLRDQGALCDVVVRGDKLAIKAHRVVLAAASEYFRACLLGPMQESSDKVIHLSGMDDGALKELVSYAYCGR
VRLAEDTVLEILVAANRLQVPQVVEQCCDFLIRRMDSSNCLTILGIANMFHCAHMRIKAEAYLHRHFESVYLGEEFLQMP
LDKLVQLLSNDSVSVTSEETIFNAAVRWISADADTRRGHMLALLRTVRLQLVPTFFIADVIHQHDLVQCCPEAERLLLQA
YHWHNSALQDLREQAYRYVRGRAIRPVIYVIGGDDGHDDRNPYSDVISLNTHLSAWRKVSPLQQKRSVCGAATLRGKMYV
VGGYDGERAVETVEEFDPTTNTWRYVANLSQRRCSCGCAVIDGLLYAVGGVCGPLALNEVERYDPVTDQWTLVPAMQDGR
SGCGVAELGGLLYAVGGINCNGDTVCTAECFDPSTQRWQSVQSMHETRRSFGLAALGGLLYACGGNDGVNDLNTLEVYNP
LTNRWKVLVPMRHARMYCSVVVLDEKLYAVGGLDGTTSLNVVEVFDPSRGVWEEILPKLDHHVCGCGLAVMDCVAPEKEL
DVKEKIKSIGGYADQNNLSGAMTVESASDEAVISVNNG
>Ngad_30562
QSRHDSMLYSWGRGEDGQCGNGSTNDQEMPMPVEGLKHLTVVQIACGSGHTVVLTEDKDVWSWGRGDDGRLGHADNHWKY
VPHLVSALTGKSIKQVTCGSYHTAAISDTGALWTWGGGMYGKLGHGNEVGHPAPCRVEAFIGREVEQIACGSRHTAALLA
GGEVYCWGDRENGVCGIGEAAEHQYLPKKLEALKGVKITQIAACGFHTAAITQDGALWSWGEGKFGRLGQGNEKNQVTPC
CVEGLQGVRVKQVACGGFHSACVTEDGDVLTFGGGEHGQLGHGDKTNRTVPCLVKALEGAGIVQVTCGWSHTVTLSEDGK
VFTFGNGDHGKLGHGANAKSALPKLVESLANMKVAKVASYNEHTVALCHAEQEQAVMSFDFLKDLRRLINNADFADVVFL
VEGQRVHAHRAVLAMRCQHFRAMFKSGMRESWEEEVAIPGTRQVIFLLLLEYLYTDLVPSDTMLPETAIELFIAADMYGV
ERLKRACENVFQTGLNIENAATLLQTAENFK
>Aano_F0YC82
MGQLEAWGVRRESAVRCLASAIVALAALSSNAPPAQKRAIVAARRRQSSHPSPAALTNEIPMAVAVEVVPLRAAPAAPPA
PPAPPPAPPARSVSLGADGCERLVAALRDQLSAGGGDVAILGDGGALFPAHGALLRASSPVLAAMLDDVADDGETDIPEL
RVGGASEPVLRAVLDYAYGGVVRLTEENVEPLLELASRLRMTALVEECCSFVAQRTRPCRACRVLAVADRHKCRLLRRDV
LLCVLRHFDEAAGLAVDPAVAVVAAGGRATKKTMAEGSRAACGAGDRAEAAAGFGDLPLHLLEEVLADDRLDARDESVVF
AAAVAWLDAPGREAQREAHADAVLRHVRFPLIDPEFLADCVEPHARMQSPAGTKLVHDAYRNLCLPPERRKVAPRAAARH
VPCSETHRPPSRPPSPEPAPAARAPSVDSGNPRTMMLNALKSVLPKGRSAATTRSVSGLLEGYGDHVFKGAVAESYLSKQ
GLAAGTLDNSEWTKDAAAADKVAAAVLEWATENDATSFCHVFQPLGASGVRHGLVGQVQNAFFKFDAAGKPVWDLKGKDI
LAGETDGSSYPNGGLRGTHCAGGYVSVDPTSPIFLRGDCIFIPSTLCSYYGDALDEKTPLLRSVDALNSEGTRLLSKLGF
DVGGLQSNIGLEQEIFLIPRDQYVQRLDLMMTGRTIMGADAPRGQEMCDHYMASPSSATAALACMQEIQAQCFRLGIPLV
TRHREVAPNQYEFAPQFGTATTQIDQNVTLMQIIEEVAPQYGLAALLQEKPFNNVNGSGKHNNWSIGTSEGVNLLNVGQV
TDKSGSPDVFPTIMAAIVGAVDDHGDLLRMAIASPGNDFRLGACEAPPAIVSTYLGEDMTSYLEAYAAGGEAEYKPGTKA
LDMGVAAMKKLDIPAQDRNRTSPFPYGGHRFEFRAVGSAQNVSLVNTVLNTITAAAFKKVADRMDAGESARDVATELIKT
HVPKVVFNGDNYDEANQDMLTKRGVWRIDSGVEAICRYSAPKNTALFEEMGVLRAEECAARQQILLDHYVGVVDMEAKCM
IDMINKHVIPSVKAAGLGPLSDLEAAVGTLEAAVHAIEESEGEAQATLARDLRLETMEAIRAHCDAAEKVCPASLWTLPT
YVDLMFLDQTSNFSYED
>Aano_F0Y9D2
MEARGDGLERFLPAAADAQKTTSQLALPDGEFGGALRSSLSALYEDRGGAELELTVEGSTQGVACHAFVLETWSPVLRAQ
LSSRWAGGGVSGPRSVEAPGGSRPAVEALVAFCYTGRCSVDDDSVLPLLRLSDFWGVAHLRELCGSFLFETSRSSDSGDL
AGLVEKLKIADEFGLARLRQRCASALAAKFDELFRAADGDSGKRSSPLLLELAPDMVGEVLCKSRLAVVDEADVLDFVRE
YAASRGDPPAVLAQLLPAVRFPFLGDKLLELRADAELMAVDGAVALVMRALEILAFPQSAKPVSAKEEETADPSTLQTLA
RTGLCCFDGTFSSSLATIGDRARALSIPVGPHVLFAVTRPGLAPRRDGAPDGPAPVVSWRVRVDALPPPRNRDGDGPANG
ARGGRGARGLGARAVQEQMRELIGVSADSWTFGLWVVAEHDVAPLSPTNNPRANPACSNFAICNFTKARNTTVLQIQGNG
RAALAGQQAGPGQQNLEGHEILVGDVYGADLRSLKRPGAPDDAPGDLVVAFSRNGALLGQPVPLSVPLLNPALFPEPHSR
FNLRKENFRLLVAGGRAQAGANLMPVASTSLGTLGCTLLPQGETTPLPAAAFANAPRLGLFFVVATCRRAAASNASEVAT
LGTWWQNVDATTDGSLNASFSSWWQGDFMASVVDWLNATNGQRPSLAYVEDEAAYRRWYEHERCAFALPWAGQESEIPNF
KGSFLGRFPLALPWDESGASFVSSHELETEILGDNTATTFRSSRTPKYDSSLRPSEWWHESLGRESADGEATGADGVQDV
FVNFALSSFDINEHRGSWSARGYIGAYWYDPRLMFALTEGCGDDAAVASGHYWLLSDGFEKKIWNPDVKPKNLAAGGFKI
RDRYVKIFPYGLVYDVKSVEMELDCDLYLRKFPYDDQACKIVLSSPRNDVNYLRFGPSVEDPVTAYDERTGDKVFNDQWR
VKDESYEVVSTTYFAQADQEFVKNTPVETDAAYALPLSGTYHEFECVFDFRRKPGFYEVEIVLPAIFFVIISYVGFFINA
SVAPARVAITVIPVLIMRTLLSQVSAKLQIISYLTCLSKFLLYCQFLTVFAVLEYGFVQICLLHESRCRDVRTHLNSMKR
FIVADSAEDAPSLFDKEDASARDVLLVEEGAKAGAELRMIKHEMKHVERLERSNPVFKELLDELRKLYRGADATGDGLME
PFELAIALRSYAVYIAPSQARKAMINQKYEHGGDDVEIDHATVAFTFAEFLHYVVHYDENPTTFNGRRSWAEHFNPSFHP
PSTNCDGLFRLLFPPVLTLLFITTLGRCHWDSR
>Aano_F0Y2T0
MGDDGWERVDRPRARSKSRGREEHKSPGRGLYQRGGDDRGPRGRSQPRYGDRYGDRPGRPDWRERRDDGRDRARTPDRRD
ARPPPSSPEASNWRSAPRAEFAPDPAPSANAPGWTEVLVWGLPATWTFRNVLAYAQKGGGPQPVSARLLRNRATHAPLGC
AVLTCRSEGDGRAVCRRVDGQAVGRNRLSARSDLRRLGASRLAVRGVDFRRTSTRSLRAFLEQVGDLRTFDVPRPGSPED
VDGVSAVVTYAEGFGAAGAFAVLDGAVLDGAALRIATLAVKALRVETPPPPPDEEDDDGDGDDDAPPPPPDQGNAATLTS
IHDSSTRWEPAVNLKWSAELARGVMDAATYSSHGAAESKDADDERPPRPTSSRLVRRAVAFQVDAATSFLGAAPARAAPP
DLDTDVVAFRALLPQRFELGGTAWCAGLWPDAVSLWEMLGKTKLIPAGGAAPPPRHVALALAMVDGDGDDPAAVSSALDK

VTVAVVAALRPADPAVSLSRSPLDPAVLVRADGSVASAHEPILLARHCGGPDKSRDILLCLFDRRLAVKRPYLHEDAMRP
ELILTLLDAAEPPADAAPRSAPAPAVPESPPDDDDEDLAAAFYPSEIGLGLLDLWEGKVGADVTIECADGVVLDAHATIL
AARSGYFYREFTAPGGEVTKAFLFNGEDSKVMAGVLCFVYGDALVDDHLDLLNDYGATAALLRAAKRLDIHGLAVWVEAC
LESTMDVAGLPALIDALGPISAPPHARPATPAEAALHAAPLAGLHSLRAAARDFAEDVSAEPDVAAMLLAAAPRDPDFRA
TLEAALAATIKATSVAALLDLATKVDSEALRVACMAFARNNLSKVMCDPAFSELLQRRSDLVFELLTALDDQPQAKA
>Aano_F0Y4D1
MRAWLRVTHDEGPCQRSLHVAAIWRDKMLCFGGYDGSNRVNDCWEFDFGKRSWSLVVPASGSPPTPRDRHVAVVWGSSFY
VFAGFDGTSRVNDFHEFSFGSSSWAPVRALSGLAPSPRHSHAAVVYHDSLYVFGGYDGSYRCDFHEFNFVTCAWSPITSD
GRVPRARYRATTVVHEHAMYLFGGHDGTRHLNDVHVFDFGARAWSGLQAEGPAPIPRDSHVAVTHGHSMFVFGGSTGSAM
NDFHELRLDARKWQPVQASGYAPGHRFCHVAVVHKDSLYVFGGYDGSNRLNDFLEFKFGFGGTDIPASSLITDLRQLVDS
ETLSDVTFLVDGLPVRAHKVLCMRCSYFRALLTGEMLESRASEIAINDVRHPIFLALLEYLYTDNVDIALDIAMELFQAA
DQFGVERLKRMCESKMLASIHVENAATIFHAADQHAAKSLREKCLNFILTNFDAVTRTASFEEMGRVNVELVFEILKSRS
N
>Aano_F0XZL5
MEPHDLGAYEDGEDHSYQPLSWRTDPATSLSDWDVVVTWEADGGGEETRTYHVHKQTLAVGPRSGAYWAKLFARWADGTA
ASEMRLDAAAAACVPALLDYQYDPRGALDVSTATAMPLLHLASYLGMRSLYDALVAFVQRDLRPETAAAYVAAADRRADR
FGLDRCRAAAVKVCASHMEHAPSLEELAPDLFLLVACSEDRCCTGAAFSRRVASYYRTRASDLEDPGDFLRAIGARWPGD
AVDPGEALFLLGHALDHGEADLAERCVAAMSRSWERTLFEADPCPDGVDPEILRGVPGFPDGAAAAAAPPPPRRRLLPAA
AAALPPAVELDVLRRALGQARDDVADARRDASLLVDELRAAKSETRALHNFVRVPLSCHPSKFGGGLDRPPTAMPPHDAE
PENGYVVTEHSWDLAHWVTKYWPVYIYKERPATTAPPSPSSPRIDDDELQAEREREAEASMARLLEEAMVDAAPLELPPR
>Ptri_XP_002185016
MADLPNTAQVLHEAILTNSLDVVQSYLGAAGVDPNQSPPLCPLAQLKRALRRPDAVAHDVVVVATQDVYRVSPLHVAIFN
CYHNHGSQEDPTPRETALAIVQALVDAGADTTLVASHIAVVQKRDSSLIRIQDRAPIGLALLLKQNARGPDEVNMVESLD
AAMQCIVPRADARDASGQDDVIETMTVPESFAQSFGSLLFSQEFSDVKFVCKDGTVLHAHQNILAAASSYFRTYFQGPWG
TLHTDGCWKTEITPDVLRAVLMFVYTGKVDDDLLEEEAKNIISVAHEYELFDLQLLAQSSCVANLCPENSKEMLQLAKLH
ESDTLKDACFDYIKENMAAVLMHPTFVSLADEDSALWAELNEFLQESPTQAGRKRSRS
>Ptri_XP_002184283
MANNGGARQANAQINEHVEFTVKPEEDLYLSGSENTIRRVTAETNYSCLMLKTQRFSSLFRHYAKYHGLRKEDLEYYFVN
LLENEDTPESVQLQRGDTIMVRKRRKHEPPEAAADDEEFIKDMRELLDDEEHMDAVFVLNKDMDCQQEIRAHKSVLTARA
EYFKALFRTNASAEKKEGTAFKESEECVIQVEKDFTEQQIRYVLEFIYTNRIQVVRDISTDDLLCLLHLSDKWLLRDLKR
LVEHELIRDHMTVNTVARLYGATEDYHASRLSRACIEFIMTNLRQLAGNTVFEEEMKSYPHLCMPVLKAAANLIPDGPAL
KKQRTDHHGTPSGIGSSPVPDSDT
>Ptri_XP_002177347
MDRMRWTEQSASRVGCPTIKNHSVTHFGDYLFCFGGYDGRRNHMTLLIYSILEQRWFRPHHAMGTEGQGSNFLGDPSFLV
QGTPPPGRNGHSATLAADPDDEENGRVIIIGGWLGTGPLAASDMHVLDISGAGRQLRWYQPPIKGTPPGPCNMHSADYVS
ALKEVFVFRGGNGREYLNDLHALHVETLTWRRVETTGAIPQQRANHSSAFLEETQELFVFGGWNGTERLNDIHILDTATN
TWTCPRVGGVLPHPRAGMTLTALRGRLYLFGGSGTSAKCFQDLQILDRQTMAWLDVTQYETGRNGHHDSEHETTGTFRFG
GSHTLDDGQSHMYGHLEETPRFTFGGAQQDNSLDANQEAVARPPQQGGGQDGASLSLGSFTSSRADWHARDMAARHRQPA
LPNVSPNPNDEDSVPAVLIDGTGPGRRAGHTATAVHRKIFVFGGSCGSDYLNDFFVLDTDPPPHALVSEPTSVQLFERRL
RHFFNDEEFSDVTFVVQGEKVYGHKMVLSIVSDCFRAMFTTGFRESEAMEIEIPDCSHASFLSVMEYVYTGALPKMDMAN
QDRDRSLTRVVEMLELSDRFFLDHLKQICESILQPAVTHDTAEYLLGIAQKTNASQLQSICEHFVRNRNEIA
>Ptri_XP_002177179
MVPPPRSGAASVVVKGRLYVFGGYGGGTGRLDDFFQYSFETGQWDEVKVLSEAKPGRRENNGVVISDSSRSIYLFGGYNG
SSWLNDLWKFDIESQRWTCIQESSDPDRADDANAASGDVPAGHQVRGKAPSRRFGYVSVVHEGKFVLFGGFDGSRWLNDM
FEFDFSTKTWSEIDAKGSLPSVRSCPAWAKDETHVYIQGGYDGVERKADFFACDLATYTWTELPCYGTPPSPRYFHSCCL
YGNKMYCYGGYSGSERLADMFAYDFETNHWVQVDCSSGDAPSGRSSLVAQVYENCLYVFGGYNGATVLNDFYKFRLKPIL
MPPPTLVNDFSRMINNPDLSDVRFLVEGKDVFAHRSVLAFRSEYFRVMLCGGMRESLARQDAGTSCVPSHDLQAIDLPNV
SHLVFLKVLEFLYTDSVKDVSLETGIYLLIASEQFMLDRLKALCEDLIRRDIQVENVIGILAAAHSHHAAGLKDIALEYI
MRNLNDPVIMAGLADLKSEPDLLLEI
>Ptri_XP_002183875
MKALHAEVAKDLLDALLDDELSDVYLTSSDDVQVPACRFVLAARSTVLKRMLYGSFREAKSSTICMLGYDSVTLQAIVDF
CAHNDISRCTAKLKQDETSIRQLVHLARAADYLELPGLEQLCECEIRARMTLHPPLACAVYDEAERSTEVSEYAMQMLEC
RPYVALDHGGDRATGGGIESLHSERLLDVVHNTQLGAGEFFLFRMMERWFQDAQLKDHNAALQTAHKCTQYLQFENIEPS
LLLKEVKSCEFVSSELIFAAVAKQALRASQDRVWSLGCRGKEHVERVLVEGAGNRDANGMYYLIAPLANNDLYAKREVAC
GQQRVYTLSCSTKEEQYECRIFCSTLLTSGAIFNLQSMQKTSVVEPVFQPVLQIIELKEPESSVPTPIRKYYRVRVSDGE
VHMAGTLATAWNSMVESKEVLARSICKILQYGLYEIDGTVCIHIREMSIVTTNPGQRFGRPIPLEEADGYSREHQSENLL
SLYSCTHPRVQGAKDIKVPRTGWQVDDQGDGPVPSCTWMPPLRKADNLNTSYTSLRSSTSPISGNTEPHNGSS
>Ptri_XP_002183770
MVPLPQEDGERRPLKQGDYGAIRVTNDHDNSLEMEEADDGQGKDQASSVPPPSSTSSDAAHRVLIKTQGSFWEDARDFAP
GSIPHSAVLALTIGCVCGVAAWLYYMFLEWALDFLWHDLPERFVIDYWPEYLYVLWIPLIGFGMAVGLGIVVKVMGEPGD
LPYTVKCVHEKGYVAMNHVMPMVMASQCSILAGGSLGPEAPLVAICAALGGFVSRSVFGMTDRNLVRKHTLMGMAGALAA
FFGCPLGGSLFAMEVNSRFGIEYFEHTLEAILCGEICLVVFRALAGLPIQAIWDISDDKLEATSVVDILYGCVLGLLGAG

VAWGFANFHWKVMHMFQHFNLLKNERAIHRAMAGAVVVVLMGMLVPQTMFWGEFEFETIATMAPTKDLMHIWPTSGLLGF
EMDSFGSAMIVGVAKLIAISFTVSGGYRGGYIFPAFAAAAALGRAISILCPFIPVQLCVLCMAAALNVALTRTALATTLI
LAYLSGEQNSISAILAASLVSLFATGYMPFIRSQMVRADLDASLYYSEDYPEEAFSMTPMADVHKMSNTTTTPGAVQGRK
SNGGVQVVSYQNDTEWPLHYACRVGNLEQVQYLVKQSKASIGSKDDIVNQHDDNDATPLYYAALTGNTDICRFLLEKAGA
RCQGHDGARVFYVALTSDLRKLLRVWSLSAATRDPYLDRWQDAFVQASESDQQGDCVYHRRSSFQHNSKPFCRSKSIYFH
RIVVQARCPALADFLHEHYPESAEDSKAASELRLDEYPEAVVCGFLEYLYTGVWYIENLLELRSQALALASKLEQQDLVQ
IIHTKWPDMAKRIEEDGKGFQTSLTMEVSSIPRLRKDLRKLAHWVSTPHHEISSFHRLNHLLTWSDATIQCRDCSWSVHR
FVTQSVSEFLDRAWSGSFLEAQEGSLNVSEMPCTPEAWSLAIQWMYADQFLTSVDSDTALDVLELATTILCPQLASYVAN
IVLVSLVELENVLDLLLLARSYGFDKLEDCCIGVVALHIDKLVGSDDLRSILTEEAAEISQGGDVRPQILFFGACQPFAP
PWQNNNGEIALGSVGNPSRRATAILVLAWAQTSVTASSCDDSVHCSAGYYLESASLRSASAPCIAIGKGFWSPSFDNNRH
SCQILPLYVAASVALPFAGTTDDVTGSERYRLREETERLSAFRDACERPGGSSLTFMETPPAMTSRRSTSHNSVYIPLEP
FLSIFIVVMLAIFFTAILDRRANDNELELETLRAQTAQEIAALRKMLLDFQLKADRNCID
>Ptri_XP_002183444
MQSLSPLQTDFSHKAQKRVAQHSAEREIHKKARPLLCWGAEPSENHSDWMIEVVRADGDKTCPALSFYVHKCVLAAGSQY
FAQLFRHGNRFTEGKTQSSRIELCEIGASAFLAFLDYLYSGNLEEESPTMTFTATPLQYLARYFECPQLQSDIRRFWTRD
LGTQNCWIYYEHSKIFDDKEIMDAVINLCIEKPSEIPTVSSLFEITDTKFWLEILEKSSKEIVVRYHLSLLIAIYLFTEE
KKGTTKREDFFRLTDEKYLPEVDYRAAMFFLRVEETFMSDRSAGLSSLQERCVRALVANWTRMSYFDQTNIGILLKQDTV
LLVRLLSLTVSQAKESHSELLLKDNMNEKIISHLYPLVKKHIQNEEFIATFHS
>Ptri_XP_002177255
MDDERSNDEDNGGEVSDQAAAAAPPDILPVEEEDHERNPPPPVVPVHAEAVSILQLHRAPPNANFHGSATSSSSCAVSAS
NQSLLRQQAHSPVSNATHGLEVSARSFQGGYRRSYEAPFQQLAFSDTSPELDMEDPNASEQSSGWSDVAHSQDHGNGGVP
PSPRSLHAGALLNGNFYTFGGYDGNQRVNTFHAFSFAEKRWSPVFPSANSSPPPTPRDRHVAVAFGNAFYVHGGFDGTSR
VADFWAFDFSTMSWREIVALQGRHPSPRHSHAAVVHGHSMYIFGGYDGSYKSDLHEFDFTTSRWNAVPAVGRRPRARYRA
TCVVHKNSMILYGGHDGTRHLSDTHVFDIDTKTWAILLTEGAPPVPRDSHVSVIHMNSMYVFGGSTGSAMNDLHELQLPS
SSSMSAKWRSINASHAEQPRHRFCHVAVVHSDAMFVFGGYDGSDRLNDFIRFDFTVYDLSFEVPQSTLIADFRSMINDVT
LSDVSFIVEGRDSPIYAHKLMLVRCPYFESLFLGSMRESRQATIYIEQVSYPIFLATLEYLYTDHVSISLKNAMELFEAA
DLFCIPRLKTMCEKRMLQSITVENAAGIFLAADMHSASALRQKVKKFILSNFEEVSKSSCFEEMGRSNIELVFELLQSR
>Ptri_XP_002185140
MADKDRYIVAQEETGYGYFDAEGERELFQSKSQDDFPLTWRGDPAENCADWTVVVVTNELKAASYHVHKNIMCFGSRASK
FFSRTMLNKTPSKKRSKRQQLSTTKVELNQQDADNFPLLLDFIYAPCGKMTSGGTVLTAASTLTSPSLLPVVNEDDGSSS
YTVENISTNNAVSLRHLAKKFEIEALVLAVNKFIQRDLNFKTGPAYLCAASEYKDERLLESSRRLCAENFEQIDMRELIR
LPLCLFRVVVRCLESFERDNKSLSCFLSEVVCRYLEKHQKEINGELLLELTDSLVMPYIAPEAAIGFTSLIKELKTENAE
KHFHALSNLSRRCAQSVVKEYGWTDFSVSAAVDEYLGHSKDFKFDDHEGWRVDSLLFATSFAAALEQAQDDYEEVLVEQE
RLSCMVGALHQTVSMMEVLNSKKDEHMTKQQRAIDEAKKQILGLKDQISMISQQQLQRTSIYPALYEQTDTEIQMGSSLD
FRHEKEHRDAPPPLLSYSEETWSPVKDLVSPCQVGLDVQQNKSRRKQELRTKAEMRSRSMLV
>Ptri_XP_002176931
MANTEGLSDENVESLLRFDSSDHTRDRPRNNPSARLRERMDAPMGERPAATVIRRSTDSGRRPRGRAIVEPTAPTRLSTS
AHGLPHADNGNAPSPRAAAASSQYLPQNRHQEQRQPELEQAELNFPVVGSIVERPRNRTPGASSSERTRPSKFAQERHVR
AGDATSLNQGFPSLNVPLGTFVKDNHQQRRNATKTTTSIPPTSPLLVESAEVPARTSNSIDELRTISQRDAQGMLQNMTP
VEIQSHVQDLRQALPSSTLAFLQSRSKKSPVAKPPTVLAEAKVEHSEPARVTRDDRVLGKEEFSQLLSSISTYEDLDAAY
ATYVPTELQEKAASTLDQQQDDSFPMACDLLRSTAPRQNLWAARAVYHRLRDDWEAGQFCSLETDRDVVWPYPVVLPVSL
RCLLDNSFQRTNGFVLHTYVLQSIYMLLLLRSSREHVLDVTGQYVSSSMVYQECALDDAVPTPPTSAYYRSNQVTPLSVG
AHENVAYSTSSSSESATMDGQAFQSDPMWTLLSKMRIVPRLASLLYCPDVPLILLPAKALVSICGILAMIGQRSPGAASA
IVQHPTLLSNLVERTCVPFDGESRCNPNVALPTILLLCTLARQSRVAALGISVDAVLLQILSRKAEHESEYRLQRWTVIL
WRTLLRYGHCLSPLSALVPLAAPHLSLGVSYRFSLASDFASAFATVQRCAWVASLMDENDKRITAEQQTILAHSGAWLAS
SIRNAIVNVSTQVAVSSIDESTSDWIARLRYGSSCFQLLASFLAIAQDNSTNTEECKSDNLTVLEERDCVSALVSIENSC
VMQRILDVSMRSAFVVEFSGNAPFNEEEEACACTFLVEYLKLLESLRERFDVDIDSTETGRNLRLLLERITRLLEDKFLF
ARLRGEVSEDGAESKKYNRQRRAWLNRCHSGIVKFLVTQHSTLSELSGLACTIIGRLENGDEALAANLLSHDCLFSSQKQ
QFPRAVSPISTMMVRELCRTQIGKTQLDHSFKLQHGLGITAGGVGQFDISSLLSQADAGPAPDQTSPDAFLPVGKLWVWQ
ILSGASALGVGQIRLQAGHEEVICILMSCLDLIQGFEKYQYIGGEGYGNSLPRGEKLYHLMNICLQTEIVLSEGIILAAA
TNLFVEYTQRNCNQSFVANFCDACLLHSTARSRREVNEKKTEEETNLLALLDEPSDTLLLTKASMRSLSDFVIDLYESFV
DHGAQYPFFTKCIRTFLLPTFPARIRCELLGRLRGLLHLLSLEGDNMSELLYFYLSAKSPLVDESSRDDPEFLDEITSAY
AIGSGTRGDEGFVSMFAVAALTKSLAISLAFPSSGQSASKRRIQRLEHSVAIKVLQSAARFASSRRTCSALVSAVMQGDA
NDDTCNLNSELVSAPIDDEKWNAIISVLRNRFADSVKIMLNEGTEIRADMDLLRNFSGYFNALLDGCFAESRSRCVELLS
LDKAAVQTLIDFAYDTNSVDWDAVEDLSLLDEAADYLQIHNEQLDVTLQALHIVQDCVEQGWVSFTKPRHVLQLPISKKN
RAWRLWVCYVLCLELLPHPETHVDQPTFTQMLQKITGDAVALRRELIEYGLVLREGDGSSYWRPHYSICMQANTSDEIRS
L
>Tpse_XP_002293822
MSAPTLAVLCEDADEETTIPIVGVRFEIFQCMLRYAYGDDIPDELWKPTNNRNNSNNNILSSSPAMELLDAANRFGVVGL
KILAESKVQQHNISIETASDLILYADAKNCPLLKEKAMDYFVLHAEEIRKSPSFERIEESTHIMVELMDALLSSRRSSTN
TLRSFSLGEQDVDYGSMSVNLLRRMLEERGLDVDGSREMLIGRLEGWNVGRNNM
>Tpse_XP_002292857

MDVHDKPPKPSSEEAATAPLSTAISSESAISNCTTNGTLLADIHSLLHVEEVTDISLIPTPSSCAASSSNDNSITSVPAV
KAILAARSAVFRRMLYGEFCETARSNSKNIAENVEVQLDYSGRVLQLLVEFCFTDQLASLNHGEDGENGGKSKDGRNNKY
DINSNNNGGPISLEEKARLLTNLSGAAHYFDIPKLESDIKKQLSTMMLDHPSLACAILDESSKILPGEEIGLLAMDRIRA
RPKAALLPWMHGVGGSGEIVTPSFGAVNNNHDSTTRSWEGKGGVIALSASLLEQVIFDEKSSASELTKFHCLRKWVQGCK
HEVSMEELELPRKQSEDDILSLDQSRAPSPPTSPPNKSSFRDGKFEDEHHQQKLAIAKHLAQKLDLSLIPASDLSSTVND
SQLLSTHDLYQAYRLQALNAERVKSKVIVEGAGLAEVNGTYIQGGVHEGTPMYNKEGVWKDREEVFRIFMCTYSNGNKSW
CISIVPKGKEPGKTTDVDFYECPVSYGKGIGGGPLYGMEMGVVPSRGWKLVSYGQPPVPRASLISVLEK
>Tpse_XP_002292823
MIPSISNAKETTEEMRKAFKNLYANAVSCSDLSAIQSITIPDGACKEGGWKLDGCKEGSGRDMEGAVTLDWRADPFESMS
DLTLVVYDGSGGVPYHVHTILMAFGGRRSGFVVDQMKQQQKKSGGSKQSSSSGSKNNTNAPDGKEYTIEIYVPALAAKYI
PTLLDYIYGSTLRFTTSNAPPIRYLSNKFDIRDLHTQVSKSFISQDLELNTAPQYCIAADELKDFELRDKALRIMAERME
RMNVNLLNGMSPRLMRSLVQCDKLECGGVVLSEKVAMWLRCRDAAEKEGSSVIPLTDEDFYWLTHVQLMPQVSPHDALFY
LNYGANYPQVMNEIGPGSIKSRCLAACSGSWALDNLTAHLDNPEKRSMKLYENLEMDMKVQVLESSLVGAKKLMGERDQL
SSSRDVRENDVQLSDEIMYQSIQKQYTSSSKVSKVIVMGAGIAPANGVYIYNPNSAANKSQQKDFPNMTIYEKEAVWNQS
SVTFVLYPTSAGQYYTQYKLAVRLNNKMIVFYNSPTVMGVNGVSSRGESGAPLPEQGWEVEGEGVHPPPQFVGKIEQPVS
IERSSLTKSV
>Tpse_XP_002291664
MSSQATAIIDVGTPPPTTLNLEKLFTFNFHNFESLDSTKGTFVISPSIKCFDHEWAVTLYPGGDKNARDGMVSLFLRKTT
KGSIAVEFALIIRDNEVTNNRTTDEFSDNVPEWGYSDLLDRSLILSPSINMLNNGTLTIELRMKSDESKLCSNLIPKNPC
STDLISLLKHNEKSPSDITFHVEVISAEKKSYKRYHAHRALLEKFAPSLASLSESYDNTNPMVVSGVDRIIFGAVLRYVY
GNSLNNVDWEKESKNLIDAADRFGVTTLKIEAEVRHLKYCSKFTVDNAVDTFLYADSKQCALLKEAATNFILENAVDILK
SDAFKSMPGDKSLMSEILMGVARNKKQRSGAKPNDYEAMSVNELRVRLYDRKLDIDGSREMLVARLKGVANERKSEEMSN
NDAEAS
>Tpse_XP_002288579
MAECWLHIFLDEASADVLFEVHPPLAIVDEADVDQQRRTLAPARFFAHRHILCNCEVLRNLSNAYNSLEPIPITDTAPDM
FRRILYYIYGGEISIVWMYDHAEEMIDAAFQYDIGTLKIEAETWYRIKVKLTIDNVVDCYQYAVSRQLKTQMKVIQAFVV
ENEAAILDMIPITDNPGFHTMVVDSLTESAEATRSSASRQLDEASQRNALSSTIDS
>Tpse_XP_002295639
MVNDDEYSDVTFIVEGQPVYALRGVLAKRCEHFAAMFRSGMRESEEGVEIPIPNISQAVFLLILEYLYTDSVKVELEHAV
ELYIAADLYQIASLRDMC
>Tpse_XP_002295574
MRWSEAAITSDDVGTAPTIKNHSVVSTSVYVVTCEKSVGLLFLQLTTNLITDTIYSQTYHRGLLYCFGGYDGRRNHQTLL
IYSLKEQRWLTPGGGRHGAEFTVGGSAPPGRNGHTATLATCRSSRQRRRLIEASIRDNMMAQPHNPNIERVPEFMERDLM
RRGGNMNIGNNMIVDDSRNNNEGGNAQGGDDHRPNPNDIDAEMAEEVNIMDSNEDDDDEDAQIIIIGGWLGSGPLAAHDM
WVLDISGGLERLRWFQPPSRGTNPGPCNMHSSDFIPSRGEVYVFRGGNGREYLNDLHALDVESYVWRQVETNGEAPQQRA
NHSSAVLEWNGTSELFVFGGWNGSERLNDIHVLDTSTSTWSTPRVTGVKPHPRAGMTLTALRGRLYLFGGSGTSSKCFDD
LQILDRAEMCWLDVNDEDAKSTAARCDNAIDNRDIHQWGSAYSGQDGMGGDDDQQGGECRGGNSRSIKFADWRSQENHQV
GSALACTSANPNDEDMVPSVFVTGKPPGKRAGHTSTAVGKHIYVFGGSCGTDYLNDFFVLDTDPPPLMKVTEPASPQLYA
RRLQHFYNDEEFSDVTFMVEGRRVYGHKLVLSTVSDCFRAMFMTGFRESGAGCTEIEIPHTTYDIFVAMMEYIYTGKAPE
IDVFSTEPGHGLDRAIALLELADQFFLYNLKQSCEQLLQPAVNAETYTFLLNVAQKTNASQLESYCRYFERNMNELMD
>Tpse_XP_002296040
MNQPRERAIDVDVGKPPFVSSDWQCINFHFHAFPSLITRPNHEVTSPEIPFGGHRWALVMYPSGFGAYRHDGVQKVDITL
KHCSENGTTARVEYTVRAKDLNGNVKAGMSQKQSFVGTMSSGFLTLVRSEILDPTSNILSDGTLTVELRIKSMESASNTS
SATKCQPFVPTNPLCRNIQRLLFDEDTADVLFEISKRQTSSDEGGNNDEIVTFRAHRAILHASAPTLATLCESCDNSTPV
PITNVTPDIFELVLRYVYGLCIQAEDWKLHSKELIDAADQYGVINLKLEAEYWYVKMTNVMIDNVVDVLLYAESKHLALL
EERVMDYFVENGDEALKLVPFHQIPESRSFFTGLLAAMNVKKGEGGGSVSISTIKHLNYKTARVDELRRRLDDAGYDVDG
SREILIGRYEKMLADGNGDENGDEEEIASLTSSRRRSSTLSDGPRH
>Tpse_XP_002295852
MDSNLTVGGVQQLVETEGHFTNRHQPVLQAVLSDGERSIEVVISTLLSHLVKSEQIAEYTLIKVKTYQYNITDDNIHIVS
ISKIEILENNPGGVIGNPASVLDSDSAHKKDVKNGYHYFNVDDGDWDNESDKEDPLTWSLEEPSDFCDWTIEVTRIGRHG
KAKDNLASSNKSQLQRYHVHKAILSVGPRRSEYFATLFRQDHLSEFSTNTSRINLEESAADVFPSVLDYIYSEKTVMFTS
KNATAVRHLAHYFGVRLLWKFASVYIKGDFGLETAATYLSEALLYHDDKLVQASVDILAECIEEVSRRTLTKLPPSSFER
IVTSPHLKCRSKKLSDIVLKYCQAQGESVDMPLLMKWTKYERMPSVSRKAALPLLKIAVSREEQAGVNLADTHNVLRTRC
VEACVEEWKETLAKPLLALESRDKNISRILGCQSVEHRDLPKEIQVELLEKALCAAKIELDDVKAELLYREEQVEELSKN
IRKPASAS
>Tpse_XP_002290420
MDEGTPPSNNSGTNNNQGRGTTSLKQAAAVTRSSTRGTSGERRQQHQSHDRRRRSNNNSAAGSVPCQQDALRRLASHKTA
AFVFPSGSSRRVPDLSVATSSSSVASGSLTTSTSAANVRSPVAAFASASPSAAGSTERAVSAETRSIGVQPQRALVSATS
SQQHAPRLQMSFHSQSSQRSSHPESSFSVSSNNDTAPQKEGWSVVNTDPLAPGRPPCQRSLHAAAVLRDSMFIFGGYDGQ
NRVNDFYQYHFPSASWKEIITLGAPLVGGVATAQQGHGGADTGGGANNHNNPAAGGNNNAAGVVGVGVHHRSFGATVTAT
GTPPTPRDRHAAVAHGSTFYIFGGFDGTSRVSDLYGFDVERLVWNEIRPRVMTTPTNNNVNEGNAGQAAGGAGRRPGFNN
VDAGGGVGGNGNNNNNNAAGAWEPLRGGGDDDRAANAHQQQAVVRQIHSPPSPRHSHAAVVYNDSMYVFGGYDGSYRSD

FHEFDFIHLSWRPVFGSGRSPRARYRSTACVHGDTMILFGGHDGTRHLADVHTFDFVSQVWSLLMTDGVPPLPRDSHVSV
VYKDSMYIFGGSTGSAMNDLHELAFPPSSQEKFATDSSAAPDYATTAGSFGLREGENGEPALAKWRQIPPIAGGGIAVHR
FCHVGAVHNGSLYVFGGYDGSSRLNDFVKYDLAIDDLFQTEIPPPTLLSDLRSILDDEGAMSLSDITLMVEGIPVRAHKL
MLMRCPYFRAMLLGDMAESSQTIVNLEIVRHPIFMSVMEYLYTDDVSIPLDSAMELFVAADLFDIPRLQAMCERKLLESI
TIDNAATIFHAADVHSASSLRNKALGYVLAHFEAVSKTPAFEDMARCNVELVFEILKNR
>Tpse_XP_002289494
MFGGYGGGTGRLDDFYSFSFDSNSWEEVEVLSEEKPGCRENNGVVIGDSSRVYLFGGYNGNSWLNDLWCFDITTQRWTCI
QESSDASEDASNSALGRLGEGAGQGPSRRFGYVSVVHNNKFVLFGGFDGSRWLNDMFEFDLNTNTWKTIQAIGQLPSVRS
CPAWAKDDTYVYIHGGYDGVERKADFFACDLSTYTWSELPCKGTPPSPRYFHSCCIHGNKLYTYGGYSGSERLADMFAYD
FETNHWSEVDCTNGECPSGRSSLVAQVYENSLYIFGGYNGVTVLNDFYKFRLKPVSIPPSALVSDLRRLMIREDMSDVTF
IVEGQEVFANRALLAVRSEYFDVMLFGGMRESMRDDAGNTNEPIELQDVSYAVFTKVIEYLYTDTVSDLSWDISIPLMIA
SEQFMLDRLKALCEDSIRKEITVDNVIGVLIASQRHNATGLKDIALEFILRNIMDPSVVAGLSELKSEPDLLVEIITRHS
HTQSSASWGSSAVAVTGAFGPSSEWSGSRR
>Tpse_XP_002288002
MAANPARQGGAGAAAQGGNNGNQNNMNEHVEFTVRAEEDLYLTGSENTIRRVTADTNYSCLMLKTQRFSSLFRHYAKYHG
LRKDDLEYYFVNPLENEDTPESVHLQRGDIIMVRKKRKAEVPEPAADDSEFFKDMKELLDDEEHMDTLFLVHANPLSRAS
VMGGGEGTKGGDAAIVPGNESKKRKIGGDSGSSNSNNKSNNSSISSDNNNAMDNDTDLVEIRAHKCILTARADYFKALFR
KSAGENSSGEKVGTFKESTECIIRVEPYFSPKIVRLMLEFLYTNRIECLKVSSRVTSSGMVTSPQNVSTDELLSILHLAD
MWQLRDLKRLVEHELIRSHMHVDNVARMFCATEDFHAKRLNKSCLEFIMENYREVTGSVSFQEELKNYPHLCIPVLKAAA
ELIPEPAYKKARLSGMGDLGSTPGSAAGGIASSPVPDSDH
>Tpse_XP_002287637
MKTTSMNRSASSPLEISPPARGVGRTSSGRRMFQSLNNAASLTRRQSSASDITMDLAGLGTDNIEAKPKKKGFFYSKTWR
EYKKRRNAKHKEISDEGSSISGEIMINRDLTRRKRSSFLKGNSVKDLNATNVTAYLAARIEAERPKISEVYIHSSAILVG
MSKSHMLDVALVGNDDIPVRASRFILGCYSKLLEEVFFKQRECSMYDEEKQTIVVDFCNSQVLKAAVHHCFSGELPSDFN
VDRPDEDVARNLAQLDHVAKTFQLRALGEVVYRAARTLINRRTVLACAVFDELSLRHGVGSVDSTCIKRYALDTMREMPM
DTLLGGGVQWMKEESVMSIMQDQDLDVDEFYMFKILKAWEGGATRTNDRLAKARKMAKHIELKFIEAELIKSQISNSGYF
EDADIAEAIKLIEETLANRDPHEMERVLVEGAGTPIVNGIYLRVEEELGMSEEEILFVKEADDGFSDVGLYLYGTKWHIA
MCADYSNCFYSCVDDPGKTAANELVPRNGWTTTYGGEDPPPACTYLPNTRAARLGLDGTVLAPNLEEMMDPTIAEKRRSG
YFEKQNHDTIEKRTMTLEQMMNLPEDRGDVFTSP
>Pinf_XP_002908277
MEREEDEEVEDSDDEEETQIPIEDQMMEMTQELPATERDTSPPPVQRRSPPASPPPMTEALAGPTTGSPAASMSPFADVE
STTPQSNEDEDSRSETSSEVSLGSGRPGRRQRARKRVSTACSDHRNLSERMKKAVACEGDGEDSFGQSMIGADVVLCVRH
NENRREANGHGNAASNGSTVRGIRRFHAHRFILAASSPPFRAMLMGNMRESSRRDVELHDIEPNIVEKMLLFMYTGDVVL
DLESVLGLLIAAEMYELVALREMCKGFVLKYAHEVFCDPQIVQLPEKILLELIPQDELQIREMALMEALVMWGESRVANA
NKPLGELLAEMMDFVRFPTMSVSDLYGKVRPLVNNGVICEHLLTEALFNHLKWGSQTGIASKRAKPRALTASLRKRKRVS
FTQHVTFETTPQGDGT
>Pinf_XP_002908044
MGLVFAYGHSLDGALGLGHDLSGYVGQPTLVDFFFDNLIVVRDISCGGDQLAGAHSAAVSQDGELYTWGVGIALGRGTLR
SASTPQRIALPPVETTANGAEATTTVGSVSCGSGFCVAQTREGHAFSWGKWGDGRLGLGRIPIIARTSRRHGGGAVRKQF
QSFQLTPRQIQSIYVTQVKPSQHEAPLFSKIACGDAHCVGLTRNGALVTWGRGNSGQQGRGDASDALHPTAVLKESAHKQ
WRDVAAGENWSMALSFDGRVWTWGACGGSVLGHGLYGSQKNALLAEMILQRHQRLLSQRKKSTTASLSCPRLPQLKWMTP
QMIPCFASPDFRICRISAGTQHAAAISDTGDLYMWGEGNSDDSDGLALSSLPKLVNSGQSHGEAGADDGNAADIGTHSVE
HVVCGGHQTIAFTSGSFLARSMTKLYRDSVARDDKTGIAGADLVLVVSGQRLPAHKLLLAQRSPVLRELILDEEQQQQNR
ITASDSDTYKTSVSETMELLLPALRADVARLLLEFIYTDNFTLDVSKSSYYLVHDVLRAAKLYKLPSLVLLCRQRLFSAS
PSSLFGASASSPEIAKDELSDSNSEGDEDELETLRRSPRMLNDDMKFALSDAVWADTVLIAEGRRIPVHRCMLIARSEYF
RAVLAFHRLAESEATKRKMAVVNVEDSYAAIVRVLRFIYYDQVALPQSKRNGETDTDVSDQLLEDLVAADKYGLERLKRL
CEHAIRVTAANCLEVLVVAELVHAAHLKQVAMRFVQTNLADVTARREDFQRFQEDFPQLLEELYARLRDASRDEFLLREW
HTEVGTSLAAQREEQELQWGKKSDTATFPWVPLSLTLAFGTMYLSMMHAQEHEYSAVPATNLVAIAAIGGAIVMGYL
>Pinf_XP_002906559
MPEEDDCDTLVSDLLQVKKLFANNHLSDVLSTTGDHLIPAHRLVLSLRSGAFQAALLRAKSSQVPGQVRFPLKIHVQDTP
YHVFHSLLRFLYTNELGADGEEGGKQRDREEFWQQLLRAAFMYLVPSLVGICRKRQLQKKTLDVLVFMDAVLTTNPLTAR
RREPPDSNTITPPSSQNGRRLINEAGDATNSVLSRCTKAVHELQSMCLKQLQHMDEAAFEELLQSDTGRQCSTERLCEIL
RMRSDTPLVVAIRYQLGRVVNELLRRGEPLDQVGDDGADLPLVAAIKTGNNAIIRRLLVDESAPFFLLTDKVPLMFLASA
SGNVQHCEILIEQNAADVNLISQLKDGDKEITAEFGHQQTPLHVASRKGHSEVVELLLQHNAASNLPDEEGNTALHYASN
IETVEVLLNSAFRTNANIPNRRGRTPLHIAAARGDVAVVAYLIRHGAEQDIVDDQGQNAFHHAAANGHTAVTLVLLHENE
AAMREEPSGFDINKEDLKGNTALHLAAMSPSERCQKMLQLLLENGADPNRANWFGYTALHLFCSHQNGPASIINAFIEHG
SNIHAQSLDGSTALHLAVGRGSQDVAVALVSAGAFVHLLDAAGRSVVDLVENTNQGSMLVPVLRNLSHPPEWVGDEQTTE
CSSCHTAFRLAMRKHHCRHCGRTVCYNCSSNKIAIPKFQVLKPDRVCDTCFDVLSFRKLL
>Pinf_XP_002904031
MNRSVRPENVEFTVKAAEDLVLTGKERSIVRVAANNMYSCLMQKTQRFSSLFRHYSKHHGLPRESLDFFFTNRLDPEDSP
ESVHLQKNDIILVRRRVAPTTLTLPSHNDDAYFATMRELFLSGIGSDITLDVGGDREILRAHRLILTTRCEIFEAMFRPG
AMKESWDGVVRIEDHSPEMVSKMLEFIYTNRVLDLAKLNSNQLIDLLTLSEQYLLLPLKHLCEVAAQDVLSIGNVGRFLC

AAEKFNAAYLKEYCLAFFMNHTSEIVDDENFREEIESCPSMALTIVRATTRHSGSSLEPVQKRRRLNMPFEETEYLSPST
>Pinf_XP_002909053
MATNHLLATPAFALPLGSSSDDEGSSNNRAGLFSFGQNTYGELALGDTNDRSTPTRVDLGRINDTKHVVDVACGNEQTTI
LFENGDVFTCGYNDSGQCAIGTTERVPSLRFSPGLSSHHTVRLFSGNGSEHLAALCANGFLYTVGFNMRGQLGLGTATTV
TEATLVEELAGKRVVDVACSYFHTAIVMDNGDLHACGCNDYGQLGMGDHSGHLVPRPVDYFFRHPVLAVACGQHYTVASL
RDGGVVAFGKNDHGQLGLDRVSEPVLLPTRLAPPLDRAVVPQLSCGYHHTAIVTEDGAVYTFGRNDYGQLGLGHKLHMAR
PTPVESLSRMRITQVACGCYHTLALSDDGKVFPFGRNNHGQLGLETSMDCLSPQLISTLRNKPVAAGFYHSVCLVGTPKS
ENPPPYTLSGDLRKMLNNPTRSDTTFVIEGRPLFAHSCILVARCEPLEKMLDGRMKDGAQPEIVIPEYSYDVFAALMEFL
YTDQVAVLASPDLTADFALELHALADQYLVTTLRSACENSLLQILSVENVVIIVESAHFRNAFTLKKRCLGFIMDHFARV
IATQAFVGLPQELLQEILLSASHQGVSIPNQANAGATTSEYLQSRYNYSTE
>Pinf_XP_002908946
MLFGSQYDEDGGTDHSGSSTTPPQSSRKKKSVVSSRKSKTSSRRDKTRPSMAGAASAVIAAGPSSPTRSFDSTNSSSANF
NYSSFVDNSVPVNGNGITAVNAPTLLPKTRRRVSKKKPERFKKPKRPVDRQCMYPNVWKQLQFRKKIVQLATNMSEKPIC
VTGVDGFLASWIVCELLSRGYHVRGTVQNGKDDISGLLQLPNASKNFSVVETSLLTPEACDMAVQGCDFVIHTGTPSSCS
VRDPLSEQQEPGVHSIGSRMHCIIPMMTNFIQACARGRIKRVVLTSSIAAMADSVTPDSIINDACWNVTSSLEKNPHFLS
LKLAEEAAWQLVDQLPSDRRIDLVTMNPGTLFGPMLSKSKLAPGNQVIYDLITGQYSALVDLNWSMIDVRDCAVAHVAAL
ENNDARGRYICVNRSVWMKDIVEMLSNNGYSGRDLPWRVGLPRWVGRLPAYSIQLGQVGASLYAYDPTSVRSSPYLGDKI
VEHLNARFRTVDSTVVESANDLLKWGLIKPWAEDHEALECGCCHNPFTFYRRKHHCRECGVIICNDCSMSRAVVEGTEGR
ARLCDKCVKGSIPDLLELLRDEDPDKKRTAVVALESLMENPLNHDYVTRFGGILLLMDAIHHPDEPISSHAAGALCALSL
SVASTLQMVLEGAVLQMLEVEETLSTWAICLQALRNIWKQINRQDFRRMLHAVARVSADANIGELRGNILLTFVHMMDAE
EVPTLLSEGLLSVLYHMLQSTAEFPRCAAAHAIKHLVPAGYDPDVRIDVPPYVVDDHEELFLNSSLSDLQFLVKGHIAPI
NAHKVVLFFRNSYFKNMVRVSIFGTATSQTAVIEVDNCSYEVFSILLRFLYTGKVDITPDVAEELLRASSFYCVYELQKR
TEAFLSGQICVENVVDLLTLSEECNADDLKKNCVPFLMRHIHEVVRLPAFEEHRVRSSEEVLKALTNMLGPEWETSYAKF
KKSVGIKDKDVEDEEPAEAEKTDEEEEMQPTGHQVNEIQKAPLSPRFLLSPVPPPSFSGNTSAFATESPRLNTVGEEKST
ENFHFMKTFGNEDVLFRRSRNISEDYSEGLSEGVC
>Pinf_XP_002907441
MYIFGGSDSHYDRESMSSRSSSEYGRLDSGRHVNDMHSFNLETNSWSLIRTTGEVPYPREGASMIVYNQSCVLFGGYDHD
LGYLNDVHVFNFETRVWSSLETKGTAPTGCHHPLVTLHNNSLIVFGGQTGSNLYELDLETNTWETIAYAGSSPKSNAPAG
CVNEDKLIVLSEHNEAASFKQIQLPQKKTKCTTEEDLKPDEEGSAIAHLRSLVNNQLMSDVTFLVDGEPIYGHKSLCVRC
NYFKAMFTGEMNESTADEVEISDVSRATFLSLLEYVYTDRLAVADDDVKELFVAADRYGIESLKRLCAQRLLKSVSVDNV
ASILQAADQHNSPSLRDECFAYTLKHFDTVSKTPSFLEMARNNIELALQILQQR
>Pinf_XP_002907439
MALQPSSRAWAPVPCENPSAAPCHRSLHVCAVRKDSLFIFGGYDGSNRINDFYEFNFKRKLWSVVLAIGSAPSPRDRHVA
VVYKDSFYVFAGFDGSSRVNDFIEYNFLTQRWSNVVVSAGLPPTARHSHAAVVYDKSMYCFGGYDGSYRNDFHEFNFETN
TWSLVAATGRVPRPRYRSSLVVHNHTCVLFGGHDGSRHLNDVHVYDFDTRVWSLLATEGPAPIARDSHVAVIHSNSMYIF
GGSTGTAVNDFYELDLEVNTWQPMQFNGQPPGQRFCHVGTAYDSSLIIFGGYDGSSRLNDFKQFRFGEEEFQLEIPESTL
INDLRMLVNNDVMSDVTFVVEGIPVYGHKILCIRCSYFNAMLTGEMLESRAREIQITDVRRPIFISLMEYLYTDYLDVAV
DVAMELFVTADRYGVERLKRICESKMLGSLCVENAASIFHAADLHNATVLRDQCVTFMLHNFDAVTKTDAFEEMGRTNVE
LVFELLKRR
>Pinf_XP_002902415
MALYVWGHNNSQQLGLDVADKEVFELRQVEALRNEVIVGFSAGERHSLALNEFGQVFAWGRGREGQLGLGNVAGVASAVS
QPRRIGGALAGQLVTKISCGESHSLALTVSGDVFMWGLLPVAKVLYDENGEATDASDRAAVELAGLSSEEMREAQRARSR
GDLRRHMDDTIMARLVRDSMQVYEDAGVVNGENVKVQTVRAPCMTPRLATGPLARLVVTNIAAGFAHSLATTNEGAVYSC
GYNDNGQLGLGSRRNSAEFQRIRALESYFIEHIACGQQHSLACSRVIDPGENSHDDDPKSRSGVCFSWGLGVLGQLGTGI
NKSWLPEEVKLARPAVSVAAGSHHSVAVTDDGKVYTWGHSEYGQHGAGETFYDLQHNAHYFFPRVQQALADDPRIKVVSA
CCSSHSTFALSRDGSLMSWGWNAFGVLGNGKYQHSVHPQRVFGLKDNAAVAVGAGSNHCAVAVRPRGAHYSLSYDRVLAN
ADFADLVFLVGGPTKVERIKAHRVVVSARSSYIKGLLRVLDLVTDSQAGGTKVAKKEERLFEVDEFQDVDAQVFRAYLAF
LYTNRLAVASHKRKALGQFASRVCCDALVVQCLDTWRKERLASLPPPSFRRAGHLTATGPASSEAQQFGEDMKRMVLSED
VADVVFLWPVEDSEPETFERLPAHKAVLSQVEYFRTMFMGGFSEGKTAQEATTGEGSDASSKQTHEIPLHYMHRDGVSLE
AFKGLLLWIYTGCFELLSARLEPSDMMDLFVGASLLGLTVLANRCELQLVELLPQLDVDSLRACEDFAERRAGTLVLGSH
VNSVYPTH
>Pinf_XP_002902385
MTVYSWGRGEDGQLGLGDTSDQHRPVPIDALAERRIVQIACGSGHTVVLTEEGEVYTWGRGDDGRLGHGDNGWKFVPRLV
EELRGKNIRQVTCGSYHTAAVTVSGDLYTWGGGMYGKLGHGNETGHSTPYLVETLKSMQVRQVACGSRHTVVLLENKDVY
TWGDKENGVSGHGDTEGHQYLPCPVEELRGKSIVQISACGFHTAALSEFGEVFTFGEGKFGRLGHNSERNQPVARFVDAL
AGKRVKQVACGGFHTAAVTETGEVYTWGGGEHGQLGHGDKVNKTVPTRVESLLEKLVLQITCGWSHTVTLTDTGEVYTWG
NGDHGKLGHNDTAKVTLPKPVDVLDGKRVISVASYNEHTVALVDPVAMLRASMLTSSYVGDMRQLIDSAEFSDVTFLIEG
RAVHSHRAILAARSDHFRAMFSSGMRESHEQEIPLSHTRVPVFLALLEYIYVDSINVGAEMALELYAAADLYTLDRLKGL
CEIIVQKNINVENAAVLFQSANDLHSYRLREICLSYMVQNFDMVTKSDGFASLSRDLILEVLQNR
>Pinf_XP_002901502
MASHMKNSAAQLPAMLSQLSSVHSDAGDQQEQRHKLFAMCQRQELTDVVLLLQPEPLQSQDEAMSDVQEVAAHRVILATA
SPFFHALFTSGMKESHEISPRVPLPGVPFAAFQEILRYVYMGELRVSGDTILSVLHAANRLELEEVVEICCKQLMLEINV

NNCVEIYMSCEQLQMRAACRLLAQAARAMIDTYFCDVYRSESFKNLSLHAVMKILRPKERNNSTAVKSWMMRHPIAKQRQ
LSEVLQLPTNTEDETAPKRRNSGNAMVNSKERRPSVSTDDAMDVYTANVLSREIEENAKRQLTPTVFVIGGFNSPGALNS
VEYLDFHRREWHPAASMATRRSYGGVAVSSDHKIFVMGGTSSSSHHHKTMEVYDPEANTWTSMPAMKNARSYLGATMVGD
FIYAVGGFNGQTHLSSVERFDIQTQHWESMPSLSTGRSGLAVAALNGLVYAIGGYDGRKHLKSVEVFDPQTNEWSTIASM
RYARNGPAAVVQERCNSILVYGGESRHGSRMNTSDRLDLDSGVWSDADAFADCRSGHVASSFLNESFVFCLGGSNKKDEY
LDAVHRFDNLSKQWTLHSQMRAQRCGLSVAVVKTSRYAECFAVQKQREQQLDSTSSSLWRKSCEGNTFAQGA
>Tgon_XP_002372052
MFLASVSSVGNDQIGTRDAGVCFHRVPSPLKEMRGASHLASACGTYRQPPPQQGNENAAAGPASYCRGPCNSSQQKQQAP
CTHGDYGACGSRGRCCGPSAADGSCGAGAVRRWRPVQCVGDERPERRSGAASVVYGDCLYLFGGYGGHARLDDLCKFDFV
QRRWTKVKAANSPSARENNGAVVYRGCMYIFGGYSGIHWLQDLHAFDFEKEEWHEVETGGQKPSARFGFVSALCGTDGLL
YLYGGYDGTSWLRDMHELNLHSSQWTETKQHGHLPSGRSCPSWAHHQNCIYMFGGYDGINRLSDFFCFHIPTRCWTLIAT
HPPPAAPFSSPSSSPPLYTLSLRQGGATTGGGDAARSSDSASATPSSPAGELASARGSWDSSASRVPLARAGRMGYSNAG
AGDAPPGITDSMVCGNGGASGGGAYVWYPIDSTASSTSSSSTTPRRGSNGGAGCCRVATLGSGRGGRLDSNDDGGCNYCR
GTHGAGGGALATGYGGSCCSCALSGSSTGAPSARYFHAAAAHGNCLYIFGGYNGQERLNDLYVFNLDTHEWQVVEGLDTP
TGRSSMVAQVYKNALYIFGGYNGHNVLNDFYEFKFPTVAVPPSTFLADLRRLLSPPNSDVTFTVEGGKRLYACKAILMTR
SEHFRALFNSGLKETEMAENGIPIRIEGVQYEVMAALLEYLHADMVSEQLSCQQTVQLMITAERFCVDRLKCLCVESLRR
MLSVDTVVEVLIAANAHNILDLKEICLDYLLDHEEEVKERDAMRPLISHSTLLYEVYMRSSGNSSRRSSDSGSGVRRGGR
RSRPDSGVGGVDEPRGGGSTFLPTCHGAQQGRGCSSCGGGGTCGGACEDSRGHDI
>Tgon_XP_002370667
MMHSQAGVVVSSGLDNSSLLSSRQAKQGNPLARLRTFQQSIHRLLTDPAFESLFDVVIVAENKELRAHQCILSACSEYFK
RLFCGPDTAKRIEFQQRWVVVQQLVRCMYGADIEPGTDPEIILEVLAEARNLEVNCLSIEDCLSLIVDQLSVVNCARVLT
HEEVAHHRELSRQVCSYIVKRFSDVVRNPTSRQQLLQMPRNQIVYMVRELCRRCDTNRDTDLIVRFVVDWSQFETACDLL
RDCKLWDWSLEPGALSVFRDEDQSALQAPSLPQAVQWRIPNLRVALREQLPMKYVVGTYFDWSVRLDHGGENKLRLVYES
ALDRNPGQPACIKRFPAALFAWQVIFRGEDVFRERPVFICFPEHVALHWSTTLPISTADVTDGDELTILVTMTEIPLVSL
ILYYFSSDLNQTLAHEDILNRLPHIEYRCLSSYTLFHSAQPQPLASVL
>Tgon_XP_002369261
MSGPEEDVSGEAASFGRCNSSSGSSSSADAASASLPVAEDPCSFASPPFFSSLSSSSALMSSALSASLPPASGAASLEGF
GTGGSVPASPSPHFWSRPNPSGTSPGPRAAHSCDVIGTKMFIFGGWNGKFALNDLFILDVQSLRWTRVEQDGCSPPEARN
NHTTAAVGDRIFVHGGHDGTQWLADLHVLDTTPAHMGRHRGLSWSSPPVSGRRPSARACHSFTRVNEKLYMFGGYDGANC
FQDIDILDLDTMAWIQPAVTGEKPQARNAHTMTVVDRKLVLFGGHSGNTHLTDLHVFDTATLTWTKPEISGSPPPGLRGH
TANLIGHKIFLFGGYDGKRRTNEIYILDTKARAWVVVSNAACSAVCDNAPPSGRQRHSAALVSNRKLFVFGGFDGNKWLN
DLHVLDASRFEEDALNDSAVRQLVENLRSLVNCPDFADVVLVVEGREIHAHKNILAANCAYFKQMFLGSMLESKQSKVVI
PGWSYDAYIAMIEFLYTGKLNETRTDVVCEVMGLADHYTLFTLKSYSENVLTALVDTDTVCSLLKSAETYQARNLKRFCL
DFVFRHADQIMNTPAFDELEAIPSLVMEIAKMSLVKSRKGGGGSGAGSDSGGVDNEQKRETSSSR
>Tgon_XP_002368765
MEAVTTKMHWETVTYTWEVKDFVDLREMAQNELTDGCTRLESTTFHDFALWLFPNGQKQHSSVNSIDISLMYRGSKDFVV
AKWSACFLDEEDQPVPRTRLGDPLKKYFRGGDGRRFSYSPSDASIQKWLVDGALRVKCEVQIAMELTSSRPLRGSGKTAD
NSFKQDIVKYCERDETRDVSINCGKEVYRASRFMLAARSEVLQAMLEGKFKEASESEINFEELSPHIAKQILDFIHYDRC
SLLEGSPKAGKDGDKRKRIHDILQLFAAADKYDIKGLGDLCQDALIQNMDHYTIADIMVFAEQISSQKLLQAGQTYLQYT
NAWTLWQALRGSASTTPVSVTTNFRLMEAPTKEVKQV
>Tgon_XP_002365587
MLEKKNMAIDLPHIQIEDFQKFKNLLYTGCLEEEPTDDQLLDLFELADHYQVQHLCEVLANHIHLRLSPQNFDRFCHFSI
THSSALLRLPCCIYAASNESVKAQFTGGKLSEPVTEELARFFGVDVNPAKKRRFSL
>Tgon_XP_002365401
MMTHQSDGAMAMQSENTVEPLFQLLVHKVTIENFDLMRDLARRKALPDCPEDSDDFFEQGTVLFLGPFGQAAGLDLQLWL
CPNGDREDWDALTITISNEGTEDLPDFTKLALLDSKDGMKEISSCETVGICRAKRGYVRFFFNWTEHNILRVGSRLVLKM
EMRFLRRMVQLGSEPTKPQVSSTFHQDVFDYLSDNFFKSTTDRVFIEVTGTRIVCSKALLSCRSPVFQRMFAGNFREATS
ETIELSDLDVDTAILLLSWIQTDRFPGASEISWAACVKLLMAADRFQIQRLIDSCNRQLRHLLARGLASLQDVVSMRKLI
QENATPITSQLGMEELMHCRPVLRIASHLSAARDCEGYLASKAALDPSRLWSVLDFVPQCAKAD
>Tgon_XP_002364367
MDFSHPLHARLRPRARPGGSGSGAVQEIYTGRYRMSYGCHFAAASAHDSSVYVVEHDFKIYRLINPSSESPSPASAGAEG
ACVSSCCTYSSDCPFSQRQSGSALRGETERHLRRASDERRHLRGGTLPGELADEMRASGELQKEWRKQLLVDVIPYLFCN
HRKEYSKLFTGNKQASRAFAVQDRKGSRAAQPTLSFSVAAETPGEPDATKQLLLSGFDVPRMRELRRSCCLMANSCDGML
YLLLHHTLFRISLSGVMTPVANLVQAATALLREHCSRPLLLCPPRDAGASEERRGPSRRNLSRAQDRERRERASRDRDAC
AGVDTGGRQPRAKKGVSASDEDWGFEPRGVRTPQEEARRGFSSRAARPSDGRARREACGSKCGVASASSRPLSPKSRDSR
HAAARREKAREETAEEEDFFSNLSFHTRLDGADPTSALEVATCCIDGRGWCFFTVQGTDDASRSTLPFVDSVRPASRSAD
EGPPSERKPEGVLLCLDTGAVTPVIHPVFLPTTVDPARVFLPSVAAHPVGGITCAVGQAAVTIRKPEARRRSRREREHAE
VEDSPDARSRARVEIRSKREEGREDGGEAAREDGGEESEEKEGEEGGATAPPQGASGSVREDLERRRGQRQRERGESKEG
EQQEEEEMEGAGEELRVQRRPAGNRAAGLPFGDGEPPGEEAKRPARRQTEEAREDTFSAELGEGSPSPRLSARGGEENCR
EIERETEQGSLSKGETRTGDAPQGLPSFFSSFTSPGCTDTRRDWRGASAGASSVAGAFAAQDEGDEERREEKETREAEEE
REEEEEREEEEERDEEEERGEEEDREEEDREEEEREEEERRDEEEREEEGTRSDWWFYPVDEDEPWSLVPMMEVMNNRDL
HELAQQTTLNFGGSLENSVCIYAFDRYSACCWLLWQPGHSVREQGRGETAVSLECAYLIQLPAELYWQEAQRCLLALRAK

QRADRRRRRELARRRREETLTLEGRGRSGEDWQRGRSDEEASDSGEEGDEAFMASAAAALFGPGAADTALVPGVSTPGEG
YSRSAGAPPALLHIISQDARHLEVREMLPTSTGHLLIMAEAGNKLKKSSRLVQVGRRVGSSPSSPGGGLARASTIAARAG
TLHDAGSEQGSRGSQPRRADSSSSSTLASGSLFASLSSTLSWAAAPSGTSPRSEGDEGVSSEAAGISSSMPTAALARRLQ
TTRRPVLGGSLGEALPIPRRGTAVSATASRRLGARASARLPPMTAAGVATAGDVPRRLLSGPGAESALQQSHIVLAAAAD
PEGDEDEDCDAVVLFLLDGLLQCEAPSFWSEGGSPGGAGRDSWGDSALSRHPGTTGSCRRGIAPLSTLLGESSASSQAVG
MPRHRETYEAHPAVAGFSRGLNPLPAREAGTQPIPASPLRASSAPSAFPLSLTQNARSLCCFCRLRREHADSLSLSDDFF
GLWTDAAAPSATSSGRLVLHRNFLDVEICCSDGVRLPSHRALLAAKSAFFEAMLTGACVWREQEPQSDCARGRRDGVGTK
MREKDIARGEEIWVLNLPSHSSRVVAPILQYLATDKLTIPSRCCCSRCCPFLSETTGAAAAVCEKAFVPKNPRQVVKLER
RRNFGYRLCLPGYSPFSPDILLVLPELSETQAGPSDRSTLLPWQDEDPADCGYDPMHWPGKDSGVRTLEDQFSGEAIHES
CGLVEIWGKALGLSRDVRRRVERELQGIIAATR
>Ptet_A0CPK9_A0CPK9
MSWITIGSQNKGPQGRWGHTSVAQDDKLYIFGGYDGNYLNDFYSYDFDNNMWQTIHQEGQVPEPRSNHIMLINSGTIYIQ
GGGGMSKTRFGDLYSFNNKTWLKQKINLIPRTYHTGCVGDNKLFIYGGESGRDLDDLEIVDLSGMTQYTVKVECQVKPEP
RRFATLQYYNNQLILIGGCTQSYINTPSIYVASFKEVDNNISVAWTQLQIQFSKWGQSCIQFDDLLYIFGGRDDKDSDEL
YSFDLQNSKIQIINNQNKIKARRKHSASIIGNSLVIFGGFNGKYQNDLCFYELPLINSVPLSVELPSKPYQEVPQYQFEN
FFQFENGIHFYKEKYDCLIQTDEKCFGVNKSVLLNTSGYFRELFSGDYAEGQLLQLDLSFVDHIAFGIVLIFSYENKLTL
PILNKEELFRLLELCDYLDMMQLKEKTQYYIAQTVDKNTIQEIYKLSVEHQNNQLQNFCAFKVSKLKMPLQEIDNIQSQQ
VNNMKKIRKLSIVNEL
>Ptet_A0CPW5_A0CPW5
MNRRLQIEHGNLKVKRALMRQQINQSHRIDRDFDIDERFRSNSMCMKNDEFEIEDPEYYDQQQTWERLDRLVQSLPNQRN
SCSWVIFEDCMYIFGGFTFNGRLDDVHRYSFQSNQWQRLNTTGTKPTARENNGAIEYKGQMYIFGGCDGLLWLNDFYSLN
LKTLIWEKIEPTGQCPSERFGIACGAYQTKMLIFGGCDGNHYLNDAYVWDFEEQVWNKLQLIGDIPSARSCPSFSVLNNQ
IYIFGGFDGVNRLNDFYKINIFTGKVKRISQHGTIPCPRYFHASEIYQNKLLLFGGFNGQARLNDLYEFDFGIKTWKKLE
VHESPKGRSSMVFQIYNDSLYIFGGYDGDELLNDIYKLEFKNAHMPRSSFLSDLHTLINNPLMSDVVFLVEDHQIYANKC
ILGARSEHFQTLFFEEFRDKEQIFIEITDCAYQTFMDMLLYIYTDQLDYSFNTNRLLNLIILSDQYLLQRLKYLCEEQLI
RKINCNNVIDIILFSKKYNCKLLLVQTMKQLIDNISTIKKRKDFIKLAQEPEILLEIIQKKC
>Ptet_A0DU98_A0DU98
MQQQRPQTAHQPSLSSNPIESPAKTLNMLTSLQLSTNRKLLEQFPKDAHADVVIQVKNKRFELQKAHLRIESPFFDCDKQ
VISIQEYDPDIFEGILKCFYGGQYIIYTYQELYLAYQICSFIKCDGLCKQIEGSFQINKKSFTFALQLSELYGLEDLRVS
CYEFLDSNPDAFLQLFDLGMLKLKKNHKKTTPSDVLGLNKACFIHLIQAHNTYKKTKSEQRQYLTNYEIFQILELYNSNE
EDLKELIVKLIDRNSLTSEEYYKIYHQLACRQSHTILDEPHIQPCHQSVKDYQLQPQPEKNYFDYFDVNKSENYFYKKRM
SELEDQNKALEQETQKLSIALETSMKNTNNFKDTLLETITQKLDNKLIEKDKEIENLRQELLTLKSQINQKDRTIIELED
SIKDINDREIITLRSQIETLKSQLIQRGQTVSELQYSEPDFNDQTLESMNQFTTSQFLQQQELAKSEYQFLKPQTVAQSQ
IQLTVQQTERFQYDAEPVTLDKINQLNYIFDHLKITIFSQIYLRRLQLGSLSGYDVDSFKNNCSGVENLLILVRVFDYNK
IIGVLVFGNEKYLLNISNRQIQELDSIFLFEDNIIEIPGQLTICNGCDSNTCYGQTSLTGNKEFVVEDVEAYSIEISHNL
>Ptet_A0D728_A0D728
MNRRLSSEQDNLRIKRALMRQQINQSHRVDREFEIDERFNSISMYIKHDELEIDDSGYYDHQTWERLDRFDQALPNQRNN
CSWVAYEDFLYIFGGFTFNGRLDDVHRYSFSSNSWQRLNTTGQKPSARENNGAIQYKDHMYIFGGCDGLLWLNDFYSLNL
KTLQWKKIEPTGQCPSERFGIACGAYQTKMLIFGGCDGNHYLNDAYVWDFEEQVWNKLQLIGDIPSARSCPSYSTFNNYI
YIFGGFDGVNRLNDFYKINIFSGKVKRISQHGTIPCPRYFHTSEVYQNKLLLFGGFNGQARLNDLYEFEFGSKTWKKLEV
HEPPKGRSSMVFQLYNDSLYVFGGYDGDELLSDIYKLEFKNAQVPRSSFIQDLRSLINNSQMSDVVFIVEDQQIYANRCI
LGARCEHFQTLFFEEFRDKDQIFIEITECTHQTFMDMLQYIYTDQLDSCLNTTRLLSLIILSDQYLMQRLKYLCEEQLIR
KINCNNVIELILFSKKYNCRLLRIQTMKQLVDNIATIKRRKDFIKLAQEPEILLEIIQKKC
>Ptet_A0DZH7_A0DZH7
MLKKRGSDQLDIIFEAGEDLMNSSKNQVIILKNNSIRLPITKPRTFLSYSLEFCGITGLNYKQLRFQSLGRDIRPETSIM
TASLVKVIHTNEFNQMVQNPLKTSLQNLFLGGIFSDVILKINDEIVLPLHKCILSCRSPKFNGMFSSNLIESTQSIIKVE
CKKPELFKLMLGWIYSGYWKEFPDNIADACDLMLLADEYMIMDLKQKCEEDIISKLDISNILQILLFVEKYCDILSPIIV
DKAHSLFIDDFDQILRLNPNLEQEITKVPGLMTKLFLNYHQKKIRKARKVHFVVEDFDQQESDSDYNSQDYVRNYTQNFY
Q
>Ptet_A0BPT8_A0BPT8
MSWITINSQNEGPGGRWGHSSIALDEKLYIFGGFGIKQNYNQDGNYLNDFYSYDFDNNLWQTILQIGQVPEPRSNHIMFI
NNGTIYVQGGGGQTKTRFGDLYSFNKNTWIKQKTNLIPRTYHAGCVGDNKLYIYGGESGKDLDELEIVDLCNNTQYTVKV
ENQKKPEPRRFATLHYHNSQLILIGGCTQSYSLTPSIYIANCKQEEDQINISWCLLKTQFPKWGQSCVQFDDLFYLFGGR
DDKDSDELYSFDLQTHQIQIIKNDIKVKARRKHCASVIGNSLVIFGGFDGKYQNDLCFYELPLVNTVPLSVELPSKPYEE
ISQHQFENYFQFENGVHFYNDQYDCLIQTDEKCFGVDKSVLINASGYFTELFNGDYVEGQLLQLDLSYVDHIAFGIVLIF
SYKNNLILPILNKEELFRLLELCDYLDMIQLKQKTQYYIAQTVDKNTIQEIYNLSIEHQNNQLQNFCAFKVSKLKMPLQE
IDNLQSQQVNNMKRIRKLSIVNEL
>Ptet_A0BMK6_A0BMK6
MYHYDGNGQSPNYCQQEKLVAKVVRKNAQQCPAIKNHTSVHYKNQIIIFGGYDSKKNHHDIHIYRDGQWTKCKANGKIPE
SRNGHSATVVDNKMFVIGGWLGSGTYASGEVYVLDLDTLTWTLVNTIGEVIAIIKQIPGPCNMHSADLIGQLIYIFRGGD
GKDYLNDLHSFDANTNIWKFIQTPDKDKPPPRANHSSTVWENKLFIFGGWDGKKRLNDLYSYDTSSNKWSELNAAYSPSA
RAGMCMTTINNNIYLFGGSGPQTTCFGDLQCYDPIKNQWTIVELQDEEHFDKARAGHSMTAMGNLIYIFGGSCGTQYFRD

FFIIDTITPPNIHILDFNNIQVNNYFKQFYNSPKYSDLIFFVEGQQFYAHKLLLSRYQNFSKLFESEQQNEEQKIYIKDT
SAAVFEQLLLYIYTGEQPSDQFSTCLQQVKSLLSTADYYMLEDLKSLCEKILCNFIDQNSVSQLKSFAELSNATQLYKYC
DWYQTHHL
>Ptet_A0ECW0_A0ECW0
MFQDNIWECVQQNGMPTPKSGHCCCVVANNFYVFGGQSNTEILNEFHRFNLEDKTWSKIKTDSQPNGRSSAGMCGVDDIK
KLYLFGGTLLIVEKTSKNDLYEYDIQKNNWTIVITQTPGPCERYGHSMIMSNYQIYIFGGVHYVKAQNRMNPEFLNDLWV
FSLIDKIWKKLDLRAQIQPQARYRHTTAVIGNELYIIGGTTNQNRYGQIVKTDFTNMQWSYLELEALKNKSKKNLFKGRY
GHACFVMENMVYIYSGMDDGQCDDALQLDVIQNKWKSKKFRGNIPSREFFSFAQTSNQLFIFGGRTQQNQRLNDLYVWNH
NLRQEEQQYCTLRQEMHSLLEIHQNCSDFTIFTKSGTQYALHKSLIKVRCPILLQFQNLQFSDILVQILIKYIYKAELAL
DLLSKVEIREALQFSAFISDELFATAIIQYFLIDSRGQSDYDFIEKELQQIQGKGNSSAQINFLLKSVLQILEQRKQKQL
VNLFQQQRIIFNNVSNQIVMKFYQDMRKLQYHSETDTIITAQDKQINVHSFVLVARSEYFREMIHFENGVIQQIQVDFDP
QILQNIINYLYCGYQALQRQNEGNFDHLINAFKASSYFMLSNQTFLYLIQEGMAEFISKDNVFDLAELAEQLNATYLLYE
CCTFISENLKGVSLSDLRKLSKEVLIRVLEVIGQKQ
>Ptet_A0DQI2_A0DQI2
MNSLATLKFVNIENNDLQSHFPIDPNADIILVLNGKETFQLQIRLLKKRSQYFETQLHNSDSKFKVIPGKHDGVVFQKIL
NCFYGYQYHIYKAEEFYSAYKISHFLQINNFITTLQDSLYENFQRIDIHIVLTLAELYEIESLREKYIDYLDENPQLWKI
ILPIQKLEFYRQNYYSICCTKQNDDIPINVLHLKEDILIKLLSFHNEQKNKKSFPEKFLTPDDILKIIQFQTQEERQENT
IKIVNSIYKVEDIYSDNDARRLQTFMGKSYSSSVNSSSCSDRSSKLIIEQQRDIEELKMKLQCSIKENNELQLKSKMLEE
RIENLQQLIENQKKTSINASNLNNSNSQDIESSQFVLQQVAESIPLESSSIDQSDNTFFKSQLHFNSTPQTVFTLSELSY
LGEILNAKIYQQIKFKLLYRGTENNFYLASEEQLTSIKKATSILILIRSVDNDSVFGIFNQNSRIQIINFQSKKRVDYKK
ELKLAINSNYLLKLGNDIEIFVDCCNKKNNICNNINGLGCLAGKTEFQVDELEIYECSIHNEIV
>Ptet_A0D9R2_A0D9R2
MDYCMRKTEMTDNKEDDVTFASYEWENIKATTASQLPTCRNCHTATTFKHYMIIFGGKEGEGRKKFCNDIHILDLKRLKQ
CNNSMISWTSQIKVSGQIPDVRMGHSAQNYYDKIVYYGGWNGYTVLDDIILMTPSEQMNVVCIDWQHLKSENTPPKRQFH
TANICGDFMYIFGGGDGKMWLSDLYKFDLVKCFWTQVETTGQKPQGRLQHSSVIYDHKIYVFGGEPDRSHQLNDLYQLDI
ENNVWTRLQPKGSTPSPRVSASAVMMNNKIYLFGGYDGQQWRNDVFMYNITENQWEYIVINEQEILPHFRCSSKENTSQQ
SSPPRPRCRHSAIAYKNTIVIFGGNDSEKSYNDVYMLKQQSTIKLAESTLKQDFSSILFSPVLSDITFFVENQEIYAHKI
ILASRCEYFKTLFLNEQFNLGDKLAITDTTINVFKAILQYIYTDEVFIDSYIVQDLLALADKYMLQRLKNLCEDHLIKNI
SLKNVIEVVNLADKFSAQELKANAMIFLLDNKQKILNTQDINMLSKEILIELLKFTK
>Ptet_A0E0E8_A0E0E8
MSAATKGLIEFVNPCNTKYIYLDKLPKFVKQVHQQMREIEGRQPFGKGLYHCNNYENLIQKLANTRQQYRQSVQIEIRKQ
IASQEYQAWANYIKDRSLELPEQHKVTGKQLNELRRSYDVFIAKGENGLRPSELLNVFNDYTRVNQFTILMDNWCVLQMV
HYSMGYPMNMNRLLTFEEIATLVQTKVLATYERSLGQDLLFREICSYGYWNLFDQSKGYMSIKEFSNFVKIFKYNVEPTL
GGILKEFGFAANLFQGEFVKEIDPKEDIVRFDFFRYLFLERNLMIWRKIDPQGKQPGDRAAHSCDLIMGKLYIFGGWNGM
NALADIHIYDLNQNQWTELQTNGELPSYRNNHTTAVYQTKLYVHGGHNGNTWLDDLYYLETNGQHGQASWYKVHPQGQIP
TARACHSLNIVSKKLYLFGGYDGQECFNEIEIYDIQENRWIQPSVIGTIPTARNAHTMTRYKENLYLFGGHSGAQHLQDL
HVFNTYKLEWTQVVTKGTLPKGLRGHTANLIQNNIYVFGGYDGSGRSNDLFIFNFLTYQWVIPNHHGTGTYLQMEEVALS
QIPQPRQRHSATATENDLIYIFGGFDGNKWLNDLYVLDVGLLENRTIQEENYQRVISNIHKNLFNNQELSDIAFEIGNQK
IYAHKIYLAAQSPQFKALFFSDTKESDQRIFIVENYTYKSFYIFLLFVYTGFINVAELDIELMGEILSLADQYLIDGLKN
LMQKSIKKYLNNETVCDLLIFAQKCSAHSLKNACMNHLLKNISIISETPKYEKLELQPSLLTEITRALLQHKE
>Ptet_A0EIL2_A0EIL2
MLKKKGSEQLDIIFEAGEDLLNFSKNQVIMIKNNSIKLPIIRPRTFLSYSLEFCGITGLNYKMLRFQSLGRDIRPETSIM
TSCLVKVIHTTEFSQMAQNPLKTSFQGLFIQGFHSDILLQINEESPIPLHKCILACRSPKFSGMFSSNMIESNQTLVKVE
HKKPDLFKVMLQWIYCGYWKEFPEEIEDTCDLMLLADEYLITDLKQKCEEDIISKLDSSNILQILLFVEKHSNIISQQLL
EKTNSMFIDDFDKIHKLNPNLEQDITRVPGLMTKLFENLHQKKIRKGRKVHFVVDDFEESDSDDYVRNYTQHFYQ
>Ptet_A0C1Z7_A0C1Z7
MFQNDGNGQSPNWCSYERQAVKVIRSNANQCPAIKNHTSIHYKKQIFIFGGYDSKKNHNDIHIYKDGNWTKCKANGRIPE
SRNGHTATVVENKMYVIGGWLGSGTYASRDVYVLDLDCLFWTLVNTMGEVPGPCNMHSADLIGQLIYIFRGGDGKDYLND
LHSFNTKTNMWKLVQTAENQRPPPRANHSSAVWQNKLFIFGGWDGSKRLNDLHCYDVTTNRWSELKPIQSPSARAGMCMT
TIDNKIYLFGGSGPQTTCFGDLQCYDPVKNAWSIIELQDDEQFDKARAGHSMTAIGNLIYIFGGSCGSHYFKDFFIIDTD
PPPNISVTDFNNISINQYFRGFFNQSKYSDIIFVVEDKQLYAHKIILSRYEMFNKMFEWEYKNQQQKVYINDCSFIIFEQ
LLYFIYSGDLQCDQNQQSVEFYRQLLQMADYYLIPDLKSLCEKSLSLLIDNTTLAKIKLYAEQMNANQLLKFCEWYENHN
N
>Ptet_A0DJU3_A0DJU3
MNRRLSSEQDNLRVKRALMRQQINQSHRVDREFEIDERFHSVSMYIKHDELEIDDCEYFDQQTWERLDRFDQVVPNQRNN
CSWVTYEDFLYIFGGFTFNGRLDDVHRYSFSSNQWQRLNTSGQKPSARENNGAIQYKGHMYVFGGCDGLLWLNDFYSLNL
KTLQWKKIEPTGQCPSERFGIACGAYQTKMLIFGGCDGSHYLNDAYVWDFEEQVWNKLQLIGDIPSARSCPSFSTFNNQI
YIFGGFDGVNRLNDFYKINIFTGKVKRISQHGTIPCPRYFHTSEVYQNKLLLFGGFNGQARLNDLYEFEFGSKTWKKLEV
HEPPKGRSSMVFQLYNDSLYVFGGYDGDELLSDIYKLEFKNAQVPRSSFIQDLRSLINNPQMSDVVFIVEDHQIYANRCI
LGARSEHFQTLFFEEFRDKEQIYIEITECCHQTLMDMLQYIYTDQLDSCLNTTRLLSLIILSDQYLMQRLKYLCEEQLIR
KINCNNVIELILFSKKYNCRLLRIQTMKQLVDNISTIKRRKDFIKLAQEPEILLEIIQKKC
>Ptet_A0C3G5_A0C3G5

MLKKKGSEQLDIIFEAGEDLLNISKNQVIMIKNNSIKLPIIRPRTFLSYSLEFCGITNLNYKMLRFQSLGRDIRFKPETS
IMTACLVKVIHTTEFSQMAQNPLKTSFQTLFNHGAHSDILLQINDEQPLLLHKCILASRSPKFNGMFSMNMIESNQSVLR
VECKKPDLFKVMLQWIYCGYWKEFPVEIEDTCDLMLLADEYLIADLKQKCEEDIISKLNSNNILQILQFVEKHSNIISSM
LLEKTNSMFIDDFDKIHKLNSDLEREITKVPGLMTKLFQNLHQKKIRKGRKVHFVVDDFDNQESDSDDYVRNYTQHFYQ
>Ptet_A0DE28_A0DE28
MFQNDGNGQSPNWCSSERQAVKVIRNNTNQCPAIKNHTSVHYKNQIFIFGGYDSKKNHNDIHIYKDGNWTKCKANGKIPE
SRNGHTATVVDNKMYVIGGWLGSGIYASRDVYVLDLDCLNWTLVNTMGEVPGPCNMHSADQIGQLIFIFRGGDGKDYLND
LHSFNTKTNMWKLVQTAENQRPPPRANHSSAVWQNKLLIFGGWDGSKRLNDLHCYDVTTNKWCELKPIQSPSARAGMCMT
TIENKIYLFGGSGPQTTCFGDLQCYDPIKNAWTTIELQDDEQFDKARAGHSMTAIGNLIYIFGGSCGSYYFKDYFIIDTD
PPPNISVTDFNNISLNQYFRAFFNSPKYSDIIFVVEDKHLYAHKIVLSRYEMFKKMFEWEYKNQQQKVYINDCSNQIFEQ
LLYFIYAGDLQCDQFQQSVEFYRQFLQLADYYLIPDLKSLCEKQLSLLIDNTTFPKIKLYAEQMNANQLLKFCEWYESHN
N
>Ptet_A0DN48_A0DN48
MLKKKGSEQLDIIFEAGEDLLNFSKNQVIMIKNNSIKLPIIKPRTFLSYSFEFCGITGLSYKMLRFQSLGRDIRPETNIM
TSCLVKVIHTTEFSQMAQNPLKTSFQGLFTQGSHSDILLQINKESPIPLHKCILACRSPKFNGMFSSNMIESNQILVKVE
HNKPELFRIMLQWIYCGYWKEFPEEIEDTCDLMLLADEYLITDLKQKCEEDIISKLGSSNILQILLFVEQHSNLVSQLLL
EKTNSMFIDDFDKIHKLNPNLEQDITRMPGLMTKLFQNLHQKKIRKGRKVHFVVDDFEESDSDDYVRNYTQHFYQ
>Ptet_A0C020_A0C020
MDYCIRKTEMTDNKEDDVTFASYEWENIKATTASQLPTCRNCHTATTFKHYMIIFGGKEGEGRKKFCNDIHILDLKRLNW
TSQIKVNGQIPDVRMGHSAQNYYDKIVYYGGWNGYTVLDDIIMMTPSEQMNIVCIDWQHLKSENTPPKRQFHTANICGDF
MYIFGGGDGKMWLSDLYKFDLVKCFWTQVETTGQKPQGRLQHSSVIYDHKIYVFGGEPDRSHQLNDLYQLDIENNLWTRL
QPKGSTPSPRVSASAVMMNNKIYLFGGYDGQQWRNDVFMYNITENQWEYIVINTLDNQSNFRGQTKDSTSQQSSPPRPRC
RHSAIAYKNTIVIFGGNDSEKSYNDVYMLKQQSIIKLAESTLKQDYSSILFSPILSDITFYIDNQEIHAHKIILASRCEY
FKTLFLNEQFNLGDKLAITDTSINVFKAILQYIYTDEVFIDSHIVYDLLALADKYMLQRLKNLCEDHLIKNISLKNVIDV
VNLADKFSAQELKANAMIFLLDNKQKILNTQDINMLSKEILIELLKFTK
>Ptet_A0DVS4_A0DVS4
MSVATKGLIEFVNPYKLPKFVKQVHLQMREIEGRQPFGQGLYHCNNYENLIKRLTPTRQQYRQSKEIETRKQLATQEFQA
WTDYIKERTLELPQQHQVTGKQLNELRRSYEVFIAKGEKGLRPSELLNVFNDYTRVNQFMIPVDNWCVLQMVHYNMGYPM
NMNRLLTFEEIATLVQTKVLATYERSLGQDLLFREICSYGYWNLFDQSKGYMNIKDFSNFVKIFKYNVEPTLGGILKEFG
FAANLFQGEFAKEIDPKEDIVRFDFFRYLFLERNLMIWRKIDPSGKQPGDRAAHSCDLIMGKLFIFGGWNGMNALADIHI
YDLNSNQWSELQTNGELPSYRNNHTTAVYQTKLYVHGGHNGNTWLDDLYYLETNGQHGQASWYKVHPQGQIPTARACHSL
NIVSKKLYLFGGYDGQECFNEIEIYDIQENRWLQPTVSGTIPTARNAHTMTRYKENLYLFGGHSGAQHLQDLHVFNTYKL
EWTQVLTKGTLPKGLRGHTANLIQNNIYVFGGYDGSGRSNDLFIFNFLTYQWVIPNHHGTGTHLQMEEVALSQIPQPRQR
HSATATENDLIYIFGGFDGNKWLNDLYVLDVGLLENRTIQEENYQRVISNIHKNLFNNEELSDIAFLIGNQKIYAHKIYL
AAQSLQFKALFFSDTKESDQETLIIENYSYKSFYIFLLFVYTGFINVAELDIELMGEILSLADQYLIDGLKNLMQKSIKK
YISNETVCDLLIFAQKCSAHSLKNACMNHLLKNINIISESPKYEKLELQPSLLTEITRALLQHKE
>Ptet_A0E4A4_A0E4A4
MLKKRGSDQLDIIFEAGEDLMNSSKNQVIILKNNSIRLPITKPRTFLSYSLEFCGITGLNYKQLRFQSLGRDIRVFDQLM
IQPETSIMTASLVKVIHTNEFNQMVQNPLKSSLQNLFLGGIFSDVILKINDEIVLPLHKCILSCRSPKFNGMFSSNLMES
TQNIIKVEYKKPELFKLMLGWIYSGYWKEFPDNIADACDLMLLADEYMIMDLKQKCEEDIISKLDITNILQVLLFVEKYC
DILSPIIVDKAHSLFIDDFDQILRLNPNLEQDITKVPGLMTKLFLNYHQKKIRKARKVHFVVEDFDQQESDSDYNSQDYV
RNYTQNFYQ
>Tthe_XP_001032224
MSYQQVQQLTSSSISSKETSSDSTPIKSSNVSIWKEAQLSHKNKEIPSARWGHSFVKANNDLLYLFGGYAESNYMNDQWV
FDLNSFQWIALPNYGNIPEKRSNHSGCYLEQKNKILIFGGGGKEKKRFNDVHLYDIDNSNWEYLKVQNSDLITPRTYHSA
NLFFDKYLVVFGGEGVGDLNDLCVLNLEQEPSWILLQPLGKVPPKRRFHSSATVQNKLYILGGCFSNYRCHDDIWELNIG
EYLQDPHNTQLEWRQIIHYNHVFQPRWGQCSQVYDDKIYIFGGRNLKDLNETIIFDPSKQADCLVKIDQTSPNPRRRGAA
VVVGSTIVVFGGFDGVYYNDIHYLNISQTENEHNSKIDTESHFKFLQNIMYSQQGYDLEIQMSSFKVGIHKVIVLERMND
PKNKAFLNRIEIADVLNLSHRDFEQRFFITTVVQYLYSGVFDYRQIRNQKDLNQLQEFAHEIKLITLLEALNQNEQWIRY
FRYKQHYKKFLSFANSDKKLKSEHNLDSKDKSNNTHQQIKNYDPTFVIVNDSIWFEDNEVFYQYSDITLQCEFKQIRANK
LLLSSQSLYFKTLFEQAFIEKSESILYIEIVSYSTLKILMNFFYYHKIILPSCFEISDWLNLIEASKYFQIPQIQEICEI
ELIKMINIDNLFNLYYSSILFNCPKLRKNCSYQYAQKLYSQSIFMEQNPNEQDDDILIQMGIEAQYIYKILRFQISQDKV
IEQNAINEAEQIDIEYDNHGDLKKVQEFLSNNINWERQEDIINRLKRCNTKQKSFSHYFNNNMIQNNTQVSNAYQSCYCS
NNCEQAFSQNFDSNTNVHIYDSGDSSCKQINNYHSAEIEKFNHSTQSSSKCLSQLFQSQDLTVFSKKNYTTSNLQNNQKT
SKNTKYKNSSNSTNEYSFCLSGRSQDSIQYGTIQNGNKTSSENCNSDFNADSEEYDIQNTAMIEETQTFVQYQSNMEYSL
>Tthe_XP_001023884
MYIFGGYSDEKALNDMYKYNILSKTWEKIEYQNSISEIPSGRASAQLCAYSEMGKIYLFGGTGMEVNEHSANDLYEFDVN
QKTWRIIETGKYSPCERYGHSMTLHNNFLILFGGARIVKQGNKLVSEYLNDLWMLSLLDKNWRKITPGGTIPSPRYRQEI
IIHNNGLYLIGGGTTNERFSSIFKYEFSQNSWTQVNLANQNSNNKLIDFYNFKLGRMGHICILDNEEILLYGGVDDVVNT
DPLSYSIDKRMWRKIFIRGKHPKPREFFSGCKFHHSIIIFGGRVGQKKVNDLYIWNISCPTPTKKHCTLKLEMLQLFYTQ
ENQGDWVITCKKDSKTISAHSYIIRHRSNRFYEHLKQSNLIFDGCSKLLTHILHFIYSGEVNFAQINPEIITVLIEFSIS
SEMKYFFRIIILHCLVTSANQVFNLKSQKYLSKYYSNYQDELNILDGILRSLQNKCNESQNPFFKSLLNWMDLRRSNQIL

SCLNLNLFYVKLDIKQNQPSQSTPTSRSQNRRESDINGEIFKSLNSFQQSFDSVFHLNTMLYYSDFNQLKLDTDTSNFCF
VYENKQYKVHTAILSARCGYFESYFNVHSLLSQQNEMVIQPFLIHSNTQLLEFFIFYLYNGMEAFTKFKLTTENLINLLD
ITNYFMIKSPTFNYLIENAIFNEGITLENCLKNMQFAEQFNAIFLRSKILSFMVQNMSKVLKLYEKDLLAMNPHFLVEMI
RMQNDQIISYNL
>Tthe_XP_001032021
MISDGTQYLYFFSLNCQNSYFSNYRYTLYRFNTQKEIFDEVNAGGIPPKERECLNSVFHNNKIYVFGSDSTFSQGETQDS
QLKQQFQQQKNIYVFDLIQEEWVSKEVSEIEIPKLCSERYCNFAENLFYLEKSLNMTVVKRNLESLQGQKHVFGYLKNRK
IQDQGLLCASDNHLYIYDIDSLYKIENIAYQANENQNTIELADQKQLQLIRLNQGVILTECMRSDIHLKGFRVLNGILYS
LVQIKNKIALLSISQTDLKDLNEEAYLLKQNSNLLQAHSDQNQINQNIIQQIQEEQQNRQNELQKQFNGQITIEQILQEQ
QKNQKISNKNVANSKNRKINQSYYGQSLEEDDISVELINYFNQPTYSDLRFKFENNNDMYCNKQLLSYYSKYFRKHLSDP
DLLIVKINDVSFTAFQLLIQQFYYQFTNDMSVYDYNTIIDLFNMANKFSIPILQEKCESLITISENNFKQIIPLIKKVST
YSLKEKCIEFMKRNKQFLNSEYMKSFEKSDLILLLTWLNNKQCQHKKTIKIQSELKSNYQYNYSNVANAKGVIGHSATSS
QDLQLTSNKNQQIEGGQQSQQNLAKSQNNQETQETFNCNKYKNFSGIIKDGALDIHICLNCKMVLNYE
>Tthe_XP_001011983
MNENFEITLEVNPNLQQYFLQDSLSDIIIKVNAPNCKNHNKEYKLQKGYLQLESGYFYDTFKNNPSIKQIILSVDSPLLE
AILKCFYGFTCQINKKNIFDALDVCEYLQIETLQDQILEIMQENIDQFDIVKTWIISQKYKDRCPAMEEVCIKRLKQEEY
CIFRNQDSFQNPVGTNQINSKTDLQLIPQKPNCNVVLMNPEYFKKALLSQGSDFQFLKRQHVFNAIKFYCEKKFSSNKEA
VNDKICKMTYELINPNDCSADEYKDIFCQQIFHNYNHTRHSSGNMNNVINDPFRQSYLLQSSQQLNLTFNQTPINSKLLL
NPYQNIQNMHRFSGTNMVSPQHRPNFSTSPNGFLYNSSIGADSFIFAQSINGGRYYQQPLNDEKSEKLINTFDALKESYQ
NFMKLSSSSQQGNQSIIESCELQVETLEQKMKRLLEEKKMRVSVLDEELKKVDEYILEQKKKHNTSMLNSNYMEDINKML
ISSNNQSNQNINSSGNGNLPQIANQLIQSQHIPFHQQNDDLNEIERQIKRKNTHQRLLCSNSSDNQDNKDILLSNNQNLD
KQKEQFFIQDNPLFNKVESDNEDNQLAEDDDEDAVIEQQNQQNQNKNMQNNKSNKYDEDIQDYEEEDDDEFGSGDVDFDE
IMQDIDEFDHAQKNIQPYQKQTVLNTPHINIEEITNSVQMINNLHPTDNIEISNSNFHLDQSKNFPILFYETESGSHDYS
KNGQLSTSKSQQNSKMEQTALQQNNILKTSPQNHEKSIKSRIFGYFLKK
>Tthe_XP_001033382
MLTTFETECPSIKNHSAIYYKERIYIFGGYNGQKNLNKLHILNLRTNVWEQPRFANDSNELPQGRNGHTSVVVNNKMYVI
GGWIGQGQHASNQLYILDLDLLKWTKMETSGQEPGPCNMHTAEHWENKIFVYRGGDGKQYFSDLHSLDIITFKWEKVEAK
GNAPPPRANHASCLIGDFIYIFGGWDGQKRLNDLYKMNLRKLEWTQIERSEWIQAPPARAGMKMISVEEIIYMFGGSGPS
STCFNDLWLFDPKCNQWQQCRVTLNESDDSHNSFEGNDQSGDSINNKQFLQNIQKSNQNGLMSSNLNVNNLELIKNKNNL
MNNNGNGQTIESYLNPRSGHSMTLYKNLLYIIGGSWGPNYYKNYIALDIDPKPELTIKYDDQNTSSGCGFFQNLKQFYNN
PQFSDVQFSFQGEILYAHKVILSLMGESFNTMFTLGMKETHKNVIEIKNIEMQIFKIIVKSLYYNNLELEEQQNGDLSLL
FEVLRVCDQFLIEKMIIIVQQKIKELITNENIEEVLQMSLQYNASYLIKYCLWLQRRNKYQESLQQIDNQTI
>Tthe_XP_001007536
MEIKALALNQFLSDIKLVHSSNEKQKEYLAHKFVLATQSQVFLKILEEHKQVEKIVLAKSIKSAKNQDLLRVQSEEDHLG
QILKLVYLSIQNPQQVRDEVVKIVNKNNCFNYYSICQSLGLDKIKIYFGEYIVSNIQKEDDSVNLLLDSIQFQSEEMKQS
ALDVIVPKFSDFLRSSFLMNELMNLPYEAFKALVQRDDICVNEESDVFNLVYKYILKREVFFKLFIYQISNCELQQSDPE
HPSVLKQAQDAKQKAEDLIGEKPNPEEAPQNIEEAPKQEGEEAPKNEGEEAPKQEGEEAQKVEQQENNENKEQPPVQVVS
GLDEKVYIMPDNLESQAKERLQNFKLTQEQKKDLLLSIRLGYVSHETLLKYSNMEVFQEFKDIFLQAISSKLNFYDSQEF
LNKYQFNLDPRASYQDRVNNLSVAYPNNTSKLNQNSVNHQRTSSINQQFPNHDKNILLNDTIKSENVQATNRAKHSPQQV
NNNILQESSRVGAANPYSLIDPKLQLQHPNNNLLSHNNISINPFQGNSALNLPSYNNFQQYQNHQQLPIHQSLYQPSGQL
GNNMRPPQHLNQSQQPYPTIQSQFYNNGQENHQNLNITNSSNLKSPTRLPPRQSHGNIEFVYKSDLDDNGVLYYLGTYGK
TKPYVNPHVLGQVQAFASTIRAGKIEYFVGREIQNFSTCNEKMSFMGVDLGEDRFIYPTAYTIRNRNSTTYVMLNWVLEA
SADGSHYIPIDKRIHYDPNDSHFNYTTEREREALKERGASSSYSIDEVSLKRILSDHQLKGFRFFRIVQISKNSHGSDNL
SLSGFELYGISYGNSWF
>Tthe_XP_977176
MHSRNANSAIISTNQYNQQFQNSMSQSGISENQQSLPRQYRSNSFYLFEETQGINRTGSILRSDSSLDENEGEVIFRTER
RTSNLNFTPIDDMIFKSREPIQYNQIQAGFQEYESYVWEKVESKGSQRPCPRSSSASVIYKECLYLFGGFTFNGRLNEIY
QFNFKTLKWTKIKATGTKPSARENNGAVVYQNKLYIYGGYDGVSWFKDFYSFDFATYEWTRLPIQGDEPTQSFGFASGSH
ENSFAIFGGFEGQNWLNDYFEYNFTTSKWKKVELKGSVPSERSCPSYCSKDEYLYVFGGYNGIDKLQDFYRINMKKGKSI
FIQQKGSIPSPRYFHSQIYYSDKIFLFGGYNGQVRLNDLYEFNVNTNKWTKIDQKDPPAGRSSMTACLPKPQLNQDIFSL
LNNQQLSDVQFLVEGQIIYANKAILSCRSEYFRMMFTNGLQENCQQQSQQPVVIKDTSYDSFVDVLVYIYTDTIDNNLSP
QRIIALLQLSDLYFLDRLKYLCEEKLVRTAQEKNIIDYLLLSQKNRCSYLKKYCMAQILDNLNEIKQQPCFQKLLIDPTL
LMEIIMNKN
>Pmar_XP_002769412
MTVVGTVCIACLLCIVQVMRYEALSVKLTGWSYEAFSVMLEFLYTGRVAHHKLDTASMAEVLGLADHYALDGLKHLCQAV
LIHMVDVDNVCTLLKISDQHQAVDLKRHCMSFVLKNFDQVTALPSFDQLSSVPSLLLEVTKAAALGHGK
>Pmar_XP_002767915
GRSNDLYILDTGYPGGFSPSTGDVSGSAVHRWIHPTETDHVPAGRQRHSACLVGSAKLYVFGGFDGVRWLNDLHVLDVTR
LEETELSEGAVAALLTNLRGLLNNPEFSDVTLIIGDTGERVYAHKAILASQCSHFRAMFTGGMKESREREVKLTGWSYEA
FSVMLEFLYTGRVAHHKLDTASMAEVLGLADHYALDGLKHLCQAVLIHMVDVDNVCTLLKISDQHQAVDLKRH
>Pmar_XP_002766771
MACMELYDLFREAARYELDRLASLCHRQIRIKLSSESVLLILRDCYDSMPCSASVRSLIETCKYFFLNNYAECAALESCE

TLDPRLLCELMRCMTVTSQGPSLGTATTGARLNSPSRGFSTASMDGIRVAPSPGGDGSAAFAYQRRCPLSPIVVEASTMQ
YDMQKLLHGDDNDGDFDVVVQGEEFRCHRFVLAARCQYFAMLMQSGMTESQENRLVLPDEATSMTATGFNTFLEYVYTGT
TNMVPQTAMYLVDASDFYQLSNSRLRYFCEIALRQSFNERDVLDVFEASAALNVGPVRRMALDFIAEHFTAVGRQEKLAS
LDKSLLVEILRYLADHHTINNAGMSGRRTPVASQDVEPVMQSLG
>Pmar_XP_002764699
MTQVALGVSELSRYYSDGGPRAAHSCDVIDGSLYIFGGWNGKKALNDLYVLDIPTFHWYEVVMPRGTPLPAARNNHTTAV
VDGRLFIHGGHDGGKWLADTHVLVNLDYPEHRLAGQQLQQRLRHDLPASPQLQRTLSKWISSRVVPSASAGISFKAWHWG
SGIDCERFIGTRCSWHQLLIKKRKMRRYVRCLECYCAICGQVLPITNSAYRSLRWIKAITSGQPPSARACHTLSRLNKKL
YMFGGYDGQKCFNDMDVLDLETMTWIQPNVCGQPPMARNAHTMTVVGTKLYLFGGHSGNKHLTDLHVFDTANLLWYQPSI
LGAPPPGLRGHTANLIGKKIFLFGGYDGKGRSNDLYILDTGYPGGFSPSTGDVSGSAVHRHSACLVGSAKLYVFGGFDGV
RWLNDLHVLDVTRLEETELSEGAVAALLTNLRGLLNNPEFSDVTLIIGDTGERVYAHKAILASQCSHFRAMFTGGMKESR
EREVKLTGWSYEAFSVMLEFLYTGRVAHHKLDTASMAEVLGLADHYALDGLKHLCQAVLIHMVDVDNVCTLLKISDQHQA
VDLKRHCMSFVLKNFDQVTALPSFDQLSSVPSLLLEVTKAAALGHGK
>Pmar_XP_002788423
MQYDMQKLLHGDDNDGDFDIIQVVVQGEEFRCHRFVLAARCQYFAMLMQSGMTESQENRLVLPDEATSMTATGFNTFLEY
VYTGTTNMVPQTAM
>Pmar_XP_002780872
MDSGAGAPISSPEPSSRGGGGSAGDVIDRASGIAANRLARHLAGGGPSAAGGCETSPSTTDPHYSFLGPCTPSSRAVVRR
WRPVQYGCTSEKAMELPGDRSGAASVVYNDALYVLGGYGGSGRLDDLFKFDFNTRLWSQVHTKGDTPTGRENNGAVVIKN
HMYLFGGYSGYNWLNDFHCFNFDTSTWAPVEVKGGSPPSTRFGYVSSVHGSVFFVFGGYDGQTWLNDMHEFDVEEGVWSQ
THVLGYIPTGRSCPSWAYHEGSVYLFGGYDGVHRMNDFHRFEMSNRKWSVVATRSSGQPPSPRYFHASVVHGNSLYLFGG
YSGQERLNDLHEFRFDLQTWFLVQTENPPSGRSSLVAQVHNNSLYVFGGYNGSIVLNDFHEFRFDPIHVPASTMEDDLRR
LMTAPEFADVAFLVEGRRLLANKALLASRSEHFCAMFYSCGLREAAAATAGRSHQGDGGSACVPITDVDYETFNLLLEYI
YTDKVSPDLNTGQALSLLITAGMFCIDRLRVSNM
>Pmar_XP_002779960
MSDVSEDRHSGDNEHSAVLLKPGPDTNASILAEINRLRRAGMWCDVVLTSSDGVEFPAHKVVLSAFSTYLATLLSSSSGF
REASTSYSAMMHDNDYDALHLRIDSLDSELLGCILDFAYGCDTTLPSARLPELLLAAHRFEMRRILVPTAEILSSVLDID
LCIRLLVECGPVMQDTDSLWMAVSKYAGKRFGKLVASPNFPRLPVSILMLLLLDDDLKCGISNSIVFREGATDRQLSEDQ
>Pmar_XP_002775873
MSTFSHADVNVDIPPSTLLEDLRDMVDDPTWSDVVFEVEGKKVYAHKMLCVRCPYFQAMFSRSMNMKESTMSIDECIPIQ
GGVTHRAFKGVLEFLYTDEVHELTVDSAMDLFVTADQFGIDRLKKICEKEILQSINIDNAPTILQAADMHAASGLRKRCL
DFILRNFDSISKTTAFEEMGRNNVELLLEILRMR
>Pmar_XP_002767908
LVLIRLMTAPEFADVAFLVEGRRLLANKALVMTRSEHFCAMFYSCGLREAAAATTGRSHQGDGGSACVPITDVDYETFNL
LLEYIYTDKVSPDLNTGQALSLLITAGMFCIDRLRVEVLITAHHHNADGLKEICLEFLLSNEEACKHHSDGFKDLIGEPQ
LM
>Pmar_XP_002765810
MPAVSIQLGSPSSKGDVRSAAVRHSTVEVSAKVSNPLLNRLWSMLTEHNGQLADIWIYCVDYVGSRSSVLPAHRCILAAG
CPNLMRKINPRRTSSCATADTSCEKMEKKGKKIEESIEVRRLDFTDVAPTWAVSCLLRYIYGQRVEVDVEKLNVLYMLAE
EMGCEGLEKACREKMIDFGERLVDAQLRADVDMVGKWQKTPEFDNGDTLHACAWEDSPAGVQPGLTSASSDQLYDDEDSQ
DSVVRALKDPGGDWKFRGAVTEGAVLKKENSKSSSTGSFLDMIRHSGSLPQLDRGRMIDTPGAFEKKYELISIEEGGILG
EGMNGAVRLAKDRFSGDRVAVKSLSLVNISSAQRDMLYDEINIYLPLSHPNIVRLEEVYEEPGEKIILVMEACTGGELYE
RLAKLNRYSERDAAKILRHILYAVSYLHSLNICHRDIKLENFVYTDDTADCRLKLCDFGFGTIVRREEDSLTASMGSVHY
IAPEVLEGKYGLQCDMWSVGVILYMLLSGTPPFDGANDGEISDAVRHAPLKLTGSRWDCISRSAKDLVRKLICRNPSERL
TATEALKHEWCGSTDILKLNTAPNEIDTDVLRKMHAFARCSAIQRAALGLLAQKRGDDFVGIDENLKLMEETFMRMDTND
DGVITLPEFEAAVRLHVGMSEYEAEKIFGEISSCGSTAAQAEPVIRYRDFLAATIYSSRNRDSKRDDTPGSISRPRGESA
MVWRWNSEDIQSAYEAFDAGRKGYVTKEDLLSILACSARELERVFEAAGFDAERTHRVDLDGFSK
>Bnat_86770
MSSGMSVLSEKRRKQDELFPCDIHLRPVAANEDLKEKTQRDIYLRHSFGHYIPSKYVLTFESGRGERTWRQSTEMQQHDL
QAISFGLSAKEDLLLHGSLERVKKGTYLHVKMEVNEPLKKAFQYFCNKHKLDINGMVFKLGNREIKVTESAENIFMKQQD
IIYIYNRRKLPESNFCEQMWSMYKNGEFTDVCLVVEGKKFNVHKAFLSARSEKFRALFRTKLKESEQKTISLIQDRPGSA
EAFALLLEYLYTDTVRSFTEIRYPPQEIEESSLSSIHIRGKAEMSSKSEIVQDSKRSCIGDGEKPDEVPLIPSDEEATGC
TTTFGSDRCEPLFHGGGVPSRPAESNVRSPAKEDADAKERSDLVKEYTYHESTKADFFANGRVRCILQILYHTDLLNAEA
LKDHCFGIIIENYGETLYGEQVKSLPKPLILELLQRFAESRNPNLKKRRSRDGDDSSGSTRGSTKRRAPYRKVRFDVAAD
PI*
>Bnat_133867
MEIDPIGALIKMYSMLSFMIFERTRADISERALLAMPPQKPGLHAGAVVWGKFGEYPWWPAVVDPPEFQSAEHYHTNKPE
DKSCRLVQFFDEDMSYAWLKPKHIRAWNHRRAWISRKSKDLGIIQGVRQAEAYLKKAGREIVIPRKPAKPSSQSHKRRSS
SMAIQIESLTGAKQTKVPQSTFAVWEGLLFSSEFSDVFFRCSDSKGLVPAHCCVLAGSSEYFRKLLREQAVAEDSTNGEA
KEKKKVVDICKPEAIVKGLLRFVYTGEISLPLLNMDQKELFEMGLKYGFPALIKVGEAGAAKTVSRENVLHFTKIAFQHK
AKMLRDECLNFIRLNSLDFLTDKDFMKQILELSHADPELWTEISASLHNKRARKARKTDRGGA*
>Bnat_86838

MNDRQSSKSTLTSLDPSDDEKRNKEAAMSLDSDVKSNILTPKGMRVSKHKQICTAYSNLLESGKFADLELVCRNKKFGVH
RVILASQSSVFAAQLYGRLKESQEDVIEVSDEYDPDIFMTFLRCCLISSLTPTPFSFLDEQDFDMATLMGMIKTWSTGGA
AIQGYLMSRYCDLVPSFSEFVKAKEFLTMNENVLIELLKLDQLCLEESEVLQAVISWGKNQIKTGNSNSTTTPPLKEVIA
TCMRYVRFELIDTKFIVKDVIPQNLISPDRFVEIMQRKYSDECPAPRRSASFLAWRGKRGNLKTTASYATIMNTYAASRI
KIHRFDDKKQGVFYFIGTSGLKTSWTNPCKGGWMTVQLSSSGGASRHCICDRRKWGTGAVENSYGSSPTWISFDLKDYQV
VPRSYYVHQDQDHFLRNWKFEVSNDNVRWIVLSNHTNDATITNDNRFGHFKVQCGRPYRYFKIHVTGPSHKGQPSFDLTQ
VVFVYLLTLSCYLDVSVRRQHCA*
>Bnat_132044
MSLSTGSPLRRAFGLDPPPVDRANSTSSVTDSNKSPRINFLAQHLLNLFDEGKECDVLFQLDDGEIVGAHKLILISVEFF
RRMLGGNFREGTKDEPIRLSGLTAREMRLVFRYIYTGNLEESLTHKMALRLLRLSQMWLFSNLRRSVVEWALGNLDKKTC
ILTFVNVSNVLLSSPEEKAMIRIPPERSVASKGARLFRAMSASSSYSRSKPALEATLDEGSQMSEYERFLKRYRSFLQTL
ATRDFRKAIREQANELARISDYRALEAFAALVSKRAYKEDEGMGKTSFVRRRGREAGTFAASSEEKECPTSQGSILKALF
DAVVKYFKVSSDAIFVRESRIMESTIRTTPSAPQPSISIALTTPPLCFSERKKTAATTETAFSPGESGKGGEEGGTGEQA
SEICTTDKYTLAVATQSIAAARSRNSATFAMATTMGERATATEKEKEEDTPTITATVNLMAGGI*
>Bnat_89400
MDAGVGGGDPKSRPKRAAWRGPKPWNGNGILRYGYMMNEIAIWSFIWLLPSGSSREQKRRDEKQLEKALKQGHVVLLGRR
RRSNVLASPSKPSFENNEPSATSMRQPEQQLRQKEHHGSIERNVTTPTAISSSSSSFESRGIGQWSRRINVKALDEHKGI
STLCPICGASMPPSKLRSHVNHCLDHQERESSVASSQDLSSIISIPPSTYSSSNGRHRRLPHRQQQQRRYSSSLASSSSA
AAGAASPPSTYVPPWRRRREQRYLNKAAAAAAAAGPSSSCFGSGRKIGQLDFDSVLAVSSKTLEERREGERRRRRLQREQ
QQEEEGVPRSSGNQHHHHHHHNGRRMGLGGGGSVDGLLWEAPTLEEKAKFVLLIEQQARDDAEREREEQERENNQEARKN
QLSQRPKYYHPPPKGSVGGGQECGVDAAAAMMAAAFYDNDPNSTTTNNTSSSMGYVVLRNRRDLEQGLKALLGSSSSTCN
GDSKNSSSRLEGARITGTTSSSDEEAAKIAAAIASSTAHTATATASFSMASLFPPKSSSERALMAAVLKADVKAVESFLL
PRRPIRTLRQALSLHVACLLGYADITRRLAKRRSMLLPVLYFWRVYDNSKNTIGILRHIFSLAAKDSRGKTALHIACIFG
RSECTKALLEAKAPAHVKDSRTYNTPLHCAAQVNATECIKTLVEAKASLEWTNKKGHTPLHTACIAQSPDAVQCILKLRR
RSRMMDGPSGSVEAAFSSLSSKQRQKREGETAILLALEHGSLECAKILIEEDGAVMDARCAAICRNTMRSIMRSWRSEAG
ETAEQKQQHNKYDIKNSSFYYCSRGVAGKNAAKENENNNNNNNNNNIPLYDTSDDLPATTREAMALLPPSLVSINMWNLL
ANQELCDVWFRLKKNRYVGAHRIVLAARSEYFRSLFNGHIGRGRRRSREEKEVVHSLNFVSVESFSALLVYLYTGRTPQC
TTSTATSSSSSSSSRRRNFELDDRKKAVSSSSSSSSSFEQLVEVLVYSNMLMLWPLRDFAEKELTSQYLDKHNARHLLNI
ASFHNADKLAAAANITLLLSAPTDDDFNSDGGWHCYDDGSSNSAIITTTTTTTTSIDRYHSDHEDCLLSVYVEEEEEEE
EEEEEETNQNEKHLRYDRDKILKSFPAQDHLHFSEFDCPQTKRTTGGNVDDGVSLSSSTTSPSIASSSSSSSIEINTSLH
TLCSSSGSKEGGEDVCFQLLVCEVDSKEKRGSANNKKKNNTNNEKGKKRNKGLPLDKKKVPNSSVEDDDRRDGEEEHLDM
LSSSSTPTTAAATSTTHYGTLLAHSAIIAAGWPEFKKYFLKIKETSQAHSTPTKHNDDYSKKSNTNAAKEKGGQSPFHSL
RKTARGKGSFSSLPSSSSSSSSSSSQSIMVIHIVGCEYELKAFSMFVRMLYAPQREIALIMRQQRLERKKQDLDNGQQHP
GERVLEEHLPESRKELLSLSLLLLLRSSYLHFTQNYFASYMEKVRQKVVGGAGEGGGRRKGTKSTNSITITGKGVSRAIERI
LASSEYFINAVTVVIALQVIRAKECPYLYRACTSFVVQHLDEIATVLSPSPKMQIAMMEKHRTTASTPVTNNESQAGNEH
IRQSVIKRSETKDGCEGDMIDALAATSARNTATNKAAARLKSLVLLPPQPVP*
>Bnat_72960
MEEDMEWNPQFDSEAKMKIRDEKEQLINCEWREISTMKGVTPPLTSGMSCVQYEGYVYIFGGFGGHSSKQQAIDSLYRFD
TFTSTWTDIKAFGQGPKARTSHSMVIDRKKGVIYIMLGSGSRFGYSNYDDLFAYDILYRRWSRIQLKMRQGEKITGRYGH
TSVFHSGCIYIFGGCFGRSLSDEMFCVNVKTKIVRRLAPGTRFPQPRYKHCMVKGGSARNRCNGNSYQPSMSEQIRGGGG
DGFVPSSRWRKTVQKDILVLYGGCSERQAMNDTWIFDPETDLWEDITQTANELTAPRRCFAHTMVNVNGRLIVHGGTDAW
SILQDVYEMDTTVGHPSKWRWRKVTDSGPVPPARYFHVMTALPDNSILIWGGKGSITHPHRFNDMFLGKIPNVKLSNYAE
IPKKNGGRAEEKEEEEEAPHNSRITNDSKNHGEDDDEKEEEDAVNNIRQGLLELLGSSRGRRAGGSNIMQEGGGEAQRRG
GGVTASRVRVKSFRPEQMAVAHQLYLSGENNHKDGMSGYNDAKLQRLQCGRTRRRRGRRRKRRGHYQHHTAAALASNVLS
RDMLKLFNSGRMCDVEFLPRTARSSSGYEEDYDDVGNGLCNGYEGAQQSEQQQPRVRRRVMTAAKRSQEAYGGSKRVLVA
ILQYIYTDDAAGLLECSLLFLIKVIRPARQQSGEVFSWALRLSLYRLAQLIRRRIALTIRIGEASLLYMHLLNDIKLLKS
KLSPRSFNILGVLLMFLSTLATMEQLHEEKSSSESSSSSGGSIYHQGKISARLLSNKPRTTPLPPPSHGAYVEKEGGQLG
GTSGAVMNNVDPDSRMVIEHSEDSKVQENYYLVYLIDIKSDSKQAAAAATVLKPMAVRRGVTDSVVEAKSKLERRSPYYF
DNNISRQSCRTEEYLLRKWHPPLISEIKEVEAATLHRDLLAILELFQKEEEEEEEAASLPATVNNLHSRSHRPLQRNDPA
ILKLKISKDRTVFVHKYILAARSAYFGCFSFISGMREAYTGKCDLTSTPFGEGALDLMIRYIYGGTAAVIRHCLESTDHE
SIRQGLEEVVASNMSDYFRLDPEFSLLCEQKCKIESGTHFALVNQQTDQI*
>Bnat_137283
MIWFICDGFETASLKRVSPGIPPLQAGAAMNVAERRKKRLKVLQTHKLVLVSASAKFRDLICDNEGCIELNCDEDLKGPD
VDLSSWKLFLRSLYPAVKVSHLIPTQNNENNEYFLSVATGLLFLASRYSVNSVVESIEELLIGKNLVDLNTLKLASRFGL
QRLERRCIRHLVVGWNNCGQVLPLHQQAEKFRQNFAQICQASQSLSKDTIISIMWMYEKLFGSFKLIPRNDYERARRAWQ
AFKLFRSGLKIRKHD*
>Bnat_76002
MHPSTRKNTPRKPQSLAGKVLPLCASTNIGLKRRAAISGNENASTRKRQKQKEEGVVKEDEEDCRFGPREIPTDFTFRVA
GHALRTHKLILLSVSPVFRAMLHGEADCEELSCNEDVKELGVDEHTWSMFLQLLYPGTRLQDVVPLHVDGCFRKEVVNAI
LYLAFKYSVTSLVDSMESLLISERSPVDVDMLRMASRYHMKRYTQYCIETLIPGVIYQRQSSGHANRLLSSGVRDGGNGS
FAREVSLICKESKSLSKETVVAVLHRCGQVFGSYRLVPRQDFERARVAWEILKETTSSRLCTKCLAEEHSVSETDNDRDS
DGGQGRDQHH*

>Bnat_88919
MHSLLGIGETSDEEYRPAVPPERAEDDPLVRAGGRYFTNTSLYYYDGPGARDRPAFEIEEDPWDETVKRLPFLRDKKKER
KDRVKPKPLKEKKTRSGKHQASNGEDESSDSNDNWAFEKPERKGQRRAARMPKERKKFFRASARPASLVTPSLAVTDLQD
RNAVSMIIREDRNRTDGVGHRLQGRKIHLYPAEGRGGGGKSTWRLVRISSVPGRNRRKYPVLPGKNMNGVSGEWSLRQRP
ISPNVSHFRCRSGQFRQMYLIFVVAFNRMDRNSGSAAEKDHLKIQPERALPSPPPPAFRKKMSKEIKESTSPPQIPLSLS
ERSDVSTILPLSNPPSSYSEESKAPSGTNGTNATNVSFKVGDWVRVKKSVTNPRCNWGGANHNMIGRVRTVGLEAVYVDW
VHPINIESKTVKLKDVYEGAAALVSAIRSLGSGAGKPFTDITLLVNDGKTDTKIGAHRAILAAWSNQFRNLLAGGGIVSQ
PYKAGDTIKLKTNNLVRGRIGAFINIIKKEAFREFIRFMYNPIARISKQTVNEMLELSLRWEIPALTKQCVAYLQETTRV
GDISKIWALGSRLFLSEITEFCEKIAAESIRSIHEELSTKFLAKVLQRDDLGVGSEQEVWKIVEKRIKRLTGSSSKEGDD
VDEQIYQLLGSLRISNLPTDFEDAEIVKLDVLVEKRTDSKQLRLKPEYFRVLSRTKRGDREGLRFQDVQELKEGMLVRIS
ANIPKVLEIFRKTRGIHFNPLMAFDYGSVQIVHEVMESRAIIELELNWPLATLEKIPRYELEAMEKERKARRFSQGDQES
KGGERGAQEVRVRFHCWDRAQDLWVESDSPRISPSKLTKKEGVNRTLRHLMKSHRDAKQNRFSKISSDDYAWPPENVDLQ
PFLIGKPDDDYSANKMGRKIMDKEMHLRRISQLADVRVGSRIQAPRFSSPRCDSAEGDQKVFEERNYIMSEEIPKLEELG
INIMESVLMLWDGETDEVKLTRNLATEGEKVVMRSILESTKTHIHRQDALEKKGLPDVLVTEYENGDERLQALNVAGLYK
FKQKFKGARPVFEAEHGKLLQFNKGPERWEIVDGSTLAVVFRMPDTVGFIPPLGEHKFRGHRINCSFGSSSPPRGGGRGN
EVKTGNGRNSEKSEDSKSTSPPSSSTNHESSDIETLELLIGIYSSFCPQEGKVATIIAKLRDMGFLDKQILKAVNTVGCL
NLIQMMRCLVNADEEEDGGVHIPIDAGRLPPDLLGPGECRSQ*
>Bnat_68368
MRIIKAIKKTFPPFAPRSDNPGHPFSAGAASAVESPRFEYLARGSTVGIRPVFGILPEEPEEKQDKKFGADKNRSEVNGG
QDEDDLLQFVAKPARRGSTDSATTGIVSPRISLSSPRADLCDLTVPALSRDASLISRAFEDRKGAGGGGEPAVAGGEPKL
GDWVRVKASVSEPRHHWGRANHSYVGRITAIDGDSCYIVWEHPESIKKPWHGVLSEMEICTPPPPPPGSRAFTELARRLG
GGGKQVVRKTSDEKIELRIAAHRAILSSWSPKFRAMFLSPRMRNLPFGAKVTIPVEDPITFREFVRFMYIPLARLSRDNV
FSMIRLSHQWRVRELEAQCVRFIQKTLTVREIAKDWELADVRGQKAFHQSCPEFAAKNVRQCHEILTREHLAKVLARDDL
GVADETEVFEIVMERVRGLLKDQKEHRQEEGEAEEKAMRRHRQGMMAQVVSLLSALRVGSLPEGYLEDIVLKQRELQGPN
GKLESDFMEVLLMSPRRAPLGRKAYPGGLLGFRSATRLGLRFTSVGDIKANMVVRVLSSVPEVARLCNEKPAGATSGAVG
FQPSMAKFCGKLVRVKSISVGPAAARIHGYMYPYTCLEQVPAVELEEYQQRLKDRVLREGELVDVLDREDDWLPARVEKI
KTDAKSGQLEALVSLVEFPSKWDEWIPVCSHRIAELHEYSRRRGEVLSVVKPMIKRGLLKQQRFSDLSPLLQGYLAFILE
HGADARQFWTGEVRKELASAFGIERLQVGDQEWAAVCVHKRLRHLSAMMMDSKGRTRRSQRDASRLNSDDPKWPPACVDV
SGLKANSLHEKVLMGQEVSLRRIAQLANARVAARIEHPRFGAAQEVEGAEAKFFAERSHVASGELSEYDNTGIHILPKVL
ELWDGERDELKLTKGLSGLDTVLMQTLLRTTMIHEVKQARITNAPPFVASGCDDKSVDGLYKIVGYQNMRPAYENASGTR
MWFDDAVWRIKAKGYDPKKAVSTSSVSASSCRAKNKFQKGDPCPICYKEMNEEEEALAFCGVKCGGGFHAECIRVWAATR
EEDNKAVSCPLCRADWSGTSVEFEHAVGFFPPLGRWPGQGAPTLAITAEAGAAAAAEAAAVAMEKETKGEPPLRRRGKEG
GGQGEKARKATKRNGLRGESKALPARSNLEDQLKTPVGICERLICGLGKSNNPNSYAALEGPEKVKGTFKELKRMGFTAQ
MLTTEANLVGCDLDAMTKRLVSAVGAAEEEKASDDEAEESKMTELLPALLRALES*
>Bnat_68424
MLVQKVSRPSQLETSVFGLFGRVRVAVWTVEAEAITKKDANVTEMCVVSFNKHARVRVGIFNDEFKISRLKIQRRPFCSM
IQGYIGRTSNVIKDYGHTADDSATPDSTGDKMARLRGSSASPTGVAYRANQSAGDMSGASPSSIAGNQTTPAPTAASSSS
SSSSSSTSNAATSTTGSSEAASTSLGQSTTTTTSTTTSTGASSSNSAPPVPVPPVPVLNSSDEKPTTKKKRYTLLPGERN
LTLVSNENTKAETEWKVDDKVEAPWTDGELYPGRIAEKHDDGTYTVQFDDGDVRTMQSDGFVRIDKFDTTTPFKGIEGTI
ITCSEKCSQSYPLSSLFDYKHGTKGSKSNEWRIKVVKQQNNADNKNGSKKNVKLTIRFSKNYYISRIHIYHTMPDQANTM
TNYSLCVIPTESASSKLAQKPVEIVKLVDTTKELPAGMKEFIIERHLRAVIVTLPYPPSKKWIGLGAIDIFHKHAEEKRN
VKQTNFREDVGNLLKNPVFADCRIIAGRGEHRRAIAAHKNILACRSDFFRKMLQSSSLSSCQSPASPMKIAKVKKEQDEG
RGRKRIRLSKTESGNPIDGKRPPSSPGNNDMKVETGPLLTPSSSIDSSTGLPPTESKAAAAPRYGSTLFDKRAKEQKSAE
NFDTAMHVLPVAEKMQEIHMPELTYSILMSVLTFIYEDRLIIKDVDEAVELWCTSERYQFLRLQEQVEKYLEHNLTVEKA
IEICRTLRELHLLGSLKELLISYLATEIDTAMVQRDFVSIPKDIMHNIFCQTQNQKQIETAFSHG*
>Bnat_67346
MTLHKGTPHGVRCVVARDRAAASAEGQTGKCSTDDTLEAPRIDVTVRVVLPQEGGGEVVPGTAAFSAADGGMRSSTDRLL
MASQMKALCREILFQARDDNDAKHEEEGGGNRFERKGGGDRAGTEVAAVTTTANQGAKAAAKSPIPPRLPRFSSEAESMA
CLRAAGSRANVYVHRAMLASRSEFFRVMWSMPMRERSSGIEQPIELKGVKSSAALRAMVLYMYTASLSYPCASEKQCAEM
LKLADQFGIPCLKQACERLATSALSKTNFSSFYFAAGEERETGAYLATQDEMVEWAPFSPVQQERAI*
>Bnat_91404
MGASLNSSSTPPTWRKMLFSEEDSDVHIVCMDSEEKEPETIYAHRCVLSAGNPFFHQKLRRLPRNKPVEWRFCESAGIMR
GVLRYVYLGDPRFLENYKPSWQVFRATCKFELEGLQEQAERLAASAVTLETLKASVLVLDNYPGKGYLREACANFIRRHS
LPVLLSHANSDNSVLKVADTHPELWKNLVLTAMGKVLPEQKADRRRRRPREQNDVPDVVEEQREDVSDILHGDAKSNQVQ
QNNQHPAKRSRIESKETVLESSSDVDVDNDENQEPDHSEKDENQQPES*
>Bnat_89153
MDIDSMSVLDLRKELGERGLNTKGRKEELLRRLNEARQKDRINTSNETFELTTTRTSTLHEIRWEIDWHVLEQIKQSQDG
EDNGEMLESAPCKIMSHDWGCYVEKFKEDKFLSFYVYGHDSAKASYSLYMKSSKTLEWKLDQDAGYYDGGWSRGWGWRCW
KKVSQLKQNHVINGKIEIKVKLHIYNDIKHTCSKVLAAIPTAPKPELKSRNERMLNEKIHTDVTLKCQDVEIEAHKAILA
SSSEVFAAMFNHKMRESLDNAVEIGGLEPEVLTLLIKYIYTEELPEEYDEIKLLLAADEYQIKRLSILCMQTLGRKLNAE
NVLDIMQTAHKLSHIPEAVSLKKMCMKLFANEAEEITSQKSFERFAGSNVSLTKELLLYMAPPRDSPSNAGGTSEEKTDC
ATASSSSSTKRRSNLKRRYNEIDTQYGSE*

>Bnat_73413
MASLMDRAPLPKPFNEWGRWRTASYHALPLKMNGLQPRQFEFNRDRAVMAIGTCSGEVVLVDWMKNQVGACIKQSEEDHS
IMSLSWLRKHRNLILTCSSAGVPSLLHTELSSTLSIAHRFPVIQDTNSVNTNADDTWFVANGLSSSIHIMDLQEGRLHRE
LKDAHNGYVNICKFSNTMPQIMLSCSLDCRAKLWDLRARASVATLFEHTAQRPIVTVSFSPDDSTFLLSAVDNDVCQLPF
TMVRHYNHNKASSTGRRRAPTPRRPLKFAIQETGSSHNYTRSYYTASGNSIISGSSNDDHLYLCDAATGRLSYAPRIYPR
KRHPSLFIQSLRGHPTLEHIHAVLVCYRQMSDPYEIVLLDWSRDHDGELLTPPRCPVARHTFDLGDDILRGALTSDSALS
IQPPPLSPQNRIADLVQRPSPPSEFPHSSSPSSSKCHSPHHRTASSIAKLVPELSAKREAPPPSRSSSSAYPRSYVLCSA
EILRARWPWFSQQASRINDGDEKAKGGEEEPDAKAEKQSVGNPHSLSSSPVASRPGPKKRAVGAADTSSSSMNRIRSLDD
DEEPEEDLPDQANGRDGCSNAIISVVVPGIDLTGVRVILGYLCSGDLFVPGVWAHSPSPIVQLCLCAKRVGLGGLSRLVE
CWLLETRLNLAEAPALLAMAREIGATDMEKVAIDFLSRTEIAYPSSPLTSLNNRGRENKRSTAAQASSAISTVSRNDDPA
SPSVSDVKTSISNRIRDYVGMRARGKEARGTEREGTEMRVYRSSLVIDDDAERGEGEGRHSARAERSSVHEAANQPSSAS
PPPSAAALQRRGHTLVMVGERGYVIGGFEPSLGGPVDLSVIPVLQLNSGKWAGTGDGDGSNAAQDTRFGDGDDSDANAEN
ADERNRTTSTEGKETFSSNAAASMPECLCFHASFYANEAVWSFGGGTDSFCSSDMWMLHLGRMMMWEKVSPRPEVKGGPT
PCARHRHTATFIDGTDKVLIFGGRGSRGVCLGDLWEFCLTSRKWRLVEPRGDSLRPSPRFGHTATYLPALDSLVVLGGQV
TPGHANGHSFDVYVFDRALEVWRLVVPSVIPSPNSSTMLAALTPSRPPARMHHSAVALPNSNQIVTMGGVLTPRTKRKQY
VFGKVQILNFTPAASPPPSQAQDGEEGKSSMVSTSRAPDGGNGRGYYEATWFTPDIQRNGDVARYLHTMFISKCDKQSSA
TKRRTSSSSSPTSSPSSSSPSKLKYTVGIFGGCRERGKLPKPGLYTGINSFSRRSRKGYCYRVCDEIIGEMRVTNRAKNT
LCRDLSGYLSDSSCDNMREAERGTTADVGAMKTSPHTVKLIASDGKGYVMASREILSWRSAYFQKMFQSKMREAVSSEIR
FPHVSYEQLVTIVHFLHCGDLPRESVRGRDPLEAHSRGAQRPLRDTRAPPAKFRNRSDRKREGGGGSEELSITRNPRCAT
AVDMGGSSSNENNRGSADSDPPSPPHPRKEEDVNSSGGASGEAKAKAASHSPVNLVSFKTARNKLRIENLYRTGSMANQK
RPGINSSRRTSSSSIRIGDSFLGMNDERRHEYIKALVDVLILCDRFQIEGLRALVEDEIWPHITPKIAPISPFKCCHLNA
LFPRTPYKGIFEIARQFNCTRLRHTALNKLVCEAIAKASTEEPPPVACNVDTDAKTPHKAEGTNEEAGGGEKKRGRTERK
SIGTCSADSFSSSSSSSNGSRSSRREKDERLRRGERTGDHSHAPKGSRELAAHIANDILNRTA*
>Ehux_349124
MKKTRGDASLPHGPVTFVVGKEEQRIEHVSKNLLCVRSEYYGKMFGIGMKERDAAEITVPKTDLASFTAFIDYLCTDQLD
LGEGEGEQARRALVLRELAQMYQVPRLELLCAQALQESVGPASAVPLLEAAHATGDGRLLAQCRRYVADHAAEVRASGGV
EQLRDLGVAKGLLGDALDQVAELKGTVAARDTELEKVRAEVAERA*
>Ehux_60979
MARTKQTARKSTGGDAPRRQLATKAARKSAPATGPARPPLHLVLKSPKLTENAREMWTRQQQGEFTDAVINVQHRAFQVH
RVVLASASPYFQNLFSCGMCRRATPAASRCPRLPPTSSKPCSASCTCRKWSSRLRMTCFSRCFSSSCRRRRISR*
>Ehux_195792
MFGIGMKERDAAEITVPKTDLASFTAFIDYLCTDQLDLGEGEGEQARRALVLRELAQMYQVPRLELLCAQALQESVGPAT
AVPLLEAAHTTGDGRLLAQCRRYVADNAAELRASGGVEQLRDLGVAKGLLGDALDQVAELKMGAARTAAGQRAAP*
>Ehux_308441
MRGDASLPHGPVTFVVGKEEQRIEHVSKNLLCVRSEYYGKMFGIGMKERDAAEITVPKTDLASFTAFIDYLCTDQLDLGE
GEGEQARRALVLRELAQMYQVPRLELLCAQALQESVGPASAVPLLEAAHTTGDGRLLAQCRRYVADNAAEVRASGGVEQL
RDLGVAKGLLGDALDQVAELKMGAARTAAGRRAAPA*
>Ehux_349742
MASAARKRARTGGGGEEEDEEPVPVADGFEAPIFGARVVGQVTTPTLEGEDGPLSLGPVGVASDHAGAWYVATKDSLLQV
KPSTCRVAALPASEAFYTVAASPDGSAVFVADSSGNQIRRVEVATGAVTTLAGSGENGDADGVGEAAQLCYPAGIAVSPD
GSALFVADVDNHKIRRVEVATGAVTTIAGSGDAGDTDGVGEAAQFSYPYGVAISPDSSGLFVGDCSNHKIRRVEVATGAV
TTFAGRGDEGDADGVGDAAQFASPYGVAISPDGGALFVADSGNDKIRRVEVATGEVTTVAGSGTAGSADGVGDAAGFASP
VEVAISPDGSTLLVADGDLRHVCVAAPPPPPSFAPIVVPPSTFSADMAKTWGDATLPQGMVTFLVGDDEERIEHVSKNNL
CARSPVFRTMFGIGMKERDAAEVTVSHTDLASFTALVRYLLTDQFNLGEEEGRAQRALDLRELAQMYQVPRLELLCAQAL
QESVGPATAVPLLEAAHTTGDGRLLAQCRRYVADHAAEVRASGGVEQLRDFGVAKGLLGDALDQVAELKGT
>Ehux_349765
MLSGGSASFADSQSAALELPELCATCFEAALEYVYTGSCSIDEAGLQTLLEVAGRLQLASLQGVVAEALEARLSVSNCVE
TWSFASDAALVRLEAAATAFVLKHFVEVSLGASLSPEMMGVLLRSEDIEASEEAVFSALVRWLRAQPESAREEGAVAALL
EHVRFAAMDQKFVRETVEQEPLMKNVPALTVLAQALREGLSKEGTARSLPRLPQGGEWVIEAPGAIAAVDGKTLTFLSRQ
DDDDDDDNERSRNGLVRLSVPLAAGRYEVRFKLLEDEVRGDNARCGFGLIKEGASEAFELQPWSHGFGVQNLWWLRRFER
TVYSEWETSGNFDDETNHQPYQQFPIPSDVRIVVDMDAGEATFYVDEVEIPHKATGITGAVFPCAMAYKMENSLGNTNVV
VSFAGVRRL*
>Ehux_350095
TRGDASLPEGKVTFLVGDDRERIEYVTKAVLCARSPVFRTMFGIGMKERDAAEVTVSHTDLASFTALIDYLLSNKFDLGE
EEGGRAQRALDLRELAQMCQVPRLELLCAQALQESVAPATAVPLLEAAHTMGDGRLLAQCRRYVADHAAEVRASGGVEHL
RDFGVAKGLLGDALDQVAELKGA
>Ehux_350107
MKKTRGDATLPTGLVTFVVGKEEQRLEHVSKNLLCVRSEYYGKMFGIGMKERDAASITVPNTDLASFTAFIDYLCTDQLD
LGEGEGEQARRALVLQELAQMYQVPRLELLCAQALHERVGPATAVPLLEAAHTTGDGRLLAQCRRFVADHAAEVRASGGV
EQLRDLGVAKGLLGDALDQVAELKGT
>Ehux_197359
MEGAELGAPSLQMLRSDEETADVVIALDDGSLLPAHRSVLALASPVFRRCLYGPMKLPADQMFRMRGKDPATVEGLLAYC

YGLPRVTPETAIRLLQIADEFCVDGLKAWCDRWIVTNLDDGRFDDEAAANAVRWACEYRCPALQRSLPQRLVKLLHDHLD
AGRSAQASVPDSEHDLSLLGVTADAIGLSSGGLRSRAAAQPARPIFGDCGRTVTRPKERTRAFSRPMANAHIPDY*
>Ehux_198332
MAELIALLRGTQLRSTAAANRAAYDDRLRQLEERLQSLKDQAGALAAVAFDADFALVAGDGTKVPARKQDLCAQSRFFKR
MMEHDDSAEARAGEVIAKTGDGVGASAAALRHLMALLYGQQLQTSTESAVLLEICELATLWEFDSILESMSTMLVASAKA
SAKTACEMLVAALRHTEADAHSEVWKRLREASAQGVAEAGRKLLDVSAFADLDLSAICEAMTHVKASTVRLPSLEVDTSS
ERWTAHQAMDGSPTDQDLSWLLGATGAKVKARLGWNGSVMLTGENTGGRAVLAKVSSWVKHPQRPTNERRASGSYRVLGS
DTQGVVLGRNLPDVLSDGKFLCGGDLEVSKYQRQYELFNLWLLASKRVASPLSPVDSLLCLRGCATGCDVDGASVAELQA
MLEQRGLSTSGAKTSLQLRLRDAAYAEVPEGSAAAKATKAFAKLVARHYKGSAVKVKDLDAASLAEVLNCDELHALNEKT
ILDNTISWAQLAGRTDEIIAKVMPLVRWPLVPLVQLKPPLKALMKRCPVVKELVEEAIGLQMKPGSEFSFTPKRHRLMDG
SEDTVVPRHKKRKHCHGDKVQLLDASALVAAMLPSGLRTLVSASHSADKPADSVRSICQDALCALSCFRGFDHPPDYHSD
DAFCEEDGIYL*
>Ehux_97052
MHAPRSPKRPAPADDAADGYVKLIGGTPFPEALQEQREAGDFCDAVIMAGGQRFVAHRAVLAGTSLYFRGMYKSMMADSH
DAHTLPAISADSFAAMLSWMYKGRCVLGSNDELVPVLEAASLLQVHPLRDTAAVAIAKRLTADSCVGAWDLAERLSLDAL
RDEARTACLACWPELRAAPEKLATIKLDRLADLLGDDCLKVQEEAEVLDAIDAWVSAQKPSAPSEEAVARLVAQVRFPLL
SAEQCTQAASQPCMQGAAAMKAAFLSLATRKATERQPCWLQEGGAPGEFVWSIAQFSALEERTDSPIFEYAGIRWCLLVF
PRGNKADSLAVYLKAVDAATKPCAASFCFTIPSAKSDKSVTKDSEHKFESDKMDWGFREMILLAELRDPECGYLAEDKLT
ISLKIRNPRLLDSA*
>Ehux_350880
RTMFGIGMKERDAAEVTVPHTDLASFTALVRYLLTDQFDLGEEGGRAQRALDLRELAQMYQVPRLELLCAQALQESVAPA
TAVPLLEAAHTMGDGRLLAQCRRYVADHAAEVRSSGGVEQLRDFGVAKGLLGDALDQVAE
>Ehux_433296
MSVSPSRKRARTESDGGGEEGGEPVAVAAGFEAPIFGARVVGQVTTLALEGEDGPLASLSLRGVAIDHAGGWYVTTKNSV
LHVSAAGRVRTVATCEGGELFGLAISQNGSALFVSDLDAHKIRRVEVATGAVTAIVGSGGNGDADGVGDAAQFGEPRGLT
ISPDGSALFVAACGNHKIRRVEVATGAVTTLAGSGNDGDADGVGGVAEFCCPFGVTVSPDGSALFLTDYGNDKMRRVEVA
TGAVTTLAGGEEGGADGVGGAAHFNGARGLAISPDGSALFVADTFNNKIRRVEVATGAVTTLAGSGASGSADGVGGAAQF
NYPMALAISADGSTLSVCAMGGLRQVCVAAPPPPPSFAPLVVPPSTFSADMANTWGDATLPTGLVTFLVGDDEERIEHVS
KCVLCVRSVFFRTMFGIGMKERDAAVVTVRKTDLATFTALVRYLLTDQLDLGEEEGGRAQRALDLRQLAQMYQVPRLELL
CAQALQESVAPATAVPLLEAAHTMGDGRLLAQCRRFVADHAAEVRASGGVEQLCDLGVAKGLLGDALDQVAELKKGATRA
AAA*
>Ehux_309158
MASCPVNTARLYMTDWDASSVIMITLPQGQATTIAGSGAASSIDGVGLDATFNRPCALAVTADGAALFVADCLGHRIRRV
EVATGAVTTVAGSGTAGRANGAGNEAQFYHPAALALAPNGDTLFVADCTGHVIRRVNVASGVVTTLAGSGAKGSADGPGP
SATFSRPQGLALSPDGAALFVADHGNHKIRLVDVSSAVVTTVAGSGEKGSADGVGGCAQLEAPRAIALSPQGSTLAVCCV
ADPSRIRFVHVAAPPPPPSFAPLVVPPSTFSADMKKTWGDASLPQGMVTFLVGDDEERVEHVSKCVLCARSVVFRTMFGI
GMKERDAAEVTVSDTNLASFTAFIEYLCADELNLGEGEQARRALDLRELAQMYQVPRLELLCAQALQESVGPATAVPLLE
AAHMTGDGRLLAQCRRFVADNAAEVRASGGVEQLSDLGVAKGLLGDALDQVSELQGAMRALGAVR*
>Ehux_351649
MSDSSRSRRKRSRHNDPDGAGPSRDAAFKVTVSGGHRYPPKLVEQWRQGTLCDCTIVVDPETFSGHRNVLAAASGYFAGL
YSGAGVVMRERNGGPAQLEGMSPEVFGAALEFMYTGECGLPETMLTELLQAASRLQVEDLLRATTQALKERLQPSNCLAI
WELAERLGLAELQEHARESATRDFEKLQSSPESLFLSLPHARLATLLADMNLNVESEDAAFQAMVAWARHQQPAPSEAEL
EQLLSLIRFPRMTRAFLVDVEPLLLGHPRGAIIYAQSFREVQFCDDTPRAKKRLVRLSASNVKYTDLKAEMAVRICGEMG
EVKQACEQRAPGASEAVEWNEDMVDYFEERHRGAGLRIRKISQELQAAELEHPSGGSDHFWFPFNVLARAARLG*
>Ehux_351789
MRPSPVFRTMFGIGMKERDAAEVTVSHTALASFTALVDYLLSDKFDLGEEEGGRAQRALDLRELAQMYQVPRLELLCAQA
LQESVGPATAVPLLEAAHTTGDGRLLAQCRRYVADHAAEVRASGGVEQLRDFGVAKGLLGDALDQVAELKG
>Ehux_352162
GKVTFLIGDDEERVEHVSKNMLCARSPVFRTMFGIGMKERDAAEVTVSHTDLASFTALVDYLLSDKFDLGEEEGGRAQRA
LDLRELAQMYQVPRLELLCAQALQESVAPATAVPLLEAAHTLGDGRLLAQCRRYVADHAAEVRASGGVEQLRDFGVAKGL
LGDALDQVAELKGKVTFLIGDDEERVEHVSKNMLCARSPVFRTMFGIGMKERDAAEVTVSHTDLASFTALVDYLLSDKFD
LGEEEGGRAQRALDLRELAQMYQVPRLELLCAQALQESVAPATAVPLLEAAHTLGDGRLLAQCRRYVADHAAEVRASGGV
EQLRDFGVAKGLLGDALDQVAELKGTVAARDAELEK
>Ehux_352226
TRGDAILPQGMVTFLVGDDEERIEHVSKCVLCARSPVFRTMFGIGMKERDAAEVTVSHTDLASFTALIDYLLSDKFDLGE
EEGGRAQRALDLRELAQMYQVPRLELLCAQALQESVAPATAVPLLEAAHTMGDGRLLAQCRRYVADHAAEVRASGGVEQL
RDFGVAKGLLGDALDQVAELKGA
>Ehux_462761
MASTEGSAVMVLPGGRSGPASRHRSPCQSPTTTTASCDAGTQPPSPEAIVSGDCGAALFNPCGLTASANGEVYVADTGHH
RICVLKQGGMRVLAGSGARGFADGAGQDAAFAHPCGLAIDSEGTLYVADCGNHRIRRVTMDGVVTTIAGNGAAAHRDGQG
RVASFYNPCGIAIDSNDVLYVADYSNNCVRVVSRGGVVATLSCHEDEAALDAPYGIAVSIFVSSYHSNSLAAISPDGQVS
VLAGCGAAKHRDGSGKDAAFHAPNGLAIDSEGTLYVADSGNHCVRRVTLAGEVTTLAGTGQAAMSESQFNSPCGLCVSLL

PGIGPALLITDRSNSCVRYLSVDALPPRRVAPSTLRQDLRRLLDGDSESVIEGEAVFEVEGRTLRASKAVMCVRSAHFRA
MFTSGMRECSDDVVKVPGVAYAAFRSLVNYLFTDDLPRGMSAAHALELMMLANAYQLTRLEQLCEQALLLLLDRENASDI
VAFAELIGACGLARASKRLIEGEQKDSSVADARAKSPRLSPPSSPLSPLDSSRMSCGETSPVEIC*
>Ehux_352708
DFATTRGDASLPQGMVTFLVGDDRERIENVSKNNLCARSPVFRTMFGIGMKERDAAEVTVSHTDLASFTALVDYLLSDKF
DLGEEEGRAQRALDLRELAQMYQVPRLELLCAQALQESVAPATAVPLLEAAHTTGDGRLLAQCRRYVADHAAEVRASGGV
EQLRDFGVAKGLLGDALDQVAELKKGALRVAAD*
>Ehux_434364
MCVYMASAARKRARTGGGGEDGDAPVPVADGFEAPIFGARVVGQVTTLTLEGEDGPLASLGPVGVASDHAGAWYVSTKDS
LLHVSAAGRVRRVAALPASGVFYTVAASPNGSALFVADFTRNKIRRVEVATGAVTTLAGSGQVGGADGVGDAAEFCSPLG
LAISPNGSALFVADFHNHKIRRVEFATGEVTTIAGSGDEGDADGVGDAAEFYFPHGISISPDGSALFVADSSNHKIRRVE
VATGAVTTLAGSGEEGGADGVGDAAQFYNPSGVAISPDGGALFVANYGNDKIRRVEVATGEVTTLAGSGTSGSVDGVGDA
AELNGPVEVDISPDGSTLLVASDGGLRQVCVAAPPPPPSFAPIVVPPSTILADLAKTRGDATLPQGMVTFLVGDDEERIE
HVSKNNLCARSPVFRTMFGIGMKERDAAEVTVAHTGLASFTALVDYLLSDQFDLGEEEGRAQRALDLRELAQMYQVPRLE
LLCAQALQESVAPATAVPLLEAARTMGDGRLLAQCRRFVADHAAEVRASGGVEQLRDFGVAKGLLGDALDQVAELKGAMH
ALRVAES*
>Ehux_353638
MAARKRARTGGGVGEDEPVPVADGFEAPIFGARVVGQVATLALEGEDGPLSLGTVGVASDHAGAWFVSTKDSLQQVSAAG
RVRRVAALPASEVNSIAASPDGSAAFVATGEMATLAGSGEEGDADGAGDAAEFYNPSGVAISRDGGTLFVADFSNNKIRR
VEVTTGAVTTIAGSGIKGSADGLGDAAQFCCPAGIAISPDGGALFVADQGNHKIRRVAVATGEVTTIAGSGEGGDADGVG
DAAEFCYPNGIAISPDGSALFVADYSNHKIRQVEVATGEVTTLAGSGTEGSADGVGDAAQFDGPDGVALSPDGSTLLVGS
DGGLRQVCVAAPPPPPSFAPIVVPPSTLAADFATTRGDASLPEGKVTFLVGDDEERIENVSKNNLCARSPVFRTMFGIGM
KERDAAEVTVSHTDLASFTALVDYLLSDKFDLGEEEGGRAQRALDLRELAQMYQVPRLELLCAQALQESVALATAVPLLE
AAHTTGDGRLLAQCRRFVADHAAEVRASGGVEQLRDLGVAKGLLGDALDQVAELKKGALRAAAD*
>Ehux_353651
GKVTFLVGDDEERIEHVSKNVLCVRSVFFRTMFGIGMKERDAAEVTVLETDLASFTALIRYLLTDQLDLGEGEGEQARRA
LVLRQLAHKYQVPRLELLCAQALQESVGPATAVPLLEAAHTTGDGRLLAQCRRFVADHAAEVRASGGVEQLRDLGVAKGL
LGDALDQVAELQQGAGKVTFLVGDDEERIEHVSKNVLCVRSVFFRTMFGIGMKERDAAEVTVLETDLASFTALIRYLLTD
QLDLGEGEGEQARRALVLRQLAHKYQVPRLELLCAQALQESVGPATAVPLLEAAHTTGDGRLLAQCRRFVADHAAEVRAS
GGVEQLRDLGVAKGLLGDALDQVAELQQGALRSHS*
>Ehux_353724
IRRVEVATGAVTTLAGSGEFCDTDGVGDAAEFCGPRGIAISPDGGALFVADFSNRKIRRVEVATGEVTTIAGSGTEGSAD
GGGDAAGFDCLEEVAISADGSTLLVSSDGGLRQVCVAAPPPPPSFAPIVVPPSTILADLAKTRGDATLPEGKVTFLQVRP
LRPVFLTMFGIGMKERDAAEVTVSHTDLASFTALVDYLLTDQFDLGEEEGRAQRALDLRELAQMYQVPRLELLCAQALQE
SVAPATAVPLLEAAHTMGDGRLLAQCRRFVADHAAEVRASGGVEQLRDFGVAKGLLGDALDQVAELKGAMRALRVAES*
>Ehux_450179
MQASLIEQELEPADAPAPGPSCAEPAGEFGPRWTPVDFVGGSPGSRVFPSAVVSGACVYIFGGHNGTTYRNDLLVLSLET
RSWTVDSEVDGDGRPTPRDAHAAVLLGGRLYVFGGYDSKRYLADFHSYGPLATAPPTGLNPDWVRYPGGMVHVGLDSGEW
RVEAASGSPPSPRGGHAAVVHGSRLIVFGGCDGWNYFNDTLAFDVHSAEWAPAAAQTVRIEGVSHAAFKQVLHYLYSGRP
REMNAEAALEVLAAANLYGVEPLKRAAAEIIGRSLTPENAAAVLSAADSYQAPALRAQATAFIVRHFAAVVQTDAFAELV
SERSRPLLLALLREVGTVVSPAGLAASDGLLGVAGGLPAGGYARRRPDARSPADAVRQLDELGIRGIADAE*
>Ehux_450242
MERPSSSSQGTLCEPPRPTFVCDALDEKQLTTSLPFKVQVRVLDEKAEAMSSFQGDIMLLAISRRTCLAEGFERKALGRW
QPASSPLHHESQYSHGFDSEVAAPGSSHSLYLSGGNDANFGMTLKLPATALEAEPPEHSPVAGGGPGESEYEDAAALNGK
FQPDAVSFYVRTDNSLADAGHFILGESNEVNRRVAQFQFTRDGKMGLLGTGGTTHGATPYESNRWYHVMLCFDWKRKTVA
FSVDGKLLQRSVPFRREASNFIGACALGNRDRCTTWFDSIRFVQETTLNRVAVSIVDGVAETWLGPLGEDEEGRSLAARD
GFVLRAEDADGQVSELAGPYHPLCRIEGAQRVAINNAALADFNTLLADDASADVTFVVGGRDVPAHRCILAARCETFRRM
FNSSMREGSREEGQRVPIAEVSHAAFSCMLQYIYSGAVAVPEELALELLGLADRYLLEGLKLLCGFTLTRMVSADTVAPI
LQAADRYDSPSSQLKAHCMQFVLSNYEAVVNSPSFEELTSSPQLLLAVTRAAAKVVAPSGSSRQISWDSDGAPGSKRARH
A*
>Ehux_100655
MAMASRSGKRVRTESDGNGGEEGEPVAVAAGFEAPIFGARTVGQVTTLALEGKDRPLASLSLRGVASDHAGGWYVATGVS
VLHVSSAGRVRTAATSEGGYLFGLTISPDGSALFVTNISNNKIQRVEVATGAVTTLAGSGEEGDADGVGDAAQFKNPMGA
VISPDGSALFVADTHNHMIRRGEVATGAVTTLAGSGEDGDADGVGGAAHFREPHSLAISSDGSALFVTDHGNNKIRRVQV
ATGAVTTLAGSGERGDADGVGGAAQFNSPAGLAASLDGSALFVADLFNEKIRRVEVATGAVTTLAGSGRRQRRLGSVDDV
GDEAQLRLPRGLAISADGSTVLVCSQNGGIRQVRVAAPPPPPSFAPLVVPPSTFSADMGKMWGDDALPQGLVTFLVGDDE
ERVEHVTKAALCVRSVFFRTMFGIGMKERDAAEVTVLETDLASFTALVRYLLTDQLDLGEGEGEQARRALDLRQLAQKYQ
AAHTTGDGRLLAQCRRYVADHAAEVRASGGVEQLRNMGVAKGLLGDALDQVAELRGAVAARDAELEKNRTQS*
>Ehux_205795
MFGIGMKERDAAEVTVLETDLASFTALVRYLLTDQLDLGEGEGEQARRALDLRQLAQKYQVPRLELLCAQALQESVGPAS
AVPLLEAAHTTGDGRLLAQCRRFVADHAAEVRASGGVEQLRDLGVAKGLLGDALDQVAELQKGRTAAA*
>Ehux_354038

MGKMWGDATLPQGMVTFLVGDNEERVEHVSKCALCVRSVFFRTMFGIGMKERDAAEVTVLETDLASFTALVRYLLTDQLD
LGEGEGEQARRALVLRQLAQKYQVPRLELLCAQALQESVGPASAVPLLEAAQTTGDGRLLAQCRRFVADHAAEVRASGGV
EQLRDLGVAKGLLGDALDQVAELKGALRSHSRS*
>Ehux_354066
MGKMWGDATLPQGMVTFLVGDDEERIEHVSKNALCVRSVFFRTMFGIGMKERDAAEVTVLETDLASFTALVRYLLTDQLD
LGEGEGEQARRALVLRQLAQKYQVPRLELLCAQALQESVGPATAVPLLEAAHTMGDGRLLAQCRRFVADHAAEVRASGGV
EQLRDLGVAKGLLGDALDQVAELQKGALRSHS
>Ehux_421393
MGKMCGDATLPQGMVTFLVGDDEERYEHVTKAILCVRSVFFRTMFGIGMKERDAAEVTVPNADLASFTALVRYLCTDQLD
LGEGEGEQARRALVLRQLAQMYQVPRLELLCAQALQERVGPASAVPLLEAAHTTGDGRLLAQCRRFVADHAAEVRASGGV
EQLRDLGVAKGLLGDALDQVAELEGAMRALRVAKD*
>Ehux_100831
MASASKRARTACDGEEEEEEEEEEEEEEEEAAAAAAAAAFCVAAGFETPIFGAPVVGQVFTLPDDGPLASLDVHGVASDG
AGAWYLATGGSVLHLSAGGEEPLDRMYEPSRHSLSVRSVATCPEGGRFEGIAVSPDGSTLFVADCRGQAIRKVEAATGTL
TTLAGSGSSGSANGVGVAAHFNGPTYLALSPDGGTLFVADTGNARIRRVDVASGAVTTLAGGVGHGSIDGVGDAARFQYP
YGLALSPDGRALFVADCGSDKVRRVEVLNGAVTTLAGCGRRGGTDGAGNTARFHAPIGIAMSPDGSALFVTDFSGHKIRR
VEVVGRAVTTLAGKGWGSADGVSKSAQLSFPGGVAMSADGSTLLVRSGAPSSALLCLVCLVPPPPPDAFAFAPIVVPPST
LPADLKKARGDATLPQGMVTFVVGRGRQRLEHVSKNVLCVRSDYFRTMFGAGMKERDAAEVTVPHADLATFTALVDYLFA
DELDLGEEEGGRVQRALALRQLAQMYQVPRLELLCAQALQQSVVAETAVPLLEAAHTMGDGRLLAQCRRYVADNATEVRA
SGGVEQLRNLGVAKGLLGDALDQVTELQEALDALRVD*
>Ehux_100839
MAGEHLTAAELRARIEEQRALRVTLKEERSALTREIEEAKERQQLRRELERESQINIWVRSGNGAKRDIRTRIDNDEAGD
VDGAQTEKKPTRTSCTLAKTTVSYASAVVKREYVWEVQGLSWLEQQLTQEGRECTSSDIFEVDPSDDCSTYLLAFSPRGG
SLKHKTDDRFEMFDDDLCGTLALIRTADNYGTKICYRFFVRDASGEFHPLGEATRVAVPARDEASCFVGGPDLETESKGL
FGLSFDELLGSEWVKDDSICFKVCIEEAAMKDHPSQGQVFVGKMDVVYERKPPSVEVPPPTLKAELLSLLTEGRHSDVTI
EAAYGEAAPVTFSAHTNILSQRSDVFAAAFAHEMRESTTRTLTVTDVPPPALKALLHFLYTDDFDEVQKVLREEGSSEGS
SSSGGASSSGASGAAEQSIAQLQAVLAAAHKYGVLRLLRWAEGQLCDKLSCEMACSLLSLAHVYGATELERNCLAFMKAN
MAQVVKRADFAALAPEALVKFHMHCAGVEPGRKRKRDEE*
>Ehux_450691
MYCGGIPMPCRSDSLTWARCRYLSARKVFSEIAFAARLKAYTDSTINARGRGSMASHPSHEATWPWHTGHSCSVVGKRVF
ILFGKHEDEEGKVICPPLQMLDTVNMTLTYPQVLQDAEGRSTTPDDREGHTASVVDQQIYIFGGTWEDEKTGVNAYLDDL
HVFDVASYSWMKPAYTGSPPEGREGHSAAVIGRLIFIFGGTRGTWVQEAERTPHRELEDEGESVYLNDLHTFDTATGAWS
MPEAHGQLPSQREGHSACVVGSDMILFGGAGLDGDDRSINLCDLHKLDTAPDEIARDCTRLHEIARARHDTPPPPILGSS
PPPERRYHTASVVEGRLLVFGGQYYDYGKDLHFECSNAVCEIDLEQLAWRIHPPSSPSPLKRACHAAGVVGKCVYVVGGR
YWDEQEDDYIFLNDVLDTRPSSSLASDWRRYFNSELLSDVCLLVGDMRIPAHRVVLAARCDFFARMFASGMREATEEELP
IVDVSPPVFQLLLEHLYTDSSAVPEESALELFAAADRFGVERLRALCSDRIEGMLSAANVCDVLTVADQHNGAELVEAAV
SHIVENFGEIHCSPPFRELSRPLLDVVHAAIAPRLVLAAPPAGLAAEAGGSRQSARRHST*
>Ehux_435579
MFAWGEDAQKQLGSGGSAPHSSPLPAAVCEDALQAAVVQVASGDGHSVACDALGGVYCWGRNREGQCGELGTARVSQPML
VEGLQHEAIVAVACGSDSSFAVSAGGSVFRWGATHRPSSGLADANVAGYGRSVDELSDRERAMLKESLLSFLYSGDSGEP
TDHSAAEVGTRREIVASPERVELPAGARACAVAAGFGFGVIALQSGGALAFGLNDRFQCGTGDRATKGIPTRVAGLDATR
LRSVACGQQHCFASDEAGGAWSWGLGSFGQLGHGGRSDERRPRVVEALRAKGAVFAVACGQQHSVALLRPGGSGGGGGGG
GSGGRGDGGDGGSGGGSGGDGGDGGGGGGGGGEGASLWGCGHAEYGQLGTGDAANVGDAERDYPEPRQIPLPADGEPCGV
SAGALHTAVLTERGEVLTFGWGAAGALGHGGFGYELEPRAVEALRSIRATSISAGARHTVAVQAAASTGGPSLARDFGRL
LRAGTGADVAVVAGKGPGRRRFLLHRAVLACRCPRLLAMAALSSRFCRGAPAAEEPREWEWHRPEGRCYEGGWPPPDCLP
ALGLPECRAPIFALLATWLYTGRLLCSERLFLGQLADAAASLRLRALVAECRRLIGSTLWPGDAASPHDDLGASFARLLL
EASSGPADDVCLRAVDGEVGASRALLCARSEFFRSALQGGFHQLPTIDLQWTGVGRHELRQLLSFVYSGRVVASGSEGRG
DLALLSPEAALSLLPLAAALLLDGLQRLSEAVLSAVVDEENASALLEVADQCYASRLKATCSEALRS*
>Ehux_354836
ESHLPGRRRRGAHRAREQESHLPGRRRRGAHRAREQKHALRPVPVFRTMFGIGMKERDAAEVTVSHTDPASFTALIDYLL
SDKFDLGEVEGGRAQRALDLRQLAQMYQVPRLELLCAQALQESVAPATAVPLLEAAHTTGDGRLLAQCRRYVADHAAEVR
ASGGVEQLRDFGVAKGLLGDA
>Ehux_354993
MANTCGDATLPQGMVTFLVGDDEERVEHVSKCVLCVRSVFFRTMFGIGMKERDAAVVTVRKTDLATFTALVRYLCTDQLD
LGEEEGEQARRALDLRQLAQMYQVPRLELLCAQALQESVAPASAVPLLEAAHTTGDGRLLAQCRRFVADHAAEVRASGGV
EQLRDLGVAKGLLGDALDQVAELKKGRRARRRPDAPLCRVA*
>Ehux_208291
MESTGALALDAEEPVPIADGFEAPIFGARVVGQVTTLTLEGEDGPLASLGPVGVASDHTGAWYVSTEDSLLHVSAAGRVR
RVAALPATSVFYAVAASPDGSALFVADFSNDKIRRVEVTTGEVSTISGSGTAGSADGVGDASQFYQPSGLAISPDGSALF
VADKGNNKIRRVEVATGEVTTIAGSGEAGDADGVGDAAQFANPAGIAISPDGGALFVADCNNNKIRRVEVATGEVRTIAG
SDKAGDADGLGDAAEFCSPTGLAISPDGSALFVTDFSNDKIRRLEVTTGEVTTISGSGTAGSADGVGDAAQFNYPEEVAI
SPDGSTLLVSSSGGLRQVCVAAPPPPPSFAPIVVPPSTILADLAKTRGDATLPEGKVTFLVGDDEERIEHVSKNVLCIRS

PVYRTMFGIGMKERDAAEVTVSHADPASFTALIDYLLSDKFDLGEEEGRAQRALDLRELAQMYQVPRLELLCAQALQESV
APATAVPLLEAAHTTGDGRLLAQCRRFVADHAAEVAELKGAMREADLEKARAEVSERA*
>Ehux_208373
MARKRARTGGGEDGDVPVPVADGFEAPIFGARVVGQVTTLTLEGVDGLSLGEVGVASDHAGAWYVATKDSLLHVSAAGRV
RRVAALPASDEFYTVAASPNGSAVFVADFSNHEIRRVEVATGAVTTLAGSGEEGAADGVGDAAEFDSPYGVALSSDASAL
FVADSSNHKIRRVEVATGAVTTLAGSGESGDADGVGDAAQFCSPLGLAISPDGSALFVADYSNNKIRRVEVATGAVTTLA
GSGEDGGADGVGDAAQFCYPHGISISPDGGALFVADVDNHKIRRVEVATGEVTTLAGSGTRGSTDGVGDASELNGPVEVA
LSPDGSTLLVGSNGGLRQVCVAAPPPPPSFAPIVVPPSTLGADFAKTRGDASLPQGMVTFLVGDDRERIEHVSKNVLCAR
SPVFRTMFGIGMKERDAAEVTVSHTDLASFTALVDYLLSDKFDLGEEEGRAQRALDLRELAQMYQVPRLELLCAQALQES
VAPATAVPLLEAAHTLGDGRLLAQCRRFVADHAAEVRASGGVEQLRDFGVAKGLLGDALDQVVELKGAMHALRVAES*
>Ehux_355087
MKERDAAEVTVTHTDLASFTALVDYLLSDKFDLGEEEGSRAQRALDLRELAQMYQVPRLELLCAQALQESVAPATAVPLL
EAAHTMGDGRLLAQCRRYVADHAAEVRASGGVEQLRDLGVAKGLLGDALDQVAELKK
>Ehux_310539
MVTFLVGDDKERIEHVSKCNLCARSPVFRTMFGIGMKERDAAEVTVSHTDLASFTALVDYLLSDQFDLGEEEGSRAQRAL
DLRELAQMYQVPRLELLCAQALQESVAPATAVPLLEAAHATGDGRLLAQCRRFVADHAAEVRASGGVEQLRDFGVAKGLL
GDALDQVAELKGAMRALRVAES*
>Ehux_208409
MASSARKRARTGGGEEDKEPVPVAAGFEAPIFGARVVGQVTTLALEGEDGPLSLGEVGVTSDHAGAWYVSTKDSLLHVSA
AGRVRRVAALPASGVNSIAISPDGSAVFVADCRNHEIRRVEVATGAVTTLAGSGESGDADGVGDAAQFCYPTGIAISPDG
SAMFVADHYNHKIRRVAVATGAVTTLAGSGEEGDAAGVGDAAEFYYPVAISPDGGAVFVTDHDNHKIRRVEVATGEVTTI
AGSGTEGSADGVGDAAEFSYPTEVAISADGSTLLVTSDGGLRQVCVAAPPPPPSFAPIVVPPSTILADLAKTRGDASLPE
GKVTFLVGDDEERVEHVSKCNLCARSPVFRTMFGIGMKERDAAEVTVAHTDLASFTALIDYLLSDQFDLGEEEGRAQRAL
DLRELAQMYQVPRLELLCAQALQESVAPATAVPLLEAAHTMGDGRLLAQCRRYVADHAAEVRASGGVEQLRDLGVAKGLL
GDALDQVAELKGTVAARDAELEKVRAVSE*
>Ehux_355105
IRRVEVATGAVTTIAGSGEDGDADGVGDTAEFCSPTGIAISPDGGALFVADHGNRKIRRVEVATGEVTTVAGSGTEGSAD
GVGDAAEFDCPTEVAISPDGSTLLVSSSGGFRQGCVAAPPPPPSFAPIVVPPSTLGADFATTRGDATLPQGMVTFLVGDD
KEHIEHVSKNNLCARSPVFRTMFGIGMKERDAAEVTVSHTDLASFTALVDYLLSDKFDLGDEEGRAQRALDLRELAQMYQ
VPRLELLCAQALQESVAPATAVPLLEAAHTLGDGRLLAQCRRYVADHAAEVRASGGVEQLRDFGVAKGLLGDALDQVAEL
KGT
>Ehux_356057
DLAKTWGDKTLPEGKVTFLVGDDLERIEHVSKNVLCARSPVFRTMFGIGMKERDAAEVTVSHTDLASFTALVRYLLTDQF
DLGEEEGGRAQRALDLRELAQMYQVPRLELFCAQALQESVAPATAVPLLEAAHTQDVGRQNPPRGESHLPGRRRPGAHRA
REQERALRPVPGLSDHVWHRHEGARRRRGHGVPHRPRQLHCPRPLPPHRPVRPRRGGGRQGAARARLAGAGADVPGAAPR
AVLRAGAAGERRAGDRRAAARGGAHAGGVEQLRDFGRRRARPGGGAQGHDARAPRRGEL
>Ehux_356388
LKKMRGDASLPQGQVTFLVGKEEQRVEHVSKNVLCVRSVFFRTMFGIGMKERDAAEITVPKTDLASFTAFIDYLCTDQLD
LGEGEGEQARRALLLRELAQMYQVPRLELLCAQALQESVGPASAVPLLEAAHTTGDGRLLAQCRRYVADHAAEVRASGGV
EQLRDLGVAKGLLGDALDQVAELKGTVAARDTELEKVRAEVA*
>Ehux_103377
MFFDSWESAVDRGRSYKRQRDEAADLISGPCWLKTNVVKAWREGRFTDALVVVEGKEYAVHRAVLAGASKFFDRAFSGSF
SEAADAVIRLNMPAAAWEAAADYIYTSAVQIDQASLVPLLEVARFLELPSLEGLLVDRIADGVSPDTFDTAWDAAGRLNL
KPLEKKLATMVATCMENVRAFAIARDMSSLPEPVRAALCGEASMQLRVKYIRICMPRDGSASGSKLAVCRRTFALLGGTE
DVDDLSMDIDKQAKVSLRFTAPPEACIGYQRFQYDYQTLVLPIAAGKVLPAWGEAAPLKVSRHIRLPVEWTIFSVRIGDG
DYPKTVHAWPGGPTLQLYKLVVTLKEGITGRMGDRVVARDFDFVTGDALVPLWGLLGPRAVLEVRTDADPATELRGLWLP
PDGIGMKTLAPA*
>Ehux_356531
MVTFIVGDDEERYEHVTKALLCIRSVFFRTMFGIGMKERDAAEVTVSNTDLATFTALVRYLCTDQLDLGEEEGGRAQRAL
DLRQLAQMYQVPRLELLCAQALQESVGPASAVPLLEAAHMTGDGRLLAQCRRYVADNAAVVRASGGVEQLRDLGLAKGLL
GDALDQVAELKMVTFIVGDDEERYEHVTKALLCIRSVFFRTMFGIGMKERDAAEVTVSNTDLATFTALVRYLCTDQLDLG
EEEGGRAQRALDLRQLAQMYQVPRLELLCAQALQESVGPASAVPLLEAAHMTGDGRLLAQCRRYVADNAAVVRASGGVEQ
LRDLGLAKGLLGDALDQVAELKGTVAPVG
>Ehux_103498
MVPTKQTARKSTGGKAPRGQLATKAARKSAPATGPLRLVLESPKLKENACEMWTRQQQGEFTDAVINVQHRAFQVHRVVL
ASASPYFQKLFSCGMQESNACSVTLPDITADIFEAVLRFMYMSEVELPIEDADMLLEMLLELVQAAAYFQVTPLLKTVES
ELCGQVNASNALQLLTFADTYEVPQLYAKALRAARRDLELVATQPAFNAVSLPRLLSLLMSVRAPRGFDIGVTQSAVTCW
ARAKRLENVDTLAVLLATTSVRGERAAVVDGRMFVLRGCVSISVSEGEAAPRLEAAPLLLRAFDPAMGTWQPSSSWQSAE
AAAGEDLIAGGSFMPALVALNGNLYAVNAGLRSQKRLLFPTPNTHPPETTDELAAMRVFIPAPPIPWDRAGSLPGRWENA
PPIPLGRSNAVVASFGATLIVAGGLSRSSADGDTFECLARCDIFHPDRGAWVEGPAMLEPRCDAAAVVFDGALYVIGGAS
SNGQPLSTVEKLTMDGGWQPAPSLKQARRNVTAFVFSGSLFAGSYSTVLSGSYSPAVEKLLPSGEWVVLPEHATAGGGAS
VGSSSAGGAQIAESAHNVRAADRRCNYMRSLDGTCFDIAYPTIPMDWAARIAPYDVLYVASAGSAGEGLFTSAPLAEGHL

IELTGRLIETAEAERAYASSFLEKYAVALDESGESCKWYLVPEPNGGGLALYFANCGDEQTPCNCEIVTLAAAGCAVPHV
RLFLRTRAIATDEELRWDYGPQYNRSWLAKKARN*
>Ehux_311149
MADDASMREGKVTFLVGDDEERIENVSKCNLCARSPVFRTMFGIGMKERDAAEVTVPHTNLASFTALVDYLLSDKFDLGE
EEGRAQRALDLRELAQMYQVPRLELLCAQALQESVGPASAVPLLEAAHTLGDGRLLAQCRRFVADHAAEVRASGGVEQLR
DFGVAKGLLGDALDQVAELKGTVAACSAELEKVRA*
>Ehux_357019
VGKTWGDATLPQGMVTFLVGDDEERIEYVTKAVLCARSPVFRTMFGIGMKERDAAEVTVSHTDLASFTALVRYLLTDQFD
LGEEEGGRAQRALDLRELAQMYQVPRLELLCAQALQESVGPASAVPLLEAAHTTGDGRLLAQCRRYVANHAAEVRASGGV
EQLRDLGVAKGLLGDALDQVAELKMGAARTAAGQRAAP*
>Ehux_212874
MPSRKRARAPSTALVVRAEPSEGSLRAGLAKLWRAGTCCDVELVVEGRSFSAHRVVLAAESPCLAALVCGGFAEAGMPRI
ELQEISSSIFERALDFLYLQECTLASQDELQPLLRAACHFQIAPLQAACEAAVAERLDASNCLRGLALADALTLSSLRQS
ATDVALASFEEAAAAEDGAALAALPAAMVGALLASDELKVAKEEAVLGAAKAWLAAQQPAASSEVARGLLEHVRFPLVDD
LAALERDELLLQHPALVMRAYREKVTGEATPRTRRRGKALSYEDLEIGMAVQVSGDAEFVKEQCKQVAPGADGIAGWTEG
GVMEAMVGHEYKIVALGSCFRAAKLIQDGGRSFWFPYTVLLRA*
>Ehux_104201
MAKRLREPDRAAGERPTVKLSGGQPCPDGQVNLWRAGKLCDATVHVDGRAFQVHRAVLAGASEFMSALFTSGMRDSDEPS
ISEVAGDVFSALLEWIYCGECRVEADLLGGMLDASSRLQLASLQSATVEALVQGIDTSNAVQYWECGDAHNIPRLVEAAK
AAALQSFGEFSTCVAFDALPIGRVCELLKSDALKADSEEIVFRAAARWAAANLSASEQEVGAVYSHVKYGLLPAAFLSSV
VEQDPPLVTHPRGGWIVARSLKAHIDQGSSPRGPLVTVLNPGSVYGGFEAMAVHLGVRDEWDRRADAVAGDKGVPLAMGQ
RSSSPDDKVVAVRILRTGSVILIGQLGLELVRGAWPASKGRPPR*
>Ehux_214578
MPARKRARAEAPVETATLTDPDEHHPTFRQLATLWRAGQLCDVTFTVEGRSFSAHKLVLAAASAYVRALVDGPRFADSSS
DALTLDEMPAAAFELLLEWIYSGSCNAPLNLLQPLLLAAGRLQVPALEMAAANALAERLDATNCSNLWAFAERAALVDLA
AAAKAFALNHFQEIAGDAAFLNLPRAQLEVLLSSDDIALKEELVFENLMLWLSAQPAEAREPDEVAKLLEHIRFATMERA
FITERVQPHELMQSMPAYKVLAEALADSCFGAGTARSAPRGGSCGWLLEGLDCGDKVTAEGRVLTFKVDAYRLVRLDKLI
SSGKWEIGFELVADNAGCGAAATVFGLVARDGGPPVDFDLKGTSITLGLDNTFMLRRHRRLVYANGTKARGGAEATRNSL
SAPAHLSWCIVSPPIRHNKT*
>Ehux_357873
MRGDASLPDGKVTFIVGDDEERIEHVSKSLLCVRSEYYGKMFGIGMKERDAAEITVPKTDLASFTAFIDYLCTDQFDLGE
GEGEQARRALVLRELAQMYQVPRLELLCAQALQESVAPATAVPLLEAAHATGNGRLLAQCRRFVADNAAEVRASGGVEQL
RDLGVAKGLLGDALDQVAELMRGDASLPDGKVTFIVGDDEERIEHVSKSLLCVRSEYYGKMFGIGMKERDAAEITVPKTD
LASFTAFIDYLCTDQFDLGEGEGEQARRALVLRELAQMYQVPRLELLCAQALQESVAPATAVPLLEAAHATGNGRLLAQC
RRFVADNAAEVRASGGVEQLRDLGVAKGLLGDALDQVAELKGT
>Ehux_214981
MPARKRARAEAPVETATLTDPDEHHPTFRQLATLWRAGQLCDVTFTVEGRSFSAHKLVLAAASAYVRALVDGPRFADSSS
DALTLDEMPAAAFELLLEWIYSGSCNAPLNLLQPLLLAAGRLQVPALEMAAAQAERLDATNCSDLWAFAERAALVDLAAA
AKAFALNHFQEIAGDAAFLNLPRAQLEVLISSDDIALKEELVFENLMLWLAAQPAEAREPDEVAKLLEHIRFATMERAFI
TARVQPHELMQSMPAYKVLADALADSCFGAGTARSAPRGGSCGWLLEGLDCGDKVTAEGRVLTFKAGAYRLVRLDKLISS
GKWEIGFELVADSAGCRRAATVFGLVARDGGPPVDFCLKTSASIVLDNAYMLRRHQRLVYANGTMVGEGDGQVGQGESES
YAQFPVGSLVRIIADMDKREATFSIDGTAVTAVARGISGPVYPFVGSYHSAGPDKVIAFKHVRRLA*
>Ehux_215577
MPPRKRARADAAAASSAPLVALKTSHDVDNPQLQAEAAMWRAGELCDLTVTVDGSAFAVHKLKLAAASDYFRALLPGDRF
ADSAEHQLKDMKASVFEGVLSFIYEGSCNVPEELLVPLLEAASRLQVAPLAEALTAALKERISVANALSTWIFADRASLP
ELAATARQHTLKHFADVAATDDFVQLSHEMLSALLASDEVEADEAAVFTALVRWLRAQAPAVREESTVAALLEHVRFSTM
SADFLTEHVETEPLMNTAAAFKVLAQAMKEKSFGAATPRTTPRTGGPLNPKWVLEGHDAANVSAAGETMTTQKGLVRVDT
LIASGTYEVVFKLTEDGADLSAAVSGFGIVRDNCDRLESNSNGMPNVWWLRRFHRMVYSENHKEGLTPRGYEFPIGAEAR
MLVDMAAGQVTFFIDGVEVPHKAKGITGPVYPCALSYGRKVSVSIVSVRRLKL*
>Ehux_358189
VRSEYYGKMFGIGMKERDAAEITVPKTDLASFTAFIDYLCTDQLDLGEGEGEQARRALDLRELAQMYQVPRLELLCAQAL
QESVGPASAVPLLEAAHTMGDGRLLAQCRRYVADHAAEVRARGGVEQLRDLGVAKGLLGDALDQVAEL
>Ehux_217037
MRLVCDDGLHVTAHRCVIALASPVLSQLLYGNMPPPSSDGFAANLQLPGKQSKAVQEFVAWCYTDQIAPSCDPVALLHLS
EEWDAPSLKAGLEPLLMDGISKLTCVDALLWGLEFQLAKLASAARVTILAEYTHVAGLQSFGRLPFSELRGMLQDDKLVV
PSEMVVFESVASWCGQQSSAVDTSDVVQLLACVRYASMETAFLRGTVAVHPLLTRCDAAGEHVSDAVLHIAEEVDAAAAV
SVGSKRPRETSIRPLPYKKRARLTGEFCTSSASRPSVRLAIASSDGVRLDFDAADASPRAPGYFYWLGTCAGTREWTNPA
QEGEMEVKLANDYEKRTFVDEHLVLLPFDNTDPERDIYATLTSREKDRHNCPISHLQFQLPHRFVLTGYAFTILSRHEDE
DSDSDEHVGSTITWDVLTSKDGNCWKKLVRKQEATQSTDPFDEVLSRCVTGAEAPTFFPIPVDKVDTSGSRFFRFQARHP
NYLDIWRVELYGSSLSSDQ*
>Ehux_311940
MHASAARKRARTGGGGEVEAVPVADGFEAPIFGARVYGQVTTLPIEGEDGPLASLGQVGVASDHAGAWYVSTKDSLLHVS

AAGRVRRVAALPASNYYTAAASPDGSAAFVADFINHEIRRVEVATGEVMTIAGSSTAGSADGVGGAAQFCYPGGIAISPG
GGALFVADFNNHKIRRVEVATGAVTTIAGSGEDGDADGVGDAAEFCGPRGLAISPDGGALFVADSENHKIRRVEVATGAV
TTISGSGEGGDADGVGDAAEFYDPRGIAISPDGSALFVADHDNRKIRRVEVATGAVTTIAGSGTTGIVDGVGDAADLASP
DEVAISSDGRALLVKTRDGRLRQVCVATPPPPPSFAPIVVPPSTFSADMAKTWGDATLPEGKVTFLVGDDEERIEHVSKC
NLCARSPVFRTMFGIGMKERDAAEVTVSHTDLASFTALVRYLLTDQFDLGEEEGGRAQRAFDLRELAQMYQVPRLELLCA
QALQESVAPATAVPLLEAAHTTGDGRLLAQCRRFVADHAAEVRASGGVEQLRDFGVAKGLLGDALDQVAELKGAMRALRV
AES*
>Ehux_217402
MTAVNVNSPDEDKIMAAKRRRGSDESSTQSSSAAITLECSTAASAAIREDGLRTAIAAQWREGTLCDVDLLVEGTAFKAH
RMLLAAESPYLSALVTTAMRERAGPIELKELSAASFAAALSFMYTRRCTLGSQDALQPLLHAAHLLRVPPLLAAAEAAVA
ERIDASTCLRALGLAEHLSLAALANTAQKLALQHFGTAAEAEDGAALHTLSAEQLGDIIASDDLAVSDESVVLEAVKGWL
AAQQPAASQATAGALLAHVRFAELSKEQKVALDDEPLLQQHFKIVATAYREKMHGEDTPRARPRSGSFRCCLQYDDLRVG
MMVKVHPDRAFVEEQCRKVVAGATKNCGWAVNMTSTCGSTYRVAELRENSCSARLDIPRVIWHLPYTVLQIVCDA*
>Ehux_217625
MASAARERARTAGEDSESDGGAVAVADGFEAPLFGLRVVGQVTTLPLKDEDTFWHRSNHSASQAITRAPVAGSAPFVADF
DGNKILQVEAANGALITLAGSGTSGSADGVAGAAQFSRPRGVAISPEAARSFPDGSALFVADFRGFKILRVEMSTGAVTA
VAGSGTSGSADGVGGAAQFHHPCDIVCVSAPPPPPSFAPIVVPPSTLVADLGKTWGDATLPIGLVTFLVGDDEQRLSVCK
CNLCARSIFFRTMFGIGMKERDFAEVTVSNTDLATFIPLVCYLLTDQLDLGEEKGGRAQRALGLRQLAQMYQVPRLELLC
AQALQESVGPASAVPLLEAAHATGDGRHCGYVADNAAEVRASGGVEQLRDLGVAKGLLGDALDQVAELKGTVAAREGSRT
QG*
>Ehux_359034
MEKTWGDATLPQGMVTFLVGDDEERVEHVSKNVLCVRSVFFRTMFGIGMKERDAAEVTVLETDLASFTALVRYLLTDQLD
LGEGEGEQARRALVLRQLAHKYQVPRLELLCAQALQESVGPATAVPLLEAAHTMGDGRLLAQCRRFVADHAAEVRASGGV
EQLRDLGVAKGLLGDALDQVAELKGARVERVDNGLVRF*
>Ehux_359044
ARAGRGWLRGAHLWRARRRPGLHAGARGRGRPSVGVASDHAGAWYVATKDSLLHVSAAGRVRRVAALPASGVFYTIAASP
DGSAAFVADCNNHKIRRVEVATGEVTTLAGSGTTGSADGVGDAAQFDCPNGIAISPDSSAVFVADCGNRKIRRVEVATGE
VTTLAGSGEGGGADGVGGAAEFYHPYGNAISPDGGALFVADVTNHKIQRVEVATGEVRTIAGSGASGSTDGVGDAAQFDC
PAGIAISPDGGALFVADNGNKKIRRVDVATGEVTTVAGSGTRGSADGVGDAAELNGPAEVAISADGSTLLVSSSGGLRQV
CVAAPPPPPSFAPIVVPPSTILADLAKTRGDASLPQGMVTFLVGDDEERVEHVTKANLCARSPVFRTMFGIGMKERDATE
VTVAHTDLASFTALIDYLLSDKFDLGEEEGRAQRALDLRELAQMYQVPRLELLCAQALQESVGPATAVPLLEAAHTMGDG
RLLAQCRRFVADHAAEVRASGGVEQLRDFGVAKGLLGDALDQVAELK
>Ehux_217900
MRDTFFFSKFFFHEKVFTLPDDGPLASLDVHGVASDGAGAWYLATGGSVLHLSAGGEEPLDRMYEPSRHSLSVRSVATCP
EGGRFEGIAVSPDGSTLFVADCRGQAIRKVEAATGALTTLAGSGSSGSANGVGVAAHFNGPTYLALSPDGGTLFVADTGN
ARIRRVDVASGAVTTLAGGVGHGSIDGVGDAARFQYPYGLALSPDGRALFVADCGSDKVRRVEVLNGAVTTLAGCGRRGG
TDGAGNTARFHAPIGIAMSPDGSALFVTDFSGHKIRRVEVVGRAVTTLAGKGWGSADGVSKSAQLSFPGGVAMSADGSTL
LVRSGAPSSALLCLVCLVPPPPPDAFAFAPIVVPPSTLPADLKKARGDATLPQGMVTFVVGRGRQRLEHVSKNVLCVRSD
YFRTMFGAGMKERDAAEVTVPHADLATFTALVDYLFADELDLGEEEAGRVQRALALRQLAQMYQVPRLELLCAQALQQSV
VAETAVPLLEAAHTMGDGRLLAQCRRYVADNATEVRASGGVEQLRNLGVAKGLLGDALDQVTELQEALDALRVD*
>Ehux_422528
MLTMAGEHLTAAELRARIEEQRALRVTLQEDRSTLTRELEEAKERQQLRRELEQESRINAGIRASNGVKGRIRIRIDNDE
AGDLDGTPQQTEQKPKPAPSSTLAKSTVSYASDVVKREYVWEVQGLSWLEQQLTQEGRECTNSELFEVDRTDECSTYMLA
FSPRGGSLQHEMDDQFEMFVDDLCGTLALIRCADNYGTNLCYRFFVRDASGEFQPLGDTTRVAVPARGESFCFVGGPDLE
TESKGLFGLSFDELLGSEWVKDDSICFKVCIEEAAMKDHPSQGLVRVGHHASIYEHTPPSVEVPPPTLKADFLSLLTEGR
HSDVTIEAVYGEASPVTFSAHTNILSQRSDVFAAAFAHEMRESTTRTLTVTDVPPPALKALLHFLYTDDFDEVQKVLREE
GSSEASSSSGGASSSGASGAAEQSIAQLQAVLAAAHKYGVLRLLRWAEGQLCDKLSCEMACSLLSLAHVYGATELERNCL
AFMKANMAQVVKRADFAALAREALVKFHMHCAGVDPAEEEPGRKRKRDGE*
>Ehux_359371
YSNNAIRRVGVATGAVTTLADSGTMGDADGVGDAAQFHSPAGIAISPDGRALFVAGCSNHKIWRVELATGTVTTLAGSGE
DGDADGVGDAAQFECPEEVALSPDGSTLSVGSRGGLRQVCVAAPPPPPSFAPIVVPPSTLGADLATTRGDASLPQGMVTF
LVGDDEERIEHVSKNNLCARSPVFRTMFGIGMKERDAAEVTVSHTDLASFTALVDYLLSDKFDLGEEEGRAQRALDLREL
AQMYQVPRLELLCAQALQESVAPATAVPLLEAAHTTGDGRLLAQCRRFVADHAAEVRASGGVEQLRDFGVAKGLLGDALD
QVAELKGAMRALRVAES*
>Ehux_359390
MRGDASLPTGLVTFIVGDGEQRIEHVSKSLLCVRSEYYGKMFGIGMKERDAAEITVPKTDLASFTAFIDYLCTDQLDLGE
GEGEQARRALVLRELAQMYQVPRLEMLCAQALEESVGPASAVPLLEAAHTTGDGRLLAQCRRYVADHAAEVRASGGVEQL
RDLGLAKGLLGDALDQVAELKGTVAARDTELEKVRAEVAERA*
>Ehux_219635
MWRSGELCDVVLSAENEKIRAHRLILAGGSSYMRSCFSSGMRDGEAAVLTLQGVTACSLRCLLDFLYLGECEATPSSIVP
LMHAASLMEVRPLLQLAADFLGKDGLLPESYLNIIELCDTLDRPETAGLRAACLAFVSEHLEALAAEEQWRRLPRPWLRA
LLESEAITLPELCVFQSAIAWLEAQPQPQRDGEACAELLGIVRYATIPADTLVDAVEPRIESLGHAGLAQRLLHAAFRFS

SLSPARQMGEERSAEFCRREGQVGDLAWRLLNTPSDDEASVLDGNVLTVAADSDEQGVSLHQVSSACVTLRSELTWSVLL
AVKEGCPALDCHDMLFVSFGTGEPVAGWINHRSLAACEEAWASVDGYEDHGSGANRDRSFEVTLYARLTRISGSEAAAEV
WLEDQAGQKHEVSDSILDSKFPAGAPCRLVLGLTYGPAEAVLLDVKAR*
>Ehux_107259
MSRKRSRSSSCSAPSASAASPAILLSGGSQALHRDAVDKFRAGELCDVELESADGETVGAHRIVLVGASKYFKTLWSGKW
PGDCRGPLDLTALSTACLKACLEFAYTGEAEVADEDALAELLAGAAFLQMDELLEAAAEAYVSRLAASNALAVWELADRI
GRLPRLAEAAAKAARANFGEVVAGGEAWTAAPASIVRSLLEDDRLRASEEDVYRAGVAWLQAHDPPPGEEEVAALLSRVR
CGTLAWAADAASTRQASRRREGQLVTLESRTLTLMAPTAEAKEMWVVGINAILSGLYF*
>Ehux_359899
LGKTWGDATLPQGMVTLLVGEDEERLEHVSKSQLCVRSVFFRTMFGIGMKERDAAEVTVRNTDLATFTALVRYLCTDQLD
LGEEEGGRAQRALDLRQLAQMYQVPRLELLCAQALQESVGPASAVPLLEAAHTMGDGRLLAQCRRCVADHAAEVRASGGV
EQLRDLGVAKGLLGDALDQVAELKGAMHALCVAES
>Ehux_359918
GIGMKERDAAEVTVRNTDLATFTALIRYLCTDQLDLGEEEGGRAQRALDLRQLAQMYQVPRLELLCAQALQESVGPASAV
PLLEAAHATGDGRLLAQCRRYVADNAAEVRASGGVEQLRDFGVAKGLLGDALDQVAELKG
>Ehux_220166
MRRVATLEGVDPESDIFHSIAVSPDGGALVVAGGNKLRLVEMDTGELTSLAGSGEAGDADGVRGAAQFHNPTGVAISPDG
SALFVADNRSHKIRRVEVATGAVATLAGSGDADDADGVGDAAHFSYPSAVAISPDGGALFVTDSGNHKIRRVEVATGSVT
TVAGSGDEGDADGVGDAAHFCNPIGIANSLDGSALFVADNENHKISGGLSGEGGNADGVGDAATFDQPVEVISADGSTLL
VSSVGDLRQVCVMASPSPPSFAPIVVPPSTILADFAKTRGDATLPTGLVTFLVGDDRERLEHVSKSLLCARSVFFRTMFG
IGMKERDTAEVTVPNTDLATFTALIDYLLSDKFDLGEEEGRAQRALDLRELAQMYQVPRLELLCAQALQESVGPATAVPL
LEAAHTMGDGRLLAQCRRYVADHAAEVRASGGVLRDFGVAKGLLGDALDQVAELKETVAELKGAVATREAEICVNAAVEP
LRRLTLDGCAGPGRGWEASSHVTYVIVLSYVCGT*
>Ehux_220182
MSSSSSRGQRASRSRKRERDAASPRAPALLRIDGGSALPARVVKLWQQGEVCDATVVAQGREFPVHRCVLMAASEFFDGA
FGSGLAESASTRVSLDSVEASALEPILEYVYTGECQVDEAALLTLLQGASFLRVGPLIAAIVARLEDRLTPDNCLDLWAF
GEAHTLLALVEAARVAALRGFDAAAASAAFLHLPHARLLELLQDPRLEASREESVHEAVLAWAKAQRPAPDEEALLPLLR
TVRYPLVARDFFEQTVMAEPLLHSGTLAIKLFGSAFVDVAFGPRIGRRPGFGPSRPQLTWSSTHKGDGIVLLDAGLLAIG
GESEGEVVRSAEPLPATGKHLVEVVYEQGLASAATTNLGACYFTGVVSADVDTFREMDGDENLAHLAHGFWGIDDIGDGG
ETTGVRRGKGGEEGAPPAALVDLGTSQERGLFVRGDRLGLLVDMDERTLTFHRNGDPIPSLVLGGLPEEVYVVATPYNTG
SRVRIVQSD*
>Ehux_359972
MAKTWGDATLPEGKVTFLVGEDEERIEHVSKNNLCARSPVFRTMFGIGMKERDAAEVTVSHTDRASFTALVRYLLTDQFD
LGEEEGRVQRALDLRELAQMYQVPRLELLCAQALQESVAPATAVPLLEAAHTMGDGRLLAQCRRFVADHAAEVRASGGVE
QLRDFGVAKGLLCDALDQVSELKGAMRALRVAES*
>Ehux_359990
MRGDATLPDGKVTFIVGDDEDRIEHVSKSLLCVRSEYYGKMFGIGMKERDAAEITVPKTDLASFTAFIDYLCTDQLDLGE
GEGEQARRALVLRELAQMYQVPRLELLCAQALQESVGPASAVPLLEAAHATGDGRLLAQCRRYVADHAAEVRASGGVEQL
RDLGVAKGLLGDALDQVVELKMGAARTAAGQRAAP*
>Ehux_220735
MASKRPRPAEPDRPAGERPTVTLSGGEPCPAGQVNLWRAGKLCDATVHADGRAFQVHRAVLAGASEFMTALFTSGMRDSD
EPTISEVAGDVFSALLEWIYCGECRVEAALLGGMLDASSRLQLPSLQNATVEALVQGIDPSNAAQYWECGDAHNFPRLVE
AAKAAALQSFGEFSTCVAFDALPVGRVCELLKSDDLMADSEEVVFRAAARWVAANSSASEKEVEAVYSQVKYGLLPAAFL
SSVVEQDPPLATHPRSGWIVARSLKAHIDQGPSPRGPIVTVLNPRYLYIRDNLMALHLGVQDEWDRRAIVVAGDKGTPLA
MGATWDFASPNFVAVRISRTGSVILIEQQKLELVRGAWPASKGRPPR*
>Ehux_360174
MANTWGDATLPTGLVTFLVGDDEERIEHVSKANLCVRSVFFRTMFGIGMKERDAAEVTVLETDLASFTALVRYLLTDQLD
LGEGEGEQARRALVLRQLAHKYQVPRLELLCAQALQESVAPATAVPLLEAAHTTGDGRLLAQCRRFVADHAAEVRASGGV
EQLRDLGVAKGLLGDALDQVAELKGALRMANTWGDATLPTGLVTFLVGDDEERIEHVSKANLCVRSVFFRTMFGIGMKER
DAAEVTVLETDLASFTALVRYLLTDQLDLGEGEGEQARRALVLRQLAHKYQVPRLELLCAQALQESVAPATAVPLLEAAH
TTGDGRLLAQCRRFVADHAAEVRASGGVEQLRDLGVAKGLLGDALDQVAELKGALRSHS
>Ehux_220814
MASRKRARTSSSSSSGVTVVLAGSPLGSKMAAFWRAGDLTDVVVTVEGRSFPAHRLVLAAGAEYFEKLFKSECRDREAPT
IKDELPAAAFEPLLAFLYEGSVAVAEELITPLLHAADYLGVEPLKEAAAGALVARLAPSNALATWALAERLSLPSLAEAA
KQNKEEATGGTGGGCSDDDGLCVLCYAQPRVLRYRPCGHCVACERCAIRHVLAQLQAGELRPRCSHGCGAEIVDVLRVER
ADPPIFERDEGNASCQPGAHSLEAFLERAAQKVLRCSVAQDASQAWLRLREAAGARRQLAFFEAVEAGDLDGCVALRRAG
ASVAQLLRERGADLAAADLDGRTALHFASVNGHLEAGCLTRNASDSPESAACLARAVARWLVGEGVAADSRDAMGRRALD
DTVPCR*
>Ehux_221122
MPKRAHDAVDDDQVAAVELSDGEPQPERQIALWRASTFCDATVVVEGRSFAAHRLVLASSDFMSTCLQSSMTEQTGRVEL
HDISASSFELVLEYLYTGKARVAVRSLVELLEAAAMLQVQPLVAELAKVLGAGLAGDNCLDLWATAARLGLPAVKPLKAA
CLKFACESFADVVSTTSFLSLSCPQLECLIRGRRVHASEETVFAAVIKWARAAQPSPGELSAILQHIHFRDMRPSFVSSE

VLTEPLVAEHMQLVAKRLLSDQPQRRIHLEATGRVASLAYTWKVPSFSVSGDSEGRVYSPIFRSGGDEWQLKLSKGGEVE
GYLGIFLICPNAVGDVTVKADFTLKLHNQTPRKSVQKWAEHEFKHTAKPHMGWINEDWGWQKFIELEKLSAGFLVDDTLT
VEVSIERRNE*
>Ehux_360359
TCGDPTLPTGLVTFIVGEDEERYEHVSKNLLCIRSVFFRTMFGIGMKERDAAEVTVRNTDLATFTALVRYLCTDQLDLGE
EEGGRAQRAFDLRQLAQMYQVPRLELFCAQALQESVGPASAVPLLEAAHATGDGRLLAQCRRYVADHAAEVRASGGVDQL
RDLGVAKGLLGDALDQVAELKGTTCGDPTLPTGLVTFIVGEDEERYEHVSKNLLCIRSVFFRTMFGIGMKERDAAEVTVR
NTDLATFTALVRYLCTDQLDLGEEEGGRAQRAFDLRQLAQMYQVPRLELFCAQALQESVGPASAVPLLEAAHATGDGRLL
AQCRRYVADHAAEVRASGGVDQLRDLGVAKGLLGDALDQVAELKGTVAALKGTQG*
>Ehux_360557
MWGDATLPEGKVTFVVGDDEERLEKVSKNLLCIRSVFFRTMFGIGMKERDAAEITVRNTDLATFTALVRYLLTDQLDLGE
GEGGRAQRALDLRQLAQMYQVPRLELLCAQALQESVGPASAVPLLEAAHAMGDGRLLAQCRRYVADNAAEVRASGGVEQL
RDLGVAKGLLGDALDQVAELKGA
>Ehux_312703
MANTWGDASLPQGMVTFLVGDDEERIEHVTKANLCARSPVFRTMFGIGMKERDAAEVTVSHTDLASFTALVRYLLTDQFD
LGEEEGRAQRALDLRELAQMYQVPRLELLCAQALQESVAPATAVPLLEAAHTMGDGRLLAQCRRFVADHAAEVRASGGVE
QLRDFGVAKGLLGDALDQVAELKGTVAARDVELKKVRA*
>Ehux_360801
GIGMKERDAGEVTVRNTDLATFTALVRYLCTDQLDLGEEGGGRAQRALDLRQLAQMYQVPRLELLCAQALQESVGPASAV
PLLEAAHATGDGRLLAQCRRYVADNAAEVRASGGVEQLRDLGVAKGLLGDALDQVAELKGTVAAR
>Ehux_108401
MCSPTDVVCVDGWLYVVEPHTVRCVSPTHHVTTIAGAHNSSGYKDMVGTDARFHNGSTGRITSDGRGLVVLTDINNKRLR
SFRVRIESSVEIPLSTLQADLEGLMSNEDAADFKIVVEGKTLYTLRGLLTTRCAHFRTMLASGFSESTSDEITLTEDSFE
SVRAVLLFLHTDRLEVDDEHAVEVLRLSMMWELPRLQSLAQDSITRRVTASSVCAFLSAANAHDAPGLRAFCVRYIVLNF
GSVQASGNFASLDKELLCEVIQSIRI*
>Ehux_222586
MPPRVLPVIVVELYRDYLTELARRGFDNLSAGGDRVSISTPRKVWATLRALAKDGESDGAAAVADGFEAPLFGAPVVGQV
TTLPLEGEDGPLASIKPLGVTSDRAGAWYVGSKGSVLHVSAAGRVRSVASLEDASLRGIAISPDASALFVADMFNHKIRR
VEVATGAVTTLAGSGTVGSAEGVGGAAQFTVPSGVAISPDSSALFVADSHNHKIRRVEVATGAVTTLVGSGTEGSTDGVG
GAAQFYSPIDVAISPDGSAMFVADFGNHKIRRVEVATGAVTTLAGSGTRGSTDGVGGAAQFHLPSDVAISPDGSALFVTD
ANNHKIRRVEVATGAVTTLAGSGTEGSADGVGGTAQFRNPLGVAVSADGSTLLVCTKDGGLRQVCVSAPPPPPTFAPIVV
PPSTLVADLGKTCGDASLPEGKVTFIVGDDEERYEHVSKCVLCVRSVFFRTMFGIGMKERDAAEVTVRNTDLATFTALVR
YLCTDQLDLGEEGGGRAQRALDLRQLAQMYQVPRLELLCAQALQESVGPASAVPLLEAAHMTGDGRLLAQCRRYVADNAA
EVRASGGVEQLRDLGVAKGLLGDALDQVAELKGNRTQG*
>Ehux_222694
MEVYFLLFLSCQRTYKREGKKNTRPAAPLPLLIMHAANKLPRRSLALPDLSRDQLLALVESLRPLGGNGLAVEDYLRTAS
SQEQAGWEPLCSDAAQLLAMGSPGELVTVRTIASPEAQSCLDAGEPADLTLLSDDQRFRVHTSAMAAASDFLAALFGAGM
TDSTAREVALREVTATELQALLELVYHGECSVRCGGLPALLITAARLQMPTAQRAVEGAIASRLTAANVLDAWELAEQHN
LPALREAAEALGRETFDFGPLDFGPLDSHETMHPGYPRDIPCKCGRLQWLKLDRDRGISFERAAAHVAELRLASGGGLFE
GEGFYVSHNSLLGREKDVLAYALLICKSRHVHDRNNEVALFRVFRPGIGPGVTVTLAEFTQKLPSGVPRFKLIAEDDPQL
AAGWTRLYDDALSICAHGPRDSWIMACADWNELKDLISVREVKELKTVARTDDYSDDSESEEEVEEVVGTALQLTRAELG
GRHVFGLPLKLNGAPTERKLRGLELRQPNPQCWLDTGYPLPKQLRRVM*
>Ehux_360994
MFGIGMKERDAAEVTVPHIDPASFTALIDYLLCDKFDLGEEEGRAQRALDLRELAQMYQVPRLELLCAQALQESVAPATA
VPLLEAAHTMGDGRLLAQCRRYVADHAAEVRASGGVEQLRDLGVAKGLLGDALDQVAELKGAMREADLEKVRAERA*
>Ehux_222729
MTRRFNVGDYGGGDEPAAKGALLSWRDDPDETLSDWRIEVNVPDAARNSKTYHVHKVVIAGGPRKSDYFVRQCRGNGASR
LAEAARGESKLDLDAEAAEAMPAFLDYVYTGELQATKENACALFHLAEYTANPSLHAEVKLYLEESLDYTCAPTYIAHAH
LFSQDKLIDAAVEAAAEQLQLYADHVFLPGDHRFDPATADNFSGCPELHDLFALHHTLFARVVTSDHRPEADHGGYYHGG
GYYHQRHGGAGVSWCVVRLVADYCTRRADDIDASFLEAVVDESFLDNLGSDVAMPLLEAAMKHPGSDASTKLRDRCIQKA
GEDWKKTLLPLAQKEKRRREEQAAAEEAAAARSRKKKAAKRTAPPAASSSETVPAMPLGPGLPADMQLKLLGNMLLHQGG
EDPFD*
>Ehux_454325
MSRKRARSSAADSSASTRDRPVHVGAGLLPQRPEVIQKFRDGDLCDVELQPAYGPAVRAHRIILVGVSQYFKALFSGTWS
GSGSHDLSALSPACLHACLEFAYTGEAEVADEAALSELVEGASYLQMDELLVSASEAYEARLSAKNALAVWQLAEAIGRL
PRLAEASAWVARVNFGEVASDEAWASAPAWIVRSLLEDDGLHASEEEVYRAGVAWVKARDPPLSETEVAELLARVRFALL
PPALLPKMASLARRRAFLLTRALLLLLASTRCAEQPPAEEQEQLWESARRALKKGLHNKAFEAYSSLSKSLGPPDAVHWE
KHPDKRLELQEGLCLSAARLRKEQVAIGACEAAGGAVRGGKRASVGMLMALGEAKFFDGKWEEAGAYFAQAEAQARRGDA
VKQEREAAEARGRAEVRMFVSFGRQRGRPAATSLLSKSRKKVEEALVECAATAACKAVPPGTASSGPLYVRDAQTRYLRL
AGSPLKSRFLPHPSPRLGGRQPMQAMSLCDSEAATHPCAAFVVEVGGGGGATPLSAGSVFRVSFWKPLPRLTTATAGVEL
PEVLSFVRLPEEAPQQQQQQSQQQQRRERKQQQQQQWHQQQQQARGQRGRGGGRFPQQQQAPKQKVRDYYAILSVKRGAS
KREIKKAYHAAAKRWHPDKNRAEGQEARLEKAERNFKLIARAYEVLSDPDTRAAYDRGENVDDPKWAQRQSAQQEAQRRG

GNVRFNAGPQGQQQFQRKGGRGGGGQRRGPRTFHF*
>Ehux_312979
MFGIGMKERDAAEVTVRNTDLASFTALIDYLLSDKFDLGEAVGGRAQRALDLRELAQMYQVPRLELLCAQALQESVAPAS
AVPLLEAAHTTGDGRLLAQCRRFVADHAAEVRASGGVEQLRDFGVAKGLLGDALDQVAELKGAVAARDAELGKVRAEVAE
RA*
>Ehux_361353
MGKMWGDATLPQGMVTFLVGDDEERVEHVSKCALCVRSVFFRTMFGIGMKERDAAEVTVLETDLASFSALVRYLLTDQLD
LGEGEGEQARRALVLRQLAHKYQVPRLELLCAQALQESVGPASAVPLLEAAHTTGDGRLLAHCRRFVADHAAEVRASGGV
EQLRDLGVAKGLLGDALDQVAELKGALRGHSRS*
>Ehux_361541
MFGIGMKERDAAEVTVPHIDLASFTALIDYLCTDELDLGEEEGRAQRALDLRELAQMYQVPRLELLCAQALQESVAPATA
VPLLEAAHTMGDGRLLAQCRRFVADHAAEVRASGGVEQLRDFGVAKGLLGDALDQVAELKGTVAKLKGTVAARDVELEKV
RAEVAERA*
>Ehux_361563
DLAKTRGDATLPQGMVTFLVGDDEERVEHVTKAVLCARSPVFRTMFGIGMKERDAAEVTVPHIDPASFTALIDYLLSDKF
DLGEEEGNRAQRALDLRELAQMYQVPRLELLCAQALQESVAPATAVPLLEAAHTLGDGRLLAQCRRFVADHAAEVRASGG
VEQLRDFGVAKGLLGDALDQVAELKGAMHALRVAES*
>Ehux_224182
MIRLYNTDSGGNKLRLVEVDTGELTSLAGSGEAGDADGVRGAAQFHNPTGVAISPDGSALFVADNRSHKIRRVEVATGAV
ATLAGSGDADDADGVGDAAHFSYPSAVAISPDGGALFVTDSGNHKIRRVEVATGSVTTVAGNGDEGDADGVGDAAHFCNP
IGIAISLDGSALFVADNENHKIRRVEVATGAVTTLAGSGEGGNADGVGDAATFDQPVEVAISADGSTLLVSSVGDLRQRL
EHVSKSLLCARSVFFRTMFGIGMKERDTAEVTVPHTDLASFTALVRYLLTDQLDLGEEEGRAQRALDLRELAQMYQVPRL
ELLCAQALQESVGPATAVPLLEAAHTMGDGRLLAQCRRYVADHAAEVRASGGVLRDFGVAKGLLGDALDQVAELKETVAE
LKGAVATREAEICVNAAVEPLRRLTLDGCAGPGRGWEASSHVTYVIVLSYVCGT*
>Ehux_361588
MAKTWGDASLPEGKVTFLVGDDEERIEYVTKANLCARSPVFRTMFGIGMKERDAAEVTVPHTDLASFTALVRYLLTDQFD
LGEEEGRAQRALDLRQLAQMYQVPRLELLCAQALQESVAPATAVPLLEAAHTLGDGRLLAQCRRFVADHAAEVRASGGVE
QLRDFGVAKGLLGDALDQVAELKGA
>Ehux_224247
MAAHRMSARLSGLSPEQLLAFIDEQAPLWSDAAVRVAEEHARLVEQPEWILSEVLLSPDLAPHILAQLPTKEHAVKGTCR
AWRRGWKQTLRARTGGGEVDEEPVPVADGFDARIFGARVVGQVSTLTLEGEDGPLSLGPVGVASDHAGAWYVATKDSLLH
VSTAGRVRRVAALPASDDYYYTAAASPDGSAVFVADFSGHEIRRVEVATGEVTTIAGSGTRGSTDGVGDAAEFNNPCGIA
ISSDASALFVADHENHKIRRVETIAGSGASGSTDGVGDAAQFYNPHNVAISPDGGALFGMVTFLVGDDEERVEHVTKANL
CARSPVFRTMFGIGMKERDAAEVTVAHTDPASFTALIDYLLSDKFDLGEEAGGRAQRALDLRELAQMYQVPRLELLCAQA
LQESVAPATAVPLLEAAHTMGDGRLLAQCRCFVADHAAEVRASGGVEQLRDFGVAKGLLGDALDQVAELKGAVAARDAEL
EKVRADNEELTAALASQF*
>Ehux_362066
MFGIGMRERDAAEVTVAHTDLASFTALVRYLLTDQFDLGEEEGRAQRALDLRELAQMYQVPRLELLCAQALQESVAPATA
VPLLEAAHTMGDGRLRAQCRRFVADHAAEVRASGGVEQLRDFGVAKGLLGDALDQVAELKGTVAVLKVTVAARDVELEKV
RAEVAERA*
>Ehux_313262
MKKTWGDASLPQGMVTFLVGDDEERVEHVSKCVLCARSVVFRTMFGIGMKERDAAEVTVSDTNLASFTAFIEYLCADELN
LGEGEQARRALELRELAQMYQVPRLELLCAQALQESVGPATAVPLLEAAHMTGDGRLLAQCRRYVADHAAEVRASGGVEQ
LSDLGVAKGLLGDALDQVSELQGAMRALGAVR*
>Ehux_225462
MAVALRTRRTEAMHDSGMFFVNEAIERQSEYVLACLDDQELLKATSQWMFEADIFRAAMGPELCVYRARADAAAASSAPL
VALKTSHDVDNPQLQAEAAMWRAGELCDLTVTVDGSAFAVHKLKLAAASDYFRALLPGDRFADSAEHQLKDMKASVFEGV
LSFIYEGSCNVPEELLVPLLEAASRLQVAPLAEALTAALKERISVANALSTWIFADRASLPELAATARQHTLKHFADVAA
TDDFVQLSHEMLSALLASDEVEADEAAVFTALVRWLRAQAPAVREESTVAALLEHVRFSTMSADFLTEHVETEPLMNTAA
AFKVLAQAMKEKSFGAATPRTTPRTGGPLNPKWVLEGHDAANVSAAGETMTTQKGLVRVDTLIASGTYEVVFKLTEDGAD
LSAAVSGFGIVRDNCDRLESNSNGMPNVWWLRRFHRMVYSENHKEGLTPRGYEFPIGAEARMLVDMAAGQVTFFIDGVEV
PHKAKGITGPVYPCALSYGRKVSVSIVSVRRLKL*
>Ehux_362186
MWGDATLPEGKVTFIVGDDEERLEKVSKNLLCIRSVFFRTMFGIGMKERDADEVTVPNTDLATFTALIRYLCTDQLDLGE
EEGEQARRAFDLRELAQMYQVPRLELLCAQALQESVGPASAVPLLEAAHTTGDGRLLAQCRRYVADNAAEVRASGGVEQL
RDLGVAKGLLGDALDQVAELKGNRTQG*
>Ehux_226236
MTETTYVSPDDLSSGVIAAMCGGMACVAVKSDGSVHAWGAPVPVADGFEAPIFGARVVGQVSTLPLEGEDGLVGVASEHA
GAWYVATMDSLLHVSAAGRVRRVAALPASEVFYTVAASPDGSAVFVADDTNEIRRVEVATGAVTTLAGSGNMGDADGVGD
AAQFDGPAGIAISPDGSAAFVADCSNHKIRRLEVATGAVTTLAGGGDVGDADGVGDAAEFDGPSGVALSSDGSALFVADS
CGNKIRRVEVATRAVTTLAGGGEDGGADGVADAAQFRYPAGIAISPDGGALYVADYENHKIRRVEVATGEVTTVAGSGTR
GREDGVGDAAQLDGPDEVAISSDGSTLLVSSSGGLREVCVAAPPPPPSFAPIVVPPSTLGADLATTRGDASLPEGKVTFL

VGDDLERIEYVTKAVLCARSPVFRTMFGIGMKERDAAEVTVRNTDLASFTALIDYLLSDKFDLGEAEGGRAQRALDLREL
AQMYQVPRLELLCAQALQESVAPASAVPLLEAAHTTGDGRLLAQCRRYVADHAAEVRASGGVEQLRDFGVAKGLLGDALD
QVAELKGAVAARDAELGKVRAEVAERA*
>Ehux_362471
MKKMRGDASLPHGPVTFVVGKEEQRIEHVSKNLLCVRSEYYGKMFGIGMKERDAAEITVPKTDLASFTAFIDYLCTDQLD
LGEGEGEQARRALVLRELAQMYQVPRLELLCAQALQESVGPASAVPLLEAAHTTGDGRLLAQCRRFVADHAAEVRASGGV
EQLRDLGVAKGLLGDALDQVAELKMGAMKKMRGDASLPHGPVTFVVGKEEQRIEHVSKNLLCVRSEYYGKMFGIGMKERD
AAEITVPKTDLASFTAFIDYLCTDQLDLGEGEGEQARRALVLRELAQMYQVPRLELLCAQALQESVGPASAVPLLEAAHT
TGDGRLLAQCRRFVADHAAEVRASGGVEQLRDLGVAKGLLGDALDQVAELKMGAARTAAGQRAAPA*
>Ehux_362661
MFGIGMKERDAADVTVAHTDLASFTALIDYLCTDELDLGEEEGRAQRALDLRELAQMYQVPRLELLCAQALQESVAPATA
VPLLEAAHTMGDGRLLAQCRRFVADHAAEVRASGGVEQLRDFGVAKGLLGDALDQVAELKGTVAKLKGTVAARDVELEKV
RAEVAERA*
>Ehux_110702
MVLDLGGFADGFETSASPLCWRDPPESSLSDWTVHVAAPELPPATYHVHRAMLATGLRRSEYFSRLFTGAGASGLAESAR
RGSSVELQPSAARAFPAFLDFVYKGDLPIDNTSAIALLHLANYFLNRALFDRVTEYVQGVLEGDAADQAAPLYLGEADRF
GLDKAANACVAVCAKHLLGRKPGYCAELAPPVYLRVVSATACATRQHSLALSQHVSAYCNAHSESVDLVLLESLTARLTS
VAADSALDLLGHAVSRHGAEALQSACARAIGEEWEDALLPSVAASLDAPGDQGAEWAPLRGRTSYPPGLQLGLLSHALVH
AGRNLEQTRAQLALREKRIEELEATLSKIKLSSNYLTANCRECGQPARRENRELRIIIANM*
>Ehux_363154
LKKTRGDPTLPTGLVTFLVGDDEERIEHVSKNLLCVRSEYYGKMLGIGMKERDAAEITVPKTDLASFTAFIDYLCTDQLD
LGEGEGEQARRALVLRELAQMYQVPRLELLCAQALQESVGPASAVPLLEAAHTTGDGRLLAQCRRYVADHAAEVRASGGV
EQLRDLGVAKGLLGDALDQVAELKGT
>Ehux_228056
MTRRFNVGDYGGGDEPAAKGASLSWRDDPDETLSDWRIEVNVPDAARNSKTYHVHKVVIAGGPRKSDYFVRQCRGNGASR
LAEAARGESKLDLDAEAAEAMPAFLDYVYTGELQATKENACALFHLAEYTANPSLHAEVKLYLEESLDYTCAPTYIAHAH
LFSQDKLIDAAVEAAAEQLQLYADHVFLPGDHRFDPATADNFSGCPELHDLFALHHTLFARVVTSDHRPEADHGGYYHGG
GYYHQRHGGAGVSWCVVRLVADYCTRRADDIDASFLEAVVDESFLDNLGSDVAMPLLEAAMKHPGSDASTKLRDRCIQKA
GEDWKKTLLPLAQKEKRRREEQAAAEEAAAARSRKKKAAKRTAPPAASSSETVPAMPLGPGLPADMQLKLLGNMLLHQGG
EDPFD*
>Ehux_111164
MPWGSFLPVSVRVCVSAPPPPPTFAPIVVPPSTLVADLGKTCGDASLPEGKVTFIVGDDEERLESVSKNVLRIRSVFFRT
MFGIGMKERDAAEVTVSNTDLATFTALVRYLCTDQLDLGEEEGGRAQRALDLRELAQMYQVRRLELLCAQALQESVGPAT
AVPLLEAAHTLGDGRLLAQCRRYVADNAAEVRASGGVEQLRDLGVAKGLLGDALDQVAELKGTVAALKGNRTQG*
>Ehux_363571
MASPSRKRPRSEEEGEPVAVAAGFEAPIFGARAVGQVTTLVLEGEDGPLASLTLRGVASDHAGAWYVATKDSVLHVSAAG
RVRTVATCEGGRFSRVAISPDGSALFVADAGNEKIRRVEVASGAVMTLAGSGEEGDADGVGDAAQFCSPVGLAISPDGSA
LFVADNDNHKIRRVEVATGAVTTLAGSGEEGDADGVGGAAQFCNPNGLTISPDGSALLVADFCNNKIRRVEVATGAVTTL
AGSGEEGDADGVGDAAQFNGPHGLAISPDGSALFVADCENNKIRRVEVATGAVTTLAGSGEEGGADGVGGAAQFEYPVAL
AISADGTTLLVRMEGSALRQVCVAAPPPPPSFAPLVVPPSTFSADMANTWGDATLPEGKVTFLVGDDKERVEHVSKNVLC
VRSVFFRTMFGIGMKERDAAEVTVLETDLASFTALVRYLLTDQLDLGEGEGEQARRALVLRQLAHKYQVPRLELLCAQAL
QERVAPASAVPLLEAAHTMGDGRLLAQCRRFVADHAAEVRASGGVEQLRDLGVAKGLLGDALDQVAELQKGAPLRSHS*
>Ehux_467644
MTAPAPHRRRAQAFCAQALLLLLLHRSLPVKSLTEGSIGVSEVGSIGEVESCTGALNPIGLVLDFSTDDISGDTNLLGGF
PGQVSTDFTDAANCTGKLPWGTPAPEGGCPCGCGCQCNNPDPAAVEQGGAMLRLRKVGRLYDGTGAAATILDLMVKNTTY
YMPWASDQTGRGTNQTFAQVSLAADQNATFLYRLIANDYACPTGADCDPPAVEVPYSRALAAYDFCFFDFDTGPQSNPSD
PNLGETFTTCGIDTSTSYGGYFMHGRKPNSENPVTKFLCDEIDTTDYMDVTEGAIDGGTDCVRVVAKKEGVGSDNPRLFS
EVLMNFPSCDDGDCAGLCASYDTCPNFTCDQLEIDDDGTTSCGDAADGTVTSGFERSPGVVPDILTCSSKEETIPGAGVY
PSSSGVADRLVWRPYRYTMPKFVCVTYPKNTGEFNVTYKVGKLFQGDSTEAHVLDLVIRNTTEYMPWSSAQNGRSDGDTF
AQISLAASQSLAMRFAFEDSVTGKETQIPYPYDMCFFDMDTGTTIDGVEVLAETLHACGVSSFFHQGKKPADRLGLPARV
ADYICNSTDIAVEDYMEINSTDSCVTVRALAKGIGKDNPRSWSDVLLLDGDISAGPWEASGRCADYNLCPPSGVSGEARG
PGVPGPIQDIITCEPGSTDGSDEAGVYPSTTTPGGNSILARLVGKPYRYTMPKFICLTYPAGTSNFTVTFTVGNNPPSPA
GYGRNMLFAGTGNAECIFDEAPPSQGTLCEPPRPTFVCDALDEKQLTTSLPFKVQVRVLDEKAEAMSSFQGDIMLLAISR
RTCLAEGFERKALGRWQPASSPLHHESQYSHGFDSEVAAPGSSHSLYLSGGNDANFGMTLKLPATALEAEPPEHSPAAGG
GPGESEYEDAAALNGKFQPDAVSFYVRTDNSLADAGHFILGESNEVNRRVAQFQFTRDGKMGLLGTGGTTHGATPYESNR
WYHVMLCFDWKRKTVAFSVDGKLLQRSVPFRREASNFIGACALGNRDRCTTWFDSIRFVQETTLNRVAVSIVDGVAETWL
GPLGEDEEGRSLAARDGFVLRAEDADGQVSELAGPYHPLCRIEGAQRVAINNAALADFNTLLADDASADVTFVVGGRDVP
AHRCILAARCETFRRMFNSSMREGSREEGQRVPIAEVSHAAFSCMLQYIYSGAVAVPEELALELLGLADRYLLEGLKLLC
GFTLTRMVSADTVAPILQAADRYDSPSSQLKAHCMQFVLSNYEAVVNSPSFEELTSSPQLLLAVTRAAAKVVAPSGSSRQ
ISWDSDGAPGSKRARHA*
>Ehux_364410
MAKTWGDASLPQGMVTFLVGDDEERIEHVSKCNLCARSPVFRTMFGIGMKERDAAEVTVRNTDLASFTALVRYLLTDQFD

LGEEEGGRAQRALDLRELAQMYQVPRLELLCAQALQESVGPATAVPLLEAAHTMGDGRLLAQCRRYVADHAAEVRASGGV
EQLRDFGVAKGLLGDALDQVAELKMAKTWGDASLPQGMVTFLVGDDEERIEHVSKCNLCARSPVFRTMFGIGMKERDAAE
VTVRNTDLASFTALVRYLLTDQFDLGEEEGGRAQRALDLRELAQMYQVPRLELLCAQALQESVGPATAVPLLEAAHTMGD
GRLLAQCRRYVADHAAEVRASGGVEQLRDFGVAKGLLGDALDQVAELKGTES*
>Ehux_231698
MIPLAPCDAGFTPTCEQAKGFGLCDATSSACDTTCVDEVKKACPVTCSLAPEETSGHELWTTGAAPPDYDQPAPAERDAA
EITVPKTDLASFTAFIDYLCTDQLDLGEGEGEQARRALVLRELAQMYQVPRLERLCAQALQESVGPASAVPLLEAAHTTG
DGRLLAQCRRYVADHAAEVRASGGVEQLRDLGVAKGLLGDALDQVAELKMGAARTAAGQRAAP*
>Ehux_231699
MSRYPKTIKKSRLVFSDDASHDGGRLLGVFGGAVQPLLRAEGDAIVHSCRLLHAVSAMTGHGRHAEEGEAVPVAGGFEAP
IFGARVVGQVTTLALEGEAGPLASLLRAVASDRAGGWYVSTKEGSLLHVSAAGRIRTVATCEDCRDIALSPDGSALFVAD
CDNHKIRRVEVATGTVSTLAGSGGEGDADGMADAAQFQDLYGVTSPDGSVLFVADFCKIRRVEVATGAVTALAGSGEGGD
ADGVGDAAQFNDIFGVTISPDGSALFVADFGNRKIRRVEVATGAVTTVAGSGAQGSADGVGGAAQFSRPATVAVSPDGSA
LFVADYSNRKIRRVEVATGVVTTLAGSGAGGSSDGVGREAQFDNPVCVAAPPPPPSFAPLVVAPSTFFADMKKMRGDPAL
PTGLVTFLLGDDEERIEHVSKNLLCVRSETEYYGKMFGIGMKERDAAEITVPKTDLASFTAFIDYLCTDQLDLGEGEGEQ
ARRALVLRELAQMYQVPRLERLCSATRSTRSECYGKMFGIGMHWVWRRGPPEVLATSYNHR*
>Ehux_456343
MESSRMPPAGRKRKRGREISAGESGAGCSAPLVDPARVRREPHGAADMLAKMWEDEELKDCCLVVQEEGCEPTELWAHRA
VLVAASRPFRAMLAGSHRERGANRLVLQEVSAAPLAELLRFVYGQEVQLTAENALPLYRTADLYEVLELATLCCAFLKRS
AGVGNCVALLAASDTAHCKPVALHCIDLLKRHFGQVAERASFVSLPGQLLLEVLQADDLSAPSEELVLSAVLSWLDADPQ
GRLGMLPQLGAEIRWPFVPPARLARLEVERSELLDAFPGLACLLREAYRFQALQASNATGELRALLSPRTTRRRSQLCDF
KRLRFHAAIGSKGNASGDFQLPGIFNSGDLNCLRRLELPLGDFNCPEGVALSSDGEPAAAADLRPRCELVVVADSLNHRV
QLFSRDGTFLHAFGRKGRSAGEFDMPGGVAVGTGNGRVQLFERDGTFVRTVSRDGSEPGCLKEPTGVAVTRSGLIVVADY
QNHRVQLFDIAGRLAPPLRPSQAARNLVRAFGRRGSGDGEFCHPSGLAITRSGEIIVADYQNDRIQFFNEDGQFLRCLGS
HGAKAGSFDRPFDVAVSPVEGHILVTDYENHRVQVFTEGGAFVHEFGRHGDGEGMFDSPGGIAVSSDGLVCVTDCGNGRF
QMFDCNPDGRVASGRAGCSSSKPAEPN*
>Ehux_365135
TRGDPTLPTGLVTFLVGDDEERIEHVSKNLLCVRSEYYGKMFGIGMKERDAAEITVPKTDLASFTAFIDYLCTDQLDLGE
GEGEQARRALVLRELAQMYQVPRLELLCAQALQESVGPASAVPLLEAAHTTGDGRLLAQCRRYVADHAAEVRASGGVEQL
RDLGVAKGLLGDALDQVAELKGTVGLVTFLVGDDEERIEHVSK
>Ehux_232263
MCRARTGGGEEDEEPVPVADGFEASIFGARVVGQVSTLALEGEDGPLASLSRVGIASDHAGAWYVATKDSLLHSAAGRVR
RVAALPASDEFYTIAASPDGSAVFVADCDCSNHKILRVAVATGAVTTLAGSRTTGSADGVGDAAQFCNPNGIAISPDGGA
LFVADCNNHKIRRVEVATGEVTTGMVTFLVGDDKERIEHVSKNVLCARSPVFRTMFGIGMKERDAAEITAPKTDLVSFTA
LIDYLLSDKFDLGEEEGGRAQRALNLREPAQMYQFYRAPPLLGVQRRALQFL*
>Ehux_365474
HVSKNNLCVRSVFFRTMFGIGMKERDAAEVTVLETDLASFTALVRYLLTDQLDLGEGEGEQARRALDLRQLAHKYQVPRL
ELLCAQALQESVGPATAVPLLEAAHTTGDGRLLAQCRRFVADHAAEVRASGGVEQLRDLGVAKGLLGDALDQVAELQKGH
VSKNNLCVRSVFFRTMFGIGMKERDAAEVTVLETDLASFTALVRYLLTDQLDLGEGEGEQARRALDLRQLAHKYQVPRLE
LLCAQALQESVGPATAVPLLEAAHTTGDGRLLAQCRRFVADHAAEVRASGGVEQLRDLGVAKGLLGDALDQVAELQKGRT
AAA*
>Ehux_365476
MGKMWGDDALPQGMVTFLVGDDEERVEHVTKAALCVRSVFFRTMFGIGMKERDAAEVTVLETDLASFTALVRYLLTDQLD
LGEGEGEQARRALDLRQLAQKYQVPRLELLCAQALQESVGPASAVPLLEAAQTTGDGRLLAQCRRYVADHAAEVRASGGV
EQLRNMGVAKGLLGDALDQVAELRGAVAA
>Ehux_365532
MVTFIVGDDEERYEHVSKSLLCIRSVFFRTMFGIGMKERDAAEVTVRNTDLATFTALVRYLCTDQLDLGDEEGGRAQRAL
DLRQLAQMYQVPRLELLCAQALQESVLPASAVALLEAAHTMGDGRLLAQCRRYVADNAAEVRASGGVEQLRHLGVAKGLL
GDALDQVAELKGAVAAREADLKKVRA*
>Ehux_365724
MKKTRGDASLPEGKVTFLVGDDEERIEHVSKCVLCVRSVFFRTMFGIGMKERDAAEVTVLETDLATFTALIDYLCTDQLD
LGEGEGEQARRALVLRQLAQKYQVPRLELLCAQALQESVAPATAVPLLEAAHATGDGRLLAQCRRFVADHAAEVRASGGV
EQLRDLGVAKGLLGDALDQVAELKGAMRALRVAKD*
>Ehux_365774
MKERDAAEVTVRETDLTTFTALVRYLCTDQLDLGEEEGGRAQRALDLRQLAQMYQVPRLELLCAQALQESVAPATAVPLL
EAAHATGDGRLLAQCRRYVADNAAEVRASGGVEQLRDFGVAKGLLGDALDQVAELKGA
>Ehux_365786
MKKMRGNASLPTGLVTFIVGDDEERIEHVSKNLLCVRSEYYGKMFGIGMKERDAAEITVPKTDLASFTAFIDYLCTDQLD
LGELEGEQARRALVLRELAQMYQVPRLELLCAQALQESVGPASAVPLLEAAHTTGDGRLLAQCRRYVADHAAEVRASGGV
EQLRDLGVAKGLLGDALDQVAELKMGAARTAAGQRAAPA*
>Ehux_365941
SKIRRVEVATGEVTTLAGSGEEGDADGVGDAAEFCHPYGVAISPDGCALFVADQCNDKIRRVEVATGEVTTVAGSGTRGS

ADGVGDAAEFEGPAGIASSPDGSTLLVASDDGLRQVCVAAPPPPPSFAPIVVPPSTLGADFAKTRGDASLPEGKVTFLVG
DDEERIEHVSKNNLCARSPVFRTMFGIGMKERDAAEVTVSHTDLVSFTALIDYLLSDKFDLGEEEGRAQRALDLRELAQM
YQVPRLELLCAQALQESVAPATAVPLLEAAHTMSDGRLLAQCRRFVADHAAEVRASGGVEQLRDLGVAKGLLGDALDQVA
ELKGTVAALNGTQG*
>Ehux_366377
MFGTGMKERDAAEVTVSNTDLASFTALVRYLLTDQFDLGEEQGGRAQRALDLRELVQMYQVPRLELLCAQALQESVAPAT
AVPLLEAAHTLGDGRLLAQCRRFVADHAAEVRASGGVEQLRDFGVAKGLLGDALDQVAELKGTVAARDVELEKIRAEVAE
RA*
>Ehux_366395
LKKTRGDASLPDGKVTFIVGDDEERIEHVSKNLLCVRSEYYGKMFGIGMKERDAAEITVPKTDLASFTAFIDYLCTDQLD
LGEGEGEQARRALVLRELAQMYQVPRLELLCAQALQESVGPASAVPLLEAAHTTGDGRLLAQCRRFVADHAAEVRASGGV
EQLRDLGVAKGLLGDALDQVAELKMGAARTAAGQRAAP*
>Ehux_366483
MKKTRGDASLPEGKVTFLVGDDEERIEHVSKCVLCVRSVFFRTMFGIGMKERDAAEVTVLETDLATFTALIDYLCTDQLD
LGEGEGEQARRALVLRQLAQKYQVPRLELLCAQALQESVAPATAVPLLEAAHTTGDGRLLAQCRRFVADHAAEVRASGGV
EQLRDLGVAKGLLGDALDQVAELKGAM
>Ehux_114540
MPGAPRPTEQKRPPSSSALAKSTVTYASNVAKHEFVWEVQGLSWLEQQLTQEGRECTNSNIFQVDPTDGYSTYMLVFSPR
GGSLEHEADDDYQVFDSIPRGTLALIRRADDYGTNLCYRFFVRDASLEFQPLGEATRVAVPAREGYEPACFVGGPDLHSD
SKGLFGLSFDELLGSEWVKDDSICFKVCIEEAVMKEHPSGGQIRVGKTDVVYEHKPPSVEVPPPTLQADFLSLLTEGRHS
DVTIEAAYGEAAPVAFSAHTIILSQRSDVFAAAFSHEMRESSTRTLTVTDVPPPALKALLHFLYTDDFDEVQKVLREEGS
SEASSSSSGAAASGGASSGAAEQSIAQLQAVLAAAHKYGVLRLLRWAEGQLCDKLSCEMACSLLSLAHVYGATELERNCL
AFMKANMANVVKRADFAALAPEALVKFNMHCAGVDPAEEEPGRKRKRDDE*
>Ehux_315403
MWGDATLPHGPVTFLVGDDEERIESVSKNLLCVRSEQFRTMFGIGMKERDAAEVTVLETDLATFTALIRYLLTDQLDLGE
EEGGRAQRALDLRQLAQMYQVPRLELLCAQALQESVGPASAVPLLEAAHMTGDGRLLAQCRRYVADNAAEVRASGGVEQL
RDLGVAKGLLGDALDQVAELKGAVARAEVAERV*
>Ehux_115974
MSWSPPAHVLDDDPTISPPPPPPPPSSALAPALSSGLAEVWANGSFSDCRLVPRSGEEFAAHRLVLSARSEHFRELLAPT
ASAVTAAAIRLSVDAERPLVEAALRHVYLEAPPPDGLSHEELLALRGLLIMNGGCDGVSPALLHASADPGAAPLVELLLS
RGVEPLRASKALLAAGASPNTADFTSGQIAIAERQTPLHRLSDHGGGAAAAETLAALLDAGAHINDGRGHTPLHLAAWRG
HFGLARSLVAAGASPNVASTPPGVRAFAAPVPDPSSTRANPFDANAGPNPFAADAASPVTEALAERAVVVTPPRAQGLAA
AWSSPDPDTPLINPFDDDFGPPPGGVAEPEGGTNGNGNPFG*
>Ehux_238895
MASKRPRPAEPDRAAGERPTVTLNGGEPCPAGQVNLWRAGKLCDATVHADGRAFQVHRAVLAGASEFMTALFTSGMRDSD
EPTISEVAGDVFSALLEWIYCGECRVEVALLGGMLDASSRLQLPSLQNATVEALVQGIGTSNAAQYWECGDAHNIPRLVE
AAKAAALQSFGEFSTGVAFDALPVGRVCELLKSDDLMADSEEVVFRAAARWAAANSSASEEEVEAVYSLVKYSLLPAAFL
SSVVEQDPPLATHPRGGWIVARSLKAHIDQAPSPRDPGLPTGWWAEERTASSGRVYKVYYGPNGEYAESGRISRNGSVIL
MQQDALKPASKGRPQR*
>Ehux_367892
LGKLWGDETLPQGMVTFLVGDDEERVEHVTKAVICVRSVVFRTMFGIGMKERDAAEVTLRKTDLATFTALVRYLLTDQLD
LGEEEGGRAQRALDLRELAQMYQVPRLELLCAQALQESVGPASAVPLLEAAHATGDGRLLAQCRRYVADNAAEVRASGSV
EQLRHLGVAKGLLGDALDQVAELKGTVAALKGGLTQG*
>Ehux_239268
MSMRSSCFCGTARRHTPSHQPALAPIFGARVYGQVATLALEGEDSPLGSVGVASDHAGAWYVSTKDSLLHVSAAGRVRRV
AALPAALLPANSIAASPDGSAAFVADYDNNAIRRVEVATGAVTTLAGSGIKGSADGVGDAAEFDEPAGIAISPGGGALFV
ADFHSHKIRRVEVATGAVTTLVGSGEAGDADGVGDAAEFCNPTGIAISPDGGALFVADSDSSKIRRVEVATGEVTTLAGS
GEEGDADGVGDAAEFCHPYGVAISPDGCALFVADQCNDKIRRVEVSTGEVTTVAGSGTRGSADGVGDAAEFEGPAGIASS
PDGSTLLVASDDGLRQVCVAAPPPPPSFAPIVVPPSTLGADFAKTRGDASLPEGKVTFLVGDDEERIEHVSKNNLCARSP
VFRTMFGIGMKERDAAEVTVSHTDLVSFTALIDYLLSDKFDLGEEEGRAQRALDLRELAQMYQVPRLELLCAQALQESVA
PATAVPLLEAAHTMSDGRLLAQCRRFVADHAAEVRASGGVEQLRDLGVAKGLLGDALDQVAELKGTVAALNGTQG*
>Ehux_444112
MCVYMASAARKRARTGGGGEDGDAPVPVADGFEAPIFGARVVGQVTTLTLEGEDGPLASLGPVGVASDHAGAWYVSTKDS
LLHVSAAGRVRRVAALPASGVFYTVAASPNGSALFVADFTRNKIRRVEVATGAVTTLAGSGQVGGADGVGDAAEFCSPLG
LAISPNGSALFVADFHNHKIRRVEVATGEVTTIAGSGDEGDADGVGDAAEFYFPHGISISPDGSALFVADSSNHKIRRVE
VATGAVTTLAGSGEEGGADGVGDAAQFYNPSGVAISPDGGALFVANYGNDKIRRVEVATGEVTTLAGSGTSGSVDGVGDA
AELNGPVEVDISPDGSTLLVASDGGLRQVCVAAPPPPPSFAPIVVPPSTILADLAKTRGDATLPQGMVTFLVGDDEERIE
HVSKNNLCARSPVFRTMFGIGMKERDAAEVTVAHTGLASFTALVDYLLSDQFDLGEEEGRAQRALDLRELAQMYQVPRLE
LLCAQALQESVAPATAVPLLEAAHTMGDGRLLAQCRRFVADHAAEVRASGGVEQLRDFGVAKGLLGDALDQVAELKGAMH
ALRVAES*
>Ehux_458337
MLDTVNMTLTYPQVLQDAEGRSTTPDDREGHTASVVDQQIYIFGGTWEDEKTGVNAYLDDLHVFDVASYSWMKPAYTGSP

PEGREGHSAAVIGRLIFIFGGTRGTWVQEAERTPHRELEDEGESAHGQLPSQREGHSACVVGCATGGVWWGPFTAGDSFL
ERRYHTASVVEGRLLVFGGQYYDYGKDLHFECSNAVCEIDLEQLAWRIHPPSSPSPLKRACHAAGVVGKCVYVVGGRYWD
EQEDDYIFLNDVLDTRPSSSLASDWRRYFNSELLSDVCLLVGDMRIPAHRVVLAARCDFFARMFASGMREATEEELPIVD
VSPPVFQLLLEHLYTDSSAVPEESALELFAAADRFGVERLRALCSDRIEGMLSAANVCDVLTVADQHNGAELVEAAVSHI
VENFGEIHCSPPFRELSRPLLDVVHAAIAPRLVLAAPPAGLAAEAGGSRQSARRHST*
>Ehux_368882
MAKTWGDATLPEGKVTFLVGDSRERIEYVTKANLCARSVVFRTMFGIGMKERDAAEVTVSHTDLASFTALVRYLLTDQLD
LGEEEGGRAQRALDLRELAQMYQVPRLELLCAQALQESVAPATAVPLLEAAHTMSDGRLLAQCRRFVADHAAEVRASGGV
EQLRDFGVAKGLLGDALDQVAELKGT
>Ehux_424715
MLSGGSASFADSQSAALELPELCATCFEAALEYVYTGSCSIDEAGLQTLLEVAGRLQLASLQGVVAEALEARLSVSNCVE
TWSFASDAALVRLEAAATAFVLKHFAEVSLGASLSPEMMGVLLRSEDIEASEEAVFSALVRWLRAQPESAREEGAVAALL
EHVRFAAMDQKFVRETVEQEPLMKNVPALTVLAQALREGLSKEGTARSLPRLSQGGEWVIEAPGAIAAVDGKTLTFRSRQ
DDDDDDDDERIRDGLVRLSVPLAAGRYEVRFKLLEDEVQGDEASCGFGLIKEGASEALEFQPRPNGFGVQNLWWLRRFER
TVYSERETSGQFEDEADDQPYRQFPIPSDVRIVVDMDAGEATFYVNEVEIPHKATGITGPVFPCAMAYGISANVVVSFAG
VQRL*
>Ehux_241795
MLGTEPACQAGFRVGVMASRKRARTAGEGEEEGEAVPVAGGFEAPIFGARVVGQVTTLALEGEDGPPQKLYAVASDRAGG
WYVTTGALVLHVSAAGRTRTVATCEDCRGIDLSPNGSALFVADYGNNKIYWVEVATGAVSTLAGSGERGDADGVGDAAQF
NGPYGVTISPDGSALFVADWGSHKIRRVEVATGAVTTVAGRGTAGRADGVGGAAEFHSPVGIAISPEGSALFVADHSNHK
IRRVEVATGAVSTLAGSGIAGSVDGVEDAAQIHKPRGIAVSPDGSALFVADFGNHKIRRVEVATGAVTTVAGSGAKGRAD
GVGDAAQFNAPLGVALSPDGSTLLVRSGAASLRQVCVAAPPPPPSFAPIVVPPSTFFADMKKTRGDASLPHGPVTFIVGR
EAQRIEHVSKNLLCVRSEYYGKMFGIGMKERDAAEITVPKTDLASFTAFIDYLCTDQLDLGEGEGEQARRALVLRELAQM
YQVPRLELLCAQALQESVGPASAVPLLEAAHMTGDGRLLAQCRRYVADHAAEVRASGGVEQLRDLGVAKGLLGDALDQVA
ELKMGAARTAAGQRAAP*
>Ehux_117587
MPGAPRPTEQKRPPSSSALAKSTVTYASNVAKHEFVWEVQGLSWLEQQLTQEGRECTNSNIFQVDPTDGYSTYMLVFSPR
GGSLEHEADDDYQVFDSIPRGTLALIRRADDYGTNLCYRFFVRDASLEFQPLGEATRVAVPAREGYEPACFVGGPDLHSD
SKGLFGLSFDELLGSEWVKDDSICFKVCIEEAVMKEHPSGGQIRVGKTDVVYEHKPPSVEVPPPTLQADFLSLLTEGRHS
DVTIEAAFGEAAPVAFSAHTIILSQRSDVFAAAFSHEMRESSTRTLTVTDVPPPALKALLHFLYTDDFDEVLKVLREEGS
SEASSSSSGAAASGGASSGAAEQSIAQLQAVLAAAHKYGVLRLLRWAEGQLCDKLSCEMACSLLSLAHVYGATELERNCL
AFMKANMANVVKRADFAALAPEALVKFNMHCAGVDPAEEEPGRKRKRDDE*
>Ehux_242292
MFGIGMKERDAAEVTVRNTDLATFTALVRYLLTDQLDLGEEEGGRAQRALDLRQLAQMYQVPRLELLCAQALQESVGPAS
AGGEILARSAMQGGRTVLAECVPPRRDALRDSADGTRLEGRLSRRHGWQPWREARLRKDQDSSTGKLLADTRAPSS*
>Ehux_369229
GIGMKERDAAEVTVSHTDLASFTALVRYLLTDNFDLGEEEGGRAQRALDLRELAQMYQVPRLELLCAQALQESVAPATAV
PLLEAAHATGDGRLLAQCRRFVADHAAEVRASGGVEQLRDLGVAKGLLGDALDQVAELKG
>Ehux_117715
MLSQSVLSLQVDELNGPALQLYRGLGYEVVGRERASTTPSRYRLVSKLLLGGVRERTLLVLQKPRPPAAAEAAEPEARPE
SSPISPMAAAARKRARTGGGGEEDEEPVPVAGGFEAPIFGARVYGQVTTLTLEGEDGPLSLGPVGVASDHAGAWYVATKD
SLLHVSAAGRVRRVAALPASNVFNSIAVSPDRSAAFVADYSNHKIRRVEVATGEVTTLAGSGGEGEADGVGDAAEFCGPT
AITINPGGGALFVADQDNHKIRRVEVTTGEVTSIAGSGEPGDADGVGDAAEFCGPRGLAISPDGGALFVADQGNDKIRRV
EVTTGEVTTVAGSVNEDEGGNADGVGDAAQIDGPSGIAISPDGGALFVADLGNNKIRRVEVATGAVTTIAGSGNEGSTDG
VGDAAEFNEPLEIAISAGGSTLLVRTKFTVGSRGGLRQVCLVAPTPPPSFAPIVVPPSTFSADMAKTWGDASLPQGMVTF
LVGDDEERIEHVSKCNLCARSVVFRTMFGIGMKERDAAEVTVSHTDLASFTALVRYLLTDNFDLGEEEGGRAQRALDLRE
LAQMYQVPRLELLCAQALQESVAPATAVPLLEAAHTTGDGRLLAQCRRYVADHAAEVRASGGVEQLRDLGVAKGLLGDAL
DQVAELKGAMRALRVAES*
>Ehux_369243
MWGDATFPQGMVTFLVGDDQQRLESVTKAILCIRSVFFRTMFGIGMKERDAGEVTVRNTDLATFTALVRYLCTDQLDLGE
EGGGRAQRALDLRQLAQMYQVPRLELLCAQALQESVGPASAVPLLEAAHATGDGRLLAQCRRYVADNAAEVRASGGVEQL
RDLGVAKGLLGDALDQVAELKGT
>Ehux_316126
MCGDPTLPEGKVTFIVGDDEERYEHVSKCVLCVRSVFFRTMFGIGMKERDAAEVTVRNTDLATFTALVRYLLTDQLDLGE
EGGGRAQRALDLRQLAQMYQVPRLELLCAQALQESVGPASAVPLLEAAHTMGDGRLLAQCRRYVADHAAEVRASGGVEQL
RDLGVAKGLLGDALDQVAELKGTVAELELELEKVRM*
>Ehux_369490
CGATLLCRPQGMVTFLVGDNEERVEHVSKCALCVRSVFFRTMFGIGMKERDAAEVTVLETDLASFTALVRYLLTDQLDLG
EGEGEQARRALVLRQLAHKYQVPRLELLCAQALQESVGPASAVPLLEAAHTTGDGRLLAHCRRYVADHAAEVRASGGVEQ
LRDMSVAKGLLGDALDQ
>Ehux_369504
MGKMWGDATLPQGMVTFLVGDDEERVEHVSKCALCVRSVFFRTMFGIGMKERDAAEVTVLETDLASFTALVRYLLTDQLD

LGEGEGEQARRALVLRQLAHKYQVPRLELLCAQALQESVGPATAVPLLEAAHTTGDGRLLAQCRRFVADHAAEVRASGGV
EQLRDLGVAKGLLGDALDQVAELQKGALRSHS
>Ehux_458743
MQASLIEQELEPADAPAPGPSCAEPAGEFGPRWTPVDFVGGSPGSRVFPSAVVSGACVYIFGGHNGTTYRNDLLVLSLET
RSWTVDSEVDGDGRPTPRDAHAAVLLGGRLYVFGGYDSKRYLADFHSYGLDSGEWRVEAASGSPPSPRGGHAAVVHGSRL
IVFGGCDGWNYFNDTLAFDVQSAEWAPVRVAGTAPGARSAPSTAVHEATNALYVFGGYDGARSLNDLFRMDLRTSEWAPV
RTSGVPPSPRGGHTAVVHGDRIYTFGGKSGRSPFNDLCCLDLTSGVWEAVRAGSPAPAPRCAHVSVVHGGSLLIFGGYDG
RHYFSDCFEWSLEADRGAASQALAGDLECMVESGQFSDIWFEVEGKTVPAHKFLLFARCEYFRRMFTSGYAEAAAQTVRI
EGVSHAAFKQVLHYLYSGRPREMNAEAALEVLAAANLYGVEPLKRAAAEIIGRSLTPENAAAVLSAADSYQAPALRAQAT
AFIVRHFAAVVQTDAFAELVSERSRPLLLALLREVGTVVSPAGLAASDGLLGVAGGLPAGGYARRRPDARSPADAVRQLD
ELGIRGIADAE*
>Ehux_243667
MVATRKRARPAGSCSLDESTLASLNTSRAPLELTGGHAQPARMVQYFREAKLTDVTIIVDGKSYAAHRNVLAAASDYLDK
LFAGPKWADQEQPVKLSQVPGAHTFANILEYIYTGSCMTNESDLISMLEAAHYLQIEPLLRSLQELVARRVDASNCLEAW
KVADTYTLEKLSATAMQEEAPARVDLSNADVLGSGVQRPVPADLLCGWECHFEEIDLHEGPGDSWMKLVYFACIS*
>Ehux_370068
MEKTWGDATLPQGMVTFLVGDDEERIEHVSKNVLCVRSVFFRTMFGIGMKERDAAEVTVLETDLASFTALVRYLLTDQLD
LGEGEGEQARRALVLRQLAHKYQVPRLELLCSQALQESVGPATAVPLLEAAHTTGDGRLLAQCRRFVADHAAEVRASGGV
EQLRDLGVAKGLLGDALDQVAELKGALRSHSRS*
>Ehux_370561
MRGDASLPDGKVTFLVGREEQRIEHVSKNLLCVRSEYYGKMFGIGMKERDAAEITVPKTDLASFTAFIDYLCTDQLDLGE
GGGEQARRALVLRELAQMYQVPRLELLCAQALQESVGPASAVPLLEAAHTTGDGRLLAQCRRYVADHAAEVRASGGVEQL
RDLGVAKGLLGDALDQVAELKMGAARTAAGRRAAPA*
>Ehux_246339
MEASRKRARTGGEEGEPVAVAAGFEAPIFGARAVGQVTTLALEGEDGPLASLNLRGVASDHAGAWYVATLVSVLHVSAAE
RVRTVATCEGGGFSGVALSPDGSALFVADAGNNKIRRVEVATGTVTATAGSGEGGDADGVGGAAEFHCPWGVTVSPDGSA
LFVADCDNNKIRRVEVATGAVTTLAGSGEEGDEDGVGGVAHFNGPAGLALSLDGSALFVADFFSHKIRRVEVATGAVTTL
AGSGEEGSADGVGGAAQFRFPHGVTISPDGSALFVADNGNHKIRRVWVATGAVTTLAGSGARGSADGVGGAAQFEYPVAL
AISADSTTLLARMEGSALRQVCVAAPPPPPSFAPLVVPPSTFSADMGKMWGDATLPQGMVTFLVGDNEERVEHVSKCALC
VRSVFFRTMFGIGMKERDAAEVTVLETDLASFTALVRYLLTDQLDLGEGEGEQARRALVLRQLAQKNP
>Ehux_371011
MSDSSRSRRKRSRHNDPDGAGPSRDAAFKVTVSGGHRYPPKLVEQWRQGTLCDCTIVVDPETFSGHRNVLAAASGYFAGL
YSGAGVVMRERNGGPAQLEGMSPEVFGAALEFMYTGECGLPETMLTELLQAASRLQVEDLLRATTQALALKERLQPSNCL
AIWELAERLGLAELQEHARESATRDFEKLQSSPESLFLSLPHARLATLLADMNLNVESEDAAFQAMVAWARHQQPAPSEA
ELEQLLSLIRFPRMTRAFLVDVEPLLLGHPRGAIIYAQSFREVQFCDDTPRAKKRLVRLSASNVKYTDLKAEMAVRICGE
MGEVKQACEQRAPGASEAVEWNEDMVDYFEERHRGAGLRIRKISQELQAAELEHPSGGSDHFWFPFNVLARAARLG*
>Ehux_246966
MVCGAGWSPATEPKKLRPGSSHAEAPQHSVRCAGPAVQLDAKAASKTAASGHEGPNVPLATPPRRPLAPPPNGPCSSATR
AAETRLSPPKQQQQQWQRDLTSRAIGDADGVGDAAEFYNPRGIVTSPDGGALFVADWSNHKIRRVEVATGAVTILAGSGT
RGREDGVGDSAEFYDPTEVALSPDGSTLFVGSYGGLRLVCVAAPPPPPSFAPIVVPRSTLGADFAKTRGDASLPQGMVTF
LVGDDRERIEHVSKNVLCARSPVFRTMFGIGMKERDAAEVTVSHTNLASFTALIDYLLSERAEAAATPPSTERYQMAQMT
KIKIV*
>Ehux_371358
DLAKTRGDASLPEGKVTFLVGDDEERVEHVSKCNLCARSPVFRTMFGIGMKERDAAEVTVSHTDLASFTALVDYLLTDKF
DLGEEEGRAQRALDLRELAQMYQVPRLELLCAQALQEGVAPATAVPLLEAAHTMGDGRLLAQCRRYVADHAAEVRASGGV
EQLRDFGVAKGLLGDALDQVAELKGAMRALRVAES*
>Ehux_371362
IRRVEVATGAVTTIAGSGEDGDADGVGDTAEFNNPSGLAISPDGDALFVADNGNRKIRRVEVATGEVTTVAGSGTEGSAD
GMGDAAEFDGPDEVALSPDGSTLLVNCNGGLRQVCVAAPPPPPSFAPIVVPPSTLAADFAKTRGDASLPEGKVAFLVGDD
EERIDDVSKCVICARSPVFRTMFGIGMKERDAAEVTVSHTDLASFTALVDYLLSDKFDLGEEGGRAQRALDLRELAQMYQ
VPRLELLCAQALQEVVAPATAVPLLEAAHTTGDGRLLAQCRRYVADHAAEVRASGGVEQLRDFGVAELKGTVAARDAELE
KVRAEVAERA*
>Ehux_371496
MGKMWGDATLPEGKVTFLVGDDEERVEHVTKAALCVRSVFFRTMFGIGMKERDAAEVTVLETDLASFTALVRYLLTDQLD
LGEEEGEQARRALVLRQLAHKYQVPRLELLCAQALQESVGPATAVPLLEAAHTTGDGRLLAQCRRFVADHAAEVRASGGV
EQLRDLGVAKGLLGDALDQVAELMGKMWGDATLPEGKVTFLVGDDEERVEHVTKAALCVRSVFFRTMFGIGMKERDAAEV
TVLETDLASFTALVRYLLTDQLDLGEEEGEQARRALVLRQLAHKYQVPRLELLCAQALQESVGPATAVPLLEAAHTTGDG
RLLAQCRRFVADHAAEVRASGGVEQLRDLGVAKGLLGDALDQVAELQKGALRSHS
>Ehux_371510
MKKMRGDATLPQGMVTFLVGDDRERIEHVSKNVLCIRSPVLRTMFGIGMKERDAAEVTVAHTGLASFTALIDYLCTDELD
LGEEEGGRAQRALDLRELAQMYQVPRLELLCAQALQESVAPATAVPLLEVAHTMGDGRLLAQCRRYVADHAAEVRASGGV
EQLRDVGVAKGLLGDALDQVVELKGAMRDEEDAGRRHPPAGHGHLPGRRRSGAHRAREQKRALHSVPGPSDHVWHRHEGA

RRRRGHGGPHRPRQLHRPHRLPLHRRARPRRGGGRQGAARARPAGAGADVPGAAPRAALRAGAAGERRAGDRRAAARGGA
HDGRRAAPRAVPPLRGRPRRRGAGERRRGAAARRGRGQGAARRRARPGGGAEGRDARGGPREGPGGARRGAPCTGSALLR
RRYV
>Ehux_371726
MANTWGDASLPQGMVTFLVGDDKERIEHVSKCNLCARSPVFRTMFGIGMKERDAAEVTVAHTDLASFTALVRYLLTDQFD
LGEAEGGRAQRALDLRELAQMYRVPRLELLCAQALQESVAPATAVPLLEAAHTMGDGRPLLAQCRRYVADHAAEVRASGG
VEQLRDFGVAKGLLGDALDQVAELKMANTWGDASLPQGMVTFLVGDDKERIEHVSKCNLCARSPVFRTMFGIGMKERDAA
EVTVAHTDLASFTALVRYLLTDQFDLGEAEGGRAQRALDLRELAQMYRVPRLELLCAQALQESVAPATAVPLLEAAHTMG
DGRPLLAQCRRYVADHAAEVRASGGVEQLRDFGVAKGLLGDALDQVAELKGT
>Ehux_371749
MWGDATLPQGMVTFIVGDDQQRLESVSKNVLCIRSVFFRTMFGIGMKERDAAEVTVSNTDLATFTALVRYLCTDQLDLGE
EEGGRAQRALDLRELAQMYQVRRLELLCAQALQESVGPATAVPLLEAAHTLGDGRLLAQCRRYVADNAAEVRASGGVEQL
RDLGVAKGLLGDALDQVAELKMWGDATLPQGMVTFIVGDDQQRLESVSKNVLCIRSVFFRTMFGIGMKERDAAEVTVSNT
DLATFTALVRYLCTDQLDLGEEEGGRAQRALDLRELAQMYQVRRLELLCAQALQESVGPATAVPLLEAAHTLGDGRLLAQ
CRRYVADNAAEVRASGGVEQLRDLGVAKGLLGDALDQVAELKGTVAALKGNRTQG*
>Ehux_248850
MAELIALLRGTQLRSTAAANRAAYDDRLRQLEERLQSLKDQAGALAAVAFDADFALVAGDGTKVPARKQDLCAQSRFFKR
MMEHDDSAEARAGEVIAKTGDGVGASAAALRHLMALLYGQQLQTSTESAVLLEICELATLWEFDSILESMSTMLVASAKA
SAKTACEMLVAALRHTEADAHSEVWKRLREASAQGVAEAGRKLLDVSAFADLDLSAICEAMTHVKASTVRLPSLEVDTSS
ERWTAHQAMDGSPTDQDLSWLLGATGAKVKARLGWNGSVMLTGENTGGRAVLAKVSSWVKHPQRPTNERRASGSYRVLGS
DTQGVVLGRNLPDVLSDGKFLCGGDLEVSKYQRQYELFNLWLLASKRVASPLSPVDSLLCLRGCATGCDVDGASVAELQA
MLEQRGLSTSGAKTSLQLRLRDAAYAEVPEGSAAAKATKAFAKLVARHYKGSAVKVKDLDAASTILDNTISWAQLAGRTD
EIIAKVMPLVRWPLVPLVQLKPPLKALMKRCPVVKELVEEAIGLQMKPGSEFSFTPKRHRLMDGSEDTVVPRHKKRKHCH
GDKVQLLDASALVAAMLPSGLRTLVSASHSADKPADSVRSICQDALCALSCFRGFDHPPDYHSDDAFCEEDGIYL*
>Ehux_371997
MAKTWGDASLPEGKVTFLVGDDEERIEHVSKNNLCARSPVFRTMFGIGMKERDAAEVTVSHTDLASFTALVRYLLTDKFD
LGEEVGGRAQRALDLRELAQMYQVPRLELLCAQALQESVAPATAVPLLEAAHTLGDGRLLAQCRRFVADHAAEVRASGGV
EQLRDFGVAKGLLGDALDQVAQLKGAMRALRVAES*
>Ehux_372475
MKERDAAEVTVPHIDPASFTALIDYLLCDKFDLGEEEGRAQRALVLRELAQMYQVPRLELLCAQALQESVAPATAVPLLE
AAHTMGDGRLLAQCRRYVADHAAEVRASGGVEQLRDLGVAKGLLGDALDQVAELKGAMREADLEKVRAERA*
>Ehux_372600
EWRPPPASAPAPAAARRAGCPCRSRMASRRPSLARAVSSDRSPRWRSRARTALYRSVTEVGIASDHAGAWYVATKDSLLH
VSAAGRVRRVAALPASKVNSIATSPNESAAFVADYSSHKIRRVEVATGAVTMLAGSGNDGAADSVGDAAQFCYPTGIAIS
PDSSALFVTDFGSHKIRRVEVATGEVTTLAGSGESGDADGVGDAAQFCYPTGIAISPDGGALFVADYGNDKIRRVEVATG
EVTTIAGSGTSGSADGVGDAAEFDEPEDVAISADGSTCLRGGASSASLLCPDRHTGRRLPPRGEVTFLCNLCARSPVFRT
MFGIGMKERDAAEVTVAHTDLASFTALVRYLLTDQFDLGEAEGGRAQRALDLRELAQMYRVPRLELLCAQALQESVAPAT
AVPLLEAAHTMGDGRPLLAQCRRYVADHAAEVRASGGVEQLRDFGVAKGLLGDALDQVAELKGT
>Ehux_372834
MAKTWGDASLPQGMVTFLVGDDEERIEHVSKCNLCARSVVFRTMFGIGMKERDAAEVTVSHTDLASFTALVRYLLTDNFD
LGEEEGGRAQRALDLRELAQMYQVPRLELLCAQALQESVAPATAVPLLEAAHTMGDGRLLAQCRRFVADHPAEVRASGGV
EQLRDFGVAKGLLGDALDQVAELKGAMRALRVAES*
>Ehux_446827
MSVSPSRKRARTESDGGGEEGGEPVAVAAGFEAPIFGARVVGQVTTLALKGEDGPLASLSLRGVAIDHAGGCYVTTKNSV
LHVSAAGRVRTVATCEGGELFGLAISQNSSALFVSDLDAHKIRRVEVATGAVTAIVGSGGNGDADGVGDAAQFGEPRGLT
ISPDGSALFVAACGNHKIRRVEVATGAVTTLAGSGNDGDADGVGGAAEFCCPFGVTVSPDGSALFVSDYGNDKMRRVEVA
TGAVTTLAGGEEGGADGVGGAAHFNGARGLAISPDGSALFVADTFNNKIRRVEVATGAVTTLAGSGASGSADGVGGAAQF
NYPMALAISADGSTLSVCAMGGLRQVCVAAPPPPPSFAPLVVPPSTFSADMANTWGDATLPTGLVTFLVGDDEERIEHVS
KCVLCVRSVFFRTMFGIGMKERDAAEVTVRKTDLATFTALVRYLLTDQLDLGEEEGGRAQRALDLRQLAQMYQVPRLELL
CAQALQESVAPATAVPLLEAAHTMGDGRLLAQCRRFVADHAAEVRASGGVEQLCDLGVAKGLLGDALDQVAELKKGATRA
AAA*
>Ehux_251852
MTAVNVNSPDEDKIMAAKRRRGSDESSTQSSSAAITLECSTAASAAIREDGLRTAIAAQWREGTLCDVDLLVEGTAFKAH
RMLLAAESPYLSALVTTAMRERAGPIELKELSAASFAAALSFMYTRRCTLGSQDALQPLLHAAHLLRVPPLLAAAEAAVA
ERIDASNCLRALGLAEHLSLAALANTAQKLALQHFGTAAEAEDGAALHTLSAEQLGDIIASDDLAVSDESVVLEAVKGWL
AAQQPAASQATAGALLAHVRFAELSKEQKVALDDEPLLQQHFKIVATAYREKMHGEDTPRARPRSGSFRCCLQYDDLRVG
MMVKVHPDRAFVEEQCRKVVAGATKNCGWAVNMTSTCGSTYRVAELRENSCSARLDIPRVIWHLPYTVLQIVCDA*
>Ehux_373118
MGKMWGDATLPEGKVTFLVGDDEERVEHVTKAALCVRSVFFRTMFGIGMKERDAAEVTVLETDLASFTALVRYLLTDQFD
LGEGEGEQARRALVLRQLAHKYQLPRLELLCAQALQESVGPETAVPLLEAAHTTGDGRLLAQCRRFVADHAAEVRASGGV
EQLRNLGVAKGLLGDALDQVAELKGTVAARDAELERNRTQS*
>Ehux_122601

MSRAAPLVAAASPVNPAASPVIPPPAPPPPPPRASPPPPPTALPPAPLSARPEVIQKFRDGDLCDVELQPAYGPAVRAHR
IILVGVSQYFKALFSGTWSGSGSHDLSALSPACLHACLEFAYTGEAEVADEAALSELVEGASYLQMDELLVSASEAYEAR
LSAKNALAVWQLAEAIGRLPRLAEASAWVARVNFGE
>Ehux_373528
MRGDASLPHGPVTFLVGNEEERIEHVSKSLLCVRSEYYGKMFGIGMKERDAAEITVPKTDLASFTAFIDYLCTDQLDLGE
GEGEQARRALVLRELAQMYQVPRLELLCAQALQESVGPASAVPLLEAAHTMGDGRLLAQCRRYVADHAAEVRASGGVEQL
RDLGVAKGLLGDALDQVAELKMGAMRGDASLPHGPVTFLVGNEEERIEHVSKSLLCVRSEYYGKMFGIGMKERDAAEITV
PKTDLASFTAFIDYLCTDQLDLGEGEGEQARRALVLRELAQMYQVPRLELLCAQALQESVGPASAVPLLEAAHTMGDGRL
LAQCRRYVADHAAEVRASGGVEQLRDLGVAKGLLGDALDQVAELKMGAARTAAGQRAAPA*
>Ehux_373642
GKVTFLVGDDRERIEHVSKNMLCARSPVFRTMFGIGMKERDAAEVTVSHTDLASFTALVDYLLSDKFDLGEEEGRAQRAL
DLRELAQMYQVPRLELLCAQALQESVAPATAVPLLEAAHTTGDGRLLAQCRRYVADHAAEVRASGGVEQLRDLGVAKGLL
GDALDQVAELKGT
>Ehux_373820
MIRRVEVATGAVTTIAGSGERGDADGVGDAAEFNNPSGITMSPDGNALFVADWSNNKIRRVEVATGAVTTLAGSGTEGNA
DGVGDAAQFDGPDEVAISPDGSTLLVSSNGGLRQVCVAAPPPPPSFAPIVVPPSTLGADFATTRGDASLPEGKVTFLVGD
DRERIENVSKNNLCARSPVFRTMFGIGMKERDAAEVTVSHTDLASFTALVDYLLSDKFDLGEEEGRAQRALDLRELAQMY
QVPRLELLCAQALQESVAPATAVPLLEAAHTTGDGRLLAQCRRYVADHAAEVRASGGVEQLRELWRGQGVASGTRFDQVA
ELKKG
>Ehux_257922
MVATRKRARPAGSCSLDESTLASLNTSRAPLELTGGHAQPARMVQYFREAKLTDVTIIVDGKSYAAHRNVLAAASDYLDK
LFAGPKWADQEQPVKLSQVPGAHTFANILEYIYTGSCMTNESDLISMLEAAHYLQIEPLLRSLQELVARRVDASNCLEAW
KVADTYTLEKLSATAMQMALRSFEQVSTSAGFSGMPLPFVRQLVSDDKLIAQEEQRVFEAAERWGGGCCVRQPTQ*
>Ehux_258654
MSRADDERPPKRRRVLSDAEDEPTRELQRDPLDIRGSLLELHQNMPSFSDVCVVVEGSKSSQEFACIGAVLASASRPLAA
MLYGESSRMVTPSAGSSRPRLVLRGTEPWCFDLLLRYVHGLQVALDVDMALTLYHAADYYEVLPLRDGCCAFLLDA
>Ehux_374581
VTFIVGDDAERIESVSKNLLCIRSVFFRTMFGIGMKERDAAEVTVRNTDLATFTALIRYLCTDQLDLGEEEGGRAQRALD
LRQLTQMYQVPRLELLCAQALQESVGPASAVPLLEAAHATGDGRLLAQCRRYVADNAAEVRASGGVEQLRDFGVAKGLLG
DALDQVAELKGAMHALRVTGS*
>Gthe_99531
MRRSGGLNISSSASRLADDPRGRSKSPGGLRNWRNAEHEGSRNGSGHFLSPSMPAREQDAAILQSVSGDDVKIRQGKALL
KPSEIRNRTPGKKRQKDWGECPLTFDKIKEPVRAMDGNVYERWAIKKWLEENGNRSPLTNVVIDPTLTPLQDSDEGTVKG
SRFVSSPPRDKDGVKTEINTVLNTVRDVVNVDESLYTDVEPVSGKQDATPTRAPLPRAASASPAPAPAPSPAPAPAPAPA
PAPSATPLLRAESLPSAGPAHTPSGGEGQDDAGNRNKMIAEIMRDTSLTMAEKNAKIQDIRAGKLKPSAPDGAATSEREK
AERVDKSQQPQVISPNEVHIPTTKTSPPGAPKVLSHRKPVQAETEERRGEEKDVKTPASSNPSRGMPKEEVERTALQTDE
GGNGADKVGVGITFGQFHKGSLHRGALYVKHLQDGSSAHESGKIRVGDVLSMVDGKDVYRSSVDLIQSSVLGPVGTSVEL
VFKRHTAGADLGNQEFEDMETIISRLIRRPPAPGQQFGIVEETRKGRKAEEPEAKASPHSSKAAAKPPPPKPRGSDTTVV
HTMERKKVELMQRHSLHAELNQSLEIRSEKPPFTLFGKENSMVTWMLESNHFLDLSIRCSSNELVWAHRVVLAASSPFIR
HALCQDAGSHTEMVLHDCPRKEVEAIVHFLYHGRLLVSNAWELIDLFLVSNSLEFECVQEASEDGLIAMITIATCCELLE
LSSKLGSARVVESCRQHVVNNFEEVSRSSSFLRLDEKTLESFLQSEQLVARGEEVVYEALVRWILRQSEGRDGEPLRGEK
LFRHIRFATMSSSYLQDIARSDLIARSSRLRSYIQEALALKKSGAQAQARPFVGDKDASPAKSDKKDEGNSSMTCDS*
>Gthe_134570
MDFFTRTGSSDKKKKGEKAKSKKGSSKDQGSAEGIASFLQSLGNSISGSVGNGSIRAKSINKNQEASRASTKSKRGAKSK
VARSGIVAKSGINAGNQFSRENVLMEYRSATLGTQLTEALADDTLADVTFIVGPERKVVKGLRIVIAARNKFFKSMLYES
KMEDGSLQATFELPSAHAVSFDLMMKFIHAGGISWEPHQTVQLLEVSIDFQVEELMKKCMELVSESMTDNLSLAELTKLI
QILLRGEGFDVNLQHPKTGTSALHVASREGGVELVIDLLSRGANLNWTDIHGATAMHLARNAEVAQLLINAKADVFAADK
GGNPPLVYSIVHKRKDVAELLLAMNCGEALFRSVQEKNDTISRKLNKGRVTPITEVKTSPTMEALLKVIEQEAELYPLMI
SPPSLIEVARAGSTWGTKSALARDEDVNQRDDREAAKLSLPGYTALHWAAAMGHFGVIQCLLSSDDIEINPTDTEEEDGY
TPLQLCINYRQRQWEACAALMRAHGAKERASFESDEDEDEDEEMEGASMSKTSINRGKTSGSSAPTTAFFSRVFNNRITS
AVSKIVARPSTSASKKWQSSKRQGGSRKVRSSHSSSKNSSRK*
>Gthe_135627
MASFRGGQIEVDSDDDRAGEAGEGEEEEEEEEEGEESLLEMRKPEAWMLEHGHFSDVTVTCQNQSYHLHKFVLCRESLRF
QEMLMDPASENDRIQVDYDDDDFELFVHLLYTGENLSGDFDADLVFKKMKKLEVNPTRSWDQPALKYFKTRSIAQIVSSF
ENYLGDDDIGLLPVEAMNEVMRISHGRKPKPPQDFLFCAISSRNIVLQDLKREVNRVNLRQAMSLYPSIGYDQVCDLLIQ
CVKHGADKEIERCIPVLASNLPEVDRGKVLKISKRAMLDTLRHEAISDMEHAYALGKEFLLTSSPEIKPLIDKMDGQGIV
SGDRTKRPRAGTEEWYLFEILEALLSLRKKIKLRKMEAARNLPSFRILLVCAETFEDYREDVDIFLAHLRNPSLEQLLTY
HSVLLWSNADFHDPEALGDVLADAVEGGVGIVVGAYALNDGEGRMCVSGRLLQFMPCHAGRVSGGQELTLRRCEAQLARF
PKCGEMLEGVRSFSGGEESMHQSLEKKVGMDCSVLVAEWSSGAPLVLVSLPEGKRKGKVVVWNCRPGSSDASFYGWRVKT
DGARLMFNMLKFATATHTDSKFVEAD*
>Gthe_105602
MLLAIPRIVLLCSLQVALIVAEHHGAANMLINDARMVHSKRIQSRFGMLYELTKDQKGPGLMGSLGLPCLQRLRGGKRKA

KHSKYDDDSDDVTDMSDSADSLTSSYTSTEYDETEYSDEDQDAKALDERDHNREEGYDDDSSAHEYFDYDHAFGKGKKPL
ALRDTETRDETEAEGQNILTISSEGHVQYLAKGLRELQESSKLIDITIKGQGGVKIAAHQAILAAASPELKKMLENLPAG
TKELDMQSMDGESLTVVFDYIYARKIKVSEEKLPDILGISQKLGISGLKDTCYFHLMKDLKPSNALRMRHLGKTLECHEL
LDAANRFIMHDFVAVSKSQSFNDLTEDALYEIIGSDDLFVESEKVIFDSVIRWVEFNEANRSSSLLRLMGQIRFGLLPLD
TLAKKVAAAPLIHKHLANNPQWQAMLNAAIAYVGSTPEQKQMLESSQTKVRLGSKGRYLVCAGGRKGKNSDALKSALMID
SITGEGFVLDEMHIARKQVSAAEADGIVYVAGGWDGHKYLRSVEMFDLSSNSWKKGPPMLTARGSFGMANLGGMICAAGG
YDGHKHLSSCEFLNRTMQVWQQMAPMPHARSGARLAVLGDHLYAVGGFDGKNVLNTVEIFDSVSMKWVEGPPLNHARRDH
AMAVLDGVLYVAGGFDSKHDLATVEKLDPKTNTWQLVAKMQKKRSGLALHVLNHKLCAIGGYDGLKYLSAIESYDPRLDQ
WNLEHDVYLPSRMAHFAAAIV*
>Gthe_136651
MKEEIYGNGELLDLEILSKKKGCDQMTAKRAHSVIMALVSPVLRRCKANRRVIELQLEEVDSNVLEAFVDFVYTGSAHRL
DVTSLRETVIRESSKMLDVDTCVAFLNASRDSGLPELEELCFQFALDHYEEDIVLSILRWLEGCHQDHEAEKGETSCSNA
RAEEDGVSADSDNQGHGMNEEMELDCDRSRRQFSSKMISSIRWNLIHHQFVEDLMLKCASSESCSLLAQFVGDLSKFPNS
YRSHRGGSGPGYDVIRRLRVTREMKHHAGAVAALAFHEGMIVSASHDGSIKLVNLTSGKMEGSLHFTPHPRESQRQLGAV
YAMLQHGDVLVTAGQMKLGIAPLNVWNLLTRRHVKELEGHKDAVMCLTRNEVDLFSGSWDRTVRQWDLSSFSCKRVISSQ
HGPVLSICSLSRAIVAAGLESAWHLVLDEPRKEEEKEEEERKESLPLVFFSGSDPVTCVAVGSGPRATTGGSSWIFRPEE
RIFSGHKSGAIRVWCSETLTCVANLQCHDDAVSALTAYGSWLVTGSMDGSVRMWNFTQAFACNKGEGGSQLAAAVDKPER
RESAKTIVIGSEVKSLHLIKTNACERGSRSADGYILCGCQDRAVRLISDGFARNEEA*
>Gthe_136945
MTLTFLAHLTDLAMAVVNDDLEEYAMEQFQNSLLRSLSENEGFWDVTLHLGREGSGTASVNCHRHMLAAHSSVFAQLLMQ
AQSQQLQGLASLSGKQSNRISLRVECDPDAFSDILTYMYSGKLSLNPEHAIELLSVSTYYQIEPLVRAYSQFAESYLKVE
TVNVLLASALQFNLSDTVDRRAICEHYILRNATPVMLNPTFEAVPEPYVCRLLENDELQIQEVDAFSAVVRWGKARYGDI
DPNVLREKLANIMQYVRFPMMDSKDMADSVEPLNLVDQSLLLEAYRFKATGGDLRNTRGDASRGNRRFIPRRAISDQAPY
ANSSSRYPMAQVPFQSRPTLSAQPTPTPISQPPSQMQSQSQMQTQTPMVGVGLGLSLHSSLPCMLVTEVAPWCQIQSGDL
QKIEVGDMLKSIDGWPVNRSVDAVKKHLLGPPNTLVTLGILRRGVDYEVVVVRGSSDRMSSRANSGRNTPYDSYRSPNSI
APSRSTMNDTRKQLEDAKNALNAFSRPSSMAPGSNYQGERERLDVV*
>Gthe_106331
MLPSTFRLVLKFLYTGEAELSAENLHGLMRAADVLGMEQLQQLCRQRVKQLLNISNCASIWGLAEDLGEEEVESKCREFV
LDNFTRLLGSEGFVRAVRGRQVLELLRSEDLLLFREEDAVGFIMRWTDLDRTRRLPDAAALLREVRMGLLSRECMSELVE
WLEQLYAPAPCDAPLLLLLKLLRDSHQQDLDAMEKKVRSLLESEARKEEHEQEEQEERAGATRSCLLSLSNSFRERGSKR
RFLLAIGGKRGDLEVAVMSRYDTRRKVWIDMEPMSGGARYAHAVCSLRDQVYVAGGHLHSPLDTLELYDARDERWEKRAT
MLQPRSDFAMLVGGEKLFAIGGETTDVEVYDPERNQWSDCCPSLPLSHRSCRGTALNGRLFVLGGFDQEEDILSAVHSFD
PIAMTWKAEPDMLEPRLACGAAVCCGAVYVVGGYNQLRGHLKAVDCLDPRVGRWQRTASCHLKRYDLSLANCGEMLYAMG
GCDVEDACSAVVEVFDPRMNRWTFDPNMPFPLQYFGLASFESR*
>Gthe_138195
MSYPRKRQRSESPDAQCSTPVHGSGEAVLDPLFHSHRSKELVSLWESRELCDVDVVVEGTRFQAHRVVLAAGSPHFRAMF
TKNFSESKAREVELHEITAEGFQGVLSFLYSGEVSLRDSTAELVLLAADRCEVLGLVNLCCSFLLDRITWRNCLHYWSLA
EQVVCLKLKRKARLVALKHFEVVLTLFLQELYHTGLLLDLDAAKMSRIIKSKKLRASNEQVVLEGLVTWLQHDFESRRHV
AESMLRCEEKKHEEEEKKLVRLPRLAENTLRQLLNTTFLRDCSTLDVLIADVQKYQTMTEEDQRNYSEVWARKRWNECGW
LYAVGGSDGLYHLDSVERYEAVEDVWYPVAPMRTARRNCGVGVLNGHLYAVGGRNENKQVMDNIERYDSKEDRWERVEHP
MQTARSLEILRVPSSRGGDKRNSSLQTAECYDAESGKWSPVASMSERRYGCGAGVLDGKLYVVGGTVEKNGDYLETVERY
DSETDKWESVAPMSTSRYCCGVAVMKGKLYAVGGVDKRYNKLSSVESFDPTTGAWSPEPPMLTARYNCGVEEAAGNLYVV
GGRDEKNRALCTVECFDGQTHQWRTVSQMSTVRSSGAVAVLPPMR*
>Gthe_138531
MEDPFDAFQSNLLRTLREGDPYVDLQLQLGRPGSSAGTFHCHKHMLAAQSPVFSHLFQQMSLPNAGKATLRVECDPDAFN
DIILYVYTGRTQVRPDHAVEILNVAQHYEIDALVRLYFNFLENYISVETVNSVLCSALQFGLGQVIQMCERFILVNATQT
FAHPSIEHLPEDALCKLISNDDLQMQEIEVFLAVVRWGQSRFKNASQQTLQAKLSNAMHYVRFPKIDVHEDPCDGKDLIR
TEQGKDMAEVVEPTGLVETSLLLEGYRFRATGGEQASHVLKQYQFPPESKVPFATLSTSQYFSSSCHSSRTSFLVTSRSC
RDPNPVKILTCMKRIEVGDVIKAIDGWTVAWNVEAVKKHLMGPKDTRVELLISRNGVEYRVYVLRCASDGHRDMNAERAM
QDFSMSSASSIKPFDLSRSVNEARESLRNLQKQTAEDSSLGQDDGPLEEV*
>Gthe_139415
MDETFNESSSASANGVVETPDNSFVLSGMNSSDRSGSSWNSRFDVKRFFNNKQYSDIVLEFTCEDGSVQIPSHKLVLSQS
PFFERAMNIGMCEAENNHLVIRDVPPQVGMAITGFFYGLDFEITEDHLLKLLTTLDRLQICELVEETSERLKKLLNANNA
LKLASGIAEGCPSMHSSWIPICSAFISGHEDEIAETDTFLDLDLDTVMFLVSVRDPPIFVSPVSFVIRWLQKQQAVSDFL
RHSLSKSSLTNNDKVLSGILRDAIFSFDSFATLAKHIGYDDAKKYMEEYLADNISPFNAFAFVSASFSFKNCDVTKELNT
RFDWHELVCMEYVRLNAKEVLENEGIVSLEADIVRTLAKEDRIFAHEDDIFRAVLRWATWQTSFGEEEDGEHEKRVKKLL
KDTGIIELIRFPTMSLNFFHQVVEKSGVIPSRLLLERYQYHHGSLTYCKENVRLHNRLPMWLGKGCYACNDPSHPASCER
WVIACSPSDNGMGFNTERICYKGPEIRTCGGDLWGVTVFPPGTDEREIRGSTLAHNEWAVNIRRLSQDTNWTLTQCSTGL
SRRFNSLNETFWDLGSAPSISPSLIVPHSAPLLSSCSFSPLLAFL*
>Gthe_110021
MSSDRPEDSNHLDASFDANASFESCTSQQLQMYASEHRDDDLTVDSPARRSSLESQVGQKRKRSTDADDCKVPILVKHQE
HAAELLQGLSQLFQDESLCDVTIRVEDEDFRAHRVILASSSDFFKILLVGGFRESKEEVIKLEGVDKRSFKWILDFIYNG

QLVLHGTEELVNLLVASEHLGFSSVRSICVQRLQQRLDLPNALKLRLFGQEVGCQELIEAASEVVFNQFTQIASESEDFL
QLDLSQLCDILASDNLAVDSEYDVFEVKANSFLIYKREPLLGQLLCNLRIGLLSEENINSILEEPMVKKRISGTSACPEI
QHLFSSVPKAKRMKLDPDNQKLLARLKNPKDQIQQDSKLDTKQCKNRKQRITKCLLALGGRPAWTKVERLDSKTSSWDEI
APMIQKRMRHGSSSVKGMVYVVGGKDETGRALASIERYNAYQNSWKLLSPMKTARTGLGVAAVAGVIYAVGGRNDSGYRL
NSVECYNVQTDNWSVCASMREARGAVRLGALNNILYAVGGRSEKDAAMASVEAYDPVTDTWCNVAPMRTCRVGAAVEVLE
GYLYAIGGKDDFGNKLRSVERYDPTTNSWTPVANMGTKRWGAGVAVMDKKLYVLGGMNGAERGLLPTVEVYDPVKNSWSE
LKEGPKLARGSCTFAAL*
>Gthe_56353
DVTILKADAIRTSNSMQLESDLLRLSEDPTMSDFTLIVEGNKLHVHKAILYARCPHLRSMFTSGMIECEKKELVLEDVPL
VHFRKVCQFIYTGSVFVDQEDAMDVMMLADRFLLYGLKQQCAAVLKSSIAMENVIHILKCADRFDAPDLK
>Gthe_113641
MPPMMAQELERLSSDMVKARKEEKLLTEQGKQQTLTFLVQGQEEARVLALRAIVAARAFDGKLEGAGSLKTVSEHGEGGE
GEAAAAAAGEAEGDEDTIVLRDLKVELLEHAIHYMYTGSFEVDEGNVHDMLKLGKRMSLPGVINICSSFLSERCSSSSVC
KALASAEASEAAELKEFCLNFMDRSAKDVLGDPSFVELPKEVLQEVLRRDSLEVEEVDLFAAVVRWGKRQAEIVEQEARR
MAEAAAEAAPSGEGNNEGNEGGGGGGGGGGGGGGGKRDNRPFLEIALSGVIDHVRFPLISPAALRDVVQPSLTVEHEGEK
RCVAERYVLEGYQHHALTKIGQPSNVEEERRRPRKNQSSSQAEVNREEEEEAGRVEEARRRAGRTVRWLENLPRQAARVS
MRGMGLGYADAVVTAEHVKRGGRSLEEMDLKNNDLDLSSMQVLLPALGGCSGLKGLALGDNPIFTVRARLSRR*
>Gthe_142949
MQGREKGCKQAVPLITSSLQGMPQDEQGYHLRMKGSKRLARSETQSNGSGKHKDWDPLQAIQDASWPWGNAKREGGEHEN
EASHKSEDLETSSRAGSNTHSIVPSVRGHQENPRPVSGQQSDEDVVRGEYLQSQHLAKFVGFPYEDTVPGEKIETTTFQL
CNEPWALWICPSGMTESESEYVSVFLAYKGSGSVRAWFSFVLLDTEGRSTVIFQDNELEGSVFEGYDDHWGKQKCILREE
LEEVAPEGEFAIQVNLRLFESDRASARNVSVMEEEEVRSVFSEATFSHKSSVKHVTIPATTLTYDLEMLLHSGMGSDVTF
VVGTECEHIAAHKAILIARCPVPILTDETMHLREINVPDFRPDYFRVLLHFIYTDKDQIEEGMLDNMADELLSAAQRFGL
TRLKALCEDQLTRSLTVENACDILAIAIDDEADQLKARPPLLSFPLTHICAGDVLQLHLQILQGSV*
>Ngru_XP_002683354
MSSSKHPGRSIKYLLSRNEINNQYPHLYDAILTTSTGDSIPFYQLFIQNNAPELQVLKKEKNGIITYKTTYDTDITIKAL
EYILTGKIDKEFPQYKKVEEFLKTSGTSTSKWIKYSMPTIYEKMPLADLALKSKYLKQPIQMHRLVLACHCTKFRNVLSQ
VSNKPTPPQSSTPYIAPENFAKYMRERKENMLSNPMEIDLDQFQPSSEKHIRAIIQYCYTEDFESSCEKQGISSDEDLTE
LFNLAERMGLIGIVGEYAKKMSTSITLKNVGRIFSLATRGEEQDIHDKAVHFIFTNWGILRKGELDGQMSSDEERHLFEK
VSGIADMFCLHKKNGMM
>Ngru_XP_002682386
MIPSQQQEQTSGKDEDEEILNLLPKERKSTNNHSQNHEIFSMCKVGNLNRIKTLLPTIRDPKNFMDSHGNSMLYYACLCG
QLNVVRYLSDLGHRDDRFGRCFLNALSLEVRRMLKLYNNYKSHRSTGGGSDEVDHDDDLEYLMKSYAMKSIAEQETSFKK
RLEEESSLVTLESFLQETENPKDDILLRMTFKGSKSVAEYKCSKWILLSRWPQLVLFGLQQKAGQNKKRTSLITEELKKS
LTHSDMDLYPFNLTATELDTLSKNKNSSRYGKKVPRLKIEQKDVKEKFKYSTIPTTQEVDQELAKEKNLEQFNKEFFTII
DFGLVPFERRMFEIVLVWLYCGKMVNMQQKNSKIFFLEENFVTCINELAQVAHSLRIRTLAEHIKNTFLSFAPQGTKFPC
QVIKTMEQEFFKIMNDQIKEEFNPLPLCKDPNNEKESSLNSLKKLTFNMAITFKSENPTETTPRYLCNKQFLMDRSIFFD
VIVNCEFSDGQEFRKQEANNEIPLITIEQCNNLEIFEQVVRYLYSESIQPTKQNVIDLWSISHLFNLQKLARICENFISY
NVSQEDADMYQLYKLAHVIGSAKLKAEWERQTLVYLKEQIKLKKLDSESILTELNELGVDPEVLHKFKIHLTGY
>Ngru_XP_002682074
MLPNNQANSGQLLNALVMMMNNNNGNNNNNNNNGNNSMNEINSLLNMMKNNSNSNNNMNSIINLFQQQQMQNNQPSPSN
NNNQQAMGIFNNLMNLIQQNQQQNNTNQQQNMLQQLLNQQNSNQQVIQVSSPPMNNQFSNMVVNQLSNNNNLQQISPNSS
SILQIQQPTQSPVSTSSSSTNNAGQAGLGQSSITESTAQTFISKSNTMGSSIKQFFNNPILSDVTFIIQGKKFYAHKLIL
CARSNYFNQLILVKCSNTSILEIEIQDASADIFYNILEFVYTDCTILRSDKIWDLYQAAKFYQLSALLSQCQEFIIGTLS
VDNVFQQWAKAQQYGTAVVAEHCLLFAKSKYEQVLKMLDLYNNINISSNPDIISRAIQSQQQMMGSMLPPSATSTSQPST
PNSTNGSANGTPKGDIYQSLTAFTFPPPKKDEETLNTYKFPPQKEVSSSDPETTSNTEEKDRHELTQTFELWGEENTPPA
AFPTRYAMDMYPSFFRIEPQEKVASSSTTPVVENSAENGGDNNNTQKKENHVKIGESTTEKGCVYRRLSVLRQDLHSVGV
FFQHIISCITRMLHAERTTLFMYDRQTDELISQVAIGEDEIRIPSDRGIAGSCFQSGKSVNIPDVRSNSKFAPEVDKTTG
FSTKSVICSPIVGSGNQFVGVLEVLNKHTGNFSERDEQILSFVCDLLSIFVQEQKLDEIENIHKTSILLQPSSYRRKSVA
PVTMARKASHRMSISVDDSFALLFDDGDFNFDLVNPDEQPHSLLSVNNDIANSLVGPNIDPLWNYVQNNDVNSNLTSSFV
SMNAQPMSNFGSSSSIVASGQVDNITDTNVKSETKSVTPPLTTTTQPVEKKRKRNQNNNSTNNKAANAGMPFMHFRIPRY
ELQFIQFDAGGVKKKKKT
>Ngru_XP_002681714
MHVIVKFHHPVYVNEVHIYETSFPGSVVKISCKVKQQNEEAVVPEKKSFLRKAIEKVIGKDGEWVVLFERPDAVFVTGAQ
DFSPELSNKGTYSDTIRIDMKLGANWSEIDAIKLVGKTKVDLPKITKLGAIYKKMFDDALFTDLNIVHKQSGNTLRVHKS
IMTSRSEYFKVICESEMIESKTSIIEFEEDVPFNTFKDVVEFIYSNHININQENIYQVYLLADQFLIGSLSTYCGRLFST
WMDGENAFEMARMAKKHCCHKLNEVALEYIVDNYTLLFLNPQFEDLEHEDVITICRRLAERHNSKDQEANPKPNTNTSET
VSECTEDPCSEEICDE
>Ngru_XP_002681708
MNLDLCILACSSTDKTQQLKMSALQPSVEPIRFIGQKSESGNTPTINVPFKSIVSHAMASTIVDPFSNKNEDIPFSLFLF
GGQINNKRSDGMFECRIEKMVAYDEKEVQDLLLETKVANNSMLTITEFITCEKRDGCWPSARSHHQMVYRPPNDEHIDGS
LVLFGGQDSQFKALGDLYEFDIHTREWKQIVEQSGQLWPTRRHGHSMIYRPSKDSLVILGGVDTLNRTIFEPFEYLFPEK

KWKKIELIAKTGSALSLTSTSSIDPLCLRPRANHSAVLYNAQNSRFVQFHTLNNAETVEDGTQDVMIIFGGNNEQDYYND
MFSINLGTLNVDIIMSSHRINVYPRMKHTCIITRGEKALVIGGNRKARYDEMKVVSGKTSKSFFMSVLGSIAPKSSIDAP
ILNGMVARNLITDFTVKNQEAELDFNDISHHTTCSVSPSCFIFVGGQFNNVGEKMTTNTKNDKIWVCNLGQYSETIEKLQ
NAEQKKEFGKEFYRRMLDNLGEYGPIGYDLRVNVLNEKTFLVHKFMLSRFEYFRKLIKEHSSHQVGGIVEIPISDASVDT
ISNVIDYIYGIEPPVFSNPEEVQILTKYCEKHSMGSIVKYTHGKVYLQRSDYESYLPEIQLLLFFKRVYMGEFPELHQYF
NSTVRLITQGENAENTNIDIQLPLQILVNNDFFHGCLRDLYIKHKLHGQDSQNYIIEIDNSIFPFLDTSYFPIIERYIAT
LYQAPVHIFSSEGEAVVSQLIQLYLVSNLLSDTYMEVLIMDSIKRRISFENAFQLLCFAYQHQSPNEVVFIYNECIRKLK
EVIPLEDVNKIINFLNQEYAKQFDI
>Ngru_XP_002681175
MLQQHKTSGLGSPLSPMSDIYSFCKVGDLNKIRSIIKTSDLDRPDRYGNTPLYYACLCGHLQVVKYLSDLGARDDKFARC
YWNALSLPVRHLLKVYNKYGGPTQVKKAQKKTEEEEDEEIEQMYEQDQFKSLDPFMMKMAKRVIYGTEKPDECDLTIRIR
FGKDEEDTIYYCNWWIILKRWPMLLYFALVNKERPIPMKVSTILTSEDLKVVDLKTNREIKKYFDSMNKEVLMVDVYNKK
IKEIKKQMEIANEKKNYQRPQDDLQAIENLIPDSDREIREFLKREELQVVDLGQVGLTKSQFDNLMVWIYTGCLLKTENQ
KKFMKLENLGVFVQRTEYCDWMETLKMYTLVDMFKDYLTKDIKVAMKESDSQFKNDLKGCIFHNNYEAISKFERLQRSLC
DVQIKIKNYLEEVFFVESTEAILCHKHFLSERSMFFNVLLNSNFDETERMKESALHEDIPTLEIQGCNFSVVQELCNFIY
SARFEQLDESNLLELFSYGHRLGFPENTINMLEDRTLTLTILDEELVAFCKACFPIFKDASKRAMEKPLYEYLSKFSEQE
RFDTLIELDYTADDIQRLDTCYQNN
>Ngru_XP_002681121
MTEKSGHDVTFQLKSGNQITAHRSFLASRSKFFEKLFETHQNTYPLEGLQDDTLQFYINFLYTGYPQISILPKPIDSKLI
QILQVDQNNSLRNYITGEAVYITDIYLITRSIDDDIRKLYNLAIQSKNQSKKAINNSSLICIQLMEENTKTDCFFVDKTF
MRRIDFFRNILDSGMSEAETGVITLEISSECCEKLIEFMYIGQISNISDSALLELFIISKQYLHCQELVHYCENNLSRII
QNLTPQVAVHIIDYFRDTCHAVDDLISRCRYYICTNFDNSREDILNLDSDSRYELSRLYFDYLKRKFKRK
>Ngru_XP_002680950
MTETKQTFYGCGQGFWSTLSALPWTKDIQPDQSTFSKIDFLEYYSFHDPIIQFEITSTYSYEFCCFLTKSGKVYTLGTRN
DCGWLGVGDLDSRQIPTPIEFEDLNNDDQIVQIRLGEKHALFLSRLGRVFSCGSNSTGQLGNPLRKECNKPVEILQSLFD
SPIRKIFTGPSQSFFVSEKTTVYACGNNNIEQCFLIDDGDFKMTFQVNTPTSIFCNLCLHTSRSFPIADDIVGGETRSVF
LEKGQKATFSNSDYMIVEYANNRPQHDPCYVKPAAQLVAEGVKKIVLGKSNIISLHSNGECFVSGAGTLLGIGTREQVYP
FEKIPLNDEYIDDVWSGPYHVILKTSSRKLYAFGSNRFGELGKNSKTVSFREPQLINLPFEWKEGEDVRVICGNNSTLVM
HETPLLSTQELIATNTVMLIDELRDCSQFFHVENSKIHVFLPLIKRISKDLHSLLINREFTERLKAEEGEILFRICKFIS
CELSLATEEEFTNNFQSNEATSNTTKEEESYSDRLFIYFDRFLSKLSEENIEKDLVTCLFILNDISDVGIYFKTFLFNEL
ANRITVENVMDILTWTSNYLSWKVLKNSETSILLLLVKKCFAFIKANYNECHNRYGSLDSFLKIKPKIDQTKKTFFAVQV
QWKEDNILMNLEQSLVTLIEDTSSDLKVIIDSNSEQVMNLHKCVLAVNSPVLSTMISNLSEDSTINLLEFILMMSDNLNE
MFFEHCKKSRCEILRIVLEMCYGKFEEYDLNEWICILFISHQFQMEFIVKHCCTELIKHLSVGNKSNIHLFFYLATVLEV
DSLCKDFVETFVIHCARLFTPEDLQNILKMYSEERFTPTFVKRVRLLQDLIYKKHVEK
>Ngru_XP_002680778
MESGTLFRYLCSLAHRSLGLDTIHNLMKKGEDEFHLWEHCDLNDSFDSLVYGACDRLISVQCRFRDQDVDSTYYFHPLVI
WQRWPMLLGYALNSELKGEIKREELAIVTENDLFLATLNTNKQIEKYFGSINDKVLDQLEFRNQIRQLKLEIAKAKSNIE
QHSSIFVKHEKKIQNLHECLKQFDETGLKSLLLPTSQLWNIHLGNIDFHKQAFDEIVFYLYTSKFVNLDNDPTESTLLPP
VRSLEDLILGKDKEYFQEILTLLNSLGIQPINSLLYKDSASLNNFESESEIRKAKIYDTLFGMLKEQLADGIFFTTTQPP
NNPLETLKKFMCNLIIEQQPPEATDDHVDEFTELPEHSEEQIFGHKSFLMKHCPFIDVLINSDFQEAFQIRETESKNEIP
VIHVECSNFQILTDIIRYIYSNRISINKSNVFELLLLSQKLGLDDLTELCEKSVPEMDFFDFELVQVISLTISLSLPHLK
NIFEQKLFSQLKTMIKGRLMGSDEAQVILEQCGYSFEEVNDLLVQLIWAD
>Ngru_XP_002680314
MSSLCGIPEPIQYLFSHRIVDCAAGEGHSAAVSEDGRLFTWGLENRHKPVMVRNSLIDSEVCASVNCGTHHGFVLTESGK
LFGYGKNSDGQVTKDGSAHISEEKIVEVKHPYAESGSRWVQVSCGMRHTMALDNLGNIYVWGRIKTAKTEKNDHLSDANS
EIMNPTMVSIPISKGNIKKIASSYSSFIVLTNDGNVYSWGHNLHFNLGHGHGKYVNDPTPVGSIAETYIGDVFVGGYNMF
LVTGMSFTASYTKLLKETQYYADCILKSKNGHEAVCHAAILSARSPLFTSLLIKEYLESNNNQSSKFKVTDTSHIERVIT
LDFVESQEELDELLNFIYTNATGRSAKNERMLLIINEHINNTQAATKILTELLKKEDISDENKKDLATMIEVVSKEMIHE
KVKGSSSLLGELSDYTRDFENLYKLAMRESSGLNDPAADTIFSFLSDVSFSLDDGTFIRSHRCILCARLEYFRAMFSIGQ
FSESFQKVIHLDSTTRDSFEILLKHIYTGGNINDNELTAEQSVPLLELATRFNIDTLKLAAERKIVLYLQPSNVLQMCQV
AEMCQAEIVKKCCIHLIARNFTNYTQEQLEAELDQNLIETITIVHEEFTLARSKQQDNEQKVFQYMKEQKDAYDSLLEDG
ERQHLVDEYDEDDVQVIKSILGGSHNPNAPVHANVNRHHQKPSSSGNKSLGMNNNNSTITSSWFKESSQYHSIVNFFPPF
IVILLNNMIHIIRSHHHHYPIHHLNHDEEDIQSVKNQNFGKCYNIHGNTLDNSLELLLKKNSNNNYDDKNYNYKNNGDED
DVCFERTEIKNDNNGVDDDGVEEGRIVDKVITSRENCDDEAFHSKLLPKLVKTEQLGLLAQTCFGKEKVLRLLLLMIISL
PIVISVFQLLPSSPSSLELLNEFRRNNLVEKFWNLTKGDALTYGNNGTTLSINTISEKFTHCRVLPLDGDKKGRAILQWK
FISNHTFQNYYPLFNDTQSSRFQNPNVNPVVRMRIIIGEANLSISAGNFKLFGFSKGITADAGFDKIYNNLQTSKPDEKG
VQYRYFFDKNIKDNRKIKVLDFMNQPQATFHAVYGDGFPSFYVGYDNSSNPYLRKYTGTITTVKNYDHYPTCNDDVNMAL
SPTAVGERVNLNGTYSEYSKFCVYQIHDVTPYTGGTYIYFEKRLNSIVRPKNLPNTRTFSEDNNNTTDHKYFIYFMPIVN
AGYKNTTMQGQQDFLNSYMNKDSLQSYYLAHEVDFSVDYYDEDCDYYVQIYQTSMFQIYYYIRAVRHTIVGIFSLLVVGF
LFIYRQKQPVKSRLHTAMISAVISVIFNALGVISDIYSVFNYDAIEYSYYLTLAMLYLVSLIRYTFVRNLYRVIRIVDKM
EDTVISVGEFNDEGLVSNYKRKTMQMRFLSKLASKRIYLSAIISLIIIVNLPTPFFLWILNSSSGDTFSVVVSIIGYVKL
AALIVGFVLPGFIALSFDLFINRKWLFYDFCKRIFNFGDSNISTYMSKVLSKTRKMEELSCGFQTYFSDYDDPLCYRSEF

LIVTSLSLLIGISTFVVGMIDIPNWRTSLKLTILGTHGTLYFLFDYCYVLLTSGFVVFMQLIVERRKWKTRTSVQDKKAT
ITGKNPITVLLIKTIGDRTGEGRELIYKRCCKVLELLKDQKYKTDPQTMNKKVCDIYNSFVKTSSSLEVAIPYELKTKIS
AFMESYIGDEYSVSTDSSTFNHNKKFYAIFKTEQQQPPVVGPNVSGVDNRSIELINYQTAAFLSDLIDQLKESTLSSLQD
TFSRLMATKAYMAYFKKKEMEAQTMKEVGFTREDTIEIDTNALAFIESQHQQQQPSQITTIDMGGTQIVASINAAQQEQV
SKRYLQAYDNNDEIAISEESSETEDEEIIEEVDTRHSLDKIDD
>Ngru_XP_002680312
MKRRATTEDKKSRLKKLKMQLLLATNANFKGPSSVFVSSNGEVYIADIGNHKIRKVLTNGKMVTIAGNGQNGFSGDNGPA
TNAMLNYPIGVFVSPYNEVYIADYFNNRIRKILTNGNIITIAGNNVNGKGVYNRLAINAMLNRPSGVFVTLNDEVYIADQ
NRNKIRKIESNGNIVTIAGNGKEGYGDDNVPAVNAMLNRPSSVFVTSNNEVYIADTGNHRIRKILRNGNIVTIAGNGLVG
FHEDNILATNTSLNFPKSVFVSSNGEVYIADTGNHRIRKILANGNIATIAGNGKEGYGDDNVPAIDTMLNRPSSVFVSSN
EVYIADQSNQRIRKILQNGNIVTIAGNGREGNSGDSFFDISRYPHIGITSTHIPKLKKQARDQINILDIYGFIPLIEKKS
PQFCKIILQNEYLLSKINLCQREIIQRLINSLIYQNDNLEFMEHEILNVLSLLGLFENGEFINLKKNLVKKLVESITVER
FTSQWEQIETHLKFCNEDLSLQNKIFEYIHEYFVEYAALRLKSTENSVPLGNSPIVLKNSVSIIQKMAIPQKTIINLDEK
VEIPPRGLVDLYNDEKTGDIKVKLGLDKYLYCHKTVLSSSSIFFDALFDSGSSFSDLSDDGVFVPDPSEDLELLEIVIKS
CYGIPFKFDATKQIELIDLALRHEISNLLEFCKNNFVLTTANYYKIAISCKNYLHLDSFKDFYRELLLFGIEHCKELFND
CEKVSFLPEQILHALFVQFSLERK
>Ngru_XP_002680301
MSSLATSAQLNRPNNVFVSSNNEVYIADCFNNRIRKILENGTIVTIAGNGTKGSSGDNGLATNAQLNRPYSVFVSSNNEV
YIADQGNNRIRKILENGNIITIAGNGIHGFSGDNGLATNAQLYTPCSVFVSSNNEVYIADQGNHRIRKILENGNIVTIAG
NGIHGFSGDNGLATNAQLNSSYSVFVSSNNEVYIADYFNNRIRKILENGNIITIAGNGTHGFNGDNENGNIITIAGNGIH
GFNGDNGLATNARLNHPFSVFVSSNNEVYIADYYNNRIRKILENGNIITIAGNGTAGFSGDSPFDIRTYPHIGNKLLTGN
GNLHSKIEFHNLKKHSRDELFGLPIYGFIPLIEKTSPKFSNILKNEKLLCLMSSSQEIIQKLIDSLMYDKNKVKFSKEEI
LDVLFILGLFKNGEFIDLKRSLTSEFVNSLTLENLTFKWETIENLLKICNEEFNVQNYILDHLNNYCIEYAVVQMRTKKG
NTLLPTLPIIMRNATAIFSKMIITQPTAINIEETIETEPQIIESLYNDKKTSDVKIKMGQDKYLYCHKTILTSFSTLFNA
LFDSGSCFSDLSDDGIFTPDESEDLELLEIVIKYCYGISTDKIDDVKIIGLLDMAMKHEIQYLVDYCKNNFNLSIDNYFT
ILEACEKYLDLQSFEQFLREIVKFSITNRKQLFRDHTNRIYKLPEKVCQEMLLRLSQDF
>Ngru_XP_002680171
MSSLATSAQLNSPFGVFVSSNNEVYIADYGNQRIRKILKNGNIVTIAGNGTAGFRGDNGPATNAQLYNPYSVFVSSNNEV
YIADFSNHRIRKILENGKIVTIAGNGTGGFSGDNGPATNAQLNNPYSVFVSSNNEVYIVDYNNHRIRKILKNGNIVTIAG
NGTGGFSGDNGPATNAQLNNPMGVFVSSNNEVYIADYYNHRIRKILENGNIVTIAGNGTAGFSGDSPFDIRTYPHIGNKL
LTGNGNLHSKIEFHNLKKHSRDELFGLPIYGFIPLIEKTSPKFSNILKNEKILCLMSSSQQEIIQKLIDSLIYDKNKVKF
NKEEMLDVLFILGLFKNGEFIDLKRSLTKNGNIVTIAGNGTAGFRGDNGPATNAQLYNPYSVFVSSNNEVYIVDYNNHRI
RKILKNGNIVTIAGNGTGGFSGDNGPATNAQLNNPMVVIHLGPATNAQLNNPYSVFVSSNNEVYIVDYNNHRIRKILENG
NIVTIAGNGTGGFSGDNGPATNAQLNNPMGVFVSSNNEVYIADYYNHRIRKILENGNIVTIAGNGTAGFSGDSPFDIRTY
PHIGNKDELFGLPIYGFIPLIEKTSPKFSNILKNEKILCLMSSSQQEIIQKLIDSLIYDKNKVKFNKEEMLDVLFILGLF
KNGEFIDLKRSLTSEFVNSLTLENLTFKWETIENLLKICKEEFNVQNYILDHLNNYCIEYAVVQMRTKEGNTVLPTLPII
MRNGVAIFSKMIITKPTAINIEETIEIKPQTIESLYNDKKTSDVKIKMGQNKYLYCHKTVLSSSSTLFNALFDSGSCFSD
LSDDGIFTPDESEDLELLEIVIKHCYGISIDKIDAVKIIGLLDMAMKHEIQYLVDYCKNNFNLSIDNYFTILEACEKYLD
LQPFEQFMRDIIKFSITNRKQLFKDRNRISNLPEKVWQEMVFKFSQDF
>Ngru_XP_002679813
MENKDPDLDTAISLCKTGKLGELRNLILSGRVKQNVIDFHGNSLLYYACVCGSLDIVHYLSALGLRDCKYHRCWWNSLSL
EVRNALKLYNSYKTNEEANTKPKKKDDYSLNGLLDRYLKRIIYQSQNNEDYDLILKLKFADQQAPIEYKCHLWIILAKWP
EIVYFVKNKIDTRVFPIFSCEDYLLTLKSNKKIEDEMKQKYPKETPKQLYSIVKKEKQKVQKLKESYSLFNMNTPIDEYI
EGIPTDMEGLKEYIQTNLTFIDFGQVPLGKSLFESILTWIYTGHVPQQLEEQSILASVALRFKIEKLFNTIFGYLKKEPI
LVNNQSPSDLSILPLIEPFEKMDIFTKIRRVTCNMGITLIENPQEIFIPCHKNFIMDRSEFFKVIINCQFEEGNKFREQE
ARKELPIIELYQSLPKETLILILNFIYCEKIELNKGNFVEFLTLGELFGMPSLVKQCDDFLFKSRFNGEEIFELYKYALN
CDSESWILHLERNALEHVKRESFKRKNPWNLEQVVDDLLKMGANPKSIAKYIGV
>Ngru_XP_002679718
MKRTLDENSDSETDGENNPRKKSSSCNTSSYLATDAKLCYPFGVFVSPTNEIYIADQYNHRVRKILESGRIVTIAGNGKG
GFSGDDGLATNAQLNCPSSVFVSNKNEVFIADQYNHRIRKILESGRIVTIAGNGEEGFSGDNGPATSARLNCPMSIFVSN
MNEVYFVDSNNNRVRKILENGIIVTIAGNEELGFSGDSILATNAKLNGPVSLHVSDKNEVYFAELKNNKIRKILRNGFLE
TVMGDGFSTTSVKVQSPTSVFVSPQNEIYFSDNTSNRIRKILENGNVVTVAGTGQQGFSGDGGPATRAQLACPTGLFVTL
NNEIYFADSANNRIRKVLENGNIVTIAGNGKHGYSGDAPFDFSLHPHIGSTNLEKQETVLLQRDIYFPKIKLKKYNRDRL
FGLDIYGFMPLLENFSPEFSKTVLRNEKLLCEMDSSQREIVQKLIDSMIYNDDKMNFNEEYTVNVLYILGFFKNAEFLDL
KQDMVTKFINSLSLTSLSSVWEKVEELLKICENLGLSNFILEKLNKYCTEYTAVQLKSPEGSKILSNISIILRNSVRIFP
LMAISEPSPIIIDEIVEKFQQGVNSLYNDKGTYDFRVKLSEDEFLYCHKTILSSSSSIFETFFQNQEIDNIKDGFMVPDP
SENVDLVKIIIQACYRCGNIEFNSNQNMSLLSTAMKHEMKYLIDCWLKNFKISVDNYFEVVSVCKNYLHLPCCEEFYQLL
LMFGSVYRAKLFTDCDRVALLPDQLLRALLVQISSDGFIFNR
>Ngru_XP_002679529
MDMIKLLLSCSQINPNIGKVEHVNDGSMVISPLGLAVKKGLTEIVKILVLDPRIEIGTGCVKYETFGATNYTCLDLACGE
SLFKVVELLLFHPYVQVNLPLLHHHTHLRRYPLTVACENNCIEIVRLLLARPEIDINVTQDPKHNNCIDQFSLNTPMFSI
EEIRKVGFNYQIIYDLIVAGLTPSNPTLLKDYSLEEIKRRKFKYFTNRIIIQKFEWDKIGDTNLTAFVPLLKIMDPELET

ILKLGVNFDKEEERFVQTVIDFLIYGSDIDFRQLFQAYSNKGHNANIKGVIGALVLCGSLPQINSLCYECISILIKDEYS
LDMVFNIWKIIHEYEKRFPSLLQSMNKSLVGKIKQHVLDCIGNFGLKKDANISEFLSDFDNYHEVIEALMKRRATPSFPK
FSKNETISFPFSSVKLFDQLALDSTTDIELQIGKERVKCHRTILMSKSTYFATLLNGNWNSQNDNYPIEMKYLIQFCYGF
LEYVEPKLCIPLMKLANMHEMKELAIQVEKQAIITRINVAEYLNSLLNDVEEQENEIIQEFIRRIVLYK
>Ngru_XP_002679477
MIKLNYDQELDMFICGGSGIEFTSQISANQPLIKTSLVELYFPSKQSSSGKTNTTQTTDSSNKDQSTNSDDKKITPPIQY
GAIPKFLQKTNQNNIVFVSTPYSEGVIFHNYKEIFLCGHVLKDSVQTLHKVPVFSETDNSIVQIVTCRHSMFVLLSNGKM
YSLRGNEMVVYLDQEKRALDPKCLLQEIPFDKPIKYVSNGGGKHFFAQTTDNELYAIGDDTFGQLCLSKSSIRSHNGFSN
NTKNVDERKELDDYESTTFYPKFVKVPIDKTISMQDNEIEKIECGLNHTFILIRNVQTGAKSLYSSGCSYYGQTAIGSTR
NRNRLSQVELNCSNEIIDIKCNNNGSYVITPRALFYAGINDQNILSVNKAYSSVLTEVPLTTQIASNDEFSIMECGYNHC
LAVTKRNRIFGFGRSSSNQFGIGLNNYICQEIETKELFDKKIVAICCGHSHSIIFSQPLVAPVEDEVQLHEDLIKLRSSS
NVWDLQIFTASKDFSVKIPLYILSKRRPELLSYNKSKAPKSKDSLLYLIDWIYFNTFIEPSLQIDSLIDVLEWMTVNNVQ
DESLTNLVLNESIIRTNEENVYDTIQYILNRKYPKEESPLSAFWHHALSFCKQYIVENSFCKIKNSSFLKQSILVEIAKL
EDPNLLECRSLSEVQESLPQTIPEIVSEIFDSPQLSDLQIVLDKSRGFVVYCHKSILETRSNYFNMKKYGFQAATINEID
VSCLFTIFKKNEDITFEDFINLKSLLKYLYTGRIEITTKNALTLMLLWEAMGMSEKSPLFETCKDFVCRGLCIENIMNVI
TKVVNREKKSTPTESPSMIGFGIFRNLIQEEESHLQPYNYSIIETECLNFCANKWKEILLIYKHDILNFLSKEQLLKITC
IQNRVSL
>Ngru_XP_002679438
MLNNRIIPTTPYINPKLNYGAAVIANLHLDQVVEVKGSHWYCDPSVVTKEKIFGPTTTLQVGGMDAIDTAFVKSVQNSMF
FRFDLDDEFVVNRNYSLIINGNTLQVDKELVCKYSSVLKQLIDSSSSSEVVIEESQGEYLQHVLTFIHEGKLGVESSKFS
SQQDAGLINLKQLIELIKIADKYQVESLLVEIANIVNDMIAMIIIEKCKTISNEGKKIVFEKCYEFIGFNPASVVAAKVY
NDQPIITNFLEDFRIKMQELQLASEDMIIDYLFSENGYDVNVDITNNKKTFAKMIGALKTDASLIAFSELDAETLMGILG
SKFLSMKTEVLLSVLVGWYLADTSRLEDACKFLLANDWSDCEREAFNLLFYNIFIFGAKKHENTELLDKLRDKLSQNMFK
KENVMGDEWHVYNENRPPRSAYRNNSGDLRVLLLGLDASGKTCILYMMKLGERITTIPTIGFNVETVNSCGKNLTVWDVG
GQDKIRPLWRHYYSNTSIVMFVVDACDRERFEDACDCIQRLGSEDDLKDCTILVFANKMDLPNSASIKEIVEKLELNRLK
NPNWMVQGCSATQNIGLEEGFKLVIKAHQYQSQNTAVIEQLTSQEHEKPPRTTTHFGLFEPKFNTF
>Ngru_XP_002679183
MAVVRLDQINDHYYKLFNLNVPKDLVNNIKKAIVSQDLVWIIDNDGKLYECKFSGTMSPCYCKLFDTREINEPILDIVGN
DNHLFLTTKSQMYGLGGNGFGQLGLGSTKFKESTCQPITFFDRINVNSSKYKDRVVKIACGEAFTLFLTNTNKVYSCGSN
YYGQQTFPMESAFLPTPQRITEIFKENQKVVNIHCGQTYSLFELDDGSFLCYGRLGSFLVKDPKIVPKLQEFDRIDVVIK
TISKANQDNFFVVHSVKEGSFQCVEKNSQEIILNCNEQHFKLFHIQYKATNSVKVYQGDKLIRTITINSDPSRLRVCFTS
GENILIYMSNSTSQRKKTIHELILGKSKEFKLPSDTINVGSSPVYAFLPFIQIRNPQFYDLLRTGDLFTSDRSPTIIQDL
IDYCFSQDCSFLRSYTSPMVAELIYLLEKMGVIYNEFTAPLIFQSSVQISASEDFAPNADAYVVKLKQEGITPKLFGNSQ
FFNNQTPAYLPPIGSVLGKLFNSESSADTSIVIDQFGLEKVKCHRSVLIAQSTFFEAFYTGHFASNEINLFNDEDTGELD
INTEDMSEEERQMLIDPESQIKTRALRNILMMFYEIEPTVEYELVIPMLDMAHKLDSENLVMKCLNMFENNLDDESYQVV
NQWLGGLEQKTDIQNMILKTADTWASQVSQKIILFENYELEEEFGKYIRKLNNFEICQEFIPNFTTHYVATKLDKKDYNF
LCAVAGNCSIVKPEYLRKCSTSLKGKHG
>Ngru_XP_002678806
MIIAIIAALSSSIVIVIVGLLFGVYSSFKIVKPFHNLIDLFASVSNMDLDNIIVLPSPFREVHQLQLHFKTMVVKIKQYR
CFIPPHLLAQIDAEGAGDENDRDFEKPSIRAAKDHNTTQSSLRSGKSSRHSGISRSGNATSNYHSTSNQSSFRPVSKSLF
KLGLEKKKITVANFYFVGLDYCMENLSDSELVTVMSDYFEVIQKVAKLTLGQLGSFEDNSMTISWNSTSEVSNHEEKGVA
SSHQILLKLSQLKETKWKKKEHLVKKDLLQNVDFRASILTQVCRCGTQEIKTYSIVGSYLHNLHKIMKHGLKLNVKLIAS
EETLFKSNDTHQHRLVGVKKVLSPDETSAEFFSDSQKKKQKQKKAKKEKELWGDDVSPMPTTMKDYFAQLSQDCSEQEAT
ILDHEPVQHCKKERNPPSEKKKEKKKNAQPASSSVAATSSDVVDNDENNETDRGKTQESKKKKQMSNSDYLELLESSLDD
EEKMIYSNSSRITTTAAMTSYIYATPYKVLPPVQPPKRIELPPTFDKIEDLLKELSLTFEYFKVQDNSTSKTIYLEKLVH
MTDSLSKKCFQKADKNAIFMLGGLNFVCVLFAYFASGENENEQYEKIINSLSYVLYRFSDHNRARSAMLTTGVFYSLWYV
GGLSKNISTLSHVNEVIDLLGGQLSIGFELSNLLTKSNSQLLDISYISTEKTVDSSSQTIYSDGFTFPDTFLGVGTDFVS
LSENNFPIQLHYSIIISRTSNFLSDFCVKWDPTTGQVVENSKNPGFYLMKDSNISWYALLKFVQLLYTDKISVCKEERER
ELKNRNSIFESKLVSEGFPRLLTTLRNQKTRENAINCMSTLTSMEDHEECDNGGLFDEFDNELVNDELNYFFGKPLNYWY
LPKPKENNFSTDFLRKKEKIRQQAILLSGLEQDLGSLYVEMISSQTFKAEEAKALSFYPNVKVMLADTLGEIDHILEEHI
NSIPVLKNIIDCKGFYLHKSILATRSEFMKKLMQYDLEQTENGMDVVYINNFSLSTLFTVFQFMYGGLSEEGFPLLFTEN
LVECFLAADMFLLFGLKKYAEQKICEQITLESVQDLYHISIIYNSDLIKDKCIEIMSKNNIIYESDIKKLNSTGSNGKSL
SDVLKNDRESLLKNPIQLIQLIHHEIDHMVEKSRKIEGELSTHFLNKDSSMFVKKEDDDKNSSSPITMISQFVEKLNGSE
GGVLASFPINTELVEKFKGSSDILLKAFDTIKDHLGDGSLVSGGCMFSIVACCVAAMLEEIENNAKEEWQKLYSEAKETI
DEFKQKISSTLVETIAKLKEIGTETLNEIKRSIQKGCELASSFPKEVREEIENIAKRHLEEFKNVTNLMKHLKEKNFLTN
QFEQFHQEFKALVTSFADRFIKELLGNIIGTIVKFFKSIAQVFRGLIKESFGIIIEIVKKIFEFVKSAPERLRKIFDFSG
NCMAML
>Ngru_XP_002678676
MSNNLISVPCFDCTFSECFNNAESFPDVIFETLEGKIYAHKFMLYQNRYFKNLFDVHEKVLTPRGSSSGTVSNSSILTIP
IQEPLKIFKSLIEYFYSGVVNCQVNDLSEFIMMAYRFKIINLITVLKKILMGEAATQIIAKTKFEKTEILNLPENTKMTM
ENALSLFGEVCGNSVLETTVFKTEITLAKRVILKSVCFSLLFSSNTTIEKMLKFSVNSMNELLLLIAEHLDLIKAVQKYL
VDVSIINTPLNMKEIIKFTLKWMCYMKSDRLISISDLYETLNAIEKSIQQPKEVAEFIKSIEVMRNMASKNQNKHQHANS

DTSDHQNATENDDSNNRKRVGSTLVKLKSMYSSPSQKNSKDEESGGGLLTTSGIQIVREEEPAQEQSSLRRMKRTSTFIQ
PKVNY
>Ngru_XP_002678441
MGQAQQKNNSTTTTTAANTSQFSSELSSPNSAHNHGDDFSKILAHNFSEKSPLTPKKGFDPFDFCDHEGFSLAKQGNWPR
VEKMLKTKQATITDIDRHGNTMLYYSCLAGHVHVAKRLLDLGARDDKFSRCYLNSLSQEMRWLLKSYHNYSFSTQKKAKE
DESGVIEDDNLVNLLLSFVKRAIFSNEEENMDGPDMTLTIKFSGEQLPATYRLHKWILISRWPEIIDFALNESSEHPIQN
SVLSALDLKTMLAYDLDPREYQREVFPAFSVLQRPTLESIRSSVLVAKKYVKEKFLPYQLPTLNEEQIDQVYERLSDLNL
GDAPFSRKPFEVILAYIYTGRIIDPRVKSIFIPLEKIIIRDASVQFMQELMDAAYSLKLTNLAKILFVEIGVQADFIARK
FKVKANLDNFQDARKKELQKDLSPLGFCLESKQPTNLLDILRRKGCDMKIKLAESIEDEEVSEQERDVILCHKKFVTKRS
AYFDIITSCGFSEGIQFREEEEKNGIATLELNCPNMEILKQLINYTYSENANITPSICIELSIQSQKLDYNALFKKCEDF
MVENLTIQDADMFELYKLANFLGSDKLKKFWKPQTIKYVANLIKNGNITLEQVKESLLNELDCDENECETLVSQIMEKVD
YC
>Ngru_XP_002678166
MVDETTFESLESKWMKIIKSTQIDFVVLVGMKNDLISDEKFQGVSEEKIRNFMRRHPRIDRFFSCSSKSELSVNNFFEYL
LNRGPIEPSNLTEFEVEDYFLVDKFSKLLNNEWKSDLIFECGVEGKLFGHSWILYCCPCPLLKRISEVIINSNGQSLDQR
EIISIQQEFQIDLSIELDSNISVVRIAFENISKKDLLDILKLVYSGDWSEGINFQNPFSFSDYEAITHLNKNLLDREHIK
NSINNAYFNNIRQSTLENSHWLDVSFMIGNNILKSNRNFLSCACEYFQIMFEGNFTESESCIVDLTNDSEGDEKFYYHFK
SIVEYISTGKVEMTEENAISLLTLSNKYMISSLSSICELYIASIVNEELETPLKDCSIDLFTIFEISETYQALQLQKVVL
YSFASHLKEIKKDPQWKTFPYKELVLLKNVKCLLQ
>Ngru_XP_002677795
MVHQLGSAVDEPNGVETPNLDDSDLNISQNYGDQIFGLVGTDLQDTSTLSTDFEQLYQMEHFRDVFFLVGSEHHDQELIS
AHKCVLAARCPYFRNMFTLGFRESNSSEENPIKKPNVLPNVFKEVLRFIYCGKVQISAANVVPLIDAAGELELPLLKKLC
LRFIEVIIIYSVALDHVDECFENDTFLTLLSEDSLIEVLQSDYLNLKEEYLFEKVVSWGMIRIDRKDSTLSVRDIIKNVV
DFIRFPLIKPEYLLNSVEPLDIVPHELLFEAYKYYSTNETSFVKNRRAVLRGDEGRINPNRKGIYVVQVFKQNAHAHYRW
RIPNYSKISKKHVSSPLIQIGGHTWKVVLYPLGDSFNTHISVFLSLVIENNNQSSAYCDFTLRVVNQKDMQNLSVEHECF
NEHFQKDSASLGRQQLLALERLNDPQSGFLVDNTLYIDVIIKML
>Ngru_XP_002677665
MSYSSEEDYQEEEPFSSSSPSSPSSSSSGDEEEESTDDRYVRAEGNNPKRKNLNVTSNSSSSNMNFIQSNFNDHHYIYPD
SDLNYGFFLNTTANRRSSSSISGGDKKNGAAAFTFTASGQPFGISGGGLGGGDQTLISSSNSNSFSSSNSTPCGGFKFDD
TSKVLFSQQKPIFGSFNTSQPFGTNSGDDHQATNNSNSSNTNNASSTSSLAASEEKLFVFGSVPSSSSTSLSHSPTKTMH
RRGINVASKNRNENIDNIFNSLPKPPTFGYQQAVDQKDSTTGDSLSEMSQSLYSILRMAKVKGVTTKISELKSRKEPRKY
QIKSYPRDLILEGGMLQVYAFKPLLKIVAPNFCKNVICNEPLLHRLESKEIETLNFFIYMIYSKSSFTNFSLFPSLPFLV
FLMDVSKEDFLQQKSYFISQLGFVYLNTKNAVDILVGTDKYIALRNELFTEDISLKFLDQVRITCLEYIGANSSCKEFDY
NQDPRVKDYVLEFYRYKHVTRDIVPPFQTPVVIGGINIIYNFKEDSDAVVKIPDSLGGVKTIYCHKTVLSSASEMLEVNF
GPNWSNEEDEDGRAIINVEHLIEVASDPSVMEFIIEYAYGIFAPIKVSSKQVIPLIKAAHFYRIQGLLDECLYLCVNRDK
LSFFEVAESLEDHFEDETMEAFEDVMVEIGTANYPFIIGASDEELMKLPKRMLIKMFRKIYKTYIPNIKTL
>Ngru_XP_002677608
MFGFVANNDQYMSLTFAEECKGKSFEEWRWEFYSVFEKDRKEERDGLLVAEYLQTRKFRKVPLTLESNNNLPLLFDSSFS
TEYFTLPTSEEENETPRVKELIITDGYRSFFNNDLYSDFTIKFMKQGKEINSLKVHKMVLAENSDFFKNMFSLGMIESNS
NELIVDLEEEKEYAFVRMIQSIYDPTILFRDMFLNYELILLFDKYGFDTHKRTLIRNVLATRDNISFFQYMFNNLFVYEI
VVDGHSNELIQAINRNPNVLASYRDLAPDLLVRYSKKSFLNEFQWNTFSFLYSAVEDKHLKEECFAIAQRKFSTPLDFKV
RRQFSNWKD
>Ngru_XP_002677587
MLPIPRRENNDSTIVLINEEEQGSSSASVNSKYRVQDIAPDYYDDYKFGDEEDEEEEEDVGPSEQWASSVISYSSSYWGVH
IYESSTPGSVTKISGRRENSNEWVTLWSGPATNGIHVARDFAPELTVKGFKTNAIRIDLSMNGGWTEIDAVKICGKGGVQ
KEEVPEISKFYRSLLKSGLHADLTVVHKSSGREIKCLKAILAARSPFLKTLIDSSVNNTIVVDTFAPFPALVAALDYMYS
SFTNVRPCHLIETFVLADQLMLPGLSEYCVNLFRKVVSSENIFVFYSAAKEHQSHTFLRSTLDYMASNYKSVFLQPDLHI
VEKSDLLTICKSLATKNN
>Ngru_XP_002677447
MEIGSNTSEDSTTTITTTPATLGATSSSIANNGEGGDIQGTTVTTTTITDQGQEASSADTQTTSSLFTTAADDTTTTLAT
TTASASDDSTTLDSATDVIASSPKGIKINKKPLEGSKLFTLAVIPNYNNATKEYKSLVQVDPTSSLITKYKSVPPPVDPT
LATTTTPTPVAAATTTLPTPTTTTTAVPTPVTPATATTEKVDVVPLEVIEQAKVIPGLFIEGFLPFYKSGLFTDATVTCG
GVQFKVHKMVLGYRSSYFREIFQTTSDCVIDEKSSNLSLCNVDQVFPTVIEYLYSGMITIDRYNVVSLKLLANFLDVDPL
KCEVENFLLSFINAENVFDVLDSAIISNDNSIKSICVDSIAYNFDILNQKLVEKVFDNDRKFPLDIFFSIIGNKNMKSNP
TEERVKIVSEIVQRFIDEFKAFDNIELFKNIINASVDSRSLDVELAIKYLQYCDSHVPELEEQSGKCSQILAANFYTLLN
SGRSTIIYDVLPNTFVELLRCDELYTKDEDQVYEIACNYIESNKDKLTQEQKVQIFKCVRYTYLSIPLLTKLKNDPLFVS
KEDILEALWSRVSRLEGKKNDESVYSTRPRKIRVFIYEKDFDTNGILYWLGTNYSQEAYTSPMDRSYLSVTASSNFEVGS
AKDLVSHEPTKCNLIGKANAFIIIKFETIRVMPTKYSLRHTMSRDGEALRHWTLSGSSDGINYSDIFTHTNDQALNLKGS
TGSWDLNCTSYYQYFKITQTANNSSNNNNYFSLAGVEFYGLISRICE
>Ngru_XP_002677434
MNLSPQHPSGELHTINSSLEILPTVSSSNIEEESETKFETVSDKNYMTPITQQEEASIDASYDYEDENIKSPPHTKQMHK
QNSENNLIISSSPSSSSILDMIIDQDSNINQEELEINRRNTINIVPTHFVHKRGSQELPELVSPYRKIATTNNLKAALKN

SSSASSSQQKLNKSGNSASYSKIESKSGDAYKSLQILTNNQKNSQVSSLNNGGNNESFSKYKYALENDDFSQIDFGLEVN
SAQTSASEITFPVPNKPNAFYGNAQNSNANGNYFFNGHHNTASVDETIMRKNSVIDDSVSDSGELEISKHYELYLICIGD
LDNCLVHKMLKTAGGQMYISNRQVSFILRNNNEKVTCTFNFYSQFNWYLMDFLKDEAKDCHGLLFMFNITHSEGWNELKQ
TISKNANNNNHDQEDVPPPLKKQANQLNLMTNVIIRTSFKDYQVHDLIVKRSGLFKIMLESHETWNLEKQDGKPIINLIT
MEKSIFNDGSEITQEDFELVMRYLYSDEIVVEELDGDRVKKIVFMARFFDLDCLNEYLLRIYGNEENLQYSGIYFQIKHL
LEREFTQEDFVAMVFNDYQDWETYTNELVDLICQQEIVPLEKKLKLLREQIKYHNQVTTITSDHLKPFEKSFPILHLQAR
SIFKNQIDHSHNCEEIAKLLGIDLSPINGTIENNAELLSSQSITNYLLVADEDNYGIIKLFDGNLVINIRCQEFDSAYSW
YLRAEDRANSNSVTIYYQYGNQPITKIRRCEFKIHCEQYKNCGLVNILVMIKK
>Ngru_XP_002677414
MKKNNNTVFFTLPIKGGCMYDNPNSFPDLQIKIIVNKLINTKNECFMEIKAHRCFIANSSEVLRGLFEQQDNQHILKLIC
STEFEAMHCEKAIKSIYTSITIETDEFSSMYDMYSKLGMKSHFEHLKQLWTLQCQNSFPVAIHTLRKFGEKNPENHMDNY
IAQLVLICIDKLITDQERAIPLHQLWLSKFIENQTTATNPERIISLIDSSLSEKYTTNEQAEKLIRCLFLKLDSKMTQQQ
FFNLISQLSCSNSIYLSFNVLPQTFKLEMVSTMLHVIKFSSFISTIPTSLFLQQNPRITFQTNSNSPSSYKLSINERELE
ELQNLYLSPSTDEDF
>Ngru_XP_002677205
MTSLFARAATFRTLAPSSGLSILTNNVNNSSLAINDDITDEYEHTNTELNVSTISGIDHEGYGSDSESSSHLTPFNSPAG
IALGERTPNGERELLVSDYGNHKIRRIKSGIITTIAGCGERGFNRDGCVATVSKLSYPTRIARCENGDLYIADRHNHRIR
KVSAVDGSISTIAGTGREGYNGDGIPAIHADLYFPTGIAIDEREQELFISDYHNNRIRRVSLRNGLIHTVAGFGEKGYDG
DGGFAFYARIRCPTGIAVNQRGDVYFSDSGNNVIRKISTCGIITTIAGTGEKGFNGDQMDALDVMLNGPSGIAISQEGEI
LFTDIYNNRICKINRDRTLVTIAGTTEEGYQDGPVRMAKFNNPSDIAIDNETGDVYVVDGGNNYIRKISTTKYLNSGGRM
KKYYWDRVDNTNIQGVSYLIKMASPQFYESVLKCDILKKMSTTQHEIVQYLIDDIIYEPVKNWQDNYDPIDILSILGLFQ
GCEFIEMKKSLVKKIKKTCCQENIHFMMEKIEQYIEILPDNIILNQLFEYCICMAASFLRDSPQILQHSNILKHITLIAY
KISERTTPNIHIQENASEKKNLSSLFNDKTSSDVTIIIGKEKFYCHKTVLSSNSHFFEELFKNKNNEKDTFYEMVECKDL
KISEMIIKYCYDLLMISAIKSPPVKNSLLDYANLYGMRKLSDYCSLTLNKQ
>Ngru_XP_002676738
MYNLKRIFGFQVLNRFDEGSSKKLLSECDFSQAQQMAEIIEYMFVKDLSKQLSNHIKVKMATIKDVTTIVNEVNQWVTTT
LAQFNNQEEIVLTPIEKSYSYFSDKPYKANQNKITKKSDNYELNTIKFMHKGSFSIEQFFSNKIFEKQCITEKQPNPLSK
RFLHKSVFIRSKNCIVTYGGFAIGKGITDDIIKFDLEQKKATQSTSRKGRIFHTFDYLPHLNKMVVYGGIEKTGSISSDL
ISISMDFSSDVRSNEFLPPLCCHSAVTLFDTLYIFGGFNGSTSKYSNTMYSLHIPTLSIKSSASKFCPSERIGSAMTIVG
DNIFVFGGMDSNGNILSDIYRYNISQDLFHPILSDEYAINPKLCFSNMFIIGNNIYLFGGWNGVEWNNSLFETSKEEFKW
KIAKGYTNSISSDFNSYNPMSSIVPVSEKIIYLLGGLDLPFPFISILDTFATLATGKIDFTISNTKPSYRIFQHKRQTDE
FLLSQTYLNWFKKQIPEECDVTILVNIPNQVEQELFYGFKLVLVRSPVLSRKILEQEGHNKWRNLKHGSHAVILLDDFPS
NVENLRRSFNSVLEYLYSGSFSTKDLKSEDYSDIYKVACYLEIPLLYKALDGTLNVDDSLETKNLLSTITIQLDHLFSPC
ETISNIEDFMTTIDENPIAPGMVKIIAPTSLDSSTYQSILVHKFILTQRCRYFNAVFEMNFLESHTRVVLIEQNTFKGLL
SILHFVYTGNIVELDLSNAVEIFMSSFQFELEELMEASKLFIREHEFDVFTTIRLLEICETVNDKIMQDYCKYSIAKRYE
IVMKHDPSALDNLSKVSRVDINDLRKSVLKH
>Ngru_XP_002676409
MTCLSSDHKYLFIAGGITANTQHDDLIVYDLTKGRLNIYRDIMRVSNEMNANSNPILAGQFMIEMNGDIYIFGDSVYKIQ
KIFKPQEICFTPGMLNSWYGGFYLYSRRTHFSFNSTIAEGGIDVFVFGGKTLANSILDDLYIIHIPNNVNNQIPMAYAKV
EKPRRGERDWPSARFGHSSCIIANLMFVYGGMSAKNQLLNDLYVFDTSTRVWTEINVESMLPPYTGTIILSNSKEVNQFL
EFVKLVSEVKHFPYIQKICSMDLRKDLSLTREILEVMHKDFTSLIDDKTYSDLVIVLDHDGSNQPPPMEEISMEDELLSP
LSDSKIPSSGIFVHKLIVSRSPFFARMFVTSGMLESKEKVVHLTDYSKEIMMDLLKYLYTDIIQLSPRNCLGILVYCLML
DLGDLASCCRRMVSSLVDNNNVWTIHEIAVLYNEKTLECSCEQYIANNYATLSTNSFFLELPDMSRIKIKQIYDRKNKKK
>Ngru_XP_002676083
MSSLATRAQLYNPVGIFVSSNNEVYIADYSNHRIRKILKNGNIATIAGKGTCGFSGDNGPATNAQIYYPSSVFVSSNNEV
YIADQSNHRIRKILENGNIVTIAGNGIGGFSGDNGPATNAQIYYPYSVFVSSNNVVYIVDYGNNRVRKILGNGNIVTIAG
NGTSGFSGDNGPATNAQLNNPVGVFVSSNNEVYIADQSNHRIRKILENGNIVTIAGNGTGGFGGDNGPATNAQLYIPYSV
FVSSNNEVYIVDYGNNRIRKILGNGNIVTIAGNGTSGFSGDNGPATNAQLNRPSSVFVSSNNEVYIADLNNHKIRKILENGN
IITIAGNGTKGFSGDSPFDIRMYPHIGNNLFTGSSILYSKIQVHNLCKHSRDQLFGLQIYGFIPFIEKISPQFSNILKNE
KILCLMSSSQQEIIQKLIDSLIYDKDKVMFSKEETLDVLFILGLFKNGEFIDLKRSLTSGFVNSLTLENLLLKWETIEIL
LKICKEEFNVQNYILDHLNNYCIEYAVVQMKTKEGNALLSTLPIIMRNGATIFPKLIITQPTAINIEETIEIKPQTIESL
YNDMKTSDVKIKMGQNKYLYCHKTVLSFSSTFFNALFDSGSCFSDLSDDGVFTLNEPEDLELLEIVIKYCYGISIDKIDP
VKSMGLLDMAMKHEIPYLVDYCKNSFNLSIDNYFNMLEVCENYFDLQLFEPCFREFVKFSIANRKQLFSDRDRISKLPEK
VLQEMVFSFSQDF
>Ngru_XP_002676026
MPPPHPLVSATITATATTTTTNSVDVSAEVKPIDTTKRYQQDNRVDINTKDAFANGFSKYFLNDLFSDISINHIPTNRSY
RCHRIILAHQSQFFESLFTCAFRDSSGSVEVHFEDTESVFEKVLEYLYTGKVNIPVTSIVALHVAADQLICPDLSNSVMN
LLDQFISNENVFSFLDDSLKLYDDGILEKACGYIALNFVQLIDEFNLIFLDLPPQVFFGIIAHENIGKGPGAKTKKCALI
TECVNEFCKKNNVLENVEMLKMCIDAMAATESITPDSAAFYLMECEKHGLVEHQSACTRILAANFNDIKDTTIINQFNPD
TFSELVDLDELNVKNEDQVFEIVLSFLVNKKGLDLEKKQVIGQKIRIPFLSYDKLQIIMEKKEPILIESIPHDVLSESLL
ERIRKLEKKGLYKICGDESPKYLRPRTARLFTHTSDFDEGGVLYWLGTLYHTETYSSPIGRGLLKVTTSIGFEAGSDEDV
ISRVPVSCNLINSPNTTITFEFVSNMILPSAYTIRHTNARDTECLRTWRFQGSIDGLTFTDLKVHNNDSTLTTKSQSATF

PITDCNEYYKYFRILQDGPNSSSNNYLSFGGFEIYGAVKLI
>Ngru_XP_002675918
MFHFATTKKTYTVEPKQINIDVDDKIIVLSVYAPSLKGYAVPTTQTYQISIPNDIVIKKAIVSQSNLFVIDSEGKLYKCK
YSESYSILKLVDTINDSVLDIVGTNTHLFLLGKSKVYGMGKNDYGQLGLGHLDTVHDFVPLNFFDRININSKKYKDKVVK
IACGDAFTLFFTNTKKIYTCGSNYYGQQTFENDSVYLSTPQRITELKENIVDVYCGRSYSLFKLSDGSYVAYGQFLGETP
LRNTKVIPTLQQFDRGNRYSTQIEYQNNDSEDVLLFCNEKTTNNMHIQTTDGMNTVMVENTSVNEPVLIVNFGTDSNARV
NFNDDELIFYKSDYSPHQVEKSIYQLVSEKAKQFKLSSDTIQIGNSNNIYTFLAFIQIRNPSFYTFLKQDNLVNSENSPN
ILQDLVDYCFSENCSFMKKYSTSMIAELLYYLERMCVDFNDFTAPLMYLSLVHISKCIGFEPIADKFIKMLKQEGIDPKI
LGKDLSTFMKNGNFATLPTTHSCLESLFNSDSSDTTIIIDQFGTEKIKCHRSVLIAQSTFFEAFYTGHFASDEINLFNDE
DVADGALINFAEMSDEEKQLLIDPESQIKTRALKNILMMFYGIEPVVENDLVIPMLDIAHKLDAEHLVMKCLKMFETSLD
EESLQVINQWLSGVEDKTDVQNMILKSADNWSNEISQRNILFQNPELENELTKDIGILTHYQINKEFVPNVTTHFVANHL
NTEDYNFLCAVAGNCAIVKPEYIQKSIESQCWIDEEPYLFNKCAHEFDSKVQTLLKVNQNHKALKTFVFSHMTGQVLLRK
HTKSRVECLIRSGGGKIDNFYANLVLINKNTFSKHKKIGAKCENCVVVDYRNLVDILMGENPAKHEIHSIHQVAIANAEK
KKRKRDPNEIIEID
>Ngru_XP_002675610
MEHSDSPIFSMCKTGDLHGVKNLLTNDQTRQLLHAKDAHGNSLLYYASLCGRISLVKYLSELGLRDDEYRRCWWNALSLQ
TRSVLKLFNDYKNIPSTSSGMNTESNVLSVMHKLLMKCLHNDLNNSSSDQLLRESNSGEDMILRFKFKDQNQPTEFKCQS
WIILSRWPQIVLFALKKVQLEKVINLPTLDKHEMNLCKLSAQDFENYVKRNNTNVIQQRLTRIITQEKNKLKELKSRYSM
ILGIPNLDMCNQALAKENFEMAQTNLKCIDFGSINANKEHFELALTYLYTTKIPPISDKYDIYCLMELGVMLNMKSMVDH
IFNMLREANDNQQEDVLLNDLDPINYLFKDCKSKIDSLKKTSCNMGLKMVNSTDGKILLCHKSIAMSRCAFFDVLGNCDF
EDGLKFKQQEATHELPVMELQQFSLESTLQTLLRYIYCEKAEITKSNAVELMHISDLYGLSSLSDKCEQFLCENTTSRDG
NLFELYKLSIEMERKKWQRSLEPKALEFIEEQTRRQKINQAEIVRDLEDLDLNPDTIKKFKNASNSAN
>Ngru_XP_002675386
MKRSLATTHSSESNNGESCDAANDENKLKKAKMMMATDAMLNCPVEVFVAPNDEIYIADRDNHRVRKILKDGTMVTIAGW
SFGGFGGDGGPATSAMLDFPTSIAVSSDNEVYIADLGIYRIRKILRNGNIVTIAGTGEEGFSGDGGPAINAQISAVWNIA
LSQNNELYFVDYSNCRIRKILRNGTIVTIAGTGEDGFSGDGGLAINAKLGYPCSVFVSKDDEVYIADSQNNRVRKILRNG
IINTIAGTGEEGFSGDGGPATNAQLNVPCGVFVSATNEVYIVDSGNCRIRKILRNGIINTIAGTGEEGYSGDGGPAINAQ
ISNVYNIFVSQNDEVYIADTNNHRIRKILKDGTIETIAGNGEKGFSGDSPFDFSSHPHIGNDYTIIPKIQLKKHSRDELF
GLELIGFIPLIEKLSPQFCKIILKNEKLLKSMNSQQIEIIQKLIDSLIYENVYHFTEIERLNSFKEYDILNVLFILGMFK
NCEFIDFKRVFVNAFINSITLETLPSKWEQSENLLKFCQEIEVENYILEYLNNYCLEYSAIQLRNPQSAKHLKTAPIIMR
NSLNIVLMLEVTTTSPMQIEDTEIKLQGMESLFNDEKSSDVKIKISKDQYLYCHKSILSSSCIFFNALFESGSSFSDLSD
DVFTPDESEDLELLEISIKSCYGIPIHNSFSFIRLIDIALKHENQCLIYHCKKNFSLTIDNYFEKALECGKYFHLDCFSS
FFDTFLSFGTRNCEKLFTDCAKVGQLPEQLRNALIIDFTLVLSFSSRPALTWRYPNVYYDDPEFGVRF
>Ngru_XP_002675262
MTKKKGGGGQYHLPKHLRNSGGGGKGSGGGSSTTTTISNSNNSSTNYNSTNADVNNMSASTLSHQPIEVLSRMSPQKLLE
TVGCSNSSMMELGEAYYLRLVYIDPDEGMKKKCKLLQEYYGNLCKLFELESFVTELEVDVNIVHQNAQLFEDIQNVLSAI
SNQITTYDIRAMLYKLGGLTHACDICCTLVFIIPHIIDKSLNAQCSKFIENSLLALTFILFKITDHSRGREILKQSGMFG
TLWNISQFYSQYIDKFLKNEGQKTEWYKVDSVVSETVTSILYHIGEILNALTEEYALEDDFEKLLITANPKWFPTIGMNE
KFGRFTSDIHFAILDLPDLLDWNPNEFNLYITGSKLRLSVEDFDIHAHYPVIRARTGSKFLHTANVLKWNPQTGLVEEDN
DNGPGYYILRGDHHPRNTLAFVKFIQVLYCGNIKKYQSDFEYLNELVPSSLGIQEQQNEIKKKEDEMCDSLHSFSIFIHN
EETKKSLRLIVPQYKNSGLSSSVSQAYSCLFDDTDKILFTLHVRGSYGSQNQFENYKKAYIRSLSYNKTISPINADFKAY
FSNQHLNLDQHYDIQLQSYGNIVDEAIASSKNVHYNKVLTTIKEESGIPSHRCILACRSEYLRKVLEYESLSHNNSTNMD
EFVVQDISATTLWIINQYMYSGTSVFERNPIPSEALIECFVVSDLYMLFGLKKFAEEKLCEVLAQWKNLFSKWKTEDVSG
SSEMAIIEELFELATVHVAVNLKSDSYDLLKEYKTKTSE
>Ngru_XP_002675177
MSDLDNMFGDLEIDDSNIPEPPQVVEGEKKEKKLVYKDMNAQGMKQIFDREEYSDFRVILQQTGKTVYVYKGILAARSEF
FKAITEGGFSEKTDNELVVDDESEEKALLPLLEFCYTGEISIPEHEVLAYYFISDKYQVLSLKSKCSDFLVGNLKPENAV
ENFVEYYTAAERTAGIADELTEKIIKRSVLYILKNFSVICKSPEKRQIMLNMDFEMLNRWLSPSIEAKELRGINSDPSTF
LRFMLEWVSQDISTRGSHLPELVQMEKKVEYGTVSILRDDGAELQSIYGEPEFIYETSATEISGAKITKKTANYSSIYVN
TMLGYGSSTAVIRWKVKIVTFNSWIGIGVAMRDVVEKKKLTFSPYDKHGTYMLSQNGYIWNQFMKGSTKYESVGCSFTVG
DVVILELVCAKKELNFYKNDTTNKLASFSNIKLPVYPSFMLCGTECIEFNWDLEEMKNPNRSQILILYRKLLRTGYQWPR
WETSADNAFMGDYILDKTKREFRKNISAVETGPLMEKARKELRFLHTLQADYFKKQFVLKRNYAEAAEEIKHD
>Ngru_XP_002674836
MSYYLNQLEGSDDDEYYSEEEEEEFDDDDEIIHDQEEEVDEEEDSEERAAAIEQQKASLSIQTLSNKKQQNKQKNKIAKT
NTSKKKDQQQESVVMSMTEEELEYYQNLKLYGISGPKNMNKKENQVIEEIKFKLDVLQSSKMFFESVRPTSLTIISGNPK
YEEQKRYIMAHQYEARKGKHYNNDDAMIEGEVVAPKFLFQDVSSSKIGEYVRNLMNRVDHLSTLSHDERSLFIYGGRTGT
LLHDDLIRYDFMTGKMVVFKNLGFVSHCDGTRVQVPCPSISNSEMVSCGGRVYIFGATYGFPNSLDSVLTRAHFGYFWGN
RPNTAWTNNELVILCMDGVIGFNSGLLGVDIPRVNTTALTERSHPVRSATSDIPPARSHFSMVKMVKGSNLVEAIIFGGK
KNTVLDDMYTFTFSDRGQTYEVKQIKKPPPSKKDGLVTWPCARYGHTACLVSNLMFVYGGCNVKGKLLNDLYMFDISTQK
WTEIICDSMLPPPLFKSQSFAVNNQSIYIYGGQSTQENSNIPPTISSSLFRFDITDKKWNIIEIITEEADDIQNNKAVPC
VGHQTQIVHGKLLRIGGLANNVGDPFVKLLNLNDPGRPIPLCSYLSSKRADGFLCDVVFRVRDVLGQEIDSLSYVLAHKA
ILKARCSSLHKMILASTETMSTTKLSGTFNDEMSEITLVDIEQVDTAVFEQYLNFLYSGSLDLKSLNEVNSFLEFAKTIS

PDNHYPAIVKICTAEEQKDLEVTKLVLSQIHKDFSTLINDHSYSDLIVVLDQGDAVAQHVGGPTEEIFEEEPFEIPETSF
ETNDATAELEHEIMSPLTRNTVPTGGIAVHRLIMSRSPFFSRMFVTSGMMESKERVVHLTDYSTEVMLEVLSYLYTDTIR
LSPRNCLGILVYCIMLDLVDMASVCRRMVVSLLEDSIVWSVIDIAMLYNDKTLESECEHYILNNFEKLRGAAGFLNLPEL
TRIKIKQLYEKKAEKKSKK
>Ngru_XP_002674680
MEEINVHIAGNGIGSEGDGGLAINAELEEEIFFLYFHPITDDILFPDSGRIRKIDSSTQVISTIAGSTKKIYDSAENENN
GDGGLAIDASFYRYSYGLVIHPITLDIYVFGGAYIRKIDSKTQIISTIAKAQNQIYNFTLDPKNNILYLAEKNRIVGLDL
STKNQHILFSTKDEITSLDWDINDQRLYFVKSYIQYCKLDPITKRITILLKSDFDEESLVTQSQNKLRNIVDMIKDPNSD
FVYFVDEGFNEMRIVRMNSLDNSIVTLTKSKDTLWLLNVDSNGDIYYHIQTGSSIFKIVRMSLLKKYERDEIDNTSLVCC
RYLLDVYAPHFHDFLFNDEIVIKSLNKNQEYIIQHLINSIIYQEEDQWFDKYDLIDILDILGKIEGNNFMKLKRYLINKF
MASINNQSCIETLEKVSKMKNNSKLMQEMFEFTIEKIADLMKFTSSELVNHEIIQQNILLISGKFNKVHNGQPSPIIIQH
SPISRKISSIESLFNDEKTSDIRIKINNTKYHCHKTILSSHSTYFESLFSNNFQDINNNDYEVTTEEFDENAHEIILKYC
YSISGNIELDTRIFKCATFYGMQKLADACVEKIVLNENNYFEIVQQFSSIENDLLNQKIIEFGFINAKRLFADEENYSKV
DEKIKDKILQKFITLARTPKRKH
>Ngru_XP_002674458
MLSLSSSSSSGGGAVQKTAIDEEAYIECTEKTLSMISSLYLDEEASDCVLISKNGKRFPALRALVGKWSTKLKELLFVPE
FEIGKELVLPQVTDLGLEIFLKVVYFGCTKDYTMDKFIDLYTFAHTYGMQEIEEMTLEIMRNAAVDADSSFLLLENCSQY
LEEFKEINSLKKTLLTKISADFDTVCESQHFVKLPIEDLKIMLAGKELICSEGNLFKSLMRWIEHDKENRLQYLDYFLGE
IRFPIVEGEVLATVDSHPLLVSKRDVIFPKLYEAMRFNLNPAMIENKEDKNFRERDCVLYDFLPKGMSIKFTNLGATGKN
GPTVIGSQYDSCKFLKNKVKLENGIQKWVVPATGFYKITAAGARGGKNTYTTTFREGYGAIVSGVFHLKRGRELSILVGQ
VGGDCTNGSSCGAGGGGGGTFVVDSDTMKPLIIGGGGNGANWYSWTVQSPGGQVYTKGPAEARSQKAAKGDRGGGGGGLF
IDGGAGDQCVGGKSFKNGGEGGTFSNACGANGGFGGGGGSLYEGGGGGGFGGGECTPQNNYGVHYVTHAAYSYNEGVKQE
GTPDSNNGDGYVELVRVIISQSEYEKEKKTMDSTVVEEPLSPSTPALIGSSGLFD
>Ngru_XP_002674389
MEPFDDIVTPTKMEAPIDDFTSDQTPQTPGSAIDFGALSGLEVDVAEIENPDAVQEKLASLYNVEECSDIVFIVRNRRFF
AIKALVCGWSPVFKSMLCGEFIESSLREVPLDDVEETSMEMFLKVIYSGKTGPYPIHNFVDLYAFANRFEVPEIEKLCLT
ALQEQNLAKHLFPLIDACEKYIDLEEIEKIRTEVVAKICCEFDEVCSRPDFVNMRIDYLLRFLKSGIIVIPEDVLFNSLM
RWIVHDKEQRIQYLDELLSFIRFPMMESVALTEIENHPLLQDKMDEMKSIIYEAMKFQLNKDAIPEERRVHENFKEREGP
PEEDGHSYKYKKVFTLTSLGCTGSVKPTSIGTHYDKHLSKVITLKNGIQEWKVHASGKYRITAVGASGGINPHTTNTKGG
KGAKVSGIFKLKRGQLIQVLVGQKGEDATGSTGGGAAGGGGGTFVVDGELQQPLIIGAGGSGANWYSFTIDGPGGQLYDP
SIESTKSVEAYAERGGGGGGFYIKGANGSSCTGGASFLEGGSGGTNSTSTYGAKGGFGGGGGSEHEGGGGGGYVGGKSLK
SNEYNTSRPDYGAYSFNSGLKPEGEPACHVGDGYVEFARVF
>Ngru_XP_002674155
MKTTNKASDSSQQKTPSRKRKYKEDNEHASSSASENTLHHDEISSTTNEQKENNSEANHSLETQELIVERIKRKKTLEPV
EPSPLVEKLQEDTFQEDEEDYDIVLNHGFTLDKQDILMAEQQTSFVPIECLLLDEGDSVPDFKTVSSNHARKINLLKLSK
KHKYILLYFFRDNKEKLKRTDLSQVFSEDLSKFKEKEIAVIGISNSKTLDKSNEYEQDIILDKDLKICREFRCLDAKLKK
PQSVTYLISKDLKVLHIFHKISEHQIVEEILSFIKEVESGKFSEQLKAEKKLTHFPLMDSIHDASSRLLACITGETLHDN
VVEFISEGEALDTLFNACHEMIRSLQVIEPKTRKTWEIIILYLKKTISVCHKIHKDYSDRVSDSFIEFWPKFWNKVFNLF
SELRNDERITSLQRHELVDKMILIEKEGNLGSRMGTCRSTFEKKEKKRYLHIVFENFKKRPTREHFLEIERNFYDHSSDS
REEILQKMIDFWIKNIETHAKDILFVSCNGKHAKIYEIMKMLGTDIESSFEEMHAAKTIVSTYPDFVKELALSRMKSICT
KMLTVLNGRMKYLMSTNKKDSSHLQLLTHFNSKKFTSTKQEELIKTIDTRFEEEIKSKTEQHHQGIDIPYYKFPPIGKVF
QDWTLILREAIQKVKKTNQFKNMEENNQAAQFEEFQVLQSIYADDIQQLSPSCSNDDNNNLDHGNNIEDNLLSFRLKIPS
LIERQIITSKKTDHLYHLFQTGLILSVEFPSGYPTSDVPQIKIQSAMEKVFIYCPPEMYEEHEEFKEMRRVAQEIQEKEI
EDLLEILNNKARDSIGQVVMFILIQDVEDFLVEKSSAIKDFDSELVNKGNDIQKPKVKQSNPTDDLEKLERTILHYKKDT
KKGYYAFVDSFYSSLSFTKECVEKKKGMQRMMMTSCNMDSKVYTWGGLTDPKTYSAEIYTGRITGAGIEWQTAGKLASPI
CGHSSCVYDNRLYTLGGYGKSGYTNDFNCYEWHGGFTSFEKVESIASDISPRAGAAITQSQGRIFIFGGHNDGQIFNDMH
YYDIETKLWTNKTTQTNFVPQLTMATLLMRDSNTLVLYGGFDGSNWNTNFFEYNIKENRWLIVPSVPDNDFDSAATAGTI
QTLTDSYVLLGGCSTRSLTFNSYGAFLNRILFQKKSITSEHSVRCFRSDKILKSQKSAVVAQSETYFKWLETTQPDDADL
VFIFTPEEEESEVLTEREKLPCCAHKLILQRFPELKKMIMVAELRKLGLDVNSSNLDSYELNHGDLTYIRINYVPEVKHL
KRAFIRVLQYIYSGMFDATDLKSEHFKDIFQMAVHFKITLLVQSLLREPSLSSFLSHNFKTLEKELPSFLKISPNEDVED
IEEFRSMLSNDPNSVPDVKQGLVRILAPDHTYNPPIYRSINVHKMILCISSKYFAATFNSQFFEAQKRILIMDSISIQSL
VVVMSFIYCPSYPLDLNSGNAVEIYVLSHLLELSLLQDKARATIKQFEFDEYTALHILEIANEFGDAVLQDYCFFLLARN
YDDILQRDPQIFDELTPEQRVNIYKKHLIKK
>Ngru_XP_002673908
MLSSSFNNMERMLDDNHNNLIQTGDMITIHVDNNISKPATSTEGPSGSCEYLTGKFSKRATTPSYYHLLYNDAPVCPPDF
IINIKLIDMNMTTVSIKVHKVFLALESLYFRTLLNSEFTESTNSVVEFELTSVEKDIFEQIILRYIYGERTLDIDKGLPY
ACTLCLAHYLQISPLECQLLQQMTKTSKYTEQHLLYYSQLRDHSMLAVNSSFLSQSLTAFGKDISKKRDEVMNLPVEFFL
WLVSLDNLNVSNEDEVFDLIQEYIIRNEKSIRQNNPEGFDQKPDIIEQVWSNVRVCFLSETKLLSFMDQPSISTDLKISM
LRKKTVYSIYKDRYKKVLATKQQASTETTAGNASMSVETNSGTNNENTVVKKEPVPATNATPTPSATIIANELMINLDDP
QFIKRGNILEHLITGSKGVIQIWDVNTTECLSTFPTDSFVRCLALYERHYLFSGGHDKKIKKWNLRTNECELVLKGHHDY
VLSFALVDDCLYSAGGVGDSTVKKWNLKSNQLEASLEGHQKAVLSLITYGDMFVFSGGEDSTIRKWNVRTNKCELTIQAS
VHGDDKLWCLATFGDSLFAGGSKGTIYQFNVNDGRLEREWSAHENIVWCLLVHEDYLFSGSKDKCVKRWNLNTPNTDMEG

ADPTNTPYVCEAIMREHTDSVRSLNVCNNYLFSGGGDEKIIKWSLLTNQKEAVVTAAEYICSALLA
>Ngru_XP_002673834
MSFDGLVFKKLSEKKQNKKKTTRKILPSNKKENRETIILIDQEQELNNDDLNVVVDEKVSNKELEINLDLDVSQPLSLFF
DALDPQVIILSGTAKYDENYKYLMKNEFTWYNYKSKQEMLEKKLEYPRFEVEHYPRAKSFSFVQQLLSRTEYLSCMSSDC
KFLFVYGGRTGNVMHDDFISYDIASNVLYVYKNLYLACHGPFYKNIRLPIGLIGYEMIELDQLIFIFGKGEIHRIEKVTD
PLNLSYLTQVERSALPNRIHFTMTKVLIENKLYLYVFGGKSDKGVLDENLYMIYFNGASYNPFTCTTYSPPKQFKELRRW
PSARYGHSACKVSNSIFIYGGVTTGDKILNDLFMLDISSKTWTEIIVQSIIPPPLFKHCSFSVNERSFFIYGGNKSEGIS
DCFYGYDIIDNKWNFIEIVSDEMKQNISGYKCLTTLKGEVFAIGSSQKEKNHRSFSKLLNVGDSFKSVSVLQYLNQKREE
GYLCDVSFQVNDLAPSSSSPYILAHKCIIKARCPILYQKIQSSKQWINTNQLEGVFLEKEMIIVDIAECDSNTLMKYLKY
LYTGTIHLKASESLPFLSFVQATSEQKHYPIIQKIYSSAESKNIETTMEILNTIQNDFNTLLGDSVDSDLTLVLDNNNET
ANQGTNEEVIIHAHRLFLSRSPFFSKMLVSSGMLETIEKTVHLKDYYKEVMIDMLKYLYTDRLQLNPNNALGLLIYSLLF
DMDELASCCRTLVASLLDKSNVCSIFEVATFHNDKALQNACENFMVDNFQSLSQLPHLLKQYPNKQEFPFNKSMKSSARK
SSKKRRNQR
>Ngru_XP_002673817
MLYGVGKGFFTPFINQNENTLQPVPFFQYLLDHPDDEIVQVEMKYNNVLIRSQMGQVLGCGLNSFGELLQPQDRQMKRFP
VPIFKKGIRHCALGYQCSIFLDFDGIVSWYYDGRVFKLDGLKLGLEKVTIIGDTPFAIAKYKSFRVKSTGFVDCTQPKGN
EEPAFYFNRGSMKYKYEKKYGMITLSGSSLPHFFYEHCYGSVHFNSVNSKIIKVEASETHLVNLHFDKYLTSFGSNQFGK
LGIGSTAQNHFISHEAVGDDNFERLYLDFDNSLELDVFVGPLCTVAVTYSNQKTTIDYHAKSLLHNLVVINEEIEDEPYL
QVYFPLIEIISPDLCKVFKNEKLIKGLSRDELVLSRELIIYICEVIPYINSGKQSCIDIFHEDNFLSKYFDDIKSLTLFL
FLLNDVRDCGIDLKETIFSKLSTFIQVENVMQIIDQISDYLSRKVLKNSHNSILMLLVKKCIAFIKSNYTQVRYLHGADE
KLLKLSSRIEESKQSFQSIEVTWNEEIFTQVFSLSDAILLCSQHNYDCTLIIDDKGTEIQVNKQIVTKSSPVLAVYFEKY
SSSRISYDVFEFVKSEEIGAETEKIEAIRILLSEACNCNYSNLRLCSDQTFIYILSMSHIFEMKEIMRECCKIMLSERLN
SSNQESFLALSPIYDDDTNMRLLVFLLKNLSKVSSIAKVLPRQWEEKLEDYAHMVNCENGVPAWMSVNLPKESLTEEELM
STISFITQYEGPEEYDRSSEIRRR
>Ngru_XP_002673756
MVENEQRSTTLSSVDASVQEPERKKIKANNVEELVEETKYWYHIIASLDNGPVNYKSFDQLEKGLTDREKVIENINQLWH
VHKARLILESLDLSNPYWNEDWFELTNNVGELPDTEYEDVKLKDKVTAEELGLTSEISKDLLKEYISKREDIESHSLFLV
EKLFKKCLVQSVEEITEMIDKCIEIDEKNVEVYFRQLMDSDLPYDMSSRKTILDQWSKYILPQIVKTQNELNMIDDPVSL
STNEESKTVSKFVLENDSEDTDAFLLETMLFGDEEHIKIVKQDEKDDEEDFNDDYVPDIFYCSYLFDMELPDEKGKIGSN
FLGLLHCEHNNSNENIYLKIIEIVMKMHMIGVAPQEHQEIEVSELRLAVSLMILDCLNKELTYLILSQSKESTYMLYRPL
VRAILDQFIVDYCNPTKYDKYMFVICTNPDIQPMINVMFEEESVVDTDNLTWDVVQTQRGSCLDEEEENNDVQQSVKEKH
PIWESTVDKLKEKYFTHQTEAHSKISRQNLDDSMYITYYTDRDNAISEPLSNQNLVSFYRYDFFDYSEEPFTYEQTADNL
IENRSIDCYYKNDKSLHLDLTRLLRRGGVASFDEGLLLEAYSTLKNYISLSLQTLEDISEDGIVEVDHVEKVAKLLAIKG
GTNGREYIRLELPYYYKLDYQQDTPVAKELDDVRDLEEDLLRGTEIVELEPVSASTNQEIPEGLGKDLYNALNNEQFSSL
CDSFIQTEDGAKVGVFTPIIKDRLPEFYSQYFDSEEKRKNGLCITSVHFEVQEGEQLIFDEVVITKWLTCIYTGIFEIES
SEEEKNILTIEKLLAKWVPREEVSLKEPKHETQPPQATAIMIDENEEEDSEEESSEEDSEEESQAYQEESDSDSEEGIYN
YSDEEEESIYYKKIKEEEEKTFKCEPLSRLFLDEEGKDFSLVVNSHIVKVHKYILSIRSSYFKVLFLGNFSDKNISEYDV
SDFLKNPHSLDLFVYFLYHDTLENENFTLEHYKYQNLIDLEEFSQFVLSERLKSECVSKLISHSTLEQTVEMLRTSLEAN
KEEVFNIAHTILLKTQYPYFNGTIIDDVTTQYYQVMCTSLTLENSLVKLDYLNKIEQTTTALHPFILNQIKSQIENLHWF
IANRASSLDNKFIRTLPQPVQELISRFFTHDNLGMASFEEVTIEE
>Ngru_XP_002673632
MGNQTARSFKSVTKSQKQKNTQHKMISDTPTSSTEELITNFTKIPVTRPSSSPMSDMVYRCHVVEQLKDLLNNEKALMKR
NLDFVENLKDVIHRISNTIGMRQNKNDVFRCGGLKMICLLFNQLASYTFDDLTEGSVKQKPWKELEETISDISYILYKMA
EHTRARAVMITTGVIYSMWTVAQRFTNILELFFTDKSTNTTERPIARFYRLRLEANQQIESAIYHMNEIIELLSSELSLS
SDLTDLFKSANRHLNSATTDEEEEVDSFGLQSCDVFLGFVDLPLTEWSQKGFEMFLENNTNPSLNLSLSDFDLNLHSSII
NARTNEFLDRFFVKWNFDKSENQKAGFYLLKRDQGIGKFVLQKFCQLLYCDSLIFTQSDIQHLKDEWKSEVDKLANAGED
TSDMEFSIDQTFEKGGIKILLESLKNASTKTNIERLLNFKEIIVLNADTSLFDEFDENLLKQEMDFFFKENQERDNVQFF
HHKKDQIKEHARHTGGLEEDFTNVFQSIARGKTDDFDVLMSTEGNELGQVDTEVMGDEKYALQSSWLISAQEMGGMACHR
CILSARSEYMRKIFKYETSPKCLVKCEDLKWTNDMELFNKEVKSIQIPNLSYSTLSLILRFIYCGENILKIDAKSEGSDS
YQLIPQTYVECLIASDLFLLYPLKKFCELQIMSQLTSMDEVDQEFAIMLLNIALTYNCSDAFKQKCSSYIK
>Ngru_XP_002673613
MLCANNSTEVEETSVKRKREEDFVGHNKNESAEEHLSTTSSSFNEVVDDQIEPQVKKIKTELELKAGLVDEDLFPKSGSK
LYTDDDETRIEDIWHVKYVRKIILDVLDADLTNWQDSEGKHLLDELIERREEIESISLFLAESMIKKVTIHDTKYIKEMI
SNLNIPDMVKTLEEELTEDYPRGSNTLKYWLKYILPEIVKKQNELNLIESNEESEKLSPEEEKEKDEILNSMQYDEKEED
GYAFLLDSCNLSVPSEIKTMIDNEEEKCEEIEETSDDYIIDNLFNEYIFNKTLPDERGEHSYNISPFKGILTEDAASLQE
DNPFLSILEKTIRMSTNNEYKEEHQDLLVSPMRLGLSLLILDCLNRELSLAFNRRYYLYFTGRPIVRAVLDHLIDFDPNK
VYFKQPPGLVEMNGRVFSESLESRITEEHIRQVKNVFDISKPIFNNDDYGFEIDESDTESESSDEVIDETDDDFENSTLF
SEYGEHARDDFKKPRYENFQYGPCGGNINVRFTRSEHPFWSSFPLSQKNLVMLNNYQLNSYCPVPLTIESSSDWLYTEEK
LDGEEEIQVPLHQQKIVSLFPNELDEENGNMTIPAVDHLIHVFSDARHQPPTQNSIHIDINRLFRRAGNMSFSSGALVEG
FLSLKSFISRIIETLMVIKRDDLVTKLDIVDALNICNYTHYPDLKISQPSEQKYIPIGELKFTTEPMVTPENFEDVQVEG
LEVFLSRTDPNGNEMSEDETITMNADYDYVVVNNSTFADHITLFLNDPNLYNDSFIELNGHRVHIYTPIIKHRCPKMYEEH
FSTPEKRNQGFILNNENDFTFDMVFHWIESYYLGSFQPMNDNNRDTENFYLLLRSYEIKYNAVYKGDYSSDDPEDLKDGE

EYELDGLDPSNTCFTLHSLYNDEETKDFQLSFVDHTTMEKKTRKVHKYILACRSPFFRQIFEGNYTDRVTDNFDIGDIFK
DEKSVDAFIYYLYHNFIVIPTLNPNEFEPRNMGYFDLKDILSEKDTSTKQDSANLCAEENEINLYLSLFISSDYLMVDGL
KNLCENVIGSLVDEENFAQLLEFSNEQSSETIFSFVDNFLLSSSLVLENPQIINQDISLYYTYLSGPITLENVAERIRLT
LELVDKSESQLYRFHKERLEEFHIPQLVSFILKNYDFFSQQEYFYQPFNEIKYSEYQSVWEHVSAKYWEMNPRY
>Ngru_XP_002673527
MQIHSQEFVDTTPINTQTHSQVISPKQLNVKPSFIPSIKHENPIISQFLTTPENTTITGEDIHSEQSLLREYSITFQINL
LGNGHGQWRVLFHHGNANTVRQPACWLCPGSNLLHCRCDSSVADNAGFDSRQEIDLNRWTHVGLVYYGPYQFLFIDGKLD
KYEKFGQYLKRNDNQNFHLFESGYGYDRANAQIKNFRMYNMALCDVDILEDMKSDFPISMSTRLEKALESKSDSFLRRDL
VQLLEMKEFANVHIACSDSDQILYANHAILVARSLYFKTLLSTQWNNSKEYTSQNPFVMKVEFEYLIMKYILEYIYTGTI
KTKDGECIPMETLLKLTQVGNQLMLTEMEDLATRFIIPNITTDNVCELFDLAETANIESLFSYCKIFISNRFKLIPTERI
TSLRTELLSIVISDLKNRLQ
>Ngru_XP_002673472
MSDLLQQIVGDEDEYYDDDDDDYDYDEEYNDEEEEGGEEEEIIEENDAEEIIEQTKANLTFKNHSERKQELKQNKHTKSK
LVASSNDNHHHSEQVLEISEEEMEYYKQLKLYGINGPPKKEDGELNEEINFKLDVLQSSKMFFESVQPTLVTIYSGSKLY
DDQYRHLRQNSYRLIREKGYQTDQEMMAAELTHPRLLVDIMPLSKSIDYVQQIMNRTEHLSCLSSDMKYLYIYGGKYRNS
LRDDFIQYEILTGKMNIYNNLSLASHIDNYQGTCIVIPPLFGGGMFEHDGKVYIIGKGEKSPSTHIYTIENLREPMKVKN
GISAGYPWSYQHVNTIFNSRTFFTIHHSYESNVLEVFLFGGKCAEKAMDEMINIHIPIQNSYVQPITYTVLTKPKVGEWP
SARYGHSSCNIANLIFIYGGINTKNQLLNDLYMFDTTTQVWTEVSVESMLPPPLFKHQSFSINNQSFYIFGGQTQHDASN
SLFRFDITDKKWYLIEVISDERNAKLSGHQCLATSNKLFIIGGVSDQENTKSMRNFSVLLNVSDPGKPIPICNYLSTKRR
EGYLCDIIFRVKDMGTDTTSHILAHRCILKARCSYLTEKFSNCVETMNTHNFEGTFLDEEIGVVDISECQTHIFEKYLEF
LYSGSLYLENLEVVSQFLEFVKNISTERHYPSLQKICTLDSRKDLSLTKQILADMHKDFATLIDDNTYSDVVVVLDQENM
FEKDSVPMEDGEFNEELLSPLTSATISSSGISVHRLIMSRSPFFSRMFVTSGMMETKEKVVHLSDYSKEVMIDLLKYLYT
DIIQLSPSNCLGVLVYSLMFDLTDLASCCRRMVISLLDNSTVWVVHEIAILYNEKSLESECEHFIANNFVTLNSSSAFLD
LPDMTRIRIKQLYERKYHKNKTK
>Ngru_XP_002672901
MSNKLFGSKQQHSSPPPAFYYNPNSENIPPSNVLTSPRKNANTPQKVSTPRKMLANMKAKVLRKPFTATEEENYQNDATS
TSSSGSAEEQVPVTPQRRNTCEDEIVSMDEALAKRSPEAIPISMDDSSHKIVQVEKKLLKKASAAQLEISSDDVNLEEVH
KDLVNMSEQLSACVFDKQFSDFVIQCGPGNATKYYVHRFILSSRSAYFRALLSSTASVTGDIQSNKQRYTWEREDIHPEI
FGRVLEYMYTGQIKLRLDNVLDVFVQSEEFGIPCLKKLCENYLIAHGDYENMAQLLEMAIENKAQELVKHCYKYIDEHIK
RVLKSETIKNVKTSTIIQIISRDSLQLEPLEEVLVFEAVQKWAELRKHLPFETPNISKIIEHVRYPLMSSEQLANVVEPT
QLVPQHPLLFEAYKYHLVKDKSSNQAPRFRARAGASSQ
>Ngru_XP_002672866
MSSSNNNSKTIRFFSDTNSNTLIRNSPSNNTNLKDNSNPILSITTTTTPNNNKSTTQSPHHLDFQLSPSTLQNTRKFSLN
KDILISMINNSSTSATSPSSSSINSPSSSSSSSQQLNNNFSSSNNLFNSVDPDSRRRSSGGENINIGSGNGSGNNNASVN
SLGGAGNNSVLFLNLDERLAMQTQNELNSSRPINHQRNRHKGSGGISEYSFTVDDLILPNSDISSPRLAISSNLNSNIVS
SNVSNAKSPTPHSKSNSTTTATFVPYISPLNTSNSLESNELGTTEFVNLDLTSILPHGKLLRFFLVNNQDYFTDTVQLRK
HSPVIAQEIDFQQRLNEFENQNTGFFSDISIRFVPSGREIRAHKFILSMYSPVLATLFSSRWKETHDNVIEVLTEVSNQS
GGGVNFEEVDDEQLFVEMILCMYSGQIYQVEQHLIVPLLELSDKYQFTELSRACEEYLRVHLNEHNCISLLYLANGISIE
NTLQSSANTRLSISSTTGSKYSKGKFDRLKERALAYIDTHFELVSKTKAFLFLDVNMLIFLLNRGELRVDSEDVILSAIL
NWCDGDLCNRRELLFSTLLEHVRLYHLSPNRLAHLGEVLERYTGTLENSSLMMPWMNRIMQAMSYQMSHTLGFQLSQSMQ
QKIALECPPRKRATQDITRISNLLSQEQFLQIAEWINEQGKQGLMKDVSERFVSIFPL
>Ngru_XP_002672856
MKRTKRDDPVALVIDLASSSSDEEEEEEEQQKEQQSNKIRCTNCRKGHSKCSSLQDGIFPCKRCKDKKKECIPVNFKRGM
KESDSKTSSKSSKSPSSKSSASGKSNNKKNSSSQTSSSSSIVQTVVKKEEEPKKKRKVVKDESDDSPTSKPLLTNLNGHS
QHDQIEPFDETTYNNFERYSERLLEVTSDDGRSNTTSFINNNNNNNNNNSMNIFLARMPNGDLNLMRAPSLLQSKIMLEK
MVAEPETAMIYQYDGPLCFGFSPTVRPLASAEQYMINQVSTLSKSRDTLLHQHFVENYKKNHMLDCKFRVYDVESKTEVD
LECHSAVVLSFSPYLRSLVKPTATQPVFKFGNKFHYVNKNALLNIIEFLYLGKKIAGSMDELFDIVLCSILMDIEEVKYA
ACLTIITKLFKEGQIAPLKPKRAAKIVGVLHFADEHFLQLLIQAIFNRIVREDVGEELKQHGLSKHLLEKLRSWQEYRDG
VVWNEEGLTKDVSMKLDNNTLSTKTWHKLTKTAYPNRQYCQSKEDLKEVIISEQIEHDLKKAKEATNSQSLSSPKKSIHK
RVVSNQLL
>Ngru_XP_002672535
MIWIARLLGSFWKSCNDNQLQRYFHSSVIINKQYMYIYGGADKAEGQHFSNSFVVYNLESGEYHCLNDVIKILRRKKAIK
PTGIRTIKPEKLNRNDYELFPNAKLPKLSQHTMTQVSKCTLYIYGGQLEKGTASNSLYRFDIESMEWLKVKCSARHSAQD
ILPALYGHTTNVIDGTKMYIFGGTDGTNYFNDLMVIDTESNTWVREKTQGVKPSPRYGHTCVHYNNSLYIFGGGNDQHLF
NDLYSLDLDTLTWKHIKIEGTTDSAKRVHHTANIIANKMIVFGGLVNAHSHSNDLMVLDLEHFRWDIEKPYVDKNSPAPP
SLVGHSAQMAGTKLWIIGGKFAENDSSTQISNNVYTLETGIRGIEPIDCGRSTLTSDLSSLIDSEDYSDTILEYNGQFYH
AHRPIIYARAPLLYRECMRLSSNDIVNVGDAADALMKKKKHKATEKYEELKQKSEKVKSYGFRGLLEYLYTDRVGLLDGE
DIPVDVDIRCCIYELIFLAVAFSLDRLFALCSKHLDSDFTTSIPAPTLYTDMKKLYESTEKSLLLEKNEDQDRLDSPPSS
SKAHFDFSQYVSASQSENDDSSDEDDNYSITSPMSVSTQDGGNIFSTVLSTTTFINSDQSNCDVLFSVGAEKERIACHRC
ILNSRSKFFRRMLSNKPQQQHSIGMTKIDEFELTGIRPQVFKLVLQYLYSGNVSINFDISVELLVAAEVYQLERLSLMCQ
SVIEKKIHPGNASTIFSIADSYDVKHLRETCTYFIVHNMSKVRKTNNYKKDLSSDLKQELKDLRKLFRQQEGSGFYEQSE
YALPFSTYYHSHFASSSYNNKQKKKHKNKQKQSAKKLAPPPTPNSAPPVPTSLGRKKIRKPNSLSSMNDIDGKEEVSYLT

SPGNILLGVSPPSPWSFPSDNSPTLADDSYSSGSVGKIQKKRKKNVKKNIYH
>Ngru_XP_002672368
MNDDEFLLDSWSHWYCKEGSGLEDGFFSDQHNEVAIRKVQNSMYYYLMRDEEDQLTSANNNSPKIYHDLFKVEQCHDDSE
NSFLIKKFNLLETKKETIYFTIHIPEIRKKLLVSRELIMLESKVFEAMLNIEMAESKSNEMTLQNCEKSWCDYFEMVIEF
IHVGKLGLSKRLFNENDARIIGWKDLLELLKIADEYQIESLIRAISRVVNQFNALTIGLRCLEFSADCKDIIIPKVCYML
GYNSSCVISDTVYRGEKLEYANDEDEKKYMENPKLLIKYYPFLDNIMISKKLTQLLLALLYVPVLKIVSSLPLELFEGLI
DSPHFSKVEGNKMAYIVMWMLQDIENRFKDAYGLLRKYDWSLCDYEILYSFCLIFIGIRKSMPQFESLIEEIQDLLHIYM
FRKERVIPDELWKKYLIQKIPEKVNYINTKLRFLLLGLDSSGKTTILNQFKSGEPGVTTPTIGFNVETLERGTAEICIWD
IGGNEKNRSPQKVWKHYLSNTDCILFVLDSNDRQRLQEATDDLNRILKDDELKNCPILVFANKQDISTSMSRVELVSLLK
LHTFRQKWYVQSCCAITGLGLSDGLEWAIQTVEKAVGFPKLLINHKKYTHFGRVSNW
>Ngru_XP_002672337
MWNIMDHISSDQAKTVTEKVIDEEDVSKYSHWYCRDESLLKEDAESKPNEEAIRKVQNSMIYYLIRDEETKSDAADQKQQ
VETETEKTNDENNQEKKEENKEKKEEKIPNDLFEMETTQGEDDLVTVNYNRLGQKEEELVYFTIHIPDIKKKLLVSRELL
LLESKVFEGMLNSEMLESKSNEMTLQKEQAQEGEKSSCEYFDLAMEFIHVGKLGVSKTSFNENVSNIIEWKDLLGLLRIA
DEYQIESLSRAISRVVNQFNALTIGITCLEFSADCKSIILPNVCDNLAFNPACVVSDMVYSGIPIETANHEEVNQYLEDP
KLALKEYPFLDSNLAPPTLHKILATPLVQPLAKIIISLPIELFEGMMTSPYCALKQDPKMAFIVLWMLQDVENRYIDTFR
LLKSVDWSLCETEILYTLIYFVFIGIGKTLPQHKSICGEAHDMLHMSMFRKNNTLSDEDWSNYLTKLFPEREQGKISHLH
KELRFLNLGIDASGKTTLLYKLKLDEVVTTIPTIGFNVETVDYKNAKITMWDVGGAYKLRALWRHYYQNTNGVVFVVDSN
DRDRLEEAADDLHKLAKEDELKDCPILILANKQDLPNAMSVAEIADKMKLTSLSQKKWFIQACCALTGEGLYEGLEWAVE
SCNVKLVNTPKGNLQKFSHFGTMTF
>Ngru_XP_002672264
MSKRYSNNSSSSSNASSSSSPRTIFETSVGGSXNTKQFSSSPTQLVLNVSNDISNNTTSSDNQEINTTITNSSSETLSGM
VLAVQNVSPSSNPLNILQATEQDSKETLLSNNSPTSLNTSSSIIATSTTDNNQTKSSPIAISNSTTSTTTSEGSNSSSLT
NQQSPSILQSINFNPPPPPSSPIMMGGQTPPQRHHTRNLSMTNTILNSSLRGFVLSENNVRLQKAKITFGKSFESNVPSL
FNNEELADFRIVMKHSNKLNLGNVLSCCENSSDKEDSSSTLPNCLHTNATEVDSLQDNLTIKTNDIPQLTSTTLYVNKAI
LSLYSPFFKEMMFGKMKTKCDFIEIMDHSVMECPDFHNNQDQENDIGYEDVRIFCIMIKTMYTLEVDCLECDSAPLLEIA
DKYMFVDLASICEAYLEKNLNEFNVLSCIFLENSKYQSLKTKASQFIAQKFFIICKNGQLNLIGDVETVLNIISRDDLNV
RTEDDILNAALLWVEYDFQVRKKYFPTLLNHIRLQYLSPECLHYLISIPEKYVADLELQVKLLKKIAKAMSCQLNTIVHK
VDKANQQLALQKTTTQQLAGIANISTSNLLSAPVMHVEQISVLSPTSIFDSSEVIGVSSDDDFFTTKPRHHVSGIVSGFD
LLMTGKDLQVSNEGKTLTKINIDGWSVNLCVEPIRFGQRKEFNLKVNKTAQGIINIGICKADVNLNGNLNYENGWCYYLY
NGHTCHNFHSLHYDDNFGGGGTGDTFKVQVDLRTNGTLSFVKNSQPLGIAFQEIVKRLNGEDDIFYFAVALYSAGDAVSI
INSPIKM
>Ngru_XP_002672199
MLTSSSNNNNTTSSTNGSTNNNSSSSITSSASTSISNTTTTSSIITSNNNNNNGSTTTGGNNVGSSSLGTLNSSSNNANN
NNNNGGSLLTTPTIATPGMNNNLMLNSGGVSLLSMAQSPPSNNSILNSGLHSSSLYGIQKNATENVPTNSTVTTRDENSV
YGNYSKIVEKSETFRTVTYANVSRLATDFGFLIDNEELSDVQLRIGSGKNQRTFHCHKVILCQRSEYFRNMFLVSRGMFL
ESGTPLSTGTSLPFSGNSEVLTNSFGTRISIVEKPNITPIVFEKVLQYIYTGRVTFTTQLKHKKIKDKTVHVTHMISTDD
IDNVISMCMHVLAAADELILLELKNFCVDYVKQYIDSESAIVLLDLASKYGSEDLIENCFDFIKHNAREVIASESLPFVS
KLTMKTFLESEYLAVDEIELFRALLRWGLSQIEHCQDEDAVETYIANADDEELSDEEEEIALGSNGLSPEDISHIKEILS
QPPEEEELIFRKKHIAMFRKKQLLNDYESPNDGDDTETIPFAVVKNPLRDDISLHTRLSLDPVPQNRHHTDLIQLANRLR
DEDKQLLRDACMDLISCIRFPLMTAHQLTDVVEIFNIVEDSLLLEAYRHVCAPDRTPLVNCKRAQKRKGATEGENTHFGI
STILKSKNRQELIKMAGETSITKGKIWTLAFKATKDGFDSNVFHKMCDQKGPSILVCRTTEGYIFGGYNSVEWNSSNQWL
KANDTFLFSLINPYNDGPRKLKVKNTQRAVYCHASSGPSFGGGTNLVFDPYDLYLDSTLKRGHTNVGGAYSTFRGGFRSD
EAQKSLAGSLNAWILNEVEVYLLQ
>Ngru_XP_002672190
MKPQKEAIWSLKLDEIFSCYSPRDLPDAIRNYFEEDCDEIYLEIGNSSAGLKHIMERHENQFIIPKLRKLNDEKGGDDDD
IERSMSSCSSSEGLEIDFTNNINDDRNENIENKEMENMKNCNENDTDSIPSLERNYQLVSEYIYNIMKRADYYYYGYKIL
PGKKIPERVTLALLYQLDFNKFLRVSMGTNGFIVTAVPQSINNYKTKQKSSSNSQQQHQHTTTSKASGSSPALEGKEKKR
NSDPPPPNDQVVAQQIVVEQSSSDEKEKKKNLEEEIPEVVYQDGEQDALDRSQEIEVLQSIYGEDFAMIDAEQCFEITIR
GKLNFELPSDSDIANNHFSISEIPFKLSHVGLKLWVKYVNGYPSQTLPKFKCIPLIDLNRIMSLVRSASQVHAQLSLKSQ
MQEEEIESIYAKIRAKSSELLGYAMVYSFIQEIEEYLESNNVSVSLAEEQFQFQQKEKKIIKTSVVLTMIGDAALTSDNV
LNVETSAKYKNKENHEEEDRKKKGCTKNNAVWENIQYFKAKNRISIESYVKVEASYSKNSISSNFWLNRYGHAVASNGNH
LWFSFGGIHEGRVTADLISFNDSGIAKPALLKETINPRIFHTFTYIVTAEIFVILGGMSENRDYYSDAYLIDCQFNHPNV
QRLPVFPSKIAGHSATNFEDKLYVFGGFSGDTYSNHLYSLTLRKPKIGNAPSNSWIRIDAPNGPSRRCGCSSVVFRDILY
VYGGNNKGAFLDDLYAFDLNLKYWTKIVTFGTSTALSHAQLFATANNTLTLIGGIYVYRNWNTTVLEFSIDSSTWSALKC
EQTKKFNDRTALCSVLQDNQNFIYTGGIKNLGFTSLAKISQSNPIMAKEMPEKVDSVRKMVKRYVGGLEYTRTLFDVYYD
MITSQIENNSDLIINYYEKDENGCTDHRKIFAHQYVVISNPVLRRLLAIQNVRDHLNQQDYQYSMEADEENVLSFKNVYI
DIQTFKGFEDLTFNGIMGCLQFVYSYSFDPELVRTDKECLELYQAAKKFKLTLLCEMLCNSITADSLQLTIGDFIEQSMQ
EFLKPAAVGPEEQYFLNTANEFETFSSEQTKDTPYVRIIAPDLYSKLNLERARYIKSMNEESLEESAIPEEMPEVESFDE
ELFEEIDSAKGEGFNWMTDVPKSADDLPELEWKYIIVHKSILVYRSKFFRTMFNMDFSESKTNEIRLNDTSMQSIEAILY
WMYSGKFPTILSENAIELFVAASQYDLPEVRDWARSFIEKFIDVNNVITVLDIADMIGDVSLKRFCIYFIANRLDIICRD
PLFNEIPHNTRFEIRSLNSKINRKKVKK

>Ngru_XP_002672188
MSSLLTSKQISQLVSLPGNNKCADCQAPNPRFADIKQFTLSCISCAGIKRTKIGTHVCQLRVVGLDTWKEEHFNAMIEQG
GNDAVNRILEAELKDQDRKEEMKDMTRFIRAKYEEMRWKGCKALNEVFMKSYWCNLKELLERNGAVGSIEIVLKSVNSND
CVRVKQGQKMYEINLRDFTMEQIENSTDSSIELSLNSYQSIDKPIYQAMIDQSDEISIFSNGRGFFKRSGTRLFDFNLEN
DFILQIISTINGIFVIDRNLKVYKYYFLKICENEKFTNGITLQKCERDETPCGLTYFKSFISRIYPNLYKFIENDNLLTN
EEIGTLNIIFNMLLYDNYDINGIDLFSLACIINNSTPDLDFFMEKLEFLTFKDMNGIQTVIEKVKNYIGENSTRSVSFLK
LYLFKCLQFISNYGNVNNIKIYLPFNYSLKPKSKENLTCLFNCQKSSDITIKIENELFYLHRTVLTSKSSVFKIILDENS
KFNDVKNNILEIDCEIFKNSKNGNVPNLMVKCFTILFNYFYDFEIDLSKFNFYLMSTLLEMSQFYQLDQFHDKINNELKF
FRF
>Ngru_XP_002672175
MENSLKLVVVGDSKVGKSCLLIASTTNSFPEDYAPTVFDSVNVIYGNRNYNVGLWDTDYDRLRALSYLRTDVFFVCFSMV
DRISFENVESKWIPELIHHCPDIPIILVGCKSDLVSNADCDSIVTKEMIEQKLQNSLASLCIITSSRAMYNIDKLFKLAI
SETIYVSKSSKKGRKKVVIGNPEDIEPTPPIVPPARKAPRIYVESSNYGKGFKNLLDSDFCCDTKFVIYNSKKDIDVEIK
AHSLVLACVPSKVIGKLFDEVLVSRDAGMNSYSHEAFEKLSAKINGGRVSGFSSVSIDENEPNILKFRLSNTIHHRAFKS
LLEIQYYGGSETMRKRCKKDQFLNKVKTASTILECEYTNNIVSNYQIVEEDVFNESIETYLNDLLGSEIKKKYFDKKTHS
DVEIVFSTTDSSKNFHGHKLFLSANCDVFRTMFEANFVEHSSRQVDLSGEIGENENRISEFHALFEYLYSAHSTINERNA
VGLLSLANEYGNLIV
>Ngru_XP_002672041
MRQSPKGMEQTIWTIAGNGIKGFYGDNGLAIEAKLSSPYGVAVNPYSNDVYIADYRNHCIRKVSALDNKITTIAGTGVAG
YSGDGGLALHAQLNCPLSVSIHPKNEELYIADYSNHRIRKISLTYGTISTVAGNNTGGTSGDGGLALFAQLYFPQCVKIH
PITFDIYIIDFVNNKIRRISNSSGIISTFAGNGTAGFCGEGGFATNAQLNGPSGLDFNPTTGDVYIADSNNHRVRKVNCK
SGIITTLAGTGKAGYSDGIDAQLNYPYDVSFCTRGQIIYVTDRSNNRICTVSPIDGRITTVCGINEKGFDGDGGLAINAK
ISSPCQIAVHAKNGDVYFSDFGNNRIRKIVGSYHATLLKPLSFKLKKYDRDLVDGTNLICCKSVFNILAPHFSKSIVFNS
FLLKHLTSTQMEIIQHLIDSIMYQQYHDSEWFDKYELVDILAILGMSKGFTSLKRKLVDEFTKSFSQSCAQLVSKNLEKV
TQAKDSAPLFIDNPLLDQLLDYCIGHAAKTMQQSASAVDIPIFQKHLVPIAKKLSTTINPITINDDETSDKTTKSVETLF
NDKATSDITIKTGTKEFYAHKTILASNSPYYEALFNSGFEYQDLKDDIYVIDTEEFSEDLHEIVLKYCYGVFDVSSVPIN
QALPLFKCFALYDVKEMIDYYLNNLQITIDNFCTLFEQLGNYNDSVYERLINFGRNTGKTFNRGQG
>Ngru_XP_002671867
MSTEEDWRSINTAGTTLSTIDGKMQITLDKRVTHKAFKEVLEYLYTDKVSWDKDTEKDLIKETQEAAKYLSLDRLDVIAE
TYLDKSKNVVVPESSWWKNMKWAFENLRTGKWDLSDLTLVCSEEDGKTVEVKCHAVILTSACKYFHGMLLGDVENEQKKT
MRIDIEDATEKQMLAILKYIYTREFEVEVDDVVGVWIAAHKFLLEDLQVECESMIMKNINKENVEEIKKIAEMVQSKRII
ELCDEIQHKN
>Ngru_XP_002671805
MYEFQSITKMATYSSYSFEKLRYEDYMGKKKQKIETTTSSDQEIATELFQRYDIKADQNDDQSIINGNVSSGRNSYLSLN
ISSIYFNIMSGDDSVEKRSSDVAIVMADSKVLCHKIILLCRSPVFKKLFEMYPDCKLISIPSIKKPIMEVLIQYIYTGQI
DNEHLIIHDYVIDVFEAAILLQMKPLIEVLAKIVSSTTRNVYLSAKIWTWTNKMYSQKASKNIFDNYFLIDEISANITLE
DSSTGLDDLISDLHTKTKNLYLESFGMEIEKSTNYLEEQGPNFLEILFRLLEEPDTNSLLSKDATSSK
>Ngru_XP_002671763
MSSNTSSSDSPLSIEEQQNQVFELFHVATICKNVITDAERLMVRNYRQRPLTASQQSLLVETCAMRMLYGQEGLPCVNEN
SRKHFTEKATNQDLYHRKKYVNDDEYSFQQRTSMNKSNWQLNDEFIRNENTRSKIFNEINECHNNSAEYKQKYNEYDKLI
SNDWMEKGSFSRVFPDVVKNLSSSNDSISILSNLKKKLMSNYNKNRIDSIVDEHYPKEKKELIECIQNNISSHQSFNYPN
FLELCKIQVNQFYFKALTQSCSTQFKGCMERNGDTVQSVSMLQSMDCSSQQVTPEEISCVQMVLTTEQEKELSLHILQCT
MKIAEKLNIDIDDILSKGQIIHRVEKEDDHNPTNLYEEGKFEMDEDSEMEDLEDEIKDLEEDSKRVEELTSKTNQGDVIL
KFKFMNGMSFEGGFKSCVLLGEVVEWLALKSGVIKEMIGLRINNQIFENRKSDIYSFCKVGDLNRVRATIKISDLHKPDR
YGNTPLYYACLCGHLNLVKYLSELGARDDKNARCYMNALSLRVRELLRVYNSYEGLKMFKMKNGDCEDIGEQTYEQDQFK
PLDLFMMKMARRVIYGTKPEESDLTIRIRFGKNEEDTIYYCHWWIILKRWPMLLYFALVNKERPIPQKTSYTLTSDELAI
VNLKTKLEIEKYFNSINKKALKIKDYDRKIEQIKEQILVVNQKKNFKRPTDEFDAMKNLIPDDDSEIRDFFKREELRHID
LGPVPLTKIQFDNLLVWIYTGCIVRTENPKKCTKLESLERFTQKQDYCDWMESLGMYNFVDIFRDHRKDINIAMKESDEL
FKKDLKGCIFHSSFESRSKFEKLQRSLCNVQIKIENYLEEVFFVESTESILCHKDFLSERSMFFNILLNSNFDETERMKE
SALHDEIPTLEIQGCNFSVIQELCNFIYTGKFEKLDENNLLELFSYGHRLGFPENTINMLEERIISLTVSDEEIVAFCKA
CFPIFKKSSKLAFVKQLCNHLVKFTPQERLEIFTDLDFAEKDKFRILNDQFRTIRLAFKRNYRDKKMKVSTWLLVLLLLS
GCVVFSAQQPSFPTQRMFGKKIRSRNLLDESIFIGKALDIGFTSPTIHPFKNNNEFVHASQTDSYGFSLFEYKHSEESLK
RRRNPHEILKEEDYMTVSQSGQAIIHGSHHIYSLYGKVISGVSMLDHDVNVWDVKCSDNQLDIDFANGHSTSKRLIPGSR
LVIGKQWQCYGQDTDTKYGNENIQAFLVLAREIRYGEDNMVRVKFHVKKIMPKDLFYDLKVDYYSVPLVRNMVDSNTLQI
SEPTNKRYSTTYRLFNTGFNYDIAEQKVKNPSIGLYKYVNGTTSFTIDCLDCFATVNLEIKAEIELGWWKVSSAKILVGG
QTLANATLLFSYQASYSKTKEGTLYKLPAPIKISLFYLFSLDITPSLEYKLNIEASGKIEYRRNFLVSYSKYVGYKYESG
TGFSDINEGDGLVWSSKSPGIFMEGNATAELAVYPKFKFELTSWLPSAEVAIIPTIKGQIHTEGTVSRSSASLFVKFDAF
YKLGLSIDVKDTFSKSFEVITYTHIPSISGTYSLQTTFFSATASNSTVSANNPIPVNYINETVITGSTTNTIEFSSTPKN
SVLISKRTIFKHLRNILNTFSFISFSTKSSIVPQLQTPLTGFSEFYDSSNSKYVYSAFLNNTDFIINPINSLTIQFEQVF
AYQPIESSETLSYPRQAIRDTSTMVTFIQKVNRKIPKFGVVMDSYGCFDAYVYEAWLQNGRFVQTDGRKYEETYPYWSIL
GNYFYPITMQNNYESADHYVVIQLIPNDPDFPLELNSIRFDPYFEFSPSDASVVQDNLNRLIGLKFGDKDVVYPYLFVRP
VNNELNVKLYTDYEIYRAFYAKPKPGVGLTYTLTQTGSYIPMLKDNQYGTEVGIATTKPVTISVFPNYKLVNGIPSSAKN

LASQEQTYFAFDCRTFYDSVALNTNSKVYLRVIDSTTGNLIVNNFELDIRIVTSDGYVYNTLTKTVDNNMFVLDTDSISN
IKGNCVVFDRFVQIRLKNLATTTKTFQLELSLVHVPKYNEPISISMNPSTFGYGYKDLSEILTREKNSGKSGIVLKQQFA
SYPIGKVYLSSSTKNIPPSFSDPSSIMTASSRVPDYIGSSNVIERLPISFKTNYGNFLFGYTHLITSQSISFSVENLPNT
DISLSFDKGLTNLERLSNINTTIINVKINGMDHYWRESLQKSLQQSDQEAFLYLKAFDQNNNVIVLRNYFSGINTLCTDS
QLTFELVPIQGAILPSKFKLTFESASSLFTTAYDIPVLNSLQVDSGDDNNGIPSVECPERCISCDISNSTCLVCQFGYFG
RNCTETCLITCNTCTNATSCETCPLGSFGIRCDIQDTQENPCSQLSETPIVSVSSSGGGYQLTYSIKIPKSLLSTTNQTV
ISHSSIDSCSKNQPSFQPEQLFECQAQYSLTTSISTLMNDSRIVKQLDSSGTVLTLNVPINIGLITNKFDSQNGDYCEIV
NYGVNHQVKLVLSSHQMIGMDVNGNMTFGDNVNVNYRKEIITIQDGLFSLSGKLFTNNVTVVSVSYLGSQPYQNIDFDII
LSEYQPNSYNLIISSKQKVSYISGIFTFSIQVRTGEKIVQVLLSISLSYTMPINPPPTELKYSTQVNLYSNNFGQPKNLF
TISDSPHVLVNIVDSNGKSSSLPSSISLSIKNAYLCCTKSGVSMPTYDPSQNSYGCQRYNSTTMSNWLHLIDNQVVSSQY
GTELIKFPLINFQLGFKFTIQSNMFPIGSSESVQVNTCFIHTESIMKSTSSNRELNLDESGTSIHNAFDIIQVFSAAQQK
PPIQPSKTSPIISQSLRLNHSNPSRQYLSHLLVVFVAAMFIFL
>Ngru_XP_002671189
MVRFSSSELFSAFSSDEESQVADPAALDLIEGELSDDEEVIQNAFDQHAIPVQFKSQKKKKIKRKQKTVKKEKQIIYEFE
EQVSAPKVVKSTVEKQNNQQEKGLLDEVRFNFPICFSKNLWKDVVPEQITTYQVDSNYISDVMDISFNYHRLSEARSKIF
KEPFKNFTEAVEELVRIPRFRERTTSRKLNSSSTNLLSQSRSIERKIFSCVAHNFSYILEKKENTLHFLIYDSARNTLVD
YLNFEKLFFLKGKHSMIKLDLEFNSDYKIAKFADNILIFCGDHSLTESEYLEQGEVENAHFSAHDRVQFRNIKADEMIIY
KIIKVDTPKEIAIVPCFSINKDGDNCITKRSDYSVNSTNGSFIYVFGGVEKNGKLSNALFMLTRSTTHNGRLDSRLQNNE
CAPCPRYKHSSCMIISDLFIYGGIGENGKVLNDLYRFKDGKWVEIIVDSLLPSPTADCSLFSDSRNVYLFQKNSDSYCIE
VNEKNPSWKILNGLIKEKRDGQPPIKIDATSSIFELSDEGLHFCPKYLFDFRSQRTSLPQFLMEKRNEYYMVDMAFKLED
SLNDLSDYILAHKCVILARCPGLYKYIEETNQELEKEEFIGVIFPNDDKATIPLVRVLPGYEPVFKTYLDFLYCGEIIVT
EAKNVPLLLDLARILSPESHYPFFLKLFNKSTKIDMSLCRESIREYEKNFENLFKNNTGSDLALVFPDSHPNIELDDCGM
VADQEKIPTGALNVHRLFLSRSPYLSRVFVTSRMSEAMSNVVYMEEYSYDAFKKMVEFFYTDKIQLDGQNCLATLIYSSL
FECEDLLSCSRSVATELIDTATVWAVMGIALHYQDPGLQIACEDYIIKNNSLMDTRDFHELPKDAKRRIKEQFKKKKPNK
KK
>Ngru_XP_002671157
MRNNLSISSINNTSTYSPSSSPRSLDSAYSSSSPSTSPSSNSERKFSLQKHERDLIAHSNVTAFRPLINEMAPEFGENVL
SEHNLSIMEEEYLEIVQHLVDQLIYKEHADKYWRNYYSALQILYVLGLSAVNDGRYLTNLKQDLINSVTKSITVKNLVVM
FEKIDGLQQNFIENFNKVNYLLDLLHNYCIEFTVVAFEHPKVGCSKKLLDSPIIIRNYAKIMAKVNLTKPTQIKLTCNDK
LPSSILQALKKLYNQESTSDVKIILGSKLSLNCHKTVLASSSSHFRIWFEKNFDSNVYTPVRDDH
>Ngru_XP_002671085
MLGSYSHTQDFAEHVEKDIRMDNYLKLFFKRKSIRSIVINEDRDVNIQNPYVKLFNHEPSHDLRILVGDQVTYYCHKTIL
ESTSLYFTDLFKSSNWSDFDTDVYKLDVNDEESIIATDYIIRYCYDLQIDIEPSHLIGVMKASHMHNIKPLITLCSKKLE
ISTDNYFSLIEGLYDYIEDPLFSKLHQNIIEFGVKHCKQLFSQDKKSCRKLPPTTLEEIFTRLASR
>Ngru_XP_002671043
MNKFIIALVATLLLASCLVNCQYSNNYGTNYGTSGGAGSNYGVTGGNYNQYGQNSQSYLNQVNAVNSQPYSSYGSSYTGP
SSYGTNYGSAYSMPFYGGYGGYGIPFYGGYGGGFGYGGGFGYGGGLGCVGGVRCFDPHDDELSERKHYEILNYTSGISLK
KFNHDSNNQITAFIPLLKVMFPTFFENVFLNEKLLSQLSKLEREELQVIVDVLMYDSIEVLPDDLDEEESIILERKLALL
FLLDKADENELTYWKKILFEGVNLGYLESFGFTEKAKQKVAYYIENFECGIYLNNLHNLLVSNNSITELDITEKEDISNP
LWELFTKECDSDIDISITSEDGLTSFTVKCHKTILMSKSLFFEAFLSSENGTWNSSEIFPYETLFIIEYIYGKEHLEIPN
RNLLNVMKKAHEHGLNDLIEYCTEKVEFTNEEEASKWAEQLEDYYEEPIFCSTILSKLAQICKSVPCKYSL
>Ngru_XP_002670970
MAPKRKPVISQKQETLVLSSDDEDEDDQPQQVTKTNNEEQEAKKRKIAEEDEEDFDIDSEDEDDADEFGDEDEEGFGDDS
IGKPVSLYVCRWPNGDFSLVSAYSKANAAEILDEISDPGHAIITPYNGPLFFDFTHKKPVFDNVAYVFSTKTAEFSKKER
NSSLFKHFEELYKKKKDFDITIKFKDTSTGKEGSLPAHKMFLSIFCNYFKKYFDKSPKKNQIVLGSSLSITEAHVKSFLD
LMYTNRCPEQSLIHFYKVAQIATIFECDAVIFSACNEMLNSIRFSYSAADMVSILNSIHESSSWNLLFRALCERVHKEVE
QLRNGGLKSQICEILSNWKGSDNYIQWSEDKEALSHNHFKLSKSTYSPEDAYTMWKTLKETVFPLSHKLKKPEPFKKAFI
QEINECQNQSEEALNQHPELAGLMNRADLTSIDVINSWMTFMGRPTL
>Ngru_XP_002670779
MGVFKLFNSPQNVEECFNKSESYPDGEIRVTFNGTGEVFKIHRIVLACKNMSFKQLFSSSSMITITKHKPSIVRKFLTFY
YSGRIDWENNEIIPLLQLSNEYFAPEIVVSLLHNLHDNFTSIIQGDNIFEDVLKLLSIFNETKTSNYCKNTFIETIVDPN
SFMNDMLKPVLITRQRSNVNTFTKAYVPPFKLKEKENLNFIERYGGKIWNEKFKSIKVKCLECLQGNFEKFIKNQVFLSI
VNIEDVKRIIMELSELNNFGGRGQFLPALLNWINHDYQNRFKDSSLLFNFLQDLSESCMSELTISKNKQVKKQEQEDDFK
LTIELPTPPKREFIEKKEENTPEIAIKKKSSISFLGIDMEEAHSASTPGSPVISLVTSPQLDELITPPSSQKSVETPSSQ
TTPTLSPTISSTDSGKKVGRSKSAFIRPRMEQEKGTFGHLKRLATPFAFSHSETDDPIHSNAPPKTTFEVEEETLSDWQE
VGRVVSYVPVKRIEEIVIKKPQE
>Ngru_XP_002670693
MLERCKKDQFLNKVKIASTIFECEYTDNIVSNYQRGEEDVFNESIETYLNDLLGSEIKKKYFDKKTHSDVEIVFSTTDSS
KNFQGHKLFLSANCDVFRTMFEGNFVEHSSRQVDLSGEIGENENRISEFHALFEYLYSAHSTINERNAVGLLSLANEYGM
SGLTSMCEIFISKNIDKSVAQGIEKADIDIIGLLLLAQTYEANQLANFCLHFISTNYGPMKARREWELLTGENLKHVTEN
KWPPDSYFKQVEEYEKKMEKYRKRLKKKQSKSGASIINLIKHNNLVFKSFWFRAKPKPKPKPKPKPKPKPKPKPKPKPKP
KPKPKPKPKPKPKPKPKPKPKPKPKPKPKPKPKPKPKPKPKPKPKPKPKPKPKPKPKPKPKPKPKPKPKPKPKPKPKPKP

KPKPKPKPKPKPKPKPKPNSKNRIILNPTFKP
>Ngru_XP_002670692
MDNSLKLVVVGDGAVGKSCLLIASTTNSFPEDYVPTVFDNYSANVTYGNRNYNVGLWDTAGQEDYDRLRPLSYPGTDVFF
VCFSMVNRASFANVKSKWIPELIHHCPDIPIILVGCKSDLVSDADRDSIITQEMIEQMVQNSPASGSIVTSSRTMYNIDK
LFNLAISQAIDESKSSKKGRKKVVIGNSEDIEPTPPVMPPAGKAPWIYVESSNYGKGFKNLLDSDFCCDTKFVIYNSTKD
IDVEIKAHSLVLACVPSKVIRKLFDDVLVCRDAGMNSYSHEAFEKLSAKINGGRVSGFSSVSIDENEPNILKFRLSNTIH
HRAFKSLLEIQYYGGSETMLERCKKDQFLNKVKIASTIFECEYTDNIVSNYQRGEEDVFNESIETYLNDLLGSEIKKKYF
DKKTHSDVEIVFSTTDSSKNFQGHKLFLSANCDVFRTMFEGNFVEHSSRQVDLSGEIGENENRISEFHALFEYLYSAHST
INERNAVGLLSLANEYGMSGLTSMCEIFISKNIDKSVAQGIEKADIDIIGLLLLAQTYEANQLANFCLHFISTNYGPMKA
RREWELLTGENLKHVTENKWPPDSYFKQVEEYEKKMEKYRKRLKKKQSKSGGADEDSQFDSEIDDEKKKCFIQ
>Ngru_XP_002670676
MKYTLPTDFNNHKIRKILKNGNIITIAGNGKRGHGGDSTFDISKNPHIGGFKYPQKLEIKLKKHSRDLLFKTSIYAFIPL
IEILSPLFSKLILKNEPLISSLNSTQQQIIQNLIDSQLYNEIKLKFNDDEILNVLFILGLFQNNEFIELKKLCVFQFIQS
LTIQNLSKKWEEIELYLSQCTNELHLKNYILEYLHNYCVEFAAIQTKTEEGSKYLPTLPIMTRYCVNIFSKIVLSQPNPI
ILNEIIEIEPLKINQLYNDKKTSDLKIQMDENNYLYCHKTILSSTSSLFNAMFDCGSTFADLNKSEKIISLLDMAMKYEV
KYLVIYCKNNFRITIDNYFDIVSICENYIDLEFFESFFKELLKFGIQNRKKLFYDSETVSRLPPKLCQAMVI
>Ngru_XP_002670611
MYPHIGNKLLTGNSSLYSKIEYNLKKHSRDQLFGFQIYGFIPLIEKISPKFSNILKNEKMLCLMSSSQQEIIQKLIDSLL
YDKNKVKFSKEEVLDVLFILGLFKNGEFIDLKRSLTSEFVNSFTLENLTFKWETIENLLKTCNEEFGIQNYILDQLNNHC
IEYAAVQMQTKEGNTLLSTLPIIMRNATAIFPKMIIAQPTAINIEETIENEPQTIESLYGDMKTSDVKIKMGQNKYLYCH
KTVLSSSSTLFNALFDSGSCFSDLSDDGIFTPDESEDLELLEIVIKYCYRISIDKIDAVKIIGLLDMAMKHEIQYLINYC
KNNFTLSIDNYFAMLEVCENYFDLQLFEQFLREILKFSIVNRKQLFIDQNRISKLPEKFWQEILKLSQDF
>Ngru_XP_002670527
MSLLASRAQLNYPRCVFVSSNNEVYIADEGNQRIRKVVENGNIVTIAGNGTAGFSGDNGPATSAQLYNPFGIFVSSNNEV
YISDSGNNRIRKVLENGNIITIAGNGTVGFSGDNGPATSAQLYNPFGIFVSANNEVYISDSNNNIIRKILENGNIVTIAG
NGTRGFSGDSPFDIRMYPHVGNSSLYSKIEFHNLKKHSRDQLFGLQIYGFIPLIEKTSPKFCNILKNEKILCLMSSSQQE
IIQKLIDSLMYDKNKVNFSKEEILDVLFILGLFKNGEFIDLKRSLTSEFENSLTLEGLTFKWETIENLLKVCKEEFNVQN
YILDHLNNYCIEYAVVQMRTKEGNTLLRTLPIIMRNGVALFSTMIFTQPTAINVEETIENEPQTIESLYNDMNTSDVKIK
MGQNKYLYCHKTVLSSSSTFFNALFESGSCFSDLSDDGEFTPD
>Ngru_XP_002670302
MNENFDLQRSIIEEDIMNVVKKNEDNVCASYLISSDLDINVTLCMHRVINAAYQETKVSTLKLLVSTCLPPPVRKCPRLP
EKREKPQPLERIGFSFENNQSLLNNSAFSDVHIIGMNYENNEIENLYAHSIILSCSCSKVLENLFTKLKYLAANDKIEEI
PELYSEYSTLFKTILYEEKIFKLELVESISTQEFITILELTYCGKTINLEKKFLKGRIDELSLLFDAPSLKNFYDKCDLF
NEGSENIMQDFYSKLNTNFFGKKEYADVQFKIGDNYRYANKCFLFVCSDVFKVMFTDGFIESSSSMTEINLFEGQELTNS
IEDQIKGCEAFLQHVYHRQPVFDSNSVCTILAIAHLYNMNHLETQCEVFISKSINEENAKNGSEEIVTLYLFSKLYNAKQ
LRKLCLYYMTNNYANYLPLIQNSLEEEDLIEIKTNVWPPSYYYTELEKYEKEKANYESYKKHLQHFTNFKRVQSLLSSST
NNCILQ
>Ngru_XP_002670287
MSMISSLYLDEEVSDCVLISQNGKKFPALRAMVGKWSTKLKELLFIPEFKIGMELVLDQVTDLGLEIFLKVVYFGSTKDY
AVDKFMDLYTFAHTYGMQEIEGRALKIMRSAAVGDSFLLLENCSQYLEEFKEINSLKKTLLTKISANFDAVCESEHFVKL
SIEDLKVMLAGEELKLIGSEGNLFKSLMRWIEHDKENRLQYLDYFLSEIRFPIIEGEVLATVDSHPLLVSKRDIISPKLY
EAMIYNLNPAMIENKEDKKFLKRDCDLFNFIPNGMPIKFTNLITGKHVTVIMNGSNDKGEVTKKKRRNQVKSPCDQCRKA
HVFCTDEKPCPRCVKLGFQCVGGKSLQ
>Ngru_XP_002670135
MLPQQVIDIVDDDDDESTTIQQHASSSIHNNNNFSTLNGHLVSSSNGESSRSRFAGAFSIDDSFSGDNDALSPYTPSKRK
LEEIKSFEEPTSEDDDDVYGDVNLDQLIMNYEKTKSASSSSSENSSSYFVTGSNMFGMLGLGNPVSDSAKISSFTRLNNP
AIDPSQVKFLACGHYHTILLTKENQLYACGKNNYGQLGMGNLTKVVNIFKFVDSSLNGMPKELITDIKCGRYHTMFITNQ
PNDNVWVCGKNKTGQMSLPSMNIYPSPQRLITQFSGKVLFASCGSEYTFIKTTDHIYACGDNSRGQCGVGIELPQIKKFT
VVRDPLFIRQDIVQIECGEFHTLFLTASGEIWGAGTTVNGQLGLDPVVLGKQNLYSPTKIPVSNNEPIKSISCGFHHSAF
LTKTGKIFICGRNGDGQLATGETVPRLFSFTHIPLSASSLGHHFVQDLYCYSYYSIAITDKREMFLTGSNKCGQLGCLET
PKIEKFSRTFKHRVMNVYCGRDTLMVEAENKEYTEAVHEDTLGKLLDFDNEDIMSLLDSYIICSDDKQYPILYDFVMARF
PFFEAFSKDVSGVPSLERPSKKRKSRGESSIRIIDLSSISTLSSKSVLKILKYIYTDVLDDFGTTIQEDLDVILWIAEKS
NKDFSSGPPSISNIARYVKLVLQNTLSGITNGTVVKTLSALQLFKNHPLIESIYRHCIALIRKIEKTYTINEIKDYNDLS
KEALIEALTAGENYHCLGSSDVSIPPSTFSEDMESLYAKTQTTNSGDVKLVLSKSTTIHAHKAILAVKCDFFRVKFTSGM
CDMDSFDISDDSEEEDKEEVQAHISFIKYLYTNQVLLNGSNAVSFLTKWNFYSMDINHQISLRCQSIIIEGLTESNVLEF
QKSFFFDRSSPYHHLRKACVQALISHWPAINQRYTKKQIQDHMTFDQYIKITQAFLKLQAKTNNTV
>Ngru_XP_002670103
MLQLDEEEETTQQLEGILMQSTIPLKSFRGKNIKDYELALDWCRKFDERESAVDYLEDLNRVKLLKNLLNIKSNLVGLTE
LFLTSCFRHVGNSMFSGKQEDYALYTRIIQDWMNLQLTMLQQSKETTGVTEKTAFDYLMEDDEQEEDEDTTATDDDFNTE
GAEEETEINFDEEKSDEPPIVNILNMITDMFEKECQFSQLFSNAVSHVDNFPLLEVNTNYYTSSLEYCVKCRHVSFVQVK
LITSQLEILSEMYSSGISKFFKRLCKIVASNSRGLETLILSITDLSSQHLDTFSNLLLEELPNILSNWKQQSKYVSRIFD
TGEISLLDCNLSHYLILLLAELSNSWTNFVMIQITKKLLSSDHDTLHSLISYLFKHGSKTLLQFLSHWQTLLPNNKLQLF

KREQLSMTLPSASTTAVTSSSSSSTPSTVTTPSSTLTPTTTSASSTLASTAASASGATSIVVTATTSVTTPTTAVPKLGV
TGLTTGTSSASSARSRTISIGKPNSSTTQKDPSFDLLLLQQYRQDIMKLFESEIMSDLTIVVGPSKLIGSMEEDKPYKTT
NSSDKFKNKYYPSDDCSVVEFKVHRAILCARCEYFSKLFHSQMKDCMQGYLKFPDKRPDDFKCLLDFFYSAVININHFNA
FGVLKLSDELGVQIVKEKCIEYVKDCVSADNVFDVLEESSFHNFDELRKYCKEYLEENANQMFFSESFLNITKETLIDLL
KSDKIHLGELDIFIAVVIWGKHQLIHRMGVQIVSPKSGANQITVQTHVASGHQNSVGSTSTPTKTVISYGMDGSSKVLSK
ENLPDHLKKSGLTESLTERNLSISEQKAPQPSPELSISELNPKHDDDDETDKDVVDEDDEYEEEVDTLAYSTLNPEIMLT
NPSASRKQDADELKQKKERILKIVNYTHDKSFCQQLAKLLYDILHNVRFPTLDPIELYEFVEISKVVPSELLLEAYRAHS
LQNRVDLFNSARLKVREGSLFYVSGVCTDVPISKLKGWCLYYHKPYHHPTSEADIQAIKGTRILIGARHKNSSALALCAM
GMKQKVLRETFENETTKENGCFFYHWKNHSFGFSGSSLVQLGTADLSEGKDKISWHLTGRGGYRCGSLTSLNESKEWEKM
LFYA
>Ngru_XP_002670022
MNTSSLNTNNAEEQFELGIAILNKAWKTKELQMLDEIEGLKKIGQKKQEEIHQINQQHSKLQFEYQSLNTAMKTIQEMNQ
DLQSERDRLSAQVKALNGELSKLKAFKKQIIQSINLEELEDPSSNGNNTSQFIPPTQTNNSMMSENRSFNSSNINNQSIM
QQQTQSQQAQALEGKEFFKQAKEVLTNRQFADFLQQIKLLNNQQQSAQQTLDNVNIIFKGEHPNLIEGFKRILTQRQQSA
AMNNSETSFIDSDELDKVEVVGNEIFIQRRELIRDIFKNWIIDIAFICLGIMSLWRIPRIILHYLTIITNEEKAGSMRNK
AMEEFVQSILDVVAITAMVVLVVPTLYRVGTTIKRIRTEDTLYQFIIISEAFAVLKDLPFILLGLLSMWRLPVIILKIWV
FHKEEYETAEKRRKLALQQFLDAMIDIPFTISLVLMILSIMHIFTIFLLFRKYKKAKKEEKELGKQRELFTFFRGKVLKQ
LYLLPLQIPLIICTYLVTFTVYRIPITVTTMKSLVGQKEPKKLAAKVIVSQALSIPLDLICTVFSAIIVLTVYRIKDLVA
VLKTHPVFKSTFAIREALEVHKQIALVFWNLVKDIPIIILFLLSSLTVWRIKHILRALANETDYKKRLILLGTNAFLTVL
DIPCILCSALLLVSWRSKRVVNIITAAYVSGKLSEAHYSILLEFYELILDIIHIALIPLLLWRIPNALKSAWIEETAKGR
RGIIRYHLSQIILDVPYMLLTLVVVLSVYRNVELYHFLSKKKSKRGYREFIRDQALQLIIDIPLVIISLCTCIVIPYRVY
FMFKYLFLYEQWKNRDEERRRELRVLIAKTLVDLLTLVFGIALLVMFWRIPFVVYRYLMLKEGVRRTLWIEFKSAILDII
CLAALFINLTFVYQFPYTVKRLYFFTVACIKHLMAFMKTKPFSTTKSKTKNTSEDNGEILPSDMFFEILTYLTPEENAAS
FDRVCKHWHELSRQDSFLWQLYLDIDKKANLIDKKFQAKYNNTNNYNSYHYYNNINLNEAYYSAIGSYKNEDFTSVFKKA
FSASDSENRNEYIKRYLEKKSRYSYKQYTFEDAVNDDYKAGFHHIVKAETQHVIEHIYKLFFIRYKLLGLVLYPFYYLIS
LFSHVEGIESDMTWDRIEFLVLLIPINLFMLLVSLTEKLFNLMDVFPFLGYRWWGDRDFQLWNYRSFYGKKYATSVFLGI
ASVLSSVGIILYGFSVAFSPFYFDIRVLVCKTVMSIGSIASVDSIILNICKKKNIDLEELRKQALVDHMNRDFASDAVNY
WHFEDNISVRKLEMDRFDRLIAFRPLLARMTPNFYKNVISNNNFISSLNEKECSIVQRILDQILYQKHSTLSNMDTDESL
CILFLLGQAHGDLIYMKSMIIQSVITKIGFDNVLDILESIESYLTRDASFFSSTSAASPLMKIKDYCLDFMGSQASEPGY
EFFIETDERVDKYLRLIIKRKTIKPVTWEEKPNNISKSPLMDLFNDHSTSDICFLVGNQKIHCHKTVLISRSVYFETLFE
GNWSIELEEYGPEMKYLIQYCYGFMNYVPSIHQIPLIKKAHQHEMKDLIPIVSKQIIVGKDNFEMLWSELEHYSDDSTFS
EIIERVIKYGFDHGLFGREEQSFNQWYEDLSSGNSDVESFCFDQQEDMIINTIQLKEVSSNAATGAAASSVIESIKGHEV
IDITEDDDSQNDQEVTNLENVMEINSSDEDEEIMHNDKADSEDDSDDEIYFLTSDEEEDYHPSKTTKRMDKILSELVPDV
LEGNFSVGGEFDIYPAIGLQLLVSNDTNVVVERRNISLPFASKEQANDIIQLTTMNEETKPWQLELNQFEITNPNWNDMI
NNLLQKEIKQGLGVGAGGEMSVKYKSNENFYPNSSKSHYSTSYVAFYHESDFKVNVLTSGLRLALVYNVIFNGPYYLMPA
ANAAEMTLNYLSKTLNEWESETPYIIYILNEQYSRHELSDGSNLKENDAVIYDWLRNYNSIHNNFYLCLGNMHKSKSGDV
TSMSVQEWKRHKRLQNEGGRNYENSDLDDVDSEDLENSYSTYTIEYLKNGNEIFSKEIKVSDISIIIPFDRINDFENDGF
HVYHDDRFLVKANFSNCKHKYFDMLVDDQLENPNKANVDKILEMMKTNWLQESALLNAVLKLKNDEIATEFSNKHLCLKS
WQDLISTFGFMNSFKQPFFKFITSNLSSKMIFDFAFETILLQENSTLTSDLCDYVFEYLSTTGYLSPYAVERILNCMQLC
AVPLMESTSVYMIKRCMVVDQTELSLILKYLENYHSYMVSALNLLLGNKEKFLRYYTVIIYYLKYFFCEEVKNFLEGIIT
EMDLNTSLKFVIHLNGLGDHEVANVMLLKLRERIINQFITDDISVENCKAIISAICYCNFPNELSEVTSAITSKIKDFVG
VAKILIPLIKYIEITYGKSAFIDVKAFAQLVTCSIELIKSILNIQEKADVRDWSLPTFVCETNCSNCRDINNFLQNSNPA
SKKYELHRTMPLLRSHIETNLHSVTGLFSYITFETNQRTASVIIQKISNPRLNIYEQCSKTKDALNYLENLLSNTPIIQQ
QPTQASSSLITHQPKRSGSFSSSYPNKKKK
>Ngru_XP_002669860
MSKRMDKREIDWLGPIIIRNEKRISNLNRNTTIIEEEKAELQNFSSECFTFHFIVDGTKISITKHIHTEIVSTFSKLISK
QVKEGEELEFTFEIAKKQFKGLVYLYNIKEKQKYLENVIDLLVVGLYCPYLIIDYFEYGRMEGGMEISPYIFDSEFYVYD
LLLISKFFDLTISKEFGSYLSGCGRNFNPTFCIDEFIGSWENCIQKWLNGSVDDKPSESLVYFTSNVDEISDSNPGKVLQ
ITNDIIRYRSPFFNTLLSSGMIECSQRIVPMDVSSSLLETILLFIMTGNIQNDSIAIELMLYCDMVGFDILSNYCAHVIK
ESTRGIDNALLVLQSLLEMDYIDSVRPLLIYLARYCFFVRTYTLSSCIEIPKEIQTKYPLEDFKLARNHKTFQSRYSYII
CVGIGNDSGANSLLHQFAYNTFHSNSHEKVNIYYQNIYHTGVYSPKVSLNYKSTSLVICFSVTDTKENVFPQIQSIIKNG
GTGIQATQFLLLATKCDLPRVCWRISREEMFLFAKQNKLMLAETSALTGITALP
>Ngru_XP_002669715
MEATSAQLNNPFSVFVSSNNEVFIADYSNQSIRKVLQNGNIVTIAGNGDKGFSGDNCLATSAQLNNPIGVFVSSIMKKIL
QNGTIVTIAGNGKPGFGGDNGLATNAQLNYPRNVYVSSNNEVYIADFCNQRIRKVLQNGNIITIAGNGTKGFSGDNGPAT
NAQLNGPAGVFVSNNEVYIADYSNHVIRKISQNGTIVTIAGNGKPGFSGDNGLATNAQLYNPSGTFVSSNNEVYISDCFN
HVIRKILQNGTIVTIAGNGKGGFSGDNGLATNAQLYSPLGVFVSSNNEVYISDCFNHRIRKVLHNGNIVTIAGNGTPGFS
GDSPFDISLYPHFGNSSSLTRRIEFHSLKKYSRDQLFGLEIYGFMPLIERISIQFSQILKNEKIIWSMNASQRAIIQKLI
NSLIYDNNQLNFTKNELLDVLFILGLFKNGEFIELKRSLTSDFINSITLKEFPAEWEKIESLIMTCKDFGNENYILECLN
NFCIEFVAVKMKTTEGSAVLSSLPIVLRNATAFFPKLVLGQPTPINIEEKIEVNPQTLESLFNDKKTSDIQIKMGEDKYL
YCHKTVLSTTSTFFAALFESGSSFSDFNNGIFTPDESEDLELLEIIIKYCYGISFDNEKISGLLDMAMKHEIKYLVDYYC
INFTLSIQNYFSIVSTCEQYLDLPVFEGFFKEIIRFGVTNKKQLFTDLSVLSKLPEKVWQVMFLNWAQDN

>Ngru_XP_002669512
MSLRDTLLEQIRSRRTLVEQGEKSILETPIFHGITSNGLMVHFTQFFNNDLCSDFKLILKRKDPSQVNELDAYGNRKLNV
IYAHKFVLAQSEWFRTIFESNMMEQLTNELIVDENEENLDTFTAMIAMMYGNYVPSFSSLNSTLVIDLLVCFDKYQATQL
FDRVIAGLVKNLARDTPNYRSVNLLHIFSMFGDRTKSKELHASILTLLVTPLWTKGVRVLRPEVFSNDEFMDFFNKQDFN
SCYFFLEEFVRATHAFDLICQSIDNWIKFDEADRFDKGFLLYRVALNAHKLIR
>Ngru_XP_002669511
MRSKLESDSEPKTTSSNNTTNGTQEAEEQQFDYVEKEIDEDLICSICLLPFVDPISHNSDACGNTFCLKCVEKVSDCPMC
REVDFKKNCSSAKTIAKLLDKLKVKCGMCKNIVLRGELKVHQEKYCPLHEAYQQVEEFKKKLEQEFQQKSEKLQREHEEL
IEIVKAEMEQHLREAKENLAKLEENLLQQFKKKEELLESNMKERESRILTREKLENSNKPIHINVRGTIMTVSLGDFIHN
ERESDNLFKKMFTGQHPCYQTPSKQFDDSIYFIDCDPNVFKHMIEWLQYGEIAELSNDMRRSVLNACKKFGLTNMSNDLS
ESINKESNSKQIMPMSQFDFMNIVNLTRSQKSALNLSGLDLRKLVINNTNLEKSEIIGSDFSGMNLKQTNFKGSNLRGCN
FSNCDLTSVNLQECELDDCNFSNAILGKTNFSKSSLLNINLSNAIYKETKFIGAKFENVTGFQNRKLGSLDFSNSSFKSI
EFCNSDLSNSNFTNCNAIDCNLESTILRKTNFEKSQFDNCRFNNKCKFEETSFKNSSMKNVSGLIGIDLVRVDLSNCKLT
KTDFTVSYIFETNFLNCNFCMEVLRSKHNIISNCDLSGLFLNGDFTGFKFENVKFTNTNIEKSKFENSTFVKCKIQMNFK
KDSLQHCNVIECDFNGSIFEEIDFTLIQANLETNSFNNCVFKNADLRELKDLSKFLGSTFDACLFNFKEFTNKHIQKIHF
QNINLQDLGGFKFSAITVDIPWFNLLSSSVIVNSCDIAIGCELYSNTKSKSIPQLLYRGSRDGFKAEHFHSKCDNQGPTL
TIIKSEHNQIFGGFTSKSWRSRSGECVTDSSAFIFKITDSNGSYQFEHFKIQREENAILVNQYYLALFGADIQIESDCNL
NYRSRSNLGSYYELPKGYQKGSNEANNYLAGSYYFKVSEIEVFKINQ
>Ngru_XP_002669462
MSVFPDLSSTSFPALKECSDSLKVWNLQELYDFSSSEYKTAWESNIDHHDKLSCVYHDCVYLQSLNNFSFNHADERDGRL
ILINLKTKTNRTIPVRGLHCTKVDRDGKVILISSDRKSLQVFHLEMKSKIADIRTEKEFHFADWLDSENIVFVEGSSLCK
LNISQQDQQQEVIIENIPGEFNKDITGVIFNMEMCAIQTVKKGEYSFYLYKSDLKVGQIVGPNNAFGVCLMEDSKKLVLL
SSNGLLFKDPTVGNITEPVLFSEKAYMTHYFKESQLVAFVGYKGDSGVIDLSSKKLIARHDFKTEIVNSFKTDDSLVIVT
KGGKVIECCFNAEFMLHTYRNNYDVSTEIMAYWKRFSESNIKDFRLSSIQSILPLQTPLNKYNRDYYIPHGGIHIFHPII
SYYFPYMYQCVIRNELSIEYTTEEQNIIKDFVSYLIYRGQDLKIDRDNYLKWMFIFDRCWFEFTELESKIVTLFNNDFGL
ILPEISPLLQTLTEYQSRNLNFENGLLQKCINYCRDTMDGKEVSQVEPFINEHIPIRLSIDCIYNRQDTHDITLVAYGRK
IGAHKTVLASTSPILEVMLDEEGSFKSEVTENQELVLGGEDLEIPEKDREKKGELFELLVKFCYTSSVDCSPEDAFLLYE
MADQYQITDLKTSLADKIIAQLNLDTFMEIVKWSDIFVQISNKHLHRGILNFASLNGMKLLELYSDQLVAYPEFLVRFLK
FSFNVE
>Ngru_XP_002669427
MEESRLSTTEDDSMVEATSPESPSSSASSSLANHQHDEEETLNNNSSPNTSKSSSNNNRNSVSLPLLHIASPNAPLNLIY
SCGKNKYGILGQLDDYSEKPVPTVTPISKNSSHHTIENPSFAPIMIKQVEAGAYHLAILTEFGEVYVSGYNSFKGAMKTN
QSGLSQMHKLHFDKKVKAISSGWFHLMLLTNEGELYSSGLNTNGQLGLEECSSSGSGSSNEDNVVEHSLEDGKEEIIESY
ELNIPIKRLCCGEHHSAFITNNGALYVAGKNNCGELGIGRKSSKPNVAYFTLVQAGFLAQDISFGKDHSVLLSQDGQIYV
SGSNESGQIGLGNVKSVKKFVKINMVRPGFKITKISCGFVHSMAITNTGGYMICSGGNINGELGLSDRVKKPQFAMLKTN
LIPKHVSCGASLSVFLTDQNAYSCGINTDGRKAIIDSLPILHFPIRHYNRDRIHDKIYGYGPLLRKVTPKFYENVVINEE
YLNSMTTIALNFAQYLVDCVQGHFVHKKEKIVDHLVSMTPIEKVHALYVMNHIETDLIIENFRRFVMYYLVEEIDGNVEL
TDQVADEIRMLLDNFQDFNANIQDTPLEKVSRVVRLKKPFAHYINHPIHEQSNHRHIFHNMFNDQATSDVKVIIDSEKKH
VLYLHKTILSSYSPLFEKIFDEQFQFSKDDNCLDLYDFDLDSRNAIEASEAKIKEKALLHLMHACYGISKQQDSVDDSFV
LLNSEITILIGMIYHASLFQMDSVIQIYLIRKLATLLSVENFLDICKWMLMFGDETLFQQQRIPSSSPIYEPEMIPPVHE
IFNLLTQFAEMNLKPLSKMYEGKLNEWDSIPHIKQLLSRKSPTNKIPQIPPYSSSEMHLQQTQDSLKEPLVQQEASNSSD
HAEYYQPTGVVNETIDQVQQHQQQQQYIYQVPSQIPPQQGMMNNLPEPIGFVPPQQPLQPQYNQQMPPVQQVYIPQSPPS
NLNSADVQVDEHGRIVMKAPKNNFAWVFFIVGLVFLWFPFLAIPFLIIALITRDTDFIFDDRKRELMSKSYGAVVKFGCL
DREVSVPYSSINDVVVEMDFSMRVNGVPSGKIIIVTSSGNVKFTKGYLNYYKAKETAQKIMNFINQRKVQY
>Ngru_XP_002669314
MKAFLKNFAKAIGGSDKVSSESQVLLENIPFEAKMREQYPCNVLGYSTFYNDQGLPNDRTQQWCPYQVIGPPTCYPNLGD
NVTAWCPAGMAGPEHIIVEYEKPVIAKEVHVYETLGPGSTCKISCKVYKGEEDKSEEWIVLWEDKTVVTNVSATAARDFS
PPISQLVLTKVIRVDLNITSTWSEIDTIKLVGNYDVDHVEITELGSEYRQLFTQAQEDGQNYSDVTISLVNSDKQINAHK
GIIASRSPYLLKQLTVQDGPQIHLGLDVSFESLKQAISFLYCNHCAVSLDTLFELYAIANIFDCDDLKEFCEHNLTLLLT
PSNLQEVYDKAVQNHHTSLTDTILRVMFKHYDRIQQLGLQQILTEKDALTLKNMRRSNSK
>Ngru_XP_002669261
MSTNATRAQLFNPFGVFVSSNNEVYIADFCNHAVRKILENGNIVTIAGNGTAGFSGDNGPATNAQFNYPSSVFVSSNNEV
CIADLHNHRIRKILENGSIITIAGNGTHNQRIRKILENGNIVTIAGNGTYGFSGDNGLATNAQLKILENGNIITIAGNGT
DGFSGDNGPATNAQLYYPSSVFVSLTNEVYIADQHNHRIRKILENGNIITIAGNGTYGFSGDNGPATNAQLNHNQRIRKI
LENGNIVTIAGNGTAGFSGDSPFNIIMYPHIGNKLLTGNSSLYSKIEYNLKKHSRDQLFGFQIYGFIPLIEKISPKFSNI
LKNEKMLCLMSSSQQEIIQKLIDSLLYDKNKVKFSKEEVLDVLFILGLFKNGEFIDLKRSLTSEFVNSFTLENLTFKWET
IENLLKTCKEEFGIQNYILDQLNNHCIEYAAVQMQTKEGNTLLSTLPIIMRNATAIFPKMIITEPTAINIEETIENEPQT
IESLYGDMKTSDVKIKMGQNKYLYCHKTVLSSSSTLFNALFDSGSCFSDLSDDGIFTPDESEDLELLEIVIKYCYRISID
KIDAVKIIGLLDMAMKHEIQYLINYCKNNFTLSIDNYFAMLEVCENFLNAIFERDSQV
>Ngru_XP_002669137
MMRRTTIPKVINTKLLPSYCGIQRQLPSSSSIITTTNNTTGASEEETDSQSLLPNSSRTLKQYHNPSIKPFERDLMNNAH
GTGFHVFRPYVNLVAPSFVKTVLDNEQLMNKLDDFERQVIQRIIQHIYQEASLEFELDDVDCLCRFLFLLQSCSGELVEV

KRNLLQCLQHKITVSNVLDVLDSIDNHIQTLQSFGNSPSNDDYNLLNAVKSYCFSFIRQHVFDEEFDIKLNPRVEKHIFH
FYNKKEQDFIEYHEKPNENSSPFIFEELFNEGEPLEYDFTIDFGDGIFIRTHKTILASNSLFFESMLSSSGASFVDVDKS
IFYPDNSINASAVLLSTAMEFSIPLTSINTIFLLSFKWLTNMTFLH
>Ngru_XP_002669008
MLLGIFSTSSVVKDLIIVINKMDLVDNSLELFDFVRIYIEKSFKQMQTFRKTTLKIIPMSLMNEKNIVRPFGSPFEQYDT
FLSCLRELKPYPYDHTAPFRMIHFDTYSMYGLGRICAGKVISGMVRNTEEFEICQSTLRKTFQCTSQGQSLMSFYDSLTV
SVAGELLACNLFRGWALINYDDYYGPMTFFNRSETICKPCTSYFTVKLSGPVCNMKFNHFIKELMVFFFGFKAHVELISK
SKPLHNQQYIYATFKFKDNEPAFTLEKFDDVPEMSRITLFLNRVIGYGIVTDITNDKYLAELDYCSDDDDEYNEEENEWS
IEEQRLKLYGREESDEEEDEVDGVDDLVDHFKSHSMVSTGEQKRIVPKNKKKKEQDDANRIIIVQVEEKKSKENVNWDSV
YSKLENRKLNQMKFTMKDIESHKLHYDKVDTSRFEVTNSVAVDNSDDEDAQQRRKFKVFEQKVIKQSVDLSLGKMAGSAH
GSLVFLSGGGKSGRIYSQLVCYEVKEKKWVDMKENMFNYYRKQNLTDMLFPPAIEGHIILILGEYLIWYDFKHTCMALKN
YLELIKPTPANKIDKSNPPIPVWMNVSVETITKAVRETTGHSLSKSNNEPKFWSFGGRDVNGVLTNQFVEYNMVRRTFWS
KVQNGVIEFSMTRDLANIANPKAIPLPRENHAQCTVGNSIFIFGGNYTQKDNLLNDLHRYDIVSKKWNEIDLTFIPPPMR
NHSLCNCIDQYIYLTGGILRGNTISDVIYRFDVKNNEFAVINPGGPSEYVLYGNGITTPRDLQLPRADHTTLYDQGQLYM
IGGMTDYAKRSYCKEVVVLKECSDIGVENSSYIYLKNEFEKQANGDLSGYDLILKVAERTSGETEYIYVHKCLVSSRSTY
FKDLLVGLNDVDMVDGKSIFELNDYHLDTMNIFIRFLYTGDIIIPDTVNTECLQELLNISKLDIDRYGIILSVCDQKRLY
LDSSTAVLNQLKHDCSALYQNIEDTMDNFIREPCAENNYADVTIQLLDPHTGELLNELRAHKLFICRSNYISTVFSSGME
ETTTGVIKFDEISLNGLLSVLKYLYTNEIIVSIEHAVEVFICSLLFQLNELANYCRTIVIQHITVDNVLELSFLVDVYND
LALKNNCIKCISQNFEEISKRDGFENLSSEIKGEARTRHFEMVKKLTKMKAKERMKVKK
>Ngru_XP_002668936
MATNAQLFNPRSIFVSSSNDEVYIADTGNHTVRKILENGNIVTIAGNGQQGYSGDNGLAVNAQLVSPSYVFVSSKNEVYI
ADYQNNRIRKILENGNIITIAGNGQQGYNGDNGLAVNAQLASPSSVCVSLKNEVYIADSGNHSIRKILENGNIVTIAGNG
QQGYNGDNGPAVNAPLFDPSSVFVTPNDEVYIADTGNHRIRKILRNGNIITIAGNGQQGYSGDNGPAVNAKLSSPVDVFV
SSKNEVYIADYQNTRIRKILENGNIITIAGNGKLGYGGDNGLAVNAKLYSPVGVFVSQDNEVYIADYQNNRIRKILENGN
IITIAGNGELGYDGDSVFDFSKYPHIGPIYSSTYGKAKLKKHSRDLLFNSSIYAFIPLVEMVSPLFCKYILKNESLISSM
NSIQQEIIQKLIDSLIYNNEIQLKFNNDEILDVLFILGFFKNNEFIQLKKLCTTQFIQSLTIQNLSFKWEQIEIYLSQCS
DFHLENYILEYLNNYCVEFAAIQMKTPGGVNSLPNLPIIVKNAANILSKHSLSQPNYIIIDEAVEIELMDIGKLYNDKKT
SDLKIQIDEDNYLYCHKTILSSSSTLFNAMFDSGSTFTDLSNDIFIPDSSDDFEFLEIIIKTCYETPVQDVKYEKLLRLL
DMAMKHEMEYLVKYCKNELSISIDNYIDIVSACENYIGLQCFEGFYNELITFGIKHRRQLFPDCETILKLPPKLLPLFFL
KFAQEFL
>Ngru_XP_002668792
MSSLVTRAQLFNPSSVFVSSNNEVYIADFCNHRIRKILENGNIVTIAGNGNYGFSGDNGPATNAQFNYPCSVFVSSKNEV
YITDYSNHSIRKILENGNIITIAGNGTVGFSGDSGPATNAQLYNPSSVFVSSKNEVYFTDQHNNRIRKILENGNIITIAG
NGTYGFSGDNGPATNAQLYNPYSVFVSSNNEVYITDYSNHRIRKILENGNIVTIAGNGNYGFSGDNGPATNAQLNRPNSV
FVSNNEVYISDQSNQRIRKILENGNIITIAGNGNYGFSGDNGPATNAQLNRPNSVFVSNNEVYISDQSNQRIRKILENGN
IVTIAGNGTAGFSGDSPFNIIMYPHIGNKLLTGNSSLYSKIEYNLKKHSRDQLFGFQIYGFIPLIEKISPKFSNILKNEK
MLCLMSSSQQEIIQKLIDSLLYDKNKVKFSKEEVLDVLFILGLFKNGEFIDLKRSLTSEFVNSFTLENLTFKWETIENLL
KTCKEEFGIQNYILDQLNNHCIEYAAVQMQTKEGNTLLSTLPIIMRNATAIFPKMIITEPTAINIEETIENEPQTIESLY
GDMKTSDVKIKMGQNKYLYCHKTVLSSSSTLFNALFDSGSCFSDLSDDGIFTPDESEDLELLEIVIKYCYRISIDKIDAV
KIIGLLDMAMKHEIQYLINYCKNNFTLSIDNYFAMLEVI
>Ngru_XP_002668691
MSYSSDEEDYQEEEEEPFSSSSPSSPSSSSSGDEEEESTDDRYVRVEGNNPKRKNLNVTSNSSSSNMNFIQSNFNDHHYI
YPDSDLNYGYFLNTTANRRSSSSGGDKKTGAAAFTFTASGQPFGISGGGLGGGDQTLISSSNSNSFSSSNSTLCGGFKFD
DTSKVLCSQQKPIFGSFNTSQPFGTNSGDDHQATNNSNSSNTHNASSTSSLAASEEKLFVFGSVPSSSSSSLSHSPTKTM
HRRGINVASKNRNENIDTIFNSLPKPPTFGYQQAVDQKDSTTGDSLTEMKDSDAVVKIPDSLGGVKTIYCHKTVLSSASE
MLEVNFGPNWSNEEDEDGRAIINVEHLIEVASDPSVMEFIIEYAYGIFAPIKVSSKQVIPLIKAAHFYRIQGLLDECLYL
CVNRDKLSFFEVAESLEDHFEDETMEAFEDVMVEIGTANYPFIIGASDEELMKLPKRMLIKMFRKIYKTYIPNIKTL
>Ngru_XP_002668650
MSSLATSAQLYSPESVFVSSNNEVYIADYGNHRIRKILENGNIVTIAGNGTAGFSGDNGIATKAQLNGPVGVFVSSNNEV
YIADYDNHRIRKILENGNIVIIAGKGTAGFSGDNGLATKEKLNFPRCVFVSSNNEVYIADQINHRIRKILENGNIVTIAG
NGPYGFCGDNGLATNAQLNSPAGVFVSSNNEIYIADYDNHRIRKILENGNIVTIAGKGTAGFSGDNGLATKEKLNFPRCV
FVSSNNEVYIADQINHRIRKILENGNIVTIAGNGPYGFCGDNGLATNAQLNSPAGVFVSSNNEIYIAEYGNHRIRKILEN
GNIVTVAGNGTAGFSGDSPFDIRMYPHFGNSSLYSKIEFHNLKKHSRDELFGLPIYGFIPLIDKISPKFCGILNNEKILC
LMSSSQQEIIQKLIDSLIYDKNKVKFNKEEMLDVLFILGLFKNGEFIDLKRSLTSEFVNSLTLENLTFKWETIENLLKIC
NEEFNVQNYILDHLNNYCIEYAVVQMRTKKGNTLLPTLPIIMRNATAIFSKMIITQPTAINIEETIETEPQIIESLYNDK
KTSDVKIKMGQDKYLYCHKTILTSFSTLFNALFDSGSCFSDLSDDGIFTPDESEDLELLEIVIKYCYGISIDIDDVKIIG
LLEMVTKHEIQYVIDYCKNNFNLSIDNYFNMLKACEKYLDHPIFEQFMGEIVTFSMVNGKQLFRDRTEISKLPEKVWQEI
FFKFSQDF
>Ngru_XP_002668147
MLSSSFNNMERMLDDNHNNLIQTGDMITIHVDNNISKPATSTEGPSGSCEYLTGKFSKRATTPSYYHLLYNDAPVCPPDF
IINIKLIDMNMTIVSIKVHKVFLALESLYFRTLLNSEFTESTNSVVEFELTSVEKDIFEQIILRYIYGERTLDIDKGLPY
ACTLCLAHYLQISPLECQLLQQMTKTSKYTEQHLLYYSQLRDHSMLAVNSSFLSQSLTAFGKDISKKRDEVMNLPVEFFL

WLVSLDNLNVSNEDEVFDLIQEYIIRNEKSIRQNNPEGFDQKPDIIEQVWSNVRVCFLSETKLLSFMDQPSISTDLKISM
RERRQ
>Ngru_XP_002668130
VLHNGNIVTIAGNGTPGFSGDSPFDISLYPHFGNSSSLTRRIEFHSLKKYSRDQLFGLEIYGFMPLIERISIQFSQILKN
EKIIWSMNASQRAIIQKLINSLIYDNNQLNFTKNELLDVLFILGLFKNGEFIELKRSLTSDFINSITLKEFPAEWEKIES
LIMTCKDFGNENYILECLNNFCIEFVAVKMKTTEGSAVLSSLPIVLRNATAFFPKLVLGQPTPINIEEKIEVNPQTLESL
FNDKKTSDIQIKMGEDKYLYCHKTVLSTTSTFFAALFESGSSFSDFNNGIFTPDESEDLELLEIIIKYCYGISFDNEKIS
GLLDMAMKHEIKYLVDYYCINFTLSIQNYFSIVSTCEQYLDLPVFEGFFKEIIRFGVTNKKQLFTDLSVLSKLPEKVWQV
MFLNWAHDN
>Tvag_XP_001583419
MSEQPSFPWASQMIEIKARNGQWSVHVYEPLIKHRWQFYAREKAQALQKLQMLPHNTIQAILEHLYANTPVARTNLPAFK
TCKVVDSIPFESTYHRDMMQLLDDESSWDFALIPRDSEDRVNVHRFMLYARSGFFRSQFETNSTMLQFRDPNMCKAALEM
FAGYIYTGRLDPTDAVALVDLFGAGKNYQLRDPLEIDFLAMNNLQKLLTPQNAAEVKARAEERKLQEVINLVQDYYPC
>Tvag_XP_001583415
MNQEVLSTDRNPFKPLPCIFKSLLYNPLFSDFTLFLPNAPPGKKQYIPVHRFIFDVRCSFFHVFFERMPNEKTLTLCFPQ
WAAPDVFTVIEYIYTSEPINITNENIAAIAFISLIWDCSELIKLATDFIIHKIDPKYSLDTLNKLCNIIKHDAPAIQKLL
NMVVNNIAIFNPDDFNELQFDLFESIINTVYSKRPEDIAAEALCNCIEHYFIANVDKLGKDEFRVLVQRFMMKTYHNGVI
TLYKHAIRLNWSIAHCEAKILHSWLHLDQNKLATIPVDRLAVILNENYLNCGSEDELFKFILLVHEVQPTADLTPIWKCL
RVPALTEECKEKLKECGIAPQFVIDAINSAGRYIDFRRLPRARTHCLIIGAADNWALEDLKEFMGFAGFNPKNITAMRAA
PDMDADFYKFHAILVFGFYKFPPSLSSKIATYVSDGGGLVVAFGAHRNDDFGIGEPILSMLPIEAFRQEIAEVCYVRAPT
DSNFNDGVDIASEEKEQIFTGCKNMRMLFSVKEGASVISSWDDGVPFVVIQNRTEKHGTIIVFNASPVSNEIFPEQWSKH
DKTMARILSNCIITASNKVFSKASKD
>Tvag_XP_001581670
MKPVEIFSLQFHPSNLKSLPCDFEIRVKGSPQTSYKVHLCVLSEFSSKVRLLILNDPCKRSIEYPGADPLGYLQQLIKYM
YGDEINITNENVCFFNTAAAFFDNQHLLDTTSNYIQKFGIQPDFFQKLRQSDPLYVLPQISLAALQFEALKQQLQLMDLS
LPILDEILRKQDLYYENEVSLFLWMNGLVEKHGPEYVQLFAHLILEDLDSSCMHLLYSKITPDLIGGALWEAICNRLVYS
VPTEESPHVRHWSQNQQQGYQNNNQTRPQFNNSINTFTPQSNPISLPKSSHFKEYPKQPGQYFNGIFQALTNTYGKNPCS
ENIVFIDGGGGPKSRFLPRLLEYEDISPWWNNYNGSGFNKNDQWIEITFYKHRVILTGYTFASPMKYPNQSQPRSWKIFG
SNDDGQTWEIIDEVVNSDDMNFSNAIASFQVKKRPEPYSKFRIVQLASQLKKPSLELYQFCLNAIEFFGYIEEIQD
>Tvag_XP_001580841
MSSSSNKNYSIIYDNKKYRVDPLIFCKMSEKFKTIYEQNPEKYKFENDEIDQEVFVAFISSCQLKTFQINPKKAQALLKL
AREWEAPSLESYATTVCRENGYPVYPRVDPIGDLKIKQDRNEDCGEDYKRAGAVFMDSLDDDRLMEFDPEPLFRIVAYAE
KLPNFDEQKYADFVMKLRERRMDAAVLLSLRLNFTKITEEDTDALFQSPEMRQQSIGFFVAYSLSQTRHKTQELINETTA
KCMDQLHEFGNDMLDANGSLKKALQKDHEKNMKLLNQEIDANADELNRLVREFTRTADILVEGPLSPNGFADPALKRIED
DAQERLNNLYNHIREEIGKNHDEKHPKMRDDVDRVEQDWENEYNDPEATQKRMEDMLNELEGVASKYRGEIDQIDKDLDH
IRGSINAKIARDYVRTDKGSRVEANIYSIFDEADFVKEESSAIEDIDARLDKQCPLKIGKK
>Tvag_XP_001328363
MTSPPWKSQMIEIKSREGVVTEVFAPLLKFRWPLYAKTPTAALKKLTVLPKSTVQAILEHLYANVPVSRTNLPAFKTCKI
VDKLPFESSYYNDLINLLHDTESCDFELIAENGEMTLPIHKYILATRSSYFRNLFKEDPGVESITMQRMNGDALTMWAKY
IYTGDFETTTVSALPELIGSGYTYGLRDPAEIDYLAVYALQSSLNSENVESVREKATDLCLPEIIELTDSIPLGTISE
>Tvag_XP_001325264
MTTLQIRIPFNPTLDTMKEFQDVVLVSPYHKVEYKANRLKLAQLSPYFRRKFNEIKPEPGNVLRFQLEKDPKNATQVVLD
GFQTFKLTINPDIAPALLCVSSIYELPHYCQFAIEVIQKAIVANSPYKTISRYLSEFQEYGIVEHVDKLIPYFAQYLIDA
IARPEIADHSLDELLDLITDGRVLSKLILYIIKERNFKLATSAILKTFDTFYDKHPNMSADEKESFMDNEIINWDQPDTF
QYLTKFKCLWLGYKKARENFKKIFVNRRKIINEFKPDESNPKQMVKRWYALSLIKDIHESDPSQDNENYNTIKFITTLGG
VAKQLDPEKFGLINPDPKVIPCGDQILDDNYKHSNIFMKNEKIFCAIGNDKHAVVDFGSEVYASSIVLDFEIAARIRKIG
DISQYFPHPKSVEITFFSGQKQTHSQKYDVKSRIFTIKQVNRAFTKIVISPSNKAATFKLKNVEIVGHFTN
>Tvag_XP_001327639
MIDLSNIRYCSTFNDFKFSHRGKDYFVNSFLLCTVSPFVHRIFEEGNFNNELEIPPVEGDFNEFINALYGQKIRIDSHNC
RFLHFISKFLHITQLETVTEKVCEETNTFENNFRWAIQLFNANQQRDEIINKLSYNLRNNLQNPCLFEAPPGLILEILNS
KFLQIDQGGKLQLVDNLIQYDSAKYQSLSTFSYSKSFLLKQFTEKPFDLNKIRSNVIRNVQYFVSNDKSKRIVFSPQPSN
LFNGILRSKTPFILGSSDTLGKEFDISNISKEKSGENIFCSKSEKNPFFIIKFQTVKFQLTHYAIKSSAFGNLSFCPKSW
KLEVRTSSNSWTQIDIVENDNSLYGSGFQSIFDIKFSISPMSEFRFTQLDSHHNTNKSLMIDLFEMYGIAFENPKRLIN
>Tvag_XP_001325335
MSTQQSQLPKVPYVFWWNEKRYTVDAELFKKLSNKFARDLEDEDKQNQQTITDVMDEDTFKAFLAACQFQHYDITKKNAH
DLLKLARKWEVPSLYEYVDEYIKKNEIQEDRKNYLNILVEKVKTDSDTKEDWTNVAFDINRYMNEEPFKTLPPEVIYQIL
AIADRKYLDQQIFNDFVLDLFKTNPTNAVPLMLRLDFSLLTDDQIASIFETKNVHQMNINFFTAASLSAVTNKARLQLQN
EEIRQKINLDVFRVKNMIHQNSLTEDIQSDYSKEVSEIRETISKQKQQIEQLSRDFDIHNDRMKKIEHKITSSRTPCDRT
VLATIQNNVRDYVDKTTTEVNKQLEKHFQKMNGFYNSVPKQTDDYFREQTMNGDNEHFKSQFLLNSTMERNHQVNAAIDK
MKEQMTDVKAMLCAKVVRDKLRYDEFLRKNLSKKFNIFDVEPSIWGIRSNQVKDQYERLQKMEAQLDAVCPTRQGDKASS
KKK
>Tvag_XP_001321987

MSTQSSHRSKRLGYYIFLRDEKIKVDPVIFAKKSSYFAEQSASNPDGFEVQENVLKESFLTFLSYCQDKKYEINTDNAND
LLKLSRNWNCTDLEGEVTDFCRKHKIVVRPKIDPLGNLIEAIDNQKESVQDLREVAKIINTLYDDDRLPDVEPELLYRIV
IIAEKEFTLDKIKFKKFVLRLFKSNPDAAVILSLRLNFNELTDEEFEMIANCPEIRSQSLNFFAASSISIIRNRIKNNLA
QFQIKNNNLLNKLNNQIRYQHEMFLYQLEEYKSQKKALIDQRLEQQKAEINDIYEQLVSVAAHLDGGILSENGLVDPRIN
QNINAAEKRFADLHQQIQEALEKDKQDRHAELKSGVYQRLKVWEAKHGDEDSQKKLLDEAFEDIGAISKDIQEKKSEIDE
DLVHIKSAVNAKIVRDKIRSDKGRRDTANIWSIFDESERESVIKEDEYIQNVEKKIEEFCPIRKH
>Tvag_XP_001318359
METFRAKTPELPRDFTLIFKGEKYKVSKFMFSMHSLKFRELLPTFKTDFLEIPVQTTENVFREFLKAIHNEEYCITTDNI
YDLYKIAKEWRIQSLIDQMDKFAQENNISKDKLNEKSREISPTLMTILAKNINKAITMSSFTNLPFDVISKILSLPEAKI
SDHQTYYDFIKLMLDIHGKKATELTKYIDLKLIPSSQLVELLENPNLDKEFIAPNLLTIAKYYVEETAKLEENYAQAEAN
AASLQNPDSALKKAVTRVKHLQDSLNECVEQQDSIEKDITEELTAIKMLLTELEKKVKLESQNQDEESSKAFAQIKDLCD
RLEQLKHKDNKRSPQRRHSIDDTASTFDQRQAHLTPFNMGENPLDGILNSLVSRGVEIKVIGSSTDHNYPSILVRRDTND
YWMSSNKVDQFVRFDFAERQIKVSQYTLKTIKFGEDSCHMKSWILEGSNDDIEYSKIDERKTDELNGSNKIVTFKCTDDC
PAYRYIKIRMTDANHRGDNTMALNSVEFFGIIIEE
>Tvag_XP_001314088
MEIRKSINLRIRVHPHGYIPDENFSTVTVKTSHYEFTCKRSILTKLSPRFKKYLDSKPNIDIINLKDIISADYSSYLAFS
NYINGESLIINDENVECIYNLATKFGFPVLLSVVVPYYFLVDQLDKLVNNSEIIAQTNPYSLAIAPIFPLFRIAYKKERF
PYEFIVKILQSSKLYPISSETQLLSLLICLKSSKQRDFSNKRFTVSEDLNSKINEYTKGLSITEKQNINRSLSFYKSVEP
YSAIERFEIQSCWNNYVSNLSSYPFSYGSSYDSTDSLKYTLEYES
>Tvag_XP_001330654
MFSAEELTNYFDNLPRDLTIIIGSKTYKANKQVMCIFSGIIQEKLQNSADNEIKLEIEDPKEMIPLVISFLHGHQINISY
ENDFYLNKISDELKISSLKEAVQESLNEPITISNIISRLEQNTSQVLENENSELLKFLVENIEEIQKDDSLFLSDPRILL
KALEKSKFENDDSKNIFKLKCASRHIDNFDDFLSLDDFQHMSIDVICEFVTNPYYSTLDQKMPSILIASRIVSAITSLNT
DINEINEKIQNYNSEIKELQEEHDRVMVEKCQSDEKYLKVAEQFKQFKLQMISTSERIESHANSLNSFKISEKTVNEIGS
KLNFLKVTCERLQFFSERLSRAVILYPETTKNVNQVYNDWKGSIAPLQQEIANLESNEKDIDSIAADFHKLVEAIRKLSN
I
>Tvag_XP_001310351
MDQKETYTIIYQNKSIPVNKEYISLQSKVFRRLIKENPKTLRLNGGYPFDSFNQFLRAIQGMEFNTTVENCDDIIKIAQE
YQIKNLVTLMEQERERLFAPENILLLAIQSNREHKSVLPYIPFLAQNISQMVKIPTFALLPVKLIDQIFSHDDCHPDNQG
DLIRFFEALLDFKQKEDPSILFNHIDFGKVSFEEMDHFLNIPKLNKKAVIQKVANASLVLMDIIRSEEEGNKDTIGKTQE
IVDDLKYQIDYITKAQEKIVKHQQSATRRLESAKEDVAKSIAYMRKVEAKLRQTCTRSSIPGMPKLQPVPPHRPNIFIEW
CQNGFSEDGPSKRPPPPKKTKTISKNVYVTSQIKIEAPAVKIRSYAEHLGNSTYSTAPSTGFRMSGQSFGSKMNLDNVKV
EMPNDRPPIKIERRTTAKAVLETGGIKIEKPVRLETTFKVPDVNEVSSSSSNPEISQSSKHEIKIEYPSRKNE
>Tvag_XP_001305641
MTNDLCSIPFLSRWQDFEINHLDKKYYVSGQILSAVSPVVKNLITKEKVFHYQLPPIPGNIQDLLDLITGKEIFISDSNV
LFLFSCASCLQIEDLIHHTNGALAAVADKNEIITLVSRLKEKDVDITNICQFISASIDKFIRLEKFKRIDISILTKIIDS
PALNCVDTGSLFTYIVSRFPESPTEFSKLLQIICNNYLNPITLSIILKSPNIDINSLKESLIPVLVNGSGCTSILYDNSQ
TIIPYEFGKEMSGLFEYFRSHHVLSTSVDISASSTFDENYSIKNLLNTNRSQYFASKSGPVEFIQIHILTGSLAPTHYTL
VSCESGISNLAPCTWTLSGSNNGKTWTLLDSQSNNNSLFFPEKAVIFPINKESWRSFSYFQFTQYDSGGIKNKKLLLAGI
ELFGAYTPGVN
>Tvag_XP_001303603
MDTIEEIQISPLFDFSSFFNTGYLSDCMIYFGAKEDHPTDSVKAHVLVLANGSRFFYDSFTSQMREDKERVFYATVNPMN
LLRRVLRYLYNGQISYEFDETMALLKIATYYMIDVLKIELQNQINSITDPNLYINLVNQCYDNQIEDQLPYLSQFIIKNY
NSYDIQTLSDSYDVMAFAAIIRQVQLPIDKLISDITAFLGDFEADEEELAALNKLLFDYNTEDKRKAFEKSKPKWIK
>Tvag_XP_001302260
MKREYQTLPIPSQNTTDFTIIYNGTDVPVSKFVLGCYSAFFRTIPDFFTATTFKYDDIPSLESFKTFIDAAHGHEFPITK
ENIYSLLRFASTWEVSTLLDTLIEYYNNNYDMTLSLKELISSKNEKYTNLLCNLISSQLDIALKDESFAKIPYSTVALIL
KSPIKVLNDSQLLYKYLCDRIPTLDTNLYELFRYVDLQTLTAEYSKQIFTNFQLMKFFSTFKEPRQPGDITNDYNEIVIE
MKSVDKRIKRLEKLKFEEKFEAISEHFGDIEERISEEIKTRLREERKELDPENKLDKKLKQMADLLAKKILDLEQKIKRQ
DFAQDAHAKEIISKSQALNSNFDPIRAEMREMQRMLRVINVKQIGITEHVNNRAAAKDKE
>Tvag_XP_001276906
MKVIQVQHSQKFQIFLEEKRFIDTVLHFLDTRIEAHSLILAQNCTWFKERFTCSEPIEKDSSSIYHITLPINPENSVQHL
IKTIYSQKIVVSIKDAIPLLKSAEFYGCQYIVEKTSEFIEKSLTEDNVLFIVQTLSKYNLYKLGIENLQ
>Tvag_XP_001314416
MSDKQDPNYNTEIDLDPLWDFLDYYKTGELADCTIVADGKELKCHQIVLANGSEFFKDNFTSGDTFKGSSERRIDITVNP
EGKLEKVIEFLYCGQITLSEDELMPLLEIAIFYRISILKALITEQLDKITDHNKIMNMFTECFDKDYSNATKVFPTYIAK
FYNKYSKKQISDCLDTDCFLNTLLLLKMKSKDMATEILSFFNDFNFFQDEATKQAHDTFMKFIQDSVKDNNDLTKILKSK
KPEWFK
>Tvag_XP_001330419
MSQEFIIDNILPYHYNKFFFVNEFTDISIHTNKEVIPAHKIFLAYNCKYFHKLFADSAESISDYTIPFNPLSEFKTFISA
LYSGQLRVTRKSLIPLISISEYYGCEYMIKILKFYFSKIFPDGPTVDCIKQFCKCGCLKFSLEYIDQIILLISQSSESQK
QKIFKHIYIEHLLEIVKSKYYKSLLSNERFLLISRVCQKYTNISQEDKEKLSSTLDFNFDDIQSYVLNYNINWIPFSVSK

KIFSNILDYRRKTMSKFFESFQKQDFYNFSYNFLVSLYSIQEFNHNEQGVCDIVQSVCKATNMKDKAFDFEVFSLDASPS
NIPELSLVKIFSNDKYYCSSDEIEGNPYFNISFNKIRAKCHSIAIQSIVNNHGTMMKAPESLFIALNGKPDLIEVPASHP
GIYNYKPDTPFLLNHLHIERHPKSKYNNILRISKVSVFGELC
>Tvag_XP_001329827
MAVSFSSLLQSKAFSDLCLVANGTRISIHRAIVAERSGYFHDLFAKDPSQKIVEITLPDPCSECLPHVLNFLYSDPNFHV
TKNSFGPILNIAVCFKIQALIDTLFEWMKANITAENTMYFYFAIKDIQDKLKAENDTQDKSKADADTQKQYIQKQLEETF
MTTIIDNIVLHFELIDRGDIWSLPYNTFYTVITHKNFNSSSFCLSSAIAYTIMNNSKLEKSEVETLLSLYLQIDWPASIT
ELVQNSAPNDPRNQNSISSLLLPFVAKHFRRIPNTEFSKFPHKFMTALVSRDDLNAPSSEYVRQQIQNLTNSTTRVNKQR
NLQMWEAFLGDGKEREYHTLPVTKENIRVLILSSVYLDVLTDIKQDLVEGGIQAQNIFLFNADTGHPTSTLLFQFDVVLA
FTHYQFENPDITCSFLYDYVQNGGGLVTCYGFCRDDEWGLGDEALDSLMPFARGTQLTKCAREKVIVEDNHPLMDGIKTI
RHGEFSPRCNVKLNEGATLVASFADGVPLVAYKETNRMNQKIVSINFYPVSSRVHRLGFPPDQPWTQIFTRAILYACGID
ISNKPEEASGDEDPAPAD
>Tvag_XP_001329125
MSLTDNDDNFTEQVGEDGQVLVKVPKSRIDSAFNEFRETGRFVDCQFQVKQGIISCHRVILSKYSDVFLELFSKLPNDEN
HIIIDIPFCLNEESFKDVIDFIYTFSIKVTPKTIADILITSYLYGIEELVAIATDYFLEALDGENVLDITEKFKFSLSNE
TIQKYPALYQNYLQLIQNSGLFASIIAKKFTNYPRQKIMDKIPTFLVADIMKNLNYETINDISQYPSINDFIIEFIDKFV
EGKQLSDLDKECLSKVIDWSDPESYQYFVYHQCDWVTSSISRRMFDKILNMRRNSIPSIENQVSNLKSNSHHFLGVLSLL
QLIVSSKGETEVSECNILKSIGTLWNTVKPFNPLTYNFVQLSADTSPPLCPHMSVQNMFVDNEEYYFAHSAYASEDIEPY
KKSLKVGITFDIHTMDGRDIHANLRNQKMDIRTPAKKKDKGLPLKISTCTDYDTSSIEIFCSQQSHYISIFGLRISFIDI
IGKFVLK
>Tvag_XP_001323955
MKQLFSNSDCQNMDSDAAKIVCRFKEFRQKGLFIDCIFTSWNKPEVKAHRIILSKNSTLLKHYFETTPYNPSNIKLNIPF
NTPDHSFSKIVNFFYNNTIELTNDNVIILLAHSIIYGIKHLEKIIMDLISDSINKTSSVNFFKQVISFTISADSYSKFPD
LAQNYDRFIRQLDFLAKYVAENYVDDELYEVFSCVNPRLLSMILKHSSIPDSKKVKIINDFVANLELTTNDMTYLTDTIH
WENPDSYTYLVKNSCEWLLPKVSRKLYSIIITNRRISANAFQHKIDSISDIDPSNSDNKQAQGTTENQPPSRKLQNWFPF
AWLTVVWDSQGKTCTPEVELSEFIGTLGGLVKFFNPVNYLLLESFASTPIHPKIFSAAYIFEDSKNYFLSNCEHNYIGFK
MRLPCFKLSSIIVTFDNRERGKPMTSNTKLMYNSLGKMKLLISDSNNKTKFPNPVLPGVPFECKQIPVLFICVIPDTAGD
GNIKCLRVSRLKSYGTFDVD
>Tvag_XP_001315684
MQSNKKVIVEKTNENDEPNTLETLIKIEIHPVESKFVEFFATKSFVDCILELSSEQIHCHRLILAKYSNVFRSYFIKHDT
TDNKLLPPPYVISDKALSQIIDFCYKSTISVDSSNIVDVFITSAMYGIKVLTALLSESLLNILDNGNILDYLPKFKIQLT
DEAIQNFPELLDNYRDLRDNIGGICDIISKNFAFISRSKFIDNTPLYMLPDILKKIDYKDLYDTAVFESKDDFVLYTIDK
IVGDSEISTLDKECLSKAIEWDQENSYLYLTKYRLNWVLPSILRPILAKVFKSRQKTIQKVDKIVGKLKSKIHSNFGVLD
LLQIIYASKGETKVESKNVLSVLSTMWGTTSITNPALSGFVQLTADTDGPLSSFYDPRNVFVQDDYSYLTNSTCDYQNPE
FTHYQQKLTLGFVFDKTVGNKVIPSNLKIDEIKYHLPESVKKETSKITIMYDTNNPGTGIKLVCKPSKDQATYNIFSMHV
KYVEIIGKFELE
>Tvag_XP_001315189
MSIQNKLNEYRLNGYFIDYELKCGEKTFKCHKILLAKNSTLFKEIFSKKSDEQVEIADCYESEFESIMESVYTNKLEFTQ
DNVVKILGFALAYGFKSITNTARTKMTEFLQKSKDKVLEYSEQMVEFNISDEESNRAIQKIICDNFDNYDIDSLLDKITS
TCLYLLLSSDKLKVRSDDYRLALIDLFHERRPITDQKIRYDLANLFDFHPETNESAYKFIINHKCMWAPEELTIKLYRIA
IKNRLQIARQYSEATNTFADTKSRWIDFQWCSQIHNSEISEDSIKIELVSFARTFGGFIDAVNPRTFGLIGCSSSTPFGF
NKNKNSEYKCQFSSSGIFMDDNRYFASMGDAYIEVNFGEDSSFKISKIEIQSLPKSVGEFKWNKFPLKFAEIKNEEKIKN
EENQVNLDETEIMKKRLGVPESLDVLVNDKCAVQNVKFERNGEEKVFVWSHTFDDETSNKVRIAMSKNNNAGGKVLRIGL
IKIYGHFV
>Tvag_XP_001307753
MTEQSQLLTRDTVFEFEKIALDQRFVDCTIIVEGKEFKAHRVLLAGCSKYFNEYFKDNSVTTCTLENYSASSFQLFITFL
YTKKILLNLDNLANAMKLAFYLKIDTLFNTINDFLQTVANPDTAIVLLKSVGFALSALTRFMKFCATLMEKMSANTDFAF
LKVPEMKLLIKKARFSNGYVRDDIIAKYCRAAQIDPEQFAEFKQFDPALPDSSLKKPTFSAVCLAAPPDPGLFQRLKGQV
GVEASGSLNGRDPATIIEEDPIKHWFTESDGNAWVLFEFKELYIQPTHYGIWSHGGTSTLKNWAFQASNDRQNWIVLSTH
NNDDSLKEPFSEKTWPIDTNGYFKYFRIIQTGHNWSGNRWLYLQKVEIWGVACSKDKF
>Tvag_XP_001305583
MQQEFDFSEFFDKSLVTDCLIKFEGSSSAEIRAHKLILANSSNFFFNMFTGQMDEERTGTVNLVYNPDNLLQEAIRWMYN
GKKFYEDYDIEKLLKLYAVSAFYTIENLQNDLKTVISQKIPQLKMLNYCDFCYKQGLSETLNYLAVLIGENFEKYDDISK
MSDILDVRVYALALQK
>Tvag_XP_001304004
MAHYISSIQWPESSIIKIQTSDGFPMILHKPFLKHRWLLYNKQPELALQTAQSLTKDELQALLLFIYSNLPAKLSYVKIF
QQCGLTNTASLQDSTFTEDMRQMMNDTETFDFKLNAAEEGKFVLCHRPVLAARSKYFKALFISHSLENETSEWRCAKPIP
FKTLQFFVEYLYTGQINAPEIMHIIPLCWLVRYLRLSNETEIENIVISNLSRELCDDNVEEISKIAEEWNIRCVKGLVEK
HQSTH
>Tvag_XP_001303054
MSIKQKFTLNHTLKNNIRSFYLDGHFFDCTLVSGTSTLQVHKIFLTNLSEWFEEKFLECSDSMKMTTLQITMDPQNLLFS
FIDSIYKGKLVLNSENYAAYLKCAVYYKFNSIIQQIEDFIMKKIVFLDATKELCNFQLNEQLIRMKFNGKDSLRLILDEF

TNATNKTKIYDILSATIFANILRISKYSDDEKVALIDEFVIYKKLEDSLSEQEKNHFASVINWHNQDAYLYLVHYDCIWV
PSEISRNLYSKILSNRRMSVKGFENDVKKVVGSSSISNPLSWIAEINNSKGASQTPEVSIFEMFSTLGTHMFINPVEYNL
LHVSASPHLGIQRPILRDYIPANVFSNNGRYYLSQAGSKSNLPYFQLSLNKKDKFVPSSITVASNESFPINTPPKANSVR
KTLQKKAPKDICIDFANSDKSVAQINCCMKQGNNLSLDADKSIQININERIEINSVRITMTSPSESGYWNFRLSGLDMKG
KFLP
>Tvag_XP_001581374
MADDQQERQIELKLDTKFDYHEFLDQNLITDCVLVIDGQEIHAHRVILANSSSYFNNIFTSGMTEDITKKVEIIKNPENV
FSLVLRWMYTGNLQFPKEKIHAIIEVAKIYGVNALLDLLENYLSKLFEAPLESITDFINATYDKRYPDGTKYENTLFIVA
KHLAHYLPSCAESDEQLNIISNLVDCKAWGIALSHYTADSEPKKDLEKKIDLTLKFLNSTNDYKLTEQDVNNLKIGFEGL
PNNTKLEKFYNKLYNKCQ
>Tvag_XP_001581269
MSAVRIKQRLISRFHEYLDRGQFVDLKLRHGVNTYQVHSLVLAKYSGWFAKTIKEMDCCPGDRMMKINLPEDPDDQFGTI
IKILYTDQLTLDIKSAPALLRIATYYEMPHLQAIVRHFIAKTTVEPIINDAVLFSYASKFIQFKLIDDAIKLAPEFAKRL
MSKSKTPSDVNKYIVRIYQEVKDGRVVAAIILDQNFLNYWSEEEKVAQLDRYHKFNPDITDDEKEALSNFNIINWKSPTA
YQYLVKYQCDWLPPRVLRPLYESIFKYRRANAHHFEKDIQNSSSTVSRWYPFSWATQIANAIESESTPVVELISFITTLG
GLIVPKDGDAVKFGLLNPNGNQKLPQEELSTFEMANIFKDDNSYYQAILYDDKIGHGPNFIVIELGKSCEFKPQNVIVDM
KYPDTISDSLSKYKNSRKQHFPEYITTSSFSSIPEFKETLFKTDNKQQPLFEYALSKPFNRIYINPPLDGSLIVFRVRLV
KIFGSFLQTHG
>Tvag_XP_001318671
MIQKPCYAPIFNFSEFLDNSIISDCEIILDDGEPPIKAHKLILANSSSFFLNIFTAQMEESATGKVPITKNPGKLLRRVI
NWMYDGKIQYNDDNKDLLRLYEISLYYGVLVLNESLQKLIREKIKTEDILDYCDFCFNEELSRTLEFLAELIGENIGEFT
DLEKLSQILDVKTFSLALKSSKLNTREKLQKISKFIGDYTMEGNDISDLKDALNKSSDKGTINKDQLKKEFPELSVLLDD
>Tvag_XP_001314915
MKVFNRDQNQNNILDLYQEKLFCDIVIHWKNEEYYAHRLILQNYSSYFASIFEKYKDTTDIPRVDHVTIEINPQNMFSKV
IDILYSKPVQITVTNVVSIYKICCYYGIYSFIENIKNFITKSLSSNTVLHYVTEFISYDMIEESKIIVPFLSQTFLQIIN
SKTPQQEAELEQLLKCVSNGILFSYVIMADPLNSALSEYDKFCLVDKYSKIKRFSEAESIELASLFNWEDPNSLNYFVEF
SCDWLPLNISRKCYSTLLSRRKNIIKVFESISENVKEQASRLHCFLWVDICSKASIVPDHPSINILDFISSLGNSNVEFD
PIKYSYLLCNVHQTTYDIFHPIQSIFKNDQSYAIIESCDDHPASISLSFGNHTVITANSFTISCDIEKNTRKPNNITTKQ
YYTVPNQLLVKTFLNGTISNEFEISSDQNKIYQLEKVPNSFDSILVSIPPSLSDIYKILRIRNISIVGTVDTQ
>Tvag_XP_001311911
MESMKKRPDHPDFLVTTNDGVPLGLHKALLFNRWPLFRESQERCRTNIQKIDSGPFKQILEYLYAGLMPSESLRPTFGTI
GIPFPSSDFREKYIADMRRLYTEKTCSDFKISAHGKIFSVHRFILASSSEFFYSLFSSGFEEDVTQTMEDLFSTSIKQIE
SMLSYIYTGEVVLSSVDECLQFLYICKKYIVKAPSPREPETMIATLITSKFMSEIDYARSKAVSYSYKVLSEILSACLE
>Tvag_XP_001306001
MFNVKHRLHRIYESFLHNDQFIDTILVSPDNAEISSHKILLSKESKWFFEYFQANPSNEKVQIVHLPFNPDNIFTDIIQF
IYSERLTITTKNMAQLYACATYYKFEKLRFIVSRLFEKSLCVENSLFLCSNLIKFGMDEGAVKIAPIFANEFSKNLKGEE
SLLSIQQIFDCCSPRVLAEILKDPCLDFVTRNQKVQLIDQFCTNDLVLDDQNKEYLASVIDWTLKDSYKIYLSSQCDWLP
SKIQRFILSDAFNARKNTLKSLSSTSNHTPRKFGNWYLLSWLTALSKGEVSEKPPEVEAIKFMTTLGISDTRYDPELFTL
IRSKSSELLDQDFSPSKLFTDTGYFCSISTEETPYLEIDFGAHAGIITSEIGISCELTNDSDIPKPVPKKLVVKGFVEGN
DQCLYEKVFRYAKHEAKDGIKTFPLVCDVPIRKIRISVPDVSAFGFEILRITKLTLTCSFIPA
>Tvag_XP_001303053
MRNLRITSQVNHFRNNNMFLDVELRHKGESIKAHRLILSQNSQWFREKLNNVKPPSYNEPVVIDLPWNEDNAFQHVLEAI
YTGHLQLTPDIAPAVLKVADFYKFRNIAYLTSDYIKDLVKTYNNEKKDIKAEYCKKFVSYQLPEYYDILVDYAARKFKQL
FIAEDQNETVDEVYKMCADGRFIAKIFMHQDTESTKGRDPSSGTLYYDIKSLFKDDDDAICEQFDAFFKNHRTNMTLEEK
KALSDVEIINWTKQEKYQLIIRHTCNWLDPSISRNLYGIVLSNRRQVCKHFDGDIQKIQEETVNRWYGAEWLSQITSAID
SETTPEINVVKFMTTLGGAVDYVDPELYGFINPESPNSKNSPEEIIQERQPKNAFRDDTICFTALQCILGDFKQYQFHVD
FGPRAIFQAHSILVDMDNMYRNLEVTDNQSCIVDPKRRKSHKLPPKVYVQSCPDNKTEAFVPDGIGVNWQEFEHTLNKFV
VTVDKDEPTIFLRYVDIKGCFLPTIQLPKK
>Tcru_XP_819374
MRDSVDASEIKALLQELEGVVGTPTTNKGENERPFSPKKRGSSNVDVAVSRYAGHLGQSKFSTYANTTNDSRRAVVPLWE
RLPPMNTVDITGDASCPVKSPVTASQKTFLRPKLVGGVVGASLQRMPLEGKVNNASPLKNDEQGLQQTKLTSQKQIQEEE
KEPPTTSFSKEPVAQGEGEDREREVNFLPIMCARYINPAGITPRARWGHTLTPMQGQKLLLFGGMEIEEGETASLFEYDT
HHISWDPVCVSGGVTPTPRTNHAACASGGRWLYISGGTTDRGTRVLGDLYCYDIFTQEWTCLWEYGGSSTRSKHEPTPRF
GHSMVSWEDRLYIFGGKTPKRDQGDSQSTQHVTVASTDVYVFSLTSRKWKRRIHAGKTMGVNGDGKNILNNRKTEETPLP
AASSVTQLTNVFSERPANRFFHAACALGDCMYINGGTGDGGVILSDTWFLDLRKQHWVLLHNGRTADSFPREKHHLFACG
EALLVVGGCSASSQSTRITLKYHNFVAVLPFEGNDSPCWIPVAMGNVSIVAPNRKSFGAAFAGGFVHVFGGVSGSEPASN
SMVRFLAADGYFETDSRSWAKSNADALTSMMRHIREASISTMFDTYAIPRSWDGETGIDTLSNRPYGVHRGFLQQRAPKF
WEDLKRCRMEHVKPAGTGSAMPSLDLTDPLKEAFTPSPVFPTTGGVATTSTAAGETHPSHAVAYFTEGNSRIQGLSVSLS
AEQLQCLLDYIYWGDVRHRILLEQEEEEYTKDANVSGQGDAMQEEAKVLQALRHAADVYELHPLMDICDALLARSRKKLK
QAHERSTRQLQADLLSLLESSNSATATVLFVDPHTKQKSVYSLHPFILMAASSFFNDLLRPLVSGEKTTFQVGPVAAKMR
SAGRGSSQTRRDILVGPVSVPYLAVKPLLRFLYTQMLTVPREAVFATLLGSHLLDVAPLQAYCESILAREEVNYDTAGHF
YSLGRKYHAPLLQETALLTAVLGYAEVCRSPAYKALSEKEAREIDAVAHELGATSWIAPPQTVSEVKPAAVYQLRWNASS

PLSK
>Tcru_XP_812911
MRDSVDASEIKALLQELEGVVGSPTTTKEENERPFSPKKRGSSNVDVAVSRYAGHLGQSKFSTYANTTNDSRRAVVPLWE
RLPPVNTVDITGDASCPVKSPVTASQKTFLRPKLVGGAVGASLQRMPLEGKVNNASPPKNDEQGLQQTKLTSQKQIQGEE
KEPPTTSFSKEPLAQGEGEGEDREREVNFLPIMCARYINPAGITPRARWGHTLTPMQGQKLLLFGGMEIEEGETASLFEY
DTHHISWDPVCVSGGVTPTPRTNHAACASGGRWLYISGGTTDRGTRILGDLYCYDIFTQEWTCLWEYGGSSTRSKHEPTP
RFGHSMVSWEDRLYIFGGKTPKRDQGDSQSTQHVTVASTDVYVFSLTSRKWKRRIHAGKTMGLNGDGKNILNNRKPEETP
LPAAPSVTQLTNVFSERPANRFFHAACVLGDCMYINGGTGDGGVILSDTWFLDLRKQHWVLLHNGRTADSFPREKHHLFA
CGEALLVVGGCSASSHSTRITLKYHNFVAVLPFEGNDSPCWIPVAMGNVSIVAPNRKSFGAAFAGGFVHVFGGVSGSEPA
SNSMVRFLAADGYFETDSRSWAKSNADALTSMMRHIREASISTMFDTYAIPRSWDGETGIDTLSNRPYGVHRGFLQQRAP
KFWEDLKRCRMEHVKPAGTGSAMPSLDLTDPLKDAFTPSPVFSTTGGVATTSTAAGETHPSHAVAYFTEGNSRIQGLSVS
LSAEQLQCLLDYIYWGDVRHRILLEQEEEEYTKDANVSGQGDAMQEEAKVLQALRHAADVYDLNPLMDICDALLARSRKK
LKQAHERSTRQLQADLLSLLESSNSATATVLFVDPHTKQKSVYSLHPFILMAASSFFNDLLRPLVSGEKTTFQVGPVAAK
MRSAGRGSSQTRRDILVGPVSVPYLAVKPLLRFLYTQRLTVPREAVFATLLGSHLLDVAPLQAYCESILAREEVNYDTAG
QFYSLGRKYHAPLLQETALLTAVLGYAEVCRSPAYKALSEKEAREIDAVAHELGATSWIAPPQAVSEVKPAAVYQLRWNA
SSPLSK
>Tcru_XP_811698
MESIETVEVVSENGARFRYHCPVKLLRNMSLDASHYSPQFTVGGYTWRLHLQRRATAASGEEFLSIHLQSCTNAAVVAQF
KLLLQCYEDPPSSKGHIFRCTFKKSGSAWGMNEFIPMWRLIQQENGFVYEDEGSGALYFDIDVLLKIVDANGQSYGNGGF
ETNVLPGRSSSPPAQLNFHQDPNRALSRMRSPMEYRGVGPTRSTSARGHLIVAPENGFIVGGGQAVVRAPLLYPFEHLEA
LTDMTFDIQGVRMKAHRCVIAARMRPLLPENVLPLQPGCVVTIAVTADVFSAFLRYVYTEECPQQGVLPPEALLDLYLLS
SACEFYELSGVCLNFVRPLLTNENILHITLTRYNAGDEVLNALYLRVLLENYDFLIQDPKFEEIPGHLFRRLSLILRDKE
PVPQVTIPTSKNTLGRQLAWLAESGEYSDMELVVGPKHLIVKAHRYILASRCVLFSQAINPRSSVAVPPFTAAEFDFSQR
SWQKLLIGIYRHHLESYRDFSAEDVAIVFKMHSVFGMDGHLKREADEAFNHQNALRLLIYAVKHQVPELHERGISYVASN
FNALIQNDPQAWELVSELPQHAVLALFRMVVESQR
>Tcru_XP_809118
MESIETVEVVSENGARFRYHCPVKLLRNMSLDASHYGPQFTVGGYTWRLHLQRRATAASGEEFLSIHLQSCTNAAVVAQF
KLLLQCYEDPPSSKGHIFRCTFKKSGSAWGMNEFIPMWRLIQQENGFVYEDEGSGALYFDIDVLLKIVDANGQSYGNGGF
ETNVLPGRSGSPPAHLNFHQDPNRALSRMRSPMEYRGVGPTRSTSARGHLIVAPENGFIVGGGQAVVRAPLLYPFEHLEA
LTDMTFDIQGVRMKAHRCVIAARMRPLLPENVLPLQPGCVVTIAVTTDVFSAFLRYVYTEECPQQGVLPPEALLDLYLLS
SACEFYELSGVCLNFVRPLLTNENILHITLTRYNAGDEVLNALYLRVLLENYDFLIQDPKFEEIPGHLFRRLSLILRDKE
PVPQVTIPTSKNTLGRQLAWLAESGEYSDMELVVGPKHLIVKAHRYILASRCVLFSQAINPRSSVAIPPFTAAEFDFSQR
SWQKLLIGIYRHHLESYRDFSAEDVAIVFKMHSVFGMDGHLKREADDAFNHQNALRLLIYAVKHQVPELHERGISYVASN
FNALIQNDPQAWELVSELPQHAVVALFRMVVESQR

*******Cullin***********
>Hsap_ENSP00000252050
MVGERHAGDLMVPLGPRLQAYPEELIRQRPGHDGHPEYLIRWSVLKCGEVGKVGVEEGKAEHILMWLSAPEVYANCPGLL
GERALSKGLQHEPAGVSGSFPRDPGGLDEVAMGEMEADVQALVRRAARQLAESGTPSLTAAVLHTIHVLSAYASIGPLTG
VFRETGALDLLMHMLCNPEPQIRRSAGKMLQALAAHDAGSRAHVLLSLSQQDGIEQHMDFDSRYTLLELFAETTSSEEHC
MAFEGIHLPQIPGKLLFSLVKRYLCVTSLLDQLNSSPELGAGDQSSPCATREKSRGQRELEFSMAVGNLISELVRSMGWA
RNLSEQGMSPPRPTRSIFQPYISGPSLLLPTIVTTPRRQGWVFRQRSEFSSRSGYGEYVQQTLQPGMRVRMLDDYEEISA
GDEGEFRQSNNGIPPVQVFWQSTGRTYWVHWHMLEILGPEEATEDKASAAVEKGAGATVLGTAFPSWDWNPMDGLYPLPY
LQPEPQKNERVGYLTQAEWWELLFFIKKLDLCEQQPIFQNLWKNLDETLGEKALGEISVSVEMAESLLQVLSSRFEGSTL
NDLLNSQIYTKYGLLSNEPSSSSTSRNHSCTPDPEEESKSEASFSEEETESLKAKAEAPKTEAEPTKTRTETPMAQSDSQ
LFNQLLVTEGMTLPTEMKEAASEMARALRGPGPRSSLDQHVAAVVATVQISSLDTNLQLSGLSALSQAVEEVTERDHPLV
RPDRSLREKLVKMLVELLTNQVGEKMVVVQALRLLYLLMTKHEWRPLFAREGGIYAVLVCMQEYKTSVLVQQAGLAALKM
LAVASSSEIPTFVTGRDSIHSLFDAQMTREIFASIDSATRPGSESLLLTVPAAVILMLNTEGCSSAARNGLLLLNLLLCN
HHTLGDQIITQELRDTLFRHSGIAPRTEPMPTTRTILMMLLNRYSEPPGSPERAALETPIIQGQDGSPELLIRSLVGGPS
AELLLDLERVLCREGSPGGAVRPLLKRLQQETQPFLLLLRTLDAPGPNKTLLLSVLRVITRLLDFPEAMVLPWHEVLEPC
LNCLSGPSSDSEIVQELTCFLHRLASMHKDYAVVLCCLGAKEILSKVLDKHSAQLLLGCELRDLVTECEKYAQLYSNLTS
SILAGCIQMVLGQIEDHRRTHQPINIPFFDVFLRHLCQGSSVEVKEDKCWEKVEVSSNPHRASKLTDHNPKTYWESNGST
GSHYITLHMHRGVLVRQLTLLVASEDSSYMPARVVVFGGDSTSCIGTELNTVNVMPSASRVILLENLNRFWPIIQIRIKR
CQQGGIDTRVRGVEVLGPKPTFWPLFREQLCRRTCLFYTIRAQAWSRDIAEDHRRLLQLCPRLNRVLRHEQNFADRFLPD
DEAAQALGKTCWEALVSPLVQNITSPDAEGVSALGWLLDQYLEQRETSRNPLSRAASFASRVRRLCHLLVHVEPPPGPSP
EPSTRPFSKNSKGRDRSPAPSPVLPSSSLRNITQCWLSVVQEQVSRFLAAWRAPDFVPRYCKLYEHLQRAGSELFGPRA
AFMLALRSGFSGALLQQSFLTAAHMSEQFARYIDQQIQGGLIGGAPGVEMLGQLQRHLEPIMVLSGLELATTFEHFYQHY
MADRLLSFGSSWLEGAVLEQIGLCFPNRLPQLMLQSLSTSEELQRQFHLFQLQRLDKLFLEQEDEEEKRLEEEEEEEEEE
EAEKELFIEDPSPAISILVLSPRCWPVSPLCYLYHPRKCLPTEFCDALDRFSSFYSQSQNHPVLDMGPHRRLQWTWLGRA
ELQFGKQILHVSTVQMWLLLKFNQTEEVSVETLLKDSDLSPELLLQALVPLTSGNGPLTLHEGQDFPHGGVLRLHEPGPQ
RSGEALWLIPPQAYLNVEKDEGRTLEQKRNLLSCLLVRILKAHGEKGLHIDQLVCLVLEAWQKGPNPPGTLGHTVAGGVA

CTSTDVLSCILHLLGQGYVKRRDDRPQILMYAAPEPMGPCRGQADVPFCGSQSETSKPSPEAVATLASLQLPAGRTMSPQ
EVEGLMKQTVRQVQETLNLEPDVAQHLLAHSHWGAEQLLQSYSEDPEPLLLAAGLCVHQAQAVPVRPDHCPVCVSPLGCD
DDLPSLCCMHYCCKSCWNEYLTTRIEQNLVLNCTCPIADCPAQPTGAFIRAIVSSPEVISKYEKALLRGYVESCSNLTWC
TNPQGCDRILCRQGLGCGTTCSKCGWASCFNCSFPEAHYPASCGHMSQWVDDGGYYDGMSVEAQSKHLAKLISKRCPSCQ
APIEKNEGCLHMTCAKCNHGFCWRCLKSWKPNHKDYYNCSAMVSKAARQEKRFQDYNERCTFHHQAREFAVNLRNRVSAI
HEVPPPRSFTFLNDACQGLEQARKVLAYACVYSFYSQDAEYMDVVEQQTENLELHTNALQILLEETLLRCRDLASSLRLL
RADCLSTGMELLRRIQERLLAILQHSAQDFRVGLQSPSVEAWEAKGPNMPGSQPQASSGPEAEEEEEDDEDDVPEWQQDE
FDEELDNDSFSYDESENLDQETFFFGDEEEDEDEAYD
>Hsap_ENSP00000264414
MSNLSKGTGSRKDTKMRIRAFPMTMDEKYVNSIWDLLKNAIQEIQRKNNSGLSFEELYRNAYTMVLHKHGEKLYTGLREV
VTEHLINKVREDVLNSLNNNFLQTLNQAWNDHQTAMVMIRDILMYMDRVYVQQNNVENVYNLGLIIFRDQVVRYGCIRDH
LRQTLLDMIARERKGEVVDRGAIRNACQMLMILGLEGRSVYEEDFEAPFLEMSAEFFQMESQKFLAENSASVYIKKVEAR
INEEIERVMHCLDKSTEEPIVKVVERELISKHMKTIVEMENSGLVHMLKNGKTEDLGCMYKLFSRVPNGLKTMCECMSSY
LREQGKALVSEEGEGKNPVDYIQGLLDLKSRFDRFLLESFNNDRLFKQTIAGDFEYFLNLNSRSPEYLSLFIDDKLKKGV
KGLTEQEVETILDKAMVLFRFMQEKDVFERYYKQHLARRLLTNKSVSDDSEKNMISKLKTECGCQFTSKLEGMFRDMSIS
NTTMDEFRQHLQATGVSLGGVDLTVRVLTTGYWPTQSATPKCNIPPAPRHAFEIFRRFYLAKHSGRQLTLQHHMGSADLN
ATFYGPVKKEDGSEVGVGGAQVTGSNTRKHILQVSTFQMTILMLFNNREKYTFEEIQQETDIPERELVRALQSLACGKPT
QRVLTKEPKSKEIENGHIFTVNDQFTSKLHRVKIQTVAAKQGESDPERKETRQKVDDDRKHEIEAAIVRIMKSRKKMQHN
VLVAEVTQQLKARFLPSPVVIKKRIEGLIEREYLARTPEDRKVYTYVA
>Hsap_ENSP00000314004
MAAAVVVAEGDSDSRPGQELLVAWNTVSTGLVPPAALGLVSSRTSGAVPPKEEELRAAVEVLRGHGLHSVLEEWFVEVLQ
NDLQANISPEFWNAISQCENSADEPQCLLLLLDAFGLLESRLDPYLRSLELLEKWTRLGLLMGTGAQGLREEVHTMLRGV
LFFSTPRTFQEMIQRLYGCFLRVYMQSKRKGEGGTDPELEGELDSRYARRRYYRLLQSPLCAGCSSDKQQCWCRQALEQF
HQLSQVLHRLSLLERVSAEAVTTTLHQVTRERMEDRCRGEYERSFLREFHKWIERVVGWLGKVFLQDGPARPASPEAGNT
LRRWRCHVQRFFYRIYASLRIEELFSIVRDFPDSRPAIEDLKYCLERTDQRQQLLVSLKAALETRLLHPGVNTCDIITLY
ISAIKALRVLDPSMVILEVACEPIRRYLRTREDTVRQIVAGLTGDSDGTGDLAVELSKTDPASLETGQDSEDDSGEPEDW
VPDPVDADPGKSSSKRRSSDIISLLVSIYGSKDLFINEYRSLLADRLLHQFSFSPEREIRNVELLKLRFGEAPMHFCEVM
LKDMADSRRINANIREEDEKRPAEEQPPFGVYAVILSSEFWPPFKDEKLEVPEDIRAALEAYCKKYEQLKAMRTLSWKHT
LGLVTMDVELADRTLSVAVTPVQAVILLYFQDQASWTLEELSKAVKMPVALLRRRMSVWLQQGVLREEPPGTFSVIEEER
PQDRDNMVLIDSDDESDSGMASQADQKEEELLLFWTYIQAMLTNLESLSLDRIYNMLRMFVVTGPALAEIDLQELQGYLQ
KKVRDQQLVYSAGVYRLPKNCS
>Hsap_ENSP00000326804
MSSTRSQNPHGLKQIGLDQIWDDLRAGIQQVYTRQSMAKSRYMELYTHVYNYCTSVHQSNQARGAGVPPSKSKKGQTPGG
AQFVGLELYKRLKEFLKNYLTNLLKDGEDLMDESVLKFYTQQWEDYRFSSKVLNGICAYLNRHWVRRECDEGRKGIYEIY
SLALVTWRDCLFRPLNKQVTNAVLKLIEKERNGETINTRLISGVVQSYVELGLNEDDAFAKGPTLTVYKESFESQFLADT
ERFYTRESTEFLQQNPVTEYMKKAEARLLEEQRRVQVYLHESTQDELARKCEQVLIEKHLEIFHTEFQNLLDADKNEDLG
RMYNLVSRIQDGLGELKKLLETHIHNQGLAAIEKCGEAALNDPKMYVQTVLDVHKKYNALVMSAFNNDAGFVAALDKACG
RFINNNAVTKMAQSSSKSPELLARYCDSLLKKSSKNPEEAELEDTLNQVMVVFKYIEDKDVFQKFYAKMLAKRLVHQNSA
SDDAEASMISKLKQACGFEYTSKLQRMFQDIGVSKDLNEQFKKHLTNSEPLDLDFSIQVLSSGSWPFQQSCTFALPSELE
RSYQRFTAFYASRHSGRKLTWLYQLSKGELVTNCFKNRYTLQASTFQMAILLQYNTEDAYTVQQLTDSTQIKMDILAQVL
QILLKSKLLVLEDENANVDEVELKPDTLIKLYLGYKNKKLRVNINVPMKTEQKQEQETTHKNIEEDRKLLIQAAIVRIMK
MRKVLKHQQLLGEVLTQLSSRFKPRVPVIKKCIDILIEKEYLERVDGEKDTYSYLA
>Hsap_ENSP00000358147
MEESMEEEEGGSYEAMMDDQNHNNWEAAVDGFRQPLPPPPPPSSIPAPAREPPGGQLLAVPAVSVDRKGPKEGLPMGPQP
PPEANGVIMMLKSCDAAAAVAKAAPAPTASSTININTSTSKFLMNVITIEDYKSTYWPKLDGAIDQLLTQSPGDYIPISY
EQIYSCVYKCVCQQHSEQMYSDLIKKITNHLERVSKELQASPPDLYIERFNIALGQYMGALQSIVPLFIYMNKFYIETKL
NRDLKDDLIKLFTEHVAEKHIYSLMPLLLEAQSTPFQVTPSTMANIVKGLYTLRPEWVQMAPTLFSKFIPNILPPAVESE
LSEYAAQDQKFQRELIQNGFTRGDQSRKRAGDELAYNSSSACASSRGYR
>Hsap_ENSP00000364589
MADEAPRKGSFSALVGRTNGLTKPAALAAAPAKPGGAGGSKKLVIKNFRDRPRLPDNYTQDTWRKLHEAVRAVQSSTSIR
YNLEELYQAVENLCSHKVSPMLYKQLRQACEDHVQAQILPFREDSLDSVLFLKKINTCWQDHCRQMIMIRSIFLFLDRTY
VLQNSTLPSIWDMGLELFRTHIISDKMVQSKTIDGILLLIERERSGEAVDRSLLRSLLGMLSDLQVYKDSFELKFLEETN
CLYAAEGQRLMQEREVPEYLNHVSKRLEEEGDRVITYLDHSTQKPLIACVEKQLLGEHLTAILQKGLDHLLDENRVPDLA
QMYQLFSRVRGGQQALLQHWSEYIKTFGTAIVINPEKDKDMVQDLLDFKDKVDHVIEVCFQKNERFVNLMKESFETFINK
RPNKPAELIAKHVDSKLRAGNKEATDEELERTLDKIMILFRFIHGKDVFEAFYKKDLAKRLLVGKSASVDAEKSMLSKLK
HECGAAFTSKLEGMFKDMELSKDIMVHFKQHMQNQSDSGPIDLTVNILTMGYWPTYTPMEVHLTPEMIKLQEVFKAFYLG
KHSGRKLQWQTTLGHAVLKAEFKEGKKEFQVSLFQTLVLLMFNEGDGFSFEEIKMATGIEDSELRRTLQSLACGKARVLI
KSPKGKEVEDGDKFIFNGEFKHKLFRIKINQIQMKETVEEQVSTTERVFQDRQYQIDAAIVRIMKMRKTLGHNLLVSELY
NQLKFPVKPGDLKKRIESLIDRDYMERDKDNPNQYHYVA
>Hsap_ENSP00000376808
MATSNLLKNKGSLQFEDKWDFMRPIVLKLLRQESVTKQQWFDLFSDVHAVCLWDDKGPAKIHQALKEDILEFIKQAQARV
LSHQDDTALLKAYIVEWRKFFTQCDILPKPFCQLEITLMGKQGSNKKSNVEDSIVRKLMLDTWNESIFSNIKNRLQDSAM

KLVHAERLGEAFDSQLVIGVRESYVNLCSNPEDKLQIYRDNFEKAYLDSTERFYRTQAPSYLQQNGVQNYMKYADAKLKE
EEKRALRYLETRRECNSVEALMECCVNALVTSFKETILAECQGMIKRNETEKLHLMFSLMDKVPNGIEPMLKDLEEHIIS
AGLADMVAAAETITTDSEKYVEQLLTLFNRFSKLVKEAFQDDPRFLTARDKAYKAVVNDATIFKLELPLKQKGVGLKTQP
ESKCPELLANYCDMLLRKTPLSKKLTSEEIEAKLKEVLLVLKYVQNKDVFMRYHKAHLTRRLILDISADSEIEENMVEWL
REVGMPADYVNKLARMFQDIKVSEDLNQAFKEMHKNNKLALPADSVNIKILNAGAWSRSSEKVFVSLPTELEDLIPEVEE
FYKKNHSGRKLHWHHLMSNGIITFKNEVGQYDLEVTTFQLAVLFAWNQRPREKISFENLKLATELPDAELRRTLWSLVAF
PKLKRQVLLYEPQVNSPKDFTEGTLFSVNQEFSLIKNAKVQKRGKINLIGRLQLTTERMREEENEGIVQLRILRTQEAII
QIMKMRKKISNAQLQTELVEILKNMFLPQKKMIKEQIEWLIEHKYIRRDESDINTFIYMA
>Hsap_ENSP00000384109
MMSQSSGSGDGNDDEATTSKDGGFSSPSPSAAAAAQEVRSATDGNTSTTPPTSAKKRKLNSSSSSSSNSSNEREDFDSTS
SSSSTPPLQPRDSASPSTSSFCLGVSVAASSHVPIQKKLRFEDTLEFVGFDAKMAEESSSSSSSSSPTAATSQQQQLKNK
SILISSVASVHHANGLAKSSTTVSSFANSKPGSAKKLVIKNFKDKPKLPENYTDETWQKLKEAVEAIQNSTSIKYNLEEL
YQAVENLCSYKISANLYKQLRQICEDHIKAQIHQFREDSLDSVLFLKKIDRCWQNHCRQMIMIRSIFLFLDRTYVLQNSM
LPSIWDMGLELFRAHIISDQKVQNKTIDGILLLIERERNGEAIDRSLLRSLLSMLSDLQIYQDSFEQRFLEETNRLYAAE
GQKLMQEREVPEYLHHVNKRLEEEADRLITYLDQTTQKSLIATVEKQLLGEHLTAILQKGLNNLLDENRIQDLSLLYQLF
SRVRGGVQVLLQQWIEYIKAFGSTIVINPEKDKTMVQELLDFKDKVDHIIDICFLKNEKFINAMKEAFETFINKRPNKPA
ELIAKYVDSKLRAGNKEATDEELEKMLDKIMIIFRFIYGKDVFEAFYKKDLAKRLLVGKSASVDAEKSMLSKLKHECGAA
FTSKLEGMFKDMELSKDIMIQFKQYMQNQNVPGNIELTVNILTMGYWPTYVPMEVHLPPEMVKLQEIFKTFYLGKHSGRK
LQWQSTLGHCVLKAEFKEGKKELQVSLFQTLVLLMFNEGEEFSLEEIKQATGIEDGELRRTLQSLACGKARVLAKNPKGK
DIEDGDKFICNDDFKHKLFRIKINQIQMKETVEEQASTTERVFQDRQYQIDAAIVRIMKMRKTLSHNLLVSEVYNQLKFP
VKPADLKKRIESLIDRDYMERDKENPNQYNYIA
>Hsap_ENSP00000438788
MSRGFWLAEPLAGTGPHPAPVAADSRGCSSVPRRHAPSRLSVSTPSRGPGARMVGELRYREFRVPLGPGLHAYPDELIRQ
RVGHDGHPEYQIRWLILRRGDEGDGGSGQVDCKAEHILLWMSKDEIYANCHKMLGEDGQVIGPSQESAGEVGALDKSVLE
EMETDVKSLIQRALRQLEECVGTIPPAPLLHTVHVLSAYASIEPLTGVFKDPRVLDLLMHMLSSPDYQIRWSAGRMIQAL
SSHDAGEGQCGEEGKAGEGLGRLRDSQDTVAGASDLIRTRTQILLSLSQQEAIEKHLDFDSRCALLALFAQATLSEHPMS
FEGIQLPQVPGRVLFSLVKRYLHVTSLLDQLNDSAAEPGAQNTSAPEELSGERGQLELEFSMAMGTLISELVQAMRWDQA
SDRPRSSARSPGSIFQPQLADVSPGLPAAQAQPSFRRSRRFRPRSEFASGNTYALYVRDTLQPGMRVRMLDDYEEISAGD
EGEFRQSNNGVPPVQVFWESTGRTYWVHWHMLEILGFEEDIEDMVEADEYQGAVASRVLGRALPAWRWRPMTELYAVPYV
LPEDEDTEECEHLTLAEWWELLFFIKKLDGPDHQEVLQILQENLDGEILDDEILAELAVPIELAQDLLLTLPQRLNDSAL
RDLINCHVYKKYGPEALAGNQAYPSLLEAQEDVLLLDAQAQAKDSEDAAKVEAKEPPSQSPNTPLQRLVEGYGPAGKILL
DLEQALSSEGTQENKVKPLLLQLQRQPQPFLALMQSLDTPETNRTLHLTVLRILKQLVDFPEALLLPWHEAVDACMACLR
SPNTDREVLQELIFFLHRLTSVSRDYAVVLNQLGARDAISKALEKHLGKLELAQELRDMVFKCEKHAHLYRKLITNILGG
CIQMVLGQIEDHRRTHQPINIPFFDVFLRYLCQGSSVEVKEDKCWEKVEVSSNPHRASKLTDHNPKTYWESNGSAGSHYI
TLHMRRGILIRQLTLLVASEDSSYMPARVVVCGGDSTSSLHTELNSVNVMPSASRVILLENLTRFWPIIQIRIKRCQQGG
IDTRIRGLEILGPKPTFWPVFREQLCRHTRLFYMVRAQAWSQDMAEDRRSLLHLSSRLNGALRQEQNFADRFLPDDEAAQ
ALGKTCWEALVSPVVQNITSPDEDGISPLGWLLDQYLECQEAVFNPQSRGPAFFSRVRRLTHLLVHVEPCEAPPPVVATP
RPKGRNRSHDWSSLATRGLPSSIMRNLTRCWRAVVEKQVNNFLTSSWRDDDFVPRYCEHFNILQNSSSELFGPRAAFLLA
LQNGCAGALLKLPFLKAAHVSEQFARHIDQQIQGSRIGGAQEMERLAQLQQCLQAVLIFSGLEIATTFEHYYQHYMADRL
LGVVSSWLEGAVLEQIGPCFPNRLPQQMLQSLSTSKELQRQFHVYQLQQLDQELLKLEDTEKKIQVGLGASGKEHKSEKE
EEAGAAAVVDVAEGEEEEEENEDLYYEGAMPEVSVLVLSRHSWPVASICHTLNPRTCLPSYLRGTLNRYSNFYNKSQSHP
ALERGSQRRLQWTWLGWAELQFGNQTLHVSTVQMWLLLYLNDLKAVSVESLLAFSGLSADMLNQAIGPLTSSRGPLDLHE
QKDIPGGVLKIRDGSKEPRSRWDIVRLIPPQTYLQAEGEDGQNLEKRRNLLNCLIVRILKAHGDEGLHIDQLVCLVLEAW
QKGPCPPRGLVSSLGKGSACSSTDVLSCILHLLGKGTLRRHDDRPQVLSYAVPVTVMEPHTESLNPGSSGPNPPLTFHTL
QIRSRGVPYASCTATQSFSTFR
>Hsap_ENSP00000444856
MYRVTWSTFWLRFQHYTCTMSLKPRVVDFDETWNKLLTTIKAVVMLEYVERATWNDRFSDIYALCVAYPEPLGERLYTET
KIFLENHVRHLHKRVLESEEQVLVMYHRYWEEYSKGADYMDCLYRYLNTQFIKKNKLTEADLQYGYGGVDMNEPLMEIGE
LALDMWRKLMVEPLQAILIRMLLREIKNDRGGEDPNQKVIHGVINSFVHVEQYKKKFPLKFYQEIFESPFLTETGEYYKQ
EASNLLQESNCSQYMEKVLGRLKDEEIRCRKYLHPSSYTKVIHECQQRMVADHLQFLHAECHNIIRQEKKNDMANMYVLL
RAVSTGLPHMIQELQNHIHDEGLRATSNLTQENMPTLFVESVLEVHGKFVQLINTVLNGDQHFMSALDKALTSVVNYREP
KSVCKAPELLAKYCDNLLKKSAKGMTENEVEDRLTSFITVFKYIDDKDVFQKFYARMLAKRLIHGLSMSMDSEEAMINKL
KQACGYEFTSKLHRMYTDMSVSADLNNKFNNFIKNQDTVIDLGISFQIYVLQAGAWPLTQAPSSTFAIPQELEKSVQMFE
LFYSQHFSGRKLTWLHYLCTGEVKMNYLGKPYVAMVTTYQMAVLLAFNNSETVSYKELQDSTQMNEKELTKTIKSLLDVK
MINHDSEKEDIDAESSFSLNMNFSSKRTKFKITTSMQKDTPQEMEQTRSAVDEDRKMYLQAAIVRIMKARKVLRHNALIQ
EVISQSRARFNPSISMIKKCIEVLIDKQYIERSQASADEYSYVA
>Cint_ENSCINP00000002432
FLRKLEQQWQDHCQQTIMIRCIFLVLDRTYVLQNSMLPSLWDLGLDLFRENVLSREHVRERCFFGLLNLIKRERSGDTID
RCLLRNLLSMLNDLHIYHTMFEKRFLHETEESYSIEGSAKRSSMEVHEYLIHTERRISEERDLCLACMDHSTLKPLTLCV
EEQLISKHTEALLSKGLSHLIVENRIDDLMRLYKLFSAVKDGIQSLCTHFNKHVKNVASLIVLDVSNDHTMVQDLLDLKE
KLSNIVTKCFSKDLKFVEALREAFESSINKRQNKPAELIAKYVDQRMKSGNKEATEVELDRTLDQIMMLFRFIHGKDVFE
AFYKKDLAKRLLVGKSASVDAEKSMLSKLKQECGGMFTGKLEGMFNDISHSKELMAQYRQHVTTKKEGKVPNIDMSVNIL

TMGYWPTYPPMEVQLPSYLVKLQDSFKDFYLSKHSGRKLSFRASLGHCVLKSKFKNGNKELQVSQFQALVLLLYNEATCF
SFLQIKSDTQIEDSELRRTLQSLACGKARILTKSPKGKDVNDGDNFNLNTEFKHKLIRIKINQIQLKESVEENTDTTERV
FQDRQYQIDAAIVRTMKTRKTLSHQLLLTELYDQLKFPLKATDIKKRIESLIERDYMERDKDNTTQYHYMA
>Cint_ENSCINP00000004540
MSRRINFDETWGRLSDTVQKVITAAPLPRPVWNDKFSDIYSLCIAYPEALGESLYKETKTFLTSHVQHLCKIISLNYQLF
HRHWKTYSQGAMYLNLLYHHFNHQYVRKSKQSDVDIEYGFVVDDKEQMLEIKDLAMEAWREGMLDPLQHRLVSTILRDIA
RDREGKLGGESVHQVALYSLVAVEQHKKRNALKYYEDVFEKLF
>Cint_ENSCINP00000004560
KMSRRINFDETWGRLSDTVQKVITAAPLPRPVWNDKFSDIYSLCIAYPEALGESLYKETKTFLTSHVQHLCKIISLNYQL
FHRHWKTYSQGAMYLNLLYHHFNHQYVRKSKQSDVDIEYGFVVDDKEQMLEIKDLAMEAWREGMLEPLQHRLVSTILRDI
ARDREGKLGGESVHQVALYSLVAVEQHKKRNALKYYEDVFEKLFLQETGDFYRAKALELRDDSTCSEYMNKILSLLTDEE
MRSRRFLHISSYKKTTLECQQRVIIDHIKFLQAGCRQMIRHNSATDLHHMYMLLRVSNGLTHMVSELEAHIKETGLDLVK
GIKEGNVPLQFVETILEVHKRFHDVIRDTFHSDKLFVSALDRACTAVVNYSDPKQQCRAPELVSFVYFSTSLRFGSLARY
NTFFFWLIITPTPTVCKYCDAILRRCTKGPSNDADEKLQSSILVFRYIDEKDVFQKVSYLTSPLPQFYSRALARRLIHSP
CAMDMEEMMINRLKGVCGYDFTSKLHCMFTDVRLSTELGKKYQETVAKTEDSSQPSINVNVLQAGAWPLTANQVEFVLPE
TLHRCLKQFEEFYNHKFNGRNLSWLHHLSQAEVRINFTSKPYLVSMSTYQLAIILLFNESIELTVSDISQNTKLKDKDLE
RNIAALTDANILLKTTQEKLEDMSVIRVNNKFSNKRTKFRVAFTQTQKEQSTEVQQTHTAVADDRKLYLQAAIVRIMKAR
KVLHHNTLMEEVINKSRIRFTPSVSAIKRSIEALIEKSYIERSPDSPDQYRYLA
>Cint_ENSCINP00000005419
MNELPENPEVLLEAMLETDTDHMFEALVLHHLKKHMQNTIIPEFCKRTSLPSFYDVTNAIGGLYTSVLWFLNYARVWRDC
ETLTSKIKTVVKSALRWHLPNHMKDTFTKMFTYVFQYHLWRDSEYESESFTVEWKGEKETLVAQDFNDFVGNSFSPLVST
WLVELDLVDVLSNESAHAMLEAEIERYVLNTCKGEFEQSWMTPEDGGLLQWLKSKVFPFLLVIFYKNVDKAKWNKTLCFM
LYGAYTKLRISEMLDIITDYPESVPTIQDLKLCLQHTRQRPRLIDSLKNDIRNRVLHPGVSTQNIITVYISAVKALRELD
STGVVMEMVLEPCTTYLTQREDAVRMIMQGLLGDEGTNDLAAELANSQETDQSKCWNERVTENPEDWIPDPREADPHKVD
KSSRTSDIVTLFIGMYGSKEKFIWEYQKLLSKRLMQTWYDEKSESTEIRNLELLKMRFGEDDMSKCAVMLKDIADSKRIN
SLVKEKHTKQDDQQSDIDVRTMIVSSHFWPTIPSQTMEPPEKIAELLQLFNKGYEGLKASRSLKWISGVGMVEIEIELNN
KKLEFKVPPLQATIISHFEETETWTLAELSDKIKVPPTVLRRKMSFWMQRGVLKQSSTDTYSTTEGSSDAPLPSDVASSS
SITGGCLVACDEEEEENSNQNIEKEKKEKDNKMFWFYIKGMLKNFQSLPIDRIHKMLKMFAVNDANMQIELPQLKQILNE
KVQQGLLVLNGGKFKLSDDAK
>Cint_ENSCINP00000006037
MSRLSQPTKKDTKMRIRAFPMTIDEKYVANIWDLLKKAIQEIQKKNNGGLSFEELYRNAYTMVLHKHGEKLYTGLHEVVT
EHLIKVREDILQSLNNNFLQVLNSAWDDHQTCMVMIRDILMYMDRVYVSQNNVDSVYNLGLKIYRDQVIRQKDIRECIQS
TLLELVAKERRGEVVDRGAVRNTCMMLMTVSLNGRDVYEEEFEKGFLNQSSEFYRRESQKFLVENSASVYLKKVEARIEE
EAERARHYLDPSTEPEIIAVLERELIQRHMKIVVEMENSGAVHMLQHDIKDDLLCMYQLFIRVPQGFETLRDCLSAYLRE
QGKSVVEDGGQKSPVEYIQSLLDLKDRMDDFHRNSFKSDPLFKKMICSDFEWFVNLNPKSPEYLSLFIDDKLKKGIKMLS
EQEVEVVLDKTMSLFRFLQEKDVFERYYKQHLGRRLLTNKSISDDSEKNMITKLKNECGCQFTSKLEGMFKDMHVSSTTN
DDFKKHVQSTSTSLQGVDLTVQVLTTGCWPTQASTPTCNLPAAPRHAFDVFRRFYLGKHSGRQLTLQHHRGAADMNASFF
AAAKPGASNEGESASVKPTTRRHILQVSTFQMVVLMLFNDREKWLFEEIQQETEIPVKDLSRALQSLACGKTNQRVLQKD
PKGKEIEKGNVFTVNDNFSSKLHRVKIQTVAQKQGESDPERKETRTKVQEDRRHEIEAAIVRIMKSRKEMQHNLLIAEVT
SQLKHRFLPSPVIIKRRIESLIEREYLSRSNTDRKVYIYVA
>Cint_ENSCINP00000006254
LYFVFDVQGNQQTFDEKWPTMQPIILKLLSQQSVTRQEWQDLFWDVHSVCLWDNQVGPEKVHTALKTNISNFIKEAQQRI
KTHHDGNALLRAYIVEWRKFFDQCVYLPEPFTQLEKSLSGTPSKKKKQEQNTIVRKLMLDSWNKNIFKDIYEQLKDRTME
LIKHEREGGELDAQLVIGVRQSFVNLCSNPDDPLEIYREHFENAYIASTEEHYKSEAETYLAEHGVRKYMKYAYQKLLEE
SQRGTRYLESSSQTRLNARCVKVLATQFSEAILAECKLMIRENEMDRLYLMFNLMNRVPNGIDPMLECLQEYIVQQGLAD
MKECCDIITTDSEKYIDLLLSLFNRFSDLVKKSFDDDPRFLTARDKAFECVVNDYSVFNVELQTRSKQNPIKLPPESRCP
ELLANFCDMLLRKTPLSKKLSSEEVDAKLKDVLLVLKYVSNKDVFMRYHKAHLTRRLILGTSADSEKEENMVEGLREVGM
PADFVNKLARMFQDIKVSEDLNTQFKEAREDRSSELANIKILNAGAWSRRSDRIPVSLPAILEDLIPDVEEFYKSKHHGR
MLNWNHLMSNGVMTFKTEVGSYDIEVTTFQMSVLHIWNSRPTESISFESLRLATQLPDTELRKTLWSLVANPKLKRQVLL
CEPKLSSPKEFQESSMLAVNHQFAVMKAGKPQKRGRVNLIGRLQLSTDKSREEDNEGITQLRILRTQEAIVKILKMRKRI
NNAQLQTELVEILKNLFLPQKKLIKQQIEWLIENKYMKRDDDDINTFIYIT
>Cint_ENSCINP00000013359
GLDQIWEDLQKGISQVYARQSMEKKRYMELYTHVYNYCTSVDQSVSARQAPNSSTPPGRVSTTKKDSKAMTGAQFVGFEL
YKKLKQNLQMYLADKLKAGENLLNEDVLLFYTNQWEDYRFSSRVLNGVFGYLNRHWVKRECDEGRKEIYEIYSLALVIWR
ENLFKPLNKQVTSAVLNLIEKERNGETINTSLISGVLRSYVALGLSENEQNRTQSLSVYKEAFESNFLADTERYFTSESQ
EFLAANPVTEYMKKAEARLQEEERRVQLYLHESTHDQLARKCEQVLIEQHLEQFHAEFQSLLNDDKNEALNRWKYKITSQ
IFTSVYYPLPILNTLGFPTCGTAAQFDPKLYVQTILDVHKKYNALVQTSFDNDSGFVAALDIACGRFINKNAVTTSAKSS
SKSPELLARYCDTLLKSAKVSEDAELEATLKEVLTVFRYIEDKDVFQTFYSKMLARRLVQHTSASDDAEAQMISRLKQTC
GFEYTSKLQRMFQDVDVSKNLNERFRTHIAASTPLDCKFRKENLDFSIQVLSSGSWPFQQSVTFRLPVELERSYQRFTTF
YSQAHNGRKLSWLYQMSKGEIVTNCFKNRYTFQASTFQMAILLQYNSATSYTVQQLAENTQLKMEILLQVLIHLLKCKIL
QCKDEPDANNLKPHNEIELFLGYRSKKLRVNINKPVKTEQKQEQEVTHKHIEEDRKMLIQAAIVRIMKMRKQQKHQQLLS
EVLSQLSSRFKPRVPIIKKCIDTLIEKEYLERVEGEKDMYQYLA

>Cint_ENSCINP00000013365
RSSMQHNGGGNPHARRYIGLDQIWEDLQKGISQVCEYLILQSMEKKRYMELYTHVYNYCTSVDQRRVSTTKKDSKAMTGA
QFVGFELYKKLKQNLQMYLADKLKVAGENLLNEDVLLFYTNQWEDYRFSSRVLNGVFGYLNRHWVKREYLFKITKYMLFV
EGQVFSNAMLLLKTLNVVTMNVLFIFLNLGLHSSVICEDFFTSFEDENEPNRTQSLSVYKEAFESNFLADTERYFTSESQ
EFLAANPVTEYMKKAEARLQEEERRVQLYLHESTHDQLARKCEQVLIEQHLEQFHAEFQSLLNDDKNEDLGRMFKLVSKI
KDGLGELKTLLEAHIHNQGDVAIKQCADTAVNDPKLYVQTILDVHKKYNALVQTSFDNDSGFVAALDIACGRFINKNAVT
TSAKSSSKSPELLARYCDTLLKSAKVSEDAELEATLKEVLTVFRYIEDKDVFQTFYSKMLARRLVQHTSASDDAEAQMIS
RLKQTCGFEYTSKLQRMFQDVDVSKNLNERFRTHIAASTPLDLDFSIQVLSSGSWPFQQSVTFRLPVEVLKSYQRFTTFY
SQAHNGRKLSWLYQMSKGEIVTNCFKNRYTFQASTFQMAILLQYNSATSYTVQQLAENTQLKMEILLQVLIHLLKCKILQ
CKDEPDANNLKPHNEIELFLGYRSKKLRVNINKPVKTEQKQEQEVTHKHIEEDRKMLIQAAIVRIMKMRKQQKHQQLLSE
VLSQLSSRFKPRVPIIKKCIDTLIEKEYLERVEGEKDMYQYLA
>Cint_ENSCINP00000014817
MDSFVCSQKTPNGLETVSTACINHATECLPHEQSIFVESVSLEKFEAECWPKLHQVVSTLLQAELNSQTNSNKVSFQDMY
SCVYQCICSRHTERLHYNLMELVSLHLQNISDKLLPGTTLQSNCFYITGCLEVVRKYGRALHEIVPIFTYLNRVYITPIL
HSNLEDQLLNLFKMHIIDKHAERLVGAMKNARKISFSVEPSVMAELAQALYSMKPNLRVSEADFFSHYIAQAKSSTKPMS
LEEDIEATRKMQLDLQRDPNFSFTCSQKRSKEDTEIEDEECSSSLLSSLLMGSS
>Skow_XP_002736663
MSLKPRVVNFDETWGKLLETVEGVITLGNVARATWNDRFSFADTKGVLSRFISVQHVHLHTCYSQGASYMNKLYGYLNSQ
FIKKQKLSDADIQYGYGIDINEQLMDIGELALDIWKRLMIEPVKDNLVKTLLXXXXRDRCGDTPNQAVIHGVILSFVNVE
EYKRKLQLKLYQDLFEAPFLAETGEYYKAEAARLLDDNDCSHYMEKVLQRLSEENLRSRKFLHPSSYTKVTNQCQQKMVA
EHLLFLHGECRDIIRKEKKEDMQRLFKLLQPIQNGLGVMIEELQKHIKEIGLEAICNLRGENVPSQFVESVLDVHSKFSK
LITSVLANDRAFTSALDKALTAVVNWKPSIKHVCKAPELLAKYCDTLLKKSSKGVSDSEVDDKLTLSIIVFKYIDDKDIF
QRFYSRMLAKRLIHGLSMSMDAEEGMINRLKQACGYEFTNKLHRMFTDMSVSNDLNNKFSSFVKKKDVELGIGFSIYVLQ
AGAWPLGQSTLTPFAIPQELEKSVSEFEIFYNTSFSGRKLTWLHHLCAGELKFTYLKKPYIVTVTTFQMAVLLLYNNCDS
MTYTELVDTTQINEKELAKTLQSLVDVKILNKDEKEKSTSDYSLNTNFVNKRTKFKITAAVQKETPQEVEQTHSAVDEDR
KLYLQAAIVRIMKARKVLKHNTLIQEVISQSKARFSPSISMIKKCIESLIDKQYLERNSSSTDEYNYVA
>Skow_XP_002742106
MSRSSGSGRGGQNPHGLKQIGLDQIWDDLKSGIQHVYTRQSMAKSRFMELYTHVYNYCTSVHQSQQSRSSSAKTKKGQNM
GGAQFVGLELYKRLRDFLKNYLVNLLKDGTELMDESVLTFYTNQWEDYQFSSKVLNGVCAYLNRHWVRRECDEGRKGIYE
IYSLALVTWRDNLFKPLNKQVTGAVLKLIERERNGETINTRLIRGVIDCYVELGLNEDDPTSKGPTLNVYKDSFESQFLA
DTERFYMSESTEFLRQNPVTEYMKKAESRLTEEQRRVQVYLHESTQDELAKTCERVLIEKHLEIFHAEFQNLLDDDKNLV
LNTKTGSTHPPTLFTNGCDFGGTLDPKLYVQTILDVHRKYNALVLSSFNNDAGFVAALDKACGKFINNNAVTKAANSSSK
SPELLARYCDSLLKKSSKNPEEAELEDTLNQVVMSFKDLNEQFKNHLKNSNEPLDIDFTIQVLSSGSWPFQQSCTFVLPS
ELERSFQRFTTFYAGQHSGRKLMWLFQMSKGELVTNCFKNRYTLQASTFQMAVLLQYNTSDTYSLLQLQEHTQLKLDILL
QVIQILVKSKLLECEDEDDCLTQHSVVKLFLGYKNKKLRVNINVPMKTEQKQEQETTHKHIEEDRKLLIQAAIVRIMKMR
KVLKHQQLLSEVLTQLSSRFKPRVPVIKKCIDILIEKEYLERVDGEKDTYSYLA
>Skow_XP_002740559
MTKIPSQRLKNDTRRAMDPQLVRFRGRSRATSGCCMPLTSTEKSPFIKTELETNLKKRRPDENNHHLQNSSKKFRDDSPN
ESSPMADSTQRRPNFSALNPTNNGFTNRTSPLANTKPGSTKKLVIKNFRVKPELPENYQERTWGKLKEAVQAIHKHTSIK
YSLEELYQAVENMCSHKMSASLYDKLKIVCEEHVKAQISLFYTDSTDSVSYLKILNNCWLDHCRQMIMIRSIFLFLDRTY
VLQNSLISSLWDMGLELFRQHIISHRIVEARTVDGLLLLIDRERNGEVVDHSLLKSLLRMLSDLQIYEEAFECKFLDATD
KLYAAEGQRLMQERDVPEYLAHCDRRLEEESQRILHYLDHSTKKSLIACVEKQLLEVHVNSIIQKGLDVLIDENRTKDLA
LMCNLFQRTKSGLQELCMNFGIYIKKTGTSIVINPEKDKTMVQELLDFKDKMDYILNHCFAKNDKFVNIVKEAFETFINK
RVNKPAELVAKYVDNIMRAGNKEATEEELEKMLDKVMVIFRFIHGKDVFEAFYKKDLAKRLLVGKSASVDAEKSMLSKLK
QECGGGFTSKLEGMFKDMELSKDIMVAFKQSIQYQQNPGNIELTVNILTMGYWPTYTPMEVHLPTEMVQFQEVFKKFYLS
KHSGRKLQWQPNLGHCVLKACFKADGELRRTLQSLACGKARVIAKTPKGKEVDDGDIFTFNEDFKHKLFRIKINQIQMKE
TAEEQSNTQERVFQDRQYQIDAAIVRIMKMRKTLSHTLLVSELYNQLKFPVKPADLKKRIESLIDRDYMERDKDNPNQYH
YVA
>Skow_XP_002737439
MASSNMLKDKNQLVFEDKWDNMRPTVLKLLRQEPVTRAEWQDLFWDVHSVCLWDDKGPGKVHKALQSDILDFINQAQGRV
MSHQDDSALLKSYIIEWRKFFTQCSYLPKPFGQLEVSLSGKTISTSQRKNQAEDSEVRKLMLDSWNSSIFSSIKKRLQDS
AMKLVHAERNGEAFDAQLVIGVRESYVNLCSNAEDKLQIYRDNFEKAYLDATESFYRAQAPSYLADNGVQNYMKYADAKL
KEEEQRAQRYLETRKGCNSVQALTECCVHVLVTAFKETILAECAVMIKTNETERLRLMFSLMDRVPDGISPMLKDLEDHI
IEAGLADMVAAAEIITTDSEKYVEQLLVLFNKFSSLVKDAFNDDPRFLTSRDKAYKHVVNDTKVFKLELPTKQKGVGSKT
QPESKCPELLANYCDMLLRKTPLSKKLTSDEIEFKLRNVLLVLKYVQNKDVFMRFHKAHLTRRLILDTSADSEKEENMVE
WLREVGMPADYVNKLARMFQDVKVSEDLNQQFKEAYRNNEHSAIADSINIKILNAGAWARSSERIPVSLPVELEDYIPEV
EEFYRKNHSGRKLQWHHLMSNGIITFKNKVGIYDLEVTTFQMAVLFAWNQRQYEKITFENLRLATELPDTELRRTLWSLI
QFPKMKRQVVICKPEVKSPKEFNDGTLFWVNQDFGVVKNSKLQKRGKINLIGRLQLSTERSSDEDKEGIVQLRILRTQEA
IVKIMKMRKKITNAQLQTELVEILKNMFLPSKKMIKEQIEWLIEHKYMKRDEDNINMFIYIA
>Skow_XP_002735887
MVNVRVDVLESLNNNFLQTLNSAWNDHQTSMVMIRDILMYMDRVYVQQNNVENVYNLGLILFRDLVVRYGCIRDHLRQTL
LDMVARERRGEVVDRGSVKNACQMLMVLGIDSRAVYEEDFESPFLDQSADFYRLESQNFLAENSASVYIKKVEARINEEA

ERATHYLDKSTEEPIVKVLELELICKHMKTIVDMENSGVVHMLKNKKTDDLACMYKLFIRVQEGLKTMCQCVSGYLREQG
KALVTEEEGSKNAIQYVQDLLDLKDRFDHFLHKSFGDDRLFKQTISGDFEYFLNLNNKSPEYLSLFIDDKLKKGVKGMSE
QEVEVVLDKAMVLFRFLQEKDVFERYYKQHLAKRLLLNKSVSDDSEKNMISKLKTECGCQFTSKLEGMFKDMTVSNTTME
EFKNHVQTSGTSLHGVDLNVRVLTTGFWPTQSATPKCTVPAQARTAFEAFRRFYLGKHSGRQLTLQPSLGSADLNASFFA
PKKDGSGGPQIRKHILQVSTYQMVILMLFNTREQCSYEEVAQETDIPTRDLIRALQSLACGKPQQRVLSKEPKSKEIEPN
DSFTVNDHFSSKLHRVKIQTVAAKGESEPERKETRSRVDEDRKHEIEAAIVRIMKSRKRRSHNLLVAEVTEQLKSRFLPS
PVVIKKRIENLIEREYLARTPDDRKMYTYVA
>Dmel_FBpp0072065
MCDFDALWADVLRIFPVLGGANAVSNDPVEENVFQNVRQHLMVLGSIDTFAAVVDLEIQSTIRNVLVPKFWKHIHDGLVS
SKLQGWINDQALAIEALPDKNGLFTQFIDAINELHDSFQSLMQVKPRLIQLVGAETKPTAKTVKLMQEEGIKNCLRDCLL
AQLPPNFSVVVGAFYWVHFKLFTKASNLALTTVDSDVFDELLCVGCNFEAEQCCCQRLTDMVNKTNMKLFEMNLIDRLTG
SALTALIKLKIKEHISDTCMGIFDRSHLKQLETWLSDVIMSWLTNIFTEWKSKDSISDIEVPESVKSFKVKLTYFMYETF
AQSVIGQFFSIIIDYPDSIPAIDDLKICMEKIDMRVYLTESLRNSLEARILHPGVNTMDILTGYVAAIKAIRHLDSTGVI
LEMVTAPIKDYLRKRNDTVRRVVTGLTEEGPTDLSEELAKGETIKECKDSGTDEFSNWENWQPDPFGIDASIMQYNSSRK
MRSADIISMVVDIYGSKELFMTEYRNLMADRLLAQLDFNSEKEIRNLELLKIRFGESLLHSCEVMLKDVTDSKRINAHIH
SDGDRTENQLFDISSLIVSAQFWPSFNKESLQLPEEIENEFKKYTKAYEAYKGNRTLNWRTVTGRVNIEIEIGDRTMEMV
VSPILAVIIYHFQTKNEWTIEDLSSITKVPASALRRRLSFWQNHGLISETTPGIFTLLEKESEKSQYEDMSLAEADEEDL
ESAMASASDQREEELQVFWSYIVGMLTNLDSMPIDRIHQMLKLFASHSGGVEFTQDELKHFLQRKVREHKLIFSGGVYQL
AK
>Dmel_FBpp0075633
MNRVKRISNYFDSMLPLIDNILQVVNQIFDDYTSFDGQKLMHLYHLIYKQCAAERNWNNPAQKNGRSLYIVLSDCLMKRL
KNIAWVMENQLDDNKPIRLIAKYLEHWVPYQRSCEKLNLACYHFNRNWVERERLKGDKETYPIYRLAMISWKELVFEPSV
TILAAIRLILSQMPREYNKSLEYQVYQVLQSIVELYANDKYQDVSLPSRIDKIFTEKVMDFYKSTTLHEFQKIVVSNDYK
DFKHFLRYACLAMPEIENGTQFKAILKRYLAARLQETRLSGKDYIQAFLGLCRGPLQRALRDHTKLAEVVDAVCKEEINR
KGQIIDPTRLLVTYANELMTKRQESEEAIIDELKRIVKCVEFLSDEYKFIDMYLKALRIRQINETSTSDGVESIMYSLLN
KKMANFVTRCIYVQLRDVDKLRTLKKEFQDHLNSKGIKLGFDFRPKCFKNDGSFENFNLTLPDELQQAWKEFRIFYEARK
LQSKAIIQLNNELSSGEITCHLNQSVYILEVSTLQMAVLMLFNRHERFTEQELVTALGVELETLQEALKQIKFLVYIEVN
KVNYIEINMDFTNRKRRLFCNEPLPRKMRKIEEKSEIELKIRRDKQVDAAIVRIMKGQKQLEYSELISLVYEELKDRVKP
QVSFIKKRLDYLVEREYLERDNYYNIYRYL
>Dmel_FBpp0084786
MLRAIGKRDRDIFEEVWPDKRRIVLKLLRQDTVSQREWQDLFFGVHFVCLWDEKGAAKIYDCLQQDIVEFIVQAQSQVQA
QRGEQALLATYIVEWRKFFTQSNYLPLPFRQLEQSPQVKPSSSGSSSSASVSASGASTSAAAAAAATSCSGQAGKSGAST
SAAAGASTSAAAAAASTSASTSSAAAAAAASGSGSSSSSTSTSKKNPTEDSPVRKLMLDSWNKHIFHDIKHRLQESAMKI
VHAERNGDAYDAQLVVGVRESYVNLSSNAEDKLEIYRENFEMAYLKATVEFYRLKSAEQQQENGVLAYMKYADSKLREEE
VRAKRYLEPSSFSILTYTLVNVLIVDHLNSIIAECPALIRDYETERLNLMFRLMDRVMHGVGVEPMMGDLQRHIMSAGLA
DMLSASEVITQDSEKYVERLLELFNKFSDLVRNAFNDDPRFLTARDIAFKTVVNDTSVFKMELPTSIANRGVKYTAPESK
CPELLANYCDMLLRRTPLSKRLTSEQIDARLRDVLLVLKYVNNKDVFMRYHKVHLTRRLILGTSADSEKEEDIVEWLREV
GMPADYVNRLARMFQDIKVSEDLNTQFRTSISRHDAINIKILNAGAWARCSERVSVSLPIELEDYIPDVEEFYKKKHSGR
KLQWYHHMSNGTITFVNNFGRYDLDVTTFQMAVLFAWNQRQHDKISYENLRLATELPDPELRRTLWSLVAFPKIKKQILL
MEPAAISSPKDFAENTMFYINQEFAIVKNGKSQRRGKLNLIGRLQLSTERSQQEDNQSIVQLRILRTQEAIIKIMKVRKR
MNNAALQGELIDILKNMFLPSKKMIKEQLEWLIENKYMRRDDDDINMFIYVA
>Dmel_FBpp0085254
MSLKPKIVEFVDVWPRLRCIAESVITLTKVERSVWNTSFSDVYTLCVAQPEPMADRLYGETKHFLEQHVQEMLAKKVLIE
GECSHSNGGPDLLQRYYITWMEYSQGIKYLHQLYIYLNQQHIKKQKITDTESFYGNLSSDAAEQMEIGELGLDIWRLYMI
EYLSSELVRHILEGIAADRASNGTLDHHRVQIINGVIHSFVEVQDYKKTGSLKLYQELFEGPMLEASGAYYTDEANKLLH
RCSVSEYMQEVIRILEYESRRAQKFLHVSSLPKLRKECEEKFINDRLGFIYSECREMVSEERRQDLRNMYVVLKPIPDNL
KSELITTFLDHIKSEGLQTVSALKGENIHIAFVENMLKVHHKYQELIADVFENDSLFLSALDKACASVINRRPTERQPCR
SAEYVAKYCDTLLKKSKTCEAEIDQKLTNNITIFKYIEDKDVYQKFYSRLLAKRLIHEQSQSMDAEEGMINRLKQACGYE
FTNKLHRMFTDISVSVDLNNKFNTHLKDSNVDLGINLAIKVLQAGAWPLGSTQVIPFAVPQEFEKSIKMFEDYYHKLFSG
RKLTWLHHMCHGELKLSHLKKSYIVTMQTYQMAIILLFETCDSLSCREIQNTLQLNDETFQKHMQPIIESKLLNASSENL
AGETRIELNLDYTNKRTKFKISSALQKETPQEVEHTINSVDEDRKLFLQAAIVRIMKARKVLKHNALIQEVLSLSKVSFT
PNIAMIKKCVESLIDKQYIERTANSGDEYSYMA
>Dmel_FBpp0087897
MSAAKKYKPMDTTELHENTENIAACLKKAENVAPTAAAASSVGRSAFAAQNMNSASTNGERLPNFSKLGGSHGEIRTAST
TSNLLNRMGAIHNSKPGDVKKIVIKNFKDKPTLPDNYSKDTYVKLEEAVIAIQLSKPIKYSLEELYQAVVNMCSHKMDAQ
LYAKLKELTEQHVKRNIKLKELTGGSMDKLILLEKINHWWLSFCQQMIMIRSIFLYMDRTYVLQNSTVHSIWDMGLDLFR
IHFAQNSVVQKRTVDGLLTLIEKERQGSTVDRGLLKSLVRMLCDLQIYTSSFEEKFLDATNQLYKAESQRKMQELEVPEY
LQHVNKRLAEENERLRHYLDSSTKHPLIYNVEKELLAEHLTSILQKGLDSLLEDNRLSDLTLLYGLLSRVKNGTSELCGN
FNGFIKKKGRTIVIDPEKDKSMVQDLLDFKDKMDVIVRTCFEHNEKFTNSLREAFEFFINQRANKPAELIAKYVDMKLRS
GNKGTTDEELEKTLDKIMVLFRFIHGKDVFEAFYKKDLAKRLLVGKSASVDSEKSMLSKLKQECGGGFTSKLEGMFKDME
LSRDINIAFRGHALSNNRDVHNLDLCVSILTMGYWPTYAPTEVTMPPQFINPQQIFNKFYLEKHSGRKLQWQPTLGNCML
RAQFDAGPKELLVSLFQALVLLLFNDKPVLSYEEILAATLIEDGELRRTLQSLACGRARVITKTPKGREILDGDQFDFNN

EFTNKLFRIKINQIQMKETNEEQKATEERVFQDRQYQIDAAIVRIMKMRKTLSHNLLITELFNQLTFPVKPADLKKRIES
LIDRDYMERDKDNQNQYNYVA
>Dmel_FBpp0087921
MNRSGNSQTTQKLVNLDDIWSELVEGIMQVFEHEKSLTRSQYMRFYTHVYDYCTSVSAAPSGRSSGKTGGAQLVGKKLYD
RLEQFLKSYLSELLTKFKAISGEEVLLSRYTKQWKSYQFSSTVLDGICNYLNRNWVKRECEEGQKGIYKIYRLALVAWKG
HLFQVLNEPVTKAVLKSIEEERQGKLINRSLVRDVIECYVELSFNEEDTDAEQQKLSVYKQNFENKFIADTSAFYEKESD
AFLSTNTVTEYLKHVENRLEEETQRVRGFNSKNGLSYLHETTADVLKSTCEEVLIEKHLKIFHTEFQNLLNADRNDDLKR
MYSLVALSSKNLTDLKSILENHILHQGTEAIAKCCTTDAANDPKTYVQTILDVHKKYNALVLTAFNNDNGFVAALDKACG
KFINSNVVTIANSASKSPELLAKYCDLLLKKSSKNPEDKELEDNLNQVMVVFKYIEDKDVFQKYYSKMLAKRLVNHTSAS
DDAEAMMISKLKQTCGYEYTVKLQRMFQDIGVSKDLNSYFKQYLAEKNLTMEIDFGIEVLSSGSWPFQLSNNFLLPSELE
RSVRQFNEFYAARHSGRKLNWLYQMCKGELIMNVNRNNSSTYTLQASTFQMSVLLQFNDQLSFTVQQLQDNTQTQQENLI
QVLQILLKAKVLTSSDNENSLTPESTVELFLDYKNKKRRININQPLKTELKVEQETVHKHIEEDRKLLIQAAIVRIMKMR
KRLNHTNLISEVLNQLSTRFKPKVPVIKKCIDILIEKEYLERMEGHKDTYSYLA
>Dmel_FBpp0291369
MQGRDPRQQQPEPLNNLNANGRYHNRMVAGSNNFNGATVGNVDSAVPCRIARQNVLGQQARSMNINVPNASSMQQHQQSQ
NRLSAQGLASASRRSYHLAASGGRVRADNRINTNHPLTPTPLSMPVPAPAAPAVVKTETSATTSGPSTSASASASESTEKR
FKEIARKYPLWLPEYKRRAFNASMDEKYVETIWASLKNAIQEIQKKNNSGLSFEQLYRNAYNMVLHKHGNRLYYGLREVV
SEHLEHKVRADVLEALHSNFLPKLNQAWTDHQTSMVMIRDILMYMDRVYVQQREVDNVYNLGLILFRDQVVRYSEIQKAL
REKLLGMVMEERHGEAINHLAIKNACSMLITLGINSRTVYEEDFEKPFLAQSAAFYKFESQNFLAENNAGVYIKKVEARI
TEESSRAALYLDKDTEPRIVRVVEEELIKKHMRPIVEMENSGVVYMIKNSKTEDLACTYKLFSRLKEEGLKVIADTMSAY
LREQGRMLVKEEENGNTNPITFVQNLLDLKDRFDQFLVHSFANDRIFKNVISSDFEHFLNLNNKSPEYLSLFIDDKLKKG
GKGMSEQEIESILDKTMVLFRFLLEKDVFERYYKTHLAKRLLLNKSVSDDFEKNMISKLKTECGCQFTSKLEGMFKDMSV
SNTIMDEFKNFVNNNNLSLGGVELTVRILTTGFWPTQTATPNCNIPAAPREAFDIFKNFYLNKHSGRQLTLQPQMGTAYI
NAVFYGRKAVESEKDKDAPSSSSSGCGVPTTTRKHILQVSTYQMCVLLLFNNRDVLTYDDIHQETDIPERELVRALQSLS
MGKPAQRLLVRNSKTKTKDIEPTDEFYVNDAFNSKFHRVKIQTVAAKGESEPERKETRGKVDEDRKHEIEAAIVRIMKAR
KRLAHNLLVSDVTSQLKSRFLPSPVFIKKRIEGLIEREYLQRSPEDRKVYNYLA
>Cele_D2045_6
MTNSEPRRMTCDSEVVWKKLQDGLDVAYRRENMAPKDYMTLYTSVYDYCTSITLSTSRRDGEDGRAESSTPARTAGADFV
GHEMYQRVEEYVKAYVIAVCEKGAELSGEDLLKYYTTEWENFRISSKVMDGIFAYLNRHWIRRELDEGHENIYMVYTLAL
VVWKRNLFNDLKDKVIDAMLELIRSERTGSMINSRYISGVVECLVELGVDDSETDAKKDAETKKLAVYKEFFEVKFLEAT
RGFYTQEAANFLSNGGNVTDYMIKVETRLNQEDDRCQLYLNSSTKTPLATCCESVLISNQLDFLQRHFGGLLVDKRDDDL
SRMFKLCDRVPNGLDELRKSLENHIAKEGHQALERVAMEAATDAKLYVKTLLEVHERYQSLVNRSFKNEPGFMQSLDKAA
TSFINNNAVTKRAPPQAQLTKSAELLARYCDQLLRKSSKMPDEAELEELQTKIMVVFKYIDDKDVFSKFYTKMFSKRLIS
ELSASDEAEANFITKLKSMCGYEYTARLSKMVNDTQVSKDLTADFKEKKADMLGQKSVEFNVLVLSSGSWPTFPTTPITL
PQQLSKTIEIFGQFYNEKFNGRRLTWVYSQSRGEITSTAFPKKYVFTATTAQMCTMLLFNEQDSYTVEQIAAATKMDEKS
APAIVGSLIKNLVLKADTELQKEDEVPMTATVSLNKAYMNKKVRVDLSKFTMKQDAVRDTENVQKNVEEDRKSVISACIV
RIMKTRKRVQHQQLMTEVITQLSGRFKPKVEMIKRCIGSLIEKEYMLRTEGQKDLYEYLA
>Cele_F45E12_3
MTSGAPPTISTEKSRSKQLRKSSKRTASTVEGTEAKQMRGDTENRSDGEMDDVSTSSEIRHGNGFIENFRSQTGNSSRTT
ATNERIKKKLVIKNFKANANQNDVAMGDTNIDSTDGSVGRDWAVLSDNVFAILEDRKTVTTLEGLFSKVRSVCDKNQSKV
LYDRLVAIVVQFAKSLKESLNAVEQVPLAEDNCEQYLEKFGQIWQAYPVKINLIRNIFLHLDRIALGATDTEILPLWECF
MQIFQKTFFPNIFKEFKATKLFNALYMAMQKIMQRYPVDSPLRSLVDMLQTVHVSEEFAKFLISQLREHYNNERIDKVPK
MNCNDYMEYCEDQINRYSQLVKVNFDEPSALKDVQATVTNCLIQQAIPEILTHDFDDLIDSDNISDIGRMFNLCRQCVGG
EDEVRTQFSKYLKKRGEKLIATCPDEDLVSELLAFKKKVDFIMTGSFKSANDPVKMRQCLSDAFESFVNKQVDRSAELIS
KHFHTLLHSSNKNVSDDTTLDQMVDEAIVLFRYLRGKDVFEAYYKRGLAKRLFLERSASVDAEKMVLCKLKTECGSAFTY
KLEGMFKDMDASENYGRLFNQYLEHMNKEKANFTARVITPEYWPTYDTYEINIPKEMRDTLTDYQDFYRVQHGNRNVKWH
HGLASAVISASFRPGCKKELIATMYQTVILLLFNKCETWTVAEIVEHTKILEIEVVKNVLALLGGRDKPKVLQRVEGGGS
EKKEGTVENLKNEKFVVNSKFTEKRCRVRIAQVNIKTAVEETKEVKEEVNSDRQYKIDAAVVRIMKARKQLNHQTLMTEL
LQQLRFPVSTADIKKRLESLIEREYISRDPEEASSYNYVA
>Cele_K06H7_6
MSIFDRMTTSKEAAMKYRQFCVSKKKEVWDALIYHAKYAKNIEKVYDMTTVLQRVQQECLMFALEKGVGQPDIRDAAELI
FPLAMQKVVIKVLRPYFMSHFTNYRLAMYLPHRKIDHGIDLNQELWTPEKKEICGQYVQDFNPFVNELKTGILNANRHMN
AALSVYIRELAATLVMHEKAAFDNELRCSMFQEIMESLRIWSDQVTMQDAFHLISDTIYKDVFQMLSKNAYEMIAVDFPV
KFRSLITMKYCLLRTNNHGRVDFTNYLIDQVNTKLLVASVDTKDILKAYAACVESLREMDNSCVVMHKVCGVIREYLKRR
PDTVQQIISYITSNKKNELEKDMSLQSKTVRSAMMDEEELKGVNDDFLPENMETLGWENWMPNPTDATVGDGAPGHQGVD
VFNMLVSVYGSKELFVKEYRNLLAERLSSSDNKDPEFEKRYLDLLKLRFQYSELQHCEVMLRDVIHSLDIDKKFEDMSER
SAGNYDVPIIPISACIISSHYWPKLETEKTEALMPQPLQAAMDVYQETYLDVKRDRKLEWLRSVGCVEVSINIDGVEVDR
TIPNMYALLLFLFLEKETWTTAEVVEKMGMSVVVTRKRLEWLVKQGFICMNPIISSDTWTLTRNPSGISIVRPGTPDLED
DEDVEPEENSDMVDALEQYWGYTRNFIANHAPNGEVKAERMHRVYRMFGSPTSAGPTLDHVSAFLQRKVALGLLTCINGS
YRIIQEKKDGE
>Cele_K08E7_7
MSIEAVWGTLQDGLNLLYRREHMSKKYYMMLYDAVYNICTTTTLANSNNNSPEFASEFLYKQLENYIRTYVIAIRDRISA

CSGDELLGKCTIEWDNFKFSTRICNCIFQYLNRNFVSKKVEDKNGEIVEIYKLALDIWKAEFFDNFKVKTIDAILELILL
ERCGSTINSTHISSVVECLTELDIYKVSFEPQFLDATKLFYKQEVLNSKETVIEYMITVENRLFQEEYRSRRYLGPSTND
LLIDSCESILISDRLKFLHSEFERLLEARKDEHLTRMYSLCRRVTHGLEDLRVYLEKRILKEGHETLQRLAKDSGLKTTP
KEYITKLLEVHEIYFNLINKAFDRNALFMQSLDKASKDFIEANAVTMLAPEKHRSTRSADYLARYCDQLLKKNSKVQDET
ALDKALTVLKYISEKDVFQLYYQNWFSERIINNSSASDDAEEKFITNLTATEGLEYTRNLVKMVEDAKISKDLTTEFKDI
KTEKSIDFNVILQTTGAWPSLDQIKIILPRELSTILKEFDTFYNASHNGRRLNWAYSQCRGEVNSKAFEKKYVFIVTASQ
LCTLYLFNEQDSFTIEQISKAIEMTAKSTSAIVGSLNPVIDPVLVVDKGNEKDGYPPDAVVSLNTKYANKKVRVDLTTAI
KKATADRETDAVQNTVESDRKYEIKACIVRIMKTRKSLTHTLLINEIISQLKSRFTPNVQMIKICIEILIEQLYIRRSEN
EHNVYEYLA
>Cele_Y108G3AL_1_1
MSGRSGNGGQQKMRIRPFMATIDEQYVTQTWELLKRAIQEIQRKNNSGLSFEELYRNAYTMVLHKHGERLYNGLKDVIQD
HMASVRIRIIESMNSGSFLETVAESWADHTVAMVMIRDILMYMDRIYVAQNNHVLPVYNLGLDAYRTEILRQNGIGDRIR
DALLELIKLDRKSNQINWHGIKNACDMLISLGIDSRTVYEDEFERPLLKETSDYYRDVCKNWLSGDNDACFYLAQVEIAM
HDEASRASRYLDKMTEAKILQVMDDVMVAEHIQTIVYMQNGGVKFMLEHKKIEDLTRIFRIFKRIGDSVTVPGGGLKALL
KAVSEYLNETGSNIVKNEDLLKNPVNFVNELLQLKDYFSSLLTTAFADDRDFKNRFQHDFETFLNSNRQSPEFVALYMDD
MLRSGLKCVSDAEMDNKLDNVMILFRYLQEKDVFEKYFKQYLAKRLLLDKSCSDDVEKALLAKLKTECGCQFTQKLENMF
RDKELWLTLATSFRDWREAQPTKMSIDISLRVLTAGVWPTVQCNPVVLPQELSVAYEMFTQYYTEKHTGRKLTINTLLGN
ADVKATFYPPPKASMSNEENGPGPSSSGESMKERKPEHKILQVNTHQMIILLQFNHHNRISCQQLMDELKIPERELKRNL
QSLALGKASQRILVRKNKGKDAIDMSDEFAVNDNFQSKLTRVKVQMVTGKVESEPEIRETRQKVEDDRKLEVEAAIVRIM
KARKKLNHNNLVAEVTQQLRHRFMPSPIIIKQRIETLIEREYLARDEHDHRAYQYIA
>Cele_ZK520_4a
MTFNIFLTFSTISPKIFSVTSTGNRSEVHRPQAKLASNTQPTSNRTTLGRRKAISDDNFPVVEPILKKIRPPRPQVMYSL
KPKVVEFDKVWVQLRPSIIDIINLRPITNVQWHHKFSDVYDICVSIPTPLSERLYNEVKACIQEHVRQKRQDIVDVDPDL
LLQEYHKMWRVFHEGAIFIHRLFGYLNKQFVKQKRCTDLDNFAQYAAFLQIPDVKEIGCLALEIWKEDLVKTILPQLVKL
LLIAIDNDRKGNFPHIANEVSGVINSFVKMEETDFDVVPAEGARYKARESTTAFYQESFEKPLLTDTEQYYSALAQKMLT
DLSCSEYMEQVIVLLEQEEMRAKKYLHESSVEKVITLCQKVMIKAHKDKLHAVCHDLITNEENKDLRNMYRLLKPIQAGL
SVMVKEFEEYVKKKGLEAVSRLTGENVPQQFVENVLRVYNKFNDMKTAVFMDDGEFSSGLDKALQGVVNSKEPGQSVPKA
SERLARYTDGLLKKSTKGLSETDLEAKLDSAIVIFRYIEDKDIFQKFYSKMLANRLIASTSISMDAEELMINKLKQACGY
EFTSKLSRMFTDIGLSQELSNNFDKHIADIKTVQPDVKFVPTQTMILQAGSWPLNAPQLSTNSNNQTAQDVANFHLPRIL
QPVIQEFEKFYTGKHNGRKLTWLFNMSQGDVRLTYLDKQYVAQMYVYQMAALLCFERRDAILVKDIGEEIGVSGDYLLKT
IRTILDVTLLTCDDQNLTADSLVRLNMSMTSKRMKFRLQAPQVNKAVEKEQEAVANTVSQDRKYYMECAIVRIMKTRKVL
KHNALVTEIMDQTKGRFSPDVPFIKKSIEDLIEKMYIQRTDQNDEYQYLA
>Cele_ZK856_1
MQFDEEWSKADPIVHALLHQKSVTPAAWQDLFYHVYKITSWVDDGPLKIRDILTRCINDYVHEANKRIRSLQTDGSLLIG
YIKEWNRFYQQANILPLPFKKIDESSRRRSVPETPEESIRTVMLEKWNEIIFMNISEQLLVEALRLVKEERDGNIIDAQN
VIGIRESFVALNDRAGEDPLLVYRQSFERQFIEQTTEYYKKICGNLLNELGVLEYMVYADKKLEEEQQRAKRYLEMNSPT
SGKHMEKAVIALVESFEDTILAECSKLIASKDVERLQRLYRLIRRTRSGIDTVLKCIDTHIRTEGLNDMRNNAENLSTDP
ERYVQQLLLMFDKFSSLVREGFCDDARLLTARDKAFRAVVNDSSIFKTEMMNKKGRTLSVESKCAELLANYCDLLLRKTQ
LSKKLTSEEIDEKLNQVLLVLKYVENKDVFMRFHRAHLSRRLILEMSADQEKEEMMVTKLRECGMPSDAVNKLSRMLQDI
ELNKDMNSSFKKALTGTNNNKSIADSINMKVLNGGAWGRGGSERIRFSLPRELEDFVPEMEAFYKKQHNGRKLCWMHHWS
SGTMVFGTANGGRFDLECTTFQMAVLFCFNDRAHDKISLETLRLATELPDAELNRTLLSLVAYPKMRYQILLCDVPSTTV
TARDFTDSTKFLINHDFNVVKNGKSQQRGKVNLIGRLQLSLEANAEKEHESIVALRELRVQEGIVKILKTRKTYTLAQLT
MELVEILKPLFIPNRKIIKEQIDWLIENKYMERRADDINTFVYIS
>Ctel_219953
MLRGDKWPSMRPTVLKLLRQETVSRAEWQDLFWLVHLQTDAVCLWDEKGASKVHQALQEDILDFIKQAQARVLSHEEDSA
LLKAYIAEWGKFFTQCDYLPKPFGQLETTLTGKAHSTMQKKNQGEESIVRKLMLDSWNQSIFNNIKHRLQDSAMKLLHAE
RNGEAFDSQLVIGVRESYVNLSSDTDDKLKIYRENFEKAYLEATESFYKTKAPEYLRLNGVQNYMRYADAKLKEEEQRAM
RYLETRKGCNSVAVLTECCVDVLVTVHKETILAECEQMIKANETDRLKLMFSLMDRVHEGIDPMLADMEKHISAEGLADM
IEAAETITTDSEQYVDKLLELFNSFSLLVKEAFKDDPRFLTSRDKAYKFVVNDTRIFKLELQLKNKNVGIKSQPESKCPE
LLANYCDMLLRKTPLSKRLSPEEVEGKLRNVLLVLKYVTNKDVFMRYHKAHLTRRLILDTSADNEKEENMVEWLREVGMP
ADYVNKLARMFQDIKVSEDLNVQFKEEHRNNNEGLADSINIKILNAGAWARSSDRISVTLPMELEDYIPQVEEFYRHKHS
GRKLQWHHLMSNGIITFNNEIGRFDMEVTTFQMAVLFAWNQRPKDRISYESLKLATELPDTELRRTLWSLVAFPKFKRQC
LLCSPEVKSSQDRAREFSDDTIFWVNQEFAPIKNGKVQKRGKINLIGRLQLSTEKTKEEDNEGIIQLRILRVQEAIVKIM
KMRKRITNAALQTELVEILKNMFLPSKKMIKEQIEWLIEHKYMRRDEENINMFIYMA*
>Ctel_150069
MLVNIYGSKELFVNEYRTLLADRILTHFNFDTEKEIRHLELLKLRFGESQLNLCEVMLKDVADSKRINTRISEEKAEAAT
SADASAAAAEEPLAVNSMVLSAQFWPAFREGKIQLPEQMQGALDNYTRSFETLKGNRTLDWKSHLGLVQVELELKDGKTL
QLSVTPVHAAIIWHFQEKVKWTVDELSSVTQMSTHALRRKIALWQSHGLLREESADTFVLVEEHKGTKDLGSVVMEEEEA
ESAMASVQDQREEELQVVWSYVHGMLTNLESLPLERIHSMLKMFAMQGPGSECTEEELRGFLDRKVKEQKLIYATGVYRL
PKHPA*
>Ctel_175514
MLVTYHKHWQHYSRGSGYLNQLYGYLNSQFIHKQQHSEADINYGGFGIELQEQLLEIGALALDIWKRVMVEPLKENLAQL

LLEEMEKDRRDGNVRQSIIHSVINSLVHVDEYKKKSPLKLYEDVFEVKFLRETGEYYHREALQLLDDCTCSEYMDKVIQR
LDDEDMRSRKFLHPSSYCKVTKECEQKMVADHLAFLHSECRNMVAKEKRHDMSNLYRLLCPISKGLDIFVQDVKEHIKQE
GLTAVASLKGDSVPSQFVESLLEVHAKYSDLITEVFKGDQHFIGALDKAFAAVINHRSNFKSNCRSPELVAKYCDSLLKK
TAKGMSETEIDDKLSQSITIFRYLDDKDIYQRFYARMLAKRLIYTQFHSMDAEESMINRLKQACGYEFTNKLHRMFTDMS
ISSDLNSKFSDFCKEDSTDLGINFSILVLQAGAWPIGQNNLPTFAIPQELEKSVRMFEVFYGRHFNGRKLTWMHSFCNAE
LKLTYLKRPYIVTLGTFHMALLLPFNSSHSVSFRDLVDISRLPEKELLKQVQVLLDAKIIVSNESTATMDGVFSLNLEYT
NKRTKFKIITSVQRETPQEVEQTMSNVDEDRKMYVQAAIVRTMKARKVLKHNALIQEVISQSRARFAPSISMIKKCIETL
IDKQYIERSSTSTDEYSYIA*
>Ctel_227595
MDASRKANFSAVSNPNNGIKTHSPLANIKPGSAKKIVIKNLREKPKLPDNYQQETWGKLKGAVEAIHQSHAIQSSLEELY
QAVQNMCSHQMASELYDELKVVCERYVSSNIQQFLTESIDSEQFLKQMDHCWQSHCRQMIMIRSIFLFLDRTYVLHNSNI
SSLWDMGLELFRLHIISNTVVQGRTVDGILVLIERERNGEAIDKQLLKSLLRMLSDLQIYEEAFEHRFLEATDQLYAGEG
QRLMQASTVPNYLHHIDRRLSEESERLLHYLDQSTRRPLIACVEKQLIEQHLKALLQKGLDLLLDQDRISDITLMHQLFS
RIRDGQKELCLSFASYIKKTGRLFMINHEHDHEKDRDMVQQILDFKERVDNVIEVCFQKNEKFVNAMKESFEHFINQRQN
KPAELIAKYVDSKLRAGNKEATEEELERLLDKVMVLFRFIHGKDVFEAFYKKDLAKRLLVGKSASVDAEKSMLSKLKQEC
GGHFTSKLEGMFKDMELSKDIMLAFKQHMTHVEAPGISELTVNILTMGYWPTYTPMEVNLPEAMVKYQAIFKKFYLGKHS
GRKLQWQPTLGHCVLKAHFAAGKKELQVSLLQTLCLLMFNDGDEFSFEEIKEFTKIGSHSEIVQRNAEIGELRRTLQSLA
CGKARVLLKSPKGKDVDDGDRFRCHDDFKHKLFRIKINQIQMKETQEENTNTTERVFQDRQYQVDAAIVRIMKMRKTLTH
NTLIAELFNQLKFPVKPADLKKRIESLIDRDYMERDKEQANQYHYVA*
>Ctel_217617
MSGLKGKKDTKMRIRAFPMTMDEKYVNNIWTLLKNAIQEIQKKNNSGLSFEELYRNAYTMVLHKHGEKLYTGLREVVIDH
LVNKVQSDVLESLNNNFLQTLNNSWNDHQTSMVMIRDILMYMDRVYVQQNSVDNVYNLGLMIFRDKVVRYPVIRSHLRDT
LLDMVAKERRGEVVDRGAVKNACQMLMILGIDSRTVYEEDFERPFLEQSADFYKMESQRFLAENSASVYIKKVEARIHEE
AERATHYLDKSTEDPIVKVLEDELICKHMKTIVEMEYSGVVHMLKNNKTEDLECMYKLFIRVVEGLKTMCGCISGYLREQ
GKALVTEEEGGKNAISFVQSLLDLKDRFDHFLHQSFSDDRQFKQMISSDFEYFININPKSPEYLSLFIDDKLRKGVKGMT
EQEIEAVLDKSMVLFRFLQEKDVFERYYKQHLAKRLLLNKSVSDDSEKNMISKLKTECGCQFTSKLEGMFKDMTVSNTTM
EEFKSHVQNATINLHGVDLLVRVLTTGFWPFQSASSKCNVPLAPRMAFEAFKKFYLGKHSGRQLSLQPQHGSADLNAIFY
GARKGESGAEGGAASEEGASCSSASSRARKHIIQVSTYQMVILMLFNNRDHWTYEEMKNESDIPERDLMRAVQSLALGKH
TQRVLMKEPKSKEIEGSHVFMVNEQFTSKLHRVKIQTVAAKGESEPERKETRNKVEEDRKHEIEAAIVRIMKARKQMKHN
VLVAEVTEQLKARFLPSPVVIKKRIEGLIERDYLARTPEDRKIYTYVA*
>Ctel_119976
MTITMEDYEQTYWPKLEGAINQLLTMSPGDYIPISYEQMYSCVYKCVCKQFSQRLYSDLLRQITAHLERLSLELQSQDSC
LYIERFYFALNQYTQALGGIVPIFNYMNRFYVESKLKSDLGVELRQLFVQYVADKHVNAIIPLMVDASSKPFSVSPNVMA
NLIKNIHALKPEYAKLRPQLFAKYLPGILPPCEERELNCYIEEAKQMQRDLSNHPEFIK*
>Ctel_180635
MATMRSNPHGLKQIGLDQIWDDLRYGIEHMYRRQSMPKTRYMELYTHVYNYCTSVHQPGQSGQSSRGQGNRKRNQPTGGA
QFVGWELYKKLRDFLENYLVDVLRDGQELMGESVLEYYTRRWEDYQFSSKVLNGVCAYLNRHWVRRECEEGRKGIYEIYS
LALLTWRDHLFRALHNQVTNAVLKLIERERNGETINTRLVSGVINCYVELGLNEEDPIAKGPTLNVYKEHFENSFLDDTE
SFYNRESSEFLRQNPVTEYMKKAESRLMEETRRVQVYLHESTHDHVAHVCEKVLIEKHLESFHSEFQNLLNDDKNDDLGR
MYQLVSRIKDGLGELKTLLETHICSQGLTAVERCGDSAVNEPKVYVQTVLNVHKKYNALVMTAFNNDAGFVAALDKACGK
FINNNAVTRMAAASSKSPELLARYCDLLLKKSSKNPEEAELEDTLNQVMIVFKYIEDKDVFQKFYSKMLAKRLVQHMSAS
DDAEASMISKLKQACGFEYTSKLQRMFQDIGVSKDLNEQFKRHLQQTTDSNDIDFSIQVLSSGSWPFQKSCSFTLPTELE
RSFQRFTSFYSGQHSGRKLNWLYHMSKGELVTNCFKNKYTLQASTFQMAVLLQYNSADDFSVQQLQESTQIKMDILLQVL
QILLKSKLLVTEDDEADLQPTAVLALYHQYKYKKLRVNINVPMKTDMRQEQEATHKHIEEDRKWLIQAAIVRIMKMRKVL
KHQQLLGEVLNQLSSRFKPKVPIIKKCIDILIEKEYLERVDGQKDTYSYLA*
>Ctel_195383
MVNKQRYNLLFIRQRQLVFEQIRSANLCGLVRKDVFEAFYKKDLAKRLLVGKSASVDAEKSMLSKLKQECGGHFTSKLEG
MFKDMELSKDIMLAFKQHMTHVEAPGISELTVNILTMGYWPTYTPMEVNLPEA
>Lgig_109593
MQFVTCYVFYSFIFCYFFTGEEKSPLCEGCTIDIDDCTCQDIIDTFHSVNRQLDEMGILERLSGAAVTSIVHNQIQRYIN
TTCKGNFETSYIDHLEKWLDTKVLGWLNSIYAGEKNQCSISAFRGRLLHFLFETYAHARIEQLFAIVIDYPESEPAILDL
KTCLEKTNLRALLMTSLRKELEDRLLHPGVNTTDILLAYISAIRSLRVLDPAGVILEIVSEPVRKYLRSREDTVRCIVLC
LTEEGSNELLDELIKGQPLILEDDTCSDQEEGDWETWMPDPVDADPSSTSKSRRGSDIISMLVNIYGSKELFVNEYRTLL
TDRILSQFNCDMERELRYLEMLKLRFGESQLHYCEVMLKDIADSKRINSRIVQEMDNAGQKMDIDINALILSAQFWPSFR
EEKITLPTEMQKSLEEYTKKFEALKGNRTLNWKPHLGMMKIEVEHKGTMLNFSVSPVHAAIIMKFQTQSRWDVEELSGVL
QMSVSALRRKITYWQSQGLLKEEVPDTFVLVDEVKGQGHEVVMAEDESESAMTSAYQQREEELQVFWTYIVAMLTNLESL
PLDRIHTMLRMFAVQGPSSSGEVSQQELKGFLDRQIKDQKLVYVSGVYRLPK*
>Lgig_114418
MLGQQGQCKFEDKWPCMRPTVLKLLRQEPVSRDEWHYLFEAVHSVCMWDEKEGPPKIYKALEEDILDFIRNAQTRVLQHK
EDSALLIRAYIAEWGKFFTQCNYLPMPFNQLEANLSGKLHNSTQKKDQESLVRKLMLDSWNQSIFCNIKKRLQDSAMKLV
HSERNGEAFDSQLVIGVRESYVNLSSDTEDKLKIYRENFEKAYLEATESFFKMKAPRYLEENGVQNYMSFADSKLKEEEL
RARRYLETGYGCNSVAALTECCVRVLVTDFKETILQECADMIKNNETEKLRLMFSLMDRVPDGITPMLSDLEKHIVEQGL

ADMMSAAQAITSDSEKYVEQLLDLFNRFSKLVKDAFLDDPRFLTSRDKAYRTVVNDTSVFRLEIPVKNKSIAGIKTQPES
KCPELLANYCDMLLRKTPLSKKLASEEVEAKLHDVLLVLKYVQNKDVFMRYHKAHLTRRLILDTSADNEKEENMVEWLRE
VGMPADYVNKLARMFQDIKVSEDLNVEFKDIHRNNNEGIADAINIKILNAGAWARSSDRVSVTLPRELEDYIPQVEEFYK
LKHNGRKLQWHHLMSNGIITFCNDSGGKFDLEVTTFQMGVLFAWNQRPYEKVSYESLRLATELPDAELRKTLWSLVAFPK
LKQQILLCSSDIKSPKDFVDVTVFWVNQKFTMIKNGKVQKRGKMNLVGRLQLSTEKSKEEDNEGIMQLRVLRVQEAVVKI
MKMRKRITNAALQTELVEILKNMFLPSKKLIKEQIEWLIEHKYMKRDGDNINMFIYMA*
>Lgig_136878
MMSLTVADYENHYWPKLQDAIDQLLTIKPGCYIPISYEQMYSCVYKCVCKQFSERLYKDLLRHMEQHVTHLTEELNNSVN
KREFVERFAFTMNQYLQALAGIVPIFNYMNRFYVENKLKTDLNEELRKIFRIQVVDNQISHILNLVDETSSQPFVISPPV
MARLIQNLYSLNPEYVNLKPNLFSRYIPNVLPSTSANELERYQVEVKDMQKDIYGTPGFERY*
>Lgig_187016
MPDNFQQETWRKLCEAVEAIHKSCSIRYSLEELYQAVENMCSYKMSANLYDQLKVVCESHVQSCLEQFKAYPFLTDTDNS
DYEQFLKKLDYCWQAHCRQMIMIRSIFLFLDRTFVLQNSTILSIWDMGLDLFRANVISDKVVEGKAVDGLLKLIERERNG
EAVNRQLLKSLLRMLSDLQIYKEAFEKKFLEATDLLYAAEGQQLMQERDVPEYLHHVDKRLNEETERLLHYLDQSTKKPL
ISCVEKQLLEQHLLQILQKGLDNLLQENRIEDLLLLYQLFSRVKNGQKELCTMFAEYIKVSGRCIVLNNENDADKDKEMV
QKLLDFKDKIDNIIDVCFGKSEKFVNVMKESFENFINQRQNKPAELIAKYVDSKLKAGNKEATEEELEQLLDKIMVIFRF
IHGKDVFEAFYKKDLAKRLLMSKSASVDAEKSMLSKLKQECGAAFTSKLEGMFKDMELSKDLSLALKPYMQNLDVAGGIE
LTVNILTMGYWPTYQPMEVHLPQEMIRYQEIFKKFYLGKHSGRKLQWQPSLGHCVLKVDFPSAKKELQVSLFQSLCLLLF
NDSTALSYEEIKAATAIEESILKRTLQSLACGKPTLRVLSKKPKGRDIEDGDKFYFNEEFKHPLFRIRINQVQMKETPEE
NTSTTEKVFHDRQYQVDAAIVRIMKTRKTLSHNLLISELYNQLKFPVKPTDLKKRIESLIDRDYMERDKDNPNSYHYVA*
>Lgig_207667
MSLKPRRVDFEETWNVLLGTIKGVITCGTVPRHVWNDRFSDVYKLCVACPEPLGDKLYQETKLFLENHVSELNQSVVGNG
DESLLSIYHKNWEEYSRGSSYLNQLYGYLNTTFIKKQKHSEADLNYGGFTVDPVDQMLEIGELALDIWKRVMIEPIKSQL
ISLILQEVKRDRKGVSVNQTVIHGVINSFVNVEEYKRKNPMQYYETAFEAPFLQETGEYYKQEAQQLKDECSCSEYMEKI
IQKLDTEDFRSRKFLHPSSYTKVTQECQQRAVADHLQFLHGECREMVQNEKRKDLTNMYMLLRPIQSGLGVLVEQVEEFI
KQTGLDAVLNVKGDNIPSQFVESMLEVHNRYKDMIKNVFNGDQQFVGALDKACAHVINYRLNPKSVCKSPELLSKYCDSL
LRKSSKGISETELDDKLASSITIFKYLDDKDIFQRFYSRMLAKRLIYGLSTSMDAEESLINRLKQACGYEFTNKLHRMFT
DMSISMELSSQFHDVVKRDNLHTGVNLNILILQAGAWPLGQNILTFHFPSELEKSIKRFEGFYNSKFSGRKLSWMHNFCT
GDLKFNFTKKTYFVTMGTAHMAILLIYNNRNVLSFKEMQLMTNIPEKELTKFLQSLIDIKILTTQGSIEDESTFRLNPDY
SNKRTKFKISVATQKDSPYEVEMTHQTVDEDRKMYLQAAIVRVMKSRKVLKHTLLINEVISQSNSRFTPNISMIKKCIET
LIDKQYLERTANSTDEYSYVA*
>Lgig_215748
MAGPSSRGQNPHGVRQIELDQIWDDLRSGIEHVYQRQYMNKPRYIALYTHVYNYCTSVHGQGQGQGSQVQGRGTGGRKPK
PPAGGAQFVGHELYKRLKEYLKTYLVKLLKDGQDLLDEQVLQFYTRQWEDYQFSSKVLNGICAYLNRHWVRRECDEGTKG
IHEVYSLALITWRDNLFRPLNKQVTNAVLKLIERERHGETVNTRLISGVINCYVELGLNEDDSSAKGPTLSVYKEHFETS
FLEDTDQFYIKESTEFLQQNPVTEYMKKGETRLKEEFKRVQLYLHESTQDVLAKVCEKVLIEKHLEIFHYEFQNLLDADK
NDDLGRMYQLVSRIQDGLGELKSLLETHIFNQGQTAIDRCGEAALNDPKIYVQTVLDVHKKYHALVMTAFNNDSGFVAAL
DKACGRFINNNSVTKLAQASSKSPELLARYCDLLLKKSSKNPEEAELEDTLNQVMIMFKYIEDKDVFQKFYSKMLGKRLV
QHMSASDDAEASMISKLKQACGFEYTSKLQRMFQDIGVSKDLNEQFKKHLQGDSLDIDFSMQVLSSGSWPFQQSCTFNLP
VELERSFQRFTTFYSAQHSGRKLNWLYHMCKGELVTNCFKNRYTLQSSTFQMAVLLQYNLMDNFTIQQLFENTQIKMELL
TQVLQILLKSKLLVTKEAETELTPQTVVSLFTMYKNKKLRVNINVPLKTEVKAEQEITHKHIEEDRKLLIQAAIVRIMKM
RKQLKHQNLLAEVLNQLSARFKPRVPVIKKCIDILIEKEYLERVEGQKDTYSYLA*
>Lgig_231265
MSSGLGRSSQNRKDTKMRIRAFPMTMDEKYVNNIWALLKNAIQEIQKKNNSGLSFEELYRNAYTMVLHKHGEKLYTGLRV
VVTDHLINKVRDDVLAALNNNFLQTLNSAWNDHQTSMVMIRDILMYMDRVYVQQNNVDNVYNLGLIIFRDQVVRNPIIRD
HLQTTLLEMVAKERRGEVVDRGAVKNACQMLMVLGIDSRCVYEEDFERPFLEQSAEFFRMESQKFLAENSASVYIKKVEA
RINEEAERASHYLDKSTEEPIVKVLEDELIGKHMKTIVEMENSGVVHMLENNKTDDLACMYKLCVRVANGLKTMCNCISK
YLREQGKALVVEEGEEAKNAITYIQSLLDLKDRFGHFLHESFSDDKLFKQMISGDFEYFINLNAKSPEYLSLFIDDKLKK
GVKGMTEQEIENVLDKSMVLFRYLQEKDVFERYYKHHLARRLLLNKSVSDDSEKNMISKLKTECGCQFTSKLEGMFKDMT
VSNTINEEFKLFVQQAQVNLSGVDLTVRVLTTGFWPTQSATPRSSIPSAAQTAFEAFRRFYLAKHSGRQLTLQPQLGSAD
LHATFYGPKKDEGESSNGASAKNPRKHILQVSTYQMCILMLFNNNREKWTFEEIKNHTDIPERELTRALQSLAVGKLAQR
ILHKDPKTKEIEAGHIFTVNDQFTSKLFRVRIQMVATNKGEAEPERKETRNKVDEDRKHEIEAALVRIMKARKKLQHNVL
IAECTEMLKPRFLPSPVVIKKRIEGLIEREYLARSADDRKVYTYVA*
>Nvec_XP_001641154
MSLKPGVVNFDETWAKILKTLKAVVTLEKIERAVWNDRFSDVYALCVAYPEPLGEKLYSEIKLFLEEHVKSLYQAVSSPD
IDILREYHSHWAKYSQGSSYMDLLFRYLNSHLKKQKQDYVEISPYTVTPICPDAIDIGPLALSIWKSIMIEPLKETLVQH
LLAEIRKDRNGEGTQQNVVHDTIKSFVGVQEYQSKGALSLYESEFEKPLLQETGEYYRREAADLLAENPCSVYIQKVDVR
IQDEDLRARKFLHPVSYSRVIRECEARMVEEHIPYLQAECRQMVRGEKCADLSRMYKLLKHIPRGLHVMVTELEQHVEET
GQNHDTQKFICFQGPFQYVDAMLDVHSKFTKLIDETFHADQAFHASLDKACTTIVNYRHDARKPSKSPELLAKYCDLILK
KSNKNLSDSELDEKLGEVIIVFKYIDDKDIFQKFYSKMLAKRLIHNLSISMDAEEAMISRLKHACGYEYTNRLHWMFTDM
SISSDLNSSFSDFLATAQVNMGINFSLLVLQSGAWPLGQTSVSPFSIPQELIRPVQMFEQFYNGKFNGRKLAWLQHLSNG
EVKLNYCKRTYFLTVSTFQMAVMLLFNDKLQFTFSELSTLTQLLNKELTRIIQSLVDVKLLNKTEDDDSKEATYSLNMNF

SSKRTKLKITSAVQRDSPQVVILLSTRGTADSDNWHLHLAEKPMVRSYHLQQTNDTLFSFCLQVISQSRARFIPSVPMIK
KCIEALIDKQYLDRQEGSKDEYSYVA
>Nvec_XP_001630824
MSLLLNSNKPGVQFEDKWHELKLTVNKLLKQEPVSTHEWQDLFWDVHKVCNWDEKGPHKVFHALKDEVSLFIHQVQERVL
QHQDDHSLLKAYISQWAKFFTQCSYLPKPFATLESNLQGKAGGGASKRPLAESITVQKLMLDTWNEGIFSSIKNRLQSSA
MKLVHAERNGEAFDSQLVIGVRESYVNLSSDASDKLMIYRENFEKAYIDATEEFYKAQASSYLQENGVQNYMRYAEMKLK
EEEQRALRYLETRKGCNSVAVLTECCVNVLVGSFKETILAEGPGMIAANETKKLLLMFQLMDRVKGGVDPMIQSLESYIV
TTGLNDMLAAAETITTDSEKYVEQLLALFNRFSAMVKEAFNDDPRFLTARDKAPQAFKTVVNDTKIFKLELPSKGKGSGA
LSVKPQPESRCPELLANYCDMLLRKTTYSKKLTSDEIESKLKDVLLVLKYVVNKDVFMRYHKAHLTRRLILDTSADSEME
ENMVEWLRDVGMPADYINKLARMFQDIKVSEDLNQEFKRTCRDKGDIADSINIKILNAGAWSRASERIPVSLPTELEDCI
PEVEEFYRKNHSGRKLQWHHLMSNGVITFSNKTGKFDLEVTTFQMSVLFAWNERPLDHISYENLRLATELPDSELRKTLW
SMVSIPKLKRQILTCEPAVRSPKDFTDSSMFWVNQDFALIKNGKVQKRGKVNLIGRLQLSTDKAKQEENEGIVALRILRI
QEAIVKIMKMRKRITNAALQTELVEILKNMFIPQKKMIKEQTEWLIEHKYLKRDEEDINTFIYLV
>Nvec_XP_001629633
MATRGANPHGLRQINLDEIWDDLKEGIQHVYNQQSMSKQRYMELYTHVYNYCTSVHQQSQSRVPKQKKAPNQGGAQFVGH
ELYKRLKEFLKSYLLNMQKDGADLMDESVLRFYSSRWEDYRFSSKVLNGVCAYLNRHWVRRECDEGRKGIYEIYQLALVT
WREHLFRPLNKQVTNAVLRLIERERNGETINTRLVSGVIQCYVELGLNEEEQSSKGPALTVYKQYFESVFLEDTERFYTA
ESVEFLRENPVTEYMKKAEARLLEEQRRVNVYLHESTQDELARKCEQVLIEKHLDIFYAEFQNLLNDDKNEDLGRMYSLG
SRIPDGLVQLRTLLENHICYQGLNALEKCGEQAYNVSTPASYVIIIIFVSKMCNYYFIMSFKNDAGFVAALDKAFGKFIN
SNAVTKQAQASSKSPELLARYCDSLLKKSSKNPEEAELEDILNSVMVVFKYIEDKDVFQKFYANMLAKRLVQHNSASDDA
EASMISKLKQACGFEYTSKLQRMFQDIGVSKDLNDKFKAHLANTGSLDLDFTIQVLSSGSWPFQQSWTFSLPAEMEKSHQ
RFTTFYSSQHSGRKLHWLYHKSKGELVTNCFKNRYTLQASTYQMGVLLMFNTADSYTVEQIQEQTQLKMELLIQVLGILL
KTKLLLCDTCEDVSGLEPSSVLKLFFGYKNKKLRVNINVPMKTEQRNEQEQTHKYIEEDRKLLIQAAIVRIMKMRKMLKH
QPLLAEVLSQLSSRFKPRVPTIKKCIDILIEKEYLERVEGEKDTYAYLA
>Nvec_XP_001627760
MVNPIVQRRTVDGLLQMIEKERHGEAVDRSLLKSLLRMLADIQMYEDAFESKFLEATDVLYSQEGNRYMQETDVPKYLAH
VDKRLKEEMDRLIHYLDQSTRKPLILCVEKQLLGQHLTSILQKGFDNLMLSNRIADLALMYQLFGRVRKGMEELCAAFSG
FIKKQGISIVLNPEKDKTMVQELLDFKEQLDTMIAEAFMKSEKFVNAMKESFESFINKRPNKPAELIAKFVDSKLRAGNK
EATEEELERLLDRIMVIFRFIHGKDVYEAFYKKDLAKRLLVGKSASVDAEKSMLSKLKQECGAAFTSKLEGMFKDMELSK
DVMVQFRQYLQHQSLPWNMDMVVSILTMGYWPTYLPMDVHLPTEMVHYQETFKKFYLAKHSGRKLQWQNTLGHCVVKADF
SEVKKELQVSLFQTLVLLMFNEGNEYSLEDIKQATGVEDGELRRTLQSLACGKARVIKKRPQSKDIEDGDIFTFNKEFKH
KLIRIKINQVQMKETPEENVNTTERVFQDRQYQIDAAIVRIMKTRKTLSHTLLVSELYTQLKFPVKPTDLKKRIESLIER
DYMERDKEIANQYHYVA
>Nvec_XP_001627330
MVLHKHGERLYNGLKQVVTEHLEDKIRKDVVASLNNNFLDTLNAAWNDHQTSMVMIRDILMYMDRVYVQQNGVDNVYNLG
LILFRDKVVRYGNIRDHLCQTLLSLVRKERRGEVVDRMAIRNACQMLVILGIDSRHVYEEDFERPFLEESAEFYKMEGQK
FLAENSASIYIQKVETRINEESERAKHYLDPSTEESVVKVVEEELIRKHMKTIVDMENSGVIHMLKHDKIEDLARMYRLF
YRVKEGLKTVCDCMRGYLREQGKAVVVDEESETARNPISCIQNLLELKDRFDHFLHNGFSSDRLFKQAIGSEFEYFLNLN
GKSPEFLSLFIDDKLKKGVKGYSEQEVEVVLDKCMVLFRFLQEKDVFERYYKQHLAKRLLLQKSVSDDSEKNMISKLKTE
CGCQFTSKLEGMFKDMTVSHTTNEEFRQHLSNSQINLLGVDLIVRVLTTGFWPTQSGNHKCNVPPQAQHAFDCFKRFYLG
NHSGRQLTLQPQLGTAELNATFPPVKKEGASLGQRKHIFQMSSYQMCILMLFNSQERWTYEDILQQTLIPERDLNRALQS
LACGKATQRVLSKEPKGKDITATDVFSVNDLFTSKLHRVKIQTVLAKGESEPERKETRTKVDEDRKHEIEAAIVRIMKAR
KKRPHNLLVAEVTEQLKARFLPSPQVIKKRIEGLIEREYLARTPEDRKVYLYVA
>Nvec_XP_001640749
DKEYSTVFWPKLEGAILLILKQNPGEFIPISYEEMYSTVYKCVCWQYSERMHSSLIELVSSYLRQITHELQNSPRQDYLE
KFHFAMAQYFQAVAGIVAIFNYMNRFYVVPKLHSDLSVQLKTLFMEYVADNPLLFSKYLIAELSVQPFAVNPQTMMSIIK
GLYSLNPGFAERNPALFAKYIPNLLPPSTPQDLPSLIEQTQQMQKDLVDNPDFTRYTITITQ
>Nvec_XP_001638137
MLIICDFLLAKPDLPDNYKEATWLKLKEAVCAIHHKTSIQYSLEELYKAVENMCSHKMAATLYSQLKAECEQHVKSNLVQ
FTGYPFQFLGHHSMDSVLYLGKLKQCWEGHCRQMIMIRSIFLYLDRTYVLQNTSILSLWDMGLHLFRSHIMVNPIVQRRT
VDGLLQMIEKERHGEAVDRSLLKSLLRMLADIQMYEDAFESKFLEATDVLYSQEGNRYMQETDVPKYLAHVDKRLKEEMD
RLIHYLDQSTRYKSGA
>Nvec_XP_001628530
QDRVYVQQNGVDNVYNLGLILFRDKVVRYGNIRDHLCQTLLSLVRKERRGKVVDRMAIRNACQMLMILGIDSRHVYEEDF
ERPFLEESAEFYKVSMALWMGQIFHMVQYILGRCIENEEYNV
>Nvec_XP_001625156
LVQFTQLNSWLNSIGLLQKVAGAAHVAVIHKMIKERIEQKCKGEFESSFLQPLENWMDTELYHWLFAILGDGCAGSQHVE
EWKPRLKYFMYKTYADLRIGELFSIIVEFPDSTPAIQDLKECLEMTNQRGQLVNSLREAFETRLLHPGANTSLILSQYVS
AIRSLHVLDPTGVILENVCEPVRQYLRTREDTVRCIVSSLTDENTTELAEELMHGEPHPIDGTGTSDSEDDEEDWVPDPV
DAPPARSSRSGKAPDIISMLVNIYGSKDLFVSEYRTLLADRILSTFNYDTDRELRYLELLKLRFGESHLHFCEIMLKDVA
DSRRINSNIHSCRGRAGKKPLPVDINAMILSAVFWPLFREEKLKVPDAVETSMAEYRGGFEALKAMRTLEWKTHLGLVEV
ELELEDRTLSLSVSPVHATAIWHFQEKERWQLDELSSVMHVTPGTLRRRLAFWVSQGVLREESPDTYIVMERQKGIHKAH

SAEVMVEAEPESATASVQQKREEELQVFWSYIVGMLTNLESLPLERIHSMLRIFAMQGPESAQCSVGDLKRFLDCKVKEQ
ELQFANGFYRLPKTGR
>Nvec_XP_001619889
QDRVYVQQNGVDNVYNLGLILFRDKVVRYGNIRDHLCQTLLSLVRKERRGEVVDRMAIRNACQMLVILGIDSRHVYEEDF
ERPFLEESAEFYKVSMALWMGQIFHMVQYIPGRCIENEEYNV
>Nvec_XP_001619887
FRNAYTMVLHKHGERLYNGLKQVVTEHLEDKIRKDVVASLNNNFLDTLNAAWNDHQTSMVMIRDILMYMV
>Adig_1446v119503
AVSGPDVEILRLYHNHWLQFSKGSSYMNQLFRYLNTHLKKHKQDYGEISPYSTSFCPDSGEEVMDLYEEVLEKPLLKETK
SYYRREAAQLHADNTSHAFIAKIDVRIQDEDLRARKFFHHVSFSRVLRECEARLVEDYIPYLQGECRQMIKDENRT
>Adig_6047v123139
LRYLELLKLRFGESHLHFCEIMLKDVADSRRINSHIQVLGCSDNANVSGTIPLTAMVLSAVFWPAFRDEKLKVPNEVQKA
MDEYTKGFEALKERWTLEELSGVMQAPAGLLRRRLGFWISQGVLKEESTDTFVVVEKQKGQQRISEVVMDAEVESVMASS
QDQREEELQVFWSYIVARMGSELIAHEAKSLIGY*
>Adig_9341v106238
MLPITKDADLSRGSNLHETESNVAENGGARKKRRLSNNVKNGTCIAASDPLQSELNFHPHKKQKTLISVEGAENKDFSMA
EPQRKANFSAYGNHFHERSPLANNSAKPGQAKKLVIKNFKAKPHLPENFKEATWQKLREAVQAIHNKKFVIYSSEELYKA
VENMCSHKMAATLYDLLKTECESHVKSNLQQFVCDSMGSELYLAKLNHCWEDHCRDMIMIRSIFLYLDRTYVLQNSSVLS
LWDMSLNLFRRHIMSNQTVQSRTVEGLLQLIENERHGEAVDRTLLKSLLRMLADLQMYEDAFEIKFLKATEALYHEEGNR
YMQETDVPKYLAHVDRRLREENDKLLHYLDQSTRKPLIPCVEKQLLGQHLGSILQKGFDSLMLSYRLSDLALLYQLFSRV
KKGQDELCLAFNEYIKRQGASIVLDPEKDKSMVQDLLDFKEQLDCIIDDAFMKSEKFVNSMRDAFEAFINRRPNKPAELI
AKFVDSKLRAGNKEATEEELEKLLDRIMVLFRFIHGKDVFEAFYKKDLAKRLLVGKSASVDAEKSMLSKLKQECGAAFTS
KLEGMFKDMELSKDILVHFKQFVQHQDLPGSIDMVVSILTMGYWPTYTPVEVHLPNEMVQYQESFKRFYIGKHGGRKLQW
QNTLGHCVVKADFGPSAERMGNCEEPFSLLPVVKHEYLPRNLRAEIELYNQLKFPVKPTDLKKRIESLIERDYMERDKEI
PNQYHYVA*
>Adig_15059v111538
MTMDDKYVEDIWNLLKNAIQEIQKKNNSGLSFEELYRNAYTMVLHKHGERLYSGLKQVVQEHLIEKIRKDVVNSLNNNFL
DTLNAAWNDHQTSMVMIRDILMYMDRVYVVQQNGVENVYNLGLTLFRDLVVRHGNIRDHLCQTLLTLVRRERKGEVVDRMA
IRNACQMLMILGIDKRVVYEEDFEKHFLEESAEFYRLEGQKFLVENSASVYIQKVEARINEESERAKHYLDPTTEEPIVK
VVEEELIHKHMKTVVEMENSGVIHMLKHNKIEDLARMHRLFIRVKDGLKTVCDCMRGYLREQGKALVVEEEGESSKNPIS
YIQSLLDLKDRFDHFLNNSFNNDRLFKQAISLEFEHFLNLNGKSPEFLSLFVDDKLKKGVKGLSEQEVETVLDKCMVLFR
FLQEKDVFERYYKQHLAKRLLLGKSISDDSEKNMISKLKTECGCQFTSKLEGMFKDMSLSQSTNDEFKQHISNNQINLMG
VDLVVRVLTTGFWPTQASSPKCTIPPQAQHAFDCFKRFYLGCHSGRQLTLQPQLGTAELNATFPPSKKEGEMDL*
>Adig_17956v114080
MSTESVEENSVLRQPRVTFVNDETFSAFYWPKLESAIVVILQQNPGQFIPISYEEMYSTVYKCVCQQYAEKMYNNLIEIV
SDYLKRVSEELKVTPRDAYLEKFNFLLSQFFQAVNGIVAIFSYMNRFYVKTKLQSDLNKELFRIFTNIVTDNVTLFDFVY
WNHLCNIIDLTQGNSQVKMFPHDLTISKY*
>Adig_18069v114175
MVLEEIHEFQVGNKTFKTLTSAVNELHIHLSSYRPFVELLDSLTASLLLGNLKAQFEVLVRAVIFCDTPEEFKSIVYAFY
SHTFQAYQYGYKTDTEKIDSSDDNATDQTTTEVSLDEWKPKLHYFMYKSFADLRIDQLFDIIVEYPDSLPAIQDLKECLE
VTDQRKELIVSLRTALCFVLLLDKMSSSRKAPDIISMLVNVYGSTDLFVSEYRTLLADRLLASFNYDTEKELRYLELLKL
RFGESHLHFCEIMLKDVADSRRINSHIQVLGCSDNANVSDTIPLTAMVLSAVFWPAFRDEKLKVPNEVQKAMDEYTKGFE
ALKVEIKDSEVGVRSELKIHCVQMK*
>Adig_20950v116470
MFLFLVQLEDYIPGVEEFYRKNHSGRKLQWHHLMSNGVITFANKSGRFDLEVTTFQMAVLFSWNERPHERISFENLRLAS
ELPDSELRRTLWVCRYLRVLTILVRTGRPTGLIKQSGTVTCQFCRTEKAAHDQTVDR*
>Adig_21006v116522
MNTMASRTQNPHGLRQINLDEIWDDLREGIQRVYKQQSMSRPRHMELYTHVYNYCTSVHQQSQSRIPKQKKAPNQGGAQF
VGHELYKRLKEFLRSYLTGMQKDGSDLMDESVLKFYTQHWEDYRFSSKVLNGVCAYLNRHWVRRECDEGRKGIYEIYSLA
LVTWRENLFRPLNRQVTNAVLRLIERERNGETINTRLVSGVIQCYVELGLNEEEPSSKGPTLNVYKSSFESVFLDDTERF
YTTESTEFLRENPVTEYMKKAEARLLEEQRRVRVYLHESTQDELARKCEQVLIEKHLDIFYAEFQSLLDDDKNEDLGRMY
NLVARIPDGLGQLRTLLENHITNQGLNALEKCGDQAFNDPKVYVETILEVHQKYNALVLTAFNNDAGFVAALDKACGKFI
NNNFVTKQAQSSSKSPELLARYCDSLLKKSSKNPEEAELEDILNSVMVVFKYIEDKDVFQKFYSKMLAKRLVQHNSASDD
AEASMISKLKQACGFEYTSKLQRMFQDIGVSKDLNDKFKTHLANTEALDVDFSIQVLSSGSWPFQQSAPFSLPAEMERSH
QRFTTFYSSQHSGRKLHWLYHMCKGELVTNCFKHRYTLQASTYQMAVLLMFNNSDSLTVEQIQEHTQLKTDILSQVLGIL
LKSKLLVSETTEDGSNLDRSSSVSLFLGYKNKKLRVNINVPMKTEQRQEQEQTHKYIEEDRKLLIQAAIVRIMKMRKMLK
HQPLLAEVLSQLSSRFKPRVPVIKKCIDILIEKEYLERVEGEKDTYAYLA*
>Adig_21376v102475
MNDRVFKHEDNSTLLRSYIREWSKFFTQCSYLPQPFNTLESNLQGKAGGGSSKRPQNEQSTVQKLMLESWNDGIFNTIKS
KLQSSAMRLVHAERNGEAFDSQLVIGVRESYVNLSSENLDKLKIYRENFEKAYLETTEEFYKTQAASYLQENGVQNYMQY
AEMKLKEEEQRAVRYLETRKNCDSVLVDLLISKKQQKAGASSKGL*
>Hmag_XP_002154151

MVMLLPRFLGNNIIMLLPRLLGNNLLAFEMWKSEIIKPLQDSLISALLQEITKDRMGERPSVAVVKGVIHSFTFEQPVLL
ETSNFYKNESNRILEFCNCSQYMEKVDSRLAEENLRVQRYLHPSTFEKVMNVAQSRLVEDHLELLTGECRRMVKEQKFAD
LKRMYKLLRPLPKGFKEMLTEFEIHITETGLDRVKSLHTESDTGQFVNVLLDLHVEYTQIIHTTFKKDQSFFGARDKACT
KVVNHKLDAKKPCKSPELLAKYCDSLLKKNTKNFPETEIDEKLNNVITIFKYLDEKDIFQKFYSILLAKRLIHNLSVSMD
AEEGMITKLKLACGYEYTNKLHRMFTDMAISKELESKFSDFLRGSNTELGINFSVLILQSGAWPLGQSVSPSVMLPSEFS
RSAQMFKTFYNSKFNGRKLTWLQNLSNGEVKLTYLKKPYLVTCTTYQMAVLLLYNDSDSLSYNDIKLSCELEEKELKRTL
QSIVDVKIFFKSPEDVEDLCLCSFSLNLRFANKRTKFKITAALQKETPQIIEQTHVAVDEDRKMYTQAAIVRIMKSRQIL
RHNILIQEVIDQSRAKFSPSTQMIKKSIEALIEKNYIERVSGSRDEYSYVA
>Hmag_XP_002160900
MSLSGSSRPKATNLEQIWDDLKEGIQQIYLKQNMPKPRYMQLYTHVYNYCTNVQNPSSNNKIPKKKQPNSGGAQLVGSEL
YKRLKDFLNNYLNNLLRSGADFMDESVLTFYTQQWEGYKFSSKVLHGICAYLNRHWIRRECGEGRKDVYEIYNLALVTWR
DVLFKGLDTRVTYAVLELIRRERNGDTINTSLISGVIDSYVHLGINEEDTRTTGPNLSVYRKQFESIFLQDTEQYYTAES
EAFLAHNPVTEYMKKAEIRLNEERRRVFVYLHESTQIELARKCEQVLIKKHLETLYGEFRHLLGDDKDEDLGRMYNLVSR
VPDGLVTLKQLLEQHIHTQGLNVIEKCGEAAINDPKMYVTTMLGVHRKYYALVVSAFSNDNGFVAALDKACGRFVNANSV
TKAAGNSSKSPELLARYCDSLLKKSAKNPEEAELEDILNSIMIIFKYVEDKDVFQKFYSKMLAKRLVQQNSASDDAEATI
ISKLREMCGYEYTNKLQRMFQDMNVSKDLNDKFKKHVSSQENGEVDFSIQVLSSGAWPFQQSPIFTLPPELERCLQRFTT
FYNAQHSGRKLHWLYQLSKGELVTNCFKNRYTLQASTHQMAVLLMYNSEDSYTVEQIQEHTQINMDILQQVLSILIKIRL
LISNPTIVENFPLTSVIHLYKEYKNKKLRVNINVPMKTEQKLEQQQTHKYIEEDRKLLVQACIVRIMKMRKVMKHQNLLT
EVLTQLSARFKPGIPVIKKCIDILIEKEYLERVEGEKDTYSYLA
>Hmag_XP_002157816
MNMKKRESNSKMRIRAFPMTMDEKYVADIWNLLENAIQEIQRKNNSGLSFEELYRNAYTMVLHKHGERLYNGVRKVVTDH
LVGKVRKDVITSMTNNFLETLNIAWNDHQIAMVMIRDILMYMDRAYVEQSKVVTVYDLGLILFKEQVVCHPPIQENLRET
LLSLIERERKGEVVNRLAIKNACQMLMTLGINGRSFYEDEFEKHFLQVSAEFYKLESERFLAENSASVYIWKVEARIAEE
RERARHCLDSSSEPAIVKVVEEELISKHMKTIVEMENSGVVHMLKNSKYEDLGCMCRLFQRVNYGLKAMCEAMSIYLREE
GRAIVSEEKEAEGKNAIIFIQLQKAYGGKSYSSILFIFD
>Hmag_XP_002165647
KSICIVQEAAAETWLSNIHKEYSAGPYKYKFKVKTTFPQHMFNNNFIFTVLSERCLMTNYTESFKQLKGMRTLDWINNLG
SVELEVELQDGVKNFNVTPAQATLLLTFQKKDEWTLSELSRETSVNKTSLRNRISFWVGQGVIVEKPNDVYVTASCFQSQ
NQKDTTLYDSEEENVAFSQNQKEEELHVYWSYVVGMLTNIGSLPLERIHSMLKMFATHGESSSHCSLEEVKAFLCSKIND
GELTYVGGMYKLPKGA
>Hmag_XP_002169634
NREDTIKCIITCITDEESGPDLVEELISSDVGGEYNSDHESDGEDWVPDPIDAKTDLSSRKQRTADIINILVNVYGSQDM
FVSQYRILLADRILTNFNYSVSNERRYLELLKRRFGENHLHFCDIMLKDIQDSQRINRLIKDDASKQCAIEQQIDFNAFI
LSSTFWPTFSDESVDPPEEVKSLMTNYTESFKQLKGMRTLDWINNLGSVELEVELQDGVKNFNVTPAQATLLLTFQKKDE
WTLSELSRETSVNKTSLRNRISFWVGQGVIVEKPNDVYVTASCFQSQNQKDTTLYDSEEENVAFSQNQKEEELHVYWSYV
VGMLTNIGSLPLERIHSMLKMFATHGESSSHCSLEEVKAFLCSKINDGELTYVGGMYKLPKGA
>Hmag_XP_002155486
MNMKKRESNSKMRIRAFPMTMDEKYVADIWNLLENAIQEIQRKNNSGLSFEELYRNAYTMVLHKHGERLYNGVRKVVTDH
LVGKVRKDVITSMTNNFLETLNIAWNDHQIAMVMIRDILMYMDRAYVEQSKVVTVYDLGLILFKEQVVCHPPIQENLRET
LLSLIERERKGEVVNRLAIKNACQMLMTLGINGRSFYEDEFEKHFLQVSAEFYKLESERFLAENSASVYIWKVEARIAEE
RERARHCLDSSSEPAIVKVVEEELISKHMKTIVEMENSGVVHMLKNSKYEDLGCMCRLFQRVNYGLKAMCEAMSIYLREE
GRAIVSEEKEAEGKNAIIFIQKLLDLKERFNMFLEKSFNQAVLFKQTVSGDFEHFLNLNSKSPEYLSLFIDDKLKKGSKG
LSEQDVETVLDKTMVLFRYLQEKFPRKIRKNR
>Hmag_XP_002170287
MENDSLYGKNGFNKENHITENGFIKENLFAENKHCNKSNTSLNEGFMSTPNHAKKLVIKNFKVIPSLPDNYKDVTFEKLK
DAVSAIHLEHSISYSLEELYKAVENLCSHNMSSQLYERLREVCEEHVKTIIAEFSSYPFNLNIFFDLHLTDIIDNFVFLK
KMDKQWESHCRQMTMIRSIFLYLDRVYVLQNSSVLSIWDVGLQLWRIHIMGHPFIQSKTLLHKKAFSINYCNSETVDRSL
LKRLIKMLADLQMYQQIFEPVFLKETDQLYLVEGNTLMSKVDVPNYLQHVERRLKEESERLFHYLEPCTRKALISSVENQ
MISCHLTNILNKGFNYLMDCSANVHLLLMYNLFSRVKNGLDSLCEYFGAYIKVKGLTIINDTERDKYMVQELLEFKEKLD
MLIEESFNKNEKFIITMKDSFEYFINKRPNKPAELIAKFVDIKLRAGNKEATEDELERRLDKIMILFRFIHGKDVFEAFY
KKDLAKRLLLGKSASVDAEKSMLSKLKQECGGAFTGKLEGMFKDMELSKDIMSSYKQMVQLQNTSSGIDLNVNILTMGYW
PTYTPIDVLLPNEMVKLQEVFHKFYLSKHSGKKLQWQTNLGSCTVLACFPSGNHELHVSLFQLLCLLQFNEGDEFLFEDL
LTATGIEEGELKRTIQSLACGKIRVLRKLPQGKEVNRGDKLIFNKKFSHNLYKIKINQVQMRETQEENQLTNEQVFQDRQ
YQIDAAIVRILKTRKSLIHNLLVTELYSQLKFSVTPSDIKKRIESLIDRDYMERDKDNSNTYHYIA
>Tadh_XP_002112916
MPTGLLLSADDSSNDDFERSWQQIEHTLLALLNQQPISKREWQDLFALTFRLSMWYESSNQIIVDNFNSVINRFVLDKKE
VLAAQSDKLVFLQEYVQIWETYYRLCDYIPQLFKRLEVASTNKSGSAALSVSKRNKCQAFRDTMLEKWKDGLLNYHIQRL
LDNVMELIIEEKRNHIYNGQLITNIKDALVHLHCSKDKDKLDTYQQFERAYMKNILEYYKLQSSMALDEMSMSDYIRYVD
AKLREEEDNCRRYLEFSSMKVVLESIENHLINSRKSILLQECFKMIKNGNTADLIIIFKLLDRVNGCIDNVLEALSSHIL
ESGMSTLLTDAEVIIWDSEKYVDKFIGLYEKFSELLKGGFNDDARFLSCRDQAFKKIINDRTIFSINIPTKIKGMGKLEP
ESRCPELLAGYCDLLLRKTNVNKKLTSGEIERRLKDALLLLKYVQNKDVFMRYYKVHLMKRLLLDITADQEMEENMIEWL
RNIEMPVEFINKLAKMFHDIHISHDVNNLFKEARSKEASDTINAKILSAAAWSRGSENTNVSLPSELAEYIKEIEGFYHQ

KYTTRKLQWHHLYSSGTITFATNYGKYDLEVTTFQMSVLFAWNLRPSDEISLDSLRIATGLNESELRKTLFSLTNNSKMK
YQILLVNPSVQSFREYTHQSIFRINRDFNIVKSGKIQKRGKVTLIGRLQLAPEKGNEEENKEIASLRMLRTQEAIIKIMK
MRKRLRFGQLQTELVNVLKNMFFPQKAVVKDQLEKLIEENYIKRDEQDLNTFIYLA
>Tadh_XP_002112179
MASNQASVKSTKLSVKNFKPKVRLPDAGKEEWWNQLREAVRAIHNRCPISYNREDLHKAVGHMCTHSLSPRLYNELKIQC
EEYTKASLHQLIDDFMDEMAYLIKLNSLWKDHCNQMIMIRGIYLTLDRTYVMQNPLVLSLWDMGLELFRKFIVSEQTVEK
KTIDGLLSLISRERNGETINKSLIKSLLRMLSELQMYQYHFENKFLQVTESLYATEGQNFSQSLEIPDYLSFVDKRIKEE
SERCLHYLEHSTKKPLLTSVEKQLIEYRKEMIINKGKTELLDTNRLDKLKLMYSLLARVNGGLDELCKRFSLYIQERGTS
MVMDTERDKTMVTELLDFKSKLDSVIELSFDHNPKFINTEKDSFETFINRRTNKPAELIAKYIDMKLRAGNKEATDEELD
KILDKIMVMFRFIQGKDVFEAFYKKDLAKRLLVGRSASVDAEMSMLLKLKQECGAGFTSKLEGMFKDIEHSKELMPHYKQ
YLNNQKIGHNLDMTVNVLMTSNWPTYHPMDVILPEYMISYQKHFQQFYLSKHSGRKLQWISTLGHCVVAANFPLGKKDIV
VSLLQTLVLLQFNKEDEISFLDLKQRTGIDDADMRRTLQSLACGKVRVLHKKPKGKEVEDNDVFAYVSDFKHKQFHIKIN
QVQMKETLEENINTTERVFQDRQYQIDAAIVRIMKTRKTLSHALLVTAVYEQLKFPIKPSDLKKRIESLIERDYMERDED
DAYQYHYVA
>Tadh_XP_002110752
MRKSIDYDATWASLRKVIFDVLTLKPVDRIIWSDSISDIYSICISSSEYIERIYNDCKEFLISHITHIKKELTGVYSQAL
LKKYYEYWIKFSSGASYLNSMFTYLNRSRNYMHGSDSSYEQMPYSSQENEELPAEIGQIAMQQWQEIIIEEYQVELVNVL
LAAIENDRKEENIPVADLVQGVIHSFGKFFYQSVFEARLLEETKNFYARELARLRESYDFSGFIKAVKNRIDAEQHRCQK
FFHVSSHSKVLKACISRLTAEQIDYLNCECARLVKNELIDDLNCLYKLLRLVDNGLKVMLQEIESHIKRIGKLLVINLFR
KTCEDYVNAMLKVSDRFSTLIQQAFEEDPEFKTIFGKAIRAVINHSNSSSTPIRAAEILARYADILLKKGSKNWSDAELD
DKQSHVINLFKYVDDKDLFQKFYSKFLAKRLIMNGSISLDAEEEMIKKLKIICGYEYTSRLHQMFTDIRLNEGVNSEFSS
YLNKKNIKLTVNFSASVLQSGAWPLNQTSSPYSIPPDLERSLTTFEEYYCTSLHRGRKLVWLPYLSTAELRMTYLKKGYY
VTTTTYQMAVLLLYNNALSYTGRDIATWTQLTEKELIRTLSSLEAAKILIKENSREGNSYKLNLEFGNKRTKFKIASSSI
RESPQEITKVHSRIEEERKLFLQATIVRIMKSRKVLNHNSLLEEVIKMSVNRFSPSIVLIKKCIELLIEKDYMKRNEGRQ
DEYNYIA
>Tadh_XP_002110161
MQHPLTARNVQLDQIWTDFKTGVEQIYDARTSMSRTRYMQLYTHVYNYCTTANRTSTASSRTRSGISLGAQFVGMELYSR
LKDFLAFYLVPKVLEANEAIGESLLTYFTAEWERFKFSSKVLDGICAYLNRHYIKRECDEGHDYFEIYCMAMTVWRDKVF
NNVHGKLCNAVIDLIMRERNGEMINTRLISGVLDAFVDLGLQRDGRSCKCKITLELYQSCFEQKFIEETESYYINESGQY
LKDNSITEYMKKAEARLHEERKRVQDYLHERTLDPLLRSCERILIEKHLEIFHSDFVRLLNDEKNEDLGRMYKLLCHIAS
GLDKFKSLLEKHIKDHGRQSIEECGDAATNDPKAYVKAILSVYSKYHNLVVSSFDQNSGFMAALDKACGDFINCNAVTQT
SAQVSSKSPELLARYCDSLLKKSAKNPEEDELDSALDDVMIVFKYIEDKDVFQKFYARMLAKRLVNQNSASDNAESAMIS
KLKEACGYEYTSKLQRMFQDMELSKGLNEDFRKLPNETNSVDFSVQVLSSGAWPFSPSPEFTVPSELERSLQKFSGFYST
RHSGRKLNWLFQLSKGELVTNCFKNRYSLQVSTFQMAILLMYNRGDIFTVNELQQHTQLKMEILQQVLAILLKCKLLVCD
DLDNSGELKYNNRLELFLGYKNKKLRVNINVPLKTEQKVERESTHRNVEEDRKLLIQAAIVRIMKMRKVLQHQKLIAEVL
TQLSSRFKPNVPVIKRCIDILIEKDYLQRVEGNKDEYEYLA
>Tadh_XP_002107600
MATGKSKKETKMRIRAFPMSMEEKYVDDIWQLLRNAIIEILNKNNSGLSFEELYRNAYTMVLHKHGDRLYTGCKDVIAEY
LRKVCQDLRNSVDNNFLTILNRAWTDHQTAMTMIRDILMYMDRVYVHGKSLDTIYNMGLILFRDLVARSGHIRDYLCKTL
LELVDKERQGEVVDRGAVKNACHMLINLSLGGRSVYEEDFEQPFLEQSAEFYQREGQKYLQENDSSTYIKKVEGRLNEEA
ERAAHYLDKSTEKRIVRVVEAELIEKHMKTVIEMENSGLVSMLRNAKMDDLARMYSMMNRVHGGVELMCDCMGVYLKSQG
KALVNDDDGKTGIAFIQSVIDLKDIYEQFLEKSFDNNRHFKQTINKEFESFLNINPRAPEYLSLYIDDKLKKGTKGLSDQ
EIELLLEKTMVLFRYLQDKDVFEKYYKQHLAKRLLLGKSSSNEMENSMIFKLKSECGCQFTSKLEGMFKDMSVSETVMEK
FKKHLDSSQTTINFDLNIRVLTAGFWPSQLSSNQCNIPTEISTCYDAFQSFYLGGHNGRKLVLQAQLGFADLHATFFGSK
KPDSVKLETRNHILQVSTFQMVILLLFNSKEKLSFEELKIATNIPDRDLIRALQSLACGKTSQRILTKNPKSKEIGPADE
FIVNDNFTSKLVRVKIQTAAIIRIMKARKQLHHSALVVETTELLTARFMPHPMVIKKRIESLIEREYLRRTDDRKMYSYV
A
>Tadh_XP_002114958
RISELFDIIIDFPESKASLEELKLCMERNDLRKTIAYSLRNTFETRLLHPGANTNDILDQYISAIHSLRIIDPSGIILDV
ACYPIRKYLSSREDTIRCIISNLTSDTDGELAQQLARGNNSLTDSFNESDTDSDEDWSPDPVDAIARKGMTSGYHKKKDV
LSMLVTIYGSKQLFSSEYRNLLAHRLLTLFDFNTDKEIRYLELLKLKFGETNLSDCEVMLKDVKDSCRISDHIQSTMNKI
VQEEKDDSLMLNTLIISENFWPKFKEEKLKLPPKIEKTMTAFAKHFEKLKAMRGLHWKYHLGLVNIQIEIANHQKQFSVT
PLQASIIYLFQEKVQWTIEEISEELQVSSTILRRKISYWINQGLLKETPVDSYNLVEDRITPEDAITEDDETTTVTSAAD
QHEEEQELYWSYIVGMLTNFPAMELDRICSSLKLFIPSAGELSSRDLKMFLDKKVQEQKLTHNNGEYALVK
>Tadh_XP_002114591
MYNDLTAVITKHLLSVSDKLKEVNDPLLHVQQFSYHLNQYKQALKAIVSLFHHLNRYYVTTVLHKDLSRELTKLFNQHIS
DNSQLFAALNALGKRSFAIGPENLSDIIQQLYALNPDYAKRAPEVFARFIPNVFGSTDESDIPALIQWTKELQEQFPLRG
VYPSANSHKRKMDSD
>Mlei_ML148525a
MDCQDDSTLNADVAWKQLVAYLSPPNVITPEIKSRCKELIEVLENFGYPVRRCIGSWFRVKFKHYFKSNVLPEVMTVLDV
YADYEAFEKITVLLGKGILKFLLSFYLVEETDSTFQKKDVYMDFTTILMEIEEVKKLFSMCCINFYKRGFDCYDNLAQDL
DCLPTEHSDELKNFDIINRYLCCLYLFEEVVYDAVLHVLNSQIRLFINDNFAGCSHDTSIIGPVMSWLNSSVITFIQFIT

QHQLVPPNMEIWKKRLKFFCYKTIATVRINDLFEIMVDYPESHAAVLDIKKCIGHVQCSTYLVKTVQDMFKERLLHPGAN
TEDIITQYSSAIKVLRFLDPSGAILEKVCSPLRSYLKNRDDTIRCILNSLMSEEENELTRELSSTTGTLFNSNEDSDLEN
DDWEKWKPQSKDMPTDLKSKASKSADIISTLCSIYGSASVFVKEYRSLIAEKLLTTSNYDVSLEMRSMELLKVGFGEDMS
QCQIMLQDVVDSKRTHANIINKVKQECPFEISTLIISHVFWPNLRKEEVNIYKPLQDYLSGEYQTAYSELKRSRTLEWNL
NLGFVELDLEYEGRTLSFTVSPVQATIILCFEKSEKCHADTISNTIGLSLSQTRKKLAFWVTNGVLKETSKDMYELEKEV
KETQQVVVEEMDSAMVKIEQVREEELEGFTNFVIGLINQSGQITVERVHKLLSLLLSDSPAGQLTPEEVKTFLEKKIRYF
KLTRDGYISYSVSKEDNNGTKGSIKIDKMILQLHKHSEVRFDIVTPTVTWYLRAHTPEDRDTWTGIIAKHRELLPQSREG
SVVMLTESSLQVVSDLNLKLDELNSFKDSLSQQLSEIRSTLNNESPKLKQKLATIQATVSGIVATSANCIEMFEEREQFW
QEQITAEREKSRKLEILYSATNRENSELIKITLR
>Mlei_ML148526a
MDCQDDSTLNADVAWKQLVAYLSPPNVITPEIKSRCKELIEVLENFGYPVRRCIGSWFRVKFKHYFKSNVLPEVMTVLDV
YADYEAFEKITVLLGKGILKFLLSFYLVEETDSTFQKKDVYMDFTTILMEIEEVKKLFSMCCINFYKRGFDCYDNLAQDL
DCLPTEHSDELKNFDIINRYLCCLYLFEEVVYDAVLHVLNSQIRLFINDNFAGCSHDTSIIGPVMSWLNSSVITFIQFIT
QHQLVPPNMEIWKKRLKFFCYKTIATVRINDLFEIMVDYPESHAAVLDIKKCIGHVQCSTYLVKTVQDMFKERLLHPGAN
TEDIITQYSSAIKVLRFLDPSGAILEKVCSPLRSYLKNRDDTIRCILNSLMSEEENELTRELSSTTGTLFNSNEDSDLEN
DDWEKWKPQSKDMPTDLKSKASKSADIISTLCSIYGSASVFVKEYRSLIAEKLLTTSNYDVSLEMRSMELLKVGFGEDMS
QCQIMLQDVVDSKRTHANIINKVKQECPFEISTLIISHVFWPNLRKEEVNIYKPLQDYLSGEYQTAYSELKRSRTLEWNL
NLGFVELDLEYEGRTLSFTVSPVQATIILCFEKSEKCHADTISNTIGLSLSQTRKKLAFWVTNGVLKETSKDMYELEKEV
KETQQVVVEEMDSAMVKIEQVREEELEGFTNFVIGLINQSGQITVERVHKLLSLLLSDSPAGQLTPEEVKTFLEKKIRYF
KLTRDGYISYSVSKEDNNGTKGSIKIDKMILQLHKHSEVRFDIVTPTVTWYLRAHTPEDRDTWTGIIAKHRELLPQSREG
SVVMLTESSLQCWVITQKFAVLL
>Mlei_ML20871a
MMRRPKQAKFEESWKNIQNVTTAVMSESPVDRDEWCEQITSVYYLCMAMPTPLAESLYNNLKEYLINHVQSCREKVLEVT
DILPMYDSLYKTYSRGAGYLNDLYMHLNQHFTKRTIKNEWSDHYMHSIEKVKKFEKVQDLAMNIWREEMIEKVHEPLVSA
ILQDITRDRRGVSAYSNILRDVIHSFVEVQSHEDPQGSQLQLYKELFEERLLDNTGNFYQKEAQKLYEQNDVSNYMIKVI
EKLDEEELRLRKFIHHTSITPCKKECVNKLIADIQDRLLDEYKNMLKNEHVDDLSRLYILIRRLNEGCKKMADIFHDHIF
ENGKGSIELMARGCSPQEYVDALLVVYRKFTNIVSNYLSNDQNFTDALNRAMTKMVNSTGDTKKYPRSSELLVRHADHIL
KRVNCKNNSLSEIEDQLRNFIDLFKYVDDKDLFQRFYSKKLANRLIHNTSVSIELEEVAISGLKDRCGYEYTSPLQRMLI
DMKVSDDLTKEFDQHLENKNEKLNVVFSVNILQSGAWPFSANTGASEEQSDKNAPDFNVPSDLAVCIDKFKTFYNKKYSG
RRLRFVFPLSNGTVILSHTPRQYTITMSVHQLAVLLLFNDKITVPKADIASSLGLEKAELEKTLSSLVDSNLIKSESDGY
TINTEYANKRRIFRLPTSSVKDHTEKEVEQTHQSVEEDRRLYIQAAIVRTMKSRKQLGHNDLIQQVINLSKDRFTPSVGM
IKKCVEVLIDKQYLERHSNQKDVYQYLA
>Mlei_ML215419a
MSMKPTSSKKESQSMRIAFEMNMDESYVQSMLDLLEDAIKTIQNKDNGGLSYEELYRNAYTLVLHKHGPRLYKLMKEVIE
EHLRGEVREIVHKSLYNNFLATLNTQWQDHKVSMQVIKDVFMYMDRVYYVKNNKVDEVQDLGLKLFKELVS
>Mlei_ML24227a
MTHSFSSNPHGQRQRDLETFWSDLRQGIDQVYNKENINKQRYMQLYSCAYDYCANVSGDPNAKANDLPKPTKKPMKATNG
KQGARFIGHELYKKIKDYLEVYLEQCRAKIVEKDLQDEVLLDFYTKQWSDFRFSSKVLNGICQYLNRHWVKRKREEGNKK
IYEISDLAFVQWKEHMLTPLSNQVTRAVLKLIEKERNGEVVNTGLIKTAVESFVQLGLKGNAPEARLLVYSQNFEHHFLA
ETTSYYTSESMLFLQQNPVPEYLKKAQKRLKDEQERCDKYLNESTRSELEKRCTQVLVEQHLEIFYSEFQNLLDNDKNED
IGRMYELVYRLEPGLKHLKQLLEEHISRVGLKAVQSCQESKESVDPKSYVQALLDVHRKYNDLVNKSFDKNSLFVQALDK
ACTGFINKNCVSEAANNTGKSPELLAKYCNLLLKKSSRINPEEEELESILNDVMVIFKYITEKDVFQKFYRNLLASRLVN
DTSVSHDLEGSMISKLKQECGFEYTSKLQKMYTDTHTSKDLNDRFQEYCSSNPAPNDSLQGFSVMVLSDGSWPYSRPSSE
FNIAPELRVIHDKFSAFYDKQHSGRKLSWLYSQSKGEVEWSRGKPKTSHRFHCTTFQLSILFQFNHRDSLTFGELQTNTM
IEKDKLDQLVRQLVSFNVLALSKQPTNSETKESEIQLSDIVQLNLSYSNRKIRVNLALPLKSEAKQETEQAHQSVKDDRM
LVIQACIVRTMKSRKQLKHSLLLAEVMQQLQNFQPSVPAIKKCIGILIEKDYIERLNGENDTYQYRA
>Mlei_ML27052a
MIKVDVKSCGKQPLSTMSRGVQQINPSSSLRSKSLSSNKKVQPKKLVIKNFVAPVVTSTQLDELWTRLQHGLSDILQTGD
TRNKSSLLDDADNLCIHGDQMELYRKIEQEVKRHMECCSKEFALKQFPNNTAFLRELADIWGKHSKQTYLIRNIFNYLER
RYVMLSHKQLLPLWEMGAATFRRELMCVNNVSNKCVEGMLSLIEQERCGDDVDGGLLNEIVSMLRKLNMYSSDFEGPFLV
QTNEFYRVKGDIQLNDSSVSFQTFFFRKKKNSIRCRRMELSTNPQTNPDLNKTYVKDIYLRNSNFTQFELNLIISFSTST
CVIGTLSNVLSLSYFLSVRSKKLGDNLLVLLNILDLSASLSGSFYLVLWKFLEVDIVLEVFILTLYLVFVECTGFVTTLL
TVVRSIASHFPFYEPNRKLLAVSSVVFLIYTTLKGSVFVFYTHVNIEDKLLQIKLSNIGNIMLLTSLLASVTVVVMTNLF
VIKKLLTSGNGAASLQSSTRINRQTNRQINRHATVTIIILSIVFCFFNLLYALVLCNFVMGKETISPVFRNIVVSTAVPL
NSALNPFVYFCRKQEMRSFLWGLISCRLDGVRQRFAVRETVFTNTATSFNINCNSSKLACSAITKLNLQDYPNNSKQPAC
LAIEQNKDYHVTDSNRLACPTIESSVDVLVMEVDK
>Mlei_ML44422a
MELSLNKRSSSSNESMAKMDDEQTFKEAKEFELMVPVYLIYVTKCLADEERRLRHYLGSSSRKQLIKTVESNLIGSHVKS
LLEKGFDSMMDEKRYSDLQVLYTLLNRVSEGAYRMCFFLYTYIKNRGLEVVMNPEKDKTMVQELLEFKDTIDSIIKQSFN
KEDCVEVKKKAFEEFINMRPNKPAELIAKFIDSKLRSGNKEQTEEELEELLDKVMVLFRFIHGKDIFEAFYKKDLAKRLL
MSKSASIDSEKSMLGKLKRECGSQFTFKLEGMFKDMAVSKDLMLSYKERESEGGVDLSVTVLTTGYWPSYPADSVIIPAQ
ISGVLDKYKTYYLNKHSGRKLTWQHSLSFCTLKATFGDDSSSERKIDLRKELNVSVYQGLVLFLFNNSNRLSYSTIAEST

AIEEEELGRVLQSLACGKTRVLKKHPPGKDINKTDNFEFNADFRHKLFKFKINQIQTKETAEENVNTTEKVFQDRQYQID
AAVVRIMKARKTMQHSLLVSELYNQLKFPVKPVDIKKRIESLIERDYVERSKEDKNVYHYVA
>Ocar_g295_t1
MTEPKSKKAKGELDSGGVFVIIGAQWGDEGKGKLVDQLASQADIVCRCQGGNNAGHTVVVGGTMFDFHLLPRHHRPFYVV
VFGMLQEVDGLLEQEKGKDSLGTTRKGIGPAYAAKAGRSGLRMCDLLGDFEDFASRFKMLAEGYQKRLNVDIEEELDNYK
ILADKVRPMVCDTVHYMNTALAENKKILVEGANATMLDIDFGEDDFIERTYPYVTSSHCSIGGVCTGLGIPPHSVSHVCG
VVKAYTTRAIGAXXXVVVKYSAVINGYTSLAVMKLDVLDTFEELKIGVGYKVNDKQLPSFPANQKVLRDVEVDYVSIPGW
KQSTKECRTYESLPGSAQAYIKKIEDIVGVKVAHVITLVLKMPRLKQKLKEALESLPKISKEDKFNPEGLLVPEGTLVVA
VKREVFLIPDELLRQRTFHGSQQVLVRWRQVTSGDQENGXXXXXASGSATSQTSTGAAANHYLIWMKRDEVSACCPHLLR
QQHQQKEGSTGGDDGGEKAGTNVEILDSEEMAEMENDVCSLVKRARRQISKGSQPVHLSNTLQVLSAYALVGSLAGVFRE
SGALDLIMELLWSKAPGIRKSAGSMLKALATHDTGSRAYVLLSLSNMGEDSLSDPTSRNMLMELFAETASKEEYGMSFEG
VTLPQASGNTLFSLMKKYLRVMSIVDNIEQHRKPSLPLKSRFQLSVATSQLIVELCNAMGWEKSQSRDQLVVRGSIFTPF
VQRSFDLKLKPKSKFKKKAEFPNKEAYGEYMKDSLESGMKVVMLEDYENVSAGDVGEFKKSNEGTPPAQFKWQEYGSTYW
VFWYMVEMLDNTADSTLGSDTVETDSVGKAAKLPYGLFERERPKNAVVRDHWWEVLGMLKMLPPANQEQICSKIEEMSGV
PSVSSLDEVIEADYNTHTISSLVEIARQSSSNHLLPVSTCFEDVQRAEQQPDSSESGEDPGHGGSSLSEDTEPTCNEGDN
AVVESLKPLNDLDSIKEFTSTVRENSSKPYVMFQAMKKLVQWAGSSTEVQMSNAPGDSVLELTQELMVSGSGVIRSCGCS
VLKAFGKKGDEMRINAATNGAIKTVLHCMGESSSSFGVQTEGLSTLKKLLGAQDQAGFDYKSSESAVLLRDVFQSVGGQD
AVFHAIREVCKYGVDHKSDDKSANAVADGLSTLHMLMSPSSTGPSTLIHSLLSSGVSDVALTCLKSYSDQLTVQQFGIQV
LHCVMQNFDVSKSKVQSPSPLQKEIFMATVSTVASFLSKFSDNSEAVELACTALADLVREERGMNAFLVEMFLEKSLLTT
FTQALEKNKTCVKAVQKGLKVLESILCVCASAGKKFAVAFNGGISLVLAVIEADLEVNVIELACQLLLTMTSDQDAAINM
AANGGKVILTNAKDKHSSEQKIRTSCDQLLSRLEGLDDQAGLHQKVLRGVLAGTIQLCISMIDEARSMNTFITGGIDTRV
HGIECEGFKASYWPFFKEIFTAHTLLHYFVEKDGRGFLAVAISPEAGRVGFSAAVQYEQLFADEFLPTPESRLMLGNACR
VSLIAPLVSALTARDFSEKGSIGQLIRQYMMSVSDDGSGVLNSVEISDEFVLLSRTRRMCRLLVDCDTGPQLDEGEGEEL
TKGKGLHPLSQCWKLLVKQQINAFLSRPLHGSNPHEIANSFCNLYEKLLKTMKELFGNHSQFAVALNEGLADAFKALNEI
KGIEICEALSLSVDSLLDVAGKDCVKFQNMTISDRELWFKRLAQPLNFLRNLDLAHIFEYFYRRHLARRLLRGRSVSLEL
EKKAFHEFDVSFRHKEMLEDIDESFAMMERFSDTQTEALDVALARGEETADELRRRAEKAFLYRQGSLVAYVLTPCYWSF
KKQSPLPHLSSLPDSLALFCQNYESFFTSDDICCLQQYEKARKLQWTLHGWAVMTYGGVELTVTTPQMIVLNAFNKNQTL
KYSSLSETTHFPDADLKQALIALTASRQRVLSVTNRGGSGSDADADDLAFSSNDIFEVNENFFDGLSDSNRVVRIRTVHL
IGDEPEIGRQNLAHVFERRKAIVDAAVIRLLKQEKSLTVDNVAMKVIKGCRDGQFANHKPFGAFECQYQDICMSVSRLID
NGYIKRDSDTSNLLHFIPDPSQPSAEDVCDSSLNNETAEEKSPSLSVEVPEEKKKQVAVLKRHSSRQKSMTGRQQSSGQA
SPSRGRAKKKTPALLRMYSSDVYAHDAPPISFRFASSTLSGEQRWLKEGKDFLIEAGLLVKEPIEASKDSSVTCPVCTEE
VAEGDWLALVCGHAICRSCWEGHISFQLEVNQVLSSTCPVPQCTAQLHPQFILLALGKEAATFQKYEQAVIRSYVEESKK
LTWCRNPQGCDQILVHSGIADIATCSKCDWTSCFSCTFGEGHFPASCEQISLWVDEGGFYEGMDEEAKSKQLARVISKKC
PKCNAPIEKSDGCLHMTCKCGHDFCWRCMKPWRPSHCDYYRCSAQVSSLAKSGKRFVDFNQQCASHHSAKVFASGLGKRV
ANVTESVPLKILTFVVDACRLLQRCRKVLSYSCIYIYYVKENELSHYLNVLADNLETETDALQALLENTIIRVSDIGSCL
KSMQDQQITQGQQIIAKIQGIIDETLIGSMQQYQLGESPKRKDRVKDKGSGNKGMDGSSKSDDGSSSTSDDNSAPDDDDN
VDDDDDEDEDEDEDEDEDDDNDDEDNDDDDDDDDDDIYSDENDYDDYDED
>Ocar_g915_t1
MIYCNIIVGVHYDVALRYVICFFMRRLLHPGACTTDILTHYVSTIKSLKLLDPTGVVLEQAGEPVREYLRLREDTVRRIV
FNLIDTSSGELSGELMQNEPVLIEESDGSDYEDAENWMPDPVDADPSTSKSRKSSDIMSLLVNIYGSKELFIVEYQSLLA
DRILASLDYSTEKELPLDVNCLVLSGEFWPALKEEKFKLPETVEKALDVYGSAFSLHKANRKLEWKEKFGLVDLELELAD
RKLECKVTSAQATVVMAFQEKAFERKESTVVFVDHWSVEELNKKMHMAPALLKKRLGFWINRGVLKKEPDGTYSVIEVEE
GGENRVKRSKTLSFPSTLYSL
>Ocar_g2394_t1
MMRVEEHLPKQAHVLKFYQEVFESPLLTETGDYYKFIAFQLLAECNCSEYMQKVVKILDDEKLRVQKFFHHSSHSKVIRE
TESRLVEDHLEFLQGECRTMVKDGKYEDLSRMYQILKPIHNGLQVILTELESHIRETGNIGYCQVIAKSNASLIGLGMIR
AVPSEKNPGEYVEALLALHGKFAEVVTQTFHNDKVFVAAMDRGCGSVVNHTENPRIPSKSAELLARYCDSLLKKGTKNAL
DQEIDESLAKVIIVFKYVEDKDIFQKFYSKMLAKRLIHGLSVSSEAEENMISRLKQMCGFEYTNKLHRMFTDMSLSDDLN
GSFGDFMKDKTQLGLSFSVQILQSGAWPLGQSAGSPFAIPEELLRSISSVSGIKHTDLQEATQLSDKEISRTLKSLVDVK
LLTPQEEVPSNGDKSYSLNQQFVNKRTKLKITAAVQRETPQEVAQTHTAIDEDRRLYLQAAIVRVMKSRKVLKHNLLIQE
VISQAQSRFSPSISSIKKSIESLIEKQYLERKEDSKDEYNYLA
>Ocar_g6319_t1
MKSTFSCPTTPIGCPRINLIRTRETIDVMAVPSGSQNPHGMKMTNLSEIWTDLESGIRQIFNRSNMSKKRYMELYTHVYN
YCTSVHSSASGVQGVTKVAKSKKSHQGGAQFVGLELYKKLRDWLKEYLAKLRKDGGDMLGESVLAFYTKRWEEYRFSSRV
LDGVCQYINRHWVKRECDEGRKEIFEIYNLALVTWKDQLFTPLHQQVTNAVLKLIERERNGDAINTRLITGVIQCY
>Ocar_g7057_t1
MTSTLLNAVARERHGEVADRASVKTACQMLMLLGVSSTNPRQVLEEELISVHMQTVVEMENSGVVFMLKNKRVEDLACMF
KLFGHVMGGLQVMVNCMSKHLREVGLALVNEDESEAAIGGRNAVTFIQ
>Ocar_g7353_t1
MLSDSGRTFEERWEKGAMKPSTKRLLLQQSVTKKEWQDLFWEVHGVTSWDERGSQKIYESLKSMIQDFVKDRVMANQDDS
ALLKAYIGEWGRFFAQSQYLPKPYTNVNIDPSRSTLTGPIAGGSASKDKEKPGGDVLIEKLMLDQWNEGIFSAIKERLQA
SAMKIVYCERQGEAFDTQLVIGVRQSYVNLCSVEEDKLQIYRENFETAYLDTTETFYRSKASGLLAERGIRAYMAFALEK

MKEEDQRAQRYLETSKYSQSAQLLHKRCVEILINEYKDQILAECPKMIAASENESKDICERGHQLVMMIYVGLYQMFMLM
MELPNGIEPMMGFLEAHIVRDGLADMEACAGTITSDPEKYVEKLLELFGRYSLVVKDAFRDDSHFLTARDKAYKTVVNDV
SVFKLELSTKPKGASKAHPESKCPELLANYCDLLLRKTSTSKRLTSDEIEGKLKDVLLVLKYVQNKDVFMRYHKAHLSRR
LVLNTSADSEKEENMVEWFREVGMPAEYVNKLARMFQDIKVSEDLNQVFQESLRGQGKSVADTVGIKILNAGAWSRSADR
VALEDFVPEVEEFYRAKHSGRKLQWHHHMSNGLVTFSNDVGKYDLEITTYQMAILFSWNKRPGEKLSLEDLKLSTELPGG
ELRRTLWSLTAFPRIKHQVLLCQPEVKSPKDFNDDSLFSINQGFVVVRNGKVQKRGKVNLIGRLQLSTESSQKEDNEAIV
ALRVLRVQEAIVKIMKMRKRITNAALQTELVEMLKNMFLPQKKMIKEQIEWLIEHKYMKRDESDINTWLISPTHRIDDVN
PRPHLHPLTSRYVIIPFITKMPLAKSNPWMDMKHQNGHEEEVSFLIHNKVSKRASLLFPKGLATDEVSNEHMEDEPCSEI
ELTESVTKSIRKKADPKQFELLNVLGQGSFGKVFLVRKVVGTDTGHLYAMKVLKKATLKVRDRARTKMERDILVAVQHPF
IVDIHYAFQTEGKLYIVLEFLRGGDLFTRLSKEVMFTEDDVKLYLAELALALDHLHGFGIIYRDLKPENILLDGEGHIKL
TDFGLSKEGMEMDSEEKKTHSFCGTVEYMAPEVVNRRGHGTTADWWSYGVLMYEMLTGSLPFQGANRKETMTMILKAKLG
MPQFLSGDAQGLLRALFKRNPQNRLGHGTNGIEAMKRHAFFGSIDWKKLLARQIKPPFKPISREDTHYFDREFTQRTPRD
SPGAPVSAKSHQLFRGFSFVAPSVIEEEPEETASSSGVSSDIIGTGSFSVCKRAIHRPTRKEYAVKIIDKAKSDPEEEIE
ILLRFGIHSNIISLKDVYDDGKNVYLVSEIMRGGELLDHILLQKYLSEKEAATIVNTVALTLEYLHSHGVVHRDLKPSNL
LYPDARHAPESLVIADFGFAKQIRAENGLLMTPLYTANFVAPEVLKKQGYDAAADIWGLGVLLYSMLVGQTPFPHGVGDN
PQQILQRIGEGNFSTGGNWKVLSSPAKDLIGQMLHVDPHRRPTASQVVHNTWIVNRDALPDKKLSVQDQAMKGHLETMFA
ALNAPQGAEKLEPVNKSLLAKRRQERTASMESGTAL
>Ocar_g10935_t1
MENDCHCQYWEIRLLLFFYAPRKHCFTSCVSLLVCELVELGFDEDEPNSKGPTLKVYKPYFEEEFLADTERYYTAESTRF
LQENPVTEYLKQAESRLTEEHRRVTLFLHESSQDELARKCEEVLIQKHMELFQTEFQSLLSDYKVEDLGRMYALCSRIPD
GLAKLRELLETHIYQQGIAAIERCEEAAVNDPRIYVQTILDSHKKYCALVLTSFENDTGFVTALDKACGKFVNTNSVTRS
AQGSSKSPELLARFCDALLKKSSKSPEEAELDDSLNQVMVVFKYIEDKDVFQKFYAKMLAKRLVQQNSASEDSEESMISK
LKQMCGFEYTSKLQRMFQDIGVSKSLNDRFKAHLQKTEPLEVDFSIQVLSSGSWPFQQSIPIALPVEVGTSL
>Aque_XP_003391090
MDQWSEDRVYVQGNEKLNVYDLGLVLYKDEVLHHHSIREHMKNLLLELVDKERKGEIVDRGAIQSTCKMLMCLSLSSSKR
DVYEEDFERPFLQMSREFYKAESQKLLAENSAPVYLRKVEARLVEELERTHHYLDPSTESRITKVVEDELIKEHMSTIVD
MENSGVIHMLKNIRVE
>Aque_XP_003390027
MSGKKDGKMRIRAFPVQMDPSYVEDILKLLRNAIREIQKKNNSGLSFEELYRNAYTLVLHKQGARLYTMLREVINSHLIN
EVRVDVEDSLEGLFLETMNRVWSEHQTAMVMIRDILMYMDRVYVQGNEKLNVYDLGLVLYKDEVLHHHSIREHMKNLLLE
LVDKERKGEIVDRGAIQSTCKMLMCLSLSSSKRDVYEEDFERPFLQMSREFYKAESQKLLAENSAPVYLRKVEARLVEEL
ERTHHYLDPSTESRITKVVEDELIKEHMSTIVDMENSGVIHMLKNIRVEDLGCVYKLFSRVEQGLQSVIDRMSMFLRETG
RGLVSVETSSDSTPGKNATVYIQSLLDLRDQYNVYLEKSFNNDPTFRQAIGVDFEYFINLNDKSPEYLSLFIDELLKRGV
KGYSEVEVEGILDKCIMLFRYLQDKDVFERYYKQHLAKRLLFNKTISDDFEKSMISKLKHECGGHFTSKLEGMFKDISLS
TSTMDKFRDFLQTSSNGLGGVDLHVRVLTTGFWPTATTNSPCILPQIVADAFAVFQKFYLSQYSGRQLTLQPHLGFADLH
AVFYPHNKNEASSGHAVVKKHILQVSTYQMTLLLLFNKKPVFTFQELVLETNIPHKELVRGLQSLSVGRASQKVLHWLNK
EPNSSSKDFSDGDQFAVNDQFTSKLVRVKIQAISAKGETEPERKETRQKVDDDRKHEIEAAIVRIMKARKRLPHNSLVAE
CVEQLKNRFPPNAMIIKRRIESLIERDYLSRSPDDRKVYIYMP
>Aque_XP_003385969
MSVARTPNLHSSKPVNLSDIWDDLEKGIFHVYSWQSMQKKRYMELYTHVYNYCTSIHQPTSSASIVKRRKTQTPSGAQFV
GIELYSKLKDFLTNHLETIKPAGEGLSGEQVLIFYTEAWEGYQFSSRVLNGICHYLNRHWVKREHDEGKKDVHEIYSLSL
LSWKKCIFQSLSKAVTNAVLELIERERNGETINTRLISGVVDCYVELGIRPDSTQSKGQQLDVYKEYFEAEFLTHTERYY
ISESAHFLENNPVTEYLKKVETRLLEEQKRVHTFLHESTQDELASKCEHVLIEKYLEMFHSVFNSLLSQEKNEDLARMYM
LVSRVSNGLAQLKELFELHVYSQGMASIEKCRDTAQNDPKVYVSALLNTHTKYSNLVKESFAGDSGFMTALDKACGRFVN
VNAVTTACNSSSKSPELLARHCDALLKKSAKNPDEAELDEALQNVMILFRYVEDKDVFQKFYSKMLAKRLVQQISASDDA
EASMISKLKQACGFEYTSKLQRMFQDMSLSKDLNDKFRQHLSAGDSALDSVDFSIMVLSSGAWPFTQGPSFSLPLELQRS
YSRFITFYTSQHNGRKLSWLYQLSRGELVTSCFKSRYTLQTSTYQMAVLLQYNTSESHTFGHLLESTQLKEDTLVQVVAM
LLKAKLLVSMNFSCDDQNITTESVINLFLGYKNKKLRVNINVPVKSEQKQEHEITHKNVEEDRKLLIQAAIVRIMKTRKE
LKHQQLLAEVLHQLSSRFKPKVPVIKKCVDILIEKEYLERVDGQKDTYRYLA
>Aque_XP_003385040
MEALSVEFLKEHLKVSLQDKLLPQFWSHFDDNKSNYASLQEVSEAVSDLTNSISFNNVIEEMKGLYPLQIISGLLVHIYG
GQDLFVNEYRTLLSDRLLLSLDYDVTRELRNLELLKLRFGESNLHYCEVMIKDITDSRRVNGLINNKLSIKKQQQGDGDD
HENDVIINSFILSHIFWPSFRIETMKLPAFIQKKLDDYNKSFQEIKGMRELKWCHDLGQVQLELEVNDQILNFQVSPSRA
SVIWQFQNKSVWSVEDLSTELESTPSVIRRHLLYWAGQGVLCEEKEGEVFKIFEKEGMDDTYHEGGVFEEESAMSSMKSQ
KENDLEVVWSYVTAMLTNLGPMSLDKIHNMLSMFMLSGPGGGGGAGQSSCTVAELKACLDKKVKGQELMCTGGVYQLCKN
N
>Aque_XP_003384071
MASVGTSSALLRGNGSSNFTDQWPKMQPIVVKLLKQQEITRQEWQDLFWDVHTVCSWDEKAPAKIQKALEEVILEFTREI
QTSVSQHKEESALLKAYIKEWTKFFTQCQYLPLPFQSIEVENSANKANKKRQEGNKVQVLMLQTWNRSIFADVRSQLLSA
AMSFLYKERSGEAYDAQLVIGVRESFVNLCTDTSNKLQIYLEYFEKSYLEATKEFYTSSSSQYLSENGVQNYMRYASDKI
SEEQRRATRYLETSKDSQSVKLVMTELDRILIKTHSDVILEECPKLISANEVEKLMVMYRLMDRIANGVDPMLKCLESHI
VNTGLSDMKAHAEVITTDSEKYIEELLKLFKLFSELVKDAFGDDSRFLTSRDIAYKKVVNDVSVFKLELGTKGKVSGASK

THPESKCPELLANYCDLLLRKTPLSKKLTSEEIDEKLRSVLLVLKYVQNKDVFMRYHKTHLTRRLILETSADSEKEENMV
EWLRDVGMPAEYVNKLARMFQDIKVSDDLNTEFKDKNKGSDTADLVNIKVLNAGAWSRSSERVPVSLPTELEDFIPEVEE
FYRKKHNGRKLTWHHIMSNGSITFQNQVGKYELEVTTFQMAVLFAWNQRPEDKISFESLRLATELPDPELRRTLWSLVAF
PKMKRQVLLAEPEVASFKEFCDLTMFYVNQEFGLVKQGKLQKRGKINLIGRLQLTTEKTKEEDNEGIVQLRILRTQEAIV
KIMKMRKRLTNAALQTELVEILKNMFIPPKKMIKEQIEWLIENKYMKRDEDNLSTFIYLA
>Aque_XP_003383186
MSLRPGVVDFDEKWKDLSATISAVVELRPVKRLTWNNNISDIYALCAALPKSFAEKLYESTRSLLEDSVTALAEVLWVGG
AAWVHLNKYRPQDSLDYNVPGIILPPTSPVDPNAPVEIRNMSLKVWRTRMIDVIEEKLISAVINEVVKEGNQSDLGMAHD
VLQSFIEVTEYDKERPYEYYQRVFEDVFLSEISSIYRKLASQLVTDLSYPQYIKEAVSLLQIAKIRGEKFLHQSSIIKFV
RECEMKLVTDQLQYLLSYVPQMMRSEQFENLSNLFILLSRIPKTLETLIKEFKEKVTNDGLVCIKAFMSSKQSVEALEFM
TIILNVYSKYKDIVFDVFGGNKSFLNALNEGCRGFVNYSEGHQQHCQSPRLLARYTDQLLKKGTKGITDTELEEKLDDTI
TVFNFIEDKDTFQKFYSRMLSRRLVYGFYVSMEAEENMVQKLKHVCGYEYTARLQRMLMDITLSQNLVTEFHEKLKSDKE
KETISFSTLVLQSGAWQLSQSPCPSIVLPQELLTSLIKFEGFYSSRHCGRHLSWLHHLATGELKLQYLKRSYFVTMTTYQ
IIILLLFETKESLDLQTMSSWTQIEEKELKPTVQSLVDAKILISTGQELLLNFNYSNKRTKFKISGQLQRETTQDIEQTH
QMVLEDRKMFIQAALVRVMKHRKILKHNQLIEEVILLIKHRFYPNISLIKKCIEALIDKQYIERDAESRDTYHYIA
>Mbre_XP_001750850
MPSQTIEECWEGLLPVVNVIFQSSNNTVNLNHKRYMEAYAAVYNFCTTNRPPSSNSHRDPSHIVGHDMYRKVADYLEQRC
ISLRKELEAYEGVELLEKHRQFWEDFLFSRRVLNNIFMYINRHCVARALENPDHKKNKMFELDRLALLKWREHLFKACEP
KLIRAMLDMVERDRNGEAVSTNLLRSAVDCLCSLQAEAMVALRPTSNAASRTSSVDTRQHEQQRLEVYANSFQQPFLDAT
LKYYKQESEQFLAENSISDYLKLVTKRVQQEEQRVQQYLHASTNEPLAEACNDALIRHQIPLFNGEIDTYLQQERNEAGL
TSCAHALIRVIDLHHLYVLLCRLPDGVNVLLEKLEAHIIAKGREAIDALGKISMSGEDSKKYVEALLQVYEQFNALVRNA
FNDNPRFIESRDKGCRTFVNSNSVTQATKLARSPELLAKYCDSLLKNSSKHPENVLENLLSELMVIFNYLDDKDVFEQFY
KKMLANRLIQDKSASDDAEASMLSKLKDACGGEYTNKLQRMFQDMATNKQTNAKFKEHLDQSGHIMVKIHGEEKPLDFNV
RVLTTTTWPFASKLKMVIPTILDNCIKRYELFYAQAHTGRKLDWVYHLCKGEILMLYTKKERVLEANTVQISLLLAFNEG
DSFTMENFMNQTELQMDIIQPQLDLLTKAKILLLEDGRYSLNFKYNYKKLRVKIDQPVRSEQKADTESTHKAAEEDRKFI
IQACIVRIMKTRKHMKHQQLMQETLEQLSRRFKPKVSAIKRNIESLIEAEYLRRREGEREVYEYLA
>Mbre_XP_001749209
MAAGWQEVAQALTAAVTPVSSNEDATVIETWALKLAQLLDPWPATQMIHVIRLIAYKMQMALLHHATAVQIQNLLHADPT
NVAAALPPFDAFLQQLHAWHTFAQSLAGALTNLDTRHQLQSALMQALETTLDTVTLQRKHDLLTRLYAHDLAQCLSALAP
SSCSDDDDDDDDEEDDDGDSRDDGPPRLSGADDTARRASLTAALQSHLYTALPVATRRQTALPALHTALSSVVATRIARF
KGDFETNVLANVDKLIKGLVPYFETTLDCSLDVRSIQRHVYHVLGRQRIAELFDIIVDYPDSIPAIRNLRTCLEQCDLRD
ALVHQLSAAIQKRLLHVGAQTADIISQYISTVHVLNFLDASGGILNRVGPLIRDYLQKRTDTVKNVIEAITDENTAEPDA
LRDEQTVVQWLPEPWDGVSPMSKLDQVQQLMNDPIQLLISIYGDPETLLSQYRNVLAKRLLESRSFATTESVQNGSTHAH
HACHSTQPPDPTTHARARPRAPARARVDSETRLLETLKVRFGEDALVNCDILIKDVNESRRVHQSFSQQPVPETPAAPLS
KDQVNVLVLSRLYWPGLQTSTVNFRLPDGLEQYLEEYKTSYSRLKAGRHLELRPNEGTVELQLEAGGKQLDLVVSPLAAC
IINLFEQQPEWSLQALAKKLECTGPVVRSKLAYWVGLGLVRETQPDKFLLDEDHFAHHHASSITQRFDMLIRNLGLVLDL
WLLIGARAAGGDGHHETHEEAAAVGDGADDPLESPQAIVVLRFVEGMLRNAASLPERIHVMLSMFMQGDQAFNGDVKVR
G
>Mbre_XP_001748477
MKTGKLLSLSQTLSLSLKLSLSLSLKLSLSQTLSNSLSLKLSLSLSLSLSLSLSLSLSLSLSLSLSLSFSLILSHSLS
FSLSPFIVIVGGEGVYGGNRVCRRNMEVLGSEESASSEVLECDGFILPDFIDEQCFRSFCWPHLSTIFAHALTLNIGQPY
IVPLRQADIYGGVYRCVTCGHAGLLHDNVSQLLREHCQMVYEELAAQCSSDCQTFLRRFRFRLRQFAQGAILIRPLVAYM
ERTYLEAELETDWITETETIFRTMVLDRDPIHDTLFEYLEVAFENDLLLDPSDMECMAERLFRINPHYRDLLPNFFDRYV
AQGYSPSCTTELTLKPTFLTQEQYEQLKLANPAILQHLPQPAAQPQTLKTTILNTSSAPTIPGTKLRLP
>Mbre_XP_001745691
MAKRVVIKPYKAVSANNEFSERNWSLLQDAMLSIERRQSSSLSFEQLYRTVYNLCLGHHEAEVYKRLNQAFAAMCLRYRA
AAETGLDSQTTLECLVGAYRDYFLVVNTVKDIFLYLDNTYCKPKHFPIVFQMALAVFRAEVVLHDMLNRQLRHEVRHLLA
RDREGNHTNRTLLRSCAVMLVSLGPTDDSWSLFHKHMARIHEDECESYYARHGTEYLDHHSVKEYVAYAHARVREEIERT
EECLDASLCPSITKLLRPLPEIQKLQEAIAAKFESDVLALGSSATDDKASAVKLIEQLVGLHEKSTIALRDAFHQDRDFA
FSMATGFERGINKIKNAAESLCQYIDDIHRRGTKELTDGEMESRLDHAVAIFRHLEEKDVFDKYYKLYLGKRLLLHKSAS
DDAERHFIARLKAECGRSWTAKMEGMFHDIEVSKTLAEDFRRACSKDKNPLSYDFDASILTFGHWPATSPSVTCILPDAM
RQATQRFEAHYHARHNGRKLIWQPTLGHGELKTTYLAKRQHVLQVTTQCMMVLLNFNGHLAVDALSYGALLEATQLPEKD
LQRTLQSLACGKHVLLTKSSSGKTIHSDDNFKLNHRFSSKAVRVKVQQVAARNEEREVTEKKVQGERRLEIEACLVRIMK
ARRQLGHNELQIETIKQLAPRFKAQPAQIKRRVEDLIEREFLERDPDDRTVYRYLA
>Mbre_XP_001745467
MKVWRETIYMPMSKQIIHACMIELEQHRETGNVELFNVQATLRSLTDMCTDRKNSEQHQLYIDEFETPWIKNTDDHYKAL
GLRLLEGEDRSAYLVRVTEILEQEEQRLRSAVPTISHEKILAVCHRCLISNHQAQLETQFQVSLERDNRKDLSACYKLLL
RADCLDGITNLFQARMTAVGMQDVQALGCSAATPPNPTDFIARVVDLHAKGRELTNDCFQSSSKFSESLDRSCATIVNTS
LPKRLSPAELLARGCDLALRKTKGAVEEELDSRLEDIMLIFSFLQDKDVFLKFFTKAFAKRLIHGTSVSEAMEANMIGRL
KAECGFEYTLKLQRMHQDVQVSTDFLRNFRQSNMGRTLPFSFEALILRTNAWPFGSNPPAILLPPPLSDAIYKFEQFYTH
AHSGRKLNWLYHVSTGDIKAKLPKGVYELSATTFQMCTLLAFNTQESFSSKDIQNRLGMSKKDADRVLQSLITCRLLIGP
ETNDKAADLADDEPLTLNLRFKNKKRKLKISTPLTKETQSETAQTHKAVDEDRRMVLEAIIVRIMKTRKALKYNELVQVW

TLAPSDMLHSSNKSTLMTS
>Mbre_XP_001743977
LQDEEWTTLRDAVQAIYAKQAINQTLQSLTLLVERLHHKRQSPEVYKRLKTLMRDHLIGEQPRLRQAVAVDSHDVANLFR
LTWEDHIQAMMMIQSIFVTLDRLYAQKTRGIDLLWLVGIQLFKEHILQEDKNLDRVTRAILDEIQKERTGQLANAQLRPL
CQMLIATKLYRVLETGLLSATQSFYRHDGIERIARDPLDQYIVHVTSRLREEEERARFMLAVATRRPLLALIEQTLLLEP
LDLVLGEGFFTLLEADDYKHLNMLFVLIERVERQTQFQSALSKYVELKGAEIVGNPDNDKEMVDNLLAFFDKMHRILAQA
CGNDADTDQAIEMSFERFINKRQNKPAEMVAKFMDAKLRAGYKDSTEEEFEASMNKVLHIFRFINGKDVFEAFYKSHLAR
RLLHDKSASTDLERAMLSKLKQECGASFTANLEGMFKDVTISQQLDAEFQNFRRDTVSDSPLELHVQVLTQSYWPAYAKL
PLNLPQKMIQAQELFQQFYCQKHSSRQLSWQTSQGDCLVKAGFKKGNKELQLSLSQALMLLCFNDAAELSVKEIADLTNL
EGKELHRTVLSMTLGKVRVLEKNTKTKEVAPEDRISINEKFSNQRKRIKINQIQLKETAEEQEATSKKVFKDRIYTIDAA
IVRIMKTRKTLRHQLLMSGVLEQLKFPVKPVDIKKRIESLIDRDYLERSADDAGVYNYLA
>Sros_PTSG_01885
MMQAALALVGQVFGVAEPLRFGVVGGSEGERKEHEVRLAQLREAVDTALSQKPAAKRLLVQFASHHIQQSLVKSLDVSRL
VVLGLDSNDTDTAGIDAALFQERERACQSFTACVTRHIQSLQSAFANHRAVADEAMETALLRVQAGLSLLFPVSTYKDLQ
AVHVHILWETEKEGGSGSDDEDEDDDDEREGGDAVSKALRSVPTPTLNAYLQAVHALLAACRLDAVITLPVLKAVAITVI
NRKVQQRCAEEYEDEMLPDLLAWHDAVIQPFFAAFAPPSATNNTAHVQREMRLEVYKALGTLRVAELFDIIIEYPSSLPA
IRDIKKWCVALLKRLLHPGPQTSDIINQYISTVKTLRTLDPSGVLLELVCSPVREYLKRRPDTVRCIVELLIAEDADDFD
GGRVGGDGAAEDWLPDPIDAYLDQNESLRKDSVLDLLTSIYGSKELLVEEYAHMLAENMLQCKGFDIDEEIRTTEVLRAR
FGEEYLRKCAVMIKDFANSRRLHTAFERAPPYESSPPYQFSSRSDSPLHWPFTAENVRVFVLSRLFWPGLKTDNLDLPKD
LETHLQYYMQKFKGQTQMQTLEFRPTMGTVDLTVELETRTLELRVPPVAAAALLKFEEQSRWDLDDLAAALNAPPAIVMD
GLRALMGHGVVVQVSRGVFEVDESGSLQQGAKM*
>Sros_PTSG_04332
MAATIDIRQTWAVLSLGLDKILNADLRASSNSGTIKALEKTDYIELYHEVFGYCTKSSSGSRPLGAMQAGARSQSADKIY
GKDIYDKLKDFLIEHNKKKLSALSQLRGEELLNTYRHEWGLYDFTRTIIDNIFSYLNKHCVPRAIEADMPGYFDIYTLTV
FVWREYLFKPLHEKLVESALEMVRQDRNGVQIRTSALKAFTASLVAMGLDDKENVQGLVGRVEFKPKVNLDVYEQYFERP
YLAETRAFYQKESISFADAGSLADFVKKALVRVEEEEDRVHQFMHESTGIKVAEECNKVLVVMHQDALNNEASVLLETEK
DEADLKRLYTLLRRVPETLKPLRDQVEKHIAQHARSAIDACGKLGASSEDARKFVETLLQVHEKYLKQINLAFENDTLFV
EAMDKALKDVVNRNAVTANGRNSTRSPELLAKYCDSLLKRGSKVEGEQLERRLAQVMTIFNYLEDRDVFEKFYKKFLAKR
LVTGGSASDDAEAMFLSKLKAASGHEYTHKLQRMFNDIGTSRELNTKFKNHLRVSGTSLKVDFYVQVLTSHSWPFTAQLN
VTLPPVLGRCLERFSMFYQNEHQGRKLMWAYQLCKGELLTHYLKKPFVFQANLIQMAVLLLFNQQLSMSRSQILEATGVD
EKSLKPQLDNLRKMKIFKEENEVMTLNEKYSYKKLKIKIDQPVKSEQKEESETTHKMAMEDRKLVMEACIVRIMKMRKRL
SHTSLVQEVIEQLQSRFKPDVGMIKKSIESLIDKEYLRRGQVRTEYEYLA*
>Sros_PTSG_08035
MFRGKYEPYAEPALATIRGLLCAGEEGDSMKRVGVVAVAIASQMSHLHEHRRLSQALTSPPPTQQPTPHRHQQPAQRQPS
RAPARPTPRAATTPARPAEPVPSTSTISGGAGDIDDSLVLRRAIDQAAATWHLTRDEEALLQDGSVYVDKSKWQFLFQDV
HTLCVGPHAPSLYLDVAATFAAKATVIRTELARSAEDGFLLRYREAFSKYRDAITYIDMACKFINDNLLHQDRAKKQTRL
SSWNRVHDLGMKMWRERVYSPLKDILLTDAFKEVANARKGLSTSEHTLAGVLQSMLDMCSDLDQPATVYTCDFEQPFLAT
TRQQFMEESRAFLQDHGARDYLKWASAQLDSELLRSRVVLRPLSQPKLLDTCADALVRDHLDTFTTHAKAMLEEEQEEDL
GRLYDLLTRTGDVQMLTSEYTQHVTATGTARMDQLKQARNLTAPTYFDAMCSLHTKFITMARDVFRGDPLFLAALDRACT
LVANFDTAKAPEYLAKVCDQTLKRSSKSAITEEDMDTRLKQIITIFSFLQDKDVFQKFYSRHLARRLIQQTSVSEEAEQD
MIGQLKAMCGFEFTSKLQRMFTDVTISGHLETEFKSAAASSDEPLPTNMHVLVLQTGAWPLSSSSDVVLRLPPALASCAQ
RFEQFYQAKHTGRKLTWLYSYSTAEMKARLAKVYDFTTTAYQMAVLLHFNHATEATVGTLQEQTQLADTTLARTLSSLVA
AHVLKPQQEGQSLAQPDAADVFKLNTRYNNKRTRVRISAAFQKETAAVDRQHTRKAVDADRHLFLKATGVRVMKMRKTLH
YNDLVQEIIEMSKSRFKPPIPAIKKCIEEMIDEQYIRREDGNRAMLSYIA*
>Sros_PTSG_09501
MQKIGSIKPYRRKAAVNDSVDTLWSKLDNAITLIQEHRESKLSFEELYRTAYQMCVQSHDQELVKRVTNKLKARANKLRQ
GIEECIGIDRVAFLKKLSSAYSMYCLGVIKMSDILMYLDKQYRARRNLPTIFTIAMGVFESEVVMAANLNSELQDALLVL
LDEDREGKAIERSVVKACTSMLVQLGADTSDEELHVYNTNIGKRYLDRIDNYYRNASQRLLEQNTCSWYVSYTVRHLQEE
EDRCDHCLHEETKEAALKLLRRRLLEDHIAEILRLPQGLTHMIDNKRDKDLRALLTVYAGTTVGIATAAAAVREHVVASG
RAAIDSHQSQSRPAVPIMQAILDLRKRFDGVVSVASFDPAVHAAIRRDVTQGFEDFVNELEQAPEYLSLFIDEQLKRGIK
ALSDSEVEALFDLTTKIFRAITDKDVFERFYKQHFARRLLLNKSASIDAEQSFLQRLQVECGSSYTKKLAAMFRDCTLKD
NMMERFREAVKMNEKRPMFDFSVNVLTLGSWPFTQQPPACVLPPQLMQACDVFEQWYHSFHTGRKLTWDFALGQAEIVGV
FQNGKKKHIFQVTTLQMIVLLQFRKGVALSTEALQSSTQLSLVRLHRILQCLASSKVRLLKKSPPTKTIAETDAFSVNEK
FSSRMVKIRIPQLVSKEATAAEAKDTMKKVTEDRKHEVEACIVRVLKNRKQLHYNDIVVEVTQQLAKRFQPPPLLIKKRL
EALIDREFVERDDKDRTLYRYLA*
>Sros_PTSG_11740
MGDGGWSLSGRLPAPQLHDIERQLSCGRAVQAYANAKVGQWLIRIGDGIVTRNHVHNNAAGRPTDIIGQVVRIFTIAGSS
VPFVTYIRLVRPSEAKIRSTDYAEDELLFPGRVGYSASRLDEIKARCALTTATDYQQYQAHRAWAARLTHSPLPPPSMAT
FFVRYVDESPGDIPVGAGRRLVWQGHAPKDKYLDLSSPIHMMAFEGDVAGLNRLLRKGVSPLARDCNNHTVLHYACWTGH
LRTITALLGRLGQGVAARTETVAVVKECEALCQSLGFVTAHRVISRFRARLVAAASTKRATRIRVDVSEHDLLRFASLSE
LSHTHAGAVGERAAKLAHGSGNSNDNSSNGATCAADDEDSAFSTFINKRSNKPAELIAGYLDSFLRKGLKGKREDEVERT
FQRVLDLFRFVQGKDMFEAFYQDSLSRRLLHQKTASTEYEKLLVSKLKLECGANFTSHLEGMLKDIDISAQLNADFKTHL

EVTRQQPSMELYVNVLTTSNWPTYPKVNVTLPPDVVRMQELFSQFYCNKHKNRKLQWQPSQGQCILGASFQRGNKELIVS
LFQAIVLLLFNDSQKLTCKEIQQATGIDMPTLKRTLQSLACGKIRVLAKSPKGKEVDETDKFIVNYKFSNERRRVKINQI
QMKQTQAETDATSEKVFQDRVFAIDAAIVRVMKARKTLKHNFLLTELFQQLKFPCKPVDIKKRVETLIERDYLERDPNDP
QTYKYLA*
>Cowc_CAOG_00038
MTLKPVSINFDDKWRELRQQMELIVGEQSSEVSGMIFHEIVYRVCTARPTPFADKLFREVSAFFSRHVTALREGILEQES
NLLPGYASRWSTFDAGTGYLHMVFEFYNKLATKHTTSGASFQPDDGSNTPMPIMTLAYKRWREHCFEPLKTRLLHNILSE
IEKDRNGEDINSSVILTVVNSLVTLSNDPKAPLDLYKTQFEAPFLQGTSSYYRREAAAYIADHDISAYMRKAEAWLDSEQ
LRARKHLDSSSYSSVIKLCEAEIVTAHREKIQAECTRFIDQDAREDLTRMYHLLRRIPGGIDPMLVAFEQNVTAAGLKEI
ERLSDAAQKPEPYVDALLVLHSKHNDIIRTSFDNDNQLIAALDKAFRSIINDTAKSKSAGKAPELLAAFCDQLLKKSNKN
QSEAEIEEKLQQVIKIFKYIEGKDIFQKFYSKFLAKRLIHGVSVSDEAESMMIAELKAVCGYDYTTKLQRMFTDMTVSED
INKTFNEFRSNNDIPLNIEFSMLVLQTGAWPLGSAVQSPFNIPAELEKSVTIFEAFYGKKYSGRKLNWLHHLSKGDLRAT
YGSKRYELQSTNYQMAILLQYNNEDVYSYSQLRQLTNLNDADLKKTVKSLVDVKLLNLDSGAEDVTESSLLKYNRAFSNK
RTKIKITTAVQAETKEESVQTHKSVNDDRSLYLQAAIVRIMKSRKTLSHNQLVQEVIVQLSSRFQPAIPMIKKSIEGLID
KAYLERVENTLDKYNYLA*
>Cowc_CAOG_01275
MSTGPRLFPSSSSSTSSSSSSSAAPTSASSKPGSLGSPFATSSSSTLPSSSSDPSPLAGGTGRPAAAAAAAGFTSSSAAA
MQSSPHHPHHHHALQASSASASSYMMSIGGSSSARKKTEGKTLSIKGFKVAPKLPPNYEQETWQKLQAAVRAVHEARPID
SYLEVLYEAVENLCLLGGGATLYERLTAECESHLRLEAEKLSVASEDPVTFLSVVDACWQAHCEQMITIRSIFLHLDRTY
VLQNPHVQSLWDVGLIYFRRQVAEVTVTQRRLITGILLLIEQERAGDSVNRSLLKSLLRMFSSLGMYTEAFEPHFLRATH
ELYAREGAALITTMPVPDYLAHVEARLQAESERIVHYLDIHTRRNLLATVERQLIEQHIRVLIERGFEELCNANRIADLS
RFYSLLGRVNGLEPLRVAFAAYIKKRGAALVCDPEKDKNMVQDLLDMKQQLDTLLSQCFGHNDRFQNCMKESFEAFINMR
QNKPAELIAKFIDAKLRAGNKEATEEELETVLDRLMILFRYIQGKDVFEAFYKNDLARRLLHNKSASVDSERAMLSKLKQ
ECGGQFTGKLEGMFKDMDLSKAIMVSFNQSKFASQMGDIELSVSVLTQGYWPTNKPTSMNVPSEMLRIQQEFQKFYLQKH
TGKQLSWDNPRGDCLVRAAFPKGTKELQVSFMQTLVLLALNAGDGVSFPTIKAETNIETEELKRLLQSLACGKIRVLNKN
PKGRDVNETDTFDFNTDFVNKHYRLKVNQIQMKETAEENADTNEKVNQNRQYQIDAAIVRIMKARKSLAHQLLLSELFNQ
LKFPMKPADLKKRIESLIDREYLERDEKDQSTYIYLA*
>Cowc_CAOG_03030
MSALAASIAAQAASRLPAVLEHCRSIVASASSSPSSPSTLVLEQQRLGAAIQAYFGCADAADDRQRRASAEAVLAGTLAD
LILSECRESLRAQAAPAFWAVLANTPQPDQSNSGAIATWLAKNMQAAFAVLEASIAAHETLFEAVLDFAQSIPSLSAAIR
AFNVNTEAGAIVFARPLPEFAAMVEQFVTLSYWAHAVVHDDVPNDWHKRICDDVPEEAIEALQQAVGAATAAVFSVCQRR
KLQHRVLMPFVLSQLDILVKAQTEAMANLFADSCHEAAGQWIDTYPAPWLRHVLGESDECDQLISYLKTQWLSQSLAALR
TTELFDIIVDYPESANALSDLRALLIHPQQRAALVQTLRASFERRLLHPGANTPDILTQYISAIRALRQLDESGVILEQV
CAPVREYLFGREDTIRCIVSSLVDDAESDLRDELANTNASANADSDVSDTEDATDSWNPVPLNADLTQPSSLRKTSDIIS
LLISIYGSKERFVDEYRALLSDRLISTLSYDIDKELRNLELLKLRFGEANMLFSEVMLRDITESKRNNTSVQERMPESVK
LDFPLNVIILSRLYWPNFKTEPTPLPKPVEDFMKAYEAAFEILKPTRALHWKHSVGIVTISLDFEDGELEFSVAPAMASL
IMLFERDDPWTLGELVEATESSTAVVRKRLAFWIAQRVVIESPADTFSVISKIADQGVPLQGIDADSDEEDALHNIDGEA
GEEAEGDEELDVTQCFMFVSMMLTNLGSLPADRMLTMMGMYAPTMPIDDRSFRRFLSNMVADGKLATSGGEYQLVK*
>Cowc_CAOG_04201
MKPTGELTPATKSVRIRPYTPMTSLEPAYVDRTWLLLRDAIIEIQHQNASGLSYEVLYRNSYNLVLHKQGGRLYNGLVQV
ITEHLRSVATRIENSIGGNFLARLTRAWSEHTTAMKMIRDILMYMDRVYVESKNRERAAAANDPPHLRREQHRLEVYDLG
LSIFGEEVARHPRIKQHLLRTLIDLIRRERDGEVIDRGSIKSATQMLIELGIHSHAVYYVDDLEGPLLADTEQYYQAESQR
LLGELTASEYMKRVEERIREELERVAHYLDALSEPPLKRVVERELIANHMTALVEMDNSGLVSALVHNRLDDLARMYSLF
SRVETGLSLIQKHLDVHLKEVGKAIVVADDEAGSAAAAPPAAASSSSSSSSSSSVPAAPGATAKDAGGVKDASRYVQQII
DLRDKYETILLKAFRGDRNFRSTINSCFEFFVNLNPKFPEYLSLYVDELLKNQKGFSEDEIDATLEKAVVVFRQVQEKDV
FERYYKQHLAKRLLLAKTVSDDLERSMIAKLKTECGYQFTTKLEGMFRDMALSRDSMERFQRFLDDSNINLGFQVNIRVL
TMGYWPASNVSAKVILPAELRHACEVFQTYHAKHHSGRRLFWQTSLGSADIRASFAARRHELSVSTFQMVVLMLFNQQDS
YTYQEIAQETEVPPGELKRALQSLACGKYKVLLKEPKTRDVTESDSFTFNDKFTCQLHRLKIQAVAVKENEAERTETRAK
VDDDRKHQIEAAIVRIMKARKVLDHNSLILEVITQLRARFAPTPNTIKARIESLIEREFLERTPEDRRMYRYVA*
>Cowc_CAOG_04624
MTHLANTTRAQSIDEVWADVQSGMDCFYYSDQGIDYNRHMKHYSNIYNYCTAPRTLPTDISRNARRPDSNNFKGAHVTGQ
DLYCRVIEYLRQYLRTRSEACKELSDETLLRYLNKQWDRYKIASKVLNHLFAYLNRYWIRREIEENVKNVHEIYKLALVT
WRDDLLLPFNKQITAACFRLIERERNGEKIETSLIHDIVDCYVSLGLGEEDYKKQRLGVYQQYFESGFIEQTTLFYTAES
SKFLASNPVTEYLKKIEARLAEEESRVQLYLSINSREPLLECCDKILVSNHLETLQAEFPNLLSHNQVDDLARMYTVLSR
VANGLDSLRVILEEHVSAQGLSAIESCSETALNDPTQYVTTLLAVHKRYAALVAGPFRGDASFVAALDKACRKFVNTNAV
TAKAKSSTKSPELLARYCDALLKKGSKNPDENELEELQQDIMVVFKYIDDKDVFQKFYTKMLAKRLVLGVSSSDDAEESF
ISKLKQTCGYEYTAKLHRMFNDIGLSKDLSSKFQEHLVASSTKLNLDFSIMVLGSGAWPLQGNTAPFSVPDDLVRALERF
TTFYQNQHSGRKLMWLYPQSKGELRTSYGKGATKYTLQASAYQMAILLLFNTNDSLTVEAIHQATLLPLPLLGSILAVLV
KAKLLNAEIEDENFAPTTEVSLNFDFKSKRLRVNVNLPLKSEQKAEQEDTQKTVEEDRKLLIQASIVRIMKTRKVLKHAL
LMNEVIAQLNNRFKPKIPTIKKCIDILLEKEYLERLPDQNDTYSYLA*
>Mvib_comp10446_c0_seq1_fr4
AFVQALLLLREKYQRILDVSFRADTEFRKSITAAFETFVNRCARAPEFISLFIDDQLKRGLKGSSEEEVEATLVNAISLF

RHLRDKDVFERYYKQHLAKRLLLAKSLSDDAERLMISKLKTECGYQFTSKLEGMFKDINLSQTTMNSFHLWLGTSAASAA
LPSPDSVPVDITVRVLTKGLWPTSAQSSKMCVLPLAVRTARDRFEKFYLSSHQNRLLAWQYMMSTADIRAEFRSGKKELN
VSTFQMCVLQLLSEAARSRDSPSPSISYEKLHADTDIAASDLKRALQALACGKYRVLNMEPTRGREITESTRFSINESFS
CRHVRIKIQNVAAKETELELKETAQKVDEDRKHQIEACIVRVMKARRQMAHANLVAEVTKLLQSRFMPNPAHVKKRIESL
IEREYLERSAAHRGPYRYLA*AVCTVPPLF*FIL*K****Y*FTSFYTK
>Mvib_comp12969_c0_seq1_fr4
KKLVIKNLSVSPRLPADFEAVTWAKLREAVRAIFSRRASGSLEELYQCVRDMCTHKLAKKLYESLCEECYTYAVSVLGEI
TGRGDVSGAAASFLPMIDERWQNFCKQIGMVRNVFLHLDRTYALQTAGVVSLWDMAVGYWRDIVVSNARVQNWLVLGLLG
QIELERRGDAVDRSVVRNLLRMLSSLHIYDAVFEVPFLSGTQQFYQIEGQERMASLNVPDYLAHVEARLASEAERAATYL
DVSTRAPLLASVEEHLIRVHVVSLLERGFVLLMDERRTPDLARLYSLLSRIGALADLRQHFQAFVKLRGLAMVKNADKDA
TLITELIAMKQMLDAFVHTAFADNEKFQTATKDAYESFINVRENKPAELIAKFIDSKLRMGNKELGEEELEAVLDDAIVM
FRFIHGKDVFEAFYKKDLAKRLLYGKSASVDAELMMLSKLKSECGGGFTLKLEGMFKDIGTSRDLMASFQASRSAIDATH
GIELSVNVLTQGYWPTYPPVSVHIPDDMSACQVAFQEFYLRIHTGRRLTWQNSLGDCVIKARLAKGTKELVVAMYQALVL
LLLNGPRDMQLSFCQIQETTNIASDELRRTLQSLACGKIDTRVLAKQPKGRDVADDDLFSVNQDFKNPRVKIKINQVMLK
ETPEENSNTREKVFQDRQYQVDAAIVRIMKTRKKLSHTLLISELFEQLKFPIKPSDLKKRIESLMDREYLERDPNNSSVY
IYLA*ETGVFPLFPPFFSV*T*MTHPHSVYVFKSALGNSQ**K
>Mvib_comp334369_c0_seq1_fr4
KRRKEAASARPTLSTPALTPAASEPPMEVAQLALHIWKVTLLDPPIKKKLVHDILREIHNDRNGEMVTDSVVQGVIQSFV
HMNKFNTKKP
>Mvib_comp13081_c0_seq1_fr5
QFFAVRVTHTFAMSLRPKQIVFEEKWEELRVPLDAILNHKESSSKLSGMDMFQVVYNCCVSHPIPYPDRLYNALSKFLTQ
HVTSIVAQLQAHQSDLLLAYDSSWQKYHLAIQRLGVPFRFLNQQWVKKKREEFKNRNCSAEQASDAPLYVYELALRKWRV
HVFDEIKPRLLHNIMETIERDRDGHQDVNLWVVQSVVESLTHLGNSSVVEASSQSSAESTKQVYKEEFESPFLVATQEYY
RRKSRAYLEANDSTSYMEKAEELLIEEEKRTRRCLDQSSYDLVIKECEKELVEEHTGLINAEFTKMVQHGEKMIGHLSRM
YRLLRRVPNGTTPMLAVLEAHVTQAGGVEIASIGQTPEPKQYVEKLLSVHSRYSALFASAFESDNAFIAALDKACRKIAN
DAVGISAKKTARAPEWLARYCDYLLKKSNREQSDHELEDKLKHVMVVFKYVDDKDVFQKFYSKMLAKRLIYGTSVSEEAE
STMISLLKQTCGNEYTSKLQRMFTDMTVSKDINVGFKEFLTKSHTVSLSHDFSILVLQTGAWPLGAVAGTNQFVVPSVLQ
PPVAAFEQFYNQKHNGRKLTWLWHLSKGDLIVNFTKRRYEINATNFQMALLFFFNNEREMTMLELLKQTGLPESELMRTA
KSLVTVKLLGVALENDTLSTNETDFPAQFRGDVTLRLNRAFTSNRVRLKISTAVQAETHVETEQTHKAIDDDRKLYVQAA
IVRIMKARKVLQHVQLVDDVITQSKSRFSPSMPMIKKCIEHLIDKQFLERIEGTRDQYSYVA*VNEREREL*NRLNKLM*
KKKLMSIHKKKKKK
>Mvib_comp14697_c0_seq1_fr5
QNFHFSSSFNICNMEAQTTAEDTWALIESGIGEVFGFDDTRSSEVLSTEKYMEYYTAVHNYCMGSNTNQTQVVVGPRAHT
SGAKYIGRDVYERLSRFIETYLTDISQSELTGHTGLALLERVKYQWSRFTFSGRVVNNICGYLNRHWISRELQEGHDNVH
KIYAMELIKWKEIVFVDLSMQCASALLEEIEKDRNGESTDSNLLRLVIETFLALGSPDSALNDPAHVGDEDEMNAYYREH
FEGRFIDMTRSYYYRESEQFLEENPVTEYVKKVNQRIEEEEKRLQLYIPKTTSVKLMSTLDDVLITAHLHRFQGELKKLL
DHDRTDDLRFLYNLLVRLPNGVDALRDAFEEHVKACGLRDIAEVANTAFTDPKLYVEKILHVYNKYDDFVSNAFRNSHLF
KQALDKACRAFINANAVTEKAGSSSNKGPELIARYCDQLLKKTARAVETELETVLFQIMIVFNYIDDKDVFQKFYSKLLS
KRLIAGVSASEDAEELMISKLKQACGIDYTAKLHRMFNDMGLSRDINSEFHSKMDGRINIDFSILVLTTSAWPPTKAQFK
SYLVPEELSFAIAKFTEYYQVKFNGRVLSWVYHMSKGELKTGFTKPQTYTLQVSTYQIAILLLFNRQDTIHSKEISQDTG
IPEPHLKNIMEILVKSSLLKDAGDGNYSINRSYKSNRVRVNLNMPIKAEQKVERDETHQHVLEDRKHYIQACVVRVMKAR
KQMVHSELVTEVINQLKSRFQPSVPMIKKNIGVLLEKEYLERAEDQKDLYRYKA*VLSPCCASTLYKHSFSFFFSAKKKK
>Mvib_comp10446_c1_seq1_fr6
LLKNAIQEIQSQNSSGLSFEQLYRSAYNMVLHKNGDRLYNGLRDVVKDHLTKVAASVERELDTNLLGALTAAWHDHNTAM
LMIRDILMYMDRVYVQNKMASVFDLGLEIFCTHVARHPRIRDALRSSMLSSVARERAGETIERLSMRDACQMLKVLGINS
LTVYVEDFMNPFLDASTKFYQMESQRFISENSAGEYLKQAERLVNEETQRVTNYLLPETEPEIKKIVYRELITNHMMAIV
EMENSGLNFMLEHERLDDLGRMFTLFKYVDGGHRLMCERVRDYLQTVGSRLVGEDAKTGEAAEDDSTTSTTTAAAAAAAG
AAGPADGVGGELIKTDHGTGSNGGR
>Mvib_comp14477_c2_seq1_fr6
AANDATVDVTAASGAASAGGDAAMAADTSAYAEMMAAGDDSDGGGDDWVPDPIDVPHGGSVAAAYASSARTAAAHAASMG
VGGSSAGAVRERRAADIVSLLVNIYRSQELFVKEYRALLADRLLSALDFSTTREARNVELLKLRFGEGNLLQCEVMLKDI
ADSKRINGTVHSMLEERGVETATINASPAPVSIPLDVTVLSRLFWPSFRDDDFQPHRRTALAMEQYNAAYGVLKAGRTLA
WKPSVGSVDLELELEDRTVALTVPASLANLIMLFEDRPSWSLAELVTTMRLPSSAMRKRVDFWIGKGVLVEKSVGVFAVV
EKVVAPAAGDTASTLGKKKKKSATRTATKTSSGAGGVGTAAAQDDDNTKEEVEKLKGDDGKEEDEDAVDDDDDDDDDLDY
DAK*RSGEEHPKQCTIQKLHFFLQKKKK
>Mvib_comp73693_c0_seq1_fr6
RKQSSSEELEKAVIADLRTLGGSQFTTRFEKMLGDMSVSRDLAAQFKDACRAAETRVVDLSVHVLTSSLWPVAAEGGENA
ACVYPEQAQAALTGFEQFYAKR
>Mvib_comp151878_c0_seq1_fr6
EHLHAKLHPKLVFT*LPHLGTAELKANFPRGAKELSVTTFAMVVLMLFNDRDALRYEDIRAETNIPEPELKRTLQSLSLG
KVKVLVKSPPTKEVAEGHRFTYNSDFKDAKYKIKVSAIAARADVDAERAQTLQKVEEERAQITDATVVRIMKARKTMAHN
E

>Sarc_SARC_03914
MHQAFVLLLLNKNDKLTIGQVQQHTGLGTVELERVLLSLACGKKNTRVLLKTPKSDSVAATDVFTVNEKFDNKLRRVKIL
QIQLKETIDEQRATQEQVFQDRQHQVDACIVRIMKTRKTMGHQLLIGAILEILKLPIKPTDLKKRIESLIDREYLERDEN
DSTKYHYMA*
>Sarc_SARC_08090
MFIYEEAYFLCTAVPKALSDKLYYSLEEFFRQTCEDACKEIMAGNLTSVYHAKWQVYSIGSNIIRSQCRYLESTWIKDEK
DKYRQHMYSSQSGSDSRRDCPSDLGPLAFQQWKTYVFEKVQERMIKDLLQDIQKDRDGEMINASEVQGVISSLVELDSTF
KAPEIRSRHGYQQPGTGQLSVYQETFEKQFLAETAQFYQRESNVYLSENNCSDYMKKAEARLQEEEIRARKLIHSSSFAK
HSRECEYQLVSAHMDMLKGECQSYMKNEDVDDLSRMYRLLRRVPEGVDVLLREFETYVTAVGREQIAAIQEMEPRSYVEC
LLAVHARYHQLVQNALQTDDVFVAGLDKACRSIVNHTMGAKIPQAPELLAKYCDMLLKRSPANKHVDEAEIMQKLMSVMT
LFKYVDDKDVFQKFYSRMLAKRLIHSQSVSEEAEEKMIDNLKNECGFEYTMKLQRMYTDVALSATVNQEFSAYVAQKNIV
MDLDFSSFVLQSAAWPLNAVDTDFDLPGPMERAVQHFETFYHEKHNGRKLQWMHHVSKVDIKLKYLKKRYEVQATSYQAS
VLLMFNERDTYSFAEIQEETQLNASELTRTLDSLVKTRLLLHATDNNTSAYELNTGFASNRTKFKITQAVQAEPAKEAKE
AYGAVQEHRVFYLQATIVRIMKARKLLGHTDLVQEVVVQSSRHGKFTASIPQVKKCIEQLIDKEYLERTGPNKDKYSYVA
*
>Sarc_SARC_08408
MTPKEYMKLYTSIYDYCTRPATSFTARMTPTQVSGANIVGRELYQRLVDYLRAYILTIYDEIKDDMDDTLLEKFGTRWGT
FQRTSKVVHGLFNYLNRYSIARDIAENRTDVYEISVMCVVQWREVIFMRLKEKLTSGLLKLIERERNGEAINTGLVTTVI
DCYINLGQSFSNTPMLMYQTHFEGKFLQETTEYYQRESEAFLRENSTAEYMKKVEKRFDEEIKRVKTQVNRDSMSNMMDA
LTTVLIDKKIDIIHAEFKPLLAGEKIEDLGRMYRIVSRSNTDCMGKLRTEFEERVKTEGLELMTQVSANGTGDDPVTYVE
ALLSVHTKYHRMLELAFESNSDFQTQLDQACKRFMNTNAVTSAVKSSHRSPELLSKYCDGLLKKSAKNADTAELDKMLEN
AMVIFFYIEDKDVFQKYYRLNLVKRLIGKTSVSDEAENDMINRLKIACGCEYTNKLQRMLQDDRMSQELASEFTQKMSNV
TEPDFQKLDFGIFVMATTNWPSEVYSEPALNLPKQTENYIKRFVDYYDGRHTGRKLHWLHKMSKGEIQMSVSGRKYTIAA
HAYQITILELFNSADTLKYSDVQQTTGLEESLLTGHLGIILKAKILLTKTEGDLKSDSVLTFNSSFKNKKLKFNINQPIK
TEQKKEAKQTDIQIEEGRKLAIQACVVRVMKMRKTLHHQELLKQVIEVLQSRFRPSVPKIKQAIEHLIEKDYMERLGDNN
SYQYKA*
>Sarc_SARC_09944
VIVNVHQHHYVMMAMTKQATIELDSAWQHLYLAIKEQHDEIKGTECQRWLLKLTENGQAGHFRAFVEVAAVNRIKSEVDA
FYASLRATGPQEISTNRIDNTTLEHVQQALFKFDNEVDRLSNIISGVNQLYRSVNAIYDCIDSELVVNNTLTFVTVHRDI
GKLRKIFSIVLDTAVAILYRRVVREERGSDDEYDSGDNLDTEDAVMYDAAESTAIFEACLSIGRLANSLGLSERVLDPQV
RSTLKSYMISYINEDFIKGCYEESVLGRIGHFVDAICRDLLGKITPPHSMERKRAEVRLLANKALCDARTEEIFDLIATP
DSEPALEDLKFCLEQTAQHSKFVAYAREILRMRLLHPGADTSPILTQYVDAIKALRILDPSSQMVHAVCEPVQHALRQRE
DTIRCIVQSLTDTDDKKGLLSDAHTPTTKTHARLQGVDIEIDDWENWQPTPMDASGDAKTARTADIMTMLAGIYGSQEHF
VNEYQLLLSARLLDALDYQIDDERRNLELLKVRFGETNLHQCEVMLKDIHNSRRINEAVHELLIQDSEMRNNDSSLMTTD
DVPVDLTIISRLFWPQLKNHTLILPEKVNRWLEEYEESYSKFKQDRRLAVKPGYGRVDLSLTFDEWELDVTVLPDQAAII
LCFESKDVLTVEELSTMVGLAQKEVRRRIGFWESLGVLVEVSHGEYRVQETKPSQDTRQRKAFHMGDEIDVDDAAESMQI
ELETCWNYIEAMLVQMESLTVENIHMRLQMFFSGDPPPLNRLKAYLDDKVLNDVLEYRLSEYHLTKQ*
>Sarc_SARC_12279
AKASWRLLKSAIQKIHKQDASGLSYEELYRNAYNMVLHKHETMLYNGLEAVLTEHLVSVCQTVRSAAENGTKADFLPTLN
NEWNKHRISMLMVRDILMYLDKQYVAKNEQVLTVYDLGLSLFRKHVIDQVQNKSFIQECLLESIAKERASELIDRSLVKD
VLEMLMEVGVSSSAVYEGVFEAQFLQTSADHYSACSQEFLSQNDSVTYVRKVEKWLSDELGRVDAIMDKQTRSRLQLVVE
TETIAQHRTAIVGMDKGGVVNLIVEHRLAPLSELFRVLKRVPGGIEEIRDSLLDHLQSTGTKLIHTVLEPLPLIKQLIDL
KVMYYDTVEKAFANEQLFTDGVQRKFAHVVNLNKR
>Sarc_SARC_12549
MYTSFQTAFLEATQEYYAVEGRRLVTTLTVPEYLQYISKRMQQEQSRIEDYLHTDTWVPVCAIMRQELLKTHVSELLEKG
FHAMVKDSKLDELKMLYRLLSTVDALEDLKVHFITYLRAACSEMVLDEARDSSMVVDLLVLRSKIDTILEKSFGNQEKLR
NACKDAFEATINARPNKPAELVAKFIDRKLRAGYKNTTEGELDELLDDVMALFRLIHVRDW*
>Sarc_SARC_14593
MMLKLKTESGTHYTQKLDQMFKDMSISEETNRAFASFCADKKDTKNEDGLLD
>Sarc_SARC_16145
MQDVLEPFKTNYCTKNKGRILTWLPIMGQCIMRANFKQVRISMFKRVSGVLHV*
>Sarc_SARC_16729
MYTLQGKDVFEAFYKKDLARRLLVQRSASVDAERSMLTKLKQECGPRFTQNLEGMFKDIDVSKDIMQAYNE
>Cfra_g602
MAGNVVSELDLAWQELYAIVRSNPADIAGAATRKHIDVLFAHKQDEHFIEFIKVATANRLKKEVDTFYMSIGCMVMLSEL
RVQAKRLADMVGELVKVVNAVCTCTVMDNDFVQRTLHTSIVVHEDFGIFRTALTIVVTTAIGIFYNRIVSEECNHDVSED
VDFVHDGSHSKILLEKYLAIGQIIDYLRLSKQLITPVVVSTLSIYLNKYINRDSIKGCYEESVLPRLYGFVSRICNELLC
KIMTADAISISEERIKLLANKELCELRTSEIFDIIATPESEPALRDLKICLEKTAQKPRFVAYAREILRMRLLHPGADTS
PILTQYVDAIKSLRVLDPSSQMVHAVCEPVQHALRQREDTIRCIVQSLTDTEGKKGLLSDADPMSHTQLPTSCNDNVISD
CENWQPTPLDILGEAKNARTADIMTMLVGIYGSHELFVNEYQLLLSARLLDSLDFQTDDERRNLELLKMRFGETNLHQCE
VMLKDIHNSRRINSMVHELLQLDANDVQDKANSQPTSDDVAIDLTIISRLFWPQLKNHTLVLPEKINKWLKLYEKCYSKY
KMDRKLDIKSAYGKVDLSLTFEDWSLDVTVLPDQAAIIMCFENEPVLSIDNLATIVGLAPKDVRRRIGFWENLGVLHEVS

HGEYKVQETKPTHDQKQRKVFHMGEEIEIDDASDSVAIELQTCWNYIEAMLVQMESLTAENIHMRLQMFFSGDTPPLNRL
KIFLEEKVANDVLDFRLGEYYINH
>Cfra_g1615
GQNQSGMPGQGGQGGQKKDEKKKKYEPPVPTRVGKKKRKGPDSANKLPAVTPHNKCRLRLLKLDREERMKVDELRGTPMG
VGNLEEIIDDNHAIVSSSVGPEYYVSILSFVDKDQLEPGCSVLLHHKALAVVGVLSDDADPMVNVMKLEKAPTESYADIG
GLDAQIQEIKEAVELPLTHPEFYEEMGIKPPKGVILYGPPGTGKTLLAKAVANQTSATFLRVVGSELIQKYLGDGPKLVR
EMFRVAEDHAPSIVFIDEIDAIGTKRYDSNSGGEKEIQRTMLELLNQLDGFDSRGDVKVIMATNRIETLDPALIRPGRID
RKIEFPLPDEKTKRRIFNIHTAKMTLSKDVQLDELIMAKDDLSGADIKAVCTEAGLLALRERRMKVTQKDFLESKENVLY
RKKEEYEKRWSHLHDLFSSIMRLDGEILKGPLTSTYYEKWQVFSLGADIVRSQCSYLQSTWIKAEKEKYRAHMYNTANSA
EVQKKCPKDLGPLAFSLWKTEVFELIKVKMVKDLLQEIERDRQGEIINTSEVQGVILSLVELDNTFAAPQKGGSVSSLSG
YAGGHNSKNGQLSVYQEAFENQFLAETAEFYKRESTLCLTNNNCSEYMKKAEARLQEEEIRARKLVHLSSLDRHIHVCET
ELILRHKEMMKTECEKYLKYEDVDDLSRMYRLLTRVPKGIDLMLDAFQNYVSTVGRQMVAAIPELEPRSYVECLLVVHEK
YLSLLKRALESDGDFIVALDKACRSIVNFKVDSKQPALAPELFAKYTDLVLKKSATNKNPNEGELLDKLQSLMTLFKYVD
DKDVFQKFYSRMLAKRLIHNQSVSEEAEEKMIDNLKQMCGFEFTMKLQRMFTDVALSSTINKSFAEYVKAKNIKLGLYFS
SFILQSAAWPITSPNTEFDLPGPMEKSLKYFEDFYHEKHNGRKLQWMHHVSKADIKLHYLDKRYEIQCTSYQVSVLIMFN
DDTSYTYAQIAEKTKLEKNELDKTIESLTKVRIITHGKAKDVYTLNKNFESKRTKLKITSAVPIEQAKETREAYGAVQEH
RVFYLQATIVRIMKSRKLLSHTDLVQEVVVQSKRHGKFTASISQVKKCIEQLIDKEYLERVAKQQKYIEDITITAVSIAH
ACISPDPVQPRFVAVETAVAVETVMITQ
>Cfra_g2538
MSSKGKQPATDYQISSFPNHSRKVINDYEVQTRTKNPISSTTKITSSNHFGKDGGAKKLVIRGLSGRKNTVALPENYEKE
SLMELRAAIQAVHSSKPASKNLEELYRMVEALCTAQKGHEVYTMVLINSIFLCLDRKYVLSTSNVTSLWELGLSMFREQV
ANQTIVRDRTIAALIKLINNERNGQNVDRSSIRDVCDMLSALSIYSTFLNPFLEASKEYYCGEGNKLVNELDVSEYLHHV
NKRIRQEGERVKKYLHIDTLRPLQAIVKHELLKEHIPTLLEKGFENLVMNNKLDELKLMYKLFSMVDGLEELKGHYLRYL
KMACSRIVMDAERDNYMVEDLLAFRTTMNKVLEHSFSNQEKLRNACKDAFEATINSRPNKPAELVAKFIDHKLRAGYKTT
TEEQLDELLDDVLALFRLIHGKDVFEAFYKKDLSRRLLINRSASVDAERSMLSKLKQECGARFTQNLEGMFKDIDISKDI
MYSYNKRKNKTNVDLDVTILTEGQWPTYPNSEGIIIPKEVMDDDVFSVNMDLENKAKKVKINQIQLKETIEENTATQEQV
FQDRQYQVDACVVRIMKTRKTLSHTLLIAGLFEALKFPIKPSDLKKRIESLIDREYLERDEHDTTTYHYLA
>Cfra_g4629
KGVSPITTTSGVVDFSSWKPLHKHLMGSHGQSTSCDLSEAWLTIRDGIEGLYRHPEKDMTPKEYMKLYTSIYDYCTRAAN
TTNINSLHTPMSGNGANIVGRELYQRLVKYLRSYIETIYNELEDDLDDTLLEKFGHRWKLFTQTSKVIHGLFNYLNRYSI
ARDITENRPEVYPIYTMCVVTWRDVMFMKLKDKLTSGLLKLIMRERNGEAINSGLVTTVIDCYVNLGQGIKSSSLVMYLT
YFEGQFIQETTEYYQRECEAFLTENSTSEFMKKVEKRIEEEMKRVKTQLNIDSMDRVMDAMTKVLIDERIDILHSEFKPL
LAEEKSEDLGRMYRLISRSNGDCMTKLRKEFEERVRNEGLEAMRQIHSSGTGDDPTTYVDVLLSVHKKYHDMLKVSFEAD
SEFQTHLDQACRSFINTNAVTQATKNSHRSPELLSKYCDGLLKKSSKNPDSAELDKMLDNTMVIFFYIEDKDVFQKYYRL
GLVKRLVQKVSVSDEAETDMINRLKVACGCEYTNKLQRMLQDNSLSGELNSVFSAKVADAFKNDPNHHRLDFTLFVMAIS
NWPTEQKAEPAVNLPSQVEQYIQQFQKFYDERYTGRRLAWLHKMSKGEIQMHASAYQIMILELFNAAESLTVAEVIVGCG
LEEPLAKGLIVSIVKTKILLVENGSGELTPDTVLKFNSSFKNKKLKFNINHTIKTDVKKEDRETNTAIEEDRKLAIQACV
VRVMKMRKTRQHQDLIQEVIQTLQTRFKPSVPKIKQAIEHLIEKDYIERMDNNQTYQ
>Cfra_g5700
MMDKQTRSRLQSVIEAETITNHLKSIVSMEKGGVVNLLSEHRLKPLQELFNVLKRVPRGTEEIRVCLLEHLKKIGTRLVD
SMIEPLPFIEQLIDLKLLYYNTVMVAFDSQTDFTDGVQKCFSDIVNQNKRTPEYLSLYLDDALRKRFRTMTDKEVEDTLK
NSLGIFRFLEEKDLFERYYKIHLAKRLLGGKTTNEEYERTMVTKLKTESGTHYTSKLDQMFKDMSISEEINKEYANYAVT
QTSTTSHATEGKSEDLSTPPLVPKMNFEVKVVTLGAWPVSDLPSCILPSEFERARDGWESFYAKKHSGRKLMWAYNHGTV
EIMAKFNRNKHTLVMTTMQGVIMSLFNSLAEGESYTYEELLSQTGAPEADLKRALQSLACNKSRVLTKTPKKSREINPGD
EFSVFLEFKSKFHTIRIPNMSAPSQESGPTNEKTRQTITENRKHMVDAAVVRVMKSRKTHRHSDLVRDVTQALQMRFVPT
PVMIKTRIEYLIEREYLKRSPQDHSVYNYVA
>Cfra_g6628
MRRSASGRHSMSGDRKKMNIIRPYRGYQANVGVEQAENSWRLLKSAIEKIHIQDASGLSYEELYRNAYNMVLHKHGALLY
KGLVDVVTEHLAGVSKAVAAAAENGAKVDFLPSLNSQWKLHRTSMFMVRDILMYLDKQYVSKQNDRSIVSVYELGLQLFR
EQVIEFPQNKTFIKECLLEMIERERSNELTDRSLFKD
>Contig57815_Abeoforma_whisleri_fr2
ENASELSFENLYRNAYILVIYSYGNILYDGVRNVVTEHLCDVSAQVEAAVNDNFLENLNEKWTKHTAAMKMIKDILMFMD
RHYVVNAGVKEVYDLGLELFHEKVISSDKIKSTLSKTITDLISRERAGEVIDSSLMRNILAMLMQLGMKHDGIYKEIFEE
TFLLNSIKYYKVESNLFISNHNASAYLKKVETWIHNEADRVYRYMDKSTEPKLRSIIERELIFVHTETFLQMEDSGIVYM
LKHNQKSDLERMYRLLSRVQGGLIVMCAALKNHLKEIGKAHVDNYDRKNPIGFVTTLLQIKDKFDILLHGPFQNDAEFLK
GQNEAFEYFLNLNQSSPEFLSMYIDDILKKGSKGSNEEEVETTLDKVIQLFRFLEEKDLFERYYKQHLAKRLLNQKTASN
DVEIYMITKLKSECGYQFTTKLEVMFKDIDISSELSSNFSEQNPLAYDFNVRVLAKSHWPVSTETYSLLLPQALQNTCEG
FEKFYVNNHSGRKLQWNHSVSSAELKICFAPRVVRQIAMSTSQMCVFLAFTHVNKLSYEELIQTTQLEPDKLDKTLSSLS
TSKYAFLIAERDSEDNFVSMTFNTKFTCKQRRLRAPAEKSRKPEHSEERKKTEDDRRQYIEAAIVRVMKTRKTSEHSQLV
SEVVSQLQSRFKPSLQVIKRCIGSLIEREYIARHETNPRIYNYIT*SPIEEEENT*KSSFVKVEKGKFYFIFFYYSSRPW
PAISMASIFKEKKGGKKKKKRH
>Contig2436_Abeoforma_whisleri_fr3

QTGSEYEKEIRTLELLKIKFSEELFSKCSVMLDDMADSKRLNTMISSRQREGDLGLDVIILSKLFWPSLNEEEFSLPLAI
KRKMDEYNQSFQRIKAARSLTYMPNLGKVNLELSFEDREVNVTVSPFLASMIVLFEEQGKWSLKEIANKMEVTTTIIRKR
IPFWINEGVLHETEEGVFEIIEIKQNTVDQSKVLYVEDSAAASAEDQESEDLERCWQFTYGMLTNLGSLPFERIHSMLSM
CVGGIVPSQSRLTSFFMSKVGEGLLEFSAGEYSLP
>Contig64987_Abeoforma_whisleri_fr3
YSEHYIRKVIQRDKEANVKHGGKRMLDSRESTEMNPKYWQDATDTFEVLLATVPPRDVTIPEYLDIMTKCGTVSSPSRGE
SQQEGDAKRDFYYKVTNFIKEYVLNITRDFEALQGDVLLVTFSKVWERYRFGVKIINDILKQALNTLFQNNQEGDDAQNL
RNIFEEGVYIWQTELLDHFKSKIVTATMHAITEDRQGNESCTKKVKGVIESIYTCSRGDQQYFCDFFQTTFINETKDYYK
KISESFLAENSVAEYLFKAEKWIAGEHNRVEAFLVASVEEPLMTCLYDALVVDHLSTLTSAFLVFLQSSQLAELTSLYNL
LSKPKCLEELWPVYESHVQRLLEEGINSLLPPENEKDSEDLATNYVAHFASVYRTQTSLIKQCVMNNSDAYQCRDKAFKV
VVNKNAITARAKGSTRSAQLVAVHVDKILKANQQLTDEQNDEQLNDIINVFRYLDDRDVFAKYYSTHLSGRLLKSTSTSD
SLEQSMISKLQLECGLDYTNKLLKMFTDVDSSNSMYSEFKSILSEENKELFKICPRILVLSSNYWQALKCLDSFSPPQQC
MDIVGVFENYYSSINSSKKLTWIHNHANGELRAKYLARPLDLQCSTVQMAILLGFEKSITLSFQNLQEITLLPEDILEGN
MKLLLKSHIIILGEPEAKEEASESKSKSKKRGKKGSSSSSSSENVEIDSNTLFSLDVTYSSRRKKLNINRNVPMRASADS
EKTEDFETSRKLQTQAAMVRIMKMRRECTHKQLLVETSKQLASRFPQTVKRQKRAIEYLLQEEYMERIENNSYRYLA
>Contig71973_Abeoforma_whisleri_fr3
VLDLIYIL*SKRRLSLCSLLHFENMHVNLNANLRPKKVDFEVLWERLHKQIVCIMKAKEIDPPISRTEMMEDVYSACVAH
PSPYSEELYRKIEIFLENHVLELYKDVNSKSLLTKYAAKWEIFQQGVE
>Contig33076_Abeoforma_whisleri_fr4
ECLNQIFNYLNKNWILVKQSDCNSFMEVHNQKSPIEIELLSLKKWRTLLFDPLKDRLLREILQEIENDRNGKMVNHSVVK
GVIGSYVQLNPNNPLMDLYITKFEEPFLAATTEYYRNESSLFVSDNSFSSFMKKACSRLEEEEIRARKFIDPYSFNKLIR
AVEKQWIKEHQSLFEHECEVYIEQEAFDDLSRLYQLLSRVPSLIEPLSDIFQALLVKKGPPKVQALLSSSDFTPTTYIEL
IQELHGIYSHLCQNAFNGDSHFIAALDKAFGEIINMSVNGKGSRASELIAKYCDSLLKKNNKRDASVDLEDKLTNVIVVF
NYVEDKDIFQKFYSRMLAKRLIQGQSVSHEAEATMISLIKQACGYEYTAKLQRMYTDIGLSNDMMGAFKAKLKEEDPRTE
SKTDFNILVLGQAAWPVVPQNVPFKLPNAIDESLQIFTEFYTMKHTGRKLTWLPHLSKCDLKMQYLKRRYEVSCSHYQMA
VLLMFNEVDSLSLEEVESVTQMNGPFLEKIMKSLVSTKIITSPEKGHFVINKSFTSKRLKFKISSGGSGSGESSKQQEEQ
EENESTMQAVVSDRKIMVQATIVRIMKTRRSSTHKDLVMEVISQTSLRFKPPVSLIKKCIEILIDKQYLARAEGSRDVYE
YIT*KKRKKLGGNIFNR*RCCRKQE
>Contig34844_Abeoforma_whisleri_fr6
ASLKRKSESLDILQFLKQMDLTWQSHCRSMRATRSIFLFLDRRYVLQQSKTIPSVWDLGLEIFRDHVFNVGNFKKKTISG
ILQLVLKERNGIVVERQLIKNLVAMIISLKMYSTEFQDPFIAASREYYSQDGKASIVSKDTQDYLYYVENRIKEENARVD
HYLDGSTKKTLINVVEKQLLEMHTQSLIKKGFVTMVVDQRYKELKLMYRLYQRVFATIELKKKFSEYIKDTGVEILEEET
QSTVVQRLLDAKTEIDRIYEDCFLENPIFKSAIKESFEHVINTKPVLSAQLIAKFIDKKLKTGTKDLSEEELGLLMDRVM
VLFRYIQSKDVFEAFYKSNLAKRLLLGKSASADAERSMLSKLKQECGVGFTHKLEVMFKDVDISRDIMSDFNQSPYAIGS
TKSFNVTVLTTGQWPSYSISPSLKIPPHMDDKQKQFESYYAKKYANRVLKWCSGNDHCSLTATYIQAKTSKDTTKEFFVS
LYQSLILLLFNDADEISFNDILTRTGIEEEELKSSLLSLACGKVKVLRKLPKGGKVLSSDSFTYNADFKSKSRKIRINQI
QIKETNQEIVETTEKVFQDRLYQVDAAIVRIMKTRRELSHTALISECIQQLNFPAKVSDLKKQVESLIEREYLERHPDKS
SCYRYLA*LKELMSFFSLLVIVFLIM*LCFAGEGEVN*SHCYIF*IGWCRSIKVFVIVLFSLHQRKTKKSQKKAKKKKSK
RGGSQKKKKEKK
>Contig34845_Abeoforma_whisleri_fr6
ASLKRKSESLDILQFLKQMDLTWQSHCRSMRATRSIFLFLDRRYVLQQSKTIPSVWDLGLEIFRDHVFNVGNFKKKTISG
ILQLVLKERNGIVVERQLIKNLVAMIISLKMYSTEFQDPFIAASREYYSQDGKASIVSKDTQDYLYYVENRIKEENARVD
HYLDGSTKKTLINVVEKQLLEMHTQSLIKKGFVTMVVDQRYKELKLMYRLYQRVFATIELKKKFSEYIKDTGVEILEEET
QSTVVQRLLDAKTEIDRIYEDCFLENPIFKSAIKESFEHVINTKPVLSAQLIAKFIDKKLKTGTKDLSEEELGLLMDRVM
VLFRYIQSKDVFEAFYKSNLAKRLLLGKSASADAERSMLSKLKQECGVGFTHKLEVMFKDVDISRDIMSDFNQSPYAIGS
TKSFNVTVLTTGQWPSYSISPSLKIPPHMDDKQKQFESYYAKKYANRVLKWCSGNDHCSLTATYIQAKTSKDTTYSTEMT
RGLRCKRSFKMTQIAYRISKGIDGKNREP
>Contig80451_Pirum_gemmata_fr1
NKTKQNKMDFRANRGALTDTQQQPSGKRMGFKTSSGARKSGSSSVKTLSIKNFKASKPALPANYKETSWATLEKAIESIH
NAQDIHISLEELYRLVESMCTHQMSTFVYGKLEVAISEHTKAVADSLGVKNDSLGNVDFLQHISQAWNGHCREMRATRSI
FLFLDRTFVIQMTGVDSIWDLGLKIFLKHIAGNVTFTKKIVSGLLELIENERKGVTIERGLVKTLIRMFISLKVYKDIIQ
KPFLDATEEFYRNEGQCCIKNKDTHGYLHFVSNRLSEENERVLHYLESSTRKPLIKVVEKELLETYSGILLERGFGSFMG
DQRFDDLKLMYELL
>Contig18698_Pirum_gemmata_fr2
FGFGFGFVS*LINSLQQSLQQLK**LTMQNAHSSKNCHLKPKPINFDKLWTRLHSRIVCVMKAQDCVPKISRMEMFEDVY
AASVANPPHYQALYKRLTEYLTSHVLELKEEIVQGNLIVEYDAKWKVYKIGADCLNHIFSYFNNNYVRKNKQKANSDPFM
ANMANSKQPPLLEIYDLCLVKWREHLFDAIRERLLKDILMEIENDRNGKLVNYSAAQGVIDSFVHINPPDEVPMQLYTSV
FEDVFLK
>Contig76153_Pirum_gemmata_fr2
KETAEYYKNESKLFVADNKFSSYMKKATQRLEEEELRTRKFIDPNSYNKLMKVCEKQLIENHQSLFQTEIVGYFVREDID
DLSRLYCLVKRVPAMIDPLCETIQQYLSTKGLFDDVKRLLASVDFTPKLYVELLLSLHAKFSSFCLK
>Contig3173_Pirum_gemmata_fr4

LLNRVDALKDLRKYFNVYVKKKGTELVGDTEREKEMVNDLLQFKSELDNVLKVSFNLNPNFKNTLKDSFESAINSRANKP
AELIAKYIDQKLRTGTKEISEENLDCLLDKVMVLFRCIQGKDVFEAFYKKDLAKRLLLGRSASIDAEVSMLSKLKQECGA
NFTYKLEGMFKDVENSKKILANFLQSKFYDPSFKNFSASVLTTGQWPPYPSNNVIILPELVLSFRKKFEEFYTSTNANRL
LTWVPTLEQCTLKAQFGKEQQTSKEVKELSVSCNQALVLLLFNNDEELTYEQVLAKTNIDEKNLKIVLITLSCGKAKVLK
KEPKGTDIPPGCKFIFRSDFKHKYKKIRINQIQAKETKQEHDDTAQKVFQDRQWQVDAAVVRIMKMRKTLSHTSLMTELI
HQLKFPAKGTDLKKRIETLIDREYIKRDETNKSIYHYED*KQK*QSIRNSTSAAQQHYSSIYKMKNGFCFESKNHS*ASS
IQWFPYSFPNNNNNN
>Contig3174_Pirum_gemmata_fr4
LLNRVDALKDLRKYFNVYVKKKGTELVGDTEREKEMVNDLLQFKSELDNVLKVSFNLNPNFKNTLKDSFESAINSRANKP
AELIAKYIDQKLRTGTKEISEENLDCLLDKVMVLFRCIQGKDVFEAFYKKDLAKRLLLGRSASIDAEVSMLSKLKQECGA
NFTYKLEGMFKDVENSKKILANFLQVTNNKQQNNKTKQQNKT
>Contig75186_Pirum_gemmata_fr4
FIINIINSVIVGFFRSFSFFFF*SAVFHFFIHTNSLLMQSDNILTNEQQWESLKSTFDKLLGEELQTLSISEYFSRVSEI
YNFCFSKEEDQSLAPCSKQKLLYDKTKQYFVDYSKRIFAAISQQSGEKLLQQYTKYWLRYKFALKLMNRILSHLNNTYVK
NNVREADSDCVEIYALGMIMWRDHLLDPLKSLITPAFKDAIRADRDGIESCTMKVKHAIESYTNLNRTERKNEYYKENFE
KFFIEETQNYYSLHVTKFLEENSIAQYLGKAESWLSAEHNRLQAFLPTSTEGILFEKMHDVLVRQQMEKLCSTFLLLLES
WRLSEIGALYRLTSLIPQDGLENLWPTYHSFIQKSLEEGIERITPPADEREPTELCGNYVEFISEIWHKHRDMIKECFRD
DSSAVKSRDVAFKTVINDNAITK
>Contig78704_Pirum_gemmata_fr4
FSSFCLKAFDNDSSFVAALDRAFGIVINTPSEQKDGKASELIAKYCDSLLKKANKHTSSNELEEKLTNVITAFKYVEDKD
VFQKFYSRMLAKRLIHDNSISHEAEVQMISLLKQSCGYEYTTKLQRMYTDIELSEKMKVSFKNHLIETNVHTKQDINILV
LGSGAWPITAQNFEFKNHQVIEEMQSRFKAFYSSQHTGRKLQWLPHMSKCHLQMNYLKRRYEVQVTHYQMSVLVMFNDSD
TVNKDDVETETLLEGKQLNNVLNSLVSAKLITKSPENPNSFCLNKTFSRKKGGVKFKIPANVSASDSTQEEANVTRDLII
ADRKIMVQATIVRIMKARKNASHKNLVSEVIAQTTLKFKPSVSLIKKCIESLIDGLYIKRTDGSRDVYEYIS*KC*TTFV
RCIFVKGGHFKSYFCSIFVRKGHISS*MYPL*WFF*YNFYKEKHNFVLKLSVGDIHK*KSHCFFSFLIKIVSVRIRVRV
>Contig57600_Pirum_gemmata_fr5
LLLIIIGC*CLCLVKYLFSYFKFFY*TFNMKSHGARIAPFQKATNSMDENQAKETWKLLEKAIKEVQNQNSSGLSFEELY
RNGYTMVLYNKGDILYEGVQKVVTEHLKEVSGKINAAINDNFLEKVNEEWNKHTAAMVMIRDFLMFMDRMYLKSFPELLP
VYDLGLKIFYNVVVCDPKAKTLLCNLLLDSIEKERNGEIINRPLMKNVLSMLTQLNIRESHVYELFFEKDFIATSIEYYR
IESNKYISGHNALDYLKQVEKWLSNEVERVFHYMDIGTEAKIKTVVEQELIGNHMSGIIEMPGSGMIYMLENNRTEDLDR
LYKLCSRVENGLPALCTCMNEHLKEKGKQHVESHDTKGPLGFVNYLLGLQDKYNNLLATAFNNDNDFRTSIYSAFEYFIN
LCETSPENMSLFIDDVLLKGVKGYNEQQLEDLLNKVVSIFRFLEDKDLFERYYKQHLAKRLLSGKSLSIEAERYMITKLR
SECGYQFTSKLEGMFKDIDISREAAETFSNEHKESLEFFMNVRVLTQSNWPVNLNPISITLHPLFENACQHFTAFYINEH
SGRKLNWLHSLGQVDVRANFSTKQKKQITMTAAQASVLLNFNENDELSFSDLCKILNNSEDFVRKTILTLITSKYPILIK
KVNKTESSSSTAITAITPTTPT
>Contig57601_Pirum_gemmata_fr5
KLFKMYPFTYFNRSQMPGSGMIYMLENNRTEDLDRLYKLCSRVENGLPALCTCMNEHLKEKGKQHVESHDTKGPLGFVNY
LLGLQDKYNNLLATAFNNDNDFRTSIYSAFEYFINLCETSPENMSLFIDDVLLKGVKGYNEQQLEDLLNKVVSIFRFLED
KDLFERYYKQHLAKRLLSGKSLSIEAERYMITKLRSECGYQFTSKLEGMFKDIDISREAAETFSNEHKESLEFFMNVRVL
TQSNWPVNLNPISITLHPLFENACQHFTAFYINEHSGRKLNWLHSLGQVDVRANFSTKQKKQITMTAAQASVLLNFNEND
ELSFSDLCKILNNSEDFVRKTILTLITSKYPILIKKVNKTESSSSTAITAITPTTPT
>Contig73138_Pirum_gemmata_fr5
DLQLLTKGICKSIEVLNKFQLVDQYQCHQQWKSEVFLNHSELFYPVCRCFLSRSFEAYALLNVSKSEISKENDSESTEEC
IVLTEREAIKTMQTLVEIVEVLSQLDLLNRVFDLCWSSIAHKAIISHTNLFRGRFDEVFLPSLLSWVDSAVLGWVQHLLG
NGVQRSQMHKQWQSRLQYFTYKTFADMRINEIFDIIVDYDNVDFQSKPALLDLKECLFHSDQRLQLIDHLKLQNKRRLLH
RGADTSDIITNFQQIIAALRLLDPTEMMLKAVQGEVQSYLRSRPDTTRCVISSLINDEDEVMQGLLNDLPHERSEIDDDK
WEPEPRFTCDHDFSEVSIYGSKESFVNDYHSILGEVLLSLPNYDTEKEIRNLELLKLRFGEEIFQKCTVMLEDIADSKRI
NSIIETQCMAKYDDFPFHSLILSKLFWPAINDDMFTPPLKVQKWMQTFNETYQKIKAARNLSYMSNFGKVTLELTFKDRS
VDVSVSPFLASLILLFEEKERWHIDELASSLQVSLSIIKKRIPFWINEGVLKEVEESVFEIVEEQGEHGGQDQVLFLEDD
GTTLTNDNQENEILERCWQFTTGMLTNLGPLTFERIHSMLSMCVGGTVPSQTRLKSFLQKKVS
>Contig10821_Pirum_gemmata_fr6
ITKQKSGSQRSASLVAKHCDKLLRKTDVADEAQENRSFDEVMIIFRYLVDVDVFMMYYQKLFSTRLLENASASITLERGM
ISRLNIECGMDYTSKLTRMLQDYETSMNSYSEYKRGLTGETKELFSICNRILILGSAQWPLSNLDTEMNIPNICKSIMDQ
YTGYYNKVNQGKRLQWLYQQCVGELRTKFTAKPIDLTCSTTQISILMCFDQNTKLAFHKLKELTLLNDNILEANMKLLLR
TRLISLEEEGEDEEAGAASGQASPAVASPAVGDKKDSKKRKRDKRQERKEKRKTRDEAEITTNTVFCLNLKFTNKKGKKL
NINRNITYTEKKGPVKSSSEQEEITTQRKLATNAAMVRIMKMRGICSHTELLRETTTQMIGRFPQVPRMQKKSIEWLIQE
KYIERAKDNDGTAVYKYLA
>Apar_comp21677_c0_seq1_fr4
CVCACVCVRARYTCTVEPVTQSFMLI*QQLET*GVAYYTAGIGRRQKVYCA*LGVAVG*ARAVQNKQRANSSGQHTQVGP
TGRHLRKQLVSCRVPS*GVRGPRHIPCRRGR*PTPQAACPGSCVCAQPQRAKGTMSKATLTRQRVDFEKTWASVRHNVEA
VITCQGVDKVSWMDVYTDIYKLCTANPSPYYEALYSRLKEFLQKHTERIREEVLAGDNLLAAYNDKWRVFQVGAECLQAI
FQYLNKNWVEKRRKDADSARTTLYTPSLTPAASEPPMEVAQLALHIWKVTLLDPPIKKKLVHDILREIHNDRNGEMVTDS

VVQGVIQSFVHMNKFNTKKPEELYQETFERPFLEATAEYYKRESALFLADNDCAAYMRKAEQRLKEEELRSQKFIDPSSY
KKLQQECEAQLIEAHKAIMEAECETYIRSNRLEELSRMYKLLKRIVGGLDPMLAVFQKYVTDAGRHEMAAIPTLEPQTYV
EALLAIHTKYSSLVKNAFDSDANFVASLDKACRCIVNFNGGAKGGPARSPELLAKYIDQLLKKSSRSGPEGDVDEQLGHI
MTVFKYVDDKDVFQKFYSRMLAKRLIHALSVSDEAEEAMISHLKQACGFEYTSKLQRMFTDMRVSADINTEFKEQRGTQL
GTDFHILVLQAGAWPLSQAGATSFSIPQELERCVQSFELFYGDKYTGRKLNWLHHLSKADIKVNYLKKRYELQATNYQMA
LLLMYNNADSYTRSEMEAQTQLSGSELDRTIESLLSVKLLTSQGDRYHLNMKFSNKRTKLKITSAVPQETQKESEQTHNQ
VSDDRNMYLQAAVVRTMKARKTLTHTELVQEVLTQAKARFIPSVPLIKKCIEQLIDKQYLERIEGSRDKYSYVA*LLFAY
VHIGR*MGVRVQICRARACLAMLTEQQQLPTLPSFGCRMLCERSLVVAM*YGKWSFAGNSGGWCVRCRMQRYI*GLHLCV
THCQVVRQCRHTWHVW*TDNSQ*A
>Apar_comp22032_c0_seq1_fr4
VLNLYPCMSHMCQVGSFWDLFNLKSHFWGLHT*PFLSQVESVREQRVRSVFGRKAKLEGGQKHRFWRAQMEE*TQSLLVN
INRI*PSISFSGSCFLPL**EGCFGFAANPKGHKASL*CLGTLFGSVDGKKAKSPFCCSACYLHGKESDTDNKLRDNLST
YTRWAEGLIISSVESTRRGICTTFS*IQ*ISTFVVRTHQPRSATPAQGMAGRAPGGVMPGGNGPAVRRRSSATDRPVKKL
VIKNFDAHAAPRLPANYESDTWQKLQQAVSAVHASKAVGMSLEELYQMVENLCNHKMAAGLYTKLRDVLDAHICSQLEPL
IQSDLNDTDFLTLIDNCWQDHCRHMLMIRSIFLYLDRTYVLQTSGIITLWDLGLHLFLQHIVSNVEVQHKTLAGILQLIE
TERLGNAVDRSLLKSLLRMLSSLQIYGKVFETPFITASQRFYANEGRQKMQELNVHGYLRHVESRLHEEGQRVLHYLDRA
TRRPLIHVLEAQLLKPHTGPLLEKGFEQLVDDDRYDDLSLLYSLLGRISAHDEIRTFFAAYVRTKGTALVMNTERDATMV
QDLLDFKAKLDRIVRDSFQNNERFRNAVKDALEHVINARPNKPAELIAKFIDSRLRSGNKESSDEELDQLLDNVMLLFRF
IQGKDIFEAFYKKDLAKRLLLQKSASADLERSMLSKLKQECGGGFTHKLEGMFKDVDISKDIMVSFEQSKFRNRTSKIEL
YVNVLTTGQWPTYQLDPEVIIPQEMTKYLAAFREFYLAKHTGRVLNYCHSLGGCTVRSVFNEGVKELSVSFYQALVLLLF
NDANELSYESILAKTKIEEGELKRTLQSLACGKPGTRILIKSPKGRDVNAGDRFVFFGDFKHKLTRIKINQIQIKETHEE
NKETQEQVFQDRQYQVDAAIVRIMKTRKTLSHTLLIAELFEQLKFPIKPQDLKKRIESLIDREYLERDENNSSIYHYLA*
SGDCKLKFRNTGIYVCVCSEKGCATESMYGIFSKFESCFGLGCWLLCLVEEGQNGTLGGLESAGCHSFIFSFLLLSCFSP
GS*ESG
>Apar_comp22586_c0_seq1_fr4
GVSNLVCLVRYLSDTDVFFRFYGMYYARRVLGGSSAGEEYEQCVLGMFKQQLHTDLHRSTGKLERMAQDVAANQALALNF
QAHVAQATAVPLLEFNAMATAVPRLEFNAMVLCSAAWPLKESGEGFVVPVAVSEFVTMFTNFYASQHSGRQLRWLHHHSR
ADVLFWPTQGSTRPYQLAATAYQAAVLLLFNDPPASTHTDKRQRTTPTRSLEDIAQETGLGADTVSCVVQSLVSAGMLVA
HGVDEFAPNGNFSNKRAKMNIAKAQTHAERQSKKGDEREAEAAMEQVLRGRRMELQATIVRIMKARQTLPHSDLVAQVLE
QTRRFQATLGFVKTCIGLCLDSEYMERVPGDIDTYRYMA*SELEAAVGGGFKIVCNQERHVDTLAVVKTRVAERLGEKCQ
KFMYIKWVRFYA*MKTT*CNIWANGTFMMQVLYLNVF*HTSKQVFFVDA*MC*FV*RYFAIALYAYNI*VSTLIFIYHNI
SM*MAIYLSIVSTHVGAHTHAHTRRTRTHTHT
>Apar_comp23739_c1_seq1_fr4
GRQAGRQD*GAIAAVH*G*IAAVHRQIIKVVSGQADRLTGRRASRKAGRQGR*AAGWQEACFFGSRKRVNSYRHFSGSLF
SGAG*NGGGQLGKQKCFVQNRADRAGAPCLLGLHLQHSPGSGPAAVASGRWRGSMANRIKPYRAQTAQQDPGMAEEAWRL
LRDAIVKIQHQDASQLSFEELYRNAYNMVLHKKGERLYKGLHEVVSAHLQTVAADLEGAIEGDLLGRMRTHWDRHKMAML
MVRDILMYMDKHYVSTAGVAPVYEAGLTLFLERVVGNGRIKASLTSTLLGLVARERGSEVVDRALVRNILAMLAELGISS
RAVYEDVFERHMLDATAQHYRLASAQFLAQNNAATYLRQVERWLADELERVEHYMLPETEPKLRGVLDAELISRHMQQVI
AMEHSGMVFMMENNRLEDLQLLYRLVQRGGEGVDRLCGRLGGHLRPLGEALGGRGG
>Apar_comp74147_c0_seq1_fr5
TLGGSPFTTPF*KMLGDMSVSRDLAAQFKDACRAAETRVVDLSVHVLTSSLWPVAAEGGENAACVYPEQAQAALTGFEQF
YAKR
>Apar_comp17682_c0_seq1_fr6
DGQKLGP*R*HKRILRMERSCTRRGRICTAKRDARGFFILFPSTYIYVLFSC*KN*VG*EEYCEVELQHRS*LCIEPRGG
EHYHLLKRQL*RKEDCAMASPGADLSTAWTGAVQLLQPDIDQGPLENGTHATLSSYVHAVKSAGMLALLQAWYEDTYQSF
LLQKLLPDFLSAFQAQVAQMAEETIERELSTDGMLVSFSGLSCAKARDEQARQMLELPQNMFLQDQLSTYELPPSAHLTL
PKYFMHVAEAHRRRAEENAGIQPLDEQEEGEEEEKEEKSQNDGRILGDFATWRDHLLELRLWERMFSNFWMEALCEIIES
YVRKKCTGNFENEYLPELREWESAVVHTWIDTCILNNDTEARQRARRQAHFSLMKSLADLRISEMFPIIAGYDETEPALI
DLRECMKVTNQRDQLVASLQKVVDTRLLHPGANTADIITLYISAIKALRLLDPSQVVLHQICDGIQWYLREREDTVRCVV
KSLTEEEGQSDLIGEIGSGPKIDDDRASDDEPDAGEDPEKWEPDPVEAKEHTGRWRNSDIISNLVGIYGSKELFVSEYRV
LLADRLLSALDYEIDKETRTVELLKLRFGEASLHYCEVMLKDVTDSRRINNNVHFTHKQKFDEEMPVHVTCISRLFWPQL
KDETFVLPEAIIKYIDRYNKGYTTLKAARRLDIKPHYGKVSLELTFEDRTLQVTVAPLAATIICLFETQATWTMDELAAK
IEATPSALRPHLAFWVSQGVLEESPAGTYTAMEIAGGAQKTRSRPVYAAEEEGSPAGKQRNEDMQMYMQFITGMLTNLGS
LPADRIHTMLGMFAGGSLPSQQQLKSYLDQQVANDVLEYSMGEYRLKQ*HDIAPYLENFRSAI*MI*IYNTTDNCGEGNE
NMSSCDVIVEALAVHING*HS**VHVFIIIIIIMIIEIYHDGFV*VAEYGHYE*TSMHTAAQ*LAGVKF*SSA
>Apar_comp17682_c0_seq2_fr6
DGQKLGP*R*HKRILRMERSCTRRGRICTAKRDARGFFILFPSTYIYVLFSC*KN*VG*EEYCEVELQHRS*LCIEPRGD
CAMASPGADLSTAWTGAVQLLQPDIDQGPLENGTHATLSSYVHAVKSAGMLALLQAWYEDTYQSFLLQKLLPDFLSAFQA
QVAQMAEETIERELSTDGMLVSFSGLSCAKARDEQARQMLELPQNMFLQDQLSTYELPPSAHLTLPKYFMHVAEAHRRRA
EENAGIQPLDEQEEGEEEEKEEKSQNDGRILGDFATWRDHLLELRLWERMFSNFWMEALCEIIESYVRKKCTGNFENEYL
PELREWESAVVHTWIDTCILNNDTEARQRARRQAHFSLMKSLADLRISEMFPIIAGYDETEPALIDLRECMKVTNQRDQL
VASLQKVVDTRLLHPGANTADIITLYISAIKALRLLDPSQVVLHQICDGIQWYLREREDTVRCVVKSLTEEEGQSDLIGE

IGSGPKIDDDRASDDEPDAGEDPEKWEPDPVEAKEHTGRWRNSDIISNLVGIYGSKELFVSEYRVLLADRLLSALDYEID
KETRTVELLKLRFGEASLHYCEVMLKDVTDSRRINNNVHFTHKQKFDEEMPVHVTCISRLFWPQLKDETFVLPEAIIKYI
DRYNKGYTTLKAARRLDIKPHYGKVSLELTFEDRTLQVTVAPLAATIICLFETQATWTMDELAAKIEATPSALRPHLAFW
VSQGVLEESPAGTYTAMEIAGGAQKTRSRPVYAAEEEGSPAGKQRNEDMQMYMQFITGMLTNLGSLPADRIHTMLGMFAG
GSLPSQQQLKSYLDQQVANDVLEYSMGEYRLKQ*HDIAPYLENFRSAI*MI*IYNTTDNCGEGNENMSSCDVIVEALAVH
ING*HS**VHVFIIIIIIMIIEIYHDGFV*VAEYGHYE*TSMHTAAQ*LAGVKF*SSA
>Apar_comp18734_c0_seq1_fr6
KL*QRGCGCSRVHGKNYCRLW*KWKAKTASV*MFCRCGFSS*DLGHPPTASPCYTLKQKKKRRGVAW*ERWGLETNRAG*
GGGKGRRECRW*GCEGPGLAENARMNARTQDPKALWATIEAGVTRMVERLEDGLSPTEYMEFYTAVYNYCTVQSNPPSYG
VGGSAGTSQTNMAGVELYKQLKVYLDRRLNAVLKEMDGLMQESLLEVYVEQWEKFARSVKCVNGAFTYLNRHWVKREKEE
GNPVHEVHTLCFVTWRDIVFQTLQTKLTSAMLELIRRERNGDTINNSLLKGTVSCYVALGIADGPEKSSNLGIYEHAFQK
DFLESTELYYKAESDSFISNNSVSEYMKKVERRLEEEEHRVLASLHDTTREPLMERLDQVLIIKHFDVIKAEFGKLLALE
KNEDLARMYKLLARTKEKLDEVKNVLEEHVCNTGMQAIERVAQGAAEDPKMYVDTLLEIHTKYDRLVQVAFAKDSKFVSA
LDKACRKFVNENAVTKHFKTAQKSPELVARYCDMLLKKSAKNPEENELEELLNKILVVFKYIEDKDVFQKYYSKMLAKRL
VGATSVSDDMEASMISKLKITCGYEYVSKLQRMVQDIDVSKSLNERFQQHLASSNLDRKGMDFEIKVLSTASWPLNSPVT
PFNLPTELEKWVERFKLFYATQHNGRKLTWLYHNSKGEVTTTYLKAKYTIQASTYQMGILLLYNNQKEYTWSELQTHTGL
NDSHVDGIMEIMVKAKILLSDCDSEASFTPATKFTLNEGYKNKRLKVNINAPIKSEIKQEQEEAKTNIDEDRKIVIQAAV
VRIMKMRKNLNHTTLITEVIEQLQGRFSPKVPLIKKCIDILIEKEYLARQEGQKDMYVYMA*LGICWDCVGFSMLQNHFS
PRDFTRVAHSCFQFPFQLHIFAGPFPPLHINDMQVGQFESCFMTYSYFFYRFITVCT*EISTLHVKLCSFHTWMYLGSVF
CSVSKHPTMLLQPWRLCMIICTYMP*QQERKRIQVIHMTLIEVKMQTSP*KKKKK
>Apar_comp23688_c1_seq1_fr6
SFQKLEMSRNVVLVGL*MKHKISMEKY*VWRVGAANEVAASIGRGHFQFGHFSFPPADGVMFKVKGSFLSYMPVFSEA*G
NSFFCAWCTLIT*WYARERKRDRHSLGLHGALF*PEQQEGT*EHTH*TGRNFNEEGQRMKMLAISLDDYREVYWPKLLQR
LELILAREPGSRAASFSHEEAYRMVYNVCCQSHHRSLYSDLKNTVSLYLRQVAETVASTPDDQFLPCYAAYVEHYMACID
VLCPVFLYLERTYLQAKLQTSLHYELALLFRECVMGHPLLRSRLSVLMRTIADTSPWAELPGLDVAFRSMHALDPGFAQQ
YPILLARFLSSNNMAGAGVGSAGRWGLARQKREGEEMDMYDQGRNSKRALLDSQPHLGVSVIMD*ISENWGQGGSKLKGM
NYLENEFECKRRFGLDVHTRFRLPDT*DS*TSLE*DLHAIMLGKGRHNLQERICVQKGMNTEFGACVYDCGCRRALGKLL
*MNQYLA*EM*LDIIHMYASCAVVHVNGRRKTLSECFSFT*FLVMPVQ*FRTRFAWLLFYLTHIITLEFLMIFFNFFPT*
TQSLLYVAVMATVDTFLPVLTIYIMRCLPDLKSGIWRGPKAIL*GIKNHRNVA*LFL*HQNR*IFLRY**KSLK*LYI**
*EN*NIVP*SWAREGVRTRGFEIKRKKPAF*ESTVNTLG*MT
>Apar_comp23739_c0_seq1_fr6
DELRERGTEGLHSMCDCLGQHLRTVGKALVTSEEAGKDPNKYVGDLLALKDTYDTLLKGAFASDPKFTQTLSAAFTQFIN
LNSKSPEFMSLFIDGQLKKGAKGASEDDMDGLLERTIMLFRYLEDKDVFERYYKNHLAKRLLQQRSASDDAERSMISKLR
VECGNLFTSKLEGMFKDMAISRDTMQQFRTHLADRSHTLPVDLSVQVLTTTYWPVHSEPATAILPPPLLDACKSFETYYL
GAHNGRKLTWQLQMGMADLRGQFDKAYVLSMSTYQMLVLLLFNDLGPADTLALEVIAERTGIPNGALRRALQSLACGKIK
VLAKMPKGKDVNDGDTFAINTQFTSKNIRVKIPMVAQRETEKESQDTRHRVDDDRKHVIEACVVRIMKARKTMDHNNLVA
EVTAQLQGRFLPNPQVIKQRIENLIEKEYLERNKDDRKRYHYKA*RRTT*CGAAGQLPCTYLHSAAGWPRGHPGQ*TNTV
CTHMHPQSGQ*TNTRTVCTHMQSPEWQVNKH*Y*
>Contig1933_Corallo_2_fr2
YDWRPGIRKLCNLVDSQYGS*ARTSVYKMSLKPKHIDVAQFEKRWESMEKDLSSIIELNGVQKIRWFDVYQDIYDLCTAH
PNPHQERVYKKLKKFIQDHVNNIRERIDAQQSDILSVYHQEWERFLRGSMYLDQIFGYVNKHYVMVKKQDEKVSAYSSQK
DNLMECGALALYTWKTQLLYAIKERLLSATLRYIEMDRDGKDVPSTMVRSVVSSYVKLNRYLTNHEFDLYNDEFERPFLT
ATADYYRRESTSFIEKHDIISYMKKAEQRLHEEDVRATKFIEPTSHRKVMKVCETEMIEEHSETMRQDFLGMIKREENDN
LRRLIVLLKRIDHGIDPLLVAFQQYVTEIGQQKIRGLGDQAYEPRNYVDALLEVHSKYTGMVDTALGNDPGLVAAIDKAC
RTVINDSLDKEKPLKSPELLAKYCDQLLRKGSKHGMDHDQIEEALGQLVKVFKYVDDKDIFQKFYSKLLAKRLIAGLSVS
DEAEERMISLLKQACGYEYTSKLQRMFTDMSVSSDVTNEFNDFVSDQGVQLGFNFSVSVLQTGAWPLAQASAADFKIPSA
LQQCVQLFNEFYNKKYNGRKISWLHHLSKGEIRANFAAKRYDFQVTNYQMGILLMYNDGLSYRKSVIRDTLQLEGSELDR
SLTSLLDVKLLVVDEKGTEADPMYNLNLQFNNKRTKIKITTAQLVETKKENKATHQAVEQDRKLAIQAAIVRIMKTRKEA
THKQLIQEVSEQLMSRFTPNIPMIKKCIEDLIEKAYLERVEDGRDRYRYLA*ILEEHRSNRIPNKFIH*RVSPSKRAI
>Contig6597_Corallo_2_fr2
IRKRLLHPGIDTERIAVMYIRNIKVLRLIDPTAALVDTVCEDIRRYLRRRDDTVRCIVRMITDENAARDIFGQDDDDEGD
EEDRADMQGSKDYADVNRLSINTWHKWNPLLPEALSKLKQQHFAGIPLRDRRMAHTGEMLGMLLGIYGTRENFVGEYRSL
LAERLLESMDMDTDREVRSLEMLKVHLGENNMQYCDVMLKDMVESRRMRQNICSHFQRTSRECSIDVSKMSMAILSHLFW
PTFRRDTMNLPVTIYRTMQVYEQGFKRQKPNRSLQFKPTVGMVELEVTLPDRQPKSFTVPVATAAILYMFQDANDEEVTA
AVDEMKRAESEDNVKDGITSRTGVTAATSKPEWTTQELSQTLGLAEATVRKRIAYWTRLEILKEYAPNRFCTAERESELR
RAMTIDATVGAMNEPSQSISAAEHDEDDDADDHGGALSSVRENQQEQLNLMWMFAQGMLRNLGPQSVDRLWNMVSMYVQN
SDTPVHCTQADFRQFLDVKVRQDELQVSGGLYSLGD*RVYFREA*RTLKR
>Contig291_Corallo_2_fr4
YIHGFKEAS*KIEKTKVTGIDHWTEF*TEHGASRDCNGTFRLFRRGTL*RTGCHSQQPSINYSWDIIPCSTYSIDPADMV
PAQPMAHEAMSLFDNGQNKYIYAKTSSREGNATWLKEIEPDLRSLFEDNKTGMSRATYMNVYTIIYSLCTNNASQKQPQD
SNQGAIFGGEEVYRSLSEFLKEHLQGIEKQAASLQDEGLLRYFNTEWNRYLSAAKCANHLFNYLNRHWIKRKKDELEPVY
FINTLCLVLWREEVFRPLADRISRALRMLIKNERDGETIDTSLVSQAVQCLVMIGANDDGGSASRNDERPLSIYRVIFEQ

ELLEETRKYYEAERDDFIDSNPVTEYMKKVEARLEEEQRRVQILLHPSTEEKLLKACKDVLISERIEKFYDEFKVLLEND
MNTDLARMYSL
>Contig3074_Corallo_2_fr4
KVLKFLFGKNPPEIMSMYRRHLLCCVRKSGKFDGP*DGLMSPSTTRMSNFQPPRRRLGKMAVNVTANIPQINPQHATSNW
ELLRKAIKEIQLKNPSKLSFEELYRYAYTIVLNKKGNVLYEGLQDVIREHLQQEAKKVEANLDDNTTLIRTLETVWKDHT
TAMRMIQDILMYFERAHIRKQEMLTAKELGLKIFREEVTSKSPIREQTREAMLDHIQRERLGEPMDHSTLRSIVQMLLHL
GINSRYYYEAEFELYYLERSQQHFHSESIRYIEENDVSSYLKKVENRLKEESDRVMQCLDVGTEDKVVRVVETEMIANHL
DRLLSPENSGLSYMLKNYQHDDVARMHGLLKRVDGGITRMLEVFSEYVISTGRGIVAIGQSSDMDPNEEGSSTPLSLTPK
DAIKYIGDILDFKSQTDDILEKGFDGDLTFQKNLHAAYRNVLALNQRLPEYLNMYIDDLLRKGSKLSIDEDLESLVEKCL
VIHRFLGEKDIFERYYKDHLSRRLLQDKVTNEDLEHLMISRLNTESSYAFTNKLEGMFKDIAVSNDLTDAFKRRTATTSP
FDFSVKILTMGCWPNTMQPPCTLPKEFQDALTSFENYYYNQHSGRKLSWQTNLGSVVIRCLVGLRWYELTLSTHQAVLLL
NFAGDEQLTPAELCRKTGLTQREVTGTLKAFCAGKHRLLKRVDVDGTTKYTFNTDFQSKVIKIRIPRMRDREAADADRQD
TRKKVDEDRKLTIQASIVRVMKARKHMEHSGLISEVAAQLQSRFKPDLSLIKREIEVLIDKEYLERDENNRRFYKYVA*I
PSYFSVVVT*YIV*FN*
>Contig606_Corallo_2_fr5
FRVPNGLNKCQESFQEHIIRQGNLAINANLENASTDEKKYINILLTTHRKYSELVQSAFNGDSSFVASLDKACRDFFNRN
EVTKKDATGYNTHKSPEMLAKYCDGLLRKSSKTNEEGDLEDALNDLMVIFQYMVDKDVFNKFYSKHLSARLINKMSASDD
AEESMISKLKVACGYEYVKKLQRMFQDVGTSRDLNTEFNGHLQRLEVKLDSAFEVQVLAAGSWPIGKQHTEFIPPPDLRR
ALERFEAFYNKKHSGRKLEWQYHRSKADIVARFNGMKYTITGWSYQTAILLMFNERDEITFDEIHQATKIQKPYLEKVIE
MLFRTKLLKGPEKLQDDSKIILNKDFKSKRIRINIAMALRHESKAESEETHRTIDQDRMHTVQAAIVRVMKTRKTMKHQQ
LIQEVIAQVSHHFKPNISVIKKQIDALLEKEYICRDEDSPGVYNYVA*TSRPRSVQSRFEIENPNRDTATMVTFSGSNVF
YVFEFCKGIVDLDSNDAQTVHQVFQRRDTIYGFCFSSLRCLFGLDFSTIRVGVRG*RFRSGHRVGKRMIVVVDLVQNVIQ
VKVCVVIFLNIAGRRLFILPGGD*SGSTKGHVPKVRSDLVDSIGDDRRDTRPHVGVLLLLLVVPVCAVVGSESLVLVPVG
IQSPELGQIVNAVSETTDRKILGEPWLQVPNLLLTVLVLAHPGHTHATNFRVTETQNERTRLALQEQVLCVLLADEAHNE
SSLPCCNQMALNDLFNAVEPFPEGEVAVLQRLLVELMPRANVIGPHSNLLTQVIQTVRFNFSAIYARKMFRQGGGQIVPC
DAVGNLPGHPGIDAEVVVPKNGGLSIRQLITVHNHNDGQGQRVERQRDVVGDGDRHAETHGELFFRHSLVALQITTSNLS
ETHVLEVRAQNLVALAVVHDRCVVENVTTVGQVLAIVARTGTSGNAVSCPTPLAAGTVGGNGSGNVPGGCAGQITGPQ*S
RKLHIQVAAVKALGHVSDFGKDGGGLIFIDHRSGDLWRVDGNVQYLTDEESTGRLELGLRLQYLRRLLIDQESPLNQLNE
NVQNKLAHDQDAILALGHGQLLHQHLHVPGLVLRAEHNLCELFEPGLVCGKHLVALFLSQSTQAHLFTELNGLVHHNVAL
QTRNQLNVLLQFAEQSVTELQTVLGLLAHTLLKLLVVLLQLLVVGLLFGHLLQNLLVLGSLLVLEDNIFLQLAELLNVIV
CIPSSALCGLNLVHNLLVVCGQDVLCASHGWSTLLENLLLALQGIRHDLDKAHGVIFAHLTVAGRNFFQPQTQQKAISRL
AGNLLQVLVHLLEELANLGCGLLLAVVTAGLDGLPVLFLKPFRKVVPQLDGE*VTVQFTHCRHLVHLRPGFSLGRHCVG*
DWQLDYLFLT*SFFLGLCVARFRYSNDSI*CGSTSDCPVHILTNGDRPEETFLAGCEPVVYR
>Contig3009_Corallo_2_fr6
LFCRILEKE*VGAHGQN*ERSRSL*ILVSINIIGILHRMAEVSPRGPFAKVGQLSDDPESRKRRRESLRVMNGLKMAKRQ
TNNNGPRKLVIKGLSNAPAISDDYAEKTWEKLTAAVSAVHQREPVGCSLEELYLGVENMCTHNKAEFIYEKLSNQLETHI
AGFREKLQVEMKADTDTFLSKLNSIWLEHIESMRMIRSIFLIMDRTYVLQQSKLLSTYDLGLKFFREHPFGVEEIQQRTI
RDLLALVCKERDGEDVERQLLKSVVRIFLSLRIYRDTLEPPLLKATNEYYENLANDRIQNLEVAEYLKEVDRRLNDETKR
TEFYLDMRTLRALRSAVENKMLKTHTDTLIKRGFEDLMNNKRYDILRLMYDLFGSVKAVPKLKEAFTEYLKKRGSIIVTV
EERDPTMVQDLLNLRKEMETAIKQAFASQSQFVQAMNTAFEHTINLRQNAPAQYIAKFIDEKLRSGLKELTDEELEQTLD
EVMVLFRFIHGKDMFESFYKNYLAKRLLLGRSASSDAEKSMLSKLKQECGASFTSKLEGMFKDIDVSKGIMASFNQTRGR
SLDGMELYVNVLTTGYWPTQQPQTLNLPAAMEKFRDTFTNFYLSKHGGHGGRQLTWTYQSDRCTLKARFPKGDKELQVSL
FQALILLQFNDRDTVSFKDLLSATGMEDKTLRTQLQSLACAKVRPLIKEPKGRDVEDDDVFHFNDKFENKHVKVKINQIQ
MKETKEEKENITEKVQQDRGLQVDAAIVRIMKMRKQLAHNNLIAEVYEQLKFPLKAIELKKRIESLIEREYLQRDAENSS
MYQYLA*TIIPCQIRYIKIFFDIRYQTT*L
>Spom_NP_001018806_1
MNDTDLSTFTGRSLLIDQLSSVTQGTPVLDFIDKLRIHFYTTIRQNLLKIDLKNICSLHDLTDQLSDFWLVYEQSVLESP
ILSPELDRILTCFRCLCRRYLPISVIESVLTEYLDQVLKVWLESKTNPCLDMEKFFQLCEKFKQLGLSSVLKERFVYVLQ
LHVGSLLTTRYAMSWEQSVYHEALEWIRTEFGVLVEHVFSLSNPAVLVQLDHLVSQILAHLRSDNILDIVLHYPNSLGAI
EDLRLVARQKQQRQYLTETFVKDCTSSILTASSDSSYILLFYVSTIRCFVALDPPGVLLDKAAKPIRSFLNEREDAYKCL
VSLLFVDGEKGLRSELSQIPTENIDSTTDRFDNYHWMPDPIDAAPDFKKPTDRDVVGSLISIFKSKEPLVKELQLLLADR
LLQLTDYHYEVEAKNIEFLKYRFGETVLQMCSVMLNDIENSRFIDQSIHMENYVSKGLHVTILSRLFWPTLSVRYFHLPG
PLKKELDAYAEEYRERKRKRELVFLPNLGSVELEIELEDRTLTLTVTPEQAAFISLFEETSTLHIEKAAELLDQPKEIVE
RHLKFWLHHRVLTDIGDDRYRVRETEAETATETVLDEIQGVSAVQSEAESSAAEMRVYWSFVVGMLTNLGALELERIHNM
LTMFIPPPNGYTRTQSELREFLALMIKEEKLEFTGGAYKLK
>Spom_NP_594259_1
MTTLNTNDKDLPIVKKYDSLNGTWDFLKTGVSQIFERLDEGMTITKYMELYTAIHNYCADASKTITVDNFNDQTANVLGE
ALYNNLVLYLEEYLARLRKECISQTNHEEQLAAYAKYWTRFTTSARFINHLFGYLNRYWVKLKNRFTETLVYDIYTLCLV
SWHHHVFSHIRDSLLQNLLYMFTKKRLYEPTDMKYVEVCVDSITSLSFDKTDMTKPNLSSYKTFFETNFIENTKNFYAKE
SSEYLASHSITDYLKKAEIRLAEEEELVRLYLHESTLKPLLEATEDVLIAQHEEVLHNDFARMLDQNCSEDIIRMYRLMS
RTPNGLQPLRQTFEEFVKRSGFAAVAKIVPQVGGEADVDPKEYMEMLLSTYKASKELVNTAFHGDTDFTKSLDTAFRELV
NRNVVCQRSSSRSPELLAKYADSILRKSNKNVDIDDVEDCLSSIIIIFRYVEDKDVFQNFYTKLLAKRLVNGTSNSQDAE

SSMLSKLKEVCGFEYTSKLQRMFQDISLSQEITEAFWQLPQSRAGNIDFSALVLGTSFWPLSPNNVNFHLPEELVPLYEG
FQNYYYSCHNGRKLSWLFHLSKGEIKARINPQTNVTYVFQVSTYQMGVLLLYNHRDSYTYEELAKITGLSTDFLTGILNI
FLKAKVLLLGDNDKLGDPNSTYKINENFRMKKIRVQLNLPIRSEQKQESLETHKTIEEDRKLLLQSAIVRIMKARRTLKH
VVLVKETIDQIKSRFTPKVSDIKQCIDMLIEKEYLERQGRDEYIYLA
>Spom_NP_594195_1
MPPEAKRIVVKGFDPRKSRQRQETYYVTMIDRLNMALQVVMAGLGLKTGYQELYSGVENLTRADQASRCFNILQHHMSSG
IQLLKDSAESFIQLEGTETDTNACTVVVGCWNKWLERVEIVQNIFYYMDKTFLSHHPDYPTIEELSLSLFREKLMAVKNI
QIPFLNSLLQSFENLHSSKSTDHAYLQDAMLMLHRTEMYSSVFVPMYLVMLSRFYDTESSQKIQELPLEEYLEYAMSSLE
REDAYVEKFDIVRDKKSIRETVQRCLITSHLDTLTKGISQFIEKRDAHSCKLLYALLQFNHETEYLIQPWSDCLVDVGFK
LVNDESKDDTLVQELLSFHKFLQVVVDESFLHDETLSYAMRKAFETFINGAKGSQREAPARLIAKYIDYLLRVGEQASGG
KPLKEVFSEILDLFRYIASKDIFEAYYKLDIAKRLLLNKSASAQNELMLLDMLKKTCGSQFTHSLEGMFRDVNISKEFTS
SFRHSKAAHNLHRDLYVNVLSQAYWPSYPESHIRLPDDMQQDLDCFEKFYLSKQVGKKISWYASLGHCIVKARFPLGNKE
LSISLFQACVLLQFNNCLGGEGISYQDLKKSTELSDIDLTRTLQSLSCARIRPLVMVPKSKKPSPDTMFYVNEKFTDKLY
RVKINQIYLKEERQENSDVQEQVVRDRQFELQASIVRVMKQKEKMKHDDLVQYVINNVKDRGIPLVSDVKTAIEKLLEKE
YLEREDNDIYTYVT
>Tmel_XP_002839867
MPEKVSDQETQSQGKKRKQNLQSPRPSQGFSQRSITDFFGDSTLGLDVPTSRPSSPKRSRKNSEQPELPKDMSTPLNEQA
WRRISENRVADLNGLPPHPRDPPNPNTNIVATKPPRPAPFQPHAGAPRKLHIKNLRKPSKIDPDVYFNQTWGSLEAALAA
IFGSRKISASLEELYRGTENICRADRAGELYIRLKACCATYVGDYLKDSIIACNSWKDDAVKCVVSAWEKWNAQLGMIRS
VFLYLDRSYLLNNANPSLQPVEPTGLELFRHHIILAQEIETKFMDGIMALFERDRQQCSIDASLLTRAVRMVDSLDLYET
NFEPRFLAMSREYYDRLGILGATSNSLAEYLDECSQQLHKEALRCERYRLDPPTKRSMGLILEEGLLKNQLLILTDQGSI
EDLLQKQDHKSLATLYSLLDRIGEPSSYLRLAWEKHILTVGRSIIEDESRENEMVQRLLELKDSLDSFVRVPFKGDDTLA
YALRESFGTFLNARTKDRSEMVNSKPAEMIAKYVDALLRGGAKGTSTGTPGDEDARLAHSLEQVLDLFRFIQGKDVFEAF
YKRDLARRLLMDRSASRDAERSMITKLKTECGSGFTQNLESMFKDIEISREAISHFKTTRNRAGNSPNVDLNVLVLSQSA
WPTYDEVPVVIPLEMAQYLESYRNVYCEKHSGRKLMWRHALSHCVLRARFAPNVNKELVLSALQAVVLLLFNDAEFGTYL
SYQQIKGGTGLDDKQLIRTLQSLACAKYRVLQKETKGKDILPTDNFCVNRHFSAPKFRIKINQIQLKETKKEKEDTFERV
AQDRQYETQAAIIRIMKSRKKLRHNDLIQMTIDQTKNRGKLDVPEIKKQIERLIDKDYMERLPGGETWYQYVA
>Tmel_XP_002839019
MQVINTLLTTYINAHFAGVWETPSKVGDDLNHWVEHVLGQLVLDVLFSSKPPNSSEGVGEDDIKRRKEDLESWKRIAVGR
LGRLRVGELFDVVVEWPESLGGVEDLKSYIISPQTRLHLTTTFTSALSTRLLHPAAATSDILRAYISLIRAFTVLDPRGV
LLDRVSRGIRRYLRERDDTVRVIVRGIMSESLPDGLEDAEDLSELAHELGKGIPATQQGGGLEELDYDDLSWVPDPVDAG
PEFRRSKGLDVVGSLISLWESREVWTKEVAAVLSSRLLGSDEWLFEREIRTIELLKLRLGETTMQGCEVMLKDIADSKRS
DAHIRAEEHLEIEGFPEIHTKILSRLFWPTMKEGGFQVPLEIKELQERYERGFENLKKKRKLTWLQTMGTVEVELELEDR
VVQFPDATTWQAAVIHQFDEEGLSDQWTIPQLADNLEMEESLVKKAVQFWQSRDILREGSNGVYAVIENLEELEANTPLH
TGVTAAEEEEEVTDEKKRERMIVEQFVVGMLTNGGAMPLERISVMLGLVPGGFSWGQEELREFLSRMKSEGKVEYEGGSW
RIPKT
>Tmel_XP_002839000
MNRSRGKIRPPRRGLATDHVDFDEYWEVLAQSLREIYAKNASHLSFEEVYRNAYKLVLKKHGDRLYGNVKKLVGEHLQMV
AVNDRRTVGTKFLERLKFVWEDHQLCMGMMKEVLMYMDRVFCADHKIPSIYVSCMGLFRDHILRHPEYNIGNALNSVIMD
QIKMERDGDIINRATIRACVYMLEGLYETEEELEDQKVYLTSFEKNFILASEVFYQKEGEQLLRDCDAATYLRKVDKRLK
EEYSRCHDTLSVLTEPKIMKVVDQQLIDANINDVMEMEGSGLQFMLDNDRYEDLKLVYELISRVDSEKRSLKKKMCARLV
TMGKESSATIVSEEKVANNITLVAIRWVDEVLALKDKYENIWERSFDRDKGIQAAMTRAFTDFINDFDRSPEFISLFIDE
NLRKGLKGKTESEVDAVLDKALTLFRYIADKDVFERYYKKHLSRRLLMNRSVSHDAEKQMIGKFKMEVGFAFTGKFEGMF
KDMNISEEMTSEFKRLSQESDNNYKKGVELSVQILTSTFWPVGGGTSDHPCIFPLEIRAVRDSFTQYYLDRHSGRRLDWR
PDMGTADVRATFKGKRHELNVTTYGMVILMAFSELSSGGTLSFEEIQTITSIPEQDLVRNLQALAVAPKTRVLIKKPMSR
DIRLTDVFAVNEEFSSKFMRIRIGVVATNRAETEQERRDTDEKTERYRGATIEAALVRIMKQRKLISHTELVNEVLTQMA
SRFNPDLTMIKKRIESLMEREYMERAEGERQVYRYIA
>Tmel_XP_002836006
MQPKAPNREDLGQTWSYLEAGVEKIMKNLQTGVDMTTYMGVYTAVHNFCTSQKAVNSSPQALHNSGAVHRGGAVAHLLGE
DLYKNLIGYLSKHLTALKDEASGHADEALLALYIRDWDRYTTAAKYINHLFRYLNRHWVKREMDEGKKNIYDVYTLHLVR
WRVDLFDHVQKYVMDGVLKLVEKQRNGETIETAMVKSIVDSFVSLGLDESDSSKSTLDVYRQFFEKPFLEATTAYYQMES
KQFVAENSVVEYMKKAETRLAEEEGRVQMYLHPDIYGPLMKTCQKVLIQEHAPLLREEFQVLLDNDRQSDLQRMYNLLSR
IPDGLEPLRTKFEAHVRRAGTSAVDKIADEGGDNLDPKVYVDALLEVHTQYQNLVNVAFKGEAEFVRSLDNACREFVNRN
KVCKSASTKSPELLAKYADSLLRKSAKSAEESDLENKLTAIMTVFKYVEDKDVFQKFYSKMLAKRLVNFTSASDDAETSM
IGKLKEACGFEYTNKLQRMFQDMQISKDLNDNYKAWLEGKGESASNGVDFSCQVLGTSFWPLNPPTTPFNIPEVIVQTYT
RFVEFYNGKHNGRKLTWLWHLCKGELKASYCKATKTPYTFQVSTYQMAMLLLFNDATKISYEEFEKSTGLSKEYMEPALA
VFLKAKVLTISPPGSKIGPGTQYSLNFDFKSKKIRVNLNMAVRAEQKQEVEETHKTIEEDRKLLMQSAIVRIMKARKVLK
HVVLVQETIGQIKSRFTPKIPDIKKCIDILLEKEYLERLDGERLGYLA
>Ncra_246NCU00272
MPSNSTANPSELSRDNNNNNNNNNNNNNNNNNNNNNITTSHLHQHTPSSPPQATPTKRRFDDLNLKDKGAQQSHLHAAASST
TNTAAPNKRSRSELFAASSAVHPTTATTPPTMSAKLKGKRPASGPPEVIDLTQGSNNSAGSSPVATSSHSDIRGPPLSSS
HSTGRTSTAPLNPKGGGMATGKTPGGTNYLQTHIGARRLVVKNLRPVATQTQTEEAHYARIRGDLDTALRAIFSGHGAGA

GAGAGAAPALGGYSPSPGGPGGQSRSGAAGNNTTTGGQPMEKLYRGVEDICRRGKKESGELYEWLKDRCKGWLNSDEVLK
SLLAAAPSIASDMGGEEDVILLRAVLAAWKRWIAQLLVIRWIFSYLDRSYLLPGGSGTSAEGKGKSASGKREGPTSVNDM
GISAFRSAMYSSRSRNGANMLTIGARVVNAVCVLVMFDRLDDSRFDSQLLRESVAMLRLWGVYGKELEPKFINESREYVR
RFAEERSESCGLKDYIVACERLLNKESERCDVYNFDSTTKRQLKDDAHDILIFNYAEKLLDSGSVAKLLDANDLDSIKAL
YELLKLSGIQKRLKGPWEQYIRKAGAAIVSDTARGDEMIIRILQLRRALDVMVRDAFGRDEDFTYGLRDAFGFFINDKSV
SSSWNTGTSKVGEMIAKHIDMLLRGGLKTLPKALLSDVKDRQDAERSGIASTADEDAELDRQLDHSLELFRFIQGKDIFE
AFYKKDLARRLLMGRSASRDAERNMLAKLKNECGSSFTHNLEIMFKDQELAKDEIASYKTWLAGRGEDSPVANSELDLSV
NVLSAAAWPTYPDVRVLLPQNVLDHITTFDTYYKSKHTGRRLTWKHNLAHCVVKARFDRGPKELLVSAFQAIVLVLFNEA
EEKSPDGILSYEQLASATGMPDPELQRTLQSLACGKTRVLNKHPKGRDVNKTDTFSINKSFTDPKFRVKINQIQLKETKE
ENKETHERVAQDRQFETQAAIVRIMKSRKKMAHAQLVAEVINQTKQRGAVDAADIKANIEKLIEKDYIEREGGNYVYLA
>Ncra_466NCU00512
MVVVMSSIRTLEAALRLYSHGFDLLVKGITQAKSAAAFGTPVVNVEILVNRFRRDIHALVGNSASQQLTKSLRAVLVRLV
GNILGIPSEDGDDDDARNFKSRRPAPPTDDDFKALAARQRLHELVEQLHNVGLAGERFQVLFAEVMNWMMSAFVCGAYAG
VWSATDRRSLSSVLNTVSKGTGSSVTSPCIISLMDWIENHFARLSFEVLSRISHGSVSPVTLSDLKTYQSLALGRLSTLR
IAELFDIVLAWPDSRGALDDLRATITTSARRLQLTSHFTQALKTRLLHPGCSTLEILQTYIDIIHTFHALDHSKVLLGHV
EPSLKLYLWQREDAVRIVVTGLLASPEEVRAARKAKEISKQQAEQKKAGKRSEASTRRGAGKGPVVTPTTTGRSSRTTST
TTRQRDDLHESSMRTPGTTPITGKSTGTGKLVELALLLNDPTKTRHTLVEDEELDWNDMNWVPDPVDAGANYKRPKSEDV
IGTLISALGSEDVFIKEFASIVAERLLSLSDPARFDQELRVLDLLKRRFGEAALQNCDVMIKDIEDSRRLDVDIRRAWDE
QRTLTAATPMMMSGHFLRSADKRKKAALQRQQQQEPTQYHARVLSRLFWPALDREHFLLPDPIIEEQKRYEQGYEHLKSN
RKLTWLNQLGTVRVELELRDRTVTVDCTTPQATVIYAFNDSDSDNGHGIEIDNKPPARRTVDELYTTLQMDEDLITSCLE
YWVSKSVLRRLRSPANTQGSHTYVVIESLSDPIVDDNDDQFFKDDDIFGNPTTSVPAQEGAQPAEDPKQESNKPAMDPKE
QERRTIYWQYIKGMLTNASASMPLAQMAMMMKMLIADGFPWTNEELGEFLAEKIAEGEMELVSGGKYRLVKK
>Ncra_2229NCU02498
MASRTNKIRPIRKPITNRDQSEFEPCWALLRDAMTDIHLQNAGRLSFEQLYRASYKIVLRKKGALLYERVRDFEQEWFRD
HIMPNIAALITKNLINISLLQHPGSSSHERREMGEKFLRGIRDSWTDHNRSMNMIADVLMYLDRVYTLETKQPSLFAVTI
GLFRNNVLRSHIGAAAEDIEQDFVVFDILCAVILDLINMERDGDIINRNLVRKITAMLESLYETDDEIENHRLYLTLFEP
RYLEASTEFYRKECEKLVQEANCSTWLRHAQRRLNEERERCGTTLSIMTTDKIASVVEKELIEAKLDVFLAMEGSGLKPM
IDNDRLDDLSILYQLISRVDSTKSALKVILQRRVRELGLEIEKALKNTDFSVAGAAAGDGEDAGEAAEKAKPQTLNPAQQ
QTAAAIKWVDDVLQLKDKFDRILSDCFCDDLLLQSAITRSFSDFINSFNRSSEYVSLFIDDNLKRGIKTKTEAEVDAVLD
KAIVLLRYLTDRDMFERYYQKHLAKRLLHGKSEIHTEKEMVSRMKSEMGNHFTSKFEGMFKDMELSKDLTDNYRDHIASL
GDADYKMVDLNINVLTTNNWPPEVMGGGTSKGEGAKLDCFYPPEIKRLQESFYKYYLKDRSGRVLTWVSSAGNADIKCVF
PKVPGKETGPLSKERRYELNVSTYGMIVLMLFNDLVDGESLSFDEIQAKTNIPAPELMRTLASLSSVPKCRVLLKEPATK
NVKNTDKFSYNAQFVSKAIRIKAPVISSISKVEGDEERKETERKNDQTRAHVIDAAVVRIMKQRKLLAHTKLVNEVISQL
MGRFKPDVPLIKKRIEDLLAREYLERVEGDSSTYRYLA
>Ncra_4621NCU05204
MPPATMMMGAYGQQPSGSDGIDSTWPYLQSSINKIMTNLQEGLDMSSYMGIYTAVHNFCTSQKANGGSSSPSNHLPGIGA
QRGAHLLGEDLYKKLANYLTDHLKHLVSEAEAHKDEALLAFYIREWQRYTNAAKYIHHLFKYLNRHWVKREMDEGKKNIY
DVYTLHLVQWRDVLFQAVSKKVMDAVLKLVERQRLGETIEYTQIKQVVDSFVSLGMDEGDNTKTTLEVYRYHFERPFLEA
TKVFYQNESKQFVAENSVVEYMKKAEARLAEEEERVRMYLHPDIALHLKRTCNQALIAEHSTLLREEFQALLDNNREDDM
RRMYSLLSRIPDGLEPLRTRFEAHVRKAGLAAVAKVAADADKLEPKVYVDALLEIHTQYQGLVERAFNKEPDFTRSLDNA
CKEFVNRNEVCKSGSNKSPELLAKYTDVLLRKSSTGVEEAELENTLTQIMTVFKYIQDKDVFQKFYSRMLARRLVHSNSN
SDDAETSMISKLKEACGFEYTNKLQRMFQDMQISKDLNTGFKEHVASLNMDGKPLDSTYSILGTGFWPLVPPNTSFVAPA
EISADCDRFTRFYKNKHEGRKLTWLWQLCKGDIKANYMKGAKMPYIFSVSAYQMAILLLFNEKDQYTFEELASITQLNAD
VLEGALGILVKAKVLTAEGGEGGKIGPGATFSLNYDFKNKKYRINLNVGMKSETKQEEAETNKTIEEDRKLLLQSAIVRI
MKARKKMKHQQLVSETINQIKARFMPKIGDIKKCIEILLDKEYLERLEDDELGYLA
>Scer_SCRT_00627
MSETLPRSDDLEATWNFIEPGINQILGNEKNQASTSKRVYKILSPTMYMEVYTAIYNYCVNKSRSSGHFSTDSRTGQSTI
LVGSEIYEKLKNYLKNYILNFKQSNSETFLQFYVKRWKRFTIGAIFLNHAFDYMNRYWVQKERSDGKRHIFDVNTLCLMT
WKEVMFDPNKDVLINELLDQVTLEREGQIIQRSNISTAIKSLVALGIDPQDLKKLNLNVYIQVFEKPFLKKTQEYYTQYT
NDYLEKHSVTEYIFEAHEIIKREEKAMTIYWDDHTKKPLSMALNKVLITDHIEKLENEFVVLLDARDIEKITSLYALIRR
DFTLIPRMASVFENYVKKTGENEISSLLAMHKHNIMKNENANPKKLALMTAHSLSPKDYIKKLLEVHDIFSKIFNESFPD
DIPLAKALDNACGAFININEFALPAGSPKSATSKTSEMLAKYSDILLKKATKPEVASDMSDEDIITIFKYLTDKDAFETH
YRRLFAKRLIHGTSTSAEDEENIIQRLQAANSMEYTGKITKMFQDIRLSKILEDDFAVALKNEPDYSKAKYPDLQPFVLA
ENMWPFSYQEVEFKLPKELVPSHEKLKESYSQKHNGRILKWLWPLCRGELKADIGKPGRMPFNFTVTLFQMAILLLYNDA
DVLTLENIQEGTSLTIQHIAAAMVPFIKFKLIQQVPPGLDALVKPETQFKLSRPYKALKTNINFASGVKNDILQSLSGGG
HDNHGNKLGNKRLTEDERIEKELNTERQIFLEACIVRIMKAKRNLPHTTLVNECIAQSHQRFNAKVSMVKRAIDSLIQKG
YLQRGDDGESYAYLA*
>Scer_SCRT_01005
MITNKKIKISVPEKLGLSGESFEESWETVKYAVDHIYSDDMAELSFEQVYKTIYTIVLNKKGPILYNRLKDYLIQKLALL
RETIVKDNTHDYEFLGTMARLWDVECHCFKITGDLMMYMDKVYCKPNRCMEVYDMCLDLFRIEILQKCSSSLISALISDI
ERIRNLGSVDSEHTSLWKVLIGMMETLHDNRDSFFLTDFEPVLITATEEYYNKAIDIELLTPIESLEKIRKLRQFESMLD
SSFLNVDSLNKLKTVLENVLIWGKLSDIIEDLTHEAMVISNGKLLQEIYDLSSEEKYRVTVIESIKSYINKNAINIPFNE

GNRKKGQNAITWSSEIVELYRSQHSFLESIDFGSVRLNNLTGDVSNAILGDVFSMYFSKEGALPSEYLSTYVDHCMKRTK
EKDAEIVKIKQDLLDSTKLIGLLTEKDIFEKIYKKQLSRRLLQQRSLVEIEKWMVQMIKKVLGTFFTSKLEIMLRDISLS
SEMYQAFQHSTTNSIEYLSFAPQVLTRTSWPFQSTNPIDEGISLPPRMSQILAGFEGYYSLKYKERVLKWAHHLSVIEIG
CQFNSGYYEISFSVYAGVIFLLFEDYEELTLGEIYELTHIPIDDVKSLVMSMSTIPRCKILKKSSSSGNMKFSVNYFFSS
PNRKVKVPVIACPLPSQKSDNLATASSVDTYDNEIVMELSAIIVRIMKTEGKLSHQQLLERTTKRTQSRLSLTPSIFKRS
IQLLIEKEYIQRNADDPSYYHYLS*
>Scer_SCRT_03625
MINESVSKREGFHESISRDTSASNALGLYNKFNDERNPRYRTMIAELHEFFHLTLAETITETDVKELECNKEKAAKFRKL
MPKMLNNCRELTQRKSYIPYNSEFNGNDEKQKKFQLLHQHQIVLSFQEFCDELAKLIIDAHVLSFLTRCDYSYEIIPKNW
TSFYKLFQYVMGAVGPIISYVPVNYPMIRKELGFETLTIFQYYDSKLFECMKSHFGREFSTLVSATIHHYIHMFPITNTM
LEKEVPMLRIMSNCNFSIEGLSPKDFYMKTLRQYYCEESNLRPRLETFKNFKVLLTRNALLASLFSPEWVSDANDLFISH
LLLNKKSISEYIEIGKDTYDEEKERYFKTETHFSLLMFRNAFEAKNMLSKFKEFCDDAVSEKLKAAYGSNHDTERLFDEV
VQLANVDHLKIYSDSIEYHLCNLLGSTSKAIEQYVKYFESHLFIIVRKIKTTKKDLPRDMKIKYLNENLPILRLKFVNLP
TFPNFFERSIFRKTILQSDQNSSFIKDILPVYKDSLMELFKQRIITNVSQEDEMRYRDQYQPYLSQFFQPVEVMADLRIK
YASFLSFYENIEAAVKFGKTYNENNSKSFFPLIFDRERIPKVFQQSNEVKKNFVLPQEMDDTWNQFLRNYHEQNKVEDSD
ASKKELYPMWNLHHCEVESPYIIQDGTNLIFELTLFQTCVLTLFNESDHLTLQVISEQTKLAYKDLALVLKSFCNYKILT
RDIDNTYSINESFKPDMKKVKNGKLRVVLPRTASLQSSNTGGERTSSAHHEGSNSQWTQELLKACITRSVKSERNGLDYD
HLFETVKQQIKGFSVGEFKDALAKLLRDKFITRDESTATYKY*
>Scer_SCRT_05107
MSFQITPTRDLKVITDELQTLSSYIFHTNIVDDLNSLLTWMSPNDAKSNHQLRPPSLRIKNIIKVLFPNNATTSPYSMIN
TSQANNSIVNEGNTNKELQLQLFSTLKEFYIFQVRYHFFLHFNNINYLKDIQRWENYYEFPLRYVPIFDVNVNDWALELN
SLRHYLLNRNIKFKNNLRTRLDKLIMDDDFDLADNLIQWLKSANGSLSSTELIVNALYSKINKFCEDNMSRVWNKRFMVM
ETFNKFINQYWSQFSKLVGCPEDDHELTTTVFNCFESKFLRIRTNEIFDICVLAYPDSKVTLLELRKIMKDFKDYTNIVT
TFLSDFKKYILNPSVTTVDALLRYVKTIKAFLVLDPTGRCLHSITTFVKPYFQERKHLVNVLLYAMLDLPEEELKEKINF
NVDMKALLSLVDTLHDSDINQDTNITKRDKNKKSPFLWNLKVKGKRELNKDLPIRHAMLYEHILNYYIAWVPEPNDMIPG
NIKSSYIKTNLFEVLLDLFESREFFISEFRNLLTDRLFTLKFYTLDEKWTRCLKLIREKIVKFTETSHSNYITNGILGLL
ETTAPAADADQSNLNSIDVMLWDIKCSEELCRKMHEVAGLDPIIFPKFISLLYWKYNCDTQGSNDLAFHLPIDLERELQK
YSDIYSQLKPGRKLQLCKDKGKVEIQLAFKDGRKLVLDVSLEQCSVINQFDSPNDEPICLSLEQLSESLNIAPPRLTHLL
DFWIQKGVLLKENGTYSVIEHSEMDFDQAQKTAPMEIENSNYELHNDSEIERKYELTLQRSLPFIEGMLTNLGAMKLHKI
HSFLKITVPKDWGYNRITLQQLEGYLNTLADEGRLKYIANGSYEIVKNGHKNS*
>Cneo_XP_572139
MHPAPADAHAQRPPKKIGPDTSIKDTWAKLAAAIREIQNHNASRLSFEEHYRYAYNLVLFKHGDQLYSGVKTLVVEHLDR
LAHDRIVPAFPRSGGTRGAGKLGGGAEAIERATEGDRFLKAVKGVWEDHTGSMRKLKDVLKYMASLDKVHAPTAGVPPVY
ELGLSLFLTHIIRQPTIHTHLISTLLSQVQLEREGFTITRSTVRECIDILLRLRVPEREGGGNVYQQDFEPEFLRRSGEW
YEYEAGEELVHGDASLYLSNVSRRLAEEHDRTIHYLSPSTLPHLESLLISSLLTPHLVTVLNMPGSGLVQMVDKDRYGDL
KRLYELFGKVPGDQGVAALKHAVAADIDLRGKAVNAGTADVDPNTANPKPTPPLTLALQWVHSILLLFDKYTLILASSFS
SSLALQSTINSSFQTVINAHPRAPEFLSLYIDETLKKGKGAKGVGIAGAGKGVTEEEMEEAKEKTIRIFRFLTDKDKFER
YYKNHLARRLLSGKSVGGDAEQEMVGRLKKEVGFQFTHRLEGMFTDMRLSDEAANIFGNDPRYNDIPFTLHVSVLTSSNW
PPSTLLSLPLTFPAPLLPALEHYQTFYDSRHSGRRLTWQGLLGSADLKVRTRKGQWEVNLSTIGMVVLLAFSDLKPGDVL
SYHELKAQTSLPDAELARTLQSLACGKHRLLVKHPKGREVEQGDTFEFNEAFSSPLARIKILQISSSSSAASTSTSSARG
VGVENAQEREETERQIEEERKHQVEACIVRIMKDRKTMRHNDLVSEVAHQLAKRFMASVPMIKKRIEGLIDREYLERTED
MGSYRYLA
>Cneo_XP_572097
MSSLVVFPSTTNAFTSYRPSVRAHVDFIKPGSGSQHSTGRIVKLELPSLVQPQATDQLEPLFKSIDAILAGQPTPLSYQH
ITAFCQTVVLQDRDAASQLSDKCTSSIDRYTEIIRRELKGGILAKDRNTLGKLCESWQVWTARTTLLSSLLVHLDKVYHS
SFSEPNILRSRAIAKFKSIVWQDSVIHAVIIDDIFRWLSEERQTGIPSSARPTILAVDKIAQTLQTFGELAIAYQDETHD
YYTSALRNAVSEIEQGKRKAGEYAPWFLAKMVEEENRVRECFHTFTLQLLARDLRKVGQSETTDKVIELAVEQAMSNDEA
KGFSELYHFSIETDKFASLVKSLENYLAKRVSTLISDPVNDPRMIDETLKLKRFAEKTVSNLFSEEDTEPLPADEDVKMI
SVEEKEEREKEKEAAKEKRQSVRMRMFELEEAVRSGFKTGMGSRQNAPAEWIAKHLDAAMRKGQGSGTEEQFNNHLDEIV
TLIGFTKDKDVFKAFYSSQLAKRLLLNKSASNDQEKTMVAKLQKELGEEFTSGDVMMKDLQLSETLVRSYQLAQSRGPAQ
YGETSNFTANVLTESAWPAYPLLKDGWNFHLTPSLQSSIDDFTSWYTTQHKNRVLSWRFQLATVTLTARFPSGRYEVGVS
LFQAVVLLLYNEIESLTFAEIKERTGIENNELVRTLQSLALGRKGTRVLLKKPSGKEVNPTDIFAWNKGFTSERIKFKIN
QIQQDMSAEESRKTNEQVALDRVSILEATIVRIMKSRKKLTLQLLIDGVVSDVSKRFPPDVKEIKKRVESLIEREFMARD
EEDRSILHYVA
>Cneo_XP_568721
MAALQAALLEWDINLPHLEQGRIAPAVEQVRKYIYPRNLHQVGSAKPQISPEVSHAFAIVKQYDMSKSIFDDFLDDVENN
FELVEYDVLAAIERLETPAEDDDDKLVLHTMSGLFERLATWQRSWGLPLRAFQDNIYVALFTRKFHHLLHSSFPPSFPDH
LLRFLHASLASLPEYLTNPPPQHDYLQSVRINYQKVLPPTPHLSRLGIFPRYAGSLSKVAHEEIERIAREEAGKGWGQRR
LGRARQRIGDGVANWLSGMFEGNEAVQGALRPMFSRFDYFLCKCFFDIRTDELFDIIIDFPDSMAALEDLKECLFKVDQR
LELVNKLNAANVKRLLHPGAETHVILNIYISTIRSLRILDPVGVLLHAVALPIREHLRKRPDTIKCIVEALVQGEELQDE
NEGGLIGEGDSEAEDFNDPKWEPEPADAAPEFRSGKTSDIVSTLVSIFGTKEVIVKELQNYLAGRLLQVKDYDVVHEVRT
IELLKLRFGESALAGCDVMVKDVADSKRIDGHVQEEKKSVVHPLVLSKVFWPNMPRSSLKLPSKLQKIHAEYESSFHIFK

PDKHLRFVPHLGTLSLTVTLSDRTVTVDATPLQASIMELFEKQDVWAVDQLVNKLAVGKGEVGKALGWWAERGVVKDEGA
KGWRLLEFAEEEFEGE
>Cneo_XP_568711
MSAAEMNWVESTEAPARDADFETCWNFIAAGVKRIVVAHNNHFRSISDQYYGLLYNTINNCCTSPPIMEGDGATAQDEEP
KRPALLLYERLQYRFSDYCRPIAEELDSLPDDQLFQVYALRWGRYTATATHLDRLFSCFNGNWTRHQHIAKRDSVFTIGM
LARVTWKQHVYQHLVERGRDSLIYKALINQIEQYRKGQDVDTVMLEQAIDSHAYFGLIRPRVLGLAPHSPYLVSSGPTVA
T
>Cneo_XP_568555
MSASASGSSWTEPTKAQAPPKDADLKQAWAFLSVGVDHIMTRLSFGMSYSYYILLYTAIYNYCTQPGKTGLPSFSPQRGG
ASLQGADLHRSLHNWLSAHCKSMREEAEKLPDQELLKYYARQWDRYTRGALYVNKLFNYLNKHWVKREKDEGRKDVYQVY
TLALVSWKNNFFDHFTDNKGTSRLTQALLRQIQQQRNGEEVDSGLLKKVIDSYVSLGLDEADAQRQNLDTYRKHFQTQFL
EATDTYYRAESSAFVGSNSVADYMKKAEARLQEEADRVNLYLHDNTRNDLKTRCEKVLIEEHQAIMWDEFQTLLDSDRVD
DLARMYGLLSRVLNGLDPLREKFGQHVRRAGRAAVEKVLPAPGAVNEAGKAESLDPKAYVEALLEVHGKYTSMVEGPFRG
EMGFNRALDQACGDFCNSNAACTVSTKSPELLASYCDLLLRKSNKDSDAESLEASLSKAMIIFNFIDDKDVFHKFYQKKL
AQRLVGSLSASDDAESSMITKLKELSGFEYTNKLSKMFTDVNLSKDLMERFNEREREKGIASDIDFQPLVLGSNSWPLHP
QQTDFAIPREIQALYDRFNAFHGEVHQGRTLNWLWHISKNELRTTYLNQKYILMTSAYQMAILTQFNVSDTLSYKDIEAG
TKLSPTVLKPQLGLLVKLKILLNTNEEYSLNTGFKSKKIRVNLNQTIKSEARAEQKEVIAAVDEDRKFVYQATIVRLMKG
RKTMQHQALIQEVTAQISSKFTPKIPEIKKAIEYLIDKEYLERAPDSNNTYNYLA
>Ccin_CC1G_02840
MSLLLESFLDQIHQNFHLIDYEINEYMVRYESSQKVELIRMLVSRLLDWYKAWAPPPEFVSLLFTRIPQALTDSRLGPVV
ASSYTLTFQTRLFSGLPNSFTQGFKSLCTWCLAPENFPPSDTLAALSPNMAVVWRDFEVLGLIDRYESVIASVGYEFIEK
HVIEVCEGNWSKPMLEELRNWMSAKVVPWMLQIYARGATSTEEARSMLQGVGSRFDFHINKTLCDLRTREIFDIIIDFPD
SMCALQDLKECLQRVDQRGPLVTALRKANAKRLLHPGADTKLILTQYVATIKCLRIVDPPGVLLFKVADPIRRYLRERPD
TIRSIVASLVGDDDSGDSLLDDNEPIQPLQQLETEDYSDVMWEPEPVDAGPEFRTNKPSDIISTLVSIYDSKDLFVKELQ
VLLAQRLLAITDGDIARVERERKNIEILKIRFGEAALQVCEVMLRDMTDSKRIDTHIQAQAPSVVRPTIISKHFWPSLES
SDFTMPGQFQALQEKYSAEFTLFKPDKKLKWLPQLGTVHLELELEDRKLDLEVPPLEAALIELFSQKDTWTLEEMLGVIG
KVDRASAIKALATWVNMGVLKEAEENVFVLLEREEEGEPRELAVPAAPAAAPVAPVQSMQQQQAEKMRVYWKFIEGMLKN
LGSLPLDRIQTMLRLAPGYDQTIEQLGMFMEAARREGMVTSRDGMWRLQ*
>Ccin_CC1G_02887
MALVASLLELPKTSNGLETFGIGIQDNSNDGSASPRRKIARLDADSDIASRSKTKTTPSGPFVIRIIGEDIASKDQAEIR
RGLLGSVNRCVRHILTVGQDATALPMSYEKIYQDCRSIVTVHSAGSELYDYVKLDLEQAMSKLASHLLTFDAQEKVKWLS
FFAQNLKWFDGRITLLQSLLTYLDQVYYVANHSLTKTTIHDLAYGLFADRIFSNPDIRDRLLSGLSSWLKYERDNVTRAEE
RPQIAELIKYLINHNQYRTFEEHYLEVTQFYYRRESKAKVESMRDNPKGFFNLIESRIKQEIERSRELLQVGTWSIALET
TETALLDGRVDFLSTSLVPLLLGESDIDTLGALYSRLNRVDALKPLAQAFKEYVQGEVKTIVTDTERDSEMVERLLDLNR
LAHKAIDQAFVKVSQPSQKPSTSATPVEPEKKPDQEFIYAMEDAFNRGFRFRRNKPAEMIAKYLDKQLRKGQKGMKDAEF
QAELSRVLPLYRFTEDKDVFRTFYHRMLSKRLLLGKSASTDIEKWMLKQLKDKYDPEFGTAEDMFKDLNLSRDLVEGFHR
KNDNPESLKLNVMVLQQSVWPFSRPQTDVDLPVEMQDQLIKFTEYYKDQHQGRTLHWDPALGTVSLRASFKAGVKELSVS
LYQAIILLLFNDQDDIPFKDIAEQTRIEDAELRRTLQSLACGKKRVLRKVPPGRDVEDGDVFKFNADFTDPHHRVHINTI
QAKVSAEESKRTNISIESDRIHTIDAAIVRIMKAKKELNYEQLKVATIDAVKNHFVPSVDLIKKSIDSLVDRDYLTRNEE
DMSKFVYVA*
>Ccin_CC1G_03473
MAAPASLPMPPPSADLATTWRYLEDGVDHIMTKLQTGVSYTKYMSLYTVAYNYCTSSKMHGSDGSIGLQNRTGANLMGSD
LYNNLIRYFNGHLQGLRESAENLQDEALLRYYAAEWDRYTIGANYINRLFTYLNRHWVRRERDEGRKSVYPVYTLALVQW
RTQLFIPIQRKQKIVNALLRLIENQRNGDTIDQGLVKKVVDSFVSLGLDEADINKACLDIYREHFEVPFIDATETYYKHE
SEAFLGSNSVSDYLKKAEERLREEEDRVERYLNTSTRKALITKCEHVLIRDHSQLMWDSFQSLLDYDKDEDLQRMYALLS
RIPEGLEPLRKRFEEHVKKSGQDAVARLMGANNEALDPKEYVDALLAVHQKNADTVNRSFRGEAGFVASLDKACREFVNR
NGATGNSNSKSPELIAKHADLLLRKNNKVAEEGDLEGALNRVMILFKYIEDKDIFQQFYTTKLSKRLIHGVSASEESESS
MISKLKEACGFEYTNKLQRMFTGKQSPDMSLSKDLTDQFKTRMEQNHDDMDINFSIMVLGTNFWPLTAPQHEFIIPEEML
TTYDRFQKYYQTKHSGRKLTWLWNYCKNELRTNYTNQKYILMTSTFQTAILTQYNRNDTLSLDELVTATSIPKDHLVQVL
ALLVKAKVLINEETDQYDLNPGFKSKKIRVNLNLPIKAETKAESKDVMKTVEEDRKYVIQATIVRIMKARKTMKNQALLD
EVISQISQRFTPKVPDIKKAIDTLLEKDYIERVEGTRDTFAYLA*
>Ccin_CC1G_03743
MPAIPNLASGSANPATSASALWAYIKPALDHIVKSPSNDPNGKAPAIDVVLYSGIHSACYNYFTAQSEAASVGASSRGKA
PANGSDIYENLDRYFQEVCQEIALGVPDDDSTLVHYLIPAFNRFSAGAQSANRLLNYINRHYVKRAVDEDRGWLRLNDVL
ESVVKNITIEDTREKISTRLKEKRAEELKKWGYSEGDSGERLAYAEACSEAASGPDRIVPVVSLAHRRFRIEVFEPLLLA
PKTKGTSKAKHRIPKAVSTAAPYRPKGRLARAVQAVMTADDIEESERLQLITRLGHALQTVGVRPDHALRKRIDKYIASP
ESRPQKRNGSESS*
>Ccin_CC1G_10644
MATTSPPVDTDFANMPPRTADLEETWAYLNGGVEHIMTNFELGLSFKGYTSLYSTVYNYCTSTKMHGKLDGNRTGANLVG
SDLYSKLSTYFVNHFKGMLEKAATLEDMDLLRYYASEWDRYTRGANYLNRLFTYLNRYWVKRERDEGKKGVYQVYTLALS
QWRNHFFMHIQKDNSKLSNAVLKLITQQRNGEIVDQGLIKKVVDSFVSLGLDNADPNKECLDIYKEQFEVAFLAATEAYY
KQESEAFLAAHSVSDYLKKAEDRLREEENRVERYLHNKTRKELVSKCEHVLIREHSELMWESFQSLLDFDKDEDLQRMYA

LLSRIPEGLEPLRKRFEGHVKAAGLSAIGRLIGEGGANVDSLDAKAYVDALLEVHHKNSETVARSFKSEAGFAASLDKAC
REFVNRNAATGSSSTKSPELIAKHADMLLRKNNKMAEEGDLEGALNRVMILFKYLEDKDVFQTFYTTKLSKRLIHGVSAS
DEAEASMISKLKEACGFEYTNKLQRMFTDMSLSKDLTDAFKERQQHAEDTDITFTVMVLGTNFWPLNPPTHEFIIPQEIT
PTYERFQRFYQNKHSGRKLTWLWNYSKNELRTNYTNQKYILMTSSYQMAVLMQYNRNDTLSLDELVAATSISKEILTQVL
AVLVKAKVLINEEPEQYDLNPGFKSKKIRVNLNLPIRAEVKAESSDVLKAVDEDRKYVIQATIVRIMKARKTMKNQALIQ
EVISQITQRFAPKIPDIKKAIETLLEKEYIERVEGSKDTFAYVA*
>Ccin_CC1G_14444
MATAARRTGRPKIHGPEFSAEKTWNALSQNIREIQNHNAASLSFEENYRYAYNMVLYKEGDMLYRGVCNLIASNLDQLAE
QHIIPRFPAGSINDRLQRSQAGELLLKALREVWDDHVSNMTKLGQLLKYMDRIYTKNANVPETWDKGVELFLKHVIRSPI
KDHLVSGILDQVQCERDGHTINRSAVKGCVDVLLWLETGNSITVYKKELEPPFLKESEAFYKDESRHLLDTCDAPEYLQR
VEARFESEDSRIHHYLSPQTSAAIKQILQDHLLTPNLSAVISMPNSGLDVMIDANKLDDLSRLYRLFMQVPTGLPVLRKS
LRESIIRRGKELNDASLGAGTADAEGDGPREEKGKGKARPVNTVLPAVTWVQDVLALKDRFDQVWKEAFQSDRDLEAAIN
EAFESFVNAHGKAPEYTSLFIDDHLKRGLKGSHLAKRLLHGRSVNDDAERGMLAKLKLESGFQFTSKLEGMFNDIKLSND
AMVEYREYIQSRTVWSPSIIAVTGLIYFAKKAPAIELSVTVMTTTFWPISPPAVPCAVPDILAEACKSFEGFYFSRHSGR
RLTWSMALGNADVRTRFKTRTHDLNVSTYALIILLLFENLAESDFLTYEEIKEGTGIEEHELKRNLQSLACAKFRILKKH
PPGRDIHEEDSFSFNHDFSEKMQRIKISTISSKPETTRERQETNERIDEERKFQIEACIVRVMKDRKHLAHNALVNEVTK
QLSSRFHPDPLAIKRRIEGLIEKEYLERCEDRKSYNYLA*
>Umay_XP_761710
MSLAEALLSRAQTKRSALTDKALSNTADAAKGGLATFLAKAGDSSKNAVAFSVASSGARAPASIQITSFHPEKTLPPANY
ASLATAQLIRSVRLILVSTWRRPDTSAARQPLQQLYSTANVLVMYCSAEELDHLYDSVKIEIERAVGNWVATLHSSASIT
DLLQDKVGWLVELRSIWFEWSDNLALVRNILYSLDRYVLNRRTADGYYRSKSFVSHDDRISIRDLGLKEFGHHILKDATL
FRTFLKCIVGAIDGKRKLLLTYGKLHHDLLTMLGQLQAEDALDDAVSKATDVFYQAESTASIASLSPTEYVEYAWGRMSE
ERDRSQWALSTGTGQHKMIAAARKQLVTQHADTILAALPDLVTSGNCDGLDKMYQLANCSDRLADLRKAFAEFIKTHGAA
IVEDRERDDKMIEGLLAFKSSIDRVVHHSFGGDDDFVLAQKQGFEFCINKRETKVAELIAKYLDAKLRSGNKTMSDLELE
NSLDEALILFRYTQAKDMFEEFYKRHFAKRLLLNRSASSDAERSMLLKLKAECGPEFTAKLETMIKDVEVSKDLMDEYVR
FAAKQRKDEPSPKDDFDLSVSVLTQAHWPTYPNIDVALPVELAAAAERFAAFYQNRNSGRRLHWQHSLGTLTMTAKFEKA
GVKELHVSTFQAIILMLFNTLEPGQKLSYADIRTHTGLNDQELKRTLQSLACGLIPTRVLRKHPQGKEVNDDDHFTFNDN
FKNDRHRIRINQIQMKETAEEQKSTEQRVFLDRELILQAATVRVLKAKKTIKHSELITEVVDQIKNRFTVDVAEIKKEFE
ILIEKEYMERVEGQRGMYRYLA
>Umay_XP_760046
MNREWLVELGLDSNPTLSHEWATESTRSSLSSPCPFSYRGHAEQLQQVGLASLCHHRLLHPDATLASANVLAIMNAPGSM
RRPGGTRGTKLKPPKKIGLDVSIEDMWLRLVHAISQIQNHNISKLSYEEHYRYAYNLVLYQQGDMLYHGVKKQVQQHLDK
LCREKIVPAFPPGGAASVSAGIILPESLLNRSQASSSSISRINPDPSTSSSSKGKARAIDSDPSSFADADTDATPAVSAA
SAAAASVSMSATQAGDHADAVARTQAGERLMTAIRDTWLDHRSCMSKLSEVLKYVDRVYVENHKVPSINRLGLEMFRDCV
IRSSKYPIQIYLYSTLLTHIQIEREGSAISRSLLKSNIDMLSDLTQPKSGAPTSQDASVYSSDFEPALLQTSAAFYSAEA
DRWLDAGDAARYLAHVARRLQEEADRVAVYLKPETHKPLQHLLETNFLGKHLSTIINMPGSGLVAMLDEERNEDLARLYT
LFSKVSGGDTTLRLALKSYIGAKGKLINDAVTSQTPAQAASTNPSQGQDDASGSGCKGRVKEKAGGDVDASTPQAATAIR
WVEEVLEFKNKFDSILQVALANDTGCETAINEAFESFINTNKRAPEFISLFIDENLKKGLKGKTEVEVEEMLRKTISVFR
FLHEKDTFERYYKQHLAKRLLQGRSVSDDAERGMMAKLKIESGHGYVAKLQGMLNDMKTSEETVEEFNRSVKNSARGMRF
GVSVNVLTSTNWPISAQPPSCTMPEMLMEARRRFEEFYQSKHNGRVLTWHANLGNADIRVAFNSRTHEINVSTFALVVLL
LFEEVPDPQQDSERQGGREGGQVLFQLGLYMSTGAIQDPASGSASGNEQGEARDKCET
>Umay_XP_759373
MSLTQAWQHALHSLTRHHKQPSARTSCSILLPSLHAYTVLMQYLEPVAPLDCRFYSFEADSFILDAEASNALHELHASDV
SVPSSSKTNLSSPSPSLIALSHHYIDRCLQRIRKVALECGVTTAHEQGTGFSTLGDDLPCTLVTISHWFRAWLSPYSEGS
IFESLSSDRNRLLKPLQAYVRSCFDARAADALRSHLEHLVSDQNDTDLHATFVAHINTAGLTQPALILLTRVVHLEISRK
VRAVIGAEDVRPQDSVLPLDYAARRVLQQWLDDEVKPRFAAIRQMCRQQHVSSVLDEEMPDVDSADQSTWQTRLDYQLDK
ALCLTRAAQLFDLVALYPESSAALDDLRLSLNNADQRLSVAKTLSQSLHMRLLHPGAHTRDIIQMYVHLVRSLREVDPTG
VVLSRVVSPLRRYLRARKDTVLVIVASMLGDDPDFTLLKDELERADQEEQHQESQSENKRRRRPRRSLQTSTAAAATRSG
NPSGKRRFANSRRAANSSHASDSDASSEEDWDDPAWVPKPVEAGSGYRMSTSKDIVSMLTSIFDDRSGFIAALEKSMADQ
LVQVKGYKAMREYRNNMILKKRFGEKNMGKCDVMLGDVTESRRIDSEVHQRRRLAAPASTSAVQGMVSRLHPLIISRQFW
PENTTKPNSGVRQTAANPGGVQMGTPAPVTEFTLPPLFLQAQEEYSKTYRQSKAMRKLHWLNHLGSVELDVQLDSGQTIS
VECSLIQASTLEVISRCKTRTASAEDASPTTGANLVTLSDVMEQLNLQREKDARDALDYWVRVGLLKQAGVADAFIVQSS
LAVPAEARDDEPL
>Umay_XP_757234
MSGSGFTSSGPSATAAGGSRLPAVHDANATWAFLEPGIDLMMTRLKEGMTYPRYMELYTVAYNHFTSSSLASSSTALGRS
SGPFGSKGGTALVGAELYNHLTVYFRTHLEQVRTDSDGLSEEALLRYYATEWDRYTTGANFVHRLFAYLNRYWVKREKDE
GHKYVYTVYILALVQWKEHMFRYIQQKGRLVQALLKQIEKQRNGEIIEASLVKKVVDSLVSLGLDESDTNRQNLDVYRQE
FEKPFIEATEIYYITESDAFVSQNAATDYMKKAETRLKEEEDRVELYLHASTRTKLVPTCDNVLVRRHSAMLWDEFQELL
DSDQADDLYRIYTLLSRIPEGLEPLRRKFEEHVKRVGHSAVEKVMGGGDVGSADTGAATNGAAGSSAATAAAPAASDSLD
PGAYVSALLKTHQSNLNTVNVAFRGEAGFLAALDKACRDFANRNKATGASTSKSPELLAKHADALLKKSNKATAENSLEE
ALADVMVVFKYIEDKDVFQKFYSKMLAKRLVNFASASDDAEASMISRLKEVCGVEYTKKLQTMFTDMGLSKELNDHFKDT
MANHYDKTELDVDFYSLVLGRSSWPLQAPTTEFSIPTELLPTYERFQRYYSVKHQGRKLIWLWQLSKNEVKTNYLSQKLQ

FQTSTFQTAVLLQFNTNDSLTKTQLQQATGLNDATIKPVLGMLSKAKVLVTSSSDPEAYELNANFKSKKLRVNLNLPVKA
EQKAESNDVLKTIDEDRRLLLQATIVRVMKSRKQLKHQALIQETVAQVSGRFNPRVSDIKKAIDQLIDKEYLERLEGSKD
TYSYLA
>Mver_MVEG_01468
MAYSLLSRIEDGIEPLLEIFEKYITTVGRDIILGLGASISKDPREYVEKLIDLHSKYLQMCLKVFANDAAFVAAVDKAFR
TVVNDTATNSAARSPEVMARYTDTLLRKKQKTGLTEAEIEDRLTRVVILFKYIDDKDLFQKFYSRVLAKRLIFDSSLSAE
AEANMISRLKSACGVEYTSKLQRMFTDMTISNDLNAGFKEWLSMNDLSNGLDFGILVLTAGSWPVNSTQPLEFQCPDELE
KSITNFTTYYDSRHSGRKLSWFWHWCRADVRVNFLDKRYELSLSLYQFAVLAVFNAGDSFTVHEIRSQIKLVEFELIRVV
KSLVEAGLLTTTATNMDLNSVIKLNFGFSNKRTKLKISGGLQADTPQETTATLKAVDEDRRLCIQASIVRIMKSRRVLSH
VQLVQEVIEQCKTRFSPNVPMIKKCIEQLLDKQYIERADDSLDRYIYVT*
>Mver_MVEG_01469
MSLRRKRIDFNAAFEQFKSDMYKMFDFSGTSNSVSGMGMHQLVYDICNSIPKPFPEKLYCAIADFLVEYTIGVRKAILSH
DEVVPTYAMYWKQYSIASYYLNMACEYLNGHITKQKKTGAIGVHPFVGQSNYPRQDIQALAHQIWREQIVVEIKLRKQNR
IMYQIFETIRQDREGRQVNYSVVQEAILSLVALNSKTDQPLELYIDEFETPYIAQTKAYYKMESNIKLSSCTISQYMRSA
IDRLAQEVSRNSRYCHPTSHARVIQECETQYISEHQTNIQSEFERMIANERTEGKRVPLK*
>Mver_MVEG_01966
MATLPGRRPIRGKIRPPTKKAAAESLFDTSWPELASAMTTIQQQKTNTLSFEEVYRLGYNMVIHKYGEKLYLGIQGLIDK
HLKTEAENKIVPLLGIADASPAEGVELLKAIQKVWMHHITCMKMFKDTLLHLDKSYVPQTRLPATYDMGLYIFRDSIIRS
STHPIQAHLQLVLLNQITQERKGDVIDRGAVKASTDMLLEMKENNGTDAVYVADFETLFVETSREFYRLESEDLVRRFDP
PDYMKKVENRLDEEKLRCNHYLTARTEPKIRLIVEDEMIAKHLKTIMEMENWGLKQLLINHRLQDLDRMYRLFLRVPDGA
KELQNGVAAYINECGKASNAGVKASTQESLEKGAVPGVTVALRWVQEVLDLKDKFDKVLVLSFAKDKAFETAINAAFERF
INLNTKAPEFMSLFIDNKLKKEFKGKTDDEVDTILNKMTTLFRFLSDKDVFERYYKQHLSSRLLLGKSVSDEVERGMITK
LKIECGYQFTSKLEGMFTDMRLLADTTSSFKDFLENAVEPPPFDLHTTVLTSTFWPIATTPITCHFPESFLTAIKVFERF
YTSRHNGRKLSWHPTMGNVDLRATFNTKKHELNVSTMAAVVLLLFNNIPADEPLTYTAIREETKLPDDHLKRTLQSVACG
KFKILIKEPKSREIEDTDQFTFNAGFSEKLMRIKIQTIASKVETVAELKETKEKVDDARNHMAEAAIVRVMKNRKQLDHN
NLVAEVVAQLQVRFNPSPTMIKRRIEALIEREYLERAPGDRKLYNYMA*
>Mver_MVEG_05228
MSSSKALSSPPPHGSDIHATWTFLEQGIDQIMNRLEDGLTYKRYMEIYTVIYNYCTTKTTNDGMSASIQTRGATLGGSDL
YSHLMRYFHKHLDVIRKDSEKFMDVSLLEYYTKQWVKFTNASTYVHHLFTYLNRHWVKREIDEGRKTVHDVGTLALVSWK
DSLFSHVERNVMTAVLQLIERQRNGESVETGLLRNVVTSFVSLGLDENDSKKTTLEIYRDFFEKPFVSATEVYYKMESEK
FLSENSVTEYMKKAEARLAEEESRVQMYLHASTHKPLIQTCETVLVKGHTEIIWEEFQNLLNQDKQDDLFRMFSLLLRIP
EGLDPLRARFEAHVRKAGLSAIEKIATDAEALEPKVYVDALLEVHKKYNDLVQTAFRGEAGFVASLDKACREFVNRNKIC
KSSTSKSPEQLARYCDSLLRKSAKNPEESDLEDILNSIMTVFKYVEDKDVFQKFYSKMLAKRLVNGTSASDDAEASMISK
LKEACGFEYTSKLQRMFTDMGLSKELNDAFRDKMAQNHDASDNTVDFSILVLGTASWPLQAPSTSFNLPDDVVKTYERFQ
RFYQDKHSGRKLNWLFQLSKADLKTNYTKGNKTGYSFQVSTYQMGILLQYNSGLVYSLEDLQTQTNLNMDILTGALGILV
KAKVLLLETGENVGDAGTRYELNTEFKSKKIRVNLNLPLKAEQKVETEETHKTIEEDRKLLMQAAIVRIMKTRKVMKHGA
LMTEVITQLQTRFKPRVPDIKKCIDVLLEKEYIERVDGQKDTFSYVA*
>Mver_MVEG_08802
MNKNIAEQWRAGLGTINENSVSSKAGDDQAQSTNAFSREKTVFTRAMDPNSAIDPGARRLGASLRTLMREGYGQWAGNWI
LDTFRRDALEKLVPLLEDTDYQYKEFIKASQDGLSKEWTLHAFRNVIRTIHARLSISRQLTHIFDEEIRAMRMDSSLEVS
AKFLAQFSAELATRLSPSFDGMAYMYFEEVFRQYELSARQHLLDSKENNPGFEISEREQQQEQQRILGKKRRRLMQDGRA
AMDLFVDDMDDEVVEQLFHGNTSISEAEAMTNQDQIQEYLTLCLQLDELDFASRMTDVIMRMLYRQVEAKILDSFQTKWD
IPTLESGKEWMIHVVLPFLRLTLMPKKDHASPTGIKRFKMWASRLEFYFFKTFGDLRIKEFFDIIVECPKSEPAVKDLKM
CIGWTGQREQLQKAILAEMDKRLLHPGAETHDLVEFYICAIKHLRILDPSGVMLDQAARAINQYLRTRDDTMQAIVKSIV
DDSNNLLLDGSTEGIQTGMEVDEDGSDDETTWVPEPANAGPDLTSARRKMADIISVLASIYDTNDRFIKEFQSILSDRLLQ
ATDFQVDREIRQLELLKLRFGEVDLHHCEVMLADIAESKRVSSNIQERNPSVVVNTLVASRFYWPAIEEKDSFELPLKYQ
ELLDSFEVEFELSRPAQKLALFPSLGLVEIELELEGREPISMRVPPIQASIIHLFESQDTLTLSEIAIKLQMMESSLEPK
IQFWVREGVLREIDRAKYQLIEGSDQSQS*
>Mver_MVEG_11468
MSNRAPTEASPRKRKSSSFARPSESGSSSSSHSPHSSSSSNALPWTVLPNSLENSFARAREIDTKRRRTLSNSSDNTSQS
THSPTPHKHNTMSIGHSNFFTDIGSVSGLGRSGPRTEGVTSSLGLSIASKSPIRRTGVQGKQPVKKLVIKNFTVTPQLPE
DYELKTWNVLQQAVQAIQQSKPVESSLEELYKTCENLCHQKFGDNLYKRLSAEVESHVQNVVDGLLNKSENKSEESFLEA
VHTAWSDHCRQMVMIRSIFLYLDRTFALQTLGVSTIWELGLDLWRRLAMNSLEIRSKVLGGILALIEQERSGDTSSHLLI
VSLLRMLTDIQMYNSVFEIPFLQASRLYYHVDGEQNVHARTVPEYLRYAQRRLVEETGRFEGHLDKSTRRSLIAAVEGEL
LERWTDMILQKGFETVMQDHNLVDLKLWYELMMRVNGLEKLSFHFGNYVKKTGKALVADPSHDDSMVEELLKFKLGLDEI
VNSCFQNSDLFLNVLKECFEAFINTRQNKPAELVAKYLDLRLRSGNKDLSDEELESTLDRVLILFRYIQGKDVFEAFYKR
DLAKRLLLNKSASFDAEKSMLSKLKAECGSGFTTKLEGMFKDMDTSKDIMINFRASKMQSKIAGLDLTVNVLTQGNWPTY
PPAEANIPAKISHCQEVFKEFYLSKHKGKRLMWQNSLGHSVVKATFPSGTKELQLSLFQAIVALLFNETGDSSLSYKEIQ
QLSNIEPKELSRTLQSLACGKYRVLVKNPKSKDVSDTDTFKFNEDFTASLTRIKINSIQLKETPEENSSTTERIFQDRQF
QVDAAVVRIMKTRKQLSHTLLISELFEQLKFAIKSSDLKKRIESLIDRDYLERDKTDSTLYKYVA*
>Pbla_Phybl2_129269
MSYRKIRIKPPTRLPNVDREQEYEDGFKLLSQAYIEIFRKNAKILSYEVLYRTAYNLTFQQFGEKLYFGVKDVIANYLEA

KAEGAIVPAFVNTSSTKESSAAGSDSSAMFMNTVKLVWDDYVTALTMIRMTLKYLDDRLPKYNLPNVTDMGLDLFRDRVI
RSDKYPIQAQLIAAMLNQIQLERHGDVIDRSVIKAAVSMLSELTDPTTKESVYIVDFENKYLETSAAFYQIESEVLSTSY
DAPEFMRKVERRLEEEYERTIHCLSLVTEPKIRHIVETHLITDNLKTVIKMKNSGLDSMLNADKYGDLLRMYKLFSRVSA
GLNELRTAMMAYILRIGAEINRSIAADIGDMNKPKSVAEKSAVGGAQVAIRWVEQVLNLQEKFDRILEFAVNNDKSFQTV
FNEAFESFINDNTKSAEFISLFIDENLKKGLKGKSEDEVDDILGKTITLFRFLRDKDVFERYYKQHLAKRLLFNRSVSDD
AERGMLAKLKRECGYQFTNKLEGMFNDIKLSAEMDVQFKDFSDRQPFVDVGVTVLTSTFWPMNISSSPKCNMPQVILGAC
ESFEKFYFDRHSGRRLTWQPQMGTADIRATFKSCRPMLNLSTFAMIVLLLFNDIHEDNGLSLEEIKGQTQIPEAELKRTL
QSLACAKYKVLTRNSKGREIHPGDIFYFNTSFSTNLAKIKIQTVASKVESEGERKNTQDKVDEERKHQIEAAIVRIMKDR
KSMEHNLLIAEVTKQLTPRFLPNPVMIKKRVEALIDREYLERSSEDRRSYRYLA*
>Pbla_Phybl2_185609
MSLRRKPINFEKTFAELKDILDAIFSFSALGGVSGMNMFQLIYDMCNAIPQPFTEKVFTGIAEYIDKHTVELCQNILKHD
DLVTAYARPFERFKLAAEDTSAGCNYLNGIITAAYSTGSRDRKPSVAGGKYKKQSVESLAMSLWKTNVLFTIRDRHQNRL
LYQVFELIRRDRDGVDAPYGTIKGVVTSLVQAHSFTGQPLQLYIEEFERPYLVHTKRYYEAEAAREIASGSVSSFMKKAS
ERLEQEIKRNNQYCHSTSHSRIVKEFEAQYITAYQDRIIDEFENMLRDERYEDCTRAYNLLSRIPDGLKPILTIYEEYIS
KIGKDMVSKLNSSALKNPRLYVDQLLILHGKFHQVNQQVFLADPLFTAAGDKAFRTIINDPNGATSSNGSETLARYCDMM
LKKNAGKKEAGVTASTTNSLDGKKKGALKKPVQDIEDGDPEEKLAKMITLFKYVDDKDVFQKFYSRMLAKRLIYSASASE
EMEANMINCLKEICGVEYTSKLNKMFTDMSLSSDLNVNFKDYLKQQSCKMNVGFDILVLTQGAWPLNQKEDPNEADINKV
QIPSELEENVTLFEDFYGKHHNGRRLLWQWNLARGEMRLNYLDRHYELQVGLYQIVILLLFNSVTEMSIADLLVHSGLSL
PDTLRSIKPLVDIHILESNASSLNKSSVVKINMKFSSKRTKIKVSAAGAQAEAQQESQATRKAVEEDRRMYIQAAVVRIM
KSRQRLSHVQLIQEIIDQANSRFSPSVIMIKKCIEQLLEKQFIARQGRDTYVYVA*
>Pbla_Phybl2_166592
MECIQYNLLDLIDDFPNSMSNVNDLRLEMEKYNDAKQRITEFQEEMKLRLLHQGASSVEIVRFYILCIRTFRYLDPSCEL
LLPVVEMMEETYRKDMVHAVVKRIREDDDNSLCPDPEDTYVFDADELGKSVSEGYYPLCEDKGEFCKDPKKEKKKESSSH
YLFLVERQWLERKSLDIVAMLFTLCETQESLAKENFVKEYQDQLGKALLKDPGYDTETEIIRLEKINERLLGGAMQSCSI
MIKDMEKSAQLNDRIRKSTSVWPTDFKALVLSRHYWMDPEDDIPEQTLRLDTPCIESMEIYEQKYPIIQPSRTLNWLPEQ
GSVTIELTFKSRTIEMSVDPITATVISQFSTKDITFTAKQIAKNIGVPSKIAFKSLVFWQQQNILAITKDHHFQLMLIMA
ITTTTTVQYCVYFDLVICCQNCTLYDLVYSDIK*
>Pbla_Phybl2_158694
MTSMGNLPPADSFKATWDFLEQGLDQIMNRLEEGLDFTRYTVLYSAVHNYCARGSPTSSHGVPDPLSNPHSRRAAQLIGG
DLYTSLSQYLEVHLQKIKTESERYMDEALLQYYTKQWERYTTAARVVNNIFMYINRYWVRRQIDEDRKSDVYDVFTLALV
SWKKNMFEYVHHNVIGAVLKLIQRQRSGETIENGLIKSIVDSFVSLGLDSSDSTKSNLDVYREYFETPFVQATEVYYKTE
SEKFISENSVPDYMKKAEARLLEEETRVSLYLHESTNKTLVPTCERVLVKNNSEVMWEGFKSLLALDKQDDLRRMYSLLA
RIPDGLDPLRKSFEAHVKKAGLSAIERIAQQEGEVVDPKTYVDALLDTHRKYNDLVQNAFSGEAGFVASLDKACGEFVNR
NQICKTSSSKSPELLARFCDSLLKKSAKNPEENELEDVLNSIMTVFKYVEDKDVFQKFYTKSLAKRLVNGTSASDDAEGS
MITKLKEACGFEYTSKLQRMLTDMSLSKELNDHFKELVQQSSQTTSSNVDFTILVLGAGSWPLSAPSTDFNIPEDLVKVY
DRFQKFYQNKHSGRKLNWLFQLSKAELKTSYLKASKAGYAFQVSAYQMGILLQYNTATSYTYQDLQKSTALTPEALHPAL
SILVKAKVLLLSDGEKVGDDGSRYSLNFDFKSKKVRINLNMQMRTEQKVEADETHKTIEEDRKYLIQAAIVRIMKTRKVM
KHSALIDEVLTQLQSRFKPRIPDIKKCIDILLEKEFIERVEGQKDMYSYVA*
>Pbla_Phybl2_148970
MDWPQQPMKRPRLDNPLTINNEPYDEVMSEPQTPSLPFSAFLSNSLHRTQRLPILSDEKLIVYDLKVERPVLPNGYEESA
WERLKQAIHAIHKNTPIKESLEVLYQLSENLCQYDLAEALYGRLRHECEEYLEAEFDKLSRQVSYSCRHESEGMEFLELV
NKLWKNHCDQILQLKCIFLHMDRIYLNSMTKSASIWNMGVELFSASLLKRNNVLEKLIRNLLLQIQEERDQQPINTGLLH
SNLRMLIDLSLYHTVFEGRLLEESRRYYKAEGDRLIETMNMSAYLIHVSTRVHQESAIRVKRYFDKSSKSALTAAVEEEL
LSTRVNAILDKSFKHFMESNKVDDLSMLYRLLEKVDKIDICVKYFVNYIKRKGSSILRDHSGGKDPIPALAYFKRKTDAI
VEHSFEEDDRFVNGMKDGVDYFVNLRQNNATELLARHTDYALRNNKVDEKSLEQSIIFFRVLQSKDIFEALYKRDLAKRL
SLDVMNRNAEKLMLAKMKKECGVAYTSKMEGMLNDLKISDELMHDFRTSINYGETQSFEFRANILTSGFWPSYTPVKINL
PQEFTHIQKLYQDFYCTKNERRCLTWQNSLSICEVLANYPFGAKEITLTLLQTVVLLLFNDPTTPSLSFSDILLETKLDE
LELRRTLKSLACGPHKLLVKTPNGLDVEPTDMFTFNTDFQAEQTKFQMNTDTLNEVIEKDSSLDQTAFNREVQIDAVIVR
IMKDKRTLRHSLLMNEVTRHVRSRVTASDVKKRVEVLIEKDFIARTEDDGYEYLC*
>Pbla_Phybl2_135478
MVIDSTPLVLNSKPIKESETPGKQARSKFMSPVHTNAKLLWDSKPSVTTGEEYLKDAWNTHEALIKSILSGKTISSGMND
AYIKCEILCRDGRSAVIYSRVKNVLSRHIDGLAKNLGEMASSYDNFLQYTNKCWNQLSFDLHQLKKLFMPMDRSYIVQST
EYDSIIDLGYSLFKDNLVKNDAIKERLVANILYYVKVERDGGTVDQPLIKSLLQMLMTLSLYSTIFEPRFIEETARYYEQ
EGRSLIQKIDIPNYLVHVTRRVKEEGEDRKQAYLDKSTEIRLVQTVKNELVYRMTNDILSKGFDSMADEMKLESLKTLYD
LLSPEKDRTELRQVFGSYIKKRGIEFIKDPSNDNAMIVTLLDRKQRADTIMEKCFDNNILFANTVKESFETFINSRNNKP
AELIAKYIDSKLRMSEKKKAEVDMEKVLDDALAIFRFTQSKDIFEAFYRRFFARRLLSTFDVSEEAEMSMITKLKSECGP
DFTKDLEIMFKDMSISSELQAKFREATINTDVYDKTMNVIILTSSAWPNTPKHEAILPPKLAESQEVFKQFYVSKFKGKK
LTWHNALGQCILRVRFPRGVKELSTSLFQSIVLFLFNDETKETISFPEIATATGIDEKELRRVLQSLACGQSQVLTKTPP
GTEVKDTDVFAYNADFSAARPRVKISPIISDTSAEENKDTENSVLFNRQCQIDAAIVRIMKAKNKLSHGALVNELFATLR
FPINASDIKKRIESLIEKEFLLRDEEDNSAYIYNT*
>Mcir_Mucci2_135986
LCRFGKSEQVYNSLKQEIERHVEEIKQSLMSKPLNKDLLSRLNAQWATFCQQLTIIRTVFMELDRYYILPNTKYKSITQL

GKDLFSELIMEKGNFLHVVIAEVIQQIKAERDGAKINIPLIRDILHMLTDQAYYKSEFEPRFLESTNLYYKEEANRRIGY
GTIPEYIYHAADRRTQESEERIKNYLNPDTKQALISVVVDRLVYAKAEVIIQEGFEAMMDTDMYEPLKIFYQLLIQSPKM
SLLRNAFGAYVKEEGMKIIKDPKSDPTMITSLLLFKRKIDKILKSCFSDDVTFVNAQKESFEQFINTRGNKPAELLAKHL
DFKLKIPVKKQRFQEIVDTVSLDSILTLFRYIQTKDAFEAYFKRFLAKRLLMDRSASSEFENHVLVNLKAECGPDFTKNL
ESMFTDMQISTDLAAEFKEFKKEKPRLPMSIKVIAQATWPSYTLLDIKLPANVSIYICLFEAFYVSKFNGRKLVWQNRLS
SCSIIANYPKGRKEIIASLAQAVILFIFNDASKKTLTFSRIQTITALAKNDLIGLLTSLCTGPYKLIINTSSTSSKDPVT
PTDSFAYNFDFESANQKVRIPPAASIEPTKDEEKDVEKKVHISRQHQLEAAIVRIMKAKKTSTHNALVDELFKELKYPVQ
VDDIKKRIETLIDRDYIIRDTEDPSSYVYQS*
>Mcir_Mucci2_106712
MLTLYHNAPKQTRALDAIRKLKAYFERQGINEKEPLPNMQKIVDYCLKYPRLLRIRDSPLKYLHDPKLPYTVVHDWYIGI
ALDYLEASIFVLLEQWKSDYMKKRTDKKQIAQLFAKAVNDIYQCYEHTGLALGIPFPVGCALAAVELKEKVRNGSRALIK
SKISYYFTDARNTFYTLAMHQIEALFKANQPNNAFAYLFSPHIMKYKEDELETKVQVFLDVLLPKLVDVFNLPDDILDSA
LISENVNDGDNQVDMNIWLTSSSSEGEEGPNDGKDALEAHIQTFSTVCEQLNNLHLTPNWTDILKETVQKRLQSMDWEEN
ADVSVVQIQLKWLHVLILPWLSHLIPKTDDIETNWYNFLREKLKAEHVLYEVIYQTKIPEIFDIVLNYPDTECIIKDLHI
IATKRGLLEDLKDSIMREIQTRLLHQGASAVDILQYYIDCIRSVSVIDPSCDIMESIIELIEHYLKTFRKDVTAGVVHLI
RNAHEYSNLQVEDADIYVFKKSELNNEELPLDAVVIKEDRPAQLRRLQQKSKDSIAMLISVCSSLKDFIKGYSDKLGHVL
LSTNNYDTTEEIQRLELLKRNFPPDTFLRCDIMLQDVTDSRRLDTSVHLNHGINPNLHAIIVSRKYWPGGNDEDEDDDLD
DDSNMEDGNNASIKMWPGQQESLEEYDQEFRKIKKSRKLKFLPTKGSVRLNLEFESRTFTVDVSPEAATILSLFEGKGQA
YTRNDIATKTKIDKAVVVECLELWLKKEVLKLSADGHYQLVEE*
>Mcir_Mucci2_138405
MQLPPGNDFNATWTFLQEGLDQIMCRFEEGLDRTRYSILYRHDAVHNYCARSDSNLHGNISAPAAPAPPLIGGEVYQHLS
NYLKLHLEKIRKESEQYMDESLLQYYTKQWTRYTAAARVVNNIFMYLNRYWVKREIDEDRKSDVYDVFTLTLVSWKKYMF
EYVNHNVIAAVLKLITKQRNGEVIETGLIKNVVDSFVSLGLDHNDSSKSNLDVYKDYFEKEFLEATEVYYKTESEKFISE
NSIPDYMKKAETRLNEEEGRVQVYLHPTTHKTLIPICETVLVKNQEESIWDGFQGLLNADKQDDLHRMYSLLARITDGLN
PLRVSFEAHVKKAGLSAIERISQNESESVDPKSYVDTLLEVHKKYNDLVQSAFSGEAGFVAALDKACGEFVNRNKVCKGS
SSKSPELLARFCDQLLKKSAKNPEEDELEDVLNNVMTVFKYVEDKDVFQKFYSKMLAKRLVNGTSASDDAEGSMISKLKE
ACGFEYTSKLQRMLTDMSLSKELNDQFKDLVSQGQEGSSGADFNILVLGAGSWPLSAPSTSFNLPDDVVKMYDRFQKFYQ
NKHSGRKLNWLFQLSKAELKTHYLKASKVGYTFMVSAYQMGVLLQFNTTDSCTYEELHKSTALAPEALNPALGILVKAKV
LLLRDAENVGDIGSRYVLNLDFKSKKVRINLNMQMRMEQKAEADETHKTIEEDRMFVMQAAIVRIMKTRKVMKHVALIEE
VITQLQSRFKPRVPAIKKCIDVLLEKEYIERVESQKDMYSYVA*
>Mcir_Mucci2_142460
MSRGCCYLDRINTSSYASGRNSGANHSGKYRKQNVEALALLLWKSNVLFTIRDRYQNKLFYQVFEWIRQDRDGDDAPHST
IKSVVTSLVQVNSFTEQPLQLYIEEFERPYLVHTKRYYEAEAAREIASGSISNFMQKANERLQQEIMRNNQYCHSTSHGR
IVKEFEAQYITAYQDRIIDEFEVMLKDERFRDCTLAYTLLSRIPEGLKPILDIYENYVAKLGKEIVSRLGTSVTKNPRSY
VDQLLNLHSKYYHVNQQVFSSDPLFTASVDKAFRTIINDTLSANVPANGPETLARYCDMMMKKNAGKRDPVADMDEGDQE
ERLMKMVTLFKYVDDKDVFQKFYSRMLAKRLIYSASSSEELELNMINRLKEICGVEYTSKLNKMFTDMSLSSDLNTKFRG
YIKGNGQGRLCSWPTVFQQLILLITAGLVNMDILVLTAGAWPLNQKDDAGPDTNKLQIPSVLENNITSFENFYGNQYSGR
RLLWQWNLTRGEIRINHLDKNYELQVSLYQMIILLLFNQGVSLPVNEIVNQSGLSVSDTMKSLKPLVDMQILTISNQHDA
LSQTSEIQVNRSFTSKRTKIKVPAAASQNESQQESQAARKSVDEDRRMYLQAAIVRIMKSRQVLPHVQLIQEIIDQANSR
FSPSVIMIKKCIEQLLEKQFIARQERDTYVYVA*
>Mcir_Mucci2_112869
MSLRKGRIRPVKRMPVMDHTQEYEEGFEILSNAITVIFQKQARELSYELLYRTAYKLTIRQFGERLYHDVAKVIADYLEA
TAEQAIVPAFINTSVETSTADEGAAFLKTVKKVWDDFKAAIDLILQVLYYLNDRLPKYNLPSVDEMGLNLFRDKVIRSKQ
YPIQQHLISAMLTQIQLERNGDVIDRSAIQSAVSMLVELTDPDTKLTVYAIDFEATYLDTTTSYYQIESQKLVSSYDAPE
FMRKVEKRLEEEYERTVHCLSMTTEIKIRTIVETQLIANNLKTLMEMKNSGLESMLNADKYGDLSRMYSLFSRVPAGLNE
MRAFISNYILTLGAEINQHIMSDLKSKAEKGSSSIAINWVMEVLALQDKFDKILDLAASKDKSFQTVFNEAFEKFINDNQ
KSAEFISLFIDENLKKGLKGKSEDEVDDVLDKTITLFRFLQDKDVFERYYKQHLAKRLLLNRSVSDDAERGMLSKLKREC
GYQFTNKLEGMFNDMKLSTEMSTAFKEYLEKSTNPATNKLSLDASVTVLTSTFWPMNLSASPKCVMPANVTQACKSFEHF
YFDRHSGRRLTWQPQMGSADIRAYFKKGKHLLNVSTYAMVVLLLFNKNDTLSWEDIKNYTQIADQDLKRTLQSLACAKYK
ILVKSSKGRDILEDDSFAFNQDFSSNMARIKIQALASRVENDGERKNTQDKVDEERKHQIEASVVRIMKDRKTMEHNLLI
SEVTKQLSSRFMPSPVMIKKRIEALIDREYLERSAEDRRAYHYLA*
>Mcir_Mucci2_114677
MTEWEAIKRPKLDNMVEQSSSMSSAGFMPLPFSRYLSNSFQHSQKHPILSDSKLVVYDLKVDRPELPTDYEETAWNRLKA
AIHAIQKNEPPSESLEVLYQLCENLCQYDKAETLYHHLYEECRRYIEIRFEALASNSKEGDEYLHDVHLLWKSYCKQMTQ
IRCLFLYLDRTYIASTTKTGSMWNMAMDLFRENFSRYQGVWHKVLHIILDMIRIERIGQAVDEMFLQSNIRMLMDLNLYH
SAFEQQLLVQTQQFYDKEGDRLLESINIFDYLEHVSTRVHQESILRVKSYFDKSTKSQLQSIVERELLTKRVESILAKCF
DYFQSIDHFQDGDFDKFSLLYRLLQKVDKLEICAKYFTNYVKTKGSSILCDKTSIQDKISMIAAFDSQNDEVVDHSFEGD
EQFVDGLKDGTDYFINLRENNTIKLLAKYTDQVLRNPVFDQELLEKGMFLFRQLQGKDEFEVLYKRDLAKRLLLNISHKS
AEKMILAKMKIECGAGYTGKMEGMVKDIEKSGELMKEFHEYNMNTQPALNINVNLLTYGFWPSYIPINVNLPAAFHQAQE
MYNQFYQTKFEKRVLTWQNSLSVCEITANYPNGTKQITLTLLQTIVLLLFNDTTRQAYSFTDILNYSQLDELELRRTLVS
LSCREYKLLQKHPQGDDIKPTDVFSFNYEFYAAKDHLSMTTATLSEVIEKNARLDTTVLFAKDQQIDAVIVRIMKSRSTA
SHGSLLNEVTGVVRFPVTAQEIKKRIEVLIDKEYLERGPDDTYSYLA*

>Rory_3579
MIIIRGSALASVELKNSLKKACIDLIQKKISFTFNDAQETYCKIALKHIEQIFKQKQSHSPKTLSFASLFVPDMKYQGKE
LERQSRLELDIELPNLADLFNLSAEFLERSSRKALLELDDDLTIEDVNSWLLDEEDDTVDVDSIQSKLQQFLDICSKLNA
LCLEPDWVKILKEIIKERLNKDDWFAFLRQKIKAEHILYEAIYQFRIPAILDIIWDIPETDEVILDLQGVFTVDILDYYA
SCIRCLRRIDPSCKILMAVTEIIQAYMSGYRKDVAKGVVEMIRDSDTYSHICRSDDDDPYVFTALELNNEQIQVEPESIK
EDMLAKLRRLQTKSTDITAMLISMCNPVKDFVTTYSNQLGKMLLSTKNYDTDAEILRLETLKLNFPPNTFIRCDIMLKDV
ADSKRIDKAVHESKNIDHSFHSIILSRKYWPGGKDDGDDDDASDNDHSDVAFQQWPTRQQNIENYMKEYTKVKASRKLKF
IPTSGTVSLELDFETHSQSFDVRPEAAAIIKLFEGDGSCFLYYCNLTT*
>Rory_3797
MQCKKFALAAEYMSRGCCYLDRMNNSNQLNNRNGGKYKRQNVEAVALSQWKQNVLFVIRDHYQNKLFYQVFEWIRQDRNG
DNAPHNIIKSVVTSLVQVNAFTDQPLQLYIEEFERPYLVHTKRYYAAEAAREIAYGSISHFMERANDRLQQEIMHRIIDE
FETMLKDERFHDCTLAYKLLSRIPDGLKTILDIYENYITKLGKDILSQLGAGVSKNPKPFVDQLLALHSKYYQVNSQVFE
SHSLFTAAVDKAFRTIVNDAANANGPETLARYCDMMLKKNVGKKEIGTGQRKKIKKEEDQEDQEARLMKMITLFKYVDDK
DVFQKFYSRMLAKRLIYNASLSEELEINMINRLKEICGVEYTSKLNKMFTDITLSSDLNSNVEALVLTAGAWPLNQKDDT
ATDTNKLMLPAILENNITWFESFYNSLYNGRKLLWQWNLTRGEVRANHFDKTYELQVSMYQMIVLLLFNQCNSMTVKEIA
DRSGLSLAGTTRSLRPLVELGLLNTLDGKFNEKSIIEINREFSNKRSKIKVGNTVAQQQSESQQESQAARKSVEEDRRMY
IQAAIVRIMKSRQTLSHVQLIQEILDQSNSRFSPSVSMIKKCIEQLMEKQFIARQEKDCYVYVA*
>Rory_4952
MKNHPILSDRKLIVHDLKVERPNLPEEYEQDAWNRLKRAISSIKQDEAQHDSLEVLYQLCENLCQYDKAQELYDLLYAEC
FQIVQDQFDSLNLDLDKDETFYLNKVNRLWKEYCEQMSQIRGLFLYLDRTFALNAKGGSIWTMAMDLLRSSFLQHEAVRH
KMLSSILELIRLERTSQEIDLGLLQSVIRMLLDLNLYHNEFEPSFLKETRAFYGAEGDHLIETIPMGDYLDHVSTRVHQE
SSLRVKRYFDKSTKAPLQAIVEEELLTKRVTTILDKCFSYFHENYLVDELSLLYRLLKKVGQLNICAKYFTNYIKTKGSS
ILCDKMFVQEKMNVLSTFKKKNEYIVDHSFEGDEQFLDGLKDGTEYFINLKENGAIKLLAKHADNVVKNEKMDEKQLDQC
MFLFHHLQGKDEFEILYKRDLAKRLLLDTTNRNAEKVMLAKMKKECGAAYTSKLEGMLRDMKQSNELMQEFNKVQDLYTN
AYMTKYPRRRLTWQNSLSVCEVDAHYPKVKHAYFDNKNH*
>Rory_6858
MSSMQLPPHNDFNATWAFLEEGLDQVMCRFEQGLTRARYSILYSAVHNYCARSDSALHSTTQYSTIQSQSSRRPAPAPPL
IGGEVYLNLCEYLKRHLENIRAESEQYMDESLLQYYTKQWTRYTAAARVVNNIFMYLNRYWVKREIDEDRKSDVYDVFSL
TLYSWKKYMFEYVHYNVISAVLKLIEKQRNGEVIETGLIKNVIDSFVSLGLDHNDSSKSNLDVYRNYFEQPFLEATEVYY
KTESEKFISENSIPDYMKKAEVRLNEEETRVQLFLHPSTHQTLVPICETVLVKNQEESIWDGFQGLLDLDKQEDLHRMYT
LLARIEEGLNPLRASFEAHVKKAGLTAIERIAQSEADGFDPKSYVDTLLDVHKKYNDLTQSAFCGEAGFVAALDKACGEF
VNRNKVCKGASNKSPELLARFCDQLLKKSAKNPEEDELEDVLNNVMTVFKYVEDKDVFQKFYSKMLAKRLVNGTSASDDA
EGSMISKLKEACGFEYTSKLQRMLTDMSLSKELNEEFKSVAQNSSETPNSSADFNILVLSAGSWPLSAPSTSFNLPDDVV
QMYDKFQQFYQTKHIGRKLNWLFQLSKAELKTHYLKSSKVSYTFMVSAYQMGILLQYNNADSYTYEELQKSTGLASEALN
PALGILVKAKVLLLRDGTNVGDAGSRYVLNQDFKSKKVRINLNMQMKMEQKAETDETHKNIEEDRMFVMQAAIVRIMKTR
KVMKHVVLIDEVITQLQSRFKPRVPAIKKCIDVLLEKEYIERVENQKDMYSYVA*
>Rory_8594
MSLKKGRIRPPKRAPGVDQTQEYDEGFEILSNAIMVIFQKQARELSYELLYRTAYKLTMRQFGEKLYHDVEKVIAEYLEK
TAQETIVPAFVQTKTDTIDAGASFLKTIKRVWDDYTTAVELILQVLTYLNDRLPKYNLPGVYDMGLNLFRDKVIRSNNYP
IQKHLISAMLTQIQFEREGDVIDRSAIQSAVAMLAELKDSATNNTVYAVDFESDYLEKSTSFYQIESQKLVSSYDASEFM
RKVEKRLEEEYERTVHCLSMTTEVKIRTIIETELIENNVKALMEMKNSGLESMLAADKYEDLLRMYNLFSRVPAGLNEMR
SFISKYILTLGSQINQHINSDLKIEKGSSQLAIRWVQEVLELQDKFDKILDQAANKDKSFQTVFNEAFERFINENPKSAE
FISLFIDENLKKGLKGKSEDEVDDILDKTITLFRYLQDKDVFERYYKQHLAKRLLLNRSVSDDAERGMLSKLKRECGYQF
TNKLEGMFNDMRLSSEMNGLFKEYLDKINERLPPEVSVTVLTSTFWPMNLSTSPRCTMPPTVIAACQSFERFYFARHSGR
RLTWQPQMGTADVRAVFSKSKHLLNVSTYAMMVLLQFNQHDTLSWQELKTLTQIADADLKRTLQSLACTKYKILNKSSKG
RDVLDDDTFSFNASFTCNLARIKIQAVASKVENDSERKNTQDKVDEERKHQIEAAIVRIMKDRKTMEHNLLIAEVTRQLS
SRFMPSPLMIKKRIEALIDREYLERSTEDRRAYHYLA*
>Rory_13748
MVIFQRKTKALSYELLYRTVYKLTTRQFGERLYYDVEKVIAECLQKTLQDTIVPVLVQTQVDSLDAGISFLNTMNCVWND
YMTAIELIMQMLMYLIILNQIEFERQGCVIDRNAIQPIIAMLLELKDVETNNTIYAVEFEVVFLEKSALFYQIKSQRLLS
NCDALEFMQKSYILEQMKRSELECMLTKDKYDAILQMYHFFSRVPTGQSDMIRFICKYILETGSKMNQEANYELKMRSSS
QPTAVGWVQQVLKLQDKADKILAQAVNNDKSFQIAFSEAFETFINENWKSAEFISLFIDEILKKGLKGKLEYEIEDTLDK
TIMIFRYLKNKDMFVRFYKQHLGKRLLLNKSVSDDAERGILSKLKRECGCQFTNKLEGMFKDMKLSVDMNSQFKDYLSTT
NQKFPFEFYVTTLTSTFWPFPSTPQVCVLPPMLLKARDSFENFYLNRHSGRRLTWQPQMGTADVRGHFSKSSHSLNVSTY
AMTVLLLFNQHDTLSFKEIKAMTRIADADLKRVFYSLACAKYKILLKSSTENREVRDSDSFSFNSNFTCHLARIKIQAGV
SKVETDKNTRDKVDEERKYQIEAAIVRVMKDRKKTL*
>Rory_16415
MPPKPITKTSKVSKKRKLEDPTQPKLEFFIQKNDPMQIDSVNDKPATLGTFRQQLIVPKMTIVNLDSARPSELPTSFYLN
AWQIQRNLILAIFTNSTKHANYAAAARLCENMCRYGKAQELYENLKVEIEEEAKKIQSVLFTVSDDELLETLNDRWESLC
NQLAIIRNVFMELDRYHILSHTKYSSIVHLGIDIFRETVMSSDKFRDGIIWQVLKLIQQDRDGMAVKDRLIKDILHMLQE
LSYYSSDFEPTFLEHTTAYYRLESDRLLNSLSAWKYIQHAFQRQQEEVGIRISRYLHIQTKQPLLNTVTDQLVYQKVNVI
LSRGFEEMMDKKMHKVLSIFHALLSGNQNMALLRTFFGEYIKKHGMALIQDPKKDANMVTSLLEFKEELDKVLHDCFQND

DQFANTLKESFEYFINTRKNKPAEMISKFLDTRLKAPTKKQARPSENISMSTIDNVLTLFRYIQGKDAFEAYYKKYLAKR
LLLDRSISLETECEVVQKLKGQCGHEFTKNFETMLKDIRLSSELNQDFKQTNPYPIYVKVVTQAIWPTYSTTSLALPLEM
AKTQEAYSQFYASKFKGRKLIWQNSLSSCVLTGHFRKGSKELTMSLSQAVVILLFNHTEKHAWSVGEMKKATSLEDGELQ
RILTTLSTGSFAILNKKSRTQGISDTDLFQFNTEFEATGSRLKIPAVQQEQAVEEKKEVESKVLINRQHQLEAAIVRIMK
ANKTMSQENLLSEVFKQVKFPVDVHDFKRRIESLIERDYVVRDPANNSGKKKNKQQINEWTKVNNCDEYEVWPFVADDLS
ARLPLRNLKWQPSSQRAECLIPTLEVDLKRFTPDLSPLPLLTTTQTVYLNLYFVTCEDNEIYKTRIRKNIKSWLELIQSK
KNQEWLIVYVAEADTKRSNNYLGLKSSVFDKIRTDFNPPKQDSFDMQVFQIQEDTRRLDMQRHMPGWNYCTFFILKEGLA
QAFEIMTLYEDALIQYDELEASFFQVLKDKALAWFGHFGGTDPGDDSGNILDFKRKNYRDMITKNMISVFDFRCYLFARQ
CRMLLKMHKVIDVTARAQLFITNFIPSIRENEDNLPINFVESWLDKIGIRCKHLPMTDPFSIYINHNVNEQINPEQSKKS
ITNTKLIEAMQSIDAFDKTYMGLSTRAIKSYDASLRSKAALNVHGDIAALKYIREKYEEAVRIYESIICRYGEQEWSSIE
NSLLIKCADSQRRLGKNNQYVESLLALLRNAAFLSEKDSIFYTDELIEHVGKLDKEIKRPFSPIFSVNVNCIIDDPNTVD
TTSVEVCIDNHLPKKICYDTISLRLVGNDPDQISFIIHSKDLVPGKNTFLLTSKTSTSGNYLVETCEMKLGKLIFGHNFL
HDGQKKRTVRLNHDIKQLYATVSQPNESERQKFSVKIDSREISVTKGILLLESQTEGTKIVQLSKVAGLLHSNGEAKEVQ
LDMLETGEISLIDLEPKQILELFVIYEGPYTEFDYRKKKVKTTIIYETNGDTHKFISSNVVKVTVPLLVTESTIFRETCV
FLKVELSCNGELPVRIFGSHAKPSTYYLVESESKLAECDLTLFPRQTVAFVYKLVKRESDEKDTESDNQVNSKIHFTAKF
LSLKDEQHLPYVFDKVKEAFLTSVDYASYGLTDVVQLDDFDAELCESFLLHRDLKTKVQLLDLIEEFFEENVAITAQTIR
QYSPNPTLHSISFPLEVPKTKILHTAELILSPKKDLLVTEAYSCILRIKQSTYWATSSTIHDENTNNEFCYDIDVDYENW
LLSGKRRLRFISKPGEVSEFPISLVPLRTGNLLLPSVRVSAVSPEIFASTVYLNSAQQVLVKPKSRTATFFVEQQQRFIQ
PTGQVPYMASSVRSGGS*
>Amac_AMAG_00688
MYSSGSSSSAGGSAGAHRRVKIRPVRKANDSEHDASMDVVLRAMDVIYRRDASSLSFEEIYRSTYTLVLHKRGDDLHRRL
LMTMQSILAADVAAKLAPLVGAGAGDFGGDQGRVFLRAVAGLWDHFATSLKLMGDVLMYMDKNHCRRPPKNLPSSWELGL
LVFRDHVLRSTTVPVRATLRACLLDQIAAERNGDVIDPSLMRRVLTMLVDLGHSTADPLGVKHPTHIFGETVPHGSVYQA
DFETAFLAATIDFYKAEAQRLVPSLDVAAYLVQSETRLQEEERRAQQYVHVSTLRPLLQRAREQLLSEHVATLLQTGLAT
LLFTPAHDPDLNRLFRLLDPVPDGHSKLLDAIAAELTARGQAIHALVTDDARDPAVPWMDAVLTLDRRVTRVLTACFSGK
PEFEKRMQTALAGVIHAQPKAAELMAQFLDVHLRRGAVKKGEDEIEAVLKDAVKVLHFLHDKDTFERFYNQHLARRLLRK
QSSSEELEKAVIADLRTLGGSQFTTRFEKMLGDMSVSRDLAAQFKDACRAAETRVVDLSVHVLTSSLWPVAAEGGENAAC
VYPEQAQAALTGFEQFYAKRHPKRVLTWLPHLGTAELKANFPRGAKELSVTTFAMVVLMLFNDRDALRYEDIRAETNIPE
PELKRTLQSLSLGKVKVLVKSPPTKEVAEGHRFTYNSDFKDAKYKIKVSAIAARADVDAERAQTLQKVEEERAQITDATV
VRIMKARKTMTHNELVTETVRQLAARFMASPALVKKRIESLIEREFLERAAGAHSVYNYLA*
>Amac_AMAG_00961
MFFKIYIPVNEINPEVELIKTAPILRDHLFSELNRYLGDCLHVADQELIAAQPDTNESIRRALAHLEHLHRAHGIVAGWL
QLLDAPATTRAEFALLFQAYGSHIFQDRVVQHQLATLIATPSTSLDYLWGRRPVVATRYVQGALQSLLSTQEARNVLLGV
AEYSRSPRPARAAVFAASAGMIPIIRDLGLLAPLRPTILRRVQDHVADYVAQTCAEVGLDVRAIDVCLYTVTEFAVPRLA
GTNCFGTKEEMIDALEQLVVAVMHAMVDARAPAIVDMVVDLGASEGARSDLKIALEATKRVADVAAIANSAMTQRFAHLG
QSSTDLLFYLSNATRLCQYLDASGELFAAATPDLAAYFRTRTDAMPAILEFVLGSGRALLAESHGRETREHDEIMFTDTI
DFSGVDEDASTLSTRGLRAPPDTLSLVLNVYSSKDVFMNVYRAGLADRILKAMSVTSAALDVKAEKDVLSKLRVRFGAKA
VVACDDMIRDLEASQALNVAYAGVCKPDEPSMIALVVSDVMWPEDEKQEYQFHPGIQRAHSHYLTTFQAHRPLRNLEWLP
HHGHIVLEVTLDDGVSHDIACMPTTADVFLHLQDLHLDAAPTREAVVTALAAATHSTAAHVRTALDFWVTEGFLDRSTYA
PLPSRLLGTRLTTISTAAAARPRPSSPIPDILHDPAFFAAPLSSMTVKSDAPVLSARDKALATVLDAPPVPGTSTARIDQ
IARFAPIVLGRGPAPSLTRLQEGLRMFGVANVTPAEMAFVVGKLVREKKVDVNEKGEYVKALPPPSP*
>Amac_AMAG_02188
MYSSGSSSSAEGSAGAHRRVKIRPVRKANDSEHDASMDVVLRAMDVIYRPDASSLSFEEIYRLRPLVGGGAGDYGGDQGR
VFLRAVAGLWEHFATSLKLMGDVLMYMDKNHCRRPPKNLPSSWELGLLVFRDHVLRSTAVPVRATLRACLLDQIAAERNG
DVIDPSLMRRVLTMLVDLGHSTADPLGVKHPTHIFGETVPQGSVYQADFEAAFLAATTDFYKAEAQRLVPSLDVATYLAQ
CETRLQEEERRVQQYVHVSTLRMLLQRAREQLLSEHVATLLQTGLAPLLFTPVHDPDLNRLFRLLDPVPDGHSTLLDAIA
AELAARGQAIHALVTDDARDPAVPWMDAVLALDRRVTRVLTACFAGKPEFEKRMQTALAGVTHAQPKAAELMAQFLDVHL
RRGAVKKGEDEIEAVLKDAVKVLHFLHDKDTFERFYNQHLARRLLRKQSASEELEKAVIADLRTLGGSQFTTRFEKMLGD
MSVSRDLAAQFKDACRATEARLVDLSVNVLTSSLWPVAAEGADKAACVYPAPAQAALTAFEQFYAKRHPKRVLTWLPHLG
TAELKANFPRGGKELSVTTFAMVVLMLFNDRDALRYEDIRAETNIPEPELKRTLQSLSLGKVKVLVKSPPTKEVAEGHQF
SYNGDFKDAKYKIKVSAIAARADVDAERAQTLQKVEEERAQITDATVVRIMKARKTMAHNELVTETVRQLAARFMASPAL
VKKRIESLIEREFLERAAGAHSVYNYLA*
>Amac_AMAG_02472
MPAILEFILCSGREFLSETHGRKTRTHDEITFNDSINLTGADEDAAMLSTLRDLANFGAGLETHSNLVSNEVTRGLRAPP
DTLSLVLNVYSSKGVFMNVYRAGLADRIFKAISATDAALDVKAETDVVSKLRVRFGAKAVVACDDMIRVLEASQALNVAY
AGVS*
>Amac_AMAG_03390
MYLDRTYALPRHERPLYDLGLHLFAEHVMLAHQYQLRNRALAAVDRALASARDLESVPSSEIKSQLAGTAALFRALHLDA
PVLSPHVVREARQTLARAAATELAVQMSDPAAYLDWIDAVLDREKVLAESMALAGAADVDAAVRNVVIAQHADVLLAGVP
AWLLDGDATALARVMDRLASVNLLAPLVDQVGAWIAEQGCAIVEREAAKARGAAVPAIAGRQAAWHNPTGTVPMIEALLV
FKQHVDDLVVAAFRSHADVQNAVKTALHKAMNVVKNKPAECMAKYVDEQLRSRRTVDEARLEQTLDQVLHLFRHVDAKDT
FEAFYKQLLAKRLLLGRVTSMDAEKSMVARLRAERGVQSTEALDKMFADVELSDQLAKAFKDSRFAAQMPGGYDLSVIVC

QSSAWPTLTTTVSALHGKAKGNGHDDDKSAPKPIGRLPEFMTRGIDLFSQFYATQYSGRQLAWQHDMDQVTLKTSFGKEL
DVALVQALVLLEFADMNAPPLSFTELHARTGIEPAELKRTLQSLACGKVRVLKKSPPPARESGGEIADTDTFTPVAKLDQ
VKKYRFRIPQIIPREVQQQDDEHTHEQVAEDRSLALQAAIVRVMKARKVLPYQSLLNEVISQCKFPVEASKFKLQIEDLT
EREFLERDKDQHNVYRYVA*
>Amac_AMAG_03826
MSLRKRKINFDEVFRRFMGVVQQMYNPIAVHAPEPVKGMDLYHDVYLMCNNQPRPMNEELFLRLADFLEAQTTNVAAVWY
LPALAYASCTRLANRATSDPWFALQRIYASDHLLQSYAAEWDIFQFATETLNTVCDMLNRTMEANLRGAPMTSAMRAFQG
QHPNVGNSRYRRQSMESLAYSLWKERVFRHLRRELIGSTMEVVRIDREGIQGPHDAAKALALSLVTLQPKKRDETATMGP
TGSLYLSEYEAVFLAETKKYYEQESRRVMRDLSISEFMKTAEDRLKQEYHRALRFLHPSSIDKVTAECHAQYIDAHKHRI
HDAFLGMLQNRVSQDCKLAYELMALIPGGIHMLTDVYEKYVTEEGRRLVSKDPKTLANNLGQLHANRMADITQMFRFDPS
FVAALDKAFRSIINDTATHAPDLLAKYCDQLLRKRSKLSETETEDQLTMATVLFHYIDDKDVFQKHYARYLAKRLIYRQS
ASDDLEYLMLTKLKVACGVEYTAKLQRMFTDVQVSQETGTEFHAEVEKPNAAEFNALVLTAGAWPFTQASAASQAIAQLP
VELANGVAAFTEFYNTKHSGRKLFWLWNLCRADVKLTGYDKRYELNLTAHQLTVLLLFNDADTIPTATIASVLGGTRSDA
DKIIQSLIDAQLLTSSPGGTVTVNKKFSSKRIKLKLTASSGESGSGGGAATPTAHETDRSLDEDRKLYLQAIIVRIMKSR
RELSHTELVPLVVDQAKARFVPSVVLVKKCIEQLIDKQYLDRAEHDHDRYLYMA*
>Amac_AMAG_04672
MPPAGRRPARPVPPSSSGPAPPAPASSSASNSSRARPAITIRQPTLDYFGQLAAGSSTTSGSSTRPPASSSSSSSSPSSS
TQSRKRSRDAADLSTTDDRPTMLHQPSNAHGSRSSASAPARQATPASSAPSQPAVSAPPRSLTFARQSTFMLGPNGSSTP
STMTAAASRPALGSKHFAKKLVIRDLNIKPTLPPDFEATTWSKLAAAITAIFARQPVADSLEELYKACEALCKHHFAANV
YARVAAAITDQVRACFTYLAAVPDDDFLAPLVRSWTDFTDALLLVRAVFMYLDRTYALPRHERPLYDLGLHLFAEHVILA
HQYQLRDRALAAVDRALAFARDLEAVPSSETKSQLAGTAALFRALHLDAPVLSPHVVREARQTLARAASTELAVQMSDPV
AYLDWIDAVLDREKGLAETMALAGAADVDAAVRNVVIAQHADVLLAGVPAWLLDGDAAALARVMDRFTSVCLLAPLVDQV
GAWIAEQGCTIVEREAAKARGAAVPAIAGRQAAWHNPTGTVPMIEALLVFKQHVDVLVVSAFRSHADVQNAVKTALHKAM
NVVKNKPAECMAKYVDEQLRSRRTVDEARLEQTLDQVLHLFRHVDAKDTFEAFYKQLLAKRLLLGRVTSMDAEKSMVARL
RAERGVQSTEALDKMFADVELSDQLAKAFKDSRFATQMPGGYDLSVIVCQSSAWPTLTTTVSALHGKSNGNGHDDDKAAP
KPIGRLPEFMARGVDLFSQFYATQYSGRQLAWQHDMDQVTLKSSFGKELDVALVQALVLLEFADMDAPPLSFTELHARTG
IELAELKRTLQSLACGKIRVLKKSPPPARESGGEIAETDTFTPVAKLDQVKKYRFRIPQIIPREVQQQDDEHTHEQVAED
RSLALQAAIVRVMKARKVLPYQALLNEVISQCKFPVEASKFKLQIEDLTEREFLERDKDQHNVYRYVA*
>Amac_AMAG_06993
MSLRKRKINFDEVFRRFMGVVQQMYNPIAVHAPEPVKGMDLYHDVYLMCNNQPRPMNEELFLRLADFLEAQTTNVAARIY
ASDHLLQSYAAEWDIFQFATETLNTVCDMLNRTMEANLRGAPMTSAMRAFQGQHPNVGNSRYRRQSMESLAYSLWKERVF
RHLRRELIGSTMEVVRIDREGIQGPHDAAKALALSLVTLQPKKRDETTTMGPTGSLYLSEYEAVFLAETKKYYEQESRRV
MRDLSISEFMKTAEDRLKQEYHRALRFLHPSSVDKVTAECHAQYIDAHKHRIHDAFLGMLQNRLSQDCKLAYELMALIPG
GIHMLTDVYEKYVTEEGRRLVSKDPKTLANNLGQLHANRMADITQMFRFDPSFVAALDKAFRSIINDTATHAPDLLAKYC
DQLLRKPSKMSEAETEDQLTMATVLFHYIDDKDVFQKHYARYLAKRLIYRQSASDDLEYLMLTKLKVACGVEYTAKLQRM
FTDVQVSQETGTEFHAQVEKPNAAEFNALVLTAGAWPFTQASAASQAIAQLPAELANGVAAFTEFYNTKHSGRKLFWLWN
LCRADVKLTGYDKRYELNLNAHQLTVLLLFNDADTIASSTIAGVLGGTSTDADKIIQSLIDTQLLTPTPGGTVTVNKKFS
SKRIKLKLTASSAESSGTGGGAATPTAHETDRSLDEDRKLYLQAIIVRIMKSRREMSHTELVPLVVDQAKARFVPSVVLV
KKCIEQLIDKQYLDRAEHDHDRYLYMA*
>Amac_AMAG_07578
MSHDAAASTPNQVWASLRPTLLSLLRDPDAGLSSAQYMSTYTAVYNHCTSKPDTTAQYSAGANVYGSDLYFHLRDLVHEH
LNVLTKQAAGLIDIDLLAFYMREWNKFSRTAKFLDHLFSYINRHWVKRAVDDHRSGVYRIHTMVLVTWKQRFFDAISDSL
IKSILDQIKLERDGEQIDRSMLKSVIDSLVALDMPNNEAAGANAAQPERVYRTVFENPFFSATSLYYKAESERFLAENSV
IEYMKKVEARLQEEADRVVKYLDASTLAPLTTLCEDRLIADHQEAFKDEFQSLLDHDKVEDLARMFVLLQRRANTLDPLR
TKLELHVRKVGQDHVTQIAAAGEGAGAAAGGGDDDEGAGGASGAAGAGVDAKVYVNTLLQVQKKYADLVNVAFKNDTGFV
ASLDKACREFVNRNAVCKTSTAKSPELLAKYSDMLLKKSAKNPEEAELDEALNQIMTVFKYIEDKDVFQKFYAKALAKRL
VNQTSASEDAESLMITKLKDACGYDYTSKLQRLFTDVGTSKDLNEAFRKQVADLGLDFSIIVMSTGVWPFQVPASAFELP
EELLPTYNRFMGFYTAKHSGRKLTWLHNQSKGEVRAQWATGNKLQYAFQVHVYQMGILLQYNNATSYTWEELLALTKLDA
PTLAGHLGTFLKAKIMVQEPEGATVGEAGTKYALNMSYKNRKVRMNLNVPIKAEQKAEADEAHKTIEEDRKLLIQAAIVR
IMKTRKVMKHQALVQETVTQLSARFKPKVSDIKKCIEILLEKEYLERVDGEKDTFSYVA*
>Amac_AMAG_16949
MSHHAAASTPNQVWASLRPTLLSLLRDPDAGLSSAQYMSTYTAVYNHCTSKPDTSAQYSAGANVYGSDLYFHLRDLVHEH
LNVLTKQAAGLIDIDLLAFYMREWNKFSQTAKFLDHLFSYINRHWVKRAVDDHRSGVYRIHTMVLVTWKQRFFDAISDSL
IKSILDQIKLERDGEQIDRSMLKSVIDSLVALDMPNNEAAGANAAQPERVYRTVFENPFFSATSLYYKAESERFLAENSV
IEYMKKVEARLQEEADRVVKYLDASTLAPLTTLCEDRLIADHQEAFKDEFQSLLDHDKVEDLARMYVLLQRRANTLDPLR
TKLELHVRKVGQDHVTQIAAAGEGAAGNAAGGGDDDEGAGGPGGAAGAGVDAKVYVNTLLQVQKKYADLVNVAFKNDTGF
VASLDKACREFVNRNAVCKTSTAKSPELLAKYSDMLLKKSAKNPEEAELDEALNQIMTVFKYIEDKDVFQKFYAKALAKR
LVNQTSASEDAESLMITKLKDACGYDYTSKLQRLFTDVGTSKDLNEAFRKQVADLGLDFSIIVMSTGVWPFQVPASAFEL
PEELLPTYNRFMGFYTAKHSGRKLTWLHNQSKGEVRAQWATGNKLQYAFQVHVYQMGILLQYNNATSYTWEELLALTKLD
APTLAGHLGTFLKAKIMVQEPEGATVGEPGTKYALNMSYKNRKVRMNLNVPIKAEQKAEADEAHKTIEEDRKLLIQAAIV
RIMKTRKVMKHQALVQETVTQLSARFKPKVSDIKKCIEILLEKEYLERVDGEKDTFSYVA*
>Bden_Batde5_19177

MLPTAGPQRQHPQQTSQQQGTNLPKPDTRHSSNKRPKLETPFTMLNANNDADQKQSQRLGGKTAKKLVIKSFKVKPKLPE
NFEADTWEKLRLAVQAIHARQPVQDSLEELYKACENLCHHNRQSNLYQKLYSVCKDHVLVELDALKSNIHTSGCNILVAV
NECWLRYCQQMMLIRSIFLYLDRTYVLQTASLKSIWSMSMDLFRSYVLDDKEIQERVVRELIQEINCERREQQISRPLMR
SLIRMMTDLSVYIRVFETTFLENTRQFYRVFSKTIVDSIDGNLALGEGANRVSSYLIQVSNRLEQETQRCSPGEGYIDPL
TRKKLVLTLEDELLRQHATLLLDVGFDQLVAAQRIDDLALFYKLLERIGMLEELKRRMSQYIQATGIFIVKDPTRDKTMV
QELLEFKMRLDDILKNAFQSTESFDHAIKESFEKFINQRQNKPAEMIAKYIDELLKHVKGMTDLEVDRRLDQCLAIFRLV
QGKDVFEAFYSKDLAKRLLLEKSTSVDAEKSMLFKLKAECGPGFTSKLEGMFKDMELSRDIKRKFEDTAGFYNRIGRIDL
NVYVLTSGLWPTYTPVDLNLPNEMTVCQEVFKEYYMSKHNGRRLVWHNSLGSCILRAQFEKPKELQLSLFQAVIMLCFNN
SKTLSFNALHTLTNLDEKELSRTLQSLSVGKSRVLLKESKGKDVELDDTFEVNEHFTHPQYRIKIGSISVRESVDEMVET
NEKVFQDRVFQVDAAIVRIMKTEKRCAHATLVSKLFQIVKFPIAAEDLKKRIESLIEREYLDRDSNDKSLYIYLA
>Bden_Batde5_84491
MSLRPKVIDFPTAFASFQAGLQEMFLLSNNHVQGMDMYQIVYDICTARPKPFVEELLQGIVQYLLGHTVRLKQLILEHED
IVSAYSAEWEQYYTASYYADRVCDFLNRHSQKRAASHTSVQAQKIFNLTVQGHAYLIWKQNILSSIKRNHANALVEQMLT
MLRRERDGEAPPSNAIRSAVNSFILVNTQSDMHLQLYTEEFEIPYIDSITSYYKNESTAAMSSLSISDFMIKAIYRLDEE
AERSKRYCHYSSLPRASFACVSEYVSAHQSKIHSDFENMIIEGRFEDCTRAYILLSKVEGGINRIIKEFEKFVTKQGKDA
MSPFAASILKDPRKYVDTMIETHSRLMKLAVDVFKGDSVFIAEIDKAFRDIVNTREFGKDNLALEAFARYCDLFLKKTPK
STLGDVDVDERLGKMANIFQKFYSRALAKRLINSASVSSDSERAMILRLKDACGYEYTCKLQRMFTDISLSEDINDRFKS
DLDAKSRKLGIDFQIFVLTAGSWPLTAGVFPDFQLPVEFENPTIEFLSFYNSIFSGRKLSWLHHLSKADVKLNFTDKRYE
LNISMHYLAVLLLFNDSLSLTVGECEQLTKIKLAEIFKTIKVFADMKLLLVDNDKIDSLSVDTVVTLNTMFTSKRLRIKV
TSGPTPTEQRHELDTTRKAVDEDRRHFLQATIVRIMKARTKLSHSGLIQEVMEQSKSRFTPSTILIKRCIEQLIEKQFLD
RSERDQYIYVT
>Bden_Batde5_13800
MNPIGLAAAWDTLRPGVDRILTKWDEGISANEYMVLYTATYDYCTNTKGISGISEQRADQKGNSLSANLIGADLYLELRR
HIETHLQQTTDLAADLIDDAIINYYTKHWTKFTVSVTTLNHIFGYLNRHWVKREIDEGHKTIYEIYILALVSWRDHIFQR
LQEKVIKAALKTITKQRNGETIDTGLLKTIVESCVSIGLDENDSRKSTLDIYKIYFEAPFIDATESYYKAESEMFTTQNP
ITEYMKKAEIRLQEEEKRVEMYLHASTQKTLITTCETVLIKNHTGLIQDEFQALLDNDRVDDLSRMYSLLHRVPEGLDRL
RVIFEAHVRKQGLMAIEKVSEKSANDSTDVDPKLYVDSLLSVHKKYADLVQVAFRGEAGFVASLDKACREFTNRNLVCKT
SSSKSPELLARYCDSLLRKSNKMAEDTEFEELLSSVMTVFKYVEDKDVFQKFYSKHLAKRLVNGTSSSDDGELLMLTKLK
DACGHEYTSKLQRMFTDMGVSKDLDDAFKEQMRRNHESEESLDFGALVLNTASWPFQPPKSGLNIPDDLLKKYERFQRFY
QSKHSGRKLTWLFQFCKGELKTNYTRGSKTGYTFQVSTYQMAVLLLYNTATLYTLDELLGTTGVVKDVLLPTVGLLVKAK
ILLVQGGALGAPSSRYVLNEDFKSKKVRINVNLPIKTEQKAESDDTHRTIEEDRKLLIQAAIVRVMKTRKTLKHVTLVTE
VIQQLQTRFKPQVSDIKKCIDILLEKEFIERADNQKDVFNYLA
>Bden_Batde5_92557
MVQDRSQIEQDRFEILQHPCIHELETLCNLLQQAGLSSCFQDIYNSVLTRHIKQHVVTLARHLEYVSVSNQLQQWLKSHI
LHLSHILGKLDSNQHAPSREKKLEFFAFQTLCVTRVQDAFEIVRDFPDSVPAVEDLYIALQKYNNIGALLKTLETSIGKR
LIHCGAFTQDILSFYIALAQVLCMLECSEDIVNGMLNPIRLYLRSRGDSLEQIITQLVNQDTEVILLVDHAVSQRLSAVR
IPQLLNIFPNKEVYAKTYIDLLATRLLSMHDFDLDDDKASLAMLERRIGNKITTCAQVMIKDMQESRRFAMQFHKQTIDA
SYFSVLVISARFWPDEKIRDAPLTLPHSIQQFQKVYQDSYALLKARRLKWRPMLGSVDIEIGQGDSSLRVSATPAQATLV
SLFSDKDSILVSEAIEATQLSKTAMYQAVGFWNHHGVLALRQDHIINIHNTVKELTQDKLAATSVTTPMQDVIPVQDPAA
ERLETLQNTVWPFVEAAMTAFDRLTVHDIQKQLAPFGLGMDEMELRKLLEWAIECGKADKVGQHYVPKLA
>Bden_Batde5_20511
MSHSYLNSSRSVKIGARPTRERNTDEQWKILSNGITQIYCKNALQLSFEELYRNAYSMCLQKAGERLYKGTQTLISEFLE
KAVAQNVVPAFPHNATPSQSKAHVFLNQVKSLWDDHIVCLGMIRDILMYLDRTYVKSANLPTVYEMGLETFRNVVLQSVQ
YQVKSHIITTLLYQIQLERDGQMIDRMLLKNIIDMLLTLPASSTSSSRTIATVYHADFERAFLQTSQTFYSRESEVLLKE
CDAIQFLKRVEKRLNEEDIRTKHYIHASTRPKIQSIFEKELLENNIKTILEMDSGLVPIVANDRFEDLQRIYSLFSRVPN
GHVELRFGLSNIIKQSNTSRPSNTNMPSETNPLSPILWVEAMISLKDKYDTMLDACFARDKTFQNDINSSLETCINLNIK
CPEFLSLFIDENLRKGIKGKLDDEIEKFLEKSVCFFRFIREKDVFERYYNQHLAKRLLYGRSVSHDTEKNMISKLKVECG
HQFISKLEGMFKDMHVSNDLTHGFKDYMASVSVCETKTPDLSIYVLTNTFWPVTVPPAMMACYLPPSLAITVDHFQKYYM
TLHSGRQLTWLKHMGTADLKAQFTTCKKELNVSTYAMVILLTLFNSLEVNEPIGYQRIMNETEIPSGDLARTLQSLSLGK
YRILLKSTKTKSIGLDDTFVVNAAFTSPLSKIKIQTVAASTVAGSTTHTGLDPTSLATSSTAANSVETEFERVKTMEQVA
QDRKHQIEACIVRVMKSRKSMRHNELVAMVISQLSLRFSPDPLVIKTRIEELFEREYLERDTENRQLYHYVA
>Spun_SPPG_01185
MSTRLRNSDTKKRKTTDTQLPISHFQQSTLDHFANRAVADGSSSLTGAKKPRLEMQSKVNSGPARNGVYLDLTSLSPSAM
EDSKYGCGFGGDAKGKGKAPAGRPDSPIAVTAPLARGRREPGQKLVIKSFKVKPKLPENFVTDTWKKLEQAVHAVHESRP
VADSLEELYKACENLCHHKKADELYSKLRDVCDQHIQKELGKIKRSFTNVGATIADRWSIFLYLDRTYVLQTSGLRSLWD
MGLDLFRERIMDDMQPVKLKTVKALLDEIERERNGEQVERELLRSLLRMFLELSIYFSGFDAAFRERTESYYREESDRRI
NALDNEGSGGGNVARYLEHVEIRLQQEVERCSAGLGYLDAGSKKGLVTVVETQLLKRHVKTLLDKGLHELMEDTRTTDLA
RMYTLFNRVGATEDLRKAFSQYIRKTGLTLVSSEARDPTMVDDLLKFKTRVDHLWEKSFAKNESFGNALREAFEFFINQR
QNKPAELIAKYVDVKLRSGKGVTEEELEVALDQCLVLFRFIHGKDVFEAFYKKDLAKRLLLQRSTSVDAEKSMLSKLKTE
CGPNFTSKLEGMFKDVEISRDYMTSFRESPRYTNQLGAIELCVNVLTQGYWPTYPPINVNLPDELSHCQDVFKDFYMHKH
SGRKLTWQNSLGTCILKSNFNKGKKELSVSLFQAIVLLLFNDETHHTYTEIREATGLEDKELERTLQSLACGKVRVLNKT
PKSKDVSHSDSFEFNASFEYALYRVKINQIQMKETSEEQKDTEERVFQDRQYQVDAAIVRIMKMRKTLSHSLLMTELFDQ

LKFPIKAADLKKRIESLIDRDYLERDAKDPTVYNYLA*
>Spun_SPPG_02066
MAARRPKVAMKIRPPAKKSNAETQWTETWERLSTAIKEIYKRNAGVLSFEELYRNAYNMVLNKQGDKLYNGVRDVVTEHL
QQVARDFVVPSFPQMAPGGGMTGGTEFLKTVKNVWEDHTTCMLMIRDILMYMDRVYVKAANLAPVYDLGLDLFRDVVQRS
PDYPISRHLIATLLYHIRLERDGEVIDRLVLKNIIDMLLGLSEPRGPTRTRNDKTVYVTDFEETFLESSREFYRLEGQVF
LQQCDATEYLKKAERRLQEEEQRIANYLASTTEPKIRQVLEEELLQRHVKAVIEMENSGLVAMLSNDKYNDLSRMYHLFS
RVSNGHAEMRHNASSYVEGLARAINETYGGMGEAADPSTIATSASLAKASEQDVEASAASTQQANPTQWVERILALKDKF
DKILEQSFANDRHFETDINAAIERSINQNRKAPEFVSLFIDENLRKGLKGKTEIEVDSLLDKTVTLFRFIEEKDVFERYY
KQHLAKRLLLGRSVSEDAEKNMIGKLKVECGCQFTVKLEGMFNDMRLAADTMGDFRSHLANSITAPKNMPELYVNVLTST
FWPMNTATSGDTCVFPTEITAAMDHFQRFYHSRHSGRRLTWLTHIGSADIKANFDKGRKEINVSTYAMVLLVGIFNKLAD
GQSISYDRIREESSIPDHDLKRTLQSLSVAKHRILLKSTKGRDISPSDHFKVNTGFTSQLHKIKIQQISATSGSSSSASN
SMETDAERADTLEKVDEARKHQVEACIVRIMKSRKRMDHNNLVAEVVKQLSARFSPSPLMVKKRVEGLIEREYLERDKSD
RMVFEIRAIGRPKEKLKQRAVRKRAYAPEKSKEEALIGKVQVQVSTGTVFVYVPVDPTYEALQERLSRIDACNIGVQRAK
SKDAATLEVETHSALFLTVRIYHDLSDDGVPARFSPVPVRPDVRHRRCLSETSAITTTPKISRPHRASPYVVSVPSSPRT
SSPQSPSTPNCGFGKNSTLRPCFPSPPRSLPASPTPIMQEYPSDTDTGATDKSAFMSEFFYNTSPPIQACFPDIHFEQLF
NNASRTPAPEGGSDVGLLGEPWLDLLPLEDSFMALDETSASVNSGVDADGFEDVFCQVDESIRTSDEFFQREDIGDTSVQ
SCVSRCVLGDNGFEDILRRAREM*
>Spun_SPPG_02377
MAAYGAQAQPIDLATTWPKLEAGVDRIMNRLRDGLSYGTWMDLYTLIYNYCTSSRMNTGLNNEPIGGSSSNRGANLMGAD
LYINLKAYLKQHLDALKTEADQQMDEDLLTFYTREWKRYTTATMYIHHIFRYLNRHWVKREIDEGHKTIYDVYTLTLVTW
RDHFFGGGPGTVQQKVMDAVLKLIERQRNGETIDTSLIYNVVESFVSLGLDENDSTKSTLDVYKRYFEVPFLAKTDAYYK
MESEKFISENTITDYMKKAEARLAEEDHRVQMYLHSSTQKGLILTCETVLIKNHTGPIQEEFQNLLDQDKVEDLSRMYGL
LCRVPEGLEKLRLIFEVHVRKQGLSAVEKVAGSSAAPVNEEGDEDDEPTPKKKTKKSAAGDVDPKVYVDALLAVHRKFSA
LVDEAFKGEAGFGASLDKACREFVNRNKVCKEGSSKSPELLAKHCDSLLKKNSKLTEEHEVEDVLNSVMTIFKYVEDKDV
FQKFYQKSLAKRLVHGSSASEDMEESMINKLKDACGFEFTNKLSRMFQDVTLSKDINDQFREQMSRTHEKDDLTDFHILV
GGTASWPLHASPSGFNIPSELLKTYERFQNFYNHKYSGRKLNWLFHLGKAELKTNYVKLGKSPYTLIVTNYQMGVLLQYN
AATTYTWEELLNSTGVAPEQLAGQIGTLVKGKILTVEGGKVGEAGSRYELNMGFKSKKLRIKLDQPVKAEQKAEADDTHK
TIEEDRKMLIQAAIVRIMKTRKVMNHQTLMGEVISQLNARFKPKVPDIKKCIDILLEKEYIERMEGQKDMFSYVA*
>Spun_SPPG_04663
MTIDPPAKRVKTDLECEMSCTISTPIAEKVPIVYTLDPFFSQEAPARPLLNVDNVSDELITALETLSTALSEYIQSSCST
VECETALSNASELTNSVHVLRAEGFQSLITEYYFVSVQEHFYKTVATHLQNFEMDYSTLNDEDVDDATYSEWCWKEYTSL
VTRLAGYMNAYTCGFDLLGNVDGTFHTRLYNFFEGHLRRSLPSCWQDVTTRIFEHSLQKYDTFLSNEDSMEDVELELDSL
PELMSQLKSIGLLSESTSALMLVARERVKRKVEECRGQYEEPALAECIQWVEQHLCGGWLRRIVQYERDSTTNLKLDHWC
RDLTVWTSREFCSMRIQEIFDIVRNYAFGLTSEATLTDLKHCLTTSVQKEELVKSLREGIEERALHAGVATEDIITILVA
TENTLRVIDPTSNMLLKTIGPMKEYLRRRPDTVQHILSRMLDEDSELAEAMRQGDSVRDAEGADDDYDNWEPEPVDAVGT
RQERAADIITRLISICESKDAFVHEYHKMLLDCLIHKLDYETDKETLEVELLKTRFEEASFNQCEIMLMDMATSRRLDHQ
LYSKAGTEETMETESNPAPLHTTVMSHLFWPPLDTTDFSLPPEIERAKRTYERHFEEQKPNRKLAWKPNAGIVEIELQME
DRMETFTVTELQATVIHLFGEQSPLTWDEIVAKLREPNSPEDFKQDVGDCLSFWMSQGILKQLDEETYAPIETAGEDISN
GPSLPMPTSRRNEEDRARAKQAAELRTKFQPMIMAMLTNQGPATVERVKNLLTMFFQFSHTQNELQTLLDQLVSEGLLLV
GRNREYSART*
>Spun_SPPG_08614
MSLRPKRVDFNASFPAFRQELLNMFHSTTARAKVSGIDMFQQVYDLCTSYPKPHTELLFQSIGDLLREHVSGVLQTILSR
DDVVTAYAREWEKYRVAANYANIICEYLNRMLLKMRPPFGDTKRPFSHGRYLKLSVEALAFHIWKETVLAGLQTHHGNRL
VFQLLDWIRQDRDGHIVLHDVIYNAINSLVQLNQHTDQPLQLYIEMFENPYLEDTRDYYARESNTQIAQDRISCYMRKIH
ERLADEGARSARFCHPTSLERVIKECELQCITAHHRELYAEFRVILENERFADCILAYSLLSRSADGISAMLDIFENFII
TIARDVIGRLGSSLTKDPREYAESLMSLQAKYMGMCDEVFAGNLAFTAAVDKAFRSIVNTAIPSTSSSAAELLARYCDLL
LKRTAKGLVESEVDDKLSRMITLFKYLDDKDVFQKFYSRMIAKRLIYGTSVSEEAELNMLSRLKVAACGVEYTSKLQRMF
TDVTISNDLNVGFSKFAQLNSINLGTEFSILVLTAGSWPLSASASGFVLPSALEKSVSQFTTFYGKHHNGRKLTWLHHLA
RADVKINHMDKRYELNVSLYQLGVLLLFNVTPSLTIHDIIHQTKLNSSEVERILKIFVDINLLTVIDTQTYIVNHGFASK
RTKIKINTALQTETPQEVTAARKAVDEDRKLYLQAAIVRIMKSRKELSHTQLVQQVIDQAKVNFAPSIPVIKRCIEQLIE
KQYIDRVPHTRDRYVYIS*
>Ecun_XP_955735
MGSCTRRSRESTAVKNFVMEYLKNGSTASTCEEIYQLLHHTLRTRVSRECLKQVKKVSRTFVNESIEKTNEMELEDVGRY
LYGFNCHWDRTMNLFEKVFRPITPIGRNLMEGLFSRLFRRFVRGVEGRLTAFMTWSLKEEIERKGDGGEAAVSSYENYES
SPNKKQKVSQSSPAILKKTSGREEDPRRFESLCRREQITAIVHILQSRGCFGIFLERVVECLIAALSSRVEDKEVEIFGI
KDLIDEYRKAEYMKDYFYAVESGLIRRVVMKYTNEEIFRMLCREDEMRLCMDFFCISNSEKRLVEALGEYCREIIENLSA
DGFITGYYWIYLKFRDVEKCKDDGYEEISEGIRRCKKDVVNSNVERVVECICGWTDSLLRGRMSIEKTTIEGMDHRIQIP
GSREEDVLETRGVLKRLGIDEVLLEGGVWKEYLLSNIFRAIGEIFDLCNDKKAFEMALQNRLGNRLISQSSVSMYWENLL
IDGIDPGRNSLGKMRCMIRDFSERELFMGHEVLLLRACKWPGYEGTSLGIPDVEEVKGKYEDALRNKKKRIRWNDLLSSC
SVEILGHAATVTLTQYLMIRQLAEKDRAAEDLKIYKGWDENLGALVKNGLVRADGRRYFLNMDWEGNCSGNRLIPDTFPL
VRLSEAARGFDKSESCKDLLDCRIIKEMKNKRVLDRDELRRLMGREYSSEALDERIESLAGREFLSIEGESIRYLP
>Ecun_NP_585844

MFIADHPRMDVQEFERLEDLTMLLLSGHRTFSSKDFMEVYDIIYSHCTETAAAFETRGAKVYECLERTMVSYVDRLRCFT
SLKTLHGQLLEFSGALDLVARAYSYLERYFIRVSLERRDGHIQDVRVLGYTVFYRRYMERMMAQAKEMIFFEIGVSRGSR
DYNFGRLGETVKLLKRMLFFCNESEEYEDMIQRYLDGVEALVDFTGEINKILKRVYLEIYIASKVFDPDNNRLYKGIAAG
VRNRFDDVLGLLVHKMERFEKFKLYVKIVHFMEPEYMARIDEKYKEILRKKVLGAGSLRELVVEYLNICRQMEENLMRED
ELVPYLREEIRKHLHMSGRLGFEDEVYRDIDRTVRNGEEMGGEMEALVMFVSLMSSKEVIVGKIARDARRRLMSCKSNLK
REEMLVLSMEKHLGASISRCMSVMYENQANYVRNSFRIAGQATEFVTETRFLTKGFYDLRASGESLPPPLRDVWRIVSQP
KMAQYPRGELLCCHSLSPMVFSMNGFNFRMNTDKVVVMLWLDVDRGVSDLERCVGGTGFRQNLEYLLSNGFVMCTDGVLS
LNRMFSCREYGYIRNKYRDRICLLDSEHNLVLDGDGGSGMLEEVVDLFEAEISEAVAKRETEATGSSSDAVLEARVMRIL
KREKRVESLRMCRMVGEEFGDDEKAVMQTVEKLIEKEYCRADGDYLEYVP
>Ecun_NP_586014
MDIGRRTLSEEDFSSIWCSIERELGKLASGGLCSALEIYNKIYVVCTSSTPRLESRLYWKIGDFIYMRAREIRREVFDAK
DWIEEYNRGFRKFREMVFAIDELCDFLNECVKGRTMRDLGFLLWERCVLQQTAKYKHTTLGFELVSQGRIEVVKEAIESF
RLIVPDAKNPMLYYTQRYEQFALQKIGCKYSEEIKSACEEVERYSFYVREKIFYEKSIQDKIFLEESWPKMEMILEEVFI
LNKRDYLLSEIHRIMSMYRLPVEILAKISGEGECDKHRTDSGGHRTNDFYELQGGSGARKAHNDVFLEKMSVLYRNLSEF
KSAYAILQEVFTRYVRDESEKHIHVYSRSIETLYVFHCMLRHVVDVGFLGDRECLKILKKEFSASCMRLKPSLEARLLEF
SGCIVGNSGNAFKFLDSEFEGADDGGRMSLPFLGDGEVCGDGPGLGQSFCGKNDFLRQVFGTLYKMVENKHVFYEMYLSG
LGKRLLEGNPNLQEERKLISLMKRKSNQDFMKKAETMIADISISLSYNKDVDRFVWILTQAYWPMQAESQSTRIPAPLEM
IKKVYDSKFNKKRLHWVWHLGSVCVEIRGVEIQMNVLQYSVVDLFNKHEEIDFEKVLGLVKMGTKTTDDVLRSLVESNLV
VRAGDWYRMGDVSKVGEDIRAFYCSESICMAHEIDRSAYYQSLVSRILKRRKRMCLGDILKEILAEHTERFEYDEAMFSE
GVRTLGDKGIIEDVEGIYVYLP
>Npar_EIJ87227
MDPPVASINTERFTTYWEVIQGEANKLASGVKCSGMKIYSSIYHIVCGGDVSYAVRLHWCIGKFFFAFCVKLREKIQGED
WVKEYAAAFNIYENTVETIDLLSLHLNEAILENKECKNVKDLGYIIWERCILRQRYEGNLPALSESLPKRRKYAEVCLRS
LSKIITNSENRFEYYKNNYEAGLFSLIIEEFKYAVNIQGEIKLASYLLKCSNCIKDSIKTYTPLLLPISLPALEEALEKV
IFSKHPHAVKEEMLRILMKKDKQEIKDLCLSARLLSKKVFPAFLECIKEFINNTWTTEKTCQAAIATYIELSDLLMPDKC
AKTLCVLKKVLSTKISLAGIGKEMGDYLESLINEKNVDKIKQMNILLDSLPIKEEKQVFYNMYTSKLAERLYSLKFDPAV
EKETIVNLNLPWLLKKKVNKIFDDMVQSTNENELFKQAYGAEHFRYLTSNSNEIFFYGIITTACVWPISEEAIQTTRFPA
EIDSILTAFSGQYLAKHARRRLAWADALSIIEVEITTDKVYNVSMSLTHYSVLDIVEKSPGMLDHISKEAGLSTKATANL
IEPFVRLSIIQCTNEIYSINSKFTSATENITIQAAESTLKRIGNRKSYYQAWISRKLKQMGECPLNALSDTLQKTHTSIF
EWNVQEYSEALRSLNDRGLVEIADTTIKYLP
>Ttra_AMSG_01998
MCRLYEKVEVRLAEEVDRVARYLHHSTLERLTKTCETVMVAQHIDKIQAEFPSMLTQDKQDDLRRLYNLLARIENGLGPL
RDIFRNHVRDDGLARIEALNNVNDDDDDGAAASSSTAAASKDEKKKRSSKKQEFKSYVETLIAVHDRYWSLIVNVFAEDN
GFAVEMNKAFDHFMNLEGNRSPEYLSLYIDDKLKKGAKGVTEDEKSAIIDKCLILFRRIEEKDMFERYYKNHLAKRLLSG
RSASNDAEREVITKLKSECGMSFTHKLEGMFKDVQLSADIMDKFGESELAKNLGITLSVQVLTTGFWPQVAGSCILPPEI
AKCQEVFKQFYLNSHSGRVLNWQSTQGTADLKATFGGKTKEISVSTTQMAILCLFNQADSMTFTEIRDATELDIVALKRD
LLSLACAKYKILTKEPKSKKIKPDDVFTFNETFKCKLFKFKIPQVVTKESSREREVTIRKVDDDRKFKIDAAIVRIMKAR
KTLDHANLVAAVIKQLKARFTPNPPVIKKQIETLIERGYLERDPKSRKQYNYLA*
>Ttra_AMSG_04777
MWRDKVLAPLKDRLVYAILEQVRKERDGEAIERELVVKPIESMVKLGLERNKPLAVYSETIELPYIAALHEYYAAETTNF
LAQNAVPDYYSHAETRMAEESARAVLFLDTSSEPRVTAALTDIFVTAHLPALQGEVIPLVAAMVADSSSESHAAKALARG
YALLARVPGAFDSVQDALGAFVVERGLAALDKLGDEPASNPRKYVEALLEHYAAFRTVVDTAFAGSGQFSLVLDKAMREI
VNAKPKTNAGSSGGASSLPPAAELLAKYCDMVLRKSSKSQLEDAELSKVLDSVMEVFQYLEDKDVFQKFYSQMLAKRLIQ
SLSVSDDAESAMISRLKKAAGYEYTSKLQKMFQDMTLSDELNASFATAVEKTGMDIGGVAFSFKVLTKGSWPLSSPSTPF
VQPPELDKLIKRFEAFYKNQHSGRELFWLPELTKGELRTAYLAKNHDLIVTNYQMAILCLFNRGTVFSVAEIGKLVGLES
KVLMRNVASLVKVKLLSLVADAGDTAVSQAEPKAEAKAKTEAKTEAETDDAAASSSAKPSKGKKGKGKKRAPIKLNSSTK
LEVNLNFSNKRIRVKVLSSLIRDSTEERAATVRGVSEERMFFLQAAVVRLMKARKKMAHSVLVGEVCEIAAARFKPRVAL
IKKAIEQLIDKGYLARVQGEKGMYEYLA*
>Ttra_AMSG_05411
MIRKGANLGRRPKARTKITIKPFKVKPTLPANYFDESWNKLRTAVASIYGNSGAGGSSLEELYSLVDNLCSYKHAAELHT
KLMAELESIHVRMGAELAARARELDGPRLLSAMDGVYTQAAEHLLLVRQVFLVLDRSYVIATPRLTSLWASGLALIRKHV
LAAHPALLSGVVGALVDGITAERSGHAVDSEIVVRLVQMLVALQLYEDHVEAKFVDATAAFYAQEGGVKLAQLGLPSYLA
HVQARITEESERLTRYGTVALSGERVLALVDEHLIRANVEVVLEQGGLSELLNASMHEPLATLYGLLGRVSALPALCTAI
RGYVESTGTAIVSDPAKDKGMVDALIELHTRVLAVVEGPFQNNPDIGYALKDAFEKFINVRQNRPAELIAKYMDKLLKSS
AGKGSDEDVLLGKLDAGLALFRFVSGKDVFEAFYKKDLAKRLLLGKSASADAEKAMVARLRDECGSTFANKLEGMMKDIE
LSRELNASWSEGGSAASVAMFAHVLTMGFWPTYPEHTVLLPPDMAGHVDDFEAFYKTKHSGRKLKWQHSLATVSLGAVFP
SAGKRCWPSRSSRPLSCCCSTTATRCLLPTSRRRPNWTTRNWCERCSHCVQASPRPASCSRAPRASLSAATTSSPSTPPL
PTRCSRSRSTRSRSARPRPRPRRPRPASSRTASTSSTQPLSAS*
>Ttra_AMSG_10992
MALEAAGTVGTGAEAEALFSAYARAIADEALPPQRARDVWSGLLEVLEAKRGCDDEAGVVVGSVIEATGKIVGLFEAVAT
ALDTLAAALGITPEAAESRQTAALAGLQAALQVALPHTLFEEVMAVVYKEALLVFGLSVVCRDDDDEEYDDEDDDAENDD
EDEAATSAGSAVLGLDSLTQLMHQLSVLGLAQASVSVVTQLLYRVVEAHILSQAKGEFEEAWLDELIRWMEADVAVAVEA

VVKGCGNATVAHLRERLWFHMYEAFGELRIRELFDIIVDFPDSAPAVEDLRRALARTWQHERLVDVLSAAFDKRLLHLGA
NTVDILGQYVSSIKVLTRLDPSGVMLDSVSHGIRNYLKNREDTIRCVVTSLTDPSPTSLLGGELVSASGPTGSGVGGGRG
GGAANEDYDNWTPAPLESGALSATTTAPAARTDILAMLVNIYGSQELFVSEYKVLLASKLLAITDYNTDDQVRNSELLKL
RFGESSMQPAEVMLKDLADSKRINAYVHSHEGGGDNELVVSGTVISRLFWPPLKEVNFQLPPAMAAQMETYAKAFHSLKT
PRTLSFLPNLGSVSLTLEFDDTELEVTVSPLLATIISYFDAKTAAAVGGASIAVDGTVTWPARALAKVVGIDVALLRKQM
VYWLAASVVVDAGDECYASVSVLGDAPSSSAGGAGGMDADSGVASLEDQKAEEMRVYQSYVNGMLGNLGALPAERIHNML
KMFVANSGYTASLNELISFLNTLVRKEELAFADGMYSLPK*
>Ttra_AMSG_11828
MPGKLRMSMREFRKVQMEPSHAEATWELLKNAIRQIHSQNASGLSFEELYRNAYNLVLHKYGSMLYNGLRDVVGAHLRKV
AEEVASLTDGSDFVLGVDKAWADHSLSMIMIRDILLYMDRAYVENKGIKPVYQLGLQLFCTTVIQHPRVEARLSAALLDI
ITAEREGQIVDRELVKRMTEMYVALGEDSLSVYKQYFETPFLLQSREYYQNEALAYIADNSAAAHLRRAAARLDEERSRV
AHCLDASTEAAILAVVDTEFIANHMKTIVNMDRFGLSAMLDGQRVDELELTYTLLERVAGGRELLLDALTEYIVSKGKLL
VADKMMEAKNGPRSAIPRRGLPASSSPRKSTTSFSATPFMPTASTRPPSTRPLRRLSTSTRARPSTSRSLLTVN*
>Ddis_XP_629756
MSMMTSTPTKRSVKLDDIWPELEEGIYKIITDLNKGFPKQKWIALYTHVYDYCAASQSKSSAKVGMPKQQASGANYVGED
LYNRLNLFLKKHMSQLLKLTETKMDEPLLNYYYTEWDRYTSAMKYINNIFQYMNRYWIKREIDDGKKEVYEIFILSLVIW
RDCLFTPLKQRLTNSLLDIIESERNGYQINTHLIKGVINGYVSLGLNREKPKETILQVYKSGFEELFLTATENYYTNESA
KFISENSVADYMKKVETRLNEEVKRVQQYLHQNTESELIAKCEKVLIEKHVEVIWNEFQTLLEKDKIPDLTRMYSLLSRI
PRGLEPLRTTLEKHVQNVGLQAVSSIATNGVIEPKVYIETLLKVFKKYNELVTGAFRSDTGFVASLDKACRRFINENAVT
IAAKSSSKSPELLARFTDFLLKKSPNNPEESEMEQLLNDVMIVFKYIEDKDVFQDFYSKMLAKRLIHGTSTSEDLEGTMI
GKLKSTCGYEYTSKLQRMFTDMSLSRELLDRFNNHIEQVERSSLNIDFSVLVLATGSWPLQPPSTNFSIPKELQACEQLF
QKFYQNQHSGRKLNWLHHLSKGELKTKYLQTSKSGYTLQCSTYQIGVLLQFNQYETLTSEEIQESTQLIDSVLKGTLTSL
AKSKILLADPPLDDEEIAKTTKFSLNKQFKNKKTKIFINVPVLTQVKEEIDSIHKTVEEDRKLQIQAAIVRIMKMRKQLA
HSGLMTEVISQLQTRFNPKVNIIKKCIDILIEKEYLMRVEGKKDHYSYVA
>Ddis_XP_629469
MNFNNNNNNNNNNNNNNNLNNNNNNVNNINNNNKKPLTNSLAGTPPAKKILVIKNLKQIPKTPDNYEDSSWNKLSSAITS
INMKQATTLTQEELYKMVENLCFDKILASNLYNKISVQIEKHITLTIKHLVLTMSSDPIIFLKSINSIWKDHTNQMIMIR
SIFLYLDRTYVIQNSNTVKSIWDLGLFYFGNNLSQQSNLERKTIDSLLYSIRCEREGDEIDRDLIHSLVKMLSSLNIYTK
FEIEFIKETNRFYDMEGNSKINEIETPMYLKYVCERLNQEGERLMRYLEQSTKKQLMAVLDRQLIERHVDVILEKGFNAM
VNGDRLEDLGKLYQLLNSVGEIKKIKESWQSYIKQTGIQMLNDKEKEATLIQDLLDYKDRLDRILSQSFSKNELLTYALK
ESFEYFINTKQNKPAELVARFIDSKLKVGGKRMSEEELETVLNKSLILFRYIQGKDVFEAFYKQDLSKRLLLDKSTSIDA
EKSMISKLKTECGTTFTAKLEEMFKDIELSNDIMNSFRDSPMTQNFKSIEMNIYVLTSGNWPIQPPIEATLPKEFLEYQE
VFNKFYLSKHNGKTLKWQNALSYCVLKANFIQGKKELSVSLFQTIILYLFNDVIDGGELSFRDIQANTGLAIPELKKNLL
SLCSSKSDILIQKKSSTSSNTSSNTSSNTSSSASGSASGGASGGATKTKVIDETDTFLFNSKFSSKLFKIKVNSIQIQET
VEENQKTNENIISDRQYQVDAAIVRIMKTRKTLAHNLLISELVSLLKFQPKPVDLKKRIEILIEKEYLCRDPENAMIYNY
MA
>Ddis_XP_643183
MFQNKEQTESILVWEKIVSIFSIPSQNEILNFKQKSPNNIDNNLKQLFNFVKLYKLESLVLDWYFESIKKYFKSTLSNEF
WKFFNNVNLAELDSSTTSGRQLKFINHQFALSINLLHKVFSFFKSNLFLFYELLFKKESYNFNFLIKKLQDLLITNIMHT
TNQQTKYFNTILFMFFERDFISFTKSFYHSKKQILKDEEENNNNSKDLEFNDQQQEEEEEEEENEEESKSYIIMEMSFED
SITDINIKEDSFMDLCKKLQDLNFIVISEEIFTQILFKKVFEYIETRCKGVFEKSFLKSILEWADQVIFKWLAMILLSST
TTTKINNYNDIINNNDDDNDDDDDDENKENSLKIFNQWKKRLEFSIYENYSQQRISELFDMIVQYPDSLPSLEDLSICFQ
KIPIEKTMITNLKRVLHNRLLHPGANTSDIITQYISTIHAMDIIDPSGMVMEKVGKPIREYLSQREDTIRCIISSFTEES
NEIYQELCNYDPQDNGGDDDSNNSLLAFGNCDLYVDEGDNFSSIDDFKFWIPNKIDGSSTTISIGNAKANNNNKKKKKKD
TISHLVNIYDGIDLFINEYRSMLSDRLLSVVDFDLDKEIKNIELLKLRFGDSVLFNCEIMIKDMVDSKRLNLQIKNSQGV
NNNNNNNNNNNNNELKEFETLILSQLFWPTLKGDEFKYPKSIEKKMQIYSKEYERIKTPRQLIWKQHLGLVDLDLEIGNNI
QSFQVSPIHATLIMLFESDDGDDDDEKELTLEYLSKQLEISKDLVKKKLIFWLNNQIIKETSHETYKINNKEKEEQKQRQ
QQIENDDQDESSSDDDDDDNNIVVEEEEEEEKSTSAKEKEEQMRVVESFIIGMLINFKTLPLERIHSMLTMFNSELYTSTI
HELKAFLSKLVNEEKIELVGNDFKIKK
>Ddis_XP_641893
MSDQITVDELWAECEQTFEDLFLNLKKGLSRKRYMEIYTKIYNYCSSANEKALIDFYIPKVKVLVAQKAVEIMSRSESYP
NDALLLFFRNQWNEWKMSSNVLKNLLSPVNKIHSSDKKTTSSSANQNESVVYSDTLNSWRETAFNPLKNKLSVSLLQIIK
NDRTGFSTNLQVLSDSLECYVQLGPEKNKLEIYQSCFEQQFLQETETFYKAESADFIEKNGVCEYMRHVYNRIEQETNRV
NQYMPISTLEKLTKILNNVLISNYKEQFASKFLDILIEDKSSDLVMMYSLLSRVNHLTPLKNIFSDFIKSEGLKEIESNL
KEAQEKPQVLISILLKIYSRFNIMIKECYGNDTDFTTAMDKSFSILVNENPASYDPKKKESNIPVVLSKFCDQILRKGPH
HISDEAELEKKLTEAVCLFKYLPDKDIFMLNYQKMLSKRLVEDLSASEDAETLMINKLKNYQGFDYCTKLTRMITDMRLC
KDININFQNHLNEKSLTLPYQFNFYVLTNGSWTLTNKQTATPFKPPSEMLSSITYFESFYKKSYQGRVLTFLYDFSRADV
DSRQAKGKIYKLTTTAYQMAILLMFNGADKITRFLINDTIGLDETSIRLPLLALIKTGIIECSEPSFKNWNNDTEFTVNS
KFSSKKMKVSCNIAVQIGETKQSEGQQTVSEQEIEKERFFKLQAAIVRIMKSKKTMTHNDLTVETTTQVSKWFTPKITAI
KKAIEYLIDQEYIRRTTDDNPSARKYEYMA
>Ddis_XP_647212
MMNGLGRQDIDFNTIWKNIADQVYKILTGSQNVSAMFLYEDVYKLCIAQPQPYCEPLYENIKKFFEQHVDQILLIILDTK

SDTISEYLKQWKLFFSGCELCNKVIFRYLNLNWINKKILDKKFGHPPDVYEIQILGLMIWKERLFFKIKDRVLKCVEILI
QKDRDGELVQHQFISQFMESLIKLDSVDKDRTLYLIEYEASYLENTRQFYTRESVAFIASSGISSYMKKAETRIDEEEQR
SQKYLNSSSHDKMRRLLHSILIEKHKELLQSECINYLKDEKLDEIYNMYKLLSRIEGGLAPVLETVQKYIQHVGIDAIKS
IPDRNNPDPKIYVETLLKIYLQFSSIIKKSFNNDVSFITVLDLACHKIFNQNHITRNTTKSPELLAKYCDMLLKKGNKQH
EEIELEEKLGQIIVLFKYVDDKDVFQKFYSKMLSRRLINGTSVSDDIEKFMITGLKQACGFEYTSKFQRMFNDITLSAET
NEEFKNHLIKNSLSIGKIDFSILVLTSGSWSLHSQTSSFIVPQELTLCISAFQQYYSTQHQGRKLNWLHHLCKAEAKSFF
AKKSYDFQVTNFQLGILLIFNTQESVSLEEITKFTNLNENELSRTLQSLIEAKILISKKKDQNSINNNNNNNNNNNNGE
GNNSLVDSANNMQPSSSSSSTQEYTVNSAYSNKRSKVKVSSSLQKETPLQNEETYKGIDEDRKLYLQASIVRIMKARKTM
NHVSLIQEVIEHSRLRFQPNIPMIKKCIEQLIEKEYITRAEGESDRYLYAA
>Ddis_XP_639922
MMAKPNGKIQFKNLQSHGVLADPDFPKRTWKLLKTAMRQIHQQNASNLSFEELYRNGYNMVLQKHGDLLYNNLKKMVDKH
LKAVAKTVSESIDEKFLLELNSSWINHKTSMLMIRDILMYMDRNYVKQNNLSSVFDLGLYLFRDNVAHCSTIKDRLLNTL
LSMVQKEREGEVIDRILIKNIVQMLIDLGVNSKNVYIEDFEKPLLLKTSSHYQAQSQTLIQTCSCPDYMKKVEICLKEEL
ERVSHYLDSSSEPKLKEVCEKQLISNHMRTLIDMENSGLISMLKDDKIEDLKRMYNLFSRVSDGLNLMKDVISSYVKEIG
RGIVMDEEKTKESGTYFQSLLDLKDKYDNLLQNALYNDKQFIHSIQQAFEYFINLNPKSPEYISLFIDEKLKKGLKGVSE
EEVDIILDKILMLFRLIQEKDVFEKYYKQHLAKRLLLGRSISDDAERNMIAKLKTECGYQFTSKLEGMFTDMRLSQDTMS
GFKTYIQNLKKALPIDLNVHVLTTGFWPTQNTANCNLPREILLCCEAFKSYYLSNHNGRLLLWQTNMGTAEIKANFPSKS
HELQVSSYQMVILLLFNDQSKLTFKEIADQTGIPTIDLKRNLLALTNPKNKILDRELPSTTSSTTTTTTATSSSTSTSP
SSSSSSISTPTPSKSIDESDVFAFNTKFKSKLFRVKVMAVVQKETPVEEKETRDKVDEDRKHQIEASIVRIMKARKTLEH
SNLVSEVIKQLQSRFVPNPVIVKKRIESLIEREYLERSKQDRKIYNYMA
>Ppal_EFA84651_1
MANNNQNITLESLWESCEKPFQDIFVHLNKGLNNQTYMAIYTIVYNYCSSQNDGVLKDFYPKIENLLASKAAEIIKGADD
LTDYDLLLYFRNKWNDWKFSAKVLKNWLEPVNRIYASDNSSRSTPVSGTGGPSQSSPPQSKIMQDGLNAWKKAAFDKLNN
KLSASLLKIIQNDREDRDSSNLQVLADTLECYVQLGPEKNKLEVYQQYFEARFLSETEKFYKVESLDYVAKNGIPGYMKH
ISKRVEEERARVQKFMHSASTLDKLDPLLNQTLIIDYKEEFANKFLDLLTENKEEELTMMYNLLLRVDHLETLRVKLQEF
IKIEGLKEIEKDLKEAQEKPQVLVKILLEIWNRFNRLIKNCFNNDSRFHSAMDQSFSIVINNNPASYDERKKESNIPVVL
SKFCDQILRKGPYHISDEAELEVKLSEAVCLFRYLPDKDIFMLNYQKMLSKRLVEDLSASEDAEASMINKLKAHQGFDYC
TKLTRMINDMRLCKDINTNFQNYLNDQGLTLPYTFNFYVLTNGSWSLSNKTSTTPFKPPTEMLPSLTYFEKFYKSSYKGR
VLTFLYDFSRADVDSRQVKGKVYKLTTTAYQMAVLLMLNSSDKITRFQILDTIGLDENTIRLPLLSLIKIGVIESDNADY
KSWTNDTTFSVNPKFTSKKMKVNCNIAVQIGETKTSEGAQTVSQTEIEKERYFKLQAAIVRIMKSKKVLSHNDLVVETTS
QVSKWFTPKIPTIKKAIEYLIDQEYIKRTSDDNQNQRKYEYIA
>Ppal_EFA83667_1
MSIASTQSVSQKNVKLDEIWPELEEGIYKIITELYKGFPKQKWMALYTHVYNYCAASQSKTGKVGVTKQSNAGANYVGEE
LYNRLNNFLKRHMKELLKVAETKMDEPLLNYYYTEWDRYTCAMKYINNIFQYLNRYWIKREIDDGKKEVYEVFVLSLVIW
RDCLFTPLKSRLTSALLDLIENERNGYQINTHLVKGVINGYVSLGLNREKPKETILQVYKTSFEELFLTATENYYTNESV
KFISENTVAEYMKKIENRLNEEVKRVQQYLHPSTETDLISRCEKVLIEKVVEVIWNEFQNLLETDKIADLTRMYSLLSRI
PKGLEPLRATLEKHVQNVGLQAVSSIGAVGATDPKLYIETLLQVFKKYNDLVTGAFRCDTGFVASLDKACRRFINENAVT
QAAKSSSKSPELLAKFTDFLLKKSPKNPEEAEMDQLLNDVMIVFKYIEDKDVFQDFYSKMLAKRLIHGTSTSEDLEGVMI
GKLKSTCGYEYTSKLQRMFTDMSLSRELLDRFNQHLEEQSALGGIDFSVLVLATGSWPLQPPATNFSIPKELQACEQLFQ
KFYQVQYSGRKLNWLHHLSKGELKTKYLPSNKSGYTLQCSTYQIGVLLQFNTDEELTAEEIQGSTQLIDHALKGTLTTLV
KSKILLADPPIEDEIPKTTKFTLNKQFKNKKTKIFINVPLAQQAKEETDTTHKTVEEDRKLQIQAAIVRIMKMRNHYIMN
YASFSDKASSTTQKPPIFITQPTHNTTQLINSTQSSFVQ
>Ppal_EFA82957_1
MVYLNKEVLSEKCHISNTNVITIRTISIFIGNMLDLKDICILKIVKLIKDGNLNVLSYNQILPLDLIEYLLTMLQKIEKW
PLNNQFLEYLSLYSIESLDLGNQELINLSQWSNYLDLLNNNNNNNNNCNNNNKSNDNNNNICNNNSLYSICNSLSQLSLS
GISMLNDQILSRLLKHFQRSLKSLKLSHCTLLDGSFIASFNQSVFKSIEYLNVRGCISMIPDNFLFVNIKELILSKTNVT
NDDLSMFGNESTLRSNASNNRYIEKDDEAGGDVFISKPFRPNLVELILKMTTSKNDNDTTTQLEHKQQNQQQQQQNLCT
QIIKQNKQYKVKMNHKKTTAPMSSAPGSRGIGGSAPKKLVIKNLKTVTNSFDNYESESWLQLENAVNCIHMKLSIQLTLE
DLYRMVENICLSGNATNLYKKLSELIEKHVKHSLKSLVGSTSDLVDYLGLLNTCWKDHSNNLILIMSIFLTLDRTYVIQN
ANTVKSIWDLGLHYFRETLLSTPELDRKLKGGLLVSIESERNGETVNRDLLSSLIKMMKSLHGNRLVVDYELPRYLKHVQ
TRLNEEYDRSLRYLDVVTRKLIVAMVEKHLIERHSNALIAKGFDQLIDLNRIDDLQLMYSILARVGVLQQLKTAWSNYIK
KTGLAMVTDTEKESTLIQDLIAFKSKLDVILSVSYQKSDLMTYSLKESFENFMNKGDNRLAELIAKYIDSKLRSGNKGMT
EDELEDTLSKALILFRYIQGKDVFEAFYKIDLSRRLLLEKSTSIDAEKSMVSKLRAECGNTFTQKLEGMFQDIELSEEIM
QNFKQSTSLPITINVFVLTAGNWPTYTPMEALLPKEFVEQQELFTQFYTKKYSNRKLLWQNPLAHCILKATFPSGKKELY
VSLFQTLILNQFNNADELTFTQIKELTGIEEETLKKNIKPLTSSKTRILNRKSKTKSKSIESDDLFSFNNDFTQKLVRIK
VNAIQSQETVEENKKTNDGVIQDRHQNIDAAVVRIMKARKSLTHNLLIAELIQQLKFSPKIIFDHIRLKRCCSHDRCSKH
FKYNEMIKNPYMMAYYNYFNLFEKFMEGAKSNSNLECTTVNSNLLFITATRVGNLPMIKYLIDYYDPFDNQYTLNNMLVA
ACQFDRFNIIKYSFEKV
>Ppal_EFA80076_1
MTDFENQRILCLESVRLVLDNSNITPKEYMPDLPETLYEEIKSTIENYLFMARSEQFNTSVSYTNEELFDSFKSLWSNFK
KSVRLLSNILEPLSRVDPYVKQNIISLGFTHFKSIIIESTSIASIVLIDQNKEM
>Ppal_EFA80064_1

MSEATMSSSLNIDLIKRLESYQVLFNDKISKVFWSYFINTNVDTDKNNSRFTNAINDVHQYFTKTRDELIEFCNINNIQN
KEIWVERLQNMLMASLIIDKEQSKRFNQILFSFFESEFVSFSKSYIGSMRKVVVESEELDDDDDDEINNNSSVANQDNEM
IDISLNDSFTDISVKELMFMEICDRLSQLNFIIVSEDLVSRILLSKIHEYIDTKCRGEYERRLLKPILNWADNVIFKWLS
MILSKSSGATFEQWKMRIEFSIYEYYSDQRIDELFNMIRLYPDSTPALQDLSICFQKISIQKTLVENLKKVFHQRLLHPG
ANTNDIITTYISCIQSMKIIDPSCIVMDQVGQPIKDYLANREDTIRCIVSSFTDETNEIYQEFSSGVENDDGTDQHALDI
ENCDVYLDDADSHMDFKSFESWQPASLESHPNQKKQKYKDTISSLVNIYESTDLFVNEYRLMLSERLLSMTDFDVDKEIE
NVELLKLRFGENSMHNCEIMIKDIQDSKRLNNQIGQQLREKQLEAIKFDTLILSELFWPSMKDEGFTYPKSLSSQMTAFS
KEYEAIKAPRKLQWKQHLGFVELEIEINGQLKQLSVSPIHATIIMLFESKDRLSLSELADQLQIKEELVKKKLGFWVANQ
VLVEVERGVYETVKPTSTADGKSSMDVDNGGMEGTGSDALDVVVEDDEDEQQSASAREKEEQLKVIENFVFGMLNNFKTL
PIDRIHTMLTMFNPENYSLTTAELKSFLQKLQNEERLEFSGGEYRIKK
>Ppal_EFA79173_1
MSTKPTKFLVKYSSYPHNNDPNTTTTAWKLLEEAIKTIHLKNASSLSFEELYRNAYNMVLHKNGEMLYNNLKNLVNGHLK
EVAKQVEIANDEAFLNELNTSWSEHKTSMLMVRDILMYMDRTYVDQAKLPTVYDMGLNLFRDNVVHSPYIKDRLLNTLLD
LIQKERNGEIIDRILIKNITQMLIDLGVNSKIVYEEDFETPFLAKTASYYQVESQQFISSCSCPDYMKKVEICLKEELER
VSHYLDSSSEPKVKDVTEKQLISNHMKTLINMENSGLISMLQEDKIEDLKRMYNLFGRVSDGLNYMKEVISNHIREIGKE
IVMDEEKTKEQTTFFQSLLDLKDKYDRLHKEAFYNDKQFQHSLFRAFEYFINLNPKSPEYISLFIDEKLKKGLKGVSEEE
VDVLLDKILMLFRFIQEKDVFEKYYKQHLAKRLLLGRSVSDDAERNMIAKLKTECGYQFTSKLEGMFTDMRLSVDTMAGF
KNYIQTLQKPMSFDLNVNVLTTGFWPTQSASNCILPREILHCCEAFKAYYLSNHNGRLLIWQTNMGTAEIKASFPSKTHE
LQVSTYQMVILLLFNESPKLTFKEISEQTGIPPVDLKRNLMALTSAKNKVLDKETKDAKEAPNKNIEESDVFFFNTKFKS
KLFRVKIMSVVQKETPVEATETRQKVDEDRKHQIEASIVRIMKARKTMDHSNLISEVIKQLQSRFVPNPIIVKKRIESLI
EREYLERSKQDRKQYIYMA
>Ppal_EFA78583_1
MASNLNRKAVDFNQIWLNLENGMQSLLNAKHINSMELIEDVYNLCVSRPQPHSEPLYENIKKFFENHVDNIRETILSTNS
DTITEYLKQWERFSLGSKACHVVYRYLNQNWIQKKMGDKKYGQVADIYEVYNLALMIWKDRLFYKLKDKVLRCAEILIYQ
NRENGTDDQDANVTKFMNSLINLDNIDKDKVLYKSEYEASYLANTEQYYARESSAYILTYGISSYMKKAEIRIDEEYLRS
QKYLNSTSHERLKRLLDSILIEKHRDLLHAECINYLKDEKLDRIIEGGLGPVLETVQNYIQHTGFEALKAIPDKSITDPK
VYVETLLEIYQRFSAVIRKSFNNDVSFITALDAACHKIFNQNHLTKNTTKSPELLAKYCDLILKKVNKQAAEEVELEEKL
NQIIILFKYVDDKDVFQKFYSKMLSRRLINGTSVSDDSERFMITGLKQACGFEYTSQFQRMLTDITLSSETNEDFKLTIQ
RNQIQIIDFSILVLTSGSWSIHSQPSSFIVPQELTACISAFQSYYQTKHQGRRLNWLHHLSKAEVKSHYLKKNYEFQVTN
FQLGVLLLFNSQESVTTDDITKFTNLNENELPRTLQSLVDAKILNQKTRPDTNIQEYQLNPTYSNKRLKVKVSSSLQKDT
TTQTEETYKGIEEDRKLYLQASIVRIMKARKSMNHVALIQEVIEHSRLRFQPHIPMIKKCIEQLIEKEYIQRVEGESDKY
NYVA
>Ehis_1_m00738
MTTTLFQLKKNNQIQFFINTKTTPTTNRKTEKLTAISTCLDDIINGRKPTLSYDIVYNYISNLCYSPEGNIIEQITSIVD
STLTSLASLTKQRLVIEPSLNQLHQQYIIFSKKLALLRNLLLPYDRISNKDPLISKAFGLFREVVLSNNTNTIIDMLIVL
INTLRANNSDNAIVSSLVSMMFDCNLQNQFKDKLLKSTSEFYSSAAQNKISETLEDYLNSVKSMFDREEHIAQSYYPTMK
IDTVAFTEKELMTETLPLILTTKLTQLKQFISQQKTSSILLFISLGSRVKKDEDIQKALIGVIVTLGKEFCSEKDEKTKI
NKIFSLYDYLSQLEKQAPMINNIIKISFEKFISSFDNDIAIALVKFADRMLRKGGMNQLWASKILQIFKFLNGKDTFEQH
YQLLLSKRLLYKGFQAEDEKIVLEQLREECGNSYTFKLEEMVKDVETGKGWYQSLNDEMNKNGMNGQYGMVVINGTSWPA
CPTIRCISLPKEIEKGEEIMRKWYIKEHPNRKVDFNPLLGIVSVKAHCFGNIEITMNVLQFEILMNCEGKTIKELKSKFE
GEEDILMLQIKSLMGKEKLICVQSPDGKIKKPEEIKEEDVVMLNSNYHNNKSKIKINTFQTKESKEEKEKVDEKIVKDRE
TRIDAVVVRVMKKEKKMTVSELLTTITPKLDFKPDVEEIKQRIDKLIEREYLEKDEKNEDVLLYLA
>Ehis_18_m00280
MAPKRKETEDPAVQNLFEELEPFFDGILVSMKSFSLAERAKYQQTFTEFLTQSGSNFYESFINKFRDRLKDGFQTSMKKM
KDVPIDENFPKIIKEDLDRWEYAKSAILVLFKPLDYAMGNGASITIDMIVKDKWNTDYLHTLNDTFELTTTLTRVFSDSR
RSGNKMSVLPVKNILMLYKRENIVTRGQVPLIKTFEDAFCKTSNEDYKIFATEFFKEGVHGFLPQAKQFMEDEEFRIENA
MDDEIGEKMKKRLQNDFLKVSLQMVYEKYDEILEQDKELEFQAITRLFGPINQLDKFAEKTKVSFMNYCQNKMENAVKQS
PEFIRNYPAVVSLLFELLDRLEKMQQNYFLSNPVFKSRLDTAFKDALITNKIVDLSDKKDEVFPRILANYSHMLLKKGSK
IEPDQEKVKENIVKLGKVLQFVGNRDVFEMVYQKNFQDRLVLGLMESSDLEMYMINELSNICDRQVSHKLHTTFQEYNQS
GDILEKFKSYCAKGNIQLPKFDFNVVVFSNSSSQNQNTTSYNMPSQLKEVFDKFSEFYKNTQNKKLTLNHTSSTGVIQFS
WLRRPIQITCRFYQIGLLMLFNDNKEITYAELGTKTALSNDDLAICMSGMVSKGLLSNSTGKEFKPDSVFKFNMSFKPKM
TKLSVMQADKEYVEKEQQKKSDAESEQLEETRNYVIQAKQVRIMKQRRTMKYVDLINETIEHLRSRFNITSRMARKNIEY
LINKEYFKRSDSDREVLEYLA
>Ehis_32_m00219
MFQSLYTQRYSEEQYTQYKNTLYSKFKEIMESKEVIDSGEVMLLYNIVYSICASQDDRLRERLYYDIYSFITRRAKEITI
TIQSNKNQELLKSYNEEFEQFENGINYIERACKYLSKHTIETEIIKNGSSAKFKEISDIGKIEWKKNIFDLLEKQIQEVV
INIIEQIRNGKSDNYEMKEIKKYCKTLIQLHSIKGSDIIGLYVSSFENDYLNKLKKYTKKWADIHIKELDMKNYLKEGIN
LLKKEQEVIGCNLDSITFDHISSIMSEELWKEKIEVLKVHFKEGFEAENFEYILLFYKALNKCSNSNELLTDLSRSFGEW
VISMFNKTLNGFTCNESNDIGIELLNRYIFCHTKVSEVLKLFNGNALFGEELKQRLNQFLNSPSNKYNELHPKFIAQYID
QLLRGVGVGKDSKEVILTKIEELMNILQAVQNKDIFMHFNSHYFARRLINKTVSIDEDIEKSSIDGIRSVCGFEYVSKMN
HMVRDINNSVNLNAHFHELYITPLYVNVLTSGIWPFSTENSLSNDMLPKSISLAQSRFEEEYLKNGKSKKLKWLNNLAKV
NVKVTIGKYKYEMILAGIYYIILDLINQYQTEWKCKCKIKEEDIERAENVLTKLGVIANNHIKTSYFTEQKRKKIVIGFQ

EINQITKENQINKELVQSRKVSLQAIIVRIMKQRKVIGYQQLKEEIMKISSYKFVPSTIMIKECIEFLIEKEYMKRKDDD
PSTYEYVY
>Ehis_80_m00162
MENLFKEVLIEQSNYINDKDNIIIPTLLSRYINLIIRKNSMVCVDSDSVNNTIEIVFKIGEFIVNKDIFCHFICKNFIER
LILMNSNEENEEILMKHMRNFDRLGVMKMIKIQTEYNQSIGYSQQYSQESKSNNFNCLVFGGLNVYLKDKYQMKIDSMTT
ERFDSFTRFYIKKKPTQKLTMCLEKSSGIIKYNQKYFITATLPQMMILLLFNTHNILKISEIALDLNLPSSFVKNAISGI
IHKQVLCQNTSLIENETQVSVNTNFNLKVRKISVMMVLKKLISQTQQEQIKKDDNDEDEILKLRENLLKCTIVRIMKHSK
VMSCQQLIQQTITVVQKTFKAHVNVIRRVIEILIDQNFLMRDGQDIQYVA
>Ehis_100_m00143
MNLQTPSQMFQTLSSFCDTVLSNQPYDSKTHMGMYSTCILYSQQTQGNAQKLFQLIHQKCIMKLKVLLKQTQQINSTLDF
LRFYITNYKLYLISCQKISHVFSYLDRFFIKQQKAINGEYSNPVFKHCMSLFWCDFLRANATNIVENTMNLLLAERIDNN
TQNRELINGIMLLFLSFSNPEIVTVSLMNLNIYDNYYQSEYIKAAQSYYKDLNNYFKNYGITEFLEKTIEIIELERQRSQ
LYVAANLQREFGEFLQKQFISEQCKFIIEKMESLLKTREKDDLKKCFTVLKDGEQVDSAIPVFEVYCKKIGMEKVKCIMS
IQQAFEIIKVIIEYYEDITNLIQECFRNHPSFNAGFGRAFREIINSNVKIANGEEMEILIPEYLAKYSDIVVKKCVGVDE
LNNKLDQIFCIYDFIENKDIFENYYIKAIAKRMLLRGNNDDLSQENIVFDRHRVKFSSTFSYKIQKMMSDLQISQSLSNQ
FLATVNVPFPFFVNVLQTSVWPLEPQQFMFELPDELKTSHIFFEKWYIKQHQRRKLQWLHQYTRGKIKTNCFNKSYNLFG
SAYQLSILVLFNQKNEITIDEIFNTIHLTMDIIKSIISSLQSVDLISVEDGNIVKINNSFKYAKTKFVLPIPKAVVFKQE
KKEEEREITAERVGLIEACIVRLMKKNRKMQHNDLMQAVVTSLSSRFRTPIPLVRRTIESLIEREYISRSDVRKDEYIYI
E
>Ehis_130_m00114
MNIEEIAQKGVSLAFERKMKLQEVVEINFQFSEYIISNPEEKARLINLIISDVSKTVKTLIEPFYSKGEHIHELIAWIKD
VKKQFEQAQDNIKGMFCILSDQRRLRVGISFEKELKKCWGESVIKELEKTDIIEWLKKQLKEIKEKDKNYPFAIGELIYF
LKEVISWQFGEKVREKLNEYIRNDFNNDFNEARQSGEYIKKINELMKKENKVLQTLLKEDEVTLRKEMNNKFVFIEEVIY
GLKKYFEHSDIHGIKQSLQLLHLVSLKEDIINSFKSLCNSGSKQYIKKLFDCFKEFNAEKVCETIMIFDEIQSDSIYGES
IISGLKEGIASSDCLLKNGDTIHVIMTKYAFQIIDNFNETKIIQFMKTLILLPSRDIFESLFWKTLYGKIVSGKIKSIDT
INVFVNYLKQYFGTSTVSRISSLIQDYITSLELVKQFKEKNTSNVEQKIKFDIVVFGLPQHEINSIVHLPPSLDAIFQPF
KQFYLEKFPSRRISIEPEHSLCVVSFSFNNKTLLYCTTIQYCILYHFHSKYECCIEELSKSIENRTFLLQVCDNLVAHGL
LIKTEDTYKINNNYQPQHTVTNITKLIKTTPQMKLLDINEDVEQKRSIQLQCSIIKIMKRERQKLISNLINEVVIESI
>Ehis_224_m00085
MTLLSPKALSTTEYNKLWKFIDSNLVLLFSSEPTKISEMELYHFVYVISIAPGELWKTLYSNLILFLNHFCESICCKMSV
KPITNLYIQEYLKYEHALKKMEKICKYLTTYYLHESIIDSGKRIWKELVFKPNSFEIISECLSYLNEIRQSKIDQSFLCT
FRDCLLSLGATSQASRENYEIFEDEYIKDFELHSSSLLKYSQRDFLEQVTIMEKIEEERVWVSLENTTKVRAKKILNDIW
LKKSDCVSQLLLQGLDKENDLVEMIFNGCKRAIDSSLLEKLRGIYAKWILSIAYKKLDNFTGDRNGWIEKLVDILRCVNK
SLDLFHNDLSFIQTAQITLKNIMSSVPHKNTPNNAMYIALYLHDLLRKHYSHSTCLQPTLNYSSVEVLELSNYLQDKDVF
EEWYLKLLAKRLLNKEATNTSMEENIINKLREMSGVSYATKLGRMFSDIASSISLTNDFNNFIRSTILDVSIGTGGTWPL
KPSENIELPPVMNDLLNSFEFFYKRKYESRKLQWVMNMSTTQIEWKYNGELKKVTLPTLAIALIISKSDGFQMKNEGEVY
TMYQALIKSEIFDSSGNITSSFLQGHHKKLGITEQKTSEKIKEDRSAWGEAVCVRLMKKMKKCRELDLINMVIKEKAPFI
PTEQFVQNVIEKLIEKEYIERDKIKGVLNYLY
>Acas_g1628
MATKLNGSSNGGLRNSAGSKPVKKLVIRGLKTSKGLPDDYEQKTWEKLQAAIAAVQNKQAVQYGEEELYKATENLCSQKL
GAGLYSKIQGECERHIRAQKAALQQLVRTQEPSSFLISVNNVWNDYCQAMFYIRSIFLYLDRTYVIQTAGVSSLWDLGLQ
LWRDNVIADSEVEKKLIVGLLSLVERERDGEMVERDLIKNLIRMLASIGVYAERFERSFVVATGKYYSQESARLLADMEM
ADYLAHAEERLVQEEQRVTHYLEPSTRRPLLTAVENALIAAHADGILQKGFDRLVDQGRVADLARLYTLFSRVQSLPLVR
VAFNTHIRAAGAEIVNDAERDKTMVPTLLELKTKLDTILRDSFHSTDIFAHAMKEAFEHFINTRENRPAELIAKFVDAKL
KAGNKAATEEELEALMDRVMVLFRFINGKDVFEAFYKKDLAKRLLLGKSASIDAEKSMISKLKTECGSGFTSKLEGMFKD
VELSKDIMISFRQSRQAQELKDLEVNVSVLTTGYWPAYTPLDIKLPPQLAHCQDVFRAFYLGKYQGRRLFWQHTLGHTVL
KAFFPKTVVMLLFNDTKSISYKDIAEATGIEQKELKRTLLSLACGKVRPLTKEPKGKEVGDDDVFNFNDDFRHKLYRIKV
NSIQMKETEEENTKTKESVFQDRQFQIDAAIVRIMKTRKTLTHNQLMAELYQQLKFPLKPADVKKRIESLIDREYLERDP
KNTAIYNYLA
>Acas_g2130
MGASKSNALPSRPSHYPDFDELELCCAELKSVTTVSYREAFSRYEGYLRSFARDYLEPQLESVGLCTYLAALVKCWEQFS
IYTKSVIMLMQQLHYSDRYGPMAKQVFMKLFLQSDRLARELQRVLQEGRFLDDEETNHHIVSTLVLLSEIGYNNELFVKY
FETPLIERCAADARLRLQTSSVTNYLAYVDSLFHRFALSAFFDSTKAKFLHTLQQRNIRTRSTTSVYQVLCKLFRNSVRR
KIEDVVAMLKGKQLGSLDTLSVVNSMIDAYRYCHGMTSNTEDAGLISALQQGTAEALTVEWSLPKEEQQGRGRLVEEYLA
HFCHRLLSKQSDKLFQADLVEEIVDGVTDLYRHCANKDIFVALYQRHLIKRLLLEGSSATEDRELAMLSRLKTATVRTIG
DLSGLHRCDMAVREVYQSAELSARFKASPFAQGLSTDINVKVISSFLFPYQFKPVALPVEFYTDLTAKSKKLFWIPALSS
AVVSASFTKGKKEFRVSLHQASILFLFNDSSALTLNDILRHTKMDKEECALLVKQLVKIKLILSVNAGGDSNAETFQVHD
AFANKRFSINLNAAQLKLAAQAAVVRIMKSRRVLTHNELVSETMAQLKFPFEIGWLKRVLEALISSGYMARTTRADQAAY
EYLP
>Acas_g3115
MASKKPKITIKPFRHQVQMDPNYAENTWKLLRGAIHEIHKQNASGLSFEELYRNAYNMVLHKYGDRLYKGLQQVVDEHLK
EVANDVAAANDDTFMDALNKAWNDHKISMLMIRDILMYMDRVYVMHHNVHTVYDLGLILFRDNIARSARIKDRLLKTILS

WIQRERTGEVINRGLLKNITQMLVDLGVNSRTVYEDDFERHFLETSATFYRVESQEFIASNSASDYMRKVSARIQEEMER
VAHYLDAGTEPKIKEVVERELIASHMKTLIEMEGSGCISMLKDDKLEDLSRMYNLFSRVSNGLAFMREKISNFVRETGKE
LVTDEEKNKNPQLYVQALLDLKDKYDRLLTGAFTNDKNFQHAINQSFEHFINANNKSPEFISLFIDDQLRKGLKGVGEEE
VETILNKVMMLFRFIQEKDVFEKYYKQHLAKRLLLGRSVSDDAERNMIAKLKTECGYQFTSKLEGMFTDMKLSADTMESF
KGYLQKADGDILHGVDLSVHVLTTGFWPTQAAAKCNLPPEILHCCEVFKKFYLSNHNGRRLTWQTNMGTADLKAFFGSRK
HELNVSTYQMVILLLFNTSSQITFKELKETSGIPVPELKRNLMALCMAKYKVLSKEPKTKQVADEDTFAFNSAFKSKLYR
VRILPLARGESKEERDATREKVDEERKHQIEAAIVRVMKARKTMEHSQLIAEVTQQLSARFMPNPMVVKKRIESLIEREY
LERSKKDRKLYNYLA
>Acas_g6186
MMDTMATNDIDQQIETNGPPTAGDREFAFHTLRSKMGHLEEVIEDIGQFICENQLEDHFINFVFRLSSVSKDLNTEISTV
ESYLEFIKRHNVTCSRNTGYFSVIPDEVTIHVFSYLNESELSSAASVCREWHSLTSDNTLWKSLYARYWGADAAARISSN
QIYRPKPLPLDMASEHNKAPIIKKPAATTVRRFSLWPSSSSSSSSSATSAAGGSTSGQATPSYGGKKMAKMYKMYRGTPE
EDEELERERRDHGKWKSYFMKYHRTKRNWDTGKYSLATLTHHKNTVRCVQFDENYNMITASDDKTVQRLDWDADKGAYVP
KMKLTGHEGGIICMQFDGNQMITGSRDKTLRLWDLEKGKTISTFKNHTGQVWCLQFDKHKIVSGSDDKRLNVWDINSGKL
ITDLQGHSWGIGCLQFDSTKIISGAADKTIKVWDLAMMRCAQTLKGHKSSVRCVQFDDTRIVSGSWDNTIKLWDVNTYRN
TDTLQGHSNKLMCLQFDETKIISGAQDKTIVVWDLHTGKQLTTLQSHTDSLCDLHFDDCKLVTGSRDKTVKGTVDKKMEA
TLLIPNFLYLGSEFNAANRELLIEYNISAIVNAAAPQTANHFIQESSCYRYHSIDLLDAAEEDLLKHIEPYSRFLDEAER
KGEVALVHCKSGNSRAAALCIAHLMRRHRWPLAKAFAFVKMKRPAAQPNKGFLEQLAIYQRYLQEEEEQAEQEENDHDDD
DKDQDGDEGAQEEEDAMELELARRRAAFMRRYAGGGEDDSSDEGEGEEYEMDLDDDQEPLVKGKEKQQPAAVANTNEDLN
WEKQRKDEEEDDEEEEREEREEEARRAKAKRDWQRWFAKPPALVDLVVRHRRAALAHRVSALFAAGNWEELKRVYASLRD
TPEFVNIVRAAYFAATKADAVAAAAAIVAAQTATGEEAKLDAVVDWRRIQQERLVSTFGTAPEPGFQRAFEQTLPYFLNP
LAKTIVAHLDRTLKSAGERSPKEVEDELWPLLESSLPYMGRNAAAFEFHFANALAGRLLSIGPGQNHRFHVERAVAQLVG
RFQSFSGFGALMEAMVSDAQFSFEMEEMQKSAAKDKVRQGEESGALVSVRLLAADLWPRRRILPPASKDLTDADMRSWRG
VPLANWPKSYVLPSSVQQKWDAFAAAYLAENPQRRLQYIPHMGTAEMQVVLGESRYFFTVTTAMMCVLHRFAFGQEFSLR
ELHEETAIPDQDLKRTLFSLISPLQGSRVGLLVKQPVKGKFEPTDRFSVNAAFASRLRKIRVPALPYPPPRKQRQPTSSS
SSSSSTGGAMME
>Acas_g6314
MAHVASKRVSLDEIWPDLEDGITSLVTNLNAGFPRKRWMELYSNVYNYCTTSRPAPGRKPGGISGANFVGEELYTRLSEF
LKNHMKKLCKQAEGKMDDDLLMYYSKEWERFTTAMKYINNIFDYLNRHWIKREADDGKKEVYDIYTLSLVIWRDHMFHAQ
KGRLINSLLGLIEKERNGEQVNTHLLGTVIQGYVKLGLNKEKPKENTLEIYRQFFEDDFITATEVYYTAESTHFIATNSV
AEYMKKVETRLDEETRRVQQYLNPTTETELVQKCDRVLIEKHLDTIRSDFQNMLSNDKIEDLTRMYKLLSRINRALDPLK
TTFEKHVSQVGKQAIQQVMKTAIKDPQQYVETILKVYKQYNALVIGSFRNDPGFVAALDKACRSFINENAVCKLAKSASK
SPELLARFVDSLLKKSAKNPEEQEMEQLLTDVMTVFKYIEDKDVFQTFYSKNLAKRLIHGTSASEDLEGTMIGKLKSACG
YEYTSKLQRMFTDMSTSRDLLERFKNHLEDSSQTSAVGLDFNVLVLATGSWPLQPPSTNFTIPKDLVACEQLFVKFYQQQ
HSGRKLNWLHQLSKGELRARYGKKYSFVTSTYQMGILLQFNSNESMTTDDLQIATQLTQSTLVNTLATLVKTKVVKMDPA
PPPEEEVPKIEKDAVFTINENFRSKKQKVNINIPMRREKEEENEETHKAVEEDRKLQIQAAIVRIMKMRKRSQHGALMSE
VIAQLQTRFKPRVAVIKKCIDILIEKEYLERVEGQKDMYSYVA
>Acas_g6460
MEGTTNFKDIAELWALLQPSLDQIISAEESGDASQRLPTHTYSQLYSVVYTVCTKAECHQAGVVDQLYKRVGQFVDGYCR
ERLAPQLRGLPPDRLVPQVLARWGRFTTVLKRITSIFSYLDRHYCQSLRLRTTKEAGVNSFRLLVVDPVVEELSNAVLNG
LQAARASSGSVAEPDQLKSVSQMFVELGLDKLFFYQENIEQPYLTQVSVNSLALRLSFTQLRSIIDKEMAIARQVLHAST
EAKVEQLLPQQTSHQ
>Acas_g7402
MRMRRSIIHAALAASSLTLAPVLDLRTHLFARFEIFSQLQLITEKPAIREALFYCLRRRFVIYSSKWIKAHEDEAEDEEA
DHDEQNGENEEMDYNDGREVNATHGIDALLAMASQEVCEEIVTEVVFEETENRVNSACKAVFDSSVLPSMLRWLAGAALQ
WMALTLRRPLSSLEQWKARLESHLYQIIATFRTNELFDIIVDYPDSVPALLDLKESLARSDQHLELVSSLTQTGASTNDI
IDTYVSTICSLRLLDPTSVLLDAIRPPIAAYLRGRDDTMRCVVSTVTGDTNPELYEELGHAEAEEFRTMLASRLLAITDY
NTDKDYHNLELLKLRFGETRLHECEIMLQDMSESRRLNTNINKTIQAKAPTGIAFVPSIPSPGSMESYEKEYQAVKASRK
LCWRPALEIWPIEELAEKLGTTSEVVRKGAGYWVANGALRASFQGRSLVYEVSFGEEVNPSGEGVVADEEGEGLAEGDEA
DEEMWTMLEEFIKAMLMNLERLPADRIQEMLAMTSDSYDKSLSELVQHLQTMIAADVVTLDVDGNYALKRN
>Acas_g8300
MHPEKQIMQEELYRTVYNVCCQRHQPRLYEDTLKLVRGHLAQMRVDLLAANEQTFFSLFSRSFVRFHRAVDILCVGFRYL
ERSYVLEKATTLMKVFNDAYTAGVLQPPDEPPEVKRRLQITLQHFTPPIPDPTTTMHLIKGLYALNKEYASFNPFLFAMY
IPCLQPPRGLEDDRQETLAHLRILTHQAIPRGEGRRKRTYDDFLSL
>Acas_g12691
MNAIDNKESLTRKEWMGHYDTCYKLCTGTDRHELGLYNCLRASFVKQVAHIINTKLADLKDVSLLKKYLETFEIYRSSAR
AIANTSQILARFWIPSQRASKKDVREIAPLSIWVWREYCYKALKEQLLSALFSYIERDRNGAMLDKSLVRDMIESLNAIA
LDDSRKFYEQEFEARFLEETKAYYVNESTNFLSSNSISDYMTKAEERIKEESSNCEASYKDTLAKLEQALNFALIENHME
SLQATFKSMLTADRQQGESHSTLTYDSGTHAHSPCAKTFEEHVAAQGVAIVKEQASKKTAQAAMKSAISFIQQTAELYTK
YSAQVDQCFLKNSSFKEAFDKAFVTIMNLSAGKFTTSRHVSAPLSLLLNFYLDYLLKGKAKIAEEEKAAQLEDIARLFSY
FRDKDEFAEYARRGLCKRLLARGSGFDETAEKNFISLLKAQCGNSFTRRLQGMFTDAEDESIARVKQRFQEWNGDSDKVA
GLQLSVQVLNDSFWPISGVDKFPLPGQSSEFVASVSKFEEFYKHETAAGVALVRGQAEKSAKGGESEADAESSAEKINDD

DVFTVKIDLKKKRRVAFPAGSAKQESKQAKQDQVAVVKQRAFEVDAAMVRVLKSRNVLSWTELQLQVVDILKVRFLPQAK
LLKARLESLIERGFLDRDANDRTLLKYIA
>Acas_g13326
MSLSPQKVDFEKIWNGLQEGVANIITLTGVKGMPMIDSAQLRLNPILRSSTFAFALSSSGMSALCAIDMLEGQGDLLADY
LKKWEAYSTGSEYCHHIFRYLNNNWIRKRLEDSRNKLGGFSAGPSSSTEVYEVFTLALVIWKDHVFSKVKDRLVRSLLEL
ITKERDGELINERVVAGVIQSFVKLGSINKNKPLEIYKDFFEGPFLEDTRDYYARESGAFISTNGVSSYMKKAKERLEEE
AGRGKKYLDSSSFEKLKRECDTALIERHKDLMQVECKTYLADDKRDDLSRMYHLLSRIPEGINPMLEVLQKYVTDVGFDA
VKSIPEASTKDPVKYVQTLLDVYVKFSDVVKTAFENDSAFVASLDKAMRQVVNDNPINKRTTKSPELLAKYSDFILSKSN
KTFEDDKLDQMLNQVLTIFKYVDDKDVFQKFYSKMLARRLIHGTSLSDDAESAMIGGLKQACGYEYTSKLQRMFNDMALS
NDINEKFKEYLEIKSLSNGLDFNILILTAGSWPLTAQSATFNVPQELERCVNNFTGYYNSQYTGRKVNWLHHLSKGDLKT
FYLKKRYEFQVTNYQMGVLLMFNKAERLTVEEISSSTNLKDRELTRTLQSLVSSKILRKEPDGATCEPTDAVTLNDRFAS
KRLRFKPAAVLQKETKEENSETHKSIEEDRKLFLQAAIVRIMKARKTLTHVNLVKETISQAKARFQPSIPMIKKCIEHLI
EKEYLQRQEGETNTYSYVA
>Acas_g15602
QQVAGLQLSVQVLNDSFWPISGVDKFPLPGQSSEFVASVSKFEEFYKHETGGSKQLRWLYNHGTVMLSTTEGKAKRELVV
TPLQASILLLFNQKDAWALNEIKAVLWPGDQSRAKLRASRSSEYDINLMEILKAAIQPLACWSFTALERSTQSPAEVQTE
GQAEKSAKGGESEVDAESSAEKINDDDVFTVKIDLKKKRRVAFPAGSAKQESKQAKQDQVAVVKQRAFEVDAAMVRVLKS
RN
>Atha_AT2G04660
MEALGSSDCNLEILETLSDDAIQEITESYDGFFTTVESLIAGTGDSLVEDEFVSHVYCLCKYGLDSLVRDHFLRSLEQAF
EKGGASSFWQHFDAYSEKKHHNYGEEIQIVLCKALEEISIEKQYHEKCLSIVVHALQSFKEQSSDDRQNSDTERVHLFSR
FQSMLSSTLMTTLPQHFPEILHWYFKERLEELSAIMDGDGIEEQEDDCMDLDEKLRYKNGEMDVDEGCSQGKRLGHDKLV
KNIGKVVRDLRSIGFTSMAENAYASAIFLLLKAKVHDLAGDDYRTSVLESIKEWIQTVPLQFLNALLSYLGDSVSYGTTS
SGLTSPLACCPSPSFSRVVTPSEGIVRWKLRLEYFAYETLQDLRIAKLFEIIVDYPESSPAIEDLKQCLEYTGQHSKLVE
SFISSLKYRLLTAGASTNDILHQYVSTIKALRAIDPAGVFLEAVGEPIRDYLRGRKDTIKCIVTMLTDGSGGNANGSGNP
GDSLLEELMRDEESQENVGFDDDFHTDDKQAWINASRWEPDPVEADPLKGSLSQRKVDILGMLVDIIGSKEQLVNEYRVM
LAEKLLNKTDYDIDTEIRTVELLKIHFGEASMQRCEIMLNDLIDSKRVNTNIKKASQTGAELRENELSVDTLTSTILSTN
FWPPIQDEPLELPGPVDKLLSDYANRYHEIKTPRKLLWKKNLGTVKLELQFEDRAMQFTVSPTHAAIIMQFQEKKSWTYK
DLAEVIGIPIDALNRRVNFWISKGVLRESTGANSNSSVLTLVESITDSGKNEGEELLTGEEEGETSIASVEDQLRKEMTI
YEKFIMGMLTNFGSMALERIHNTLKMFCVADPSYDKSLQQLQSFLSGLVSEEKLEFRDGMYLLKK*
>Atha_AT4G02570
MERKTIDLEQGWDYMQTGITKLKRILEGLNEPAFDSEQYMMLYTTIYNMCTQKPPHDYSQQLYDKYREAFEEYINSTVLP
ALREKHDEFMLRELFKRWSNHKVMVRWLSRFFYYLDRYFIARRSLPPLNEVGLTCFRDLVYNELHSKVKQAVIALVDKER
EGEQIDRALLKNVLDIYVEIGMGQMERYEEDFESFMLQDTSSYYSRKASSWIQEDSCPDYMLKSEECLKKERERVAHYLH
SSSEPKLVEKVQHELLVVFASQLLEKEHSGCRALLRDDKVDDLSRMYRLYHKILRGLEPVANIFKQHVTAEGNALVQQAE
DTATNQVANTASVQEQVLIRKVIELHDKYMVYVTECFQNHTLFHKALKEAFEIFCNKTVAGSSSAELLATFCDNILKKGG
SEKLSDEAIEDTLEKVVKLLAYISDKDLFAEFYRKKLARRLLFDRSANDDHERSILTKLKQQCGGQFTSKMEGMVTDLTL
ARENQNSFEDYLGSNPAANPGIDLTVTVLTTGFWPSYKSFDINLPSEMIKCVEVFKGFYETKTKHRKLTWIYSLGTCHIN
GKFDQKAIELIVSTYQAAVLLLFNTTDKLSYTEILAQLNLSHEDLVRLLHSLSCAKYKILLKEPNTKTVSQNDAFEFNSK
FTDRMRRIKIPLPPVDERKKVVEDVDKDRRYAIDAAIVRIMKSRKVLGHQQLVSECVEQLSRMFKPDIKAIKKRMEDLIT
RDYLERDKENPNMFRYLA*
>Atha_AT4G12100
MKRSISPDPFSSTKSPKLVHHSPDDGGAEGNPYRLPFTLSDENLSCLPISQAREPPPIFNLDPNSVKDTQDRNKLYLAKA
WDLLKPAIKIILDDDEYKKPGDVLCFTTIFRAVKRACLGDPRQSELVFNLVKHECEPHIAELIQSLEKNCSGSDDPSVFL
PHVYNRWLDFKRKMSLVSDVAMYQTLNGLTLWDVGQKLFHKQLSMAPQLQDQVITGILRLITDERLGKAANNTSDLLKNL
MDMFRMQWQCTYVYKDPFLDSTSKFYAEEAEQVLQRSDISHYLKYVERTFLAEEEKCDKHYFFFSSSRSRLMKVLKSQLL
EAHSSFLEEGFMLLMDESLIDDLRRMYRLFSMVDSEDYIDRILRAYILAKGEGARQEGSLQELHTSIDKIWHQCFGQDDL
LDKTIRDCFEGFGLHVPGEFSDQLQWIDDDDDED*
>Atha_AT1G26830
MSNQKKRNFQIEAFKHRVVVDPKYADKTWQILERAIHQIYNQDASGLSFEELYRNAYNMVLHKFGEKLYTGFIATMTSHL
KEKSKLIEAAQGGSFLEELNKKWNEHNKALEMIRDILMYMDRTYIESTKKTHVHPMGLNLWRDNVVHFTKIHTRLLNTLL
DLVQKERIGEVIDRGLMRNVIKMFMDLGESVYQEDFEKPFLDASSEFYKVESQEFIESCDCGDYLKKSEKRLTEEIERVA
HYLDAKSEEKITSVVEKEMIANHMQRLVHMENSGLVNMLLNDKYEDLGRMYNLFRRVTNGLVTVRDVMTSHLREMGKQLV
TDPEKSKDPVEFVQRLLDERDKYDKIINTAFGNDKTFQNALNSSFEYFINLNARSPEFISLFVDDKLRKGLKGITDVDVE
VILDKVMMLFRYLQEKDVFEKYYKQHLAKRLLSGKTVSDDAERSLIVKLKTECGYQFTSKLEGMFTDMKTSEDTMRGFYG
SHPELSEGPTLIVQVLTTGSWPTQPAVPCNLPAEVSVLCEKFRSYYLGTHTGRRLSWQTNMGTADIKAIFGKGQKHELNV
STFQMCVLMLFNNSDRLSYKEIEQATEIPAADLKRCLQSLACVKGKNVIKKEPMSKDIGEEDLFVVNDKFTSKFYKVKIG
TVVAQKETEPEKQETRQRVEEDRKPQIEAAIVRIMKSRKILDHNNIIAEVTKQLQPRFLANPTEIKKRIESLIERDFLER
DSTDRKLYRYLA*
>Atha_AT1G43140
MATILFKVIMMKELILLEEGWSVMKTGVAKLQRILEDLSEPPFDPGQYINLYTIIYDMCLQQPPNDYSQELYNKYRGVVD
HYNKETVLPSMRERHGEYMLRELVKRWANHKILVRWLSRFCFYLDRFYVARRGLPTLNDVGFTSFHDLVYQEIQSEAKDV

LLALIHKEREGEQIDRTLVKNVIDVYCGNGVGQMVIYEEDFESFLLQDTASYYSRKASRWSQEDSCPDYMLKAEECLKLE
KERVTNYLHSTTEPKLVEKVQNELLVVVAKQLIENEHSGCLALLRDDKMGDLSRMYRLYRLIPQGLEPIADLFKQHVTAE
GNALIKQAADAATNQDASASQVLVRKEIELHDKYMVYVDECFQKHSLFHKLLKEAFEVFCNKTVAGASSAEILATYCDNI
LKTRGGSEKLSDEATEITLEKVVNLLVYISDKDLFAEFYRKKQARRLLFDRSGIMKEVTDITLARELQTNFVDYLSANMT
TKLGIDFTVTVLTTGFWPSYKTTDLNLPTEMVNCVEAFKVFYGTKTNSRRLSWIYSLGTCHILGKFEKKTMELVVSTYQA
AVLLLFNNAERLSYTEISEQLNLSHEDLVRLLHSLSCLKYKILIKEPMSRTISKTDTFEFNSKFTDKMRKIRVPLPPMDE
RKKVVEDVDKDRRYAIDAALVRIMKSRKVLAHQQLVSECVEHLSKMFKPDIKMIKKRIEDLINRDYLERDTENANTFKYV
A*
>Atha_AT1G02980
MAKKDSVLEAGWSVMEAGVAKLQKILEEVPDEPPFDPVQRMQLYTTVHNLCTQKPPNDYSQQIYDRYGGVYVDYNKQTVL
PAIREKHGEYMLRELVKRWANQKILVRWLSHFFEYLDRFYTRRGSHPTLSAVGFISFRDLVYQELQSKAKDAVLALIHKE
REGEQIDRALLKNVIDVYCGNGMGELVKYEEDFESFLLEDSASYYSRNASRWNQENSCPDYMIKAEESLRLEKERVTNYL
HSTTEPKLVAKVQNELLVVVAKQLIENEHSGCRALLRDDKMDDLARMYRLYHPIPQGLDPVADLFKQHITVEGSALIKQA
TEAATDKAASTSGLKVQDQVLIRQLIDLHDKFMVYVDECFQKHSLFHKALKEAFEVFCNKTVAGVSSAEILATYCDNILK
TGGGIEKLENEDLELTLEKVVKLLVYISDKDLFAEFFRKKQARRLLFDRNGNDYHERSLLTKFKELLGAQFTSKMEGMLT
DMTLAKEHQTNFVEFLSVNKTKKLGMDFTVTVLTTGFWPSYKTTDLNLPIEMVNCVEAFKAYYGTKTNSRRLSWIYSLGT
CQLAGKFDKKTIEIVVTTYQAAVLLLFNNTERLSYTEILEQLNLGHEDLARLLHSLSCLKYKILIKEPMSRNISNTDTFE
FNSKFTDKMRRIRVPLPPMDERKKIVEDVDKDRRYAIDAALVRIMKSRKVLGHQQLVSECVEHLSKMFKPDIKMIKKRIE
DLISRDYLERDTDNPNTFKYLA*
>Atha_AT1G69670
MSNQKKRNFQIEAFKQRVVVDPKYADKTWKILEHAIHEIYNHNASGLSFEELYRNAYNMVLHKYGDKLYTGLVTTMTFHL
KEICKSIEEAQGGAFLELLNRKWNDHNKALQMIRDILMYMDRTYVSTTKKTHVHELGLHLWRDNVVYSSKIQTRLLNTLL
DLVHKERTGEVIDRVLMRNVIKMFMDLGESVYQDDFEKPFLEASAEFYKVESMEFIESCDCGEYLKKAEKPLVEEVERVV
NYLDAKSEAKITSVVEREMIANHVQRLVHMENSGLVNMLLNDKYEDMGRMYSLFRRVANGLVTVRDVMTLHLREMGKQLV
TDPEKSKDPVEFVQRLLDERDKYDRIINMAFNNDKTFQNALNSSFEYFVNLNTRSPEFISLFVDDKLRKGLKGVGEEDVD
LILDKVMMLFRYLQEKDVFEKYYKQHLAKRLLSGKTVSDDAERNLIVKLKTECGYQFTSKLEGMFTDMKTSHDTLLGFYN
SHPELSEGPTLVVQVLTTGSWPTQPTIQCNLPAEVSVLCEKFRSYYLGTHTGRRLSWQTNMGTADIKAVFGKGQKHELNV
STFQMCVLMLFNNSDRLSYKEIEQATEIPTPDLKRCLQSMACVKGKNVLRKEPMSKEIAEEDWFVVNDRFASKFYKVKIG
TVVAQKETEPEKQETRQRVEEDRKPQIEAAIVRIMKSRRVLDHNNIIAEVTKQLQTRFLANPTEIKKRIESLIERDFLER
DNTDRKLYRYLA*
>Atha_AT1G59800
MVAPTEIKFEVEWSNIQQGFTKLIRMIEGESEPAFNQEIMMMMHTATYRICAYKNPQQLYDKYRELIENYAIQTVLPSLR
EKHDECMLRELAKRWNAHKLLVRLFSRRLVYLDDSFLSKKGLPSLREVGLNCFRDQVYREMQSMAAEAILALIHKEREGE
QIDRELVRNVIDVFVENGMGTLKKYEEDFERLMLQDTASYYSSKASRWIQEESCLDYTLKPQQCLQRERERVTHYLHPTT
EPKLFEVRYGIIIWN*
>Atha_AT1G59790
MDSRRMSRPRQIKFEEGWSNIQKGITKLIRILEGEPEPTFYFSECFKLYTIIYDMCVQRSDYSQQLYEKYRKVIEDYTIQ
TVLPSLREKHDEDMLRELVKRWNNHKIMVKWLSKFFVYIDRHLVRRSKIPIPSLDEVGLTCFLDLVYCEMQSTAKEVVIA
LIHKEREGEQIDRALVKNVLDIYVENGMGTLEKYEEDFESFMLQDTASYYSRKASRWTEEDSCPDYMIKVEECLKMERER
VTHYLHSITEPKLVEKIQNELLVMVTKNRLENEHSGFSALLRDDKKNDLSRIYRLYLPIPKRLGRVADLFKKHITEEGNA
LIKQADDKTTNQLLIELHNKFIVYVIECFQNHTLFHKVRVLFMCVYLFQQYYIE*
>Atha_AT3G46910
MKTTPGLRIFKPIIPHTPKPDSRIYVEDAWTMLKPAIRAIFLDEPQDFACSGLFNAVNKSWCEKSSGEALYKLILEECEI
YISAAIQSLESQCDTDPSLFLSLLEKCWLDFRRKLQFLCSIAGGEGQTVGPHSVWDLGSELSPKHLFSAQKVRDKLLSII
LQLIRDQRSFMSVDMTQLKNTTRPVMSVHMTQLNNLRGLFYGQSLYKSPFFKKPFIDCAVEFYSAEAMQFKEQSDIPLYL
KRVEYVA*
>Atha_AT5G46210
MSLPTKRSTFSAASASDDSSYSSPPMKKAKNDLHHSPQHPNTADKVVGFHMEEDPTPAAANLSRKKATLPQPTKKFVIKL
NKAKPTLPTNFEENTWEKLQSAIRAIFLKKKISFDLESLYQAVDNLCLHKLDGKLYDQIEKECEEHISAALQSLVGQNTD
LTVFLSRVEKCWQDFCDQMLMIRSIALTLDRKYVIQNPNVRSLWEMGLQLFRKHLSLAPEVEQRTVKGLLSMIEKERLAE
AVNRTLLSHLLKMFTALGIYMESFEKPFLEGTSEFYAAEGMKYMQQSDVPEYLKHVEGRLHEENERCILYIDAVTRKPLI
TTVERQLLERHILVVLEKGFTTLMDGRRTEDLQRMQTLFSRVNALESLRQALSSYVRKTGQKIVMDEEKDKDMVQSLLDF
KASLDIIWEESFYKNESFGNTIKDSFEHLINLRQNRPAELIAKFLDEKLRAGNKGTSEEELESVLEKVLVLFRFIQGKDV
FEAFYKKDLAKRLLLGKSASIDAEKSMISKLKTECGSQFTNKLEGMFKDIELSKEINESFKQSSQARTKLPSGIEMSVHV
LTTGYWPTYPPMDVKLPHELNVYQDIFKEFYLSKYSGRRLMWQNSLGHCVLKADFSKGKKELAVSLFQAVVLMLFNDAMK
LSFEDIKDSTSIEDKELRRTLQSLACGKVRVLQKNPKGRDVEDGDEFEFNDEFAAPLYRIKVNAIQMKETVEENTSTTER
VFQDRQYQIDAAIVRIMKTRKVLSHTLLITELFQQLKFPIKPADLKKRIESLIDREYLEREKSNPQIYNYLA*
>Mgut_mgv1a024606m
MTNNRPKIIEFDEGWDFVQKGITKLKKILEGLPEPQFTSEERMSLYMTIYNMCTQKPPHDYSEELYVKYRELFEEYIAST
VIPSLSEKHGELMLREFVIMWSNVKIMGDKLSRFFHYLDRYYIARRYLPPCNKVGLVCFRNLVYKQMKGNVIGAVLSLIH
RERLGDQIDRVLLKNVLDIFVEIGMKYYENDFEEAMLKDTAAFYSHKASIWILNNSCPDYMLKARECLKQEKERVSHYLQ
PSTELKLLEKVQQEFSSVVTGHSAQLLEKMHLEDSCDDM

>Mgut_mgv1a002528m
MEEGWAIMEDGIAKVKNILEELESQFSSEEYMRLYTLGNFVYKKNGMRLCLYNNYKESVQAYIMSTVNPSLRDKHDEFML
RELVRRWSDHKVMVKWLSRFLSYLDRYFVVRKKIESIKDHGFSCFRDMVDAMLKDTAHYYSRKASQWIFQDSCPDYMLKV
EECLKTEKNRVSHYLHSSSETKLLEQVENELLVVYANQLLEKETGCRALLRDDKVDELSRMYRLFHNIPKGLEEVAKIFK
EHVHSEGLMAIQMIEDATCNKALMTTFIELHNKYSAYTTGCFCNNAVLHKALKQAFESFCNQVVCGTSNADHLASYCDYI
LKKGGSEKISDEAIDESMDKIVKLLAYISDKDLFAEFSRKKLSRRLLFDRNVNDDHERLFLTKLKQQCGGQFTSKMEGMV
TDLALAKENQNQFHEYLINNNAAIPGMDLTVNVLTTGFWPTYKTCDLRLPAEMVKCVQVFKEHYEAKTKHRKLTWIYSLG
TCSINGKFDSKTIELVVGTYQGAVLLLFNDAQRLSYSDIKTQLNFEDDDLMRVLHSLSCSKYKILKKDPDSKFVSPNDYF
EFNSKFTDKMRRIRIPLPVVDDRKKVIQDVDNDRRYSIDAALVRIMKGRKILGHQQLILECVEMLSNMFKPDVKVIKKRI
EDLLARDYLERDAENPNLYKYVA*
>Mgut_mgv1a001310m
MSQRTNSSSTNPNKRSSSTNPNANTPIAAAAAASPIFPALKKAKSQGVSCSLDGNMNGQQQQQPTPHVHFAETPALSPMI
EDDPNDAVLDASSPSSAFGRVGATSCGGITSNLSRKKATPPQPTKKLVIKLFRAKPTLPSNFEETTWEILKSAISTIFLK
QPNPCDLEKLYQAVNNLCLHKLGGNLYQRIEKECEFHISAALQSLVGQSEDLVVFLSLVENCWQDFCDQMLMIRGIALFL
DRTYVKQTPNVRSLWDMGLQLFHKHLALAAEVEHKTVFGLLKMIESERLGESVDRTLLNHLLKMFTALGIYPESFEKPFL
ERTSEFYAAEGVKYMQQADVPDYLKHVEIRLQEENERCLLYIDASTRKPLVATAERQLLERHISAILDKGFMMLMDGKRI
DDLQRMYLLFSRVNALESLRQSLNQYIRKTGQSIVMDEEKDKDMVSSLLDFKANLDRIWEESFYKNDSFSNTIKDAFEHL
INIRQNRPAELIAKFVDEKLRAGNKGTSEEELEGTLDKVLVLFRFIQGKDVFEAFYKKDLAKRLLLGKSASIDAEKSMIS
KLKTECGSQFTNKLEGMFKDIELSKEINESFKQSSQARTKLPSGIEMSVHVLTTGYWPTYPPMDVRLPHELNVYQDIFKE
FYLSKYSGRRLMWQNSLGHCVLKAEFPKGRKELAVSLFQTVVLMLFNDAQKLSFQDIKESTGIEDKELRRTLQSLACGKF
RVLQKMPKGRDVEDDDTFVFNDQFAAPLYRIKVNAIQMKETIEENTSTTERVFQDRQYQVDAAIVRIMKTRKMLSHTLLI
TELFQQLKFPIKPADLKKRIESLIDREYLERDKNNPQVYNYLA*
>Mgut_mgv1a022204m
MSSGHKKRNFQIEAFKHKVVVDPKYAEKTWKILEHAIHEIYNHNASGLSFEELYRVCPWRFILEELNTKWSDHNKALQMI
RDILMYMDRTFIPSTRKTPVHELGLNLWRDNVIHSDKIQTRLLNTVLELILRERTGEVINRGLMRNIIKMLMDLGPLVYQ
DDFEKPFLEVSADFYRAESQEFIECSDCGDYLKKAERRLNEEIERVSHYLDAKSEAKITNVVEKEMVANHMLRLVHMENS
GLVKMLLDDKLEDLGRMYALFKRVPIGLPTIRDVMTSHIRDTGKQLVTDPEKSKNPVEFVESLLDKKDKYDKVIASSFNN
DKTFQNALNSSFEYFINLNPRSPEYISLFVDDKLRKGLKGVKEEEIETMLDKVMMLFRYLQEKDVFEKYYKQHLAKRLLS
GKTVSDDAERSLIVKLKTECGYQFTSKLEGMFTDMKTSQDTMQGFYSAHGSDLASGPTLVVQVLTTGSWPTQSSITCNLP
SEMLALCDKFRSYYLGTHTGRRLSWQTNMGTADLRATFGNGQKYELNVSTYQMCVLMLFNNADCLSYREIEQATEIISSD
LKRCLQSLACVKGKNVLRKEPMSKDIGEDDVFSVNDKFTSKLRKVKIGTVAAQKETEPEKQETRQRVEEDRKPQIEAAVV
RIMKARRVLDHNNIIAEVTKQLQSRFLANPSEIKKRIESLIERDFLERDNVDRRLYRYLA*
>Mgut_mgv1a001885m
MTMNQRNTIDLEQGWDFMQKGILKLKNILEGLPEPQFSSEDYMMLYTTIYNMCTQKPPHDYSQQLYDKYRESFEEYITST
VLPSLREKHDEFMLRELVKRWQNHKIMVRWLSRFFHYLDRYFIARRSLPPLKEVGLTCFRNLVYQEINGKVRDAVISLID
QEREGEQIDRALLKNVLDIFVEIGMGQMDYYENDFEEAMLKDTAAYYSRKASIWILDDSCPDYMLKAEECLKREKDRVSN
YLHSSSEAKLLEKVQHELLSVYATQLLEKEHSGCHALLRDDKVEDLSRMYRLFSKIPRGLEPVANIFKQHVTAEGTALVK
QAEDAASNKKADKKDVIGLQEQVFVRKVIELHDKFMAYVNECFLNHTLFHKALKEAFEIFCNKGVAGSSSAELLATFCDN
ILKKGGSEKLSDEAIEETLEKVVKLLAYISDKDLFAEFYRKKLARRLLFDKSANDEHERSILTKLKQQCGGQFTSKMEGM
VTDLTLARENQTSFEEYLGNNPNANPGIDLTVTVLTTGFWPSYKSFDLNLPAEMVKCVEVFREFYQTKTKHRKLTWIYSL
GTCNINGKFEAKTIELIVTTYQAAALLLFNSSDRLSYQEIMTQLNLSDDDVVRLLHSLSCAKYKILNKEPSTKIISPTDV
FEFNSKFTDKMRRIKIPLPPVDEKKKVIEDVDKDRRYAIDASIVRIMKSRKVLGYQQLVMECVEQLGRMFKPDVKAIKKR
IEDLITRDYLERDKDNPNLFKYLA*
>Mgut_mgv1a001887m
MTMHQRNTIELDKGWDFMERGIVKLKNILEGLPEPQFNSEDYILLYTTIYNMCTQKPPHDYSQQLYDKYREAFEDYITRT
VLPSLREKHDEFMLRELVRRWLNHKVMVRWLSRFFHYLDRYFIARRSLPPLKEVGLTCFRDLVYHEVNGKVRDAVISLID
QEREGEQIDRALLKNVLDIFVEIGMGQMEQYENDFEEAMLKDTAAYYSRKASNWILDDSCPDYMLKAEECLKREKDRVSH
YLHSSSETKLLEKVQHELLSVYATQLLEKEHSGCHALLRDDKVEDLSRMYRLFSKVPRGLEPVANIYKQHVTAEGTALVK
QAEDAASNKKAERKDVVGLQEQVFVRKVIELHDKFMAYVNECFLNHTLFHKALKEAFEIFCNKGVAGSSSAELLATFCDN
ILKKGGSEKLSDEAIEDTLEKVVKLLAYISDKDLFAEFYRKKLARRLLFDKSANDEHERSILTKLKQQCGGQFTSKMEGM
VTDLTLARENQTSFEEYLSNNANANPGIDLTVTVLTTGFWPSYKSFDLNLPAEMVKCVEVFREFYQTKTKHRKLTWIYSL
GTCNINGKFEQKTIELIVTTYQAAALLLFNSSDRLSYQEIMTQLNLSDDDVVRLLHSLSCAKYKILSKEPNTKTISPTDV
FEFNSKFTDKMRRIKIPLPPVDEKKKVIEDVDKDRRYAIDASIVRIMKSRKVLGYQQLVMECVEQLGRMFKPDVKAIKKR
IEDLITRDYLERDKDNPNLFKYLA*
>Mgut_mgv1a025238m
MNNPRNTIQFDEGWNFVQKAITKLTNILEGLPEPQFTTQENMMIYETIYNMCTQKPPYDHTARLYEKYQESYVEYILTTV
LPSLNEKNGELMLRELVIRWSNHKIMVDCLSKFFHYIDRYYVVRLYLIPCKEVGLLTFRDLVYYKIKGKVRDAVICLINR
EREGEQIDRVLLKNVLDFFVEIGMGEMDYYEDFEEAMINDAAAYYSQKASIWVLDSSCYDYMLKAHECFRQEKDRVSCYL
HPSTETKLLKVRLKRLMVQFYAKFS
>Mgut_mgv1a002087m
MVISQIKAMEEGWAIMEDGIAKVKNILEELESQFSSEEYMRLYTYPSRIFYKIYYVNPSLRDKHDEFMLRELVRRWSDHK
VMVKWLSRFLSYLDRYFVVRKKIESVKDHGFSCFRDMAYGEVKHKAKDAVIALVNKEREGGQIDRALLKNVLDVFVEMGK

GKIEFYTDDFEDAMLKDTAHYYSRKASQWIFQDSCPDYMLKVEECLKTEKNRVSHYLHSSSETKLLEQVENELLVVYANQ
LLEKETGCRALLRDDKVDELSRMYRLFHNIPKGLEEVAKIFKEHVHSEGLMAIQMIEDATFNKTEKAGGARGEHALMTTF
IELHNKYSAYTTGCFCNNAVLHKALKQAFESFCNQVVCGTSNADHLASYCDYILKKGGSEKISDEAIDESMDKIVKLLAY
ISDKDLFAEFSRKKLSRRLLFDRNVNDDHERLFLTKLKQQCGGQCTSKMEGMVTDLALAKENQNQFHEYLINNNAAIPGM
DLTVNVLTTGFWPTYKTCDLRLPAEMVKCVQVFKEHYEAKTKHRKLTWIYSLGTCSINGKFDSKTIELVVGTYQGAVLLL
FNDAQRLSYSDIKTQLNFEDDDLMRVLHSLSCSKYKILKKDPDSKFVSPNDYFEFNSKFTDKMRRIRIPLPVVDDRKKVI
QDVDNDRRYSIDAALVRIMKGRKILGHQQLILECVEMLSHMFKPDVKVIKKRIEDLLARDYLERDAENPNLYKYVA*
>Mgut_mgv1a023949m
MEQGWAIMEEGIAKLKNILEELESQFSSEEYMRLYTLVNFVYKKNGTRLCQLYNNYKESVQAYIMSTVNPSLRDKHDEFM
LRELVRRWSDHKVMVKWLSRFLSYLDRYFVVRKKVESVKDLGFSCFRDMAYGEVKHKAKDAVIALINKEREGGQIDRALL
KNVLDVFVEMGKGEIEFYTDDFEDAMLKDTAHYYSRKASQWIFQDSCPDYMLKVEECLKTEKNRVSHYLHSSSETKLLEQ
VENELLVVHANQLLEKETGCRALLRDDKVDELSRMYRLFHNIPKGLEEVAKIFKEHVHSEGLMAIQMIEDATCNKALMTT
FIELHNKYSAYTTGCFCNNAVLHKALKQAFESFCNQVVCGTSNADHLASYCDYILKKGGSEKISDEAIDESMDKIVKLLA
YISDKDLFAEFSRKKLSRRLLFDRNVNDDHERLFLTKLKQQCGGQFTSKMEGMVTDLALAKENQNQFHEYLINNNAAIPG
MDLTVNVLTTGFWPTYKTCDLRLPAEMVKCVQ
>Mgut_mgv1a014605m
MMVRLLSIFFHYLDQYFTVRTLLPSCKEVGLICFRDLVYKEMKKKVRDAVISLIDREREGEEIDRVLLKNVLDIFVELGM
GYYRDDFEEAMLKDTAVYYSRKASVWVSNNSCRDYMLKAEECLKREKDRVSHYLHASSETKLLQKVRHELLSVSSVHATK
LLEKMHLGSHPLLRDEKAEDFMDI*
>Mgut_mgv11b018794m
MKLYDQYKRTFEDYISSKVFEEMNDQVGVIIVSLLNMAGAAAPQKDITFDECWSILQEEAINKMVNIFEAEEQPNDKIFD
SEEYMRFYTPDNWNQKLYDQYKKTFEDYISSKVYREIHEQVHDITMSMIDRERNGEQIDQAMVSEVIKICEEMGSGNASK
HDANNIDEALVLLGEATYSFTGLSEYLSLQIRLPFSAVSELSMAGGAAALKYPMNFEEAWPILEEEAINKFIDKLELEEH
LPSHQYINSEEYMRFYTLVYNVCSPNPIGPEVLQLYEQYKRIFEDYVSSKVYWEVNVQVSEIIISLRQKYAQIDKERKGE
EIDQALVKEVLNIYDEIESGDASKHHAKYIEDAIIEATSKFYSRKAVNWIAMESYDDYMLKIHILKQHDHKMRLFTCKYQ
IVVH*
>Mgut_mgv1a001957m
MSSGHKKRNFQIEAFKHKVVVDPKYAEKTWKILDHAIHEIYNHNASGLSFEELYRNAYNMVLHKFGEKLYSGLVSTMTLH
LQTMSKSIESAHGGLFLEELNTKWSDHNKALQMIRDILMYMDRTFIPSTHKTPVHELGLNLWRDNVIHSDKIQTRLLNTL
LELILRERMGEVINRGLMRNIIKMLMDLGPSVYQDDFEKPFLEVSADFYRAESQEFIECSDCGDYLKKAERRLNEEIERV
SHYLDAKSEAKITNVVEKEMVANHMLRLVHMDNSGLVKMLLDDKLEDLGRMYTLFKRVPIGLPTIRDVLTSHIRNTGKQL
VTDPEKSKNPVEFVEALLDKKDKYDKIIASSFSNDKTFQNALSSSFEYFINLNPRSPEYISLFVDDKLRKGLKGVKEEDI
EIMLDKVMMLFRYLQEKDVFEKYYKQHLAKRLLSGKTVSDDAERSLIVKLKTECGYQFTSKLEGMFTDMKTSQDTMQGFY
SAHGGDLASGPTLVVQVLTTGSWPTQSSISCNLPSELLTLCEKFRSYYLGTHTGRRLSWQTNMGTADLRATFGNGQKYEL
NVSTYQMCVLMLFNNSDCLSYREIEQATEIISSDLKRCLQSLACVKGKNVLRKEPMSKEIGEDDVFSVNDKFTSKLRKVK
IGTVVAQKESEPEKQETRQRVEEDRKPQIEAAVVRIMKSRRVLDHNNIIAEVTKQLQPRFLANPSEIKKRIESLIERDFL
ERDNVDRRLYRYLA*
>Mgut_mgv1a001129m
MATTEGSLTSPICNLAILDSLSANSTGQMADSWNAFCVATESVIGGGGGDLLLAPDFVSHLRNLCNLGLKSLIVEHFLCS
LEEIFEKRGASRFWKHFDAYSNSSVLHMEDLDEQDGMQDLLHKALEEISSEKQYQEKCLLKLVQALEMCRQSKPNGPTYS
DERNYLLSKYQLTVSSVLMVSLPRHFPDILRWYFRRRLEELSAMMAVGCEYDNKLHIDDGMDLDCKTGEMDADEIHQDRT
ILGNNNLVRNIGEIVRDLRNLGFTSMAEDAYASAIFLLLKSKVHELAGDEFRFSVLESIKGWIQAVPLQFLHALLAYLGD
SRSHEGLSSGLKSPLASHPSFKYYGTGVPSEGLVRWQLRLEYFAYETLQDLRIAKLFEIIVDYPDSSPAIEDLKQCLEYT
GQHSKLVDSFIAALKYRLLTAGASTNDILHQYVSTIKALRTIDPAGVFLEAVGEPIREYLRGRKDTIKSIVTMLTDGAGG
NPSGPGSTGDSLLEELNRDEENQESSSYDDDVNTDDKQAWINAQSWEPDPVEADPLKGGRYRRKVDILGMIVGIIGSKDQ
LVNEYRVMLAEKLLNKSDYDIDSEIRTLELLKIHFGEGSMQKCEIMLNDLIDSKRTNTNVKATIKQQPQPASDVGEHELS
LDNLNATIISSNFWPPIQDEALNIPGRMEKILSDYAKRFNEIKTPRKLLWKKNLGTVKLELQFEDRVLPFTVTPLHASII
SQFEDQTSWTSKSLAAAVGVPVEILNRRIYFWINKGILAESVEESGDNKFSLVEAMVENGKNGVNSGGCDELLAGDDDGE
RSIASVEDQLLKEMTVYEKFITGMLTNFGSMALDRIHNTLKMFCRGDPAYDKSLQQLQSFLAGLVAEEKLELKDGMYLLK
K*
>Mgut_mgv1a013613m
MSCGGGECCKQRITVEACWTLLHEVGIDRMIAAFEAGKPLGDDSSNPRTYDAYTAVYNVCTSYTTNAQTQKLYERYRTTL
EDYISAKVLPSLREKSNETLLGEFFRRWRNHKIMTKWLSVFFQYLSRTYIQMKNLPSLLEVSYSTFYKLVYLEMKDRVRY
AVTSMINSEQVDEAMLKEVLAFCEEIKGGDASKNYAKDMEEAINEATFKFKSLSL*
>Acoe_002_00570
MSAPKKRNFHIEAFKHKVVVDPKYAEKTWKVLEHAINEIYNHNASGLSFEELYRNAYNMVLHKFGDKLYSGLVATLTMHL
KEISKSIEAAQGGLFLEELDRKWTDHNKALRMIRDILMYMDRTYIPNTHKTPVHELGLNLWRDHIIHSSEIQTRLLTMLL
ELVQRERTGEVINRGLMRNVIKMLMDLGLSVYQEEFEKPFIEVSSNFYSLESQQFIECCDCGDYLKKAERRLNEEMERVS
HYLIDRTESKITNVVENEMISNHMQRLIHMENSGLVSMLVDDKYEDLGRMYSLFHRVPDGLSSIRDVMTSHLRETGKQLV
MDPERLKDPVDFVQRLLDKKDKYDNIISLALNNDKTFQNALNSSFEHFINLNSRSPEFISLFVDDKLRKGLKGVSEEDVE
VILDKVMMLFRYLQEKDVFEKYYKQHLAKRLLSGKSISEDVERSMIVKLKTECGYQFTSKLEGMFTDMKTSQDTVQEFYA
NQAADTAESPVLAIQVLTTGSWPTQPSTPCNLPTEILEVCEKFRTYYLGKHTGRRLTWQTNMGTADLKATFGKGQKHELN

VSTYQMCILMLFNNADRLTYREIEQATEIPASDLKRCLQSLACVKGKNVLRKEPMSKDIGEEDAFFFNDKFTSKFYKVKI
GTVAAQKETEPENQETRQKVEEDRKPQIEAAIVRIMKSRRVLDHNNLVAEVTKQLQSRFMPNPVLIKKRIESLIEREFLE
RDKADRKLYRYLA
>Acoe_002_00666
MATHSACSNYYNLGILDSITDDSIEDILDSWSGFCNTTESLLKDKGDLSVGSEFISHVHILCENRLTSLVQDHFFKALEE
TFERNGRSKFWHHFDAFSDVSTSELNESHKDWVQQVLCKALEEICIEKQYQEKCLLILVHALQSYNEGISKGRYNSDAER
VTLFSRYRLMVSSVLMTTLPRHFPEILRFYFKERLEELSDLMSGNSEDDDEFQPKDGMDIDERTKLSPEVSEMDIGETFS
GRKLSENNKLVKNIGKVVRDLRSLGFMSMTEDAYASAIFLLLKTKVHDLAGDDYRTSVLESIKEWIRAVPLQFLYALLAY
LGDSVSYNSPSSGLKSPLASHPSSFYPGIDIPSEGLVRWQLRLEYFAYETLQDLRIAKLFEIIVDYPDSSPAIEDLKQCL
KYSGQHSKLVDSFISSLRYRLLTAGASTNDILHQYVSTIKALRIIDPTGVFLEAVGEPIRDYLKGRKDTMKCIVTMLTDG
PGGNPNGPGNTGDSLLEELNRDEENQEDDVCDDDFNSDDKQAWINAERWEPDPVEADPLRGSRNRRKFDILGMIVGVIGS
KEQLVNEYRVMLAEKLLNKSDYDIDSEIRTLELLKIHFGESSMQKCEIMLNDLINSKRINTNIKASISDVSQKGSDRGEI
GVSLDLLDATIISSNFWPPVQDESINIPETVDQLFSDYAKRFYEIKTPRKLLWKKNLGTVKLELQFGDRAAQFTVTPVLA
AIIMNFQDQPRWTSKDLAAAIGVPVDILNRRINFWITKGVIAESIGAESDDNIFDLVDAMVDSSRNGVNNASCEDLAADE
EGETSVASLEEQLLIEMTVYEKYIMGMLTNFGNMSLDRIHNTLKMFCSADPVYDKSLQQLQSFLSGLLSEEKLEFRDGMY
YLKR
>Acoe_003_00808
MSTSPEPPGPVKWSDGWKEMEIGFTKLVRILDGYDEPDFTAKEYIQLYTVVYNMCTQKPPDDFSEELYNGYIQLIHDYVP
NKVLPSINSKHDVFLLEELEKRWLNHKVMLKWLSRFFQYLERYHIPRNSLRTLKDAGHNCFLEMVYEKIQVQVRNVILSL
IDREREGEQIDRSAVKNVLSMFVELEREKGTKPYENDFEAFMLQNTDAYYSRKASKHILVDSCPDYLLMAEECLKNEKDR
ATHYLHSSSESKLVEKTQDVLLFQHLNQIIEKENSGLHIMLRDDKIEDLSRMYRLFKKIDKGLEPVAAIFKKHVTSAGVA
LFKEIEDAAKSKKAYVNKVLEFHEKYLSYCNDCFMGDINFHKALKEGFESFFNKDIVGMSCAEMLSTFCDTFLQKGSAVE
KLGDDAIEKTIEKVALLLAYLQEKDLFAEFSRKKLARRLLTDRSSNLEHEKHFISKLKQQFGSYFTSKMEGMITDLSLAR
ETQAEFETYIKDNPHVTQGADLSVTVLTTGFWPTYKTVDFNIPPDVFYCIDAFKTYYKCKANSRKLTWIFSLGGCNLIGR
FDSKIIELQVSTYQAAALLLFNTTDRFSYQDIATELKLPNDELIRVLHSLSCGKYKILLKQPNSKAISHSDVFEFNSKFT
DKMRRIKVPLPHIEEKRKVIEDVDKERKFSIDAALVRIMKSRKVLGHQQLVLECVNILNRMFKPDIKVIKKRIEDLILRD
YLARDEENPNSYKYLA
>Acoe_003_00810
MATKVTLNLKDFEHGWSIMQIGIQKLINMLEGIPSNAFTSEEYMQYYTVVYELCSPQELEPSQKFYDKYKETFQDYITSK
VLPSLRENKDEEFVREIVKRWSNHKIMVRWLSRFFHYLDRYYLNKQKLPSTQEVGFMIFYDMVYLEMKDQLRLVLISMIN
RECKGEKIDQNLLKDALDIYVEIGRGSLKHYEDDMEKAMLENAAAYYSQKASSWIMVDSYADYMLKAKECMEQEKDRVAS
YLPSSSQKKLLEIVDHELISSYVSKLQEMKQIRSVVLI
>Acoe_003_00814
MSMNERKIIDLDQGWEFMYKGITKLKRILEGQPEPQFNSEDYMMLYTTIYNMCTQKPPNDYSQQLYDKYREAFEEYINLA
VLPSLREKHDEFMLRELVERWMNHKVMVRWLSRFFHYLDRYFIARRSLPALKDVGLTCFRDLVYQELNVKVKDAVIFLID
QEREGEQIDRALLKNILDIFVEIGMGQMERYENDFETPMLLDTSDYYSRKASIWIEEDSCPDYMLKAEECLKREKDRVAH
YLHSSSETKLLDKVQYELLTVYTTQLLEKEHSGCHALLRDDKVDDLSRMYRLFSKIPHGLEPVSNMFKQHVTAEGTALVN
QAEDAASNKKVCFPQAEKRDVVGLPEQVFVKKVLDLHDKYLAYVNDCFLNHTLFHKALKEAFEVFCNKGVSGSSSAELLA
TFCDNILKKGGSDKLSDEAIEETLEKVVKLLAYVSDKDLFAEFYRKKLARRLLFDKSANDDHERSILTKLKQQCGGQFTS
KMEGMVTDLTLAREHQTNFEEYLTINPATNPGIDLTVTVLTTGFWPSYKSFDLNLPAEMVKCVEVFKEFYQTKTKHRKLT
WIYSLGTCNINGKFDQKTIELIVTTYQASLLLLFNSSDRLSYSEIKEQLNLTDDDIIRLLHSLSCAKYKILNKVPNTKTI
SQKDVFEFNSKFTDKMRRIKIPLPPVDEKKKVIEDVDKDRRYAIDASIVRIMKSRKVLGHQQLVMECVDQLGRMFKPDFK
AIKKRIEDLITRDYLERDKDNPNLYKYLAMSMNERKIIDLDQGWEFMYKGITKLKRILEGQPEPQFNSEDYMMLYTTIYN
MCTQKPPNDYSQQLYDKYREAFEEYINLAVLPSLREKHDEFMLRELVERWMNHKVMVRWLSRFFHYLDRYFIARRSLPAL
KDVGLTCFRDLVYQELNVKVKDAVIFLIDQEREGEQIDRALLKNILDIFVEIGMGQMERYENDFETPMLLDTSDYYSRKA
SIWIEEDSCPDYMLKAEECLKREKDRVAHYLHSSSETKLLDKVQYELLTVYTTQLLEKEHSGCHALLRDDKVDDLSRMYR
LFSKIPHGLEPVSNMFKQHVTAEGTALVNQAEDAASNKKAEKRDVVGLPEQVFVKKVLDLHDKYLAYVNDCFLNHTLFHK
ALKEAFEVFCNKGVSGSSSAELLATFCDNILKKGGSDKLSDEAIEETLEKVVKLLAYVSDKDLFAEFYRKKLARRLLFDK
SANDDHERSILTKLKQQCGGQFTSKMEGMVTDLTLAREHQTNFEEYLTINPATNPGIDLTVTVLTTGFWPSYKSFDLNLP
AEMVKCVEVFKEFYQTKTKHRKLTWIYSLGTCNINGKFDQKTIELIVTTYQASLLLLFNSSDRLSYSEIKEQLNLTDDDI
IRLLHSLSCAKYKILNKVPNTKTISQKDVFEFNSKFTDKMRRIKIPLPPVDEKKKVIEDVDKDRRYAIDASIVRIMKSRK
VLGHQQLVMECVDQLGRMFKPDFKAIKKRIEDLITRDYLERDKDNPNLYKYLA
>Acoe_006_00278
MNTQKRRNFQIEAFKHRVVVDPKYAEKTWKVLEHAIHEIYNHNASGLSFEELYRNAYNMVLHKFGDKLYSGLVSTMTMHL
KDISQSIEIAQGGLFLEEMNNKWAEHNKALQMIRDILMYMDRTFIPSTHKTPVHELGLNLWRDNIIHSGNIQTRLLNTLL
ELVQRERMGEVINRGLMRNIVKMLMDLGSAVYQEDFEKPFLEVSANFYSVESQQFIECCDCGDYLKKAERRLNEEMERVS
HYLDARSEVKITNVVEKEMIANHMQRLVHMENSGLVNMLVDDKYEDLGRMYNLFRRVPDGLSTIRDVMTSHIRENGKQLV
TDPEKLRDPVDFVQRLLDEKDKNDKIISLAFNNDKTFQNALNSSFEYFINLNPRSPEFISLFVDDKLRKGLKGVSEEDVE
LVLDKVMMLFRYLQEKDVFEKYYKQHLAKRLLSGKTVSDDAERSLIVKLKTECGYQFTSKLEGMFTDMKTSQDTMQGFYT
SKAAEVGDSPALAVQVLTTGSWPTQPTNTCNLPAEILGVCEKFRMYYLGTHTGRRLTWQTNMGTADLKATFGNGQKHELN
VSTYQMCILMLFNNADRLSYREIEQATEIPASDLKRCLQSLACVKGKNILRKEPMSKDIGEDDAFFFNYKFSSKFYKVKI
GTVVAQKESEPEKLETRQRVEEDRKPQIEAAIVRIMKSRRVLDHNNIVAEVTKQLQSRFLPNPVIIKKRIESLIEREFLE

RDKVDRKLYRYLA
>Acoe_009_00641
MTDREEIIEFEVGAKFIKNGIAKFIYLQENFPEEKLSPHEVMKLYSCVYNMCIQNYPHSYDSELYNLYKEIIEEYVKGTV
LSSLREKHDEFFLLELIKRWKNHKILVRLMSSQIFQYLNRYHIGRLPWEPSLDKFGFTCFYDAVSQDINMKARDIVISLI
EKEREGAQIDKVLLKNVTDYFVEIGAGWDHYFLEAGPGFYYQKQYEEVILNSTVSYYLGKAMSWISEEDYIEKAKECLKR
EEENVSHYLAFVSKDKLLATVQQQLLSVYEKHQLDKDKVEELSRNLSSLLV
>Acoe_017_00270
MAMHERKTIDLEQGWEFMQKGITKLKNILEGLPEPQFSSEDYMMLYTTIYNMCTQKPPHDYSQQLYDKYRESFEEYITST
VLPSLREKHDEFMLRELVKRWANHKVMVRWLSRFFHYLDRYFIARRSLPALNEVGLTCFRDLVYREINGKVRDAVISLID
QEREGEQIDRALLKNVLDIFVEIGMGQMDCYENDFEVSMLKDTASYYSRKASNWILEDSCPDYMLKAEECLKREKDRVSH
YLHSSSEQKLLEKVQHELLYVYASQLLEKEHSGCHALLRDDKVEDLSRMYRLFCRIPRGLDPVSTIFKQHVTAEGTALVK
QAEDAASNKKAEKRDVVGLQEQVFVRKVIELHDKYLAYVNDCFMNHSLFHKALKEAFEVFCNKGVAGSSSAELLATFCDN
ILKKGGSEKLSDEAIEETLEKVVKLLAYISDKDLFAEFYRKKLARRLLFDKSANDDHERSILTKLKQQCGGQFTSKMEGM
VTDLTLARENQTSFEEYLNSNPQVNPGIDLTVTVLTTGFWPSYKSFDLNLPEEMVKCVEVFREFYQTKTKHRKLTWIYSL
GTCNIIGKFEAKTMELIVTTYQASVLLLFNASDRLSYSEIMTQLNLTDDDIVRLLHSLSCAKYKILNKTPSTKTITPTDY
FEFNSKFTDKMRRIKIPLPPVDEKKKVIEDVDKDRRYAIDASIVRIMKSRKVLGHQQLVMECVEQLGRMFKPDFKAIKKR
IEDLITRDYLERDKDNPNLFRYLA
>Acoe_022_00213
MSHPKRSYNNTSSSSSSSSGSGSGSGVGGGVGGIGGNPFQTTSNTNTSSMKKAKSQAISCSMEKNGQNNHHYEDFGTSMI
VDDNNLKSESQFGVIDCTSQNPTTIVAANLSRKKATPPQPAKKPLVIKFTKGKPKPPTNFEETTWIVLKSAINAIFLKQR
TPSHFEDLYQAVDSLCIHKMGGDLYQRIEKECEAHICRVLQSLVGQSPDLVVFLSLVENCWQDLCNQIMMIRGIALVLDT
TYVKQTSNVLSLWDMGLHLFRRHLSLCPEVEHRTVTGMLRLIEKERLGEAIDRTLLNHLLKMFTALGIYSESFEKPFLEC
TTEFYAAEGVKFMQLSDVPDYLKHVELRLHEEHERCLLYLDSSTRKPLVATTERQLLERHTSAILDKGFTMLMDGNRIED
LRRLYALFSRVNAFELLRQALSSYIRGTGQGLIMDEEKDKDLVSSLLEFKASLDMIWEESFSRNDVFSNTIKDAFEHLIN
LRQNRPAELIAKFLDEKLRAGNKGTSEEELEGLLDKVLVLFRFIQGKDVFEAFYKKDLAKRLLLGKSASIDAEKSMISKL
KTECGSQFTNKLEGMFKDIELSKEINESFRQSSQARTKLPSGIEMSVHVLTTGYWPTYPPMDVRLPHELNVYQDIFKEFY
LSKYSGRRLMWQNSLGHCVLKAEFPKGRKELAVSLFQTVVLMLFNDAPKLTFQDIKDSTGIEDKELRRTLQSLACGKVRV
LLKIPKGREVDDNDYFLFNDDFNAPLYRIKVNAIQLKETVEENTSTTERVFQDRQYQVDAAIVRIMKTRKVLSHTLLITE
LFQQLKFPIKPADLKKRIESLIDREYLERDKSNPQIYNYLAMSHPKRSYNNTSSSSSSSSGSGSGSGVGGGVGGIGGNPF
QTTSNTNTSSMKKAKSQAISCSMEKNGQNNHHYEDFGTSMIVDDNNLKSESQFGVIDCTSQNPTTIVAANLSRKKATPPQ
PAKKPLVIKFTKGKPKPPTNFEETTWIVLKSAINAIFLKQRTPSHFEDLYQAVDSLCIHKMGGDLYQRIEKECEAHICRV
LQSLVGQSPDLVVFLSLVENCWQDLCNQIMMIRGIALVLDTTYVKQTSNVLSLWDMGLHLFRRHLSLCPEVEHRTVTGML
RLIEKERLGEAIDRTLLNHLLKMFTALGIYSESFEKPFLECTTEFYAAEGVKFMQLSDVPDYLKHVELRLHEEHERCLLY
LDSSTRKPLVATTERQLLERHTSAILDKGFTMLMDGNRIEDLRRLYALFSRVNAFELLRQALSSYIRGTGQGLIMDEEKD
KDLVSSLLEFKASLDMIWEESFSRNDVFSNTIKDAFEHLINLRQNRPAELIAKFLDEKLRAGNKGTSEEELEGLLDKVLV
LFRFIQGKDVFEAFYKKDLAKRLLLGKSASIDAEKSMISKLKTECGSQFTNKLEGMFKDIELSKEINESFRQSSQARTKL
PSGIEMSVHVLTTGYWPTYPPMDVRLPHELNVYQDIFKEFYLSKYSGRRLMWQNSLGHCVLKAEFPKGRKELAVSLFQLT
FQDIKDSTGIEDKELRRTLQSLACGKVRVLLKIPKGREVDDNDYFLFNDDFNAPLYRIKVNAIQLKETVEENTSTTERVF
QDRQYQVDAAIVRIMKTRKVLSHTLLITELFQQLKFPIKPADLKKRIESLIDREYLERDKSNPQIYNYLAMSHPKRSYNN
TSSSSSSSSGSGSGSGVGGGVGGIGGNPFQTTSNTNTSSMKKAKSQAISCSMEKNGQNNHHYEDFGTSMIVDDNNLKSES
QFGVIDCTSQNPTTIVAANLSRKKATPPQPAKKPLVIKFTKGKPKPPTNFEETTWIVLKSAINAIFLKQRTPSHFEDLYQ
AVDSLCIHKMGGDLYQRIEKECEAHICRVLQSLVGQSPDLVVFLSLVENCWQDLCNQIMMIRGIALVLDTTYVKQTSNVL
SLWDMGLHLFRRHLSLCPEVEHRTVTGMLRLIEKERLGEAIDRTLLNHLLKMFTALGIYSESFEKPFLECTTEFYAAEGV
KFMQLSDVPDYLKHVELRLHEEHERCLLYLDSSTRKPLVATTERQLLERHTSAILDKGFTMLMDGNRIEDLRRLYALFSR
VNAFELLRQALSSYIRGTGQGLIMDEEKDKDLVSSLLEFKASLDMIWEESFSRNDVFSNTIKDAFEHLINLRQNRPAELI
AKFLDEKLRAGNKGTSEEELEGLLDKVLVLFRFIQGKDVFEAFYKKDLAKRLLLGKSASIDAEKSMISKLKTECGSQFTN
KLEGMFKDIELSKEINESFRQSSQARTKLPSGIEMSVHVLTTGYWPTYPPMDVRLPHELNVYQDIFKEFYLSKYSGRRLM
WQNSLGHCVLKAEFPKGRKELAVSLFQLTFQDIKDSTGIEDKELRRTLQSLACGKVRVLLKIPKGREVDDNDYFLFNDDF
NAPLYRIKVNAIQLKETVEENTSTTERVFQDRQYQVQPHLFLLCIFFEAFNLQQYLYVWIWMFGRLMLPLSASMSHPKRS
YNNTSSSSSSSSGSGSGSGVGGGVGGIGGNPFQTTSNTNTSSMKKAKSQAISCSMEKNGQNNHHYEDFGTSMIVDDNNLK
SESQFGVIDCTSQNPTTIVAANLSRKKATPPQPAKKPLVIKFTKGKPKPPTNFEETTWIVLKSAINAIFLKQRTPSHFED
LYQAVDSLCIHKMGGDLYQRIEKECEAHICRVLQSLVGQSPDLVVFLSLVENCWQDLCNQIMMIRGIALVLDTTYVKQTS
NVLSLWDMGLHLFRRHLSLCPEVEHRTVTGMLRLIEKERLGEAIDRTLLNHLLKMFTALGIYSESFEKPFLECTTEFYAA
EGVKFMQLSDVPDYLKHVELRLHEEHERCLLYLDSSTRKPLVATTERQLLERHTSAILDKGFTMLMDGNRIEDLRRLYAL
FSRVNAFELLRQALSSYIRGTGQGLIMDEEKDKDLVSSLLEFKASLDMIWEESFSRNDVFSNTIKDAFEHLINLRQNRPA
ELIAKFLDEKLRAGNKGTSEEELEGLLDKVLVLFRFIQGKDVFEAFYKKDLAKRLLLGKSASIDAEKSMISKLKTECGSQ
FTNKLEGMFKDIELSKEINESFRQSSQARTKLPSGIEMSVHVLTTGYWPTYPPMDVRLPHELNVYQDIFKEFYLSKYSGR
RLMWQNSLGHCVLKAEFPKGRKELAVSLFQVGLLKFLMSHPKRSYNNTSSSSSSSSGSGSGSGVGGGVGGIGGNPFQTTS
NTNTSSMKKAKSQAISCSMEKNGQNNHHYEDFGTSMIVDDNNLKSESQFGVIDCTSQNPTTIVAANLSRKKATPPQPAKK
PLVIKFTKGKPKPPTNFEETTWIVLKSAINAIFLKQRTPSHFEDLYQAVDSLCIHKMGGDLYQRIEKECEAHICRVLQSL
VGQSPDLVVFLSLVENCWQDLCNQIMMIRGIALVLDTTYVKQTSNVLSLWDMGLHLFRRHLSLCPEVEHRTVTGMLRLIE
KERLGEAIDRTLLNHLLKMFTALGIYSESFEKPFLECTTEFYAAEGVKFMQLSDVPDYLKHVELRLHEEHERCLLYLDSS

TRKPLVATTERQLLERHTSAILDKGFTMLMDGNRIEDLRRLYALFSRVNAFELLRQALSSYIRGTGQGLIMDEEKDKDLV
SSLLEFKASLDMIWEESFSRNDVFSNTIKDAFEHLINLRQNRPAELIAKFLDEKLRAGNKGTSEEELEGLLDKVLVLFRF
IQVSASVDLILFVIFKDPSCLSLLFLLCRPIFFHISAYNCH
>Acoe_033_00008
MTDLDEEIIEFEEGVEIIKNGIAKFIDLQENFPKEKLTPRELMKLYSCIYDLCAIHSYSPQFYDLYEQTIKEYIKGIVLS
SLREKQDVCFLSELVKRWKNYKVMCSWMSSHLFQYLDRYYTSDERWLPSLAKVGFTYFYDAVSQDINMKARDIVISLIEK
EREGTQIDRVLLKNVTDYFVEVGAGWDNYFFKHGPGFYYEKHYEEGILSNTASYYLGKAMSWISEEDNGDQYYIERAKEC
LKREEENVSHYLTYVSKDKLLATVQQQLLSVYEKHQLDKAKVEELSRNLSFS
>Acoe_044_00028
MAMATHPASSNRYNLGILDSISDSAIVDILESWIGFCTTTESLLKDEGNLSVGSDFVSYVHILCESRFSSLVQDHFLKAL
EETFEKHLARSFWCHFDPQGDGLLPKWNAYHDKEDWGEDAFSEALEEICLEKQYQEKCLLVLVHALQTYRESMPANKHNS
DVEMTSLMCRYRSMVCSVITTILPRQFSDTLRFYFTERLEKLSTTRSADDDGVYENQAKDGMDIDERTSFSSSVTDMDID
EIYHKRNVLENSKLMKSIGKVVRDLRSLGLASLTEDAYASSIFSLLKTKVHALADDDYRSSVLESIKAWIKTVPLQFLYA
LLAYLGESVSYDSPSSGLKSPLASHPPSFYPGIDIPSEGLVRWQLRLEYFAYETLQELRIGKLFEIIVDYPDSCPAIDDL
KQCLKYTGQHSKLVESFISSLRCRLLTAGASTSDILHQYVSTIKVLRIIDPTGVFLEAVGEPIREYLKGRKDTRKCIVTM
FTDGTGGNPNGPGNTADSLLEESQENDNCEDYCNSDEKQAWINADCWEPDPIETDPLKGSSTRRKTDILGMIVGIIGSKD
PLIDEYSVMLAEKLLNKSDYDIDSEIRTLELLKLHFGESSMQTCEIMLNDLINSKRINTNIKASSAHPPKKGANLGEIGM
SLDFLDATIISSNFWPPVQEESINLPEPVDQLLSDYAKRFNEIKTPRKLLWKKNLGTVKLELQFGDRATQFIVTPVNAAI
IMHFQSQTSWTSKDLAAAVGVPVDALNWRMNFWISKGVITEATDGKADDYTYTLVDLVDSSENGIDNPPCEDLRSDEEGE
TSVASQEQLKKEHAVYENFIMGMLTNLGSMSVDHCYSILKRRCSYDKSIQELRIFLSSFVSEEKLEFRDGIYSLRRKMAT
HPASSNRYNLGILDSISDSAIVDILESWIGFCTTTESLLKDEGNLSVGSDFVSYVHILCESRFSSLVQDHFLKALEETFE
KHLARSFWCHFDPQGDGLLPKWNAYHDKEDWGEDAFSEALEEICLEKQYQEKCLLVLVHALQTYRESMPANKHNSDVEMT
SLMCRYRSMVCSVITTILPRQFSDTLRFYFTERLEKLSTTRSADDDGVYENQAKDGMDIDERTSFSSSVTDMDIDEIYHK
RNVLENSKLMKSIGKVVRDLRSLGLASLTEDAYASSIFSLLKQTKVHALADDDYRSSVLESIKAWIKTVPLQFLYALLAY
LGESVSYDSPSSGLKSPLASHPPSFYPGIDIPSEGLVRWQLRLEYFAYETLQELRIGKLFEIIVDYPDSCPAIDDLKQCL
KYTGQHSKLVESFISSLRCRLLTAGASTSDILHQYVSTIKVLRIIDPTGVFLEAVGEPIREYLKGRKDTRKCIVTMFTDG
TGGNPNGPGNTADSLLEESQENDNCEDYCNSDEKQAWINADCWEPDPIETDPLKGSSTRRKTDILGMIVGIIGSKDPLID
EYSVMLAEKLLNKSDYDIDSEIRTLELLKLHFGESSMQTCEIMLNDLINSKRINTNIKASSAHPPKKGANLGEIGMSLDF
LDATIISSNFWPPVQEESINLPEPVDQLLSDYAKRFNEIKTPRKLLWKKNLGTVKLELQFGDRATQFIVTPVNAAIIMHF
QSQTSWTSKDLAAAVGVPVDALNWRMNFWISKGVITEATDGKADDYTYTLVDLVDSSENGIDNPPCEDLRSDEEGETSVA
SQEQLKKEHAVYENFIMGMLTNLGSMSVDHCYSILKRRCSYDKSIQELRIFLSSFVSEEKLEFRDGIYSLRRKMATHPAS
SNRYNLGILDSISDSAIVDILESWIGFCTTTESLLKDEGNLSVGSDFVSYVHILCESRFSSLVQDHFLKALEETFEKHLA
RSFWCHFDPQGDGLLPKWNAYHDKEDWGEDAFSEALEEICLEKQYQEKCLLVLVHALQTYRESMPANKHNSDVEMTSLMC
RYRSMVCSVITTILPRQFSDTLRFYFTERLEKLSTTRSADDDGVYENQAKDGMDIDERTSFSSSVTDMDIDEIYHKRNVL
ENSKLMKSIGKVVRDLRSLGLASLTEDAYASSIFSLLKQTKVHALADDDYRSSVLESIKAWIKTVPLQFLYALLAYLGES
VSYDSPSSGLKSPLASHPPSFYPGIDIPSEGLVRWQLRLEYFAYETLQELRIGKLFEIIVDYPDSCPAIDDLKQCLKYTG
QHSKLVESFISSLRCRLLTAGASTSDILHQYVSTIKVLRIIDPTGVFLEAVGEPIREYLKGRKDTRKCIVTMFTDGTGGN
PNGPGNTADSLLEESQENDNCEDYCNSDEKQAWINADCWEPDPIETDPLKGSSTRRKTDILGMIVGIIGSKDPLIDEYSV
MLAEKLLNKSDYDIDSEIRTLELLKLHFGESSMQTCEIMLNDLINSKRINTNIKASSAHPPKKGANLGEIGMSLDFLDAT
IISSNFWPPVQEESINLPEPVDQLLSDYAKRFNEIKTPRKLLWKKNLGTVKLELQFGDRATQFIVTPVNAAIIMHFQSQT
SWTSKDLAAAVGVPVDALNWRMNFWISKGVITEATDGKADDYTYTLVDLVDSSENGIDNPPCEDLRSDEEGETSVASQEQ
LKKEHAVYENFIMGMLTNLGSMSVDHCYSILKLIAEALFLMAMATHPASSNRYNLGILDSISDSAIVDILESWIGFCTTT
ESLLKDEGNLSVGSDFVSYVHILCESRFSSLVQDHFLKALEETFEKHLARSFWCHFDPQGDGLLPKWNAYHDKEDWGEDA
FSEALEEICLEKQYQEKCLLVLVHALQTYRESMPANKHNSDVEMTSLMCRYRSMVCSVITTILPRQFSDTLRFYFTERLE
KLSTTRSADDDGVYENQAKDGMDIDERTSFSSSVTDMDIDEIYHKRNVLENSKLMKSIGKVVRDLRSLGLASLTEDAYAS
SIFSLLKTKVHALADDDYRSSVLESIKAWIKTVPLQFLYALLAYLGESVSYDSPSSGLKSPLASHPPSFYPGIDIPSEGL
VRWQLRLEYFAYETLQELRIGKLFEIIVDYPDSCPAIDDLKQCLKYTGQHSKLVESFISSLRCRLLTAGASTSDILHQYV
STIKVLRIIDPTGVFLEAVGEPIREYLKGRKDTRKCIVTMFTDGTGGNPNGPGNTADSLLEESQENDNCEDYCNSDEKQA
WINADCWEPDPIETDPLKGSSTRRKTDILGMIVGIIGSKDPLIDEYSVMLAEKLLNKSDYDIDSEIRTLELLKLHFGESS
MQTCEIMLNDLINSKRINTNIKASSAHPPKKGANLGEIGMSLDFLDATIISSNFWPPVQEESINLPEPVDQLLSDYAKRF
NEIKTPRKLLWKKNLGTVKLELQFGDRATQFIVTPVNAAIIMHFQSQTSWTSKDLAAAVGVPVDALNWRMNFWISKGVIT
EATDGKADDYTYTLVDLVDSSENGIDNPPCEDLRSDEEGETSVASQEQLKKEHAVYEVCIVSTCIASCCQQHLGLVPAIM
AMATHPASSNRYNLGILDSISDSAIVDILESWIGFCTTTESLLKDEGNLSVGSDFVSYVHILCESRFSSLVQDHFLKALE
ETFEKHLARSFWCHFDPQGDGLLPKWNAYHDKEDWGEDAFSEALEEICLEKQYQEKCLLVLVHALQTYRESMPANKHNSD
VEMTSLMCRYRSMVCSVITTILPRQFSDTLRFYFTERLEKLSTTRSADDDGVYENQAKDGMDIDERTSFSSSVTDMDIDE
IYHKRNVLENSKLMKSIGKVVRDLRSLGLASLTEDAYASSIFSLLKQTKVHALADDDYRSSVLESIKAWIKTVPLQFLYA
LLAYLGESVSYDSPSSGLKSPLASHPPSFYPGIDIPSEGLVRWQLRLEYFAYETLQELRIGKLFEIIVDYPDSCPAIDDL
KQCLKYTGQHSKLVESFISSLRCRLLTAGASTSDILHQYVSTIKVLRIIDPTGVFLEAVGEPIREYLKGRKDTRKCIVTM
FTDGTGGNPNGPGNTADSLLEESQENDNCEDYCNSDEKQAWINADCWEPDPIETDPLKGSSTRRKTDILGMIVGIIGSKD
PLIDEYSVMLAEKLLNKSDYDIDSEIRTLELLKLHFGESSMQTCEIMLNDLINSKRINTNIKASSAHPPKKGANLGEIGM
SLDFLDATIISSNFWPPVQVNITILILIVKIHQMAMATHPASSNRYNLGILDSISDSAIVDILESWIGFCTTTESLLKDE
GNLSVGSDFVSYVHILCESRFSSLVQDHFLKALEETFEKHLARSFWCHFDPQGDGLLPKWNAYHDKEDWGEDAFSEALEE

ICLEKQYQEKCLLVLVHALQTYRESMPANKHNSDVEMTSLMCRYRSMVCSVITTILPRQFSDTLRFYFTERLEKLSTTRS
ADDDGVYENQAKDGMDIDERTSFSSSVTDMDIDEIYHKRNVLENSKLMKSIGKVVRDLRSLGLASLTEDAYASSIFSLLK
TKVHALADDDYRSSVLESIKAWIKTVPLQFLYALLAYLGESVSYDSPSSGLKSPLASHPPSFYPGIDIPSEGLVRWQLRL
EYFAYETLQELRIGKLFEIIVDYPDSCPAIDDLKQCLKYTGQHSKLVESFISSLRCRLLTAGASTSDILHQYVSTIKVLR
IIDPTGVFLEAVGEPIREYLKGRKDTRKCIVTMFTDGTGGNPNGPGNTADSLLEESQENDNCEDYCNSDEKQAWINADCW
EPDPIETDPLKGSSTRRKTDILGMIVGIIGSKDPLIDEYSVMLAEKLLNKSDYDIDSEIRTLELLKLHFGESSMQTCEIM
LNDLINSKRINTNIKASSAHPPKKGANLGEIGMSLDFLDATIISSNFWPPVQVNITILILIVKIHQ
>Bdis_1g06387
MSHPHATAPKRPFSSASPSPTSPAPPHMKKAKLPASSSSSAGPTEKNGLHLDPTAAAAVRGSGRTNGEEDAEMLLADQDE
LRAPSASVPGGVTANLFRKKATLPQPSATSATRKPLRIKIGQPKLPKNFEEDTWAILKDAITAIFLKQKLSCDVEKLYQA
AGDLCLHKLGANLYERVKKECEIHIAAKISALVGQSPDLVVFLSLVHRTWQDFCDQMLIIRGIALLLDVKYVKNIANLCS
VWDMGLQLFRKHVSLSPEIEHKTVTGLLRLIESERLGEAIDKTLLSHLLKMFTDLGMYSETFEKPFLECTSQFYATEGVK
YLQQSDIPDYLKHVESRLQEEHERCIMYLEANTRKPLIATTEKQLLHRHTSAIIEKGFTMLMEANRVKDLWRMYTLFQRV
DAIEMLKQALSLYIRGTGQGIIMDEEKDKDLVPFLLEFKASLDKILEESFAKNESFSNTIKESFEHLINLRQNRPAELIA
KFLDEKLRAGNKGTSEEELEGILDKVLVLFRYIQGKDVFEAFYKKDLAKRLLLGKSASIDAEKSMITKLKTECGSQFTNK
LEGMFKDIELSKEINDSFKQSSQARTKLPTGIEMSVHVLTTGYWPTYPPMDVKLPHELNVYQDIFKEFYLSKYSGRRLMW
QNSLGHCVLKVEFPKGRKELSVSLFQSVVLMLFNDAQKLSFLDIKDSTGIEDKELRRTLQSLACGKVRVLQKTPKGRDVD
DKDEFVFNDEFSAPLYRIKVNAIQMKETVEENTSTTERVFQDRQYQVDAAIVRIMKTRKTLSHTLLITELFQQLKFPIKP
ADMKKRIESLIDREYLERDRSNPQIYNYLA*MSHPHATAPKRPFSSASPSPTSPAPPHMKKAKLPASSSSSAGPTEKNGL
HLDPTAAAAVRGSGRTNGEEDAEMLLADQDELRAPSASVPGGVTANLFRKKATLPQPSATSATRKPLRIKIGQPKLPKNF
EEDTWAILKDAITAIFLKQKLSCDVEKLYQAAGDLCLHKLGANLYERVKKECEIHIAAKISALVGQSPDLVVFLSLVHRT
WQDFCDQMLIIRGIALLLDVKYVKNIANLCSVWDMGLQLFRKHVSLSPEIEHKTVTGLLRLIESERLGEAIDKTLLSHLL
KMFTDLGMYSETFEKPFLECTSQFYATEGVKYLQQSDIPDYLKHVESRLQEEHERCIMYLEANTRKPLIATTEKQLLHRH
TSAIIEKGFTMLMEANRVKDLWRMYTLFQRVDAIEMLKQALSLYIRGTGQGIIMDEEKDKDLVPFLLEFKASLDKILEES
FAKNESFSNTIKESFEHLINLRQNRPAELIAKFLDEKLRAGNKGTSEEELEGILDKVLVLFRYIQGKDVFEAFYKKDLAK
RLLLGKSASIDAEKSMITKLKTECGSQFTNKLEGMFKDIELSKEINDSFKQSSQARTKLPTGIEMSVHVLTTGYWPTYPP
MDVKLPHELNVYQDIFKEFYLSKYSGRRLMWQNSLGHCVLKVEFPKGRKELSVSLFQSVVLMLFNDAQKLSFLDIKDSTG
IEDKELRRTLQSLACGKVRVLQKTPKGRDVDDKDEFVFNDEFSAPLYRIKMKETVEENTSTTERVFQDRQYQVDAAIVRI
MKTRKTLSHTLLITELFQQLKFPIKPADMKKRIESLIDREYLERDRSNPQIYNYLA*
>Bdis_2g12970
MAQIPIELEVGWRSMVAGISKLKSILGGDGGVCFSSKEYIDLYTTIFNMCTQKPPNDYSKQLYERYKEALDDYIKSVVVP
SLKGKHGEFLLRELVGRWKNHKVMVRWLSRFFHYLDRYYVSRKLLLPLNELGQSCFHDLVFKELKTTLTLTLIDMIDAER
EGQLIDRALVKDVIDIYVEIGWGSLGLYEGDFEQDFLNSTTDYYSKKAQAWIVEDSCPEYLLKAEECLQKEKERVGHYLH
SKTETKLLEDSLLELISRRAEQILNKENSGCRVLLLDGKTEDLSRMCRLFSRVEDGLFQLSKVFKEHVNEEGMSLLKSAT
DAANSKNERKEIVGALDQDFVRKVIELHDKQQSYVINCFQNNTVFHKAIKEAFEIICNKEVAGCTSAESLATFCDNILRK
GGSEKLGDEALEETLEKVVTILTYISDRDLFVEFHRKKLGKRLLFDKSVNDEHERSLLSKLKQYFGGQLTSKMEGMLGDM
TRARDQQANFDEYMSQITESNPRVDFSVTVLTAGRWPTYKSSNINLPSEMIKCVEAFKNYYDSKEKCKKLSWLYSMGNCN
IVAKFDTKPIELIVTTYQAALLLLFNGADRLSYSEIVTQLNLPDDDALRLLHSLSCAKYKILNKEPLNRTISPNDIFLFN
HKFTDKMRRIKVPLPPTDEKKKVVEDVNKDRRFSIDAAIVRIMKSRKVMGHQQLVVECVEQLSRMFKPDVKLIKRRIEDL
ISREYLERDLETTNSYRYLA*
>Bdis_2g35820
MATHERKTVDLEEGWAFMQKGITKLKNILEGKPEPQFSSEDYMMLYTTIYNMCTQKPPHDYSQQLYDKYRESFEEYISSM
VLPSLREKHDEFMLRELVKRWSNHKVMVRWLSRFFHYLDRYFISRRSLPALREVGLSCFRDLVYQEIKGKVKSAVISLID
QEREGEQIDRALLKNVLDIFVEIGLGSMECYENDFEDFLLKDTADYYSIKAQTWIVEDSCPDYMLKAEECLKREKERVAH
YLHSSSEQKLLEKVQHELLTQYANQLLEKEHSGCHALLRDDKVEDLSRMYRLFSRITRGLEPVSQIFKQHVTNEGTALVK
QAEDAASNKKPEKKDIVGLQEQVFVRKIIELHDKYVAYVTDCFQGHTLFHKALKEAFEVFCNKGVSGSSSAELLATFCDN
ILKKGGSEKLSDEAIEDTLEKVVRLLAYISDKDLFAEFYRKKLARRLLFDKSANDEHERSILTKLKQQCGGQFTSKMEGM
VTDLTVARDHQTKFEEFISAHTELNPGIDLAVTVLTTGFWPTYKSFDINLPAEMVKCVEVFKEFYQTRTKHRKLTWIYSL
GICHITAKFEAKTIELIVTTYQAALLLLFNGADRLSYSEIVTQLNLSDDDVVRLLHSLSCAKYKILNKEPAGRTISPTDV
FEFNSKFTDKMRRIKIPLPPVDEKKKVVEDVDKDRRYAIDASIVRIMKSRKVLGHQTLVMECVEQLGRMFKPDFKAIKKR
IEDLITRDYLERDKENPNVYRYLA*
>Bdis_2g35830
MTGQERKTIDLDEGWSFMQRGIVKLINILEGKPEPQFSSEDYMMLYTTIYNMCTQKPPHDYSQQLYDKYKDSFQEYINAM
VLPSLREKHDEFMLRELVQRWANHKVMVRWLSRFFHYLDRYFITRRSLVALKDVGLICFRDLIFQEIKGKVKDAVIALID
QEREGEQIDRALLKNVLDIFVEIGLGIMECYENDFEDFLLKDTTDYYSVKAQSWIVEDSCPDYMIKAEECLKREKERVGH
YLHINSEPKLLEKVQNELLAQYATQLLEKEHSGCFALLRDDKVEDLSRMYRLFSKVTRGLEPISNMFKKHVTNEGTALVK
QAEDSANNKKPEKKDMVGMQEQVFVWKIIELHDKYVAYVTDCFQGHTLFHKALKEAFEVFCNKGVSGSSSAELLATFCDN
ILKKGCSEKLSDEAIEDALEKVVRLLAYISDKDLFAEFYRKKLARRLLFDKSANDEHERSILTKLKQQCGGQFTSKMEGM
VTDLTVARDHQTKFEEFVAEKSELNPGVDLAVTVLTTGFWPTYKTFDINLPSEMVKCVEVFKEFYQTRTKHRKLTWIYSL
GTCNINAKFDTKVIELIVTTYQAALLLLFNGSDRLSYSEIVTQLNLSDDDVVRLLHSLSCAKYKILTKEPAGRSISPNDV
FEFNSKFTDRMRRIKIPLPPVDEKKKVVEDVDKDRRYAIDASIVRIMKSRKVMAHTQLVAECVEQLSRMFKPDFKAIKKR
IEDLITRDYLERDKDNANTYRYLA*MTGQERKTIDLDEGWSFMQRGIVKLINILEGKPEPQFSSEDYMMLYTTIYNMCTQ

KPPHDYSQQLYDKYKDSFQEYINAMVLPSLREKHDEFMLRELVQRWANHKVMVRWLSRFFHYLDRYFITRRSLVALKDVG
LICFRDLIFQEIKGKVKDAVIALIDQEREGEQIDRALLKNVLDIFVEIGLGIMECYENDFEDFLLKDTTDYYSVKAQSWI
VEDSCPDYMIKAEECLKREKERVGHYLHINSEPKLLEKVQNELLAQYATQLLEKEHSGCFALLRDDKVEDLSRMYRLFSK
VTRGLEPISNMFKKHVTNEGTALVKQAEDSANNKKPEKKDMVGMQEQVFVWKIIELHDKYVAYVTDCFQGHTLFHKALKE
AFEVFCNKGVSGSSSAELLATFCDNILKKGCSEKLSDEAIEDALEKVVRLLAYISDKDLFAEFYRKKLARRLLFDKSAND
EHERSILTKLKQQCGGQFTSKMEGMVTDLTVARDHQTKFEEFVAEKSELNPGVDLAVTVLTTGFWPTYKTFDINLPSEMV
KCVEVFKEFYQTRTKHRKLTWIYSLGTCNINAKFDTKVIELIVTTYQAALLLLFNGSDRLSYSEIVTQLNLSDDDVVRLL
HSLSCAKYKILTKEPAGRSISPNDVFEFNSKFTDRMRRIKVCPILCSLLGWLL*
>Bdis_2g60057
MDAHLSFEDGWRVLEQGIVTCSKILGGETRTRPTVAEYMNCYDCAYRMAVQKTHYCEEMYVGYKNILADCVRAMVLPHLS
DKRGGHFLAQLVKMWSNYCTMVKCVSGFFSYLDRCFIEQRKLPSLEDTAATSFFAPVFNFFSNEITDALLTSIRQEREGS
KVDMDLLRGIMRGICRSEVKTAMQNTVIEDTYSYYSRKSSDWIVLYPLPDYLAKVQDTMEKEIERLMHYLCITERDSTEL
CLKVVSAPLMQTYDNYAREKNIGGELLIQTYKTVEDDLLARCSRLTIDSGSDNSSASYMG*
>Bdis_3g15900
MSSKKRPARIEPFRHRVETDPKFFDKSWKKLHDAIREIYNHNASGLSFEELYRTAYNMVLYKHGPKLYENLIETMTGHLQ
EMRRSIEAAQGGLFLEELQRKWDDHNKALQMIRDVLMYMDRTYIPTHKKTAVFDLGLELWRDNIVRSSNIRVRLLNTLLD
LIHSERMGEVINRSLMRSTTKMLMDLGSSVYQDDFERPFLKVSASFYSGESQQFIESCACGEYLKKAQKRLDEEAERVAQ
YLDAKTDEKITAVVVKEALTNHMQRLFLMEDSGLVNMLVEDKYEDLTMMYNLFQRVPDGHSTIKSVMTSHVKESGRSLVT
DPERLKDPVDFVQRLLNEKDKYDNIISISFGNDKSFQNALTSSFEYFINLNNRSPEFISLYVDDKLRKGMKDANEEDVET
VLDKVMMLFRYLQEKDLFEKYYKQHLAKRLLSGRAASDDSERSMLVKLKTECGYQFTSKLEGMFTDLKTSQDTTQGFYGA
SSSDAGDAPLISVQILTTGSWPTQPCSTCNLPPEILSVSEKFRAYYLGTHNGRRLTWQTNMGTADIKVTFGNGSKHELNV
STYQMCVLMLFNSADCLTYNEIEQATAIPPVDLKRCLQSLALVKGKNVLRKEPMSRDISPNDNFYVNDKFTSKLFKVKIG
TVAVQKESEPEKMETRHRVEEDRKPQIEAAIVRIMKARRVLDHNSIVTEVTKQLQPRFLPNPVVIKKRIESLIEREFLER
DKTDRKMYRYLA*
>Bdis_3g59330
MSGGGPPRKRNFKIEAFKHRVELDPKYAERTWKVLEHAIHEIYNHNASGLSFEELYRSAYNMVLHKYGEKLYTGLQSTMT
WRLKEISKSIEAAQGGLFLEELNAKWMDHNKALQMIRDILMYMDRTYVPTSHKTPVHELGLNLWRDHIIHYDMIHDRLLH
TLLDIIHRERMGEVINRGLMRSITKMLMDLGPVVYQDDFEKPFLEVSASFYSGESQEFIECCDCGNYLKKAERRLNEEME
RVSHYLDAGSDAKITSVVEKEMIANHMHRLVHMENSGLVNMLVDDKYEDLGRMYTLFRRVPDGLSTIRDMMTSYLRETGK
QLVTDPERLKDPVEFVQCLLNEKDKHDKIIHVAFGNDKTFQNALNSSFEFFINLNNRSPEFISLYVDDKLRKGLKGATEE
DVEAILDKVMMLFRYLQEKDVFEKYYKQHLAKRLLSSKTVSDDAERSMIVKLKTECGYQFTSKLEGMFTDMKTSQDTMRD
FYAKKSEELGDGPTLDVHILTTGSWPTQPSPPCSLPPEILAVCEKFRAYYLGTHNGRRLTWQTNMGTADIKATFGKGQKH
ELNVSTYQMCILMLFNSADGLTYKDIEQGTEIPAVDLKRCLQSLACVKGKNVLRKEPMSKDISEDDTFYFNDKFTSKLVK
VKIGTVVAQKESEPEKQETRQRVEEDRKPQIEAAIVRIMKSRRVLDHNSIVAEVTKQLQARFLPNPVIIKKRIESLIERE
FLERDKADRKLYRYLA*
>Bdis_5g13780
MHLDDADGALDSWARFCSLSGELVGGAGDLSVGPRLAPVVADLCARGLATLVRDYFLHNLEETFRNNAVKMFWQKFHPYC
NSSAVERIKFCVQESWPEDVLSIALEDICLEKSYQEKCVLVLVHVLQSYEEKTENKLKTVEFSSSLMPRYQLMVSSVLLT
TLPLSFPEILNIYFKKKLEELNTMMDGSDETDLACHELFGRSKVSAWDSKMDIDGQETVISESGNLVRNIGKVVRDLRCL
GFTSMTEDSYSSAIIWLLKSKVYELAGDDYRIPVLGRVKKWIQAVPLQFLHSLLAYLGDSVDYESGLSGLKSPLASRPSS
FPGIGVPSEALLRWHMRLEYFAYETLQDLRIGKLFEIIVDYPESSPAIEDLKQCLEYTGQHSKLVDSFISSLRYRLLTAG
ASTNDILHQYVSTIKALRSIDPTGVFLEAVGEPIRDYLRGRKDTIKCIVTMLTDGSGGNTNGSGNAGDNLLEELNRDAEN
QENADYDDHTNIDEKQAWLNSESWEPDPVEADPLKGSRNRRKIDILGLMVSIIGSKDQLVNEYRVMLAEKLLSKSDFDID
SDIRTLELLKIHFGESSMQKCEIMLNDLIDSKRTNSNIKTSLLRTFETVAGQEETEMSHDVLDATIISSNFWPPIQTEDL
AVPASVDQMLSDFAKRFHQIKTPRKLLWKKNLGMVKLELQFEDRNMQFTVVPVHAAIVMRFQEKPSWTSKTLATEIGIPV
DSLNRRIGFWTSKGVLTESVGPDADDHIFTVVDSMSDVNKNSIVNESCEAFQMTEDEGESSVASVEEQLKKEMTVYEKFI
IGMLTNFGNMTLDKIHNTLKMFCAEPSYDKSLQQLQSFLSGLVSDEKLEMRDGLYLLKK*
>Bdis_5g23640
MFPAPAHATGQPPRRRQGLKIQPFRRRADVAPDFAVNSCLALSSAFRDCYAGNARDLDFETLYRCAYDVVLFGHGEMLYT
QVATTMASEAEKLAASLLADHAAQTDDGAFLPELLGKWKTHSTAVAMINDVVRYMDAVFVPKRGKVPVRELGHRAWRKAV
VGSDESTPVGATLRAALLEISRRERAGGAPVDAEICGLMAAAAKMLVDLGGGAYEETLEAPFLDEAARFYAVESVRLRLE
LGSCSSCGEYLGEVESMADAERARAARCGLDAAQMGEKAAAVVLWEMVEKDGVAMARLVGGLAAMLAEGRCSDMARMHRL
LGRVPGGVAAIRSAMEAHFREIRERAGDDERMLSGEKDRYREMIDGVFHGEVSFHAALDSCFG*
>Bdis_5g23650
MEQPEMEMEMEQPAAVSEVLGLDHLLDQILLRLDIPVFLVLASQVNKSWRRRATDRDFLVRFRDRLASRPRPRPLGFYVS
FVDALGLQHSAQDRTKFVPMSQHPKLADVMNDAFFGNFGDNLDPDEKPVVMDCRNGRVLLNQSRRDAGPYVVLGPLDPAP
APALLPPLPPHPLRNKMFWFLGDYRRDGTLCVYVGIPTQLHQPKDMVRLALLQGDNWVSNVSYVTGLSATLGFPFDQLLV
GDNLYMLSSNGYIVRLDLVSMRFRVIQLPDWWTDLDADKQILSRAYNDFGINFIEIKGLHLHVWVCKSDGGNAGTWMLEA
DISLPAAFGDLANLIADEDGDVVDEPDDGRYELLRLLSAGDNADFVFMVDGGTHFQHIFYLDIRTRNVEEIVIFQVGIVI
FLPGTTSFSSGTVCNSAKHRVEADPKFKSWKNLDDAIREIHNHNASGLSFEELYRTAYNMVLHKHGPKLYEKLIENLKGH
LRERCRLVEAAQGGLFLEELQRRWADHNKALQMIRDILMYMDRTFIPTSKKTPIFELGLELWRDIVIRSPKIHARLFDTL
LEHIHRERMVEMINRGLMRNTTKMLMELGSSVYQADFERPFLEVSASFYSCESQQFIECCDCGEYLKKAERRLAEESERV

SQYMDAKTADKIANVVDKEMLSNHMQRLIHMEHSGLVNMLIDDRHEDLTRMYDLFKSVPDWHSTIRSIMASHVKETGKTL
VTDLERLKDPVDFVQRLINAKDKYDEIVSVSFSNDKVFQNALNSSFENFINLNNRSPEFISLYVDDKLRKGVKGANEEDI
EAVLDKLMMLFKYLQEKDVFEKYYKQHLAKRLLSGKTTSDEAERSMLVKLKTECGYQFTSKLEGMFTDLKTSQDTMQSFY
ANLSADIDAPTISVQILTTGSWPTQPCATCNLPPEILGQTNMGDADIKATFGGRRHELNVSTYQMCVLMLFNSVDSLTYN
EIQQATAIPHVDLKRCLQSLACVKGKNVLRKEPMSKDISESDTFHFNDKFTSKLVKVKIGTVVAQKETEPEKMETRHRVE
EDRKPQIEAAIVRVMKSRRVLDHNSVVTEVTKQLQARFLPNPVVIKKRIESLIEREFLERDKSDRKLCRYLA*
>Sbic_01g005920
MSQPHAAAAASKRPFSSTTTSPSPTSSPATPLMKKAKHPAASSSSAGTVEKNGIQLDTAVAAAAATGGGRTNGEEDAEMV
LADQDELPAPSAPASAGVAANLFRKKATLPQPSTSARKPLRIKIGQPKLPKNFEEDTWAILKDAITAIFLKQKLSCDVEK
LYQAAGDLCLHKLGANLYERIKKECEIHIAEKISALVGQSPDLVVFLSLVQRTWQDFCDQMLIIRGIALLLDVKYVKNVA
NICSVWDMGLQLFRKHLSLSPEIEHKTVTGLLRLIESERLGEAIDRTLLSHLLKMLTALGMYSESFEKPFLECTSEFYAT
EGVKYMQQSDIPDYLKHVESRLQEEHERCILYLEANTRKPLIATTEKQLLERHTSAIIEKGFSMLMDANRINDLSRMYDL
FQRVNAVELLKLALSSYIRATGQGIIMDEEKDRELVPFLLDFKASLDKILEESFAKNEAFSNTIKDSFEHLINLRQNRPA
ELIAKFLDEKLRAGNKGTSEEELEGILDRVLVLFRFIQGKDVFEAFYKKDLAKRLLLGKSASIDAEKSMITKLKTECGSQ
FTNKLEGMFKDIELSKEINDSFRQSSQARTKLPSGIEMSVHVLTTGYWPTYPPMDVKLPHELNVYQDIFKEFYLSKYSGR
RLMWQNSLGHCVLKADFPKGKKELAVSLFQSVVLMLFNDAQKLSFLDIKDSTGIEDKELRRTLQSLACGKVRVLQKIPKG
RDVEDKDEFVFNEDFSAPLYRIKVNAIQMKETVEENTSTTERVFQDRQYQVDAAIVRIMKTRKVLSHTLLITELYQQLKF
PVKPADIKKRIESLIDREYLERDRSNPQIYNYLA*
>Sbic_02g013030
MEQGDKKIVGLEEGWSFVATGLSKIRRAIDCGGEGLSSGEYMQVFTTVYCMCTQASPHNYSEQLYQRYKEDLDDYIKSNV
LTFLRELRGETLLRELVERWRNHNLIVRSETNIFRYLNRYYISKRSLPSIQQVSSSSFHDLVFNELKSSVTRTVLGMIDD
EREHKLIDRDLLKNVLAIYVEIGSGSLRIYRVDFEQAFLESTKNYYSRKFQSWNLDYSDDHDYKIKVVKSELITKNTA*
>Sbic_03g015000
MATHERKTIDLEQGWEFMQKGITKLKNILEGKPEPQFSSEDYMMLYTTIYNMCTQKPPHDYSQQLYEKYRESFEEYITSM
VLPSLREKHDEFMLRELVKRWSNHKVMVRWLSRFFHYLDRYFISRRSLPPLREVGLSCFRDLVYQEIKGKVKSAVISLID
REREGEQIDRALLKNVLDIFVEIGLGTMECYENDFEDFLLKDTADYYSIKAQSWILEDSCPDYMLKAEECLKREKERVSH
YLHSSSEQKLLEKVQHELLTQYASQLLEKEHSGCHALLRDDKVEDLSRMYRLFSRITRGLEPVSQIFKQHVTNEGTALVK
QAEDAASNKKPEKKDMVGLQEQIFVRKIIELHDKYVAYVTECFQGHTLFHKALKEAFEVFCNKGVSGSSSAELLATFCDN
ILKKGGSEKLSDEAIEDTLEKVVRLLAYISDKDLFAEFYRKKLARRLLFDKSANDEHERSILTKLKQQCGGQFTSKMEGM
VTDLTVARDHQTKFEEFISSHPELNPGIDLAVTVLTTGFWPSYKSFDINLPSEMVKCVEVFKEFYQTRTKHRKLTLIYSL
GTCNISAKFEAKTIELIVTTYQAALLLLFNGADRLSYSEIVTQLNLSDDDVVRLLHSLSCAKYDILNKEPNNRSIAPNDV
FEYNSKFTNKMRRIKIPLPPVDEKKKVVEDVDKDRRYAIDASIVRIMKSRKVLGHQQLVMECVEQLGRMFKPDFKAIKKR
IEDLITRDYLERDKDNPNVYRYLA*
>Sbic_03g015010
MAHSREPILLDEGWAHMRAGFEKLKLILAGEPGVAFVSVEYMHLYTTIYNMCTQKPPNDYSGLLYQRYQEVLNDYITATD
KHGEFLLKELVFRWKNHKLMVRWLSRFFYYLDRYFISRRSLVPLKNVGWDSFKTLVFDNHKATVTSILIAMVDEDREGQI
IDRTLVKNVLDIYIEIDSDSGSKLYNEDFEDAFLKATVDYYSKKAQAWIVEDTCPEYMVKAEECLQKEKQRVAQYLHANT
EPRLMEDVQEELLTSHMEQILRKQNSGCKVLLCDEKVEDLSRMFSLFSRINGGLTPVSKIFQEHVNEVGMSLLKQAIDAA
TSKKNEKKDVVSTLELEFFLLVLIGSLLQDYVRKILDLHDKYKAYVINCFQNHTLFHKALKEAFEVVCNKTVAGCSSAEL
FAAYCDSILKKGGCEKLSDEAIEENLDKAWKKLGRRLIFDRSGNSDQERSLLSKLKQYFGAQFTSKMEGMINDVTVAKDK
HTDLENYIRENPELNPRVDLSVQVLTTGYWPTYKSTDINLPSEMVKCVEVFSKFYHSNTDRKRKLNWIYSLGNCTVVGNF
KAQKIDLIVTTYQAALLLLFNESERLSFSEIVTQLNLSEDDTVRLLHSLSCAKYKILNKEPNSRTISPKDVFEFNHRFTD
KMRRIKVPLPPSDEKKKVIEDVNKDRRFAIDAALVRIMKSRKIMTHQNLVAECVQQLSRMFKPDIKMIKRRIEDLITRDY
LERDRDAPNSYRYVA*
>Sbic_03g045160
MNSHLSFEEGWKVLEQGILKCSKILECTSIRPTVNEYMNYYDCAYKMAVQKQHYCQEMYNGFRMTLAECVRTMVLPHLMH
KQNDSFFRELVKMWSNYCIMIRCVTGFFNYLDRCFVEQYKLPSLSDAAATAFFGPVFSYFNDEARTALLTLVEESMETET
KRLAYYLEISSGDSYPLCLQAVNAPLMETYVNYVTEQQISGQLMLETYKTVEEELLGRCRSLTLG*
>Sbic_04g027970
MSGGGPPRKRNFKIEAFKNRVELDPKYAERTWKVLEHAIHEIYNHNASGLSFEELYRSAYNMVLHKYGEKLYDGLQNTMT
WRLKEISKSIEAAQGGLFLEELNAKWMDHNKALQMIRDILMYMDRTYVPTSHRTPVHELGLNLWRDHIIHSPMIHSRLVN
TLLDLIKGERMGEVINRGLMRSITKMLMDLGPAVYQDDFEKPFLDVSASFYSVESQEFIECCDCGNYLKKAERRLNEEME
RVSHYLDAGSEAKITSVVEKEMIANHMHRLVHMENSGLVNMLVDDRYEDLGRMYSLFRRVPDGLSTIRDVMTYYLRETGK
QLVTDPESLKDPVEFVQRLLNEKDKHDKIISVAFGNDKTFQNALNSSFEYFINLNNRSPEFISLYVDDKLRKGLKGATEE
DVEVILDKVMMLFRYLQEKDVFEKYYKQHLAKRLLSGKTVSDDAERSMIVKLKTECGYQFTSKLEGMFTDMKTSQDTMQD
FYAKKSEELGDGPTLDVHILTTGSWPTQPSPPCNLPTEILTVCEKFRAYYLGTHNGRRLTWQTNMGTADIKATFGKGQKH
ELNVSTYQMCVLMLFNNADGLTYKDIERDTEIPASDLKRCLQSLACVKGKNVLRKEPMSKDISEDDTFYFNDKFTSKLVK
VKIGTVVAQKESEPEKQETRQRVEEDRKPQIEAAIVRIMKSRRVLDHNSIVAEVTKQLQARFLPNPVVIKKRIESLIERE
FLERDKVDRKLYRYLA*
>Sbic_07g004560
MNGGGGGQKKRGTRIEPFRHRVETDPKFFDKSWRKLHDAIREIYNHNASGLSFEELYRTAYNMVLNKFAPQLYEKLTENM
KEHLEDMRTCIDAAQGGLFLEELQRKWNDHNKALTMIRDILMYMDRTYIPTNKKTPVFDHGLELWRDTIVRSPTIQRRLS

DTLLELIHSERTGDVINRGLMRTTTKMLMDLGLSVYQDDFERPFLEVSASFYSGESQQFIECCACGEYLKQAERRLSEES
ERVSQYLDVKTHEKITAVVVNEMLANHMQRLILMENSGLVNMLVEDRYEDLTRMYTLFNHVPDGLTTIRSVMASHIKDTG
KSLVTDPERLKDPVDFVQRLLNMKDKYDNIINVSFSNDKSFLNALNSSFEHFINLNNRSPEFISLFVDDKLRKGVKEANE
EDLETVLDKVMMLFRYLQEKDLFEKYYKQHLAKRLLSGKNASDDSERSMLVKLKTECGYQFTSKLEGMFNDLKTSEDTTQ
GFYASTSSELLADAPTISVQILTTGSWPTQTCNTCNLPPEIVPISEKFRAYYLGTHNGRRLTWQTNMGHADIKATFGNGS
KHELNVSTYQMCVLMLFNSSDVLTYREIEQSTAIPATDLKRCLQSLALVKGKQVLRKEPMSRDIADDDSFFVNDKFTSKL
FKVKIGTVAAQKETDPEKLETRQRVEEDRKPQIEAAIVRIMKSRRVLDHNSIMMEVTKQLQPRFMPNPVVIKKRIESLIE
REFLERDKTDRKMYRYLA*
>Sbic_09g003820
MAGQERRTIDLEEGWAFMQKGITKLKNILEGKPEPQFSSEDYMMLYTTIYNMCTQKPPHDYSQQLYDKYRESFEEYITSM
VLPSLREKHDEFMLRELVQRWSNHKVMVRWLSRFFHYLDRYFISRRSLTPLKEVGLTCFRELIYQEIKGQVKDAVIALID
KEREGEQIDRALLKNVLDIFVEIGLGQMECYENDFEDFLLKDTTEYYSVKAQSWILEDSCPDYMIKAEECLKREKERVGH
YLHISSEQKLLEKVQNELLAQYATPLLEKEHSGCSALLRDDKVEDLSRMYRLFSKITRGLEPISNMFKTHVTNEGTALVK
QAEDSASNKKPEKKDMVGMQEQVFVWKIIELHDKYVAYVTECFQGHTLFHKALKEAFEVFCNKGVSGSSSAELLATFCDN
ILKKGCSEKLSDEAIEDALEKVVRLLAYISDKDLFAEFYRKKLARRLLFDKSANDEHERSILTKLKQQCGGQFTSKMEGM
VTDLTVARDHQTKFEEFVAGHPELNPGIDLAVTVLTTGFWPSYKTFDINLPAEMVKCVEVFKEFYQTRTKHRKLTWIYSL
GTCNINAKFDAKPIELIVTTYQAALLLLFNGSDRLSYSEIVTQLNLSDDDVVRLLHSLSCAKYKILTKEPANRSISPNDV
FEFNSKFTDRMRRIKIPLPPVDEKKKVVEDVDKDRRYAIDASIVRIMKSRKVMGHQQLVAECVEQLSRMFKPDFKAIKKR
IEDLITRDYLERDKDNANMYKYLA*
>Sbic_10g029080
MEREDADGALDSWAQFCSLSNELLAGDGDLTVGPRLAPVVADLCTRGLATLVRDYFLYSLEETFRNNAVKKFWQHFHPYC
SASAVDRIKFCVKENWPEEILSKALEDICLEKGYQEKCVLVLVQAFQSYEDRAPQKKFKAVDCISSLMARYQLMVSSVLL
TTLPLSFPDVLNIYFKKKLEELNTIMAGSYESDQLVDIEPFQSNTSDWHSGMDIDGSEVSESSSLVKNIGKVVRDLRCIG
FTSMTEDAYSSAIIWLLKSKVYELAGDDYRVPVLGCVKKWIQAVPLQFLHALLTYLGDSVDYDSGSSSLKSPLASRPSSF
PGIGVPSEALVRWHMRLEYFAYETLQDLRIGKLFEIIVDYPESSPAIEDLKLCLEYTGQHSKLVDSFISSLKYRLLTAGA
STNDILHQYVSTIKALRTIDPTGVFLEAVGEPIRDYLRGRKDTIKCIVTMLTDGSGGSASGTGNAGDNLLEELNRDAENQ
ENADYDDHANIDEKQAWLNSESWEPDPVEADPLKGSRNRRKVDILGLMVSIIGSKDQLVNEYRVMLAEKLLNKSDFEIDS
DIRTLELLKIHFGESSMQKCEIMLNDLIDSKRTNSNIKTSLLKTSQTVPGQEEAEVSHDVLDATIISSNFWPPIQTEDLV
VPASVDQLLSDYAKRFHQIKTPRKLLWKKNLGTVKLELQFEGRSMQFTVAPVHAAIIMRFQEKFSWTSKTLATEIGIPVD
SLNRRISFWTSKGVLTESAGPYADDRTFTVVDSMSDVNKNSIVNERLTEYQMTEEEGESSVASVEEQLKKEMTVYEVWLK
RKKY*
>Osat_LOC_Os01g27150
MATHERKTIDLEQGWEFMQKGITKLKNILEGKPEPQFSSEDYMMLYTTIYNMCTQKPPHDYSQQLYEKYRESFEEYITSM
VLPSLREKHDEFMLRELVKRWSNHKVMVRWLSRFFHYLDRYFISRRSLPQLSEVGLSCFRDLVYQEIKGKVKSAVISLID
QEREGEQIDRALLKNVLDIFVEIGLTSMDYYENDFEDFLLKDTADYYSIKAQTWILEDSCPDYMLKAEECLKREKERVAH
YLHSSSEQKLLEKVQHELLTQYASQLLEKEHSGCHALLRDDKVDDLSRMYRLFSRITRGLEPVSQIFKQHVTNEGTALVK
QAEDAASNKKPEKKEIVGLQEQVFVRKIIELHDKYVAYVTDCFQGHTLFHKALKEAFEVFCNKGVSGSSSAELLATFCDN
ILKKGGSEKLSDEAIEDTLEKVVRLLAYISDKDLFAEFYRKKLARRLLFDKSANDEHERSILTKLKQQCGGQFTSKMEGM
VTDLTVARDHQAKFEEFISTHSELNPGIALAVTVLTTGFWPSYKSFDINLPAEMVKCVEVFKEFYQTRTKHRKLTWIYSL
GTCNINAKFEAKTIELIVTTYQAALLLLFNGVDRLSYSEIVTQLNLSDDDVVRLLHSLSCAKYKILSKEPNNRSISPNDV
FEFNSKFTDKLRRLKIPLPPVDEKKKVVEDVDKDRRYAIDASIVRIMKSRKVLGHQQLVMECVEQLGRMFKPDFKAIKKR
IEDLITRDYLERDKDNPNVYRYLA*MATHERKTIDLEQGWEFMQKGITKLKNILEGKPEPQFSSEDYMMLYTTIYNMCTQ
KPPHDYSQQLYEKYRESFEEYITSMVLPSLREKHDEFMLRELVKRWSNHKVMVRWLSRFFHYLDRYFISRRSLPQLSEVG
LSCFRDLVYQEIKGKVKSAVISLIDQEREGEQIDRALLKNVLDIFVEIGLTSMDYYENDFEDFLLKDTADYYSIKAQTWI
LEDSCPDYMLKAEECLKREKERVAHYLHSSSEQKLLEKVQHELLTQYASQLLEKEHSGCHALLRDDKVDDLSRMYRLFSR
ITRGLEPVSQIFKQHVTNEGTALVKQAEDAASNKKVFVRKIIELHDKYVAYVTDCFQGHTLFHKALKEAFEVFCNKGVSG
SSSAELLATFCDNILKKGGSEKLSDEAIEDTLEKVVRLLAYISDKDLFAEFYRKKLARRLLFDKSANDEHERSILTKLKQ
QCGGQFTSKMEGMVTDLTVARDHQAKFEEFISTHSELNPGIALAVTVLTTGFWPSYKSFDINLPAEMVKCVEVFKEFYQT
RTKHRKLTWIYSLGTCNINAKFEAKTIELIVTTYQAALLLLFNGVDRLSYSEIVTQLNLSDDDVVRLLHSLSCAKYKILS
KEPNNRSISPNDVFEFNSKFTDKLRRLKIPLPPVDEKKKVVEDVDKDRRYAIDASIVRIMKSRKVLGHQQLVMECVEQLG
RMFKPDFKAIKKRIEDLITRDYLERDKDNPNVYRYLA*
>Osat_LOC_Os01g27160
MNMSPRIPVVDLEDGWRDVLAGVAKLKCILDGSNVVHFVPDEYMHLYTTVYNMCTQKPPNDYSQVLYDRYKQALDDHIES
VVLPSLNEKHGVFLLREIVQRWEKHKLMVRWLRRFFDYLDRYYVTRRSLDSLKDLGWSSFRDLVFDKLKSTVATIMIGMI
DDEREGNLIDRPLLKNALDIYVEIGDSQLNYYSDDFEQSFLNGTTDYYSKKAQTWILENSCPEYMLKAEECLQKEKDRVA
NYLHSTTEPKLFAAALFELIDRRAEEILNKENSGCKVLLCDEKTEDLARMFRLFSRITDGLLPVSKIFKEHVIAEGMSLL
KHATDAANSRKDEKKGVVVGLPEQDFVRSVIELHDKYMAYVTNCFQSNSVFHKALKEAFEVICNKDVVGCSSAELFAAYC
DSILKRGGSEKLSDEAIDESLEKVVKLLTYLSDKDLFVEFHRKKLGRRLLFDKNTNDEHERILLSKLKQFFGGQFTSKME
GMLKDITLAKEHQSSFEEYVSNNPESNPLIDLNVTVLTTGYWPTYKNSDINLPLEMVKCVEVFKEYYRSDKQHRKLTWIF
SLGNCVVIGNFDAKPVEFVLNTYQAALLLLFNEADKLSYSDIVSQLKLSDDDAVRLLHSLSCAKYKILNKEPSNRVISPE
DEFEFNSKFTDRMRRIKVPLPQIDEKKKVVDDVNKDRRFAIDASLVRIMKSRKVLGHQQLVAECVEQLSRMFKPDIKIIK
RRIEDLISREYLERDSENAQTYKYLA*

>Osat_LOC_Os01g50980
MDIEDGWRRLAAGFQKLLRILDGEERLSFSGAEYSELLQYGTLFFSTSFCFFLFSLGFNLTHIDMGRLPRRRITYKLCYE
SPAGHAAEMYDRWDKTIRHHIVYQVLPSLQDMQGEPLLKNFVHHWENHKVLMKWLKSVCMYLRLAFTNQRSLPPIMDIAL
NLFKNVVIKSIQEEEERIQNYLKPWTEARLSKTVLLELVSRQAEWLLDDDKSGFRGILAAENDLLDDGKSFLQDFWWFIV
MAIAFQQHIRDILQQAVGAAHMEKGKEPSNSIVEVFVLRIMKVLQKYEAHVINNFDNHILFRKALDENFRMICNRNIADF
SDGEFFIIFLERLIEQRTCGKLDDDSVEDTLAKVVKLLPYLHSKDYLVELYRNRLLGRLSIGCNIEVETSFITKLKLVLD
VSILEDMLEDYSISKELQKFFKDYMSMNPESNTLVDMDTMVLKQGHFPSQQKQHLSLPPDMLNCAEAFEKFYQEFHGQAT
GNRRGRTLTWIYSLGNCNIVGNFEGKSVEMIVSPMQAALLLLFNEDDRLSYNDIVAKLEIMDNDAKVMLYSLSCGKYSIL
KKEPSNKTIAPDDIFEFNNNFSVKTGKIKVPLHHVDRGDFRASETMEDVRRYRKQNVDCAIVRIMKDRKTLDHEKLVEEC
KKLCDPYFKVDDDLIQMRIDHLVAENYLARKEGCTYEYLP*
>Osat_LOC_Os01g70920
MNEHLRFEDGWKILEQGIVKCSKLLEDCPGGRPTVNEYMNYYDCAYRMAVQKDQYCQEMYNSYKATHESCVCAMVLPHLM
HKQGDLFWRELVKMWSNYCAMIRFTTGFLAYLDRCFVTHKKLPSLEDAAATSFFSPVFSYFNNEISDVLLTLIRQERDGC
NVDMDLLMGIMRGICRSEVKTKLKSAVIQDTYLYYSRKSYEWIVQYPLQDYLAKVQETVQKEMKRLIHYLCISEEEGSGL
CLKAVSAPLMQAYENYTKEKHIGGQVLLQTYKQTVEDDLLDRCSRLTIDNGLDNNSFSHME*MNEHLRFEDGWKILEQGI
VKCSKLLEDCPGGRPTVNEYMNYYDCAYRMAVQKDQYCQEMYNSYKATHESCVCAMVLPHLMHKQGDLFWRELVKMWSNY
CAMIRFTTGFLAYLDRCFVTHKKLPSLEDAAATSFFSPVFSYFNNEISDVLLTLIRQERDGCNVDMDLLMGIMRGICRSE
VKTKLKSAVIQDTYLYYSRKSYEWIVQYPLQDYLAKVQETVQKEMKRLIHYLCISEEEGSGLCLKAVSAPLMQAYENYTK
EKHIGGQVLLQTYKTVEDDLLDRCSRLTIDNGLDNNSFSHME*MNEHLRFEDGWKILEQGIVKCSKLLEDCPGGRPTVNE
YMNYYDCAYRMAVQKDQYCQEMYNSYKATHESCVCAMVFSYFNNEISDVLLTLIRQERDGCNVDMDLLMGIMRGICRSEV
KTKLKSAVIQDTYLYYSRKSYEWIVQYPLQDYLAKVQETVQKEMKRLIHYLCISEEEGSGLCLKAVSAPLMQAYENYTKE
KHIGGQVLLQTYKTVEDDLLDRCSRLTIDNGLDNNSFSHME*MNEHLRFEDGWKILEQGIVKCSKLLEDCPGGRPTVNEY
MNYYDCAYRMAVQKDQYCQEMYNSYKATHESCVCAMVLQY*
>Osat_LOC_Os02g51180
MSGGGPPKKRNFKIELFKHRVELDPKYAERTWKVLEHAIHEIYNHNASGLSFEELYRSAYNMVLHKYGEKLYDGLERTMT
WRLKEISKSIEAAQGGLFLEELNAKWMDHNKALQMIRDILMYMDRTYVPQSRRTPVHELGLNLWRDHIIHSPMIHSRLLD
TLLDLIHRERMGEMINRGLMRSITKMLMDLGAAVYQDDFEKPFLDVTASFYSGESQEFIECCDCGNYLKKSERRLNEEME
RVSHYLDSGTEAKITSVVEKEMIANHMHRLVHMENSGLVNMLVDDKYDDLARMYNLFRRVFDGLSTIRDVMTSYLRETGK
QLVTDPERLKDPVEFVQRLLNEKDKHDKIINVAFGNDKTFQNALNSSFEYFINLNNRSPEFISLYVDDKLRKGLKGATEE
DVEVILDKVMMLFRYLQEKDVFEKYYKQHLAKRLLSGKTVSDDAERSMIVKLKTECGYQFTSKLEGMFTDMKTSQDTMID
FYAKKSEELGDGPTLDVHILTTGSWPTQPCPPCNLPTEILAICDKFRTYYLGTHSGRRLTWQTNMGTADIKATFGKGQKH
ELNVSTYQMCVLMLFNSTDGLTYKDIEQDTAIPASDLKRCLQSLACVKGKNVLRKEPMSKDISEDDTFYFNDKFTSKLVK
VKIGTVVAQKESEPEKQETRQRVEEDRKPQIEAAIVRIMKSRRVLDHNSIVAEVTKQLQARFMPNPVVIKKRIESLIERE
FLERDKADRKLYRYLA*
>Osat_LOC_Os03g57290
MSHPHATAPKRPGHFSSSSAAASSPTSPAQPHMKKAKFPGSSSSSSSAAAPGVTEKNGLHVDPTAAAARTGGRTNGEEDA
EMVLADQEELAAPSASAPAGVAANLFRKKATLPQPSAARKPLRIKIGQPKLPTNFEEDTWAILKDAITAIFLKQKLSCDV
EKLYQAAGDLCLHKLGANLYERIKKECEVHISAKISALVGQSPDLVVFLSLVQRTWQDFCDQMLIIRGIALLLDVKYVKN
VANICSVWDMGLKLFRKHLSLSPEIEHKTVTGLLRLIESERLGEAIDRTLLSHLLKMFTALGMYSESFEKPFLECTSEFY
ATEGVKYLQQSDIPDYLKHVETRLQEEHERCILYLEANTRKPLITATEKQLLQRHTSAILEKGFTMLMEANRVKDLSRMY
TLFQRVDAIELLKQALSSYIRGTGQGIIMDEEKDKELVPFLLEFKASLDRILEESFAKNEAFSNTIKESFEHLINLRQNR
PAELIAKFLDEKLRAGNKGTSEEELEGILDKVLVLFRFIQGKDVFEAFYKKDLAKRLLLGKSASIDAEKSMITKLKTECG
SQFTNKLEGMFKDIELSKEINESFKQSSQARTKLPSGIEMSVHVLTTGYWPTYPPMDVKLPHELNVYQDIFKEFYLSKYS
GRRLMWQNSLGHCVLKAEFPKGKKELAVSLFQSVVLMLFNDAQKLSFLDIKESTGIEDKELRRTLQSLACGKVRVLQKMP
KGRDVEDKDEFVFNEEFSAPLYRIKVNAIQMKETVEENTSTTERVFQDRQYQVDAAIVRIMKTRKTLSHTLLITELFQQL
KFPIKPSDIKKRIESLIDREYLERDRSNPQIYNYLA*
>Osat_LOC_Os04g40830
MQLVDDADGALDSWARFCDLSDELFGGAGDLSAGPRLAPVVADLCARGLAELLRDQFIRSLEGIFRSNAVKKFWQQFHPY
CNSSAVERIKFCVQENWPEDILSKALEDICLEKNYQEKCVLALVHSLQSYEDRSPHRKSKALDCSSSLMPRYQLMVSSVL
LTTLPLSFPEILNVYFKKKLEELNIMMAGLDGSDPFDNHDLFERNSTSAWHSEMDIDGQEPGISESRNLVKNIGKVVRDL
RYLGFTSMTEDSYSSAIIWLLKSKVHELAGDDYRIPVLGCVKKWIQAVPLKFLHAQLTYLGDSLDNESGSSGLKSPLASR
PSSFPGIGVPSEALVRWHMRLEYFAYETLQDLRIGKLFEIIVDYPESSPAIEDLKQCLEYTGQHSKLVDSFISSLRYRLL
TAGASTNDILHQYVSTIKALRTIDPTGVFLEAVGEPIRDYLRGRKDTIKCIVTMLTDGSGGNANGTGNAGDNLLEELNRD
AENQENVDYDDHTNIDEKQAWLNAESWEPDPVEADPLKGSRNRRKIDILGLIVSIIGSKDQLVNEYRVMLAEKLLNKSDF
DIDSDIRTLELLKIHFGESSMQKCEIMLNDLIDSKRTNSNIKTSLSKTSQTVGTVQEETELSHEVLDATIISSNFWPPIQ
TEDLTVPASVDQLLSDYAKRFHQIKTPRKLLWKKNLGTVKLELQFEDRSMQFTVAPVHAAIIMQFQEKPSWTSKTLATAI
GVPVDSLNRKISFWTSKGVLTESVGPDADDPTFTVVDSTSDFNKNSTVNQLSERFQITEEEGESSIASVEEQLRKEMTVY
EKFIIGMLTNFGSMTLDRIHNTLKMFCIAEPSYDKSLQQLQSFLSGLVSDEKLEMRDGSYLLRK*
>Osat_LOC_Os04g55000
MEKLNFEQLYRRVYEVVLNKHGELMYSEVATALTAEVEGLRTSLVAVADGGGGGGAFLRELLSKWRRHTEAVAAVRDMVM
YMERTFIVTYRKVSVQELGVKLWRDGVVCSGDVMPRLVEAVRRERAAAAEPGELMAGVAEMLTKLRDKVLSQVMDASSVD
DYSSASLEKSVSEYQ*

>Osat_LOC_Os04g55030
MNSQKKRSPKIEPFRHRVDADPKSFDKSWKKLEDAIREIYNHNASGLSFEELYRTAYNLVLHKHGLKLYDKLTENLKGHL
KEMCRSIEDAQGSLFLEELQRRWADHNKALQMIRDILMYMDRTFIATNKKTPVFDLGLELWRDIVVRTPKIHGRLLDTLL
ELIHRERMGEMINRGLMRSTTKMLMDLGSSVYHDDFEKPFLEVSASFYSGESQQFIECCDCGEYLKKAERRLAEELERVS
QYMDAKTADKITSVVDTEMLANHMQRLILMENSGLVNMLVDDKHEDLSRMYNLFKRVPDGHSTIRSVMASHVKESGKALV
SDPEKIKDPVEFVQRLLNEKDKYDEIISISFSNDKAFQNALNSSFENFINLNNRSPEFISLFVDDKLRKGVKGANEEDVE
TVLDKVMMLFRYLQEKDVFEKYYKQHLAKRLLSGKTTSDEAERSMLVKLKTECGYQFTSKLEGMFNDLKTSHDTMQSFYA
NLSGDTDSPTISVQILTTGSWPTQPCTPCKLPPEIVDISEKFRAFYLGTHNGRRLTWQTNMGNADIKATFGGRRHELNVS
TYQMCVLMLFNSADGLTYGDIEQATGIPHADLKRCLQSLACVKGKNVLRKEPMSKDISEDDTFYYNDKFTSKLVKVKIGT
VVAQKETEPEKLETRQRVEEDRKPQIEAAIVRIMKSRRVLDHNSIITEVTKQLQSRFLPNPVVIKKRIESLIEREFLERD
KVDRKMYRYLA*
>Osat_LOC_Os05g05700
MAGQERRTIDLEEGWAFMQKGITKLKNILEGKPEPQFSSEDYMMLYTTIYNMCTQKPPHDYSQQLYDKYRESFEEYITSM
VLPSLRDKHDEFMLRELVKRWSNHKIMVRWLSRFFFYLDRYFISRRSLIPLEQVGLTCFRDLIYQEIKGQVKGAVIALID
KEREGEQIDRALLKNVLGIFVEIGLGSMECYENDFEDFLLKDTTDYYSLKAQSWILEDSCPDYMIKAEECLKKEKERVGH
YLHISSEQKLLEKVQNELLAQYATPLLEKEHSGCFALLRDDKEEDLSRMYRLFSKINRGLEPIANMFKTHVTNEGTALVK
QAEDSASNKKPEKKDMVGMQEQVFVWKIIELHDKYVAYVTECFQGHTLFHKALKEAFEVFCNKGVSGSSSAELLATFCDN
ILKKGCSEKLSDEAIEDALEKVVRLLAYISDKDLFAEFYRKKLARRLLFDKSANDEHERSILTKLKQQCGGQFTSKMEGM
VTDLTVARDHQTKFEEFVAAHQELNPGIDLAVTVLTTGFWPSYKTFDINLPAEMVKCVEVFKEFYQTRTKHRKLTWIYSL
GTCNINAKFEAKTIELIVTTYQAALLLLFNGSDRLTYSEIVTQLNLSDDDVVRLLHSLSCAKYKILNKEPANRSISPNDV
FEFNSKFTDRMRRIKIPLPPVDEKKKVVEDVDKDRRYAIDASIVRIMKSRKVMGHQQLVAECVEQLSRMFKPDFKAIKKR
IEDLITRDYLEREKDNANVYRYLA*MGVHAEGHHQAEEHPRGQARATVQLRGLHDALHVRPPPPPPPPPGTIYNMCTQKP
PHDYSQQLYDKYRESFEEYITSMVLPSLRDKHDEFMLRELVKRWSNHKIMVRWLSRFFFYLDRYFISRRSLIPLEQVGLT
CFRDLIYQEIKGQVKGAVIALIDKEREGEQIDRALLKNVLGIFVEIGLGSMECYENDFEDFLLKDTTDYYSLKAQSWILE
DSCPDYMIKAEECLKKEKERVGHYLHISSEQKLLEKVQNELLAQYATPLLEKEHSGCFALLRDDKEEDLSRMYRLFSKIN
RGLEPIANMFKTHVTNEGTALVKQAEDSASNKKPEKKDMVGMQEQVFVWKIIELHDKYVAYVTECFQGHTLFHKALKEAF
EVFCNKGVSGSSSAELLATFCDNILKKGCSEKLSDEAIEDALEKVVRLLAYISDKDLFAEFYRKKLARRLLFDKSANDEH
ERSILTKLKQQCGGQFTSKMEGMVTDLTVARDHQTKFEEFVAAHQELNPGIDLAVTVLTTGFWPSYKTFDINLPAEMVKC
VEVFKEFYQTRTKHRKLTWIYSLGTCNINAKFEAKTIELIVTTYQAALLLLFNGSDRLTYSEIVTQLNLSDDDVVRLLHS
LSCAKYKILNKEPANRSISPNDVFEFNSKFTDRMRRIKIPLPPVDEKKKVVEDVDKDRRYAIDASIVRIMKSRKVMGHQQ
LVAECVEQLSRMFKPDFKAIKKRIEDLITRDYLEREKDNANVYRYLA*
>Osat_LOC_Os05g10580
MSTGQVHRGREGAAVESHGEDLLSVLPDEILLHIRLARWRGARGGGVLDLEEGWRDVLAGVAKLKSIHTDSDFGGFSPDE
YMHIYTLVYYMCTQKGHKDYPKELYHLCKQALDDHLDSIVLPSLNEKHGNFLLAEMLQSWEKHKLMVRWLRRFFDYLDRV
SITWKSLHSLEHMGWIGFRDMVFNKLKSTLTTTVIGMINDERNGLLIDRALLKNVIHMCNEFGDSELNSYPEYILKAEEC
LQKEKEQVYSHSTTEPKDTSDKGMALVKHGTDTAKSRKDKKNEVMVGFPINLISGSRVVDEILGRVPVKAPFTDSTFEFG
NSSCNPPDSFGYIPTTSKSFAERLMAPENEDLVVMLSLDLAEELETIDEVYQQISRKGERGSSKLWKFIDHGVKKCTQIR
RSITLLGGSPQEIPNYLIR*
>Osat_LOC_Os08g07400
MSSRKKPSRIEPFRHKVETDPRFFEKAWRKLDDAIREIYNHNASGLSFEELYRTAYNLVLHKHGPKLYDKLTENMEDHLQ
EMRVSIEAAQGGLFLVELQRKWDDHNKALQMIRDILMYMDRVFIPTNKKTPVFDLGLDLWRDTIVRSPKIHGRLLDTLLD
LIHRERTGEVINRSLMRSTTKMLMDLGSSVYQDDFERPFLEVSASFYSGESQKFIECCSCGEYLKKAQQRLDEEAERVSQ
YMDAKTDEKITAVVVKEMLANHMQRLILMENSGLVNMLVEDKYEDLTMMYSLFQRVPDGHSTIKSVMNSHVKETGKDMVM
DPERLKDPVDFVQRLLNEKDKYDSIVTTSFSNDKSFQNALNSSFEHFINLNNRCPEFISLYVDDKLRKGMKEANEEDVET
VLDKVMMLFRYLQEKDLFEKYYKQHLAKRLLSGKAASDDSERSMLVKLKTECGYQFTSKLEGMFNDLKTSHDTTQRFYAG
TPDLGDAPTISVQILTTGSWPTQPCNTCNLPPEILGVSEMFRGFYLGTHNGRRLTWQTNMGTADIKAVFGNGSKHELNVS
TYQMCVLMLFNSADCLSYRDIEQTTAIPSADLKRCLQSLALVKGKNVLRKEPMSRDISDDDNFYVNDKFTSKLFKVKIGT
VATQKESEPEKMETRQRVEEDRKPQIEAAIVRIMKSRRVLDHNSIVTEVTKQLQPRFMPNPVVIKKRVESLIEREFLERD
KTDRKLYRYLA*
>Osat_LOC_Os12g13360
MAGIVDFEEGWRLLATSLAKQRSIIDGSMSKSSSEDDNMQLYMYVLPSMRQINGETLLKGLVDRWRNHKKIVISETRFFF
YLDRYYILRKSLVPLEQLNLCSFRDQVYSELKDKITRTVVDMINDERDGKVIDRDLLKDVLDVYVQIGLGMECYEVDFEN
AFRESTRNYYSNKAQTSILECNGADSPEYMLKAVECLQAELERVSHYLHSSTEPKLMQDLQSELMITPVETHTEEAD*
>Smol_XP_002993378
MSQPKKKPFKIEPFKHKVEMDPRYAEKTWKILEDAIHEIYNRNASGLSFEELYRNAYNMVLHKYGEKLYAGLETTMTQHL
QEFSRVIEAAQGGLFLEELNGKWSEHNKALQMIRDILMYMDRTYVQNSSKTPVHELGLNLWRDTIVRCPTIKDRLRDTLL
DLVHRERTGEVINRGLMRNITKMLMDLGVAVYEEEFEKPFLDAAADFYRIESQQFLESSDCADYLKKAERRLNEEMDRVT
HYLFPRSEPKITSVVDREMIGHHMKLLVEMENSGLVSMLTDDKYDDLARMYSLFRRVTTGLQTIRDLMTSHLREVGKNLV
VDPERLKDPVEFVQRLLDEKDKYDRIIRSSFSNDKTFQNALNSAFEYFINLNARSPEFISLFVDDKLRKGLKGVSEEDIE
TVLDKVMMLFRYLQEKDVFEKYYKQHLAKRLLSGRTISDDAERSLIVKLKTECGYQFTSKLEGMFTDMKTSRDTMQGFSS
MMANCEQPGEAPTLSVQVLTTGSWPTQSGARCNLPTEILSVCDKFKTYYLSTHTGRRLTWQTNMGTADLKATFGNGARHE
LNVSTYQMCVLMLFNMADKVTYREIEQATDIPAADLKRCLQSLALVKGKNVLRKEPMSKDINEDDVFLFNDKFASKLYKV

KISTVVAQKESEPEKQETRQKVEEDRKPQIEAAIVRIMKSRRVLDHNNIVSEVTKQLQARFLPNPAVIKKRIESLIEREF
LERDKVDRKLYRYLA
>Smol_XP_002993347
MAAAKRGVRSKTAAVAIEAPAPKKIRINPVVRRCPIDVQGSSRVLQNAIGEIFRRSNASGLSFEELYRHAFNLCQGNHAA
KLYQMFREELVRNLAVYRDGVFAAADTGSMFEVLDEKWLEFSRALQLIRALLNCMDRTYVIRYRERSVYDLGLELWKVEV
VSSPKLQAALTGFLLGEIHKERSGEMIDRSKMRRAVQMLIELDYKIYLLVVEEPFIAASKDFYSIESQQLMACGDCSAML
KRVERRLKEESVRVSRYLSEKTGPKISRVVVDIFVGKNIKQLVDMENTGLEFMLSQDRLDDLARMYEFLQHWDEGGKEIL
DGLTRHIKANGAQLVQDPERQKDPVAFIQLLLSFREKYDAIVSSSFKRNKAVAAGLEVAFVEVVNLNRRLPEFLSLFLDN
KLRQGGKSDSGGSDDPEAFMDKAMLIFRYINEKDMFEKYYKHHLAKRLLLSKFAEDELERSLILKIKTVCGYQFTSKIET
MLKDMRTSEDLMQRFRNMQANINAAMNINVQVLTTGSWPAYASSSQCILPREVHGLCERFKTFYLMEHRGRRLTWQGNLG
SADLKLTIDDTTKTLSCSTYQMCILMLFNDSDRLSYKEIKDATGIQQASELKRNLQSLALVRGKNVLRKEPMSKEIGEMD
VFVFNEAFTSKLAKIKICTVAAQKETGEENSRTREKIESDRNPQIEAAIVRVMKSRQRMEHNNLVSEVIAQLQSRFTPNP
AVIKKRIEALIERDYLERDRDDRRTYCYLA
>Smol_XP_002986737
MTMNDRKVIELEQGWAFMQKGITKLINLLEGVAEQQFNSEEYAMLYTTIYNMCTQKPPQDYSQQLYDRYREAFEEYINSM
VMPALREKHNEFMLRELVQRWDNHKIMVRWLSRFFNYLDRYFIARRSLPALGEVGLMCFRDLVYQEMKNNVKDAVITLID
REREGEQIDRALLKNVLGIFVEIGMGSMEAYEADFEAPMLQDTAAYYSRKAASWIEEDSCPDYMLKAEECLKREKERVGH
YLHSSSESKLLEKVQQELLSQYEQQLLEKEHSGCHALLRDDKVEDLSRMYRLFCRIPKGLEPVAAIFRMHVTEEGTTLVK
QAEDAASSKKADKKDTVGVQEQAFVRKVIELHDKYLQYVSECFVNHSLFHKALKEAFEVFCNKGVGGSTSAELLATFCDN
LLKKGGSEKLSDEAIEDTLEKVVKLLAYISDKDLFAEFYRKKLARRLLFDKSANDDHERSILTKLKQQCGGQFTSKMEGM
VTDLTLARENQTLFEEYLSENPQSNPGIDLTVTVLTTGFWPSYKSSDLALPSEMVKCVETFKEFYQTKTKHRKLTWIYSL
GTCNIVGKFEPKQIELVVTTYQAAVLLLFNAAERLSYSDIKGQLNLTDEDIVRLLHSLSCAKYKILNKEPNTKTVSGSDT
FEFNNKFTDKMRRIKASCLKYLAIPLPPMDEKKKVIEDVDKDRRYAIDASIVRIMKSRKVLPHQQLVLECVEQLGRMFKP
DFKIIKKRMEDLIAREYLERDKDNPNMFRYLA
>Smol_XP_002984123
MVVDVKATGHGLSVASPVTAVGLAGGSNSTGTTANLSRKKATPPQPTKKLVIKPFKDKPKLPANFEDATWDKIRAAVSAI
HVKQPVSCSLEQLYQNVEDLCLHKLSGNLYQRLQQECETHISAKLNALVGQSPDPVVFLSHVERCWQDHCDQMLMIRSIA
LYLDRTYVIQNSSVRSLWDMGLQLFRKHLTSCPEVEHKTVTGILRLIEKERTGETVDRTLLKHLLRMFSALGTYSESFEK
PFIDCTAEFYAAEGTRYMQQTDVPDYLRHVEARLHEENERCLLYLDANTRKHLISTSEKQLLERHSPTILDKGFGMLMDA
NRVADLHRMYLLLARVGALESLKQALSAYIKATGHSIIVDEEKDKDMVSTLLDFKARLDMIWEESFSKNEPFANTIKEAF
EHLINLRQNRPAELIAKFIDGKLRAGNKGTSEEELESMLDKVLVLFRYIQGKDVFEAFYKKDLAKRLLLGKSASIDAEKS
MISKLKTECGSQFTNKLEGMFKDIELSREINESFKQSSQARTKLPAGIEMNVHVLTTGYWPTYPPMDIRLPHELNVYQDI
FKQFYLSKHSGRRLMWQNSLGHCVLKAEFPKGKRELSVSLFQTVVLMLFNDSLRLSFQDIKDSTGIEDKELRRTLQSLAC
GKVRILQKQPKGREVEDDDVFTFNEDFTAPLFRIKVNAIQLKETVEENASTTERVFQDRQYQIDAAIVRIMKTRKVLSHT
LLITELFQQLKFPIKPADLKKRIESLIDREYLERDKNNPQVYNYLA
>Smol_XP_002980495
MAQLREIAQAWKIYCASALDSPIQQQRPGRRKGSAGFKGQDVSRSLRFLCERGLGEVVMQHFMDELERSLKENYIPEFWS
HFTDFLSVKENSDTKQLNEWIHSSLPVALEKLCSKKVSQEDGLNALIKALETNASVNGSQILTSHQTTVTALLLTTSPSY
FSDLLKLYFTARLEEFSGSFRKRPCNSDDEDCEASTDVEDMDVLGVESPCKGEAEATEVSEDSVSVRNVVKRLRDLGFAA
LCEEAYASAILSLLKKKIYSIADKRYEKPVLGPIRHWIEAVPMRFLSAILESSAGSSSLSPLPATPSPLASSSTLSDLSK
ERIIRWRLRLQFFTYETLAIEDLRQCLANTGEHSKLVKSFRLALKQRLLTAGAATTDILTQYVSTIKALRTMDPTGVILE
AVGEPIREYLRGRKDTIRCIVTMLTDDTATTSGTTVAGGGEGLLEELSRGTTTVENADSDEEGELDGEEAWAAAQRWEPD
PVEADLSRTSKSRRSMDIISMLVGIYGSKELFVNEYRVMLAEKLLNKSDYDTDREIRTLELLKLRFGENNMHSCEIMLKD
LADSKRINSNIKAKGKPQETFQKGEELTLHNVDATVISSLFWPPFQTETLQVPDFVDKLLDDYAQQYHTVKAPRKLQWKK
HLGTVKLELQFEDRSAQFVVSPMQASIILKFESCSRWSASELAAASGIPVTTLRRRIVLWINQGVLVESHGDKDGEVFYQ
VVETIGDGGQRGASAPTEAAVPLLEEEDGQSAVTPMEDQWQQEMNVYESYIVGMLTNLESLPLDRIHNMLKMFVSDPPYD
KTLQQLQGFLGSLIADEKLEFREGVYRRKQ
>Smol_XP_002973273
MTMNDRKVIELEQGWAFMQKGITKLINLLEGVAEQQFNSEEYAMLYTTIYNMCTQKPPQDYSQQLYDRYREAFEEYINSM
VMPALREKHNEFMLRELVQRWDNHKIMVRWLSRFFNYLDRYFIARRSLPALGEVGLMCFRDLVYQEMKNNVKDAVITLID
REREGEQIDRALLKNVLGIFVEIGMGSMEAYEADFEAPMLQDTAAYYSRKAASWIEEDSCPDYMLKAEECLKREKERVGH
YLHSSSESKLLEKVQQELLSQYEQQLLEKEHSGCHALLRDDKVEDLSRMYRLFCRIPKGLEPVAAIFRMHVTEEGTTLVK
QAEDAASSKKADKKDTVGVQEQAFVRKVIELHDKYLQYVSECFVNHSLFHKALKEAFEVFCNKGVGGSTSAELLATFCDN
LLKKGGSEKLSDEAIEDTLEKVVKLLAYISDKDLFAEFYRKKLARRLLFDKSANDDHERSILTKLKQQCGGQFTSKMEGM
VTDLTLARENQTLFEEYLSENPQSNPGIDLTVTVLTTGFWPSYKSSDLALPSEMVKCVETFKEFYQTKTKHRKLTWIYSL
GTCNIVGKFEPKQIELVVTTYQAAVLLLFNAAERLSYSDIKGQLNLTDEDIVRLLHSLSCAKYKILNKEPNTKTVSGSDT
FEFNYKFTDKMRRIKASCLKYLAIPLPPMDEKKKVIEDVDKDRRYAIDASIVRIMKSRKVLPHQQLVLECVEQLGRMFKP
DFKIIKKRMEDLIAREYLERDKDNPNMFRYLA
>Smol_XP_002972211
MVVDVKATGHGLSVASPVTAVGLAGGSNSTGTTANLSRKKATPPQPTKKLVIKPFKDKPKLPANFEDATWDKIRAAVSAI
HVKQPVSCSLEQLYQNVEDLCLHKLSGNLYQRLQQECETHISAKLYALVGQSPDPVVFLSHVERCWQDHCDQMLMIRSIA
LYLDRTYVIQNSSVRSLWDMGLQLFRKHLTSCPEVEHKTVTGILRLIEKERTGETVDRTLLKHLLRMFSALGTYSESFEK

PFIDCTAEFYAAEGTRYMQQTDVPDYLRHVEARLHEENERCLLYLDANTRKHLISTSEKQLLERHSPTILDKGFGMLMDA
NRVADLHRMYLLLARVGALESLKQALSAYIKATGHSIIVDEEKDKDMVSTLLDFKARLDMIWEESFSKNEPFANTIKEAF
EHLINLRQNRPAELIAKFIDGKLRAGNKGTSEEELESMLDKVLVLFRYIQGKDVFEAFYKKDLAKRLLLGKSASIDAEKS
MISKLKTECGSQFTNKLEGMFKDIELSREINESFKQSSQARTKLPAGIEMNVHVLTTGYWPTYPPMDIRLPHELNVYQDI
FKQFYLSKHSGRRLMWQNSLGHCVLKAEFPKGKRELSVSLFQTVVLMLFNDSLRLSFQDIKDSTGIEDKELRRTLQSLAC
GKVRILQKQPKGREVEDDDVFTFNEDFTAPLFRIKVNAIQLKETVEENASTTERVFQDRQYQIDAAIVRIMKTRKVLSHT
LLITELFQQLKFPIKPADLKKRIESLIDREYLERDKNNPQVYNYLA
>Smol_XP_002962592
NASVNGSQILTSHQTTVTALLLTTSPSYFSDLLKLYFTARLEEFSGSFRKRPCNSDDEDCEASTDVEDMDVLGVESPCKG
EAEATEVSEDSVSVRNVVKRLRDLGFAALCEEAYASAILSLLKKKIYSIADKRYEKPVLGPIRHWIEAVPMRFLSAILES
SAGSSSLSPLPATPSPLASSSTLSDLSKERIIRWRLRLQFFTYETLGDLRISELFDVIVDYPDRQVTRYFLPAIEDLRQC
LANTGEHSKLVKSFRLALKQRLLTAGAATTDILTQYVSTIKALRTMDPTGVILEAVGEPIREYLRGRKDTIRCIVTMLTD
DTATTSGTTVAGGGEGLLEELSRGTTTVENADSDEEGELDGEEAWAAAQRWEPDPVEADLSRTSKSRRSMDIISMLVGIY
GSKELFVNEYRVMLAEKLLNKSDYDTDREIRTLELLKLRFGENNMHSCEIMLKDLADSKRINSNIKAKGKPQETFQKGEE
LTLHNVDATVISSLFWPPFQTETLQVPDFVDKLLDDYAQQYHTVKAPRKLQWKKHLGTVKLELQFEDRSAQFVVSPMQAS
IILKFESCSRWSASELAAASGIPVTTLRRRIVLWINQGVLVESHGDKDGEVFYQVVETIGDGGQRGASAPTEAAVPLLEE
EDGQSAVTPMEDQWQQEMNVYESYIVGMLTNLESLPLDRIHNMLKMFVSDPPYDKTLQQLQGFLGSLIADEKLEFREGVY
RRKQ
>Smol_XP_002972732
MAAAKRGARSKAAAVAIEAPAPKKIRINPVVRRYPIDVQGSSRVLQNAIGEIFRRSNASGLSFEELYRHAFNLCQGNHAA
KLYQMFREELIRNLAVYRDGVFAAADTGSMFEVLDEKWLEFSRALQLIRALLSCMDRTYVIRYRERSVYDLGLELWKVEV
VSSPKLQAALTAFLLGEIHKERSGEMIDRSKMRRAVQMLIELDYKIYLLVVEEPFISASKDFYSIESQQLLACGDCSAML
KRVERRLKEESMRVSRYLSEKTGPKISRVVVDIFVGKNIKQLVDMENTGLEFMLSQDRLDDLARMYEFLQHWEEGGKEIL
DGLTRHIKANGAQLVQDPERQKDPVAFIQLLLSFKEKYDAIVSSSFKRNKAVAAGLEVAFAEVVNLNRRLPEFLSLFLDN
KLRQGGKSDSGGSDDPEAFMDKAMLIFRYINEKDMFEKYYKHHLAKRLLLSKFAEDELERSLILKIKTVCGYQFTSKIET
MLKDMRTSEDLMQRFRNMQANINAAVNINVQVLTTGSWPAYASSSQCILPREVHGLCERFKTFYLMQHRGRRLTWQGNLG
SADLKLTIDDTTKTLSCSTYQMCILMLFNDSDRLSYKEIKDATGIQQASELKRNLQSLALVRGKNVLRKEPMSKEIGETD
VFVFNEAFTSKLAKIKICTVAAQKETGEENSRTRETIESDRNPQIEAAIVRVMKSRQRMEHNNLVSEVIAQLQSRFTPNP
AVIKKRIEALIERDYLERDRDDRRTYCYLA
>Php_XP_001781780
MNPAKKKNFKIEPFKHKVEMDPKYAEKTWKILEDAIHEIYNHNASGLSFEELYRNAYNMVLHKYGEKLYSGVVQTMTQHL
REIAKIIEGAQGGLFLEELDVKWREHNKSLQMIRDILMYMDRTFVNNFNKTPVHELGLNLWRDHIVRSPQIRDRLLNTLL
DLVRRERTGEVINRGLMRNITKMLVELGTNVYQEDFERPFLDAASDFYRLESQQLIETSDCPDYLRKAEKRLNEEIERVA
HYLDSKSEPKITQVVEREVIGNRMRLLVEMENSGLISMLIDDKYDDLGRMYNLFRRISTGLQTMRELMTAHLRETGRQLV
MDPERLKDPVEFVQRLLDEKDKYDRIIQQSFHNDKMFQNALNSSFEYFINLNIRSPEFISLFVDDKLRKGLKGASEEDVE
LVLDKVMMLFRYLQEKDVFEKYYKQHLAKRLLSGRTVSDDAERSLIVKLKTECGYQFTSKLEGMFTDMKTSRDTMQGFNA
TGAGAEGNEGPTLTVQVLTTGSWPTQSGARCNMPTEILAMCDKFKMYYLSTHTGRRLTWQTNMGTADLKATFGDGNKHEL
NVSTYQMCILCLFNQADRLSYREIEQATDIPAPDLKRSLQSLACVKGKNVLRKEPMSKDISEDDTFVFNDKFSSKFYKVK
ISTVVAQKESEPEKQETRQKVEEDRKPQIEAAIVRIMKSRRLLDHNNIISEVTKQLQARFMPNPAVIKKRIESLIEREFL
ERDRMDRKLYRYLA
>Php_XP_001776334
MYVDGVEDHLASTVSYPAGSVSAVTLSNNPGVGTTANLSRKKATPPPPAKKLVIKPFKDKPKLPANFEEVTWVKIKEAVT
AIHLKQPVNCSLEELYRAVEDLCVHKMAGNLYKRLQLECESHISIKLRDLAGRSPDAVVFLSHVERCWQDHCNQMLVIRS
IALYLDRTYVIQNSGVRSLWDMGLLLFRRHLSACPEVQSKTVSGLLRLIEEERMGESVDRSLLKHLLRMFSALGIYAESF
ERQFLDCTSDFYAAEGTRFMQQTDVPDYLKHVETRLHEENERCLLYLDGSTRKPLVATAEKQLLSRHTAAILEKGFGMLM
DANRVADLQRMYMLFTRVNALESLKMALSTYIKTTGNSTVMDEEKDKDMVSWLLDLKARLDAIWDESFSRNETFANTLKD
AFEHLINLRQNRPAELIAKFIDGKLRAGNKGTSEEELEGILDKVLVLFRFIQGKDVFEAFYKKDLAKRLLLGKSASIDAE
KSMISKLKTECGSQFTNKLEGMFKDIELSREINESFRQSAQARMKLPSGIEMNVHVLTTGYWPTYPPMEVRLPHELNVYQ
DIFKEFYLSKHSGRRLMWQNSLGHCVLKANFPKGKKELSVSLFQTVVLMLFNDAQSQSFQEIKDTTAIEDKELRRTLQSL
ACGKVRVLNKQPKGREVEDDDIFVFNEDFVAPLFRIKVNAIQLKETVEENTSTTERVFQDRQYQIDAAIVRIMKTRKVLS
HTLLITELFQQLKFPIKPADLKKRIESLIDREYLERDKANPQIYNYLA
>Php_XP_001774461
MINERRVIELEQGWNFMQKGITKLKNLLEGVPEQQFSSEEYMLLYTTIYNMCTQKPPQDYSQQLYDRYRESFEGYINSKV
LPALREKHEEFMLKELVKRWDNHKIMVRWLSRFFNYLDRYFIARRSLPALSEVGLMRFRDLVYEEMKVNVKDAVIALIDR
EREGEQIDRALLKNVLGIFVEIGMGNMDAYETDFEAFMLEDTASYYKRKASSWIQEDSCPDYMLKAEECLKREREREVGHY
LHASSEQKLLEKVQHELLTQYETQLLEKEHSGCHTLLRDDKVDDLSRMYRLFCRILKGLDPVAAIFREHVTGEGTALVKQ
AEDAASNKKAERKDIVGVQEQAFVRKVIELHDKYLQYVSDCFLNHSLFHKALKEAFEVFCNKGVAGSTSAELLATFCDNL
LKKGGSEKLSDEAIEDTLEKVVKLLAYISDKDLFAEFYRKKLARRLLFDKSANDDHERSILTKLKQQCGGQFTSKMEGMV
TDLTLARENQINFEEYLSDNTQSNPGIDLTVTVLTTGFWPSYKSSDLALPAEMVKCVEVFKEFYQTKTKHRKLTWIYSLG
TCNITGKFDAKPIELIVTTYQAAVLLLFNAADRLSYNDIKSQLNLTDEDIVRLLHSLSCAKYKILNKDPITKTVGQSDIF
EFNTKFTDKMRRIKIPLPPMDEKKKVIEDVDKDRRYAIDASIVRIMKSRKMLPHQQLVLECVEQLGRMFKPDFKVIKKRV
EDLIAREYLERDKDNPNVFKYVA

>Php_XP_001773913
MTMNNERKVIELEQGWSFMQIGITKLKNLLEGVPEQQFSSEEYMLLYTTIYNMCTQKPPQDYSQQLYDGYRVSFEEYINS
KVLPALREKHEEFMLKELVKRWYNHKIMVRWLSRFFNYLDRYFIARRSLPALSEVGLICFRNLVYAETKINVKDAVVALI
DREREGEQIDRALLKNVLGIFVEIGMGNMDAYDTDFEQFMLEDTAAYYRRKASSWIQEDSCPDYMLKAEECLKREKERVG
HYLHASSEQKLLEKVQQELLTQYETQLLEKEHSGCHTLLRDDKVDDLSRMYRLFYRIPKGLEPVASIFKQHVTEEGTALV
KQAEDAVSNKRAEKKDTVGVQEQVFVRKVIELHDKYLQYVSECFANHSLFHKALKEAFEVFCNKGVAGSTSAELLATFCD
NLLKKGGSEKLSDEAIEDTLEKVVKLLAYISDKDLFAEFYRKKLARRLLFDKSANDDHERSILTKLKQQCGGQFTSKMEG
MVTDLTLARENQSNFEDYLSDNTKSNPGIDLTVTVLTTGFWPSYKSSDLALPAEMVKCVEVFKEFYQTKTKHRKLTWIYS
LGTCNITGKFDAKPIELIVTTYQAAVLLLFNAEDRLSYNDIKNQLNLTDEDIVRLLHSLSCAKYKILNKDPNTKAVGQND
IFEFNTKFTDKMRRIKIPLPPMDEKKKVIEDVDKDRRYAIDASIVRIMKSRKMLPHQQLVLECVEQLGRMFKPDFKVIKK
RVEDLIARDYLERDKDNPNMFKYVA
>Php_XP_001771962
MNNERKVIELEQGWSFMKKGITKLKNILEGVPEQQFSSEEYMLLYTTIYNMCTQKPPQDYSQQLYDRYRESFEDYIKSKV
LPALREKHEEFMLKELVKRWDNHKIMVRWLSRFFNYLDRYFIARRSLPALSEVGLMCFRDLVYAEIKINVKDAVIALIDR
EREGEQIDRALLKNVLGIFVEIGMGNMDAYESDFETFMLQDTASYYSRKAASWIEEDSCPDYMLKAEECLKREKERVGHY
LHASSEHKLLERVQHELLTQYEPQLLEKEHSGCHTLLRDDKVEDLSRMYRLFLRIPKGLEPVAAIFKLHVTEEGTALVKQ
AEDAAGNKKAEKKDTVGVQEQAFVRKVIELHDKYLQYVSECFVNHSLFHKALKEAFEVFCNKGVAGSTSAELLATFCDNL
LKKGGSEKLSDEAIEDTLEKVVKLLAYISDKDLFAEFYRKKLARRLLFDKSANDDHERSILTKLKQQCGGQFTSKMEGMV
TDLTLARENQTNFEEYLTENIQSSPGIDLTVTVLTTGFWPSYKSSDLALPAEMVKCVEVFKEFYQTKTKHRKLTWIYSLG
TCNITGKFDAKPIELIVTTYQAAVLLLFNAEDRLSYNDIKNQLNLTDEDIVRLLHSLSCAKYKILNKDPHTKTVGQTDTF
EFNTKFTDKMRRIKIPLPPMDEKKKVIEDVDKDRRYAIDASIVRIMKSRKVLPHQQLVLECVEQLGRMFKPDFKVIKKRV
EDLIAREYLERDKDNPNMFKYVA
>Php_XP_001764780
MTVSSSPGIGTTANLSRKKATPPQPARKLVIKPFKEKPKLPKDFEEVTWAKLREAVTAIHLKQPVNCSLEELYRAVEDLC
LHKMAGNLYRRLQQECESHISVKLRDLVGRSPDSVVFLSHVESCWQDHCDQMLLIRSIALYLDRTYVIPNSGVRSLWDMG
LQLFRRHLSACPEVESKTVSGLLTLIEKERMGETVDRSLLKHLLRMFSALCIYSESFERRFLDCTADFYAAEGIRFMQQT
DVPDYLKHVENRLHEENERCLLYLDGSTRKSLVATAEKQLLSRHTTAILEKGFSMLMDANRLADLQRMYMLFARVNTLES
LKMALSTYIKATGNSTVMDEEKDKDMVSWLLDLKARLDAIWEESFFRNETFSNTLKDAFEHLINLRQNRPAELIAKFIDG
KLRSGNKGTSEEELEGILDKVLVLFRFIQGKDVFEAFYKKDLAKRLLLGKSASIDAEKSMISKLKTECGSQFTNKLEGMF
KDIELSREINESFRQSAQARLKLPSGIEMNVHVLTTGYWPTYPPMEVRLPRELNVYQDIFKEFYLSKHSGRRLMWQNSLG
HCVLKANFPKGKKELSVSLFQTLVLMLFNDAQSLTFHDIKDTSAIEDKELRRTLQSLACGKIRVLNKIPKGREVEDEDTF
VFNEDFVAPLFRIKVNAIQLKETVEENTTTTERVFQDRQYQIDAAIVRIMKTRKVLSHTLLITELFQQLKFPIKPADLKK
RIESLIDREYLERDKANPQIYNYLA
>Php_XP_001758274
MNQAKKKNFKIEPFKHKVEMDPKYAEKTWKILEDAIHEIYNHNASGLSFEELYRNAYNMVLHKYGEKLYSGVVTTMTQHL
REIARIVEAAQGGLFLEELDVKWREHNKSLQMIRDILMYMDRTFVNNFNKTPVHELGLNLWRDHIVRSPKIRDRLLRTLL
DLVHRERTGEVINRGLMRNITKMLVELGTNVYQEDFERPFLDAASDFYRLESQQLIETSDCPDYLRKAEKRLNEEIERVA
HYLDSKSEAKITQVVEREVIGNRMKLLVEMENSGLISMLIDDKYDDLGRMYSLFRRISTGLQTMRELMTAHLRETGRQLV
TDPERLKDPVEFVQRLLDEKDKYDRIIQQSFNNDKMFQNALNSSFEYFINLNIRSPEFISLFVDDKLRKGLKGVSEEDVE
LVLDKVMMLFRYLQEKDVFEKYYKQHLAKRLLSGRTVSDDAERSLIVKLKTECGYQFTSKLEGMFTDMKTSRDTMQGFNA
TSAGTEGNEGPTLTVQVLTTGSWPTQSGARCNMPTEILAMCDKFKMYYLSTHTGRRLTWQTNMGTADLKATFGDGNKHEL
NVSTYQMCILYLFNQADRLTYKEIEQATDIPALDLKRSLQSLACVKGKNVLRKEPMSKDISEDDVFVFNDKFSSKFYKVK
ISTVVAQKESEPEKQETRQKVEEDRKPQIEAAIVRIMKSRRLLDHNNIISEVTKQLQARFMPNPAVIKKRIESLIEREFL
ERDRVDRKLYRYLA
>Php_XP_001758242
MQLNEWVQSTFPKVLSEVSSAKLAQEESLRLLTQLIFHGTSGHSTEVERYLSHYHTAISALLLTSAPQQFPEILNLYFKA
RLEEFSKAMRQHNRSTQSCGSEGSDDDDVSEESEVIKGEDDEMESIRSIAVVAGEEWVTGLGTVIRNLRRIGFAALSEEG
YASSIYSLLKVKIHAITNKRYDKPVLESIRSWIEAVPLRFLFVILASSGAPPSKTSLATFPSPLASLSIPTTGADEAVKE
GVVRWRLRLHFFAYETLGDLRITELFDIIVDYPESLPAIEDLRQCLTNTRHHSKLIDSFRTSLRQRLLTAGAATTDILLQ
YVSTIKALRTMDPTGVVLEAVSEPIREYLRGRKDTIRCIVTMLTDDTGAGGSAGLGGVGESLFEELSRGVTSLENVGSDD
DADLDGNDAWAAAERWEPDPVEADPSRTTKSRRLMDIISMLVGIYGSKELFVNEYRVMLAEKLLNKSDFDTDRDIRTLEL
LKLRFGENNMHGCEIMLKDLADSKRINTNIKANDEIVSVDGDKKLPLDVLEATIISSLFWPPFQQVETLKVPAVVDELLE
EYAQRYHAVKAPRKLQWRKNLGTVKLELQFEDRSAQFVVSPMHAAIIMHFENQPRWTATALAEAVGLPVATLRRRIMLWM
NQGVLVESHNTKDGDIEYSIVETMGDAGSRAGAGAPSMDSALPLLGDEERESAVASMEDQWHQEMNVYESYVIGMLTNFD
SLPLDRIHNMLKMFVSDPPYDKTLQQLQGFLGRLVGEEKLEFREGVYRRRQI
>Crei_XP_001703219
MSAVRGKGGAPLGGLAPVAGSANTQQAKKLVIKPLKVKPELPANFEESTWSKLRDCIIAVHCKRPVSNSLEELYTAVQDM
CMHKMADKLYTRLQKECDAHIAAHVGSLGDCLGLDAVPYLDRVDSVWQDHCSQMLLTRQIFLYLDRTHVLQLSSSATPVK
SIFDMGLALFRTHLAERPQIKERTVEGLLELVQRERCGEGVNRALLQRLLRMLSSLGIYTDAFHEPFMKASGQFYRSEGE
RLVAELDVPAYLKHCETRLGEEFERCSEYLDASSRRPLIAAVEGALVARHTGPLLDRGLGPLLDGHRVGDLGRLYGLLGR
VGASEALRAAFREYVRSTGLALVKDEEKDKEMVERLLDLKSRLDEVVAGAFGRSEAFAATLKESFEYFINQRANKPAELI
AKFIDARLRAGGRGAAAGAAGAGGSGAGTGGSEEELEAALDRALILFRYIQGKDVFEAFYKKDLAKRLLLGRSASVDAEK

AMIAKLKARRRSGGRARARARGRLLQPSQFTAKLEGMFKDVELSDDVMAAFRGAPAAAGLPSGVDVAVSVLTSGYWPTYP
VTEVKLPEVLDRAGAVFRDFYLSKYSGRRLVWQHSLGSCMLRASFPKGMKELSVSTFQAAVLMLFNDTDTLSYKDVLAGT
GLEEKELKRTLQSLACGKVRVLTKEPKGRDVNDDDSFSFNTGFTEKLFRIKINSIQMKETEEENKKTNEQVLQDRQYQID
AALVRIMKTRKTLSHKLLVAEALQQLKFPLKAADLKKRIESLIDREYLARDANDANVYNYLA
>Crei_XP_001700877
MKKGPIKIEPFRHPVTVDPQYADKTWKVLEDAIREIHNQNASGLSFEELYRNAYNMVLHKYGPRLYEGLIRTLTAHLTEV
AKKIEEKEGAPFLRELKKRWDEHTKSSQMIRDILMYMDRTFVVQQQKTPVFTLGLELWRDVVVRNRAISERLLAIVSSLI
MKERQGEVIERGLIKSVTQMLGELGHAVYVEDFEKPFLAAAAEFYRKEAQEYITSSDCPEYLRKAEARLGEEAERCGAYL
DANSTEPKITRVVETELLKAQAARTTLAITSNAPFLVHPVPPLLLPMPPQVRHMLCEHVKEVGRALVSDPERSKDPVEYV
QALLDMRDKYERIITQAFADDKTFRNALNQAFEHFVNLNVRSPEFISLFIDDKLRRGIKGLSDTDVEGVLDKVMALFRYL
QEKDVFEKYYKQHLAKRLLSGRTTSDDAERNLLVKLKTECGYQFTSKLESMFTDIKTSRDTMNEFRTRLVETGKLEAELG
GIDLQVQVLTTGSWPTQAPSKCNLPRELEAACESFRNFYLSTHSGRRLTFQPNMGTADLRAVFGAGRRHELNVSTYQMCV
LLLFNEADSLSYRDIAQATEIPAPDLKRALQSLACVKGRNVLRKEPAGKDVADSDVFFYNDKFTSKLIKVKISTVAATKE
GESEKAETRQKVEEDRKPQIEAAIVRIMKARQRLDHNTIITEVTRQLQARFVPNPATIKKRIESLIEREFLARDEADRKF
YTYVA
>Crei_XP_001692935
MADRKPIELAEGWSFMEQKGIQKLIRLLEGEPEDQFNAEQYMHLYTTIYNMCTQKPPHDYSEQLYSKYRDAFNKYINEKV
LPSLREHRDEVLLKELYQRWGNHKLMVRWLSRFFNYLDRYYVLRHSLHPLKDVGLLCFKDLVYVEIKKRTKDGVLLLVEK
EREGELVDRALVKNILGIFIELGMSNMDCYEKDFEEYLLTETSAFYRRKASQWIEQDSCPDYMLKAEECLRLEEERVDNY
LHATTRNKLLKEVETELLSNYETRLLTKEHSGCAALLRDDKTEDLARMYRLFQRIPKGLDPVADIFKEHVDSEGMKLVKE
AVELAKEKQAKTGPSRDTGTSAEQQYVRAVIDLHDKYLQYVSTCFCNSSLFHKSLKEAFENFVNKSVAGSTSAELMASFC
DNLLKKGGSEKLSDEAIEETLEKVVKLLAYVSDKDMFAEFYRKKLSRRLLQDKSASDDHERSLLSRLKQQCGAQFTSKME
GMVTDLQLAKEKQQHFDDWLKKGSKLPIDLSVTVLTTGFWPTYKSIDVALPREMVEGVEVYRSYYDSDSKHRKLTWIYTL
GTAVLRGNFDSKPIEMQMNTLQAALCMLLNDVDELSYGEIQERLRLPDDDLQRLLHSLVCAKYKIIKKDPDGKSIGKSDK
FAFNNRFTDKMRRIKIPLPPLDEKKKVMEDVDKDRRYAIDAAIVRIMKSRKVLQHQTLVMEVIQQLQRMFKPDLKLIKKR
IEDLIQREYLERDKDNPTLFKYLA
>Vcar_XP_002955111
MNVKKPQVRATWKACHFLWPNSYNAGPTQIKIEPFRHPISVDPQYADKTWKVLEDAIREIHNQNASGLSFEELYRNAYNM
VLHKYGPRLYDGLIVTLSSHLTDIASKVEGKEGSPFLKELKKRWDEHNKSTQMIRDILMYMDRTFVVQQQKTPVFALGLE
LWRDVVVRNRKISERLLSILMQLITKERQGEVIERGLIKSVTQMLVELGHQVYVEDFEKPFLAAAAEFYRTEAHAFITTS
DCPDYLRKAEQRLHEEQERCAAYLDASTEPKITRVVEAELLKSQMTALLEMENSGLIALLRDDKYDDLSRLYCLMRRVDH
GLATVRSMLCEHVKDVGRALVTDPERTKDPVEYVQALLDMRDKYEKIITQALELRTRLLPNSLQQAFEHFVNLNVRSPEF
ISLFIDDKLRRGIKGLSDTDVEGVLDKVMALFRYLQEKDVFEKYYKQHLAKRLLSGRTTSDDAERNLLVKLKTECGYQFT
SKLESMFTDIKTSRDTMADFRTKLVESGRLDELGGIDLQVQVLTTGSWPTQTPSKCNLPRELEAACEAFRNFYLTTHSGR
RLTFQPNMGTADLRAVFGAGRRHELNVSTYQMCILLLFNEQDSLMYREIAQATEIPTTDLKRALQSLACVKGRNVLRKEP
ASKDVLDTDVFYFNDKFTSKLIKVKISTVAATKEGESEKAETRQKVEEDRKPQIEAAIVRIMKARQRLDHNTIITEVTRQ
LSARFVPNPATIKKRIESLIEREFLARDENDRKFYTYVA
>Vcar_XP_002950020
MNNSSSNADTTAPASMAANANAANTAAATTVAHSASTPAADVAAVGLAEPGTEARTLTEWRLRLSYLVYETLGRLRISQM
FDVVVDYPDSLPAIGDLAACLRHTNLQSLFVCSFKAALQQRLLHAGASATGIIHHYVATIRTMREIDPSGSLLHAVAQPI
RDYLRGRSDTIKCLVSMVTQDGGGPNGESLLEELQAATAASGAAAAAAMEVDVATEDADERVLRVATATAAAGFPGVAPP
PPGVAVGGGGGSGAIIAAAGAAPGGAATAAAAAAAGSSAADVVSMLTGVFGSPEVFINEYRNLLCGQLLYRSDYDTAREV
RTLELLKMRFGENALHACEVMLKDVADSKRINHRVHAMPPAGSALPQPASPIGSAFHLLSAAIISYLFWPRQEDPTGLVL
PAEIRTAMETYEQRYHHLKTPRKLRWRPHLGSVDLDVSAGGVTASFSVSPLMATLLIAFKDRPVMSAGELAAAVGLPPAL
VRRKMLFWVNHGVIVEGRTLPTLMSSMCAVGAKLTDYGAEDEAPAQLPSAADRAVEEVGPFENYIKGMLQNYGGLPLDRL
NHMLRLFVVSTPKYDKTPEQLQVWHPTLVNEAHNWNKRIVHDCIMMFLVLAAIIP
>Vcar_XP_002947918
KPELPANFEEATWSKLRDCIMAVHCKRPVSCSLEELYTAVQDMCMHKMADKLYSRLQQECDSHISAHVSSLSDCLSLEAV
PFLDRVAAVWQDHCSQMLMTRQIFLYLDRTHVLQLTSSAAPIKSIFDMGLALFRVHLATRPEIQHRTVEGLLELIGRERC
GEAVNRPLIKGLVRMLTSLAIYTDAFHEPFMKAASRFYRAEGERLVAELDVPAYLRHCETRLFEEYERSSEYLDSSSRRP
LISAVEAQLVGRHTGPLLDRGLGPLLDGHRVADLARLYGLMGRVGAVEPLRAAFREYVRATGLALVKDEEKDKEMVERLL
DLKGRLDEVVGSAFVRSENFLATLKESFEYFINQRANKPAELIAKFIDARLRAGGRAAGAEELEAALDRALTLFRFIQGK
DVFEAFYKKDLAKRLLLGRSASVDAEKAMIAKLKVECGSQFTAKLEGMFKDVELSDDVMAAFRASSAAAGLPAGVDVTVS
VLTSGYWPTYPVLDVKLPEALDRASTVFRDFYLSKYSGRRLVWQHSLGSCVLRAAFPRGLKELSVSTFQTAVLLLFNDAD
TLSYKEIAAGCGLEEKELKRTLQSLACGKVRVLVKDPKGRDVADTDSFSFNSTFSEKLFRIKINSIQMKETEEENKKTNE
QVLQDRQYQIDAALVRIMKTRKTLSHKLLVVEALQQLKFPLKAADLKKRIESLIDREYMARDPSDANVSCRCRPASMAWG
RSLTFPAAHAAQCTNPKVKSTVCCLHMGKANGYAS
>Vcar_XP_002947131
MMADRKPIELAEGWSFMEKGIQKLIRLLEGEPEDQFNAEQYMHLYTTIYNMCTQKPPHDYSEQLYGKYREAFNKYINEKV
LPSLREHRDEVLLKELYQRWGNHKLMVRWLSRFFNYLDRYYVLRHTLHPLKDVGLLCFKDHVYAETKKRTKDAVLMLIEK
EREGELVDRALVKNILGIFIELGMGNMDCYEKDFEEFLLAETSAFYRRKASEWIEQDSCPDYMLKAEECLRLEEERVENY
LHASTKPKLLKEVEAELLSNYETRLLTKEHSGCAALLKDDKTEDLARMYRLFQRIPKGLDPVAEIFKEHVDSEGMKLVKE

VTEAVELAKEKQAKAGPSRDTGTSHEQQYVRAVIDLHDKYLLYVSTCFCNSSLFHKSLKEAFENFVNKSVAGSTSAELMA
SFCDNLLKKGGSEKLSDEAIEETLEKVVKLLAYVSDKDMFAEFYRKKLSRRLLQDKSASDDHERSLLSRLKQQCGAQFTS
KMEGMVTDLQLAKEKQQNFDDWLKEKGKKLAIDLSVTVLTTGFWPTYKSIEVALPREMVEGVEVYRQYYDSDSKHRKLTW
IYTLGTAVLRGNFQSKPIEMQMNTLQAALCMLLNDVDELSYQEVQERLRLPDDDLQRLLHSLVCAKYKIIKKDPEGKTIS
KSDKFSFNHGFTDKLRRIKIPLPPLDEKKKVMEDVDKDRRYAIDAAIVRIMKSRKVLQHQTLVMEVIQQLQRMFKPDLKL
IKKRIEDLIQREYLERDKDNPTLFKYLA
>Cvar_EFN59544
LQAWRARLGYYVYETVGSLRIGDMFDIVVDYPDSLPAIEDTRDCLQNTNLHRKFITLFRKALMDRLLHPGAATADIIQQY
VSAIKALSHVDPGGAILSAVGSPIAAYLRGRRDTIRCIVTMLTADEEDAAAQSLFAELGNAEGAGAEVGMRWVEAGGRLK
PAGLHPLDADPARGASAAAAAAAGDVISKLVGIYGSKELFINEYRSMLADRLLAKGGDYECDRELRTLELLKVRFGEGNL
HNAEVMLKDLADSKRINANVKSVPNTATPLRRRRQLVNIDGLSATIVSQLFWPTLPQDEFNLPPEVQAMLATYGAKYRSL
KAPRKLQWKPNLGTVQLELAIGDQSLEFNVSPFHASVLMHFHTRPEWPAAELAEQMGVAPDALRRKIIYWINQGKARLGI
RHAKAPVASPPLQEVPTVQGVSPCASDLFLRPPPPTHTGGMTAYEPFIMGMLTNFDALPLDRVHNMLKMFVSDPPYDKTL
EQLGAYMGRLVADEKLSLDGGTYRKRT
>Cvar_EFN52532
MAGKPKKFKIEPFKHPLKLDPNYADNTWLLLESAIHEINNHNASGLSFEELYRNAYNMVVNKYGERLYRGLVDTETAHLR
KVAARIEAAQGEGFLRAIKAEWESHNKSVQMIRDILMYMDRIYVKQQNKTTVHQLGLDLWRDVVVRNRRIRDRLLGMLLD
MVGRERAGDVVDKGLVRAMTQMLVDLGHQVYCEDFETPFLERTAEFYAAEAAEFVSSCDCPTYLAHAERRLGEEVERVGA
YLDPSTEAKVVKVVERELISRQMRGLVDMENSGLVPQLVQDKYGDLSRMYCLFRRVEGGVDLLRQTMGDHLKEGGKALVL
DPERQKDPVEWVQRLLQEKEKYDALISRAFSHDKLFVAALNSAFEHFLNLNPRSPEYISLFMDDKLRKGLKGMSEDDIEV
VLDKGIMLFRFLQARAGLPFPALGLAWWSCPAWPALEKDVFEKYYKQHLAKRLLHGRSTSEDSEQLLLTKLKTECGYQFT
SKLETMFSDIKLSREKMADFKGYLEGQGRRLDVEMTMQVLTSGMWPQTSSAPTCVLPRELEQCTSEFVAYYLHANSGRRL
TWQTGLGTADIKAMFGGGARKYEISCSTYQMAVLMLFNDAESLVYEEIEAATSIPEDDLKRVLQSLACVKGKAVLRKEPM
SKDVRPGDRFSVNDAFTSKSYKVKIGMVTAQVGGEGRAAGGSTGQGCGDCGDGDREFLERDATDRSTYYVYLA
>Cvar_EFN56087
MALQGHADRRPIELEAGWQYMEDGITKLKHILEGDKPEAFTAEHYMMLYTTIYNMCTQKPPHDHSEQLYARYTEAFQVYI
QEKVLPSLRDHHDEHLLKQLKQRWDNHKIMVRWLSRFFNYLDRYYIQRHNLHPLNDVGLLVFRDHVYAEIKRASRDAMLK
LVEAEREGEQIDRSLLKNVLAIFQEVGMGLMECYERDFEEAMLKDTAEYYRRRAAVWIQEDSSPDYLVKAEECLRDEEER
VNSYFHVSTKPKLLKEAENELLKVHQMQLLEKEHSGCAALLRDDKKADLGRMYRMFNRLPKGLEPMAEIFRKHVEEEGMK
LVREATEAAESKKEKEREAGDSPENAFIRGVIALHDKYMEYVQDSFGNSSLFHKALKEAFESFCNKQVSGASVAELMASF
CDNLLKKVGGEGACCSCRCCCCVWGRRCCRCCRCCRAAPLACSLSLAQAAPARGPSRTTPPLHPQHAFPFFRIRSNQSCL
SIPILQMEGMVNDLQLAKEREKAFEEWRERKGFAGGMEMNVTVLTTGFWPTYKGVEQYTSYFDETTSKTRRLSWQFTNGT
VHVKATYDKNYELILMPLQAAVLLPFNDSDSLSYGELKEATKLPDEDLTRCLASLTLSKYKLLAKEAASKGIGPADSFRI
NPKFTDRMRRIRVPLPPVDDRKKVQEDVDKDRKHAIEAAIVRIMKSRKALKHQQLLVEVVQQLQRMFTPDVKVIKRAIDS
LIERDYLERDANDQQLYKYLA
>Cvar_EFN51797
MLQLVEAERSGEAVNRYLLKHTVAMLTNLRLYEDGARDMLLSSAIQYYNREGSSLINELELAAYLVHCERRLAEEFNRCE
AYLGFALRKPLKDILDQCLLEAHMSSILDSSMRLLASCQEQDLGRLYSMCARIGALQGLRLVFRDYIRTAGSAVVMDEHK
EEEMVSRMLKFRADMLSVLRNSFANHAEFAQALKEGFEACLNSRTDKPAELIARYLDSILRRGSKAGAQESSLEEVLDAA
LALFRYVQGKDIFAAYFKRIMSRRLLMGRSASMDAEKLCISKIKAECGPQFTNQLEGMLKDIEISSDIMSGFKHYIAAKP
GSIVDMNVLVLTSGFWPSYRAFDCLLPTELVRAQKEFAEYYLSKHGGRKLAWHSTSSNCVVRAQFPMGVKELQASLHQAT
VLLLFNESEQLTFSEIQAALKLEDSELRRTLASLSLAKEKVLRKEPASAEIGPQDVFKFNEAYTSRLFRVKINNLQMHDS
DEDSKKTNEQVLQDRFHQIDAAIVRIMKMRKSLSHNLLLGELASQLRFPTGQADVKKRIESLIDREYLQRVEHGYEYLA
>Otau_XP_003081880
MAVDAWCLARSLAANDPSTSALSAKDVDEAFATLRRSNCSDVLERAFLSAFASGVGKDALEAWCKSCEVPATLVRALNDG
VSDERLDAWTRERAAPATLKFAEIVESERCRARAFSSRARSENETSASVEAFVRVFDAEVGAMVCARGPPRLSAILSAWY
RVQAKEYDRAVRRRWGETSDDEENDLMETNMLERQGTISMDIAGCAEVMGLLWSAATAEMRTALAKCHESLGRVAENAAA
RALGGCVQQHVVRRCEGAFDAPKLRATLRWVRAVPLEFFKTALSLSANDGAAIDSWRGRLEYAVYEHLGALRIHELFDII
VEYPDSLPAITDLRTCLQNTMLHSVLVDSFVDATRSRLLHAGASTVDIVQQYIGTIKTLLELDPSGVVLELVSGPIKEYL
RERKDTIRCVVTMLTDDGGGGEGDGEGALYAELGRLARGESMEFAESSVPAPNPSVGLMDADNLDRMDSITAEMVAIAAT
ITEDAHEDDIVQTQQVLSGWDAWEPEPVETEAAASRGRRRKGGDIIGLLVGIYGSKELFINEYRTMLAEKLLAKTTYDTD
REMHALELLKLRFGEGSLHNCEVMLKDFADSKRTNANIKVQPTTGTPSAKDRRANDILMHTPVEATIVSSMFWPAFSTDV
TDFKLPQEIQEQMDLYAKRYHQIKAPRKMEWRPALGIVVMDVTHNDRTFEVSVNPLQATILHHFQRAESWRASDLAHEVG
VSVDALRRRIAVWINHGVLIERNDGQGVVYALTELTDEVDAMDGVHNDDEHISGVVTAEESAAAGMMVYEQYVMGMLTNF
PSLSLDRIHNMLKMFVVDPVYDKSIEDLEQFLLGLVAQDKLSADGTSFSKK
>Otau_XP_003080123
MTDRRPVALEEGWGRMQDGIMKLRRILDTDDAEPFTSEEYMNLYTYVSKSAGRPEIHDTSDDDIPYSDSHFSLNTLVDYF
SALSDASSGFWLQLRSAALRVTSSFNLCTRSTIYNMCTQKAPYDFSEQLYERYEAAFNQYINAKVLPTLVEKKGEYMLKS
LVMRWENHKIMVRWLSKFFNYLDRYYVQRHHFPPLKDVGVNCFRRLVYDEIKLSVKTAVLELIDKEREGEKTDRTLIKNI
TSIFVEMGLGTMDAYQNDFEADLLAHTASFYSRKALQWIAEDSCPAYLIKAEECLNSERERVQLYLHQTTESKLISKVEQ
QLLEQYENELLEKENSGCAALLVEDKTEDLARMYRLFRAVPSGLKPIAEIFKAHVKKDGMNLVSVAEQTASNMKSKKPDK
DAASTSVEQVFTRSAIELYDKYSTYVNECFDSSALFNRALTEAFENFCNKGIAGNSTAQLLADFSDKLLRKGGSEKLSDE

KMEETLEKVVKLLAFISDKDMFGEFYRKKLARRLLTDSSASQDYERSILSKLKTQCGAQFTGKMEGMLNDLQSARETQDT
FERWMEEDAANRKPPLDFSVTILTHGFWPQHKPVEFQLNDELAKCVDTFRSFYDKRMGQRKLTWIHHLGTATVVGKFETK
SIEMLMQTTQCAVLLLFGAKTELTMQNVIDLTKLPPDDAKRALYSLSCAKYKILNKSPEGKTIGPDDVFAFNEKFTDRSR
RIKIGLPPVDEKKVTIEHVEHDRRHAIDAAIVRTMKARKSLAYNQLIIEVVSQLKQKFVPEPKQIKIRVEELINKEFIER
DKENPQVFKYMA
>Otau_XP_003078573
MSSSRRGDHSRSATGEPSTRSNDGRRSAHGAPGTARGLTIKPFKRAPTVPTEFATRARETLREAIRRVQAKEVTGVSHEA
LYRHVENLCVHRRAAEAFEDFQSGADRRAREVLRGLEGRKIEDSGVFLTKFDETWGDYCAQALTLRSIFLYLDRAQANGG
GKSSTLWDVSLRVFHEHLEGTAKSVKGKVVRGLLDLVERERMGEKIDRALAKRVLRALSALGVYQEAFENVFIEASQEFY
RKEGNEYSVQTDVSDYLKHCERRLEEEAERRSCGRASQGLIEAHIGDILDKGFVDLMRQHRLEDLRRLHSLLARMDGLAR
LCSAFVTYLKQQGTAIVKDEARDKDMVDRLLTMKTAVDEVVSKSFGRTIADGSNDIFINGVKESFESFINCRQNVPAELI
AKYIDSKLKSGSKGLSEEELERTLDKALTLFRYIVGKDVFEVFYKKELSKRLLHGKSASIDAERSMIQKLKAECGSQFTQ
HLEGMFKDIDLSREIMQSFRQTFENDPIIEMNVNVITAGCWPSYPSVDVKLPEELANLQEKFMSFYLGKHSGRKLTWQNS
EGHCVLKARFDGGMKELSVSLFQCVILMLFNDSKKLSYTEIAQKTGMEEKELKRALQSLACAKVRILNKEPKSREINDDD
SFEVNTALNERLFRIKVNSIQVKETAEENKQTMERVFQDRQQQIDAAIVRVMKTRKSLTHALLISELMAQLKFPTKASDL
KKRIESLIEREYLERDREDAQTYNYLA
>Otau_XP_003078189
MDSPHRDKSNDIRRARALEPHVSAHARASRAMGDDEAIERARAASTSAIESTDARERDGESDGRGSAALGKRRADGSTGR
ALEPFRHRVRQDPEFVESTLRALRDGTTKLLRLETSGLSFEALYGSAYALVLRKQGDALYDAIFGAVTDHLCQHVAISVA
NVAAEGDVEFLKALETGFLTHRKGTQMLVDVFNYLDRVHLPRSGKANLEPVGKLSMTLWRECVVRNPRIKRRMRSCVLDL
IRRERDGERIDRDTLRQVTDMLLGLGESVYVEEFESNVLEETRSYYKALAQKRIDIDDCPTYLKLAETRIDQERDRSEAY
MAPTTTTLLVAEVRQQLLKEMSQSLLHNTTSGMVHMLRTSQLDSLSCLYKLFSAMDDLEGIRDLMFEHIKDVGKGIVNDS
ENEKNPAQFVEELLKYKGKYDDILRVAFANSRVIESQCNQAYQYVANLNPRSPEYMSLYLDQVLRKSPKEMSQNELENIF
NRSMGLFRLFHEKDVFEGYYRLHLSRRLLNKRSASDDNELAFIARLKDECGYTFTSKMESMFSDMLTSGDLNREFHETKF
ASGTPLDASFSVLTTGVWPMRMQKSHPFLPSECEAACAAFEAFYLGRHAGRKIYWQSAMGQAEIKFTVASGEYDLITSTR
HMCVLMLFNRHNVLTTAQISQLTLMHDDELKACLQALSCVKGKNVLKRTPDGKEVLPTDTFEVNEDFSSKSSRVKISTIS
SRRENDHERASKSRQLSDDRKYQVEATIVRVMKTKKRLSHNDIVVEVTAQVKNRFMPTPADIKKYIEGLVEKDYIRRDPN
DRRLYEYVA
>Mpus_Micpu213862
MINDRKVISLDEGWEFMQNGIVKLRNILEGEKNEENFNPEEYINLYTTIYNMCTQKPPHDYSQQLYERYREAFNEYITTK
VLPALREKQGEYMLKELVKRWDNHKIMVRWLSRFFNYLDRYYIQRHNLAQLKDVGMLCFRDLVFAEIKRTVKDAVLQLVE
KERDGEQVDRALMKNILGIFVEMGMGGMDAYENDFECHLLTNTAAFYAKKATIWIEEDSCPDYLVKAEECLRREKERVGH
YLHASSETKILKECEKEVLAQYETQLLEKEHSGAAVLLRDDKTEDLGRMYRLFKRIPAGLPPVADIFKKYVEREGVTLVK
AAEEAATQKKEAKAAGGAGKDASNAASASTEQMFVRNVIELHDKYLAYVGDCFSNDSLFHRALKEAFEVFCNKGVAGSTS
AELLATFCDNLLKKGSSEKLSDDAVEETLEKVVRLLAYISDKDLFAEFYRKKLSRRLLFDKSANDDHERSILTKLKQQCG
AQFTSKMEGMVTDLQLARDNHRPAFEKWMSEDEDKRRPKVDFQVTVLTTGFWPTYKFMELALPKEMVECVETFKDFYEAH
FVHRKLTWIYALGMCHVKAAFTAKPIELQISTFQAACLLLFNETDSLTFEEVKERLNLPNEDVIRSLHSLSCAKYKILTK
IPEGKTIDAGDVFSFNAKFTDRLRRIKVPLPPVDEKKKTVEDVDKDRRYAIDAAIVRTMKSRKVLPHQQLVLEVVQQLNR
MFKPDFKMIKKRIEDLIARDYLERDKDDANVFKYLA*
>Mpus_Micpu49280
MEEWAQRAEGSGGVGNLARKVVKPASAGRKLTIKPFKEKPKLPADFEAKAWDALSGAIDAIHAKRAVAASFEELYRRVED
ACSHKLADSLYQKLRAAMKARAIAQLKALRSRSCPDPIAFLSRVDECWSDHCASTLTTRSIFLYLDRAYCAKTPGVKGVW
DLGLMLFRASLVGGDEEGGGGGGVGNASNSGVTVVEEDVGEIVRKTTRGLLASIQRERDGEAVDRARIKRLTAALVNLGL
YADHFERAFLDHSAAYYRAEGTRAAQSSDAAGFLTHCEARLAEEEDRASTYLDASTRRTLTRCVEQNLVETHVIGVLDKG
FDALCAENRIEDLRRLHALCARVDKVDKLRDAFAARAKRVGAAIVQDEENDKDMVQNLLDVKESLERIVSDAFGGSLELF
SNALKEAFESFVNSRRNRPAELIAKYVDGKLRAGSKSGHIQGKDVFEAFYKKDLAKRLLLSKSASVDAEKSMISRLKAEC
GSQFTTKLEGMFKDVETSRDIMRGFAADEKIAKELPENVDVFVHVLTAGYWPTYAPCEVKLPRELDHLQRVFSEYYLSKH
GGRRLVWQNALGHVLLRAEFPKCGVKELAVSLFQAVVLMLFNDAETMSFEELKDATGIEDKELRRTLQSLACGKANQRVL
SKTPKGKDVDDGDVFAVNDDFNERLTRIKVNSIQMKETKEDNDATNERVFQDRQYQIDAAIVRVMKTRKTLSHQARSGYT
GPHTTASAW*
>Mpus_Micpu35295
MDLDRPTPPIFSCKDALGAPWADAVAAVATGVGGGREWTERHEARDDGGCALGAVAEAAVAKALELSVRKTVSAKAKGVF
DRRALPRLLRWLDAVPLQFLATTLGMTHGAGGPSGRTDADAAATASKLAEWRSRLEYAIHEHLGALRVSEFFDVIVEFPD
SVPAVRDLRACLRRTTLHAQLVRRLRSAMQSRLLVAGAPTADIIEQYRLTIRALRALDPSGVVLRVVSGPLKEYLRERKD
TIRCVVTMLMGGGGGGGDGDGGDDVDALLGADGDDAVEGSDDEIDRDDWEPEPVESEAASSRGRRRRAADELGHLIGIYG
SKELLVNEYRNMLAERLLSKVGYDVDREMHTLELLKLRFGESSLHKCEVMLKDVLDSKRINANVKAPPAPGTPAAADVES
SNLLRESPLDATIVSALFWPPFACEAPEFKPPPTMQSMMDAYGKRYHHLKAPRQMRWKPTLGTVVVEVMRGDIEIELSVS
PIEAAVLTQFQTKERWGMDELAAELGITKATLRRKTVVWVNRGLVAEEKAPGAVGGSETSYYVLTSGKDGGDNKFGAAAA
DDEGGGGGAVASAEDQAAAGMKVYEQYVVGMLTNFPSLPLDRIHNMLKMFVSEPPYDKSIDQLEAFLTQLVGEDKLVLEG
TQYRRRTSA*
>Mpus_Micpu192537
MSGKARGFRIEPFKHKVELEPGYGDKIWAVLKDAIHEINHRNASGLSFEELYRNAYNMVLHRHGRTLYDGLAETIADHLK

DVASQIDATLGDGFLPELQRRWREHVKSMSMIRDIMMYMDRIYAVPNGLQPVHELGLALWRDHVARRPSIKNRVRATLVN
SVNRERRGEQIDQGLVRATCGMLMDLGEDVYVNDFEEPYVSSTKDFYRAESQTFLSSNDCRDYLLRSESRLEEEQLRVKE
YLQKRTEGAAVSCVVNELLAKPMRAVLSLGTGGVVSSLRDDKHEQLALMHKLFSHVKDGVKTLKEMMAEHVKEEGKALVL
DPEKGKDPNAYVEGLLKLKDKYDGVIKHAFGGCRSFVNALHGAFETFINMNNRSPEYISLYVDDKLRKGLKGASEDDVEV
VLDKVMILFRFLQEKDVFEKYYKHHLAKRLLGGKTTSDDAERSFIVKLKTDCGYQFTSKIEGMFNDIRISRDTMASFRTH
VEEKAKGGGGDGSVSVSTTGLSPMDAQESGGSGGGGGGGGGGGGAGGGGAGAGAGAWTRGGGLAAHLGGVDLDVQVLTT
GSWPMTGNTGATTCTIPAQLQPACEAFKNFYLASHSGRRLYWLTSMGSADLRATFADGVKRELSVSTYAMCVLLLFNDQD
EIGYKDIATATAIPAVDLKRTLQSLACVKGRNVLRKEPMSRDVNDDDVFAFNDKFTSKLLKVKIGTVSAQKESEPEKMQT
RRRIDDDRKPQIEAAIVRIMKARRRLDHNSVVQEVTKQLSSRFIPDLADIKKHLENLIEREFIERDRNDRRLYIYMA*
>Cmer_CMC102C
MQSSQELPETSRSLPDAWETAVHHLQEALLERRHPTQHSTVERQQLKDALRWLVSTSQRASVPFCRYAVLFLKEFVREQV
WEALLEPVLGCTHSTCAPWNDSRKRLTGTADCDTPTSFDPAATRACIAEAAELLEQALQVMDVVAERLDPSRRLYWRGAL
SVDAQAVILQQVTQTAMRRLVRSMFVWALQDAEEQVVTGNSDELGHVPSQYDSLRAQQRHLRSEGDCAQAVPLLLADLER
QLAVLGLLDQWRDLVVVQIYESIEERAYRLARDELHEGVLDDLLAWVQKLLQPWVAVMPLDPPDAEISMREGVQSTSGSA
FERKVGYFTHETLLHARLSQLFDLIVEYPASKAPLLDIRRCLESTDALQETTQSLCSQLERRLLHPGASTSDILHHFVNT
IQSLLLVDPHGYLLARATTLVRQYLRRQCPEAMTRLVELFTSPEYAELFALNGQQQQQQQIDHCPGPRFHAYTHLEDAS
SDDTDDSRSETDADPASSDGMFSVSGLETRGQIAHRFAQHDILHILMGVCGGRERFIAEFQFYLARKLTTVMDYEFELLV
RNLEVLKVRLGETSLNDADIMLKDISDSRRFMNFIRQEKHMELDSKQQTELVFISYLYWPLLCPEAGTHFGPPERTQSLL
APNLTGSEYFATLKSYAHHLNADLEQFIRLCQESFARQRAPRVLHWIPVAGVCDLELEFADGRHVEFTNVPLLNAMLLEL
FAEQPRWSRNALRQKLFLLEPGGDATPDAASSLPPYDALLQGALRFWSRHGILRLVDEGDFYEIVEHGGTEPNTREWQAS
PIDATQIDPIADALSAVPGPATEKIQDKDDNTEEVIGNYILGMLANFESLTLERMHQMLTMFCTDPPFRGTLGDVAACLN
RLLDAGCVRYERGLYARTG
>Cmer_CMM291C
MTSRKTRLTIQPFKYVSMSEVTLEQAQKSWEKLEKALVRIFARDHQDLSFEELYRTAYNLVLHRYGELLYEGLERGFHYQ
AQLVRSTVQSRAHRSEVLLESLRTRWQLYKNAVRTVRDVFMYADRSFIRSTGRTPVYELGMSAFRDDVLRDQTFATQLVD
AMLDVVTRSRLGEQPPLTLLRDTLDMYIELDMYAEAFEGRFLRVSREFYTLAAARVLQTCSGRSYLQHVREWFLFEEKLA
NECLAVRTTTRTALQTLLGEILLSEHLNEILDTTDTGFLQLLQSQRLEDLQMMYDLLMRIPEGVDAMRGRLAPYVREKVRA
DLVRGASALEQPLEWVARAMHLKDEFYNLLAALQMDPKFVRAVQEGFEEALNECPCAQEFLSLYIDKALRESPSISREAT
RAALEHASGLFRLLRNKDVFEQHYKVHLSRRLLRRFASNTVVPPSANDDAEQHFISKLREECGAVYTAKLEAILQDMRTS
DELNAAYRRHQEMIQLYGSRARPMLQVCVITNGIWPVAEAPTAQIPACHPELVEITQEYEKFYHERHAKRRLQWLLHQGS
AELCMRLSDEGPALSLKVTSLQMLILLLFNNAEALTVQEMLRLLQFPGLSPELMRALRALCDSRHAILVRAANGPVICNR
DVFRVNKAFQSTTLDIDLTDTDHVDENAGVERAARSSELLERDRRPEITACVMRILKRRQELEHSQLVAQVIDELHNRFV
PTVAEVKREIEALLEREYIARESDELTVYRYIP
>Cmer_CMN113C
MESNQRAPKKLIIRPLRREPKLPQEFYQESWTHLARALDQVFSPSSRRPLSYEEHYRRVEDLCLHHLAKQLYADLQREVD
GYLERVFREQLSDPVLAAMLHVAQGSAQAVEPTERPAFAPVLDVWRAFCERFRDIQAIFLYLDRTYAAESPQVPSLWEMA
LELFAKHMLAEQQRVLLPTLGAVLLEIELDRKGKQRGDCSDLRAILREMFAPLGFYRTHFEPLFFQQTRAFYVAKGTEFV
TQGTLSDYLAYFEHSTQAEAERCRLHLEPSTEKPLFAILHDVLGRHCVSVMMDRGLESLLDAVSLTDLRRLYVLLRSIDE
LWRLVPRYSDYIRVRGTQIVNDRARDHEMIERLLALLDRAEQTEREAFQADVRFHNAAQTAFEAALNTRQPTPAERLARS
FHDLLRVSSTSIPEPDIRRKIGKLLELFRYLSGKDSFLAFYKKYLAQRLLFDLTASTACEQYVVELLRAECGAVYVNHLV
NMLKDMDLSQDLLQEYRMGPGSGSSRLPSFTCKVITQAYWNSSESFLLQVPPGLESQQRSFHRFYHARFSGRRLIWEPSL
SSAELRVQFANGEKVLHVTLSQAVVLLLFNERDAWTLAELQQQTGIADSAELERILDSLSSSQCPVLIRERGAEAASSGP
VTAPARYRFNADLQTSRRRLRLLDIALLERPPEQVPSGPVASPDRQHQIDAAIVRLLKKQKSLSHADLVQQLGAELCFGP
SVSDVKQRVESLLQREYIGRDERDPNLYHYVS
>Cmer_CMT046C
MAALTTPPASRHAVSTAPFHETREAELVGEESAPPGSPVSSGGGDLADDFEPSRSLAETRQSALTRPTIHTVHVPAPRGI
LSASVWMELYRTVLLLCTKPGPCAVKRALYEQVEREMGAFCTSKILPDMERRIPTREGANRAMSAMLSYAPSGCSPMREL
EEARSGRSALRAGRARTSMDTQSSHFSHSKSTVGDSDRDQFPVSHQSMEEGSLQGIELMDMASDDVSDTSEENNGAGASI
TSIEELLHAFCAWWMAYQHLVQFVCKIFSYLDRCYTDKENGPPPLEHLGRILFRTKVLDKMRDVLRTAILTLIARDRSGE
VVDRALIHSAVVVFTATDWVSYYTDEIETPYLAAFQTHYAEASERWLRDCSFPEYMREAEAALRQEIAIAQAVLHPSSMD
RVKDAIENVILLAHEEKLLAAETSGFVALLSQRQFDDLKRVYWLYSGKWIHELCGQPMDGNGGLARSNASGRLVPESQAH
QERALRPIAQQFARCAEAEGAALFAHYDAITTEMNPNGDSVGYEDSDRSVARKRHADGSEKQETGPMSSALSCVLSPKFD
APYASCGPEAEISPTEQAGMWLVRELVNLHETYQLVLRTCFENHEVFSQHFRMAFESILNNPRDMSMIPKLLASYVDRVL
QRIYMIEPPGADLSGHLRVIVQLLEYVYDKDVFADLYQFLLCRRLIFDLSIARDLEITFVEELKAVIGPLFTARVEGMLR
DMRTSLRFQERFNQWNAILDNRERQRSTGAVTLEHATPSHTPVFQAILTTTSIWPSFPSDDDLRLPPALASCLTEYECFY
AHQTQYRQLRWIHVLSKGLVECTRAAFPYMTRYDQVELELNTYQLCMLLLFEDRNEMTFEQICDALNIQAPEILDLFKRC
VLSLCNKKHCLFIKSPAGAELRPTDTLQLNRYFDPQQRRLSFAYLADGFKREECQATRQNGHDDQVPLLEATIVRIMKMH
RQFAHQKLVAGVQNALSTRFTPDVHSIKERIESLIAREYLERDPQDPSLYHYLA
>Cpar_1137Contig11716
MENSGLVAMLKDDKIELARMYDLFRRGLNTMRDVLGTHLRAVGKAVVTDEERLKDPVAFVTSLLELKDKHDRVIQQAFGN
DKSFQNTLNQAFEHFINLNNRSPEYISLYVDDKLRKGLKGVSEEEVETTLDKVMMLFRYLQEKDVFDKYYKQHLAKRLLT
GRSTECGYQFTSKLEGMFNDMRLSHDAMDQFQAHRANASAQGQGDLLQGVELQCTLPQEIVRCCEEFAKFYHTAHTGRRL

TWQTNMGTADLRAYFDTKRHELTVNTYQMCILLLFNSAERLSYGEIKQATGIQPADLKRNLQSLACAKYRVLNKEPKGKD
VAETDSFSFNGGFQCKLFRVKIGTVDEDRKPQIEAAIVRVMKSRKTMEHSALVAEVTKQLQARFLPNPTIIKKRIESLIE
REFLERAGEGGRTYRYLA*
>Cpar_1991Contig13428
MSTTKKAKFVIAPFKQQVKIDPNFAEKTWATLKEAIEIFKHNASGLSFEELYRKAYNLVLHKYGEMLYDGLYKTVREQLN
TVQVELANCNDDRLLTDLNKKWQDHKLSMTMIRDILMYMDRTYVMTQKKTPVYELGLEVFRDVVTRAERIQSRVLHLILS
LIHKERTMEMVDRPLLKQVMQMFVELGRETVYVEDFESYFLAESCNFYQIESQQFIASNSCPDYLKKPARRSRHGGAERA
EARIKEETARVAHYLDPSTEPKIRNTVETELIAKHMRHLVEMENSGLVAMLKDDKIE
>Cpar_13013Contig6938
MTLLHTRPVSFDSTWAKVEGAVFGVDLARGRGLSQSDWMRMYGDVYKLCTSPGKPDAVFFRLKHALQQHVQGIQRVRKRQ
AVLRRLIAGSLHVGGGELIDEYLQRWNEFADVWGSLQHLFSYLDRYWIRQNTDDMAPEPPSAGGICPVHGLAMSTWKESL
FDKVKGHLFKRAMELIGRDRRGEAVNLSCVRALVDSYNLGTFVGAGLGVYREDLERGFLEETRAWYLSESQRVLSSASVD
EYVLRAEEWLQREERRAQLVLDASSQLEVKRACEQGLITPHKEIIYGEADRLLEENRVALRKLFL
>Cpar_25964Contig52765
MHGAATSSGGKRKSATAAATASTGAPSAAVSKRIKPDEMEDGSSGSGTSPVGGMSTGSSNLSRKKTPAPAPVKKMVIKPF
KKPKLPEQFEAETWEKLKGAVAAVHTKQPVSCSLEELYRAVEDLCLHKMAENLYKQLQAECESFAEKKLKELLKYPCNCE
RLPSVLADERSLLDPRRTQTPDHVVFLAQVESAWQDHCNQMLTIRSIFLYLDRTYVIQTANLFRKYLTQHKEVEEKTIQG
LLALIEKERCGEVVDRPLLKNLLRMLSSLQARAPAPCARWIRAA*
>Cpar_25965Contig52765
MYADCFEKVFLEETNAFYRSEGERFKSQTDVPDYLKHVETRLKEENERVNNYLDASTKKPLIQALEKQLLTSHVEHILTA
FDGLMDETRKEDLRRMYTLLKSVMSVEELKKTWHKYIRTTGLKMVEDEQKDKQMDAFESFINTRQNKPAELIKFIDGLLR
AGNKGQSEEELESAMDKVMTLFRFIQGKDVFEAFYKKDLAKRLLLGKSASTDAEKSMISKLKNECGSTFTNKLEGMFKDI
DLSKDIMGTFRQSSRHAELLGDIELHVSVLTTGSWPPYTADKVNLPQELGKVQEVFSQFYLAKHSGRRLHWQNSLGHCVL
KAWFPLGKKELSVSLFQAVVLMLFNDTDSIGFQEIKEASGIDKELRRTLQSLACAKIRGKDVADEDVFQFNAQFQQKLFR
IKINSIQMKETQEESASTNERVDAAIARKTLAHNQLLTELLPQLKFPVKPADCKKRIESLIEREYLERSKDNPQARRFSS
FPAVYNYLA*
>Cpar_28677Contig58946
MLAERLLLAPSYETHRELANVELLKLRFGEAPLHSCDIMLKDVLDSRRVNASIHHDFKATEAAPPPHLDATIISAEFWPP
VQSQD
>Cpar_29415Contig7137
MSHASKRAKGTNMPSTTNIEEDWNKIKNGGIVKLQEVVDSGFTATLTSAEFMALYTLVYNLCAKPGEEGKKNSQELYDRY
KEAVNAYLVERVLPAVRDKHDSYLLEEFVKRWDNHKVMVKWYLKFFAYLDRFHIPREKLKPLNDLTHLFHETVFDAVKKD
VRLVLLGMVRKERDGERVDRTLMKNTIQIFVDMDANNMSTYASDFEEAFLQETAEFYSRESARWIEECSCPEYMRKAEAC
LEEEKGRIGHYLNQSSETKLMKVCEDQLLERTEEKLLEMENSGCEALLRDNKTDLSRMFRLFSRLQSGLQPIARIFKKHI
SDEGLALVESQQNSEDTQYIMKLIAMHDKYNGLVNKCFASNPAFIVFVNKEGVGKKSTAELLASFCDNMLKKGTEKARPS
PAGRRRWPR*
>Cpar_29417Contig7137
MQKSISQDIEKSMISKLKTCGSQFTNKLEVMLRDLDLSKDITTNFRQHAKNQAKAGDVELNVQVLTTGFWPNLGEFQEMF
KTYYLGKHSGRKLTWHYAWSICMLKANYPKGRRELQVSLYQAVILLLFNDTDTLSLKDLSEQTGMEKELKRNMIGMTMSK
VKLLNKSPNTKELKDDDVFSFNTEFTAKMYRIKVNSIQMKETQEENQQTNKRVFQDRQYQARLAMRPCAALGIDAAIVRI
MKTRKTLSHTLLVSEIYNQLKFPFKPADIKKRIESLIEREYLQRDKDNPQVYIYMP*
>Esil_CBN77335
MLADKLVGNLSYDTDKEVQNLELLKLRFGETSMHHCEIMVRDLEESKRVNANVQDRLAQRQTGEAQKEDARQHFWPHLLG
EDMRLHPRMQDRLAAFGKAYSVIKNPRLLVWKKQLGVVELNLDFNGVERSFSVSPLHATLIMHFEGKAETWRLSDLAREV
ELPPSVVQKRMMLWVNQGVVSESNPGPVYRLVKDQTQNRSGGEGMESMVLEDNTEAAVSADANDAESDKIIVQFLTGMLT
NFSKLPLDRIHNNLKMFMSGGDNKYDKSLPELQQLLWRLCSDGKLEHADGEYRLVK
>Esil_CBJ32430
MAREAQKIISLEDGWCNNIKPNAINVLEDHLNRGFDKRTSQLFTPKDFMSTYTTCYDMCTQRSPYNWSEQLYDRHGETIS
QYLSGTVVNALREQHGEFLLKELVRRWSNHKIMNQWMQKFFQYLDRYYVKHHSLPSLKEAGLKHFKTLVYDVVKSTVVNA
MLDVINKEREGTIIDRPLIGSCVELFESMGMGTLDSYVADLEEALLANTKDHYARKSQEWIETDSTPDYMIKAENALEAE
KLRVANYLNPSTEAKLLRVCDDEMLEKREKILLEKEGSGCKVLLANDKSDDLSRMYRLFNRLPKGLEPMAEIIKDHITEM
GNEIIKRREAKIEGGEKDTNQDPNFVKELLALHDKYMAVVNDQFAGNSLLQKALKEAFVDFVNRDVGKFKNADLMCSFCD
RILKTGGEKLGDAEVEEYLAKVVQLFSYLTDKDLFAEIYRNQLARRLLNSRSASDDMERLMIGKLKLKCGSQFTSKMEGM
MNDLAIGGDHEAAFSAYLKDGQETRKIDVAKIDFNVQVLTTGYWPAYKPMEVTLPSTMKKCTEVFKKYYAETTSKRRLGW
SHTLGNVTIRAKYQKSYDLQVTTLQAAIVRIMKARKTIGHPQLVAEVLSQLSFFRPNPKVIKARIHGLIEREYLERDASQ
ANHYNYLA
>Esil_CBJ31794
MTLLTPEPIDFDDTWVLLESSLITILQDLGQGFPNDLWISLYTGVYKLCTKPMDPQHSKLYARLKETLELYVAGVLKGLL
AFDNATAHSYTELLVRYRTSFHNYSVGMHYGSDWFKYLDRHWINTNHCETGVSPKEGVYFVREMSLMVWKERVFEALKER
LRHNTMKVINAARERNLSNLDDNDAVRSLLKTYETLGFEKRDRSHLFQTELEDFLVTDTGRYYSRRGSDLLQHMSVPRYL
QEVERYLQEEASRCESFVGSYSARRILVAAQEALIKAHSSRLVEEARGMLSASPEKRVDLRRLFTLISYLEDGETRGGGG
AVENNAMKALEEVVRDHVRSVGLTVVKDLQAKGAMDVSDDEAGVRDNDLLPAGGGKAAVVTSDDVVAAMLEVYEHYKGLM

ADSFDSSPGFRAVLDEACKSFVNAVPQASEWLARYAHCLLDKGFKESKVDEGTRQESLDHVGFLFAYIADKDIFHKYYSK
LLSKRIIQLTSVSDEAEERMLKNMRKISGFEYTSKLQRMFVDKTLSRDLHTGYVEWQVRGGGIGGGGGGGRGGGGGWGGG
GLGAGAEGEGGFVYPAGLVQLPMHFEAFTFVLTAGCWPLQSVSSSFKPPAPIEDYVVSFLDFYSEVHSGRKLEWLYHLGH
GEMVTHCFDKTYRIYSSTFQMGLLHQFNDSTEIAVSELARGINVSDTEIVRHLFPLIKAGLLRLHGADGADLKPTSCDDI
PPGARARLNASFSHKRTRVKINIVDHSRQPLKEAKPSTEIIQDRKMSVQAAISRVMKIRKEASHRDLVVQVREQLRHLFD
VDPPFVKRNIEDLINKDYLHRLEGREPGKEGYKYVA
>Esil_CBJ49233
MSQSKGKFVIKPPKPRNHMDERAALVIWDLLSTAIGEIHNKNASSLSFEELYRNAYNLVLHKHGDLLYAGVRESVQAHLD
EVGEIIATATDDRLLHDLSQQWGDHQVTMQMVRDILMYMDRTYVSFNKKMPVYEMGLVVFRDTVARHDKVKGRLQSLLLQ
NIADERASRLIDRDLMKTSLSMLSGLGVDGVAVYEEDFENEFLATTRAFYRAESQEFIARNTCPAYMKKAEDRLGEEAAR
SINYLAAGTEPKLKHIVETELIRNHAKVLVEMENSGCTSMFRDDKIEDLRRMYDLFSRVPVTLDDLRRSMCEYVKATGKA
LVTDQESAKDPVAFVQGLLSLRGKYDSIVNDAFRGEKRSQKRLKEAFEDFINTDSRCASYLATYIDDLLKSGLRGMAEDQ
AEAMLEKVIVIFRYLQDKDVFENFYKTHLSKRLLGGRSVSDEMEKNMIVKLKNECGYQFTSKLEGMFTDMKISKDVMEEY
RKTGRHTNHGMELVVEMLTTGYWPAQSGPKCRLPKQVLRCCEDFEEFYLKKHTGRKVTWHTSQGNADLKSTFGKNRHDLN
VSTQQMCILLLFNSADTLSYADIQEATQIGDPELKRHLISLCTPKFRILRKASKVKGKGISGPGDTFSFNADFTSKLKRV
RIPLVSIKDSASGPAASASLPPAVEEDRRHLTEAAVVRIMKARKSLRHNDLVAEVTRQLSSRFVPSPTVIKSRIESLIDR
EYLERDRNDRRAYNYLA
>Esil_CBJ49182
MMGKQGRGSFSSGKAAGSSTGVKKKKMVIKPFKVQPKLPEQFEDSTWEMLQRAVVAIQTKQPIDTSREELYRAVEDLCVH
KMGANLYDRLRDECGSHTRREMESLVGQTPDCNAFLQLVDRNWQDHCSSMLTLRNVFLYLDRSFVLQAPNLRSIWDMGLE
HFRNHFQALEEVEAKTVAGILTLIERERTGVDVNRPLLRSLLRMLSALQVYEELFEGRFLRETEEFYAAEGVRYMATADV
PHFLQHVEERLQQEADRASLYLDSSTRKLLVTTAESQLLKPHTQALLERGFGSLMDSQRLPELKVMYQLFQRVQALDELK
AAMTAYVQSKGLYIVHDKDNDKQMISNLLAFRAKLDECINTACDGNESYRYKLKEAWEAFLNARHNRPAELMAKFLDVKL
KGEKGTSDDEVEAVLERVMVLFRYLQGKDVFEAFYKKDLAKRLLLGKSSSFDLERSMISKLKTECGSAFTSKLEGMFKDI
DLSRDLMTTYSHHLKTKLHDRTVFKLDKSREMDLHVQVLTTGYWPGYPAMEVGMPDEMKEHVECFRCYYQNKYQGRRLVW
QPVLGQCVLKVAFPKGRKELAVSQLQTLVLWCFSTDDEVSFAEVKAKTAIEDGELRRTLQSLACGKVRVLHKEPRGREVN
DGDNFLFNKDFTAKLHRIRINSIQLKETSEENEKTHEAVFRDRQYQVDAAIVRIMKARKNLAHTMLMSELFSQVKFPATP
VDLKKRIESLIERDYLERDPNKPGDYRYLA
>Esil_CBJ29134
MLEKREKILLEKAGSGCKVLLANDKSDDLSRTYRLFNRLPKGLEPMAEIIKDHITEMRNEIIKRPGAKIEGGEKDINQDP
NFVKELLALHGKYMAVVNDQFVGNSLLQKALAFVGFVNRDVGKFKNADLMCSFCDRIMKTGGEKLGDVEEYLAKVVQLFS
YLADKDLFAKIYRNQLARRLLNSRSASDDMERLMIGKLKLKCGSQFTFKMEGMMNDLAIGGDHEAAFSAYLKDGQETRKI
DVAKIDFNVQVLTTDYWPAYKPMEVTPPSTMKEVHRGLQEVLRRDHVTFKRRLGWSHTLGNVTIRAKYQKSYDLQVTTLQ
ACYLMVFSKETNTLVLGEVSQRLHLPDDTVKRILHSLSCGKCKVLKREGQGGRIKATDKFAFNASVNCPLRKFRIPMASL
EESHNPKRVEEDRGIAIEAAIVRIMKARKTIGHPQLVAEVLSQLSFFSPNPKVIKARIHGLIEREYLERDASQVNHYNYL
A
>Esil_CBJ29133
MPRKLLSRPTVPLLVLGIWILPPRHEYLSGTVVNALREQHGEFLLKELVRRWSNHKIMNQWMQKFFQYLDRYYVKHHSLP
SLKEAGLKHFKTLVYDAVKSTVVNAMLDVINKEREGTIIDRPLIGSCVELFESMGMATLD
>Ngad_01071
MLDLYSHYLTLVRVTFKDDSRFAKAFKTACEHFINALPNMSESLAIHAHYFLDKNCPESRMENVDVALDSIVLLFNFIQD
KDLFHRSYARLLAARIISFSSVSDEAERRMMKHLMQVCGFQYAMTFQRMFMDKTLSFELSCEFAEWEREHRAQQQRLLLM
RKKQLAEGRGVCLEGGDEVGGADGVTGLEEGGRGGAGGAGGAIGEVGRGGHGRRKSDSAIEFVETESGHVTPRSVSPKLS
HGMRFIGLHDSSPLQVLLSKRVFADKVKKYKERGLVSTPASSALAPKGDLPHPSSASPQAAAQGGGGTIGTYSVMSGRTP
SYHRGNTFFFGDDDMNSPGTAGAGGGGGGSGGAGFSWSHSVLDAPNAKKGIRQTSGAGGEAEVEFFVFILTGGSWPYPST
GQLVQLPAPLMRCVESFSTFYDAKYKRRRLTWLHELGHGEVLFRCPASDRSYTLLVSTSQICILLLFNSRSAWSVEELQR
ALDLDLGELVLCLYPLLRSQVLTCRPEVAVASKLTKHHTVVVNEGLSRRGCRLAVPRKCDEAELPSALDSPRSMDPARIS
PTLLNERKSAIEARVVHLMKKAGSMRHDKLIEAVLQHTRQMNFLPPVDFICEVIRADIEKDFLELAEDGPNPLYRYLA
>Ngad_03094
MATNKKFVIKPYKQHAQMDRQRATTVWGELSGAIDEIFRKNASNLSFEHLYRNAYNLVLHKHAEILYQGVQNSVQTYLNT
VSTFVANASDERLLEEINLRWGEHQVTMSMVRDILMYMDRTYVPQHNKTKVYDLGLRIFRDAIARHPQVKDRLRSLLLGS
IRAEREGQLVDRSLLRSTLSMLMELGIEGTAVYEEDFEREFLRTTRAFYRAESLDVISKNTCPVYMRKAEQRLLEEQERV
AQYLDRSTDPKLRAILEEELVKAHASSLIEMEGSGCRAMFRDAKTEDLERMYLLFARVPSTLEELRGAMAEYVKSAGREL
VADQERVREPVVFVKGLLDMRDKFDRIVSDAFQGEKAAQKKLQEAFEDFINMDTRCAHYLALYVDDLLKVGLKGMTEEEA
DVALSKFIVIFKFLQDKDVFENYYKQHLSKRLLGGRSVNDEFEKQMIVKLKTECGYHFTSKLEGMFNDMRLSKDAMEEFR
KAGVSRVGGVELEVNMLTMGYWPSQALTTCNLPPEIKACCQVFQNFYLNKHSGRKVTWQAGMGSADIRATFGNGRKHELN
VHTYSMCILMLFNAADTLTLEDIQKATQIPENELRRNLISLCTNKHRILRKASKGKGVTDNDRFTFNANFTSKLRRIKVP
LVSMKEAVVDGRAGEGEGGGGGLGPGGERLVNGIPAAVEEDRRHLVEAAIVRIMKARRKLGHNELIAEVTRQLSMRFPAS
PPFIKNRIESLIERDYLERGANDRKTYAYLA
>Ngad_04210
MSAAASSSQSRPAGLVMSSRNATVQGNNRGNNGAAGGSNKKMVIRPFKVQPKLPENFEDATWEVLRQAVVAVQSKTAVAI
SYEELYRAVENLCVHKMGARLYDRLRMECARHVAAVVGTLASGGVEGFMDPALFLAKVDDVWQDHCEHMLTIRNIFLYMD

RSFVMQTSALQPIWDMGLTLFRLELAAQGRGLQRPGAAGGVQAAAAGAGEGGVAGNTGLDIKIVKGMLRVMERQRNGEAV
NLALLRSLVRMLGSLGLYKPLFLRAFLEDTERYFAVEGTKKMESLDVVAFLLHVERRLGEAEESVVHYLEPGTRKALITA
VESRLLAPHVTALIDKGLTPLMDAQRVEDVRRLYQLACRKPGGAEELKAAFNAYIRRRGGELVGDDAREKEMIEDLLAFK
DKCDVILSEALGGRSNPACLAAMKDGFEFALNVRPSRPAELLAKFLDAKLKGEKGVSEGEMEGLLDRIMVLFRYVGSKDV
FEAFYKKDLAKRLLLGRSASADLERSMVAKLKAECGATFTNKFEGMFKDMDLSRELMGNYAQFLGTTAAGEKAGGREVET
SVHVLTTGYWPAYPSSDVVVPPVLEPHMARFSAYYTGKYQGRRLVWQHSLGTCVIKARFPLGTKELSVSFFQAIVLLCFN
DGESPRVAFTDIKAATRIEDGELRRTLQSLACGKINQRVLQKEPKGKDVEDNDVFSLNAGFTHKLYRIKINTIQLKETVE
ENDKTHAEVHRDRQYQIDAAIVRIMKARKKLSHNLLLSELFGQLKFSVVPVDVKRRVESLIERDYLCRDDINPSVYNYVA
>Ngad_30764
MSSRITRSSSGGGVSSPPSVSFSERAGVAGPLEASPVGPRCAGGSNSTLAIRFQIQDKEQVDALWERVRIEVLDPLEAAL
ANFDPRATEVVRKKYYEVYSSVYHVVNDPNRDDLDRHPLILAKHLETLTSYLERVTLPSLAAAHDGYLLRAVGEQWGRYV
VLNNWLHKLLIFVERSYLGRQPGKMGLKERGLRQFSKIVFSRSGQGRLLPLVLAIDNVVTRWRSQGEGDNGGGAGGRTGP
TVDADEKEACRACVELLVETGVTTELKDRLLASARKYFAGRAALWMGGLGGPEEGGREDASRVGWYMRRVETALLHEEGL
LREFDLSRALGEESLVVAVQELVQAHGESVVTREVGGCAWLLRQRRYEELGLMYRLFRREMLTVAAAQAAASAAAAVAVK
EMSAQAAAAAAAALGGPSRGHDTPPSTPPSVSYASRTSSTPHAPPPHPSFSPASPELASLSTLSLAPGPLTPADPSASAS
PPSLVPTHSSSSEHPLLDRMGVILEAHVISLGEALQEDRNARLAKDRRLEAHDPLYIEALLEMHAEYVELTTSVLGAHRV
FKSRLHAAFVAILNDQTSKPTASSASRHLPRQARTQGSRPLGGNLSLRGQEEKPRLITSACDRLLRVGGTREEAKVKEIL
GLVLYLRDKDFFEGCYRTELMRRLLAHRYEEGAEKSAIARLRVEHGVHYAQHSQRMLNETATSKELVHSFLHSYPPALAL
RRESCVDFSVTVMAQGGWPVPAAFAQFSCRPPPVLVSYMECFSSFYLGLDIGRGKKLDWVLREGLLVVQATYPSSSGGSR
RFEVSSNPHMLLPLLAFSIDESKSLSLAALQERTGMTKGNLDIVLGELRKAGFLRMETRKGGREGGGEG
>Aano_F0YHJ9
MSTKGKFVIKPFRPHCQMDAEQAQRIWAQLRLAVSEIYNKNASVLSFEELYRNAYNLVLHKHGDLLYDGVQETVEMRLRS
VAEAVASSPDEQLLSQICEQWKEHQVTMVMVRDILMYMDRTYVPQNKKMAVYDVGLRAFRETITRHDHVRDRLRCVLLEN
VRIERAGRLIDQTGMRCALYMLADLGIESSSVYEEDFECFFLEETRSFYRNESRAFLAANTCPDYLKKVESRLNEEQDRV
PNYLHASTRPKLEHIVESELISAHAASLINSRDGGFMSLLDMSEDRMSDLARMYALFSRVPATLDLLRGALFEHVYDAGR
RLVDTAVEMPVDFLEGLLLLRSKYDAVVTLAFRGETAAQKRLKEAFEQFLNADARCASCLVIYVDELMRRGFKGATERDV
ERQLDQVILIFRYLNDKDVFEAYYKQHLAKRLLHARSMPSDAERSMLAKLKSECGYQFTTKLEGMFTDIRFSKDAMDKYR
AHTTRTSPGSEVHAVVRPTILALDLDVTTLTAGYWPMQATNTCRLPAAAQAVCEPFESFYLKQHTGRKLTWLTSTGSAEI
RATFSQAAKHELTVSTYMMCILVLFNDLDHGAEITFAALAAQTKIPRNELKRHVVSLCTPKHRILLKKSKGKGVSDDDAF
KVNIKYSSKLKRVRVPLVAMKEAGAHPDSSDKVPAAVEEDRRHLCEATVVRIMKARKHAKHNDLIAEVTRQLSQRFFPQP
QFIKKCIESLLEREYLERDASDSKMYIYMA
>Aano_F0YKY7
MCSNAGTCDHSKALYDRTKSEIENVLQNHVVPELKKNLTDGGHMILSRFSHHWENHKVFVKWMQQLFRHLDNGYVANSSI
STITSVGLKLFFDIVFDRFKGEICDSLINAIDKERDGADIDPQLLRSCVEVFPVMGLCSKCTDLKTVQSVLNTQPDLTVY
EADFETLLLERTSDYYARKSIDWLGAKSTPSYLRKAEAALDSERERVSRYLHMSSQQKLLGTCERELLQKHKEVLIEREQ
SGLIALLAEDRAEDLKRMFDLFRRISDGLTPMATTTKKFVQIQGGKLLQQRRDLVQALKSEGKKVTADDPSMINCLLALH
AKMSTLVFDLFDGENQFQRALKEAFQDVINTDATPDISNVEMLVMHTDRVLSGKVRLAEEEVESCLEQIIQLFQFLSDKD
LYAELYREQLAKRLLSRRSTAIHTEKSMIVKMKTQQGAPFTTKLEGMVNDFTLGKELDQTWSSHLNKLRVEGLPADQLKM
NFSVQVLTQGFWPSQKQRELQLSREMSNAQSMFDKWYKERHSHRILSWIYALGDVIVKGGFSDRSYDMTMTAFQAMALLG
LSSRTDAMSFHEIRDQMAIDETTGKRVLHSLSCGKYRLLKKTGHPRTINCLMDSFHSNASFSSKLKRFLVQMSALDGEGK
KKVDVEIQQQRGFSIDATIVRILKARKRLSHQELVGEVIHQVQNFAPESKLIRQRVEGLIERERAPLSQALVECSSFLAF
AQVLKKR
>Aano_F0YL58
MSACGAKRAAASFWCNCGLSIETSKQRKSNSLAHRIVRATETARTSLQRLSASSGDRKTLATCDYFAALIFAHAPNEFII
CIEDMIEDSLTEVHDSFFSGDDLSDLGSAMQPISSKVFVSFWNSIQELQWCHFFDETVHKVFYRGIERCVFGLCSAKYDS
PMTEILDRWKELVLLPWLRVIEGRRLEGQCKCAKGRGIESLTGLVNLVIDENYVRARINELYDIIADFPESMCAVNELRA
TLSRTQQHQLLARSLRSTLQRRLLHSGANSSQIIDVYISTIKVLRILDPRDILLDVVARPVRVYLQRRKDTVRCIVANLT
DELSGELYEELHRIDAPQVDYVGDSEDEIAEAGIDWAPRSNKSRLANSSSVGDILSMLISIYGTKEIFVNEYRFMLADKL
IAKTGLYETARELKNLELLKFRFGDASLHHCEIMLRDVEDSKRLNKHVSSEFLQATVVSQVFWPPLSDDVITIHPRMAGQ
VDLSLRSFAEMYNALKAPRKLVWKKQVGLARLKLDFDAGISKVFDVSLIHATIIMHFHDCDMNTLQDLVAATSIPESLMQ
KKIAFWINHGVIEEIAPRVYAISSQYFNTDLSNFNPATLDEEHLYTSTHSARSVPDDHGLCSQYILGLLTNLGLLPFLRI
HDLLRVLMSRDNISSRRLSRVLEQMCIRHEIEYSRGLYKLTRRDSA
>Aano_F0YNZ8
MLERGEGDNSTILPRFHSDGGLGGRPARITQNALYCVLCTSHGWGKIKLNGIDVLLEVLRGNAMETQPFGNKGYVSLYTI
SYRMCSNAGSCDHSKALYDRSKAEMEKVLRSHVLPELQRLKGISTTAKGGEYLLRRFSHHWTCHKIFLKWMQQLFRHLDN
GYVANSSIATLTSVGLELFHNIIFSEFKREVRDSLVHVIERERDNKCIDPELIRTCVSVFPTMGLCSKTSDLRTIQSALL
MQPDLDIYETDFESYLLKRTSDYYARQSRQWLEVDSIPIYLKKTELALKHELGRVRSYLHSSSESKLLTVCEYELLQTHK
DALVDRENSGMIVLLAQDQNDDLMRMFNLFRRIPQGLVPMASTFKKFVLAQGTCVLKERLNEQEQTNGERKRPSSDDPLT
VEKLLSMHRKMKTMVAELFGQDNRFQRALKEALQDVINTDLSRGLSNVEMLVMYTDRVLSGKLKLCEEDLEKILDELLDL
FLFISDKDLYSELYREHLAKRLLSKKCTLLHVEKSLIVKMKTQQGEDLERVWRAHQNAGRGEKSSGDVHGKLEFSVQVLT
QGFWPTQRYRELHLSREMIIAKMAFDGWYRDRHSHRILSWIYALGDVTIKGIFGARTYDINMITFQAMVLLHFSEFGGQI
TFDEYKVLKKSGNSRTINSTVDQFYADPLFTSKLKRFCIQMSSLDGETKKKVDQEVIQQRSYNIDATCVSPFPYEIRLAH

QELMGEVIHQIQHFKPDSKLVRQRIEGLIEREYLQRDTNDPKMYVYLP
>Aano_F0XVV7
MLSPKIIALEEGWNNEIKAKAIDVLEEMLNNGFDQKSQRLFAPKEYVQTYTTCYNMCTQRSPYNWSEQLYQRHGETICDY
LTKTVLPALRHQHNDFLLTELTKRWANHKIMNKWMRLFFMYLDRYYVKHHSLPTLDVAGLKHFKTLVYNEVKKDVVNAMI
GLIDAERDEKLIDRGLVKNCVELLEAMGMGSLDAYVTDFEDQLLGSTKEYYARKSQEWVETDDTPTYLAKAEVALDAEKA
RVAHYLNSASEPKLLRVCEHEILELRETVLLEKEGSGCRALLANDKAADLSRMYRLFSRVPNGLPPMAALVRAHIEAMGN
EVINRREARLEAGEKDSNQDPAFVKELLALHDKYMAVVSAQFAGNALFQKALKEAFVEFTNRDVGKFTNAELMSSFCDRI
LKSGGEKLSDEDVESYLEKTVQLFSYLTDKDLFAEIYRNQLAKRLLNQRSASDDAERLMIGKLKLRCGSQFTGKMEGMLN
DLAIGVDHQSDFDQTVKEDKSKSLGKLDFAVQVLTTGYWPSFAAIDAHLPPEIVQCTRVFKDYYDTKNSKRRLTWMFSLG
NASVKGAFGKKSYDFQVSTLQAIALLAFNADGDGAAPSLAYDAVRERINLPDEHLKRVLHSLACGKYKVITKTPAGNTIK
NTDAFKVNADFKCQMRKIRVPMANLDESHNPKRVEEDRTVAIEAAIVRIMKARKTLSHQQLLAEVLSQLAFFRPNPKVIK
RRIEALIDREYLERDPDVANSYRYLA
>Aano_F0YK28
MPSDAERSMLAKLKSECGYQFTTKLEGMFTDIRFSKDAMDNPGSEVHAVVRPTILAVDLDVTTLTAGYWPMQATNTCRLP
AAAQAVCEPFESFYLKQHTGRKLTWLTSTGSAEIRATFSQAAKHELTVSTYMMCILVLFNDLDHGAEITFAALAAQTQIP
RNELKRHVVSLCTPKHRVLLKKSKGKGVSDDDAFKVNIKYSSKLKRVRVPLVAMKEAGAHPDSSDKVPAAVEEDRRHLCE
ATVVRIMKARKHAKHNDLIAEVTRQLSQRFFPQPQFIKKCIESLLEREYLERDASDSKMYIYMA
>Aano_F0YK27
MSTKGKFVIKPFRPHCQMDAEQAQRIWAQLRLAVSEIYNKNASVLSFEELYRNAYNLVLHKHGDLLYDGVQETVEMRLRS
ARPRCVCTLYEQLLSQICEQWKEHQVTMVMVRDILMYMDRTRGAPGYVPQNKKMAVYDVGLRAFRETITRHDHVRDRLRC
VLLENVRIERAGRLIDQTGMRCALYMLADLGIESSSVYEEDFEC
>Ptri_XP_002182902
MADSKKRFNIRPFRTHQPMDRADANATWEILEHAMDEIANRNASQLSFEELYRAAYNLVLHKHGALLYEGVTEKLNAILL
QSVETLAAQPNETLLETMATVWNEHMITMTMIRDILMYMDRTYVIQQRRRVVYELGLHLFRITVWEHPAVGPRVMELTLD
LINLQRIGKIPDDRDARLQNVVRMLLELGRADFQANVYHEFETAFLSTTLEFYQQESLSFLSNNTAIDYAAKAASRLEAE
ARRAKTLQLPVTTEGPLMTTLETEWIQRHSRVLVDMEPSGFSAMLQDDTKVQSLRDMYDLFVRVPSSVDHLREALAARIK
QDGAALVQDQEKGASDPSAFCRGVLVMKAKYDRIVNEAFRDEKKAQKRMKESFEDFLNQDARAASCLATYVDELLRVGLR
GATEVQILDSLNQAIVIFRFLSDKDVFESFYKQQLAKRLLGGRSVSDDAERSMVSLLKAECGYQFTTKLEGMFNDMRISR
ETRDKYKSFKRQEGEKNMVDIEVDVLTTGYWPSQNVPPCTLPVPIQESIDRFSKFYLDKHTGRKLKWQTNTGAAELKVTF
GTGPDKYRRHELCVSTYQMCILLLFNDKETLTLAQIRQQTQIPDQELRRHLISLCTPKNRILKKGSKGRGIISDEDTFTY
NMDFTSKLKRVRIPLVKEASMVRPETAAGLIGADGKDAHVAPGSVPVSVEEDRRHLVEAAIVRIMKARKALNHNDLIAEV
TRQLTNRFQPTPQFIKKRIESLIDREYLERSEREHRVYNYVA
>Ptri_XP_002178608
MPRNKSLLKGGHTSRKIVIRPYSKPPTLPENYYDDTVRSLLQSISEASSHRTFTGTAPSPNSTGVSLQNAYKAVVYLVSH
QYGPRLYRDLMDHMKQVAARILPEEREASASRASLLMYIPKQYQLYLEYLLLCKHVFLPLDRTHAWQPETKTVVVASTQT
PGGLLTLWQVGLEMLQTRMQELTLDRELYQEWLAALLQDWNPASNNNLDAANRQDLQSVWYLWQDLGQLAVLPLQEDLEE
YWKNQSQQMMEGYRAGSFLQFAYDKHVHVTIWQPWLPSQWLRSVLENCFFQPHLNDQYLLKPENLHPILQSELFAIKTVV
GVSSTAMEKLSSTQQLWTLAGRIAGGQRLVATSIANFAKTQGLACVQPAVELSDGAGKAAAGQHILDKSPIPATNNVQIV
SDLLDTQQRISRLIQSLPHGPELIIILKNVWEEVLNVETTPALAELLAKFLDQILRSNKKMDQYQSESEQWLQRIISGLFI
PLQAKDIFEAFYKRDLAKRLLWNRVVNMDVEKQVCSLLKAECGAGFTSKMEGMFQDVDWSRETMMVYKQSTADILPTENS
VEMEAQVLTTGFLPRIPGYWPVYPQYPNLHLPESLKEPQERFGNHYKIKYQGRRMTWQYALGHCVVRSSGFPKTYEFVVS
LCQALVLIQFEEADTKLSLPTLMQAIGLEDRDEMERVLQSLALGKDGTRILRKLDYDSEPNKKKKIRMNVDNRDEFTINR
KFESNQRRIRINNIMMKESKEEREKTVEAVSRDRLYLIDAVLVRIMKARKTILHQTLIPQVVEQVKVPAQPGDIKQRIES
LIEREYMERDAKDRNRYNYLA
>Ptri_XP_002182637
MSGNASGIIPLEEGWNDEIKAKAIDKLEAMLNGGLKSGETNMFGPREYVQIYTTCYDMCTQRSPYNWSRELYQRHGETIE
RYLASTVIPALRDKTGQGGTTLLTELQHRWGDHQIMNKWLKKFFTYLDRYYVKHHSLPTLSQAGLRCFRTHVYDEMKRET
TAAILGLINDEREGQIIDKSLVKSIVELYENMGMGSLDAYNGDLEEPLLQSTREFYAKRREEWINDSTPDYLVKAEEALQ
EERSRVADYLSSSSEPKILRVVEEEILEKVELVLLEKETSGCRALLQNDKSEDLSRMFRLFQRLENGLTPIAAIVQEFIT
SMGQEILKRRQARLDGGEKDKNDDPKFVKAIIELHEKYLGVVKKDFSGHSLFQKALKDAFVEIVNKNVGSFTNAELMSTF
CDRILKSGGEKLSEAEVEESLDRIVQLFSYLTDKDLFAEIYRNQLSKRLLNQRSTSDDAEKLMIAKLKVQCGTQFTSKME
GMLADLAVGSQQRTEFEQRMRQVETSLDFSVQVLTTGFWPTYKSPQVTLTEEMNKCMKVFREWHELKHQKRKLGWVLTQG
SATVRGTFGKKSYEIQVSTLQAIALDALSGGETLSFEDLSQRLNLEETILKPLMHSLSCGKYKVIAKTPASNKINTTDKF
TANAKFSSNMRKIRIPMASLDANFNTKKVEEDRSIAIEAAIVRIMKARKTLQHQQLLSEVLAQLSFFNPNPRVVKKRIEA
LIDREYLERGTDNPGVYNYLA
>Ptri_XP_002181074
MLLELGRADFQANVYHEFETAFLSTTLEFYQQDSLSFLSNNTASDYVAKAASRLEAEARRAKSLQLPVTAEGPLLATLET
EWIQRHSRVLVDMEPSGFSAMLQDDTKVQSLRDIYDLFVRVLSSVDHLREALAARIKQDGVALVQDQEKGASDPSAFCRG
VLVMKA
>Ptri_XP_002181491
MATVTSSADRAPEALWRDEFRHGFGSADAAHAVRTRPTTVAAVEFAADGGDWCTLPERVAALAAHVQRWDSLGAIPENSS
EWTYAFGSTPQESRIMALTAAVRVTVPQQILNRQFSCYLQASLDYCCRTRLGEEMEGEEESVNGDEKYLNDALLFNSLRV

LGWLRVPRRIFWTEELKLALRRKMKQYVRDVITGDYEASGRFETLRCWKDQTILPWLQSALIISSLTDPEEEQEMERWSQ
RLQHWLADTYCEVRNEEIFELVADYPDSHEAVLELRYVLQQSNCGSHEFPTLQLPYERLRNALRSALVRRVCHAGATTSQ
IIDVYIATIKVMRVMDPSDRLLQVVAEPVRLYLQTRLDTVRCITTSLTDADQGGELYEELRRQDAKPLEHVTVDSDDEEE
PPTMDWQPRPSIHQPKGTFLETLSTNNNADGDILAMLVSIYGSKDLFVNEYRLMLADKLLASVDYNTDKEVHTLELLKLR
FGESSMRNCEVMIKDMDDSKRANTNIQGSLKSRGTTSSTVDAAIISHIFWPTLHSDSLVHHPRIQVKLDDFSTMYANLKN
PRKLVWMHQLGTVQLELDVIEDGPNGEPVIMTKEFSCSPLLATLISHFEDRTEWTAEDLSNETGVASHAIKKRMIYWINH
RVITVHITPAGVTVYNLATLASHEENDAHGSPQHLASEDDDLHDHTVSISAQQDQEMDVFLSYVVGMLTNLGQLPLKTIH
NNLKTFVTGSDVRYDKTPQQLSAFLQNLCRQERLECGPDGMYKLFKK
>Tpse_XP_002291443
MSTTAAPPAPPPAYQHLYHRRLSRAIQRANATQNRPTTKLAREFFKDVHQFDLRDATGWRSLPRRVATLASYLNDPTSVI
CDTEESFSIRVSHDVHETRRVALTAAIFANSPPTFLNEFYYLFEALLVARQRQWKKSNPLDESMETDDGDDDDDDDGGEE
ELMLLQEEEELYQSLSILGWLPDQLRRPFREALHSVMMNVVKSLISGDFESEGMLERTLQWKTDVLIPWVHSVVGSEAYN
RDQWDAQLEYAASECFVRVRMNELFDLVTDYPDSLPAVRELSVALDRTGRLFYHSLANEWRGALVKRLIHPGAQTSQIID
VYINTIKVLREMDPSGELLQVVTQPVREYLRGRADTVRCIITSLTDEEGGGDLYEELRRQDAKPLDEAQLDEEDDEEPPT
FDWVPPPSILQRRGVITGQVGRVTSSSRRAGDILSMLVGIYGSKELFVNEYRIMLADKLLSNLEYDTDKDVHNLELLKLR
FGEASMRQCEVMIKDIDDSKRIHNNIRSTLEAIALSSNPPVVDAAIVSHIFWPALQKEPLKNHPRIQAELDQFSKEYAKL
KNPRRLVWMKQLGTVELEVEFYDGDEDSIFTSHVKYITCTPAHATLLAHFEDKPQWTADELSGETEMPEEIVRKRMSFWA
SHRVVQVRRSGSDFVYTLSSLADVNELSFADHDDDDHEHAVSLGAHEKEEIKVFESYIVGMLSNLGQLPLERIHTMLKSF
TTGSDHNYNRTPQQLAVFLQQLCKEEKLECSPDGLYKLLKKRS
>Tpse_XP_002294721
MDTPSASSTFQSLSSAMDEIHNRNASTLSFEELYRNAYNLVLHKHGGLLYEGVTERLTWHLRRSGGRLVSSGREYKLLEE
LSTVWKEHRITMVMVRDIFMYMDRTYVPQNRRRPVYDLGLYLFRRVSPTLGNVTSLLLLRVVHQDRMDRLDDAPQRIALC
KSLIHMLLELAHATSAGANAIPVYERDFEEVFLGESQDFYRMESASRLSQGAAMEYVHHAQARLVEEKARAALLDLPAQT
RVNLMNIVETELIERHAKTLVEMEGSGFADNNGVVVDHARIADLAAMYELFSRVPSSVNHLRDALSERIRLDGRSLVRDQ
ETNVAPPAAFVKGVLAMRERFHAVVTEAMKGEKKAQKRMKEAFEDFLNADARAANCLAVYVDELLRVGLRGADERKVSSE
LDRAIVIFRFLADKDVFEAYYKSHLAKRLLGNKSGNEDAERAMVSLLKAECGYQFTSKLEGMFNDIRISKETAEKYRSHK
KKSNNEPVDVEVSVLTTGYWPSQNVPPCILPPPVRAAMDRFQKYYLNTYTGRKLSWQTLAGTAEIRATFPPPKGSSKPRR
HDLTVTTYQMCILVLFNNRDTLTLKQIREEIQIPEDELRRHLVSLCTPKHRILKKGSKGKAISGDDDTFTYNSDYTSKMT
KVKVPMVSMRDATASAKSDGAAGSLPASVEEDRRHLLEAAIVRIMKARKMLNHNDLVAEVTKQLAGRFIPPPQFVKKRVE
SLIEREYLERDEADRRVYLYMA
>Tpse_XP_002290076
MDCVLRHEALVASSNEKELYRSVEDLCVHKFGNKLYLDVVEIMNGAAGEVVQSSNQQRYALLKKMHTICRLSYAEEYLTF
VRSIFLALDRAFVASNTRIWGLWDIGIACLRKHMMRSPQLTVSDDVRPLVRNCVRTCIDLGALSSLFEELIVVASVRFEK
EGKSWGRALSDSKKSAPEFLRHAENRLKQSSALTSYYLPSTNNSTRRILPTIIETQLLAPHLVPSGVLHPRHLYLMLDDD
DGSNTKDFCKCQAIYGRLRGAEIVKQSLSSGADASVSNIKEMEKKVIPDLLAFKSHLNSLHVIAFRSDELFGATVRSILE
DVLNGSTTNNDGDGGRRIAELLAKYVDARFKDAKAQATSSVEAFQNEVMALFRHVNSKDVFEAFYKRNLAKRLLTGRSVS
SDMERSFLSKLKAECGAGYTSKMEGMFKDMELSRDIMSSYSAYSSGATSAQAQPMPVPHLGSGKATEMDVQVLTTGYWPV
YPKYPGILLPPEIVAHQAKFESYYKNKYQGRRIAWQYSLGNCIVKANFPKQPAGKELLVNLCQTLVLLCFQYEDGPDGKG
LTIGDIVKKTGIDDRDEVERVLQSLSLGRDGTRVLIKKKKIRRNVGPHDRFLFNASFVSNQRRIRITNITMKETSEERKE
THEAVSKDRLYIIDATVVRIMKARKTIDHRLLMGEVMTQLKFPASAADVKKRVESLIEREYMERVEGDRSRYNYLA
>Tpse_XP_002286654
MCTQRSPYNWSRDLYQRHGETIEQYLRTTVLPALENKTGQGGTILLNELKHRWSNHQIMNKWLKKFFTYLDRYYVKHHSL
PTLEQAGLSHFKTEIYMHVKDNSTSAIISLIDEEREGEIIEKTLVKSIVELYESMGMGDLNSYTNDLEQPLLDATRSYYG
RKREDWIAKDSTPDYLIKVEKALNEEKVRVVEYLNPASEPKLRRVVEDEILQKVQMNLLEKEGSGCRVLLANDKSEDLQR
MFQLFSRLENGLQPMATIVENFITAQGNACIDKRQARLDSGEKDKNDDPEFVKSLIDLHEKYLGVIREVFASHHLFQKAL
KNSFEEIINNDVGQFSNAELMSTFCDRVLKSGGEKLSETEVEQSLDRIVQLFSFLTDKDLFAEIYRNQLAKRLLNQRSAS
DDAEKLMIAKLKVQCGTQFTSKMEGMLNDLAVGSDQKSEFDARMSQQGSSLSFGVQVLTTGFWPSYKAPEVALPTQMTEC
MEVFKEWHDNKHQKRKLTWVHSLGNATVRATFGKKSYDLQVTTLQAVVLNAFNEGKTLGLEDLKKTLNLDDQTLKPLMHS
LSCGKHKVLLKSPASNKINSTDTFTSNAKFTCNMRKIRIPMASIEASHNKNRVEEDRSIAIEAAIVRIMKARKTLKHQQL
IAEVLSQLAFFKPQPRVIKKRIEALIDREYLERSSEDQQQYNYLA
>Pinf_XP_002906175
MSLLKASTVNFENEWKDMQPRLASLVTGTPQTLTNEKWLKMYSGIYKICTNPGAPQAEMLFFRLRGLLVKHVEAILMELN
AINGEPEFLNHYCTSFEAFTTGTSYISELFRYLNRYWISYSHCETGQAPVPGVYPVTELSLHIWHDIAFSKLKKRLVKAI
IHIFHAARRDRSECFEDGDCIASTVQTYFSLGLCRQDQMSLYREELEQPFLEDAARSYSTKAITLLSRVTISGYLREAEL
LCAQEQRRCESRLHRTTVIQVRQACCRVLVDEHADRICEDAENFLINNQKEDLHRMFSLFSELANENALISFKNILKRYI
ERSGMEVVQKFQQEETTKNPEGYIEALVQVRNKYFELIKDAFGFHPLMRTALDQACRTFANSHPRLPELLARYTHYLMSR
DKKHGCSRTLLLPGSPRSVLPPLLDDLLEQKIENISVVFCLIDDKDVFKKYYSKFLAKRLIKGTSASDDMEILLIQKLRD
ICGCDFVSKLQKMMKDKMLSKELMDSFTAWLEEKDIELRSEHVANASAIDLHHAVTYHCDVLTAGAWPISSVAAEHKIFL
PPAMEAHTNLFTQFYTGRSTGRKLLWIHHLSFGMIQSHCFGKRYEFLLSFYQMLVLVQFNTAKDINRSDIVQLTNIPEQD
CTHHLASLVKAKILTSNGDTANPRYEINFGFSSRKLRISAVPNSPVESPKVAKTPTREVEEDRKMSLQAAIVRVLKTRRD
IRQAQLVHEVAEMLVNQFVPTTTAIKQNVENLIQKEYLRRHEEDKTRFLYVA
>Pinf_XP_002998391

MSGSLHVDRELLWRKLFAFDHQPEDALTGIQDSTDHLCGVIQTAVAHGRHEPLRQMLLQRLGYWLRSDVVPPFWSFFDAL
PIEIGRSRDVRRGRRHVALLCAQQLTLALQFAEDAFAHCVQIASLFDDHVQLKDGQPTMLQELRTTFRCLVFEDTRTVVK
FEELLLIFFSMSFQKFRGKEKSQTFDSRPIRQTLLQLEWLHVAEPALLRVLHSQIKKVVKSTCGEVYDELFLTEVEQWAC
SELLPWLEEIMQTKDEASTGKWREILSRHVRQEFGSQRIKQLFEIIKEFPDSVPALEDLRLCLERTQQHGELLQEFRGAL
QSRLLQPGANTSAILDIYVSTIKAFRLLDPKGVLLEALSCPVKEYLRKRKDTVRCIVQSLTDEQSGDLFEELRRDNMRII
QHDDDSDDDEDMSPDAWEPDPIEADPTKTSRSRSSDDILRILVNIYGSRDFFVNEYRMMLADKLLQNLEFDTDRDVQTLE
LLKLRFGEDSLQQCEIMVRDIEDSKRLNGNQFWPSLQGDDFVLHPKVSKDIDAFKNAYHVLRNPRSLEWNTSLGSVQLSI
ELEGEEREFNVSPLQATMILYFEEKDRWSVEELATKLEISADVLLKHVSLWVNHGLVSFASGRRELVASVSFQDTRCNDD
SMVEELETAVSSDAQAEEDFKVLETYIVGMLSNFGSLSIQRIHNMLSTFARSGAQPYTKTISGLSVVLGKLVNTNKLEFV
GGQYQLAK
>Pinf_XP_002907623
MSGKNMISLEEGWDQEIKPKAIDVLLDILDKGFDQVKVSPFPPNAFMPIYTTCYNMCTQRSPYNFSEQLYDRHGQTFDTY
LEQKVLPSLEQAHDEFFLQELVKRWTNHKLMMKWMTRFFMYLDRYYVKHHSLPTLDDAGLQSFDRMVFQKVKVRVKDAMI
ELIEKERNGEIIDTTLMKNCVEIFEVMGMKSLDVYQSCLEADLVSTSAIYYERKSKGWLSEDSTPVVFVFSYLTDKDLFA
EIYRNQLAKRLLNQRSASADAEVLMIGKLKLRCGAQFTGKMEGMMNDLAIGSDHHQEFEGFLKKQRESDSNEAALNLEFS
VQVLTTGYWPSYRILEVTMPPLMVRCMNLFKVYYDSKTSHRRLQWVHSLGNATIRANFPKKKWYDLQVTTLQAVALLLFN
EGEGALSFEAVHESLNVTVDVVKRIMHSLSCGKYKLLAKTPAGKTISTSDQFAVNKTFASPMRKLRIPMASLEESHSQKN
VEEDRSIAIEAAIVRIMKARKTLQHQQLISEVLSQLAFFKPNLKVIKRRIEALIDREYLERDPDQANTYRYLA
>Pinf_XP_002998367
MASAGFTSSANGRTGFNRKQERSNSGFGLKLGAKKKKMTIKPFKVTPKLPEAFEHDTWTKLEAAVHAVHGKQMSTLSREE
LYRSVEDMCTWKMAARLYTRLEETCSLHIRERVEDLAQYTGGDMNLFLEAVHRLWEDHCEDMLVIRTIFLYLDRTYVMQT
PHIASIWDMGLNLVRDNLVQRRSLETKLIDALLELVEHERKGEAINRSYLYNLLRMLLSLHLYHADFETPFLMASERFYL
QEGAAKVECVSVQQFLVHAEKRLHEETERVNHYLDASTKKQLVSVVENKLLKPHVATLLERGFETLMEEGRLDDLKRMYA
LFARVEAINDLKTAFSSYIQKNVSKLVMDDQQEKTFVEKILKLKADLDAVLSDSFQANSKFAFAMKSAMENAINVRANRP
AELVAKFVDSKLRTGNKGGSEAEVESLLDRVMVIFRYIQGKDVFEAFYKKDLAKRLLVGKSASFDLEKLMLSKLKTECGS
SFTNKLEGMFKDIDLSQNVMTQFQQHAASRNALEALHGNRGVPDMQVQVLTTGFWPPYAAVEINLPAALLPLKEIFDKFY
SSKYQGRQLQWQHSLAQCVVKATFPSGKKELVVSLYQTVVLLCFNGADSLGFKEIKEQARIEDGELRRTLQSLACGKTRV
LQKQPKGREINDDDTFEFNSKFANQLIRIKINSIQMKETKKENEDTHERVFRDRQYQVDAAIVRIMKARKKLSHALLMTE
IFTQVRFPAKAADIKRRIESLIDREYLERDSSNAQMYNYLATDTQFGNGLFSLGLARLDAVLFLRPISKMPPSKDIKASA
TRPAKFSKAKVTSKTKTKAKAKIREPEKIICFMAASNGNDPNFDTAFSGSLSKIKLKAKVSPTKVKCAASSYTQNYRYTK
AHIVTRHRAAASKSSTSEDLVTIYVLALTKGKYYVGKTARTNVDERLEEHKKGKGSAWTRLHRPLRIVEIIRDADPFDED
KFTKKCMLKYGVENVRGGSYSRVDLTRSELDAVSQQLRGSTDQQAFRNNHCIAASIQ
>Pinf_XP_002998018
MKKLVIKPYRQNMGMDQARAQEIWTSLRTAIYEIFSHNASLLSFEELYRNSYNLVLHKHGDLLYNGVVDVITEHLQSVTQ
QVAAVSDDMLLVALNDQWVDHQIVMTMVRDILMYMDRTYVTQKRKLPVYDNGLYIFRDVIVRHDSIRDRLRARLLLSIER
ERHGELIDRDLVKSVLRMLVDLGVHSNTVYENDFEKFFLDTTLDFYRAEAQSMLDVATCPEYLEKAEQRLNEEGARVLHY
LSPSTEHKLKTIVETQLIKNQAKALVEMEHSGAVALFRDGKSQALRRMYSLFRRVPSTLPEISESVFQYIKTLGDEVVKT
QSNSETALDASQFVEKLLALREKFVGFLSDCFFDDPQLHKSIKQGFEAFMNTNTVCAGYLAHYLDELLRSKKRFEEEMDT
RVTQVIALFRYLQDKDVFEEFYKVLLAKRLLNSRGTSDEAEKLVISKLKAECGYQFTSKLEGMFKDMSISKDLMELYRKS
GHDTRGTGFGIDMSVAPMPLSVHVLTSGFWPTEMAPMCALPLELVQMTQAFESFYYARHNGRKLAWMANMGTVDVRAMFS
AGLEDAKRRHELNVSTYQAVILMLFNQRSEWRFKELLERTRIDVKDLKRHLISLCTPKYKILIKSSKGKRIDEETDTFSV
NDSYKSKLLRVRIPLVSQKETSLLPAVASSTNNAADALPPTVAEDRKHLVEASIVRIMKTRKQMQHNQLIAEVTRQMTGR
FTPSPQLIKLRIESLIEREYLQRSITDRRMYNYLA
>Tgon_XP_002369193
MQKEVSGENAARKERKEDVFRREEMEGKTIKGRCGSISPRTKPKAFQTVLNGSNFASILSTYQGPPVTPSDPQFTASLSS
SSSSSAVLPSLPSFSDARSCAPASSQTPPLTPSSSSFSTSSSSRVSESTASDLHASLLKAKENLPLSLVLQAPSASLSSF
FLLLPALFPLSTASSTPPPSLLLSAAVVDYIDQTLRETPRASNVHALTADSQRARDGTGRISRVEREAKKRDCITGNTGR
LGTSQGHWLASLCQVVQHLQDRETFCSLFRLRLARRLLSNALRVPDVPSCRRDDFSSLVSSSVSPRFSRGTSLLSPPTFG
GPSEPGDSREQLRKEHVEEKEALREKGEGFNEREGRDEHVTSCLELDNGPSRRPKRMELPRAHAKSRNSCATIVHPGTGE
DSARAGAEEGEAKTTLKDHGERDKQLEARETERLNGDPECLKRRLVPSTSNAYTFVRSSRHFGRRGEDVEPCKVRRTYKE
INEQGNTGEGASSQLEARTPEEVEAKEAVCCLNSEVHLRGEERRSNGRVDTRSPGSRSLEQREKARLKTEQRVAQAVLEA
AVGDFSRQQESWKLRQMFEDLAVAVRVKEAFEQGQRDREERREKEDRRNTQTSWKPKLHAVQQEETTCTAEERGKSQCVA
MHTEAGERGNLPRNSMAPLNPRPGRNTQLLASASTVTSFNSSVPLPSSNVLFHPLILTQHAWAHALPASDLYLPSSSFVD
ESGRSASVPSSSGSTSTLSSSSPTPARRGASPRAATVAHACRPLRDFQEQLIAIGQTEPGGTGLLDPGVEAKKNDDAKKP
EKTRDYQVLSVMRVGDLWLPRYLGIPLKKFTEFYKARFAERRLTWHIRLSRMQLYCYDTNATKNPKRTPNAQNVASSSTL
LNVPAVIGCILLLFNTRTTLSMQEISTHTGLAEWEVKKVLVSLLAPPQQLLLLLATDVKALDVAGKGHCFSSAEENQDLS
RFVSTGGGRDKGEIDEEKEMQGNGQRLSCEAEQRPQEKKAMKTITEECRVASPSVSFLQLWRQMRENAETQFSLNEGFFA
IPRGNCEVVDIHPIEVRMRVKPGT
>Tgon_XP_002368366
MTATRACYLPSSSSSSSSSSSSSSSSSSSSSSFASAEERMPPAMGVARAMGGPQGVVDLEAGWAMLRDCAIRRLEHFLRTG
EALGRRKDGKLCIFTRKEYSDMYTTVYNMCTQRYPNNWSAQLYQRYGEALASYVNREVVPRLEGLTEEELLRELLHRWKN
HKIYVSWLERFFVYLDRYYVKLQSEEPLHHKGILIFKELVFNRVRIPLREAILRAIQRQREGERVDEELLGDVVFMYIGL

DANGLSLYQRELEDYLLPESRDYYARVSASWVVNLSFTDYMHHAQEALLAEQNRCAMILHRSTKLKLQAVVFDALLSARQ
DQLLEKNTAIEFLLARDRRQELRLAHRLFTYVDGGVEAIALTFKNFVTSQGNKIVDQRLAQLEPASSPPSASPPASPSPA
SRPSGSASIAEAQQTDAAFVQQLLDLHERSKKVLEECFLLSSPASSSRSLLGVSPQQKLFRPCEETPWSDAGRDSGALSS
APDPLFQKSLKEAFEHFVNRDMGRQSFAHLLAIFCDRLLKKTSEKRSEEQIESMLVRVVEMFNYVTEKDVFAECYRSQLA
RRLLHETSASEDLEKSVIGKLKLKCGAHFTSKLEGMLHDLNGAADTYRKFLAWISEEKQKRPAEAEAGEAQASVSGENEE
KTVEKLLERVSVSSASAHMQTGATGHGVAFVDGIEFSVQILTTGYWPTYPTAPVNLPASMQLCQTVFEKFYASQTQHRRV
TWIPALGTASVSAMFQKRHDFICNTYQACVLLLFNWESAHQRSEAEQNGPRVDPQLSLSTITAALGLDDATAKKMLASFF
LGRFKIIKKLSDDAYAVNSGFTCLNRKIKIPTPVQEEVQSRERVEEDRSIAIEAAIVRIMKARKTMQHQQLLAEVLSQLS
FFKPNPKLIKKRLEHLIEREFLERDAENTNLYRYVA
>Tgon_XP_002367473
MRSLASAGNAGRPPGGPAASLLRPSSSSSSSSSSSSSSSFSSSSSSFSFSSSSDFGLFGHKAMTGDEAARRWEFLRAAFQ
HIFAKKAGALSYEEVYRYAYLLVINKRGRLLYDGARQCVEGHLQAVASEIAEKDDDDLFLAALLEKWRDHRVNMQMIKDV
LLYLDKHYVEMHHLTPSFSMGMSLFCSTVLQHPNIQLRFRHLLLDRIRKEREGHQIDRMVMREATAMLSQLRLHAHRPVY
TEEFEAPFLAATREFYTREAADFIAYNSSPEFLQKAESRIREEARRVEEYLDLETTAPLRALMEDVWLGQHFKTLVYNPN
SGCTHLFQADKIADLARMHRLFSSVPGALEEVRQVMKESITKYGEAIVRDPEKTRDPVTFVQNFLALKAKFDQIVVRAFA
ESKEAFGALVAAFENVLNKDTRAARFLSLYLDDLFRKSMRGLSEAEAEQKLDEVLVLFRYLRDKDIFEALYKQHLARRLL
GGRSASEEEEKKMIVKLKAECGQQYTSKLEVMYRDMQASDDIMRQYHAREDLQRDTACPVSLLDARLLQLGRAIARRAGT
FASASGSFSASALHQPVDDTVRSPNDPNASDSPESPAFTTPLPSSSSASSSSSASSSSSASSSSSSSSSSSASSFAPCPP
SSSVWGCEVPQGRGEFEFSVRVITQGTWPVDAQTLISETQLLPSLLAEEATRFEAFYLGRHNGRVLRWNLAQGKASVRGQ
LLHSRHEFECTTLQMIFLLAFNFFPSTPVSVSDLLQPFFAVSSSLSRAELKRHLISLTTPRCKILLRASSGAGAANAAEL
KDSDTLTVNLSYTNKLRRVRVPLVAVPAAAGSGEAADAWATPHLEAQAGADVPSSVEQDRNYLVEAAIVRVMKTRRRMIH
NDLLVEVTRHLAQRFRPSPALIKQRVEKLIEREFLERDAADRRMYNYLA
>Tgon_XP_002364830
MPSATRDGSLWPPESSEVVKVSIQGLRTEKPEPWPETFKECEKQVSVLMEDVFERTGPLRNRQALSLMIEELVLEGQGAQ
LLSLFVTHVDCRVAKILSCIYGALSPDLAFLHTVADGWTSFRRALHLVLQVFAFLEQHFVAYSNEGSLIDVSEALWLSRQ
NELGKDFEASLVNALLRAIELHRTGDVAWQDDIRTVTSMLSSLGVYHDLFERRFLEASAEFYARESAELSVALTVGEYSS
YAERRLREEDARASAFLSEASREPLLDLVRHHLVGEQVDVLSAVPSLRQLAETRQTAQLGRLYTLMSQVGQLDLLRLRFV
DAVRESGCELLRQLRESANSAEKKDRELKGREFTAQLFALKDSHDLAWMQAFNRNPQFSLGIKEAWEKFLNQDVESSRKI
TRFLAKRCDCLLRETKSAEDLEKEMAKVMVIFRYLDGKDYFEEFYRTDLCKRLLTRKSASDDAEKAMVKQLKDECGQQYT
HKMESMFNDVHLSRQTMALFNADSASQEEVAKTGVSFDVATCAAGVWPPSAPTEKLILPPIAETLRQVFSNFYKAKHPGR
NLSWISSLGACEVRANFSVNCFGSSSSTSSSSSTSSSSSTSSSSLTSSSSSASPAHAQEQSEEEDSPGSVHARAWPDYLF
KVSESQAWILLLFNERDSMTVQEIAEATGFGSDELQRQLLALYVNKATRILLRYKDEHEERYSVNCDFQSKLRCMHVAQI
QLTTHPKEEIAKVEARVSQDRDHQIDACVVRIMKAKRELRHSLLVAEVSSQLSFKCDPAMLKKRIEALIHREYLKRDDSD
HNIYHYVA
>Pfal_C6KTD3_C6KTD3
MDPTAVNNYPVFYVKNKDFLNVDNEHIRCSLKRFEEYIHECFSILPYNKIKIQNEEKISVQHFCDVLVFNQCEQIICAII
EKECNEKTKQFFEEVEIRIKNEEFKNGEEEFLKLFVSIWNNFSLVLIHISDILQSFIAYTKNMCCNFFDPTQIFNYLWKK
YINQYVIIKNMFISSICNYLTYDKIYCETYFYEYVCVNICNKYATKRYNLKKNQINYIERLQNIQKNYQVNNEATNKQTN
KINKKDCINKKEIPTENITYKTKEEEHIQRGDHNNNNNYNNSYDNNNYSDDIYNSNPYLDDTNHEYKNTQKKKKINDKVL
SLSIKISDILNLYDDFERIYKNETYTFYKEKIEKYKKDMETLGYEECNFDFPIIVENYLCHEQIRSRKFLKEHTEITILN
MLKDLLLVKNRDVLFKDSYIKYCIIKEKYDPLRSVYLYSLNLDDLENFSNIFFNVVDNIGTELVNDVINKRNNSSLLNNA
IIKLINFKLNVDRIIIMSFRYSSYFMKKWREVFEHFINKGIQAETYMPIILSMYLNNLLTMYNACLRKLKKYYILNKKIK
NNRSLFNNLLFEVDWNNYCTQPISETIDFYDISTDSDNMLGIKKYLRKKRKNTKKDLINFEHEEEYVKRKHMDNNIHNND
HNDNIYFNNNLYFNNDYHNNLYYDSLNINKRSDEKIFFHLLKIDKLFKKYHIIGKYIMNMITVILSLFRYVSDKEKFEKY
YRTYMCKRLINDSSFNIILDVKVFKTLKKECGAQFTKKIEIILKDMKFTSKTLMKFYKELPNNVNHFLKRKKYAVNIIFN
ESWEYVNTENNVIYPPMIKMCNEAFFLFYQKYNKSKNIKFLPLLGLCTLRVHFKNFKEEGSFDLGKRDDNNDEKRDDNND
EKRDHNNIEKRDDNNIEKRDDNNIEKRDDNNIEKRDDNNDEKRDENNDKKRDENNNYSDDNDDDLNNEDSSSTYNKKGKK
KIYITVTILQALVLLLFNKKNEYHINEIKELTGISTENIIRYLKTIYTIGETHILIYDKANEILKLNFSFRSKKKFLIVN
YNDVLYKDDMGPTTSLLNEDNEIEDKTLHIDAAIVKFLKTNGKSSERKICSFIKDKMDISSNEQIKNRISSLLSREFIFF
KNNNYHYEL
>Pfal_Q8IAU5_Q8IAU5
MDIVNINFESGWKIIREEAIEKIEKYLENEHIEKNKNLFSATEYTRLYTVVYNMCAKKNPFCYSKEVYRKYGESLSMYTI
DKIKPLLKNSDELNKTKILIDAWFKYSFYTKWMNKFLRYLDRYYVEYNSSLCLSAYTKNIFKITLFNELREDIKNIIYEI
YNNLRLQEEIDQKELFCNIVELYKELDNESNEKMYEHDIEKKIVENVNNFYKKKAEEWINDYPFDDYIISIENAIEKEYE
KNKSLNLNDDTCEKVTNIIVKILIYEKLNTLIDNKNNIFHLLKNNNLSSLRRTYILFSYFPEALTGLKKIIGEYIKMEGN
ELKDKYVQLSRKMHMSKNVNNMQNVDDNTMEDNDNYIDISNDDNNNNDNSNNNNIMNTEQNDDIILCDYIEQLIKLHNHY
DNIFKLSFFNISNKNIDPNFNECLKDYFESFVEHEDEYFSTVKLLVIYTDNILKRDLNNENSVDEKQKIHMNKEEKNDEQ
TKFLMKKMSEIVEIFNYTSNKETFFEYYRVYLANRLINNIYISLHIEKKFIEQLYYLCGSQYTSKLGGMIQDLINNTNMN
NKFYDHMKNVFNNNEIVNNNNNNSIYNDHFSNYHNVKDFFSVKILNKGYWPTLEKIHIQLNEPFNKYIQVFEEYYKSENK
NRKLEWIYELSEVILHYEFNNILYNLYCNVVNAQILLLFNKYKYINYDIVKNELQIDLKSFTDHMYTSFYHFKLITSNDQ
IPDWTNSNFYINNNFSYPNKNVYIKKTTALLSKEHEKTKEDRTMAIEAAIVRVMKMHKKLFYDQIFDYVKKSLSSFSPTN
QVIEKKIDLLVEREYIQKEENSQIYVYIP

>Ptet_A0CT81_A0CT81
MKKIIQENLSPEEAKTLWKYLDDACYLIAEHQTKQLSFQELYNHAYKLILNKFGDFAYTQLQVSIHNIVDKLIHPLNISN
DDQLLNDFVKAYEEVQYFSKLLAGILLYMEKSYIIQKSLKTIKIICNESFKTKCFEKDQNLASKLLNCFLAQIRNHRNSQ
VIELFNLKNALQILVIIFTLLQIDINQEQQIKSKDYSYTIEYRNNDDFYKYFLERKLIQDSTRYFKEESQLNLNKMTIEE
YILFVEKRYQNEVERVQSCIPKISHQKVLDSFIKNYITSNSQYISQGLQDFIENDKTTIFIKIFQLFVKSEEFDQFIKTL
TQIIIDDLQLLNQNDNTLQGFIKLYEKIFSMYDKIAQFGNNQHQYKFHKAIKDAFEQVINKDNFIMFELNKYFDYIMRME
SLRDDEKRVQIEKGFLIFKLVQSKDEFEQIYRRHLCVRLLDQASSSSEVEHDLLKKLKLECGSVLTHKMETMFSDLQRSN
EESQKFRQKLSQSQRELIDLDVLVLTSEQWPIADYQPIIVHNELLQWQQQFTSYYQSKNQKRKLAFNYGLGSVSLKATFD
LNCKKDFVCSVLQATILMHFNKQRIYKLDELIKLTNTDQEVMQSELENLLQFKLLIQNQEDNSLQLNYKFQHRSYKIKVQ
PKKQALLFNQKSKNKYQEYKIDQKEIALDRRVYLESLIVRVLKTKKQLDHKDLFKYIDKDAKTRHFPIEIPFFKECIENL
IQKEYLIRQEGQLDTYIYKA
>Ptet_A0C548_A0C548
MVFKILTDSPIQLENVPYMKIFYELNEIIDGNILKGKIYDAVYQDLLKVINNEDKQQTISCIFDKKEYYEQKLRDLFNSN
EKIKISLSSAIESAIQIKVNWLSEQLSSYFDQLLIKKQENKDFEKVKERIKQSLQLMPYFSAKDIFEHFYTQRMTRRLLL
ELSISQELETEILINLKQQCGDQYVRKAEEVLKEYKKCYQFQHAGVEVKLVVISQNSWTLKQDQIPKFFLKEQQEYFQED
KKQDFKQKILFWMLECSNCSVKGFKKYIFIVSVVQAMILDLFNKQKSYKASDFIQLSGLSKDEIIRNLIPLEKEKILLLN
QGVYSVNHEFESKKTHIKINQMQKIEKKEEIEENTQKLLHDRKYVIDASIVKIMKRDKQHTLQEIVNLVLKDLGLPLKAV
DIKQQIEVLTEKEYLQRDQNDMSLFIYLA
>Ptet_A0DHG1_A0DHG1
MIIENEDSDLEIISKYFKNEADEFEVYEVLSHCNLKATRFLSYHHRQDEILELISDHYSQYKEKIDTLMKQNVCSISSIE
EDSILFDMTIDLWVKFKQKSQCLKDVLKYLDIKTPVQNYITNILLKLIKVEVENRLQMVTLLEIKRFRQVIVTLSNQQAI
EDPPLQLLDLLILLPNYQQFERRFYEQTQIYYKEFILDTNPSQSVQIANIIFQAEKRLSSKFQNEFQLRLNELLKQLIFN
QVNMTDLDSLYFNTEIMLHYVRFAQEINALPKLLSALRKHIYKEVICILQQQPTSAIENMLNLEKTCQTLFEQLPMEHTN
ATIITHAIEDAVNENYEKYSFTLASVINLQNQHSIKKYKHLIKYLTDKESFQNYYVKFLIKRLLKEYNQNELELLNELQP
YCNSEWISESLEFIKNSKESQNLTNQFLQKLRQKKSPIQFHFHLIPKSIWPYQIENLILPSPLNQFADNFIKNQSEDLLF
QKKKIIFQSKLSKLNLVVKIDHQFELELPLAQGLELLRFNAQKSINQISNYKLYNGLIVRSENNTFKINYQYKHANQIMI
INEEIQIEMEKNQQIISDYLYVVDAAIVKIIKQNKKINSESLIQATFDLIKKEYPRFIDLCEYDIIEKQIVSLLEKEMIK
QRFDDFELL
>Ptet_A0CBV9_A0CBV9
MNKKIEGNFEKQKQYQKDLQDIQDKCKKATTDIRQRLLDGCAIKLKNEDVMYIYNKIYRLTEDAGTAQALKNYSKNQNNR
GEKVEEQLIQYFKDILNEFIADIYMRLNEFQGEKESVLFIVSRTYHDYRSYSYWLYKMFLYLDKFAMPHVGTSLCSTSLK
LFKEGYFDKCNKLILNTILQFIQETRKNGILLNKQIKHLINLFSVMGSREVEFTKALGEYDYICKKQEDAQKYYKDNFQY
YLLLETSKFYKDEIEDKQKFTAPEFVSWGNSIFKLELDMCIECYPKSQQAIENKLKIILVKDQATRLVDSPTGVGYMLQY
DRTDELKQLFQFIFRTKECITHIAKAYQTYFEQQGQLINNSIEQEQQQVKDGRQIHEAELYFTRMLEVMEKAQSILKNQL
QDEPEIQKSYSGAFMLVINKNEKSPMWLAIYTDIIIRSEKGVNEMETDKRLSKIVSLFQLLYQRDVFFRHYQKFLSNRLL
NQQLQNIQLEKQLLQKFKGETGTNVLTQLSSMINDIEQSNRFASDQQISKDTKFDLNVFLLSQGCWPITTILDNVIKPIQ
IQNVLENYEKIYLGKHNGRILTWCFNMGQGELIYKLQADKYYLNVNTMQMIAFLLFNQATQFSIKNILEMTKIDKIDLEN
SLIPFVCLKIIQREKQEIEDFSDENEILKLNLGFNNRAKKMKLLPNPKMQPKRISKVKEQNQEELQQMEQINKQREFVVD
SQLVRLMKSRKTIKHHELLENCQQMISIFKPDILFIKKRIENLIEREYIRRDEKDWNIYHYLN
>Ptet_A0BJU9_A0BJU9
MQNDVKLISAYETAFTTAFNEIQDSHQYLAIYADVSIRSEKGVNEIETDKKLNKIFSLFQLLYSRDKFFQQHQRLLSSRL
LNQQTQNINLEKNLLQKFKGETQTNVLSQLQKMVNDIQQSYRFATDQMIHKNQKFDLTVYLLSQGCWPINNIQEQIIAPQ
EMLSSLNLYEQIYLIKNNGRILLWTYNLGQGELNYKIQNDKYYITATTFQMITFFLFNKENELTIQQIQERTQIKIIDLE
NSLIPFICLKVLSRQKEDLDEFSDKNEVIKLNLNYNNKQKKLRVLPNPKMQPKRQRKVGELTQEEREYEEQLIKQRELVV
DSQLVRTMKSKKSVTHSDLIAQCAQMITIFKPDIKFVKKRIENLIDREYIKRDERDCNLYHYLN
>Ptet_A0BLH1_A0BLH1
MENLIIFDQLKYQVNIKINKQKIARMLPSKTSLYENIEEACTNILEKNIDVSLQQKSKINREIDEYIKNQNNDQQQQEAK
MFIKVALDCIKKHYAKNILPKFQDKYKDALYHVETFLQIHTTFVKSMKSFKFLLYQAAQNLEQESIPGFYSSLFDNIFVQ
ELQMVFKSVLFKCVVDMIKTACEEEQKQLKISDLLYKFGNVMSQLQKSTEELLDFKIEQEFKQKIFDYFEEKYQQNYKSW
HQSLNTQMYLRKVQQQKEINEKVLKFGDKTILEQVNRILNSHLLTYYKPYLLADTNPNNFEHLLNEFHKEPVLQLQNLKL
IGDLYGSLDDHYDQITTNFKNIITQEGQAIMQKKNHQIVETNQDQIFSDPAFIGQFYSLYQKYSLLIESCFAQKSDFIMA
LNNAFESFINLPIGNHEFADYLVTFIGEQIELLRNSPKNATKSPEQIVKLFVFINQKARFLKLYKISLAQRLIKYQRQVE
AKKNKQACTIEMNIVEEMEKKCGAQDLESLKIMIKDFQKTENEVTKIQEEKGLYKLQIQPPIIINKQHWPEIDEIQLNLQ
SEIITQQKEIIAKRQQQKTLIWLDLISYVEIQSTEKALTFNLSVPQAVILLLYQYRNDILDVNRISSLTGLKEQYVIHNC
KMMKDAKILDEQIVNDQICYQFNENLTKQKKGKSKKIVTETYFPQNQVEQVVSVYDKDLAIEAAIVKIMKKQKEMQFSDL
VIQVQGHMKKNQNVEIPFAQIKQMIERLQRNEYLERDANNMQLIKYK
>Ptet_A0BJA6_A0BJA6
MSNTKDKELFESYTNEVCQLMRSDKNVQMSKARYLPLYNWTYQFKNDQVMGLKQIFEDWLLKYLNTQLIPELLNSQDFIS
TFIFEQALFKSLLRYLSILFFNYDAERGSPQLYIVELGLLNFDKLVLSNDTLKQRYKESFINRLNQIRLSNNQNQKQQLL
TFLEIFDLNTLAQNQRLEITSIDQQVIIQVRQKTPKMVNLPSPQNVCDNFLFDVIQEATKKFYSELSQTWIARGSSTEYV
KQAANQILIEESLREQLYQKFSRSNDIMKVFICEMLEQNVDQIIQNPQDGLYQQFLQLRNSSDLSPILKLYQLYKNTPNY
IENQLKEFQKFVQNQVTQMLTESEEPGQPGQQQQLQQKQAQQQQANRSLQIHIAQIDNLQKTYEYCLRLVQNCFEGSHRF

RNNMELAFKEKLNQADRFMDKLSIYIHVSLKDKLKETNNEEDRQKFEKFNKNILAFIQFINSKGKLFQKITDNLKNRIFR
GEIKNFGYEEEFLKSLRREHPEHLPSDLLTVWKDYKYYRNLDNEIQTLLSNKKLLQNAQAQFTIFTRTNSLKEFNTQNKK
DNFIPPKMIKDAISQMELFYQTKQQTEKKQLVWNFRIGQTLINYKFGPSVQQVGKIIVSNLQLFVILYLKQFGSRTKSEL
LNDTGINYDEELTQQMENLLDLRIIVENEGKFYLQTDQTKLKVQLVPNRPITLVPKKVGENTEDQIMLKKERDLKIQSSI
VRLMKTNKEMYYHQICAGVQKGLLGYYDFFSQDILAQIDELINSNYIKRDERINNKFLYTPV
>Ptet_A0BKZ0_A0BKZ0
MNIFDILNSQFVSQTQYDTIIAQQKAYQQYYSQLQSDPNKIAEIWPIVRQAGILDICYENYKYFAKENKLSSLEPIQKLM
SSALEEEGKIDYLADFVKFMGIKQDYSLFDQVRTVVEKYELEYDIQDRLKSLNNLNFKEQYQTITIELLKLYAEKVYSEE
GFLNELLEFGAALESIEPLAKNIIQQVHADRTIKNMFEAVLYYPDTSDHLHEFKSSMEVTKMYANMDSCLQQQMKKRLLI
SGVNTDTILKFYVNLLKVLQFVDSDYMVFDKVTKPIKEYLLQRSDFLRCIIGILTQQNYSTDKVMIFEQQDSSDEESSQE
KDLTSVLVSLYGSQEAFISEYQNMVAEKILTPKDFSLEQEIANIELMKIRFGEQSMNTCSIMMKDIYESKRIDTNIQSLT
TQFNMLKPLFLSKTFWPISYEFKPTFKLPPAIEQLFNDYQKRFEKIKTMRSLLWHHDLGSVTLDLTFDNGDFEFKCLPIH
ACIIGYFNDDDSKGLYSDALAQQLQMNHEDLKRRMQFWVQKGVIREQKGENDVICYTSVKIYVPQSCDDLLIEEIPEDLF
VSSTNFLQSNQLIQQISHNLIELLKQTGTLKIEKIVQLFKTTFKSEFSTPITENNIKEAIKILIQKGKLQGSEYIELKQ
>Ptet_A0BJV0_A0BJV0
MLKDFQNRFDILEEKCLKNARLLKNSLFSGAQFQLPKDEIMYLNREILDLSSEWSIHFPRTAKKQSKEESNLGIEFKMIN
LYKLCINEFVAEMYEELKKTQNQKETTLQLVAKHYHDYKAFSYWLYKIFYHLDTNTLSTLGADLHSLSLETLKTVYFKEA
QEKLFRTILDVLLESRQKQLLLDKKIKSLCELFVVMGANTVKLKYEKEQGMYDYICQNFLETEKFYKENFEKRFLEETQK
FYKYQISERQNLGVIEFVRWADRVISLEEQMCLESYSKSFDQIKQLFNQLFVKEQAERLSAGGLGELFNWENYTVQYLGR
VSVYNFLLKNSKFTIQLLFVK
>Ptet_A0CFE8_A0CFE8
MNTFEKIRSGLQVKINNLRDHIEKVLKGEKKVYNTKETMTFYTEIFNYIVQGEQQGQKMYDLFKGYNEDFLKESEKIARQ
SLKSDKNFIEEILYQHEIYEIHTRILQSYFTYLDRSFVEVLNNTSQYPNLKQQAKKQFYEQFYNKLFFSIKEEFKAKLKL
DRILLDKESQLLLQRVYKIIYGMKTFQDIGNNQLIQKEIKEDSKTYYQQQYSNFQQQSFLDQIKSLVQFKQEELGRIRLI
FGCEQSELQKELIDLFNNYIINSYASDLNSDNNDTLKNIIIQDYYEQFGQLVKFFDHFKSNTHIFVRKFQEIIIQQGQEI
IVEQKNRISQTKIKQEKEDQKSKFLEQLISLQQSYKKLIYDHAENNQFLLANLRVAFEMITANAEQHFIMESLLQKLHTF
TLKRIDIGEEKQLKEVMQLLECFQAKDLLLRNYQNKLAQRILTLFDYHSDFDKQIIDQFRKTYGPEHTKQLESMIKDYEQ
QKNEEKITISDIEIQAKILQKEYWPEIRPQLNLDNILILNQLKAAYREKFNSQQEKNQLIDISWQDQLSMVELMFRTNQE
YKVIIGVVGGAILLQFNHQNNPQTSEQLAQSIGVNNQYFLNQIKLLESKKLLTRDGQAYKFNEEFSNPKLKFRVGIILEQ
FVEYQYLIEEDIQGDRKIAIQAAIVRIMKGKKTQTFQQLIQLVKEQLKMFKPNDIDIKDVIESLMNQEYLKRNQQNMNEL
IYVS
>Ptet_A0BYN1_A0BYN1
MKKIISENLSPEEAKTLWKYLDDACYLIAEHQTKQLSFAELYQHAYKLILNKFGDFAYSQLQISIHNVVDKLISPLNISN
DDQLLYDFVQAFEEVQHFSKLLAGILLYMEKSYILQKSLKTIKTICNESFKIKCFEKDQNLANKLLNCFLSQIRNDRNSQ
FIEHFKLKKALQILIDLNQEQQIKNKEYNYTIDYRINDDFYKHFLERKLIQDSRSYFKEESQSNLNKMTIEEYILFVEKR
YLDEVDRVQHYIPKISHQKVLDCFIKIYITSNAQHISQGLYDFIENEKTAIIIIIFQLFVKSEEFDLFVKTFSQIIIDDL
QILNQNANTLQGFIKLYEKIFSLYDKIAQYGNNEHQYKFHKAIKNAFEQVINKDNFIMMELNIYFDFLMKKETLRDEEKK
LQIDKGFLIFKLVQSKDEFEQIYRKHLCVRLLDQTYSSLEVEKDLLKKLRLECGSVLTHKMETMFSDLERSSEDSQKFRQ
KLSQNQRDSIDLDILVLTSEQWPITDSQPIIIHTELLLWQQQFTQYYQSKNSKRKLSFNYGLGSVSLKATFDQNSKKDFV
CSVFQATILMHFNKQRVYKVDELIKLTNSDKEIMQYELENLLQFKLLIQNEEENSLQLNQKFQNRSYKIKVQPKKQQSMA
LCSKQKVKCQELQLDSKEIALDRRFSTTKKQLDHKDLFKYIDKDAKTRHFPVEIPFFKECIENLIQKEYLIRQEGQLDTY
IYKA
>Tthe_XP_001025745
MRTIQIYERQIAMSVFQPISEEATKYTLNQATKLLEKVTCSFRTGKIDENSFYEFFKLSAQIRSSNYFKLILAWHYEQIL
NFVNAIKADLQSNINLEVEEQIKLISKYYSCFQIFVVQIKQFLLSIEDFLYKTQLWTTVFKKSFEYFRKEFEGLKEIMIE
KIFKLIEKARTNYVIPNEELSRVIQIIIDLDFKKDIKIERDLKNNELRVISDSSTDNSKNYIQLFKNPFLEKTKNHFQKL
ASLWAGMNDVEYFNLALQYIKREEEICYPLLINNTKETIMDALQLKSRLQLYYQQKIFKPVFESAQKKITDYQSEEKNKA
YSKKNFRDLNQKIATEWVHDLIQTKKYVEKQIPNEDLLFNLAHQEAIDKTFQYCLTEFKNASQLLLLYYLSDEEYERRQA
DFNTIFVYMQSKDEFLQYYQQALCKRLICFNFKSIDAEYKMVTQFKNSIGVHTTVIRFQNMLTDLKINEQYNKEKFQNDV
LKIYILSSVAWPLLRNEQDNIIFPSSFVGLINQFTYQYNQIYNKRKIYWLLNEGSAEINFISDNMDNFGGKYILRTTSYQ
MLILMKMNEKDQFTVKDLMDQTGINKYSFDSNLKQLIKLKILNCSENDKYTEFSEIKINTQFYSTKKSILCLPHASVSTQ
QDDSNISSIQTTVEQERTQIIKAYLVKIMKIRKVMAHNDLIKECVELIKKCSRTFEPCFLHIKNCIENLIEQEYIKRIEN
DYKKYQYVQ
>Tthe_XP_001019597
MENQQMSDLQQAISKIEATQQKLIDFLSSGQDIQISNKEYMHCYEQNREEMQLFDWFSQQMEQYLDNQFQRKIQVLTGEE
YLEKVKEEWQNLQLYLFWLKFIFTKFDQSYKALHGQSLSGKTFTLLRERIFIRNKEIIYQIIFDFLNKHRDDLQVPFQLI
VKILQYFVELGYAENVQLVKYKDLQGFSKFGYVSQQCKEDLSYYREHFEQRLIDNAKEYWNKKSAQWLEDFSVPEFVKNA
KYALEREKERSQQCFKWSYKLYMDTCEKEIISNNAKELIGNKSSGLANMIKQDKYQEIQELYNLMNYRREDTISILSSEL
NSYIKTVGNDINNNEQLNKDSKEYIREVISFYKKIDGIIKEQLNKEQDIQMARDRGFREFLNKNDKYIFFLAKHCDQELR
VGMKGNSETEIDEKLQDIIDIFLCFDSRDSFITHYQKFLALRLLQGNTLSDENEKKLITLIKKEMGKSTTNKLTEMCTDM
VVNQETMNDIKKKMKEIDSRKCDEIDWKITILTSGHWTIQKDILDVPNELIWIADTYIKLYKEKHKGRKIDWAFSQGNAE
ISTKFDKKYTLDVTNYQLLILLMFNKADSYTFKNISDVLKIPLPELHLNLITLCKHKLFNRDSKNDLSEFKDDEVISVNT

KFSSKIVKIKMHPNPELKKALKNQEKTVNVNENEIEGELKKLREHQLDACIVRIMKSRKTLALNDLNAEVVKLIGHIFKP
QPQQIKQRIESLIERDYLIRHIDDRTKFIYKP
>Tthe_XP_001031380
MLQPKRDSLQLEQLENKFEKHMLKEYKDEFYSILNLDRTESEINTQNFIKALLSLNKKYEIHMNFLQDLDLECEEMQKKQ
NLSVNLSQIFSLNLIEIANLNENIYAFYRKELIKFMKFVLKEHSSQIDQSLITESRRTLIENEPENFQVDIYQNFFQISI
ELGFYTKSLQILNEVLVKDIQEYIESQQEALQNNTPILKRCISYFSNFHLNFLQVIAQSVKTISSKEIQLEQLQGELKIL
QDQLLSKIYEIYGKICIQNIFRIVLEFPESYELVQELKEIVEKTNLLNQLKIANTWSYNNKHFKLIHKLSLNSLNYRSKS
SYFGQFNISICHNQITNQINLNQFIEKVTFPIKNYLLKRNDTLRCIIEHLTDDKNTNKLVQERLKIPSKFQSYDLSSDED
ENEAEKWEESDLLSILVNLYGSQESFISEYQNMLAEKMMGTRNFDIDEEIKNLELLKLRCGDYSLQTCNIIVKDVKDSKR
IDNLIHSYKKPINPEASLKNPLLSIDQLHCMFVSKGYWPINYEVEGFPLPSYLEPLFQEYAKRFERNRAMRKLIWHTNLG
HVNLTLTFDNGEFEFKCLPIHAILIGYFDETSIKKLKLYLEQKYFIIKQIGFNPEKGVSSDFLSQETQLSQNQVKQKMAF
WVHKGVVREFKKQVPKAQSSNFKNFQGYNEEGAENADNDVNVYFPVRYFIFKIDLIYCEDEENEMILRDQSYIGFENTQF
IKYIENLIISILNKTGPRSASQLHCLLKTLYKTDLEYPVQEDQIKDVLRNMSQKHKLIFNGEDYSLPISQEKVQ
>Tthe_XP_001026152
MQIEYPLTSQVSIPNSFSFDIEAEIEKPNQNYFEKNWLEIEQFLKSIYESEDINCYCFQHFYDMISSICDNEFDELLYKK
LEEFYRLKFNVILNDIALQPEDFINKIEKEWIKINRCLIILSEIFKQFEGSYLYKTKTPSFDNFILKILSDQFNQEFNSL
LDAIITSLLDTFRNIRDQNPYNHEQLKHLMGMIIKIGSYENQFKSHFFEQSNKYYLDLQQKHRANFDLRVYLQEIEHRLQ
QETGLIEAYLSKATGKILIDLIQKHLIAENLETIFSNGFDDLLNQRDYQNLQRLFHNMRNIDKLDFLKKNWNQYIRRKGE
EIVNEENQDDITQHLLDFFHDLDIIIQKCFENTNLLKQAKNYALEHVLSIKVNTIAELTSKHIDTKLKKQNKTMQDHDQI
EKDVDDALELFRYLPAKDIFEAFYNKRLARRLLMNLAYSYELERKVLDRLRSECGDQYTMKADEILKDVNESKQLNKDFN
DYLSSQGLDYNKKNIFSCIVVSSSAWPMKNQQLPILFEPFDKFQKEFKKFYELKHKGVCINWQHETSTCDIQGNYNKEKY
IFQVQLIQGLILLCFNLKNKLSYTEIHNLVQIDEEELKKNLVSLYAMKDTQKLLNKSGEAKRVDETDVFEVNEAYQSKKK
LIKVNSIFKKETKEDVKETTDRVLTERGFVLDASIVKILKSKKNIYHQELMKELFNDLMLPINASEVKKRIEGLIDREYM
KRDPENHSLYHYVA
>Tthe_XP_001014520
MSKAQSSYKPLSEEATKQVLNNANQLLDRVRRNLKTGEAIKISNEEYCKLQSGEMSVVVFELDMDAKIFSEEAQMSLRIE
IRNTDQFNLIGNWHKEQILNYVNSIKIDLDSKTNVPEEEYIKLISKHYYDFQILVMWIQKFLMSHETFYFKAQLQTTCFK
KSFEFFRDEYFLGLKDRLIEKVFKFVQKARQNVDVPKEELKRVVQIYDDQDFGNDIRIEEDRQSHKLRVISDSSSNKQSN
NYVDLFEKPYLVEVFKTILFQIYCISKVAYQNTSNILEKTIEEFQFAQDTSQIILYFLSQISYFYFDLRPYLILQANSDR
FLILNFVQFLHPFYPIFSKHQQIEKHFQKQASLWTDKTTPEYVQEALIALNKEEQLCQDYYPRSMNPINQTINGIIIKDQ
TQYLLEHSTGIENMLKNGKDNEMKDLYKLLCRVKESLAQLGQKVRTYYQSCISQKCKDAKEKISAFKPDKTKNIRDFTKE
TAIEWVKGLIEFKIFVDNQISTLFSNDLELQKARDNAFQISLREFKESPHFLSQYCDDLMKQQLTDHESNDRQIEVNAIF
DYMNTSRDEFLLHYQQALCRRLIGFTSKSMAAEKQIVTKFKNSVGALPTLNRLQNMLTDVETNEQYNKEKVQNDILKIYV
LNSVAWPLKRNELEKIIFPSSFAGIIDQFTKQYNQTYKQRKIFWLFNEGSAEINFTSANKKTFAGKYILRTTSYQMLILM
KMNEKASITIQDLMDQTGINKDSFDNNLKQLIKLKILSCAEEDKYTESSTVQVNAQFDFPKKVVPCLPRAPTSGPGANNP
NIQAIVEQERTYIIKAYLVRIMKGRKVMAHNDLIKECVELIKTCARTFEPSFPHIRKCTESLIEEEYIKRQENDYNKYEY
IP
>Tthe_XP_001012772
MSSKTFMNKENTEGKSINIKGSVTSQGQIPQQQQAQQQQQKQGFVYKDPKALQSWDVIEQAIQMIYEKKTHTLSYQELYH
HAYILAFYGHGDQAYSRLKSLLEKYSLKMLNTLGYYDSEEQILVKMVAVWQDYKQMINILKGIFLYLDNNYVTTKNLQPV
LVLGYEVFQQILFIQKSEIYSKVLEIILTMIDKQRLGENIEGQLIQKIIQMSVDLGMKANIQKCNQYSRQIQYEYIPELT
IYEKYFEKGYLDCSEKYFSEESQRLILQLSPKDYIIAVERRFLEEKKRVHSYFYQTSKNKIQSLFFQKYINDFAKAIVDN
QENGLFNWLDNDEVVIIKKSHTLFQNTKESYTIFLNIFYKYVAHQTSALAARQSTPVKEYIEDIILFRLKIINIMQEAFF
SLLKTDDQLTIQRQTQKSFEDSLNDSNFPTEQLNCYIDEAFQKDFKNKSEQQIEEVLNSVFEIFCLIRNKDYFASIYMKS
LAKRILYNKSLDENNEKQIISKFKLECGTVYTKKMETMFLDMQQSLEYYNEFKQLFIKNLNTKSAYKSLDFDIKVLTCGN
WPYKLNQFSQVKIPNQLEPYISEFEKHYKSKFKGRKLKWIYSVGKSIVKGYYSFNNSKKEFQVSNVQMVILLLFNNKIKY
TQQELTALLQMPYEEYKLHLLPLVQLKILKSENNIDFEYNDQFSYKNMRVQVNVMKKEASKTESSKNLEQICLERKFVVE
SLIVKIMKSRRKMEHQELYQEVARISQQHLFLPDQQTVKQCVNSLINRDYIRLSENKDYLQYVV
>Tthe_XP_001012757
MHQSIQKYDFQTGWKEIDPVIQKLNKMLDQQSGSGNSEEKLMINKIEYSNTYTLIYNLCLEKDSNNSEQLYEQYKKQIQS
FVKDKRKGTQRRGFAQENNIKVGYLSNLLKIHDQILFIFRQVIHRAQQPLEAQINRQINEQNVDENLKFVFFAKGYYLYL
KQVHDHLKDNIILAFQQEIEKDRNNYKSDLDMLRKISEIFVEINKSDNKDENYQNLGKAILSHAKAYYQSKFQEWSESCS
CEEYISNIIIVQKEENRRFEKCFTKFTDTQKQISQLFNEEMVLKYRPILLVKDHGFTFMLENNKSHVLQQIYQLYKNLND
NFEELRQTFGDWVYKIGSNIFEEKIKQNIEEKPDQKKKKHATDHEFVEKLFNEYKEMNNMIVQNFNSDANFYMSFKAALD
KFLNVPIDDYGLPELLANYTDFILKKENLKQEELDSKLNQIIEIFNHLTEKDIYLILYQKQLATRLLKAELKAGSRYDDE
SLMITKIKGCCGQGQETKNYEGMISDIKYTMDLKKNFDEYMNQKIKVVDLNVKVLQEANWPSYKSTTEIILPPLYENLKE
DFKKFYQLQKKSQKLNWINTLGSFDIKMNITFDKSNFFLVNVSPLQLCILLQFEKVDTWIPISTILATIGMKKDNIAEFN
KEVAQLIGKKILLASKSQTQKQEEKKDESDEVSFKFNTQFTYQKKKFSTVPEESQQKFKRDIVNENREHVIEATIVRIMK
SRKTMKYGELVSEVNVLLRKFQTQPHQIKKRIESLVEREYIERDKNDMNLYHYKQ
>Tthe_XP_001012576
MLHTAKVNEELYQKARELVTDVHAKVLKILKRGENVVVSNGEYMPCYNAVIKACDSSQEPTANKDEKIQNNEERLFNWFK
QLIKEYLLGDVQESNLIMDQEQYIEKVYKQYKNFQIYLHWLSRLFYYLDQFFLKNKNSTLHVEGFKIYRDDYFQIINNKL

FNHIVNFQKMAREDQSIPRETVKRLIQIYQEVGFKKTVKLKKIANSSEFAYEVDDGSKYYEDNFQAKFADEMEQYYLKRV
NEWSNLSTPEYVEQALKSLQKEEEIAQYFYSKSQKIIVNRIEQIVIQQQSETLANNEQTGLYSMLKEKKENEMKNLYKLF
KRVAETLDHVAKKLGQYINYHGNIFNEQSDSRKSEGVTQKDIAVEFVQKVFALKKECDHLVQDVFNQDITIQKARDNAFQ
NFLNKNDKSTFFLATHADIILKQEGLQNEQEIEDRVQEIVGIFVYFYSRDTFFKHYQKFFSNRLLNATSRNKEAEKSLIA
RFKTEAGQTGVNKIETMLKDINNSEEFNQDNRKHISPLGIELNVSVLTTGSWPIANTQEKVSTPQVLKSSIEKFEQLYKS
KYKGRNINWLYSQGTAEMQFRSKEKYLLIVNSYQMVALLSIQSQANTISYQKLVQISGIPENELEFLLMPFVKLKILNKS
DEGDAFNNNSELSINSQFQNKLKKIKCIPGGKQAQQKKQKEDDEGRTQFMEEMNREREFIVDACIVRIMKSRKTMKHNDL
FPEVIKLINNFKPEIPLIKRRIESLLDRDYLKRDENDRNTFIYVP
>Tthe_XP_001007602
MKILKKIPKIFYKIRKLLANNQYEDINKLFSQDKYANLFQNVYQLDGKQVQLLVDEIMINILDSSNQQLKLIFKGKSSDD
DSTILNQFQKILRDQLIQIYFISVFFYQFDELIFKGLHQVSLQQYCLQKVLDSSNGKFSMKIVDQIVKDLNKRKQQSYDF
QISVLFNCDFTEEEAEKLIRYQEKKRIEEEKFKQLMEDDSSSDENKQVSSSQKSLDQQEIEQEQEEGDQSLEIELENKKS
KKKKRVIESKSKKEKKKKEKVEFESIFSLIEEKYIQKLKSDSEEFIDRIKDEKLEDQITQIEEYIFREQQFISYYMNRIS
IKVSDLLQKMCIYQNYPKLINHQKYGLTYCFNNQNYKELKLIYNFVQNDNESIKTLSQKFNSIVESVIQKVEKEQEIMFE
DYKKEQQNQAENYMQNSESKDENLEKDIQYIQNCQQKWIKQIYEIYLIYYQNGLVQAFDNNPTLNTALSRAFESQFNSSE
LAVDYLVNSCHQYLLNEKFRQTSLELKEDEFENFISKIFSYIFQKDQFLEIYRKLLSRRLIYKQSQGEKQELKYLEYFEK
SIGLITQIDHMKQMIQDLNKSDLIKFDYYEQMKKINKDNQDQKSKNSLSSRSRSRSRSRSKSIESRSEKSNKRRNNGIQI
DISDDNSEEEIEIISQNNQIVIDDNAEIEQSRQVIYYSLPKNNSQRNQEVGQMRSNSRTERSDSNQLIQIEDSDQNNSDE
DLIISETKPTKQKGKRYLLKKNQIIIDEKEENEEEGGKHNNNSQEIKNSLIDVMILTNMKWPLKLSKTNANSQIILPKAL
QRYYQEYSESFRKVFKNQRKLDLLHHVSYSTLQFKSDSKLQFGGTYLLQVSSYSMIILLCFNKNNSISVQELKEKTGINK
KIIKEKLNILSKFNILKLKITEDNKDQQSEKNKKNYEVNTDFYAKTQDLEIDSIPQEQFYSQQILQNLRADAQTQKSQKE
INSNIINHRKIMKDKETLHKDELIKLVQQKLKDNKQIFIITDSFVKDQIQSCIDQDYLKNSQEQDILLYVK
>Pmar_XP_002781751
MSHSRAFNTNTSSSSSSSAAAAGFRHFRAGEPIDEGEAARTWESLKSAIQQIHNHNASHLSFEELYRNGYNLVLHKYGLK
LYKGVEETVSLHLMEVSKRCIESPDEDLLARLKVEWEDHKMTMGMIRDILMYMDRNYVRQHPEQCVPVYDMGLRLFRDTV
IGHARVRDRAIGQILAELRRELHGETITDPHLIKTVLSMLVELSSIQTLPSQRSVPETGYRADQSLQATSPSPSDMRPVI
YVEVPSPMQSANDYYSWFEINYLALIRDFYTREANEYINRHTVGEYLEKANSRMRQEKRRVETYMDRNQTMPKVQEVLDS
VWLGRHYKSLIQQESSGCKVMFAQARVSELRLMYSLFSRIPDALSDIATVMQQSISTAIADLIADESTVNAPVSFVEKLL
SLRERFERIVSQAFRGSLEFSNHMKMAFENSLNNGPKCAHYLSLYLDELLRKKLRDMTDADFHSNVDQVISVFRYLIDKD
VFESYYRSSLCRRLLNSKPSSANVEEAEKLVVTKLRAECGQQYTSKLEGMLKDVSLSQVGDNILNLFRNLSVSSSCQVCT
SGFWPTHTPPRCDIPVDMKSLIDRFETFYLSKHSGRKLTWLFNYGTGELRGIARGTIPILFAVVVTDYFASVDIRSRAGR
SPHVLTVSTYQAMILLMFNSLESLSVNEKSEREDDAEIKRHLMSLYVNPRVRVLLRESPQSSKEPISGDIFRVNTEFESR
VRSVKVPLIALANNKDGAAESSSSGNAIPQVVEEDRKHIVEAVLVRIMKSRKQLDHNSLVVEATEQLSQRFRPTPQLIKQ
RIEHLIERDFLERCPHDHKTYNYLA
>Pmar_XP_002775084
NSSYRGHELEKSMLQKLRTECGGGFTSKLEGMYRDMDLSCALNKEFTEKRNQQLQGDDNQSPQFEAMVLTSGIWPTYTQW
PKD
>Pmar_XP_002774654
MSAHGETVPLDEGWTLIREKAIDKLEYYLDTGEVPKDVVQVEGKAPRIFGAGDYAQLYTTVYNMCTQRSPNNWSEELYQR
YGESMSSYVTRRVVPRIEGLEGKPLLEELLLRWNNHKLYSKWMERFFTYLDRYYVKLQSVDTLAVRSVTIFKTLAFDHGH
VPARCRAAILEMINKEREGTEIEQSLLRGIVDMLFDLGNASRSTSAAEGSSSSSSNRPSLGAAPSHGNDELSTLWVYQQE
LEEFLLPETARFYERQAKAWLVSDSLPEYLVKTESALMAEQKRVETYLHPSSMQKIKNVIWNQLVDYCQSQALEKDTSVT
WMLDNDRREDLSRLWRMFGLVNNGLVPIAASFKQYVQDLGNSVVDALLDQLTKLGPQPSPQAKAEILADPSFVQKLIDMH
DRFKTIVAECFQSDGLFQKSLKEAFETFINRDLGRFSIAAMMSSFCDKVLRRGGEKRSEEQVDALMSKLVDLFSFLTDKD
VFAEIYRNQLAKRLLYDTSASDEAEKNVIQKLKMKCGAQFTSKLEGMITDISLAADMQKQFREYLSHRDSQADYDNIDFS
VTVLTTGFWPTYHPIDNVILPAPMTRCLGVFTDFYNGRTQHRKLSWIHTLGQAVVGARFGSRKHDLHCSTLQALILLLFN
NPAAHGGDNEGWISFQEIHTATGCGDDTLCKKLLATLSIARYKVLEKSGSNPRIIDVEEKFRVNPKFSCPQRKIKIPPPA
QDETHKAERVQEDRSISIEAAIVRIMKTRKTCSHQQLVSEVLEQLSFFKPNPKVIKQRIEHLIEREYLERDENQPNIYRY
LA
>Pmar_XP_002771836
MSAHGETVPLDEGWTLIREKAIDRLEYYLNTGKVPKDVVQVEGKPPRIFGAGDYAQLYTTVYNMCTQRSPNNWSEELYQR
YGESMASYVTKKVVPKIEGLEGEALLKELLLRWNNHKLYSKWMERFFTYLDRYYVKLQSVDTLAVRSVTIFKTLAFDHGH
VPVRCRTAILEMINIEREGTEVDQGLLRGVVDMLFDLGNASRASSASDGQGSSSSSSTSNRPTLGAAPVHANDELGSTLW
VYQQELEEYLLPETDRFYERQAKAWLVGDSLPEYLVKAESALMAEQKRVETYLHPSSMQKIKNVVIKATLANTMSQTLEK
DTSITWMLDNDRREDLSRLWRLFGLVNNGLVPIAASFKQYVQDYGNSVVDVLLEQLKALGPQPSPQAKAEILADPSFVQK
LVDMHDRFKSIVAECFQSDGLFQKSLKEAFETFINRDLGRFSVSAMMSSFCDKVLRKGGQRGSCWHMGWVRIHNGVSGEE
QSRFDLPLGQAGGSPETVLYTLDRFCQHFCSCPIQRLQWLCNDGDRVTRERCDVRMLVSALEARHSDAVAATPLPPLLLC
TVPMTPACEMRVFIPRQGEGEPIRLKSLYPRRVAGTGPVELRMSTSYLPEGHVYSVVFTSQWDSGQTFTADCAVNTDRMG
VVLMAPSELLHTAWFQYGNDLYDVHLVIDRKYRSENRRALTVQSPSEIGSSSSGTEGSLSFEVFDIERGSNDIDGTSPYR
NTPTNWTPLSE
>Pmar_XP_002768880
PDEDLLARLKVEWEDHKMTMGMIRDILMYMDRNYVRQHPEQCVPVYDMGLRLFRDTVIGHARVRDRAIGQILAELRRELH

GETITDPHLIKTVLSMLVELSSIQTLPSQRSVPETGYRADQSLQATSPSISDMRPVIYVEGPSPMQSANDYYSWFEINYL
ALIRDFYTREANDYIDRHTVGEYLEKAHSRLRQEKRRVETYMDRNQTMPKVEVLDSVWLGRHYKSLIQQESSGCKVMFAQ
ARVSELRLMYSLFSRIPDALSDIATVMQQSISTAIADLIVDETTVNAPVSFVEKLLSLRERFERIVSQAFRGSLEFSNHM
KMAFENSLNNDPKCAHYLSLYLDELLRKKLRDMTDADFHSNVDQVISVFRYLIDKDVFESYYRSSLCRRLLNSK
>Pmar_XP_002767085
MQVIYPLKSSIGMGWAVRYNLVLHKYGMKLYKGVEETVALHLMEVSKRCIESADEDLLARLKIEWEDHKMSMGMIRDILM
YMDRNYVRQHTQQCVPVYDMGLRLFRDTVIGDARVRGRAIGQILAELRRELHGETITDPQLVKSALSMLVELSDIQTLSG
HTETDSENVYYSWFEVNYLALIRDFYTQEANEYIERHTVGEYLEKADSRMRQERERVETYMDRTLTMAKVEVLDSVWIGR
HYKMLIQQESSGCKVMFAEARVSELRLMYSLFSRIPETLTDIAEIMQQSIGAAIADLIADEATVNAPVCFVEKLLALRQK
FEGIVSQAFRGSLEFSNQMKVAFEKSLNNSPKCAYYLSLYLDELLRKRLKDMTDAEFHSNVDQVISVFRYLIDKDVFESY
YRTSLCRRLLNSKYESDSLCHIRHYGSPRPSSANVEEAEKLVVSKLRAECGQQYTSKLEGMLKDVSLTRCEGEGVYAFRA
VLGAYVYSVSLAQVCTSGFWPTHSPPGCEIPVEMKCLIDRFETFYLSKHSGRRLTWMFNYGTADVRSRVGRHPYVLTVST
YQAMILLLFNSSDALSVDERSEREDNAEIKRHLMSLYVNPKVRVLLRESLNASKEPTAGDIFRVNAEFESRVRSVKVHAG
GGSASSVEVGSAVPQAVEEDRKHIVEAVLVRIMKSRKQLDHNSLVVEATRQLSQRFLPAPQLIKQRIEHLIEREFLERCP
HDHKTYNYLA
>Pmar_XP_002787261
FKSIVAECFQSDGLFQKSLKEAFETFINRDLGRFSVAAMMSSFCDKVLRKGGEKRSEEQVDALMSKLVDLFSFLTDKDVF
AEIYRNQLAKRLLYDTSASDEAEKNVIQKLKMKCGAQFTSKLEGMITDISLAADMQKQFREYLSHRDSQADYGK
>Pmar_XP_002786385
MKSLIDRFETFYLSKHSGRKLTWLFNYGTVDIRSRAGRSPHVLTVSTYQAMILLMFNSLESLRVLLRESSQSSKEPTSGD
IFRVNTEFESRVRSVKVPLIALANNKDGAAESSSSGNAIPQVVEEDRKHIVEAVLVRIMKSRKQLDHNLLVVEATEQLSQ
RFRPTPQLIKQRIEHLIERDFLERCPHDHKT
>Pmar_XP_002784071
MDYLNLMSSSSRLSQLPVELSGDVRSMGSISSSDDDIESVEVVRGEAEPQGQPSASSGSKKRARKPSTSAHAAPMVDDSS
NDTSHPEGNPPRTARRRIMSSDNHHCIATLPDRLPPMRRYSKLNKSIAYRPGQAAVYHPMLERKLILKPFQVHPSELPPD
FVTDMWERSLKEAVRASYGRDGDGIEEMRGNRSLEERYRIVDTICRNGRELELFDLLRSELKNDIFSIVKSISECKVDDS
ATNEAVECLKQVQDVWNRHRKDLGLVRNIFLPLDNFLAATGGMLEGEKDSSRTRKNLPVGLREMGLGMLKKYMCSWEKKN
NERSIISRACECLLTCIQHSRSVCVGEDPSWDLYGDVIVMIRSLDMYKDMFERPFEEATKEYYEDESASKIDTLSTEGYL
DYVEGVWEKERSLHDACKLHSHTWQDTDRILRDQLLVMHSASLTSTERLLELLDAKPEPKTEAAKTLLRFLEVSEEYDMV
HVTSEVKLSWSQAIRTIGEALMKKYLAFNTLAEGVGLIKELLTLKENLDLILADTFVDPDLAVSFRIAVKEAFETFMNSC
DQVNKCAHLLAKYCDDSIGSYVRNMKSSLTGEEVEARMDSVMTLFRFISAKDIFEAHYKNDLAKRLLSAHTMRGPDEPLT
ENSSYRGHELEKSMLQKLRTECGGGFTSKLEGMYRDMDLSCALNKEFTEKRNQQLQGDDSQSPQFEAMVLTSGIWPTYTH
SVPTLPAPIASLQQSFTQFYIGKHSGRTLTWMPSLGGALVRYNMRSTARPVVKDLVVSAAQAIVLTYVFNDSSTATAAKV
CEVTGLPVEELRRILFPMVYRVRILNRSTGGPEDKQVAASESYTLNRELQEKKRRIIVPQMAVRDEKAEVRATDSRVNED
RQFLLDAVLVRIMKSKKTLSHNSLVAETLKECSATFTAEIGEVKKRIESLIEREYLERDLSSPSTYHYLA
>Bnat_78854
MFLSLSLSLSLSLSLCTVFFLPVSAHWKDARELAFTYAVSCCCPRIARGGRSSSSRNSAAAAGGDGVVAELVRLHEHCLGLV
EGPFQGSPLFHKSLKEALEDVVNSRVNDVSVGEMLAGRFHHLLKKGGASKGAPPEKLNQEISQLVALFGYLTDKDHFAEF
YRNDLAWRLLHNKSASMDMERVVIGKLKYSCGAQYTSKIEGMVSDAKTSIEFASGATRREQASPLLFDLGGDFNARVLTS
GFWPSHPEENLVLPDYMTATLGEFEKYYMDKFRRRRLKWIHSVATVTIGATFKSRAYGEIATHITQRTTIKHCTTIHQLL
VVPSPSHILTYHYNTVGTQYEFSVNGFQAAVLLALNEHGETGATIATIRRRTQLEAGVLKRVLLCLVVKSKILRKSPAKD
YNDSDRIFPNRSFASAQRRLKVPSHIPAKEKKSKSRKAMMAVVSEDRKGALECAIVRIMKSRVKIRHKSRDGRFCGSDPA
SNLVGSNKMMMIVSDNSYSLYRSSFHG*
>Bnat_90538
MDMLSTELKVRRGASNYIPKQRVTIEPFKRRPEPPSDFEQKALETLTEAVNRIHRKVPIGFSREELYRLVESLCIHEKAS
KLYSCLHDLCKQFVQQQVRSLGGESINKGIGGKLEVGAVAVAVTQQDEKILMKASSIWQDHRKQMNAIRSIFLYLDRSYV
VSHPRLTSLKGMGLSLLRTTLTSQHPEVLRDIVRAVLSYIKRDRSRKDKVTADTVLLKSVLDMLSHLEIYVQEFEGVFLK
ATKEFYAYEGQRFAMVKGVPAYMKHISERLIEENKRVEDYLAESTRKPLLKALEEALIASHVDTIVRRGVDELVEKYATE
DLARMYHLLSRVDALASLKKYFLDHLKVAGRNLVMMGDESQVLKDKQPGERLNPHEIKLLEQEEKKVIPELLMYKKRLDK
LVHDAFENDQNFTYALKQGFEYFVGGADYRVAELLSRHVDALLRGAKARTLTTEPQVNEIFNPLLTLFGFVQSKDVFKGF
YQKELATRLLTGKSASRNVEKIMLQKLRALCGAAFTKHMESMFGDISLSEDLAREFHRSGSSSSVAHQSGTQLSIDFSVR
VLTEGSWPKSDDNGSMQSISLNLPQEIKDSQASFEAFYAKRHK
>Bnat_84566
MFLSLSLSLSLSLSLCTRGGSGKGETRFGAAVQALQPVRPIPPWPISPNSWGARILADARDPRGRSSSSRNSAAAAGGDGVV
AELVRLHEHCLGLVEGPFQGSPLFHKSLKEALEDVVNSRVNDVSVGEMLAGRFHHLLKKGGASKGAPPEKLNQEISQLVA
LFGYLTDNLIEVLFYRNDLAWRLLHNKSASMDMERVVIGKLKYSCGAQYTSK
>Bnat_84567
MAGRHQYSRVMEGSSTSALDGSPAGGRRRKPRTITLEEGWGRIYREGIQKLHRVIKEKGSGRQFSNEEHVKLYTLVYNMC
NQQAPHCYTGKLYTLFGKSVRHYLNKVSLPAIQERPPGAYLVREVVSQWDDHCLISRWMCNFFDYVNRFYVRRGSLATLE
QVCTTAFKTAIFDPIHRKLTSTLMALILADREAVAVQHDRRSMPLLVQGEGTAAMGLFGTNISF*
>Bnat_73813
MAASSTEATVDAWTELSTTLGDLHIWAGPNASCSASAFKSNQKIRSAAVQLKKAGINQVFFEWFLEKCLDKIKKKYVPEF

HEDCEFLMKSRKEEGEDDEGSQEECTNQNQQEKKEMKILPNTSGEDLLRMLRGWNVEKYSDSLDKVLGILLKTRTFILNV
SVLLMLDGGRMNMSMLSVSSYSSVREKPPNTPFWQRVDAQFFTKMPTGFQDGMMAFANMRFLQYQKLQKIRKVTIQGNAT
LDAKQSVGEGGQRKADTRKMRREGKMKKKIRRRSFRVPPPLATCYDHQLREIGWMRFVEKAFSRVIYARIESRLANICAG
VYNVPQLDNAISWMKLTVLRWLQCFTNSDSSTTTTTAAAAAAAATPTVSLTKRRREKAYLDPSAASSTTTTSSSSSSSTS
GVPDATSAAAESSWHQRVVMFTYRKFAAMRIDEMWDIILEFPDTEAALCDLRKALAFTGDHHLLIQSLKHTFNNRLLTPG
ANTQDILLQYSYTIGALCVVDPTRIILKAIGPRICGYLRSRSDTVRCIVGSLIDPKGDEKERDGLAELAADLDPLGNTEE
RHSDDETGDPFEWEPDAILADSSKVRGATTAHKSDILQMLMTIYGSNALFIKEYRLLLADLLVNKIDYDTDKQVQQLELL
KIRFGENAMHACDVMLRDLANSKRINSHIRPALVAKDAKMSSSSSRASRPTTPAETPIKRGRVTRQTRQSTISFRSRKSR
ASPTIHIHSDTSGSKENDAKSSQQGRGLKDTAPVTPPGATTRSHIASSSSSSSSIDYDKKFLEATIISKEFWPERDENELK
LPAQVARVAELYSEQFTKLKAPRVLRLMPSLGKVHLEIELEDRQLDLEVTPIQASCLAQFGETESDQLTLNDIAEGMACD
LDLCRRALAFWLQKKVIKEVKSEDGSVISYRPLEKLTGEEDDDHNMTISLADDNEDLESALRSQRATESKKVESYIMGVV
VNFKTVTLQHIKNMLKLLTSDEAIRLTDRQIMDTLERMAKSGKIECRDGRYRSIKKRR*
>Bnat_46233
MIETESNNFFRAKEFVELYDHIFKMCIQRDPYNWSEELYRRYSDSIKNYLRENVKPALEKSRTRYERAYLEQWKTRYANH
GRIVFGLSKLFMYLDRFYTPSTEGALSLKKQGYKLFDDIVFQKYSQYARGAILNAINHERNQKEQDRKLLKDSVNVFVDL
GYNIGENTAVRLRYYEQQLQQDIVKDAGNFYRREATTWLREDSTSAYLIKCEEALDAETDRVRAYLHRSTLEPLVKKCYT
ELLKVHQSALLNKTTGLYYLLEEKQNEDLKRVHDLYSRYPQDLPPIGEMFRTQVQKEGKAIVKRTNEAKGKSQSDEKHVL
VTDLITLHRHYDTVVRDCFGHHQVFQKALKTAFESFINENDSVSKLLALYAHDVLKKGSKVTVENVEKTLDHVVFLYGYI
RDKDVFDHDYQQLLQERLLKRKCSSEANERAMIAKLKSEAGYQWTNKLEGMFKDVGVSKNLTGLFKNFFNTEEKCGLTFN
VNVCTHGFWPSTNYTSSKTPDALKMATDNFKDFYHRRYAEREIQWRMDMGEAEVLVRFNTKTEVRLSVSSYQMAFLLVFN
KKKIASFQDILDATGLKSDTTLQNHLLSLAHPKLGVLLKRPNSRTLDRKHKFQLNSNFKCKMRRITVPIINVIRHSVNPT
VDPSIAIQRRCQMDAAIVRAMKTKKRMQHMELVNEVTAQLRARFSAQPGMIKKRIEHLIEQEYLERDSSARGVYKYLA*
>Bnat_40690
MSKSVLNINNAGLTNDQAEKIWSGLRTAIIEIQNQNASELRFEELYRNSYTLVLHKHGDLLYNGVRRTVAENLVKISNEV
SNTVDGQLLEKIVEAWENHKIVMTMINDVLMYMDKTYCLLKKKEPAYNMGVLLFKDHVIMEGQVANRLKSLLLENIRLER
TGRTVDRILLKNVLCMLVEINVRNTNIYKAVFESDLLSSTAVFYQQESQNFMAQNTVSDYLRKIERRIRQEEDRALEYLD
KSTHAKLRHVVQTELISNYAQQLVENKSTGCVHMFHNDMVGDLRRMFVLFSRVPGTLTHIRSCMSSLVKKTGTNIDLENL
KHPKFFVQKVLNCRKKFHIFVKESFMEDRSFTRDLKDSLEYFINLDSRAARYLSLYLDDMMRKGLKGKSPDEREKILNDV
ISIFRYLSDKDVFEDFYKKHLASRLLIDNSGDREDEKMMISKLKAECGHQFTSRLEGMFADIQISSDLMASFKNQHQSNV
DINVTVLTTGFWPVHNMEHCTLPPEAKKATDEFATFYKTRNSGRRLTWQTQSGTAEVLCNFKHGNKLLAVQTYQMCILML
YNRAETYTYAQIAKLTNIPDKVVTAAA*
>Bnat_54551
MSKSVLNINNAGLTNDQAEKIWSGLRTAIIEIQNQNASELRFEELYRNSYTLVLHKHGDLLYNGVRRTVAENLVKISNEV
SNTVDGQLLEKIVEAWENHKIVMTMINDVLMYMDKTYCLLKKKEPAYNMGVLLFKDHVIMEGQVANRLKSLLLENIRLER
TGRTVDRILLKNVLCMLVEVNVRNTDIYKAVFESDLLSSTAVFYQQESQNFMAQNTVSDYLRKIERRIRQEEDRALEYLD
KSTHAKLRHVVQTELISNYAQQLVENKSTGCVHMFHNDMVGDLRRMFVLFSRVPGTLTHIRSCMSSLVKKTGTNIDPENL
KHPKFFVQKVLNCRKKFHIFVKRSFMEDRSFTRDLKDSLEYFINLDSRAARYLSLYLDDMMRKGLKGKSPDEREKILNDV
ISIFRYLSDKDVFEDFYKKHLASRLLTDNSGDREDEKMMISKLKAECGHQFTSRLEGMFADIQISSDLMASFKNQHQSNV
DINVTVLTTGFWPVHNMEHCTLPPEAKKATDEFATFYKTRNSGRRLTWQTQSGTAEVLCHFKHGNKLLAVQTYQMCILML
YNRAETYTYAQIAKLTNIPDKELRRHLLSLAHPRVKVLKKFPNKKSLAPSHTFTYNYGKQSPLFRIKVPLLKVENDSKEI
KVPPSVEEERKNRIEVMIVRVMKTRKTLDHNTLMSEVMKQSLRFSVKPIFIKKRIESLIEREYIERDKNKR*
>Bnat_128991
MQYPPLEILPLNEGWEKIYKMGVLPLQQSVEGQGTELWGANKFIMVYDLVFKLCIQRDPYNYSEQIYKNYCKVIGEYIHS
GSEHLSRAQKDSDSKYLEEFYLRWTRFSIYINRMTKVFGYLDRFYTPNFRVRSTSEEGTFQFRKWVLSPSLGNVLKASSN
LFRSESAEDVDV*
>Ehux_432479
MESGGEPMDTDDEDGGGGGGLRALWGGGPAPVQLRRLSEALRGLRWMPLAEASVSAMLQAHLRLSLRERCAGRFESCVME
RQLDWIDASVLPWVSAALESGADQWRARDLLTAASAPLKQWRARLRFFFMQSLAGMRIAELFDILVDFPDSTPALDDLHA
CLHHTHQHADIVQGLGEALEKRLLKPGAETHQILEVYIGMIRALRRLDPSGLTLDAVSERVRAYLKLRPDTVKQIVTTLT
DPDSSDLLEPVGGGAAAEVVQEAAADGDMAPDPDESRGDEAAMLKWEPEPVTADGASSGGAAARRAPDVLAILVQIYGSK
ATFVNEFRKMLSEKLLASASHDTEKEVRNLELLKKRFGDAALSACEVMLTDVAESRRISRSIQQHFGGVSGGGEGATLID
ATVVSRLCWPSLPTDTFTLPAGVKKEMARFEKQFAHIKAPRKLVWKPSLGTVTLDVTFPDGSVGDVQCKPLQATILLRFG
AQQRWALADLASELGIATDVVRKNLSIWLNRGFISEVRDAGGGAVFEAATSLGAGDESRHDAGEDEDGDAVASLAEEQRI
AEMKVYEQYVIGMLTNLESLPVQRIHNMLKMFVPASGGDRGYDCSEQELQRFLMHMVEEAKLEHSAGQYRIAGRH*
>Ehux_448976
MQGAAQGATAQRVPKRLLDDVWRVRATDDDCCAICLEAPAQPLILLCGHMFCLDCFQTYATHAREYDDECTRCPTCRSPH
APPPVGQVEAEQVERGDALLARAMRMDRSRPNFLPLLEGATRFYVAALDLHPYCSRAHAMIGWCRKVAGEDSLDAFEDSA
RACHGALPAFAIVTLWNQTILDWLGESESAQRIELVRAGVRACSWPQAVQREAAGTELPVAENAYRKACSAFEDLTLPRV
RELLENLLAHYHRMVELFSLVPIYLPLIANIVRKRITDAGLALVKKQSANAEGDLMAYVQELLDLDGVYSEIIGSTPGEG
YFNGDATFLKAAREAFEVFVNKDIGMSATAELLSTFCDNLLKNAAEEAAADRHLSSDPELRRLYQSVVGGGALSTADFWA
QRRGLLRAAMQREGLSTRRQQPTDAESSAATAAAAGCEQPEANAATVAFTLTPAMIARIFEKYPRVQRSFLDNDKLEKVV
RLFGYLSDKDIFVEFYRKQLAKRLLLARSVGASAERSMIAKLKLRCGAQFTSKLEGMVTDMNLSQDIQSAFAEHVKDKEL

ELDTDLTVQVLTTGFWPQYKSDELKLPKEMLQCVETFKAFYDSHTSHRRLRWVHSLGSATVVGKFTASGKSKEHDLLVST
YQACILLLFNEADSMTFSDIQGHLNLPAEELKRYVLSLCGGKYKILTKDPAGREVAPTDTLSCNATFSDRHQRIKVPMIA
PKMTQEEKDAALKQAELERKHVVEWAIIKVMMREKRVEHEDLAEQAQAWIRTRPSTGFHMARDYFDAMVVDLIRREYLEP
EAPERPPGSGSFYKYLA*
>Ehux_434769
MAAGGKTKFVIPPFRQSQAMDEEYANRTWQTLHSAIHEIHRQNASGLSFEELYRNAYNMVLHKFGDKLYNGLVETITLHL
RGVAQEVAVAADGDFLPQLKEKWEKHRLSSIMIRDILMYMDRTYVVAQKKVAVFERGLQIFRDEVCRNDRIKGRLLATVL
DLIHRERCSETIERGVIKTVTSMLVELGQDVYQRDFEVPFLEATADFYRKESHECLAQNSASEYMRKAEARLGEEHERVT
HYLDKASEGRLNEVVERQLIAEHMRTLAEMEHSGVVPMLEDDRIPDLKRAYELFGRVTSPDGLGVIRELMSGHVKGRGQQ
LVAEEPGAARDPVEFVKGLLALRDKYQSIIQGAFSGDKTFFNALNSTFEYFINLNQRSPEYISLFVDEQMRKGMRGTSED
EVEAMLDKVVMLFRYLQEKDVFEKYYKQHLAKRLLGGRSISDDVERSMIQKLKQECGYQFTSKLEGMFMDMKVSADTQDK
FRAANGSSKVESIDLSVQVLTTGFWPTQVSGGCNLPAPVARCCEVFREHYLKQHSGRRLQWQTSMGNADLKAQFGASRHE
INVSTYMMCVLLLFNDNDRLSYADIARDTAIAPAELKRTLQSLACGKFKLLLKEPKGREVDESDAFSFNASFSSKQLRFK
VGTVSAQRETEAEKHETRQKVDEDRKPQIEAAIVRIMKSRKEMEHNALIADVTAQLTARFMPHPNVIKKRIESLIEREFL
ERDKNNWRMYKYLA*
>Ehux_427672
MQQKAGPSAAGAPRKLTIKPLRTAPKLPANFEQATWAKLLDAVRAVHGKRAVNHSLEELYRGVEDMCVQHMAARTYDKLQ
AECEAHVEASIEALQTPDTSAFLSLVHGCWSAHCEEMLTLRSIFLYLDRTYVMQSAARRSLWEMGLHSFRAQLSARPELL
AKLRDGVLASIERERGGDQVERSLLAELLRMLYDLGLYQRHFEEQFLAASSDLRRQRPPPPPLPPPAPFPKARDVPSYLE
HVASRLSREEARVAHYLHGSSRKPLMAAVRQTLLAVHVRPLLERGFADLVQHARLDDLRRMYSLFAQARVNALPELRQAF
AAHIKSVGGAMVSDQERERGLVQERGGCRSGGLVGESFEQFINIRQNRPAELVARFLDSKLRAGNKGSSEGELEEVLDKA
MTLFRYIDGKDLFEAFYKKASAPPASSPRRARDGCATRPPPRLLFDKSASIDTEKAMISKLKAECGSAFTSKLEGMFKDI
DLSQDVMASFRTSPQAAKVSPSLELSVHVLTQGYWPTYPPVELKLPGEILELQEIFSTYYMSKHNGRRLQWHPCLGHCTL
KANFPLGKKEISVSLLQTIVLLLFNDGDGLSYAHVLQATGIEDRELKVTLQSLACGKVRVLRKEPKGREVDGSDRFFLDA
SFKHPLFRIKINSIQTRESEAENEQTSERVFQDRQYQIDAAIVRVMKARKTLSHSLLMSELFQQGPPPPPPRPLLPRPPPP
RLTSRRLPRGQLKFPLKPPDLKKRIESLIDREYLERDPKSASTYIYLA*
>Ehux_224320
MLLATPPPQPADPPLAPLYVAGDSHALSPSWRVVRWRGQPRVLVTGLKLWHLRDGADFFPKANFEAAAASLPDGADVVFA
FGEIDCREGLLLAVERARYRDLAQAIDTVVDIYIRTLRRLAARRRFNILVPPVLPETRAVVKQFNAALRRAVERASPVLA
WLDFFEQLLTPAGDALADGLALDGTHLNPQYVRLTAPRKLTIKPLRTAPKLPANFEQATWAKLLDAVRAVHGKRAVNHSL
EELYRGVEDMCVQHMAARTYDKLQAECEAHVEASIEALQTPDTSAFLSLVHGCWSAHCEEMLTLHSIFLYLDRTYVMQSA
ARRSLWEMGLHSFRAQLSARPEVLAKLRDGVLASIERERGGDQVERSLLAELLRMLYDLGLYQRHFEEQFPSVKT*
>Ehux_224974
MAAVRREAAGTELSVAENAYRKACSAFEDLTLPRHYHRMVELFSLVPIGLPPIANIVRKHITDAGLALELLDLDGVYSEI
IGSTLGEGYFNGDATFLKAAREAFEEFVNKDIGKSATAELLSNFCDNLLKNSGALLSDEALEDKLEKVVRLFGYLFDKEA
AREALLLAPSVGASAERSMIAKLKLRGGAQFTHDMEGMMTDMRLSQDIQSAFAEHVKDKELEHDKVFSLETKKDKELDLT
VQVLTTKFWPQYKSDELKLQKEMLQCVEAFKAFYDSHTSHRRLRWACILLLFNEADSMTFSDIQGRLNLPAEELKRYDPA
GREVAPTDTLSYATFSDRQQRIKVRRIAAEMTQEEKDAALKEAEQERKHVVEWGIIKVMMSEKRVEHEDLAEKAQALIRT
RPRWTTSWTNFTLARDYFDAMVVDLIRRDIKRKNTMAKIV*
>Ehux_229261
MQGATAQRVPKRLLDDVWRARATDDDCAICLEAPAQPLILLCGHMFCLDCFQTYATHTREYDDECTRCPTCRSPHAPPPV
GQVEAEQVEHGDALLARAMRMDRSRPNFLPLLEGATRFYVAALDLHPYCSRAHAMIGWCRQLAGEDSLDAFEDSARACHG
ALPAFAIVTLWNQTILDWLGESESAQRIELVRAGARACSWPQAVQREAAGTELSVAENAYRKACSAFEDLTLPRVHELHY
HRMVELFSLVPIGLPPIANIVHKHITDAGLALVKKQSANAEGDLMAYVQELLDLDGVYSEIIGSTPGEGYFNGDATFLKA
AREAFEEFVNKDIGKSATAELLSNFCDNLLKKSGERLSDEALEDKLEKVVRLFGYLFDKNIFSQFYRNQLAKRLLLAPSE
VDSAERSMIAKLKLRGGSQFTHDMEGMMTDMRLSQDIQSAFAEHVKDKELGTDLSVQVLTTKFWPQYKSDELKLPKEMLQ
CVEAFKAFYDSHTKHRRLRWVHSLGSATVVGKFTASGKSKEHDLLVSTYQACILLLFNEADSMAFSDIQGRLNLPAEELK
RCVLSLCGGKYKILTKDPAGREVAPTDTLSYNATFSDRARRIKVPMIAAKMTQEEKDAALKQAELERKHVVEWGIIKVMM
REKRVEHEDLAEKAQAVIRTRPSWTTSWTNFTLARDYFDAMVVDLIRREHLEPEAPEHPPGSGSFYKTSSDTGGSATQEW
SGGLPS*
>Ehux_468368
MQQKAGPSAAGAPRKLTIKPLRTAPKLPANFEQATWAKLLDAVRAVHGKRAVNHSLEELYRGVEDMCVQHMAARTYDKLQ
AECEAHVEASIEALQTPDTSAFLSLVHGCWSAHCEEMLTLRSIFLYLDRTYVMQSAARRSLWEMGLHSFRAQLSARPELL
AKLRDGVLASIERERGGDQVERSLLAELLRMLYDLGLYQRHFEEQFLAASSAYYLAEGAAEMNARDVPSYLEHVASRLSR
EEARVAHYLHGSSRKPLMAAVRQTLLAVHVRPLLERGFADLVQHARLDDLRRMYSLFAQARVNALPELRQAFAAHIKSVG
GAMELISFKEKLDTESFEQFINIRQNRPAELVARFLDSKLRAGNKGSSEGELEEDLSKRLLFDKSASIDTEKAMISKLKA
ECGSAFTSKLEGMFKDIDLSQDVMASFRTSPQAAKVSPSLELSVHVLTQGYWPTYPPVELKLPGEILELQEIFSTYYMSK
HNGRRLQWHPCLGHCTLKANFPLGKKEISVSLLQTIVLLLFNDGDGLSYAHVLQATGIEDRELKVTLQSLACGKVRVLRK
EPKGREVDGSDRFFLDASFKHPLFRIKINSIQTRESEAENEQTSERVFQDRQYQIDAAIVRVMKARKTLSHSLLMSELFQ
QGPPPPPPRPPPPRLTSRRLPRGQLKFPLKPPDLKKRIESLIDREYLERDPKSASTYIYLA*
>Ehux_443003
MNAQVIQLEDGWNKLKTGGVLKIEEILEDASGGVYKHRISTDEYSQLYTCALPPLRPAPPQQRAARFARSSFAPASAVRR

TVYTMCTQKPPNNWSEPLYNNYCEAVIDYLTARILPRIKEKHDEAMLRELVRRWENHKTIIRFLSHVFKYLDRFYVKRLS
LPELAEVGSQKFLEIVFNAVKREVRTAILELVRRERECEQVDKQLIKSVIDIFVEMGGSRASLETYATDFEEMLLSTTAD
YYSRESAKWAGNDSFPEYMRKAEDRLKQEQDRVANYLHSSTEEKLLKVCEEQLLQNPEQQLLEKEHSGCEKLLLDNKTED
LSRMFRLFSRIPTGLPPIANIVRKHITDVGLALVKKQSVNAEGDLMPYVQELLDIHDKYTELVGSGPEKGCFQGHNIFHK
AMKEAFEVFVNKDIGKSATAELLSNFCDNLLKKSGERLSDEALEDKLEKVVRLFGYLSDKDIFAEFYKKQLAKRLLLARS
SSDDAERSMIAKLKLRCGAQFTSKLEGMVTDMNLSQDIQSAFAEHVKDKELELDTDLTVQVLTTGFWPTYKSDELNLPKE
MLQCIETFKAFYDLRTSHRRLRWVHSLGSATVVGKFTPSGKSKEHDLLVSTYQACILLLFNEADSMTFSDIQGHLNLPAE
ELKRYVLSLCVGKYKILTKDPAGKEVAPTDKITYNAAFSDRARRIKVPMIAAKITQEEKDATRATVDEDRKHAIEAAIVR
IMKTRKSLDHQKLVLEASTQLMRHFKPDPKQIKKRIEDLIHREYLERDPSNPNLYKYLA*
>Ehux_470695
MQGAAQGATAQRVPKRLLDDVWRVRATDDDCCAICLEAPAQPLILLCGHMFCLDCFQTYATHAREYDDECTRCPTCRSPH
APPPVGQVEAEQVERGDALLARAMRMDRSRPNFLPLLEGATRFYVAALDLHPYCSRAHAMIGWCRKVAGEDSLDAFEDSA
RACHGALPAFAIVTLWNQTILDWLGESESAQRIELVRAGARACSWPQAVQREAAGTELPVAENAYRKACSAFEDLTLPRV
RELLENLLAVSTRGGCGSSSGGGGGSSGGGGGGSGGGGSSSSGGGGSSTTTLSRQHYHRMVELFSLVPIYLPLIANIVRK
HITDAGLALVKKQSANAEGDLMAYVQELLDLDGVYSEIIGSTPGEGYFNGDATFLKAAREAFEVFVNKDIGMSATAELLS
TFCDNLLKKWRSSPRAAEEAAADRHLSSDPELRRLYQSVVGGGALSTADFWAQRRGLLRAAMQREGLSTRRQQPTDAESS
AATAAAAGCEQPEANAATVAFTLTPGMIARIFEKYPRVQRSFLDNLSDEALEDKLEKVVRLFGYLSDKDIFVEFYRKQLA
KRLLLARSVGASAERSMIAKLKLRCGAQFTSKLEGMVTDMNLSQDIQSAFAEHVKDKELELDTDLTVQVLTTGFWPQYKS
DELKLPKEMLQCVETFKAFYDSHTSHRRLRWVHSLGSATVVGKFTASGKSKEHDLLVSTYQACILLLFNEADSMTFSDIQ
GHLNLPAEELKRYVLSLCGGKYKILTKDPAGREVAPTDTLSCNATFSDRHQRIKVPMIAPKMTQEEKDAALKQAELERKH
VVEWAIIKVMMREKRVEHEGLAEQAQDWIRTRPSTGFHMARDYFDAMVVDLIRREYLEPEAPERPPGSGSFYKYLA*
>Ehux_254675
MVELFSLVPIGLPPIANIVRKHITDAGLALELLDLDGVYSEIIGSTLGEGYFNGDATFLKAAREAFEEFVNKDIGKSATA
ELLSNFCDNLLKNSGALLSDEALEDKLEKVVRLFGYLFDKVIFSQFYRRQLAKRCSWRPQWALAQSAR*
>Gthe_101196
MTSYQIDKELHNLELLKIRFGEGSLHNCEVMLKDLGDSKRLDAYVHNESKKGALDAEVASWLNGKILSASFWPSLNSENI
KMHPMIDQQLQSYGRYYSLLKKPRALKWKPTLGVVKLNLSFEDGREVPFTVSPVLANLILHFQDKPSWKLAELAEVTELA
VDAVRKRMVFWVGNGVCQEQPPPSGAGIGETVYTVVEKATGPVMQQTTDFAMEEDDAAAQANDAQDEVIRSYVFGMLTNF
KMLPLERIHNMLKMFVSDPPYTKKIQELESLLNALIASEKLEFDGSNYSLKSS*
>Gthe_152166
MSQQKKSAFVIHAFKTQPPKDADMPKRSWEKLEGAIIQIFNENAGELSFEELYRTGYNMVLHKHGDMLYNNVDATLKRRS
MELCERVEKNTDETFLSSLKKIWTEYKRSLQMVQDILMYMDRTYVKQNQKKPVYDMGLGIFCQHCVRAAGVKDRLRRLTL
ELIRRERDGEKIERDILRSISQMLQEMGKSVFHEDLEKPFIESSQQYYMVQSESLITGSSTPEYLRYVEAKLLEESERVS
SCLSIDYNAGDSGIKQTVENELIGRHMMSLVEKEGSGLIRLLEDFRIQELKSMFDLFSRVQGGTDIIEGKVADHVGQKGR
EIVMSLENQADPLQFVHQLLELKENYDRMVREAFRKEKSLINKLHKAFEVFINLNSRSPEYISLAMDTHLRGTKTKSSGP
SNISEEQTEGVLERTLQLFRFLQEKDMFEKYFKQHLAKRLLGDRSQSEDLERKVIQMLKTECGYQFTAKLEGMFKDMHTS
ADLHQSFSRHLSQGDGNSLSLDLQVKVLTTGFWPTQPAQQCRLPPEIDHACMVFQRFYLAQHNGRQLTWQTNMGNADLKA
KYDKTYQINVPTFHMVVLLLFSPEGSNQLSFKEIEAGTNIPKADLQRTLQSLACAHHKLLVKEPKSKNVSEDDVFFYNSK
FTNRLIKFKVSTIAASKESNEEVQASRNKMNEDRNPQIDAAIVRVMKARRVMEHNLLVAEVTKQLQSRFNPNPVIIKKRI
EGLIERDFLQRQRGDIKKYEYLA*
>Gthe_160035
MQRASRAQVTDKDKWNKPVGEPAKKRLRTLTNEQEPLNSSVSSSEKMETDKDGISASGRNLPSTRPQIGENRAHPKKMII
SLKKEKPKLPETFEEDTWKKLKMSVHAVHREQPVEQSFEELYKAVEDLCIHKLGPNLYSRLQNDCEEHIKSEIESLVGQP
DDATIFLETVEACWQKHCNQMSLIRSIFLYLDRTYVIQSSNVCSLWAMGLQSFRKHLNSAPEVQNKIVSGMLSLILQERS
GDMVNRSLLRNLLRMLAQLQLYSSFETAFLADTESFYRQEGSDKLQDLDIPNYLLFVERRIEEEHDRIGHYLDIQTKKPL
ISKLDAQLLEAHAQTIVDKGFEILMTQHRIKDLQRLYNLLLRVNGLSNIRQAFSAYIKKTGVEIVMNDERGLEMVQDLLD
FKARLDELLEQAFASNDELSHALKDAFETLINARQNKPAELIAKFVDQQLRSGGKGISEQESELILERVLILFRYLQGKD
VFEAFFKKDLAKRLLLNKSASIDAEKAIISKLKQECGSSFTNKLEGMFKDMELSKDIMTAYSNSSVTSELSVHVLTTGYW
PAYPPAPLNLPKEILDHQEAFEKFYLSKHQGRRLTWQNSLAHCSLKATFRPNAAGRKELLVSLYQAAVLLLFNGSDELSF
SEIAGAVGMDDKELRVTLQSLACAKIKILNKSPKGRDVEDGDSFTFNSKFESKQLRIKVNSIQLKETQEENDKTTESVFQ
DRQYQVDAAIVRVMKARKSLSHTLLISELFKILKFPVTPPDLKKRIESLIEREYLERDRDSPSVYKYLA*
>Gthe_158687
MQEGFDKFIEIMEGGFREPFALSLHSELYTNCYAMCTQKAPNNYADQLYQKYGMIYETYLHATVLPAIKSKKGEAMLHEF
AKRWKNHKLLVRQMWKLFVYLDRFYIKRISGLPLKAVGVQKFEQVVFNAVKEDVRAGILGMIEKEREGEDVDRELLKSVV
NVKLGDIGAARFNVYNKELEQNLLATTSEFYARESAQWIATDSCPEYMKKAENRLQQEVERVHAYLHSVSEEKLLKECEN
QLLAVHQTALLDKEETGCRALLREGKTEDLARMYKLFTRLPNSPDCGLQPISQIVREHIVDVGMSLVRKQEGEKDHSNYA
QQLIELHDQYLALVNGPFGNNTLFQKVLKEAFEVFVNKDIGSTTTAELLSSFCDNIMKTGGDKIEGEIDSILDKIVMLFS
YLSDKDMFAEYYRKQLAKRLLLNRSASDDDERSLITKLKYRCGAQFTSKLEGMLTDMNVSKDGQNNFTQWMKNNDINLGM
ECSVTVLTTGFWPTYKVDEVNLPNELVKCVDKFTQFYESRTSHRKLKWIHTLGTCVVLGRFDPKPIDLVISTYQACILML
YNQQEEYTTQEIANATKLPMEELKKYLQTLALSKYQILTKTPKGKEIADSDVFTFNRKFTDRQRKIKMSLLVTKDEKLST
KQTVDEDRKHAVEASIVRVMKARKTMAHQQLVMEVSQQLMKLFKPDPKVIKNRIESLISREYLERDKDNNGVYKYLA*
>Gthe_148610

MSLLNARPLDFDTTWKLLQSEVSRLVTDPTVGISNQKFVQCHTYIYKICTIPQGREGKPLADRLYYELKGCLERVCKQIA
RELLVSSNLLPLYLAKWKNFAIGLRCVNAIFDYLNRFWIRSHCQEGLIPEDGVYPSVLEMGTAVWRDNVQVIPVAQQMLE
AMWSHICEGRKGALLEFSLIREILGVYRELGCRPDSGRVYDSDFAGPFVQLTFQFYSQEALELMAAQDFDAYALRAHESI
SSELAAASRFLLPETLPELCMACERAFIEEQKPWLQSIFEALLLQDDMREQLWRLYALLRRVDGALDGGRTSLFELISRE
GTAAMAALEEGEDGEGRQGIDPSMFLSTFTERLEYYYTLVHDSLEDDAEFLNAVAKALVVVVNSIKSCALLLAQHAHSVL
DKHGARERGIGCDTVSAMTDVVTCLKYVLDKDIFQENYVRLYAQRLILDSSISQEAEEFMNDSLRSVCSPDFISRLQRML
ADKVRSKELEDDFYDDYDADTSVCGMHVLLLTAGTWPIAIPSPIPIPDCLRQGIETFEVFYGSKYKGRRLSWMLQLGKAE
VRGFFSRTYEFSVSMAQLCVLMAFNTAPSLSFPELLTMTGFEEEELDQVLRSLAKSKLLLQVKTSDAAGTEEGDRSGEES
EGSERRREEMKMLVNDKYSNKKTRVRIVPHMAMGKKEEASNAAAEELPQDRNPQIQAVIVRIMKHRKILSHNDLTKEVCS
LLPRH*
>Ngru_XP_002683001
MNSFTEYYPKLYNGRHLTWLNILSKGVVRGHFTNSSMPYYDITVSLIQLSIFLQFNNGENSKTIQDLSLAVGIPIEELIQ
TLVPIINFKIFTSSSAITSLDQSSIITINDSFNYQRNKISLFQHSIKQYYEEGKSNPANQKILAQIKEDRKYTLQAAIVR
ILKTRKQIEHKQLVNLLLAQLSSNFKPTNSDIKRAIDSLLQQEYIKRVSEDSSWYQYIA
>Ngru_XP_002681494
MEDIISNFRQFCDKLRLLRANFMNSICSNSWIAFMFSAIKEKITEVSTTDFEQEILTYIQQWKEQIIYSYMEIYFCGDSS
HEMFNTLKSQVDYFVFENLAKIRISQIFDIVIDFPNSQQAITDLKDCLNHCGMHNELVKELSKGVKSRLLLASVETKNII
MAFIRIIKSLLIIDPTGISLESVSFHIKEYLREKRPDAVRCIINEMTDEQNDSALYRELLTSEPQSSLDYDSDDASENWN
PKPIHPSSSKSKMEKPDIIKIFIEMYRGNKEIFVNEYRMILAESLLKNETYDTNNEAKMAELLKIRFGDKNMQNCEIMLK
DISDSKFLNSGIQSRLANRSDYIELSNSQIAEISIQADSQIPSVFMLSKNYWPQVVFNQGAPPGQQLMTDENKPTILLHP
FIEQLMEEYANQYNAIKAPRKLLWEKQMGSIDFDIEIGGEELELTNIHPVAASILFHFSDRNVWTMDEIMEMMKIDDKTF
QKRINTWVKRGIIIDEGNKTYRLVDSLTSAATEQGEDENMEEMTTAQSQEEELRVYQDIIINMIENFDSMPLAKIHNMLA
MFALDPLPPFDKSLSELSSFLNILVKEEVLECDGGEYKIKR
>Ngru_XP_002680365
MTTTIPSTSGFTKNNTTASTGGGLTNPSPSVTPTKKKITCLKIKPLSLKPTVSNFEEETWNKLNNAVVAIQKKEKICTGQ
EELYQLCSDLARHKKSESTYSKLKLLCSKHIENVIYDLGHKATTDHTTFLNIVVKSWEEFTDQINMIRSIFLYLDRSYVM
TIPDKSIWDMNLQIFKQNLKINEHLLKKIISGILILIKHERSGESIDKSVVQRLIRMLTSLHLYEDEFEKSFLEETRSFY
SNDGLNNIDKLNVPEYLQYVESRLRQEVDRVTNYLSKLTKKPLIQIVENELIKKHVKTILDKGFEELMDLNRIMDLNRMY
GLFKLVNELDAIKEAFTVYLKIRGKRIVDDDQNDKNMVQDTLQFKSKIDQLHEQSFHKNEEFKHAIRKAFEYFLNIVPNK
PSELIAKYIDGKLKNSKGLTDDELERCMDNALTIFKYINGKDIFEAFYKKDLGKRLLFGKTSSYDAEKTMISKLRAECGT
QFSNKLEGMFKDIDISAELMKGYETSAEFKKFINEVGEEKDRALQIASSLGVKVLTLSYWPNYTPDTLNLPMELSLLQDS
FRDFYTHKYSGRILKWVSNLGQCSMKALFPCGKKELIISFYQAVVLLQFNSKEKISVRELKQSTGIQDEKQLILTLQSLA
FHKEKILKKETKGTQVEENDIFFVNEDYSQSKTKIKIDSFQLKETKKEREETTEKVLLDRSYVIDAAIVRIMKTRKQLTH
QQLLTEVLSQVRFSIQGQDVKKRIESLIDREYLERDNNSQAGSANCIYHYVA
>Ngru_XP_002680364
KRESQNLNELKPWENDEHVLLFTTIYNLCILPDSVYYEEQLYNLYSIYIKKYLQTTILPKLKDKYGEDLLNEIVFIWINF
RDIFVKFCSYIFKYLDQFYVASNTKRTLKYEAYYLFKTSIFDHCKVQLRQILLDKITQDQYLLFAEECIQQEQTRIQAIF
PTSFSQNKLMKLCDLELLKNMQKRLLEMSGSGVKILIRDEKFDDLKRLYRLMNRLEGGLDPIADLFKQYLIFVGNELFVK
YENASSEMIKSNDNNLNNNNNNDLLELHQKMKNITNGSFSRNVIFHKAMSEGFKQFVNKNITLGQFEIRIVQLFAYYTDD
VLRKKSDEKKLDCIVDFIQFFSDRDMFIEEHRKLFAIRLLVTDYQELEERMMISKLKYHYRGVADTYKLEKMLTDKTMAN
DMKLEFQNYITTNQLQLSYDVNVTVLTMGMWPLKAKEHMLLPKEFLESQHLFKQFYDSRNGKRVLKWVYSKSMAQIHAHY
INGNHLFELSTLQASILLLFNDQLQLSVKQIEDLTGLNFDDIDLKQSIISLSSTKFPILIFNQKEMTLSLNENFTSRSFK
LKIPLPRITQKDTQGTQTSVSTDRVHILDACVVRIMKTRKTMNIQSLFNEVSSQLIPIFTPDVKQIKKRIESLLERDFLK
RDEQNNSILHYVA
>Ngru_XP_002680175
MSQGGKTPSLGGTNKLLIPSYIQRKGQMGKSDVDDKLEKIIHAIDQIYQENQSQLSFQVLYTSGYQIVLHKNGDSLYDAV
KNKLSEYIQGVREKTMEFTDDGFLKELLKQWEKHRTSVSMVRDILMYMDRNYVKQFKKTPVYELGIKLFGTEVFHKSTLE
RIQRLIMDIILKDRCGEVVADRFLMKSLTQMMIEISKKDIYETHFEKKLLDETRQFYTKESNEYFESSTATDYLKKVTLR
LKEERERVDRCMDPDTKPKIEAVLKNVMIDKYKHRIIEKEGSGCIAMLQTWKVDDLRLVFDVLSLVEGALDPCVDLVENF
CRSEGYQIVKDKNKEENPVDFIADLIVLKEKYEGLLDRAFSVKKGKQSARDSKFQACVKKAFDDTINANERFPEFLSLYV
DSKLKKGKTQVSESEFDVLFEQVITLFRHLREKDIFEKYYKTHLAKRLLNQRSQSDDAEKAFIGKLKQEFGYQFTAKLEG
MFNDMRLSRETNESFKSYIDRFPNKKPAIDLSVQVLTTGYWPVTQSIAITVPETIDKSANIFKEFYIDSHNGRKLTWQYN
MGSADIKANGYDKKYEINVSTFQMVVLLLFNEKETISYGDILQTTKIPMNELKKNLLALTVKTATHQKLLTSSTDKTLTK
ESVFTVNNEFESKLIKVKIAPIVLKETKEQQEETKQKIDEERKWLLDATIVRIMKARKTLEHRDLVIEVTKQLQQRFMPS
PDMIKKRIESLIEREYLERSQESRSKYNYVA
>Ngru_XP_002679430
MPLDEANKTIEILTSAIDEIYEERAAVMDYQKLFDLAYKLVTKKYGEKLYDKVRQTISKHTKGVCNDINQQKEITFLPHL
LTVWKKYRKAACTIRDLLLFLDEQWVERQSTHDNKIKTVFELGIFIFREEVLIKLTDRVQSIMLSIIRKERDNIEPADKF
LLRSLTQMMVEIDKEKVYIPVFESKFLSESHIYYKIEAEKIFDSCTAVDYLKKIQQRLKEETDRADRCLDPETRNKIENV
VKEEFITRYKDSVVNKEGSGVLVMLKDKKETELRLVYDVLGLVEGALEPTINIYREYVTEQGLAIVTSEEKNNDYITLVT
EIIQLRVYYDELLLRISKTRKTNTFIRDKDFSKATKDAFDRVVNQNEKFSEYLSLLLDKKLKKGKQQIEEEQLDTFFDQV
IMIFRHVKDKDIFEKYYKEHLAVRLLEERCASDDAEKLFLSKLKTEFGVQFTTRLENMFKDIKLSKDLMGQWNEYRTRPP

IDMNIQVLTQGSWPGTTSYKIEFSEQDINKSMNVFNDFYQGQHNGRKLTWQYQLGNASIIMNGFTQKFEITASTFQMAVL
LLFNDNEKLTYKEIETSTKIPAAELKKNLIQLTKPLDDGEQYKKVAKVLTVKASEDQQQSTAEGDKKKFTISATTIFATN
NLFKSRKLKMNAMPPMTKQTEEGASKINQQVEEERKMVVDAVIVRIMKSRKVMTHRDLVLEATSQLQQRFMPAPNLIKKR
IENLIEREYLERDENDRQTYKYLA
>Ngru_XP_002672641
MPFFQSFIGKTDAKKDVQTIEKATGSGKQEAPDFEGKWKYLQQGFNKLIDFLDKNMSKPFDYNEYADLYSTVFNLCTQKV
DTNKKGGATELLYDRYRTCISDYLKSLVVVALKEKQGDGLLMEAVKRWRDHQLVVRYMVKLYNYLDRYYTKHNNRDDLRN
VGLKCYQELVYGSIKKDMAQALLDKIYKEREGDLIDRSMMKDGITLFIEMGLGSLNAYDEDFERTLLQNTQSYYSIQSSK
WIAEDSCPDYMKKTEEKLESEERRATAYLHTNTKPKLISKVQDELIRKHQTTLLNMDGSGLVALLKTGDKHEDLSRMYTL
FDRIESLQPMSEKLRDFITEEGVKIHTNQCQQENIDAKGYIEELLKLHLTYSKLVNIQFKQNPLFLDALRDAFTHFVNLE
VVSPGDKNRSTTAELISTYCDSIMKEVDKVGEENLDELLENIVKLFGYLKDKDMFLAFYREHLSKRLLVASRLNLDAERN
FIGKLKMRMGMSFTQKLEGMIKDKSISENLRNDFKNYTTNKSITLPFDFNPEVLTLGCWPQMKIDKMTIPQELSVCLDTF
KKFYDSITQQRKLDWIHSLGTAIVTGRFSAGTKEISTNTYQACILLLFNNQAEMTFQDIQNSLNLPPTEIKRNLLSLCAT
KAANLLSTDGNKKAVNPTDKFTVNADFESPQRRIKIPNVVVHVTQQQKQDISQKAQEERKYVIDAALVRIMKTRKILKYQ
ELMTETIKQLSSHFQPDPKLIKRRVEDLIAREYLERDAKDSSTIQYVA
>Ngru_XP_002671343
MITKQSTNQQAPRRRTRATLAIKSVDFNKLWISLEYDLAQVLTDPLNATLKNDTEKLHNIVYKLSTGQCFNLPDGSILSQ
NPHSPSIPQDGDIQPYTLYFMLKRLISNHLKLFISKLNNLTGIPLLKQYFTLWNNYLIACDFIMKVFHYLNSDWITEQEI
KRKSKANQPEKYTERSANQIVHFSESNHVCNTKLTCLLLWREWFFERCENLSKPLGYQEADSPLIEEKEVTFDKFGFYDV
IMKIIEMDRNYNNPYLKNSFCVEEFYPILKSVIGSLLYLSLDSEEDFYFSVKYKYYPSMNLFESLNVHIEDYYSKILDQT
MTWKYPIFYLYDFERIFLETSKRYYCAEIDSLLITYLSGENHHDSVRKYIQHVSARLDQETNKNILHYLPQITRDNIQKI
MCDLFIEGIKENMSSLNFILSDIEAWINEPEVYKDNLELLYKVITKSNRKIALNELGTRIRNRILFSLNSDMRKVKMISQ
DTYDPIQFIDVIIDNYNIFSSIIRQYFSNNYELNICKDKTFKEFFNNAPIFTSDPNMKCAEYLSRYINHLLKGSSRPTLN
CYSDNLDIESRMDYAVYLCRLLNDVDVFIQYYKVLLAKRLLIFKYEGEATQEELEYTMITKLKKVCTFGHIYKIQTMLND
LNNAKSLAERFTKWNENGEARIQFTAKVLTKGYWPLNNSSLITPSNAFCIPTTISERMNSFTEYYSKLYNGRHLTWLNIL
SKGVVRGHFTNSSMPYYDITVSLIQLSIFLQFNNGENSKTIQDLSLAVGIPIEELIQTLVPIINFKIFTSSSAITSLDQS
SIITINDSFNYQRNKISLFQHSIKQYYEEGKSNPANQKILAQIKEDRKYTLQAAIVRILKTRKQIEHKQLVNLLLAQLSS
NFKPTNSDIKRAIDSLLQQEYIKRVSEDSSWYQYIA
>Ngru_XP_002669996
MRKVQTGKKFEIKAPRAESTTTEAFFSTTWTLLKQAIQQIYHNKSSELSYEELYRNTYKIVLNRHGESLYENVEACMLEQ
VEFILSSVLKKCPDELFLKTICNVWEEYKTETSVVSSVLMYLNTNYALKQLQQQSQSSGNGANQTPVKHTLFVYDNGVE
IFKRVVIYQSQSGVKIKNIVIEMIGKERGGEYVDRLLLKKVVRMLCEMNCYNDVLEEPFLETSAQYYLQLSRDLLAQTSI
TDYLKLVDERLREEDNRVQYYLSFTTKPKISKILRQEMITKHLDTITESPSGYISFLKDDKISELHRMYNLFLGNEEEHL
SIMIKLYKQYITDVGIAYVMDEEKLQGSAVTFIEGLLEQKRKYDRITRESFKSNSKFEQAQKEGFSIFSDGTRQKRVSEY
LSLYLDNTIRTLGDSEQELEPIMEDAMALFRFLRDKDIFENYYKVHLSKRLLSKGHQANSEKMFILKMKKECGYSFTSKI
EGMFNDMKISAQTNEQYQQHDAFKLKPERMDFNVNILTHSFWPAYTLNNIILPADLNLCCESFAKFYNHIKELTQIPEKD
LKKTLTILCMNKTKILSKEPKTKNLEDNHKFVLNQDFKNANYRVRLAITSTKETVEEVQETESKIELERKPVIEAVIVRV
MKARKKLHHNELMSEVVKQLQSRFVPNPQEVKRRIENLIERDFLSREVEDHKTYNYVA
>Tvag_XP_001583635
MHKLQIDKQNQLSAFKPCQIYTDSAKERPTSVPSSITVQVAQKEEWTVFIDEKLTDLFEKDQTPKFSIVDQRFVYSLAIY
STPSSILSYINEKLTSYMPKIMEKFGSSKDTNSFIALIKNIQLKFSYLQFTFSSLLPNQESFYNILINGLIFGETEIKFF
VGFITGILEKLFKDSNEEDIRALITYMKDTKLLEVHGEKIFTDIFTKLSKELSAVEIYQAFRILYTVAGNDIETMVLYNI
ASMFAKDFVANISSQPDLINADPVNTNILAIIFKSLSTISESYRKFTDFITKNSKDALKDDKLFENFIKFQSQLLSLEHI
GQVGRNNLTRVLERQIGATSEQVTRLLASEVSRIADIGPSAVTDDAIEAISNAFAWMGNTKLFEELYIADLVKRLIENKD
IQADCLLAQRIESLVPELATKIIYILEDFKQNKDAVQEFTKKYPAQFNLSFSIIQSYILKNEQNLNVKPPEEVLKAMTEF
TKYYTDKVNKRALNWHAGLSRCNLEVKNCGSLRKVRCDGIVASILTVIGEGKQEEELVELVGLEKPKLEEILTALSSSKY
GSLITKTKSTYKVNVDCKPDENGIIDIPTISEKTPTSQVFESKESYASRESQIDAAITRALKGGGPMTEKDLISVVQKEV
EFPVSEDLFQRRLKILITRQFLEREGAKIAYLP
>Tvag_XP_001320654
MEKIFQETNNYIDTLRISQEKETKLQWIIDFIIKIGLEKEFLQSFEENSKNFLFRNPITDYQSTTIISQVDEIISNNKSI
FQSINSSFLEKIDNLYRSLAYNTFIVSELSTIFDNAFDLQDKNAFKTITNFINSSNIEGLVDKYKNLWISRLSTYLNSLF
SMTNNSCIPKLISVYNFIKSFNDEIPSGLQGDLLLLLQRFLNDSNTFGIYMIARYIHNIIVTEEVTDQKMKDVVSILSLF
DDTDQFSQFYHHFLCNRLLRYFTPPPAIEKNSVISFSPFIEESIMKQINRMMDEMENVEIFEHSSEMNIKFIAVTSGLWI
VPLSKQLFVPPEVEIARESYRKHYKEKFPKRALNFTPLVDHVTFKYKGVSWSGFYPYLAVLMAISEDKNIFEIGLTQKCI
PEVVNALLKLGLIQRMAGKYILASNFKPQRKLNKLPQLSIFLPQVPRKLDHKELFSKKKPKLEFLITRTMKNYEGVSADE
LYQQIANTSQFSIHRKEFDCCIASLIATEQITMDCNQKLHYNPF
>Tvag_XP_001306780
MQEILPLILSLEPDNIKSVLTNYRDNENYKLFTRLFLVQVRNSISKLLKEEDYGSISIAAQKTFDKMKGLMEVVKPIDED
LEQEIVTLIRSLLIHKYPYGMQEFESKDLDSILEISDPKFKALFSCGLKQYLSEIVLNKILDQCKEKINEFNDEYEVSVL
SATMKTLSDNIFLFSDKFPELSNKLPSMKATIYSLVVDFRAKQIFDIVASYPDSTPSLYDLRESCLHAHAHKKVAQIATE
IFCNRLLHLGASTTDIVTQYISAIQALNIVDESGGLTRAISPPIQSYLTTRPDLLTTIVEKILEDNTLMDNSRPSAQKPN
DDDVLREASAQRELDEKWNPEPLHSHIRDLQSLVRDASDSDALALLLNVYGSISSFVSQLEREIAARVESRPGYNFDSEV

RAIEILKRRFGAQIFLNCEVILQDVADSKRLQQGSGVMHFQPLVASHMYWPELNGDEFTLPQGLAEEVSAFEKEFDRVRK
QRKLKWYHTIGCVEIVLDFDDDETLSITVPPLAAATIALFNDKTEFDTSYVSKKLSISKRAAEHAISFWMSGGVIVRGSS
EGSFRMSSKKPAAAVELAESARSDMIGLLFEEKKNPEEEENGEEEEDFSSPVVLSKRLRPFALTILQARPREKFTIDSYY
SLLMRFVRIPKLRIEKAEFLKIIPIWVSENMITVDGENVALVK
>Tvag_XP_001305732
MSQGFRRQIRHDSSPLSSESDSPWSPHQLAIQNRHQQQIRHIDGTNFSQFPLQCNEHKLEFDKEWPKILQIVDSIVNEKE
VVGYATASSIISELTEDSHNIPKITSYVRELLIKPTKKLIESIESANDITEVANIIKVCAKKHVLISSIFQQWEKIENIT
ISSIIYTLLKSEISTNPSILDHVLLIVSQAFTKSRQDEKLYRQEIKRSVHYFSQIDRLSSLTDSIAKEVTSYYRCHPIDP
QLGPFLFITNARRIISSETAHLSVFPPNIAIPALSAARKYIYVDYFKYSMQQIIEAVLIEKPSFISDLRSLCEDSGDASL
VASLHDHLGAGIKDRVSAFIKQNQSSGVAQILELLSMLRKYRDDAGAELTQIAIQAVRETLNKSLFQVSFMIAKYTHTRI
MDSLTEFTSNKNKIIELLQLLENTDVFLDYHRKFLGQRLLSNSEVPEVETQLLKDLSQFAKYGSMNPLYTMISDLEESKK
DTSDFDAKKSFLFTVTTITAYSWPHYPFINANVPDDVRDAREKFVVFYKQLRKCMIRWIDPLENVTFTYKKVTFWSSTLQ
FLVLKTIVEHGDLKSTGIQQCYINEIANQLVKVGLIAKKGGKFVPQNFKVTKSSMKLPPLGLLIASKKKDRLEADVFQGR
KESIRTSIVCTLKKFGELPFDALFEETKQAFRYPLEEGDFINFVDELISTDVIVKGTNGNYRFST
>Tvag_XP_001313897
MPRKTSNTYSSHKDLEEISKSITSILTHKHSSLSLSYLYNCMNNLVNNSCTNKLRDTLQNHLDKQFSKQYNSLSKTALVN
PLVIKFSKHCSYFASFLKILPKLYSRYDESLAENSEYRTEKILFTKFKEKVLNQTKVVDLIIKSMIDVIEDARTNRIVSG
TANLRQIIELFYEYKNSNCDRSIFENFMASLKESTKKKYENEYNSSENFVEFLKRIKELLDFEENLLLKAIFKDEEDVNE
ILMIAHESAIFKNTETFANQKDFQVPPIYSALQDKNLLRWLIRDSYLRFHQKNHIMCEFIPKICEFMTKDIKSFEPKFSD
HMKLNDKITQIHEIIKKVLSQTEPFEFVFCSQEHMRKKSDDPNFDMEKEAREKFEKAITAGWNIPAFNIVENFVQYIDFH
IKNEKKRLSKTENENFERTCARFFRYTEDKVGFCTTYEQALLQRLIKFLTKFDAEERMMIETISKEKQPEFMKGYKDLKA
CIDNSAQIDDDFKKQIMNTKIEWPDGMREVQFSPIVFNKNHYKLYIIEQTKIPEFLSPVHDRFIQFYQEKNKSMTLMLLA
DTSTVQSKWRIPKNSKSNKPETYVLTTDILTSKILSYLHKNQTEKVSVQDIIMYTQEEKPLVKKCIAKLLGLQICVREKT
VKTTEKQQQQQLSDTDLIMMNKEFHMNGDIKRIVIPTCISVQKQNKQIVTAETESEKVNTTKAFIVRTLKQMNTIDTDDL
INLTIDYLRRFFVADKEFIKKILGELDNDGTYFKKLDKGMLEYIR
>Tvag_XP_001306671
MPRVKAKVDLTPDVKRVEEGLDSIIHRQPCKHDFNSLYSSTDKIINNDNQAKLFDLLNNKINEEIAKWEKQLKEVTSNSL
LSLFSTLYDNFKIYSDIIPKIYSSYDSKSSPENKSRMIIRRIYFKLVISDRSIISSVTKALLKQIDYFRDTPTDTNYSIP
KNIVDSFYQFNPDCDENNKILATFKIDFTGGVYAYFNQFFEKRNECDIAKYTSDSFTHLTLEENIISTIFRKVEADLMFK
ESVCALFDYGDFFDIDKSINVAPLLEKDETDLLDRLNKLTRLYMNGRVEPDINRVEPLIDLLCNYIKNRMLSFRPQFEDG
PAKSENKEEGAKEGGDEEKAAPAKKTVAKKTVAKKIIKKKVVAKKEDNEEKEDDEEKENEEKPTTAEPKMRIVKKATPKA
GTASAAEKSSKSKIGKDEKITAMKDLIFTALKLYSNFNKIFQDSSDKKKMSNAVEQAWNAKEFRPHYNLVQFIHYSVVNN
LENIPDEYKSKFPQICKLFFDFTTDKAVFVPLFMNYQIQRIIKNRESEQDTRALEFEIVKQLKSIGASSNTEDFGKHLEE
IYNQVRISIDTTKNFKATIPSDSQVDFIATVLNHNIVEKFATDNAFKIMPFLQDLHNRFVGFYQQNHQNSDLKIIPDISQ
LQFTFRVPKNKKSANLKVYTINTDAWGGSILMAIYEAKETGLKKADINAITAVKEDLVDAYLKMMYHMKLIAMKDKGASN
KMEDIEWVLNKNFFNQQKNITIPTVDVQKDKASDVAAERKQQLITIHIIRVGKGNKDGISKTMLIEEVARACRKYFEPSI
EEITTKMDKMAVPGPENYLEIKSDGTVLYKR
>Tvag_XP_001303424
MLFSSNVAKILANCKNEEKAFSKEKDTPFFHNVTNKHSSANQSFLIKPEIIFQSKTPSADYSTIISEAMNNVILHNSLEN
YNKAFQCIQKLSRNRNQSNDVVNFAKSFITEDLEIILSQFSSLDSMEKINLKLIELKKAIEYITSLLYSPIRSYSFSREP
NQIITDVLIDFVKYNPDIIKTNMEFALNYAVQSILNTPENIDDLTLDFIKNLSDIFTQSQFITEFSSFLSKGDYDYMIYD
QEIDQVLYHLYNTRAQIKKLSSKLGETLVASIIDAVTTSLFKNRFENENVEIIQQIFTSDNRELINILLEISDARNDTTL
SDLLSSGYQSYTKSVLKKYLSYGDLGGIDQILLFFQSLLDLNLPQIHPVMKAFQQGLNESPSLISFEFARFCNTMILFHQ
EDFKKNIDLIIHFFGELYSIDNFLTYYMQFLTLRLLEYKEFIPDAEKIMLKHINDACGYNTNASKLEIMIKDAENNSQQA
NSNSIATFYLIKEDAWPYFSEVEIETPDMINNAFEEFKNQYIEKNPKHQIKWLKILDKVHFTCNSIEIECSAFHYSILLQ
ITQQKEIELKTERDKSAIEDLIKANLIVKHENTYLFTPPTKPVKVSNLPELIPVAEKESEANQINQIRQTALEAGIIKIA
KKHQIIKVDDLFNETSSYVKFPLTREDFDIRLGKILKMELLSTTKDGRIQFTP
>Tvag_XP_001302864
MQNPSVSQILDRLNQDSQTSTTKKSTKTIKIKKPVKKNITSPSINRTSSVSEQQTEENFDIQWESILECVKQTFSSVKSA
NYETTYQLIEKLCKNQVTLQKLADKYTNTIDEYSNNSIKSILQLQNYESFENFYIELQQNIEKLETLFSTFEKSYLYPVT
ISKSIQNQFIKYISQNSAQQDFIITFLADGFKQNRLKYVNGSTDIKHFQIIANCSHFFEVESDLQAVIIEDATQYYQTLE
VPQLDNENLINWFEKQINYEIDMYQYFKTTIGIEIIDTSRQTLYGMKFYMYLQNIISDLVFSKKYDSIVKVSKLCDYSPE
MKELFTDEFKKFAKNCVSQVKKGTNVAIISDIFGKLLVFRDLKEGIENETINDAFKAFCTEMRSKYRKLQLMYAEFINQK
IQEDDFPQHLSDYILFFRQLSSIDEFVLENRSYLVNRLLSPNYSHISNEEIFGKAIEEIIGSDGIKSTFQMISDVKYSRS
MDIKLSIPGFVTLIKYDTWPEYQEIFPEVPRQIEVLRKEFENQFHEENKKISWVSPLETVDFEYGNAKISGSLTHLCALM
KISEGNDKIPKDIKEQLVAAKILDENNMISTEGNFILPLPKYRIPDFHKEENSYEAQQNRKDRIIARIVLIAKKTKEVPK
HDLEEQLKQFISELTEEEIINSINNAVKREYISFDNDIIRFLP
>Tvag_XP_001302856
MIIDSLEVYQTNLDNFIGKIVKDLEYMPSVSEYTNVAAACFNLSTKYAGFYKEENSKIDNPFIMLNRYIEKLLFKHENKS
YSTLNDEMKIKIIENASKRIHLLQNLCLFGETMNKSIYKNKKFDDIQSIYSRFLMTSIVQANLGIFLDLSIKQFTKAFET
TENTDLSMIISLFQINYFGAPTQEICYNNSSFFIAEIEKSIEKLVPDLQKLPTVMKQVKTFLDLIQNDTKTKERFVKVFG
EKIILPLMPKITQSIKSADIFNSIYPSVTFDGELLISVSLIKSDFSQQFIESVFEERLDKFISSLQKETKIKKLCETLLD

LMKFLETFNKIIYLESFREFSILKTKSKLQDAKFKDVFGGIWPIALSFIFDSLMQRGNPLQGMLTTNTIIKMLSFVNDKD
IFMKQYENHLFNRLATRATIGKEKEIEAINALKVATSYENLKNTENLLNNYEVYIEPGFQKFTYVMFPKTSIPELGHEFF
IKLPSEYSEIITQITPNLKKKWPQRHFDWRYSLVSCESRLKTKAGVSNLTISLAQAAVLQCLCDSDGVTFDTLSSVTQID
AERLNKMINKLLKQKIIKISDKNDIEKAKISINNNFHQTKAIIADNWKIEPAKFEQISETRMLVLQSITMRIMKMVKIIK
ISVLKEKVMEEVSLTFKARPEDITRCFNDLTIKGFIELYGEDRFIFNK
>Tvag_XP_001320004
MSDNANAGTMNPNQKRIVVGMYKNTKSSTKVFKSPFQDIPQPTFDLFKDEIQDNLNKILVDGDLSLPGKSFIDISRQLAI
PTETSPTEGHQLYDFTSQHVVDFANDVTTHISKFKNVDQIGSFWTDISSKINEISISFSPLSTKSNRLPLLRSKFEDVIK
TNLESTLPEFDRVCDIIIKEYNNNRTPENFETEKLAFNFVKDSGLFDEVFLPKLIQSVVDRIRDQVDAAFEGPLHEYLSQ
AVSLVQSEINDAEPFISPSSLRKLQRELYNLIFTSKFQQIVKVGLPVIVKEKNADSIKICVNLAKETDKINEFVRRLSHV
FEAEADCFNLENPIAHILDLHRTMIMFNEAAQFSPEHIKTIRSSFDKGFNNLPDVAARLLAIEVNREFITGQVSDETLEH
LLDVFKILSYKDVFVSYHAYFLAKRILLMKKHTVDDDLKFMDNLRVLCGPEYTKPLRMMFEGLKQSLEVMDALRSEKSIP
KWFSCVMFSQESWPGIEPCDAIIPSQVLPHLQAFENKAVAEKKKRIMHSLQLTRVKLEVKGVKGIKTIKCNGLYASYLLA
FNDVPSMLVSQISKATKIDQKDVEEMTEILKRKRAGNLILLVHQMVRINPEASVESGALNIAFSFPPISQKDDEHTKNAI
MQNRDNQIDAMVVRILKFEKSLDREDLKLRLKDALKFRMDDEMYEKRLNSLNKRQFIKLDASGCVNYI
>Tvag_XP_001313683
MKRKSPAGTIDSFFNASKEKKQASIDELMPIPRRQLTVQKTQQQKGVIHIQIPSLSDESSINEDKIKEIMAYLKEGIHLL
FDDKIQNANFAKLFTNVEYICRNKTPEYLYKSISEDMQEAALNFINSISEEFDITLISQKISLFEKCLDYFLRTFLYLDR
SYIFPLRIDGFTSLSEIVYSYIRNGMSKELADLISDQILITISKYRLHEEIKLSELKTGLNFAKSTKFYNDHLEPTLLIQ
TKDYFEDISSHLSLKDLINWNYSSKEIETQLQECGLEKSTYLSIDSMQNEIVLKKAGQRVLFSQEFYDMIDQFDKEELKR
VVSTFDKEELRKLLLKSVSSYFSTKIKSTIQSSTDQTLIPDILRIISKISDFDQFIFENSRDAVKTINESVSASMSSQRD
HIAEYLARYIDSGNPLVGPSLILFKLIDASEVFESTYFHLFGRRFLSWNVDVQRENELIDTLQMNASKEYTDRLVKTIND
YNQSKRILQPTSDGMEYKAMALYERDWSTNEGEIKFPEQIEILNEEFKKRYMKQQKTDSHIVLHFSAALSNCVLKVNNKS
EIKMSGEQAIIVLLLSEKGPMNVDDIHNHTGINESQIKINLDILSKKPLNLLIKGDDDKWSVNKDAQFPENYSYPSTRSG
NLQLQRAVIDNDINYIRTNMIMSCMYKILKEFKTLKLEELISRVKKQLNFFPETEKLMFVINDLKNKDVLTQDENHVIRY
VP
>Tvag_XP_001308718
MRTKQLRAPLHDEFEIPNIAHITRGFAVKSMRPPNLIKAPFEIPEITKFANYQDQFQDAITHCLTLDQVGFQVQKIARIS
QSLSLSKDCAEAHEFISKQIIEFAKSIPTEISNCRTYEEIGQFWEKIQSKLSITTLSFSYLCVKSLGLRTYRDIFLEALR
NEYTQNHELFETVANIIIEAYNESRNDCSSEKLKIHFIFLNDTGLFDTLFIEMLINSVLSIITPVIKESLNNGISSYLHQ
AQELSIREEKLASPLLTQTQLRKLTSSVNTAIFTSNLNNFVAGGLHELVTNNDKESISICAHFSTATNTTSTFTQDLARV
FEEEVSKCFEKEDPIPDIINLYKALSSFNAAAFGTSSARVLTSGFERGFKVDDENTARKLELSIHKCFPLQVCDISPYYG
LFKMLRCKDTFQSYHSLYLNRRSLECKPEQLENDKKFADNLRESCGTRYTEPIDQTFADINLSSSLKKKFFGKIPNSPVK
SIFSPLILKREQWPTIEPITGIVPADIKQCMDDFETYFKVECRGKSIQWTMQLTKVSLECKGFSNLTELTCSGVVALVLL
SIASGNDSVEKIIENTKLTEKDIQNAIAKMSQRKNGKVLLKPLALNPEASVEGGKLDVFYYVPATTSSSAKIPVNYDKQI
EAAIMKILKEKQTLEKEELKGEVSKYLPFKFEDKDFDLRIKALDAQAYIKVDPSGKIHYIP
>Tcru_XP_819883
MTLSSIRQTTNMIEDEKWEIIRETVLSILNKVMSQYSFQQVHHSVFQMCQQQGSGLLFDHLEAILATHVNSICDRLLSVT
SENFFKSLKTEWDDFSSAVNFISKTLLYLNNNYTCHRQSISQMGERLFCEVVLRNTQISEMFSICVRGIFTCGQTSKDVL
KELASRLSQSYRHNIFEPYIEDPFIAALVDQYRQEMGIKLNELGTKNYLEWVVGVIEESKTNVSNILGESTAQRVEKVLG
ILLIKENTTKLLYNEAGGGSSMFRGMETQSLHLLAEALGKVNESEAFLDMIVGTAKKMGSELLADVDNASPISAVEKILN
LRDRIKNLILNLPFISKSNQSPVSQAITEIIKDSTEFAENLAYYYDAKVKSKISEAELERLVADVFGLFRLLKTKEVFDH
AFKLLLAARLINSKPEDPLSNETFLIDQIRNECGESVNHLEIMIKDGRMRREMNKGFIRSLGTGNKLPHEFYVTVITAGV
WPEYTDLAPDLPESMQNCMQLFCSYYLPHHNGRKLSFNTTLGTVFFTLNHGKIYEIVAPTAFASTILCFQNSEQNNEYLS
LQEICDKTKLIERDASFQLESLSQLGLITVDRVDDVLRYAFNQAFNNPRSKLRVRTTNGNRILEKNRETRAYCEPLGDSI
SFSIQAAAIQIIKSKKKIGHTEFCNAITKSLQTAGFVPSVADIKKGLETLMSKGLISRGGNSNIYIYES
>Tcru_XP_818330
MPTSLGDLISIRGSSSGSSNGSRSGSKHDSGGCKSDAKRVDCSCIRERDVFSRLQDRPSVDDLWPGVESYLVHAAHTIRT
GSAEMMDRLKDVRHRMNWYAVIYSVCSGHPQKSSEVYHRLSLFLLNDLEVNVLRQFLRRNAPCGSPPSSSLCLQFVQQYK
LFMAFRRVVLSCFSYLDQYFTAKFRLDPVTALCVKLFYVVVYEPMKEALIAEVLELADAARTAYLNEKAVSSACSDVQQT
LLSIVEILSTVKAFSAPYDANMQSALTHQKKDTSNSCNYKRGVGEGRNMLAAFAAKVVRMQSPSTAPNRDPPRTMGESPR
SPLNKSHNSDHSRVEQQTGSEGHSISSSPLAKDWDAMAYRSLDGIRMLRERLERRSFGDLMAAHVMRSSSRNKSSHHQHN
NGAGIGITSGGCEGSAKCSVDSAGVVIVEITQPLHFLIFAEFGLRYVQAAETYYRNRREMQFSTPEGWMDYIPWVQGCLA
IERSLMRDVNAPLLLSALRERIQHVLLVEVHRSIILAKEVGFRAQLEAWDSKTEQGAGDMWKDGSSSSSSVPPMGAAMSG
TCSDVDAVTSPAAFFSAQSTNPWEQLSREELGDRIKEFVATFVSTQDEACWTLLASEFASKVVMDTAALFFTYMSQLEAL
APQQLQQGEKKRPGTQQSASSPPVQPRKTVIPMRRRRSLRGDGLSSDRNFLTQQDLAMALIKGLVDTSVHYNKLICEKFN
EHPPMQVAMGDAMREILNPERWGQLVVGGAMHGGVAAVNRGELPSVLLNHDAAKKTVAVLNRLATMRGSREISVPQFLAM
YCDQFCRREMDDGVTDPTDHILYLITLVDDKDVFLEHYKLLLARRLLFLPSAQRNMDREHVMMHKMHHALGRTLTYGLEA
MLRDHEVTASMNEEFSRSDVALALPTRLRVQVLTSVHWPTYRVVPLTPCDSLALVMKTFTDYYIGVHPSRMLHWIHTLGT
ATLHAVFPKGDKEVIANNLQAHILILISDAYNMDRGGAASSGANDSHTAMAIGRSLSGREIATAMGMEFCDIYMYLAPLV
KHKLYNLLVRVDPSAVAGTGQTTQSSSPLSPPNARNAAAAAAAAAVDDDAAAAAAPSGTAPGQLLPEDHFTLNVEYTHKL
RKFGLPVARPSRGEGFKGSVVSSTHVEKTKAALSDEIEASRRIQMDAAIVRIMKSRRSLRYYELFDLTVQQLSKQFVPHP

RSVKMQVEDLVCRGFLRRSEKDTSVFEYMA
>Tcru_XP_821644
MAEREAFANLFGILEVIRRDGILAFADTRVAEKCRCNETLAILATPPFNTAVGELLVRKVFEAFASSSLAEYHQNMCDGD
ELLALRSLHEAYRRTLLLLNNWCKQLHAMEFVVRFTVSLRCHLLQPDLLTGEHPPGKEVLLQFLRNHGRKLLGDIEGTEG
RATLMRGILGHRDEVLQRLHGLTLSSTPDIKALWKSVLVECTRERIEMLDDDLTVGRIISCMQWKDDVVDTFVRMALSPD
PNNSDVRTEVNRWCEELEQLLLVTYGRKRIAALWDVVVEYPDSTPTLQDLRLCLLRCSDDTLQNELIQTAKKMLASRLHR
AGTRTEDILAILIGTIHSLCVLFSRNDQGSVIFTIVGDTLEHLKKRRDCVSAVVQAITQPSADSVLHVELQNFSPNSFTS
VAADDDEVNEDDLLSVAGQSPLSMHERPDVLRVLLSAISVTSLVEEYKRLLASQLLGKKLHDFDTTAEEEVLERLKCAFG
EDVLAPCVVMIRDLQASRRHTLHMKEAYRRAAAEGLPQEKRRDWPLSIDVLSTTSWPKLSAFLPAGETQPVPERYNPHPL
FASAMEDVMEEYKRMKANQRLEWIPSHGYVSLELTQKEASTNTMVAATYDLSLFSASLVLYVGDISTELGSPAPLKTVAE
RMGVKPHVLQQRLSHLFPSLLVSTDENRSLSLQRNYVSTANFTFDEADEREGQPVGLSPDQVKMLLSMMTAMLKARGPCG
VNDIFNSMKMFGQFPGNIDDMKKLLHMFVTQGRLVLNEAKLFTLPGS
>Tcru_XP_813446
MTLSSIRQTTNMIEDEKWEIIRETVLSILNKVMSQYSFQQVHHSVFQMCQQQGSGILFDHLEAILATHVNSIRDRLLSVT
SENFFKSLKTEWDDFSSAVNFISKTLLYLNNNYTCHRQSISQMGESIFCEVVLRNTQISEMFSICVRRSFTSDQTSKNVL
KELASRLSQSYRHNIFEPYIEDPFISALADQYRQEMGIKLNELGTKNYLEWVVGVIEESKTNVSNILGESTAQRVEKILG
ILLIKENTTKLLYNEAGGGSSMFRGMETQSLHLLAEALGKVNESEAFLDMIVGTAKKMGSELLADVDNASPVSAVEKILN
LRDRIKNLISNLPFISNSNQSPVSQAITEIVKDSAVFAEKLAYYYDAKVKSKISEAELERLVADVFGLFRLLRTKEVFDH
AFKLLLAARLINSKPEDPLSNETFLIDQIRNECGESANHLEIMIKDGRMRREMNKGFIRSLGTGNKLPHEFYVTVITAGV
WPEYTDLAPDLPESMQNCMQLFRSYYLPHHNGRKLSFNTTLGTVFFTLNHGKIYELVAPTAFASTILCFQNSDQNNEYLS
LQEICDKTKLIERDASFQLESLSQLGLITVDRVDDVLRYAFNQAFDNPRSKLRVRATSGNRILEKNRETRAHCEPLGASI
SFSIQAAAIQIIKSKKKIGHTEFCNAITKSLQKAGFAPSVADIKKGLETLMSKGLLSRKNSNIYIYES
>Tcru_XP_811550
NPLHVVEEVRQKLTVGNPSVDISELIQLFSHPARDAEERLVITVLQSVVHGCRGFFQREALLSLLEPGISEESRHVFLLK
IWPQYWNNELEVMRELPAVEEACVKGILVKLVFVWETGNDVSVQETSNAPCGLRPYYIFHLMRATLQPLLDALFALDAAA
EEGETRSTLELLLDIANSMACIEAPPRRTLRLWDQVGAGAALPSSIPREVGYVVMADVCRAFLEVRLPSAVDHFLSGGEI
PEEQRWPRLIAYLIRTEHLFHRLCEMRLLKAKLLDNCSAVPCFYGIVKNAIANHLQRKRNADLFVETLLGIMQTYLLYGS
HEGAEVRRVGGGGREKMVLVSLHNEDEVMSAVRLAFSVSSIDRFLRLYKELLAYRLLFYAVGRASDGSVDAMEARREKLQ
TEKLVCEYLHELLPFERNAIQQMIQMCMDMVTGNNTPEAYDPQALQPEQCGHGADAFSVRPTMRLLSRLAWPSYPLLTDV
PKPLHNVMDQFARFYHAQYSNRRVVWLRTSMETITFTVAYPQAGKVIVGSLELFNIFHFLSEAGTSGTTWTLLAQRIGKD
KTLLQRGLKKLIHDGFFKIQMGSGEEIVALNAAFKSHKPRYHFLQPPPRVGALLEENGIINEEVHRSRIASIQAVIIKHM
KTVRTCLYDELFCWTRQQNPQFQLQNSDFKLALEMLIEKEFVQRDPSQKQRFVYKA
>Tcru_XP_809668
MGRVPASDDFRRDVVKAYSGPLFATTFDFGVLWNKVDSVCNKILGWTDSPEFRGQTVIQQVVRAYTIVYRLVSAPCLDCP
KLVRGTNAQQDTIGGGSEGTQALVVYYLLRETIRKRLSSVVTHRLKMSLTAEPPTLLQVYLTEWRTFIIAVNHLKVVFSY
LHSPWQKHELPSEQPLLPTEVVALVQWSDIIMSPEICEGLAEQIFNLISRDRLHRLDEGSTSLVRDISHSLAMLNDPRHT
AYSSLIEESYLVHLQRFYKENINVLKAGGILSYIERSLIIFEDEMERVNRILNKCTMLPMLQRLSDVLIDSEIPYIKSYL
PTWILNDRGYFLNKMYQLLSKNSTGLLVLKEIFEKCVFEKGVEDISRICEESIRANENVYKAVLEGIISVHTYFLRVSES
FEGNEVLEKAMLSGLEKILTTLTYVKSYHILGEELARFAHTKLKSNESKEQCEGILREIVTVFQLLPSKSSFLESYPKFL
SVRLLFEPYSEEQERFAIRTISQATECTSDFVYRCEVMLMDVTENSVSLRDMFDTKYGNSHNKSSEWLFQPSVLTSYSWP
DASCDVNLPTPSILLPKMREFQLFYSQVRPKRHISFVSRCSRGVVRMNLPRDSRVPSIDLCVGQSQLLLAECFNNRTDWS
IGNLLQTMELTNNEIFLRSLNAFAGCGILEVYDEGGPVDLPLRSVMAGKYVRLGSNPDTRKRKLNLFPCDWEGGFQIATV
SSNDFVSDPVDASHHMVDQLKAPAVQATLVRVFKASGTLSFYELMEKVKDESLQKFMPSVQQVKVALEFLISRGFVIRDN
SNGGTFSYIC
>Tcru_XP_807714
PGILNDRGYFLNKMYQLLSKNSTGLLVLKEIFEKCVFEKGVEDISRICEASIRANENVYKAVLEGIISVHTYFLRVSESF
EGNEVLEKAMLSGLEKILTTLAYVKSYHILGEELARFAHTKLKSNESKEQCEGILREIVTVFQLLPSKSSFLESYPKFLS
VRLLFEPYSEEQERFAIRTISQATECTSDFVYRCEVMLMDVTENSVSLRDMFDAKYGSSHNKSSEWLFQPSVLTSYSWPD
ASCDVNLPTPSILLPKMREFQLFYSQVRPKRHISFVSRCSRGVVRMNLPRDSRVPSIDLCVGQSQLLLAECFNNRTDWSI
GNLLQTMEVTNNEIFLRSLNAFAGCGILEVYDEGGPVDLPLRSVMAGKYVRLGSNPDTRKRKLNLFPCDWEGGFQIATVS
SNDFVSDPVDASHHMVDQLKAPAVQATLVRVFKASGTLSFYELMEKVKDESPQKFMPSVQQVKVALEFLISRGFVIRDNS
NGGTFSYIC
>Tcru_XP_807387
MLEEDKEAIRRMKADFEAIAELTESEFKLNTGFARRMNHYSAVYTAATRTTNMRSFSDAFGYYVDELLYTDFQEMLTRYL
LKYRDLQDSSQMRLFVKILKLWNHYKVLMKWNMRAFAYLSRFYIVNCSKPSLQQVALNIFLEQILKKNVHVISRVTQDLL
CLERNGESVNRDQIRGAIELMSSVSVEKKDEIYTEQFLRPYMALTKSHYEGLVTEWSKSFAPSELLRQIEQAHNEEKARC
MCYFSPDDRKIIMTHVEEVLLESPATVEKLLKSDDGFVAALRGRDEALLEKYYNLLSCRTKCLAYLSNLMRDEIIAEGKE
RLLQHDSQKKEMDFRSCVGDMLQLQDDFMQLLARCFNSNAIMMKSMREGLEKVFGGSVHASRGPQRSVPFSELLAYYVDA
VLQGNEGSTVEENLEKAVAALAYVTDRDTFLAHSRELLAQRILFPRKKMDEATERSLIQRISQRCGVSSTSYLEGMLHDV
DIAEGFGVTEKLEAVGKAPNFAFSVLVLKKGIWPPRIQGECFVPPRVIQEKLSAFQKIYLEGTTGRVLTWSYSNSSGDVC
AVFKKGVKILSMTGIQCWVVLAFNELNELTPNDMMSLFGMSLEDAKPALLPLLKCSILRGESDATTIQPQAKFFINEDFS
SKWKKVRVPMPTCRRDGLLHGEEIAKKIEEDRRPAIDACLVRIMKSRRVLSHSSLVEECHQKLSQLFSADQKLIKQRIEE

LIRKEYIERDHKNPSVYQYTA
>Tcru_XP_806684
MASRTLYDPPPTALQWDVGPSQEKPCITVKLLTHDAEMHRLACEKEKEKAWTVAEGMLDALLVRLAAQSENVISSTPPFE
HSTSLLSFLISSSPSASSMSSISVRIPPVNVSNRGYAADAACRSVRTLVELDAASAVYEKLKQSLEAYVMHILQFLTTAC
EPDGEGCLTFMRLVHVWGHYRLAVAELQEVWVYLDRWYISRMHAVRSIDGLAVAIMKEALLQHPWLLSRAQLGFLTFLRQ
DFENESNVRREILLFTNLCSSIEVYFLRVEPDVLAVAKEFYTEVADRMWHDGESAEVFFQQVGCFLQEGRRRVQACLEAN
TMSKLEEIAQTSLLAAHGTDVLARDFERLVKDEKYDCIRLAWQFLALGKYVQLGKECGAVFREYILHEGVTIIRQLTSRP
ADREGFVAVKAMIALIRRGESVIDECFAENSMVFTVQLNDALVEVLQENQTEFARQIARYIDFLMREEENDTLMSAPANT
KGDDGSGDNCLYADATATANDGGSDEVAAAATATATAVSPCGGLLNYIGRIYALLPSRGIFETFYWHDLARRLLQYQRPR
RLDAERSFIQELKKACGVETSKFEGMFNDLKVSQELNERYQAWVTGERDDASAQWPPVMRVDNEEEEEGEAMSLIAELPW
NTEVKLHILTSGFWPTQSTLAVRLPSPMRLLAKSVQDFYRQCFTDRQLLWQHQLSSAVVRCSIGTVRRNLTGTLLQAAIL
LTLQEMMDTTPSSSSSQRVEAVTVGALCDRLAVDILVPAVTGSIYGLCHPKFSLLLREPAMGGSSAAAFPMLAGTDRLRF
NTHFAVSSMRCRIPFYNTPNSGEDVSTGRNDADSMLAVDDVLKEHKHVIEAAVVRFMKEKRCATHEDLVTAVSTLVQFPV
TMATLKCVIERLIDRGFLERNGATAYTYTL
>Tcru_XP_805928
MAAGCSAMRCPCLFSLPHSLRLQHSVSLALFYFSLHSVQAVLVSGLFSARIKFVKRRRRRGWKKHQRTTMVEESCKYEEK
EDERDRPCEEVVAASRLREAYAAVDRESFGALGVQALSPRIPCASSLLETFPSLRELFTVTLNLEVLLRRREGDALRTIR
EKHLKWYKRIFHTLHGVHDIHALLRYWVKGMVLYEFCEINRHMEKEDDEEEEEINNDTGRQNCERGGVMRAADYAMELRE
ALRRFPKLVAFLFLSKSQEYWAQTLSEVIREDVGSSLSDRLKLNVLQRLLMKVLQCQRQRRYGVLRCEHFATVHPHCHNN
GNPLHVVEEVRQKLTVGNPSVDISELIQLFSHPARDAEERLVITVLQSVVHGCRGFFQREALLSLLEPGISEESRHVFLL
KIWPQYWNNELEVMRELPAVEEACVKGILVKLVFFWETGNDVSVQETSNAPCGLRPYYIFHLMRATLQPLLDALFALDAA
AEESETRSTLELLLDIANSMACIEAPPRRTLRLWDQVGAGAALPSSIPREVGYVVMADVCRAFLEVRLPTAVDHFLSGGE
IPEEQRWPRLIAYLIRTEHVFHRLCEMRLLKAKLLDNCSAVPCFYGIAKNAIANQLQRKRSADLFVETLLGIMQAYLLHG
SHEEAEVRRVGGGGRAKTVVVSLHNEDEVMSAVRLAFSVSSIDRFLRLYKELLAYRLLFYAVGRARDGSVDAMEARREKL
QTEKLVCKYLHELLPLERNAIQQMIQMCMDMETGKNAPEGYDPQALQPEQCGHGAETVTVRPTLRLLSRLAWPSYPLLTD
VPQPLHNAMDQFARFYHAQYSNRRVVWLRTSMETITFTVAYPQAGKVIVGSLELFNIFQFLSEAGTSGTTWTLLAQRIGK
DKTLLQRGLKKLIHDGFFKIQMGSGEESVALNAAFKSHKPRYNFLQPPPRVGALLEENGIINEEVHRSRIASIQAVIIKH
MKSVRTCLYDELFCWTRQQNPQFQLQNSDFKLALEMLIEKEFVQRDPSQKQRFVYKA
>Tcru_XP_805723
MPTSLGDLISIRGSSSGSSSSNGSRSGSKHDSGGCRSDAKRVDCSCIRERDVFSRLQDRPSVDDLWPGVESYLVHAAHTI
RTGSAEMMDRLRDVRHRMNWYAVIYSVCSGHPQKSSEVYHRLSLFLLNDLEVNVLRQFLRRNAPCGSPPSSSLCLQFVQQ
YKLFMAFRRVVLSCFSYLDQYFTAKFRLDPVTALCVKLFYVVVYEPMKEALIAEVLELADAARTAYLNDKAVSSACSDVQ
QALLSIVEILSTVKAFSAPYDANTQSALTHQKKDNSNPCNYKRGVGEGRNMLAAFAAKVGRMQSPSTAPNRDPPRTMGES
PRSPLNKSHNSDHSRVEQQNGSEGHSISSSPLAKDWDAMAYRSLDGIRMLRERLERRSFGDLLAAHVMRSSSRNKSSHHQ
HNNGAGIGITSAGCEGSAKCSVDSAGVVIVEITQPLHFLIFAEFGLRYVQAAETYYRNRREMQFSTPEGWMDYIPWVQGC
LAIERSLMRDVNAPLLLSALRERIHHVLLVEVHRSIILAKEVGFRAQLDAWDSNTEQGAGDMWKDGSSSSSSIPPMGAAM
SGTCSDVDAATSPAAFFSAQSTNPWEQLSREELGDRIKEFVATFVSTQDEACWTLLASEFASKVVVDTAALFFTYMSQLE
ALAPQQLQQGEKEPPGTQQSASSPPVQPRKTVIPMRRRRSLRADGLPSDKNFLTQQDLAMALIKGLVDTSIHYNKLICEK
FNEHPPMQVAMGDAMREILNPERWGQLVVGGAMHGGVAAVNRGELPSVLLNHDAAKKTVAVLNRLATMRGSREIAVPQFL
AMYCDLLCRREMDDGVTDPTDHILYLITLVDDKDVFLEHYKLLLARRLLFLPSAQRNMDREHVMMHKMHHALGRTLTYGL
EAMLRDHEVTASMNEEFSRSDVALALPTRLRVQVLTSVHWPTYRVVPLTPCDSLALVMKNFTDYYIGVHPSRMLHWIHTL
GTATLHAVFPKGDKEVIANNLQAHILILISDAYNMDRGGAASSGANDSHTAMASRRSLSGREIATAMGMEFCDIYMYLAP
LVKHKLYNLLVRVDPSAVAGTGQTTQSSPPLSPPNARNAAAAAVDDDDDAAATPSGTAPGQLLPEDHFTLNVEYTHKLRK
FGLPVARPSRGEGFKGSVVSSTHVERTTAALSDEIEASRRIQMDAAIVRIMKSRRSLRYYELFDLTVQQLSKQFVPRPRS
VKMQVEDLVCRGFLRRSEKDTSVFEYMA
>Tcru_XP_805473
MASRTLYDPPPTALQWDVGPPQEKPCITVKSLTHDAEMHRLACEKEKEKAWTVAEGMLDALLVRVAAQSENVISSTPPFE
HSTSLLSFLLSSSPSASSMSSICVRIPPVNVSNRGYAADAACRSVRTLVELDAASAIYEKLKQSLEAYVMHILQFLTTAC
EPDGEGCLTFMRLVHVWGHYRLAVAELQEVWVYLDRWYISRMHAVRSIDGLAVAIMKEALLQHQWLLSRAQMGFLTFLRQ
DFEKESNVRRELLLFTNLCSSIEVYFLRVEPDVLAVAKEFYTGVADRMWHDGESAEVFFQQVGCFLQEGRRRVQACLEAN
TMPKLEEIAQTSLLAAHGTDVLARDFERLVRDEKYDCIRLAWQFLALGKYVQLGKECGAVFREYILHEGVTIIRQLTSRP
ADREGFVAVKAMIALIRRGESVIDECFAENSMVFTVQLNDALVEVLQENQTEFARQIARYIDFLMREEENDTLVSAPANT
KGDDGDGDNCLYADATATANDGGSDEVAATATSTATAVSPCGGLLNYIGRIYALLPSRGIFETFYWHDLARRLLQYQRPR
RLDAERSFIQELKKTCGVETSKFEGMFNDLKVSQELNERYQAWVTGKRDDTSAQWPPVMRVDNEEEEEEAMSLIAEFPWN
TEVKLHILTSGFWPTQSTLAVRLPSPMRLLAKCVQDFYRQCFTDRQLVWQHQLSSAVVRCSMGTVRRNLMGTLLQAAILL
TLQEMMDTTPSSSSSQRVEAVTVGALCDRLAVDISIPAVSGAIYGLCHPKFSLLLREPATGGPSATALSMLAGTDRLRFN
THFAVSSMRCRIPFYNTPNSGEDVSTGRNDADSMLAVDDVLKEHKHVIEAAVVRFMKEKRCATHEDLVTAVSTLVQFPVT
MATLKCVIERLIDRGFLERNGATAYTYTL
>Lmaj_XP_001686885
MSSLVSSRKRMAEYSTFSDKWSIIEETIRCIFKKEVSKNSFQRIHHSVFQLCQAHYGSLVLVQLEEQFFAQVSIIVNRID
GSSTYVADFLREWQDYNDSVSHISNILLYFNKNYMYACHHSSIEHLGERIFCSSTLGNPEVSTCLIASIKQLIHVPAAES
TIRDIGQELYTAEGSKYFARCMETPFVDAMVDKYVVQGEQQKQALTTSGYLQWVQNVVCTESHKYADTFFYILLDKLTSA

LYSSLVLSDPSSVNEMLMGPTGLVHMLEEWDVASISTFVQVFCAMKREKDVTDIIAHVLKEKCEDIINDRDTSVFPFPGV
QKMLQLIERAKALDSLFPSEEGHSQSPFLRVIRNVLGNDTRFMETLSVYYDNGIRQGKDDIVSTVGNNVLTILQLTPDLE
AFEVAFRSHLAVRLIYAKPHAVNMECLFIDRLFNIYGSSVVNRFQKMVEDIRSATAAQEKLIADMKTKRISPPLEFNVLV
LTSGLWPQYTNIPLSIPQSMEACKRVFQEYYRRRHNGRKLVFQMSLGSIAFQLCHGDRTYHVSAHTHFVNSIMALNSDEN
LSVAVVAQQGALSCDEVLTQFNTLCHVGLAERNGTEFRFNRNFYSSKPKVKVSAGVQKSAHDGGSASAATRKGMDGARTM
SLDATIVKLLKEHNSMDYQTLCGAIESELRDVCSPTRTDVKLRIDALIEKGLMTRGESAHCYFYCS
>Lmaj_XP_001684494
MAEQESRTWNSFPPLWKEVAGVCNTAFQKFKKRPSQNLCKDYNKLISQICEAHGLVYNLTAYQCSNYPALVRGSNSTPVV
GETVESTQVLVVYYLLRELLKKQVNSVKSTIQKDSILSYLDAYEKYTAGGNVVKSIFTYLHWSWQKRGLPAEHIIQPTEI
VVGHLWMDVILTSELKESFRAKVNEIVCRVRSGGPTSVGEMESVKRFSLNLGCSTDVRLTLYSSMVEETYLKSLASFYCA
KRPGFKALGVVEYINQCVKAVKKEDVLARCFLIRSSYEQVSESVLRILIHEEKNYLLPFCKKCIDPSDGNDPLSSKKSLS
ALYDLLGGGAEESWMEDLLTETVSEYADRELKKTSDSSEASSILRILKKAQQTFEMTISGIFGHQPRLIRAVYDGIQANL
AALERPTATEDNAAKIAKRLAKYAAEEIKSMPIDELRRTNNWIAVIYRLCSRQDVYHLSYTRFLQERLLTNIFGTGKQSE
IAVREESMLSSLLIKDKNFAFIFNCMRMLNDARRNDSKRAERVQQLLVKPYILTAFAWGESRRSPDMGRTSVPLELQSVI
THDIDAYSALRNGRKLLFSPEHSGARVRMSVSNKAASFLLLVSLLQMRYLLVMNKMSEWSADELCSQTRTSLEECKRALT
PFVQTGLLQETAQHVFKLDPNIFALQSGEEKMYDLRYIELGSQQSNPEKNTLFLRGEKSHVLSIEGAVMRLLKELGPLSL
DVIIGKVSASLGKLSVQRRDIKKVLEKLVEREYVERSEDNCFSFIP
>Lmaj_XP_001683747
MLEEDRQALRKMKADFEALADRANSDFQGYNTFERRMNHYNTVYTAATRNTSKAAEYPGYDAGELLYMEYNEMLTTYLWR
YRDLSGDSEQELFQKILDVWDHYKILMKWNMRTFGYLSRYYIVYHSKPSLQQVGLSIFLEQVFKKNADVVSSIMQKLLLK
ERADRVVSTDAKQISTAIGLFSSMNIEDTQSIYVTAFLEPYLDKTKRDYERFLQEWDAVADAAVFLRSVHDALTHERNIC
RRYFSTQDEERIMTCVEAALVDSPVTRRKLVDSPSGVLAMLRSRDEAQLQMCNSFFSRRESGIALLAGLIKKEIEAEGLE
LCERFKGEEASVDIVAYTTGMMHLQEVYPQLLSRCFQLDFTLSKAVREGLEACYNHGVAVTSSCGGGESRIVPFCEWLSH
YVNHLFQQEAGPASAEMDRIVATLAYVTERDRFIATSREHMADRILLPIKKFSEANERALIQRFRQRCGPTSTACLESML
HDWETSNSFCAMEVLASKNVPRTFEVELLVAKKGIWPSRLSDEPDIVVPSFLQRVLEPLRDDYLAGKSGRVLTWSHECSS
GELQGSFKKGTQLFFACGIQVLILLCFNSSPVCTPQQIGDRCHVTFASLQRYLPPLIRCRILTRKSGKALLQMDDELTVN
DDFVCRARRMRIPPSLARVSVGESEHISKQVEEDRKPAIDGALVRIMKGRRSLDHAELVLQCQQQLSSRFSPDVKLIKQR
IEELIRREYISRDPRSKGVYHYIV
>Lmaj_XP_001682203
MRDAAERRHSSVELQRPQSQQRQPEEHHHQQHEDAAHNQRHGSRTTSGGGAALEEAASFQARPLARTPPQHPGFVETFGG
MPAALVLSRSIRNQAHARDMGGLSCVASARSAADKDESATLEVPQSLCRHTAAFPTHHLSTGTPCSSHATASTAELEMPM
QFESLWGDIDRYLFAVYTTLQTGDEMSLRPLRTPKHRMGWYTVIYNACSGDPTKSRELYARLALLLLHILESHVLFPVLQ
NVDHSRVHFLTGAEDSRGACGSGSDQRGGQSSRSAPTGSGIDGCSSGGWSRFKSIAIAATSGSVHRSPLKETSRNSSFTW
SSRGYRNSDGSLALMKRLFHPSPHPTATTTAARASPVGGGGVKADEEEDGAGQPPQSPKAPAPVNGDSMASSGVGSRGTS
WFAQQGSGGSVAVPKSPAAANEFQTAFAGFDSTVHRSPENRVSLKLPRQQQRTTGFSIVTSTQSRPTTSPPRSPAIRMHT
PRTPSSVTSLHVTQHAGSAPTESGRSTLTARSPTSIGGRALPPATHSAVSPHHARVASKTGRVVVEDDEEVLYAVDTVPA
AARHSLRRRQHRGSSSDSNSVAAPVSFNDTEEGSAGLRAGADNDDDDDSLHTMNSFSSESVGRESRARLGKCAGEGATAT
AAREDARGRDAARAGEPPRTDHSDEGGTAEPPASRSSPFTTTLSSINDALSPLLAPEAPATMRSTGTNAPPSLLRATAAV
DASIRCTSSPDAKTVSETSVTATGYSAREASSSSPSATVALAPPAASVTTSTGTTSPATPAYSAAETASSVARTASTARP
DERRFSLCYTFLQEWHTFVVFRSVVLSCFRYLDQYYTRRFGMDTITLMCFKVFYVVVYEPLRPLLVIELCYLMQYVREVF
ETTGQVAREQLRLIQETYSVVTELLLIVQSTSSSMVAQQQQQPQAGSGVSGGGGATAAAASAATASTAGSSNATNGPPSV
VAPSAAGLSSMVGAFSAAEAAVAETPVRSHASSRARSTSVHNLSTASSAVSTPHVSALDRHNSSSTVAAPSPAAADGEQS
GAHPQVSRRRRLISLFTSGRLGDGVKGGGGNSARDQRATSRASSTPASPWVGLASSTSSAARLPSTFEGTPRPPAGRGGD
SGRLSARDEAEGCSDKQQGGAAKADYISAPAVVVDLRGTVELRDRLEGHTTAHHLTTQALKRWEGMSADRRLRLGTIVKE
IAKPLSTIVMEDVGNEYVAGIYAFYRYTRDARRSTEEGRRGYVSWAGDVKELEKLVWRRVQLPFLQASLRSALNEVLVVE
PHRSILLDTRFGLRALLDTWSASVESADLSLAPPMSAAAARPGDAATQADGSSTTAVAAFHRRTGSTMTSTSLGGGLGEA
DPLARSGVQRGGGKYPESLILFGVHGSHPTHPLSFPHQRTPRSFPFDGGERDEVVSQTSCSVFSGEKEKEKDDGRHGSSG
GDVVSAVRWTTAGSNDSHSTVITVAPAIDPSSNDRLMEHAGAWTGTRRGEGKPSTVATASTPRFTSRGDSSTCVPPMEPA
THAALAPHYRLGEQASLSSGTAAAAETGRTVSGEAEGDSASAWAALKSLYGLFDDVTEDECFLLIAAILITKLIRDAADI
IERYLRAMSSPASATPSQPHPQAPGIHLMASLVGLVERYTDLVEGVFRNHATVLRALSNAIEELMCPFRWTRKGALQASQ
QRAQEALTAAASATPGAYGRSWSEVLSSTGADLPSVLLSRDARQATLPLVSLAANYPQAVQQLKFSVLIARYTDVLLQQE
RGGGRVLSGNLHKRLRLVARLVSLLEDKDTFLEHHRLCLARRLLGYASGPAAPQSEAALAVVGGNAKQPLAVGRASQGSS
ASASLNLEAERQLVSFLQSYLGVTATHAFEAMLRDYEGAERTRDLFEQEAAYLELLTKIRVQLITSAQWPTYAMPPLQVH
ASLQRGMEVFRAYYARQHPSRTLLWVFSLGSATLIAEFVSGTKQVAASTLLATLLLVVSDAYNAEASDAAQAGALTGAQL
ARTLGLPFHALRAHLQLLTQHRAFNLLRWIPANSSGGGTGAAAATASITENDSFSLNPCYAHKLRKIRLPLPKARPTGLP
SGDAQAFDGGPEPATQREPSATLSSPGATTAAATVTAIACEDAVVAQHVQATRRVLLDAALVRIFKSRRVVLFEDLFQTV
TAHLSRQFMPSRRDVKAQLEGLIDRDYVKRSPDNPNTFVYLS
>Lmaj_XP_003722769
MMPYQHSINEDGAMRILAEMLDALDTHGLALFSESGHGAAWPLSSPTTWSSALAVLATSAPLNAMVADTLLERVSAALSV
EIDVYHSAVLCCSGPLSTEVALPALERLGEVYRQVHSHLLRWCSALHTPGVAALLAVRLRCALLQTALVTGEEPPGRQAL
FSFLWRHGRFVLAKQRKAAGDGDEEDAGDSGCDGTLGEAGDANESASAPASASPAPDSVMTCLHRLALNRSAEVKQLWRS
VLEEGIRDRLSDLSDDFSTRILHKHLSWLENSVVPFVRMVLPDSDGESDPLCRSSGSLRDETSANRRCEDVGTTSEGCHM

VPGHVQEGDACPSSQTNEAVRAEREAWCADLRLLLLHTYARRRLDGFWDILSDYPDSVPALEDMHYCLQSTPESGLKTEL
VQTVRHLLSSRLHRAGTRTDDILAILINTIHAFCILFPRNEQSSVVFSVTSDTLEHLRRRKDCVPAVVQAVMQPGSAVNV
APPPLTQAASDGLVGNLDFDGDAGSKEGELHRAASRGRPDVVRVLLTSIPRRALVDEYQQMLAEQLLQKPMHQFDTTPEE
EVLERLRHVFGETALDRCAVMVRDMQMSRRISQQLREQLGSSREDRLPQLRPTAASATAVSPAASVSVLSMTAWPPLPRC
SSANGGDSGPASAPLKFCPHPALQEEMDALAEAYKRLKDNQRMTWLPSLGRVELELKQKDPSKGNALVAVPQVLSLFDAS
FVLHVKEMASRGGFSTPVHLKEVAVVMEVQLEELRAHAQRLSPAVLMFQPATDLVTMQLGVAASADFVFMEDRETDEESG
KPAGLPPERVKLMERMVTSLLKTRGAQSSTAIHSSLKLLGKFEGSNADVVELLRSFVGRGLIVLNDARQYALPPK
>Lmaj_XP_001684495
MCNTVMDAYLLVYHLISHPCSNTPLVKVNGKEIWEGQQIMAVYSLQGAIFEKHLLNNVMPAFSQVSNGSTIQTYVRSWRS
FLVAVVNIKTVFSSLADKWSLLGLEDNPLDRTEDIALRKWSSVVLTPRMVSQLRKELRALLADERAGHGAPDLSFAVEIK
DELSMLPDSNYYRSVVEVDYIRDMCDYCWSKVGGVVESDLFTYAKLCLGLIEEEVKRAERFLTSKDHAVDRLVETLVDDR
ISAFECGRLSQWLGSIGQRETDEKLQTVFHLLWWSKCKGAPLMEGMFKESVAQHTSSALTGTVRAAGEGTDVYAAVIECF
ASIIRKYRDVVMTIFDYNGCMLEAMDDGLRCGFTSLRSFNYKKLADRLAAFSNAILGNTGSTKLRLEDVVSVYYFLPDAE
NAAKDVFLVSYQKHLAKRLLLHHYDEAREQRSMEQLVQIKQSPILFCCRSMLKATTTQSIYVGASSVNGVKVNPALLSRG
TWPSLPHTLASSSVSDSVLRQIEGAQRICSTRRHGQRIEFSAPYSSAVIRMFRPAGSTAAGDSVQLKVSFLQMCIIDHFN
AKSQCTVQELCESLQVSEVECAFALNPLVSATVLKLSGAMEPSSIVSLGPCDSSIGDVTNVMPLEFHSFAHRAVVKSHEQ
RTLQSAAKANPQRMESQVVHTLKQSGSKTAEELMIYLTSAMQPLIVSRGELKRVLEKLIERGLLVRDDSQRKFVYSP
>Lmaj_XP_001683883
MRCVCARLALLRISCLPSWTSPALMWLYRMSACVHVRVSLSVYPSPPPLPFRGISLRLPSFRFSLPLCVSGHVRRSEHLD
TRSRAHVWWASPRTSGSRLRSSAALAALYPLTGIKMTMFPRPPLPSFQVTLLPELPPGKAHSASSPPSLSCSSRASVQMH
DEAAAKAMLHAVLDMAYDCTTPQRRFCSYSPEAVLRSVRALVDSDLCAPLVEALQAATRAYAQRCVEVLLGQQQQLPPGA
GTPQGMANGDTESDEEATALSANATLGVVQILAQLVTYWERLQQGLAELSSVWVYADQWCAMQVGSAMPRSLIAAATQEV
REVFSGYPELCDTALAGYVAVLKAELTADTAALLSASRSARMSYAAALPDKRAAASPAGSEQPVASTPPLPRPDATPLAA
LACPPSLASLDKPLRQRLLRLFTQLTLATQQYTTRVEPTVVRVVQGHYEEVAHRLWAENVSARVFFRFCDGVLRHLGPMS
ASLLHPGTSLMLEEAIQGSLISRVGSEVLKRDFVALMEWQDYRSLRLAWRLLSVSARVRSSPLVADLFHEYLMDCGQRIM
EELVPSSTKGTPAAALVPPCERPFLAVRHLLGLLEQGSRTVAKCFAECERDFKVALHEDLQTILEAYPQAFSEQLAAYQD
AIMREVEVGHVPVGSSKPTAPVALEKNGDDDNDDEEDVLSSPLRRPRPEGTLPSSRPPVAAPSPGFTRAEEDEGVSSGRE
ESAEVEDAAEGDAVMGAAALHHAAPLPTEVREATSVLRRIAHIYSYHPKKDLFEAAYWRDFARRCLHAHRKLNSAPENTF
IGFLRDICGLSFTSKFEGMLTDLTSSTELTTQYNTWVQEQEQQQQQPSGEAGVDTAAVGRLVSEAKGLDAHLFILTQSY
WPECPSMPGNFYVPPALRAVTQLVEKFYTGRFPNRHLSWQHPLSHATLLVRLTPQSSVLTLTGTYTQCVLLMQLDALYDR
GVTTITAKALCDAVGMDMTNTELLWSLQGLCHPRFRLVLRAEAASSPPSGGGAQRKSAELDNEDDESAARVEESAASCTS
PQSVSPGAAAGASAGCTLAATDLLQLNLSFTSATSKVRIPFQTRMRTTASAGLDGPSRGGRTADNGGHLVEVAIVSCLKS
QRTMTRTDLASAVAARVPFPVTTTVLKKAIDKLIERGFMIRGKGSTYVYSA

*******DUF2042***********
>Hsap_ENSP00000358283
MADAWEEIRRLAADFQRAQFAEATQRLSERNCIEIVNKLIAQKQLEVVHTLDGKEYITPAQISKEMRDELHVRGGRVNIV
DLQQVINVDLIHIENRIGDIIKSEKHVQLVLGQLIDENYLDRLAEEVNDKLQESGQVTISELCKTYDLPGNFLTQALTQR
LGRIISGHIDLDNRGVIFTEAFVARHKARIRGLFSAITRPTAVNSLISKYGFQEQLLYSVLEELVNSGRLRGTVVGGRQD
KAVFVPDIYSRTQSTWVDSFFRQNGYLEFDALSRLGIPDAVSYIKKRYKTTQLLFLKAACVGQGLVDQVEASVEEAISSG
TWVDIAPLLPTSLSVEDAAILLQQVMRAFSKQASTVVFSDTVVVSEKFINDCTELFRELMHQKAEKEMKNNPVHLITEED
LKQISTLESVSTSKKDKKDERRRKATEGSGSMRGGGGGNAREYKIKKVKKKGRKDDDSDDESQSSHTGKKKPEISFMFQD
EIEDFLRKHIQDAPEEFISELAEYLIKPLNKTYLEVVRSVFMSSTTSASGTGRKRTIKDLQEEVSNLYNNIRLFEKGMKF
FADDTQAALTKHLLKSVCTDITNLIFNFLASDLMMAVDDPAAITSEIRKKILSKLSEETKVALTKLHNSLNEKSIEDFIS
CLDSAAEACDIMVKRGDKKRERQILFQHRQALAEQLKVTEDPALILHLTSVLLFQFSTHSMLHAPGRCVPQIIAFLNSKI
PEDQHALLVKYQGLVVKQLVSQSKKTGQGDYPLNNELDKEQEDVASTTRKELQELSSSIKDLVLKSRKSSVTEE
>Cint_ENSCINP00000007324
MADWKDIEQLQAEFKTAQLSSAAQKLSERNCIEILNKLQEMKLLDVLHSVDGKEYLTPTHLAKEIRDELYMQGGRINLVD
LQQALNVDFSHVEAKSNELVKSDRNLQLIYGQMIDSTYLDRLAEEINENLQSTGQLTIAELSKQYDVPGDFLIPELISRI
GKQIDGKLDEINRNAIFTEAYIAKHKARIRGVLSAVTRPIQLYPLSHDHGLSMQLLFSIVEELIKEGRIKGQIAGGRSEK
SMFLPEIHTQTQNQWIDNFYAQNNYIEFDAVSRLGISDPKSYVRRRLTTNPPLFLKSVCVGQSIVDQIMATVDETIANGD
WVDMMNFLPTSFLDEDCNQLLHHVMRERKKIKSSVPGIIFANSIVANEAFVNSCLKYFDGMMREKAEKDVKKSSVFALTE
EERKEYLHQTTGDPKKKSEKKDDRRKKATEGSGSVKQGFEGASGSGKGAREVKTKGKDKKRLKWKKEHETQTETKPSKPS
SSQDDLPFMDVGEITDVLHNRIRDCPPDFLQELSERIHRPLHTSYQAVVKSVFQISAQQSKEAGESGQDASAGGCAEKKS
LAKSRKQSIEEISTMWANTRLFMEAIKLFEGDTAVQLEKHLLKTICLEITNSIFALVAIESVSGADANTEMTANVRIKML
SLFPDELKPSLTRLNSSVTGKDLHEFTEALQEATSPKTCDIFLRSADKKKDRQVMHGHKMSLLKQLENESNGAMALHLAV
TIVFQSVTGCMIHTPGKCVPEVLGKIQPLVDKDVHRTLNRYQDLVMKEMITRKSSTPDNDHLAELQSDLINDLPVIQTIA
ATTKKQTT
>Skow_XP_002733949
MSTEDWNEVKRLAADFQRAQLSSAAQRLSERNCIEIVNKLIEDKSLEVIHTNDGKEYLTHAQLMREIRDELIVHCGRINL
VELQQIINVDFSHIEAKTNDIVKSDKNLNLILGKLIDSEYLDLIADEINDKLQESGQVSIAELTKHYDLPGDFIIEVIEQ

RLGTIIKGQMDQYDRDIIFTESYVARQTAIIRGVFSAITRPTPVISLLNQYKFHEKLFYNVIEKLVEQGRLKGSVVGGKQ
DKSSYVPDIYAKTQNNWVDSFYQQNGYLEYDTLARLGIQDGKSYLKRRYKSQDVLYLHTVCVGRSIQDQMDASIDEALNT
NTWVDISPLLPSPFTDTDANQLLNKIMKDKPNARVFCDSIVASQSFLTKCKSPFENLMKKKAEKDAKNAPAMLKEEKAVA
SGSGKKDKRDERRKRATGGAGSTHSGVGGNAREVKIKKAKKGRGGRFVDAEDEEDMDDFTQGSRDRPQELKFMSQEEIED
VLSENLTDCSDNFISEIASELVRPLTKSYQEVARSIFMQTTGTSDSVDRRKTHSELQDRINGLWTNIKLFEKGIKIVSED
LQGQLYRYILKTLCSDISNLLFEALANDNMLRVEDSSTLTPELRAKLLNKFPANIQSKMTKLHNSLTEKNLDDFNSHFDD
ACSLCDVMLKKSDKKKDRQLIFNHRQALAEQIRNEEEPAMLLHLCSVMLFQTYTNCMIHVPGRCVPQLIAFLADEIPSEH
HEQLLKFQGLVQKRLGTDRAEKLDDCSEKSDVEINSDTEVVAEMSSFVTVIREIALSKKHVPNKED
>Dmel_FBpp0081157
MGSDWDEIKRLAADFQKAQLTSTLQKLSERNCVEIVTLLLEKQMLEVVFTNDGKEYITPDHLEREIQDELYVNGGRANLV
EVSKTLNVDLSRIELLAERISAENQSVHLVLGQLIDEDYISHIAQEINEKLVQRGEVSISELASQFDLPSDFLQHDVVEK
HLGKIIKGRQDASNPRVFFTQAYIQRCKAKIRGALAAITRPINVAVILQKIGVQEKIFYSLLDEIAPAGQVTSKQANSQY
VPHIYAKTQADWVNSFYKQNSFLEYDAIQKLGISDAKSYIRKQFPNEEFLYLKRVALGARLVELTVVTALNECSATKQYL
DLTTILPSNLSEEDIEEVFSTIMAQKHSNPSNFVYLDGIVFSQPYLAQLVQPCQALAESQAKAAIDGGVYQQYIVEKTLA
QKGNVSTQELEDEGKVDKRDERRKKASSGKAGGGAQGRETKTKSTKKHQRGKAAAQFDSDDEDDAQQGSRGGGGASKKAV
KPLELVKTADIVKLITASLEEEGLEHLSKSIASLYTNQFNQTALARAQELFEATPQTNRRQTHAAIQDRINTLLIDIRLY
EKGLKLFPQDTQTQLVKYLLKSLGNEICNELSLYVASECNLTVKNTNLNVDQRNKLAQECEAQYRAALLEQNKALNKSID
DFELATETVLKTCSMIIKKVDKKKDRLLIADHKKKLQKQLLECNEPALLLHLAALILFTTITGSILHASGKFVSAILQHI
RGSLNEDQNALLLRYHDLVLQVLQAIPDSNESKLANEHLQTMQNQVVELAQNFSRASISKAD
>Cele_C06G3_9_1
MTSWADIQKLASDLQRVQLSQSSKKLSEVNCIEVLQKLIASHRIDVVYTRDGHSYVTKNHLETEIKNECIAAGGRASLTD
IAVALNIDFDHIEKTSRLIVSTDDEFTISNAEIFATEYVHRLRNELRTLLDEQGNQTTAALCKHWNLSSELLQSLLIEKL
GSSDFQGVVDGDTIYTSSFLNARQLVLRAILIALTKITPISTIQKRVGLTPKRFWIAFENLQSLGEIPGTLIGSRTSPSC
SYRPMMYDHLVKSCVLNQYRQNEFLEISTLKTLGVDAKPALEEVLGSSEFKKLVSMRSMFMTKELMDQCINAVQEDLQKS
GISDVHLALQSLNLPLDTADEDEIGSKVANVEKDSHFAEGFVFKGAVLTEALRSIDKLLDVRAHEEVDRLEAEKKKQGGA
KAAVKVQEETDDWGDGKKGGKGGKKNAKSVKGGSKSSAPSTSSNLSANISINSEELEIWLRESQSVPEEILSVIVEKLNQ
ETTTLLRKKVQDIQAHQLVASVANSKKSLSAIGDKCRQLYDSFNTFETATSTFADPLGSDLRQYLLKTVGNEIALALLSY
VMGVDNAHQLKEKQREETIENLPEMLRDPIRSVFASLKSTDDDALDKFHDAVYNCSAPSATSLALKKVDKKGRAEVGAKI
TAELHEQLCSQTEPATTLLLSVLYILAKAGRPTTASGKFVSQLVAQIKDLCPENVFDLLQACQKGVVTCIKNKGDEVAKE
MLVNDISSLKQSIMP
>Ctel_148455
MADWEEIKRLAADFQRAQLSTSVQRLLSERNCIEIVQHLQKLNLINVVYTIDGREYLTPHELHKEILEELAVHGGKINVV
ELQQLLNVDLHHIETVVTQIVKSQQNLNLVLGQLIDKSYLDNLAESINDQLQKNGQLFISELTKAYDLPSDFLSDAIFAR
LGKIIKGQVDAQDRNIIFTRAFVARRQAKIRGALSALTKPVTLMSIMNSLNIQENMFFGILDELIKSKRLAGTIVGGHHE
RSQYVPDIYTRSQNEYVDCFYKQNGYLEYDALSRLGITDCKSYIQKRLKGEPLLMLSSCCIGKAIRESIEAEVDEALSTA
SWVDIVHLLPSICDDADAAKLLSTIMKTKNNAHVLCETIAVSESFISSSKQPFDSLLSSKAKHVAVSDPAAFNPATSTKK
EDAMEERKEQRRKKAAGGGKGGGGTQGREGKTKSTKKKGGRGRDQDEDDDDEASAPKGKVVDMEFMSIELLADELKKVDK
FSECPDSMLEEISAKLYRPLNKEFTEMAKSIFLESQGSGTSAARKKTHSQLSDKIVGLWTNAKLFEKGLKLFPDEAQVAL
SKHLLHTVCSDIANLMLNSVAMDNMISVQDESSITAAIRQKLLNRLPDSKTKDYLTKLNLSLTGKAESLEDFFEQMEVLC
GSEHLEIALKKVDKKKERQLSFGHRQALAEQLRAEGDLALGLHLACVILLQVFTGAMVHAPGRCVPHLVALLKKHVTEEQ
HALLIRAQEAVIKQMKQSEGSVEKEEVKVEAKEEVKEEAKEEGEEEAKEEGEGEEVVGQQEDLLPIIKDLALNARKSNNN
QD*
>Lgig_229220
MADLEAVRREFQEVQLSAAKQKLSERNVVEIVAKLIELNYIDVVYTCDGKEYLTHDEINKEIKEELQVHGGRINLVELQE
ILNVDLSVIEGQVGKIIKQGTFSLVLGQLIDKTYQNQLAEEVNDKLQEQGYIKIADIVRQYDLPFEFVSKVLISRIGSTI
KGRVDKFDRDVIFTEAYVRRMKAKIRGALSAVTSPFTVTAIQNHLGLQENLFTSILEDLIKSSRLCGNLMGNIYTPDIYV
KSQNDWVDTFYQQNGYLEYAPLHRLGITEVKEYIKQRFHQEDIVYLSSSCAGKGLQDLVEASVDETLSQDTWLDISTILP
SVFTTNDAQQLLTAYLKNHNGAIICGKTIIASEKIITQCHEIFKDRVIKKAKKDALADPKKFAPDEGKGRSSKDDDFVDM
KEERREQRRKKAAAGSGSTKSGGGTQGREIKTKSTKKKGGRGRDNDRGGDSDDDNNKPSQVEVIEFLSIEELSEIIRDQP
FLRDCPEKLIKEIATQLYRPLNKEYLEVVKSTLSEIVGTTSSVDRKKTHNVLQEKILGLWTNVKLFEKGLKNFKDDTQTQ
LSKHLLNTVCNDMCNIIVNAVAGDHQMSVQDESTLTNEERLKLIMKLPSKDQVPLTKLHQAVRGKSLDEFFNQFENLCGP
EHLGLMLKKPDKKKERQLTFSHRQSLAAQLKDENDPAMTLHLASVILFYTFTQTILDTPGRLVPQIVQFLSTYLDTEKQT
ILTDFQELVIKSAKLKSSEEEIEDLDSIEKEMKDLLPQVKEIALTTRKSAGGNED*
>Nvec_XP_001634100
MADWDEIKKLAADFHRVQLTSTAHKLSERNCIEIVQKLINTGLLEVIYTTDGKEYLTPQQLEREIKDELFVHGGRFFLLN
LVDLQQIINVDLSHVDNKVSEILRHDKSLVLIQGDLIDKDYLDRMAEEINETLQEAGQVFIAELSKTFSLPTDFMLEVVE
SRLDIQIMGKLDQMDRNVLYTHAFIARQTARMRGVLSAITRPTQFSNIISKYGFQEKLFYSVIGDLLSSKRIMGSTQGRQ
DKISFVPDVYTQAQNNWVDDFFKQNGYVEYDALTRLGITDGKQFLKRRYHGNEIFLPTCCVGERVLDQADAAIDDALSSG
TWVDIMPHLPTPFTPDDAEQLLQHCLKTPGRSTAHVYCSSIVASDKFVEACKAPFETRMHAKAKKDAAESPALFAELSKK
DLASLKGGGGGADRREARKEDRRKTASSGGKGGGSVGGRGGRETKTKKVKKKDFRNRNDEDDTNEADSRSNPDELQFMTT
EEIAEVLRKDRPDIDDDFLEELAQEMHRPLTRAYQQVARAVLQSSGDKKRKSHAEIQEKVNALWANAKLFDKSINLFPED
VQVHLSRHLLRTVCTDITNLMVNMLAADHMLAVTDETTITPETRLKIIKKMPEEIKAPLTKLNSSLTAKETEEFFAQFDL

VAGQGLCELMIKKLDKKKERQISIERRAALQEQLRHEDEPAMTLHLAVSLLFQHFTSSLLHAPGRCVPQIVALLAEHVTP
EQHEILLRCQSLVVKQLTRSGSDEVEEEAGDEHENEAGVEKSTAVLLEEGMREIKKMVLELKKDNSKNES
>Adig_10811v107606
MLIGVDFSHVEKKVIEMVKHSSSLKLIQGDLIDKNYLDQVAEDINDFLQESGQISIAELSKKFTFPTDFLLEIIESHLGT
VIHGQLDMLDRGVLFTESFVAQQTACIRGVFSAITKPTTLSSLIAEYGFHEKLLYGDAARLLQDIFKSSVHSLAHVFCES
IAVSDRFLQNCKDPFQQLMQDKAKTDSLTAPALFSELKMKDLAAMGVXXXXXXXXXXXXXXXXXXXXXXXXXXXXXXXXX
XXXXXXXXXXSKTQKVRDKKKDRSTKDEGLDDESKSKQNQSELPFMSVEEISEELQKEMSKLFRRLNRRNRQLSLQTADP
VLPGSCQVSFSLHWRQTAKEPLGSAEQLSLERRCALQEQLRQENDPAMALHLVSAVLFQHHTGCMLHAPGRCVPQIISFL
EDKLSPEHYASLNQYVTLVIKQLSGSEDHESTEGQEGKSVTDQLEEGLDNIKKLALELKKAKDTDS*
>Adig_22370v117572
MKVNEGERDKAPAVSQLVEHRVVMREVLIGVDFSHVEKKVIEMVKHSSTLKLIQGDLIDKNYLDQVAEDINDFLQESGQI
SIAELSKKFTFPTDFLLEIIESHLGTVIHGQLDMLDRGVLFTESFVAQQTACIRGVFSAITKYVYQHT
>Hmag_XP_002160620
MSASDWEEIKRLAADFQRAQLCESLHKLSERNCIEIVNKLIKLKLLDVIYTIDGKEYLTPQQVEKEIRDELSVHGGRINL
VDLQQILNIDLSHIECRVNDLVRGDRHFILIQGQLLDKDYLDRVAEEINETLQEAGQVAIADIATSFNLSADFLLSVIET
KLGTIIRGSLDPINRDVLFTSAFIARNIAKIRGIFSAVTRPVHVIQLIAQYGLPEKFFYSVLETLMNTHRLCGSIQGYHE
KAIFTPDIYSKTQTNYIDSFFNQNGYIEYNTLVKLGINDGQQFMKKRFSGNILLLSSCCVNHHIIDQVDAAIQDSLLNKS
FIDIKXXXXXXXXXDERNKKANSGSGQKGGGGRGARESSTKKTKNKYRERLKGNEKENDENEEFADSKKISTSSQLVNFFS
VEQIEEVLKSELKDCEDNEFVHEIASYLFRPLTREYQNVVKDVFVSTAGSVSLTKKNSHQDYQEKVNGLLANMKLFEKGL
NLFEGETRILLIKHLLKTIGTDIVNVICELVASEHLMSLSENEVFTQDTRLKLLNKLSGDWKEVMTKLNSSLGGKDLEDF
FIQLDRATDINHCDLAIKKMDKKKERQILFNHRQSLIDQLDNETDPAMGLHLACVLLFQYQTGMLLHAPGRCVPQLIAYL
EKRISDSDHKILHEYQQLVIKHLVFKNKTPESECLDSTDSQDNSSKCLSEGIILIKQMVKNFKKTHQEDL
>Tadh_XP_002112924
MSDWNEIQRLAADFQRIQLTASAHQLSERNCIEIITKLIAMNKVQVMYTIDGKEYLTPQQLEREIRDELFVHSGRINLVE
LQQVINVDLTHIDSKVKEMLRGDRSLYLIQGDLIDRDYIDRLAEEINDILQESGQISVSELGKTFNLPTDFLQTNIEKRM
GIYIHGQVNPLERGTIYTEAYVARHAAKIRGVFSAITRPTSVSSIIYQYSFPEALLHDILRKLLDEKRLAGSVQGHQSKA
IYTPNIYSRTQSNWVLSFFRQNDYIEYDSLTRLDITDPKNYLKKCLNKNVIFLESCVSGQNILGQVQAAIEDVVATPSWV
DIMTLLPSPFTTGDASVLLQKHCLKSNKNNTSVQCLCDKFVVSNKFIQNCLQLFDDHMKTKAEKVYFHLLFIDAGKVAAK
FASTSSTNPNHSTLTKHDDSNVTGGKKKKGDDSTSSKRKGKGKDRSTPDDLESTRSHIKQNKQDLEFMAIPEIIEVLQRE
HSNCEDQFLEEIASQLFSPLKRKYQEVAKSVFLASTSSVTSEKRKLHSDAQDKINGLLTNAKLFGKGLQHFSGDAHTTLG
KHLLHTLCTEITNIVFSLLISEHIMVDSDPNSLNPETRSSALEKFPNNVKKAASALEKSLSGKDVEQFFDALDVVLGPSI
CQIMIKKLDKKKERQIIFNHRQSLIEQLNKESKPAMCLHLCTLLLFQRHTQCMIHAPGRCIPQIISFLKQHLTDEQYKTI
YEYQQLIIQSIQQSSDKQKPEMSEEPKDSDNSNDNQNIDLQLQEKMTTIKAIALENKKQS
>Mlei_ML212011a
MSAWAEVRQLQEELYNVQLEATKNKLSERNCVAVIQRLIGLGWLEVVFSCDGKEYITPKQLEKDILDELYVCGGRVAMTD
LAQTLNVDYSHVDKKVNELLSHDTKLSIHYGELISRQFFDNLAIEINETLQETGLLPLAQLSREVSLPVSTLAKELAPRV
GDLINGTYDKKTDTLFTTAFVERQAAKLRGACSAATRPVHLGKIMEIHGITASLGHSCVKDMIASGRLQGQLQGHSTNPV
FIPSLYISAQNQWADSFYKQNGYITLEMMQKKGIDNPQSVMSRLENSVVLSSVCVSDNSIASAQSIVEEAIVTDSYIELI
DWMFDWCNEADESKLLELVLANSKSEDYRILPSKSVVSVNLLNKLLNSFDSKIAEQVKSDAVNPKILASLKKFDSASVSV
SESKPAKKAKGGKKGKKGRAKDDDDLDYTAPKPVLGPTYLSTQQIEEELKSFRFFLDNEPKEETTIEEFSELLRKELDDK
YCKALDEEVRKKTLKSSKSQQKADEETINNLYNTVILCEKGLKVFEDSTTSAQIEKHLLKTLCLDLTKATFSKLAEEHNC
PFQGDNMKLDSIVAYLPKNAKQFATSIITLVTSGTSVQEFIDNYTELTDPQYTQLLVKKLDKKRERNMVFAQKQALLEQL
KNEQKSAELLNISCNLIFIKQHSTLLHVPGRCISSVVKLISKDFPGPQLEVINSSLSSVVAELKGEGEMSDEQKKTLRDL
VSEVLFGKQSQEEE
>Ocar_g8408_t1
MQGRMEKAIFVPEVYTRTQSDWISSFFKQNGYIEYDTLKRLGVAEPKQYCKRKLGSEALVLLPTCCLGKSLQDQVEASID
GALASESWIDVLPLLPSPCSVADTTEFVQLIIQRRGMATLHLFCDGCVVVSEQFLQKCFKIFDDIMAQKAEKAAISSVHK
STDAGAGDGKTASSALTKESGEKALTKKEQRKQKMASGPSSAAQGGKGGFSREKRIPKSKDRRKERAEVDEDSMLTSKKT
RFLSVSEIEEILSEKFEECSDDLIQDLSAYLVRPLSAKYEELMLSVFVTANVTRDEKGSAHKRKHGEYEEKLNGFYTNAR
LFRKGQDLFSDDAHVQLSKHLLRTLCTDMVNLCLATLTEEHLLLTVDPQTLSTEDRAKVLLKLPPSIKPTMVRLHNTLTK
STDEFFEALEIACKPEVCQFLIKKVDKKKERQLVFTHRQSLMDQLRRELEPAMALHLTAVILFIRHSHCLIHCPGRCVPF
VISFLEAKMSPSDFTVISDYQKLVMKQFQKGDDELKEGDETNISQTLSNDLESLKKLAISPEKEAV
>Ocar_g8409_t1
MSSGDWEEVLALQKDFARTQLSSTVHRLAERNVVEVVSKLVQMGLLEVIYTIDGREYLTPGQLDKEIRDEQFLHGGRINL
VDLHQIINVDLKHIEARVAELVRRDRHVIIVQGQLIDKDYLDHIAEEINEMLQGSGQVSIADLSKTFNLPAEILLEVH
>Aque_XP_003386214
MSDWNEVRRLQAELERVQSASTAFRLSEVNCVEIVKKLTELKLISLLYTTDGKEFIVQEHLEKEIRNEIEANKGRIEVIE
LQQILNVDINHINRGVDNIVRYDESFERIPGEVITSSYKVALCTEINEMLLQNGFVSLSDLSSTFSLPLYFITKCVEDSL
STLINGQLDKSGSGLLYTESYVSRHRSRLRGLFSAMTQPTPIPALVTEGKYHERLFHSTLNELISSGDLSGSVHGRGMRA
IFIPAVYSQMQNQWVDRFLDDNGYLEYDALVRMGIESPQVYIKTRYKDRTGDLMMLKSCCIGQSVISEIEANVEDTVVSQ
SQWIDIMSVLPSPCTDEDGELLLQRLYSNEERFLISETILSHQDFVRNCLDIFKSLIEEKAAKAAATSGSFLRQVSQESK
KDGGAVGGGGGGKKGGKGKKGKANHSNDCIVLHIRPLQERFESEARSMLAQRAEGEAGSQRKRHSELQERLQQLWNRLRL

FEKGIEVFEGDTKVQLSKYLLHTICTDLTNQLLRYIAQDYKITVPEDEGAFNKERTKLVGKLPDAVRKLAQAVNSSLSEK
EVAVFVSSFEALCDTESCGVMLKKLDKKKEKQVIINHVEELKANLKDEQDPAMSLHLVTLLLFQAHTGCLLHVPGRLIPR
VISLLRSHMTSNDFAILDTYQSLVVLDLKKSTASQSEGEGDENEDKSKLEIDSNLFEEEAESIDQSKEKVSESSDDKGGV
PVGDAPAAGGSIREQLDLKMGQLKDLVFNLKK
>Mbre_XP_001743247
MLAPPLPPAHVAPQSVLLPCSSIWIAIGGEDSVAVDNIAAEVNELLQEAGKQLTQRVGTIINGKLDRDGHAIHTDAYLNR
QNAILRGFFAALASPIALSAVRQRFQTEDVRLSDLVSEMIASGAVTGTLEGKGEKGRFVPALFQQAQQQYLESFFSANNY
IDVVDLPQAVAEQVQPRLQASFEQKVAEVVASHGDDQVDQRKLREELIGQYRLELVAKHINRTLGLDALHIIVKFMVRLD
LTPSSLLKLQLD
>Cowc_CAOG_04387
MDEIRRLQEEFQRAQQSTSVSRLSERSVVEIVMKLTELSLIDVLFTADGKEYITPSHLQKEVRDELYVRGGRIALVELPH
LLNVDLTHIEARVQGIIAAEPHIQLVAGELIESRYLDGVAQEINEYLQAIGQITVADMCKRFNLPVELLQLEIERRLGSQ
IHGQLDAQDRGVVFTQAFVARQTAAIRGAFTGITRPTPVTQVLSAYKFPERIFYVVVARLVETKQLAGSLQGRNERAVYF
PDVFSRTQHHFAEQFFAQNNYIDHDTLKRIEISDPRTFCATQLSNAVPLVSVHVNQSVIDHVNNSIVELVETAAQSPKDA
FLDISPLIPAPCSVADATVILEKCSSLSENSDTLRVLADTFAVSKHFLEFCSGLLTKFLDGKAHHAPSILALAAPSTHVL
EGAEDDASAVPATIINTGKKAPAASAPSAKGKGKRGAAASSTAGKATGDAAAVSEPFDEAEVLIQLQLNLRHCEDALLEQ
VAHALMPSLLAQYHEFLKQVFIVKTAELEAQQQQQQQQQQQQQHQHRHQQQQQQQSSSSVDTHTPSAGALGDFDSVLQF
TLQHIRLFEAGIRYFDAAAAGILSKHLLKTHCTELVNLITLRAAGKPFDAEAAAALTPEARAALAKSASPAAQTALKRLN
ETLNGSSVPAFLENIAEAAEACEYFIKKQDKRGSVRRQVAAEYLAVLEQRLRAADDPALVLHLAVVILFSHTAQALLHAP
GRAVPQIITWLGKHLQGMPDTAAASVAEASKKGDDTRGKKRDKKNADREEASSAAASGSDAAAAPPAGFRLSKDDYANVL
AYQKLVIDLIKSEGSSEGKTSAGIIRNDLMSGMQAIKELAPPSSQLEWSD*
>Mvib_comp14877_c1_seq1_fr4
TSFCLLKLEYMDDELRALQAQLASAQAATSSVRMSERNCIEIVAKLIAMKLLEVIYTVDGKEYLTPQQLEREIRDEVDAH
QGRINVTDLPQLLNVEFYHIEGKVGTVVKNDPRLQLVNGEIITTDYLDSMAMEVNELVQESGNISVADVCKRFQFATEFV
LPHLQSRMGTLIHGSLELSRLPMIVTEAYRAREQARMRGALCGVTRPTNTVSLATSLGFDADLLGAYALELTSSTHALLS
GTVQGRQGKGKYTPNVFMKTQTDWINSFYSQNALIGYDTLARLDIGEDPAGYTKKRFASALQLESCAVAPSLVARADAAV
EHAVETRTWCNVEPLLPPSLTPDDIRCLLTTHCPAFSSSACHILCDTYVVPKTFVETNMNIFEGSIKERVHHAAKAKTVT
VVGTASRNQSTSTDDSAAVASQSQSQSQSQSQSQSQQRRKQRGQQGDALVIGTVASDDDDDWGSTSRAKGKKGKKGGGQGTG
KSKGKGGGNSTSTTPAPTPDASTTFTLTEICSLVRAHLASAQSMSESDADEFSDEFLEALAALFERPLRAALDRAMHEVV
LSTAMQSESALKKARKETLERVPPLFESLVLFQKGIASVTAPDKIIGLLERHLLKTIGTDIATLLVRHAAVDAAIELPGT
FGDPLSAEQRLSAIQQMGHDFRKALDAVTDALTGKTCDAFVECLEEHDELFSISLRRPDKKKERQILFHQLQSLSEKVAH
EESPALLFHLVAVVLFQQHVGTMVNAPGSCVPALVEQLKGIVGAEAHTLLVEYQQAVVAG
>Sarc_SARC_04953
MLLAINTLLHANGCLTLKDLYEEYSLPLVYIQKMIEDDNGANVECILDITHGWSFMHTAQYTEQIAYRIKDALKDLTKPT
QLKLVFKQVDTIPAIFQSTLRQLVTSAAVEGSVKGPYDSSGMYIPTAFRDYQRSWTSEFFGQNGYLDFREMKAMLMDDPA
THTAALPGHVLGHVFVGLGILAKVDCACDEALSTQGFYDLMVRYG*
>Cfra_g6276
SHIRELVKILQERGFADVVLTRDGSEVVTHDRLLLEMKDALVHNNGRISLEDLNTQLQVSSDVIHNTAVEVMREVPTTHL
ISRVLVTRGYLDSLLVMINSRLQSVGYLTLREIYQEWNLPIDYLQKVHMNYAYDILIEDNAQSMECFFDTPNGHGWSYMY
TRMYMAEISNRIRESLCFATAPTTLKSILDLVPTAPVIFQDAFRSLLDSGAVAGSVKGGVSATGIYVPSVFVETQSKWVA
QSFSQNGYLGACCIY
>Contig76800_Abeoforma_whisleri_fr5
EKIVRKICDETYGKKLMYLLVSGEVLQSCLMEKYLQEVNDWLQLHRKVTILEIQERFSLPRDFLIKNIEDNLENIINGYF
LDSTTLITTTYEDEQREKILGIFTAIVGHPVALSSIASRHGFEMSKMTTYVQELISQDLIQGVLTGGTYIPTLFTIKQQE
WLSSFIDQ
>Contig5072_Pirum_gemmata_fr2
KDFEERIEEKILEVLLPLSESVLLSDIQNEHDIKNVGFAAAVQNLIDKGKLSGSIKGIYDSATYIPEVVRLEQEKWLIDF
LRQNQYLEYSKAVTNGISLSVVKQKCENF
>Contig57415_Pirum_gemmata_fr2
KFCTSKYKITYIN*K*ILILNNNFT*L*NFASLKESFLFFAF*NSW*RRKK*AKLHWWR*IDMFVNFVPSL*P*IELKNS
KSTISN*QN***QIK*IDK*NAY*QQKTINNK**TINYKLYKQ*TILKLFLSFPFSGFFYYFQNCIIVSTMSTEQSSPTL
YNDSILQDVENQCNIEKITENQGENAFLTKSNLIDLITEMKKLGLFDDIISSISGEDYMTKDHLEKQIIEALLSTGRIKL
SCLSQQLKVDNKHVDKIITKIIKENSDGFHNVNGDIISRECIDGYLAESNSILQEKGDVCISDLSRRFDLPREFFLKMVQ
QNIDVIIHGSFLDQNTIVTKDFEE
>Apar_comp17119_c2_seq1_fr5
SVRSLGLSLGHNTLYIMDEIKWLEEQLQAVQLESTLQKLSESNVVEIVSKLRALGLVTVIHTQDGKEYLTPQQLEREVRD
GIYIHQGRVNVTTLQEEIGVDLRHVDDVVQRLVRSEPNLHLVTGDVIDDGYLDQMAEQINEDLQAKGHLTIAAICHKFNL
PVDFVLQVVGARLGTLIHGRHNSDDRGYLYTRAFLDGQRALVRGVLTGVTRPTP
>Contig5491_Corallo_2_fr1
VHMTQTYIK*RWRCVWGSSWTRSMDELKELQRQLRDLQTPGDPGVVKKDTAAQRQARPSQQYAFSDHVAVEIVVSALLNG
SIPSLEKLKRKAATVPDTALERLASGEGFITWARLRHDLYSMLGQRRRCTFNEAATTLDVDVSSVKAMIAAELAKPQSDR
IIDFLMSGDEIFAAEYLDRVASRINELLYRRGMLSFEDVCRSLVDVPVKIVRAVMESWRIGMLIKGQYNTSNRTLYTESF

MQRQEAMVTGSLRGTLMKTPIVDVLVEDPVLGKSLNRKQATDILLSMIRAQPTGNPQLFPSGDLVRHGGGQTVRLDFTPE
VYHNKVRDRKLANLKSNGYMAVDDLGDINKDLLPHGRVLGGALYVMDVRLDSFCRNIGDRLERSGAVAVRYALQTDESVL
SPAFGTEVIGRVDILEALMIVLRDRRLIKEGILELPGPVLVKDSLVLSLVEPIQASAKSFFEVEMQSHKGKRKAKKGQSP
TYVSPNGMLDAGLRKHLTQDLVNAGIDQAVLSAILSQPIDASSQVTGSSRKKSKTAAKRDFTVKEEIENGSMARLLNSIL
SIRDEILDQTSVVNRIQGLLVAQSWHALLCLSETLALLAMADSLVASLLPKDQKDLRKFQVACGEYMQTVTVPEVNALMH
CVASLLYAVPDADAVEKFLSTDLGPLVEPDATPSDENMLRQFGVGLLPQIRQSSLYAQAAEDPEHRRVLRSYVPNDAIAQ
ALETLEIAALSVARNAVDASFSTTSTVVAQATRVEETAQTLMQESYAQSFAGWKRHYAEVRGLESTVDSAGDIAVHGQLL
MGQWLTNFATASRAAAGYSVQDATYCLHLGICMAFYQCSQPQLVHIGGGKALSAALKALKVIKSDATSVEDDVLVGLQQL
QTFIKDAIKSGQGEVRDTGLQPLLKHLFRVYEQRHVSSASWSP*AKPELIESLFHK
>Mver_MVEG_05430
MTSSIWTSIFGTGISQEDLKGPASLSEDACNDLVHSLVRLGYLGDIATSLDGKTFITQEQLTKDITSLLEKRNGRVSILD
LPRALNANSDDIQARIVDLMKTNPGRYVQAQDELLKMEYLDEMVSQLNDELTKRGFWTVADQCRKYKFSLEFMKQLLRDK
VGTVIKGQWDTADRGAVYTSWFIENEKSALSTVLQKLKEPTPLHALQARHVVQDQFFYALCEALSKDQDVPGVFMGMNDQ
GVFVPRPYKEQQAEMIEIFFRNNGFIEYTTIKKHGVTDPRAYLQTNHPSALLLDTHAVKESIWSIVDASVEDSISNLSWI
DIKPLVPTPLTREDLSSLLRQLPSLKEPSRIAVSPEQDHSLTGLGGVAPQETTVVQDSILVTAGQFQKCLLNMGPLLDRK
AKALVSWRLSFGGNARIEGEEDLEEMHVLGHVGGKTTSPALARANSKFAATVAKQQKKKFQDFLTIQDVKDEIRNLEPDF
ESVLVNAVAGTLYKDLLLNLRDRNRSVVLNQAEDEDEASDTEADVDSKEPARESNIGLIQLAAKGLSDKIRLASKGIDLF
EDVIVRNSLSKYLLQSWCVEMLDILLLSCSLVETTTLSSPSDTATQARDRIRKSFHDCEQGSITSEVTQGPFIITAEDQQ
HLLTFVAADAVTSLQKLRKIVGGSCKYKSLVEFQTISQPFVDALPKTAVLGRDESQLLAGHLTELNHNLSGIKPQTGDAL
MLHIVTLIIFQSWTGNMLHASGKFVPRILRQLRVIVDKSSSSGSDIRLEQLDLLDKMLITVLAAAKQELDPLEGTSQPCQ
DVYNLGMELSAPQA*
>Pbla_Phybl2_179417
MVNSVLLTSTLFKKLIDQGIQRGYLNDFLPTLDGESYLTKQHIQLSIQNHVMKHGRASIAELADLLNLEQSCIIQALNDI
NEFIKVDDLVFTSKYIDGFINQIKDRVDQTGWVSVLAQAQSISLPYAFLQTILDEKLLCNEDYIKYSTLPDLILSEEYYN
KSIETIKGALEEASDKPIHIVMVDGLAKENVLKGHFRGRRERAVFEPTVYRDRQVELIFSLLEAAGYIEYETVVRHYPYM
NPTDLLKKNYPNILLLDGCAATEDLVKKLEAKIQETYDTSTWLDASAWAPFVFTPADATGLLEHTMTRLTHLTSPTTSRR
SLGSLADPENRLIVLESRFVTSTRYLEELIKSSTDFLNKRALSEIQQQKKTHSSKGKKHRADDKGLVLTEQEICKYLIEE
CGLEKSFARSVSTTLRRPMTDAFQRAIQTVYLPSSNNGSGTLLSPESQWIEALKKRQQTLLSTLHQTIVYTTQAIQIFKD
ESGNKSLEKYVVVRVLCLEWLYNFLVLQTLSEAYDIAGVEDAVGVSEKDLENQKKISEDHQKMAIQRMLEVHSSDVQLKDL
HQAALAGKKPDLFLSYFESSPPNFFKPLTENVKKQAWKDMVNSLGRLLNTCSLSESTGPSILHITSLICFEYIYHNTPFS
VSGKYVPLIIRHCTPQLEAKDQSKEAKLLNDAHAMIMAHVKSKQPVDLDLLAKVKKQGQQWVSKLQDI*
>Mcir_Mucci2_112731
MTAIPANLFVQFIQQGLEKGLINDITKTLDGEYYCNDQYIEKRISALVEQNGKCKIDVERLAEHLCIEQHNVKRVIQHMP
ADNTWTVCDDLILTQECVDGIKKDLELKIDASGSLSIIAQTQQMKLPYSLLKSILDDTVIADGRYSRYAQIPDVIFTSTY
IDGCKKKIVDALKSYEEPFPMFKLQKSQNIQEDLFYLLLEQIVKDASFDLGSLRGKRSRAIFEPSSFKERQLSLIKSIFE
SNDCISYNSIENLYTFSSPADLIADSYDKDSFLSLNSCFIKTTVKDKTKSTLEGISEYCDVNDLLPAILTFEDVNKVIDT
ILSEMSSANSTTKLIDLDGGYVVNAAYVQRLIAESDNYLRNLSRTLKQKLSKNYASNSINLGDNDIIKAFECLECPRNIA
ERLLPFSRNAMLTQFSDILQTPYIETEAITSGDAWILEQKTRALKKLESLRQSIYFNDQAKKLFQDAAAQKSLEKYILKN
QCTEFLFHLVIYLVLDQSYNKAEVSQSTSLCIKLEDIENQSIVDAKQQKCVISYFIRENDHKYDNTYVVEIEEMLKKKKL
DQFIATILIKDKQHMFGSQLPLTSEQIKKSANEITNKQLYHQLQQLPVSTQTVPQMLHLVSLLIFQKVFQLPLYVTGKFV
PVILDEIRPHLSSNEQELLNTVHLSIINDQRQEHLEDYERLHSLGMKYKLI*
>Amac_AMAG_05809
MANTVSAATFDALLGLLVDRGELPPVYHTFTGDLVTLAHLCSTLRDKIAADGFLPTTHLEQILGVNQRTGEHVVETVVGE
SQGDLEIVQAFLLSRKGRDQLRASAVDFILQAEITAGDAVALALRVTTNLVYELLFSANPSDQVFRDKYESRVFYSASFR
ERLRNLLAEGLQTDRPISIAHILKGTECPPGLREELLHDILHPAASIHSPTAPIRGKLRGDIFTPDAYIAAQAAHLASQL
SSIGVLPLSDIASMRPPPSGLHLATVFIASTHIDRLRDAVLAMTDAADPARPIANVVDMAALDVTGTVAAALGDAHDAAA
FLQAMFPPQKRTTRGEDPVVVAGTVVARRARLVNACLAECDAALVAPLLKPTAVAAVTATGAVPTSREVSREAVAHRLLW
VPADLRDAVVSDILPELRRRVDAARSLRMDPQRALLHWVSRFHVYCDGLDQLAGWGLPSSTVKTLGRHLLKTVGPNVAEA
LLAFVDDAQLTTIRDMMNQIIQSKSVAMFRKTIRTIAQHCKVDLPVTEEEDAELTAMLDEAKTNPLADPLVVELASQHGI
MVLVDPAVHGEAIRRVLEKRQTASS*
>Amac_AMAG_12226
MANTVSAATFDALLGLLVDRGELPPVYHTFSGDLVTLAHLCSTLRDKIAADGFVPTTHLEQILGVNQRTGEQVVETVVGE
SQGDQEVVQTFLLSRKGRDQLRTSAIDFVQQAEITAGDAVALALRVTTKLVYELLFAANPSDQVFRDKYESRVLYSASFR
ERLRALLAQGLQIDRPISIAHILKGTDCPPGLREELLHDLLHPSTSTPSPTAPIRGKLQSDIFTPDAYVAAQAAHLASQL
TSIGVLPLADIATMRPPPTGLRLATVFVASTHIDRLRDAVLAMADAADPARPIANVVEMAALDVTARSGRPARPERIRCG
AGTVVARPARLVNACLAECDAVLVAPQLKPSAVAAVTATGAAPTPREVSKEAVAHRLLWVPSDLHDVVVPEILPELRRRV
DAARALCMDPQRALLHWVGRFHVYCDGLDQLAGWGLPSSTVKSLGRHLLKTVGPNVAESLLAFVDDAQLMTIRDMMNQII
KSKLTVDVHFAQSVAMFRKTIRTIAQHCKVDLPVTEEEDAVLTAMLDETKTNPLADPLVVELASQYGIMVLLDPAVHGEA
IRRVLEKRQTASS*
>Ttra_AMSG_04610
MATELAQLQAQLQSTFAAPSAVRITQSNAVELVQRLKELQLVDVLFTRDGSAFVTPQHLEDEVADEVALAGRVRIAEVAD
TLLVEYVEVEAAVRRRLARNPTALVLVGDEILHPPYLEELMSEVLDEAIAHGVLPLATAVVRVALPSSFLRQQLEALVSR

DSRLVLLDAALYSQVFLDRIMARVRGRLRAALLPTRLNVVLAASGAEGHAERIEALVAREAGSGKLDGTLAGSGVAATWV
PDVYTQARVDAVERQLEVNSVVLYSVLAECGFDGGAAFAHARWGESGVALESVFVASSHLDVVDAIAGAALAPDGPGFVS
LRSALHPDIGPDDTALVLERGLLPCLGGDVQVLDERGAAVAIAGRLMEVLRAAVSDAVETALEAGAYESVFLALVQLEEG
KAGARSKRGRSDHASAAAKGLVAELTLPVDAVEAAVSTASPELGREAECLDAVTAALVGESKAMIAGALRSALERRSVLR
AAAPEFSGGSSEPSPEQAMVSEVAGELEVYAAGVRYVAELASGASTSPGVAHALEAHVLKTVALPLVDALVASALAATGS
ELPQPVTASKRKKAIKTLTEPPRDTLLALVKAGTPGKAAKSPEAQAALEAHPAGERIDKFLAALMAAAPVLDLALEAGSC
AEPSAQDGRRQAHMAVVADALGAATDPADALQLVLVYLFASDHHALVNVAGRDVGRMVSLYTSVADSAEQLARAELLTSL
YDVTYEWLIGRRADAALGARAVELVRDMGLLLGVTAER*
>Ddis_XP_638953
MEELEILRKKLYSIQRQDNTQKLSERNCIEIIVKMIDLKMIDIIHSLDGKEYITPKQLENEIKDEILKCGGRVVVCDLQS
ILSIDIIHIEEALEKLKKRDKSIQIYQGEIMTRYYLDSILQEIQEQLQEVGKLHINDLSNRFSIGVDFLINLIEQNLKKF
NRSLNNSTNNNNNNNNILLKNIIFDNQEVLYTQSHINRHYHKVIGLFSSITQPILINNVIKQHQNHYQLNERLVRQQLQE
LIENKRINGFIQGKASNAEFIPTIFSQSRLKWIDSFYHQNQFITFDSVSKLQINEPQSFLKNTFTDGLLLHSCFIHKSLI
DKIDDNIIEIIENSNWLDLIPLLPPLTDKDISMIVSNCPNMKTNNAILLGNSFIISKSFVDRCFTLLQKTIQERMEKQQL
ILENIASSNIKKQQQSKSEIIDQKIETTTTTTTTTQPSKKKDNLINSDDDDNQDNNKKSSKGKNKKSKQQQSSIQKLIND
SEDDYKVYNKKQENNNNNKKVDHLKEINQLLSKWYENMEEELVESLSQYLRPLVNQSWESMVKEAKEKLENETQKQRKQQ
LQQLNSQFFSLYNSFLLFRKGLQSLNDADDDNGKTIIALEKHLLKTVGINITNLLIEINANYHMLDKTTFETPSERSLIL
SQFPPQLSKSFEKLIQSLNKSTLNDFIDSLENVCSQSQIKLKSLDKKLEKQLLIENQNELEEQFLNEIDVGNQFQIIVNL
MYIRYKNNYIFSPPRLIGKLVSTLVMDTSIDKEIITRLTKLHQEIVKLITNNKLSNQDNDNDSTELNDHIKFFKDLILN
>Ppal_EFA84337_1
MDELELLRQRLVSVQKEQTSQKLSERNCIEIISKMIELKMIDMINTSGGKEFVTPKQLENEIRDEILASGGRVVVSDLQP
LLKVDLTYIQEKVNYIVSRDRSMQLYYGEILTRYYLDSVVEEIHETLQEVGRLDINDLSQRFNLNVEILSDAINLRLGKL
IQGVFDSDILYTQAHVNRHKSKVCGLFTSVTKPTNISSLCKQYQMNERLFHSQLAELIQSKRINGQIQGKASQAEFIPTI
FSQSRSKWIDSFFTQNSYIQYSTLTQLQIPDPTSYLKNTYKNGVALSSCFVSGYIIENVDSFITEIIANSGWMDVSTLVP
SSLGNKDVAMVIDSCPAMKDKNNRQAIVLNDFFIVSQSFVDRCFVLLEKMIQTKVEKQAIIQESIATSSGTTATSQSQSK
LSDSTDSTSSKSSGKQSGGGGGKKSKQQQIEDERPTKQKGGKKGGSKKGGKRTNDDSDDEDDFTSSKQQQQSKKKTSQTP
KVDHLSDIIQILTKSFENMEEELIQALAQYLRPKVNQIWETLVKEAKEKLETETIKSRKGQQQELANAFNTLYQNALLFK
KGLEDLDPASTVLHKHLLKTVCTNLANLVVEINGSYHMVENCNCDTPAQRSAVISQLPTAIAKNLEKLIKCLTKDTVNDF
MDTLESVCEQSQIHLKSLDKKSEKNLLESHQKELYEQLQNDQDIGNQFQAIVLLLYIKYKKHLVHISPRSIGTLVQTLSQ
QELIPDQQLLKTLTEAQQDVVNFIINKSEKKEDEESLTKKLDILKNALHVPTSTTTTSS
>Acas_g7809
MDEILALQEELRKLQEQSSIHRLSERNCVELIMRLVKLGKLTVYFTQDGKEYVTPEQLESEIRDELMQHGGKVGVLNLPT
LLNIDLNHIEARLAEMLKRDKRLQIMNGEVMTKYYLDGVAQEINEALAESGQIILTELAPRFSLTTDFVVQIVQERLGRT
IKGQLEQGVLYTHLFIDRCKAQMRAAFSAITRPTPISQIVSTYSFNERLFYGTYYVPHIYSYTRKKWIESFLSQNGFIEYN
KLSKINIPEPRKYLLVNFPEGLPLETCFVASSVIQQVDASVSDALANNAWLDPLVEFQLSAKDAMVLLKSCPSFIAASQE
KKESPVILDDHYLVSRGLLERCVASLKDKVTTETPKKAPLIIQALGKHIPASKPAAADEEEETKGKGRGGKGGKGGKAAA
SGGGGKGGRGGNQKGKRRGKGRDDDEDEEEEEEKVVERPSARGRNAAAAAASAKSRAAGGVQQEVLTEAEVRACLKQWYE
EAEADFLDVIYQQIRPIVRELNKAAVESIQQNASSGKKKDHKALEQQFSVLYQNIQLFLRSAEEVTDSAVSEALRRHLLA
TLCTEATNLLVYMMALHHGVSMEAEKITTANERKEVIAQLPLTAATPLAALGDSLRGSGVEEFLKQLESLSEEAQLHLKP
LDKKKQRLLTHTHKLDFLEQLKEVTEPPLAFHLAAVVLYAQVFNLALHIPGMRGQCLCQPGWGTANCSVKLDHTPCFGVD
CGFYGECDTASAEAGGRKCKCMAGYTGELCTVPPADSPCRGVECGPHGICKGGVCECSAGFKGCACDVAPNCSFVADADY
AGFDLPASSGGGGVASSSAQECCAKCRQAKGCQGFTQSGGRCYLKSSTDQGTSSAPGQTAGSLPNTVTLAPESCLGTYGG
AAGLVSPLGQLLAQLGL
>Atha_AT3G46220
MDDELLELQRQFEFAQQVKSSVRLSDRNVVELVQKLQELGVIDFDLLHTVTGKEYITQEQLRNEITREISKLGRVSVIDL
ADTIGVDLYHVEKQAQDVVLNDPGLMLVQGEIISQSYWDSIAEEINERLQECSQIAVAELAGQLQVGSELVQSVLEPRLG
TLVKARLEGGQLYTPAYVERVTAMVRGASRGIFVPSNLSALWAPLQQLVQETNGASGVAVENSFFQSIFNRLLKEEEMLG
SLRAGTHWTPSAFATAQKECVDSSFSQNSYISYESMQKLGISQAVQFLQSRYPDGTPLAAVFIHSSMIEMLDSATEDAIE
QNSWIDSLSVLPSSFTSQDANKMLLLCPSVQSALKAEKALILGESYVLSSGFIKGIYDQIEKEADAFSIQASTATLIHPS
SKSSESTESIPANTDKGSKKKKGKSASTKAATVETVPDDEEDARPKSKRNQKKGRDSSSSQKLDSKAGGKKESVKAQESN
NIIPPDEWVMKKIVDSVPEFEDDGTSENFFLCTENPDSILKHLADHMKPMLINSLKERRKKIFTENADRMRRLIDDLQKK
LDESFLNMQLYEKALDLFEDDQSTAVVLHRHLLRTTAATIADTLLHGLDIHNKMKNGTEVEESKTQDLVLLDSSERTALA
KNLNGSLSKKALALVEALEGKRVDTFMVTFRDLAEESGLVLKKLDKKLERTLLHSYRKDLISQVSTESDPIALLAKVVSL
LFIKIHNKALQAPGRAIAAAISHLKEKLDESAYKTLTDYQTATVTLLALMSASSGEEHDCSADRILTKRELLESQMPLLR
TLVLGDSQPQQS*
>Mgut_mgv1a001428m
MDEELLELQRQFESAQQAKSSIRLSERNVVELVQKLQQLQIIDFDLLYTTSGKEYITPEQLRSEIVSEINKRGRASLIDL
ADTTGLDLYHVEKQSQHVVSNDSSLMLINGEIISNSYWDTVSEEINERLQECSQISLAEIAAQLQVGSELLVSVLEPRLG
TLVKGRLEGGQLYTPAYVARVSAMVRGAARGIAVPMNLSAWWSSLQILLQDMDGFSGVAVESSFFQSLFNGLVKGGEILG
SLRAGVHWTPSVFAVAQKECVDSFFSQNSFISYDTLHKLGIPQPIQFLQSRYPEGKPLVTVFAHASMIEMLDSAVEDAVE
RGTWIDSLTILPISFGSQDASKILSLCPSVEKALKSSKAHLLGESYIFSDTFVKGLFDSIEKELENLNTTGLTAAGSSDI
PHVIKDSKQGHDDSSSQADLDEYDTQSGTGKSASEKGSKKKKGKATGSAKAGTDESVPEFQESTATKSKKKQKKGKVIPS

AQVSDSKPGAKRDTDRMETPSFLSEESLIQRIMSLIPDLEEQGMDDPETVLAPLATHLRPMLLNSWMERRKVAFTDNAQK
MRRVLDNIQRKLDEALLNIQLYEKALDLFEDDPSTAALLHKHLLRTAATPIVEHLLVNLDMYNKLKNGIQLEELQNPETV
SMSSSDRIALAKGLPRSLSLKAVGLVETLEGKRIELFINAVRELAEESGLMLKKLDKKLERTLLHSYRKDLTSQVSAETD
PVALLPKVVSLLYVQIHGKALQAPGRAISVAISKLKDKLDDIAFKTLTDYQSAAVGLLSLISAGTGDEEDCTSDRILSKR
ELLEASMPALKSLVLGGNSQF*
>Acoe_010_00661
MDAELLELQRQFELAQQAKSSIRLSDRNVVELVQKLQQLQIIDYDLLHTITGKEYITPDQLRHEMVTEINKTGRVSLIDL
SDVIGVDLYHIEKQAQYIVADDSGLMFNQGEIIAQSYWDSVAEEINERLQECSQIALAELAAQLHIGSELISSVLETRLG
TIVKGRLEGGQLYTPAYVARVNAMVRGATRAVTVPTNLSIVWNSLQQLLQDVDGANGIAVESTFFQSLFNGLVKEGEILG
SVRAGIHWTPSIFAHAQRESVDSFFSQNSYISYQALQKLAIPQPKQYLQSRYPEGIPLDTVFVHSSMIEMLDSAAEDAIE
QNSWIDSLSVLPAFFGTQDASKLLSLCPSIQKALKSSNALILGDSCVFSNAFVKDVFEHIDKEMAKTSGQGLNDNLHIVD
EAKGRNDPDKFVESNEVGLETGGNKSALEKGSKKKRGKPTGNTKTGGAESGTGNNDQEPTKAKKTQRKSKDQLSDTKVGA
KKDFDKAKVDNLNVYSEELIMEKILILVPDLEGLGGMDDAHSVVRLLATHLRPMLVTSLRERRKALLNENAERMRRLLDN
LQKKVDEAFLNMQLNEKALDLFEDDPSASVILHKHLLRTAATPIVDMLLINLDIHNKLKNGIEVEESDSPESASLSSGDR
LSLAKGLTGPLSVKAVAVVETLEGKRVENFMAALTSIAEESGLLIKKLDKKLERTLLHSYRKELTSQISSETDPVALLPK
VVSLLYLQVHNKALQAPGRSISVAVSRLKGKMEDAAFKILIDYHTATVALLALLSNATDDEEDCTTDRIMSKREFLESLV
PNLKGLVMGATQSMDAELLELQRQFELAQQAKSSIRLSDRNVVELVQKLQQLQIIDYDLLHTITGKEYITPDQLRHEMVT
EINKTGRVSLIDLSDVIGVDLYHIEKQAQYIVADDSGLMFNQGEIIAQSYWDSVAEEINERLQECSQIALAELAAQLHIG
SELISSVLETRLGTIVKGRLEGGQLYTPAYVARVNAMVRGATRAVTVPTNLSIVWNSLQQLLQDVDGANGIAVESTFFQS
LFNGLVKEGEILGSVRAGIHWTPSIFAHAQRESVDSFFSQNSYISYQALQKLAIPQPKQYLQSRYPEGIPLDTVFVHSSM
IEMLDSAAEDAIEQNSWIDSLSVLPAFFGTQDASKLLSLCPSIQKALKSSNALILGDSCVFSNAFVKDVFEHIDKEMAKT
SGQGLNDNLHIVDEAKGRNDPDKFVESNEVGLETGGNKSALEKGSKKKRGKPTGNTKTGGAESGTGNNDQEPTKAKKTQR
KSKDQLSDTKVGAKKDFDKAKVDNLNVYSEELIMEKILILVPDLEGLGGMDDAHSVVRLLATHLRPMLVTSLRERRKALL
NENAERMRRLLDNLQKKVDEAFLNMQLNEKALDLFEDDPSASVILHKHLLRTAATPIVDMLLINLDIHNKLKNGIEVEES
DSPESASLSSGDRLSLAKGLTGPLSVKAVAVVETLEGKVSA
>Bdis_2g37530
MDAELLELQRQLEAAQSARSSVRLSERNVVELVQKLQERGLIDFELLHTVSGKEYITSDHLKHEIKMEIKNRGRASLVDL
SDILGVDLYHIERQAQKVVTEDPALMLINGEIMSQSYWDTVTEEINEKLQDRSQIALAEIAAQLHIGSELVINILEPRLG
TIVKGRLEGGQLYTPAYVSRITAMVRGAARGITVPTNLSSVWNSLQQQLQEMHGANGVSVEGSFFQSIFVSLLKEGAVLG
SVRAGVHWTPAVFAHAQKESVDAFFSQNSYIGYEVLQKLAIPQPKQYLEARYPDGIALEAVFIRPSVVDMLDAAVGDAIE
NGHWIDSLSVLPSYISGPDATKILSLCPSLQKAVKSSKAVLFGESCVFSNVFIKGIFDRLEKEIDSFGIKHSSVQGTSMN
VNPSSEHRSGSVQYTDTKDIGDNDTSNTGVSSERGTKKKRGKAAGSAKGGPLEKDDDSEEIIPVKGKKAHRKNKDAGSSG
DAKRGGKKASEKSKDENTNIFPDELIEQKVLAAAPELEEVAGSDDLNDPIKLLSSHLRPMLIDSWMKKRNTMLSENAERR
RSVLDNMQKQLDEAVLDMQLYEKALDVFEDDPATSGILHKHLLRTMGTPIVDKILLTLDKDNKLKNGVELEDNEEQHVPL
STADRTFLAKDLPGSLSPKAQALVEALDGKRFDSFMHTLRDITEESGLLFKKLDKRLERSMLHAYRKDLTEQVSSESDPV
SFLPKVVALLFLQAYNKALQAPGRAVGAVITLLKDKLPASTFKVLTDYHSTTVKLLALQAAATGDEQDCTSDRMLEKKED
LVERLMPEMKSLALGTSKE*
>Bdis_4g35665
MRSCKNTARSPWLRLLHIGSELVVNILEPRLGTNVQGRLEGGQIYSPAYVSRITAMVRGAARGITVLSNLPSVWNSLQQQ
LQGVSVEGLFFQSIFVSLLKEGAVLGSVRGRVQ*
>Sbic_09g001810
MDAELLELQRQLEAAQSAKSSVRLSERNVVELVQKLQERGIIDFDLLHTVSGKEYITSDHLKHEIKMEIKKRGRASLVDL
SDTLGVDLYHVERQSQKVVSDDPTLMLINGEIMSQSYWDTVTEEINEKLQERSQIALAEIAAQLHIGSELVISILEPRLG
TIVKGRLEGGQLYTPAYVSRITAMVRGAARGITVPTNLPSVWNSLQQQLHEMHGASGVSVEGSFFQSIFNGLLKDGAVLG
SVRAGGQWTPAVFAHAQKESVDAFFSQNSYIGYDVLQKLAIPQPKQFLEARYPDGIALDAVFVHPSVVDMLDTAVGDAIE
NGHWIDSLSVLPSYISGPDATKILSICPSLQKAIKSSKAVVFGESCVFSNMFIKSIFDRLEKDMDSFGIRHSVGQGRPLN
ANLGSEHKTGCGQYSDTKDLGDNDTSSTGVSSDRGSKKKRGKGTGSTKGGSLEKDDDNEESIPIKGKKSHRKNKDAGASG
DVKHGGKKASEKIKEESTNIFPDELIEQKVLAAAPELEELGGSDDSNAPLKLLSSHLRPMLVDSWTKKRNTMLSGNAERR
RRLLDNLQKQIDEAVLDMQLYEKALDVFEDDPSTSGILHKHLLRSMGTPIVDKVLITLDKDSKLKNGMEVEDSEDHVQLS
TADRSSLAKDLPGALSLKAQALIEALEGKRFDSFMDALRDVLEESGLTFKKLDKRLERTMLHSYRKDLTAQVSSENDPVS
FLPKVVALLFLQAYNKALQAPGRAVGAVITLLKDKLPASTYKVLADYHSTTVKLLALQAAATDDEEDCTSDRTRERKEDL
EERLMPELKSLVLGTNKE*
>Osat_LOC_Os05g02650
MDAELLELQRQLEAAQSARSSVRLSERNVVELVQKLQERGIIDFELLHTTSGKEYITSDHLKHEIKMEIKKRGRASLVDL
SDILGVDLYHVERQSQKVVADDPSLMLINGEIMSQSYWDTVTEEINEKLQERSQIALAEIAAQLHIGSELVVNILEPRLG
TIVKGRLEGGQLYTPAYVSRITAMVRGAARGITVPTNLPSVWNSLQLQLQEMHGASGVSVEGSFFQSIFNGLLKEGVVLG
SVRAGVQWTPAVFAHAQKESVDAFFSQNSYIGYEVLQKLAIPQPKQYLEVMEAVNVPLLFKSSARYPDGIALEAVFVHPS
VVDMLDAAVGDTIENGQWIDALSVLPSYITGPDATKILSLCPSLQKAIKSSKAVVFGESCVFSNAFIKGIFDRLEKEMDS
FGIKHSAGQGKPSNMSSEHRIGSDGKDLGDNDTSSIGASSDKGPKKKRGKVSGSAKGAAVEKDDDNEESIPVKGKKAHRK
NKDAGSSGDAKHGGKKASEKTKEDNTNIFPDDLIEQKVLTVAPELEELGGSDDLNGPLKLLSSHLRPMLMDAWMKKRNTM
LSENAERRRRLLDNLQKQLDEAVLDMQLYEKSLDVFEDDPATSAILHKHLLRTMGAPVVDKILLTLHKDNKLKNGMDVED
SEENVQLSTADRTSLAKDLPGSLSVKAQALAETLEGKRFDSFMDALRDTAEESGLLFKKLDKRLERSMLHSYRKDLTAQV

SSENDPISFLPKVVALLFLQAYNKALQAPGRAVGAVIALLKDKIPAPTYKVLADYHSTTVKVLALQAAATEDGEDCATDR
MLERKEDLEERLMPELKSLVLGTSKE*
>Smol_XP_002982462
MDAELRELERLFEAAQQAKPTARLSERSVVELVVKLQQLGILDADLLHTVSGKEYITQDRLRQEMEAEINRLGRVSLVDL
ASLVGVDLFHCERQADQIVSSSPELMLVQGEVVSTKYWDNLAEEVNETLQEASQVSLGELARRYGVGAELLTGMLSSRIG
TIIHAKLEGGQLYTPAYVARVKATVRGALRAITVPTSLQSVWNLLQGLLRDRDDGGNVTGEHSLFHMVLNELVAEEAVKG
TLRGSVWTPAVFAHSQRESVEAFYSQNSYITYDALQKLNISQPRQYLQTKYPEGIALDNVFVHNSFISLLDSAAEESVKG
GGWLDALDVLPSTFGCSDSGKLLLLCPFVQRALKDSTATLVAETCIVGTGFVKALLEKFQNMLREIQDKTFSTLEVKLEA
EPEPELDENETKPFASRSKKNDKKQKDNSIRKEEENEEKSGKSKKKTARHKSSKTPSVKEAVPSKARKGSPADEMDLLAE
EKLSKKIIEWYPDLEADGTEDGDTGVLAKCLADQLRPQLISYMTSLKRAYLGANTEERRKRIDALQRKIDEDYANLQLFS
KAIELFEDDKGMEAVLHKHLLRSTASEIADVFLETQALEHRIENAESAGAQPCKPFSMAVRMSLAKNLPEPLSSAATEMV
ASLEGKNIGAFDSALEASVTESGLRVRKLDKKGERSLFFAYRKSLSEQVNQEQDPIALLPKVVALLYVQVHGRALQAPGR
AMSSAVSRLKSAIPDKAFSTLDQYLSETVSLLSSSATDVRYNLPAGPLSHTAVPAGFQKGSSRGAHG
>Smol_XP_002980981
MDAELRELERLFEAAQQAKPTARLSERSVVELVVKLQQLGILDADLLHTVSGKEYITQDRLRQEMEAEINRLGRVSLVDL
ASLVGVDLFHCERQADQIVSSSPELMLVQGEVVSTKYWDNLAEEVNETLQEASQVSLGELARRYGVGAELLTGMLSSRIG
TIIHAKLEGGQLYTPAYVARVKATVRGALRAITVPTSLQSVWNLLQGLLRDRDDGGNVTGEHSLFHMVLNELVAEEAVKG
TLRGSIWTPAVFAHSQRESVEAFYSQNSYITYDALQKLNISQPRQYLQTKYPEGIALDNVFVHNSFISLLDSAAEESVKG
GGWLDALDVLPSTFGCSDSGKLLLLCPFVQRALKDSTATLVAETCIVGTGFVKALLEKFQNMLREIQDKTFSTLEVKLEA
EPEPELDENETKPFASRSKKNDKKQKDNSIRKEEENEEKSGKSKKKTARHKSSKTPSVKEAVPSKARKGSPADEMDLLAE
EKLSKKIIEWYPDLEAAGTEDGDTGVLAKCLADQLRPQLISYMTSLKRAYLGAKTEERRKRIDALQRKIDEDYANLQLFS
KAIELFEDDKGMEAVLHKHLLRSTASEITDVFLETQALEHRIENAESAGAQPCKPFSMAVRMSLAKNLPEPLSSAATEMV
ASLEGKNIGAFDSALEASVTESGLRVRKLDKKGERSLFFAYRKNLSEQVDQEQDPIALLPKVVALLYVQVHGRALQAPGR
AMSSAVARLKSAIPDKAFSTLDQYLSETVSLLSSSAADVRYNLPAGPLSHTAVPAGFQKGSSRGAHG
>Php_XP_001780735
MDLELEELQKLFEQTQNAKAKVRLSERNVVELVSKLQALKLLDNDLLHTVSGKEYITQDELKREIESEIQRLGRVSLVDL
SGTLGVELVHCERQAQQIVQNNPSLSLMQGEILSSGYWDSIAEEINESLEVAGQISIVELARRFTVGAELMTNVLSARLG
TSIHGKLEGGQLYTPAFVARIRATVRGAVRAIMVPTSVSTLSSFLQQQMREVDEGPITKVTADGGLFQSVLNQLIADGEV
KGSLRGGNAVYTPAVFAGAQRGSVEAFFSQNGYITFDTLRKLNVPQPKQYLQEMYKDGVALEDVFVHSSLISQLDAAAEE
AIAGKSWFDVLPLVPSSIGPSDAEKLVVLCPTVQKALKDGTAVILADTCVLSATFVKKRVENKKFNKVSTQSLSEGVTGI
STTDTGTEDASGQLKGGRRKKGNQSNGATRPAPEIEAVVPKEGKGKRRAGKSKGEKVSGEAVAGVSKGFKSGTGPNSAND
EDNIFTVEFLTSKILEWFPDLESAGIVEEGILVKALADLLKPKVMAAAIHAKQALFSAQAEDRRRRLDTLQHKIDEASTP
FFCLPWLFSKALNLFEDNEKTGVLLHRHLLRTTGAELVDLFLIAQAEDDIDDKEVGITATKETELLNTSQRTAMAKSLPA
RNLSSKALELVESLEAKNVDAFERTFEAAVTESGLRVKKLDKKSERALMFSYRKGLTSQVGDETDSVALLPKVVGLLFVQ
VHSRALQAPGRAMLAAIMRLKGSIPDELFEVLMAYHNGTVQLLSLQTSANLEETDASNETITKQEELKTLIPQLKSMVLP
SSSSASTTNDKQQQ
>Crei_XP_001691945
MEAALGLDLGSLLSQLTATQQAQSAAKLSERNVVELINKLRQLGILSEGELLHTLNGKEYMTVERLRAEVQKALRQAGGR
LALVELPTIVGVDLIHCERQVAAIIAQSAGTVQEVNGELITTQYLDTIASEINDQLQESGQINVGELAAQYGLATELVMN
VV
>Vcar_XP_002952984
MEAALGLDLSSLLTQLAATQQALSAAKLSERNVVELINKLRQLGILSEGELLHTLNGKEYLTVECLRSEIQNALRQAGGR
LSLVELPTIVGVDYVHCERQASVIVSESGGTVMEVQGELITTQYFDAIAAEINDQLHEAGQLNIGELAAQYGLATEMVLN
MVSMRLGSTITGRLEGGLLYTQSYLRAIKAQLRGALRAAASPALVLNLMRDAGLGATGDAASAGGGGGGAGLAAALASSG
GGGVIATLIEELVQEGALRGALKGGGTSWAPAVYGEVQQDAVASFYSQNGWVAYDTVRRKFTPILIRDVPEPSYAPSLGI
SNERSYLRGTFPDGLPLESAFVSPSLLAALEAAVEDVAAAASTGSGVGSGGGGWVDVAPLLPSVLSEADVAALLGRIPLL
RDQERRPAKERKVHVEKADADARTAAHKALQERKASSGAAGTAGAGGGGSGAGAAALPDWFPEMDWGGVDDGKAEAVAQL
LLPGAVAAYEEALQAAFTAGAEERRRAKESLARQFEESCTRLLLYGAGVETYADDEATYTALQRHALRTTGAECVDLLYR
WARVEHPPEGAEQSAAAGGPASVSGNGPGFLTAAEVKQVLRSVPPDVAEGLAAAADAPGAATVRDAEVLLDRAAAAVGVR
AKKLDKKGEKAALAALRERLLSSLASEVDPPAALTLMVPLANMRIMGKAVTLPGKALGQVLSRLQQAAEEGSELAALVRG
LGELHEGVVEYLKAQSGGSAAATTTGAMLERLSAMLPQLKAGLGLQEQHQ
>Cvar_EFN57486
MELSLDDLLSGLQEAQQKKSKARLSERNVVELVNKLKQLGLLGDDLLYTSNGKEYVTRERVAAEVRAAVRAAGGRIPLVR
RSLEAQQAGERACLPREACVDLPSQLGLDLVHCERAADAVVAASSGGIQLAQGELFSSAYFDGLAAEVDEGLQEAGVVSV
GDLARRFGLGAEMIGGVLGERVGTRIHGRMDAGVIYTQAYLTRIKAQLRGALRGAVSPVSIPALVRELGIEGLASLKALV
PSLVDELAAEGSVAGKLAPGASSWVPATYVQSQQDAVTRFFQQNGHIAYDTVRQYGIPAPRPFLAHHFPDGLALDSAYLS
PAVVEAAAEEALAAGGWCDVAPHLPSILSPGDTAALLAQCRAVAGAAAATGGRAHVMAGSCVVAAALLESIRAQLLEAAR
QAADDAHRRQKKGGEEGGGGGGAAGRGKVTVTVLPEGGGKKAAAAAAAADSGSDNGWDVGGKKGKKGGRGKKGKAAAGGS
GGGKPARSGGGGGGKAAAGQGASAGGGGGGGGGGGPSSGALSLDSLARRVAQMHPDTEGAGADGDLPRAIAAGKPPPLGAP
LWLRPAIVAEYERALHAIFTAGAERRRRLRDAAVAALDAAHERLLLYAHGAELFADDEAAVPRAAIIKRLQPDVKPAVSA
AVDKLTGSSLEQFQSELDAAAEAAGLRLRRLDKRTERTLVFAQRKLWESQAAAAPDASTLLMLVVPLLLARQHGRAVSLP
GRAVSAALDLLQGGQLPQDSLQLLADFHAAVVDQLKLQSGGRDEEARDEATQKLERLMPHVWELAAAGGGGAGGEAGGEA

>Otau_XP_003074345
MTPMHGEDADALTAEDVVALVSKMRELDILDFGSLIVSDAKRGVVTRDRLREDARAELLRRGGRATTTAIAMALEVDVGD
[truncated: 711,515 more chars]
